# Aberrant NSUN2-mediated m^5^C modification of H19 lncRNA is associated with poor differentiation of hepatocellular carcinoma

**DOI:** 10.1038/s41388-020-01475-w

**Published:** 2020-09-25

**Authors:** Zhen Sun, Songlei Xue, Meiying Zhang, Hui Xu, Xuming Hu, Shihao Chen, Yangyang Liu, Mingzhou Guo, Hengmi Cui

**Affiliations:** 1 Institute of Epigenetics and Epigenomics and College of Animal Science and Technology, Yangzhou University, 48 East Wenhui Road, Yangzhou, 225009 Jiangsu China; 2 The General Hospital of the People’s Liberation Army (PLAGH), Beijing, China; 3 Joint International Research Laboratory of Agricultural and Agri-Product Safety, Ministry of Education of China, Yangzhou University, Yangzhou, 225009 Jiangsu China; 4 Jiangsu Co-Innovation Center for Prevention and Control of Important Animal Infectious Diseases and Zoonoses, 225009 Yangzhou, China; 5 Institute of Comparative Medicine, Yangzhou University, 225009 Yangzhou, China; 6 Ministry of Education Key Lab for Avian Preventive Medicine, Yangzhou University, Yangzhou, 225009 Jiangsu China

**Keywords:** Cancer genetics, Epigenetics

## Abstract

RNA methylation is an important epigenetic modification. Recent studies on RNA methylation mainly focus on the m^6^A modification of mRNA, but very little is known about the m^5^C modification. NSUN2 is an RNA methyltransferase responsible for the m^5^C modification of multiple RNAs. In this study, we knocked down the NSUN2 gene in HepG2 cells by CRISPR/Cas9 technology and performed high-throughput RNA-BisSeq. An important tumor-related lncRNA H19 was identified to be targeted by NSUN2. Studies have shown that the expression of *H19* lncRNA is abnormally elevated and has a carcinogenic effect in many types of tumors. Our results demonstrated that m^5^C modification of *H19* lncRNA can increase its stability. Interestingly, m^5^C-modified *H19* lncRNA can be specifically bound by G3BP1, a well-known oncoprotein which further leads to MYC accumulation. This may be a novel mechanism by which lncRNA H19 exerts its oncogenic effect. Besides, both the m^5^C methylation level and the expression level of *H19* lncRNA in hepatocellular carcinoma tissues were significantly higher than those in adjacent non-cancer tissues, which were closely associated with poor differentiation of hepatocellular carcinoma (HCC). In conclusion, we found that *H19* RNA is a specific target for the NSUN2 modifier. The m^5^C-modified *H19* lncRNA may promote the occurrence and development of tumors by recruiting the G3BP1 oncoprotein. Our findings may provide a potential target and biomarker for the diagnosis and treatment of HCC.

## Introduction

In recent years, the association between abnormal RNA modifications and tumorigenesis has attracted widespread attention. Several studies have clarified that N6-methyladenosine (m^6^A) modification of RNA plays an important role in the development of cancers, such as lung cancer [1], hepatocellular carcinoma [2], and glioblastoma [3]. As a widespread modification, 5-methylcytosine (m^5^C) in DNA and RNA has been found for decades. The function of DNA m^5^C modification in carcinogenesis has been studied extensively. However, very little is known about the function of RNA m^5^C modification in tumorigenesis. NSUN2(NOP2/Sun domain family, member 2), also named Misu [4], is one of the major m^5^C-modifying methyltransferases in mammals and plays an important role in RNA metabolic processes, such as RNA processing [5], RNA stability [6–8], RNA export [9], and mRNA translation [10, 11].

A previous study demonstrated that *NSUN2* is a downstream target gene of MYC, and is essential for MYC-induced proliferation and cell-cycle progression [4]. Similar to MYC, NSUN2 is highly expressed in a variety of tumors. Immunohistochemical studies confirmed that *NSUN2* protein levels are significantly increased in various +tumors, including the esophagus, liver, pancreas, cervix, prostate, kidney, bladder, thyroid, and breast cancers compared to normal controls [12]. In breast epithelial cells, overexpression of NSUN2 was shown to promote cell proliferation, migration, and invasion while NSUN2 knockdown inhibited these processes in vitro and in vivo [13]. The close association of NSUN2 with tumor features suggests that it may be a key factor in the study of the relationship between RNA m^5^C modification and tumorigenesis.

NSUN2 was shown to account for the formation of 5-methylcytosine (m^5^C) in a variety of RNA species, including tRNA, rRNA, mRNA, lncRNA, and mitochondrial RNAs [6, 14–16]. Long non-coding RNAs (lncRNAs) are a class of RNA molecules that are longer than 200 nucleotides in length and lack protein-coding capacity. As the most abundant non-coding RNA species, a large number of m^5^C modifications are detected in lncRNAs [14]. In two well-studied lncRNAs, XIST and HOTAIR, methylated cytosines were found to be located within or near functionally important regions that are known to mediate interactions with chromatin-associated protein complexes [17].

Functional studies have revealed that lncRNAs are involved in multiple biological processes and disease-related pathways, such as proliferation [18], differentiation [19], stem cell pluripotency [20], tumorigenesis, and metastasis [21]. A recent study on esophageal squamous cell carcinoma (ESCC) identified lncRNA NMR (NSun2 methylated lncRNA), which is highly expressed in ESCC and plays a key regulatory role in the tumorigenesis and drug resistance of ESCC [22]. NMR is methylated by NSUN2 and might competitively inhibit the methylation of potential mRNAs. Thus, it was speculated that NSUN2 may be implicated in tumorigenesis through its methylated lncRNAs.

In this study, we constructed an NSUN2-deficient hepatocellular carcinoma cell line and investigated the effect of NSUN2 on cell proliferation, migration, and invasion in vitro and in vivo. Furthermore, we provide a landscape of NSUN2-mediated m^5^C modification in hepatocellular carcinoma cells, and identified *H19* lncRNA as a target of NSUN2. Recent studies have confirmed that *H19* lncRNA has an oncogenic function in a variety of adult cancers, such as hepatocellular carcinoma [23], colorectal cancer [24], pancreatic cancer [25], and glioma cell [26]. Our further results show that methylation of *H19* RNA by NSUN2 can affect the stability of H19 and its interaction with the G3BP1 oncoprotein. In addition, hypermethylation and overexpression of lncRNA H19 were identified in liver cancer tissues compared to matched non-cancerous liver tissues, and a higher level of *H19* RNA m^5^C methylation was correlated with HCC progression. Thus, our findings demonstrate a regulatory mechanism involving NSUN2 and its mediation of RNA m^5^C modification in the progression of human HCC, indicating that this interaction may serve as a prognostic biomarker in HCC patients.

## Results

### NSUN2 deficiency inhibits the proliferation of HCC cells

**Fig. 1:**
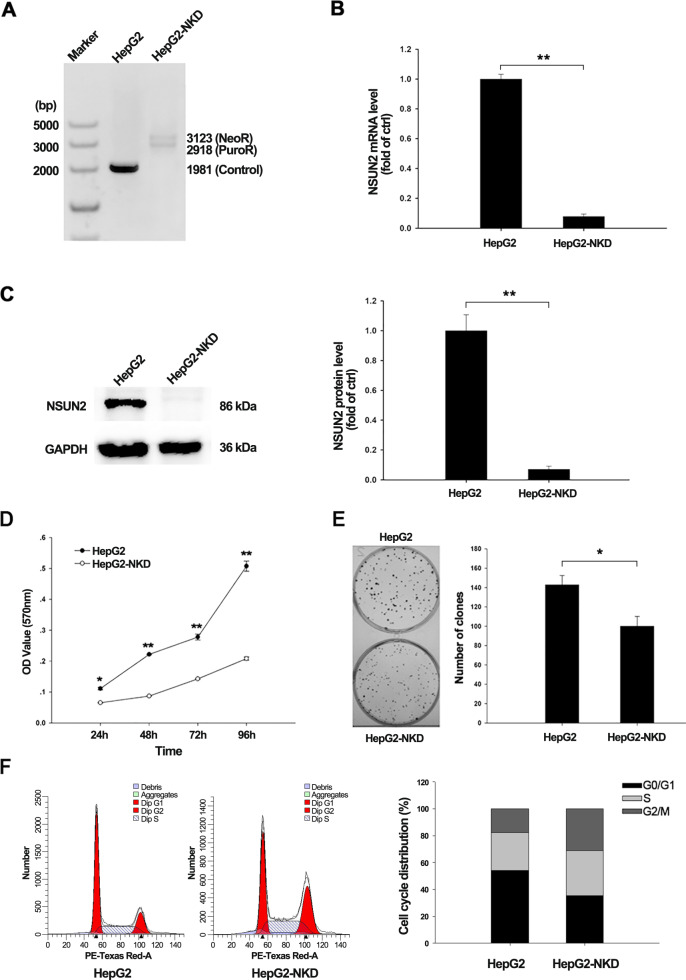
Identification and characterization of NSUN2-deficient HepG2 cells. **a** Genotyping of NSUN2-deficient HepG2 cells based on the PCR method. Because of the insertion of *Neo* and *Puro* into the double allele, two longer bands could be amplified in NSUN2-deficient cells compared with normal cells. **b** *NSUN2* mRNA level in HepG2 and NSUN2-deficient HepG2 cells were assessed by real-time qPCR. Data are represented as the mean ± SEM (*n* = 3 independent experiments). **c** *NSUN2* protein level in HepG2 and NSUN2-deficient HepG2 cells were assessed by western blotting. Three independent experiments were performed for quantification (mean ± SEM). **d** Cell proliferation was determined by CCK-8 assay. Data are represented as the mean ± SEM (*n* = 6 independent experiments). **e** Representative picture of the colony formation assay result. Three independent experiments were performed for quantification (mean ± SEM). **f** FACS analysis was subjected to assess the cell-cycle distribution. OD optical density; HepG2-NKD, NSUN2-deficient HepG2 cells. *P* values were calculated by Student’s *t* test. *p < 0.05, **p < 0.01.

To explore the functional role of NSUN2 in hepatocellular carcinoma, NSUN2-deficient HepG2 cells were generated. The genotype of NSUN2-deficient cells was assessed by PCR, and the gene silencing efficiency of cells was determined using RT-qPCR and western blotting. As a result, we obtained an NSUN2-deficient HepG2 cell line with a biallelic insertion of a transcriptional terminator signal (Fig. 1a). The results of RT-qPCR and western blotting showed that over 90% of the NSUN2 gene expression was repressed (Fig. 1b, c).

To further characterize NSUN2-knockdown phenotypes, CCK-8 and colony formation assays were employed to detect the impact of NSUN2 depletion on the proliferation of HepG2 cells. The CCK-8 assay was performed at 24, 48, 72, and 96 h after cell seeding. Compared to normal HepG2 cells, a significantly decreased viability was observed in NSUN2-deficient HepG2 cells after 24 h (Fig. 1d). The results of the colony formation assay were similar. As shown in Fig. 1e, the clone number in NSUN2-deficient HepG2 cells was significantly lower than that in wild-type HepG2 cells. Quantification of clone number revealed that NSUN2 deficiency inhibited the colony formation ratio by an average of 14% compared to that in wild-type HepG2 cells. To address whether NSUN2 deficiency affects the cell division cycle of HepG2 cells, the cell-cycle distribution of the cells was analyzed by FACS. As shown in Fig. 1f, NSUN2-deficient HepG2 cells had smaller G1 and S populations and larger G2/M populations than wild-type cells.

### NSUN2 deficiency inhibits the migration, invasion, and angiogenesis of HCC cells

**Fig. 2:**
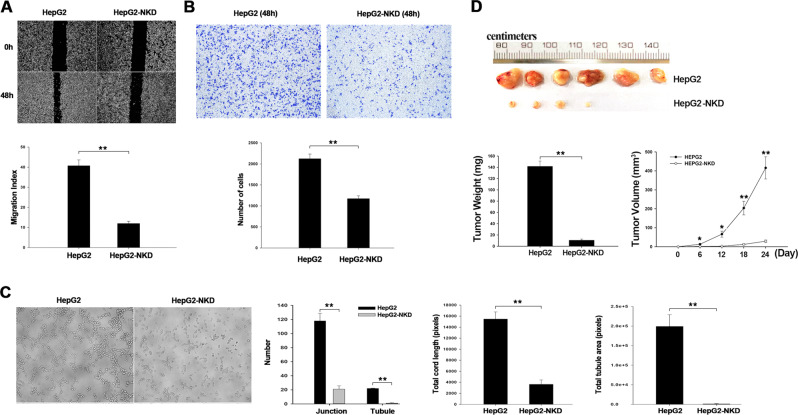
Deficiency of NSUN2 decreased migration, invasion, and angiogenesis of HepG2 cells. **a** Cell migration was determined by wound healing assay. **b** Trans-well invasion assay. Three independent experiments were performed for quantification (mean ± SEM). **c** Tubule formation assay was used to test the effect of NSUN2 deficiency on angiogenesis ability of cells in vitro. **d** Xenograft nude mouse model was inoculated with NSUN2-deficient HepG2 cells or the normal HepG2 cells (*n* = 6/group), at day 24 after inoculation. Three independent experiments were performed for quantification (mean ± SEM). HepG2-NKD, NSUN2-deficient HepG2 cells. The data are represented as mean ± SEM, obtained in three independent experiments. *P* values were calculated by Student’s *t* test. *p < 0.05, **p < 0.01.

To investigate whether NSUN2 deficiency affects the metastasis and invasion of HepG2 cells, a wound healing assay was used to analyze the effect of NSUN2 depletion on the migration ability of HepG2 cells. As shown in Fig. 2a, NSUN2-deficient HepG2 cells displayed significant delays in wound closure resulting from diminished cell migration. Quantification of wound size revealed that wound closure rate was inhibited by an average of 72.5% in NSUN2-deficient cells compared to wild-type HepG2 cells. A transwell invasion assay was used to detect the invasion ability of NSUN2-deficient HepG2 cells in vitro. NSUN2 depletion significantly reduced the number of invaded cells (Fig. 2b). The inhibition of HepG2 cell invasion in response to NSUN2 deficiency was higher than 50%. Moreover, a tube formation assay was used to detect the effect of NSUN2 knockout on the angiogenesis of HepG2 cells in vitro. The results indicated that NSUN2 depletion decreased cell cord formation on Matrigel, with significantly reduced junctions, cord length, tubules, and tubule area (Fig. 2c).

To further determine whether NSUN2 deficiency inhibits tumorigenicity in hepatocellular carcinoma in vivo, we established a xenograft model by inoculating nude mice with NSUN2-deficient HepG2 cells. As shown in Fig. 2d, the tumors formed by NSUN2-deficient HepG2 cells were significantly smaller than those formed by wild-type HepG2 cells. These results are consistent with the results in vitro and further confirm that NSUN2 plays an important role in promoting the tumorigenicity of hepatocellular carcinoma cells.

### NSUN2 deficiency significantly influences the expression profiles of HepG2 cells

**Fig. 3:**
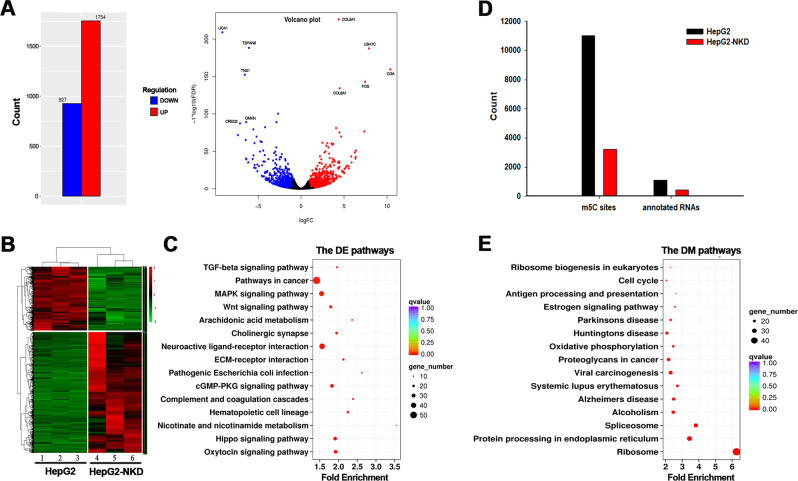
Overview of RNA expression and m^5^C modification pattern in normal and NSUN2-deficient HepG2 cells. **a** Volcano plot of differentially expressed genes (DEGs) after NSUN2 depletion. **b** Heat-map expression profile of 2681 significantly dysregulated RNAs between HepG2 and NSUN2-deficient HepG2 cells. **c** Statistical analysis of KEGG pathway enrichment for the DEGs. **d** Histogram showed the number of total m^5^C sites and annotated RNAs in HepG2 cells and NSUN2-deficient HepG2 cells. **e** Statistical analysis of KEGG pathway enrichment for the differentially methylated genes (DMGs).

Based on the cell phenotype alteration after NSUN2 depletion, further experiments were performed to determine the molecular basis for the phenotype alterations. Genome-wide RNA-sequencing (RNA-Seq) was employed to detect NSUN2 deficiency induced genome-wide expression alterations. In total, 2681 differentially expressed genes (DEGs) were found between NSUN2-deficient HepG2 and wild-type HepG2 cells (Fig. 3a, b, Additional file 1). Among them, 1754 genes were upregulated and 927 genes were downregulated. Functional enrichment analysis indicated that the DEGs were mainly involved in biological processes such as cell migration, angiogenesis, cell adhesion, and cell communication. KEGG analysis showed that these DEGs were enriched in 32 pathways, most of which were related to carcinogenesis, including the pathways in cancer, Rap1 signaling pathway, PI3K-Akt signaling pathway, proteoglycans in cancer, and TGF-beta signaling pathway (Fig. 3c).

### NSUN2 depletion decreases RNA m^5^C abundance in HepG2 cells

Genome-wide RNA bisulfite sequencing (RNA-BisSeq) analysis was performed to map and compare transcriptome 5-methylcytosine sites between NSUN2-deficient HepG2 cells and wild-type HepG2 cells (Additional file 2, Fig S1). RNA-BisSeq analysis identified a total of 11,027 m^5^C candidate sites in wild-type cells, while the number of m^5^C candidate sites in NSUN2-deficient HepG2 cells was considerably reduced to only 3182 m^5^C candidate sites (Fig. 3d, Additional file 3). The methylation levels of the candidate methylation sites ranged from 20 to 30%. Sequence context analysis of m^5^C sites revealed no preferred motif (Additional file 2, Fig S2).

A total of 11,788 m^5^C candidate sites within 1208 genes were shown to be differentially methylated between HepG2 and NSUN2-deficient HepG2 cells. Differentially methylated genes (DMGs) screened by RNA-BisSeq were further subjected to GO and KEGG pathway enrichment analyses to identify the biological role of NSUN2-mediated RNA m^5^C methylation. The significantly enriched GO terms of DMGs between NSUN2-deficient HepG2 and wild-type cells were associated with translational initiation (ontology: biological process), membrane (ontology: cellular component) and poly(A) RNA binding (ontology: molecular function). KEGG pathway analysis show enrichment of these DMGs in 20 pathways, including spliceosome, proteoglycans in cancer, cell cycle, estrogen signaling pathway, etc. (Fig. 3e).

To verify the candidate m^5^C sites identified by RNA-BisSeq, several differentially methylated m^5^C sites (DMSs) were randomly selected for subsequent bisulfite-PCR amplification and Sanger sequencing. The results indicated that all of the Cs were converted to Ts except the sites identified by RNA-BisSeq (Additional file 2, Fig S3). The methylation levels of the candidate DMSs were different when comparing NSUN2-deficient HepG2 cells and wild-type HepG2 cells, which was consistent with the RNA-BisSeq results.

### *H19* lncRNA is a substrate of NSUN2 and its m^5^C modification affects the half-life of *H19* RNA

**Fig. 4:**
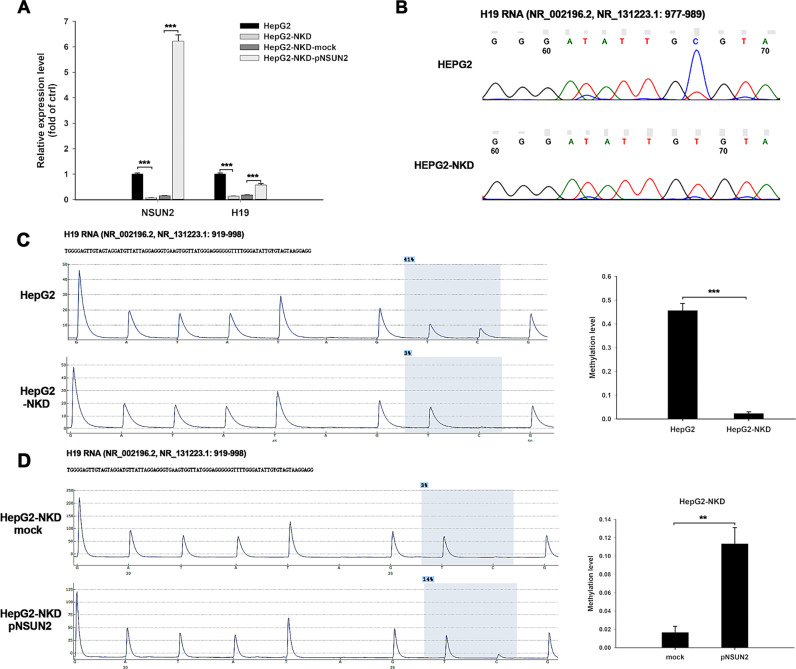
NSUN2 methylated *H19* RNA and regulated its expression. **a** Deficiency of NSUN2 decreased the level of *H19* RNA in HepG2 cells, while restore NSUN2 could recover the level of *H19* RNA in NSUN2-deficient HepG2 cells. **b** Sanger-based validation of m^5^C sites within *H19* RNA. cDNA was amplified by PCR using specific primers for bisulfite-treated RNAs. **c** Quantitative determination of methylation level of *H19* RNA C986 site in normal and NSUN2-deficient HepG2 cells through pyrosequencing. **d** Pyrosequencing result demonstrated restore of NSUN2 could recover the methylation level of H19 C986 site in NSUN2-deficient HepG2 cells. Mock, cells transfected with empty pcDNA3.1 vector; pNSUN2, cells transfected with pcDNA3.1-NSUN2 vector; HepG2-NKD, NSUN2-deficient HepG2 cells. The results are expressed as the mean ± SEM from three independent experiments, **p < 0.01, ***p < 0.001.

Compared to the results of the transcriptome-wide RNA-BisSeq and RNA-Seq analyses, 52 RNAs showed a significant change in both m^5^C methylation and RNA expression following NSUN2 depletion, including *H19* lncRNA (Additional file 2, Table S1). NSUN2 deficiency resulted in a complete loss of methylation at the *H19* RNA C986 site, and this was accompanied a decrease in the *H19* RNA level. RT-qPCR results confirmed that H19 expression was reduced by 80% after NSUN2 depletion (Fig. 4a). Bisulfite-PCR sequencing (BPS) using H19-specific primers revealed that methylation of the H19 C986 site was completely ablated in NSUN2-deficient HepG2 cells (Fig. 4b). Additional results from the BPS of *H19* RNA confirmed that the methylation level of *H19* RNA C986 was reduced from 45 to 1% after NSUN2 depletion (Fig. 4c). Moreover, the re-expression of NSUN2 restored the expression and methylation of *H19* RNA to some degree in NSUN2-deficient HepG2 cells (Fig. 4d).

**Fig. 5:**
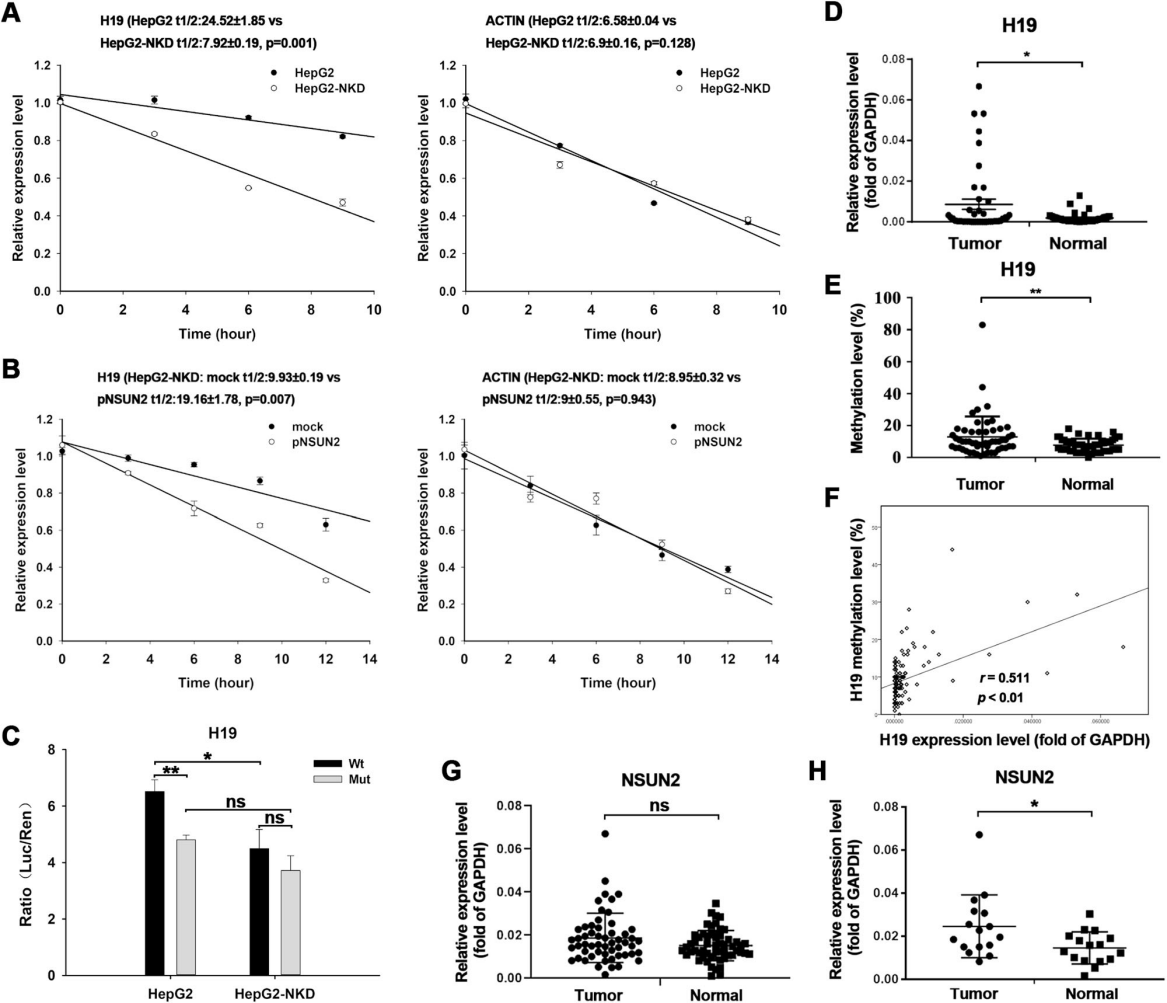
The expression and methylation status of *H19* RNA in liver cancer tissues. **a** NSUN2 deficiency decreased the half-life of *H19* RNA in HepG2 cells. Cells were exposed to actinomycin D (5 μg/ml), whereupon the cellular RNA was isolated at times indicated. Real-time qPCR against *GAPDH* was performed to assess the half-lives of *H19* RNA and *β-actin* mRNA **b** Restore of NSUN2 could recover the half-life of *H19* RNA at a certain level in NSUN2-deficient HepG2 cells. Three independent experiments were performed for quantification (mean ± SEM). **c** HepG2 cells or NSUN2-deficient HepG2 cells were transfected with plasmids pimrGLO-H19-wt or pimrGLO-H19-mut. After 48 h, Firefly luciferase activity was determined and normalized against Renilla luciferase activity. Data are represented as the mean ± SEM from three independent experiments. **d** *H19* expression in 55 pairs of liver cancer tissues and matched no-cancerous liver tissues. The relative expression levels were normalized to the expression amount of the GAPDH gene. **e** *H19* RNA methylation status in 55 pairs of liver cancer tissues and matched no-cancerous liver tissues. **f** Pearson’s correlation analysis demonstrating that *H19* expression is positively correlated with the m^5^C level in 110 samples (55 liver cancer tissues and 55 matched no-cancerous liver tissues). **g** NSUN2 mRNA level in 55 pairs of liver cancer tissues and matched no-cancerous liver tissues. **h** NSUN2 mRNA level in 16 pairs of liver cancer tissues and matched no-cancerous liver tissues with H19 C986 site methylation level difference over 10%. The relative expression levels were normalized to the expression amount of the GAPDH gene. mock, cells transfected with empty pcDNA3.1 vector; pNSUN2, cells transfected with pcDNA3.1-NSUN2 vector; HepG2-NKD, NSUN2-deficient HepG2 cells. *p < 0.05, **p < 0.01.

NSUN2-mediated RNA methylation has been reported to affect RNA stability. Thus, we wondered whether *H19* lncRNA methylation mediated by NSUN2 could affect *H19* lncRNA levels. To test this notion, NSUN2-deficient HepG2 cells were subjected to Actinomycin D (ActD) treatment followed by RT-qPCR to analyze the *H19* RNA half-life. As shown in Fig. 5a, the half-life of *H19* RNA was markedly shortened after NSUN2 depletion. Furthermore, NSUN2 re-expression restored the half-life of *H19* RNA to a certain extent in NSUN2-deficient HepG2 cells (Fig. 5b). As a negative control, the depletion or re-expression of NSUN2 did not affect the half-life of *ACT-B* mRNA. Furthermore, the specific role of the H19 m^5^C site in regulating its stability was determined using a *luciferase* reporter assay with a gene construct containing the wild-type H19 fragment (H19-Wt) or fragment with a mutated m^5^C site (H19-Mut). As expected, mutation of m^5^C site significantly inhibited *luciferase* activity in normal HepG2 cells but not NSUN2-deficient HepG2 cells (Fig. 5c). And, the *luciferase* activity of H19-Wt was significantly higher in normal HepG2 cells than that in NSUN2-deficient HepG2 cells. Thus, these results suggest that NSUN2-mediated RNA methylation may stabilize *H19* lncRNA.

### H19 expression and RNA m^5^C modification are linked to poor differentiation of HCC

We next determined the expression and methylation levels of *H19* RNA in HCC tissues and their matched normal tissues derived from 55 HCC patients. The results of the RT-qPCR showed that H19 expression was significantly increased in HCC tissues compared to matched non-cancerous liver tissues (Fig. 5d). Bisulfite-PCR pyrosequencing (BPP) results demonstrated that the methylation level at the H19 C986 site in HCC tissues was significantly higher than that in matched non-cancerous liver tissues (Fig. 5e). Moreover, the *H19* RNA level was significantly correlated (*p* < 0.01, *r* = 0.511) with its RNA methylation level in these tissues (Fig. 5f). The mRNA level of NSUN2 was also determined by RT-qPCR. The result showed no significant change in NSUN2 mRNA level between HCC tissues and matched non-cancerous liver tissues (Fig. 5g). However, when compared the tissues with H19 C986 methylation level difference over 10%, the NSUN2 mRNA level was significantly higher in HCC tissues than in matched non-cancerous liver tissues (Fig. 5h).

Further analysis revealed that the m^5^C methylation level and expression level of *H19* RNA in HCC patients are significantly (*p* < 0.001) associated with the differentiation stages of tumors (Tables 1 and 2), indicating that abnormal NSUN2-mediated m^5^C modification of *H19* lncRNA links to poorly differentiated hepatocellular carcinoma. Meanwhile, no significant differences (*p* > 0.05) were identified between the RNA m^5^C modification of H19 and other clinicopathological characteristics, including patient age, sex, extrahepatic metastasis, liver cirrhosis, and tumor size (Table 1 and Table 2). Taken together, our findings suggest that RNA methylation level of H19 is increased in HCC and is positively correlated with its expression, which significantly links to malignant HCC.

**Table 1:** Correlation between H19 RNA level and clinicopathologic characteristics in HCC patients.

		H19 expression			
	*n*	High	Low	*χ*^2^	*p* value
Age					
≥55	28	11	17	0.012	1
<55	27	11	16		
Gender					
Male	44	17	27	0.17	0.739
Female	11	5	6		
Metastasis					
Yes	14	5	9	0.144	0.762
No	41	17	24		
Liver cirrhosis					
Yes	43	17	24	0	1
No	12	5	7		
AFP					
≤25	18	7	11	0.077	1
>25	35	15	20		
Tumor size					
≤8	30	15	15	2.75	0.166
>8	25	7	18		
Anti-HCV					
Positive	4	0	4	2.876	0.141
Negative	51	22	29		
HBsAg					
Positive	45	19	26	0.509	0.723
Negative	10	3	7		
Differentiation stage					
High	15	0	15	15.574	0.000^*^
Moderate	18	8	10		
Low	18	12	6		

The patients were divided into two groups (high/low) according to the expression level of H19.

*p < 0.05 indicates statistical significance by *χ*^2^ test.

**Table 2:** Correlation between H19 RNA methylation level and clinicopathologic characteristics in HCC patients.

		Methylation level			
	*n*	+	−	*χ*^2^	*p* value
Age					
≥55	28	8	20	0.007	1
<55	27	8	19		
Gender					
Male	44	13	31	0.022	1
Female	11	3	8		
Metastasis					
Yes	14	3	11	0.535	0.734
No	41	13	28		
Liver cirrhosis					
Yes	43	12	31	0.134	0.73
No	12	4	8		
AFP					
≤25	18	4	14	0.821	0.53
>25	35	12	23		
Tumor size					
≤8	30	10	20	0.576	0.556
>8	25	6	19		
Anti-HCV					
Positive	4	1	3	0.035	1
Negative	51	15	36		
HBsAg					
Positive	45	15	30	2.159	0.25
Negative	10	1	9		
Differentiation					
High	15	1	14	18.675	0.000^*^
Moderate	18	2	16		
Low	18	12	6		

The patients were separated into two groups according to the difference of the methylation level of H19 RNA between the HCC tissues and matched non-cancerous liver tissues. The patients with difference of methylation rate over 10% were considered as positive (+), while others are negative (−).

*p < 0.05 indicates statistical significance by *χ*^2^ test.

### Methylated *H19* lncRNA interacts with oncoprotein G3BP1

**Fig. 6:**
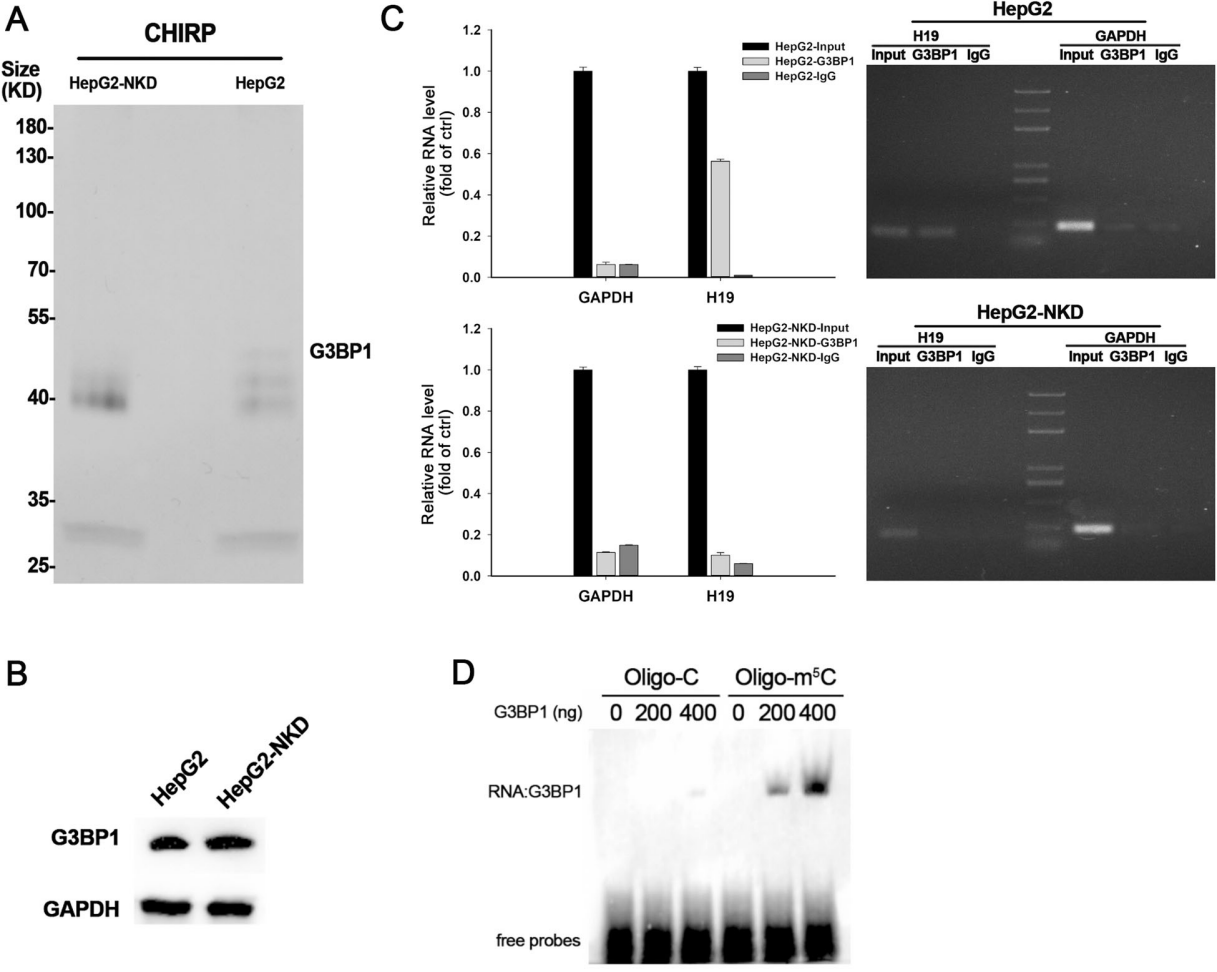
Methylated H19 lncRNA interacts with oncoprotein G3BP1. **a** Proteins retrieved by H19 probes visualized by silver staining. **b** *G3BP1* protein level in HepG2 and NSUN2-deficient HepG2 cells were assessed by western blotting. **c** RIP was performed using HepG2 or NSUN2-deficient HepG2 cell lysate and either anti-G3BP1 or Normal Rabbit IgG as the immunoprecipitating antibody. Purified RNA was analyzed by real-time qPCR or RT-PCR using primers specific for *H19* or *GAPDH*. Data are represented as means ± SEM from three independent experiments. **d** In vitro protein-RNA-binding assays (rEMSA) were performed using H19 oligonucleotides with or without m^5^C as well as the indicated amounts of purified *G3BP1* protein (200–400 ng/reaction). HepG2-NKD, NSUN2-deficient HepG2 cells.

The m^5^C modification within *XIST* and *HOTAIR* lncRNAs was shown to modulate their interaction with PRC2. Thus, we hesitated that m^5^C-modified *H19* lncRNA may regulate its interaction with other proteins. An *H19* lncRNA-binding protein, G3BP1 was identified by chromatin isolation by RNA purification (CHIRP) using H19-specific probes and subsequent mass spectrometry analysis (Fig. 6a, Additional file 2, Fig S4, Additional file 4). Moreover, G3BP1 pulled down by the *H19* RNA probe was detectable in wild-type HepG2 cells but not in NSUN2-deficient HepG2 cells, meanwhile, no significant change was detected in G3BP1 protein level between wild-type HepG2 cells and NSUN2-deficient HepG2 cells (Fig. 6b), suggesting that the interaction between *H19* RNA and *G3BP1* protein might be influenced by NSUN2. This notion was confirmed by RNA-binding protein immunoprecipitation (RIP) assay using a G3BP1 antibody (Fig. 6c). The fact that G3BP1 bound specifically to *H19* lncRNA in wild-type HepG2 cells but not in NSUN2-deficient HepG2 cells suggests that the interaction of *H19* lncRNA with G3BP1 protein may depend on NSUN2-mediated RNA methylation. Further rEMSA experiment was done using purified recombinant human G3BP1 protein and H19 RNA probes with or without m^5^C modification. The result showed that G3BP1 recombinant protein preferably binds to m^5^C-modified oligonucleotides (Fig. 6d), indicating the binding of G3BP1 protein and H19 RNA was regulated by m^5^C modification.

## Discussion

Dysregulation of epigenomic modifications, including DNA methylation, histone modifications, and RNA methylation, plays an important role in tumorigenesis. In recent years, accumulating evidence has demonstrated that RNA m^6^A modification is implicated in the initiation, progression and drug response of multiple human cancers [27–29]. NSUN2, an m^5^C methyltransferase of mammalian RNA, was reported to be highly expressed in multiple tumors. In the present study, we found that depletion of NSUN2 led to inhibition of the proliferation, migration, and invasion of hepatocellular carcinoma cells in vitro. Consistently, the in vivo orthotopic tumor xenograft results also confirmed that NSUN2 deficiency suppressed HCC growth in nude mice. Furthermore, NSUN2 was shown to methylate *H19* lncRNA and affect its stability. The Ras-GTPase-activating protein-binding protein 1 (G3BP1) was confirmed to bind methylated *H19* RNA based on the presence of NSUN2. Moreover, lncRNA H19 was shown to be overexpressed, and its overexpression accompanied by hypermethylation in HCC tissues compared to matched normal liver tissues; in addition, high levels of *H19* RNA methylation and expression were correlated with HCC poor differentiation. It is well known that tumor poor differentiation is an important feature of malignant tumors, which directly affects tumor progression and prognosis. Taken together, these results suggest that abnormal NSUN2-mediated RNA m^5^C modification represents a mechanism for the progression of human maglinant HCC, and it may serve as a prognostic biomarker to distinguish HCC patients.

Interestingly, NSUN2 is a direct target gene of MYC and is upregulated upon MYC activation. RNAi of NSUN2 blocked MYC-induced keratinocyte proliferation and cell-cycle progression. The growth of human squamous cell carcinoma xenografts was also inhibited by NSUN2 knockdown [4]. Here, we showed that NSUN2 deficiency decreased the proliferation, migration, and invasion of HepG2 cells. Moreover, NSUN2 deficiency resulted in smaller G1 and S populations and a larger G2/M population than those observed in normal cells, suggesting that NSUN2 depletion might block cells in the G2 phase and therefore suppress the proliferation of HepG2 cells. Previous reports have shown that NSUN2 is dynamically expressed during the cell division cycle, with the lowest level in the G1 phase and the highest level in the S phase [4, 30]. The dynamic distribution and expression of NSUN2 during the cell-cycle imply that NSUN2 may play an important role in regulating cell division. Wang et al. found that NSUN2-mediated methylation of *CDK1* mRNA regulates CDK1 translation and thus affects the cell cycle [10]. Furthermore, in our study, functional enrichment analysis of DMGs revealed that many DMGs, including HSP90AA1, CKAP5, TUBB, and CDK2, were enriched in the cell-cycle pathway (hsa04110) and related processes (such as GO:0000086-G2/M transition of mitotic cell cycle). These results suggest that NSUN2 may regulate the cell cycle through RNA methylation, thereby affecting the proliferation of cancer cells.

Transcriptome sequencing identified 2681 DEGs and 1208 DMGs in HepG2 cells. Among them, 52 genes including the lncRNA H19 had changes in both RNA expression and methylation level. In many recent studies, H19 was identified as an onco-lncRNA and to promote tumorigenesis, such as in glioblastoma [31], colorectal cancer [32], and gastric cancer [33]. In HepG2 cells, H19 RNA was methylated at C986 site and this methylation was completely lost after NSUN2 depletion. Moreover, restore of NSUN2 could recover this methylation to some degree, but not the original level. We speculated this may be mainly caused by the NSUN2 overexpression method we used, the transient transfection efficiency of HepG2 cells is relatively low, and a large number of cells with no restore of NSUN2 pulled down the recovery level of H19 methylation.

Depletion of NSUN2 lead to reduced methylation of H19 RNA, which was also accompanied by decreased expression of H19. Many previous reports have shown that NSUN2-mediated RNA m^5^C modification may exert its function mainly by affecting translation of the RNA rather than by altering the RNA level [10, 11]. A recent study showed that in human urothelial carcinoma of the bladder (UBC), NSUN2-mediated m^5^C modification stabilizes oncogene RNA through the “reader” YBX1, and hypermethylated m^5^C levels correlate with oncogene mRNA overexpression in UBC cells [8]. NSUN2 may also play a similar role in lncRNAs. Here, we showed that NSUN2 deficiency significantly decreased the half-life of *H19* RNA, which might be regulated by NSUN2-mediated m^5^C modification, since mutation of the methylation site could significantly inhibit *luciferase* activity of reporter gene in normal HepG2 cells but not NSUN2-deficient HepG2 cells.

*H19* lncRNA has been confirmed to be highly expressed in many organs from early embryonic development to the fetal stage, but not after birth [34]. However, reactivation of H19 expression has been detected in many adult malignant tumors, including breast cancer [35], lung cancer [36], and bladder cancer [37]. Similarly, our results demonstrated that H19 is frequently upregulated in HCC tissues compared to matched non-cancerous liver tissues, and a higher level of H19 expression is correlated with HCC poor differentiation. Moreover, hypermethylation of *H19* RNA is observed in HCC tissues compared to matched normal liver tissues, which is also correlated with HCC malignancy. Unexpectedly, the expression and methylation level of H19 RNA did not show significant correlation with clinical indicators such as tumor size and metastasis. It seems that H19 may not be the best target for the function of NSUN2 in tumor which NSUN2 deficiency significantly inhibited cancer cell proliferation and migration. We speculate that this may be due to improper sample selection. For example, in 55 clinical cases, 41 cases did not show metastasis, while only 14 cases were metastatic. In addition, the limited sample size may also be one of the reasons for this phenomenon. Moreover, NSUN2 may participate in regulating tumor progression through multiple mechanisms, which needs more research to explore. Maybe, further study in more tumor samples and multiple types of tumors can solve this. Abnormally high level of lncRNA and DNA m^5^C hypermethylation have been used as diagnostic tools in many types of tumors. Our study provided a mechanism by which RNA m^5^C methylation may act as a new diagnostic marker for tumors.

Most lncRNAs function by binding to proteins. A previous study demonstrated that methylated cytosine of the lncRNAs XIST and HOTAIR affects their recruitment of the chromatin-modifying complex PRC2 [17]. We tested whether NSUN2 influenced the ability of *H19* RNA to bind with other proteins. Through ChIRP assays, we found that G3BP1 specifically binds to *H19* RNA in normal HepG2 cells but not in NSUN2-deficient HepG2 cells, and RIP assays also confirmed this specific interaction. Ras-GTPase-activating protein SH3 domain-binding protein 1 (G3BP1), a known oncoprotein, is overexpressed in a variety of human cancers, such as breast cancer [38], renal cell carcinoma [39], and hepatocellular carcinoma [40]. G3BP1 was shown to be repelled by m^6^A and to positively regulate mRNA stability in an m^6^A-regulated manner [41]. Besides, a previous study found that G3BP1 protein was also pulled by the affinity chromatography of RNA m^5^C [9]. Thus, we hypothesized that G3BP1 may also bind to *H19* RNA by recognizing m^5^C, and this was confirmed by further rEMSA assay. G3BP1 specific binds m^5^C modified H19 oligos, but not oligos without m^5^C. These results therefore suggested that NSUN2-mediated methylation of H19 RNA regulated its interaction with G3BP1 protein.

G3BP1 regulates a variety of carcinogenesis-related pathways, including Ras [42], Wnt/β-catenin, PI3K/AKT [43], and NF-κB/Her2 signaling pathways [44]. NSUN2 may affect these signaling pathways by regulating the binding of *H19* RNA to G3BP1 and then affect the progression and malignancy of cancer cells. Moreover, G3BP1 was shown to bind *MYC* mRNA and promote its decay [45]. Methylated *H19* RNA may compete with *MYC* mRNA for binding to G3BP1, promoting tumor cell progression. Hypomethylated H19 RNA will lose binding ability of G3BP1, and lead to an increase in binding ability between G3BP1 and *MYC* mRNA, which increases the decay rate and decreases the mRNA level of *MYC*, thus suppressing cell proliferation. In addition, MYC has been shown to bind to the promoter region of H19 and promote the transcription of *H19* RNA [31]. The reduction of MYC expression may further decrease the *H19* RNA level. Thus, we propose a regulatory axis: MYC-NSUN2-H19-G3BP1-MYC (Additional file 2, Fig. S5). In tumors, high expression of NSUN2 leads to increased methylation levels of *H19* RNA. Hypermethylated *H19* RNA may competitively bind to G3BP1, which further delays *MYC* mRNA decay and leads to a further increase in MYC levels, resulting in consequent deterioration of tumors. Nevertheless, this axis remains to be fully characterized in future studies.

In conclusion, our results revealed that the NSUN2-mediated m^5^C modification of H19 lncRNA exert an important function in the progression and malignancy of hepatocellular carcinoma. H19 RNA m^5^C methylation might be a new target and biomarker for HCC treatment and diagnosis.

## Materials and methods

### Cell culture and transfection

The human hepatocellular carcinoma cell line HepG2 was purchased from ATCC (HB-8065). For the details of cell culture, see the Supplementary of Materials and methods.

### Plasmid construction

All primers used for plasmid construction are shown in Additional file 2, Table S2. For the details, see the Supplementary of Materials and methods.

### Generation of NSUN2-deficient HepG2 cells

The NSUN2-deficient HepG2 cell line was generated via a CRISPR-Cas9-mediated method as previously described [46]. For the details, see the Supplementary of Materials and methods.

### Human liver cancer specimens

Fifty-five pairs of HCC tissues and matched non-cancerous liver tissues were surgically removed following the guidelines approved by the Institutional Review Board of Chinese PLA General Hospital. For the details, see the Supplementary of Materials and methods.

### RNA preparation and real-time PCR

The specific primers used for amplification are shown in Additional file 2, Table S3. For the details of qRT-PCR, see the Supplementary of Materials and methods.

### CCK-8 assay

Cell viability was determined using the CCK-8 assay. For the details, see the Supplementary of Materials and methods.

### Colony formation assay

Colony formation assay was performed as previously described with several modifications [47]. For the details, see the Supplementary of Materials and methods.

### Wound healing assay

A wound healing assay was performed as previously described with several modifications [48]. For the details, see the Supplementary of Materials and methods.

### Transwell invasion assay

Transwell invasion assay was performed as previously described with several modifications [13]. For the details, see the Supplementary of Materials and methods.

### Xenografted tumor model

All mice experiments were performed according to the institutional guidelines and were approved by the Institutional Animal Care and Use Committee. For the details, see the Supplementary of Materials and methods.

### Tube formation assay

The impact of NSUN2 deletion on in vitro angiogenesis of HepG2 was determined by tube formation assay. For the details, see the Supplementary of Materials and methods.

### Gene expression profiling

Gene-expression profiling were described in previous studies [47]. For the details, see the Supplementary of Materials and methods.

### Library construction and sequencing of bisulfite-converted RNAs

RNA-BisSeq were described in previous studies [47]. Primers for the validation of the candidate m^5^C sites are listed in Additional file 2 and Table S4. For the details, see the Supplementary of Materials and methods.

### Bisulfite-PCR pyrosequencing

Primers for amplification and sequencing and nucleotide dispensation orders are listed in Additional file 2 and Table S5. For the details, see the Supplementary of Materials and methods.

### RNA half-life measurement

The stability of the *H19* RNA was assessed by the addition of actinomycin D (ActD, 5 μg/ml) into the cell culture medium. For the details, see the Supplementary of Materials and methods.

### Luciferase reporter assay

HepG2 cells and NSUN2-deficient HepG2 cells were seeded into 24-well plates 24 h before transfection with pmirGLO-derived vectors. Forty-eight hours after transfection, cell lysates were collected and the firefly and Renilla luciferase activities were measured with a Dual Luciferase Reporter Assay system (Vazyme) following the manufacturer’s instructions. All firefly luciferase measurements were normalized to Renilla luciferase measurements from the same sample.

### Antibodies and western blot analysis

Anti-NSUN2 (Abcam, UK, ab272624, 1:1000), anti-G3BP1 (Santa Cruz, America, sc-365338, 1:500), and anti-GAPDH (CWBIO, China, CW0101, 1:5000) antibodies were used. For the details of western blot, see the Supplementary of Materials and methods.

### Chromatin isolation by RNA purification and mass spectrometry analysis (ChIRP-MS)

CHIRP was performed as previously described with slight modifications [49]. For the details, see the Supplementary of Materials and methods.

### RNA-binding protein immunoprecipitation (RIP)

The RIP experiment was performed as described by Tenenbaum et al. [50] For the details, see the Supplementary of Materials and methods.

### RNA electrophoretic mobility shift assay

rEMSA was performed as previously described with some modifications [9]. For the details, see the Supplementary of Materials and methods.

### Statistical analysis

All statistical results were analyzed using IBM SPSS Statistics 21 software. For the details, see the Supplementary of Materials and methods.

## Supplementary information

Supplementary Materials and Methods

Additional file 1

Additional file 2

Additional file 3A

Additional file 3B

Additional file 4

## References

[1] Choe J, Lin S, Zhang W, Liu Q, Wang L, Ramirez-Moya J (2018). mRNA circularization by METTL3-eIF3h enhances translation and promotes oncogenesis. Nature.

[2] Zhao X, Chen Y, Mao Q, Jiang X, Jiang W, Chen J (2018). Overexpression of YTHDF1 is associated with poor prognosis in patients with hepatocellular carcinoma. Cancer Biomark.

[3] Zhang S, Zhao BS, Zhou A, Lin K, Zheng S, Lu Z (2017). m^6^A demethylase ALKBH5 maintains tumorigenicity of glioblastoma stem-like cells by sustaining FOXM1 expression and cell proliferation program. Cancer Cell.

[4] Frye M, Watt FM (2006). The RNA methyltransferase Misu (NSun2) mediates Myc-induced proliferation and is upregulated in tumors. Curr Biol.

[5] Hussain S, Sajini AA, Blanco S, Dietmann S, Lombard P, Sugimoto Y (2013). NSun2-mediated cytosine-5 methylation of vault noncoding RNA determines its processing into regulatory small RNAs. Cell Rep.

[6] Tuorto F, Liebers R, Musch T, Schaefer M, Hofmann S, Kellner S (2012). RNA cytosine methylation by Dnmt2 and NSun2 promotes tRNA stability and protein synthesis. Nat Struct Mol Biol.

[7] Zhang X, Liu Z, Yi J, Tang H, Xing J, Yu M (2012). The tRNA methyltransferase NSun2 stabilizes p16INK(4) mRNA by methylating the 3’-untranslated region of p16. Nat Commun.

[8] Chen X, Li A, Sun BF, Yang Y, Han YN, Yuan X (2019). 5-methylcytosine promotes pathogenesis of bladder cancer through stabilizing mRNAs. Nat Cell Biol.

[9] Yang X, Yang Y, Sun BF, Chen YS, Xu JW, Lai WY (2017). 5-methylcytosine promotes mRNA export–NSUN2 as the methyltransferase and ALYREF as an m(5)C reader. Cell Res.

[10] Xing J, Yi J, Cai X, Tang H, Liu Z, Zhang X (2015). NSun2 promotes cell growth via elevating cyclin-dependent kinase 1 translation. Mol Cell Biol.

[11] Li Q, Li X, Tang H, Jiang B, Dou Y, Gorospe M (2017). NSUN2-mediated m5C methylation and METTL3/METTL14-mediated m6A methylation cooperatively enhance p21 translation. J Cell Biochem.

[12] Okamoto M, Hirata S, Sato S, Koga S, Fujii M, Qi G (2012). Frequent increased gene copy number and high protein expression of tRNA (cytosine-5-)-methyltransferase (NSUN2) in human cancers. DNA Cell Biol.

[13] Yi J, Gao R, Chen Y, Yang Z, Han P, Zhang H (2017). Overexpression of NSUN2 by DNA hypomethylation is associated with metastatic progression in human breast cancer. Oncotarget.

[14] Squires JE, Patel HR, Nousch M, Sibbritt T, Humphreys DT, Parker BJ (2012). Widespread occurrence of 5-methylcytosine in human coding and non-coding RNA. Nucleic Acids Res.

[15] Huang T, Chen W, Liu J, Gu N, Zhang R (2019). Genome-wide identification of mRNA 5-methylcytosine in mammals. Nat Struct Mol Biol.

[16] Shinoda S, Kitagawa S, Nakagawa S, Wei FY, Tomizawa K, Araki K (2019). Mammalian NSUN2 introduces 5-methylcytidines into mitochondrial tRNAs. Nucleic Acids Res.

[17] Amort T, Souliere MF, Wille A, Jia XY, Fiegl H, Worle H (2013). Long non-coding RNAs as targets for cytosine methylation. RNA Biol.

[18] Bian Z, Jin L, Zhang J, Yin Y, Quan C, Hu Y (2016). LncRNA—UCA1 enhances cell proliferation and 5-fluorouracil resistance in colorectal cancer by inhibiting miR-204-5p. Sci Rep.

[19] Lin Z, Pei-Cheng X (2013). Downregulated LncRNA-ANCR promotes osteoblast differentiation by targeting EZH2 and regulating Runx2 expression. Biochem. Biophys. Res. Commun.

[20] Yang YW, Flynn RA, Yong C, Qu K, Wan B, Wang KC (2013). Essential role of lncRNA binding for WDR5 maintenance of active chromatin and embryonic stem cell pluripotency. eLife.

[21] Li SP, Xu HX, Yu Y, He JD, Wang Z, Xu YJ (2016). LncRNA HULC enhances epithelial-mesenchymal transition to promote tumorigenesis and metastasis of hepatocellular carcinoma via the miR-200a-3p/ZEB1 signaling pathway. Oncotarget.

[22] Li Y, Li J, Luo M, Zhou C, Shi X, Yang W (2018). Novel long noncoding RNA NMR promotes tumor progression via NSUN2 and BPTF in esophageal squamous cell carcinoma. Cancer Lett.

[23] Ding K, Liao Y, Gong D, Zhao X, Ji W (2018). Effect of long non-coding RNA H19 on oxidative stress and chemotherapy resistance of CD133+ cancer stem cells via the MAPK/ERK signaling pathway in hepatocellular carcinoma. Biochem Biophys Res Commun.

[24] Han D, Gao X, Wang M, Qiao Y, Xu Y, Yang J (2016). Long noncoding RNA H19 indicates a poor prognosis of colorectal cancer and promotes tumor growth by recruiting and binding to eIF4A3. Oncotarget.

[25] Ma C, Nong K, Zhu H, Wang W, Huang X, Yuan Z (2014). H19 promotes pancreatic cancer metastasis by derepressing let-7’s suppression on its target HMGA2-mediated EMT. Tumour Biol.

[26] Yan S, Yingyi W, Wenkang L, Ping W, Tao T, Junxia Z (2014). Long non-coding RNA H19 promotes glioma cell invasion by deriving miR-675. PLoS ONE.

[27] Lin S, Choe J, Peng D, Triboulet R, Gregory RI (2016). METTL3 promotes translation in human cancer cells. Mol Cell.

[28] Wang X, Li Z, Kong B, Song C, Cong J, Hou J (2017). Reduced m6A mRNA methylation is correlated with the progression of human cervical cancer. Oncotarget.

[29] Taketo K, Konno M, Asai A, Koseki J, Toratani M, Satoh T (2018). The epitranscriptome m6A writer METTL3 promotes chemo- and radioresistance in pancreatic cancer cells. Int J Oncol.

[30] Hussain S, Benavente SB, Nascimento E, Dragoni I, Kurowski A, Gillich A (2009). The nucleolar RNA methyltransferase Misu (NSun2) is required for mitotic spindle stability. J Cell Biol.

[31] Barsyte-Lovejoy D, Lau SK, Boutros PC, Khosravi F, Jurisica I, Andrulis IL (2006). The c-Myc oncogene directly induces the H19 noncoding RNA by allele-specific binding to potentiate tumorigenesis. Cancer Res.

[32] Tsang WP, Ng EK, Ng SS, Jin H, Yu J, Sung JJ (2010). Oncofetal H19-derived miR-675 regulates tumor suppressor RB in human colorectal cancer. Carcinogenesis.

[33] Zhuang M, Gao W, Xu J, Wang P, Shu Y (2014). The long non-coding RNA H19-derived miR-675 modulates human gastric cancer cell proliferation by targeting tumor suppressor RUNX1. Biochem Biophys Res Commun.

[34] Zhang Y, Tycko B (1992). Monoallelic expression of the human H19 gene. Nat Genet.

[35] Lottin S, Adriaenssens E, Dupressoir T, Berteaux N, Montpellier C, Coll J (2002). Overexpression of an ectopic H19 gene enhances the tumorigenic properties of breast cancer cells. Carcinogenesis.

[36] Zhang E, Li W, Yin D, De W, Zhu L, Sun S (2016). c-Myc-regulated long non-coding RNA H19 indicates a poor prognosis and affects cell proliferation in non-small-cell lung cancer. Tumor Biol.

[37] Luo M, Li Z, Wang W, Zeng Y, Liu Z, Qiu J (2013). Long non-coding RNA H19 increases bladder cancer metastasis by associating with EZH2 and inhibiting E-cadherin expression. Cancer Lett.

[38] Winslow S, Leandersson K, Larsson C (2013). Regulation of PMP22 mRNA by G3BP1 affects cell proliferation in breast cancer cells. Mol Cancer.

[39] Wang Y, Fu D, Chen Y, Su J, Wang Y, Li X (2018). G3BP1 promotes tumor progression and metastasis through IL-6/G3BP1/STAT3 signaling axis in renal cell carcinomas. Cell Death Dis.

[40] Dou N, Chen J, Yu S, Gao Y, Li Y (2016). G3BP1 contributes to tumor metastasis via upregulation of Slug expression in hepatocellular carcinoma. Am J Cancer Res.

[41] Edupuganti RR, Geiger S, Lindeboom RGH, Shi H, Hsu PJ, Lu Z (2017). N(6)-methyladenosine (m(6)A) recruits and repels proteins to regulate mRNA homeostasis. Nat Struct Mol Biol.

[42] Parker F, Maurier F, Delumeau I, Duchesne M, Faucher D, Debussche L (1996). A Ras-GTPase-activating protein SH3-domain-binding protein. Mol Cell Biol.

[43] Zhang LN, Zhao L, Yan XL, Huang YH (2019). Loss of G3BP1 suppresses proliferation, migration, and invasion of esophageal cancer cells via Wnt/beta-catenin and PI3K/AKT signaling pathways. J Cell Physiol.

[44] Barnes CJ, Feng L, Mahitosh M, Zhibo Y, Sahin AA, Rakesh K (2002). Heregulin induces expression, ATPase activity, and nuclear localization of G3BP, a Ras signaling component, in human breast tumors. Cancer Res.

[45] Tourriere H, Gallouzi IE, Chebli K, Capony JP, Mouaikel J, van der Geer P (2001). RasGAP-associated endoribonuclease G3Bp: selective RNA degradation and phosphorylation-dependent localization. Mol Cell Biol.

[46] Liu Y, Han X, Yuan J, Geng T, Chen S, Hu X (2017). Biallelic insertion of a transcriptional terminator via the CRISPR/Cas9 system efficiently silences expression of protein-coding and non-coding RNA genes. J Biol Chem.

[47] Zhen S, Songlei X, Hui X, Xuming H, Shihao C, Zhe Y (2018). Effects of NSUN2 deficiency on the mRNA 5-methylcytosine modification and gene expression profile in HEK293 cells. Epigenomics.

[48] Nobes CD, Hall A (1999). Rho GTPases control polarity, protrusion, and adhesion during cell movement. J Cell Biol.

[49] Chu C, Quinn J, Chang HY (2012). Chromatin isolation by RNA purification (ChIRP). J Vis Exp.

[50] Tenenbaum SA, Lager PJ, Carson CC, Keene JD (2002). Ribonomics: identifying mRNA subsets in mRNP complexes using antibodies to RNA-binding proteins and genomic arrays. Methods.

