# Supplementary material for: Aberrant NSUN2-mediated m5C modification of H19 lncRNA is associated with poor differentiation of hepatocellular carcinoma
Source: Oncogene. 2020 Sep 25;39(45):6906–19. doi: 10.1038/s41388-020-01475-w (PMC7644462; doi:10.1038/s41388-020-01475-w)
Supplement: Supplementary file 1 — Supplementary Materials and Methods [file 41388_2020_1475_MOESM1_ESM.pdf]

Materials and methods

Cell culture and transfection

The human hepatocellular carcinoma cell line HepG2 was purchased from ATCC (HB-8065). Cells were maintained in Dulbecco's modified Eagle's medium (DMEM) supplemented with 10% fetal bovine serum (HyClone, America), 100 units/ml penicillin, and 100 mg/ml streptomycin at 37℃ with 5% CO2. For transfection, cells were seeded into 6 well plates at a density of 5×105 cells/well and cultured in an antibiotic-free medium. Plasmids were transfected with Lipofectamine 2000 (Life Technologies, America) at a ratio of 1:2 (m/v) in serum- and antibiotic-free DMEM. After incubation for 20 minutes at room temperature, the mixture was added to serum- and antibiotic-free culture medium. Six hours after transfection, the medium was replaced with complete DMEM.

Plasmid construction

For generation of NSUN2-deficient HepG2 cells, a single guide RNA (sgRNA) sequence (GACGCGGAGGATGGCGCCGA) was designed using CHOPCHOP (http://chopchop.cbu.uib.no/). The sgRNA oligonucleotide was synthesized and cloned into the Cas9 expression plasmid (Addgene, 42230). The Neo/Puro-BGH polyA donors were constructed as previously described [46].

The H19 fragment containing the WT (H19-WT) m5C site (chr11: 1996890) was amplified by PCR using the primers and inserted downstream of the pmirGLO vector (Promega, E1330), named pmirGLO-H19-WT. The cytosine (methylated site) was mutated to adenosine using pmirGLO-H19-WT as a template to generate pmirGLO-H19-Mut.

For the construction of vectors expressing NSUN2, the full-length coding region of NSUN2 was amplified by PCR using primers and inserted between EcoRI and XbaI site of pcDNA3.1+ vector.

All primers used were shown in Additional file 3, Table S2.

Generation of NSUN2-deficient HepG2 cells

In briefly, a double strand break was created at the first exon of NSUN2 using CRISPR/Cas9. Then, two screening markers (PuroR and NeoR) and a poly(A) signal sequences were integrated into the genome through homologous recombination repair. Last, double marker selection was employed for screening

clonal cell lines with successful biallelic integration of poly(A) signal. The screened cell colonies were subjected to limiting dilution to get monoclonal cells. Genotype and gene silencing efficiency of cells were assessed using PCR and Western blot.

Human liver cancer specimens

Fifty-five pairs of HCC tissues and matched non-cancerous liver tissues were surgically removed following the guidelines approved by the Institutional Review Board of Chinese PLA General Hospital with written informed consent from patients. Before RNA extraction, all the samples were frozen in liquid nitrogen and stored at -80 ℃ once tissues were isolated. Clinical parameters including age, gender, metastasis, liver cirrhosis, AFP, tumor size, and differentiation stage were collected. For HCC pathological classification, all samples were double-blind reviewed by two independent pathologists.

RNA preparation and real-time PCR

Total RNA of cells was extracted using TRIzol according to the manufacturer's instructions. Real-time quantitative RT-PCR was performed using a CFX ConnectTM real-time system (Bio-Rad) and RNA levels were quantified using SYBR Green Master Mix (Vazyme, China). Gene expression was normalized to GAPDH. The specific primers used for amplification are shown in Additional file 3, Table S3.

CCK-8 assay

Cell viability was determined using the CCK-8 assay. Cells were seeded in 96-well plates at a density of 2×10^3 cells/well and cultured for 24, 48, 72 and 96 hours. Then, 10 μl CCK-8 (Beyotime, China) was added to each well and incubated at 37°C for 1 hour. Cell viability was evaluated by measuring the optical density at 450 nm.

Colony formation assay

Cells were seeded in 6-well plates at a density of 400 cells/well and cultured for 14 days. The colonies were fixed with methyl alcohol for 30 minutes and stained with crystal violet for 15 minutes. Cells were washed with phosphate-buffered saline, photographed and counted using a light microscope to determine colony formation rates.

Wound healing assay

In brief, cells were seeded in 6-well plates at a total of 10^5 cells per well. When cellular confluence reached ~90%, a 200 μl pipette was used to create wounds in the confluent cells. Wells were then rinsed with medium to remove any free-floating cells and debris, and medium without serum was added. Forty-eight hours after the scrape line was made, wound healing was observed and representative scrape lines were imaged. The percentage of wound area was calculated as follows: wound area = (original wound area-recovered wound area)/original wound area.

Transwell invasion assay

Matrigel (Corning, America) was diluted 1:5 with chilled serum-free growth medium just prior to coating. One hundred microliters of the chilled diluted Matrigel was placed directly onto the centre of the upper chamber (24-well Transwell) and incubated at 37°C for 2 hours. A total of 10^5 cells in 100 μl serum-free growth medium were added to the chamber containing Matrigel, and 600 μl culture medium supplemented with 10% fetal bovine serum added to the lower chamber. The plates were incubated at 37°C for 48 hours. The chambers were submerged in methyl alcohol for 30 minutes and stained with crystal violet for 15 minutes, washed by PBS. Cells on the top of the Transwell were swabbed, and the remaining cells were counted by using a light microscope to determine cell invasion.

Xenografted tumor model

Male BALB/c-nude mice (4-5 weeks old) were purchased from the Institute of Comparative Medicine of Yangzhou University and were housed in the Comparative Medicine Institute of Yangzhou University. All mice experiments were performed according to the institutional guidelines and were approved by the Institutional Animal Care and Use Committee. Mice were randomly divided into two groups (n=6/group). For tumor cell implantation, 3×10^6 NSUN2-deficient HepG2 cells and control cells in 150 μl PBS were injected subcutaneously into the mice. Tumour growth was monitored by measuring tumor length (L) and width (W) once every 3 days, and tumor volume was estimated using the formula (tumor volume =0.5× (L × W2)). At the end of the experiment (24 days after injection), animals were euthanized, and tumors were excised and weighed.

Tube formation assay

The impact of NSUN2 deletion on in vitro angiogenesis of HepG2 was determined

by tube formation assay. 2×10^4 cells were seeded into 96-well plate coated with 5mg/ml Matrigel matrix and incubated at 37%. After 3h, images were captured using a microscope and analyzed using an angiogenesis analyzer script in image J software.

Gene expression profiling

Total RNA was isolated from HepG2 cells and NSUN2 -deficient HepG2 cells, each with three replicates. mRNA expression profiling on total RNA was performed by GENEWIZ. Next-generation sequencing libraries were constructed using the NEBNextR Ultra™ RNA Library Prep Kit for IlluminaR and sequenced on an Illumina HiSeq instrument according to manufacturer's instructions (Illumina). The raw reads filtered using Trimmomatic (v0.30) were mapped to the hg19 genome via software Hisat2 (v2.0.1). Gene expression level were counted using HTSeq (v0.6.1). Differentially expressed genes were determined using the DESeq Bioconductor package with a fold-change >1 and P-value <0.05.

Library construction and sequencing of bisulfite-converted RNAs

High-throughput RNA m^5C sequencing was provided by Cloudseq Biotech Inc. (Shanghai, China). Briefly, 2 μg of total RNA for each sample was rRNA depleted using NEBNext rRNA Depletion Kit (New England Biolabs, America). rRNA-depleted RNA was bisulfite-converted and purified using the EZ RNA Methylation Kit (Zymo Research, America). RNA libraries were later constructed with the TruSeq Stranded Total RNA Library Prep Kit (Illumina, America) according to the manufacturer's instructions. The library quality was evaluated with the BioAnalyzer 2100 system (Agilent Technologies, America). Library sequencing was performed on an Illumina Hiseq instrument with 150 bp paired-end reads.

Paired-end reads were obtained from the Illumina HiSeq 4000 sequencer and were quality controlled by Q30. After 3' adaptor-trimming and removal of low-quality reads by cutadapt software (v1.9.3), the high-quality trimmed reads (clean reads) were aligned to the human reference genome (UCSC HG19) using meRanGh (one component of meRanTK) software with default parameters. The methylation status of each C within the genome was extracted by meRanCall (one component of meRankTK) software. MeRanCompare (one component of meRanTK) software was used to identify differentially methylated sites (DMSs). The methylated sites and DMSs were annotated using Ensembl genome features. Next, gene ontology (GO)

and pathway analysis were conducted on those DMS-related genes.

For the validation of the candidate m^5C sites identified by RNA-BisSeq, several differentially methylated m^5C sites (DMSs) were randomly selected for subsequent bisulfite-PCR amplification and Sanger sequencing. Primers for amplification are listed in Additional file 3, Table S4.

Bisulfite PCR Pyrosequencing

For the validation of H19 RNA methylated sites, bisulfite converted RNA was reverse transcribed into cDNA using the PrimeScript™ II 1st Strand cDNA Synthesis Kit (Takara, Japan) according to the manufacturer's instructions. cDNA was amplified by PCR using specific primers for bisulfite-treated RNAs and the PyroMark PCR Kit (Qiagen, Germany). Pyrosequencing reactions were performed according to the manufacturer's instructions using the PyroMark Q24 Advanced system and CpG reagents kit (Qiagen, Germany). The degree of methylation was determined by PyroMark Q24 Advanced Software (Qiagen, Germany). Primers for amplification and sequencing and nucleotide dispensation orders are listed in Additional file 3, Table S5.

RNA half-life measurement

The stability of the H19 RNA was assessed by the addition of actinomycin D (ActD, 5 μg/ml) into the cell culture medium. Total RNA was extracted at 0, 3, 6, and 9 hours after ActD treatment. Real-time qPCR against GAPDH was performed to assess the half-lives of H19 RNA and β-Actin mRNA.

Luciferase reporter assay

HepG2 cells and NSUN2-deficient HepG2 cells were seeded into twenty-four well plates 24h before transfection with pmirGLO-derived vectors. Forty-eight hours after transfection, cell lysates were collected and the firefly and Renilla luciferase activities were measured with a Dual Luciferase Reporter Assay system (Vazyme) following the manufacturer's instructions. All firefly luciferase measurements were normalized to Renilla luciferase measurements from the same sample.

Antibodies and Western blot analysis

Cells were harvested and lysed in RIPA lysis buffer (Beyotime, China) containing phenylmethylsulfonyl fluoride (PMSF) and protease inhibitor cocktail (Beyotime, China). The concentration of total protein was determined by a BCA protein assay

kit (Vazyme, China). Equal amounts of each protein sample (40 μg) were separated by 12% SDS-PAGE and transferred to PVDF membranes (Merck Millipore, Germany). After blocking with nonfat skim milk and incubating with the indicated primary and secondary antibodies, the membranes were visualized with enhanced chemiluminescence (ECL) chemiluminescent substrate and scanned by a FluorChem Q imaging system. Anti-NSUN2 (Abcam, UK, ab272624, 1:1000), anti-G3BP1 (Santa Cruz, America, sc-365338, 1:500), and anti-GAPDH (CWBIO, China, CW0101, 1:5000) antibodies were used.

Chromatin isolation by RNA purification and mass spectrometry analysis (ChIRP-MS)

Briefly, antisense DNA probes against H19 lncRNA were designed by ChIRP Probe Designer and are listed in Additional file 3, Table S6. The 3'-end Biotin-TEG-labelled probes were synthesized by GENEWIZ. A total of 4×10^7 cells were harvested and fixed with 1% glutaraldehyde for 10 minutes at room temperature and quenched with 1/10 volume of 1.25 M glycine at room temperature for 5 minutes. The cross-linked cells were lysed with lysis buffer (50 mM Tris-Cl, pH 7.0, 10 mM EDTA, 1% SDS, PMSF, protease inhibitors, and SUPERase-In). Cell lysates were sonicated in a 4 ℃ water bath at the highest setting with 30 seconds ON, 45 seconds OFF pulse intervals for a total of 1 hour. The sonicated cell lysates were hybridized with a mixture of biotinylated DNA probes against human H19 lncRNA in hybridization buffer (750 mM NaCl, 1% SDS, 50 mM Tris-Cl, pH 7.0, 1 mM EDTA, 15% formamide, PMSF, protease inhibitors and SUPERase-In) for 4 hours at 37 ℃. Then the binding complexes were recovered by streptavidin-conjugated C1 magnetic beads (Invitrogen, America) and eluted with biotin elution buffer (12.5 mM biotin, 7.5 mM HEPES, pH 7.5, 75 mM NaCl, 1.5 mM EDTA, 0.15% SDS, 0.075% sarkosyl, and 0.02% Na-deoxycholate). Proteins were precipitated with 25% total volume TCA. Final protein samples were size-separated in SDS-PAGE gels and stained using the Fast Silver Stain Kit (Beyotime, China). The unique protein band was cut for MS.

RNA Binding Protein Immunoprecipitation (RIP)

A total of 10^7 cells were harvested and lysed with ice-cold polysome lysis buffer (100 mM KCl, 5 mM MgCl2, 10 mM HEPES-NaOH pH 7.0, 0.5% NP-40, 1 mM DTT, 200 units/ml RNase OUT, and protease inhibitors). The cell lysate was split

into three fractions (input, mock, and IP) and then centrifuged at 4°C and 20000 g for 10 minutes. Antibodies against normal mouse IgG (ABclonal, China, AC011) and G3BP1 (Santa Cruz Biotechnology, America, sc-365338) were added to the supernatant and incubated overnight at 4°C with gentle rotation. Next, 50 μl of protein A/G magnetic beads (Merck Millipore, Germany, 16-663) were washed and resuspended in 900 μl NT2 buffer (50 mM Tris-HCl pH 7.4, 150 mM NaCl, 1 mM MgCl2, 0.05% NP-40, 20 mM EDTA pH 8.0, 1 mM DTT, and 200 units/ml RNase OUT) supplemented with 5% BSA. The beads were added and the mixture was incubated at 4°C for an additional hour. The beads were separated by a magnetic strip, washed five times with 1 ml of NT2 buffer, and then resuspended in 1 ml of RNAiso Plus for RNA extraction. The co-precipitated RNAs of H19 were detected by qRT-PCR. Primers are listed in Supplemental Table S1.

RNA electrophoretic mobility shift assay

1μl synthesized biotin-labeled RNA probe with or without m^5C (1mM final concentration) and purified recombinant human G3BP1 protein (0, 200, or 400ng) were mixed with binding buffer and incubated at room temperature for 20 min. The entire 10μl RNA-protein mixture was mixed with 1μl 10×loading buffer without bromophenol blue and separated on 6% TBE gel on ice for 45 min at 80 V. The gel was transferred on positive charged nylon transfer membrane (GE Healthcare). Nucleic acids were detected by the Chemiluminescent EMSA Kit (Beyotime, GS009) following the manufacturer's instructions.

Statistical analysis

All statistical results were analyzed using IBM SPSS Statistics 21 software. A two-tailed Student's test was used to calculated the significance of data analysis between two independent groups. The correlation between the expression level and methylation level of H19 RNA was analyzed using Pearson test. $\chi 2$ test was used for statistical analysis of the correlations between clinic pathologic parameters and H19 RNA methylation and expression levels. All data were presented as mean ± standard error of mean. Differences with $p<0.05$ were considered as statistically significant.
