# Supplementary material for: Aberrant NSUN2-mediated m5C modification of H19 lncRNA is associated with poor differentiation of hepatocellular carcinoma
Source: Oncogene. 2020 Sep 25;39(45):6906–19. doi: 10.1038/s41388-020-01475-w (PMC7644462; doi:10.1038/s41388-020-01475-w)
Supplement: Supplementary file 2 — Additional file 1 [file 41388_2020_1475_MOESM2_ESM.pdf]

gene_id	GeneSym	length	HEPG2-SE	HEPG2-SE	HEPG2-SE	HEPG2-SE	HEPG2-SE	HEPG2-SE	HEPG2NK	HEPG2NK	HEPG2NK	HEPG2NK	HEPG2NK	HEPG2NK	baseMean	lfcSE	stat	log2FoldC	Regulatio	pvalue	padj	GO	KO
ENSG000	MARCH3	163095	45	0.17	28	0.11	34	0.13	15	0.06	19	0.07	14	0.05	26.33737	0.348214	-3.56397	**-1.241**	**Down**	**0.00037**	**0.00269**	cellular_cc	K10658
ENSG000	SEPT14	69246	164	1.01	222	1.43	136	0.85	0	0	0	0	0	0	92.97474	0.476066	-12.9361	**-6.1584**	**Down**	**2.8E-38**	**8.1E-36**	molecular	K16941
ENSG000	5S_rRNA	13248	12	0.1	4	0.04	5	0.04	0	0	2	0.02	2	0.02	4.293591	0.570839	-2.65488	**-1.5155**	**Down**	**0.00793**	**0.03359**	_	_
ENSG000	A2M	48566	73	0.2	99	0.29	80	0.23	1160	3.39	2009	5.44	1301	3.45	718.764	0.223404	16.81915	**3.75746**	**Ups**	**1.8E-63**	**1.3E-60**	biological	K03910
ENSG000	A2MP1	47285	15	0.07	20	0.1	14	0.06	132	0.63	292	1.3	162	0.71	96.28624	0.328311	9.236463	**3.03243**	**Ups**	**2.6E-20**	**2.4E-18**	_	K03910
ENSG000	A4GALT	29178	574	2.68	457	2.22	591	2.77	310	1.5	213	0.96	306	1.35	417.7557	0.244971	-4.5373	**-1.1115**	**Down**	**5.7E-06**	**7.2E-05**	cellular_cc	K01988
ENSG000	AASS	68634	11	0.04	6	0.02	9	0.03	30	0.1	146	0.47	51	0.16	37.6822	0.459101	4.966118	**2.27995**	**Ups**	**6.8E-07**	**1.1E-05**	cellular_cc	K14157
ENSG000	AATK	48783	1241	2.44	1125	2.31	1257	2.48	17	0.03	307	0.58	173	0.32	716.6591	0.567037	-3.37603	**-1.9143**	**Down**	**0.00074**	**0.00488**	molecular	K17480
ENSG000	ABAT	110011	0	0	2	0	2	0	12	0.02	17	0.03	15	0.03	7.397427	0.530903	4.062118	**2.15659**	**Ups**	**4.9E-05**	**0.00047**	biological	K13524
ENSG000	ABCA4	128296	9	0.02	17	0.05	12	0.03	31	0.09	41	0.11	40	0.1	23.81983	0.359102	3.311498	**1.18917**	**Ups**	**0.00093**	**0.00592**	biological	K05644
ENSG000	ABCA8	88101	244	0.46	331	0.66	205	0.39	591	1.17	1747	3.21	780	1.4	600.0596	0.320925	4.949403	**1.58839**	**Ups**	**7.4E-07**	**1.2E-05**	molecular	K05650
ENSG000	ABCB5	161829	1	0	3	0.01	1	0	9	0.02	75	0.18	25	0.06	16.59047	0.544893	4.817005	**2.62475**	**Ups**	**1.5E-06**	**2.1E-05**	cellular_cc	K05660
ENSG000	ABCC3	57476	2164	3.81	1724	3.17	1905	3.38	295	0.54	506	0.86	353	0.59	1204.752	0.183365	-13.6331	**-2.4998**	**Down**	**2.5E-42**	**8.5E-40**	molecular	K05667
ENSG000	ABCG2	141059	321	1.14	295	1.1	280	1	125	0.46	166	0.57	123	0.41	222.5024	0.183715	-7.10129	**-1.3046**	**Down**	**1.2E-12**	**5.4E-11**	molecular	K05681
ENSG000	ABHD12B	32811	7	0.03	18	0.08	5	0.02	23	0.11	43	0.18	38	0.16	20.98388	0.426699	3.051215	**1.30195**	**Ups**	**0.00228**	**0.01245**	molecular	K13705
ENSG000	ABLIM3	119060	69	0.14	58	0.12	71	0.15	12	0.03	22	0.04	23	0.04	43.83155	0.307055	-6.10891	**-1.8758**	**Down**	**1E-09**	**2.8E-08**	molecular	K07520
ENSG000	ABR	225558	1038	1.29	922	1.2	1004	1.26	445	0.58	404	0.48	374	0.44	716.6258	0.181929	-7.95312	**-1.4469**	**Down**	**1.8E-15**	**1.1E-13**	molecular	K08878
ENSG000	AC00012	6844	0	0	1	0.02	0	0	2	0.03	16	0.25	2	0.03	3.040965	0.580327	2.500358	**1.45103**	**Ups**	**0.01241**	**0.04747**	_	_
ENSG000	AC00206	261206	3	0.06	4	0.09	1	0.02	19	0.42	20	0.41	15	0.3	9.722661	0.492921	3.870308	**1.90776**	**Ups**	**0.00011**	**0.00094**	_	_
ENSG000	AC00242	59620	6	0.06	2	0.02	3	0.03	41	0.43	16	0.15	46	0.43	18.14762	0.491718	4.663794	**2.29327**	**Ups**	**3.1E-06**	**4.2E-05**	_	_
ENSG000	AC00245	6735	119	4.14	131	4.76	107	3.75	43	1.56	38	1.28	48	1.58	83.4643	0.250179	-6.39904	**-1.6009**	**Down**	**1.6E-10**	**5E-09**	_	_
ENSG000	AC00310	15742	16	0.06	31	0.12	28	0.1	64	0.24	75	0.26	71	0.24	45.49674	0.30633	3.80948	**1.16696**	**Ups**	**0.00014**	**0.00117**	_	_
ENSG000	AC00423	54497	0	0	1	0.03	4	0.13	10	0.33	18	0.55	23	0.68	8.57462	0.532691	3.918244	**2.08722**	**Ups**	**8.9E-05**	**0.00079**	_	_
ENSG000	AC00444	433	9	0.53	7	0.43	6	0.36	23	1.42	16	0.92	25	1.4	13.84914	0.430066	2.595972	**1.11644**	**Ups**	**0.00943**	**0.03853**	_	_
ENSG000	AC00446	1445	199	3.54	148	2.75	168	3.01	53	0.98	63	1.08	69	1.16	119.8381	0.225813	-7.22765	**-1.6321**	**Down**	**4.9E-13**	**2.3E-11**	_	_
ENSG000	AC00451	8809	5	0.16	2	0.07	3	0.09	0	0	0	0	0	0	1.773388	0.571087	-2.68499	**-1.5334**	**Down**	**0.00725**	**0.03131**	_	_
ENSG000	AC00454	100382	14	0.05	12	0.05	12	0.04	106	0.41	30	0.11	89	0.31	42.57172	0.440208	4.494589	**1.97855**	**Ups**	**7E-06**	**8.6E-05**	_	_
ENSG000	AC00483	2956	0	0	0	0	0	0	2	0.07	11	0.37	3	0.1	2.319433	0.576312	2.829542	**1.6307**	**Ups**	**0.00466**	**0.02212**	_	_
ENSG000	AC00498	11736	29	0.64	17	0.39	23	0.51	78	1.78	49	1.04	48	0.99	39.71544	0.359375	2.87051	**1.03159**	**Ups**	**0.0041**	**0.02**	_	K17630
ENSG000	AC00515	1393	5	0.09	0	0	6	0.11	19	0.36	21	0.37	14	0.24	10.2454	0.501489	3.091537	**1.55037**	**Ups**	**0.00199**	**0.01115**	_	K17985
ENSG000	AC00525	690	44	1.65	30	1.17	49	1.84	12	0.47	9	0.32	17	0.6	27.77429	0.371953	-4.53308	**-1.6861**	**Down**	**5.8E-06**	**7.3E-05**	_	_
ENSG000	AC00528	45482	10	0.16	9	0.15	4	0.06	17	0.28	32	0.49	23	0.35	14.90266	0.424868	2.789492	**1.18517**	**Ups**	**0.00528**	**0.02449**	_	_
ENSG000	AC00561	3148	2	0.01	3	0.02	2	0.01	10	0.07	18	0.12	18	0.12	8.170604	0.506429	3.575162	**1.81057**	**Ups**	**0.00035**	**0.00259**	_	_
ENSG000	AC00568	17477	23	0.4	10	0.18	14	0.25	70	1.28	23	0.39	73	1.21	34.56463	0.44616	3.028604	**1.35124**	**Ups**	**0.00246**	**0.01328**	_	K14685
ENSG000	AC00574	957	1	0.03	0	0	1	0.03	9	0.25	7	0.18	7	0.18	3.928782	0.573455	3.065449	**1.7579**	**Ups**	**0.00217**	**0.01197**	_	_
ENSG000	AC00604	1917	13	0.23	11	0.2	4	0.07	33	0.6	27	0.46	26	0.43	18.30303	0.417651	2.873128	**1.19996**	**Ups**	**0.00406**	**0.01986**	_	K12735
ENSG000	AC00615	9252	0	0	0	0	0	0	7	0.48	4	0.26	1	0.06	1.911577	0.566733	2.496608	**1.41491**	**Ups**	**0.01254**	**0.04787**	_	_
ENSG000	AC00626	16755	48	1.31	40	1.13	42	1.15	5	0.14	23	0.6	18	0.46	29.88539	0.378551	-4.07271	**-1.5417**	**Down**	**4.7E-05**	**0.00045**	_	_
ENSG000	AC00626	13918	5601	55.87	5739	59.71	6356	63.77	1923	19.95	1460	14.05	2096	19.72	4002.525	0.20844	-8.78587	**-1.8313**	**Down**	**1.6E-18**	**1.3E-16**	_	_
ENSG000	AC00626	11412	117	0.48	109	0.46	111	0.46	61	0.26	72	0.28	54	0.21	88.60925	0.226965	-4.5307	**-1.0283**	**Down**	**5.9E-06**	**7.4E-05**	_	_
ENSG000	AC00646	31773	12	0.17	16	0.23	12	0.17	6	0.09	2	0.03	4	0.05	9.04167	0.487928	-3.04235	**-1.4844**	**Down**	**0.00235**	**0.01278**	_	_
ENSG000	AC00694	2855	8	0.07	11	0.1	6	0.05	23	0.22	37	0.32	24	0.2	17.14979	0.402892	3.233953	**1.30293**	**Ups**	**0.00122**	**0.00743**	_	_
ENSG000	AC00719	95096	92	7.12	103	8.32	150	11.68	27	2.17	35	2.61	34	2.48	76.20324	0.289475	-6.68853	**-1.9362**	**Down**	**2.3E-11**	**8.3E-10**	_	_
ENSG000	AC00739	156128	1	0.01	3	0.03	0	0	13	0.14	12	0.12	14	0.14	6.720341	0.540292	3.701333	**1.9998**	**Ups**	**0.00021**	**0.0017**	_	_
ENSG000	AC00740	1745	18	0.29	10	0.17	26	0.42	7	0.12	11	0.17	6	0.09	13.25966	0.451727	-2.52571	**-1.1409**	**Down**	**0.01155**	**0.0449**	_	_
ENSG000	AC00762	2231	35	0.3	68	0.6	20	0.17	67	0.59	476	3.9	123	0.99	117.883	0.482395	3.444128	**1.66143**	**Ups**	**0.00057**	**0.00395**	_	_
ENSG000	AC00774	12237	8	0.03	24	0.11	11	0.05	2	0.01	6	0.02	3	0.01	9.309718	0.513832	-3.13268	**-1.6097**	**Down**	**0.00173**	**0.0099**	_	_
ENSG000	AC00775	13155	11	0.07	21	0.13	17	0.1	4	0.02	3	0.02	4	0.02	10.45817	0.480153	-3.82403	**-1.8361**	**Down**	**0.00013**	**0.00111**	_	_
ENSG000	AC00787	6238	34	0.49	30	0.45	30	0.44	1	0.02	8	0.11	1	0.01	18.16006	0.46227	-5.93945	**-2.7456**	**Down**	**2.9E-09**	**7.4E-08**	_	_
ENSG000	AC00797	144488	33	0.47	41	0.6	38	0.54	104	1.53	110	1.5	104	1.39	68.76903	0.25125	4.874895	**1.22482**	**Ups**	**1.1E-06**	**1.6E-05**	_	_
ENSG000	AC00875	14527	3	0.18	2	0.12	2	0.12	40	2.44	10	0.56	22	1.21	12.83296	0.528734	4.152569	**2.1956**	**Ups**	**3.3E-05**	**0.00033**	_	_
ENSG000	AC00912	782	0	0	1	0.06	0	0	5	0.28	9	0.47	11	0.56	3.941098	0.578293	3.48783	**2.01699**	**Ups**	**0.00049**	**0.00345**	_	K02889
ENSG000	AC00912	2298	1	0.05	1	0.05	1	0.05	6	0.31	17	0.82	4	0.19	4.524996	0.572016	2.830038	**1.61883**	**Ups**	**0.00465**	**0.02209**	_	_
ENSG000	AC00922	6310	11	0.81	3	0.23	11	0.81	3	0.23	1	0.07	0	0	5.099974	0.570149	-2.83986	**-1.6191**	**Down**	**0.00451**	**0.02156**	_	_
ENSG000	AC00940	7722	29	0.17	35	0.21	31	0.18	96	0.57	120	0.66	95	0.51	64.3854	0.257601	5.474779	**1.41031**	**Ups**	**4.4E-08**	**9E-07**	_	_
ENSG000	AC01012	344386	1	0.01	0	0	2	0.01	86	0.51	157	0.86	95	0.51	51.38993	0.460991	10.41378	**4.80066**	**Ups**	**2.2E-25**	**3E-23**	_	_
ENSG000	AC01123	17302	7	0.31	3	0.14	8	0.35	43	1.97	67	2.85	32	1.33	24.70902	0.42041	5.487733	**2.3071**	**Ups**	**4.1E-08**	**8.4E-07**	_	_
ENSG000	AC01174	15228	11	0.07	15	0.11	12	0.08	54	0.38	38	0.25	34	0.22	26.4605	0.378104	3.562032	**1.34682**	**Ups**	**0.00037**	**0.00271**	_	_
ENSG000	AC01206	1437	3	0.05	4	0.07	3	0.05	11	0.2	13	0.22	18	0.3	8.184802	0.492396	2.847087	**1.4019**	**Ups**	**0.00441**	**0.0212**	_	_
ENSG000	AC01207	6673	0	0	0	0	1	0.06	7	0.45	6	0.36	9	0.52	3.571074	0.580048	3.287824	**1.9071**	**Ups**	**0.00101**	**0.00637**	_	_
ENSG000	AC01214	2912	2078	41.25	1765	36.54	1957	39.07	924	19.08	578	11.07	1173	21.96	1448.114	0.268294	-4.61972	**-1.2394**	**Down**	**3.8E-06**	**5.1E-05**	_	_
ENSG000	AC01230	1411	3	0.39	0	0	2	0.26	9	1.22	19	2.39	10	1.23	6.571738	0.541005	3.275479	**1.77205**	**Ups**	**0.00105**	**0.00659**	_	_
ENSG000	AC01236	15802	65	0.59	67	0.64	70	0.64	171	1.62	148	1.3	182	1.56	113.3319	0.23553	4.475712	**1.05416**	**Ups**	**7.6E-06**	**9.3E-05**	_	_
ENSG000	AC01251	718	2	0.07	0	0	0	0	7	0.26	16	0.55	15	0.51	6.027105	0.561116	4.012789	**2.25164**	**Ups**	**0.00006**	**0.00056**	_	K09113
ENSG000	AC01326	3109	7	0.08	7	0.08	10	0.11	17	0.2	43	0.46	26	0.27	17.07643	0.42137	3.182647	**1.34107**	**Ups**	**0.00146**	**0.00861**	_	_
ENSG000	AC01629	408	65	4.1	72	4.73	63	3.99	34	2.23	35	2.13	29	1.72	50.69496	0.272162	-4.31544	**-1.1745**	**Down**	**1.6E-05**	**0.00018**	_	K00236
ENSG000	AC01671	7973	1	0.06	0	0	1	0.06	28	1.61	3	0.16	19	0.99	8.454661	0.575034	3.922948	**2.25583**	**Ups**	**8.8E-05**	**0.00078**	_	_
ENSG000	AC01683	74930	41	0.65	27	0.45	35	0.56	11	0.18	13	0.2	4	0.06	22.61573	0.402473	-4.47716	**-1.8019**	**Down**	**7.6E-06**	**9.2E-05**	_	_
ENSG000	AC01690	92739	0	0	0	0	1	0.01	6	0.04	4	0.02	7	0.04	2.821044	0.583324	2.779212	**1.62118**	**Ups**	**0.00545**	**0.02512**	_	_
ENSG000	AC01874	23190	0	0	0	0	0	0	4	0.05	13	0.15	13	0.15	4.444044	0.581183	4.113156	**2.3905**	**Ups**	**3.9E-05**	**0.00039**	_	_
ENSG000	AC01875	11590	2	0.03	4	0.05	0	0	10	0.13	9	0.11	16	0.19	6.441919	0.541862	2.858873	**1.54911**	**Ups**	**0.00425**	**0.0206**	_	_
ENSG000	AC02101	31078	4	0.19	6	0.3	1	0.05	10	0.5	27	1.25	23	1.04	10.87545	0.495421	3.343536	**1.65646**	**Ups**	**0.00083**	**0.00538**	_	_
ENSG000	AC02259	4597	5	0.03	3	0.02	7	0.04	0	0	2	0.01	0	0	2.950986	0.582887	-2.56051	**-1.4925**	**Down**	**0.01045**	**0.0416**	_	K07604
ENSG000	AC02416	770	0	0	0	0	0	0	3	0.19	21	1.21	1	0.06	3.576561	0.576522	3.004309	**1.73205**	**Ups**	**0.00266**	**0.01413**	_	_
ENSG000	AC02647	1123	1	0.04	2	0.09	1	0.04	13	0.6	7	0.3	11	0.46	5.578089	0.551598	3.14522	**1.7349**	**Ups**	**0.00166**	**0.00955**	_	_
ENSG000	AC06199	32070	10	0.18	6	0.11	9	0.16	3	0.06	1	0.02	0	0	5.1021	0.558851	-3.0977	**-1.7312**	**Down**	**0.00195**	**0.01096**	_	_
ENSG000	AC06795	749785	1	0.03	3	0.08	2	0.05	33	0.89	44	1.1	39	0.95	18.7195	0.461935	6.774931	**3.12958**	**Ups**	**1.2E-11**	**4.8E-10**	_	_
ENSG000	AC06858	2139	36	0.46	34	0.45	32	0.41	17	0.23	10	0.12	12	0.14	24.27647	0.363855	-3.88843	**-1.4148**	**Down**	**0.0001**	**0.00088**	_	_
ENSG000	AC06927	245651	77	0.61	95	0.79	76	0.61	230	1.9	292	2.24	252	1.89	162.1696	0.199508	6.888716	**1.37435**	**Ups**	**5.6E-12**	**2.3E-10**	_	_
ENSG000	AC06951	2363	32	0.49	21	0.33	20	0.31	10	0.16	12	0.18	12	0.17	18.15342	0.386528	-2.98931	**-1.1555**	**Down**	**0.0028**	**0.0147**	_	_
ENSG000	AC07313	11129	0	0	6	0.05	3	0.02	13	0.1	20	0.15	16	0.12	9.032851	0.510347	3.155898	**1.6106**	**Ups**	**0.0016**	**0.00926**	_	_
ENSG000	AC07313	89968	2	0.04	4	0.08	2	0.04	13	0.24	26	0.45	14	0.24	9.374287	0.499166	3.699823	**1.84683**	**Ups**	**0.00022**	**0.00171**	_	_
ENSG000	AC07325	31024	10	0.04	15	0.06	16	0.06	43	0.16	54	0.19	39	0.13	28.07762	0.345117	3.933917	**1.35766**	**Ups**	**8.4E-05**	**0.00075**	_	_
ENSG000	AC07421	4709	40	0.63	36	0.59	40	0.63	134	2.19	76	1.15	123	1.82	72.7765	0.318945	3.832242	**1.22228**	**Ups**	**0.00013**	**0.00108**	_	_
ENSG000	AC07438	3662	1214	24.51	909	19.14	1236	25.1	679	14.26	292	5.69	702	13.37	861.0275	0.326898	-3.31548	**-1.0838**	**Down**	**0.00091**	**0.00585**	_	_
ENSG000	AC07946	8542	128	0.74	205	1.23	157	0.91	0	0	5	0.03	3	0.02	88.5663	0.409829	-12.4321	**-5.095**	**Down**	**1.8E-35**	**4.4E-33**	_	_
ENSG000	AC07977	15097	0	0	3	0.07	0	0	12	0.3	9	0.21	5	0.11	4.605144	0.574901	2.749059	**1.58044**	**Ups**	**0.00598**	**0.02695**	_	_
ENSG000	AC08729	15705	34	0.58	25	0.44	26	0.44	7	0.12	10	0.16	19	0.3	20.55928	0.395415	-3.21397	**-1.2709**	**Down**	**0.00131**	**0.00787**	_	_
ENSG000	AC09341	116349	1	0	4	0.01	0	0	33	0.11	9	0.03	24	0.07	11.43587	0.547872	4.106912	**2.25006**	**Ups**	**4E-05**	**0.0004**	_	_
ENSG000	AC09362	941	5	0.22	6	0.28	2	0.09	0	0	0	0	1	0.04	2.460112	0.582817	-2.64303	**-1.5404**	**Down**	**0.00822**	**0.03456**	_	_
ENSG000	AC09666	26840	21	0.25	24	0.3	18	0.22	248	3.13	242	2.83	234	2.68	122.6718	0.263069	11.89443	**3.12906**	**Ups**	**1.3E-32**	**2.7E-30**	_	_
ENSG000	AC09882	42974	7	0.24	12	0.43	8	0.28	0	0	1	0.03	0	0	4.955192	0.571566	-4.22026	**-2.4122**	**Down**	**2.4E-05**	**0.00026**	_	_
ENSG000	AC10295	773	0	0	0	0	1	0.11	13	1.44	7	0.72	11	1.1	5.044896	0.573126	3.953783	**2.26602**	**Ups**	**7.7E-05**	**0.0007**	_	_
ENSG000	AC10505	10262	0	0	2	0.03	1	0.02	1	0.02	23	0.33	9	0.13	5.249533	0.582065	2.64237	**1.53803**	**Ups**	**0.00823**	**0.03462**	_	_
ENSG000	AC10708	17651	6	0.19	12	0.4	5	0.16	41	1.37	13	0.4	30	0.91	17.4805	0.479825	2.759637	**1.32414**	**Ups**	**0.00579**	**0.0263**	_	_
ENSG000	AC11222	3130	7	0.06	9	0.08	5	0.04	15	0.13	35	0.28	15	0.12	13.42714	0.451156	2.495958	**1.12607**	**Ups**	**0.01256**	**0.04793**	_	_
ENSG000	AC11360	5236	1	0.03	0	0	0	0	1	0.03	23	0.57	9	0.22	4.885587	0.583456	3.204262	**1.86955**	**Ups**	**0.00135**	**0.00809**	_	_
ENSG000	AC11813	23457	63	1.48	38	0.93	54	1.28	32	0.78	23	0.52	26	0.58	40.17157	0.327405	-3.17853	**-1.0407**	**Down**	**0.00148**	**0.00872**	_	_
ENSG000	AC12376	8743	14	0.37	16	0.44	9	0.24	24	0.66	44	1.13	41	1.03	23.33217	0.368771	2.995061	**1.10449**	**Ups**	**0.00274**	**0.01448**	_	_
ENSG000	AC13378	6017	64	0.48	40	0.31	60	0.45	23	0.18	28	0.2	21	0.15	40.17591	0.314096	-4.13106	**-1.2975**	**Down**	**3.6E-05**	**0.00036**	_	_
ENSG000	AC13793	3978	26	0.38	38	0.58	35	0.51	22	0.33	16	0.22	10	0.14	25.17925	0.381411	-2.872	**-1.0954**	**Down**	**0.00408**	**0.01992**	_	_
ENSG000	AC13865	2398	0	0	1	0.01	0	0	6	0.07	4	0.04	6	0.06	2.669639	0.583449	2.68323	**1.56553**	**Ups**	**0.00729**	**0.03145**	_	K09228
ENSG000	AC13910	30509	11	0.05	13	0.06	12	0.05	35	0.16	45	0.19	31	0.13	23.34416	0.356873	3.508934	**1.25224**	**Ups**	**0.00045**	**0.00322**	_	_
ENSG000	AC14091	1916	0	0	0	0	0	0	5	0.11	13	0.26	10	0.19	4.165711	0.581024	4.083877	**2.37283**	**Ups**	**4.4E-05**	**0.00043**	_	_
ENSG000	AC14158	27145	55	0.41	66	0.52	69	0.52	37	0.29	34	0.25	29	0.21	49.33273	0.285578	-3.73666	**-1.0671**	**Down**	**0.00019**	**0.00151**	_	_
ENSG000	AC14192	4643	1	0.01	1	0.01	0	0	9	0.05	4	0.02	17	0.09	5.014731	0.575078	3.321369	**1.91005**	**Ups**	**0.0009**	**0.00575**	_	_
ENSG000	AC15645	5311	9	0.08	8	0.08	11	0.1	58	0.56	20	0.18	38	0.33	23.47373	0.446713	3.443401	**1.53821**	**Ups**	**0.00057**	**0.00396**	_	_
ENSG000	AC15954	18025	0	0	1	0	3	0.01	9	0.02	25	0.05	10	0.02	7.238013	0.550018	3.647211	**2.00603**	**Ups**	**0.00027**	**0.00204**	_	_
ENSG000	ACBD7	11254	2	0.03	3	0.05	2	0.03	13	0.22	82	1.26	21	0.32	18.00403	0.530675	4.78341	**2.53844**	**Ups**	**1.7E-06**	**2.5E-05**	molecular	_
ENSG000	ACOT1	6681	265	2.45	238	2.29	233	2.17	604	5.81	499	4.45	665	5.8	404.2035	0.208903	4.903528	**1.02436**	**Ups**	**9.4E-07**	**1.4E-05**	biological	K01068
ENSG000	ACPL2	66181	181	0.27	209	0.32	169	0.25	241	0.37	912	1.29	452	0.63	335.3807	0.337112	3.353496	**1.1305**	**Ups**	**0.0008**	**0.00521**	molecular	_
ENSG000	ACPT	4810	4	0.07	1	0.02	3	0.06	24	0.46	7	0.12	22	0.38	9.833945	0.531424	3.3057	**1.75673**	**Ups**	**0.00095**	**0.00603**	molecular	K19284
ENSG000	ACSM3	187339	10	0.03	7	0.03	6	0.02	32	0.12	50	0.17	36	0.12	21.93867	0.384815	4.825461	**1.85691**	**Ups**	**1.4E-06**	**2.1E-05**	molecular	K01896
ENSG000	ACTBP7	1124	2	0.05	4	0.1	4	0.09	0	0	0	0	0	0	1.787746	0.571908	-2.70759	**-1.5485**	**Down**	**0.00678**	**0.02971**	_	K05692
ENSG000	ACTC1	8044	245	1.24	227	1.2	256	1.3	1316	6.92	4062	19.82	3266	15.57	1410.061	0.292946	10.51322	**3.07981**	**Ups**	**7.5E-26**	**1.1E-23**	molecular	K12314
ENSG000	ACTG1P1	1129	1	0.02	1	0.02	1	0.02	8	0.19	6	0.13	7	0.15	3.797	0.569244	2.611147	**1.48638**	**Ups**	**0.00902**	**0.03715**	_	K05692
ENSG000	ACTG2	27552	590	2.83	567	2.83	725	3.49	627	3.12	5588	25.83	3754	16.96	1781.756	0.530149	2.805747	**1.48747**	**Ups**	**0.00502**	**0.02347**	molecular	K12315
ENSG000	ACTL8	71751	133	1.84	116	1.67	147	2.04	27	0.39	50	0.67	45	0.59	88.88906	0.255862	-7.17684	**-1.8363**	**Down**	**7.1E-13**	**3.2E-11**	cellular_cc	_
ENSG000	ACTR3C	79810	161	0.56	139	0.51	148	0.52	37	0.13	97	0.33	71	0.23	110.2271	0.276939	-4.68068	**-1.2963**	**Down**	**2.9E-06**	**3.9E-05**	molecular	K18584
ENSG000	ADAM21	7749	66	0.34	29	0.15	57	0.29	69	0.36	211	1.03	126	0.6	87.07341	0.365945	2.807492	**1.02739**	**Ups**	**0.00499**	**0.02337**	molecular	K08610
ENSG000	ADAM22	268747	220	0.37	240	0.42	192	0.32	440	0.76	985	1.59	504	0.79	404.1463	0.263916	4.702344	**1.24102**	**Ups**	**2.6E-06**	**3.5E-05**	molecular	K16068
ENSG000	ADAM23	177589	16	0.05	7	0.02	7	0.02	86	0.28	88	0.27	82	0.25	44.65664	0.347712	7.442681	**2.58791**	**Ups**	**9.9E-14**	**5E-12**	molecular	K06837
ENSG000	ADAM33	14282	109	0.3	62	0.18	88	0.25	300	0.87	232	0.62	316	0.83	177.3916	0.273276	5.206091	**1.4227**	**Ups**	**1.9E-07**	**3.5E-06**	molecular	K08616
ENSG000	ADAM8	14466	4	0.01	5	0.02	14	0.04	24	0.08	37	0.11	57	0.16	21.94933	0.447874	3.86116	**1.72931**	**Ups**	**0.00011**	**0.00097**	biological	K06540
ENSG000	ADAMTS	9663	434	1.01	386	0.94	358	0.84	1730	4.2	3861	8.7	2205	4.86	1374.11	0.238041	9.94157	**2.3665**	**Ups**	**2.7E-23**	**3.2E-21**	biological	K08617
ENSG000	ADAMTS	368658	1	0	0	0	2	0	14	0.03	13	0.03	8	0.02	5.952814	0.554426	3.641729	**2.01907**	**Ups**	**0.00027**	**0.00208**	molecular	K08626
ENSG000	ADAMTS	45031	6	0.02	4	0.01	9	0.03	72	0.26	60	0.2	71	0.23	34.78586	0.382619	7.301218	**2.79359**	**Ups**	**2.9E-13**	**1.4E-11**	molecular	K08627
ENSG000	ADAMTS	179975	20	0.08	10	0.04	16	0.06	3	0.01	8	0.03	4	0.02	10.39991	0.474099	-3.07662	**-1.4586**	**Down**	**0.00209**	**0.0116**	molecular	K08630
ENSG000	ADAMTS2	198056	0	0	2	0.01	0	0	4	0.01	44	0.14	17	0.05	9.697607	0.572834	4.189908	**2.40012**	**Ups**	**2.8E-05**	**0.00029**	molecular	K09609
ENSG000	ADAMTS3	287831	4	0.02	13	0.05	9	0.03	63	0.25	113	0.42	81	0.29	43.34109	0.368289	7.344655	**2.70495**	**Ups**	**2.1E-13**	**1E-11**	molecular	K08619
ENSG000	ADAMTS4	14749	19	0.04	23	0.05	36	0.08	137	0.3	58	0.12	109	0.22	61.97521	0.389743	3.984058	**1.55276**	**Ups**	**6.8E-05**	**0.00063**	biological	K07764
ENSG000	ADAMTS6	333185	57	0.1	63	0.12	48	0.09	158	0.3	232	0.4	158	0.27	113.0365	0.237278	5.949517	**1.41169**	**Ups**	**2.7E-09**	**7E-08**	molecular	K08621
ENSG000	ADAMTSL	437057	3	0	1	0	2	0	88	0.1	84	0.09	71	0.08	38.54536	0.437982	9.292017	**4.06973**	**Ups**	**1.5E-20**	**1.5E-18**	cellular_cc	_
ENSG000	ADCY7	71999	429	0.72	395	0.69	370	0.62	121	0.21	368	0.59	140	0.22	305.3763	0.322905	-3.3816	**-1.0919**	**Down**	**0.00072**	**0.0048**	molecular	K08047
ENSG000	ADHFE1	41417	17	0.05	11	0.04	25	0.08	79	0.26	98	0.3	76	0.23	48.07072	0.321279	5.793384	**1.86129**	**Ups**	**6.9E-09**	**1.7E-07**	cellular_cc	K11173
ENSG000	ADM2	4885	68	0.19	46	0.14	64	0.18	362	1.07	150	0.41	286	0.77	157.8558	0.353643	5.054279	**1.78741**	**Ups**	**4.3E-07**	**7.1E-06**	biological	_
ENSG000	ADORA1	76752	81	0.18	58	0.14	101	0.23	37	0.09	24	0.05	34	0.07	57.58077	0.325122	-4.33024	**-1.4079**	**Down**	**1.5E-05**	**0.00017**	biological	K04265
ENSG000	ADRA2C	2177	31	0.21	40	0.29	41	0.28	177	1.27	30	0.2	158	1.03	78.40373	0.462215	2.68861	**1.24272**	**Ups**	**0.00718**	**0.03106**	molecular	K04140
ENSG000	AF00154	6320	11	0.34	4	0.13	8	0.25	26	0.83	24	0.71	24	0.7	15.49708	0.419973	2.967787	**1.24639**	**Ups**	**0.003**	**0.01559**	_	_
ENSG000	AF12189	213733	20	0.22	23	0.27	17	0.19	2	0.02	2	0.02	5	0.05	12.05653	0.473962	-4.84314	**-2.2955**	**Down**	**1.3E-06**	**1.9E-05**	_	_
ENSG000	AGMAT	12758	1176	12.09	939	10.07	1099	11.37	548	5.86	622	6.17	603	5.85	843.2975	0.15111	-7.0044	**-1.0584**	**Down**	**2.5E-12**	**1.1E-10**	cellular_cc	K01480
ENSG000	AGO4	49719	229	0.75	249	0.85	219	0.72	339	1.16	971	3.07	488	1.51	390.4392	0.293445	3.500858	**1.02731**	**Ups**	**0.00046**	**0.00331**	cellular_cc	K11593
ENSG000	AGPAT4	144083	61	0.09	69	0.1	72	0.1	10	0.01	11	0.01	11	0.01	40.91292	0.328715	-7.97844	**-2.6226**	**Down**	**1.5E-15**	**9.2E-14**	molecular	K13523
ENSG000	AHNAK2	41114	2445	1.52	2268	1.47	2307	1.44	1104	0.71	1900	1.14	1026	0.6	1857.597	0.209274	-4.8035	**-1.0052**	**Down**	**1.6E-06**	**2.3E-05**	cellular_cc	_

ENSG000	AHRR	134116	2340	4.45	2069	4.1	2113	4.04	3999	7.91	10592	19.43	5522	9.9	4147.476	0.261541	4.936547	1.29111	Ups	8E-07	1.2E-05	biological_	K09094
ENSG000	AIM1	58597	68	0.19	80	0.23	64	0.18	6	0.02	27	0.07	15	0.04	44.79247	0.360235	-5.94127	-2.1403	Down	2.8E-09	7.4E-08	molecular_	K04257
ENSG000	AIM1L	32272	36	0.14	32	0.13	28	0.11	4	0.02	7	0.03	11	0.04	20.38888	0.399113	-5.07719	-2.0264	Down	3.8E-07	6.4E-06	_	_
ENSG000	AJ003147	2786	0	0	0	0	2	0.11	10	0.59	22	1.2	12	0.64	6.935907	0.555324	4.356238	2.41913	Ups	1.3E-05	0.00015	_	_
ENSG000	AK5	277916	9	0.02	7	0.02	9	0.02	17	0.04	32	0.08	22	0.05	15.1209	0.408658	2.654566	1.08481	Ups	0.00794	0.03361	molecular_	K00939
ENSG000	AKAP11	51108	272	0.71	445	1.2	208	0.54	332	0.9	1829	4.58	666	1.63	575.4844	0.412639	2.725073	1.12447	Ups	0.00643	0.02854	cellular_cc	K16527
ENSG000	AKAP6	502089	114	0.16	97	0.14	86	0.12	362	0.52	719	0.96	340	0.45	265.9349	0.276007	6.858733	1.89306	Ups	7E-12	2.7E-10	biological_	K16523
ENSG000	AKNA	60250	64	0.1	65	0.1	74	0.11	35	0.06	27	0.04	21	0.03	49.11169	0.305713	-4.53046	-1.385	Down	5.9E-06	7.4E-05	molecular_	_
ENSG000	AKR1B1	16935	35238	114.08	48125	162.5	41797	136.1	9326	31.4	5300	16.55	9265	28.29	26055.77	0.273691	-8.87336	-2.4286	Down	7.1E-19	5.9E-17	molecular_	K00011
ENSG000	AKR1B10	13817	33	0.35	33	0.37	51	0.55	6	0.07	11	0.11	7	0.07	24.48946	0.391798	-5.57177	-2.183	Down	2.5E-08	5.4E-07	biological_	K00011
ENSG000	AKR1B15	30740	4	0.03	7	0.05	5	0.03	1	0.01	0	0	0	0	3.027735	0.582766	-3.08243	-1.7963	Down	0.00205	0.01142	molecular_	K00011
ENSG000	AKR1C3	72333	15730	59.83	18734	74.32	18193	69.6	6942	27.47	2940	10.79	6177	22.16	11920.22	0.312181	-5.58461	-1.7434	Down	2.3E-08	5.1E-07	biological_	K04119
ENSG000	AKT3	362847	49	0.12	57	0.15	43	0.11	514	1.32	1064	2.54	540	1.26	343.7071	0.278995	12.04216	3.3597	Ups	2.1E-33	4.9E-31	biological_	K04456
ENSG000	AL022344	1049	1	0.02	3	0.08	3	0.07	14	0.36	9	0.21	21	0.49	8.070117	0.523061	3.281796	1.71658	Ups	0.00103	0.00647	_	K04614
ENSG000	AL022344	17527	28	1.39	15	0.77	32	1.59	51	2.63	76	3.63	61	2.85	41.84229	0.320362	3.136527	1.00482	Ups	0.00171	0.0098	_	_
ENSG000	AL035610	112574	4	0.02	3	0.02	2	0.01	11	0.06	12	0.06	14	0.07	7.260477	0.502986	2.691683	1.35388	Ups	0.00711	0.03081	_	_
ENSG000	AL590822	2261	18	0.2	17	0.2	12	0.14	56	0.66	30	0.33	52	0.56	29.98399	0.381612	3.134368	1.19611	Ups	0.00172	0.00985	_	K17985
ENSG000	AL592284	181321	32	0.77	25	0.62	26	0.63	86	2.14	37	0.86	92	2.08	48.54355	0.38088	2.741943	1.04435	Ups	0.00611	0.02742	_	K15258
ENSG000	AL592494	66370	10	0.07	11	0.08	11	0.08	24	0.18	77	0.52	53	0.35	28.472	0.401594	4.295705	1.72513	Ups	1.7E-05	0.00019	_	_
ENSG000	AL603965	23910	164	2.14	145	1.97	163	2.14	52	0.71	97	1.22	82	1.01	118.7717	0.229366	-5.30944	-1.2178	Down	1.1E-07	2.1E-06	_	_
ENSG000	ALDH3A1	10960	336	1.09	279	0.94	301	0.98	171	0.58	113	0.35	187	0.57	236.2529	0.257769	-4.25888	-1.0978	Down	2.1E-05	0.00022	biological_	K00129
ENSG000	ALG9	89387	866	1.92	827	1.92	838	1.87	1648	3.81	2661	5.7	2030	4.25	1408.55	0.154125	6.933983	1.0687	Ups	4.1E-12	1.7E-10	cellular_cc	K03846
ENSG000	ALOX12B	15068	33	0.24	30	0.23	33	0.24	13	0.1	26	0.18	12	0.08	24.74209	0.357822	-2.87471	-1.0286	Down	0.00404	0.01978	molecular_	K08021
ENSG000	ALPL	69048	0	0	2	0.01	1	0	10	0.04	18	0.07	11	0.04	6.409624	0.547981	3.914651	2.14515	Ups	9.1E-05	0.0008	biological_	K01077
ENSG000	ALS2CL	24708	509	0.73	462	0.69	565	0.81	160	0.24	380	0.52	235	0.32	389.1959	0.25106	-4.70376	-1.1809	Down	2.6E-06	3.5E-05	molecular_	_
ENSG000	AMIGO2	4245	542	1.17	689	1.55	457	0.99	173	0.39	312	0.65	205	0.42	404.4804	0.233469	-6.28547	-1.4675	Down	3.3E-10	1E-08	molecular_	_
ENSG000	AMT	5976	53	0.1	46	0.09	59	0.11	186	0.36	159	0.29	191	0.34	110.9504	0.24817	5.964909	1.48031	Ups	2.5E-09	6.4E-08	molecular_	K00605
ENSG000	AMY2B	25720	17	0.05	35	0.1	21	0.06	338	0.97	248	0.66	321	0.84	153.9106	0.312371	10.12302	3.16214	Ups	4.4E-24	5.7E-22	molecular_	K01176
ENSG000	ANK2	565632	20	0.02	33	0.03	19	0.02	78	0.07	167	0.14	86	0.07	62.34349	0.340779	5.18122	1.76565	Ups	2.2E-07	3.9E-06	biological_	K10380
ENSG000	ANKRD13	24993	1627	6.2	1178	4.68	1382	5.3	911	3.61	489	1.8	683	2.45	1072.196	0.280729	-3.96487	-1.1131	Down	7.3E-05	0.00067	_	_
ENSG000	ANKRD19	79306	5	0.03	1	0.01	9	0.06	17	0.11	14	0.08	30	0.17	12.0553	0.49857	2.732352	1.36227	Ups	0.00629	0.02807	_	K15410
ENSG000	ANKRD30	124467	1	0	4	0.01	2	0.01	1	0	57	0.18	12	0.04	11.12352	0.578756	2.757886	1.59614	Ups	0.00582	0.02641	molecular_	K17299
ENSG000	ANKRD31	168604	3	0.01	4	0.02	2	0.01	7	0.03	20	0.07	14	0.05	7.683191	0.511225	2.767294	1.41471	Ups	0.00565	0.02582	_	_
ENSG000	ANKRD34	5139	98	0.7	88	0.65	64	0.46	178	1.32	233	1.6	208	1.4	138.6695	0.219231	4.762737	1.04414	Ups	1.9E-06	2.7E-05	cellular_cc	K03024
ENSG000	ANKRD36	142955	55	0.15	75	0.21	59	0.16	122	0.34	318	0.82	162	0.41	123.1262	0.313053	4.154996	1.30073	Ups	3.3E-05	0.00033	molecular_	K17299
ENSG000	ANKRD44	344157	54	0.13	66	0.16	53	0.12	96	0.23	415	0.93	127	0.28	123.9496	0.399704	3.451933	1.37975	Ups	0.00056	0.00387	molecular_	K15503
ENSG000	ANKRD45	60302	0	0	1	0.01	2	0.02	9	0.07	17	0.13	12	0.09	6.250047	0.548735	3.85697	2.11646	Ups	0.00011	0.00099	molecular_	K14726
ENSG000	ANTXR2	224306	113	0.22	81	0.16	88	0.17	1	0	8	0.01	4	0.01	52.01845	0.390468	-10.2631	-4.0074	Down	1E-24	1.4E-22	molecular_	_
ENSG000	ANXA3	58925	7316	24.78	7374	26.05	6954	23.69	1997	7.03	5581	18.24	4094	13.08	5586.961	0.260272	-4.19836	-1.0927	Down	2.7E-05	0.00028	molecular_	K17089
ENSG000	ANXA8	23948	234	0.66	246	0.73	113	0.32	18	0.05	110	0.3	40	0.11	129.7153	0.429424	-4.10868	-1.7644	Down	4E-05	0.00039	molecular_	K17096
ENSG000	ANXA8L1	16111	521	3.28	558	3.66	551	3.49	25	0.16	35	0.21	34	0.2	304.697	0.212988	-19.6723	-4.19	Down	3.7E-86	5.8E-83	molecular_	K17096
ENSG000	ANXA8L2	16106	528	3.32	513	3.37	610	3.86	58	0.38	132	0.8	73	0.43	333.4846	0.250377	-11.0179	-2.7586	Down	3.1E-28	5.1E-26	molecular_	K17096
ENSG000	AOC2	6108	76	0.38	69	0.36	71	0.36	200	1.05	211	1.03	204	0.97	132.8273	0.204419	6.126626	1.2524	Ups	9E-10	2.6E-08	molecular_	K00276
ENSG000	AOC3	6947	38	0.13	50	0.18	40	0.14	137	0.51	151	0.52	120	0.4	85.39126	0.250352	5.527798	1.3839	Ups	3.2E-08	6.8E-07	molecular_	K00276
ENSG000	AOX1	91197	281	0.97	301	1.08	288	1	21	0.08	141	0.47	41	0.13	184.3079	0.417888	-4.86184	-2.0317	Down	1.2E-06	1.7E-05	molecular_	K00157
ENSG000	AP000472	89516	4	0.05	2	0.03	0	0	5	0.07	27	0.33	8	0.09	6.862029	0.563906	2.646104	1.49215	Ups	0.00814	0.0343	_	_
ENSG000	AP000473	1537	1	0.02	0	0	0	0	8	0.14	6	0.1	2	0.03	2.686137	0.582886	2.554315	1.48887	Ups	0.01064	0.04215	_	_
ENSG000	AP000569	1472	9	0.39	7	0.32	5	0.22	1	0.05	0	0	0	0	3.901703	0.578217	-3.63894	-2.1041	Down	0.00027	0.0021	_	_
ENSG000	AP000662	14767	14	0.07	13	0.07	6	0.03	50	0.26	49	0.24	51	0.24	28.93281	0.360423	4.876009	1.75743	Ups	1.1E-06	1.6E-05	_	_
ENSG000	AP001046	4203	65	0.6	60	0.57	69	0.64	20	0.19	3	0.03	18	0.16	41.12659	0.42675	-4.74368	-2.0244	Down	2.1E-06	2.9E-05	_	_
ENSG000	AP001048	1990	23	2.11	13	1.24	23	2.12	4	0.38	7	0.62	8	0.69	13.35927	0.441264	-3.47946	-1.5354	Down	0.0005	0.00354	_	_
ENSG000	AP001065	4712	16	0.45	13	0.38	16	0.45	3	0.09	2	0.05	2	0.05	9.106689	0.497576	-4.31521	-2.1471	Down	1.6E-05	0.00018	_	_
ENSG000	AP001505	824	314	9.8	251	8.17	335	10.51	109	3.54	115	3.46	116	3.41	212.437	0.203958	-7.70025	-1.5705	Down	1.4E-14	7.6E-13	_	_
ENSG000	AP001631	3212	53	1.3	40	1.02	40	0.98	6	0.15	6	0.14	11	0.25	27.16445	0.37929	-6.35041	-2.4086	Down	2.2E-10	6.8E-09	_	_
ENSG000	AP001631	570	345	21.02	299	19	261	15.99	74	4.69	14	0.82	74	4.25	186.7743	0.422652	-5.27712	-2.2304	Down	1.3E-07	2.5E-06	_	_
ENSG000	AP003733	1303	29	0.57	28	0.58	41	0.81	7	0.14	6	0.11	18	0.33	22.22366	0.416893	-3.8266	-1.5953	Down	0.00013	0.0011	_	K06115
ENSG000	AP1S3	86342	187	0.64	179	0.64	166	0.57	236	0.84	823	2.72	332	1.07	299.2901	0.337608	2.991207	1.00986	Ups	0.00278	0.01463	cellular_cc	K12395
ENSG000	APBB2	406688	455	0.38	540	0.47	462	0.39	852	0.75	2252	1.83	1129	0.9	888.1485	0.276508	4.339365	1.19987	Ups	1.4E-05	0.00016	molecular_	K04530
ENSG000	APCDD1	35321	9	0.03	5	0.02	8	0.03	53	0.19	68	0.22	76	0.24	33.8918	0.363392	7.163025	2.60298	Ups	7.9E-13	3.5E-11	biological_	K17985
ENSG000	APEX2	8701	5414	37.9	4488	32.77	5395	37.98	2444	17.8	2875	19.42	3064	20.23	4003.667	0.142128	-7.54806	-1.0728	Down	4.4E-14	2.3E-12	molecular_	K10772
ENSG000	APLP1	11893	60	0.25	46	0.2	42	0.17	112	0.48	158	0.63	178	0.69	94.25404	0.252815	5.148831	1.3017	Ups	2.6E-07	4.6E-06	molecular_	K05639
ENSG000	APOBEC3I	6844	7	0.08	2	0.02	9	0.1	17	0.2	17	0.19	30	0.33	12.99964	0.467543	2.738555	1.28039	Ups	0.00617	0.02765	cellular_cc	K18750
ENSG000	APOE	3640	16	0.11	12	0.08	29	0.19	132	0.91	94	0.6	170	1.07	71.50779	0.363411	6.340068	2.30405	Ups	2.3E-10	7.2E-09	biological_	K04524
ENSG000	AQP6	9946	33	0.12	20	0.08	24	0.09	83	0.32	42	0.15	71	0.25	44.48131	0.365461	2.832053	1.03501	Ups	0.00463	0.02198	molecular_	K09868
ENSG000	ARC	4429	5	0.03	1	0.01	8	0.05	44	0.27	7	0.04	26	0.14	14.96074	0.542459	2.86526	1.55429	Ups	0.00417	0.02026	cellular_cc	_
ENSG000	AREG	9876	19	0.19	14	0.15	17	0.17	6	0.06	2	0.02	7	0.07	11.25932	0.466015	-3.3093	-1.5422	Down	0.00094	0.00596	molecular_	K09782
ENSG000	ARHGAP2	136147	11	0.02	25	0.05	20	0.04	3	0.01	2	0	0	0	10.80395	0.520588	-4.89186	-2.5466	Down	1E-06	1.5E-05	molecular_	_
ENSG000	ARHGAP2	29749	29	0.32	13	0.15	31	0.34	4	0.05	18	0.19	5	0.05	16.92367	0.474974	-2.8189	-1.3389	Down	0.00482	0.0227	_	_
ENSG000	ARHGAP2	458628	126	0.15	120	0.14	132	0.15	303	0.36	566	0.63	351	0.38	250.7338	0.227438	6.110534	1.38977	Ups	9.9E-10	2.8E-08	;molecular	_
ENSG000	ARHGAP2	40513	606	0.92	534	0.85	583	0.89	282	0.45	333	0.49	276	0.4	442.9902	0.156616	-7.34452	-1.1503	Down	2.1E-13	1E-11	molecular_	_
ENSG000	ARHGAP2	126081	229	0.36	418	0.68	177	0.28	499	0.8	1283	1.92	618	0.9	503.4961	0.344484	3.35861	1.15699	Ups	0.00078	0.00514	;cellular_cc	K17516
ENSG000	ARHGAP4	27632	34	0.07	23	0.05	27	0.05	115	0.23	63	0.12	99	0.18	58.36984	0.342388	4.053485	1.38787	Ups	5.1E-05	0.00048	molecular_	_
ENSG000	ARHGAP5	83615	1140	1.31	1979	2.37	881	1.02	1709	2.04	6924	7.68	2533	2.75	2347.248	0.38134	2.757992	1.05173	Ups	0.00582	0.0264	biological_	K13709
ENSG000	ARHGDIB	19712	148	0.86	160	0.97	196	1.14	16	0.1	71	0.4	35	0.19	107.7416	0.356664	-5.82162	-2.0764	Down	5.8E-09	1.4E-07	molecular_	K12462
ENSG000	ARHGEF12	152859	2655	2.29	2868	2.58	2302	2	3871	3.48	14195	11.84	5190	4.23	4806.924	0.334626	3.514702	1.17611	Ups	0.00044	0.00316	molecular_	K07532
ENSG000	ARHGEF25	9200	36	0.17	31	0.15	40	0.19	175	0.87	101	0.46	162	0.73	87.57558	0.319048	5.302299	1.69169	Ups	1.1E-07	2.2E-06	molecular_	_
ENSG000	ARHGEF4	210348	1018	1.41	754	1.09	823	1.15	355	0.51	610	0.82	392	0.51	666.101	0.206909	-5.48159	-1.1342	Down	4.2E-08	8.7E-07	molecular_	K05769
ENSG000	ARID5B	195645	624	1.55	844	2.18	663	1.65	1353	3.49	2971	7.11	1592	3.72	1263.985	0.253155	4.57678	1.15863	Ups	4.7E-06	6.1E-05	biological_	_
ENSG000	ARMC4	223863	421	1.53	450	1.71	477	1.75	871	3.3	2014	7.07	1267	4.35	859.8283	0.238896	5.472621	1.30739	Ups	4.4E-08	9.1E-07	_	_
ENSG000	ARMCX1	4170	0	0	0	0	0	0	20	0.25	23	0.27	15	0.17	8.889162	0.558345	5.845851	3.264	Ups	5E-09	1.2E-07	cellular_cc	_
ENSG000	ARNT2	193587	138	0.26	115	0.23	131	0.25	14	0.03	33	0.06	23	0.04	78.79573	0.28076	-8.99659	-2.5259	Down	2.3E-19	2E-17	biological_	K15589
ENSG000	ARRDC4	54285	44	0.22	33	0.17	30	0.15	96	0.51	188	0.92	100	0.48	76.63921	0.302999	4.89899	1.48439	Ups	9.6E-07	1.5E-05	_	_
ENSG000	ART5	3814	77	0.45	76	0.47	96	0.57	213	1.3	141	0.8	252	1.4	138.5233	0.290368	3.48122	1.01083	Ups	0.0005	0.00352	molecular_	K00775
ENSG000	ASAP3	56006	188	0.63	181	0.63	179	0.6	447	1.55	569	1.83	480	1.51	325.6124	0.156874	7.662759	1.20209	Ups	1.8E-14	1E-12	molecular_	K12488
ENSG000	ASB14	24336	19	0.09	17	0.08	20	0.09	55	0.26	52	0.23	48	0.21	33.87541	0.316214	3.65358	1.15531	Ups	0.00026	0.002	biological_	K10336
ENSG000	ASNS	20425	1832	4.85	1663	4.59	1817	4.84	4160	11.45	4685	11.97	4873	12.17	3044.555	0.124233	9.147446	1.13641	Ups	5.8E-20	5.4E-18	biological_	K01953
ENSG000	ASPHD2	15743	4	0.03	9	0.07	10	0.08	32	0.25	23	0.17	25	0.18	16.57673	0.427072	3.126247	1.33513	Ups	0.00177	0.0101	cellular_cc	_
ENSG000	ASTN1	307672	3	0.01	1	0	1	0	100	0.18	18	0.03	67	0.11	30.69122	0.529537	6.272071	3.3213	Ups	3.6E-10	1.1E-08	biological_	_
ENSG000	ATAD3B	26086	3938	9.13	3234	7.82	3885	9.06	1736	4.19	1343	3	2156	4.72	2777.887	0.222949	-5.5339	-1.2338	Down	3.1E-08	6.6E-07	molecular_	K17681
ENSG000	ATF3	55444	214	0.74	212	0.76	267	0.93	18379	65.93	3081	10.25	10679	34.74	5324.684	0.53196	6.917186	3.67967	Ups	4.6E-12	1.9E-10	molecular_	K09032
ENSG000	ATG16L2	29367	224	0.3	172	0.24	162	0.22	544	0.75	488	0.62	473	0.59	331.6833	0.215018	5.50733	1.18417	Ups	3.6E-08	7.6E-07	cellular_cc	K17890
ENSG000	ATOH8	36723	1022	3.42	853	2.98	1090	3.67	4087	14.24	3451	11.15	4808	15.19	2433.423	0.203545	8.808729	1.79297	Ups	1.3E-18	1E-16	molecular_	K09084
ENSG000	ATP11B	128136	881	1.83	1163	2.52	698	1.46	1112	2.4	5244	10.51	1713	3.36	1663.857	0.382694	2.90634	1.11224	Ups	0.00366	0.01826	molecular_	K01530
ENSG000	ATP6AP1L	107516	56	0.15	53	0.15	46	0.13	303	0.87	284	0.76	246	0.64	156.1423	0.237196	8.946672	2.12212	Ups	3.7E-19	3.1E-17	biological_	K17985
ENSG000	ATP6V0E2	12914	3	0.01	3	0.01	1	0	13	0.06	18	0.07	20	0.08	8.982973	0.500092	3.894641	1.94768	Ups	9.8E-05	0.00087	_	_
ENSG000	ATP6V1G	3966	54	0.27	45	0.24	40	0.2	105	0.55	124	0.61	115	0.55	77.28183	0.237412	4.38021	1.03991	Ups	1.2E-05	0.00014	cellular_cc	K02152
ENSG000	ATP8A2	653781	5	0.01	4	0.01	3	0.01	0	0	0	0	0	0	2.131787	0.579088	-3.01887	-1.7482	Down	0.00254	0.0136	molecular_	K14802
ENSG000	ATRNL1	855380	14	0.03	17	0.03	13	0.02	64	0.12	40	0.07	61	0.11	33.58243	0.361374	4.216049	1.52357	Ups	2.5E-05	0.00026	biological_	K03177
ENSG000	ATXN1	462380	380	0.5	435	0.59	388	0.51	709	0.97	2317	2.93	907	1.12	793.8608	0.323858	4.059102	1.31457	Ups	4.9E-05	0.00047	molecular_	_
ENSG000	AVPR2	4636	55	0.22	33	0.14	37	0.15	2	0.01	5	0.02	2	0.01	23.53787	0.444326	-7.27506	-3.2325	Down	3.5E-13	1.6E-11	biological_	K04228
ENSG000	B3GNT3	18255	59	0.35	47	0.29	55	0.33	20	0.12	2	0.01	7	0.04	33.43204	0.468455	-4.42676	-2.0737	Down	9.6E-06	0.00011	cellular_cc	K07970
ENSG000	B4GALT6	63590	48	0.12	78	0.21	35	0.09	88	0.23	474	1.17	221	0.53	142.723	0.420829	4.00413	1.68505	Ups	6.2E-05	0.00058	cellular_cc	K07553
ENSG000	BAGE2	102955	25	0.13	38	0.2	11	0.06	0	0	0	0	1	0	13.31344	0.547141	-6.25282	-3.4212	Down	4E-10	1.2E-08	cellular_cc	K09188
ENSG000	BAI2	37777	81	0.15	83	0.16	107	0.19	318	0.6	355	0.62	310	0.53	198.9277	0.207003	7.662553	1.58617	Ups	1.8E-14	1E-12	molecular_	K04597
ENSG000	BBC3	11943	257	1.13	215	0.99	244	1.08	1206	5.53	286	1.22	831	3.46	499.9287	0.399487	3.345368	1.33643	Ups	0.00082	0.00534	biological_	K10132
ENSG000	BCAM	12346	647	2.02	484	1.57	550	1.73	1716	5.57	1369	4.12	1921	5.65	1073.248	0.216895	6.099631	1.32298	Ups	1.1E-09	3E-08	molecular_	K06578
ENSG000	BCAR3	285360	1985	7	1948	7.16	1841	6.53	637	2.34	1190	4.05	872	2.9	1433.78	0.181927	-7.18413	-1.307	Down	6.8E-13	3.1E-11	molecular_	_
ENSG000	BCL2	196783	274	0.53	226	0.45	240	0.46	451	0.9	1141	2.12	481	0.87	439.7827	0.295507	3.866631	1.14262	Ups	0.00011	0.00096	biological_	K02161
ENSG000	BCYRN1	13458	58	0.11	45	0.09	45	0.09	11	0.02	14	0.03	23	0.04	33.60976	0.340806	-4.90839	-1.6728	Down	9.2E-07	1.4E-05	_	_
ENSG000	BDKRB1	13144	1	0.01	1	0.01	2	0.01	60	0.44	53	0.36	49	0.33	25.7669	0.471139	8.066318	3.80036	Ups	7.2E-16	4.6E-14	biological_	K18047
ENSG000	BDKRB2	39651	67	0.1	87	0.14	89	0.14	756	1.23	1117	1.68	885	1.3	461.4437	0.196575	16.15018	3.17473	Ups	1.1E-58	7.2E-56	molecular_	K03916
ENSG000	BDNF-AS	191337	6	0.03	3	0.02	5	0.02	26	0.13	22	0.1	29	0.13	14.37718	0.441578	4.173505	1.84293	Ups	0.00003	0.00031	_	_
ENSG000	BEGAIN	50265	3102	7.7	2270	5.88	2940	7.34	1467	3.79	991	2.37	1301	3.05	2064.962	0.237517	-5.38509	-1.279	Down	7.2E-08	1.4E-06	molecular_	_
ENSG000	BEST1	15695	3	0	5	0.01	5	0.01	27	0.05	24	0.04	14	0.02	12.40673	0.468617	3.562808	1.66959	Ups	0.00037	0.0027	molecular_	K13878
ENSG000	BEST3	56117	66	0.15	67	0.15	60	0.13	19	0.04	49	0.1	21	0.04	47.58296	0.331349	-3.75701	-1.2449	Down	0.00017	0.00141	molecular_	K13880
ENSG000	BEX2	1701	48	0.39	32	0.27	45	0.37	162	1.37	98	0.77	181	1.39	90.95147	0.323329	4.601451	1.48778	Ups	4.2E-06	5.5E-05	cellular_cc	_
ENSG000	BEX5	2350	0	0	0	0	0	0	4	0.06	3	0.04	4	0.06	1.706466	0.571156	2.554423	1.45897	Ups	0.01064	0.04215	cellular_cc	_
ENSG000	BHLHE41	5102	26	0.15	37	0.22	24	0.14	79	0.46	89	0.48	74	0.39	52.57552	0.284524	4.134907	1.17648	Ups	3.6E-05	0.00036	biological_	K03730
ENSG000	BIK	18965	83	2.24	58	1.63	78	2.12	38	1.07	23	0.6	37	0.94	54.26728	0.315695	-3.96319	-1.2512	Down	7.4E-05	0.00067	molecular_	K18452
ENSG000	BIRC7	4625	100	1.23	78	1	77	0.96	36	0.46	6	0.07	27	0.31	56.44002	0.433326	-3.93091	-1.7034	Down	8.5E-05	0.00076	molecular_	K16061
ENSG000	BMP4	9026	36	0.12	42	0.15	61	0.21	99	0.35	238	0.77	156	0.5	98.30238	0.314593	4.594187	1.4453	Ups	4.3E-06	5.7E-05	biological_	K04662
ENSG000	BMP5	121920	2	0.01	6	0.03	8	0.04	198	1.02	73	0.35	174	0.81	73.27299	0.44865	8.267656	3.70929	Ups	1.4E-16	9.5E-15	biological_	K04663
ENSG000	BMP6	154626	1712	15.83	1327	12.8	1376	12.8	632	6.08	755	6.74	738	6.44	1109.666	0.152797	-8.19977	-1.2529	Down	2.4E-16	1.6E-14	biological_	K16620
ENSG000	BMPER	250962	105	0.32	113	0.36	99	0.3	386	1.22	316	0.93	351	1.01	219.542	0.222997	6.594159	1.47048	Ups	4.3E-11	1.5E-09	biological_	_
ENSG000	BMPR1B	400481	54	0.18	88	0.3	62	0.21	158	0.54	231	0.73	198	0.62	125.3889	0.245176	5.025189	1.23206	Ups	5E-07	8.2E-06	biological_	K13578
ENSG000	BMPR2	190816	500	1.06	661	1.46	397	0.84	863	1.9	2463	5.02	1204	2.4	948.3699	0.307583	3.831691	1.17856	Ups	0.00013	0.00108	biological_	K04671
ENSG000	BOLA2B	1373	35	0.41	29	0.35	26	0.31	16	0.19	10	0.11	12	0.13	21.95117	0.373126	-3.42693	-1.2787	Down	0.00061	0.00416	_	_
ENSG000	BRSK1	30462	27	0.15	25	0.14	32	0.17	155	0.87	74	0.39	130	0.66	71.48678	0.353691	4.850368	1.71553	Ups	1.2E-06	1.8E-05	molecular_	K08796
ENSG000	BSCL2	19571	425	0.92	334	0.75	317	0.69	207	0.46	194	0.4	207	0.42	284.985	0.201858	-4.95751	-1.0007	Down	7.1E-07	1.1E-05	molecular_	K19365
ENSG000	BTC	49928	14	0.12	14	0.12	13	0.11	1	0.01	7	0.06	5	0.04	9.193749	0.484246	-3.04267	-1.4734	Down	0.00234	0.01277	molecular_	K09783
ENSG000	BTG2	4112	284	1.83	249	1.67	279	1.81	5922	39.71	1096	6.82	4099	24.93	1933.65	0.520384	5.005554	2.60481	Ups	5.6E-07	9E-06	molecular_	K14443
ENSG000	BTN3A3	12944	141	0.25	148	0.28	165	0.3	230	0.43	627	1.09	311	0.53	254.1739	0.295931	3.459716	1.02384	Ups	0.00054	0.00377	biological_	K06712
ENSG000	BVES	40353	6	0.02	9	0.04	6	0.02	40	0.16	50	0.18	41	0.15	23.72891	0.380104	5.563733	2.1148	Ups	2.6E-08	5.6E-07	biological_	K17985
ENSG000	BZRAP1	27561	36	0.05	26	0.04	34	0.05	45	0.07	121	0.17	84	0.11	54.22721	0.331261	3.097526	1.02609	Ups	0.00195	0.01096	molecular_	_
ENSG000	C10orf10	7830	16	0.11	6	0.04	13	0.09	36	0.27	34	0.23	37	0.25	22.69441	0.381691	3.21798	1.22827	Ups	0.00129	0.00777	_	_
ENSG000	C10orf11	958928	1	0	0	0	0	0	14	0.04	11	0.03	30	0.08	8.62092	0.560535	5.033218	2.82129	Ups	4.8E-07	7.9E-06	biological_	_
ENSG000	C10orf118	53359	263	0.64	380	0.96	223	0.54	378	0.95	1519	3.54	614	1.4	522.1468	0.365205	3.056008	1.11607	Ups	0.00224	0.01228	_	_
ENSG000	C10orf90	245514	52	0.16	41	0.13	47	0.14	2	0.01	11	0.03	17	0.05	29.33558	0.410332	-5.1295	-2.1048	Down	2.9E-07	5E-06	cellular_cc	K16773
ENSG000	C11orf53	30420	30	0.64	41	0.91	35	0.75	261	5.79	42	0.86	171	3.44	95.42859	0.462173	3.502896	1.61894	Ups	0.00046	0.00329	_	_
ENSG000	C11orf86	1728	6312	136.69	6140	138.68	6796	148.02	2592	58.39	660	13.79	2067	42.22	4278.216	0.379284	-4.67156	-1.7718	Down	3E-06	4.1E-05	_	_
ENSG000	C12orf23	23060	1668	3.49	2792	6.1	1354	2.85	3351	7.3	6590	13.32	4904	9.69	3263.724	0.275931	3.76484	1.03883	Ups	0.00017	0.00137	cellular_cc	_
ENSG000	C12orf39	11071	2	0.01	3	0.02	5	0.03	16	0.1	90	0.53	40	0.23	23.02562	0.501472	5.240328	2.62788	Ups	1.6E-07	2.9E-06	cellular_cc	K00693
ENSG000	C12orf5	31968	1665	24.64	1719	26.53	1535	22.85	706	10.87	1197	17.09	922	12.87	1302.008	0.165067	-6.16407	-1.0175	Down	7.1E-10	2.1E-08	_	K14634
ENSG000	C12orf75	160808	1774	9.34	1519	8.35	1536	8.14	580	3.18	634	3.22	651	3.23	1144.962	0.141102	-11.0977	-1.5659	Down	1.3E-28	2.2E-26	_	_
ENSG000	C15orf37	2082	1	0.01	4	0.05	1	0.01	8	0.1	23	0.27	7	0.08	6.689431	0.549506	2.828907	1.5545	Ups	0.00467	0.02215	_	K16580

ENSG000	C15orf48	18233	3	0.03	0	0	0	0	10	0.12	3	0.03	12	0.13	4.467737	0.580304	2.533705	1.47032	Ups	0.01129	0.04411	_	_
ENSG000	C15orf56	2306	9	0.07	10	0.08	14	0.11	4	0.03	0	0	5	0.04	7.331071	0.530661	-2.71311	-1.4397	Down	0.00667	0.02937	_	_
ENSG000	C16orf86	1943	5	0.03	10	0.05	13	0.07	55	0.29	8	0.04	48	0.23	22.81666	0.515415	2.583113	1.33137	Ups	0.00979	0.03963	_	_
ENSG000	C16orf89	21989	1	0.01	0	0	2	0.01	12	0.07	3	0.02	14	0.07	5.124283	0.574479	2.910202	1.67185	Ups	0.00361	0.01807	cellular_cc	_
ENSG000	C17orf10	14540	93	0.25	69	0.19	87	0.23	251	0.7	182	0.47	205	0.52	143.613	0.263148	4.175469	1.09877	Ups	3E-05	0.00031	_	K06975
ENSG000	C17orf72	5954	6	0.01	5	0.01	5	0.01	0	0	1	0	0	0	2.98498	0.582547	-3.14483	-1.832	Down	0.00166	0.00956	_	_
ENSG000	C17orf89	2043	7855	69.99	6216	57.76	7154	64.11	2685	24.88	1520	13.07	2344	19.7	4802.893	0.260333	-6.84483	-1.7819	Down	7.7E-12	3E-10	cellular_cc	_
ENSG000	C19orf25	18414	455	0.78	325	0.58	395	0.68	225	0.4	151	0.25	227	0.37	302.8358	0.257377	-4.26865	-1.0986	Down	2E-05	0.00021	molecular	K08671
ENSG000	C19orf38	33216	0	0	0	0	2	0.03	20	0.27	12	0.15	30	0.36	9.980098	0.547787	5.072162	2.77846	Ups	3.9E-07	6.5E-06	_	_
ENSG000	C19orf57	24105	43	0.17	37	0.15	40	0.16	156	0.63	213	0.8	192	0.7	106.6197	0.224609	8.548641	1.9201	Ups	1.3E-17	9.6E-16	molecular	_
ENSG000	C19orf66	7131	110	0.25	76	0.18	99	0.23	269	0.64	402	0.89	369	0.8	208.225	0.211113	7.486836	1.58057	Ups	7.1E-14	3.7E-12	_	_
ENSG000	C1orf100	37029	0	0	2	0.03	4	0.07	15	0.25	5	0.08	15	0.23	6.613259	0.557573	2.642149	1.47319	Ups	0.00824	0.03463	_	_
ENSG000	C1orf116	14236	21	0.06	27	0.09	31	0.09	5	0.02	12	0.04	8	0.02	17.82626	0.40513	-4.01984	-1.6286	Down	5.8E-05	0.00055	_	_
ENSG000	C1orf132	67633	63	0.09	63	0.09	61	0.09	20	0.03	38	0.05	28	0.04	46.22272	0.284555	-4.46181	-1.2696	Down	8.1E-06	9.8E-05	_	_
ENSG000	C1orf198	32471	1318	3.24	1260	3.23	1282	3.17	2571	6.58	4136	9.82	2831	6.57	2129.082	0.162597	6.443334	1.04767	Ups	1.2E-10	3.8E-09	cellular_cc	_
ENSG000	C1orf21	241963	520	1.17	482	1.13	501	1.13	140	0.33	173	0.37	175	0.37	342.0222	0.159027	-11.3916	-1.8116	Down	4.6E-30	8.6E-28	_	_
ENSG000	C1orf222	81881	8	0.02	6	0.01	1	0	0	0	1	0	0	0	2.792407	0.582309	-2.71925	-1.5834	Down	0.00654	0.02893	_	_
ENSG000	C1orf228	50900	11	0.04	4	0.01	8	0.03	35	0.12	31	0.1	51	0.16	22.07603	0.411874	4.406842	1.81506	Ups	1.1E-05	0.00012	_	K14572
ENSG000	C1QBP	16054	28319	200.71	25256	186.69	28369	202.22	12733	93.86	10082	68.94	13398	89.56	20197.67	0.195535	-6.8547	-1.3403	Down	7.2E-12	2.8E-10	biological	K15414
ENSG000	C1QL4	4772	24	0.3	8	0.1	12	0.15	154	1.99	28	0.34	117	1.37	55.81141	0.48795	4.1268	2.01367	Ups	3.7E-05	0.00037	cellular_cc	_
ENSG000	C1QTNF1	26975	232	0.46	226	0.47	229	0.46	112	0.23	87	0.17	109	0.2	170.0949	0.222868	-5.88366	-1.3113	Down	4E-09	1E-07	cellular_cc	K19470
ENSG000	C1QTNF1	8447	221	3.92	224	4.15	236	4.21	64	1.18	54	0.92	77	1.29	151.4184	0.228489	-8.47346	-1.9361	Down	2.4E-17	1.8E-15	_	_
ENSG000	C1QTNF2	22891	26	0.28	23	0.26	31	0.33	83	0.92	102	1.05	133	1.34	62.68532	0.287761	5.732296	1.64953	Ups	9.9E-09	2.3E-07	biological	K09228
ENSG000	C1QTNF3	24385	27	0.09	27	0.09	35	0.11	98	0.33	106	0.33	98	0.3	62.18002	0.264996	5.507225	1.45939	Ups	3.7E-08	7.6E-07	molecular	_
ENSG000	C1QTNF9	8375	18	0.24	16	0.22	23	0.31	1	0.01	3	0.04	1	0.01	10.89959	0.503487	-5.33356	-2.6854	Down	9.6E-08	1.9E-06	_	_
ENSG000	C1R	57691	643	1.73	688	1.93	821	2.22	275	0.77	276	0.71	332	0.84	519.2872	0.19155	-7.62541	-1.4606	Down	2.4E-14	1.3E-12	molecular	K01330
ENSG000	C1S	81986	778	1.43	831	1.59	838	1.55	96	0.18	273	0.48	189	0.33	518.8015	0.268975	-8.3968	-2.2585	Down	4.6E-17	3.3E-15	molecular	K01331
ENSG000	C20orf14	847	5	0.1	2	0.04	7	0.14	0	0	0	0	1	0.02	2.645012	0.583182	-2.74783	-1.6025	Down	0.006	0.02704	cellular_cc	_
ENSG000	C20orf24	6824	6088	46.52	5779	46.06	5531	42.51	2672	21.24	2514	18.53	3356	24.19	4410.544	0.171963	-7.03873	-1.2104	Down	1.9E-12	8.3E-11	molecular	_
ENSG000	C21orf59	20788	2287	6.36	2043	5.92	2283	6.38	993	2.87	988	2.65	1184	3.1	1663.6	0.157995	-7.93273	-1.2533	Down	2.1E-15	1.3E-13	cellular_cc	_
ENSG000	C22orf34	243015	32	0.08	31	0.08	25	0.06	97	0.25	139	0.34	116	0.28	69.07083	0.256678	6.546549	1.68036	Ups	5.9E-11	2E-09	_	_
ENSG000	C2orf48	70343	24	0.33	18	0.25	23	0.31	61	0.86	84	1.1	69	0.88	44.09255	0.289243	4.817114	1.39332	Ups	1.5E-06	2.1E-05	_	K11447
ENSG000	C2orf54	10842	18	0.09	6	0.03	7	0.03	0	0	0	0	1	0	5.634129	0.573539	-4.25482	-2.4403	Down	2.1E-05	0.00023	_	K00757
ENSG000	C2orf62	11244	9	0.04	7	0.03	6	0.03	24	0.11	19	0.08	25	0.11	14.43716	0.419907	2.856783	1.19958	Ups	0.00428	0.02072	_	K14693
ENSG000	C3	52859	425	0.91	411	0.92	378	0.81	14	0.03	46	0.1	26	0.05	228.8001	0.278148	-13.7286	-3.8186	Down	6.9E-43	2.5E-40	biological	K03990
ENSG000	C3orf14	17241	205	0.91	288	1.33	214	0.96	61	0.28	107	0.46	97	0.41	165.9524	0.242231	-6.5211	-1.5796	Down	7E-11	2.4E-09	_	_
ENSG000	C3orf58	76922	342	0.8	341	0.83	231	0.54	679	1.65	1014	2.29	1002	2.21	570.6709	0.207661	6.187316	1.28486	Ups	6.1E-10	1.8E-08	cellular_cc	_
ENSG000	C4B	20657	24	0.05	16	0.04	41	0.09	3	0.01	8	0.02	5	0.01	16.83305	0.460526	-4.4825	-2.0643	Down	7.4E-06	9E-05	molecular	K03989
ENSG000	C4BPA	40711	45	0.4	35	0.33	35	0.32	12	0.11	24	0.21	13	0.11	27.79706	0.347167	-3.80486	-1.3209	Down	0.00014	0.00119	cellular_cc	K04002
ENSG000	C4orf19	169555	0	0	4	0.04	6	0.05	11	0.1	63	0.55	38	0.33	18.13271	0.526546	4.216883	2.22038	Ups	2.5E-05	0.00026	_	_
ENSG000	C4orf21	97660	213	0.47	278	0.63	178	0.39	430	0.98	1094	2.31	446	0.92	411.8662	0.315598	3.784183	1.19428	Ups	0.00015	0.00128	molecular	_
ENSG000	C5	97939	19	0.07	15	0.06	16	0.06	114	0.45	197	0.72	121	0.43	74.02557	0.304085	8.742319	2.65841	Ups	2.3E-18	1.8E-16	biological	K03994
ENSG000	C5AR2	11203	101	0.49	100	0.51	85	0.42	3	0.02	8	0.04	4	0.02	53.12963	0.368231	-10.6723	-3.9299	Down	1.4E-26	2.1E-24	molecular	K04171
ENSG000	C5orf46	25813	144	2.46	103	1.83	138	2.37	10	0.18	23	0.38	16	0.26	75.82957	0.305052	-9.72859	-2.9677	Down	2.3E-22	2.5E-20	cellular_cc	_
ENSG000	C5orf56	65409	14	0.07	18	0.09	18	0.09	30	0.15	53	0.25	47	0.21	28.49666	0.336443	3.079155	1.03596	Ups	0.00208	0.01152	_	_
ENSG000	C6orf100	661	8	0.44	8	0.46	6	0.33	42	2.41	20	1.06	39	2.03	19.78811	0.4347	3.835086	1.66711	Ups	0.00013	0.00107	_	_
ENSG000	C6orf132	41502	51	0.19	60	0.23	72	0.27	7	0.03	18	0.06	15	0.05	38.60549	0.343965	-6.38658	-2.1968	Down	1.7E-10	5.4E-09	_	K17985
ENSG000	C6orf89	57095	3045	8.49	3145	9.14	3336	9.35	7383	21.4	8484	22.81	6767	17.79	5162.772	0.1434	7.069733	1.0138	Ups	1.6E-12	6.7E-11	cellular_cc	K12733
ENSG000	C7orf50	141275	6815	20.88	5423	17.32	6125	18.87	3222	10.27	2443	7.22	4475	12.93	4834.112	0.242966	-4.16769	-1.0126	Down	3.1E-05	0.00032	_	_
ENSG000	C8orf22	21780	22	0.16	19	0.15	34	0.25	1090	8.41	530	3.79	1001	7	425.1958	0.346943	12.80893	4.44397	Ups	1.5E-37	3.9E-35	biological	_
ENSG000	C8orf46	58522	13	0.03	11	0.03	12	0.03	32	0.07	43	0.09	39	0.08	23.74304	0.348368	3.711952	1.29313	Ups	0.00021	0.00164	_	K06519
ENSG000	C8orf59	6340	2581	10.7	2258	9.77	1928	8.04	1176	5.07	1162	4.65	1414	5.53	1779.72	0.177049	-5.86132	-1.0377	Down	4.6E-09	1.1E-07	_	_
ENSG000	C9orf116	6554	223	1.91	217	1.94	231	1.99	82	0.73	57	0.47	79	0.64	153.4323	0.243659	-7.14641	-1.7413	Down	8.9E-13	4E-11	_	_
ENSG000	C9orf169	1677	235	2.97	186	2.45	208	2.64	116	1.52	44	0.54	83	0.99	150.4908	0.34388	-4.05496	-1.3944	Down	5E-05	0.00048	_	_
ENSG000	C9orf170	10913	12	0.1	17	0.14	12	0.1	82	0.68	60	0.46	54	0.4	37.87536	0.358407	5.129306	1.83838	Ups	2.9E-07	5E-06	_	_
ENSG000	C9orf173	2222	4	0.06	3	0.05	7	0.11	27	0.43	25	0.37	19	0.27	13.47365	0.451828	3.815866	1.72411	Ups	0.00014	0.00114	_	_
ENSG000	C9orf47	5278	6	0.03	11	0.05	9	0.04	0	0	3	0.01	1	0	5.208842	0.557467	-3.25904	-1.8168	Down	0.00112	0.0069	cellular_cc	_
ENSG000	C9orf72	27321	254	0.77	355	1.12	195	0.59	384	1.21	1238	3.62	509	1.46	457.3771	0.346118	2.958157	1.02387	Ups	0.00309	0.01599	cellular_cc	_
ENSG000	CA11	8371	87	0.84	82	0.82	94	0.91	1207	12.06	358	3.32	937	8.49	445.4032	0.378365	7.178628	2.71614	Ups	7E-13	3.2E-11	molecular	_
ENSG000	CA12	60784	76	0.17	43	0.1	55	0.12	178	0.41	168	0.36	147	0.31	106.967	0.269654	4.533934	1.22259	Ups	5.8E-06	7.3E-05	molecular	K01672
ENSG000	CABYR	22626	13	0.05	20	0.08	13	0.05	36	0.14	52	0.18	35	0.12	26.87165	0.347389	3.085349	1.07182	Ups	0.00203	0.01133	biological	_
ENSG000	CACNA1H	68531	19	0.03	21	0.04	16	0.03	3	0.01	7	0.01	5	0.01	12.20806	0.446547	-3.90089	-1.7419	Down	9.6E-05	0.00085	molecular	K04855
ENSG000	CACNA2D	497355	172	0.4	278	0.67	148	0.34	401	0.96	1452	3.23	637	1.38	472.5786	0.359792	4.421624	1.59086	Ups	9.8E-06	0.00012	molecular	K04858
ENSG000	CACNA2D	952011	218	0.79	206	0.78	187	0.68	29	0.11	93	0.32	63	0.21	136.0941	0.299353	-6.15082	-1.8413	Down	7.7E-10	2.2E-08	molecular	K04860
ENSG000	CACNB2	401193	73	0.16	93	0.21	73	0.16	428	0.98	1744	3.71	834	1.73	483.3029	0.352932	8.572272	3.02543	Ups	1E-17	7.9E-16	molecular	K04863
ENSG000	CACNG4	68489	47	0.36	45	0.36	41	0.31	329	2.6	315	2.31	316	2.27	171.5306	0.22419	11.32422	2.53878	Ups	1E-29	1.8E-27	molecular	K04869
ENSG000	CADM2	1115448	0	0	1	0	0	0	24	0.06	26	0.06	25	0.05	11.67642	0.534206	6.308356	3.36996	Ups	2.8E-10	8.7E-09	cellular_cc	_
ENSG000	CALB1	36868	10	0.02	20	0.05	8	0.02	2	0	1	0	3	0.01	7.708889	0.531864	-3.6849	-1.9599	Down	0.00023	0.0018	molecular	K14757
ENSG000	CALD1	226477	7204	9.64	10231	14.28	7126	9.59	14799	20.61	40194	51.91	21061	26.59	15662.35	0.283979	4.506488	1.27975	Ups	6.6E-06	8.2E-05	molecular	K12327
ENSG000	CALHM3	6437	103	1.6	88	1.43	91	1.42	20	0.32	3	0.05	15	0.22	56.30303	0.406898	-6.4302	-2.6164	Down	1.3E-10	4.1E-09	biological	K19740
ENSG000	CALM1P2	448	11	0.63	15	0.9	12	0.69	0	0	0	0	0	0	6.774806	0.566211	-5.43376	-3.0767	Down	5.5E-08	1.1E-06	_	K02183
ENSG000	CAMK1D	486065	711	1.95	585	1.67	702	1.94	1876	5.35	1846	4.88	1648	4.26	1182.591	0.17409	6.829101	1.18888	Ups	8.5E-12	3.3E-10	molecular	K08794
ENSG000	CAMK1G	30222	3	0.03	13	0.12	2	0.02	56	0.5	9	0.07	46	0.37	21.03139	0.542163	3.06376	1.66106	Ups	0.00219	0.01202	cellular_cc	K08794
ENSG000	CAMK2D	310896	80	0.15	107	0.2	83	0.15	349	0.66	1149	2.03	529	0.91	346.9643	0.331814	7.234246	2.40042	Ups	4.7E-13	2.2E-11	biological	K04515
ENSG000	CAMK2N1	3830	153	1.25	135	1.15	137	1.13	512	4.35	438	3.45	569	4.38	310.1147	0.211123	7.462936	1.5756	Ups	8.5E-14	4.3E-12	cellular_cc	_
ENSG000	CAMK4	271234	54	0.07	70	0.1	55	0.07	314	0.43	409	0.52	301	0.37	187.9388	0.218966	10.07695	2.20651	Ups	7E-24	8.9E-22	biological	K05869
ENSG000	CAPG	23685	6665	32.37	6122	31.01	6530	31.9	4107	20.75	1981	9.28	4064	18.62	5033.065	0.290303	-3.5712	-1.0367	Down	0.00036	0.00263	molecular	K10368
ENSG000	CAPN12	39718	3	0.01	3	0.01	3	0.01	10	0.02	14	0.03	11	0.02	6.919806	0.503988	2.552757	1.28656	Ups	0.01069	0.0423	molecular	K04740
ENSG000	CAPN3	64216	2	0	1	0	5	0.01	21	0.05	25	0.06	15	0.03	10.76915	0.494464	4.095724	2.02519	Ups	4.2E-05	0.00041	biological	K08573
ENSG000	CAPN6	25421	0	0	2	0.01	1	0.01	33	0.22	6	0.04	13	0.08	9.014547	0.567634	3.880628	2.20278	Ups	0.0001	0.00091	biological	K08575
ENSG000	CAPS	4171	47	0.18	29	0.12	60	0.24	126	0.51	87	0.33	129	0.47	77.39806	0.32675	3.163415	1.03365	Ups	0.00156	0.00905	molecular	K17985
ENSG000	CAPSL	34485	7	0.1	10	0.15	11	0.16	1	0.02	5	0.07	2	0.03	6.164881	0.529495	-2.71448	-1.4373	Down	0.00664	0.02927	molecular	_
ENSG000	CARD10	29150	3184	9	2360	6.96	2775	7.89	773	2.27	511	1.39	715	1.91	1791.434	0.238548	-9.01569	-2.1507	Down	2E-19	1.7E-17	molecular	_
ENSG000	CARD6	18990	1526	8.25	1717	9.68	1543	8.39	566	3.18	453	2.36	447	2.28	1080.217	0.203939	-9.05827	-1.8473	Down	1.3E-19	1.2E-17	cellular_cc	K12797
ENSG000	CARF	74850	37	0.05	59	0.09	34	0.05	128	0.19	275	0.38	163	0.22	107.8329	0.308669	5.602766	1.7294	Ups	2.1E-08	4.6E-07	molecular	_
ENSG000	CASC10	4605	771	5.21	759	5.35	793	5.39	317	2.23	238	1.55	308	1.96	547.7077	0.210551	-7.48027	-1.575	Down	7.4E-14	3.8E-12	_	K09228
ENSG000	CASC4	127030	819	1.71	1508	3.29	663	1.39	1826	3.97	5412	10.91	3232	6.37	2083.194	0.336558	4.157873	1.39937	Ups	3.2E-05	0.00033	cellular_cc	K16467
ENSG000	CASC8	192323	67	0.63	54	0.53	40	0.38	30	0.29	26	0.24	26	0.23	41.28381	0.314684	-3.45888	-1.0885	Down	0.00054	0.00378	_	_
ENSG000	CASKIN1	19343	18	0.07	8	0.03	13	0.05	89	0.38	71	0.28	72	0.28	42.92738	0.350829	6.067048	2.1285	Ups	1.3E-09	3.6E-08	cellular_cc	_
ENSG000	CASP1	75989	11	0.04	18	0.07	12	0.05	1	0	9	0.03	5	0.02	9.482866	0.495089	-2.62784	-1.301	Down	0.00859	0.03576	biological	K01370
ENSG000	CASP4	26571	1264	2.99	1259	3.1	1237	2.94	518	1.27	702	1.6	582	1.3	943.6623	0.129535	-9.8231	-1.2724	Down	9E-23	1E-20	molecular	K04394
ENSG000	CASZ1	160047	16	0.03	20	0.04	24	0.04	5	0.01	11	0.02	1	0	13.24514	0.479021	-3.35779	-1.6085	Down	0.00079	0.00515	molecular	_
ENSG000	CATSPER	43797	13	0.19	6	0.09	11	0.16	33	0.5	19	0.27	30	0.41	18.16232	0.421319	2.517945	1.06086	Ups	0.0118	0.04564	biological	K16891
ENSG000	CBR1	3226	7356	42.76	6444	39.07	7101	41.52	3862	23.35	3179	17.83	4743	26	5540.247	0.202051	-4.95271	-1.0007	Down	7.3E-07	1.1E-05	molecular	K00079
ENSG000	CBX3P2	3226	44	0.55	32	0.42	24	0.3	77	1	111	1.34	86	1.02	59.33149	0.283633	4.053526	1.14972	Ups	5.1E-05	0.00048	_	K11586
ENSG000	CBX7	32508	316	0.84	255	0.71	332	0.89	743	2.05	771	1.97	776	1.94	512.3469	0.176787	6.229604	1.10131	Ups	4.7E-10	1.4E-08	biological	K11454
ENSG000	CBY3	2392	3	0.1	3	0.1	1	0.03	13	0.44	12	0.38	15	0.46	7.400533	0.513489	3.229334	1.65823	Ups	0.00124	0.00753	_	_
ENSG000	CCDC103	6249	27	0.13	16	0.08	34	0.17	15	0.08	5	0.02	12	0.05	18.80089	0.442606	-2.70669	-1.198	Down	0.0068	0.02979	biological	_
ENSG000	CCDC114	25438	6	0.02	5	0.02	1	0	23	0.08	28	0.09	29	0.1	14.32795	0.452097	4.485201	2.02775	Ups	7.3E-06	8.9E-05	cellular_cc	K12581
ENSG000	CCDC120	16409	1293	2.09	1070	1.81	1262	2.05	418	0.7	938	1.46	614	0.94	939.6239	0.230292	-4.71486	-1.0858	Down	2.4E-06	3.3E-05	_	_
ENSG000	CCDC122	55783	18	0.09	31	0.16	14	0.07	46	0.23	66	0.31	58	0.27	37.01714	0.334904	3.262405	1.09259	Ups	0.0011	0.00685	_	_
ENSG000	CCDC126	47330	136	0.48	179	0.65	114	0.4	328	1.2	1037	3.51	695	2.3	381.0537	0.318082	5.767305	1.83447	Ups	8.1E-09	1.9E-07	cellular_cc	_
ENSG000	CCDC136	31376	14	0.03	10	0.02	12	0.03	26	0.06	54	0.12	49	0.11	25.73023	0.367813	3.856334	1.41841	Ups	0.00012	0.00099	cellular_cc	_
ENSG000	CCDC137	7541	3361	16.28	3044	15.38	3439	16.75	1759	8.86	1287	6.01	2080	9.5	2547.341	0.224354	-4.89758	-1.0988	Down	9.7E-07	1.5E-05	_	K09228
ENSG000	CCDC146	207100	2	0.01	2	0.01	0	0	17	0.06	33	0.11	16	0.05	10.61522	0.520687	4.968979	2.58728	Ups	6.7E-07	1.1E-05	_	_
ENSG000	CCDC147	101327	7	0.05	18	0.13	12	0.08	46	0.33	44	0.29	38	0.24	26.3514	0.376158	3.690657	1.38827	Ups	0.00022	0.00177	_	_
ENSG000	CCDC151	15332	32	0.2	30	0.2	29	0.19	113	0.75	57	0.35	105	0.63	59.36087	0.345528	3.67956	1.27139	Ups	0.00023	0.00183	_	K17639
ENSG000	CCDC153	7133	39	0.3	44	0.35	41	0.31	154	1.22	51	0.37	106	0.76	71.6474	0.389668	2.59699	1.01196	Ups	0.0094	0.03844	_	_
ENSG000	CCDC159	10261	67	0.2	57	0.18	97	0.3	291	0.92	125	0.37	237	0.68	142.5327	0.357596	3.466833	1.23973	Ups	0.00053	0.00369	_	K07884
ENSG000	CCDC180	140042	15	0.02	12	0.01	15	0.02	51	0.06	57	0.06	52	0.05	31.98235	0.324222	4.82479	1.5643	Ups	1.4E-06	2.1E-05	cellular_cc	_
ENSG000	CCDC183	11392	20	0.08	23	0.09	26	0.1	73	0.3	38	0.15	62	0.23	39.47619	0.360453	2.813934	1.01429	Ups	0.00489	0.02299	_	_
ENSG000	CCDC64	104626	18	0.05	29	0.08	23	0.06	10	0.03	9	0.02	6	0.01	16.36378	0.413722	-3.49584	-1.4463	Down	0.00047	0.00337	cellular_cc	K16756
ENSG000	CCDC85A	202051	25	0.16	19	0.13	13	0.08	1	0.01	5	0.03	0	0	10.98412	0.516078	-4.76412	-2.4587	Down	1.9E-06	2.7E-05	_	_
ENSG000	CCDC86	9011	6452	45.22	5698	41.65	6363	44.85	2909	21.2	2654	17.94	3065	20.26	4626.236	0.161964	-7.92827	-1.2841	Down	2.2E-15	1.3E-13	cellular_cc	K14822
ENSG000	CCER2	3179	13	0.21	12	0.2	8	0.13	41	0.67	27	0.41	36	0.54	22.08594	0.389264	3.26151	1.26959	Ups	0.00111	0.00686	_	_
ENSG000	CCL26	20364	25	0.75	23	0.72	47	1.42	9	0.28	13	0.38	13	0.37	22.27377	0.399997	-3.60477	-1.4419	Down	0.00031	0.00235	biological	K05514
ENSG000	CCL28	35747	5	0.02	4	0.02	1	0	46	0.19	60	0.23	64	0.24	27.63248	0.42126	7.595154	3.19953	Ups	3.1E-14	1.6E-12	cellular_cc	K05513
ENSG000	CCNE1	12411	562	2.55	509	2.41	598	2.73	229	1.08	166	0.73	218	0.93	392.5358	0.225579	-7.00957	-1.5812	Down	2.4E-12	1E-10	biological	K06626
ENSG000	CCNG2	276239	281	0.73	454	1.23	226	0.59	746	2.01	1367	3.42	1034	2.53	644.6172	0.272412	5.075086	1.38251	Ups	3.9E-07	6.4E-06	biological	K10146
ENSG000	CCNL1	14253	1677	1.69	2030	2.13	1695	1.72	4082	4.28	5858	5.7	4423	4.21	3142.639	0.155955	7.425416	1.15803	Ups	1.1E-13	5.6E-12	biological	_
ENSG000	CCR1	6688	0	0	0	0	0	0	28	0.27	3	0.03	17	0.15	7.798998	0.581373	4.44169	2.58228	Ups	8.9E-06	0.00011	biological	K04176
ENSG000	CCR3	103102	3	0.01	6	0.02	1	0	18	0.05	11	0.03	15	0.04	8.661923	0.509669	2.791146	1.42256	Ups	0.00525	0.02441	biological	K04178
ENSG000	CCRL2	5835	402	1.54	399	1.6	454	1.75	2	0.01	2	0.01	4	0.01	224.8651	0.36755	-17.5241	-6.441	Down	9.4E-69	9.1E-66	molecular	K08373
ENSG000	CCT5	16492	72583	184.6	74740	198.26	80335	205.5	38828	102.72	44170	108.39	47820	114.71	60568.47	0.135403	-7.43254	-1.0064	Down	1.1E-13	5.3E-12	cellular_cc	K09497
ENSG000	CD160	19817	0	0	3	0.01	2	0.01	9	0.04	21	0.09	6	0.03	6.260936	0.556006	2.913592	1.61998	Ups	0.00357	0.01792	molecular	K06542
ENSG000	CD163L1	133213	80	0.27	63	0.22	78	0.26	28	0.1	25	0.08	43	0.14	54.0929	0.298501	-4.41716	-1.3185	Down	0.00001	0.00012	molecular	K06545
ENSG000	CD180	14525	16	0.13	13	0.11	10	0.08	0	0	0	0	0	0	6.929695	0.565899	-5.46839	-3.0946	Down	4.5E-08	9.3E-07	biological	K06555
ENSG000	CD1D	4950	2	0.02	2	0.02	0	0	47	0.56	53	0.58	61	0.66	25.32139	0.468553	8.117433	3.80344	Ups	4.8E-16	3.1E-14	molecular	K06448
ENSG000	CD24P4	243	2693	284.88	2852	314.66	2697	286.94	768	84.5	1975	201.57	1071	106.85	2035.134	0.25449	-5.12166	-1.3034	Down	3E-07	5.2E-06	_	K06469
ENSG000	CD27-AS	12567	55	0.17	60	0.19	81	0.25	187	0.6	128	0.38	196	0.57	114.406	0.291967	3.774925	1.10215	Ups	0.00016	0.00133	_	_
ENSG000	CD2AP	149475	566	1.93	1289	4.58	404	1.38	1435	5.08	3865	12.7	2214	7.11	1518.326	0.372027	3.496702	1.30087	Ups	0.00047	0.00336	cellular_cc	K13738
ENSG000	CD33	18796	57	0.16	44	0.13	57	0.16	0	0	0	0	6	0.02	29.02899	0.462539	-8.19861	-3.7922	Down	2.4E-16	1.6E-14	molecular	K06473
ENSG000	CD37	8165	31	0.08	34	0.09	22	0.06	48	0.13	146	0.36	54	0.13	52.0952	0.383618	2.847524	1.09236	Ups	0.00441	0.02118	cellular_cc	K06475
ENSG000	CD38	74956	0	0	1	0	1	0	4	0.01	28	0.08	11	0.03	6.580846	0.571768	3.78367	2.16338	Ups	0.00015	0.00129	biological	K01242
ENSG000	CD47	47728	2167	4.42	2076	4.42	1944	3.99	3325	7.05	7568	14.89	5338	10.27	3525.243	0.220392	4.972901	1.09599	Ups	6.6E-07	1.1E-05	molecular	K09228
ENSG000	CD70	20921	8885	99.39	8323	97.1	9212	103.64	2785	32.4	839	9.05	2213	23.35	5634.873	0.357196	-5.86602	-2.0953	Down	4.5E-09	1.1E-07	molecular	K05470
ENSG000	CD72	37278	7	0.02	2	0.01	4	0.01	18	0.07	13	0.04	18	0.06	9.913151	0.481817	2.755667	1.32773	Ups	0.00586	0.02655	molecular	K06504
ENSG000	CD83	19278	573	5.96	519	5.63	534	5.59	2874	31.1	2822	28.32	3184	31.24	1654.464	0.153242	14.34249	2.19788	Ups	1.2E-46	5.2E-44	cellular_cc	K06510
ENSG000	CDC20P1	1500	29	0.26	29	0.27	29	0.26	165	1.54	90	0.78	167	1.41	81.51288	0.333583	5.692033	1.89876	Ups	1.3E-08	2.9E-07	_	K03363
ENSG000	CDCP1	64145	571	2.1	455	1.75	504	1.87	175	0.67	85	0.3	102	0.35	329.5164	0.308259	-6.75756	-2.0831	Down	1.4E-11	5.4E-10	cellular_cc	K06732
ENSG000	CDH12	1102950	10	0.03	17	0.04	17	0.04	336	0.88	789	1.91	412	0.97	237.0083	0.333656	13.21978	4.41086	Ups	6.8E-40	2.1E-37	molecular	K06804
ENSG000	CDH5	38154	133	0.4	182	0.57	153	0.46	49	0.15	80	0.23	59	0.17	111.8517	0.242279	-6.11065	-1.4805	Down	9.9E-10	2.8E-08	biological	K06533
ENSG000	CDHR3	159636	30	0.05	26	0.04	17	0.03	62	0.1	57	0.08	65	0.09	41.32425	0.310866	3.335128	1.03678	Ups	0.00085	0.00551	molecular	K16503
ENSG000	CDK18	28199	492	0.61	411	0.53	480	0.6	1772	2.3	1482	1.78	1605	1.89	998.2764	0.194455	8.044931	1.56438	Ups	8.6E-16	5.5E-14	molecular	K15596
ENSG000	CDK5R2	2500	41	0.42	16	0.17	29	0.3	12	0.13	8	0.08	3	0.03	18.89209	0.461954	-3.63105	-1.6774	Down	0.00028	0.00215	biological	K11716

ENSG000	CDKN1C	2669	1175	4.14	953	3.5	1183	4.19	217	0.8	272	0.92	374	1.24	720.8026	0.213018	-9.81761	-2.0913	Down	9.5E-23	1E-20	biological_	K09993
ENSG000	CDNF	19326	9	0.07	23	0.18	16	0.12	66	0.52	76	0.55	66	0.47	40.40763	0.337283	5.08875	1.71635	Ups	3.6E-07	6E-06	cellular_cc	_
ENSG000	CDR2L	18169	3490	25.37	3103	23.53	3215	23.51	2229	16.85	1228	8.61	1773	12.16	2566.819	0.26177	-3.93911	-1.0311	Down	8.2E-05	0.00074	_	_
ENSG000	CEACAM1	22087	24	0.07	20	0.06	24	0.07	168	0.48	182	0.49	176	0.46	92.73798	0.255657	10.14453	2.59352	Ups	3.5E-24	4.6E-22	cellular_cc	K16825
ENSG000	CEBPA	2631	95	0.93	90	0.92	82	0.81	9	0.09	30	0.28	21	0.19	56.38455	0.318543	-6.91926	-2.2041	Down	4.5E-12	1.9E-10	biological_	K09055
ENSG000	CELSR1	176337	1276	1.88	1156	1.77	1103	1.63	421	0.64	612	0.87	402	0.56	846.8935	0.175399	-8.52045	-1.4945	Down	1.6E-17	1.2E-15	biological_	K04600
ENSG000	CENPV	11123	6865	43.69	5566	36.95	6426	41.13	3540	23.43	2654	16.3	3570	21.43	4870.84	0.208316	-5.32991	-1.1103	Down	9.8E-08	1.9E-06	cellular_cc	_
ENSG000	CEP152	98219	728	1.74	875	2.18	757	1.82	1196	2.97	3304	7.61	1507	3.4	1310.949	0.287466	3.520977	1.01216	Ups	0.00043	0.0031	biological_	K16728
ENSG000	CEP170B	31491	1763	3.04	1495	2.69	1543	2.68	616	1.11	1278	2.13	874	1.42	1268.925	0.209676	-4.80474	-1.0074	Down	1.6E-06	2.3E-05	cellular_cc	K16463
ENSG000	CEP97	46638	376	0.52	463	0.66	347	0.48	496	0.71	1940	2.57	819	1.06	688.6057	0.344542	3.084927	1.06289	Ups	0.00204	0.01134	molecular_	K16717
ENSG000	CERKL	143990	2	0.01	3	0.01	1	0	19	0.07	22	0.08	16	0.05	9.795971	0.500635	4.421897	2.21376	Ups	9.8E-06	0.00012	molecular_	K19602
ENSG000	CES3	13912	20	0.06	27	0.08	26	0.07	646	1.89	1169	3.17	815	2.16	408.8287	0.257397	18.13201	4.66713	Ups	1.8E-73	1.9E-70	;cellular_c	K15743
ENSG000	CFB	24387	87	0.2	109	0.27	100	0.24	47	0.11	59	0.13	42	0.09	75.3633	0.250198	-4.65707	-1.1652	Down	3.2E-06	4.3E-05	molecular_	K01335
ENSG000	CGA	9609	10	0.36	6	0.23	11	0.4	17530	660.15	74005	2585.05	54727	1868.76	21502.74	0.380505	27.27616	10.3787	Ups	8E-164	3E-160	molecular_	K08522
ENSG000	CGNL1	174761	4	0.01	8	0.02	7	0.02	19	0.06	54	0.15	23	0.06	17.60807	0.452413	3.719993	1.68297	Ups	0.0002	0.0016	molecular_	_
ENSG000	CHAC1	3551	176	1.17	131	0.91	129	0.86	1760	12.18	214	1.37	1159	7.27	584.836	0.554589	3.161861	1.75353	Ups	0.00157	0.0091	molecular_	K07232
ENSG000	CHAF1B	33638	1738	8.46	1679	8.52	1923	9.41	950	4.81	1024	4.81	1034	4.75	1412.518	0.141141	-7.29861	-1.0301	Down	2.9E-13	1.4E-11	molecular_	K10751
ENSG000	CHCHD2F	759	0	0	0	0	0	0	8	0.49	15	0.86	11	0.61	5.110401	0.57573	4.578436	2.63594	Ups	4.7E-06	6E-05	_	K00901
ENSG000	CHCHD2F	462	1	0.03	6	0.18	4	0.11	23	0.68	11	0.3	16	0.43	9.86593	0.509509	2.871993	1.46331	Ups	0.00408	0.01992	_	K00901
ENSG000	CHMP1B	3054	2388	17.59	2373	18.23	2569	19.04	6171	47.29	5636	40.06	6643	46.16	4149.11	0.162744	6.759247	1.10003	Ups	1.4E-11	5.3E-10	biological_	K12197
ENSG000	CHN2	392055	65	0.08	58	0.08	47	0.06	136	0.18	174	0.21	140	0.17	98.82545	0.228313	4.967524	1.13415	Ups	6.8E-07	1.1E-05	molecular_	_
ENSG000	CHRDL2	34957	13	0.07	15	0.09	10	0.06	6	0.03	2	0.01	2	0.01	8.376054	0.504679	-3.10772	-1.5684	Down	0.00189	0.01064	biological_	K17280
ENSG000	CHRM1	13129	2	0.01	0	0	3	0.01	9	0.04	14	0.06	8	0.03	5.584157	0.54739	2.846405	1.55809	Ups	0.00442	0.02122	molecular_	K04129
ENSG000	CHRM3	528886	33	0.07	40	0.08	31	0.06	216	0.45	300	0.57	190	0.36	125.8847	0.257675	9.328371	2.40368	Ups	1.1E-20	1.1E-18	biological_	K04131
ENSG000	CHRNA9	19889	4	0.03	8	0.06	6	0.05	0	0	0	0	0	0	3.214812	0.583397	-3.72493	-2.1731	Down	0.0002	0.00157	molecular_	K04810
ENSG000	CHRNB4	103636	3	0.01	3	0.01	1	0	15	0.06	10	0.04	12	0.04	7.017624	0.52229	2.979185	1.556	Ups	0.00289	0.01509	biological_	_
ENSG000	CHST11	306720	678	1.97	665	2.01	725	2.12	136	0.41	243	0.68	149	0.41	447.9289	0.204954	-10.4353	-2.1388	Down	1.7E-25	2.4E-23	cellular_cc	_
ENSG000	CHST2	3628	133	0.97	99	0.75	124	0.91	34	0.26	36	0.25	51	0.35	81.8644	0.265118	-6.333	-1.679	Down	2.4E-10	7.5E-09	cellular_cc	K04745
ENSG000	CICP16	2882	0	0	1	0.01	1	0.01	6	0.05	38	0.29	2	0.02	6.958583	0.582567	3.261867	1.90026	Ups	0.00111	0.00685	_	_
ENSG000	CITED2	2365	1830	12.44	1699	12.05	1779	12.17	6737	47.65	4521	29.66	5807	37.25	3605.869	0.223156	6.43613	1.43626	Ups	1.2E-10	4E-09	biological_	_
ENSG000	CLCF1	10010	452	3.39	393	3.08	496	3.74	244	1.9	112	0.81	170	1.2	322.0208	0.311756	-4.51566	-1.4078	Down	6.3E-06	7.8E-05	biological_	K05421
ENSG000	CLCNKA	15176	13	0.07	19	0.11	14	0.08	52	0.3	41	0.22	51	0.27	30.5127	0.345445	3.757199	1.29791	Ups	0.00017	0.00141	molecular_	_
ENSG000	CLCNKB	13532	91	0.72	61	0.5	78	0.62	256	2.1	118	0.9	201	1.49	131.6027	0.337188	3.08547	1.04038	Ups	0.00203	0.01133	molecular_	K05018
ENSG000	CLDN4	33143	150	0.34	130	0.31	121	0.28	59	0.14	49	0.11	72	0.15	99.11793	0.251199	-5.17155	-1.2991	Down	2.3E-07	4.1E-06	molecular_	K06087
ENSG000	CLEC2L	21129	0	0	0	0	0	0	2	0.02	3	0.03	8	0.07	1.962601	0.572364	2.641395	1.51184	Ups	0.00826	0.03468	cellular_cc	_
ENSG000	CLGN	39514	339	1.9	437	2.55	292	1.64	566	3.29	1152	6.21	914	4.82	584.7546	0.239947	4.194783	1.00652	Ups	2.7E-05	0.00028	molecular_	K09551
ENSG000	CLIC6	48838	0	0	0	0	1	0.01	9	0.06	7	0.04	3	0.02	3.152929	0.583262	2.890853	1.68613	Ups	0.00384	0.019	molecular_	K05026
ENSG000	CLIP4	91939	59	0.14	81	0.21	59	0.15	118	0.3	474	1.12	234	0.54	156.6388	0.364738	4.329393	1.57909	Ups	1.5E-05	0.00017	_	K10423
ENSG000	CLPB	142224	2177	5.78	1986	5.5	2223	5.94	1051	2.9	956	2.45	1114	2.79	1618.389	0.166487	-7.30482	-1.2162	Down	2.8E-13	1.3E-11	molecular_	K03695
ENSG000	CLSTN3	29248	648	1.47	593	1.4	637	1.45	422	1	312	0.68	310	0.66	496.9874	0.226956	-4.41295	-1.0015	Down	1E-05	0.00012	cellular_cc	_
ENSG000	CLUH	23278	8621	20.55	6723	16.72	7912	18.97	4071	10.1	3576	8.23	4035	9.07	5942.263	0.179309	-6.52117	-1.1693	Down	7E-11	2.4E-09	cellular_cc	K03255
ENSG000	CMC4	9741	22	0.3	13	0.18	11	0.15	7	0.1	4	0.05	2	0.03	10.22064	0.490905	-3.13561	-1.5393	Down	0.00171	0.00982	cellular_cc	_
ENSG000	CMTM8	131647	11	0.18	11	0.18	13	0.21	4	0.07	4	0.06	6	0.09	8.383861	0.477162	-2.53443	-1.2093	Down	0.01126	0.04405	molecular_	_
ENSG000	CNGA1	80696	1	0	1	0	6	0.02	10	0.03	28	0.09	14	0.04	9.142906	0.527427	3.223858	1.70035	Ups	0.00126	0.00764	molecular_	K04948
ENSG000	CNTN5	1337934	0	0	1	0	0	0	1	0	10	0.02	7	0.01	2.78871	0.582212	2.540064	1.47886	Ups	0.01108	0.04352	cellular_cc	K06763
ENSG000	COBLL1	190056	658	0.57	998	0.9	722	0.63	1981	1.79	4722	3.95	2642	2.16	1816.797	0.268536	6.030813	1.61949	Ups	1.6E-09	4.4E-08	molecular_	_
ENSG000	COL10A1	39825	2	0.01	2	0.01	1	0	13	0.05	32	0.11	16	0.05	9.967194	0.516896	4.535988	2.34463	Ups	5.7E-06	7.2E-05	biological_	K19479
ENSG000	COL14A1	312257	5	0.01	29	0.07	10	0.02	77	0.18	56	0.12	77	0.16	40.48122	0.440803	3.835871	1.69086	Ups	0.00013	0.00106	biological_	K08133
ENSG000	COL25A1	491937	0	0	2	0.01	0	0	2	0.01	56	0.18	11	0.03	10.11333	0.582339	3.635938	2.11735	Ups	0.00028	0.00212	molecular_	K16617
ENSG000	COL3A1	38427	1845	5.3	1993	5.98	1625	4.7	43064	128.76	51175	141.93	45263	122.72	22289.77	0.135289	32.47233	4.39314	Ups	3E-231	5E-227	biological_	K19720
ENSG000	COL4A5	257702	1604	3.37	1681	3.69	1278	2.7	2144	4.69	9160	18.58	3689	7.31	3005.002	0.347687	3.716084	1.29203	Ups	0.0002	0.00162	molecular_	K06237
ENSG000	COL5A2	147984	154	0.55	181	0.67	142	0.51	11	0.04	31	0.11	23	0.08	94.60893	0.292708	-9.92409	-2.9049	Down	3.3E-23	3.8E-21	biological_	K19721
ENSG000	COL6A1	23314	112	0.44	90	0.37	96	0.38	26	0.11	75	0.29	60	0.22	76.90636	0.306253	-3.44835	-1.0561	Down	0.00056	0.00391	cellular_cc	K06238
ENSG000	COL6A2	34753	38	0.11	72	0.22	74	0.22	26	0.08	35	0.1	25	0.07	45.97851	0.334681	-3.60648	-1.207	Down	0.00031	0.00234	molecular_	K06238
ENSG000	COL7A1	31195	7163	13.63	5559	11.03	6459	12.36	3522	6.97	2873	5.27	3522	6.32	4947.644	0.193959	-5.77754	-1.1206	Down	7.6E-09	1.8E-07	molecular_	K16628
ENSG000	COL8A1	160752	86	0.19	92	0.21	75	0.16	2364	5.35	2574	5.4	2195	4.5	1139.125	0.179326	25.03558	4.48954	Ups	3E-138	6E-135	biological_	K09228
ENSG000	COLEC10	111131	0	0	2	0.03	0	0	4	0.06	9	0.13	8	0.11	3.497041	0.579421	2.721375	1.57682	Ups	0.0065	0.02881	molecular_	K10065
ENSG000	COLGALT:	108068	7	0.02	3	0.01	4	0.01	11	0.03	32	0.08	21	0.05	11.96778	0.474166	3.212883	1.52344	Ups	0.00131	0.00789	cellular_cc	K11703
ENSG000	COLQ	71619	12	0.03	21	0.06	25	0.07	102	0.3	81	0.22	78	0.21	50.90108	0.33814	5.267498	1.78115	Ups	1.4E-07	2.6E-06	biological_	_
ENSG000	COPS3	34467	8055	30.98	7871	31.57	8558	33.1	4290	17.16	4750	17.62	5018	18.2	6513.652	0.12597	-7.99716	-1.0074	Down	1.3E-15	8E-14	biological_	K12177
ENSG000	COTL1	52484	14028	33.71	10756	26.96	13337	32.23	6282	15.7	6096	14.13	7891	17.88	9898.232	0.183647	-5.96367	-1.0952	Down	2.5E-09	6.5E-08	molecular_	_
ENSG000	CPE	137127	387	2.92	427	3.36	330	2.51	1797	14.11	2778	20.23	2525	17.98	1277.816	0.171393	13.68825	2.34608	Ups	1.2E-42	4.2E-40	biological_	K01294
ENSG000	CPEB3	244396	135	0.32	151	0.37	85	0.2	212	0.52	509	1.15	291	0.65	216.7806	0.300835	3.683406	1.1081	Ups	0.00023	0.00181	molecular_	K02602
ENSG000	CPEB4	73697	961	1.23	1332	1.78	828	1.07	1901	2.53	7166	8.86	3092	3.74	2341.092	0.34052	4.477479	1.52467	Ups	7.6E-06	9.2E-05	molecular_	K02602
ENSG000	CPED1	308768	609	1.54	713	1.88	592	1.51	924	2.44	3115	7.62	1342	3.21	1133.479	0.317769	3.520993	1.11886	Ups	0.00043	0.0031	cellular_cc	_
ENSG000	CPLX1	41242	91	0.47	54	0.29	83	0.43	36	0.19	7	0.03	29	0.14	52.08717	0.435077	-3.49095	-1.5188	Down	0.00048	0.00341	molecular_	K15294
ENSG000	CPNE5	99227	1	0	0	0	2	0.01	17	0.08	10	0.04	21	0.08	8.008084	0.544766	4.337884	2.36313	Ups	1.4E-05	0.00016	cellular_cc	_
ENSG000	CPPED1	140956	8	0.03	8	0.03	17	0.06	0	0	1	0	0	0	6.036245	0.568263	-4.55983	-2.5912	Down	5.1E-06	6.5E-05	molecular_	_
ENSG000	CPQ	504428	57	0.25	54	0.24	63	0.27	445	2	365	1.52	423	1.72	221.8687	0.235523	10.65153	2.50868	Ups	1.7E-26	2.6E-24	molecular_	K01302
ENSG000	CPT1C	22834	30	0.06	27	0.06	26	0.05	357	0.74	132	0.25	310	0.58	141.2067	0.376699	7.230371	2.72368	Ups	4.8E-13	2.2E-11	molecular_	K19524
ENSG000	CPVL	200221	898	4.28	942	4.68	841	4.03	2715	13.46	2205	10.14	2862	12.87	1681.165	0.192724	6.726733	1.2964	Ups	1.7E-11	6.5E-10	molecular_	K09645
ENSG000	CPZ	27102	30	0.07	25	0.06	24	0.06	142	0.36	164	0.39	137	0.32	81.84505	0.255213	8.435705	2.15291	Ups	3.3E-17	2.4E-15	molecular_	K13022
ENSG000	CRABP2	6211	77	1.02	93	1.29	89	1.19	556	7.68	329	4.22	569	7.13	273.1374	0.288185	7.446528	2.14598	Ups	9.6E-14	4.8E-12	molecular_	K17289
ENSG000	CRB1	276994	48	0.07	52	0.07	54	0.07	599	0.86	1039	1.38	863	1.12	404.3519	0.218257	16.68646	3.64194	Ups	1.6E-62	1.2E-59	molecular_	K16681
ENSG000	CRB3	3445	114	1.15	111	1.16	103	1.04	35	0.37	17	0.17	38	0.36	72.49373	0.318529	-5.94518	-1.8937	Down	2.8E-09	7.2E-08	cellular_cc	K06090
ENSG000	CREB5	526572	3	0.01	3	0.01	3	0.01	28	0.05	54	0.09	43	0.07	20.37338	0.443707	6.455695	2.86444	Ups	1.1E-10	3.5E-09	molecular_	K09047
ENSG000	CREBRF	82937	105	0.13	141	0.18	97	0.12	524	0.65	684	0.79	437	0.49	311.8966	0.234131	8.333688	1.95117	Ups	7.8E-17	5.6E-15	biological_	K12581
ENSG000	CREG2	42045	764	2.54	701	2.43	738	2.47	1	0	7	0.02	1	0	393.5446	0.351101	-20.2562	-7.112	Down	3.1E-91	5.3E-88	cellular_cc	_
ENSG000	CRIP2	7201	947	2.41	757	2.01	979	2.51	3336	8.83	859	2.11	2726	6.55	1581.68	0.384814	2.749456	1.05803	Ups	0.00597	0.02693	cellular_cc	_
ENSG000	CRYAB	15158	134	0.48	118	0.44	131	0.47	315	1.17	513	1.76	559	1.88	277.5765	0.219045	7.122436	1.56013	Ups	1.1E-12	4.7E-11	biological_	K09542
ENSG000	CRYBB3	7514	25	0.53	36	0.79	28	0.6	15	0.33	9	0.18	17	0.34	22.25866	0.380821	-3.05713	-1.1642	Down	0.00223	0.01225	molecular_	_
ENSG000	CSAG1	6291	82	0.86	71	0.77	98	1.03	1305	14.2	837	8.45	1344	13.26	587.6892	0.273364	12.40034	3.38981	Ups	2.6E-35	6.5E-33	_	_
ENSG000	CSAG4	7161	3	0.08	1	0.03	0	0	38	1.02	30	0.75	24	0.59	15.01908	0.505395	5.987503	3.02605	Ups	2.1E-09	5.7E-08	_	_
ENSG000	CSDC2	16979	1201	9.15	997	7.92	1233	9.45	329	2.61	75	0.55	221	1.59	711.286	0.396647	-5.69425	-2.2586	Down	1.2E-08	2.8E-07	molecular_	K17497
ENSG000	CSF1R	60082	74	0.29	79	0.32	65	0.25	25	0.1	5	0.02	9	0.03	45.17014	0.428428	-5.14821	-2.2056	Down	2.6E-07	4.6E-06	biological_	K05090
ENSG000	CSMD2	651835	0	0	1	0	0	0	118	0.13	370	0.39	156	0.16	95.22324	0.489571	11.36368	5.56332	Ups	6.3E-30	1.2E-27	cellular_cc	K17495
ENSG000	CSPG4	38527	3304	10.25	2832	9.16	3168	9.88	221	0.71	620	1.85	354	1.04	1833.519	0.264818	-11.5058	-3.0469	Down	1.2E-30	2.4E-28	biological_	K08115
ENSG000	CSTB	3934	6784	61.49	5691	53.8	7367	67.16	3175	29.93	2314	20.24	4018	34.35	5006.055	0.241074	-5.00842	-1.2074	Down	5.5E-07	8.9E-06	molecular_	K13907
ENSG000	CTA-14H!	342	91	6.84	101	7.92	97	7.33	321	25.1	172	12.47	389	27.58	189.137	0.318068	4.094404	1.3023	Ups	4.2E-05	0.00041	_	_
ENSG000	CTA-228/	1433	7	0.13	11	0.21	9	0.16	26	0.49	42	0.73	29	0.49	19.47732	0.3863	3.639266	1.40585	Ups	0.00027	0.0021	_	_
ENSG000	CTA-250I	1078	12	0.29	11	0.27	14	0.34	44	1.09	52	1.2	66	1.48	31.3023	0.341814	5.040462	1.7229	Ups	4.6E-07	7.6E-06	_	_
ENSG000	CTA-292E	47877	26	0.38	27	0.42	36	0.53	77	1.18	42	0.6	106	1.48	50.90073	0.371343	2.715042	1.00821	Ups	0.00663	0.02923	_	_
ENSG000	CTA-941F	1190	116	5.44	110	5.38	120	5.66	45	2.2	32	1.45	66	2.92	83.73972	0.295126	-4.68087	-1.3814	Down	2.9E-06	3.9E-05	_	_
ENSG000	CTB-129(	2009	107	7	108	7.37	108	7.1	48	3.27	33	2.08	72	4.44	81.20159	0.304366	-3.91367	-1.1912	Down	9.1E-05	0.00081	_	_
ENSG000	CTB-13F3	625	20	0.82	12	0.51	9	0.37	36	1.54	52	2.06	41	1.59	26.84183	0.357326	3.572858	1.27667	Ups	0.00035	0.00261	_	_
ENSG000	CTB-175F	16843	358	1.31	350	1.33	393	1.44	87	0.33	352	1.24	165	0.57	284.7264	0.342515	-3.02336	-1.0355	Down	0.0025	0.01344	_	_
ENSG000	CTB-1I21	19760	26	1.19	23	1.09	18	0.83	48	2.28	93	4.09	64	2.75	42.68979	0.314154	4.025747	1.2647	Ups	5.7E-05	0.00054	_	_
ENSG000	CTB-36H:	2178	10	0.12	28	0.34	5	0.06	63	0.77	73	0.83	77	0.86	40.21465	0.418702	4.214074	1.76444	Ups	2.5E-05	0.00026	_	_
ENSG000	CTB-43E1	22250	6	0.06	6	0.06	6	0.06	2	0.02	0	0	1	0.01	3.701189	0.572464	-2.70903	-1.5508	Down	0.00675	0.02963	_	_
ENSG000	CTB-50L1	1273	11	0.55	10	0.53	16	0.81	78	4.09	24	1.17	44	2.09	29.98833	0.448265	3.314983	1.48599	Ups	0.00092	0.00586	_	_
ENSG000	CTB-55O(	34089	79	0.51	70	0.47	61	0.39	221	1.48	130	0.81	159	0.96	117.3672	0.300057	3.389893	1.01716	Ups	0.0007	0.00468	_	_
ENSG000	CTB-61M	73738	23	0.46	17	0.35	9	0.18	0	0	0	0	0	0	8.68971	0.562712	-5.81184	-3.2704	Down	6.2E-09	1.5E-07	_	_
ENSG000	CTC-203F	2017	2	0.03	4	0.05	8	0.1	9	0.12	25	0.31	17	0.2	10.07875	0.493053	2.485702	1.22558	Ups	0.01293	0.04902	_	_
ENSG000	CTC-205N	3088	15	0.12	15	0.13	18	0.15	63	0.55	58	0.47	55	0.43	35.71982	0.321963	4.731139	1.52325	Ups	2.2E-06	3.1E-05	_	_
ENSG000	CTC-303L	6217	26	1.63	37	2.43	31	1.96	32	2.09	271	16.43	59	3.5	68.68876	0.481207	2.653421	1.27684	Ups	0.00797	0.03371	_	_
ENSG000	CTC-338N	760	1	0.06	1	0.06	2	0.11	11	0.64	12	0.65	8	0.43	5.476108	0.547361	3.197789	1.75034	Ups	0.00138	0.00823	_	_
ENSG000	CTC-366E	1167	12	0.26	7	0.16	5	0.11	26	0.6	28	0.6	42	0.87	18.9273	0.412711	3.672371	1.51563	Ups	0.00024	0.00188	_	_
ENSG000	CTC-425(	1991	7	0.12	8	0.15	3	0.05	15	0.27	50	0.85	12	0.2	14.51426	0.499851	2.755102	1.37714	Ups	0.00587	0.02657	_	_
ENSG000	CTC-479(	6404	217	0.89	154	0.66	177	0.73	81	0.35	62	0.25	106	0.41	135.9329	0.271112	-4.67292	-1.2669	Down	3E-06	4E-05	_	K02733
ENSG000	CTC-490E	5990	3	0.16	6	0.33	2	0.11	15	0.83	18	0.93	21	1.06	10.1961	0.476459	3.388784	1.61462	Ups	0.0007	0.00469	_	_
ENSG000	CTC-490(	2136	349	29.8	304	27.08	435	37.36	144	12.79	175	14.42	221	17.8	276.2115	0.225547	-5.26878	-1.1884	Down	1.4E-07	2.6E-06	_	_
ENSG000	CTC-510F	23665	4	0.1	2	0.05	3	0.07	21	0.53	7	0.16	23	0.53	9.646023	0.521751	3.152524	1.64483	Ups	0.00162	0.00935	_	_
ENSG000	CTD-200(	736	14	0.49	14	0.51	17	0.6	61	2.22	38	1.28	80	2.64	35.82679	0.374974	4.20812	1.57794	Ups	2.6E-05	0.00027	_	_
ENSG000	CTD-2007	5466	2	0.16	0	0	0	0	3	0.25	16	1.22	6	0.45	3.984874	0.581644	2.778043	1.61583	Ups	0.00547	0.02517	_	_
ENSG000	CTD-2008	130485	7	0.18	2	0.05	9	0.23	0	0	1	0.02	0	0	3.343534	0.58324	-3.14834	-1.8362	Down	0.00164	0.00946	_	_
ENSG000	CTD-2008	187650	15	0.12	16	0.14	24	0.2	9	0.08	2	0.02	5	0.04	12.39778	0.476952	-3.22637	-1.5388	Down	0.00125	0.00759	_	_
ENSG000	CTD-2008	2736	9	0.08	7	0.07	6	0.06	22	0.21	31	0.28	22	0.19	15.30368	0.408566	3.226867	1.31839	Ups	0.00125	0.00758	_	_
ENSG000	CTD-2013	42682	21	0.1	14	0.07	16	0.07	44	0.21	79	0.35	51	0.22	35.25978	0.331477	4.214272	1.39693	Ups	2.5E-05	0.00026	_	_
ENSG000	CTD-2062	1471	38	1.77	24	1.16	29	1.36	7	0.34	7	0.31	8	0.35	19.5518	0.399034	-4.88885	-1.9508	Down	1E-06	1.5E-05	_	_
ENSG000	CTD-2078	4278	61	1.51	49	1.26	45	1.12	117	3.01	299	7.13	298	6.95	133.9328	0.315732	5.683156	1.79435	Ups	1.3E-08	3E-07	_	_
ENSG000	CTD-2095	1029	3	0.07	4	0.1	5	0.13	0	0	0	0	1	0.02	2.293144	0.582757	-2.57802	-1.5024	Down	0.00994	0.04004	_	_
ENSG000	CTD-2108	269	4	0.38	2	0.2	4	0.38	24	2.39	11	1.01	12	1.08	9.244915	0.511027	2.933238	1.49896	Ups	0.00335	0.01703	_	K02901
ENSG000	CTD-2139	8405	10	0.11	3	0.03	8	0.09	19	0.22	23	0.25	29	0.3	14.54866	0.433874	2.941725	1.27634	Ups	0.00326	0.01666	_	_
ENSG000	CTD-2165	2797	75	0.49	76	0.51	71	0.46	21	0.14	47	0.29	46	0.28	56.57714	0.290284	-3.87055	-1.1236	Down	0.00011	0.00094	_	_
ENSG000	CTD-2223	749	14	0.78	11	0.64	19	1.06	31	1.79	36	1.93	47	2.47	25.24024	0.357467	2.876899	1.0284	Ups	0.00402	0.01967	_	_
ENSG000	CTD-2227	747	8	0.28	14	0.5	9	0.31	0	0	6	0.2	3	0.1	6.812117	0.534886	-2.64465	-1.4146	Down	0.00818	0.03441	_	_
ENSG000	CTD-2228	407	12	0.76	1	0.07	0	0	8	0.53	52	3.17	24	1.43	14.46579	0.569632	2.519449	1.43516	Ups	0.01175	0.04548	_	_
ENSG000	CTD-2231	47713	12	0.44	12	0.46	8	0.29	29	1.1	94	3.31	29	1	28.07055	0.439798	3.718748	1.6355	Ups	0.0002	0.00161	_	_
ENSG000	CTD-2235	347	0	0	2	0.15	0	0	3	0.23	16	1.14	6	0.42	3.993686	0.581647	2.76864	1.61037	Ups	0.00563	0.02578	_	K02976
ENSG000	CTD-2235	16760	0	0	0	0	0	0	4	0.2	3	0.14	4	0.18	1.706466	0.571156	2.554423	1.45897	Ups	0.01064	0.04215	_	_
ENSG000	CTD-2235	483	6	0.32	3	0.17	2	0.11	9	0.5	19	0.98	14	0.7	8.234777	0.4972	2.571778	1.27869	Ups	0.01012	0.04057	_	_
ENSG000	CTD-2263	7413	811	26.29	736	24.88	707	23.05	318	10.72	350	10.95	521	15.93	582.7617	0.212539	-5.19472	-1.1041	Down	2.1E-07	3.7E-06	_	_
ENSG000	CTD-2269	106339	51	0.64	58	0.76	27	0.34	133	1.73	230	2.78	190	2.24	107.5245	0.292332	5.697722	1.66563	Ups	1.2E-08	2.8E-07	_	_
ENSG000	CTD-2311	13853	181	0.98	289	1.63	223	1.22	5	0.03	13	0.07	10	0.05	127.8079	0.334088	-13.1701	-4.4	Down	1.3E-39	4E-37	_	K03767
ENSG000	CTD-2341	195064	4	0.06	2	0.03	2	0.03	11	0.16	35	0.47	18	0.24	10.86828	0.504603	3.983129	2.0099	Ups	6.8E-05	0.00063	_	_
ENSG000	CTD-2354	24938	3789	34.9	4431	42.56	3609	33.43	1922	18.41	2000	17.77	2050	17.81	3023.728	0.150743	-7.83379	-1.1809	Down	4.7E-15	2.8E-13	_	_
ENSG000	CTD-241(	58902	7	0.37	13	0.72	8	0.43	1	0.06	3	0.15	2	0.1	5.884422	0.537797	-3.08714	-1.6603	Down	0.00202	0.01128	_	K03094
ENSG000	CTD-2514	3363	1	0.01	1	0.01	2	0.01	8	0.06	4	0.03	12	0.08	4.451625	0.566138	2.491098	1.41031	Ups	0.01273	0.04845	_	_
ENSG000	CTD-251(	85250	1	0	0	0	1	0	8	0.02	18	0.05	6	0.02	5.12977	0.569257	3.555599	2.02405	Ups	0.00038	0.00277	_	_
ENSG000	CTD-2517	9827	10	0.06	20	0.12	7	0.04	41	0.25	54	0.31	31	0.17	25.80352	0.398075	3.348604	1.333	Ups	0.00081	0.00529	_	_
ENSG000	CTD-2528	2058	6	0.11	9	0.18	8	0.15	16	0.31	26	0.47	36	0.64	15.87471	0.424007	3.027039	1.28349	Ups	0.00247	0.01332	_	_
ENSG000	CTD-2535	5907	11	0.64	19	1.14	9	0.52	36	2.16	39	2.17	27	1.47	22.61863	0.381959	2.686765	1.02623	Ups	0.00721	0.03119	_	_
ENSG000	CTD-2541	89735	3	0.02	0	0	6	0.04	18	0.13	18	0.12	11	0.07	8.857001	0.521102	2.966087	1.54563	Ups	0.00302	0.01567	_	_
ENSG000	CTD-2555	10573	273	4.38	181	3.03	264	4.26	106	1.77	40	0.62	101	1.53	166.7621	0.357279	-4.30034	-1.5364	Down	1.7E-05	0.00019	_	_
ENSG000	CTD-2561	19668	0	0	1	0	0	0	2	0.01	33	0.11	14	0.05	7.198974	0.580548	3.91566	2.27323	Ups	9E-05	0.0008	_	_

ENSG000	CTD-258	791	150	4.87	98	3.32	182	5.95	60	2.03	30	0.94	50	1.53	98.61904	0.341712	-4.78449	-1.6349	Down	1.7E-06	2.5E-05	_	_
ENSG000	CTD-261	17727	31	1.45	12	0.58	30	1.41	14	0.68	6	0.27	11	0.48	17.8777	0.449724	-2.58794	-1.1639	Down	0.00966	0.03921	_	K03521
ENSG000	CTD-261	5402	8	0.04	10	0.05	6	0.03	24	0.12	24	0.11	28	0.13	15.94336	0.403519	3.078503	1.24223	Ups	0.00208	0.01154	_	_
ENSG000	CTD-261	4008	4	0.13	7	0.24	3	0.1	22	0.74	11	0.35	20	0.61	10.81777	0.484203	2.756846	1.33487	Ups	0.00584	0.02648	_	_
ENSG000	CTD-305	1045	3	0.07	7	0.18	3	0.07	1	0.03	0	0	0	0	2.492261	0.582913	-2.62724	-1.5315	Down	0.00861	0.0358	_	_
ENSG000	CTD-308	260	0	0	2	0.21	1	0.1	8	0.82	11	1.05	8	0.75	4.644269	0.561997	3.089451	1.73626	Ups	0.00201	0.01121	_	K02881
ENSG000	CTD-308	163743	5	0.01	2	0	5	0.01	60	0.12	48	0.09	48	0.09	26.34344	0.422865	6.870906	2.90547	Ups	6.4E-12	2.5E-10	_	_
ENSG000	CTD-311	745	15	0.63	13	0.57	3	0.13	13	0.57	58	2.35	50	1.98	23.2819	0.483253	2.733136	1.3208	Ups	0.00627	0.02802	_	_
ENSG000	CTD-320	1684	4	0.06	3	0.05	6	0.09	23	0.37	16	0.24	19	0.27	11.35678	0.466682	3.320174	1.54946	Ups	0.0009	0.00577	_	_
ENSG000	CTD-322	2192	88	0.65	72	0.56	81	0.6	37	0.29	25	0.18	42	0.29	59.04696	0.293964	-4.49409	-1.3211	Down	7E-06	8.6E-05	_	_
ENSG000	CTF1	6954	2	0.02	0	0	2	0.02	130	1.06	22	0.17	104	0.77	41.8053	0.531452	6.998916	3.71959	Ups	2.6E-12	1.1E-10	molecular	K05422
ENSG000	CTGF	3198	964	10.59	821	9.41	883	9.76	30158	344.74	19274	204.37	27362	283.61	12457.34	0.236927	18.80797	4.45612	Ups	6.5E-79	7.8E-76	biological	K06827
ENSG000	CTNND2	932204	0	0	1	0	0	0	18	0.03	84	0.14	42	0.07	21.23027	0.538486	6.967161	3.75172	Ups	3.2E-12	1.4E-10	biological	_
ENSG000	CTRB1	5925	0	0	0	0	0	0	2	0.04	4	0.08	8	0.15	2.10112	0.575873	2.770542	1.59548	Ups	0.0056	0.02565	molecular	K01310
ENSG000	CTRB2	3090	2	0.01	2	0.01	1	0.01	13	0.08	19	0.11	15	0.09	8.016176	0.516494	4.085663	2.11022	Ups	4.4E-05	0.00043	molecular	K01310
ENSG000	CTSB	26925	5973	7.78	5667	7.7	6017	7.89	2590	3.51	3586	4.51	3273	4.02	4579.897	0.116934	-9.57773	-1.12	Down	9.9E-22	1E-19	biological	K01363
ENSG000	CTSF	5379	276	1.3	228	1.12	257	1.22	1690	8.28	1466	6.66	1795	7.98	899.7037	0.192628	12.59284	2.42574	Ups	2.3E-36	5.9E-34	molecular	K01373
ENSG000	CTSO	29800	14	0.12	10	0.09	6	0.05	86	0.78	79	0.66	69	0.57	41.4642	0.35421	6.958341	2.46472	Ups	3.4E-12	1.4E-10	molecular	K01374
ENSG000	CXCL10	2378	0	0	0	0	1	0.02	2	0.05	15	0.32	7	0.14	3.65491	0.583272	3.038336	1.77218	Ups	0.00238	0.01292	biological	K12671
ENSG000	CXCL16	6397	681	3.58	519	2.85	656	3.47	243	1.33	452	2.29	310	1.54	481.4433	0.212987	-5.10765	-1.0879	Down	3.3E-07	5.5E-06	molecular	K10035
ENSG000	CXCL2	2259	54	0.61	38	0.45	43	0.49	246	2.89	68	0.74	228	2.43	110.1084	0.411984	3.811598	1.57032	Ups	0.00014	0.00116	biological	K05505
ENSG000	CXCR4	3817	2985	15.16	3164	16.76	3247	16.58	1440	7.61	552	2.7	1177	5.64	2175.981	0.32375	-4.92039	-1.593	Down	8.6E-07	1.3E-05	biological	K04189
ENSG000	CXCR6	7421	10	0.03	8	0.03	18	0.06	3	0.01	5	0.02	2	0.01	7.938166	0.510967	-2.97861	-1.522	Down	0.0029	0.0151	molecular	K04191
ENSG000	CXorf57	67513	85	0.26	108	0.35	80	0.25	130	0.42	452	1.34	196	0.57	163.121	0.348643	3.196207	1.11433	Ups	0.00139	0.00827	_	_
ENSG000	CXXC4	26590	14	0.04	16	0.04	10	0.03	170	0.46	253	0.64	191	0.47	100.1823	0.290364	11.89458	3.45376	Ups	1.3E-32	2.7E-30	molecular	K03344
ENSG000	CYB5D1	4537	2610	9.39	2540	9.53	2719	9.83	1232	4.61	1816	6.3	1223	4.15	2049.522	0.161068	-6.77779	-1.0917	Down	1.2E-11	4.7E-10	molecular	K11642
ENSG000	CYB5R2	12123	4	0.01	2	0	1	0	19	0.04	14	0.03	19	0.03	9.308997	0.504847	3.892813	1.96527	Ups	9.9E-05	0.00087	molecular	K00326
ENSG000	CYBRD1	35887	995	2.47	1160	3	963	2.4	1840	4.74	3707	8.86	2262	5.29	1726.18	0.216469	4.792984	1.03753	Ups	1.6E-06	2.4E-05	molecular	K08370
ENSG000	CYCSP6	302	6	0.51	2	0.18	4	0.34	0	0	0	0	0	0	2.128521	0.577384	-2.94571	-1.7008	Down	0.00322	0.01648	_	K08738
ENSG000	CYP11A1	29982	383	1.38	376	1.42	416	1.51	70	0.26	191	0.66	158	0.54	271.6179	0.274311	-5.98686	-1.6423	Down	2.1E-09	5.7E-08	biological	K00498
ENSG000	CYP17A1	7003	3	0.02	4	0.02	3	0.02	92	0.57	16	0.09	61	0.34	29.03117	0.514566	5.421674	2.78981	Ups	5.9E-08	1.2E-06	molecular	K00512
ENSG000	CYP1A1	6069	31	0.12	24	0.1	32	0.12	7	0.03	9	0.03	3	0.01	18.39498	0.4167	-4.90138	-2.0424	Down	9.5E-07	1.5E-05	biological	K07408
ENSG000	CYP24A1	20525	6	0.02	4	0.02	6	0.02	1	0	1	0	0	0	3.158582	0.579582	-2.79046	-1.6173	Down	0.00526	0.02445	biological	K07436
ENSG000	CYP2S1	14491	585	3.25	477	2.76	587	3.28	216	1.25	242	1.3	173	0.91	390.3614	0.200482	-7.74989	-1.5537	Down	9.2E-15	5.3E-13	molecular	K07420
ENSG000	CYP2W1	6442	1	0.01	3	0.02	0	0	11	0.06	16	0.08	9	0.04	6.178171	0.547259	3.425347	1.87455	Ups	0.00061	0.00417	molecular	K07423
ENSG000	CYP3A7	30160	59	0.34	112	0.67	77	0.44	17	0.1	16	0.09	14	0.08	51.52125	0.344724	-6.84454	-2.3595	Down	7.7E-12	3E-10	molecular	K17691
ENSG000	CYP4F12	24418	8	0.02	10	0.02	2	0	0	0	2	0	1	0	3.978331	0.578121	-2.72838	-1.5773	Down	0.00636	0.02832	biological	K17730
ENSG000	CYR61	3202	6851	61.99	6866	64.79	8106	73.77	60836	572.54	30525	266.47	51589	440.25	26361.95	0.28403	8.344861	2.37019	Ups	7.1E-17	5.1E-15	biological	K06829
ENSG000	CYS1	24165	5	0.04	4	0.03	1	0.01	20	0.17	16	0.12	22	0.17	10.74249	0.483671	3.697671	1.78846	Ups	0.00022	0.00172	biological	_
ENSG000	DAAM1	182760	796	1.37	1036	1.86	811	1.41	204	0.37	601	1	291	0.47	633.2019	0.298764	-4.78088	-1.4284	Down	1.8E-06	2.5E-05	cellular_cc	K04512
ENSG000	DACT1	14355	21	0.05	6	0.01	8	0.02	5	0.01	3	0.01	2	0	7.766983	0.5317	-2.61704	-1.3915	Down	0.00887	0.03663	biological	_
ENSG000	DACT2	26925	10	0.03	9	0.03	14	0.04	5	0.02	93	0.28	55	0.16	27.89468	0.523428	2.612372	1.36739	Ups	0.00899	0.03707	biological	_
ENSG000	DAPP1	53322	15	0.05	28	0.1	16	0.06	94	0.35	680	2.36	277	0.94	162.553	0.451144	6.929073	3.12601	Ups	4.2E-12	1.7E-10	molecular	K12229
ENSG000	DCP2	44269	626	0.84	882	1.24	486	0.66	1096	1.53	2386	3.09	1687	2.14	1128.143	0.268059	3.953427	1.05975	Ups	7.7E-05	0.0007	biological	K12613
ENSG000	DCST2	15262	21	0.12	17	0.1	23	0.13	61	0.35	51	0.27	75	0.39	39.71942	0.322775	3.988689	1.28745	Ups	6.6E-05	0.00062	cellular_cc	_
ENSG000	DDR2	156028	26	0.06	20	0.04	24	0.05	135	0.3	264	0.54	125	0.25	91.09141	0.319548	7.633824	2.43938	Ups	2.3E-14	1.2E-12	biological	K05125
ENSG000	DDX43	22822	0	0	0	0	0	0	5	0.04	1	0.01	9	0.06	2.353213	0.573687	2.74198	1.57304	Ups	0.00611	0.02742	molecular	K17043
ENSG000	DDX53	2118	0	0	3	0.04	1	0.01	8	0.1	171	2	28	0.32	29.99188	0.572974	2.881533	1.65104	Ups	0.00396	0.01943	molecular	K17043
ENSG000	DEF6	23954	8	0.06	4	0.03	11	0.08	40	0.29	43	0.29	47	0.31	24.01615	0.386281	5.13937	1.98524	Ups	2.8E-07	4.8E-06	molecular	_
ENSG000	DEFB1	7448	3	0.16	6	0.34	3	0.16	0	0	0	0	0	0	2.140599	0.578222	-2.98123	-1.7238	Down	0.00287	0.01501	biological	_
ENSG000	DEGS2	13745	0	0	0	0	0	0	8	0.07	6	0.05	5	0.04	2.962137	0.583406	3.430339	2.00128	Ups	0.0006	0.00411	molecular	K04712
ENSG000	DENND1	15351	42	0.11	42	0.11	30	0.08	24	0.06	15	0.04	10	0.02	27.99745	0.381904	-3.24289	-1.2385	Down	0.00118	0.00723	cellular_cc	_
ENSG000	DEPTOR	177196	147	1.22	178	1.54	135	1.12	813	7	715	5.71	823	6.42	444.8591	0.208593	9.949074	2.0753	Ups	2.6E-23	3E-21	molecular	_
ENSG000	DERL3	4626	21	0.06	23	0.07	34	0.09	189	0.54	106	0.28	167	0.43	86.29643	0.342109	6.277769	2.14768	Ups	3.4E-10	1.1E-08	molecular	K13989
ENSG000	DFNB59	9955	43	0.25	49	0.3	39	0.23	84	0.51	154	0.87	125	0.69	77.93208	0.261394	4.457082	1.16505	Ups	8.3E-06	0.0001	biological	_
ENSG000	DGAT2	42023	94	0.2	64	0.14	85	0.18	153	0.33	259	0.52	209	0.41	136.8969	0.235584	4.540208	1.0696	Ups	5.6E-06	7.1E-05	molecular	K11160
ENSG000	DGCR10	495	0	0	1	0.05	0	0	5	0.27	12	0.6	7	0.34	3.755597	0.579571	3.37658	1.95697	Ups	0.00073	0.00487	_	_
ENSG000	DGCR9	2415	4	0.04	6	0.07	5	0.05	22	0.24	38	0.39	30	0.3	16.24192	0.425147	4.6156	1.96231	Ups	3.9E-06	5.2E-05	_	_
ENSG000	DGKH	216539	236	0.29	255	0.32	177	0.22	375	0.48	924	1.09	413	0.48	373.5921	0.297772	3.436594	1.02332	Ups	0.00059	0.00404	molecular	K00901
ENSG000	DGKI	466056	2	0	4	0.01	5	0.01	12	0.02	21	0.03	11	0.01	8.599453	0.49304	2.715037	1.33862	Ups	0.00663	0.02923	molecular	K00901
ENSG000	DHRS2	15525	31	0.06	15	0.03	33	0.06	19	0.04	154	0.29	104	0.19	54.29413	0.469629	2.57567	1.20961	Ups	0.01	0.04023	molecular	K11164
ENSG000	DHX37	42298	2522	5.67	2029	4.75	2390	5.4	1276	2.98	1198	2.6	1211	2.57	1803.417	0.168964	-6.49656	-1.0977	Down	8.2E-11	2.8E-09	molecular	K14780
ENSG000	DHX58	11330	34	0.15	22	0.1	43	0.19	81	0.37	99	0.43	90	0.38	58.84668	0.287619	3.978492	1.14429	Ups	6.9E-05	0.00064	molecular	K12649
ENSG000	DIO3OS	8211	81	0.17	65	0.14	104	0.21	39	0.08	32	0.06	36	0.07	61.1298	0.295429	-4.52064	-1.3355	Down	6.2E-06	7.7E-05	_	_
ENSG000	DIRAS3	5670	2	0.01	1	0.01	0	0	9	0.07	8	0.06	12	0.08	4.992279	0.560236	3.245741	1.81838	Ups	0.00117	0.00717	biological	K07842
ENSG000	DISC1	414458	58	0.08	53	0.07	52	0.07	15	0.02	14	0.02	18	0.02	36.24426	0.315349	-5.86681	-1.8501	Down	4.4E-09	1.1E-07	biological	K16534
ENSG000	DKFZP43	3659	1	0.01	0	0	0	0	4	0.03	8	0.05	4	0.03	2.573857	0.583441	2.632137	1.5357	Ups	0.00848	0.03546	_	K17985
ENSG000	DKK3	46664	161	0.42	155	0.42	154	0.41	655	1.79	775	1.96	670	1.66	404.6828	0.157493	12.08303	1.90299	Ups	1.3E-33	3E-31	cellular_cc	_
ENSG000	DLC1	432298	2	0	7	0.01	7	0.01	50	0.08	105	0.15	72	0.1	36.85308	0.404046	7.530672	3.04274	Ups	5.1E-14	2.6E-12	biological	_
ENSG000	DLG4	29813	134	0.45	89	0.31	133	0.45	407	1.43	288	0.94	354	1.13	226.6357	0.267949	4.806202	1.28782	Ups	1.5E-06	2.2E-05	biological	K11828
ENSG000	DLG5-AS	2453	8	0.42	4	0.22	8	0.42	37	2.03	16	0.81	35	1.74	17.41454	0.457327	3.43819	1.57238	Ups	0.00059	0.00402	_	_
ENSG000	DMBX1	7231	50	0.24	41	0.2	54	0.26	13	0.06	37	0.17	24	0.11	36.79881	0.338288	-3.27042	-1.1063	Down	0.00107	0.00669	biological	K11447
ENSG000	DMC1	51338	9	0.06	7	0.05	6	0.04	28	0.2	21	0.14	33	0.22	16.60617	0.41675	3.436883	1.43232	Ups	0.00059	0.00404	cellular_cc	_
ENSG000	DMGDH	238424	5	0.02	13	0.05	8	0.03	30	0.12	23	0.08	23	0.08	16.46219	0.424111	2.660855	1.1285	Ups	0.00779	0.03316	molecular	K00544
ENSG000	DMKN	16439	654	0.77	635	0.78	734	0.87	7	0.01	8	0.01	3	0	363.2426	0.313097	-20.4534	-6.4039	Down	5.6E-93	1.1E-89	cellular_cc	_
ENSG000	DNAAF3	8060	42	0.14	29	0.1	36	0.12	15	0.05	13	0.04	8	0.03	24.62017	0.371455	-4.24055	-1.5752	Down	2.2E-05	0.00024	cellular_cc	K19752
ENSG000	DNAH1	84173	22	0.04	17	0.03	22	0.04	71	0.12	202	0.32	72	0.11	61.90414	0.382508	5.120917	1.95879	Ups	3E-07	5.2E-06	biological	K10408
ENSG000	DNAH10	8561	3	0.02	0	0	0	0	3	0.02	41	0.2	13	0.06	8.674508	0.578457	3.448681	1.99491	Ups	0.00056	0.0039	_	K10408
ENSG000	DNAH6	303135	15	0.02	11	0.02	14	0.02	45	0.08	67	0.11	35	0.05	29.41873	0.359042	4.099361	1.47184	Ups	4.1E-05	0.00041	biological	K10408
ENSG000	DNAH7	331110	65	0.07	58	0.07	59	0.07	31	0.04	29	0.03	24	0.03	45.36497	0.285899	-4.34991	-1.2436	Down	1.4E-05	0.00015	biological	K10408
ENSG000	DNAJB9	5283	382	2.7	566	4.17	314	2.23	905	6.64	1498	10.2	1114	7.42	755.8003	0.243157	4.868101	1.18371	Ups	1.1E-06	1.7E-05	molecular	K09515
ENSG000	DNAJC12	41498	1188	8.82	1196	9.26	1040	7.76	592	4.57	621	4.45	705	4.94	903.5861	0.15497	-6.66822	-1.0334	Down	2.6E-11	9.4E-10	_	K09532
ENSG000	DNAJC22	10610	65	0.34	45	0.24	53	0.28	195	1.05	154	0.77	183	0.9	111.4442	0.262884	5.417347	1.42413	Ups	6.1E-08	1.2E-06	cellular_cc	K19370
ENSG000	DNER	356930	451	2.95	402	2.74	330	2.17	387	2.63	2343	14.77	1172	7.22	777.7867	0.389568	3.204947	1.24854	Ups	0.00135	0.00807	biological	_
ENSG000	DNM1	51870	24	0.08	28	0.09	26	0.08	70	0.23	63	0.19	60	0.18	43.71644	0.294481	3.467556	1.02113	Ups	0.00053	0.00369	molecular	K01528
ENSG000	DNM3	576986	85	0.12	111	0.16	85	0.12	223	0.33	377	0.51	235	0.31	176.0706	0.240397	5.296914	1.27336	Ups	1.2E-07	2.2E-06	molecular	K01528
ENSG000	DNMT3A	109615	173	0.42	134	0.34	154	0.38	258	0.66	683	1.61	275	0.63	262.4513	0.3141	3.339217	1.04885	Ups	0.00084	0.00544	biological	K17398
ENSG000	DNMT3L	15878	10	0.1	7	0.08	13	0.14	0	0	1	0.01	3	0.03	5.936067	0.551134	-3.58102	-1.9736	Down	0.00034	0.00254	cellular_cc	K17400
ENSG000	DOC2A	17762	29	0.06	13	0.03	24	0.05	106	0.23	60	0.12	88	0.17	51.54222	0.367763	4.241057	1.55971	Ups	2.2E-05	0.00024	molecular	_
ENSG000	DOCK2	446136	19	0.03	16	0.03	14	0.02	2	0	3	0	3	0	9.924311	0.486249	-4.43378	-2.1559	Down	9.3E-06	0.00011	biological	K12367
ENSG000	DOCK3	708958	48	0.14	45	0.14	36	0.11	12	0.04	27	0.08	17	0.05	31.31039	0.336347	-3.89421	-1.3098	Down	9.9E-05	0.00087	molecular	K05727
ENSG000	DOCK4	480301	263	0.34	322	0.43	192	0.25	519	0.69	1724	2.14	638	0.77	562.4827	0.349086	4.172225	1.45647	Ups	3E-05	0.00031	molecular	K17697
ENSG000	DOK7	38168	261	0.82	192	0.63	208	0.66	49	0.16	95	0.29	92	0.27	152.9726	0.24836	-6.61263	-1.6423	Down	3.8E-11	1.3E-09	molecular	_
ENSG000	DPF3	274806	20	0.06	25	0.07	31	0.09	10	0.03	13	0.03	7	0.02	18.14363	0.397555	-3.40727	-1.3546	Down	0.00066	0.00442	cellular_cc	_
ENSG000	DPP3	29647	1905	6.44	1519	5.36	1759	5.98	887	3.12	952	3.11	952	3.04	1350.301	0.150066	-7.26353	-1.09	Down	3.8E-13	1.8E-11	molecular	K01277
ENSG000	DPPA2	22730	0	0	1	0.02	0	0	338	6.53	251	4.5	328	5.75	142.5266	0.456579	13.84917	6.32323	Ups	1.3E-43	4.9E-41	molecular	_
ENSG000	DPYSL3	119246	860	1.79	744	1.62	750	1.57	91	0.2	148	0.3	114	0.22	472.2272	0.179185	-16.1712	-2.8976	Down	8.1E-59	5.3E-56	cellular_cc	_
ENSG000	DQX1	8206	9	0.04	8	0.04	9	0.04	39	0.2	17	0.08	23	0.1	17.16674	0.44242	2.650906	1.17281	Ups	0.00803	0.03389	molecular	K14433
ENSG000	DTX4	37158	649	2.49	503	2.01	570	2.2	32	0.13	336	1.24	128	0.46	377.6675	0.541241	-2.65224	-1.4355	Down	0.008	0.0338	cellular_cc	K06058
ENSG000	DUOX2	21695	0	0	0	0	0	0	2	0	2	0	8	0.01	1.824081	0.566856	2.488839	1.41081	Ups	0.01282	0.04869	molecular	K13411
ENSG000	DUSP1	3106	4928	62.74	4288	56.94	4079	52.23	58635	776.49	26313	323.22	47603	571.62	23275.63	0.300426	9.716816	2.91918	Ups	2.6E-22	2.8E-20	biological	K04459
ENSG000	DUSP13	14788	26	0.1	23	0.09	24	0.09	12	0.05	5	0.02	9	0.03	17.10891	0.411701	-3.492	-1.4377	Down	0.00048	0.0034	molecular	K14165
ENSG000	DUSP2	2275	477	4.98	363	3.95	468	4.92	5285	57.42	1127	11.36	4051	39.91	1909.121	0.508118	4.156395	2.11194	Ups	3.2E-05	0.00033	biological	K04459
ENSG000	DUSP4	17605	739	2.34	574	1.89	611	1.94	136	0.45	283	0.86	205	0.61	435.681	0.224065	-8.02309	-1.7977	Down	1E-15	6.5E-14	biological	K04459
ENSG000	DUSP5	13707	382	3.11	328	2.79	352	2.88	2705	22.92	1146	9.01	2250	17.28	1152.377	0.315052	6.864885	2.1628	Ups	6.7E-12	2.6E-10	biological	K04459
ENSG000	DUSP6	6040	138	0.43	171	0.56	121	0.38	1122	3.67	1477	4.49	1181	3.51	652.1442	0.184252	15.38789	2.83524	Ups	2E-53	1.2E-50	biological	K04459
ENSG000	DUSP8	17877	264	0.8	199	0.63	232	0.7	765	2.4	651	1.89	758	2.15	459.6555	0.207559	6.71662	1.3941	Ups	1.9E-11	7E-10	biological	K04459
ENSG000	DUSP9	8836	816	4.89	679	4.25	813	4.9	197	1.23	184	1.06	286	1.62	513.3629	0.22136	-8.71924	-1.9301	Down	2.8E-18	2.2E-16	biological	K18498
ENSG000	DYRK4	51956	654	1.58	632	1.59	699	1.7	256	0.64	358	0.83	397	0.9	507.0223	0.175753	-6.69092	-1.1759	Down	2.2E-11	8.2E-10	molecular	K18669
ENSG000	DYX1C1	97710	58	0.33	76	0.46	61	0.35	29	0.17	42	0.23	35	0.19	50.83643	0.274679	-3.79794	-1.0432	Down	0.00015	0.00122	biological	K19758
ENSG000	EAF1-AS1	15432	0	0	1	0	0	0	6	0.02	13	0.04	6	0.02	3.916314	0.579043	3.444935	1.99476	Ups	0.00057	0.00394	_	_
ENSG000	EBLN2	1679	5	0.08	11	0.18	3	0.05	7	0.11	102	1.51	22	0.32	22.02823	0.548153	2.971718	1.62896	Ups	0.00296	0.01542	_	_
ENSG000	EBNA1BP	106762	12057	51.45	11003	48.97	13735	58.95	5189	23.03	4901	20.18	6848	27.56	9158.994	0.193308	-6.70025	-1.2952	Down	2.1E-11	7.7E-10	cellular_cc	K14823
ENSG000	ECEL1	8002	10	0.06	18	0.11	11	0.07	54	0.34	25	0.14	51	0.29	27.39671	0.414563	3.175786	1.31656	Ups	0.00149	0.00878	biological	K09610
ENSG000	ECM1	5728	226	0.76	229	0.81	261	0.89	27	0.09	44	0.14	34	0.11	143.4692	0.233005	-12.3378	-2.8748	Down	5.7E-35	1.4E-32	biological	_
ENSG000	ECM2	42573	30	0.14	48	0.24	20	0.1	5	0.03	15	0.07	15	0.07	22.64627	0.424677	-3.43112	-1.4571	Down	0.0006	0.00411	molecular	K08119
ENSG000	EDA2R	43630	0	0	2	0.01	2	0.01	30	0.11	21	0.07	10	0.03	10.3045	0.539674	4.456197	2.40489	Ups	8.3E-06	0.0001	molecular	K05163
ENSG000	EDIL3	444239	969	2.78	1567	4.69	765	2.21	2950	8.81	10332	28.61	4789	12.96	3247.706	0.346872	5.66031	1.9634	Ups	1.5E-08	3.4E-07	molecular	_
ENSG000	EDN2	5899	33	0.37	36	0.42	34	0.38	138	1.6	262	2.82	279	2.94	120.3667	0.281548	8.246932	2.32191	Ups	1.6E-16	1.1E-14	biological	K16367
ENSG000	EDNRA	64038	290	0.98	337	1.18	268	0.91	565	1.98	1602	5.2	780	2.47	595.9674	0.293706	4.613996	1.35516	Ups	4E-06	5.2E-05	biological	K04197
ENSG000	EEF1A2	11140	793	11.06	542	7.88	761	10.68	113	1.64	125	1.68	160	2.1	433.8218	0.222673	-11.304	-2.5171	Down	1.3E-29	2.3E-27	molecular	K03231
ENSG000	EEF1DP5	772	0	0	1	0.03	0	0	8	0.28	6	0.19	4	0.13	2.991073	0.583138	2.867325	1.67205	Ups	0.00414	0.02017	_	K15410
ENSG000	EEPD1	148395	263	0.84	211	0.7	217	0.7	84	0.28	141	0.43	97	0.29	171.5094	0.215896	-5.96577	-1.288	Down	2.4E-09	6.4E-08	molecular	_
ENSG000	EFEMP2	7152	15	0.03	19	0.04	32	0.06	109	0.22	88	0.17	112	0.21	59.61531	0.330389	5.566464	1.8391	Ups	2.6E-08	5.5E-07	molecular	_
ENSG000	EFHD1	76725	206	0.75	160	0.61	203	0.75	620	2.36	911	3.22	759	2.62	448.4572	0.178026	9.746597	1.73515	Ups	1.9E-22	2.1E-20	molecular	_
ENSG000	EFTUD2	49720	12424	18.7	11699	18.37	13165	19.93	6789	10.63	6465	9.39	6519	9.25	9688.219	0.153565	-7.19617	-1.1051	Down	6.2E-13	2.8E-11	biological	K12852
ENSG000	EGF	99383	5	0.01	3	0.01	6	0.01	15	0.04	105	0.23	42	0.09	25.93621	0.500855	4.795704	2.40195	Ups	1.6E-06	2.3E-05	biological	K04357
ENSG000	EGFEM1P	581078	34	0.1	26	0.08	30	0.09	69	0.21	200	0.56	95	0.26	69.89131	0.3484	4.533631	1.57952	Ups	5.8E-06	7.3E-05	_	_
ENSG000	EGLN3	538544	2660	7.11	3001	8.37	2630	7.07	1376	3.83	1137	2.93	1509	3.8	2098.578	0.191888	-6.32906	-1.2145	Down	2.5E-10	7.7E-09	biological	_
ENSG000	EGR1	3826	320	2.62	257	2.2	280	2.31	6846	58.33	5259	41.56	6040	46.67	2969.232	0.208363	19.52334	4.06793	Ups	7E-85	1E-81	biological	K09203
ENSG000	EGR2	107905	1	0	0	0	1	0	204	0.8	44	0.16	152	0.54	64.4725	0.520435	8.807561	4.58377	Ups	1.3E-18	1E-16	molecular	K12496
ENSG000	EGR3	5644	0	0	2	0.01	1	0	43	0.13	2	0.01	41	0.11	14.3925	0.576226	4.102663	2.36406	Ups	4.1E-05	0.0004	biological	K12497
ENSG000	EGR4	2776	0	0	0	0	0	0	8	0.05	0	0	8	0.04	2.581815	0.563414	2.525111	1.42268	Ups	0.01157	0.04493	molecular	K12498
ENSG000	EHF	39965	588	1.72	1008	3.08	789	2.32	3	0.01	0	0	2	0.01	426.5181	0.400174	-18.3724	-7.3521	Down	2.2E-75	2.5E-72	molecular	K17102
ENSG000	EIF3EP1	1316	33	0.64	34	0.69	29	0.57	106	2.15	136	2.56	110	2.03	70.7396	0.249866	6.284465	1.57027	Ups	3.3E-10	1E-08	_	K03250
ENSG000	EIF4E3	79358	220	0.29	269	0.37	227	0.3	371	0.51	1050	1.34	568	0.71	422.3758	0.289872	3.895326	1.12915	Ups	9.8E-05	0.00086	molecular	_
ENSG000	EIF5A	5457	63808	310.06	51670	261.86	63015	307.97	35240	178.11	21059	98.73	33532	153.68	45809.93	0.256494	-4.36306	-1.1191	Down	1.3E-05	0.00015	molecular	K03263
ENSG000	EIF5AL1	3832	1211	8.12	972	6.8	1359	9.17	703	4.91	374	2.42	638	4.04	899.8553	0.286404	-4.02233	-1.152	Down	5.8E-05	0.00054	molecular	K03263
ENSG000	EIF5AP4	465	674	37.26	591	34.07	692	38.47	333	19.15	225	12	365	19.03	491.9566	0.245621	-4.97861	-1.2229	Down	6.4E-07	1E-05	_	K03263
ENSG000	ELAC2	25797	11000	22.22	10082	21.24	10566	21.46	5538	11.63	5582	10.88	5683	10.83	8217.159	0.13493	-8.23123	-1.1106	Down	1.9E-16	1.3E-14	molecular	K00784
ENSG000	ELANE	5229	3	0.07	1	0.02	0	0	8	0.19	8	0.18	11	0.24	4.844346	0.559041	2.785454	1.55718	Ups	0.00535	0.02473	biological	K01327
ENSG000	ELFN2	59506	240	0.64	168	0.47	206	0.56	3	0.01	48	0.12	22	0.06	119.6782	0.445099	-6.19899	-2.7592	Down	5.7E-10	1.7E-08	molecular	_
ENSG000	ELSPBP1	30503	0	0	1	0.01	2	0.02	23	0.28	5	0.06	9	0.1	6.551488	0.571947	3.312039	1.89431	Ups	0.00093	0.00591	cellular_cc	_
ENSG000	EMC6	854	2403	43.65	2241	42.46	2413	44.09	1247	23.56	913	16	1479	25.34	1820.546	0.224299	-4.96006	-1.1125	Down	7.1E-07	1.1E-05	cellular_cc	_
ENSG000	EMG1	33113	5332	22.55	4975	21.94	5622	23.91	2427	10.67	1570	6.41	2726	10.87	3883.052	0.244136	-5.62925	-1.3743	Down	1.8E-08	4E-07	molecular	K14568

ENSG000	EMP1	20059	2839	8.04	3186	9.42	2954	8.42	1147	3.38	1509	4.13	1320	3.53	2205.083	0.125093	-11.0799	**-1.386**	**Down**	**1.6E-28**	**2.6E-26**	cellular_cc	_
ENSG000	EMR2	46149	416	0.96	381	0.92	382	0.89	50	0.12	62	0.14	49	0.11	234.4534	0.195808	-15.3451	**-3.0047**	**Down**	**3.8E-53**	**2.1E-50**	molecular_	K08443
ENSG000	ENPP1	87140	469	1.27	540	1.52	477	1.3	799	2.25	2845	7.43	1332	3.4	996.8061	0.319499	4.218201	**1.34771**	**Ups**	**2.5E-05**	**0.00026**	molecular_	K01513
ENSG000	ENPP3	118972	2	0.01	0	0	1	0	11	0.05	17	0.06	9	0.03	6.134224	0.55116	3.778188	**2.08239**	**Ups**	**0.00016**	**0.00131**	molecular_	K01513
ENSG000	EOGT	38748	382	0.75	445	0.91	379	0.75	648	1.32	1877	3.55	968	1.79	732.0767	0.288394	4.109084	**1.18503**	**Ups**	**4E-05**	**0.00039**	cellular_cc	K18134
ENSG000	EOMES	6767	25	0.08	26	0.09	18	0.06	8	0.03	17	0.06	3	0.01	16.46048	0.448491	-2.81079	**-1.2606**	**Down**	**0.00494**	**0.02318**	biological_	K10173
ENSG000	EPB42	115059	5	0.02	8	0.03	16	0.05	45	0.16	35	0.11	56	0.18	26.21681	0.411113	4.180278	**1.71857**	**Ups**	**2.9E-05**	**0.0003**	biological_	_
ENSG000	EPHA1	18604	6	0.02	4	0.02	3	0.01	28	0.11	15	0.05	20	0.07	12.21861	0.474572	3.444242	**1.63454**	**Ups**	**0.00057**	**0.00395**	biological_	K05102
ENSG000	EPHA10	51254	56	0.13	46	0.11	37	0.09	8	0.02	11	0.03	19	0.04	30.46265	0.3644	-5.11276	**-1.8631**	**Down**	**3.2E-07**	**5.4E-06**	molecular_	K08897
ENSG000	EPHB2	204487	85	0.26	73	0.23	78	0.24	4	0.01	11	0.03	8	0.02	45.42124	0.347569	-9.26366	**-3.2198**	**Down**	**2E-20**	**1.9E-18**	biological_	K05111
ENSG000	EPHX4	33555	202	2.13	190	2.09	159	1.69	53	0.58	87	0.88	68	0.68	129.44	0.221928	-7.10035	**-1.5758**	**Down**	**1.2E-12**	**5.5E-11**	cellular_cc	K12581
ENSG000	EPS8	262172	1091	2.63	1294	3.25	941	2.28	409	1.02	949	2.2	473	1.07	865.3515	0.266188	-3.962	**-1.0546**	**Down**	**7.4E-05**	**0.00068**	molecular_	K17277
ENSG000	EPSTI1	105884	0	0	0	0	1	0	2	0.01	22	0.07	6	0.02	4.474282	0.583346	3.188477	**1.85999**	**Ups**	**0.00143**	**0.00847**	_	_
ENSG000	ERBB4	1163120	18	0.03	18	0.03	22	0.04	101	0.19	273	0.47	124	0.21	84.20668	0.354166	7.180497	**2.54309**	**Ups**	**7E-13**	**3.1E-11**	biological_	K05085
ENSG000	EREG	23609	72	0.29	146	0.61	45	0.18	1	0	2	0.01	2	0.01	47.61364	0.483435	-8.93372	**-4.3189**	**Down**	**4.1E-19**	**3.5E-17**	biological_	K09784
ENSG000	ERG	281756	26	0.05	31	0.06	23	0.04	74	0.14	89	0.16	58	0.1	48.05343	0.297593	3.890883	**1.1579**	**Ups**	**1E-04**	**0.00088**	molecular_	K09435
ENSG000	ERO1LB	66465	96	0.38	113	0.46	86	0.34	89	0.36	583	2.21	227	0.84	182.7551	0.41814	2.660159	**1.11232**	**Ups**	**0.00781**	**0.0332**	molecular_	K10976
ENSG000	ERP27	25048	7	0.06	15	0.14	11	0.1	1	0.01	3	0.03	4	0.03	7.084371	0.522825	-3.09423	**-1.6177**	**Down**	**0.00197**	**0.01107**	cellular_cc	_
ENSG000	ERRFI1	21905	449	1.72	442	1.76	419	1.61	1290	5.13	2273	8.38	1598	5.76	1010.705	0.181152	9.353185	**1.69435**	**Ups**	**8.5E-21**	**8.4E-19**	;molecular	_
ENSG000	ERVMER6	2635	3	0.03	0	0	0	0	5	0.06	7	0.07	11	0.11	4.00924	0.576403	2.537853	**1.46283**	**Ups**	**0.01115**	**0.04371**	_	_
ENSG000	ESR1	472929	1	0	2	0	1	0	3	0	22	0.03	5	0.01	5.029672	0.574848	2.52206	**1.4498**	**Ups**	**0.01167**	**0.04522**	molecular_	K08550
ENSG000	ESR2	253881	12	0.02	6	0.01	14	0.03	34	0.07	30	0.05	37	0.07	21.27657	0.387118	3.260639	**1.26225**	**Ups**	**0.00111**	**0.00687**	biological_	K08551
ENSG000	ETNPPL	21015	457	2.08	648	3.07	509	2.33	2111	9.98	1943	8.52	2295	9.83	1265.864	0.202024	8.465116	**1.71016**	**Ups**	**2.6E-17**	**1.9E-15**	cellular_cc	K14286
ENSG000	ETV4	51777	1103	3.56	752	2.53	847	2.75	121	0.41	290	0.9	236	0.72	576.8046	0.260137	-8.42309	**-2.1912**	**Down**	**3.7E-17**	**2.7E-15**	molecular_	K15592
ENSG000	EVA1A	100421	1427	5.33	1563	6.09	1598	6	716	2.78	514	1.85	704	2.48	1118.171	0.21868	-6.36876	**-1.3927**	**Down**	**1.9E-10**	**6E-09**	cellular_cc	_
ENSG000	EVA1C	103394	1728	5.28	1491	4.75	1776	5.45	764	2.43	675	1.99	889	2.56	1248.361	0.188748	-6.75561	**-1.2751**	**Down**	**1.4E-11**	**5.4E-10**	cellular_cc	_
ENSG000	EVI2B	10347	24	0.17	19	0.14	12	0.08	1	0.01	3	0.02	0	0	10.35194	0.522681	-5.18958	**-2.7125**	**Down**	**2.1E-07**	**3.8E-06**	cellular_cc	K16854
ENSG000	EVPLL	11986	82	0.79	72	0.72	75	0.72	0	0	0	0	1	0.01	40.91122	0.480397	-10.5981	**-5.0913**	**Down**	**3E-26**	**4.6E-24**	_	_
ENSG000	EXPH5	88308	83	0.06	124	0.09	103	0.07	273	0.2	427	0.29	315	0.21	208.867	0.225994	6.271108	**1.41723**	**Ups**	**3.6E-10**	**1.1E-08**	biological_	_
ENSG000	EYS	1987243	21	0.02	12	0.01	9	0.01	22	0.03	59	0.06	44	0.05	26.02259	0.400792	2.848946	**1.14183**	**Ups**	**0.00439**	**0.02112**	molecular_	K19601
ENSG000	F8	191153	73	0.15	82	0.18	68	0.14	211	0.46	323	0.66	237	0.47	156.4615	0.213304	7.045665	**1.50287**	**Ups**	**1.9E-12**	**8E-11**	biological_	K03899
ENSG000	FA2H	61877	278	1.61	247	1.49	272	1.58	29	0.17	30	0.17	44	0.24	157.6761	0.237636	-12.7631	**-3.033**	**Down**	**2.6E-37**	**7E-35**	biological_	K19703
ENSG000	FABP5	4415	866	8.45	715	7.28	793	7.79	387	3.93	273	2.57	466	4.29	597.0907	0.245037	-4.96787	**-1.2173**	**Down**	**6.8E-07**	**1.1E-05**	molecular_	K08754
ENSG000	FABP5P7	410	276	8.73	252	8.31	282	8.97	156	5.13	95	2.9	168	5.01	209.5652	0.271908	-3.97437	**-1.0807**	**Down**	**7.1E-05**	**0.00065**	_	K08754
ENSG000	FABP6	51369	15	0.21	5	0.07	12	0.17	56	0.81	41	0.55	86	1.13	33.94193	0.409785	4.806813	**1.96976**	**Ups**	**1.5E-06**	**2.2E-05**	molecular_	K08755
ENSG000	FAM105A	33233	627	3.82	690	4.38	653	4	1393	8.83	2194	12.9	1843	10.59	1172.095	0.154386	7.802891	**1.20466**	**Ups**	**6.1E-15**	**3.5E-13**	_	_
ENSG000	FAM106A	2281	3	0.03	6	0.07	4	0.05	4	0.05	77	0.84	9	0.1	15.02917	0.566027	2.63719	**1.49272**	**Ups**	**0.00836**	**0.03506**	_	K14993
ENSG000	FAM109B	5191	46	0.42	42	0.4	51	0.47	8	0.08	2	0.02	6	0.05	27.32256	0.405315	-6.96771	**-2.8241**	**Down**	**3.2E-12**	**1.4E-10**	biological_	K01204
ENSG000	FAM110C	8057	183	0.98	154	0.86	155	0.84	313	1.75	549	2.85	357	1.81	271.209	0.213907	4.829093	**1.03297**	**Ups**	**1.4E-06**	**2E-05**	cellular_cc	_
ENSG000	FAM115A	50824	374	1.02	484	1.38	329	0.91	689	1.96	1509	3.99	967	2.5	684.5957	0.250586	4.414965	**1.10633**	**Ups**	**1E-05**	**0.00012**	_	_
ENSG000	FAM117A	78849	6	0.02	11	0.05	8	0.03	24	0.1	30	0.12	28	0.11	16.96477	0.396074	3.250686	**1.28751**	**Ups**	**0.00115**	**0.00707**	_	_
ENSG000	FAM124A	61875	158	0.61	109	0.44	126	0.49	390	1.55	964	3.56	537	1.94	351.3582	0.281032	6.712253	**1.88636**	**Ups**	**1.9E-11**	**7.1E-10**	_	K17675
ENSG000	FAM126A	72872	710	1.2	842	1.49	572	0.98	2719	4.8	8179	13.38	4435	7.09	2646.444	0.292728	8.233016	**2.41003**	**Ups**	**1.8E-16**	**1.2E-14**	molecular_	_
ENSG000	FAM126B	97954	165	0.24	269	0.41	132	0.19	259	0.39	1139	1.61	505	0.7	379.1757	0.385491	3.326511	**1.28234**	**Ups**	**0.00088**	**0.00565**	cellular_cc	K07560
ENSG000	FAM129B	73651	13953	43.5	10830	35.22	12561	39.39	6360	20.62	5482	16.49	7127	20.96	9572.039	0.189928	-6.05821	**-1.1506**	**Down**	**1.4E-09**	**3.8E-08**	molecular_	_
ENSG000	FAM133A	38262	50	0.2	83	0.34	42	0.17	16	0.07	18	0.07	30	0.11	40.94823	0.363191	-4.10779	**-1.4919**	**Down**	**4E-05**	**0.00039**	_	_
ENSG000	FAM13C	117050	15	0.02	7	0.01	6	0.01	54	0.09	84	0.13	60	0.09	34.92282	0.365968	6.276287	**2.29692**	**Ups**	**3.5E-10**	**1.1E-08**	_	_
ENSG000	FAM160B	78089	385	1.39	652	2.46	367	1.34	587	2.21	2446	8.54	949	3.24	832.9737	0.373958	2.881292	**1.07748**	**Ups**	**0.00396**	**0.01944**	_	_
ENSG000	FAM169A	89378	182	0.37	309	0.66	133	0.27	535	1.13	2118	4.17	725	1.39	605.7638	0.393531	4.754396	**1.871**	**Ups**	**2E-06**	**2.8E-05**	cellular_cc	_
ENSG000	FAM171A	159420	47	0.27	50	0.29	60	0.34	0	0	6	0.03	1	0.01	28.97707	0.449228	-8.31095	**-3.7335**	**Down**	**9.5E-17**	**6.7E-15**	cellular_cc	_
ENSG000	FAM171B	71988	35	0.15	57	0.26	33	0.15	57	0.26	256	1.09	131	0.55	87.2594	0.398828	3.353338	**1.33741**	**Ups**	**0.0008**	**0.00521**	molecular_	_
ENSG000	FAM179A	104763	58	0.07	58	0.07	94	0.11	384	0.47	420	0.48	485	0.54	234.7656	0.244988	9.327953	**2.28523**	**Ups**	**1.1E-20**	**1.1E-18**	_	_
ENSG000	FAM184A	189625	14	0.03	11	0.03	14	0.03	52	0.13	85	0.2	60	0.14	36.70114	0.329591	5.811696	**1.91548**	**Ups**	**6.2E-09**	**1.5E-07**	molecular_	_
ENSG000	FAM198B	48845	0	0	0	0	2	0	4	0.01	21	0.03	8	0.01	5.161556	0.577096	3.28636	**1.89655**	**Ups**	**0.00101**	**0.00639**	cellular_cc	_
ENSG000	FAM19A2	570892	14	0.02	12	0.02	12	0.02	577	0.98	235	0.37	568	0.88	224.1747	0.383821	11.3435	**4.35387**	**Ups**	**8E-30**	**1.5E-27**	cellular_cc	_
ENSG000	FAM19A5	361453	5	0.02	11	0.05	7	0.03	0	0	2	0.01	0	0	4.384584	0.574348	-3.4352	**-1.973**	**Down**	**0.00059**	**0.00406**	cellular_cc	_
ENSG000	FAM207A	36980	3093	35.96	2542	30.82	3148	36.81	1539	18.61	693	7.77	1635	17.93	2170.969	0.311732	-4.04136	**-1.2598**	**Down**	**5.3E-05**	**0.00051**	_	_
ENSG000	FAM207B	505	88	4.48	85	4.51	96	4.91	56	2.96	27	1.33	47	2.26	68.3868	0.321919	-3.52712	**-1.1354**	**Down**	**0.00042**	**0.00304**	_	_
ENSG000	FAM212B	75195	110	0.22	138	0.29	148	0.3	235	0.49	444	0.85	336	0.63	223.1674	0.233704	4.560275	**1.06576**	**Ups**	**5.1E-06**	**6.5E-05**	_	K09228
ENSG000	FAM221A	23120	0	0	0	0	0	0	4	0.01	14	0.04	8	0.02	3.831239	0.582766	3.84163	**2.23877**	**Ups**	**0.00012**	**0.00104**	_	_
ENSG000	FAM222A	56280	81	0.32	96	0.4	125	0.5	50	0.21	39	0.15	42	0.16	74.24044	0.289443	-4.56078	**-1.3201**	**Down**	**5.1E-06**	**6.5E-05**	_	_
ENSG000	FAM222A	39343	46	0.61	55	0.76	43	0.57	6	0.08	8	0.1	17	0.21	30.34854	0.375194	-5.7306	**-2.1501**	**Down**	**1E-08**	**2.3E-07**	_	_
ENSG000	FAM25A	4425	34	2.53	26	2.02	20	1.5	1	0.08	0	0	0	0	14.38144	0.527496	-7.05359	**-3.7207**	**Down**	**1.7E-12**	**7.6E-11**	_	_
ENSG000	FAM26D	29858	1	0.01	1	0.01	2	0.01	2	0.01	16	0.09	13	0.07	5.229351	0.569116	2.719256	**1.54757**	**Ups**	**0.00654**	**0.02893**	biological_	_
ENSG000	FAM26E	13147	4	0.01	7	0.02	9	0.02	5	0.01	75	0.19	45	0.11	21.58982	0.52931	3.102622	**1.64225**	**Ups**	**0.00192**	**0.0108**	biological_	_
ENSG000	FAM3B	53220	0	0	1	0.01	3	0.02	26	0.17	3	0.02	17	0.1	8.174293	0.573474	3.218305	**1.84561**	**Ups**	**0.00129**	**0.00777**	molecular_	_
ENSG000	FAM43A	3141	121	0.99	97	0.83	91	0.75	322	2.74	374	2.95	333	2.57	212.3206	0.191857	7.686757	**1.47476**	**Ups**	**1.5E-14**	**8.4E-13**	_	_
ENSG000	FAM50B	1932	59	0.43	32	0.24	43	0.31	144	1.08	69	0.48	149	1.01	80.58367	0.363098	3.069392	**1.11449**	**Ups**	**0.00214**	**0.01183**	cellular_cc	K13119
ENSG000	FAM57B	28552	2	0.01	2	0.01	3	0.01	27	0.1	32	0.11	36	0.12	15.74764	0.463904	5.939211	**2.75522**	**Ups**	**2.9E-09**	**7.4E-08**	cellular_cc	K18034
ENSG000	FAM63B	90709	200	0.28	269	0.39	182	0.25	352	0.51	1842	2.45	654	0.85	530.1181	0.3931	4.06096	**1.59636**	**Ups**	**4.9E-05**	**0.00047**	molecular_	K12581
ENSG000	FAM66C	35943	7	0.03	4	0.02	2	0.01	1	0	0	0	0	0	2.473497	0.582604	-2.59196	**-1.5101**	**Down**	**0.00954**	**0.03886**	_	_
ENSG000	FAM71F2	14588	9	0.04	12	0.06	7	0.03	22	0.11	48	0.23	27	0.12	19.48617	0.404456	3.284332	**1.32837**	**Ups**	**0.00102**	**0.00643**	_	K09131
ENSG000	FAM72C	20800	10	0.17	35	0.6	12	0.2	62	1.07	55	0.88	96	1.5	42.92065	0.417828	3.417794	**1.42805**	**Ups**	**0.00063**	**0.00428**	_	_
ENSG000	FAM78A	18472	582	2.39	527	2.26	525	2.17	341	1.46	260	1.03	307	1.19	431.8026	0.211301	-4.78882	**-1.0119**	**Down**	**1.7E-06**	**2.4E-05**	_	K12581
ENSG000	FAM83A	31115	826	1.71	710	1.53	759	1.58	43	0.09	25	0.05	23	0.04	422.8243	0.266467	-17.1796	**-4.5778**	**Down**	**3.8E-66**	**3.3E-63**	_	K09228
ENSG000	FAM83A-	1572	9	0.12	8	0.11	5	0.07	0	0	0	0	0	0	3.908441	0.581453	-4.14432	**-2.4097**	**Down**	**3.4E-05**	**0.00034**	_	_
ENSG000	FAM83H	9869	6415	17.8	5381	15.57	5713	15.94	2433	7.02	2567	6.87	2258	5.91	4230.335	0.146349	-9.97498	**-1.4598**	**Down**	**2E-23**	**2.4E-21**	biological_	K19603
ENSG000	FAM83H-	12198	116	0.35	121	0.38	94	0.29	60	0.19	62	0.18	55	0.16	86.11168	0.238314	-4.49063	**-1.0702**	**Down**	**7.1E-06**	**8.7E-05**	_	_
ENSG000	FAM86B2	11003	58	0.6	39	0.42	49	0.51	22	0.24	17	0.17	22	0.22	35.4183	0.325462	-4.12591	**-1.3428**	**Down**	**3.7E-05**	**0.00037**	_	K12354
ENSG000	FAM86C1	13727	693	2.26	566	1.93	786	2.58	356	1.21	410	1.29	393	1.21	541.5498	0.180461	-5.62065	**-1.0143**	**Down**	**1.9E-08**	**4.2E-07**	_	K03842
ENSG000	FAM92A1	32967	57	0.12	153	0.35	54	0.12	201	0.45	357	0.75	331	0.68	180.9731	0.361705	3.717092	**1.34449**	**Ups**	**0.0002**	**0.00162**	_	_
ENSG000	FAP	74468	555	1.46	660	1.81	505	1.34	7	0.02	11	0.03	14	0.03	311.1886	0.287688	-19.3146	**-5.5566**	**Down**	**4.1E-83**	**5.5E-80**	molecular_	K08674
ENSG000	FAR2	191878	38	0.13	33	0.12	38	0.13	85	0.3	119	0.38	90	0.28	64.07559	0.253776	4.50623	**1.14357**	**Ups**	**6.6E-06**	**8.2E-05**	cellular_cc	K13356
ENSG000	FARP1	307212	1215	0.84	1003	0.72	1151	0.8	2605	1.87	3338	2.22	2624	1.71	1905.619	0.144627	7.642119	**1.10526**	**Ups**	**2.1E-14**	**1.2E-12**	molecular_	K17985
ENSG000	FBLN7	49830	277	0.69	237	0.62	228	0.57	2014	5.21	1846	4.43	2026	4.76	1039.488	0.179022	15.1574	**2.71351**	**Ups**	**6.8E-52**	**3.5E-49**	molecular_	K17342
ENSG000	FBRSL1	95638	2152	4.83	1600	3.75	1979	4.47	1207	2.82	824	1.78	1118	2.37	1510.066	0.235039	-4.30724	**-1.0124**	**Down**	**1.7E-05**	**0.00018**	_	_
ENSG000	FBXO25	64798	31	0.11	31	0.11	33	0.12	0	0	1	0	0	0	17.06353	0.515729	-7.75047	**-3.9971**	**Down**	**9.2E-15**	**5.2E-13**	cellular_cc	K10305
ENSG000	FBXO30	21252	767	2.19	1244	3.7	663	1.9	2329	6.91	6766	18.63	3226	8.68	2300.198	0.321224	5.529248	**1.77613**	**Ups**	**3.2E-08**	**6.8E-07**	molecular_	K10307
ENSG000	FBXO32	43318	20	0.05	20	0.06	28	0.08	93	0.26	201	0.53	148	0.38	78.24996	0.308665	7.330835	**2.26277**	**Ups**	**2.3E-13**	**1.1E-11**	cellular_cc	K10305
ENSG000	FBXO43	12441	57	0.27	54	0.27	51	0.25	176	0.88	313	1.45	210	0.95	134.1029	0.240455	7.429465	**1.78645**	**Ups**	**1.1E-13**	**5.4E-12**	cellular_cc	K10318
ENSG000	FCHO2	134542	210	0.54	433	1.16	137	0.35	456	1.22	1717	4.25	890	2.15	589.2231	0.399762	3.664736	**1.46502**	**Ups**	**0.00025**	**0.00193**	molecular_	K12581
ENSG000	FCN2	7271	3	0.04	0	0	3	0.04	81	1.04	52	0.62	64	0.75	31.85479	0.462719	8.027265	**3.71436**	**Ups**	**1E-15**	**6.3E-14**	biological_	K10104
ENSG000	FER1L4	48978	22	0.04	17	0.03	30	0.05	201	0.35	253	0.41	258	0.41	120.7807	0.260947	11.39141	**2.97255**	**Ups**	**4.6E-30**	**8.6E-28**	cellular_cc	K09537
ENSG000	FES	12082	14	0.04	11	0.03	13	0.04	34	0.1	25	0.07	44	0.12	22.70107	0.380385	2.841738	**1.08095**	**Ups**	**0.00449**	**0.02148**	molecular_	K07527
ENSG000	FGD2	23425	0	0	1	0	0	0	5	0.01	3	0	8	0.01	2.659187	0.583356	2.613697	**1.52472**	**Ups**	**0.00896**	**0.03695**	cellular_cc	K05721
ENSG000	FGF1	105875	5	0.01	3	0.01	4	0.01	19	0.05	33	0.08	21	0.05	13.13641	0.453002	4.215671	**1.90971**	**Ups**	**2.5E-05**	**0.00026**	biological_	K18496
ENSG000	FGF12	628370	69	0.14	68	0.14	92	0.19	204	0.43	541	1.06	310	0.59	197.541	0.300811	5.992347	**1.80256**	**Ups**	**2.1E-09**	**5.6E-08**	molecular_	_
ENSG000	FGF20	10013	1	0.01	2	0.03	1	0.01	6	0.08	18	0.21	11	0.13	5.894569	0.549527	3.311896	**1.81998**	**Ups**	**0.00093**	**0.00592**	molecular_	K04358
ENSG000	FGF21	2772	4	0.05	4	0.05	4	0.05	47	0.6	5	0.06	24	0.28	14.542	0.542072	3.124171	**1.69353**	**Ups**	**0.00178**	**0.01015**	molecular_	K04358
ENSG000	FGFBP1	3172	1068	10.82	1241	13.11	1173	11.95	5636	59.37	1758	17.18	4641	44.33	2533.498	0.356007	4.087066	**1.45503**	**Ups**	**4.4E-05**	**0.00043**	molecular_	_
ENSG000	FGFR2	120125	14	0.02	9	0.01	13	0.02	44	0.06	89	0.11	70	0.08	36.83943	0.348975	5.80114	**2.02446**	**Ups**	**6.6E-09**	**1.6E-07**	biological_	K05093
ENSG000	FGL1	45986	9	0.04	7	0.03	8	0.04	0	0	0	0	1	0	4.420525	0.573723	-3.99846	**-2.294**	**Down**	**6.4E-05**	**0.00059**	cellular_cc	_
ENSG000	FHDC1	43345	150	0.32	149	0.33	141	0.3	512	1.12	849	1.73	560	1.11	368.3996	0.200134	9.15248	**1.83172**	**Ups**	**5.6E-20**	**5.2E-18**	_	K10408
ENSG000	FHOD3	482342	7	0.02	11	0.03	3	0.01	18	0.05	35	0.08	32	0.07	16.49674	0.434609	3.394114	**1.47511**	**Ups**	**0.00069**	**0.00462**	molecular_	_
ENSG000	FIGF	38786	2	0.02	3	0.03	1	0.01	9	0.09	29	0.27	17	0.16	9.191252	0.521046	3.943577	**2.05479**	**Ups**	**8E-05**	**0.00072**	biological_	K05449
ENSG000	FIGN	142617	207	0.5	239	0.6	171	0.41	380	0.95	1400	3.24	561	1.27	453.6106	0.346804	4.282292	**1.48511**	**Ups**	**1.9E-05**	**0.0002**	molecular_	_
ENSG000	FKBP9L	32179	36	0.23	33	0.22	36	0.23	17	0.11	18	0.11	12	0.07	25.92678	0.341273	-3.65343	**-1.2468**	**Down**	**0.00026**	**0.002**	molecular_	K09575
ENSG000	FKSG48	375	1	0.07	1	0.07	0	0	9	0.64	4	0.26	10	0.65	3.962878	0.57692	2.959814	**1.70758**	**Ups**	**0.00308**	**0.01594**	_	_
ENSG000	FLNC	28898	59	0.14	71	0.17	69	0.16	17	0.04	59	0.13	21	0.05	49.73953	0.372115	-3.0976	**-1.1527**	**Down**	**0.00195**	**0.01096**	molecular_	K04437
ENSG000	FLRT2	98547	22	0.04	34	0.06	33	0.06	286	0.53	834	1.44	366	0.62	235.7358	0.346389	9.857891	**3.41467**	**Ups**	**6.3E-23**	**7.2E-21**	molecular_	K16362
ENSG000	FLRT3	14629	277	0.76	370	1.06	227	0.63	58	0.17	277	0.73	120	0.31	222.0634	0.381021	-2.82656	**-1.077**	**Down**	**0.00471**	**0.02227**	molecular_	_
ENSG000	FLT3LG	12025	4	0.02	1	0.01	4	0.02	59	0.34	18	0.1	41	0.22	20.4292	0.503492	5.139424	**2.58766**	**Ups**	**2.8E-07**	**4.8E-06**	molecular_	K05454
ENSG000	FLVCR2	84598	175	0.59	178	0.63	186	0.64	65	0.23	131	0.43	107	0.34	141.4641	0.231638	-4.44567	**-1.0298**	**Down**	**8.8E-06**	**0.0001**	cellular_cc	K08220
ENSG000	FMO3	26942	0	0	0	0	0	0	5	0.02	35	0.13	12	0.04	7.51367	0.57798	4.723497	**2.73008**	**Ups**	**2.3E-06**	**3.2E-05**	molecular_	K00485
ENSG000	FMR1NB	45339	0	0	0	0	0	0	4	0.08	10	0.19	13	0.24	4.028485	0.582261	3.937946	**2.29291**	**Ups**	**8.2E-05**	**0.00074**	cellular_cc	_
ENSG000	FN1	75733	905	0.76	1033	0.9	1076	0.91	2081	1.81	6110	4.93	2692	2.12	2147.108	0.295096	4.996168	**1.47435**	**Ups**	**5.9E-07**	**9.4E-06**	biological_	K05717
ENSG000	FOLH1	62036	15	0.05	24	0.09	15	0.05	5	0.02	2	0.01	2	0.01	11.06768	0.488932	-4.31541	**-2.1099**	**Down**	**1.6E-05**	**0.00018**	molecular_	K14592
ENSG000	FOS	3457	270	0.76	248	0.73	207	0.59	85651	251.68	43546	118.69	76857	204.79	32481.38	0.28829	25.81627	**7.44258**	**Ups**	**6E-147**	**2E-143**	biological_	K04379
ENSG000	FOSB	7185	21	0.03	31	0.05	29	0.05	16787	27.47	4781	7.26	11166	16.57	5249.695	0.387266	18.98489	**7.35221**	**Ups**	**2.3E-80**	**2.9E-77**	biological_	K09029
ENSG000	FOSL1	8525	653	3.26	471	2.45	583	2.93	338	1.75	155	0.75	321	1.51	431.6792	0.315847	-3.63727	**-1.1488**	**Down**	**0.00028**	**0.00211**	molecular_	K04502
ENSG000	FOXF1	3944	473	4.73	389	4.06	374	3.77	989	10.3	1022	9.87	982	9.27	679.5252	0.166537	6.253271	**1.0414**	**Ups**	**4E-10**	**1.2E-08**	biological_	K09399
ENSG000	FOXN1	32654	82	0.45	83	0.48	76	0.42	2	0.01	3	0.02	4	0.02	44.27404	0.401582	-10.3913	**-4.1729**	**Down**	**2.7E-25**	**3.7E-23**	biological_	K09407
ENSG000	FOXN2	64658	187	0.85	302	1.44	158	0.73	417	1.98	1480	6.53	558	2.41	476.0717	0.367654	3.976615	**1.46202**	**Ups**	**7E-05**	**0.00064**	molecular_	K09407
ENSG000	FOXP2	607446	66	0.05	82	0.07	48	0.04	862	0.73	975	0.77	716	0.55	426.1955	0.238414	13.98239	**3.3336**	**Ups**	**2E-44**	**8E-42**	biological_	K09409
ENSG000	FPGS	19731	5261	12.24	4011	9.73	4578	10.71	2926	7.08	1505	3.38	2720	5.97	3585.772	0.281273	-3.78657	**-1.0651**	**Down**	**0.00015**	**0.00127**	biological_	K01930
ENSG000	FRAT1	2651	127	0.99	122	1	121	0.95	429	3.49	193	1.46	339	2.5	217.5425	0.321151	3.430725	**1.10178**	**Ups**	**0.0006**	**0.00411**	biological_	_
ENSG000	FREM2	198809	1	0	1	0	0	0	17	0.03	46	0.08	21	0.03	12.81331	0.533505	5.886427	**3.14044**	**Ups**	**4E-09**	**1E-07**	biological_	_
ENSG000	FRK	129610	27	0.05	54	0.11	20	0.04	70	0.14	108	0.2	90	0.16	58.55929	0.343873	3.083429	**1.06031**	**Ups**	**0.00205**	**0.01138**	molecular_	K08892
ENSG000	FRMD3	295557	186	0.61	270	0.93	155	0.51	93	0.32	72	0.23	95	0.3	149.1358	0.28199	-4.78008	**-1.3479**	**Down**	**1.8E-06**	**2.5E-05**	cellular_cc	_
ENSG000	FRMD7	51028	2	0.01	2	0.01	2	0.01	6	0.02	15	0.06	9	0.03	5.533614	0.539016	2.596307	**1.39945**	**Ups**	**0.00942**	**0.0385**	molecular_	_
ENSG000	FRY	265358	141	0.2	241	0.36	210	0.3	392	0.58	786	1.08	393	0.53	341.2399	0.289214	3.736889	**1.08076**	**Ups**	**0.00019**	**0.00151**	cellular_cc	_
ENSG000	FSBP	58576	3	0.02	0	0	4	0.03	23	0.17	61	0.42	32	0.21	18.47053	0.488393	5.79012	**2.82786**	**Ups**	**7E-09**	**1.7E-07**	molecular_	K10877
ENSG000	FSIP2	94663	2	0	3	0	0	0	11	0.01	21	0.02	9	0.01	7.045562	0.540082	3.420415	**1.84731**	**Ups**	**0.00063**	**0.00424**	cellular_cc	_
ENSG000	FSTL4	416109	154	0.46	154	0.48	159	0.48	5	0.02	48	0.14	25	0.07	94.4569	0.410179	-5.96744	**-2.4477**	**Down**	**2.4E-09**	**6.4E-08**	molecular_	_
ENSG000	FTH1	7943	157274	688.61	127672	583.01	160644	707.41	63216	287.89	37481	158.33	67226	277.61	105535.7	0.268208	-5.62705	**-1.5092**	**Down**	**1.8E-08**	**4E-07**	molecular_	K00522
ENSG000	FTH1P10	930	43	0.75	34	0.62	40	0.7	18	0.33	5	0.08	11	0.18	26.27234	0.408291	-4.11139	**-1.6786**	**Down**	**3.9E-05**	**0.00039**	_	K00522
ENSG000	FTH1P11	551	13	0.61	11	0.54	15	0.7	7	0.34	4	0.18	3	0.13	9.16072	0.479492	-2.73463	**-1.3112**	**Down**	**0.00624**	**0.02792**	_	K00522
ENSG000	FTH1P2	413	251	7.84	171	5.57	237	7.45	120	3.9	44	1.33	109	3.21	160.4261	0.358861	-3.60259	**-1.2928**	**Down**	**0.00032**	**0.00237**	_	K00522
ENSG000	FTH1P7	552	84	3.91	72	3.5	106	4.96	60	2.91	25	1.12	50	2.2	68.02503	0.354884	-2.8799	**-1.022**	**Down**	**0.00398**	**0.0195**	_	K00522
ENSG000	FTH1P8	514	39	1.95	36	1.88	36	1.81	22	1.14	8	0.39	13	0.61	26.61614	0.390733	-3.45747	**-1.3509**	**Down**	**0.00055**	**0.0038**	_	K00522
ENSG000	FTLP12	449	7	0.4	9	0.54	1	0.06	0	0	0	0	0	0	3.016694	0.58095	-3.2694	**-1.8994**	**Down**	**0.00108**	**0.00671**	_	_
ENSG000	FUT1	7380	15	0.07	13	0.06	10	0.05	56	0.27	31	0.14	51	0.22	28.37038	0.386673	3.768242	**1.45708**	**Ups**	**0.00016**	**0.00136**	cellular_cc	K00718
ENSG000	FYN	213121	685	1.11	577	0.98	607	0.99	6817	11.53	5804	9.1	6388	9.79	3272.131	0.18076	16.96242	**3.06613**	**Ups**	**1.6E-64**	**1.2E-61**	biological_	K05703
ENSG000	FZD10	3282	63	0.25	39	0.16	47	0.19	1	0	0	0	0	0	26.64923	0.502199	-8.93897	**-4.4891**	**Down**	**3.9E-19**	**3.3E-17**	biological_	K02842
ENSG000	FZD10-AS	11908	18	0.05	21	0.07	20	0.06	1	0	1	0	0	0	10.82726	0.525818	-6.09586	**-3.2053**	**Down**	**1.1E-09**	**3E-08**	_	_

ENSG000	FZD4	9713	221	0.77	183	0.66	214	0.75	840	3.04	1504	5.05	913	3	600.4089	0.215447	9.655997	2.08035	Ups		4.6E-22	4.9E-20	biological	K02354
ENSG000	GABRG3	561945	1	0.01	0	0	1	0.01	121	0.7	100	0.54	98	0.51	49.80094	0.471207	10.44725	4.92282	Ups		1.5E-25	2.2E-23	molecular	K05186
ENSG000	GAGE2D	7261	0	0	0	0	0	0	0	0	8	0.38	8	0.38	2.310274	0.563157	2.495077	1.40512	Ups		0.01259	0.04802	molecular	_
ENSG000	GAL	7397	6629	116.4	5034	92.19	6829	120.6	3608	65.89	1869	31.66	3213	53.21	4656.277	0.283126	-4.20891	-1.1917	Down		2.6E-05	0.00027	molecular	_
ENSG000	GAL3ST1	19953	4	0.01	0	0	2	0	100	0.2	40	0.07	96	0.17	38.27226	0.489062	7.641727	3.73728	Ups		2.1E-14	1.2E-12	cellular_cc	K01019
ENSG000	GALNT13	581936	32	0.08	39	0.1	36	0.09	0	0	1	0	4	0.01	19.814	0.473923	-7.35694	-3.4866	Down		1.9E-13	9.2E-12	cellular_cc	K00710
ENSG000	GALNT16	95190	1	0	0	0	1	0	12	0.03	9	0.02	3	0.01	4.122148	0.578805	2.932121	1.69713	Ups		0.00337	0.01708	cellular_cc	K00710
ENSG000	GALNT5	56614	16	0.06	12	0.05	14	0.05	105	0.4	200	0.71	92	0.32	67.10863	0.346887	7.773371	2.69648	Ups		7.6E-15	4.4E-13	cellular_cc	K00710
ENSG000	GALNTL6	1229306	486	1.15	554	1.37	410	0.98	1330	3.28	1445	3.31	1295	2.9	882.2939	0.172309	7.224354	1.24482	Ups		5E-13	2.3E-11	cellular_cc	K00710
ENSG000	GALR2	2748	58	1.09	46	0.9	40	0.76	28	0.55	14	0.25	21	0.37	35.51859	0.353643	-3.51215	-1.242	Down		0.00044	0.00319	molecular	K04231
ENSG000	GAS1	2826	903	8.21	1049	9.95	749	6.85	3920	37.09	1892	16.6	2770	23.76	1835.257	0.298774	4.646259	1.38818	Ups		3.4E-06	4.5E-05	biological	K06232
ENSG000	GAS2L3	54604	233	0.53	320	0.76	196	0.45	386	0.91	1414	3.09	642	1.37	492.3244	0.346413	3.720122	1.2887	Ups		0.0002	0.0016	biological	K14404
ENSG000	GATA3	21595	5	0.02	8	0.04	5	0.02	106	0.5	118	0.52	109	0.47	54.21441	0.352176	10.08958	3.55331	Ups		6.1E-24	7.9E-22	cellular_cc	K17895
ENSG000	GBP2	44325	156	0.55	201	0.74	229	0.82	70	0.26	62	0.21	67	0.22	135.3131	0.250341	-6.73326	-1.6856	Down		1.7E-11	6.3E-10	cellular_cc	_
ENSG000	GBP3	16229	25	0.06	29	0.07	14	0.03	2	0.01	15	0.03	3	0.01	14.9649	0.495846	-3.11997	-1.547	Down		0.00181	0.01027	molecular	_
ENSG000	GCA	52756	134	0.48	140	0.52	124	0.45	232	0.87	496	1.72	398	1.35	239.3947	0.240492	4.972111	1.19575	Ups		6.6E-07	1.1E-05	molecular	_
ENSG000	GCGR	9882	42	0.21	37	0.2	43	0.22	140	0.74	91	0.44	142	0.68	79.8139	0.299416	4.390158	1.31448	Ups		1.1E-05	0.00013	molecular	K04583
ENSG000	GCNT3	45365	302	0.88	298	0.91	319	0.94	3	0.01	21	0.06	20	0.06	170.1492	0.322242	-13.143	-4.2352	Down		1.9E-39	5.5E-37	cellular_cc	_
ENSG000	GCOM1	122838	40	0.11	66	0.18	15	0.04	7	0.02	31	0.08	6	0.01	27.92696	0.501945	-2.60306	-1.3066	Down		0.00924	0.03787	_	_
ENSG000	GDF3	5995	0	0	1	0.02	0	0	16	0.35	1	0.02	15	0.29	5.331084	0.583434	3.272859	1.9095	Ups		0.00106	0.00664	biological	K05495
ENSG000	GDPD3	9047	103	0.94	83	0.79	119	1.1	64	0.61	30	0.27	42	0.36	75.84205	0.337926	-3.61846	-1.2228	Down		0.0003	0.00225	biological	_
ENSG000	GEM	13098	46	0.21	38	0.18	30	0.14	108	0.51	168	0.74	107	0.46	78.23581	0.272611	5.241997	1.42903	Ups		1.6E-07	2.9E-06	molecular	K07846
ENSG000	GET4	19885	938	3.08	693	2.37	691	2.28	525	1.79	334	1.06	332	1.03	599.4528	0.272918	-3.95608	-1.0797	Down		7.6E-05	0.00069	cellular_cc	_
ENSG000	GFI1	12115	25	0.13	22	0.12	16	0.09	55	0.31	61	0.32	60	0.3	38.14866	0.302902	3.868237	1.17169	Ups		0.00011	0.00095	biological	K09223
ENSG000	GFPT2	52698	186	0.78	190	0.84	182	0.77	50	0.22	87	0.35	77	0.31	131.6261	0.216403	-7.20837	-1.5599	Down		5.7E-13	2.6E-11	molecular	K00820
ENSG000	GGACT	57973	29	0.2	45	0.32	24	0.17	115	0.82	106	0.7	123	0.79	70.45993	0.294278	5.063703	1.49013	Ups		4.1E-07	6.8E-06	molecular	K19761
ENSG000	GGT5	25489	300	1.43	199	0.99	282	1.35	5	0.02	1	0	4	0.02	140.5566	0.38136	-14.5397	-5.5449	Down		6.8E-48	3.1E-45	molecular	K18592
ENSG000	GGT7	28141	39	0.27	40	0.29	46	0.32	277	1.98	155	1.03	209	1.35	122.9284	0.313666	6.380977	2.00149	Ups		1.8E-10	5.6E-09	molecular	K00681
ENSG000	GJA3	22795	8	0.04	12	0.06	6	0.03	0	0	0	0	0	0	4.630786	0.578801	-4.46827	-2.5862	Down		7.9E-06	9.6E-05	molecular	K07612
ENSG000	GJB3	5181	854	5.47	726	4.85	769	4.95	62	0.41	126	0.78	102	0.62	461.5273	0.209657	-15.0218	-3.1494	Down		5.3E-51	2.7E-48	biological	K07622
ENSG000	GJB4	3984	3	0.03	10	0.09	2	0.02	0	0	1	0.01	0	0	2.815578	0.582475	-2.73741	-1.5945	Down		0.00619	0.02772	molecular	K07623
ENSG000	GJB5	3466	347	6.58	327	6.47	341	6.51	8	0.16	16	0.29	12	0.21	186.1468	0.273663	-17.3006	-4.7345	Down		4.7E-67	4.3E-64	cellular_cc	K07624
ENSG000	GKN1	6402	175	3.35	149	2.98	170	3.28	8	0.16	35	0.65	32	0.58	98.98379	0.333474	-8.11261	-2.7053	Down		5E-16	3.2E-14	molecular	_
ENSG000	GKN2	7739	26	0.34	19	0.26	15	0.19	0	0	4	0.05	0	0	11.20861	0.522858	-5.36126	-2.8032	Down		8.3E-08	1.6E-06	cellular_cc	_
ENSG000	GLB1L3	45320	19	0.03	25	0.04	31	0.05	80	0.14	60	0.1	65	0.1	45.26696	0.324105	3.522174	1.14155	Ups		0.00043	0.00309	molecular	K16467
ENSG000	GLRB	96034	192	0.94	230	1.18	169	0.83	426	2.17	595	2.82	529	2.45	340.6318	0.183779	6.159895	1.13206	Ups		7.3E-10	2.1E-08	biological	K05196
ENSG000	GLUL	10503	1258	1.54	1374	1.75	1387	1.71	4281	5.45	4346	5.13	4267	4.93	2697.738	0.144288	10.01572	1.44514	Ups		1.3E-23	1.6E-21	molecular	K01915
ENSG000	GNA15	27577	7	0.06	6	0.05	4	0.03	0	0	3	0.02	0	0	3.435672	0.579708	-2.49021	-1.4436	Down		0.01277	0.04854	molecular	K04637
ENSG000	GNAO1	166055	167	0.26	141	0.23	137	0.21	64	0.1	111	0.17	63	0.09	115.0442	0.247409	-4.38236	-1.0842	Down		1.2E-05	0.00014	molecular	K04534
ENSG000	GNAS-AS	31985	230	2.17	188	1.85	226	2.14	131	1.28	52	0.47	97	0.86	159.0201	0.33763	-3.6962	-1.2479	Down		0.00022	0.00173	_	_
ENSG000	GNAZ	54685	60	0.34	66	0.39	69	0.39	135	0.79	372	2.02	241	1.28	145.7837	0.299734	5.200006	1.55862	Ups		2E-07	3.6E-06	molecular	K04535
ENSG000	GNB4	52389	348	1.85	470	2.6	274	1.46	620	3.42	3534	18.1	915	4.58	928.4119	0.517454	2.699066	1.39664	Ups		0.00695	0.03029	molecular	K04538
ENSG000	GNMT	3123	13	0.31	26	0.65	25	0.6	46	1.15	71	1.64	60	1.36	38.22403	0.327247	3.439398	1.12553	Ups		0.00058	0.00401	molecular	K00552
ENSG000	GOLGA2P	17303	529	0.62	474	0.58	582	0.69	1273	1.55	1613	1.83	1170	1.29	901.1503	0.172326	6.421713	1.10663	Ups		1.4E-10	4.3E-09	cellular_cc	_
ENSG000	GOLGA6L	9376	9	0.02	16	0.04	14	0.03	24	0.06	38	0.09	50	0.11	23.88117	0.38129	2.959153	1.1283	Ups		0.00308	0.01596	_	K10595
ENSG000	GOLGA6L	9116	10	0.06	7	0.04	5	0.03	20	0.12	41	0.22	27	0.14	17.08972	0.416344	3.58141	1.4911	Ups		0.00034	0.00254	_	_
ENSG000	GOLGA8A	209436	1220	2.62	1240	2.77	1063	2.29	2141	4.78	4959	10.26	3147	6.37	2156.2	0.231046	5.338265	1.23338	Ups		9.4E-08	1.8E-06	cellular_cc	K10595
ENSG000	GOLGA8H	10436	1	0.01	1	0.01	1	0.01	7	0.1	10	0.13	5	0.06	3.878087	0.569037	2.660025	1.51365	Ups		0.00781	0.03321	cellular_cc	K10595
ENSG000	GOLGA8K	10414	2	0.03	5	0.07	0	0	7	0.1	44	0.58	13	0.17	10.50112	0.556602	3.258536	1.81371	Ups		0.00112	0.00691	cellular_cc	K10595
ENSG000	GP1BA	2734	14	0.14	20	0.21	10	0.1	19	0.2	99	0.98	42	0.41	31.13584	0.451666	2.849303	1.28693	Ups		0.00438	0.02111	biological	K06261
ENSG000	GPC1	32406	3873	10.52	2994	8.48	3572	9.76	707	2	1317	3.45	1182	3.03	2339.648	0.198731	-9.49838	-1.8876	Down		2.1E-21	2.2E-19	biological	K08107
ENSG000	GPLD1	70641	187	0.78	175	0.76	159	0.67	299	1.3	1575	6.36	495	1.95	436.8358	0.396066	4.141668	1.64037	Ups		3.5E-05	0.00035	biological	K01127
ENSG000	GPR1	42732	45	0.16	41	0.16	50	0.18	3	0.01	25	0.09	13	0.04	30.16372	0.419639	-4.03498	-1.6932	Down		5.5E-05	0.00052	molecular	K04310
ENSG000	GPR114	49261	10	0.02	14	0.04	10	0.03	3	0.01	5	0.01	3	0.01	7.720911	0.494502	-2.84877	-1.4087	Down		0.00439	0.02113	molecular	K08459
ENSG000	GPR115	36158	4	0.03	5	0.04	4	0.03	0	0	0	0	0	0	2.316533	0.581227	-3.17348	-1.8445	Down		0.00151	0.00883	molecular	K08457
ENSG000	GPR124	60706	85	0.21	82	0.21	73	0.18	311	0.8	299	0.71	238	0.56	173.5327	0.229872	6.731444	1.54737	Ups		1.7E-11	6.3E-10	biological	K08461
ENSG000	GPR133	187563	2	0	3	0	2	0	16	0.02	94	0.11	41	0.05	23.18895	0.510742	5.746475	2.93496	Ups		9.1E-09	2.1E-07	molecular	K08465
ENSG000	GPR137C	84566	33	0.1	79	0.25	37	0.11	107	0.33	171	0.49	146	0.41	90.67648	0.32884	3.52624	1.15957	Ups		0.00042	0.00304	cellular_cc	_
ENSG000	GPR143	60952	28	0.2	42	0.32	43	0.31	5	0.04	18	0.13	6	0.04	24.43248	0.415538	-4.52675	-1.881	Down		6E-06	7.5E-05	molecular	K08470
ENSG000	GPR155	54857	124	0.28	136	0.32	129	0.3	282	0.67	781	1.72	363	0.78	280.6736	0.301842	4.948448	1.49365	Ups		7.5E-07	1.2E-05	cellular_cc	K02927
ENSG000	GPR162	8426	11	0.04	10	0.04	4	0.01	69	0.25	23	0.08	50	0.16	27.03516	0.461884	4.053471	1.87223	Ups		5.1E-05	0.00048	molecular	K08440
ENSG000	GPR35	25829	7	0.03	4	0.02	9	0.03	34	0.13	26	0.09	30	0.11	17.53657	0.419329	3.951741	1.65708	Ups		7.8E-05	0.0007	molecular	K04276
ENSG000	GPR37	19631	424	3.61	388	3.44	382	3.27	983	8.7	2107	17.3	1412	11.33	886.0965	0.220302	7.276286	1.60298	Ups		3.4E-13	1.6E-11	molecular	K04243
ENSG000	GPR39	229986	122	0.65	108	0.6	142	0.77	1	0.01	4	0.02	2	0.01	67.31374	0.408507	-12.019	-4.9099	Down		2.8E-33	6.4E-31	molecular	K08412
ENSG000	GPR56	54381	781	0.51	527	0.36	687	0.45	426	0.29	100	0.06	235	0.14	477.4787	0.404444	-3.26353	-1.3199	Down		0.0011	0.00682	biological	K08450
ENSG000	GPR64	133329	448	0.76	499	0.88	509	0.87	12	0.02	66	0.11	24	0.04	274.3394	0.365891	-9.98133	-3.6521	Down		1.8E-23	2.2E-21	molecular	K08451
ENSG000	GPR87	22850	438	7	554	9.24	497	7.99	6	0.1	18	0.28	13	0.2	270.9468	0.286334	-18.1178	-5.1877	Down		2.3E-73	2.3E-70	cellular_cc	K08389
ENSG000	GPR97	21877	12	0.05	8	0.04	7	0.03	1	0	1	0	2	0.01	5.405012	0.549436	-3.43322	-1.8863	Down		0.0006	0.00408	molecular	K08452
ENSG000	GPR98	634878	2	0	3	0	3	0	5	0	24	0.02	10	0.01	7.117632	0.536867	2.559923	1.37434	Ups		0.01047	0.04165	cellular_cc	K18263
ENSG000	GPRC5B	28874	81	0.17	76	0.16	101	0.21	35	0.08	49	0.1	28	0.05	63.02284	0.282667	-4.74693	-1.3418	Down		2.1E-06	2.9E-05	molecular	K04619
ENSG000	GPRC5D	11373	57	0.98	56	1	84	1.45	33	0.59	34	0.56	42	0.68	51.85901	0.295838	-3.38053	-1.0001	Down		0.00072	0.00481	molecular	K04621
ENSG000	GPRIN1	14332	1126	6.87	974	6.2	992	6.09	616	3.91	321	1.89	470	2.71	771.5813	0.279364	-4.42091	-1.235	Down		9.8E-06	0.00012	cellular_cc	_
ENSG000	GPRIN3	71625	1	0	1	0	0	0	9	0.01	15	0.02	8	0.01	5.186063	0.56347	3.734245	2.10413	Ups		0.00019	0.00152	_	_
ENSG000	GPS2	4241	702	2.06	791	2.42	558	1.64	340	1.04	271	0.77	264	0.73	500.9276	0.23203	-5.90181	-1.3694	Down		3.6E-09	9.2E-08	biological	K15307
ENSG000	GPX1	1425	17301	157.04	14527	137.52	17498	159.74	7497	70.78	5240	45.89	10457	89.52	12373.04	0.259822	-4.70778	-1.2232	Down		2.5E-06	3.5E-05	biological	K00432
ENSG000	GPX1P1	606	742	31.47	650	28.76	728	31.06	240	10.59	224	9.17	388	15.52	508.1862	0.239293	-6.11393	-1.463	Down		9.7E-10	2.8E-08	_	K00432
ENSG000	GPX2	3662	5	0.04	9	0.07	6	0.05	2	0.02	2	0.01	1	0.01	4.341034	0.557432	-2.50093	-1.3941	Down		0.01239	0.0474	biological	K00432
ENSG000	GPX3	8431	2	0.01	1	0.01	3	0.02	10	0.05	14	0.07	13	0.06	6.687142	0.522852	3.215317	1.68113	Ups		0.0013	0.00783	molecular	K00432
ENSG000	GRAMD2	37979	31	0.08	30	0.08	25	0.07	212	0.59	280	0.72	251	0.63	128.367	0.232477	11.889	2.76392	Ups		1.4E-32	2.9E-30	cellular_cc	_
ENSG000	GRB14	129037	84	0.42	84	0.44	82	0.41	320	1.66	220	1.06	309	1.45	176.6173	0.258588	5.744806	1.48554	Ups		9.2E-09	2.1E-07	cellular_cc	_
ENSG000	GRIK2	671295	221	0.56	255	0.68	179	0.46	571	1.52	870	2.14	543	1.31	417.1878	0.220248	5.974407	1.31585	Ups		2.3E-09	6.1E-08	biological	K05202
ENSG000	GRIN3A	169228	4	0.01	7	0.02	4	0.01	19	0.06	43	0.13	32	0.09	16.71652	0.437932	4.511985	1.97594	Ups		6.4E-06	8E-05	molecular	K05213
ENSG000	GRIP2	52970	53	0.07	50	0.07	46	0.06	342	0.49	620	0.82	450	0.58	239.0029	0.231946	12.40965	2.87836	Ups		2.3E-35	5.8E-33	cellular_cc	_
ENSG000	GRM7-AS	173761	3	0.02	5	0.03	1	0.01	17	0.1	26	0.15	18	0.1	10.83885	0.482692	4.018677	1.93978	Ups		5.9E-05	0.00055	_	_
ENSG000	GRM8	814697	6	0.01	3	0.01	3	0.01	23	0.05	19	0.04	13	0.03	10.67932	0.479957	3.224878	1.5478	Ups		0.00126	0.00762	molecular	K04608
ENSG000	GS1-122H	184752	0	0	1	0.01	0	0	4	0.03	10	0.07	4	0.03	2.855302	0.583411	2.778787	1.62117	Ups		0.00546	0.02514	_	_
ENSG000	GSTM2	41528	16	0.04	8	0.02	13	0.04	44	0.13	50	0.13	31	0.08	25.74723	0.373156	3.644534	1.35998	Ups		0.00027	0.00206	molecular	K00799
ENSG000	GTF2IRD2	57365	63	0.15	35	0.09	48	0.11	203	0.5	175	0.4	203	0.45	115.6948	0.271064	6.216716	1.68513	Ups		5.1E-10	1.5E-08	molecular	K03121
ENSG000	GTF2IRD2	57260	110	0.22	102	0.21	93	0.19	316	0.66	268	0.52	390	0.74	204.5018	0.23505	5.977456	1.405	Ups		2.3E-09	6E-08	molecular	K14404
ENSG000	GTF2IRD2	37242	6	0.04	4	0.03	3	0.02	26	0.17	28	0.17	29	0.17	15.02682	0.435423	4.650899	2.02511	Ups		3.3E-06	4.4E-05	_	K03121
ENSG000	GTF3C6	9331	4483	108	4070	102.26	4620	111.94	2373	59.46	1839	42.74	2976	67.62	3457.291	0.21923	-4.73874	-1.0389	Down		2.2E-06	3E-05	cellular_cc	K15203
ENSG000	GUCY1B2	86352	19	0.09	21	0.1	14	0.07	58	0.29	59	0.27	61	0.27	36.95063	0.311872	4.44953	1.38768	Ups		8.6E-06	0.0001	molecular	K12319
ENSG000	GUSBP3	216302	4	0.04	6	0.06	6	0.06	31	0.33	19	0.19	14	0.14	12.93831	0.470536	3.037802	1.42939	Ups		0.00238	0.01294	_	K01195
ENSG000	GUSBP4	10519	2	0.06	2	0.06	0	0	10	0.31	11	0.32	4	0.11	4.557381	0.567109	2.523644	1.43118	Ups		0.01161	0.04508	_	K01195
ENSG000	GXYLT2	110066	26	0.16	17	0.11	19	0.12	97	0.64	309	1.88	164	0.97	95.19226	0.361917	7.219804	2.61297	Ups		5.2E-13	2.4E-11	cellular_cc	K13676
ENSG000	GYG2	54031	1	0	1	0	4	0.01	8	0.03	19	0.06	16	0.05	7.491156	0.528337	3.383661	1.78771	Ups		0.00072	0.00477	cellular_cc	K00750
ENSG000	H19	6295	379833	674.58	301852	559.11	373144	666.51	78415	144.85	59243	101.51	75555	126.56	220860.1	0.217669	-11.0921	-2.4144	Down		1.4E-28	2.3E-26	_	_
ENSG000	H1FX-AS1	10834	96	0.51	72	0.4	117	0.62	296	1.63	141	0.72	267	1.34	161.4831	0.336562	3.02905	1.01946	Ups		0.00245	0.01326	_	_
ENSG000	HACE1	131827	191	0.29	265	0.42	165	0.25	264	0.42	941	1.38	462	0.66	355.9288	0.341998	3.042766	1.04062	Ups		0.00234	0.01277	cellular_cc	K12166
ENSG000	HAPLN2	6432	2	0.01	4	0.03	0	0	17	0.11	7	0.04	12	0.07	6.771055	0.550065	2.819042	1.55066	Ups		0.00482	0.0227	molecular	K06851
ENSG000	HAPLN3	18339	25	0.11	20	0.09	20	0.09	5	0.02	0	0	9	0.04	13.77533	0.493071	-3.70339	-1.826	Down		0.00021	0.00169	molecular	K06852
ENSG000	HCLS1	29529	0	0	1	0	1	0	2	0.01	10	0.03	12	0.04	3.892836	0.580202	2.816856	1.63435	Ups		0.00485	0.02283	biological	K06106
ENSG000	HDAC9	915468	11	0.01	22	0.02	16	0.02	53	0.06	175	0.17	80	0.08	54.15315	0.398098	5.162336	2.05512	Ups		2.4E-07	4.3E-06	cellular_cc	K11409
ENSG000	HDHD1	99271	1741	8.34	1765	8.82	1856	8.94	551	2.74	1176	5.43	893	4.03	1347.426	0.211004	-5.87815	-1.2403	Down		4.2E-09	1.1E-07	molecular	K17623
ENSG000	HEATR4	80463	4	0.01	8	0.03	6	0.02	19	0.07	44	0.15	25	0.08	16.34307	0.436815	3.859884	1.68606	Ups		0.00011	0.00098	_	_
ENSG000	HECA	45691	202	0.92	226	1.08	131	0.6	620	2.95	1047	4.63	735	3.17	461.833	0.240056	7.428808	1.78333	Ups		1.1E-13	5.4E-12	molecular	K12581
ENSG000	HEPHL1	92391	1	0	5	0.03	6	0.03	56	0.28	14	0.06	32	0.15	18.55845	0.516911	4.052687	2.09488	Ups		5.1E-05	0.00048	molecular	_
ENSG000	HES1	2588	194	1.93	207	2.15	206	2.06	3425	35.4	361	3.46	2280	21.37	1091.446	0.560367	3.559736	1.99476	Ups		0.00037	0.00273	biological	K06054
ENSG000	HEY2	13606	29	0.27	28	0.27	47	0.44	209	2.04	109	0.99	170	1.5	95.25079	0.348752	5.276737	1.84027	Ups		1.3E-07	2.5E-06	biological	K09091
ENSG000	HIPK3	100352	887	2.33	1477	4.04	570	1.5	1322	3.61	5594	14.17	2135	5.29	1846.202	0.392081	2.976409	1.16699	Ups		0.00292	0.0152	molecular	K08826
ENSG000	HIST1H2/	531	70	3.39	74	3.74	48	2.34	129	6.5	249	11.63	117	5.34	108.4726	0.303898	3.425429	1.04098	Ups		0.00061	0.00417	_	K11251
ENSG000	HIST1H2I	17165	227	1.12	198	1.02	186	0.92	452	2.32	579	2.76	539	2.51	347.8513	0.160174	6.964435	1.11552	Ups		3.3E-12	1.4E-10	cellular_cc	K11252
ENSG000	HIST1H4I	4480	174	3.13	201	3.77	197	3.56	788	14.72	403	6.98	655	11.1	392.1071	0.295425	4.752295	1.40395	Ups		2E-06	2.8E-05	_	K11254
ENSG000	HIST2H2I	2223	468	5.41	334	4.03	392	4.56	1276	15.35	625	6.97	1009	11	670.6028	0.30115	3.418217	1.0294	Ups		0.00063	0.00427	cellular_cc	K11252
ENSG000	HIVEP2	193735	208	0.36	324	0.59	220	0.39	346	0.63	1125	1.9	530	0.87	429.2392	0.331868	3.127532	1.03793	Ups		0.00176	0.01006	molecular	_
ENSG000	HKDC1	47257	53	0.28	51	0.28	64	0.33	3	0.02	18	0.09	15	0.07	35.20971	0.383195	-5.65678	-2.1676	Down		1.5E-08	3.4E-07	biological	K00844
ENSG000	HLA-B	3317	672	3.02	671	3.14	768	3.47	1180	5.52	2539	11.01	2847	12.07	1359.215	0.257942	5.096306	1.31455	Ups		3.5E-07	5.8E-06	cellular_cc	K06751
ENSG000	HLA-DM/	20482	116	0.6	116	0.62	124	0.64	438	2.34	421	2.09	514	2.49	274.5166	0.197697	8.510865	1.68258	Ups		1.7E-17	1.3E-15	biological	K06752
ENSG000	HLA-DRA	5205	8	0.13	3	0.05	4	0.06	0	0	0	0	0	0	2.657308	0.582229	-3.3183	-1.932	Down		0.00091	0.0058	cellular_cc	K06752
ENSG000	HMCN1	456403	12	0.01	13	0.02	7	0.01	53	0.07	100	0.12	49	0.06	36.04491	0.376155	5.651408	2.12581	Ups		1.6E-08	3.5E-07	molecular	K17341
ENSG000	HMGB1P1	636	9	0.36	7	0.3	3	0.12	18	0.76	32	1.25	18	0.69	13.61027	0.445062	2.95761	1.31632	Ups		0.0031	0.01601	molecular	K10802
ENSG000	HMGB3P1	937	0	0	3	0.14	1	0.05	14	0.65	9	0.39	14	0.59	6.48279	0.547742	3.483529	1.90807	Ups		0.00049	0.00349	_	K11296
ENSG000	HMGB3P2	15750	3	0.04	5	0.08	1	0.01	16	0.24	21	0.3	20	0.28	10.27432	0.48348	3.862993	1.86768	Ups		0.00011	0.00097	_	K11296
ENSG000	HMGCR	25776	2210	5.33	3163	7.96	2169	5.26	4616	11.58	13086	30.45	6057	13.78	4863.165	0.298172	4.329258	1.29086	Ups		1.5E-05	0.00017	molecular	K00021
ENSG000	HMGCS1	24118	1907	7.1	2405	9.33	1695	6.34	2884	11.16	10063	36.13	4590	16.11	3651.001	0.319052	3.657806	1.16703	Ups		0.00025	0.00197	biological	K01641
ENSG000	HMGN1	7333	17277	28.7	16886	29.26	17769	29.69	7714	13.33	9360	15	9877	15.48	13361.42	0.12154	-9.52078	-1.1572	Down		1.7E-21	1.8E-19	biological	K11299
ENSG000	HMGN1P:	1129	309	5.55	330	6.18	288	5.2	121	2.26	104	1.8	190	3.22	228.9093	0.257378	-5.05857	-1.302	Down		4.2E-07	7E-06	_	K11299
ENSG000	HMGN2P4	75155	18	0.08	18	0.09	28	0.13	65	0.32	109	0.49	82	0.36	50.05177	0.305477	5.338603	1.63082	Ups		9.4E-08	1.8E-06	_	K11300
ENSG000	HNF1B	58803	205	0.66	182	0.61	200	0.65	0	0	0	0	0	0	104.515	0.463529	-13.8524	-6.421	Down		1.2E-43	4.8E-41	biological	K08034
ENSG000	HNF4A	77146	16	0.04	9	0.02	7	0.02	0	0	4	0.01	3	0.01	6.676748	0.539157	-3.05671	-1.648	Down		0.00224	0.01226	molecular	K07292
ENSG000	HNRNPA1	561	1144	52.42	1122	53.62	1402	64.61	576	27.45	591	26.13	620	26.79	928.23	0.170954	-7.17175	-1.226	Down		7.4E-13	3.3E-11	_	K12741
ENSG000	HOGA1	28480	149	0.75	130	0.68	139	0.71	434	2.28	586	2.85	557	2.65	314.1259	0.166978	9.898392	1.65281	Ups		4.2E-23	4.9E-21	cellular_cc	K18123
ENSG000	HOMER2F	964	0	0	1	0.03	0	0	12	0.33	6	0.15	8	0.2	4.28198	0.578086	3.56511	2.06094	Ups		0.00036	0.00268	_	K15010
ENSG000	HOTTIP	8685	372	1.09	323	0.99	297	0.87	112	0.34	112	0.32	105	0.29	227.0735	0.195714	-8.9585	-1.7533	Down		3.3E-19	2.8E-17	_	_
ENSG000	HOXA1	3004	2	0.01	2	0.01	5	0.03	17	0.1	19	0.1	9	0.05	8.525795	0.509273	3.022129	1.53909	Ups		0.00251	0.01348	molecular	K09301
ENSG000	HOXA2	2710	2	0.02	5	0.06	4	0.05	23	0.3	10	0.12	18	0.21	10.02353	0.501576	3.009255	1.50937	Ups		0.00262	0.01393	biological	K09302
ENSG000	HOXA3	46398	32	0.11	27	0.1	34	0.12	277	0.98	75	0.25	206	0.66	105.6788	0.413682	4.988634	2.06371	Ups		6.1E-07	9.7E-06	biological	K09303
ENSG000	HOXA4	2293	15	0.09	17	0.11	26	0.16	66	0.41	32	0.18	52	0.29	33.98603	0.38995	2.644772	1.03133	Ups		0.00817	0.03441	molecular	K09304
ENSG000	HOXA5	2617	99	1.28	77	1.04	78	1.02	337	4.54	144	1.8	265	3.24	163.0562	0.339719	3.685924	1.25218	Ups		0.00023	0.0018	biological	K09305
ENSG000	HOXA-AS	26526	75	0.21	65	0.19	84	0.23	541	1.54	152	0.4	376	0.97	210.7626	0.393536	4.627169	1.82096	Ups		3.7E-06	4.9E-05	_	_
ENSG000	HOXB6	10716	1394	6.04	1205	5.45	1352	5.89	681	3.07	576	2.41	633	2.59	995.7691	0.185094	-6.6732	-1.2352	Down		2.5E-11	9.1E-10	molecular	_
ENSG000	HOXB8	3740	127	1	91	0.74	116	0.92	74	0.6	36	0.27	53	0.39	85.13833	0.32588	-3.41739	-1.1137	Down		0.00063	0.00428	molecular	K09308
ENSG000	HOXC4	39099	9	0.06	3	0.02	0	0	21	0.15	42	0.28	15	0.1	13.80424	0.522533	3.349062	1.75	Ups		0.00081	0.00529	molecular	_
ENSG000	HOXD8	3002	124	0.41	105	0.36	109	0.36	239	0.82	313	1	372	1.16	200.621	0.208984	5.662663	1.18341	Ups		1.5E-08	3.3E-07	biological	K09308
ENSG000	HOXD-AS	15764	21	0.06	12	0.03	14	0.04	53	0.15	86	0.23	71	0.18	40.06779	0.322341	5.474581	1.76468	Ups		4.4E-08	9E-07	_	_

ENSG000	HPD	24070	90	0.67	78	0.61	106	0.8	2481	19.26	746	5.37	1894	13.33	864.6405	0.374996	9.549644	3.58108	Ups	1.3E-21	1.4E-19	molecular_	K00457
ENSG000	HPSE2	776745	0	0	0	0	0	0	193	1.83	5	0.04	103	0.89	49.45505	0.548538	2.561842	1.40527	Ups	0.01041	0.04149	cellular_cc	K07965
ENSG000	HR	18970	1017	1.86	871	1.66	984	1.81	189	0.36	507	0.9	329	0.57	663.5644	0.260981	-6.33506	-1.6533	Down	2.4E-10	7.4E-09	molecular_	K00478
ENSG000	HRASLS2	10614	451	15.62	445	16.08	502	17.49	115	4.14	133	4.45	166	5.42	312.3067	0.192298	-10.0026	-1.9235	Down	1.5E-23	1.8E-21	cellular_cc	K16817
ENSG000	HRCT1	950	899	24.33	640	18.06	958	26.07	393	11.06	293	7.65	523	13.35	631.5453	0.267268	-4.4192	-1.1811	Down	9.9E-06	0.00012	cellular_cc	_
ENSG000	HRH1	126465	293	0.65	366	0.84	304	0.68	146	0.34	195	0.42	131	0.27	243.5004	0.208176	-5.83161	-1.214	Down	5.5E-09	1.3E-07	molecular_	K04149
ENSG000	HS3ST5	287460	0	0	1	0.01	0	0	4	0.03	11	0.06	9	0.05	3.745145	0.579989	3.351366	1.94375	Ups	0.0008	0.00525	cellular_cc	K08104
ENSG000	hsa-mir-4	496549	1	0	0	0	0	0	9	0.03	5	0.02	11	0.03	4.072462	0.579025	3.476604	2.01304	Ups	0.00051	0.00357	_	_
ENSG000	HSD17B1	23662	6	0.06	4	0.04	4	0.04	82	0.85	21	0.2	103	0.97	35.01499	0.492236	5.652392	2.78231	Ups	1.6E-08	3.5E-07	molecular_	_
ENSG000	HSD17B1	1943	71	1.91	42	1.18	51	1.38	123	3.44	153	3.97	127	3.22	90.62402	0.251879	4.063481	1.0235	Ups	4.8E-05	0.00047	_	K00044
ENSG000	HSPA12A	71383	626	2.19	593	2.16	614	2.16	75	0.27	589	1.99	222	0.73	454.2964	0.420704	-2.71603	-1.1426	Down	0.00661	0.02916	molecular_	_
ENSG000	HSPA2	7333	127	0.57	127	0.6	157	0.71	318	1.49	354	1.54	386	1.65	235.1516	0.192477	5.774551	1.11147	Ups	7.7E-09	1.8E-07	cellular_cc	K03283
ENSG000	HSPB1P2	546	24	1.13	14	0.69	18	0.85	6	0.29	6	0.27	13	0.58	13.76929	0.437417	-2.62133	-1.1466	Down	0.00876	0.03632	_	K04455
ENSG000	HSPE1	3464	20522	128.73	17209	112.58	17093	107.84	9430	61.53	8525	51.59	12537	74.17	14444.58	0.198477	-5.16208	-1.0246	Down	2.4E-07	4.3E-06	molecular_	K04078
ENSG000	HTR1D	4230	366	2.33	343	2.28	436	2.79	127	0.84	218	1.34	171	1.03	281.8634	0.205336	-6.54479	-1.3439	Down	6E-11	2.1E-09	molecular_	K04153
ENSG000	HTR2C	326074	1	0	1	0	2	0.01	128	0.46	262	0.87	179	0.58	85.97932	0.437847	12.11939	5.30644	Ups	8.3E-34	2E-31	molecular_	K04157
ENSG000	ICA1	149504	125	0.32	90	0.24	110	0.29	651	1.75	321	0.8	507	1.23	290.7364	0.312773	5.921179	1.85199	Ups	3.2E-09	8.2E-08	cellular_cc	_
ENSG000	ICAM1	15781	108	0.51	102	0.5	130	0.61	369	1.8	550	2.48	408	1.8	261.7325	0.200057	8.393645	1.6792	Ups	4.7E-17	3.4E-15	biological_	K06490
ENSG000	ICAM2	18041	46	0.16	52	0.19	41	0.15	0	0	0	0	1	0	24.9031	0.500829	-8.8971	-4.4559	Down	5.7E-19	4.8E-17	cellular_cc	K06523
ENSG000	ICAM5	6798	6	0.03	7	0.03	6	0.03	20	0.09	24	0.1	22	0.09	13.46473	0.420033	3.161683	1.32801	Ups	0.00157	0.0091	molecular_	K06769
ENSG000	ICOSLG	17976	204	0.41	112	0.23	157	0.32	9	0.02	40	0.08	35	0.07	96.39385	0.369765	-6.5922	-2.4376	Down	4.3E-11	1.5E-09	molecular_	K06710
ENSG000	ID1	1233	13789	159.95	10898	131.85	12937	150.93	47027	567.41	15955	178.56	44452	486.33	23696.23	0.349835	3.458242	1.20981	Ups	0.00054	0.00379	biological_	K04680
ENSG000	ID2	5609	711	4.12	698	4.22	714	4.16	3881	23.39	3003	16.79	4092	22.36	2078.305	0.20115	10.45023	2.10206	Ups	1.5E-25	2.1E-23	biological_	K17693
ENSG000	IER2	4494	3907	15.92	2907	12.35	3715	15.22	40209	170.38	8487	33.36	25907	99.54	13876.87	0.403988	5.704174	2.30442	Ups	1.2E-08	2.7E-07	cellular_cc	_
ENSG000	IFI16	55188	1716	4.58	2238	6.23	1624	4.36	216	0.6	1080	2.78	523	1.32	1259.308	0.364226	-4.65412	-1.6951	Down	3.3E-06	4.4E-05	biological_	K12966
ENSG000	IFI27	11852	9	0.03	4	0.02	12	0.04	116	0.44	1226	4.29	1254	4.29	382.7165	0.57099	5.913625	3.37662	Ups	3.4E-09	8.6E-08	molecular_	_
ENSG000	IFI44L	26224	13	0.02	6	0.01	5	0.01	39	0.07	763	1.28	286	0.47	159.6413	0.580045	4.407517	2.55656	Ups	1.1E-05	0.00012	cellular_cc	_
ENSG000	IFI6	6158	435	5.49	352	4.64	395	5.02	2288	30.06	7688	93.69	9049	107.81	3029.625	0.338422	10.03328	3.39549	Ups	1.1E-23	1.4E-21	biological_	_
ENSG000	IFIH1	51625	195	0.88	241	1.14	211	0.96	463	2.18	2866	12.53	1173	5.01	768.431	0.39693	5.457559	2.16627	Ups	4.8E-08	9.9E-07	molecular_	K12647
ENSG000	IFNAR2	35775	908	2.32	830	2.21	764	1.96	368	0.98	474	1.17	422	1.02	637.7642	0.142511	-8.35683	-1.1909	Down	6.4E-17	4.6E-15	molecular_	K05131
ENSG000	IGF2	20492	30	0.05	11	0.02	31	0.05	2	0	7	0.01	6	0.01	15.02169	0.477386	-4.09075	-1.9529	Down	4.3E-05	0.00042	biological_	K13769
ENSG000	IGFALS	4559	5	0.03	5	0.03	2	0.01	20	0.11	16	0.08	12	0.06	9.599378	0.485862	2.846874	1.38319	Ups	0.00442	0.02121	molecular_	K17256
ENSG000	IGFBP4	14282	716	5.95	644	5.58	796	6.65	2191	18.93	1253	10.04	2103	16.48	1251.544	0.267787	4.142751	1.10937	Ups	3.4E-05	0.00035	biological_	_
ENSG000	IGFBP5	23421	9	0.03	9	0.03	11	0.04	95	0.34	238	0.79	131	0.42	74.2059	0.364797	9.079873	3.31231	Ups	1.1E-19	9.7E-18	biological_	K10138
ENSG000	IGFBP6	4910	1134	10.31	948	8.99	1138	10.41	716	6.77	183	1.61	572	4.91	808.1076	0.386103	-2.92	-1.1274	Down	0.0035	0.01764	biological_	_
ENSG000	IGFBP7-A	95749	3	0.04	4	0.05	4	0.05	38	0.46	16	0.18	38	0.42	16.44247	0.480602	4.603271	2.21234	Ups	4.2E-06	5.4E-05	_	_
ENSG000	IGFL1P1	1086	400	28.56	445	33.14	452	32.46	124	9.21	136	9.37	121	8.15	289.581	0.179218	-10.8181	-1.9388	Down	2.8E-27	4.5E-25	_	_
ENSG000	IGFN1	38128	32	0.05	22	0.04	21	0.04	90	0.16	1060	1.75	454	0.73	243.8954	0.555227	4.682148	2.59966	Ups	2.8E-06	3.9E-05	cellular_cc	_
ENSG000	IGIP	2871	99	0.89	100	0.93	89	0.8	266	2.48	315	2.72	345	2.91	192.6247	0.190406	7.471384	1.4226	Ups	7.9E-14	4.1E-12	cellular_cc	_
ENSG000	IKZF2	152723	35	0.04	45	0.06	32	0.04	117	0.15	432	0.51	154	0.18	123.1119	0.382856	5.429369	2.07867	Ups	5.7E-08	1.1E-06	molecular_	K09220
ENSG000	IL11	6075	72	0.51	61	0.45	54	0.39	14	0.1	10	0.07	17	0.11	39.60885	0.331729	-6.58328	-2.1839	Down	4.6E-11	1.6E-09	molecular_	K05417
ENSG000	IL11RA	11191	90	0.3	63	0.22	96	0.32	340	1.17	322	1.03	376	1.17	204.0736	0.232269	7.627283	1.77158	Ups	2.4E-14	1.3E-12	molecular_	K05056
ENSG000	IL17RD	80325	87	0.16	82	0.16	93	0.17	241	0.47	249	0.45	249	0.44	160.1437	0.198693	6.250606	1.24195	Ups	4.1E-10	1.2E-08	cellular_cc	K05167
ENSG000	IL1B	7153	2	0.01	1	0.01	1	0	7	0.04	18	0.09	8	0.04	5.611831	0.552318	3.175425	1.75385	Ups	0.0015	0.00879	biological_	K04519
ENSG000	IL1R1	115331	140	0.27	181	0.37	148	0.29	11	0.02	89	0.17	25	0.05	101.5785	0.440439	-4.14274	-1.8246	Down	3.4E-05	0.00035	molecular_	K04386
ENSG000	IL20RB	64856	2438	11.82	2255	11.4	2495	12.16	116	0.58	420	1.96	340	1.55	1409.464	0.310652	-9.82181	-3.0512	Down	9.1E-23	1E-20	biological_	K05137
ENSG000	IL4R	51111	426	0.87	336	0.71	347	0.71	16	0.03	147	0.29	84	0.16	232.9952	0.426256	-4.84826	-2.0666	Down	1.3E-06	1.9E-05	biological_	K05071
ENSG000	IL6R	64258	226	0.6	202	0.56	217	0.58	85	0.23	145	0.37	85	0.21	162.3526	0.227172	-5.36844	-1.2196	Down	7.9E-08	1.6E-06	biological_	K05055
ENSG000	INADL	421444	841	1.35	895	1.5	856	1.39	1703	2.85	2754	4.28	1921	2.92	1425.6	0.166067	6.262765	1.04004	Ups	3.8E-10	1.1E-08	molecular_	K06092
ENSG000	ING1	8339	839	2.92	684	2.48	721	2.53	1843	6.68	1616	5.43	2002	6.58	1241.775	0.185377	5.659283	1.0491	Ups	1.5E-08	3.4E-07	cellular_cc	K19197
ENSG000	INHA	6552	253	3.08	201	2.55	246	3.01	1163	14.72	489	5.74	975	11.19	539.4231	0.325574	4.873354	1.58664	Ups	1.1E-06	1.7E-05	biological_	K05500
ENSG000	INHBB	5666	49	0.39	35	0.29	49	0.4	6	0.05	31	0.24	9	0.07	30.3548	0.427948	-3.53346	-1.5121	Down	0.00041	0.00297	biological_	K04667
ENSG000	INMT	59618	2	0.01	0	0	0	0	63	0.34	61	0.31	81	0.4	31.83583	0.484817	9.045541	4.38543	Ups	1.5E-19	1.3E-17	molecular_	K04579
ENSG000	INPP4B	824273	16	0.02	21	0.03	23	0.03	4	0.01	12	0.02	3	0	13.51059	0.456892	-3.36957	-1.5395	Down	0.00075	0.00497	cellular_cc	K01109
ENSG000	INSIG1	12460	4219	13.43	4130	13.71	3649	11.68	10776	35.68	12406	38.1	11691	35.1	7469.279	0.120702	10.82124	1.30615	Ups	2.7E-27	4.4E-25	molecular_	_
ENSG000	INSL4	3886	273	3.6	346	4.75	329	4.36	864	11.83	556	7.06	926	11.5	534.2229	0.263175	3.992734	1.05079	Ups	6.5E-05	0.00061	molecular_	K05255
ENSG000	IQCH-AS1	118226	81	0.27	57	0.2	91	0.31	166	0.58	276	0.9	161	0.51	131.8363	0.268981	4.069452	1.09461	Ups	4.7E-05	0.00046	_	_
ENSG000	IQCJ-SCH	3226	0	0	2	0.07	0	0	5	0.18	7	0.24	7	0.23	3.2422	0.580139	2.591599	1.50349	Ups	0.00955	0.03888	_	_
ENSG000	IQSEC2	88465	885	2.07	830	2.03	978	2.3	315	0.77	665	1.5	554	1.22	709.4883	0.221793	-4.60559	-1.0215	Down	4.1E-06	5.4E-05	molecular_	K12495
ENSG000	IRAK1	9481	17471	40.35	14265	34.36	16190	37.61	8837	21.23	6802	15.16	8159	17.77	12222.07	0.200601	-5.83886	-1.1713	Down	5.3E-09	1.3E-07	biological_	K04730
ENSG000	IRF2BP2	5257	2439	9.83	1918	8.06	1910	7.74	6593	27.64	3694	14.37	5579	21.21	3601.537	0.264864	4.133509	1.09482	Ups	3.6E-05	0.00036	cellular_cc	_
ENSG000	IRF5	12424	49	0.14	22	0.07	26	0.08	4	0.01	21	0.06	17	0.05	23.34938	0.442643	-2.72971	-1.2083	Down	0.00634	0.02825	molecular_	K09446
ENSG000	IRF7	3447	251	0.72	164	0.49	220	0.63	381	1.13	642	1.77	810	2.18	389.2888	0.263975	4.627361	1.22151	Ups	3.7E-06	4.9E-05	biological_	K09447
ENSG000	IRF9	5513	69	0.23	89	0.3	69	0.23	119	0.4	265	0.83	187	0.58	125.7832	0.271771	3.744713	1.0177	Ups	0.00018	0.00147	molecular_	K04693
ENSG000	IRS1	68443	230	0.58	241	0.63	212	0.54	426	1.12	1275	3.1	597	1.42	461.411	0.303172	4.535347	1.37499	Ups	5.8E-06	7.2E-05	biological_	K16172
ENSG000	ISCA1P4	134	0	0	2	0.4	0	0	19	3.79	10	1.85	20	3.62	8.025669	0.553758	4.589415	2.54143	Ups	4.4E-06	5.8E-05	_	K13628
ENSG000	ISG20	20331	113	0.23	91	0.19	105	0.21	289	0.61	426	0.83	559	1.07	247.8434	0.245661	7.027164	1.7263	Ups	2.1E-12	9E-11	molecular_	K12579
ENSG000	ISM1	78881	31	0.31	17	0.18	26	0.26	110	1.13	123	1.18	111	1.04	65.84195	0.280186	6.693744	1.87549	Ups	2.2E-11	8.1E-10	cellular_cc	_
ENSG000	ISM2	24471	3	0.01	1	0	1	0	14	0.05	5	0.02	11	0.04	5.6439	0.55608	2.711206	1.50765	Ups	0.0067	0.02949	cellular_cc	_
ENSG000	ITGA10	18978	5	0.02	7	0.02	8	0.03	46	0.16	15	0.05	32	0.1	18.39061	0.470283	3.435672	1.61574	Ups	0.00059	0.00405	molecular_	K06586
ENSG000	ITGA2B	17326	26	0.09	16	0.05	34	0.11	257	0.87	117	0.37	214	0.66	106.2296	0.375325	6.50886	2.44294	Ups	7.6E-11	2.6E-09	biological_	K06476
ENSG000	ITGA5	24200	4757	9.59	3888	8.18	4217	8.55	989	2.07	1068	2.08	848	1.61	2734.644	0.16304	-14.1876	-2.3132	Down	1.1E-45	4.5E-43	biological_	K06484
ENSG000	ITGB2-AS	8628	12	0.08	12	0.09	13	0.09	2	0.01	1	0.01	1	0.01	7.226048	0.530064	-4.31968	-2.2897	Down	1.6E-05	0.00017	_	_
ENSG000	ITGB3	90447	5	0.02	2	0.01	2	0.01	5	0.02	35	0.16	8	0.03	8.505659	0.552795	2.488232	1.37548	Ups	0.01284	0.04874	biological_	K06493
ENSG000	ITGB4	36492	1191	2.92	835	2.14	938	2.32	454	1.16	463	1.1	403	0.93	729.9589	0.194252	-6.88836	-1.3381	Down	5.6E-12	2.3E-10	molecular_	K06525
ENSG000	ITGBL1	270491	1	0	1	0.01	2	0.01	6	0.03	15	0.07	5	0.02	4.578562	0.564059	2.599914	1.4665	Ups	0.00932	0.03817	cellular_cc	_
ENSG000	JAK3	23292	15	0.04	9	0.02	7	0.02	35	0.1	45	0.11	43	0.11	24.22802	0.367312	4.262074	1.56551	Ups	2E-05	0.00022	biological_	K11218
ENSG000	JMY	91027	508	1.38	608	1.72	426	1.17	2275	6.44	4629	12.15	2318	5.95	1656.445	0.253695	8.732639	2.21543	Ups	2.5E-18	2E-16	molecular_	_
ENSG000	JPH1	86629	453	2.34	461	2.49	418	2.18	872	4.69	1998	9.98	1127	5.5	833.672	0.243064	5.227289	1.27057	Ups	1.7E-07	3.1E-06	molecular_	K19530
ENSG000	JPH3	96322	549	1.67	416	1.32	573	1.76	129	0.41	142	0.42	149	0.43	338.1504	0.199478	-10.1685	-2.0284	Down	2.7E-24	3.6E-22	molecular_	K19530
ENSG000	JUN	3321	286	2.21	267	2.16	318	2.48	19248	154.97	4607	34.4	13352	97.47	6119.23	0.491073	8.132224	3.99352	Ups	4.2E-16	2.7E-14	cellular_cc	K04448
ENSG000	JUNB	1815	4650	65.86	3806	56.22	4264	60.74	31667	466.5	7983	109.08	24719	330.19	12545.35	0.382197	5.012407	1.91573	Ups	5.4E-07	8.7E-06	cellular_cc	K09028
ENSG000	KAL1	203313	29	0.1	19	0.07	32	0.11	0	0	8	0.03	2	0.01	15.65337	0.490163	-5.02724	-2.4642	Down	5E-07	8.1E-06	molecular_	_
ENSG000	KALRN	646303	156	0.1	132	0.09	124	0.08	1	0	8	0.01	4	0	75.16601	0.371801	-12.2189	-4.543	Down	2.5E-34	5.8E-32	molecular_	K15048
ENSG000	KANK3	20679	3	0.02	2	0.01	3	0.02	14	0.1	19	0.13	20	0.13	9.475435	0.487684	3.890846	1.8975	Ups	1E-04	0.00088	_	_
ENSG000	KANK4	82435	7	0.02	4	0.01	7	0.02	59	0.21	78	0.26	59	0.19	33.04814	0.374416	7.53593	2.82158	Ups	4.9E-14	2.5E-12	cellular_cc	_
ENSG000	KAT2B	114382	351	1.43	425	1.8	210	0.86	544	2.3	1653	6.49	766	2.94	613.2788	0.337852	3.547298	1.19846	Ups	0.00039	0.00284	cellular_cc	K06062
ENSG000	KAZN	519340	11	0.02	13	0.02	16	0.02	286	0.44	90	0.13	198	0.27	98.6811	0.41808	7.420754	3.10247	Ups	1.2E-13	5.8E-12	cellular_cc	_
ENSG000	KB-1460A	2825	21	0.19	13	0.12	15	0.14	35	0.33	50	0.44	59	0.51	30.53313	0.340054	3.534112	1.20179	Ups	0.00041	0.00297	_	_
ENSG000	KB-1991C	13113	22	1.04	21	1.03	30	1.42	67	3.28	127	5.77	77	3.42	53.73617	0.311087	4.891219	1.52159	Ups	1E-06	1.5E-05	_	_
ENSG000	KBTBD7	4734	129	0.7	281	1.59	109	0.6	286	1.62	999	5.23	490	2.51	353.8954	0.386891	3.384573	1.30946	Ups	0.00071	0.00476	molecular_	K10474
ENSG000	KCNAB3	7945	57	0.22	65	0.26	48	0.19	24	0.1	15	0.06	18	0.07	39.18919	0.329444	-4.92552	-1.6227	Down	8.4E-07	1.3E-05	molecular_	K04884
ENSG000	KCND1	9338	380	1.27	278	0.97	311	1.04	72	0.25	205	0.66	124	0.39	231.7714	0.289298	-4.94408	-1.4303	Down	7.7E-07	1.2E-05	molecular_	K04891
ENSG000	KCND3	218494	6	0.02	7	0.02	6	0.02	73	0.21	590	1.54	184	0.47	125.3502	0.479752	8.237797	3.9521	Ups	1.8E-16	1.2E-14	molecular_	K04893
ENSG000	KCNE2	7366	14	0.34	9	0.23	9	0.22	29	0.74	25	0.59	31	0.72	18.80556	0.385367	2.721397	1.04874	Ups	0.0065	0.02881	molecular_	K04896
ENSG000	KCNH1-IT	787	1	0.04	0	0	0	0	3	0.13	8	0.32	8	0.31	3.002454	0.583076	2.902076	1.69213	Ups	0.00371	0.01847	_	_
ENSG000	KCNH5	395469	20	0.08	20	0.08	14	0.05	1	0	1	0	6	0.02	10.8172	0.50077	-4.38726	-2.197	Down	1.2E-05	0.00013	molecular_	K04908
ENSG000	KCNIP3	88774	239	0.58	182	0.46	197	0.48	760	1.93	375	0.89	644	1.49	389.7035	0.303811	4.10854	1.24822	Ups	4E-05	0.00039	biological_	_
ENSG000	KCNJ11	3473	6	0.02	3	0.01	5	0.02	30	0.12	32	0.12	19	0.07	14.94958	0.446834	4.221894	1.88648	Ups	2.4E-05	0.00026	molecular_	K05004
ENSG000	KCNJ12	43671	706	2.42	510	1.82	574	1.98	78	0.28	91	0.3	91	0.29	358.0427	0.199507	-14.5969	-2.9122	Down	2.9E-48	1.4E-45	molecular_	K19468
ENSG000	KCNJ15	150152	7	0.01	5	0.01	5	0.01	20	0.02	17	0.02	17	0.02	11.37983	0.44565	2.680913	1.19475	Ups	0.00734	0.03162	molecular_	K05008
ENSG000	KCNJ2	11376	13	0.05	4	0.02	11	0.04	90	0.34	404	1.42	142	0.49	97.79392	0.44732	7.655475	3.42445	Ups	1.9E-14	1.1E-12	molecular_	K04996
ENSG000	KCNJ2-AS	2442	9	0.09	0	0	6	0.06	17	0.19	42	0.43	38	0.38	17.11492	0.4915	3.782841	1.85927	Ups	0.00016	0.00129	_	_
ENSG000	KCNJ4	28874	19	0.24	31	0.4	11	0.14	3	0.04	3	0.04	2	0.02	12.09349	0.500078	-4.6408	-2.3208	Down	3.5E-06	4.6E-05	molecular_	K04998
ENSG000	KCNJ8	10627	7	0.06	8	0.07	14	0.12	0	0	1	0.01	0	0	5.320432	0.570764	-4.32672	-2.4695	Down	1.5E-05	0.00017	biological_	K05001
ENSG000	KCNK15	5255	400	7.79	379	7.7	406	7.95	156	3.16	44	0.83	150	2.76	266.5908	0.380732	-4.45124	-1.6947	Down	8.5E-06	0.0001	molecular_	K04923
ENSG000	KCNK5	40478	511	3.5	484	3.45	551	3.79	266	1.89	234	1.55	245	1.58	390.5229	0.190505	-6.4394	-1.2267	Down	1.2E-10	3.9E-09	molecular_	K04916
ENSG000	KCNMB2	571498	5	0.02	10	0.04	5	0.02	21	0.08	21	0.07	25	0.08	13.8549	0.42937	2.969592	1.27505	Ups	0.00298	0.01552	biological_	K04938
ENSG000	KCNQ1	404426	143	0.66	143	0.68	141	0.65	11	0.05	13	0.06	21	0.09	82.90238	0.288955	-11.1975	-3.2356	Down	4.2E-29	7.3E-27	molecular_	K04926
ENSG000	KCNQ2	66452	87	0.27	84	0.27	68	0.21	1	0	6	0.02	1	0	43.67679	0.418945	-10.063	-4.2158	Down	8.1E-24	1E-21	molecular_	K04927
ENSG000	KCNQ4	56441	91	0.3	76	0.26	106	0.35	47	0.16	43	0.14	47	0.15	69.76593	0.263577	-4.33804	-1.1434	Down	1.4E-05	0.00016	molecular_	K04929
ENSG000	KCNT2	383447	27	0.06	26	0.06	18	0.04	33	0.07	464	0.97	121	0.25	100.7711	0.568963	2.94592	1.67612	Ups	0.00322	0.01648	molecular_	K04947
ENSG000	KCNU1	151805	10	0.05	4	0.02	10	0.05	63	0.31	28	0.13	45	0.2	25.77371	0.440271	4.34604	1.91343	Ups	1.4E-05	0.00016	molecular_	K05274
ENSG000	KCP	48269	40	0.1	34	0.09	42	0.11	7	0.02	5	0.01	9	0.02	23.91103	0.386177	-6.04022	-2.3326	Down	1.5E-09	4.2E-08	cellular_cc	_
ENSG000	KCTD19	37336	1	0	3	0.01	2	0	10	0.02	10	0.02	8	0.02	5.385007	0.538717	2.513751	1.3542	Ups	0.01195	0.04609	biological_	_
ENSG000	KDM1A	64242	17723	53.73	18910	59.79	19598	59.76	7429	23.43	7909	23.13	7811	22.33	13571.33	0.131864	-11.2057	-1.4776	Down	3.8E-29	6.7E-27	biological_	K11450
ENSG000	KIAA0040	36013	307	1.04	280	0.99	347	1.18	571	2.01	1336	4.36	747	2.38	562.2012	0.25683	4.621107	1.18684	Ups	3.8E-06	5.1E-05	_	_
ENSG000	KIAA0355	101050	346	0.84	369	0.93	350	0.85	813	2.05	1306	3.06	763	1.75	625.4924	0.205496	5.652883	1.16164	Ups	1.6E-08	3.5E-07	_	_
ENSG000	KIAA0930	50432	6511	10.26	5324	8.75	6177	9.79	3345	5.48	3100	4.71	3676	5.46	4764.77	0.167949	-6.0654	-1.0187	Down	1.3E-09	3.6E-08	_	_
ENSG000	KIAA1109	210426	613	0.61	708	0.73	594	0.59	845	0.87	3741	3.58	1226	1.15	1189.3	0.367846	3.172705	1.16707	Ups	0.00151	0.00884	biological_	_
ENSG000	KIAA1199	172434	209	0.61	204	0.62	149	0.44	33	0.1	105	0.3	54	0.15	128.3094	0.321036	-5.17227	-1.6605	Down	2.3E-07	4.1E-06	cellular_cc	K19031
ENSG000	KIAA1211	142414	66	0.28	54	0.24	62	0.26	122	0.54	219	0.9	170	0.68	109.3154	0.239656	4.986363	1.19501	Ups	6.2E-07	9.8E-06	_	_
ENSG000	KIAA1324	93101	362	0.71	401	0.82	399	0.79	64	0.13	39	0.07	52	0.1	231.3436	0.254502	-11.5851	-2.9484	Down	4.9E-31	9.8E-29	molecular_	_
ENSG000	KIAA1430	49840	321	1.05	504	1.72	263	0.87	606	2.06	2075	6.55	911	2.81	722.686	0.350218	3.720998	1.30316	Ups	0.0002	0.0016	_	_
ENSG000	KIAA1644	69185	342	1.3	302	1.2	301	1.15	57	0.23	279	1.02	112	0.4	233.487	0.373625	-3.22041	-1.2032	Down	0.00128	0.00773	cellular_cc	_
ENSG000	KIAA1683	17412	17	0.03	14	0.02	15	0.02	91	0.14	77	0.11	72	0.1	45.3645	0.321917	6.195182	1.99433	Ups	5.8E-10	1.7E-08	cellular_cc	_
ENSG000	KIAA2026	126306	252	0.35	237	0.35	190	0.27	380	0.55	1028	1.39	467	0.62	398.8902	0.299671	3.732503	1.11852	Ups	0.00019	0.00153	_	_
ENSG000	KIF17	54004	20	0.08	16	0.07	16	0.06	7	0.03	7	0.03	10	0.04	12.92803	0.421066	-2.67641	-1.1269	Down	0.00744	0.03196	molecular_	K10394
ENSG000	KIF5C	250455	0	0	0	0	1	0	4	0.01	7	0.02	4	0.01	2.440884	0.583333	2.539955	1.48164	Ups	0.01109	0.04352	molecular_	K10396
ENSG000	KIF9	55426	387	1.09	339	0.99	403	1.14	219	0.64	142	0.38	238	0.63	294.2728	0.259281	-4.06449	-1.0538	Down	4.8E-05	0.00046	molecular_	K10397
ENSG000	KLF12	448169	209	0.46	278	0.63	213	0.47	368	0.84	1575	3.33	603	1.25	497.0057	0.365444	3.840754	1.40358	Ups	0.00012	0.00105	biological_	K09205
ENSG000	KLF15	14808	9	0.07	17	0.15	19	0.16	40	0.34	77	0.61	71	0.55	36.27928	0.355318	4.580752	1.62762	Ups	4.6E-06	6E-05	molecular_	K09210
ENSG000	KLF2	3058	45	0.45	25	0.26	50	0.5	1605	16.62	234	2.25	1053	9.89	488.8067	0.552716	5.127483	2.83404	Ups	2.9E-07	5.1E-06	biological_	K17845
ENSG000	KLF4	5631	336	1.22	286	1.08	232	0.85	1906	7.18	1249	4.36	1670	5.7	904.4864	0.253573	8.652968	2.19416	Ups	5E-18	4E-16	biological_	K17846
ENSG000	KLF6	9286	897	1.7	909	1.8	849	1.62	3550	7.02	3562	6.53	3165	5.67	2054.026	0.153831	11.12082	1.71072	Ups	9.9E-29	1.7E-26	molecular_	K09207
ENSG000	KLF9	30038	355	1.76	414	2.14	308	1.54	1604	8.27	2211	10.58	1841	8.61	1051.318	0.164643	12.87744	2.12018	Ups	6E-38	1.7E-35	molecular_	K09208
ENSG000	KLHL1	407866	0	0	0	0	0	0	2	0.01	8	0.04	7	0.04	2.504933	0.580924	3.069722	1.78327	Ups	0.00214	0.01183	molecular_	K10442
ENSG000	KLHL13	219528	1214	3.05	1631	4.27	1209	3.06	2454	6.41	8239	19.97	3822	9.06	2861.301	0.313748	4.599065	1.44295	Ups	4.2E-06	5.6E-05	biological_	K10447
ENSG000	KLHL15	43467	516	2.11	864	3.69	411	1.69	793	3.38	3728	14.74	1237	4.78	1158.182	0.398352	3.029237	1.2067	Ups	0.00245	0.01326	biological_	K10452
ENSG000	KLHL24	48910	346	0.61	453	0.83	301	0.53	1129	2.07	1927	3.28	1348	2.24	860.1321	0.216129	7.86035	1.69885	Ups	3.8E-15	2.3E-13	cellular_cc	K10461
ENSG000	KLHL3	118591	30	0.04	38	0.06	31	0.05	95	0.14	260	0.36	100	0.14	85.06938	0.356007	4.872369	1.7346	Ups	1.1E-06	1.7E-05	molecular_	K10443
ENSG000	KLHL35	8237	105	0.51	88	0.44	106	0.52	223	1.12	221	1.03	300	1.37	167.3902	0.22604	4.673883	1.05649	Ups	3E-06	4E-05	_	K10461
ENSG000	KLHL38	7424	0	0	1	0.01	0	0	2	0.03	15	0.19	8	0.1	3.804034	0.583015	3.121112	1.81965	Ups	0.0018	0.01024	molecular_	K13959
ENSG000	KLHL41	16561	1	0.01	1	0.01	0	0	23	0.22	19	0.17	16	0.14	9.356728	0.537946	5.261742	2.83053	Ups	1.4E-07	2.6E-06	cellular_cc	K10473
ENSG000	KLK2	19000	6	0.01	9	0.02	7	0.01	21	0.04	30	0.05	25	0.04	15.45785	0.4059	3.290695	1.33569	Ups	0.001	0.00631	molecular_	K01351

ENSG000	KLRC2	15447	146	1.27	101	0.91	91	0.79	667	6.02	213	1.78	464	3.8	274.2897	0.380373	4.237567	**1.61185**	**Ups**	**2.3E-05**	**0.00024**	molecular_	K06541
ENSG000	KLRC3	8284	4	0.06	3	0.05	6	0.09	29	0.44	38	0.53	28	0.38	16.79137	0.430353	5.088112	**2.18969**	**Ups**	**3.6E-07**	**6.1E-06**	molecular_	K06541
ENSG000	KLRC4	2374	3	0.08	1	0.03	2	0.06	19	0.55	30	0.8	11	0.29	10.14954	0.515745	4.235826	**2.18461**	**Ups**	**2.3E-05**	**0.00024**	biological_	K06541
ENSG000	KLRC4-KL	37794	1	0.01	0	0	1	0.01	10	0.06	13	0.08	10	0.06	5.383155	0.55963	3.883561	**2.17336**	**Ups**	**0.0001**	**0.0009**	_	K06728
ENSG000	KPNA7	33933	17	0.25	9	0.14	12	0.18	2	0.03	4	0.06	4	0.06	8.24843	0.497901	-3.25017	**-1.6183**	**Down**	**0.00115**	**0.00708**	cellular_cc	_
ENSG000	KRT12	5908	4	0.06	4	0.06	3	0.04	11	0.16	49	0.65	14	0.18	12.74524	0.513109	3.494927	**1.79328**	**Ups**	**0.00047**	**0.00338**	molecular_	K07604
ENSG000	KRT14	4643	52	0.46	68	0.63	59	0.52	2	0.02	7	0.06	17	0.14	35.78397	0.415143	-6.16502	**-2.5594**	**Down**	**7.1E-10**	**2E-08**	molecular_	K07604
ENSG000	KRT15	8787	66	0.12	58	0.11	43	0.08	7	0.01	2	0	2	0	31.46939	0.425986	-7.97164	**-3.3958**	**Down**	**1.6E-15**	**9.6E-14**	molecular_	K07604
ENSG000	KRT16	6122	101	0.74	83	0.63	126	0.92	20	0.15	44	0.31	76	0.52	76.21479	0.366862	-3.37394	**-1.2378**	**Down**	**0.00074**	**0.00491**	molecular_	K07604
ENSG000	KRT16P1	10845	18	0.08	10	0.05	19	0.08	2	0.01	1	0	0	0	8.848185	0.535254	-4.98969	**-2.6708**	**Down**	**6.1E-07**	**9.7E-06**	_	K07604
ENSG000	KRT17	5406	11125	58.61	10934	60.08	10958	58.07	5237	28.7	6897	35.06	6820	33.89	8763.473	0.115031	-8.84927	**-1.0179**	**Down**	**8.8E-19**	**7.2E-17**	biological_	K07604
ENSG000	KRT18P7	1219	0	0	0	0	1	0.02	4	0.09	9	0.18	4	0.08	2.717923	0.583453	2.706511	**1.57912**	**Ups**	**0.0068**	**0.02979**	_	K07604
ENSG000	KRT4	8003	3	0.01	16	0.08	23	0.12	50	0.26	168	0.81	155	0.73	62.72481	0.461284	4.995451	**2.30432**	**Ups**	**5.9E-07**	**9.4E-06**	molecular_	K07605
ENSG000	KRT40	9420	0	0	0	0	0	0	8	0.07	4	0.03	7	0.05	2.985628	0.583305	3.397342	**1.98169**	**Ups**	**0.00068**	**0.00457**	molecular_	K07604
ENSG000	KRT7	19667	103714	390.23	82282	322.89	102950	389.58	30089	117.75	25573	92.83	34907	123.87	65416.32	0.202049	-9.01607	**-1.8217**	**Down**	**2E-19**	**1.7E-17**	molecular_	K07605
ENSG000	KRT83	7098	6	0.08	11	0.16	9	0.12	3	0.04	1	0.01	1	0.01	5.449189	0.548266	-3.04057	**-1.667**	**Down**	**0.00236**	**0.01284**	molecular_	K07605
ENSG000	KRT8P42	1699	10	0.18	7	0.13	9	0.17	31	0.59	30	0.53	16	0.28	16.53154	0.422228	2.710255	**1.14435**	**Ups**	**0.00672**	**0.02955**	_	K07605
ENSG000	KRTAP5-1	942	2	0.05	3	0.09	1	0.03	11	0.31	19	0.5	19	0.49	8.45151	0.51103	3.923442	**2.005**	**Ups**	**8.7E-05**	**0.00078**	cellular_cc	_
ENSG000	KRTAP5-/	27832	2	0.01	1	0.01	5	0.03	7	0.05	43	0.28	11	0.07	10.24697	0.549057	3.119548	**1.71281**	**Ups**	**0.00181**	**0.01028**	_	_
ENSG000	KYNU	164824	14998	107.12	16012	119.28	13570	97.48	1380	10.25	4315	29.73	2747	18.5	9186.601	0.281452	-8.89205	**-2.5027**	**Down**	**6E-19**	**5E-17**	cellular_cc	K01556
ENSG000	L1CAM	47709	14232	28.3	11718	24.3	12905	25.81	3113	6.44	8430	16.17	4409	8.27	9281.924	0.266457	-5.48331	**-1.4611**	**Down**	**4.2E-08**	**8.6E-07**	molecular_	K06550
ENSG000	LA16c-60	1049	22	0.54	17	0.43	5	0.12	61	1.55	44	1.04	52	1.2	32.22237	0.40772	3.446521	**1.40522**	**Ups**	**0.00057**	**0.00392**	_	K11518
ENSG000	LACC1	14649	78	0.26	96	0.33	55	0.18	96	0.33	399	1.28	154	0.48	135.7222	0.387624	2.754873	**1.06785**	**Ups**	**0.00587**	**0.02658**	_	K05810
ENSG000	LAD1	26365	9	0.05	7	0.04	16	0.09	35	0.21	60	0.33	65	0.35	29.82753	0.378145	4.838384	**1.82961**	**Ups**	**1.3E-06**	**1.9E-05**	molecular_	_
ENSG000	LAMA4	146179	21	0.03	14	0.02	29	0.04	2	0	7	0.01	12	0.02	14.52706	0.467582	-3.1508	**-1.4733**	**Down**	**0.00163**	**0.0094**	biological_	K06241
ENSG000	LAMB3	37597	5253	21.72	4809	20.74	5286	21.99	1269	5.46	813	3.24	1103	4.3	3230.452	0.231812	-10.1725	**-2.3581**	**Down**	**2.6E-24**	**3.5E-22**	molecular_	K06244
ENSG000	LCA5	52468	52	0.2	60	0.24	24	0.09	84	0.34	314	1.19	116	0.43	99.58206	0.4097	3.431231	**1.40578**	**Ups**	**0.0006**	**0.00411**	molecular_	_
ENSG000	LCN2	4385	9	0.06	8	0.06	21	0.15	2	0.01	2	0.01	1	0.01	7.566107	0.539482	-3.84613	**-2.0749**	**Down**	**0.00012**	**0.00103**	molecular_	_
ENSG000	LDB2	397269	26	0.06	27	0.06	19	0.04	4	0.01	11	0.02	1	0	15.17331	0.469932	-4.0551	**-1.9056**	**Down**	**5E-05**	**0.00048**	biological_	_
ENSG000	LDB3	67620	45	0.1	24	0.05	41	0.09	131	0.29	122	0.25	127	0.25	78.13598	0.283862	5.210818	**1.47915**	**Ups**	**1.9E-07**	**3.4E-06**	molecular_	_
ENSG000	LDHA	14038	125341	202.97	128519	217.06	135222	220.23	68689	115.7	76935	120.2	77224	117.94	103432.6	0.125512	-8.05763	**-1.0113**	**Down**	**7.8E-16**	**5E-14**	molecular_	K00016
ENSG000	LDHAP4	998	77	1.98	74	1.99	89	2.31	47	1.26	44	1.09	41	1	63.13158	0.259594	-3.93144	**-1.0206**	**Down**	**8.4E-05**	**0.00076**	_	K00016
ENSG000	LEPREL1	165710	2002	6.12	1707	5.44	1714	5.27	4686	14.89	6789	20.01	5279	15.21	3506.747	0.140567	9.79479	**1.37682**	**Ups**	**1.2E-22**	**1.3E-20**	molecular_	_
ENSG000	LEPREL1-/	23883	2	0.07	1	0.04	6	0.21	25	0.92	21	0.71	23	0.76	12.28738	0.483354	4.312563	**2.08449**	**Ups**	**1.6E-05**	**0.00018**	_	_
ENSG000	LEPREL2	11447	12	0.03	6	0.02	3	0.01	48	0.14	25	0.07	39	0.1	21.31524	0.450744	4.02259	**1.81316**	**Ups**	**5.8E-05**	**0.00054**	molecular_	_
ENSG000	LFNG	16649	200	0.95	190	0.94	226	1.08	83	0.41	49	0.22	76	0.34	142.2854	0.276024	-6.00355	**-1.6571**	**Down**	**1.9E-09**	**5.2E-08**	biological_	K05948
ENSG000	LGALS12	10691	18	0.11	24	0.16	22	0.14	9	0.06	5	0.03	4	0.02	14.26037	0.438536	-3.82677	**-1.6782**	**Down**	**0.00013**	**0.0011**	cellular_cc	K10095
ENSG000	LGALS2	12369	8	0.23	6	0.18	5	0.14	20	0.59	20	0.54	36	0.96	15.00441	0.437222	3.370159	**1.47351**	**Ups**	**0.00075**	**0.00496**	molecular_	K10090
ENSG000	LGALS3	21299	7954	49.55	7042	45.76	7397	46.35	2743	17.78	2438	14.65	3109	18.27	5264.155	0.17213	-9.32379	**-1.6049**	**Down**	**1.1E-20**	**1.1E-18**	biological_	K06831
ENSG000	LGI4	17939	0	0	1	0	0	0	4	0.01	3	0.01	9	0.02	2.636989	0.583143	2.569972	**1.49866**	**Ups**	**0.01017**	**0.04071**	cellular_cc	_
ENSG000	LGSN	44027	18	0.07	20	0.08	14	0.05	74	0.29	249	0.9	148	0.52	78.74777	0.371161	6.929399	**2.57192**	**Ups**	**4.2E-12**	**1.7E-10**	molecular_	K09228
ENSG000	LIMD1-A!	10092	0	0	1	0.01	1	0.01	9	0.1	8	0.08	14	0.14	5.123561	0.564853	3.666669	**2.07113**	**Ups**	**0.00025**	**0.00192**	_	_
ENSG000	LIMK1	39593	1394	4.4	1054	3.47	1283	4.07	634	2.08	768	2.34	705	2.1	985.5756	0.156677	-6.55122	**-1.0264**	**Down**	**5.7E-11**	**2E-09**	molecular_	K05743
ENSG000	LINC0002	1797	0	0	0	0	0	0	5	0.23	3	0.13	11	0.45	2.930781	0.582389	3.252625	**1.89429**	**Ups**	**0.00114**	**0.00703**	_	_
ENSG000	LINC0016	5521	2884	101	2495	91.13	3039	107.04	742	27.03	879	29.7	1266	41.82	1939.222	0.207682	-8.23751	**-1.7108**	**Down**	**1.8E-16**	**1.2E-14**	_	_
ENSG000	LINC0017	3097	0	0	0	0	0	0	12	0.11	1	0.01	5	0.04	2.959388	0.576464	2.917975	**1.68211**	**Ups**	**0.00352**	**0.01772**	_	_
ENSG000	LINC0020	10796	0	0	1	0.01	0	0	22	0.11	31	0.15	10	0.05	9.770118	0.555529	5.357005	**2.97597**	**Ups**	**8.5E-08**	**1.7E-06**	_	_
ENSG000	LINC0027	22895	1	0	8	0.03	5	0.02	73	0.25	7	0.02	39	0.12	21.92979	0.551631	3.447733	**1.90188**	**Ups**	**0.00057**	**0.00391**	_	_
ENSG000	LINC0028	121748	2	0.01	0	0	0	0	1	0	17	0.05	9	0.03	4.229264	0.583447	2.628313	**1.53348**	**Ups**	**0.00858**	**0.03573**	_	_
ENSG000	LINC0030	5100	0	0	0	0	0	0	1	0.01	4	0.02	9	0.04	2.078923	0.570695	2.622616	**1.49671**	**Ups**	**0.00873**	**0.03622**	_	_
ENSG000	LINC0031	17441	203	1.91	152	1.49	174	1.65	24	0.23	30	0.27	32	0.28	107.2038	0.251746	-10.736	**-2.7027**	**Down**	**6.9E-27**	**1.1E-24**	_	_
ENSG000	LINC0032	9623	9	0.33	16	0.61	19	0.7	46	1.76	49	1.74	28	0.97	26.79492	0.383095	2.895213	**1.10914**	**Ups**	**0.00379**	**0.0188**	_	_
ENSG000	LINC0032	3402	43	0.53	58	0.75	47	0.58	156	2	144	1.72	142	1.65	94.57418	0.2503	5.198961	**1.3013**	**Ups**	**2E-07**	**3.6E-06**	_	_
ENSG000	LINC0034	9098	11	0.1	13	0.12	18	0.17	108	1.02	200	1.76	149	1.28	76.2177	0.313473	9.387178	**2.94263**	**Ups**	**6.2E-21**	**6.1E-19**	_	_
ENSG000	LINC0046	127243	1	0.01	1	0.01	1	0.01	6	0.07	14	0.15	13	0.14	5.461821	0.555037	3.510921	**1.94869**	**Ups**	**0.00045**	**0.0032**	_	_
ENSG000	LINC0047	556875	423	1.23	514	1.55	395	1.15	223	0.67	205	0.57	198	0.54	333.8886	0.204937	-6.13572	**-1.2574**	**Down**	**8.5E-10**	**2.4E-08**	_	_
ENSG000	LINC0048	7093	0	0	0	0	0	0	4	0.04	8	0.08	4	0.04	2.399063	0.581432	3.079623	**1.79059**	**Ups**	**0.00207**	**0.01151**	_	_
ENSG000	LINC0050	417705	3	0.01	3	0.01	3	0.01	20	0.09	28	0.12	17	0.07	11.48529	0.473613	4.309167	**2.04088**	**Ups**	**1.6E-05**	**0.00018**	_	_
ENSG000	LINC0051	317348	130	0.28	141	0.32	148	0.32	14	0.03	2	0	13	0.03	79.3256	0.373009	-9.51885	**-3.5506**	**Down**	**1.8E-21**	**1.8E-19**	_	_
ENSG000	LINC0051	6313	14	0.08	28	0.18	49	0.3	13	0.08	2	0.01	4	0.02	19.42147	0.514717	-3.41405	**-1.7573**	**Down**	**0.00064**	**0.00433**	_	_
ENSG000	LINC0051	5620	40	0.19	35	0.18	41	0.2	4	0.02	4	0.02	8	0.04	23.10372	0.402674	-6.53046	**-2.6297**	**Down**	**6.6E-11**	**2.3E-09**	_	_
ENSG000	LINC0052	5297	35	0.38	25	0.28	36	0.39	5	0.06	6	0.06	7	0.07	19.83529	0.406338	-5.53707	**-2.2499**	**Down**	**3.1E-08**	**6.5E-07**	_	_
ENSG000	LINC0053	781	1	0.05	0	0	1	0.05	3	0.15	11	0.51	6	0.27	3.297823	0.580575	2.608512	**1.51444**	**Ups**	**0.00909**	**0.03734**	_	_
ENSG000	LINC0060	8011	12	0.26	10	0.23	14	0.31	1	0.02	5	0.11	3	0.06	7.730134	0.506218	-3.26872	**-1.6547**	**Down**	**0.00108**	**0.00672**	_	_
ENSG000	LINC0060	94862	2	0.09	0	0	0	0	13	0.6	13	0.55	14	0.58	6.496054	0.553673	4.307802	**2.38512**	**Ups**	**1.7E-05**	**0.00018**	_	_
ENSG000	LINC0067	93073	2	0.01	7	0.04	3	0.02	5	0.03	57	0.33	29	0.16	15.26061	0.539779	3.280087	**1.77052**	**Ups**	**0.00104**	**0.00651**	_	_
ENSG000	LINC0067	2890	3	0.03	5	0.06	2	0.02	14	0.16	16	0.17	14	0.14	8.515547	0.488751	2.995619	**1.46411**	**Ups**	**0.00274**	**0.01446**	_	_
ENSG000	LINC0067	2161	0	0	0	0	0	0	2	0.07	6	0.18	5	0.15	1.927365	0.575032	2.711067	**1.55895**	**Ups**	**0.00671**	**0.02949**	_	_
ENSG000	LINC0070	27970	7	0.08	15	0.18	3	0.03	0	0	0	0	0	0	4.452572	0.582809	-4.05399	**-2.3627**	**Down**	**5E-05**	**0.00048**	_	_
ENSG000	LINC0070	63309	609	5.1	608	5.31	671	5.65	18	0.16	145	1.17	43	0.34	366.0619	0.423777	-6.94529	**-2.9433**	**Down**	**3.8E-12**	**1.6E-10**	_	_
ENSG000	LINC0088	30826	5	0.04	7	0.06	0	0	16	0.14	23	0.18	32	0.25	12.88218	0.493541	3.578964	**1.76637**	**Ups**	**0.00034**	**0.00256**	_	_
ENSG000	LINC0088	69717	26	0.15	34	0.21	40	0.24	132	0.81	107	0.61	85	0.47	68.21011	0.312343	4.408604	**1.377**	**Ups**	**1E-05**	**0.00012**	_	_
ENSG000	LINC0089	285970	29	0.07	26	0.07	23	0.06	37	0.1	141	0.35	69	0.17	50.15664	0.383837	3.176657	**1.21932**	**Ups**	**0.00149**	**0.00876**	_	_
ENSG000	LINC0091	17840	3	0.01	2	0.01	6	0.02	20	0.07	12	0.04	19	0.06	9.931326	0.492585	3.09877	**1.52641**	**Ups**	**0.00194**	**0.01093**	_	_
ENSG000	LINC0092	7397	20	0.11	16	0.09	25	0.14	92	0.53	49	0.26	104	0.54	49.15307	0.371014	4.322325	**1.60364**	**Ups**	**1.5E-05**	**0.00017**	_	_
ENSG000	LINC0093	3632	79	0.56	63	0.47	51	0.36	275	2.02	217	1.48	296	1.98	156.2598	0.26294	6.577676	**1.72953**	**Ups**	**4.8E-11**	**1.7E-09**	_	_
ENSG000	LINC0094	7669	46	0.29	36	0.23	42	0.26	10	0.06	19	0.11	8	0.05	27.62459	0.361597	-4.8733	**-1.7622**	**Down**	**1.1E-06**	**1.7E-05**	_	_
ENSG000	LINC0095	3136	10	0.07	10	0.07	11	0.07	46	0.31	52	0.33	48	0.3	27.87265	0.345785	5.24947	**1.81519**	**Ups**	**1.5E-07**	**2.8E-06**	_	_
ENSG000	LINC0096	2204	73	1.16	69	1.15	68	1.09	13	0.22	34	0.52	21	0.32	47.49506	0.309771	-5.57373	**-1.7266**	**Down**	**2.5E-08**	**5.4E-07**	_	_
ENSG000	LINC0098	3037	153	1.6	159	1.73	161	1.69	464	5.05	546	5.51	453	4.47	307.9949	0.169177	8.145502	**1.37803**	**Ups**	**3.8E-16**	**2.5E-14**	_	_
ENSG000	LINC0109	1727	0	0	1	0.02	0	0	7	0.11	9	0.14	2	0.03	2.933639	0.583392	2.711925	**1.58212**	**Ups**	**0.00669**	**0.02944**	_	_
ENSG000	LINC0111	8343	14	0.17	11	0.14	8	0.1	112	1.39	146	1.68	175	1.97	71.69696	0.320036	9.968708	**3.19034**	**Ups**	**2.1E-23**	**2.5E-21**	_	_
ENSG000	LINC0111	18249	0	0	0	0	0	0	11	0.42	18	0.64	13	0.45	6.343876	0.569081	5.081793	**2.89195**	**Ups**	**3.7E-07**	**6.3E-06**	_	_
ENSG000	LINC0112	38848	13	0.02	24	0.04	28	0.04	80	0.13	79	0.12	66	0.1	46.28011	0.328067	4.384775	**1.4385**	**Ups**	**1.2E-05**	**0.00013**	_	_
ENSG000	LINC0113	7602	36	0.24	47	0.33	39	0.27	142	1	88	0.58	125	0.8	77.21064	0.308218	4.038959	**1.24488**	**Ups**	**5.4E-05**	**0.00051**	_	_
ENSG000	LINGO2	722208	22	0.1	30	0.14	28	0.12	86	0.39	59	0.25	74	0.31	48.39495	0.319406	3.601028	**1.15019**	**Ups**	**0.00032**	**0.00238**	cellular_cc	_
ENSG000	LMCD1	66413	0	0	1	0.01	2	0.01	2	0.01	19	0.1	8	0.04	4.718792	0.578904	2.67003	**1.54569**	**Ups**	**0.00758**	**0.03241**	biological_	_
ENSG000	LMOD1	50136	1	0.01	4	0.03	5	0.03	21	0.14	89	0.56	64	0.39	27.36018	0.483406	6.029673	**2.91478**	**Ups**	**1.6E-09**	**4.4E-08**	molecular_	_
ENSG000	LNPEP	102122	457	0.7	542	0.87	420	0.65	848	1.35	3392	5.01	1120	1.62	1037.152	0.360021	4.054941	**1.45986**	**Ups**	**5E-05**	**0.00048**	biological_	K01257
ENSG000	LNX1	242105	11	0.03	22	0.07	14	0.04	35	0.11	64	0.18	44	0.12	29.90295	0.356822	3.421845	**1.22099**	**Ups**	**0.00062**	**0.00422**	molecular_	K10692
ENSG000	LOX	15091	689	2.11	793	2.53	597	1.83	1411	4.48	3693	10.89	2130	6.14	1445.162	0.265735	5.470694	**1.45375**	**Ups**	**4.5E-08**	**9.2E-07**	biological_	K00277
ENSG000	LOXL2	128140	8951	23.9	8748	24.36	8224	22.08	5325	14.79	4125	10.63	4180	10.53	6733.193	0.200538	-5.43429	**-1.0898**	**Down**	**5.5E-08**	**1.1E-06**	biological_	K00280
ENSG000	LOXL4	20561	16	0.11	14	0.1	20	0.14	54	0.4	44	0.3	53	0.35	32.28414	0.335297	3.75538	**1.25917**	**Ups**	**0.00017**	**0.00142**	molecular_	K00280
ENSG000	LPAR1	165439	19	0.04	20	0.05	19	0.04	81	0.19	147	0.32	105	0.22	60.44113	0.295478	7.182629	**2.12231**	**Ups**	**6.8E-13**	**3.1E-11**	biological_	K04289
ENSG000	LPAR4	9386	2	0.01	3	0.02	1	0.01	6	0.04	34	0.19	8	0.04	8.01408	0.555288	3.072429	**1.70608**	**Ups**	**0.00212**	**0.01174**	molecular_	_
ENSG000	LPAR5	17613	61	0.36	39	0.24	39	0.23	12	0.07	3	0.02	10	0.05	28.67221	0.414815	-5.43311	**-2.2537**	**Down**	**5.5E-08**	**1.1E-06**	molecular_	K08390
ENSG000	LPHN2	686276	2	0	3	0	4	0.01	83	0.12	86	0.11	55	0.07	36.10014	0.428339	8.435302	**3.61317**	**Ups**	**3.3E-17**	**2.4E-15**	molecular_	K04593
ENSG000	LPL	65542	129	0.56	105	0.47	110	0.48	493	2.21	2894	12.03	1059	4.3	706.2314	0.397581	7.389331	**2.93786**	**Ups**	**1.5E-13**	**7.3E-12**	biological_	K01059
ENSG000	LPP	737389	778	0.69	764	0.71	631	0.56	822	0.76	3829	3.29	1377	1.16	1265.762	0.366612	2.873326	**1.05339**	**Ups**	**0.00406**	**0.01986**	molecular_	K16676
ENSG000	LPPR4	45638	0	0	1	0	0	0	8	0.02	32	0.08	19	0.05	8.846551	0.559788	5.096959	**2.85321**	**Ups**	**3.5E-07**	**5.8E-06**	molecular_	K19582
ENSG000	LRFN4	3829	6718	40.59	5489	34.58	6242	37.93	3870	24.32	1673	9.75	3586	20.43	4721.594	0.308517	-3.56948	**-1.1012**	**Down**	**0.00036**	**0.00264**	cellular_cc	K16357
ENSG000	LRG1	4066	39	0.28	19	0.14	33	0.24	6	0.04	10	0.07	4	0.03	19.19399	0.42941	-4.68427	**-2.0115**	**Down**	**2.8E-06**	**3.8E-05**	molecular_	K17985
ENSG000	LRGUK	137292	15	0.11	16	0.12	12	0.09	5	0.04	8	0.06	4	0.03	10.22471	0.456275	-2.77631	**-1.2668**	**Down**	**0.0055**	**0.02527**	molecular_	_
ENSG000	LRIG1	122467	294	0.72	226	0.58	229	0.57	1192	3.04	944	2.24	944	2.19	611.3735	0.225134	7.869932	**1.77179**	**Ups**	**3.6E-15**	**2.1E-13**	cellular_cc	_
ENSG000	LRP2	235577	42	0.06	45	0.07	44	0.07	136	0.21	299	0.42	187	0.26	116.312	0.278346	6.737229	**1.87528**	**Ups**	**1.6E-11**	**6.1E-10**	biological_	K06233
ENSG000	LRRC16B	17732	1	0	3	0.01	2	0.01	10	0.03	13	0.03	16	0.04	7.002683	0.522086	3.324765	**1.73581**	**Ups**	**0.00088**	**0.00568**	cellular_cc	_
ENSG000	LRRC17	31959	2	0.01	1	0.01	0	0	5	0.03	10	0.06	12	0.07	4.579469	0.565997	2.996709	**1.69613**	**Ups**	**0.00273**	**0.01442**	biological_	_
ENSG000	LRRC20	83654	70	0.28	69	0.29	66	0.26	259	1.07	157	0.6	231	0.86	137.6292	0.285502	4.810121	**1.3733**	**Ups**	**1.5E-06**	**2.2E-05**	_	K04240
ENSG000	LRRC3	3371	59	0.6	40	0.42	54	0.55	12	0.13	24	0.24	20	0.19	35.61848	0.328029	-4.68886	**-1.5381**	**Down**	**2.8E-06**	**3.8E-05**	molecular_	K17985
ENSG000	LRRC56	17390	36	0.33	19	0.18	21	0.2	86	0.83	40	0.36	65	0.57	43.62423	0.386396	2.608255	**1.00782**	**Ups**	**0.0091**	**0.03736**	_	K09228
ENSG000	LRRC61	15512	7504	32.17	7189	32.14	7671	33.07	3077	13.72	1496	6.19	2780	11.24	5138.933	0.28462	-5.87467	**-1.672**	**Down**	**4.2E-09**	**1.1E-07**	_	_
ENSG000	LRRC8C	136832	46	0.1	56	0.12	46	0.1	9	0.02	20	0.04	14	0.03	32.79759	0.336844	-5.44094	**-1.8327**	**Down**	**5.3E-08**	**1.1E-06**	cellular_cc	_
ENSG000	LRRN2	68564	322	0.91	273	0.8	354	1	892	2.61	743	2.02	897	2.38	560.5891	0.208951	5.603611	**1.17088**	**Ups**	**2.1E-08**	**4.6E-07**	molecular_	_
ENSG000	LSMEM2	9088	7	0.12	10	0.18	8	0.14	17	0.3	23	0.38	30	0.49	15.08402	0.411684	2.602642	**1.07147**	**Ups**	**0.00925**	**0.03789**	cellular_cc	_
ENSG000	LSP1	39298	47	0.18	48	0.19	33	0.12	8	0.03	21	0.08	16	0.06	29.46095	0.356592	-4.38879	**-1.565**	**Down**	**1.1E-05**	**0.00013**	molecular_	K14957
ENSG000	LTBP1	452538	170	0.3	177	0.32	184	0.32	1078	1.96	3037	5.13	1577	2.6	938.1812	0.283636	10.42529	**2.95698**	**Ups**	**1.9E-25**	**2.7E-23**	molecular_	K19559
ENSG000	LTBP3	20126	953	1.43	714	1.12	907	1.37	2951	4.61	1603	2.32	2737	3.88	1600.352	0.278036	4.453508	**1.23824**	**Ups**	**8.5E-06**	**0.0001**	molecular_	K08023
ENSG000	LUM	9203	379	2.03	598	3.34	353	1.9	13	0.07	17	0.09	12	0.06	243.4683	0.311844	-15.3479	**-4.7861**	**Down**	**3.7E-53**	**2.1E-50**	molecular_	K08122
ENSG000	LY6D	1713	23	0.17	18	0.14	32	0.23	0	0	3	0.02	3	0.02	13.88308	0.492995	-5.7455	**-2.8325**	**Down**	**9.2E-09**	**2.1E-07**	cellular_cc	K06846
ENSG000	LYPD1	26727	19	0.15	11	0.09	19	0.15	2	0.02	0	0	0	0	9.063659	0.541614	-5.32894	**-2.8862**	**Down**	**9.9E-08**	**1.9E-06**	cellular_cc	_
ENSG000	LYPD3	4874	455	3.05	387	2.7	432	2.91	82	0.57	106	0.69	127	0.8	274.6968	0.197086	-11.0391	**-2.1757**	**Down**	**2.5E-28**	**4.1E-26**	biological_	_
ENSG000	LYPD6	144164	115	0.33	134	0.4	137	0.39	313	0.92	704	1.93	417	1.12	282.9794	0.258538	6.011928	**1.55431**	**Ups**	**1.8E-09**	**5E-08**	cellular_cc	_
ENSG000	LYRM9	16439	198	0.63	178	0.59	245	0.78	617	2.04	349	1.07	538	1.62	346.2848	0.287427	3.541863	**1.01803**	**Ups**	**0.0004**	**0.00289**	_	_
ENSG000	LYSMD3	13974	271	0.75	744	2.15	239	0.67	864	2.49	2932	7.84	1347	3.52	981.348	0.404044	3.746519	**1.51376**	**Ups**	**0.00018**	**0.00146**	cellular_cc	_
ENSG000	MAFF	14630	406	1.2	295	0.91	404	1.21	1425	4.4	369	1.06	933	2.61	633.7567	0.391129	2.563702	**1.00274**	**Ups**	**0.01036**	**0.04132**	biological_	K09037
ENSG000	MAGEA1	4594	23	0.35	21	0.33	25	0.38	819	12.82	423	6.14	810	11.5	333.8466	0.331744	12.89559	**4.27803**	**Ups**	**4.8E-38**	**1.3E-35**	biological_	_
ENSG000	MAGEA12	3892	138	1.01	170	1.3	126	0.93	1812	13.78	1496	10.55	1925	13.27	886.2944	0.21866	14.92381	**3.26323**	**Ups**	**2.3E-50**	**1.1E-47**	molecular_	_
ENSG000	MAGEA2	3978	7	0.03	8	0.04	6	0.03	19	0.09	26	0.12	20	0.09	13.62277	0.416706	2.854877	**1.18964**	**Ups**	**0.00431**	**0.02082**	biological_	_
ENSG000	MAGEA8	4669	0	0	1	0.01	0	0	21	0.11	57	0.28	40	0.2	17.7071	0.527721	7.124076	**3.75952**	**Ups**	**1.1E-12**	**4.6E-11**	molecular_	_
ENSG000	MAGEB17	3984	32	0.31	47	0.47	25	0.24	1	0.01	14	0.13	10	0.09	22.13867	0.455842	-4.11931	**-1.8778**	**Down**	**3.8E-05**	**0.00038**	_	_
ENSG000	MAGEB18	2393	0	0	0	0	1	0.01	1	0.01	12	0.16	7	0.09	3.066889	0.582699	2.655129	**1.54714**	**Ups**	**0.00793**	**0.03358**	molecular_	_
ENSG000	MAGEB6	3207	29	0.38	23	0.32	19	0.25	42	0.58	137	1.74	87	1.08	51.91068	0.366641	3.980852	**1.45954**	**Ups**	**6.9E-05**	**0.00063**	molecular_	_
ENSG000	MAGEH1	1461	1	0.02	1	0.02	1	0.02	35	0.64	106	1.8	85	1.41	34.02608	0.491186	8.440256	**4.14574**	**Ups**	**3.2E-17**	**2.4E-15**	cellular_cc	_
ENSG000	MAGI2	1436498	4	0.01	2	0	4	0.01	8	0.02	24	0.04	21	0.04	9.638658	0.498542	3.235303	**1.61293**	**Ups**	**0.00122**	**0.0074**	biological_	K05629
ENSG000	MALL	32697	0	0	0	0	0	0	4	0.02	19	0.11	5	0.03	4.073043	0.583409	3.724363	**2.17283**	**Ups**	**0.0002**	**0.00158**	cellular_cc	_
ENSG000	MAMDC2	183390	450	2.82	752	4.92	420	2.65	3819	24.9	8967	54.23	5360	31.69	2995.314	0.284541	10.61752	**3.02112**	**Ups**	**2.5E-26**	**3.7E-24**	molecular_	_
ENSG000	MAMDC2	90073	3	0.01	0	0	5	0.01	40	0.1	64	0.15	37	0.09	22.74961	0.458133	6.695116	**3.06725**	**Ups**	**2.2E-11**	**8E-10**	_	_
ENSG000	MAN1A1	172553	442	1.99	626	2.94	330	1.49	1459	6.83	5767	25.05	2411	10.24	1661.702	0.359036	6.235431	**2.23874**	**Ups**	**4.5E-10**	**1.3E-08**	cellular_cc	K01230
ENSG000	MANEAL	7336	1178	3.45	868	2.65	1094	3.23	360	1.1	622	1.76	476	1.32	778.5157	0.195452	-6.68281	**-1.3062**	**Down**	**2.3E-11**	**8.6E-10**	cellular_cc	_
ENSG000	MAOA	90602	7	0.03	4	0.02	5	0.02	38	0.18	78	0.33	38	0.16	25.9102	0.420852	6.015682	**2.53171**	**Ups**	**1.8E-09**	**4.8E-08**	cellular_cc	K00274
ENSG000	MAOB	115836	682	4.16	609	3.88	657	4.03	166	1.05	556	3.28	339	1.95	503.4106	0.301361	-3.52766	**-1.0631**	**Down**	**0.00042**	**0.00303**	cellular_cc	K00274
ENSG000	MAP2	310061	11	0.01	17	0.02	4	0.01	113	0.15	162	0.2	101	0.12	62.7956	0.362126	8.155794	**2.95343**	**Ups**	**3.5E-16**	**2.3E-14**	biological_	K10430
ENSG000	MAP2K6	128634	2226	5.99	2729	7.67	2386	6.46	971	2.72	1757	4.57	1275	3.24	1910.845	0.187911	-5.79321	**-1.0886**	**Down**	**6.9E-09**	**1.7E-07**	biological_	K04433
ENSG000	MAP3K14	53927	1055	2.7	845	2.26	1020	2.63	407	1.08	717	1.77	513	1.24	766.3742	0.191396	-5.45919	**-1.0449**	**Down**	**4.8E-08**	**9.8E-07**	molecular_	_
ENSG000	MAP7D2	110205	3	0.01	3	0.01	2	0.01	5	0.01	17	0.04	16	0.04	7.044038	0.525691	2.672765	**1.40505**	**Ups**	**0.00752**	**0.03224**	_	_

ENSG000	MAPK4	171747	685	1.94	482	1.42	598	1.7	1591	4.69	4735	12.94	2352	6.28	1597.651	0.295388	6.404843	1.89192	Ups	1.5E-10	4.8E-09	biological_	_
ENSG000	MAPKAPK	37770	2579	9.5	2109	8.11	2332	8.64	1010	3.87	913	3.25	1100	3.82	1715.224	0.174527	-7.96971	-1.3909	Down	1.6E-15	9.7E-14	biological_	K04444
ENSG000	MAPRE3	56585	453	2.01	360	1.67	444	1.98	215	0.99	224	0.96	230	0.96	326.433	0.181434	-6.05014	-1.0977	Down	1.5E-09	4E-08	;molecular	K10436
ENSG000	MARCO	52495	4	0.03	6	0.05	3	0.02	0	0	0	0	0	0	2.315393	0.580592	-3.13264	-1.8188	Down	0.00173	0.0099	biological_	K13884
ENSG000	MAST4	573248	88	0.07	84	0.07	66	0.05	211	0.18	686	0.55	304	0.24	219.4314	0.336069	5.583608	1.87648	Ups	2.4E-08	5.1E-07	molecular_	K08789
ENSG000	MATN2	167877	480	0.92	462	0.93	460	0.89	736	1.47	2299	4.27	1219	2.21	878.2088	0.292655	4.246371	1.24272	Ups	2.2E-05	0.00023	biological_	_
ENSG000	MBLAC2	16566	328	1.36	410	1.77	244	1.01	502	2.16	1637	6.53	824	3.21	611.9577	0.325673	3.716033	1.21021	Ups	0.0002	0.00162	molecular_	K14026
ENSG000	MBOAT1	111736	766	5.16	676	4.75	687	4.65	1593	11.15	1884	12.23	1622	10.3	1158.357	0.133331	7.698824	1.02649	Ups	1.4E-14	7.6E-13	cellular_cc	K13517
ENSG000	MCOLN3	30418	67	0.15	68	0.16	53	0.12	357	0.82	835	1.79	485	1.02	283.5659	0.276335	9.875082	2.72883	Ups	5.3E-23	6.1E-21	cellular_cc	K04994
ENSG000	MDFIC	97048	487	2.02	861	3.72	382	1.59	963	4.15	2183	8.74	1802	7.05	1047.551	0.310989	3.745483	1.16481	Ups	0.00018	0.00147	molecular_	_
ENSG000	MDK	3070	15	0.06	7	0.03	18	0.08	84	0.37	34	0.14	62	0.25	35.63531	0.427766	3.906346	1.671	Ups	9.4E-05	0.00083	biological_	K06828
ENSG000	MED12L	351377	18	0.03	25	0.05	11	0.02	2	0	6	0.01	0	0	10.78606	0.518814	-4.0984	-2.1263	Down	4.2E-05	0.00041	molecular_	K15162
ENSG000	MED13L	319433	454	0.72	491	0.82	342	0.55	783	1.3	2626	4.04	1076	1.62	889.4946	0.329397	4.234521	1.39484	Ups	2.3E-05	0.00024	molecular_	K15164
ENSG000	MED28P7	530	0	0	2	0.1	1	0.05	7	0.35	6	0.28	9	0.41	3.929474	0.570622	2.632929	1.50241	Ups	0.00847	0.03541	_	K15141
ENSG000	MED31	8320	1485	12.32	1389	12.02	1292	10.78	675	5.82	615	4.92	763	5.97	1057.73	0.174061	-6.90688	-1.2022	Down	5E-12	2E-10	molecular_	K15153
ENSG000	MEF2A	239302	962	1.02	1090	1.21	900	0.96	1406	1.55	5062	5.18	1948	1.95	1762.168	0.330887	3.399796	1.12495	Ups	0.00067	0.00453	biological_	K09260
ENSG000	MEF2C	185948	8	0.01	4	0	0	0	46	0.04	42	0.03	37	0.03	21.42602	0.455295	5.576197	2.53882	Ups	2.5E-08	5.3E-07	biological_	K04454
ENSG000	MEIS3	16400	52	0.14	71	0.2	70	0.19	192	0.54	173	0.45	210	0.54	123.0684	0.245491	5.293092	1.29941	Ups	1.2E-07	2.3E-06	molecular_	K16671
ENSG000	MEX3B	4364	58	0.19	60	0.2	54	0.18	128	0.44	214	0.68	147	0.45	104.4356	0.237436	5.121885	1.21612	Ups	3E-07	5.2E-06	cellular_cc	K15686
ENSG000	MFAP2	6334	71	0.39	54	0.31	87	0.48	526	3.03	96	0.51	370	1.93	197.3876	0.443713	3.8563	1.71109	Ups	0.00012	0.00099	cellular_cc	_
ENSG000	MFNG	17339	41	0.16	21	0.08	42	0.16	3	0.01	7	0.03	6	0.02	20.89262	0.43279	-5.60692	-2.4266	Down	2.1E-08	4.5E-07	cellular_cc	K05948
ENSG000	MFSD4	34034	20	0.06	15	0.05	18	0.06	37	0.12	52	0.16	55	0.17	31.27866	0.32132	3.463787	1.11298	Ups	0.00053	0.00373	cellular_cc	K17985
ENSG000	MGAT4A	112021	190	0.37	235	0.48	172	0.34	343	0.7	942	1.77	476	0.88	367.5071	0.296939	4.064621	1.20695	Ups	4.8E-05	0.00046	cellular_cc	K00738
ENSG000	MGC4294	945	0	0	0	0	0	0	8	0.23	1	0.03	4	0.1	2.119275	0.569084	2.580793	1.46869	Ups	0.00986	0.0398	_	_
ENSG000	MGLL	134143	5895	9.78	5576	9.65	6255	10.44	2793	4.82	4006	6.41	3157	4.94	4668.634	0.13382	-7.83914	-1.049	Down	4.5E-15	2.7E-13	;molecular	K01054
ENSG000	MGMT	300824	84	0.57	70	0.49	88	0.6	303	2.13	86	0.56	310	1.98	153.8473	0.402167	2.914933	1.17229	Ups	0.00356	0.01786	molecular_	K00567
ENSG000	MGP	4746	10	0.07	12	0.09	11	0.08	30	0.22	34	0.23	38	0.25	21.47566	0.362182	3.458457	1.25259	Ups	0.00054	0.00379	biological_	K19481
ENSG000	MGST1	262118	7167	20.95	8201	25	7630	22.43	2208	6.71	2741	7.73	3094	8.53	5323.754	0.149133	-11.4851	-1.7128	Down	1.6E-30	3E-28	molecular_	K00799
ENSG000	MICALCL	83065	3	0.02	9	0.06	8	0.05	40	0.28	62	0.4	21	0.13	22.22215	0.452362	4.268222	1.93078	Ups	2E-05	0.00021	cellular_cc	_
ENSG000	MINOS1-	61077	20	0.19	16	0.16	24	0.23	2	0.02	6	0.05	2	0.02	12.1678	0.473611	-4.6418	-2.1984	Down	3.5E-06	4.6E-05	_	K19558
ENSG000	MIPEP	159231	781	5.36	774	5.54	755	5.21	238	1.7	252	1.67	266	1.72	527.2944	0.150773	-11.9244	-1.7979	Down	8.8E-33	2E-30	molecular_	K01410
ENSG000	MIPEPP3	8235	18	0.58	14	0.47	27	0.88	7	0.24	6	0.19	9	0.28	13.91499	0.436141	-3.12087	-1.3611	Down	0.0018	0.01024	_	K01410
ENSG000	MIR1249	66	5	1.95	3	1.22	4	1.57	1	0.41	0	0	0	0	2.305389	0.582828	-2.55591	-1.4897	Down	0.01059	0.04204	_	_
ENSG000	MIR137H	61864	79	0.22	125	0.37	108	0.31	23	0.07	91	0.25	55	0.15	80.52184	0.362203	-2.84782	-1.0315	Down	0.0044	0.02117	_	_
ENSG000	MIR23A	73	3	1.06	0	0	0	0	14	5.13	5	1.7	46	15.28	10.54363	0.575278	3.816305	2.19544	Ups	0.00014	0.00114	_	_
ENSG000	MIR24-2	1844	858	11.94	735	10.67	919	12.86	5347	77.4	1506	20.22	3727	48.92	2138.219	0.369836	4.59716	1.70019	Ups	4.3E-06	5.6E-05	_	_
ENSG000	MIR296	594	53	2.02	24	0.95	56	2.15	18	0.71	10	0.37	21	0.76	31.30896	0.404421	-3.4853	-1.4095	Down	0.00049	0.00348	_	_
ENSG000	MIR3153	82	2	0.63	2	0.65	3	0.95	4	1.3	54	16.33	9	2.66	10.77129	0.565469	3.105251	1.75592	Ups	0.0019	0.01072	_	_
ENSG000	MIR3197	73	36	12.68	15	5.51	19	6.73	1	0.37	1	0.34	7	2.32	13.76986	0.510553	-4.49935	-2.2972	Down	6.8E-06	8.4E-05	_	_
ENSG000	MIR3671	88	0	0	3	0.91	0	0	6	1.82	12	3.38	7	1.93	4.286459	0.573106	2.713672	1.55522	Ups	0.00665	0.02933	_	_
ENSG000	MIR378I	76	5	1.69	1	0.35	1	0.34	14	4.93	15	4.89	20	6.38	8.731065	0.512405	3.640573	1.86545	Ups	0.00027	0.00209	_	_
ENSG000	MIR4645	77	0	0	6	2.09	2	0.67	14	4.86	31	9.98	10	3.15	9.6471	0.538066	3.170337	1.70585	Ups	0.00152	0.0089	_	_
ENSG000	MIR4712	82	5	1.57	9	2.94	3	0.95	10	3.26	54	16.33	28	8.28	16.43987	0.496187	3.285697	1.63032	Ups	0.00102	0.0064	_	_
ENSG000	MIR519A:	2239	6	0.07	6	0.07	2	0.02	17	0.2	19	0.2	24	0.25	11.65472	0.458675	3.290667	1.50935	Ups	0.001	0.00631	_	_
ENSG000	MIR568	95	10	2.71	5	1.41	7	1.91	21	5.91	46	12.01	21	5.36	17.05548	0.433477	3.367266	1.45963	Ups	0.00076	0.00501	_	_
ENSG000	MIR589	99	17	4.41	9	2.44	13	3.39	2	0.54	6	1.5	4	0.98	8.705809	0.490612	-3.01723	-1.4803	Down	0.00255	0.01366	_	_
ENSG000	MIR616	97	3	0.8	3	0.83	4	1.07	16	4.41	15	3.84	15	3.75	8.874497	0.483606	3.163967	1.53011	Ups	0.00156	0.00904	_	_
ENSG000	MLC1	26512	2	0.01	3	0.01	4	0.02	15	0.06	11	0.04	15	0.05	7.973163	0.501765	2.934517	1.47244	Ups	0.00334	0.01699	molecular_	_
ENSG000	MLF1	36090	154	0.41	169	0.46	111	0.29	389	1.07	410	1.04	487	1.21	274.2804	0.218042	5.970961	1.30192	Ups	2.4E-09	6.3E-08	biological_	K15622
ENSG000	MLXIPL	31350	35	0.11	22	0.07	25	0.08	186	0.59	195	0.58	200	0.58	103.7109	0.256594	9.647876	2.47558	Ups	5E-22	5.3E-20	biological_	K09113
ENSG000	MME	159585	4	0.01	2	0	3	0.01	20	0.04	84	0.16	30	0.06	21.19142	0.496262	5.398594	2.67912	Ups	6.7E-08	1.3E-06	biological_	K01389
ENSG000	MMP11	16091	24	0.06	19	0.05	16	0.04	88	0.25	63	0.16	110	0.28	50.9178	0.337838	5.213625	1.76136	Ups	1.9E-07	3.4E-06	molecular_	K07993
ENSG000	MMP13	12740	15	0.13	34	0.3	20	0.17	10	0.09	7	0.06	14	0.11	17.11946	0.433103	-2.63303	-1.1404	Down	0.00846	0.03541	molecular_	K07994
ENSG000	MMP17	23391	32	0.1	25	0.08	35	0.11	169	0.53	48	0.14	156	0.44	75.62162	0.41815	3.752088	1.56893	Ups	0.00018	0.00143	molecular_	K07997
ENSG000	MMP24	50345	573	3.34	490	2.98	507	2.97	290	1.76	348	1.96	258	1.42	416.3841	0.174933	-5.74681	-1.0053	Down	9.1E-09	2.1E-07	molecular_	K08002
ENSG000	MMP7	10246	6	0.05	5	0.05	5	0.04	0	0	0	0	2	0.02	3.14699	0.580999	-2.72954	-1.5859	Down	0.00634	0.02826	biological_	K01397
ENSG000	MOB1B	120124	216	0.55	411	1.1	184	0.47	427	1.13	1199	2.96	614	1.48	476.5778	0.346435	3.124699	1.0825	Ups	0.00178	0.01014	molecular_	K06685
ENSG000	MPDU1	9261	5784	10.67	5294	10.18	6296	11.68	3534	6.78	2444	4.35	3606	6.27	4584.987	0.225079	-4.50585	-1.0142	Down	6.6E-06	8.2E-05	cellular_cc	K09660
ENSG000	MPHOSPI	22429	1070	6.57	1200	7.69	940	5.81	432	2.76	675	4	642	3.72	836.0627	0.177988	-6.09286	-1.0845	Down	1.1E-09	3.1E-08	biological_	K12593
ENSG000	MPP1	42324	556	1.24	499	1.16	562	1.26	45	0.1	169	0.36	91	0.19	332.8017	0.317774	-7.74196	-2.4602	Down	9.8E-15	5.6E-13	molecular_	_
ENSG000	MPZL2	11134	649	2.29	908	3.34	592	2.1	120	0.44	414	1.41	268	0.89	501.2292	0.32261	-4.80786	-1.5511	Down	1.5E-06	2.2E-05	molecular_	_
ENSG000	MRAP2	57126	12	0.14	7	0.09	14	0.17	207	2.57	47	0.54	167	1.88	73.18096	0.461193	6.085216	2.80646	Ups	1.2E-09	3.2E-08	cellular_cc	_
ENSG000	MRM1	7407	919	8.46	711	6.82	909	8.41	281	2.69	237	2.1	347	3.01	585.4082	0.221149	-7.6679	-1.6957	Down	1.8E-14	9.6E-13	molecular_	K15507
ENSG000	MROH1	113925	450	0.85	417	0.82	390	0.74	233	0.46	231	0.42	249	0.44	333.3136	0.172059	-5.86235	-1.0087	Down	4.6E-09	1.1E-07	_	_
ENSG000	MROH6	6785	836	1.74	576	1.25	715	1.5	286	0.62	205	0.41	246	0.48	492.9754	0.241923	-6.8064	-1.6466	Down	1E-11	3.9E-10	_	_
ENSG000	MRP63	2440	3202	36.65	2646	31.58	2857	32.89	1362	16.21	740	8.17	1551	16.74	2119.541	0.282046	-4.79275	-1.3518	Down	1.7E-06	2.4E-05	molecular_	K17985
ENSG000	MRPL19	44069	4438	19.61	4512	20.8	3891	17.29	1825	8.39	3132	13.35	2278	9.49	3376.876	0.168095	-6.21299	-1.0444	Down	5.2E-10	1.5E-08	molecular_	K02884
ENSG000	MRPL23-,	6684	412	5.65	317	4.53	349	4.81	156	2.22	144	1.9	157	2.03	262.2024	0.200755	-6.99243	-1.4038	Down	2.7E-12	1.1E-10	_	_
ENSG000	MRPL27	5358	4487	20.12	4163	19.47	4900	22.1	2269	10.58	1498	6.48	2714	11.48	3420.611	0.24862	-4.83439	-1.2019	Down	1.3E-06	2E-05	molecular_	K02899
ENSG000	MRPL33	216371	7451	69.98	6116	59.91	6931	65.47	3558	34.76	2610	23.65	4322	38.28	5272.909	0.228804	-4.89736	-1.1205	Down	9.7E-07	1.5E-05	molecular_	K02913
ENSG000	MRPL36	2981	6384	60.58	5914	58.53	6323	60.34	2728	26.92	1910	17.49	3378	30.23	4558.603	0.237002	-5.71783	-1.3551	Down	1.1E-08	2.5E-07	molecular_	K02919
ENSG000	MRPL39	21862	2496	48.64	2473	50.27	2298	45.04	879	17.82	1008	18.95	1009	18.55	1736.706	0.121363	-12.6091	-1.5303	Down	1.9E-36	4.9E-34	molecular_	K17420
ENSG000	MRPS17	4681	6008	63.79	5319	58.9	6165	65.84	3001	33.14	1947	19.95	3069	30.73	4363.538	0.23698	-5.32767	-1.2626	Down	1E-07	1.9E-06	molecular_	K15120
ENSG000	MRPS23	10576	5461	63.09	5059	60.96	5137	59.69	2571	30.9	2070	23.07	2994	32.62	3967.554	0.197419	-6.09782	-1.2038	Down	1.1E-09	3E-08	molecular_	K17402
ENSG000	MRPS28	111573	3274	19.27	2964	18.2	2982	17.65	1565	9.58	1332	7.56	2086	11.58	2409.06	0.20728	-5.11545	-1.0603	Down	3.1E-07	5.3E-06	molecular_	K17407
ENSG000	MSH5	24898	171	0.39	191	0.46	166	0.38	364	0.87	536	1.18	452	0.98	298.9956	0.173391	6.339971	1.09929	Ups	2.3E-10	7.2E-09	biological_	K08741
ENSG000	MSH5-SA	24832	22	0.1	19	0.09	20	0.09	85	0.4	40	0.17	85	0.36	43.82962	0.375103	3.755315	1.40863	Ups	0.00017	0.00142	molecular_	K08741
ENSG000	MSL2	48320	531	1.58	539	1.67	427	1.28	1161	3.59	1681	4.82	1317	3.69	897.3877	0.163719	7.446482	1.21913	Ups	9.6E-14	4.8E-12	molecular_	K13164
ENSG000	MSMO1	15538	1404	7.68	1648	9.4	1491	8.21	3101	17.65	5344	28.21	4002	20.65	2686.031	0.172461	6.875638	1.18578	Ups	6.2E-12	2.5E-10	molecular_	K07750
ENSG000	MSRB2	26508	872	8.85	731	7.74	1005	10.26	3605	38.05	1132	11.08	2950	28.24	1686.468	0.360876	3.450139	1.24507	Ups	0.00056	0.00388	molecular_	K07305
ENSG000	MST1	5555	46	0.07	35	0.05	44	0.06	163	0.25	200	0.28	197	0.27	107.6648	0.227153	8.20367	1.86349	Ups	2.3E-16	1.6E-14	molecular_	_
ENSG000	MST1L	15328	32	0.13	25	0.1	31	0.13	92	0.38	104	0.4	100	0.38	60.96294	0.260353	5.56778	1.44959	Ups	2.6E-08	5.5E-07	molecular_	_
ENSG000	MST1P2	4846	56	0.37	53	0.36	48	0.32	122	0.83	199	1.26	159	0.98	100.4402	0.232103	5.690029	1.32067	Ups	1.3E-08	2.9E-07	_	_
ENSG000	MSTN	7033	1	0.01	1	0.01	0	0	13	0.12	17	0.15	31	0.27	9.609039	0.543157	5.165899	2.80589	Ups	2.4E-07	4.2E-06	molecular_	K05497
ENSG000	MTUS2	479145	166	0.58	159	0.58	167	0.59	36	0.13	58	0.2	27	0.09	105.9252	0.26677	-7.98402	-2.1299	Down	1.4E-15	8.8E-14	molecular_	_
ENSG000	MUC1	4408	528	2.13	471	1.98	642	2.61	179	0.75	147	0.57	213	0.81	375.7118	0.233062	-7.46331	-1.7394	Down	8.4E-14	4.3E-12	cellular_cc	K06568
ENSG000	MUC15	13201	3	0.02	6	0.03	5	0.03	0	0	1	0	0	0	2.639799	0.583454	-2.8469	-1.661	Down	0.00441	0.02121	cellular_cc	K19498
ENSG000	MUC20	20242	5	0.01	1	0	2	0	23	0.04	20	0.03	29	0.05	12.50854	0.484089	4.64734	2.24973	Ups	3.4E-06	4.5E-05	biological_	K17985
ENSG000	MUC3A	63932	20	0.06	16	0.05	24	0.07	5	0.01	10	0.03	1	0	13.089	0.474783	-3.54243	-1.6819	Down	0.0004	0.00289	molecular_	_
ENSG000	MVP	27641	3143	7.55	2618	6.56	3023	7.31	1074	2.68	825	1.91	1000	2.27	2013.457	0.201528	-8.64965	-1.7431	Down	5.2E-18	4.1E-16	molecular_	K17266
ENSG000	MYBBP1A	16735	7328	15.68	5561	12.41	6933	14.92	3392	7.55	3149	6.5	2882	5.82	4981.967	0.18478	-6.74188	-1.2458	Down	1.6E-11	5.9E-10	molecular_	K02331
ENSG000	MYBL1	52073	303	0.87	539	1.62	278	0.81	493	1.48	2072	5.77	920	2.51	709.9654	0.375716	3.180033	1.19479	Ups	0.00147	0.00868	molecular_	K09421
ENSG000	MYBPC3	21297	13	0.08	6	0.04	6	0.04	2	0.01	1	0.01	4	0.02	5.514058	0.545945	-2.48334	-1.3558	Down	0.01302	0.0493	molecular_	K12568
ENSG000	MYCL	6831	122	0.34	98	0.28	80	0.22	331	0.95	191	0.51	271	0.7	177.5775	0.298811	3.789286	1.13228	Ups	0.00015	0.00126	_	K09110
ENSG000	MYEOV	120890	416	0.85	341	0.73	360	0.74	72	0.15	230	0.45	164	0.32	267.6638	0.299349	-4.73036	-1.416	Down	2.2E-06	3.1E-05	_	_
ENSG000	MYH15	148954	17	0.06	20	0.07	23	0.07	10	0.03	7	0.02	8	0.02	14.59968	0.414498	-3.02362	-1.2533	Down	0.0025	0.01343	molecular_	K10352
ENSG000	MYH3	28784	25	0.08	29	0.1	21	0.07	128	0.45	104	0.34	105	0.33	65.61275	0.294687	6.205397	1.82865	Ups	5.5E-10	1.6E-08	molecular_	K10352
ENSG000	MYL3	24298	1	0.01	0	0	1	0.01	25	0.34	6	0.08	15	0.19	7.751774	0.566377	4.15198	2.35159	Ups	3.3E-05	0.00033	biological_	K12749
ENSG000	MYLIP	19124	247	1.82	320	2.46	285	2.11	817	6.26	642	4.56	682	4.74	484.2264	0.225408	4.811929	1.08465	Ups	1.5E-06	2.2E-05	molecular_	K10637
ENSG000	MYLK3	83429	275	0.98	280	1.04	205	0.73	541	2	3013	10.32	848	2.84	773.2996	0.40648	4.726282	1.92114	Ups	2.3E-06	3.2E-05	biological_	K00907
ENSG000	MYLK4	87338	12	0.03	15	0.04	11	0.03	39	0.1	90	0.21	38	0.09	31.6721	0.387862	4.235484	1.64278	Ups	2.3E-05	0.00024	molecular_	K00907
ENSG000	MYLK-AS	2764	0	0	0	0	0	0	4	0.1	6	0.14	2	0.04	1.821495	0.572014	2.603011	1.48896	Ups	0.00924	0.03787	_	_
ENSG000	MYO15B	38791	77	0.09	55	0.07	64	0.08	22	0.03	32	0.04	33	0.04	48.04238	0.286332	-4.56976	-1.3085	Down	4.9E-06	6.3E-05	molecular_	K10361
ENSG000	MYO16-A	37582	10	0.44	18	0.83	13	0.58	58	2.67	80	3.42	51	2.13	36.06581	0.341724	5.226734	1.7861	Ups	1.7E-07	3.1E-06	_	_
ENSG000	MYO1A	22682	4	0.02	6	0.03	8	0.03	10	0.04	45	0.18	16	0.06	13.57933	0.493164	2.630788	1.29741	Ups	0.00852	0.03553	molecular_	K10356
ENSG000	MYO1G	16437	56	0.09	33	0.06	55	0.09	4	0.01	16	0.03	4	0.01	29.12795	0.42599	-5.57357	-2.3743	Down	2.5E-08	5.4E-07	molecular_	K10356
ENSG000	MYO7A	86975	66	0.12	104	0.2	82	0.15	399	0.76	246	0.44	314	0.54	195.0323	0.296879	5.444473	1.61635	Ups	5.2E-08	1.1E-06	molecular_	K10359
ENSG000	MYO7B	101927	123	0.23	100	0.2	105	0.2	17	0.03	34	0.06	17	0.03	68.55131	0.29271	-7.97465	-2.3343	Down	1.5E-15	9.4E-14	molecular_	K10359
ENSG000	MYOCD	103060	2	0	3	0.01	0	0	11	0.02	101	0.2	31	0.06	21.43295	0.547329	5.157407	2.8228	Ups	2.5E-07	4.4E-06	biological_	_
ENSG000	MYOM1	153302	7	0.02	12	0.04	17	0.05	37	0.12	89	0.27	52	0.15	32.96283	0.389667	4.57697	1.78349	Ups	4.7E-06	6.1E-05	molecular_	_
ENSG000	MYOZ1	10104	1	0.02	3	0.05	4	0.07	36	0.62	3	0.05	26	0.41	11.96483	0.561283	3.133326	1.75868	Ups	0.00173	0.00988	molecular_	_
ENSG000	MYRFL	133794	2	0.01	3	0.02	0	0	34	0.2	8	0.04	35	0.18	13.11832	0.54595	4.389245	2.39631	Ups	1.1E-05	0.00013	molecular_	_
ENSG000	MYSM1	45354	244	0.39	373	0.62	199	0.32	278	0.46	1499	2.3	500	0.75	476.0964	0.409266	2.482	1.0158	Ups	0.01306	0.04945	molecular_	K11865
ENSG000	MYT1L	542148	100	0.13	85	0.12	74	0.1	202	0.28	222	0.28	217	0.27	144.2526	0.2059	5.125794	1.0554	Ups	3E-07	5.1E-06	molecular_	_
ENSG000	N6AMT1	13181	445	2.04	442	2.12	480	2.22	188	0.9	293	1.3	267	1.16	356.6824	0.17284	-6.24425	-1.0793	Down	4.3E-10	1.3E-08	molecular_	K02493
ENSG000	N6AMT2	45228	217	3.07	195	2.88	253	3.6	107	1.57	95	1.3	128	1.71	169.3469	0.229896	-5.10658	-1.174	Down	3.3E-07	5.6E-06	molecular_	_
ENSG000	NAA11	100357	0	0	1	0.01	1	0.01	52	0.38	49	0.33	36	0.24	21.52455	0.502143	7.710609	3.87183	Ups	1.3E-14	7E-13	molecular_	K00670
ENSG000	NAALADL	1367066	14	0.04	8	0.02	5	0.01	18	0.05	36	0.09	33	0.08	17.83217	0.41851	2.93408	1.22794	Ups	0.00335	0.017	biological_	_
ENSG000	NAIP	56632	10	0.02	13	0.02	13	0.02	46	0.09	70	0.12	69	0.12	34.41984	0.334434	5.778775	1.93262	Ups	7.5E-09	1.8E-07	molecular_	K12807
ENSG000	NALCN	362714	140	0.34	120	0.31	139	0.34	211	0.54	1008	2.39	331	0.77	296.7971	0.38822	3.752877	1.45694	Ups	0.00017	0.00143	molecular_	K17985
ENSG000	NAT1	53213	76	0.25	97	0.34	81	0.27	46	0.16	40	0.13	49	0.15	66.11124	0.264743	-4.01896	-1.064	Down	5.9E-05	0.00055	molecular_	K00622
ENSG000	NCALD	438365	4	0	3	0	0	0	6	0.01	19	0.02	16	0.02	7.307653	0.538468	2.883166	1.55249	Ups	0.00394	0.01936	molecular_	K05069
ENSG000	NCAM1	317162	18	0.02	10	0.01	18	0.02	0	0	4	0	8	0.01	9.940598	0.515551	-3.07844	-1.5871	Down	0.00208	0.01154	cellular_cc	K06491
ENSG000	NCCRP1	4924	292	3.79	228	3.09	230	3.01	34	0.46	72	0.9	54	0.66	157.3269	0.246508	-9.54215	-2.3522	Down	1.4E-21	1.4E-19	cellular_cc	K12606
ENSG000	NCK2	149377	204	0.99	178	0.9	189	0.93	29	0.15	154	0.72	73	0.34	138.9424	0.379108	-3.34281	-1.2673	Down	0.00083	0.00539	molecular_	K07365
ENSG000	NCLN	24013	7513	22.91	5761	18.32	7190	22.05	3906	12.39	2823	8.3	4089	11.76	5321.349	0.223961	-4.79773	-1.0745	Down	1.6E-06	2.3E-05	molecular_	_
ENSG000	NCOA2	294044	311	0.58	526	1.03	256	0.48	440	0.86	1901	3.44	691	1.22	637.8288	0.389536	2.655369	1.03436	Ups	0.00792	0.03356	biological_	K11255
ENSG000	NCS1	64727	4547	22.4	3465	17.81	4034	19.99	2383	12.21	1782	8.47	2376	11.04	3158.053	0.213298	-4.8828	-1.0415	Down	1.1E-06	1.6E-05	molecular_	_
ENSG000	NDUFA4L	5813	744	3.24	674	3.06	926	4.06	3566	16.17	2151	9.05	3829	15.74	1906.141	0.269818	6.406711	1.72865	Ups	1.5E-10	4.8E-09	_	K03948
ENSG000	NDUFA9	40194	4977	18.39	5061	19.5	5598	20.8	2722	10.46	2531	9.02	3205	11.17	4088.054	0.170689	-6.29366	-1.0743	Down	3.1E-10	9.5E-09	molecular_	K03953
ENSG000	NDUFB8P	538	21	1	14	0.7	16	0.77	2	0.1	2	0.09	2	0.09	9.987395	0.499179	-4.84816	-2.4201	Down	1.3E-06	1.9E-05	_	K03964
ENSG000	NEB	249152	26	0.02	33	0.03	25	0.02	55	0.04	127	0.09	51	0.04	49.70762	0.355021	3.078598	1.09297	Ups	0.00208	0.01154	molecular_	K18267
ENSG000	NEBL	394215	79	0.12	91	0.14	62	0.09	148	0.22	802	1.13	270	0.37	218.4855	0.408842	4.401494	1.79952	Ups	1.1E-05	0.00013	cellular_cc	_
ENSG000	NEIL1	8297	91	0.15	72	0.13	103	0.18	239	0.42	211	0.34	268	0.43	158.1006	0.23985	4.873001	1.16879	Ups	1.1E-06	1.7E-05	molecular_	K10567
ENSG000	NELL2	413574	232	0.47	230	0.49	202	0.41	22	0.05	24	0.05	26	0.05	129.2223	0.244019	-13.3832	-3.2658	Down	7.6E-41	2.4E-38	molecular_	_
ENSG000	NETO1	125833	5	0.02	5	0.02	8	0.03	20	0.07	103	0.35	45	0.15	27.69135	0.472324	4.875479	2.30281	Ups	1.1E-06	1.6E-05	cellular_cc	_
ENSG000	NEU4	8820	0	0	0	0	2	0	3	0	10	0.01	7	0.01	3.315114	0.581735	2.543475	1.47963	Ups	0.01098	0.04321	molecular_	K12357
ENSG000	NEXN	55383	292	0.79	338	0.95	280	0.76	340	0.96	1480	3.86	692	1.76	529.7336	0.352119	3.016586	1.0622	Ups	0.00256	0.01368	cellular_cc	_
ENSG000	NEXN-AS	8192	6	0.05	7	0.06	10	0.09	31	0.27	43	0.35	35	0.28	20.66849	0.385252	4.563903	1.75825	Ups	5E-06	6.4E-05	_	_
ENSG000	NFATC1	133470	15	0.03	4	0.01	5	0.01	0	0	0	0	1	0	4.390668	0.580721	-3.62856	-2.1072	Down	0.00029	0.00217	biological_	K04446
ENSG000	NFATC4	13932	4	0	4	0	10	0.01	57	0.07	13	0.01	31	0.03	19.50867	0.507434	3.393056	1.72175	Ups	0.00069	0.00464	molecular_	K17334
ENSG000	NFKBIZ	33032	227	0.67	258	0.8	211	0.63	5159	15.89	2682	7.66	3971	11.09	1981.906	0.282126	12.97639	3.66098	Ups	1.7E-38	4.8E-36	cellular_cc	K14242
ENSG000	NINL	132813	217	0.75	221	0.8	214	0.74	584	2.1	471	1.57	514	1.68	359.3227	0.209126	4.923545	1.02964	Ups	8.5E-07	1.3E-05	biological_	K16477
ENSG000	NIPSNAP:	13301	18	0.17	19	0.19	14	0.13	40	0.39	71	0.64	53	0.47	33.77324	0.325407	4.070101	1.32444	Ups	4.7E-05	0.00045	_	_
ENSG000	NKAIN1	59810	21	0.08	21	0.08	25	0.09	6	0.02	6	0.02	9	0.03	15.16063	0.414018	-3.88662	-1.6091	Down	0.0001	0.00089	cellular_cc	_

ENSG000	NKG2-E	23690	3	0.08	0	0	0	0	11	0.29	5	0.12	13	0.32	5.067503	0.572913	2.956912	1.69405	Ups	0.00311	0.01603	_	K06541
ENSG000	NKPD1	10401	0	0	0	0	0	0	4	0.01	10	0.02	9	0.02	3.427426	0.583147	3.704706	2.16039	Ups	0.00021	0.00168	cellular_cc	K08857
ENSG000	NKX3-2	4221	43	0.39	32	0.31	46	0.42	466	4.45	248	2.2	362	3.13	190.6622	0.321119	8.47957	2.72295	Ups	2.3E-17	1.7E-15	biological_	K09995
ENSG000	NLGN3	26371	8	0.03	9	0.03	9	0.03	39	0.13	80	0.25	43	0.13	28.90317	0.383335	5.455334	2.09122	Ups	4.9E-08	1E-06	biological_	K07378
ENSG000	NLGN4X	388227	0	0	0	0	1	0	21	0.03	54	0.07	22	0.03	14.58792	0.539389	6.439075	3.47316	Ups	1.2E-10	3.9E-09	biological_	K07378
ENSG000	NLK	154645	1113	3.66	1109	3.8	801	2.65	2608	8.92	3896	12.36	2763	8.57	1942.365	0.187323	7.199383	1.34861	Ups	6.1E-13	2.8E-11	molecular_	K04468
ENSG000	NLRC4	41402	18	0.1	4	0.02	17	0.09	36	0.2	31	0.16	46	0.23	24.34378	0.422681	2.645389	1.11816	Ups	0.00816	0.03436	molecular_	K12805
ENSG000	NMNAT1	42074	145	0.56	106	0.43	115	0.45	281	1.14	312	1.17	335	1.23	207.098	0.198366	5.500788	1.09117	Ups	3.8E-08	7.9E-07	molecular_	K06210
ENSG000	NMNAT2	170366	102	0.34	64	0.23	68	0.23	322	1.13	267	0.87	266	0.85	174.0488	0.260949	6.061235	1.58167	Ups	1.4E-09	3.7E-08	cellular_cc	K06210
ENSG000	NMNAT3	117838	2	0	0	0	1	0	10	0.02	28	0.06	20	0.04	9.138389	0.537082	4.763953	2.55863	Ups	1.9E-06	2.7E-05	molecular_	K06210
ENSG000	NNMT	55499	8300	37.83	7699	36.6	9631	44.15	4622	21.91	1852	8.14	4286	18.42	6264.993	0.322872	-4.05287	-1.3086	Down	5.1E-05	0.00048	cellular_cc	K00541
ENSG000	NOP2	11829	8502	18.57	8590	19.57	9696	21.3	4165	9.46	4027	8.49	3932	8.1	6640.961	0.156782	-8.48168	-1.3298	Down	2.2E-17	1.7E-15	molecular_	K14835
ENSG000	NOX5	132220	12	0.02	17	0.03	12	0.02	0	0	6	0.01	5	0.01	8.89044	0.507261	-3.13857	-1.5921	Down	0.0017	0.00974	biological_	_
ENSG000	NOXA1	11057	108	1.27	97	1.19	112	1.32	353	4.3	161	1.82	381	4.21	196.8898	0.333943	3.577879	1.19481	Ups	0.00035	0.00257	molecular_	_
ENSG000	NOXO1	5276	4	0.02	5	0.03	3	0.02	1	0.01	0	0	0	0	2.308655	0.582826	-2.55634	-1.4899	Down	0.01058	0.04202	molecular_	K17934
ENSG000	NPIPB6	20954	4	0.05	6	0.08	11	0.13	42	0.53	43	0.5	44	0.5	23.56951	0.394327	5.257717	2.07326	Ups	1.5E-07	2.7E-06	_	_
ENSG000	NPIPB8	21029	0	0	1	0.01	1	0.01	10	0.11	8	0.08	6	0.06	4.093905	0.573044	3.125908	1.79128	Ups	0.00177	0.01011	_	K01896
ENSG000	NPIPB9	21021	0	0	3	0.03	1	0.01	11	0.1	9	0.08	5	0.04	4.613022	0.567237	2.525079	1.43232	Ups	0.01157	0.04493	_	K01896
ENSG000	NPPB	1468	1	0.04	1	0.04	3	0.11	12	0.46	42	1.5	47	1.64	15.84482	0.517376	5.427777	2.8082	Ups	5.7E-08	1.1E-06	biological_	K12335
ENSG000	NPR3	102644	35	0.07	36	0.08	37	0.08	122	0.26	1983	3.89	363	0.7	369.5122	0.575889	3.977297	2.29048	Ups	7E-05	0.00064	biological_	K12325
ENSG000	NPY1R	20872	2	0.01	5	0.02	3	0.01	280	1.09	1536	5.54	592	2.09	351.7987	0.455849	12.89995	5.88043	Ups	4.5E-38	1.3E-35	molecular_	K04204
ENSG000	NPY4R	4787	996	12.92	991	13.4	860	11.22	291	3.93	555	6.94	237	2.9	669.4513	0.264118	-5.86686	-1.5495	Down	4.4E-09	1.1E-07	molecular_	K04206
ENSG000	NR1D2	35359	544	1.61	909	2.8	334	0.99	1377	4.23	3200	9.12	1731	4.82	1259.065	0.335663	4.235237	1.42161	Ups	2.3E-05	0.00024	molecular_	K08531
ENSG000	NR3C2	365938	38	0.06	38	0.06	18	0.03	78	0.12	144	0.21	101	0.15	65.27346	0.31768	4.460746	1.41709	Ups	8.2E-06	9.9E-05	molecular_	K08555
ENSG000	NR4A1	36676	1515	1.9	1098	1.43	1365	1.72	25651	33.38	8205	9.9	16268	19.19	8712.622	0.35538	8.823477	3.13569	Ups	1.1E-18	9.1E-17	molecular_	K04465
ENSG000	NR4A2	17917	252	0.59	245	0.6	218	0.51	2345	5.71	2167	4.89	2116	4.67	1149.822	0.17811	16.47248	2.93391	Ups	5.8E-61	3.9E-58	biological_	K08558
ENSG000	NR4A3	45037	166	0.54	141	0.48	148	0.48	400	1.35	1185	3.71	598	1.83	403.962	0.300414	6.171394	1.85397	Ups	6.8E-10	2E-08	molecular_	K08559
ENSG000	NRCAM	309094	44	0.07	52	0.08	38	0.06	112	0.18	237	0.35	125	0.18	94.79019	0.297338	4.923773	1.46403	Ups	8.5E-07	1.3E-05	biological_	K06756
ENSG000	NREP	334844	589	0.71	568	0.71	571	0.69	1807	2.26	2841	3.3	2199	2.49	1343.318	0.150696	11.42314	1.72142	Ups	3.2E-30	6.1E-28	molecular_	_
ENSG000	NRG1	1125647	532	0.85	565	0.94	572	0.92	127	0.21	114	0.18	96	0.14	349.5118	0.205306	-11.8689	-2.4368	Down	1.7E-32	3.6E-30	biological_	K05455
ENSG000	NRG2	196521	2	0.01	5	0.01	3	0.01	16	0.04	71	0.17	41	0.1	20.54174	0.488675	5.249075	2.56509	Ups	1.5E-07	2.8E-06	molecular_	K05456
ENSG000	NRP1	158771	566	0.81	605	0.91	641	0.93	222	0.33	160	0.22	187	0.25	411.4962	0.225672	-7.96495	-1.7975	Down	1.7E-15	1E-13	biological_	K06724
ENSG000	NRP2	116144	832	1.02	863	1.11	912	1.13	567	0.73	387	0.46	406	0.47	676.9483	0.239379	-4.51536	-1.0809	Down	6.3E-06	7.8E-05	biological_	K06819
ENSG000	NRTN	4523	10	0.23	10	0.24	18	0.42	55	1.33	27	0.6	53	1.16	27.97553	0.411768	3.389998	1.39589	Ups	0.0007	0.00468	biological_	_
ENSG000	NRXN3	1622029	68	0.08	101	0.12	48	0.05	181	0.21	344	0.38	192	0.21	146.3586	0.308797	4.429499	1.36782	Ups	9.5E-06	0.00011	biological_	K07377
ENSG000	NSUN2	34053	13924	34.11	13809	35.28	15148	37.32	2342	5.97	2630	6.22	2634	6.09	8804.195	0.12375	-21.6871	-2.6838	Down	3E-104	6E-101	molecular_	K15335
ENSG000	NT5E	45692	669	2.54	709	2.8	700	2.67	910	3.59	3649	13.35	1674	5.99	1284.169	0.33286	3.567345	1.18743	Ups	0.00036	0.00266	molecular_	K01081
ENSG000	NTN3	2647	14	0.18	10	0.13	11	0.14	41	0.55	71	0.89	43	0.52	29.59006	0.35567	4.807765	1.70998	Ups	1.5E-06	2.2E-05	molecular_	K06844
ENSG000	NTN5	11675	0	0	0	0	1	0.01	7	0.05	8	0.06	4	0.03	3.09679	0.582664	2.963162	1.72653	Ups	0.00304	0.0158	cellular_cc	_
ENSG000	NTS	8698	71	0.59	96	0.83	63	0.53	7	0.06	10	0.08	11	0.09	45.22028	0.349246	-8.38076	-2.9269	Down	5.3E-17	3.8E-15	molecular_	K05235
ENSG000	NTSR1	53935	39	0.24	33	0.21	34	0.21	18	0.11	19	0.11	15	0.09	26.85226	0.331641	-3.42072	-1.1345	Down	0.00062	0.00424	molecular_	K04211
ENSG000	NUAK1	76694	633	2.05	534	1.8	607	1.98	1573	5.3	2952	9.23	1661	5.08	1245.586	0.218041	6.887058	1.50166	Ups	5.7E-12	2.3E-10	molecular_	K08800
ENSG000	NUDT6	34272	546	1.31	630	1.58	518	1.25	246	0.61	259	0.6	318	0.72	427.8357	0.185384	-6.61911	-1.2271	Down	3.6E-11	1.3E-09	cellular_cc	_
ENSG000	NUDT7	19747	101	0.66	95	0.65	103	0.68	340	2.32	194	1.23	292	1.81	182.6403	0.28696	4.167139	1.1958	Ups	3.1E-05	0.00032	molecular_	K17879
ENSG000	NYNRIN	20503	8	0.03	11	0.04	8	0.03	40	0.13	32	0.1	25	0.08	19.89999	0.40149	3.517791	1.41236	Ups	0.00044	0.00313	molecular_	_
ENSG000	OAS2	33329	3	0.01	4	0.01	0	0	14	0.04	129	0.33	49	0.12	28.88764	0.536855	5.467628	2.93532	Ups	4.6E-08	9.3E-07	molecular_	K14216
ENSG000	OCLN	65813	9	0.03	9	0.03	9	0.03	45	0.14	50	0.14	71	0.2	30.16457	0.365639	5.814029	2.12584	Ups	6.1E-09	1.5E-07	molecular_	K06088
ENSG000	ODAM	8081	23	0.27	36	0.45	18	0.22	2	0.02	1	0.01	3	0.03	14.6518	0.491165	-5.85464	-2.8756	Down	4.8E-09	1.2E-07	cellular_cc	_
ENSG000	OFD1P17	3018	3	0.03	2	0.02	0	0	9	0.08	15	0.12	9	0.07	5.865115	0.543947	3.004792	1.63445	Ups	0.00266	0.01411	_	K16480
ENSG000	OGDHL	27737	3	0.01	16	0.05	6	0.02	216	0.73	170	0.53	174	0.53	91.41981	0.383353	9.709296	3.72209	Ups	2.8E-22	2.9E-20	molecular_	K00164
ENSG000	OLAH	41626	1	0.01	1	0.01	0	0	8	0.06	24	0.15	12	0.08	6.861335	0.557432	4.292457	2.39275	Ups	1.8E-05	0.00019	molecular_	K01071
ENSG000	OLFM1	45758	538	1.07	460	0.95	475	0.95	520	1.07	3371	6.46	1947	3.65	1111.327	0.391913	3.765737	1.47584	Ups	0.00017	0.00137	molecular_	_
ENSG000	OLFML1	25990	951	3.28	1061	3.82	1271	4.41	2046	7.34	8114	27	3898	12.68	2648.108	0.33207	4.983938	1.65502	Ups	6.2E-07	9.9E-06	cellular_cc	_
ENSG000	OLFML2A	37728	428	0.91	343	0.76	333	0.71	1437	3.19	1826	3.76	1316	2.65	894.8434	0.178561	10.00672	1.78681	Ups	1.4E-23	1.8E-21	cellular_cc	K12581
ENSG000	OPLAH	12569	224	1.01	220	1.03	224	1.01	814	3.81	576	2.5	773	3.28	455.2999	0.231302	6.21192	1.43683	Ups	5.2E-10	1.5E-08	molecular_	K01469
ENSG000	OR1F12	918	0	0	0	0	0	0	2	0.06	8	0.22	3	0.08	1.903874	0.572306	2.632278	1.50647	Ups	0.00848	0.03546	molecular_	K04257
ENSG000	OR2B6	942	32	0.87	45	1.28	50	1.37	112	3.18	92	2.42	146	3.76	76.67258	0.299998	3.871869	1.16155	Ups	0.00011	0.00094	molecular_	K04257
ENSG000	OR51A9P	911	14	0.2	12	0.18	12	0.17	39	0.58	75	1.04	57	0.77	32.44166	0.34304	5.07993	1.74262	Ups	3.8E-07	6.3E-06	_	K04257
ENSG000	OR51E1	12069	4	0.03	3	0.02	3	0.02	12	0.08	49	0.3	23	0.14	14.09088	0.495535	4.231493	2.09685	Ups	2.3E-05	0.00025	molecular_	K04257
ENSG000	OSBP2	214043	5937	8.93	5058	7.94	5202	7.87	2742	4.29	3147	4.57	2634	3.74	4186.88	0.136561	-8.2591	-1.1279	Down	1.5E-16	1E-14	molecular_	K15286
ENSG000	OSBPL6	204953	133	0.35	165	0.46	158	0.42	236	0.65	585	1.5	338	0.85	253.8337	0.27522	3.716237	1.02278	Ups	0.0002	0.00162	molecular_	_
ENSG000	OSGIN1	18051	408	1.9	313	1.52	395	1.85	102	0.49	143	0.64	147	0.65	258.1279	0.199381	-8.45313	-1.6854	Down	2.8E-17	2.1E-15	cellular_cc	_
ENSG000	OTOG	99778	0	0	0	0	1	0	42	0.09	46	0.1	54	0.11	21.90994	0.508361	8.138854	4.13748	Ups	4E-16	2.6E-14	molecular_	_
ENSG000	OTOGL	169638	1	0	3	0.01	0	0	31	0.07	145	0.29	54	0.11	34.25834	0.514003	7.280428	3.74216	Ups	3.3E-13	1.6E-11	cellular_cc	_
ENSG000	OVGP1	13464	14	0.1	13	0.1	24	0.17	86	0.64	36	0.25	74	0.5	40.0429	0.408655	3.684299	1.50561	Ups	0.00023	0.0018	molecular_	K17526
ENSG000	P2RX3	32226	5	0.04	5	0.05	3	0.03	8	0.07	27	0.23	17	0.14	9.985211	0.488937	2.733672	1.33659	Ups	0.00626	0.02799	molecular_	K05217
ENSG000	P2RX4	24250	235	0.48	221	0.47	269	0.55	585	1.23	499	0.98	637	1.22	394.9213	0.208872	4.812232	1.00514	Ups	1.5E-06	2.2E-05	molecular_	K05218
ENSG000	P2RX5	24206	1216	3.54	945	2.87	1175	3.44	564	1.71	488	1.37	633	1.74	853.776	0.20049	-5.76672	-1.1562	Down	8.1E-09	1.9E-07	molecular_	K05219
ENSG000	P2RX6P	9775	26	0.92	32	1.18	46	1.64	7	0.26	3	0.1	4	0.13	20.79852	0.437745	-5.80349	-2.5404	Down	6.5E-09	1.6E-07	_	K05221
ENSG000	P2RY11	3835	146	1.5	151	1.62	198	2.05	558	5.97	228	2.26	464	4.51	285.8255	0.341277	3.079668	1.05102	Ups	0.00207	0.01151	molecular_	K08387
ENSG000	P2RY6	34113	185	0.37	153	0.32	160	0.32	338	0.7	945	1.82	677	1.27	379.5311	0.282766	5.688061	1.60839	Ups	1.3E-08	2.9E-07	molecular_	K04272
ENSG000	P4HA3	75857	49	0.2	40	0.17	38	0.16	3	0.01	20	0.08	14	0.06	27.97728	0.402748	-4.35238	-1.7529	Down	1.4E-05	0.00015	molecular_	K00472
ENSG000	PADI1	40880	61	0.16	45	0.12	53	0.14	22	0.06	17	0.04	10	0.02	35.93606	0.349809	-4.88896	-1.7102	Down	1E-06	1.5E-05	molecular_	K01481
ENSG000	PAGE1	8544	568	21.96	515	20.76	506	19.67	4767	191.66	3087	115.13	6054	220.72	2442.262	0.260533	10.6628	2.77801	Ups	1.5E-26	2.4E-24	biological_	_
ENSG000	PALM2	310688	2	0	4	0.01	1	0	74	0.13	106	0.17	77	0.12	40.26237	0.425007	9.492073	4.0342	Ups	2.3E-21	2.3E-19	cellular_cc	K16519
ENSG000	PAN2	17831	668	1.01	640	1.01	623	0.95	1402	2.21	2224	3.25	1453	2.08	1111.996	0.175617	6.453067	1.13327	Ups	1.1E-10	3.6E-09	molecular_	K12571
ENSG000	PANX2	9564	578	3	410	2.22	599	3.13	214	1.16	180	0.9	217	1.06	376.9741	0.229635	-6.61514	-1.5191	Down	3.7E-11	1.3E-09	biological_	K03443
ENSG000	PAOX	12504	17	0.09	11	0.06	10	0.05	69	0.37	20	0.1	57	0.28	29.98145	0.450318	3.216194	1.44831	Ups	0.0013	0.00781	molecular_	K00308
ENSG000	PAPPA2	382429	0	0	0	0	1	0	11	0.02	68	0.14	19	0.04	14.35178	0.560925	5.593056	3.13729	Ups	2.2E-08	4.8E-07	biological_	K08647
ENSG000	PAQR5	108834	1035	2.22	1196	2.67	1084	2.33	184	0.41	558	1.15	309	0.62	746.1815	0.286062	-6.29364	-1.8004	Down	3.1E-10	9.5E-09	molecular_	K10408
ENSG000	PAQR6	4676	125	0.42	106	0.37	121	0.41	698	2.45	445	1.45	662	2.1	344.1605	0.263086	7.790557	2.04958	Ups	6.7E-15	3.9E-13	cellular_cc	_
ENSG000	PAQR8	46357	26	0.07	37	0.1	22	0.06	142	0.38	105	0.26	125	0.3	72.95976	0.307854	5.792497	1.78325	Ups	6.9E-09	1.7E-07	molecular_	K14993
ENSG000	PARD6B	25252	52	0.27	61	0.33	29	0.15	108	0.59	165	0.83	237	1.17	102.3447	0.329427	4.46148	1.46973	Ups	8.1E-06	9.8E-05	molecular_	K06093
ENSG000	PARP10	35620	75	0.13	69	0.13	86	0.15	160	0.29	218	0.37	239	0.39	134.688	0.220541	5.214281	1.14996	Ups	1.9E-07	3.3E-06	molecular_	_
ENSG000	PAX6	33170	13	0.01	25	0.03	14	0.01	57	0.06	56	0.05	40	0.04	32.87509	0.357524	3.362992	1.20235	Ups	0.00077	0.00508	cellular_cc	K08031
ENSG000	PCAT1	173960	4	0.04	0	0	3	0.03	25	0.25	7	0.06	16	0.14	8.925619	0.54776	3.105122	1.70086	Ups	0.0019	0.01072	_	_
ENSG000	PCDH7	426386	851	1.29	825	1.31	685	1.05	1424	2.25	3383	4.96	1728	2.48	1393.977	0.257375	4.471893	1.15095	Ups	7.8E-06	9.4E-05	molecular_	K16498
ENSG000	PCDHB10	3274	8	0.06	14	0.11	7	0.06	15	0.12	53	0.4	44	0.33	21.70964	0.438593	3.181269	1.39528	Ups	0.00147	0.00865	molecular_	K16494
ENSG000	PCDHB12	3428	20	0.1	25	0.12	22	0.11	57	0.28	71	0.33	50	0.23	39.12181	0.302564	3.635136	1.09986	Ups	0.00028	0.00212	molecular_	K16494
ENSG000	PCDHB13	3485	42	0.31	39	0.3	32	0.24	90	0.69	156	1.11	75	0.52	68.50149	0.305392	3.848192	1.17521	Ups	0.00012	0.00102	molecular_	K16494
ENSG000	PCDHB16	4814	29	0.15	32	0.18	22	0.12	92	0.51	190	0.98	131	0.66	76.64079	0.296151	6.499957	1.92497	Ups	8E-11	2.7E-09	molecular_	K16494
ENSG000	PCDHB7	3715	5	0.03	6	0.04	5	0.03	20	0.14	33	0.22	28	0.18	15.07866	0.424059	4.160754	1.76441	Ups	3.2E-05	0.00032	molecular_	K16494
ENSG000	PCDHB9	4219	29	0.18	24	0.15	26	0.16	84	0.53	65	0.38	61	0.35	46.71538	0.310429	3.596366	1.11642	Ups	0.00032	0.00242	molecular_	K16494
ENSG000	PCDHGA4	157779	1	0.01	0	0	0	0	12	0.07	28	0.15	6	0.03	7.024473	0.571371	4.393766	2.51047	Ups	1.1E-05	0.00013	molecular_	K16495
ENSG000	PCDHGA5	148649	1	0.01	2	0.01	1	0.01	5	0.03	16	0.09	6	0.03	4.693744	0.563838	2.651258	1.49488	Ups	0.00802	0.03387	molecular_	K16495
ENSG000	PCDHGB3	142716	0	0	1	0.01	0	0	3	0.02	10	0.05	4	0.02	2.68284	0.583344	2.632775	1.53581	Ups	0.00847	0.03542	molecular_	K16496
ENSG000	PCK1	5378	2	0	1	0	2	0	132	0.3	557	1.19	339	0.71	151.7496	0.461317	11.9748	5.52418	Ups	4.8E-33	1.1E-30	molecular_	K01596
ENSG000	PCLO	408918	59	0.05	79	0.08	52	0.05	145	0.14	508	0.45	197	0.17	158.8243	0.365416	4.590937	1.6776	Ups	4.4E-06	5.7E-05	molecular_	K16882
ENSG000	PCMTD1	81596	402	1.05	582	1.58	326	0.85	830	2.25	1895	4.76	937	2.3	779.7882	0.294176	3.889303	1.14414	Ups	0.0001	0.00088	molecular_	_
ENSG000	PCNX	207978	1413	1.72	1706	2.17	1405	1.72	2119	2.68	6647	7.81	2827	3.25	2517.061	0.306025	3.28504	1.0053	Ups	0.00102	0.00641	cellular_cc	_
ENSG000	PCOLCE	5999	127	0.49	95	0.39	127	0.5	3517	14.22	209	0.78	2035	7.46	1003.414	0.578562	3.619697	2.09422	Ups	0.00029	0.00224	molecular_	_
ENSG000	PCOLCE-A	14805	1	0.01	0	0	1	0.01	4	0.03	7	0.04	15	0.09	4.26859	0.576623	3.076888	1.77421	Ups	0.00209	0.01159	_	_
ENSG000	PCP2	2138	1	0.03	2	0.05	5	0.13	24	0.65	10	0.25	12	0.29	8.762355	0.531665	3.038936	1.61569	Ups	0.00237	0.0129	molecular_	_
ENSG000	PCSK2	258472	0	0	0	0	0	0	36	0.1	5	0.01	21	0.05	10.05679	0.576841	4.995891	2.88184	Ups	5.9E-07	9.4E-06	molecular_	K01360
ENSG000	PCSK4	9325	33	0.12	30	0.11	42	0.15	96	0.35	69	0.23	109	0.36	61.21152	0.308493	3.533612	1.0901	Ups	0.00041	0.00297	cellular_cc	K08671
ENSG000	PCSK5	471696	0	0	2	0	0	0	3	0.01	28	0.05	10	0.02	6.256979	0.578664	3.433861	1.98705	Ups	0.0006	0.00408	biological_	K08654
ENSG000	PCYOX1L	11647	91	0.27	77	0.24	79	0.23	386	1.18	305	0.87	352	0.98	205.6635	0.234613	7.663178	1.79788	Ups	1.8E-14	1E-12	molecular_	K05906
ENSG000	PDCD4-A	2366	139	4.59	134	4.62	161	5.35	452	15.53	240	7.65	420	13.09	251.6526	0.29778	3.656465	1.08882	Ups	0.00026	0.00198	_	_
ENSG000	PDCL3	14046	1569	18.76	1458	18.18	1665	20.02	696	8.66	868	10.01	1024	11.55	1229.93	0.164368	-6.46295	-1.0623	Down	1E-10	3.4E-09	molecular_	_
ENSG000	PDE10A	659316	298	0.41	234	0.33	198	0.27	634	0.9	1437	1.89	754	0.97	551.4217	0.270702	5.924999	1.60391	Ups	3.1E-09	8E-08	molecular_	K18438
ENSG000	PDE11A	485087	0	0	3	0	1	0	105	0.17	145	0.22	98	0.14	53.63773	0.444065	10.71403	4.75773	Ups	8.8E-27	1.4E-24	molecular_	K13298
ENSG000	PDE2A	98451	11	0.02	9	0.02	10	0.02	3	0.01	4	0.01	2	0	6.710921	0.509956	-2.81957	-1.4379	Down	0.00481	0.02267	biological_	K18283
ENSG000	PDE4C	47459	11	0.02	13	0.02	22	0.03	113	0.17	80	0.11	80	0.11	50.81076	0.357301	5.928692	2.11833	Ups	3.1E-09	7.9E-08	molecular_	K13293
ENSG000	PDE4D	1553083	1503	1.43	1823	1.8	1442	1.38	3206	3.16	5768	5.28	3443	3.08	2718.702	0.20813	5.287948	1.10058	Ups	1.2E-07	2.3E-06	biological_	K13293
ENSG000	PDE5A	134597	190	0.32	301	0.53	164	0.28	856	1.5	2400	3.89	1088	1.73	760.2881	0.326333	6.901176	2.25208	Ups	5.2E-12	2.1E-10	molecular_	K13762
ENSG000	PDE6C	53423	10	0.07	5	0.04	4	0.03	23	0.17	28	0.19	19	0.12	14.0655	0.435229	3.168985	1.37923	Ups	0.00153	0.00893	cellular_cc	K13757
ENSG000	PDE8B	219359	947	2.27	1410	3.53	935	2.26	2503	6.25	5143	11.91	3035	6.87	2186.951	0.249878	5.513225	1.37763	Ups	3.5E-08	7.4E-07	molecular_	K18437
ENSG000	PDK4	12993	221	0.82	232	0.9	159	0.59	473	1.83	1561	5.6	702	2.46	512.1672	0.328106	5.259968	1.72582	Ups	1.4E-07	2.7E-06	molecular_	K00898
ENSG000	PDLIM4	15784	3	0.01	4	0.02	8	0.04	27	0.14	7	0.03	24	0.11	11.91637	0.51489	2.509608	1.29217	Ups	0.01209	0.04654	molecular_	_
ENSG000	PDXK	43214	17682	27.47	14604	23.66	15967	24.95	6386	10.32	5420	8.12	6402	9.38	11401.34	0.179906	-8.72912	-1.5704	Down	2.6E-18	2E-16	molecular_	K00868
ENSG000	PDZD7	23451	48	0.16	37	0.13	41	0.13	182	0.62	110	0.35	146	0.45	90.97832	0.306586	4.85459	1.48835	Ups	1.2E-06	1.8E-05	molecular_	_
ENSG000	PDZK1IP1	7452	41	0.45	46	0.53	40	0.44	4	0.05	7	0.07	9	0.09	25.63519	0.382545	-6.60125	-2.5253	Down	4.1E-11	1.4E-09	cellular_cc	_
ENSG000	PEAR1	22737	215	0.51	178	0.44	192	0.46	69	0.17	142	0.32	95	0.21	149.9483	0.238815	-4.71616	-1.1263	Down	2.4E-06	3.3E-05	cellular_cc	_
ENSG000	PER3	60858	141	0.36	132	0.35	114	0.29	218	0.57	521	1.27	264	0.63	218.2943	0.280817	3.739826	1.05021	Ups	0.00018	0.00149	biological_	K02633
ENSG000	PFAS	22874	4814	11.68	3992	10.1	4141	10.1	2328	5.87	2980	6.97	2100	4.8	3433.446	0.161456	-6.24165	-1.0078	Down	4.3E-10	1.3E-08	molecular_	K01952
ENSG000	PFKP	71380	17188	69.82	14518	61.51	16203	66.2	9820	41.49	6660	26.1	7779	29.8	12313.05	0.226552	-4.97028	-1.126	Down	6.7E-07	1.1E-05	molecular_	K00850
ENSG000	PFN1P2	418	0	0	1	0.06	1	0.06	5	0.32	12	0.71	3	0.17	3.334878	0.581538	2.5606	1.48909	Ups	0.01045	0.0416	_	K05759
ENSG000	PGAM4	1678	52	0.8	49	0.78	50	0.77	22	0.35	33	0.49	26	0.38	39.15925	0.287606	-3.66357	-1.0537	Down	0.00025	0.00193	molecular_	K01834
ENSG000	PGAM5	11824	3804	17.19	3259	15.36	3192	14.51	2068	9.72	1468	6.4	1886	8.04	2667.97	0.215334	-4.98946	-1.0744	Down	6.1E-07	9.7E-06	molecular_	K15637
ENSG000	PGAP1	94793	117	0.11	240	0.23	90	0.08	288	0.28	1191	1.06	449	0.39	361.8041	0.409852	3.807873	1.56066	Ups	0.00014	0.00118	molecular_	K05294
ENSG000	PGBD5	104084	139	0.56	121	0.51	102	0.42	5	0.02	26	0.1	20	0.08	71.84327	0.348826	-7.94142	-2.7702	Down	2E-15	1.2E-13	cellular_cc	_
ENSG000	PGF	13951	24	0.06	11	0.03	23	0.06	72	0.19	32	0.08	65	0.16	36.93117	0.407227	2.865556	1.16693	Ups	0.00416	0.02025	biological_	K16859
ENSG000	PHACTR1	570753	2	0.01	3	0.01	0	0	4	0.01	12	0.03	14	0.03	5.342975	0.559516	2.581765	1.44454	Ups	0.00983	0.03975	molecular_	K17594
ENSG000	PHB	10833	20462	88.58	19175	86.57	22801	99.27	10968	49.39	8126	33.94	12293	50.19	15989.09	0.219302	-5.2427	-1.1497	Down	1.6E-07	2.9E-06	biological_	K17080
ENSG000	PHBP4	837	1	0.03	7	0.22	2	0.06	0	0	0	0	0	0	1.789872	0.562414	-2.49969	-1.4059	Down	0.01243	0.04753	_	K17080
ENSG000	PHEX	218869	29	0.09	45	0.14	26	0.08	13	0.04	26	0.07	15	0.04	25.91933	0.369262	-2.70916	-1.0004	Down	0.00675	0.02963	biological_	K08636
ENSG000	PHIP	142370	1050	1.89	2028	3.8	810	1.47	1866	3.49	7162	12.43	2710	4.6	2414.134	0.385346	2.981276	1.14882	Ups	0.00287	0.01501	biological_	K11797
ENSG000	PHOSPHO	7405	9	0.03	6	0.02	11	0.04	2	0.01	1	0	4	0.01	5.716583	0.538192	-2.65965	-1.4314	Down	0.00782	0.03322	biological_	K06124
ENSG000	PHYHIP	12633	372	1.28	279	1	398	1.37	206	0.74	129	0.43	211	0.68	271.8973	0.27632	-3.86861	-1.069	Down	0.00011	0.00095	_	_
ENSG000	PIGZ	22718	42	0.18	27	0.12	21	0.09	307	1.37	222	0.92	281	1.14	141.8884	0.305146	9.022258	2.75311	Ups	1.8E-19	1.6E-17	molecular_	K08098
ENSG000	PIH1D2	10265	107	0.62	138	0.83	105	0.61	60	0.36	53	0.29	74	0.4	91.17703	0.258763	-4.09632	-1.06	Down	4.2E-05	0.00041	_	_
ENSG000	PIK3IP1	10942	17	0.04	16	0.04	11	0.03	93	0.22	85	0.19	126	0.27	54.56892	0.326195	7.208153	2.35126	Ups	5.7E-13	2.6E-11	biological_	_
ENSG000	PIKFYVE	92485	495	1	678	1.43	463	0.94	721	1.52	4163	8.14	1330	2.54	1192.372	0.401466	3.497713	1.40421	Ups	0.00047	0.00335	cellular_cc	K00921
ENSG000	PILRA	32567	26	0.19	12	0.09	10	0.07	67	0.51	92	0.65	106	0.73	48.72525	0.348247	5.786923	2.01528	Ups	7.2E-09	1.7E-07	molecular_	K15411
ENSG000	PIM1	5224	2346	15.92	1923	13.61	2043	13.94	6866	48.45	3475	22.75	5516	35.29	3617.442	0.279016	3.874114	1.08094	Ups	0.00011	0.00093	molecular_	K04702

ENSG000	PIP5K1B	303518	106	0.44	128	0.56	105	0.44	479	2.08	762	3.07	630	2.48	343.2294	0.1938	11.17079	2.1649	Ups	5.7E-29	9.8E-27	cellular_cc	_
ENSG000	PITPNC1	319798	189	0.46	142	0.36	173	0.42	423	1.07	325	0.76	448	1.02	274.9701	0.237819	4.204897	1.00001	Ups	2.6E-05	0.00027	molecular_	K05725
ENSG000	PITPNM3	105231	624	1.01	565	0.95	520	0.84	76	0.13	224	0.35	121	0.18	366.4134	0.285387	-7.48541	-2.1362	Down	7.1E-14	3.7E-12	molecular_	_
ENSG000	PITRM1	35084	3474	8.86	3462	9.21	3753	9.63	1247	3.31	2211	5.44	1536	3.7	2656.572	0.175399	-7.46205	-1.3088	Down	8.5E-14	4.3E-12	molecular_	K06972
ENSG000	PJA2	75286	1646	5.06	2830	9.07	1235	3.82	2353	7.52	9698	28.77	3502	10.15	3292.958	0.383532	2.654095	1.01793	Ups	0.00795	0.03365	cellular_cc	K10634
ENSG000	PKD1L1	173839	0	0	1	0	2	0.01	6	0.02	11	0.03	15	0.04	5.352338	0.560254	3.353806	1.87898	Ups	0.0008	0.00521	biological_	K04987
ENSG000	PKD1L3	70437	10	0.05	4	0.02	4	0.02	19	0.1	17	0.08	26	0.12	12.72459	0.453821	2.810328	1.27539	Ups	0.00495	0.02321	biological_	_
ENSG000	PKD2	70110	587	1.78	739	2.34	428	1.31	950	3	2459	7.2	1075	3.08	978.211	0.308508	3.275688	1.01058	Ups	0.00105	0.00659	biological_	K04986
ENSG000	PKIA	89129	72	0.3	69	0.3	36	0.15	165	0.72	252	1.02	172	0.68	120.6509	0.282336	4.995052	1.41028	Ups	5.9E-07	9.4E-06	biological_	K15985
ENSG000	PKIB	254443	49	0.19	43	0.17	35	0.13	9	0.04	32	0.12	26	0.09	32.47404	0.366266	-2.85195	-1.0446	Down	0.00435	0.02097	molecular_	K09228
ENSG000	PLA2G16	43689	18797	112.37	17641	109.99	21131	127.05	9798	60.92	6040	34.84	10769	60.72	14402.26	0.255833	-4.85173	-1.2412	Down	1.2E-06	1.8E-05	molecular_	K16817
ENSG000	PLA2G3	5799	129	1.23	132	1.31	114	1.09	636	6.31	418	3.84	588	5.29	322.7042	0.255993	7.183982	1.83905	Ups	6.8E-13	3.1E-11	molecular_	K01047
ENSG000	PLA2G6	94196	147	0.29	135	0.28	126	0.25	498	1.02	378	0.72	516	0.96	288.3926	0.232007	6.487282	1.50509	Ups	8.7E-11	2.9E-09	biological_	K16343
ENSG000	PLAC1	198485	10	0.08	11	0.09	16	0.13	58	0.48	53	0.41	62	0.47	33.26532	0.342907	5.295898	1.816	Ups	1.2E-07	2.2E-06	biological_	_
ENSG000	PLAC9	13678	3	0.04	1	0.01	3	0.04	15	0.21	22	0.29	21	0.27	10.03452	0.49187	4.29291	2.11156	Ups	1.8E-05	0.00019	cellular_cc	_
ENSG000	PLCB1	836180	144	0.32	165	0.38	91	0.2	446	1.02	1513	3.21	638	1.32	453.5162	0.350578	6.224377	2.18213	Ups	4.8E-10	1.4E-08	biological_	K05858
ENSG000	PLCB4	412480	903	2.3	1149	3.05	874	2.24	1614	4.27	4887	12	2185	5.24	1804.983	0.302936	3.979672	1.20559	Ups	6.9E-05	0.00064	molecular_	K05858
ENSG000	PLCE1-AS	7795	0	0	2	0.02	2	0.01	3	0.02	30	0.21	15	0.1	7.646022	0.567436	3.330276	1.88972	Ups	0.00087	0.00559	_	_
ENSG000	PLCXD3	203675	8	0.01	12	0.02	7	0.01	0	0	3	0.01	0	0	5.226684	0.564659	-3.51155	-1.9828	Down	0.00045	0.0032	molecular_	_
ENSG000	PLEKHG4I	49713	16	0.03	20	0.04	21	0.04	278	0.56	527	0.98	352	0.64	184.0053	0.283582	13.51268	3.83196	Ups	1.3E-41	4.3E-39	molecular_	K06115
ENSG000	PLEKHG5	53970	68	0.18	62	0.17	65	0.17	1413	3.8	607	1.51	1069	2.61	523.1217	0.328318	10.59766	3.47941	Ups	3.1E-26	4.6E-24	molecular_	K19464
ENSG000	PLEKHH2	130715	36	0.08	58	0.13	35	0.08	185	0.41	642	1.33	184	0.37	171.4818	0.398072	5.844202	2.32641	Ups	5.1E-09	1.3E-07	molecular_	_
ENSG000	PLEKHN1	9369	80	0.48	46	0.29	63	0.38	22	0.14	25	0.14	30	0.17	45.35319	0.312725	-4.46531	-1.3964	Down	8E-06	9.7E-05	molecular_	_
ENSG000	PLG	51078	1	0	1	0	1	0	13	0.03	5	0.01	6	0.01	4.370527	0.571667	2.756216	1.57564	Ups	0.00585	0.02651	biological_	K01315
ENSG000	PLGLB1	19294	2	0.01	2	0.01	2	0.01	17	0.09	7	0.03	24	0.12	8.576513	0.534186	3.547462	1.895	Ups	0.00039	0.00284	cellular_cc	K01315
ENSG000	PLK2	6279	3305	9.25	3937	11.49	4075	11.47	1550	4.51	2510	6.78	1957	5.17	2927.156	0.169381	-6.64431	-1.1254	Down	3.1E-11	1.1E-09	biological_	K08861
ENSG000	PLLP	28596	147	1.71	101	1.22	105	1.23	550	6.65	239	2.68	448	4.91	258.0085	0.336118	4.424118	1.48702	Ups	9.7E-06	0.00011	biological_	_
ENSG000	PLP2	3316	19312	361.04	17282	336.97	22383	420.86	7889	153.4	10248	184.84	11137	196.37	14962.69	0.164698	-7.36733	-1.2134	Down	1.7E-13	8.5E-12	molecular_	_
ENSG000	PLTP	13396	14	0.11	24	0.2	23	0.18	96	0.8	55	0.42	82	0.62	47.39237	0.359818	4.317361	1.55346	Ups	1.6E-05	0.00018	cellular_cc	K08761
ENSG000	PLXDC2	473618	92	0.18	121	0.25	108	0.21	1	0	0	0	0	0	57.41336	0.471655	-11.6091	-5.4755	Down	3.7E-31	7.5E-29	molecular_	_
ENSG000	PLXNA2	222079	314	0.49	236	0.38	287	0.45	42	0.07	107	0.16	44	0.06	177.6106	0.304343	-7.213	-2.1952	Down	5.5E-13	2.5E-11	biological_	K06820
ENSG000	PMEL	19213	51	0.13	54	0.15	51	0.13	225	0.61	149	0.38	190	0.47	115.7823	0.279783	5.568086	1.55785	Ups	2.6E-08	5.5E-07	molecular_	K17304
ENSG000	PNMA2	9407	22	0.07	28	0.09	38	0.12	80	0.26	140	0.42	99	0.29	63.78188	0.296359	5.076247	1.50439	Ups	3.9E-07	6.4E-06	molecular_	_
ENSG000	PNRC1	4410	1277	7.77	1261	8	1119	6.85	3035	19.2	3149	18.48	3103	17.8	2076.875	0.139264	8.003259	1.11457	Ups	1.2E-15	7.6E-14	molecular_	K18774
ENSG000	POLN	170204	83	0.2	89	0.23	82	0.2	221	0.56	323	0.76	258	0.59	166.8688	0.196041	7.071814	1.38636	Ups	1.5E-12	6.7E-11	molecular_	K16618
ENSG000	POMP	19822	9555	172	8867	166.47	9155	165.75	4668	87.4	3955	68.69	5714	97.01	7121.64	0.19239	-5.81455	-1.1187	Down	6.1E-09	1.5E-07	cellular_cc	K11599
ENSG000	POU2AF1	103379	0	0	0	0	0	0	7	0.02	1	0	9	0.02	2.698137	0.57727	2.91539	1.68297	Ups	0.00355	0.01784	molecular_	_
ENSG000	POU5F1B	5777	32	0.12	25	0.1	43	0.16	131	0.5	135	0.48	138	0.48	79.85721	0.269156	6.300763	1.69589	Ups	3E-10	9.1E-09	molecular_	K09367
ENSG000	PPARG	146989	1100	4.06	1051	4.05	1163	4.32	24	0.09	88	0.31	59	0.21	615.5417	0.290052	-14.6012	-4.2351	Down	2.8E-48	1.3E-45	biological_	K08530
ENSG000	PPDPF	1483	4894	57.5	3024	37.05	4546	53.72	2361	28.85	1579	17.9	2862	31.71	3273.128	0.27302	-3.69248	-1.0081	Down	0.00022	0.00176	biological_	_
ENSG000	PPFIA4	52243	123	0.15	116	0.15	116	0.14	560	0.71	792	0.93	623	0.71	363.1074	0.171351	12.82214	2.19709	Ups	1.2E-37	3.3E-35	molecular_	_
ENSG000	PPFIBP2	143830	173	0.27	167	0.28	177	0.28	772	1.27	672	1.03	698	1.05	423.1965	0.196685	9.106933	1.7912	Ups	8.5E-20	7.7E-18	molecular_	_
ENSG000	PPM1H	291056	1092	3.62	1021	3.53	1000	3.34	2410	8.32	3532	11.31	2567	8.03	1844.792	0.147232	8.150679	1.20004	Ups	3.6E-16	2.4E-14	molecular_	K17503
ENSG000	PPP1R12/	161898	1464	1.73	2081	2.56	1151	1.37	2317	2.84	7706	8.77	3012	3.35	2756.007	0.340337	3.191316	1.08612	Ups	0.00142	0.00839	biological_	K06270
ENSG000	PPP1R15/	3666	1350	8.46	1070	6.99	1320	8.32	7061	46.01	2784	16.83	5530	32.68	3100.121	0.322421	5.317305	1.71441	Ups	1.1E-07	2E-06	molecular_	K14019
ENSG000	PPP1R1C	177158	1	0	2	0	0	0	11	0.02	8	0.02	6	0.01	4.440021	0.568134	2.875648	1.63375	Ups	0.00403	0.01974	molecular_	K17549
ENSG000	PPP1R3C	4613	869	8.85	961	10.21	903	9.25	1792	18.98	2801	27.52	2123	20.39	1503.011	0.154464	6.749333	1.04253	Ups	1.5E-11	5.7E-10	molecular_	K07189
ENSG000	PPP2R2C	243023	25	0.05	16	0.04	13	0.03	5	0.01	3	0.01	2	0	11.15986	0.484744	-4.16039	-2.0167	Down	3.2E-05	0.00032	cellular_cc	K04354
ENSG000	PPTC7	52006	1065	3.36	1203	3.96	777	2.47	1877	6.16	3897	11.87	2166	6.45	1730.852	0.250651	4.304583	1.07895	Ups	1.7E-05	0.00018	molecular_	K17508
ENSG000	PRAP1	5538	0	0	0	0	1	0.02	5	0.08	6	0.09	5	0.07	2.625091	0.583406	2.695225	1.57241	Ups	0.00703	0.03058	cellular_cc	_
ENSG000	PRC1-AS1	22280	11	0.13	11	0.14	11	0.14	33	0.42	24	0.28	39	0.45	20.75371	0.385429	3.023718	1.16543	Ups	0.0025	0.01343	_	_
ENSG000	PRDM16	369454	1	0	3	0	2	0	16	0.02	11	0.01	19	0.02	8.211211	0.518945	3.708175	1.92434	Ups	0.00021	0.00166	biological_	K04462
ENSG000	PREX1	203631	403	1.12	339	0.98	338	0.95	27	0.08	120	0.32	51	0.13	221.0878	0.352209	-6.94271	-2.4453	Down	3.9E-12	1.6E-10	molecular_	K12365
ENSG000	PRICKLE1	132018	25	0.05	38	0.08	36	0.07	168	0.34	576	1.09	233	0.43	161.4442	0.369303	7.257101	2.68007	Ups	4E-13	1.9E-11	biological_	K04511
ENSG000	PRKAG2-/	2173	157	2.81	153	2.85	164	2.95	76	1.41	30	0.52	56	0.94	110.1134	0.336455	-4.66009	-1.5679	Down	3.2E-06	4.3E-05	_	_
ENSG000	PRKAR1B	178454	1854	5.82	1553	5.09	2001	6.32	961	3.14	633	1.92	1098	3.25	1381.634	0.252782	-4.526	-1.1441	Down	6E-06	7.5E-05	molecular_	K04739
ENSG000	PRKAR2B	117163	49	0.28	75	0.45	43	0.25	98	0.59	359	1.99	208	1.13	127.6443	0.364799	4.195539	1.53053	Ups	2.7E-05	0.00028	biological_	K04739
ENSG000	PRKCE	536646	92	0.2	68	0.15	87	0.19	147	0.33	307	0.64	203	0.41	142.3373	0.260599	4.233381	1.10321	Ups	2.3E-05	0.00025	biological_	K18050
ENSG000	PRKCH	363418	503	1.01	562	1.18	541	1.09	1123	2.34	2107	4.08	1483	2.81	994.5735	0.194739	6.524403	1.27056	Ups	6.8E-11	2.3E-09	molecular_	K18051
ENSG000	PRKD1	615418	49	0.18	48	0.19	59	0.22	157	0.61	303	1.09	206	0.72	127.809	0.25617	6.850928	1.755	Ups	7.3E-12	2.9E-10	biological_	K06070
ENSG000	PRKG1	1307166	0	0	2	0.01	2	0.01	14	0.04	24	0.06	16	0.04	8.862253	0.523228	4.56209	2.38701	Ups	5.1E-06	6.5E-05	biological_	K07376
ENSG000	PROC	10820	103	0.34	92	0.32	116	0.39	78	0.27	27	0.09	50	0.16	80.11486	0.369172	-2.82101	-1.0414	Down	0.00479	0.0226	molecular_	K01344
ENSG000	PROCR	5290	1824	34.78	1598	31.78	1564	30	1053	20.89	402	7.4	909	16.35	1261.115	0.328781	-3.46402	-1.1389	Down	0.00053	0.00373	molecular_	K06557
ENSG000	PROM2	16856	44	0.09	27	0.05	30	0.06	7	0.01	16	0.03	16	0.03	23.76729	0.378	-3.74386	-1.4152	Down	0.00018	0.00147	cellular_cc	K15602
ENSG000	PRR16	223055	63	0.32	64	0.34	45	0.23	5	0.03	4	0.02	3	0.01	32.46326	0.406107	-8.43418	-3.4252	Down	3.3E-17	2.5E-15	_	_
ENSG000	PRR25	8419	33	0.7	15	0.33	21	0.45	10	0.22	14	0.29	5	0.1	16.65854	0.435967	-2.805	-1.2229	Down	0.00503	0.02351	_	_
ENSG000	PRR5	68969	888	1.98	643	1.5	845	1.9	448	1.04	170	0.37	411	0.87	585.3996	0.34221	-3.64993	-1.249	Down	0.00026	0.00202	biological_	_
ENSG000	PRR7-AS1	9197	122	1.42	78	0.95	101	1.18	36	0.44	56	0.63	45	0.49	74.24438	0.266698	-4.83427	-1.2893	Down	1.3E-06	2E-05	_	_
ENSG000	PRRT2	4025	8	0.03	10	0.03	11	0.04	47	0.16	44	0.14	47	0.15	26.43711	0.360136	5.038289	1.81447	Ups	4.7E-07	7.7E-06	cellular_cc	_
ENSG000	PRSS8	4328	111	0.56	127	0.66	130	0.66	74	0.39	28	0.14	50	0.24	89.75863	0.351533	-3.70943	-1.304	Down	0.00021	0.00166	molecular_	K08664
ENSG000	PRTFDC1	103998	27	0.26	30	0.31	20	0.2	133	1.35	101	0.95	123	1.13	69.11271	0.299928	6.225693	1.86726	Ups	4.8E-10	1.4E-08	molecular_	_
ENSG000	PRTG	131545	232	0.4	280	0.5	226	0.39	469	0.83	1502	2.47	633	1.02	515.5435	0.322272	4.400892	1.41828	Ups	1.1E-05	0.00013	biological_	_
ENSG000	PRTN3	7213	11	0.22	6	0.12	9	0.18	40	0.82	16	0.3	32	0.59	18.54426	0.447514	2.887386	1.29215	Ups	0.00388	0.01915	molecular_	K01350
ENSG000	PSCA	12417	2730	23.02	2579	22.68	2924	24.79	147	1.29	71	0.58	143	1.14	1523.331	0.282863	-15.559	-4.4011	Down	1.4E-54	8.5E-52	cellular_cc	_
ENSG000	PSMA7	6706	23306	210.73	20563	193.91	24826	225.76	12297	115.65	8082	70.5	14715	125.49	17687.17	0.25158	-4.40985	-1.1094	Down	1E-05	0.00012	biological_	K02731
ENSG000	PSMB6	2352	12470	137.52	11300	129.97	13409	148.72	6749	77.41	4169	44.36	8014	83.35	9568.468	0.261613	-4.23097	-1.1069	Down	2.3E-05	0.00025	biological_	K02738
ENSG000	PSMB9	15450	30	0.18	26	0.16	25	0.15	25	0.15	90	0.51	108	0.6	47.4184	0.402178	2.570896	1.03396	Ups	0.01014	0.04066	biological_	K02741
ENSG000	PSMD11	39058	10033	23.75	10010	24.72	11672	27.79	4249	10.46	6362	14.53	5203	11.62	8048.302	0.147112	-8.28069	-1.2182	Down	1.2E-16	8.6E-15	biological_	K03036
ENSG000	PSMD3	17164	13201	54.98	11918	51.77	14101	59.07	6974	30.21	6385	25.66	7291	28.64	10168.9	0.166015	-6.69138	-1.1109	Down	2.2E-11	8.2E-10	biological_	K03033
ENSG000	PSMD6-A	8127	10	0.1	5	0.05	4	0.04	12	0.13	57	0.58	15	0.15	15.58442	0.505948	2.743829	1.38823	Ups	0.00607	0.02731	_	_
ENSG000	PTCH2	23220	8	0.04	16	0.08	15	0.07	69	0.35	63	0.29	44	0.2	34.20891	0.368782	4.718011	1.73992	Ups	2.4E-06	3.3E-05	molecular_	K11101
ENSG000	PTGES	14717	1487	18.84	1327	17.53	1349	17.19	310	4.09	200	2.44	323	3.86	870.6975	0.241314	-9.96034	-2.4036	Down	2.3E-23	2.7E-21	biological_	K15729
ENSG000	PTGES3L-	30003	22	0.14	15	0.1	24	0.16	4	0.03	14	0.09	12	0.07	15.29391	0.426323	-2.50562	-1.0682	Down	0.01222	0.04695	molecular_	K07050
ENSG000	PTGFR	235867	23	0.11	20	0.1	9	0.04	0	0	0	0	0	0	9.227309	0.561209	-5.93383	-3.3301	Down	3E-09	7.7E-08	molecular_	K04262
ENSG000	PTGS1	25159	1221	3.88	1527	5.07	1412	4.52	9307	30.79	1533	4.7	6113	18.34	3477.725	0.531902	2.488496	1.32364	Ups	0.01283	0.04872	biological_	K00509
ENSG000	PTGS2	8637	260	0.75	315	0.94	208	0.6	1124	3.36	405	1.12	917	2.49	527.1455	0.354648	3.730668	1.32307	Ups	0.00019	0.00154	biological_	K11987
ENSG000	PTH1R	26052	22	0.12	16	0.09	15	0.08	79	0.44	52	0.27	92	0.47	44.06963	0.351026	4.79554	1.68336	Ups	1.6E-06	2.3E-05	biological_	K04585
ENSG000	PTK6	8946	508	4.13	416	3.53	369	3.02	50	0.42	83	0.65	103	0.79	265.4852	0.246406	-10.393	-2.5609	Down	2.7E-25	3.7E-23	cellular_cc	K08894
ENSG000	PTK7	85452	252	0.36	200	0.3	183	0.26	851	1.26	376	0.52	633	0.85	406.8563	0.322611	3.919516	1.26448	Ups	8.9E-05	0.00079	biological_	K05127
ENSG000	PTN	116524	0	0	4	0.04	0	0	289	2.8	403	3.62	430	3.78	170.9955	0.412264	15.15731	6.24881	Ups	6.8E-52	3.5E-49	molecular_	K16642
ENSG000	PTP4A3	39528	37	0.26	34	0.25	44	0.31	206	1.5	106	0.72	166	1.1	95.64932	0.332005	5.133276	1.70427	Ups	2.9E-07	4.9E-06	molecular_	K18041
ENSG000	PTPN14	203754	1441	1.89	1502	2.05	1445	1.9	4393	5.98	8919	11.26	4572	5.64	3461.719	0.229541	7.432836	1.70614	Ups	1.1E-13	5.3E-12	biological_	K18025
ENSG000	PTPRD	2298478	0	0	0	0	1	0	8	0.01	8	0.01	6	0.01	3.569781	0.579856	3.29804	1.91239	Ups	0.00097	0.00617	molecular_	K06777
ENSG000	PTPRG-AS	108466	14	0.04	12	0.04	12	0.04	4	0.01	1	0	4	0.01	8.191014	0.502081	-3.37191	-1.693	Down	0.00075	0.00494	_	_
ENSG000	PTPRN	20026	4	0.01	8	0.02	3	0.01	65	0.12	12	0.02	43	0.07	22.00745	0.514726	3.995673	2.05668	Ups	6.5E-05	0.0006	biological_	K07817
ENSG000	PTPRO	275003	109	0.22	145	0.31	122	0.25	26	0.06	36	0.07	23	0.04	79.96475	0.271939	-8.2173	-2.2346	Down	2.1E-16	1.4E-14	biological_	K18035
ENSG000	PVRL3	205493	332	1.07	635	2.14	275	0.89	601	2.02	1955	6.1	1081	3.3	758.3084	0.356746	3.208914	1.14477	Ups	0.00133	0.00798	biological_	K06592
ENSG000	PVRL3-AS	177846	2	0.03	1	0.01	2	0.03	12	0.16	10	0.12	15	0.18	6.598178	0.531862	3.412628	1.81505	Ups	0.00064	0.00434	_	_
ENSG000	PVRL4	18605	4	0.02	3	0.02	6	0.03	8	0.04	28	0.14	25	0.12	11.33367	0.489069	3.08434	1.50845	Ups	0.00204	0.01136	molecular_	K06593
ENSG000	PWP2	23893	3019	9.11	2292	7.22	2696	8.18	1618	5.08	1112	3.24	1514	4.31	2085.202	0.229554	-4.63746	-1.0645	Down	3.5E-06	4.7E-05	molecular_	K14558
ENSG000	PYGO1	50058	127	0.3	158	0.38	99	0.23	140	0.34	724	1.62	295	0.65	237.1269	0.394896	2.867812	1.13249	Ups	0.00413	0.02015	biological_	_
ENSG000	PYY2	1503	12	0.23	12	0.24	23	0.44	59	1.16	29	0.53	51	0.91	30.25157	0.406415	2.901258	1.17912	Ups	0.00372	0.01849	molecular_	K05233
ENSG000	QKI	164597	722	0.53	1107	0.85	537	0.4	1117	0.85	3897	2.75	1681	1.16	1406.463	0.352502	3.122389	1.10065	Ups	0.00179	0.0102	biological_	K14945
ENSG000	QPRT	35421	6597	26.58	5747	24.15	6385	25.87	19441	81.47	11109	43.18	19279	73.26	11123.05	0.26067	4.447758	1.1594	Ups	8.7E-06	0.0001	molecular_	K00767
ENSG000	R3HDM2	181397	322	0.76	301	0.74	351	0.83	755	1.85	962	2.19	724	1.61	545.7783	0.167187	6.44861	1.07812	Ups	1.1E-10	3.7E-09	molecular_	_
ENSG000	RAB26	13363	832	3.29	631	2.61	702	2.8	2569	10.58	997	3.81	2258	8.43	1305.559	0.331355	3.457223	1.14557	Ups	0.00055	0.0038	cellular_cc	K07913
ENSG000	RAB33B	23378	119	0.6	180	0.94	84	0.42	209	1.09	451	2.18	400	1.89	226.8277	0.309248	3.641095	1.126	Ups	0.00027	0.00209	cellular_cc	K07920
ENSG000	RAB3A	7291	98	0.71	74	0.56	107	0.78	254	1.92	243	1.7	267	1.83	167.2732	0.225399	5.293226	1.19309	Ups	1.2E-07	2.3E-06	cellular_cc	K07882
ENSG000	RAB3B	82809	27	0.05	36	0.08	28	0.06	36	0.07	156	0.3	60	0.11	53.0537	0.406634	2.508537	1.02006	Ups	0.01212	0.04665	molecular_	K06108
ENSG000	RAB43	35233	114	0.44	95	0.38	95	0.37	37	0.15	48	0.18	37	0.13	72.67258	0.246143	-5.9659	-1.4685	Down	2.4E-09	6.4E-08	molecular_	K07930
ENSG000	RAB5C	30042	6928	34.33	6675	34.5	7453	37.15	3746	19.31	4008	19.16	4237	19.8	5589.103	0.132436	-7.67155	-1.016	Down	1.7E-14	9.4E-13	molecular_	K07889
ENSG000	RAC2	19188	34	0.21	42	0.28	28	0.18	0	0	1	0.01	1	0.01	18.80768	0.503351	-7.73695	-3.8944	Down	1E-14	5.8E-13	molecular_	K07860
ENSG000	RAG1	82448	2	0	4	0.01	4	0.01	8	0.02	26	0.06	11	0.03	8.421862	0.51375	2.70173	1.38801	Ups	0.0069	0.0301	biological_	K10628
ENSG000	RAP1GAP	73094	481	1.35	370	1.08	458	1.29	198	0.58	124	0.34	161	0.43	308.4919	0.260762	-5.91801	-1.5432	Down	3.3E-09	8.4E-08	cellular_cc	K17700
ENSG000	RAP2C-AS	215716	13	0.09	3	0.02	4	0.03	26	0.18	77	0.49	41	0.26	24.84215	0.460525	4.504294	2.07434	Ups	6.7E-06	8.2E-05	_	_
ENSG000	RASA3	150893	1220	6.32	1003	5.42	1092	5.69	407	2.19	751	3.75	571	2.79	849.9382	0.190137	-6.03984	-1.1484	Down	1.5E-09	4.2E-08	molecular_	K12380
ENSG000	RASA4CP	14942	2	0.01	5	0.02	3	0.01	9	0.04	18	0.07	16	0.06	8.236352	0.497059	2.822043	1.40272	Ups	0.00477	0.02255	_	K17630
ENSG000	RASD1	1959	6	0.05	9	0.08	6	0.05	35	0.3	14	0.11	25	0.19	15.47566	0.460476	2.854632	1.31449	Ups	0.00431	0.02083	molecular_	K07843
ENSG000	RASD2	13134	156	1.18	119	0.94	130	0.99	70	0.55	34	0.25	50	0.36	96.33202	0.317262	-4.57306	-1.4509	Down	4.8E-06	6.2E-05	biological_	K07844
ENSG000	RASGEF1E	617851	0	0	0	0	0	0	12	0.03	30	0.06	17	0.03	8.779631	0.561792	5.695596	3.19974	Ups	1.2E-08	2.8E-07	molecular_	_
ENSG000	RASGRP1	77473	3	0.01	3	0.01	6	0.02	5	0.02	46	0.14	16	0.05	11.78246	0.536849	2.784046	1.49461	Ups	0.00537	0.02482	cellular_cc	K04350
ENSG000	RASGRP3	128427	21	0.03	19	0.03	23	0.03	111	0.16	330	0.43	168	0.21	101.3225	0.349831	7.723196	2.70181	Ups	1.1E-14	6.4E-13	biological_	K12362
ENSG000	RBL1	99647	552	2.14	632	2.55	485	1.89	793	3.2	2557	9.56	1153	4.21	961.4173	0.312027	3.431139	1.07061	Ups	0.0006	0.00411	biological_	K04681
ENSG000	RBM44	44420	9	0.03	8	0.03	8	0.03	15	0.05	43	0.13	25	0.07	16.74935	0.425376	2.917614	1.24108	Ups	0.00353	0.01774	molecular_	_
ENSG000	RBM47	207621	464	0.53	510	0.61	433	0.5	122	0.14	275	0.3	128	0.14	328.9504	0.269494	-5.86046	-1.5794	Down	4.6E-09	1.1E-07	molecular_	_
ENSG000	RBP2	27864	2	0.05	3	0.08	3	0.07	68	1.72	56	1.31	78	1.78	32.63337	0.432863	8.322107	3.60234	Ups	8.6E-17	6.1E-15	biological_	K14622
ENSG000	RCN3	19345	66	0.53	45	0.38	55	0.44	895	7.46	732	5.66	1206	9.11	466.4882	0.265509	13.82869	3.67164	Ups	1.7E-43	6.4E-41	molecular_	_
ENSG000	RDH10	30670	956	3.71	903	3.65	952	3.71	1823	7.35	3468	12.98	2636	9.64	1691.486	0.186146	6.542952	1.21794	Ups	6E-11	2.1E-09	biological_	K11151
ENSG000	RDH16	7940	0	0	2	0.01	1	0	22	0.11	3	0.01	14	0.06	6.85217	0.574421	3.292028	1.89101	Ups	0.00099	0.00629	molecular_	K11154
ENSG000	REEP6	6762	299	2.61	201	1.83	275	2.41	128	1.16	62	0.52	122	1	186.8716	0.319322	-4.30501	-1.3747	Down	1.7E-05	0.00018	cellular_cc	K17279
ENSG000	REPS2	206582	320	0.65	413	0.87	331	0.67	79	0.17	251	0.49	108	0.21	254.2575	0.32332	-4.39043	-1.4195	Down	1.1E-05	0.00013	molecular_	K12756
ENSG000	RGCC	13324	0	0	1	0.02	4	0.09	27	0.66	2	0.05	21	0.46	8.989634	0.577797	2.915534	1.68459	Ups	0.00355	0.01783	biological_	K04257
ENSG000	RGS16	5786	30	0.32	22	0.24	34	0.36	164	1.81	82	0.84	145	1.45	76.74853	0.352161	5.099689	1.79591	Ups	3.4E-07	5.7E-06	molecular_	K16449
ENSG000	RGS17	126791	22	0.06	46	0.14	26	0.08	10	0.03	23	0.06	2	0.01	21.98859	0.489209	-2.64147	-1.2922	Down	0.00825	0.03468	molecular_	K16449
ENSG000	RGS19	6790	804	6.29	732	5.97	871	6.85	422	3.43	229	1.73	435	3.21	598.6495	0.282532	-4.43709	-1.2536	Down	9.1E-06	0.00011	molecular_	K16449
ENSG000	RGS2	3235	2599	21.87	2358	20.69	2241	18.96	6396	55.98	4310	34.99	6303	50.02	3928.189	0.226172	4.448558	1.00614	Ups	8.6E-06	0.0001	molecular_	K18154
ENSG000	RGS5	210667	19	0.05	25	0.07	24	0.07	356	1.03	1486	3.97	720	1.88	387.5563	0.374609	11.62495	4.35481	Ups	3.1E-31	6.3E-29	molecular_	K16449
ENSG000	RHBDF2	30900	705	1.18	496	0.87	611	1.03	258	0.45	264	0.43	239	0.38	439.2779	0.196676	-7.22471	-1.4209	Down	5E-13	2.3E-11	molecular_	_
ENSG000	RHBDL2	55993	18	0.11	34	0.21	22	0.13	11	0.07	15	0.09	8	0.05	18.38355	0.405982	-2.8519	-1.1578	Down	0.00435	0.02097	molecular_	K02857
ENSG000	RHOB	2366	6175	67.09	4608	52.21	5408	59.09	16246	183.59	10384	108.85	15760	161.49	9488.76	0.244843	4.667	1.14268	Ups	3.1E-06	4.1E-05	biological_	K07856
ENSG000	RHOBTB3	110862	1539	3.04	2047	4.21	1087	2.16	2842	5.83	12038	22.92	4395	8.18	3649.907	0.369154	4.229934	1.5615	Ups	2.3E-05	0.00025	molecular_	K07869
ENSG000	RIMS1	516440	24	0.03	20	0.03	23	0.03	70	0.09	185	0.22	80	0.09	61.64649	0.356034	5.186754	1.84666	Ups	2.1E-07	3.8E-06	;molecular	K15291
ENSG000	RIMS4	58531	74	0.32	74	0.34	65	0.29	24	0.11	39	0.16	33	0.14	52.4177	0.268955	-4.84274	-1.3025	Down	1.3E-06	1.9E-05	biological_	K15299
ENSG000	RIN1	6599	1116	2.81	786	2.06	951	2.41	443	1.16	243	0.59	382	0.91	674.8861	0.28077	-5.35017	-1.5022	Down	8.8E-08	1.7E-06	molecular_	K17638
ENSG000	RIN3	175222	299	0.61	218	0.46	256	0.53	48	0.1	170	0.33	120	0.23	187.3541	0.323726	-4.12704	-1.336	Down	3.7E-05	0.00037	molecular_	_

ENSG000	RN7SKP2	304	0	0	0	0	0	0	7	0.62	9	0.73	2	0.16	2.75444	0.581448	3.143002	**1.82749**	**Ups**	**0.00167**	**0.00961**	_	_
ENSG000	RN7SL51	246	0	0	0	0	0	0	15	1.63	36	3.63	18	1.77	10.2784	0.557122	6.043906	**3.3672**	**Ups**	**1.5E-09**	**4.1E-08**	_	_
ENSG000	RNASEK	2116	850	3.63	723	3.22	798	3.43	465	2.07	352	1.45	490	1.98	624.6307	0.215897	-4.73697	**-1.0227**	**Down**	**2.2E-06**	**3E-05**	molecular	K19770
ENSG000	RNASEK-	4886	45	0.33	37	0.28	28	0.21	11	0.08	9	0.06	12	0.08	24.49256	0.371515	-4.74182	**-1.7617**	**Down**	**2.1E-06**	**3E-05**	molecular	K19770
ENSG000	RNF112	6152	30	0.15	31	0.16	37	0.19	126	0.65	91	0.44	119	0.56	69.68858	0.29352	5.002993	**1.46848**	**Ups**	**5.6E-07**	**9.1E-06**	molecular	_
ENSG000	RNF150	353071	1751	2.87	2030	3.47	1851	3.05	4824	8.21	9075	14.33	4906	7.57	3829.869	0.218508	6.587233	**1.43936**	**Ups**	**4.5E-11**	**1.6E-09**	molecular	K15701
ENSG000	RNF157	97921	62	0.15	57	0.14	49	0.12	476	1.19	505	1.17	333	0.75	231.9707	0.250222	10.4787	**2.622**	**Ups**	**1.1E-25**	**1.6E-23**	molecular	_
ENSG000	RNF165	136332	291	0.42	266	0.4	311	0.45	15	0.02	54	0.07	34	0.05	169.7938	0.299401	-10.3318	**-3.0934**	**Down**	**5.1E-25**	**6.9E-23**	molecular	K17821
ENSG000	RNF182	55857	51	0.14	77	0.23	69	0.19	108	0.32	449	1.22	224	0.59	149.6368	0.371606	4.062279	**1.50957**	**Ups**	**4.9E-05**	**0.00047**	molecular	K11983
ENSG000	RNF43	65096	348	0.67	291	0.59	320	0.62	37	0.07	96	0.18	40	0.07	196.3737	0.295255	-8.58771	**-2.5356**	**Down**	**8.9E-18**	**6.9E-16**	molecular	K15694
ENSG000	RNMTL1	10237	2289	16.98	2274	17.59	2687	20.05	1314	10.14	927	6.64	1404	9.82	1858.171	0.228265	-5.01147	**-1.1439**	**Down**	**5.4E-07**	**8.7E-06**	molecular	_
ENSG000	RNU2-27	142	14	2.53	11	2.08	6	1.09	16	3.01	42	7.34	31	5.29	18.73577	0.417929	2.582519	**1.07931**	**Ups**	**0.00981**	**0.03967**	_	_
ENSG000	RNU5A-1	116	11	2.44	16	3.7	6	1.34	44	10.14	20	4.28	48	10.03	23.4434	0.440608	2.95018	**1.29987**	**Ups**	**0.00318**	**0.0163**	_	_
ENSG000	RNU5D-1	116	1	0.22	1	0.23	0	0	8	1.84	3	0.64	7	1.46	3.201102	0.581588	2.494313	**1.45066**	**Ups**	**0.01262**	**0.0481**	_	_
ENSG000	RNU6-11	102	42	10.58	62	16.3	51	12.93	7	1.83	11	2.67	5	1.19	31.13133	0.377979	-6.91095	**-2.6122**	**Down**	**4.8E-12**	**2E-10**	_	_
ENSG000	RNU6-41	97	17	4.51	15	4.15	18	4.8	68	18.74	24	6.14	55	13.75	32.22206	0.415841	2.821407	**1.17326**	**Ups**	**0.00478**	**0.02259**	_	_
ENSG000	RNU6-58	92	1	0.28	1	0.29	0	0	3	0.87	21	5.66	4	1.05	4.381348	0.582125	2.843845	**1.65547**	**Ups**	**0.00446**	**0.02137**	_	_
ENSG000	RNU7-11	62	54	22.39	42	18.16	55	22.93	109	47.01	173	69.2	126	49.27	88.57954	0.24556	4.661226	**1.14461**	**Ups**	**3.1E-06**	**4.3E-05**	_	_
ENSG000	RNU7-16	63	1	0.41	4	1.7	0	0	19	8.06	29	11.42	20	7.7	11.19073	0.507915	4.944082	**2.51117**	**Ups**	**7.7E-07**	**1.2E-05**	_	_
ENSG000	RNVU1-1	164	2	0.31	1	0.16	6	0.95	27	4.4	8	1.21	22	3.25	10.68128	0.533282	3.199864	**1.70643**	**Ups**	**0.00137**	**0.00818**	_	_
ENSG000	RNVU1-6	164	4	0.63	7	1.14	4	0.63	40	6.52	12	1.81	42	6.21	17.54677	0.492538	3.800029	**1.87166**	**Ups**	**0.00014**	**0.00121**	_	_
ENSG000	ROPN1B	14311	0	0	0	0	0	0	2	0.01	7	0.02	3	0.01	1.765355	0.570155	2.551796	**1.45492**	**Ups**	**0.01072**	**0.04238**	biological	_
ENSG000	ROR1	407489	123	0.34	159	0.45	98	0.27	148	0.42	722	1.9	281	0.72	235.4256	0.391947	2.911042	**1.14097**	**Ups**	**0.0036**	**0.01804**	molecular	K05122
ENSG000	RORA	741036	20	0.03	16	0.02	28	0.04	30	0.04	120	0.15	53	0.06	41.17282	0.412027	2.876736	**1.18529**	**Ups**	**0.00402**	**0.01968**	molecular	K08532
ENSG000	RP1-102D	12881	1	0.05	0	0	0	0	3	0.15	7	0.32	6	0.27	2.563405	0.583442	2.631082	**1.53508**	**Ups**	**0.00851**	**0.03551**	_	_
ENSG000	RP1-102H	77137	5	0.02	5	0.02	10	0.05	16	0.08	21	0.09	27	0.12	13.29882	0.437682	2.740455	**1.19945**	**Ups**	**0.00614**	**0.02751**	_	K08857
ENSG000	RP11-100	16982	0	0	0	0	1	0.03	1	0.03	8	0.22	8	0.22	2.663076	0.581918	2.48023	**1.44329**	**Ups**	**0.01313**	**0.04966**	_	_
ENSG000	RP11-102	1394	6	0.11	14	0.27	19	0.35	48	0.92	81	1.44	36	0.63	31.89181	0.406578	3.875276	**1.5756**	**Ups**	**0.00011**	**0.00093**	_	_
ENSG000	RP11-103	13457	8	0.27	12	0.42	18	0.61	5	0.17	2	0.06	6	0.19	8.835803	0.500673	-2.6315	**-1.3175**	**Down**	**0.0085**	**0.03549**	_	K02957
ENSG000	RP11-103	18893	25	0.26	21	0.23	29	0.3	51	0.55	167	1.68	93	0.91	59.26584	0.363694	4.369436	**1.58914**	**Ups**	**1.3E-05**	**0.00014**	_	_
ENSG000	RP11-105	2127	57	0.74	64	0.87	74	0.97	129	1.75	188	2.36	219	2.69	115.9744	0.247143	4.724088	**1.16753**	**Ups**	**2.3E-06**	**3.2E-05**	_	_
ENSG000	RP11-105	690	6	0.22	8	0.31	2	0.07	17	0.66	31	1.11	24	0.84	13.67535	0.449131	3.502901	**1.57326**	**Ups**	**0.00046**	**0.00329**	_	_
ENSG000	RP11-106	3732	1	0.02	1	0.02	3	0.06	5	0.11	42	0.85	28	0.55	11.78256	0.548337	4.166346	**2.28456**	**Ups**	**3.1E-05**	**0.00032**	_	_
ENSG000	RP11-108	69631	5	0.01	11	0.02	3	0	40	0.06	67	0.09	47	0.06	26.62792	0.405327	5.857034	**2.37401**	**Ups**	**4.7E-09**	**1.2E-07**	_	K13912
ENSG000	RP11-108	2984	0	0	1	0.04	5	0.17	15	0.54	9	0.3	13	0.43	6.867948	0.548312	2.866256	**1.5716**	**Ups**	**0.00415**	**0.02021**	_	_
ENSG000	RP11-109	110	10	2.34	4	0.97	15	3.53	55	13.37	19	4.28	66	14.55	27.20459	0.476157	3.439645	**1.63781**	**Ups**	**0.00058**	**0.00401**	_	_
ENSG000	RP11-109	3359	0	0	0	0	0	0	8	0.52	3	0.18	4	0.24	2.396314	0.579301	2.949239	**1.7085**	**Ups**	**0.00319**	**0.01632**	_	_
ENSG000	RP11-10A	16395	21	0.3	18	0.27	29	0.42	12	0.18	13	0.18	8	0.11	17.19854	0.399601	-2.72803	**-1.0901**	**Down**	**0.00637**	**0.02834**	_	_
ENSG000	RP11-10L	46480	0	0	0	0	0	0	3	0.05	5	0.07	7	0.1	2.261837	0.580178	2.976118	**1.72668**	**Ups**	**0.00292**	**0.01521**	_	_
ENSG000	RP11-10N	3342	10	0.49	7	0.35	10	0.49	4	0.2	1	0.05	2	0.09	5.934631	0.534208	-2.75201	**-1.4701**	**Down**	**0.00592**	**0.02677**	_	_
ENSG000	RP11-110	1045	2	0.04	2	0.04	2	0.04	658	14.32	421	8.5	529	10.44	252.3555	0.41026	15.51755	**6.36623**	**Ups**	**2.6E-54**	**1.6E-51**	_	_
ENSG000	RP11-111	819	0	0	0	0	0	0	2	0.07	3	0.09	7	0.21	1.812336	0.570263	2.56052	**1.46017**	**Ups**	**0.01045**	**0.0416**	_	_
ENSG000	RP11-114	3003	1	0.03	1	0.04	0	0	25	0.89	5	0.16	25	0.8	9.114762	0.566152	4.366478	**2.47209**	**Ups**	**1.3E-05**	**0.00014**	_	_
ENSG000	RP11-115	175148	241	1.69	308	2.25	201	1.41	427	3.11	1095	7.39	754	4.97	472.1869	0.279939	4.502866	**1.26053**	**Ups**	**6.7E-06**	**8.3E-05**	_	_
ENSG000	RP11-116	6165	11	0.32	4	0.12	10	0.29	16	0.48	53	1.48	46	1.26	21.45603	0.445681	3.571198	**1.59162**	**Ups**	**0.00036**	**0.00263**	_	_
ENSG000	RP11-120	111327	0	0	2	0.02	0	0	4	0.05	12	0.13	5	0.05	3.461806	0.581402	2.616178	**1.52105**	**Ups**	**0.00889**	**0.0367**	_	_
ENSG000	RP11-124	3026	49	0.42	56	0.5	48	0.41	139	1.23	137	1.12	122	0.98	88.53809	0.240827	4.626465	**1.11418**	**Ups**	**3.7E-06**	**4.9E-05**	_	_
ENSG000	RP11-125	218694	7	0.04	9	0.05	8	0.04	14	0.08	29	0.15	29	0.15	15.06829	0.418332	2.718876	**1.13739**	**Ups**	**0.00655**	**0.02895**	_	_
ENSG000	RP11-126	1398	68	2.37	63	2.29	63	2.21	27	0.98	28	0.94	37	1.22	48.63179	0.277252	-4.39233	**-1.2178**	**Down**	**1.1E-05**	**0.00013**	_	_
ENSG000	RP11-127	2651	12	0.06	12	0.06	19	0.09	8	0.04	0	0	5	0.02	9.8054	0.527095	-2.53463	**-1.336**	**Down**	**0.01126**	**0.04403**	_	_
ENSG000	RP11-127	665	3	0.12	8	0.32	1	0.04	44	1.77	8	0.3	39	1.42	16.69513	0.539796	3.444643	**1.85941**	**Ups**	**0.00057**	**0.00394**	_	_
ENSG000	RP11-127	3636	0	0	2	0.06	2	0.05	12	0.34	11	0.29	7	0.18	5.364192	0.554447	3.025372	**1.67741**	**Ups**	**0.00248**	**0.01338**	_	_
ENSG000	RP11-128	25856	10	0.09	6	0.06	3	0.03	17	0.16	37	0.32	22	0.19	14.72706	0.447635	3.208781	**1.43636**	**Ups**	**0.00133**	**0.00798**	_	K12807
ENSG000	RP11-131	34060	12	0.06	3	0.01	2	0.01	30	0.15	84	0.38	16	0.07	22.20954	0.520406	3.680906	**1.91557**	**Ups**	**0.00023**	**0.00183**	_	K12807
ENSG000	RP11-134	637	0	0	0	0	0	0	3	0.13	7	0.27	2	0.08	1.787552	0.570216	2.555917	**1.45743**	**Ups**	**0.01059**	**0.04204**	_	_
ENSG000	RP11-134	26368	14	1.06	29	2.29	29	2.21	60	4.72	71	5.18	69	4.92	43.42464	0.319283	3.590379	**1.14635**	**Ups**	**0.00033**	**0.00247**	_	_
ENSG000	RP11-134	1101	125	2.92	120	2.92	145	3.4	37	0.9	93	2.09	78	1.72	100.4865	0.28257	-3.84148	**-1.0855**	**Down**	**0.00012**	**0.00104**	_	K00802
ENSG000	RP11-135	681	7	0.47	5	0.35	7	0.47	16	1.12	20	1.3	31	1.96	13.56992	0.43766	3.009481	**1.31713**	**Ups**	**0.00262**	**0.01393**	_	_
ENSG000	RP11-145	2089	61	0.75	235	3.02	68	0.84	921	11.79	766	9.09	1166	13.53	505.1897	0.402561	5.89992	**2.37508**	**Ups**	**3.6E-09**	**9.3E-08**	_	_
ENSG000	RP11-150	2379	52	0.48	51	0.49	39	0.36	26	0.25	7	0.06	18	0.16	33.42013	0.406818	-3.47927	**-1.4154**	**Down**	**0.0005**	**0.00354**	_	_
ENSG000	RP11-152	833	80	2.47	98	3.15	113	3.51	53	1.7	45	1.34	56	1.63	75.71217	0.268915	-3.96926	**-1.0674**	**Down**	**7.2E-05**	**0.00066**	_	_
ENSG000	RP11-154	4312	6	0.24	7	0.3	4	0.16	0	0	0	0	0	0	3.02452	0.583457	-3.63663	**-2.1218**	**Down**	**0.00028**	**0.00212**	_	_
ENSG000	RP11-157	1592	1	0.02	3	0.05	1	0.02	11	0.18	7	0.11	19	0.29	6.614483	0.546333	3.174336	**1.73424**	**Ups**	**0.0015**	**0.00881**	_	K09428
ENSG000	RP11-158	1902	24	0.37	5	0.08	13	0.2	2	0.03	1	0.02	8	0.12	9.121027	0.547306	-2.59458	**-1.42**	**Down**	**0.00947**	**0.03864**	_	_
ENSG000	RP11-15E	24506	16	0.37	23	0.56	15	0.35	11	0.26	7	0.16	7	0.15	13.54196	0.430489	-2.55581	**-1.1002**	**Down**	**0.01059**	**0.04204**	_	_
ENSG000	RP11-15F	24772	9	0.35	16	0.65	16	0.63	2	0.08	8	0.3	5	0.18	9.530182	0.484005	-2.72302	**-1.318**	**Down**	**0.00647**	**0.02868**	_	_
ENSG000	RP11-162	1371	3	0.09	1	0.03	3	0.09	10	0.32	21	0.63	9	0.26	7.230514	0.525297	3.031633	**1.59251**	**Ups**	**0.00243**	**0.01318**	_	_
ENSG000	RP11-164	64215	0	0	3	0.02	2	0.01	10	0.05	25	0.13	14	0.07	8.189594	0.531315	3.862563	**2.05224**	**Ups**	**0.00011**	**0.00097**	_	_
ENSG000	RP11-167	2306	1	0.05	0	0	5	0.26	257	13.66	327	16.12	425	20.48	154.5576	0.407574	14.30917	**5.83205**	**Ups**	**1.9E-46**	**8.3E-44**	_	_
ENSG000	RP11-168	1231	1	0.02	2	0.04	3	0.06	8	0.17	16	0.32	14	0.28	6.773928	0.525914	3.203848	**1.68495**	**Ups**	**0.00136**	**0.0081**	_	K11584
ENSG000	RP11-169	2570	23	0.24	28	0.31	31	0.33	0	0	0	0	0	0	14.62838	0.536341	-7.46082	**-4.0015**	**Down**	**8.6E-14**	**4.3E-12**	_	_
ENSG000	RP11-169	490	132	6.92	119	6.51	164	8.65	45	2.46	59	2.99	85	4.21	102.6792	0.272106	-4.72191	**-1.2849**	**Down**	**2.3E-06**	**3.2E-05**	_	K02870
ENSG000	RP11-173	31295	13	0.47	3	0.11	17	0.62	54	2.02	46	1.6	48	1.63	28.77325	0.41377	4.023763	**1.66491**	**Ups**	**5.7E-05**	**0.00054**	_	_
ENSG000	RP11-175	26588	13	0.16	8	0.1	7	0.09	26	0.34	88	1.07	18	0.21	24.34679	0.47883	3.212322	**1.53816**	**Ups**	**0.00132**	**0.0079**	_	K16580
ENSG000	RP11-180	1733	1	0.03	3	0.09	1	0.03	13	0.38	10	0.27	14	0.37	6.623642	0.534126	3.378473	**1.80453**	**Ups**	**0.00073**	**0.00484**	_	_
ENSG000	RP11-181	2881	5	0.27	4	0.22	1	0.05	5	0.28	34	1.76	15	0.76	9.597052	0.538567	2.719083	**1.46441**	**Ups**	**0.00655**	**0.02894**	_	_
ENSG000	RP1-118J	1196	32	0.69	27	0.61	33	0.71	4	0.09	23	0.48	16	0.32	22.66304	0.412884	-2.80742	**-1.1591**	**Down**	**0.00499**	**0.02337**	_	_
ENSG000	RP11-180	507	0	0	0	0	0	0	6	0.32	2	0.1	5	0.24	2.063135	0.575058	2.729067	**1.56937**	**Ups**	**0.00635**	**0.02828**	_	_
ENSG000	RP11-190	732	23	0.81	15	0.55	16	0.57	33	1.21	44	1.49	63	2.09	30.84647	0.350078	2.949464	**1.03254**	**Ups**	**0.00318**	**0.01632**	_	_
ENSG000	RP11-190	504	9	0.46	12	0.64	10	0.51	5	0.27	2	0.1	2	0.1	6.966818	0.515127	-2.78939	**-1.4369**	**Down**	**0.00528**	**0.0245**	_	_
ENSG000	RP11-195	18969	6	0.23	9	0.36	5	0.19	36	1.42	46	1.68	18	0.64	18.84856	0.440968	3.916668	**1.72713**	**Ups**	**9E-05**	**0.0008**	_	K06088
ENSG000	RP11-204	1297	0	0	0	0	0	0	8	0.39	1	0.05	6	0.27	2.419805	0.574454	2.772382	**1.5926**	**Ups**	**0.00556**	**0.02553**	_	_
ENSG000	RP11-207	1224	0	0	0	0	0	0	13	0.64	3	0.14	4	0.18	3.258625	0.581771	3.23949	**1.88464**	**Ups**	**0.0012**	**0.0073**	_	_
ENSG000	RP11-20I	397	9	0.58	5	0.34	5	0.33	2	0.13	0	0	0	0	3.715765	0.578609	-2.98086	**-1.7248**	**Down**	**0.00287**	**0.01502**	_	_
ENSG000	RP11-20I	2894	10	0.56	4	0.23	3	0.17	1	0.06	1	0.05	1	0.05	3.467002	0.577633	-2.51413	**-1.4522**	**Down**	**0.01193**	**0.04606**	_	_
ENSG000	RP11-211	1735	8	0.38	5	0.25	4	0.19	31	1.55	16	0.74	13	0.59	12.53179	0.482141	2.644674	**1.27511**	**Ups**	**0.00818**	**0.03441**	_	_
ENSG000	RP11-215	26566	20	0.4	25	0.52	27	0.54	1	0.02	15	0.29	2	0.04	15.39583	0.50876	-3.32198	**-1.6901**	**Down**	**0.00089**	**0.00573**	_	_
ENSG000	RP11-216	23510	27	0.45	27	0.47	24	0.4	62	1.07	53	0.85	79	1.24	43.79108	0.306358	3.331584	**1.02066**	**Ups**	**0.00086**	**0.00557**	_	_
ENSG000	RP11-218	44676	2	0.04	6	0.12	1	0.02	17	0.34	10	0.19	18	0.33	8.626942	0.518322	2.934456	**1.52099**	**Ups**	**0.00334**	**0.01699**	_	_
ENSG000	RP11-222	196204	37	0.1	43	0.12	32	0.08	107	0.29	150	0.38	112	0.28	76.00485	0.252042	5.628092	**1.41852**	**Ups**	**1.8E-08**	**4E-07**	_	_
ENSG000	RP11-22F	174935	2	0.07	1	0.04	1	0.04	9	0.35	8	0.29	7	0.25	4.421295	0.558142	2.62781	**1.46669**	**Ups**	**0.00859**	**0.03576**	_	_
ENSG000	RP11-23J	58866	32	0.21	32	0.22	31	0.21	10	0.07	22	0.14	12	0.07	23.49355	0.359024	-3.35696	**-1.2052**	**Down**	**0.00079**	**0.00516**	_	_
ENSG000	RP11-242	506	10	0.51	30	1.59	12	0.61	58	3.06	74	3.63	67	3.21	39.60899	0.371092	4.073303	**1.51157**	**Ups**	**4.6E-05**	**0.00045**	_	_
ENSG000	RP11-244	3354	49	0.26	36	0.2	40	0.22	80	0.45	163	0.85	101	0.51	73.78206	0.286413	3.973913	**1.13818**	**Ups**	**7.1E-05**	**0.00065**	_	_
ENSG000	RP11-245	2314	22	2	37	3.51	45	4.11	14	1.32	19	1.67	22	1.88	26.94331	0.368502	-2.76946	**-1.0206**	**Down**	**0.00561**	**0.02572**	_	_
ENSG000	RP1-124C	4882	1	0.02	3	0.08	3	0.07	14	0.36	13	0.31	15	0.35	7.722607	0.510366	3.363746	**1.71674**	**Ups**	**0.00077**	**0.00507**	_	_
ENSG000	RP11-24F	42105	345	13.52	374	15.28	406	16	3	0.12	6	0.23	12	0.44	203.6942	0.319988	-16.9684	**-5.4297**	**Down**	**1.4E-64**	**1.1E-61**	_	_
ENSG000	RP11-250	2123	1	0.01	0	0	2	0.02	15	0.19	21	0.25	16	0.18	8.43555	0.531004	4.757567	**2.52628**	**Ups**	**2E-06**	**2.8E-05**	_	_
ENSG000	RP11-252	21592	2	0.05	1	0.02	0	0	3	0.07	17	0.38	12	0.26	5.204182	0.570925	3.069444	**1.75242**	**Ups**	**0.00214**	**0.01183**	_	_
ENSG000	RP11-254	1572	86	1.41	86	1.47	76	1.25	302	5.14	194	3.06	279	4.3	165.0295	0.271965	5.026242	**1.36696**	**Ups**	**5E-07**	**8.1E-06**	_	_
ENSG000	RP11-259	21002	2	0.06	1	0.03	4	0.12	25	0.76	21	0.59	27	0.74	12.52776	0.484714	4.940056	**2.39451**	**Ups**	**7.8E-07**	**1.2E-05**	_	_
ENSG000	RP11-263	240	12	1.29	13	1.45	11	1.18	67	7.46	28	2.89	36	3.64	27.2539	0.422509	3.373655	**1.4254**	**Ups**	**0.00074**	**0.00491**	_	K10104
ENSG000	RP11-267	196163	58	0.46	35	0.29	36	0.29	3	0.03	20	0.15	10	0.08	27.69268	0.424754	-4.40815	**-1.8724**	**Down**	**1E-05**	**0.00012**	_	_
ENSG000	RP11-269	5277	1	0.09	3	0.27	6	0.52	14	1.25	16	1.32	20	1.62	9.430509	0.493571	3.209909	**1.58432**	**Ups**	**0.00133**	**0.00795**	_	_
ENSG000	RP11-269	245818	54	2.38	45	2.07	46	2.04	210	9.63	159	6.76	223	9.27	117.5492	0.26625	6.484433	**1.72648**	**Ups**	**8.9E-11**	**3E-09**	_	_
ENSG000	RP11-274	585	3	0.13	2	0.09	3	0.13	18	0.82	12	0.51	20	0.83	9.195647	0.499911	3.620509	**1.80993**	**Ups**	**0.00029**	**0.00223**	_	_
ENSG000	RP11-274	1267	2	0.04	2	0.04	5	0.1	25	0.53	12	0.23	19	0.36	10.4385	0.504682	3.569293	**1.80136**	**Ups**	**0.00036**	**0.00264**	_	_
ENSG000	RP11-276	53335	112	0.66	69	0.42	80	0.47	395	2.41	421	2.38	383	2.12	230.3595	0.223748	8.539315	**1.91065**	**Ups**	**1.4E-17**	**1E-15**	_	_
ENSG000	RP11-277	34738	1	0.01	3	0.03	2	0.02	13	0.12	21	0.18	19	0.16	9.07902	0.504699	4.184414	**2.11187**	**Ups**	**2.9E-05**	**0.0003**	_	_
ENSG000	RP11-284	3222	67	0.53	59	0.49	68	0.55	508	4.22	232	1.79	359	2.7	208.2394	0.329305	6.449878	**2.12398**	**Ups**	**1.1E-10**	**3.7E-09**	_	_
ENSG000	RP11-285	5640	46	3.4	28	2.16	31	2.3	169	12.98	87	6.2	160	11.15	83.88829	0.348026	4.665568	**1.62374**	**Ups**	**3.1E-06**	**4.2E-05**	_	_
ENSG000	RP11-288	809	3	0.16	1	0.05	0	0	5	0.27	51	2.59	13	0.64	10.58383	0.568648	3.768062	**2.1427**	**Ups**	**0.00016**	**0.00136**	_	_
ENSG000	RP11-289	704	226	4.75	203	4.45	204	4.31	93	2.03	108	2.19	86	1.7	156.5921	0.196291	-6.73131	**-1.3213**	**Down**	**1.7E-11**	**6.3E-10**	_	_
ENSG000	RP11-290	12310	2	0.01	5	0.03	6	0.04	0	0	1	0.01	0	0	2.466145	0.582773	-2.65911	**-1.5497**	**Down**	**0.00783**	**0.03326**	_	_
ENSG000	RP11-290	243	1	0.11	3	0.33	1	0.11	12	1.32	6	0.61	9	0.9	5.145778	0.554344	2.558576	**1.41833**	**Ups**	**0.01051**	**0.04178**	_	_
ENSG000	RP11-293	12540	0	0	0	0	0	0	8	0.19	4	0.09	4	0.09	2.534834	0.581267	3.085058	**1.79324**	**Ups**	**0.00204**	**0.01134**	_	_
ENSG000	RP11-293	2208	0	0	2	0.07	0	0	21	0.73	5	0.16	10	0.32	6.175348	0.575662	3.531371	**2.03287**	**Ups**	**0.00041**	**0.003**	_	_
ENSG000	RP11-294	49732	5	0.03	4	0.03	1	0.01	15	0.11	28	0.19	20	0.13	11.24188	0.478819	3.908749	**1.87158**	**Ups**	**9.3E-05**	**0.00082**	_	_
ENSG000	RP11-295	6169	1537	24.49	1279	21.26	1547	24.8	600	9.95	334	5.14	609	9.15	1018.094	0.273938	-5.8015	**-1.5893**	**Down**	**6.6E-09**	**1.6E-07**	_	_
ENSG000	RP11-297	1026	9	0.33	9	0.34	16	0.58	0	0	1	0.04	3	0.1	6.660693	0.546826	-3.86569	**-2.1139**	**Down**	**0.00011**	**0.00096**	_	_
ENSG000	RP11-298	25954	8	0.19	2	0.05	9	0.21	17	0.41	36	0.81	19	0.42	14.1534	0.460748	2.923788	**1.34713**	**Ups**	**0.00346**	**0.01747**	_	_
ENSG000	RP11-299	3751	13	0.13	13	0.14	14	0.15	29	0.31	45	0.45	31	0.3	23.01966	0.352722	2.95414	**1.04199**	**Ups**	**0.00314**	**0.01615**	_	_
ENSG000	RP11-29H	45520	94	0.96	96	1.03	93	0.96	35	0.37	43	0.43	45	0.44	69.15982	0.241651	-5.63556	**-1.3618**	**Down**	**1.7E-08**	**3.9E-07**	_	_
ENSG000	RP11-301	6095	0	0	0	0	0	0	6	0.09	26	0.36	9	0.12	5.988662	0.578572	4.522257	**2.61645**	**Ups**	**6.1E-06**	**7.6E-05**	_	_
ENSG000	RP11-305	508	27	1.37	25	1.32	29	1.48	85	4.47	41	2	104	4.96	50.39539	0.373491	3.108036	**1.16082**	**Ups**	**0.00188**	**0.01063**	_	_
ENSG000	RP11-305	2348	2	0.06	4	0.13	3	0.1	19	0.62	15	0.46	26	0.77	10.86886	0.485898	3.960371	**1.92434**	**Ups**	**7.5E-05**	**0.00068**	_	_
ENSG000	RP11-305	5072	1	0.01	1	0.01	0	0	10	0.08	6	0.04	12	0.08	4.712908	0.569617	3.419654	**1.94789**	**Ups**	**0.00063**	**0.00425**	_	_
ENSG000	RP11-310	21723	2	0.03	8	0.11	7	0.09	0	0	1	0.01	0	0	3.184084	0.583379	-3.0685	**-1.7901**	**Down**	**0.00215**	**0.01185**	_	K17299
ENSG000	RP11-311	1228	4	0.08	4	0.09	1	0.02	7	0.15	23	0.46	15	0.3	8.243468	0.517392	2.874091	**1.48703**	**Ups**	**0.00405**	**0.01981**	_	K11110
ENSG000	RP11-312	1097	255	5.98	271	6.62	363	8.56	94	2.29	236	5.34	157	3.47	231.0925	0.279965	-3.77494	**-1.0568**	**Down**	**0.00016**	**0.00133**	_	K00802
ENSG000	RP11-313	1852	9	0.58	4	0.27	14	0.9	20	1.33	32	1.98	25	1.51	16.45318	0.42222	2.552594	**1.07776**	**Ups**	**0.01069**	**0.04231**	_	_
ENSG000	RP11-313	36109	20	0.05	29	0.08	9	0.02	68	0.18	192	0.48	104	0.25	64.26643	0.394165	5.253366	**2.07069**	**Ups**	**1.5E-07**	**2.8E-06**	_	_
ENSG000	RP11-313	708	18	0.65	14	0.53	23	0.84	7	0.26	5	0.18	7	0.24	12.75459	0.439831	-3.27617	**-1.441**	**Down**	**0.00105**	**0.00658**	_	_
ENSG000	RP11-314	5141	362	1.81	691	3.6	286	1.44	628	3.27	2730	13.17	1325	6.25	924.2448	0.389229	3.41207	**1.32808**	**Ups**	**0.00064**	**0.00435**	_	_
ENSG000	RP11-315	35918	94	3.37	65	2.43	68	2.45	23	0.86	20	0.69	34	1.15	52.18772	0.311364	-5.25226	**-1.6354**	**Down**	**1.5E-07**	**2.8E-06**	_	K12359
ENSG000	RP11-315	3526	0	0	0	0	0	0	11	0.13	1	0.01	2	0.02	2.336132	0.564882	2.521517	**1.42436**	**Ups**	**0.01168**	**0.04526**	_	K02998
ENSG000	RP11-316	712	2	0.09	4	0.19	0	0	15	0.69	10	0.43	16	0.67	7.442749	0.533197	3.279285	**1.7485**	**Ups**	**0.00104**	**0.00652**	_	_
ENSG000	RP11-317	1376	2	0.04	3	0.06	7	0.13	23	0.45	32	0.58	27	0.48	14.60597	0.449729	4.568786	**2.05471**	**Ups**	**4.9E-06**	**6.3E-05**	_	_
ENSG000	RP1-131F	2433	7	0.07	3	0.03	2	0.02	18	0.2	13	0.13	16	0.16	9.431142	0.489539	2.7646	**1.35338**	**Ups**	**0.0057**	**0.02599**	_	_
ENSG000	RP11-31F	2215	74	2.33	64	2.1	70	2.22	22	0.72	26	0.79	33	0.98	49.38172	0.280529	-5.29529	**-1.4855**	**Down**	**1.2E-07**	**2.2E-06**	_	_
ENSG000	RP11-324	335	4	0.31	7	0.56	11	0.85	21	1.68	24	1.78	26	1.88	14.79037	0.423667	2.910465	**1.23307**	**Ups**	**0.00361**	**0.01807**	_	_
ENSG000	RP11-324	813	0	0	0	0	0	0	3	0.1	3	0.09	6	0.18	1.834533	0.573175	2.634669	**1.51013**	**Ups**	**0.00842**	**0.03527**	_	_
ENSG000	RP11-325	699	9	0.33	8	0.31	5	0.18	50	1.91	40	1.42	46	1.6	24.9845	0.388685	5.41395	**2.10432**	**Ups**	**6.2E-08**	**1.2E-06**	_	_
ENSG000	RP11-325	1065	6	0.26	5	0.22	3	0.13	28	1.25	38	1.57	33	1.33	17.5372	0.420749	5.172021	**2.17612**	**Ups**	**2.3E-07**	**4.1E-06**	_	_
ENSG000	RP11-325	1241	0	0	2	0.04	0	0	5	0.11	3	0.06	13	0.25	3.58971	0.582991	2.524176	**1.47157**	**Ups**	**0.0116**	**0.04503**	_	K11090

ENSG000	RP11-326	6992	27	0.27	24	0.25	26	0.26	87	0.91	58	0.56	84	0.8	49.36963	0.3179	3.970259	**1.26215**	**Ups**	**7.2E-05**	**0.00066**	_	_
ENSG000	RP11-326	565	13	0.59	25	1.19	12	0.55	65	3.08	36	1.58	51	2.19	32.77663	0.393867	3.115908	**1.22725**	**Ups**	**0.00183**	**0.01039**	_	_
ENSG000	RP11-329	526	1	0.05	0	0	2	0.1	23	1.17	1	0.05	9	0.41	5.993004	0.583083	2.674023	**1.55918**	**Ups**	**0.00749**	**0.03214**	_	_
ENSG000	RP11-329	4875	7	0.26	9	0.34	11	0.41	33	1.26	16	0.57	32	1.11	17.53613	0.433246	2.674365	**1.15866**	**Ups**	**0.00749**	**0.03212**	_	_
ENSG000	RP11-32D	560	2	0.09	3	0.14	16	0.74	28	1.34	30	1.33	50	2.16	20.27039	0.493841	3.282585	**1.62108**	**Ups**	**0.00103**	**0.00646**	_	K16941
ENSG000	RP11-331	2122	1	0.01	2	0.02	0	0	6	0.07	14	0.15	12	0.12	5.310416	0.558875	3.382924	**1.89063**	**Ups**	**0.00072**	**0.00478**	_	K01310
ENSG000	RP11-333	2463	0	0	0	0	3	0.03	9	0.1	7	0.07	9	0.09	4.415198	0.571434	2.782941	**1.59027**	**Ups**	**0.00539**	**0.02488**	_	_
ENSG000	RP11-336	31894	0	0	0	0	0	0	1	0.02	13	0.19	4	0.06	2.574275	0.575331	2.837877	**1.63272**	**Ups**	**0.00454**	**0.02169**	_	_
ENSG000	RP11-336	4188	39	0.46	45	0.56	47	0.56	20	0.25	16	0.18	33	0.37	33.98121	0.342233	-3.01537	**-1.032**	**Down**	**0.00257**	**0.01372**	_	_
ENSG000	RP11-336	238	1	0.11	0	0	0	0	6	0.67	18	1.88	10	1.02	5.205565	0.572638	4.031961	**2.30886**	**Ups**	**5.5E-05**	**0.00052**	_	K02219
ENSG000	RP11-337	4200	50	2.28	45	2.14	33	1.51	19	0.9	29	1.28	10	0.43	31.55138	0.369103	-3.2895	**-1.2142**	**Down**	**0.001**	**0.00633**	_	_
ENSG000	RP11-343	1326	185	3.59	373	7.54	259	5.05	763	15.39	880	16.46	1049	19.18	556.9998	0.262108	5.399215	**1.41518**	**Ups**	**6.7E-08**	**1.3E-06**	_	_
ENSG000	RP11-344	133844	0	0	0	0	0	0	2	0	10	0.02	4	0.01	2.331178	0.578175	2.901841	**1.67777**	**Ups**	**0.00371**	**0.01847**	_	_
ENSG000	RP11-346	19660	2	0.04	5	0.1	5	0.09	0	0	0	0	1	0.02	2.29755	0.582109	-2.52708	**-1.471**	**Down**	**0.0115**	**0.04478**	_	_
ENSG000	RP11-347	4756	16	0.11	29	0.21	26	0.18	4	0.03	0	0	4	0.03	13.97324	0.492041	-5.08073	**-2.4999**	**Down**	**3.8E-07**	**6.3E-06**	_	K11364
ENSG000	RP11-347	51270	24	0.56	21	0.51	26	0.61	4	0.1	2	0.05	4	0.09	14.21503	0.455157	-5.33321	**-2.4274**	**Down**	**9.7E-08**	**1.9E-06**	_	_
ENSG000	RP11-348	1649	0	0	4	0.07	2	0.03	30	0.49	26	0.39	28	0.41	14.06026	0.488099	5.477408	**2.67352**	**Ups**	**4.3E-08**	**8.9E-07**	_	_
ENSG000	RP11-348	1953	38	0.5	57	0.78	62	0.82	165	2.26	136	1.73	200	2.48	105.3855	0.286777	4.77209	**1.36852**	**Ups**	**1.8E-06**	**2.6E-05**	_	_
ENSG000	RP11-351	1048	22	0.7	24	0.8	19	0.61	3	0.1	12	0.37	4	0.12	14.35339	0.446007	-3.71333	**-1.6562**	**Down**	**0.0002**	**0.00164**	_	_
ENSG000	RP11-352	556	0	0	0	0	0	0	4	0.19	3	0.13	5	0.22	1.856731	0.574395	2.670717	**1.53405**	**Ups**	**0.00757**	**0.03236**	_	_
ENSG000	RP11-362	1015	42	1.6	33	1.31	48	1.84	8	0.32	5	0.18	3	0.11	24.43433	0.411615	-6.47293	**-2.6644**	**Down**	**9.6E-11**	**3.2E-09**	_	_
ENSG000	RP11-368	18631	1	0.04	2	0.08	0	0	15	0.56	31	1.08	30	1.02	11.92217	0.519863	5.64425	**2.93423**	**Ups**	**1.7E-08**	**3.7E-07**	_	_
ENSG000	RP11-368	445	0	0	0	0	0	0	1	0.06	7	0.39	5	0.27	1.893422	0.570001	2.574881	**1.46769**	**Ups**	**0.01003**	**0.04029**	_	_
ENSG000	RP1-136E	701	0	0	0	0	0	0	2	0.08	6	0.21	5	0.17	1.927365	0.575032	2.711067	**1.55895**	**Ups**	**0.00671**	**0.02949**	_	_
ENSG000	RP11-375	1375	12	0.22	11	0.21	15	0.28	8	0.16	3	0.05	3	0.05	9.019869	0.490335	-2.5496	**-1.2502**	**Down**	**0.01078**	**0.0426**	_	_
ENSG000	RP11-37E	559	0	0	1	0.06	2	0.12	25	1.53	19	1.08	18	1	10.18807	0.527048	5.164198	**2.72178**	**Ups**	**2.4E-07**	**4.3E-06**	_	_
ENSG000	RP11-380	1736	2	0.1	3	0.16	0	0	5	0.27	19	0.94	15	0.73	6.635339	0.550586	3.13216	**1.72452**	**Ups**	**0.00174**	**0.00991**	_	_
ENSG000	RP11-380	7835	45	0.25	43	0.25	40	0.22	0	0	1	0.01	2	0.01	23.22395	0.479332	-8.41041	**-4.0314**	**Down**	**4.1E-17**	**3E-15**	_	_
ENSG000	RP11-382	404	0	0	0	0	0	0	3	0.2	3	0.18	7	0.42	1.984798	0.575258	2.725351	**1.56778**	**Ups**	**0.00642**	**0.02853**	_	K08754
ENSG000	RP11-383	1488	1	0.06	1	0.06	0	0	6	0.39	26	1.56	1	0.06	5.140538	0.583433	2.819575	**1.64503**	**Ups**	**0.00481**	**0.02267**	_	_
ENSG000	RP11-383	290227	11	0.03	11	0.03	6	0.02	0	0	2	0.01	1	0	5.403271	0.557981	-3.71102	**-2.0707**	**Down**	**0.00021**	**0.00165**	_	K04984
ENSG000	RP11-383	3360	1	0.01	0	0	1	0.01	8	0.06	12	0.09	4	0.03	3.998123	0.576102	3.010825	**1.73454**	**Ups**	**0.00261**	**0.01388**	_	_
ENSG000	RP11-383	5902	20	0.93	18	0.87	12	0.56	2	0.1	12	0.54	3	0.13	11.3435	0.48953	-2.85047	**-1.3954**	**Down**	**0.00437**	**0.02104**	_	_
ENSG000	RP11-384	3528	19	0.14	27	0.21	18	0.13	39	0.3	81	0.57	50	0.34	36.86493	0.336684	3.140364	**1.05731**	**Ups**	**0.00169**	**0.00969**	_	_
ENSG000	RP11-386	209	2	0.25	8	1.03	0	0	15	1.92	12	1.42	19	2.2	8.887381	0.534858	2.563579	**1.37115**	**Ups**	**0.01036**	**0.04133**	_	_
ENSG000	RP11-38L	3086	14	0.65	9	0.44	23	1.08	43	2.09	33	1.49	50	2.2	27.70798	0.385504	2.824704	**1.08893**	**Ups**	**0.00473**	**0.02239**	_	_
ENSG000	RP11-390	1122	3	0.07	9	0.22	6	0.14	0	0	1	0.02	1	0.02	3.508003	0.579568	-2.94329	**-1.7058**	**Down**	**0.00325**	**0.01659**	_	K05692
ENSG000	RP11-394	1884	2	0.03	5	0.07	2	0.03	15	0.21	12	0.16	11	0.14	7.508344	0.509767	2.688396	**1.37046**	**Ups**	**0.00718**	**0.03107**	_	_
ENSG000	RP11-395	10884	2	0.09	0	0	1	0.04	8	0.37	8	0.34	5	0.21	3.769104	0.572647	2.531412	**1.44961**	**Ups**	**0.01136**	**0.0443**	_	_
ENSG000	RP11-396	50625	197	1.88	241	2.4	200	1.92	388	3.86	656	6.05	508	4.58	347.8081	0.197442	5.128592	**1.0126**	**Ups**	**2.9E-07**	**5E-06**	_	K03121
ENSG000	RP11-399	178014	12	0.12	9	0.09	8	0.08	43	0.43	33	0.31	29	0.26	21.49773	0.391973	3.667435	**1.43753**	**Ups**	**0.00024**	**0.00191**	_	_
ENSG000	RP11-399	7013	0	0	0	0	4	0.12	3	0.09	36	1.01	16	0.44	8.629684	0.577664	3.15284	**1.82128**	**Ups**	**0.00162**	**0.00935**	_	_
ENSG000	RP11-400	2311	659	7.33	578	6.71	632	7.07	357	4.13	322	3.46	351	3.68	491.6565	0.179127	-5.81901	**-1.0423**	**Down**	**5.9E-09**	**1.4E-07**	_	_
ENSG000	RP11-403	24803	46	1.11	37	0.93	42	1.02	105	2.65	180	4.21	198	4.52	95.03962	0.27042	5.96991	**1.61438**	**Ups**	**2.4E-09**	**6.3E-08**	_	_
ENSG000	RP11-406	64206	1	0.01	0	0	0	0	11	0.15	13	0.16	13	0.16	5.826072	0.563726	4.471013	**2.52042**	**Ups**	**7.8E-06**	**9.4E-05**	_	_
ENSG000	RP11-407	65534	2	0	0	0	2	0	14	0.03	6	0.01	11	0.02	5.608766	0.558812	3.020271	**1.68776**	**Ups**	**0.00253**	**0.01355**	_	_
ENSG000	RP11-410	2202	0	0	2	0.02	1	0.01	13	0.16	6	0.07	7	0.08	4.663717	0.569944	2.893767	**1.64928**	**Ups**	**0.00381**	**0.01887**	_	_
ENSG000	RP11-412	3428	0	0	0	0	0	0	4	0.27	15	0.95	10	0.62	4.270288	0.581766	4.032039	**2.3457**	**Ups**	**5.5E-05**	**0.00052**	_	_
ENSG000	RP11-416	3077	66	0.55	73	0.64	68	0.57	10	0.09	30	0.24	13	0.1	44.71473	0.340926	-5.87676	**-2.0035**	**Down**	**4.2E-09**	**1.1E-07**	_	_
ENSG000	RP11-416	23471	2	0.15	0	0	0	0	8	0.61	5	0.36	11	0.76	4.074794	0.577049	2.999241	**1.73071**	**Ups**	**0.00271**	**0.01433**	_	_
ENSG000	RP11-410	4452	36	0.35	33	0.34	32	0.31	6	0.06	2	0.02	0	0	19.28885	0.464034	-6.47019	**-3.0024**	**Down**	**9.8E-11**	**3.2E-09**	_	_
ENSG000	RP11-420	26746	250	2.73	248	2.83	304	3.34	601	6.83	826	8.71	640	6.59	457.1995	0.175389	6.331096	**1.1104**	**Ups**	**2.4E-10**	**7.6E-09**	_	_
ENSG000	RP11-423	13626	13	0.2	17	0.28	14	0.22	1	0.02	6	0.09	6	0.09	9.74864	0.481084	-3.23624	**-1.5569**	**Down**	**0.00121**	**0.00737**	_	_
ENSG000	RP11-426	30474	102	0.98	109	1.09	136	1.32	60	0.6	23	0.21	64	0.58	85.03857	0.363399	-3.49413	**-1.2698**	**Down**	**0.00048**	**0.00339**	_	_
ENSG000	RP11-430	25782	2	0.02	3	0.03	8	0.07	13	0.12	20	0.17	14	0.12	9.446009	0.487215	2.556487	**1.24556**	**Ups**	**0.01057**	**0.04201**	_	_
ENSG000	RP11-430	2938	1	0.01	0	0	0	0	5	0.06	9	0.1	6	0.07	3.185368	0.581806	3.078908	**1.79133**	**Ups**	**0.00208**	**0.01153**	_	_
ENSG000	RP11-431	14359	288	14.1	408	20.84	251	12.36	85	4.33	174	8.22	74	3.42	218.6007	0.30598	-5.30982	**-1.6247**	**Down**	**1.1E-07**	**2.1E-06**	_	_
ENSG000	RP11-431	17929	50	0.94	51	1	50	0.95	1	0.02	6	0.11	4	0.07	28.5005	0.412645	-8.15232	**-3.364**	**Down**	**3.6E-16**	**2.4E-14**	_	_
ENSG000	RP11-434	1553	2	0.03	3	0.05	0	0	38	0.65	23	0.37	36	0.56	16.03623	0.498328	5.897259	**2.93877**	**Ups**	**3.7E-09**	**9.4E-08**	_	_
ENSG000	RP11-434	58751	3	0.02	4	0.03	7	0.05	18	0.12	48	0.3	27	0.17	16.31396	0.457321	4.339643	**1.98461**	**Ups**	**1.4E-05**	**0.00016**	_	_
ENSG000	RP11-435	476	27	1.46	13	0.73	24	1.3	5	0.28	5	0.26	9	0.46	14.28447	0.446087	-3.61452	**-1.6124**	**Down**	**0.0003**	**0.00228**	_	_
ENSG000	RP11-439	3472	21	0.16	18	0.14	13	0.1	29	0.22	104	0.74	38	0.27	34.35817	0.417457	2.942899	**1.22854**	**Ups**	**0.00325**	**0.01661**	_	_
ENSG000	RP11-439	16373	49	0.21	53	0.23	67	0.28	210	0.91	114	0.46	191	0.75	110.8541	0.3196	4.074451	**1.30219**	**Ups**	**4.6E-05**	**0.00045**	_	_
ENSG000	RP11-440	719	3	0.11	0	0	5	0.18	6	0.22	23	0.79	15	0.51	7.900773	0.540905	2.729025	**1.47614**	**Ups**	**0.00635**	**0.02828**	_	_
ENSG000	RP11-443	20927	54	0.3	61	0.36	62	0.35	4	0.02	49	0.27	27	0.14	43.08557	0.455729	-2.56113	**-1.1672**	**Down**	**0.01043**	**0.04156**	_	_
ENSG000	RP11-445	51130	87	1.08	90	1.16	80	1	48	0.62	33	0.39	46	0.54	65.52372	0.277502	-4.1353	**-1.1476**	**Down**	**3.5E-05**	**0.00036**	_	_
ENSG000	RP11-446	111758	0	0	0	0	0	0	7	0.26	3	0.1	3	0.1	2.073587	0.575275	2.736845	**1.57444**	**Ups**	**0.0062**	**0.02777**	_	_
ENSG000	RP11-44I	27743	6	0.35	1	0.06	1	0.06	24	1.47	8	0.45	12	0.66	8.458725	0.547643	2.755753	**1.50917**	**Ups**	**0.00586**	**0.02654**	_	_
ENSG000	RP11-454	271274	3	0.06	3	0.06	3	0.06	7	0.14	18	0.32	12	0.21	7.106763	0.511117	2.565661	**1.31135**	**Ups**	**0.0103**	**0.04111**	_	_
ENSG000	RP11-457	1724	29	0.43	43	0.67	31	0.46	106	1.64	76	1.09	110	1.55	63.70273	0.307819	3.905766	**1.20227**	**Ups**	**9.4E-05**	**0.00083**	_	_
ENSG000	RP11-458	9381	20	0.11	20	0.12	11	0.06	51	0.3	123	0.66	54	0.28	43.01137	0.377866	4.442211	**1.67856**	**Ups**	**8.9E-06**	**0.00011**	_	_
ENSG000	RP11-458	367	1	0.07	1	0.07	1	0.07	6	0.44	6	0.41	17	1.12	4.954723	0.567421	3.068365	**1.74105**	**Ups**	**0.00215**	**0.01185**	_	_
ENSG000	RP11-462	15089	27	0.9	26	0.9	36	1.2	75	2.59	95	3.04	90	2.82	55.4887	0.269645	4.620538	**1.24591**	**Ups**	**3.8E-06**	**5.1E-05**	_	_
ENSG000	RP11-463	3515	0	0	2	0.02	2	0.01	3	0.02	16	0.11	15	0.1	5.706749	0.565872	2.922492	**1.65376**	**Ups**	**0.00347**	**0.01753**	_	_
ENSG000	RP11-474	979	12	0.32	20	0.55	21	0.55	5	0.14	5	0.13	8	0.2	12.22568	0.450089	-3.22316	**-1.4507**	**Down**	**0.00127**	**0.00766**	_	_
ENSG000	RP11-476	137915	0	0	0	0	0	0	63	2.04	92	2.76	103	3.02	39.08617	0.502281	9.996824	**5.02121**	**Ups**	**1.6E-23**	**1.9E-21**	_	_
ENSG000	RP11-477	3586	20	0.17	15	0.13	19	0.16	43	0.37	63	0.51	40	0.31	31.76352	0.327413	3.364934	**1.10172**	**Ups**	**0.00077**	**0.00505**	_	_
ENSG000	RP11-477	2513	3	0.11	0	0	1	0.04	13	0.47	6	0.2	12	0.4	5.581022	0.56046	2.987518	**1.67439**	**Ups**	**0.00281**	**0.01477**	_	_
ENSG000	RP11-47I	14341	22	0.1	17	0.08	19	0.09	4	0.02	4	0.02	7	0.03	12.61409	0.444326	-3.99763	**-1.7763**	**Down**	**6.4E-05**	**0.0006**	_	_
ENSG000	RP11-480	657	239	9.35	217	8.85	229	9.01	1240	50.46	929	35.07	1353	49.93	667.8057	0.224709	9.247675	**2.07804**	**Ups**	**2.3E-20**	**2.2E-18**	_	_
ENSG000	RP11-480	2143	3	0.04	4	0.06	2	0.03	13	0.19	30	0.41	19	0.25	10.85448	0.485389	3.990776	**1.93708**	**Ups**	**6.6E-05**	**0.00061**	_	K09040
ENSG000	RP11-480	13488	12	0.13	3	0.03	4	0.04	21	0.24	20	0.21	29	0.3	14.10625	0.459864	2.920684	**1.34312**	**Ups**	**0.00349**	**0.01761**	_	_
ENSG000	RP11-482	15945	0	0	1	0.03	0	0	17	0.55	5	0.15	2	0.06	4.104182	0.583278	2.944431	**1.71742**	**Ups**	**0.00324**	**0.01654**	_	_
ENSG000	RP11-489	710	4	0.14	8	0.3	10	0.36	149	5.61	14	0.49	100	3.41	46.59877	0.525917	4.583735	**2.41067**	**Ups**	**4.6E-06**	**5.9E-05**	_	_
ENSG000	RP11-48E	1805	1	0.01	9	0.13	2	0.03	14	0.21	15	0.21	24	0.32	10.24689	0.513706	2.719265	**1.3969**	**Ups**	**0.00654**	**0.02893**	_	_
ENSG000	RP11-492	9148	49	0.51	41	0.45	46	0.49	0	0	5	0.05	7	0.07	25.95217	0.425423	-7.28602	**-3.0996**	**Down**	**3.2E-13**	**1.5E-11**	_	_
ENSG000	RP11-493	40064	25	0.19	22	0.18	17	0.13	62	0.5	52	0.39	73	0.53	40.243	0.319134	3.849505	**1.22851**	**Ups**	**0.00012**	**0.00101**	_	_
ENSG000	RP11-495	14159	1	0.01	3	0.04	0	0	9	0.12	12	0.15	13	0.15	5.880228	0.547286	3.336639	**1.82609**	**Ups**	**0.00085**	**0.00548**	_	_
ENSG000	RP11-496	1045	6	0.15	5	0.13	8	0.2	30	0.77	21	0.5	39	0.9	17.33058	0.431193	3.929949	**1.69457**	**Ups**	**8.5E-05**	**0.00076**	_	K11518
ENSG000	RP11-496	2513	10	0.09	12	0.11	7	0.06	18	0.16	25	0.21	35	0.29	16.98728	0.4063	2.530046	**1.02796**	**Ups**	**0.0114**	**0.04446**	_	_
ENSG000	RP11-497	38880	1	0.06	0	0	9	0.56	14	0.9	35	2.08	17	0.99	11.61501	0.544982	2.991974	**1.63057**	**Ups**	**0.00277**	**0.01461**	_	K01195
ENSG000	RP11-498	221	8	0.93	6	0.73	10	1.17	87	10.53	6	0.67	43	4.72	26.57365	0.538868	3.010478	**1.62225**	**Ups**	**0.00261**	**0.01389**	_	_
ENSG000	RP11-49K	2117	1	0.01	2	0.03	0	0	7	0.09	23	0.27	4	0.05	5.527435	0.574014	3.05242	**1.75213**	**Ups**	**0.00227**	**0.01241**	_	_
ENSG000	RP11-503	776	3	0.11	1	0.04	5	0.18	7	0.26	19	0.67	12	0.41	7.247562	0.521726	2.498926	**1.30375**	**Ups**	**0.01246**	**0.04761**	_	_
ENSG000	RP11-506	16602	13	0.25	12	0.24	7	0.14	36	0.72	25	0.47	45	0.82	22.11863	0.399813	3.288037	**1.3146**	**Ups**	**0.00101**	**0.00636**	_	_
ENSG000	RP11-509	1944	88	1.16	51	0.7	84	1.12	434	5.97	458	5.84	496	6.19	252.4914	0.234714	9.883841	**2.31988**	**Ups**	**4.9E-23**	**5.6E-21**	_	_
ENSG000	RP11-50D	3843	3	0.02	3	0.02	2	0.01	9	0.06	11	0.07	16	0.1	6.902769	0.513992	2.742464	**1.40961**	**Ups**	**0.0061**	**0.0274**	_	_
ENSG000	RP11-50E	16501	43	0.08	48	0.1	40	0.08	95	0.19	180	0.34	104	0.19	80.27625	0.27841	4.353363	**1.21202**	**Ups**	**1.3E-05**	**0.00015**	_	_
ENSG000	RP11-513	560	3	0.14	4	0.19	4	0.18	10	0.48	16	0.71	20	0.87	8.908767	0.485013	2.906268	**1.40958**	**Ups**	**0.00366**	**0.01826**	_	_
ENSG000	RP11-519	65341	51	0.32	47	0.31	56	0.35	105	0.69	165	1	166	0.98	93.34408	0.242596	4.98557	**1.20948**	**Ups**	**6.2E-07**	**9.8E-06**	_	_
ENSG000	RP1-151F	1515	20	0.46	31	0.74	26	0.6	41	0.97	113	2.49	63	1.36	45.93023	0.354579	3.122981	**1.10734**	**Ups**	**0.00179**	**0.01019**	_	_
ENSG000	RP1-151F	2538	63	0.64	64	0.68	35	0.36	130	1.37	279	2.73	176	1.68	116.3063	0.303103	4.909684	**1.48814**	**Ups**	**9.1E-07**	**1.4E-05**	_	_
ENSG000	RP11-521	1142	8	0.18	5	0.12	7	0.16	1	0.02	0	0	2	0.04	4.029719	0.56989	-2.92819	**-1.6687**	**Down**	**0.00341**	**0.01725**	_	_
ENSG000	RP11-523	649	2	0.08	6	0.25	1	0.04	17	0.7	14	0.53	8	0.3	7.678372	0.528067	2.539693	**1.34113**	**Ups**	**0.01109**	**0.04354**	_	_
ENSG000	RP11-526	2387	1	0.01	1	0.01	3	0.03	13	0.15	13	0.14	7	0.07	5.989627	0.543772	3.023931	**1.64433**	**Ups**	**0.0025**	**0.01342**	_	_
ENSG000	RP11-529	5872	14	0.33	8	0.19	9	0.21	29	0.7	21	0.47	44	0.97	20.02573	0.414687	2.856845	**1.1847**	**Ups**	**0.00428**	**0.02072**	_	_
ENSG000	RP11-531	10884	6	0.04	13	0.1	11	0.08	27	0.2	72	0.5	35	0.24	25.25124	0.415596	3.878434	**1.61186**	**Ups**	**0.00011**	**0.00092**	_	_
ENSG000	RP11-538	3562	0	0	3	0.07	1	0.02	15	0.35	11	0.24	19	0.4	7.683615	0.53864	3.969546	**2.13816**	**Ups**	**7.2E-05**	**0.00066**	_	_
ENSG000	RP11-539	3072	31	0.44	28	0.41	33	0.47	17	0.25	10	0.14	10	0.13	22.20711	0.37768	-3.53131	**-1.3337**	**Down**	**0.00041**	**0.003**	_	_
ENSG000	RP11-540	1894	104	1.41	121	1.71	94	1.28	207	2.92	344	4.5	245	3.14	176.9789	0.218378	4.776181	**1.04301**	**Ups**	**1.8E-06**	**2.5E-05**	_	_
ENSG000	RP11-545	71674	4	0.02	10	0.04	3	0.01	42	0.17	106	0.4	41	0.15	31.11952	0.449466	5.78604	**2.60063**	**Ups**	**7.2E-09**	**1.7E-07**	_	_
ENSG000	RP11-546	407	0	0	1	0.07	1	0.06	13	0.85	9	0.55	14	0.83	5.951929	0.558626	4.037698	**2.25556**	**Ups**	**5.4E-05**	**0.00051**	_	_
ENSG000	RP11-548	8832	31	0.16	35	0.19	26	0.14	80	0.44	82	0.42	71	0.35	52.2038	0.27719	3.816998	**1.05803**	**Ups**	**0.00014**	**0.00114**	_	_
ENSG000	RP11-549	3157	7	0.06	5	0.04	6	0.05	21	0.18	15	0.12	21	0.16	12.05665	0.444223	2.693402	**1.19647**	**Ups**	**0.00707**	**0.03068**	_	_
ENSG000	RP11-540	2086	9	0.11	1	0.01	8	0.1	78	1	11	0.13	63	0.73	27.6375	0.53587	3.738885	**2.00356**	**Ups**	**0.00018**	**0.0015**	_	_
ENSG000	RP11-552	3175	67	0.54	59	0.5	73	0.59	23	0.19	18	0.14	22	0.17	45.21458	0.300943	-5.76945	**-1.7363**	**Down**	**8E-09**	**1.9E-07**	_	_
ENSG000	RP11-552	2023	9	0.11	2	0.03	12	0.15	44	0.58	39	0.48	40	0.48	23.0968	0.420007	4.434505	**1.86252**	**Ups**	**9.2E-06**	**0.00011**	_	_
ENSG000	RP11-553	1679	90	1.38	130	2.08	118	1.82	405	6.45	212	3.13	399	5.76	219.4764	0.315467	4.082095	**1.28777**	**Ups**	**4.5E-05**	**0.00043**	_	_
ENSG000	RP11-554	1645	246	6.9	212	6.2	214	6.03	39	1.14	101	2.73	78	2.06	152.0195	0.267432	-6.61779	**-1.7698**	**Down**	**3.7E-11**	**1.3E-09**	_	_
ENSG000	RP11-556	212644	0	0	0	0	0	0	1	0.02	12	0.27	11	0.24	3.487608	0.582189	3.324705	**1.93561**	**Ups**	**0.00089**	**0.00568**	_	_
ENSG000	RP11-558	1488	1	0.06	0	0	0	0	6	0.39	12	0.72	3	0.17	3.322594	0.583077	2.990195	**1.74351**	**Ups**	**0.00279**	**0.01467**	_	_
ENSG000	RP11-55K	2693	7	0.07	9	0.09	14	0.13	3	0.03	4	0.04	1	0.01	6.582841	0.527675	-2.83374	**-1.4953**	**Down**	**0.0046**	**0.0219**	_	_
ENSG000	RP11-560	1078	42	1	25	0.62	21	0.5	85	2.11	80	1.84	106	2.38	57.27737	0.313277	4.155537	**1.30183**	**Ups**	**3.3E-05**	**0.00033**	_	_
ENSG000	RP11-566	3148	158	0.71	138	0.64	167	0.75	450	2.09	405	1.75	446	1.88	283.1901	0.201452	6.168351	**1.24262**	**Ups**	**6.9E-10**	**2E-08**	_	_
ENSG000	RP11-572	5109	6	0.03	22	0.12	13	0.07	41	0.21	47	0.23	29	0.14	25.27462	0.410947	2.681855	**1.1021**	**Ups**	**0.00732**	**0.03157**	_	_
ENSG000	RP11-573	2400	17	0.18	21	0.23	14	0.15	26	0.29	212	2.19	41	0.41	49.27045	0.494658	3.257208	**1.6112**	**Ups**	**0.00113**	**0.00694**	_	_
ENSG000	RP11-57H	70764	2	0.02	1	0.01	2	0.02	7	0.07	11	0.1	9	0.08	4.972799	0.545574	2.646598	**1.44391**	**Ups**	**0.00813**	**0.03426**	_	_
ENSG000	RP11-57H	4237	61	0.37	69	0.44	71	0.43	146	0.92	215	1.26	133	0.76	110.7777	0.242628	4.194499	**1.0177**	**Ups**	**2.7E-05**	**0.00028**	_	_
ENSG000	RP11-595	70039	0	0	0	0	1	0.02	27	0.54	1	0.02	30	0.54	9.483278	0.582573	3.913658	**2.27999**	**Ups**	**9.1E-05**	**0.00081**	_	_
ENSG000	RP11-599	16497	32	2.06	43	2.88	35	2.26	101	6.75	91	5.64	75	4.55	60.90469	0.286458	3.496615	**1.00163**	**Ups**	**0.00047**	**0.00336**	_	_
ENSG000	RP1-159A	4757	1	0.09	6	0.57	5	0.46	25	2.36	18	1.58	27	2.31	13.01374	0.473966	3.851013	**1.82525**	**Ups**	**0.00012**	**0.00101**	_	_
ENSG000	RP11-59D	15807	6	0.11	12	0.22	7	0.12	0	0	2	0.03	0	0	4.738578	0.571378	-3.633	**-2.0758**	**Down**	**0.00028**	**0.00214**	_	_
ENSG000	RP11-61L	586	8	0.35	8	0.37	9	0.4	0	0	3	0.13	3	0.12	5.321359	0.544631	-2.80533	**-1.5279**	**Down**	**0.00503**	**0.02349**	_	_
ENSG000	RP11-624	170633	0	0	0	0	0	0	6	0.04	3	0.02	4	0.02	2.05139	0.576639	2.778075	**1.60195**	**Ups**	**0.00547**	**0.02517**	_	_
ENSG000	RP11-626	11255	68	2.08	49	1.56	42	1.29	221	7.03	112	3.3	177	5.1	108.4662	0.335564	4.069378	**1.36554**	**Ups**	**4.7E-05**	**0.00046**	_	_
ENSG000	RP11-62F	711	72	4.32	60	3.76	74	4.47	33	2.06	17	0.99	39	2.21	50.58869	0.337519	-3.78944	**-1.279**	**Down**	**0.00015**	**0.00126**	_	_
ENSG000	RP11-63A	6265	25	1.63	16	1.09	20	1.31	1	0.07	2	0.13	2	0.12	11.59387	0.49887	-5.55752	**-2.7725**	**Down**	**2.7E-08**	**5.8E-07**	_	_
ENSG000	RP11-65J	7158	9	0.45	3	0.16	3	0.15	0	0	0	0	0	0	2.651762	0.581339	-3.24266	**-1.8851**	**Down**	**0.00118**	**0.00724**	_	_
ENSG000	RP11-65J	24066	267	5.21	244	4.96	238	4.67	124	2.52	99	1.86	95	1.75	182.6894	0.22733	-6.07877	**-1.3819**	**Down**	**1.2E-09**	**3.4E-08**	_	_
ENSG000	RP11-661	196504	0	0	2	0.02	1	0.01	11	0.11	5	0.05	14	0.13	5.232126	0.566745	3.150021	**1.78526**	**Ups**	**0.00163**	**0.00942**	_	_
ENSG000	RP11-665	422973	0	0	2	0.05	0	0	4	0.11	11	0.27	5	0.12	3.323286	0.581608	2.555453	**1.48627**	**Ups**	**0.0106**	**0.04206**	_	_
ENSG000	RP11-66E	12148	6	0.09	4	0.06	5	0.07	16	0.24	24	0.33	19	0.26	11.60615	0.444935	3.213116	**1.42963**	**Ups**	**0.00131**	**0.00789**	_	_
ENSG000	RP11-66N	6977	44	0.35	31	0.26	47	0.37	118	0.97	195	1.49	118	0.88	86.81516	0.277934	5.357121	**1.48893**	**Ups**	**8.5E-08**	**1.7E-06**	_	_
ENSG000	RP11-66N	49019	9	0.03	16	0.06	9	0.03	21	0.08	47	0.17	30	0.1	20.70346	0.397548	2.785071	**1.1072**	**Ups**	**0.00535**	**0.02475**	_	_
ENSG000	RP11-67L	5456	105	0.49	88	0.43	115	0.54	235	1.15	347	1.58	256	1.14	181.9246	0.208242	5.631605	**1.17273**	**Ups**	**1.8E-08**	**4E-07**	_	_

ENSG000	RP11-680	1451	6	0.11	6	0.11	8	0.14	16	0.29	22	0.38	29	0.48	13.73118	0.428206	2.959987	1.26748	Ups	0.00308	0.01594	_	_
ENSG000	RP11-686	2129	3	0.2	2	0.14	0	0	9	0.61	20	1.26	9	0.55	6.557712	0.544146	3.221105	1.75275	Ups	0.00128	0.00771	_	_
ENSG000	RP11-689	30341	2	0.01	0	0	5	0.03	10	0.07	22	0.14	10	0.06	7.525984	0.537791	2.93669	1.57933	Ups	0.00332	0.0169	_	_
ENSG000	RP11-690	5113	46	0.23	90	0.47	64	0.32	191	1	256	1.24	212	1.01	135.9676	0.26411	5.337033	1.40957	Ups	9.5E-08	1.8E-06	_	_
ENSG000	RP11-694	20766	15	0.21	15	0.22	8	0.11	67	1	38	0.52	55	0.74	31.83588	0.388055	4.233321	1.64276	Ups	2.3E-05	0.00025	_	K06243
ENSG000	RP11-698	3676	45	0.31	39	0.28	54	0.38	140	1.02	142	0.96	122	0.8	86.73962	0.251723	5.045213	1.27	Ups	4.5E-07	7.4E-06	_	_
ENSG000	RP11-69I	126448	0	0	2	0.11	1	0.05	67	3.62	40	2	50	2.45	25.14772	0.495802	7.688956	3.8122	Ups	1.5E-14	8.2E-13	_	_
ENSG000	RP11-6O2	6054	13	0.12	4	0.04	6	0.06	19	0.19	45	0.42	23	0.21	17.0374	0.447842	3.055397	1.36833	Ups	0.00225	0.0123	_	_
ENSG000	RP11-70F	18368	31	0.14	37	0.18	34	0.16	192	0.92	133	0.59	167	0.72	94.81058	0.289902	6.673465	1.93465	Ups	2.5E-11	9.1E-10	_	_
ENSG000	RP11-713	2206	0	0	1	0.01	0	0	2	0.02	23	0.26	15	0.16	5.964044	0.580248	3.77243	2.18895	Ups	0.00016	0.00134	_	_
ENSG000	RP11-715	3264	7	0.06	9	0.07	5	0.04	29	0.24	18	0.14	16	0.12	13.63704	0.449966	2.523334	1.13542	Ups	0.01162	0.0451	_	_
ENSG000	RP11-715	504	17	0.87	13	0.69	12	0.62	84	4.46	71	3.49	83	3.99	44.25884	0.324534	6.464411	2.09792	Ups	1E-10	3.4E-09	_	_
ENSG000	RP11-71H	699	34	1.25	32	1.23	31	1.15	93	3.56	119	4.22	104	3.61	65.41824	0.248721	5.663152	1.40854	Ups	1.5E-08	3.3E-07	_	_
ENSG000	RP11-731	1684	25	0.38	23	0.37	19	0.29	66	1.05	86	1.27	60	0.86	44.22895	0.295742	4.525106	1.33826	Ups	6E-06	7.5E-05	_	_
ENSG000	RP11-736	45341	5	0.06	10	0.13	8	0.1	33	0.44	33	0.41	48	0.58	21.58379	0.398467	4.517942	1.80025	Ups	6.2E-06	7.8E-05	_	_
ENSG000	RP11-737	18393	28	1.27	16	0.76	23	1.05	4	0.19	5	0.22	1	0.04	13.44195	0.472727	-4.88096	-2.3074	Down	1.1E-06	1.6E-05	_	_
ENSG000	RP11-753	144028	4	0.1	4	0.11	2	0.05	0	0	0	0	0	0	1.776654	0.571874	-2.70551	-1.5472	Down	0.00682	0.02984	_	_
ENSG000	RP11-755	2268	3	0.14	2	0.1	0	0	7	0.35	15	0.69	10	0.45	5.670455	0.547265	2.89246	1.58294	Ups	0.00382	0.01892	_	_
ENSG000	RP11-750	9494	1	0.05	0	0	0	0	12	0.59	15	0.68	5	0.22	5.073455	0.575085	3.89099	2.23765	Ups	1E-04	0.00088	_	_
ENSG000	RP11-760	4022	9	0.23	10	0.26	3	0.08	69	1.82	46	1.13	72	1.72	32.997	0.406332	6.065363	2.46455	Ups	1.3E-09	3.6E-08	_	_
ENSG000	RP11-767	510	4	0.2	1	0.05	3	0.15	25	1.31	7	0.34	18	0.86	9.405348	0.533641	3.174961	1.69429	Ups	0.0015	0.0088	_	_
ENSG000	RP11-770	146943	9	0.1	7	0.08	7	0.08	20	0.23	20	0.21	30	0.31	14.81749	0.416378	2.826817	1.17702	Ups	0.0047	0.02226	_	_
ENSG000	RP11-774	3249	5	0.04	10	0.08	18	0.14	77	0.63	63	0.48	72	0.54	38.73746	0.382003	5.665624	2.16428	Ups	1.5E-08	3.3E-07	_	_
ENSG000	RP11-779	4331	5	0.27	0	0	0	0	21	1.19	4	0.21	17	0.87	7.604251	0.577278	2.731923	1.57708	Ups	0.0063	0.0281	_	_
ENSG000	RP11-779	738	0	0	1	0.04	0	0	22	0.8	4	0.13	16	0.53	6.93168	0.576568	4.114265	2.37215	Ups	3.9E-05	0.00039	_	_
ENSG000	RP11-783	2254	211	6.6	191	6.23	226	7.11	71	2.31	76	2.29	131	3.86	154.3224	0.264218	-5.00289	-1.3219	Down	5.7E-07	9.1E-06	_	_
ENSG000	RP11-793	285	29	2.62	20	1.88	27	2.45	7	0.66	22	1.91	11	0.94	19.42976	0.401058	-2.52733	-1.0136	Down	0.01149	0.04476	_	K09481
ENSG000	RP11-796	2086	15	0.6	13	0.55	16	0.65	1	0.04	6	0.23	4	0.15	9.441578	0.485311	-3.55596	-1.7257	Down	0.00038	0.00276	_	_
ENSG000	RP11-797	672	27	1.03	26	1.04	11	0.42	77	3.06	41	1.51	61	2.2	39.48739	0.391798	2.887494	1.13131	Ups	0.00388	0.01915	_	_
ENSG000	RP11-798	7489	6	0.3	5	0.26	4	0.2	17	0.87	39	1.86	12	0.56	12.80341	0.479642	3.135916	1.50412	Ups	0.00171	0.00981	_	_
ENSG000	RP11-798	6667	95	0.69	114	0.86	92	0.67	329	2.48	263	1.84	320	2.19	194.8808	0.235061	5.685133	1.33635	Ups	1.3E-08	3E-07	_	_
ENSG000	RP1-179N	50508	13	0.21	13	0.22	8	0.13	18	0.3	94	1.46	45	0.69	28.9317	0.451279	3.476694	1.56896	Ups	0.00051	0.00357	_	_
ENSG000	RP11-7F1	28440	1	0.01	3	0.03	3	0.03	9	0.08	11	0.09	12	0.1	6.132463	0.52502	2.636902	1.38443	Ups	0.00837	0.03509	_	_
ENSG000	RP11-800	3361	4	0.03	8	0.06	4	0.03	17	0.14	46	0.34	29	0.21	16.51556	0.44968	4.096279	1.84201	Ups	4.2E-05	0.00041	_	_
ENSG000	RP11-803	670	0	0	0	0	0	0	4	0.16	4	0.15	6	0.22	2.145515	0.579364	2.909247	1.68551	Ups	0.00362	0.01812	_	_
ENSG000	RP11-804	163261	0	0	0	0	0	0	7	0.22	4	0.12	2	0.06	2.061842	0.574257	2.705003	1.55337	Ups	0.00683	0.02988	_	_
ENSG000	RP11-806	1126	2	0.16	3	0.25	2	0.16	11	0.9	15	1.14	7	0.52	6.274595	0.527879	2.661199	1.40479	Ups	0.00779	0.03313	_	_
ENSG000	RP11-809	6017	4	0.12	3	0.09	5	0.15	0	0	0	0	0	0	2.138473	0.579087	-3.01957	-1.7486	Down	0.00253	0.01358	_	_
ENSG000	RP11-814	57840	2	0.02	2	0.02	1	0.01	8	0.07	17	0.13	9	0.07	5.975238	0.539768	3.107492	1.67732	Ups	0.00189	0.01064	_	_
ENSG000	RP11-818	18409	319	11.7	284	10.86	327	12.06	74	2.82	227	8.03	170	5.88	235.3742	0.295116	-3.9249	-1.1583	Down	8.7E-05	0.00078	_	_
ENSG000	RP11-83E	1592	7	0.14	3	0.06	8	0.16	24	0.5	14	0.27	22	0.42	12.58806	0.461237	2.678766	1.23555	Ups	0.00739	0.03178	_	_
ENSG000	RP11-847	19303	0	0	1	0.03	0	0	2	0.07	15	0.47	3	0.09	3.052711	0.582144	2.606788	1.51753	Ups	0.00914	0.03751	_	_
ENSG000	RP11-849	660	37	1.44	16	0.65	34	1.33	105	4.25	38	1.43	117	4.3	56.41935	0.424664	2.778631	1.17998	Ups	0.00546	0.02515	_	_
ENSG000	RP11-85N	448464	177	1.76	160	1.66	140	1.4	6	0.06	70	0.67	26	0.24	99.49605	0.447379	-4.62035	-2.067	Down	3.8E-06	5.1E-05	_	_
ENSG000	RP11-865	4022	1	0.05	2	0.1	1	0.05	4	0.21	9	0.43	18	0.85	5.354822	0.562938	2.88171	1.62222	Ups	0.00396	0.01943	_	_
ENSG000	RP11-867	7980	0	0	0	0	2	0.03	4	0.05	9	0.11	11	0.13	3.950116	0.577572	2.941514	1.69894	Ups	0.00327	0.01667	_	_
ENSG000	RP11-869	700	0	0	3	0.11	3	0.11	12	0.46	19	0.67	20	0.69	8.785329	0.5142	3.944891	2.02846	Ups	8E-05	0.00072	_	_
ENSG000	RP11-873	1609	17	0.27	4	0.07	10	0.16	40	0.66	29	0.45	39	0.59	22.26756	0.423356	3.187319	1.34937	Ups	0.00144	0.00849	_	_
ENSG000	RP11-875	1387	13	0.87	11	0.77	10	0.67	3	0.21	3	0.19	6	0.38	7.881447	0.491123	-2.68069	-1.3166	Down	0.00735	0.03164	_	_
ENSG000	RP11-889	11043	3	0.16	9	0.51	5	0.27	0	0	0	0	1	0.05	3.189143	0.583083	-3.11581	-1.8168	Down	0.00183	0.01039	_	_
ENSG000	RP11-88H	9073	0	0	0	0	1	0.01	2	0.01	33	0.17	35	0.18	10.35567	0.577479	4.445015	2.5669	Ups	8.8E-06	0.00011	_	_
ENSG000	RP11-890	3994	46	0.3	25	0.17	39	0.25	131	0.88	278	1.73	135	0.82	100.9405	0.324547	5.80352	1.88352	Ups	6.5E-09	1.6E-07	_	_
ENSG000	RP11-893	6279	3	0.1	5	0.18	2	0.07	0	0	0	0	0	0	1.78106	0.571112	-2.68647	-1.5343	Down	0.00722	0.0312	_	_
ENSG000	RP11-902	24655	24	0.12	30	0.15	33	0.16	50	0.25	169	0.79	94	0.43	61.68004	0.363884	3.859557	1.40443	Ups	0.00011	0.00098	_	_
ENSG000	RP11-91J	3512	2	0.01	4	0.03	4	0.03	0	0	0	0	0	0	1.787746	0.571908	-2.70759	-1.5485	Down	0.00678	0.02971	_	_
ENSG000	RP11-932	8776	7	0.19	9	0.25	5	0.13	13	0.36	44	1.12	16	0.4	14.47915	0.470014	2.58728	1.21606	Ups	0.00967	0.03925	_	_
ENSG000	RP11-956	2093	2	0.11	0	0	1	0.05	10	0.56	12	0.62	11	0.56	5.569694	0.55239	3.591499	1.98391	Ups	0.00033	0.00246	_	_
ENSG000	RP11-95F	7965	52	0.39	50	0.39	52	0.39	157	1.22	127	0.91	148	1.04	94.33464	0.252809	4.824111	1.21958	Ups	1.4E-06	2.1E-05	_	_
ENSG000	RP11-96H	72590	83	2.41	82	2.49	52	1.52	245	7.41	113	3.17	195	5.35	125.7875	0.34483	3.074042	1.06002	Ups	0.00211	0.01169	_	_
ENSG000	RP11-970	52813	3	0.06	4	0.08	4	0.08	18	0.38	23	0.45	19	0.36	11.10784	0.461256	3.837839	1.77023	Ups	0.00012	0.00106	_	_
ENSG000	RP11-981	2860	0	0	1	0.01	1	0.01	14	0.13	20	0.17	17	0.14	8.0989	0.540338	4.983115	2.69256	Ups	6.3E-07	9.9E-06	_	_
ENSG000	RP11-982	3787	70	1.38	72	1.48	67	1.33	21	0.43	32	0.61	18	0.33	47.97981	0.291622	-5.71249	-1.6659	Down	1.1E-08	2.6E-07	_	_
ENSG000	RP11-983	1818	1	0.05	0	0	1	0.05	7	0.35	4	0.19	9	0.41	3.468829	0.578991	2.71719	1.57323	Ups	0.00658	0.02906	_	_
ENSG000	RP11-998	8661	31	2.23	25	1.87	36	2.6	4	0.3	3	0.21	4	0.27	18.0973	0.437719	-6.12306	-2.6802	Down	9.2E-10	2.6E-08	_	_
ENSG000	RP1-239E	2883	366	3.26	240	2.23	361	3.24	1334	12.37	409	3.52	1109	9.33	625.4476	0.376641	3.270429	1.23178	Ups	0.00107	0.00669	_	_
ENSG000	RP1-251I	4631	28	0.88	14	0.46	18	0.57	43	1.4	94	2.84	47	1.39	38.14828	0.365223	3.3397	1.21973	Ups	0.00084	0.00543	_	_
ENSG000	RP1-257A	30726	7	0.06	7	0.06	6	0.05	25	0.21	32	0.25	22	0.17	15.60999	0.411263	3.639404	1.49675	Ups	0.00027	0.0021	_	_
ENSG000	RP1-261D	29090	97	2.55	100	2.74	97	2.57	308	8.43	311	7.89	367	9.11	203.713	0.19791	7.501855	1.48469	Ups	6.3E-14	3.3E-12	_	_
ENSG000	RP1-276N	3514	204	11.23	165	9.47	217	12.01	0	0	0	0	0	0	104.3596	0.466531	-13.6761	-6.3803	Down	1.4E-42	4.8E-40	_	_
ENSG000	RP1-286D	531	36	1.74	33	1.67	35	1.7	13	0.65	19	0.89	14	0.64	25.49564	0.336922	-3.7822	-1.2743	Down	0.00016	0.00129	_	_
ENSG000	RP1-313L	819	5	0.18	6	0.22	6	0.22	17	0.63	32	1.11	26	0.88	14.30257	0.428581	3.705247	1.588	Ups	0.00021	0.00168	_	_
ENSG000	RP13-20L	680	14	0.53	2	0.08	5	0.19	0	0	1	0.04	0	0	3.84573	0.58335	-3.23856	-1.8892	Down	0.0012	0.00732	_	_
ENSG000	RP13-270	6415	15	0.23	15	0.24	20	0.31	51	0.81	57	0.84	54	0.78	33.72217	0.316007	4.293695	1.35684	Ups	1.8E-05	0.00019	_	_
ENSG000	RP1-32I1	734	29	1.23	13	0.58	14	0.6	4	0.18	1	0.04	9	0.36	12.10412	0.50605	-3.21973	-1.6293	Down	0.00128	0.00774	_	_
ENSG000	RP13-360	2080	23	0.65	22	0.65	17	0.49	39	1.15	80	2.19	87	2.33	41.90904	0.337683	4.001507	1.35124	Ups	6.3E-05	0.00059	_	_
ENSG000	RP13-383	611	70	2.95	50	2.19	55	2.33	21	0.92	16	0.65	48	1.9	44.16496	0.378991	-2.91429	-1.1045	Down	0.00356	0.01789	_	K15203
ENSG000	RP13-463	1128	8	0.3	3	0.12	10	0.37	0	0	0	0	3	0.1	4.190142	0.578953	-2.73008	-1.5806	Down	0.00633	0.02822	_	_
ENSG000	RP13-487	3194	0	0	0	0	0	0	3	0.03	7	0.05	8	0.06	2.689141	0.582719	3.25167	1.89481	Ups	0.00115	0.00705	_	K19409
ENSG000	RP13-735	30154	5	0.03	5	0.03	3	0.02	19	0.12	21	0.12	23	0.13	11.95277	0.450033	3.707394	1.66845	Ups	0.00021	0.00167	_	_
ENSG000	RP1-37N7	9130	28	0.15	18	0.1	17	0.09	0	0	0	0	0	0	11.1856	0.549111	-6.6424	-3.6474	Down	3.1E-11	1.1E-09	_	_
ENSG000	RP1-63G5	87857	7	0.07	9	0.1	9	0.1	3	0.03	3	0.03	0	0	5.392357	0.546694	-2.73355	-1.4944	Down	0.00627	0.02799	_	_
ENSG000	RP1-63G5	2650	10	0.13	6	0.08	1	0.01	0	0	1	0.01	1	0.01	3.292259	0.583365	-2.59186	-1.512	Down	0.00955	0.03887	_	_
ENSG000	RP1-90J2	9409	14	0.67	10	0.5	10	0.48	2	0.1	4	0.19	5	0.23	7.692834	0.496029	-2.83249	-1.405	Down	0.00462	0.02195	_	_
ENSG000	RP3-337H	545	25	1.18	11	0.54	18	0.85	56	2.75	52	2.37	38	1.69	32.15811	0.361961	3.0276	1.09587	Ups	0.00247	0.01331	_	_
ENSG000	RP3-402C	478	5	0.27	8	0.45	2	0.11	17	0.95	16	0.83	16	0.81	10.22065	0.471266	2.50723	1.18157	Ups	0.01217	0.04677	_	_
ENSG000	RP3-406A	374	49	3.37	35	2.51	73	5.05	253	18.09	86	5.7	180	11.67	110.5949	0.401351	3.348254	1.34382	Ups	0.00081	0.0053	_	_
ENSG000	RP3-416H	2401	691	10.49	543	8.6	666	10.17	246	3.89	190	2.78	217	3.11	439.5461	0.219602	-7.63825	-1.6774	Down	2.2E-14	1.2E-12	_	_
ENSG000	RP3-439F	6702	14	0.12	19	0.17	21	0.18	5	0.05	1	0.01	0	0	10.63987	0.517688	-4.54383	-2.3523	Down	5.5E-06	7E-05	_	_
ENSG000	RP3-466F	1649	18	0.28	17	0.28	21	0.33	83	1.35	76	1.14	87	1.28	47.89469	0.300319	5.94052	1.78405	Ups	2.8E-09	7.4E-08	_	_
ENSG000	RP3-477C	427	7	0.42	3	0.19	12	0.73	39	2.44	10	0.58	31	1.76	16.69466	0.504858	2.502871	1.2636	Ups	0.01232	0.04723	_	_
ENSG000	RP3-508I	10039	2	0.1	1	0.05	2	0.1	17	0.91	4	0.2	9	0.44	5.727783	0.562291	2.619623	1.47299	Ups	0.0088	0.03642	_	_
ENSG000	RP3-508I	491	39	2.04	26	1.42	48	2.53	157	8.55	50	2.53	119	5.88	72.01649	0.409455	2.846474	1.1655	Ups	0.00442	0.02122	_	_
ENSG000	RP3-523C	14196	0	0	0	0	0	0	2	0.05	7	0.17	3	0.07	1.765355	0.570155	2.551796	1.45492	Ups	0.01072	0.04238	_	_
ENSG000	RP4-569D	484	0	0	0	0	0	0	6	0.33	6	0.31	7	0.35	2.917743	0.583451	3.457335	2.01719	Ups	0.00055	0.0038	_	_
ENSG000	RP4-584D	837	127	3.9	103	3.3	132	4.08	64	2.04	57	1.69	71	2.06	94.06322	0.244024	-4.40834	-1.0757	Down	1E-05	0.00012	_	_
ENSG000	RP4-607J	2497	12	0.21	9	0.17	17	0.3	49	0.9	32	0.55	32	0.53	24.46784	0.393475	3.036944	1.19496	Ups	0.00239	0.01296	_	_
ENSG000	RP4-612J	31985	3	0.13	0	0	3	0.13	6	0.27	19	0.8	13	0.54	6.685485	0.542907	2.937967	1.59504	Ups	0.0033	0.01684	_	_
ENSG000	RP4-717I	83840	72	0.17	95	0.24	60	0.15	195	0.49	298	0.69	223	0.51	148.8475	0.238129	5.720046	1.36211	Ups	1.1E-08	2.5E-07	_	_
ENSG000	RP4-740C	2407	21	0.22	23	0.26	16	0.17	68	0.76	43	0.44	54	0.54	36.47576	0.347896	3.275452	1.13952	Ups	0.00105	0.00659	_	_
ENSG000	RP4-758J	1523	174	2.94	117	2.06	127	2.16	46	0.81	95	1.55	77	1.23	106.9466	0.268526	-4.14725	-1.1136	Down	3.4E-05	0.00034	_	_
ENSG000	RP4-791N	2700	5	0.15	8	0.25	4	0.12	15	0.46	27	0.77	13	0.36	11.30933	0.461001	2.537202	1.16965	Ups	0.01117	0.04379	_	_
ENSG000	RP4-802A	470	0	0	0	0	1	0.06	8	0.46	3	0.16	8	0.41	3.177713	0.583247	2.89916	1.69093	Ups	0.00374	0.0186	_	_
ENSG000	RP4-803A	531	0	0	1	0.03	0	0	10	0.25	9	0.21	13	0.3	5.103938	0.570044	4.093081	2.33324	Ups	4.3E-05	0.00042	_	K02882
ENSG000	RP4-803A	1282	2	0.06	1	0.03	4	0.12	19	0.59	3	0.09	19	0.53	7.797515	0.559419	2.646028	1.48024	Ups	0.00814	0.0343	_	K02981
ENSG000	RP4-806M	2721	13	0.29	13	0.3	13	0.29	4	0.09	6	0.13	2	0.04	8.767828	0.482772	-3.0897	-1.4916	Down	0.002	0.01121	_	_
ENSG000	RP5-1021	15911	32	0.74	23	0.56	58	1.35	140	3.37	75	1.67	129	2.82	74.09251	0.378948	3.287207	1.24568	Ups	0.00101	0.00637	_	_
ENSG000	RP5-1028	9977	71	1.52	58	1.3	40	0.86	4	0.09	11	0.23	6	0.12	33.13268	0.391585	-7.16059	-2.804	Down	8E-13	3.6E-11	_	_
ENSG000	RP5-1042	1359	5	0.09	7	0.14	11	0.21	16	0.31	22	0.4	29	0.52	14.27661	0.429862	2.550052	1.09617	Ups	0.01077	0.04255	_	_
ENSG000	RP5-1092	1945	13	0.17	8	0.11	6	0.08	22	0.3	25	0.32	28	0.35	16.25252	0.403464	2.674697	1.07914	Ups	0.00748	0.03211	_	_
ENSG000	RP5-1139	7088	5	0.17	3	0.11	4	0.14	8	0.28	49	1.59	10	0.32	11.80273	0.534228	2.830303	1.51203	Ups	0.00465	0.02208	_	_
ENSG000	RP5-1159	2337	1	0.02	2	0.03	0	0	6	0.1	12	0.18	12	0.18	5.033377	0.560434	3.262539	1.82844	Ups	0.0011	0.00684	_	_
ENSG000	RP5-850C	279	4	0.37	2	0.19	5	0.46	37	3.55	3	0.27	26	2.26	12.66281	0.554524	2.816203	1.56165	Ups	0.00486	0.02286	_	_
ENSG000	RP5-884M	20554	4	0.03	11	0.07	3	0.02	0	0	0	0	0	0	3.211392	0.583109	-3.52428	-2.055	Down	0.00042	0.00307	_	_
ENSG000	RP5-983L	6454	22	0.15	21	0.15	13	0.09	0	0	0	0	1	0.01	10.10334	0.543032	-6.05295	-3.2869	Down	1.4E-09	3.9E-08	_	_
ENSG000	RP6-201G	1432	0	0	1	0.02	0	0	13	0.24	4	0.07	15	0.25	5.229256	0.577926	3.772131	2.18001	Ups	0.00016	0.00134	_	_
ENSG000	RP6-99M	25039	352	2.88	443	3.79	294	2.42	88	0.75	286	2.26	229	1.77	283.1367	0.320562	-3.20006	-1.0258	Down	0.00137	0.00818	_	_
ENSG000	RPL12P2	495	0	0	0	0	0	0	3	0.08	3	0.08	6	0.15	1.834533	0.573175	2.634669	1.51013	Ups	0.00842	0.03527	_	K02870
ENSG000	RPL13AP2	612	23	0.48	16	0.35	23	0.49	57	1.25	38	0.77	70	1.39	36.64788	0.354022	3.06485	1.08503	Ups	0.00218	0.01199	_	K02872
ENSG000	RPL17	4056	3359	11.41	3008	10.66	3002	10.26	1691	5.97	1156	3.79	1700	5.45	2374.757	0.223663	-5.32357	-1.1907	Down	1E-07	1.9E-06	biological_	K02880
ENSG000	RPL3L	13633	2	0.02	1	0.01	9	0.07	28	0.24	17	0.14	25	0.19	13.09223	0.503904	3.446713	1.73681	Ups	0.00057	0.00392	biological_	K02925
ENSG000	RPRM	1471	261	4.56	199	3.63	237	4.17	1429	25.97	678	11.43	1390	22.91	673.255	0.310116	6.390582	1.98182	Ups	1.7E-10	5.3E-09	cellular_cc	K10128
ENSG000	RPRML	1092	5	0.12	1	0.02	2	0.05	14	0.34	10	0.23	10	0.22	6.71616	0.52686	2.520305	1.32785	Ups	0.01173	0.04538	cellular_cc	K10128
ENSG000	RPS10P7	11772	72	0.79	49	0.56	64	0.71	138	1.58	182	1.94	196	2.04	111.3699	0.234904	5.111134	1.20063	Ups	3.2E-07	5.4E-06	_	_
ENSG000	RPS27AP2	465	1	0.06	2	0.12	0	0	123	7.07	124	6.61	132	6.88	58.75739	0.447839	11.25405	5.04	Ups	2.2E-29	3.9E-27	_	K02977
ENSG000	RPS6KA4	13068	3497	13.5	2776	11.18	3337	12.96	1454	5.84	1327	4.94	1686	6.14	2398.428	0.18201	-7.04178	-1.2817	Down	1.9E-12	8.2E-11	molecular_	K16510
ENSG000	RRP1	15781	3260	14.28	2576	11.77	3615	15.93	1911	8.71	877	3.71	1898	7.84	2419.634	0.309693	-3.55952	-1.1024	Down	0.00037	0.00273	cellular_cc	K14849
ENSG000	RRP12	45013	3434	9.26	2737	7.69	3259	8.84	1920	5.38	1605	4.17	1769	4.5	2497.708	0.188903	-5.33227	-1.0073	Down	9.7E-08	1.9E-06	cellular_cc	K14794
ENSG000	RSPO3	79162	144	1.02	117	0.86	120	0.85	0	0	1	0.01	2	0.01	68.2165	0.440678	-12.2923	-5.4169	Down	1E-34	2.4E-32	molecular_	_
ENSG000	RTKN2	85673	239	0.62	364	0.99	256	0.67	476	1.29	1307	3.28	699	1.71	521.3434	0.300009	3.919425	1.17586	Ups	8.9E-05	0.00079	molecular_	_
ENSG000	RTN2	11773	409	1.51	336	1.3	388	1.45	1318	5.08	563	2.01	1279	4.47	699.1537	0.323026	3.693637	1.19314	Ups	0.00022	0.00175	molecular_	_
ENSG000	RUNX2	336193	9	0.01	9	0.01	8	0.01	55	0.09	78	0.11	63	0.09	34.38528	0.345679	6.987154	2.41531	Ups	2.8E-12	1.2E-10	cellular_cc	K09278
ENSG000	RYR1	153866	1	0	3	0	4	0.01	21	0.03	24	0.03	26	0.04	12.28681	0.474784	4.758443	2.25923	Ups	2E-06	2.8E-05	biological_	K04961
ENSG000	S100A11	15402	17978	679.61	14970	590.21	19714	749.53	6334	249.05	5678	207.09	8115	289.33	12478.57	0.204171	-7.58035	-1.5477	Down	3.5E-14	1.8E-12	cellular_cc	_
ENSG000	S100A14	2732	137	1.32	119	1.19	130	1.26	25	0.25	4	0.04	15	0.14	75.83529	0.404685	-6.97449	-2.8225	Down	3.1E-12	1.3E-10	molecular_	_
ENSG000	S100A16	6260	14995	117.41	13147	107.36	15774	124.22	2350	19.14	1535	11.6	2723	20.11	8848.736	0.247884	-11.2564	-2.7903	Down	2.2E-29	3.8E-27	molecular_	_
ENSG000	S100P	4102	8989	145.14	7431	125.14	9081	147.47	1427	23.97	955	14.88	1815	27.64	5191.64	0.254801	-10.4449	-2.6614	Down	1.6E-25	2.2E-23	molecular_	_
ENSG000	S1PR1	4631	21	0.13	25	0.16	28	0.17	10	0.06	6	0.04	9	0.05	17.1083	0.405204	-3.7559	-1.5219	Down	0.00017	0.00141	biological_	K04288
ENSG000	S1PR3	13564	627	1.27	686	1.45	617	1.25	63	0.13	456	0.89	160	0.31	441.8688	0.415286	-3.70313	-1.5379	Down	0.00021	0.00169	biological_	K04290
ENSG000	S1PR4	7986	9	0.12	14	0.19	9	0.12	4	0.05	2	0.02	4	0.05	7.272945	0.507644	-2.74357	-1.3928	Down	0.00608	0.02732	molecular_	K04293
ENSG000	S1PR5	4985	93	0.85	79	0.75	76	0.7	278	2.65	139	1.23	288	2.49	154.5933	0.322176	3.761918	1.212	Ups	0.00017	0.00138	cellular_cc	K04295
ENSG000	SAA1	3804	34	0.62	33	0.63	37	0.68	8	0.15	1	0.02	8	0.14	21.24949	0.433558	-5.35277	-2.3207	Down	8.7E-08	1.7E-06	molecular_	K17310
ENSG000	SALL4	18479	57	0.22	62	0.25	57	0.22	370	1.49	638	2.38	428	1.56	247.8527	0.22623	11.84242	2.67911	Ups	2.4E-32	4.9E-30	biological_	_
ENSG000	SAMD11	19696	512	2.42	361	1.78	518	2.46	1116	5.48	982	4.47	1317	5.86	773.994	0.221369	4.729891	1.04705	Ups	2.3E-06	3.1E-05	biological_	_
ENSG000	SAMD13	91592	27	0.09	31	0.11	23	0.08	85	0.3	63	0.21	90	0.29	51.33227	0.309023	4.038803	1.24808	Ups	5.4E-05	0.00051	_	_
ENSG000	SAMD15	14809	52	0.46	42	0.39	34	0.31	18	0.17	17	0.15	26	0.22	32.11325	0.33449	-3.49295	-1.1684	Down	0.00048	0.0034	_	_

ENSG000	SAMD5	228621	23	0.09	27	0.11	17	0.07	413	1.7	1653	6.32	712	2.66	419.1125	0.377248	11.84228	**4.46748**	**Ups**	**2.4E-32**	**4.9E-30**	_	_
ENSG000	SAPCD1	2053	32	0.36	22	0.26	34	0.39	103	1.21	64	0.7	115	1.23	59.57664	0.336544	4.000989	**1.34651**	**Ups**	**6.3E-05**	**0.00059**	_	_
ENSG000	SATB1	100202	201	0.24	202	0.25	173	0.2	447	0.55	728	0.82	486	0.54	353.4921	0.19839	6.319061	**1.25364**	**Ups**	**2.6E-10**	**8.2E-09**	biological_	_
ENSG000	SATB2-AS	19236	3	0.01	0	0	1	0	11	0.04	16	0.05	11	0.03	6.471028	0.542171	3.612077	**1.95836**	**Ups**	**0.0003**	**0.00229**	_	_
ENSG000	SBK1	31331	5	0.03	2	0.01	3	0.02	16	0.09	12	0.06	13	0.06	8.148457	0.498191	2.762863	**1.37643**	**Ups**	**0.00573**	**0.02612**	molecular_	K08858
ENSG000	SBSPON	59549	272	1.32	231	1.17	216	1.05	669	3.37	1535	7.17	1040	4.75	612.1723	0.242932	7.549906	**1.83411**	**Ups**	**4.4E-14**	**2.3E-12**	molecular_	K16467
ENSG000	SC5D	16242	2010	4.61	3090	7.38	1747	4.03	4505	10.74	11740	25.95	6782	14.66	4642.373	0.290934	4.773847	**1.38887**	**Ups**	**1.8E-06**	**2.6E-05**	molecular_	K00227
ENSG000	SCAPER	557260	234	0.38	214	0.36	153	0.25	377	0.63	857	1.34	502	0.77	366.0048	0.269316	4.481306	**1.20689**	**Ups**	**7.4E-06**	**9.1E-05**	cellular_cc	_
ENSG000	SCG5	55423	22	0.12	17	0.09	17	0.09	127	0.69	114	0.58	145	0.72	69.43993	0.293484	8.168187	**2.39723**	**Ups**	**3.1E-16**	**2.1E-14**	molecular_	_
ENSG000	SCHIP1	57500	22	0.05	28	0.07	18	0.04	47	0.11	123	0.27	64	0.14	46.86973	0.354798	3.856358	**1.36823**	**Ups**	**0.00012**	**0.00099**	molecular_	_
ENSG000	SCN1A	138854	23	0.04	42	0.07	21	0.04	391	0.68	639	1.04	463	0.73	240.853	0.276704	13.16606	**3.6431**	**Ups**	**1.4E-39**	**4.2E-37**	cellular_cc	K04833
ENSG000	SCN4B	19512	1	0	1	0	0	0	22	0.05	26	0.06	23	0.05	11.20576	0.524595	5.924247	**3.10783**	**Ups**	**3.1E-09**	**8.1E-08**	cellular_cc	K04848
ENSG000	SCN9A	180809	79	0.12	91	0.14	66	0.1	839	1.27	3221	4.53	1207	1.66	814.2549	0.356298	10.546	**3.75752**	**Ups**	**5.3E-26**	**7.8E-24**	cellular_cc	K04841
ENSG000	SCNN1D	11594	50	0.18	49	0.18	58	0.21	116	0.43	130	0.44	149	0.5	88.38277	0.234473	4.533683	**1.06303**	**Ups**	**5.8E-06**	**7.3E-05**	molecular_	K04826
ENSG000	SCO1	18039	3549	19.59	3573	20.56	3448	19.14	1633	9.37	1982	10.55	2074	10.79	2750.261	0.119947	-9.21397	**-1.1052**	**Down**	**3.1E-20**	**3E-18**	molecular_	K07152
ENSG000	SDAD1P1	3695	28	0.14	31	0.16	31	0.15	97	0.5	72	0.34	90	0.42	56.26601	0.295357	4.166243	**1.23053**	**Ups**	**3.1E-05**	**0.00032**	_	K14856
ENSG000	SDC2	118422	2718	8.98	3542	12.21	2506	8.33	7204	24.76	10623	33.87	9396	29.28	5687.544	0.177684	7.700623	**1.36828**	**Ups**	**1.4E-14**	**7.6E-13**	biological_	K16336
ENSG000	SDPR	12954	34	0.27	51	0.42	28	0.22	518	4.29	2223	17.07	1240	9.31	603.7242	0.373707	11.45274	**4.27997**	**Ups**	**2.3E-30**	**4.4E-28**	molecular_	K19387
ENSG000	SEC14L5	60842	3	0.01	5	0.02	6	0.02	25	0.1	22	0.08	25	0.09	13.61802	0.443019	3.969509	**1.75857**	**Ups**	**7.2E-05**	**0.00066**	molecular_	_
ENSG000	SEC31B	43230	101	0.14	88	0.13	117	0.17	290	0.42	283	0.38	252	0.33	181.6052	0.219304	5.356088	**1.17461**	**Ups**	**8.5E-08**	**1.7E-06**	cellular_cc	K14005
ENSG000	SEC61G	7725	34071	237.93	31597	230.13	30251	212.47	10085	73.25	8244	55.54	12555	82.69	21840.84	0.205576	-8.66518	**-1.7814**	**Down**	**4.5E-18**	**3.6E-16**	biological_	K07342
ENSG000	SECTM1	13051	770	4.09	625	3.46	729	3.9	113	0.62	283	1.45	235	1.18	472.0602	0.257291	-7.39221	**-1.9019**	**Down**	**1.4E-13**	**7.1E-12**	molecular_	_
ENSG000	SEL1L3	116328	2835	6.19	2802	6.38	2671	5.87	800	1.82	2168	4.57	1160	2.39	2091.933	0.262753	-4.57967	**-1.2033**	**Down**	**4.7E-06**	**6E-05**	cellular_cc	_
ENSG000	SELM	15298	260	1.06	210	0.89	255	1.04	1872	7.93	826	3.25	2023	7.77	870.3167	0.325218	7.093414	**2.30691**	**Ups**	**1.3E-12**	**5.7E-11**	cellular_cc	_
ENSG000	SEMA3D	191303	45	0.13	79	0.24	39	0.11	63	0.19	562	1.55	148	0.4	140.008	0.475209	3.210314	**1.52557**	**Ups**	**0.00133**	**0.00795**	molecular_	K06840
ENSG000	SEMA4A	30387	0	0	0	0	0	0	10	0.02	27	0.05	8	0.02	6.666765	0.574943	4.826254	**2.77482**	**Ups**	**1.4E-06**	**2.1E-05**	biological_	K06521
ENSG000	SEMA5B	119412	5	0.01	4	0.01	0	0	10	0.03	22	0.06	23	0.06	9.818906	0.511798	3.363517	**1.72144**	**Ups**	**0.00077**	**0.00507**	molecular_	K06841
ENSG000	SEMA6D	590123	50	0.06	72	0.09	44	0.05	123	0.15	611	0.67	176	0.19	161.8719	0.420112	4.354837	**1.82952**	**Ups**	**1.3E-05**	**0.00015**	molecular_	_
ENSG000	SENP7	189037	137	0.36	177	0.48	96	0.25	191	0.52	1066	2.7	373	0.92	309.6285	0.412322	3.514295	**1.44902**	**Ups**	**0.00044**	**0.00317**	cellular_cc	K17985
ENSG000	SERPINB3	6767	4	0.04	3	0.03	3	0.03	0	0	0	0	0	0	1.777794	0.573036	-2.73677	**-1.5683**	**Down**	**0.0062**	**0.02777**	molecular_	K13963
ENSG000	SERPINB7	52436	9	0.04	13	0.06	8	0.04	0	0	2	0.01	0	0	5.622498	0.562371	-4.13107	**-2.3232**	**Down**	**3.6E-05**	**0.00036**	molecular_	K13964
ENSG000	SERPINB8	35120	554	1.65	587	1.83	575	1.73	93	0.29	226	0.65	153	0.43	376.0563	0.243325	-8.29729	**-2.0189**	**Down**	**1.1E-16**	**7.5E-15**	molecular_	K13963
ENSG000	SERPINB9	16015	31	0.19	40	0.26	24	0.15	0	0	0	0	0	0	16.91475	0.532945	-7.77145	**-4.1418**	**Down**	**7.8E-15**	**4.5E-13**	molecular_	K13963
ENSG000	SERPINF2	12433	258	1.54	237	1.47	292	1.75	51	0.32	9	0.05	38	0.21	155.9787	0.413407	-6.50994	**-2.6913**	**Down**	**7.5E-11**	**2.5E-09**	molecular_	K03983
ENSG000	SERPING1	17467	42	0.11	31	0.09	42	0.11	247	0.69	137	0.35	203	0.51	112.5499	0.320816	6.192493	**1.98665**	**Ups**	**5.9E-10**	**1.7E-08**	biological_	K04001
ENSG000	SERPINI1	90326	1093	7.39	1021	7.2	947	6.44	2017	14.18	5027	32.79	3479	22.18	2111.759	0.241383	6.032297	**1.4561**	**Ups**	**1.6E-09**	**4.4E-08**	molecular_	_
ENSG000	SESN2	22965	456	3.39	361	2.8	414	3.1	1975	15.29	841	6.04	1434	10.07	891.6449	0.317348	4.687823	**1.48767**	**Ups**	**2.8E-06**	**3.8E-05**	cellular_cc	K10141
ENSG000	SEZ6	51540	0	0	0	0	0	0	5	0.01	3	0.01	4	0.01	1.878928	0.574415	2.67411	**1.53605**	**Ups**	**0.00749**	**0.03214**	cellular_cc	_
ENSG000	SFRP1	47536	0	0	0	0	0	0	3	0.01	13	0.04	3	0.01	2.768934	0.580639	3.099076	**1.79945**	**Ups**	**0.00194**	**0.01092**	biological_	K02166
ENSG000	SFTPD	44875	1	0.02	1	0.02	0	0	10	0.19	8	0.14	11	0.19	4.839683	0.56567	3.569518	**2.01917**	**Ups**	**0.00036**	**0.00264**	biological_	K10068
ENSG000	SGK1	148867	5990	5.84	6845	6.96	6779	6.64	16262	16.48	24720	23.24	19628	18.04	12674.34	0.147474	9.288912	**1.36987**	**Ups**	**1.6E-20**	**1.5E-18**	biological_	K13302
ENSG000	SGK2	29270	0	0	0	0	0	0	6	0.02	11	0.04	6	0.02	3.460076	0.582926	3.749989	**2.18596**	**Ups**	**0.00018**	**0.00144**	biological_	K13303
ENSG000	SGPP1	43826	316	2.45	862	6.98	204	1.59	874	7.06	2886	21.61	2353	17.22	1150.566	0.527169	2.531776	**1.33467**	**Ups**	**0.01135**	**0.04428**	cellular_cc	K04716
ENSG000	SGPP2	136432	30	0.23	18	0.14	19	0.15	128	1.03	54	0.4	89	0.65	54.82463	0.388897	4.135739	**1.60838**	**Ups**	**3.5E-05**	**0.00036**	cellular_cc	K04717
ENSG000	SGSH	14208	948	1.64	741	1.34	963	1.68	463	0.83	526	0.88	438	0.71	690.6859	0.176041	-6.16979	**-1.0861**	**Down**	**6.8E-10**	**2E-08**	molecular_	K01565
ENSG000	SH2B2	33774	193	1.25	151	1.02	171	1.11	127	0.85	51	0.32	91	0.55	134.2737	0.340138	-2.9553	**-1.0052**	**Down**	**0.00312**	**0.0161**	cellular_cc	K07193
ENSG000	SH2D5	13106	307	1.34	195	0.89	262	1.15	116	0.53	111	0.47	115	0.47	188.5164	0.233802	-5.61443	**-1.3127**	**Down**	**2E-08**	**4.3E-07**	_	_
ENSG000	SH3BGRL2	72373	92	0.51	110	0.63	83	0.46	439	2.52	533	2.84	410	2.14	261.9115	0.201798	9.874372	**1.99263**	**Ups**	**5.4E-23**	**6.1E-21**	cellular_cc	_
ENSG000	SH3BP4	103742	1021	2.38	961	2.34	847	1.99	2137	5.18	4816	10.83	2602	5.72	1930.073	0.240549	5.970189	**1.43612**	**Ups**	**2.4E-09**	**6.3E-08**	molecular_	_
ENSG000	SH3BP5	86516	1056	3.13	937	2.9	846	2.53	1985	6.13	3121	8.94	1789	5.01	1548.54	0.20453	4.956468	**1.01375**	**Ups**	**7.2E-07**	**1.1E-05**	molecular_	_
ENSG000	SH3GL2	218048	836	6.48	894	7.23	777	6.06	1403	11.32	3177	23.77	1832	13.4	1403.782	0.235881	4.467683	**1.05384**	**Ups**	**7.9E-06**	**9.6E-05**	cellular_cc	K11247
ENSG000	SH3RF3	516404	348	1.37	246	1.01	265	1.05	23	0.09	77	0.29	38	0.14	173.0441	0.318162	-8.36357	**-2.661**	**Down**	**6.1E-17**	**4.4E-15**	molecular_	K12171
ENSG000	SH3RF3-A	1604	27	0.43	26	0.43	20	0.32	0	0	9	0.14	1	0.02	14.38236	0.506896	-4.53289	**-2.2977**	**Down**	**5.8E-06**	**7.3E-05**	_	_
ENSG000	SH3TC1	59732	904	1.2	668	0.92	822	1.1	442	0.61	441	0.56	470	0.59	633.8981	0.181451	-5.56571	**-1.0099**	**Down**	**2.6E-08**	**5.6E-07**	cellular_cc	_
ENSG000	SH3YL1	48669	90	0.1	111	0.12	76	0.08	215	0.24	436	0.45	298	0.3	191.5812	0.25556	5.682658	**1.45226**	**Ups**	**1.3E-08**	**3E-07**	biological_	_
ENSG000	SHANK3	58884	225	0.55	163	0.42	183	0.45	19	0.05	58	0.14	45	0.1	119.6132	0.300961	-7.63983	**-2.2993**	**Down**	**2.2E-14**	**1.2E-12**	biological_	K15009
ENSG000	SHF	33962	25	0.06	18	0.04	15	0.04	64	0.16	63	0.14	65	0.15	39.83205	0.311124	4.489821	**1.39689**	**Ups**	**7.1E-06**	**8.7E-05**	biological_	_
ENSG000	SHOX2	9345	49	0.16	39	0.14	45	0.15	111	0.38	151	0.48	98	0.31	78.45466	0.256922	4.471519	**1.14883**	**Ups**	**7.8E-06**	**9.4E-05**	molecular_	K09331
ENSG000	SHROOM	162988	71	0.18	43	0.11	45	0.12	1	0	13	0.03	2	0	30.50498	0.46356	-6.11641	**-2.8353**	**Down**	**9.6E-10**	**2.7E-08**	biological_	K18625
ENSG000	SIGLEC22	1009	8	0.23	8	0.24	4	0.12	1	0.03	1	0.03	0	0	3.864289	0.575222	-3.20733	**-1.8449**	**Down**	**0.00134**	**0.00801**	_	K06473
ENSG000	SIK2	128463	2438	6.27	2411	6.47	2267	5.87	4808	12.86	7520	18.66	4902	11.89	3874.49	0.167037	6.103366	**1.01949**	**Ups**	**1E-09**	**2.9E-08**	molecular_	K16311
ENSG000	SIPA1L1	420781	655	1.02	677	1.1	697	1.09	1524	2.47	3330	5.01	1575	2.32	1322.274	0.253688	5.283226	**1.34029**	**Ups**	**1.3E-07**	**2.4E-06**	molecular_	K17701
ENSG000	SKAP2	328178	314	1.18	424	1.67	335	1.27	742	2.91	974	3.55	840	2.99	580.387	0.176718	5.662725	**1.0007**	**Ups**	**1.5E-08**	**3.3E-07**	molecular_	_
ENSG000	SLC13A4	48022	10	0.02	7	0.02	11	0.02	20	0.05	33	0.07	24	0.05	16.61281	0.395242	2.688236	**1.0625**	**Ups**	**0.00718**	**0.03108**	cellular_cc	K14444
ENSG000	SLC15A3	15447	1	0	3	0.01	1	0	2	0.01	16	0.04	15	0.04	5.70794	0.56669	2.565914	**1.45408**	**Ups**	**0.01029**	**0.0411**	molecular_	K14638
ENSG000	SLC16A12	126348	0	0	0	0	1	0	5	0.02	9	0.03	7	0.02	3.341179	0.580963	3.176118	**1.84521**	**Ups**	**0.00149**	**0.00878**	cellular_cc	K11810
ENSG000	SLC16A3	32733	35564	52.19	27727	42.43	31555	46.57	15540	23.72	8698	12.31	14634	20.25	22959.54	0.266943	-5.20847	**-1.3904**	**Down**	**1.9E-07**	**3.4E-06**	cellular_cc	K08180
ENSG000	SLC16A4	28235	41	0.17	34	0.14	35	0.14	239	1.01	211	0.83	236	0.9	125.4798	0.247534	9.361847	**2.31738**	**Ups**	**7.8E-21**	**7.7E-19**	cellular_cc	K08181
ENSG000	SLC16A8	5960	8	0.09	9	0.1	8	0.09	15	0.17	74	0.77	13	0.13	19.24468	0.503013	2.592098	**1.30386**	**Ups**	**0.00954**	**0.03886**	cellular_cc	K08185
ENSG000	SLC16A9	85238	27	0.15	31	0.18	29	0.16	0	0	2	0.01	0	0	15.78152	0.509204	-7.25115	**-3.6923**	**Down**	**4.1E-13**	**1.9E-11**	cellular_cc	K08186
ENSG000	SLC17A7	12960	2	0.01	0	0	0	0	6	0.02	8	0.03	6	0.02	3.394104	0.579018	2.704827	**1.56614**	**Ups**	**0.00683**	**0.02989**	molecular_	K12302
ENSG000	SLC18A2	38338	163	0.97	313	1.95	110	0.66	465	2.89	517	2.98	613	3.45	348.34	0.32515	3.444791	**1.12007**	**Ups**	**0.00057**	**0.00394**	biological_	K08155
ENSG000	SLC19A1	50840	3028	5.3	2392	4.37	2896	5.1	1289	2.35	636	1.07	1208	1.99	1972.107	0.289706	-5.12537	**-1.4849**	**Down**	**3E-07**	**5.1E-06**	molecular_	K06823
ENSG000	SLC19A2	22095	311	1.31	604	2.66	208	0.88	700	3.07	1470	5.99	837	3.33	650.2269	0.344986	3.062548	**1.05654**	**Ups**	**0.00219**	**0.01205**	molecular_	K14610
ENSG000	SLC1A2	168858	1	0	0	0	6	0.01	18	0.02	27	0.03	13	0.01	10.05462	0.528376	3.67449	**1.94151**	**Ups**	**0.00024**	**0.00187**	molecular_	K05613
ENSG000	SLC20A1	17971	5980	16.56	5423	15.66	5801	16.16	3149	9.07	3288	8.79	3165	8.27	4537.341	0.130974	-7.95556	**-1.042**	**Down**	**1.8E-15**	**1.1E-13**	molecular_	K14640
ENSG000	SLC22A1	36930	27	0.19	34	0.24	38	0.26	22	0.16	18	0.12	9	0.06	25.30504	0.383348	-2.79204	**-1.0703**	**Down**	**0.00524**	**0.02436**	molecular_	K08198
ENSG000	SLC22A18	15961	30	0.27	12	0.11	25	0.22	8	0.07	5	0.04	8	0.07	15.17712	0.448587	-3.41703	**-1.5328**	**Down**	**0.00063**	**0.00428**	molecular_	_
ENSG000	SLC22A2	106578	8	0.03	10	0.03	11	0.04	0	0	0	0	0	0	5.174086	0.574777	-4.7938	**-2.7554**	**Down**	**1.6E-06**	**2.4E-05**	molecular_	K08199
ENSG000	SLC22A3	106715	432	1.41	429	1.46	372	1.22	21	0.07	120	0.38	69	0.21	250.0863	0.370382	-6.77748	**-2.5103**	**Down**	**1.2E-11**	**4.7E-10**	molecular_	K08200
ENSG000	SLC24A1	49630	123	0.19	117	0.19	128	0.2	231	0.37	419	0.62	264	0.38	203.0978	0.225407	4.567085	**1.02945**	**Ups**	**5E-06**	**6.3E-05**	molecular_	K13749
ENSG000	SLC25A42	48890	125	0.6	117	0.58	101	0.49	378	1.88	286	1.32	366	1.65	220.8341	0.237955	5.574047	**1.32637**	**Ups**	**2.5E-08**	**5.4E-07**	molecular_	K15085
ENSG000	SLC27A2	54200	4190	23.93	4105	24.45	3923	22.53	1138	6.76	2830	15.59	2041	10.99	3070.436	0.236862	-5.17544	**-1.2259**	**Down**	**2.3E-07**	**4E-06**	biological_	K08746
ENSG000	SLC27A6	495630	10	0.04	34	0.15	11	0.05	162	0.72	365	1.5	237	0.95	123.9357	0.386415	7.982242	**3.08446**	**Ups**	**1.4E-15**	**8.9E-14**	biological_	K08749
ENSG000	SLC29A4	32502	30	0.09	34	0.11	34	0.1	117	0.37	83	0.25	113	0.33	66.12326	0.295293	4.646822	**1.37218**	**Ups**	**3.4E-06**	**4.5E-05**	molecular_	K03323
ENSG000	SLC2A13	351069	215	0.5	266	0.64	192	0.45	277	0.67	1224	2.75	436	0.96	402.7083	0.374873	2.919168	**1.09432**	**Ups**	**0.00351**	**0.01768**	cellular_cc	K08150
ENSG000	SLC2A4RG	3645	4244	24.55	3115	18.8	3819	22.22	2227	13.4	1437	8.02	2206	12.04	2903.359	0.245555	-4.35298	**-1.0689**	**Down**	**1.3E-05**	**0.00015**	molecular_	_
ENSG000	SLC2A6	8043	211	1.13	206	1.15	217	1.17	769	4.27	340	1.75	603	3.04	383.26	0.316099	3.662854	**1.15783**	**Ups**	**0.00025**	**0.00194**	molecular_	K08144
ENSG000	SLC30A4	43197	106	0.36	112	0.4	99	0.34	178	0.63	420	1.38	225	0.72	179.1382	0.276225	3.819588	**1.05506**	**Ups**	**0.00013**	**0.00113**	molecular_	K14691
ENSG000	SLC35C1	8944	695	1.93	506	1.46	601	1.68	188	0.54	258	0.69	222	0.58	422.0606	0.181288	-8.91924	**-1.617**	**Down**	**4.7E-19**	**3.9E-17**	cellular_cc	K15279
ENSG000	SLC35F3	419584	406	2.22	463	2.64	337	1.85	182	1.04	163	0.86	189	0.98	297.0771	0.211889	-6.3122	**-1.3375**	**Down**	**2.8E-10**	**8.5E-09**	biological_	K15288
ENSG000	SLC38A11	59340	1	0	2	0.01	0	0	8	0.02	7	0.02	6	0.02	3.784115	0.571792	2.552071	**1.45925**	**Ups**	**0.01071**	**0.04236**	biological_	K14997
ENSG000	SLC38A4	67646	10	0.05	16	0.08	15	0.07	4	0.02	7	0.03	5	0.02	9.73104	0.465073	-2.73025	**-1.2698**	**Down**	**0.00633**	**0.02822**	cellular_cc	K14991
ENSG000	SLC39A11	446764	316	0.79	247	0.64	247	0.62	812	2.1	609	1.46	777	1.83	485.1944	0.228409	5.22419	**1.19325**	**Ups**	**1.8E-07**	**3.2E-06**	biological_	K14717
ENSG000	SLC43A1	31253	242	1	187	0.8	203	0.84	958	4.11	551	2.19	831	3.23	478.8324	0.27558	5.815022	**1.6025**	**Ups**	**6.1E-09**	**1.5E-07**	cellular_cc	K08228
ENSG000	SLC44A2	42103	1804	3.74	1382	2.99	1594	3.32	5087	10.97	4608	9.22	4969	9.72	3112.722	0.176328	7.816713	**1.3783**	**Ups**	**5.4E-15**	**3.2E-13**	molecular_	K15377
ENSG000	SLC45A1	26342	6	0.03	6	0.03	9	0.04	20	0.1	22	0.1	36	0.16	15.65322	0.426754	3.277089	**1.39851**	**Ups**	**0.00105**	**0.00656**	biological_	K15378
ENSG000	SLC45A3	22609	361	2.51	328	2.38	279	1.95	149	1.08	167	1.12	147	0.96	243.1114	0.186867	-6.67715	**-1.2477**	**Down**	**2.4E-11**	**8.9E-10**	cellular_cc	K15379
ENSG000	SLC4A3	14654	58	0.19	31	0.11	57	0.19	121	0.42	140	0.45	103	0.33	81.71051	0.287056	3.584648	**1.029**	**Ups**	**0.00034**	**0.00251**	molecular_	K13856
ENSG000	SLC4A4	384802	1	0	1	0	0	0	3	0.01	8	0.02	7	0.01	3.031389	0.581005	2.483287	**1.4428**	**Ups**	**0.01302**	**0.0493**	molecular_	K13575
ENSG000	SLC52A2	7138	5633	15.87	4338	12.74	5074	14.37	3167	9.28	1523	4.14	3030	8.05	3889.481	0.293936	-3.62353	**-1.0651**	**Down**	**0.00029**	**0.00221**	molecular_	_
ENSG000	SLC52A3	8408	39	0.2	41	0.21	37	0.19	9	0.05	3	0.01	11	0.05	24.45735	0.399377	-5.49192	**-2.1933**	**Down**	**4E-08**	**8.3E-07**	cellular_cc	K17985
ENSG000	SLC5A12	56409	0	0	0	0	0	0	8	0.02	4	0.01	5	0.01	2.685098	0.582376	3.202814	**1.86524**	**Ups**	**0.00136**	**0.00812**	molecular_	K14388
ENSG000	SLC5A2	7859	10	0.03	7	0.02	5	0.02	16	0.06	20	0.07	41	0.13	15.59467	0.451766	2.847747	**1.28652**	**Ups**	**0.0044**	**0.02117**	molecular_	_
ENSG000	SLC6A12	24494	625	1.46	1012	2.47	782	1.84	85	0.21	24	0.05	76	0.17	461.0259	0.377902	-9.03064	**-3.4127**	**Down**	**1.7E-19**	**1.5E-17**	molecular_	K05039
ENSG000	SLC6A14	24836	19	0.1	23	0.13	14	0.08	29	0.17	113	0.6	69	0.36	40.9898	0.400379	3.541483	**1.41794**	**Ups**	**0.0004**	**0.00289**	biological_	K05038
ENSG000	SLC6A16	35588	37	0.17	19	0.09	32	0.15	105	0.51	75	0.33	70	0.31	54.65905	0.338779	3.514249	**1.19055**	**Ups**	**0.00044**	**0.00317**	molecular_	K05048
ENSG000	SLC7A8	58380	311	0.53	270	0.48	354	0.61	1156	2.05	858	1.41	959	1.54	628.9049	0.231311	6.102681	**1.41162**	**Ups**	**1E-09**	**2.9E-08**	molecular_	K13781
ENSG000	SLC8A3	144854	1	0	3	0.01	0	0	9	0.02	15	0.03	13	0.02	6.295786	0.54382	3.527658	**1.91841**	**Ups**	**0.00042**	**0.00303**	molecular_	K05849
ENSG000	SLCO1B3	279405	382	2.29	497	3.11	269	1.62	94	0.59	317	1.84	223	1.26	297.9761	0.336952	-3.02846	**-1.0204**	**Down**	**0.00246**	**0.01328**	molecular_	K05043
ENSG000	SLCO3A1	318741	925	1.67	842	1.58	901	1.63	3966	7.43	3606	6.27	3661	6.22	2208.662	0.164886	11.09425	**1.82928**	**Ups**	**1.3E-28**	**2.3E-26**	molecular_	K14353
ENSG000	SLCO4A1	43341	4450	18.12	4046	17.18	4146	16.98	1941	8.22	1687	6.63	1794	6.89	3088.57	0.168968	-8.25722	**-1.3952**	**Down**	**1.5E-16**	**1E-14**	molecular_	K14354
ENSG000	SLFN13	13742	12	0.01	10	0.01	3	0	0	0	1	0	0	0	4.569061	0.578932	-3.79755	**-2.1985**	**Down**	**0.00015**	**0.00122**	molecular_	_
ENSG000	SLITRK6	6699	7	0.04	17	0.11	7	0.04	38	0.24	28	0.16	40	0.22	21.97503	0.411805	3.244063	**1.33592**	**Ups**	**0.00118**	**0.00721**	biological_	_
ENSG000	SLPI	2326	4429	191.02	4272	192.17	4304	186.7	1756	78.78	1502	62.5	2359	95.96	3181.26	0.205428	-6.69287	**-1.3749**	**Down**	**2.2E-11**	**8.1E-10**	molecular_	_
ENSG000	SMAD3	131433	1531	3	1292	2.64	1407	2.77	3197	6.51	4793	9.05	3474	6.41	2490.178	0.153666	7.712688	**1.18518**	**Ups**	**1.2E-14**	**6.9E-13**	biological_	K04500
ENSG000	SMAD9	75935	24	0.09	32	0.13	26	0.1	113	0.44	624	2.27	193	0.69	149.5438	0.427082	6.291632	**2.68704**	**Ups**	**3.1E-10**	**9.6E-09**	biological_	K16791
ENSG000	SMC1B	69557	1	0.01	2	0.01	3	0.02	21	0.12	15	0.08	7	0.04	7.825563	0.537349	3.303333	**1.77504**	**Ups**	**0.00096**	**0.00608**	cellular_cc	K06636
ENSG000	SMC5-AS	64870	5	0.07	7	0.1	9	0.13	24	0.35	49	0.67	19	0.25	17.533	0.440301	3.527654	**1.55323**	**Ups**	**0.00042**	**0.00303**	_	_
ENSG000	SMIM6	1735	19	0.27	11	0.16	21	0.3	1	0.01	1	0.01	0	0	9.390396	0.538782	-5.49755	**-2.962**	**Down**	**3.9E-08**	**8E-07**	cellular_cc	_
ENSG000	SMPDL3A	20551	225	1.75	241	1.96	170	1.33	332	2.69	706	5.31	577	4.24	354.9284	0.247692	4.215674	**1.04419**	**Ups**	**2.5E-05**	**0.00026**	molecular_	K01128
ENSG000	SMS	67108	20673	154.8	18792	146.76	22845	172.04	8205	63.9	14118	101.99	13599	96.04	16515.02	0.185819	-5.42922	**-1.0088**	**Down**	**5.7E-08**	**1.1E-06**	molecular_	K00802
ENSG000	SNAI2	4051	524	3.6	499	3.57	503	3.47	1378	9.84	1034	6.85	1219	7.89	835.7782	0.209636	4.855305	**1.01785**	**Ups**	**1.2E-06**	**1.8E-05**	biological_	K05706
ENSG000	SNAI3-AS	23889	56	0.14	53	0.14	62	0.16	163	0.44	124	0.31	146	0.35	97.6935	0.261406	4.123348	**1.07787**	**Ups**	**3.7E-05**	**0.00037**	_	_
ENSG000	SNAP25	88589	102	0.71	74	0.53	100	0.7	404	2.91	827	5.52	592	3.86	322.3108	0.245874	9.600516	**2.36052**	**Ups**	**8E-22**	**8.4E-20**	molecular_	K18211
ENSG000	SNAP25-A	346805	4	0.02	1	0	7	0.03	22	0.09	30	0.11	19	0.07	12.94553	0.469652	3.916382	**1.83934**	**Ups**	**9E-05**	**0.0008**	_	_
ENSG000	SNCAIP	152866	6	0.01	3	0.01	2	0	42	0.08	1	0	32	0.06	14.13744	0.571905	2.672598	**1.52847**	**Ups**	**0.00753**	**0.03225**	molecular_	K04558
ENSG000	SNED1	96729	62	0.07	51	0.06	54	0.07	74	0.09	298	0.35	110	0.13	100.2849	0.381214	2.881618	**1.09851**	**Ups**	**0.00396**	**0.01943**	molecular_	_
ENSG000	SNORA26	122	12	2.53	14	3.08	14	2.97	2	0.44	6	1.22	6	1.19	9.208709	0.474044	-2.91036	**-1.3796**	**Down**	**0.00361**	**0.01807**	_	_
ENSG000	SNORA40	121	2	0.42	0	0	4	0.85	9	1.99	17	3.48	14	2.81	7.081644	0.531201	3.221163	**1.71109**	**Ups**	**0.00128**	**0.00771**	_	_
ENSG000	SNORA75	137	10	1.88	7	1.37	5	0.94	33	6.44	16	2.9	19	3.36	14.66663	0.456865	2.560178	**1.16966**	**Ups**	**0.01046**	**0.04163**	_	_
ENSG000	SNORA76	507	199	9.89	227	11.77	180	9	93	4.81	51	2.45	111	5.21	147.7063	0.306993	-4.35152	**-1.3359**	**Down**	**1.4E-05**	**0.00015**	_	_
ENSG000	SNORA80	136	1	0.19	1	0.2	1	0.19	6	1.18	8	1.46	7	1.25	3.729115	0.568518	2.61007	**1.48387**	**Ups**	**0.00905**	**0.03723**	_	_
ENSG000	SNORD10	76	16	5.41	26	9.17	17	5.78	57	20.05	34	11.1	58	18.5	33.77703	0.368138	2.744664	**1.01042**	**Ups**	**0.00606**	**0.02725**	_	_
ENSG000	SNORD11	88	1	0.29	6	1.83	1	0.29	17	5.17	14	3.95	9	2.48	7.653844	0.53348	2.730147	**1.45648**	**Ups**	**0.00633**	**0.02822**	_	_
ENSG000	SNORD12	91	7	1.98	7	2.06	12	3.41	29	8.52	40	10.9	18	4.8	17.88898	0.422282	3.023015	**1.27657**	**Ups**	**0.0025**	**0.01345**	_	_
ENSG000	snoU13	86	0	0	0	0	0	0	4	1.24	3	0.87	7	1.97	2.15726	0.578241	2.862816	**1.6554**	**Ups**	**0.0042**	**0.02039**	_	_
ENSG000	snoU13	107	0	0	0	0	0	0	4	1	6	1.39	3	0.68	1.97176	0.576658	2.768365	**1.5964**	**Ups**	**0.00563**	**0.02578**	_	_
ENSG000	SNRPGP1	225	359	41.01	314	37.41	294	33.78	190	22.58	118	13.01	213	22.95	253.1591	0.271481	-3.7752	**-1.0249**	**Down**	**0.00016**	**0.00133**	_	K11099
ENSG000	SNX19	41074	628	1.01	504	0.85	564	0.92	1060	1.78	2651	4.13	1309	1.99	1048.52	0.268926	4.587648	**1.23374**	**Ups**	**4.5E-06**	**5.8E-05**	molecular_	K17930
ENSG000	SOBP	170196	29	0.11	24	0.09	34	0.13	283	1.1	122	0.44	186	0.66	109.1568	0.364695	6.318927	**2.30448**	**Ups**	**2.6E-10**	**8.2E-09**	biological_	_
ENSG000	SOCS2	13674	179	0.3	217	0.38	160	0.27	94	0.16	105	0.17	90	0.14	143.3086	0.217637	-5.15097	**-1.121**	**Down**	**2.6E-07**	**4.5E-06**	biological_	K04695
ENSG000	SOD3	10931	4	0.04	2	0.02	5	0.05	0	0	0	0	0	0	1.959274	0.575165	-2.83502	**-1.6306**	**Down**	**0.00458**	**0.02182**	biological_	K16627
ENSG000	SOHLH2	129635	37	0.22	48	0.3	42	0.25	244	1.5	111	0.63	228	1.27	114.36	0.348025	5.217526	**1.81583**	**Ups**	**1.8E-07**	**3.3E-06**	molecular_	_
ENSG000	SORBS2	371209	105	0.08	136	0.11	115	0.09	813	0.68	2338	1.81	1088	0.82	691.0219	0.304989	10.03683	**3.06112**	**Ups**	**1.1E-23**	**1.3E-21**	molecular_	_

ENSG000	**SORL1**	181491	134	0.15	146	0.17	124	0.14	838	1	4618	5.11	1387	1.5	1064.571	0.398298	8.211489	**3.27062**	**Ups**	**2.2E-16**	**1.5E-14**	molecular_	_
ENSG000	**SOS1-IT1**	1579	11	0.12	28	0.31	8	0.09	45	0.5	77	0.8	74	0.75	37.92943	0.395044	3.992457	**1.5772**	**Ups**	**6.5E-05**	**0.00061**	_	_
ENSG000	**SOS2**	114430	357	1.1	521	1.67	293	0.91	732	2.34	1789	5.31	850	2.46	710.3827	0.304276	3.857428	**1.17372**	**Ups**	**0.00011**	**0.00099**	molecular_	K03099
ENSG000	**SOWAHC**	4653	445	2.46	621	3.58	335	1.86	1144	6.57	1369	7.3	1195	6.23	815.9763	0.23045	4.925007	**1.13497**	**Ups**	**8.4E-07**	**1.3E-05**	_	K12581
ENSG000	**SOX18**	1919	63	0.94	46	0.72	69	1.04	18	0.28	7	0.1	11	0.15	37.42551	0.377311	-5.81409	**-2.1937**	**Down**	**6.1E-09**	**1.5E-07**	biological_	K09270
ENSG000	**SOX6**	773144	34	0.04	45	0.05	39	0.04	167	0.2	737	0.82	244	0.26	188.5949	0.396796	6.527754	**2.59019**	**Ups**	**6.7E-11**	**2.3E-09**	biological_	K09269
ENSG000	**SOX7**	116080	44	0.23	62	0.33	60	0.31	7	0.04	7	0.03	15	0.07	34.05254	0.371316	-6.51231	**-2.4181**	**Down**	**7.4E-11**	**2.5E-09**	biological_	K09270
ENSG000	**SP110**	58436	24	0.06	23	0.06	27	0.07	68	0.18	273	0.66	176	0.42	89.17566	0.377922	5.886936	**2.22481**	**Ups**	**3.9E-09**	**1E-07**	molecular_	_
ENSG000	**SP4**	86789	44	0.11	170	0.43	52	0.13	202	0.51	585	1.38	285	0.66	206.2293	0.428622	3.404744	**1.45935**	**Ups**	**0.00066**	**0.00446**	molecular_	K09194
ENSG000	**SP6**	10962	11	0.04	13	0.05	15	0.05	4	0.01	8	0.03	3	0.01	9.206224	0.47512	-2.67124	**-1.2692**	**Down**	**0.00756**	**0.03234**	molecular_	K09196
ENSG000	**SPAG16**	1126113	48	0.12	64	0.17	44	0.11	0	0	1	0	0	0	27.93236	0.499474	-9.14636	**-4.5684**	**Down**	**5.9E-20**	**5.4E-18**	cellular_cc	_
ENSG000	**SPAG4**	5158	164	1.03	141	0.92	206	1.3	470	3.06	322	1.94	520	3.07	294.8815	0.271616	4.028411	**1.09418**	**Ups**	**5.6E-05**	**0.00053**	molecular_	_
ENSG000	**SPATA13**	327269	54	0.06	47	0.05	24	0.03	1	0	2	0	1	0	22.78917	0.489577	-7.59053	**-3.7162**	**Down**	**3.2E-14**	**1.7E-12**	molecular_	_
ENSG000	**SPATA17**	240373	133	0.45	163	0.58	135	0.46	85	0.3	40	0.13	67	0.22	107.0708	0.319256	-3.88638	**-1.2407**	**Down**	**0.0001**	**0.00089**	molecular_	_
ENSG000	**SPATC1L**	23329	574	4.95	368	3.31	576	4.99	303	2.72	141	1.17	284	2.31	384.6153	0.326107	-3.47961	**-1.1347**	**Down**	**0.0005**	**0.00354**	molecular_	_
ENSG000	**SPHK1**	11277	2252	6.22	1665	4.79	2004	5.56	581	1.67	320	0.85	688	1.79	1301.313	0.290103	-6.72053	**-1.9496**	**Down**	**1.8E-11**	**6.8E-10**	molecular_	K04718
ENSG000	**SPINK13**	18075	2073	36.7	2161	39.9	1839	32.74	125	2.3	735	12.55	450	7.51	1272.232	0.376279	-5.85805	**-2.2043**	**Down**	**4.7E-09**	**1.2E-07**	molecular_	_
ENSG000	**SPINK6**	12344	184	4.91	166	4.62	192	5.15	0	0	3	0.08	2	0.05	97.25054	0.410252	-13.7389	**-5.6364**	**Down**	**5.9E-43**	**2.2E-40**	molecular_	_
ENSG000	**SPINT1**	14190	7	0.02	6	0.02	10	0.03	34	0.1	22	0.06	32	0.09	17.82177	0.418025	3.509127	**1.4669**	**Ups**	**0.00045**	**0.00322**	molecular_	K15619
ENSG000	**SPINT2**	48580	83	0.22	53	0.15	57	0.15	227	0.62	167	0.42	278	0.69	138.3401	0.291203	5.125151	**1.49246**	**Ups**	**3E-07**	**5.1E-06**	molecular_	_
ENSG000	**SPNS2**	40198	83	0.29	80	0.29	66	0.23	28	0.1	32	0.11	29	0.1	54.36553	0.269215	-5.54951	**-1.494**	**Down**	**2.9E-08**	**6.1E-07**	biological_	_
ENSG000	**SPOCK1**	623082	127	0.43	120	0.42	128	0.43	4	0.01	52	0.17	16	0.05	77.08337	0.454762	-4.77908	**-2.1733**	**Down**	**1.8E-06**	**2.5E-05**	biological_	K08136
ENSG000	**SPON2**	42031	1808	3.69	1444	3.07	1796	3.68	752	1.6	361	0.71	683	1.31	1181.01	0.296199	-5.25183	**-1.5556**	**Down**	**1.5E-07**	**2.8E-06**	cellular_cc	_
ENSG000	**SPRED1**	104924	199	0.59	330	1.02	117	0.35	293	0.9	1118	3.19	599	1.67	410.4244	0.388552	3.058209	**1.18827**	**Ups**	**0.00223**	**0.0122**	biological_	K04703
ENSG000	**SPRY1**	6961	86	0.28	60	0.2	62	0.2	199	0.67	258	0.81	200	0.61	137.0763	0.224664	6.14698	**1.381**	**Ups**	**7.9E-10**	**2.3E-08**	biological_	K04704
ENSG000	**SPTBN5**	45931	15	0.03	14	0.03	18	0.04	51	0.11	97	0.19	51	0.1	38.27228	0.34509	4.809303	**1.65964**	**Ups**	**1.5E-06**	**2.2E-05**	molecular_	K06115
ENSG000	**SPTLC3**	157785	141	0.46	127	0.43	137	0.45	310	1.06	468	1.48	333	1.03	240.4394	0.191722	6.206354	**1.18989**	**Ups**	**5.4E-10**	**1.6E-08**	molecular_	K00654
ENSG000	**SRGAP3**	382463	59	0.06	40	0.04	43	0.04	114	0.12	148	0.14	106	0.1	81.32506	0.255738	4.273448	**1.09288**	**Ups**	**1.9E-05**	**0.00021**	molecular_	K07526
ENSG000	**SRM**	5441	8191	62.78	6136	49.05	8285	63.86	3839	30.6	2750	20.33	4558	32.95	5753.338	0.24215	-4.81189	**-1.1652**	**Down**	**1.5E-06**	**2.2E-05**	molecular_	K00797
ENSG000	**SRMS**	6695	20	0.34	14	0.25	26	0.44	2	0.04	2	0.03	5	0.08	12.06679	0.483361	-4.67669	**-2.2605**	**Down**	**2.9E-06**	**4E-05**	molecular_	K08895
ENSG000	**SRP68P2**	905	0	0	0	0	0	0	2	0.21	9	0.88	4	0.38	2.192659	0.577178	2.836881	**1.63739**	**Ups**	**0.00456**	**0.02173**	_	K03107
ENSG000	**SRPX2**	27082	31	0.28	31	0.29	33	0.3	8	0.08	0	0	5	0.04	19.05603	0.463546	-5.20329	**-2.412**	**Down**	**2E-07**	**3.5E-06**	biological_	K19408
ENSG000	**SRRM3**	85390	2	0.01	4	0.01	1	0	8	0.02	14	0.04	14	0.04	6.669402	0.523819	2.862297	**1.49932**	**Ups**	**0.00421**	**0.02041**	_	_
ENSG000	**SRRM4**	181557	79	0.23	92	0.28	67	0.19	230	0.69	539	1.5	487	1.33	229.8851	0.281335	7.156501	**2.01337**	**Ups**	**8.3E-13**	**3.7E-11**	molecular_	_
ENSG000	**SRXN1**	6756	497	4.74	518	5.15	341	3.27	154	1.53	452	4.15	145	1.3	352.1522	0.349152	-2.88496	**-1.0073**	**Down**	**0.00391**	**0.01927**	molecular_	K12260
ENSG000	**SSBP2**	338777	195	0.29	225	0.35	177	0.27	500	0.78	857	1.24	668	0.95	411.644	0.197872	7.467463	**1.4776**	**Ups**	**8.2E-14**	**4.2E-12**	molecular_	_
ENSG000	**SSTR1**	5069	713	4.27	607	3.79	568	3.42	171	1.06	276	1.59	176	0.99	430.0042	0.200309	-8.87101	**-1.7769**	**Down**	**7.3E-19**	**6E-17**	molecular_	K04217
ENSG000	**SSTR2**	6035	5	0.04	2	0.02	1	0.01	38	0.3	9	0.07	30	0.21	13.72089	0.532163	3.978949	**2.11745**	**Ups**	**6.9E-05**	**0.00064**	molecular_	K04218
ENSG000	**SSTR5**	8699	5	0.02	4	0.02	9	0.04	1	0	0	0	1	0	3.536553	0.578542	-2.95225	**-1.708**	**Down**	**0.00315**	**0.01622**	molecular_	K04221
ENSG000	**SSUH2**	125641	3	0.01	6	0.01	5	0.01	24	0.05	21	0.04	40	0.08	15.55987	0.453069	4.245891	**1.92368**	**Ups**	**2.2E-05**	**0.00023**	cellular_cc	_
ENSG000	**ST18**	350121	23	0.03	44	0.06	17	0.02	5	0.01	11	0.01	5	0.01	18.10817	0.452575	-4.01288	**-1.8161**	**Down**	**0.00006**	**0.00056**	molecular_	_
ENSG000	**ST5**	217601	251	0.21	211	0.19	233	0.2	931	0.82	425	0.35	630	0.5	437.8033	0.313622	3.947226	**1.23794**	**Ups**	**7.9E-05**	**0.00072**	molecular_	_
ENSG000	**STAB1**	29158	6	0.01	4	0.01	2	0	12	0.02	19	0.03	12	0.02	8.630833	0.486769	2.549343	**1.24094**	**Ups**	**0.01079**	**0.04261**	molecular_	K19020
ENSG000	**STARD4**	16558	720	1.33	1159	2.23	686	1.27	2094	4.01	6929	12.32	3284	5.71	2271.664	0.32869	5.523631	**1.81556**	**Ups**	**3.3E-08**	**7E-07**	biological_	_
ENSG000	**STARD9**	145323	190	0.16	222	0.2	213	0.18	370	0.33	867	0.72	387	0.31	353.4654	0.284765	3.684767	**1.04929**	**Ups**	**0.00023**	**0.0018**	molecular_	K16491
ENSG000	**STK10**	146314	1993	3.53	1654	3.05	1804	3.21	847	1.56	901	1.54	791	1.32	1359.834	0.152633	-8.47379	**-1.2934**	**Down**	**2.4E-17**	**1.8E-15**	molecular_	K08837
ENSG000	**STON1**	69504	77	0.11	110	0.17	106	0.15	1460	2.19	1392	1.94	1415	1.93	709.5255	0.208858	16.90409	**3.53055**	**Ups**	**4.2E-64**	**3.2E-61**	biological_	K12581
ENSG000	**STON2**	175810	241	0.61	231	0.61	247	0.62	103	0.27	125	0.3	105	0.25	178.9207	0.183163	-7.09401	**-1.2994**	**Down**	**1.3E-12**	**5.7E-11**	molecular_	_
ENSG000	**STRC**	118863	0	0	0	0	0	0	11	0.02	2	0	10	0.02	3.676769	0.583118	3.462279	**2.01892**	**Ups**	**0.00054**	**0.00375**	cellular_cc	_
ENSG000	**STRCP1**	18697	4	0.02	0	0	1	0	9	0.05	15	0.07	7	0.03	5.561319	0.555677	2.718088	**1.51038**	**Ups**	**0.00657**	**0.029**	cellular_cc	_
ENSG000	**STX11**	37845	19	0.25	13	0.18	18	0.24	63	0.87	222	2.86	153	1.93	73.50374	0.37948	6.618868	**2.51173**	**Ups**	**3.6E-11**	**1.3E-09**	molecular_	K08487
ENSG000	**STXBP5L**	516690	2	0	1	0	0	0	4	0.01	17	0.03	4	0.01	4.174526	0.57878	2.523168	**1.46036**	**Ups**	**0.01163**	**0.04511**	cellular_cc	K08518
ENSG000	**SUGCT**	725788	20	0.09	22	0.1	24	0.11	146	0.69	98	0.43	153	0.66	73.51131	0.317853	6.901452	**2.19365**	**Ups**	**5.2E-12**	**2.1E-10**	_	K18703
ENSG000	**SULF1**	194292	12	0.01	15	0.02	9	0.01	65	0.08	98	0.12	69	0.08	41.56179	0.330172	6.77309	**2.23629**	**Ups**	**1.3E-11**	**4.9E-10**	biological_	K14607
ENSG000	**SUSD2**	7852	738	2.64	572	2.13	653	2.35	269	1	216	0.75	266	0.9	465.5449	0.211019	-7.27572	**-1.5353**	**Down**	**3.5E-13**	**1.6E-11**	molecular_	_
ENSG000	**SUSD5**	69171	49	0.23	45	0.22	42	0.2	134	0.66	255	1.16	165	0.74	107.4292	0.260043	6.499775	**1.69022**	**Ups**	**8E-11**	**2.7E-09**	molecular_	_
ENSG000	**SV2B**	201360	25	0.03	37	0.04	30	0.03	206	0.22	314	0.32	220	0.22	128.491	0.256007	10.27934	**2.63158**	**Ups**	**8.7E-25**	**1.2E-22**	cellular_cc	K06258
ENSG000	**SVEP1**	214630	1018	1.5	899	1.38	1049	1.55	1790	2.74	6021	8.55	2407	3.34	2032.637	0.31835	4.359367	**1.38781**	**Ups**	**1.3E-05**	**0.00015**	molecular_	K17495
ENSG000	**SVOPL**	107068	0	0	0	0	0	0	39	0.14	24	0.08	38	0.12	15.76055	0.541764	7.117638	**3.85608**	**Ups**	**1.1E-12**	**4.8E-11**	cellular_cc	_
ENSG000	**SYCE2**	20491	28	0.35	20	0.26	26	0.33	97	1.26	87	1.05	107	1.26	58.0254	0.288152	5.722067	**1.64882**	**Ups**	**1.1E-08**	**2.4E-07**	cellular_cc	K19535
ENSG000	**SYCP2**	70093	11	0.03	22	0.07	12	0.04	26	0.08	122	0.36	44	0.13	36.02425	0.450642	3.272129	**1.47456**	**Ups**	**0.00107**	**0.00666**	cellular_cc	K19529
ENSG000	**SYDE2**	44174	85	0.28	112	0.38	83	0.27	272	0.93	776	2.46	286	0.89	247.2726	0.33902	5.308734	**1.79977**	**Ups**	**1.1E-07**	**2.1E-06**	;cellular_c	K17985
ENSG000	**SYNE2**	373483	1319	0.82	1519	0.99	1563	0.98	2552	1.65	6237	3.75	2568	1.51	2474.578	0.282186	3.678439	**1.038**	**Ups**	**0.00023**	**0.00184**	molecular_	K19346
ENSG000	**SYNE3**	58343	59	0.15	46	0.12	43	0.11	11	0.03	29	0.07	30	0.07	36.73272	0.342761	-3.49076	**-1.1965**	**Down**	**0.00048**	**0.00342**	molecular_	_
ENSG000	**SYNGR1**	35664	301	0.89	279	0.86	298	0.88	966	2.97	608	1.73	951	2.65	550.0709	0.253537	4.991108	**1.26543**	**Ups**	**6E-07**	**9.6E-06**	biological_	K04403
ENSG000	**SYT11**	25691	5	0.02	5	0.02	4	0.02	15	0.06	23	0.09	16	0.06	10.66844	0.455268	3.045876	**1.38669**	**Ups**	**0.00232**	**0.01264**	molecular_	K17985
ENSG000	**SYT15**	15957	70	0.22	75	0.25	85	0.27	274	0.9	565	1.72	272	0.81	207.3946	0.280964	6.75115	**1.89683**	**Ups**	**1.5E-11**	**5.6E-10**	molecular_	_
ENSG000	**SYTL4**	57623	554	1.3	618	1.52	552	1.31	110	0.27	119	0.27	124	0.28	361.2163	0.173235	-14.1479	**-2.4509**	**Down**	**1.9E-45**	**7.8E-43**	molecular_	K17598
ENSG000	**SYTL5**	122238	223	0.75	288	1.02	202	0.69	924	3.25	1799	5.87	994	3.17	684.9319	0.248174	8.195521	**2.03391**	**Ups**	**2.5E-16**	**1.7E-14**	molecular_	K17598
ENSG000	**TAC3**	18884	2	0.02	2	0.02	1	0.01	14	0.16	17	0.17	16	0.16	8.061863	0.515496	4.111036	**2.11922**	**Ups**	**3.9E-05**	**0.00039**	molecular_	K05240
ENSG000	**TACO1**	7495	3419	23.97	2860	20.92	3547	25.01	1833	13.37	1377	9.32	1995	13.19	2556.439	0.216296	-4.98264	**-1.0777**	**Down**	**6.3E-07**	**1E-05**	cellular_cc	K18189
ENSG000	**TAX1BP3**	5781	1652	30.84	1236	24.06	1537	28.86	859	16.68	586	10.55	848	14.93	1144.174	0.238579	-4.58181	**-1.0931**	**Down**	**4.6E-06**	**6E-05**	molecular_	_
ENSG000	**TBC1D12**	133427	335	1.12	323	1.12	227	0.76	647	2.25	1327	4.27	873	2.75	583.954	0.241969	5.679472	**1.37426**	**Ups**	**1.4E-08**	**3.1E-07**	_	_
ENSG000	**TBC1D2**	56605	2782	10.86	2283	9.3	2442	9.59	330	1.34	435	1.64	397	1.46	1512.603	0.140584	-20.4419	**-2.8738**	**Down**	**7.1E-93**	**1.3E-89**	;molecular	_
ENSG000	**TBC1D32**	255252	82	0.23	128	0.38	79	0.23	233	0.69	473	1.3	294	0.79	201.3987	0.278576	5.199552	**1.44847**	**Ups**	**2E-07**	**3.6E-06**	;cellular_c	_
ENSG000	**TBC1D8B**	73466	184	0.4	217	0.5	136	0.3	226	0.52	981	2.08	366	0.76	325.4356	0.377403	2.958257	**1.11645**	**Ups**	**0.00309**	**0.01599**	;molecular	_
ENSG000	**TBC1D9**	135356	531	2.28	571	2.55	431	1.86	1359	6.06	2540	10.5	1553	6.28	1092.442	0.217099	7.047374	**1.52998**	**Ups**	**1.8E-12**	**7.9E-11**	;molecular	_
ENSG000	**TBX19**	33387	24	0.09	18	0.07	16	0.06	68	0.27	58	0.21	54	0.19	38.18193	0.32382	4.038471	**1.30774**	**Ups**	**5.4E-05**	**0.00051**	molecular_	K10184
ENSG000	**TBX6**	6095	23	0.12	30	0.17	31	0.17	93	0.51	66	0.34	81	0.41	52.33949	0.311687	3.885955	**1.2112**	**Ups**	**0.0001**	**0.00089**	biological_	K10180
ENSG000	**TBXAS1**	243276	42	0.09	45	0.1	45	0.1	8	0.02	4	0.01	5	0.01	26.20572	0.392669	-6.95155	**-2.7297**	**Down**	**3.6E-12**	**1.5E-10**	molecular_	K01832
ENSG000	**TC2N**	87786	76	0.15	128	0.27	68	0.14	10	0.02	38	0.07	10	0.02	56.97601	0.415451	-5.10524	**-2.121**	**Down**	**3.3E-07**	**5.6E-06**	cellular_cc	K17287
ENSG000	**TCAP**	2369	12	0.11	10	0.1	8	0.08	28	0.28	38	0.35	37	0.33	20.98472	0.368905	3.730368	**1.37615**	**Ups**	**0.00019**	**0.00154**	biological_	_
ENSG000	**TCEB1P2**	409	121	9.37	110	8.88	96	7.48	49	3.95	55	4.11	70	5.11	84.76239	0.242558	-4.45028	**-1.0795**	**Down**	**8.6E-06**	**0.0001**	_	K03872
ENSG000	**TCF12**	380659	1147	1.2	1500	1.64	1132	1.19	1795	1.95	5681	5.73	2248	2.22	2107.727	0.318421	3.148358	**1.0025**	**Ups**	**0.00164**	**0.00946**	molecular_	K15603
ENSG000	**TCF4**	442457	38	0.03	47	0.03	30	0.02	601	0.43	1327	0.88	649	0.42	405.4617	0.298886	13.14649	**3.9293**	**Ups**	**1.8E-39**	**5.3E-37**	biological_	K15603
ENSG000	**TCF7L1**	176979	43	0.24	25	0.14	33	0.18	110	0.63	133	0.7	128	0.66	74.57515	0.261953	5.983938	**1.56751**	**Ups**	**2.2E-09**	**5.8E-08**	molecular_	K04490
ENSG000	**TCL6**	29336	2	0	8	0.01	1	0	25	0.04	23	0.04	27	0.04	13.51817	0.480332	4.104802	**1.97167**	**Ups**	**4.1E-05**	**0.0004**	_	_
ENSG000	**TCP11L2**	45087	35	0.11	59	0.19	36	0.11	98	0.32	157	0.48	115	0.34	79.11209	0.282091	4.23063	**1.19342**	**Ups**	**2.3E-05**	**0.00025**	_	_
ENSG000	**TDRD6**	16445	20	0.03	30	0.05	15	0.02	93	0.16	107	0.17	79	0.12	54.30844	0.311657	5.569947	**1.73592**	**Ups**	**2.6E-08**	**5.5E-07**	cellular_cc	K18405
ENSG000	**TEFM**	9485	1185	5.65	1219	6.06	1032	4.95	609	3.02	645	2.97	621	2.79	899.3747	0.150103	-7.13386	**-1.0708**	**Down**	**9.8E-13**	**4.3E-11**	molecular_	K17658
ENSG000	**TEK**	121035	15	0.05	17	0.06	8	0.03	4	0.01	3	0.01	1	0	8.366692	0.514091	-3.54542	**-1.8227**	**Down**	**0.00039**	**0.00286**	biological_	K05121
ENSG000	**TEKT2**	4201	4	0.04	2	0.02	4	0.04	0	0	0	0	0	0	1.778934	0.571881	-2.70594	**-1.5475**	**Down**	**0.00681**	**0.02982**	biological_	K18629
ENSG000	**TENM1**	587914	27	0.04	46	0.07	37	0.05	285	0.41	1407	1.89	407	0.53	324.8416	0.416858	8.039863	**3.35148**	**Ups**	**9E-16**	**5.7E-14**	cellular_cc	_
ENSG000	**TENM2**	979359	7	0.01	7	0.01	3	0	18	0.03	30	0.05	18	0.03	12.98364	0.444585	3.178872	**1.41328**	**Ups**	**0.00148**	**0.00871**	biological_	_
ENSG000	**TENM4**	788117	6	0.01	0	0	5	0.01	26	0.04	18	0.02	29	0.04	13.2855	0.491287	3.898887	**1.91547**	**Ups**	**9.7E-05**	**0.00085**	biological_	_
ENSG000	**TESC**	60557	58	0.36	48	0.31	40	0.25	5	0.03	4	0.02	10	0.06	28.87225	0.39288	-6.9376	**-2.7256**	**Down**	**4E-12**	**1.6E-10**	molecular_	K17612
ENSG000	**TESK2**	147318	96	0.31	79	0.27	91	0.29	330	1.1	229	0.71	244	0.74	172.646	0.265494	4.989107	**1.32458**	**Ups**	**6.1E-07**	**9.7E-06**	molecular_	K08842
ENSG000	**TEX11**	379792	0	0	0	0	0	0	34	0.24	3	0.02	20	0.13	9.284565	0.580778	4.614092	**2.67976**	**Ups**	**4E-06**	**5.2E-05**	biological_	_
ENSG000	**TEX14**	135378	3	0.01	8	0.03	10	0.04	28	0.12	24	0.1	14	0.06	14.01849	0.461736	2.509973	**1.15895**	**Ups**	**0.01207**	**0.04651**	cellular_cc	K17540
ENSG000	**TEX37**	4934	0	0	1	0.03	0	0	6	0.2	6	0.19	5	0.15	2.796413	0.583151	2.808065	**1.63753**	**Ups**	**0.00498**	**0.02334**	cellular_cc	_
ENSG000	**TGFA**	106914	117	0.42	164	0.61	99	0.36	1	0	14	0.05	9	0.03	71.15739	0.391195	-9.36398	**-3.6631**	**Down**	**7.7E-21**	**7.6E-19**	biological_	K08774
ENSG000	**TGFBI**	34924	4332	7.3	4214	7.41	4927	8.35	1047	1.84	1054	1.71	1119	1.78	2895.602	0.151201	-14.8181	**-2.2405**	**Down**	**1.1E-49**	**5.4E-47**	biological_	K19519
ENSG000	**TGM2**	38118	1827	5.32	1358	4.12	1458	4.27	549	1.66	734	2.06	620	1.7	1115.156	0.162208	-9.13555	**-1.4819**	**Down**	**6.5E-20**	**6E-18**	biological_	K05625
ENSG000	**THAP5**	15208	280	0.83	660	2.04	268	0.8	613	1.89	1786	5.12	1260	3.53	757.9938	0.362073	3.281074	**1.18799**	**Ups**	**0.00103**	**0.00649**	molecular_	_
ENSG000	**THBD**	4109	73	0.46	61	0.4	52	0.33	76	0.49	362	2.18	186	1.1	124.2692	0.384267	3.341128	**1.28389**	**Ups**	**0.00083**	**0.00541**	molecular_	K03907
ENSG000	**THBS1**	18388	749	1.4	835	1.63	694	1.31	4677	9.12	5830	10.54	4635	8.19	2716.359	0.144841	17.05646	**2.47047**	**Ups**	**3.1E-65**	**2.7E-62**	biological_	K16857
ENSG000	**THBS3**	13464	233	0.81	179	0.65	216	0.75	1023	3.69	580	1.94	823	2.69	492.195	0.278652	5.953731	**1.65902**	**Ups**	**2.6E-09**	**6.9E-08**	biological_	K04659
ENSG000	**THNSL2**	16312	121	0.34	126	0.36	119	0.33	347	1	358	0.96	315	0.82	221.9574	0.1904	6.459697	**1.22993**	**Ups**	**1.1E-10**	**3.5E-09**	molecular_	_
ENSG000	**THY1**	7606	33	0.08	31	0.08	37	0.09	8	0.02	19	0.04	16	0.04	24.41176	0.356962	-3.68789	**-1.3164**	**Down**	**0.00023**	**0.00178**	biological_	K06514
ENSG000	**TIGIT**	33376	0	0	0	0	0	0	3	0.01	5	0.01	5	0.01	1.961307	0.577073	2.781505	**1.60513**	**Ups**	**0.00541**	**0.02496**	molecular_	K16350
ENSG000	**TIMM21**	10452	2141	8.7	2042	8.66	2144	8.77	1110	4.69	1233	4.84	1250	4.79	1676.866	0.123454	-8.28867	**-1.0233**	**Down**	**1.2E-16**	**8E-15**	molecular_	K17796
ENSG000	**TIMM23B**	16379	222	2.73	229	2.94	206	2.55	124	1.59	109	1.29	131	1.52	173.1596	0.207602	-4.9505	**-1.0277**	**Down**	**7.4E-07**	**1.2E-05**	molecular_	K17794
ENSG000	**TIMP4**	6301	175	2.73	135	2.19	165	2.59	6	0.1	26	0.39	22	0.32	92.47905	0.326233	-9.47592	**-3.0914**	**Down**	**2.6E-21**	**2.7E-19**	cellular_cc	_
ENSG000	**TLE1**	105623	457	1.73	357	1.41	375	1.43	199	0.79	124	0.45	181	0.65	290.1768	0.260534	-5.19881	**-1.3545**	**Down**	**2E-07**	**3.6E-06**	molecular_	_
ENSG000	**TLR4**	12540	95	0.21	138	0.31	71	0.15	146	0.33	1167	2.44	317	0.65	288.6047	0.543981	2.630239	**1.4308**	**Ups**	**0.00853**	**0.03557**	biological_	K10160
ENSG000	**TLR6**	33103	353	0.92	439	1.19	337	0.88	106	0.29	255	0.64	97	0.24	269.3245	0.309162	-4.65653	**-1.4396**	**Down**	**3.2E-06**	**4.3E-05**	biological_	K10169
ENSG000	**TM4SF19**	19162	77	0.67	75	0.68	82	0.72	7	0.06	18	0.15	16	0.13	47.79179	0.318751	-7.91179	**-2.5219**	**Down**	**2.5E-15**	**1.5E-13**	cellular_cc	K02927
ENSG000	**TM7SF2**	4540	378	0.81	307	0.69	333	0.72	1500	3.36	751	1.56	1236	2.51	729.588	0.291703	5.109821	**1.49055**	**Ups**	**3.2E-07**	**5.5E-06**	cellular_cc	K00222
ENSG000	**TMC4**	13099	12	0.04	11	0.04	9	0.03	31	0.12	25	0.09	40	0.14	20.51168	0.385783	3.113514	**1.20114**	**Ups**	**0.00185**	**0.01045**	biological_	_
ENSG000	**TMCC3**	83439	45	0.11	68	0.18	53	0.13	3	0.01	4	0.01	6	0.01	31.58237	0.406935	-8.10293	**-3.2974**	**Down**	**5.4E-16**	**3.5E-14**	cellular_cc	_
ENSG000	**TMED10P**	612	0	0	0	0	2	0.08	1	0.04	12	0.49	11	0.44	3.848288	0.583448	2.515774	**1.46782**	**Ups**	**0.01188**	**0.0459**	_	_
ENSG000	**TMEM12**	4009	2	0.02	0	0	1	0.01	13	0.13	5	0.05	6	0.05	4.36612	0.574478	2.686481	**1.54333**	**Ups**	**0.00722**	**0.0312**	molecular_	_
ENSG000	**TMEM13**	1853	43	0.6	57	0.82	50	0.7	64	0.92	206	2.76	125	1.64	85.10319	0.338576	3.026728	**1.02478**	**Ups**	**0.00247**	**0.01333**	cellular_cc	_
ENSG000	**TMEM13**	285915	533	1.38	894	2.41	401	1.04	1107	2.97	2290	5.71	1327	3.23	1033.212	0.302882	3.427671	**1.03818**	**Ups**	**0.00061**	**0.00415**	cellular_cc	_
ENSG000	**TMEM15**	66178	7	0.05	7	0.05	3	0.02	0	0	2	0.01	0	0	3.296013	0.581569	-2.78682	**-1.6207**	**Down**	**0.00532**	**0.02464**	cellular_cc	K09228
ENSG000	**TMEM17**	45820	282	0.83	493	1.51	206	0.61	612	1.87	1618	4.59	965	2.68	649.4642	0.334942	3.914161	**1.31102**	**Ups**	**9.1E-05**	**0.00081**	cellular_cc	K11447
ENSG000	**TMEM17**	52982	1	0.01	0	0	0	0	5	0.04	15	0.11	2	0.01	3.415426	0.583395	2.855691	**1.666**	**Ups**	**0.00429**	**0.02078**	cellular_cc	_
ENSG000	**TMEM17**	406149	30	0.07	16	0.04	18	0.04	24	0.06	221	0.5	56	0.12	54.52385	0.491167	3.013985	**1.48037**	**Ups**	**0.00258**	**0.01378**	cellular_cc	_
ENSG000	**TMEM19**	24113	1021	3.42	961	3.36	1172	3.95	586	2.04	533	1.72	608	1.92	828.2883	0.182226	-5.77244	**-1.0519**	**Down**	**7.8E-09**	**1.8E-07**	cellular_cc	_
ENSG000	**TMEM22**	31302	1	0	1	0	0	0	9	0.04	5	0.02	6	0.03	3.500338	0.578428	2.746969	**1.58892**	**Ups**	**0.00601**	**0.0271**	cellular_cc	_
ENSG000	**TMEM23**	5354	191	4.91	254	6.81	83	2.15	128	3.42	27	0.67	44	1.07	126.2974	0.470846	-2.59545	**-1.2221**	**Down**	**0.00945**	**0.03856**	cellular_cc	_
ENSG000	**TMEM25**	16240	369	0.45	296	0.37	343	0.42	753	0.95	846	0.99	862	0.98	555.9782	0.15778	6.650994	**1.0494**	**Ups**	**2.9E-11**	**1.1E-09**	cellular_cc	_
ENSG000	**TMEM25**	32053	22	0.09	16	0.07	16	0.06	46	0.19	69	0.26	55	0.21	35.35376	0.31285	4.197821	**1.31329**	**Ups**	**2.7E-05**	**0.00028**	_	_
ENSG000	**TMEM45**	44185	71	0.44	67	0.44	73	0.46	18	0.12	16	0.1	21	0.12	46.05774	0.29714	-6.74615	**-2.0046**	**Down**	**1.5E-11**	**5.8E-10**	cellular_cc	_
ENSG000	**TMEM51**	67949	330	1.15	297	1.08	357	1.26	100	0.36	88	0.3	110	0.36	221.2506	0.210791	-8.87033	**-1.8698**	**Down**	**7.3E-19**	**6E-17**	cellular_cc	_
ENSG000	**TMEM56**	80270	589	1.97	878	3.06	528	1.77	1072	3.72	3019	9.73	1775	5.59	1225.3	0.298925	4.014023	**1.19989**	**Ups**	**6E-05**	**0.00056**	molecular_	_
ENSG000	**TMEM59L**	13610	8	0.04	5	0.03	7	0.03	56	0.28	51	0.24	56	0.26	28.69392	0.378221	6.515305	**2.46422**	**Ups**	**7.3E-11**	**2.5E-09**	cellular_cc	_
ENSG000	**TMEM61**	11502	8	0.19	4	0.1	3	0.07	17	0.41	16	0.36	25	0.55	11.56095	0.463665	2.991579	**1.38709**	**Ups**	**0.00278**	**0.01462**	cellular_cc	_
ENSG000	**TMEM74**	4855	0	0	0	0	2	0.02	10	0.08	9	0.07	4	0.03	3.933035	0.578944	2.866318	**1.65944**	**Ups**	**0.00415**	**0.02021**	cellular_cc	_
ENSG000	**TMEM86**	11664	10	0.05	7	0.03	9	0.04	46	0.23	63	0.29	40	0.18	27.29597	0.36917	5.490195	**2.02682**	**Ups**	**4E-08**	**8.3E-07**	cellular_cc	_
ENSG000	**TMEM92**	10078	71	0.52	67	0.51	87	0.64	18	0.14	8	0.06	18	0.12	47.02354	0.346289	-6.6458	**-2.3014**	**Down**	**3E-11**	**1.1E-09**	cellular_cc	_
ENSG000	**TMOD1**	100569	547	1.89	538	1.94	475	1.65	325	1.17	176	0.59	273	0.89	399.135	0.277278	-4.05237	**-1.1236**	**Down**	**5.1E-05**	**0.00048**	molecular_	K10370
ENSG000	**TMOD2**	64808	4	0.01	18	0.03	13	0.02	19	0.03	86	0.14	34	0.06	26.56765	0.481107	2.761808	**1.32873**	**Ups**	**0.00575**	**0.02619**	molecular_	K10370
ENSG000	**TMPRSS5**	18824	79	0.37	52	0.25	56	0.26	31	0.15	25	0.11	39	0.17	47.89576	0.310932	-3.52072	**-1.0947**	**Down**	**0.00043**	**0.0031**	molecular_	K09636
ENSG000	**TMSB4X**	2120	131080	1158.3	126066	1161.85	132581	1178.31	61724	567.32	56000	477.43	82165	684.79	100161.2	0.196398	-5.80659	**-1.1404**	**Down**	**6.4E-09**	**1.5E-07**	biological_	K05764

ENSG000	TMSB4XP	612	1571	57.69	1587	60.78	1804	66.63	753	28.76	672	23.81	1033	35.78	1262.497	0.211082	-5.61105	**-1.1844**	**Down**	**2E-08**	**4.4E-07**	_	K05764
ENSG000	TMTC1	283920	372	0.46	422	0.55	331	0.41	610	0.79	2957	3.54	1161	1.36	889.5995	0.369859	4.273543	**1.58061**	**Ups**	**1.9E-05**	**0.00021**	cellular_cc	_
ENSG000	TNC	97731	273	0.48	279	0.51	261	0.46	25	0.05	107	0.18	43	0.07	170.3786	0.35229	-6.35008	**-2.2371**	**Down**	**2.2E-10**	**6.8E-09**	cellular_cc	K06252
ENSG000	TNF	2770	0	0	2	0.03	3	0.05	10	0.16	14	0.21	12	0.17	6.36649	0.536253	3.270585	**1.75386**	**Ups**	**0.00107**	**0.00669**	biological	K03156
ENSG000	TNFAIP3	16099	473	2.16	435	2.07	502	2.31	1546	7.34	2531	11.15	1654	7.13	1116.917	0.184782	9.414802	**1.73969**	**Ups**	**4.7E-21**	**4.7E-19**	biological	K11859
ENSG000	TNFRSF12	3939	5800	42.62	5047	38.68	5626	41.58	1815	13.87	1836	13.02	2321	16.09	3848.922	0.16287	-10.1033	**-1.6455**	**Down**	**5.3E-24**	**6.9E-22**	biological	K05149
ENSG000	TNFRSF13	1778	24	0.67	18	0.52	20	0.56	63	1.82	75	2.01	66	1.73	42.19899	0.291723	4.786291	**1.39627**	**Ups**	**1.7E-06**	**2.4E-05**	biological	K05151
ENSG000	TNFRSF25	5045	11	0.03	10	0.03	10	0.03	621	1.82	117	0.32	519	1.38	206.8113	0.458874	8.989078	**4.12486**	**Ups**	**2.5E-19**	**2.2E-17**	molecular	K05160
ENSG000	TNFSF10	18000	24	0.11	43	0.21	22	0.1	309	1.48	65	0.29	199	0.86	108.0654	0.451766	4.569802	**2.06448**	**Ups**	**4.9E-06**	**6.3E-05**	molecular	K04721
ENSG000	TNFSF15	21492	793	2.15	1006	2.85	769	2.1	405	1.14	133	0.35	303	0.78	591.3682	0.35703	-4.45243	**-1.5896**	**Down**	**8.5E-06**	**0.0001**	molecular	K05478
ENSG000	TNFSF4	23580	11	0.04	18	0.07	16	0.06	61	0.22	84	0.28	64	0.21	39.80653	0.318465	5.725085	**1.82324**	**Ups**	**1E-08**	**2.4E-07**	biological	K05469
ENSG000	TNFSF9	4922	7929	123.01	7021	113.6	7962	124.23	3186	51.41	1731	25.91	2964	43.37	5314.593	0.266542	-6.11328	**-1.6294**	**Down**	**9.8E-10**	**2.8E-08**	molecular	K05472
ENSG000	TNNC2	10532	2	0.06	0	0	2	0.06	27	0.82	3	0.08	7	0.19	6.834155	0.578993	2.78295	**1.61131**	**Ups**	**0.00539**	**0.02488**	biological	K12042
ENSG000	TNNT1	16561	181	1.12	189	1.22	231	1.44	114	0.73	37	0.22	99	0.58	146.827	0.369971	-3.44699	**-1.2753**	**Down**	**0.00057**	**0.00392**	biological	K10372
ENSG000	TNNT2	18755	144	0.31	125	0.28	213	0.46	309	0.69	817	1.68	975	1.96	398.952	0.338285	5.000362	**1.69155**	**Ups**	**5.7E-07**	**9.2E-06**	molecular	K12045
ENSG000	TNS1	203207	1785	1.97	1510	1.74	1502	1.67	15	0.02	21	0.02	11	0.01	860.6176	0.245872	-26.6594	**-6.5548**	**Down**	**1E-156**	**5E-153**	molecular	K18080
ENSG000	TNXB	74181	157	0.21	148	0.21	144	0.19	33	0.05	73	0.09	47	0.06	102.7987	0.255934	-6.6604	**-1.7046**	**Down**	**2.7E-11**	**9.9E-10**	molecular	K06252
ENSG000	TOMM5	9997	6246	29.01	5412	26.22	5158	24.1	3098	14.97	2627	11.77	3760	16.47	4454.957	0.19656	-5.09724	**-1.0019**	**Down**	**3.5E-07**	**5.8E-06**	cellular_cc	K17773
ENSG000	TOR4A	4893	1915	11.87	1535	9.92	1726	10.76	945	6.09	763	4.56	878	5.13	1321.668	0.196591	-5.93563	**-1.1669**	**Down**	**2.9E-09**	**7.6E-08**	molecular	K15696
ENSG000	TOX2	154753	127	0.65	88	0.47	100	0.51	15	0.08	17	0.08	13	0.06	62.89757	0.305077	-9.16979	**-2.7975**	**Down**	**4.7E-20**	**4.4E-18**	molecular	_
ENSG000	TP53AIP1	8415	2	0.01	0	0	2	0.01	11	0.04	18	0.05	15	0.04	7.354672	0.532136	4.037679	**2.14859**	**Ups**	**5.4E-05**	**0.00051**	molecular	K13773
ENSG000	TP53I11	65387	70	0.1	50	0.08	60	0.09	3	0	36	0.05	32	0.04	42.3285	0.455222	-2.8914	**-1.3162**	**Down**	**0.00384**	**0.01897**	biological	_
ENSG000	TP63	265864	2	0	2	0	4	0.01	12	0.03	41	0.08	12	0.02	10.98137	0.521591	3.719958	**1.9403**	**Ups**	**0.0002**	**0.0016**	biological	K10149
ENSG000	TPSG1	3607	7	0.12	3	0.05	3	0.05	0	0	1	0.02	0	0	2.440694	0.582843	-2.66118	**-1.5511**	**Down**	**0.00779**	**0.03313**	molecular	K09615
ENSG000	TPTE	123519	101	0.55	110	0.62	93	0.51	0	0	1	0.01	1	0.01	54.42653	0.458419	-11.5215	**-5.2817**	**Down**	**1E-30**	**2E-28**	molecular	K18079
ENSG000	TRANK1	118238	61	0.13	52	0.12	72	0.15	121	0.27	559	1.15	179	0.36	158.163	0.400995	4.144832	**1.66206**	**Ups**	**3.4E-05**	**0.00034**	molecular	_
ENSG000	TRAPPC6	15310	104	1.5	122	1.84	140	2.03	403	6.05	142	1.98	372	5.06	210.359	0.367414	2.781646	**1.02202**	**Ups**	**0.00541**	**0.02496**	molecular	_
ENSG000	TRGC1	7800	41	0.39	32	0.31	29	0.27	227	2.22	645	5.86	495	4.4	221.0058	0.319335	10.00886	**3.19618**	**Ups**	**1.4E-23**	**1.7E-21**	cellular_cc	_
ENSG000	TRGC2	9993	0	0	0	0	1	0.03	18	0.48	55	1.35	51	1.22	18.56673	0.531725	7.042697	**3.74477**	**Ups**	**1.9E-12**	**8.1E-11**	molecular	_
ENSG000	TRGV10	512	1	0.05	0	0	1	0.05	10	0.52	17	0.82	5	0.24	5.18591	0.56976	3.549488	**2.02236**	**Ups**	**0.00039**	**0.00282**	_	_
ENSG000	TRGV9	1845	0	0	0	0	0	0	5	0.08	13	0.19	7	0.1	3.714917	0.582614	3.843137	**2.23907**	**Ups**	**0.00012**	**0.00104**	_	_
ENSG000	TRIB1	8085	453	1.77	379	1.55	375	1.47	1299	5.28	1236	4.66	1215	4.48	792.5357	0.174441	7.99542	**1.39473**	**Ups**	**1.3E-15**	**8.1E-14**	molecular	K08814
ENSG000	TRIM16	56352	3774	7.61	3492	7.35	4384	8.89	1819	3.82	2273	4.42	2155	4.1	3028.431	0.142981	-7.74264	**-1.1071**	**Down**	**9.7E-15**	**5.5E-13**	molecular	K12006
ENSG000	TRIM16L	38121	4875	12.51	4689	12.55	5924	15.29	1642	4.38	2093	5.18	2072	5.01	3645.173	0.151669	-10.6408	**-1.6139**	**Down**	**1.9E-26**	**2.9E-24**	cellular_cc	K12006
ENSG000	TRIM2	186979	370	0.74	613	1.28	295	0.59	929	1.94	3002	5.81	1300	2.46	999.1204	0.350292	4.521751	**1.58393**	**Ups**	**6.1E-06**	**7.6E-05**	molecular	K11997
ENSG000	TRIM22	47401	0	0	4	0.01	3	0.01	11	0.03	72	0.2	30	0.08	17.63625	0.53461	4.645604	**2.48359**	**Ups**	**3.4E-06**	**4.5E-05**	molecular	K11999
ENSG000	TRIM46	11575	10	0.03	8	0.02	15	0.04	56	0.17	20	0.06	42	0.12	24.62602	0.443185	3.086994	**1.36811**	**Ups**	**0.00202**	**0.01128**	cellular_cc	K12022
ENSG000	TRIM47	4415	2346	9.03	1834	7.36	2458	9.52	1285	5.15	562	2.09	1083	3.93	1644.192	0.311243	-4.01838	**-1.2507**	**Down**	**5.9E-05**	**0.00055**	cellular_cc	K12023
ENSG000	TRIM54	25048	15	0.09	28	0.17	18	0.11	8	0.05	4	0.02	10	0.06	14.32204	0.447092	-3.07303	**-1.3739**	**Down**	**0.00212**	**0.01172**	molecular	K10653
ENSG000	TRIM6	16850	4	0.01	11	0.02	8	0.02	82	0.17	84	0.17	78	0.15	41.61127	0.358962	7.900551	**2.836**	**Ups**	**2.8E-15**	**1.7E-13**	molecular	K11999
ENSG000	TRIM62	36658	229	1.05	203	0.97	203	0.93	730	3.47	754	3.33	744	3.21	455.1523	0.163922	9.546902	**1.56494**	**Ups**	**1.3E-21**	**1.4E-19**	cellular_cc	K12030
ENSG000	TRIM73	15943	4	0.01	3	0.01	4	0.01	14	0.05	14	0.04	17	0.05	8.866376	0.479506	2.941402	**1.41042**	**Ups**	**0.00327**	**0.01667**	cellular_cc	K12024
ENSG000	TRIM74	9982	4	0.05	3	0.04	2	0.03	25	0.35	25	0.33	30	0.38	13.87994	0.460515	5.042709	**2.32224**	**Ups**	**4.6E-07**	**7.5E-06**	cellular_cc	K12024
ENSG000	TRIML2	18331	65	0.32	69	0.35	102	0.5	346	1.76	440	2.08	457	2.11	231.4125	0.228699	9.103311	**2.08192**	**Ups**	**8.8E-20**	**7.9E-18**	cellular_cc	K12039
ENSG000	TRNP1	7192	283	2.87	281	2.97	275	2.81	87	0.92	105	1.03	122	1.17	197.2963	0.189773	-8.41468	**-1.5969**	**Down**	**3.9E-17**	**2.9E-15**	molecular	_
ENSG000	TRPC1	83815	77	0.31	84	0.35	48	0.19	116	0.49	326	1.27	176	0.67	128.7778	0.330746	3.593288	**1.18847**	**Ups**	**0.00033**	**0.00244**	molecular	K04964
ENSG000	TRPM2	92919	767	2.28	609	1.88	806	2.41	244	0.75	161	0.46	272	0.76	493.8078	0.25908	-6.90181	**-1.7881**	**Down**	**5.1E-12**	**2.1E-10**	molecular	K04977
ENSG000	TRPM6	165600	3	0.01	2	0.01	5	0.01	2	0.01	59	0.15	21	0.05	13.45761	0.563339	2.947804	**1.66061**	**Ups**	**0.0032**	**0.01638**	molecular	K04981
ENSG000	TRPS1	401176	177	0.23	207	0.28	193	0.25	411	0.56	784	0.99	437	0.54	347.9853	0.237791	5.045843	**1.19986**	**Ups**	**4.5E-07**	**7.4E-06**	biological	_
ENSG000	TSACC	9682	59	0.64	52	0.59	62	0.67	33	0.37	25	0.26	37	0.38	45.52632	0.296509	-3.37264	**-1**	**Down**	**0.00074**	**0.00493**	cellular_cc	K06580
ENSG000	TSC22D1	143629	8641	16.47	7885	15.67	7609	14.58	16957	33.61	24546	45.13	21435	38.53	13841.05	0.126367	9.007977	**1.13831**	**Ups**	**2.1E-19**	**1.8E-17**	molecular	_
ENSG000	TSC22D3	64122	2735	6.24	3266	7.77	3200	7.34	10062	23.88	5241	11.54	9848	21.19	5581.516	0.28059	4.228747	**1.18654**	**Ups**	**2.4E-05**	**0.00025**	biological	_
ENSG000	TSHZ2	522924	66	0.1	78	0.12	93	0.14	457	0.7	728	1.04	448	0.63	289.2616	0.237911	10.23972	**2.43614**	**Ups**	**1.3E-24**	**1.8E-22**	molecular	K09236
ENSG000	TSKS	23578	0	0	3	0.03	1	0.01	16	0.15	2	0.02	12	0.1	5.557549	0.578534	2.515743	**1.45544**	**Ups**	**0.01188**	**0.0459**	molecular	K16492
ENSG000	TSPAN12	71081	1185	6	1435	7.58	1105	5.63	2539	13.38	2931	14.33	3475	16.6	2029.61	0.173671	5.862236	**1.0181**	**Ups**	**4.6E-09**	**1.1E-07**	biological	K17355
ENSG000	TSPAN2	41490	1	0.01	2	0.01	3	0.02	13	0.08	27	0.16	19	0.11	9.911277	0.504089	4.3958	**2.21588**	**Ups**	**1.1E-05**	**0.00013**	biological	K17354
ENSG000	TSPAN8	316814	1995	13.37	1978	13.82	1732	11.67	24	0.17	33	0.21	29	0.18	1028.587	0.204938	-29.6173	**-6.0697**	**Down**	**9E-193**	**6E-189**	molecular	K17349
ENSG000	TSPAN9	209210	303	0.8	246	0.68	328	0.87	110	0.3	137	0.35	138	0.34	214.8816	0.198398	-6.90509	**-1.37**	**Down**	**5E-12**	**2E-10**	cellular_cc	K17350
ENSG000	TSPY26P	4051	10	0.04	7	0.03	8	0.03	39	0.16	41	0.16	25	0.09	20.60699	0.39805	4.024933	**1.60213**	**Ups**	**5.7E-05**	**0.00054**	cellular_cc	K11286
ENSG000	TSR1	15005	7080	26.38	7415	28.81	7535	28.23	3169	12.28	5164	18.56	3618	12.71	5730.672	0.16005	-6.86463	**-1.0987**	**Down**	**6.7E-12**	**2.6E-10**	cellular_cc	K14799
ENSG000	TSSC2	28018	9	0.05	5	0.03	0	0	31	0.16	28	0.14	35	0.17	16.95328	0.471728	4.227541	**1.99425**	**Ups**	**2.4E-05**	**0.00025**	_	K18134
ENSG000	TTBK2	182076	289	0.55	367	0.73	301	0.58	572	1.13	1305	2.4	608	1.09	541.3412	0.2758	3.822846	**1.05434**	**Ups**	**0.00013**	**0.00112**	molecular	K08815
ENSG000	TTC16	15535	23	0.06	22	0.06	18	0.05	11	0.03	1	0	5	0.01	13.99569	0.487667	-3.23491	**-1.5776**	**Down**	**0.00122**	**0.0074**	_	_
ENSG000	TTC30A	5975	102	0.44	148	0.66	83	0.36	193	0.86	476	1.98	271	1.1	199.2609	0.307635	3.727026	**1.14656**	**Ups**	**0.00019**	**0.00156**	cellular_cc	K19683
ENSG000	TTC39B	143739	58	0.1	57	0.1	38	0.06	118	0.2	264	0.42	163	0.26	108.6182	0.29389	5.020182	**1.47538**	**Ups**	**5.2E-07**	**8.4E-06**	_	K12581
ENSG000	TTLL1	49913	65	0.58	62	0.58	86	0.78	549	5.14	293	2.54	557	4.73	256.9466	0.315067	7.380759	**2.32543**	**Ups**	**1.6E-13**	**7.7E-12**	biological	K16599
ENSG000	TTLL11-I	79084	2	0.05	7	0.18	2	0.05	0	0	0	0	0	0	1.964666	0.57127	-2.72298	**-1.5556**	**Down**	**0.00647**	**0.02868**	_	_
ENSG000	TTLL6	54980	16	0.05	18	0.06	10	0.03	1	0	6	0.02	3	0.01	9.280067	0.498658	-3.57902	**-1.7847**	**Down**	**0.00034**	**0.00256**	biological	K16582
ENSG000	TUBA1A	4529	569	1.79	560	1.83	639	2.02	2250	7.35	665	2.01	1656	4.91	1044.04	0.366499	2.93491	**1.07564**	**Ups**	**0.00334**	**0.01697**	biological	K07374
ENSG000	TUBA1B	3616	59751	188.6	57981	190.87	61252	194.45	23684	77.76	38147	116.17	30654	91.26	45855.37	0.147635	-7.92797	**-1.1704**	**Down**	**2.2E-15**	**1.3E-13**	biological	K07374
ENSG000	TUBA4A	28460	13943	53.56	13049	52.28	16513	63.8	6964	27.82	3605	13.36	6571	23.81	10441.26	0.281985	-5.08293	**-1.4333**	**Down**	**3.7E-07**	**6.2E-06**	biological	K07374
ENSG000	TUBAL3	11733	6	0.05	11	0.09	5	0.04	2	0.02	0	0	0	0	4.266583	0.575949	-3.25326	**-1.8737**	**Down**	**0.00114**	**0.00702**	molecular	K07374
ENSG000	TUBB2A	3858	551	6.84	508	6.57	578	7.21	145	1.87	356	4.26	270	3.16	406.4727	0.24954	-5.11534	**-1.2765**	**Down**	**3.1E-07**	**5.3E-06**	molecular	K07375
ENSG000	TUBB3	17370	110	0.2	90	0.17	84	0.15	20	0.04	27	0.05	24	0.04	61.29946	0.27477	-7.61251	**-2.0917**	**Down**	**2.7E-14**	**1.4E-12**	molecular	K07375
ENSG000	TUBBP1	1646	53	0.46	49	0.44	38	0.33	21	0.19	22	0.18	20	0.16	34.5722	0.312568	-4.03832	**-1.2623**	**Down**	**5.4E-05**	**0.00051**	_	K07375
ENSG000	TULP2	17769	0	0	1	0.01	0	0	18	0.15	4	0.03	6	0.04	4.739184	0.582245	3.380479	**1.96827**	**Ups**	**0.00072**	**0.00481**	cellular_cc	_
ENSG000	TULP4	199169	301	0.6	301	0.62	307	0.61	699	1.45	992	1.9	695	1.3	524.3127	0.170364	6.676002	**1.13735**	**Ups**	**2.5E-11**	**9E-10**	molecular	_
ENSG000	TUSC3	349435	17	0.05	21	0.06	9	0.02	4	0.01	5	0.01	3	0.01	10.19099	0.484345	-3.48484	**-1.6879**	**Down**	**0.00049**	**0.00348**	molecular	K12669
ENSG000	TXN	12830	29558	518.99	26289	481.42	26922	475.43	13647	249.24	11043	187.07	17226	285.27	21204.37	0.210196	-5.45673	**-1.147**	**Down**	**4.9E-08**	**9.9E-07**	biological	K03671
ENSG000	TXNDC17	3784	4498	15.59	4121	14.89	4380	15.27	2145	7.73	1416	4.73	2617	8.55	3273.911	0.247678	-4.88865	**-1.2108**	**Down**	**1E-06**	**1.5E-05**	molecular	_
ENSG000	TXNIP	4167	4910	17.83	4870	18.44	4303	15.72	9035	34.13	14309	50.13	10880	37.26	7682.093	0.14984	6.861932	**1.02819**	**Ups**	**6.8E-12**	**2.7E-10**	biological	_
ENSG000	TYRP1	24852	6	0.02	2	0.01	4	0.01	30	0.1	26	0.08	23	0.07	14.35998	0.45378	4.451795	**2.02013**	**Ups**	**8.5E-06**	**0.0001**	molecular	K00506
ENSG000	U52111.1	30854	43	0.95	46	1.06	36	0.8	6	0.14	5	0.11	3	0.06	24.42972	0.407018	-7.02859	**-2.8608**	**Down**	**2.1E-12**	**8.9E-11**	_	_
ENSG000	UBA7	8740	7	0.02	9	0.03	3	0.01	18	0.05	73	0.2	56	0.15	25.00844	0.461547	4.641753	**2.14239**	**Ups**	**3.5E-06**	**4.6E-05**	molecular	K10698
ENSG000	UBASH3B	158799	910	2.23	871	2.23	761	1.88	148	0.38	721	1.7	311	0.72	624.5132	0.363816	-3.40988	**-1.2406**	**Down**	**0.00065**	**0.00438**	molecular	K18993
ENSG000	UBE2L6	16629	11	0.07	20	0.14	7	0.05	42	0.3	89	0.58	91	0.58	40.01484	0.387269	5.175306	**2.00424**	**Ups**	**2.3E-07**	**4E-06**	molecular	K04553
ENSG000	UBE2Q2P	64959	2	0.02	0	0	1	0.01	5	0.06	16	0.19	6	0.07	4.510138	0.57157	2.843535	**1.62528**	**Ups**	**0.00446**	**0.02138**	_	K10582
ENSG000	UCA1	7360	5443	44.17	6278	53.13	6849	55.89	2	0.02	14	0.11	6	0.05	3314.752	0.293741	-31.2083	**-9.1672**	**Down**	**8E-214**	**8E-210**	_	_
ENSG000	UCN2	2047	116	2.01	129	2.34	133	2.32	69	1.25	29	0.49	51	0.83	90.95849	0.334195	-4.14883	**-1.3865**	**Down**	**3.3E-05**	**0.00034**	molecular	K05257
ENSG000	UGDH	29557	2962	7.75	3433	9.37	3281	8.64	705	1.92	1804	4.56	1030	2.54	2250.875	0.252857	-6.43549	**-1.6273**	**Down**	**1.2E-10**	**4E-09**	biological	K00012
ENSG000	UGT1A7	91362	4	0.02	5	0.03	4	0.02	0	0	0	0	0	0	2.316533	0.581227	-3.17348	**-1.8445**	**Down**	**0.00151**	**0.00883**	molecular	K00699
ENSG000	UGT3A1	50019	4	0.01	8	0.02	4	0.01	198	0.39	1100	2.02	501	0.9	264.6557	0.446207	11.65999	**5.20277**	**Ups**	**2E-31**	**4.2E-29**	molecular	_
ENSG000	UGT8	79770	156	0.59	202	0.79	159	0.6	572	2.24	795	2.89	621	2.21	394.2259	0.186128	8.965702	**1.66877**	**Ups**	**3.1E-19**	**2.6E-17**	biological	K04628
ENSG000	ULBP1	9704	40	0.33	28	0.24	37	0.3	123	1.05	172	1.36	125	0.96	82.5032	0.255816	6.57657	**1.68239**	**Ups**	**4.8E-11**	**1.7E-09**	cellular_cc	K07986
ENSG000	ULK4P1	28396	8	0.09	6	0.07	4	0.04	26	0.29	57	0.6	38	0.39	21.28459	0.415846	5.087589	**2.11565**	**Ups**	**3.6E-07**	**6.1E-06**	_	K17545
ENSG000	ULK4P3	28038	1	0.01	4	0.03	2	0.02	7	0.06	20	0.15	12	0.09	7.033074	0.529504	2.911918	**1.54187**	**Ups**	**0.00359**	**0.01799**	_	K17545
ENSG000	UNC13A	87265	3	0	3	0	7	0.01	9	0.01	22	0.03	25	0.03	10.68057	0.487764	2.923072	**1.42577**	**Ups**	**0.00347**	**0.0175**	biological	K15293
ENSG000	UNC13D	17493	4145	9.17	3452	7.96	3942	8.77	1164	2.68	1282	2.74	1287	2.68	2625.736	0.135892	-13.3934	**-1.8201**	**Down**	**6.6E-41**	**2.1E-38**	biological	K19728
ENSG000	UNC5B	90295	25	0.09	27	0.1	18	0.07	4	0.02	9	0.03	12	0.04	16.19405	0.418343	-3.4799	**-1.4558**	**Down**	**0.0005**	**0.00354**	molecular	K07521
ENSG000	UPB1	61153	1	0	0	0	0	0	8	0.02	1	0	11	0.02	3.345922	0.582556	2.699786	**1.57278**	**Ups**	**0.00694**	**0.03024**	molecular	K01431
ENSG000	UPK1A	11653	2	0.03	2	0.04	0	0	16	0.28	8	0.13	10	0.16	6.078184	0.553589	3.261195	**1.80536**	**Ups**	**0.00111**	**0.00686**	cellular_cc	K17347
ENSG000	UPK1A-A	5344	0	0	1	0.03	0	0	23	0.77	3	0.09	11	0.34	6.214299	0.581016	3.730265	**2.16734**	**Ups**	**0.00019**	**0.00154**	_	_
ENSG000	USH1C	50522	6	0.02	11	0.05	18	0.07	3620	14.9	5295	20.22	5096	19.03	2129.789	0.267182	29.55085	**7.89545**	**Ups**	**6E-192**	**3E-188**	biological	_
ENSG000	USP53	82931	163	0.21	333	0.45	140	0.18	308	0.42	1000	1.26	462	0.57	374.4726	0.373886	2.844777	**1.06362**	**Ups**	**0.00444**	**0.02131**	;cellular_cc	_
ENSG000	USP6	58597	8	0.01	8	0.01	7	0.01	14	0.02	40	0.05	60	0.07	21.06546	0.454586	3.660657	**1.66409**	**Ups**	**0.00025**	**0.00195**	molecular	K11837
ENSG000	UTRN	567334	436	0.73	711	1.23	478	0.8	672	1.16	3023	4.85	886	1.39	957.5971	0.390859	2.684521	**1.04927**	**Ups**	**0.00726**	**0.03135**	biological	_
ENSG000	UTS2B	63369	7	0.03	8	0.04	2	0.01	14	0.06	31	0.13	20	0.08	12.7317	0.461988	2.933288	**1.35514**	**Ups**	**0.00335**	**0.01703**	molecular	_
ENSG000	VAC14-A	18154	11	0.04	9	0.04	11	0.04	4	0.02	3	0.01	4	0.01	7.225733	0.497261	-2.59548	**-1.2906**	**Down**	**0.00945**	**0.03856**	_	_
ENSG000	VLDLR	38220	364	0.85	307	0.75	309	0.73	797	1.94	2074	4.68	1175	2.59	775.6671	0.26587	6.349854	**1.68824**	**Ups**	**2.2E-10**	**6.8E-09**	biological	K17599
ENSG000	VOPP1	136933	18106	38.03	16580	36.32	18122	38.29	9803	21.42	7310	14.82	9501	18.82	13534.95	0.202902	-5.66716	**-1.1499**	**Down**	**1.5E-08**	**3.3E-07**	molecular	_
ENSG000	VPREB3	1726	2	0.05	6	0.14	7	0.16	37	0.89	8	0.18	21	0.46	13.33198	0.522995	2.697113	**1.41058**	**Ups**	**0.00699**	**0.03044**	cellular_cc	K06553
ENSG000	VPS13C	208085	816	0.92	1174	1.37	752	0.85	1202	1.4	4738	5.13	1840	1.95	1628.72	0.353111	3.10902	**1.09783**	**Ups**	**0.00188**	**0.0106**	_	K19525
ENSG000	VPS37D	4288	8	0.07	7	0.06	8	0.07	27	0.25	22	0.19	40	0.33	17.80996	0.415032	3.556199	**1.47594**	**Ups**	**0.00038**	**0.00276**	cellular_cc	K12185
ENSG000	VPS9D1-	6310	269	2.58	137	1.37	254	2.45	119	1.19	112	1.03	128	1.16	172.6473	0.280998	-3.63364	**-1.021**	**Down**	**0.00028**	**0.00213**	_	_
ENSG000	VSTM2A	28756	278	0.55	275	0.57	280	0.56	33	0.07	43	0.08	45	0.08	166.7772	0.212297	-13.6813	**-2.9045**	**Down**	**1.3E-42**	**4.5E-40**	cellular_cc	_
ENSG000	WAS	14834	13	0.08	8	0.05	11	0.07	4	0.03	3	0.02	3	0.02	7.245856	0.503495	-2.79391	**-1.4067**	**Down**	**0.00521**	**0.02424**	molecular	K05747
ENSG000	WASL	67133	1018	5.9	1212	7.33	841	4.9	1990	12	3523	19.7	2302	12.58	1723.909	0.210553	5.077567	**1.0691**	**Ups**	**3.8E-07**	**6.4E-06**	molecular	K05747
ENSG000	WDFY1	89672	708	2.72	1023	4.11	557	2.16	758	3.03	3823	14.19	2040	7.4	1374.351	0.377658	2.908176	**1.0983**	**Ups**	**0.00364**	**0.01817**	molecular	_
ENSG000	WDR25	153886	788	3.2	724	3.07	785	3.21	411	1.74	302	1.18	464	1.78	591.4825	0.226143	-4.9556	**-1.1207**	**Down**	**7.2E-07**	**1.1E-05**	_	_
ENSG000	WDR4	36475	2104	14.53	1530	11.02	1825	12.68	1021	7.33	607	4.04	1074	7	1392.611	0.267803	-4.24665	**-1.1373**	**Down**	**2.2E-05**	**0.00023**	molecular	K15443
ENSG000	WDR64	149856	3	0.01	2	0.01	3	0.01	12	0.05	34	0.12	20	0.07	11.2083	0.495019	4.207839	**2.08296**	**Ups**	**2.6E-05**	**0.00027**	_	_
ENSG000	WEE1	19777	995	2.16	1056	2.39	797	1.74	2217	5	3317	6.93	2744	5.61	1761.03	0.164245	7.797326	**1.28067**	**Ups**	**6.3E-15**	**3.7E-13**	biological	K06632
ENSG000	WFDC1	35199	2305	9.34	1970	8.33	1899	7.74	5250	22.13	6196	24.22	7999	30.57	4064.049	0.177458	7.883539	**1.399**	**Ups**	**3.2E-15**	**1.9E-13**	molecular	_
ENSG000	WFIKKN1	4878	10	0.04	12	0.05	7	0.03	43	0.17	13	0.05	32	0.11	19.18581	0.463163	2.477486	**1.14748**	**Ups**	**0.01323**	**0.04997**	molecular	_
ENSG000	WIPI1	36566	308	1.15	323	1.26	354	1.33	706	2.74	816	2.94	845	2.98	537.3222	0.154275	6.640766	**1.0245**	**Ups**	**3.1E-11**	**1.1E-09**	biological	K17908
ENSG000	WNK2	135657	165	0.21	111	0.15	146	0.19	45	0.06	59	0.07	75	0.09	102.2651	0.259951	-5.33208	**-1.3861**	**Down**	**9.7E-08**	**1.9E-06**	molecular	K08867
ENSG000	WNT2B	63625	18	0.03	19	0.03	18	0.03	60	0.1	135	0.21	61	0.09	48.01121	0.3519	5.019879	**1.7665**	**Ups**	**5.2E-07**	**8.4E-06**	biological	K00182
ENSG000	WNT5A	24231	3087	5.79	3882	7.59	2976	5.61	912	1.78	2814	5.09	1390	2.46	2527.88	0.295201	-3.87577	**-1.1441**	**Down**	**0.00011**	**0.00093**	biological	K00444
ENSG000	WNT5B	117353	21	0.07	13	0.04	12	0.04	114	0.38	76	0.24	111	0.34	55.03189	0.347277	6.51723	**2.26329**	**Ups**	**7.2E-11**	**2.4E-09**	biological	K00444
ENSG000	WNT6	14412	79	0.96	77	0.98	71	0.87	357	4.52	104	1.22	268	3.08	156.6572	0.389175	3.411638	**1.32772**	**Ups**	**0.00065**	**0.00436**	biological	K00445
ENSG000	WNT7A	63864	54	0.28	40	0.22	31	0.16	0	0	0	0	2	0.01	22.49792	0.500358	-8.14564	**-4.0757**	**Down**	**3.8E-16**	**2.5E-14**	biological	K00572
ENSG000	WNT7B	56768	2728	10.48	2362	9.46	2751	10.63	742	2.96	953	3.53	803	2.91	1776.861	0.134749	-13.7263	**-1.8496**	**Down**	**7.1E-43**	**2.5E-40**	biological	K00572
ENSG000	WSCD1	352194	234	0.33	206	0.3	214	0.3	35	0.05	77	0.1	59	0.08	141.9774	0.242103	-8.58615	**-2.0787**	**Down**	**9E-18**	**7E-16**	molecular	_
ENSG000	WWTR1	219480	979	1.96	1004	2.09	863	1.73	1582	3.29	3309	6.38	2232	4.21	1573.26	0.211883	4.896723	**1.03753**	**Ups**	**9.7E-07**	**1.5E-05**	biological	K16820
ENSG000	XAF1	20201	0	0	1	0	0	0	1	0	28	0.04	13	0.02	6.18365	0.583054	3.517075	**2.05064**	**Ups**	**0.00044**	**0.00314**	cellular_cc	_
ENSG000	XAGE2	6651	2	0.08	5	0.22	4	0.17	25	1.08	79	3.17	59	2.31	26.08716	0.456614	6.305685	**2.87926**	**Ups**	**2.9E-10**	**8.8E-09**	_	_
ENSG000	XAGE2B	6651	13	0.54	8	0.35	2	0.08	114	4.93	167	6.7	266	10.44	86.83045	0.405213	9.112506	**3.69251**	**Ups**	**8.1E-20**	**7.3E-18**	_	_
ENSG000	XKR4	439665	0	0	0	0	0	0	3	0	4	0	6	0.01	1.973053	0.576649	2.768492	**1.59645**	**Ups**	**0.00563**	**0.02578**	cellular_cc	_
ENSG000	XXbac-BP	1138	43	1.56	31	1.17	46	1.68	170	6.41	66	2.31	168	5.74	85.07228	0.380453	3.632591	**1.38203**	**Ups**	**0.00028**	**0.00214**	_	_
ENSG000	XXbac-BP	5006	3	0.02	0	0	0	0	20	0.12	31	0.18	21	0.12	11.42329	0.521011	5.540831	**2.88684**	**Ups**	**3E-08**	**6.4E-07**	_	_
ENSG000	XXbac-BP	6589	6	0.51	3	0.27	5	0.43	20	1.78	21	1.73	20	1.61	11.85151	0.448613	3.457957	**1.55128**	**Ups**	**0.00054**	**0.00379**	_	_
ENSG000	XXbac-BP	5352	23	0.11	38	0.19	24	0.12	73	0.36	231	1.07	115	0.52	77.02619	0.367656	4.906158	**1.80378**	**Ups**	**9.3E-07**	**1.4E-05**	_	_
ENSG000	Y_RNA	1059	1	0.02	7	0.16	2	0.04	36	0.83	21	0.45	34	0.71	16.01642	0.486551	4.664256	**2.2694**	**Ups**	**3.1E-06**	**4.2E-05**	_	_
ENSG000	Y_RNA	96	0	0	1	0.28	0	0	4	1.11	15	3.88	6	1.52	3.848429	0.5814	3.2884	**1.91188**	**Ups**	**0.00101**	**0.00636**	_	_
ENSG000	Y_RNA	99	5	1.3	1	0.27	1	0.26	5	1.35	35	8.77	18	4.41	9.648766	0.549478	3.257022	**1.78966**	**Ups**	**0.00113**	**0.00694**	_	_

ENSG000(	Y_RNA	108	1	0.24	0	0	0	0	3	0.74	12	2.76	8	1.8	3.556532	0.582008	3.173314	**1.8469**	**Ups**	**0.00151**	**0.00883**	_	_
ENSG000(	Y_RNA	91	0	0	2	0.59	1	0.28	12	3.53	2	0.55	17	4.53	5.439824	0.578399	2.850548	**1.64875**	**Ups**	**0.00436**	**0.02104**	_	_
ENSG000(	YARS2	28413	3119	23.43	2894	22.67	2998	22.65	1251	9.77	1311	9.5	1482	10.5	2224.486	0.138245	-9.78332	**-1.3525**	**Down**	**1.3E-22**	**1.5E-20**	molecular_	K01866
ENSG000(	YPEL1	38291	5	0.02	2	0.01	2	0.01	20	0.09	30	0.13	16	0.07	11.60211	0.482451	4.203665	**2.02806**	**Ups**	**2.6E-05**	**0.00028**	cellular_cc	K17985
ENSG000(	YPEL2	70041	48	0.13	58	0.16	41	0.11	155	0.42	231	0.58	167	0.41	110.0015	0.240705	6.668374	**1.60511**	**Ups**	**2.6E-11**	**9.4E-10**	cellular_cc	_
ENSG000(	YPEL3	4602	374	1.11	299	0.93	361	1.08	1984	6.15	543	1.56	1431	4.02	816.466	0.379685	4.123406	**1.56559**	**Ups**	**3.7E-05**	**0.00037**	cellular_cc	_
ENSG000(	YPEL4	4858	12	0.03	7	0.02	5	0.01	30	0.07	27	0.06	18	0.04	15.87228	0.430925	2.788668	**1.20171**	**Ups**	**0.00529**	**0.02453**	cellular_cc	_
ENSG000(	YPEL5	13593	1552	5.84	1975	7.76	1467	5.56	4722	18.5	3534	12.84	4488	15.94	2868.04	0.220585	5.031309	**1.10983**	**Ups**	**4.9E-07**	**8E-06**	_	_
ENSG000(	Z83851.1	4837	308	3.3	314	3.51	283	3.05	123	1.37	58	0.6	126	1.27	209.3217	0.310468	-5.19239	**-1.6121**	**Down**	**2.1E-07**	**3.7E-06**	_	_
ENSG000(	Z83851.3	5444	31	0.35	36	0.43	46	0.52	18	0.21	31	0.34	10	0.11	29.0665	0.38154	-2.70209	**-1.031**	**Down**	**0.00689**	**0.03009**	_	_
ENSG000(	ZBED3-AS	80170	30	0.07	32	0.08	35	0.09	76	0.19	99	0.23	94	0.21	58.23553	0.256972	4.596972	**1.18129**	**Ups**	**4.3E-06**	**5.6E-05**	_	_
ENSG000(	ZBED6CL	2871	2308	20.66	2529	23.62	2413	21.73	696	6.48	448	3.87	631	5.33	1568.688	0.23422	-9.092	**-2.1295**	**Down**	**9.7E-20**	**8.7E-18**	_	_
ENSG000(	ZBTB10	40647	98	0.14	122	0.18	64	0.09	576	0.84	840	1.14	675	0.89	367.6572	0.23921	10.58824	**2.53281**	**Ups**	**3.4E-26**	**5E-24**	molecular_	K10497
ENSG000(	ZBTB18	6194	103	0.69	108	0.75	88	0.59	219	1.52	440	2.83	314	1.98	199.1281	0.238311	5.845081	**1.39295**	**Ups**	**5.1E-09**	**1.2E-07**	biological_	_
ENSG000(	ZBTB20	809178	37	0.07	23	0.05	26	0.05	81	0.16	95	0.17	74	0.13	53.52617	0.28544	4.3317	**1.23644**	**Ups**	**1.5E-05**	**0.00017**	molecular_	K10501
ENSG000(	ZBTB41	46863	161	0.37	377	0.89	137	0.31	329	0.78	1554	3.41	646	1.39	489.477	0.4204	3.254238	**1.36808**	**Ups**	**0.00114**	**0.007**	molecular_	K10513
ENSG000(	ZC3H6	64470	122	0.14	135	0.17	77	0.09	278	0.34	504	0.57	283	0.31	219.6862	0.276649	4.868653	**1.34691**	**Ups**	**1.1E-06**	**1.7E-05**	molecular_	K12581
ENSG000(	ZCCHC24	63303	373	1.75	364	1.78	309	1.46	1111	5.43	1544	7	1164	5.16	766.5394	0.161538	9.957564	**1.60852**	**Ups**	**2.3E-23**	**2.8E-21**	molecular_	_
ENSG000(	ZCWPW2	188977	10	0.06	10	0.07	6	0.04	18	0.12	38	0.23	23	0.14	16.44612	0.412972	2.807541	**1.15944**	**Ups**	**0.00499**	**0.02337**	molecular_	_
ENSG000(	ZDBF2	39762	99	0.25	202	0.53	90	0.23	229	0.6	1534	3.7	447	1.05	388.8846	0.539785	2.787237	**1.50451**	**Ups**	**0.00532**	**0.02462**	molecular_	K11447
ENSG000(	ZDHHC11	140631	36	0.08	47	0.11	69	0.15	122	0.28	244	0.52	163	0.34	106.4907	0.301867	4.762166	**1.43754**	**Ups**	**1.9E-06**	**2.7E-05**	molecular_	K18932
ENSG000(	ZDHHC11	56593	1	0.01	1	0.01	6	0.03	4	0.02	97	0.51	32	0.17	20.37074	0.5675	3.815828	**2.16548**	**Ups**	**0.00014**	**0.00114**	molecular_	K18932
ENSG000(	ZDHHC14	297014	286	0.34	268	0.34	349	0.42	1022	1.28	598	0.69	965	1.09	565.0515	0.274871	4.544321	**1.2491**	**Ups**	**5.5E-06**	**7E-05**	molecular_	K16675
ENSG000(	ZEB2P1	36638	0	0	0	0	0	0	6	0.08	3	0.04	2	0.02	1.750861	0.56781	2.487638	**1.41251**	**Ups**	**0.01286**	**0.04881**	_	_
ENSG000(	ZFHX4	186068	1	0	3	0	1	0	5	0	17	0.01	7	0.01	5.161728	0.558154	2.539521	**1.41744**	**Ups**	**0.0111**	**0.04355**	molecular_	K09380
ENSG000(	ZFP36	2600	79	0.32	77	0.32	87	0.35	2142	9	988	3.85	1756	6.69	813.4326	0.312914	12.22467	**3.82527**	**Ups**	**2.3E-34**	**5.5E-32**	biological_	K15308
ENSG000(	ZFYVE16	71338	960	1.14	1626	2.01	767	0.91	1551	1.91	5387	6.15	2316	2.58	1959.208	0.355311	2.999038	**1.06559**	**Ups**	**0.00271**	**0.01433**	molecular_	K04679
ENSG000(	ZIC2	4993	307	1.97	252	1.69	270	1.74	666	4.45	682	4.22	749	4.53	469.3901	0.169825	6.480196	**1.1005**	**Ups**	**9.2E-11**	**3.1E-09**	biological_	_
ENSG000(	ZKSCAN1	26109	850	1.37	986	1.66	765	1.24	1955	3.27	4400	6.83	2144	3.25	1732.042	0.257848	5.348269	**1.37904**	**Ups**	**8.9E-08**	**1.7E-06**	molecular_	K09229
ENSG000(	ZMAT1	49743	8	0.01	6	0.01	1	0	19	0.04	126	0.22	38	0.07	29.09419	0.517786	4.577309	**2.37007**	**Ups**	**4.7E-06**	**6.1E-05**	molecular_	_
ENSG000(	ZMIZ1-AS	124568	5	0.03	7	0.04	8	0.04	0	0	1	0.01	2	0.01	4.010135	0.569985	-2.9585	**-1.6863**	**Down**	**0.00309**	**0.01598**	_	_
ENSG000(	ZNF136	26186	189	0.5	262	0.72	189	0.5	293	0.81	885	2.26	491	1.23	360.9717	0.30877	3.33784	**1.03062**	**Ups**	**0.00084**	**0.00546**	biological_	K09228
ENSG000(	ZNF185	59039	278	0.59	241	0.54	258	0.55	617	1.37	702	1.45	587	1.18	430.1627	0.162918	6.477445	**1.05529**	**Ups**	**9.3E-11**	**3.1E-09**	cellular_cc	_
ENSG000(	ZNF192P1	7814	22	0.12	23	0.13	34	0.18	45	0.25	123	0.63	60	0.3	47.91319	0.362114	3.122559	**1.13072**	**Ups**	**0.00179**	**0.0102**	_	K09229
ENSG000(	ZNF233	25153	15	0.04	18	0.05	14	0.04	64	0.19	75	0.21	75	0.2	41.06865	0.304641	5.968879	**1.81836**	**Ups**	**2.4E-09**	**6.3E-08**	molecular_	K09228
ENSG000(	ZNF252P-	3236	23	0.18	19	0.16	23	0.18	5	0.04	16	0.12	4	0.03	15.25255	0.443923	-3.01793	**-1.3397**	**Down**	**0.00255**	**0.01363**	_	_
ENSG000(	ZNF280B	24739	4	0.01	4	0.01	2	0	0	0	0	0	0	0	1.776654	0.571874	-2.70551	**-1.5472**	**Down**	**0.00682**	**0.02984**	molecular_	_
ENSG000(	ZNF295-A	15758	18	0.1	27	0.15	23	0.12	6	0.03	6	0.03	8	0.04	15.20051	0.420341	-3.9858	**-1.6754**	**Down**	**6.7E-05**	**0.00062**	_	_
ENSG000(	ZNF311	10532	115	0.78	101	0.72	92	0.63	230	1.63	362	2.38	286	1.84	187.5778	0.201451	6.14944	**1.23881**	**Ups**	**7.8E-10**	**2.2E-08**	molecular_	K09228
ENSG000(	ZNF32	4998	39	0.38	37	0.37	50	0.48	143	1.43	225	2.09	242	2.2	114.6574	0.258343	7.483141	**1.93321**	**Ups**	**7.3E-14**	**3.7E-12**	molecular_	K09228
ENSG000(	ZNF32-AS	2078	0	0	0	0	0	0	5	0.15	13	0.36	0	0	2.663064	0.56365	2.544226	**1.43405**	**Ups**	**0.01095**	**0.04314**	_	_
ENSG000(	ZNF337-A	54030	147	0.56	133	0.53	125	0.48	311	1.22	434	1.59	301	1.07	231.0536	0.196099	5.652062	**1.10836**	**Ups**	**1.6E-08**	**3.5E-07**	_	_
ENSG000(	ZNF34	14232	66	0.34	76	0.41	83	0.43	216	1.15	182	0.9	191	0.92	131.2867	0.24086	4.687533	**1.12904**	**Ups**	**2.8E-06**	**3.8E-05**	molecular_	K09228
ENSG000(	ZNF365	297821	5	0.01	11	0.03	6	0.02	17	0.05	76	0.19	22	0.05	20.69237	0.48658	3.329625	**1.62013**	**Ups**	**0.00087**	**0.0056**	molecular_	K16737
ENSG000(	ZNF385B	419524	35	0.1	33	0.09	28	0.08	58	0.16	133	0.35	67	0.17	55.57452	0.323676	3.328774	**1.07745**	**Ups**	**0.00087**	**0.00561**	molecular_	_
ENSG000(	ZNF391	29290	93	0.42	107	0.51	84	0.38	129	0.61	402	1.76	198	0.85	158.2636	0.324209	3.085539	**1.00036**	**Ups**	**0.00203**	**0.01133**	molecular_	K09228
ENSG000(	ZNF425	23563	18	0.1	13	0.08	12	0.07	73	0.43	56	0.3	43	0.23	34.44817	0.365361	4.385228	**1.60219**	**Ups**	**1.2E-05**	**0.00013**	molecular_	K09228
ENSG000(	ZNF433	21010	9	0.03	7	0.02	6	0.02	18	0.06	71	0.21	51	0.15	24.51228	0.442027	4.464827	**1.97357**	**Ups**	**8E-06**	**9.7E-05**	molecular_	K09228
ENSG000(	ZNF460	13519	39	0.18	40	0.19	27	0.12	83	0.39	100	0.44	85	0.36	59.79293	0.265031	3.984719	**1.05607**	**Ups**	**6.8E-05**	**0.00062**	molecular_	K09228
ENSG000(	ZNF488	18843	234	0.45	195	0.39	171	0.33	25	0.05	55	0.1	42	0.08	124.925	0.261538	-9.18363	**-2.4019**	**Down**	**4.2E-20**	**3.9E-18**	molecular_	_
ENSG000(	ZNF501	7488	6	0.03	15	0.09	9	0.05	34	0.2	57	0.31	29	0.16	23.47682	0.403731	3.747721	**1.51307**	**Ups**	**0.00018**	**0.00146**	molecular_	_
ENSG000(	ZNF503	4077	106	0.48	105	0.49	123	0.56	923	4.32	323	1.4	805	3.42	384.4133	0.359036	6.092176	**2.18731**	**Ups**	**1.1E-09**	**3.1E-08**	molecular_	_
ENSG000(	ZNF530	12838	10	0.03	8	0.02	8	0.02	55	0.15	57	0.14	39	0.09	27.86561	0.373155	5.488861	**2.04819**	**Ups**	**4.1E-08**	**8.4E-07**	molecular_	K09228
ENSG000(	ZNF567	40090	2	0	4	0.01	8	0.02	0	0	1	0	0	0	2.647625	0.58287	-2.72643	**-1.5892**	**Down**	**0.0064**	**0.02846**	molecular_	K09228
ENSG000(	ZNF595	34921	0	0	0	0	1	0	270	0.83	328	0.94	264	0.74	131.8494	0.447285	14.21525	**6.35827**	**Ups**	**7.4E-46**	**3.1E-43**	molecular_	K09228
ENSG000(	ZNF600	22597	15	0.07	3	0.01	7	0.03	0	0	2	0.01	1	0	4.849187	0.574087	-3.12977	**-1.7968**	**Down**	**0.00175**	**0.00999**	molecular_	_
ENSG000(	ZNF654	5562	172	0.89	263	1.42	109	0.57	266	1.43	830	4.15	390	1.9	316.3005	0.362261	2.907219	**1.05317**	**Ups**	**0.00365**	**0.01822**	molecular_	_
ENSG000(	ZNF658	75716	14	0.04	10	0.03	8	0.02	27	0.08	55	0.15	21	0.05	21.11244	0.417024	2.937833	**1.22515**	**Ups**	**0.00331**	**0.01684**	molecular_	K09228
ENSG000(	ZNF670	133265	116	0.55	170	0.84	111	0.53	48	0.24	99	0.45	56	0.25	101.1642	0.29201	-3.8756	**-1.1317**	**Down**	**0.00011**	**0.00093**	molecular_	K09228
ENSG000(	ZNF671	7874	0	0	0	0	0	0	4	0.02	10	0.04	3	0.01	2.525838	0.580982	3.075595	**1.78687**	**Ups**	**0.0021**	**0.01164**	molecular_	K09228
ENSG000(	ZNF699	15564	15	0.1	18	0.12	12	0.08	27	0.18	83	0.52	26	0.16	28.07206	0.425564	2.629986	**1.11923**	**Ups**	**0.00854**	**0.03559**	molecular_	K09228
ENSG000(	ZNF772	10908	29	0.07	35	0.09	17	0.04	74	0.19	201	0.48	89	0.21	68.38496	0.370147	4.565812	**1.69002**	**Ups**	**5E-06**	**6.4E-05**	molecular_	K09228
ENSG000(	ZNF773	18490	3	0.01	4	0.01	6	0.01	90	0.21	120	0.26	92	0.2	48.2915	0.37931	9.847835	**3.73538**	**Ups**	**7E-23**	**7.9E-21**	molecular_	K09228
ENSG000(	ZNF788	44973	2	0.01	1	0	3	0.01	11	0.03	18	0.05	9	0.02	6.812624	0.527907	3.185246	**1.68151**	**Ups**	**0.00145**	**0.00855**	molecular_	K09228
ENSG000(	ZNF812	10853	0	0	0	0	0	0	11	0.07	23	0.14	13	0.08	7.036473	0.567607	5.239487	**2.97397**	**Ups**	**1.6E-07**	**2.9E-06**	molecular_	K09228
ENSG000(	ZNF831	68094	4	0.01	4	0.01	3	0.01	22	0.06	43	0.11	13	0.03	13.66094	0.487262	4.042733	**1.96987**	**Ups**	**5.3E-05**	**0.0005**	molecular_	_
ENSG000(	ZNF85	27476	13	0.03	28	0.06	9	0.02	0	0	6	0.01	7	0.01	10.79594	0.529562	-2.92354	**-1.5482**	**Down**	**0.00346**	**0.01748**	molecular_	K09228
ENSG000(	ZNF862	29113	117	0.31	94	0.26	92	0.25	298	0.83	476	1.23	340	0.86	222.3059	0.209525	7.587237	**1.58972**	**Ups**	**3.3E-14**	**1.7E-12**	molecular_	_
ENSG000(	ZNF90	49083	4	0.02	4	0.02	3	0.01	0	0	0	0	0	0	1.956993	0.57675	-2.88882	**-1.6661**	**Down**	**0.00387**	**0.01908**	molecular_	K02995
ENSG000(	ZXDA	5204	256	1.26	245	1.26	169	0.84	468	2.4	1109	5.29	592	2.76	442.4157	0.279856	4.833046	**1.35256**	**Ups**	**1.3E-06**	**2E-05**	molecular_	_
ENSG000(	ZXDB	5638	1663	7.58	1677	7.97	1478	6.78	2676	12.69	11510	50.63	4612	19.83	3606.631	0.34492	4.39995	**1.51763**	**Ups**	**1.1E-05**	**0.00013**	molecular_	_
ENSG000(	ZYG11B	100889	506	1.45	663	1.98	416	1.2	725	2.16	2713	7.48	1114	3	950.5098	0.346502	3.238092	**1.12201**	**Ups**	**0.0012**	**0.00733**	_	K10350
