# Supplementary material for: Aberrant NSUN2-mediated m5C modification of H19 lncRNA is associated with poor differentiation of hepatocellular carcinoma
Source: Oncogene. 2020 Sep 25;39(45):6906–19. doi: 10.1038/s41388-020-01475-w (PMC7644462; doi:10.1038/s41388-020-01475-w)
Supplement: Supplementary file 3 — Additional file 2 [file 41388_2020_1475_MOESM3_ESM.pdf]

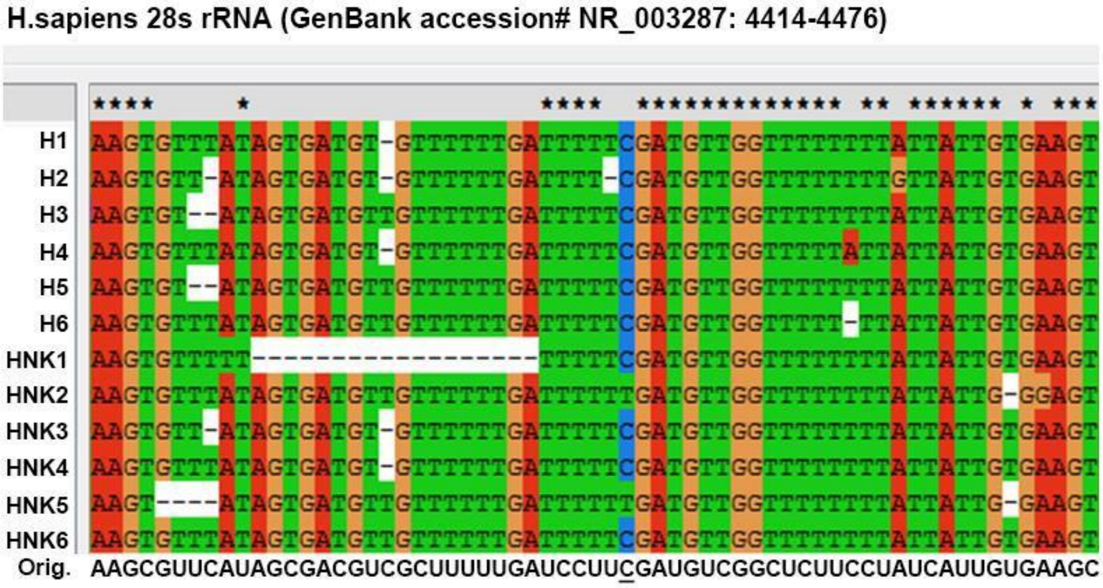

Fig S1: Identification conversion efficiency of RNA bisulfite treatment. Human 28s rRNA is usually 100% methylated at position 4447C, while other cytosines are not. We use it to detect the conversion efficiency of bisulfite treatment through the BSP methods.

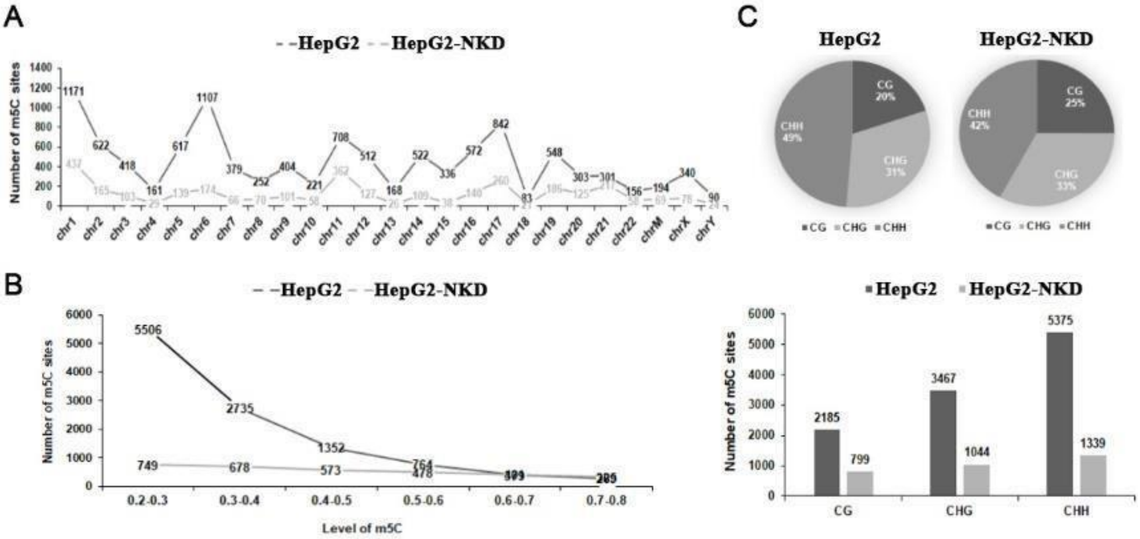

Fig S2: Distribution profile of m⁵C in *NSUN2*-deficient HepG2 cells and normal HepG2 cells. (A) RNA m⁵C abundance along the chromosome. (B) Line graph showed the methylation level of m⁵C sites. (C) Proportions of RNA m⁵C sites identified in each sequence context: CG, CHG and CHH, where H=A, C, or U. HepG2-NKD, *NSUN2*-deficient HepG2 cells.

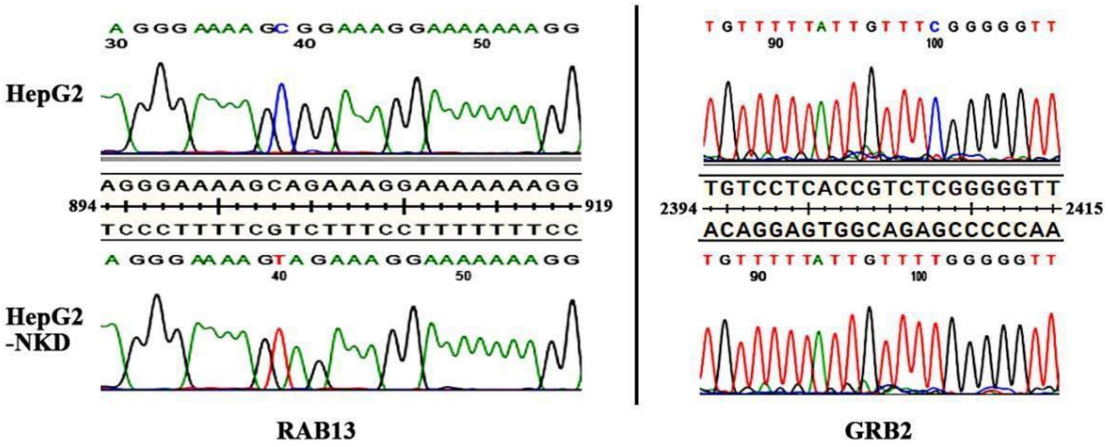

Fig S3: Validation of representative m^{5}C sites by Sanger sequencing. m^{5}C sites within *RAB13* and *GRB2* mRNA identified by RNA-BisSeq were validated. cDNA was amplified by PCR using specific primers for bisulfite-treated mRNAs.

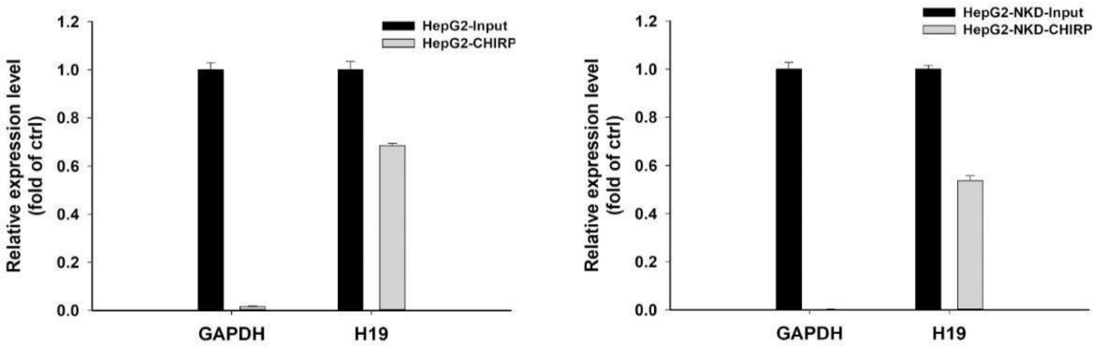

Fig S4: Enrichment efficiency of H19 RNA pulled by CHIRP. *H19* RNA Enrichment efficiency was identification by real-time qPCR using primers specific for *H19* or *GAPDH*. The real-time qPCR data are represented as means ±SEM from 3 independent experiments. HepG2-NKD, *NSUN2*- deficient HepG2 cells.

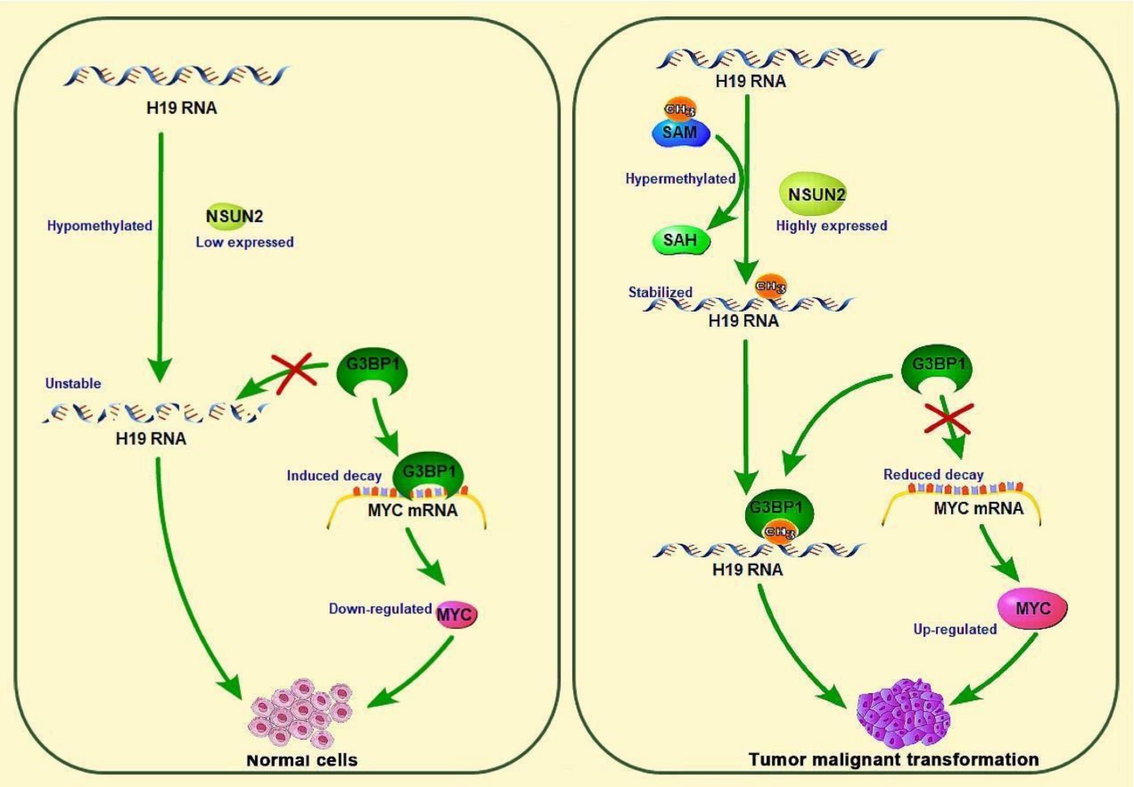

Fig S5: Graphical Abstract. Effect of aberrant H19 lncRNA m^5C methylation on tumor malignant transformation.

Table S1: RNAs with significant change in both m^{5}C methylation and RNA expression.

Transcript ID	Gene Name	Transcript ID	Gene Name	Transcript ID	Gene Name
ENST00000578966	RPL17	NM_005514	HLA-B	NM_004489	GPS2
NR_002196	H19	NM_004040	RHOB	NM_018669	WDR4
ENST00000580695	PDE10A	NM_020843	SCAPER	NM_002998	SDC2
ENST00000429798	LIMD1-AS1	NM_003437	ZNF136	NM_001946	DUSP6
ENST00000575282	SGSH	NM_001554	CYR61	NM_012073	CCT5
NR_026951	LINC00324	NM_021197	WFDC1	NM_005709	USH1C
NM_004965	HMGN1	ENST00000567976	KCTD19	NM_030627	CPEB4
NM_017755	NSUN2	NM_004595	SMS	NM_003329	TXN
NM_002480	PPP1R12A	NM_014330	PPP1R15A	NM_006918	SC5D
NM_000985	RPL17	ENST00000555686	FOS	NM_004089	TSC22D3
NM_004342	CALD1	NM_004247	EFTUD2	NM_017705	PAQR5
NM_003509	HIST1H2AI	NM_003520	HIST1H2BN	NM_138408	GTF3C6
NM_002599	PDE2A	NM_003785	PAGE1	NM_020724	RNF150
ENST00000555672	FOS	NM_003105	SORL1	NM_002032	FTH1
NM_004417	DUSP1	NM_152405	JMY	NM_014520	MYBBP1A
NM_018128	TSR1	NM_005556	KRT7	NM_000094	COL7A1
NM_003205	TCF12	NM_031220	PITPNM3	NM_015229	CLUH
NM_005566	LDHA				

Table S2: Specific primers used for vector construction.

Primer	Sequence
NSUN2-CDS-F	GGAATTCCTGGGCTATGGGGCGGCGGTC
NSUN2-CDS-R	GCTCTAGACCTGCTCACCGGGGTGGATG
H19-WT-F	CGAGCTCGCTAGCCTCGAGTTTTAGTAGCAGGCACAGGGG
H19-WT-R	TGCCTGCAGGTCGACTCTAGATACAGCGTCACCAAGTCCA
H19-Mut-F	GGCCCCGGGACATTGAGCAGCAAGGA
H19-Mut-R	TCAATGTCCCGGGGCCCCCCTCCCGT
HAL-BsaI-F	TTTGCTCAGGTCTCACTCTGTGGCTGGGCAGCGCG
HAL-BsaI-R	ATGTAACGGGTCTCGAATTCGGCGCCATCCTCCGCGTCC
HAR-BsaI-F	TTTGCTCAGGTCTCTCTAGAAGGGTGGTGGAAAGCGCGG
HAR-BsaI-R	ATGTAACGGGTCTCACTCTAGTCACTACTTTTACACTGTATGCCTTC
T2A-EcoR1-F	CGAATTCGAGGGCAGAGGAAGTCTGCTAACATGCGGTGACGTCGAGGAGA
BGH-Xba1-R	GCTCTAGACCATAGAGCCCACCGCAT
T2A-heper-PURO-F	ATGCGGTGACGTCGAGGAGAATCCTGGCCCAATGACCGAGTACAAGCCCAC
PURO-R	CGAGGCTGATCAGCGGGTTTTCATCCTGCAGTCAGGCACC
PURO-BGH-F	GGTGCCTGACTGCAGGATGAAAACCCGCTGATCAGCCTCG
T2A-heper-NEO-F	ATGCGGTGACGTCGAGGAGAATCCTGGCCCAATGATTGAACAAGATGGAT
NEO-R	CGAGGCTGATCAGCGGGTTTAGGCGTCGCTTGGTCGGTCAT
NEO-BGH-F	ATGACCGACCAAGCGACGCCTAAACCCGCTGATCAGCCTCG

Table S3: Gene specific primers used for qRT-PCR.

Gene	Forward primer (5'-3')	Reverse primer (5'-3')
NSUN2	ATCTTGAGAAAATCGCCACAC	ATCATTCGCAATAACAAATCCCT
H19	CATGACATGGTCCGGTGTGA	CACCTTCCAGAGCCGATTCC
ACTB	AAGACCTGTACACCAACACAG	AGGGCAGTGATCTCCTTCT
GAPDH	AAGGTGAAGGTCGGAGTCAA	GGAAGATGGTGATGGGATTT

Table S4: Specific primers used for validating representative m^{5}C sites.

Gene	Forward primer (5'-3')	Reverse primer (5'-3')
28s rRNA	GGGGTTTTAYGATTTTTTTGATTTTTTGGG	CCAACTCACRTTCCCTATTAATAAATAAAC
GRB2	TGTTTAGGGTGTAGTGTGAGTGT	ACTTCCTCCTCCACTCTCCTTATCTA
RAB13	GAGGGGTTTGGAGGGTTATAT	CTCCTCCCTCTCTTCCTAC

Table S5: Specific primers used for Bisulfite PCR Pyrosequencing of H19 RNA.

Primer	Sequence (5'-3')
Forward Primer	AGAGTGGGAAGATAGGTAGTGTT
Reverse Primer	AAACATACAACATCACCAAATCCACTAT (5'-Biotin labelled)
Sequencing Primer	GTTTGGGGAGTTGTAGTAG

Table S6: DNA probes against H19 RNA (3'-end Biotin-TEG-labelled).

Probe	Sequence (5'-3')
P1	TCATCCCGGTCACTTTTGGT
P2	CTTCCCCAGCCTTCTGAAAG
P3	GCAAAGGTGACATCTTCTCG
P4	TCATTTAGTAGCAGGCACAG
P5	CTGTATGCCCTCACCGCTCA
P6	GAGTGAATGAGCTCTCAGGA
P7	TGACAAGCAGGACATGACAT
P8	TTTACTTCCTCCACGGAGTC
P9	GACACAAAACCCTCTAGCTT
P10	CGACTCCATCTTCATGGCCA
P11	AAGTCATTTGCACTGGTTGG
P12	ATTCCATTACGCCCCATCTC
