# Supplementary material for: Aberrant NSUN2-mediated m5C modification of H19 lncRNA is associated with poor differentiation of hepatocellular carcinoma
Source: Oncogene. 2020 Sep 25;39(45):6906–19. doi: 10.1038/s41388-020-01475-w (PMC7644462; doi:10.1038/s41388-020-01475-w)
Supplement: Supplementary file 4 — Additional file 3A [file 41388_2020_1475_MOESM4_ESM.pdf]

SeqID	refPos	refStran	refBase	cov	C_count	methRat	mut_cou	mutRate	CalledB	CB_cou	state	c95_CI_	c95_CI_	p_value_	p_value_	p_value_	score	seqContext	candidateName
chr1	564976	+	C	34	14	0.412	0	0	T	20	M	0.264	0.578	0	9.2E-06	0	73.8241	aataagctatCgggcccatac	m5C_658
chr1	564980	+	C	39	9	0.231	0	0	T	30	M	0.126	0.383	4.9E-12	1.2E-06	5.7E-12	12.8739	agctatcgggCccataccccg	m5C_669
chr1	564988	+	C	38	11	0.289	0	0	T	27	M	0.17	0.448	2.1E-15	3.1E-06	2.7E-15	27.4488	ggcccataccCcgaaaatgtt	m5C_666
chr1	565007	+	C	37	17	0.459	0	0	T	20	M	0.31	0.616	0	2.1E-05	0	105.53	ttggttatatCcttcccgtac	m5C_656
chr1	565013	+	C	39	11	0.282	0	0	T	28	M	0.165	0.438	3.1E-15	3.1E-06	4E-15	26.4001	atatccttccCgtactaatta	m5C_652
chr1	565017	+	C	33	8	0.242	0	0	T	25	M	0.128	0.41	3.1E-11	6.8E-07	3.6E-11	10.7866	ccttcccgtaCtaattaatcc	m5C_653
chr1	1756907	-	C	32	7	0.219	0	0	T	25	M	0.11	0.388	8.5E-10	3.4E-07	9.5E-10	6.99971	cattgaagagCactaagatcg	m5C_711
chr1	1756917	-	C	32	10	0.312	0	0	T	22	M	0.18	0.486	1.1E-14	2E-06	1.3E-14	25.0836	ttgaaggtggCattgaagagc	m5C_680
chr1	2246740	+	C	37	13	0.351	0	0	T	24	M	0.218	0.512	0	6.7E-06	0	56.7461	ggcggcggtgCgaccccccagc	m5C_488
chr1	6271531	+	C	40	20	0.5	0	0	CT	20	M	0.352	0.648	0	1.4E-06	0	140.797	tcataggggggCctgattcctg	m5C_745
chr1	6271539	+	C	39	24	0.615	0	0	C	24	M	0.459	0.751	0	9.1E-05	0	220.314	ggcctgattcCtggactcatc	m5C_754
chr1	7279545	+	C	52	12	0.286	10	0.192	T	30	M	0.172	0.436	2.2E-16	7.5E-08	2.9E-16	32.2467	ctcactgcatCatttgtggta	m5C_772
chr1	7279582	+	C	89	20	0.225	0	0	T	69	M	0.15	0.322	0	1.5E-13	0	60.1775	gtgctctctcCtgacatttga	m5C_773
chr1	8030958	+	C	38	8	0.211	0	0	T	30	M	0.111	0.363	1.3E-10	6.8E-07	1.4E-10	8.77246	cactagtctgCtgctgtgaag	m5C_1048
chr1	8030975	+	C	136	32	0.235	0	0	T	104	M	0.172	0.313	0	0	0	110.006	gaaggagataCtgaaggagca	m5C_1049
chr1	8266602	-	C	84	30	0.357	0	0	T	54	M	0.263	0.464	0	1.2E-11	0	157.795	cagggcgaggCtcatgccctg	m5C_1081
chr1	8266607	-	C	113	70	0.619	0	0	C	70	M	0.527	0.704	0	6.2E-12	0	738.384	tttcccagggCgaggctcatg	m5C_1113
chr1	8266613	-	C	120	29	0.242	0	0	T	91	M	0.174	0.326	0	0	0	100.837	aggtggtttcCcagggcgagg	m5C_1102
chr1	8923296	-	C	35	12	0.343	0	0	T	23	M	0.208	0.508	0	4.6E-06	0	49.9956	gtgcactgggCaggtaagaat	m5C_1068
chr1	8923301	-	C	41	12	0.293	0	0	T	29	M	0.176	0.445	1.1E-16	7.5E-08	1.5E-16	33.7134	gggctgtgcaCtgggcaggta	m5C_1075
chr1	8923303	-	C	45	20	0.444	0	0	T	25	M	0.309	0.588	0	1.4E-06	0	123.755	tggggctgtgCactgggcagg	m5C_1062
chr1	8923308	-	C	43	29	0.674	0	0	C	29	M	0.525	0.795	0	9.7E-06	0	304.592	ggttgtggggCtgtgcactgg	m5C_1094
chr1	8924018	-	C	31	18	0.581	0	0	C	18	M	0.408	0.736	0	2.7E-05	0	146.757	aggccgtgaaCgagaagtcct	m5C_1143
chr1	8924024	-	C	33	10	0.303	0	0	T	23	M	0.174	0.473	1.6E-14	2E-06	2E-14	23.982	tcgccaaggcCgtgaacgaga	m5C_1092
chr1	8924059	-	C	34	27	0.794	0	0	C	27	M	0.632	0.897	0	0.00015	0	341.287	gggggatgatCtcacagtgac	m5C_1083
chr1	8924078	-	C	51	23	0.451	0	0	T	28	M	0.323	0.586	0	8.7E-08	0	148.43	gtgcaggaatCcaggtagtgg	m5C_1076
chr1	8925480	-	C	32	9	0.281	0	0	T	23	M	0.156	0.454	5.3E-13	1.2E-06	6.4E-13	17.1983	tggtcatcggCatggacgtag	m5C_1131
chr1	8931963	-	C	31	19	0.613	0	0	C	19	M	0.438	0.763	0	3.4E-05	0	166.53	ataagactcgCtatatgggga	m5C_1073
chr1	8931965	-	C	32	10	0.312	0	0	T	22	M	0.18	0.486	1.1E-14	2E-06	1.3E-14	25.0836	tgataagactCgctatatggg	m5C_1139
chr1	8931967	-	C	31	12	0.387	0	0	T	19	M	0.237	0.562	0	4.6E-06	0	56.9589	aatgataagaCtcgctatatg	m5C_1100
chr1	8931984	-	C	33	12	0.364	0	0	T	21	M	0.222	0.534	0	4.6E-06	0	53.2487	ccctagagctCcgggacaatg	m5C_1071
chr1	8931986	-	C	31	21	0.677	0	0	C	21	M	0.501	0.814	0	5.2E-05	0	210.591	ggccctagagCtccgggacaa	m5C_1085
chr1	9211728	-	C	40	10	0.25	0	0	T	30	M	0.142	0.402	1.8E-13	2.4E-08	2.2E-13	18.0914	gttgtggggcCcaagagggaa	m5C_1112
chr1	9211806	-	C	88	37	0.42	0	0	T	51	M	0.323	0.525	0	9.7E-11	0	238.849	ttggcagtgtCttagctggtt	m5C_1079
chr1	10665285	+	C	60	14	0.233	0	0	T	46	M	0.144	0.354	0	3.2E-11	0	40.4304	tgtggacgagCtgccctggtg	m5C_442
chr1	10665289	+	C	59	19	0.322	0	0	T	40	M	0.217	0.449	0	2.4E-08	0	82.4122	gacgagctgcCctggtggtcg	m5C_431
chr1	10665290	+	C	58	14	0.241	0	0	T	44	M	0.15	0.365	0	2.8E-09	0	41.8845	acgagctgccCtggtggtcgg	m5C_435
chr1	11968246	+	C	37	15	0.405	0	0	T	22	M	0.263	0.565	0	1.2E-05	0	79.0389	aaatctttcgCcttttactaa	m5C_444
chr1	11968287	+	C	926	268	0.289	0	0	T	658	M	0.261	0.319	0	0	0	1399.63	gagtgtgagtCtgaaaccaat	m5C_430
chr1	11968308	+	C	799	176	0.22	0	0	T	623	M	0.193	0.25	0	0	0	679.06	tttttgaggcCttgcgtttct	m5C_427
chr1	11968317	+	C	802	224	0.28	1	0.001	T	577	M	0.25	0.312	0	0	0	1118.55	ccttgcgtttCttagcagggc	m5C_443
chr1	11968327	+	C	639	251	0.393	0	0	T	388	M	0.356	0.431	0	0	0	1785.53	cttagcagggCttattttaag	m5C_440
chr1	11969899	-	C	35	26	0.743	0	0	C	26	M	0.579	0.858	0	0.00013	0	301.238	agttttaaacCaattttttag	m5C_458
chr1	11969900	-	C	36	27	0.75	0	0	C	27	M	0.589	0.862	0	0.00015	0	318.218	gagttttaaaCcaatttttta	m5C_451
chr1	11969912	-	C	98	47	0.48	0	0	T	51	M	0.383	0.577	0	3.7E-11	0	360.33	aggaataattCtgagttttaa	m5C_469
chr1	11969929	-	C	87	27	0.31	0	0	T	60	M	0.223	0.414	0	4E-12	0	120.361	taaagatttcCgtggagagga	m5C_461
chr1	11969930	-	C	80	25	0.312	0	0	T	55	M	0.222	0.421	0	1.8E-12	0	110.751	ctaaagatttCcgtggagagg	m5C_472
chr1	12306131	+	C	44	20	0.455	0	0	T	24	M	0.317	0.599	0	1.4E-06	0	126.82	tccaggctgaCttgtgtggag	m5C_244
chr1	12630066	-	C	50	10	0.2	0	0	T	40	M	0.112	0.33	2.6E-12	2E-10	3.1E-12	13.024	cagcactcccCtcgacaagga	m5C_266
chr1	12630067	-	C	46	11	0.239	0	0	T	35	M	0.139	0.379	2.8E-14	4.4E-08	3.5E-14	20.7303	tcagcactccCctcgacaagg	m5C_272
chr1	12630068	-	C	50	13	0.26	0	0	T	37	M	0.159	0.396	0	1.6E-09	0	41.2656	gtcagcactcCctcgacaag	m5C_267
chr1	12630071	-	C	47	10	0.213	0	0	T	37	M	0.12	0.349	1.3E-12	2.4E-08	1.5E-12	14.271	aaggtcagcaCtcccctcgac	m5C_256
chr1	12630073	-	C	48	18	0.375	0	0	T	30	M	0.252	0.516	0	7.7E-07	0	90.7743	agaaggtcagCactcccctcg	m5C_275
chr1	13949751	-	C	66	22	0.333	0	0	T	44	M	0.232	0.453	0	1.5E-09	0	101.888	ctggatgcgtCtcagaagcta	m5C_269
chr1	13949761	-	C	140	50	0.357	0	0	T	90	M	0.283	0.439	0	0	0	282.56	ccataccattCtggatgcgtc	m5C_264
chr1	13949771	-	C	137	56	0.409	0	0	T	81	M	0.33	0.492	0	4.4E-16	0	369.621	gcgcctacggCcataccattc	m5C_258
chr1	13949774	-	C	136	28	0.206	0	0	T	108	M	0.146	0.281	0	0	0	82.0171	ccggcgcctaCggccatacca	m5C_276
chr1	15755140	+	C	33	18	0.545	0	0	C	18	M	0.38	0.702	0	2.7E-05	0	136.748	caaggcagagCaggaggaaag	m5C_40
chr1	16134106	-	C	31	14	0.452	0	0	T	17	M	0.292	0.622	0	9.2E-06	0	81.6522	ttaccgaatcCggagatcctg	m5C_8005
chr1	16134112	-	C	31	11	0.355	0	0	T	20	M	0.211	0.531	1.1E-16	3.1E-06	1.5E-16	37.0595	agatgcttacCgaatccggag	m5C_7996
chr1	16861784	-	C	652	137	0.21	1	0.002	T	514	M	0.181	0.243	0	0	0	495.602	gttcgattccCggtcagggaa	m5C_8039
chr1	16861785	-	C	666	136	0.205	2	0.003	T	528	M	0.176	0.237	0	0	0	478.345	ggttcgattcCcggtcaggga	m5C_8032
chr1	16861797	-	C	672	167	0.249	0	0	T	505	M	0.217	0.283	0	0	0	725.878	cgccgcggccCgggttcgatt	m5C_8035
chr1	16861799	-	C	653	162	0.248	0	0	T	491	M	0.216	0.283	0	0	0	701.447	accgccgcggCccgggttcga	m5C_8002
chr1	16861814	-	C	594	124	0.209	0	0	T	470	M	0.178	0.243	0	0	0	441.423	ggattcggcgCtttcaccgcc	m5C_8020
chr1	16861834	-	C	402	93	0.232	1	0.002	T	308	M	0.193	0.276	0	0	0	359.486	ccctggtggtCtagtggctag	m5C_8050
chr1	16872426	-	C	179	43	0.24	0	0	T	136	M	0.184	0.308	0	0	0	157.824	gcaagagggtCtttttcaccc	m5C_8001
chr1	16872435	-	C	233	48	0.206	0	0	T	185	M	0.159	0.263	0	0	0	152.687	ccggccaatgCaagagggtct	m5C_8017
chr1	16872444	-	C	252	51	0.202	0	0	T	201	M	0.157	0.256	0	0	0	160.563	gttcaattccCggccaatgca	m5C_8000
chr1	16872451	-	C	243	50	0.206	0	0	T	193	M	0.16	0.261	0	0	0	159.701	gacccgggttCaattcccggc	m5C_8015
chr1	16872457	-	C	247	75	0.306	2	0.008	T	170	M	0.252	0.366	0	0	0	377.66	gcgggagaccCgggttcaatt	m5C_8038
chr1	16872468	-	C	236	63	0.267	0	0	T	173	M	0.215	0.327	0	0	0	270.361	tcgcctcccaCgcgggagacc	m5C_8006
chr1	16872472	-	C	233	57	0.245	0	0	T	176	M	0.194	0.304	0	0	0	221.015	attctcgcctCccacgcggga	m5C_8009
chr1	16872479	-	C	226	81	0.358	0	0	T	145	M	0.299	0.423	0	0	0	483.948	tggtagaattCtcgcctccca	m5C_8040
chr1	16872492	-	C	224	58	0.259	0	0	T	166	M	0.206	0.32	0	0	0	238.923	attggtggttCagtggtagaa	m5C_8013
chr1	17004765	-	C	80	18	0.225	0	0	T	62	M	0.147	0.328	0	4.5E-14	0	53.0391	ggccactgcaCgtggttgttt	m5C_8008
chr1	17004777	-	C	126	35	0.278	0	0	T	91	M	0.207	0.362	0	0	0	144.921	ggttcaattcCcggccactgc	m5C_8004
chr1	17004789	-	C	213	76	0.357	0	0	T	137	M	0.296	0.423	0	0	0	449.206	gcgggagaccCgggttcaatt	m5C_8007
chr1	17004790	-	C	240	53	0.221	0	0	T	187	M	0.173	0.277	0	0	0	183.359	tgcgggagacCcgggttcaat	m5C_8049
chr1	17004802	-	C	373	79	0.212	0	0	T	294	M	0.173	0.256	0	0	0	273.935	tctcgcctccCatgcgggaga	m5C_8027
chr1	17004806	-	C	498	113	0.227	0	0	T	385	M	0.192	0.266	0	0	0	434.584	gaattctcgcCtcccatgcgg	m5C_7999
chr1	17004811	-	C	612	154	0.252	0	0	T	458	M	0.219	0.287	0	0	0	674.133	tggtagaattCtcgcctccca	m5C_8018
chr1	17004835	-	C	431	98	0.227	0	0	T	333	M	0.19	0.269	0	0	0	373.022	tccagagaggCgttggtggtt	m5C_8011
chr1	17052065	+	C	41	13	0.317	0	0	T	28	M	0.196	0.47	0	1.2E-07	0	50.8675	tgtgcagtttCtgtagtatag	m5C_7959
chr1	17052083	+	C	53	15	0.283	0	0	T	38	M	0.18	0.416	0	4.6E-09	0	53.8984	tagtggttatCatgtttgcct	m5C_7911
chr1	17053782	+	C	200	39	0.267	54	0.27	T	107	M	0.202	0.344	0	0	0	157.558	gtgtagaggcCttggtggtgc	m5C_7989
chr1	17053792	+	C	234	69	0.295	0	0	T	165	M	0.24	0.356	0	0	0	331.392	cttggtggtgCagtggtagaa	m5C_7905
chr1	17053809	+	C	172	55	0.32	0	0	T	117	M	0.255	0.393	0	0	0	280.123	agaattctcgCctcccacgtg	m5C_7903
chr1	17053810	+	C	177	37	0.209	0	0	T	140	M	0.156	0.275	0	0	0	115.169	gaattctcgcCtcccacgtgg	m5C_7900
chr1	17053812	+	C	176	41	0.233	0	0	T	135	M	0.177	0.301	0	0	0	144.824	attctcgcctCccacgtggga	m5C_7933
chr1	17053827	+	C	131	31	0.237	0	0	T	100	M	0.172	0.316	0	0	0	106.649	gtgggagaccCgggttcaatt	m5C_7950
chr1	17053840	+	C	133	32	0.241	0	0	T	101	M	0.176	0.32	0	0	0	112.573	gttcaattccCggccaatgca	m5C_7976
chr1	17053869	+	C	31	10	0.323	0	0	T	21	M	0.186	0.499	7.1E-15	2E-06	9E-15	26.2725	ttcttcatttCattatggcct	m5C_7995
chr1	17067034	+	C	38	8	0.211	0	0	T	30	M	0.111	0.363	1.3E-10	6.8E-07	1.4E-10	8.77246	aggggagataCcatgatcacg	m5C_7893
chr1	17067035	+	C	38	8	0.211	0	0	T	30	M	0.111	0.363	1.3E-10	6.8E-07	1.4E-10	8.77246	ggggagatacCatgatcacga	m5C_7939
chr1	17067056	+	C	38	17	0.447	0	0	T	21	M	0.301	0.603	0	2.1E-05	0	102.497	aggtggttttCccagggcgag	m5C_7970
chr1	17067083	+	C	139	31	0.223	0	0	T	108	M	0.162	0.299	0	0	0	100.315	ccattgcactCcggatgtgct	m5C_7921
chr1	17067096	+	C	150	32	0.213	0	0	T	118	M	0.155	0.286	0	0	0	99.4313	gatgtgctgaCccctgcgatt	m5C_7948
chr1	17067098	+	C	147	32	0.218	0	0	T	115	M	0.159	0.291	0	0	0	101.522	tgtgctgaccCctgcgatttc	m5C_7926
chr1	17067102	+	C	141	32	0.227	0	0	T	109	M	0.166	0.303	0	0	0	105.98	ctgacccctgCgatttcccca	m5C_7897

chr1	17067108	+	C	129	27	0.209	0	0	T	102	M	0.148	0.287	0	0	0	79.9307	cctgcgatttCcccaaatgtg	m5C_7953
chr1	17067109	+	C	132	37	0.28	0	0	T	95	M	0.211	0.362	0	0	0	155.94	ctgcgatttcCccaaatgtgg	m5C_7978
chr1	17067124	+	C	97	24	0.247	0	0	T	73	M	0.172	0.342	0	1.7E-14	0	82.6914	atgtgggaaaCtcgactgcat	m5C_7924
chr1	17067129	+	C	90	22	0.244	0	0	T	68	M	0.167	0.342	0	5.8E-15	0	73.6237	ggaaactcgaCtgcataattt	m5C_7971
chr1	17067154	+	C	65	20	0.308	0	0	T	45	M	0.209	0.428	0	6.9E-10	0	83.5475	tagtgggggaCtgcgttcgcg	m5C_7932
chr1	17067161	+	C	56	16	0.296	2	0.036	T	38	M	0.191	0.428	0	7.3E-09	0	61.2393	ggactgcgttCgcgctttccc	m5C_7896
chr1	17067163	+	C	56	20	0.357	0	0	T	36	M	0.245	0.488	0	3.4E-08	0	97.8256	actgcgttcgCgctttcccct	m5C_7910
chr1	17067169	+	C	49	10	0.204	0	0	T	39	M	0.115	0.336	2.1E-12	2.4E-08	2.4E-12	13.4191	ttcgcgctttCccctgacttt	m5C_7972
chr1	17067170	+	C	46	13	0.283	0	0	T	33	M	0.173	0.425	0	1.2E-07	0	45.0425	tcgcgctttcCcctgactttc	m5C_7981
chr1	17067171	+	C	44	18	0.409	0	0	T	26	M	0.277	0.556	0	7.7E-07	0	99.6755	cgcgctttccCctgactttct	m5C_7960
chr1	17067172	+	C	41	10	0.244	0	0	T	31	M	0.138	0.393	2.4E-13	2.4E-08	2.9E-13	17.4472	gcgctttcccCtgactttctg	m5C_7931
chr1	17186721	+	C	54	22	0.407	0	0	T	32	M	0.287	0.54	0	6.5E-08	0	126.183	ttattatgttCgcttcacata	m5C_7928
chr1	17186723	+	C	62	24	0.387	0	0	T	38	M	0.276	0.512	0	3E-09	0	132.405	attatgttcgCttcacatatg	m5C_7918
chr1	17186726	+	C	46	15	0.333	1	0.022	T	30	M	0.214	0.479	0	2.8E-07	0	64.0761	atgttcgcttCacatatgaaa	m5C_7943
chr1	17186728	+	C	42	25	0.595	0	0	C	25	M	0.445	0.73	0	4.5E-06	0	222.47	gttcgcttcaCatatgaaagg	m5C_7901
chr1	17186740	+	C	44	13	0.295	0	0	T	31	M	0.182	0.442	0	1.2E-07	0	47.2039	tatgaaaggtCtctggttcga	m5C_7916
chr1	17186742	+	C	56	23	0.411	0	0	T	33	M	0.292	0.541	0	8.7E-08	0	134.166	tgaaaggtctCtggttcgaga	m5C_7942
chr1	17186753	+	C	81	21	0.259	0	0	T	60	M	0.176	0.364	0	2.6E-13	0	74.0352	tggttcgagaCtgcgtgggaa	m5C_7890
chr1	17186767	+	C	82	30	0.366	0	0	T	52	M	0.27	0.474	0	1.2E-11	0	161.871	gtgggaacatCgtgttttgtt	m5C_7979
chr1	17188445	+	C	290	58	0.2	0	0	T	232	M	0.158	0.25	0	0	0	183.301	agaattctcgCctcccacgcg	m5C_7982
chr1	17188449	+	C	285	77	0.27	0	0	T	208	M	0.222	0.325	0	0	0	341.773	ttctcgcctcCcacgcgggag	m5C_7975
chr1	17188452	+	C	292	62	0.212	0	0	T	230	M	0.169	0.263	0	0	0	209.948	tcgcctcccaCgcgggagacc	m5C_7949
chr1	17188462	+	C	216	70	0.324	0	0	T	146	M	0.265	0.389	0	0	0	371.284	cgcgggagacCcgggttcaat	m5C_7930
chr1	17188463	+	C	211	46	0.218	0	0	T	165	M	0.168	0.278	0	0	0	154.201	gcgggagaccCgggttcaatt	m5C_7951
chr1	17188494	+	C	155	39	0.252	0	0	T	116	M	0.19	0.325	0	0	0	148.098	gcaaaagggtCtttttcaccc	m5C_7917
chr1	17199109	+	C	771	159	0.206	1	0.001	T	611	M	0.179	0.237	0	0	0	570.469	ggattcggcgCtttcaccgcc	m5C_7919
chr1	17199118	+	C	799	175	0.219	0	0	T	624	M	0.192	0.249	0	0	0	671.046	gctttcaccgCcgcggcccgg	m5C_7983
chr1	17199119	+	C	793	184	0.232	1	0.001	T	608	M	0.204	0.263	0	0	0	751.624	ctttcaccgcCgcggcccggg	m5C_7909
chr1	17199124	+	C	806	180	0.226	8	0.01	T	618	M	0.198	0.256	0	0	0	712.51	accgccgcggCccgggttcga	m5C_7894
chr1	17199125	+	C	794	212	0.271	13	0.016	T	569	M	0.241	0.304	0	0	0	1023.67	ccgccgcggcCcgggttcgat	m5C_7969
chr1	17199126	+	C	790	161	0.207	11	0.014	T	618	M	0.18	0.237	0	0	0	578.675	cgccgcggccCgggttcgatt	m5C_7920
chr1	17199132	+	C	788	159	0.204	9	0.011	T	620	M	0.177	0.234	0	0	0	563.777	ggcccgggttCgattcccggc	m5C_7925
chr1	17199139	+	C	748	157	0.21	2	0.003	T	589	M	0.183	0.241	0	0	0	573.755	gttcgattccCggccagggaa	m5C_7955
chr1	17216188	+	C	33	8	0.242	0	0	T	25	M	0.128	0.41	3.1E-11	6.8E-07	3.6E-11	10.7866	gtggcgcaatCggttagcgcg	m5C_7914
chr1	17222532	+	C	34	16	0.471	0	0	T	18	M	0.315	0.633	0	1.7E-05	0	100.644	cagggcgaggCttatccattg	m5C_7904
chr1	17222538	+	C	35	7	0.2	0	0	T	28	M	0.1	0.359	1.9E-09	3.4E-07	2.1E-09	6.13884	gaggcttatcCattgcactcc	m5C_7967
chr1	17222547	+	C	61	13	0.213	0	0	T	48	M	0.129	0.331	1.4E-15	1.6E-11	1.9E-15	24.8871	ccattgcactCcggatgtgct	m5C_7956
chr1	17222560	+	C	63	16	0.254	0	0	T	47	M	0.163	0.373	0	1.1E-10	0	52.0969	gatgtgctgaCccctgcgatt	m5C_7973
chr1	17222563	+	C	59	14	0.237	0	0	T	45	M	0.147	0.36	0	2.8E-09	0	41.1446	gtgctgacccCtgcgatttcc	m5C_7938
chr1	17222566	+	C	43	10	0.233	0	0	T	33	M	0.132	0.377	4.3E-13	2.4E-08	5.2E-13	16.2683	ctgacccctgCgatttcccca	m5C_7991
chr1	17222572	+	C	43	23	0.535	0	0	C	23	M	0.389	0.675	0	2.9E-06	0	179.011	cctgcgatttCcccaaatgtg	m5C_7944
chr1	17222573	+	C	38	11	0.289	0	0	T	27	M	0.17	0.448	2.1E-15	3.1E-06	2.7E-15	27.4488	ctgcgatttcCccaaatgtgg	m5C_7987
chr1	17222588	+	C	34	9	0.265	0	0	T	25	M	0.146	0.431	1.1E-12	1.2E-06	1.3E-12	15.7394	atgtgggaaaCtcgactgcat	m5C_7980
chr1	17222590	+	C	33	11	0.333	0	0	T	22	M	0.198	0.504	2.2E-16	3.1E-06	2.9E-16	34.0074	gtgggaaactCgactgcataa	m5C_7986
chr1	17222593	+	C	35	10	0.286	0	0	T	25	M	0.163	0.451	3.4E-14	2E-06	4.2E-14	21.9991	ggaaactcgaCtgcataattt	m5C_7977
chr1	17439797	-	C	89	43	0.483	0	0	T	46	M	0.382	0.585	0	4.1E-10	0	328.684	ccctggtggtCtagtggttag	m5C_8053
chr1	17946016	+	C	65	29	0.446	0	0	T	36	M	0.332	0.567	0	1.3E-08	0	192.382	ttctctggggCcacgcttgtc	m5C_7946
chr1	17946019	+	C	68	15	0.221	0	0	T	53	M	0.138	0.333	0	6E-11	0	41.5467	tctggggccaCgcttgtctgg	m5C_7994
chr1	17946021	+	C	61	16	0.262	0	0	T	45	M	0.168	0.384	0	1.1E-10	0	53.8837	tggggccacgCttgtctggat	m5C_7940
chr1	18055888	+	C	60	12	0.2	0	0	T	48	M	0.118	0.318	3.3E-14	7.6E-12	4.2E-14	19.13	aatgccacgcCtgcccgggtg	m5C_52
chr1	18055892	+	C	59	29	0.492	0	0	T	30	M	0.368	0.616	0	3.9E-07	0	213.693	ccacgcctgcCcgggtgctgc	m5C_64
chr1	18055902	+	C	57	22	0.386	0	0	T	35	M	0.271	0.516	0	6.5E-08	0	119.075	ccgggtgctgCgtttgctcac	m5C_65
chr1	21012796	+	C	48	22	0.458	0	0	T	26	M	0.326	0.597	0	2.3E-06	0	143.33	agggagagaaCagggaagaga	m5C_391
chr1	21103141	-	C	43	14	0.326	0	0	T	29	M	0.205	0.475	0	1.9E-07	0	57.3807	aagggggaacCtgagaatgaa	m5C_401
chr1	21103142	-	C	45	18	0.4	0	0	T	27	M	0.27	0.545	0	7.7E-07	0	97.2889	aaagggggaaCctgagaatga	m5C_411
chr1	21103153	-	C	32	18	0.562	0	0	C	18	M	0.393	0.718	0	2.7E-05	0	141.571	gcagagcagcCaaagggggaa	m5C_412
chr1	24019161	+	C	41	20	0.488	0	0	T	21	M	0.343	0.635	0	1.4E-06	0	137.017	tctgtctcaaCatctgtgttg	m5C_154
chr1	24019185	+	C	74	17	0.23	0	0	T	57	M	0.149	0.337	0	2.2E-12	0	50.548	agagtggagaCagactgacgc	m5C_144
chr1	24019189	+	C	36	8	0.222	0	0	T	28	M	0.117	0.381	7.4E-11	6.8E-07	8.4E-11	9.49676	tggagacagaCtgacgcgagc	m5C_170
chr1	24019193	+	C	34	10	0.294	0	0	T	24	M	0.168	0.462	2.3E-14	2E-06	2.9E-14	22.9564	gacagactgaCgcgagcagcc	m5C_134
chr1	24019203	+	C	36	9	0.25	0	0	T	27	M	0.138	0.411	2E-12	1.2E-06	2.4E-12	14.4765	cgcgagcagcCaaggtgttgg	m5C_151
chr1	24022338	+	C	41	21	0.512	0	0	C	21	M	0.365	0.657	0	1.8E-06	0	153.234	gcaggacaggCtgcattgggg	m5C_149
chr1	24022350	+	C	31	12	0.387	0	0	T	19	M	0.237	0.562	0	4.6E-06	0	56.9589	gcattggggcCaaacacagaa	m5C_138
chr1	24402611	+	C	53	42	0.792	0	0	C	42	M	0.665	0.88	0	3.9E-06	0	558.953	agtggcgtagCccagtggcag	m5C_168
chr1	24402624	+	C	40	17	0.425	0	0	T	23	M	0.285	0.578	0	5.6E-07	0	96.9312	agtggcagagCtctggcctct	m5C_147
chr1	26232882	-	C	39	16	0.41	0	0	T	23	M	0.271	0.566	0	1.7E-05	0	86.6531	tcggactgagCaggtgggtgc	m5C_334
chr1	26232891	-	C	46	12	0.261	0	0	T	34	M	0.156	0.403	6.7E-16	7.5E-08	8.7E-16	28.4116	tcaggcggctCggactgagca	m5C_353
chr1	26737926	+	C	50	20	0.4	0	0	T	30	M	0.276	0.538	0	1.4E-06	0	110.433	cgccggaggaCgcggcccggg	m5C_300
chr1	26737932	+	C	51	32	0.627	0	0	C	32	M	0.49	0.747	0	7.2E-07	0	313.76	aggacgcggcCcgggcggcgg	m5C_292
chr1	26737940	+	C	35	14	0.4	0	0	T	21	M	0.256	0.564	0	9.2E-06	0	71.5414	gcccgggcggCggacgagcct	m5C_277
chr1	27269155	+	C	32	8	0.25	0	0	T	24	M	0.133	0.421	2.3E-11	6.8E-07	2.6E-11	11.2828	aagcaggataCtgaggaagat	m5C_301
chr1	28160933	+	C	41	22	0.537	0	0	C	22	M	0.387	0.679	0	2.3E-06	0	170.483	actaaccgagCtgtctatatc	m5C_4217
chr1	28160943	+	C	33	9	0.273	0	0	T	24	M	0.151	0.442	7.5E-13	1.2E-06	9E-13	16.4418	ctgtctatatCctagccttgt	m5C_4152
chr1	28160956	+	C	36	11	0.306	0	0	T	25	M	0.18	0.469	1E-15	3.1E-06	1.3E-15	29.7077	agccttgtgtCaaaggcttgt	m5C_4252
chr1	28160982	+	C	60	17	0.283	0	0	T	43	M	0.185	0.408	0	1.8E-10	0	62.9227	gttgtgatagCtgggtttaag	m5C_4219
chr1	28160998	+	C	62	15	0.242	0	0	T	47	M	0.152	0.362	0	6E-11	0	45.7373	ttaagaagtgCaatattaaaa	m5C_4165
chr1	28358523	-	C	34	7	0.206	0	0	T	27	M	0.103	0.368	1.4E-09	3.4E-07	1.6E-09	6.4054	cgccaggttgCctaaggaagg	m5C_4320
chr1	28358533	-	C	42	19	0.452	0	0	T	23	M	0.312	0.601	0	1E-06	0	118.648	ggtgggggaaCgccaggttgc	m5C_4294
chr1	28358534	-	C	42	10	0.238	0	0	T	32	M	0.135	0.385	3.2E-13	2.4E-08	3.9E-13	16.8407	gggtgggggaCcgccaggttg	m5C_4298
chr1	28358593	-	C	78	28	0.359	0	0	T	50	M	0.261	0.47	0	2.6E-10	0	146.414	gtgcgctatgCtgattgggta	m5C_4271
chr1	28358600	-	C	74	15	0.203	0	0	T	59	M	0.127	0.308	0	6E-13	0	38.0609	ggctgtagtgCgctatgctga	m5C_4306
chr1	28562913	+	C	38	8	0.211	0	0	T	30	M	0.111	0.363	1.3E-10	6.8E-07	1.4E-10	8.77246	tccgggaagcCggtggggcct	m5C_4144
chr1	28562921	+	C	58	26	0.448	0	0	T	32	M	0.327	0.575	0	1.9E-07	0	170.298	gccggtggggCcttcggaaag	m5C_4153
chr1	28562922	+	C	61	18	0.295	0	0	T	43	M	0.196	0.419	0	2.9E-10	0	70.3975	ccggtggggcCttcggaaaga	m5C_4216
chr1	28833882	+	C	892	212	0.238	0	0	T	680	M	0.211	0.267	0	0	0	894.201	gattgtccaaCgtggatacac	m5C_4145
chr1	28833890	+	C	1576	322	0.204	0	0	T	1254	M	0.185	0.225	0	0	0	1192.29	aacgtggataCacccgggagg	m5C_4209
chr1	28833893	+	C	1627	624	0.384	0	0	T	1003	M	0.36	0.407	0	0	0	4495.31	gtggatacacCcgggaggtca	m5C_4190
chr1	28833894	+	C	1627	346	0.213	0	0	T	1281	M	0.193	0.233	0	0	0	1338.79	tggatacaccCgggaggtcac	m5C_4174
chr1	28833906	+	C	1035	248	0.24	2	0.002	T	785	M	0.215	0.267	0	0	0	1066.52	ggaggtcactCtccccgggct	m5C_4139
chr1	28833908	+	C	993	221	0.223	0	0	T	772	M	0.198	0.249	0	0	0	874.198	aggtcactctCcccgggctct	m5C_4140
chr1	28833909	+	C	973	233	0.239	0	0	T	740	M	0.214	0.267	0	0	0	995.88	ggtcactctcCccgggctctg	m5C_4199
chr1	28833910	+	C	948	234	0.247	1	0.001	T	713	M	0.221	0.276	0	0	0	1032.8	gtcactctccCcgggctctgt	m5C_4189
chr1	28833915	+	C	783	235	0.3	0	0	T	548	M	0.269	0.333	0	0	0	1264.6	tctccccgggCtctgtccaag	m5C_4168
chr1	28833917	+	C	746	209	0.28	0	0	T	537	M	0.249	0.313	0	0	0	1041.34	tccccgggctCtgtccaagtg	m5C_4159
chr1	28833922	+	C	695	158	0.227	0	0	T	537	M	0.198	0.26	0	0	0	624.817	gggctctgtcCaagtggcgta	m5C_4228
chr1	28833929	+	C	496	137	0.278	3	0.006	T	356	M	0.24	0.319	0	0	0	658.094	gtccaagtggCgtaggggagc	m5C_4160
chr1	28833939	+	C	363	101	0.278	0	0	T	262	M	0.235	0.326	0	0	0	473.976	cgtaggggagCatagggctct	m5C_4205
chr1	28833946	+	C	245	68	0.278	0	0	T	177	M	0.225	0.337	0	0	0	306.328	gagcatagggCtctgccccat	m5C_4183
chr1	28833948	+	C	196	79	0.403	0	0	T	117	M	0.337	0.473	0	0	0	532.287	gcatagggctCtgccccatga	m5C_4247
chr1	28833951	+	C	157	41	0.263	1	0.006	T	115	M	0.2	0.337	0	0	0	164.037	tagggctctgCcccatgatgt	m5C_4211
chr1	28833952	+	C	144	30	0.208	0	0	T	114	M	0.15	0.282	0	0	0	90.006	agggctctgcCccatgatgta	m5C_4224

chr1	28833954	+	C	135	28	0.207	0	0	T	107	M	0.148	0.283	0	0	0	82.6429	ggctctgcccCatgatgtaca	m5C_4248
chr1	28833963	+	C	47	17	0.362	0	0	T	30	M	0.24	0.505	0	5.6E-07	0	81.4845	ccatgatgtaCaagtcccttt	m5C_4163
chr1	28833968	+	C	47	13	0.277	0	0	T	34	M	0.169	0.418	0	1.2E-07	0	44.0347	atgtacaagtCcctttccaca	m5C_4172
chr1	28833969	+	C	55	11	0.2	0	0	T	44	M	0.116	0.324	2.9E-13	4.4E-10	3.6E-13	15.9231	tgtacaagtcCctttccacaa	m5C_4154
chr1	28833970	+	C	50	17	0.34	0	0	T	33	M	0.224	0.478	0	5.6E-07	0	76.285	gtacaagtccCtttccacaac	m5C_4170
chr1	28833974	+	C	64	13	0.203	0	0	T	51	M	0.123	0.317	3E-15	1.6E-11	3.9E-15	23.1722	aagtccctttCcacaacgttg	m5C_4213
chr1	28833977	+	C	77	23	0.299	0	0	T	54	M	0.208	0.408	0	4.3E-11	0	95.6973	tccctttccaCaacgttggaa	m5C_4156
chr1	28834007	+	C	166	40	0.241	0	0	T	126	M	0.182	0.311	0	0	0	145.79	ggcctcgtgtCtgcgcctgca	m5C_4212
chr1	28834012	+	C	177	50	0.282	0	0	T	127	M	0.221	0.353	0	0	0	221.326	cgtgtctgcgCctgcatattc	m5C_4207
chr1	28834016	+	C	178	39	0.219	0	0	T	139	M	0.165	0.285	0	0	0	128.405	tctgcgcctgCatattcctac	m5C_4184
chr1	28834022	+	C	140	68	0.486	0	0	T	72	M	0.404	0.568	0	2.2E-16	0	550.007	cctgcatattCctacagcttc	m5C_4234
chr1	28834029	+	C	171	41	0.24	0	0	T	130	M	0.182	0.309	0	0	0	149.192	attcctacagCttccccagagt	m5C_4202
chr1	28834040	+	C	357	115	0.322	0	0	T	242	M	0.276	0.372	0	0	0	634.271	ttcccagagtCctgtcgacaa	m5C_4157
chr1	28834041	+	C	382	82	0.215	0	0	T	300	M	0.176	0.259	0	0	0	289.351	tcccagagtcCtgtcgacaat	m5C_4223
chr1	28834045	+	C	451	130	0.289	1	0.002	T	320	M	0.249	0.332	0	0	0	647.237	agagtcctgtCgacaattact	m5C_4255
chr1	28834054	+	C	674	165	0.246	3	0.004	T	506	M	0.215	0.28	0	0	0	708.926	tcgacaattaCtggggagaca	m5C_4178
chr1	28834068	+	C	950	195	0.205	0	0	T	755	M	0.181	0.232	0	0	0	705.083	ggagacaaacCatgcaggaaa	m5C_4158
chr1	28834082	+	C	687	197	0.287	0	0	T	490	M	0.254	0.322	0	0	0	1001.53	caggaaacagCcttctagagc	m5C_4151
chr1	28834083	+	C	574	145	0.253	0	0	T	429	M	0.219	0.29	0	0	0	634.495	aggaaacagcCttctagagca	m5C_4148
chr1	28835075	+	C	659	190	0.288	0	0	T	469	M	0.255	0.324	0	0	0	969.136	agcattccaaCgtggataccc	m5C_4167
chr1	28835083	+	C	1299	297	0.229	0	0	T	1002	M	0.207	0.252	0	0	0	1227.32	aacgtggataCcctgggaggt	m5C_4137
chr1	28835085	+	C	1301	344	0.264	0	0	T	957	M	0.241	0.289	0	0	0	1659.22	cgtggataccCtgggaggtca	m5C_4208
chr1	28835094	+	C	1353	351	0.259	0	0	T	1002	M	0.237	0.283	0	0	0	1662.14	cctgggaggtCactctcccca	m5C_4195
chr1	28835096	+	C	1201	262	0.218	1	0.001	T	938	M	0.196	0.243	0	0	0	1026.4	tgggaggtcaCtctccccagg	m5C_4173
chr1	28835098	+	C	1097	278	0.253	0	0	T	819	M	0.229	0.28	0	0	0	1270.85	ggaggtcactCtccccaggct	m5C_4176
chr1	28835102	+	C	890	223	0.251	1	0.001	T	666	M	0.223	0.28	0	0	0	996.633	gtcactctccCcaggctctgt	m5C_4169
chr1	28835107	+	C	860	233	0.271	1	0.001	T	626	M	0.243	0.302	0	0	0	1130.42	tctccccaggCtctgtccaag	m5C_4240
chr1	28835109	+	C	765	229	0.299	0	0	T	536	M	0.268	0.333	0	0	0	1227.26	tccccaggctCtgtccaagtg	m5C_4250
chr1	28835113	+	C	680	192	0.283	1	0.001	T	487	M	0.25	0.318	0	0	0	960.73	caggctctgtCcaagtggcat	m5C_4242
chr1	28835114	+	C	665	145	0.218	0	0	T	520	M	0.188	0.251	0	0	0	546.154	aggctctgtcCaagtggcata	m5C_4200
chr1	28835121	+	C	512	122	0.238	0	0	T	390	M	0.203	0.277	0	0	0	496.328	gtccaagtggCataggggagc	m5C_4232
chr1	28835131	+	C	359	77	0.214	0	0	T	282	M	0.175	0.26	0	0	0	269.753	cataggggagCttagggctct	m5C_4214
chr1	28835138	+	C	249	75	0.301	0	0	T	174	M	0.248	0.361	0	0	0	371.391	gagcttagggCtctgccccat	m5C_4251
chr1	28835160	+	C	61	28	0.459	0	0	T	33	M	0.34	0.583	0	9.8E-09	0	190.471	atgtacagtcCctttccacaa	m5C_4254
chr1	28835165	+	C	44	14	0.318	0	0	T	30	M	0.2	0.466	0	1.9E-07	0	55.9976	cagtccctttCcacaacgttg	m5C_4229
chr1	28835190	+	C	112	28	0.25	0	0	T	84	M	0.179	0.338	0	0	0	100.241	tgaagctgggCctcgtgtctg	m5C_4253
chr1	28835191	+	C	93	24	0.258	0	0	T	69	M	0.18	0.355	0	1.7E-14	0	86.3923	gaagctgggcCtcgtgtctgc	m5C_4193
chr1	28835193	+	C	81	18	0.222	0	0	T	63	M	0.145	0.324	0	4.5E-14	0	52.3602	agctgggcctCgtgtctgcgc	m5C_4225
chr1	28835204	+	C	80	22	0.275	0	0	T	58	M	0.189	0.381	0	2.8E-11	0	83.2375	gtgtctgcgcCtgcatattcc	m5C_4162
chr1	28835213	+	C	70	17	0.243	0	0	T	53	M	0.158	0.355	0	2.2E-12	0	53.5559	cctgcatattCctacagcttc	m5C_4142
chr1	28835224	+	C	131	27	0.206	0	0	T	104	M	0.146	0.283	0	0	0	78.6736	ctacagcttcCcagagtcctg	m5C_4177
chr1	28835232	+	C	318	64	0.201	0	0	T	254	M	0.161	0.249	0	0	0	205.92	tcccagagtcCtgtggacaat	m5C_4239
chr1	28835245	+	C	761	184	0.242	0	0	T	577	M	0.213	0.273	0	0	0	782.779	tggacaatgaCtggggagaca	m5C_4203
chr1	28835258	+	C	929	264	0.284	0	0	T	665	M	0.256	0.314	0	0	0	1352.25	gggagacaaaCcatgcaggaa	m5C_4191
chr1	28905266	-	C	453	143	0.316	0	0	T	310	M	0.275	0.36	0	0	0	785.282	gggactgagaCcagctcctag	m5C_4277
chr1	28905272	-	C	960	228	0.237	0	0	T	732	M	0.212	0.265	0	0	0	965.169	ggatatgggaCtgagaccagc	m5C_4331
chr1	28905299	-	C	1042	217	0.209	5	0.005	T	820	M	0.186	0.235	0	0	0	805.48	gagtggacatCcatggatgag	m5C_4282
chr1	28906291	-	C	1061	267	0.252	2	0.002	T	792	M	0.227	0.279	0	0	0	1211.64	ggcatgtgggCtagtttcaga	m5C_4301
chr1	28906378	-	C	404	89	0.22	0	0	T	315	M	0.183	0.263	0	0	0	325.067	cccatcggatCtgaacactgg	m5C_4283
chr1	28906383	-	C	380	88	0.232	0	0	T	292	M	0.192	0.277	0	0	0	337.882	cctttcccatCggatctgaac	m5C_4341
chr1	28906387	-	C	363	78	0.215	0	0	T	285	M	0.176	0.26	0	0	0	274.129	gatccctttcCcatcggatct	m5C_4258
chr1	28906392	-	C	355	112	0.315	0	0	T	243	M	0.269	0.366	0	0	0	603.333	ctcctgatccCtttcccatcg	m5C_4273
chr1	28906394	-	C	347	77	0.222	0	0	T	270	M	0.181	0.269	0	0	0	279.294	tcctcctgatCcctttcccat	m5C_4260
chr1	28906905	-	C	101	25	0.248	0	0	T	76	M	0.174	0.34	0	4.4E-16	0	86.8236	tgccactcagCtttcctataa	m5C_4304
chr1	28906912	-	C	111	28	0.252	0	0	T	83	M	0.181	0.34	0	0	0	101.178	tgcaagctgcCactcagcttt	m5C_4344
chr1	28906934	-	C	227	58	0.256	0	0	T	169	M	0.203	0.316	0	0	0	235.671	cagcgcaggtCagtacaatac	m5C_4280
chr1	28906944	-	C	288	68	0.236	0	0	T	220	M	0.191	0.288	0	0	0	259.4	aagactttgaCagcgcaggtc	m5C_4328
chr1	28906960	-	C	430	110	0.256	0	0	T	320	M	0.217	0.299	0	0	0	477.096	gcaagagcaaCctggaaagac	m5C_4303
chr1	28906976	-	C	615	134	0.218	0	0	T	481	M	0.187	0.252	0	0	0	501.335	ggatctccaaCtgcatgcaag	m5C_4259
chr1	28906980	-	C	654	157	0.24	0	0	T	497	M	0.209	0.274	0	0	0	655.959	catgggatctCcaactgcatg	m5C_4287
chr1	28906996	-	C	878	206	0.235	0	0	T	672	M	0.208	0.264	0	0	0	856.082	tgtggctggtCatagccatgg	m5C_4285
chr1	28907001	-	C	964	255	0.265	0	0	T	709	M	0.238	0.293	0	0	0	1212.03	agggctgtggCtggtcatagc	m5C_4308
chr1	28907436	-	C	286	63	0.22	0	0	T	223	M	0.176	0.272	0	0	0	221.925	tgggaaaataCagttccattg	m5C_4329
chr1	28907451	-	C	350	78	0.223	0	0	T	272	M	0.182	0.269	0	0	0	284.543	tgatctgcaaCattttgggaa	m5C_4326
chr1	28907454	-	C	341	69	0.202	0	0	T	272	M	0.163	0.248	0	0	0	225.118	aactgatctgCaacattttgg	m5C_4312
chr1	28907457	-	C	348	131	0.376	0	0	T	217	M	0.327	0.428	0	0	0	857.113	tgtaactgatCtgcaacattt	m5C_4300
chr1	28907480	-	C	430	105	0.244	0	0	T	325	M	0.206	0.287	0	0	0	432.52	ttgacggtcgCcgttctgttt	m5C_4343
chr1	28907492	-	C	400	83	0.207	0	0	T	317	M	0.171	0.25	0	0	0	283.252	tgacagttttCcttgacggtc	m5C_4281
chr1	28907508	-	C	489	126	0.258	0	0	T	363	M	0.221	0.298	0	0	0	556.664	aaaaaggagcCcagagtgaca	m5C_4274
chr1	28907526	-	C	562	115	0.205	0	0	T	447	M	0.173	0.24	0	0	0	398.659	cgcatagctgCtgtggtcaaa	m5C_4295
chr1	28907540	-	C	659	152	0.231	1	0.002	T	506	M	0.2	0.265	0	0	0	609.264	gctgcagctgCttccgcatag	m5C_4265
chr1	28907549	-	C	727	146	0.201	0	0	T	581	M	0.173	0.231	0	0	0	506.066	cttatcgaagCtgcagctgct	m5C_4269
chr1	28907554	-	C	718	147	0.205	0	0	T	571	M	0.177	0.236	0	0	0	519.875	tggcccttatCgaagctgcag	m5C_4266
chr1	28907560	-	C	599	129	0.215	0	0	T	470	M	0.184	0.25	0	0	0	475.514	gccccttggcCcttatcgaag	m5C_4290
chr1	28975123	+	C	289	88	0.304	0	0	T	201	M	0.254	0.36	0	0	0	447.554	aaaagggcttCtgtcgtgagt	m5C_4231
chr1	28975127	+	C	345	95	0.275	0	0	T	250	M	0.231	0.325	0	0	0	438.699	gggcttctgtCgtgagtggca	m5C_4218
chr1	28975136	+	C	378	82	0.217	0	0	T	296	M	0.178	0.261	0	0	0	292.479	tcgtgagtggCacacgtaggg	m5C_4180
chr1	28975150	+	C	644	147	0.228	0	0	T	497	M	0.198	0.262	0	0	0	580.685	cgtagggcaaCtcgattgctc	m5C_4238
chr1	28975163	+	C	1001	227	0.227	1	0.001	T	773	M	0.202	0.254	0	0	0	917.58	gattgctctgCgtgcggaatc	m5C_4182
chr1	28975195	+	C	822	180	0.219	0	0	T	642	M	0.192	0.249	0	0	0	691.377	atttcggaagCataatttttt	m5C_4164
chr1	28975234	+	C	1574	351	0.223	1	0.001	T	1222	M	0.203	0.244	0	0	0	1426.84	gatcgttggtCccggcgccct	m5C_4241
chr1	28975235	+	C	1564	332	0.212	0	0	T	1232	M	0.193	0.233	0	0	0	1279.71	atcgttggtcCcggcgccctt	m5C_4171
chr1	28975241	+	C	1353	304	0.225	0	0	T	1049	M	0.203	0.248	0	0	0	1235.72	ggtcccggcgCcctttcttta	m5C_4175
chr1	28975242	+	C	1189	281	0.237	3	0.003	T	905	M	0.214	0.262	0	0	0	1200.46	gtcccggcgcCctttctttac	m5C_4146
chr1	28975243	+	C	1074	420	0.391	1	0.001	T	653	M	0.363	0.421	0	0	0	3046.34	tcccggcgccCtttctttact	m5C_4249
chr1	29475240	-	C	65	17	0.262	0	0	T	48	M	0.17	0.38	0	1.8E-10	0	57.8614	gcaaggcgggCagcagcaaga	m5C_4257
chr1	29475244	-	C	52	19	0.365	0	0	T	33	M	0.248	0.501	0	2.4E-08	0	94.242	gacagcaaggCgggcagcagc	m5C_4291
chr1	29475327	-	C	45	21	0.467	0	0	T	24	M	0.329	0.609	0	1.8E-06	0	138.327	ggaagagaagCagagaggaga	m5C_4324
chr1	29475351	-	C	57	28	0.491	0	0	T	29	M	0.366	0.617	0	3.1E-07	0	205.05	agagcaaggaCaagaggaagg	m5C_4338
chr1	29475366	-	C	41	12	0.293	0	0	T	29	M	0.176	0.445	1.1E-16	7.5E-08	1.5E-16	33.7134	gcaggagccgCagcaagagca	m5C_4336
chr1	29475368	-	C	38	14	0.368	0	0	T	24	M	0.234	0.527	0	9.2E-06	0	65.4752	gagcaggagcCgcagcaagag	m5C_4293
chr1	29475369	-	C	36	15	0.417	0	0	T	21	M	0.271	0.578	0	1.2E-05	0	81.421	ggagcaggagCcgcagcaaga	m5C_4313
chr1	29793163	+	C	90	33	0.367	0	0	T	57	M	0.274	0.47	0	7.5E-13	0	181.151	ctcagaggttCaggacactgt	m5C_4181
chr1	29793170	+	C	87	55	0.632	0	0	C	55	M	0.527	0.726	0	4.2E-09	0	580.001	gttcaggacaCtgttgacaac	m5C_4141
chr1	31836897	+	C	32	10	0.312	0	0	T	22	M	0.18	0.486	1.1E-14	2E-06	1.3E-14	25.0836	gaaaaagaaaCatagagatag	m5C_597
chr1	32620269	+	C	44	16	0.364	0	0	T	28	M	0.238	0.511	0	4E-07	0	76.1067	agaaatgagaCgaagaagaga	m5C_1378
chr1	33245660	-	C	32	10	0.312	0	0	T	22	M	0.18	0.486	1.1E-14	2E-06	1.3E-14	25.0836	gagtggggcaCaggacctggg	m5C_1440
chr1	33245662	-	C	32	20	0.625	0	0	C	20	M	0.453	0.771	0	4.3E-05	0	181.016	tggagtggggCacaggacctg	m5C_1420
chr1	35650119	-	C	70	17	0.243	0	0	T	53	M	0.158	0.355	0	2.2E-12	0	53.5559	gcctggaactCcagcaggata	m5C_844
chr1	35653616	-	C	31	14	0.452	0	0	T	17	M	0.292	0.622	0	9.2E-06	0	81.6522	aaatgaggcgCcaaagagagg	m5C_849
chr1	35653627	-	C	40	18	0.45	0	0	T	22	M	0.307	0.602	0	2.7E-05	0	110.538	gatggaagaaCaaatgaggcg	m5C_861

chr1	35653642	-	C	60	19	0.317	0	0	T	41	M	0.213	0.442	0	4.5E-10	0	80.9617	gattcgtcaaCgtgagatgga	m5C_865
chr1	35653675	-	C	126	28	0.222	0	0	T	98	M	0.158	0.302	0	0	0	88.7373	ggaggaacgaCgtagaagaga	m5C_860
chr1	35653678	-	C	146	42	0.288	0	0	T	104	M	0.22	0.366	0	0	0	185.16	agaggaggaaCgacgtagaag	m5C_852
chr1	35661572	+	C	32	9	0.281	0	0	T	23	M	0.156	0.454	5.3E-13	1.2E-06	6.4E-13	17.1983	aaacccatggCtggtctgagt	m5C_838
chr1	35661577	+	C	76	31	0.408	0	0	T	45	M	0.304	0.52	0	6.4E-10	0	188.751	catggctggtCtgagtgcggc	m5C_835
chr1	35661599	+	C	75	24	0.32	0	0	T	51	M	0.225	0.432	0	6.4E-11	0	108.206	gtgtttacaaCtaattgatca	m5C_831
chr1	35661610	+	C	75	16	0.213	0	0	T	59	M	0.136	0.319	0	1.2E-12	0	43.4563	taattgatcaCaaccagttac	m5C_827
chr1	35661614	+	C	73	21	0.288	0	0	T	52	M	0.197	0.4	0	1.8E-11	0	82.5367	tgatcacaacCagttacagat	m5C_821
chr1	35893487	-	C	304	69	0.227	0	0	T	235	M	0.183	0.277	0	0	0	253.181	atatgttggtCtgagtgttgt	m5C_854
chr1	36766568	+	C	34	13	0.382	0	0	T	21	M	0.239	0.55	0	6.7E-06	0	62.1399	cagaagagtaCactgaagaga	m5C_1605
chr1	36766570	+	C	38	22	0.579	0	0	C	22	M	0.422	0.721	0	6.3E-05	0	185.645	gaagagtacaCtgaagagaca	m5C_1607
chr1	36766579	+	C	38	13	0.342	0	0	T	25	M	0.212	0.501	0	6.7E-06	0	55.1518	actgaagagaCagaggaaaga	m5C_1612
chr1	36884144	-	C	34	9	0.265	0	0	T	25	M	0.146	0.431	1.1E-12	1.2E-06	1.3E-12	15.7394	ctgttaagtgCtgtattgtca	m5C_1624
chr1	36884154	-	C	107	47	0.439	0	0	T	60	M	0.349	0.534	0	1.3E-12	0	328.003	tacaatatagCtgttaagtgc	m5C_1621
chr1	37499850	-	C	38	15	0.395	0	0	T	23	M	0.256	0.553	0	1.2E-05	0	76.7936	ccgcggggcgCcccccacttg	m5C_1633
chr1	38034606	-	C	81	18	0.222	0	0	T	63	M	0.145	0.324	0	4.5E-14	0	52.3602	ggaacaggagCaacaaagaga	m5C_1245
chr1	38034612	-	C	83	24	0.289	0	0	T	59	M	0.203	0.394	0	1.1E-12	0	97.2831	ggaggaggaaCaggagcaaca	m5C_1222
chr1	38034674	-	C	40	11	0.275	0	0	T	29	M	0.161	0.428	4.3E-15	3.1E-06	5.5E-15	25.4502	gacctggttcCtgtggaggtg	m5C_1223
chr1	38034675	-	C	42	16	0.381	0	0	T	26	M	0.25	0.532	0	4E-07	0	79.9985	tgacctggttCctgtggaggt	m5C_1239
chr1	38435326	-	C	55	30	0.545	0	0	C	30	M	0.415	0.67	0	4.8E-07	0	249.135	ggaagaagagCagatcagtga	m5C_1255
chr1	38435347	-	C	160	36	0.225	0	0	T	124	M	0.167	0.296	0	0	0	120.371	aggagaagagCgagaagaaga	m5C_1232
chr1	38435358	-	C	164	35	0.213	0	0	T	129	M	0.158	0.282	0	0	0	110.35	caagccaggaCaggagaagag	m5C_1217
chr1	38435368	-	C	78	17	0.218	0	0	T	61	M	0.141	0.322	0	2.2E-12	0	47.8608	acagcgcaagCaagccaggac	m5C_1252
chr1	38435374	-	C	41	16	0.39	0	0	T	25	M	0.257	0.543	0	4E-07	0	82.099	aaatgtacagCgcaagcaagc	m5C_1235
chr1	39500091	+	C	48	10	0.208	0	0	T	38	M	0.117	0.343	1.6E-12	2.4E-08	1.9E-12	13.8343	catcaggaagCagcgggataa	m5C_1209
chr1	39500094	+	C	50	10	0.208	2	0.04	T	38	M	0.117	0.343	1.6E-12	2.4E-08	1.9E-12	13.8343	caggaagcagCgggataagct	m5C_1206
chr1	39619930	-	C	33	22	0.667	0	0	C	22	M	0.496	0.802	0	6.3E-05	0	218.273	ctgatctcgtCtgattttgga	m5C_1226
chr1	39905998	-	C	48	12	0.25	0	0	T	36	M	0.149	0.388	1.3E-15	7.5E-08	1.7E-15	26.6338	cccaggagttCtggacaagcc	m5C_1211
chr1	39906006	-	C	53	16	0.302	0	0	T	37	M	0.195	0.435	0	7.3E-09	0	62.4581	tgcttgagccCaggagttctg	m5C_1231
chr1	39906025	-	C	38	15	0.395	0	0	T	23	M	0.256	0.553	0	1.2E-05	0	76.7936	gaggccgaggCtggaggattg	m5C_1218
chr1	40033085	-	C	36	18	0.5	0	0	CT	18	M	0.345	0.655	0	2.7E-05	0	124.107	ggttgttttcCatttgtcttg	m5C_915
chr1	40033097	-	C	49	14	0.286	0	0	T	35	M	0.178	0.424	0	1.9E-07	0	49.9784	gaaatccagaCaggttgtttt	m5C_938
chr1	40033101	-	C	49	17	0.347	0	0	T	32	M	0.229	0.487	0	5.6E-07	0	77.9423	agaggaaatcCagacaggttg	m5C_908
chr1	40033102	-	C	48	15	0.319	1	0.021	T	32	M	0.204	0.462	0	2.8E-07	0	61.1856	cagaggaaatCcagacaggtt	m5C_916
chr1	40033112	-	C	70	14	0.2	0	0	T	56	M	0.123	0.308	4.4E-16	2.8E-13	5.8E-16	26.447	acagaatgagCagaggaaatc	m5C_920
chr1	40033124	-	C	99	29	0.293	0	0	T	70	M	0.212	0.389	0	1.7E-13	0	123.172	acagatggtgCtacagaatga	m5C_942
chr1	40033144	-	C	175	49	0.28	0	0	T	126	M	0.219	0.351	0	0	0	214.375	tgcctcgtgcCggtgtggga	m5C_921
chr1	40033145	-	C	167	38	0.228	0	0	T	129	M	0.171	0.297	0	0	0	129.584	ctgcctcgtgCcggtgtgggg	m5C_911
chr1	40033149	-	C	157	48	0.306	0	0	T	109	M	0.239	0.382	0	0	0	229.459	cttgctgcctCgtgccggtgt	m5C_927
chr1	40033164	-	C	149	31	0.208	0	0	T	118	M	0.151	0.28	0	0	0	93.3852	gaatctttccCattccttgct	m5C_917
chr1	40033166	-	C	151	32	0.212	0	0	T	119	M	0.154	0.284	0	0	0	98.7534	ctgaatctttCccattccttg	m5C_940
chr1	40033177	-	C	82	17	0.207	0	0	T	65	M	0.134	0.307	0	2.3E-14	0	45.4454	actcagagcaCctgaatcttt	m5C_924
chr1	41220052	+	C	33	17	0.515	0	0	C	17	M	0.352	0.675	0	2.1E-05	0	119.742	ctgtaaacatCcttgactgga	m5C_889
chr1	41220053	+	C	33	16	0.485	0	0	T	17	M	0.325	0.648	0	1.7E-05	0	104.012	tgtaaacatcCttgactggaa	m5C_878
chr1	41270684	+	C	147	31	0.211	0	0	T	116	M	0.153	0.284	0	0	0	94.6934	atcaagttgcCtagggagggg	m5C_883
chr1	41270699	+	C	179	42	0.235	0	0	T	137	M	0.179	0.302	0	0	0	149.969	gagggtgaaCtggcccaggt	m5C_900
chr1	41270705	+	C	180	52	0.289	0	0	T	128	M	0.228	0.359	0	0	0	236.738	tgaactggccCaggtcagaaa	m5C_888
chr1	43162471	+	C	113	31	0.274	0	0	T	82	M	0.201	0.363	0	1.1E-16	0	124.318	gggaaaagaaCgagggatcgg	m5C_950
chr1	44587888	-	C	85	23	0.271	0	0	T	62	M	0.188	0.373	0	7.2E-13	0	86.2936	caggaagaggCagaggcagag	m5C_6950
chr1	45187459	-	C	124	82	0.661	0	0	C	82	M	0.574	0.739	0	2.3E-12	0	941.786	ctaggcaaggCtgaatgttct	m5C_6922
chr1	45196744	-	C	106	35	0.33	0	0	T	71	M	0.248	0.424	0	3.4E-14	0	173.585	ttttttgaagCcctatgttgg	m5C_6909
chr1	45196774	-	C	34	19	0.559	0	0	C	19	M	0.395	0.711	0	3.4E-05	0	149.924	ggagagaaacCgttttgagtt	m5C_6927
chr1	45218845	+	C	36	13	0.361	0	0	T	23	M	0.225	0.524	0	6.7E-06	0	58.436	ggctgaaataCcattgaggat	m5C_6827
chr1	45218862	+	C	52	13	0.25	0	0	T	39	M	0.152	0.382	1.1E-16	1.6E-09	1.5E-16	31.5947	ggatggtcagCgaggagatgg	m5C_6844
chr1	45241605	+	C	1207	272	0.225	0	0	T	935	M	0.203	0.25	0	0	0	1102.54	ccttggagagCtgagcgtgcg	m5C_6886
chr1	45242185	+	C	2776	631	0.227	0	0	T	2145	M	0.212	0.243	0	0	0	2676.68	aaaaagaatcCttaggcgtgg	m5C_6851
chr1	45242191	+	C	2740	638	0.242	101	0.037	T	2001	M	0.226	0.258	0	0	0	2881.28	aatccttaggCgtggttgtgg	m5C_6890
chr1	45242202	+	C	2594	582	0.226	17	0.007	T	1995	M	0.21	0.242	0	0	0	2445.74	gtggttgtggCcgtcttggtc	m5C_6887
chr1	45242241	+	C	2001	401	0.2	0	0	T	1600	M	0.183	0.219	0	0	0	1471.2	aatgcaaggaCttgtcatagt	m5C_6839
chr1	45243531	+	C	1359	302	0.222	1	0.001	T	1056	M	0.201	0.245	0	0	0	1214.45	gatgaaaactCtgtccagttc	m5C_6856
chr1	45243536	+	C	1364	280	0.205	0	0	T	1084	M	0.185	0.228	0	0	0	1034.24	aaactctgtcCagttctgcta	m5C_6877
chr1	45243568	+	C	1181	417	0.354	2	0.002	T	762	M	0.327	0.381	0	0	0	2726.46	gagatgagagCcttttaggct	m5C_6896
chr1	45243577	+	C	942	441	0.468	0	0	T	501	M	0.436	0.5	0	0	0	3849.77	gccttttaggCtggaggaaggc	m5C_6848
chr1	45244095	+	C	1213	254	0.209	0	0	T	959	M	0.187	0.233	0	0	0	952.174	agttctgctaCtgacagtaag	m5C_6900
chr1	45244122	+	C	1413	641	0.454	0	0	T	772	M	0.428	0.48	0	0	0	5485	taaagtgtgtCtgaggagaca	m5C_6891
chr1	45244252	+	C	47	10	0.213	0	0	T	37	M	0.12	0.349	1.3E-12	2.4E-08	1.5E-12	14.271	ttgcagcgtgCatcgcttcaa	m5C_6867
chr1	45244257	+	C	50	16	0.32	0	0	T	34	M	0.208	0.458	0	7.3E-09	0	66.4257	gcgtgcatcgCttcaaggccg	m5C_6855
chr1	45244265	+	C	55	19	0.345	0	0	T	36	M	0.234	0.477	0	2.4E-08	0	88.7776	cgcttcaaggCcgggacagtg	m5C_6880
chr1	45244271	+	C	47	10	0.213	0	0	T	37	M	0.12	0.349	1.3E-12	2.4E-08	1.5E-12	14.271	aaggccgggaCagtgtggccg	m5C_6832
chr1	45244280	+	C	45	18	0.4	0	0	T	27	M	0.27	0.545	0	7.7E-07	0	97.2889	acagtgtggcCgacagatgg	m5C_6899
chr1	45976792	-	C	39	12	0.308	0	0	T	27	M	0.186	0.464	0	4.6E-06	0	44.5578	gggcaggctaCtggtttgtat	m5C_6946
chr1	45976795	-	C	37	12	0.324	0	0	T	25	M	0.196	0.485	0	4.6E-06	0	47.1194	tgtgggcaggCtactggtttg	m5C_6923
chr1	45976799	-	C	49	26	0.531	0	0	C	26	M	0.394	0.663	0	5.5E-06	0	204.779	gcgttgtgggCaggctactgg	m5C_6937
chr1	45976808	-	C	43	22	0.512	0	0	C	22	M	0.368	0.654	0	2.3E-06	0	161.709	cctgagttggCgttgtgggca	m5C_6925
chr1	46034890	+	C	35	19	0.543	0	0	C	19	M	0.382	0.695	0	3.4E-05	0	145.12	atatattgtgCctatgcttac	m5C_1005
chr1	46034891	+	C	35	25	0.714	0	0	C	25	M	0.549	0.837	0	0.00011	0	274.724	tatattgtgcCtatgcttacg	m5C_1008
chr1	51210383	-	C	51	35	0.686	0	0	C	35	M	0.55	0.797	0	1.3E-06	0	384.809	tgttaaaaggCtggaagacgg	m5C_1526
chr1	51822486	-	C	96	26	0.271	0	0	T	70	M	0.192	0.367	0	4.6E-14	0	99.859	tcgaatgggcCaagagggaaa	m5C_1536
chr1	51822487	-	C	101	32	0.317	0	0	T	69	M	0.234	0.413	0	1.1E-14	0	149.91	atcgaatgggCcaagagggaa	m5C_1538
chr1	52306043	-	C	35	18	0.514	0	0	C	18	M	0.356	0.67	0	2.7E-05	0	128.047	gattctggagCtgaaatagaa	m5C_1313
chr1	52306049	-	C	34	14	0.412	0	0	T	20	M	0.264	0.578	0	9.2E-06	0	73.8241	gatgaagattCtggagctgaa	m5C_1318
chr1	52439149	+	C	41	17	0.415	0	0	T	24	M	0.278	0.566	0	5.6E-07	0	94.3715	ggggttgggcCtggttagtac	m5C_1286
chr1	52615983	+	C	36	11	0.306	0	0	T	25	M	0.18	0.469	1E-15	3.1E-06	1.3E-15	29.7077	tgaactggccCaggctgggaa	m5C_1306
chr1	55048330	+	C	41	17	0.415	0	0	T	24	M	0.278	0.566	0	5.6E-07	0	94.3715	ggactgtgccCgtgttccctt	m5C_1559
chr1	55048338	+	C	37	14	0.378	0	0	T	23	M	0.241	0.539	0	9.2E-06	0	67.3786	cccgtgttccCttgttggtga	m5C_1557
chr1	55522033	+	C	40	8	0.2	0	0	T	32	M	0.105	0.348	2.1E-10	5.5E-09	2.3E-10	8.13503	ccccgccatgCgggaggctgg	m5C_1556
chr1	55522040	+	C	40	24	0.6	0	0	C	24	M	0.446	0.737	0	3.6E-06	0	214.059	atgcgggaggCtggggaggaa	m5C_1545
chr1	58551769	-	C	31	9	0.29	0	0	T	22	M	0.161	0.466	3.7E-13	1.2E-06	4.4E-13	18.0154	cctgtagtccCagctacttgg	m5C_1769
chr1	58551771	-	C	33	9	0.273	0	0	T	24	M	0.151	0.442	7.5E-13	1.2E-06	9E-13	16.4418	cgcctgtagtCccagctactt	m5C_1787
chr1	60440481	-	C	31	8	0.258	0	0	T	23	M	0.137	0.432	1.7E-11	6.8E-07	1.9E-11	11.8182	tgggattgtgCctgtggatag	m5C_2005
chr1	63000630	+	C	40	12	0.3	0	0	T	28	M	0.181	0.454	1.1E-16	7.5E-08	1.5E-16	34.6049	ggtgtggtgaCgtgtgcctgt	m5C_1465
chr1	63000637	+	C	38	20	0.526	0	0	C	20	M	0.373	0.675	0	4.3E-05	0	149.035	tgacgtgtgcCtgtagtccca	m5C_1448
chr1	63000645	+	C	40	13	0.325	0	0	T	27	M	0.201	0.48	0	1.2E-07	0	52.2192	gcctgtagtcCcagctttggg	m5C_1450
chr1	63000649	+	C	39	20	0.513	0	0	C	20	M	0.362	0.661	0	4.3E-05	0	144.796	gtagtcccagCtttgggaggc	m5C_1467
chr1	63000659	+	C	36	14	0.389	0	0	T	22	M	0.248	0.551	0	9.2E-06	0	69.397	ctttgggaggCtgaggcagga	m5C_1447
chr1	64121534	+	C	42	11	0.262	0	0	T	31	M	0.153	0.411	8.4E-15	4.4E-08	1.1E-14	23.69	tgtcgggtgtCtgcactaagt	m5C_1830
chr1	64121537	+	C	42	9	0.214	0	0	T	33	M	0.117	0.359	1.1E-11	1.2E-08	1.3E-11	11.5454	cgggtgtctgCactaagtttg	m5C_1809
chr1	65330575	-	C	49	20	0.408	0	0	T	29	M	0.282	0.548	0	1.4E-06	0	112.86	aggagaaaaaCaagatccggg	m5C_1838
chr1	65524129	-	C	88	31	0.352	0	0	T	57	M	0.261	0.456	0	1.7E-11	0	161.548	actgtgataaCtgaaggatgg	m5C_1843
chr1	67878916	-	C	32	9	0.281	0	0	T	23	M	0.156	0.454	5.3E-13	1.2E-06	6.4E-13	17.1983	tgtggatgacCcagaggcatt	m5C_1953
chr1	67878931	-	C	33	19	0.576	0	0	C	19	M	0.408	0.728	0	3.4E-05	0	155.067	tgcttctgctCctgatgtgga	m5C_1951

chr1	68153623	+	C	45	9	0.2	0	0	T	36	M	0.109	0.338	2.3E-11	1.2E-08	2.7E-11	10.4289	agatagatgaCtttgcagatg	m5C_1703
chr1	70716106	+	C	75	27	0.36	0	0	T	48	M	0.261	0.473	0	1.9E-10	0	140.75	tgaaagagaaCgatcaacaag	m5C_1666
chr1	70716110	+	C	73	24	0.329	0	0	T	49	M	0.232	0.443	0	6.4E-11	0	111.333	agagaacgatCaacaagcaag	m5C_1653
chr1	70716113	+	C	72	31	0.431	0	0	T	41	M	0.323	0.546	0	6.4E-10	0	199.998	gaacgatcaaCaagcaagaag	m5C_1665
chr1	70716117	+	C	70	21	0.3	0	0	T	49	M	0.205	0.415	0	1.8E-11	0	86.2531	gatcaacaagCaagaagaaga	m5C_1651
chr1	74272642	-	C	55	18	0.327	0	0	T	37	M	0.218	0.459	0	1.7E-08	0	78.5264	gtggttatcaCattcgcctca	m5C_1755
chr1	74272644	-	C	61	32	0.525	0	0	C	32	M	0.402	0.645	0	2.7E-08	0	257.004	tagtggttatCacattcgcct	m5C_1760
chr1	74272661	-	C	77	24	0.312	0	0	T	53	M	0.219	0.422	0	6.4E-11	0	105.25	tagttgtttcCatagtgtagt	m5C_1748
chr1	74272662	-	C	34	15	0.441	0	0	T	19	M	0.289	0.605	0	1.2E-05	0	86.6497	ctagttgtttCcatagtgtag	m5C_1753
chr1	76252760	+	C	250	62	0.248	0	0	T	188	M	0.199	0.305	0	0	0	246.203	ttttttgggtCaatgatgagt	m5C_3694
chr1	76252774	+	C	657	146	0.226	10	0.015	T	501	M	0.195	0.259	0	0	0	569.751	gatgagttggCatgtattctg	m5C_3675
chr1	76252782	+	C	649	179	0.276	0	0	T	470	M	0.243	0.311	0	0	0	869.294	ggcatgtattCtgaatctaaa	m5C_3681
chr1	76252788	+	C	601	133	0.221	0	0	T	468	M	0.19	0.256	0	0	0	505.233	tattctgaatCtaaagttgat	m5C_3676
chr1	76252804	+	C	516	158	0.306	0	0	T	358	M	0.268	0.347	0	0	0	846.834	ttgattattaCtactttagct	m5C_3678
chr1	76252807	+	C	498	125	0.252	1	0.002	T	372	M	0.215	0.291	0	0	0	538.42	attattactaCtttagctcta	m5C_3688
chr1	76252813	+	C	467	103	0.221	0	0	T	364	M	0.185	0.26	0	0	0	381.75	actactttagCtctagaatta	m5C_3673
chr1	76252815	+	C	478	132	0.276	0	0	T	346	M	0.238	0.318	0	0	0	628.253	tactttagctCtagaattact	m5C_3680
chr1	76252824	+	C	446	134	0.3	0	0	T	312	M	0.26	0.345	0	0	0	696.135	tctagaattaCtctgagacct	m5C_3684
chr1	76252826	+	C	412	132	0.321	1	0.002	T	279	M	0.278	0.368	0	0	0	733.552	tagaattactCtgagacctga	m5C_3693
chr1	76252832	+	C	273	67	0.245	0	0	T	206	M	0.198	0.3	0	0	0	265.505	tactctgagaCctgaaaatta	m5C_3682
chr1	76252833	+	C	252	56	0.222	0	0	T	196	M	0.175	0.278	0	0	0	196.312	actctgagacCtgaaaattac	m5C_3695
chr1	76253607	+	C	1427	296	0.207	0	0	T	1131	M	0.187	0.229	0	0	0	1108.16	ttatctgaatCtattgctgat	m5C_3689
chr1	76253637	+	C	1109	328	0.296	1	0.001	T	780	M	0.27	0.324	0	0	0	1770.48	cactttagctCtagaattact	m5C_3691
chr1	76253648	+	C	879	186	0.212	0	0	T	693	M	0.186	0.24	0	0	0	691.498	tagaattactCtgagaccttg	m5C_3687
chr1	76253655	+	C	542	120	0.222	1	0.002	T	421	M	0.189	0.259	0	0	0	453.196	actctgagacCttgaaaaagt	m5C_3677
chr1	76255224	+	C	301	117	0.389	0	0	T	184	M	0.335	0.445	0	0	0	784.761	taaaattacaCtgagaccttg	m5C_3674
chr1	78098494	-	C	68	17	0.25	0	0	T	51	M	0.162	0.364	0	1.8E-10	0	55.1986	aggtgaatgtCagtgaggaag	m5C_1869
chr1	81195313	-	C	33	14	0.424	0	0	T	19	M	0.272	0.592	0	9.2E-06	0	76.259	gactagcttgCtgctcacccc	m5C_2033
chr1	81195321	-	C	31	17	0.548	0	0	C	17	M	0.378	0.708	0	2.1E-05	0	128.424	caatgtttgaCtagcttgctg	m5C_2026
chr1	81768880	-	C	31	10	0.323	0	0	T	21	M	0.186	0.499	7.1E-15	2E-06	9E-15	26.2725	agagtaggagCtgtttgggca	m5C_2035
chr1	85598309	+	C	33	10	0.303	0	0	T	23	M	0.174	0.473	1.6E-14	2E-06	2E-14	23.982	gaaaaagggaCtttttttga	m5C_2047
chr1	86048562	+	C	33	16	0.485	0	0	T	17	M	0.325	0.648	0	1.7E-05	0	104.012	ccccagctgaCcaggactgtg	m5C_1893
chr1	86048563	+	C	36	8	0.222	0	0	T	28	M	0.117	0.381	7.4E-11	6.8E-07	8.4E-11	9.49676	cccagctgacCaggactgtga	m5C_1883
chr1	86048584	+	C	42	16	0.381	0	0	T	26	M	0.25	0.532	0	4E-07	0	79.9985	agatgcggttCcgctgcgaag	m5C_1894
chr1	86048585	+	C	42	13	0.31	0	0	T	29	M	0.191	0.46	0	1.2E-07	0	49.5844	gatgcggttcCgctgcgaaga	m5C_1881
chr1	86048587	+	C	45	24	0.533	0	0	C	24	M	0.391	0.671	0	3.6E-06	0	187.571	tgcggttccgCtgcgaagatg	m5C_1889
chr1	86048590	+	C	38	12	0.316	0	0	T	26	M	0.191	0.475	0	4.6E-06	0	45.8026	ggttccgctgCgaagatgggg	m5C_1880
chr1	87918970	-	C	112	25	0.223	0	0	T	87	M	0.156	0.309	0	0	0	78.0053	actcggatggCagaaaaatga	m5C_1911
chr1	87918989	-	C	122	39	0.32	0	0	T	83	M	0.244	0.407	0	0	0	189.938	agggtcgggtCtggttagtac	m5C_1901
chr1	87918994	-	C	108	29	0.269	0	0	T	79	M	0.194	0.359	0	2.9E-15	0	112.49	taagcagggtCgggtctggtt	m5C_1908
chr1	87919000	-	C	82	17	0.207	0	0	T	65	M	0.134	0.307	0	2.3E-14	0	45.4454	ggaagctaagCagggtcgggt	m5C_1898
chr1	89260169	+	C	43	11	0.256	0	0	T	32	M	0.149	0.402	1.2E-14	4.4E-08	1.5E-14	22.8887	agcctgtagtCctagctactg	m5C_1917
chr1	89260170	+	C	48	15	0.319	1	0.021	T	32	M	0.204	0.462	0	2.8E-07	0	61.1856	gcctgtagtcCtagctactgg	m5C_1913
chr1	89260177	+	C	46	17	0.37	0	0	T	29	M	0.245	0.514	0	5.6E-07	0	83.3801	gtcctagctaCtggggagact	m5C_1919
chr1	89260186	+	C	48	12	0.25	0	0	T	36	M	0.149	0.388	1.3E-15	7.5E-08	1.7E-15	26.6338	actggggagaCtgaggcaaga	m5C_1912
chr1	91852821	-	C	683	290	0.425	1	0.001	T	392	M	0.389	0.463	0	0	0	2254.09	attcaccaagCgttggattgt	m5C_2834
chr1	91852879	-	C	656	137	0.212	11	0.017	T	508	M	0.183	0.246	0	0	0	500.292	atagcgacgtCgctttttgat	m5C_2788
chr1	91852946	-	C	42	33	0.786	0	0	C	33	M	0.641	0.883	0	1.9E-05	0	422.795	gggttttaagCaggaggtgtc	m5C_2833
chr1	91852961	-	C	64	51	0.797	0	0	C	51	M	0.683	0.877	0	7.9E-07	0	696.519	tccttctgacCttttgggttt	m5C_2798
chr1	91853017	-	C	139	43	0.309	0	0	T	96	M	0.239	0.39	0	0	0	205.114	ctcgcttgatCttgattttca	m5C_2840
chr1	91853023	-	C	139	49	0.353	0	0	T	90	M	0.278	0.435	0	0	0	272.474	caaaagctcgCttgatcttga	m5C_2808
chr1	91853033	-	C	95	44	0.463	0	0	T	51	M	0.366	0.563	0	1.8E-11	0	322.322	agcagaagggCaaaagctcgc	m5C_2757
chr1	92135819	+	C	41	9	0.22	0	0	T	32	M	0.12	0.367	8.4E-12	1.2E-08	9.9E-12	11.9621	ccattcagctCgcttcccggt	m5C_2928
chr1	92135825	+	C	52	13	0.25	0	0	T	39	M	0.152	0.382	1.1E-16	1.6E-09	1.5E-16	31.5947	agctcgcttcCcggtcaggga	m5C_2931
chr1	92135826	+	C	50	19	0.38	0	0	T	31	M	0.259	0.518	0	2.4E-08	0	98.2779	gctcgcttccCggtcagggaa	m5C_2913
chr1	92135830	+	C	53	14	0.264	0	0	T	39	M	0.164	0.396	0	2.8E-09	0	46.0242	gcttcccggtCagggaaccaa	m5C_2918
chr1	92135837	+	C	47	19	0.404	0	0	T	28	M	0.276	0.547	0	1E-06	0	105.031	ggtcagggaaCcaagaaatgt	m5C_2898
chr1	93302869	+	C	1068	219	0.205	0	0	T	849	M	0.182	0.23	0	0	0	796.806	atcccactaaCtgagcagtca	m5C_2919
chr1	93302930	+	C	1011	319	0.316	1	0.001	T	691	M	0.288	0.345	0	0	0	1836.91	caagacgggaCtgatggcagc	m5C_2914
chr1	93306285	+	C	936	231	0.247	0	0	T	705	M	0.22	0.275	0	0	0	1017.54	tgtgcaaactCgatcactagc	m5C_2927
chr1	93306297	+	C	934	224	0.24	0	0	T	710	M	0.214	0.268	0	0	0	956.687	atcactagctCtgcgtgatgt	m5C_2909
chr1	93306325	+	C	789	212	0.269	0	0	T	577	M	0.239	0.301	0	0	0	1013.1	gcgaagggaaCcaggtttgca	m5C_2920
chr1	93306343	+	C	640	184	0.287	0	0	T	456	M	0.254	0.324	0	0	0	933.927	gcaaaagtaaCtgtggtgatg	m5C_2903
chr1	93306366	+	C	508	158	0.311	0	0	T	350	M	0.272	0.353	0	0	0	860.509	aatgtgttagCctcagacact	m5C_2938
chr1	93306373	+	C	468	98	0.219	21	0.045	T	349	M	0.183	0.26	0	0	0	359.397	tagcctcagaCactactgagg	m5C_2916
chr1	93306378	+	C	281	58	0.206	0	0	T	223	M	0.163	0.258	0	0	0	189.309	tcagacactaCtgaggtggtt	m5C_2905
chr1	93306393	+	C	230	54	0.235	0	0	T	176	M	0.185	0.294	0	0	0	199.409	gtggttctttCtatcctagta	m5C_2935
chr1	93306397	+	C	211	44	0.209	0	0	T	167	M	0.159	0.268	0	0	0	140.068	ttctttctatCctagtacagt	m5C_2907
chr1	93306404	+	C	195	40	0.205	0	0	T	155	M	0.154	0.267	0	0	0	123.529	tatcctagtaCagtctaagta	m5C_2932
chr1	93621922	-	C	51	31	0.608	0	0	C	31	M	0.471	0.73	0	5.9E-07	0	291.926	ggcacaagaaCaagaagattg	m5C_2948
chr1	93953891	-	C	51	24	0.471	0	0	T	27	M	0.341	0.605	0	1.2E-07	0	163.453	gtgctacaggCtttttctttt	m5C_2945
chr1	93953898	-	C	56	18	0.321	0	0	T	38	M	0.214	0.452	0	1.7E-08	0	77.043	atactgggtgCtacaggcttt	m5C_2950
chr1	93953905	-	C	69	23	0.333	0	0	T	46	M	0.234	0.451	0	2.1E-09	0	107.414	cttgggtataCtgggtgctac	m5C_2968
chr1	93953918	-	C	74	27	0.365	0	0	T	47	M	0.264	0.479	0	1.9E-10	0	142.767	ggatgggagaCtgcttgggta	m5C_2947
chr1	93981875	-	C	41	13	0.317	0	0	T	28	M	0.196	0.47	0	1.2E-07	0	50.8675	gagcatttgaCtgcagatcaa	m5C_2956
chr1	93981882	-	C	51	12	0.235	0	0	T	39	M	0.14	0.368	3.3E-15	8.8E-10	4.3E-15	24.3305	ggtggtagagCatttgactgc	m5C_2958
chr1	93981894	-	C	54	27	0.5	0	0	CT	27	M	0.371	0.629	0	2.5E-07	0	200.418	gggtatagctCaggtggtaga	m5C_2952
chr1	93981896	-	C	55	11	0.2	0	0	T	44	M	0.116	0.324	2.9E-13	4.4E-10	3.6E-13	15.9231	gggggtatagCtcaggtggta	m5C_2951
chr1	94058374	-	C	45	30	0.667	0	0	C	30	M	0.521	0.786	0	1.2E-05	0	312.421	ctttgggcctCactgcccaga	m5C_2359
chr1	94313131	+	C	191	51	0.267	0	0	T	140	M	0.209	0.334	0	0	0	213.506	caacaggtggCtccgtggcgc	m5C_2289
chr1	94313139	+	C	218	55	0.252	0	0	T	163	M	0.199	0.314	0	0	0	219.194	ggctccgtggCgcaatggata	m5C_2333
chr1	94313141	+	C	214	59	0.276	0	0	T	155	M	0.22	0.339	0	0	0	259.815	ctccgtggcgCaatggatagc	m5C_2328
chr1	94313151	+	C	174	36	0.207	0	0	T	138	M	0.153	0.273	0	0	0	110.416	caatggatagCgcattggact	m5C_2307
chr1	94313153	+	C	154	50	0.325	0	0	T	104	M	0.256	0.402	0	0	0	255.767	atggatagcgCattggacttc	m5C_2292
chr1	94313160	+	C	128	56	0.438	0	0	T	72	M	0.355	0.524	0	1.3E-14	0	397.176	gcgcattggaCttctagaggc	m5C_2337
chr1	94313163	+	C	132	38	0.288	0	0	T	94	M	0.218	0.37	0	0	0	165.3	cattggacttCtagaggctga	m5C_2314
chr1	94313196	+	C	120	34	0.283	0	0	T	86	M	0.21	0.37	0	0	0	143.075	gttccgggttCgagtcccggc	m5C_2320
chr1	94313201	+	C	113	55	0.487	0	0	T	58	M	0.397	0.578	0	2.9E-13	0	436.184	gggttcgagtCccggcggagt	m5C_2331
chr1	94313203	+	C	138	48	0.348	0	0	T	90	M	0.273	0.43	0	0	0	262.519	gttcgagtccCggcggagtcg	m5C_2308
chr1	94313206	+	C	138	38	0.275	0	0	T	100	M	0.208	0.355	0	0	0	157.838	cgagtcccggCggagtcgtaa	m5C_2324
chr1	94341946	-	C	41	15	0.366	0	0	T	26	M	0.236	0.519	0	2.8E-07	0	70.7689	gtgaggttgcCattgaggaag	m5C_2355
chr1	94341947	-	C	38	9	0.237	0	0	T	29	M	0.13	0.392	3.7E-12	1.2E-06	4.3E-12	13.3739	agtgaggttgCcattgaggaa	m5C_2358
chr1	95554762	+	C	32	7	0.219	0	0	T	25	M	0.11	0.388	8.5E-10	3.4E-07	9.5E-10	6.99971	ctgaatgcgcCtgatcttgtc	m5C_2326
chr1	95554772	+	C	45	16	0.356	0	0	T	29	M	0.232	0.502	0	4E-07	0	74.3005	ctgatcttgtCtggttttgga	m5C_2329
chr1	95621358	-	C	64	14	0.219	0	0	T	50	M	0.135	0.334	1.1E-16	3.2E-11	1.5E-16	30.1591	ctggttgatcCtgccagtagg	m5C_2369
chr1	95621359	-	C	66	24	0.364	0	0	T	42	M	0.258	0.484	0	3E-09	0	123.873	cctggttgatCctgccagtag	m5C_2350
chr1	95621368	-	C	31	12	0.387	0	0	T	19	M	0.237	0.562	0	4.6E-06	0	56.9589	tctgtaatacCtggttgatcc	m5C_2365
chr1	96691577	+	C	31	8	0.258	0	0	T	23	M	0.137	0.432	1.7E-11	6.8E-07	1.9E-11	11.8182	aattcgctgCataatttgtg	m5C_2008
chr1	108113565	-	C	187	88	0.471	0	0	T	99	M	0.4	0.542	0	0	0	704.635	gtcttgaagcCcaggctggg	m5C_2275
chr1	108113566	-	C	191	38	0.2	1	0.005	T	152	M	0.149	0.263	0	0	0	113.484	agtcttgaagCccagggctgg	m5C_2270
chr1	108113573	-	C	199	70	0.352	0	0	T	129	M	0.289	0.42	0	0	0	404.307	tgcggtgagtCttgaagccca	m5C_2239

chr1	108113581	-	C	208	63	0.303	0	0	T	145	M	0.244	0.368	0	0	0	308.041	agggccgctgCggtgagtctt	m5C_2265
chr1	108113584	-	C	211	84	0.398	0	0	T	127	M	0.334	0.465	0	0	0	561.866	aggagggccgCtgcggtgagt	m5C_2267
chr1	108113586	-	C	176	44	0.25	0	0	T	132	M	0.192	0.319	0	0	0	168.809	gtaggagggcCgctgcggtga	m5C_2274
chr1	108113587	-	C	132	36	0.273	0	0	T	96	M	0.204	0.354	0	0	0	146.869	agtaggagggCcgctgcggtg	m5C_2232
chr1	109642871	+	C	59	14	0.237	0	0	T	45	M	0.147	0.36	0	2.8E-09	0	41.1446	gctgtggcgtCgcgtgtgagg	m5C_2176
chr1	109642882	+	C	98	24	0.316	22	0.224	T	52	M	0.222	0.427	0	6.4E-11	0	106.707	gcgtgtgaggCgcgtgcaggg	m5C_2177
chr1	109642888	+	C	108	37	0.343	0	0	T	71	M	0.26	0.436	0	6.7E-14	0	192.31	gaggcgcgtgCagggtgagtg	m5C_2211
chr1	109642907	+	C	145	66	0.455	0	0	T	79	M	0.376	0.536	0	2.2E-16	0	496.739	tgtgagtggaCgcgtgagtgt	m5C_2227
chr1	109642909	+	C	156	54	0.346	0	0	T	102	M	0.276	0.424	0	0	0	298.085	tgagtggacgCgtgagtgtgt	m5C_2228
chr1	109642927	+	C	121	35	0.289	0	0	T	86	M	0.216	0.376	0	0	0	151.158	tgtgagtgtgCgcgcttggag	m5C_2200
chr1	109642929	+	C	119	28	0.235	0	0	T	91	M	0.168	0.319	0	0	0	94.1382	tgagtgtgcgCgcttggagcg	m5C_2195
chr1	109642931	+	C	123	34	0.276	0	0	T	89	M	0.205	0.361	0	0	0	139.447	agtgtgcgcgCttggagcgtg	m5C_2209
chr1	109642938	+	C	101	33	0.327	0	0	T	68	M	0.243	0.423	0	1.6E-14	0	160.431	gcgcttggagCgtgttaggcg	m5C_2184
chr1	109642947	+	C	55	13	0.236	0	0	T	42	M	0.144	0.363	2.2E-16	1.6E-09	2.9E-16	29.2353	gcgtgttaggCgagtgcgtgc	m5C_2222
chr1	109642953	+	C	35	11	0.314	0	0	T	24	M	0.186	0.48	6.7E-16	3.1E-06	8.7E-16	30.9686	taggcgagtgCgtgcgcccac	m5C_2204
chr1	109643087	+	C	48	10	0.208	0	0	T	38	M	0.117	0.343	1.6E-12	2.4E-08	1.9E-12	13.8343	cgctgtgcagCgaggcccctt	m5C_2225
chr1	109643093	+	C	34	20	0.588	0	0	C	20	M	0.422	0.736	0	4.3E-05	0	168.885	gcagcgaggcCccttaggcgg	m5C_2202
chr1	109643104	+	C	44	10	0.227	0	0	T	34	M	0.128	0.37	5.7E-13	2.4E-08	6.8E-13	15.7274	ccttaggcggCggccacgctg	m5C_2170
chr1	109643110	+	C	46	16	0.348	0	0	T	30	M	0.227	0.492	0	4E-07	0	72.5786	gcggcggccaCgctgtggtcc	m5C_2171
chr1	109643112	+	C	44	19	0.432	0	0	T	25	M	0.297	0.578	0	1E-06	0	112.794	ggcggccacgCtgtggtcccc	m5C_2193
chr1	109643120	+	C	38	20	0.526	0	0	C	20	M	0.373	0.675	0	4.3E-05	0	149.035	cgctgtggtcCccgaggtccc	m5C_2174
chr1	109643121	+	C	36	16	0.444	0	0	T	20	M	0.295	0.604	0	1.7E-05	0	94.5313	gctgtggtccCcgaggtcccg	m5C_2160
chr1	109643122	+	C	39	25	0.641	0	0	C	25	M	0.484	0.773	0	0.00011	0	242.089	ctgtggtcccCgaggtcccgg	m5C_2198
chr1	109643140	+	C	42	17	0.405	0	0	T	25	M	0.27	0.555	0	5.6E-07	0	91.945	cggagctggcCctgcggggcc	m5C_2208
chr1	109643150	+	C	63	27	0.429	0	0	T	36	M	0.314	0.551	0	7.5E-09	0	169.542	cctgcggggcCcggcgctcag	m5C_2196
chr1	109643156	+	C	76	16	0.211	0	0	T	60	M	0.134	0.315	0	1.2E-12	0	42.8641	gggcccggcgCtcagaagtga	m5C_2203
chr1	109643158	+	C	97	31	0.32	0	0	T	66	M	0.235	0.418	0	3.7E-13	0	145.815	gcccggcgctCagaagtgatg	m5C_2182
chr1	109643184	+	C	115	43	0.374	0	0	T	72	M	0.291	0.465	0	1.2E-14	0	250.176	atcagatagaCgaggccgggc	m5C_2226
chr1	109643189	+	C	93	23	0.247	0	0	T	70	M	0.171	0.344	0	1E-14	0	78.5777	atagacgaggCcgggcttgtc	m5C_2215
chr1	109643190	+	C	75	25	0.333	0	0	T	50	M	0.237	0.446	0	9.3E-11	0	118.54	tagacgaggcCgggcttgtcc	m5C_2223
chr1	109643199	+	C	88	24	0.273	0	0	T	64	M	0.191	0.374	0	1.1E-12	0	91.5137	ccgggcttgtCcccggccact	m5C_2220
chr1	109643226	+	C	206	100	0.485	0	0	T	106	M	0.418	0.553	0	0	0	836.159	cgaggcgattCtgatctgggc	m5C_2216
chr1	109643231	+	C	180	36	0.2	0	0	T	144	M	0.148	0.264	0	0	0	106.638	cgattctgatCtgggcacggc	m5C_2172
chr1	109772140	+	C	32	16	0.5	0	0	CT	16	M	0.336	0.664	0	1.7E-05	0	107.618	ggtttgagaaCctccgagaga	m5C_2173
chr1	110279373	-	C	35	9	0.257	0	0	T	26	M	0.142	0.421	1.5E-12	1.2E-06	1.7E-12	15.0859	actggagtccCtgtgaagact	m5C_2461
chr1	110279375	-	C	37	20	0.541	0	0	C	20	M	0.384	0.69	0	4.3E-05	0	153.535	ggactggagtCcctgtgaaga	m5C_2462
chr1	110279382	-	C	39	12	0.308	0	0	T	27	M	0.186	0.464	0	4.6E-06	0	44.5578	ttaaatgggaCtggagtccct	m5C_2455
chr1	113456651	-	C	229	59	0.258	0	0	T	170	M	0.205	0.318	0	0	0	242.282	agaaagcaaaCgagcagaaaa	m5C_2637
chr1	115033373	+	C	34	7	0.206	0	0	T	27	M	0.103	0.368	1.4E-09	3.4E-07	1.6E-09	6.4054	tgagttatctCaacagattgt	m5C_2537
chr1	115263273	-	C	56	25	0.446	0	0	T	31	M	0.324	0.576	0	1.5E-07	0	161.933	ggattgcctgCagaaagggga	m5C_2583
chr1	115263289	-	C	80	24	0.3	0	0	T	56	M	0.211	0.408	0	1.1E-12	0	101.109	ggatggccaaCaaaggggatt	m5C_2561
chr1	115263304	-	C	36	14	0.389	0	0	T	22	M	0.248	0.551	0	9.2E-06	0	69.397	catttggcatCgttggatgg	m5C_2586
chr1	115270343	+	C	36	14	0.389	0	0	T	22	M	0.248	0.551	0	9.2E-06	0	69.397	aatctaagggCtggtctgaag	m5C_2534
chr1	115270348	+	C	93	26	0.28	0	0	T	67	M	0.199	0.378	0	4.6E-14	0	103.221	aagggctggtCtgaaggtagt	m5C_2536
chr1	115270366	+	C	35	15	0.429	0	0	T	20	M	0.28	0.591	0	1.2E-05	0	83.953	agtgggttatCtcgattgttc	m5C_2527
chr1	119874116	-	C	48	12	0.25	0	0	T	36	M	0.149	0.388	1.3E-15	7.5E-08	1.7E-15	26.6338	accatgatcaCgaagatttc	m5C_2601
chr1	119874118	-	C	45	13	0.289	0	0	T	32	M	0.177	0.434	0	1.2E-07	0	46.0978	ataccatgatCacgaagattt	m5C_2606
chr1	119874125	-	C	47	16	0.34	0	0	T	31	M	0.222	0.483	0	4E-07	0	70.9352	aggggagataCcatgatcacg	m5C_2598
chr1	120468397	-	C	41	10	0.244	0	0	T	31	M	0.138	0.393	2.4E-13	2.4E-08	2.9E-13	17.4472	cagctgtggaCaagtgaaatg	m5C_2507
chr1	120468404	-	C	38	21	0.553	0	0	C	21	M	0.397	0.699	0	5.2E-05	0	166.765	gccagagcagCtgtggacaag	m5C_2513
chr1	120543996	-	C	97	33	0.34	0	0	T	64	M	0.254	0.439	0	7.5E-13	0	167.39	ccgtagcagtCctgatgtgca	m5C_2516
chr1	120544006	-	C	186	55	0.296	0	0	T	131	M	0.235	0.365	0	0	0	258.262	tggtggaggtCcgtagcagtc	m5C_2521
chr1	144056373	+	C	164	34	0.207	0	0	T	130	M	0.152	0.276	0	0	0	103.578	ttgcatgagcCcaggtgtttg	m5C_3425
chr1	144056374	+	C	168	34	0.204	1	0.006	T	133	M	0.15	0.271	0	0	0	101.666	tgcatgagccCaggtgtttga	m5C_3431
chr1	144056397	+	C	391	88	0.225	0	0	T	303	M	0.186	0.269	0	0	0	328.167	atgcagtgagCcgtgatcatg	m5C_3438
chr1	144056404	+	C	327	74	0.226	0	0	T	253	M	0.184	0.275	0	0	0	272.729	gagccgtgatCatgccactgc	m5C_3403
chr1	144056414	+	C	132	42	0.323	2	0.015	T	88	M	0.249	0.408	0	0	0	208.962	catgccactgCtctccagcct	m5C_3387
chr1	144056423	+	C	91	24	0.264	0	0	T	67	M	0.184	0.363	0	1.7E-14	0	88.3703	gctctccagcCtgggcaacag	m5C_3434
chr1	144056428	+	C	80	17	0.212	0	0	T	63	M	0.137	0.314	0	2.3E-14	0	46.6217	ccagcctgggCaacagaatga	m5C_3399
chr1	144056431	+	C	71	18	0.254	0	0	T	53	M	0.167	0.366	0	4E-12	0	60.0495	gcctgggcaaCagaatgagac	m5C_3447
chr1	144056441	+	C	40	9	0.225	0	0	T	31	M	0.123	0.375	6.4E-12	1.2E-08	7.6E-12	12.4041	cagaatgagaCcctgatagtt	m5C_3424
chr1	144056443	+	C	43	17	0.395	0	0	T	26	M	0.264	0.544	0	5.6E-07	0	89.6414	gaatgagaccCtgatagtttg	m5C_3419
chr1	145277369	+	C	37	17	0.459	0	0	T	20	M	0.31	0.616	0	2.1E-05	0	105.53	tggtggaggtCcgtagcagtc	m5C_3405
chr1	145277418	+	C	116	86	0.741	0	0	C	86	M	0.655	0.812	0	7.7E-11	0	1126.36	ggtgtaggggCgaaagactaa	m5C_3441
chr1	145277425	+	C	126	93	0.738	0	0	C	93	M	0.655	0.807	0	1.2E-11	0	1218.48	gggcgaaagaCtaattgaacc	m5C_3398
chr1	145277434	+	C	90	61	0.678	0	0	C	61	M	0.576	0.765	0	1.1E-08	0	702.313	actaattgaaCcatctagtag	m5C_3416
chr1	145277470	-	C	84	61	0.726	0	0	C	61	M	0.623	0.81	0	1.1E-08	0	759.479	gccagctatcCcgagggaaac	m5C_3521
chr1	145277471	-	C	79	60	0.759	0	0	C	60	M	0.655	0.84	0	1.6E-07	0	785.569	tgccagctatCccgagggaaa	m5C_3466
chr1	145277475	-	C	82	58	0.707	0	0	C	58	M	0.601	0.795	0	6.8E-09	0	697.555	agagtgccagCtatcccgagg	m5C_3533
chr1	145277478	-	C	78	56	0.718	0	0	C	56	M	0.61	0.806	0	9.3E-08	0	682.851	gcgagagtgcCagctatcccg	m5C_3506
chr1	145277479	-	C	77	58	0.753	0	0	C	58	M	0.646	0.836	0	1.2E-07	0	749.899	tgcgagagtgCcagctatccc	m5C_3470
chr1	145395523	-	C	442	109	0.247	0	0	T	333	M	0.209	0.289	0	0	0	455.008	ccacgttgggCgctgtgtttt	m5C_3497
chr1	145395533	-	C	469	99	0.212	1	0.002	T	369	M	0.177	0.251	0	0	0	350.384	ggttcgagccCcacgttgggc	m5C_3452
chr1	145395539	-	C	479	189	0.395	0	0	T	290	M	0.352	0.439	0	0	0	1329.83	gtcgtggggtCgagccccacg	m5C_3472
chr1	145395555	-	C	384	82	0.214	0	0	T	302	M	0.175	0.257	0	0	0	287.811	gactcttaatCtcagggtcgt	m5C_3519
chr1	145396856	-	C	65	13	0.2	0	0	T	52	M	0.121	0.313	3.8E-15	1.6E-11	4.8E-15	22.646	gctggggtggCggttttttt	m5C_3520
chr1	145396873	-	C	67	16	0.239	0	0	T	51	M	0.153	0.353	0	1.1E-10	0	48.8576	gcaggtggttCtaacttgctg	m5C_3489
chr1	145396944	-	C	71	26	0.366	0	0	T	45	M	0.264	0.482	0	1.3E-10	0	137.124	tcgccgtgatCgtatagtggt	m5C_3460
chr1	145396950	-	C	69	22	0.319	0	0	T	47	M	0.221	0.436	0	1.5E-09	0	97.2163	atggctcgcCgtgatcgtat	m5C_3510
chr1	145396951	-	C	69	23	0.333	0	0	T	46	M	0.234	0.451	0	2.1E-09	0	107.414	tagtggctcgCcgtgatcgta	m5C_3468
chr1	145397859	-	C	218	56	0.257	0	0	T	162	M	0.203	0.319	0	0	0	227.855	aacgcagtgtCagtttttctc	m5C_3459
chr1	145397870	-	C	252	53	0.212	2	0.008	T	197	M	0.166	0.267	0	0	0	175.842	gattcccggcCaacgcagtgt	m5C_3495
chr1	145397871	-	C	251	53	0.211	0	0	T	198	M	0.165	0.266	0	0	0	175.124	cgattcccggCcaacgcagtg	m5C_3458
chr1	145397881	-	C	225	59	0.262	0	0	T	166	M	0.209	0.323	0	0	0	246.721	gacccgggttCgattcccggc	m5C_3485
chr1	145397888	-	C	234	55	0.235	0	0	T	179	M	0.185	0.293	0	0	0	203.789	agcagttgacCcgggttcgat	m5C_3531
chr1	145397889	-	C	234	58	0.248	0	0	T	176	M	0.197	0.307	0	0	0	228.417	aagcagttgaCccgggttcga	m5C_3508
chr1	145397904	-	C	237	56	0.236	0	0	T	181	M	0.187	0.294	0	0	0	209.079	gcatagctgcCttccaagcag	m5C_3513
chr1	145399257	-	C	185	39	0.211	0	0	T	146	M	0.158	0.275	0	0	0	123.412	ccgccgcggcCcgggttcgat	m5C_3488
chr1	145399258	-	C	178	48	0.27	0	0	T	130	M	0.21	0.339	0	0	0	201.449	accgccgcggCccgggttcga	m5C_3482
chr1	145399261	-	C	178	40	0.225	0	0	T	138	M	0.17	0.291	0	0	0	135.671	ctcaccgccgCggcccgggtt	m5C_3490
chr1	145399266	-	C	167	34	0.204	0	0	T	133	M	0.15	0.271	0	0	0	101.666	gcgctctcacCgccgcggccc	m5C_3451
chr1	145399302	-	C	58	14	0.241	0	0	T	44	M	0.15	0.365	0	2.8E-09	0	41.8845	tagcagcttcCctggtggtct	m5C_3484
chr1	145509172	+	C	61	16	0.262	0	0	T	45	M	0.168	0.384	0	1.1E-10	0	53.8837	ctagaggtggCcgaagacgca	m5C_3395
chr1	145509173	+	C	60	22	0.367	0	0	T	38	M	0.256	0.493	0	1.5E-09	0	112.731	tagaggtggcCgaagacgcag	m5C_3417
chr1	145509181	+	C	59	20	0.339	0	0	T	39	M	0.231	0.466	0	3.4E-08	0	92.5499	gccgaagacgCagcagaagtc	m5C_3391
chr1	145509191	+	C	54	31	0.574	0	0	C	31	M	0.442	0.697	0	5.9E-07	0	273.807	cagcagaagtCcagaccggag	m5C_3427
chr1	146556166	-	C	76	22	0.289	0	0	T	54	M	0.2	0.4	0	2.8E-11	0	87.8279	gctcttttccCccgtttttt	m5C_2897
chr1	146556167	-	C	85	25	0.294	0	0	T	60	M	0.208	0.398	0	1.8E-12	0	103.927	cgctcttttcCcccgtttttt	m5C_2892
chr1	146556168	-	C	91	24	0.264	0	0	T	67	M	0.184	0.363	0	1.7E-14	0	88.3703	tcgctcttttCccccgttttt	m5C_2889
chr1	146556177	-	C	101	24	0.238	0	0	T	77	M	0.165	0.329	0	2.2E-16	0	79.2953	agactgcgttCgctcttttcc	m5C_2884
chr1	146556181	-	C	124	27	0.218	0	0	T	97	M	0.154	0.298	0	0	0	83.2571	tgggagactgCgttcgctctt	m5C_2883

chr1	146556184	-	C	124	54	0.435	0	0	T	70	M	0.351	0.523	0	7.5E-15	0	379.567	tagtgggagaCtgcgttcgct	m5C_2887
chr1	146556212	-	C	292	86	0.295	0	0	T	206	M	0.245	0.349	0	0	0	421.705	gtgggaagctCgactgcataa	m5C_2888
chr1	147753495	+	C	34	13	0.382	0	0	T	21	M	0.239	0.55	0	6.7E-06	0	62.1399	gtggttagtaCtctgcgttgt	m5C_2861
chr1	147753497	+	C	31	11	0.355	0	0	T	20	M	0.211	0.531	1.1E-16	3.1E-06	1.5E-16	37.0595	ggttagtactCtgcgttgtgg	m5C_2854
chr1	147825693	+	C	37	14	0.378	0	0	T	23	M	0.241	0.539	0	9.2E-06	0	67.3786	acagctggttCcatgggttaa	m5C_2866
chr1	147825694	+	C	37	11	0.297	0	0	T	26	M	0.175	0.458	1.4E-15	3.1E-06	1.9E-15	28.5508	cagctggttcCatgggttaat	m5C_2869
chr1	147825711	+	C	31	15	0.484	0	0	T	16	M	0.32	0.652	0	1.2E-05	0	95.91	taatggtggagCaccctggact	m5C_2851
chr1	148241547	+	C	83	18	0.217	0	0	T	65	M	0.142	0.317	0	4.5E-14	0	51.0532	tccggatgtgCtgacccctgc	m5C_4441
chr1	148241551	+	C	82	17	0.207	0	0	T	65	M	0.134	0.307	0	2.3E-14	0	45.4454	gatgtgctgaCccctgcgatt	m5C_4398
chr1	148241554	+	C	86	25	0.291	0	0	T	61	M	0.205	0.394	0	1.8E-12	0	102.662	gtgctgacccCtgcgatttcc	m5C_4444
chr1	148241563	+	C	77	18	0.234	0	0	T	59	M	0.153	0.34	0	4E-12	0	55.1862	cctgcgatttCcccaagtgtg	m5C_4403
chr1	148248119	+	C	184	42	0.228	0	0	T	142	M	0.174	0.294	0	0	0	145.773	gtacctgtctCtgtggcgcaa	m5C_4425
chr1	148248147	+	C	226	53	0.235	0	0	T	173	M	0.184	0.294	0	0	0	195.034	gcgcgttcggCtgttaaccga	m5C_4451
chr1	148248175	+	C	213	54	0.254	0	0	T	159	M	0.2	0.316	0	0	0	215.806	gtggttcgagCccacccaggg	m5C_4392
chr1	148248176	+	C	211	54	0.256	0	0	T	157	M	0.202	0.319	0	0	0	217.915	tggttcgagcCcacccaggga	m5C_4452
chr1	148248189	+	C	182	49	0.282	8	0.044	T	125	M	0.22	0.353	0	0	0	215.651	cccagggacgCctctttgagc	m5C_4453
chr1	148248192	+	C	117	36	0.456	38	0.325	T	43	M	0.35	0.565	0	2.4E-09	0	252.348	agggacgcctCtttgagcttt	m5C_4435
chr1	148248199	+	C	91	37	0.407	0	0	T	54	M	0.311	0.509	0	2.7E-12	0	230.47	cctctttgagCttttaaagta	m5C_4411
chr1	149173923	+	C	54	41	0.759	0	0	C	41	M	0.631	0.854	0	3.3E-06	0	517.028	attggcggtaCagtggcagaa	m5C_4426
chr1	149173936	+	C	31	22	0.71	0	0	C	22	M	0.534	0.839	0	6.3E-05	0	234.992	tggcagaattCtcgcctccca	m5C_4406
chr1	149194108	-	C	475	124	0.261	0	0	T	351	M	0.224	0.302	0	0	0	554.487	gcgctttcccCtgaattttcc	m5C_4524
chr1	149194109	-	C	528	148	0.28	0	0	T	380	M	0.244	0.32	0	0	0	721.305	cgcgctttccCctgaattttc	m5C_4520
chr1	149194111	-	C	589	150	0.255	0	0	T	439	M	0.221	0.291	0	0	0	663.455	ttcgcgctttCccctgaattt	m5C_4539
chr1	149194115	-	C	645	141	0.221	7	0.011	T	497	M	0.191	0.255	0	0	0	537.293	tgcgttcgcgCtttcccctga	m5C_4474
chr1	149194117	-	C	655	169	0.258	0	0	T	486	M	0.226	0.293	0	0	0	763.832	actgcgttcgCgctttcccct	m5C_4536
chr1	149194119	-	C	643	151	0.235	0	0	T	492	M	0.204	0.269	0	0	0	615.192	ggactgcgttCgcgctttccc	m5C_4517
chr1	149194126	-	C	760	169	0.223	1	0.001	T	590	M	0.195	0.254	0	0	0	657.415	tagtgggggaCtgcgttcgcg	m5C_4462
chr1	149194151	-	C	358	72	0.201	0	0	T	286	M	0.163	0.246	0	0	0	234.529	ggaaactcgaCtgcgtaattt	m5C_4535
chr1	149194166	-	C	66	16	0.432	29	0.439	A	22	M	0.287	0.591	0	1.7E-05	0	91.7488	atttccccaaCtgtgggaaac	m5C_4532
chr1	149194169	-	C	59	16	0.271	0	0	T	43	M	0.174	0.396	0	7.3E-09	0	55.7978	gcgatttcccCaactgtggga	m5C_4484
chr1	149194181	-	C	37	10	0.27	0	0	T	27	M	0.154	0.43	6.7E-14	2E-06	8.3E-14	20.2815	gtgttgacctCtgcgatttcc	m5C_4463
chr1	149194184	-	C	34	7	0.206	0	0	T	27	M	0.103	0.368	1.4E-09	3.4E-07	1.6E-09	6.4054	gatgtgttgaCctctgcgatt	m5C_4548
chr1	149239535	+	C	76	17	0.224	0	0	T	59	M	0.145	0.329	0	2.2E-12	0	49.1676	tctggcattcCtatcagagcc	m5C_4407
chr1	149294676	-	C	495	118	0.238	0	0	T	377	M	0.203	0.278	0	0	0	478.967	gttcaattccCggtcaaggca	m5C_4496
chr1	149294677	-	C	501	151	0.301	0	0	T	350	M	0.263	0.343	0	0	0	793.813	ggttcaattcCcggtcaaggc	m5C_4470
chr1	149294689	-	C	462	99	0.215	2	0.004	T	361	M	0.18	0.255	0	0	0	356.599	gcgggacaccCgggttcaatt	m5C_4559
chr1	149294691	-	C	459	128	0.279	0	0	T	331	M	0.24	0.322	0	0	0	613.904	acgcgggacaCccgggttcaa	m5C_4480
chr1	149294706	-	C	643	206	0.32	0	0	T	437	M	0.285	0.357	0	0	0	1176.11	gaattctcgcCtcacacgcgg	m5C_4471
chr1	149294711	-	C	616	142	0.231	1	0.002	T	473	M	0.199	0.266	0	0	0	566.071	tggtagaattCtcgcctcaca	m5C_4545
chr1	149298562	-	C	465	103	0.222	0	0	T	362	M	0.186	0.261	0	0	0	383.426	cgaaactgggCggaaacagag	m5C_4460
chr1	149298577	-	C	276	96	0.348	0	0	T	180	M	0.294	0.406	0	0	0	564.619	gaaaggtcccCggttcgaaac	m5C_4491
chr1	149298578	-	C	281	68	0.242	0	0	T	213	M	0.196	0.295	0	0	0	266.033	cgaaaggtccCcggttcgaaa	m5C_4538
chr1	149298580	-	C	279	76	0.272	0	0	T	203	M	0.224	0.327	0	0	0	339.745	cgcgaaaggtCcccggttcga	m5C_4521
chr1	149298590	-	C	376	86	0.229	0	0	T	290	M	0.189	0.274	0	0	0	325.319	tcgcctcacaCgcgaaaggtc	m5C_4476
chr1	149298596	-	C	468	94	0.201	0	0	T	374	M	0.167	0.24	0	0	0	314.069	tcacgttcgcCtcacacgcga	m5C_4549
chr1	149298623	-	C	913	236	0.258	0	0	T	677	M	0.231	0.288	0	0	0	1091	cgtgtggtttCcgtagtgtag	m5C_4475
chr1	149326281	-	C	35	13	0.371	0	0	T	22	M	0.232	0.537	0	6.7E-06	0	60.2306	ttcgaacacaCccagaggcgt	m5C_4550
chr1	149326289	-	C	33	10	0.303	0	0	T	23	M	0.174	0.473	1.6E-14	2E-06	2E-14	23.982	gttggtggttCgaacacaccc	m5C_4522
chr1	149514107	-	C	31	7	0.226	0	0	T	24	M	0.114	0.398	6.4E-10	3.4E-07	7.2E-10	7.33205	tgggggactgCgttcgcgctt	m5C_4541
chr1	149514140	-	C	31	8	0.258	0	0	T	23	M	0.137	0.432	1.7E-11	6.8E-07	1.9E-11	11.8182	acgtgggaaaCtcgactgcat	m5C_4515
chr1	149514155	-	C	37	15	0.405	0	0	T	22	M	0.263	0.565	0	1.2E-05	0	79.0389	ctgcgatttcCccaaacgtgg	m5C_4472
chr1	149514156	-	C	46	14	0.304	0	0	T	32	M	0.191	0.448	0	1.9E-07	0	53.4231	tctgcgatttCcccaaacgtg	m5C_4518
chr1	149514162	-	C	53	14	0.264	0	0	T	39	M	0.164	0.396	0	2.8E-09	0	46.0242	ctgacctctgCgatttcccca	m5C_4494
chr1	149514165	-	C	59	16	0.271	0	0	T	43	M	0.174	0.396	0	7.3E-09	0	55.7978	gtgctgacctCtgcgatttcc	m5C_4527
chr1	149514181	-	C	90	21	0.233	0	0	T	69	M	0.158	0.331	0	3.1E-15	0	66.3519	ccattgtgttCcggatgtgct	m5C_4490
chr1	149514196	-	C	63	15	0.238	0	0	T	48	M	0.15	0.356	0	6E-11	0	44.981	cagggcgaggCttatccattg	m5C_4487
chr1	149514201	-	C	40	24	0.6	0	0	C	24	M	0.446	0.737	0	3.6E-06	0	214.059	tttctcagggCgaggcttatc	m5C_4552
chr1	149606060	+	C	32	12	0.375	0	0	T	20	M	0.229	0.547	0	4.6E-06	0	55.0408	cagtggggaaCtgtgtctgtg	m5C_4443
chr1	149680216	-	C	886	206	0.233	1	0.001	T	679	M	0.206	0.262	0	0	0	849.202	aattcccggtCaagataagag	m5C_4554
chr1	149680220	-	C	885	192	0.217	0	0	T	693	M	0.191	0.245	0	0	0	733.625	gtttaattccCggtcaagata	m5C_4458
chr1	149680244	-	C	929	193	0.21	10	0.011	T	726	M	0.185	0.238	0	0	0	713.754	tcgcctcccaCgcgggagacc	m5C_4528
chr1	149680250	-	C	929	192	0.207	0	0	T	737	M	0.182	0.234	0	0	0	698.376	gaattctcgcCtcccacgcgg	m5C_4542
chr1	149684139	-	C	58	19	0.328	0	0	T	39	M	0.221	0.456	0	2.4E-08	0	83.9159	tagtggttatCacgttcgcct	m5C_4511
chr1	149728329	-	C	47	18	0.383	0	0	T	29	M	0.258	0.526	0	7.7E-07	0	92.8457	atttgtggtcCagtggtagaa	m5C_4534
chr1	149783789	-	C	37	9	0.243	0	0	T	28	M	0.134	0.401	2.7E-12	1.2E-06	3.2E-12	13.907	gcaagaagcgCaagcgcagcc	m5C_4482
chr1	149783801	-	C	63	22	0.349	0	0	T	41	M	0.243	0.472	0	1.5E-09	0	107.034	agaagaaggaCggcaagaagc	m5C_4466
chr1	149783812	-	C	58	25	0.431	0	0	T	33	M	0.312	0.559	0	1.5E-07	0	155.908	tacgaaagtgCagaagaagga	m5C_4519
chr1	150193024	-	C	45	21	0.467	0	0	T	24	M	0.329	0.609	0	1.8E-06	0	138.327	aaacgagatgCtgaagacgat	m5C_3197
chr1	150199001	-	C	36	9	0.25	0	0	T	27	M	0.138	0.411	2E-12	1.2E-06	2.4E-12	14.4765	gaagcaggttCagagttggga	m5C_3200
chr1	150199079	-	C	81	32	0.395	0	0	T	49	M	0.296	0.504	0	2.3E-11	0	189.235	gctggtccacCggaaggatat	m5C_3214
chr1	150199080	-	C	81	40	0.494	0	0	T	41	M	0.388	0.6	0	2.1E-10	0	310.175	agctggtccaCcggaaggata	m5C_3233
chr1	150199082	-	C	64	17	0.266	0	0	T	47	M	0.173	0.385	0	1.8E-10	0	58.8072	gaagctggtcCaccggaagga	m5C_3196
chr1	150199088	-	C	58	16	0.276	0	0	T	42	M	0.178	0.402	0	7.3E-09	0	56.8071	gaaaatgaagCtggtccaccg	m5C_3182
chr1	150199123	-	C	67	24	0.511	20	0.299	C	24	M	0.372	0.647	0	3.6E-06	0	178.763	ttaaagatggCgatgaagatg	m5C_3199
chr1	150208154	-	C	33	9	0.273	0	0	T	24	M	0.151	0.442	7.5E-13	1.2E-06	9E-13	16.4418	agagtagtagCtgaagagcaa	m5C_3230
chr1	150208180	-	C	32	12	0.375	0	0	T	20	M	0.229	0.547	0	4.6E-06	0	55.0408	ttgggaaggCaggaaaggga	m5C_3166
chr1	150209446	+	C	102	40	0.392	0	0	T	62	M	0.303	0.489	0	1.8E-13	0	242.379	taggagcttgCttgttcactt	m5C_3144
chr1	150209452	+	C	166	48	0.289	0	0	T	118	M	0.226	0.362	0	0	0	216.551	cttgcttgttCacttcacgca	m5C_3152
chr1	150209468	+	C	401	81	0.202	0	0	T	320	M	0.166	0.244	0	0	0	268.29	acgcatcgatCtggtattgca	m5C_3163
chr1	150209499	+	C	207	46	0.222	0	0	T	161	M	0.171	0.284	0	0	0	157.263	gaacagtgcaCcccctcaggg	m5C_3156
chr1	150297431	+	C	46	23	0.5	0	0	CT	23	M	0.361	0.639	0	2.9E-06	0	166.146	gctggatgagCtgaaaccatg	m5C_3142
chr1	150488091	-	C	32	11	0.344	0	0	T	21	M	0.204	0.517	2.2E-16	3.1E-06	2.9E-16	35.1442	cgctcattggCtcgcgtgctg	m5C_3231
chr1	150549703	-	C	75	54	0.72	0	0	C	54	M	0.61	0.809	0	6.9E-08	0	658.358	ttatggctaaCaagaataaat	m5C_3216
chr1	150549707	-	C	77	33	0.429	0	0	T	44	M	0.324	0.54	0	1.1E-09	0	213.863	aggattatggCtaacaagaat	m5C_3217
chr1	150549721	-	C	62	13	0.21	0	0	T	49	M	0.127	0.326	1.8E-15	1.6E-11	2.3E-15	24.3221	aagcaagtggCaagaggatta	m5C_3178
chr1	150549728	-	C	48	35	0.729	0	0	C	35	M	0.59	0.834	0	2.5E-05	0	413.019	gccagaaaagCaagtggcaag	m5C_3227
chr1	153507183	-	C	38	16	0.421	0	0	T	22	M	0.279	0.578	0	1.7E-05	0	89.1273	caatgaagccCtcaagggctg	m5C_3383
chr1	153507185	-	C	38	11	0.289	0	0	T	27	M	0.17	0.448	2.1E-15	3.1E-06	2.7E-15	27.4488	tacaatgaagCcctcaagggc	m5C_3373
chr1	153507250	-	C	36	20	0.556	0	0	C	20	M	0.396	0.705	0	4.3E-05	0	158.323	ggaacaaggaCcaggaggtga	m5C_3334
chr1	153507262	-	C	53	17	0.321	0	0	T	36	M	0.211	0.455	0	1.1E-08	0	71.713	aagacttggaCcggaacaagg	m5C_3361
chr1	153507268	-	C	63	13	0.206	0	0	T	50	M	0.125	0.322	2.4E-15	1.6E-11	3.1E-15	23.6977	tgatggaagaCttggaccgga	m5C_3381
chr1	153507279	-	C	41	19	0.463	0	0	T	22	M	0.321	0.613	0	1E-06	0	121.813	aattgcaaggCtgatggaaga	m5C_3345
chr1	153507284	-	C	35	8	0.229	0	0	T	27	M	0.121	0.39	5.6E-11	6.8E-07	6.4E-11	9.89691	gctgaaattgCaaggctgatg	m5C_3368
chr1	153591391	-	C	161	46	0.286	0	0	T	115	M	0.222	0.36	0	0	0	203.838	gaagaaagacCtgaagatcag	m5C_3337
chr1	153591417	-	C	114	60	0.526	0	0	C	60	M	0.435	0.616	0	9E-13	0	522.362	ggggagctggCcaaggaaatc	m5C_3382
chr1	153591421	-	C	115	23	0.2	0	0	T	92	M	0.137	0.282	0	0	0	63.0797	gattggggagCtggccaagga	m5C_3377
chr1	153591440	-	C	35	13	0.371	0	0	T	22	M	0.232	0.537	0	6.7E-06	0	60.2306	tcaatgagtaCtggagattga	m5C_3346
chr1	153643751	+	C	335	69	0.207	2	0.006	T	264	M	0.167	0.254	0	0	0	230.645	cggaagcgtgCtgggcccata	m5C_3287
chr1	153643764	+	C	316	68	0.216	1	0.003	T	247	M	0.174	0.265	0	0	0	236.648	ggcccataacCcagaggtcga	m5C_3285
chr1	153643765	+	C	316	80	0.253	0	0	T	236	M	0.208	0.304	0	0	0	333.412	gcccataaccCagaggtcgat	m5C_3292
chr1	153643772	+	C	295	59	0.2	0	0	T	236	M	0.158	0.249	0	0	0	186.842	acccagaggtCgatggatcga	m5C_3290

chr1	153643780	+	C	292	60	0.207	2	0.007	T	230	M	0.164	0.257	0	0	0	197.104	gtcgatggatCgaaaccatcc	m5C_3299
chr1	153643790	+	C	264	68	0.258	0	0	T	196	M	0.209	0.314	0	0	0	283.65	cgaaaccatcCtctgctatcg	m5C_3282
chr1	153643792	+	C	265	65	0.245	0	0	T	200	M	0.197	0.3	0	0	0	256.571	aaaccatcctCtgctatcgga	m5C_3276
chr1	153643799	+	C	247	53	0.215	0	0	T	194	M	0.168	0.27	0	0	0	178.032	cctctgctatCggagtttttt	m5C_3258
chr1	153758253	-	C	32	8	0.25	0	0	T	24	M	0.133	0.421	2.3E-11	6.8E-07	2.6E-11	11.2828	tgatcacaacCagttacagat	m5C_3303
chr1	153758254	-	C	35	8	0.229	0	0	T	27	M	0.121	0.39	5.6E-11	6.8E-07	6.4E-11	9.89691	ttgatcacaaCcagttacaga	m5C_3329
chr1	153758259	-	C	47	12	0.255	0	0	T	35	M	0.153	0.395	1E-15	7.5E-08	1.3E-15	27.4561	actcattgatCacaaccagtt	m5C_3379
chr1	153874360	+	C	43	9	0.209	0	0	T	34	M	0.114	0.352	1.4E-11	1.2E-08	1.6E-11	11.1522	gtgtctacagCcataccaccc	m5C_3251
chr1	153874368	+	C	43	13	0.302	0	0	T	30	M	0.186	0.451	0	1.2E-07	0	48.3648	agccataccaCcctgaacgtg	m5C_3281
chr1	153874370	+	C	41	18	0.439	0	0	T	23	M	0.299	0.59	0	7.7E-07	0	107.604	ccataccaccCtgaacgtgcc	m5C_3271
chr1	153874379	+	C	44	15	0.341	0	0	T	29	M	0.219	0.489	0	2.8E-07	0	65.6269	cctgaacgtgCccggtcctgt	m5C_3250
chr1	153874381	+	C	42	20	0.476	0	0	T	22	M	0.334	0.623	0	1.4E-06	0	133.437	tgaacgtgccCggtcctgtct	m5C_3265
chr1	153874390	+	C	37	8	0.216	0	0	T	29	M	0.114	0.372	9.7E-11	6.8E-07	1.1E-10	9.12273	ccggtcctgtCtaaaatggcc	m5C_3298
chr1	153947190	-	C	32	16	0.5	0	0	CT	16	M	0.336	0.664	0	1.7E-05	0	107.618	cagaaaggctCtggaaaaggt	m5C_3320
chr1	153954356	-	C	72	30	0.417	0	0	T	42	M	0.31	0.532	0	4.8E-10	0	185.91	tgaaagaaggCaaggaggtag	m5C_3335
chr1	153954425	-	C	120	30	0.25	0	0	T	90	M	0.181	0.334	0	0	0	108.658	aagggaaaagCagaaaggaaa	m5C_3321
chr1	154232224	+	C	344	83	0.241	0	0	T	261	M	0.199	0.289	0	0	0	330.475	tagaccttgtCtgactgtgct	m5C_4017
chr1	154232228	+	C	344	78	0.227	0	0	T	266	M	0.186	0.274	0	0	0	289.621	ccttgtctgaCtgtgctcatg	m5C_4000
chr1	154232233	+	C	344	81	0.235	0	0	T	263	M	0.194	0.283	0	0	0	313.797	tctgactgtgCtcatggccag	m5C_4006
chr1	154232235	+	C	340	78	0.229	0	0	T	262	M	0.188	0.277	0	0	0	293.108	tgactgtgctCatggccaggc	m5C_3987
chr1	154232240	+	C	339	113	0.333	0	0	T	226	M	0.285	0.385	0	0	0	644.7	gtgctcatggCcaggcagggg	m5C_3995
chr1	154232245	+	C	224	54	0.243	2	0.009	T	168	M	0.191	0.304	0	0	0	206.802	catggccaggCaggggggaca	m5C_3988
chr1	154232254	+	C	157	49	0.312	0	0	T	108	M	0.245	0.388	0	0	0	239.944	gcaggggggaCagtgtatgca	m5C_3968
chr1	154232285	+	C	86	34	0.395	0	0	T	52	M	0.299	0.501	0	4.2E-11	0	203.063	ggagtttgtgCtaactctagc	m5C_4009
chr1	154232291	+	C	71	22	0.31	0	0	T	49	M	0.214	0.425	0	2.8E-11	0	94.3341	tgtgctaactCtagccagctt	m5C_4008
chr1	154232311	+	C	69	48	0.696	0	0	C	48	M	0.579	0.792	0	5.1E-07	0	555.996	taattagtgaCtggataaatt	m5C_3960
chr1	154232328	+	C	57	17	0.298	0	0	T	40	M	0.195	0.427	0	1.1E-08	0	66.4101	aattgcacaaCtctcacattc	m5C_4004
chr1	154232330	+	C	59	22	0.373	0	0	T	37	M	0.261	0.5	0	6.5E-08	0	114.769	ttgcacaactCtcacattctt	m5C_3979
chr1	154232334	+	C	52	15	0.288	0	0	T	37	M	0.183	0.423	0	4.6E-09	0	54.9894	acaactctcaCattcttctgt	m5C_3973
chr1	154938975	-	C	35	9	0.257	0	0	T	26	M	0.142	0.421	1.5E-12	1.2E-06	1.7E-12	15.0859	gctttgatggCtcagcatggg	m5C_4058
chr1	155164997	+	C	57	27	0.474	0	0	T	30	M	0.35	0.601	0	2.5E-07	0	188.93	agggacgggaCgcggtgcagt	m5C_3992
chr1	155164999	+	C	55	12	0.218	0	0	T	43	M	0.129	0.344	9.8E-15	8.8E-10	1.2E-14	21.7648	ggacgggacgCggtgcagtgt	m5C_3989
chr1	155288572	+	C	31	14	0.452	0	0	T	17	M	0.292	0.622	0	9.2E-06	0	81.6522	acagcgcgaaCcatgtctgga	m5C_3962
chr1	155649001	-	C	47	13	0.277	0	0	T	34	M	0.169	0.418	0	1.2E-07	0	44.0347	caagctgtatCtgtttgggaa	m5C_4061
chr1	155649011	-	C	107	43	0.402	0	0	T	64	M	0.314	0.497	0	4.3E-13	0	269.983	agctgtaggcCaagctgtatc	m5C_4053
chr1	155649012	-	C	112	24	0.214	0	0	T	88	M	0.148	0.299	0	0	0	71.251	cagctgtaggCcaagctgtat	m5C_4105
chr1	155649019	-	C	106	25	0.236	0	0	T	81	M	0.165	0.325	0	4.4E-16	0	82.5797	ggcagtgcagCtgtaggccaa	m5C_4090
chr1	155649022	-	C	104	41	0.394	0	0	T	63	M	0.306	0.49	0	2.4E-13	0	250.664	tggggcagtgCagctgtaggc	m5C_4062
chr1	155708087	+	C	31	10	0.323	0	0	T	21	M	0.186	0.499	7.1E-15	2E-06	9E-15	26.2725	atgctggaccCagtaagatga	m5C_3994
chr1	155708104	+	C	51	19	0.373	0	0	T	32	M	0.253	0.51	0	2.4E-08	0	96.2173	atgaggaagtCgggcagtaca	m5C_3967
chr1	155708108	+	C	50	21	0.42	0	0	T	29	M	0.294	0.558	0	4.8E-08	0	123.374	ggaagtcgggCagtacacagg	m5C_4015
chr1	155889704	-	C	547	118	0.216	0	0	T	429	M	0.183	0.252	0	0	0	432.582	gggggttgcaCagtgtgtaac	m5C_4026
chr1	155889728	-	C	351	106	0.302	0	0	T	245	M	0.256	0.352	0	0	0	543.395	tacctgtgggCtgtgagcact	m5C_4072
chr1	155889743	-	C	128	33	0.258	0	0	T	95	M	0.19	0.34	0	0	0	125.313	attgcccagtCcccttacctg	m5C_4103
chr1	155889748	-	C	89	19	0.213	0	0	T	70	M	0.141	0.31	0	8.4E-14	0	53.6358	gaaccattgcCcagtcccctt	m5C_4073
chr1	155889754	-	C	80	24	0.3	0	0	T	56	M	0.211	0.408	0	1.1E-12	0	101.109	agcctcgaacCattgcccagt	m5C_4100
chr1	155889777	-	C	59	13	0.22	0	0	T	46	M	0.134	0.341	8.9E-16	1.6E-09	1.2E-15	26.1299	gtacccatgcCatagcaaatg	m5C_4020
chr1	155889793	-	C	128	63	0.492	0	0	T	65	M	0.407	0.578	0	6.6E-14	0	512.916	gtgggcctctCatagtgtacc	m5C_4066
chr1	155889795	-	C	149	65	0.436	0	0	T	84	M	0.359	0.516	0	1.1E-16	0	466.967	ctgtgggcctCtcatagtgta	m5C_4095
chr1	155889797	-	C	160	48	0.3	0	0	T	112	M	0.234	0.375	0	0	0	224.988	ctctgtgggcCtctcatagtg	m5C_4046
chr1	155889798	-	C	158	41	0.259	0	0	T	117	M	0.197	0.333	0	0	0	161.889	tctctgtgggCctctcatagt	m5C_4080
chr1	155889807	-	C	251	69	0.275	0	0	T	182	M	0.223	0.333	0	0	0	308.254	gtgggtccttCtctgtgggcc	m5C_4083
chr1	155889811	-	C	304	78	0.258	2	0.007	T	224	M	0.212	0.31	0	0	0	330.98	tatggtgggtCcttctctgtg	m5C_4089
chr1	155895749	-	C	88	27	0.307	0	0	T	61	M	0.22	0.41	0	4E-12	0	118.926	acccaacactCtaccccagtt	m5C_4091
chr1	155895751	-	C	109	58	0.532	0	0	C	58	M	0.439	0.623	0	1.5E-11	0	509.177	ctacccaacaCtctaccccag	m5C_4029
chr1	155895753	-	C	128	59	0.461	0	0	T	69	M	0.377	0.547	0	2.6E-14	0	444.836	tactacccaaCactctacccc	m5C_4096
chr1	155895756	-	C	131	39	0.298	0	0	T	92	M	0.226	0.381	0	0	0	176.342	ctgtactaccCaacactctac	m5C_4077
chr1	155895757	-	C	148	41	0.277	0	0	T	107	M	0.211	0.354	0	0	0	173.234	actgtactacCcaacactcta	m5C_4041
chr1	155895758	-	C	153	35	0.232	2	0.013	T	116	M	0.172	0.305	0	0	0	120.151	gactgtactaCccaacactct	m5C_4081
chr1	155895761	-	C	143	39	0.273	0	0	T	104	M	0.206	0.351	0	0	0	160.987	gctgactgtaCtacccaacac	m5C_4049
chr1	155895766	-	C	163	37	0.227	0	0	T	126	M	0.169	0.297	0	0	0	125.361	acagggctgaCtgtactaccc	m5C_4070
chr1	155895770	-	C	164	38	0.233	1	0.006	T	125	M	0.175	0.304	0	0	0	132.863	tatcacagggCtgactgtact	m5C_4104
chr1	155895777	-	C	270	71	0.263	0	0	T	199	M	0.214	0.319	0	0	0	303.935	tgaagagtatCacagggctga	m5C_4068
chr1	155895788	-	C	269	56	0.209	1	0.004	T	212	M	0.165	0.262	0	0	0	184.31	gcagccttcaCtgaagagtat	m5C_4051
chr1	155895790	-	C	277	91	0.329	0	0	T	186	M	0.276	0.386	0	0	0	502.109	gagcagccttCactgaagagt	m5C_4076
chr1	155895797	-	C	356	81	0.228	0	0	T	275	M	0.187	0.274	0	0	0	302.976	ttggagagagCagccttcact	m5C_4033
chr1	155895823	-	C	1005	205	0.204	0	0	T	800	M	0.18	0.23	0	0	0	738.886	tgatacattcCtcctagaaaa	m5C_4021
chr1	155895853	-	C	1510	326	0.216	3	0.002	T	1181	M	0.196	0.238	0	0	0	1279.68	aaggctgagtCtgatgaagta	m5C_4101
chr1	155895859	-	C	1558	408	0.262	3	0.002	T	1147	M	0.241	0.285	0	0	0	1967.52	actaagaaggCtgagtctgat	m5C_4097
chr1	155895868	-	C	1567	374	0.239	1	0.001	T	1192	M	0.218	0.261	0	0	0	1633.35	aactggaggaCtaagaaggct	m5C_4047
chr1	156104258	+	C	37	14	0.378	0	0	T	23	M	0.241	0.539	0	9.2E-06	0	67.3786	cgggtggatgCtgagaacagg	m5C_3027
chr1	156107492	+	C	31	10	0.323	0	0	T	21	M	0.186	0.499	7.1E-15	2E-06	9E-15	26.2725	tggttgaggaCgacgaggatg	m5C_2987
chr1	156186232	+	C	42	14	0.333	0	0	T	28	M	0.21	0.484	0	1.9E-07	0	58.8344	tagagagcagCtgacacgctg	m5C_2997
chr1	156186236	+	C	53	12	0.226	0	0	T	41	M	0.135	0.355	5.8E-15	8.8E-10	7.3E-15	22.9888	gagcagctgaCacgctgtcct	m5C_3028
chr1	156186238	+	C	121	53	0.438	0	0	T	68	M	0.353	0.527	0	5.8E-15	0	374.042	gcagctgacaCgctgtcctct	m5C_3009
chr1	156186240	+	C	119	35	0.294	0	0	T	84	M	0.22	0.381	0	6.7E-16	0	153.806	agctgacacgCtgtcctctgg	m5C_2986
chr1	156186245	+	C	113	44	0.389	0	0	T	69	M	0.305	0.482	0	1.6E-14	0	267.984	acacgctgtcCtctggcgacc	m5C_3022
chr1	156186247	+	C	116	27	0.233	0	0	T	89	M	0.165	0.317	0	0	0	89.1977	acgctgtcctCtggcgacctg	m5C_3020
chr1	156186251	+	C	112	27	0.241	0	0	T	85	M	0.171	0.328	0	0	0	92.4985	tgtcctctggCgacctgtcgc	m5C_3011
chr1	156186254	+	C	110	32	0.291	0	0	T	78	M	0.214	0.382	0	2.2E-16	0	137.11	cctctggcgaCctgtcgctgg	m5C_3016
chr1	156186255	+	C	116	31	0.267	0	0	T	85	M	0.195	0.354	0	1.1E-16	0	120.976	ctctggcgacCtgtcgctgga	m5C_3041
chr1	156186261	+	C	96	39	0.406	0	0	T	57	M	0.313	0.506	0	4.9E-12	0	244.492	cgacctgtcgCtggagaggtt	m5C_3018
chr1	156186276	+	C	74	25	0.338	0	0	T	49	M	0.24	0.451	0	9.3E-11	0	120.232	gaggttgggaCtctggatgcg	m5C_2982
chr1	156186285	+	C	79	24	0.304	0	0	T	55	M	0.213	0.412	0	6.4E-11	0	102.453	actctggatgCgtgcggggct	m5C_3029
chr1	156186301	+	C	52	25	0.481	0	0	T	27	M	0.351	0.613	0	1.5E-07	0	175.519	gggctctggcCtaccggtgac	m5C_3024
chr1	156186304	+	C	35	13	0.371	0	0	T	22	M	0.232	0.537	0	6.7E-06	0	60.2306	ctctggcctaCcggtgacccg	m5C_3037
chr1	156287293	-	C	84	20	0.238	0	0	T	64	M	0.16	0.339	0	1.5E-13	0	63.8983	ccgaattctcCagatggagga	m5C_3091
chr1	156287303	-	C	96	26	0.271	0	0	T	70	M	0.192	0.367	0	4.6E-14	0	99.859	aggacttcacCcgaattctcc	m5C_3051
chr1	156287304	-	C	97	27	0.278	0	0	T	70	M	0.199	0.375	0	7.2E-14	0	107.412	gaggacttcaCccgaattctc	m5C_3094
chr1	156287309	-	C	97	49	0.505	0	0	C	49	M	0.407	0.603	0	5.8E-11	0	399.226	gagaggaggaCttcacccgaa	m5C_3085
chr1	156287320	-	C	61	22	0.361	0	0	T	39	M	0.252	0.486	0	1.5E-09	0	110.765	tgagattacaCgagaggagga	m5C_3080
chr1	156287322	-	C	50	14	0.28	0	0	T	36	M	0.175	0.417	0	1.9E-07	0	48.9272	attgagattaCacgagaggag	m5C_3099
chr1	156712861	-	C	48	30	0.625	0	0	C	30	M	0.484	0.748	0	1.2E-05	0	290.175	gggttgggatCaggggtagga	m5C_3082
chr1	156713997	-	C	70	40	0.571	0	0	C	40	M	0.455	0.681	0	5.8E-09	0	363.821	aggagaagaaCgagaaaggag	m5C_3059
chr1	159758157	-	C	99	25	0.253	0	0	T	74	M	0.177	0.346	0	2.9E-14	0	88.6463	gggacgtgtgCatctgtaaag	m5C_3134
chr1	160278931	+	C	48	25	0.521	0	0	C	25	M	0.383	0.655	0	4.5E-06	0	191.645	ggatggtgtaCaggtcatagg	m5C_7014
chr1	160874836	+	C	51	23	0.451	0	0	T	28	M	0.323	0.586	0	8.7E-08	0	148.43	agaatgtggtCtgagtgattt	m5C_7086
chr1	161173262	+	C	31	9	0.29	0	0	T	22	M	0.161	0.466	3.7E-13	1.2E-06	4.4E-13	18.0154	gtggaatgggCacagcagttt	m5C_7029
chr1	161369558	-	C	63	14	0.222	0	0	T	49	M	0.137	0.339	0	3.2E-11	0	38.4297	cgcctggtttCcgtagtgtag	m5C_7200
chr1	161369565	-	C	52	30	0.577	0	0	C	30	M	0.442	0.701	0	4.8E-07	0	265.165	ctgccaccgcCtggtttccgt	m5C_7161
chr1	161397865	-	C	110	40	0.364	0	0	T	70	M	0.28	0.457	0	1.8E-13	0	223.801	ccagggacggCtgaaatttta	m5C_7110

chr1	161397868	-	C	110	33	0.3	0	0	T	77	M	0.222	0.391	0	2.2E-16	0	146.716	cacccaggaCggctgaaatt	m5C_7164
chr1	161397875	-	C	110	29	0.264	0	0	T	81	M	0.19	0.353	0	0	0	110.364	tcgagcccacCcagggacggc	m5C_7113
chr1	161397911	-	C	68	35	0.515	0	0	C	35	M	0.398	0.629	0	5.3E-08	0	278.842	ttagcgcgttCggctgttaac	m5C_7106
chr1	161397915	-	C	57	15	0.263	0	0	T	42	M	0.166	0.39	0	4.6E-09	0	49.9369	tcggttagcgCgttcggctgt	m5C_7111
chr1	161397930	-	C	65	15	0.231	0	0	T	50	M	0.145	0.346	0	6E-11	0	43.5411	gtctctgtggCgcaatcggtt	m5C_7167
chr1	161397936	-	C	67	28	0.418	0	0	T	39	M	0.307	0.537	0	9.8E-09	0	172.156	gtgtcagtctCtgtggcgcaa	m5C_7117
chr1	161413106	+	C	35	11	0.314	0	0	T	24	M	0.186	0.48	6.7E-16	3.1E-06	8.7E-16	30.9686	atgggtggttCagtggtagaa	m5C_7090
chr1	161417385	-	C	57	13	0.228	0	0	T	44	M	0.138	0.352	4.4E-16	1.6E-09	5.8E-16	27.6258	gttcgattccCggccaacgca	m5C_7166
chr1	161417386	-	C	53	12	0.226	0	0	T	41	M	0.135	0.355	5.8E-15	8.8E-10	7.3E-15	22.9888	ggttcgattcCcggccaacgc	m5C_7173
chr1	161417398	-	C	54	13	0.241	0	0	T	41	M	0.146	0.369	2.2E-16	1.6E-09	2.9E-16	29.8003	gcagttgaccCgggttcgatt	m5C_7163
chr1	161417399	-	C	60	14	0.233	0	0	T	46	M	0.144	0.354	0	3.2E-11	0	40.4304	agcagttgacCcgggttcgat	m5C_7174
chr1	161417415	-	C	31	8	0.258	0	0	T	23	M	0.137	0.432	1.7E-11	6.8E-07	1.9E-11	11.8182	gcatagctgcCttccaagcag	m5C_7207
chr1	161417419	-	C	34	7	0.206	0	0	T	27	M	0.103	0.368	1.4E-09	3.4E-07	1.6E-09	6.4054	gtgagcatagCtgccttccaa	m5C_7180
chr1	161417424	-	C	38	10	0.263	0	0	T	28	M	0.15	0.42	9.4E-14	2E-06	1.2E-13	19.5037	tagtggtgagCatagctgcct	m5C_7134
chr1	161420479	+	C	259	58	0.224	0	0	T	201	M	0.177	0.279	0	0	0	205.799	atgggtggttCagtggtagaa	m5C_7072
chr1	161420492	+	C	223	52	0.233	0	0	T	171	M	0.182	0.293	0	0	0	189.788	tggtagaattCtcgcctgcca	m5C_7026
chr1	161420494	+	C	208	44	0.212	0	0	T	164	M	0.162	0.272	0	0	0	142.142	gtagaattctCgcctgccacg	m5C_6998
chr1	161420496	+	C	210	45	0.214	0	0	T	165	M	0.164	0.275	0	0	0	147.764	agaattctcgCctgccacgcg	m5C_7042
chr1	161420497	+	C	215	62	0.288	0	0	T	153	M	0.232	0.352	0	0	0	287.621	gaattctcgcCtgccacgcgg	m5C_7035
chr1	161420505	+	C	154	63	0.409	0	0	T	91	M	0.335	0.488	0	0	0	421.556	gcctgccacgCgggaggcccg	m5C_6993
chr1	161420512	+	C	112	23	0.205	0	0	T	89	M	0.141	0.289	0	0	0	64.8224	acgcgggaggCccgggttcga	m5C_7064
chr1	161420513	+	C	114	41	0.36	0	0	T	73	M	0.277	0.451	0	6E-15	0	227.514	cgcgggaggcCcgggttcgat	m5C_7060
chr1	161420514	+	C	115	49	0.426	0	0	T	66	M	0.34	0.517	0	6.6E-14	0	332.745	gcgggaggccCgggttcgatt	m5C_7050
chr1	161420520	+	C	84	25	0.298	0	0	T	59	M	0.21	0.402	0	1.8E-12	0	105.223	ggcccgggttCgattcccggc	m5C_7046
chr1	161420527	+	C	59	17	0.288	0	0	T	42	M	0.188	0.414	0	1.1E-08	0	64.0436	gttcgattccCggcccatgca	m5C_7030
chr1	161420530	+	C	67	16	0.239	0	0	T	51	M	0.153	0.353	0	1.1E-10	0	48.8576	cgattcccggCccatgcagca	m5C_6996
chr1	161420531	+	C	64	23	0.359	0	0	T	41	M	0.253	0.482	0	2.1E-09	0	116.326	gattcccggcCcatgcagcac	m5C_6980
chr1	161427943	+	C	31	7	0.233	1	0.032	T	23	M	0.118	0.409	4.8E-10	3.4E-07	5.4E-10	7.69147	acgcgggaggCccgggttcga	m5C_7017
chr1	161427945	+	C	32	13	0.406	0	0	T	19	M	0.255	0.577	0	6.7E-06	0	66.3505	gcgggaggccCgggttcgatt	m5C_6975
chr1	161435287	+	C	50	11	0.22	0	0	T	39	M	0.128	0.352	8.5E-14	4.4E-10	1.1E-13	18.3342	agaattctcgCctgccacgcg	m5C_7008
chr1	161435288	+	C	48	11	0.229	0	0	T	37	M	0.133	0.365	5E-14	4.4E-08	6.2E-14	19.4712	gaattctcgcCtgccacgcgg	m5C_6991
chr1	161435303	+	C	46	19	0.413	0	0	T	27	M	0.283	0.557	0	1E-06	0	107.496	acgcgggaggCccgggttcga	m5C_7059
chr1	161435304	+	C	45	9	0.2	0	0	T	36	M	0.109	0.338	2.3E-11	1.2E-08	2.7E-11	10.4289	cgcgggaggcCcgggttcgat	m5C_6997
chr1	161435305	+	C	42	21	0.5	0	0	CT	21	M	0.355	0.645	0	1.8E-06	0	149.208	gcgggaggccCgggttcgatt	m5C_7005
chr1	161435317	+	C	41	10	0.244	0	0	T	31	M	0.138	0.393	2.4E-13	2.4E-08	2.9E-13	17.4472	ggttcgattcCcggcccatgc	m5C_7057
chr1	161435322	+	C	34	11	0.324	0	0	T	23	M	0.191	0.492	4.4E-16	3.1E-06	5.8E-16	32.3088	gattcccggcCcatgcagcac	m5C_7028
chr1	161435327	+	C	33	11	0.333	0	0	T	22	M	0.198	0.504	2.2E-16	3.1E-06	2.9E-16	34.0074	ccggcccatgCagcacgccct	m5C_7001
chr1	161450368	+	C	148	84	0.568	0	0	C	84	M	0.487	0.645	0	7.8E-15	0	818.226	ataggtggttCagtggtagaa	m5C_7066
chr1	161450381	+	C	113	44	0.389	0	0	T	69	M	0.305	0.482	0	1.6E-14	0	267.984	tggtagaattCttgcctgcca	m5C_7000
chr1	161450385	+	C	113	34	0.301	0	0	T	79	M	0.224	0.391	0	4.4E-16	0	152.327	agaattcttgCctgccacgca	m5C_6995
chr1	161450386	+	C	106	28	0.264	0	0	T	78	M	0.19	0.355	0	1.8E-15	0	106.141	gaattcttgcCtgccacgcag	m5C_7081
chr1	161450390	+	C	83	22	0.265	0	0	T	61	M	0.182	0.369	0	4.4E-13	0	80.0989	tcttgcctgcCacgcaggagg	m5C_7058
chr1	161450394	+	C	68	14	0.206	0	0	T	54	M	0.127	0.316	2.2E-16	3.2E-11	2.9E-16	27.787	gcctgccacgCaggaggccca	m5C_7068
chr1	161493636	-	C	988	302	0.306	0	0	T	686	M	0.278	0.335	0	0	0	1677.55	ggccaatgcaCtttcattttt	m5C_7118
chr1	161493647	-	C	1195	289	0.242	0	0	T	906	M	0.218	0.267	0	0	0	1262.44	gttcgattccCggccaatgca	m5C_7155
chr1	161493648	-	C	1204	254	0.211	1	0.001	T	949	M	0.189	0.235	0	0	0	960.192	ggttcgattcCcggccaatgc	m5C_7168
chr1	161493654	-	C	1178	326	0.277	0	0	T	852	M	0.252	0.303	0	0	0	1642.7	ggcccgggttCgattcccggc	m5C_7098
chr1	161493660	-	C	1156	317	0.275	4	0.003	T	835	M	0.25	0.302	0	0	0	1586.03	gcgggaggccCgggttcgatt	m5C_7158
chr1	161493661	-	C	1146	262	0.229	3	0.003	T	881	M	0.206	0.254	0	0	0	1078.31	cgcgggaggcCcgggttcgat	m5C_7151
chr1	161493662	-	C	1122	315	0.281	1	0.001	T	806	M	0.255	0.308	0	0	0	1609.45	acgcgggaggCccgggttcga	m5C_7178
chr1	161493678	-	C	921	195	0.212	2	0.002	T	724	M	0.187	0.24	0	0	0	729.217	agaattctcgCctgccacgcg	m5C_7116
chr1	161493695	-	C	830	175	0.211	1	0.001	T	654	M	0.185	0.24	0	0	0	646.389	attggtggttCagtggtagaa	m5C_7149
chr1	161500157	-	C	389	134	0.345	1	0.003	T	254	M	0.3	0.394	0	0	0	803.39	cccctggaggCgtgggttcga	m5C_7156
chr1	161500165	-	C	347	70	0.202	0	0	T	277	M	0.163	0.247	0	0	0	228.028	cgcagtctccCctggaggcgt	m5C_7128
chr1	161500166	-	C	339	95	0.283	3	0.009	T	241	M	0.237	0.333	0	0	0	450.778	tcgcagtctcCcctggaggcg	m5C_7133
chr1	161500167	-	C	331	68	0.206	1	0.003	T	262	M	0.166	0.253	0	0	0	225.655	gtcgcagtctCccctggaggc	m5C_7101
chr1	161500169	-	C	323	67	0.207	0	0	T	256	M	0.167	0.255	0	0	0	223.48	aggtcgcagtCtcccctggag	m5C_7153
chr1	161500195	-	C	317	125	0.394	0	0	T	192	M	0.342	0.449	0	0	0	855.241	gccgagcggtCtaaggcgctg	m5C_7199
chr1	161500199	-	C	337	98	0.291	0	0	T	239	M	0.245	0.341	0	0	0	479.982	gatggccgagCggtctaaggc	m5C_7191
chr1	161500203	-	C	330	107	0.324	0	0	T	223	M	0.276	0.377	0	0	0	590.664	tcaggatggcCgagcggtcta	m5C_7170
chr1	161500925	+	C	1058	218	0.206	0	0	T	840	M	0.183	0.231	0	0	0	796.839	tagtggtgagCatagctgcct	m5C_7055
chr1	161500930	+	C	1017	218	0.214	0	0	T	799	M	0.19	0.241	0	0	0	829.419	gtgagcatagCtgccttccaa	m5C_7033
chr1	161500934	+	C	997	261	0.262	0	0	T	736	M	0.235	0.29	0	0	0	1229.04	gcatagctgcCttccaagcag	m5C_7038
chr1	161500937	+	C	1009	211	0.209	1	0.001	T	797	M	0.185	0.236	0	0	0	782.123	tagctgccttCcaagcagttg	m5C_7079
chr1	161500942	+	C	995	209	0.21	0	0	T	786	M	0.186	0.236	0	0	0	776.973	gccttccaagCagttgacccg	m5C_7027
chr1	161500949	+	C	810	166	0.205	0	0	T	644	M	0.179	0.234	0	0	0	592.829	aagcagttgaCccgggttcga	m5C_6977
chr1	161500950	+	C	835	311	0.372	0	0	T	524	M	0.34	0.406	0	0	0	2116.77	agcagttgacCcgggttcgat	m5C_7025
chr1	161500951	+	C	775	173	0.224	1	0.001	T	601	M	0.196	0.254	0	0	0	676.675	gcagttgaccCgggttcgatt	m5C_7088
chr1	161500957	+	C	427	134	0.314	0	0	T	293	M	0.272	0.359	0	0	0	727.962	gacccgggttCgattcccggc	m5C_7054
chr1	161500964	+	C	342	103	0.301	0	0	T	239	M	0.255	0.352	0	0	0	525.251	gttcgattccCggccaacgca	m5C_7022
chr1	161501962	+	C	95	44	0.463	0	0	T	51	M	0.366	0.563	0	1.8E-11	0	322.322	cgcgggagagCggggttcgct	m5C_7047
chr1	161501969	+	C	86	37	0.43	0	0	T	49	M	0.331	0.536	0	9.7E-11	0	244.786	gagcggggttCgctctcccga	m5C_6982
chr1	161501975	+	C	83	17	0.205	0	0	T	66	M	0.132	0.304	0	2.3E-14	0	44.8792	ggttcgctctCccgacgggga	m5C_6973
chr1	161501980	+	C	79	32	0.405	0	0	T	47	M	0.304	0.515	0	8.5E-10	0	194.343	gctctcccgaCggggagatta	m5C_7021
chr1	161510060	+	C	96	38	0.396	0	0	T	58	M	0.304	0.496	0	3.7E-12	0	230.912	ttagcgcgttCggctgttaac	m5C_7039
chr1	161510063	+	C	104	28	0.269	0	0	T	76	M	0.193	0.362	0	1.8E-15	0	108.266	gcgcgttcggCtgttaaccga	m5C_6976
chr1	161510087	+	C	103	43	0.417	0	0	T	60	M	0.327	0.514	0	4.3E-13	0	281.122	gttggtggttCgatcccaccc	m5C_6978
chr1	161510091	+	C	111	29	0.261	0	0	T	82	M	0.189	0.35	0	0	0	109.33	gtggttcgatCccacccaggg	m5C_7065
chr1	161510110	+	C	82	21	0.256	0	0	T	61	M	0.174	0.36	0	2.6E-13	0	73.0945	ggacggaagtCgcattttgtg	m5C_7087
chr1	161582507	+	C	591	147	0.249	0	0	T	444	M	0.216	0.285	0	0	0	633.796	aaaccggggaCgcgttggtgg	m5C_7052
chr1	161582530	+	C	1244	400	0.322	1	0.001	T	843	M	0.296	0.348	0	0	0	2371.31	tagtggtgagCacagctgcct	m5C_7083
chr1	161582539	+	C	995	287	0.289	2	0.002	T	706	M	0.262	0.318	0	0	0	1502.06	gcacagctgcCtttcaagcag	m5C_6989
chr1	161582567	+	C	589	129	0.22	2	0.003	T	458	M	0.188	0.255	0	0	0	485.413	gggttcgattCccgggtaacg	m5C_7010
chr1	161591475	+	C	49	11	0.224	0	0	T	38	M	0.13	0.359	6.6E-14	4.4E-08	8.1E-14	18.8888	gtttctgtagCgcgatcggtt	m5C_6986
chr1	161591490	+	C	48	20	0.417	0	0	T	28	M	0.288	0.557	0	1.4E-06	0	115.398	tcggttagcgCcttcggctgt	m5C_7069
chr1	165728791	-	C	48	10	0.208	0	0	T	38	M	0.117	0.343	1.6E-12	2.4E-08	1.9E-12	13.8343	ataacagagtCagctggtcga	m5C_3627
chr1	166975286	+	C	635	157	0.247	0	0	T	478	M	0.215	0.282	0	0	0	675.965	agatgagagaCtgcctgggaa	m5C_4568
chr1	166975289	+	C	1013	262	0.259	0	0	T	751	M	0.233	0.286	0	0	0	1218.93	tgagagactgCctgggaatac	m5C_4590
chr1	166975290	+	C	1087	373	0.343	0	0	T	714	M	0.316	0.372	0	0	0	2353.77	gagagactgcCtgggaatact	m5C_4575
chr1	166975299	+	C	1246	354	0.284	0	0	T	892	M	0.26	0.31	0	0	0	1839.11	cctgggaataCtgggtgctgt	m5C_4560
chr1	166975306	+	C	1144	437	0.382	0	0	T	707	M	0.354	0.41	0	0	0	3096.38	atactggggtgCtgtaggcttt	m5C_4579
chr1	166975313	+	C	868	414	0.477	0	0	T	454	M	0.444	0.51	0	0	0	3675.53	gtgctgtaggCttttactgtg	m5C_4574
chr1	167683964	+	C	146	38	0.26	0	0	T	108	M	0.196	0.337	0	0	0	148.88	ccgagagcggCtcgttggtct	m5C_4596
chr1	167683973	+	C	147	41	0.279	0	0	T	106	M	0.213	0.356	0	0	0	174.457	gctcgttggtCtaggggtatg	m5C_4572
chr1	167683987	+	C	121	45	0.372	0	0	T	76	M	0.291	0.461	0	5.6E-16	0	261.872	gggtatgattCtcgcttcggg	m5C_4578
chr1	167684022	+	C	109	24	0.22	0	0	T	85	M	0.153	0.307	0	2.2E-16	0	73.2781	ggttcaaatcCcggacgagcc	m5C_4571
chr1	167684033	+	C	97	30	0.309	0	0	T	67	M	0.226	0.407	0	2.5E-13	0	135.624	cggacgagccCaggcttttac	m5C_4592
chr1	167684037	+	C	91	38	0.418	0	0	T	53	M	0.322	0.52	0	3.7E-12	0	244.425	cgagcccaggCttttacccac	m5C_4565
chr1	167684785	-	C	54	23	0.426	0	0	T	31	M	0.303	0.558	0	8.7E-08	0	139.525	gctcgttggtCtaggggtatg	m5C_4618
chr1	167684794	-	C	51	13	0.255	0	0	T	38	M	0.155	0.389	1.1E-16	1.6E-09	1.5E-16	32.2431	tttgtgtcggCtcgttggtct	m5C_4613
chr1	167971790	+	C	64	15	0.234	0	0	T	49	M	0.147	0.351	0	6E-11	0	44.2493	agagcaaggcCggagagtgaa	m5C_4585

chr1	167971801	+	C	79	30	0.38	0	0	T	49	M	0.281	0.49	0	4.8E-10	0	168.398	ggagagtgaaCgagaacgaga	m5C_4581
chr1	171461713	+	C	32	12	0.375	0	0	T	20	M	0.229	0.547	0	4.6E-06	0	55.0408	gagtgggggaCtaacaggttg	m5C_3657
chr1	171461717	+	C	50	14	0.28	0	0	T	36	M	0.175	0.417	0	1.9E-07	0	48.9272	gggggactaaCaggttgcctg	m5C_3635
chr1	171461725	+	C	101	25	0.248	0	0	T	76	M	0.174	0.34	0	4.4E-16	0	86.8236	aacaggttgcCtgaggagggg	m5C_3640
chr1	171461740	+	C	115	36	0.313	0	0	T	79	M	0.235	0.403	0	1E-15	0	169.555	gagggtgaaCtggtccaggt	m5C_3659
chr1	171461746	+	C	99	32	0.323	0	0	T	67	M	0.239	0.42	0	5.3E-13	0	153.087	tgaactggtcCaggttggcaa	m5C_3652
chr1	171501776	+	C	35	8	0.229	0	0	T	27	M	0.121	0.39	5.6E-11	6.8E-07	6.4E-11	9.89691	agaacgggagCgtgagaaaga	m5C_3656
chr1	171501788	+	C	44	26	0.591	0	0	C	26	M	0.444	0.723	0	5.5E-06	0	230.929	tgagaaagaaCttgaaaaaga	m5C_3633
chr1	171501800	+	C	44	16	0.364	0	0	T	28	M	0.238	0.511	0	4E-07	0	76.1067	tgaaaaagaaCaagaacagga	m5C_3655
chr1	171501812	+	C	43	29	0.744	4	0.093	C	29	M	0.589	0.854	0	0.00019	0	341.725	agaacaggagCgagagaagga	m5C_3651
chr1	172157598	-	C	48	27	0.562	0	0	C	27	M	0.423	0.693	0	6.7E-06	0	228.284	accctgtggtCtagtggttag	m5C_18500
chr1	173761003	+	C	93	20	0.215	0	0	T	73	M	0.144	0.309	0	1.7E-15	0	57.4997	tgagttgctgCatgtggcagt	m5C_18490
chr1	173761014	+	C	118	46	0.39	0	0	T	72	M	0.307	0.48	0	2.8E-14	0	282.103	atgtggcagtCtgcctttctt	m5C_18497
chr1	173761018	+	C	115	55	0.478	0	0	T	60	M	0.389	0.569	0	2.9E-13	0	428.065	ggcagtctgcCtttctttaaa	m5C_18489
chr1	173761022	+	C	106	34	0.321	0	0	T	72	M	0.24	0.415	0	2.3E-14	0	162.865	gtctgcctttCtttaaaacaa	m5C_18487
chr1	173833294	-	C	494	146	0.296	0	0	T	348	M	0.257	0.337	0	0	0	750.473	aataaaatatCtgatattctg	m5C_18541
chr1	173833305	-	C	622	212	0.341	0	0	T	410	M	0.305	0.379	0	0	0	1291.78	tacttgatgaCaataaaatat	m5C_18550
chr1	173833313	-	C	603	123	0.204	0	0	T	480	M	0.174	0.238	0	0	0	427.397	tcactgattaCttgatgacaa	m5C_18566
chr1	173833320	-	C	584	150	0.257	0	0	T	434	M	0.223	0.294	0	0	0	669.252	aactctctcaCtgattacttg	m5C_18536
chr1	173833324	-	C	599	123	0.205	0	0	T	476	M	0.175	0.24	0	0	0	430.3	cttgaactctCtcactgatta	m5C_18571
chr1	173833326	-	C	608	198	0.326	0	0	T	410	M	0.29	0.364	0	0	0	1146.83	aacttgaactCtctcactgat	m5C_18580
chr1	173833328	-	C	612	332	0.543	1	0.002	C	332	M	0.504	0.582	0	0	0	3344.74	ccaacttgaaCtctctcactg	m5C_18501
chr1	173833334	-	C	630	250	0.397	0	0	T	380	M	0.359	0.436	0	0	0	1796.79	ctcaatccaaCttgaactctc	m5C_18574
chr1	173833338	-	C	645	183	0.284	0	0	T	462	M	0.25	0.32	0	0	0	916.062	tgatctcaatCcaacttgaac	m5C_18516
chr1	173833344	-	C	637	339	0.532	0	0	C	339	M	0.493	0.571	0	0	0	3344.96	acatgatgatCtcaatccaac	m5C_18555
chr1	173833353	-	C	265	53	0.2	0	0	T	212	M	0.156	0.252	0	0	0	165.656	ttgcagaataCatgatgatct	m5C_18551
chr1	173833514	-	C	460	203	0.441	0	0	T	257	M	0.397	0.487	0	0	0	1610.19	cattttgattCtgaggttact	m5C_18573
chr1	173833530	-	C	492	160	0.327	3	0.006	T	329	M	0.287	0.37	0	0	0	918.718	cactgtaaaaCcgttccattt	m5C_18544
chr1	173833560	-	C	520	134	0.258	0	0	T	386	M	0.222	0.297	0	0	0	594.888	atgattctgcCaaatgaaata	m5C_18570
chr1	173833561	-	C	523	139	0.266	0	0	T	384	M	0.23	0.305	0	0	0	638.628	aatgattctgCcaaatgaaat	m5C_18557
chr1	173833564	-	C	520	257	0.495	1	0.002	T	262	M	0.452	0.538	0	0	0	2325.14	tgtaatgattCtgccaaatga	m5C_18565
chr1	173833976	-	C	176	42	0.239	0	0	T	134	M	0.182	0.307	0	0	0	152.604	tcgctcctatCtgatgtatct	m5C_18528
chr1	173833981	-	C	217	61	0.284	2	0.009	T	154	M	0.228	0.347	0	0	0	277.756	gtctttcgctCctatctgatg	m5C_18579
chr1	173833983	-	C	223	99	0.444	0	0	T	124	M	0.38	0.51	0	0	0	752.858	aagtctttcgCtcctatctga	m5C_18552
chr1	173833985	-	C	218	75	0.344	0	0	T	143	M	0.284	0.409	0	0	0	426.248	ttaagtctttCgctcctatct	m5C_18517
chr1	173834000	-	C	272	82	0.301	0	0	T	190	M	0.25	0.358	0	0	0	410.016	cgctgatgagCaatattaagt	m5C_18513
chr1	173834008	-	C	310	105	0.339	0	0	T	205	M	0.288	0.393	0	0	0	605.399	cagactaacgCtgatgagcaa	m5C_18525
chr1	173834014	-	C	334	68	0.204	0	0	T	266	M	0.164	0.25	0	0	0	222.894	gttcagcagaCtaacgctgat	m5C_18518
chr1	173834502	-	C	1303	481	0.369	1	0.001	T	821	M	0.344	0.396	0	0	0	3305.76	ggagattaatCttaaactgaa	m5C_18527
chr1	173834526	-	C	1820	416	0.229	0	0	T	1404	M	0.21	0.248	0	0	0	1746.06	gggaatctctCtgagaaagaa	m5C_18509
chr1	173834769	-	C	1025	220	0.215	0	0	T	805	M	0.191	0.241	0	0	0	838.592	acaaaggtaaCactgaagaac	m5C_18548
chr1	173834796	-	C	1281	430	0.336	0	0	T	851	M	0.31	0.362	0	0	0	2668.93	aatgtctgacCtgaaatgagc	m5C_18559
chr1	173835100	-	C	45	18	0.4	0	0	T	27	M	0.27	0.545	0	7.7E-07	0	97.2889	tgactaaaggCatttgttagt	m5C_18558
chr1	173835111	-	C	753	215	0.286	0	0	T	538	M	0.254	0.319	0	0	0	1093.99	ttagctctaaCtgactaaagg	m5C_18520
chr1	173835140	-	C	831	182	0.222	13	0.016	T	636	M	0.195	0.252	0	0	0	710.984	caaatgctgaCtgaacatgaa	m5C_18577
chr1	173835453	-	C	1206	335	0.278	0	0	T	871	M	0.253	0.304	0	0	0	1696.67	gatgtactatCtgtctgatgt	m5C_18538
chr1	173835799	-	C	1724	815	0.473	0	0	T	909	M	0.449	0.496	0	0	0	7322.89	tgatgaggatCttaaccacca	m5C_18519
chr1	173835815	-	C	1753	429	0.246	6	0.003	T	1318	M	0.226	0.266	0	0	0	1938.67	ctcttgagtgCtagaatgatg	m5C_18554
chr1	173835839	-	C	1656	362	0.219	0	0	T	1294	M	0.199	0.239	0	0	0	1443.34	acaaatgatgaCagtttatttg	m5C_18549
chr1	173836049	-	C	589	120	0.207	8	0.014	T	461	M	0.176	0.241	0	0	0	421.444	gagtagtggaCagaagggatt	m5C_18578
chr1	173836809	-	C	35	13	0.371	0	0	T	22	M	0.232	0.537	0	6.7E-06	0	60.2306	gatggtggcaCatgccgagtc	m5C_18542
chr1	173836811	-	C	46	13	0.283	0	0	T	33	M	0.173	0.425	0	1.2E-07	0	45.0425	ctgatggtggCacatgccgag	m5C_18514
chr1	173836821	-	C	2102	441	0.21	0	0	T	1661	M	0.193	0.228	0	0	0	1701.65	gccaaccgctCtgatggtggc	m5C_18529
chr1	173836823	-	C	2285	498	0.218	0	0	T	1787	M	0.201	0.235	0	0	0	2006.9	atgccaaccgCtctgatggtg	m5C_18505
chr1	173836829	-	C	2646	533	0.202	1	0	T	2112	M	0.187	0.217	0	0	0	1989.83	tgatgaatgcCaaccgctctg	m5C_18567
chr1	173836849	-	C	2914	605	0.208	0	0	T	2309	M	0.193	0.223	0	0	0	2338.7	gtagggacatCtgagagtaat	m5C_18515
chr1	174701208	+	C	35	9	0.257	0	0	T	26	M	0.142	0.421	1.5E-12	1.2E-06	1.7E-12	15.0859	ggtgttgtggCgcgtgcctgt	m5C_3801
chr1	174701210	+	C	36	17	0.472	0	0	T	19	M	0.32	0.63	0	2.1E-05	0	108.752	tgttgtggcgCgtgcctgtag	m5C_3796
chr1	174701215	+	C	39	29	0.744	0	0	C	29	M	0.589	0.854	0	0.00019	0	341.725	tggcgcgtgcCtgtagtccca	m5C_3797
chr1	174701222	+	C	33	11	0.333	0	0	T	22	M	0.198	0.504	2.2E-16	3.1E-06	2.9E-16	34.0074	tgcctgtagtCccagcttggg	m5C_3807
chr1	175937533	-	C	40	17	0.425	0	0	T	23	M	0.285	0.578	0	5.6E-07	0	96.9312	tccgtacagcCtttaaaactg	m5C_3817
chr1	175937537	-	C	48	16	0.333	0	0	T	32	M	0.217	0.475	0	4E-07	0	69.3651	cccctccgtaCagcctttaaa	m5C_3812
chr1	175937541	-	C	65	14	0.215	0	0	T	51	M	0.133	0.33	1.1E-16	3.2E-11	1.5E-16	29.6776	cgttcccctcCgtacagcctt	m5C_3814
chr1	175937542	-	C	64	16	0.25	0	0	T	48	M	0.16	0.368	0	1.1E-10	0	51.2473	tcgttcccctCcgtacagcct	m5C_3824
chr1	175937544	-	C	68	17	0.25	0	0	T	51	M	0.162	0.364	0	1.8E-10	0	55.1986	gatcgttcccCtccgtacagc	m5C_3816
chr1	175937546	-	C	68	25	0.368	0	0	T	43	M	0.263	0.486	0	4.1E-09	0	131.501	ctgatcgttcCcctccgtaca	m5C_3823
chr1	175937566	-	C	72	17	0.236	0	0	T	55	M	0.153	0.346	0	2.2E-12	0	52.0084	cttaggacagCtggctaagtc	m5C_3826
chr1	175937576	-	C	84	28	0.333	0	0	T	56	M	0.242	0.439	0	5.8E-12	0	135.392	ctctgctggCttaggacagc	m5C_3829
chr1	175937580	-	C	84	25	0.298	0	0	T	59	M	0.21	0.402	0	1.8E-12	0	105.223	aggtctctgCtggcttagga	m5C_3830
chr1	175937586	-	C	80	20	0.25	0	0	T	60	M	0.168	0.355	0	1.5E-13	0	67.2244	atatggaggtCtctgtctggc	m5C_3825
chr1	175937603	-	C	49	11	0.224	0	0	T	38	M	0.13	0.359	6.6E-14	4.4E-08	8.1E-14	18.8888	agcgatcctaCataaatatat	m5C_3828
chr1	175937606	-	C	44	10	0.227	0	0	T	34	M	0.128	0.37	5.7E-13	2.4E-08	6.8E-13	15.7274	tgcagcgatcCtacataaata	m5C_3811
chr1	175937607	-	C	58	14	0.241	0	0	T	44	M	0.15	0.365	0	2.8E-09	0	41.8845	ttgcagcgatCctacataaat	m5C_3820
chr1	175937614	-	C	52	15	0.288	0	0	T	37	M	0.183	0.423	0	4.6E-09	0	54.9894	ctacagattgCagcgatccta	m5C_3819
chr1	175937621	-	C	81	26	0.321	0	0	T	55	M	0.229	0.429	0	2.7E-12	0	119.299	cttttgctaCagattgcagc	m5C_3827
chr1	175937632	-	C	131	28	0.214	0	0	T	103	M	0.152	0.292	0	0	0	85.2447	tgaaaaaatgCcttttgctta	m5C_3822
chr1	175937654	-	C	190	40	0.211	0	0	T	150	M	0.159	0.274	0	0	0	126.868	tggaaccagcCatgtggacaa	m5C_3815
chr1	175937665	-	C	219	58	0.265	0	0	T	161	M	0.211	0.327	0	0	0	244.548	atggaagctgCtggaaccagc	m5C_3809
chr1	182913548	-	C	34	16	0.471	0	0	T	18	M	0.315	0.633	0	1.7E-05	0	100.644	ctggttagtgCttggatggga	m5C_3873
chr1	182913558	-	C	35	8	0.235	1	0.029	T	26	M	0.124	0.4	4.2E-11	6.8E-07	4.8E-11	10.3259	agggtcgggcCtggttagtgc	m5C_3870
chr1	182913559	-	C	35	9	0.257	0	0	T	26	M	0.142	0.421	1.5E-12	1.2E-06	1.7E-12	15.0859	cagggtcgggCctggttagtg	m5C_3869
chr1	182913563	-	C	34	8	0.235	0	0	T	26	M	0.124	0.4	4.2E-11	6.8E-07	4.8E-11	10.3259	taagcagggtCgggcctggtt	m5C_3871
chr1	184555230	-	C	36	28	0.778	0	0	C	28	M	0.619	0.883	0	0.00017	0	346.724	aagttgtggaCttatacactg	m5C_3924
chr1	184764314	-	C	38	13	0.342	0	0	T	25	M	0.212	0.501	0	6.7E-06	0	55.1518	ttctgaggaaCaagaagagat	m5C_3925
chr1	184764322	-	C	38	9	0.237	0	0	T	29	M	0.13	0.392	3.7E-12	1.2E-06	4.3E-12	13.3739	agccaggtttCtgaggaacaa	m5C_3940
chr1	184764329	-	C	34	13	0.382	0	0	T	21	M	0.239	0.55	0	6.7E-06	0	62.1399	cagtgagagcCaggtttctga	m5C_3947
chr1	184984123	-	C	31	9	0.29	0	0	T	22	M	0.161	0.466	3.7E-13	1.2E-06	4.4E-13	18.0154	ctggttagtaCtggatgggag	m5C_3923
chr1	185276170	-	C	57	15	0.263	0	0	T	42	M	0.166	0.39	0	4.6E-09	0	49.9369	agaatggagaCagtctggaag	m5C_3942
chr1	185276185	-	C	81	47	0.58	0	0	C	47	M	0.472	0.682	0	9.7E-10	0	443.243	agcgtagcatCtgggagaatg	m5C_3941
chr1	185276196	-	C	52	15	0.288	0	0	T	37	M	0.183	0.423	0	4.6E-09	0	54.9894	caactgggtgCagcgtagcat	m5C_3920
chr1	187249535	+	C	45	12	0.267	0	0	T	33	M	0.16	0.41	5.6E-16	7.5E-08	7.3E-16	29.2263	ctaaagggcaCtatcaggagt	m5C_4110
chr1	187249539	+	C	46	10	0.217	0	0	T	36	M	0.123	0.356	9.7E-13	2.4E-08	1.2E-12	14.7309	agggcactatCaggagtaaaa	m5C_4109
chr1	192999211	-	C	61	22	0.361	0	0	T	39	M	0.252	0.486	0	1.5E-09	0	110.765	gagttgggttCagaatttctc	m5C_4378
chr1	198828061	-	C	43	16	0.372	0	0	T	27	M	0.244	0.521	0	4E-07	0	78.0036	tcattgctgtCggtgggttga	m5C_4642
chr1	201465392	-	C	31	14	0.452	0	0	T	17	M	0.292	0.622	0	9.2E-06	0	81.6522	tggggtgtgtCagaagacggt	m5C_4910
chr1	202129921	-	C	33	13	0.394	0	0	T	20	M	0.247	0.563	0	6.7E-06	0	64.1755	tcgggaaggcCtggcccctca	m5C_4700
chr1	202129922	-	C	33	15	0.455	0	0	T	18	M	0.298	0.62	0	1.2E-05	0	89.5281	gtcgggaaggCctggcccctc	m5C_4696
chr1	202469305	+	C	66	17	0.258	0	0	T	49	M	0.167	0.374	0	1.8E-10	0	56.9456	ccctggtggtCtagtggttag	m5C_4643
chr1	202469329	+	C	108	27	0.25	0	0	T	81	M	0.178	0.339	0	1.1E-15	0	96.0537	ttggtgctctCgctgtctctc	m5C_4651
chr1	202469331	+	C	103	32	0.311	0	0	T	71	M	0.229	0.406	0	1.1E-14	0	146.862	ggtgctctcgCtgtctctctc	m5C_4660
chr1	202469335	+	C	81	27	0.333	0	0	T	54	M	0.24	0.441	0	4E-12	0	129.755	ctctcgctgtCtctctctctt	m5C_4672

chr1	202920103	-	C	48	10	0.208	0	0	T	38	M	0.117	0.343	1.6E-12	2.4E-08	1.9E-12	13.8343	aactgggaccCctgctagaag	m5C_4699
chr1	202920104	-	C	50	18	0.36	0	0	T	32	M	0.241	0.499	0	1.7E-08	0	86.8988	gaactgggacCcctgctagaa	m5C_4706
chr1	202920105	-	C	52	30	0.577	0	0	C	30	M	0.442	0.701	0	4.8E-07	0	265.165	tgaactgggaCccctgctaga	m5C_4691
chr1	204475657	+	C	962	211	0.219	0	0	T	751	M	0.194	0.247	0	0	0	820.074	tcgggagcgcCcggatagctc	m5C_6080
chr1	204475702	+	C	43	14	0.326	0	0	T	29	M	0.205	0.475	0	1.9E-07	0	57.3807	atctgaggggtCcaggggttcaa	m5C_6100
chr1	204475710	+	C	39	8	0.205	0	0	T	31	M	0.108	0.355	1.6E-10	6.8E-07	1.8E-10	8.44384	gtccaggggttCaagtccctgt	m5C_6103
chr1	204475715	+	C	41	16	0.39	0	0	T	25	M	0.257	0.543	0	4E-07	0	82.099	gggttcaagtCcctgttcggg	m5C_6089
chr1	204475717	+	C	38	19	0.5	0	0	CT	19	M	0.348	0.652	0	3.4E-05	0	132.429	gttcaagtccCtgttcgggcg	m5C_6111
chr1	204475722	+	C	33	7	0.212	0	0	T	26	M	0.107	0.378	1.1E-09	3.4E-07	1.2E-09	6.69165	agtccctgttCgggcgtttgt	m5C_6083
chr1	204476199	-	C	98	31	0.316	0	0	T	67	M	0.233	0.414	0	3.7E-13	0	144.255	gagcatcagaCttttaatctg	m5C_6155
chr1	204476220	-	C	154	32	0.208	0	0	T	122	M	0.151	0.279	0	0	0	96.774	gcccggatagCtcagtcggta	m5C_6116
chr1	204476227	-	C	148	40	0.27	0	0	T	108	M	0.205	0.347	0	0	0	164.164	tacgggagccCggatagctca	m5C_6146
chr1	204531542	-	C	79	35	0.443	0	0	T	44	M	0.339	0.553	0	1.9E-09	0	237.073	gtgctgtaggCtttttggctc	m5C_6150
chr1	204531549	-	C	97	45	0.464	0	0	T	52	M	0.368	0.563	0	2.3E-11	0	331.152	atacctggtgCtgtaggcttt	m5C_6160
chr1	204531555	-	C	94	28	0.298	0	0	T	66	M	0.215	0.397	0	1.1E-13	0	120.312	ctgggaatacCtggtgctgta	m5C_6134
chr1	204531556	-	C	94	20	0.213	0	0	T	74	M	0.142	0.306	0	1.7E-15	0	56.8671	cctgggaataCctggtgctgt	m5C_6126
chr1	204531565	-	C	66	17	0.262	1	0.015	T	48	M	0.17	0.38	0	1.8E-10	0	57.8614	gggagaccgcCtgggaatacc	m5C_6142
chr1	204531566	-	C	60	18	0.3	0	0	T	42	M	0.199	0.425	0	2.9E-10	0	71.6328	tgggagaccgCctgggaatac	m5C_6133
chr1	204531568	-	C	48	22	0.458	0	0	T	26	M	0.326	0.597	0	2.3E-06	0	143.33	gatgggagacCgcctgggaat	m5C_6129
chr1	204531569	-	C	53	23	0.434	0	0	T	30	M	0.31	0.567	0	8.7E-08	0	142.371	ggatgggagaCcgcctgggaa	m5C_6120
chr1	205683303	-	C	35	13	0.371	0	0	T	22	M	0.232	0.537	0	6.7E-06	0	60.2306	tgggtgtgtaCgtgtgagtgt	m5C_6165
chr1	205689662	-	C	225	126	0.56	0	0	C	126	M	0.495	0.623	0	0	0	1246.57	tgaggaagaaCaagaagagga	m5C_6144
chr1	205689675	-	C	350	130	0.371	0	0	T	220	M	0.322	0.423	0	0	0	838.393	aagatgtgggCagtgaggaag	m5C_6124
chr1	205689704	-	C	61	27	0.443	0	0	T	34	M	0.325	0.567	0	7.5E-09	0	175.541	agcttctaaaCagagagagat	m5C_6148
chr1	205698728	-	C	72	18	0.25	0	0	T	54	M	0.164	0.361	0	4E-12	0	59.1801	ttgttgattaCtcacagtttc	m5C_6151
chr1	206519107	+	C	38	8	0.211	0	0	T	30	M	0.111	0.363	1.3E-10	6.8E-07	1.4E-10	8.77246	gatcatgccaCtgcactccag	m5C_4795
chr1	207262836	+	C	39	15	0.385	0	0	T	24	M	0.249	0.541	0	1.2E-05	0	74.6735	ggtgtctgagCtgggtgaatt	m5C_4817
chr1	207262848	+	C	38	8	0.211	0	0	T	30	M	0.111	0.363	1.3E-10	6.8E-07	1.4E-10	8.77246	gggtgaattcCagcctgggga	m5C_4815
chr1	207271514	+	C	41	9	0.257	6	0.146	T	26	M	0.142	0.421	1.5E-12	1.2E-06	1.7E-12	15.0859	agtgggcgtgCaggagcagca	m5C_4778
chr1	207271523	+	C	38	10	0.263	0	0	T	28	M	0.15	0.42	9.4E-14	2E-06	1.2E-13	19.5037	gcaggagcagCaatgcgttga	m5C_4808
chr1	207495889	+	C	31	15	0.484	0	0	T	16	M	0.32	0.652	0	1.2E-05	0	95.91	agtcaatggtCagatattgaa	m5C_4806
chr1	212272995	-	C	135	38	0.281	0	0	T	97	M	0.212	0.363	0	0	0	161.483	tgtgcttggtCgaaggaggaca	m5C_5308
chr1	212411710	+	C	43	17	0.395	0	0	T	26	M	0.264	0.544	0	5.6E-07	0	89.6414	gtttggtggcCtaggtaggcc	m5C_5289
chr1	212454147	+	C	52	28	0.538	0	0	C	28	M	0.405	0.667	0	3.1E-07	0	226.819	ggatttggtgCtctctcctgc	m5C_5269
chr1	212783135	+	C	37	21	0.568	0	0	C	21	M	0.409	0.713	0	5.2E-05	0	171.838	gctttgtggtCtagtgccagg	m5C_5271
chr1	212823106	+	C	32	10	0.312	0	0	T	22	M	0.18	0.486	1.1E-14	2E-06	1.3E-14	25.0836	gtttgtctgaCtagcttgctg	m5C_5296
chr1	214815447	+	C	34	11	0.324	0	0	T	23	M	0.191	0.492	4.4E-16	3.1E-06	5.8E-16	32.3088	catgcagtcaCaagaaattag	m5C_5167
chr1	214815461	+	C	62	20	0.323	0	0	T	42	M	0.22	0.446	0	6.9E-10	0	87.8173	aaattagtggCcttaaagact	m5C_5153
chr1	214815462	+	C	64	21	0.328	0	0	T	43	M	0.226	0.45	0	1E-09	0	94.7957	aattagtggcCttaaagactg	m5C_5141
chr1	214818586	+	C	68	22	0.324	0	0	T	46	M	0.224	0.442	0	1.5E-09	0	98.7248	aggtgaatgaCagctggaagg	m5C_5159
chr1	214818589	+	C	54	14	0.259	0	0	T	40	M	0.161	0.389	0	2.8E-09	0	45.1318	tgaatgacagCtggaaggaga	m5C_5166
chr1	214818605	+	C	73	26	0.356	0	0	T	47	M	0.256	0.471	0	1.3E-10	0	133.143	ggagagatttCttgatgtgga	m5C_5175
chr1	214818623	+	C	74	24	0.324	0	0	T	50	M	0.229	0.437	0	6.4E-11	0	109.747	ggaaaatgagCtgagtaggat	m5C_5147
chr1	214819764	+	C	39	10	0.256	0	0	T	29	M	0.146	0.411	1.3E-13	2E-06	1.6E-13	18.7753	attatgaaggCcacagaacag	m5C_5180
chr1	214819765	+	C	38	19	0.5	0	0	CT	19	M	0.348	0.652	0	3.4E-05	0	132.429	ttatgaaggcCacagaacaga	m5C_5163
chr1	214819778	+	C	33	21	0.636	0	0	C	21	M	0.466	0.778	0	5.2E-05	0	195.787	agaacagagtCtagacccacc	m5C_5142
chr1	214825101	+	C	36	23	0.639	0	0	C	23	M	0.476	0.775	0	7.6E-05	0	218.844	gaagaagagcCtagattgcat	m5C_5151
chr1	214825115	+	C	63	20	0.317	0	0	T	43	M	0.216	0.44	0	6.9E-10	0	86.3461	attgcatgcaCaaagaccagg	m5C_5177
chr1	214825121	+	C	66	23	0.348	0	0	T	43	M	0.245	0.469	0	2.1E-09	0	112.588	tgcacaaagaCcaggtggaaa	m5C_5144
chr1	214825122	+	C	68	15	0.221	0	0	T	53	M	0.138	0.333	0	6E-11	0	41.5467	gcacaaagacCaggtggaaaa	m5C_5150
chr1	220112837	+	C	36	12	0.333	0	0	T	24	M	0.202	0.497	0	4.6E-06	0	48.5146	tggccattatCttggatcgct	m5C_5002
chr1	222461186	+	C	55	17	0.309	0	0	T	38	M	0.203	0.44	0	1.1E-08	0	68.9592	caacttatgcCtgcttgagta	m5C_4969
chr1	222638357	+	C	70	21	0.3	0	0	T	49	M	0.205	0.415	0	1.8E-11	0	86.2531	ggctccatagCtcagtggtta	m5C_4955
chr1	222638386	+	C	62	17	0.274	0	0	T	45	M	0.179	0.396	0	1.8E-10	0	60.7951	gcttgtaaaCcaggggtcgc	m5C_4970
chr1	222638402	+	C	59	17	0.288	0	0	T	42	M	0.188	0.414	0	1.1E-08	0	64.0436	gtcgcgagttCgatcctcgct	m5C_4947
chr1	222638406	+	C	72	26	0.361	0	0	T	46	M	0.26	0.476	0	1.3E-10	0	135.104	cgagttcgatCctcgctgggg	m5C_4960
chr1	222638409	+	C	76	24	0.316	0	0	T	52	M	0.222	0.427	0	6.4E-11	0	106.707	gttcgatcctCgctggggcct	m5C_4966
chr1	222638411	+	C	83	18	0.217	0	0	T	65	M	0.142	0.317	0	4.5E-14	0	51.0532	tcgatcctcgCtggggcctcc	m5C_4963
chr1	222638417	+	C	87	33	0.379	0	0	T	54	M	0.285	0.484	0	3.1E-11	0	187.772	ctcgctggggCctcctgttgg	m5C_4957
chr1	222638418	+	C	85	20	0.235	0	0	T	65	M	0.158	0.336	0	1.5E-13	0	63.1177	tcgctggggcCtcctgttggc	m5C_4953
chr1	222638420	+	C	82	39	0.476	0	0	T	43	M	0.371	0.582	0	1.6E-10	0	289.417	gctggggcctCctgttggctt	m5C_4964
chr1	222638421	+	C	84	27	0.321	0	0	T	57	M	0.231	0.427	0	4E-12	0	124.88	ctggggcctcCtgttggctta	m5C_4944
chr1	222638432	+	C	51	27	0.529	0	0	C	27	M	0.395	0.659	0	2.5E-07	0	213.425	tgttggcttaCttttatttta	m5C_4968
chr1	223943207	+	C	33	9	0.273	0	0	T	24	M	0.151	0.442	7.5E-13	1.2E-06	9E-13	16.4418	accctcagtaCctgatcaagc	m5C_4967
chr1	223943213	+	C	45	19	0.422	0	0	T	26	M	0.29	0.567	0	1E-06	0	110.08	agtacctgatCaagctggagg	m5C_4965
chr1	223943217	+	C	75	16	0.213	0	0	T	59	M	0.136	0.319	0	1.2E-12	0	43.4563	cctgatcaagCtggaggagga	m5C_4961
chr1	223943237	+	C	112	25	0.223	0	0	T	87	M	0.156	0.309	0	0	0	78.0053	aggatgaggaCgaggaggatg	m5C_4952
chr1	225706984	-	C	73	26	0.356	0	0	T	47	M	0.256	0.471	0	1.3E-10	0	133.143	gagactggaaCgggagaggca	m5C_5116
chr1	225706990	-	C	81	35	0.432	0	0	T	46	M	0.33	0.541	0	5.6E-11	0	230.803	gctggagagaCtggaacggga	m5C_5134
chr1	225706999	-	C	90	36	0.4	0	0	T	54	M	0.305	0.503	0	7.4E-11	0	219.52	acgagagaggCtggagagact	m5C_5133
chr1	225707008	-	C	96	44	0.458	0	0	T	52	M	0.362	0.558	0	1.8E-11	0	318.724	aagacaagaaCgagagaggct	m5C_5106
chr1	225707014	-	C	77	38	0.494	0	0	T	39	M	0.385	0.603	0	3.7E-09	0	292.47	gcaagaaagaCaagaacgaga	m5C_5125
chr1	225707032	-	C	46	12	0.261	0	0	T	34	M	0.156	0.403	6.7E-16	7.5E-08	8.7E-16	28.4116	acgcctggatCgggagaggca	m5C_5113
chr1	226109621	-	C	36	15	0.417	0	0	T	21	M	0.271	0.578	0	1.2E-05	0	81.421	tgggcttctgCactgaggtgg	m5C_5467
chr1	226109627	-	C	37	16	0.432	0	0	T	21	M	0.287	0.591	0	1.7E-05	0	91.7488	gcagcgtgggCttctgcactg	m5C_5469
chr1	226349338	-	C	39	9	0.231	0	0	T	30	M	0.126	0.383	4.9E-12	1.2E-06	5.7E-12	12.8739	agaacgctcgCaaaaggagga	m5C_5454
chr1	226349341	-	C	41	22	0.537	0	0	C	22	M	0.387	0.679	0	2.3E-06	0	170.483	aagagaacgtCtgcaaaagga	m5C_5447
chr1	226349365	-	C	33	14	0.424	0	0	T	19	M	0.272	0.592	0	9.2E-06	0	76.259	gcgaaggcggCgtgaagagga	m5C_5476
chr1	226613236	-	C	47	22	0.468	0	0	T	25	M	0.333	0.608	0	2.3E-06	0	146.658	gaggggtgaaCtgactcaggt	m5C_5485
chr1	226613252	-	C	35	8	0.229	0	0	T	27	M	0.121	0.39	5.6E-11	6.8E-07	6.4E-11	9.89691	caccaggttgCttaaggaggg	m5C_5461
chr1	227748974	+	C	32	7	0.219	0	0	T	25	M	0.11	0.388	8.5E-10	3.4E-07	9.5E-10	6.99971	actgggagacCgcctgggaat	m5C_5423
chr1	227748976	+	C	33	10	0.303	0	0	T	23	M	0.174	0.473	1.6E-14	2E-06	2E-14	23.982	tgggagaccgCctgggaataa	m5C_5436
chr1	227748977	+	C	33	7	0.212	0	0	T	26	M	0.107	0.378	1.1E-09	3.4E-07	1.2E-09	6.69165	gggagaccgcCtgggaataac	m5C_5434
chr1	227748987	+	C	47	12	0.255	0	0	T	35	M	0.153	0.395	1E-15	7.5E-08	1.3E-15	27.4561	ctgggaataaCgggtgctgta	m5C_5427
chr1	227748993	+	C	43	15	0.357	1	0.023	T	27	M	0.23	0.508	0	2.8E-07	0	68.967	ataacgggtgCtgtaggcttt	m5C_5426
chr1	227749000	+	C	34	11	0.324	0	0	T	23	M	0.191	0.492	4.4E-16	3.1E-06	5.8E-16	32.3088	gtgctgtaggCtttttggttt	m5C_5443
chr1	228743561	+	C	35	13	0.371	0	0	T	22	M	0.232	0.537	0	6.7E-06	0	60.2306	ctggttagtaCttggatggga	m5C_5241
chr1	228743574	+	C	44	30	0.682	0	0	C	30	M	0.534	0.8	0	1.2E-05	0	320.657	ggatgggagaCcgcctgagaa	m5C_5216
chr1	229673633	+	C	34	26	0.765	0	0	C	26	M	0.6	0.876	0	0.00013	0	312.018	agaattctcaCctgctaattc	m5C_5233
chr1	229685741	+	C	70	17	0.243	0	0	T	53	M	0.158	0.355	0	2.2E-12	0	53.5559	ggatgggagaCtgcctgggaa	m5C_5238
chr1	229685744	+	C	70	15	0.214	0	0	T	55	M	0.134	0.324	0	6E-13	0	40.3158	tgggagactgCctgggaatac	m5C_5211
chr1	229685745	+	C	72	29	0.403	0	0	T	43	M	0.297	0.518	0	3.6E-10	0	172.381	gggagactgcCtgggaataca	m5C_5240
chr1	229685754	+	C	69	30	0.435	0	0	T	39	M	0.324	0.552	0	1.7E-08	0	194.596	cctgggaataCaaggtgctgc	m5C_5223
chr1	231030057	+	C	33	17	0.515	0	0	C	17	M	0.352	0.675	0	2.1E-05	0	119.742	gaggggtgcaCtggcccaggt	m5C_5355
chr1	232510865	-	C	48	16	0.333	0	0	T	32	M	0.217	0.475	0	4E-07	0	69.3651	ccttgtagaaCtggatctgtg	m5C_5763
chr1	232746424	-	C	36	21	0.583	0	0	C	21	M	0.422	0.729	0	5.2E-05	0	177.24	ttctggcggcCtagtgtcagg	m5C_5772
chr1	235291119	-	C	89	24	0.27	0	0	T	65	M	0.188	0.37	0	1.1E-12	0	90.4412	cctgcgacatCttttaagaga	m5C_5539
chr1	235291140	-	C	111	24	0.216	0	0	T	87	M	0.15	0.302	0	0	0	71.9141	gtggctcagtCttatttcata	m5C_5554
chr1	235291146	-	C	118	25	0.214	1	0.008	T	92	M	0.149	0.296	0	0	0	74.5642	tctcccgtggCtcagtcttat	m5C_5538
chr1	235291152	-	C	133	34	0.256	0	0	T	99	M	0.189	0.336	0	0	0	128.58	tgcgcttctcCcgtggctcag	m5C_5515
chr1	235291155	-	C	149	40	0.268	0	0	T	109	M	0.204	0.345	0	0	0	163.022	ggctgcgcttCtcccgtggct	m5C_5549

chr1	235291163	-	C	173	69	0.399	0	0	T	104	M	0.329	0.473	0	0	0	453.796	gtccaaagggCtgcgcttctc	m5C_5528
chr1	235291220	-	C	90	26	0.289	0	0	T	64	M	0.205	0.39	0	4.6E-14	0	106.818	tagcttcgttCtaagtgcttc	m5C_5553
chr1	235291224	-	C	84	23	0.274	0	0	T	61	M	0.19	0.377	0	7.2E-13	0	87.3664	ttggtagcttCgttctaagtg	m5C_5551
chr1	235291239	-	C	92	37	0.402	0	0	T	55	M	0.308	0.504	0	2.7E-12	0	227.808	cattcttaaaCcctcttggta	m5C_5559
chr1	236431196	+	C	487	139	0.285	0	0	T	348	M	0.247	0.327	0	0	0	686.971	ggaataggagCttgctccatc	m5C_5790
chr1	236431200	+	C	273	91	0.333	0	0	T	182	M	0.28	0.391	0	0	0	509.722	taggagcttgCtccatccact	m5C_5789
chr1	236431202	+	C	176	43	0.244	0	0	T	133	M	0.187	0.313	0	0	0	160.599	ggagcttgctCcatccactcc	m5C_5775
chr1	236431203	+	C	153	41	0.268	0	0	T	112	M	0.204	0.343	0	0	0	167.369	gagcttgctcCatccactcct	m5C_5774
chr1	236431209	+	C	84	21	0.25	0	0	T	63	M	0.17	0.352	0	2.6E-13	0	71.2832	gctccatccaCtcctcgcatc	m5C_5788
chr1	236431219	+	C	540	118	0.219	0	0	T	422	M	0.186	0.255	0	0	0	438.296	ctcctcgcatCgacctggtat	m5C_5778
chr1	236431237	+	C	1402	298	0.213	0	0	T	1104	M	0.192	0.235	0	0	0	1143.95	tattgcagtaCttcaggaacg	m5C_5791
chr1	236431246	+	C	1358	308	0.227	0	0	T	1050	M	0.205	0.25	0	0	0	1264.77	acttcaggaaCggtgcacccc	m5C_5784
chr1	236431253	+	C	845	169	0.2	0	0	T	676	M	0.174	0.228	0	0	0	589.52	gaacggtgcaCccctccgggg	m5C_5787
chr1	236431255	+	C	146	42	0.288	0	0	T	104	M	0.22	0.366	0	0	0	185.16	acggtgcaccCctccgggga	m5C_5780
chr1	236431256	+	C	54	14	0.259	0	0	T	40	M	0.161	0.389	0	2.8E-09	0	45.1318	cggtgcacccCtccgggggaa	m5C_5783
chr1	237766560	+	C	31	22	0.71	0	0	C	22	M	0.534	0.839	0	6.3E-05	0	234.992	ggaaagaagaCcctgttgagc	m5C_5792
chr1	238193497	-	C	31	11	0.355	0	0	T	20	M	0.211	0.531	1.1E-16	3.1E-06	1.5E-16	37.0595	tcatggctgaCaggatccatc	m5C_5417
chr1	239528131	-	C	49	17	0.347	0	0	T	32	M	0.229	0.487	0	5.6E-07	0	77.9423	agtggcagagCgtttgactgc	m5C_5421
chr1	239528142	-	C	49	19	0.388	0	0	T	30	M	0.264	0.528	0	1E-06	0	100.429	gggtgtagctCagtggcagag	m5C_5412
chr1	239528144	-	C	41	12	0.293	0	0	T	29	M	0.176	0.445	1.1E-16	7.5E-08	1.5E-16	33.7134	gggggtgtagCtcagtggcag	m5C_5420
chr1	239847440	+	C	48	30	0.625	0	0	C	30	M	0.484	0.748	0	1.2E-05	0	290.175	atgggtggttCagtggtagaa	m5C_5409
chr1	240293920	-	C	41	21	0.512	0	0	C	21	M	0.365	0.657	0	1.8E-06	0	153.234	tgattgtttaCtaaaaacaca	m5C_5734
chr1	241793554	-	C	61	34	0.557	0	0	C	34	M	0.433	0.675	0	4.2E-08	0	294.461	aaaagtagtaCatggggagat	m5C_5723
chr1	242247832	-	C	165	35	0.212	0	0	T	130	M	0.157	0.281	0	0	0	109.662	gccgagtggtCtgaggctcca	m5C_5572
chr1	242247840	-	C	206	82	0.398	0	0	T	124	M	0.334	0.466	0	0	0	547.214	tcaggacggcCgagtggtctg	m5C_5573
chr1	242247841	-	C	202	41	0.203	0	0	T	161	M	0.153	0.264	0	0	0	125.696	gtcaggacggCcgagtggtct	m5C_5579
chr1	243589817	+	C	31	15	0.484	0	0	T	16	M	0.32	0.652	0	1.2E-05	0	95.91	attggaggaaCagtgtgtcca	m5C_5563
chr1	245021336	-	C	47	17	0.362	0	0	T	30	M	0.24	0.505	0	5.6E-07	0	81.4845	accaaaggggCctgaagagaa	m5C_5657
chr1	245021344	-	C	35	11	0.314	0	0	T	24	M	0.186	0.48	6.7E-16	3.1E-06	8.7E-16	30.9686	gttagaggacCaaaggggcct	m5C_5641
chr1	245021345	-	C	34	12	0.353	0	0	T	22	M	0.215	0.521	0	4.6E-06	0	51.5705	agttagaggaCcaaaggggcc	m5C_5634
chr1	245021357	-	C	33	8	0.242	0	0	T	25	M	0.128	0.41	3.1E-11	6.8E-07	3.6E-11	10.7866	cttagaggatCgagttagagg	m5C_5674
chr1	245027364	-	C	172	49	0.285	0	0	T	123	M	0.223	0.356	0	0	0	218.25	cggccggcggCgatgaagagg	m5C_5620
chr1	245027367	-	C	166	60	0.361	0	0	T	106	M	0.292	0.437	0	0	0	350.711	ccgcggccggCggcgatgaag	m5C_5659
chr1	245027370	-	C	163	69	0.423	0	0	T	94	M	0.35	0.5	0	0	0	483.117	aggccgcggcCggcggcgatg	m5C_5642
chr1	245027376	-	C	129	53	0.411	0	0	T	76	M	0.33	0.497	0	5.8E-15	0	349.51	agcaggaggcCgcggccggcg	m5C_5638
chr1	245027388	-	C	48	20	0.417	0	0	T	28	M	0.288	0.557	0	1.4E-06	0	115.398	gagcaggcctCgagcaggagg	m5C_5629
chr1	245027390	-	C	50	19	0.38	0	0	T	31	M	0.259	0.518	0	2.4E-08	0	98.2779	gggagcaggcCtcgagcagga	m5C_5636
chr1	245027391	-	C	41	9	0.22	0	0	T	32	M	0.12	0.367	8.4E-12	1.2E-08	9.9E-12	11.9621	cgggagcaggCctcgagcagg	m5C_5653
chr1	245027395	-	C	31	12	0.387	0	0	T	19	M	0.237	0.562	0	4.6E-06	0	56.9589	cgctcgggagCaggcctcgag	m5C_5626
chr1	248160309	+	C	44	11	0.25	0	0	T	33	M	0.146	0.394	1.6E-14	4.4E-08	2E-14	22.132	attggctggtCtgagtgcagt	m5C_7568
chr1	249168064	+	C	217	54	0.249	0	0	T	163	M	0.196	0.31	0	0	0	211.709	gtcaggatggCcgagtggtct	m5C_7584
chr1	249168099	+	C	50	25	0.5	0	0	CT	25	M	0.366	0.634	0	1.5E-07	0	183.221	aaggtaagcaCcttgcctgcg	m5C_7575
chr1	249168100	+	C	45	13	0.289	0	0	T	32	M	0.177	0.434	0	1.2E-07	0	46.0978	aggtaagcacCttgcctgcgg	m5C_7557
chr1	249168105	+	C	42	24	0.571	0	0	C	24	M	0.422	0.709	0	3.6E-06	0	202.589	agcaccttgcCtgcgggcttt	m5C_7573
chr1	249168121	+	C	223	52	0.233	0	0	T	171	M	0.182	0.293	0	0	0	189.788	gctttctggtCtccggatgga	m5C_7579
chr1	249168134	+	C	373	132	0.354	0	0	T	241	M	0.307	0.404	0	0	0	810.676	cggatggaggCgtgggttcga	m5C_7570
chr1	249168142	+	C	182	66	0.365	1	0.005	T	115	M	0.298	0.437	0	0	0	393.369	ggcgtgggttCgaatcccact	m5C_7559
chr1	249168147	+	C	94	19	0.202	0	0	T	75	M	0.133	0.294	0	8.9E-16	0	50.6896	gggttcgaatCccacttctga	m5C_7569
chr1	249168148	+	C	80	17	0.212	0	0	T	63	M	0.137	0.314	0	2.3E-14	0	46.6217	ggttcgaatcCcacttctgac	m5C_7582
chr1	249168151	+	C	84	19	0.226	0	0	T	65	M	0.15	0.326	0	8.4E-14	0	56.9464	tcgaatcccaCttctgacaca	m5C_7564
chr1	249168445	+	C	40	16	0.4	0	0	T	24	M	0.263	0.554	0	1.7E-05	0	84.314	cgcatcatgtCttccctggtg	m5C_7576
chr1	249168449	+	C	466	96	0.206	0	0	T	370	M	0.172	0.245	0	0	0	329.785	tcatgtcttcCctggtggtct	m5C_7563
chr1	249168458	+	C	1059	252	0.238	0	0	T	807	M	0.213	0.265	0	0	0	1074.97	ccctggtggtCtagtggttag	m5C_7560
chr1	249168476	+	C	1170	234	0.2	0	0	T	936	M	0.178	0.224	0	0	0	833.404	taggattcggCgctctcaccg	m5C_7562
chr1	249168478	+	C	1169	253	0.216	0	0	T	916	M	0.194	0.241	0	0	0	980.458	ggattcggcgCtctcaccgcc	m5C_7577
chr1	249168480	+	C	1168	291	0.249	0	0	T	877	M	0.225	0.275	0	0	0	1310.6	attcggcgctCtcaccgccgc	m5C_7556
chr1	249168482	+	C	1163	249	0.214	1	0.001	T	913	M	0.192	0.239	0	0	0	954.439	tcggcgctctCaccgccgcgg	m5C_7580
chr1	249168488	+	C	1115	250	0.224	1	0.001	T	864	M	0.201	0.25	0	0	0	1004.44	ctctcaccgcCgcggcccggg	m5C_7574
chr1	249168494	+	C	967	223	0.231	0	0	T	744	M	0.205	0.258	0	0	0	915.004	ccgccgcggcCcgggttcgat	m5C_7558
chr1	249168495	+	C	934	209	0.224	0	0	T	725	M	0.198	0.252	0	0	0	828.486	cgccgcggccCgggttcgatt	m5C_7578
chr1	249168506	+	C	734	172	0.235	2	0.003	T	560	M	0.206	0.267	0	0	0	707.576	gggttcgattCccggtcagga	m5C_7565
chr10	200088	+	C	36	10	0.278	0	0	T	26	M	0.158	0.44	4.8E-14	2E-06	5.9E-14	21.1115	tactgtagcaCttttgggaat	m5C_37019
chr10	1700034	-	C	33	19	0.576	0	0	C	19	M	0.408	0.728	0	3.4E-05	0	155.067	gaatcagagaCagagatagag	m5C_37040
chr10	1700040	-	C	39	27	0.692	0	0	C	27	M	0.536	0.814	0	0.00015	0	289.324	gagagagaatCagagacagag	m5C_37084
chr10	1700067	-	C	34	14	0.412	0	0	T	20	M	0.264	0.578	0	9.2E-06	0	73.8241	cagagagaaaCtgagagcaga	m5C_37082
chr10	1759668	-	C	33	9	0.273	0	0	T	24	M	0.151	0.442	7.5E-13	1.2E-06	9E-13	16.4418	aagaaaagaaCagaaaagaaa	m5C_37070
chr10	5794938	-	C	82	45	0.549	0	0	C	45	M	0.441	0.652	0	6.4E-10	0	397.175	ctctggcggtCtagtggttag	m5C_36733
chr10	5858445	-	C	46	36	0.783	0	0	C	36	M	0.644	0.877	0	2.9E-05	0	463.867	ggttttggcaCcaaaaaaataa	m5C_36727
chr10	5895691	-	C	55	21	0.382	0	0	T	34	M	0.265	0.514	0	4.8E-08	0	111.366	gtcctgggttCaagccccagt	m5C_36721
chr10	5895698	-	C	149	39	0.262	0	0	T	110	M	0.198	0.338	0	0	0	154.273	gcagaaggtcCtgggttcaag	m5C_36712
chr10	5895716	-	C	164	51	0.311	0	0	T	113	M	0.245	0.385	0	0	0	250.041	atcacatctgCtttacacgca	m5C_36711
chr10	5895741	-	C	168	48	0.286	0	0	T	120	M	0.223	0.358	0	0	0	213.878	cctgtggttcCatagtgtagt	m5C_36728
chr10	6252991	-	C	35	10	0.286	0	0	T	25	M	0.163	0.451	3.4E-14	2E-06	4.2E-14	21.9991	gagcatttgaCtgcagaacaa	m5C_36636
chr10	6253009	-	C	68	18	0.265	0	0	T	50	M	0.174	0.38	0	2.9E-10	0	62.8186	ggatatagctCaggggtagag	m5C_36628
chr10	6287768	-	C	64	16	0.25	0	0	T	48	M	0.16	0.368	0	1.1E-10	0	51.2473	gtagtgcttaCaactaattga	m5C_36631
chr10	6287787	-	C	70	28	0.4	0	0	T	42	M	0.293	0.517	0	9.8E-09	0	164.271	tcaggctggtCtgagtgcagt	m5C_36630
chr10	13522425	+	C	43	18	0.419	0	0	T	25	M	0.284	0.567	0	7.7E-07	0	102.184	ggtttgatgtCacagtaaggt	m5C_37089
chr10	13769798	+	C	75	15	0.2	0	0	T	60	M	0.125	0.304	0	6E-13	0	37.5361	actctcaggtCtatggccata	m5C_37110
chr10	13769804	+	C	199	59	0.296	0	0	T	140	M	0.237	0.363	0	0	0	280.094	aggtctatggCcatactgccc	m5C_37086
chr10	13769812	+	C	217	71	0.327	0	0	T	146	M	0.268	0.392	0	0	0	380.901	ggccatactgCcctgaatgcg	m5C_37113
chr10	13769814	+	C	207	46	0.222	0	0	T	161	M	0.171	0.284	0	0	0	157.263	ccatactgccCtgaatgcgcc	m5C_37101
chr10	13769829	+	C	159	38	0.239	0	0	T	121	M	0.179	0.311	0	0	0	136.313	tgcgcctgatCttgtctgatt	m5C_37095
chr10	13769834	+	C	134	34	0.254	0	0	T	100	M	0.188	0.334	0	0	0	127.586	ctgatcttgtCtgatttcaga	m5C_37111
chr10	16518041	+	C	65	31	0.477	0	0	T	34	M	0.36	0.596	0	2.1E-08	0	223.334	agtgtagtgtCtgttcttatc	m5C_36943
chr10	16518046	+	C	63	27	0.429	0	0	T	36	M	0.314	0.551	0	7.5E-09	0	169.542	agtgtctgttCttatcagttt	m5C_36952
chr10	17271977	+	C	51	13	0.255	0	0	T	38	M	0.155	0.389	1.1E-16	1.6E-09	1.5E-16	32.2431	catgcgcctcCgggagaagta	m5C_36939
chr10	17272668	+	C	56	16	0.286	0	0	T	40	M	0.184	0.415	0	7.3E-09	0	58.9396	ggagatgcttCagagagagga	m5C_36950
chr10	19850305	+	C	51	14	0.275	0	0	T	37	M	0.171	0.409	0	2.8E-09	0	47.9195	tactgcctgaCtggtttgctg	m5C_36984
chr10	19850313	+	C	53	26	0.491	0	0	T	27	M	0.361	0.621	0	1.9E-07	0	187.845	gactggtttgCtgccctctga	m5C_36985
chr10	19887384	+	C	39	16	0.41	0	0	T	23	M	0.271	0.566	0	1.7E-05	0	86.6531	agaatgcagtCtgagtggttt	m5C_36990
chr10	22518433	-	C	139	39	0.281	0	0	T	100	M	0.213	0.36	0	0	0	165.798	gggacgcgctCtccttttatg	m5C_37143
chr10	22518439	-	C	169	48	0.284	0	0	T	121	M	0.221	0.356	0	0	0	212.567	cacccagggaCgcgctctcct	m5C_37141
chr10	22518446	-	C	185	42	0.227	0	0	T	143	M	0.173	0.293	0	0	0	144.962	tcgagcccacCcagggacgcg	m5C_37140
chr10	22518479	-	C	165	50	0.303	0	0	T	115	M	0.238	0.377	0	0	0	238.046	gcgcgttcggCtgttaaccga	m5C_37147
chr10	22518482	-	C	158	58	0.367	0	0	T	100	M	0.296	0.445	0	0	0	343.261	ttagcgcgttCggctgttaac	m5C_37148
chr10	22518507	-	C	114	25	0.219	0	0	T	89	M	0.153	0.304	0	0	0	76.5914	accgaagtctCtgggcgcaa	m5C_37154
chr10	22518515	-	C	45	22	0.489	0	0	T	23	M	0.35	0.63	0	2.3E-06	0	153.81	aaaatgacacCgaagtctctg	m5C_37145
chr10	23286259	+	C	31	11	0.355	0	0	T	20	M	0.211	0.531	1.1E-16	3.1E-06	1.5E-16	37.0595	ggatgggagaCtgcccgggaa	m5C_37130
chr10	23286262	+	C	34	18	0.529	0	0	C	18	M	0.367	0.685	0	2.7E-05	0	132.251	tgggagactgCccgggaaaac	m5C_37134
chr10	29741203	+	C	34	24	0.706	0	0	C	24	M	0.538	0.832	0	9.1E-05	0	258.388	ctcctgggctCaatctgtcct	m5C_37177
chr10	30844745	-	C	40	14	0.35	0	0	T	26	M	0.221	0.505	0	1.9E-07	0	61.9761	gagttttgggCtgcagtgcgc	m5C_37419

chr10	31986697	-	C	52	11	0.212	0	0	T	41	M	0.122	0.34	1.4E-13	4.4E-10	1.7E-13	17.3019	ctgcgcagccCagcaggaagc	m5C_37411
chr10	31986699	-	C	54	13	0.241	0	0	T	41	M	0.146	0.369	2.2E-16	1.6E-09	2.9E-16	29.8003	ggctgcgcagCccagcaggaa	m5C_37414
chr10	32308822	-	C	46	14	0.304	0	0	T	32	M	0.191	0.448	0	1.9E-07	0	53.4231	ttgaaagccaCagatcaggaa	m5C_37285
chr10	32308824	-	C	39	8	0.205	0	0	T	31	M	0.108	0.355	1.6E-10	6.8E-07	1.8E-10	8.44384	agttgaaagcCacagatcagg	m5C_37301
chr10	32308841	-	C	43	23	0.535	0	0	C	23	M	0.389	0.675	0	2.9E-06	0	179.011	aagagtagaaCatgagaagtt	m5C_37279
chr10	32308856	-	C	36	9	0.25	0	0	T	27	M	0.138	0.411	2E-12	1.2E-06	2.4E-12	14.4765	agagcaggaaCgtctaagagt	m5C_37281
chr10	32699352	-	C	32	10	0.312	0	0	T	22	M	0.18	0.486	1.1E-14	2E-06	1.3E-14	25.0836	agttgggttgCttgggaatgc	m5C_37270
chr10	33200919	-	C	35	26	0.743	0	0	C	26	M	0.579	0.858	0	0.00013	0	301.238	cgtctgcggaCagtgtgtttg	m5C_37274
chr10	33201027	-	C	43	15	0.349	0	0	T	28	M	0.224	0.498	0	2.8E-07	0	67.2552	tgttggtagaCattgtgaatg	m5C_37269
chr10	33201039	-	C	43	22	0.512	0	0	C	22	M	0.368	0.654	0	2.3E-06	0	161.709	caatgaagggCgtgttggtag	m5C_37255
chr10	33208819	-	C	41	14	0.341	0	0	T	27	M	0.216	0.495	0	1.9E-07	0	60.3641	gagtgtggcgCgtgcaggtaa	m5C_37297
chr10	33208821	-	C	37	13	0.351	0	0	T	24	M	0.218	0.512	0	6.7E-06	0	56.7461	ttgagtgtggCgcgtgcaggt	m5C_37271
chr10	34490972	-	C	43	15	0.349	0	0	T	28	M	0.224	0.498	0	2.8E-07	0	67.2552	gttatcagaaCttattaatgt	m5C_37569
chr10	34490994	-	C	45	15	0.333	0	0	T	30	M	0.214	0.479	0	2.8E-07	0	64.0761	taggctggttCgatggtagtg	m5C_37588
chr10	34814285	-	C	33	10	0.303	0	0	T	23	M	0.174	0.473	1.6E-14	2E-06	2E-14	23.982	ccagtaggaaCattcgctagt	m5C_37593
chr10	35841398	-	C	52	12	0.231	0	0	T	40	M	0.137	0.361	4.4E-15	8.8E-10	5.7E-15	23.6371	gtggttaccaCattcacctga	m5C_37586
chr10	35841400	-	C	55	12	0.218	0	0	T	43	M	0.129	0.344	9.8E-15	8.8E-10	1.2E-14	21.7648	tggtggttacCacattcacct	m5C_37599
chr10	35841418	-	C	33	16	0.485	0	0	T	17	M	0.325	0.648	0	1.7E-05	0	104.012	tgtgttgtttCtgcagtgtgg	m5C_37592
chr10	37555512	-	C	92	53	0.576	0	0	C	53	M	0.474	0.672	0	1.3E-10	0	502.496	cgtgccgggcCtataaaaagc	m5C_37360
chr10	37555513	-	C	102	21	0.206	0	0	T	81	M	0.139	0.294	0	0	0	58.2909	gcgtgccgggCctataaaaag	m5C_37353
chr10	37555518	-	C	205	74	0.361	0	0	T	131	M	0.298	0.429	0	0	0	441.548	cggaagcgtgCcgggcctata	m5C_37358
chr10	43292245	+	C	53	22	0.415	0	0	T	31	M	0.293	0.549	0	6.5E-08	0	128.747	gaaggggaagCggaggaagct	m5C_37369
chr10	43292254	+	C	50	18	0.36	0	0	T	32	M	0.241	0.499	0	1.7E-08	0	86.8988	gcggaggaagCtgatgaaagc	m5C_37376
chr10	43882365	-	C	32	13	0.406	0	0	T	19	M	0.255	0.577	0	6.7E-06	0	66.3505	gagattggccCagatggaaga	m5C_37385
chr10	43882367	-	C	32	16	0.5	0	0	CT	16	M	0.336	0.664	0	1.7E-05	0	107.618	ttgagattggCccagatggaa	m5C_37386
chr10	43882381	-	C	40	13	0.325	0	0	T	27	M	0.201	0.48	0	1.2E-07	0	52.2192	tgtgagagtcCatattgagat	m5C_37395
chr10	50468365	+	C	31	19	0.613	0	0	C	19	M	0.438	0.763	0	3.4E-05	0	166.53	ctttctagtgCtggactacct	m5C_37427
chr10	52279619	-	C	32	20	0.625	0	0	C	20	M	0.453	0.771	0	4.3E-05	0	181.016	aaagttggaaCacagcagagg	m5C_37549
chr10	59455358	-	C	58	13	0.224	0	0	T	45	M	0.136	0.347	6.7E-16	1.6E-09	8.7E-16	26.8192	ttggctggttCtgctagtatc	m5C_37647
chr10	65325780	-	C	45	16	0.356	0	0	T	29	M	0.232	0.502	0	4E-07	0	74.3005	tgcctgtagtCccagctgctg	m5C_38000
chr10	66499053	+	C	38	12	0.316	0	0	T	26	M	0.191	0.475	0	4.6E-06	0	45.8026	gtagcagcttCctggtggtct	m5C_37654
chr10	66499054	+	C	42	9	0.214	0	0	T	33	M	0.117	0.359	1.1E-11	1.2E-08	1.3E-11	11.5454	tagcagcttcCtggtggtctc	m5C_37657
chr10	66499062	+	C	94	54	0.574	0	0	C	54	M	0.474	0.67	0	1.6E-10	0	511.413	tcctggtggtCtcgtggttag	m5C_37661
chr10	66499064	+	C	95	21	0.339	33	0.347	T	41	M	0.233	0.463	0	1E-09	0	98.0345	ctggtggtctCgtggttagga	m5C_37655
chr10	69524265	+	C	122	35	0.287	0	0	T	87	M	0.214	0.373	0	0	0	149.867	ctcgtggcagCgatggccgag	m5C_37685
chr10	69524272	+	C	160	63	0.394	0	0	T	97	M	0.321	0.471	0	0	0	404.947	cagcgatggcCgagtggttaa	m5C_37670
chr10	69524292	+	C	91	27	0.297	0	0	T	64	M	0.213	0.397	0	7.2E-14	0	114.822	aggcgttggaCttgaaatcca	m5C_37678
chr10	69524312	+	C	64	18	0.281	0	0	T	46	M	0.186	0.401	0	2.9E-10	0	66.9356	aatggggtctCcccgcgcagg	m5C_37682
chr10	69524313	+	C	59	24	0.414	1	0.017	T	34	M	0.296	0.542	0	1.2E-07	0	142.214	atggggtctcCccgcgcaggt	m5C_37677
chr10	69524314	+	C	59	16	0.271	0	0	T	43	M	0.174	0.396	0	7.3E-09	0	55.7978	tggggtctccCcgcgcaggtt	m5C_37684
chr10	69524347	+	C	75	21	0.28	0	0	T	54	M	0.191	0.39	0	1.8E-11	0	80.2327	gctgcggaagCgggtgctctt	m5C_37676
chr10	69524353	+	C	81	20	0.247	0	0	T	61	M	0.166	0.351	0	1.5E-13	0	66.3608	gaagcgggtgCtcttattttt	m5C_37689
chr10	69524355	+	C	65	18	0.277	0	0	T	47	M	0.183	0.396	0	2.9E-10	0	65.8564	agcgggtgctCttatttttc	m5C_37679
chr10	69676933	+	C	36	18	0.5	0	0	CT	18	M	0.345	0.655	0	2.7E-05	0	124.107	agaaggaagtCaacaatatgt	m5C_37681
chr10	69676936	+	C	35	8	0.229	0	0	T	27	M	0.121	0.39	5.6E-11	6.8E-07	6.4E-11	9.89691	aggaagtcaaCaatatgtggg	m5C_37688
chr10	69767584	-	C	85	17	0.2	0	0	T	68	M	0.129	0.297	0	2.3E-14	0	43.7882	tgaactggctCaggttggaaa	m5C_37712
chr10	69767586	-	C	92	22	0.239	0	0	T	70	M	0.164	0.336	0	5.8E-15	0	71.9621	ggtgaactggCtcaggttgga	m5C_37701
chr10	69767590	-	C	98	48	0.495	1	0.01	T	49	M	0.397	0.593	0	4.6E-11	0	381.558	gaggggtgaaCtggctcaggt	m5C_37698
chr10	69767613	-	C	133	47	0.353	0	0	T	86	M	0.277	0.438	0	0	0	260.663	ggggggccacCaggttgccta	m5C_37696
chr10	69767614	-	C	136	29	0.213	0	0	T	107	M	0.153	0.289	0	0	0	88.6066	tggggggccaCcaggttgcct	m5C_37690
chr10	69767616	-	C	139	38	0.273	0	0	T	101	M	0.206	0.353	0	0	0	156.66	agtggggggcCaccaggttgc	m5C_37693
chr10	69767617	-	C	129	33	0.258	1	0.008	T	95	M	0.19	0.34	0	0	0	125.313	gagtgggggggCcaccaggttg	m5C_37710
chr10	69767631	-	C	59	17	0.288	0	0	T	42	M	0.188	0.414	0	1.1E-08	0	64.0436	tggtgacttcCtgggagtggg	m5C_37692
chr10	69767635	-	C	50	11	0.22	0	0	T	39	M	0.128	0.352	8.5E-14	4.4E-10	1.1E-13	18.3342	aatatggtgaCttcctgggag	m5C_37691
chr10	70098955	+	C	103	21	0.204	0	0	T	82	M	0.137	0.292	0	0	0	57.7068	ggaatgatggCtttgatgaca	m5C_39799
chr10	70221254	+	C	57	28	0.491	0	0	T	29	M	0.366	0.617	0	3.1E-07	0	205.05	ggatgggagaCcacctgggaa	m5C_39778
chr10	70221255	+	C	56	25	0.446	0	0	T	31	M	0.324	0.576	0	1.5E-07	0	161.933	gatgggagacCacctgggaat	m5C_39798
chr10	70221257	+	C	73	22	0.301	0	0	T	51	M	0.208	0.414	0	2.8E-11	0	91.6188	tgggagaccaCctgggaatat	m5C_39805
chr10	70221258	+	C	73	37	0.507	0	0	C	37	M	0.395	0.618	0	3E-09	0	292.093	gggagaccacCtgggaatatt	m5C_39801
chr10	70221274	+	C	115	54	0.47	0	0	T	61	M	0.381	0.56	0	2.3E-13	0	411.279	atattgggtgCtgtagcttta	m5C_39809
chr10	70221280	+	C	97	27	0.278	0	0	T	70	M	0.199	0.375	0	7.2E-14	0	107.412	ggtgctgtagCtttaagaaag	m5C_39808
chr10	70514955	+	C	747	151	0.202	0	0	T	596	M	0.175	0.232	0	0	0	528.197	aaatcctattCcattgctgaa	m5C_39769
chr10	70514956	+	C	769	164	0.213	0	0	T	605	M	0.186	0.244	0	0	0	609.342	aatcctattcCattgctgaaa	m5C_39797
chr10	70520897	+	C	39	10	0.256	0	0	T	29	M	0.146	0.411	1.3E-13	2E-06	1.6E-13	18.7753	ttagagaaatCtgaaaaagaa	m5C_39782
chr10	70723193	+	C	35	19	0.543	0	0	C	19	M	0.382	0.695	0	3.4E-05	0	145.12	tcatggggaaCtgcaagacag	m5C_39771
chr10	70723196	+	C	33	7	0.212	0	0	T	26	M	0.107	0.378	1.1E-09	3.4E-07	1.2E-09	6.69165	tggggaactgCaagacaggaa	m5C_39800
chr10	71982329	+	C	33	15	0.455	0	0	T	18	M	0.298	0.62	0	1.2E-05	0	89.5281	attggtggttCaggggtagaa	m5C_39768
chr10	74885942	-	C	59	17	0.288	0	0	T	42	M	0.188	0.414	0	1.1E-08	0	64.0436	agcagggggcCtaaagaatgg	m5C_39137
chr10	76781507	+	C	43	14	0.326	0	0	T	29	M	0.205	0.475	0	1.9E-07	0	57.3807	tgagatgatgCagctggaatg	m5C_37883
chr10	76781520	+	C	44	14	0.318	0	0	T	30	M	0.2	0.466	0	1.9E-07	0	55.9976	ctggaatgtgCttggctggct	m5C_37890
chr10	76781525	+	C	43	27	0.628	0	0	C	27	M	0.479	0.756	0	6.7E-06	0	258.442	atgtgcttggCtggctggctg	m5C_37888
chr10	76781533	+	C	45	13	0.289	0	0	T	32	M	0.177	0.434	0	1.2E-07	0	46.0978	ggctggctggCtgtcctgatc	m5C_37897
chr10	76781537	+	C	40	14	0.35	0	0	T	26	M	0.221	0.505	0	1.9E-07	0	61.9761	ggctggctgtCctgatcagaa	m5C_37906
chr10	76781543	+	C	31	11	0.355	0	0	T	20	M	0.211	0.531	1.1E-16	3.1E-06	1.5E-16	37.0595	ctgtcctgatCagaaccgact	m5C_37898
chr10	76990664	+	C	31	10	0.323	0	0	T	21	M	0.186	0.499	7.1E-15	2E-06	9E-15	26.2725	aggtgtgaagCttacactctc	m5C_37884
chr10	76990668	+	C	38	10	0.263	0	0	T	28	M	0.15	0.42	9.4E-14	2E-06	1.2E-13	19.5037	gtgaagcttaCactctctgct	m5C_37908
chr10	76990670	+	C	37	9	0.243	0	0	T	28	M	0.134	0.401	2.7E-12	1.2E-06	3.2E-12	13.907	gaagcttacaCtctctgctct	m5C_37910
chr10	76990672	+	C	36	18	0.5	0	0	CT	18	M	0.345	0.655	0	2.7E-05	0	124.107	agcttacactCtctgctctgg	m5C_37895
chr10	76990674	+	C	33	8	0.242	0	0	T	25	M	0.128	0.41	3.1E-11	6.8E-07	3.6E-11	10.7866	cttacactctCtgctctggta	m5C_37903
chr10	76990677	+	C	32	8	0.25	0	0	T	24	M	0.133	0.421	2.3E-11	6.8E-07	2.6E-11	11.2828	acactctctgCtctggtagat	m5C_37904
chr10	85686860	-	C	45	13	0.289	0	0	T	32	M	0.177	0.434	0	1.2E-07	0	46.0978	tagtggactaCagatgctggt	m5C_37963
chr10	88386120	-	C	39	22	0.564	0	0	C	22	M	0.41	0.707	0	6.3E-05	0	180.292	tgaatgctggCctcagcaagg	m5C_39209
chr10	88386124	-	C	44	33	0.75	0	0	C	33	M	0.606	0.854	0	1.9E-05	0	399.69	aggatgaatgCtggcctcagc	m5C_39226
chr10	88649360	+	C	37	13	0.351	0	0	T	24	M	0.218	0.512	0	6.7E-06	0	56.7461	accacggtcaCgaaggtggtt	m5C_39179
chr10	88649373	+	C	32	18	0.562	0	0	C	18	M	0.393	0.718	0	2.7E-05	0	141.571	aggtggttttCccagggcgag	m5C_39191
chr10	88649470	+	C	129	31	0.24	0	0	T	98	M	0.175	0.321	0	0	0	108.359	tggtgggggaCtgcgttcatg	m5C_39204
chr10	88649477	+	C	71	27	0.38	0	0	T	44	M	0.276	0.497	0	1.9E-10	0	149.186	ggactgcgttCatgcttttcc	m5C_39182
chr10	88649481	+	C	53	13	0.245	0	0	T	40	M	0.149	0.376	2.2E-16	1.6E-09	2.9E-16	30.3876	tgcgttcatgCttttccctgg	m5C_39200
chr10	88649487	+	C	37	8	0.216	0	0	T	29	M	0.114	0.372	9.7E-11	6.8E-07	1.1E-10	9.12273	catgcttttcCctggttaaat	m5C_39207
chr10	88649488	+	C	39	15	0.385	0	0	T	24	M	0.249	0.541	0	1.2E-05	0	74.6735	atgcttttccCtggttaaatt	m5C_39202
chr10	89513002	-	C	43	13	0.302	0	0	T	30	M	0.186	0.451	0	1.2E-07	0	48.3648	taacaaaaagCaagttatgga	m5C_39221
chr10	89513009	-	C	40	12	0.3	0	0	T	28	M	0.181	0.454	1.1E-16	7.5E-08	1.5E-16	34.6049	caatggataaCaaaaagcaag	m5C_39232
chr10	89513019	-	C	41	22	0.537	0	0	C	22	M	0.387	0.679	0	2.3E-06	0	170.483	ttcaggtgaaCaatggataac	m5C_39211
chr10	91973242	+	C	43	22	0.512	0	0	C	22	M	0.368	0.654	0	2.3E-06	0	161.709	tagtctgtggCcataccaccc	m5C_38079
chr10	91973243	+	C	43	20	0.465	0	0	T	23	M	0.325	0.611	0	1.4E-06	0	130.043	agtctgtggcCataccaccct	m5C_38077
chr10	91973250	+	C	46	24	0.522	0	0	C	24	M	0.381	0.659	0	3.6E-06	0	183.058	ggccataccaCcctgagcgtg	m5C_38073
chr10	91973252	+	C	47	23	0.489	0	0	T	24	M	0.353	0.628	0	2.9E-06	0	162.266	ccataccaccCtgagcgtgcc	m5C_38064
chr10	91973262	+	C	43	10	0.233	0	0	T	33	M	0.132	0.377	4.3E-13	2.4E-08	5.2E-13	16.2683	ctgagcgtgcCtgatcttgtc	m5C_38070
chr10	91973267	+	C	40	11	0.275	0	0	T	29	M	0.161	0.428	4.3E-15	3.1E-06	5.5E-15	25.4502	cgtgcctgatCttgtctgaaa	m5C_38071
chr10	95057904	-	C	32	7	0.219	0	0	T	25	M	0.11	0.388	8.5E-10	3.4E-07	9.5E-10	6.99971	acgaggtggcCaagtggttaa	m5C_39167
chr10	95057905	-	C	31	17	0.548	0	0	C	17	M	0.378	0.708	0	2.1E-05	0	128.424	gacgaggtggCcaagtggtta	m5C_39176

chr10	95270409	-	C	35	12	0.343	0	0	T	23	M	0.208	0.508	0	4.6E-06	0	49.9956	ctgaacgcgcCtgatcttggc	m5C_39158
chr10	95270414	-	C	35	16	0.457	0	0	T	19	M	0.305	0.618	0	1.7E-05	0	97.4905	ccaccctgaaCgcgcctgatc	m5C_39159
chr10	95270419	-	C	35	8	0.229	0	0	T	27	M	0.121	0.39	5.6E-11	6.8E-07	6.4E-11	9.89691	ccataccaccCtgaacgcgcc	m5C_39168
chr10	95270420	-	C	34	10	0.294	0	0	T	24	M	0.168	0.462	2.3E-14	2E-06	2.9E-14	22.9564	gccataccacCctgaacgcgc	m5C_39173
chr10	95270421	-	C	36	11	0.306	0	0	T	25	M	0.18	0.469	1E-15	3.1E-06	1.3E-15	29.7077	ggccataccaCcctgaacgcg	m5C_39178
chr10	95270429	-	C	33	8	0.242	0	0	T	25	M	0.128	0.41	3.1E-11	6.8E-07	3.6E-11	10.7866	aagtctacggCcataccaccc	m5C_39165
chr10	95270432	-	C	34	16	0.471	0	0	T	18	M	0.315	0.633	0	1.7E-05	0	100.644	ttgaagtctaCggccatacca	m5C_39171
chr10	95707876	-	C	37	18	0.486	0	0	T	19	M	0.334	0.641	0	2.7E-05	0	120.406	gtagggtggcCgagtggtcta	m5C_39156
chr10	95707877	-	C	40	20	0.5	0	0	CT	20	M	0.352	0.648	0	1.4E-06	0	140.797	ggtagggtggCcgagtggtct	m5C_39160
chr10	101996820	-	C	138	35	0.254	0	0	T	103	M	0.188	0.332	0	0	0	131.868	tgtttctccaCccctgtgatg	m5C_39286
chr10	101996823	-	C	131	34	0.26	0	0	T	97	M	0.192	0.341	0	0	0	130.616	gggtgtttctCcacccctgtg	m5C_39264
chr10	101996838	-	C	101	44	0.436	0	0	T	57	M	0.343	0.533	0	5.7E-13	0	301.891	atattctgtgCtgagggggtgt	m5C_39287
chr10	101996854	-	C	38	12	0.316	0	0	T	26	M	0.191	0.475	0	4.6E-06	0	45.8026	cttgtctggcCtgaggatatt	m5C_39247
chr10	101996859	-	C	38	17	0.447	0	0	T	21	M	0.301	0.603	0	2.1E-05	0	102.497	acaggcttgtCtggcctgagg	m5C_39250
chr10	101996864	-	C	33	8	0.242	0	0	T	25	M	0.128	0.41	3.1E-11	6.8E-07	3.6E-11	10.7866	ctgacacaggCttgtctggcc	m5C_39268
chr10	103047419	-	C	38	10	0.263	0	0	T	28	M	0.15	0.42	9.4E-14	2E-06	1.2E-13	19.5037	gctgggtgggCgcagctcagt	m5C_41184
chr10	103067471	+	C	72	15	0.208	0	0	T	57	M	0.131	0.316	0	6E-13	0	39.1558	aatggtgcatCaccagggtgc	m5C_41153
chr10	103124093	-	C	114	47	0.412	0	0	T	67	M	0.326	0.504	0	3.8E-14	0	306.645	ttcagggcatCactgtggaat	m5C_41201
chr10	103124592	-	C	54	13	0.241	0	0	T	41	M	0.146	0.369	2.2E-16	1.6E-09	2.9E-16	29.8003	tccggggataCaaaaaaaaaa	m5C_41202
chr10	103124600	-	C	132	51	0.386	0	0	T	81	M	0.308	0.472	0	1.1E-16	0	313.784	gcactcgctcCggggataca a	m5C_41185
chr10	103124603	-	C	150	31	0.207	0	0	T	119	M	0.15	0.278	0	0	0	92.7446	ggtgcactcgCtccggggata	m5C_41203
chr10	103124605	-	C	268	70	0.262	1	0.004	T	197	M	0.213	0.318	0	0	0	298.279	acggtgcactCgctccgggga	m5C_41207
chr10	103124607	-	C	2064	571	0.277	1	0	T	1492	M	0.258	0.296	0	0	0	2945.25	gaacggtgcaCtcgctccggg	m5C_41182
chr10	103124614	-	C	3161	709	0.226	23	0.007	T	2429	M	0.212	0.241	0	0	0	3001.17	cctccaggaaCggtgcactcg	m5C_41180
chr10	103124638	-	C	3845	791	0.206	3	0.001	T	3051	M	0.193	0.219	0	0	0	3059.48	atgcatcgacCtggtattgca	m5C_41199
chr10	103124716	-	C	204	47	0.23	0	0	T	157	M	0.178	0.293	0	0	0	167.237	tgtcctctatCtgaggacaat	m5C_41183
chr10	103124732	-	C	61	20	0.328	0	0	T	41	M	0.223	0.453	0	6.9E-10	0	89.3398	agtttagtatCtgatatgtcc	m5C_41212
chr10	103541601	-	C	32	22	0.688	0	0	C	22	M	0.514	0.82	0	6.3E-05	0	226.305	ttacgatgagCaatgatgttt	m5C_41193
chr10	103920371	+	C	33	9	0.273	0	0	T	24	M	0.151	0.442	7.5E-13	1.2E-06	9E-13	16.4418	acgagaaaggCtgacagcagc	m5C_41143
chr10	103920384	+	C	37	17	0.459	0	0	T	20	M	0.31	0.616	0	2.1E-05	0	105.53	acagcagctcCagtgaggaag	m5C_41164
chr10	104849089	-	C	64	27	0.422	0	0	T	37	M	0.309	0.544	0	7.5E-09	0	166.696	aggaaagagtCtcagagtgga	m5C_39559
chr10	105154065	-	C	255	93	0.365	0	0	T	162	M	0.308	0.425	0	0	0	572.952	ggcgtggcgtCggtcgtggta	m5C_39554
chr10	105154073	-	C	238	51	0.214	0	0	T	187	M	0.167	0.271	0	0	0	170.248	atcgactcggCgtggcgtcgg	m5C_39571
chr10	105154106	-	C	72	30	0.417	0	0	T	42	M	0.31	0.532	0	4.8E-10	0	185.91	gaccggacctCgaccggctcg	m5C_39569
chr10	105154109	-	C	68	24	0.353	0	0	T	44	M	0.25	0.472	0	3E-09	0	120.009	ctcgaccggaCctcgaccggc	m5C_39530
chr10	105154114	-	C	32	7	0.219	0	0	T	25	M	0.11	0.388	8.5E-10	3.4E-07	9.5E-10	6.99971	ccaatctcgaCcggacctcga	m5C_39572
chr10	111629625	+	C	51	15	0.294	0	0	T	36	M	0.187	0.43	0	4.6E-09	0	56.1257	aagctttgcgCagtggcagta	m5C_39377
chr10	111629656	+	C	318	82	0.258	0	0	T	236	M	0.213	0.309	0	0	0	349.108	tgaggtctatCcgaggcgtga	m5C_39380
chr10	111629662	+	C	281	81	0.289	1	0.004	T	199	M	0.239	0.345	0	0	0	387.682	ctatccgaggCgtgattattg	m5C_39383
chr10	112360292	+	C	33	12	0.364	0	0	T	21	M	0.222	0.534	0	4.6E-06	0	53.2487	aacgcttggaCcaagtagaac	m5C_39398
chr10	112360293	+	C	33	9	0.273	0	0	T	24	M	0.151	0.442	7.5E-13	1.2E-06	9E-13	16.4418	acgcttggacCaagtagaaca	m5C_39405
chr10	112360783	+	C	34	10	0.294	0	0	T	24	M	0.168	0.462	2.3E-14	2E-06	2.9E-14	22.9564	gttacaggaaCttaatgagct	m5C_39397
chr10	112641254	+	C	38	15	0.395	0	0	T	23	M	0.256	0.553	0	1.2E-05	0	76.7936	gctggataggCgatccagatc	m5C_39407
chr10	112641259	+	C	37	11	0.297	0	0	T	26	M	0.175	0.458	1.4E-15	3.1E-06	1.9E-15	28.5508	ataggcgatcCagatctggga	m5C_39415
chr10	115933916	-	C	87	21	0.241	0	0	T	66	M	0.164	0.341	0	2.6E-13	0	68.729	gaaacggccgCtgagtgaggc	m5C_39463
chr10	115933919	-	C	91	23	0.253	0	0	T	68	M	0.175	0.351	0	1E-14	0	80.3739	ggggaaacggCcgctgagtga	m5C_39469
chr10	115933922	-	C	94	45	0.479	0	0	T	49	M	0.381	0.579	0	2.3E-11	0	342.513	tttggggaaaCggccgctgag	m5C_39464
chr10	120797794	-	C	37	10	0.27	0	0	T	27	M	0.154	0.43	6.7E-14	2E-06	8.3E-14	20.2815	gagaaagggaCagagagagag	m5C_39897
chr10	120797810	-	C	65	31	0.477	0	0	T	34	M	0.36	0.596	0	2.1E-08	0	223.334	gacaaggaccCtgagagagaa	m5C_39903
chr10	120797812	-	C	63	37	0.587	0	0	C	37	M	0.464	0.7	0	7.9E-08	0	343.472	atgacaaggaCcctgagagag	m5C_39938
chr10	120797818	-	C	65	22	0.338	0	0	T	43	M	0.235	0.46	0	1.5E-09	0	103.547	aggagaatgaCaaggaccctg	m5C_39882
chr10	120797838	-	C	80	29	0.362	0	0	T	51	M	0.266	0.472	0	8.4E-12	0	154.11	cagagataatCaagatcggga	m5C_39925
chr10	120797848	-	C	72	31	0.431	0	0	T	41	M	0.323	0.546	0	6.4E-10	0	199.998	aagaaagggaCagagataatc	m5C_39917
chr10	120797866	-	C	56	22	0.393	0	0	T	34	M	0.276	0.524	0	6.5E-08	0	121.353	gtgaatgggaCagagaaaaag	m5C_39907
chr10	120802262	-	C	32	12	0.375	0	0	T	20	M	0.229	0.547	0	4.6E-06	0	55.0408	tggagaagggCgagatgagga	m5C_39929
chr10	120809363	-	C	299	112	0.375	0	0	T	187	M	0.322	0.431	0	0	0	720.464	agaacgagaaCggcgtagaga	m5C_39932
chr10	120809369	-	C	324	88	0.272	0	0	T	236	M	0.226	0.323	0	0	0	397.864	aattgaagaaCgagaacggcg	m5C_39884
chr10	120809393	-	C	199	57	0.286	0	0	T	142	M	0.228	0.353	0	0	0	260.067	gaaaaaacgcCaaagggagtt	m5C_39921
chr10	120809396	-	C	174	63	0.362	0	0	T	111	M	0.294	0.436	0	0	0	370.881	aaggaaaaaaCgccaaaggga	m5C_39910
chr10	120810053	-	C	63	20	0.317	0	0	T	43	M	0.216	0.44	0	6.9E-10	0	86.3461	cagagaagggCagaagaacaa	m5C_39909
chr10	120810088	-	C	37	10	0.27	0	0	T	27	M	0.154	0.43	6.7E-14	2E-06	8.3E-14	20.2815	ggataacataCtatagagaaa	m5C_39920
chr10	120810092	-	C	35	12	0.343	0	0	T	23	M	0.208	0.508	0	4.6E-06	0	49.9956	cgcaggataaCatactataga	m5C_39934
chr10	120817632	-	C	57	34	0.596	0	0	C	34	M	0.467	0.714	0	1.1E-06	0	317.54	ccagaaagtgCggaaggctga	m5C_39899
chr10	120817642	-	C	63	41	0.651	0	0	C	41	M	0.528	0.757	0	1.7E-07	0	432.56	aagctgaactCcagaaagtgc	m5C_39935
chr10	120817644	-	C	63	19	0.302	0	0	T	44	M	0.202	0.424	0	4.5E-10	0	76.9026	ggaagctgaaCtccagaaagt	m5C_39923
chr10	120817649	-	C	56	12	0.214	0	0	T	44	M	0.127	0.338	1.3E-14	8.8E-10	1.6E-14	21.1897	cagagggaagCtgaactccag	m5C_39908
chr10	120817659	-	C	32	8	0.25	0	0	T	24	M	0.133	0.421	2.3E-11	6.8E-07	2.6E-11	11.2828	agaattggaaCagagggaagc	m5C_39892
chr10	120972547	+	C	32	10	0.312	0	0	T	22	M	0.18	0.486	1.1E-14	2E-06	1.3E-14	25.0836	gaggggtgaaCtggcttggtg	m5C_39852
chr10	122892823	-	C	31	23	0.742	0	0	C	23	M	0.568	0.863	0	7.6E-05	0	261.066	cagcctgtacCtgaaggtgaa	m5C_39678
chr10	124562225	+	C	50	10	0.2	0	0	T	40	M	0.112	0.33	2.6E-12	2E-10	3.1E-12	13.024	agagccatgaCtctcagtggg	m5C_39698
chr10	125965940	-	C	35	20	0.571	0	0	C	20	M	0.409	0.72	0	4.3E-05	0	163.429	gatcctggccCcattccttat	m5C_39725
chr10	125965942	-	C	44	16	0.364	0	0	T	28	M	0.238	0.511	0	4E-07	0	76.1067	ctgatcctggCcccattcctt	m5C_39730
chr10	125965946	-	C	49	28	0.571	0	0	C	28	M	0.433	0.7	0	8.1E-06	0	242.313	gagtctgatcCtggccccatt	m5C_39727
chr10	126454772	+	C	33	11	0.333	0	0	T	22	M	0.198	0.504	2.2E-16	3.1E-06	2.9E-16	34.0074	cttggctggtCtgagtgcagt	m5C_39982
chr10	126490693	-	C	35	13	0.371	0	0	T	22	M	0.232	0.537	0	6.7E-06	0	60.2306	gccgggacggCcgagcggctg	m5C_40004
chr10	130910219	+	C	66	21	0.318	0	0	T	45	M	0.218	0.438	0	1E-09	0	91.7652	ctccgggggaCtgtgcttgtg	m5C_39968
chr10	130910235	+	C	56	23	0.411	0	0	T	33	M	0.292	0.541	0	8.7E-08	0	134.166	ttgtgttcttCcaggttcctt	m5C_39965
chr10	133754603	-	C	33	10	0.303	0	0	T	23	M	0.174	0.473	1.6E-14	2E-06	2E-14	23.982	cctgaatgtaCctgtggccca	m5C_40031
chr10	134276716	+	C	54	18	0.333	0	0	T	36	M	0.222	0.466	0	1.7E-08	0	80.0685	gtagcctgcgCtgctctgccg	m5C_40134
chr10	134539927	+	C	116	27	0.233	0	0	T	89	M	0.165	0.317	0	0	0	89.1977	ctgagctctgCggtggattat	m5C_40124
chr11	252525	+	C	33	14	0.424	0	0	T	19	M	0.272	0.592	0	9.2E-06	0	76.259	tgaaggaccgCctggagttct	m5C_40229
chr11	532453	-	C	44	17	0.386	0	0	T	27	M	0.257	0.534	0	5.6E-07	0	87.4514	gaaggaaggaCggaagcaagg	m5C_40288
chr11	532475	-	C	34	17	0.5	0	0	CT	17	M	0.341	0.659	0	2.1E-05	0	115.835	gaggtgcagaCggaaggagga	m5C_40286
chr11	532479	-	C	44	14	0.318	0	0	T	30	M	0.2	0.466	0	1.9E-07	0	55.9976	gaaggaggtgCagacggaagg	m5C_40276
chr11	568115	-	C	134	27	0.201	0	0	T	107	M	0.142	0.277	0	0	0	76.8604	gtgacagcggCtgatctgtgc	m5C_40291
chr11	811685	+	C	57	14	0.246	0	0	T	43	M	0.152	0.371	0	2.8E-09	0	42.6516	ttttcatggtCcatcctaatc	m5C_40228
chr11	811695	+	C	150	47	0.313	0	0	T	103	M	0.245	0.391	0	0	0	229.875	ccatcctaatCcctgccggtc	m5C_40213
chr11	811705	+	C	145	32	0.221	0	0	T	113	M	0.161	0.295	0	0	0	102.966	ccctgccggtCcatctgtggc	m5C_40239
chr11	811706	+	C	143	44	0.308	0	0	T	99	M	0.238	0.388	0	0	0	209.354	cctgccggtcCatctgtggcc	m5C_40242
chr11	811709	+	C	141	31	0.22	0	0	T	110	M	0.159	0.295	0	0	0	98.8482	gccggtccatCtgtggcctgc	m5C_40252
chr11	811716	+	C	141	57	0.404	0	0	T	84	M	0.327	0.487	0	0	0	372.589	catctgtggcCtgccaggttt	m5C_40186
chr11	811720	+	C	140	37	0.264	0	0	T	103	M	0.198	0.343	0	0	0	146.701	tgtggcctgcCaggtttcgct	m5C_40208
chr11	811729	+	C	125	27	0.216	0	0	T	98	M	0.153	0.296	0	0	0	82.5698	ccaggtttcgCttgtggacca	m5C_40188
chr11	811738	+	C	85	20	0.235	0	0	T	65	M	0.158	0.336	0	1.5E-13	0	63.1177	gcttgtggacCagagcaccct	m5C_40245
chr11	811747	+	C	63	18	0.286	0	0	T	45	M	0.189	0.407	0	2.9E-10	0	68.051	ccagagcaccCtagaagcctc	m5C_40174
chr11	811759	+	C	55	11	0.2	0	0	T	44	M	0.116	0.324	2.9E-13	4.4E-10	3.6E-13	15.9231	agaagcctcaCccgaggagtg	m5C_40234
chr11	811760	+	C	58	13	0.224	0	0	T	45	M	0.136	0.347	6.7E-16	1.6E-09	8.7E-16	26.8192	gaagcctcacCcgaggagtga	m5C_40251
chr11	811761	+	C	75	16	0.213	0	0	T	59	M	0.136	0.319	0	1.2E-12	0	43.4563	aagcctcaccCgaggagtgag	m5C_40201
chr11	811772	+	C	85	19	0.224	0	0	T	66	M	0.148	0.323	0	8.4E-14	0	56.2519	gaggagtgagCagggctccag	m5C_40211
chr11	811777	+	C	93	27	0.29	0	0	T	66	M	0.208	0.389	0	7.2E-14	0	112.24	gtgagcagggCtccagtgggc	m5C_40187
chr11	811787	+	C	89	24	0.27	0	0	T	65	M	0.188	0.37	0	1.1E-12	0	90.4412	ctccagtgggCtcacgtcatg	m5C_40240

chr11	811791	+	C	92	20	0.217	0	0	T	72	M	0.145	0.312	0	1.7E-15	0	58.1465	agtgggctcaCgtcatgggca	m5C_40216
chr11	811800	+	C	82	21	0.256	0	0	T	61	M	0.174	0.36	0	2.6E-13	0	73.0945	acgtcatgggCacttctagac	m5C_40175
chr11	811810	+	C	78	23	0.295	0	0	T	55	M	0.205	0.404	0	4.3E-11	0	94.4108	cacttctagaCactctcagga	m5C_40184
chr11	812623	+	C	43	12	0.279	0	0	T	31	M	0.167	0.427	2.2E-16	7.5E-08	2.9E-16	31.457	ctggttctgcCcctgctgcag	m5C_40189
chr11	812628	+	C	69	25	0.362	0	0	T	44	M	0.259	0.48	0	4.1E-09	0	129.478	tctgcccctgCtgcaggtaag	m5C_40244
chr11	812760	+	C	123	25	0.203	0	0	T	98	M	0.142	0.283	0	0	0	70.8161	tgttccacagCagaggagaag	m5C_40203
chr11	812793	+	C	92	42	0.457	0	0	T	50	M	0.359	0.558	0	1.1E-11	0	301.161	aaggaggagtCtgaagagtca	m5C_40256
chr11	812802	+	C	66	25	0.379	0	0	T	41	M	0.271	0.499	0	4.1E-09	0	135.746	tctgaagagtCagatgatgac	m5C_40236
chr11	812812	+	C	43	12	0.279	0	0	T	31	M	0.167	0.427	2.2E-16	7.5E-08	2.9E-16	31.457	cagatgatgaCatgggatttg	m5C_40235
chr11	833458	+	C	41	17	0.415	0	0	T	24	M	0.278	0.566	0	5.6E-07	0	94.3715	cgggcggggtCggcggcggcg	m5C_40207
chr11	833464	+	C	38	19	0.5	0	0	CT	19	M	0.348	0.652	0	3.4E-05	0	132.429	gggtcggcggCggcggtgacc	m5C_40233
chr11	833473	+	C	39	13	0.333	0	0	T	26	M	0.206	0.49	0	6.7E-06	0	53.6452	gcggcggtgaCctcattgcag	m5C_40182
chr11	1389073	-	C	36	12	0.333	0	0	T	24	M	0.202	0.497	0	4.6E-06	0	48.5146	gtaccccccacCcctgggtcct	m5C_40281
chr11	1389086	-	C	39	12	0.308	0	0	T	27	M	0.186	0.464	0	4.6E-06	0	44.5578	ggggggcctcCtggtacccccc	m5C_40280
chr11	1900659	+	C	34	27	0.794	0	0	C	27	M	0.632	0.897	0	0.00015	0	341.287	gtgagccgacCctgtaaggtg	m5C_40246
chr11	2017551	-	C	34	18	0.529	0	0	C	18	M	0.367	0.685	0	2.7E-05	0	132.251	tccggtgtgaCggcgaggaca	m5C_40623
chr11	2017559	-	C	38	9	0.237	0	0	T	29	M	0.13	0.392	3.7E-12	1.2E-06	4.3E-12	13.3739	tgacatggtcCggtgtgacgg	m5C_40565
chr11	2018038	-	C	195	74	0.379	0	0	T	121	M	0.314	0.449	0	0	0	465.203	gcggagagggCccacagtgga	m5C_40610
chr11	2018794	-	C	70	36	0.514	0	0	C	36	M	0.4	0.628	0	2.4E-09	0	287.67	tgagctagggCtggaaagaag	m5C_40591
chr11	2018805	-	C	71	22	0.31	0	0	T	49	M	0.214	0.425	0	2.8E-11	0	94.3341	gacgatgggcCtgagctaggg	m5C_40636
chr11	2018806	-	C	61	27	0.443	0	0	T	34	M	0.325	0.567	0	7.5E-09	0	175.541	ggacgatgggCctgagctagg	m5C_40583
chr11	2050029	-	C	57	12	0.211	0	0	T	45	M	0.125	0.333	1.6E-14	8.8E-10	2E-14	20.643	ggagatggcgCaggggcacgg	m5C_40602
chr11	2246174	-	C	210	58	0.276	0	0	T	152	M	0.22	0.34	0	0	0	255.373	ttgcaggacaCatgtatcatc	m5C_40585
chr11	2246176	-	C	369	96	0.26	0	0	T	273	M	0.218	0.307	0	0	0	418.621	aattgcaggaCacatgtatca	m5C_40597
chr11	2985005	-	C	109	37	0.339	0	0	T	72	M	0.257	0.432	0	6.7E-14	0	190.457	gtcttggagaCaaatgctgca	m5C_40660
chr11	2985013	-	C	133	35	0.263	0	0	T	98	M	0.196	0.344	0	0	0	137.009	agtttcaggtCttggagacaa	m5C_40642
chr11	2985028	-	C	195	56	0.287	0	0	T	139	M	0.228	0.354	0	0	0	255.661	tgactggtggCtttcagtttc	m5C_40577
chr11	2985050	-	C	166	47	0.283	0	0	T	119	M	0.22	0.356	0	0	0	206.898	tttccagcgaCacatgactga	m5C_40589
chr11	2985053	-	C	191	41	0.23	13	0.068	T	137	M	0.175	0.297	0	0	0	143.148	gcatttccagCgacacatgac	m5C_40568
chr11	2985056	-	C	198	59	0.298	0	0	T	139	M	0.239	0.365	0	0	0	281.559	atagcatttcCagcgacacat	m5C_40574
chr11	2985079	-	C	234	71	0.303	0	0	T	163	M	0.248	0.365	0	0	0	352.274	ctaatgggttCcattccggtg	m5C_40582
chr11	2985099	-	C	424	90	0.212	0	0	T	334	M	0.176	0.254	0	0	0	316.826	acccgttagcCtggctgtagc	m5C_40626
chr11	2985100	-	C	432	93	0.215	0	0	T	339	M	0.179	0.256	0	0	0	333.16	aacccgttagCctggctgtag	m5C_40581
chr11	3765861	-	C	35	10	0.286	0	0	T	25	M	0.163	0.451	3.4E-14	2E-06	4.2E-14	21.9991	gctggacaggCatctttgttt	m5C_40652
chr11	3765865	-	C	32	8	0.25	0	0	T	24	M	0.133	0.421	2.3E-11	6.8E-07	2.6E-11	11.2828	tggtgctggaCaggcatcttt	m5C_40629
chr11	3810678	-	C	58	23	0.397	0	0	T	35	M	0.281	0.525	0	8.7E-08	0	129.207	ggaataaagaCagagatgtga	m5C_40647
chr11	3877461	-	C	40	14	0.35	0	0	T	26	M	0.221	0.505	0	1.9E-07	0	61.9761	atgtggacacCtcgggatccc	m5C_40620
chr11	3877462	-	C	37	17	0.459	0	0	T	20	M	0.31	0.616	0	2.1E-05	0	105.53	gatgtggacaCctcgggatcc	m5C_40595
chr11	3943904	+	C	31	8	0.258	0	0	T	23	M	0.137	0.432	1.7E-11	6.8E-07	1.9E-11	11.8182	tgctcggtagCtggtgattgg	m5C_40547
chr11	3943921	+	C	67	14	0.209	0	0	T	53	M	0.129	0.321	2.2E-16	3.2E-11	2.9E-16	28.2168	ttggaagacaCtctgtgacag	m5C_40563
chr11	3943929	+	C	67	37	0.552	0	0	C	37	M	0.434	0.665	0	7.9E-08	0	320.845	cactctgtgaCagcgttcagt	m5C_40542
chr11	4142864	+	C	31	8	0.258	0	0	T	23	M	0.137	0.432	1.7E-11	6.8E-07	1.9E-11	11.8182	ttacctggagCcttggcattt	m5C_40404
chr11	4142878	+	C	46	25	0.543	0	0	C	25	M	0.402	0.678	0	4.5E-06	0	200.9	ggcatttagaCatctttgaat	m5C_40402
chr11	4142881	+	C	50	18	0.36	0	0	T	32	M	0.241	0.499	0	1.7E-08	0	86.8988	atttagacatCtttgaattcc	m5C_40385
chr11	4142908	+	C	124	62	0.5	0	0	CT	62	M	0.413	0.587	0	5.3E-14	0	512.524	taaagaagaaCacaggaaagg	m5C_40400
chr11	4142910	+	C	129	33	0.256	0	0	T	96	M	0.188	0.337	0	0	0	124.306	aagaagaacaCaggaaaggaa	m5C_40405
chr11	4159525	+	C	321	70	0.218	0	0	T	251	M	0.176	0.266	0	0	0	246.923	gaaaaggtatCaaaagaggaa	m5C_40396
chr11	4159558	+	C	145	39	0.269	0	0	T	106	M	0.203	0.346	0	0	0	158.684	aggaacacagCagccatggtg	m5C_40398
chr11	4159561	+	C	166	38	0.229	0	0	T	128	M	0.172	0.299	0	0	0	130.389	aacacagcagCcatggtgtgc	m5C_40403
chr11	4159562	+	C	170	55	0.324	0	0	T	115	M	0.258	0.397	0	0	0	283.553	acacagcagcCatggtgtgct	m5C_40383
chr11	4159571	+	C	246	79	0.321	0	0	T	167	M	0.266	0.382	0	0	0	420.162	ccatggtgtgCtctttggaga	m5C_40381
chr11	4159573	+	C	287	65	0.226	0	0	T	222	M	0.182	0.278	0	0	0	236.411	atggtgtgctCtttggagaat	m5C_40388
chr11	4159596	+	C	766	164	0.214	0	0	T	602	M	0.187	0.245	0	0	0	611.767	agatgaatgtCtgatgtgtgg	m5C_40379
chr11	4159609	+	C	771	173	0.225	2	0.003	T	596	M	0.197	0.256	0	0	0	681.147	atgtgtggatCctgaggaaag	m5C_40377
chr11	4159610	+	C	750	221	0.295	0	0	T	529	M	0.263	0.328	0	0	0	1163.13	tgtgtggatcCtgaggaaaga	m5C_40386
chr11	4159621	+	C	38	20	0.526	0	0	C	20	M	0.373	0.675	0	4.3E-05	0	149.035	tgaggaaagaCttggaaagaga	m5C_40384
chr11	4621850	-	C	32	9	0.281	0	0	T	23	M	0.156	0.454	5.3E-13	1.2E-06	6.4E-13	17.1983	tgagtggggcCtgggagtatg	m5C_40411
chr11	6022856	-	C	106	35	0.33	0	0	T	71	M	0.248	0.424	0	3.4E-14	0	173.585	ggctacctggCttgtgtgcga	m5C_40109
chr11	6038336	+	C	58	37	0.638	0	0	C	37	M	0.509	0.749	0	1.8E-06	0	376.862	atattgggtgCtgtaggaaaa	m5C_40090
chr11	8231253	-	C	118	83	0.703	0	0	C	83	M	0.616	0.778	0	5E-11	0	1021.93	ggacttcaggCagcctgactg	m5C_42920
chr11	8231257	-	C	44	15	0.341	0	0	T	29	M	0.219	0.489	0	2.8E-07	0	65.6269	tgcaggacttCaggcagcctg	m5C_42938
chr11	8705811	+	C	1508	305	0.202	1	0.001	T	1202	M	0.183	0.223	0	0	0	1115.52	cggctattgcCtgagtgtgct	m5C_42900
chr11	8705820	+	C	1366	324	0.237	0	0	T	1042	M	0.215	0.26	0	0	0	1395.72	cctgagtgtgCtagagtcctc	m5C_42882
chr11	8705867	+	C	651	158	0.243	0	0	T	493	M	0.211	0.277	0	0	0	667.84	tggctgtgggCcttatggcac	m5C_42862
chr11	8705877	+	C	441	101	0.229	0	0	T	340	M	0.192	0.27	0	0	0	388.336	ccttatggcaCagtcagtcac	m5C_42903
chr11	8705899	+	C	359	101	0.282	1	0.003	T	257	M	0.238	0.331	0	0	0	480.776	aggttagagaCatgcttcaca	m5C_42868
chr11	8705903	+	C	264	65	0.246	0	0	T	199	M	0.198	0.302	0	0	0	257.569	tagagacatgCttcacattca	m5C_42879
chr11	8706993	+	C	342	96	0.281	0	0	T	246	M	0.236	0.331	0	0	0	452.577	tttgccgagaCtagagtcaca	m5C_42856
chr11	8707006	+	C	356	84	0.236	0	0	T	272	M	0.195	0.283	0	0	0	327.285	gagtcacatcCtgacacaact	m5C_42902
chr11	8707017	+	C	267	55	0.206	0	0	T	212	M	0.162	0.259	0	0	0	177.999	tgacacaactCttgtcctggt	m5C_42853
chr11	8707031	+	C	280	59	0.211	0	0	T	221	M	0.167	0.262	0	0	0	197.089	tcctggtgtgCtagagtactc	m5C_42869
chr11	8707082	+	C	81	17	0.21	0	0	T	64	M	0.135	0.311	0	2.3E-14	0	46.026	tgggggcagtCggtgcccccg	m5C_42870
chr11	8707087	+	C	53	16	0.302	0	0	T	37	M	0.195	0.435	0	7.3E-09	0	62.4581	gcagtcggtgCccccgttagt	m5C_42891
chr11	8707089	+	C	36	8	0.222	0	0	T	28	M	0.117	0.381	7.4E-11	6.8E-07	8.4E-11	9.49676	agtcggtgccCccgttagtgc	m5C_42889
chr11	8707090	+	C	38	10	0.263	0	0	T	28	M	0.15	0.42	9.4E-14	2E-06	1.2E-13	19.5037	gtcggtgcccCcgttagtgcc	m5C_42892
chr11	8707106	+	C	32	9	0.281	0	0	T	23	M	0.156	0.454	5.3E-13	1.2E-06	6.4E-13	17.1983	gtgcccagatCagaaacatac	m5C_42855
chr11	9450333	+	C	811	168	0.207	0	0	T	643	M	0.181	0.236	0	0	0	607.056	gtaatgtgtgCataggtttca	m5C_42873
chr11	9450345	+	C	636	155	0.244	0	0	T	481	M	0.212	0.279	0	0	0	657.038	taggtttcatCtgtgtctggt	m5C_42861
chr11	9450351	+	C	546	161	0.297	3	0.005	T	382	M	0.26	0.336	0	0	0	835.989	tcatctgtgtCtggtagcagt	m5C_42875
chr11	9450358	+	C	377	88	0.233	0	0	T	289	M	0.194	0.279	0	0	0	340.632	tgtctggtagCagtgtctgtc	m5C_42884
chr11	9450364	+	C	275	95	0.345	0	0	T	180	M	0.292	0.403	0	0	0	554.285	gtagcagtgtCtgtctgtgtt	m5C_42872
chr11	9450382	+	C	129	59	0.457	0	0	T	70	M	0.374	0.543	0	2.6E-14	0	441.167	gtttttcattCagatcttgct	m5C_42886
chr11	9450391	+	C	64	19	0.297	0	0	T	45	M	0.199	0.418	0	4.5E-10	0	75.639	tcagatcttgCtatccacaca	m5C_42898
chr11	9450396	+	C	37	15	0.405	0	0	T	22	M	0.263	0.565	0	1.2E-05	0	79.0389	tcttgctatcCacacaaacat	m5C_42850
chr11	9450400	+	C	36	17	0.472	0	0	T	19	M	0.32	0.63	0	2.1E-05	0	108.752	gctatccacaCaaacatcatg	m5C_42851
chr11	9450404	+	C	42	9	0.214	0	0	T	33	M	0.117	0.359	1.1E-11	1.2E-08	1.3E-11	11.5454	tccacacaaaCatcatgcggc	m5C_42864
chr11	10529707	-	C	65	13	0.2	0	0	T	52	M	0.121	0.313	3.8E-15	1.6E-11	4.8E-15	22.646	cgtgaagaggCgggcatagta	m5C_40456
chr11	10529717	-	C	83	23	0.277	0	0	T	60	M	0.192	0.382	0	7.2E-13	0	88.4663	ttgacctgccCgtgaagaggc	m5C_40459
chr11	10530739	-	C	292	107	0.366	0	0	T	185	M	0.313	0.423	0	0	0	670.332	cttgacctgctCtgagccaaac	m5C_40458
chr11	10530743	-	C	313	133	0.425	0	0	T	180	M	0.371	0.48	0	0	0	987.9	tcaacttgacCgctctgagcc	m5C_40455
chr11	10530744	-	C	322	111	0.345	0	0	T	211	M	0.295	0.398	0	0	0	654.706	ctcaacttgaCcgctctgagc	m5C_40466
chr11	10530749	-	C	310	82	0.265	0	0	T	228	M	0.219	0.316	0	0	0	358.363	ttcaactcaaCttgaccgctc	m5C_40465
chr11	10530754	-	C	311	70	0.225	0	0	T	241	M	0.182	0.275	0	0	0	255.053	gagatttcaaCtcaacttgac	m5C_40495
chr11	10530775	-	C	87	27	0.31	0	0	T	60	M	0.223	0.414	0	4E-12	0	120.361	aagcacccaaCttacacttag	m5C_40498
chr11	10530780	-	C	77	19	0.247	0	0	T	58	M	0.164	0.354	0	6.9E-12	0	62.335	acacaaagcaCccaacttaca	m5C_40496
chr11	10823016	-	C	176	54	0.307	0	0	T	122	M	0.243	0.378	0	0	0	262.889	gatatgagggCaagtctccta	m5C_40482
chr11	10823038	-	C	217	59	0.272	0	0	T	158	M	0.217	0.335	0	0	0	256.108	gtcctgtgagCgaagattatg	m5C_40457
chr11	10823045	-	C	102	37	0.363	0	0	T	65	M	0.276	0.459	0	6.7E-14	0	204.235	gtccagcgtcCtgtgagcgaa	m5C_40471
chr11	10823049	-	C	61	15	0.246	0	0	T	46	M	0.155	0.367	0	6E-11	0	46.5196	gaatgtccagCgtcctgtgag	m5C_40464
chr11	10823052	-	C	31	11	0.355	0	0	T	20	M	0.211	0.531	1.1E-16	3.1E-06	1.5E-16	37.0595	tttgaatgtcCagcgtcctgt	m5C_40481
chr11	10823090	-	C	38	13	0.342	0	0	T	25	M	0.212	0.501	0	6.7E-06	0	55.1518	tgacgtttttCtctctttct	m5C_40488

chr11	10823097	-	C	84	51	0.607	0	0	C	51	M	0.5	0.705	0	2.1E-09	0	510.22	gagttcttgaCgtttttctct	m5C_40454
chr11	10823102	-	C	126	62	0.492	0	0	T	64	M	0.406	0.578	0	5.3E-14	0	503.818	tgctggagttCttgacgtttt	m5C_40489
chr11	10823110	-	C	199	49	0.246	0	0	T	150	M	0.192	0.311	0	0	0	187.724	ggactttatgCtggagttctt	m5C_40480
chr11	10823117	-	C	231	71	0.307	0	0	T	160	M	0.251	0.37	0	0	0	357.008	attaagaggaCtttatgctgg	m5C_40468
chr11	10823133	-	C	279	95	0.341	0	0	T	184	M	0.287	0.398	0	0	0	546.058	tataaaaagaCgcgttattaa	m5C_40501
chr11	10823150	-	C	126	35	0.278	0	0	T	91	M	0.207	0.362	0	0	0	144.921	atttattgccCgatgattata	m5C_40474
chr11	10823152	-	C	127	27	0.213	0	0	T	100	M	0.15	0.292	0	0	0	81.2288	aaatttattgCccgatgatta	m5C_40492
chr11	10825053	-	C	34	18	0.529	0	0	C	18	M	0.367	0.685	0	2.7E-05	0	132.251	gacagtgggaCctagattaga	m5C_40462
chr11	10953017	+	C	48	31	0.646	0	0	C	31	M	0.504	0.766	0	1.4E-05	0	312.721	gaggaggcggCcgggcggcca	m5C_40447
chr11	10953018	+	C	47	13	0.277	0	0	T	34	M	0.169	0.418	0	1.2E-07	0	44.0347	aggaggcggcCgggcggccaa	m5C_40436
chr11	12962684	+	C	33	14	0.424	0	0	T	19	M	0.272	0.592	0	9.2E-06	0	76.259	aatgtgtggaCaggtcagctt	m5C_40337
chr11	13347807	-	C	31	6	0.2	1	0.032	T	24	M	0.095	0.373	1.7E-08	1.5E-07	1.8E-08	4.4363	gagttatctcCattgatagtt	m5C_40360
chr11	13347828	-	C	48	10	0.208	0	0	T	38	M	0.117	0.343	1.6E-12	2.4E-08	1.9E-12	13.8343	tgaggctggtCcgaaggtagt	m5C_40364
chr11	17096208	-	C	120	38	0.317	0	0	T	82	M	0.24	0.404	0	0	0	182.573	ccttggatgtCtgagtgagca	m5C_40885
chr11	17096217	-	C	132	42	0.318	0	0	T	90	M	0.245	0.402	0	0	0	205.656	ccagttcttcCttggatgtct	m5C_40900
chr11	17096237	-	C	136	36	0.265	0	0	T	100	M	0.198	0.345	0	0	0	142.389	aatgtttttcCttatgttggc	m5C_40890
chr11	17096238	-	C	145	48	0.331	0	0	T	97	M	0.26	0.411	0	0	0	249.281	aaatgtttttCcttatgttgg	m5C_40886
chr11	17096255	-	C	181	52	0.287	0	0	T	129	M	0.226	0.357	0	0	0	235.383	ttcgcagtttCcaccagaaat	m5C_40881
chr11	17096267	-	C	298	62	0.208	0	0	T	236	M	0.166	0.258	0	0	0	205.624	ggttgccagaCattcgcagtt	m5C_40882
chr11	17097346	-	C	1024	238	0.232	0	0	T	786	M	0.208	0.259	0	0	0	988.083	ttgggtaaacCttccttggat	m5C_40922
chr11	17097363	-	C	1030	216	0.21	1	0.001	T	813	M	0.186	0.236	0	0	0	804.087	aaaggttttcCttagtgttgg	m5C_40925
chr11	17542894	-	C	53	12	0.226	0	0	T	41	M	0.135	0.355	5.8E-15	8.8E-10	7.3E-15	22.9888	ggagatggaaCagtgagtacc	m5C_40878
chr11	17542909	-	C	68	37	0.544	0	0	C	37	M	0.427	0.657	0	7.9E-08	0	315.657	tgagagatacCggaaggagat	m5C_40916
chr11	17542910	-	C	70	19	0.271	0	0	T	51	M	0.181	0.385	0	6.9E-12	0	68.854	atgagagataCcggaaggaga	m5C_40869
chr11	17661115	+	C	32	16	0.5	0	0	CT	16	M	0.336	0.664	0	1.7E-05	0	107.618	tgcaaggaacCcggggttgca	m5C_40855
chr11	19852909	+	C	31	9	0.29	0	0	T	22	M	0.161	0.466	3.7E-13	1.2E-06	4.4E-13	18.0154	atcctcggagCaggcctactg	m5C_40672
chr11	19852913	+	C	32	13	0.406	0	0	T	19	M	0.255	0.577	0	6.7E-06	0	66.3505	tcggagcaggCctactggttg	m5C_40669
chr11	20630553	+	C	34	10	0.294	0	0	T	24	M	0.168	0.462	2.3E-14	2E-06	2.9E-14	22.9564	gcaggctgagCatgttgactg	m5C_40748
chr11	21022428	-	C	146	91	0.623	0	0	C	91	M	0.542	0.698	0	2.7E-14	0	987.305	gtgctgtaggCtgtctacgac	m5C_40761
chr11	21022435	-	C	177	93	0.525	0	0	C	93	M	0.452	0.598	0	0	0	840.908	ataccgggtgCtgtaggctgt	m5C_40767
chr11	21022441	-	C	164	81	0.494	0	0	T	83	M	0.418	0.57	0	0	0	677.813	ctgggaatacCgggtgctgta	m5C_40757
chr11	21022442	-	C	158	63	0.399	0	0	T	95	M	0.326	0.477	0	0	0	410.335	cctgggaataCcgggtgctgt	m5C_40758
chr11	21022451	-	C	72	47	0.653	0	0	C	47	M	0.538	0.752	0	2.2E-08	0	505.354	gggagacctcCtgggaatacc	m5C_40772
chr11	21022452	-	C	65	45	0.692	0	0	C	45	M	0.572	0.791	0	3.3E-07	0	514.819	tgggagacctCctgggaatac	m5C_40764
chr11	21022454	-	C	50	38	0.76	0	0	C	38	M	0.626	0.857	0	2.1E-06	0	475.662	gatgggagacCtcctgggaat	m5C_40771
chr11	21022455	-	C	52	38	0.731	0	0	C	38	M	0.597	0.832	0	2.1E-06	0	454.081	ggatgggagaCctcctgggaa	m5C_40760
chr11	24477486	+	C	48	23	0.479	0	0	T	25	M	0.345	0.617	0	2.9E-06	0	158.567	gtgtttacaaCtaactgatca	m5C_40509
chr11	24477507	+	C	49	26	0.531	0	0	C	26	M	0.394	0.663	0	5.5E-06	0	204.779	caaccagttaCagacttcttt	m5C_40505
chr11	24477528	+	C	44	17	0.386	0	0	T	27	M	0.257	0.534	0	5.6E-07	0	87.4514	gtcttttccaCtctcactgct	m5C_40510
chr11	24477530	+	C	46	16	0.348	0	0	T	30	M	0.227	0.492	0	4E-07	0	72.5786	cttttccactCtcactgcttc	m5C_40508
chr11	24477540	+	C	48	15	0.312	0	0	T	33	M	0.199	0.453	0	2.8E-07	0	59.8365	ctcactgcttCatctgactag	m5C_40507
chr11	26863850	-	C	55	37	0.673	0	0	C	37	M	0.541	0.782	0	1.8E-06	0	400.362	ttcctgaggcCttgttctggc	m5C_40728
chr11	29902121	+	C	124	35	0.282	0	0	T	89	M	0.211	0.367	0	0	0	147.352	agttttgattCtggtcaggga	m5C_40519
chr11	32379152	-	C	32	7	0.219	0	0	T	25	M	0.11	0.388	8.5E-10	3.4E-07	9.5E-10	6.99971	gacttggggtCagaagacctg	m5C_40966
chr11	32379160	-	C	34	16	0.471	0	0	T	18	M	0.315	0.633	0	1.7E-05	0	100.644	gagcactggaCttggggtcag	m5C_40969
chr11	35250851	+	C	33	8	0.242	0	0	T	25	M	0.128	0.41	3.1E-11	6.8E-07	3.6E-11	10.7866	aaggaacctgCagaatgtgga	m5C_41074
chr11	35250862	+	C	35	9	0.257	0	0	T	26	M	0.142	0.421	1.5E-12	1.2E-06	1.7E-12	15.0859	agaatgtggaCatgaagattg	m5C_41063
chr11	35685022	+	C	59	31	0.525	0	0	C	31	M	0.4	0.647	0	5.9E-07	0	248.245	aggatgagagCgatgaggaga	m5C_41062
chr11	36320701	-	C	38	12	0.316	0	0	T	26	M	0.191	0.475	0	4.6E-06	0	45.8026	tggccaacctCtgggatgtgt	m5C_40808
chr11	43964837	+	C	41	14	0.341	0	0	T	27	M	0.216	0.495	0	1.9E-07	0	60.3641	gcgccgcccgCggggggacg	m5C_40819
chr11	45848241	-	C	35	9	0.257	0	0	T	26	M	0.142	0.421	1.5E-12	1.2E-06	1.7E-12	15.0859	agattgatagCtctttctcaa	m5C_41126
chr11	46265717	+	C	154	33	0.214	0	0	T	121	M	0.157	0.286	0	0	0	103.52	atgggctggtCcgagtgcagt	m5C_41234
chr11	46265718	+	C	162	34	0.21	0	0	T	128	M	0.154	0.279	0	0	0	104.894	tgggctggtcCgagtgcagtg	m5C_41233
chr11	46450360	-	C	43	19	0.442	0	0	T	24	M	0.304	0.589	0	1E-06	0	115.646	ggtgagcgacCtgcgagactc	m5C_41316
chr11	46450361	-	C	40	9	0.225	0	0	T	31	M	0.123	0.375	6.4E-12	1.2E-08	7.6E-12	12.4041	gggtgagcgaCctgcgagact	m5C_41320
chr11	46450376	-	C	47	29	0.617	0	0	C	29	M	0.474	0.742	0	9.7E-06	0	275.073	gcctaaaggtCtggagggtga	m5C_41302
chr11	46450385	-	C	37	9	0.243	0	0	T	28	M	0.134	0.401	2.7E-12	1.2E-06	3.2E-12	13.907	caggcctcggCctaaaggtct	m5C_41245
chr11	46771875	-	C	36	22	0.611	0	0	C	22	M	0.449	0.752	0	6.3E-05	0	197.402	aagaggccttCgggtgattga	m5C_41309
chr11	46783944	-	C	47	15	0.319	0	0	T	32	M	0.204	0.462	0	2.8E-07	0	61.1856	ggccatctgaCtcacaggcat	m5C_41308
chr11	46783952	-	C	109	45	0.413	0	0	T	64	M	0.325	0.507	0	7.4E-13	0	292.428	tgcacaggggCcatctgactc	m5C_41331
chr11	46783960	-	C	126	27	0.214	0	0	T	99	M	0.152	0.294	0	0	0	81.8938	aaatcaagtgCacaggggcca	m5C_41272
chr11	46783981	-	C	135	43	0.319	0	0	T	92	M	0.246	0.401	0	0	0	211.456	ctcagtatcaCcctggtgaca	m5C_41278
chr11	46783993	-	C	209	54	0.258	0	0	T	155	M	0.204	0.322	0	0	0	220.065	atacaggcacCactcagtatc	m5C_41289
chr11	46784000	-	C	279	74	0.265	0	0	T	205	M	0.217	0.32	0	0	0	320.974	tcaggtgataCaggcaccact	m5C_41253
chr11	46784009	-	C	339	97	0.286	0	0	T	242	M	0.241	0.336	0	0	0	466.824	gccatggtatCaggtgataca	m5C_41283
chr11	46784018	-	C	466	147	0.316	1	0.002	T	318	M	0.276	0.36	0	0	0	810.03	actggtggagCcatggtatca	m5C_41287
chr11	46800062	-	C	44	34	0.773	0	0	C	34	M	0.63	0.872	0	2.2E-05	0	428.45	tggagatgaaCcagatgacgg	m5C_41263
chr11	47193245	-	C	37	14	0.378	0	0	T	23	M	0.241	0.539	0	9.2E-06	0	67.3786	cagcaggcagCcgatgccaag	m5C_41327
chr11	47193261	-	C	34	15	0.441	0	0	T	19	M	0.289	0.605	0	1.2E-05	0	86.6497	agagaagctcCgtgagcagca	m5C_41317
chr11	47193262	-	C	35	10	0.286	0	0	T	25	M	0.163	0.451	3.4E-14	2E-06	4.2E-14	21.9991	cagagaagctCcgtgagcagc	m5C_41271
chr11	47748515	-	C	40	8	0.2	0	0	T	32	M	0.105	0.348	2.1E-10	5.5E-09	2.3E-10	8.13503	gtgtttacaaCtaattaatca	m5C_41277
chr11	47748537	-	C	39	15	0.385	0	0	T	24	M	0.249	0.541	0	1.2E-05	0	74.6735	tctggctggtCcgagtgcagt	m5C_41333
chr11	56898454	+	C	47	16	0.34	0	0	T	31	M	0.222	0.483	0	4E-07	0	70.9352	gagaagggtgCcaccaggagt	m5C_41010
chr11	56898458	+	C	43	13	0.302	0	0	T	30	M	0.186	0.451	0	1.2E-07	0	48.3648	agggtgccacCaggagtaggc	m5C_41011
chr11	57094201	-	C	61	22	0.361	0	0	T	39	M	0.252	0.486	0	1.5E-09	0	110.765	agaacaagagCaaaaagaaga	m5C_41046
chr11	57094217	-	C	48	15	0.312	0	0	T	33	M	0.199	0.453	0	2.8E-07	0	59.8365	gagagctcttCgggagagaac	m5C_41025
chr11	57095779	-	C	32	7	0.219	0	0	T	25	M	0.11	0.388	8.5E-10	3.4E-07	9.5E-10	6.99971	ccgcaagaagCctgtggaggt	m5C_41055
chr11	57095786	-	C	31	13	0.419	0	0	T	18	M	0.264	0.592	0	6.7E-06	0	68.6798	gcaagagccgCaagaagcctg	m5C_41040
chr11	57095789	-	C	33	14	0.424	0	0	T	19	M	0.272	0.592	0	9.2E-06	0	76.259	accgcaagagCcgcaagaagc	m5C_41054
chr11	57095795	-	C	31	7	0.226	0	0	T	24	M	0.114	0.398	6.4E-10	3.4E-07	7.2E-10	7.33205	ccaaggaccgCaagagccgca	m5C_41027
chr11	57529343	+	C	51	20	0.392	0	0	T	31	M	0.27	0.529	0	3.4E-08	0	108.109	ggtgttggatCtgagggggaa	m5C_41015
chr11	59318103	-	C	118	27	0.229	0	0	T	91	M	0.162	0.312	0	0	0	87.6343	ccagtggaacCatgagatgtt	m5C_41932
chr11	59318113	-	C	153	34	0.222	0	0	T	119	M	0.164	0.294	0	0	0	111.252	ggttcgagccCcagtggaacc	m5C_41921
chr11	59318127	-	C	733	169	0.231	2	0.003	T	562	M	0.202	0.263	0	0	0	683.032	cgcagaaggtCctgggttcga	m5C_41917
chr11	59318139	-	C	45	15	0.333	0	0	T	30	M	0.214	0.479	0	2.8E-07	0	64.0761	gtctgctttaCacgcagaagg	m5C_41934
chr11	59318144	-	C	58	13	0.224	0	0	T	45	M	0.136	0.347	6.7E-16	1.6E-09	8.7E-16	26.8192	atcacgtctgCtttacacgca	m5C_41923
chr11	59318147	-	C	59	12	0.203	0	0	T	47	M	0.12	0.323	2.6E-14	8.8E-10	3.3E-14	19.6113	gttatcacgtCtgctttacac	m5C_41935
chr11	59318444	-	C	83	19	0.229	0	0	T	64	M	0.152	0.33	0	8.4E-14	0	57.6583	tgattcatacCtttttctttt	m5C_41912
chr11	59318449	-	C	103	28	0.272	0	0	T	75	M	0.195	0.365	0	1.8E-15	0	109.36	cggcgtgattCatacctttttt	m5C_41902
chr11	59318459	-	C	146	37	0.253	0	0	T	109	M	0.19	0.33	0	0	0	140.462	agtggaaccaCggcgtgattc	m5C_41905
chr11	59318472	-	C	184	53	0.288	0	0	T	131	M	0.227	0.357	0	0	0	241.12	gggttcgagcCcccagtggaac	m5C_41922
chr11	59318495	-	C	909	182	0.203	12	0.013	T	715	M	0.178	0.23	0	0	0	647.458	ctgctttacaCgcagaaggtc	m5C_41896
chr11	59318505	-	C	846	227	0.268	0	0	T	619	M	0.24	0.299	0	0	0	1087.6	gttatcacgtCtgctttacac	m5C_41906
chr11	59318510	-	C	833	175	0.21	0	0	T	658	M	0.184	0.239	0	0	0	643.237	tagcggttatCacgtctgctt	m5C_41897
chr11	59318535	-	C	95	26	0.274	0	0	T	69	M	0.194	0.371	0	4.6E-14	0	100.955	ccattccgcaCgcggttccat	m5C_41913
chr11	59319238	+	C	48	17	0.354	0	0	T	31	M	0.234	0.496	0	5.6E-07	0	79.6738	accagaatggCcgagtggtta	m5C_41854
chr11	59319239	+	C	44	13	0.295	0	0	T	31	M	0.182	0.442	0	1.2E-07	0	47.2039	ccagaatggcCgagtggttaa	m5C_41882
chr11	59323904	+	C	222	89	0.401	0	0	T	133	M	0.339	0.467	0	0	0	602.791	cttcaggggcCcggatagctc	m5C_41867
chr11	59323905	+	C	228	60	0.263	0	0	T	168	M	0.21	0.324	0	0	0	252.316	ttcaggggccCggatagctca	m5C_41872
chr11	59323933	+	C	186	38	0.204	0	0	T	148	M	0.153	0.268	0	0	0	115.99	gagcatcagaCttttaatctg	m5C_41850
chr11	59323941	+	C	31	19	0.613	0	0	C	19	M	0.438	0.763	0	3.4E-05	0	166.53	gacttttaatCtgagggtccg	m5C_41883

chr11	59323949	+	C	40	13	0.325	0	0	T	27	M	0.201	0.48	0	1.2E-07	0	52.2192	atctgagggtCcgggggttcaa	m5C_41859
chr11	59323950	+	C	42	17	0.405	0	0	T	25	M	0.27	0.555	0	5.6E-07	0	91.945	tctgagggtcCgggggttcaag	m5C_41869
chr11	59327809	-	C	56	22	0.393	0	0	T	34	M	0.276	0.524	0	6.5E-08	0	121.353	cctgttcgggCggtatctttt	m5C_41903
chr11	59327813	-	C	66	14	0.212	0	0	T	52	M	0.131	0.325	2.2E-16	3.2E-11	2.9E-16	28.6601	agtccctgttCgggcggtatc	m5C_41937
chr11	59327819	-	C	85	20	0.235	0	0	T	65	M	0.158	0.336	0	1.5E-13	0	63.1177	ggttcaagtcCctgttcgggc	m5C_41933
chr11	59327820	-	C	77	41	0.532	0	0	C	41	M	0.422	0.64	0	7.1E-09	0	346.162	gggttcaagtCcctgttcggg	m5C_41919
chr11	59327833	-	C	87	61	0.701	0	0	C	61	M	0.598	0.787	0	1.1E-08	0	729.713	atctgagggtCcagggttcaa	m5C_41918
chr11	59327841	-	C	50	14	0.28	0	0	T	36	M	0.175	0.417	0	1.9E-07	0	48.9272	gactttaatCtgagggtcca	m5C_41899
chr11	59327849	-	C	118	24	0.203	0	0	T	94	M	0.141	0.285	0	0	0	67.5163	gagcatcagaCttttaatctg	m5C_41894
chr11	59327856	-	C	176	36	0.205	0	0	T	140	M	0.152	0.27	0	0	0	109.127	gtcggtagagCatcagacttt	m5C_41929
chr11	59327864	-	C	184	59	0.321	0	0	T	125	M	0.257	0.391	0	0	0	303.819	atagctcagtCggtagagcat	m5C_41914
chr11	59327877	-	C	176	58	0.33	0	0	T	118	M	0.264	0.402	0	0	0	306.693	accgtgagccCggatagctca	m5C_41931
chr11	59333901	-	C	44	23	0.523	0	0	C	23	M	0.379	0.662	0	2.9E-06	0	174.502	gttgggagagCgttagactga	m5C_41927
chr11	59333915	-	C	44	12	0.273	0	0	T	32	M	0.163	0.418	4.4E-16	7.5E-08	5.8E-16	30.1147	gccgaaatagCtcagttggga	m5C_41898
chr11	59333924	-	C	40	9	0.225	0	0	T	31	M	0.123	0.375	6.4E-12	1.2E-08	7.6E-12	12.4041	tccacggcagCcgaaatagct	m5C_41915
chr11	59394451	-	C	36	12	0.333	0	0	T	24	M	0.202	0.497	0	4.6E-06	0	48.5146	tagggcaactCgactgctctg	m5C_41893
chr11	59394453	-	C	55	18	0.327	0	0	T	37	M	0.218	0.459	0	1.7E-08	0	78.5264	cgtagggcaaCtcgactgctc	m5C_41928
chr11	59394463	-	C	150	39	0.26	0	0	T	111	M	0.196	0.336	0	0	0	153.208	gagtggcacaCgtagggcaac	m5C_41901
chr11	59394465	-	C	157	51	0.329	2	0.013	T	104	M	0.26	0.406	0	0	0	265.179	gtgagtggcaCacgtagggca	m5C_41930
chr11	59394476	-	C	132	51	0.386	0	0	T	81	M	0.308	0.472	0	1.1E-16	0	313.784	gggcttctgtCgtgagtggca	m5C_41924
chr11	59394483	-	C	75	26	0.347	0	0	T	49	M	0.249	0.459	0	1.3E-10	0	129.387	caaaaaagggCttctgtcgtg	m5C_41925
chr11	61520163	+	C	31	12	0.387	0	0	T	19	M	0.237	0.562	0	4.6E-06	0	56.9589	tgccgcgcccCcgggccgggc	m5C_41603
chr11	61520164	+	C	32	12	0.375	0	0	T	20	M	0.229	0.547	0	4.6E-06	0	55.0408	gccgcgccccCgggccgggct	m5C_41616
chr11	61569555	-	C	40	13	0.325	0	0	T	27	M	0.201	0.48	0	1.2E-07	0	52.2192	ctactgaaccCagagtcagga	m5C_41677
chr11	61569557	-	C	40	18	0.45	0	0	T	22	M	0.307	0.602	0	2.7E-05	0	110.538	acctactgaaCccagagtcag	m5C_41651
chr11	61569562	-	C	43	12	0.279	0	0	T	31	M	0.167	0.427	2.2E-16	7.5E-08	2.9E-16	31.457	gagggacctaCtgaacccaga	m5C_41637
chr11	61569565	-	C	46	30	0.652	0	0	C	30	M	0.508	0.773	0	1.2E-05	0	304.619	gaggagggacCtactgaaccc	m5C_41663
chr11	61569566	-	C	49	10	0.204	0	0	T	39	M	0.115	0.336	2.1E-12	2.4E-08	2.4E-12	13.4191	agaggagggaCctactgaacc	m5C_41650
chr11	61582689	-	C	46	12	0.261	0	0	T	34	M	0.156	0.403	6.7E-16	7.5E-08	8.7E-16	28.4116	gcggggacggCgattggtccg	m5C_41667
chr11	61582692	-	C	42	26	0.619	0	0	C	26	M	0.468	0.75	0	5.5E-06	0	243.432	gcggcggggaCggcgattggt	m5C_41666
chr11	62294664	-	C	47	11	0.234	0	0	T	36	M	0.136	0.372	3.8E-14	4.4E-08	4.7E-14	20.0856	tgtagatgttCccaaattgga	m5C_58086
chr11	62301485	-	C	69	17	0.246	0	0	T	52	M	0.16	0.36	0	1.8E-10	0	54.3648	cggctgaagtCggaagatgga	m5C_58121
chr11	62301495	-	C	42	11	0.262	0	0	T	31	M	0.153	0.411	8.4E-15	4.4E-08	1.1E-14	23.69	gatcaagccaCggctgaagtc	m5C_58184
chr11	62303945	-	C	58	16	0.276	0	0	T	42	M	0.178	0.402	0	7.3E-09	0	56.8071	cggaatcctgCaaaggtcagt	m5C_58073
chr11	62303948	-	C	58	13	0.224	0	0	T	45	M	0.136	0.347	6.7E-16	1.6E-09	8.7E-16	26.8192	gagcggaatcCtgcaaaggtc	m5C_58068
chr11	62303961	-	C	34	10	0.294	0	0	T	24	M	0.168	0.462	2.3E-14	2E-06	2.9E-14	22.9564	ggcattcgctCctgagcggaa	m5C_58149
chr11	62334898	-	C	35	10	0.286	0	0	T	25	M	0.163	0.451	3.4E-14	2E-06	4.2E-14	21.9991	cgaagtgaaaCtgtgtgagaa	m5C_58012
chr11	62334908	-	C	39	27	0.692	0	0	C	27	M	0.536	0.814	0	0.00015	0	289.324	ctgtcttgggCgaagtgaaac	m5C_58087
chr11	62360198	+	C	81	32	0.395	0	0	T	49	M	0.296	0.504	0	2.3E-11	0	189.235	tagggtggttCagtggtagaa	m5C_57997
chr11	62360216	+	C	55	11	0.2	0	0	T	44	M	0.116	0.324	2.9E-13	4.4E-10	3.6E-13	15.9231	gaattctcgtCtgccaaatac	m5C_57994
chr11	62400706	-	C	87	25	0.287	0	0	T	62	M	0.203	0.39	0	1.8E-12	0	101.428	aaagatgagcCaggagcctgg	m5C_58018
chr11	62400707	-	C	83	44	0.53	0	0	C	44	M	0.424	0.634	0	5.2E-10	0	372.951	gaaagatgagCcaggagcctg	m5C_58140
chr11	62400731	-	C	59	14	0.237	0	0	T	45	M	0.147	0.36	0	2.8E-09	0	41.1446	agaggagactCaggggaaggc	m5C_58143
chr11	62400733	-	C	56	13	0.232	0	0	T	43	M	0.141	0.358	4.4E-16	1.6E-09	5.8E-16	28.1397	ccagaggagaCtcaggggaag	m5C_58069
chr11	62609076	-	C	137	34	0.248	0	0	T	103	M	0.183	0.327	0	0	0	124.695	ggggagagaaCaaccgttgtt	m5C_58152
chr11	62609094	-	C	1146	240	0.209	0	0	T	906	M	0.187	0.234	0	0	0	896.907	acggtgcactCtcccttcggg	m5C_58072
chr11	62609096	-	C	3784	824	0.218	0	0	T	2960	M	0.205	0.231	0	0	0	3376.73	gaacggtgcaCtctcccttcg	m5C_58021
chr11	62609127	-	C	6487	1535	0.237	4	0.001	T	4948	M	0.227	0.247	0	0	0	6956.09	acgcatcgacCtggtattgca	m5C_58188
chr11	62609150	-	C	4651	1030	0.222	5	0.001	T	3616	M	0.21	0.234	0	0	0	4325.67	taggagcttgCtccgtccact	m5C_58126
chr11	62609198	-	C	672	225	0.335	0	0	T	447	M	0.3	0.371	0	0	0	1350.75	tatccgaggaCaatatattaa	m5C_58176
chr11	62609204	-	C	611	166	0.272	0	0	T	445	M	0.238	0.308	0	0	0	789.901	gtcctctatcCgaggacaata	m5C_58161
chr11	62609205	-	C	619	197	0.319	2	0.003	T	420	M	0.284	0.357	0	0	0	1117.84	cgtcctctatCcgaggacaat	m5C_58056
chr11	62609209	-	C	604	213	0.353	0	0	T	391	M	0.316	0.392	0	0	0	1344.39	gatacgtcctCtatccgagga	m5C_58165
chr11	62609211	-	C	562	192	0.342	0	0	T	370	M	0.304	0.382	0	0	0	1165.9	ctgatacgtcCtctatccgag	m5C_58090
chr11	62609212	-	C	557	176	0.316	0	0	T	381	M	0.279	0.356	0	0	0	981.17	tctgatacgtCctctatccga	m5C_58110
chr11	62609215	-	C	487	171	0.351	0	0	T	316	M	0.31	0.395	0	0	0	1060.37	atatctgataCgtcctctatc	m5C_58168
chr11	62609221	-	C	404	200	0.495	0	0	T	204	M	0.447	0.544	0	0	0	1786.29	agtttaatatCtgatacgtcc	m5C_58074
chr11	62609232	-	C	330	112	0.339	0	0	T	218	M	0.29	0.392	0	0	0	650.529	ctgttcttatCagtttaatat	m5C_58055
chr11	62609237	-	C	269	106	0.394	0	0	T	163	M	0.338	0.454	0	0	0	715.59	agtatctgttCttatcagttt	m5C_58091
chr11	62609242	-	C	250	115	0.46	0	0	T	135	M	0.399	0.522	0	0	0	918.366	agtgtagtatCtgttcttatc	m5C_58180
chr11	62609254	-	C	184	82	0.446	0	0	T	102	M	0.376	0.518	0	0	0	616.106	tggctaagatCaagtgtagta	m5C_58020
chr11	62609261	-	C	140	53	0.379	0	0	T	87	M	0.302	0.461	0	0	0	320.632	ggccttttggCtaagatcaag	m5C_58172
chr11	62609268	-	C	88	45	0.511	0	0	C	45	M	0.409	0.613	0	6.4E-10	0	367.788	gcttctcggcCttttggctaa	m5C_58046
chr11	62609269	-	C	84	53	0.631	0	0	C	53	M	0.524	0.726	0	3E-09	0	555.599	cgcttctcggCcttttggcta	m5C_58113
chr11	62609272	-	C	59	47	0.797	0	0	C	47	M	0.677	0.88	0	7.6E-06	0	636.678	tatcgcttctCggccttttgg	m5C_58170
chr11	62609274	-	C	55	42	0.764	0	0	C	42	M	0.637	0.856	0	3.9E-06	0	534.672	ggtatcgcttCtcggcctttt	m5C_58013
chr11	62620390	-	C	543	194	0.357	0	0	T	349	M	0.318	0.398	0	0	0	1234.23	aaggaactgtCtgagggatga	m5C_58028
chr11	62620403	-	C	592	224	0.378	0	0	T	368	M	0.34	0.418	0	0	0	1524.15	agatttgttcCagaaggaact	m5C_58151
chr11	62620404	-	C	577	230	0.399	0	0	T	347	M	0.359	0.439	0	0	0	1653.52	aagatttgttCcagaaggaac	m5C_58162
chr11	62620429	-	C	511	197	0.387	2	0.004	T	312	M	0.346	0.43	0	0	0	1362.12	ctgggactggCtgggggagaa	m5C_58146
chr11	62620433	-	C	507	201	0.397	1	0.002	T	305	M	0.356	0.44	0	0	0	1429.21	cccactgggaCtggctggggg	m5C_58061
chr11	62620443	-	C	488	109	0.223	0	0	T	379	M	0.189	0.262	0	0	0	411.254	taaaggtgaaCccactgggac	m5C_58034
chr11	62620456	-	C	371	77	0.213	9	0.024	T	285	M	0.174	0.258	0	0	0	267.469	gtcccagagcCtgtaaaggtg	m5C_58131
chr11	62620487	-	C	288	62	0.215	0	0	T	226	M	0.172	0.266	0	0	0	212.934	gaaactttcaCatgtcttact	m5C_58147
chr11	62620794	-	C	47	24	0.511	0	0	C	24	M	0.372	0.647	0	3.6E-06	0	178.763	gagctgtgagCctgccttcca	m5C_58050
chr11	62620840	-	C	1047	223	0.213	0	0	T	824	M	0.189	0.239	0	0	0	844.108	gaataccgccCcagtctgatc	m5C_58155
chr11	62620841	-	C	1021	220	0.215	0	0	T	801	M	0.191	0.242	0	0	0	841.924	tgaataccgcCccagtctgat	m5C_58105
chr11	62621135	-	C	86	21	0.244	0	0	T	65	M	0.166	0.345	0	2.6E-13	0	69.5597	atctgattttCtgagaatgat	m5C_58065
chr11	62621384	-	C	360	177	0.492	0	0	T	183	M	0.44	0.543	0	0	0	1558.96	acatgaacacCtgagaaactg	m5C_58122
chr11	62621385	-	C	358	78	0.218	1	0.003	T	279	M	0.179	0.264	0	0	0	278.839	tacatgaacaCctgagaaact	m5C_58137
chr11	62621393	-	C	443	94	0.212	0	0	T	349	M	0.177	0.253	0	0	0	332.146	agtgaaaataCatgaacacct	m5C_58179
chr11	62621404	-	C	433	144	0.333	0	0	T	289	M	0.29	0.378	0	0	0	834.711	ctatgaccgaCagtgaaaata	m5C_58130
chr11	62621407	-	C	445	136	0.306	0	0	T	309	M	0.265	0.35	0	0	0	719.796	tcactatgacCgacagtgaaa	m5C_58098
chr11	62621408	-	C	438	125	0.285	0	0	T	313	M	0.245	0.329	0	0	0	612.759	ctcactatgaCcgacagtgaa	m5C_58175
chr11	62621418	-	C	392	112	0.286	0	0	T	280	M	0.243	0.332	0	0	0	544.861	atcaaactagCtcactatgac	m5C_58024
chr11	62621422	-	C	378	92	0.243	0	0	T	286	M	0.203	0.289	0	0	0	373.24	atgaatcaaaCtagctcacta	m5C_58029
chr11	62622094	-	C	270	110	0.407	0	0	T	160	M	0.35	0.467	0	0	0	771.089	gttctgatggCagttgttgtt	m5C_58128
chr11	62622136	-	C	620	148	0.239	0	0	T	472	M	0.207	0.274	0	0	0	612.209	agctgatgttCtgtgaggtac	m5C_58026
chr11	62622492	-	C	125	92	0.736	0	0	C	92	M	0.653	0.805	0	1.1E-11	0	1200.64	catcttactaCtgagaagtga	m5C_58088
chr11	62622495	-	C	158	62	0.392	0	0	T	96	M	0.32	0.47	0	0	0	396.411	tgtcatcttaCtactgagaag	m5C_58139
chr11	62622499	-	C	164	33	0.201	0	0	T	131	M	0.147	0.269	0	0	0	97.0335	gtgatgtcatCttactactga	m5C_58145
chr11	62622512	-	C	211	75	0.355	0	0	T	136	M	0.294	0.422	0	0	0	440.967	gctgaactttCaagtgatgtc	m5C_58066
chr11	62622516	-	C	246	105	0.427	0	0	T	141	M	0.367	0.489	0	0	0	769.884	tatggctgaaCtttcaagtga	m5C_58079
chr11	62622521	-	C	243	127	0.523	0	0	C	127	M	0.46	0.585	0	0	0	1168.32	aagcatatggCtgaactttca	m5C_58010
chr11	62622528	-	C	218	49	0.225	0	0	T	169	M	0.174	0.285	0	0	0	170.911	aaatgacaagCatatggctga	m5C_58045
chr11	62622532	-	C	222	71	0.32	0	0	T	151	M	0.262	0.384	0	0	0	372.008	cacaaaatgaCaagcatatgg	m5C_58033
chr11	62622540	-	C	217	74	0.341	0	0	T	143	M	0.281	0.406	0	0	0	416.169	atgatgaacaCaaaatgacaa	m5C_58022
chr11	62622542	-	C	220	64	0.292	1	0.005	T	155	M	0.236	0.356	0	0	0	302.078	ccatgatgaaCacaaaatgac	m5C_58051
chr11	62622552	-	C	91	24	0.264	0	0	T	67	M	0.184	0.363	0	1.7E-14	0	88.3703	gataaccactCcatgatgaac	m5C_58042
chr11	62622762	-	C	33	12	0.364	0	0	T	21	M	0.222	0.534	0	4.6E-06	0	53.2487	ctgagtagcaCtgtactgtac	m5C_58076
chr11	62622821	-	C	2015	496	0.247	3	0.001	T	1516	M	0.228	0.266	0	0	0	2263.58	gatgattttaCgaactgaact	m5C_58017

chr11	62623045	-	C	973	202	0.208	0	0	T	771	M	0.183	0.234	0	0	0	740.508	ataaataactCtgaggagatg	m5C_58040
chr11	62623062	-	C	1106	273	0.247	0	0	T	833	M	0.222	0.273	0	0	0	1213.92	tactgagctcCgtgaggataa	m5C_58135
chr11	62623065	-	C	1115	259	0.232	1	0.001	T	855	M	0.209	0.258	0	0	0	1080.73	ctgtactgagCtccgtgagga	m5C_58031
chr11	62623087	-	C	1141	256	0.224	0	0	T	885	M	0.201	0.249	0	0	0	1029.67	tgatgaggacCttttcacaga	m5C_58118
chr11	62623088	-	C	1144	231	0.202	2	0.002	T	911	M	0.18	0.227	0	0	0	831.574	atgatgaggaCcttttcacag	m5C_58153
chr11	62659095	-	C	78	38	0.487	0	0	T	40	M	0.379	0.596	0	3.7E-09	0	288.411	ccctggtggtCtagtggttag	m5C_58173
chr11	65190963	+	C	32	16	0.5	0	0	CT	16	M	0.336	0.664	0	1.7E-05	0	107.618	gaggggtggtCtgaggagtga	m5C_43218
chr11	65266576	+	C	33	9	0.273	0	0	T	24	M	0.151	0.442	7.5E-13	1.2E-06	9E-13	16.4418	cttggaggaaaCcgcagataag	m5C_43029
chr11	65266577	+	C	32	11	0.344	0	0	T	21	M	0.204	0.517	2.2E-16	3.1E-06	2.9E-16	35.1442	ttgaggaaacCgcagataagt	m5C_43158
chr11	65266721	+	C	71	54	0.761	0	0	C	54	M	0.65	0.845	0	6.9E-08	0	701.552	aatatgaagaCttagaagagt	m5C_43111
chr11	65267137	+	C	561	168	0.299	0	0	T	393	M	0.263	0.339	0	0	0	883.786	aagtggaaaaCtggaagacag	m5C_42993
chr11	65267145	+	C	526	120	0.228	0	0	T	406	M	0.194	0.266	0	0	0	466.373	aactggaagaCagaagtacgg	m5C_43230
chr11	65267153	+	C	335	130	0.389	1	0.003	T	204	M	0.338	0.442	0	0	0	880.031	gacagaagtaCgggaaggcga	m5C_43142
chr11	65267161	+	C	419	130	0.316	7	0.017	T	282	M	0.273	0.362	0	0	0	708.599	tacgggaaggCgaagaaaaga	m5C_43242
chr11	65267285	+	C	35	11	0.314	0	0	T	24	M	0.186	0.48	6.7E-16	3.1E-06	8.7E-16	30.9686	aaacaaaaagCtaagggcaaa	m5C_43214
chr11	65267292	+	C	51	22	0.431	0	0	T	29	M	0.305	0.567	0	6.5E-08	0	134.205	aagctaagggCaaaatgtaca	m5C_43091
chr11	65267305	+	C	57	27	0.474	0	0	T	30	M	0.35	0.601	0	2.5E-07	0	188.93	aatgtacaaaCttagaagaaa	m5C_43018
chr11	65267563	+	C	64	28	0.438	0	0	T	36	M	0.323	0.559	0	9.8E-09	0	180.845	agggtgtttaCgtagaccaga	m5C_42980
chr11	65267569	+	C	55	24	0.436	0	0	T	31	M	0.314	0.567	0	1.2E-07	0	150.592	tttacgtagaCcagaaccaat	m5C_43074
chr11	65267575	+	C	52	22	0.423	0	0	T	30	M	0.299	0.558	0	6.5E-08	0	131.419	tagaccagaaCcaatttagaa	m5C_42970
chr11	65267576	+	C	49	16	0.327	0	0	T	33	M	0.212	0.466	0	4E-07	0	67.8634	agaccagaacCaatttagaag	m5C_43026
chr11	65267591	+	C	34	9	0.265	0	0	T	25	M	0.146	0.431	1.1E-12	1.2E-06	1.3E-12	15.7394	tagaagaataCttgaagctag	m5C_42961
chr11	65267598	+	C	44	16	0.364	0	0	T	28	M	0.238	0.511	0	4E-07	0	76.1067	atacttgaagCtagaaggggga	m5C_43054
chr11	65267647	+	C	48	24	0.5	0	0	CT	24	M	0.364	0.636	0	3.6E-06	0	174.668	actaaaaggaCtggtgtaatt	m5C_42946
chr11	65267667	+	C	55	18	0.327	0	0	T	37	M	0.218	0.459	0	1.7E-08	0	78.5264	ttaaaaaaaaCtaaggcagaa	m5C_43103
chr11	65267673	+	C	63	18	0.286	0	0	T	45	M	0.189	0.407	0	2.9E-10	0	68.051	aaaactaaggCagaaggcttt	m5C_42945
chr11	65267757	+	C	36	13	0.361	0	0	T	23	M	0.225	0.524	0	6.7E-06	0	58.436	actttcgtaaCggaagtaatt	m5C_43187
chr11	65267898	+	C	46	14	0.304	0	0	T	32	M	0.191	0.448	0	1.9E-07	0	53.4231	ttcagtgaatCtaggaagaca	m5C_43003
chr11	65267913	+	C	52	15	0.288	0	0	T	37	M	0.183	0.423	0	4.6E-09	0	54.9894	aagacagcagCagacaggatt	m5C_43240
chr11	65267925	+	C	38	8	0.211	0	0	T	30	M	0.111	0.363	1.3E-10	6.8E-07	1.4E-10	8.77246	gacaggattcCaggaaccagt	m5C_42972
chr11	65267931	+	C	65	19	0.292	0	0	T	46	M	0.196	0.412	0	4.5E-10	0	74.4164	attccaggaaCcagtgtttga	m5C_43178
chr11	65267932	+	C	66	15	0.227	0	0	T	51	M	0.143	0.342	0	6E-11	0	42.8553	ttccaggaacCagtgtttgat	m5C_43009
chr11	65267947	+	C	170	40	0.237	1	0.006	T	129	M	0.179	0.306	0	0	0	143.121	tttgatgaagCtaggactgag	m5C_43227
chr11	65267953	+	C	230	68	0.296	0	0	T	162	M	0.24	0.358	0	0	0	326.976	gaagctaggaCtgaggagcaa	m5C_43060
chr11	65267961	+	C	307	64	0.208	0	0	T	243	M	0.167	0.257	0	0	0	213.465	gactgaggagCaagcgagcaa	m5C_43122
chr11	65267973	+	C	375	117	0.312	0	0	T	258	M	0.267	0.361	0	0	0	625.277	agcgagcaagCagcagttcgt	m5C_43151
chr11	65267976	+	C	366	86	0.236	1	0.003	T	279	M	0.195	0.282	0	0	0	335.352	gagcaagcagCagttcgtggt	m5C_43193
chr11	65267981	+	C	375	105	0.28	0	0	T	270	M	0.237	0.327	0	0	0	497.62	agcagcagttCgtggtgaaga	m5C_43105
chr11	65268004	+	C	178	47	0.264	0	0	T	131	M	0.205	0.333	0	0	0	192.477	ggaaaagagtCcaggagccag	m5C_43189
chr11	65268005	+	C	181	75	0.414	0	0	T	106	M	0.345	0.487	0	0	0	517.658	gaaaagagtcCaggagccagt	m5C_43062
chr11	65268012	+	C	214	77	0.36	0	0	T	137	M	0.299	0.426	0	0	0	459.694	gtccaggagcCagtgcgattt	m5C_43022
chr11	65268017	+	C	249	67	0.269	0	0	T	182	M	0.218	0.327	0	0	0	291.863	ggagccagtgCgatttggtga	m5C_43097
chr11	65268034	+	C	232	69	0.297	0	0	T	163	M	0.242	0.359	0	0	0	334.345	gtgaaggaagCtaggaagaag	m5C_43143
chr11	65268052	+	C	41	9	0.22	0	0	T	32	M	0.12	0.367	8.4E-12	1.2E-08	9.9E-12	11.9621	aaggaaggagCgctaacgatt	m5C_43087
chr11	65268074	+	C	63	24	0.387	1	0.016	T	38	M	0.276	0.512	0	3E-09	0	132.405	ggtggtgaagCtaggaaaaag	m5C_42987
chr11	65268089	+	C	50	20	0.4	0	0	T	30	M	0.276	0.538	0	1.4E-06	0	110.433	aaaaaggattCcaggaaggag	m5C_43019
chr11	65268168	+	C	36	8	0.222	0	0	T	28	M	0.117	0.381	7.4E-11	6.8E-07	8.4E-11	9.49676	ggcgagcaggCgttgtgcgta	m5C_43241
chr11	65268175	+	C	43	28	0.651	0	0	C	28	M	0.502	0.776	0	8.1E-06	0	280.96	aggcgttgtgCgtagaggatc	m5C_43017
chr11	65268185	+	C	44	19	0.432	0	0	T	25	M	0.297	0.578	0	1E-06	0	112.794	cgtagaggatCctagaccagc	m5C_43212
chr11	65268186	+	C	39	19	0.487	0	0	T	20	M	0.339	0.638	0	3.4E-05	0	128.687	gtagaggatcCtagaccagca	m5C_43089
chr11	65268191	+	C	31	9	0.29	0	0	T	22	M	0.161	0.466	3.7E-13	1.2E-06	4.4E-13	18.0154	ggatcctagaCcagcatgcca	m5C_43156
chr11	65268192	+	C	31	10	0.323	0	0	T	21	M	0.186	0.499	7.1E-15	2E-06	9E-15	26.2725	gatcctagacCagcatgccag	m5C_43077
chr11	65268448	+	C	32	10	0.312	0	0	T	22	M	0.18	0.486	1.1E-14	2E-06	1.3E-14	25.0836	actgccaagtCctggagaaat	m5C_43192
chr11	65268449	+	C	31	16	0.516	0	0	C	16	M	0.348	0.68	0	1.7E-05	0	111.488	ctgccaagtcCtggagaaata	m5C_43005
chr11	65268671	+	C	36	17	0.472	0	0	T	19	M	0.32	0.63	0	2.1E-05	0	108.752	ggatgtgtaaCtgaggcgggg	m5C_43118
chr11	65269107	+	C	49	21	0.429	0	0	T	28	M	0.3	0.567	0	1.8E-06	0	126.097	aaggagtgtaCcgctgtgctg	m5C_43239
chr11	65269115	+	C	47	31	0.66	0	0	C	31	M	0.517	0.778	0	1.4E-05	0	320.358	taccgctgtgCtgttggcacg	m5C_43233
chr11	65269124	+	C	37	12	0.324	0	0	T	25	M	0.196	0.485	0	4.6E-06	0	47.1194	gctgttggcaCgaacaccttc	m5C_43013
chr11	65269134	+	C	43	15	0.349	0	0	T	28	M	0.224	0.498	0	2.8E-07	0	67.2552	cgaacaccttCagggactgga	m5C_43145
chr11	65269140	+	C	35	11	0.314	0	0	T	24	M	0.186	0.48	6.7E-16	3.1E-06	8.7E-16	30.9686	ccttcagggaCtggagctgct	m5C_42994
chr11	65269548	+	C	36	12	0.333	0	0	T	24	M	0.202	0.497	0	4.6E-06	0	48.5146	taaatatgagCcactgggtgt	m5C_42967
chr11	65269708	+	C	46	10	0.217	0	0	T	36	M	0.123	0.356	9.7E-13	2.4E-08	1.2E-12	14.7309	atgggaaatgCaaaagttgtt	m5C_43162
chr11	65269740	+	C	83	35	0.422	0	0	T	48	M	0.321	0.529	0	5.6E-11	0	224.86	gtgtgtggttCtcttttggaa	m5C_43043
chr11	65269758	+	C	33	10	0.303	0	0	T	23	M	0.174	0.473	1.6E-14	2E-06	2E-14	23.982	gaatttttttCaggtgattta	m5C_43028
chr11	65269795	+	C	36	17	0.472	0	0	T	19	M	0.32	0.63	0	2.1E-05	0	108.752	actatagaaaCtgcagagcaa	m5C_43085
chr11	65269798	+	C	44	10	0.227	0	0	T	34	M	0.128	0.37	5.7E-13	2.4E-08	6.8E-13	15.7274	atagaaactgCagagcaaagg	m5C_42962
chr11	65269803	+	C	62	36	0.581	0	0	C	36	M	0.457	0.695	0	6.5E-08	0	328.791	aactgcagagCaaaggaagtg	m5C_42995
chr11	65269815	+	C	118	52	0.441	0	0	T	66	M	0.354	0.531	0	1.4E-13	0	368.548	aaggaagtggCttaatgatcc	m5C_42975
chr11	65270066	+	C	69	39	0.565	0	0	C	39	M	0.448	0.676	0	1.2E-07	0	349.348	agagggtgggCttttgttgat	m5C_43232
chr11	65270177	+	C	60	12	0.2	0	0	T	48	M	0.118	0.318	3.3E-14	7.6E-12	4.2E-14	19.13	gcagagaaaaCagctccttgg	m5C_42955
chr11	65270183	+	C	62	33	0.532	0	0	C	33	M	0.41	0.651	0	3.4E-08	0	270.493	aaaacagctcCttggtgaatt	m5C_42971
chr11	65270511	+	C	41	20	0.488	0	0	T	21	M	0.343	0.635	0	1.4E-06	0	137.017	taaaagaagcCgaaataaatg	m5C_43064
chr11	65270538	+	C	144	44	0.306	0	0	T	100	M	0.236	0.385	0	0	0	207.84	gagttgggatCaagtggattg	m5C_43216
chr11	65270555	+	C	159	49	0.308	0	0	T	110	M	0.242	0.384	0	0	0	236.804	attgaggaggCtgtgctgtgt	m5C_43114
chr11	65270560	+	C	122	57	0.471	1	0.008	T	64	M	0.384	0.56	0	1.6E-14	0	438.213	ggaggctgtgCtgtgtgccaa	m5C_43190
chr11	65270568	+	C	91	30	0.33	0	0	T	61	M	0.242	0.431	0	2.5E-13	0	145.025	tgctgtgtgcCaatgtttcgt	m5C_43109
chr11	65270576	+	C	63	19	0.302	0	0	T	44	M	0.202	0.424	0	4.5E-10	0	76.9026	gccaatgtttCgtttgcctca	m5C_43197
chr11	65270585	+	C	42	16	0.381	0	0	T	26	M	0.25	0.532	0	4E-07	0	79.9985	tcgtttgcctCagacaggtat	m5C_43146
chr11	65270953	+	C	32	16	0.5	0	0	CT	16	M	0.336	0.664	0	1.7E-05	0	107.618	atttgtttagCattgaatctc	m5C_43115
chr11	65270970	+	C	40	15	0.375	0	0	T	25	M	0.242	0.53	0	2.8E-07	0	72.6683	tctctgaaggCtctatgaaag	m5C_43182
chr11	65271220	+	C	35	17	0.486	0	0	T	18	M	0.33	0.644	0	2.1E-05	0	112.18	tgcgaacactCtttaatggac	m5C_43069
chr11	65271255	+	C	189	55	0.291	0	0	T	134	M	0.231	0.359	0	0	0	254.016	agcggaagaaCgaatgtaact	m5C_43154
chr11	65271281	+	C	41	8	0.2	1	0.024	T	32	M	0.105	0.348	2.1E-10	5.5E-09	2.3E-10	8.13503	gcaggaaagaCaaattttatt	m5C_43166
chr11	65271392	+	C	39	8	0.205	0	0	T	31	M	0.108	0.355	1.6E-10	6.8E-07	1.8E-10	8.44384	agattatgatCagagtaaaag	m5C_43086
chr11	65271646	+	C	31	16	0.516	0	0	C	16	M	0.348	0.68	0	1.7E-05	0	111.488	ttttgaattgCatatgagtgc	m5C_43120
chr11	65271901	+	C	54	14	0.259	0	0	T	40	M	0.161	0.389	0	2.8E-09	0	45.1318	cattgtgaaaCgactggagta	m5C_43191
chr11	65271904	+	C	55	14	0.255	0	0	T	41	M	0.158	0.383	0	2.8E-09	0	44.2735	tgtgaaacgaCtggagtatga	m5C_43081
chr11	65271968	+	C	39	10	0.256	0	0	T	29	M	0.146	0.411	1.3E-13	2E-06	1.6E-13	18.7753	ggaaaaaaatcCagctgagtga	m5C_43205
chr11	65271971	+	C	38	20	0.526	0	0	C	20	M	0.373	0.675	0	4.3E-05	0	149.035	aaaaatccagCtgagtgataa	m5C_43099
chr11	65272021	+	C	31	9	0.29	0	0	T	22	M	0.161	0.466	3.7E-13	1.2E-06	4.4E-13	18.0154	tttaagcagtCgtatttgtga	m5C_43245
chr11	65272408	+	C	72	19	0.264	0	0	T	53	M	0.176	0.376	0	6.9E-12	0	66.8558	gattgggaacCactagttctt	m5C_43138
chr11	65272434	+	C	31	11	0.355	0	0	T	20	M	0.211	0.531	1.1E-16	3.1E-06	1.5E-16	37.0595	tggtattcttCagactataga	m5C_42953
chr11	65272696	+	C	38	18	0.474	0	0	T	20	M	0.325	0.627	0	2.7E-05	0	116.924	taaagtaggaCaaccatggag	m5C_43219
chr11	65273102	+	C	52	34	0.654	0	0	C	34	M	0.518	0.768	0	1.1E-06	0	352.262	tagcttggatCcttgtgggca	m5C_43112
chr11	65273117	+	C	32	13	0.406	0	0	T	19	M	0.255	0.577	0	6.7E-06	0	66.3505	tgggcatgatCcataatcggt	m5C_43153
chr11	65273147	+	C	104	47	0.452	0	0	T	57	M	0.36	0.548	0	1.3E-12	0	338.106	acgatggtgtCgaggtctttg	m5C_43058
chr11	65273153	+	C	125	45	0.36	0	0	T	80	M	0.281	0.447	0	5.6E-16	0	253.067	gtgtcgaggtCtttggtgggt	m5C_43184
chr11	65273168	+	C	76	34	0.447	0	0	T	42	M	0.341	0.559	0	1.4E-09	0	231.747	gtggggttgaaCtatgttagaa	m5C_42944
chr11	65273183	+	C	58	14	0.241	0	0	T	44	M	0.15	0.365	0	2.8E-09	0	41.8845	ttagaaaaggCcattaatttg	m5C_43117
chr11	65273184	+	C	52	16	0.308	0	0	T	36	M	0.199	0.443	0	7.3E-09	0	63.7266	tagaaaaggcCattaatttgc	m5C_43217
chr11	65273258	+	C	47	16	0.34	0	0	T	31	M	0.222	0.483	0	4E-07	0	70.9352	aatacttatcCagtgactaaa	m5C_43207

chr11	65273264	+	C	46	23	0.5	0	0	CT	23	M	0.361	0.639	0	2.9E-06	0	166.146	tatccagtgaCtaaaaccaac	m5C_43231
chr11	65273274	+	C	57	18	0.316	0	0	T	39	M	0.21	0.445	0	1.7E-08	0	75.6149	ctaaaaccaaCttaaaccagt	m5C_43208
chr11	65273330	+	C	588	188	0.32	0	0	T	400	M	0.283	0.358	0	0	0	1065.23	tgggtgggaaCatgtaacttg	m5C_43052
chr11	65273337	+	C	573	185	0.323	1	0.002	T	387	M	0.286	0.363	0	0	0	1059.6	gaacatgtaaCttgtagactg	m5C_43235
chr11	65273345	+	C	127	46	0.362	0	0	T	81	M	0.284	0.449	0	6.7E-16	0	261.091	aacttgtagaCtggagaagat	m5C_43130
chr11	65273359	+	C	52	15	0.288	0	0	T	37	M	0.183	0.423	0	4.6E-09	0	54.9894	agaagataggCatttgagtgg	m5C_43236
chr11	65273370	+	C	69	29	0.42	0	0	T	40	M	0.311	0.538	0	1.3E-08	0	180.414	atttgagtggCtgagagggct	m5C_43023
chr11	65273379	+	C	81	44	0.543	0	0	C	44	M	0.435	0.647	0	5.2E-10	0	383.01	gctgagagggCttttgggtgg	m5C_43010
chr11	65273605	+	C	59	33	0.559	0	0	C	33	M	0.433	0.678	0	8.8E-07	0	285.708	gtggttggcaCtcctggtttc	m5C_43032
chr11	65273608	+	C	62	20	0.323	0	0	T	42	M	0.22	0.446	0	6.9E-10	0	87.8173	gttggcactcCtggtttccag	m5C_42940
chr11	65273615	+	C	61	19	0.311	0	0	T	42	M	0.209	0.436	0	4.5E-10	0	79.5616	ctcctggtttCcaggacgggg	m5C_42960
chr11	65273621	+	C	48	21	0.438	0	0	T	27	M	0.307	0.577	0	1.8E-06	0	128.944	gtttccaggaCgggggttcaaa	m5C_43045
chr11	65364151	-	C	31	15	0.484	0	0	T	16	M	0.32	0.652	0	1.2E-05	0	95.91	gtctgcagttCaggggcaggg	m5C_43302
chr11	65661664	+	C	70	23	0.329	0	0	T	47	M	0.23	0.445	0	4.3E-11	0	105.794	catggtggccCagtggtagga	m5C_43049
chr11	65831149	+	C	31	10	0.323	0	0	T	21	M	0.186	0.499	7.1E-15	2E-06	9E-15	26.2725	ggaatggagaCcccctgaactc	m5C_43048
chr11	65831159	+	C	31	17	0.548	0	0	C	17	M	0.378	0.708	0	2.1E-05	0	128.424	cccctgaactCattgagctga	m5C_43206
chr11	66115593	+	C	353	127	0.36	0	0	T	226	M	0.311	0.411	0	0	0	791.117	tcacgtaggaCgaggtggccg	m5C_41445
chr11	66115601	+	C	426	129	0.303	0	0	T	297	M	0.261	0.348	0	0	0	673.653	gacgaggtggCcgagtggtta	m5C_41427
chr11	66115602	+	C	425	92	0.216	0	0	T	333	M	0.18	0.258	0	0	0	331.105	acgaggtggcCgagtggttaa	m5C_41452
chr11	66115615	+	C	386	89	0.231	0	0	T	297	M	0.191	0.275	0	0	0	340.566	gtggttaaggCgatggactgc	m5C_41470
chr11	66115622	+	C	329	78	0.237	0	0	T	251	M	0.194	0.286	0	0	0	303.148	aggcgatggaCtgctaatcca	m5C_41426
chr11	66115630	+	C	267	59	0.222	1	0.004	T	207	M	0.176	0.275	0	0	0	207.722	gactgctaatCcattgtgctt	m5C_41411
chr11	66115647	+	C	83	27	0.325	0	0	T	56	M	0.234	0.432	0	4E-12	0	126.464	gctttgcacgCgtggttcga	m5C_41485
chr11	66115655	+	C	90	22	0.244	0	0	T	68	M	0.167	0.342	0	5.8E-15	0	73.6237	cgcgtgggttCgaatcccatc	m5C_41438
chr11	66115665	+	C	92	21	0.231	1	0.011	T	70	M	0.156	0.327	0	3.1E-15	0	65.5958	cgaatcccatCctcgtcggcc	m5C_41403
chr11	66115668	+	C	88	22	0.25	0	0	T	66	M	0.171	0.35	0	4.4E-13	0	75.3641	atcccatcctCgtcggccctg	m5C_41432
chr11	67353889	+	C	36	11	0.306	0	0	T	25	M	0.18	0.469	1E-15	3.1E-06	1.3E-15	29.7077	acctgctggaCttgctgctga	m5C_41430
chr11	67353917	+	C	39	11	0.282	0	0	T	28	M	0.165	0.438	3.1E-15	3.1E-06	4E-15	26.4001	ggtcctagccCctggctgcct	m5C_41439
chr11	67353925	+	C	38	27	0.711	0	0	C	27	M	0.552	0.83	0	0.00015	0	298.31	cccctggctgCctggatgcgt	m5C_41418
chr11	67353926	+	C	34	24	0.706	0	0	C	24	M	0.538	0.832	0	9.1E-05	0	258.388	ccctggctgcCtggatgcgtt	m5C_41480
chr11	67353933	+	C	34	7	0.206	0	0	T	27	M	0.103	0.368	1.4E-09	3.4E-07	1.6E-09	6.4054	tgcctggatgCgttccccctg	m5C_41434
chr11	68227660	-	C	32	22	0.688	0	0	C	22	M	0.514	0.82	0	6.3E-05	0	226.305	gaggagcgttCgtttagtatg	m5C_41400
chr11	68227664	-	C	35	13	0.371	0	0	T	22	M	0.232	0.537	0	6.7E-06	0	60.2306	agtggaggagCgttcgtttag	m5C_41395
chr11	70275224	+	C	32	13	0.406	0	0	T	19	M	0.255	0.577	0	6.7E-06	0	66.3505	agcaggaggaCaggcggaagg	m5C_41554
chr11	72287334	-	C	44	10	0.227	0	0	T	34	M	0.128	0.37	5.7E-13	2.4E-08	6.8E-13	15.7274	attctgtgtgCgttgtggggt	m5C_41735
chr11	72287341	-	C	40	16	0.4	0	0	T	24	M	0.263	0.554	0	1.7E-05	0	84.314	ctgtttcattCtgtgtgcgtt	m5C_41738
chr11	72287345	-	C	44	9	0.205	0	0	T	35	M	0.112	0.345	1.8E-11	1.2E-08	2.1E-11	10.7806	ttctctgtttCattctgtgtg	m5C_41747
chr11	72287351	-	C	31	13	0.419	0	0	T	18	M	0.264	0.592	0	6.7E-06	0	68.6798	ctctttttctCtgtttcattc	m5C_41761
chr11	72351666	-	C	63	18	0.286	0	0	T	45	M	0.189	0.407	0	2.9E-10	0	68.051	gtgtttgcagCctaggtggct	m5C_41745
chr11	74860448	-	C	40	16	0.4	0	0	T	24	M	0.263	0.554	0	1.7E-05	0	84.314	gcttgtctggCtaagtttatc	m5C_42241
chr11	75111455	+	C	255	88	0.345	0	0	T	167	M	0.289	0.405	0	0	0	509.406	gagatgatgaCgagtctgact	m5C_42230
chr11	75111460	+	C	185	58	0.314	0	0	T	127	M	0.251	0.384	0	0	0	291.195	gatgacgagtCtgacttgggg	m5C_42215
chr11	75111464	+	C	173	45	0.26	0	0	T	128	M	0.2	0.33	0	0	0	180.412	acgagtctgaCttggggatgt	m5C_42169
chr11	75111484	+	C	74	24	0.324	0	0	T	50	M	0.229	0.437	0	6.4E-11	0	109.747	ttctctttgcCcaggtggcct	m5C_42186
chr11	75111485	+	C	69	29	0.42	0	0	T	40	M	0.311	0.538	0	1.3E-08	0	180.414	tctctttgccCaggtggccta	m5C_42154
chr11	75111492	+	C	57	17	0.298	0	0	T	40	M	0.195	0.427	0	1.1E-08	0	66.4101	gcccaggtggCctactctgtg	m5C_42202
chr11	75111493	+	C	57	12	0.211	0	0	T	45	M	0.125	0.333	1.6E-14	8.8E-10	2E-14	20.643	cccaggtggcCtactctgtgc	m5C_42231
chr11	75111498	+	C	38	18	0.474	0	0	T	20	M	0.325	0.627	0	2.7E-05	0	116.924	gtggcctactCtgtgctgcgt	m5C_42152
chr11	75111506	+	C	34	15	0.441	0	0	T	19	M	0.289	0.605	0	1.2E-05	0	86.6497	ctctgtgctgCgttctgtggc	m5C_42192
chr11	75111510	+	C	35	18	0.514	0	0	C	18	M	0.356	0.67	0	2.7E-05	0	128.047	gtgctgcgttCtgtggcacag	m5C_42173
chr11	75111516	+	C	34	9	0.265	0	0	T	25	M	0.146	0.431	1.1E-12	1.2E-06	1.3E-12	15.7394	cgttctgtggCacagtttaaa	m5C_42183
chr11	75111518	+	C	35	17	0.486	0	0	T	18	M	0.33	0.644	0	2.1E-05	0	112.18	ttctgtggcaCagtttaaaga	m5C_42208
chr11	75115465	+	C	92	19	0.207	0	0	T	73	M	0.136	0.3	0	8.9E-16	0	51.8283	tctgtgtaccCttcagtgatg	m5C_42200
chr11	75115479	+	C	358	75	0.209	0	0	T	283	M	0.171	0.255	0	0	0	255.805	agtgatgacaCgatgacgagt	m5C_42178
chr11	75115485	+	C	354	124	0.35	0	0	T	230	M	0.302	0.401	0	0	0	750.038	gacacgatgaCgagtcagaaa	m5C_42172
chr11	75115510	+	C	123	28	0.228	0	0	T	95	M	0.162	0.309	0	0	0	90.974	acgtcctgctCttggtccttg	m5C_42181
chr11	75115517	+	C	89	19	0.213	0	0	T	70	M	0.141	0.31	0	8.4E-14	0	53.6358	gctcttggtcCttgtcagtgc	m5C_42193
chr11	75115528	+	C	76	24	0.316	0	0	T	52	M	0.222	0.427	0	6.4E-11	0	106.707	ttgtcagtgcCatgttctgtg	m5C_42180
chr11	75115534	+	C	81	21	0.259	0	0	T	60	M	0.176	0.364	0	2.6E-13	0	74.0352	gtgccatgttCtgtggtgctg	m5C_42177
chr11	75115542	+	C	113	33	0.292	0	0	T	80	M	0.216	0.382	0	2.2E-16	0	142.654	ttctgtggtgCtgtgcacgag	m5C_42203
chr11	75115555	+	C	138	35	0.255	1	0.007	T	102	M	0.19	0.334	0	0	0	132.865	tgcacgagttCcttggcaga	m5C_42164
chr11	75115572	+	C	130	50	0.385	0	0	T	80	M	0.305	0.47	0	2.4E-15	0	305.438	cagaagtgtcCtatttattga	m5C_42201
chr11	75115584	+	C	214	45	0.21	0	0	T	169	M	0.161	0.27	0	0	0	144.93	atttattgatCgatttagagg	m5C_42198
chr11	75115602	+	C	230	62	0.27	0	0	T	168	M	0.216	0.33	0	0	0	268.275	aggcatttgtCtgagaagggt	m5C_42151
chr11	75221890	-	C	36	21	0.583	0	0	C	21	M	0.422	0.729	0	5.2E-05	0	177.24	gagagtgaggCaattccagga	m5C_42235
chr11	75380203	+	C	37	25	0.676	0	0	C	25	M	0.515	0.804	0	0.00011	0	257.317	cacctgctgtCgtcagcttgc	m5C_42195
chr11	75380206	+	C	38	27	0.711	0	0	C	27	M	0.552	0.83	0	0.00015	0	298.31	ctgctgtcgtCagcttgcgct	m5C_42224
chr11	75380209	+	C	37	28	0.757	0	0	C	28	M	0.599	0.866	0	0.00017	0	335.341	ctgtcgtcagCttgcgctgct	m5C_42179
chr11	75528484	+	C	43	9	0.209	0	0	T	34	M	0.114	0.352	1.4E-11	1.2E-08	1.6E-11	11.1522	tggattggtgCcgagggatat	m5C_42189
chr11	75946561	+	C	67	16	0.239	0	0	T	51	M	0.153	0.353	0	1.1E-10	0	48.8576	agcgacggctCgttggtctag	m5C_42150
chr11	75946568	+	C	91	23	0.253	0	0	T	68	M	0.175	0.351	0	1E-14	0	80.3739	gctcgttggtCtaggggtatg	m5C_42209
chr11	75946582	+	C	96	24	0.25	0	0	T	72	M	0.174	0.345	0	1.7E-14	0	83.5865	gggtatgattCtcgcttaggg	m5C_42232
chr11	75946595	+	C	119	37	0.311	0	0	T	82	M	0.235	0.399	0	1.4E-15	0	173.729	gcttagggtgCgagaggtccc	m5C_42157
chr11	75946603	+	C	149	48	0.324	1	0.007	T	100	M	0.254	0.403	0	0	0	244.01	tgcgagaggtCccgggttcaa	m5C_42188
chr11	75946605	+	C	152	59	0.388	0	0	T	93	M	0.314	0.467	0	0	0	370.935	cgagaggtccCgggttcaaat	m5C_42156
chr11	75946627	+	C	177	37	0.209	0	0	T	140	M	0.156	0.275	0	0	0	115.169	ccggacgagcCctcattttct	m5C_42160
chr11	75946628	+	C	159	33	0.208	0	0	T	126	M	0.152	0.277	0	0	0	100.172	cggacgagccCtcattttctt	m5C_42182
chr11	75946913	-	C	59	29	0.492	0	0	T	30	M	0.368	0.616	0	3.9E-07	0	213.693	gtatgattctCggtttgggtc	m5C_42249
chr11	75946915	-	C	58	44	0.759	0	0	C	44	M	0.635	0.85	0	5.1E-06	0	558.534	gggtatgattCtcggtttggg	m5C_42240
chr11	77354073	+	C	31	13	0.419	0	0	T	18	M	0.264	0.592	0	6.7E-06	0	68.6798	gagctcggtgCtgtagctcag	m5C_42283
chr11	77354081	+	C	32	7	0.219	0	0	T	25	M	0.11	0.388	8.5E-10	3.4E-07	9.5E-10	6.99971	tgctgtagctCagcagctaag	m5C_42277
chr11	77354087	+	C	34	8	0.235	0	0	T	26	M	0.124	0.4	4.2E-11	6.8E-07	4.8E-11	10.3259	agctcagcagCtaaggctttg	m5C_42281
chr11	77354099	+	C	31	13	0.419	0	0	T	18	M	0.264	0.592	0	6.7E-06	0	68.6798	aaggctttgcCattttacagt	m5C_42288
chr11	85195031	+	C	53	35	0.66	0	0	C	35	M	0.526	0.773	0	1.3E-06	0	368.162	ttccaggagtCgggttgcttg	m5C_41967
chr11	85195038	+	C	64	25	0.391	0	0	T	39	M	0.281	0.513	0	4.1E-09	0	140.277	agtcgggttgCttgggaatgc	m5C_42008
chr11	85195048	+	C	63	42	0.667	0	0	C	42	M	0.544	0.771	0	2E-07	0	456.683	cttgggaatgCagcccaaagc	m5C_41981
chr11	85195052	+	C	55	32	0.582	0	0	C	32	M	0.45	0.703	0	7.2E-07	0	288.21	ggaatgcagcCcaaagcgggt	m5C_42001
chr11	85195053	+	C	54	36	0.667	0	0	C	36	M	0.534	0.778	0	1.5E-06	0	384.197	gaatgcagccCaaagcgggtg	m5C_42019
chr11	85195058	+	C	57	36	0.632	0	0	C	36	M	0.502	0.745	0	1.5E-06	0	361.278	cagcccaaagCgggtggtaaa	m5C_41987
chr11	85195204	+	C	88	28	0.318	0	0	T	60	M	0.23	0.421	0	5.8E-12	0	128.926	tccgcgcagtCcgcccggagg	m5C_41998
chr11	85195205	+	C	92	21	0.228	0	0	T	71	M	0.154	0.324	0	3.1E-15	0	64.8567	ccgcgcagtcCgcccggagga	m5C_41959
chr11	85195207	+	C	110	41	0.373	0	0	T	69	M	0.288	0.466	0	2.4E-13	0	236.238	gcgcagtccgCccggaggatt	m5C_41950
chr11	85195209	+	C	103	29	0.287	2	0.019	T	72	M	0.208	0.382	0	2.9E-15	0	120.626	gcagtccgccCggaggattca	m5C_41962
chr11	85195218	+	C	55	13	0.236	0	0	T	42	M	0.144	0.363	2.2E-16	1.6E-09	2.9E-16	29.2353	ccggaggattCaacccggtgg	m5C_41997
chr11	85195221	+	C	55	27	0.491	0	0	T	28	M	0.364	0.619	0	2.5E-07	0	196.433	gaggattcaaCccggtggcgg	m5C_41943
chr11	85195228	-	C	35	17	0.486	0	0	T	18	M	0.33	0.644	0	2.1E-05	0	112.18	gaaagatccgCcaccgggttg	m5C_42091
chr11	85195231	-	C	50	20	0.4	0	0	T	30	M	0.276	0.538	0	1.4E-06	0	110.433	cgggaaagatCcgccaccggg	m5C_42040
chr11	85195248	-	C	33	11	0.333	0	0	T	22	M	0.198	0.504	2.2E-16	3.1E-06	2.9E-16	34.0074	tcgggaggaaCgggggggcggg	m5C_42086
chr11	87524701	-	C	49	36	0.735	0	0	C	36	M	0.597	0.838	0	2.9E-05	0	430.11	tttgtgcagCcaccaccgtc	m5C_41820
chr11	87606646	-	C	51	18	0.353	0	0	T	33	M	0.236	0.49	0	1.7E-08	0	85.0835	cagtggccagCacaaagtggg	m5C_41822
chr11	87833170	+	C	55	31	0.564	0	0	C	31	M	0.433	0.686	0	5.9E-07	0	268.271	ttaggctggtCtgaaggtaat	m5C_41808

chr11	91818562	-	C	31	8	0.258	0	0	T	23	M	0.137	0.432	1.7E-11	6.8E-07	1.9E-11	11.8182	agttaggattCagtgctctcc	m5C_42131
chr11	91818577	-	C	110	42	0.382	0	0	T	68	M	0.296	0.475	0	3.2E-13	0	249.035	ccctggtggtCtagtagttag	m5C_42129
chr11	91818587	-	C	34	10	0.294	0	0	T	24	M	0.168	0.462	2.3E-14	2E-06	2.9E-14	22.9564	ctaggcatttCcctggtggtc	m5C_42133
chr11	93274701	-	C	33	25	0.758	0	0	C	25	M	0.59	0.872	0	0.00011	0	294.875	attggtcctgCtggggtttgt	m5C_44485
chr11	93274704	-	C	35	18	0.514	0	0	C	18	M	0.356	0.67	0	2.7E-05	0	128.047	tggattggtcCtgctggggtt	m5C_44474
chr11	93274705	-	C	35	16	0.457	0	0	T	19	M	0.305	0.618	0	1.7E-05	0	97.4905	ttggattggtCctgctggggt	m5C_44503
chr11	93463694	-	C	50	29	0.58	0	0	C	29	M	0.442	0.706	0	3.9E-07	0	256.553	ctatgttttcCtgaggagata	m5C_44476
chr11	93463704	-	C	49	34	0.694	0	0	C	34	M	0.555	0.805	0	2.2E-05	0	377.209	atgtttggtgCtatgttttcc	m5C_44496
chr11	93463720	-	C	44	13	0.295	0	0	T	31	M	0.182	0.442	0	1.2E-07	0	47.2039	agccatagcaCtcctaatgtt	m5C_44464
chr11	93463754	-	C	590	126	0.214	0	0	T	464	M	0.182	0.248	0	0	0	459.644	acgctgtgacCaaagatggaa	m5C_44471
chr11	93463763	-	C	794	189	0.238	0	0	T	605	M	0.21	0.269	0	0	0	792.733	gagctcttaaCgctgtgacca	m5C_44493
chr11	93463768	-	C	889	180	0.203	1	0.001	T	708	M	0.178	0.23	0	0	0	639.243	acccagagctCttaacgctgt	m5C_44488
chr11	93463770	-	C	946	286	0.306	10	0.011	T	650	M	0.277	0.336	0	0	0	1583.81	aaacccagagCtcttaacgct	m5C_44449
chr11	93463785	-	C	1265	434	0.344	4	0.003	T	827	M	0.318	0.371	0	0	0	2764.21	gcttatgaggCtgtgaaaccc	m5C_44461
chr11	93463794	-	C	1274	264	0.207	1	0.001	T	1009	M	0.186	0.231	0	0	0	982.125	tcaaagagggCttatgaggct	m5C_44470
chr11	93464179	-	C	32	17	0.531	0	0	C	17	M	0.364	0.691	0	2.1E-05	0	123.927	tttctatctgCtactttgcta	m5C_44492
chr11	93464186	-	C	39	13	0.333	0	0	T	26	M	0.206	0.49	0	6.7E-06	0	53.6452	agtaggttttCtatctgctac	m5C_44456
chr11	93464212	-	C	57	17	0.298	0	0	T	40	M	0.195	0.427	0	1.1E-08	0	66.4101	tggacatgacCataaaaaaat	m5C_44500
chr11	93464250	-	C	129	36	0.279	0	0	T	93	M	0.209	0.362	0	0	0	150.419	attaccaaggCttttagaatg	m5C_44467
chr11	93464255	-	C	124	28	0.226	0	0	T	96	M	0.161	0.307	0	0	0	90.216	tggtcattacCaaggctttta	m5C_44491
chr11	93464672	-	C	405	88	0.218	1	0.002	T	316	M	0.18	0.261	0	0	0	317.383	cctttctgaaCatccatttac	m5C_44489
chr11	93464681	-	C	912	232	0.254	0	0	T	680	M	0.227	0.284	0	0	0	1054.17	tgaaaatggcCtttctgaaca	m5C_44450
chr11	93464696	-	C	1055	216	0.209	22	0.021	T	817	M	0.185	0.235	0	0	0	800.93	tctgaaggggCtgaatgaaaa	m5C_44487
chr11	93464705	-	C	1107	241	0.218	2	0.002	T	864	M	0.195	0.243	0	0	0	938.695	ttcaactgctCtgaaggggct	m5C_44453
chr11	93465214	-	C	72	17	0.236	0	0	T	55	M	0.153	0.346	0	2.2E-12	0	52.0084	atgcttccatCacagtatgca	m5C_44497
chr11	93465241	-	C	156	35	0.224	0	0	T	121	M	0.166	0.296	0	0	0	116.182	gttaatgtggCtaaattgaga	m5C_44475
chr11	93465268	-	C	366	94	0.258	1	0.003	T	271	M	0.215	0.305	0	0	0	404.88	cactgttgatCatggtgtcca	m5C_44504
chr11	93465276	-	C	376	108	0.287	0	0	T	268	M	0.244	0.335	0	0	0	526.676	agaatgggcaCtgttgatcat	m5C_44477
chr11	93465549	-	C	33	13	0.394	0	0	T	20	M	0.247	0.563	0	6.7E-06	0	64.1755	cttgttaaacCtggtatttct	m5C_44447
chr11	93465559	-	C	37	16	0.432	0	0	T	21	M	0.287	0.591	0	1.7E-05	0	91.7488	tggctttgtgCttgttaaacc	m5C_44448
chr11	93465566	-	C	47	15	0.319	0	0	T	32	M	0.204	0.462	0	2.8E-07	0	61.1856	gttttcttggCtttgtgcttg	m5C_44484
chr11	93465571	-	C	75	20	0.267	0	0	T	55	M	0.18	0.376	0	1.1E-11	0	71.9047	cgttggttttCttggctttgt	m5C_44457
chr11	93465594	-	C	167	37	0.222	0	0	T	130	M	0.165	0.29	0	0	0	122.269	tatagaaattCagtatctggc	m5C_44495
chr11	93465605	-	C	257	81	0.316	1	0.004	T	175	M	0.263	0.376	0	0	0	425.261	aaggtcagtgCtatagaaatt	m5C_44451
chr11	93465610	-	C	257	90	0.35	0	0	T	167	M	0.294	0.41	0	0	0	530.041	gttcaaaggtCagtgctatag	m5C_44436
chr11	93465640	-	C	395	93	0.235	0	0	T	302	M	0.196	0.28	0	0	0	365.07	ctgcactcagCagtttacacc	m5C_44455
chr11	93465650	-	C	413	116	0.281	0	0	T	297	M	0.24	0.326	0	0	0	556.102	tgcatggtatCtgcactcagc	m5C_44501
chr11	93465661	-	C	374	87	0.233	0	0	T	287	M	0.193	0.278	0	0	0	335.211	cataattgcaCtgcatggtat	m5C_44459
chr11	93466398	-	C	112	24	0.214	0	0	T	88	M	0.148	0.299	0	0	0	71.251	ttaattctgaCagagctggat	m5C_44438
chr11	93466409	-	C	145	40	0.276	0	0	T	105	M	0.21	0.354	0	0	0	167.688	tgaactaaaaCttaattctga	m5C_44479
chr11	93466415	-	C	158	52	0.329	0	0	T	106	M	0.261	0.406	0	0	0	271.088	cgggcatgaaCtaaaacttaa	m5C_44462
chr11	93466439	-	C	174	46	0.264	0	0	T	128	M	0.204	0.334	0	0	0	188.088	aactgttcaaCtgctgaatga	m5C_44444
chr11	93466636	-	C	374	105	0.281	0	0	T	269	M	0.238	0.328	0	0	0	498.986	aattacaagaCagttgctaaa	m5C_44458
chr11	93466678	-	C	300	62	0.207	0	0	T	238	M	0.165	0.256	0	0	0	204.222	ttttactttaCtcacaggact	m5C_44463
chr11	93466683	-	C	268	55	0.206	1	0.004	T	212	M	0.162	0.259	0	0	0	177.999	tgtggttttaCtttactcaca	m5C_44494
chr11	93466720	-	C	525	123	0.234	0	0	T	402	M	0.2	0.272	0	0	0	492.16	gcctgcacatCgttggaaacg	m5C_44486
chr11	93466725	-	C	593	142	0.239	0	0	T	451	M	0.207	0.275	0	0	0	587.479	ctgtagcctgCacatcgttgg	m5C_44469
chr11	93466741	-	C	667	171	0.256	0	0	T	496	M	0.225	0.291	0	0	0	768.461	cccgatggggCttttcctgta	m5C_44445
chr11	93466750	-	C	699	167	0.241	6	0.009	T	526	M	0.211	0.274	0	0	0	703.479	gaggtctatcCcgatggggct	m5C_44446
chr11	93466755	-	C	658	170	0.258	0	0	T	488	M	0.226	0.293	0	0	0	769.7	tggttgaggtCtatcccgatg	m5C_44441
chr11	93468403	-	C	52	11	0.212	0	0	T	41	M	0.122	0.34	1.4E-13	4.4E-10	1.7E-13	17.3019	catttgggaCtgcacttatg	m5C_44490
chr11	102076746	+	C	34	8	0.235	0	0	T	26	M	0.124	0.4	4.2E-11	6.8E-07	4.8E-11	10.3259	gatgcgactgCagcaactgca	m5C_42484
chr11	102076755	+	C	33	12	0.364	0	0	T	21	M	0.222	0.534	0	4.6E-06	0	53.2487	gcagcaactgCagatggagaa	m5C_42481
chr11	102100519	+	C	75	35	0.467	0	0	T	40	M	0.358	0.578	0	1.9E-09	0	250.709	aaatgtggacCttggaacact	m5C_42488
chr11	102100526	+	C	102	24	0.235	0	0	T	78	M	0.164	0.326	0	2.2E-16	0	78.4895	gaccttggaaCactggaagga	m5C_42492
chr11	102100528	+	C	119	24	0.202	0	0	T	95	M	0.139	0.283	0	0	0	66.9316	ccttggaacaCtggaaggaga	m5C_42487
chr11	103475564	+	C	66	24	0.364	0	0	T	42	M	0.258	0.484	0	3E-09	0	123.873	tcatggctgtCtgagtgctgc	m5C_42490
chr11	103475574	+	C	65	17	0.262	0	0	T	48	M	0.17	0.38	0	1.8E-10	0	57.8614	ctgagtgctgCtttaagaaag	m5C_42496
chr11	107779393	+	C	51	14	0.275	0	0	T	37	M	0.171	0.409	0	2.8E-09	0	47.9195	gcagcccctgCtgctggttcc	m5C_42461
chr11	107779396	+	C	64	19	0.297	0	0	T	45	M	0.199	0.418	0	4.5E-10	0	75.639	gcccctgctgCtggttccgcc	m5C_42460
chr11	107779402	+	C	82	26	0.317	0	0	T	56	M	0.226	0.424	0	2.7E-12	0	117.769	gctgctggttCcgcccctgct	m5C_42458
chr11	107779405	+	C	90	18	0.2	0	0	T	72	M	0.13	0.294	0	4.4E-16	0	46.9523	gctggttccgCccctgctgca	m5C_42463
chr11	107779406	+	C	94	24	0.255	0	0	T	70	M	0.178	0.352	0	1.7E-14	0	85.4363	ctggttccgcCcctgctgcag	m5C_42457
chr11	107779414	+	C	116	30	0.259	0	0	T	86	M	0.188	0.345	0	0	0	112.548	gcccctgctgCagcagaggag	m5C_42459
chr11	108100324	-	C	48	18	0.375	0	0	T	30	M	0.252	0.516	0	7.7E-07	0	90.7743	tcaggctggcCtgagtgcagt	m5C_42823
chr11	109473925	+	C	66	27	0.409	0	0	T	39	M	0.299	0.53	0	7.5E-09	0	161.283	aagggcacggCcgtgatcgta	m5C_42808
chr11	109473926	+	C	70	27	0.386	0	0	T	43	M	0.28	0.503	0	7.5E-09	0	151.457	agggcacggcCgtgatcgtat	m5C_42805
chr11	109473932	+	C	150	89	0.593	0	0	C	89	M	0.513	0.669	0	1.9E-14	0	913.754	cggccgtgatCgtatagtggt	m5C_42795
chr11	109473948	+	C	115	59	0.513	0	0	C	59	M	0.423	0.602	0	7.2E-13	0	498.851	gtggttagtaCtctgtgttgt	m5C_42811
chr11	109473950	+	C	96	48	0.5	0	0	CT	48	M	0.402	0.598	0	4.6E-11	0	385.845	ggttagtactCtgtgttgtgt	m5C_42796
chr11	109512231	+	C	34	14	0.412	0	0	T	20	M	0.264	0.578	0	9.2E-06	0	73.8241	agggtctgatCcctgtctctc	m5C_42791
chr11	109709718	+	C	33	11	0.333	0	0	T	22	M	0.198	0.504	2.2E-16	3.1E-06	2.9E-16	34.0074	tttgatatgcCcggctagctt	m5C_42799
chr11	109709722	+	C	72	25	0.347	0	0	T	47	M	0.248	0.462	0	9.3E-11	0	123.766	atatgcccggCtagcttggtt	m5C_42814
chr11	109937246	-	C	52	17	0.327	0	0	T	35	M	0.215	0.462	0	1.1E-08	0	73.1745	gtagtgttatCatgtttgcct	m5C_42818
chr11	110122651	-	C	32	9	0.281	0	0	T	23	M	0.156	0.454	5.3E-13	1.2E-06	6.4E-13	17.1983	gctagactctCttgggcaaga	m5C_43362
chr11	110122653	-	C	33	10	0.303	0	0	T	23	M	0.174	0.473	1.6E-14	2E-06	2E-14	23.982	ctgctagactCtcttgggcaa	m5C_43363
chr11	110122655	-	C	33	11	0.333	0	0	T	22	M	0.198	0.504	2.2E-16	3.1E-06	2.9E-16	34.0074	tgctgctagaCtctcttgggc	m5C_43354
chr11	110122672	-	C	33	10	0.303	0	0	T	23	M	0.174	0.473	1.6E-14	2E-06	2E-14	23.982	cagaggtatgCtgcctgtgct	m5C_43350
chr11	110122683	-	C	35	8	0.229	0	0	T	27	M	0.121	0.39	5.6E-11	6.8E-07	6.4E-11	9.89691	gggagaatgcCcagaggtatg	m5C_43330
chr11	110122684	-	C	35	13	0.371	0	0	T	22	M	0.232	0.537	0	6.7E-06	0	60.2306	tgggagaatgCccagaggtat	m5C_43359
chr11	110122695	-	C	35	14	0.4	0	0	T	21	M	0.256	0.564	0	9.2E-06	0	71.5414	gcagatatacCtgggagaatg	m5C_43349
chr11	110122696	-	C	34	10	0.294	0	0	T	24	M	0.168	0.462	2.3E-14	2E-06	2.9E-14	22.9564	agcagatataCctgggagaat	m5C_43343
chr11	110122707	-	C	35	11	0.314	0	0	T	24	M	0.186	0.48	6.7E-16	3.1E-06	8.7E-16	30.9686	gcaaagtagcCagcagatata	m5C_43348
chr11	110122722	-	C	32	9	0.281	0	0	T	23	M	0.156	0.454	5.3E-13	1.2E-06	6.4E-13	17.1983	tgattaagccCattggcaaag	m5C_43329
chr11	112097770	+	C	38	21	0.553	0	0	C	21	M	0.397	0.699	0	5.2E-05	0	166.765	ccctggtggtCtagtggctag	m5C_42749
chr11	112103910	+	C	32	10	0.312	0	0	T	22	M	0.18	0.486	1.1E-14	2E-06	1.3E-14	25.0836	gcccccttgatCataagaatct	m5C_42751
chr11	114314649	+	C	32	12	0.375	0	0	T	20	M	0.229	0.547	0	4.6E-06	0	55.0408	aggagcatcaCgggaaggtaa	m5C_42836
chr11	114315268	+	C	31	8	0.258	0	0	T	23	M	0.137	0.432	1.7E-11	6.8E-07	1.9E-11	11.8182	tgcagtctggCcttaccaagg	m5C_42835
chr11	114315274	+	C	34	15	0.441	0	0	T	19	M	0.289	0.605	0	1.2E-05	0	86.6497	ctggccttacCaaggcagtga	m5C_42831
chr11	116100244	+	C	42	30	0.714	0	0	C	30	M	0.564	0.828	0	1.2E-05	0	338.595	tttgaaacacCaccaatgtgt	m5C_43394
chr11	116126362	-	C	43	11	0.256	0	0	T	32	M	0.149	0.402	1.2E-14	4.4E-08	1.5E-14	22.8887	ttgctagtaaCtgtaacacaa	m5C_43415
chr11	116126369	-	C	46	34	0.739	0	0	C	34	M	0.597	0.844	0	2.2E-05	0	406.236	tttggaattgCtagtaactgt	m5C_43411
chr11	116886620	+	C	51	26	0.51	0	0	C	26	M	0.377	0.641	0	1.9E-07	0	195.94	acaggctggtCctatggtagt	m5C_43389
chr11	116886621	+	C	49	11	0.224	0	0	T	38	M	0.13	0.359	6.6E-14	4.4E-08	8.1E-14	18.8888	caggctggtcCtatggtagtg	m5C_43376
chr11	116886643	+	C	39	17	0.436	0	0	T	22	M	0.293	0.59	0	2.1E-05	0	99.6354	gttatcagaaCttattaactt	m5C_43392
chr11	117930115	-	C	33	26	0.788	0	0	C	26	M	0.622	0.893	0	0.00013	0	323.69	atgctggggtgCtcttccctcc	m5C_43403
chr11	118621711	-	C	34	22	0.647	0	0	C	22	M	0.479	0.785	0	6.3E-05	0	210.82	attatgggagCagctaaagat	m5C_43529
chr11	118622539	-	C	51	20	0.392	0	0	T	31	M	0.27	0.529	0	3.4E-08	0	108.109	ggaaaggggaCaaggaaggaa	m5C_43544
chr11	118841215	+	C	92	39	0.424	0	0	T	53	M	0.328	0.526	0	4.9E-12	0	255.823	gatggcttgtCtgatggtagt	m5C_43438
chr11	118841238	+	C	65	17	0.262	0	0	T	48	M	0.17	0.38	0	1.8E-10	0	57.8614	gttatcagaaCttattattaa	m5C_43445
chr11	118919511	-	C	78	34	0.436	0	0	T	44	M	0.331	0.546	0	1.4E-09	0	225.373	caggaagcggCgaatggtaga	m5C_43545

chr11	118919514	-	C	77	26	0.338	0	0	T	51	M	0.242	0.449	0	1.3E-10	0	125.839	cgccaggaagCggcgaatggt	m5C_43514
chr11	118919521	-	C	40	15	0.385	1	0.025	T	24	M	0.249	0.541	0	1.2E-05	0	74.6735	agaagcccgcCaggaagcggc	m5C_43466
chr11	118919522	-	C	40	28	0.7	0	0	C	28	M	0.546	0.819	0	8.1E-06	0	305.59	cagaagcccgCcaggaagcgg	m5C_43513
chr11	119181070	-	C	97	48	0.495	0	0	T	49	M	0.397	0.593	0	4.6E-11	0	381.558	caagagggctCcgggagacca	m5C_43537
chr11	119181072	-	C	94	27	0.287	0	0	T	67	M	0.206	0.386	0	7.2E-14	0	110.993	gacaagagggCtccgggagac	m5C_43522
chr11	119181080	-	C	94	36	0.383	0	0	T	58	M	0.291	0.484	0	2E-12	0	209.621	gcagcggtgaCaagagggctc	m5C_43546
chr11	119181086	-	C	73	21	0.292	1	0.014	T	51	M	0.199	0.405	0	1.8E-11	0	83.7392	agggcagcagCggtgacaaga	m5C_43530
chr11	119181089	-	C	49	14	0.286	0	0	T	35	M	0.178	0.424	0	1.9E-07	0	49.9784	tgcagggcagCagcggtgaca	m5C_43532
chr11	119181092	-	C	36	14	0.389	0	0	T	22	M	0.248	0.551	0	9.2E-06	0	69.397	tcctgcagggCagcagcggtg	m5C_43486
chr11	119182847	-	C	38	18	0.474	0	0	T	20	M	0.325	0.627	0	2.7E-05	0	116.924	cccgcagcccCtgagagacag	m5C_43526
chr11	119182848	-	C	39	17	0.436	0	0	T	22	M	0.293	0.59	0	2.1E-05	0	99.6354	tcccgcagccCctgagagaca	m5C_43459
chr11	119182853	-	C	41	16	0.39	0	0	T	25	M	0.257	0.543	0	4E-07	0	82.099	gtgagtcccgCagcccctgag	m5C_43456
chr11	119182855	-	C	43	12	0.279	0	0	T	31	M	0.167	0.427	2.2E-16	7.5E-08	2.9E-16	31.457	gagtgagtccCgcagcccctg	m5C_43461
chr11	119182857	-	C	40	24	0.6	0	0	C	24	M	0.446	0.737	0	3.6E-06	0	214.059	ccgagtgagtCccgcagcccc	m5C_43489
chr11	119366361	+	C	37	18	0.486	0	0	T	19	M	0.334	0.641	0	2.7E-05	0	120.406	atgagggcagCaggcatgatg	m5C_43448
chr11	119366372	+	C	36	13	0.361	0	0	T	23	M	0.225	0.524	0	6.7E-06	0	58.436	aggcatgatgCcaggatgccc	m5C_43422
chr11	119366373	+	C	37	9	0.243	0	0	T	28	M	0.134	0.401	2.7E-12	1.2E-06	3.2E-12	13.907	ggcatgatgcCaggatgcccc	m5C_43440
chr11	119366381	+	C	36	8	0.222	0	0	T	28	M	0.117	0.381	7.4E-11	6.8E-07	8.4E-11	9.49676	gccaggatgcCccctgactcc	m5C_43433
chr11	119366382	+	C	36	10	0.278	0	0	T	26	M	0.158	0.44	4.8E-14	2E-06	5.9E-14	21.1115	ccaggatgccCcctgactcct	m5C_43428
chr11	119366383	+	C	37	12	0.324	0	0	T	25	M	0.196	0.485	0	4.6E-06	0	47.1194	caggatgcccCctgactcctg	m5C_43441
chr11	120356317	+	C	70	40	0.571	0	0	C	40	M	0.455	0.681	0	5.8E-09	0	363.821	ggaagtgagaCggggaaggga	m5C_43710
chr11	121178115	+	C	31	15	0.5	1	0.032	CT	15	M	0.332	0.668	0	1.2E-05	0	99.4616	gggaagggacCgctcagttat	m5C_43707
chr11	121503826	+	C	36	12	0.333	0	0	T	24	M	0.202	0.497	0	4.6E-06	0	48.5146	actttctatgCaagactgaga	m5C_43717
chr11	121970486	-	C	53	12	0.226	0	0	T	41	M	0.135	0.355	5.8E-15	8.8E-10	7.3E-15	22.9888	gggttaggctCttgggagctg	m5C_43738
chr11	121970488	-	C	55	39	0.709	0	0	C	39	M	0.579	0.812	0	2.5E-06	0	451.3	acgggttaggCtcttgggagc	m5C_43731
chr11	121970497	-	C	35	13	0.371	0	0	T	22	M	0.232	0.537	0	6.7E-06	0	60.2306	tttaaatccaCgggttaggct	m5C_43740
chr11	121970528	-	C	34	19	0.559	0	0	C	19	M	0.395	0.711	0	3.4E-05	0	149.924	tccctgagacCctaacttgtg	m5C_43734
chr11	122022988	-	C	76	25	0.329	0	0	T	51	M	0.234	0.441	0	9.3E-11	0	116.896	tagatccgaaCttgtggtatt	m5C_44909
chr11	122022992	-	C	80	25	0.312	0	0	T	55	M	0.222	0.421	0	1.8E-12	0	110.751	cccgtagatcCgaacttgtgg	m5C_44904
chr11	122022993	-	C	75	39	0.52	0	0	C	39	M	0.409	0.629	0	4.7E-09	0	318.817	acccgtagatCcgaacttgtg	m5C_44893
chr11	122023000	-	C	37	11	0.297	0	0	T	26	M	0.175	0.458	1.4E-15	3.1E-06	1.9E-15	28.5508	gccacaaaccCgtagatccga	m5C_44860
chr11	122023001	-	C	34	11	0.324	0	0	T	23	M	0.191	0.492	4.4E-16	3.1E-06	5.8E-16	32.3088	tgccacaaacCcgtagatccg	m5C_44877
chr11	122023002	-	C	36	8	0.222	0	0	T	28	M	0.117	0.381	7.4E-11	6.8E-07	8.4E-11	9.49676	ttgccacaaaCccgtagatcc	m5C_44892
chr11	122874335	+	C	41	10	0.244	0	0	T	31	M	0.138	0.393	2.4E-13	2.4E-08	2.9E-13	17.4472	gatggtttttCatatcattgg	m5C_44827
chr11	122874347	+	C	83	32	0.386	0	0	T	51	M	0.288	0.493	0	2.3E-11	0	184.39	tatcattggtCgtggttgtag	m5C_44839
chr11	122874360	+	C	74	23	0.311	0	0	T	51	M	0.217	0.423	0	4.3E-11	0	99.7772	ggttgtagccCgtgcgagaat	m5C_44833
chr11	122928783	-	C	38	15	0.395	0	0	T	23	M	0.256	0.553	0	1.2E-05	0	76.7936	tctgagtgacCcaagatgtta	m5C_44897
chr11	122928784	-	C	331	79	0.239	0	0	T	252	M	0.196	0.287	0	0	0	309.541	gtctgagtgaCccaagatgtt	m5C_44873
chr11	122928802	-	C	752	177	0.235	0	0	T	575	M	0.206	0.267	0	0	0	730.809	caactaccttCcttggatgtc	m5C_44901
chr11	122928805	-	C	729	151	0.207	0	0	T	578	M	0.179	0.238	0	0	0	541.447	tggcaactacCttccttggat	m5C_44869
chr11	122928809	-	C	738	166	0.226	5	0.007	T	567	M	0.198	0.258	0	0	0	656.158	gtgttggcaaCtaccttcctt	m5C_44879
chr11	122928842	-	C	690	147	0.213	0	0	T	543	M	0.184	0.245	0	0	0	541.323	ccaaaacattCgcggtttcca	m5C_44895
chr11	122929619	-	C	85	22	0.259	0	0	T	63	M	0.178	0.361	0	4.4E-13	0	78.1351	gatgtttgagCgaccattctt	m5C_44923
chr11	122929633	-	C	107	27	0.252	0	0	T	80	M	0.18	0.342	0	1.1E-15	0	96.9857	cagttccttcCttggatgttt	m5C_44919
chr11	122929634	-	C	104	41	0.394	0	0	T	63	M	0.306	0.49	0	2.4E-13	0	250.664	ccagttccttCcttggatgtt	m5C_44920
chr11	122929637	-	C	104	28	0.269	0	0	T	76	M	0.193	0.362	0	1.8E-15	0	108.266	tggccagttcCttccttggat	m5C_44875
chr11	122929643	-	C	106	27	0.255	0	0	T	79	M	0.181	0.345	0	1.1E-15	0	97.9361	ttatgttggcCagttccttcc	m5C_44885
chr11	122929644	-	C	104	24	0.231	0	0	T	80	M	0.16	0.32	0	2.2E-16	0	76.9262	cttatgttggCcagttccttc	m5C_44883
chr11	122929662	-	C	100	23	0.23	0	0	T	77	M	0.158	0.322	0	1.1E-16	0	72.8785	tagtttccacCagaaagtctt	m5C_44900
chr11	122929663	-	C	105	32	0.305	0	0	T	73	M	0.225	0.398	0	1.1E-14	0	143.936	gtagtttccaCcagaaagtct	m5C_44894
chr11	122929666	-	C	104	46	0.442	0	0	T	58	M	0.351	0.538	0	9.7E-13	0	322.553	tcgtagtttcCaccagaaag	m5C_44884
chr11	122929674	-	C	113	29	0.257	0	0	T	84	M	0.185	0.344	0	0	0	107.321	caaaaccattCgtagtttcca	m5C_44886
chr11	122929679	-	C	117	26	0.222	0	0	T	91	M	0.156	0.306	0	0	0	81.3295	gattccaaaaCcattcgtagt	m5C_44902
chr11	122929684	-	C	127	71	0.559	0	0	C	71	M	0.472	0.642	0	3.4E-13	0	670.564	tgatggattcCaaaaccattc	m5C_44868
chr11	122929685	-	C	121	37	0.311	2	0.017	T	82	M	0.235	0.399	0	1.4E-15	0	173.729	atgatggattCcaaaaccatt	m5C_44912
chr11	122929700	-	C	59	19	0.322	0	0	T	40	M	0.217	0.449	0	2.4E-08	0	82.4122	gactgactcgCtatgatgatg	m5C_44917
chr11	122929702	-	C	61	19	0.311	0	0	T	42	M	0.209	0.436	0	4.5E-10	0	79.5616	ttgactgactCgctatgatga	m5C_44863
chr11	122930050	-	C	735	192	0.261	0	0	T	543	M	0.231	0.294	0	0	0	886.137	ccttggatgtCtgagcgagtg	m5C_44861
chr11	122930059	-	C	785	169	0.216	2	0.003	T	614	M	0.188	0.246	0	0	0	636.942	caacaccttcCttggatgtct	m5C_44914
chr11	122930084	-	C	926	189	0.204	0	0	T	737	M	0.179	0.231	0	0	0	678.098	ccaccaaaagCttggctaatg	m5C_44921
chr11	122930093	-	C	963	206	0.214	0	0	T	757	M	0.189	0.241	0	0	0	779.414	tcgtagtttcCaccaaaagct	m5C_44899
chr11	122930102	-	C	1080	261	0.242	2	0.002	T	817	M	0.217	0.269	0	0	0	1135.29	gttaaacattCgtagtttcca	m5C_44880
chr11	123172188	+	C	271	60	0.221	0	0	T	211	M	0.176	0.275	0	0	0	211.267	actaatgtctCtgtgtaatca	m5C_44841
chr11	123172202	+	C	426	117	0.275	0	0	T	309	M	0.234	0.319	0	0	0	548.539	gtaatcaggaCttgcaacacc	m5C_44855
chr11	123172211	+	C	509	115	0.226	0	0	T	394	M	0.192	0.264	0	0	0	440.987	acttgcaacaCcctgattgct	m5C_44831
chr11	123172227	+	C	479	101	0.211	0	0	T	378	M	0.177	0.25	0	0	0	356.931	ttgctcctatCtgattctttc	m5C_44832
chr11	123634722	+	C	47	15	0.319	0	0	T	32	M	0.204	0.462	0	2.8E-07	0	61.1856	gtgctgactcCtgtgatttcc	m5C_44848
chr11	123634731	+	C	58	16	0.276	0	0	T	42	M	0.178	0.402	0	7.3E-09	0	56.8071	cctgtgatttCcccaaatgtg	m5C_44853
chr11	123634732	+	C	57	13	0.228	0	0	T	44	M	0.138	0.352	4.4E-16	1.6E-09	5.8E-16	27.6258	ctgtgatttcCccaaatgtgg	m5C_44844
chr11	123634733	+	C	55	11	0.2	0	0	T	44	M	0.116	0.324	2.9E-13	4.4E-10	3.6E-13	15.9231	tgtgatttccCcaaatgtggt	m5C_44843
chr11	123995006	+	C	31	20	0.645	0	0	C	20	M	0.469	0.789	0	4.3E-05	0	187.791	aagttaggatCaacagacaga	m5C_44858
chr11	125026913	-	C	39	21	0.538	0	0	C	21	M	0.386	0.684	0	5.2E-05	0	161.991	tggataaattCtccagctatt	m5C_43638
chr11	125445250	+	C	31	21	0.677	0	0	C	21	M	0.501	0.814	0	5.2E-05	0	210.591	cgaagaagggCaagtagtgtc	m5C_43593
chr11	128840454	+	C	32	17	0.531	0	0	C	17	M	0.364	0.691	0	2.1E-05	0	123.927	cctggggccaCatatgtgtta	m5C_43791
chr11	129991609	+	C	34	8	0.235	0	0	T	26	M	0.124	0.4	4.2E-11	6.8E-07	4.8E-11	10.3259	tgccctcagaCaaagattatt	m5C_43780
chr11	129991624	+	C	184	52	0.283	0	0	T	132	M	0.223	0.352	0	0	0	231.41	attattggatCtgtgtcaaaa	m5C_43784
chr11	129991630	+	C	198	44	0.222	0	0	T	154	M	0.17	0.285	0	0	0	149.522	ggatctgtgtCaaaagaagag	m5C_43787
chr11	131588720	-	C	51	11	0.216	0	0	T	40	M	0.125	0.346	1.1E-13	4.4E-10	1.4E-13	17.8061	gagagcatttCcaggagtggt	m5C_43697
chr11	134303827	+	C	37	10	0.27	0	0	T	27	M	0.154	0.43	6.7E-14	2E-06	8.3E-14	20.2815	ccttctggggCcatgtctgtc	m5C_43809
chr11	134406168	-	C	56	40	0.714	0	0	C	40	M	0.585	0.816	0	2.9E-06	0	468.196	aggatgaatgCtctcctgtcc	m5C_43823
chr11	134406181	-	C	57	41	0.719	0	0	C	41	M	0.592	0.819	0	3.3E-06	0	485.19	cgtctcctggCccaggatgaa	m5C_43827
chr12	2698772	+	C	32	16	0.5	0	0	CT	16	M	0.336	0.664	0	1.7E-05	0	107.618	atgcgggaggCtgggtctgat	m5C_43862
chr12	3563417	+	C	157	42	0.268	0	0	T	115	M	0.204	0.342	0	0	0	171.723	ttctgactccCtgacggggag	m5C_43857
chr12	3563421	+	C	151	47	0.311	0	0	T	104	M	0.243	0.389	0	0	0	228.29	gactccctgaCggggagcctg	m5C_43854
chr12	3563428	+	C	132	43	0.326	0	0	T	89	M	0.252	0.41	0	0	0	216.478	tgacggggagCctgcggatgg	m5C_43867
chr12	5320707	-	C	32	7	0.219	0	0	T	25	M	0.11	0.388	8.5E-10	3.4E-07	9.5E-10	6.99971	gtaaatatttCtgattggaac	m5C_43907
chr12	5534279	-	C	43	27	0.628	0	0	C	27	M	0.479	0.756	0	6.7E-06	0	258.442	agcaggctgtCgtcaggcccc	m5C_43917
chr12	6167705	-	C	36	11	0.306	0	0	T	25	M	0.18	0.469	1E-15	3.1E-06	1.3E-15	29.7077	ccacacctggCagcctgctgg	m5C_44755
chr12	6619403	+	C	285	65	0.228	0	0	T	220	M	0.183	0.28	0	0	0	238.111	atgatgatatCaaggctgttg	m5C_44679
chr12	6619408	+	C	282	153	0.543	0	0	C	153	M	0.484	0.6	0	0	0	1481.73	gatatcaaggCtgttgtgatt	m5C_44696
chr12	6619419	+	C	234	97	0.415	0	0	T	137	M	0.353	0.479	0	0	0	685.374	tgttgtgattCagttggtttg	m5C_44656
chr12	6619431	+	C	130	40	0.308	0	0	T	90	M	0.235	0.392	0	0	0	187.859	gttggtttggCtaagcccagg	m5C_44687
chr12	6619436	+	C	93	22	0.237	0	0	T	71	M	0.162	0.332	0	5.8E-15	0	71.1592	tttggctaagCccagggacct	m5C_44661
chr12	6619437	+	C	73	17	0.233	0	0	T	56	M	0.151	0.342	0	2.2E-12	0	51.2678	ttggctaagcCcagggacctt	m5C_44641
chr12	6619438	+	C	68	22	0.324	0	0	T	46	M	0.224	0.442	0	1.5E-09	0	98.7248	tggctaagccCagggaccttt	m5C_44650
chr12	6619445	+	C	44	15	0.341	0	0	T	29	M	0.219	0.489	0	2.8E-07	0	65.6269	gcccagggacCtttggcctgt	m5C_44683
chr12	6619598	+	C	54	14	0.259	0	0	T	40	M	0.161	0.389	0	2.8E-09	0	45.1318	attgtggtaaCtggagtgaga	m5C_44659
chr12	6619618	+	C	40	11	0.275	0	0	T	29	M	0.161	0.428	4.3E-15	3.1E-06	5.5E-15	25.4502	agagtaggaaCagggcatgtt	m5C_44711
chr12	6619623	+	C	37	9	0.243	0	0	T	28	M	0.134	0.401	2.7E-12	1.2E-06	3.2E-12	13.907	aggaacagggCatgttcaggg	m5C_44691
chr12	6619664	+	C	43	21	0.488	0	0	T	22	M	0.346	0.632	0	1.8E-06	0	145.392	aaggacttagCttgtgttatg	m5C_44692
chr12	6619677	+	C	86	18	0.209	0	0	T	68	M	0.137	0.307	0	4.5E-14	0	49.211	gtgttatggcCactgagagat	m5C_44662

chr12	6619679	+	C	96	37	0.385	0	0	T	59	M	0.294	0.485	0	2.7E-12	0	217.75	gttatggccaCtgagagatga	m5C_44726
chr12	6619699	+	C	142	49	0.345	0	0	T	93	M	0.272	0.426	0	0	0	266.45	aaacacagatCtttggtaatc	m5C_44705
chr12	6619716	+	C	129	35	0.271	0	0	T	94	M	0.202	0.354	0	0	0	141.42	aatctgatggCtgcaagtgct	m5C_44708
chr12	6647455	+	C	34	19	0.559	0	0	C	19	M	0.395	0.711	0	3.4E-05	0	149.924	tcacagttgcCatgtagaccc	m5C_44645
chr12	6647463	+	C	32	17	0.531	0	0	C	17	M	0.364	0.691	0	2.1E-05	0	123.927	gccatgtagaCcccttgaaga	m5C_44701
chr12	6647465	+	C	35	18	0.514	0	0	C	18	M	0.356	0.67	0	2.7E-05	0	128.047	catgtagaccCcttgaagagg	m5C_44660
chr12	6647483	+	C	41	32	0.78	0	0	C	32	M	0.633	0.88	0	1.6E-05	0	405.086	aggggaggggCctagggagcc	m5C_44674
chr12	6647495	+	C	39	17	0.436	0	0	T	22	M	0.293	0.59	0	2.1E-05	0	99.6354	tagggagccgCaccttgtcat	m5C_44717
chr12	6647497	+	C	41	16	0.39	0	0	T	25	M	0.257	0.543	0	4E-07	0	82.099	gggagccgcaCcttgtcatgt	m5C_44725
chr12	6690643	-	C	219	60	0.274	0	0	T	159	M	0.219	0.337	0	0	0	263.018	cacgagaggaCaatttaagat	m5C_44790
chr12	6690679	-	C	150	55	0.367	0	0	T	95	M	0.294	0.446	0	0	0	323.151	gagggccgtcCtaagaatagg	m5C_44742
chr12	6690684	-	C	128	29	0.23	2	0.016	T	97	M	0.165	0.311	0	0	0	95.8737	tttaggagggCcgtcctaaga	m5C_44795
chr12	6690703	-	C	100	41	0.41	0	0	T	59	M	0.319	0.508	0	2.4E-13	0	261.311	cacagcaattCcctctcagtt	m5C_44817
chr12	6690713	-	C	134	35	0.261	0	0	T	99	M	0.194	0.341	0	0	0	135.949	tggcaggacaCacagcaattc	m5C_44740
chr12	6690733	-	C	142	30	0.211	0	0	T	112	M	0.152	0.286	0	0	0	91.3118	tcagttctaaCagggtagtct	m5C_44757
chr12	6690744	-	C	132	29	0.22	0	0	T	103	M	0.158	0.298	0	0	0	91.3768	ctcccctgtgCtcagttctaa	m5C_44810
chr12	6690750	-	C	130	42	0.326	1	0.008	T	87	M	0.251	0.41	0	2.2E-16	0	210.655	tttctgctccCctgtgctcag	m5C_44780
chr12	6690752	-	C	126	33	0.262	0	0	T	93	M	0.193	0.345	0	0	0	127.377	ttttctgctCccctgtgctc	m5C_44822
chr12	6690757	-	C	125	27	0.216	0	0	T	98	M	0.153	0.296	0	0	0	82.5698	gcctatttttCtgctcccctg	m5C_44805
chr12	6690765	-	C	122	38	0.311	0	0	T	84	M	0.236	0.398	0	0	0	179.446	ttgtcctggcCtatttttctg	m5C_44774
chr12	6690770	-	C	125	30	0.24	0	0	T	95	M	0.174	0.322	0	0	0	104.16	tctttttgtcCtggcctattt	m5C_44801
chr12	6690771	-	C	94	26	0.277	0	0	T	68	M	0.196	0.374	0	4.6E-14	0	102.075	ctctttttgtCctggcctatt	m5C_44750
chr12	6709138	-	C	32	22	0.688	0	0	C	22	M	0.514	0.82	0	6.3E-05	0	226.305	gaggacaattCggagggtgag	m5C_44728
chr12	6711199	-	C	38	14	0.368	0	0	T	24	M	0.234	0.527	0	9.2E-06	0	65.4752	aagcttggacCtaagaaagag	m5C_44788
chr12	6875912	+	C	33	20	0.606	0	0	C	20	M	0.437	0.753	0	4.3E-05	0	174.733	gagttgagcgCcaaggtacgg	m5C_44690
chr12	6875913	+	C	47	26	0.553	0	0	C	26	M	0.412	0.686	0	5.5E-06	0	214.475	agttgagcgcCaaggtacggg	m5C_44642
chr12	6878772	+	C	63	29	0.46	0	0	T	34	M	0.343	0.582	0	1.3E-08	0	198.99	tttgcaggacCtgaaggagaa	m5C_44688
chr12	6878806	+	C	40	10	0.25	0	0	T	30	M	0.142	0.402	1.8E-13	2.4E-08	2.2E-13	18.0914	gaggagaaggCaagccggaaa	m5C_44715
chr12	6976778	+	C	54	18	0.333	0	0	T	36	M	0.222	0.466	0	1.7E-08	0	80.0685	ggaagatgaaCgggcggaagc	m5C_44658
chr12	6976782	+	C	52	11	0.212	0	0	T	41	M	0.122	0.34	1.4E-13	4.4E-10	1.7E-13	17.3019	gatgaacgggCggaagcagag	m5C_44657
chr12	6976788	+	C	48	16	0.333	0	0	T	32	M	0.217	0.475	0	4E-07	0	69.3651	cgggcggaagCagagtctggg	m5C_44722
chr12	7076726	-	C	44	24	0.545	0	0	C	24	M	0.401	0.683	0	3.6E-06	0	192.316	tgctctctggCccttggctcc	m5C_44731
chr12	7076732	-	C	53	35	0.66	0	0	C	35	M	0.526	0.773	0	1.3E-06	0	368.162	actccgtgctCtctggccctt	m5C_44820
chr12	7076738	-	C	82	25	0.312	2	0.024	T	55	M	0.222	0.421	0	1.8E-12	0	110.751	aatgcgactcCgtgctctctg	m5C_44764
chr12	7076739	-	C	96	39	0.406	0	0	T	57	M	0.313	0.506	0	4.9E-12	0	244.492	gaatgcgactCcgtgctctct	m5C_44797
chr12	7076741	-	C	118	29	0.246	0	0	T	89	M	0.177	0.331	0	0	0	102.608	gcgaatgcgaCtccgtgctct	m5C_44739
chr12	7076750	-	C	188	52	0.277	0	0	T	136	M	0.218	0.345	0	0	0	226.317	gagactaaggCgaatgcgact	m5C_44793
chr12	7076765	-	C	103	22	0.214	0	0	T	81	M	0.145	0.302	0	0	0	64.0184	catgggcaggCtgatgagact	m5C_44735
chr12	7076769	-	C	88	19	0.216	0	0	T	69	M	0.143	0.313	0	8.4E-14	0	54.2667	ccttcatgggCaggctgatga	m5C_44784
chr12	7368321	+	C	33	9	0.273	0	0	T	24	M	0.151	0.442	7.5E-13	1.2E-06	9E-13	16.4418	gaacggcagaCactgcagact	m5C_44664
chr12	7368330	+	C	32	8	0.25	0	0	T	24	M	0.133	0.421	2.3E-11	6.8E-07	2.6E-11	11.2828	acactgcagaCtaccagagag	m5C_44669
chr12	9315672	+	C	32	11	0.344	0	0	T	21	M	0.204	0.517	2.2E-16	3.1E-06	2.9E-16	35.1442	attgaagaacCagggagggtg	m5C_44035
chr12	9392848	-	C	41	9	0.22	0	0	T	32	M	0.12	0.367	8.4E-12	1.2E-08	9.9E-12	11.9621	gtggaagaggCggaaaatgga	m5C_44065
chr12	9392880	-	C	36	8	0.222	0	0	T	28	M	0.117	0.381	7.4E-11	6.8E-07	8.4E-11	9.49676	ccaccgaggaCttaaaggaga	m5C_44066
chr12	10659849	+	C	82	26	0.317	0	0	T	56	M	0.226	0.424	0	2.7E-12	0	117.769	agttgaaaaaCactggcgttt	m5C_43951
chr12	10659851	+	C	100	29	0.29	0	0	T	71	M	0.21	0.385	0	2.9E-15	0	121.885	ttgaaaaacaCtggcgtttaa	m5C_43934
chr12	10659873	+	C	103	32	0.311	0	0	T	71	M	0.229	0.406	0	1.1E-14	0	146.862	tggttggggtCagataagaag	m5C_43940
chr12	12227237	+	C	36	8	0.222	0	0	T	28	M	0.117	0.381	7.4E-11	6.8E-07	8.4E-11	9.49676	ccggatagctCagtcagtaga	m5C_44126
chr12	12227241	+	C	36	18	0.5	0	0	CT	18	M	0.345	0.655	0	2.7E-05	0	124.107	atagctcagtCagtagagcat	m5C_44130
chr12	17143740	+	C	56	41	0.732	0	0	C	41	M	0.604	0.83	0	3.3E-06	0	495.323	gagaaggaggCtgcttagatg	m5C_44078
chr12	17143743	+	C	47	10	0.213	0	0	T	37	M	0.12	0.349	1.3E-12	2.4E-08	1.5E-12	14.271	aaggaggctgCttagatggga	m5C_44080
chr12	17144521	+	C	49	12	0.245	0	0	T	37	M	0.146	0.381	1.9E-15	7.5E-08	2.4E-15	25.8006	acaatgtgggCttcaatgtca	m5C_44072
chr12	17144524	+	C	49	13	0.265	0	0	T	36	M	0.162	0.403	0	1.2E-07	0	42.1489	atgtgggcttCaatgtcaaga	m5C_44071
chr12	17144530	+	C	44	9	0.205	0	0	T	35	M	0.112	0.345	1.8E-11	1.2E-08	2.1E-11	10.7806	gcttcaatgtCaagaacgtgt	m5C_44067
chr12	17144541	+	C	37	9	0.243	0	0	T	28	M	0.134	0.401	2.7E-12	1.2E-06	3.2E-12	13.907	aagaacgtgtCtgtcaaggat	m5C_44070
chr12	19877482	+	C	36	8	0.222	0	0	T	28	M	0.117	0.381	7.4E-11	6.8E-07	8.4E-11	9.49676	atgcaagaaaCttgactgcat	m5C_43968
chr12	19877513	+	C	398	153	0.384	0	0	T	245	M	0.338	0.433	0	0	0	1034.13	agtggggggaCtgtgtttgcg	m5C_43970
chr12	19877522	+	C	345	74	0.214	0	0	T	271	M	0.174	0.261	0	0	0	258.182	actgtgtttgCgctttccctg	m5C_43971
chr12	19877524	+	C	340	68	0.2	0	0	T	272	M	0.161	0.246	0	0	0	218.877	tgtgtttgcgCtttccctgtg	m5C_43967
chr12	19877530	+	C	266	88	0.331	0	0	T	178	M	0.277	0.389	0	0	0	487.597	tgcgctttccCtgtgtatatt	m5C_43975
chr12	20704359	+	C	35	17	0.486	0	0	T	18	M	0.33	0.644	0	2.1E-05	0	112.18	aaaagatttaCggacaggatt	m5C_44327
chr12	20704363	+	C	58	29	0.5	0	0	CT	29	M	0.375	0.625	0	3.9E-07	0	217.721	gatttacggaCaggattgaca	m5C_44304
chr12	20704372	+	C	137	103	0.752	0	0	C	103	M	0.673	0.817	0	3E-12	0	1386.97	acaggattgaCagattgatag	m5C_44329
chr12	20704385	+	C	296	229	0.774	0	0	C	229	M	0.723	0.818	0	0	0	3309.72	attgatagctCtttctcgatt	m5C_44314
chr12	20704391	+	C	435	141	0.324	0	0	T	294	M	0.282	0.369	0	0	0	794.84	agctctttctCgattccgtgg	m5C_44323
chr12	20704396	+	C	634	405	0.639	0	0	C	405	M	0.601	0.675	0	0	0	4865.49	tttctcgattCcgtgggtggt	m5C_44293
chr12	20704397	+	C	664	216	0.334	17	0.026	T	431	M	0.299	0.371	0	0	0	1289.89	ttctcgattcCgtgggtggtg	m5C_44330
chr12	20704435	+	C	1089	650	0.597	0	0	C	650	M	0.567	0.626	0	0	0	7376.88	gttggtggagCgatttgtctg	m5C_44333
chr12	20704499	+	C	90	52	0.578	0	0	C	52	M	0.475	0.675	0	1.1E-10	0	493.594	acgcgaccccCgagcgggtct	m5C_44328
chr12	21791338	-	C	47	20	0.426	0	0	T	27	M	0.295	0.567	0	1.4E-06	0	118.054	gaaatgggaaCtgacaatgat	m5C_44347
chr12	21794985	-	C	34	12	0.353	0	0	T	22	M	0.215	0.521	0	4.6E-06	0	51.5705	tggatgtaatCtggattctgc	m5C_44395
chr12	21796986	-	C	311	64	0.206	0	0	T	247	M	0.165	0.254	0	0	0	210.658	agtccgtcagCaagaaggggga	m5C_44361
chr12	21796989	-	C	337	90	0.267	0	0	T	247	M	0.223	0.317	0	0	0	400.76	aggagtccgtCagcaagaagg	m5C_44393
chr12	21796993	-	C	350	100	0.286	0	0	T	250	M	0.241	0.335	0	0	0	481.825	ctgcaggagtCcgtcagcaag	m5C_44384
chr12	21807556	-	C	33	8	0.242	0	0	T	25	M	0.128	0.41	3.1E-11	6.8E-07	3.6E-11	10.7866	gaagaagaggCaacagttcca	m5C_44391
chr12	22348466	-	C	53	18	0.34	0	0	T	35	M	0.227	0.474	0	1.7E-08	0	81.6727	gcgatttcccCaaatgttgga	m5C_44003
chr12	22348469	-	C	53	12	0.226	0	0	T	41	M	0.135	0.355	5.8E-15	8.8E-10	7.3E-15	22.9888	cctgcgatttCcccaaatgtt	m5C_43999
chr12	22348478	-	C	53	11	0.208	0	0	T	42	M	0.12	0.335	1.8E-13	4.4E-10	2.2E-13	16.8213	gtgctggcccCtgcgatttcc	m5C_44004
chr12	22348485	-	C	35	11	0.324	1	0.029	T	23	M	0.191	0.492	4.4E-16	3.1E-06	5.8E-16	32.3088	acctgatgtgCtggcccctgc	m5C_43996
chr12	27127112	-	C	39	10	0.256	0	0	T	29	M	0.146	0.411	1.3E-13	2E-06	1.6E-13	18.7753	agtggaattaCgttacagatt	m5C_45263
chr12	27127131	-	C	45	19	0.422	0	0	T	26	M	0.29	0.567	0	1E-06	0	110.08	atggggcatgCtggctgtaag	m5C_45254
chr12	27908142	+	C	104	39	0.375	0	0	T	65	M	0.288	0.471	0	1.3E-13	0	224.618	aaaagtaagaCtgaagcagac	m5C_45228
chr12	31479281	+	C	32	9	0.281	0	0	T	23	M	0.156	0.454	5.3E-13	1.2E-06	6.4E-13	17.1983	agaagctccgCggcgcgggaa	m5C_44204
chr12	31479284	+	C	45	20	0.444	0	0	T	25	M	0.309	0.588	0	1.4E-06	0	123.755	agctccgcggCgcgggaaacg	m5C_44193
chr12	34356840	+	C	208	46	0.221	0	0	T	162	M	0.17	0.282	0	0	0	156.486	ctgggaatgcCgggtgctgta	m5C_44241
chr12	34356846	+	C	213	56	0.263	0	0	T	157	M	0.208	0.326	0	0	0	233.371	atgccgggtgCtgtaggcttt	m5C_44227
chr12	34356853	+	C	180	87	0.483	0	0	T	93	M	0.411	0.556	0	0	0	715.911	gtgctgtaggCttttgccttc	m5C_44220
chr12	34358737	+	C	131	27	0.206	0	0	T	104	M	0.146	0.283	0	0	0	78.6736	actgggaataCcgggtgctgt	m5C_44234
chr12	34358744	+	C	128	68	0.531	0	0	C	68	M	0.445	0.616	0	1.9E-13	0	605.407	ataccgggtgCtgtaggcttt	m5C_44225
chr12	34358751	+	C	106	48	0.453	0	0	T	58	M	0.361	0.548	0	1.6E-12	0	346.921	gtgctgtaggCttttggcttc	m5C_44232
chr12	38555269	-	C	63	28	0.444	0	0	T	35	M	0.328	0.567	0	9.8E-09	0	183.943	gtgctgtaggCttttggcttc	m5C_44276
chr12	38555276	-	C	72	26	0.361	0	0	T	46	M	0.26	0.476	0	1.3E-10	0	135.104	ataccgggtgCtgtaggcttt	m5C_44286
chr12	38555282	-	C	75	16	0.213	0	0	T	59	M	0.136	0.319	0	1.2E-12	0	43.4563	ctgggaatacCgggtgctgta	m5C_44279
chr12	38555283	-	C	74	20	0.27	0	0	T	54	M	0.182	0.381	0	1.1E-11	0	72.9204	cctgggaataCcgggtgctgt	m5C_44275
chr12	38555292	-	C	84	21	0.25	0	0	T	63	M	0.17	0.352	0	2.6E-13	0	71.2832	gggagaccgcCtgggaatacc	m5C_44280
chr12	38555293	-	C	82	20	0.244	0	0	T	62	M	0.164	0.347	0	1.5E-13	0	65.5191	agggagaccgCctgggaatac	m5C_44283
chr12	38555295	-	C	82	28	0.341	0	0	T	54	M	0.248	0.449	0	5.8E-12	0	138.875	gaagggagacCgcctgggaat	m5C_44289
chr12	38555296	-	C	81	21	0.259	0	0	T	60	M	0.176	0.364	0	2.6E-13	0	74.0352	ggaagggagaCcgcctgggaa	m5C_44288
chr12	38557213	-	C	162	33	0.204	0	0	T	129	M	0.149	0.272	0	0	0	98.2649	atgctgggtgCtgtagggttt	m5C_44277
chr12	38557229	-	C	121	37	0.306	0	0	T	84	M	0.231	0.393	0	0	0	170.732	gggaggccgcCtgggaatgct	m5C_44282
chr12	38557233	-	C	101	28	0.277	0	0	T	73	M	0.199	0.371	0	1.8E-15	0	111.617	ggatgggaggCcgcctgggaa	m5C_44278

chr12	39538805	+	C	31	7	0.226	0	0	T	24	M	0.114	0.398	6.4E-10	3.4E-07	7.2E-10	7.33205	gttccgcggcCcttatcacta	m5C_44258
chr12	39538811	+	C	32	13	0.406	0	0	T	19	M	0.255	0.577	0	6.7E-06	0	66.3505	cggcccttatCactacagccc	m5C_44265
chr12	42707694	-	C	34	11	0.324	0	0	T	23	M	0.191	0.492	4.4E-16	3.1E-06	5.8E-16	32.3088	aagaaagctcCtgaaccagaa	m5C_44635
chr12	42707697	-	C	33	8	0.242	0	0	T	25	M	0.128	0.41	3.1E-11	6.8E-07	3.6E-11	10.7866	aaaaagaaagCtcctgaacca	m5C_44638
chr12	42848527	+	C	31	8	0.286	3	0.097	T	20	M	0.153	0.471	5.8E-12	5.4E-05	6.8E-12	13.7105	aaatcggctgCttcgagtgca	m5C_44627
chr12	42848562	+	C	101	21	0.208	0	0	T	80	M	0.14	0.297	0	0	0	58.8869	taattgatcaCaaccagttac	m5C_44626
chr12	42848579	+	C	96	26	0.271	0	0	T	70	M	0.192	0.367	0	4.6E-14	0	99.859	ttacagatttCtttgttcctt	m5C_44623
chr12	42848586	+	C	95	33	0.347	0	0	T	62	M	0.259	0.447	0	7.5E-13	0	171.102	tttctttgttCcttctccact	m5C_44618
chr12	42848587	+	C	94	26	0.277	0	0	T	68	M	0.196	0.374	0	4.6E-14	0	102.075	ttctttgttcCttctccactc	m5C_44624
chr12	42848592	+	C	93	20	0.215	0	0	T	73	M	0.144	0.309	0	1.7E-15	0	57.4997	tgttccttctCcactcccact	m5C_44625
chr12	42848593	+	C	99	27	0.273	0	0	T	72	M	0.195	0.368	0	7.2E-14	0	105.15	gttccttctcCactcccactg	m5C_44630
chr12	42848598	+	C	96	22	0.229	0	0	T	74	M	0.156	0.323	0	5.8E-15	0	68.8547	ttctccactcCcactgcttca	m5C_44617
chr12	42848607	+	C	94	23	0.245	0	0	T	71	M	0.169	0.34	0	1E-14	0	77.7094	cccactgcttCacttgactag	m5C_44619
chr12	45274708	-	C	125	33	0.264	0	0	T	92	M	0.195	0.347	0	0	0	128.434	tggtcacaacCaattacagat	m5C_45212
chr12	45274712	-	C	170	40	0.235	0	0	T	130	M	0.178	0.304	0	0	0	142.253	taattggtcaCaaccaattac	m5C_45207
chr12	45274714	-	C	179	62	0.346	0	0	T	117	M	0.281	0.419	0	0	0	347.885	actaattggtCacaaccaatt	m5C_45215
chr12	45274745	-	C	201	68	0.338	0	0	T	133	M	0.276	0.406	0	0	0	376.008	cctggctggtCtgagtgcagt	m5C_45219
chr12	45581245	-	C	407	98	0.241	0	0	T	309	M	0.202	0.285	0	0	0	395.518	ccaagtggtCcgagtgttgt	m5C_45209
chr12	46752851	-	C	32	18	0.562	0	0	C	18	M	0.393	0.718	0	2.7E-05	0	141.571	gatactgtggCaaccaggaag	m5C_44602
chr12	46756092	-	C	69	19	0.275	0	0	T	50	M	0.184	0.39	0	4.5E-10	0	69.8987	aagaaagaacCtatgaaatct	m5C_44565
chr12	48358095	+	C	56	21	0.375	0	0	T	35	M	0.26	0.506	0	4.8E-08	0	109.242	ggacggtttgCtggctgaacg	m5C_45674
chr12	48358099	+	C	54	11	0.204	0	0	T	43	M	0.118	0.329	2.3E-13	4.4E-10	2.8E-13	16.3622	ggtttgctggCtgaacgagag	m5C_45694
chr12	48358104	+	C	53	12	0.226	0	0	T	41	M	0.135	0.355	5.8E-15	8.8E-10	7.3E-15	22.9888	gctggctgaaCgagagcagga	m5C_45676
chr12	49048166	-	C	485	101	0.208	0	0	T	384	M	0.174	0.247	0	0	0	352.432	gaagaaacaaCctacagctca	m5C_45709
chr12	49048182	-	C	579	127	0.219	0	0	T	452	M	0.188	0.255	0	0	0	476.371	atggctatttCatttggaaga	m5C_45748
chr12	49048203	-	C	785	159	0.203	1	0.001	T	625	M	0.176	0.232	0	0	0	560.126	gttctgggacCtcagtaggga	m5C_45763
chr12	49048229	-	C	630	142	0.225	0	0	T	488	M	0.194	0.26	0	0	0	552.349	gactgtcttcCtgtggtctgt	m5C_45721
chr12	49048233	-	C	652	168	0.258	0	0	T	484	M	0.226	0.293	0	0	0	757.968	aaaggactgtCttcctgtggt	m5C_45716
chr12	49048246	-	C	725	151	0.208	0	0	T	574	M	0.18	0.239	0	0	0	544.482	caagcagaggCctaaaggact	m5C_45734
chr12	49048252	-	C	784	189	0.241	0	0	T	595	M	0.212	0.272	0	0	0	803.02	tggtttcaagCagaggcctaa	m5C_45747
chr12	49048266	-	C	941	214	0.228	1	0.001	T	726	M	0.202	0.256	0	0	0	864.531	tgtactgtggCtgttggtttc	m5C_45736
chr12	49048272	-	C	931	211	0.227	0	0	T	720	M	0.201	0.255	0	0	0	847.799	agcagttgtaCtgtggctgtt	m5C_45702
chr12	49048280	-	C	1101	226	0.206	2	0.002	T	873	M	0.183	0.231	0	0	0	826.214	tgaagaccagCagttgtactg	m5C_45722
chr12	49048291	-	C	1135	298	0.263	0	0	T	837	M	0.238	0.289	0	0	0	1417.21	gtggccctgaCtgaagaccag	m5C_45743
chr12	49050486	-	C	126	37	0.294	0	0	T	89	M	0.221	0.378	0	0	0	163.673	tccttggtggtCtgttggctat	m5C_45724
chr12	49050498	-	C	105	21	0.2	0	0	T	84	M	0.135	0.286	0	0	0	56.5732	agagtgatgtCttccttgtgg	m5C_45761
chr12	49050511	-	C	177	45	0.254	0	0	T	132	M	0.196	0.323	0	0	0	176.202	caaacaggagCtaagagtgat	m5C_45732
chr12	49050521	-	C	220	56	0.255	0	0	T	164	M	0.202	0.316	0	0	0	225.721	ctgttggtttCaaacaggagc	m5C_45719
chr12	49050548	-	C	335	68	0.207	7	0.021	T	260	M	0.167	0.254	0	0	0	227.061	tgaatcaagaCcaatggtttg	m5C_45704
chr12	49050560	-	C	269	66	0.245	0	0	T	203	M	0.198	0.3	0	0	0	261.036	tgccttaggcCctgaatcaag	m5C_45701
chr12	49061309	-	C	32	14	0.438	0	0	T	18	M	0.282	0.607	0	9.2E-06	0	78.8622	aaagaattgtCtttctatggt	m5C_45698
chr12	49061321	-	C	80	22	0.275	0	0	T	58	M	0.189	0.381	0	2.8E-11	0	83.2375	aagcaggagcCtaaagaattg	m5C_45752
chr12	49061364	-	C	315	94	0.298	0	0	T	221	M	0.251	0.351	0	0	0	471.049	ggccctgaatCaaggccagca	m5C_45765
chr12	49061370	-	C	302	72	0.238	0	0	T	230	M	0.194	0.29	0	0	0	279.111	tcctttggccCtgaatcaagg	m5C_45707
chr12	49061371	-	C	244	57	0.234	0	0	T	187	M	0.185	0.291	0	0	0	210.78	ttcctttggcCctgaatcaag	m5C_45705
chr12	49231782	-	C	33	13	0.394	0	0	T	20	M	0.247	0.563	0	6.7E-06	0	64.1755	gcagaggatgCttgaagaaga	m5C_45729
chr12	49237776	-	C	36	11	0.306	0	0	T	25	M	0.18	0.469	1E-15	3.1E-06	1.3E-15	29.7077	ataagaaggaCcgagatcgag	m5C_45703
chr12	49237796	-	C	41	11	0.268	0	0	T	30	M	0.157	0.419	6E-15	4.4E-08	7.6E-15	24.5536	agataaggagCgggatcggaa	m5C_45766
chr12	49237808	-	C	31	18	0.581	0	0	C	18	M	0.408	0.736	0	2.7E-05	0	146.757	agaacgagaaCgagataagga	m5C_45754
chr12	49333527	-	C	31	18	0.581	0	0	C	18	M	0.408	0.736	0	2.7E-05	0	146.757	tgatcgggagCgagtaaatga	m5C_45758
chr12	49666979	+	C	65	34	0.523	0	0	C	34	M	0.404	0.64	0	4.2E-08	0	274.58	gcagatagtgCtgacggagag	m5C_45669
chr12	49666983	+	C	64	42	0.656	0	0	C	42	M	0.534	0.761	0	2E-07	0	448.589	atagtgctgaCggagaggatg	m5C_45697
chr12	50571162	-	C	60	13	0.217	0	0	T	47	M	0.131	0.336	1.1E-15	1.6E-11	1.4E-15	25.5122	cctggcaaaaCaaagaatcta	m5C_45015
chr12	50571171	-	C	48	23	0.479	0	0	T	25	M	0.345	0.617	0	2.9E-06	0	158.567	gaaaaacaacCtggcaaaaca	m5C_45013
chr12	50571172	-	C	45	10	0.222	0	0	T	35	M	0.125	0.363	7.4E-13	2.4E-08	8.9E-13	15.2157	ggaaaaacaaCctggcaaaac	m5C_44994
chr12	50571175	-	C	41	9	0.22	0	0	T	32	M	0.12	0.367	8.4E-12	1.2E-08	9.9E-12	11.9621	gtgggaaaaaCaacctggcaa	m5C_45001
chr12	50571266	-	C	35	14	0.4	0	0	T	21	M	0.256	0.564	0	9.2E-06	0	71.5414	catgtcagagCagagtgaaga	m5C_45014
chr12	50571276	-	C	37	16	0.444	1	0.027	T	20	M	0.295	0.604	0	1.7E-05	0	94.5313	aaggctggagCatgtcagagc	m5C_44989
chr12	50850369	+	C	41	9	0.22	0	0	T	32	M	0.12	0.367	8.4E-12	1.2E-08	9.9E-12	11.9621	cagtgatgatCactattccta	m5C_44970
chr12	50850534	+	C	46	13	0.283	0	0	T	33	M	0.173	0.425	0	1.2E-07	0	45.0425	ttaagtccctCtcagtgaagg	m5C_44946
chr12	50850536	+	C	49	19	0.388	0	0	T	30	M	0.264	0.528	0	1E-06	0	100.429	aagtccctctCagtgaaggt	m5C_44935
chr12	50850560	+	C	227	58	0.256	0	0	T	169	M	0.203	0.316	0	0	0	235.671	attatgagatCtgagggctgt	m5C_44964
chr12	50850567	+	C	202	152	0.752	0	0	C	152	M	0.689	0.807	0	0	0	2093.4	gatctgagggCtgtataaatg	m5C_44952
chr12	52627360	+	C	38	22	0.579	0	0	C	22	M	0.422	0.721	0	6.3E-05	0	185.645	ggagagcgagCagatcaagac	m5C_45426
chr12	53345386	+	C	142	54	0.38	0	0	T	88	M	0.305	0.462	0	0	0	328.95	gacgagctggCtcggaagaac	m5C_45402
chr12	53345397	+	C	159	37	0.233	0	0	T	122	M	0.174	0.304	0	0	0	128.614	tcggaagaacCgagaggagct	m5C_45353
chr12	53345411	+	C	158	48	0.324	10	0.063	T	100	M	0.254	0.403	0	0	0	244.01	aggagctagaCaagtactggt	m5C_45421
chr12	53345422	+	C	148	36	0.243	0	0	T	112	M	0.181	0.318	0	0	0	130.455	aagtactggtCtcagcaggtg	m5C_45341
chr12	53345427	+	C	140	29	0.207	0	0	T	111	M	0.148	0.282	0	0	0	85.9997	ctggtctcagCaggtgcgtga	m5C_45419
chr12	53345981	+	C	33	8	0.242	0	0	T	25	M	0.128	0.41	3.1E-11	6.8E-07	3.6E-11	10.7866	cgggatcctgCtgcaccttga	m5C_45382
chr12	53345984	+	C	31	22	0.71	0	0	C	22	M	0.534	0.839	0	6.3E-05	0	234.992	gatcctgctgCaccttgagtc	m5C_45366
chr12	53345987	+	C	35	20	0.571	0	0	C	20	M	0.409	0.72	0	4.3E-05	0	163.429	cctgctgcacCttgagtcaga	m5C_45423
chr12	53345999	+	C	34	18	0.529	0	0	C	18	M	0.367	0.685	0	2.7E-05	0	132.251	tgagtcagagCtggcacagac	m5C_45376
chr12	53346003	+	C	31	7	0.226	0	0	T	24	M	0.114	0.398	6.4E-10	3.4E-07	7.2E-10	7.33205	tcagagctggCacagacccgg	m5C_45347
chr12	53346005	+	C	34	7	0.206	0	0	T	27	M	0.103	0.368	1.4E-09	3.4E-07	1.6E-09	6.4054	agagctggcaCagacccgggc	m5C_45362
chr12	53428422	+	C	63	27	0.429	0	0	T	36	M	0.314	0.551	0	7.5E-09	0	169.542	agtgaagaaaCtcaggaacgg	m5C_45368
chr12	53428424	+	C	63	15	0.238	0	0	T	48	M	0.15	0.356	0	6E-11	0	44.981	tgaagaaactCaggaacggga	m5C_45405
chr12	53428436	+	C	60	14	0.233	0	0	T	46	M	0.144	0.354	0	3.2E-11	0	40.4304	ggaacgggaaCggtcgaggac	m5C_45388
chr12	53432172	+	C	36	10	0.278	0	0	T	26	M	0.158	0.44	4.8E-14	2E-06	5.9E-14	21.1115	gctcaaccatCtgaggaagga	m5C_45327
chr12	53432183	+	C	46	10	0.217	0	0	T	36	M	0.123	0.356	9.7E-13	2.4E-08	1.2E-12	14.7309	tgaggaaggaCcaggaaggaa	m5C_45429
chr12	53433092	+	C	73	27	0.37	0	0	T	46	M	0.268	0.485	0	1.9E-10	0	144.844	gtccaggcgaCggagggaaca	m5C_45394
chr12	53433101	+	C	97	38	0.392	0	0	T	59	M	0.301	0.491	0	3.7E-12	0	228.389	acggagggaaCagagaccact	m5C_45418
chr12	53433107	+	C	99	42	0.424	0	0	T	57	M	0.332	0.523	0	1.1E-11	0	278.468	ggaacagagaCcactggaagg	m5C_45367
chr12	53433108	+	C	108	26	0.241	0	0	T	82	M	0.17	0.329	0	6.7E-16	0	88.3536	gaacagagacCactggaagga	m5C_45383
chr12	53433110	+	C	105	38	0.362	0	0	T	67	M	0.276	0.457	0	9.4E-14	0	210.038	acagagaccaCtggaaggagt	m5C_45432
chr12	53433121	+	C	97	24	0.247	0	0	T	73	M	0.172	0.342	0	1.7E-14	0	82.6914	tggaaggagtCagataggtat	m5C_45380
chr12	53435134	+	C	32	13	0.406	0	0	T	19	M	0.255	0.577	0	6.7E-06	0	66.3505	aatggatggtCttggatgatg	m5C_45384
chr12	53693160	+	C	33	15	0.455	0	0	T	18	M	0.298	0.62	0	1.2E-05	0	89.5281	cagagggacaCcctttgggcg	m5C_45439
chr12	53693161	+	C	44	15	0.341	0	0	T	29	M	0.219	0.489	0	2.8E-07	0	65.6269	agagggacacCctttgggcgt	m5C_45406
chr12	53693162	+	C	40	14	0.35	0	0	T	26	M	0.221	0.505	0	1.9E-07	0	61.9761	gagggacaccCtttgggcgtg	m5C_45357
chr12	53693169	+	C	49	32	0.653	0	0	C	32	M	0.513	0.771	0	1.6E-05	0	328.39	accctttgggCgtggcttcct	m5C_45425
chr12	53693177	+	C	53	13	0.245	0	0	T	40	M	0.149	0.376	2.2E-16	1.6E-09	2.9E-16	30.3876	ggcgtggcttCctggtgatgg	m5C_45395
chr12	53693178	+	C	55	28	0.509	0	0	C	28	M	0.381	0.636	0	3.1E-07	0	213.226	gcgtggcttcCtggtgatggg	m5C_45369
chr12	54161745	-	C	39	10	0.256	0	0	T	29	M	0.146	0.411	1.3E-13	2E-06	1.6E-13	18.7753	cgagtgctgcCcttctttaaa	m5C_45102
chr12	54161746	-	C	36	22	0.611	0	0	C	22	M	0.449	0.752	0	6.3E-05	0	197.402	ccgagtgctgCccttctttaa	m5C_45110
chr12	54427222	+	C	33	8	0.242	0	0	T	25	M	0.128	0.41	3.1E-11	6.8E-07	3.6E-11	10.7866	ggcggctcgcCcggcgctgga	m5C_45093
chr12	54427223	+	C	32	14	0.438	0	0	T	18	M	0.282	0.607	0	9.2E-06	0	78.8622	gcggctcgccCggcgctggag	m5C_45082
chr12	54427241	+	C	33	16	0.485	0	0	T	17	M	0.325	0.648	0	1.7E-05	0	104.012	gaggagcgagCtaagagcagt	m5C_45089
chr12	54741599	+	C	62	24	0.387	0	0	T	38	M	0.276	0.512	0	3E-09	0	132.405	aggggctgttCttggctgtgg	m5C_45079
chr12	54741604	+	C	66	30	0.455	0	0	T	36	M	0.34	0.574	0	1.7E-08	0	204.144	ctgttcttggCtgtggatgaa	m5C_45091
chr12	56119678	-	C	49	34	0.694	0	0	C	34	M	0.555	0.805	0	2.2E-05	0	377.209	accttcagggCtgtgtggaga	m5C_46007

chr12	56119913	-	C	41	10	0.244	0	0	T	31	M	0.138	0.393	2.4E-13	2.4E-08	2.9E-13	17.4472	gaaggcgatcCataaggaggt	m5C_45889
chr12	56119918	-	C	41	10	0.244	0	0	T	31	M	0.138	0.393	2.4E-13	2.4E-08	2.9E-13	17.4472	aacgagaaggCgatccataag	m5C_46005
chr12	56120017	-	C	73	15	0.205	0	0	T	58	M	0.129	0.312	0	6E-13	0	38.6006	gctaactacaCagattgggag	m5C_45945
chr12	56120029	-	C	92	24	0.261	0	0	T	68	M	0.182	0.359	0	1.7E-14	0	87.3701	tgctgtggggCtgctaactac	m5C_45930
chr12	56120037	-	C	65	15	0.231	0	0	T	50	M	0.145	0.346	0	6E-11	0	43.5411	agtttaagtgCtgtggggctg	m5C_45926
chr12	56320745	+	C	37	23	0.622	0	0	C	23	M	0.461	0.759	0	7.6E-05	0	212.061	gaaggcggtcCggggggcggg	m5C_45876
chr12	56360805	+	C	36	12	0.333	0	0	T	24	M	0.202	0.497	0	4.6E-06	0	48.5146	ggagaacttcCaaaaggtgga	m5C_45835
chr12	56510625	+	C	60	20	0.333	0	0	T	40	M	0.227	0.459	0	6.9E-10	0	90.9164	taggagttggCgagtagtagc	m5C_45859
chr12	56554066	+	C	34	11	0.324	0	0	T	23	M	0.191	0.492	4.4E-16	3.1E-06	5.8E-16	32.3088	atgctggtggCagggcatgag	m5C_45806
chr12	56584152	+	C	46	17	0.37	0	0	T	29	M	0.245	0.514	0	5.6E-07	0	83.3801	ttggaagtcaCggtggccgag	m5C_45827
chr12	56584158	+	C	78	35	0.449	0	0	T	43	M	0.343	0.559	0	1.9E-09	0	240.339	gtcacggtggCcgagtggtta	m5C_45875
chr12	56584159	+	C	78	38	0.487	0	0	T	40	M	0.379	0.596	0	3.7E-09	0	288.411	tcacggtggcCgagtggttaa	m5C_45858
chr12	56817464	-	C	47	16	0.34	0	0	T	31	M	0.222	0.483	0	4E-07	0	70.9352	gaacgtggggCagaggaagaa	m5C_45909
chr12	57032144	-	C	40	9	0.225	0	0	T	31	M	0.123	0.375	6.4E-12	1.2E-08	7.6E-12	12.4041	gaagctgtggCaaaagctgat	m5C_45969
chr12	57032163	-	C	40	12	0.3	0	0	T	28	M	0.181	0.454	1.1E-16	7.5E-08	1.5E-16	34.6049	tatggtgggaCccattgaaga	m5C_45891
chr12	57037472	-	C	377	112	0.297	0	0	T	265	M	0.253	0.345	0	0	0	567.14	gatgcaatttCtgagaaggaa	m5C_45978
chr12	57037500	-	C	477	222	0.465	0	0	T	255	M	0.421	0.51	0	0	0	1869.68	tttcgttcttCtgagtttgct	m5C_45975
chr12	57037503	-	C	483	149	0.31	2	0.004	T	332	M	0.27	0.352	0	0	0	804.872	gactttcgttCttctgagttt	m5C_45927
chr12	57037507	-	C	483	101	0.21	1	0.002	T	381	M	0.176	0.248	0	0	0	354.667	ttctgactttCgttcttctga	m5C_45999
chr12	57037515	-	C	524	114	0.218	0	0	T	410	M	0.184	0.255	0	0	0	420.329	tgagtacgttCtgactttcgt	m5C_46020
chr12	57037529	-	C	302	90	0.298	0	0	T	212	M	0.249	0.352	0	0	0	448.607	ctattcctcaCtgatgagtac	m5C_45995
chr12	57038819	-	C	304	120	0.395	0	0	T	184	M	0.341	0.451	0	0	0	819.435	catttaggtaCtgagaaggag	m5C_45906
chr12	57038829	-	C	334	73	0.219	0	0	T	261	M	0.178	0.266	0	0	0	259.264	gctgaagccaCatttaggtac	m5C_46024
chr12	57038832	-	C	341	80	0.235	0	0	T	261	M	0.193	0.282	0	0	0	308.38	tttgctgaagCcacatttagg	m5C_46000
chr12	57038838	-	C	341	100	0.293	0	0	T	241	M	0.247	0.344	0	0	0	494.905	tctgagtttgCtgaagccaca	m5C_45944
chr12	57038847	-	C	333	89	0.267	0	0	T	244	M	0.223	0.317	0	0	0	396.206	tttcgttcttCtgagtttgct	m5C_46008
chr12	57038850	-	C	341	75	0.22	0	0	T	266	M	0.179	0.267	0	0	0	268.848	gactttcgttCttctgagttt	m5C_46015
chr12	57038854	-	C	334	115	0.344	0	0	T	219	M	0.295	0.397	0	0	0	679.383	aaatgactttCgttcttctga	m5C_45896
chr12	57038858	-	C	329	73	0.222	0	0	T	256	M	0.18	0.27	0	0	0	263.295	atcaaaatgaCtttcgttctt	m5C_46013
chr12	57038866	-	C	314	67	0.213	0	0	T	247	M	0.172	0.262	0	0	0	230.033	atgattttatCaaaatgactt	m5C_45967
chr12	57038964	-	C	32	22	0.688	0	0	C	22	M	0.514	0.82	0	6.3E-05	0	226.305	ggaggtggccCagcatttggg	m5C_45937
chr12	57038966	-	C	38	20	0.526	0	0	C	20	M	0.373	0.675	0	4.3E-05	0	149.035	ttggaggtggCccagcatttg	m5C_45914
chr12	57038986	-	C	39	9	0.231	0	0	T	30	M	0.126	0.383	4.9E-12	1.2E-06	5.7E-12	12.8739	gcagggagacCagactggttt	m5C_45903
chr12	57038987	-	C	36	11	0.306	0	0	T	25	M	0.18	0.469	1E-15	3.1E-06	1.3E-15	29.7077	ggcagggagaCcagactggtt	m5C_45976
chr12	57108175	-	C	78	20	0.256	0	0	T	58	M	0.173	0.363	0	1.1E-11	0	69.0212	taaagcaaaaCagagtcggag	m5C_45933
chr12	57108192	-	C	41	10	0.244	0	0	T	31	M	0.138	0.393	2.4E-13	2.4E-08	2.9E-13	17.4472	gatgaagaacCagtcagtaaa	m5C_45996
chr12	57108193	-	C	36	16	0.444	0	0	T	20	M	0.295	0.604	0	1.7E-05	0	94.5313	tgatgaagaaCcagtcagtaa	m5C_45954
chr12	57118810	-	C	32	13	0.406	0	0	T	19	M	0.255	0.577	0	6.7E-06	0	66.3505	ggagcagttgCagtgttgagg	m5C_45941
chr12	57118816	-	C	35	15	0.429	0	0	T	20	M	0.28	0.591	0	1.2E-05	0	83.953	gagatgggagCagttgcagtg	m5C_45957
chr12	58112013	+	C	50	14	0.28	0	0	T	36	M	0.175	0.417	0	1.9E-07	0	48.9272	aaggggtgatCcagaacggca	m5C_45045
chr12	58112014	+	C	51	17	0.333	0	0	T	34	M	0.22	0.47	0	1.1E-08	0	74.6972	aggggtgatcCagaacggcag	m5C_45046
chr12	58112019	+	C	51	17	0.333	0	0	T	34	M	0.22	0.47	0	1.1E-08	0	74.6972	tgatccagaaCggcagagaga	m5C_45023
chr12	58218442	-	C	69	46	0.667	0	0	C	46	M	0.549	0.766	0	3.8E-07	0	505.322	ccaggatagg Ctgtttccatc	m5C_45049
chr12	58218451	-	C	75	23	0.307	0	0	T	52	M	0.214	0.418	0	4.3E-11	0	98.3789	tcaagtaatcCaggataggct	m5C_45059
chr12	58218452	-	C	76	36	0.474	0	0	T	40	M	0.365	0.584	0	2.4E-09	0	263.102	ttcaagtaatCcaggataggc	m5C_45063
chr12	58218460	-	C	38	9	0.237	0	0	T	29	M	0.13	0.392	3.7E-12	1.2E-06	4.3E-12	13.3739	tggctggattCaagtaatcca	m5C_45051
chr12	58388803	-	C	37	21	0.568	0	0	C	21	M	0.409	0.713	0	5.2E-05	0	171.838	ttgccatctgCtcatccccat	m5C_45065
chr12	58388810	-	C	43	12	0.279	0	0	T	31	M	0.167	0.427	2.2E-16	7.5E-08	2.9E-16	31.457	ctgggagttgCcatctgctca	m5C_45061
chr12	58388820	-	C	43	11	0.256	0	0	T	32	M	0.149	0.402	1.2E-14	4.4E-08	1.5E-14	22.8887	tgcgggatgcCtgggagttgc	m5C_45062
chr12	62997488	+	C	94	19	0.202	0	0	T	75	M	0.133	0.294	0	8.9E-16	0	50.6896	gtagtttgtgCtgttggtcgg	m5C_47723
chr12	62997515	+	C	31	10	0.323	0	0	T	21	M	0.186	0.499	7.1E-15	2E-06	9E-15	26.2725	acattgcccgCtgtggagata	m5C_47711
chr12	63244563	-	C	111	30	0.27	0	0	T	81	M	0.196	0.36	0	0	0	117.821	cgaaactcgaCtgcatgattt	m5C_47739
chr12	63244568	-	C	40	10	0.25	0	0	T	30	M	0.142	0.402	1.8E-13	2.4E-08	2.2E-13	18.0914	aagtgcgaaaCtcgactgcat	m5C_47730
chr12	63244593	-	C	70	17	0.243	0	0	T	53	M	0.158	0.355	0	2.2E-12	0	53.5559	gtgctgacccCtgtgatttcc	m5C_47738
chr12	63244595	-	C	75	27	0.36	0	0	T	48	M	0.261	0.473	0	1.9E-10	0	140.75	gtgtgctgacCcctgtgattt	m5C_47734
chr12	63244596	-	C	85	18	0.212	0	0	T	67	M	0.138	0.31	0	4.5E-14	0	49.8101	ggtgtgctgaCccctgtgatt	m5C_47744
chr12	63244609	-	C	120	27	0.225	0	0	T	93	M	0.159	0.308	0	0	0	86.1249	ccattgcactCtgggtgtgct	m5C_47743
chr12	63244629	-	C	42	12	0.286	0	0	T	30	M	0.172	0.436	2.2E-16	7.5E-08	2.9E-16	32.2467	tttcccagggCgaggtttatc	m5C_47742
chr12	69675982	-	C	32	13	0.406	0	0	T	19	M	0.255	0.577	0	6.7E-06	0	66.3505	atgcgggaggCttgagcccag	m5C_45541
chr12	79197227	-	C	35	15	0.429	0	0	T	20	M	0.28	0.591	0	1.2E-05	0	83.953	gtaaccgggaCatgcggtgca	m5C_45505
chr12	80214601	-	C	32	11	0.344	0	0	T	21	M	0.204	0.517	2.2E-16	3.1E-06	2.9E-16	35.1442	tctagctgctCtagtgaagaa	m5C_45649
chr12	80214606	-	C	34	15	0.441	0	0	T	19	M	0.289	0.605	0	1.2E-05	0	86.6497	atgagtctagCtgctctagtg	m5C_45654
chr12	80214610	-	C	33	8	0.242	0	0	T	25	M	0.128	0.41	3.1E-11	6.8E-07	3.6E-11	10.7866	aaggatgagtCtagctgctct	m5C_45667
chr12	84683582	+	C	33	7	0.212	0	0	T	26	M	0.107	0.378	1.1E-09	3.4E-07	1.2E-09	6.69165	tagcagttatCacctttgcct	m5C_46090
chr12	88824226	+	C	37	20	0.541	0	0	C	20	M	0.384	0.69	0	4.3E-05	0	153.535	tgtagatggtCcgaaggtagt	m5C_46035
chr12	88824227	+	C	37	12	0.324	0	0	T	25	M	0.196	0.485	0	4.6E-06	0	47.1194	gtagatggtcCgaaggtagtg	m5C_46039
chr12	89746239	-	C	42	20	0.476	0	0	T	22	M	0.334	0.623	0	1.4E-06	0	133.437	gtgtgcgaggCtgagtccaag	m5C_46058
chr12	89984445	-	C	35	12	0.343	0	0	T	23	M	0.208	0.508	0	4.6E-06	0	49.9956	tatctcagtcCtggatgggag	m5C_46052
chr12	89984446	-	C	35	15	0.429	0	0	T	20	M	0.28	0.591	0	1.2E-05	0	83.953	ttatctcagtCctggatggga	m5C_46057
chr12	89984450	-	C	31	18	0.581	0	0	C	18	M	0.408	0.736	0	2.7E-05	0	146.757	tagtttatctCagtcctggat	m5C_46051
chr12	93750936	-	C	59	25	0.424	0	0	T	34	M	0.306	0.551	0	1.5E-07	0	153.063	ccctgatggtCtagtggctag	m5C_45799
chr12	95868056	+	C	37	11	0.297	0	0	T	26	M	0.175	0.458	1.4E-15	3.1E-06	1.9E-15	28.5508	gctgaggaagCagccaagaaa	m5C_46125
chr12	95868059	+	C	43	10	0.233	0	0	T	33	M	0.132	0.377	4.3E-13	2.4E-08	5.2E-13	16.2683	gaggaagcagCcaagaaaaaa	m5C_46107
chr12	95868060	+	C	41	18	0.439	0	0	T	23	M	0.299	0.59	0	7.7E-07	0	107.604	aggaagcagcCaagaaaaaaa	m5C_46123
chr12	95877001	+	C	34	10	0.294	0	0	T	24	M	0.168	0.462	2.3E-14	2E-06	2.9E-14	22.9564	atggagatggCgatggagatg	m5C_46127
chr12	95877015	+	C	34	13	0.382	0	0	T	21	M	0.239	0.55	0	6.7E-06	0	62.1399	ggagatggagCaactggaaag	m5C_46126
chr12	95877018	+	C	37	24	0.649	0	0	C	24	M	0.488	0.782	0	9.1E-05	0	234.042	gatggagcaaCtggaaagaag	m5C_46106
chr12	96429801	+	C	205	60	0.293	0	0	T	145	M	0.235	0.358	0	0	0	281.603	cgtgcggttcCtcgttagtat	m5C_46409
chr12	96429827	+	C	638	134	0.21	0	0	T	504	M	0.18	0.243	0	0	0	482.953	tgagtatcccCgcctgtcacg	m5C_46408
chr12	96429845	+	C	888	220	0.249	3	0.003	T	665	M	0.221	0.278	0	0	0	973.456	acgcgggagaCcgggggttcga	m5C_46396
chr12	96429853	+	C	903	196	0.217	0	0	T	707	M	0.191	0.245	0	0	0	750.268	gaccggggttCgattccccga	m5C_46402
chr12	96429858	+	C	890	194	0.218	0	0	T	696	M	0.192	0.246	0	0	0	745.33	gggttcgattCcccgacgggg	m5C_46413
chr12	96429860	+	C	905	182	0.201	0	0	T	723	M	0.176	0.228	0	0	0	641.652	gttcgattccCcgacggggag	m5C_46398
chr12	98850879	+	C	43	34	0.791	0	0	C	34	M	0.648	0.886	0	2.2E-05	0	440.6	aggggagcgcCgcgtcaccag	m5C_46964
chr12	98897283	+	C	167	39	0.234	0	0	T	128	M	0.176	0.303	0	0	0	137.124	ctaacgactcCtcgttagtat	m5C_46946
chr12	98897285	+	C	169	49	0.29	0	0	T	120	M	0.227	0.362	0	0	0	222.268	aacgactcctCgttagtatag	m5C_46931
chr12	98897306	+	C	633	137	0.22	9	0.014	T	487	M	0.189	0.254	0	0	0	517.428	tggttagtatCcccgcctgtc	m5C_46976
chr12	98897307	+	C	649	138	0.213	0	0	T	511	M	0.183	0.246	0	0	0	504.784	ggttagtatcCccgcctgtca	m5C_46937
chr12	98897308	+	C	650	133	0.205	0	0	T	517	M	0.175	0.237	0	0	0	466.51	gttagtatccCcgcctgtcac	m5C_46973
chr12	98897309	+	C	650	149	0.229	0	0	T	501	M	0.199	0.263	0	0	0	591.718	ttagtatcccCgcctgtcacg	m5C_46938
chr12	98897327	+	C	950	235	0.247	0	0	T	715	M	0.221	0.276	0	0	0	1038.62	acgcgggagaCcggggttcaa	m5C_46925
chr12	98897328	+	C	972	235	0.242	0	0	T	737	M	0.216	0.27	0	0	0	1014.74	cgcgggagacCggggttcaat	m5C_46941
chr12	98897341	+	C	788	191	0.242	0	0	T	597	M	0.214	0.274	0	0	0	816.569	ggttcaattcCccgacgggga	m5C_46962
chr12	98897366	+	C	41	18	0.439	0	0	T	23	M	0.299	0.59	0	7.7E-07	0	107.604	tgtagctgcaCttttttggcg	m5C_46968
chr12	98898040	+	C	57	13	0.228	0	0	T	44	M	0.138	0.352	4.4E-16	1.6E-09	5.8E-16	27.6258	gacctcgtggCgcaacggtag	m5C_46940
chr12	98898051	+	C	47	20	0.426	0	0	T	27	M	0.295	0.567	0	1.4E-06	0	118.054	gcaacggtagCgcgtctgact	m5C_46935
chr12	98898075	+	C	33	10	0.303	0	0	T	23	M	0.174	0.473	1.6E-14	2E-06	2E-14	23.982	gatcagaaggCtgcgtgttcg	m5C_46970
chr12	98993430	+	C	144	59	0.41	0	0	T	85	M	0.333	0.491	0	0	0	392.656	ttccttagatCcacctttgtg	m5C_46948
chr12	98993434	+	C	140	32	0.229	0	0	T	108	M	0.167	0.305	0	0	0	106.762	ttagatccacCtttgtggatg	m5C_46930
chr12	98993454	+	C	87	27	0.31	0	0	T	60	M	0.223	0.414	0	4E-12	0	120.361	gaatcttgaaCtgagttccac	m5C_46957
chr12	98993461	+	C	60	20	0.333	0	0	T	40	M	0.227	0.459	0	6.9E-10	0	90.9164	gaactgagttCcacttgtaaa	m5C_46959

chr12	98993462	+	C	57	22	0.386	0	0	T	35	M	0.271	0.516	0	6.5E-08	0	119.075	aactgagttcCacttgtaaac	m5C_46958
chr12	98993464	+	C	52	14	0.269	0	0	T	38	M	0.168	0.403	0	2.8E-09	0	46.9527	ctgagttccaCttgtaaactt	m5C_46966
chr12	98993611	+	C	36	10	0.278	0	0	T	26	M	0.158	0.44	4.8E-14	2E-06	5.9E-14	21.1115	aaatgacatgCatttatgctg	m5C_46927
chr12	98993623	+	C	62	13	0.21	0	0	T	49	M	0.127	0.326	1.8E-15	1.6E-11	2.3E-15	24.3221	tttatgctgcCatttgttact	m5C_46933
chr12	98993648	+	C	207	43	0.208	0	0	T	164	M	0.158	0.268	0	0	0	135.911	ggactcgactCgtgtgcggac	m5C_46955
chr12	98993658	+	C	186	47	0.253	0	0	T	139	M	0.196	0.32	0	0	0	183.934	cgtgtgcggaCatttctgtta	m5C_46975
chr12	101776964	+	C	38	17	0.447	0	0	T	21	M	0.301	0.603	0	2.1E-05	0	102.497	aaggcggagtCtgacggagaa	m5C_46071
chr12	102183762	-	C	33	19	0.576	0	0	C	19	M	0.408	0.728	0	3.4E-05	0	155.067	actgaaggaaCtacagcaggt	m5C_46199
chr12	102321584	-	C	38	8	0.211	0	0	T	30	M	0.111	0.363	1.3E-10	6.8E-07	1.4E-10	8.77246	actaattgatCgcaaccagtt	m5C_46195
chr12	102321593	-	C	46	11	0.239	0	0	T	35	M	0.139	0.379	2.8E-14	4.4E-08	3.5E-14	20.7303	gtgtttacaaCtaattgatcg	m5C_46201
chr12	102321615	-	C	49	13	0.265	0	0	T	36	M	0.162	0.403	0	1.2E-07	0	42.1489	cctggcttgtCcgagtgcagt	m5C_46190
chr12	104325338	+	C	163	48	0.294	0	0	T	115	M	0.23	0.369	0	0	0	220.689	gtggatggtaCagtagaagag	m5C_46589
chr12	104325352	+	C	356	82	0.23	0	0	T	274	M	0.19	0.277	0	0	0	310.971	agaagaggatCtgggtaaaag	m5C_46550
chr12	104325374	+	C	857	193	0.225	1	0.001	T	663	M	0.199	0.255	0	0	0	767.115	agagaaggatCaaggacggat	m5C_46553
chr12	104332136	+	C	46	17	0.37	0	0	T	29	M	0.245	0.514	0	5.6E-07	0	83.3801	tgttgaggagCccatggagga	m5C_46587
chr12	104332138	+	C	57	14	0.246	0	0	T	43	M	0.152	0.371	0	2.8E-09	0	42.6516	ttgaggagccCatggaggaag	m5C_46570
chr12	104332158	+	C	63	17	0.27	0	0	T	46	M	0.176	0.39	0	1.8E-10	0	59.7846	gaagaagcagCcaaagaagag	m5C_46574
chr12	104332179	+	C	51	37	0.725	0	0	C	37	M	0.591	0.829	0	1.8E-06	0	436.993	aaagaagaatCtgatgatgaa	m5C_46588
chr12	104336932	+	C	32	12	0.375	0	0	T	20	M	0.229	0.547	0	4.6E-06	0	55.0408	tggatgaataCtgtattcagg	m5C_46586
chr12	104336982	+	C	34	11	0.324	0	0	T	23	M	0.191	0.492	4.4E-16	3.1E-06	5.8E-16	32.3088	cagaatgttgCcaaggaagga	m5C_46566
chr12	104337001	+	C	32	25	0.781	0	0	C	25	M	0.612	0.89	0	0.00011	0	306.223	gagtgaagttCgatgaaagtg	m5C_46615
chr12	104340715	+	C	68	35	0.515	0	0	C	35	M	0.398	0.629	0	5.3E-08	0	278.842	gaccctgatgCaaaggtttgt	m5C_46561
chr12	104341101	+	C	166	46	0.277	0	0	T	120	M	0.215	0.35	0	0	0	197.477	ggaagaagagCccgaagaaga	m5C_46559
chr12	104341102	+	C	173	80	0.462	0	0	T	93	M	0.39	0.537	0	0	0	623.606	gaagaagagcCcgaagaagaa	m5C_46577
chr12	104341133	+	C	64	28	0.438	0	0	T	36	M	0.323	0.559	0	9.8E-09	0	180.845	cagcagaagaCacaacagaag	m5C_46617
chr12	104341138	+	C	53	13	0.245	0	0	T	40	M	0.149	0.376	2.2E-16	1.6E-09	2.9E-16	30.3876	gaagacacaaCagaagacaca	m5C_46548
chr12	104341145	+	C	46	10	0.217	0	0	T	36	M	0.123	0.356	9.7E-13	2.4E-08	1.2E-12	14.7309	caacagaagaCacagagcaag	m5C_46608
chr12	104341147	+	C	57	11	0.229	9	0.158	T	37	M	0.133	0.365	5E-14	4.4E-08	6.2E-14	19.4712	acagaagacaCagagcaagac	m5C_46558
chr12	104341152	+	C	78	35	0.449	0	0	T	43	M	0.343	0.559	0	1.9E-09	0	240.339	agacacagagCaagacgaaga	m5C_46571
chr12	104341157	+	C	107	45	0.421	0	0	T	62	M	0.331	0.515	0	7.4E-13	0	298.234	cagagcaagaCgaagatgaag	m5C_46549
chr12	104341201	+	C	67	20	0.303	1	0.015	T	46	M	0.206	0.422	0	6.9E-10	0	82.2155	gaagaagaaaCagcaaaggta	m5C_46611
chr12	104341204	+	C	50	39	0.78	0	0	C	39	M	0.648	0.872	0	2.5E-06	0	505.114	gaagaaacagCaaaggtatgg	m5C_46598
chr12	104720154	+	C	32	7	0.219	0	0	T	25	M	0.11	0.388	8.5E-10	3.4E-07	9.5E-10	6.99971	gagatgcttgCacaagaaaaa	m5C_46564
chr12	104720156	+	C	34	11	0.324	0	0	T	23	M	0.191	0.492	4.4E-16	3.1E-06	5.8E-16	32.3088	gatgcttgcaCaagaaaaatt	m5C_46593
chr12	104720169	+	C	119	28	0.235	0	0	T	91	M	0.168	0.319	0	0	0	94.1382	gaaaaattggCttagaaaccg	m5C_46594
chr12	104720178	+	C	180	65	0.361	0	0	T	115	M	0.295	0.434	0	0	0	382.875	gcttagaaacCgtaggggtga	m5C_46596
chr12	105245609	+	C	34	11	0.324	0	0	T	23	M	0.191	0.492	4.4E-16	3.1E-06	5.8E-16	32.3088	agaggctggtCcaatggtagt	m5C_46616
chr12	105245610	+	C	36	10	0.278	0	0	T	26	M	0.158	0.44	4.8E-14	2E-06	5.9E-14	21.1115	gaggctggtcCaatggtagtg	m5C_46580
chr12	106529141	-	C	56	27	0.482	0	0	T	29	M	0.357	0.61	0	2.5E-07	0	192.607	ctggtgcgacCctgtgtgtcg	m5C_46219
chr12	106529142	-	C	60	22	0.367	0	0	T	38	M	0.256	0.493	0	1.5E-09	0	112.731	gctggtgcgaCcctgtgtgtc	m5C_46225
chr12	106529151	-	C	76	16	0.211	0	0	T	60	M	0.134	0.315	0	1.2E-12	0	42.8641	ttggagtcagCtggtgcgacc	m5C_46241
chr12	106529162	-	C	114	37	0.325	0	0	T	77	M	0.246	0.415	0	1.4E-15	0	181.707	gaatgagatgCttggagtcag	m5C_46248
chr12	107983494	-	C	43	25	0.581	0	0	C	25	M	0.433	0.716	0	4.5E-06	0	216.642	caagtccgggCtgtgaacctt	m5C_46242
chr12	107983504	-	C	43	18	0.419	0	0	T	25	M	0.284	0.567	0	7.7E-07	0	102.184	gctggaataCaagtccgggc	m5C_46217
chr12	108954645	-	C	31	10	0.323	0	0	T	21	M	0.186	0.499	7.1E-15	2E-06	9E-15	26.2725	gaaaaaccagCtggagattga	m5C_46314
chr12	109011138	-	C	57	12	0.211	0	0	T	45	M	0.125	0.333	1.6E-14	8.8E-10	2E-14	20.643	gagggatggcCgggggcaagc	m5C_46324
chr12	109011139	-	C	56	22	0.393	0	0	T	34	M	0.276	0.524	0	6.5E-08	0	121.353	agagggatggCcgggggcaag	m5C_46309
chr12	110812101	-	C	69	15	0.217	0	0	T	54	M	0.136	0.328	0	6E-11	0	40.9219	agaggggatgCagaagatgga	m5C_46361
chr12	112298049	-	C	31	15	0.484	0	0	T	16	M	0.32	0.652	0	1.2E-05	0	95.91	gggtatagctCagcagtagag	m5C_47131
chr12	112460337	+	C	36	14	0.389	0	0	T	22	M	0.248	0.551	0	9.2E-06	0	69.397	agagatgacaCggatcgccag	m5C_47064
chr12	112460342	+	C	51	16	0.314	0	0	T	35	M	0.203	0.45	0	7.3E-09	0	65.048	tgacacggatCgccaggctga	m5C_47112
chr12	112460345	+	C	54	11	0.204	0	0	T	43	M	0.118	0.329	2.3E-13	4.4E-10	2.8E-13	16.3622	cacggatcgcCaggctgattg	m5C_47072
chr12	112460375	+	C	96	33	0.344	0	0	T	63	M	0.256	0.443	0	7.5E-13	0	169.226	agatgagtgaCgggaagaagg	m5C_47092
chr12	112460691	+	C	103	24	0.233	0	0	T	79	M	0.162	0.323	0	2.2E-16	0	77.7	aatgacacctCagaaggaatg	m5C_47113
chr12	112460706	+	C	106	54	0.509	0	0	C	54	M	0.416	0.603	0	6.4E-12	0	448.862	ggaatgagtgCtatagagagg	m5C_47098
chr12	112590564	+	C	46	27	0.587	0	0	C	27	M	0.443	0.717	0	6.7E-06	0	239.432	caacagggagCtggggatgca	m5C_47103
chr12	112590573	+	C	49	15	0.306	0	0	T	34	M	0.195	0.445	0	2.8E-07	0	58.546	gctggggatgCagaagaggaa	m5C_47078
chr12	112771888	+	C	37	16	0.432	0	0	T	21	M	0.287	0.591	0	1.7E-05	0	91.7488	gtggtgcgtgCtgtagtccca	m5C_47059
chr12	112771895	+	C	37	15	0.405	0	0	T	22	M	0.263	0.565	0	1.2E-05	0	79.0389	gtgctgtagtCccagccactt	m5C_47091
chr12	112771896	+	C	38	9	0.237	0	0	T	29	M	0.13	0.392	3.7E-12	1.2E-06	4.3E-12	13.3739	tgctgtagtcCcagccacttg	m5C_47095
chr12	112771897	+	C	40	25	0.625	0	0	C	25	M	0.47	0.758	0	4.5E-06	0	235.161	gctgtagtccCagccacttgg	m5C_47096
chr12	112771903	+	C	35	19	0.543	0	0	C	19	M	0.382	0.695	0	3.4E-05	0	145.12	gtcccagccaCttgggaggct	m5C_47108
chr12	113112490	-	C	33	17	0.515	0	0	C	17	M	0.352	0.675	0	2.1E-05	0	119.742	atccctggagCagggtcacag	m5C_47124
chr12	113329111	-	C	35	21	0.6	0	0	C	21	M	0.436	0.744	0	5.2E-05	0	183.004	cggccaactcCtgggagtaca	m5C_47132
chr12	113500330	+	C	80	23	0.287	0	0	T	57	M	0.2	0.395	0	7.2E-13	0	91.9392	gtggatttggCctgtgtgctg	m5C_47074
chr12	114394891	+	C	39	12	0.308	0	0	T	27	M	0.186	0.464	0	4.6E-06	0	44.5578	gtgtgttgacCggggctgctc	m5C_46267
chr12	117213989	-	C	41	25	0.61	0	0	C	25	M	0.457	0.743	0	4.5E-06	0	228.633	gggtgggtgcCaaagtgtttt	m5C_46381
chr12	117213990	-	C	47	26	0.553	0	0	C	26	M	0.412	0.686	0	5.5E-06	0	214.475	ggggtgggtgCcaaagtgttt	m5C_46385
chr12	117908959	+	C	32	11	0.344	0	0	T	21	M	0.204	0.517	2.2E-16	3.1E-06	2.9E-16	35.1442	ctgtgtctccCctcatccccc	m5C_46367
chr12	117908960	+	C	35	10	0.286	0	0	T	25	M	0.163	0.451	3.4E-14	2E-06	4.2E-14	21.9991	tgtgtctcccCtcatcccca	m5C_46375
chr12	118583223	+	C	31	9	0.29	0	0	T	22	M	0.161	0.466	3.7E-13	1.2E-06	4.4E-13	18.0154	acatggcaatCtagaccggta	m5C_46430
chr12	119364945	-	C	40	27	0.675	0	0	C	27	M	0.52	0.799	0	0.00015	0	280.894	ataaggagggCtgtgtggagt	m5C_46471
chr12	120574620	-	C	33	10	0.303	0	0	T	23	M	0.174	0.473	1.6E-14	2E-06	2E-14	23.982	gggaggacagCtgtcaggacc	m5C_46728
chr12	120574642	-	C	41	9	0.22	0	0	T	32	M	0.12	0.367	8.4E-12	1.2E-08	9.9E-12	11.9621	caatatctccCatggagttga	m5C_46737
chr12	120574643	-	C	40	9	0.225	0	0	T	31	M	0.123	0.375	6.4E-12	1.2E-08	7.6E-12	12.4041	tcaatatctcCcatggagttg	m5C_46696
chr12	120574658	-	C	42	10	0.238	0	0	T	32	M	0.135	0.385	3.2E-13	2.4E-08	3.9E-13	16.8407	gagattcagcCcaaatcaata	m5C_46702
chr12	120574671	-	C	41	19	0.463	0	0	T	22	M	0.321	0.613	0	1E-06	0	121.813	aggctgtgatCctgagattca	m5C_46703
chr12	120729565	-	C	214	103	0.481	0	0	T	111	M	0.415	0.548	0	0	0	855.488	tctacggagaCtgaattttta	m5C_46699
chr12	120729626	-	C	167	34	0.204	0	0	T	133	M	0.15	0.271	0	0	0	101.666	tttcccaataCcccgccatga	m5C_46725
chr12	120729633	-	C	113	23	0.204	0	0	T	90	M	0.14	0.287	0	0	0	64.2309	gaaaacttttCccaataccc	m5C_46771
chr12	120729638	-	C	93	22	0.237	0	0	T	71	M	0.162	0.332	0	5.8E-15	0	71.1592	taattgaaaaCttttcccaat	m5C_46768
chr12	120729660	-	C	89	27	0.303	0	0	T	62	M	0.218	0.405	0	4E-12	0	117.526	ttatccgaggCgcgattattg	m5C_46686
chr12	120730899	-	C	220	105	0.477	0	0	T	115	M	0.412	0.543	0	0	0	865.677	tctacggagaCtgaattttct	m5C_46713
chr12	120730905	-	C	335	69	0.206	0	0	T	266	M	0.166	0.252	0	0	0	229.238	acagtctctaCggagactgaa	m5C_46689
chr12	120730914	-	C	365	92	0.252	0	0	T	273	M	0.21	0.299	0	0	0	386.861	caatttttgaCagtctctacg	m5C_46764
chr12	120730924	-	C	372	80	0.215	0	0	T	292	M	0.176	0.26	0	0	0	282.122	tcggcactggCaatttttgac	m5C_46724
chr12	120730928	-	C	381	82	0.215	0	0	T	299	M	0.177	0.259	0	0	0	290.126	atagtcggcaCtggcaatttt	m5C_46773
chr12	120730965	-	C	70	23	0.329	0	0	T	47	M	0.23	0.445	0	4.3E-11	0	105.794	aaacttttccCaataccccgc	m5C_46715
chr12	120730999	-	C	66	18	0.273	0	0	T	48	M	0.18	0.39	0	2.9E-10	0	64.8115	gaggtctatcCgaggcgcgat	m5C_46747
chr12	120731000	-	C	66	16	0.242	0	0	T	50	M	0.155	0.358	0	1.1E-10	0	49.629	tgaggtctatCcgaggcgcga	m5C_46677
chr12	120731019	-	C	52	13	0.25	0	0	T	39	M	0.152	0.382	1.1E-16	1.6E-09	1.5E-16	31.5947	gtggcagtatCgtagccaatg	m5C_46716
chr12	121111560	-	C	39	27	0.692	0	0	C	27	M	0.536	0.814	0	0.00015	0	289.324	agaattcctgCctccctataa	m5C_46701
chr12	123001789	-	C	32	7	0.219	0	0	T	25	M	0.11	0.388	8.5E-10	3.4E-07	9.5E-10	6.99971	aggagtaggaCaaggagtagg	m5C_46513
chr12	123466278	+	C	83	21	0.256	1	0.012	T	61	M	0.174	0.36	0	2.6E-13	0	73.0945	gggggaagtaCaaggacaaga	m5C_46495
chr12	123466284	+	C	156	36	0.231	0	0	T	120	M	0.172	0.303	0	0	0	123.554	agtacaaggaCaagaggagga	m5C_46487
chr12	123466318	+	C	66	40	0.606	0	0	C	40	M	0.485	0.715	0	1.4E-07	0	388.385	gaggaagaagCtgaagaagaa	m5C_46477
chr12	123466332	+	C	49	21	0.429	0	0	T	28	M	0.3	0.567	0	1.8E-06	0	126.097	agaagaagggCaaggagaagg	m5C_46475
chr12	123686758	-	C	47	18	0.383	0	0	T	29	M	0.258	0.526	0	7.7E-07	0	92.8457	caaaagaaatCtctggagttg	m5C_46504
chr12	123686768	-	C	48	11	0.229	0	0	T	37	M	0.133	0.365	5E-14	4.4E-08	6.2E-14	19.4712	aagctggaggCaaaagaaatc	m5C_46542
chr12	124074940	+	C	38	27	0.711	0	0	C	27	M	0.552	0.83	0	0.00015	0	298.31	gtggcgatgaCagctgtaaag	m5C_47401
chr12	124074957	+	C	56	37	0.661	0	0	C	37	M	0.53	0.771	0	1.8E-06	0	392.194	aaagcacgaaCaggaatacat	m5C_47403

chr12	124074965	+	C	53	19	0.358	0	0	T	34	M	0.243	0.493	0	2.4E-08	0	92.3467	aacaggaataCatggaagtcc	m5C_47419
chr12	124101263	+	C	138	47	0.343	1	0.007	T	90	M	0.269	0.426	0	0	0	252.696	tatagcaagcCtccagcgtgc	m5C_47417
chr12	124101273	+	C	148	35	0.236	0	0	T	113	M	0.175	0.311	0	0	0	122.666	ctccagcgtgCttgggtctgc	m5C_47422
chr12	124101280	+	C	142	51	0.359	0	0	T	91	M	0.285	0.441	0	0	0	290.612	gtgcttgggtCtgcagtgacc	m5C_47427
chr12	124101290	+	C	117	26	0.222	0	0	T	91	M	0.156	0.306	0	0	0	81.3295	ctgcagtgacCccgtggattc	m5C_47397
chr12	124101291	+	C	103	24	0.233	0	0	T	79	M	0.162	0.323	0	2.2E-16	0	77.7	tgcagtgaccCcgtggattcc	m5C_47426
chr12	124101292	+	C	98	24	0.245	0	0	T	74	M	0.17	0.339	0	1.7E-14	0	81.8153	gcagtgacccCgtggattcct	m5C_47418
chr12	124101300	+	C	55	12	0.218	0	0	T	43	M	0.129	0.344	9.8E-15	8.8E-10	1.2E-14	21.7648	cccgtggattCctacagggct	m5C_47407
chr12	124101301	+	C	46	23	0.5	0	0	CT	23	M	0.361	0.639	0	2.9E-06	0	166.146	ccgtggattcCtacagggctt	m5C_47395
chr12	124101352	+	C	35	8	0.229	0	0	T	27	M	0.121	0.39	5.6E-11	6.8E-07	6.4E-11	9.89691	ttgccgtgctCcatgtagagc	m5C_47392
chr12	124101383	+	C	66	26	0.394	0	0	T	40	M	0.285	0.515	0	5.6E-09	0	148.215	aaatttcagaCaatgagagaa	m5C_47399
chr12	125397095	-	C	31	13	0.419	0	0	T	18	M	0.264	0.592	0	6.7E-06	0	68.6798	aatgtcaaggCaaagatccaa	m5C_47477
chr12	125397100	-	C	39	10	0.256	0	0	T	29	M	0.146	0.411	1.3E-13	2E-06	1.6E-13	18.7753	ttgagaatgtCaaggcaaaga	m5C_47454
chr12	125397115	-	C	36	15	0.417	0	0	T	21	M	0.271	0.578	0	1.2E-05	0	81.421	agccgagtgaCaccattgaga	m5C_47503
chr12	125397123	-	C	39	18	0.462	0	0	T	21	M	0.316	0.614	0	2.7E-05	0	113.64	cgaggtggagCcgagtgacac	m5C_47488
chr12	125398012	-	C	34	9	0.265	0	0	T	25	M	0.146	0.431	1.1E-12	1.2E-06	1.3E-12	15.7394	tcgagaacgtCaaagcaaaga	m5C_47510
chr12	125406302	-	C	259	53	0.205	0	0	T	206	M	0.16	0.258	0	0	0	169.586	ccggcatctcCattgtagttt	m5C_47450
chr12	125406303	-	C	281	61	0.217	0	0	T	220	M	0.173	0.269	0	0	0	210.9	cccggcatctCcattgtagtt	m5C_47502
chr12	125406312	-	C	288	77	0.268	1	0.003	T	210	M	0.22	0.322	0	0	0	339.323	ggttcgatccCcggcatctcc	m5C_47484
chr12	125406314	-	C	289	99	0.343	0	0	T	190	M	0.29	0.399	0	0	0	574.66	cgggttcgatCcccggcatct	m5C_47446
chr12	125406318	-	C	287	77	0.268	0	0	T	210	M	0.22	0.322	0	0	0	339.323	gccccgggttCgatccccggc	m5C_47528
chr12	125406325	-	C	234	79	0.338	0	0	T	155	M	0.28	0.4	0	0	0	442.514	gtatgaggccCcgggttcgat	m5C_47470
chr12	125406338	-	C	83	17	0.205	0	0	T	66	M	0.132	0.304	0	2.3E-14	0	44.8792	gcatgctttgCatgtatgagg	m5C_47486
chr12	125406343	-	C	119	39	0.328	0	0	T	80	M	0.25	0.416	0	3.1E-15	0	194.951	agagcgcatgCtttgcatgta	m5C_47479
chr12	125406349	-	C	136	46	0.338	0	0	T	90	M	0.264	0.421	0	0	0	243.007	agtggtagagCgcatgctttg	m5C_47473
chr12	125406360	-	C	131	32	0.244	0	0	T	99	M	0.179	0.324	0	0	0	114.351	ggatgtagctCagtggtagag	m5C_47496
chr12	125411882	-	C	56	16	0.286	0	0	T	40	M	0.184	0.415	0	7.3E-09	0	58.9396	aggccaagtaCgtttttacca	m5C_47519
chr12	125411888	-	C	68	15	0.221	0	0	T	53	M	0.138	0.333	0	6E-11	0	41.5467	acggggaggcCaagtacgttt	m5C_47506
chr12	125411889	-	C	69	23	0.333	0	0	T	46	M	0.234	0.451	0	2.1E-09	0	107.414	gacggggaggCcaagtacgtt	m5C_47455
chr12	125411897	-	C	67	18	0.269	0	0	T	49	M	0.177	0.385	0	2.9E-10	0	63.7994	gattccccgaCggggaggcca	m5C_47461
chr12	125411902	-	C	86	22	0.256	0	0	T	64	M	0.175	0.357	0	4.4E-13	0	77.189	ggttcgattcCccgacgggga	m5C_47491
chr12	125411903	-	C	91	22	0.242	0	0	T	69	M	0.165	0.339	0	5.8E-15	0	72.7834	gggttcgattCcccgacgggg	m5C_47507
chr12	125411908	-	C	84	26	0.31	0	0	T	58	M	0.221	0.415	0	2.7E-12	0	114.825	gaccgggggttCgattccccga	m5C_47437
chr12	125411916	-	C	80	22	0.275	0	0	T	58	M	0.189	0.381	0	2.8E-11	0	83.2375	acgcgggagaCcgggggttcga	m5C_47509
chr12	125411925	-	C	87	29	0.333	0	0	T	58	M	0.243	0.438	0	8.4E-12	0	141.047	ccgcctgtcaCgcgggagacc	m5C_47463
chr12	125411932	-	C	86	20	0.233	0	0	T	66	M	0.156	0.332	0	1.5E-13	0	62.356	agtatccccgCctgtcacgcg	m5C_47440
chr12	125411936	-	C	84	40	0.476	0	0	T	44	M	0.373	0.582	0	2.1E-10	0	298.226	ggtgagtatcCccgcctgtca	m5C_47521
chr12	125411937	-	C	90	27	0.3	0	0	T	63	M	0.215	0.401	0	7.2E-14	0	116.158	tggtgagtatCcccgcctgtc	m5C_47513
chr12	125411958	-	C	109	23	0.211	0	0	T	86	M	0.145	0.297	0	1.1E-16	0	66.6644	gggcggtcctCgttagtatag	m5C_47456
chr12	125411960	-	C	107	28	0.262	0	0	T	79	M	0.188	0.352	0	1.8E-15	0	105.11	atgggcggtcCtcgttagtat	m5C_47443
chr12	125411965	-	C	101	39	0.386	0	0	T	62	M	0.297	0.484	0	1.3E-13	0	231.677	gttttatgggCggtcctcgtt	m5C_47457
chr12	125412388	-	C	88	54	0.614	0	0	C	54	M	0.509	0.709	0	3.5E-09	0	549.92	ggtttcggcaCttcctctttt	m5C_47460
chr12	125412390	-	C	90	52	0.578	0	0	C	52	M	0.475	0.675	0	1.1E-10	0	493.594	cgggtttcggCacttcctctt	m5C_47439
chr12	125412393	-	C	95	26	0.274	0	0	T	69	M	0.194	0.371	0	4.6E-14	0	100.955	tcccgggtttCggcacttcct	m5C_47468
chr12	125412400	-	C	96	38	0.396	0	0	T	58	M	0.304	0.496	0	3.7E-12	0	230.912	ggttcgatccCgggtttcggc	m5C_47526
chr12	125412401	-	C	103	35	0.34	0	0	T	68	M	0.256	0.436	0	3.4E-14	0	178.898	tggttcgatcCcgggtttcgg	m5C_47458
chr12	125412402	-	C	95	30	0.316	0	0	T	65	M	0.231	0.415	0	2.5E-13	0	138.619	ctggttcgatCccgggtttcg	m5C_47492
chr12	125412406	-	C	105	31	0.295	0	0	T	74	M	0.216	0.388	0	7.1E-15	0	134.208	gtccctggttCgatcccgggt	m5C_47495
chr12	125412412	-	C	110	27	0.245	0	0	T	83	M	0.175	0.334	0	0	0	94.2425	ctaaaggtccCtggttcgatc	m5C_47489
chr12	125412413	-	C	114	62	0.544	0	0	C	62	M	0.452	0.632	0	1.4E-12	0	561.087	tctaaaggtcCctggttcgat	m5C_47523
chr12	125412414	-	C	107	31	0.29	0	0	T	76	M	0.212	0.382	0	7.1E-15	0	131.59	atctaaaggtCcctggttcga	m5C_47481
chr12	125412422	-	C	52	12	0.231	0	0	T	40	M	0.137	0.361	4.4E-15	8.8E-10	5.7E-15	23.6371	gactgaagatCtaaaggtccc	m5C_47471
chr12	125412451	-	C	44	10	0.227	0	0	T	34	M	0.128	0.37	5.7E-13	2.4E-08	6.8E-13	15.7274	gccgaaatagCtcagttggga	m5C_47475
chr12	125412463	-	C	39	17	0.436	0	0	T	22	M	0.293	0.59	0	2.1E-05	0	99.6354	gtcgtaacggCtgccgaaata	m5C_47525
chr12	125424180	-	C	32	9	0.281	0	0	T	23	M	0.156	0.454	5.3E-13	1.2E-06	6.4E-13	17.1983	cgggtactttCgtatttttaa	m5C_47452
chr12	125424184	-	C	33	24	0.727	0	0	C	24	M	0.558	0.849	0	9.1E-05	0	267.754	aggccgggtaCtttcgtattt	m5C_47474
chr12	125424191	-	C	40	26	0.65	0	0	C	26	M	0.495	0.779	0	5.5E-06	0	257.429	gacggggaggCcgggtacttt	m5C_47517
chr12	125424199	-	C	35	12	0.343	0	0	T	23	M	0.208	0.508	0	4.6E-06	0	49.9956	gattccccgaCggggaggccg	m5C_47482
chr12	125424202	-	C	35	19	0.543	0	0	C	19	M	0.382	0.695	0	3.4E-05	0	145.12	ttcgattcccCgacggggagg	m5C_47520
chr12	125424203	-	C	47	10	0.213	0	0	T	37	M	0.12	0.349	1.3E-12	2.4E-08	1.5E-12	14.271	gttcgattccCcgacgggggag	m5C_47438
chr12	125424204	-	C	47	21	0.447	0	0	T	26	M	0.314	0.588	0	1.8E-06	0	131.925	ggttcgattcCccgacggggga	m5C_47441
chr12	125424217	-	C	42	19	0.463	1	0.024	T	22	M	0.321	0.613	0	1E-06	0	121.813	cgcgggagacCgggggttcgat	m5C_47500
chr12	125424227	-	C	44	18	0.409	0	0	T	26	M	0.277	0.556	0	7.7E-07	0	99.6755	ccgcctgtcaCgcgggagacc	m5C_47512
chr12	125424260	-	C	40	16	0.4	0	0	T	24	M	0.263	0.554	0	1.7E-05	0	84.314	ggcttgtcctCgttagtatag	m5C_47518
chr12	125424263	-	C	38	9	0.237	0	0	T	29	M	0.13	0.392	3.7E-12	1.2E-06	4.3E-12	13.3739	tacggcttgtCctcgttagta	m5C_47449
chr12	125424524	+	C	42	16	0.381	0	0	T	26	M	0.25	0.532	0	4E-07	0	79.9985	ggatgtagctCagtggtagag	m5C_47402
chr12	125424541	+	C	44	13	0.295	0	0	T	31	M	0.182	0.442	0	1.2E-07	0	47.2039	agagcgcatgCtttgcacgta	m5C_47414
chr12	127650598	-	C	37	9	0.243	0	0	T	28	M	0.134	0.401	2.7E-12	1.2E-06	3.2E-12	13.907	cggccgccctCtcgcccgtca	m5C_46872
chr12	127650600	-	C	59	24	0.407	0	0	T	35	M	0.291	0.534	0	1.2E-07	0	139.626	ggcggccgccCtctcgcccgt	m5C_46875
chr12	127650602	-	C	102	27	0.265	0	0	T	75	M	0.189	0.358	0	1.1E-15	0	101.932	aaggcggccgCcctctcgccc	m5C_46894
chr12	127650608	-	C	298	91	0.317	11	0.037	T	196	M	0.266	0.373	0	0	0	484.037	gccggaaaggCggccgccctc	m5C_46907
chr12	127650616	-	C	369	85	0.23	0	0	T	284	M	0.19	0.276	0	0	0	323.51	ggggcgcggcCggaaaggcgg	m5C_46867
chr12	127650617	-	C	366	117	0.32	0	0	T	249	M	0.274	0.369	0	0	0	641.111	cggggcgcggCcggaaaggcg	m5C_46859
chr12	127650622	-	C	372	91	0.245	0	0	T	281	M	0.204	0.291	0	0	0	370.727	ggaaacggggCgcggccggaa	m5C_46914
chr12	127650636	-	C	115	32	0.278	0	0	T	83	M	0.205	0.366	0	2.2E-16	0	130.904	tgccctttgtCctgggaaacg	m5C_46898
chr12	127650642	-	C	113	35	0.31	0	0	T	78	M	0.232	0.4	0	6.7E-16	0	162.341	gcggagtgccCtttgtcctgg	m5C_46881
chr12	127650644	-	C	113	26	0.23	0	0	T	87	M	0.162	0.316	0	0	0	84.3081	gtgcggagtgCcctttgtcct	m5C_46896
chr12	127650651	-	C	109	27	0.248	0	0	T	82	M	0.176	0.336	0	1.1E-15	0	95.1394	gggtccggtgCggagtgccct	m5C_46861
chr12	127650657	-	C	66	32	0.485	0	0	T	34	M	0.368	0.603	0	2.7E-08	0	235.819	ggaccagggtCcggtgcggag	m5C_46863
chr12	127650895	+	C	59	37	0.627	0	0	C	37	M	0.5	0.739	0	1.8E-06	0	369.655	cgcggggctgCcgtatcgttc	m5C_46805
chr12	130663966	-	C	32	18	0.562	0	0	C	18	M	0.393	0.718	0	2.7E-05	0	141.571	tttctaggaaCggtgcactca	m5C_47153
chr12	130663973	-	C	32	10	0.312	0	0	T	22	M	0.18	0.486	1.1E-14	2E-06	1.3E-14	25.0836	aaaggtatttCtaggaacggt	m5C_47157
chr12	132515775	+	C	34	9	0.265	0	0	T	25	M	0.146	0.431	1.1E-12	1.2E-06	1.3E-12	15.7394	cctgcttcctCagccttactc	m5C_47272
chr12	132515783	+	C	37	8	0.216	0	0	T	29	M	0.114	0.372	9.7E-11	6.8E-07	1.1E-10	9.12273	ctcagccttaCtccagggact	m5C_47273
chr12	132515786	+	C	37	8	0.216	0	0	T	29	M	0.114	0.372	9.7E-11	6.8E-07	1.1E-10	9.12273	agccttactcCagggactttt	m5C_47275
chr12	132515803	+	C	40	11	0.275	0	0	T	29	M	0.161	0.428	4.3E-15	3.1E-06	5.5E-15	25.4502	tttttgttgcCtgtaaagtgc	m5C_47304
chr12	132515813	+	C	36	10	0.278	0	0	T	26	M	0.158	0.44	4.8E-14	2E-06	5.9E-14	21.1115	ctgtaaagtgCtctggcattg	m5C_47291
chr12	132515815	+	C	34	9	0.265	0	0	T	25	M	0.146	0.431	1.1E-12	1.2E-06	1.3E-12	15.7394	gtaaagtgctCtggcattgcc	m5C_47279
chr13	20567826	+	C	47	10	0.213	0	0	T	37	M	0.12	0.349	1.3E-12	2.4E-08	1.5E-12	14.271	ttagaaaataCtgacgggcga	m5C_47024
chr13	21186395	+	C	128	26	0.203	0	0	T	102	M	0.143	0.281	0	0	0	74.1294	atttttggagCagggagattg	m5C_47035
chr13	23665541	-	C	71	28	0.394	0	0	T	43	M	0.289	0.511	0	2.6E-10	0	161.802	gcatgcctgcCtgagtgtctc	m5C_47214
chr13	23665550	-	C	41	12	0.293	0	0	T	29	M	0.176	0.445	1.1E-16	7.5E-08	1.5E-16	33.7134	tgggaggggGCatgcctgcct	m5C_47212
chr13	24269908	+	C	37	14	0.378	0	0	T	23	M	0.241	0.539	0	9.2E-06	0	67.3786	gggccgcgtcCgcgcccctcc	m5C_47179
chr13	24269912	+	C	35	10	0.286	0	0	T	25	M	0.163	0.451	3.4E-14	2E-06	4.2E-14	21.9991	cgcgtccgcgCccctccctgc	m5C_47178
chr13	24269917	+	C	33	11	0.333	0	0	T	22	M	0.198	0.504	2.2E-16	3.1E-06	2.9E-16	34.0074	ccgcgccccctCcctgccgcca	m5C_47169
chr13	24269918	+	C	33	7	0.212	0	0	T	26	M	0.107	0.378	1.1E-09	3.4E-07	1.2E-09	6.69165	cgcgcccctcCctgccgccag	m5C_47177
chr13	25269265	-	C	57	25	0.439	0	0	T	32	M	0.318	0.567	0	1.5E-07	0	158.862	acactgggtgCtgtgtgactc	m5C_47195
chr13	26487068	-	C	34	11	0.324	0	0	T	23	M	0.191	0.492	4.4E-16	3.1E-06	5.8E-16	32.3088	aaaggctactCagttgaccaa	m5C_47829
chr13	26524952	-	C	228	64	0.281	0	0	T	164	M	0.226	0.342	0	0	0	289.767	ggaggctggtCtggaggcagc	m5C_47831
chr13	27829214	+	C	496	140	0.283	1	0.002	T	355	M	0.245	0.324	0	0	0	685.839	ttaatgatgaCtgttttttt	m5C_47812
chr13	27829258	+	C	411	146	0.355	0	0	T	265	M	0.31	0.403	0	0	0	906.653	acacatttcaCcggctctgaa	m5C_47823

chr13	27829262	+	C	375	124	0.331	0	0	T	251	M	0.285	0.38	0	0	0	706.746	atttcaccggCtctgaaagct	m5C_47811
chr13	27829264	+	C	366	131	0.358	0	0	T	235	M	0.311	0.408	0	0	0	813.559	ttcaccggctCtgaaagctct	m5C_47816
chr13	27829271	+	C	178	44	0.247	0	0	T	134	M	0.19	0.315	0	0	0	166.852	gctctgaaagCtcttgagttg	m5C_47821
chr13	27829565	+	C	130	52	0.4	0	0	T	78	M	0.32	0.486	0	4.4E-15	0	332.616	gccagttggaCttatgtcttt	m5C_47819
chr13	27829608	+	C	89	45	0.506	0	0	C	45	M	0.404	0.607	0	6.4E-10	0	363.315	aggaaaatatcCttttaaaact	m5C_47818
chr13	27829619	+	C	97	22	0.227	0	0	T	75	M	0.155	0.32	0	5.8E-15	0	68.1195	ttttaaaactCaggcaaactg	m5C_47817
chr13	27829627	+	C	99	22	0.222	0	0	T	77	M	0.152	0.314	0	5.8E-15	0	66.6952	ctcaggcaaaCtgggtgtttg	m5C_47825
chr13	27829639	+	C	112	37	0.33	0	0	T	75	M	0.25	0.422	0	1.4E-15	0	185.108	gggtgtttgtCtgtatcctgt	m5C_47809
chr13	27829646	+	C	83	23	0.277	0	0	T	60	M	0.192	0.382	0	7.2E-13	0	88.4663	tgtctgtatcCtgtcagagga	m5C_47827
chr13	27829659	+	C	72	15	0.208	0	0	T	57	M	0.131	0.316	0	6E-13	0	39.1558	tcagaggaaaCaaattgaaat	m5C_47814
chr13	29197497	+	C	37	27	0.73	0	0	C	27	M	0.57	0.846	0	0.00015	0	307.916	ctctggattgCagtttgcaat	m5C_47552
chr13	29940650	+	C	149	36	0.242	0	0	T	113	M	0.18	0.316	0	0	0	129.55	cccgctagctCagttggtaga	m5C_47554
chr13	29940662	+	C	119	35	0.294	0	0	T	84	M	0.22	0.381	0	6.7E-16	0	153.806	gttggtagagCatgacactta	m5C_47549
chr13	31248093	-	C	67	35	0.522	0	0	C	35	M	0.405	0.637	0	5.3E-08	0	283.395	acggtcagtgCttcttttccc	m5C_47240
chr13	31248102	-	C	86	22	0.256	0	0	T	64	M	0.175	0.357	0	4.4E-13	0	77.189	cacccagggaCggtcagtgct	m5C_47223
chr13	31248118	-	C	68	31	0.456	0	0	T	37	M	0.343	0.573	0	2.1E-08	0	212.689	gttggtggttCgagcccaccc	m5C_47222
chr13	31248134	-	C	38	12	0.316	0	0	T	26	M	0.191	0.475	0	4.6E-06	0	45.8026	ggctgttaacCgaaaggttgg	m5C_47229
chr13	40295721	+	C	64	35	0.547	0	0	C	35	M	0.426	0.663	0	5.3E-08	0	298.014	ttatgcttgtCagagtgctgt	m5C_48002
chr13	40589450	-	C	67	14	0.209	0	0	T	53	M	0.129	0.321	2.2E-16	3.2E-11	2.9E-16	28.2168	acctgtgactCtcagtggtgg	m5C_48028
chr13	41634884	-	C	166	35	0.212	1	0.006	T	130	M	0.157	0.281	0	0	0	109.662	gttcgactccCggtatgggaa	m5C_48018
chr13	41634885	-	C	172	44	0.259	2	0.012	T	126	M	0.199	0.329	0	0	0	174.967	ggttcgactcCcggtatggga	m5C_48032
chr13	41634886	-	C	185	47	0.255	1	0.005	T	137	M	0.198	0.323	0	0	0	185.998	gggttcgactCccggtatggg	m5C_48034
chr13	41634891	-	C	197	49	0.249	0	0	T	148	M	0.194	0.314	0	0	0	189.688	ggcccgggttCgactcccggt	m5C_48015
chr13	41634897	-	C	215	84	0.391	0	0	T	131	M	0.328	0.457	0	0	0	550.946	ccaggtggccCgggttcgact	m5C_48037
chr13	41634898	-	C	214	64	0.302	2	0.009	T	148	M	0.244	0.367	0	0	0	312.405	cccaggtggcCcgggttcgac	m5C_48042
chr13	41634899	-	C	207	45	0.217	0	0	T	162	M	0.167	0.278	0	0	0	149.964	acccaggtggCccgggttcga	m5C_48038
chr13	41634906	-	C	200	64	0.32	0	0	T	136	M	0.259	0.388	0	0	0	331.858	ggttttcaccCaggtggcccg	m5C_48036
chr13	41634908	-	C	191	43	0.225	0	0	T	148	M	0.172	0.289	0	0	0	147.622	ctggttttcaCccaggtggcc	m5C_48023
chr13	41634910	-	C	193	39	0.202	0	0	T	154	M	0.151	0.264	0	0	0	118.162	tcctggttttCacccaggtgg	m5C_48044
chr13	41634918	-	C	196	61	0.311	0	0	T	135	M	0.251	0.379	0	0	0	305.68	gttaggattcCtggttttcac	m5C_48024
chr13	41634919	-	C	197	43	0.218	0	0	T	154	M	0.166	0.281	0	0	0	143.001	ggttaggattCctggttttca	m5C_48026
chr13	41634930	-	C	201	58	0.289	0	0	T	143	M	0.23	0.355	0	0	0	267.203	tatggtctagCggttaggatt	m5C_48016
chr13	41634934	-	C	194	56	0.289	0	0	T	138	M	0.229	0.356	0	0	0	257.025	cccatatggtCtagcggttag	m5C_48041
chr13	41634943	-	C	86	20	0.235	1	0.012	T	65	M	0.158	0.336	0	1.5E-13	0	63.1177	atagcacttcCcatatggtct	m5C_48027
chr13	42030101	-	C	44	23	0.523	0	0	C	23	M	0.379	0.662	0	2.9E-06	0	174.502	ggattcggtgCtctcaccgct	m5C_47366
chr13	42030106	-	C	60	19	0.317	0	0	T	41	M	0.213	0.442	0	4.5E-10	0	80.9617	gcttaggattCggtgctctca	m5C_47372
chr13	42030115	-	C	99	18	0.237	23	0.232	T	58	M	0.155	0.344	0	4E-12	0	55.9412	tggtctagtgCttaggattcg	m5C_47375
chr13	42030121	-	C	92	50	0.543	0	0	C	50	M	0.442	0.641	0	7.1E-11	0	441.996	ccctggtggtCtagtgcttag	m5C_47364
chr13	42030129	-	C	40	12	0.3	0	0	T	28	M	0.181	0.454	1.1E-16	7.5E-08	1.5E-16	34.6049	ctaagttcccCtggtggtcta	m5C_47371
chr13	42030130	-	C	37	13	0.351	0	0	T	24	M	0.218	0.512	0	6.7E-06	0	56.7461	tctaagttccCctggtggtct	m5C_47374
chr13	42030131	-	C	33	11	0.333	0	0	T	22	M	0.198	0.504	2.2E-16	3.1E-06	2.9E-16	34.0074	ttctaagttcCcctggtggtc	m5C_47368
chr13	45443818	-	C	39	8	0.205	0	0	T	31	M	0.108	0.355	1.6E-10	6.8E-07	1.8E-10	8.44384	aggcgtgaacCactaagcctg	m5C_48879
chr13	45492072	-	C	469	114	0.243	0	0	T	355	M	0.206	0.284	0	0	0	470.68	gttcgactccCggtgtgggaa	m5C_48852
chr13	45492085	-	C	444	110	0.249	2	0.005	T	332	M	0.211	0.291	0	0	0	463.85	ccaggcggccCgggttcgact	m5C_48870
chr13	45492086	-	C	423	132	0.312	0	0	T	291	M	0.27	0.358	0	0	0	712.166	cccaggcggcCcgggttcgac	m5C_48894
chr13	45492090	-	C	347	72	0.217	15	0.043	T	260	M	0.176	0.264	0	0	0	253.312	ttcacccaggCggcccgggtt	m5C_48855
chr13	45492096	-	C	322	78	0.242	0	0	T	244	M	0.199	0.292	0	0	0	309.903	ctggttttcaCccaggcggcc	m5C_48854
chr13	45492098	-	C	311	77	0.248	0	0	T	234	M	0.203	0.298	0	0	0	312.455	tcctggttttCacccaggcgg	m5C_48845
chr13	45492106	-	C	311	74	0.238	0	0	T	237	M	0.194	0.288	0	0	0	287.111	gttaggattcCtggttttcac	m5C_48890
chr13	45492107	-	C	322	70	0.217	0	0	T	252	M	0.176	0.266	0	0	0	246.138	ggttaggattCctggttttca	m5C_48848
chr13	45492118	-	C	309	65	0.21	0	0	T	244	M	0.169	0.259	0	0	0	219.192	catggtctagCggttaggatt	m5C_48899
chr13	45492122	-	C	297	66	0.222	0	0	T	231	M	0.179	0.273	0	0	0	235.826	cccacatggtCtagcggttag	m5C_48903
chr13	45492134	-	C	46	10	0.217	0	0	T	36	M	0.123	0.356	9.7E-13	2.4E-08	1.2E-12	14.7309	ctattgccgtCtcccacatgg	m5C_48859
chr13	45910547	-	C	45	13	0.289	0	0	T	32	M	0.177	0.434	0	1.2E-07	0	46.0978	cagttgctatCtgctgctatg	m5C_48856
chr13	45910551	-	C	65	21	0.323	0	0	T	44	M	0.222	0.444	0	1E-09	0	93.2557	ggagcagttgCtatctgctgc	m5C_48850
chr13	45910568	-	C	65	17	0.262	0	0	T	48	M	0.17	0.38	0	1.8E-10	0	57.8614	atctatttgaCagacctggag	m5C_48857
chr13	45911372	-	C	31	10	0.323	0	0	T	21	M	0.186	0.499	7.1E-15	2E-06	9E-15	26.2725	ggagtggaggCattgttttta	m5C_48838
chr13	45911655	-	C	79	16	0.203	0	0	T	63	M	0.129	0.304	0	1.2E-12	0	41.1808	cgctttctgtCtgtctttgtg	m5C_48897
chr13	45911659	-	C	75	15	0.2	0	0	T	60	M	0.125	0.304	0	6E-13	0	37.5361	cctgcgctttCtgtctgtctt	m5C_48905
chr13	45911663	-	C	65	14	0.215	0	0	T	51	M	0.133	0.33	1.1E-16	3.2E-11	1.5E-16	29.6776	aatccctgcgCtttctgtctg	m5C_48893
chr13	45911668	-	C	65	26	0.4	0	0	T	39	M	0.29	0.521	0	5.6E-09	0	150.654	aaagaaatccCtgcgctttct	m5C_48872
chr13	45911669	-	C	68	22	0.324	0	0	T	46	M	0.224	0.442	0	1.5E-09	0	98.7248	gaaagaaatcCctgcgctttc	m5C_48843
chr13	45911694	-	C	50	19	0.38	0	0	T	31	M	0.259	0.518	0	2.4E-08	0	98.2779	ttttgggtttgCactaggatgc	m5C_48844
chr13	45911705	-	C	51	15	0.294	0	0	T	36	M	0.187	0.43	0	4.6E-09	0	56.1257	tactgtttgttCttttggtttg	m5C_48895
chr13	45911720	-	C	71	31	0.437	0	0	T	40	M	0.327	0.552	0	6.4E-10	0	203.025	agaccttgaaCaatttactgt	m5C_48851
chr13	45911726	-	C	90	49	0.544	0	0	C	49	M	0.442	0.643	0	5.8E-11	0	433.008	actgatagacCttgaacaatt	m5C_48881
chr13	45911727	-	C	90	51	0.567	0	0	C	51	M	0.464	0.664	0	8.8E-11	0	472.912	cactgatagaCcttgaacaat	m5C_48873
chr13	45911735	-	C	88	27	0.307	0	0	T	61	M	0.22	0.41	0	4E-12	0	118.926	tctgcatccaCtgatagacct	m5C_48883
chr13	45911737	-	C	84	28	0.333	0	0	T	56	M	0.242	0.439	0	5.8E-12	0	135.392	gttctgcatcCactgatagac	m5C_48878
chr13	45911738	-	C	88	51	0.58	0	0	C	51	M	0.475	0.677	0	2.1E-09	0	484.71	tgttctgcatCcactgataga	m5C_48865
chr13	45912861	-	C	42	18	0.429	0	0	T	24	M	0.291	0.578	0	7.7E-07	0	104.823	ctctattggaCtaccgtgagg	m5C_48880
chr13	45912869	-	C	35	13	0.371	0	0	T	22	M	0.232	0.537	0	6.7E-06	0	60.2306	catggttgctCtattggacta	m5C_48874
chr13	45912871	-	C	33	16	0.485	0	0	T	17	M	0.325	0.648	0	1.7E-05	0	104.012	ggcatggttgCtctattggac	m5C_48847
chr13	45913724	-	C	51	34	0.667	0	0	C	34	M	0.53	0.78	0	1.1E-06	0	360.183	caaagggaaaCttgaagaaca	m5C_48885
chr13	45914864	-	C	35	18	0.514	0	0	C	18	M	0.356	0.67	0	2.7E-05	0	128.047	cgggttgtgcCtggaggtgga	m5C_48896
chr13	45914874	-	C	38	26	0.684	0	0	C	26	M	0.525	0.809	0	0.00013	0	273.229	agatcgcggaCgggttgtgcc	m5C_48853
chr13	46125635	-	C	52	17	0.327	0	0	T	35	M	0.215	0.462	0	1.1E-08	0	73.1745	cctgggaataCtgggtgccag	m5C_48108
chr13	46948540	-	C	95	27	0.284	0	0	T	68	M	0.203	0.382	0	7.2E-14	0	109.773	aacggcgcacCcctctgggtg	m5C_48118
chr13	46948548	-	C	124	28	0.226	0	0	T	96	M	0.161	0.307	0	0	0	90.216	cccctaggaaCggcgcacccc	m5C_48123
chr13	46948618	-	C	1565	471	0.301	1	0.001	T	1093	M	0.279	0.324	0	0	0	2627.47	atttttggagCagggacatgg	m5C_48112
chr13	46948643	-	C	1637	428	0.262	2	0.001	T	1207	M	0.241	0.284	0	0	0	2063.31	tatccggaggaCaatatattaa	m5C_48121
chr13	46948649	-	C	1259	288	0.229	0	0	T	971	M	0.206	0.253	0	0	0	1188.85	gtcctctatcCgaggacaata	m5C_48126
chr13	46948650	-	C	1166	287	0.246	0	0	T	879	M	0.222	0.272	0	0	0	1275.86	tgtcctctatCcgaggacaat	m5C_48115
chr13	48397523	-	C	37	25	0.676	0	0	C	25	M	0.515	0.804	0	0.00011	0	257.317	gcccattggaCtgatgccttt	m5C_47581
chr13	50623164	-	C	38	9	0.237	0	0	T	29	M	0.13	0.392	3.7E-12	1.2E-06	4.3E-12	13.3739	taaatattggCgttaagattc	m5C_47614
chr13	50623176	-	C	74	39	0.527	0	0	C	39	M	0.415	0.637	0	4.7E-09	0	323.522	cttagcagcaCgtaaatattg	m5C_47612
chr13	50623178	-	C	72	16	0.222	0	0	T	56	M	0.142	0.331	0	1.2E-12	0	45.3354	gccttagcagCacgtaaatat	m5C_47615
chr13	52974146	+	C	32	15	0.484	1	0.031	T	16	M	0.32	0.652	0	1.2E-05	0	95.91	gcaggctggtCcgatggtagc	m5C_47636
chr13	52974147	+	C	33	8	0.242	0	0	T	25	M	0.128	0.41	3.1E-11	6.8E-07	3.6E-11	10.7866	caggctggtcCgatggtagca	m5C_47637
chr13	52974169	+	C	32	7	0.219	0	0	T	25	M	0.11	0.388	8.5E-10	3.4E-07	9.5E-10	6.99971	gttatcagaaCttattaacat	m5C_47639
chr13	53832322	+	C	46	12	0.261	0	0	T	34	M	0.156	0.403	6.7E-16	7.5E-08	8.7E-16	28.4116	gattgggtgtCtgcactaagt	m5C_47633
chr13	58458610	-	C	56	28	0.5	0	0	CT	28	M	0.373	0.627	0	3.1E-07	0	209.057	ccctggtggtCtaatggttag	m5C_47755
chr13	60413550	-	C	86	26	0.302	0	0	T	60	M	0.215	0.406	0	2.7E-12	0	112.026	gaatttgaggCagatgggaag	m5C_47972
chr13	60413568	-	C	105	22	0.21	0	0	T	83	M	0.143	0.297	0	0	0	62.7592	tgtagaaacgCtggaaaagaa	m5C_47970
chr13	60435549	-	C	36	21	0.583	0	0	C	21	M	0.422	0.729	0	5.2E-05	0	177.24	ttagacaaagCtagtaaaggt	m5C_47973
chr13	60761957	-	C	83	26	0.313	0	0	T	57	M	0.224	0.419	0	2.7E-12	0	116.279	caaggctggtCtgatggtagt	m5C_47960
chr13	60761962	-	C	37	10	0.27	0	0	T	27	M	0.154	0.43	6.7E-14	2E-06	8.3E-14	20.2815	aaaaacaaggCtggtctgatg	m5C_47974
chr13	70598230	-	C	37	12	0.324	0	0	T	25	M	0.196	0.485	0	4.6E-06	0	47.1194	tgtgcccgatCtcctctgatc	m5C_47796
chr13	70598235	-	C	54	18	0.333	0	0	T	36	M	0.222	0.466	0	1.7E-08	0	80.0685	ctgaatgtgcCcgatctcctc	m5C_47801
chr13	70598245	-	C	96	33	0.344	0	0	T	63	M	0.256	0.443	0	7.5E-13	0	169.226	ccataccatcCtgaatgtgcc	m5C_47799
chr13	70598246	-	C	97	34	0.351	0	0	T	63	M	0.263	0.45	0	1.1E-12	0	178.778	gccataccatCctgaatgtgc	m5C_47793

chr13	70598250	-	C	102	41	0.402	0	0	T	61	M	0.312	0.499	0	2.4E-13	0	255.876	ctacgccataCcatcctgaat	m5C_47807
chr13	70598254	-	C	105	44	0.419	0	0	T	61	M	0.329	0.515	0	5.7E-13	0	289.665	tcgtctacgcCataccatcct	m5C_47802
chr13	70598257	-	C	100	28	0.28	0	0	T	72	M	0.201	0.375	0	1.1E-13	0	112.782	caatcgtctaCgccataccat	m5C_47804
chr13	70598260	-	C	43	21	0.488	0	0	T	22	M	0.346	0.632	0	1.8E-06	0	145.392	caacaatcgtCtacgccatac	m5C_47800
chr13	73765774	+	C	303	64	0.211	0	0	T	239	M	0.169	0.261	0	0	0	216.348	atatagttggCattggcaatt	m5C_47898
chr13	73765794	+	C	302	63	0.21	2	0.007	T	237	M	0.168	0.26	0	0	0	211.329	ttttgacagtCtctatggaga	m5C_47912
chr13	73765796	+	C	304	66	0.219	2	0.007	T	236	M	0.176	0.269	0	0	0	231.829	ttgacagtctCtatggagact	m5C_47902
chr13	73765805	+	C	246	119	0.484	0	0	T	127	M	0.422	0.546	0	0	0	1004.41	tctatggagaCtgaatatttc	m5C_47913
chr13	76415986	+	C	80	23	0.287	0	0	T	57	M	0.2	0.395	0	7.2E-13	0	91.9392	gaaatgggagCaacagcttca	m5C_47876
chr13	76709296	+	C	46	11	0.239	0	0	T	35	M	0.139	0.379	2.8E-14	4.4E-08	3.5E-14	20.7303	ttgtgctgatCagtagtggga	m5C_47878
chr13	77511064	+	C	34	8	0.235	0	0	T	26	M	0.124	0.4	4.2E-11	6.8E-07	4.8E-11	10.3259	ctgagtgttgCaacaacaggt	m5C_47877
chr13	84172446	+	C	41	10	0.244	0	0	T	31	M	0.138	0.393	2.4E-13	2.4E-08	2.9E-13	17.4472	ccacgtgtgcCtgagtgctgc	m5C_48252
chr13	84172456	+	C	43	16	0.372	0	0	T	27	M	0.244	0.521	0	4E-07	0	78.0036	ctgagtgctgCtctgctgaga	m5C_48250
chr13	89494706	+	C	61	30	0.492	0	0	T	31	M	0.371	0.614	0	1.7E-08	0	222.361	attggtggttCagtagtagaa	m5C_48056
chr13	89494719	+	C	43	14	0.326	0	0	T	29	M	0.205	0.475	0	1.9E-07	0	57.3807	tagtagaattCtcgcctgcca	m5C_48048
chr13	89494721	+	C	42	17	0.405	0	0	T	25	M	0.27	0.555	0	5.6E-07	0	91.945	gtagaattctCgcctgccaac	m5C_48062
chr13	89494723	+	C	37	16	0.432	0	0	T	21	M	0.287	0.591	0	1.7E-05	0	91.7488	agaattctcgCctgccaacta	m5C_48061
chr13	89494724	+	C	38	25	0.658	0	0	C	25	M	0.499	0.788	0	0.00011	0	249.459	gaattctcgcCtgccaactat	m5C_48058
chr13	92003020	+	C	95	25	0.263	0	0	T	70	M	0.185	0.36	0	2.9E-14	0	92.532	taaggtgcatCtagtgcagat	m5C_48210
chr13	92003407	+	C	31	18	0.581	0	0	C	18	M	0.408	0.736	0	2.7E-05	0	146.757	agaactccagCttcggcctgt	m5C_48223
chr13	92003599	+	C	32	15	0.469	0	0	T	17	M	0.309	0.636	0	1.2E-05	0	92.6074	ggttgcaatgCtgtgtttctg	m5C_48206
chr13	92238195	+	C	42	16	0.381	0	0	T	26	M	0.25	0.532	0	4E-07	0	79.9985	cttggctggcCtgagggtagt	m5C_48216
chr13	92238218	+	C	37	12	0.324	0	0	T	25	M	0.196	0.485	0	4.6E-06	0	47.1194	attatcagaaCttattaacat	m5C_48220
chr13	92822821	-	C	43	10	0.233	0	0	T	33	M	0.132	0.377	4.3E-13	2.4E-08	5.2E-13	16.2683	gctgagtggcCtagaatccca	m5C_48232
chr13	92822830	-	C	50	31	0.62	0	0	C	31	M	0.482	0.741	0	5.9E-07	0	298.531	ctgggcgtggCtgagtggcct	m5C_48237
chr13	95303665	-	C	54	38	0.704	0	0	C	38	M	0.572	0.809	0	2.1E-06	0	434.509	ctgggaatacCaggtgctgta	m5C_48195
chr13	95303675	-	C	55	40	0.727	0	0	C	40	M	0.598	0.827	0	2.9E-06	0	478.14	gggagatcgcCtgggaatacc	m5C_48187
chr13	95303678	-	C	56	37	0.661	0	0	C	37	M	0.53	0.771	0	1.8E-06	0	392.194	gatgggagatCgcctgggaat	m5C_48201
chr13	95988448	-	C	90	23	0.256	0	0	T	67	M	0.177	0.354	0	1E-14	0	81.3033	tgggactggtCcgatggtagt	m5C_48189
chr13	95988453	-	C	46	13	0.283	0	0	T	33	M	0.173	0.425	0	1.2E-07	0	45.0425	ctatgtgggaCtggtccgatg	m5C_48188
chr13	97537407	-	C	88	24	0.273	0	0	T	64	M	0.191	0.374	0	1.1E-12	0	91.5137	gatgatgaggCagaggaagag	m5C_48319
chr13	98655128	+	C	46	11	0.239	0	0	T	35	M	0.139	0.379	2.8E-14	4.4E-08	3.5E-14	20.7303	gtgattgcagCtctgctgcag	m5C_48273
chr13	98655136	+	C	35	17	0.486	0	0	T	18	M	0.33	0.644	0	2.1E-05	0	112.18	agctctgctgCagaccatgga	m5C_48272
chr13	100384300	+	C	94	21	0.223	0	0	T	73	M	0.151	0.318	0	3.1E-15	0	63.4276	ttccttggggCtatgtttgtt	m5C_48142
chr13	107090670	-	C	44	11	0.25	0	0	T	33	M	0.146	0.394	1.6E-14	4.4E-08	2E-14	22.132	acgtggacacCtgggagacct	m5C_48377
chr13	107090671	-	C	49	25	0.51	0	0	C	25	M	0.375	0.644	0	4.5E-06	0	187.337	aacgtggacaCctgggagacc	m5C_48376
chr13	107090679	-	C	49	21	0.429	0	0	T	28	M	0.3	0.567	0	1.8E-06	0	126.097	cttccttcaaCgtggacacct	m5C_48371
chr13	107209466	-	C	47	16	0.34	0	0	T	31	M	0.222	0.483	0	4E-07	0	70.9352	gaagaacgtgCaaaacgtgag	m5C_48369
chr13	107211917	-	C	32	9	0.281	0	0	T	23	M	0.156	0.454	5.3E-13	1.2E-06	6.4E-13	17.1983	ggaggaagaaCtggagaaaag	m5C_48362
chr13	107219967	-	C	49	18	0.367	0	0	T	31	M	0.247	0.507	0	7.7E-07	0	88.7939	gaagcagaagCgagaggagga	m5C_48375
chr13	107219980	-	C	55	29	0.527	0	0	C	29	M	0.398	0.653	0	3.9E-07	0	230.79	gcagcctggaCgagaagcaga	m5C_48374
chr13	107219986	-	C	40	12	0.3	0	0	T	28	M	0.181	0.454	1.1E-16	7.5E-08	1.5E-16	34.6049	agcgcagcagCctggacgaga	m5C_48379
chr13	109480869	+	C	35	13	0.371	0	0	T	22	M	0.232	0.537	0	6.7E-06	0	60.2306	tgggtgggaaCcaaatccttg	m5C_48466
chr13	110372170	-	C	36	13	0.361	0	0	T	23	M	0.225	0.524	0	6.7E-06	0	58.436	ttgttggagtCattccgtgtt	m5C_48413
chr13	110372181	-	C	37	11	0.297	0	0	T	26	M	0.175	0.458	1.4E-15	3.1E-06	1.9E-15	28.5508	gagtctgaccCttgttggagt	m5C_48420
chr13	110997031	-	C	36	19	0.528	0	0	C	19	M	0.37	0.68	0	3.4E-05	0	140.622	agtatgtggcCtgagcgaggc	m5C_48411
chr13	111545380	-	C	32	16	0.5	0	0	CT	16	M	0.336	0.664	0	1.7E-05	0	107.618	aaagctagaaCtgaaggtgag	m5C_48441
chr13	113143995	-	C	34	8	0.235	0	0	T	26	M	0.124	0.4	4.2E-11	6.8E-07	4.8E-11	10.3259	tggggagtgaCggcagcagag	m5C_48542
chr13	113144008	-	C	43	29	0.674	0	0	C	29	M	0.525	0.795	0	9.7E-06	0	304.592	tgttcagggcCagtggggagt	m5C_48553
chr13	113144009	-	C	35	7	0.2	0	0	T	28	M	0.1	0.359	1.9E-09	3.4E-07	2.1E-09	6.13884	ttgttcagggCcagtggggag	m5C_48535
chr13	114133232	-	C	112	37	0.33	0	0	T	75	M	0.25	0.422	0	1.4E-15	0	185.108	gtgttctctcCtggtgtttaa	m5C_48663
chr13	114133233	-	C	116	29	0.25	0	0	T	87	M	0.18	0.336	0	0	0	104.443	cgtgttctctCctggtgttta	m5C_48660
chr13	114133250	-	C	176	50	0.284	0	0	T	126	M	0.223	0.355	0	0	0	222.629	tagtgggggaCtatgttcgtg	m5C_48665
chr13	114203825	+	C	32	10	0.312	0	0	T	22	M	0.18	0.486	1.1E-14	2E-06	1.3E-14	25.0836	aggtgtgtgcCcagaccggag	m5C_48656
chr14	20791342	-	C	188	54	0.287	0	0	T	134	M	0.227	0.356	0	0	0	245.51	cggagtcaaaCaattactaaa	m5C_52728
chr14	20791375	-	C	197	61	0.31	0	0	T	136	M	0.249	0.377	0	0	0	304.071	ggctggctgtCcgttagtgcc	m5C_52753
chr14	20791403	-	C	100	23	0.23	0	0	T	77	M	0.158	0.322	0	1.1E-16	0	72.8785	tatcttcactCagggcaccat	m5C_52571
chr14	20791405	-	C	113	28	0.248	0	0	T	85	M	0.177	0.335	0	0	0	99.3211	cttatcttcaCtcagggcacc	m5C_52576
chr14	20791415	-	C	115	30	0.261	0	0	T	85	M	0.189	0.348	0	0	0	113.564	tcaaaaatggCttatcttcac	m5C_52610
chr14	20791434	-	C	154	33	0.214	0	0	T	121	M	0.157	0.286	0	0	0	103.52	gattttgatcCatcatatatc	m5C_52614
chr14	20791435	-	C	173	39	0.225	0	0	T	134	M	0.17	0.293	0	0	0	132.226	ggattttgatCcatcatatat	m5C_52574
chr14	20791456	-	C	285	61	0.217	4	0.014	T	220	M	0.173	0.269	0	0	0	210.9	tgcttgaagcCttaggcagtg	m5C_52559
chr14	20791457	-	C	298	71	0.238	0	0	T	227	M	0.193	0.29	0	0	0	274.642	ctgcttgaagCcttaggcagt	m5C_52644
chr14	20791471	-	C	325	72	0.222	0	0	T	253	M	0.18	0.27	0	0	0	258.895	ggctgttcctCtcactgcttg	m5C_52643
chr14	20791473	-	C	327	67	0.205	0	0	T	260	M	0.165	0.252	0	0	0	220.687	atggctgttcCtctcactgct	m5C_52603
chr14	20791474	-	C	322	84	0.261	0	0	T	238	M	0.216	0.311	0	0	0	362.757	gatggctgttCctctcactgc	m5C_52740
chr14	20794621	-	C	92	22	0.239	0	0	T	70	M	0.164	0.336	0	5.8E-15	0	71.9621	taaacacatgCtctgagcaga	m5C_52697
chr14	20794627	-	C	99	20	0.202	0	0	T	79	M	0.135	0.292	0	1.7E-15	0	53.9025	cctgattaaaCacatgctctg	m5C_52708
chr14	20794655	-	C	98	22	0.224	0	0	T	76	M	0.153	0.317	0	5.8E-15	0	67.3998	atgttaagtcCgtgtttcagc	m5C_52679
chr14	20794679	-	C	34	10	0.294	0	0	T	24	M	0.168	0.462	2.3E-14	2E-06	2.9E-14	22.9564	ttacagtttgCcatgatgaaa	m5C_52642
chr14	20811233	-	C	885	235	0.266	0	0	T	650	M	0.237	0.296	0	0	0	1116.25	ctcctccgccCatttttggaa	m5C_52748
chr14	20811234	-	C	955	199	0.209	1	0.001	T	755	M	0.184	0.236	0	0	0	732.345	tctcctccgcCcatttttgga	m5C_52640
chr14	20811237	-	C	1010	254	0.251	0	0	T	756	M	0.226	0.279	0	0	0	1146.57	tactctcctcCgcccattttt	m5C_52716
chr14	20811240	-	C	1209	249	0.206	0	0	T	960	M	0.184	0.23	0	0	0	916.859	gactactctcCtccgcccatt	m5C_52711
chr14	20811243	-	C	1222	279	0.228	0	0	T	943	M	0.206	0.253	0	0	0	1147.54	tcagactactCtcctccgccc	m5C_52612
chr14	20811248	-	C	1288	288	0.224	0	0	T	1000	M	0.202	0.247	0	0	0	1161.72	ccaattcagaCtactctcctc	m5C_52594
chr14	20811277	-	C	1568	388	0.248	2	0.001	T	1178	M	0.227	0.27	0	0	0	1761.65	gtgaggtaccCcgcaggggac	m5C_52688
chr14	20811278	-	C	1567	326	0.208	3	0.002	T	1238	M	0.189	0.229	0	0	0	1232.51	ggtgaggtacCccgcaggggа	m5C_52746
chr14	20811292	-	C	1214	326	0.269	0	0	T	888	M	0.244	0.294	0	0	0	1593.24	agttcaatggCtgaggtgagg	m5C_52570
chr14	20811298	-	C	885	198	0.224	0	0	T	687	M	0.197	0.252	0	0	0	782.097	gaagtgagttCaatggctgag	m5C_52629
chr14	20811312	-	C	497	105	0.212	1	0.002	T	391	M	0.178	0.25	0	0	0	373.858	gactcacggcCagcgaagtga	m5C_52615
chr14	20811316	-	C	393	87	0.221	0	0	T	306	M	0.183	0.265	0	0	0	318.653	aacagactcaCggccagcgaa	m5C_52754
chr14	20811331	-	C	336	80	0.238	0	0	T	256	M	0.196	0.286	0	0	0	313.08	tttgcccggagCttggaacaga	m5C_52634
chr14	20811337	-	C	300	82	0.273	0	0	T	218	M	0.226	0.326	0	0	0	370.648	gcctcctttgCcggagcttgg	m5C_52662
chr14	20811342	-	C	293	60	0.205	0	0	T	233	M	0.163	0.255	0	0	0	195.04	cggatgcctcCtttgccggag	m5C_52613
chr14	20811343	-	C	304	66	0.218	1	0.003	T	237	M	0.175	0.268	0	0	0	231.046	gcggatgcctCctttgccgga	m5C_52661
chr14	20811345	-	C	321	97	0.302	0	0	T	224	M	0.255	0.355	0	0	0	493.784	cggcggatgcCtcctttgccg	m5C_52582
chr14	20811352	-	C	396	87	0.22	0	0	T	309	M	0.182	0.263	0	0	0	316.187	aggggcccggCggatgcctcc	m5C_52701
chr14	20811355	-	C	379	107	0.284	2	0.005	T	270	M	0.241	0.331	0	0	0	515.027	gggaggggccCggcggatgcc	m5C_52682
chr14	20811356	-	C	399	98	0.246	1	0.003	T	300	M	0.206	0.291	0	0	0	404.67	ggggaggggcCcggcggatgc	m5C_52592
chr14	20811357	-	C	425	171	0.402	0	0	T	254	M	0.357	0.45	0	0	0	1220.28	cggggaggggCccggcggatg	m5C_52596
chr14	20811367	-	C	632	198	0.313	0	0	T	434	M	0.278	0.35	0	0	0	1102.26	ccccgcccttCggggaggggc	m5C_52668
chr14	20811370	-	C	609	170	0.279	0	0	T	439	M	0.245	0.316	0	0	0	832.959	ggaccccgccCttcggggagg	m5C_52681
chr14	20811371	-	C	633	222	0.351	1	0.002	T	410	M	0.315	0.389	0	0	0	1398.82	tggaccccgcCcttcggggag	m5C_52651
chr14	20811375	-	C	709	181	0.255	0	0	T	528	M	0.225	0.289	0	0	0	812.95	gccgtggaccCcgcccttcgg	m5C_52689
chr14	20811376	-	C	820	173	0.211	2	0.002	T	645	M	0.185	0.241	0	0	0	639.712	tgccgtggacCccgcccttcg	m5C_52647
chr14	20811377	-	C	859	250	0.292	3	0.003	T	606	M	0.263	0.323	0	0	0	1312.89	atgccgtggaCcccgcccttc	m5C_52633
chr14	20811384	-	C	1167	242	0.208	1	0.001	T	924	M	0.185	0.232	0	0	0	896.599	gcaggagatgCcgtggacccc	m5C_52758
chr14	20811393	-	C	1248	299	0.24	0	0	T	949	M	0.217	0.264	0	0	0	1296.01	tcagactgggCaggagatgcc	m5C_52616
chr14	20811398	-	C	1168	312	0.267	0	0	T	856	M	0.243	0.293	0	0	0	1513.46	cgaggtcagaCtgggcaggag	m5C_52562
chr14	20811402	-	C	1241	283	0.228	1	0.001	T	957	M	0.206	0.252	0	0	0	1164.41	cgcgcgaggtCagactgggca	m5C_52733

chr14	20811413	-	C	984	341	0.347	0	0	T	643	M	0.317	0.377	0	0	0	2165.08	gaacggggctCcgcgcgaggt	m5C_52558
chr14	20811415	-	C	930	336	0.361	0	0	T	594	M	0.331	0.393	0	0	0	2224.62	gagaacggggCtccgcgcgag	m5C_52627
chr14	20811420	-	C	831	233	0.28	0	0	T	598	M	0.251	0.312	0	0	0	1169.23	tcccagagaaCggggctccgc	m5C_52557
chr14	20811427	-	C	603	151	0.25	0	0	T	452	M	0.217	0.287	0	0	0	656.811	ggtgagttccCagagaacggg	m5C_52705
chr14	20811428	-	C	580	169	0.292	2	0.003	T	409	M	0.257	0.331	0	0	0	867.893	aggtgagttcCcagagaacgg	m5C_52737
chr14	20811429	-	C	558	116	0.208	0	0	T	442	M	0.176	0.243	0	0	0	408.943	gaggtgagttCccagagaacg	m5C_52674
chr14	20811443	-	C	270	82	0.308	4	0.015	T	184	M	0.256	0.366	0	0	0	419.569	ccctgagcttCggggaggtga	m5C_52636
chr14	20811451	-	C	263	64	0.243	0	0	T	199	M	0.195	0.299	0	0	0	250.141	agggctctccCtgagcttcgg	m5C_52738
chr14	20811452	-	C	263	60	0.228	0	0	T	203	M	0.182	0.283	0	0	0	217.859	cagggctctcCctgagcttcg	m5C_52669
chr14	20811455	-	C	268	90	0.336	0	0	T	178	M	0.282	0.394	0	0	0	507.51	taacagggctCtccctgagct	m5C_52590
chr14	20811457	-	C	283	72	0.254	0	0	T	211	M	0.207	0.308	0	0	0	298.362	cctaacagggCtctccctgag	m5C_52622
chr14	20811466	-	C	356	118	0.331	0	0	T	238	M	0.285	0.382	0	0	0	671.628	agaggcggccCtaacagggct	m5C_52641
chr14	20811467	-	C	348	92	0.264	0	0	T	256	M	0.221	0.313	0	0	0	406.251	cagaggcggcCctaacagggc	m5C_52650
chr14	20811468	-	C	410	85	0.207	0	0	T	325	M	0.171	0.249	0	0	0	290.499	ccagaggcggCcctaacaggg	m5C_52572
chr14	20811471	-	C	565	168	0.297	0	0	T	397	M	0.261	0.336	0	0	0	877.382	gggccagaggCggccctaaca	m5C_52678
chr14	20811478	-	C	657	136	0.207	0	0	T	521	M	0.178	0.24	0	0	0	483.526	gagactagggCcagaggcggc	m5C_52670
chr14	20811484	-	C	781	201	0.258	1	0.001	T	579	M	0.228	0.29	0	0	0	917.518	aggtctgagaCtagggccaga	m5C_52675
chr14	20811490	-	C	797	266	0.334	0	0	T	531	M	0.302	0.367	0	0	0	1606	ttgggaaggtCtgagactagg	m5C_52620
chr14	20811503	-	C	500	116	0.235	6	0.012	T	378	M	0.2	0.274	0	0	0	463.008	actcccatgcCcttgggaagg	m5C_52755
chr14	20811514	-	C	705	145	0.206	0	0	T	560	M	0.177	0.237	0	0	0	514.664	cctgtcactcCactcccatgt	m5C_52656
chr14	20811519	-	C	1024	213	0.208	0	0	T	811	M	0.184	0.234	0	0	0	784.953	tgcgtcctgtCactccactcc	m5C_52744
chr14	20811523	-	C	1315	340	0.259	0	0	T	975	M	0.236	0.283	0	0	0	1602.2	tgagtgcgtcCtgtcactcca	m5C_52724
chr14	20811524	-	C	1481	348	0.235	1	0.001	T	1132	M	0.214	0.257	0	0	0	1491.05	ctgagtgcgtCctgtcactcc	m5C_52680
chr14	20811534	-	C	3074	1060	0.345	3	0.001	T	2011	M	0.329	0.362	0	0	0	6965.31	gggccacgagCtgagtgcgtc	m5C_52608
chr14	20811538	-	C	3484	725	0.208	0	0	T	2759	M	0.195	0.222	0	0	0	2826.62	agtggggccaCgagctgagtg	m5C_52749
chr14	20811540	-	C	3586	875	0.244	7	0.002	T	2704	M	0.231	0.259	0	0	0	4036.9	tcagtggggcCacgagctgag	m5C_52555
chr14	20811541	-	C	3638	1747	0.48	1	0	T	1890	M	0.464	0.497	0	0	0	16216.8	atcagtggggCcacgagctga	m5C_52719
chr14	20811549	-	C	3770	1013	0.269	2	0.001	T	2755	M	0.255	0.283	0	0	0	5164.82	ggaagctcatCagtggggcca	m5C_52672
chr14	20811552	-	C	3793	963	0.254	1	0	T	2829	M	0.24	0.268	0	0	0	4629.24	gaggggaagctCatcagtgggg	m5C_52565
chr14	20811554	-	C	3772	992	0.263	3	0.001	T	2777	M	0.249	0.277	0	0	0	4947.83	cggagggaagCtcatcagtgg	m5C_52586
chr14	20811564	-	C	3183	1488	0.468	1	0	T	1694	M	0.45	0.485	0	0	0	13402.2	tcccatagggCggagggaagc	m5C_52632
chr14	20883162	+	C	57	14	0.246	0	0	T	43	M	0.152	0.371	0	2.8E-09	0	42.6516	ggccataccaCcccgaacgcg	m5C_52499
chr14	20883173	+	C	65	30	0.462	0	0	T	35	M	0.346	0.581	0	1.7E-08	0	207.541	cccgaacgcgCccgatctcct	m5C_52501
chr14	20883175	+	C	68	14	0.206	0	0	T	54	M	0.127	0.316	2.2E-16	3.2E-11	2.9E-16	27.787	cgaacgcgccCgatctcctct	m5C_52475
chr14	20883179	+	C	65	24	0.369	0	0	T	41	M	0.262	0.491	0	3E-09	0	125.9	cgcgcccgatCtcctctgatc	m5C_52484
chr14	20883197	+	C	98	22	0.224	0	0	T	76	M	0.153	0.317	0	5.8E-15	0	67.3998	atcttggaagCtgagcagggt	m5C_52478
chr14	20883223	+	C	75	18	0.24	0	0	T	57	M	0.158	0.348	0	4E-12	0	56.7171	ttggttagtaCttggatggga	m5C_52543
chr14	21078302	+	C	66	15	0.231	1	0.015	T	50	M	0.145	0.346	0	6E-11	0	43.5411	gtagcgtggcCgagcggtcta	m5C_52516
chr14	21078316	+	C	59	24	0.407	0	0	T	35	M	0.291	0.534	0	1.2E-07	0	139.626	cggtctaaggCgctggattaa	m5C_52486
chr14	21078318	+	C	61	34	0.557	0	0	C	34	M	0.433	0.675	0	4.2E-08	0	294.461	gtctaaggcgCtggattaagg	m5C_52517
chr14	21078332	+	C	53	13	0.245	0	0	T	40	M	0.149	0.376	2.2E-16	1.6E-09	2.9E-16	30.3876	attaaggctcCagtctcttcg	m5C_52530
chr14	21078336	+	C	42	12	0.286	0	0	T	30	M	0.172	0.436	2.2E-16	7.5E-08	2.9E-16	32.2467	aggctccagtCtcttcggggg	m5C_52476
chr14	21081558	-	C	32	11	0.344	0	0	T	21	M	0.204	0.517	2.2E-16	3.1E-06	2.9E-16	35.1442	gacgagccctCgctcttttca	m5C_52732
chr14	21081562	-	C	34	10	0.294	0	0	T	24	M	0.168	0.462	2.3E-14	2E-06	2.9E-14	22.9564	cccggacgagCcctcgctctt	m5C_52683
chr14	21081571	-	C	34	23	0.676	0	0	C	23	M	0.508	0.809	0	7.6E-05	0	233.876	ggttcaaatcCcggacgagcc	m5C_52750
chr14	21081583	-	C	33	10	0.303	0	0	T	23	M	0.174	0.473	1.6E-14	2E-06	2E-14	23.982	cgagaggtccCgggttcaaat	m5C_52593
chr14	21081950	-	C	105	49	0.467	0	0	T	56	M	0.374	0.562	0	2.1E-12	0	366.627	tcgctggggcCtgtgtgtttt	m5C_52741
chr14	21081951	-	C	105	39	0.371	0	0	T	66	M	0.285	0.467	0	1.3E-13	0	222.36	ctcgctggggCctgtgtgttt	m5C_52601
chr14	21081959	-	C	100	26	0.26	0	0	T	74	M	0.184	0.354	0	6.7E-16	0	95.7038	gttcaattctCgctggggcct	m5C_52624
chr14	21081961	-	C	100	21	0.21	0	0	T	79	M	0.142	0.3	0	0	0	59.4953	gagttcaattCtcgctggggc	m5C_52588
chr14	21093539	+	C	104	23	0.221	0	0	T	81	M	0.152	0.31	0	1.1E-16	0	69.979	ggtagtgtggCcgagcggtct	m5C_52534
chr14	21093540	+	C	103	31	0.301	0	0	T	72	M	0.221	0.395	0	7.1E-15	0	136.932	gtagtgtggcCgagcggtcta	m5C_52472
chr14	21093548	+	C	113	52	0.46	0	0	T	61	M	0.371	0.552	0	1.4E-13	0	385.947	gccgagcggtCtaaggcgctg	m5C_52510
chr14	21093554	+	C	115	49	0.426	0	0	T	66	M	0.34	0.517	0	6.6E-14	0	332.745	cggtctaaggCgctggattta	m5C_52508
chr14	21093556	+	C	110	34	0.309	0	0	T	76	M	0.23	0.401	0	4.4E-16	0	156.671	gtctaaggcgCtggatttagg	m5C_52524
chr14	21093567	+	C	66	22	0.333	0	0	T	44	M	0.232	0.453	0	1.5E-09	0	101.888	tggatttaggCtccagtctct	m5C_52468
chr14	21093569	+	C	63	13	0.206	0	0	T	50	M	0.125	0.322	2.4E-15	1.6E-11	3.1E-15	23.6977	gatttaggctCcagtctcttc	m5C_52492
chr14	21093570	+	C	58	30	0.517	0	0	C	30	M	0.392	0.641	0	4.8E-07	0	234.972	atttaggctcCagtctcttcg	m5C_52503
chr14	21093574	+	C	55	17	0.309	0	0	T	38	M	0.203	0.44	0	1.1E-08	0	68.9592	aggctccagtCtcttcggggg	m5C_52481
chr14	21093576	+	C	61	13	0.213	0	0	T	48	M	0.129	0.331	1.4E-15	1.6E-11	1.9E-15	24.8871	gctccagtctCttcgggggcg	m5C_52490
chr14	21093579	+	C	69	23	0.333	0	0	T	46	M	0.234	0.451	0	2.1E-09	0	107.414	ccagtctcttCgggggcgtgg	m5C_52548
chr14	21093585	+	C	87	31	0.356	0	0	T	56	M	0.264	0.461	0	1.7E-11	0	163.51	tcttcgggggCgtggggttcga	m5C_52547
chr14	21093599	+	C	78	20	0.256	0	0	T	58	M	0.173	0.363	0	1.1E-11	0	69.0212	ggttcgaatcCcaccactgcc	m5C_52536
chr14	21093609	+	C	76	20	0.263	0	0	T	56	M	0.177	0.372	0	1.1E-11	0	70.9171	ccaccactgcCagtggtacgt	m5C_52509
chr14	21099329	-	C	40	14	0.35	0	0	T	26	M	0.221	0.505	0	1.9E-07	0	61.9761	gttcaaatctCgctggggcct	m5C_52583
chr14	21099331	-	C	35	11	0.314	0	0	T	24	M	0.186	0.48	6.7E-16	3.1E-06	8.7E-16	30.9686	gagttcaaatCtcgctggggc	m5C_52735
chr14	21099342	-	C	127	36	0.283	0	0	T	91	M	0.212	0.367	0	0	0	152.883	ccaggggtcgCgagttcaaat	m5C_52657
chr14	21099344	-	C	125	27	0.216	0	0	T	98	M	0.153	0.296	0	0	0	82.5698	aaccaggggtCgcgagttcaa	m5C_52692
chr14	21099351	-	C	117	29	0.248	0	0	T	88	M	0.178	0.333	0	0	0	103.517	tcttgtaaacCaggggtcgcg	m5C_52630
chr14	21099352	-	C	112	34	0.304	0	0	T	78	M	0.226	0.394	0	4.4E-16	0	153.748	gtcttgtaaaCcaggggtcgc	m5C_52573
chr14	21099360	-	C	108	33	0.306	0	0	T	75	M	0.227	0.398	0	1.6E-14	0	149.557	gagcactggtCttgtaaacca	m5C_52581
chr14	21099365	-	C	108	45	0.425	2	0.019	T	61	M	0.335	0.52	0	7.4E-13	0	301.225	ggttagagcaCtggtcttgta	m5C_52718
chr14	21099379	-	C	115	40	0.348	0	0	T	75	M	0.267	0.439	0	4.3E-15	0	213.578	ctccatagctCaggggttaga	m5C_52597
chr14	21099381	-	C	109	43	0.398	1	0.009	T	65	M	0.311	0.492	0	4.3E-13	0	267.336	ggctccatagCtcaggggtta	m5C_52757
chr14	21099387	-	C	73	15	0.205	0	0	T	58	M	0.129	0.312	0	6E-13	0	38.6006	tgtggcggctCcatagctcag	m5C_52710
chr14	21101169	+	C	153	36	0.235	0	0	T	117	M	0.175	0.308	0	0	0	126.054	agttctggctCgttggtctag	m5C_52511
chr14	21101176	+	C	239	98	0.41	0	0	T	141	M	0.35	0.473	0	0	0	685.191	gctcgttggtCtagtggtatg	m5C_52529
chr14	21101190	+	C	162	54	0.333	0	0	T	108	M	0.265	0.409	0	0	0	286.571	tggtatgattCtcgctttggg	m5C_52489
chr14	21101192	+	C	144	50	0.347	0	0	T	94	M	0.274	0.428	0	0	0	274.346	gtatgattctCgctttgggtg	m5C_52527
chr14	21101194	+	C	143	40	0.28	0	0	T	103	M	0.213	0.358	0	0	0	170.123	atgattctcgCtttgggtgcg	m5C_52469
chr14	21101203	+	C	91	24	0.264	0	0	T	67	M	0.184	0.363	0	1.7E-14	0	88.3703	gctttgggtgCgagaggtccc	m5C_52522
chr14	21101211	+	C	64	23	0.359	0	0	T	41	M	0.253	0.482	0	2.1E-09	0	116.326	tgcgagaggtCccgggttcaa	m5C_52549
chr14	21101212	+	C	64	40	0.625	0	0	C	40	M	0.503	0.733	0	1.4E-07	0	402.001	gcgagaggtcCcgggttcaaa	m5C_52507
chr14	21101213	+	C	62	22	0.355	0	0	T	40	M	0.247	0.479	0	1.5E-09	0	108.867	cgagaggtccCgggttcaaat	m5C_52491
chr14	21101219	+	C	54	11	0.204	0	0	T	43	M	0.118	0.329	2.3E-13	4.4E-10	2.8E-13	16.3622	gtcccgggttCaaatcccgga	m5C_52520
chr14	21101230	+	C	31	14	0.452	0	0	T	17	M	0.292	0.622	0	9.2E-06	0	81.6522	aaatcccggaCgagccccttt	m5C_52515
chr14	21101236	+	C	33	9	0.273	0	0	T	24	M	0.151	0.442	7.5E-13	1.2E-06	9E-13	16.4418	cggacgagccCcttttactttc	m5C_52473
chr14	21131339	-	C	45	13	0.289	0	0	T	32	M	0.177	0.434	0	1.2E-07	0	46.0978	gtgcccgatgCttttgcatgc	m5C_52684
chr14	21131344	-	C	51	14	0.275	0	0	T	37	M	0.171	0.409	0	2.8E-09	0	47.9195	aggaagtgccCgatgcttttg	m5C_52720
chr14	21131345	-	C	52	19	0.365	0	0	T	33	M	0.248	0.501	0	2.4E-08	0	94.242	aaggaagtgcCcgatgctttt	m5C_52645
chr14	21131346	-	C	52	14	0.269	0	0	T	38	M	0.168	0.403	0	2.8E-09	0	46.9527	gaaggaagtgCccgatgcttt	m5C_52607
chr14	21131362	-	C	37	19	0.514	0	0	C	19	M	0.359	0.666	0	3.4E-05	0	136.4	ggttcgattcCggctcgaagg	m5C_52742
chr14	21147572	+	C	33	10	0.303	0	0	T	23	M	0.174	0.473	1.6E-14	2E-06	2E-14	23.982	aggaggctggCtggtctgagt	m5C_52544
chr14	21147599	+	C	54	14	0.259	0	0	T	40	M	0.161	0.389	0	2.8E-09	0	45.1318	gtgtttacaaCtaattgatca	m5C_52550
chr14	21147610	+	C	50	12	0.24	0	0	T	38	M	0.143	0.374	2.4E-15	8.8E-10	3.1E-15	25.0697	taattgatcaCaagcagttac	m5C_52538
chr14	21147614	+	C	47	14	0.298	0	0	T	33	M	0.187	0.44	0	1.9E-07	0	52.223	tgatcacaagCagttacaggt	m5C_52474
chr14	21147620	+	C	42	13	0.31	0	0	T	29	M	0.191	0.46	0	1.2E-07	0	49.5844	caagcagttaCaggtctcttt	m5C_52525
chr14	21147635	+	C	60	14	0.233	0	0	T	46	M	0.144	0.354	0	3.2E-11	0	40.4304	ctctttgttcCttctccactc	m5C_52518
chr14	21147638	+	C	61	14	0.23	0	0	T	47	M	0.142	0.349	0	3.2E-11	0	39.7407	tttgttccttCtccactccca	m5C_52542
chr14	21147646	+	C	60	19	0.317	0	0	T	41	M	0.213	0.442	0	4.5E-10	0	80.9617	ttctccactcCcactgcttca	m5C_52488
chr14	21147657	+	C	58	15	0.259	0	0	T	43	M	0.163	0.384	0	4.6E-09	0	49.0362	cactgcttcaCttgactagcc	m5C_52519

chr14	21149873	+	C	47	18	0.391	1	0.021	T	28	M	0.264	0.535	0	7.7E-07	0	95.0149	ggggttagagCactggtcttg	m5C_52487
chr14	21149875	+	C	49	12	0.245	0	0	T	37	M	0.146	0.381	1.9E-15	7.5E-08	2.4E-15	25.8006	ggttagagcaCtggtcttgta	m5C_52512
chr14	21149880	+	C	43	11	0.256	0	0	T	32	M	0.149	0.402	1.2E-14	4.4E-08	1.5E-14	22.8887	gagcactggtCttgtaaacca	m5C_52506
chr14	21151456	+	C	125	32	0.256	0	0	T	93	M	0.188	0.339	0	0	0	120.042	gctggtagagCggaggactgt	m5C_52467
chr14	21151471	+	C	110	33	0.3	0	0	T	77	M	0.222	0.391	0	2.2E-16	0	146.716	gactgtagtaCttaatgtgtg	m5C_52540
chr14	21151484	+	C	100	23	0.23	0	0	T	77	M	0.158	0.322	0	1.1E-16	0	72.8785	aatgtgtggtCatccttaggt	m5C_52535
chr14	21151497	+	C	179	45	0.251	0	0	T	134	M	0.194	0.32	0	0	0	174.17	ccttaggtcgCtggttcgatt	m5C_52531
chr14	21151514	+	C	245	53	0.216	0	0	T	192	M	0.169	0.272	0	0	0	179.522	gattccggctCgaaggagacg	m5C_52551
chr14	21151523	+	C	249	88	0.353	0	0	T	161	M	0.297	0.415	0	0	0	522.149	tcgaaggagaCgctgctgttt	m5C_52504
chr14	21151528	+	C	213	50	0.235	0	0	T	163	M	0.183	0.296	0	0	0	182.832	ggagacgctgCtgttttgggg	m5C_52533
chr14	21152186	+	C	113	48	0.425	0	0	T	65	M	0.338	0.517	0	5E-14	0	324.084	gctcgttggtCtaggggtatg	m5C_52528
chr14	21152200	+	C	55	22	0.4	0	0	T	33	M	0.281	0.532	0	6.5E-08	0	123.721	gggtatgattCtcgctttggg	m5C_52521
chr14	21152202	+	C	59	22	0.373	0	0	T	37	M	0.261	0.5	0	6.5E-08	0	114.769	gtatgattctCgctttgggtg	m5C_52465
chr14	21152204	+	C	55	19	0.345	0	0	T	36	M	0.234	0.477	0	2.4E-08	0	88.7776	atgattctcgCtttgggtgcg	m5C_52485
chr14	21152213	+	C	38	18	0.474	0	0	T	20	M	0.325	0.627	0	2.7E-05	0	116.924	gctttgggtgCgagaggtccc	m5C_52514
chr14	21675030	-	C	32	7	0.219	0	0	T	25	M	0.11	0.388	8.5E-10	3.4E-07	9.5E-10	6.99971	aagataaatgCagataggaag	m5C_52575
chr14	21675042	-	C	45	29	0.644	0	0	C	29	M	0.498	0.768	0	9.7E-06	0	289.046	gagttagaacCcaagataaat	m5C_52722
chr14	21675043	-	C	42	14	0.333	0	0	T	28	M	0.21	0.484	0	1.9E-07	0	58.8344	ggagttagaaCccaagataaa	m5C_52745
chr14	21679454	-	C	58	16	0.276	0	0	T	42	M	0.178	0.402	0	7.3E-09	0	56.8071	tggagttgatCaaggatgatg	m5C_52699
chr14	21679465	-	C	66	18	0.273	0	0	T	48	M	0.18	0.39	0	2.9E-10	0	64.8115	tttctcacagCtggagttgat	m5C_52653
chr14	21679567	-	C	65	27	0.415	0	0	T	38	M	0.304	0.537	0	7.5E-09	0	163.944	gggggatgacCaggtgaaaac	m5C_52702
chr14	21679579	-	C	60	30	0.5	0	0	CT	30	M	0.377	0.623	0	1.7E-08	0	226.409	taatgaagatCggggggatga	m5C_52618
chr14	21679704	-	C	45	17	0.378	0	0	T	28	M	0.251	0.524	0	5.6E-07	0	85.3668	aatgataagtCagaagaggag	m5C_52635
chr14	21679971	-	C	77	25	0.325	0	0	T	52	M	0.231	0.435	0	9.3E-11	0	115.296	agcaaacaagCaggtaaatgg	m5C_52700
chr14	21679979	-	C	59	32	0.542	0	0	C	32	M	0.417	0.663	0	7.2E-07	0	266.609	aggaacagagCaaacaagcag	m5C_52707
chr14	21819989	-	C	50	27	0.54	0	0	C	27	M	0.404	0.67	0	6.7E-06	0	218.153	ctagaataccCtgatgagaaa	m5C_52646
chr14	21819990	-	C	48	21	0.438	0	0	T	27	M	0.307	0.577	0	1.8E-06	0	128.944	cctagaatacCctgatgagaa	m5C_52739
chr14	21820000	-	C	32	12	0.375	0	0	T	20	M	0.229	0.547	0	4.6E-06	0	55.0408	atgccttgggCctagaatacc	m5C_52713
chr14	21820942	-	C	42	17	0.405	0	0	T	25	M	0.27	0.555	0	5.6E-07	0	91.945	agaagaagaaCaaagtcgaag	m5C_52598
chr14	21820963	-	C	31	10	0.323	0	0	T	21	M	0.186	0.499	7.1E-15	2E-06	9E-15	26.2725	ccgagaaagtCgttacgagga	m5C_52736
chr14	21820977	-	C	42	14	0.333	0	0	T	28	M	0.21	0.484	0	1.9E-07	0	58.8344	ttctatttagCggaccgagaa	m5C_52566
chr14	21821653	-	C	43	9	0.209	0	0	T	34	M	0.114	0.352	1.4E-11	1.2E-08	1.6E-11	11.1522	ggaagaagccCgaaaaggttt	m5C_52638
chr14	21821655	-	C	40	9	0.225	0	0	T	31	M	0.123	0.375	6.4E-12	1.2E-08	7.6E-12	12.4041	gaggaagaagCccgaaaaggt	m5C_52631
chr14	21827737	-	C	32	11	0.344	0	0	T	21	M	0.204	0.517	2.2E-16	3.1E-06	2.9E-16	35.1442	aagcggcacaCggatgtgcag	m5C_52654
chr14	21827744	-	C	31	10	0.323	0	0	T	21	M	0.186	0.499	7.1E-15	2E-06	9E-15	26.2725	tgggaagaagCggcacacgga	m5C_52585
chr14	21860309	-	C	51	13	0.255	0	0	T	38	M	0.155	0.389	1.1E-16	1.6E-09	1.5E-16	32.2431	tgctgaggggCactaggtgaa	m5C_52564
chr14	21860317	-	C	120	32	0.267	0	0	T	88	M	0.196	0.352	0	0	0	125.237	ctcgtaaatgCtgaggggcac	m5C_52676
chr14	21860331	-	C	119	48	0.403	0	0	T	71	M	0.32	0.493	0	5E-14	0	306.794	gaaataaactCtttctcgtaa	m5C_52604
chr14	21860377	-	C	144	37	0.257	0	0	T	107	M	0.193	0.334	0	0	0	142.482	tacggagacaCcaggtaggga	m5C_52664
chr14	21860391	-	C	178	39	0.219	0	0	T	139	M	0.165	0.285	0	0	0	128.405	agttgccatgCtaatacggag	m5C_52666
chr14	21860396	-	C	179	41	0.229	0	0	T	138	M	0.174	0.296	0	0	0	142.324	tgatgagttgCcatgctaata	m5C_52580
chr14	21896109	-	C	40	15	0.375	0	0	T	25	M	0.242	0.53	0	2.8E-07	0	72.6683	aagaagagtgCtggggagagg	m5C_52625
chr14	21897872	-	C	43	9	0.209	0	0	T	34	M	0.114	0.352	1.4E-11	1.2E-08	1.6E-11	11.1522	ctgtggtgtgCtatgccaatc	m5C_52703
chr14	21897882	-	C	44	10	0.227	0	0	T	34	M	0.128	0.37	5.7E-13	2.4E-08	6.8E-13	15.7274	gagttctgggCtgtggtgtgc	m5C_52605
chr14	23321910	-	C	43	9	0.209	0	0	T	34	M	0.114	0.352	1.4E-11	1.2E-08	1.6E-11	11.1522	tgtgttatctCaactgattgc	m5C_48985
chr14	23321912	-	C	54	13	0.25	2	0.037	T	39	M	0.152	0.382	1.1E-16	1.6E-09	1.5E-16	31.5947	agtgtgttatCtcaactgatt	m5C_48973
chr14	23321930	-	C	61	32	0.525	0	0	C	32	M	0.402	0.645	0	2.7E-08	0	257.004	gatggttggtCtgaaggtagt	m5C_48961
chr14	23398992	+	C	167	35	0.21	0	0	T	132	M	0.155	0.277	0	0	0	108.311	ggtggggttcCtcgcagcttc	m5C_48945
chr14	23398994	+	C	163	41	0.252	0	0	T	122	M	0.191	0.323	0	0	0	156.757	tggggttcctCgcagcttcgc	m5C_48930
chr14	23399002	+	C	161	33	0.205	0	0	T	128	M	0.15	0.274	0	0	0	98.8924	ctcgcagcttCgctgcgtgag	m5C_48939
chr14	23399004	+	C	165	33	0.2	0	0	T	132	M	0.146	0.268	0	0	0	96.4293	cgcagcttcgCtgcgtgagca	m5C_48920
chr14	23399013	+	C	135	39	0.289	0	0	T	96	M	0.219	0.37	0	0	0	170.907	gctgcgtgagCattttgtaat	m5C_48947
chr14	24606623	+	C	66	18	0.273	0	0	T	48	M	0.18	0.39	0	2.9E-10	0	64.8115	gaaagaggagCggaagaaaca	m5C_48610
chr14	24606632	+	C	69	19	0.275	0	0	T	50	M	0.184	0.39	0	4.5E-10	0	69.8987	gcggaagaaaCagcaggaggc	m5C_48596
chr14	24606635	+	C	69	24	0.348	0	0	T	45	M	0.246	0.466	0	3E-09	0	118.166	gaagaaacagCaggaggcaag	m5C_48588
chr14	24606737	+	C	60	14	0.233	0	0	T	46	M	0.144	0.354	0	3.2E-11	0	40.4304	agaaggaagaCaaggatgaaa	m5C_48595
chr14	28507646	+	C	38	10	0.263	0	0	T	28	M	0.15	0.42	9.4E-14	2E-06	1.2E-13	19.5037	gtcaggatggCcgagtggtct	m5C_48563
chr14	28507647	+	C	40	17	0.425	0	0	T	23	M	0.285	0.578	0	5.6E-07	0	96.9312	tcaggatggcCgagtggtcta	m5C_48571
chr14	31727818	-	C	49	15	0.306	0	0	T	34	M	0.195	0.445	0	2.8E-07	0	58.546	cttgactagcCtttaaaataa	m5C_49048
chr14	31727828	-	C	143	33	0.231	0	0	T	110	M	0.169	0.306	0	0	0	111.74	cactgcttcaCttgactagcc	m5C_49041
chr14	31727858	-	C	150	39	0.26	0	0	T	111	M	0.196	0.336	0	0	0	153.208	ttacagatttCtttgttcctt	m5C_49025
chr14	31727872	-	C	151	36	0.238	0	0	T	115	M	0.177	0.312	0	0	0	127.778	ttgatcacaaCtagttacaga	m5C_49037
chr14	31727877	-	C	152	37	0.243	0	0	T	115	M	0.182	0.317	0	0	0	134.733	actaattgatCacaactagtt	m5C_49039
chr14	31727886	-	C	150	31	0.207	0	0	T	119	M	0.15	0.278	0	0	0	92.7446	gtgtttacaaCtaattgatca	m5C_49040
chr14	31727908	-	C	147	40	0.272	0	0	T	107	M	0.207	0.349	0	0	0	165.322	ataggctggtCtgagtgcagt	m5C_49047
chr14	32784163	+	C	46	22	0.478	0	0	T	24	M	0.341	0.619	0	2.3E-06	0	150.148	accctgtggtCtagtggcttg	m5C_48673
chr14	32954038	+	C	66	20	0.303	0	0	T	46	M	0.206	0.422	0	6.9E-10	0	82.2155	ctgagtgaagCattggactgt	m5C_48681
chr14	32954297	+	C	42	14	0.333	0	0	T	28	M	0.21	0.484	0	1.9E-07	0	58.8344	tcagttgatgCagagtggggt	m5C_48679
chr14	35016005	+	C	34	7	0.206	0	0	T	27	M	0.103	0.368	1.4E-09	3.4E-07	1.6E-09	6.4054	gatgtgctgaCccctgcgatt	m5C_49097
chr14	35016063	+	C	118	25	0.212	0	0	T	93	M	0.148	0.294	0	0	0	73.9121	tagtgggggaCtgcgttcgcg	m5C_49100
chr14	35016070	+	C	131	35	0.267	0	0	T	96	M	0.199	0.349	0	0	0	139.18	ggactgcgttCgcgctttccc	m5C_49094
chr14	35016072	+	C	130	51	0.392	0	0	T	79	M	0.313	0.478	0	1.1E-16	0	318.872	actgcgttcgCgctttcccct	m5C_49084
chr14	35016081	+	C	122	29	0.238	0	0	T	93	M	0.171	0.321	0	0	0	99.1265	gcgctttcccCtgccttactg	m5C_49093
chr14	35025443	-	C	119	40	0.336	0	0	T	79	M	0.258	0.425	0	4.3E-15	0	206.052	actgcgttcgCgctttcccct	m5C_49138
chr14	35025452	-	C	112	42	0.375	0	0	T	70	M	0.291	0.467	0	8.4E-15	0	244.345	tagtgggggaCtgcgttcgcg	m5C_49135
chr14	35037917	-	C	32	20	0.625	0	0	C	20	M	0.453	0.771	0	4.3E-05	0	181.016	ggacaggtggCagttgccaac	m5C_49101
chr14	35037924	-	C	50	10	0.2	0	0	T	40	M	0.112	0.33	2.6E-12	2E-10	3.1E-12	13.024	aatcgctggaCaggtggcagt	m5C_49103
chr14	35037929	-	C	43	21	0.488	0	0	T	22	M	0.346	0.632	0	1.8E-06	0	145.392	gacagaatcgCtggacaggtg	m5C_49106
chr14	35037931	-	C	48	21	0.438	0	0	T	27	M	0.307	0.577	0	1.8E-06	0	128.944	tggacagaatCgctggacagg	m5C_49117
chr14	35233996	-	C	34	8	0.235	0	0	T	26	M	0.124	0.4	4.2E-11	6.8E-07	4.8E-11	10.3259	aagttgatggCgatgaagaag	m5C_49113
chr14	35402742	-	C	84	25	0.298	0	0	T	59	M	0.21	0.402	0	1.8E-12	0	105.223	atgggctggtCcgagtgcagt	m5C_49134
chr14	36232022	-	C	54	31	0.574	0	0	C	31	M	0.442	0.697	0	5.9E-07	0	273.807	caagtccgacCgagaactctg	m5C_48722
chr14	36232027	-	C	54	16	0.296	0	0	T	38	M	0.191	0.428	0	7.3E-09	0	61.2393	gttttcaagtCcgaccgagaa	m5C_48727
chr14	36232032	-	C	54	18	0.333	0	0	T	36	M	0.222	0.466	0	1.7E-08	0	80.0685	aaagtgttttCaagtccgacc	m5C_48736
chr14	36232050	-	C	49	24	0.49	0	0	T	25	M	0.356	0.625	0	3.6E-06	0	170.759	ttgccaaggaCttatttaaaa	m5C_48733
chr14	36232077	-	C	47	16	0.34	0	0	T	31	M	0.222	0.483	0	4E-07	0	70.9352	taaaggtcacCaagaataatt	m5C_48719
chr14	36232080	-	C	47	14	0.298	0	0	T	33	M	0.187	0.44	0	1.9E-07	0	52.223	atataaaggtCaccaagaata	m5C_48726
chr14	36232107	-	C	48	16	0.333	0	0	T	32	M	0.217	0.475	0	4E-07	0	69.3651	ccttctgatcCcaagtatggc	m5C_48738
chr14	36232116	-	C	42	13	0.31	0	0	T	29	M	0.191	0.46	0	1.2E-07	0	49.5844	tttatatgccCttctgatccc	m5C_48724
chr14	36232117	-	C	41	11	0.275	1	0.024	T	29	M	0.161	0.428	4.3E-15	3.1E-06	5.5E-15	25.4502	ttttatatgcCcttctgatcc	m5C_48717
chr14	36232118	-	C	40	11	0.275	0	0	T	29	M	0.161	0.428	4.3E-15	3.1E-06	5.5E-15	25.4502	tttttatatgCccttctgatc	m5C_48728
chr14	39318929	+	C	31	14	0.452	0	0	T	17	M	0.292	0.622	0	9.2E-06	0	81.6522	aaaaaaaaagCctgcagctga	m5C_48752
chr14	39649914	+	C	61	17	0.279	0	0	T	44	M	0.182	0.402	0	1.8E-10	0	61.8406	atagaggaagCaggagaggaa	m5C_48753
chr14	39649941	+	C	40	8	0.2	0	0	T	32	M	0.105	0.348	2.1E-10	5.5E-09	2.3E-10	8.13503	aaggaaatagCgattgttcat	m5C_48746
chr14	39649959	+	C	62	16	0.258	0	0	T	46	M	0.166	0.379	0	1.1E-10	0	52.9752	catagtgatgCagagaaagaa	m5C_48765
chr14	39649970	+	C	66	15	0.227	0	0	T	51	M	0.143	0.342	0	6E-11	0	42.8553	agagaaagaaCaggaggagga	m5C_48769
chr14	39650813	+	C	58	21	0.362	0	0	T	37	M	0.251	0.491	0	4.8E-08	0	105.23	gagtagaagtCggagtagggg	m5C_48748
chr14	39650825	+	C	73	28	0.384	0	0	T	45	M	0.281	0.498	0	2.6E-10	0	157.082	gagtaggggcCggggacataa	m5C_48770
chr14	39650831	+	C	81	17	0.21	0	0	T	64	M	0.135	0.311	0	2.3E-14	0	46.026	gggccggggaCataatagaga	m5C_48766
chr14	39650849	+	C	109	54	0.495	0	0	T	55	M	0.403	0.588	0	6.4E-12	0	435.583	agatagaaagCacagaaggag	m5C_48768
chr14	39650851	+	C	114	36	0.316	0	0	T	78	M	0.238	0.406	0	1E-15	0	171.11	atagaaagcaCagaaggagcg	m5C_48740

chr14	39650860	+	C	88	32	0.364	0	0	T	56	M	0.271	0.468	0	2.3E-11	0	173.307	acagaaggagCgtggatcgga	m5C_48763
chr14	45580092	+	C	200	42	0.21	0	0	T	158	M	0.159	0.272	0	0	0	133.798	actgtggcaaCtgtgatgaaa	m5C_49152
chr14	45580111	+	C	477	172	0.361	0	0	T	305	M	0.319	0.405	0	0	0	1096.56	aagatttggtCtgtatgtaat	m5C_49148
chr14	45580152	+	C	289	58	0.201	0	0	T	231	M	0.159	0.251	0	0	0	183.95	gacaacagtcCctctaaactg	m5C_49153
chr14	45580153	+	C	284	131	0.461	0	0	T	153	M	0.404	0.519	0	0	0	1058.99	acaacagtccCtctaaactga	m5C_49151
chr14	45580155	+	C	293	61	0.208	0	0	T	232	M	0.166	0.258	0	0	0	202.062	aacagtccctCtaaactgatg	m5C_49149
chr14	45580160	+	C	222	61	0.275	0	0	T	161	M	0.22	0.337	0	0	0	268.716	tccctctaaaCtgatgttgcc	m5C_49150
chr14	50053299	+	C	37	11	0.297	0	0	T	26	M	0.175	0.458	1.4E-15	3.1E-06	1.9E-15	28.5508	tatccgaccgCcgggcgcggt	m5C_49944
chr14	50053300	+	C	60	30	0.5	0	0	CT	30	M	0.377	0.623	0	1.7E-08	0	226.409	atccgaccgcCgggcgcggtg	m5C_49915
chr14	50053304	+	C	100	31	0.31	0	0	T	69	M	0.228	0.406	0	7.1E-15	0	141.233	gaccgccgggCgcggtggcgc	m5C_49916
chr14	50053306	+	C	98	41	0.423	1	0.01	T	56	M	0.329	0.522	0	8.4E-12	0	269.915	ccgccgggcgCggtggcgcgt	m5C_49923
chr14	50053312	+	C	97	28	0.289	0	0	T	69	M	0.208	0.386	0	1.1E-13	0	116.425	ggcgcggtggCgcgtgcctgt	m5C_49909
chr14	50053318	+	C	99	29	0.293	0	0	T	70	M	0.212	0.389	0	1.7E-13	0	123.172	gtggcgcgtgCctgtagtccc	m5C_49940
chr14	50053319	+	C	94	20	0.213	0	0	T	74	M	0.142	0.306	0	1.7E-15	0	56.8671	tggcgcgtgcCtgtagtccca	m5C_49907
chr14	50053326	+	C	99	27	0.273	0	0	T	72	M	0.195	0.368	0	7.2E-14	0	105.15	tgcctgtagtCccagctactc	m5C_49928
chr14	50053336	+	C	101	38	0.376	0	0	T	63	M	0.288	0.474	0	9.4E-14	0	218.827	cccagctactCgggaggctga	m5C_49917
chr14	50053343	+	C	103	26	0.252	0	0	T	77	M	0.178	0.344	0	6.7E-16	0	92.8081	actcgggaggCtgaggctgga	m5C_49945
chr14	50053358	+	C	89	39	0.438	0	0	T	50	M	0.34	0.542	0	1.6E-10	0	265.043	gctggaggatCgcttgagtcc	m5C_49929
chr14	50053367	+	C	46	21	0.457	0	0	T	25	M	0.322	0.598	0	1.8E-06	0	135.049	tcgcttgagtCcaggagttct	m5C_49934
chr14	50053486	+	C	108	23	0.213	0	0	T	85	M	0.146	0.299	0	1.1E-16	0	67.3019	ggtgaaccggCccaggtcgga	m5C_49943
chr14	50053487	+	C	105	42	0.404	1	0.01	T	62	M	0.315	0.5	0	3.2E-13	0	264.264	gtgaaccggcCcaggtcggaa	m5C_49947
chr14	50053514	+	C	137	48	0.35	0	0	T	89	M	0.276	0.433	0	0	0	264.526	caggtcaaaaCtcccgtgctg	m5C_49939
chr14	50053517	+	C	82	30	0.366	0	0	T	52	M	0.27	0.474	0	1.2E-11	0	161.871	gtcaaaactcCcgtgctgatc	m5C_49937
chr14	50053518	+	C	86	20	0.233	0	0	T	66	M	0.156	0.332	0	1.5E-13	0	62.356	tcaaaactccCgtgctgatca	m5C_49938
chr14	50053522	+	C	84	28	0.337	1	0.012	T	55	M	0.245	0.444	0	5.8E-12	0	137.111	aactcccgtgCtgatcagtag	m5C_49935
chr14	50053527	+	C	104	28	0.269	0	0	T	76	M	0.193	0.362	0	1.8E-15	0	108.266	ccgtgctgatCagtagtggga	m5C_49950
chr14	50053539	+	C	138	39	0.283	0	0	T	99	M	0.214	0.363	0	0	0	167.046	gtagtgggatCgcgcctgtga	m5C_49913
chr14	50053544	+	C	137	49	0.358	0	0	T	88	M	0.282	0.441	0	0	0	276.645	gggatcgcgcCtgtgaatagc	m5C_49910
chr14	50053554	+	C	135	48	0.356	0	0	T	87	M	0.28	0.439	0	0	0	268.635	ctgtgaatagCcactgcactc	m5C_49927
chr14	50053555	+	C	125	31	0.248	0	0	T	94	M	0.181	0.33	0	0	0	111.951	tgtgaatagcCactgcactcc	m5C_49933
chr14	50053560	+	C	125	35	0.28	0	0	T	90	M	0.209	0.364	0	0	0	146.126	atagccactgCactccagcct	m5C_49948
chr14	50053565	+	C	122	25	0.205	0	0	T	97	M	0.143	0.285	0	0	0	71.4143	cactgcactcCagcctgggca	m5C_49906
chr14	50053588	+	C	110	33	0.3	0	0	T	77	M	0.222	0.391	0	2.2E-16	0	146.716	atagcgagacCccgtctcttt	m5C_49924
chr14	50053590	+	C	93	20	0.215	0	0	T	73	M	0.144	0.309	0	1.7E-15	0	57.4997	agcgagacccCgtctcttttg	m5C_49919
chr14	50053595	+	C	90	26	0.289	0	0	T	64	M	0.205	0.39	0	4.6E-14	0	106.818	gaccccgtctCttttgaacaa	m5C_49921
chr14	50313367	-	C	46	25	0.543	0	0	C	25	M	0.402	0.678	0	4.5E-06	0	200.9	gaccctgtctCtacaaataat	m5C_50017
chr14	50313375	-	C	54	15	0.278	0	0	T	39	M	0.176	0.409	0	4.6E-09	0	52.85	catagtgagaCcctgtctcta	m5C_49951
chr14	50320335	-	C	527	187	0.355	0	0	T	340	M	0.315	0.397	0	0	0	1178.75	gaccctgcctCttttgagttc	m5C_50040
chr14	50320337	-	C	626	148	0.236	0	0	T	478	M	0.205	0.271	0	0	0	606.231	gagaccctgcCtcttttgagt	m5C_50053
chr14	50320341	-	C	688	146	0.213	2	0.003	T	540	M	0.184	0.245	0	0	0	536.816	tagtgagaccCtgcctcttt	m5C_49987
chr14	50320343	-	C	726	191	0.263	0	0	T	535	M	0.232	0.296	0	0	0	887.628	catagtgagaCcctgcctctt	m5C_50034
chr14	50320361	-	C	782	222	0.284	0	0	T	560	M	0.253	0.316	0	0	0	1125.1	gcactccagcCtgggcaacat	m5C_50055
chr14	50320362	-	C	775	196	0.258	14	0.018	T	565	M	0.228	0.29	0	0	0	892.813	tgcactccagCctgggcaaca	m5C_49981
chr14	50320365	-	C	770	195	0.254	1	0.001	T	574	M	0.224	0.285	0	0	0	874.003	cactgcactcCagcctgggca	m5C_50066
chr14	50320366	-	C	786	181	0.23	0	0	T	605	M	0.202	0.261	0	0	0	731.967	ccactgcactCcagcctgggc	m5C_50033
chr14	50320368	-	C	780	204	0.262	0	0	T	576	M	0.232	0.293	0	0	0	946.229	agccactgcaCtccagcctgg	m5C_50041
chr14	50320370	-	C	784	166	0.212	0	0	T	618	M	0.185	0.242	0	0	0	612.799	atagccactgCactccagcct	m5C_50083
chr14	50320375	-	C	759	160	0.211	0	0	T	599	M	0.183	0.241	0	0	0	586.492	tgtgaatagcCactgcactcc	m5C_49996
chr14	50320386	-	C	788	252	0.32	0	0	T	536	M	0.288	0.353	0	0	0	1452.4	gggattgcgcCtgtgaatagc	m5C_49988
chr14	50320387	-	C	766	197	0.257	0	0	T	569	M	0.227	0.289	0	0	0	896.32	tgggattgcgCctgtgaatag	m5C_49959
chr14	50320408	-	C	332	80	0.241	0	0	T	252	M	0.198	0.29	0	0	0	316.946	aactcccgtgCtgatcagtag	m5C_50008
chr14	50320421	-	C	93	21	0.226	0	0	T	72	M	0.153	0.321	0	3.1E-15	0	64.1342	cggagcaggtCaaaactcccg	m5C_50005
chr14	50320431	-	C	35	7	0.2	0	0	T	28	M	0.1	0.359	1.9E-09	3.4E-07	2.1E-09	6.13884	aggtcggaaaCggagcaggtc	m5C_49952
chr14	50320442	-	C	159	47	0.296	0	0	T	112	M	0.23	0.371	0	0	0	216.357	tgaaccggccCaggtcggaaa	m5C_49983
chr14	50320448	-	C	181	61	0.341	2	0.011	T	118	M	0.275	0.413	0	0	0	335.919	gaggggtgaaCcggcccaggt	m5C_50011
chr14	50320471	-	C	100	30	0.3	0	0	T	70	M	0.219	0.396	0	4.7E-15	0	131.369	gggggaccacCaggttgccta	m5C_49971
chr14	50320475	-	C	116	30	0.259	0	0	T	86	M	0.188	0.345	0	0	0	112.548	gagcgggggaCcaccaggttg	m5C_50024
chr14	50320482	-	C	112	38	0.4	17	0.152	T	57	M	0.307	0.501	0	3.7E-12	0	233.493	ctccccgggagCgggggaccac	m5C_50068
chr14	50320488	-	C	88	29	0.33	0	0	T	59	M	0.24	0.433	0	8.4E-12	0	139.361	ggtgacctccCgggagcgggg	m5C_49999
chr14	50320489	-	C	89	25	0.281	0	0	T	64	M	0.198	0.382	0	1.8E-12	0	99.0468	tggtgacctcCcgggagcggg	m5C_50031
chr14	50320490	-	C	88	31	0.352	0	0	T	57	M	0.261	0.456	0	1.7E-11	0	161.548	atggtgacctCccgggagcgg	m5C_50009
chr14	50320492	-	C	87	35	0.402	0	0	T	52	M	0.306	0.507	0	5.6E-11	0	213.855	atatggtgacCtcccgggagc	m5C_50014
chr14	50320493	-	C	91	21	0.231	0	0	T	70	M	0.156	0.327	0	3.1E-15	0	65.5958	aatatggtgaCctcccgggag	m5C_50051
chr14	50320529	-	C	85	38	0.447	0	0	T	47	M	0.346	0.553	0	1.3E-10	0	262.91	ctatgccgatCgggtgtccgc	m5C_50065
chr14	50320533	-	C	82	20	0.244	0	0	T	62	M	0.164	0.347	0	1.5E-13	0	65.5191	tgcgctatgcCgatcgggtgt	m5C_50018
chr14	50320534	-	C	76	28	0.368	0	0	T	48	M	0.269	0.481	0	2.6E-10	0	150.501	gtgcgctatgCcgatcgggtg	m5C_50019
chr14	50320539	-	C	79	17	0.215	0	0	T	62	M	0.139	0.318	0	2.2E-12	0	47.2331	ctgtagtgcgCtatgccgatc	m5C_49966
chr14	50320541	-	C	71	32	0.451	0	0	T	39	M	0.34	0.566	0	8.5E-10	0	217.906	ggctgtagtgCgctatgccga	m5C_49993
chr14	50320549	-	C	69	23	0.333	0	0	T	46	M	0.234	0.451	0	2.1E-09	0	107.414	gagttctgggCtgtagtgcgc	m5C_49989
chr14	50320554	-	C	53	22	0.415	0	0	T	31	M	0.293	0.549	0	6.5E-08	0	128.747	ttcaggagttCtgggctgtag	m5C_50026
chr14	50320562	-	C	42	13	0.31	0	0	T	29	M	0.191	0.46	0	1.2E-07	0	49.5844	cccttgagttCaggagttctg	m5C_50035
chr14	50320570	-	C	43	28	0.651	0	0	C	28	M	0.502	0.776	0	8.1E-06	0	280.96	tggaggatccCttgagttcag	m5C_49963
chr14	50320572	-	C	46	13	0.283	0	0	T	33	M	0.173	0.425	0	1.2E-07	0	45.0425	gctggaggatCccttgagttc	m5C_49961
chr14	50320581	-	C	55	21	0.382	0	0	T	34	M	0.265	0.514	0	4.8E-08	0	111.366	gaggctgaggCtggaggatcc	m5C_49954
chr14	50320587	-	C	57	23	0.404	0	0	T	34	M	0.286	0.533	0	8.7E-08	0	131.64	actcgggaggCtgaggctgga	m5C_50050
chr14	50320599	-	C	52	12	0.231	0	0	T	40	M	0.137	0.361	4.4E-15	8.8E-10	5.7E-15	23.6371	gtagtcccagCtactcgggag	m5C_49982
chr14	50320602	-	C	54	19	0.352	0	0	T	35	M	0.238	0.485	0	2.4E-08	0	90.5268	cctgtagtccCagctactcgg	m5C_50029
chr14	50320611	-	C	54	12	0.222	0	0	T	42	M	0.132	0.349	7.5E-15	8.8E-10	9.6E-15	22.3614	tggcgcgtgcCtgtagtccca	m5C_49976
chr14	50320612	-	C	50	16	0.32	0	0	T	34	M	0.208	0.458	0	7.3E-09	0	66.4257	gtggcgcgtgCctgtagtccc	m5C_49985
chr14	50320616	-	C	53	22	0.415	0	0	T	31	M	0.293	0.549	0	6.5E-08	0	128.747	cgcggtggcgCgtgcctgtag	m5C_50006
chr14	50320624	-	C	50	10	0.2	0	0	T	40	M	0.112	0.33	2.6E-12	2E-10	3.1E-12	13.024	gcgccgggcgCggtggcgcgt	m5C_49980
chr14	50329270	-	C	610	161	0.264	0	0	T	449	M	0.231	0.3	0	0	0	742.248	gaccccgtctCttttgccccc	m5C_49997
chr14	50329276	-	C	799	178	0.223	1	0.001	T	620	M	0.196	0.253	0	0	0	696.12	tagcgagaccCcgtctctttt	m5C_50047
chr14	50329310	-	C	946	196	0.207	1	0.001	T	749	M	0.183	0.234	0	0	0	716.444	tgtgaatagcCactgcactcc	m5C_50070
chr14	50329321	-	C	908	217	0.239	1	0.001	T	690	M	0.213	0.268	0	0	0	922.778	gggatcgcgcCtgtgaatagc	m5C_50073
chr14	50329326	-	C	863	174	0.202	0	0	T	689	M	0.176	0.23	0	0	0	613.186	gtagtgggatCgcgcctgtga	m5C_50081
chr14	50329347	-	C	348	75	0.216	0	0	T	273	M	0.176	0.262	0	0	0	263.319	tcaaaactccCgtgctgatca	m5C_49967
chr14	50329356	-	C	161	35	0.217	0	0	T	126	M	0.161	0.287	0	0	0	112.467	cggagcaggtCaaaactcccg	m5C_50032
chr14	50329366	-	C	105	35	0.333	0	0	T	70	M	0.25	0.428	0	3.4E-14	0	175.32	aggtcggaaaCggagcaggtc	m5C_49990
chr14	50329372	-	C	48	11	0.229	0	0	T	37	M	0.133	0.365	5E-14	4.4E-08	6.2E-14	19.4712	cggcccaggtCggaaacggag	m5C_50064
chr14	50329377	-	C	95	29	0.305	0	0	T	66	M	0.222	0.404	0	1.7E-13	0	128.602	tgaaccggccCaggtcggaaa	m5C_49953
chr14	50329378	-	C	109	28	0.257	0	0	T	81	M	0.184	0.346	0	1.8E-15	0	103.106	gtgaaccggcCcaggtcggaa	m5C_50037
chr14	50329382	-	C	113	29	0.257	0	0	T	84	M	0.185	0.344	0	0	0	107.321	aggggtgaacCggcccaggtc	m5C_50059
chr14	50329383	-	C	105	32	0.305	0	0	T	73	M	0.225	0.398	0	1.1E-14	0	143.936	gaggggtgaaCcggcccaggt	m5C_50036
chr14	50329407	-	C	181	42	0.232	0	0	T	139	M	0.177	0.299	0	0	0	148.262	cgggggaccaCcaggttgcct	m5C_50062
chr14	50329410	-	C	187	38	0.203	0	0	T	149	M	0.152	0.267	0	0	0	115.353	gagcgggggaCcaccaggttg	m5C_49956
chr14	50329423	-	C	142	66	0.465	0	0	T	76	M	0.385	0.547	0	2.2E-16	0	507.887	ggtgacctccCgggagcgggg	m5C_49974
chr14	50329425	-	C	142	39	0.275	0	0	T	103	M	0.208	0.353	0	0	0	162.163	atggtgacctCccgggagcgg	m5C_49968
chr14	50329427	-	C	145	52	0.359	0	0	T	93	M	0.285	0.439	0	0	0	296.539	atatggtgacCtcccgggagc	m5C_50046
chr14	50329439	-	C	180	76	0.425	1	0.006	T	103	M	0.355	0.498	0	0	0	538.843	agttcggcatCaatatggtga	m5C_50084
chr14	50329445	-	C	200	80	0.4	0	0	T	120	M	0.335	0.469	0	0	0	535.367	gcactaagttCggcatcaata	m5C_49991

chr14	50329452	-	C	240	48	0.2	0	0	T	192	M	0.154	0.255	0	0	0	148.126	ggtgtccgcaCtaagttcggc	m5C_50007
chr14	50329456	-	C	218	63	0.289	0	0	T	155	M	0.233	0.352	0	0	0	293.432	atcgggtgtcCgcactaagtt	m5C_49986
chr14	50329457	-	C	226	67	0.296	0	0	T	159	M	0.241	0.359	0	0	0	322.566	gatcgggtgtCcgcactaagt	m5C_50028
chr14	50329464	-	C	212	90	0.427	1	0.005	T	121	M	0.362	0.494	0	0	0	651.071	ctatgccgatCgggtgtccgc	m5C_50039
chr14	50329468	-	C	233	70	0.303	2	0.009	T	161	M	0.247	0.365	0	0	0	346.34	tgcgctatgcCgatcgggtgt	m5C_50023
chr14	50329469	-	C	268	67	0.25	0	0	T	201	M	0.202	0.305	0	0	0	270.595	gtgcgctatgCcgatcgggtg	m5C_50048
chr14	50329474	-	C	292	64	0.221	2	0.007	T	226	M	0.177	0.272	0	0	0	226.282	ctgtagtgcgCtatgccgatc	m5C_50044
chr14	50329476	-	C	296	65	0.22	0	0	T	231	M	0.176	0.27	0	0	0	229.05	ggctgtagtgCgctatgccga	m5C_50003
chr14	50329484	-	C	301	88	0.301	9	0.03	T	204	M	0.252	0.356	0	0	0	442.812	gagttctgggCtgtagtgcgc	m5C_50077
chr14	50329489	-	C	294	93	0.316	0	0	T	201	M	0.266	0.372	0	0	0	494.439	cccaggagttCtgggctgtag	m5C_50052
chr14	50329497	-	C	269	76	0.283	0	0	T	193	M	0.232	0.339	0	0	0	352.759	cgcttgagccCaggagttctg	m5C_49960
chr14	50329498	-	C	280	58	0.207	0	0	T	222	M	0.164	0.258	0	0	0	190.001	tcgcttgagcCcaggagttct	m5C_49965
chr14	50329499	-	C	283	78	0.276	0	0	T	205	M	0.227	0.33	0	0	0	353.849	atcgcttgagCccaggagttc	m5C_50069
chr14	50329505	-	C	252	97	0.385	0	0	T	155	M	0.327	0.446	0	0	0	634.379	gggaggatcgCttgagcccag	m5C_50078
chr14	50329507	-	C	252	60	0.24	2	0.008	T	190	M	0.191	0.297	0	0	0	229.498	gtgggaggatCgcttgagccc	m5C_49979
chr14	50329522	-	C	254	53	0.209	0	0	T	201	M	0.163	0.263	0	0	0	173.005	actcgggaggCtgaggtggga	m5C_50022
chr14	50329529	-	C	264	71	0.269	0	0	T	193	M	0.219	0.325	0	0	0	311.047	cccagctactCgggaggctga	m5C_50045
chr14	50329531	-	C	273	57	0.209	0	0	T	216	M	0.165	0.261	0	0	0	187.853	gtcccagctaCtcgggaggct	m5C_50038
chr14	50329539	-	C	291	64	0.22	0	0	T	227	M	0.176	0.271	0	0	0	225.485	tgcctgtagtCccagctactc	m5C_49973
chr14	50329546	-	C	292	73	0.252	2	0.007	T	217	M	0.205	0.305	0	0	0	299.65	tggcgcgtgcCtgtagtccca	m5C_50016
chr14	50329547	-	C	297	94	0.318	1	0.003	T	202	M	0.267	0.373	0	0	0	502.26	gtggcgcgtgCctgtagtccc	m5C_49962
chr14	50329551	-	C	294	80	0.273	1	0.003	T	213	M	0.225	0.327	0	0	0	360.331	cgcggtggcgCgtgcctgtag	m5C_50043
chr14	50329553	-	C	291	73	0.251	0	0	T	218	M	0.205	0.304	0	0	0	298.593	ggcgcggtggCgcgtgcctgt	m5C_50013
chr14	50329559	-	C	294	79	0.27	1	0.003	T	214	M	0.222	0.323	0	0	0	350.815	ccgccgggcgCggtggcgcgt	m5C_49992
chr14	50329561	-	C	290	75	0.26	1	0.003	T	214	M	0.212	0.313	0	0	0	318.544	gaccgccgggCgcggtggcgc	m5C_50025
chr14	50329566	-	C	141	30	0.213	0	0	T	111	M	0.153	0.287	0	0	0	91.979	tctccgaccgCcgggcgcggt	m5C_50079
chr14	50329569	-	C	42	9	0.214	0	0	T	33	M	0.117	0.359	1.1E-11	1.2E-08	1.3E-11	11.5454	gcgtctccgaCcgccgggcgc	m5C_50056
chr14	50553707	+	C	45	9	0.2	0	0	T	36	M	0.109	0.338	2.3E-11	1.2E-08	2.7E-11	10.4289	tttggctggtCtgagtgcatt	m5C_49932
chr14	50553726	+	C	39	9	0.231	0	0	T	30	M	0.126	0.383	4.9E-12	1.2E-06	5.7E-12	12.8739	ttggtgtttaCaactaattga	m5C_49903
chr14	51865683	-	C	52	19	0.365	0	0	T	33	M	0.248	0.501	0	2.4E-08	0	94.242	gggcccgatcCtggagagact	m5C_49978
chr14	51865688	-	C	56	20	0.357	0	0	T	36	M	0.245	0.488	0	3.4E-08	0	97.8256	ggctagggccCgatcctggag	m5C_50000
chr14	56118254	+	C	60	14	0.233	0	0	T	46	M	0.144	0.354	0	3.2E-11	0	40.4304	agaagtatgaCtggtctgagt	m5C_49225
chr14	56118259	+	C	168	51	0.304	0	0	T	117	M	0.239	0.377	0	0	0	243.856	tatgactggtCtgagtgcagt	m5C_49220
chr14	56118290	+	C	128	45	0.352	0	0	T	83	M	0.274	0.438	0	5.6E-16	0	246.844	actgattgatCacaaccagtt	m5C_49227
chr14	56122798	+	C	207	45	0.217	0	0	T	162	M	0.167	0.278	0	0	0	149.964	aggttagaagCcatgctaaaa	m5C_49210
chr14	56122803	+	C	195	41	0.21	0	0	T	154	M	0.159	0.273	0	0	0	130.33	agaagccatgCtaaaagagag	m5C_49212
chr14	58706602	-	C	230	64	0.278	0	0	T	166	M	0.224	0.339	0	0	0	287.166	cgggttgtaaCttttttcctg	m5C_49479
chr14	58706612	-	C	402	112	0.279	0	0	T	290	M	0.237	0.324	0	0	0	530.958	acgttgggcgCgggttgtaac	m5C_49487
chr14	58706614	-	C	413	134	0.324	0	0	T	279	M	0.281	0.371	0	0	0	753.347	ccacgttgggCgcgggttgta	m5C_49466
chr14	58706623	-	C	392	89	0.227	0	0	T	303	M	0.188	0.271	0	0	0	335.238	gttcgagcccCacgttgggcg	m5C_49480
chr14	58706625	-	C	382	89	0.233	0	0	T	293	M	0.193	0.278	0	0	0	344.213	gggttcgagcCccacgttggg	m5C_49478
chr14	58706630	-	C	373	106	0.284	0	0	T	267	M	0.241	0.332	0	0	0	510.477	gtcgtgggttCgagccccacg	m5C_49472
chr14	58706638	-	C	355	103	0.29	0	0	T	252	M	0.245	0.339	0	0	0	505.477	atcccagggtCgtgggttcga	m5C_49483
chr14	58706646	-	C	361	75	0.208	0	0	T	286	M	0.169	0.253	0	0	0	253.634	gactcttaatCccagggtcgt	m5C_49473
chr14	58706652	-	C	428	106	0.248	0	0	T	322	M	0.209	0.291	0	0	0	443.363	gcatgggactCttaatcccag	m5C_49488
chr14	58706654	-	C	453	137	0.302	0	0	T	316	M	0.262	0.346	0	0	0	717.71	gagcatgggaCtcttaatccc	m5C_49470
chr14	58706669	-	C	592	130	0.22	2	0.003	T	460	M	0.189	0.256	0	0	0	490.783	ctagctcagtCggtagagcat	m5C_49485
chr14	58706673	-	C	574	158	0.276	2	0.003	T	414	M	0.241	0.314	0	0	0	762.083	ccggctagctCagtcggtaga	m5C_49463
chr14	58706675	-	C	575	141	0.245	0	0	T	434	M	0.212	0.282	0	0	0	597.331	gcccggctagCtcagtcggta	m5C_49476
chr14	58706679	-	C	564	119	0.211	0	0	T	445	M	0.179	0.247	0	0	0	426.809	aggcgcccggCtagctcagtc	m5C_49482
chr14	58706682	-	C	358	80	0.223	0	0	T	278	M	0.183	0.269	0	0	0	293.404	aacaggcgccCggctagctca	m5C_49484
chr14	58706683	-	C	339	70	0.206	0	0	T	269	M	0.167	0.253	0	0	0	233.526	taacaggcgcCcggctagctc	m5C_49469
chr14	60749921	-	C	60	18	0.3	0	0	T	42	M	0.199	0.425	0	2.9E-10	0	71.6328	cagcggattaCttggtttgtg	m5C_49198
chr14	61011152	-	C	42	22	0.524	0	0	C	22	M	0.377	0.666	0	2.3E-06	0	165.978	atctgtggacCtgaatttgca	m5C_49194
chr14	62647822	+	C	41	19	0.463	0	0	T	22	M	0.321	0.613	0	1E-06	0	121.813	aagtatagggCctgatgcttg	m5C_49372
chr14	62647833	+	C	38	27	0.711	0	0	C	27	M	0.552	0.83	0	0.00015	0	298.31	ctgatgcttgCttctgtgcct	m5C_49392
chr14	64315003	-	C	126	26	0.206	0	0	T	100	M	0.145	0.285	0	0	0	75.3419	tcttcactccCactgcttcac	m5C_49447
chr14	64315015	-	C	129	54	0.419	0	0	T	75	M	0.337	0.505	0	7.5E-15	0	363.995	ttctttgttcCttcttcactc	m5C_49441
chr14	64315054	-	C	124	38	0.306	0	0	T	86	M	0.232	0.392	0	0	0	176.426	gtggtgtttaCaactaattga	m5C_49442
chr14	64315073	-	C	140	45	0.321	0	0	T	95	M	0.25	0.403	0	0	0	224.754	cttggctggtCtgagtgcagt	m5C_49452
chr14	65591077	-	C	56	15	0.268	0	0	T	41	M	0.17	0.396	0	4.6E-09	0	50.8715	aggaacagtgCacccaaacct	m5C_49451
chr14	65591082	-	C	71	19	0.268	0	0	T	52	M	0.179	0.381	0	6.9E-12	0	67.8401	cttccaggaaCagtgcaccca	m5C_49445
chr14	65591106	-	C	47	15	0.319	0	0	T	32	M	0.204	0.462	0	2.8E-07	0	61.1856	acacattgacCtgatattgca	m5C_49453
chr14	66480007	-	C	133	46	0.346	0	0	T	87	M	0.27	0.43	0	0	0	248.749	gggaaaagaaCgagggatcgg	m5C_49366
chr14	67726961	+	C	41	11	0.268	0	0	T	30	M	0.157	0.419	6E-15	4.4E-08	7.6E-15	24.5536	ccataccaccCtgactgtgcc	m5C_49347
chr14	67726965	+	C	42	8	0.276	13	0.31	T	21	M	0.147	0.457	8.4E-12	5.4E-05	9.8E-12	13.0262	accaccctgaCtgtgcctgat	m5C_49331
chr14	67726971	+	C	35	12	0.343	0	0	T	23	M	0.208	0.508	0	4.6E-06	0	49.9956	ctgactgtgcCtgatcttgtc	m5C_49350
chr14	69775734	-	C	75	15	0.2	0	0	T	60	M	0.125	0.304	0	6E-13	0	37.5361	gttgattctgCcagtaataaa	m5C_49320
chr14	69775737	-	C	77	20	0.26	0	0	T	57	M	0.175	0.367	0	1.1E-11	0	69.9563	ctggttgattCtgccagtaat	m5C_49319
chr14	70444886	+	C	126	27	0.214	0	0	T	99	M	0.152	0.294	0	0	0	81.8938	tcccagatttCccaagggagg	m5C_49267
chr14	70505707	-	C	53	13	0.245	0	0	T	40	M	0.149	0.376	2.2E-16	1.6E-09	2.9E-16	30.3876	gggtttgaagCccagctctac	m5C_49298
chr14	70505722	-	C	66	20	0.303	0	0	T	46	M	0.206	0.422	0	6.9E-10	0	82.2155	ctccagcgttCtgtggggttt	m5C_49297
chr14	71915548	+	C	33	17	0.515	0	0	C	17	M	0.352	0.675	0	2.1E-05	0	119.742	gagtgggggaCcaccagattg	m5C_49253
chr14	73429691	+	C	36	17	0.472	0	0	T	19	M	0.32	0.63	0	2.1E-05	0	108.752	gggtatagctCaggggtagag	m5C_49405
chr14	74373090	-	C	38	19	0.5	0	0	CT	19	M	0.348	0.652	0	3.4E-05	0	132.429	ctgatgttcaCagtcagttac	m5C_49891
chr14	74373092	-	C	37	9	0.243	0	0	T	28	M	0.134	0.401	2.7E-12	1.2E-06	3.2E-12	13.907	aactgatgttCacagtcagtt	m5C_49883
chr14	74373123	-	C	37	10	0.27	0	0	T	27	M	0.154	0.43	6.7E-14	2E-06	8.3E-14	20.2815	atgggctggtCtgaaggtagt	m5C_49861
chr14	75746362	+	C	180	43	0.239	0	0	T	137	M	0.182	0.306	0	0	0	156.92	gaagggataaCgggaacgcag	m5C_49812
chr14	75746368	+	C	174	43	0.247	0	0	T	131	M	0.189	0.316	0	0	0	162.504	ataacgggaaCgcagcggcag	m5C_49846
chr14	75746373	+	C	159	62	0.39	0	0	T	97	M	0.318	0.467	0	0	0	393.794	gggaacgcagCggcaggatgg	m5C_49790
chr14	75746376	+	C	153	39	0.255	0	0	T	114	M	0.192	0.329	0	0	0	150.1	aacgcagcggCaggatggaag	m5C_49843
chr14	77987488	+	C	32	13	0.406	0	0	T	19	M	0.255	0.577	0	6.7E-06	0	66.3505	tttattatgtCtgtctgactg	m5C_49493
chr14	77987496	+	C	32	21	0.656	0	0	C	21	M	0.483	0.796	0	5.2E-05	0	202.905	gtctgtctgaCtgttgccccc	m5C_49501
chr14	78270041	+	C	31	10	0.323	0	0	T	21	M	0.186	0.499	7.1E-15	2E-06	9E-15	26.2725	attggttgatCttgtgacatc	m5C_51491
chr14	81430225	-	C	49	11	0.224	0	0	T	38	M	0.13	0.359	6.6E-14	4.4E-08	8.1E-14	18.8888	gggttcaagaCattctatccc	m5C_49620
chr14	81669148	-	C	124	28	0.226	0	0	T	96	M	0.161	0.307	0	0	0	90.216	gccttcagacCtggcattagg	m5C_49629
chr14	81669149	-	C	140	46	0.329	0	0	T	94	M	0.256	0.41	0	0	0	235.754	tgccttcagaCctggcattag	m5C_49616
chr14	81669156	-	C	155	62	0.4	0	0	T	93	M	0.326	0.479	0	0	0	404.48	ttcctgttgcCttcagacctg	m5C_49619
chr14	81669163	-	C	156	53	0.34	0	0	T	103	M	0.27	0.417	0	0	0	286.274	actgttcttcCtgttgccttc	m5C_49632
chr14	86327548	-	C	48	13	0.271	0	0	T	35	M	0.166	0.41	0	1.2E-07	0	43.0711	gcagtggcatCttctaaatga	m5C_49586
chr14	90117239	-	C	33	16	0.485	0	0	T	17	M	0.325	0.648	0	1.7E-05	0	104.012	ttggttggtcCgaagatagtg	m5C_49713
chr14	90117240	-	C	35	17	0.486	0	0	T	18	M	0.33	0.644	0	2.1E-05	0	112.18	attggttggtCcgaagatagt	m5C_49712
chr14	90341394	-	C	65	20	0.308	0	0	T	45	M	0.209	0.428	0	6.9E-10	0	83.5475	ccttagatgtCcacggctgca	m5C_49730
chr14	90460777	+	C	31	10	0.323	0	0	T	21	M	0.186	0.499	7.1E-15	2E-06	9E-15	26.2725	aatttaatgtCtgtttgggtg	m5C_49683
chr14	91737066	+	C	34	9	0.265	0	0	T	25	M	0.146	0.431	1.1E-12	1.2E-06	1.3E-12	15.7394	tggtggtgacCcattagaaaa	m5C_49690
chr14	92492945	+	C	40	9	0.225	0	0	T	31	M	0.123	0.375	6.4E-12	1.2E-08	7.6E-12	12.4041	gctccgaaatCaccaccaagg	m5C_49648
chr14	92492947	+	C	53	19	0.358	0	0	T	34	M	0.243	0.493	0	2.4E-08	0	92.3467	tccgaaatcaCcaccaaggac	m5C_49653
chr14	92492948	+	C	69	26	0.377	0	0	T	43	M	0.272	0.495	0	5.6E-09	0	141.353	ccgaaatcacCaccaaggact	m5C_49659
chr14	92492951	+	C	93	29	0.312	0	0	T	64	M	0.227	0.412	0	1.7E-13	0	131.502	aaatcaccacCaaggacttaa	m5C_49654
chr14	95257801	-	C	36	12	0.333	0	0	T	24	M	0.202	0.497	0	4.6E-06	0	48.5146	gtcagctactCaggaggctga	m5C_50435
chr14	95257803	-	C	36	12	0.333	0	0	T	24	M	0.202	0.497	0	4.6E-06	0	48.5146	tagtcagctaCtcaggaggct	m5C_50462

chr14	95257806	-	C	48	21	0.438	0	0	T	27	M	0.307	0.577	0	1.8E-06	0	128.944	ctgtagtcagCtactcaggag	m5C_50437
chr14	95257809	-	C	43	13	0.302	0	0	T	30	M	0.186	0.451	0	1.2E-07	0	48.3648	tgcctgtagtCagctactcag	m5C_50431
chr14	95257823	-	C	46	20	0.435	0	0	T	26	M	0.302	0.578	0	1.4E-06	0	120.836	ggtgtggtggCgtgtgcctgt	m5C_50448
chr14	95999696	-	C	106	50	0.472	0	0	T	56	M	0.379	0.566	0	2.6E-12	0	379.326	agtactgccaCaagttacttt	m5C_50456
chr14	95999702	-	C	111	52	0.468	0	0	T	59	M	0.378	0.561	0	1.4E-13	0	393.381	acactaagtaCtgccacaagt	m5C_50460
chr14	95999709	-	C	111	32	0.288	0	0	T	79	M	0.212	0.379	0	2.2E-16	0	135.822	ctgttgaacaCtaagtactgc	m5C_50458
chr14	95999719	-	C	101	29	0.287	0	0	T	72	M	0.208	0.382	0	2.9E-15	0	120.626	ggagtgttttCtgttgaacac	m5C_50444
chr14	95999884	-	C	37	17	0.459	0	0	T	20	M	0.31	0.616	0	2.1E-05	0	105.53	ccagaggaagCtgaggcatgc	m5C_50443
chr14	95999900	-	C	80	16	0.2	0	0	T	64	M	0.127	0.3	0	1.1E-14	0	40.6488	gcagaaaaagCatactccaga	m5C_50447
chr14	95999917	-	C	139	32	0.23	0	0	T	107	M	0.168	0.307	0	0	0	107.555	tcgagcacacCagacttgcag	m5C_50451
chr14	95999922	-	C	176	53	0.301	0	0	T	123	M	0.238	0.373	0	0	0	252.493	taccctcgagCacaccagact	m5C_50464
chr14	95999929	-	C	229	82	0.358	0	0	T	147	M	0.299	0.422	0	0	0	490.021	ttggtggtacCctcgagcaca	m5C_50441
chr14	95999930	-	C	229	53	0.231	0	0	T	176	M	0.182	0.29	0	0	0	192.409	gttggtggtaCcctcgagcac	m5C_50452
chr14	95999949	-	C	386	94	0.244	0	0	T	292	M	0.203	0.289	0	0	0	382.334	agtcttggagCcgcacagggt	m5C_50449
chr14	95999956	-	C	382	101	0.265	1	0.003	T	280	M	0.223	0.312	0	0	0	451.016	aatctgtagtCttggagccgc	m5C_50459
chr14	96487697	-	C	58	18	0.31	0	0	T	40	M	0.206	0.438	0	1.7E-08	0	74.239	tgatggattaCtatgctaatt	m5C_50280
chr14	96751261	-	C	32	11	0.344	0	0	T	21	M	0.204	0.517	2.2E-16	3.1E-06	2.9E-16	35.1442	aggtatagtgCaggcttgtta	m5C_50281
chr14	96851044	+	C	242	54	0.223	0	0	T	188	M	0.175	0.28	0	0	0	189.26	tatctaaggaCaatatattaa	m5C_50235
chr14	96851069	+	C	621	171	0.275	0	0	T	450	M	0.242	0.312	0	0	0	826.582	atttttggagCagggagatag	m5C_50236
chr14	97294903	+	C	97	35	0.361	0	0	T	62	M	0.272	0.46	0	1.5E-12	0	190.57	ttgtctatggCtataccaccc	m5C_50256
chr14	97294911	+	C	101	23	0.228	0	0	T	78	M	0.157	0.319	0	1.1E-16	0	72.1313	ggctataccaCcctgagtgtg	m5C_50248
chr14	97294913	+	C	99	46	0.465	0	0	T	53	M	0.37	0.562	0	2.9E-11	0	339.998	ctataccaccCtgagtgtgcc	m5C_50244
chr14	97294922	+	C	82	28	0.341	0	0	T	54	M	0.248	0.449	0	5.8E-12	0	138.875	cctgagtgtgCccgatctcgt	m5C_50241
chr14	97294924	+	C	70	15	0.214	0	0	T	55	M	0.134	0.324	0	6E-13	0	40.3158	tgagtgtgccCgatctcgtct	m5C_50255
chr14	98285508	+	C	67	25	0.373	0	0	T	42	M	0.267	0.493	0	4.1E-09	0	133.59	aacatattgaCactgagagat	m5C_49749
chr14	99439254	-	C	35	20	0.571	0	0	C	20	M	0.409	0.72	0	4.3E-05	0	163.429	catgggaccaCtgaagaaaga	m5C_49771
chr14	99439256	-	C	37	8	0.216	0	0	T	29	M	0.114	0.372	9.7E-11	6.8E-07	1.1E-10	9.12273	ttcatgggacCactgaagaaa	m5C_49759
chr14	99439257	-	C	37	12	0.324	0	0	T	25	M	0.196	0.485	0	4.6E-06	0	47.1194	attcatgggaCcactgaagaa	m5C_49754
chr14	99439268	-	C	31	19	0.613	0	0	C	19	M	0.438	0.763	0	3.4E-05	0	166.53	gagaagaaagCattcatggga	m5C_49757
chr14	100049361	+	C	52	18	0.346	0	0	T	34	M	0.232	0.482	0	1.7E-08	0	83.343	ctaggctggtCtgagtgcagt	m5C_50298
chr14	100049392	+	C	31	11	0.355	0	0	T	20	M	0.211	0.531	1.1E-16	3.1E-06	1.5E-16	37.0595	actaattgatCacaatcagtt	m5C_50312
chr14	100052927	-	C	59	41	0.695	0	0	C	41	M	0.569	0.797	0	3.3E-06	0	466.198	tcggggctgcCtgagtgttgt	m5C_50382
chr14	100576017	+	C	163	51	0.313	0	0	T	112	M	0.247	0.388	0	0	0	251.637	gtgaggggtgCtatctgtgat	m5C_50305
chr14	100576021	+	C	160	64	0.4	0	0	T	96	M	0.327	0.477	0	0	0	418.935	ggggtgctatCtgtgattgag	m5C_50360
chr14	100743063	+	C	36	19	0.528	0	0	C	19	M	0.37	0.68	0	3.4E-05	0	140.622	ggtgatgaggCaggaggcgcc	m5C_50341
chr14	102499719	+	C	39	16	0.41	0	0	T	23	M	0.271	0.566	0	1.7E-05	0	86.6531	agcagatgatCcgagacctgg	m5C_50095
chr14	102549471	-	C	145	45	0.31	0	0	T	100	M	0.241	0.39	0	0	0	216.679	ctggaacttcCagaggatgaa	m5C_50161
chr14	102549481	-	C	123	29	0.236	0	0	T	94	M	0.169	0.318	0	0	0	98.2927	caaagaaggcCtggaacttcc	m5C_50204
chr14	102549482	-	C	118	43	0.364	0	0	T	75	M	0.283	0.454	0	1.2E-14	0	243.483	ccaaagaaggCctggaacttc	m5C_50170
chr14	102551180	-	C	259	61	0.236	0	0	T	198	M	0.188	0.291	0	0	0	229.29	aggatggtgaCaagaagaaga	m5C_50168
chr14	102551279	-	C	171	45	0.265	1	0.006	T	125	M	0.204	0.336	0	0	0	183.705	aaaaggaagaCaaagaagaag	m5C_50185
chr14	102551295	-	C	189	59	0.312	0	0	T	130	M	0.25	0.381	0	0	0	295.474	gatgatgaggCtgaagaaaag	m5C_50206
chr14	102551306	-	C	155	35	0.229	2	0.013	T	118	M	0.169	0.301	0	0	0	118.531	aagaagtaagCgatgatgagg	m5C_50181
chr14	102551697	-	C	283	88	0.311	0	0	T	195	M	0.26	0.367	0	0	0	457.35	cttggaggaaCgaagaataaa	m5C_50178
chr14	102551707	-	C	226	93	0.412	0	0	T	133	M	0.349	0.477	0	0	0	649.782	aaactgagtaCttggaggaac	m5C_50198
chr14	102772418	-	C	40	8	0.2	0	0	T	32	M	0.105	0.348	2.1E-10	5.5E-09	2.3E-10	8.13503	tttggcaaggCtacttatctg	m5C_50167
chr14	102772434	-	C	56	14	0.25	0	0	T	42	M	0.155	0.377	0	2.8E-09	0	43.4474	ttttctgaggCttcgttttgg	m5C_50214
chr14	102783432	+	C	58	20	0.345	0	0	T	38	M	0.236	0.473	0	3.4E-08	0	94.2437	gtcaggcggcCggttagctca	m5C_50119
chr14	102783439	+	C	53	22	0.415	0	0	T	31	M	0.293	0.549	0	6.5E-08	0	128.747	ggccggttagCtcagttggtt	m5C_50110
chr14	102783454	+	C	55	12	0.218	0	0	T	43	M	0.129	0.344	9.8E-15	8.8E-10	1.2E-14	21.7648	ttggttagagCgtggtgctaa	m5C_50139
chr14	102783468	+	C	64	13	0.203	0	0	T	51	M	0.123	0.317	3E-15	1.6E-11	3.9E-15	23.1722	gtgctaataaCgccaaggtcg	m5C_50155
chr14	102783470	+	C	65	22	0.338	0	0	T	43	M	0.235	0.46	0	1.5E-09	0	103.547	gctaataacgCcaaggtcgcg	m5C_50101
chr14	102783471	+	C	64	13	0.203	0	0	T	51	M	0.123	0.317	3E-15	1.6E-11	3.9E-15	23.1722	ctaataacgcCaaggtcgcgg	m5C_50099
chr14	102783477	+	C	67	29	0.433	0	0	T	38	M	0.321	0.552	0	1.3E-08	0	186.204	acgccaaggtCgcgggttcga	m5C_50108
chr14	102783479	+	C	68	18	0.269	1	0.015	T	49	M	0.177	0.385	0	2.9E-10	0	63.7994	gccaaggtcgCgggttcgatc	m5C_50151
chr14	102783485	+	C	42	9	0.214	0	0	T	33	M	0.117	0.359	1.1E-11	1.2E-08	1.3E-11	11.5454	gtcgcgggttCgatccccgta	m5C_50114
chr14	102783491	+	C	48	10	0.208	0	0	T	38	M	0.117	0.343	1.6E-12	2.4E-08	1.9E-12	13.8343	ggttcgatccCcgtacgggcc	m5C_50149
chr14	102783492	+	C	44	23	0.523	0	0	C	23	M	0.379	0.662	0	2.9E-06	0	174.502	gttcgatcccCgtacgggcca	m5C_50126
chr14	102783505	+	C	44	10	0.227	0	0	T	34	M	0.128	0.37	5.7E-13	2.4E-08	6.8E-13	15.7274	acgggccaggCtaactttttt	m5C_50097
chr14	103352180	+	C	33	7	0.212	0	0	T	26	M	0.107	0.378	1.1E-09	3.4E-07	1.2E-09	6.69165	gtggccatgcCtgcctgagca	m5C_50134
chr14	103352184	+	C	32	9	0.281	0	0	T	23	M	0.156	0.454	5.3E-13	1.2E-06	6.4E-13	17.1983	ccatgcctgcCtgagcattgc	m5C_50140
chr14	103804189	+	C	74	17	0.23	0	0	T	57	M	0.149	0.337	0	2.2E-12	0	50.548	atattgcaagCaacactctgt	m5C_50145
chr14	103804192	+	C	77	21	0.273	0	0	T	56	M	0.186	0.381	0	1.8E-11	0	78.0543	ttgcaagcaaCactctgtggc	m5C_50092
chr14	103804194	+	C	75	18	0.24	0	0	T	57	M	0.158	0.348	0	4E-12	0	56.7171	gcaagcaacaCtctgtggcag	m5C_50098
chr14	103804202	+	C	74	24	0.324	0	0	T	50	M	0.229	0.437	0	6.4E-11	0	109.747	cactctgtggCagatgatcaa	m5C_50111
chr14	103804210	+	C	48	18	0.375	0	0	T	30	M	0.252	0.516	0	7.7E-07	0	90.7743	ggcagatgatCaaaactgtct	m5C_50103
chr14	103804223	+	C	32	15	0.469	0	0	T	17	M	0.309	0.636	0	1.2E-05	0	92.6074	aactgtctgaCacaatttgag	m5C_50156
chr14	104092590	+	C	34	9	0.265	0	0	T	25	M	0.146	0.431	1.1E-12	1.2E-06	1.3E-12	15.7394	ttgagggttgCcctttgcagct	m5C_50693
chr14	104480072	+	C	43	14	0.326	0	0	T	29	M	0.205	0.475	0	1.9E-07	0	57.3807	cttgagtgcgCcatgctgatt	m5C_50718
chr14	104480077	+	C	51	12	0.235	0	0	T	39	M	0.14	0.368	3.3E-15	8.8E-10	4.3E-15	24.3305	gtgcgccatgCtgattgggtg	m5C_50694
chr14	105040128	-	C	31	21	0.677	0	0	C	21	M	0.501	0.814	0	5.2E-05	0	210.591	ctggcagtggCtgagtggccc	m5C_50774
chr15	25227153	+	C	113	24	0.212	0	0	T	89	M	0.147	0.297	0	0	0	70.6001	ttcatgatgaCacaggacctt	m5C_50650
chr15	25227160	+	C	110	28	0.255	0	0	T	82	M	0.182	0.343	0	0	0	102.133	tgacacaggaCcttgtctgaa	m5C_50638
chr15	25227161	+	C	109	34	0.312	0	0	T	75	M	0.233	0.404	0	2.3E-14	0	158.175	gacacaggacCttgtctgaac	m5C_50610
chr15	25227171	+	C	101	22	0.218	0	0	T	79	M	0.148	0.308	0	0	0	65.3293	cttgtctgaaCataatgattt	m5C_50583
chr15	25230303	+	C	85	20	0.235	0	0	T	65	M	0.158	0.336	0	1.5E-13	0	63.1177	aacgtgattaCtctgaagtcc	m5C_50578
chr15	25315594	+	C	57	15	0.263	0	0	T	42	M	0.166	0.39	0	4.6E-09	0	49.9369	gatgatgagtCctccaaaaaa	m5C_50588
chr15	25315595	+	C	55	21	0.389	1	0.018	T	33	M	0.27	0.522	0	4.8E-08	0	113.575	atgatgagtcCtccaaaaaaa	m5C_50600
chr15	25315597	+	C	54	13	0.241	0	0	T	41	M	0.146	0.369	2.2E-16	1.6E-09	2.9E-16	29.8003	gatgagtcctCcaaaaaaaca	m5C_50618
chr15	25315611	+	C	53	13	0.245	0	0	T	40	M	0.149	0.376	2.2E-16	1.6E-09	2.9E-16	30.3876	aaaaacattcCttggaaaagc	m5C_50626
chr15	25315621	+	C	46	23	0.5	0	0	CT	23	M	0.361	0.639	0	2.9E-06	0	166.146	cttggaaaagCtgaacaaaat	m5C_50643
chr15	25315647	+	C	40	11	0.275	0	0	T	29	M	0.161	0.428	4.3E-15	3.1E-06	5.5E-15	25.4502	agaactcataCcgtcgttctc	m5C_50579
chr15	25315648	+	C	35	12	0.343	0	0	T	23	M	0.208	0.508	0	4.6E-06	0	49.9956	gaactcatacCgtcgttctca	m5C_50632
chr15	25315655	+	C	39	16	0.41	0	0	T	23	M	0.271	0.566	0	1.7E-05	0	86.6531	taccgtcgttCtcatcggaac	m5C_50589
chr15	25315660	+	C	33	8	0.242	0	0	T	25	M	0.128	0.41	3.1E-11	6.8E-07	3.6E-11	10.7866	tcgttctcatCggaactgagg	m5C_50587
chr15	25315665	+	C	32	7	0.219	0	0	T	25	M	0.11	0.388	8.5E-10	3.4E-07	9.5E-10	6.99971	ctcatcggaaCtgaggtccag	m5C_50602
chr15	25321089	+	C	48	14	0.292	0	0	T	34	M	0.182	0.432	0	1.9E-07	0	51.076	tcaatgatgaCttccatacgt	m5C_50642
chr15	25321092	+	C	47	14	0.298	0	0	T	33	M	0.187	0.44	0	1.9E-07	0	52.223	atgatgacttCcatacgtggg	m5C_50639
chr15	25321105	+	C	36	22	0.611	0	0	C	22	M	0.449	0.752	0	6.3E-05	0	197.402	tacgtggggtCcttggaaagt	m5C_50592
chr15	25321135	+	C	35	17	0.486	0	0	T	18	M	0.33	0.644	0	2.1E-05	0	112.18	tgagtgaaaaCtttatactgt	m5C_50577
chr15	25321142	+	C	35	12	0.343	0	0	T	23	M	0.208	0.508	0	4.6E-06	0	49.9956	aaactttataCtgtcatcctc	m5C_50564
chr15	25321149	+	C	34	8	0.235	0	0	T	26	M	0.124	0.4	4.2E-11	6.8E-07	4.8E-11	10.3259	atactgtcatCctcttcaaac	m5C_50656
chr15	25321150	+	C	33	10	0.303	0	0	T	23	M	0.174	0.473	1.6E-14	2E-06	2E-14	23.982	tactgtcatcCtcttcaaact	m5C_50645
chr15	25335086	+	C	43	11	0.256	0	0	T	32	M	0.149	0.402	1.2E-14	4.4E-08	1.5E-14	22.8887	atgatgacttCcatatgtaca	m5C_50635
chr15	25335095	+	C	43	12	0.279	0	0	T	31	M	0.167	0.427	2.2E-16	7.5E-08	2.9E-16	31.457	tccatatgtaCattccttgga	m5C_50596
chr15	25335109	+	C	42	21	0.5	0	0	CT	21	M	0.355	0.645	0	1.8E-06	0	149.208	ccttggaaagCtgaacaaaat	m5C_50625
chr15	25335129	+	C	34	7	0.206	0	0	T	27	M	0.103	0.368	1.4E-09	3.4E-07	1.6E-09	6.4054	tgagtgaaaaCtctataccgt	m5C_50573
chr15	25335131	+	C	32	16	0.5	0	0	CT	16	M	0.336	0.664	0	1.7E-05	0	107.618	agtgaaaactCtataccgtca	m5C_50595
chr15	25335137	+	C	36	14	0.389	0	0	T	22	M	0.248	0.551	0	9.2E-06	0	69.397	aactctatacCgtcatcctcg	m5C_50606
chr15	25335143	+	C	35	8	0.229	0	0	T	27	M	0.121	0.39	5.6E-11	6.8E-07	6.4E-11	9.89691	ataccgtcatCctcgtcgaac	m5C_50601
chr15	25335144	+	C	34	12	0.353	0	0	T	22	M	0.215	0.521	0	4.6E-06	0	51.5705	taccgtcatcCtcgtcgaact	m5C_50630

chr15	25342823	+	C	45	15	0.333	0	0	T	30	M	0.214	0.479	0	2.8E-07	0	64.0761	tcgatgatgaCtttaaaatgg	m5C_50591
chr15	25342836	+	C	48	12	0.25	0	0	T	36	M	0.149	0.388	1.3E-15	7.5E-08	1.7E-15	26.6338	taaaatggatCtcatcggaat	m5C_50652
chr15	25342852	+	C	46	12	0.261	0	0	T	34	M	0.156	0.403	6.7E-16	7.5E-08	8.7E-16	28.4116	ggaatctgaaCaaaatgagtg	m5C_50667
chr15	25342864	+	C	47	13	0.277	0	0	T	34	M	0.169	0.418	0	1.2E-07	0	44.0347	aaatgagtgaCcaaatcactt	m5C_50584
chr15	25342865	+	C	40	19	0.475	0	0	T	21	M	0.329	0.625	0	1E-06	0	125.154	aatgagtgacCaaatcacttc	m5C_50648
chr15	25342872	+	C	41	12	0.293	0	0	T	29	M	0.176	0.445	1.1E-16	7.5E-08	1.5E-16	33.7134	gaccaaatcaCttctgtgcca	m5C_50620
chr15	25342875	+	C	42	13	0.31	0	0	T	29	M	0.191	0.46	0	1.2E-07	0	49.5844	caaatcacttCtgtgccactt	m5C_50611
chr15	25342880	+	C	40	10	0.25	0	0	T	30	M	0.142	0.402	1.8E-13	2.4E-08	2.2E-13	18.0914	cacttctgtgCcacttctgtg	m5C_50663
chr15	25342881	+	C	41	17	0.415	0	0	T	24	M	0.278	0.566	0	5.6E-07	0	94.3715	acttctgtgcCacttctgtga	m5C_50653
chr15	25342883	+	C	42	19	0.452	0	0	T	23	M	0.312	0.601	0	1E-06	0	118.648	ttctgtgccaCttctgtgagc	m5C_50616
chr15	25351672	+	C	66	14	0.212	0	0	T	52	M	0.131	0.325	2.2E-16	3.2E-11	2.9E-16	28.6601	gtgcctggatCgatgatgact	m5C_50619
chr15	25351681	+	C	160	36	0.225	0	0	T	124	M	0.167	0.296	0	0	0	120.371	tcgatgatgaCttaaaaaaat	m5C_50662
chr15	25351707	+	C	154	34	0.221	0	0	T	120	M	0.163	0.293	0	0	0	110.508	ccttggaaatCtgaacaaaat	m5C_50646
chr15	25351724	+	C	118	24	0.203	0	0	T	94	M	0.141	0.285	0	0	0	67.5163	aaatgagtgaCcaagacactt	m5C_50627
chr15	25351730	+	C	120	34	0.283	0	0	T	86	M	0.21	0.37	0	0	0	143.075	gtgaccaagaCacttctgtga	m5C_50670
chr15	25351732	+	C	122	28	0.23	0	0	T	94	M	0.164	0.312	0	0	0	91.7449	gaccaagacaCttctgtgagc	m5C_50562
chr15	25351742	+	C	92	38	0.413	0	0	T	54	M	0.318	0.515	0	3.7E-12	0	241.596	cttctgtgagCtgaggtccag	m5C_50637
chr15	26063137	+	C	57	21	0.368	0	0	T	36	M	0.255	0.498	0	4.8E-08	0	107.198	ttggctggtCagatggtagt	m5C_50957
chr15	26063160	+	C	50	18	0.36	0	0	T	32	M	0.241	0.499	0	1.7E-08	0	86.8988	gttatcagaaCttattaacat	m5C_50960
chr15	26327371	-	C	94	33	0.351	0	0	T	61	M	0.262	0.452	0	7.5E-13	0	173.021	atgactggacCtttcttttgg	m5C_50966
chr15	26327393	-	C	205	54	0.266	2	0.01	T	149	M	0.21	0.331	0	0	0	226.779	gggttcgactCccggtgtggg	m5C_50973
chr15	26327404	-	C	153	50	0.327	0	0	T	103	M	0.258	0.405	0	0	0	257.51	ccaggcggccCgggttcgact	m5C_50972
chr15	26327405	-	C	145	29	0.201	1	0.007	T	115	M	0.144	0.274	0	0	0	83.542	cccaggcggcCcgggttcgac	m5C_50971
chr15	26327406	-	C	146	56	0.392	3	0.021	T	87	M	0.315	0.473	0	0	0	353.297	acccaggcggCccgggttcga	m5C_50962
chr15	26327409	-	C	139	48	0.345	0	0	T	91	M	0.271	0.428	0	0	0	260.542	ttcacccaggCggcccgggtt	m5C_50981
chr15	26327417	-	C	113	33	0.292	0	0	T	80	M	0.216	0.382	0	2.2E-16	0	142.654	tcctggttttCacccaggcgg	m5C_50967
chr15	26327425	-	C	107	30	0.28	0	0	T	77	M	0.204	0.372	0	4.7E-15	0	122.41	gttaggattcCtggttttcac	m5C_50977
chr15	26327441	-	C	98	35	0.357	0	0	T	63	M	0.269	0.456	0	1.5E-12	0	188.52	cccacatggtCtagcggttag	m5C_50963
chr15	26327449	-	C	46	16	0.348	0	0	T	30	M	0.227	0.492	0	4E-07	0	72.5786	tgtcatctccCacatggtcta	m5C_50969
chr15	26327450	-	C	43	18	0.419	0	0	T	25	M	0.284	0.567	0	7.7E-07	0	102.184	ttgtcatctcCcacatggtct	m5C_50980
chr15	28857868	+	C	38	8	0.211	0	0	T	30	M	0.111	0.363	1.3E-10	6.8E-07	1.4E-10	8.77246	gttgaaatagCtcagttggga	m5C_50784
chr15	28857870	+	C	37	13	0.351	0	0	T	24	M	0.218	0.512	0	6.7E-06	0	56.7461	tgaaatagctCagttgggaga	m5C_50790
chr15	29142359	-	C	39	15	0.385	0	0	T	24	M	0.249	0.541	0	1.2E-05	0	74.6735	ctttagtggaCgtgtgtgtgt	m5C_50794
chr15	33721698	-	C	40	8	0.2	0	0	T	32	M	0.105	0.348	2.1E-10	5.5E-09	2.3E-10	8.13503	cgatcatcagCgtctgccttt	m5C_51025
chr15	33721708	-	C	38	8	0.211	0	0	T	30	M	0.111	0.363	1.3E-10	6.8E-07	1.4E-10	8.77246	agtgcaaatcCgatcatcagc	m5C_51026
chr15	34393846	-	C	55	20	0.364	0	0	T	35	M	0.249	0.496	0	3.4E-08	0	99.7214	tctcggcggcCcgagtgctgg	m5C_50484
chr15	34393850	-	C	57	21	0.368	0	0	T	36	M	0.255	0.498	0	4.8E-08	0	107.198	tggatctcggCggcccgagtg	m5C_50498
chr15	36237131	+	C	35	24	0.686	0	0	C	24	M	0.52	0.814	0	9.1E-05	0	249.696	ccattttcgaCatggtatttg	m5C_50515
chr15	40491846	+	C	49	15	0.306	0	0	T	34	M	0.195	0.445	0	2.8E-07	0	58.546	gagaagagagCagaaatgcag	m5C_50902
chr15	40491854	+	C	51	14	0.275	0	0	T	37	M	0.171	0.409	0	2.8E-09	0	47.9195	agcagaaatgCagaaacagat	m5C_50895
chr15	40886009	-	C	83	45	0.542	0	0	C	45	M	0.435	0.645	0	6.4E-10	0	391.949	tggtggcttaCttttgcttgt	m5C_50939
chr15	40886013	-	C	90	33	0.367	0	0	T	57	M	0.274	0.47	0	7.5E-13	0	181.151	gatgtggtggCttacttttgc	m5C_50912
chr15	40886024	-	C	89	28	0.315	0	0	T	61	M	0.228	0.417	0	5.8E-12	0	127.405	ccatcctcgtCgatgtggtgg	m5C_50931
chr15	40886029	-	C	73	21	0.288	0	0	T	52	M	0.197	0.4	0	1.8E-11	0	82.5367	gaatcccatcCtcgtcgatgt	m5C_50919
chr15	40886030	-	C	68	31	0.456	0	0	T	37	M	0.343	0.573	0	2.1E-08	0	212.689	cgaatcccatCctcgtcgatg	m5C_50911
chr15	40886033	-	C	66	20	0.303	0	0	T	46	M	0.206	0.422	0	6.9E-10	0	82.2155	gttcgaatccCatcctcgtcg	m5C_50920
chr15	40886035	-	C	65	15	0.231	0	0	T	50	M	0.145	0.346	0	6E-11	0	43.5411	gggttcgaatCccatcctcgt	m5C_50927
chr15	40886070	-	C	197	47	0.239	0	0	T	150	M	0.184	0.303	0	0	0	173.356	cgatggactgCtaatccattg	m5C_50926
chr15	40886073	-	C	218	49	0.225	0	0	T	169	M	0.174	0.285	0	0	0	170.911	aggcgatggaCtgctaatcca	m5C_50929
chr15	40886080	-	C	239	50	0.215	6	0.025	T	183	M	0.167	0.272	0	0	0	166.732	gtggttaaggCgatggactgc	m5C_50930
chr15	40886094	-	C	316	120	0.38	0	0	T	196	M	0.328	0.434	0	0	0	787.159	gacgaggtggCcgagtggtta	m5C_50923
chr15	40886102	-	C	193	83	0.43	0	0	T	110	M	0.362	0.501	0	0	0	601.319	cttaccgggaCgaggtggccg	m5C_50924
chr15	41164742	-	C	33	10	0.303	0	0	T	23	M	0.174	0.473	1.6E-14	2E-06	2E-14	23.982	tggataaggcCtggaaagaag	m5C_50932
chr15	41164743	-	C	34	23	0.676	0	0	C	23	M	0.508	0.809	0	7.6E-05	0	233.876	gtggataaggCctggaaagaa	m5C_50936
chr15	42005639	+	C	39	17	0.436	0	0	T	22	M	0.293	0.59	0	2.1E-05	0	99.6354	aagaaggaatCagggaggagg	m5C_50827
chr15	42058543	+	C	34	7	0.206	0	0	T	27	M	0.103	0.368	1.4E-09	3.4E-07	1.6E-09	6.4054	gagagggattCagtataaatg	m5C_50837
chr15	43773156	-	C	48	15	0.312	0	0	T	33	M	0.199	0.453	0	2.8E-07	0	59.8365	agagttggaaCagaaggagaa	m5C_50875
chr15	44084540	+	C	31	14	0.452	0	0	T	17	M	0.292	0.622	0	9.2E-06	0	81.6522	aacgaggggaCgtaacggagg	m5C_51212
chr15	44084545	+	C	33	19	0.576	0	0	C	19	M	0.408	0.728	0	3.4E-05	0	155.067	ggggacgtaaCggaggcaggt	m5C_51216
chr15	44106847	-	C	39	16	0.41	0	0	T	23	M	0.271	0.566	0	1.7E-05	0	86.6531	tcagcgagcaCaggagagaaa	m5C_51259
chr15	44106849	-	C	42	12	0.286	0	0	T	30	M	0.172	0.436	2.2E-16	7.5E-08	2.9E-16	32.2467	cgtcagcgagCacaggagaga	m5C_51295
chr15	44107176	-	C	43	22	0.512	0	0	C	22	M	0.368	0.654	0	2.3E-06	0	161.709	gagaagacagCagtgaagaag	m5C_51298
chr15	44107179	-	C	40	18	0.45	0	0	T	22	M	0.307	0.602	0	2.7E-05	0	110.538	aacgagaagaCagcagtgaag	m5C_51302
chr15	44107187	-	C	39	17	0.436	0	0	T	22	M	0.293	0.59	0	2.1E-05	0	99.6354	gcgcatggaaCgagaagacag	m5C_51268
chr15	44218575	+	C	36	20	0.556	0	0	C	20	M	0.396	0.705	0	4.3E-05	0	158.323	tgaggaatttCtgtagtgtag	m5C_51198
chr15	44218593	+	C	53	18	0.34	0	0	T	35	M	0.227	0.474	0	1.7E-08	0	81.6727	tagtggttatCacgtttgtct	m5C_51173
chr15	44218595	+	C	43	13	0.302	0	0	T	30	M	0.186	0.451	0	1.2E-07	0	48.3648	gtggttatcaCgtttgtctca	m5C_51255
chr15	45010008	+	C	92	27	0.293	0	0	T	65	M	0.21	0.393	0	7.2E-14	0	113.517	acaggtagctCtaggagggct	m5C_51197
chr15	45010017	+	C	142	104	0.732	0	0	C	104	M	0.654	0.798	0	2.1E-13	0	1360.66	tctaggagggCtggcaactta	m5C_51192
chr15	45010021	+	C	145	37	0.255	0	0	T	108	M	0.191	0.332	0	0	0	141.464	ggagggctggCaacttagagg	m5C_51147
chr15	45010024	+	C	145	77	0.531	0	0	C	77	M	0.45	0.61	0	2E-15	0	693.088	gggctggcaaCttagaggtgg	m5C_51217
chr15	45010299	+	C	35	21	0.6	0	0	C	21	M	0.436	0.744	0	5.2E-05	0	183.004	tgtggttaatCtggtttattt	m5C_51207
chr15	45295203	-	C	33	18	0.545	0	0	C	18	M	0.38	0.702	0	2.7E-05	0	136.748	aggggagatgCcatgatcatg	m5C_51288
chr15	45490776	-	C	153	36	0.235	0	0	T	117	M	0.175	0.308	0	0	0	126.054	cggcaaggggCtcggtatttt	m5C_51258
chr15	45490783	-	C	172	54	0.314	0	0	T	118	M	0.249	0.387	0	0	0	269.245	aatggcacggCaaggggctcg	m5C_51283
chr15	45490786	-	C	171	56	0.327	0	0	T	115	M	0.262	0.401	0	0	0	293.002	aacaatggcaCggcaaggggc	m5C_51256
chr15	45490788	-	C	164	41	0.25	0	0	T	123	M	0.19	0.321	0	0	0	155.769	ggaacaatggCacggcaaggg	m5C_51292
chr15	45490867	-	C	61	22	0.361	0	0	T	39	M	0.252	0.486	0	1.5E-09	0	110.765	tcgccgtgatCgtatagtggt	m5C_51304
chr15	45490876	-	C	52	13	0.25	0	0	T	39	M	0.152	0.382	1.1E-16	1.6E-09	1.5E-16	31.5947	cccggtagctCgccgtgatcg	m5C_51293
chr15	45490878	-	C	47	12	0.255	0	0	T	35	M	0.153	0.395	1E-15	7.5E-08	1.3E-15	27.4561	tccccggtagCtcgccgtgat	m5C_51300
chr15	45490887	-	C	33	7	0.212	0	0	T	26	M	0.107	0.378	1.1E-09	3.4E-07	1.2E-09	6.69165	ccctacgggtCcccggtagct	m5C_51296
chr15	45492593	-	C	101	44	0.436	0	0	T	57	M	0.343	0.533	0	5.7E-13	0	301.891	gacaatggcaCggcaaggggа	m5C_51281
chr15	45492595	-	C	95	24	0.253	0	0	T	71	M	0.176	0.348	0	1.7E-14	0	84.5012	aggacaatggCacggcaaggg	m5C_51267
chr15	45492601	-	C	86	24	0.279	0	0	T	62	M	0.195	0.382	0	1.1E-12	0	93.737	attgtgaggaCaatggcacgg	m5C_51303
chr15	45492680	-	C	44	23	0.523	0	0	C	23	M	0.379	0.662	0	2.9E-06	0	174.502	agtagctcgcCgtgatcgtat	m5C_51291
chr15	45492683	-	C	44	10	0.227	0	0	T	34	M	0.128	0.37	5.7E-13	2.4E-08	6.8E-13	15.7274	cctagtagctCgccgtgatcg	m5C_51280
chr15	45492693	-	C	33	15	0.455	0	0	T	18	M	0.298	0.62	0	1.2E-05	0	89.5281	gtcctgtgtcCctagtagctc	m5C_51261
chr15	45493338	+	C	55	14	0.255	0	0	T	41	M	0.158	0.383	0	2.8E-09	0	44.2735	gtcgtggcgtCccggtagctc	m5C_51222
chr15	45493340	+	C	59	13	0.22	0	0	T	46	M	0.134	0.341	8.9E-16	1.6E-09	1.2E-15	26.1299	cgtggcgtccCggtagctcgc	m5C_51181
chr15	45493348	+	C	49	11	0.224	0	0	T	38	M	0.13	0.359	6.6E-14	4.4E-08	8.1E-14	18.8888	cccggtagctCgccgtgatcg	m5C_51174
chr15	45493350	+	C	42	10	0.238	0	0	T	32	M	0.135	0.385	3.2E-13	2.4E-08	3.9E-13	16.8407	cggtagctcgCcgtgatcgta	m5C_51143
chr15	45493351	+	C	42	10	0.238	0	0	T	32	M	0.135	0.385	3.2E-13	2.4E-08	3.9E-13	16.8407	ggtagctcgcCgtgatcgtat	m5C_51161
chr15	49288212	-	C	128	59	0.461	0	0	T	69	M	0.377	0.547	0	2.6E-14	0	444.836	aagggctggtCtgaaggtagt	m5C_51094
chr15	53140882	-	C	37	8	0.216	0	0	T	29	M	0.114	0.372	9.7E-11	6.8E-07	1.1E-10	9.12273	ggtggggggtCcggatatctg	m5C_51335
chr15	55665154	-	C	72	16	0.222	0	0	T	56	M	0.142	0.331	0	1.2E-12	0	45.3354	gagtggcagtCgaagggaagg	m5C_51425
chr15	57565262	+	C	34	12	0.353	0	0	T	22	M	0.215	0.521	0	4.6E-06	0	51.5705	gaaccctgaaCagaagataga	m5C_51126
chr15	57838251	-	C	65	21	0.323	0	0	T	44	M	0.222	0.444	0	1E-09	0	93.2557	agaatgtgggCtgagtgccgg	m5C_51139
chr15	57956003	-	C	31	13	0.419	0	0	T	18	M	0.264	0.592	0	6.7E-06	0	68.6798	ttttcaaagtCttgcttatta	m5C_51141
chr15	60649424	-	C	31	21	0.677	0	0	C	21	M	0.501	0.814	0	5.2E-05	0	210.591	ggctgggaacCgacgaggact	m5C_51474
chr15	61325251	-	C	39	29	0.744	0	0	C	29	M	0.589	0.854	0	0.00019	0	341.725	aattgcagatCtgaatctgtt	m5C_51468
chr15	63335112	+	C	35	8	0.229	0	0	T	27	M	0.121	0.39	5.6E-11	6.8E-07	6.4E-11	9.89691	cggaggccgaCaagaaggcgg	m5C_51690

chr15	63353462	+	C	159	43	0.27	0	0	T	116	M	0.207	0.344	0	0	0	178.378	ttaatggctgCagaggataag	m5C_51693
chr15	63354416	+	C	260	75	0.288	0	0	T	185	M	0.237	0.346	0	0	0	355.181	aattacagtaCtcgcagaagg	m5C_51689
chr15	63933598	-	C	42	15	0.357	0	0	T	27	M	0.23	0.508	0	2.8E-07	0	68.967	tagccttgaaCttatccctta	m5C_51705
chr15	63933605	-	C	44	12	0.273	0	0	T	32	M	0.163	0.418	4.4E-16	7.5E-08	5.8E-16	30.1147	gtgattatagCcttgaactta	m5C_51729
chr15	63970324	-	C	32	11	0.344	0	0	T	21	M	0.204	0.517	2.2E-16	3.1E-06	2.9E-16	35.1442	ggttcagagcCgagaggaaaa	m5C_51710
chr15	64448264	-	C	87	20	0.23	0	0	T	67	M	0.154	0.329	0	1.5E-13	0	61.6125	cagactgcggCaagatcgagg	m5C_52081
chr15	64448270	-	C	106	39	0.368	0	0	T	67	M	0.282	0.463	0	1.3E-13	0	220.147	tcatcgcagaCtgcggcaaga	m5C_52082
chr15	64448276	-	C	113	26	0.23	0	0	T	87	M	0.162	0.316	0	0	0	84.3081	atgtgatcatCgcagactgcg	m5C_52090
chr15	64945094	+	C	111	38	0.342	0	0	T	73	M	0.261	0.435	0	2.1E-15	0	198.111	tggggctggtCtgagtgcagt	m5C_52055
chr15	64945125	+	C	75	19	0.253	0	0	T	56	M	0.169	0.362	0	6.9E-12	0	64.0677	actagttgatCacaaccaatt	m5C_52044
chr15	65577889	-	C	41	10	0.244	0	0	T	31	M	0.138	0.393	2.4E-13	2.4E-08	2.9E-13	17.4472	ggcagtctccCttccttgcca	m5C_52094
chr15	65577891	-	C	40	13	0.325	0	0	T	27	M	0.201	0.48	0	1.2E-07	0	52.2192	gtggcagtctCccttccttgc	m5C_52098
chr15	65577910	-	C	80	19	0.237	0	0	T	61	M	0.158	0.341	0	8.4E-14	0	59.9053	tctttgggacCtgtcaagtgt	m5C_52076
chr15	65577926	-	C	34	9	0.265	0	0	T	25	M	0.146	0.431	1.1E-12	1.2E-06	1.3E-12	15.7394	cactgaactcCatgtatcttt	m5C_52100
chr15	65588403	+	C	34	18	0.529	0	0	C	18	M	0.367	0.685	0	2.7E-05	0	132.251	tctggtttctCttcagatcgc	m5C_52031
chr15	65588406	+	C	63	36	0.571	0	0	C	36	M	0.449	0.686	0	6.5E-08	0	323.002	ggtttctcttCagatcgcata	m5C_52010
chr15	65588411	+	C	83	52	0.627	0	0	C	52	M	0.519	0.723	0	2.5E-09	0	539.775	ctcttcagatCgcataaatct	m5C_52011
chr15	65588420	+	C	121	60	0.496	0	0	T	61	M	0.408	0.584	0	3.3E-14	0	489.946	tcgcataaatCtttcgccttt	m5C_52003
chr15	65588424	+	C	130	71	0.546	0	0	C	71	M	0.46	0.629	0	1.4E-14	0	653.869	ataaatctttCgccttttact	m5C_52047
chr15	65588426	+	C	140	75	0.536	0	0	C	75	M	0.453	0.616	0	1.3E-15	0	679.878	aaatctttcgCcttttactaa	m5C_51987
chr15	65588427	+	C	144	74	0.514	0	0	C	74	M	0.433	0.594	0	1.1E-15	0	640.78	aatctttcgcCttttactaaa	m5C_51993
chr15	65588433	+	C	191	82	0.429	0	0	T	109	M	0.361	0.5	0	0	0	592.362	tcgccttttaCtaaagatttc	m5C_52062
chr15	65588443	+	C	427	168	0.393	0	0	T	259	M	0.348	0.441	0	0	0	1170.13	ctaaagatttCcgtggagagg	m5C_52036
chr15	65588444	+	C	475	212	0.446	0	0	T	263	M	0.402	0.491	0	0	0	1705.41	taaagatttcCgtggagagga	m5C_52013
chr15	65588456	+	C	715	230	0.324	5	0.007	T	480	M	0.291	0.359	0	0	0	1336.52	tggagaggaaCaactctgagt	m5C_52032
chr15	65588459	+	C	739	198	0.269	2	0.003	T	539	M	0.238	0.302	0	0	0	942.142	agaggaacaaCtctgagtctt	m5C_52023
chr15	65588461	+	C	750	320	0.427	1	0.001	T	429	M	0.392	0.463	0	0	0	2510.52	aggaacaactCtgagtcttaa	m5C_52037
chr15	65588467	+	C	801	302	0.377	0	0	T	499	M	0.344	0.411	0	0	0	2078.53	aactctgagtCttaacccaat	m5C_51989
chr15	65588472	+	C	823	217	0.305	111	0.135	T	495	M	0.272	0.34	0	0	0	1180.85	tgagtcttaaCccaatttttt	m5C_52049
chr15	65588473	+	C	832	353	0.424	0	0	T	479	M	0.391	0.458	0	0	0	2761.3	gagtcttaacCcaattttttg	m5C_51988
chr15	65588474	+	C	778	289	0.371	0	0	T	489	M	0.338	0.406	0	0	0	1954.91	agtcttaaccCaattttttga	m5C_51998
chr15	65588487	+	C	768	261	0.34	1	0.001	T	506	M	0.308	0.375	0	0	0	1605.8	tttttgaggCcttgctttgg	m5C_52041
chr15	65588488	+	C	762	357	0.469	0	0	T	405	M	0.433	0.504	0	0	0	3093.9	tttttgaggcCttgctttggc	m5C_52021
chr15	65588492	+	C	678	245	0.361	0	0	T	433	M	0.326	0.398	0	0	0	1597.75	tgaggccttgCtttggcaagg	m5C_52054
chr15	65588498	+	C	577	141	0.244	0	0	T	436	M	0.211	0.281	0	0	0	595.219	cttgctttggCaaggctatat	m5C_52034
chr15	65588503	+	C	499	158	0.317	0	0	T	341	M	0.277	0.359	0	0	0	876.432	tttggcaaggCtatatgtggt	m5C_52046
chr15	65597037	+	C	44	27	0.614	0	0	C	27	M	0.466	0.743	0	6.7E-06	0	251.766	ctcttcagatCgtataaatct	m5C_52000
chr15	65597046	+	C	57	32	0.561	0	0	C	32	M	0.433	0.682	0	7.2E-07	0	276.978	tcgtataaatCtttcgccttt	m5C_52015
chr15	65597050	+	C	72	40	0.556	0	0	C	40	M	0.441	0.665	0	5.8E-09	0	352.697	ataaatctttCgccttttact	m5C_52050
chr15	65597052	+	C	72	38	0.528	0	0	C	38	M	0.414	0.639	0	3.7E-09	0	314.644	aaatctttcgCcttttactaa	m5C_52061
chr15	65597053	+	C	78	44	0.571	1	0.013	C	44	M	0.46	0.676	0	1.3E-08	0	404.891	aatctttcgcCttttactaaa	m5C_51990
chr15	65597059	+	C	117	55	0.47	0	0	T	62	M	0.382	0.56	0	2.9E-13	0	420.246	tcgccttttaCtaaagatttc	m5C_51991
chr15	65597069	+	C	285	74	0.26	0	0	T	211	M	0.212	0.314	0	0	0	314.028	ctaaagatttCcgtggagagg	m5C_52006
chr15	65597070	+	C	339	111	0.327	0	0	T	228	M	0.28	0.379	0	0	0	620.833	taaagatttcCgtggagagga	m5C_52014
chr15	65597082	+	C	589	143	0.243	1	0.002	T	445	M	0.21	0.279	0	0	0	601.344	tggagaggaaCaactctgagt	m5C_52007
chr15	65597085	+	C	604	219	0.363	1	0.002	T	384	M	0.326	0.402	0	0	0	1426.9	agaggaacaaCtctgagtctt	m5C_52002
chr15	65597087	+	C	622	170	0.273	0	0	T	452	M	0.24	0.31	0	0	0	815.182	aggaacaactCtgagtcttaa	m5C_52009
chr15	65597093	+	C	666	198	0.297	0	0	T	468	M	0.264	0.333	0	0	0	1044.75	aactctgagtCttaagctaat	m5C_51999
chr15	65597099	+	C	675	287	0.425	0	0	T	388	M	0.388	0.463	0	0	0	2229.51	gagtcttaagCtaatttttg	m5C_52039
chr15	65597113	+	C	724	202	0.279	0	0	T	522	M	0.248	0.313	0	0	0	1000.17	ttttttgaggCcttgttccga	m5C_52035
chr15	65597114	+	C	718	279	0.389	0	0	T	439	M	0.354	0.425	0	0	0	1973.14	tttttgaggcCttgttccgac	m5C_52022
chr15	65597120	+	C	696	234	0.336	0	0	T	462	M	0.302	0.372	0	0	0	1413.8	aggccttgttCcgacaaggct	m5C_52033
chr15	65597129	+	C	526	163	0.31	1	0.002	T	362	M	0.272	0.351	0	0	0	888.004	tccgacaaggCtatataaagc	m5C_52027
chr15	66161401	-	C	170	47	0.276	0	0	T	123	M	0.215	0.348	0	0	0	201.856	tcggtggaacCtgcattggtt	m5C_55643
chr15	66161402	-	C	169	62	0.367	0	0	T	107	M	0.298	0.442	0	0	0	369.41	ctcggtggaaCctgcattggt	m5C_55561
chr15	66161432	-	C	122	64	0.525	0	0	C	64	M	0.437	0.611	0	8.2E-14	0	558.825	gactctgaatCcagcgatccg	m5C_55589
chr15	66161440	-	C	124	26	0.21	0	0	T	98	M	0.147	0.29	0	0	0	76.5949	gcactctggaCtctgaatcca	m5C_55605
chr15	66161445	-	C	134	40	0.299	0	0	T	94	M	0.228	0.381	0	0	0	182.019	ggttagcactCtggactctga	m5C_55573
chr15	66161467	-	C	171	38	0.222	0	0	T	133	M	0.166	0.29	0	0	0	126.463	cgtggcggttCcatggtgtaa	m5C_55624
chr15	66277453	-	C	39	10	0.256	0	0	T	29	M	0.146	0.411	1.3E-13	2E-06	1.6E-13	18.7753	tgtagcacttCtaggatggtg	m5C_55607
chr15	66277456	-	C	38	10	0.263	0	0	T	28	M	0.15	0.42	9.4E-14	2E-06	1.2E-13	19.5037	gtatgtagcaCttctaggatg	m5C_55579
chr15	66639553	-	C	47	20	0.426	0	0	T	27	M	0.295	0.567	0	1.4E-06	0	118.054	tgcgatggctCcatacatgtt	m5C_55569
chr15	66639555	-	C	47	29	0.617	0	0	C	29	M	0.474	0.742	0	9.7E-06	0	275.073	tgtgcgatggCtccatacatg	m5C_55585
chr15	66639566	-	C	41	19	0.463	0	0	T	22	M	0.321	0.613	0	1E-06	0	121.813	atcagattttCtgtgcgatgg	m5C_55606
chr15	66639599	-	C	43	11	0.256	0	0	T	32	M	0.149	0.402	1.2E-14	4.4E-08	1.5E-14	22.8887	cagatggttcCtgccgacagg	m5C_55622
chr15	66639617	-	C	81	20	0.247	0	0	T	61	M	0.166	0.351	0	1.5E-13	0	66.3608	cagtcacacaCattaaaacag	m5C_55583
chr15	66639634	-	C	147	32	0.218	0	0	T	115	M	0.159	0.291	0	0	0	101.522	tacagtgcaaCcatagacagt	m5C_55567
chr15	66639637	-	C	165	42	0.255	0	0	T	123	M	0.194	0.326	0	0	0	163.117	ttgtacagtgCaaccatagac	m5C_55580
chr15	66639642	-	C	180	38	0.211	0	0	T	142	M	0.158	0.276	0	0	0	119.964	gttagttgtaCagtgcaacca	m5C_55635
chr15	66639657	-	C	226	49	0.218	1	0.004	T	176	M	0.169	0.276	0	0	0	165.453	gtattgattgCatttgttagt	m5C_55609
chr15	66639671	-	C	258	68	0.264	0	0	T	190	M	0.214	0.321	0	0	0	290.439	tgtgactgttCctggtattga	m5C_55565
chr15	66791902	-	C	128	36	0.281	0	0	T	92	M	0.211	0.365	0	0	0	151.641	ggcaagaagcCtgtggtaggt	m5C_55625
chr15	66791910	-	C	126	46	0.368	1	0.008	T	79	M	0.289	0.455	0	6.7E-16	0	265.483	cggttgcaggCaagaagcctg	m5C_55584
chr15	66791914	-	C	124	25	0.202	0	0	T	99	M	0.14	0.281	0	0	0	70.2278	gcggcggttgCaggcaagaag	m5C_55631
chr15	66791923	-	C	112	45	0.433	8	0.071	T	59	M	0.342	0.529	0	7.4E-13	0	307.394	gatgagaaggCggcggttgca	m5C_55599
chr15	66791935	-	C	69	15	0.217	0	0	T	54	M	0.136	0.328	0	6E-11	0	40.9219	caagccaaatCagatgagaag	m5C_55596
chr15	66791941	-	C	43	10	0.233	0	0	T	33	M	0.132	0.377	4.3E-13	2.4E-08	5.2E-13	16.2683	gcactacaagCcaaatcagat	m5C_55612
chr15	66791950	-	C	40	10	0.25	0	0	T	30	M	0.142	0.402	1.8E-13	2.4E-08	2.2E-13	18.0914	gcagcagcggCactacaagcc	m5C_55637
chr15	66791959	-	C	40	14	0.35	0	0	T	26	M	0.221	0.505	0	1.9E-07	0	61.9761	gcagctgctgCagcagcggca	m5C_55639
chr15	66793628	-	C	530	165	0.311	0	0	T	365	M	0.273	0.352	0	0	0	902.14	acttaaggtcCgtgtttctga	m5C_55623
chr15	66793629	-	C	527	174	0.33	0	0	T	353	M	0.291	0.371	0	0	0	1013.98	cacttaaggtCcgtgtttctg	m5C_55572
chr15	66794365	-	C	300	64	0.213	0	0	T	236	M	0.171	0.263	0	0	0	218.562	tgtggaaattCtgacttggca	m5C_55603
chr15	66794400	-	C	368	118	0.322	1	0.003	T	249	M	0.276	0.371	0	0	0	650.891	cttaattggtCcgtgtttctg	m5C_55608
chr15	66795151	-	C	47	13	0.277	0	0	T	34	M	0.169	0.418	0	1.2E-07	0	44.0347	tcttactgagCaagaaataac	m5C_55633
chr15	66795156	-	C	65	30	0.462	0	0	T	35	M	0.346	0.581	0	1.7E-08	0	207.541	aaaattcttaCtgagcaagaa	m5C_55636
chr15	66795179	-	C	145	41	0.301	9	0.062	T	95	M	0.231	0.383	0	0	0	189.149	taagctggtaCagaaggttga	m5C_55638
chr15	66795192	-	C	160	44	0.275	0	0	T	116	M	0.212	0.349	0	0	0	186.296	tgcctgctgtCagtaagctgg	m5C_55617
chr15	66795196	-	C	164	47	0.287	0	0	T	117	M	0.223	0.36	0	0	0	209.515	cagttgcctgCtgtcagtaag	m5C_55581
chr15	66795199	-	C	170	40	0.235	0	0	T	130	M	0.178	0.304	0	0	0	142.253	cgacagttgcCtgctgtcagt	m5C_55574
chr15	66795200	-	C	184	52	0.283	0	0	T	132	M	0.223	0.352	0	0	0	231.41	gcgacagttgCctgctgtcag	m5C_55640
chr15	66795212	-	C	226	69	0.305	0	0	T	157	M	0.249	0.368	0	0	0	343.53	actctgttctCagcgacagtt	m5C_55562
chr15	66795214	-	C	221	91	0.412	0	0	T	130	M	0.349	0.478	0	0	0	635.042	ttactctgttCtcagcgacag	m5C_55610
chr15	66795221	-	C	242	84	0.35	2	0.008	T	156	M	0.292	0.412	0	0	0	491.314	ttgcgtcttaCtctgttctca	m5C_55616
chr15	66795588	-	C	1494	417	0.279	0	0	T	1077	M	0.257	0.302	0	0	0	2143.04	ctcggatgttCtgatgctgtt	m5C_55611
chr15	66795596	-	C	1796	385	0.214	0	0	T	1411	M	0.196	0.234	0	0	0	1509.24	atgattttctCggatgttctg	m5C_55576
chr15	66795615	-	C	1782	390	0.219	0	0	T	1392	M	0.2	0.239	0	0	0	1562.13	gtccgtgtttCtgaaccacat	m5C_55582
chr15	66795623	-	C	1727	438	0.254	3	0.002	T	1286	M	0.234	0.275	0	0	0	2050.48	cttcattggtCcgtgtttctg	m5C_55560
chr15	66797161	-	C	39	8	0.205	0	0	T	31	M	0.108	0.355	1.6E-10	6.8E-07	1.8E-10	8.44384	agcagccgggCtgagaggagc	m5C_55575
chr15	67393901	+	C	32	10	0.312	0	0	T	22	M	0.18	0.486	1.1E-14	2E-06	1.3E-14	25.0836	atgatgatgcCactggaacag	m5C_55546
chr15	67393903	+	C	36	14	0.389	0	0	T	22	M	0.248	0.551	0	9.2E-06	0	69.397	gatgatgccaCtggaacaggc	m5C_55539
chr15	69008194	-	C	38	8	0.211	0	0	T	30	M	0.111	0.363	1.3E-10	6.8E-07	1.4E-10	8.77246	ggttgtaagaCttttagtggt	m5C_51598

chr15	69072774	-	C	231	56	0.242	0	0	T	175	M	0.192	0.302	0	0	0	214.665	aggtagatgaCgaggaagatg	m5C_51593
chr15	69072792	-	C	186	47	0.253	0	0	T	139	M	0.196	0.32	0	0	0	183.934	aaggttataaCgatggagagg	m5C_51582
chr15	69075313	-	C	59	16	0.271	0	0	T	43	M	0.174	0.396	0	7.3E-09	0	55.7978	aagaggaggaCgtgagtggag	m5C_51584
chr15	69075346	-	C	60	23	0.383	0	0	T	37	M	0.271	0.51	0	8.7E-08	0	124.605	acgaggaggaCgaggatgagg	m5C_51596
chr15	69698805	+	C	34	7	0.212	1	0.029	T	26	M	0.107	0.378	1.1E-09	3.4E-07	1.2E-09	6.69165	agtgagttagCtcaattgatt	m5C_51529
chr15	69747788	+	C	65	14	0.215	0	0	T	51	M	0.133	0.33	1.1E-16	3.2E-11	1.5E-16	29.6776	aaagtggaagCaaagaaagaa	m5C_51562
chr15	69747803	+	C	70	19	0.271	0	0	T	51	M	0.181	0.385	0	6.9E-12	0	68.854	aaagaagaatCcgaggagtct	m5C_51561
chr15	70080515	+	C	56	28	0.5	0	0	CT	28	M	0.373	0.627	0	3.1E-07	0	209.057	gtactgggtgCtgcaggcccc	m5C_52186
chr15	70080522	+	C	51	21	0.412	0	0	T	30	M	0.288	0.548	0	4.8E-08	0	120.768	gtgctgcaggCccctgtcggg	m5C_52181
chr15	70528736	+	C	47	27	0.574	0	0	C	27	M	0.433	0.705	0	6.7E-06	0	233.721	gaactgggtgCtgcaggccag	m5C_52175
chr15	70528743	+	C	38	12	0.316	0	0	T	26	M	0.191	0.475	0	4.6E-06	0	45.8026	gtgctgcaggCcaggttcacc	m5C_52178
chr15	70618284	-	C	31	7	0.226	0	0	T	24	M	0.114	0.398	6.4E-10	3.4E-07	7.2E-10	7.33205	tcgtgcagccCagctggaact	m5C_52202
chr15	70618286	-	C	32	11	0.344	0	0	T	21	M	0.204	0.517	2.2E-16	3.1E-06	2.9E-16	35.1442	ggtcgtgcagCccagctggaa	m5C_52203
chr15	72122460	-	C	36	9	0.25	0	0	T	27	M	0.138	0.411	2E-12	1.2E-06	2.4E-12	14.4765	tgagaacctaCagaaggagaa	m5C_52959
chr15	72122464	-	C	38	9	0.237	0	0	T	29	M	0.13	0.392	3.7E-12	1.2E-06	4.3E-12	13.3739	agattgagaaCctacagaagg	m5C_52966
chr15	72337971	+	C	172	37	0.215	0	0	T	135	M	0.16	0.282	0	0	0	118.613	acacatcgacCtggtattgca	m5C_52918
chr15	72337985	+	C	413	88	0.213	0	0	T	325	M	0.176	0.255	0	0	0	310.324	tattgcagtaCctccaggaat	m5C_52901
chr15	72337988	+	C	407	97	0.238	0	0	T	310	M	0.2	0.282	0	0	0	387.038	tgcagtacctCcaggaatggt	m5C_52889
chr15	72441576	+	C	64	15	0.234	0	0	T	49	M	0.147	0.351	0	6E-11	0	44.2493	ggtggtggatCatttggagga	m5C_52906
chr15	72491726	-	C	345	100	0.29	0	0	T	245	M	0.245	0.34	0	0	0	489.004	gcccctggagCcagatggcaa	m5C_52991
chr15	72491733	-	C	384	82	0.214	0	0	T	302	M	0.175	0.257	0	0	0	287.811	actggaggccCctggagccag	m5C_52936
chr15	72491734	-	C	406	82	0.202	0	0	T	324	M	0.166	0.244	0	0	0	271.904	gactggaggcCcctggagcca	m5C_52937
chr15	72491735	-	C	404	97	0.24	0	0	T	307	M	0.201	0.284	0	0	0	389.977	ggactggaggCccctggagcc	m5C_52929
chr15	72491742	-	C	397	95	0.239	0	0	T	302	M	0.2	0.284	0	0	0	379.906	gaatgctggaCtggaggcccc	m5C_52942
chr15	72491772	-	C	57	18	0.316	0	0	T	39	M	0.21	0.445	0	1.7E-08	0	75.6149	gtggccccacCcaatcaaggg	m5C_52994
chr15	72491773	-	C	48	27	0.562	0	0	C	27	M	0.423	0.693	0	6.7E-06	0	228.284	tgtggccccaCccaatcaagg	m5C_52999
chr15	72499483	-	C	82	19	0.232	0	0	T	63	M	0.154	0.334	0	8.4E-14	0	58.3883	ggaagcctgtCatctgtgcta	m5C_52967
chr15	72499487	-	C	88	39	0.443	0	0	T	49	M	0.344	0.547	0	1.6E-10	0	268.268	gctgggaagcCtgtcatctgt	m5C_52962
chr15	72499488	-	C	100	22	0.22	0	0	T	78	M	0.15	0.311	0	0	0	66.0052	agctgggaagCctgtcatctg	m5C_52950
chr15	72499500	-	C	81	31	0.383	0	0	T	50	M	0.284	0.492	0	1.7E-11	0	176.368	acggtgcaacCgagctgggaa	m5C_52928
chr15	72499501	-	C	83	22	0.265	0	0	T	61	M	0.182	0.369	0	4.4E-13	0	80.0989	gacggtgcaaCcgagctggga	m5C_53013
chr15	72499504	-	C	75	54	0.72	0	0	C	54	M	0.61	0.809	0	6.9E-08	0	658.358	ttggacggtgCaaccgagctg	m5C_52995
chr15	72499509	-	C	70	31	0.443	0	0	T	39	M	0.332	0.559	0	6.4E-10	0	206.146	gatgattggaCggtgcaaccg	m5C_52922
chr15	73212641	-	C	48	21	0.438	0	0	T	27	M	0.307	0.577	0	1.8E-06	0	128.944	tcagtgctctCaaatgttaat	m5C_53003
chr15	73212643	-	C	152	98	0.645	0	0	C	98	M	0.566	0.716	0	4.4E-15	0	1109.25	attcagtgctCtcaaatgtta	m5C_52940
chr15	73212645	-	C	206	85	0.413	0	0	T	121	M	0.348	0.481	0	0	0	590.905	ggattcagtgCtctcaaatgt	m5C_52945
chr15	73212650	-	C	288	98	0.34	0	0	T	190	M	0.288	0.397	0	0	0	564.44	ggttaggattCagtgctctca	m5C_52981
chr15	73212665	-	C	380	129	0.339	0	0	T	251	M	0.294	0.388	0	0	0	757.695	ccctcgtggtCtagtggttag	m5C_52958
chr15	73212674	-	C	129	28	0.217	0	0	T	101	M	0.155	0.296	0	0	0	86.6081	ccctcgtctcCctcgtggtct	m5C_52932
chr15	75275710	-	C	55	16	0.291	0	0	T	39	M	0.188	0.421	0	7.3E-09	0	60.0673	gtgggctggtCcgagtgcagt	m5C_51830
chr15	75910050	-	C	54	12	0.222	0	0	T	42	M	0.132	0.349	7.5E-15	8.8E-10	9.6E-15	22.3614	gtcccacctaCtgtggaagct	m5C_51844
chr15	75910053	-	C	54	11	0.204	0	0	T	43	M	0.118	0.329	2.3E-13	4.4E-10	2.8E-13	16.3622	gtagtcccacCtactgtggaa	m5C_51810
chr15	76486453	-	C	66	15	0.227	0	0	T	51	M	0.143	0.342	0	6E-11	0	42.8553	gcacgtgaggCtgggtcagct	m5C_51624
chr15	76486460	-	C	72	17	0.236	0	0	T	55	M	0.153	0.346	0	2.2E-12	0	52.0084	gtggggagcaCgtgaggctgg	m5C_51647
chr15	76674829	-	C	51	22	0.431	0	0	T	29	M	0.305	0.567	0	6.5E-08	0	134.205	tgcctggctaCctcagttggt	m5C_51645
chr15	77020949	-	C	32	7	0.219	0	0	T	25	M	0.11	0.388	8.5E-10	3.4E-07	9.5E-10	6.99971	ggatgcagccCgggaaagagc	m5C_51628
chr15	77020950	-	C	33	8	0.242	0	0	T	25	M	0.128	0.41	3.1E-11	6.8E-07	3.6E-11	10.7866	aggatgcagcCcgggaaagag	m5C_51626
chr15	77020951	-	C	36	19	0.528	0	0	C	19	M	0.37	0.68	0	3.4E-05	0	140.622	gaggatgcagCccgggaaaga	m5C_51649
chr15	77269453	-	C	43	24	0.558	0	0	C	24	M	0.411	0.696	0	3.6E-06	0	197.315	gtgtgctgtgCtgattggatg	m5C_51636
chr15	77269458	-	C	43	31	0.721	0	0	C	31	M	0.573	0.833	0	1.4E-05	0	355.314	ctgcagtgtgCtgtgctgatt	m5C_51627
chr15	77955360	-	C	31	8	0.258	0	0	T	23	M	0.137	0.432	1.7E-11	6.8E-07	1.9E-11	11.8182	ggggtgacccCcggcatcccc	m5C_51643
chr15	79152905	+	C	53	24	0.453	0	0	T	29	M	0.327	0.585	0	1.2E-07	0	156.755	tctctaactgCccggctagct	m5C_51732
chr15	79152906	+	C	78	18	0.231	0	0	T	60	M	0.151	0.336	0	4E-12	0	54.4514	ctctaactgcCcggctagctc	m5C_51765
chr15	79152914	+	C	86	20	0.233	0	0	T	66	M	0.156	0.332	0	1.5E-13	0	62.356	gcccggctagCtcagtcggta	m5C_51747
chr15	79152916	+	C	84	22	0.262	0	0	T	62	M	0.18	0.365	0	4.4E-13	0	79.1047	ccggctagctCagtcggtaga	m5C_51746
chr15	79152959	+	C	40	9	0.225	0	0	T	31	M	0.123	0.375	6.4E-12	1.2E-08	7.6E-12	12.4041	gtcgtgggttCgagccccacg	m5C_51769
chr15	79152963	+	C	48	10	0.208	0	0	T	38	M	0.117	0.343	1.6E-12	2.4E-08	1.9E-12	13.8343	tgggttcgagCcccacgttgg	m5C_51762
chr15	79152964	+	C	44	10	0.227	0	0	T	34	M	0.128	0.37	5.7E-13	2.4E-08	6.8E-13	15.7274	gggttcgagcCccacgttggg	m5C_51756
chr15	79152966	+	C	45	31	0.689	0	0	C	31	M	0.543	0.805	0	1.4E-05	0	336.877	gttcgagcccCacgttgggcg	m5C_51772
chr15	79152968	+	C	47	11	0.234	0	0	T	36	M	0.136	0.372	3.8E-14	4.4E-08	4.7E-14	20.0856	tcgagccccaCgttgggcggg	m5C_51764
chr15	79152975	+	C	31	17	0.548	0	0	C	17	M	0.378	0.708	0	2.1E-05	0	128.424	ccacgttgggCgggtttgttt	m5C_51774
chr15	79789556	-	C	48	17	0.354	0	0	T	31	M	0.234	0.496	0	5.6E-07	0	79.6738	cgggtgtgttCagggtggcgt	m5C_51791
chr15	80037009	+	C	214	55	0.257	0	0	T	159	M	0.203	0.319	0	0	0	223.417	gggtatagctCagtgggtaga	m5C_51924
chr15	80037021	+	C	211	61	0.29	1	0.005	T	149	M	0.233	0.355	0	0	0	284.596	gtgggtagagCatttgactgc	m5C_51927
chr15	80037028	+	C	128	53	0.414	0	0	T	75	M	0.332	0.501	0	5.8E-15	0	352.397	gagcatttgaCtgcagatcaa	m5C_51901
chr15	82340394	+	C	44	9	0.205	0	0	T	35	M	0.112	0.345	1.8E-11	1.2E-08	2.1E-11	10.7806	gaggatggtgCcgagtcctcc	m5C_51866
chr15	82340395	+	C	42	14	0.333	0	0	T	28	M	0.21	0.484	0	1.9E-07	0	58.8344	aggatggtgcCgagtcctccg	m5C_51859
chr15	83424704	+	C	69	31	0.449	0	0	T	38	M	0.338	0.566	0	2.1E-08	0	209.366	cttctggagaCtaagaaaata	m5C_51858
chr15	83424728	+	C	50	13	0.26	0	0	T	37	M	0.159	0.396	0	1.6E-09	0	41.2656	tccttgaaatCaagctgactc	m5C_51861
chr15	83424732	+	C	41	10	0.244	0	0	T	31	M	0.138	0.393	2.4E-13	2.4E-08	2.9E-13	17.4472	tgaaatcaagCtgactctgct	m5C_51857
chr15	83424736	+	C	40	23	0.575	0	0	C	23	M	0.422	0.715	0	2.9E-06	0	194.096	atcaagctgaCtctgctttta	m5C_51863
chr15	83424738	+	C	39	11	0.282	0	0	T	28	M	0.165	0.438	3.1E-15	3.1E-06	4E-15	26.4001	caagctgactCtgcttttagc	m5C_51864
chr15	83424774	+	C	46	16	0.348	0	0	T	30	M	0.227	0.492	0	4E-07	0	72.5786	gtagatagaaCaggtcttgtt	m5C_51865
chr15	83424779	+	C	35	10	0.286	0	0	T	25	M	0.163	0.451	3.4E-14	2E-06	4.2E-14	21.9991	tagaacaggtCttgtttgcaa	m5C_51860
chr15	83623658	-	C	31	14	0.452	0	0	T	17	M	0.292	0.622	0	9.2E-06	0	81.6522	gtcccagctaCttgggaggct	m5C_51899
chr15	83623661	-	C	44	14	0.318	0	0	T	30	M	0.2	0.466	0	1.9E-07	0	55.9976	gtagtcccagCtacttgggag	m5C_51889
chr15	83623665	-	C	57	18	0.316	0	0	T	39	M	0.21	0.445	0	1.7E-08	0	75.6149	gcctgtagtcCcagctacttg	m5C_51875
chr15	83623666	-	C	57	28	0.491	0	0	T	29	M	0.366	0.617	0	3.1E-07	0	205.05	cgcctgtagtCccagctactt	m5C_51873
chr15	83623678	-	C	54	15	0.278	0	0	T	39	M	0.176	0.409	0	4.6E-09	0	52.85	gtggtggtgCgcgcctgtag	m5C_51871
chr15	83826267	-	C	54	24	0.444	0	0	T	30	M	0.32	0.576	0	1.2E-07	0	153.611	actgcagatgCaagcagtgag	m5C_51874
chr15	83826276	-	C	51	22	0.431	0	0	T	29	M	0.305	0.567	0	6.5E-08	0	134.205	ggtggaaataCtgcagatgca	m5C_51870
chr15	85176879	-	C	33	11	0.333	0	0	T	22	M	0.198	0.504	2.2E-16	3.1E-06	2.9E-16	34.0074	agaacgcggcCtgtgcggcga	m5C_52150
chr15	88758575	-	C	35	10	0.286	0	0	T	25	M	0.163	0.451	3.4E-14	2E-06	4.2E-14	21.9991	cagtggggggCcgtgtttgcc	m5C_52368
chr15	89365449	-	C	38	23	0.605	0	0	C	23	M	0.447	0.744	0	7.6E-05	0	205.701	atgtgggaggCttgggccatg	m5C_52354
chr15	89843078	+	C	38	21	0.553	0	0	C	21	M	0.397	0.699	0	5.2E-05	0	166.765	gtggaagagtCgggaaagaaa	m5C_52329
chr15	89878306	+	C	209	50	0.239	0	0	T	159	M	0.186	0.301	0	0	0	186.434	aaccgacgggCcgcgtggcct	m5C_52343
chr15	89878307	+	C	215	51	0.237	0	0	T	164	M	0.185	0.298	0	0	0	188.981	accgacgggcCgcgtggccta	m5C_52349
chr15	89878328	+	C	215	40	0.202	17	0.079	T	158	M	0.152	0.263	0	0	0	121.608	atggataaggCgtctgacttc	m5C_52350
chr15	89878335	+	C	222	72	0.324	0	0	T	150	M	0.266	0.388	0	0	0	383.307	aggcgtctgaCttcggatcag	m5C_52313
chr15	89878359	+	C	154	32	0.208	0	0	T	122	M	0.151	0.279	0	0	0	96.774	attgcaggttCgagtcctgcc	m5C_52319
chr15	89878369	+	C	102	26	0.255	0	0	T	76	M	0.18	0.347	0	6.7E-16	0	93.7536	cgagtcctgcCgcggtcgaag	m5C_52331
chr15	89878375	+	C	93	20	0.215	0	0	T	73	M	0.144	0.309	0	1.7E-15	0	57.4997	ctgccgcggtCgaagggaggt	m5C_52347
chr15	89878395	+	C	82	23	0.28	0	0	T	59	M	0.195	0.386	0	7.2E-13	0	89.5943	ttatgattaaCttttagttta	m5C_52320
chr15	90983788	+	C	31	13	0.419	0	0	T	18	M	0.264	0.592	0	6.7E-06	0	68.6798	atccagatgcCtgcctttagc	m5C_52419
chr15	91017805	+	C	60	29	0.483	0	0	T	31	M	0.362	0.607	0	1.3E-08	0	209.814	aggagcttgaCcttatgaaga	m5C_52413
chr15	91021093	+	C	37	9	0.243	0	0	T	28	M	0.134	0.401	2.7E-12	1.2E-06	3.2E-12	13.907	ttgggttaatCagatggagtc	m5C_52389
chr15	91517867	-	C	50	16	0.32	0	0	T	34	M	0.208	0.458	0	7.3E-09	0	66.4257	gagtatgtggCagaacaatgg	m5C_52442
chr15	91524789	-	C	32	9	0.281	0	0	T	23	M	0.156	0.454	5.3E-13	1.2E-06	6.4E-13	17.1983	tgtgtgaagaCgaagatgcct	m5C_52450
chr15	91646527	+	C	36	15	0.417	0	0	T	21	M	0.271	0.578	0	1.2E-05	0	81.421	ggatggaggaCtgtgtttgtg	m5C_52391
chr15	92783185	-	C	45	10	0.222	0	0	T	35	M	0.125	0.363	7.4E-13	2.4E-08	8.9E-13	15.2157	tgttgaagatCagttgggtgt	m5C_52248
chr15	93760119	+	C	48	13	0.271	0	0	T	35	M	0.166	0.41	0	1.2E-07	0	43.0711	tggtggtcacCacttctgtag	m5C_52231

chr15	96289186	+	C	31	11	0.355	0	0	T	20	M	0.211	0.531	1.1E-16	3.1E-06	1.5E-16	37.0595	catgcatcgaCctggtattgc	m5C_52259
chr15	99610028	+	C	97	42	0.433	0	0	T	55	M	0.339	0.532	0	1.1E-11	0	284.591	gagaagcggcCtgagtggctt	m5C_52813
chr15	99610036	+	C	53	16	0.302	0	0	T	37	M	0.195	0.435	0	7.3E-09	0	62.4581	gcctgagtggCttggtgggaa	m5C_52820
chr16	580835	+	C	32	13	0.406	0	0	T	19	M	0.255	0.577	0	6.7E-06	0	66.3505	tctgaggggcCtgcctgagtg	m5C_55142
chr16	580839	+	C	37	18	0.486	0	0	T	19	M	0.334	0.641	0	2.7E-05	0	120.406	aggggcctgcCtgagtgctgc	m5C_55132
chr16	686735	-	C	908	353	0.389	0	0	T	555	M	0.358	0.421	0	0	0	2524.59	gggcggcgcaCttagtttttt	m5C_55150
chr16	686742	-	C	1132	257	0.227	1	0.001	T	874	M	0.204	0.253	0	0	0	1047.31	gattcccgggCggcgcactta	m5C_55164
chr16	686759	-	C	1197	289	0.241	0	0	T	908	M	0.218	0.266	0	0	0	1260.3	tcttgcgaccCgggttcgatt	m5C_55198
chr16	686760	-	C	1203	269	0.224	2	0.002	T	932	M	0.201	0.248	0	0	0	1083.01	ttcttgcgacCcgggttcgat	m5C_55192
chr16	686761	-	C	1184	308	0.26	0	0	T	876	M	0.236	0.286	0	0	0	1453.45	attcttgcgaCccgggttcga	m5C_55160
chr16	2012339	-	C	74	18	0.243	0	0	T	56	M	0.16	0.352	0	4E-12	0	57.5149	aggagtgcgaCaatttttggg	m5C_54967
chr16	2012351	-	C	81	18	0.222	0	0	T	63	M	0.145	0.324	0	4.5E-14	0	52.3602	ctctctgttcCtaggagtgcg	m5C_55054
chr16	2012422	-	C	35	8	0.229	0	0	T	27	M	0.121	0.39	5.6E-11	6.8E-07	6.4E-11	9.89691	gtgtgcttggCgtgttttcag	m5C_54985
chr16	2012427	-	C	31	7	0.226	0	0	T	24	M	0.114	0.398	6.4E-10	3.4E-07	7.2E-10	7.33205	ccagtgtgtgCttggcgtgtt	m5C_55057
chr16	2012974	-	C	225	64	0.284	0	0	T	161	M	0.229	0.347	0	0	0	293.757	gtgcaacaggCcctgggcggt	m5C_55071
chr16	2013002	-	C	243	63	0.259	0	0	T	180	M	0.208	0.318	0	0	0	262.342	cttactgtggCctctctttcc	m5C_55073
chr16	2013008	-	C	382	146	0.382	0	0	T	236	M	0.335	0.432	0	0	0	977.824	atgggccttaCtgtggcctct	m5C_54991
chr16	2013012	-	C	387	97	0.253	3	0.008	T	287	M	0.212	0.298	0	0	0	410.778	tcggatgggcCttactgtggc	m5C_54971
chr16	2013023	-	C	385	89	0.231	0	0	T	296	M	0.192	0.276	0	0	0	341.47	tcctcaggacCtcggatgggc	m5C_55007
chr16	2013024	-	C	386	87	0.227	3	0.008	T	296	M	0.188	0.272	0	0	0	327.158	ctcctcaggaCctcggatggg	m5C_55018
chr16	2013054	-	C	465	95	0.205	1	0.002	T	369	M	0.171	0.244	0	0	0	323.989	actttcgtaaCggggagagag	m5C_55042
chr16	2013071	-	C	591	130	0.22	1	0.002	T	460	M	0.189	0.256	0	0	0	490.783	ttggcttcacCcgtgtgactt	m5C_55014
chr16	2013077	-	C	549	125	0.228	0	0	T	424	M	0.195	0.265	0	0	0	486.43	ttgcacttggCttcacccgtg	m5C_55026
chr16	2013082	-	C	643	147	0.229	0	0	T	496	M	0.198	0.263	0	0	0	581.605	catagttgcaCttggcttcac	m5C_55049
chr16	2013095	-	C	702	174	0.248	0	0	T	528	M	0.217	0.281	0	0	0	756.387	tctctcggctCtgcatagttg	m5C_55105
chr16	2013100	-	C	686	198	0.289	0	0	T	488	M	0.256	0.324	0	0	0	1013.65	gggactctctCggctctgcat	m5C_54981
chr16	2015184	+	C	50	15	0.3	0	0	T	35	M	0.191	0.438	0	4.6E-09	0	57.3102	tcgagaccggCgttggttgaa	m5C_54826
chr16	2015200	+	C	135	41	0.304	0	0	T	94	M	0.232	0.386	0	0	0	190.609	ttgaaaatcgCccccggcttt	m5C_54790
chr16	2015203	+	C	129	43	0.333	0	0	T	86	M	0.258	0.418	0	2.2E-16	0	221.746	aaaatcgcccCcggctttggc	m5C_54836
chr16	2015219	+	C	131	37	0.282	0	0	T	94	M	0.212	0.365	0	0	0	157.177	ttggccgtggCcgcgggtgag	m5C_54934
chr16	2015220	+	C	136	48	0.353	0	0	T	88	M	0.278	0.436	0	0	0	266.565	tggccgtggcCgcgggtgaga	m5C_54807
chr16	2015222	+	C	121	40	0.331	0	0	T	81	M	0.253	0.418	0	1.1E-16	0	202.485	gccgtggccgCgggtgagatt	m5C_54941
chr16	2015233	+	C	80	22	0.275	0	0	T	58	M	0.189	0.381	0	2.8E-11	0	83.2375	gggtgagattCggcgcccaga	m5C_54938
chr16	2015238	+	C	80	20	0.25	0	0	T	60	M	0.168	0.355	0	1.5E-13	0	67.2244	agattcggcgCccagagcccc	m5C_54852
chr16	2015239	+	C	76	31	0.408	0	0	T	45	M	0.304	0.52	0	6.4E-10	0	188.751	gattcggcgcCcagagccccc	m5C_54774
chr16	2015240	+	C	74	17	0.23	0	0	T	57	M	0.149	0.337	0	2.2E-12	0	50.548	attcggcgccCagagcccccg	m5C_54770
chr16	2015248	+	C	59	13	0.22	0	0	T	46	M	0.134	0.341	8.9E-16	1.6E-09	1.2E-15	26.1299	cccagagcccCcgggggcctc	m5C_54843
chr16	2015249	+	C	59	13	0.22	0	0	T	46	M	0.134	0.341	8.9E-16	1.6E-09	1.2E-15	26.1299	ccagagccccCgggggcctca	m5C_54794
chr16	2015258	+	C	51	16	0.314	0	0	T	35	M	0.203	0.45	0	7.3E-09	0	65.048	ccgggggcctCagctcaccgc	m5C_54799
chr16	2015261	+	C	54	14	0.259	0	0	T	40	M	0.161	0.389	0	2.8E-09	0	45.1318	ggggcctcagCtcaccgcgcg	m5C_54869
chr16	2015263	+	C	58	13	0.224	0	0	T	45	M	0.136	0.347	6.7E-16	1.6E-09	8.7E-16	26.8192	ggcctcagctCaccgcgcgct	m5C_54778
chr16	2015266	+	C	66	18	0.273	0	0	T	48	M	0.18	0.39	0	2.9E-10	0	64.8115	ctcagctcacCgcgcgctgcc	m5C_54876
chr16	2015270	+	C	78	17	0.218	0	0	T	61	M	0.141	0.322	0	2.2E-12	0	47.8608	gctcaccgcgCgctgccccat	m5C_54845
chr16	2015272	+	C	92	22	0.239	0	0	T	70	M	0.164	0.336	0	5.8E-15	0	71.9621	tcaccgcgcgCtgccccatgt	m5C_54894
chr16	2015301	+	C	113	30	0.265	0	0	T	83	M	0.193	0.354	0	0	0	115.653	gaaacccaggCcccgacaggc	m5C_54923
chr16	2015307	+	C	97	31	0.32	0	0	T	66	M	0.235	0.418	0	3.7E-13	0	145.815	caggccccgaCaggcgctgcc	m5C_54800
chr16	2015311	+	C	73	25	0.342	0	0	T	48	M	0.244	0.457	0	9.3E-11	0	121.974	ccccgacaggCgctgccgcct	m5C_54820
chr16	2205026	-	C	363	90	0.248	0	0	T	273	M	0.206	0.295	0	0	0	371.351	aagactgaggCttgagggtgc	m5C_55024
chr16	2205032	-	C	774	157	0.203	0	0	T	617	M	0.176	0.233	0	0	0	552.679	tttagcaagaCtgaggcttga	m5C_55022
chr16	2205049	-	C	922	205	0.223	1	0.001	T	716	M	0.197	0.251	0	0	0	807.297	cacgtgcagtCtgattattta	m5C_55028
chr16	2205057	-	C	919	281	0.306	0	0	T	638	M	0.277	0.336	0	0	0	1555.83	gaaaactgcaCgtgcagtctg	m5C_54969
chr16	2205059	-	C	904	181	0.2	0	0	T	723	M	0.175	0.228	0	0	0	635.049	atgaaaactgCacgtgcagtc	m5C_55069
chr16	2205062	-	C	891	192	0.215	0	0	T	699	M	0.19	0.244	0	0	0	728.61	cgtatgaaaaCtgcacgtgca	m5C_55075
chr16	2205074	-	C	897	182	0.203	0	0	T	715	M	0.178	0.23	0	0	0	647.458	cttctgacacCtcgtatgaaa	m5C_55051
chr16	2205081	-	C	1041	276	0.265	0	0	T	765	M	0.239	0.293	0	0	0	1320.46	gctttgacttCtgacacctcg	m5C_55053
chr16	2205084	-	C	1051	307	0.292	0	0	T	744	M	0.265	0.32	0	0	0	1629.6	attgctttgaCttctgacacc	m5C_54978
chr16	2264780	-	C	32	22	0.688	0	0	C	22	M	0.514	0.82	0	6.3E-05	0	226.305	cggcgggcggCgatggcggcg	m5C_55009
chr16	2264783	-	C	45	29	0.644	0	0	C	29	M	0.498	0.768	0	9.7E-06	0	289.046	ggccggcgggCggcgatggcg	m5C_55082
chr16	2264787	-	C	47	24	0.511	0	0	C	24	M	0.372	0.647	0	3.6E-06	0	178.763	cggcggccggCgggcggcgat	m5C_55003
chr16	2264794	-	C	42	21	0.5	0	0	CT	21	M	0.355	0.645	0	1.8E-06	0	149.208	cgggcggcggCggccggcggg	m5C_55029
chr16	2264797	-	C	32	19	0.594	0	0	C	19	M	0.423	0.745	0	3.4E-05	0	160.587	cggcgggcggCggcggccggc	m5C_55098
chr16	2321780	+	C	32	10	0.312	0	0	T	22	M	0.18	0.486	1.1E-14	2E-06	1.3E-14	25.0836	ggagcggggcCtgggcagccc	m5C_54909
chr16	2321788	+	C	32	18	0.562	0	0	C	18	M	0.393	0.718	0	2.7E-05	0	141.571	gcctgggcagCcccgtgtgtt	m5C_54961
chr16	2952922	+	C	46	11	0.239	0	0	T	35	M	0.139	0.379	2.8E-14	4.4E-08	3.5E-14	20.7303	atgcatcgacCtggtattgca	m5C_54848
chr16	2959203	+	C	37	10	0.27	0	0	T	27	M	0.154	0.43	6.7E-14	2E-06	8.3E-14	20.2815	taacttgagcCtaggaatgta	m5C_54830
chr16	3132189	+	C	71	28	0.394	0	0	T	43	M	0.289	0.511	0	2.6E-10	0	161.802	aggggagataCcatgatcacg	m5C_54804
chr16	3132190	+	C	71	15	0.211	0	0	T	56	M	0.132	0.32	0	6E-13	0	39.7273	ggggagatacCatgatcacgg	m5C_54915
chr16	3132198	+	C	78	23	0.295	0	0	T	55	M	0.205	0.404	0	4.3E-11	0	94.4108	accatgatcaCggaggtgggt	m5C_54949
chr16	3200677	+	C	133	34	0.256	0	0	T	99	M	0.189	0.336	0	0	0	128.58	agccgcagggCcgcgtggcct	m5C_54913
chr16	3200678	+	C	140	34	0.243	0	0	T	106	M	0.179	0.32	0	0	0	121.932	gccgcagggcCgcgtggccta	m5C_54944
chr16	3200686	+	C	218	64	0.294	0	0	T	154	M	0.237	0.357	0	0	0	303.511	gccgcgtggcCtaatggataa	m5C_54842
chr16	3200708	+	C	200	66	0.33	0	0	T	134	M	0.269	0.398	0	0	0	354.517	gcgtctgattCcggatcagaa	m5C_54917
chr16	3200714	+	C	179	68	0.38	0	0	T	111	M	0.312	0.453	0	0	0	424.339	gattccggatCagaagattga	m5C_54936
chr16	3200737	+	C	175	51	0.295	2	0.011	T	122	M	0.232	0.367	0	0	0	236.544	gttcgagtccCttcgtggtcg	m5C_54779
chr16	3200740	+	C	165	49	0.297	0	0	T	116	M	0.233	0.371	0	0	0	227.862	cgagtcccttCgtggtcgttg	m5C_54773
chr16	3200746	+	C	156	40	0.256	0	0	T	116	M	0.194	0.33	0	0	0	155.455	ccttcgtggtCgttgccatgt	m5C_54803
chr16	3200752	+	C	130	33	0.254	0	0	T	97	M	0.187	0.335	0	0	0	123.315	tggtcgttgcCatgttaacgt	m5C_54777
chr16	3200760	+	C	116	26	0.224	0	0	T	90	M	0.158	0.308	0	0	0	82.0542	gccatgttaaCgttttcttcc	m5C_54864
chr16	3203052	+	C	34	13	0.382	0	0	T	21	M	0.239	0.55	0	6.7E-06	0	62.1399	tgcggagtttCcgaagggtta	m5C_54776
chr16	3203053	+	C	32	23	0.719	0	0	C	23	M	0.546	0.844	0	7.6E-05	0	251.276	gcggagtttcCgaagggttac	m5C_54940
chr16	3203063	+	C	33	12	0.364	0	0	T	21	M	0.222	0.534	0	4.6E-06	0	53.2487	cgaagggttaCcaaaaaggct	m5C_54896
chr16	3207407	-	C	76	17	0.224	0	0	T	59	M	0.145	0.329	0	2.2E-12	0	49.1676	ccacgttgggCgtgagatttt	m5C_54982
chr16	3207414	-	C	75	31	0.413	0	0	T	44	M	0.309	0.526	0	6.4E-10	0	191.441	tcgagccccaCgttgggcgtg	m5C_55015
chr16	3207419	-	C	73	21	0.288	0	0	T	52	M	0.197	0.4	0	1.8E-11	0	82.5367	tgggttcgagCcccacgttgg	m5C_55084
chr16	3207466	-	C	52	11	0.212	0	0	T	41	M	0.122	0.34	1.4E-13	4.4E-10	1.7E-13	17.3019	ccggctagctCagtcggtaga	m5C_55066
chr16	3207475	-	C	34	10	0.294	0	0	T	24	M	0.168	0.462	2.3E-14	2E-06	2.9E-14	22.9564	catagccgccCggctagctca	m5C_54977
chr16	3208927	+	C	31	14	0.452	0	0	T	17	M	0.292	0.622	0	9.2E-06	0	81.6522	cgtcctggctCgttggtctag	m5C_54955
chr16	3208934	+	C	35	14	0.4	0	0	T	21	M	0.256	0.564	0	9.2E-06	0	71.5414	gctcgttggtCtaggggtatg	m5C_54937
chr16	3208950	+	C	34	13	0.382	0	0	T	21	M	0.239	0.55	0	6.7E-06	0	62.1399	gtatgattctCgctttgggtg	m5C_54853
chr16	3208969	+	C	32	18	0.562	0	0	C	18	M	0.393	0.718	0	2.7E-05	0	141.571	tgcgagaggtCccgggttcaa	m5C_54866
chr16	3208983	+	C	32	10	0.312	0	0	T	22	M	0.18	0.486	1.1E-14	2E-06	1.3E-14	25.0836	ggttcaaatcCcggacgagcc	m5C_54898
chr16	3208984	+	C	33	14	0.424	0	0	T	19	M	0.272	0.592	0	9.2E-06	0	76.259	gttcaaatccCggacgagccc	m5C_54797
chr16	3208993	+	C	31	11	0.355	0	0	T	20	M	0.211	0.531	1.1E-16	3.1E-06	1.5E-16	37.0595	ccggacgagcCcgcttttttt	m5C_54859
chr16	3208996	+	C	31	10	0.323	0	0	T	21	M	0.186	0.499	7.1E-15	2E-06	9E-15	26.2725	gacgagcccgCttttttttt	m5C_54821
chr16	3214945	+	C	42	11	0.262	0	0	T	31	M	0.153	0.411	8.4E-15	4.4E-08	1.1E-14	23.69	atcagcctggCtagctcagtc	m5C_54814
chr16	3214951	+	C	43	11	0.256	0	0	T	32	M	0.149	0.402	1.2E-14	4.4E-08	1.5E-14	22.8887	ctggctagctCagtcggcaaa	m5C_54796
chr16	3214955	+	C	45	11	0.244	0	0	T	34	M	0.142	0.387	2.1E-14	4.4E-08	2.7E-14	21.4112	ctagctcagtCggcaaagcat	m5C_54822
chr16	3225702	+	C	70	22	0.314	0	0	T	48	M	0.218	0.43	0	2.8E-11	0	95.7534	gcccggctagCtcagtcggta	m5C_54890
chr16	3225704	+	C	72	18	0.25	0	0	T	54	M	0.164	0.361	0	4E-12	0	59.1801	ccggctagctCagtcggtaga	m5C_54914
chr16	3225716	+	C	70	21	0.3	0	0	T	49	M	0.205	0.415	0	1.8E-11	0	86.2531	gtcggtagagCatgagactct	m5C_54958
chr16	3225723	+	C	47	13	0.277	0	0	T	34	M	0.169	0.418	0	1.2E-07	0	44.0347	gagcatgagaCtcttaatctc	m5C_54925

chr16	3225725	+	C	39	16	0.41	0	0	T	23	M	0.271	0.566	0	1.7E-05	0	86.6531	gcatgagactCttaatctcag	m5C_54865
chr16	3225739	+	C	37	7	0.25	9	0.243	T	21	M	0.127	0.434	2.6E-10	3.4E-05	2.9E-10	8.50516	atctcagggtCgtgggttcga	m5C_54902
chr16	3230615	-	C	35	15	0.429	0	0	T	20	M	0.28	0.591	0	1.2E-05	0	83.953	ccggctagctCagtcgataga	m5C_55096
chr16	3230617	-	C	33	7	0.212	0	0	T	26	M	0.107	0.378	1.1E-09	3.4E-07	1.2E-09	6.69165	gcccggctagCtcagtcgata	m5C_55093
chr16	3232652	-	C	38	16	0.421	0	0	T	22	M	0.279	0.578	0	1.7E-05	0	89.1273	gtcccgggttCaaatcccgga	m5C_55035
chr16	3232659	-	C	123	28	0.228	0	0	T	95	M	0.162	0.309	0	0	0	90.974	gcgagaggtcCcgggttcaaa	m5C_55000
chr16	3232660	-	C	116	42	0.362	0	0	T	74	M	0.28	0.453	0	8.4E-15	0	235.477	tgcgagaggtCccgggttcaa	m5C_54979
chr16	3232695	-	C	33	9	0.273	0	0	T	24	M	0.151	0.442	7.5E-13	1.2E-06	9E-13	16.4418	gctcgttggtCtaggggtatg	m5C_54995
chr16	3234175	-	C	93	19	0.204	0	0	T	74	M	0.135	0.297	0	8.9E-16	0	51.2526	atgattctcgCtttgggtgcg	m5C_55103
chr16	3234193	-	C	40	13	0.325	0	0	T	27	M	0.201	0.48	0	1.2E-07	0	52.2192	gctcgttggtCtaggggtatg	m5C_55055
chr16	3241502	+	C	64	17	0.266	0	0	T	47	M	0.173	0.385	0	1.8E-10	0	58.8072	cccagggaagCccggctagct	m5C_54931
chr16	3241503	+	C	76	24	0.316	0	0	T	52	M	0.222	0.427	0	6.4E-11	0	106.707	ccagggaagcCcggctagctc	m5C_54837
chr16	3241504	+	C	75	19	0.253	0	0	T	56	M	0.169	0.362	0	6.9E-12	0	64.0677	cagggaagccCggctagctca	m5C_54910
chr16	3241507	+	C	124	35	0.282	0	0	T	89	M	0.211	0.367	0	0	0	147.352	ggaagcccggCtagctcagtc	m5C_54885
chr16	3241513	+	C	152	43	0.283	0	0	T	109	M	0.217	0.359	0	0	0	186.902	ccggctagctCagtcggtaga	m5C_54813
chr16	3241517	+	C	167	68	0.407	0	0	T	99	M	0.336	0.483	0	0	0	456.374	ctagctcagtCggtagagcat	m5C_54792
chr16	3241532	+	C	120	27	0.225	0	0	T	93	M	0.159	0.308	0	0	0	86.1249	gagcatgggaCtcttaatctc	m5C_54928
chr16	3241534	+	C	71	30	0.423	0	0	T	41	M	0.315	0.538	0	4.8E-10	0	188.717	gcatgggactCttaatctcag	m5C_54872
chr16	3241540	+	C	39	11	0.282	0	0	T	28	M	0.165	0.438	3.1E-15	3.1E-06	4E-15	26.4001	gactcttaatCtcagggtcgt	m5C_54924
chr16	3241572	+	C	35	21	0.6	0	0	C	21	M	0.436	0.744	0	5.2E-05	0	183.004	ccacgttgggCgacttgtttt	m5C_54880
chr16	3246165	+	C	31	13	0.419	0	0	T	18	M	0.264	0.592	0	6.7E-06	0	68.6798	ccagctagctCagccggtaga	m5C_54858
chr16	3587267	+	C	48	13	0.271	0	0	T	35	M	0.166	0.41	0	1.2E-07	0	43.0711	aaaaaaacaaCaacacaggat	m5C_54903
chr16	3587272	+	C	44	10	0.227	0	0	T	34	M	0.128	0.37	5.7E-13	2.4E-08	6.8E-13	15.7274	aacaacaacaCaggattgaat	m5C_54833
chr16	11933683	-	C	41	22	0.537	0	0	C	22	M	0.387	0.679	0	2.3E-06	0	170.483	aaagaaggaaCagaccccaga	m5C_53282
chr16	11933779	-	C	57	19	0.333	0	0	T	38	M	0.225	0.463	0	2.4E-08	0	85.4758	gaagaagaggCagcaggctag	m5C_53275
chr16	11933803	-	C	71	23	0.324	0	0	T	48	M	0.227	0.439	0	4.3E-11	0	104.222	ttttgaaaaaCaaaaggagag	m5C_53256
chr16	11990525	-	C	99	23	0.232	0	0	T	76	M	0.16	0.325	0	1E-14	0	73.6414	gaggaaagtgCccatgaaatg	m5C_53276
chr16	11990538	-	C	57	12	0.211	0	0	T	45	M	0.125	0.333	1.6E-14	8.8E-10	2E-14	20.643	tggaaggccgCcagaggaaag	m5C_53267
chr16	11990540	-	C	53	19	0.358	0	0	T	34	M	0.243	0.493	0	2.4E-08	0	92.3467	gatggaaggcCgccagaggaa	m5C_53254
chr16	11990541	-	C	52	24	0.462	0	0	T	28	M	0.333	0.595	0	1.2E-07	0	160.033	agatggaaggCcgccagagga	m5C_53261
chr16	11990557	-	C	43	12	0.279	0	0	T	31	M	0.167	0.427	2.2E-16	7.5E-08	2.9E-16	31.457	ggggtggttcCttgggagatg	m5C_53270
chr16	11990570	-	C	40	10	0.25	0	0	T	30	M	0.142	0.402	1.8E-13	2.4E-08	2.2E-13	18.0914	gaagcagagcCagggggtggt	m5C_53258
chr16	11990571	-	C	37	9	0.243	0	0	T	28	M	0.134	0.401	2.7E-12	1.2E-06	3.2E-12	13.907	tgaagcagagCcagggggtgg	m5C_53253
chr16	11990576	-	C	43	14	0.326	0	0	T	29	M	0.205	0.475	0	1.9E-07	0	57.3807	ataagtgaagCagagccaggg	m5C_53260
chr16	14379754	+	C	43	16	0.372	0	0	T	27	M	0.244	0.521	0	4E-07	0	78.0036	aacgcaggcgCggtggccaag	m5C_53113
chr16	14379760	+	C	47	21	0.447	0	0	T	26	M	0.314	0.588	0	1.8E-06	0	131.925	ggcgcggtggCcaagtggtaa	m5C_53111
chr16	14379761	+	C	51	16	0.314	0	0	T	35	M	0.203	0.45	0	7.3E-09	0	65.048	gcgcggtggcCaagtggtaag	m5C_53117
chr16	14379773	+	C	42	17	0.405	0	0	T	25	M	0.27	0.555	0	5.6E-07	0	91.945	agtggtaaggCgtcggtctcg	m5C_53112
chr16	14379776	+	C	42	13	0.31	0	0	T	29	M	0.191	0.46	0	1.2E-07	0	49.5844	ggtaaggcgtCggtctcgtaa	m5C_53115
chr16	14379780	+	C	54	20	0.37	0	0	T	34	M	0.254	0.504	0	3.4E-08	0	101.693	aggcgtcggtCtcgtaaaccg	m5C_53118
chr16	14379788	+	C	71	20	0.282	0	0	T	51	M	0.19	0.395	0	1.1E-11	0	76.1478	gtctcgtaaaCcgaagatcac	m5C_53109
chr16	14379838	+	C	35	10	0.286	0	0	T	25	M	0.163	0.451	3.4E-14	2E-06	4.2E-14	21.9991	caaccttcagCtataggtgg	m5C_53125
chr16	14379852	+	C	36	14	0.389	0	0	T	22	M	0.248	0.551	0	9.2E-06	0	69.397	agggtggagaCtttttaggag	m5C_53108
chr16	19510059	+	C	63	20	0.317	0	0	T	43	M	0.216	0.44	0	6.9E-10	0	86.3461	ttttgacagtCtctacggaga	m5C_53167
chr16	19510061	+	C	66	18	0.273	0	0	T	48	M	0.18	0.39	0	2.9E-10	0	64.8115	ttgacagtctCtacggagact	m5C_53174
chr16	19510070	+	C	51	32	0.627	0	0	C	32	M	0.49	0.747	0	7.2E-07	0	313.76	tctacggagaCtgaataagaa	m5C_53176
chr16	22207039	-	C	336	73	0.217	0	0	T	263	M	0.176	0.264	0	0	0	257.685	cgaatcccacCgctgccacac	m5C_53526
chr16	22207042	-	C	342	92	0.269	0	0	T	250	M	0.225	0.318	0	0	0	413.567	gttcgaatccCaccgctgcca	m5C_53552
chr16	22207049	-	C	357	87	0.244	1	0.003	T	269	M	0.203	0.292	0	0	0	352.571	ggcgtgggttCgaatcccacc	m5C_53547
chr16	22207057	-	C	363	84	0.238	10	0.028	T	269	M	0.197	0.285	0	0	0	330.133	atttcgatggCgtgggttcga	m5C_53518
chr16	22207063	-	C	395	89	0.225	0	0	T	306	M	0.187	0.269	0	0	0	332.636	ccagtcatttCgatggcgtgg	m5C_53560
chr16	22207075	-	C	625	137	0.219	0	0	T	488	M	0.189	0.253	0	0	0	516.585	tggatttaggCtccagtcatt	m5C_53532
chr16	22207086	-	C	874	238	0.273	2	0.002	T	634	M	0.244	0.303	0	0	0	1163.4	gtctaaggcgCtggatttagg	m5C_53567
chr16	22207094	-	C	958	245	0.256	2	0.002	T	711	M	0.23	0.285	0	0	0	1125.11	gccgagtggtCtaaggcgctg	m5C_53556
chr16	22207103	-	C	985	277	0.282	1	0.001	T	707	M	0.254	0.31	0	0	0	1408.8	ggtagcgtggCcgagtggtct	m5C_53527
chr16	22207118	-	C	40	21	0.525	0	0	C	21	M	0.375	0.671	0	5.2E-05	0	157.488	gccctacgatCgggaggtagc	m5C_53538
chr16	22308476	+	C	148	59	0.399	0	0	T	89	M	0.323	0.479	0	0	0	381.484	cgtggccgagCggtctaaggc	m5C_53484
chr16	22308488	+	C	90	18	0.2	0	0	T	72	M	0.13	0.294	0	4.4E-16	0	46.9523	gtctaaggcgCtggattaagg	m5C_53489
chr16	22308502	+	C	78	17	0.218	0	0	T	61	M	0.141	0.322	0	2.2E-12	0	47.8608	attaaggctcCagtctcttcg	m5C_53487
chr16	22308506	+	C	74	18	0.243	0	0	T	56	M	0.16	0.352	0	4E-12	0	57.5149	aggctccagtCtcttcggggg	m5C_53490
chr16	22308511	+	C	33	8	0.242	0	0	T	25	M	0.128	0.41	3.1E-11	6.8E-07	3.6E-11	10.7866	ccagtctcttCgggggcgtgg	m5C_53498
chr16	22308530	+	C	48	13	0.271	0	0	T	35	M	0.166	0.41	0	1.2E-07	0	43.0711	gggttcgaatCccaccgctgc	m5C_53506
chr16	22308532	+	C	69	18	0.261	0	0	T	51	M	0.172	0.375	0	2.9E-10	0	61.8675	gttcgaatccCaccgctgcca	m5C_53510
chr16	22308534	+	C	69	15	0.217	0	0	T	54	M	0.136	0.328	0	6E-11	0	40.9219	tcgaatcccaCcgctgccagg	m5C_53505
chr16	22308540	+	C	98	31	0.316	0	0	T	67	M	0.233	0.414	0	3.7E-13	0	144.255	cccaccgctgCcagggttgct	m5C_53483
chr16	22308541	+	C	97	27	0.278	0	0	T	70	M	0.199	0.375	0	7.2E-14	0	107.412	ccaccgctgcCagggttgctg	m5C_53486
chr16	22308553	+	C	87	27	0.31	0	0	T	60	M	0.223	0.414	0	4E-12	0	120.361	gggttgctgtCtttttgtccc	m5C_53499
chr16	23443739	-	C	44	34	0.773	0	0	C	34	M	0.63	0.872	0	2.2E-05	0	428.45	gggagctgctCtgctatgtac	m5C_53568
chr16	23443744	-	C	44	35	0.795	0	0	C	35	M	0.655	0.888	0	2.5E-05	0	458.494	agtgaggggagCtgctctgcta	m5C_53545
chr16	25247808	-	C	40	9	0.225	0	0	T	31	M	0.123	0.375	6.4E-12	1.2E-08	7.6E-12	12.4041	ggctgtggagCgtgatataga	m5C_53317
chr16	28391960	-	C	37	8	0.216	0	0	T	29	M	0.114	0.372	9.7E-11	6.8E-07	1.1E-10	9.12273	ccgcaccatgCtggttaggtg	m5C_53472
chr16	28391964	-	C	36	9	0.25	0	0	T	27	M	0.138	0.411	2E-12	1.2E-06	2.4E-12	14.4765	aagtccgcacCatgctggtta	m5C_53463
chr16	28391967	-	C	37	10	0.27	0	0	T	27	M	0.154	0.43	6.7E-14	2E-06	8.3E-14	20.2815	acaaagtccgCaccatgctgg	m5C_53447
chr16	28391970	-	C	36	11	0.306	0	0	T	25	M	0.18	0.469	1E-15	3.1E-06	1.3E-15	29.7077	ctgacaaagtCcgcaccatgc	m5C_53450
chr16	28548798	-	C	35	18	0.514	0	0	C	18	M	0.356	0.67	0	2.7E-05	0	128.047	gagtgagaggCtgggaatgga	m5C_53449
chr16	28548817	-	C	37	16	0.432	0	0	T	21	M	0.287	0.591	0	1.7E-05	0	91.7488	agcaaaagtgCaagatgggga	m5C_53467
chr16	28549371	-	C	86	19	0.221	0	0	T	67	M	0.146	0.319	0	8.4E-14	0	55.5742	ctgcagaattCagagaggaag	m5C_53474
chr16	28549385	-	C	88	35	0.398	0	0	T	53	M	0.302	0.502	0	5.6E-11	0	211.271	aactggtgacCaagctgcaga	m5C_53446
chr16	28549393	-	C	47	11	0.234	0	0	T	36	M	0.136	0.372	3.8E-14	4.4E-08	4.7E-14	20.0856	cgagaggaaaCtggtgaccaa	m5C_53455
chr16	28550279	-	C	36	21	0.583	0	0	C	21	M	0.422	0.729	0	5.2E-05	0	177.24	aaagcagagaCagacaaagcg	m5C_53478
chr16	28744703	-	C	33	14	0.424	0	0	T	19	M	0.272	0.592	0	9.2E-06	0	76.259	caaggttggtCtgggcgcagt	m5C_53480
chr16	28856114	-	C	127	29	0.228	0	0	T	98	M	0.164	0.309	0	0	0	95.0937	gatggtggaaCtggtggaact	m5C_53482
chr16	28856128	-	C	64	23	0.359	0	0	T	41	M	0.253	0.482	0	2.1E-09	0	116.326	gtccaggactCtgagatggtg	m5C_53451
chr16	28856130	-	C	54	12	0.222	0	0	T	42	M	0.132	0.349	7.5E-15	8.8E-10	9.6E-15	22.3614	ctgtccaggaCtctgagatgg	m5C_53461
chr16	30430950	-	C	53	25	0.472	0	0	T	28	M	0.344	0.603	0	1.5E-07	0	171.91	gtggggttgcaCagtagaaaac	m5C_53407
chr16	30431056	-	C	37	23	0.622	0	0	C	23	M	0.461	0.759	0	7.6E-05	0	212.061	ttatgatgggCtggattccct	m5C_53416
chr16	30465374	-	C	47	11	0.234	0	0	T	36	M	0.136	0.372	3.8E-14	4.4E-08	4.7E-14	20.0856	tgatcacaacCagttacagat	m5C_53399
chr16	30465389	-	C	50	13	0.26	0	0	T	37	M	0.159	0.396	0	1.6E-09	0	41.2656	gtgtttacagCgaattgatca	m5C_53380
chr16	30465392	-	C	52	12	0.231	0	0	T	40	M	0.137	0.361	4.4E-15	8.8E-10	5.7E-15	23.6371	gtggtgtttaCagcgaattga	m5C_53396
chr16	30465411	-	C	52	12	0.231	0	0	T	40	M	0.137	0.361	4.4E-15	8.8E-10	5.7E-15	23.6371	aggggctggcCcgagtgcagt	m5C_53378
chr16	30721868	+	C	48	16	0.333	0	0	T	32	M	0.217	0.475	0	4E-07	0	69.3651	ctggcactttCacagttcctt	m5C_53366
chr16	30721879	+	C	51	13	0.255	0	0	T	38	M	0.155	0.389	1.1E-16	1.6E-09	1.5E-16	32.2431	acagttccttCcccaggcagt	m5C_53364
chr16	30721881	+	C	50	12	0.24	0	0	T	38	M	0.143	0.374	2.4E-15	8.8E-10	3.1E-15	25.0697	agttccttccCcaggcagtgg	m5C_53377
chr16	30721882	+	C	53	14	0.264	0	0	T	39	M	0.164	0.396	0	2.8E-09	0	46.0242	gttccttcccCaggcagtggg	m5C_53350
chr16	30721894	+	C	60	29	0.483	0	0	T	31	M	0.362	0.607	0	1.3E-08	0	209.814	ggcagtggggCcaggatttgg	m5C_53376
chr16	30721895	+	C	66	25	0.379	0	0	T	41	M	0.271	0.499	0	4.1E-09	0	135.746	gcagtggggcCaggatttggt	m5C_53356
chr16	30721914	+	C	48	15	0.312	0	0	T	33	M	0.199	0.453	0	2.8E-07	0	59.8365	gtagctggtgCtgagagaaaa	m5C_53369
chr16	30721927	+	C	47	23	0.489	0	0	T	24	M	0.353	0.628	0	2.9E-06	0	162.266	agagaaaaccCttgattgtat	m5C_53330
chr16	30721939	+	C	34	7	0.206	0	0	T	27	M	0.103	0.368	1.4E-09	3.4E-07	1.6E-09	6.4054	tgattgtattCttgccctggg	m5C_53345
chr16	30721943	+	C	33	19	0.576	0	0	C	19	M	0.408	0.728	0	3.4E-05	0	155.067	tgtattcttgCcctgggatta	m5C_53349
chr16	30721944	+	C	34	10	0.294	0	0	T	24	M	0.168	0.462	2.3E-14	2E-06	2.9E-14	22.9564	gtattcttgcCctgggattat	m5C_53341

chr16	30721957	+	C	40	8	0.2	0	0	T	32	M	0.105	0.348	2.1E-10	5.5E-09	2.3E-10	8.13503	gggattatacCagtggcaact	m5C_53374
chr16	30721966	+	C	32	9	0.281	0	0	T	23	M	0.156	0.454	5.3E-13	1.2E-06	6.4E-13	17.1983	ccagtggcaaCtgtcactcaa	m5C_53340
chr16	33961278	+	C	37	18	0.486	0	0	T	19	M	0.334	0.641	0	2.7E-05	0	120.406	tactgtgtggCcgcgcctcc	m5C_56014
chr16	33961279	+	C	36	11	0.306	0	0	T	25	M	0.18	0.469	1E-15	3.1E-06	1.3E-15	29.7077	actgtgtggcCcgcgcctccc	m5C_56050
chr16	33961284	+	C	36	10	0.278	0	0	T	26	M	0.158	0.44	4.8E-14	2E-06	5.9E-14	21.1115	gtggcccgcgCctcccccttc	m5C_55952
chr16	33961285	+	C	36	11	0.306	0	0	T	25	M	0.18	0.469	1E-15	3.1E-06	1.3E-15	29.7077	tggcccgcgcCtcccccttcc	m5C_56024
chr16	33961287	+	C	37	15	0.405	0	0	T	22	M	0.263	0.565	0	1.2E-05	0	79.0389	gcccgcgcctCccccttccga	m5C_55794
chr16	33961291	+	C	41	10	0.244	0	0	T	31	M	0.138	0.393	2.4E-13	2.4E-08	2.9E-13	17.4472	gcgcctccccCttccgagtcg	m5C_55861
chr16	33961294	+	C	46	22	0.478	0	0	T	24	M	0.341	0.619	0	2.3E-06	0	150.148	cctcccccttCcgagtcgggg	m5C_56031
chr16	33961300	+	C	57	23	0.404	0	0	T	34	M	0.286	0.533	0	8.7E-08	0	131.64	ccttccgagtCgggggaggat	m5C_55966
chr16	33962647	+	C	40	16	0.4	0	0	T	24	M	0.263	0.554	0	1.7E-05	0	84.314	acttggaaaaCtgtggtaatt	m5C_55957
chr16	33962658	+	C	49	13	0.265	0	0	T	36	M	0.162	0.403	0	1.2E-07	0	42.1489	tgtggtaattCtagagctaat	m5C_55896
chr16	33962664	+	C	53	24	0.453	0	0	T	29	M	0.327	0.585	0	1.2E-07	0	156.755	aattctagagCtaatgcatgc	m5C_55858
chr16	33962951	+	C	40	19	0.475	0	0	T	21	M	0.329	0.625	0	1E-06	0	125.154	tgagaaatggCtaccacattc	m5C_55827
chr16	33962954	+	C	53	27	0.509	0	0	C	27	M	0.379	0.639	0	2.5E-07	0	204.57	gaaatggctaCcacattcaag	m5C_55886
chr16	33962955	+	C	62	34	0.548	0	0	C	34	M	0.425	0.666	0	4.2E-08	0	289.22	aaatggctacCacattcaagg	m5C_56011
chr16	33962957	+	C	65	32	0.492	0	0	T	33	M	0.375	0.611	0	2.7E-08	0	239.767	atggctaccaCattcaaggaa	m5C_55882
chr16	33962961	+	C	91	43	0.473	0	0	T	48	M	0.373	0.574	0	1.4E-11	0	320.914	ctaccacattCaaggaaggca	m5C_55981
chr16	33962970	+	C	118	30	0.254	0	0	T	88	M	0.184	0.34	0	0	0	110.569	tcaaggaaggCagcaggcatg	m5C_55910
chr16	33962973	+	C	114	36	0.316	0	0	T	78	M	0.238	0.406	0	1E-15	0	171.11	aggaaggcagCaggcatgcaa	m5C_55989
chr16	33962977	+	C	104	36	0.346	0	0	T	68	M	0.262	0.442	0	4.8E-14	0	188.406	aggcagcaggCatgcaaatta	m5C_55844
chr16	33963047	+	C	49	34	0.694	0	0	C	34	M	0.555	0.805	0	2.2E-05	0	377.209	ctttcgaggtCctgtaattgg	m5C_56036
chr16	33963065	+	C	129	26	0.202	0	0	T	103	M	0.141	0.279	0	0	0	73.5376	tggaatgagtCcactttaaat	m5C_55928
chr16	33963066	+	C	126	28	0.222	0	0	T	98	M	0.158	0.302	0	0	0	88.7373	ggaatgagtcCactttaaatc	m5C_55847
chr16	33963068	+	C	138	42	0.304	0	0	T	96	M	0.234	0.386	0	0	0	196.339	aatgagtccaCtttaaatcct	m5C_55922
chr16	33963076	+	C	137	39	0.285	0	0	T	98	M	0.216	0.365	0	0	0	168.313	cactttaaatCctttaacgaa	m5C_55950
chr16	33963083	+	C	125	32	0.256	0	0	T	93	M	0.188	0.339	0	0	0	120.042	aatcctttaaCgaagatccat	m5C_56051
chr16	33963090	+	C	89	58	0.652	0	0	C	58	M	0.548	0.743	0	6.8E-09	0	636.018	taacgaagatCcattggaggg	m5C_55947
chr16	33963091	+	C	91	25	0.275	0	0	T	66	M	0.194	0.374	0	2.9E-14	0	96.7753	aacgaagatcCattggagggc	m5C_55832
chr16	33963101	+	C	95	21	0.221	0	0	T	74	M	0.149	0.314	0	3.1E-15	0	62.7364	cattggagggCaagtttagtg	m5C_55942
chr16	33963112	+	C	79	31	0.392	0	0	T	48	M	0.292	0.503	0	6.4E-10	0	181.119	aagtttagtgCcagcagccgc	m5C_55824
chr16	33963113	+	C	70	19	0.271	0	0	T	51	M	0.181	0.385	0	6.9E-12	0	68.854	agtttagtgcCagcagccgcg	m5C_55859
chr16	33963116	+	C	71	17	0.239	0	0	T	54	M	0.155	0.35	0	2.2E-12	0	52.7708	ttagtgccagCagccgcggta	m5C_55834
chr16	33963119	+	C	77	30	0.39	0	0	T	47	M	0.288	0.501	0	4.8E-10	0	173.053	gtgccagcagCcgcggtaatt	m5C_55935
chr16	33963120	+	C	81	27	0.333	0	0	T	54	M	0.24	0.441	0	4E-12	0	129.755	tgccagcagcCgcggtaattc	m5C_56063
chr16	33963122	+	C	81	27	0.333	0	0	T	54	M	0.24	0.441	0	4E-12	0	129.755	ccagcagccgCggtaattcca	m5C_56042
chr16	33963130	+	C	32	21	0.656	0	0	C	21	M	0.483	0.796	0	5.2E-05	0	202.905	cgcggtaattCcagctccaat	m5C_55980
chr16	33963134	+	C	33	15	0.455	0	0	T	18	M	0.298	0.62	0	1.2E-05	0	89.5281	gtaattccagCtccaatagcg	m5C_55990
chr16	33963161	+	C	52	24	0.462	0	0	T	28	M	0.333	0.595	0	1.2E-07	0	160.033	aaagttgctgCagttaaaaag	m5C_55932
chr16	33963172	+	C	238	54	0.227	0	0	T	184	M	0.178	0.284	0	0	0	192.526	agttaaaaagCtcctagttgg	m5C_56019
chr16	33963174	+	C	268	71	0.265	0	0	T	197	M	0.216	0.321	0	0	0	306.269	ttaaaaagctCctagttggat	m5C_55891
chr16	33963185	+	C	518	171	0.33	0	0	T	347	M	0.291	0.372	0	0	0	995.218	ctagttggatCttgggagcgg	m5C_55801
chr16	33963193	+	C	496	164	0.331	0	0	T	332	M	0.291	0.373	0	0	0	953.441	atcttgggagCgggcgggcag	m5C_56044
chr16	33963197	+	C	406	192	0.473	0	0	T	214	M	0.425	0.521	0	0	0	1631.32	tgggagcgggCgggcagtccg	m5C_55982
chr16	33963201	+	C	126	45	0.357	0	0	T	81	M	0.279	0.444	0	5.6E-16	0	250.958	agcgggcgggCagtccgccat	m5C_55863
chr16	33963205	+	C	31	9	0.29	0	0	T	22	M	0.161	0.466	3.7E-13	1.2E-06	4.4E-13	18.0154	ggcgggcagtCcgccatgagg	m5C_55968
chr16	33963209	+	C	43	24	0.558	0	0	C	24	M	0.411	0.696	0	3.6E-06	0	197.315	ggcagtccgcCatgaggcgag	m5C_55841
chr16	33963216	+	C	135	52	0.385	0	0	T	83	M	0.307	0.469	0	1.1E-16	0	319.646	cgccatgaggCgagcactgc	m5C_55994
chr16	33963220	+	C	175	50	0.286	0	0	T	125	M	0.224	0.357	0	0	0	223.947	atgaggcgagCcactgcccgt	m5C_56020
chr16	33963221	+	C	178	107	0.601	0	0	C	107	M	0.528	0.67	0	0	0	1129.46	tgaggcgagcCactgcccgtc	m5C_56015
chr16	33963223	+	C	203	129	0.635	0	0	C	129	M	0.567	0.699	0	0	0	1463.66	aggcgagccaCtgcccgtccc	m5C_55871
chr16	33963226	+	C	223	135	0.605	0	0	C	135	M	0.54	0.667	0	0	0	1457.91	cgagccactgCccgtccccgc	m5C_56004
chr16	33963227	+	C	231	173	0.749	0	0	C	173	M	0.689	0.8	0	0	0	2384.75	gagccactgcCcgtccccgcc	m5C_55960
chr16	33963228	+	C	232	129	0.558	1	0.004	C	129	M	0.494	0.621	0	0	0	1274.43	agccactgccCgtccccgccc	m5C_55899
chr16	33963231	+	C	261	176	0.677	1	0.004	C	176	M	0.618	0.731	0	0	0	2174.86	cactgcccgtCcccgcccctt	m5C_55944
chr16	33963232	+	C	267	155	0.581	0	0	C	155	M	0.521	0.638	0	0	0	1613.86	actgcccgtcCccgccccttg	m5C_55835
chr16	33963233	+	C	271	163	0.601	0	0	C	163	M	0.542	0.658	0	0	0	1767.43	ctgcccgtccCcgccccttgc	m5C_56008
chr16	33963234	+	C	281	183	0.651	0	0	C	183	M	0.594	0.705	0	0	0	2173.39	tgcccgtcccCgccccttgcc	m5C_55892
chr16	33963236	+	C	272	172	0.632	0	0	C	172	M	0.574	0.687	0	0	0	1973.11	cccgtccccgCcccttgcctc	m5C_56018
chr16	33963237	+	C	276	166	0.601	0	0	C	166	M	0.543	0.657	0	0	0	1801.68	ccgtccccgcCccttgcctct	m5C_56016
chr16	33963238	+	C	261	155	0.594	0	0	C	155	M	0.533	0.652	0	0	0	1653.37	cgtccccgccCcttgcctctt	m5C_56000
chr16	33963239	+	C	260	149	0.573	0	0	C	149	M	0.512	0.632	0	0	0	1526.71	gtccccgcccCttgcctcttg	m5C_55997
chr16	33963243	+	C	266	140	0.526	0	0	C	140	M	0.466	0.586	0	0	0	1305.82	ccgccccttgCctcttggcgc	m5C_55880
chr16	33963244	+	C	272	148	0.544	0	0	C	148	M	0.485	0.602	0	0	0	1434.78	cgccccttgcCtcttggcgcc	m5C_56053
chr16	33963246	+	C	288	161	0.559	0	0	C	161	M	0.501	0.615	0	0	0	1614.12	ccccttgcctCttggcgcccc	m5C_55787
chr16	33963251	+	C	339	159	0.469	0	0	T	180	M	0.417	0.522	0	0	0	1324.62	tgcctcttggCgcccctcga	m5C_55883
chr16	33963253	+	C	319	156	0.489	0	0	T	163	M	0.435	0.544	0	0	0	1356.04	cctcttggcgCcccctcgatg	m5C_55889
chr16	33963254	+	C	315	152	0.483	0	0	T	163	M	0.428	0.538	0	0	0	1300.82	ctcttggcgcCccctcgatgc	m5C_55951
chr16	33963255	+	C	307	154	0.502	0	0	C	154	M	0.446	0.557	0	0	0	1373.75	tcttggcgccCcctcgatgct	m5C_55809
chr16	33963256	+	C	298	153	0.513	0	0	C	153	M	0.457	0.57	0	0	0	1398.01	cttggcgcccCctcgatgctc	m5C_56029
chr16	33963257	+	C	296	122	0.412	0	0	T	174	M	0.358	0.469	0	0	0	872.449	ttggcgccccCtcgatgctct	m5C_55972
chr16	33963259	+	C	258	117	0.453	0	0	T	141	M	0.394	0.514	0	0	0	921.648	ggcgcccccctCgatgctcta	m5C_55979
chr16	33963264	+	C	218	70	0.321	0	0	T	148	M	0.263	0.386	0	0	0	367.752	cccctcgatgCtcttagctga	m5C_55884
chr16	33963266	+	C	218	92	0.422	0	0	T	126	M	0.358	0.488	0	0	0	659.388	cctcgatgctCttagctgagt	m5C_55873
chr16	33963271	+	C	168	47	0.28	0	0	T	121	M	0.217	0.352	0	0	0	204.346	atgctcttagCtgagtgttcc	m5C_56002
chr16	33963280	+	C	99	39	0.394	0	0	T	60	M	0.303	0.492	0	4.9E-12	0	236.637	gctgagtgttCcgcggcccaa	m5C_55933
chr16	33963281	+	C	89	30	0.337	0	0	T	59	M	0.247	0.44	0	1.2E-11	0	148.456	ctgagtgttcCgcggcccaaa	m5C_55872
chr16	33963283	+	C	84	30	0.357	0	0	T	54	M	0.263	0.464	0	1.2E-11	0	157.795	gagtgttccgCggcccaaagc	m5C_55867
chr16	33963293	+	C	204	57	0.279	0	0	T	147	M	0.222	0.345	0	0	0	253.478	cggcccaaagCgtttactttg	m5C_55796
chr16	33963299	+	C	359	118	0.329	0	0	T	241	M	0.282	0.379	0	0	0	665.842	aaagcgtttaCtttgaaaaaa	m5C_55843
chr16	33963330	+	C	765	204	0.267	0	0	T	561	M	0.237	0.299	0	0	0	965.132	caaagcaggtCcaagccgcct	m5C_55913
chr16	33963331	+	C	781	214	0.274	0	0	T	567	M	0.244	0.306	0	0	0	1043.85	aaagcaggtcCaagccgcctg	m5C_55792
chr16	33963336	+	C	1211	258	0.213	0	0	T	953	M	0.191	0.237	0	0	0	985.101	aggtccaagcCgcctggatac	m5C_55934
chr16	33963339	+	C	1434	354	0.247	0	0	T	1080	M	0.225	0.27	0	0	0	1594.7	tccaagccgcCtggataccgc	m5C_55943
chr16	33963347	+	C	1931	397	0.206	0	0	T	1534	M	0.188	0.224	0	0	0	1493.99	gcctggatacCgcagctagga	m5C_56037
chr16	33963352	+	C	2157	556	0.258	0	0	T	1601	M	0.24	0.277	0	0	0	2666	gataccgcagCtaggaataat	m5C_56027
chr16	33963372	+	C	1824	609	0.334	0	0	T	1215	M	0.313	0.356	0	0	0	3807.56	tggaataggaCcgcggttcta	m5C_55878
chr16	33963373	+	C	1759	399	0.227	0	0	T	1360	M	0.208	0.247	0	0	0	1658.8	ggaataggacCgcggttctat	m5C_56068
chr16	33963380	+	C	884	248	0.281	0	0	T	636	M	0.252	0.311	0	0	0	1249.55	gaccgcggttCtattttgttg	m5C_55953
chr16	33963401	+	C	305	92	0.302	0	0	T	213	M	0.253	0.355	0	0	0	465.26	gtttatggaaCtgaggccatg	m5C_56045
chr16	33963407	+	C	285	126	0.442	0	0	T	159	M	0.386	0.5	0	0	0	971.7	ggaactgaggCcatgattaag	m5C_55890
chr16	33963408	+	C	286	77	0.269	0	0	T	209	M	0.221	0.323	0	0	0	340.544	gaactgaggcCatgattaaga	m5C_56056
chr16	33963423	+	C	175	97	0.554	0	0	C	97	M	0.48	0.626	0	0	0	931.709	ttaagagggaCagctgggggc	m5C_56047
chr16	33963433	+	C	142	59	0.415	0	0	T	83	M	0.338	0.498	0	0	0	398.493	cagctgggggCatttgtattg	m5C_55799
chr16	33963447	+	C	375	100	0.267	0	0	T	275	M	0.224	0.314	0	0	0	448.878	tgtattgcacCgctagaggtg	m5C_55993
chr16	33963469	+	C	398	97	0.244	0	0	T	301	M	0.204	0.288	0	0	0	395.993	aattcttggaCcggcgcagtt	m5C_55798
chr16	33963470	+	C	376	81	0.215	0	0	T	295	M	0.177	0.26	0	0	0	286.511	attcttggacCggcgcagttt	m5C_55866
chr16	33963473	+	C	315	102	0.324	0	0	T	213	M	0.275	0.377	0	0	0	560.033	cttggaccggCgcagtttgga	m5C_56001
chr16	33963484	+	C	219	110	0.502	0	0	C	110	M	0.437	0.568	0	0	0	960.51	gcagtttggaCcagagtgaaa	m5C_55931
chr16	33963485	+	C	235	120	0.511	0	0	C	120	M	0.447	0.574	0	0	0	1072.97	cagtttggacCagagtgaaag	m5C_55828
chr16	33963496	+	C	243	122	0.502	0	0	C	122	M	0.44	0.564	0	0	0	1072.75	agagtgaaagCatttgccaag	m5C_55991

chr16	33963502	+	C	179	113	0.631	0	0	C	113	M	0.559	0.699	0	1.1E-16	0	1262.3	aaagcatttgCcaagaatgtt	m5C_55959
chr16	33963503	+	C	177	89	0.503	0	0	C	89	M	0.43	0.576	0	0	0	765.206	aagcatttgcCaagaatgttt	m5C_55978
chr16	33963515	+	C	119	92	0.773	0	0	C	92	M	0.69	0.839	0	1.7E-10	0	1269.62	agaatgttttCattaatcaag	m5C_55946
chr16	33963522	+	C	145	104	0.717	0	0	C	104	M	0.639	0.784	0	2.1E-13	0	1329.26	tttcattaatCaagaatgaaa	m5C_55807
chr16	33963535	+	C	1322	371	0.282	7	0.005	T	944	M	0.258	0.307	0	0	0	1917.82	gaatgaaagtCggaggttcga	m5C_55927
chr16	33963543	+	C	1282	382	0.298	0	0	T	900	M	0.274	0.324	0	0	0	2090.07	gtcggaggttCgaagacgatc	m5C_55803
chr16	33963549	+	C	1015	416	0.41	0	0	T	599	M	0.38	0.44	0	0	0	3161.52	ggttcgaagaCgatcagatac	m5C_55845
chr16	33963553	+	C	609	227	0.373	0	0	T	382	M	0.335	0.412	0	0	0	1522.03	cgaagacgatCagataccgtg	m5C_55919
chr16	33963559	+	C	214	89	0.416	0	0	T	125	M	0.352	0.483	0	0	0	626.386	cgatcagataCcgtggtagtt	m5C_56049
chr16	33963560	+	C	171	59	0.345	0	0	T	112	M	0.278	0.419	0	0	0	327.907	gatcagatacCgtggtagttc	m5C_56033
chr16	33963570	+	C	157	55	0.35	0	0	T	102	M	0.28	0.428	0	0	0	308.086	cgtggtagttCtgaccataaa	m5C_55995
chr16	33963574	+	C	185	56	0.303	0	0	T	129	M	0.241	0.372	0	0	0	269.994	gtagttctgaCcataaacgat	m5C_56043
chr16	33963575	+	C	185	55	0.297	0	0	T	130	M	0.236	0.367	0	0	0	259.709	tagttctgacCataaacgatg	m5C_55930
chr16	33963586	+	C	206	65	0.316	0	0	T	141	M	0.256	0.382	0	0	0	332.723	ataaacgatgCcgactggcaa	m5C_55926
chr16	33963587	+	C	204	49	0.24	0	0	T	155	M	0.187	0.303	0	0	0	182.986	taaacgatgcCgactggcaac	m5C_55974
chr16	33963590	+	C	217	55	0.253	0	0	T	162	M	0.2	0.315	0	0	0	220.235	acgatgccgaCtggcaacgtg	m5C_55918
chr16	33963594	+	C	170	67	0.394	0	0	T	103	M	0.324	0.469	0	0	0	433.863	tgccgactggCaacgtggcgg	m5C_56065
chr16	33963602	+	C	150	53	0.353	0	0	T	97	M	0.281	0.433	0	0	0	298.254	ggcaacgtggCggtgttattc	m5C_55849
chr16	33963612	+	C	56	14	0.25	0	0	T	42	M	0.155	0.377	0	2.8E-09	0	43.4474	cggtgttattCccatgacccg	m5C_55887
chr16	33963613	+	C	44	13	0.295	0	0	T	31	M	0.182	0.442	0	1.2E-07	0	47.2039	ggtgttattcCcatgacccgc	m5C_55819
chr16	33963614	+	C	45	13	0.289	0	0	T	32	M	0.177	0.434	0	1.2E-07	0	46.0978	gtgttattccCatgacccgct	m5C_55814
chr16	33963666	+	C	287	101	0.352	0	0	T	186	M	0.299	0.409	0	0	0	603.882	tttttaggttCcggggggagt	m5C_55958
chr16	33963667	+	C	333	115	0.347	2	0.006	T	216	M	0.298	0.4	0	0	0	685.747	ttttaggttcCggggggagta	m5C_55908
chr16	33963684	+	C	624	142	0.228	0	0	T	482	M	0.196	0.262	0	0	0	557.757	agtacggttgCaaagctgaaa	m5C_55831
chr16	33963689	+	C	554	183	0.33	0	0	T	371	M	0.292	0.371	0	0	0	1070.35	ggttgcaaagCtgaaacttaa	m5C_55888
chr16	33963723	+	C	656	163	0.248	0	0	T	493	M	0.217	0.283	0	0	0	707.209	agggcaccacCaggagtggag	m5C_55909
chr16	33963734	+	C	857	281	0.328	0	0	T	576	M	0.297	0.36	0	0	0	1670.75	aggagtggagCctgcggctta	m5C_55808
chr16	33963735	+	C	841	233	0.277	0	0	T	608	M	0.248	0.308	0	0	0	1155.07	ggagtggagcCtgcggcttaa	m5C_55848
chr16	33963738	+	C	791	208	0.263	0	0	T	583	M	0.233	0.295	0	0	0	971.263	gtggagcctgCggcttaattt	m5C_55897
chr16	33963741	+	C	653	199	0.305	0	0	T	454	M	0.271	0.341	0	0	0	1077.26	gagcctgcggCttaatttgac	m5C_55854
chr16	33963910	+	C	148	101	0.682	0	0	C	101	M	0.604	0.752	0	1.3E-13	0	1219.32	taactagttaCgtgacccccg	m5C_55907
chr16	33963915	+	C	196	101	0.515	0	0	C	101	M	0.446	0.584	0	0	0	900.354	agttacgtgaCccccgagcgg	m5C_55829
chr16	33963916	+	C	209	139	0.665	0	0	C	139	M	0.599	0.726	0	0	0	1664.15	gttacgtgacCcccgagcggt	m5C_55833
chr16	33963917	+	C	226	125	0.553	0	0	C	125	M	0.488	0.616	0	0	0	1219.82	ttacgtgaccCccgagcggtc	m5C_55842
chr16	33963918	+	C	229	157	0.686	0	0	C	157	M	0.623	0.742	0	0	0	1955.63	tacgtgacccCcgagcggtcg	m5C_56059
chr16	33963919	+	C	243	129	0.531	0	0	C	129	M	0.468	0.593	0	0	0	1207.76	acgtgaccccCgagcggtcgg	m5C_56058
chr16	33963923	+	C	274	156	0.569	0	0	C	156	M	0.51	0.627	0	0	0	1591.67	gaccccgagCggtcggcgtc	m5C_55838
chr16	33963927	+	C	281	161	0.573	0	0	C	161	M	0.515	0.629	0	0	0	1656.74	cccgagcggtCggcgtccccc	m5C_55874
chr16	33963930	+	C	264	140	0.53	0	0	C	140	M	0.47	0.59	0	0	0	1316.27	gagcggtcggCgtcccccaac	m5C_55893
chr16	33963933	+	C	240	114	0.475	0	0	T	126	M	0.413	0.538	0	0	0	940.985	cggtcggcgtCcccccaacttc	m5C_56040
chr16	33963934	+	C	203	110	0.542	0	0	C	110	M	0.473	0.609	0	0	0	1041.01	ggtcggcgtcCcccaacttct	m5C_55945
chr16	33963935	+	C	174	112	0.644	0	0	C	112	M	0.57	0.711	0	1.1E-16	0	1277.07	gtcggcgtccCccaacttctt	m5C_56025
chr16	33963936	+	C	166	97	0.584	0	0	C	97	M	0.508	0.657	0	2.2E-16	0	986.073	tcggcgtcccCcaacttctta	m5C_55986
chr16	33963937	+	C	156	59	0.378	0	0	T	97	M	0.306	0.456	0	0	0	360.957	cggcgtccccCaacttcttag	m5C_55921
chr16	33963940	+	C	130	45	0.346	0	0	T	85	M	0.27	0.431	0	0	0	242.864	cgtccccccaaCttcttagagg	m5C_55905
chr16	33963943	+	C	120	45	0.375	0	0	T	75	M	0.294	0.464	0	5.6E-16	0	264.171	cccccaacttCttagagggac	m5C_55826
chr16	33963953	+	C	67	29	0.433	0	0	T	38	M	0.321	0.552	0	1.3E-08	0	186.204	cttagagggaCaagtggcctt	m5C_55789
chr16	33964053	+	C	35	26	0.743	0	0	C	26	M	0.579	0.858	0	0.00013	0	301.238	tcagcgtgtgCctaccctacg	m5C_55894
chr16	33964080	+	C	34	26	0.765	0	0	C	26	M	0.6	0.876	0	0.00013	0	312.018	aggcgggtaaCctgttgaacc	m5C_55900
chr16	33964081	+	C	37	9	0.243	0	0	T	28	M	0.134	0.401	2.7E-12	1.2E-06	3.2E-12	13.907	ggcgggtaacCtgttgaacct	m5C_55911
chr16	33964089	+	C	35	9	0.257	0	0	T	26	M	0.142	0.421	1.5E-12	1.2E-06	1.7E-12	15.0859	acctgttgaaCctcattcgtg	m5C_55813
chr16	33964090	+	C	35	8	0.229	0	0	T	27	M	0.121	0.39	5.6E-11	6.8E-07	6.4E-11	9.89691	cctgttgaacCtcattcgtga	m5C_56006
chr16	33964092	+	C	35	10	0.286	0	0	T	25	M	0.163	0.451	3.4E-14	2E-06	4.2E-14	21.9991	tgttgaacctCattcgtgatg	m5C_55812
chr16	33964509	+	C	35	10	0.286	0	0	T	25	M	0.163	0.451	3.4E-14	2E-06	4.2E-14	21.9991	ccctgagagcCggagaactcg	m5C_55973
chr16	33964518	+	C	52	13	0.25	0	0	T	39	M	0.152	0.382	1.1E-16	1.6E-09	1.5E-16	31.5947	ccggagaactCgggagggaga	m5C_55855
chr16	33964952	+	C	119	30	0.252	0	0	T	89	M	0.183	0.337	0	0	0	109.605	aggtacctagCgcgttctggc	m5C_55825
chr16	33964954	+	C	117	44	0.376	0	0	T	73	M	0.294	0.466	0	1.6E-14	0	258.325	gtacctagcgCgttctggcgc	m5C_55996
chr16	33964958	+	C	118	31	0.263	0	0	T	87	M	0.192	0.349	0	1.1E-16	0	118.847	ctagcgcgttCtggcgcggag	m5C_55939
chr16	33964979	+	C	34	11	0.324	0	0	T	23	M	0.191	0.492	4.4E-16	3.1E-06	5.8E-16	32.3088	gtttaaggacCccttgggggg	m5C_55800
chr16	33965196	+	C	54	13	0.241	0	0	T	41	M	0.146	0.369	2.2E-16	1.6E-09	2.9E-16	29.8003	ggcctgtgcaCcgtgctgtgg	m5C_55917
chr16	33965197	+	C	63	30	0.476	0	0	T	33	M	0.358	0.597	0	1.7E-08	0	214.692	gcctgtgcacCgtgctgtggg	m5C_56052
chr16	33965210	+	C	193	61	0.316	0	0	T	132	M	0.255	0.385	0	0	0	310.613	gctgtgggggCgggaatcccc	m5C_55820
chr16	33965217	+	C	234	60	0.256	0	0	T	174	M	0.205	0.316	0	0	0	245.654	gggcgggaatCcccgggcgcc	m5C_55925
chr16	33965219	+	C	227	66	0.291	0	0	T	161	M	0.236	0.353	0	0	0	310.922	gcgggaatccCcgggcgcccg	m5C_56041
chr16	33965220	+	C	229	58	0.254	1	0.004	T	170	M	0.202	0.315	0	0	0	234.606	cgggaatcccCgggcgcccgt	m5C_55914
chr16	33965224	+	C	196	51	0.26	0	0	T	145	M	0.204	0.326	0	0	0	207.884	aatccccgggCgcccgtgggg	m5C_55853
chr16	33965226	+	C	173	40	0.231	0	0	T	133	M	0.175	0.299	0	0	0	139.711	tccccgggcgCccgtggggtg	m5C_55912
chr16	33965227	+	C	153	45	0.294	0	0	T	108	M	0.228	0.371	0	0	0	204.905	ccccgggcgcCcgtggggtgc	m5C_56046
chr16	33965459	+	C	1011	216	0.214	1	0.001	T	794	M	0.19	0.24	0	0	0	819.428	gcttctgtgtCgatgaagaac	m5C_55788
chr16	33965469	+	C	1012	208	0.206	0	0	T	804	M	0.182	0.232	0	0	0	756.174	cgatgaagaaCgcagctagct	m5C_55915
chr16	33965505	+	C	241	67	0.278	0	0	T	174	M	0.225	0.338	0	0	0	301.855	aattgcaggaCagattgatca	m5C_55936
chr16	33965514	+	C	31	14	0.452	0	0	T	17	M	0.292	0.622	0	9.2E-06	0	81.6522	acagattgatCatcgacactt	m5C_55876
chr16	33965553	+	C	41	12	0.293	0	0	T	29	M	0.176	0.445	1.1E-16	7.5E-08	1.5E-16	33.7134	gggttcctccCagggctttgc	m5C_55851
chr16	33965564	+	C	163	56	0.344	0	0	T	107	M	0.275	0.419	0	0	0	308.01	agggctttgcCtgtctgagcg	m5C_56054
chr16	33965568	+	C	185	57	0.308	0	0	T	128	M	0.246	0.378	0	0	0	280.489	ctttgcctgtCtgagcgtcgc	m5C_55987
chr16	33965578	+	C	170	45	0.265	0	0	T	125	M	0.204	0.336	0	0	0	183.705	ctgagcgtcgCttgccgatca	m5C_55860
chr16	33965582	+	C	44	13	0.295	0	0	T	31	M	0.182	0.442	0	1.2E-07	0	47.2039	gcgtcgcttgCcgatcagtag	m5C_56032
chr16	46920348	+	C	142	29	0.204	0	0	T	113	M	0.146	0.278	0	0	0	84.753	atgatgaggaCagaagggcac	m5C_53668
chr16	46920367	+	C	125	29	0.232	0	0	T	96	M	0.167	0.313	0	0	0	96.6667	actgtctttgCcttgaggaat	m5C_53676
chr16	46920382	+	C	91	39	0.429	0	0	T	52	M	0.332	0.531	0	4.9E-12	0	258.823	aggaatctgaCtgccacgttg	m5C_53687
chr16	47005296	-	C	32	16	0.5	0	0	CT	16	M	0.336	0.664	0	1.7E-05	0	107.618	gtcgaaatggCagaagaagag	m5C_53723
chr16	47005304	-	C	34	16	0.471	0	0	T	18	M	0.315	0.633	0	1.7E-05	0	100.644	gagtagaagtCgaaatggcag	m5C_53717
chr16	47538740	+	C	60	22	0.367	0	0	T	38	M	0.256	0.493	0	1.5E-09	0	112.731	gaggtgggatCccgaggcctc	m5C_53680
chr16	47538741	+	C	59	12	0.203	0	0	T	47	M	0.12	0.323	2.6E-14	8.8E-10	3.3E-14	19.6113	aggtgggatcCcgaggcctct	m5C_53661
chr16	47538747	+	C	63	15	0.238	0	0	T	48	M	0.15	0.356	0	6E-11	0	44.981	gatcccgaggCctctccagtc	m5C_53674
chr16	47538748	+	C	66	21	0.318	0	0	T	45	M	0.218	0.438	0	1E-09	0	91.7652	atcccgaggcCtctccagtcc	m5C_53675
chr16	47538750	+	C	47	18	0.383	0	0	T	29	M	0.258	0.526	0	7.7E-07	0	92.8457	cccgaggcctCtccagtcccc	m5C_53662
chr16	47538753	+	C	33	8	0.242	0	0	T	25	M	0.128	0.41	3.1E-11	6.8E-07	3.6E-11	10.7866	gaggcctctcCagtcccctga	m5C_53658
chr16	49854070	+	C	42	20	0.476	0	0	T	22	M	0.334	0.623	0	1.4E-06	0	133.437	gaaggatggaCagtgagctga	m5C_53594
chr16	50132182	+	C	259	78	0.301	0	0	T	181	M	0.249	0.36	0	0	0	387.701	tgcagctggtCtgatggtagt	m5C_53641
chr16	50132200	+	C	268	56	0.21	1	0.004	T	211	M	0.165	0.263	0	0	0	185.017	agtgggttatCaggacttatt	m5C_53646
chr16	50132205	+	C	227	55	0.242	0	0	T	172	M	0.191	0.302	0	0	0	210.254	gttatcaggaCttattaacat	m5C_53636
chr16	50132213	+	C	58	17	0.293	0	0	T	41	M	0.192	0.42	0	1.1E-08	0	65.2053	gacttattaaCattagtgtca	m5C_53643
chr16	50268892	+	C	35	23	0.657	0	0	C	23	M	0.492	0.792	0	7.6E-05	0	226.098	aggtttgaatCatcttgaaaa	m5C_53648
chr16	57333874	+	C	168	39	0.232	0	0	T	129	M	0.175	0.302	0	0	0	136.283	tcaggatggcCgagcggtcta	m5C_54676
chr16	57333878	+	C	174	59	0.339	0	0	T	115	M	0.273	0.412	0	0	0	322.014	gatggccgagCggtctaaggc	m5C_54727
chr16	57333882	+	C	166	44	0.265	0	0	T	122	M	0.204	0.337	0	0	0	179.329	gccgagcggtCtaaggcgctg	m5C_54691
chr16	57333888	+	C	147	64	0.435	0	0	T	83	M	0.358	0.516	0	1.1E-16	0	458.085	cggtctaaggCgctgcgttca	m5C_54735
chr16	57333893	+	C	115	25	0.217	0	0	T	90	M	0.152	0.301	0	0	0	75.9035	taaggcgctgCgttcaggtcg	m5C_54683
chr16	57333897	+	C	97	28	0.289	0	0	T	69	M	0.208	0.386	0	1.1E-13	0	116.425	gcgctgcgttCaggtcgcagt	m5C_54745
chr16	57333911	+	C	54	25	0.463	0	0	T	29	M	0.337	0.594	0	1.5E-07	0	168.448	tcgcagtctcCctggaggcg	m5C_54693

chr16	57333913	+	C	55	25	0.455	0	0	T	30	M	0.33	0.585	0	1.5E-07	0	165.125	gcagtctcccCtggaggcgtg	m5C_54697
chr16	57333920	+	C	57	18	0.316	0	0	T	39	M	0.21	0.445	0	1.7E-08	0	75.6149	cccctggaggCgtgggttcga	m5C_54674
chr16	57333928	+	C	55	19	0.345	0	0	T	36	M	0.234	0.477	0	2.4E-08	0	88.7776	ggcgtgggttCgaatccccact	m5C_54716
chr16	57333935	+	C	57	17	0.298	0	0	T	40	M	0.195	0.427	0	1.1E-08	0	66.4101	gttcgaatccCacttctgaca	m5C_54744
chr16	57334380	-	C	150	33	0.22	0	0	T	117	M	0.161	0.293	0	0	0	106.365	agctcaattaCttttcctcat	m5C_54762
chr16	57334400	-	C	205	45	0.22	0	0	T	160	M	0.168	0.281	0	0	0	151.467	tcgaatcccaCttctgacaga	m5C_54765
chr16	57334417	-	C	206	53	0.257	0	0	T	153	M	0.202	0.321	0	0	0	214.557	cccctggaggCgtgggttcga	m5C_54750
chr16	57334424	-	C	186	42	0.226	0	0	T	144	M	0.172	0.291	0	0	0	144.16	gcagtctcccCtggaggcgtg	m5C_54748
chr16	57334425	-	C	184	58	0.317	1	0.005	T	125	M	0.254	0.388	0	0	0	294.501	cgcagtctccCctggaggcgt	m5C_54749
chr16	57334426	-	C	188	51	0.271	0	0	T	137	M	0.213	0.339	0	0	0	217.028	tcgcagtctcCctggaggcg	m5C_54769
chr16	57334433	-	C	169	39	0.231	0	0	T	130	M	0.174	0.3	0	0	0	135.452	gttcaggtcgCagtctcccct	m5C_54764
chr16	57334435	-	C	167	40	0.24	0	0	T	127	M	0.181	0.31	0	0	0	144.889	gcgttcaggtCgcagtctccc	m5C_54759
chr16	57697472	-	C	35	7	0.2	0	0	T	28	M	0.1	0.359	1.9E-09	3.4E-07	2.1E-09	6.13884	ggacgaggtgCtgcccgagct	m5C_54747
chr16	57697479	-	C	42	13	0.31	0	0	T	29	M	0.191	0.46	0	1.2E-07	0	49.5844	tgcggctggaCgaggtgctgc	m5C_54752
chr16	58194475	-	C	39	15	0.385	0	0	T	24	M	0.249	0.541	0	1.2E-05	0	74.6735	agctgggtgcCgcggggctcg	m5C_60164
chr16	58194483	-	C	43	19	0.442	0	0	T	24	M	0.304	0.589	0	1E-06	0	115.646	ccagagagagCtgggtgccgc	m5C_60161
chr16	58233655	-	C	46	34	0.739	0	0	C	34	M	0.597	0.844	0	2.2E-05	0	406.236	ctgtagtgtgCaatgccaatc	m5C_60159
chr16	58233665	-	C	47	16	0.34	0	0	T	31	M	0.222	0.483	0	4E-07	0	70.9352	gagttctgggCtgtagtgtgc	m5C_60171
chr16	58233670	-	C	51	24	0.471	0	0	T	27	M	0.341	0.605	0	1.2E-07	0	163.453	cccaggagttCtgggctgtag	m5C_60156
chr16	58233679	-	C	32	8	0.25	0	0	T	24	M	0.133	0.421	2.3E-11	6.8E-07	2.6E-11	11.2828	tcgtttgagcCcaggagttct	m5C_60151
chr16	58265881	-	C	77	16	0.208	0	0	T	61	M	0.132	0.311	0	1.2E-12	0	42.2879	ggggttatgcCtgtgtgttac	m5C_60173
chr16	58265892	-	C	75	24	0.32	0	0	T	51	M	0.225	0.432	0	6.4E-11	0	108.206	gctgtcccctCggggttatgc	m5C_60148
chr16	58582425	-	C	51	12	0.235	0	0	T	39	M	0.14	0.368	3.3E-15	8.8E-10	4.3E-15	24.3305	tctgctgttgCtgtatagagt	m5C_60178
chr16	58582434	-	C	33	10	0.303	0	0	T	23	M	0.174	0.473	1.6E-14	2E-06	2E-14	23.982	ttgtgccattCtgctgttgct	m5C_60183
chr16	58582479	-	C	36	8	0.222	0	0	T	28	M	0.117	0.381	7.4E-11	6.8E-07	8.4E-11	9.49676	cgctgtagtgCaaaagaattc	m5C_60157
chr16	58582487	-	C	34	15	0.441	0	0	T	19	M	0.289	0.605	0	1.2E-05	0	86.6497	cttggttacgCtgtagtgcaa	m5C_60146
chr16	58582489	-	C	37	9	0.243	0	0	T	28	M	0.134	0.401	2.7E-12	1.2E-06	3.2E-12	13.907	ttcttggttaCgctgtagtgc	m5C_60150
chr16	58582501	-	C	56	21	0.375	0	0	T	35	M	0.26	0.506	0	4.8E-08	0	109.242	gaatattcccCattcttggtt	m5C_60175
chr16	58582502	-	C	52	22	0.423	0	0	T	30	M	0.299	0.558	0	6.5E-08	0	131.419	ggaatattccCcattcttggt	m5C_60170
chr16	58582503	-	C	60	19	0.317	0	0	T	41	M	0.213	0.442	0	4.5E-10	0	80.9617	gggaatattcCccattcttgg	m5C_60180
chr16	58582504	-	C	57	17	0.298	0	0	T	40	M	0.195	0.427	0	1.1E-08	0	66.4101	tgggaatattCcccattcttg	m5C_60186
chr16	58582521	-	C	125	40	0.32	0	0	T	85	M	0.245	0.406	0	1.1E-16	0	195.712	atatttaaacCtgtggatggg	m5C_60166
chr16	58582533	-	C	134	27	0.201	0	0	T	107	M	0.142	0.277	0	0	0	76.8604	ggaaccagcaCtatatttaaa	m5C_60160
chr16	58593738	-	C	32	12	0.375	0	0	T	20	M	0.229	0.547	0	4.6E-06	0	55.0408	ggctcttcagCtctcaactga	m5C_60185
chr16	58593744	-	C	42	11	0.262	0	0	T	31	M	0.153	0.411	8.4E-15	4.4E-08	1.1E-14	23.69	aactggggctCttcagctctc	m5C_60172
chr16	58593746	-	C	42	16	0.381	0	0	T	26	M	0.25	0.532	0	4E-07	0	79.9985	ataactggggCtcttcagctc	m5C_60189
chr16	58593752	-	C	63	20	0.317	0	0	T	43	M	0.216	0.44	0	6.9E-10	0	86.3461	cttgttataaCtggggctctt	m5C_60179
chr16	58593762	-	C	74	17	0.233	1	0.014	T	56	M	0.151	0.342	0	2.2E-12	0	51.2678	agcataacgaCttgttataac	m5C_60162
chr16	58593765	-	C	80	16	0.2	0	0	T	64	M	0.127	0.3	0	1.1E-14	0	40.6488	tagagcataaCgacttgttat	m5C_60167
chr16	58593798	-	C	323	79	0.245	0	0	T	244	M	0.201	0.294	0	0	0	317.401	ttgtttgtagCttcacgggcc	m5C_60152
chr16	58593809	-	C	361	118	0.327	0	0	T	243	M	0.281	0.377	0	0	0	662.04	cctgatgtgtCttgtttgtag	m5C_60188
chr16	58613626	-	C	34	8	0.235	0	0	T	26	M	0.124	0.4	4.2E-11	6.8E-07	4.8E-11	10.3259	ggagagagcgCggcctgaatt	m5C_60190
chr16	58633481	+	C	156	54	0.346	0	0	T	102	M	0.276	0.424	0	0	0	298.085	gaggctggtcCaggcgcagtg	m5C_60141
chr16	58633511	+	C	84	32	0.381	0	0	T	52	M	0.284	0.488	0	2.3E-11	0	182.061	actaattgatCacaactagtt	m5C_60138
chr16	58804583	+	C	47	14	0.298	0	0	T	33	M	0.187	0.44	0	1.9E-07	0	52.223	gagctggagaCtgccaggctg	m5C_60134
chr16	58804595	+	C	97	21	0.216	0	0	T	76	M	0.146	0.308	0	3.1E-15	0	61.3984	gccaggctgcCtaaggagggg	m5C_60137
chr16	58804610	+	C	93	48	0.516	0	0	C	48	M	0.416	0.615	0	4.6E-11	0	399.316	gaggggtgaaCtggcccaggt	m5C_60139
chr16	58804616	+	C	88	31	0.352	0	0	T	57	M	0.261	0.456	0	1.7E-11	0	161.548	tgaactggccCaggtcagtaa	m5C_60135
chr16	67355569	-	C	141	40	0.284	0	0	T	101	M	0.216	0.363	0	0	0	172.63	tggcagtctgCctttaaaaaa	m5C_55354
chr16	67355572	-	C	139	31	0.223	0	0	T	108	M	0.162	0.299	0	0	0	100.315	atgtggcagtCtgcctttaaa	m5C_55361
chr16	67355607	-	C	37	9	0.243	0	0	T	28	M	0.134	0.401	2.7E-12	1.2E-06	3.2E-12	13.907	tccaagacttCagacacatcc	m5C_55367
chr16	67355615	-	C	46	18	0.391	0	0	T	28	M	0.264	0.535	0	7.7E-07	0	95.0149	gtcaagcttcCaagacttcag	m5C_55389
chr16	67355619	-	C	45	31	0.689	0	0	C	31	M	0.543	0.805	0	1.4E-05	0	336.877	ggtagtcaagCttccaagact	m5C_55349
chr16	67355769	-	C	31	19	0.613	0	0	C	19	M	0.438	0.763	0	3.4E-05	0	166.53	tgcgtccctcCtgaagctgcg	m5C_55344
chr16	67355770	-	C	31	12	0.387	0	0	T	19	M	0.237	0.562	0	4.6E-06	0	56.9589	gtgcgtccctCctgaagctgc	m5C_55356
chr16	67355773	-	C	32	12	0.375	0	0	T	20	M	0.229	0.547	0	4.6E-06	0	55.0408	cacgtgcgtcCctcctgaagc	m5C_55346
chr16	67671677	+	C	32	11	0.344	0	0	T	21	M	0.204	0.517	2.2E-16	3.1E-06	2.9E-16	35.1442	agatgctgagCccgcagaggg	m5C_55310
chr16	67671679	+	C	32	13	0.406	0	0	T	19	M	0.255	0.577	0	6.7E-06	0	66.3505	atgctgagccCgcagagggag	m5C_55303
chr16	67671681	+	C	31	15	0.484	0	0	T	16	M	0.32	0.652	0	1.2E-05	0	95.91	gctgagcccgCagagggagag	m5C_55325
chr16	67859757	-	C	31	18	0.581	0	0	C	18	M	0.408	0.736	0	2.7E-05	0	146.757	cctcctggccCtgcctgcctg	m5C_55350
chr16	67859763	-	C	31	9	0.29	0	0	T	22	M	0.161	0.466	3.7E-13	1.2E-06	4.4E-13	18.0154	tcctaccctcCtggccctgcc	m5C_55339
chr16	67859764	-	C	31	10	0.323	0	0	T	21	M	0.186	0.499	7.1E-15	2E-06	9E-15	26.2725	gtcctaccctCctggccctgc	m5C_55399
chr16	69225033	+	C	67	17	0.254	0	0	T	50	M	0.165	0.369	0	1.8E-10	0	56.0585	ttgcgctggtCcgagtgcagt	m5C_55679
chr16	69225055	+	C	70	19	0.271	0	0	T	51	M	0.181	0.385	0	6.9E-12	0	68.854	gtgtttacaaCtaattgatca	m5C_55647
chr16	69225064	+	C	72	22	0.306	0	0	T	50	M	0.211	0.42	0	2.8E-11	0	92.9565	actaattgatCacaactagtt	m5C_55700
chr16	69225090	+	C	50	12	0.24	0	0	T	38	M	0.143	0.374	2.4E-15	8.8E-10	3.1E-15	25.0697	ttatttgttcCttctccactc	m5C_55693
chr16	69225093	+	C	46	12	0.261	0	0	T	34	M	0.156	0.403	6.7E-16	7.5E-08	8.7E-16	28.4116	tttgttccttCtccactctca	m5C_55690
chr16	69225095	+	C	46	10	0.217	0	0	T	36	M	0.123	0.356	9.7E-13	2.4E-08	1.2E-12	14.7309	tgttccttctCcactctcact	m5C_55680
chr16	69225104	+	C	49	10	0.204	0	0	T	39	M	0.115	0.336	2.1E-12	2.4E-08	2.4E-12	13.4191	tccactctcaCtgcttcactt	m5C_55651
chr16	69225121	+	C	40	8	0.2	0	0	T	32	M	0.105	0.348	2.1E-10	5.5E-09	2.3E-10	8.13503	acttgactagCtttaaaaaaa	m5C_55698
chr16	69529788	-	C	38	11	0.289	0	0	T	27	M	0.17	0.448	2.1E-15	3.1E-06	2.7E-15	27.4488	gaacttggggCccaggaggag	m5C_55713
chr16	69790828	-	C	31	22	0.71	0	0	C	22	M	0.534	0.839	0	6.3E-05	0	234.992	aactggagctCaggcaggagg	m5C_55729
chr16	69814947	+	C	122	44	0.361	0	0	T	78	M	0.281	0.449	0	4.4E-16	0	247.194	acaggttggtCctgtggtagt	m5C_55681
chr16	69814948	+	C	129	30	0.233	0	0	T	99	M	0.168	0.313	0	0	0	100.821	caggttggtcCtgtggtagtg	m5C_55684
chr16	69814978	+	C	35	7	0.2	0	0	T	28	M	0.1	0.359	1.9E-09	3.4E-07	2.1E-09	6.13884	aacttattaaCattagtgtca	m5C_55702
chr16	69967047	+	C	35	10	0.286	0	0	T	25	M	0.163	0.451	3.4E-14	2E-06	4.2E-14	21.9991	tgctgttctaCcacagggtag	m5C_55663
chr16	69967048	+	C	36	11	0.306	0	0	T	25	M	0.18	0.469	1E-15	3.1E-06	1.3E-15	29.7077	gctgttctacCacagggtaga	m5C_55672
chr16	69967050	+	C	38	9	0.237	0	0	T	29	M	0.13	0.392	3.7E-12	1.2E-06	4.3E-12	13.3739	tgttctaccaCagggtagaac	m5C_55669
chr16	69967060	+	C	78	21	0.269	0	0	T	57	M	0.183	0.377	0	1.8E-11	0	77.009	cagggtagaaCcacggacagg	m5C_55675
chr16	69967061	+	C	70	30	0.429	0	0	T	40	M	0.319	0.545	0	4.8E-10	0	191.611	agggtagaacCacggacagga	m5C_55664
chr16	69967063	+	C	73	38	0.521	0	0	C	38	M	0.408	0.631	0	3.7E-09	0	309.939	ggtagaaccaCggacaggata	m5C_55692
chr16	70286627	-	C	33	17	0.515	0	0	C	17	M	0.352	0.675	0	2.1E-05	0	119.742	atgtaaagaaCtgagtgggga	m5C_56408
chr16	70296397	-	C	111	23	0.207	0	0	T	88	M	0.142	0.292	0	0	0	65.425	acggtgatggCtctgcgcagg	m5C_56377
chr16	70296406	-	C	77	26	0.338	0	0	T	51	M	0.242	0.449	0	1.3E-10	0	125.839	acagtggctaCggtgatggct	m5C_56384
chr16	70301602	-	C	43	13	0.302	0	0	T	30	M	0.186	0.451	0	1.2E-07	0	48.3648	gcatcctggaCaggaaaattc	m5C_56381
chr16	70301607	-	C	44	10	0.227	0	0	T	34	M	0.128	0.37	5.7E-13	2.4E-08	6.8E-13	15.7274	gcgtcgcatcCtggacaggaa	m5C_56425
chr16	70301611	-	C	55	35	0.636	0	0	C	35	M	0.504	0.751	0	1.3E-06	0	352.958	gagggcgtcgCatcctggaca	m5C_56413
chr16	70301616	-	C	46	11	0.239	0	0	T	35	M	0.139	0.379	2.8E-14	4.4E-08	3.5E-14	20.7303	cagcagagggCgtcgcatcct	m5C_56441
chr16	70301623	-	C	38	8	0.211	0	0	T	30	M	0.111	0.363	1.3E-10	6.8E-07	1.4E-10	8.77246	agactctcagCagagggcgtc	m5C_56451
chr16	70301628	-	C	36	10	0.278	0	0	T	26	M	0.158	0.44	4.8E-14	2E-06	5.9E-14	21.1115	tctcaagactCtcagcagagg	m5C_56396
chr16	70301635	-	C	36	10	0.278	0	0	T	26	M	0.158	0.44	4.8E-14	2E-06	5.9E-14	21.1115	tgcagtttctCaagactctca	m5C_56400
chr16	70563441	+	C	58	17	0.293	0	0	T	41	M	0.192	0.42	0	1.1E-08	0	65.2053	acattcatgtCtcttctctga	m5C_56357
chr16	70563485	+	C	59	20	0.339	0	0	T	39	M	0.231	0.466	0	3.4E-08	0	92.5499	tgtgtctgatCaggcctctag	m5C_56365
chr16	70571926	+	C	54	11	0.204	0	0	T	43	M	0.118	0.329	2.3E-13	4.4E-10	2.8E-13	16.3622	aatgatgactCtttaaaaaat	m5C_56373
chr16	70571939	+	C	52	18	0.346	0	0	T	34	M	0.232	0.482	0	1.7E-08	0	83.343	taaaaaatttCatgtctcttc	m5C_56359
chr16	70571946	+	C	57	15	0.263	0	0	T	42	M	0.166	0.39	0	4.6E-09	0	49.9369	tttcatgtctCttctctgaca	m5C_56356
chr16	70571951	+	C	57	16	0.281	0	0	T	41	M	0.181	0.408	0	7.3E-09	0	57.8536	tgtctcttctCtgacattttt	m5C_56335
chr16	70571962	+	C	58	24	0.414	0	0	T	34	M	0.296	0.542	0	1.2E-07	0	142.214	tgacatttttCtctggacaca	m5C_56374
chr16	70571964	+	C	58	29	0.5	0	0	CT	29	M	0.375	0.625	0	3.9E-07	0	217.721	acatttttctCtggacacagt	m5C_56354
chr16	70571969	+	C	55	19	0.345	0	0	T	36	M	0.234	0.477	0	2.4E-08	0	88.7776	tttctctggaCacagtttttg	m5C_56355
chr16	70571980	+	C	43	31	0.721	0	0	C	31	M	0.573	0.833	0	1.4E-05	0	355.314	acagtttttgCcttatgaatc	m5C_56347

chr16	70571981	+	C	48	21	0.467	3	0.062	T	24	M	0.329	0.609	0	1.8E-06	0	138.327	cagtttttgcCttatgaatct	m5C_56372
chr16	70571990	+	C	44	28	0.636	0	0	C	28	M	0.489	0.762	0	8.1E-06	0	273.65	ccttatgaatCtgatcaggct	m5C_56360
chr16	70812104	-	C	53	15	0.283	0	0	T	38	M	0.18	0.416	0	4.6E-09	0	53.8984	agcctatctaCctttttagat	m5C_56387
chr16	70812107	-	C	57	16	0.281	0	0	T	41	M	0.181	0.408	0	7.3E-09	0	57.8536	tgcagcctatCtaccttttta	m5C_56403
chr16	70812111	-	C	59	17	0.288	0	0	T	42	M	0.188	0.414	0	1.1E-08	0	64.0436	ccagtgcagcCtatctacctt	m5C_56437
chr16	70812124	-	C	189	43	0.228	0	0	T	146	M	0.174	0.292	0	0	0	149.229	gtttgattccCggccagtgca	m5C_56415
chr16	70812126	-	C	185	39	0.211	0	0	T	146	M	0.158	0.275	0	0	0	123.412	gggtttgattCccggccagtg	m5C_56393
chr16	70812137	-	C	206	76	0.369	0	0	T	130	M	0.306	0.437	0	0	0	465.122	gcgggaggccCgggtttgatt	m5C_56383
chr16	70812138	-	C	232	64	0.277	1	0.004	T	167	M	0.223	0.338	0	0	0	285.884	cgcgggaggcCcgggtttgat	m5C_56385
chr16	70812139	-	C	245	70	0.286	0	0	T	175	M	0.233	0.345	0	0	0	325.913	acgcgggaggCccgggtttga	m5C_56379
chr16	70812151	-	C	137	36	0.263	0	0	T	101	M	0.196	0.342	0	0	0	141.311	ttctcgcctgCcacgcgggag	m5C_56423
chr16	70812154	-	C	145	31	0.214	0	0	T	114	M	0.155	0.287	0	0	0	96.0389	gaattctcgcCtgccacgcgg	m5C_56382
chr16	70812157	-	C	135	41	0.304	0	0	T	94	M	0.232	0.386	0	0	0	190.609	gtagaattctCgcctgccacg	m5C_56439
chr16	70812159	-	C	140	33	0.236	0	0	T	107	M	0.173	0.312	0	0	0	114.213	tggtagaattCtcgcctgcca	m5C_56452
chr16	70812949	-	C	109	29	0.266	0	0	T	80	M	0.192	0.356	0	2.9E-15	0	111.416	cgattcccggCcaatgcagca	m5C_56445
chr16	70812954	-	C	88	24	0.273	0	0	T	64	M	0.191	0.374	0	1.1E-12	0	91.5137	gggttcgattCccggccaatg	m5C_56431
chr16	70812965	-	C	86	22	0.256	0	0	T	64	M	0.175	0.357	0	4.4E-13	0	77.189	gcgggaggccCgggttcgatt	m5C_56410
chr16	70812966	-	C	87	27	0.31	0	0	T	60	M	0.223	0.414	0	4E-12	0	120.361	cgcgggaggcCcgggttcgat	m5C_56429
chr16	70812967	-	C	89	18	0.202	0	0	T	71	M	0.132	0.297	0	4.5E-14	0	47.4972	acgcgggaggCccgggttcga	m5C_56450
chr16	70812974	-	C	252	52	0.206	0	0	T	200	M	0.161	0.261	0	0	0	167.413	gcctgccacgCgggaggcccg	m5C_56378
chr16	70812978	-	C	245	56	0.229	0	0	T	189	M	0.18	0.285	0	0	0	202.07	tctcgcctgcCacgcgggagg	m5C_56401
chr16	70812982	-	C	243	83	0.342	0	0	T	160	M	0.285	0.403	0	0	0	472.796	gaattctcgcCtgccacgcgg	m5C_56419
chr16	70812983	-	C	242	104	0.43	0	0	T	138	M	0.369	0.493	0	0	0	767.431	agaattctcgCctgccacgcg	m5C_56389
chr16	70812985	-	C	237	54	0.228	0	0	T	183	M	0.179	0.285	0	0	0	193.36	gtagaattctCgcctgccacg	m5C_56438
chr16	70813000	-	C	239	67	0.28	0	0	T	172	M	0.227	0.34	0	0	0	304.461	attggtggttCagtggtagaa	m5C_56404
chr16	70813011	-	C	103	34	0.33	0	0	T	69	M	0.247	0.426	0	2.3E-14	0	167.843	tcagctggagCattggtggtt	m5C_56416
chr16	70822609	+	C	190	75	0.395	0	0	T	115	M	0.328	0.466	0	0	0	491.97	attggtggttCagtggtagaa	m5C_56324
chr16	70822622	+	C	156	67	0.429	0	0	T	89	M	0.354	0.508	0	0	0	474.928	tggtagaattCtcgcctgcca	m5C_56366
chr16	70822624	+	C	160	52	0.325	0	0	T	108	M	0.257	0.401	0	0	0	267.557	gtagaattctCgcctgccatg	m5C_56328
chr16	70822626	+	C	151	50	0.331	0	0	T	101	M	0.261	0.41	0	0	0	261.069	agaattctcgCctgccatgcg	m5C_56348
chr16	70822627	+	C	144	46	0.319	0	0	T	98	M	0.249	0.399	0	0	0	228.923	gaattctcgcCtgccatgcgg	m5C_56349
chr16	70822635	+	C	106	34	0.321	0	0	T	72	M	0.24	0.415	0	2.3E-14	0	162.865	gcctgccatgCgggcggccgg	m5C_56333
chr16	70822639	+	C	90	21	0.356	31	0.344	T	38	M	0.246	0.483	0	4.8E-08	0	103.333	gccatgcgggCggccgggctt	m5C_56352
chr16	70822642	+	C	35	9	0.257	0	0	T	26	M	0.142	0.421	1.5E-12	1.2E-06	1.7E-12	15.0859	atgcgggcggCcgggcttcga	m5C_56336
chr16	70822643	+	C	33	13	0.394	0	0	T	20	M	0.247	0.563	0	6.7E-06	0	64.1755	tgcgggcggcCgggcttcgat	m5C_56375
chr16	70823411	+	C	130	43	0.331	0	0	T	87	M	0.256	0.415	0	0	0	219.961	acatctggggCattggtggtt	m5C_56329
chr16	70823422	+	C	184	52	0.283	0	0	T	132	M	0.223	0.352	0	0	0	231.41	attggtggttCagtggtagaa	m5C_56367
chr16	70823435	+	C	163	56	0.344	0	0	T	107	M	0.275	0.419	0	0	0	308.01	tggtagaattCtcgcctgcca	m5C_56330
chr16	70823437	+	C	158	66	0.418	0	0	T	92	M	0.344	0.496	0	0	0	453.639	gtagaattctCgcctgccacg	m5C_56362
chr16	70823439	+	C	130	48	0.369	0	0	T	82	M	0.291	0.455	0	1.3E-15	0	279.492	agaattctcgCctgccacgcg	m5C_56344
chr16	70823440	+	C	126	29	0.23	0	0	T	97	M	0.165	0.311	0	0	0	95.8737	gaattctcgcCtgccacgcgg	m5C_56332
chr16	70823455	+	C	122	56	0.459	0	0	T	66	M	0.373	0.547	0	1.3E-14	0	417.975	acgcgggaggCccgggttcga	m5C_56327
chr16	70823456	+	C	125	27	0.216	0	0	T	98	M	0.153	0.296	0	0	0	82.5698	cgcgggaggcCcgggttcgat	m5C_56369
chr16	70823457	+	C	122	27	0.221	0	0	T	95	M	0.157	0.303	0	0	0	84.6667	gcgggaggccCgggttcgatt	m5C_56334
chr16	70823463	+	C	74	16	0.222	2	0.027	T	56	M	0.142	0.331	0	1.2E-12	0	45.3354	ggcccgggttCgattcccggc	m5C_56368
chr16	70823470	+	C	75	16	0.213	0	0	T	59	M	0.136	0.319	0	1.2E-12	0	43.4563	gttcgattccCggccaatgca	m5C_56350
chr16	71792310	-	C	105	36	0.343	0	0	T	69	M	0.259	0.438	0	4.8E-14	0	186.52	ctgctgacagCacacgttttt	m5C_56427
chr16	71792317	-	C	461	135	0.293	0	0	T	326	M	0.253	0.336	0	0	0	683.502	actcaaactgCtgacagcaca	m5C_56417
chr16	71792355	-	C	684	165	0.241	0	0	T	519	M	0.211	0.275	0	0	0	695.204	agtgatccatCggctaagtgt	m5C_56407
chr16	71792359	-	C	703	146	0.21	8	0.011	T	549	M	0.181	0.242	0	0	0	529.75	acggagtgatCcatcggctaa	m5C_56392
chr16	71792368	-	C	787	180	0.23	6	0.008	T	601	M	0.202	0.261	0	0	0	728.279	gatgaaagtaCggagtgatcc	m5C_56414
chr16	73512264	-	C	115	35	0.304	0	0	T	80	M	0.228	0.394	0	6.7E-16	0	159.392	gttggtagagCatcagacttt	m5C_55295
chr16	73512286	-	C	125	47	0.376	0	0	T	78	M	0.296	0.463	0	1E-15	0	278.217	gggaagaagcCtggatagctc	m5C_55294
chr16	74339510	+	C	42	11	0.262	0	0	T	31	M	0.153	0.411	8.4E-15	4.4E-08	1.1E-14	23.69	aaccgggatgCagagagaaaa	m5C_55226
chr16	74339527	+	C	110	45	0.409	0	0	T	65	M	0.322	0.503	0	7.4E-13	0	289.609	gaaagaagggCaggagaaaga	m5C_55235
chr16	74339544	+	C	121	36	0.298	0	0	T	85	M	0.223	0.384	0	0	0	160.786	aagaagagagCaaaaaggata	m5C_55232
chr16	74339565	+	C	105	56	0.533	0	0	C	56	M	0.438	0.626	0	9.8E-12	0	491.034	ggaaagaggaCaaggagaaag	m5C_55223
chr16	84360495	+	C	41	18	0.439	0	0	T	23	M	0.299	0.59	0	7.7E-07	0	107.604	cagaaggtgaCtcaaagaatg	m5C_56260
chr16	84360497	+	C	40	14	0.35	0	0	T	26	M	0.221	0.505	0	1.9E-07	0	61.9761	gaaggtgactCaaagaatgtg	m5C_56264
chr16	84360509	+	C	36	11	0.306	0	0	T	25	M	0.18	0.469	1E-15	3.1E-06	1.3E-15	29.7077	aagaatgtggCagaacctgga	m5C_56274
chr16	84363389	+	C	39	10	0.256	0	0	T	29	M	0.146	0.411	1.3E-13	2E-06	1.6E-13	18.7753	accggatgtgCtgaggcctaa	m5C_56283
chr16	84363395	+	C	35	16	0.457	0	0	T	19	M	0.305	0.618	0	1.7E-05	0	97.4905	tgtgctgaggCctaaatgtta	m5C_56282
chr16	84363396	+	C	35	15	0.429	0	0	T	20	M	0.28	0.591	0	1.2E-05	0	83.953	gtgctgaggcCtaaatgttag	m5C_56266
chr16	85840465	+	C	33	10	0.303	0	0	T	23	M	0.174	0.473	1.6E-14	2E-06	2E-14	23.982	acgaaaagaaCgagtggaaga	m5C_56292
chr16	87902988	-	C	34	7	0.206	0	0	T	27	M	0.103	0.368	1.4E-09	3.4E-07	1.6E-09	6.4054	ctagcggcgcCggcggccgag	m5C_55513
chr16	88560893	-	C	37	19	0.514	0	0	C	19	M	0.359	0.666	0	3.4E-05	0	136.4	tgattggtccCtgtgacggga	m5C_59191
chr16	88560894	-	C	49	21	0.429	0	0	T	28	M	0.3	0.567	0	1.8E-06	0	126.097	gtgattggtcCctgtgacggg	m5C_59200
chr16	89627872	+	C	1897	468	0.247	2	0.001	T	1427	M	0.228	0.267	0	0	0	2134.77	gctgtacggcCttgatgaaag	m5C_59157
chr16	89699571	+	C	50	10	0.204	1	0.02	T	39	M	0.115	0.336	2.1E-12	2.4E-08	2.4E-12	13.4191	agcgatgaggCtgctgcccgg	m5C_59185
chr16	89737169	+	C	40	26	0.65	0	0	C	26	M	0.495	0.779	0	5.5E-06	0	257.429	gtgtgctgggCacatgggaca	m5C_59159
chr17	1303477	-	C	45	10	0.222	0	0	T	35	M	0.125	0.363	7.4E-13	2.4E-08	8.9E-13	15.2157	ggacgcggagCgagaggctga	m5C_56772
chr17	1303484	-	C	44	25	0.568	0	0	C	25	M	0.422	0.703	0	4.5E-06	0	211.119	gctgcccggaCgcggagcgag	m5C_56760
chr17	1340169	-	C	48	14	0.292	0	0	T	34	M	0.182	0.432	0	1.9E-07	0	51.076	atgcggaggaCagcgaaggca	m5C_56734
chr17	1466758	-	C	64	23	0.359	0	0	T	41	M	0.253	0.482	0	2.1E-09	0	116.326	ctgagtgctgCattacaggca	m5C_56731
chr17	1466768	-	C	61	19	0.311	0	0	T	42	M	0.209	0.436	0	4.5E-10	0	79.5616	atgcgcccgcCtgagtgctgc	m5C_56748
chr17	1466772	-	C	44	9	0.205	0	0	T	35	M	0.112	0.345	1.8E-11	1.2E-08	2.1E-11	10.7806	ggtgatgcgcCcgcctgagtg	m5C_56763
chr17	1585310	-	C	32	14	0.438	0	0	T	18	M	0.282	0.607	0	9.2E-06	0	78.8622	tatgatgcgcCgagaaaaaag	m5C_56741
chr17	1617211	-	C	45	17	0.378	0	0	T	28	M	0.251	0.524	0	5.6E-07	0	85.3668	agttgaagaaCtgttgccctc	m5C_56745
chr17	1617222	-	C	53	23	0.434	0	0	T	30	M	0.31	0.567	0	8.7E-08	0	142.371	ctaaagctgcCagttgaagaa	m5C_56775
chr17	1617223	-	C	52	17	0.327	0	0	T	35	M	0.215	0.462	0	1.1E-08	0	73.1745	gctaaagctgCcagttgaaga	m5C_56758
chr17	1858370	+	C	40	24	0.6	0	0	C	24	M	0.446	0.737	0	3.6E-06	0	214.059	ctctgtcagcCtgagcgccgc	m5C_56710
chr17	1858375	+	C	43	12	0.279	0	0	T	31	M	0.167	0.427	2.2E-16	7.5E-08	2.9E-16	31.457	tcagcctgagCgccgcccttt	m5C_56701
chr17	1858380	+	C	38	14	0.368	0	0	T	24	M	0.234	0.527	0	9.2E-06	0	65.4752	ctgagcgccgCccttttcacta	m5C_56714
chr17	2092255	+	C	31	8	0.258	0	0	T	23	M	0.137	0.432	1.7E-11	6.8E-07	1.9E-11	11.8182	tgccataccaCcctgaacgcg	m5C_56483
chr17	2092267	+	C	37	11	0.297	0	0	T	26	M	0.175	0.458	1.4E-15	3.1E-06	1.9E-15	28.5508	ctgaacgcgcCcgatctcgtc	m5C_56464
chr17	2092272	+	C	38	13	0.342	0	0	T	25	M	0.212	0.501	0	6.7E-06	0	55.1518	cgcgcccgatCtcgtctaaaa	m5C_56475
chr17	2233586	-	C	183	46	0.251	0	0	T	137	M	0.194	0.319	0	0	0	178.541	ctgtgtggttCtgagacttct	m5C_56514
chr17	2233610	-	C	177	42	0.237	0	0	T	135	M	0.181	0.305	0	0	0	151.715	tgaagcatctCagtgatgcaa	m5C_56501
chr17	2233631	-	C	173	45	0.26	0	0	T	128	M	0.2	0.33	0	0	0	180.412	ttcatatcgtCtgaacctgtc	m5C_56532
chr17	2235553	-	C	51	15	0.312	3	0.059	T	33	M	0.199	0.453	0	2.8E-07	0	59.8365	gaagctgaggCaaaaatgttg	m5C_56520
chr17	2236259	-	C	56	18	0.321	0	0	T	38	M	0.214	0.452	0	1.7E-08	0	77.043	gaggaggaatCtcaggtaagg	m5C_56536
chr17	4096070	+	C	44	12	0.273	0	0	T	32	M	0.163	0.418	4.4E-16	7.5E-08	5.8E-16	30.1147	attggctggtCcgggtgcagt	m5C_56553
chr17	4927585	+	C	35	22	0.629	0	0	C	22	M	0.463	0.768	0	6.3E-05	0	203.881	tgatagaagaCaagggggaga	m5C_56572
chr17	5391719	+	C	71	20	0.282	0	0	T	51	M	0.19	0.395	0	1.1E-11	0	76.1478	gtgtaggaggCagtagtcaaa	m5C_56549
chr17	5391726	+	C	72	42	0.583	0	0	C	42	M	0.468	0.69	0	8.7E-09	0	393.183	aggcagtagtCaaaaagtaga	m5C_56579
chr17	6504320	-	C	35	23	0.676	1	0.029	C	23	M	0.508	0.809	0	7.6E-05	0	233.876	atctccgaagCtaagcagggt	m5C_57149
chr17	6504333	-	C	37	15	0.405	0	0	T	22	M	0.263	0.565	0	1.2E-05	0	79.0389	ccgatctcgtCtgatctccga	m5C_57152
chr17	6504336	-	C	37	28	0.757	0	0	C	28	M	0.599	0.866	0	0.00017	0	335.341	tgcccgatctCgtctgatctc	m5C_57115
chr17	6504343	-	C	35	12	0.343	0	0	T	23	M	0.208	0.508	0	4.6E-06	0	49.9956	ctgaatgtgcCcgatctcgtc	m5C_57116
chr17	6504344	-	C	35	8	0.229	0	0	T	27	M	0.121	0.39	5.6E-11	6.8E-07	6.4E-11	9.89691	cctgaatgtgCccgatctcgt	m5C_57124
chr17	7126675	-	C	33	14	0.424	0	0	T	19	M	0.272	0.592	0	9.2E-06	0	76.259	cccgcatcccCtagggcattg	m5C_57147

chr17	7139506	-	C	34	14	0.412	0	0	T	20	M	0.264	0.578	0	9.2E-06	0	73.8241	gatacagactCtgaagaggag	m5C_57095
chr17	7154690	-	C	61	28	0.459	0	0	T	33	M	0.34	0.583	0	9.8E-09	0	190.471	ctcccggcggCggggggctgc	m5C_57141
chr17	7154693	-	C	58	14	0.241	0	0	T	44	M	0.15	0.365	0	2.8E-09	0	41.8845	cccctcccggCggcggggggc	m5C_57133
chr17	7154697	-	C	56	19	0.339	0	0	T	37	M	0.229	0.47	0	2.4E-08	0	87.0952	gttccccctcCcggcggcggg	m5C_57153
chr17	7156261	+	C	50	15	0.3	0	0	T	35	M	0.191	0.438	0	4.6E-09	0	57.3102	atatcctgggCtgtgaagtga	m5C_56965
chr17	7217869	-	C	134	40	0.299	0	0	T	94	M	0.228	0.381	0	0	0	182.019	gaaggaagaaCaggagagaag	m5C_57108
chr17	7217887	-	C	95	33	0.347	0	0	T	62	M	0.259	0.447	0	7.5E-13	0	171.102	gatgatggaaCagaagatgaa	m5C_57138
chr17	7321614	-	C	34	26	0.765	0	0	C	26	M	0.6	0.876	0	0.00013	0	312.018	cctctgtggtCtggtggccaa	m5C_57111
chr17	7449224	-	C	49	36	0.735	0	0	C	36	M	0.597	0.838	0	2.9E-05	0	430.11	atgtaaatatCtttgactgga	m5C_57107
chr17	7478041	+	C	910	203	0.223	0	0	T	707	M	0.197	0.251	0	0	0	800.729	tgtccctgacCtgggtagagt	m5C_57087
chr17	7478057	+	C	907	234	0.258	1	0.001	T	672	M	0.231	0.288	0	0	0	1080.33	agagtggcatCtggttggtga	m5C_57032
chr17	7478072	+	C	633	129	0.204	0	0	T	504	M	0.174	0.237	0	0	0	449.543	tggtgatgccCatctcatatc	m5C_57088
chr17	7478077	+	C	442	113	0.256	0	0	T	329	M	0.217	0.298	0	0	0	490.903	atgcccatctCatatcagcca	m5C_57014
chr17	7478085	+	C	269	59	0.219	0	0	T	210	M	0.174	0.273	0	0	0	205.348	ctcatatcagCcagggacaaa	m5C_56992
chr17	7478086	+	C	261	58	0.222	0	0	T	203	M	0.176	0.276	0	0	0	204.182	tcatatcagcCagggacaaag	m5C_57010
chr17	7478109	+	C	110	35	0.318	0	0	T	75	M	0.239	0.41	0	6.7E-16	0	166.975	actccttgttCatcccagctt	m5C_57065
chr17	7478114	+	C	83	19	0.229	0	0	T	64	M	0.152	0.33	0	8.4E-14	0	57.6583	ttgttcatccCagcttggctt	m5C_57018
chr17	7478122	+	C	81	18	0.222	0	0	T	63	M	0.145	0.324	0	4.5E-14	0	52.3602	cccagcttggCttttgatccg	m5C_56988
chr17	7478130	+	C	87	34	0.391	0	0	T	53	M	0.295	0.496	0	4.2E-11	0	200.584	ggcttttgatCcgtgcccatg	m5C_57047
chr17	7478135	+	C	75	18	0.24	0	0	T	57	M	0.158	0.348	0	4E-12	0	56.7171	ttgatccgtgCccatgcctgg	m5C_57009
chr17	7478136	+	C	78	18	0.231	0	0	T	60	M	0.151	0.336	0	4E-12	0	54.4514	tgatccgtgcCcatgcctggt	m5C_57023
chr17	7478142	+	C	74	22	0.297	0	0	T	52	M	0.205	0.409	0	2.8E-11	0	90.3191	gtgcccatgcCtggttcatgc	m5C_56962
chr17	7478148	+	C	71	23	0.324	0	0	T	48	M	0.227	0.439	0	4.3E-11	0	104.222	atgcctggttCatgccttgga	m5C_56964
chr17	7478152	+	C	73	25	0.342	0	0	T	48	M	0.244	0.457	0	9.3E-11	0	121.974	ctggttcatgCcttggacaca	m5C_57059
chr17	7479909	+	C	44	17	0.386	0	0	T	27	M	0.257	0.534	0	5.6E-07	0	87.4514	atcgggggcaCcaacgtgcgt	m5C_57002
chr17	7479910	+	C	44	10	0.227	0	0	T	34	M	0.128	0.37	5.7E-13	2.4E-08	6.8E-13	15.7274	tcgggggcacCaacgtgcgtg	m5C_57046
chr17	7479917	+	C	84	24	0.286	0	0	T	60	M	0.2	0.39	0	1.1E-12	0	96.0715	caccaacgtgCgtgctgaggt	m5C_57056
chr17	7479921	+	C	89	19	0.213	0	0	T	70	M	0.141	0.31	0	8.4E-14	0	53.6358	aacgtgcgtgCtgaggtgcag	m5C_56991
chr17	7479938	+	C	84	31	0.369	0	0	T	53	M	0.274	0.476	0	1.7E-11	0	169.694	gcagaaactgCagatggaagc	m5C_57012
chr17	7480157	+	C	1008	215	0.213	0	0	T	793	M	0.189	0.24	0	0	0	813.209	tgcgtgtcatCtgagcctctg	m5C_57034
chr17	7480162	+	C	893	191	0.214	0	0	T	702	M	0.188	0.242	0	0	0	719.102	gtcatctgagCctctggcttc	m5C_57069
chr17	7480163	+	C	856	195	0.228	0	0	T	661	M	0.201	0.257	0	0	0	783.741	tcatctgagcCtctggcttcc	m5C_56987
chr17	7480165	+	C	773	159	0.206	0	0	T	614	M	0.179	0.236	0	0	0	568.221	atctgagcctCtggcttccct	m5C_57077
chr17	7480178	+	C	518	120	0.232	0	0	T	398	M	0.197	0.27	0	0	0	473.72	gcttccctgcCagtgcagccc	m5C_57013
chr17	7480188	+	C	341	89	0.261	0	0	T	252	M	0.217	0.31	0	0	0	386.668	cagtgcagccCtggcagtgtc	m5C_56999
chr17	7480199	+	C	243	80	0.329	0	0	T	163	M	0.273	0.391	0	0	0	437.106	tggcagtgtcCtacttcccag	m5C_57072
chr17	7480206	+	C	247	84	0.34	0	0	T	163	M	0.284	0.401	0	0	0	476.872	gtcctacttcCcagggctgtt	m5C_57075
chr17	7480212	+	C	284	63	0.222	0	0	T	221	M	0.177	0.274	0	0	0	223.526	cttcccagggCtgttgtctgc	m5C_57020
chr17	7480219	+	C	316	82	0.259	0	0	T	234	M	0.214	0.311	0	0	0	351.377	gggctgttgtCtgcctggcgg	m5C_57017
chr17	7480227	+	C	384	81	0.213	4	0.01	T	299	M	0.175	0.257	0	0	0	283.431	gtctgcctggCgggaaggtcc	m5C_56986
chr17	7480236	+	C	467	102	0.219	2	0.004	T	363	M	0.184	0.259	0	0	0	375.617	gcgggaaggtCctgggcaaag	m5C_57045
chr17	7480254	+	C	737	186	0.252	0	0	T	551	M	0.222	0.285	0	0	0	827.152	aaggatcagtCtttgtactct	m5C_56994
chr17	7480263	+	C	676	202	0.299	0	0	T	474	M	0.266	0.334	0	0	0	1072.72	tctttgtactCtgagagcaga	m5C_57057
chr17	7480270	+	C	440	134	0.305	0	0	T	306	M	0.263	0.349	0	0	0	705.88	actctgagagCagactacttg	m5C_56972
chr17	7481290	+	C	188	44	0.234	0	0	T	144	M	0.179	0.3	0	0	0	157.711	gtgattccctCtccaagggga	m5C_57071
chr17	7481301	+	C	185	51	0.276	0	0	T	134	M	0.216	0.344	0	0	0	220.669	tccaaggggaCatcagtgcct	m5C_57005
chr17	7481309	+	C	180	48	0.271	3	0.017	T	129	M	0.211	0.341	0	0	0	202.626	gacatcagtgCctctcaggaa	m5C_57089
chr17	7481310	+	C	181	40	0.221	0	0	T	141	M	0.167	0.287	0	0	0	133.357	acatcagtgcCtctcaggaaa	m5C_57041
chr17	7481325	+	C	196	46	0.235	0	0	T	150	M	0.181	0.299	0	0	0	166.348	aggaaagtagCagcttggaat	m5C_57050
chr17	7481341	+	C	166	37	0.223	0	0	T	129	M	0.166	0.292	0	0	0	123.028	ggaatagaatCtggcatgcct	m5C_57048
chr17	7481349	+	C	78	24	0.308	0	0	T	54	M	0.216	0.417	0	6.4E-11	0	103.833	atctggcatgCctaaggcctt	m5C_57031
chr17	7481357	+	C	31	14	0.452	0	0	T	17	M	0.292	0.622	0	9.2E-06	0	81.6522	tgcctaaggcCtttggggaac	m5C_56978
chr17	7809396	+	C	47	28	0.596	0	0	C	28	M	0.453	0.724	0	8.1E-06	0	253.914	tatgatgtgaCcttactcaac	m5C_57082
chr17	7809401	+	C	47	19	0.404	0	0	T	28	M	0.276	0.547	0	1E-06	0	105.031	tgtgaccttaCtcaactgatt	m5C_57051
chr17	7809406	+	C	38	10	0.263	0	0	T	28	M	0.15	0.42	9.4E-14	2E-06	1.2E-13	19.5037	ccttactcaaCtgattatcac	m5C_57001
chr17	7809628	+	C	112	29	0.259	0	0	T	83	M	0.187	0.347	0	0	0	108.317	ggatgaagagCtgactgatgg	m5C_57064
chr17	7809632	+	C	107	30	0.28	0	0	T	77	M	0.204	0.372	0	4.7E-15	0	122.41	gaagagctgaCtgatggggcc	m5C_56989
chr17	8022476	+	C	177	44	0.25	1	0.006	T	132	M	0.192	0.319	0	0	0	168.809	gtgaagagccCggatagctca	m5C_57163
chr17	8022489	+	C	171	47	0.275	0	0	T	124	M	0.213	0.346	0	0	0	200.634	atagctcagtCggtagagcat	m5C_57162
chr17	8022520	+	C	63	41	0.651	0	0	C	41	M	0.528	0.757	0	1.7E-07	0	432.56	atctgagggtCcagggttcaa	m5C_57217
chr17	8022535	+	C	63	28	0.444	0	0	T	35	M	0.328	0.567	0	9.8E-09	0	183.943	gttcaagtccCtgttcgggcg	m5C_57223
chr17	8022552	+	C	36	17	0.472	0	0	T	19	M	0.32	0.63	0	2.1E-05	0	108.752	ggcggcatgtCtttgcttttg	m5C_57236
chr17	8023094	+	C	79	31	0.397	1	0.013	T	47	M	0.296	0.508	0	6.4E-10	0	183.593	atggttagcaCtctggactct	m5C_57166
chr17	8023110	+	C	74	23	0.311	0	0	T	51	M	0.217	0.423	0	4.3E-11	0	99.7772	actctgaatcCagcgatccga	m5C_57158
chr17	8023113	+	C	63	32	0.508	0	0	C	32	M	0.388	0.627	0	2.7E-08	0	248.081	ctgaatccagCgatccgagtt	m5C_57199
chr17	8023129	+	C	42	14	0.333	0	0	T	28	M	0.21	0.484	0	1.9E-07	0	58.8344	gagttcaaatCtcggtggaac	m5C_57208
chr17	8023633	-	C	289	64	0.221	0	0	T	225	M	0.177	0.273	0	0	0	227.084	ccaccgctgcCaggctggagt	m5C_57361
chr17	8023634	-	C	297	67	0.226	0	0	T	230	M	0.182	0.276	0	0	0	243.521	cccaccgctgCcaggctggag	m5C_57323
chr17	8023637	-	C	288	61	0.212	0	0	T	227	M	0.169	0.263	0	0	0	205.653	aatcccaccgCtgccaggctg	m5C_57351
chr17	8023640	-	C	280	60	0.214	0	0	T	220	M	0.17	0.266	0	0	0	204.312	tcgaatcccaCcgctgccagg	m5C_57279
chr17	8023643	-	C	280	57	0.204	1	0.004	T	222	M	0.161	0.255	0	0	0	183.719	ggttcgaatcCcaccgctgcc	m5C_57365
chr17	8023663	-	C	227	60	0.264	0	0	T	167	M	0.211	0.325	0	0	0	253.462	ccagtctcttCggaggcgtgg	m5C_57262
chr17	8023666	-	C	199	43	0.216	0	0	T	156	M	0.165	0.278	0	0	0	141.524	gctccagtctCttcggaggcg	m5C_57339
chr17	8023668	-	C	191	46	0.241	0	0	T	145	M	0.186	0.306	0	0	0	170.835	aggctccagtCtcttcggagg	m5C_57287
chr17	8023673	-	C	199	47	0.236	0	0	T	152	M	0.183	0.3	0	0	0	171.562	gatttaggctCcagtctcttc	m5C_57297
chr17	8023686	-	C	256	52	0.203	0	0	T	204	M	0.158	0.257	0	0	0	164.734	gtctaaggcgCtggatttagg	m5C_57286
chr17	8023694	-	C	285	86	0.302	0	0	T	199	M	0.251	0.357	0	0	0	432.389	gccgagcggtCtaaggcgctg	m5C_57256
chr17	8023702	-	C	302	66	0.219	0	0	T	236	M	0.176	0.269	0	0	0	231.829	gtagcgtggcCgagcggtcta	m5C_57294
chr17	8023708	-	C	311	98	0.315	0	0	T	213	M	0.266	0.369	0	0	0	521.366	gtgcaggtagCgtggccgagc	m5C_57369
chr17	8024245	+	C	172	67	0.39	0	0	T	105	M	0.32	0.464	0	0	0	428.573	cgtcgggtggCtctgtggcgc	m5C_57227
chr17	8024253	+	C	170	40	0.235	0	0	T	130	M	0.178	0.304	0	0	0	142.253	ggctctgtggCgcaatggata	m5C_57183
chr17	8024255	+	C	168	46	0.274	0	0	T	122	M	0.212	0.346	0	0	0	195.043	ctctgtggcgCaatggatagc	m5C_57168
chr17	8024265	+	C	142	46	0.324	0	0	T	96	M	0.252	0.405	0	0	0	232.288	caatggatagCgcattggact	m5C_57213
chr17	8024313	+	C	79	22	0.278	0	0	T	57	M	0.192	0.386	0	2.8E-11	0	84.3394	gttgtgggttCgaatcccacc	m5C_57193
chr17	8029061	+	C	33	14	0.424	0	0	T	19	M	0.272	0.592	0	9.2E-06	0	76.259	atctttaagcCgcgcattggt	m5C_57242
chr17	8029076	+	C	476	123	0.258	0	0	T	353	M	0.221	0.3	0	0	0	543.958	attggtggttCagtggtagaa	m5C_57240
chr17	8029093	+	C	444	117	0.264	1	0.002	T	326	M	0.225	0.307	0	0	0	526.99	agaattctcgCctgccacgcg	m5C_57216
chr17	8029100	+	C	441	332	0.753	0	0	C	332	M	0.711	0.791	0	0	0	4717.75	tcgcctgccaCgcgggaggcc	m5C_57203
chr17	8029109	+	C	424	118	0.279	1	0.002	T	305	M	0.238	0.324	0	0	0	562.518	acgcgggaggCccgggttcga	m5C_57160
chr17	8029110	+	C	429	134	0.313	1	0.002	T	294	M	0.271	0.359	0	0	0	726.215	cgcgggaggcCcgggttcgat	m5C_57198
chr17	8029111	+	C	418	108	0.258	0	0	T	310	M	0.219	0.302	0	0	0	472.484	gcgggaggccCgggttcgatt	m5C_57215
chr17	8029117	+	C	358	146	0.408	0	0	T	212	M	0.358	0.459	0	0	0	1045.81	ggcccgggttCgattcccggc	m5C_57161
chr17	8029123	+	C	412	105	0.255	0	0	T	307	M	0.215	0.299	0	0	0	451.863	ggttcgattcCcggccaatgc	m5C_57185
chr17	8029124	+	C	400	80	0.2	0	0	T	320	M	0.164	0.242	0	0	0	261.978	gttcgattccCggccaatgca	m5C_57184
chr17	8029135	+	C	363	99	0.273	0	0	T	264	M	0.229	0.321	0	0	0	454.35	ggccaatgcaCgagtacagtt	m5C_57233
chr17	8029141	+	C	133	37	0.278	0	0	T	96	M	0.209	0.36	0	0	0	154.722	tgcacgagtaCagttttcttt	m5C_57219
chr17	8042247	-	C	35	14	0.4	0	0	T	21	M	0.256	0.564	0	9.2E-06	0	71.5414	gcgttggactCgaaatccaat	m5C_57284
chr17	8042256	-	C	72	17	0.236	0	0	T	55	M	0.153	0.346	0	2.2E-12	0	52.0084	gtggttaaggCgttggactcg	m5C_57345
chr17	8042269	-	C	84	28	0.333	0	0	T	56	M	0.242	0.439	0	5.8E-12	0	135.392	ctgtgatggcCgagtggttaa	m5C_57285
chr17	8042270	-	C	86	18	0.209	0	0	T	68	M	0.137	0.307	0	4.5E-14	0	49.211	gctgtgatggCcgagtggtta	m5C_57312
chr17	8042282	-	C	31	11	0.355	0	0	T	20	M	0.211	0.531	1.1E-16	3.1E-06	1.5E-16	37.0595	acagcgcacgCggctgtgatg	m5C_57261

chr17	8076776	-	C	81	51	0.63	0	0	C	51	M	0.521	0.727	0	2.1E-09	0	531.252	ttgctcctgtCtgattctttc	m5C_57250
chr17	8076781	-	C	110	24	0.218	0	0	T	86	M	0.151	0.304	0	0	0	72.5897	cctgattgctCctgtctgatt	m5C_57364
chr17	8076791	-	C	121	38	0.314	0	0	T	83	M	0.238	0.401	0	0	0	180.996	cttgcaacacCctgattgctc	m5C_57358
chr17	8076794	-	C	121	48	0.397	0	0	T	73	M	0.314	0.486	0	1.3E-15	0	301.437	ggacttgcaaCaccctgattg	m5C_57374
chr17	8076797	-	C	120	52	0.433	0	0	T	68	M	0.348	0.523	0	1.4E-13	0	362.024	tcaggacttgCaacaccctga	m5C_57319
chr17	8076801	-	C	119	66	0.555	0	0	C	66	M	0.465	0.641	0	3E-12	0	613.808	gtaatcaggaCttgcaacacc	m5C_57270
chr17	8076812	-	C	116	32	0.276	0	0	T	84	M	0.203	0.363	0	2.2E-16	0	129.73	aacgtctctgCgtaatcagga	m5C_57305
chr17	8076815	-	C	132	28	0.212	0	0	T	104	M	0.151	0.289	0	0	0	84.579	attaacgtctCtgcgtaatca	m5C_57310
chr17	8076817	-	C	127	42	0.331	0	0	T	85	M	0.255	0.416	0	2.2E-16	0	214.126	tgattaacgtCtctgcgtaat	m5C_57300
chr17	8076820	-	C	130	62	0.477	0	0	T	68	M	0.393	0.562	0	5.3E-14	0	487.275	acgtgattaaCgtctctgcgt	m5C_57271
chr17	8076853	-	C	294	60	0.204	0	0	T	234	M	0.162	0.254	0	0	0	194.361	cagattagaaCatgatgattg	m5C_57338
chr17	8076870	-	C	177	50	0.282	0	0	T	127	M	0.221	0.353	0	0	0	221.326	gttcctcctcCggagggcaga	m5C_57266
chr17	8076883	-	C	106	28	0.264	0	0	T	78	M	0.19	0.355	0	1.8E-15	0	106.141	ataatccttaCctgttcctcc	m5C_57246
chr17	8076901	-	C	63	23	0.365	0	0	T	40	M	0.257	0.489	0	2.1E-09	0	118.29	tctgtatcgtCaggtgggata	m5C_57298
chr17	8089679	+	C	34	11	0.324	0	0	T	23	M	0.191	0.492	4.4E-16	3.1E-06	5.8E-16	32.3088	gccggccgacCtcgtggcgca	m5C_57239
chr17	8089681	+	C	38	10	0.263	0	0	T	28	M	0.15	0.42	9.4E-14	2E-06	1.2E-13	19.5037	cggccgacctCgtggcgcaac	m5C_57159
chr17	8089686	+	C	36	20	0.556	0	0	C	20	M	0.396	0.705	0	4.3E-05	0	158.323	gacctcgtggCgcaacggtag	m5C_57238
chr17	8089691	+	C	37	8	0.216	0	0	T	29	M	0.114	0.372	9.7E-11	6.8E-07	1.1E-10	9.12273	cgtggcgcaaCggtagcgcgt	m5C_57176
chr17	8089699	+	C	39	9	0.231	0	0	T	30	M	0.126	0.383	4.9E-12	1.2E-06	5.7E-12	12.8739	aacggtagcgCgtctgactcc	m5C_57194
chr17	8089702	+	C	39	9	0.231	0	0	T	30	M	0.126	0.383	4.9E-12	1.2E-06	5.7E-12	12.8739	ggtagcgcgtCtgactccaga	m5C_57224
chr17	8089706	+	C	39	8	0.205	0	0	T	31	M	0.108	0.355	1.6E-10	6.8E-07	1.8E-10	8.44384	gcgcgtctgaCtccagatcag	m5C_57200
chr17	8090186	+	C	296	61	0.206	0	0	T	235	M	0.164	0.256	0	0	0	199.968	tgtctgcagaCgaggtggccg	m5C_57237
chr17	8090194	+	C	469	159	0.341	3	0.006	T	307	M	0.3	0.385	0	0	0	952.759	gacgaggtggCcgagtggtta	m5C_57226
chr17	8090195	+	C	464	105	0.227	1	0.002	T	358	M	0.191	0.267	0	0	0	401.053	acgaggtggcCgagtggttaa	m5C_57231
chr17	8090208	+	C	339	92	0.271	0	0	T	247	M	0.227	0.321	0	0	0	417.326	gtggttaaggCgatggactgc	m5C_57207
chr17	8090223	+	C	180	42	0.233	0	0	T	138	M	0.178	0.3	0	0	0	149.11	gactgctaatCcattgtgctc	m5C_57180
chr17	8090238	+	C	44	19	0.432	0	0	T	25	M	0.297	0.578	0	1E-06	0	112.794	gtgctctgcaCgcgtgggttc	m5C_57220
chr17	8090248	+	C	41	9	0.22	0	0	T	32	M	0.12	0.367	8.4E-12	1.2E-08	9.9E-12	11.9621	cgcgtgggttCgaatcccatc	m5C_57187
chr17	8090259	+	C	40	20	0.5	0	0	CT	20	M	0.352	0.648	0	1.4E-06	0	140.797	gaatcccatcCtcgtcggcta	m5C_57211
chr17	8090261	+	C	38	10	0.263	0	0	T	28	M	0.15	0.42	9.4E-14	2E-06	1.2E-13	19.5037	atcccatcctCgtcggctaag	m5C_57243
chr17	8090267	+	C	42	23	0.548	0	0	C	23	M	0.399	0.688	0	2.9E-06	0	183.765	tcctcgtcggCtaaggaagtc	m5C_57196
chr17	8090283	+	C	44	18	0.409	0	0	T	26	M	0.277	0.556	0	7.7E-07	0	99.6755	aagtcctgtgCtcagttttgt	m5C_57218
chr17	8090285	+	C	45	10	0.222	0	0	T	35	M	0.125	0.363	7.4E-13	2.4E-08	8.9E-13	15.2157	gtcctgtgctCagttttgtag	m5C_57204
chr17	8090978	+	C	34	9	0.265	0	0	T	25	M	0.146	0.431	1.1E-12	1.2E-06	1.3E-12	15.7394	gatccccgtaCgggccaggat	m5C_57190
chr17	8090982	+	C	49	20	0.408	0	0	T	29	M	0.282	0.548	0	1.4E-06	0	112.86	cccgtacgggCcaggattgaa	m5C_57171
chr17	8124220	-	C	38	9	0.237	0	0	T	29	M	0.13	0.392	3.7E-12	1.2E-06	4.3E-12	13.3739	gactccagatCagaaggttgc	m5C_57359
chr17	8124226	-	C	80	27	0.338	0	0	T	53	M	0.243	0.446	0	1.9E-10	0	131.465	gcgtctgactCcagatcagaa	m5C_57340
chr17	8124237	-	C	103	30	0.291	0	0	T	73	M	0.212	0.385	0	4.7E-15	0	127.373	gcaacggtagCgcgtctgact	m5C_57342
chr17	8124246	-	C	109	27	0.248	0	0	T	82	M	0.176	0.336	0	1.1E-15	0	95.1394	cctcgtggcgCaacggtagcg	m5C_57352
chr17	8124253	-	C	99	34	0.343	0	0	T	65	M	0.257	0.441	0	1.1E-12	0	174.978	ggacaggcctCgtggcgcaac	m5C_57356
chr17	8124893	+	C	670	162	0.242	0	0	T	508	M	0.211	0.276	0	0	0	683.318	gtaagcatagCtgccttccaa	m5C_57164
chr17	8124900	+	C	636	142	0.223	0	0	T	494	M	0.193	0.257	0	0	0	547.044	tagctgccttCcaagcagttg	m5C_57205
chr17	8124912	+	C	524	130	0.249	1	0.002	T	393	M	0.213	0.287	0	0	0	554.968	aagcagttgaCccgggttcga	m5C_57169
chr17	8124913	+	C	531	197	0.371	0	0	T	334	M	0.331	0.413	0	0	0	1304.04	agcagttgacCcgggttcgat	m5C_57222
chr17	8124914	+	C	492	124	0.252	0	0	T	368	M	0.216	0.292	0	0	0	534.908	gcagttgaccCgggttcgatt	m5C_57175
chr17	8124920	+	C	221	87	0.394	0	0	T	134	M	0.332	0.459	0	0	0	576.975	gacccgggttCgattcccggc	m5C_57244
chr17	8124941	+	C	57	33	0.579	0	0	C	33	M	0.45	0.698	0	8.8E-07	0	296.867	caacgcaagtCgttttgggtg	m5C_57179
chr17	8129555	-	C	37	21	0.568	0	0	C	21	M	0.409	0.713	0	5.2E-05	0	171.838	cccagcggtgCctttattcaa	m5C_57311
chr17	8129560	-	C	41	19	0.463	0	0	T	22	M	0.321	0.613	0	1E-06	0	121.813	cgaatcccagCggtgccttta	m5C_57327
chr17	8129563	-	C	40	13	0.325	0	0	T	27	M	0.201	0.48	0	1.2E-07	0	52.2192	gttcgaatccCagcggtgcct	m5C_57288
chr17	8129564	-	C	40	28	0.7	0	0	C	28	M	0.546	0.819	0	8.1E-06	0	305.59	ggttcgaatcCcagcggtgcc	m5C_57274
chr17	8129565	-	C	43	19	0.442	0	0	T	24	M	0.304	0.589	0	1E-06	0	115.646	gggttcgaatCccagcggtgc	m5C_57331
chr17	8280903	-	C	44	18	0.409	0	0	T	26	M	0.277	0.556	0	7.7E-07	0	99.6755	aggaagaaacCattgagaaga	m5C_57268
chr17	8280904	-	C	36	16	0.444	0	0	T	20	M	0.295	0.604	0	1.7E-05	0	94.5313	aaggaagaaaCcattgagaag	m5C_57370
chr17	8280915	-	C	36	9	0.25	0	0	T	27	M	0.138	0.411	2E-12	1.2E-06	2.4E-12	14.4765	agggcaaataCaaggaagaaa	m5C_57326
chr17	8280921	-	C	36	19	0.528	0	0	C	19	M	0.37	0.68	0	3.4E-05	0	140.622	aggaaaagggCaaatacaagg	m5C_57269
chr17	8413187	-	C	87	28	0.322	0	0	T	59	M	0.233	0.426	0	5.8E-12	0	130.483	aggccaagatCaagaagatgg	m5C_57282
chr17	8413203	-	C	88	31	0.352	0	0	T	57	M	0.261	0.456	0	1.7E-11	0	161.548	gaaaaggtgaCagcagaggcc	m5C_57249
chr17	11985233	+	C	41	22	0.537	0	0	C	22	M	0.387	0.679	0	2.3E-06	0	170.483	aggtgcggggCtagggctaac	m5C_56625
chr17	15373779	-	C	42	15	0.357	0	0	T	27	M	0.23	0.508	0	2.8E-07	0	68.967	cggtggactaCctggtttgat	m5C_56693
chr17	15583574	-	C	35	12	0.343	0	0	T	23	M	0.208	0.508	0	4.6E-06	0	49.9956	atgtgtctggCtgagagagat	m5C_56690
chr17	15942782	-	C	59	17	0.288	0	0	T	42	M	0.188	0.414	0	1.1E-08	0	64.0436	agtggtgagaCacgaagagag	m5C_56688
chr17	15942793	-	C	56	15	0.268	0	0	T	41	M	0.17	0.396	0	4.6E-09	0	50.8715	cagttgtgacCagtggtgaga	m5C_56689
chr17	16342848	+	C	481	205	0.426	0	0	T	276	M	0.383	0.471	0	0	0	1569.3	ataggaagtgCcgtcagaagc	m5C_60263
chr17	16342871	+	C	467	101	0.216	0	0	T	366	M	0.181	0.256	0	0	0	366.285	taactgacgaCgtctaatgtc	m5C_60259
chr17	16342881	+	C	416	85	0.205	2	0.005	T	329	M	0.169	0.247	0	0	0	287.636	cgtctaatgtCtatctgaccg	m5C_60249
chr17	16342885	+	C	349	97	0.278	0	0	T	252	M	0.234	0.327	0	0	0	453.083	taatgtctatCtgaccgcagt	m5C_60252
chr17	16342890	+	C	212	52	0.246	1	0.005	T	159	M	0.193	0.309	0	0	0	200.903	tctatctgacCgcagtcgctg	m5C_60280
chr17	16343354	+	C	188	45	0.239	0	0	T	143	M	0.184	0.305	0	0	0	165.577	cttgactgctCtgatgaaatc	m5C_60285
chr17	16343379	+	C	995	312	0.314	1	0.001	T	682	M	0.286	0.343	0	0	0	1783.37	ataggaagtgCcgtcagaagc	m5C_60256
chr17	16343415	+	C	695	194	0.279	0	0	T	501	M	0.247	0.314	0	0	0	958.632	ctactcctgtCtgattgcagt	m5C_60254
chr17	16344617	+	C	35	8	0.229	0	0	T	27	M	0.121	0.39	5.6E-11	6.8E-07	6.4E-11	9.89691	ctgaagatatCtcttaccctg	m5C_60295
chr17	16344619	+	C	31	9	0.29	0	0	T	22	M	0.161	0.466	3.7E-13	1.2E-06	4.4E-13	18.0154	gaagatatctCttaccctgtg	m5C_60288
chr17	16613618	+	C	78	43	0.551	0	0	C	43	M	0.441	0.657	0	1.1E-08	0	379.339	gtagtggggtCgtgcctgtga	m5C_60269
chr17	16613622	+	C	69	15	0.217	0	0	T	54	M	0.136	0.328	0	6E-11	0	40.9219	tggggtcgtgCctgtgaatag	m5C_60251
chr17	17120021	+	C	38	8	0.211	0	0	T	30	M	0.111	0.363	1.3E-10	6.8E-07	1.4E-10	8.77246	cttgtctggaCcgccagtcac	m5C_60289
chr17	17120022	+	C	36	8	0.222	0	0	T	28	M	0.117	0.381	7.4E-11	6.8E-07	8.4E-11	9.49676	ttgtctggacCgccagtcaca	m5C_60262
chr17	17717209	-	C	35	9	0.257	0	0	T	26	M	0.142	0.421	1.5E-12	1.2E-06	1.7E-12	15.0859	ttgcattgcaCgtgtgtgagg	m5C_60303
chr17	17717216	-	C	38	15	0.395	0	0	T	23	M	0.256	0.553	0	1.2E-05	0	76.7936	attgctgttgCattgcacgtg	m5C_60297
chr17	18508370	-	C	38	8	0.211	0	0	T	30	M	0.111	0.363	1.3E-10	6.8E-07	1.4E-10	8.77246	gagacctcatCtcttactaaa	m5C_67805
chr17	18508386	-	C	84	30	0.357	0	0	T	54	M	0.263	0.464	0	1.2E-11	0	157.795	gcctgggcaaCatagtgagac	m5C_67788
chr17	18508395	-	C	87	32	0.368	0	0	T	55	M	0.274	0.473	0	2.3E-11	0	175.415	tgcattccagCctgggcaaca	m5C_67780
chr17	18508398	-	C	87	22	0.253	0	0	T	65	M	0.173	0.353	0	4.4E-13	0	76.2656	tgctgcattcCagcctgggca	m5C_67762
chr17	18508406	-	C	102	24	0.235	0	0	T	78	M	0.164	0.326	0	2.2E-16	0	78.4895	tgaatagctgCtgcattccag	m5C_67709
chr17	18508419	-	C	106	28	0.264	0	0	T	78	M	0.19	0.355	0	1.8E-15	0	106.141	gggatcgtgcCtgtgaatagc	m5C_67760
chr17	18508424	-	C	101	30	0.297	0	0	T	71	M	0.217	0.392	0	4.7E-15	0	130.009	gtagtgggatCgtgcctgtga	m5C_67742
chr17	18965333	+	C	215	43	0.2	0	0	T	172	M	0.152	0.259	0	0	0	130.727	ttctcctgagCgtgaagccgg	m5C_67685
chr17	18965340	+	C	174	40	0.23	0	0	T	134	M	0.174	0.298	0	0	0	138.884	gagcgtgaagCcggctttctg	m5C_67654
chr17	18965344	+	C	162	36	0.226	3	0.019	T	123	M	0.168	0.297	0	0	0	121.151	gtgaagccggCtttctggcgt	m5C_67664
chr17	18965352	+	C	234	53	0.226	0	0	T	181	M	0.178	0.284	0	0	0	188.187	ggctttctggCgttgcttggc	m5C_67668
chr17	18965371	+	C	312	64	0.205	0	0	T	248	M	0.164	0.253	0	0	0	209.968	gctgcaactgCcgtcagccat	m5C_67661
chr17	18965379	+	C	323	81	0.251	0	0	T	242	M	0.207	0.301	0	0	0	334.723	tgccgtcagcCattgatgatc	m5C_67645
chr17	18965396	+	C	258	77	0.298	0	0	T	181	M	0.246	0.357	0	0	0	378.691	gatcgttcttCtctccgtatt	m5C_67673
chr17	18965398	+	C	265	53	0.2	0	0	T	212	M	0.156	0.252	0	0	0	165.656	tcgttcttctCtccgtattgg	m5C_67703
chr17	18965400	+	C	288	70	0.243	0	0	T	218	M	0.197	0.296	0	0	0	275.954	gttcttctctCcgtattgggg	m5C_67636
chr17	18965427	+	C	392	97	0.247	0	0	T	295	M	0.207	0.292	0	0	0	402.197	agggagagaaCgcggtctgag	m5C_67692
chr17	18965433	+	C	215	119	0.553	0	0	C	119	M	0.487	0.618	0	0	0	1158.29	agaacgcggtCtgagtggttt	m5C_67669
chr17	18967241	-	C	508	169	0.333	0	0	T	339	M	0.293	0.375	0	0	0	990.658	agaacgcggtCtgagtggttt	m5C_67792
chr17	18967247	-	C	595	120	0.202	2	0.003	T	473	M	0.172	0.237	0	0	0	412.77	agggagagaaCgcggtctgag	m5C_67726
chr17	18967273	-	C	221	64	0.29	0	0	T	157	M	0.234	0.353	0	0	0	299.253	ttcttctctcCgtattgggga	m5C_67728
chr17	18967278	-	C	206	46	0.223	0	0	T	160	M	0.172	0.285	0	0	0	158.048	gatcgttcttCtctccgtatt	m5C_67812

chr17	18967281	-	C	207	44	0.213	0	0	T	163	M	0.162	0.273	0	0	0	142.847	gatgatcgttCttctctccgt	m5C_67795
chr17	18978458	+	C	129	29	0.225	0	0	T	100	M	0.161	0.304	0	0	0	93.5712	gaggagcaggCggctgagtga	m5C_67634
chr17	18978461	+	C	130	41	0.315	0	0	T	89	M	0.242	0.4	0	0	0	198.261	gagcaggcggCtgagtgagag	m5C_67653
chr17	19015741	-	C	416	167	0.401	0	0	T	249	M	0.355	0.449	0	0	0	1187.19	agaacgcggtCtgagtggttt	m5C_67790
chr17	19015773	-	C	284	79	0.278	0	0	T	205	M	0.229	0.333	0	0	0	362.26	ttcttctctcCgtattgggga	m5C_67723
chr17	19015774	-	C	277	65	0.235	0	0	T	212	M	0.189	0.288	0	0	0	245.166	gttcttctctCcgtattgggg	m5C_67815
chr17	19015778	-	C	229	76	0.332	0	0	T	153	M	0.274	0.395	0	0	0	416.638	gatcgttcttCtctccgtatt	m5C_67758
chr17	19015781	-	C	179	46	0.257	0	0	T	133	M	0.199	0.326	0	0	0	182.661	gatgatcgttCttctctccgt	m5C_67817
chr17	19015796	-	C	330	86	0.261	0	0	T	244	M	0.216	0.311	0	0	0	371.85	ctgccgtcagCcattgatgat	m5C_67719
chr17	19015803	-	C	242	49	0.202	0	0	T	193	M	0.157	0.258	0	0	0	153.547	gctgcaactgCcgtcagccat	m5C_67767
chr17	19015817	-	C	234	61	0.261	0	0	T	173	M	0.209	0.32	0	0	0	254.519	tctggcgttgCttggctgcaa	m5C_67710
chr17	19015834	-	C	235	71	0.302	0	0	T	164	M	0.247	0.364	0	0	0	350.723	gagcgtgaagCcggctttctg	m5C_67757
chr17	19015841	-	C	282	63	0.223	0	0	T	219	M	0.179	0.276	0	0	0	225.151	ttctcctgagCgtgaagccgg	m5C_67789
chr17	19015846	-	C	263	76	0.289	0	0	T	187	M	0.238	0.346	0	0	0	361.059	gcgttttctcCtgagcgtgaa	m5C_67753
chr17	19015849	-	C	282	57	0.202	0	0	T	225	M	0.159	0.253	0	0	0	181.72	gtagcgttttCtcctgagcgt	m5C_67731
chr17	19015892	-	C	33	8	0.242	0	0	T	25	M	0.128	0.41	3.1E-11	6.8E-07	3.6E-11	10.7866	tttctttgaaCgtgtagagca	m5C_67751
chr17	19015911	-	C	51	16	0.314	0	0	T	35	M	0.203	0.45	0	7.3E-09	0	65.048	tagtgtgttaCtagagaagtt	m5C_67752
chr17	19091379	+	C	108	28	0.259	0	0	T	80	M	0.186	0.349	0	1.8E-15	0	104.098	agagaagtttCtctgaacgtg	m5C_67651
chr17	19091386	+	C	203	60	0.296	0	0	T	143	M	0.237	0.362	0	0	0	284.475	tttctctgaaCgtgtagagca	m5C_67681
chr17	19091397	+	C	383	116	0.303	0	0	T	267	M	0.259	0.351	0	0	0	600.874	gtgtagagcaCcgaaaaccac	m5C_67635
chr17	19091398	+	C	391	91	0.233	0	0	T	300	M	0.194	0.277	0	0	0	352.304	tgtagagcacCgaaaaccacg	m5C_67675
chr17	19091431	+	C	1489	385	0.259	0	0	T	1104	M	0.237	0.281	0	0	0	1824.63	agcgttttctCctgagcgtga	m5C_67706
chr17	19091432	+	C	1479	350	0.237	0	0	T	1129	M	0.216	0.259	0	0	0	1509.79	gcgttttctcCtgagcgtgaa	m5C_67672
chr17	19091437	+	C	1429	343	0.24	0	0	T	1086	M	0.219	0.263	0	0	0	1499.59	ttctcctgagCgtgaagccgg	m5C_67705
chr17	19091445	+	C	1214	285	0.237	11	0.009	T	918	M	0.214	0.262	0	0	0	1218.33	agcgtgaagcCggctttctgg	m5C_67704
chr17	19091448	+	C	1191	343	0.288	2	0.002	T	846	M	0.263	0.315	0	0	0	1807.19	gtgaagccggCtttctggcgt	m5C_67658
chr17	19091469	+	C	819	166	0.203	3	0.004	T	650	M	0.177	0.232	0	0	0	588.405	tgcttggctgCaactgccgtc	m5C_67698
chr17	19091472	+	C	769	172	0.224	0	0	T	597	M	0.196	0.254	0	0	0	672.966	ttggctgcaaCtgccgtcagc	m5C_67637
chr17	19091479	+	C	640	151	0.237	2	0.003	T	487	M	0.205	0.271	0	0	0	620.103	caactgccgtCagccattgat	m5C_67682
chr17	19091500	+	C	458	119	0.26	0	0	T	339	M	0.222	0.302	0	0	0	527.83	gatcgttcttCtctccgtatt	m5C_67686
chr17	19091537	+	C	1986	790	0.398	0	0	T	1196	M	0.376	0.419	0	0	0	5948.31	agaacgcggtCtgagtggttt	m5C_67683
chr17	19093350	-	C	104	40	0.385	0	0	T	64	M	0.297	0.481	0	1.8E-13	0	237.449	agaacgcggtCtgagtggttt	m5C_67716
chr17	19093356	-	C	117	34	0.291	0	0	T	83	M	0.216	0.378	0	4.4E-16	0	146.899	agggagagaaCgcggtctgag	m5C_67797
chr17	19093382	-	C	32	15	0.469	0	0	T	17	M	0.309	0.636	0	1.2E-05	0	92.6074	ttcttctctcCgtattgggga	m5C_67743
chr17	19093383	-	C	31	9	0.29	0	0	T	22	M	0.161	0.466	3.7E-13	1.2E-06	4.4E-13	18.0154	gttcttctctCcgtattgggg	m5C_67771
chr17	19093385	-	C	40	12	0.3	0	0	T	28	M	0.181	0.454	1.1E-16	7.5E-08	1.5E-16	34.6049	tcgttcttctCtccgtattgg	m5C_67708
chr17	19093387	-	C	38	11	0.289	0	0	T	27	M	0.17	0.448	2.1E-15	3.1E-06	2.7E-15	27.4488	gatcgttcttCtctccgtatt	m5C_67786
chr17	19093394	-	C	35	7	0.2	0	0	T	28	M	0.1	0.359	1.9E-09	3.4E-07	2.1E-09	6.13884	cattgatgatCgttcttctct	m5C_67754
chr17	19093442	-	C	34	8	0.235	0	0	T	26	M	0.124	0.4	4.2E-11	6.8E-07	4.8E-11	10.3259	aacgtgaagcCggctttctgg	m5C_67783
chr17	19093443	-	C	31	8	0.258	0	0	T	23	M	0.137	0.432	1.7E-11	6.8E-07	1.9E-11	11.8182	gaacgtgaagCcggctttctg	m5C_67821
chr17	19093456	-	C	238	52	0.218	0	0	T	186	M	0.171	0.275	0	0	0	177.515	agcgttttctCctgaacgtga	m5C_67761
chr17	19093480	-	C	446	91	0.208	8	0.018	T	347	M	0.172	0.248	0	0	0	313.747	ccgaaaaccaCgaggaagaga	m5C_67808
chr17	19093489	-	C	311	78	0.251	0	0	T	233	M	0.206	0.302	0	0	0	321.151	tgtagagcacCgaaaaccacg	m5C_67741
chr17	19093501	-	C	200	41	0.205	0	0	T	159	M	0.155	0.266	0	0	0	126.986	tttctctgaaCgtgtagagca	m5C_67820
chr17	19093506	-	C	106	23	0.217	0	0	T	83	M	0.149	0.305	0	1.1E-16	0	68.6143	agaagtttctCtgaacgtgta	m5C_67759
chr17	19093508	-	C	77	22	0.286	0	0	T	55	M	0.197	0.395	0	2.8E-11	0	86.6333	agagaagtttCtctgaacgtg	m5C_67774
chr17	19206542	-	C	45	21	0.467	0	0	T	24	M	0.329	0.609	0	1.8E-06	0	138.327	gtgacagtggCtgtgtgactc	m5C_67777
chr17	19247843	-	C	31	8	0.258	0	0	T	23	M	0.137	0.432	1.7E-11	6.8E-07	1.9E-11	11.8182	tggttgtttcCggctcgcgtg	m5C_67799
chr17	19411517	+	C	160	48	0.304	2	0.013	T	110	M	0.237	0.379	0	0	0	227.949	aatggtagcgCgtctgactcc	m5C_67665
chr17	19411524	+	C	145	36	0.248	0	0	T	109	M	0.185	0.324	0	0	0	133.246	gcgcgtctgaCtccagatcag	m5C_67684
chr17	19411564	+	C	56	32	0.571	0	0	C	32	M	0.441	0.692	0	7.2E-07	0	282.479	acgtcggggtCaaggttgggt	m5C_67657
chr17	19412444	-	C	34	20	0.588	0	0	C	20	M	0.422	0.736	0	4.3E-05	0	168.885	atctgtggatCtgattctgct	m5C_67727
chr17	19764187	+	C	52	36	0.692	0	0	C	36	M	0.557	0.801	0	1.5E-06	0	401.257	attggtggttCaatggtagaa	m5C_67693
chr17	20130927	+	C	43	10	0.233	0	0	T	33	M	0.132	0.377	4.3E-13	2.4E-08	5.2E-13	16.2683	catcagtgagCtagaaagtaa	m5C_56848
chr17	20135094	+	C	100	22	0.22	0	0	T	78	M	0.15	0.311	0	0	0	66.0052	gctggaggaaCagaagtcaga	m5C_56847
chr17	20135101	+	C	95	37	0.389	0	0	T	58	M	0.298	0.49	0	2.7E-12	0	220.179	gaacagaagtCagacctggag	m5C_56849
chr17	26648530	+	C	32	10	0.312	0	0	T	22	M	0.18	0.486	1.1E-14	2E-06	1.3E-14	25.0836	gtgatgcagtCatggcttacc	m5C_59023
chr17	26648553	+	C	31	11	0.355	0	0	T	20	M	0.211	0.531	1.1E-16	3.1E-06	1.5E-16	37.0595	agccttgaacCtgagcacaag	m5C_59005
chr17	26942180	-	C	31	11	0.355	0	0	T	20	M	0.211	0.531	1.1E-16	3.1E-06	1.5E-16	37.0595	cctgaacatgCaacagcagga	m5C_59062
chr17	26942184	-	C	32	10	0.312	0	0	T	22	M	0.18	0.486	1.1E-14	2E-06	1.3E-14	25.0836	cggacctgaaCatgcaacagc	m5C_59054
chr17	26942190	-	C	36	20	0.556	0	0	C	20	M	0.396	0.705	0	4.3E-05	0	158.323	ctaaatcggaCctgaacatgc	m5C_59055
chr17	26942194	-	C	35	18	0.514	0	0	C	18	M	0.356	0.67	0	2.7E-05	0	128.047	gaaactaaatCggacctgaac	m5C_59107
chr17	27041428	-	C	44	19	0.432	0	0	T	25	M	0.297	0.578	0	1E-06	0	112.794	tccagggcgcCcagtggtagt	m5C_59056
chr17	27041431	-	C	46	29	0.63	0	0	C	29	M	0.486	0.755	0	9.7E-06	0	281.879	ctgtccagggCgcccagtggt	m5C_59065
chr17	27041436	-	C	44	11	0.25	0	0	T	33	M	0.146	0.394	1.6E-14	4.4E-08	2E-14	22.132	ggggactgtcCagggcgccca	m5C_59069
chr17	27041437	-	C	44	24	0.545	0	0	C	24	M	0.401	0.683	0	3.6E-06	0	192.316	aggggactgtCcagggcgccc	m5C_59068
chr17	27047596	+	C	741	205	0.277	0	0	T	536	M	0.246	0.31	0	0	0	1007.2	ttttaatctcCtgacacttgt	m5C_59001
chr17	27047612	+	C	808	166	0.206	1	0.001	T	641	M	0.179	0.235	0	0	0	595.067	cttgtgatgtCttcaaaggaa	m5C_59048
chr17	27047623	+	C	721	211	0.293	0	0	T	510	M	0.261	0.327	0	0	0	1099.76	ttcaaaggaaCcactgatgca	m5C_59046
chr17	27047626	+	C	617	173	0.28	0	0	T	444	M	0.246	0.317	0	0	0	852.501	aaaggaaccaCtgatgcacct	m5C_59028
chr17	27047634	+	C	61	24	0.393	0	0	T	37	M	0.281	0.519	0	3E-09	0	134.727	cactgatgcaCctgtggccag	m5C_59043
chr17	27049614	+	C	234	67	0.289	2	0.009	T	165	M	0.234	0.35	0	0	0	313.949	cagatgatgaCactgtaaagc	m5C_59022
chr17	27049624	+	C	227	79	0.348	0	0	T	148	M	0.289	0.412	0	0	0	456.692	cactgtaaagCgaccaaagtc	m5C_59008
chr17	27049627	+	C	237	79	0.333	0	0	T	158	M	0.276	0.396	0	0	0	436.705	tgtaaagcgaCcaaagtctga	m5C_59006
chr17	27049634	+	C	222	82	0.369	0	0	T	140	M	0.309	0.435	0	0	0	506.113	cgaccaaagtCtgaacaaagt	m5C_58989
chr17	27049653	+	C	190	48	0.254	1	0.005	T	141	M	0.197	0.321	0	0	0	189.349	gtgattggtaCctcgttgtct	m5C_58994
chr17	27049654	+	C	191	57	0.298	0	0	T	134	M	0.238	0.367	0	0	0	271.355	tgattggtacCtcgttgtctg	m5C_58991
chr17	27049656	+	C	196	47	0.24	0	0	T	149	M	0.185	0.304	0	0	0	174.267	attggtacctCgttgtctgat	m5C_59045
chr17	27049662	+	C	192	58	0.302	0	0	T	134	M	0.242	0.37	0	0	0	280.186	acctcgttgtCtgatgcacct	m5C_59041
chr17	27049668	+	C	93	19	0.204	0	0	T	74	M	0.135	0.297	0	8.9E-16	0	51.2526	ttgtctgatgCacctaggctc	m5C_58988
chr17	27049670	+	C	46	22	0.478	0	0	T	24	M	0.341	0.619	0	2.3E-06	0	150.148	gtctgatgcaCctaggctctc	m5C_59042
chr17	27049877	+	C	33	23	0.697	0	0	C	23	M	0.527	0.826	0	7.6E-05	0	242.243	tgtgaagaagCtgtatgacat	m5C_58996
chr17	27049885	+	C	38	19	0.5	0	0	CT	19	M	0.348	0.652	0	3.4E-05	0	132.429	agctgtatgaCattgatgtgg	m5C_58993
chr17	27050491	+	C	2021	425	0.219	81	0.04	T	1515	M	0.201	0.238	0	0	0	1710.47	atgacatgaaCaaaggaacca	m5C_59038
chr17	27050499	+	C	1869	458	0.245	0	0	T	1411	M	0.226	0.265	0	0	0	2070.95	aacaaaggaaCcactgaagtg	m5C_59010
chr17	27050502	+	C	1697	474	0.279	0	0	T	1223	M	0.258	0.301	0	0	0	2450.45	aaaggaaccaCtgaagtgccg	m5C_59050
chr17	27050726	+	C	713	188	0.271	18	0.025	T	507	M	0.239	0.305	0	0	0	897.909	atgttagcgaCcaaagcctga	m5C_58992
chr17	27050733	+	C	703	347	0.494	0	0	T	356	M	0.457	0.53	0	0	0	3170.02	cgaccaaagcCtgatctttgc	m5C_59009
chr17	27050738	+	C	700	163	0.234	2	0.003	T	535	M	0.204	0.266	0	0	0	663.894	aaagcctgatCtttgctgatt	m5C_59017
chr17	28444120	+	C	88	22	0.25	0	0	T	66	M	0.171	0.35	0	4.4E-13	0	75.3641	ggctgaggggCagagagcgag	m5C_57469
chr17	28444132	+	C	73	16	0.219	0	0	T	57	M	0.14	0.327	0	1.2E-12	0	44.6912	gagagcgagaCttttctattt	m5C_57460
chr17	29877123	+	C	44	26	0.591	0	0	C	26	M	0.444	0.723	0	5.5E-06	0	230.929	aggcgtcggtCtcgtaaaccg	m5C_57449
chr17	29877125	+	C	62	14	0.226	0	0	T	48	M	0.14	0.344	0	3.2E-11	0	39.0742	gcgtcggtctCgtaaaccgaa	m5C_57466
chr17	29877139	+	C	107	30	0.28	0	0	T	77	M	0.204	0.372	0	4.7E-15	0	122.41	aaccgaagatCgcgggttcga	m5C_57450
chr17	29877141	+	C	104	22	0.212	0	0	T	82	M	0.144	0.3	0	0	0	63.3825	ccgaagatcgCgggttcgaac	m5C_57462
chr17	29877181	+	C	806	291	0.361	0	0	T	515	M	0.329	0.395	0	0	0	1912.54	cgaggtagggCtttggctgtg	m5C_57447
chr17	29877187	+	C	786	216	0.276	4	0.005	T	566	M	0.246	0.309	0	0	0	1062.84	agggctttggCtgtggggaag	m5C_57444
chr17	29877199	+	C	766	155	0.205	11	0.014	T	600	M	0.178	0.236	0	0	0	551.837	gtggggaagtCgggttttctc	m5C_57467
chr17	29877218	+	C	628	151	0.242	5	0.008	T	472	M	0.21	0.278	0	0	0	635.32	tccacgtacgCcgtcccttct	m5C_57436
chr17	29877222	+	C	630	163	0.259	0	0	T	467	M	0.226	0.294	0	0	0	736.979	cgtacgccgtCccttctacgt	m5C_57470
chr17	29877223	+	C	630	126	0.2	0	0	T	504	M	0.171	0.233	0	0	0	429.974	gtacgccgtcCcttctacgtg	m5C_57454

chr17	29877227	+	C	628	143	0.228	0	0	T	485	M	0.197	0.262	0	0	0	562.339	gccgtcccttCtacgtggcat	m5C_57478
chr17	29887049	+	C	36	10	0.278	0	0	T	26	M	0.158	0.44	4.8E-14	2E-06	5.9E-14	21.1115	tctttgcgggCgagatgaggg	m5C_57441
chr17	29887076	+	C	35	21	0.6	0	0	C	21	M	0.436	0.744	0	5.2E-05	0	183.004	atcaactggcCtacaaagtcc	m5C_57471
chr17	29887079	+	C	37	19	0.514	0	0	C	19	M	0.359	0.666	0	3.4E-05	0	136.4	aactggcctaCaaagtcccag	m5C_57464
chr17	30290885	-	C	68	16	0.235	0	0	T	52	M	0.15	0.349	0	1.1E-10	0	48.11	ctggttggtaCttggatggga	m5C_56918
chr17	30290895	-	C	57	14	0.246	0	0	T	43	M	0.152	0.371	0	2.8E-09	0	42.6516	agggttgggcCtggttggtac	m5C_56954
chr17	30290896	-	C	55	29	0.527	0	0	C	29	M	0.398	0.653	0	3.9E-07	0	230.79	cagggttgggCctggttggta	m5C_56896
chr17	31149420	-	C	142	66	0.465	0	0	T	76	M	0.385	0.547	0	2.2E-16	0	507.887	gcgctggacgCgttatccctc	m5C_56960
chr17	31149422	-	C	153	78	0.51	0	0	C	78	M	0.431	0.588	0	1.1E-16	0	672.869	cggcgctggaCgcgttatccc	m5C_56939
chr17	31149427	-	C	161	83	0.516	0	0	C	83	M	0.439	0.591	0	0	0	728.528	acggacggcgCtggacgcgtt	m5C_56935
chr17	31149429	-	C	164	65	0.396	0	0	T	99	M	0.325	0.473	0	0	0	422.078	ggacggacggCgctggacgcg	m5C_56928
chr17	31149432	-	C	245	158	0.645	0	0	C	158	M	0.583	0.702	0	0	0	1842.79	aacggacggaCggcgctggac	m5C_56921
chr17	31149436	-	C	186	50	0.602	103	0.554	A	103	M	0.495	0.701	0	1.7E-09	0	494.846	gacgaacggaCggacggcgct	m5C_56907
chr17	31149440	-	C	858	350	0.408	0	0	T	508	M	0.376	0.441	0	0	0	2628.66	ggaagacgaaCggacggacgg	m5C_56938
chr17	31149444	-	C	1042	416	0.401	4	0.004	T	622	M	0.371	0.431	0	0	0	3089.85	gggaggaagaCgaacggacgg	m5C_56927
chr17	31149459	-	C	1128	479	0.425	0	0	T	649	M	0.396	0.454	0	0	0	3794.67	cgggagaggcCgggagggagg	m5C_56951
chr17	31149460	-	C	1119	565	0.505	0	0	C	565	M	0.476	0.534	0	0	0	5374.89	gcgggagaggCcgggagggag	m5C_56888
chr17	31149469	-	C	657	274	0.417	0	0	T	383	M	0.38	0.455	0	0	0	2082.03	ccgtggtcggCgggagaggcc	m5C_56904
chr17	31149472	-	C	558	273	0.489	0	0	T	285	M	0.448	0.531	0	0	0	2446	tgcccgtggtCggcgggagag	m5C_56944
chr17	31149478	-	C	229	75	0.328	0	0	T	154	M	0.27	0.391	0	0	0	405.013	caccactgccCgtggtcggcg	m5C_56932
chr17	31149479	-	C	176	41	0.233	0	0	T	135	M	0.177	0.301	0	0	0	144.824	ccaccactgcCcgtggtcggc	m5C_56936
chr17	31149480	-	C	130	27	0.208	0	0	T	103	M	0.147	0.285	0	0	0	79.2972	cccaccactgCccgtggtcgg	m5C_56912
chr17	31149524	-	C	604	196	0.325	0	0	T	408	M	0.288	0.363	0	0	0	1130.43	accgaagaagCggcgcgccga	m5C_56899
chr17	31149532	-	C	1041	401	0.385	0	0	T	640	M	0.356	0.415	0	0	0	2856.06	aggcgggaacCgaagaagcgg	m5C_56959
chr17	31149533	-	C	1065	373	0.35	0	0	T	692	M	0.322	0.379	0	0	0	2403.38	gaggcgggaaCcgaagaagcg	m5C_56903
chr17	31149539	-	C	951	400	0.421	0	0	T	551	M	0.39	0.452	0	0	0	3116.92	ggggaggaggCgggaaccgaa	m5C_56950
chr17	31149550	-	C	288	216	0.75	0	0	C	216	M	0.697	0.796	0	0	0	3010.69	cggcagtgaaCggggaggagg	m5C_56956
chr17	31149605	-	C	246	59	0.24	0	0	T	187	M	0.191	0.297	0	0	0	225.074	gcggccgcctCcgcggttccc	m5C_56923
chr17	31149607	-	C	262	71	0.271	0	0	T	191	M	0.221	0.328	0	0	0	313.492	gcgcggccgcCtccgcggttc	m5C_56929
chr17	31149616	-	C	413	104	0.252	0	0	T	309	M	0.212	0.296	0	0	0	441.732	cccggctcggCgcggccgcct	m5C_56906
chr17	31149619	-	C	414	111	0.268	0	0	T	303	M	0.228	0.313	0	0	0	505.537	gggcccggctCggcgcggccg	m5C_56902
chr17	31149624	-	C	361	130	0.36	0	0	T	231	M	0.312	0.411	0	0	0	811.99	gccacgggccCggctcggcgc	m5C_56952
chr17	31149630	-	C	260	78	0.3	0	0	T	182	M	0.248	0.358	0	0	0	386.161	cggcgggccaCgggcccggct	m5C_56911
chr17	31149632	-	C	203	48	0.236	0	0	T	155	M	0.183	0.299	0	0	0	175.906	gccggcgggcCacgggcccgg	m5C_56926
chr17	31149633	-	C	175	46	0.263	0	0	T	129	M	0.203	0.333	0	0	0	186.977	ggccggcgggCcacgggcccg	m5C_56955
chr17	31149637	-	C	76	17	0.224	0	0	T	59	M	0.145	0.329	0	2.2E-12	0	49.1676	cgggggccggCgggccacggg	m5C_56949
chr17	31149640	-	C	48	13	0.271	0	0	T	35	M	0.166	0.41	0	1.2E-07	0	43.0711	ggacgggggcCggcgggccac	m5C_56917
chr17	31149641	-	C	31	20	0.645	0	0	C	20	M	0.469	0.789	0	4.3E-05	0	187.791	gggacgggggCcggcgggcca	m5C_56909
chr17	31149647	-	C	74	26	0.351	0	0	T	48	M	0.252	0.465	0	1.3E-10	0	131.238	ccccccgggaCgggggccggc	m5C_56914
chr17	31149652	-	C	98	31	0.316	0	0	T	67	M	0.233	0.414	0	3.7E-13	0	144.255	ggccaccccccCgggacggggg	m5C_56890
chr17	31149653	-	C	96	24	0.25	0	0	T	72	M	0.174	0.345	0	1.7E-14	0	83.5865	cggccaccccCcgggacgggg	m5C_56915
chr17	31149655	-	C	94	26	0.277	0	0	T	68	M	0.196	0.374	0	4.6E-14	0	102.075	cgcggccaccCcccgggacgg	m5C_56942
chr17	31149660	-	C	161	43	0.267	0	0	T	118	M	0.205	0.34	0	0	0	176.084	gcccgcgcggCcacccccccgg	m5C_56948
chr17	31149665	-	C	223	82	0.368	0	0	T	141	M	0.307	0.433	0	0	0	503.75	ccggggcccgCgcggccaccc	m5C_56920
chr17	31149667	-	C	235	56	0.238	0	0	T	179	M	0.188	0.297	0	0	0	210.908	caccggggccCgcgcggccac	m5C_56925
chr17	31149668	-	C	241	69	0.286	0	0	T	172	M	0.233	0.346	0	0	0	321.455	ccaccggggcCcgcgcggcca	m5C_56908
chr17	31149669	-	C	254	81	0.319	0	0	T	173	M	0.265	0.379	0	0	0	428.725	cccaccggggCccgcgcggcc	m5C_56905
chr17	31149674	-	C	260	67	0.258	0	0	T	193	M	0.208	0.314	0	0	0	279.159	gccgccccacCggggcccgcg	m5C_56898
chr17	31149675	-	C	247	70	0.283	0	0	T	177	M	0.231	0.343	0	0	0	323.19	ggccgccccaCcggggcccgc	m5C_56958
chr17	31149677	-	C	247	67	0.271	0	0	T	180	M	0.22	0.33	0	0	0	294.298	gtggccgcccCaccggggccc	m5C_56893
chr17	31149679	-	C	198	46	0.232	0	0	T	152	M	0.179	0.296	0	0	0	164.619	gggtggccgcCccaccggggc	m5C_56894
chr17	31149680	-	C	191	50	0.262	0	0	T	141	M	0.205	0.328	0	0	0	204.571	cgggtggccgCcccaccgggg	m5C_56953
chr17	31149682	-	C	173	35	0.202	0	0	T	138	M	0.149	0.268	0	0	0	104.452	cccgggtggcCgccccaccgg	m5C_56933
chr17	31149683	-	C	157	39	0.248	0	0	T	118	M	0.187	0.321	0	0	0	146.148	ccccgggtggCcgccccaccg	m5C_56941
chr17	31149690	-	C	87	41	0.471	0	0	T	46	M	0.37	0.575	0	2.6E-10	0	303.253	gccggaacccCgggtggccgc	m5C_56946
chr17	31149691	-	C	71	18	0.254	0	0	T	53	M	0.167	0.366	0	4E-12	0	60.0495	ggccggaaccCcgggtggccg	m5C_56892
chr17	31149692	-	C	64	38	0.594	0	0	C	38	M	0.471	0.705	0	9.6E-08	0	358.302	gggccggaacCccgggtggcc	m5C_56901
chr17	31149693	-	C	59	31	0.525	0	0	C	31	M	0.4	0.647	0	5.9E-07	0	248.245	agggccggaaCcccgggtggc	m5C_56931
chr17	31149698	-	C	59	12	0.203	0	0	T	47	M	0.12	0.323	2.6E-14	8.8E-10	3.3E-14	19.6113	gcgcgagggcCggaaccccgg	m5C_56900
chr17	31149699	-	C	65	27	0.415	0	0	T	38	M	0.304	0.537	0	7.5E-09	0	163.944	cgcgcgagggCcggaaccccg	m5C_56945
chr17	31149707	-	C	54	15	0.278	0	0	T	39	M	0.176	0.409	0	4.6E-09	0	52.85	aggagggacgCgcgagggccg	m5C_56924
chr17	32953270	-	C	36	20	0.556	0	0	C	20	M	0.396	0.705	0	4.3E-05	0	158.323	ccgcgggtgaCgggggccgcg	m5C_57672
chr17	32953277	-	C	36	8	0.222	0	0	T	28	M	0.117	0.381	7.4E-11	6.8E-07	8.4E-11	9.49676	ttggcgaccgCgggtgacggg	m5C_57770
chr17	32953279	-	C	38	23	0.605	0	0	C	23	M	0.447	0.744	0	7.6E-05	0	205.701	tgttggcgacCgcgggtgacg	m5C_57729
chr17	33478133	-	C	52	15	0.288	0	0	T	37	M	0.183	0.423	0	4.6E-09	0	54.9894	cggtggggcgCgggaaatgtg	m5C_57699
chr17	33478135	-	C	50	36	0.72	0	0	C	36	M	0.583	0.825	0	1.5E-06	0	420.009	cgcggtggggCgcgggaaatg	m5C_57775
chr17	33478143	-	C	70	33	0.471	0	0	T	37	M	0.359	0.587	0	1.1E-09	0	236.971	ccccgccccgCggtggggcgc	m5C_57688
chr17	33478145	-	C	94	67	0.713	0	0	C	67	M	0.614	0.794	0	1.5E-09	0	823.251	atccccgcccCgcggtggggc	m5C_57700
chr17	33478146	-	C	98	44	0.518	13	0.133	C	44	M	0.413	0.621	0	5.2E-10	0	363.417	aatccccgccCcgcggtgggg	m5C_57674
chr17	33478147	-	C	171	105	0.614	0	0	C	105	M	0.539	0.684	0	0	0	1132.58	gaatccccgcCccgcggtggg	m5C_57685
chr17	33478148	-	C	219	133	0.607	0	0	C	133	M	0.541	0.67	0	0	0	1439.88	cgaatccccgCcccgcggtgg	m5C_57718
chr17	33478150	-	C	217	154	0.71	0	0	C	154	M	0.646	0.766	0	0	0	1989.84	gccgaatcccCgccccgcggt	m5C_57756
chr17	33478151	-	C	224	168	0.75	0	0	C	168	M	0.689	0.802	0	0	0	2316.39	cgccgaatccCcgccccgcgg	m5C_57697
chr17	33478152	-	C	229	149	0.651	0	0	C	149	M	0.587	0.709	0	0	0	1748.9	gcgccgaatcCccgccccgcg	m5C_57761
chr17	33478153	-	C	238	163	0.685	0	0	C	163	M	0.623	0.741	0	0	0	2032	agcgccgaatCcccgccccgc	m5C_57709
chr17	33478158	-	C	244	172	0.705	0	0	C	172	M	0.645	0.759	0	0	0	2218.36	agcccagcgcCgaatccccgc	m5C_57734
chr17	33478159	-	C	237	170	0.717	0	0	C	170	M	0.657	0.771	0	0	0	2233.31	gagcccagcgCcgaatccccg	m5C_57738
chr17	33478161	-	C	247	171	0.692	0	0	C	171	M	0.632	0.747	0	0	0	2162.02	aagagcccagCgccgaatccc	m5C_57683
chr17	33478164	-	C	246	159	0.646	0	0	C	159	M	0.585	0.703	0	0	0	1859.55	gggaagagccCagcgccgaat	m5C_57706
chr17	33478235	-	C	887	459	0.517	0	0	C	459	M	0.485	0.55	0	0	0	4448.49	tgaatttaagCatatttgtca	m5C_57725
chr17	33478246	-	C	1277	805	0.631	2	0.002	C	805	M	0.605	0.657	0	0	0	9732.99	tggcgacccgCtgaatttaag	m5C_57721
chr17	33478247	+	C	49	39	0.796	0	0	C	39	M	0.664	0.885	0	4.3E-05	0	517.629	ttaaattcagCgggtcgccac	m5C_57644
chr17	33478248	-	C	1333	853	0.64	0	0	C	853	M	0.614	0.665	0	0	0	10471	cgtggcgaccCgctgaattta	m5C_57676
chr17	33478249	-	C	1372	960	0.701	2	0.001	C	960	M	0.676	0.724	0	0	0	12978.2	acgtggcgacCcgctgaattt	m5C_57690
chr17	33478250	-	C	1398	951	0.68	0	0	C	951	M	0.655	0.704	0	0	0	12464.6	gacgtggcgaCccgctgaatt	m5C_57762
chr17	33478252	+	C	66	50	0.758	0	0	C	50	M	0.642	0.845	0	6.9E-07	0	641.906	ttcagcgggtCgccacgtctg	m5C_57624
chr17	33478253	-	C	1500	1005	0.674	9	0.006	C	1005	M	0.65	0.697	0	0	0	13061.6	tcagacgtggCgacccgctga	m5C_57777
chr17	33478254	+	C	65	45	0.692	0	0	C	45	M	0.572	0.791	0	3.3E-07	0	514.819	cagcgggtcgCcacgtctgat	m5C_57653
chr17	33478255	+	C	65	50	0.769	0	0	C	50	M	0.654	0.855	0	6.9E-07	0	653.551	agcgggtcgcCacgtctgatc	m5C_57623
chr17	33478257	+	C	69	50	0.725	0	0	C	50	M	0.61	0.816	0	6.9E-07	0	609.525	cgggtcgccaCgtctgatctg	m5C_57647
chr17	33478258	-	C	1542	1042	0.679	7	0.005	C	1042	M	0.655	0.702	0	0	0	13651.2	tcagatcagaCgtggcgaccc	m5C_57743
chr17	33478260	+	C	61	48	0.787	0	0	C	48	M	0.669	0.871	0	5.1E-07	0	642.023	gtcgccacgtCtgatctgagg	m5C_57666
chr17	33478262	-	C	1483	1004	0.678	2	0.001	C	1004	M	0.654	0.701	0	0	0	13126.1	gacctcagatCagacgtggcg	m5C_57684
chr17	33478265	+	C	51	30	0.588	0	0	C	30	M	0.452	0.712	0	4.8E-07	0	270.991	cacgtctgatCtgaggtcgcg	m5C_57645
chr17	33478267	-	C	1378	908	0.659	0	0	C	908	M	0.633	0.683	0	0	0	11504.1	ccgcgacctCagatcagacg	m5C_57686
chr17	33478269	-	C	1322	860	0.651	0	0	C	860	M	0.624	0.676	0	0	0	10740.1	gcccgcgacCtcagatcaga	m5C_57723
chr17	33478270	-	C	1227	822	0.67	0	0	C	822	M	0.643	0.696	0	0	0	10572.9	aggcccgcgaCctcagatcag	m5C_57741
chr17	33478273	-	C	546	342	0.626	0	0	C	342	M	0.585	0.666	0	0	0	4001.71	tcgaggcccgCgacctcagat	m5C_57687
chr17	33478275	-	C	443	290	0.655	0	0	C	290	M	0.609	0.697	0	0	0	3533.3	gatcgaggccCgcgacctcag	m5C_57694
chr17	33478281	+	C	32	15	0.469	0	0	T	17	M	0.309	0.636	0	1.2E-05	0	92.6074	tcgcgggcctCgatcagaagg	m5C_57671
chr17	33478293	+	C	34	22	0.647	0	0	C	22	M	0.479	0.785	0	6.3E-05	0	210.82	atcagaaggaCttgggcccca	m5C_57658

chr17	33478297	-	C	42	16	0.381	0	0	T	26	M	0.25	0.532	0	4E-07	0	79.9985	cgtgtggggcCcaagtccttc	m5C_57759
chr17	33478298	-	C	64	21	0.328	0	0	T	43	M	0.226	0.45	0	1E-09	0	94.7957	tcgtgtggggcCcaagtcctt	m5C_57720
chr17	33478306	+	C	42	12	0.286	0	0	T	30	M	0.172	0.436	2.2E-16	7.5E-08	2.9E-16	32.2467	gggcccccacaCgagcggcgcc	m5C_57663
chr17	33478309	-	C	158	60	0.38	0	0	T	98	M	0.308	0.457	0	0	0	369.329	cccggcgccgCtcgtgtgggg	m5C_57773
chr17	33478310	+	C	104	32	0.308	0	0	T	72	M	0.227	0.402	0	1.1E-14	0	145.385	cccacacgagCggcgccgggg	m5C_57630
chr17	33478311	-	C	163	81	0.497	0	0	T	82	M	0.421	0.573	0	0	0	682.237	tccccggcgcCgctcgtgtgg	m5C_57742
chr17	33478312	-	C	168	73	0.435	0	0	T	95	M	0.362	0.51	0	0	0	528.313	ctccccggcgCcgctcgtgtg	m5C_57724
chr17	33478314	-	C	178	89	0.5	0	0	CT	89	M	0.427	0.573	0	0	0	760.64	cactccccggCgccgctcgtg	m5C_57751
chr17	33478315	+	C	201	61	0.303	0	0	T	140	M	0.244	0.37	0	0	0	297.8	acgagcggcgCcggggagtgg	m5C_57638
chr17	33478316	+	C	207	76	0.367	0	0	T	131	M	0.304	0.435	0	0	0	462.779	cgagcggcgcCggggagtggg	m5C_57662
chr17	33478317	-	C	184	96	0.522	0	0	C	96	M	0.45	0.593	0	0	0	863.725	acccactcccCggcgccgctc	m5C_57693
chr17	33478318	-	C	191	86	0.45	0	0	T	105	M	0.381	0.521	0	0	0	655.967	gacccactccCcggcgccgct	m5C_57752
chr17	33478319	-	C	185	75	0.405	0	0	T	110	M	0.337	0.477	0	0	0	505.915	agacccactcCccggcgccgc	m5C_57740
chr17	33478320	-	C	184	77	0.418	0	0	T	107	M	0.35	0.491	0	0	0	538.351	aagacccactCcccggcgccg	m5C_57717
chr17	33478322	-	C	174	86	0.494	0	0	T	88	M	0.421	0.568	0	0	0	723.939	ggaagacccaCtccccggcgc	m5C_57744
chr17	33478324	-	C	189	90	0.476	0	0	T	99	M	0.406	0.547	0	0	0	731.11	acggaagaccCactccccggc	m5C_57765
chr17	33478325	-	C	207	113	0.546	0	0	C	113	M	0.478	0.612	0	0	0	1079.93	tacggaagacCcactccccgg	m5C_57731
chr17	33478326	-	C	193	103	0.534	0	0	C	103	M	0.463	0.603	0	0	0	954.453	gtacggaagaCccactccccg	m5C_57728
chr17	33478328	+	C	326	104	0.319	0	0	T	222	M	0.271	0.371	0	0	0	563.224	gggagtgggtCttccgtacgc	m5C_57670
chr17	33478333	-	C	144	89	0.618	0	0	C	89	M	0.537	0.693	0	1.9E-14	0	955.162	atgtggcgtaCggaagaccca	m5C_57767
chr17	33478337	-	C	113	55	0.487	0	0	T	58	M	0.397	0.578	0	2.9E-13	0	436.184	ggaaatgtggCgtacggaaga	m5C_57739
chr17	33478349	-	C	104	57	0.548	0	0	C	57	M	0.452	0.64	0	1.2E-11	0	515.754	cggtggggcgCgggaaatgtg	m5C_57705
chr17	33478351	-	C	108	76	0.704	0	0	C	76	M	0.612	0.782	0	3.2E-10	0	929.921	cgcggtggggCgcgggaaatg	m5C_57708
chr17	33478359	-	C	39	26	0.667	0	0	C	26	M	0.51	0.794	0	0.00013	0	265.081	ccccgccccgCggtggggcgc	m5C_57778
chr17	33900699	+	C	157	32	0.204	0	0	T	125	M	0.148	0.274	0	0	0	94.8725	tgaagtagagCctgacctggt	m5C_57655
chr17	33900700	+	C	149	30	0.201	0	0	T	119	M	0.145	0.273	0	0	0	86.8996	gaagtagagcCtgacctggta	m5C_57643
chr17	33900705	+	C	135	35	0.259	0	0	T	100	M	0.193	0.339	0	0	0	134.905	agagcctgacCtggtattgcc	m5C_57660
chr17	33900714	+	C	58	15	0.259	0	0	T	43	M	0.163	0.384	0	4.6E-09	0	49.0362	cctggtattgCcattgcttca	m5C_57628
chr17	33900720	+	C	35	21	0.6	0	0	C	21	M	0.436	0.744	0	5.2E-05	0	183.004	attgccattgCttcactgttg	m5C_57635
chr17	33900725	+	C	35	10	0.286	0	0	T	25	M	0.163	0.451	3.4E-14	2E-06	4.2E-14	21.9991	cattgcttcaCtgttggcttt	m5C_57639
chr17	33900750	+	C	34	16	0.471	0	0	T	18	M	0.315	0.633	0	1.7E-05	0	100.644	agggtatgatCtcttaatctt	m5C_57661
chr17	33900752	+	C	32	12	0.375	0	0	T	20	M	0.229	0.547	0	4.6E-06	0	55.0408	ggtatgatctCttaatcttct	m5C_57669
chr17	34940372	+	C	45	35	0.778	0	0	C	35	M	0.637	0.875	0	2.5E-05	0	446.113	ttgaatagatCttttactcat	m5C_57386
chr17	35310400	+	C	47	20	0.426	0	0	T	27	M	0.295	0.567	0	1.4E-06	0	118.054	gaatggatgaCcttgggagca	m5C_57399
chr17	35310401	+	C	46	29	0.63	0	0	C	29	M	0.486	0.755	0	9.7E-06	0	281.879	aatggatgacCttgggagcag	m5C_57396
chr17	35310409	+	C	41	10	0.244	0	0	T	31	M	0.138	0.393	2.4E-13	2.4E-08	2.9E-13	17.4472	accttgggagCagtgaggagg	m5C_57380
chr17	36642906	+	C	31	10	0.323	0	0	T	21	M	0.186	0.499	7.1E-15	2E-06	9E-15	26.2725	gtggctaacaCtttctgagca	m5C_57834
chr17	36642915	+	C	32	11	0.344	0	0	T	21	M	0.204	0.517	2.2E-16	3.1E-06	2.9E-16	35.1442	actttctgagCattagagtgt	m5C_57796
chr17	36908026	-	C	209	72	0.344	0	0	T	137	M	0.283	0.411	0	0	0	408.097	acgctatgacCttttttttt	m5C_57940
chr17	36908033	-	C	312	67	0.215	0	0	T	245	M	0.173	0.264	0	0	0	231.541	cccagggacgCtatgaccttt	m5C_57886
chr17	36908035	-	C	320	76	0.237	0	0	T	244	M	0.194	0.287	0	0	0	295.124	cacccagggaCgctatgacct	m5C_57927
chr17	36908042	-	C	305	61	0.2	0	0	T	244	M	0.159	0.248	0	0	0	193.936	tcgagcccacCcagggacgct	m5C_57864
chr17	36908046	-	C	323	97	0.3	0	0	T	226	M	0.253	0.352	0	0	0	490.636	tggttcgagcCcacccaggga	m5C_57876
chr17	36908051	-	C	318	84	0.264	0	0	T	234	M	0.219	0.315	0	0	0	367.443	gttggtggttCgagcccaccc	m5C_57873
chr17	36908091	-	C	233	60	0.258	0	0	T	173	M	0.206	0.317	0	0	0	246.739	gtggcgcaatCggttagcgcg	m5C_57900
chr17	36908097	-	C	218	62	0.284	0	0	T	156	M	0.229	0.348	0	0	0	283.531	gtctctgtggCgcaatcggtt	m5C_57917
chr17	36908103	-	C	191	44	0.23	0	0	T	147	M	0.176	0.295	0	0	0	155.161	gactcggtctCtgtggcgcaa	m5C_57907
chr17	36908105	-	C	190	57	0.3	0	0	T	133	M	0.239	0.369	0	0	0	272.836	tcgactcggtCtctgtggcgc	m5C_57859
chr17	37006417	-	C	38	18	0.474	0	0	T	20	M	0.325	0.627	0	2.7E-05	0	116.924	cttgtggccccCggattgcatc	m5C_57923
chr17	37006419	-	C	38	9	0.237	0	0	T	29	M	0.13	0.392	3.7E-12	1.2E-06	4.3E-12	13.3739	gacttgtggcCccggattgca	m5C_57865
chr17	37006420	-	C	35	8	0.229	0	0	T	27	M	0.121	0.39	5.6E-11	6.8E-07	6.4E-11	9.89691	agacttgtggCcccggattgc	m5C_57863
chr17	37006427	-	C	40	16	0.4	0	0	T	24	M	0.263	0.554	0	1.7E-05	0	84.314	agtgtgcagaCttgtggcccc	m5C_57926
chr17	37006625	-	C	36	20	0.556	0	0	C	20	M	0.396	0.705	0	4.3E-05	0	158.323	acaataaaggCgagatgaaag	m5C_57899
chr17	37006669	-	C	41	12	0.293	0	0	T	29	M	0.176	0.445	1.1E-16	7.5E-08	1.5E-16	33.7134	tggcgtgtttCtttattttga	m5C_57901
chr17	37006691	-	C	50	20	0.4	0	0	T	30	M	0.276	0.538	0	1.4E-06	0	110.433	gaaagtcataCcgtagaaaag	m5C_57911
chr17	37006695	-	C	39	19	0.487	0	0	T	20	M	0.339	0.638	0	3.4E-05	0	128.687	caacgaaagtCataccgtaga	m5C_57937
chr17	37009131	-	C	357	129	0.361	0	0	T	228	M	0.313	0.412	0	0	0	808.138	ttgcttcaatCggtggtgtga	m5C_57935
chr17	37009151	-	C	196	40	0.204	0	0	T	156	M	0.154	0.266	0	0	0	122.882	gaagtggcaaCatgtgttgtt	m5C_57902
chr17	37009154	-	C	182	45	0.247	0	0	T	137	M	0.19	0.315	0	0	0	171.208	ggtgaagtggCaacatgtgtt	m5C_57867
chr17	37009172	-	C	108	25	0.234	1	0.009	T	82	M	0.164	0.322	0	4.4E-16	0	81.7803	gtgaaaacatCgcttttgggt	m5C_57924
chr17	37009175	-	C	110	25	0.227	0	0	T	85	M	0.159	0.314	0	0	0	79.4726	agagtgaaaaCatcgcttttg	m5C_57879
chr17	37009196	-	C	567	154	0.272	0	0	T	413	M	0.237	0.31	0	0	0	728.794	attgagccgtCaagaaagggg	m5C_57880
chr17	37009200	-	C	648	165	0.255	0	0	T	483	M	0.223	0.29	0	0	0	734.58	acctattgagCcgtcaagaaa	m5C_57861
chr17	37009216	-	C	927	305	0.33	3	0.003	T	619	M	0.301	0.361	0	0	0	1833.19	gggcctgtggCtaatgaccta	m5C_57904
chr17	37009222	-	C	895	193	0.218	8	0.009	T	694	M	0.192	0.246	0	0	0	739.896	ctcaatgggcCtgtggctaat	m5C_57921
chr17	37009223	-	C	885	249	0.284	8	0.009	T	628	M	0.255	0.315	0	0	0	1270.26	actcaatgggCctgtggctaa	m5C_57931
chr17	37023908	+	C	165	43	0.261	0	0	T	122	M	0.2	0.332	0	0	0	171.67	gggggtatagCtcagtggtag	m5C_57809
chr17	37023910	+	C	165	45	0.273	0	0	T	120	M	0.211	0.345	0	0	0	189.469	gggtatagctCagtggtagag	m5C_57806
chr17	37023921	+	C	159	36	0.226	0	0	T	123	M	0.168	0.297	0	0	0	121.151	agtggtagagCatttgactgc	m5C_57835
chr17	37023928	+	C	128	39	0.305	0	0	T	89	M	0.232	0.389	0	0	0	180.651	gagcatttgaCtgcagatcaa	m5C_57821
chr17	37023931	+	C	98	29	0.296	0	0	T	69	M	0.215	0.393	0	1.7E-13	0	124.486	catttgactgCagatcaagag	m5C_57819
chr17	37023936	+	C	40	14	0.35	0	0	T	26	M	0.221	0.505	0	1.9E-07	0	61.9761	gactgcagatCaagaggtccc	m5C_57822
chr17	37023947	+	C	34	10	0.294	0	0	T	24	M	0.168	0.462	2.3E-14	2E-06	2.9E-14	22.9564	aagaggtcccCggttcaaatc	m5C_57850
chr17	37025548	-	C	31	10	0.323	0	0	T	21	M	0.186	0.499	7.1E-15	2E-06	9E-15	26.2725	tccgggtgccCcctcaatggc	m5C_57883
chr17	37025550	-	C	31	7	0.226	0	0	T	24	M	0.114	0.398	6.4E-10	3.4E-07	7.2E-10	7.33205	aatccgggtgCccccctcaatg	m5C_57929
chr17	37025569	-	C	37	11	0.297	0	0	T	26	M	0.175	0.458	1.4E-15	3.1E-06	1.9E-15	28.5508	tcaagaggtcCctggttcaaa	m5C_57866
chr17	37025570	-	C	36	11	0.306	0	0	T	25	M	0.18	0.469	1E-15	3.1E-06	1.3E-15	29.7077	atcaagaggtCcctggttcaa	m5C_57870
chr17	37025604	-	C	104	45	0.433	0	0	T	59	M	0.342	0.529	0	7.4E-13	0	307.394	gggtatagctCagtggtagag	m5C_57905
chr17	37025617	-	C	70	21	0.3	0	0	T	49	M	0.205	0.415	0	1.8E-11	0	86.2531	tgtagaggcgCgggggtatag	m5C_57938
chr17	37310035	-	C	64	15	0.234	0	0	T	49	M	0.147	0.351	0	6E-11	0	44.2493	agtggtagagCatttgactgc	m5C_57869
chr17	37310046	-	C	72	17	0.236	0	0	T	55	M	0.153	0.346	0	2.2E-12	0	52.0084	gggtatagctCagtggtagag	m5C_57909
chr17	37310048	-	C	64	17	0.266	0	0	T	47	M	0.173	0.385	0	1.8E-10	0	58.8072	gggggtatagCtcagtggtag	m5C_57898
chr17	37310726	-	C	140	38	0.271	0	0	T	102	M	0.205	0.35	0	0	0	155.499	ccggagttacCtcgttttgtt	m5C_57932
chr17	37310743	-	C	131	32	0.244	0	0	T	99	M	0.179	0.324	0	0	0	114.351	ggtgccccctCtgtgctccgg	m5C_57936
chr17	37310745	-	C	128	41	0.32	0	0	T	87	M	0.246	0.405	0	1.1E-16	0	201.498	cgggtgccccCtctgtgctcc	m5C_57903
chr17	37310746	-	C	117	26	0.222	0	0	T	91	M	0.156	0.306	0	0	0	81.3295	ccgggtgcccCctctgtgctc	m5C_57888
chr17	37310748	-	C	116	27	0.233	0	0	T	89	M	0.165	0.317	0	0	0	89.1977	atccgggtgcCcccctctgtgc	m5C_57916
chr17	37310766	-	C	98	26	0.265	0	0	T	72	M	0.188	0.36	0	4.6E-14	0	97.7371	aagaggtcccCggttcaaatc	m5C_57896
chr17	37310767	-	C	102	25	0.245	0	0	T	77	M	0.172	0.337	0	4.4E-16	0	85.9402	caagaggtccCcggttcaaat	m5C_57871
chr17	37310768	-	C	102	47	0.461	0	0	T	55	M	0.367	0.557	0	1.3E-12	0	345.199	tcaagaggtcCccggttcaaa	m5C_57872
chr17	37310769	-	C	99	23	0.232	0	0	T	76	M	0.16	0.325	0	1E-14	0	73.6414	atcaagaggtCcccggttcaa	m5C_57930
chr17	37310792	-	C	304	86	0.283	0	0	T	218	M	0.235	0.336	0	0	0	404.572	agtggtagagCatttgactgc	m5C_57892
chr17	37310803	-	C	348	112	0.322	0	0	T	236	M	0.275	0.373	0	0	0	615.841	gggtatagctCagtggtagag	m5C_57881
chr17	37310816	-	C	177	49	0.277	0	0	T	128	M	0.216	0.347	0	0	0	211.868	agtggcgtggCgggggtatag	m5C_57862
chr17	37358664	+	C	63	24	0.381	0	0	T	39	M	0.271	0.504	0	3E-09	0	130.163	acaccttggcCcgccggaagg	m5C_57795
chr17	37358665	+	C	69	17	0.246	0	0	T	52	M	0.16	0.36	0	1.8E-10	0	54.3648	caccttggccCgccggaaggg	m5C_57808
chr17	37358668	+	C	93	36	0.387	0	0	T	57	M	0.294	0.489	0	2E-12	0	212.01	cttggcccgcCggaagggcag	m5C_57855
chr17	37358680	+	C	91	24	0.264	0	0	T	67	M	0.184	0.363	0	1.7E-14	0	88.3703	gaagggcaggCacatgggcat	m5C_57803
chr17	37358682	+	C	89	19	0.213	0	0	T	70	M	0.141	0.31	0	8.4E-14	0	53.6358	agggcaggcaCatgggcatag	m5C_57798
chr17	37358688	+	C	82	17	0.207	0	0	T	65	M	0.134	0.307	0	2.3E-14	0	45.4454	ggcacatgggCataggtaagt	m5C_57783
chr17	37359313	+	C	66	34	0.515	0	0	C	34	M	0.397	0.632	0	4.2E-08	0	270.031	gaggattttgCgccggctgct	m5C_57849

chr17	37359315	+	C	66	17	0.258	0	0	T	49	M	0.167	0.374	0	1.8E-10	0	56.9456	ggattttgcgCcggctgctca	m5C_57827
chr17	37359319	+	C	70	16	0.267	10	0.143	T	44	M	0.171	0.39	0	1.1E-10	0	54.824	tttgcgccggCtgctcagaag	m5C_57807
chr17	37359322	+	C	69	14	0.203	0	0	T	55	M	0.125	0.312	3.3E-16	3.2E-11	4.4E-16	27.0622	gcgccggctgCtcagaagata	m5C_57810
chr17	37359333	+	C	53	15	0.283	0	0	T	38	M	0.18	0.416	0	4.6E-09	0	53.8984	tcagaagataCcgtgaatcta	m5C_57829
chr17	37360426	+	C	36	9	0.25	0	0	T	27	M	0.138	0.411	2E-12	1.2E-06	2.4E-12	14.4765	acaaggcccgCaagaagctcc	m5C_57820
chr17	37360435	+	C	59	26	0.441	0	0	T	33	M	0.322	0.567	0	1.9E-07	0	167.18	gcaagaagctCctggcgtaag	m5C_57825
chr17	37360440	+	C	61	15	0.246	0	0	T	46	M	0.155	0.367	0	6E-11	0	46.5196	aagctcctggCgtaagtttct	m5C_57826
chr17	37360782	+	C	71	29	0.408	0	0	T	42	M	0.302	0.525	0	3.6E-10	0	174.978	gaccagtgacCaggctgaggc	m5C_57814
chr17	37360793	+	C	108	46	0.426	0	0	T	62	M	0.337	0.52	0	9.7E-13	0	309.847	aggctgaggcCcgcaggtcta	m5C_57831
chr17	37360801	+	C	103	47	0.456	0	0	T	56	M	0.363	0.552	0	1.3E-12	0	341.616	gcccgcaggtCtaagaccaag	m5C_57793
chr17	37360807	+	C	77	20	0.26	0	0	T	57	M	0.175	0.367	0	1.1E-11	0	69.9563	aggtctaagaCcaaggaagca	m5C_57842
chr17	37360842	+	C	52	12	0.231	0	0	T	40	M	0.137	0.361	4.4E-15	8.8E-10	5.7E-15	23.6371	agagcgcctcCaggccaagaa	m5C_57782
chr17	37360865	+	C	33	9	0.273	0	0	T	24	M	0.151	0.442	7.5E-13	1.2E-06	9E-13	16.4418	aggagatcatCaagactttat	m5C_57858
chr17	37360870	+	C	33	18	0.545	0	0	C	18	M	0.38	0.702	0	2.7E-05	0	136.748	atcatcaagaCtttatccaag	m5C_57833
chr17	38183806	-	C	34	11	0.324	0	0	T	23	M	0.191	0.492	4.4E-16	3.1E-06	5.8E-16	32.3088	ccctgaccttCctagaggcaa	m5C_57604
chr17	38183809	-	C	186	52	0.28	0	0	T	134	M	0.22	0.348	0	0	0	228.835	ggtccctgacCttcctagagg	m5C_57557
chr17	38183815	-	C	249	59	0.237	0	0	T	190	M	0.188	0.294	0	0	0	222.288	ccacttggtcCctgaccttcc	m5C_57615
chr17	38183822	-	C	265	77	0.291	0	0	T	188	M	0.239	0.348	0	0	0	368.373	gaaggagccaCttggtccctg	m5C_57601
chr17	38183825	-	C	268	80	0.299	0	0	T	188	M	0.247	0.356	0	0	0	395.011	tttgaaggagCcacttggtcc	m5C_57549
chr17	38183844	-	C	343	76	0.222	0	0	T	267	M	0.181	0.268	0	0	0	274.879	ggattctgagCctcagagctt	m5C_57582
chr17	38399477	+	C	62	13	0.21	0	0	T	49	M	0.127	0.326	1.8E-15	1.6E-11	2.3E-15	24.3221	taatctggggCtggtctgatg	m5C_57522
chr17	38399482	+	C	181	78	0.431	0	0	T	103	M	0.361	0.504	0	0	0	563.112	tggggctggtCtgatggtagt	m5C_57506
chr17	38399505	+	C	167	96	0.575	0	0	C	96	M	0.499	0.647	0	2.2E-16	0	958.11	gttatcagagCttattaacat	m5C_57513
chr17	38399513	+	C	45	12	0.267	0	0	T	33	M	0.16	0.41	5.6E-16	7.5E-08	7.3E-16	29.2263	agcttattaaCattagtgtca	m5C_57536
chr17	39624274	+	C	36	12	0.333	0	0	T	24	M	0.202	0.497	0	4.6E-06	0	48.5146	atatctgataCgtcctccatc	m5C_57523
chr17	39624284	+	C	139	62	0.446	0	0	T	77	M	0.366	0.529	0	1.8E-15	0	453.794	cgtcctccatCtgaggacaac	m5C_57548
chr17	39624291	+	C	206	50	0.243	0	0	T	156	M	0.189	0.306	0	0	0	189.229	catctgaggaCaacatattaa	m5C_57503
chr17	39624316	+	C	111	41	0.369	0	0	T	70	M	0.285	0.462	0	6E-15	0	233.994	attttgggagCagggagatgg	m5C_57518
chr17	39624335	+	C	45	16	0.356	0	0	T	29	M	0.232	0.502	0	4E-07	0	74.3005	ggaataagagCttgctctgtt	m5C_57519
chr17	39624362	+	C	39	18	0.462	0	0	T	21	M	0.316	0.614	0	2.7E-05	0	113.64	acatgttgacCtggtattgca	m5C_57505
chr17	39624376	+	C	80	16	0.2	0	0	T	64	M	0.127	0.3	0	1.1E-14	0	40.6488	tattgcagtaCctccaggaat	m5C_57539
chr17	39624377	+	C	83	24	0.289	0	0	T	59	M	0.203	0.394	0	1.1E-12	0	97.2831	attgcagtacCtccaggaatg	m5C_57545
chr17	39624388	+	C	56	16	0.356	11	0.196	T	29	M	0.232	0.502	0	4E-07	0	74.3005	tccaggaatgCtgcacccctt	m5C_57546
chr17	39886498	-	C	34	10	0.294	0	0	T	24	M	0.168	0.462	2.3E-14	2E-06	2.9E-14	22.9564	gaggggtgaaCtggctcaggt	m5C_57570
chr17	39913880	-	C	33	9	0.273	0	0	T	24	M	0.151	0.442	7.5E-13	1.2E-06	9E-13	16.4418	cactgggtgagCtgggggaggg	m5C_57562
chr17	39974719	+	C	46	16	0.348	0	0	T	30	M	0.227	0.492	0	4E-07	0	72.5786	gggcatgtgtCctggagagag	m5C_57511
chr17	39974720	+	C	43	16	0.372	0	0	T	27	M	0.244	0.521	0	4E-07	0	78.0036	ggcatgtgtcCtggagagaga	m5C_57515
chr17	41084883	-	C	129	33	0.256	0	0	T	96	M	0.188	0.337	0	0	0	124.306	ccccccattgCtaaatttcac	m5C_58369
chr17	41084891	-	C	156	39	0.25	0	0	T	117	M	0.189	0.323	0	0	0	147.116	gtatacaaccCcccattgcta	m5C_58400
chr17	41084896	-	C	170	34	0.2	0	0	T	136	M	0.147	0.266	0	0	0	99.8234	agttggtataCaaccccccat	m5C_58385
chr17	41084929	-	C	174	51	0.293	0	0	T	123	M	0.231	0.365	0	0	0	235.134	gtcatcagaaCttattaacat	m5C_58431
chr17	41084952	-	C	170	59	0.347	0	0	T	111	M	0.28	0.421	0	0	0	329.92	ttaggctggtCcgatggtagt	m5C_58387
chr17	41105507	-	C	32	15	0.469	0	0	T	17	M	0.309	0.636	0	1.2E-05	0	92.6074	agcttctgagCtcctttcatc	m5C_58363
chr17	41150016	-	C	82	28	0.341	0	0	T	54	M	0.248	0.449	0	5.8E-12	0	138.875	gtggctggtcCgatagtagtg	m5C_58407
chr17	41150017	-	C	78	24	0.308	0	0	T	54	M	0.216	0.417	0	6.4E-11	0	103.833	ggtggctggtCcgatagtagt	m5C_58418
chr17	41226414	-	C	33	9	0.273	0	0	T	24	M	0.151	0.442	7.5E-13	1.2E-06	9E-13	16.4418	tgtggaggagCaacagctgga	m5C_58423
chr17	41464601	-	C	116	24	0.207	0	0	T	92	M	0.143	0.289	0	0	0	68.7169	aggaatggggCtccctctcct	m5C_58426
chr17	41464630	-	C	963	287	0.298	0	0	T	676	M	0.27	0.328	0	0	0	1549.72	acgcgtcgacCtggtattgca	m5C_58417
chr17	41464634	-	C	806	181	0.225	0	0	T	625	M	0.197	0.255	0	0	0	713.508	ctccacgcgtCgacctggtat	m5C_58390
chr17	41464647	-	C	304	64	0.211	1	0.003	T	239	M	0.169	0.261	0	0	0	216.348	cttgctctgtCcactccacgc	m5C_58412
chr17	41893902	+	C	42	25	0.595	0	0	C	25	M	0.445	0.73	0	4.5E-06	0	222.47	cagcccagtcCagctggcaga	m5C_58356
chr17	41893914	+	C	39	14	0.359	0	0	T	25	M	0.227	0.516	0	9.2E-06	0	63.6773	gctggcagagCaggaagaggt	m5C_58313
chr17	42088964	-	C	85	33	0.388	0	0	T	52	M	0.292	0.495	0	3.1E-11	0	192.464	gagtgggagaCagaggaagga	m5C_58707
chr17	42756247	-	C	56	18	0.321	0	0	T	38	M	0.214	0.452	0	1.7E-08	0	77.043	aaaaaggacaCagagagggga	m5C_58742
chr17	42756272	-	C	50	17	0.34	0	0	T	33	M	0.224	0.478	0	5.6E-07	0	76.285	agcaggagcgCaaggaaaagg	m5C_58734
chr17	42756274	-	C	55	23	0.418	0	0	T	32	M	0.297	0.55	0	8.7E-08	0	136.793	caagcaggagCgcaaggaaaa	m5C_58739
chr17	42932288	-	C	58	20	0.345	0	0	T	38	M	0.236	0.473	0	3.4E-08	0	94.2437	ttacgtggaaCaggtgagaac	m5C_58726
chr17	42932302	-	C	66	22	0.333	0	0	T	44	M	0.232	0.453	0	1.5E-09	0	101.888	tgaggtggtcCagattacgtg	m5C_58702
chr17	43148817	+	C	88	28	0.318	0	0	T	60	M	0.23	0.421	0	5.8E-12	0	128.926	ggtggctggtCcaagtgcagt	m5C_58673
chr17	43303048	+	C	78	38	0.487	0	0	T	40	M	0.379	0.596	0	3.7E-09	0	288.411	tggaaggtggCttggggggcc	m5C_58696
chr17	43303057	+	C	65	31	0.477	0	0	T	34	M	0.36	0.596	0	2.1E-08	0	223.334	gcttggggggCctttgagggg	m5C_58681
chr17	43303058	+	C	64	20	0.312	0	0	T	44	M	0.212	0.434	0	6.9E-10	0	84.9236	cttggggggcCtttgagggga	m5C_58689
chr17	46114544	-	C	89	27	0.303	0	0	T	62	M	0.218	0.405	0	4E-12	0	117.526	catgacagaaCttgggcccgg	m5C_58494
chr17	46114549	-	C	93	22	0.237	0	0	T	71	M	0.162	0.332	0	5.8E-15	0	71.1592	cagtgcatgaCagaacttggg	m5C_58487
chr17	46114554	-	C	90	21	0.233	0	0	T	69	M	0.158	0.331	0	3.1E-15	0	66.3519	gcagtcagtgCatgacagaac	m5C_58516
chr17	46556502	+	C	99	30	0.303	0	0	T	69	M	0.221	0.4	0	2.5E-13	0	132.757	ctgatcacaaCcagttacaga	m5C_58478
chr17	46556523	+	C	94	35	0.372	0	0	T	59	M	0.281	0.473	0	1.5E-12	0	197.001	tttctttgttCcttctccact	m5C_58484
chr17	46556530	+	C	91	25	0.275	0	0	T	66	M	0.194	0.374	0	2.9E-14	0	96.7753	gttccttctcCactcccactg	m5C_58467
chr17	46556546	+	C	92	24	0.261	0	0	T	68	M	0.182	0.359	0	1.7E-14	0	87.3701	cactgcttcgCttgactagcc	m5C_58454
chr17	46556555	+	C	62	21	0.339	0	0	T	41	M	0.233	0.463	0	1E-09	0	98.0345	gcttgactagCcttaagaaaa	m5C_58459
chr17	46657275	-	C	123	33	0.268	0	0	T	90	M	0.198	0.353	0	0	0	130.603	cctgtagatcCgaatttgtgt	m5C_58514
chr17	46657276	-	C	123	43	0.35	0	0	T	80	M	0.271	0.437	0	2.2E-16	0	233.093	ccctgtagatCcgaatttgtg	m5C_58512
chr17	46657285	-	C	51	14	0.275	0	0	T	37	M	0.171	0.409	0	2.8E-09	0	47.9195	ctgtatatacCctgtagatcc	m5C_58505
chr17	46685176	-	C	62	17	0.274	0	0	T	45	M	0.179	0.396	0	1.8E-10	0	60.7951	gaggaagagaCatgagaaagg	m5C_58495
chr17	46685188	-	C	67	29	0.433	0	0	T	38	M	0.321	0.552	0	1.3E-08	0	186.204	ggagaaagggCagaggaagag	m5C_58492
chr17	46709905	-	C	101	26	0.257	0	0	T	75	M	0.182	0.35	0	6.7E-16	0	94.7186	taggtagtttCatgttgttgg	m5C_58525
chr17	46976477	+	C	43	17	0.395	0	0	T	26	M	0.264	0.544	0	5.6E-07	0	89.6414	tctttggtctCggctgcagaa	m5C_58439
chr17	46976480	+	C	43	10	0.233	0	0	T	33	M	0.132	0.377	4.3E-13	2.4E-08	5.2E-13	16.2683	ttggtctcggCtgcagaagca	m5C_58433
chr17	47026730	-	C	117	31	0.265	0	0	T	86	M	0.193	0.351	0	1.1E-16	0	119.902	tgcgtttgcgCtctagtaaaa	m5C_58489
chr17	47026732	-	C	117	24	0.205	0	0	T	93	M	0.142	0.287	0	0	0	68.1113	actgcgtttgCgctctagtaa	m5C_58493
chr17	47026741	-	C	147	35	0.238	0	0	T	112	M	0.176	0.313	0	0	0	123.528	tagcggtggaCtgcgtttgcg	m5C_58500
chr17	47026763	-	C	151	31	0.205	0	0	T	120	M	0.149	0.277	0	0	0	92.1127	aactcgactgCgtaatttgtg	m5C_58502
chr17	47026784	-	C	182	60	0.33	0	0	T	122	M	0.266	0.401	0	0	0	318.614	gcgatttcccCaaatgtggga	m5C_58513
chr17	47026787	-	C	179	52	0.291	0	0	T	127	M	0.229	0.361	0	0	0	238.109	ctcgcgatttCcccaaatgtg	m5C_58507
chr17	47026793	-	C	149	32	0.215	0	0	T	117	M	0.156	0.287	0	0	0	100.119	tgccgactcgCgatttcccca	m5C_58523
chr17	47026797	-	C	139	51	0.367	0	0	T	88	M	0.291	0.45	0	1.1E-16	0	297.193	gatgtgccgaCtcgcgatttc	m5C_58498
chr17	47026801	-	C	123	28	0.228	0	0	T	95	M	0.162	0.309	0	0	0	90.974	tccggatgtgCcgactcgcga	m5C_58488
chr17	47026809	-	C	65	14	0.215	0	0	T	51	M	0.133	0.33	1.1E-16	3.2E-11	1.5E-16	29.6776	cattgcactcCggatgtgccg	m5C_58496
chr17	47026810	-	C	38	9	0.237	0	0	T	29	M	0.13	0.392	3.7E-12	1.2E-06	4.3E-12	13.3739	ccattgcactCcggatgtgcc	m5C_58526
chr17	47269912	+	C	40	11	0.275	0	0	T	29	M	0.161	0.428	4.3E-15	3.1E-06	5.5E-15	25.4502	taatggttagCactctggact	m5C_58464
chr17	47269914	+	C	40	10	0.25	0	0	T	30	M	0.142	0.402	1.8E-13	2.4E-08	2.2E-13	18.0914	atggttagcaCtctggacttt	m5C_58460
chr17	47269929	+	C	51	13	0.255	0	0	T	38	M	0.155	0.389	1.1E-16	1.6E-09	1.5E-16	32.2431	gactttgaatCcagcgatccg	m5C_58440
chr17	47269933	+	C	52	36	0.692	0	0	C	36	M	0.557	0.801	0	1.5E-06	0	401.257	ttgaatccagCgatccgagtt	m5C_58476
chr17	47269949	+	C	52	30	0.577	0	0	C	30	M	0.442	0.701	0	4.8E-07	0	265.165	gagttcaaatCtcggtgggac	m5C_58474
chr17	47269951	+	C	51	14	0.275	0	0	T	37	M	0.171	0.409	0	2.8E-09	0	47.9195	gttcaaatctCggtgggacct	m5C_58455
chr17	47269962	+	C	45	18	0.4	0	0	T	27	M	0.27	0.545	0	7.7E-07	0	97.2889	ggtgggacctCacgagacttt	m5C_58446
chr17	48462587	-	C	227	88	0.389	1	0.004	T	138	M	0.328	0.454	0	0	0	577.566	actgcggaagCgggagaaggc	m5C_58265
chr17	48462596	-	C	264	80	0.303	0	0	T	184	M	0.251	0.361	0	0	0	401.191	ggagcgggaaCtgcggaagcg	m5C_58251
chr17	48462608	-	C	146	59	0.404	0	0	T	87	M	0.328	0.485	0	0	0	386.989	taaggaagctCaggagcggga	m5C_58277
chr17	48462610	-	C	131	34	0.26	0	0	T	97	M	0.192	0.341	0	0	0	130.616	gctaaggaagCtcaggagcgg	m5C_58262
chr17	48462619	-	C	71	24	0.338	0	0	T	47	M	0.239	0.454	0	6.4E-11	0	114.647	aagcagcgagCtaaggaagct	m5C_58252

chr17	48462623	-	C	54	15	0.278	0	0	T	39	M	0.176	0.409	0	4.6E-09	0	52.85	ggctaagcagCgagctaagga	m5C_58292
chr17	48465473	-	C	33	18	0.545	0	0	C	18	M	0.38	0.702	0	2.7E-05	0	136.748	acatgaaggcCgtgcaggcag	m5C_58274
chr17	48465484	-	C	33	15	0.455	0	0	T	18	M	0.298	0.62	0	1.2E-05	0	89.5281	ggtgttacagCacatgaaggc	m5C_58253
chr17	48823123	+	C	38	15	0.395	0	0	T	23	M	0.256	0.553	0	1.2E-05	0	76.7936	tcgtgatgagCgtctaaaaaa	m5C_58211
chr17	48823126	+	C	41	13	0.317	0	0	T	28	M	0.196	0.47	0	1.2E-07	0	50.8675	tgatgagcgtCtaaaaaagga	m5C_58250
chr17	48823168	+	C	42	25	0.595	0	0	C	25	M	0.445	0.73	0	4.5E-06	0	222.47	agaaaaagaaCgggagagaga	m5C_58207
chr17	49239092	+	C	42	18	0.429	0	0	T	24	M	0.291	0.578	0	7.7E-07	0	104.823	cacccaggaaCattatacatg	m5C_58210
chr17	49239099	+	C	45	13	0.289	0	0	T	32	M	0.177	0.434	0	1.2E-07	0	46.0978	gaacattataCatggcagtga	m5C_58221
chr17	49239104	+	C	48	12	0.25	0	0	T	36	M	0.149	0.388	1.3E-15	7.5E-08	1.7E-15	26.6338	ttatacatggCagtgattctg	m5C_58242
chr17	49239112	+	C	46	12	0.261	0	0	T	34	M	0.156	0.403	6.7E-16	7.5E-08	8.7E-16	28.4116	ggcagtgattCtgtggagagt	m5C_58230
chr17	55951180	-	C	34	11	0.324	0	0	T	23	M	0.191	0.492	4.4E-16	3.1E-06	5.8E-16	32.3088	atgggaatctCtgagcaggat	m5C_58585
chr17	55951191	-	C	34	11	0.324	0	0	T	23	M	0.191	0.492	4.4E-16	3.1E-06	5.8E-16	32.3088	cagtggaggcCatgggaatct	m5C_58566
chr17	55951201	-	C	36	13	0.361	0	0	T	23	M	0.225	0.524	0	6.7E-06	0	58.436	gttctggaggCagtggaggcc	m5C_58575
chr17	55951208	-	C	35	13	0.371	0	0	T	22	M	0.232	0.537	0	6.7E-06	0	60.2306	ggcctttgttCtggaggcagt	m5C_58573
chr17	55951215	-	C	36	22	0.611	0	0	C	22	M	0.449	0.752	0	6.3E-05	0	197.402	agtgacgggcCtttgttctgg	m5C_58558
chr17	55951216	-	C	34	12	0.353	0	0	T	22	M	0.215	0.521	0	4.6E-06	0	51.5705	gagtgacgggCctttgttctg	m5C_58580
chr17	55951228	-	C	33	13	0.394	0	0	T	20	M	0.247	0.563	0	6.7E-06	0	64.1755	agtgatgagaCagagtgacgg	m5C_58557
chr17	56736519	-	C	139	34	0.245	0	0	T	105	M	0.181	0.322	0	0	0	122.839	tgcttttgcgCttttctgtcc	m5C_58793
chr17	56736530	-	C	293	74	0.253	0	0	T	219	M	0.206	0.305	0	0	0	305.222	tagtgggggaCtgcttttgcg	m5C_58818
chr17	56736560	-	C	39	9	0.231	0	0	T	30	M	0.126	0.383	4.9E-12	1.2E-06	5.7E-12	12.8739	aatgagaaaaCtcggctgcat	m5C_58781
chr17	57338116	+	C	40	11	0.275	0	0	T	29	M	0.161	0.428	4.3E-15	3.1E-06	5.5E-15	25.4502	ggtggctgatCtgagtgtaat	m5C_58750
chr17	57918637	+	C	67	28	0.418	0	0	T	39	M	0.307	0.537	0	9.8E-09	0	172.156	tgtcgggtagCttatcagact	m5C_58778
chr17	57918642	+	C	160	69	0.434	1	0.006	T	90	M	0.359	0.512	0	0	0	495.945	ggtagcttatCagactgatgt	m5C_58755
chr17	57918646	+	C	153	91	0.595	0	0	C	91	M	0.516	0.669	0	1.3E-15	0	938.358	gcttatcagaCtgatgttgac	m5C_58757
chr17	57918656	+	C	90	48	0.533	0	0	C	48	M	0.431	0.633	0	4.6E-11	0	413.779	ctgatgttgaCtgttgaatct	m5C_58773
chr17	58162491	-	C	54	12	0.222	0	0	T	42	M	0.132	0.349	7.5E-15	8.8E-10	9.6E-15	22.3614	atgggtgtggCcatgagtgtg	m5C_58651
chr17	58162503	-	C	43	13	0.302	0	0	T	30	M	0.186	0.451	0	1.2E-07	0	48.3648	actgtgtcagCaatgggtgtg	m5C_58641
chr17	58162506	-	C	35	21	0.6	0	0	C	21	M	0.436	0.744	0	5.2E-05	0	183.004	accactgtgtCagcaatgggt	m5C_58628
chr17	58198044	-	C	157	34	0.217	0	0	T	123	M	0.159	0.287	0	0	0	108.333	gcctgtatgaCccccggcggc	m5C_58644
chr17	58308881	-	C	48	19	0.396	0	0	T	29	M	0.27	0.537	0	1E-06	0	102.678	ctgcctcagaCatgtgtagtt	m5C_58638
chr17	58308887	-	C	49	20	0.408	0	0	T	29	M	0.282	0.548	0	1.4E-06	0	112.86	gttttcctgcCtcagacatgt	m5C_58630
chr17	58308891	-	C	50	19	0.38	0	0	T	31	M	0.259	0.518	0	2.4E-08	0	98.2779	ccttgttttcCtgcctcagac	m5C_58640
chr17	58308892	-	C	51	20	0.392	0	0	T	31	M	0.27	0.529	0	3.4E-08	0	108.109	gccttgttttCctgcctcaga	m5C_58624
chr17	58308901	-	C	51	20	0.392	0	0	T	31	M	0.27	0.529	0	3.4E-08	0	108.109	agtggggcagCcttgttttcc	m5C_58655
chr17	58308904	-	C	49	18	0.367	0	0	T	31	M	0.247	0.507	0	7.7E-07	0	88.7939	acgagtggggCagccttgttt	m5C_58653
chr17	58308913	-	C	40	8	0.2	0	0	T	32	M	0.105	0.348	2.1E-10	5.5E-09	2.3E-10	8.13503	aggactgccaCgagtggggca	m5C_58626
chr17	58308919	-	C	37	8	0.216	0	0	T	29	M	0.114	0.372	9.7E-11	6.8E-07	1.1E-10	9.12273	gtcgtcaggaCtgccacgagt	m5C_58635
chr17	58308964	-	C	63	19	0.302	0	0	T	44	M	0.202	0.424	0	4.5E-10	0	76.9026	ttctccttagCcaaggctgga	m5C_58625
chr17	58308969	-	C	61	15	0.246	0	0	T	46	M	0.155	0.367	0	6E-11	0	46.5196	ccctgttctcCttagccaagg	m5C_58639
chr17	58308972	-	C	71	34	0.479	0	0	T	37	M	0.367	0.593	0	1.4E-09	0	249.406	ggtccctgttCtccttagcca	m5C_58646
chr17	58308977	-	C	72	40	0.556	0	0	C	40	M	0.441	0.665	0	5.8E-09	0	352.697	tggggggtccCtgttctcctt	m5C_58633
chr17	58308979	-	C	77	16	0.208	0	0	T	61	M	0.132	0.311	0	1.2E-12	0	42.2879	tttggggggtCcctgttctcc	m5C_58627
chr17	58308990	-	C	93	23	0.247	0	0	T	70	M	0.171	0.344	0	1E-14	0	78.5777	ctcccatgggCtttgggggt	m5C_58649
chr17	58308996	-	C	92	31	0.337	0	0	T	61	M	0.249	0.438	0	3.7E-13	0	154.153	gcctctctccCatgggctttg	m5C_58654
chr17	58308997	-	C	93	26	0.28	0	0	T	67	M	0.199	0.378	0	4.6E-14	0	103.221	tgcctctctcCcatgggcttt	m5C_58637
chr17	58309000	-	C	94	22	0.234	0	0	T	72	M	0.16	0.329	0	5.8E-15	0	70.3741	tcgtgcctctCtcccatgggc	m5C_58636
chr17	58309004	-	C	69	24	0.348	0	0	T	45	M	0.246	0.466	0	3E-09	0	118.166	cctttcgtgcCtctctcccat	m5C_58656
chr17	58863596	+	C	50	10	0.2	0	0	T	40	M	0.112	0.33	2.6E-12	2E-10	3.1E-12	13.024	tgagggagccCggatagttca	m5C_58617
chr17	58863617	+	C	49	10	0.204	0	0	T	39	M	0.115	0.336	2.1E-12	2.4E-08	2.4E-12	13.4191	gttggtagagCatcagactta	m5C_58614
chr17	58863624	+	C	41	22	0.537	0	0	C	22	M	0.387	0.679	0	2.3E-06	0	170.483	gagcatcagaCttaatcagag	m5C_58618
chr17	60199688	+	C	248	50	0.202	0	0	T	198	M	0.156	0.256	0	0	0	156.404	tttggctggtCtgagtgcagt	m5C_58849
chr17	60199710	+	C	260	66	0.254	0	0	T	194	M	0.205	0.31	0	0	0	270.327	gtgtttacaaCtaattgatca	m5C_58869
chr17	60199725	+	C	251	62	0.247	0	0	T	189	M	0.198	0.304	0	0	0	245.194	tgatcacaacCggttacagat	m5C_58839
chr17	60199738	+	C	181	37	0.204	0	0	T	144	M	0.152	0.269	0	0	0	112.555	ttacagatttCtttgttcctt	m5C_58829
chr17	60199746	+	C	145	44	0.303	0	0	T	101	M	0.234	0.383	0	0	0	206.348	ttctttgttcCttctccactc	m5C_58832
chr17	60199749	+	C	143	37	0.259	0	0	T	106	M	0.194	0.336	0	0	0	143.513	tttgttccttCtccactccca	m5C_58840
chr17	60199757	+	C	124	27	0.218	0	0	T	97	M	0.154	0.298	0	0	0	83.2571	ttctccactcCcactgcttca	m5C_58826
chr17	60754400	+	C	43	19	0.442	0	0	T	24	M	0.304	0.589	0	1E-06	0	115.646	gaagcaggctCggtgaggtta	m5C_58852
chr17	62223440	+	C	31	16	0.516	0	0	C	16	M	0.348	0.68	0	1.7E-05	0	111.488	ggggggccggCctgctgtgat	m5C_61874
chr17	62223485	+	C	1728	364	0.217	48	0.028	T	1316	M	0.198	0.237	0	0	0	1438.71	agactgctgaCgcgggtgatg	m5C_61875
chr17	62223500	+	C	1612	386	0.239	0	0	T	1226	M	0.219	0.261	0	0	0	1692.66	gtgatgcgaaCtggagtctga	m5C_61888
chr17	62223512	+	C	913	184	0.202	0	0	T	729	M	0.177	0.229	0	0	0	650.578	ggagtctgagCctgcccgagc	m5C_61881
chr17	62223513	+	C	639	155	0.243	0	0	T	484	M	0.211	0.277	0	0	0	653.895	gagtctgagcCtgcccgagcg	m5C_61885
chr17	62223698	+	C	262	59	0.225	0	0	T	203	M	0.179	0.28	0	0	0	210.975	tcgttggcgaCgcgctgtctt	m5C_61886
chr17	62223706	+	C	703	227	0.324	3	0.004	T	473	M	0.291	0.36	0	0	0	1319.54	gacgcgctgtCtttgagcccc	m5C_61901
chr17	62223713	+	C	697	246	0.353	0	0	T	451	M	0.318	0.389	0	0	0	1566.32	tgtctttgagCccccgccgag	m5C_61899
chr17	62223717	+	C	701	194	0.277	0	0	T	507	M	0.245	0.311	0	0	0	950.262	tttgagccccCgccgagcttc	m5C_61890
chr17	62223728	+	C	528	117	0.222	0	0	T	411	M	0.188	0.259	0	0	0	440.498	gccgagcttcCtcgtggcgcc	m5C_61867
chr17	62223730	+	C	484	102	0.211	0	0	T	382	M	0.177	0.249	0	0	0	360.588	cgagcttcctCgtggcgccgg	m5C_61887
chr17	62223737	+	C	460	106	0.23	0	0	T	354	M	0.194	0.271	0	0	0	411.871	cctcgtggcgCcgggggtcaa	m5C_61865
chr17	62223738	+	C	430	92	0.214	0	0	T	338	M	0.178	0.255	0	0	0	327.177	ctcgtggcgcCgggggtcaat	m5C_61906
chr17	62223755	+	C	241	53	0.222	2	0.008	T	186	M	0.174	0.279	0	0	0	184.147	caatctgcagCgctagagcat	m5C_61889
chr17	62223757	+	C	220	67	0.306	1	0.005	T	152	M	0.249	0.37	0	0	0	333.24	atctgcagcgCtagagcatgt	m5C_61878
chr17	62223763	+	C	177	43	0.243	0	0	T	134	M	0.186	0.311	0	0	0	159.663	agcgctagagCatgtgcttgc	m5C_61894
chr17	62223769	+	C	188	53	0.282	0	0	T	135	M	0.222	0.35	0	0	0	235.811	agagcatgtgCttgcgcataa	m5C_61873
chr17	62223787	+	C	211	48	0.227	0	0	T	163	M	0.176	0.289	0	0	0	169.049	taactggggcCgcctggcctc	m5C_61877
chr17	62223789	+	C	208	53	0.255	0	0	T	155	M	0.2	0.318	0	0	0	212.43	actggggccgCctggcctccc	m5C_61891
chr17	62223798	+	C	221	51	0.232	1	0.005	T	169	M	0.181	0.292	0	0	0	184.565	gcctggcctcCcgcgggcggc	m5C_61868
chr17	62223805	+	C	240	61	0.254	0	0	T	179	M	0.203	0.313	0	0	0	247.969	ctcccgcgggCggccttttta	m5C_61880
chr17	62223809	+	C	243	78	0.322	1	0.004	T	164	M	0.267	0.384	0	0	0	415.899	cgcgggcggcCtttttaaccg	m5C_61898
chr17	62223817	+	C	251	55	0.219	0	0	T	196	M	0.172	0.274	0	0	0	189.633	gcctttttaaCcgcgagcgac	m5C_61896
chr17	62223827	+	C	246	62	0.252	0	0	T	184	M	0.202	0.31	0	0	0	250.321	ccgcgagcgaCaagaagtaac	m5C_61871
chr17	63043191	+	C	47	30	0.638	0	0	C	30	M	0.495	0.76	0	1.2E-05	0	297.214	aaggaactgaCggaaatttta	m5C_61869
chr17	63193100	+	C	36	8	0.222	0	0	T	28	M	0.117	0.381	7.4E-11	6.8E-07	8.4E-11	9.49676	caagtgattaCttggcttgtg	m5C_61895
chr17	63193105	+	C	36	8	0.222	0	0	T	28	M	0.117	0.381	7.4E-11	6.8E-07	8.4E-11	9.49676	gattacttggCttgtgtgtta	m5C_61882
chr17	63730475	+	C	40	24	0.6	0	0	C	24	M	0.446	0.737	0	3.6E-06	0	214.059	gatgtctagaCaagtggttct	m5C_61903
chr17	64263903	+	C	31	10	0.323	0	0	T	21	M	0.186	0.499	7.1E-15	2E-06	9E-15	26.2725	tctgtctgatCttggaagcta	m5C_58961
chr17	64263911	+	C	72	19	0.264	0	0	T	53	M	0.176	0.376	0	6.9E-12	0	66.8558	atcttggaagCtaggcagggt	m5C_58966
chr17	64263916	+	C	91	30	0.33	0	0	T	61	M	0.242	0.431	0	2.5E-13	0	145.025	ggaagctaggCagggtcgggc	m5C_58949
chr17	64263922	+	C	96	63	0.656	0	0	C	63	M	0.557	0.744	0	7.9E-10	0	701.667	taggcagggtCgggcctggtt	m5C_58948
chr17	64263926	+	C	83	17	0.205	0	0	T	66	M	0.132	0.304	0	2.3E-14	0	44.8792	cagggtcgggCctggttagta	m5C_58951
chr17	64263927	+	C	89	25	0.281	0	0	T	64	M	0.198	0.382	0	1.8E-12	0	99.0468	agggtcgggcCtggttagtac	m5C_58963
chr17	64263937	+	C	37	10	0.27	0	0	T	27	M	0.154	0.43	6.7E-14	2E-06	8.3E-14	20.2815	ctggttagtaCttggatggga	m5C_58958
chr17	65211896	-	C	62	25	0.403	0	0	T	37	M	0.29	0.527	0	4.1E-09	0	145.123	actgtggtggCtagtgactga	m5C_58979
chr17	65211905	-	C	77	16	0.208	0	0	T	61	M	0.132	0.311	0	1.2E-12	0	42.2879	gcccaccttaCtgtggtggct	m5C_58973
chr17	65211914	-	C	77	19	0.247	0	0	T	58	M	0.164	0.354	0	6.9E-12	0	62.335	gtttgaaaagCccaccttact	m5C_58980
chr17	65736790	+	C	104	32	0.308	0	0	T	72	M	0.227	0.402	0	1.1E-14	0	145.385	atttaccctcCtacaaaggca	m5C_58947
chr17	65736804	+	C	107	36	0.336	0	0	T	71	M	0.254	0.43	0	4.8E-14	0	182.859	aaaggcatgtCtatagttcct	m5C_58955
chr17	65736813	+	C	118	24	0.203	0	0	T	94	M	0.141	0.285	0	0	0	67.5163	tctatagttcCttgtctttgg	m5C_58959
chr17	65736818	+	C	126	27	0.214	0	0	T	99	M	0.152	0.294	0	0	0	81.8938	agttccttgtCtttggacatg	m5C_58954
chr17	66016012	-	C	146	37	0.253	0	0	T	109	M	0.19	0.33	0	0	0	140.462	atctgggtcgCgtgagattag	m5C_58931

chr17	66016020	-	C	174	47	0.27	0	0	T	127	M	0.21	0.341	0	0	0	197.055	cgagtcccatCtgggtcgcgt	m5C_58924
chr17	66016025	-	C	182	60	0.33	0	0	T	122	M	0.266	0.401	0	0	0	318.614	gggttcgagtCccatctgggt	m5C_58932
chr17	66016030	-	C	192	95	0.495	0	0	T	97	M	0.425	0.565	0	0	0	807.252	attgtgggttCgagtcccatc	m5C_58943
chr17	66016046	-	C	186	50	0.269	0	0	T	136	M	0.21	0.337	0	0	0	210.255	gcctccggagCtggggattgt	m5C_58941
chr17	66016054	-	C	150	41	0.273	0	0	T	109	M	0.208	0.35	0	0	0	170.839	aggcatcagcCtccggagctg	m5C_58936
chr17	66016061	-	C	178	43	0.242	0	0	T	135	M	0.185	0.309	0	0	0	158.738	atggataaggCatcagcctcc	m5C_58937
chr17	66016074	-	C	188	45	0.239	0	0	T	143	M	0.184	0.305	0	0	0	165.577	acccagtggcCtaatggataa	m5C_58923
chr17	66016075	-	C	196	45	0.23	0	0	T	151	M	0.176	0.293	0	0	0	158.623	gacccagtggCctaatggata	m5C_58935
chr17	67117460	-	C	35	11	0.314	0	0	T	24	M	0.186	0.48	6.7E-16	3.1E-06	8.7E-16	30.9686	taaagcaggtCtggggctgca	m5C_58944
chr17	70325202	+	C	33	13	0.394	0	0	T	20	M	0.247	0.563	0	6.7E-06	0	64.1755	gaggggtgaaCcggccccggt	m5C_59440
chr17	72832641	-	C	31	20	0.645	0	0	C	20	M	0.469	0.789	0	4.3E-05	0	187.791	gcgggggatgaCgtactggatg	m5C_59276
chr17	73030086	+	C	38	28	0.737	0	0	C	28	M	0.58	0.85	0	0.00017	0	324.749	ggtgaaagttCcttttacgga	m5C_59238
chr17	73030510	-	C	178	39	0.219	0	0	T	139	M	0.165	0.285	0	0	0	128.405	aggggctgtgCtcgcaaggtt	m5C_59302
chr17	73030533	-	C	188	55	0.293	0	0	T	133	M	0.232	0.361	0	0	0	255.416	cgagtcccacCtggggtgtcg	m5C_59320
chr17	73030536	-	C	185	59	0.321	1	0.005	T	125	M	0.257	0.391	0	0	0	303.819	gttcgagtccCacctggggtg	m5C_59288
chr17	73030537	-	C	188	62	0.33	0	0	T	126	M	0.267	0.4	0	0	0	330.559	ggttcgagtcCcacctggggt	m5C_59312
chr17	73030538	-	C	183	61	0.333	0	0	T	122	M	0.269	0.404	0	0	0	328.276	gggttcgagtCccacctgggg	m5C_59300
chr17	73030543	-	C	251	66	0.263	0	0	T	185	M	0.212	0.321	0	0	0	280.305	attgtgggttCgagtcccacc	m5C_59314
chr17	73030559	-	C	222	52	0.234	0	0	T	170	M	0.183	0.294	0	0	0	190.667	gcctcctaagCcaggattgt	m5C_59289
chr17	73030564	-	C	121	34	0.281	0	0	T	87	M	0.209	0.367	0	0	0	141.845	cactggcctcCtaagccaggg	m5C_59299
chr17	73030568	-	C	80	19	0.237	0	0	T	61	M	0.158	0.341	0	8.4E-14	0	59.9053	aaggcactggCctcctaagcc	m5C_59310
chr17	73030574	-	C	62	13	0.21	0	0	T	49	M	0.127	0.326	1.8E-15	1.6E-11	2.3E-15	24.3221	atggataaggCactggcctcc	m5C_59278
chr17	73031211	+	C	254	60	0.236	0	0	T	194	M	0.188	0.292	0	0	0	225.786	agccggagacCgcgtggccta	m5C_59261
chr17	73031235	+	C	255	55	0.216	0	0	T	200	M	0.17	0.27	0	0	0	186.584	gataaggcgtCtgacttcgga	m5C_59253
chr17	73031242	+	C	241	51	0.212	0	0	T	190	M	0.165	0.268	0	0	0	168.076	cgtctgacttCggatcagaag	m5C_59254
chr17	73031269	+	C	191	71	0.374	1	0.005	T	119	M	0.308	0.444	0	0	0	437.411	ggttcgagtcCcttcgtggtc	m5C_59234
chr17	73031273	+	C	168	45	0.268	0	0	T	123	M	0.207	0.339	0	0	0	185.968	cgagtcccttCgtggtcggaa	m5C_59246
chr17	73031279	+	C	138	29	0.212	1	0.007	T	108	M	0.152	0.287	0	0	0	87.9401	ccttcgtggtCggaacgtttt	m5C_59239
chr17	73031284	+	C	132	31	0.235	0	0	T	101	M	0.171	0.314	0	0	0	105.814	gtggtcggaaCgttttaatcc	m5C_59264
chr17	73035328	-	C	134	35	0.263	1	0.007	T	98	M	0.196	0.344	0	0	0	137.009	tctcaaaggcCaggattgtag	m5C_59282
chr17	73035329	-	C	124	33	0.266	0	0	T	91	M	0.196	0.35	0	0	0	129.51	ctctcaaaggCcaggattgta	m5C_59283
chr17	73035337	-	C	65	21	0.323	0	0	T	44	M	0.222	0.444	0	1E-09	0	93.2557	gggtgtctctCtcaaaggcca	m5C_59286
chr17	73314711	-	C	81	20	0.247	0	0	T	61	M	0.166	0.351	0	1.5E-13	0	66.3608	ttggggaaggCaagattggaa	m5C_59317
chr17	74222339	-	C	93	35	0.376	0	0	T	58	M	0.285	0.478	0	1.5E-12	0	199.243	tagagctgggCagtgaagtgg	m5C_60882
chr17	74554932	+	C	1749	666	0.381	0	0	T	1083	M	0.358	0.404	0	0	0	4772.77	gtgaggaggtCtgacttgctg	m5C_60876
chr17	74554936	+	C	1671	456	0.273	2	0.001	T	1213	M	0.252	0.295	0	0	0	2301.68	ggaggtctgaCttgctgaggc	m5C_60862
chr17	74554948	+	C	297	65	0.219	0	0	T	232	M	0.176	0.269	0	0	0	228.26	tgctgaggctCagctgtttaa	m5C_60828
chr17	74557219	+	C	971	209	0.215	1	0.001	T	761	M	0.191	0.242	0	0	0	797.289	caagttatccCtgtctgaagg	m5C_60856
chr17	74557241	+	C	1019	218	0.214	0	0	T	801	M	0.19	0.24	0	0	0	827.768	aaagaaaggcCtttctgtgtg	m5C_60796
chr17	74557264	+	C	1034	252	0.244	0	0	T	782	M	0.219	0.271	0	0	0	1101.37	atttgaatatCtgaaactcag	m5C_60838
chr17	74557740	+	C	145	34	0.234	0	0	T	111	M	0.173	0.31	0	0	0	117.59	gttagttatcCctgtctgaaa	m5C_60807
chr17	74557753	+	C	147	55	0.374	0	0	T	92	M	0.3	0.455	0	0	0	330.071	gtctgaaaatCtggactgagg	m5C_60865
chr17	74557758	+	C	156	41	0.263	0	0	T	115	M	0.2	0.337	0	0	0	164.037	aaaatctggaCtgagggaaat	m5C_60823
chr17	74557772	+	C	135	27	0.2	0	0	T	108	M	0.141	0.275	0	0	0	76.2744	gggaaataatCtattctgagg	m5C_60866
chr17	74557777	+	C	134	34	0.254	0	0	T	100	M	0.188	0.334	0	0	0	127.586	ataatctattCtgaggcttaa	m5C_60873
chr17	74557783	+	C	102	45	0.441	0	0	T	57	M	0.349	0.538	0	7.4E-13	0	313.823	tattctgaggCttaagggttt	m5C_60857
chr17	74647936	-	C	45	14	0.311	0	0	T	31	M	0.195	0.457	0	1.9E-07	0	54.6799	gtggaggaggCggaaatggaa	m5C_60902
chr17	74967518	-	C	31	19	0.613	0	0	C	19	M	0.438	0.763	0	3.4E-05	0	166.53	ccggggttcctCctaggacctc	m5C_60931
chr17	75085390	+	C	206	47	0.228	0	0	T	159	M	0.176	0.29	0	0	0	165.567	tcaggtcagaCggtctgggga	m5C_60801
chr17	75085394	+	C	226	69	0.305	0	0	T	157	M	0.249	0.368	0	0	0	343.53	gtcagacggtCtggggaaagg	m5C_60872
chr17	75085407	+	C	495	138	0.279	0	0	T	357	M	0.241	0.32	0	0	0	665.449	gggaaaggctCctgtgttgtt	m5C_60852
chr17	75085408	+	C	492	113	0.23	0	0	T	379	M	0.195	0.269	0	0	0	439.991	ggaaaggctcCtgtgttgttg	m5C_60821
chr17	75085421	+	C	409	151	0.369	0	0	T	258	M	0.324	0.417	0	0	0	978.004	tgttgttgagCctgccaagtt	m5C_60808
chr17	75085422	+	C	402	92	0.229	0	0	T	310	M	0.19	0.272	0	0	0	350.464	gttgttgagcCtgccaagttg	m5C_60816
chr17	75085426	+	C	363	76	0.209	0	0	T	287	M	0.171	0.254	0	0	0	259.409	ttgagcctgcCaagttgtggg	m5C_60818
chr17	75085449	+	C	114	33	0.289	0	0	T	81	M	0.214	0.379	0	2.2E-16	0	141.349	cagcatggtaCcaggcagccc	m5C_60861
chr17	75085450	+	C	107	58	0.542	0	0	C	58	M	0.448	0.633	0	1.5E-11	0	519.486	agcatggtacCaggcagccca	m5C_60805
chr17	75085458	+	C	67	21	0.313	0	0	T	46	M	0.215	0.432	0	1E-09	0	90.3218	accaggcagcCcaaccctgac	m5C_60836
chr17	75085553	+	C	36	12	0.333	0	0	T	24	M	0.202	0.497	0	4.6E-06	0	48.5146	gtttgatgatCatgtatgata	m5C_60829
chr17	75438198	+	C	38	11	0.289	0	0	T	27	M	0.17	0.448	2.1E-15	3.1E-06	2.7E-15	27.4488	cgtgactggtCcgaaggtagt	m5C_60842
chr17	75438199	+	C	39	8	0.205	0	0	T	31	M	0.108	0.355	1.6E-10	6.8E-07	1.8E-10	8.44384	gtgactggtcCgaaggtagtg	m5C_60868
chr17	75438230	+	C	38	10	0.263	0	0	T	28	M	0.15	0.42	9.4E-14	2E-06	1.2E-13	19.5037	gttgattgttCacagtcagtt	m5C_60811
chr17	75599402	+	C	96	36	0.375	0	0	T	60	M	0.285	0.475	0	2E-12	0	205.002	cccttggggcCcggtggccag	m5C_60798
chr17	75599422	+	C	70	42	0.6	0	0	C	42	M	0.483	0.707	0	8.7E-09	0	405.666	ggacttgggtgCaaaagccccc	m5C_60814
chr17	75916320	+	C	125	36	0.288	0	0	T	89	M	0.216	0.373	0	0	0	155.429	gtggggggggaCtgtgtttgcg	m5C_60831
chr17	75916329	+	C	122	31	0.254	0	0	T	91	M	0.185	0.338	0	0	0	114.806	actgtgtttgCgctttcccct	m5C_60799
chr17	75916337	+	C	109	38	0.349	0	0	T	71	M	0.266	0.442	0	9.4E-14	0	201.933	tgcgctttccCctgtgggttt	m5C_60809
chr17	75916338	+	C	97	38	0.392	0	0	T	59	M	0.301	0.491	0	3.7E-12	0	228.389	gcgctttcccCtgtgggtttt	m5C_60875
chr17	78137047	-	C	50	15	0.3	0	0	T	35	M	0.191	0.438	0	4.6E-09	0	57.3102	ggtttgcctcCtgatcaggga	m5C_59551
chr17	78137048	-	C	53	11	0.208	0	0	T	42	M	0.12	0.335	1.8E-13	4.4E-10	2.2E-13	16.8213	gggtttgcctCctgatcaggg	m5C_59519
chr17	78137061	-	C	49	19	0.388	0	0	T	30	M	0.264	0.528	0	1E-06	0	100.429	ctcactgcggCttgggtttgc	m5C_59563
chr17	78185297	-	C	31	11	0.355	0	0	T	20	M	0.211	0.531	1.1E-16	3.1E-06	1.5E-16	37.0595	gtgaggggctCggggtgaccc	m5C_59534
chr17	78309253	+	C	43	15	0.349	0	0	T	28	M	0.224	0.498	0	2.8E-07	0	67.2552	gtggtggactCtctggtccac	m5C_59455
chr17	79054817	+	C	37	10	0.27	0	0	T	27	M	0.154	0.43	6.7E-14	2E-06	8.3E-14	20.2815	gctgagggagCactggccagg	m5C_59470
chr17	79540523	+	C	75	36	0.48	0	0	T	39	M	0.371	0.591	0	2.4E-09	0	266.896	tctggctggtCcgaaggtggt	m5C_59471
chr17	79540524	+	C	75	25	0.333	0	0	T	50	M	0.237	0.446	0	9.3E-11	0	118.54	ctggctggtcCgaaggtggtg	m5C_59509
chr17	79687839	+	C	34	14	0.412	0	0	T	20	M	0.264	0.578	0	9.2E-06	0	73.8241	gcctgcgtccCtcgggcacct	m5C_59486
chr17	79801937	-	C	60	14	0.233	0	0	T	46	M	0.144	0.354	0	3.2E-11	0	40.4304	gcagaggagcCagacatggag	m5C_59632
chr17	79801938	-	C	70	39	0.557	0	0	C	39	M	0.441	0.668	0	1.2E-07	0	343.85	agcagaggagCcagacatgga	m5C_59565
chr17	79801946	-	C	83	18	0.217	0	0	T	65	M	0.142	0.317	0	4.5E-14	0	51.0532	ctggaagaagCagaggagcca	m5C_59550
chr17	79801956	-	C	82	33	0.402	0	0	T	49	M	0.303	0.511	0	3.1E-11	0	199.962	tctcgaggacCtggaagaagc	m5C_59544
chr17	79801957	-	C	80	23	0.287	0	0	T	57	M	0.2	0.395	0	7.2E-13	0	91.9392	atctcgaggaCctggaagaag	m5C_59594
chr17	79801963	-	C	64	23	0.359	0	0	T	41	M	0.253	0.482	0	2.1E-09	0	116.326	cccaggatctCgaggacctgg	m5C_59545
chr17	79803020	-	C	40	13	0.325	0	0	T	27	M	0.201	0.48	0	1.2E-07	0	52.2192	gggatgatgaCgtgagtgggg	m5C_59586
chr17	79890515	-	C	31	17	0.548	0	0	C	17	M	0.378	0.708	0	2.1E-05	0	128.424	acttctgtggCcaccacctcc	m5C_59557
chr17	79890542	-	C	33	17	0.515	0	0	C	17	M	0.352	0.675	0	2.1E-05	0	119.742	ggaaagggctCcttgggctgc	m5C_59612
chr17	79890544	-	C	31	15	0.484	0	0	T	16	M	0.32	0.652	0	1.2E-05	0	95.91	ggggaaagggCtccttgggct	m5C_59606
chr17	79890565	-	C	31	15	0.484	0	0	T	16	M	0.32	0.652	0	1.2E-05	0	95.91	acgcagatccCatggacattt	m5C_59558
chr17	79890566	-	C	31	16	0.516	0	0	C	16	M	0.348	0.68	0	1.7E-05	0	111.488	cacgcagatcCcatggacatt	m5C_59539
chr17	79892877	-	C	51	11	0.216	0	0	T	40	M	0.125	0.346	1.1E-13	4.4E-10	1.4E-13	17.8061	tgctgagcagCgtgggcttct	m5C_59518
chr17	79987440	-	C	53	19	0.358	0	0	T	34	M	0.243	0.493	0	2.4E-08	0	92.3467	ctgagtgccgCtgctttcact	m5C_59628
chr17	79987442	-	C	53	19	0.358	0	0	T	34	M	0.243	0.493	0	2.4E-08	0	92.3467	cgctgagtgcCgctgctttca	m5C_59555
chr17	80452588	-	C	41	9	0.22	0	0	T	32	M	0.12	0.367	8.4E-12	1.2E-08	9.9E-12	11.9621	tatagactccCttttctttcc	m5C_59377
chr17	80452589	-	C	44	9	0.205	0	0	T	35	M	0.112	0.345	1.8E-11	1.2E-08	2.1E-11	10.7806	ctatagactcCcttttctttc	m5C_59348
chr17	80452590	-	C	46	11	0.239	0	0	T	35	M	0.139	0.379	2.8E-14	4.4E-08	3.5E-14	20.7303	gctatagactCccttttcttt	m5C_59350
chr17	80452602	-	C	58	12	0.207	0	0	T	46	M	0.123	0.328	2.1E-14	8.8E-10	2.6E-14	20.1159	aaaccatcctCtgctatagac	m5C_59357
chr17	80452604	-	C	62	17	0.274	0	0	T	45	M	0.179	0.396	0	1.8E-10	0	60.7951	cgaaaccatcCtctgctatag	m5C_59353
chr17	80452608	-	C	71	21	0.296	0	0	T	50	M	0.202	0.41	0	1.8E-11	0	84.9775	ggatcgaaacCatcctctgct	m5C_59359
chr17	80452629	-	C	66	33	0.508	1	0.015	C	33	M	0.389	0.625	0	3.4E-08	0	256.847	gcccataaccCagaggtcgat	m5C_59356
chr17	80452636	-	C	62	21	0.339	0	0	T	41	M	0.233	0.463	0	1E-09	0	98.0345	gtgctgggccCataacccaga	m5C_59366
chr17	80452637	-	C	61	31	0.508	0	0	C	31	M	0.386	0.629	0	2.1E-08	0	239.335	cgtgctgggcCcataacccag	m5C_59345

chr17	80452638	-	C	63	24	0.381	0	0	T	39	M	0.271	0.504	0	3E-09	0	130.163	gcgtgctgggCccataaccca	m5C_59369
chr17	80452656	-	C	69	20	0.29	0	0	T	49	M	0.196	0.406	0	6.9E-10	0	78.4637	cagagtggcgCagcggaagcg	m5C_59361
chr17	80452669	-	C	49	11	0.224	0	0	T	38	M	0.13	0.359	6.6E-14	4.4E-08	8.1E-14	18.8888	cccttatagcCagcagagtgg	m5C_59362
chr18	252571	-	C	65	14	0.215	0	0	T	51	M	0.133	0.33	1.1E-16	3.2E-11	1.5E-16	29.6776	tagaagaaggCgaaatgggag	m5C_59425
chr18	703707	-	C	33	13	0.394	0	0	T	20	M	0.247	0.563	0	6.7E-06	0	64.1755	ccaaaaggctCagggaaggct	m5C_59410
chr18	3262208	+	C	34	10	0.294	0	0	T	24	M	0.168	0.462	2.3E-14	2E-06	2.9E-14	22.9564	tcgcgcagggCggccccggtt	m5C_59686
chr18	3262212	+	C	31	9	0.29	0	0	T	22	M	0.161	0.466	3.7E-13	1.2E-06	4.4E-13	18.0154	gcagggcggcCcccggttctgg	m5C_59682
chr18	7301125	-	C	109	56	0.514	0	0	C	56	M	0.421	0.606	0	9.8E-12	0	471.6	agagtggcatCtggctgtgac	m5C_59666
chr18	7301128	-	C	148	30	0.203	0	0	T	118	M	0.146	0.275	0	0	0	87.5036	ggtagagtggCatctggctgt	m5C_59659
chr18	7301141	-	C	153	50	0.327	0	0	T	103	M	0.258	0.405	0	0	0	257.51	tgcctctgacCtgggtagagt	m5C_59681
chr18	8476926	-	C	66	16	0.242	0	0	T	50	M	0.155	0.358	0	1.1E-10	0	49.629	caggagggtgCgtgggctggg	m5C_59933
chr18	9518199	-	C	72	21	0.292	0	0	T	51	M	0.199	0.405	0	1.8E-11	0	83.7392	atttctggagCagggagatgg	m5C_59927
chr18	10229616	+	C	84	22	0.262	0	0	T	62	M	0.18	0.365	0	4.4E-13	0	79.1047	tcctgagcacCtctgggaatg	m5C_59827
chr18	12353095	-	C	46	18	0.391	0	0	T	28	M	0.264	0.535	0	7.7E-07	0	95.0149	tcgatgaaatCgatgcggtgg	m5C_61039
chr18	12760723	+	C	49	16	0.327	0	0	T	33	M	0.212	0.466	0	4E-07	0	67.8634	acaggcttgtCcgagtgcagt	m5C_61013
chr18	12760724	+	C	47	19	0.404	0	0	T	28	M	0.276	0.547	0	1E-06	0	105.031	caggcttgtcCgagtgcagtg	m5C_61005
chr18	13279525	-	C	31	17	0.548	0	0	C	17	M	0.378	0.708	0	2.1E-05	0	128.424	gggcatagtcCagtggtgtgt	m5C_61029
chr18	18548813	-	C	48	10	0.208	0	0	T	38	M	0.117	0.343	1.6E-12	2.4E-08	1.9E-12	13.8343	agaatataaaCtggagaagga	m5C_59886
chr18	18549084	-	C	55	13	0.236	0	0	T	42	M	0.144	0.363	2.2E-16	1.6E-09	2.9E-16	29.2353	atgaagaaggCagaggaaggt	m5C_59889
chr18	18586524	-	C	33	25	0.758	0	0	C	25	M	0.59	0.872	0	0.00011	0	294.875	aggacagaatCggacacagct	m5C_59884
chr18	19203898	+	C	134	31	0.233	1	0.007	T	102	M	0.169	0.312	0	0	0	104.992	aaaagggaagCtggtaagttt	m5C_59877
chr18	19397760	+	C	49	12	0.245	0	0	T	37	M	0.146	0.381	1.9E-15	7.5E-08	2.4E-15	25.8006	tggacagtgaCtggggagaca	m5C_59865
chr18	19397773	+	C	58	32	0.552	0	0	C	32	M	0.425	0.673	0	7.2E-07	0	271.692	gggagacaaaCcatgcaggaa	m5C_59880
chr18	30050990	-	C	68	23	0.338	0	0	T	45	M	0.237	0.457	0	2.1E-09	0	109.085	gcgctggcggCcccggccggg	m5C_60067
chr18	30050997	-	C	75	16	0.213	0	0	T	59	M	0.136	0.319	0	1.2E-12	0	43.4563	gccgcgggcgCtggcggcccc	m5C_60068
chr18	30050999	-	C	64	21	0.328	0	0	T	43	M	0.226	0.45	0	1E-09	0	94.7957	gagccgcgggCgctggcggcc	m5C_60065
chr18	43123364	+	C	33	11	0.333	0	0	T	22	M	0.198	0.504	2.2E-16	3.1E-06	2.9E-16	34.0074	tagaagccagCtaactcagtt	m5C_60216
chr18	43123370	+	C	53	18	0.34	0	0	T	35	M	0.227	0.474	0	1.7E-08	0	81.6727	ccagctaactCagttggtaga	m5C_60220
chr18	43669902	-	C	73	36	0.493	0	0	T	37	M	0.382	0.605	0	2.4E-09	0	274.829	aaatgataaaCtaattaagga	m5C_60231
chr18	43669921	-	C	43	12	0.279	0	0	T	31	M	0.167	0.427	2.2E-16	7.5E-08	2.9E-16	31.457	ttggtgttgtCgtgtttggaa	m5C_60244
chr18	45374860	-	C	54	18	0.333	0	0	T	36	M	0.222	0.466	0	1.7E-08	0	80.0685	gtagaaatgaCaagaaggcat	m5C_60425
chr18	45374872	-	C	46	24	0.522	0	0	C	24	M	0.381	0.659	0	3.6E-06	0	183.058	cgaaatgccaCggtagaaatg	m5C_60420
chr18	45423063	-	C	48	12	0.25	0	0	T	36	M	0.149	0.388	1.3E-15	7.5E-08	1.7E-15	26.6338	aagaagtcagCtggtgggtct	m5C_60426
chr18	45423066	-	C	49	22	0.449	0	0	T	27	M	0.319	0.587	0	2.3E-06	0	140.152	tggaagaagtCagctggtggg	m5C_60421
chr18	46769537	-	C	41	16	0.39	0	0	T	25	M	0.257	0.543	0	4E-07	0	82.099	gaaggcagccCgctcacctga	m5C_61157
chr18	46769538	-	C	47	20	0.426	0	0	T	27	M	0.295	0.567	0	1.4E-06	0	118.054	agaaggcagcCcgctcacctg	m5C_61178
chr18	46769539	-	C	42	10	0.238	0	0	T	32	M	0.135	0.385	3.2E-13	2.4E-08	3.9E-13	16.8407	gagaaggcagCccgctcacct	m5C_61189
chr18	46769542	-	C	45	12	0.267	0	0	T	33	M	0.16	0.41	5.6E-16	7.5E-08	7.3E-16	29.2263	caggagaaggCagcccgctca	m5C_61184
chr18	47015623	-	C	283	87	0.307	0	0	T	196	M	0.257	0.363	0	0	0	446.376	gaaaactattCtgagcaaccc	m5C_61163
chr18	47015641	-	C	372	78	0.21	0	0	T	294	M	0.171	0.254	0	0	0	267.356	tttggaataaCtatgaaagaa	m5C_61167
chr18	47015652	-	C	437	103	0.236	0	0	T	334	M	0.198	0.278	0	0	0	408.525	cttaggacacCtttggaataa	m5C_61148
chr18	47015653	-	C	456	128	0.281	0	0	T	328	M	0.241	0.324	0	0	0	618.042	tcttaggacaCctttggaata	m5C_61155
chr18	47015666	-	C	485	205	0.423	0	0	T	280	M	0.379	0.467	0	0	0	1555.92	gctgtgatgaCtatcttagga	m5C_61190
chr18	47015675	-	C	220	78	0.355	0	0	T	142	M	0.294	0.42	0	0	0	459.144	tttgtggttgCtgtgatgact	m5C_61191
chr18	47016908	-	C	42	18	0.429	0	0	T	24	M	0.291	0.578	0	7.7E-07	0	104.823	agcaatggggCtggacacaag	m5C_61142
chr18	47017656	-	C	529	135	0.255	0	0	T	394	M	0.22	0.294	0	0	0	593.738	aaattctgagCagccacctta	m5C_61171
chr18	47017680	-	C	794	199	0.251	0	0	T	595	M	0.222	0.282	0	0	0	882.503	tttggatttaCcgtgaaaatt	m5C_61186
chr18	47017692	-	C	835	177	0.213	3	0.004	T	655	M	0.186	0.242	0	0	0	659.448	tcttaggacaCcttttggattt	m5C_61139
chr18	47017701	-	C	865	185	0.214	1	0.001	T	679	M	0.188	0.243	0	0	0	695.837	tgatgactttCttaggacacc	m5C_61187
chr18	47017705	-	C	853	200	0.234	0	0	T	653	M	0.207	0.264	0	0	0	829.057	gcagtgatgaCtttcttagga	m5C_61161
chr18	47018034	-	C	73	23	0.315	0	0	T	50	M	0.22	0.429	0	4.3E-11	0	101.216	ctctgagcagCtgttcgaatc	m5C_61173
chr18	47018037	-	C	180	54	0.3	0	0	T	126	M	0.238	0.371	0	0	0	256.828	actctctgagCagctgttcga	m5C_61140
chr18	47018042	-	C	235	63	0.268	0	0	T	172	M	0.216	0.328	0	0	0	271.547	caactactctCtgagcagctg	m5C_61146
chr18	47018052	-	C	289	66	0.228	0	0	T	223	M	0.184	0.28	0	0	0	242.517	ataatgaaaaCaactactctc	m5C_61185
chr18	47018082	-	C	326	145	0.445	0	0	T	181	M	0.392	0.499	0	0	0	1136.2	gatggcatttCttaggacacc	m5C_61182
chr18	47018096	-	C	143	39	0.273	0	0	T	104	M	0.206	0.351	0	0	0	160.987	agtatggctgCgatgatggca	m5C_61176
chr18	47340761	+	C	40	8	0.2	0	0	T	32	M	0.105	0.348	2.1E-10	5.5E-09	2.3E-10	8.13503	actgcgcaatCtgagttctgg	m5C_61120
chr18	47340794	+	C	39	13	0.333	0	0	T	26	M	0.206	0.49	0	6.7E-06	0	53.6452	ggagtatgttCtgagaacaga	m5C_61128
chr18	47340801	+	C	42	12	0.286	0	0	T	30	M	0.172	0.436	2.2E-16	7.5E-08	2.9E-16	32.2467	gttctgagaaCagactgaggc	m5C_61126
chr18	47340811	+	C	36	14	0.389	0	0	T	22	M	0.248	0.551	0	9.2E-06	0	69.397	cagactgaggCcgggcaagca	m5C_61119
chr18	47398029	-	C	34	9	0.265	0	0	T	25	M	0.146	0.431	1.1E-12	1.2E-06	1.3E-12	15.7394	gctggttggtCtgaaggtagt	m5C_61147
chr18	47733545	-	C	35	10	0.286	0	0	T	25	M	0.163	0.451	3.4E-14	2E-06	4.2E-14	21.9991	ttatgctgacCgggtgtctgc	m5C_61162
chr18	47733550	-	C	55	14	0.255	0	0	T	41	M	0.158	0.383	0	2.8E-09	0	44.2735	gtgtgttatgCtgaccgggtg	m5C_61143
chr18	47733578	-	C	46	11	0.239	0	0	T	35	M	0.139	0.379	2.8E-14	4.4E-08	3.5E-14	20.7303	tgcttgggttCaggagttctg	m5C_61181
chr18	47811125	-	C	70	14	0.2	0	0	T	56	M	0.123	0.308	4.4E-16	2.8E-13	5.8E-16	26.447	tgtgaagcgtCgggagaagaa	m5C_61152
chr18	47811128	-	C	60	31	0.517	0	0	C	31	M	0.393	0.638	0	5.9E-07	0	243.707	gcatgtgaagCgtcgggagaa	m5C_61150
chr18	48810105	-	C	55	21	0.382	0	0	T	34	M	0.265	0.514	0	4.8E-08	0	111.366	gtgctttcccCtgatgtttat	m5C_60341
chr18	48810106	-	C	81	18	0.222	0	0	T	63	M	0.145	0.324	0	4.5E-14	0	52.3602	tgtgctttccCctgatgttta	m5C_60337
chr18	48810107	-	C	76	19	0.25	0	0	T	57	M	0.166	0.358	0	6.9E-12	0	63.1894	gtgtgctttcCcctgatgttt	m5C_60340
chr18	48810112	-	C	86	29	0.337	0	0	T	57	M	0.246	0.442	0	8.4E-12	0	142.775	tgtgtgtgtgCtttcccctga	m5C_60327
chr18	48810123	-	C	182	53	0.291	0	0	T	129	M	0.23	0.361	0	0	0	243.866	tagtgggggaCtgtgtgtgtg	m5C_60338
chr18	51748689	-	C	315	66	0.21	0	0	T	249	M	0.168	0.258	0	0	0	222.044	tgccctgggaCtgtgccagtg	m5C_60583
chr18	51748701	-	C	301	66	0.22	1	0.003	T	234	M	0.177	0.27	0	0	0	233.411	tgttggcatcCttgccctggg	m5C_60594
chr18	51748715	-	C	329	75	0.228	0	0	T	254	M	0.186	0.276	0	0	0	278.886	tgagagaaaaCcttttgttggc	m5C_60592
chr18	51748726	-	C	332	72	0.217	0	0	T	260	M	0.176	0.264	0	0	0	253.312	gtagctggtgCtgagagaaaa	m5C_60591
chr18	51748745	-	C	406	111	0.273	0	0	T	295	M	0.232	0.319	0	0	0	515.749	gctgtggggcCaggatttggt	m5C_60590
chr18	51748754	-	C	388	112	0.29	2	0.005	T	274	M	0.247	0.337	0	0	0	553.559	ctccctcaggCtgtggggcca	m5C_60587
chr18	51748760	-	C	284	99	0.349	0	0	T	185	M	0.296	0.406	0	0	0	585.138	caggtcctccCtcaggctgtg	m5C_60589
chr18	51748761	-	C	270	55	0.204	0	0	T	215	M	0.16	0.256	0	0	0	175.975	acaggtcctcCctcaggctgt	m5C_60595
chr18	51748765	-	C	269	63	0.234	0	0	T	206	M	0.188	0.288	0	0	0	236.316	tttcacaggtCctccctcagg	m5C_60586
chr18	58072219	+	C	168	69	0.411	0	0	T	99	M	0.339	0.486	0	0	0	467.994	gatgttcagaCaggtgaaaag	m5C_60512
chr18	58141587	-	C	33	24	0.727	0	0	C	24	M	0.558	0.849	0	9.1E-05	0	267.754	catagttataCatgtgccata	m5C_60538
chr18	59998052	+	C	31	10	0.323	0	0	T	21	M	0.186	0.499	7.1E-15	2E-06	9E-15	26.2725	gcaggcaggtCtgagtgcaat	m5C_60523
chr18	59998071	+	C	31	10	0.323	0	0	T	21	M	0.186	0.499	7.1E-15	2E-06	9E-15	26.2725	atggtgtttaCaaccaattaa	m5C_60499
chr18	60984427	-	C	69	23	0.333	0	0	T	46	M	0.234	0.451	0	2.1E-09	0	107.414	tgattcttagCtgtggattat	m5C_60470
chr18	71894928	+	C	58	12	0.207	0	0	T	46	M	0.123	0.328	2.1E-14	8.8E-10	2.6E-14	20.1159	ggtgaattggCgcaggtcaga	m5C_60547
chr19	926318	+	C	63	50	0.794	0	0	C	50	M	0.678	0.875	0	6.9E-07	0	678.3	tggtgggggtCccggactggg	m5C_60672
chr19	926319	+	C	48	20	0.417	0	0	T	28	M	0.288	0.557	0	1.4E-06	0	115.398	ggtgggggtcCcggactggga	m5C_60706
chr19	926320	+	C	39	24	0.615	0	0	C	24	M	0.459	0.751	0	9.1E-05	0	220.314	gtgggggtccCggactgggaa	m5C_60683
chr19	1383566	+	C	41	17	0.415	0	0	T	24	M	0.278	0.566	0	5.6E-07	0	94.3715	agtgtcgtctCtgtggcgcaa	m5C_60687
chr19	1383572	+	C	43	10	0.233	0	0	T	33	M	0.132	0.377	4.3E-13	2.4E-08	5.2E-13	16.2683	gtctctgtggCgcaatcggtt	m5C_60698
chr19	1383574	+	C	41	9	0.22	0	0	T	32	M	0.12	0.367	8.4E-12	1.2E-08	9.9E-12	11.9621	ctctgtggcgCaatcggttag	m5C_60719
chr19	1383594	+	C	84	17	0.202	0	0	T	67	M	0.13	0.3	0	2.3E-14	0	44.327	gcgcgttcggCtgttaaccga	m5C_60714
chr19	1383623	+	C	166	60	0.361	0	0	T	106	M	0.292	0.437	0	0	0	350.711	tggttcgagcCcaacccaggga	m5C_60709
chr19	1383628	+	C	186	46	0.247	0	0	T	140	M	0.191	0.314	0	0	0	175.571	cgagcccaccCagggacgcgt	m5C_60723
chr19	1383634	+	C	182	46	0.253	0	0	T	136	M	0.195	0.321	0	0	0	179.553	cacccagggaCgcgtggtgac	m5C_60670
chr19	1383644	+	C	151	37	0.245	0	0	T	114	M	0.183	0.319	0	0	0	135.655	cgcgtggtgaCttttgccctc	m5C_60676
chr19	1749125	-	C	59	37	0.627	0	0	C	37	M	0.5	0.739	0	1.8E-06	0	369.655	ccgttggaatCgtctccccgc	m5C_60763
chr19	1749134	-	C	64	45	0.703	0	0	C	45	M	0.582	0.801	0	3.3E-07	0	524.066	cactggacgcCgttggaatcg	m5C_60779
chr19	1749135	-	C	61	43	0.705	0	0	C	43	M	0.581	0.804	0	2.4E-07	0	499.749	tcactggacgCcgttggaatc	m5C_60733
chr19	1749137	-	C	59	38	0.644	0	0	C	38	M	0.517	0.754	0	2.1E-06	0	392.58	cctcactggaCgccgttggaa	m5C_60760

chr19	2273074	+	C	33	12	0.364	0	0	T	21	M	0.222	0.534	0	4.6E-06	0	53.2487	cctacacgttCgagagagagt	m5C_61298
chr19	3976138	-	C	86	26	0.302	0	0	T	60	M	0.215	0.406	0	2.7E-12	0	112.026	tgatttctagCaggcaggaag	m5C_61385
chr19	3976156	-	C	110	23	0.209	0	0	T	87	M	0.144	0.294	0	0	0	66.0388	aaggaacacgCgggtagatga	m5C_61378
chr19	3976160	-	C	92	19	0.207	0	0	T	73	M	0.136	0.3	0	8.9E-16	0	51.8283	gggaaaggaaCacgcgggtag	m5C_61346
chr19	3979369	-	C	87	21	0.241	0	0	T	66	M	0.164	0.341	0	2.6E-13	0	68.729	tggagatctgCctgaaggacc	m5C_61338
chr19	3979372	-	C	85	26	0.306	0	0	T	59	M	0.218	0.41	0	2.7E-12	0	113.408	acctggagatCtgcctgaagg	m5C_61442
chr19	3979380	-	C	88	39	0.443	0	0	T	49	M	0.344	0.547	0	1.6E-10	0	268.268	cgagctgcacCtggagatctg	m5C_61371
chr19	3979381	-	C	91	19	0.209	0	0	T	72	M	0.138	0.303	0	8.9E-16	0	52.4171	gcgagctgcaCctggagatct	m5C_61351
chr19	3979386	-	C	76	32	0.421	0	0	T	44	M	0.316	0.533	0	8.5E-10	0	202.55	cgccggcgagCtgcacctgga	m5C_61332
chr19	3979426	-	C	46	22	0.489	1	0.022	T	23	M	0.35	0.63	0	2.3E-06	0	153.81	agtgcatcatCgaggagtcgg	m5C_61439
chr19	3979432	-	C	56	26	0.464	0	0	T	30	M	0.34	0.593	0	1.9E-07	0	176.902	cctttcagtgCatcatcgagg	m5C_61399
chr19	3979818	-	C	55	19	0.345	0	0	T	36	M	0.234	0.477	0	2.4E-08	0	88.7776	ccaagtccgaCcccatggtgc	m5C_61392
chr19	3979821	-	C	49	16	0.327	0	0	T	33	M	0.212	0.466	0	4E-07	0	67.8634	tggccaagtcCgaccccatgg	m5C_61391
chr19	3979822	-	C	47	10	0.213	0	0	T	37	M	0.12	0.349	1.3E-12	2.4E-08	1.5E-12	14.271	ctggccaagtCcgaccccatg	m5C_61440
chr19	3979828	-	C	38	9	0.237	0	0	T	29	M	0.13	0.392	3.7E-12	1.2E-06	4.3E-12	13.3739	aagcggctggCcaagtccgac	m5C_61403
chr19	3979835	-	C	38	13	0.342	0	0	T	25	M	0.212	0.501	0	6.7E-06	0	55.1518	ggggctgaagCggctggccaa	m5C_61430
chr19	3979841	-	C	49	18	0.367	0	0	T	31	M	0.247	0.507	0	7.7E-07	0	88.7939	ggtggaggggCtgaagcggct	m5C_61429
chr19	3982510	-	C	417	103	0.247	0	0	T	314	M	0.208	0.291	0	0	0	428.568	aagcctgcgtCtgaggcccat	m5C_61369
chr19	3982517	-	C	497	124	0.249	0	0	T	373	M	0.213	0.289	0	0	0	529.41	ctgaagaaagCctgcgtctga	m5C_61397
chr19	3982527	-	C	515	117	0.227	0	0	T	398	M	0.193	0.265	0	0	0	451.837	cagatgtctaCtgaagaaagc	m5C_61330
chr19	3982544	-	C	497	149	0.3	0	0	T	348	M	0.261	0.341	0	0	0	778.312	catttcttcaCtttgaccaga	m5C_61380
chr19	3982559	-	C	488	128	0.262	0	0	T	360	M	0.225	0.303	0	0	0	576.593	ttcgtgatgaCtgatcatttc	m5C_61422
chr19	3982567	-	C	211	45	0.213	0	0	T	166	M	0.163	0.273	0	0	0	147.045	cccccctattCgtgatgactg	m5C_61402
chr19	3983005	-	C	50	19	0.38	0	0	T	31	M	0.259	0.518	0	2.4E-08	0	98.2779	cgtgtgcgtgCagacggagac	m5C_61414
chr19	3983009	-	C	59	20	0.339	0	0	T	39	M	0.231	0.466	0	3.4E-08	0	92.5499	caggcgtgtgCgtgcagacgg	m5C_61405
chr19	3983118	-	C	67	16	0.239	0	0	T	51	M	0.153	0.353	0	1.1E-10	0	48.8576	tggtggtggaCtgcgtgtcag	m5C_61413
chr19	4510603	-	C	53	20	0.377	0	0	T	33	M	0.259	0.512	0	3.4E-08	0	103.744	tggcgacgttCacccaagggg	m5C_62624
chr19	4510607	-	C	442	119	0.269	0	0	T	323	M	0.23	0.312	0	0	0	547.394	gacgtggcgaCgttcacccaa	m5C_62584
chr19	4724128	+	C	945	239	0.253	0	0	T	706	M	0.226	0.282	0	0	0	1081.4	aagcagttgaCccgggttcga	m5C_62545
chr19	4724129	+	C	923	257	0.278	0	0	T	666	M	0.25	0.308	0	0	0	1287.5	agcagttgacCcgggttcgat	m5C_62559
chr19	4724130	+	C	871	203	0.233	0	0	T	668	M	0.206	0.262	0	0	0	837.159	gcagttgaccCgggttcgatt	m5C_62543
chr19	4724136	+	C	545	140	0.257	0	0	T	405	M	0.222	0.295	0	0	0	621.571	gacccgggttCgattcccggc	m5C_62537
chr19	4724156	+	C	96	22	0.229	0	0	T	74	M	0.156	0.323	0	5.8E-15	0	68.8547	ccaacgcatgCggtaccactt	m5C_62536
chr19	4724164	+	C	75	25	0.333	0	0	T	50	M	0.237	0.446	0	9.3E-11	0	118.54	tgcggtaccaCttttgcgagt	m5C_62544
chr19	4724669	-	C	399	90	0.226	0	0	T	309	M	0.187	0.269	0	0	0	337.108	gaaaggtcccCggttcgatcc	m5C_62600
chr19	4724670	-	C	414	110	0.266	0	0	T	304	M	0.225	0.31	0	0	0	495.983	cgaaaggtccCcggttcgatc	m5C_62575
chr19	4724672	-	C	412	148	0.359	0	0	T	264	M	0.314	0.407	0	0	0	930.6	cgcgaaaggtCcccggttcga	m5C_62577
chr19	4724686	-	C	465	91	0.21	32	0.069	T	342	M	0.174	0.251	0	0	0	317.442	acattcgcctCacacgcgaaa	m5C_62604
chr19	4724688	-	C	540	175	0.325	1	0.002	T	364	M	0.287	0.365	0	0	0	1002.77	tcacattcgcCtcacacgcga	m5C_62606
chr19	4724689	-	C	578	147	0.254	0	0	T	431	M	0.221	0.291	0	0	0	648.343	atcacattcgCctcacacgcg	m5C_62605
chr19	4724691	-	C	591	156	0.264	0	0	T	435	M	0.23	0.301	0	0	0	717.69	ttatcacattCgcctcacacg	m5C_62628
chr19	4724697	-	C	699	245	0.351	0	0	T	454	M	0.316	0.387	0	0	0	1548.57	tagcggttatCacattcgcct	m5C_62597
chr19	4724714	-	C	665	173	0.26	0	0	T	492	M	0.228	0.295	0	0	0	789.747	taccggtttcCgtagtgtagc	m5C_62588
chr19	4724715	-	C	371	101	0.272	0	0	T	270	M	0.229	0.32	0	0	0	463.489	ctaccggtttCcgtagtgtag	m5C_62562
chr19	5244195	+	C	33	10	0.303	0	0	T	23	M	0.174	0.473	1.6E-14	2E-06	2E-14	23.982	cgttccgcggCgcgctggccg	m5C_62560
chr19	5244204	+	C	32	18	0.562	0	0	C	18	M	0.393	0.718	0	2.7E-05	0	141.571	gcgcgctggcCggggcctgct	m5C_62548
chr19	7951863	-	C	34	15	0.441	0	0	T	19	M	0.289	0.605	0	1.2E-05	0	86.6497	gtgctgtaggCttgggctggt	m5C_60990
chr19	7951870	-	C	45	18	0.4	0	0	T	27	M	0.27	0.545	0	7.7E-07	0	97.2889	ataccgggtgCtgtaggcttg	m5C_60979
chr19	7951876	-	C	45	15	0.333	0	0	T	30	M	0.214	0.479	0	2.8E-07	0	64.0761	ctaggaatacCgggtgctgta	m5C_60994
chr19	8793669	-	C	51	19	0.373	0	0	T	32	M	0.253	0.51	0	2.4E-08	0	96.2173	tctcgcctgcCacaatggtgt	m5C_61227
chr19	8793670	-	C	57	15	0.263	0	0	T	42	M	0.166	0.39	0	4.6E-09	0	49.9369	ttctcgcctgCcacaatggtg	m5C_61241
chr19	8793673	-	C	94	48	0.511	0	0	C	48	M	0.411	0.609	0	4.6E-11	0	394.72	gaattctcgcCtgccacaatg	m5C_61237
chr19	8793674	-	C	108	48	0.444	0	0	T	60	M	0.354	0.538	0	1.6E-12	0	340.07	agaattctcgCctgccacaat	m5C_61228
chr19	8793676	-	C	119	66	0.555	0	0	C	66	M	0.465	0.641	0	3E-12	0	613.808	gtagaattctCgcctgccaca	m5C_61226
chr19	8793678	-	C	136	87	0.64	0	0	C	87	M	0.556	0.716	0	2.7E-13	0	967.795	tggtagaattCtcgcctgcca	m5C_61230
chr19	8793691	-	C	169	98	0.58	0	0	C	98	M	0.505	0.652	0	2.2E-16	0	988.82	atcagtggttCagtggtagaa	m5C_61239
chr19	8793702	-	C	70	42	0.6	0	0	C	42	M	0.483	0.707	0	8.7E-09	0	405.666	tgcagtcaggCatcagtggtt	m5C_61232
chr19	10218336	+	C	67	19	0.284	0	0	T	48	M	0.19	0.401	0	4.5E-10	0	72.0865	tcccctatctCtcatgatgaa	m5C_62130
chr19	10218356	+	C	153	42	0.275	0	0	T	111	M	0.21	0.35	0	0	0	176.377	acacatatgcCtctgagctgc	m5C_62141
chr19	10218358	+	C	158	52	0.329	0	0	T	106	M	0.261	0.406	0	0	0	271.088	acatatgcctCtgagctgctg	m5C_62102
chr19	10218366	+	C	166	43	0.259	0	0	T	123	M	0.198	0.331	0	0	0	170.6	ctctgagctgCtgtgatttct	m5C_62123
chr19	10218375	+	C	180	73	0.406	0	0	T	107	M	0.337	0.479	0	0	0	491.327	gctgtgatttCtggcttcaaa	m5C_62093
chr19	10218382	+	C	180	43	0.239	0	0	T	137	M	0.182	0.306	0	0	0	156.92	tttctggcttCaaagtaaacg	m5C_62083
chr19	10218393	+	C	175	54	0.312	2	0.011	T	119	M	0.248	0.385	0	0	0	267.627	aaagtaaacgCtctgaagaag	m5C_62105
chr19	10218395	+	C	170	52	0.306	0	0	T	118	M	0.242	0.379	0	0	0	251.202	agtaaacgctCtgaagaagag	m5C_62122
chr19	10220443	+	C	374	79	0.211	0	0	T	295	M	0.173	0.255	0	0	0	273.187	ccacatgcggCtgatgacagc	m5C_62098
chr19	10220461	+	C	860	175	0.203	0	0	T	685	M	0.178	0.232	0	0	0	622.745	agcacttctgCtgagacgctg	m5C_62129
chr19	10220467	+	C	840	210	0.25	0	0	T	630	M	0.222	0.28	0	0	0	931.978	tctgctgagaCgctgtgattg	m5C_62127
chr19	10220504	+	C	414	87	0.21	0	0	T	327	M	0.174	0.252	0	0	0	302.159	cgccctgacgCactgtgggaa	m5C_62115
chr19	10220506	+	C	197	45	0.228	0	0	T	152	M	0.175	0.292	0	0	0	157.794	ccctgacgcaCtgtgggaagg	m5C_62135
chr19	10277280	-	C	33	9	0.273	0	0	T	24	M	0.151	0.442	7.5E-13	1.2E-06	9E-13	16.4418	aagatggagaCgagaaagtaa	m5C_62183
chr19	10277292	-	C	46	10	0.217	0	0	T	36	M	0.123	0.356	9.7E-13	2.4E-08	1.2E-12	14.7309	ctgacgaggaCgaagatggag	m5C_62155
chr19	10291292	-	C	56	23	0.411	0	0	T	33	M	0.292	0.541	0	8.7E-08	0	134.166	tgttctctgaCacttgtttac	m5C_62170
chr19	10291305	-	C	59	18	0.305	0	0	T	41	M	0.203	0.431	0	1.7E-08	0	72.9125	acatgtgtttCcttgttctct	m5C_62175
chr19	10291314	-	C	64	14	0.219	0	0	T	50	M	0.135	0.334	1.1E-16	3.2E-11	1.5E-16	30.1591	gaaggaaacaCatgtgtttcc	m5C_62182
chr19	10506666	-	C	64	28	0.438	0	0	T	36	M	0.323	0.559	0	9.8E-09	0	180.845	ggaggagatgCgcaagaagga	m5C_62193
chr19	10506678	-	C	72	16	0.222	0	0	T	56	M	0.142	0.331	0	1.2E-12	0	45.3354	ggagcagaagCtggaggagat	m5C_62236
chr19	10506691	-	C	94	26	0.277	0	0	T	68	M	0.196	0.374	0	4.6E-14	0	102.075	aggagcggagCtggagcaga	m5C_62240
chr19	10506696	-	C	100	21	0.21	0	0	T	79	M	0.142	0.3	0	0	0	59.4953	caaggaggagCggagctggga	m5C_62172
chr19	10670331	-	C	50	10	0.2	0	0	T	40	M	0.112	0.33	2.6E-12	2E-10	3.1E-12	13.024	aggatgagcgCagaaaggaga	m5C_62213
chr19	10673114	-	C	43	19	0.442	0	0	T	24	M	0.304	0.589	0	1E-06	0	115.646	gaggattgagCcaggagatgg	m5C_62231
chr19	10673127	-	C	51	13	0.255	0	0	T	38	M	0.155	0.389	1.1E-16	1.6E-09	1.5E-16	32.2431	gaggctgaggCaggaggattg	m5C_62157
chr19	11122524	+	C	35	24	0.686	0	0	C	24	M	0.52	0.814	0	9.1E-05	0	249.696	atatgatgacCttccaggagt	m5C_62085
chr19	11122528	+	C	33	11	0.333	0	0	T	22	M	0.198	0.504	2.2E-16	3.1E-06	2.9E-16	34.0074	gatgaccttcCaggagtgggg	m5C_62092
chr19	11122539	+	C	35	11	0.314	0	0	T	24	M	0.186	0.48	6.7E-16	3.1E-06	8.7E-16	30.9686	aggagtggggCaccaccaggt	m5C_62108
chr19	11122541	+	C	35	25	0.714	0	0	C	25	M	0.549	0.837	0	0.00011	0	274.724	gagtggggcaCcaccaggtgg	m5C_62124
chr19	11122544	+	C	36	15	0.417	0	0	T	21	M	0.271	0.578	0	1.2E-05	0	81.421	tggggcaccaCcaggtggcct	m5C_62091
chr19	11122545	+	C	32	13	0.406	0	0	T	19	M	0.255	0.577	0	6.7E-06	0	66.3505	ggggcaccacCaggtggccta	m5C_62103
chr19	11122569	+	C	35	14	0.4	0	0	T	21	M	0.256	0.564	0	9.2E-06	0	71.5414	aggagcgaatCtgcccaggtc	m5C_62118
chr19	11122572	+	C	31	9	0.29	0	0	T	22	M	0.161	0.466	3.7E-13	1.2E-06	4.4E-13	18.0154	agcgaatctgCccaggtcaga	m5C_62140
chr19	11122573	+	C	34	9	0.265	0	0	T	25	M	0.146	0.431	1.1E-12	1.2E-06	1.3E-12	15.7394	gcgaatctgcCcaggtcagaa	m5C_62088
chr19	11553265	+	C	32	10	0.312	0	0	T	22	M	0.18	0.486	1.1E-14	2E-06	1.3E-14	25.0836	atgctgcggaCagtgaaggag	m5C_62096
chr19	12299144	+	C	34	16	0.471	0	0	T	18	M	0.315	0.633	0	1.7E-05	0	100.644	gtggagagaaCcttggtaaat	m5C_63472
chr19	12814420	-	C	1284	282	0.22	0	0	T	1002	M	0.198	0.243	0	0	0	1115.8	ttcgtactgtCtgatggggcc	m5C_63631
chr19	12814428	-	C	1551	347	0.225	9	0.006	T	1195	M	0.205	0.247	0	0	0	1421.91	gggatgtattCgtactgtctg	m5C_63623
chr19	12814445	-	C	1690	444	0.265	12	0.007	T	1234	M	0.244	0.286	0	0	0	2167.16	atgactgtgaCcacattggga	m5C_63701
chr19	12814451	-	C	1705	421	0.247	0	0	T	1284	M	0.227	0.268	0	0	0	1911.64	ccggggatgaCtgtgaccaca	m5C_63643
chr19	12817265	-	C	539	215	0.399	0	0	T	324	M	0.358	0.441	0	0	0	1541.13	ctggctgatcCtgagttgccc	m5C_63684
chr19	12845270	-	C	39	12	0.308	0	0	T	27	M	0.186	0.464	0	4.6E-06	0	44.5578	cttcctggagCtgttcaagcg	m5C_63672
chr19	12845276	-	C	36	22	0.611	0	0	C	22	M	0.449	0.752	0	6.3E-05	0	197.402	cggcagcttcCtggagctgtt	m5C_63666
chr19	12845286	-	C	34	14	0.412	0	0	T	20	M	0.264	0.578	0	9.2E-06	0	73.8241	tcgccaacgaCggcagcttcc	m5C_63646

chr19	13050369	+	C	33	10	0.303	0	0	T	23	M	0.174	0.473	1.6E-14	2E-06	2E-14	23.982	actgtgggggCggctatgtga	m5C_63577
chr19	13050372	+	C	36	21	0.583	0	0	C	21	M	0.422	0.729	0	5.2E-05	0	177.24	gtgggggcggCtatgtgaagc	m5C_63543
chr19	13051196	+	C	36	21	0.583	0	0	C	21	M	0.422	0.729	0	5.2E-05	0	177.24	ataaaggatcCtgatgcttca	m5C_63508
chr19	13051202	+	C	33	11	0.333	0	0	T	22	M	0.198	0.504	2.2E-16	3.1E-06	2.9E-16	34.0074	gatcctgatgCttcaaaaccg	m5C_63545
chr19	13051205	+	C	34	7	0.206	0	0	T	27	M	0.103	0.368	1.4E-09	3.4E-07	1.6E-09	6.4054	cctgatgcttCaaaaccggaa	m5C_63496
chr19	13051211	+	C	39	10	0.256	0	0	T	29	M	0.146	0.411	1.3E-13	2E-06	1.6E-13	18.7753	gcttcaaaacCggaagactgg	m5C_63494
chr19	13051402	+	C	119	36	0.303	0	0	T	83	M	0.227	0.39	0	1E-15	0	163.606	ctaagaagccCggaggactggg	m5C_63547
chr19	13051408	+	C	129	38	0.295	0	0	T	91	M	0.223	0.378	0	0	0	169.303	agcccgaggaCtgggatgaag	m5C_63567
chr19	13051426	+	C	66	25	0.379	0	0	T	41	M	0.271	0.499	0	4.1E-09	0	135.746	aagagatggaCggagagtggg	m5C_63515
chr19	13051465	+	C	36	22	0.611	0	0	C	22	M	0.449	0.752	0	6.3E-05	0	197.402	accctgagtaCaaggtgagtt	m5C_63586
chr19	13051570	+	C	84	19	0.226	0	0	T	65	M	0.15	0.326	0	8.4E-14	0	56.9464	tgagtggaagCcccggcagat	m5C_63553
chr19	13051572	+	C	82	21	0.256	0	0	T	61	M	0.174	0.36	0	2.6E-13	0	73.0945	agtggaagccCcggcagatcg	m5C_63473
chr19	13051573	+	C	83	40	0.482	0	0	T	43	M	0.378	0.588	0	2.1E-10	0	302.104	gtggaagcccCggcagatcga	m5C_63455
chr19	13051588	+	C	44	11	0.25	0	0	T	33	M	0.146	0.394	1.6E-14	4.4E-08	2E-14	22.132	gatcgacaacCcagattacaa	m5C_63570
chr19	13054400	+	C	34	11	0.324	0	0	T	23	M	0.191	0.492	4.4E-16	3.1E-06	5.8E-16	32.3088	aacgatgaggCatacgctgag	m5C_63460
chr19	13054404	+	C	38	9	0.237	0	0	T	29	M	0.13	0.392	3.7E-12	1.2E-06	4.3E-12	13.3739	atgaggcataCgctgaggagt	m5C_63481
chr19	13054406	+	C	41	9	0.22	0	0	T	32	M	0.12	0.367	8.4E-12	1.2E-08	9.9E-12	11.9621	gaggcatacgCtgaggagttt	m5C_63564
chr19	13054427	+	C	76	31	0.408	0	0	T	45	M	0.304	0.52	0	6.4E-10	0	188.751	ggcaacgagaCgtggggcgta	m5C_63479
chr19	13054434	+	C	81	18	0.225	1	0.012	T	62	M	0.147	0.328	0	4.5E-14	0	53.0391	agacgtggggCgtaacaaagg	m5C_63493
chr19	13054439	+	C	86	18	0.209	0	0	T	68	M	0.137	0.307	0	4.5E-14	0	49.211	tggggcgtaaCaaaggtgagg	m5C_63505
chr19	13054539	+	C	184	44	0.239	0	0	T	140	M	0.183	0.306	0	0	0	161.244	agcagagaaaCaaatgaagga	m5C_63503
chr19	13054550	+	C	346	80	0.232	1	0.003	T	265	M	0.19	0.279	0	0	0	304.72	aaatgaaggaCaaacaggacg	m5C_63480
chr19	13054559	+	C	428	155	0.362	0	0	T	273	M	0.318	0.409	0	0	0	985.887	acaaacaggaCgaggagcaga	m5C_63454
chr19	13054566	+	C	457	112	0.245	0	0	T	345	M	0.208	0.287	0	0	0	465.634	ggacgaggagCagaggcttaa	m5C_63589
chr19	13054572	+	C	387	168	0.435	1	0.003	T	218	M	0.387	0.485	0	0	0	1299.15	ggagcagaggCttaaggagga	m5C_63574
chr19	13054599	+	C	315	122	0.387	0	0	T	193	M	0.335	0.442	0	0	0	817.818	agacaagaaaCgcaaagagga	m5C_63528
chr19	13054601	+	C	320	86	0.272	4	0.013	T	230	M	0.226	0.324	0	0	0	388.78	acaagaaacgCaaagaggagg	m5C_63529
chr19	13054618	+	C	248	99	0.401	1	0.004	T	148	M	0.342	0.463	0	0	0	676.493	gaggaggaggCagaggacaag	m5C_63469
chr19	13054625	+	C	351	140	0.399	0	0	T	211	M	0.349	0.451	0	0	0	977.184	aggcagaggaCaaggaggatg	m5C_63587
chr19	13054643	+	C	581	145	0.253	9	0.015	T	427	M	0.22	0.291	0	0	0	636.759	atgatgaggaCaaagatgagg	m5C_63610
chr19	13054673	+	C	800	321	0.401	0	0	T	479	M	0.368	0.436	0	0	0	2361.5	atgaggaggaCaaggaggaag	m5C_63558
chr19	13054700	+	C	37	12	0.324	0	0	T	25	M	0.196	0.485	0	4.6E-06	0	47.1194	aggaagatgtCcccggccagg	m5C_63557
chr19	13054701	+	C	39	15	0.385	0	0	T	24	M	0.249	0.541	0	1.2E-05	0	74.6735	ggaagatgtcCccggccaggc	m5C_63471
chr19	13054702	+	C	31	14	0.452	0	0	T	17	M	0.292	0.622	0	9.2E-06	0	81.6522	gaagatgtccCcggccaggcc	m5C_63600
chr19	13055115	+	C	35	14	0.4	0	0	T	21	M	0.256	0.564	0	9.2E-06	0	71.5414	aggagggcagCagaagggggt	m5C_63451
chr19	13065108	-	C	47	21	0.447	0	0	T	26	M	0.314	0.588	0	1.8E-06	0	131.925	ggaggaaaaaCagaaacggaa	m5C_63644
chr19	13065121	-	C	40	10	0.25	0	0	T	30	M	0.142	0.402	1.8E-13	2.4E-08	2.2E-13	18.0914	gcaagcgcctCaaggaggaaa	m5C_63682
chr19	13065123	-	C	38	8	0.211	0	0	T	30	M	0.111	0.363	1.3E-10	6.8E-07	1.4E-10	8.77246	gcgcaagcgcCtcaaggagga	m5C_63626
chr19	13065124	-	C	37	17	0.459	0	0	T	20	M	0.31	0.616	0	2.1E-05	0	105.53	agcgcaagcgCctcaaggagg	m5C_63702
chr19	13065132	-	C	33	9	0.273	0	0	T	24	M	0.151	0.442	7.5E-13	1.2E-06	9E-13	16.4418	gaagaaggagCgcaagcgcct	m5C_63639
chr19	13067700	-	C	87	36	0.414	0	0	T	51	M	0.316	0.519	0	7.4E-11	0	227.586	agcagctggcCgaagagcaga	m5C_63634
chr19	13067701	-	C	85	24	0.282	0	0	T	61	M	0.198	0.386	0	1.1E-12	0	94.8899	aagcagctggCcgaagagcag	m5C_63619
chr19	13067717	-	C	41	19	0.463	0	0	T	22	M	0.321	0.613	0	1E-06	0	121.813	ggagtcgctgCgggtgaagca	m5C_63694
chr19	13067720	-	C	37	8	0.216	0	0	T	29	M	0.114	0.372	9.7E-11	6.8E-07	1.1E-10	9.12273	gcaggagtcgCtgcgggtgaa	m5C_63658
chr19	13947121	-	C	66	24	0.375	2	0.03	T	40	M	0.267	0.497	0	3E-09	0	127.996	tgtacactggCtcagttcagc	m5C_63641
chr19	13947150	-	C	59	30	0.508	0	0	C	30	M	0.384	0.632	0	4.8E-07	0	230.61	gcctactgagCtgaaacacag	m5C_63673
chr19	13947155	-	C	62	27	0.435	0	0	T	35	M	0.319	0.559	0	7.5E-09	0	172.489	cccgtgcctaCtgagctgaaa	m5C_63653
chr19	13947313	-	C	58	17	0.293	0	0	T	41	M	0.192	0.42	0	1.1E-08	0	65.2053	cagggcttagCtgcttgtgag	m5C_63678
chr19	13947331	-	C	67	15	0.224	0	0	T	52	M	0.141	0.337	0	6E-11	0	42.1908	tgtgcctggcCtgaggagcag	m5C_63700
chr19	13947337	-	C	69	23	0.333	0	0	T	46	M	0.234	0.451	0	2.1E-09	0	107.414	gaagcctgtgCctggcctgag	m5C_63676
chr19	13947342	-	C	73	18	0.247	0	0	T	55	M	0.162	0.356	0	4E-12	0	58.3356	gccccgaagcCtgtgcctggc	m5C_63695
chr19	13947343	-	C	68	37	0.544	0	0	C	37	M	0.427	0.657	0	7.9E-08	0	315.657	ggccccgaagCctgtgcctgg	m5C_63627
chr19	13947348	-	C	74	16	0.222	2	0.027	T	56	M	0.142	0.331	0	1.2E-12	0	45.3354	agagaggcccCgaagcctgtg	m5C_63697
chr19	13947359	-	C	70	18	0.257	0	0	T	52	M	0.169	0.37	0	4E-12	0	60.9449	gacggggtggCagagaggccc	m5C_63681
chr19	13947367	-	C	67	43	0.642	0	0	C	43	M	0.522	0.746	0	2.4E-07	0	449.077	tgcccggggaCggggtggcag	m5C_63640
chr19	13947374	-	C	72	15	0.208	0	0	T	57	M	0.131	0.316	0	6E-13	0	39.1558	aggatgctgcCcggggacggg	m5C_63668
chr19	13947375	-	C	71	38	0.535	0	0	C	38	M	0.42	0.646	0	3.7E-09	0	319.497	gaggatgctgCccggggacgg	m5C_63650
chr19	13947386	-	C	69	46	0.667	0	0	C	46	M	0.549	0.766	0	3.8E-07	0	505.322	gctctgccacCgaggatgctg	m5C_63699
chr19	13947389	-	C	71	27	0.38	0	0	T	44	M	0.276	0.497	0	1.9E-10	0	149.186	tgagctctgcCaccgaggatg	m5C_63622
chr19	13947410	-	C	104	47	0.452	0	0	T	57	M	0.36	0.548	0	1.3E-12	0	338.106	ccagggatttCcaaccgaccc	m5C_63660
chr19	13947419	-	C	105	25	0.238	0	0	T	80	M	0.167	0.328	0	4.4E-16	0	83.3948	atcacattgcCagggatttcc	m5C_63647
chr19	13947420	-	C	103	22	0.214	0	0	T	81	M	0.145	0.302	0	0	0	64.0184	aatcacattgCcagggatttc	m5C_63656
chr19	13947427	-	C	60	24	0.4	0	0	T	36	M	0.286	0.526	0	1.2E-07	0	137.133	tgtcacaaatCacattgccag	m5C_63664
chr19	14677016	-	C	35	27	0.771	0	0	C	27	M	0.61	0.879	0	0.00015	0	329.305	gaagaagcggCgggagaagaa	m5C_61515
chr19	15276647	+	C	54	12	0.222	0	0	T	42	M	0.132	0.349	7.5E-15	8.8E-10	9.6E-15	22.3614	caggggagtgCggcctgagtg	m5C_61470
chr19	15276651	+	C	54	37	0.685	0	0	C	37	M	0.553	0.793	0	1.8E-06	0	408.897	ggagtgcggcCtgagtggtcc	m5C_61473
chr19	15479114	-	C	44	9	0.205	0	0	T	35	M	0.112	0.345	1.8E-11	1.2E-08	2.1E-11	10.7806	aggacgaggaCgaggatgtga	m5C_61483
chr19	15479120	-	C	40	16	0.4	0	0	T	24	M	0.263	0.554	0	1.7E-05	0	84.314	agaaggaggaCgaggacgagg	m5C_61513
chr19	15509693	-	C	38	10	0.263	0	0	T	28	M	0.15	0.42	9.4E-14	2E-06	1.2E-13	19.5037	gatggcagcaCctgtgggctg	m5C_61530
chr19	15509695	-	C	38	20	0.526	0	0	C	20	M	0.373	0.675	0	4.3E-05	0	149.035	gagatggcagCacctgtgggc	m5C_61505
chr19	16197260	+	C	62	19	0.306	0	0	T	43	M	0.206	0.43	0	4.5E-10	0	78.2094	aaaaccgggcCatgaaggatg	m5C_62322
chr19	17515204	-	C	101	32	0.317	0	0	T	69	M	0.234	0.413	0	1.1E-14	0	149.91	ggcccaaggaCaaaagaaagt	m5C_62375
chr19	17515210	-	C	70	25	0.357	0	0	T	45	M	0.255	0.474	0	4.1E-09	0	127.516	agagaaggccCaaggacaaaa	m5C_62368
chr19	17515221	-	C	36	9	0.25	0	0	T	27	M	0.138	0.411	2E-12	1.2E-06	2.4E-12	14.4765	tccctggatgCagagaaggcc	m5C_62380
chr19	17973403	+	C	269	58	0.216	0	0	T	211	M	0.171	0.269	0	0	0	197.96	cctgattgcaCctaaacccaa	m5C_62358
chr19	17973404	+	C	402	90	0.224	0	0	T	312	M	0.186	0.267	0	0	0	334.538	ctgattgcacCtaaacccaag	m5C_62327
chr19	17973409	+	C	431	104	0.241	0	0	T	327	M	0.203	0.284	0	0	0	422.871	tgcacctaaaCccaagaatca	m5C_62334
chr19	17973418	+	C	444	98	0.221	0	0	T	346	M	0.185	0.262	0	0	0	361.875	acccaagaatCactgtttctt	m5C_62351
chr19	17973426	+	C	424	90	0.213	1	0.002	T	333	M	0.176	0.254	0	0	0	317.59	atcactgtttCttatagcggt	m5C_62312
chr19	17973453	+	C	658	136	0.207	2	0.003	T	520	M	0.178	0.24	0	0	0	484.276	aacagaggtgCaaacagcaag	m5C_62317
chr19	17973469	+	C	735	184	0.25	0	0	T	551	M	0.22	0.283	0	0	0	810.975	gcaagcggatCttgtcgcctt	m5C_62314
chr19	17973477	+	C	774	162	0.21	1	0.001	T	611	M	0.182	0.24	0	0	0	590.821	atcttgtcgcCtttggggggc	m5C_62345
chr19	17973493	+	C	929	203	0.223	17	0.018	T	709	M	0.197	0.251	0	0	0	798.946	ggggctgtggCcgtgcccctc	m5C_62316
chr19	17973499	+	C	753	156	0.207	0	0	T	597	M	0.18	0.238	0	0	0	560.806	gtggccgtgcCccctcaaagtg	m5C_62329
chr19	17973501	+	C	702	157	0.224	0	0	T	545	M	0.194	0.256	0	0	0	610.332	ggccgtgcccCtcaaagtgaa	m5C_62337
chr19	17973503	+	C	672	135	0.201	2	0.003	T	535	M	0.173	0.234	0	0	0	466.723	ccgtgcccctCaaagtgaatt	m5C_62310
chr19	17973523	+	C	582	117	0.201	0	0	T	465	M	0.17	0.236	0	0	0	398.92	ttggaggttcCacaactctag	m5C_62349
chr19	17973525	+	C	539	115	0.213	0	0	T	424	M	0.181	0.25	0	0	0	415.985	ggaggttccaCaactctagtg	m5C_62361
chr19	18353651	+	C	78	26	0.333	0	0	T	52	M	0.239	0.444	0	1.3E-10	0	124.137	gagggaagttCattgcttcag	m5C_62658
chr19	18418198	-	C	51	19	0.373	0	0	T	32	M	0.253	0.51	0	2.4E-08	0	96.2173	gacggatctgCtcagaaagga	m5C_62698
chr19	18418210	-	C	63	15	0.238	0	0	T	48	M	0.15	0.356	0	6E-11	0	44.981	tccacagtctCggacggatct	m5C_62720
chr19	18418212	-	C	61	27	0.443	0	0	T	34	M	0.325	0.567	0	7.5E-09	0	175.541	gctccacagtCtcggacggat	m5C_62713
chr19	18418218	-	C	42	16	0.381	0	0	T	26	M	0.25	0.532	0	4E-07	0	79.9985	gattccgctcCacagtctcgg	m5C_62723
chr19	18418295	-	C	38	8	0.211	0	0	T	30	M	0.111	0.363	1.3E-10	6.8E-07	1.4E-10	8.77246	agaagcctggCagacaggcgg	m5C_62704
chr19	18418299	-	C	39	9	0.231	0	0	T	30	M	0.126	0.383	4.9E-12	1.2E-06	5.7E-12	12.8739	gagagaagccCtggcagacag	m5C_62727
chr19	18418300	-	C	44	21	0.477	0	0	T	23	M	0.338	0.621	0	1.8E-06	0	141.77	agagaagaagCctggcagaca	m5C_62699
chr19	18418316	-	C	41	9	0.22	0	0	T	32	M	0.12	0.367	8.4E-12	1.2E-08	9.9E-12	11.9621	caggcagaggCagccagaga	m5C_62728
chr19	18418322	-	C	35	11	0.314	0	0	T	24	M	0.186	0.48	6.7E-16	3.1E-06	8.7E-16	30.9686	cgggcacaggCagaggccagc	m5C_62690
chr19	19017873	-	C	31	22	0.71	0	0	C	22	M	0.534	0.839	0	6.3E-05	0	234.992	ggagcgtggaCgtgaccaaca	m5C_62688
chr19	19017885	-	C	32	10	0.312	0	0	T	22	M	0.18	0.486	1.1E-14	2E-06	1.3E-14	25.0836	gagagatgagCaggagcgtgg	m5C_62702
chr19	19638512	+	C	34	10	0.294	0	0	T	24	M	0.168	0.462	2.3E-14	2E-06	2.9E-14	22.9564	ccccacaggaCcttgcagatg	m5C_62640

chr19	19638513	+	C	32	16	0.5	0	0	CT	16	M	0.336	0.664	0	1.7E-05	0	107.618	cccacaggacCttgcagatgc	m5C_62679
chr19	19638523	+	C	39	12	0.308	0	0	T	27	M	0.186	0.464	0	4.6E-06	0	44.5578	cttgcagatgCttcgggagaa	m5C_62647
chr19	19638564	+	C	44	25	0.568	0	0	C	25	M	0.422	0.703	0	4.5E-06	0	211.119	tcatgaaggaCgtgcccgact	m5C_62667
chr19	19638568	+	C	53	17	0.321	0	0	T	36	M	0.211	0.455	0	1.1E-08	0	71.713	gaaggacgtgCccgactggaa	m5C_62642
chr19	19638569	+	C	46	21	0.457	0	0	T	25	M	0.322	0.598	0	1.8E-06	0	135.049	aaggacgtgcCcgactggaag	m5C_62661
chr19	19638573	+	C	45	24	0.533	0	0	C	24	M	0.391	0.671	0	3.6E-06	0	187.571	acgtgcccgaCtggaaggtgg	m5C_62676
chr19	19852260	+	C	44	14	0.318	0	0	T	30	M	0.2	0.466	0	1.9E-07	0	55.9976	agcccaggttCgattcctggt	m5C_62675
chr19	19852278	+	C	36	10	0.278	0	0	T	26	M	0.158	0.44	4.8E-14	2E-06	5.9E-14	21.1115	ggtcagggaaCcagtgccact	m5C_62680
chr19	21416413	+	C	62	19	0.306	0	0	T	43	M	0.206	0.43	0	4.5E-10	0	78.2094	gcattttgaaCtgggagtaga	m5C_61251
chr19	22877626	-	C	59	44	0.746	0	0	C	44	M	0.622	0.839	0	5.1E-06	0	547.392	tggttaattcCgataataaat	m5C_61547
chr19	22877627	-	C	61	45	0.738	0	0	C	45	M	0.616	0.832	0	3.3E-07	0	554.067	ctggttaattCcgataataaa	m5C_61563
chr19	22877637	-	C	83	55	0.663	0	0	C	55	M	0.556	0.755	0	4.2E-09	0	611.328	agcgatttgtCtggttaattc	m5C_61549
chr19	22877645	-	C	68	47	0.691	0	0	C	47	M	0.574	0.788	0	4.4E-07	0	539.212	gttggtggagCgatttgtctg	m5C_61564
chr19	22877668	-	C	42	28	0.667	0	0	C	28	M	0.516	0.79	0	8.1E-06	0	288.692	cttagagggaCaagtggcgtt	m5C_61551
chr19	24184428	-	C	33	20	0.606	0	0	C	20	M	0.437	0.753	0	4.3E-05	0	174.733	ccattagaccCcagaaaatgt	m5C_61695
chr19	24184445	-	C	34	26	0.765	0	0	C	26	M	0.6	0.876	0	0.00013	0	312.018	gtgcctgatgCtgatgcccat	m5C_61706
chr19	24184696	-	C	74	56	0.757	0	0	C	56	M	0.648	0.84	0	9.3E-08	0	725.687	gtagctggttCcctctgaagt	m5C_61705
chr19	24184768	-	C	87	42	0.483	0	0	T	45	M	0.381	0.586	0	3.3E-10	0	319.809	ctgtagtggtCctgacgtgca	m5C_61816
chr19	24184778	-	C	41	22	0.537	0	0	C	22	M	0.387	0.679	0	2.3E-06	0	170.483	tggtgggggtCtgtagtggtc	m5C_61735
chr19	24184916	-	C	35	18	0.514	0	0	C	18	M	0.356	0.67	0	2.7E-05	0	128.047	tgaggcctctCcagtttgcca	m5C_61703
chr19	24184918	-	C	40	13	0.325	0	0	T	27	M	0.201	0.48	0	1.2E-07	0	52.2192	cctgaggcctCtccagtttgc	m5C_61779
chr19	24184920	-	C	52	15	0.288	0	0	T	37	M	0.183	0.423	0	4.6E-09	0	54.9894	atcctgaggcCtctccagttt	m5C_61733
chr19	24184921	-	C	56	14	0.25	0	0	T	42	M	0.155	0.377	0	2.8E-09	0	43.4474	gatcctgaggCctctccagtt	m5C_61777
chr19	24184927	-	C	62	17	0.274	0	0	T	45	M	0.179	0.396	0	1.8E-10	0	60.7951	aggcgggatcCtgaggcctct	m5C_61725
chr19	24184928	-	C	66	18	0.273	0	0	T	48	M	0.18	0.39	0	2.9E-10	0	64.8115	gaggcgggatCctgaggcctc	m5C_61783
chr19	24184971	-	C	39	9	0.231	0	0	T	30	M	0.126	0.383	4.9E-12	1.2E-06	5.7E-12	12.8739	cactgtggcgCaatgaaggtg	m5C_61792
chr19	24184973	-	C	35	8	0.229	0	0	T	27	M	0.121	0.39	5.6E-11	6.8E-07	6.4E-11	9.89691	gccactgtggCgcaatgaagg	m5C_61772
chr19	24185132	-	C	49	32	0.653	0	0	C	32	M	0.513	0.771	0	1.6E-05	0	328.39	gctgtcgggtCtggggggact	m5C_61728
chr19	24185137	-	C	46	19	0.413	0	0	T	27	M	0.283	0.557	0	1E-06	0	107.496	gttgtgctgtCgggtctgggg	m5C_61808
chr19	24185141	-	C	44	15	0.341	0	0	T	29	M	0.219	0.489	0	2.8E-07	0	65.6269	gctggttgtgCtgtcgggtct	m5C_61811
chr19	24185454	-	C	31	11	0.355	0	0	T	20	M	0.211	0.531	1.1E-16	3.1E-06	1.5E-16	37.0595	ctcggggggtCatgtcctccc	m5C_61741
chr19	24185462	-	C	49	13	0.265	0	0	T	36	M	0.162	0.403	0	1.2E-07	0	42.1489	gaaggtggctCggggggtcat	m5C_61694
chr19	24185464	-	C	50	28	0.56	0	0	C	28	M	0.423	0.688	0	8.1E-06	0	236.912	gggaaggtggCtcggggggtc	m5C_61704
chr19	24185476	-	C	33	17	0.515	0	0	C	17	M	0.352	0.675	0	2.1E-05	0	119.742	aaaggcccggCagggaaggtg	m5C_61814
chr19	24185479	-	C	67	33	0.493	0	0	T	34	M	0.377	0.609	0	3.4E-08	0	248.503	tggaaaggccCggcagggaag	m5C_61682
chr19	24185480	-	C	72	34	0.472	0	0	T	38	M	0.361	0.586	0	1.4E-09	0	245.659	ctggaaaggcCcggcagggaa	m5C_61750
chr19	24185481	-	C	79	21	0.266	0	0	T	58	M	0.181	0.372	0	1.8E-11	0	75.9915	gctggaaaggCccggcaggga	m5C_61818
chr19	24185490	-	C	339	118	0.348	0	0	T	221	M	0.299	0.4	0	0	0	706.421	tctgggacggCtggaaaggcc	m5C_61800
chr19	24185493	-	C	387	112	0.289	0	0	T	275	M	0.246	0.336	0	0	0	552.09	ggctctgggaCggctggaaag	m5C_61709
chr19	24185499	-	C	398	158	0.397	0	0	T	240	M	0.35	0.446	0	0	0	1106.38	gtgactggctCtgggacggct	m5C_61768
chr19	24185501	-	C	395	167	0.423	0	0	T	228	M	0.375	0.472	0	0	0	1252.63	ccgtgactggCtctgggacgg	m5C_61727
chr19	24185505	-	C	356	84	0.236	0	0	T	272	M	0.195	0.283	0	0	0	327.285	tgcgccgtgaCtggctctggg	m5C_61726
chr19	24185510	-	C	241	55	0.228	0	0	T	186	M	0.18	0.285	0	0	0	197.712	ggtgctgcgcCgtgactggct	m5C_61680
chr19	24185675	-	C	41	32	0.78	0	0	C	32	M	0.633	0.88	0	1.6E-05	0	405.086	tcctcccgacCcctccaaccg	m5C_61689
chr19	24185680	-	C	48	38	0.792	0	0	C	38	M	0.657	0.883	0	3.8E-05	0	499.63	cccgttcctcCcgacccctcc	m5C_61801
chr19	24185684	-	C	51	38	0.745	0	0	C	38	M	0.611	0.845	0	2.1E-06	0	464.599	gcctcccgttCctcccgaccc	m5C_61759
chr19	24185688	-	C	49	37	0.755	0	0	C	37	M	0.619	0.854	0	3.3E-05	0	458.159	tcctgcctccCgttcctcccg	m5C_61785
chr19	24185689	-	C	50	39	0.78	0	0	C	39	M	0.648	0.872	0	2.5E-06	0	505.114	ttcctgcctcCcgttcctccc	m5C_61677
chr19	24185690	-	C	50	38	0.76	0	0	C	38	M	0.626	0.857	0	2.1E-06	0	475.662	tttcctgcctCccgttcctcc	m5C_61796
chr19	24185693	-	C	49	39	0.796	0	0	C	39	M	0.664	0.885	0	4.3E-05	0	517.629	atctttcctgCctcccgttcc	m5C_61782
chr19	24185696	-	C	50	39	0.78	0	0	C	39	M	0.648	0.872	0	2.5E-06	0	505.114	cggatctttcCtgcctcccgt	m5C_61669
chr19	24185697	-	C	50	37	0.74	0	0	C	37	M	0.604	0.841	0	1.8E-06	0	447.305	gcggatctttCctgcctcccg	m5C_61701
chr19	24185701	-	C	51	39	0.765	0	0	C	39	M	0.632	0.86	0	2.5E-06	0	493.253	tctggcggatCtttcctgcct	m5C_61718
chr19	24185706	-	C	45	32	0.711	0	0	C	32	M	0.566	0.823	0	1.6E-05	0	362.44	agtggtctggCggatctttcc	m5C_61746
chr19	24187180	+	C	132	65	0.492	0	0	T	67	M	0.409	0.577	0	3.8E-15	0	531.134	acaggtgtagCcctgggagga	m5C_61617
chr19	24187182	+	C	127	43	0.339	0	0	T	84	M	0.262	0.425	0	2.2E-16	0	225.403	aggtgtagccCtgggaggaac	m5C_61633
chr19	24187218	-	C	62	15	0.242	0	0	T	47	M	0.152	0.362	0	6E-11	0	45.7373	cactgatcatCatttcaaatg	m5C_61696
chr19	24187221	-	C	152	58	0.382	0	0	T	94	M	0.308	0.461	0	0	0	357.481	acacactgatCatcatttcaa	m5C_61778
chr19	24187230	-	C	393	117	0.298	1	0.003	T	275	M	0.255	0.346	0	0	0	597.412	agttgcaggaCacactgatca	m5C_61794
chr19	24187257	-	C	1149	243	0.212	2	0.002	T	904	M	0.189	0.236	0	0	0	919.466	atgcagccagCtgtgagaatt	m5C_61780
chr19	24187279	-	C	53	41	0.774	0	0	C	41	M	0.645	0.865	0	3.3E-06	0	528.682	tccactcctgCattgatgaag	m5C_61807
chr19	30498487	+	C	38	15	0.395	0	0	T	23	M	0.256	0.553	0	1.2E-05	0	76.7936	tgataaagaaCtgtgggctcg	m5C_61570
chr19	30498494	+	C	55	21	0.382	0	0	T	34	M	0.265	0.514	0	4.8E-08	0	111.366	gaactgtgggCtcgacttgaa	m5C_61578
chr19	30498508	+	C	53	12	0.226	0	0	T	41	M	0.135	0.355	5.8E-15	8.8E-10	7.3E-15	22.9888	acttgaagaaCtagagagaca	m5C_61580
chr19	32734871	-	C	140	63	0.45	0	0	T	77	M	0.37	0.533	0	2.4E-15	0	466.228	gcgctgtaggCttgaccaggc	m5C_61861
chr19	32734878	-	C	160	71	0.444	0	0	T	89	M	0.369	0.521	0	0	0	523.954	atactgggcgCtgtaggcttg	m5C_61852
chr19	32734885	-	C	162	48	0.296	0	0	T	114	M	0.231	0.371	0	0	0	222.103	tctgggaataCtgggcgctgt	m5C_61856
chr19	32734894	-	C	134	50	0.373	0	0	T	84	M	0.296	0.457	0	0	0	295.853	gggagactgtCtgggaatact	m5C_61842
chr19	32734898	-	C	111	39	0.351	0	0	T	72	M	0.269	0.444	0	3.1E-15	0	209.718	ggatgggagaCtgtctgggaa	m5C_61858
chr19	33668045	+	C	42	13	0.31	0	0	T	29	M	0.191	0.46	0	1.2E-07	0	49.5844	ctcaaccgagCgtccaagctc	m5C_61825
chr19	33668048	+	C	44	33	0.75	0	0	C	33	M	0.606	0.854	0	1.9E-05	0	399.69	aaccgagcgtCcaagctcttt	m5C_61831
chr19	33668059	+	C	41	13	0.317	0	0	T	28	M	0.196	0.47	0	1.2E-07	0	50.8675	caagctctttCcatttttgct	m5C_61838
chr19	33770616	-	C	52	16	0.308	0	0	T	36	M	0.199	0.443	0	7.3E-09	0	63.7266	aagccccggtCccggtgtgtg	m5C_61860
chr19	35477020	-	C	34	12	0.353	0	0	T	22	M	0.215	0.521	0	4.6E-06	0	51.5705	ttgctagttgCaaaaagaatc	m5C_62076
chr19	35477027	-	C	43	12	0.279	0	0	T	31	M	0.167	0.427	2.2E-16	7.5E-08	2.9E-16	31.457	gttgatcttgCtagttgcaaa	m5C_62074
chr19	35477031	-	C	47	21	0.447	0	0	T	26	M	0.314	0.588	0	1.8E-06	0	131.925	tctggttgatCttgctagttg	m5C_62081
chr19	36066545	+	C	59	44	0.746	0	0	C	44	M	0.622	0.839	0	5.1E-06	0	547.392	ttgatatagaCagcaggacgg	m5C_63296
chr19	36066559	+	C	50	34	0.68	0	0	C	34	M	0.542	0.792	0	2.2E-05	0	368.488	aggacggtggCcatggaagtc	m5C_63328
chr19	36066657	-	C	37	14	0.378	0	0	T	23	M	0.241	0.539	0	9.2E-06	0	67.3786	tggcgatggcCgggtatgggc	m5C_63342
chr19	36066658	-	C	40	11	0.275	0	0	T	29	M	0.161	0.428	4.3E-15	3.1E-06	5.5E-15	25.4502	ctggcgatggCcgggtatggg	m5C_63336
chr19	36066706	+	C	32	22	0.688	0	0	C	22	M	0.514	0.82	0	6.3E-05	0	226.305	ggtcttggggCcgaaacgatc	m5C_63300
chr19	36066707	+	C	44	34	0.773	0	0	C	34	M	0.63	0.872	0	2.2E-05	0	428.45	gtcttggggcCgaaacgatct	m5C_63271
chr19	36066823	+	C	73	19	0.26	0	0	T	54	M	0.173	0.371	0	6.9E-12	0	65.8998	atgctgggtgCcagatgaaga	m5C_63323
chr19	36106877	+	C	31	16	0.516	0	0	C	16	M	0.348	0.68	0	1.7E-05	0	111.488	tgatgtgttgCgattttaccc	m5C_63301
chr19	36540055	+	C	493	105	0.213	0	0	T	388	M	0.179	0.251	0	0	0	376.177	tggagctggtCcgagtgttgt	m5C_63276
chr19	36540095	+	C	97	33	0.34	0	0	T	64	M	0.254	0.439	0	7.5E-13	0	167.39	ttaacattgtCtccccacaca	m5C_63293
chr19	36540097	+	C	81	23	0.284	0	0	T	58	M	0.197	0.39	0	7.2E-13	0	90.7515	aacattgtctCcccacacaac	m5C_63308
chr19	36540099	+	C	62	16	0.258	0	0	T	46	M	0.166	0.379	0	1.1E-10	0	52.9752	cattgtctccCcacacaacca	m5C_63286
chr19	38795565	+	C	49	20	0.408	0	0	T	29	M	0.282	0.548	0	1.4E-06	0	112.86	aggagaagggCaagaaggaga	m5C_61973
chr19	39215182	+	C	50	14	0.28	0	0	T	36	M	0.175	0.417	0	1.9E-07	0	48.9272	tgttgtggggCcctggatcca	m5C_61967
chr19	39215191	+	C	47	17	0.362	0	0	T	30	M	0.24	0.505	0	5.6E-07	0	81.4845	gccctggatcCagaccaagat	m5C_61965
chr19	39215195	+	C	42	12	0.286	0	0	T	30	M	0.172	0.436	2.2E-16	7.5E-08	2.9E-16	32.2467	tggatccagaCcaagatggag	m5C_61975
chr19	39215196	+	C	49	14	0.286	0	0	T	35	M	0.178	0.424	0	1.9E-07	0	49.9784	ggatccagacCaagatggagg	m5C_61953
chr19	39859979	+	C	34	17	0.5	0	0	CT	17	M	0.341	0.659	0	2.1E-05	0	115.835	gtgaactggcCcagtttggaa	m5C_61976
chr19	39877178	-	C	33	7	0.212	0	0	T	26	M	0.107	0.378	1.1E-09	3.4E-07	1.2E-09	6.69165	aaccacgaacCggaggaggaa	m5C_62009
chr19	39902807	-	C	126	44	0.355	2	0.016	T	80	M	0.276	0.442	0	4.4E-16	0	243.008	acctggagcaCtttttttccc	m5C_62016
chr19	39902809	-	C	137	30	0.219	0	0	T	107	M	0.158	0.295	0	0	0	94.7485	tcacctggagCacttttttttc	m5C_62013
chr19	39902815	-	C	146	33	0.226	0	0	T	113	M	0.166	0.3	0	0	0	109.371	cgatcctcacCtggagcactt	m5C_62001
chr19	39902816	-	C	145	49	0.338	0	0	T	96	M	0.266	0.418	0	0	0	260.688	tcgatcctcaCctggagcact	m5C_62028
chr19	39902818	-	C	153	39	0.255	0	0	T	114	M	0.192	0.329	0	0	0	150.1	gttcgatcctCacctggagca	m5C_62022
chr19	39902821	-	C	159	48	0.302	0	0	T	111	M	0.236	0.377	0	0	0	226.459	tgagttcgatCctcacctgga	m5C_62018

chr19	39902825	-	C	156	70	0.449	0	0	T	86	M	0.373	0.527	0	0	0	521.969	gttgtgagttCgatcctcacc	m5C_62026
chr19	39902873	-	C	143	31	0.217	0	0	T	112	M	0.157	0.291	0	0	0	97.4233	ggttagcgcgCggtacttata	m5C_62029
chr19	39902877	-	C	145	41	0.285	1	0.007	T	103	M	0.217	0.363	0	0	0	178.232	aatcggttagCgcgcggtact	m5C_62005
chr19	39902888	-	C	121	44	0.364	0	0	T	77	M	0.283	0.452	0	4.4E-16	0	249.342	tccagtggcgCaatcggttag	m5C_62020
chr19	40848243	+	C	34	9	0.265	0	0	T	25	M	0.146	0.431	1.1E-12	1.2E-06	1.3E-12	15.7394	aaaggctggtCcgaaggtagt	m5C_62418
chr19	42373207	+	C	39	14	0.359	0	0	T	25	M	0.227	0.516	0	9.2E-06	0	63.6773	gccacttcagCcgaggctcca	m5C_62280
chr19	42373208	+	C	43	19	0.442	0	0	T	24	M	0.304	0.589	0	1E-06	0	115.646	ccacttcagcCgaggctccaa	m5C_62267
chr19	42373228	+	C	53	11	0.208	0	0	T	42	M	0.12	0.335	1.8E-13	4.4E-10	2.2E-13	16.8213	agagtgtggcCcgccgggtcc	m5C_62284
chr19	42373231	+	C	59	26	0.441	0	0	T	33	M	0.322	0.567	0	1.9E-07	0	167.18	gtgtggcccgCcgggtcctcc	m5C_62273
chr19	42373240	+	C	31	12	0.387	0	0	T	19	M	0.237	0.562	0	4.6E-06	0	56.9589	gccgggtcctCcaagccctgg	m5C_62275
chr19	42373241	+	C	35	10	0.286	0	0	T	25	M	0.163	0.451	3.4E-14	2E-06	4.2E-14	21.9991	ccgggtcctcCaagccctgga	m5C_62265
chr19	42373794	+	C	41	27	0.659	0	0	C	27	M	0.505	0.784	0	6.7E-06	0	272.968	acctcagggaCaaagagatct	m5C_62244
chr19	42373803	+	C	38	29	0.763	0	0	C	29	M	0.608	0.87	0	0.00019	0	352.599	acaaagagatCtggacagaat	m5C_62260
chr19	42373808	+	C	35	16	0.457	0	0	T	19	M	0.305	0.618	0	1.7E-05	0	97.4905	gagatctggaCagaatcgccg	m5C_62248
chr19	44112210	-	C	32	18	0.562	0	0	C	18	M	0.393	0.718	0	2.7E-05	0	141.571	agccggactcCgaagaggaag	m5C_62487
chr19	45981860	-	C	132	45	0.341	0	0	T	87	M	0.266	0.425	0	0	0	239.011	acctttcgggCggtagtaact	m5C_62494
chr19	45981864	-	C	156	62	0.397	0	0	T	94	M	0.324	0.476	0	0	0	401.754	ttccacctttCgggcggtagt	m5C_62476
chr19	45981868	-	C	152	38	0.25	0	0	T	114	M	0.188	0.324	0	0	0	142.803	tcaattccacCtttcgggcgg	m5C_62496
chr19	45981869	-	C	156	89	0.571	0	0	C	89	M	0.492	0.646	0	8.9E-16	0	875.867	ttcaattccaCctttcgggcg	m5C_62488
chr19	45981871	-	C	158	49	0.31	0	0	T	109	M	0.243	0.386	0	0	0	238.363	ggttcaattcCacctttcggg	m5C_62520
chr19	45981872	-	C	153	38	0.248	0	0	T	115	M	0.187	0.322	0	0	0	141.838	tggttcaattCcacctttcgg	m5C_62491
chr19	45981877	-	C	174	43	0.247	0	0	T	131	M	0.189	0.316	0	0	0	162.504	cagagtggttCaattccacct	m5C_62508
chr19	45981887	-	C	179	73	0.408	0	0	T	106	M	0.338	0.481	0	0	0	494.208	tgtctagcgaCagagtggttc	m5C_62504
chr19	45981890	-	C	167	49	0.293	0	0	T	118	M	0.23	0.366	0	0	0	225.03	agctgtctagCgacagagtgg	m5C_62492
chr19	45981898	-	C	185	53	0.286	0	0	T	132	M	0.226	0.355	0	0	0	239.771	aaacctgtagCtgtctagcga	m5C_62505
chr19	45981904	-	C	171	58	0.339	0	0	T	113	M	0.272	0.413	0	0	0	316.041	ggcttcaaacCtgtagctgtc	m5C_62517
chr19	45981905	-	C	160	39	0.244	0	0	T	121	M	0.184	0.316	0	0	0	143.318	aggcttcaaaCctgtagctgt	m5C_62489
chr19	45981909	-	C	156	36	0.231	0	0	T	120	M	0.172	0.303	0	0	0	123.554	gtgcaggcttCaaacctgtag	m5C_62516
chr19	45981912	-	C	163	50	0.307	0	0	T	113	M	0.241	0.381	0	0	0	241.082	ggggtgcaggCttcaaacctg	m5C_62513
chr19	45981916	-	C	202	43	0.213	0	0	T	159	M	0.162	0.274	0	0	0	139.366	gtctggggtgCaggcttcaaa	m5C_62483
chr19	45981924	-	C	235	49	0.209	0	0	T	186	M	0.161	0.265	0	0	0	158.235	cctcagtggtCtggggtgcag	m5C_62500
chr19	45981931	-	C	239	57	0.238	0	0	T	182	M	0.189	0.296	0	0	0	215.312	ggatgatcctCagtggtctgg	m5C_62507
chr19	45981934	-	C	242	69	0.285	0	0	T	173	M	0.232	0.345	0	0	0	320.085	cccggatgatCctcagtggtc	m5C_62480
chr19	46142275	-	C	228	84	0.368	0	0	T	144	M	0.308	0.433	0	0	0	518.229	agcacacggcCtgcagagagg	m5C_63845
chr19	46142279	-	C	228	47	0.206	0	0	T	181	M	0.159	0.263	0	0	0	149.187	gcaaagcacaCggcctgcaga	m5C_63884
chr19	46154922	+	C	58	17	0.293	0	0	T	41	M	0.192	0.42	0	1.1E-08	0	65.2053	gagtgggggaCcaccagttgc	m5C_63785
chr19	46154923	+	C	56	18	0.321	0	0	T	38	M	0.214	0.452	0	1.7E-08	0	77.043	agtgggggacCaccagttgct	m5C_63789
chr19	46154925	+	C	62	19	0.306	0	0	T	43	M	0.206	0.43	0	4.5E-10	0	78.2094	tgggggaccaCcagttgctta	m5C_63787
chr19	46154926	+	C	68	17	0.25	0	0	T	51	M	0.162	0.364	0	1.8E-10	0	55.1986	gggggaccacCagttgcttaa	m5C_63829
chr19	46154948	+	C	80	26	0.325	0	0	T	54	M	0.232	0.434	0	2.7E-12	0	120.869	gaggggtgaaCtggcccaggt	m5C_63792
chr19	46154954	+	C	76	33	0.434	0	0	T	43	M	0.329	0.546	0	1.1E-09	0	216.881	tgaactggccCaggtcagaaa	m5C_63804
chr19	47111773	+	C	35	9	0.257	0	0	T	26	M	0.142	0.421	1.5E-12	1.2E-06	1.7E-12	15.0859	agttcctgacCatgatggcca	m5C_63797
chr19	47111797	+	C	82	22	0.268	0	0	T	60	M	0.184	0.373	0	4.4E-13	0	81.1184	agatgaaggaCacagacagtg	m5C_63776
chr19	47111803	+	C	80	21	0.263	0	0	T	59	M	0.179	0.368	0	2.6E-13	0	75.0005	aggacacagaCagtgaggagg	m5C_63772
chr19	47333870	-	C	215	102	0.474	0	0	T	113	M	0.409	0.541	0	0	0	833.762	ccaacctggtCcacatgggtt	m5C_63849
chr19	47333875	-	C	157	52	0.331	0	0	T	105	M	0.262	0.408	0	0	0	272.889	cgaggccaacCtggtccacat	m5C_63892
chr19	47333879	-	C	121	40	0.331	0	0	T	81	M	0.253	0.418	0	1.1E-16	0	202.485	agttcgaggcCaacctggtcc	m5C_63863
chr19	47333885	-	C	107	53	0.495	0	0	T	54	M	0.402	0.589	0	5.2E-12	0	426.554	ggcacgagttCgaggccaacc	m5C_63856
chr19	47333907	-	C	43	12	0.279	0	0	T	31	M	0.167	0.427	2.2E-16	7.5E-08	2.9E-16	31.457	ctcagggaggCgagggttcga	m5C_63874
chr19	47333918	-	C	37	12	0.324	0	0	T	25	M	0.196	0.485	0	4.6E-06	0	47.1194	gccctcggacCctcagggagg	m5C_63895
chr19	47333926	-	C	34	14	0.412	0	0	T	20	M	0.264	0.578	0	9.2E-06	0	73.8241	ttgcgcgggcCctcggaccct	m5C_63862
chr19	47333927	-	C	37	11	0.297	0	0	T	26	M	0.175	0.458	1.4E-15	3.1E-06	1.9E-15	28.5508	gttgcgcgggCcctcggaccc	m5C_63886
chr19	47333933	-	C	35	10	0.286	0	0	T	25	M	0.163	0.451	3.4E-14	2E-06	4.2E-14	21.9991	cagccggttgCgcgggccctc	m5C_63875
chr19	47609548	-	C	41	32	0.78	0	0	C	32	M	0.633	0.88	0	1.6E-05	0	405.086	ctgggtgctgCcatgttctct	m5C_63891
chr19	47609558	-	C	51	12	0.235	0	0	T	39	M	0.14	0.368	3.3E-15	8.8E-10	4.3E-15	24.3305	ccatgctgcCtgggtgctgc	m5C_63885
chr19	47609562	-	C	47	10	0.213	0	0	T	37	M	0.12	0.349	1.3E-12	2.4E-08	1.5E-12	14.271	gccaccatgtCtgcctgggtg	m5C_63853
chr19	47634148	+	C	50	17	0.34	0	0	T	33	M	0.224	0.478	0	5.6E-07	0	76.285	gggcggttggCttgagcggga	m5C_63813
chr19	47634154	+	C	73	32	0.451	2	0.027	T	39	M	0.34	0.566	0	8.5E-10	0	217.906	ttggcttgagCgggaccggag	m5C_63770
chr19	47634165	+	C	58	22	0.379	0	0	T	36	M	0.266	0.508	0	6.5E-08	0	116.882	gggaccggagCtgaggcagga	m5C_63812
chr19	48421707	+	C	47	20	0.426	0	0	T	27	M	0.295	0.567	0	1.4E-06	0	118.054	gtggctcaggCcggttgcgcc	m5C_66190
chr19	48421756	+	C	32	7	0.219	0	0	T	25	M	0.11	0.388	8.5E-10	3.4E-07	9.5E-10	6.99971	ttccagggcaCgagttcgagg	m5C_66337
chr19	48427051	+	C	131	27	0.206	0	0	T	104	M	0.146	0.283	0	0	0	78.6736	gccattgtggCtcaggccggt	m5C_66283
chr19	48427066	+	C	43	11	0.256	0	0	T	32	M	0.149	0.402	1.2E-14	4.4E-08	1.5E-14	22.8887	gccggttgcgCctgccctcgg	m5C_66234
chr19	48427072	+	C	52	14	0.28	2	0.038	T	36	M	0.175	0.417	0	1.9E-07	0	48.9272	tgcgcctgccCtcgggccctc	m5C_66209
chr19	48427084	+	C	60	12	0.2	0	0	T	48	M	0.118	0.318	3.3E-14	7.6E-12	4.2E-14	19.13	cgggccctcaCggaggcgggg	m5C_66173
chr19	48427098	+	C	47	23	0.489	0	0	T	24	M	0.353	0.628	0	2.9E-06	0	162.266	ggcggggttCcagggcacga	m5C_66260
chr19	48427104	+	C	49	14	0.286	0	0	T	35	M	0.178	0.424	0	1.9E-07	0	49.9784	ggttccagggCacgagttcga	m5C_66338
chr19	48427106	+	C	56	22	0.393	0	0	T	34	M	0.276	0.524	0	6.5E-08	0	121.353	ttccagggcaCgagttcgagg	m5C_66292
chr19	48427117	+	C	129	26	0.202	0	0	T	103	M	0.141	0.279	0	0	0	73.5376	gagttcgaggCcagcctggtc	m5C_66205
chr19	48427127	+	C	195	64	0.33	1	0.005	T	130	M	0.268	0.399	0	0	0	342.527	ccagcctggtCcacatgggtc	m5C_66258
chr19	48427137	+	C	271	90	0.332	0	0	T	181	M	0.279	0.39	0	0	0	501.696	ccacatgggtCggaaaaaagg	m5C_66227
chr19	48437459	+	C	109	46	0.422	0	0	T	63	M	0.334	0.516	0	9.7E-13	0	306.827	gtggctcaggCcggttgcgcc	m5C_66236
chr19	48437468	+	C	41	17	0.415	0	0	T	24	M	0.278	0.566	0	5.6E-07	0	94.3715	gccggttgcgCctgccctcgg	m5C_66222
chr19	48437469	+	C	43	9	0.209	0	0	T	34	M	0.114	0.352	1.4E-11	1.2E-08	1.6E-11	11.1522	ccggttgcgcCtgccctcggg	m5C_66200
chr19	48448233	+	C	56	31	0.574	2	0.036	C	31	M	0.442	0.697	0	5.9E-07	0	273.807	ctcacggaggCgggggttcca	m5C_66294
chr19	48448280	+	C	165	57	0.345	0	0	T	108	M	0.277	0.421	0	0	0	315.96	ccacatgggtCggaaaaaagg	m5C_66300
chr19	48453575	+	C	301	76	0.252	0	0	T	225	M	0.207	0.304	0	0	0	314.247	tggctccggcCggttgcgcgg	m5C_66303
chr19	48453583	+	C	306	82	0.27	2	0.007	T	222	M	0.223	0.322	0	0	0	365.634	gccggttgcgCgggccctcgg	m5C_66286
chr19	48453587	+	C	324	99	0.306	0	0	T	225	M	0.258	0.358	0	0	0	510.677	gttgcgcgggCcctcggaccc	m5C_66329
chr19	48453588	+	C	333	82	0.246	0	0	T	251	M	0.203	0.295	0	0	0	332.986	ttgcgcgggcCctcggaccct	m5C_66324
chr19	48453589	+	C	344	69	0.201	1	0.003	T	274	M	0.162	0.247	0	0	0	223.778	tgcgcgggccCtcggaccctc	m5C_66251
chr19	48453591	+	C	337	70	0.208	0	0	T	267	M	0.168	0.254	0	0	0	234.942	cgcgggccctCggaccctcag	m5C_66183
chr19	48453597	+	C	361	75	0.208	1	0.003	T	285	M	0.17	0.253	0	0	0	254.354	ccctcggaccCtcagagaggc	m5C_66165
chr19	48453607	+	C	336	88	0.263	2	0.006	T	246	M	0.219	0.313	0	0	0	385.636	ctcagagaggCgagggttcga	m5C_66344
chr19	48453615	+	C	364	115	0.316	0	0	T	249	M	0.27	0.365	0	0	0	621.71	ggcgagggttCgaggcacga	m5C_66156
chr19	48453629	+	C	290	89	0.307	0	0	T	201	M	0.257	0.362	0	0	0	456.795	ggcacgagttCgaggccaacc	m5C_66213
chr19	48453635	+	C	171	54	0.316	0	0	T	117	M	0.251	0.389	0	0	0	270.882	agttcgaggcCaacctggtcc	m5C_66199
chr19	48453639	+	C	171	68	0.398	0	0	T	103	M	0.327	0.472	0	0	0	445.167	cgaggccaacCtggtccacat	m5C_66225
chr19	48458993	+	C	123	34	0.276	0	0	T	89	M	0.205	0.361	0	0	0	139.447	ctcagagaggCgagggttcga	m5C_66197
chr19	48459021	+	C	114	23	0.202	0	0	T	91	M	0.138	0.285	0	0	0	63.6501	agttcgaggcCaacctggtcc	m5C_66218
chr19	48459024	+	C	104	25	0.24	0	0	T	79	M	0.168	0.331	0	4.4E-16	0	84.2263	tcgaggccaaCctggtccaca	m5C_66306
chr19	48459025	+	C	107	28	0.262	0	0	T	79	M	0.188	0.352	0	1.8E-15	0	105.11	cgaggccaacCtggtccacat	m5C_66202
chr19	48459030	+	C	95	35	0.368	0	0	T	60	M	0.278	0.469	0	1.5E-12	0	194.81	ccaacctggtCcacatgggtt	m5C_66174
chr19	48459031	+	C	93	32	0.344	0	0	T	61	M	0.255	0.445	0	5.3E-13	0	163.488	caacctggtcCacatgggttg	m5C_66185
chr19	48744245	-	C	36	18	0.5	0	0	CT	18	M	0.345	0.655	0	2.7E-05	0	124.107	aaaagagtagCagcagtgagg	m5C_66387
chr19	48744258	-	C	39	21	0.538	0	0	C	21	M	0.386	0.684	0	5.2E-05	0	161.991	aaggagtgtcCagaaaagagt	m5C_66361
chr19	49120025	-	C	48	17	0.354	0	0	T	31	M	0.234	0.496	0	5.6E-07	0	79.6738	ttgtggggacCataactgatg	m5C_66358
chr19	49120026	-	C	47	24	0.511	0	0	C	24	M	0.372	0.647	0	3.6E-06	0	178.763	gttgtggggaCcataactgat	m5C_66374
chr19	49377573	+	C	33	19	0.576	0	0	C	19	M	0.408	0.728	0	3.4E-05	0	155.067	aagatgaggaCagtgactctg	m5C_66327
chr19	49377581	+	C	35	10	0.286	0	0	T	25	M	0.163	0.451	3.4E-14	2E-06	4.2E-14	21.9991	gacagtgactCtggatcagat	m5C_66253
chr19	49377587	+	C	36	9	0.25	0	0	T	27	M	0.138	0.411	2E-12	1.2E-06	2.4E-12	14.4765	gactctggatCagatgaggaa	m5C_66166

chr19	49377947	+	C	33	16	0.485	0	0	T	17	M	0.325	0.648	0	1.7E-05	0	104.012	ggatatcgacCtggaaaagag	m5C_66296
chr19	49422322	+	C	48	10	0.208	0	0	T	38	M	0.117	0.343	1.6E-12	2.4E-08	1.9E-12	13.8343	atgaggaggaCgacatgcggg	m5C_66187
chr19	49422325	+	C	48	22	0.458	0	0	T	26	M	0.326	0.597	0	2.3E-06	0	143.33	aggaggacgaCatgcgggaga	m5C_66189
chr19	49422329	+	C	49	23	0.469	0	0	T	26	M	0.337	0.606	0	2.9E-06	0	155.035	ggacgacatgCgggagatgga	m5C_66256
chr19	49993247	+	C	593	127	0.214	0	0	T	466	M	0.183	0.249	0	0	0	464.923	caacattcacCatctttcgtt	m5C_66348
chr19	49993250	+	C	566	139	0.246	2	0.004	T	425	M	0.213	0.284	0	0	0	591.256	cattcaccatCtttcgtttga	m5C_66347
chr19	49993254	+	C	560	168	0.3	0	0	T	392	M	0.264	0.339	0	0	0	885.402	caccatctttCgtttgagtct	m5C_66302
chr19	49993270	+	C	596	153	0.258	2	0.003	T	441	M	0.224	0.294	0	0	0	685.576	agtctcacggCcatgagatca	m5C_66326
chr19	49993279	+	C	633	172	0.272	0	0	T	461	M	0.239	0.308	0	0	0	820.513	gccatgagatCaaccccatgc	m5C_66181
chr19	49993282	+	C	599	129	0.218	8	0.013	T	462	M	0.187	0.253	0	0	0	482.068	atgagatcaaCcccatgcacc	m5C_66254
chr19	49993283	+	C	604	128	0.212	0	0	T	476	M	0.181	0.246	0	0	0	463.876	tgagatcaacCccatgcaccg	m5C_66219
chr19	49993284	+	C	609	221	0.363	0	0	T	388	M	0.326	0.402	0	0	0	1439.46	gagatcaaccCcatgcaccgc	m5C_66280
chr19	49993291	+	C	618	153	0.248	0	0	T	465	M	0.215	0.283	0	0	0	658.429	accccatgcaCcgctctgaga	m5C_66295
chr19	49993292	+	C	617	136	0.22	0	0	T	481	M	0.189	0.255	0	0	0	515.434	ccccatgcacCgctctgagac	m5C_66171
chr19	49993294	+	C	605	208	0.345	2	0.003	T	395	M	0.308	0.384	0	0	0	1281.65	ccatgcaccgCtctgagacct	m5C_66259
chr19	49993296	+	C	551	170	0.309	0	0	T	381	M	0.271	0.348	0	0	0	922.758	atgcaccgctCtgagacctgc	m5C_66284
chr19	49993303	+	C	356	88	0.247	0	0	T	268	M	0.205	0.295	0	0	0	361.216	gctctgagacCtgccagccac	m5C_66194
chr19	49993889	+	C	591	173	0.293	0	0	T	418	M	0.257	0.331	0	0	0	890.854	gtgatgagaaCttctcccact	m5C_66312
chr19	49993929	+	C	359	90	0.251	0	0	T	269	M	0.209	0.298	0	0	0	375.59	catgagatgaCtccacatgca	m5C_66277
chr19	49993931	+	C	353	86	0.244	0	0	T	267	M	0.202	0.291	0	0	0	347.029	tgagatgactCcacatgcact	m5C_66308
chr19	49993947	+	C	312	86	0.276	0	0	T	226	M	0.229	0.328	0	0	0	393.905	gcactaccatCtgaggccacc	m5C_66178
chr19	49994177	+	C	692	153	0.221	0	0	T	539	M	0.192	0.254	0	0	0	586.802	ccatgatgttCcgcaactacc	m5C_66279
chr19	49994178	+	C	691	150	0.217	0	0	T	541	M	0.188	0.249	0	0	0	563.843	catgatgttcCgcaactacct	m5C_66285
chr19	49994187	+	C	700	152	0.217	1	0.001	T	547	M	0.188	0.25	0	0	0	572.922	ccgcaactacCtacattgttt	m5C_66316
chr19	49994201	+	C	788	171	0.217	1	0.001	T	616	M	0.19	0.247	0	0	0	649.386	attgtttgatCctcatgaaag	m5C_66315
chr19	49994217	+	C	853	190	0.223	0	0	T	663	M	0.196	0.252	0	0	0	745.172	gaaagcagcaCtggctgagac	m5C_66331
chr19	49994221	+	C	817	267	0.327	0	0	T	550	M	0.296	0.36	0	0	0	1578.07	gcagcactggCtgagacgcca	m5C_66231
chr19	49994227	+	C	546	148	0.271	0	0	T	398	M	0.235	0.31	0	0	0	696.998	ctggctgagaCgccagtccag	m5C_66233
chr19	49994505	+	C	1885	436	0.231	0	0	T	1449	M	0.213	0.251	0	0	0	1855.81	aatggcttagCtgatgccagg	m5C_66163
chr19	49994511	+	C	1112	312	0.281	0	0	T	800	M	0.255	0.308	0	0	0	1590.94	ttagctgatgCcaggaggctt	m5C_66193
chr19	49995323	+	C	47	20	0.426	0	0	T	27	M	0.295	0.567	0	1.4E-06	0	118.054	tgaaagggagCcagaagactg	m5C_66179
chr19	49995331	+	C	47	26	0.553	0	0	C	26	M	0.412	0.686	0	5.5E-06	0	214.475	agccagaagaCtgattggagg	m5C_66255
chr19	49995413	+	C	33	9	0.273	0	0	T	24	M	0.151	0.442	7.5E-13	1.2E-06	9E-13	16.4418	ttagaatgtgCaagcacttgg	m5C_66269
chr19	49995417	+	C	33	17	0.515	0	0	C	17	M	0.352	0.675	0	2.1E-05	0	119.742	aatgtgcaagCacttggggac	m5C_66328
chr19	49995427	+	C	51	18	0.353	0	0	T	33	M	0.236	0.49	0	1.7E-08	0	85.0835	cacttggggaCagcatgagct	m5C_66203
chr19	49995436	+	C	69	18	0.261	0	0	T	51	M	0.172	0.375	0	2.9E-10	0	61.8675	acagcatgagCttgctgttgt	m5C_66223
chr19	49995448	+	C	111	23	0.207	0	0	T	88	M	0.142	0.292	0	0	0	65.425	tgctgttgtaCacagggtatt	m5C_66345
chr19	50000996	+	C	277	71	0.256	0	0	T	206	M	0.208	0.311	0	0	0	296.04	tgtttgttttCacgatggtct	m5C_63145
chr19	50001005	+	C	279	108	0.387	0	0	T	171	M	0.332	0.445	0	0	0	716.782	tcacgatggtCttcagatgcc	m5C_63141
chr19	50001026	+	C	315	84	0.267	0	0	T	231	M	0.221	0.318	0	0	0	371.038	cacgtgggcaCtgctgagaaa	m5C_63143
chr19	50001041	+	C	256	66	0.258	0	0	T	190	M	0.208	0.315	0	0	0	274.672	gagaaagccaCttggtaaaac	m5C_63138
chr19	50001051	+	C	224	56	0.25	0	0	T	168	M	0.198	0.311	0	0	0	221.571	cttggtaaaaCtgatgccgga	m5C_63123
chr19	50037974	-	C	40	10	0.25	0	0	T	30	M	0.142	0.402	1.8E-13	2.4E-08	2.2E-13	18.0914	gagcattggaCttttaatttg	m5C_63187
chr19	50037981	-	C	73	21	0.288	0	0	T	52	M	0.197	0.4	0	1.8E-11	0	82.5367	gttggtagagCattggacttt	m5C_63158
chr19	50037995	-	C	62	18	0.29	0	0	T	44	M	0.192	0.413	0	2.9E-10	0	69.2043	acctgggtagCttagttggta	m5C_63249
chr19	50183048	-	C	51	32	0.627	0	0	C	32	M	0.49	0.747	0	7.2E-07	0	313.76	tgggctctggCcggggggtgg	m5C_63235
chr19	50637029	-	C	95	47	0.5	1	0.011	CT	47	M	0.401	0.599	0	3.7E-11	0	376.869	ccaacctggtCcacatgggtt	m5C_63256
chr19	50637044	-	C	53	12	0.226	0	0	T	41	M	0.135	0.355	5.8E-15	8.8E-10	7.3E-15	22.9888	ggcacgagttCgaggccaacc	m5C_63229
chr19	50637050	-	C	47	16	0.34	0	0	T	31	M	0.222	0.483	0	4E-07	0	70.9352	ttcgagggcaCgagttcgagg	m5C_63206
chr19	50637052	-	C	46	13	0.283	0	0	T	33	M	0.173	0.425	0	1.2E-07	0	45.0425	ggttcgagggCacgagttcga	m5C_63207
chr19	50637087	-	C	33	13	0.394	0	0	T	20	M	0.247	0.563	0	6.7E-06	0	64.1755	gttgcgcgggCcctcggaccc	m5C_63239
chr19	50637099	-	C	34	8	0.235	0	0	T	26	M	0.124	0.4	4.2E-11	6.8E-07	4.8E-11	10.3259	tggctccggcCggttgcgcgg	m5C_63219
chr19	50642448	-	C	232	58	0.25	0	0	T	174	M	0.199	0.309	0	0	0	230.443	ctcggaccctCagggtaggcg	m5C_63243
chr19	50642458	-	C	352	77	0.22	2	0.006	T	273	M	0.18	0.266	0	0	0	276.846	tgcgcgggccCtcggaccctc	m5C_63201
chr19	50642459	-	C	349	89	0.256	2	0.006	T	258	M	0.213	0.305	0	0	0	379.811	ttgcgcgggcCctcggaccct	m5C_63164
chr19	50642460	-	C	346	90	0.26	0	0	T	256	M	0.217	0.309	0	0	0	390.066	gttgcgcgggCcctcggaccc	m5C_63258
chr19	50642472	-	C	469	116	0.247	0	0	T	353	M	0.21	0.288	0	0	0	488.228	tggctccggcCggttgcgcgg	m5C_63254
chr19	50642477	-	C	467	106	0.227	0	0	T	361	M	0.191	0.267	0	0	0	405.57	cattgtggctCcggccggttg	m5C_63189
chr19	51004491	+	C	44	13	0.295	0	0	T	31	M	0.182	0.442	0	1.2E-07	0	47.2039	ccagatggggCagagagcgag	m5C_63151
chr19	51004503	+	C	34	14	0.412	0	0	T	20	M	0.264	0.578	0	9.2E-06	0	73.8241	gagagcgagaCccacaccagc	m5C_63130
chr19	51302291	-	C	32	14	0.438	0	0	T	18	M	0.282	0.607	0	9.2E-06	0	78.8622	ttctgaggggCcccactccgg	m5C_63165
chr19	51302299	-	C	66	22	0.333	0	0	T	44	M	0.232	0.453	0	1.5E-09	0	101.888	atccggggttCtgaggggccc	m5C_63173
chr19	51302307	-	C	69	27	0.391	0	0	T	42	M	0.285	0.509	0	7.5E-09	0	153.798	cctttgacatCcggggttctg	m5C_63195
chr19	51302310	-	C	71	18	0.254	0	0	T	53	M	0.167	0.366	0	4E-12	0	60.0495	ggacctttgaCatccggggtt	m5C_63217
chr19	51302316	-	C	63	22	0.349	0	0	T	41	M	0.243	0.472	0	1.5E-09	0	107.034	accccgggacCtttgacatcc	m5C_63202
chr19	51302323	-	C	57	13	0.228	0	0	T	44	M	0.138	0.352	4.4E-16	1.6E-09	5.8E-16	27.6258	acggtgcaccCcgggaccttt	m5C_63248
chr19	51302325	-	C	55	11	0.204	1	0.018	T	43	M	0.118	0.329	2.3E-13	4.4E-10	2.8E-13	16.3622	acacggtgcaCcccgggacct	m5C_63241
chr19	51302332	-	C	50	12	0.24	0	0	T	38	M	0.143	0.374	2.4E-15	8.8E-10	3.1E-15	25.0697	cctgaggacaCggtgcacccc	m5C_63166
chr19	51302341	-	C	75	39	0.52	0	0	C	39	M	0.409	0.629	0	4.7E-09	0	318.817	ctgactgcccCtgaggacacg	m5C_63181
chr19	51302343	-	C	78	16	0.205	0	0	T	62	M	0.13	0.308	0	1.2E-12	0	41.727	ctctgactgcCcctgaggaca	m5C_63246
chr19	51302347	-	C	83	25	0.301	0	0	T	58	M	0.213	0.407	0	1.8E-12	0	106.553	tgggctctgaCtgcccctgag	m5C_63162
chr19	51302351	-	C	84	28	0.333	0	0	T	56	M	0.242	0.439	0	5.8E-12	0	135.392	gcactgggctCtgactgcccc	m5C_63210
chr19	51302353	-	C	94	33	0.351	0	0	T	61	M	0.262	0.452	0	7.5E-13	0	173.021	cagcactgggCtctgactgcc	m5C_63191
chr19	51302363	-	C	94	21	0.223	0	0	T	73	M	0.151	0.318	0	3.1E-15	0	63.4276	ccgtgatgtcCagcactgggc	m5C_63245
chr19	51302374	-	C	34	9	0.265	0	0	T	25	M	0.146	0.431	1.1E-12	1.2E-06	1.3E-12	15.7394	acttggggacCccgtgatgtc	m5C_63178
chr19	51302701	-	C	117	41	0.35	0	0	T	76	M	0.27	0.44	0	6E-15	0	221.386	gtctgaggggCcctggctctg	m5C_63159
chr19	51302709	-	C	1088	303	0.279	1	0.001	T	784	M	0.253	0.306	0	0	0	1532.62	acccgggggtCtgaggggccc	m5C_63168
chr19	51302718	-	C	1255	306	0.244	3	0.002	T	946	M	0.221	0.269	0	0	0	1355.03	acctttgacaCccgggggtct	m5C_63228
chr19	51302726	-	C	1275	270	0.212	2	0.002	T	1003	M	0.191	0.235	0	0	0	1028.83	cccccgggacCtttgacaccc	m5C_63259
chr19	51302733	-	C	1193	383	0.321	0	0	T	810	M	0.295	0.348	0	0	0	2260.9	acggtgccccCcgggaccttt	m5C_63161
chr19	51302734	-	C	1210	254	0.21	0	0	T	956	M	0.188	0.234	0	0	0	954.565	cacggtgcccCccgggacctt	m5C_63175
chr19	51302736	-	C	1149	239	0.208	0	0	T	910	M	0.186	0.232	0	0	0	886.833	gacacggtgcCccccgggacc	m5C_63231
chr19	51302742	-	C	1177	321	0.273	0	0	T	856	M	0.248	0.299	0	0	0	1592.5	actgaggacaCggtgcccccc	m5C_63242
chr19	51302744	-	C	1176	249	0.212	0	0	T	927	M	0.189	0.236	0	0	0	942.926	ccactgaggaCacggtgcccc	m5C_63251
chr19	51302751	-	C	1194	253	0.212	3	0.003	T	938	M	0.19	0.237	0	0	0	962.109	ccgactgccaCtgaggacacg	m5C_63216
chr19	51302753	-	C	1165	233	0.2	0	0	T	932	M	0.178	0.224	0	0	0	829.634	ctccgactgcCactgaggaca	m5C_63221
chr19	51302757	-	C	1135	233	0.206	3	0.003	T	899	M	0.183	0.23	0	0	0	854.137	tgggctccgaCtgccactgag	m5C_63204
chr19	51302768	-	C	1061	216	0.204	0	0	T	845	M	0.18	0.229	0	0	0	779.509	atgtccagcaCtgggctccga	m5C_63208
chr19	51302774	-	C	1067	225	0.211	2	0.002	T	840	M	0.188	0.237	0	0	0	845.148	tccatgatgtCcagcactggg	m5C_63163
chr19	51305586	-	C	31	12	0.387	0	0	T	19	M	0.237	0.562	0	4.6E-06	0	56.9589	tctgaggggcCccagttttaa	m5C_63255
chr19	51305587	-	C	82	32	0.39	0	0	T	50	M	0.292	0.498	0	2.3E-11	0	186.781	gtctgaggggCcccagtttta	m5C_63211
chr19	51305604	-	C	843	191	0.227	0	0	T	652	M	0.2	0.256	0	0	0	762.434	acctttgacaCctgggggtct	m5C_63188
chr19	51305606	-	C	855	175	0.205	2	0.002	T	678	M	0.179	0.234	0	0	0	627.931	ggacctttgaCacctgggggt	m5C_63224
chr19	51305637	-	C	761	162	0.213	0	0	T	599	M	0.185	0.243	0	0	0	600.286	ctgatcacccCtgaggacaca	m5C_63182
chr19	51305638	-	C	740	152	0.206	1	0.001	T	587	M	0.178	0.236	0	0	0	541.419	tctgatcaccCctgaggacac	m5C_63212
chr19	51305654	-	C	689	157	0.228	0	0	T	532	M	0.198	0.261	0	0	0	622.05	atgtccagcaCtgggctctga	m5C_63220
chr19	51305669	-	C	299	72	0.241	0	0	T	227	M	0.196	0.292	0	0	0	281.984	gctggggctcCcatgatgtcc	m5C_63194
chr19	52195883	+	C	47	13	0.277	0	0	T	34	M	0.169	0.418	0	1.2E-07	0	44.0347	cccgtagaacCgaccttgcgg	m5C_62819
chr19	52195886	+	C	46	10	0.217	0	0	T	36	M	0.123	0.356	9.7E-13	2.4E-08	1.2E-12	14.7309	gtagaaccgaCcttgcggggc	m5C_62818
chr19	52195887	+	C	45	10	0.222	0	0	T	35	M	0.125	0.363	7.4E-13	2.4E-08	8.9E-13	15.2157	tagaaccgacCttgcggggcc	m5C_62817
chr19	52196531	+	C	40	23	0.575	0	0	C	23	M	0.422	0.715	0	2.9E-06	0	194.096	tccctgagacCctttaacctg	m5C_62806

chr19	52196571	+	C	32	10	0.312	0	0	T	22	M	0.18	0.486	1.1E-14	2E-06	1.3E-14	25.0836	aggtgaggttCttgggagcct	m5C_62816
chr19	53284658	+	C	74	40	0.541	0	0	C	40	M	0.428	0.649	0	5.8E-09	0	342.249	ggaggaaggaCgtgtggctga	m5C_62807
chr19	54617935	-	C	36	12	0.333	0	0	T	24	M	0.202	0.497	0	4.6E-06	0	48.5146	cggctcagggCtccgggagcg	m5C_62778
chr19	54617942	-	C	38	13	0.342	0	0	T	25	M	0.212	0.501	0	6.7E-06	0	55.1518	gtctgggcggCtcagggctcc	m5C_62765
chr19	54631746	+	C	31	9	0.29	0	0	T	22	M	0.161	0.466	3.7E-13	1.2E-06	4.4E-13	18.0154	gtatgagcttCggagaggtca	m5C_62759
chr19	54692882	-	C	43	14	0.326	0	0	T	29	M	0.205	0.475	0	1.9E-07	0	57.3807	gctggggggtCcggatatctg	m5C_62782
chr19	54711401	+	C	41	20	0.488	0	0	T	21	M	0.343	0.635	0	1.4E-06	0	137.017	ccaagaagggCcagggtgggg	m5C_62760
chr19	54711402	+	C	42	18	0.429	0	0	T	24	M	0.291	0.578	0	7.7E-07	0	104.823	caagaagggcCagggtggggc	m5C_62746
chr19	54711418	+	C	47	28	0.596	0	0	C	28	M	0.453	0.724	0	8.1E-06	0	253.914	ggggctggggCtggagacgac	m5C_62755
chr19	55390060	-	C	117	69	0.59	0	0	C	69	M	0.499	0.675	0	5.2E-12	0	688.817	tcggaatctgCtaaggagtgt	m5C_62791
chr19	55390063	-	C	118	74	0.627	0	0	C	74	M	0.537	0.709	0	1.2E-11	0	794.985	aagtcggaatCtgctaaggag	m5C_62783
chr19	55390069	-	C	82	58	0.707	0	0	C	58	M	0.601	0.795	0	6.8E-09	0	697.555	ccacggaagtCggaatctgct	m5C_62772
chr19	55913970	-	C	32	10	0.323	1	0.031	T	21	M	0.186	0.499	7.1E-15	2E-06	9E-15	26.2725	tcagtgatatCtgctgagcat	m5C_62763
chr19	55913982	-	C	34	12	0.353	0	0	T	22	M	0.215	0.521	0	4.6E-06	0	51.5705	tcctggtcacCatcagtgata	m5C_62776
chr19	55913985	-	C	32	12	0.387	1	0.031	T	19	M	0.237	0.562	0	4.6E-06	0	56.9589	gtgtcctggtCaccatcagtg	m5C_62764
chr19	55913991	-	C	32	11	0.344	0	0	T	21	M	0.204	0.517	2.2E-16	3.1E-06	2.9E-16	35.1442	gaacctgtgtCctggtcacca	m5C_62766
chr19	55913997	-	C	32	22	0.688	0	0	C	22	M	0.514	0.82	0	6.3E-05	0	226.305	gggtgggaacCtgtgtcctgg	m5C_62779
chr19	56623194	-	C	49	15	0.306	0	0	T	34	M	0.195	0.445	0	2.8E-07	0	58.546	ccggtgatgaCgccaaggact	m5C_62915
chr19	56623203	-	C	69	31	0.449	0	0	T	38	M	0.338	0.566	0	2.1E-08	0	209.366	gtggcagagcCggtgatgacg	m5C_62920
chr19	56623216	-	C	63	18	0.286	0	0	T	45	M	0.189	0.407	0	2.9E-10	0	68.051	gccatttccgCgggtggcaga	m5C_62911
chr19	56623230	-	C	62	16	0.258	0	0	T	46	M	0.166	0.379	0	1.1E-10	0	52.9752	gcagctgaggCtcagccattt	m5C_62913
chr19	58874911	+	C	95	21	0.221	0	0	T	74	M	0.149	0.314	0	3.1E-15	0	62.7364	acttgggagaCcgtctgggaa	m5C_63010
chr19	58874912	+	C	105	36	0.343	0	0	T	69	M	0.259	0.438	0	4.8E-14	0	186.52	cttgggagacCgtctgggaac	m5C_63023
chr19	58874915	+	C	124	38	0.306	0	0	T	86	M	0.232	0.392	0	0	0	176.426	gggagaccgtCtgggaacacc	m5C_63029
chr19	58906088	+	C	91	36	0.396	0	0	T	55	M	0.301	0.498	0	2E-12	0	216.958	gaaggacgagCtggagcgtgt	m5C_63004
chr19	58906094	+	C	88	38	0.432	0	0	T	50	M	0.333	0.536	0	1.3E-10	0	253.327	cgagctggagCgtgtggccaa	m5C_63030
chr19	58906101	+	C	70	36	0.514	0	0	C	36	M	0.4	0.628	0	2.4E-09	0	287.67	gagcgtgtggCcaagtccaac	m5C_63001
chr19	58906102	+	C	55	21	0.382	0	0	T	34	M	0.265	0.514	0	4.8E-08	0	111.366	agcgtgtggcCaagtccaacc	m5C_63015
chr2	1565646	-	C	31	18	0.581	0	0	C	18	M	0.408	0.736	0	2.7E-05	0	146.757	cctgcggggcCcacagaagcg	m5C_5949
chr2	1565647	-	C	31	7	0.226	0	0	T	24	M	0.114	0.398	6.4E-10	3.4E-07	7.2E-10	7.33205	tcctgcggggCccacagaagc	m5C_5943
chr2	1565656	-	C	36	15	0.417	0	0	T	21	M	0.271	0.578	0	1.2E-05	0	81.421	tgccgagtgtCctgcggggcc	m5C_5946
chr2	1607499	-	C	118	28	0.237	0	0	T	90	M	0.17	0.322	0	0	0	94.964	tggggatcggCggggggatcc	m5C_5948
chr2	1607502	-	C	133	27	0.203	0	0	T	106	M	0.143	0.279	0	0	0	77.4554	ggatggggatCggcgggggga	m5C_5950
chr2	1607517	-	C	34	7	0.206	0	0	T	27	M	0.103	0.368	1.4E-09	3.4E-07	1.6E-09	6.4054	gggaccccgtCtttgggatgg	m5C_5951
chr2	10586844	-	C	251	53	0.212	1	0.004	T	197	M	0.166	0.267	0	0	0	175.842	gtggggttgcaCagtggaaaag	m5C_6450
chr2	10586868	-	C	202	42	0.208	0	0	T	160	M	0.158	0.269	0	0	0	132.438	tgcccgtaggCtgctggcact	m5C_6419
chr2	10586875	-	C	156	45	0.288	0	0	T	111	M	0.223	0.364	0	0	0	200.814	gcctctgtgcCcgtaggctgc	m5C_6413
chr2	10586881	-	C	137	30	0.219	0	0	T	107	M	0.158	0.295	0	0	0	94.7485	tgcctggcctCtgtgcccgta	m5C_6437
chr2	10586888	-	C	89	18	0.202	0	0	T	71	M	0.132	0.297	0	4.5E-14	0	47.4972	gaaccattgcCtggcctctgt	m5C_6448
chr2	10586889	-	C	83	37	0.446	0	0	T	46	M	0.344	0.553	0	9.7E-11	0	254.272	cgaaccattgCctggcctctg	m5C_6414
chr2	10586895	-	C	52	11	0.212	0	0	T	41	M	0.122	0.34	1.4E-13	4.4E-10	1.7E-13	17.3019	tgtccccgaaCcattgcctgg	m5C_6424
chr2	10586900	-	C	47	12	0.255	0	0	T	35	M	0.153	0.395	1E-15	7.5E-08	1.3E-15	27.4561	aactgtgtccCcgaaccattg	m5C_6433
chr2	10586902	-	C	44	13	0.295	0	0	T	31	M	0.182	0.442	0	1.2E-07	0	47.2039	caaactgtgtCcccgaaccat	m5C_6451
chr2	10586908	-	C	67	18	0.269	0	0	T	49	M	0.177	0.385	0	2.9E-10	0	63.7994	ccagagcaaaCtgtgtccccg	m5C_6447
chr2	10586924	-	C	75	15	0.2	0	0	T	60	M	0.125	0.304	0	6E-13	0	37.5361	gtcatagtacCcatgccaga	m5C_6411
chr2	10586942	-	C	130	27	0.208	0	0	T	103	M	0.147	0.285	0	0	0	79.2972	gcccgtcaccCtgggcctgtc	m5C_6406
chr2	10586946	-	C	136	39	0.287	0	0	T	97	M	0.217	0.368	0	0	0	169.6	acaggcccgtCaccctgggcc	m5C_6467
chr2	10586949	-	C	148	38	0.257	0	0	T	110	M	0.193	0.333	0	0	0	146.797	atgacaggccCgtcaccctgg	m5C_6408
chr2	10586951	-	C	163	36	0.221	0	0	T	127	M	0.164	0.291	0	0	0	118.089	ttatgacaggCccgtcaccct	m5C_6478
chr2	10924405	-	C	40	8	0.2	0	0	T	32	M	0.105	0.348	2.1E-10	5.5E-09	2.3E-10	8.13503	agcttgatgaCttagggaaag	m5C_6455
chr2	10932012	-	C	35	8	0.229	0	0	T	27	M	0.121	0.39	5.6E-11	6.8E-07	6.4E-11	9.89691	tgtgattgagCtgacagacga	m5C_6468
chr2	11724907	+	C	35	16	0.457	0	0	T	19	M	0.305	0.618	0	1.7E-05	0	97.4905	cagccgggcgCggtggcgcgt	m5C_6363
chr2	11724913	+	C	31	9	0.29	0	0	T	22	M	0.161	0.466	3.7E-13	1.2E-06	4.4E-13	18.0154	ggcgcggtggCgcgtgcctgt	m5C_6396
chr2	11724919	+	C	37	9	0.243	0	0	T	28	M	0.134	0.401	2.7E-12	1.2E-06	3.2E-12	13.907	gtggcgcgtgCctgtagtccc	m5C_6384
chr2	11724920	+	C	34	12	0.353	0	0	T	22	M	0.215	0.521	0	4.6E-06	0	51.5705	tggcgcgtgcCtgtagtccca	m5C_6370
chr2	11724928	+	C	35	17	0.486	0	0	T	18	M	0.33	0.644	0	2.1E-05	0	112.18	gcctgtagtcCcagctacttg	m5C_6364
chr2	11724935	+	C	38	10	0.27	1	0.026	T	27	M	0.154	0.43	6.7E-14	2E-06	8.3E-14	20.2815	gtcccagctaCttgggaggct	m5C_6391
chr2	11724944	+	C	35	8	0.296	8	0.229	T	19	M	0.159	0.485	4E-12	5.4E-05	4.7E-12	14.4575	acttgggaggCtgaggctgga	m5C_6390
chr2	11724959	+	C	42	10	0.244	1	0.024	T	31	M	0.138	0.393	2.4E-13	2.4E-08	2.9E-13	17.4472	gctggaggatCgcttgagtcc	m5C_6376
chr2	11724961	+	C	46	11	0.239	0	0	T	35	M	0.139	0.379	2.8E-14	4.4E-08	3.5E-14	20.7303	tggaggatcgCttgagtccag	m5C_6392
chr2	11724968	+	C	44	13	0.295	0	0	T	31	M	0.182	0.442	0	1.2E-07	0	47.2039	tcgcttgagtCcaggagttct	m5C_6344
chr2	11724969	+	C	45	15	0.333	0	0	T	30	M	0.214	0.479	0	2.8E-07	0	64.0761	cgcttgagtcCaggagttctg	m5C_6378
chr2	11724977	+	C	41	12	0.3	1	0.024	T	28	M	0.181	0.454	1.1E-16	7.5E-08	1.5E-16	34.6049	tccaggagttCtgggctgtag	m5C_6366
chr2	11724990	+	C	37	10	0.27	0	0	T	27	M	0.154	0.43	6.7E-14	2E-06	8.3E-14	20.2815	ggctgtagtgCgctatgccga	m5C_6383
chr2	11724992	+	C	39	10	0.256	0	0	T	29	M	0.146	0.411	1.3E-13	2E-06	1.6E-13	18.7753	ctgtagtgcgCtatgccgatc	m5C_6371
chr2	11724997	+	C	35	12	0.343	0	0	T	23	M	0.208	0.508	0	4.6E-06	0	49.9956	gtgcgctatgCcgatcgggtg	m5C_6361
chr2	11725042	+	C	31	16	0.516	0	0	C	16	M	0.348	0.68	0	1.7E-05	0	111.488	tggtgacctcCcgggagtgga	m5C_6385
chr2	11725057	+	C	34	13	0.382	0	0	T	21	M	0.239	0.55	0	6.7E-06	0	62.1399	agtggaggacCaccaggttgc	m5C_6353
chr2	11725060	+	C	35	15	0.429	0	0	T	20	M	0.28	0.591	0	1.2E-05	0	83.953	ggaggaccacCaggttgccta	m5C_6339
chr2	13287217	+	C	51	20	0.392	0	0	T	31	M	0.27	0.529	0	3.4E-08	0	108.109	tgggattgtgCctgtggacag	m5C_5968
chr2	13287218	+	C	49	14	0.286	0	0	T	35	M	0.178	0.424	0	1.9E-07	0	49.9784	gggattgtgcCtgtggacagc	m5C_5954
chr2	15768796	+	C	35	8	0.229	0	0	T	27	M	0.121	0.39	5.6E-11	6.8E-07	6.4E-11	9.89691	aaaagcaaaaCtacgtacatc	m5C_6063
chr2	15768799	+	C	31	11	0.355	0	0	T	20	M	0.211	0.531	1.1E-16	3.1E-06	1.5E-16	37.0595	agcaaaactaCgtacatcgaa	m5C_6054
chr2	15768803	+	C	32	11	0.344	0	0	T	21	M	0.204	0.517	2.2E-16	3.1E-06	2.9E-16	35.1442	aaactacgtaCatcgaattgg	m5C_6056
chr2	15768806	+	C	34	13	0.382	0	0	T	21	M	0.239	0.55	0	6.7E-06	0	62.1399	ctacgtacatCgaattggcag	m5C_6061
chr2	20646877	+	C	32	7	0.219	0	0	T	25	M	0.11	0.388	8.5E-10	3.4E-07	9.5E-10	6.99971	cttgtatgctCagcgaggccc	m5C_6263
chr2	20646880	+	C	36	7	0.241	7	0.194	T	22	M	0.122	0.421	3.6E-10	3.4E-05	4E-10	8.08124	gtatgctcagCgaggcccgga	m5C_6271
chr2	23413153	+	C	68	15	0.221	0	0	T	53	M	0.138	0.333	0	6E-11	0	41.5467	gccccgtgatCgcacggtggt	m5C_6169
chr2	25467974	-	C	85	22	0.259	0	0	T	63	M	0.178	0.361	0	4.4E-13	0	78.1351	aagcagagtcCtgatgacacg	m5C_6230
chr2	25467981	-	C	102	24	0.235	0	0	T	78	M	0.164	0.326	0	2.2E-16	0	78.4895	cctctggaagCagagtcctga	m5C_6236
chr2	25467994	-	C	110	40	0.364	0	0	T	70	M	0.28	0.457	0	1.8E-13	0	223.801	gacttcagagCttcctctgga	m5C_6227
chr2	25467999	-	C	105	25	0.238	0	0	T	80	M	0.167	0.328	0	4.4E-16	0	83.3948	atgtagacttCagagcttcct	m5C_6242
chr2	25551520	-	C	34	12	0.353	0	0	T	22	M	0.215	0.521	0	4.6E-06	0	51.5705	ggagtgacttCacacagtcct	m5C_6229
chr2	25551537	-	C	58	22	0.379	0	0	T	36	M	0.266	0.508	0	6.5E-08	0	116.882	gatgttgcagCtgcctgggag	m5C_6239
chr2	26357827	+	C	39	10	0.256	0	0	T	29	M	0.146	0.411	1.3E-13	2E-06	1.6E-13	18.7753	aagagcccaaCagtgaaaatg	m5C_6546
chr2	26357845	+	C	70	26	0.371	0	0	T	44	M	0.268	0.489	0	5.6E-09	0	139.207	atgtagatatCagcagtggag	m5C_6543
chr2	26357865	+	C	40	15	0.375	0	0	T	25	M	0.242	0.53	0	2.8E-07	0	72.6683	ggaggcgtgaCaggctggaag	m5C_6582
chr2	27273650	+	C	144	37	0.257	0	0	T	107	M	0.193	0.334	0	0	0	142.482	cgcagcggagCcttcgatagc	m5C_6571
chr2	27273654	+	C	145	39	0.269	0	0	T	106	M	0.203	0.346	0	0	0	158.684	gcggagccttCgatagctcag	m5C_6581
chr2	27273660	+	C	142	34	0.241	1	0.007	T	107	M	0.178	0.318	0	0	0	121.038	ccttcgatagCtcagttggta	m5C_6586
chr2	27273674	+	C	92	28	0.304	0	0	T	64	M	0.22	0.405	0	1.1E-13	0	123.052	gttggtagagCggaggactgt	m5C_6585
chr2	27273701	+	C	139	43	0.309	0	0	T	96	M	0.239	0.39	0	0	0	205.114	tagggcgtggCaatccttagg	m5C_6545
chr2	27273715	+	C	307	77	0.251	0	0	T	230	M	0.206	0.302	0	0	0	316.633	ccttaggtcgCtggttcgatt	m5C_6588
chr2	27273727	+	C	362	104	0.287	0	0	T	258	M	0.243	0.336	0	0	0	505.656	ggttcgattcCggctcgaagg	m5C_6575
chr2	27273730	+	C	372	100	0.269	0	0	T	272	M	0.226	0.316	0	0	0	452.591	tcgattccggCtcgaaggact	m5C_6574
chr2	27274092	+	C	119	53	0.445	0	0	T	66	M	0.359	0.535	0	1.8E-13	0	380.729	gggggattagCtcaaatggta	m5C_6573
chr2	27274094	+	C	117	56	0.479	0	0	T	61	M	0.39	0.568	0	3.7E-13	0	437.07	gggattagctCaaatggtaga	m5C_6567
chr2	27274106	+	C	74	40	0.541	0	0	C	40	M	0.428	0.649	0	5.8E-09	0	342.249	aatggtagagCgctcgcttag	m5C_6554
chr2	27351843	-	C	32	8	0.25	0	0	T	24	M	0.133	0.421	2.3E-11	6.8E-07	2.6E-11	11.2828	ctagcaggagCtggcctccat	m5C_6623
chr2	27606883	-	C	86	38	0.442	0	0	T	48	M	0.342	0.547	0	1.3E-10	0	259.636	accgaggaggCtgaagaggac	m5C_6590
chr2	27606892	-	C	56	23	0.411	0	0	T	33	M	0.292	0.541	0	8.7E-08	0	134.166	gaggatgacaCcgaggaggct	m5C_6616

chr2	27606951	-	C	36	12	0.333	0	0	T	24	M	0.202	0.497	0	4.6E-06	0	48.5146	aggaatgcagCgaggaagagg	m5C_6598
chr2	27606954	-	C	119	40	0.336	0	0	T	79	M	0.258	0.425	0	4.3E-15	0	206.052	ggcaggaatgCagcgaggaag	m5C_6608
chr2	27607546	-	C	129	51	0.395	0	0	T	78	M	0.315	0.482	0	3.3E-15	0	321.48	aagaggaagaCagtgaggtaa	m5C_6591
chr2	27607588	-	C	57	16	0.281	0	0	T	41	M	0.181	0.408	0	7.3E-09	0	57.8536	tctttgaggaCagtgaggatg	m5C_6628
chr2	29136545	+	C	507	102	0.201	0	0	T	405	M	0.169	0.238	0	0	0	343.931	gtgatgatgcCttaatattgt	m5C_6713
chr2	29136561	+	C	478	134	0.28	0	0	T	344	M	0.242	0.322	0	0	0	648.405	attgtggtttCgactcactga	m5C_6702
chr2	29136564	+	C	464	98	0.211	0	0	T	366	M	0.177	0.251	0	0	0	345.971	gtggtttcgaCtcactgagag	m5C_6696
chr2	29136568	+	C	413	117	0.283	0	0	T	296	M	0.242	0.329	0	0	0	566.249	tttcgactcaCtgagagtaaa	m5C_6714
chr2	29136586	+	C	346	96	0.277	0	0	T	250	M	0.233	0.327	0	0	0	447.202	aaaatgaggaCctacaattcc	m5C_6710
chr2	29136587	+	C	333	91	0.273	0	0	T	242	M	0.228	0.324	0	0	0	415.32	aaatgaggacCtacaattcct	m5C_6689
chr2	29136607	+	C	252	57	0.226	0	0	T	195	M	0.179	0.282	0	0	0	203.914	ttggctgtgtCtgagcaccca	m5C_6711
chr2	29136615	+	C	39	9	0.231	0	0	T	30	M	0.126	0.383	4.9E-12	1.2E-06	5.7E-12	12.8739	gtctgagcacCcaaagtatgc	m5C_6688
chr2	29149959	+	C	196	49	0.25	0	0	T	147	M	0.195	0.315	0	0	0	190.686	catatggtttCgctgctggct	m5C_6704
chr2	29149968	+	C	191	41	0.215	0	0	T	150	M	0.162	0.278	0	0	0	133.134	tcgctgctggCtgagtttcag	m5C_6709
chr2	29149976	+	C	196	45	0.23	0	0	T	151	M	0.176	0.293	0	0	0	158.623	ggctgagtttCagagatgaca	m5C_6707
chr2	29149994	+	C	182	40	0.22	0	0	T	142	M	0.166	0.285	0	0	0	132.604	acaccttctCttggctgtct	m5C_6712
chr2	29149999	+	C	181	49	0.271	0	0	T	132	M	0.211	0.34	0	0	0	207.025	tttctcttggCtgtctgagca	m5C_6697
chr2	29150891	+	C	269	68	0.253	0	0	T	201	M	0.205	0.308	0	0	0	278.23	ttgctgagttCcagtgatgcc	m5C_6691
chr2	29150901	+	C	204	52	0.256	1	0.005	T	151	M	0.201	0.32	0	0	0	209.067	ccagtgatgcCtcttttctct	m5C_6692
chr2	29150908	+	C	200	52	0.26	0	0	T	148	M	0.204	0.325	0	0	0	212.303	tgcctcttttCtcttggctgt	m5C_6705
chr2	29169349	+	C	43	10	0.233	0	0	T	33	M	0.132	0.377	4.3E-13	2.4E-08	5.2E-13	16.2683	gatgaaatagCagataaggat	m5C_6706
chr2	29169361	+	C	40	10	0.25	0	0	T	30	M	0.142	0.402	1.8E-13	2.4E-08	2.2E-13	18.0914	gataaggattCtgaagtgagt	m5C_6687
chr2	30410416	+	C	47	19	0.404	0	0	T	28	M	0.276	0.547	0	1E-06	0	105.031	ctctctgttcCtaggagtgtg	m5C_6756
chr2	30410428	+	C	41	10	0.244	0	0	T	31	M	0.138	0.393	2.4E-13	2.4E-08	2.9E-13	17.4472	aggagtgtgaCaatttttggg	m5C_6748
chr2	31045660	-	C	97	21	0.216	0	0	T	76	M	0.146	0.308	0	3.1E-15	0	61.3984	tctgtggggtCtgatggggac	m5C_6758
chr2	31045669	-	C	107	28	0.262	0	0	T	79	M	0.188	0.352	0	1.8E-15	0	105.11	cctgagtgttCtgtggggtct	m5C_6763
chr2	32773028	+	C	38	12	0.316	0	0	T	26	M	0.191	0.475	0	4.6E-06	0	45.8026	ggatgtggaaCaagccttaac	m5C_6291
chr2	32773033	+	C	39	9	0.231	0	0	T	30	M	0.126	0.383	4.9E-12	1.2E-06	5.7E-12	12.8739	tggaacaagcCttaactaagc	m5C_6297
chr2	37439051	-	C	65	33	0.508	0	0	C	33	M	0.389	0.625	0	3.4E-08	0	256.847	gaagaatttgCtgaagttgat	m5C_6515
chr2	39356039	-	C	76	17	0.224	0	0	T	59	M	0.145	0.329	0	2.2E-12	0	49.1676	gtgtatacaaCtaattgatta	m5C_6684
chr2	39356042	-	C	77	20	0.267	2	0.026	T	55	M	0.18	0.376	0	1.1E-11	0	71.9047	gtggtgtataCaactaattga	m5C_6650
chr2	42511825	+	C	36	14	0.389	0	0	T	22	M	0.248	0.551	0	9.2E-06	0	69.397	agatagctggCgtggataaag	m5C_6777
chr2	42896614	+	C	46	11	0.239	0	0	T	35	M	0.139	0.379	2.8E-14	4.4E-08	3.5E-14	20.7303	gatggggattCgtataactta	m5C_6790
chr2	43037708	+	C	221	70	0.317	0	0	T	151	M	0.259	0.381	0	0	0	362.578	gcgcgcggtaCttatacagca	m5C_6776
chr2	43037717	+	C	198	65	0.328	0	0	T	133	M	0.267	0.396	0	0	0	346.69	acttatacagCagtacatgca	m5C_6794
chr2	43037722	+	C	218	46	0.211	0	0	T	172	M	0.162	0.27	0	0	0	149.12	tacagcagtaCatgcagagca	m5C_6770
chr2	43037737	+	C	277	72	0.26	0	0	T	205	M	0.212	0.315	0	0	0	305.007	agagcaatgcCgaggttgtga	m5C_6789
chr2	43037767	+	C	227	63	0.278	0	0	T	164	M	0.223	0.339	0	0	0	281.423	tcacctggagCaagacccttt	m5C_6798
chr2	43037774	+	C	160	32	0.2	0	0	T	128	M	0.145	0.269	0	0	0	93.0444	gagcaagaccCttttggatgt	m5C_6796
chr2	46362195	-	C	32	11	0.344	0	0	T	21	M	0.204	0.517	2.2E-16	3.1E-06	2.9E-16	35.1442	ttgatcccgcCaatagaaaaa	m5C_7391
chr2	46362199	-	C	34	12	0.353	0	0	T	22	M	0.215	0.521	0	4.6E-06	0	51.5705	ctggttgatcCcgccaataga	m5C_7403
chr2	46362200	-	C	34	12	0.353	0	0	T	22	M	0.215	0.521	0	4.6E-06	0	51.5705	cctggttgatCccgccaatag	m5C_7388
chr2	47507895	-	C	76	52	0.684	0	0	C	52	M	0.573	0.778	0	5.1E-08	0	595.921	tcgcctgacaCaattacaacg	m5C_7401
chr2	47507897	-	C	104	44	0.423	0	0	T	60	M	0.333	0.519	0	5.7E-13	0	292.627	gttcgcctgaCacaattacaa	m5C_7382
chr2	47507901	-	C	130	63	0.485	0	0	T	67	M	0.4	0.57	0	2.4E-15	0	504.487	tcacgttcgcCtgacacaatt	m5C_7405
chr2	47507902	-	C	132	51	0.386	0	0	T	81	M	0.308	0.472	0	1.1E-16	0	313.784	atcacgttcgCctgacacaat	m5C_7397
chr2	47507904	-	C	133	77	0.579	0	0	C	77	M	0.494	0.659	0	4.7E-14	0	760.724	ttatcacgttCgcctgacaca	m5C_7402
chr2	47507908	-	C	139	68	0.489	0	0	T	71	M	0.408	0.571	0	7.4E-15	0	554.228	gtggttatcaCgttcgcctga	m5C_7377
chr2	47507910	-	C	137	66	0.482	0	0	T	71	M	0.4	0.565	0	4.8E-15	0	527.638	tagtggttatCacgttcgcct	m5C_7383
chr2	47507927	-	C	119	71	0.597	0	0	C	71	M	0.507	0.68	0	7.4E-12	0	719.671	gatatgtttcCgtagtgtagt	m5C_7385
chr2	47507928	-	C	59	31	0.525	0	0	C	31	M	0.4	0.647	0	5.9E-07	0	248.245	cgatatgtttCcgtagtgtag	m5C_7398
chr2	47798893	-	C	38	26	0.684	0	0	C	26	M	0.525	0.809	0	0.00013	0	273.229	ggcagtccgcCgggcctgggc	m5C_7384
chr2	48234195	-	C	34	13	0.382	0	0	T	21	M	0.239	0.55	0	6.7E-06	0	62.1399	acctgtagacCtgaatttgaa	m5C_7242
chr2	53697749	-	C	40	14	0.35	0	0	T	26	M	0.221	0.505	0	1.9E-07	0	61.9761	gggaaaggctCctgtgtggct	m5C_7612
chr2	53697762	-	C	45	21	0.467	0	0	T	24	M	0.329	0.609	0	1.8E-06	0	138.327	ctctcatggtCtggggaaagg	m5C_7611
chr2	54872378	+	C	143	35	0.245	0	0	T	108	M	0.182	0.321	0	0	0	127.1	gctggagaatCagatggaagt	m5C_7479
chr2	54877033	+	C	32	9	0.281	0	0	T	23	M	0.156	0.454	5.3E-13	1.2E-06	6.4E-13	17.1983	acaagaaactCcctgaggagc	m5C_7442
chr2	54877034	+	C	31	9	0.29	0	0	T	22	M	0.161	0.466	3.7E-13	1.2E-06	4.4E-13	18.0154	caagaaactcCctgaggagct	m5C_7507
chr2	54877035	+	C	31	10	0.323	0	0	T	21	M	0.186	0.499	7.1E-15	2E-06	9E-15	26.2725	aagaaactccCtgaggagctt	m5C_7449
chr2	54886309	+	C	32	9	0.281	0	0	T	23	M	0.156	0.454	5.3E-13	1.2E-06	6.4E-13	17.1983	actggaagtgCgcagacagca	m5C_7503
chr2	55277299	-	C	39	15	0.385	0	0	T	24	M	0.249	0.541	0	1.2E-05	0	74.6735	aggacgaggaCgaagacctgg	m5C_7549
chr2	55277338	-	C	70	29	0.42	1	0.014	T	40	M	0.311	0.538	0	1.3E-08	0	180.414	agcccgaggaCgaggaggaag	m5C_7531
chr2	55461971	+	C	33	10	0.303	0	0	T	23	M	0.174	0.473	1.6E-14	2E-06	2E-14	23.982	taacaggagtCtactcttcat	m5C_7506
chr2	55461974	+	C	31	9	0.29	0	0	T	22	M	0.161	0.466	3.7E-13	1.2E-06	4.4E-13	18.0154	caggagtctaCtcttcatctt	m5C_7518
chr2	55461979	+	C	32	10	0.312	0	0	T	22	M	0.18	0.486	1.1E-14	2E-06	1.3E-14	25.0836	gtctactcttCatcttgtgtt	m5C_7446
chr2	55513161	+	C	53	29	0.547	0	0	C	29	M	0.415	0.673	0	3.9E-07	0	240.432	ataggctggcCtgatggtagt	m5C_7466
chr2	55513179	+	C	39	8	0.205	0	0	T	31	M	0.108	0.355	1.6E-10	6.8E-07	1.8E-10	8.44384	agtgagttatCtcaactgatt	m5C_7486
chr2	55762869	+	C	58	17	0.293	0	0	T	41	M	0.192	0.42	0	1.1E-08	0	65.2053	aggaatatgaCcaggaagaag	m5C_7481
chr2	55844470	-	C	36	10	0.278	0	0	T	26	M	0.158	0.44	4.8E-14	2E-06	5.9E-14	21.1115	ggagcgcggaCgaggtaagcg	m5C_7544
chr2	55844476	-	C	35	21	0.6	0	0	C	21	M	0.436	0.744	0	5.2E-05	0	183.004	gagtgaggagCgcggacgagg	m5C_7527
chr2	59775648	+	C	31	12	0.387	0	0	T	19	M	0.237	0.562	0	4.6E-06	0	56.9589	gttcacatggCtcattcattg	m5C_7282
chr2	59819734	-	C	54	43	0.796	0	0	C	43	M	0.671	0.882	0	4.4E-06	0	577.038	tgaatttcttCtctgataatt	m5C_7306
chr2	61225895	+	C	51	16	0.314	0	0	T	35	M	0.203	0.45	0	7.3E-09	0	65.048	ctgtctgtggCcacaccaccc	m5C_7326
chr2	61225896	+	C	48	10	0.208	0	0	T	38	M	0.117	0.343	1.6E-12	2.4E-08	1.9E-12	13.8343	tgtctgtggcCacaccaccct	m5C_7327
chr2	61225903	+	C	53	16	0.302	0	0	T	37	M	0.195	0.435	0	7.3E-09	0	62.4581	ggccacaccaCccctgaatgtg	m5C_7312
chr2	61225904	+	C	52	14	0.269	0	0	T	38	M	0.168	0.403	0	2.8E-09	0	46.9527	gccacaccacCctgaatgtgc	m5C_7321
chr2	61225905	+	C	50	10	0.2	0	0	T	40	M	0.112	0.33	2.6E-12	2E-10	3.1E-12	13.024	ccacaccaccCtgaatgtgcc	m5C_7320
chr2	64282613	+	C	71	15	0.211	0	0	T	56	M	0.132	0.32	0	6E-13	0	39.7273	atatctgtggCccctagtggt	m5C_7713
chr2	68617298	-	C	59	46	0.78	0	0	C	46	M	0.659	0.866	0	6.6E-06	0	605.977	caatccttgtCcctattgtct	m5C_7785
chr2	69814054	+	C	36	12	0.333	0	0	T	24	M	0.202	0.497	0	4.6E-06	0	48.5146	aggcagttagCtgtgagaacc	m5C_7763
chr2	70476118	-	C	127	53	0.417	0	0	T	74	M	0.335	0.504	0	5.8E-15	0	355.333	ggcgcataggCgttttgcttc	m5C_8519
chr2	70476129	-	C	254	96	0.378	0	0	T	158	M	0.321	0.439	0	0	0	615.472	gattcccgggCggcgcatagg	m5C_8514
chr2	70476133	-	C	229	47	0.205	0	0	T	182	M	0.158	0.262	0	0	0	148.519	gttcgattccCgggcggcgca	m5C_8496
chr2	70476134	-	C	216	50	0.231	0	0	T	166	M	0.18	0.292	0	0	0	180.221	ggttcgattcCcgggcggcgc	m5C_8525
chr2	70476146	-	C	222	45	0.203	0	0	T	177	M	0.155	0.26	0	0	0	139.576	tcttgcgaccCgggttcgatt	m5C_8523
chr2	70476147	-	C	236	58	0.246	0	0	T	178	M	0.195	0.304	0	0	0	226.425	ttcttgcgacCcgggttcgat	m5C_8516
chr2	70476148	-	C	241	55	0.228	0	0	T	186	M	0.18	0.285	0	0	0	197.712	attcttgcgaCccgggttcga	m5C_8503
chr2	71172663	+	C	41	32	0.78	0	0	C	32	M	0.633	0.88	0	1.6E-05	0	405.086	tgtgtctgtgCattttataat	m5C_8455
chr2	71654036	+	C	38	18	0.474	0	0	T	20	M	0.325	0.627	0	2.7E-05	0	116.924	aacaggaacgCttggtaactg	m5C_8464
chr2	71654044	+	C	59	21	0.356	0	0	T	38	M	0.246	0.483	0	4.8E-08	0	103.333	cgcttggtaaCtgtggatgaa	m5C_8454
chr2	72802661	-	C	38	24	0.632	0	0	C	24	M	0.473	0.766	0	9.1E-05	0	226.962	gaacaggattCaggaattctg	m5C_7641
chr2	72949092	+	C	47	10	0.213	0	0	T	37	M	0.12	0.349	1.3E-12	2.4E-08	1.5E-12	14.271	cttcatgcatCaacctggcat	m5C_7623
chr2	72949125	+	C	383	84	0.219	0	0	T	299	M	0.181	0.263	0	0	0	303.709	aggaatggtgCtctaattgaa	m5C_7620
chr2	72949127	+	C	112	24	0.214	0	0	T	88	M	0.148	0.299	0	0	0	71.251	gaatggtgctCtaattgaaaa	m5C_7632
chr2	74593033	-	C	47	21	0.447	0	0	T	26	M	0.314	0.588	0	1.8E-06	0	131.925	ttgaagaagtCactcaagatt	m5C_8095
chr2	74598180	-	C	37	15	0.405	0	0	T	22	M	0.263	0.565	0	1.2E-05	0	79.0389	ccagctggagCaggtgcagga	m5C_8087
chr2	74598186	-	C	38	19	0.5	0	0	CT	19	M	0.348	0.652	0	3.4E-05	0	132.429	caaaatccagCtggagcaggt	m5C_8090
chr2	74598190	-	C	38	9	0.237	0	0	T	29	M	0.13	0.392	3.7E-12	1.2E-06	4.3E-12	13.3739	aacacaaaatCcagctggagc	m5C_8086
chr2	74685549	+	C	89	20	0.225	0	0	T	69	M	0.15	0.322	0	1.5E-13	0	60.1775	attggtagggCagagcaatct	m5C_8074
chr2	74685564	+	C	55	11	0.2	0	0	T	44	M	0.116	0.324	2.9E-13	4.4E-10	3.6E-13	15.9231	caatctggatCctagttggtg	m5C_8077
chr2	74685565	+	C	45	17	0.378	0	0	T	28	M	0.251	0.524	0	5.6E-07	0	85.3668	aatctgagtcCtagttggtgg	m5C_8073

chr2	75124055	+	C	49	23	0.469	0	0	T	26	M	0.337	0.606	0	2.9E-06	0	155.035	agcctgtggtCtagtggttag	m5C_8076
chr2	76672384	-	C	84	18	0.214	0	0	T	66	M	0.14	0.313	0	4.5E-14	0	50.424	aagaggacgaCcatccccgat	m5C_7749
chr2	76672387	-	C	94	28	0.298	0	0	T	66	M	0.215	0.397	0	1.1E-13	0	120.312	tcgaagaggaCgaccatcccc	m5C_7748
chr2	76672409	-	C	80	16	0.2	0	0	T	64	M	0.127	0.3	0	1.1E-14	0	40.6488	cctcccgaagCtgcgcgctcg	m5C_7750
chr2	81694457	-	C	54	19	0.352	0	0	T	35	M	0.238	0.485	0	2.4E-08	0	90.5268	ccgctggccgCcgggtctctc	m5C_7679
chr2	85097404	-	C	120	44	0.367	0	0	T	76	M	0.286	0.456	0	4.4E-16	0	251.529	cagactggggCagtggaaaag	m5C_7876
chr2	85097410	-	C	120	31	0.258	0	0	T	89	M	0.188	0.343	0	1.1E-16	0	116.791	aggaaacagaCtggggcagtg	m5C_7846
chr2	85097414	-	C	109	23	0.211	0	0	T	86	M	0.145	0.297	0	1.1E-16	0	66.6644	gctgaggaaaCagactggggc	m5C_7848
chr2	85097423	-	C	72	25	0.347	0	0	T	47	M	0.248	0.462	0	9.3E-11	0	123.766	ggctgagcggCtgaggaaaca	m5C_7882
chr2	85097426	-	C	63	13	0.206	0	0	T	50	M	0.125	0.322	2.4E-15	1.6E-11	3.1E-15	23.6977	ggaggctgagCggctgaggaa	m5C_7847
chr2	85132841	+	C	42	18	0.429	0	0	T	24	M	0.291	0.578	0	7.7E-07	0	104.823	cgagactgcaCggtgagtgcg	m5C_7840
chr2	85133146	+	C	48	10	0.208	0	0	T	38	M	0.117	0.343	1.6E-12	2.4E-08	1.9E-12	13.8343	aagaaaatggCagacaaacca	m5C_7819
chr2	85133150	+	C	49	19	0.388	0	0	T	30	M	0.264	0.528	0	1E-06	0	100.429	aaatggcagaCaaaccagaca	m5C_7827
chr2	85133530	+	C	43	9	0.209	0	0	T	34	M	0.114	0.352	1.4E-11	1.2E-08	1.6E-11	11.1522	gcaggagaagCggagtgaaat	m5C_7841
chr2	85133577	+	C	34	8	0.235	0	0	T	26	M	0.124	0.4	4.2E-11	6.8E-07	4.8E-11	10.3259	cccccgtcctCttcgagaccc	m5C_7843
chr2	85133580	+	C	51	13	0.255	0	0	T	38	M	0.155	0.389	1.1E-16	1.6E-09	1.5E-16	32.2431	ccgtcctcttCgagaccccag	m5C_7815
chr2	85133585	+	C	65	13	0.2	0	0	T	52	M	0.121	0.313	3.8E-15	1.6E-11	4.8E-15	22.646	ctcttcgagaCcccagtcgtg	m5C_7814
chr2	85133586	+	C	67	31	0.463	0	0	T	36	M	0.349	0.581	0	2.1E-08	0	216.121	tcttcgagacCccagtcgtga	m5C_7821
chr2	85133592	+	C	119	46	0.387	0	0	T	73	M	0.304	0.476	0	2.8E-14	0	279.602	agaccccagtCgtgatgtgga	m5C_7805
chr2	85133610	+	C	175	46	0.263	0	0	T	129	M	0.203	0.333	0	0	0	186.977	ggaggaagagCcacctgcaag	m5C_7839
chr2	85133614	+	C	154	33	0.214	0	0	T	121	M	0.157	0.286	0	0	0	103.52	gaagagccacCtgcaagatgg	m5C_7844
chr2	85657611	-	C	33	8	0.242	0	0	T	25	M	0.128	0.41	3.1E-11	6.8E-07	3.6E-11	10.7866	agagggtgttCaaagcaggct	m5C_7863
chr2	86362997	+	C	297	68	0.229	0	0	T	229	M	0.185	0.28	0	0	0	251.345	tacaaccaggCtgtgatgatt	m5C_8209
chr2	86363010	+	C	723	217	0.3	0	0	T	506	M	0.268	0.335	0	0	0	1162.5	tgatgattggCgcaggggtac	m5C_8220
chr2	86363012	+	C	730	194	0.266	1	0.001	T	535	M	0.235	0.299	0	0	0	913.058	atgattggcgCaggggtacgg	m5C_8221
chr2	86363024	+	C	577	141	0.245	1	0.002	T	435	M	0.211	0.282	0	0	0	596.273	ggggtacggaCctcagctgag	m5C_8214
chr2	86363025	+	C	575	140	0.243	0	0	T	435	M	0.21	0.28	0	0	0	588.492	gggtacggacCtcagctgagt	m5C_8198
chr2	86363036	+	C	421	94	0.223	0	0	T	327	M	0.186	0.265	0	0	0	349.87	tcagctgagtCatgggagctg	m5C_8215
chr2	86363044	+	C	324	114	0.352	0	0	T	210	M	0.302	0.405	0	0	0	688.251	gtcatgggagCtgaatgtatg	m5C_8228
chr2	86363063	+	C	206	49	0.239	1	0.005	T	156	M	0.186	0.302	0	0	0	182.067	tgtgtttctcCtttgtcctgc	m5C_8216
chr2	86363069	+	C	165	43	0.261	0	0	T	122	M	0.2	0.332	0	0	0	171.67	tctcctttgtCctgcatgtgg	m5C_8229
chr2	86363073	+	C	156	38	0.245	1	0.006	T	117	M	0.184	0.319	0	0	0	139.947	ctttgtcctgCatgtggcagg	m5C_8211
chr2	86363084	+	C	120	31	0.258	0	0	T	89	M	0.188	0.343	0	1.1E-16	0	116.791	atgtggcaggCtgatggggag	m5C_8205
chr2	86363115	+	C	122	31	0.254	0	0	T	91	M	0.185	0.338	0	0	0	114.806	agactgttgcCtcaatctgag	m5C_8202
chr2	86363126	+	C	106	54	0.509	0	0	C	54	M	0.416	0.603	0	6.4E-12	0	448.862	tcaatctgagCctgcacttca	m5C_8217
chr2	86363127	+	C	92	29	0.315	0	0	T	63	M	0.229	0.416	0	1.7E-13	0	133.002	caatctgagcCtgcacttcat	m5C_8207
chr2	86650935	-	C	37	20	0.541	0	0	C	20	M	0.384	0.69	0	4.3E-05	0	153.535	aactgaggatCtgaggaaggg	m5C_8245
chr2	86650943	-	C	37	16	0.432	0	0	T	21	M	0.287	0.591	0	1.7E-05	0	91.7488	catgatggaaCtgaggatctg	m5C_8230
chr2	88315885	-	C	175	49	0.282	1	0.006	T	125	M	0.22	0.353	0	0	0	215.651	cttccagaaaCggtgcacctg	m5C_8122
chr2	88529114	-	C	32	20	0.625	0	0	C	20	M	0.453	0.771	0	4.3E-05	0	181.016	tatacaacacCccactgctaa	m5C_8128
chr2	88529120	-	C	44	11	0.25	0	0	T	33	M	0.146	0.394	1.6E-14	4.4E-08	2E-14	22.132	agttggtataCaacaccccac	m5C_8120
chr2	88529136	-	C	41	12	0.293	0	0	T	29	M	0.176	0.445	1.1E-16	7.5E-08	1.5E-16	33.7134	acattagtgtCattgaagttg	m5C_8119
chr2	88529153	-	C	43	20	0.465	0	0	T	23	M	0.325	0.611	0	1.4E-06	0	130.043	gctatcagaaCttattaacat	m5C_8130
chr2	95774038	-	C	31	21	0.677	0	0	C	21	M	0.501	0.814	0	5.2E-05	0	210.591	cagacatgatCcagcagagag	m5C_8152
chr2	95774048	-	C	40	19	0.475	0	0	T	21	M	0.329	0.625	0	1E-06	0	125.154	aaggtggaggCagacatgatc	m5C_8134
chr2	99985879	+	C	34	12	0.353	0	0	T	22	M	0.215	0.521	0	4.6E-06	0	51.5705	gaattatgtgCtgctgtagaa	m5C_8377
chr2	99985899	+	C	45	21	0.467	0	0	T	24	M	0.329	0.609	0	1.8E-06	0	138.327	agttatggaaCaaggagtacc	m5C_8372
chr2	99985908	+	C	40	8	0.2	0	0	T	32	M	0.105	0.348	2.1E-10	5.5E-09	2.3E-10	8.13503	acaaggagtaCcaggaaaagga	m5C_8353
chr2	101620646	+	C	32	18	0.562	0	0	C	18	M	0.393	0.718	0	2.7E-05	0	141.571	gcacctcgggCactcaaagag	m5C_8740
chr2	101889426	-	C	37	10	0.27	0	0	T	27	M	0.154	0.43	6.7E-14	2E-06	8.3E-14	20.2815	acaaaaatatcCtgaagcgaac	m5C_8760
chr2	101889427	-	C	39	14	0.359	0	0	T	25	M	0.227	0.516	0	9.2E-06	0	63.6773	gacaaaaatatCctgaagcgaa	m5C_8759
chr2	101889451	-	C	48	10	0.208	0	0	T	38	M	0.117	0.343	1.6E-12	2.4E-08	1.9E-12	13.8343	gtttgctctcCatgggcacac	m5C_8762
chr2	101889466	-	C	64	15	0.234	0	0	T	49	M	0.147	0.351	0	6E-11	0	44.2493	gtgtctccatCagcagtttgc	m5C_8778
chr2	101889472	-	C	65	24	0.369	0	0	T	41	M	0.262	0.491	0	3E-09	0	125.9	attgcagtgtCtccatcagca	m5C_8782
chr2	101889485	-	C	130	29	0.223	0	0	T	101	M	0.16	0.302	0	0	0	92.8281	aagaaaaggcCgaattgcagt	m5C_8774
chr2	101889486	-	C	148	30	0.203	0	0	T	118	M	0.146	0.275	0	0	0	87.5036	caagaaaaggCcgaattgcag	m5C_8769
chr2	101889496	-	C	207	55	0.266	0	0	T	152	M	0.21	0.33	0	0	0	231.214	ggaatgatgaCaagaaaaggc	m5C_8761
chr2	105982017	+	C	41	11	0.268	0	0	T	30	M	0.157	0.419	6E-15	4.4E-08	7.6E-15	24.5536	ggggagagaaCagggaagagg	m5C_8303
chr2	111808588	+	C	46	14	0.304	0	0	T	32	M	0.191	0.448	0	1.9E-07	0	53.4231	atttgcgtgaCctccagtggc	m5C_8322
chr2	118617577	+	C	37	21	0.568	0	0	C	21	M	0.409	0.713	0	5.2E-05	0	171.838	tcgcgctcgcCcgggtgccgt	m5C_8657
chr2	120288535	+	C	35	25	0.714	0	0	C	25	M	0.549	0.837	0	0.00011	0	274.724	atcctgtggtCtagtggcaag	m5C_8903
chr2	121065530	+	C	34	26	0.765	0	0	C	26	M	0.6	0.876	0	0.00013	0	312.018	tactgcttggCtgggtcctct	m5C_8901
chr2	127599429	+	C	32	11	0.344	0	0	T	21	M	0.204	0.517	2.2E-16	3.1E-06	2.9E-16	35.1442	tagcggtgagCataaccacct	m5C_8793
chr2	128321408	-	C	55	27	0.491	0	0	T	28	M	0.364	0.619	0	2.5E-07	0	196.433	ggtggtgtggCtgagtggtta	m5C_9106
chr2	128556564	-	C	36	23	0.639	0	0	C	23	M	0.476	0.775	0	7.6E-05	0	218.844	ttggctggtcCgatggtagtg	m5C_9117
chr2	128556565	-	C	39	20	0.513	0	0	C	20	M	0.362	0.661	0	4.3E-05	0	144.796	tttggctggtCcgatggtagt	m5C_9102
chr2	128825400	+	C	98	24	0.245	0	0	T	74	M	0.17	0.339	0	1.7E-14	0	81.8153	gtggtgtttaCaaccaattga	m5C_9062
chr2	128825414	+	C	98	20	0.204	0	0	T	78	M	0.136	0.294	0	1.7E-15	0	54.4704	caattgatcaCagccagttac	m5C_9075
chr2	128825417	+	C	90	18	0.2	0	0	T	72	M	0.13	0.294	0	4.4E-16	0	46.9523	ttgatcacagCcagttacaga	m5C_9083
chr2	128825418	+	C	88	37	0.42	0	0	T	51	M	0.323	0.525	0	9.7E-11	0	238.849	tgatcacagcCagttacagat	m5C_9079
chr2	131058804	+	C	34	7	0.206	0	0	T	27	M	0.103	0.368	1.4E-09	3.4E-07	1.6E-09	6.4054	ttggtggtgaCgtcggacact	m5C_8832
chr2	131094691	-	C	185	78	0.422	0	0	T	107	M	0.353	0.494	0	0	0	550.314	acaaaagtggCttttttttt	m5C_8878
chr2	131094713	-	C	222	48	0.216	0	0	T	174	M	0.167	0.275	0	0	0	160.451	gggttcgactCccggtatggg	m5C_8863
chr2	131094715	-	C	210	59	0.281	0	0	T	151	M	0.225	0.345	0	0	0	264.929	ccgggttcgaCtcccggtatg	m5C_8889
chr2	131094718	-	C	194	63	0.325	0	0	T	131	M	0.263	0.393	0	0	0	331.132	ggcccgggttCgactcccggt	m5C_8854
chr2	131094724	-	C	187	58	0.31	0	0	T	129	M	0.248	0.38	0	0	0	287.962	ccaggtggccCgggttcgact	m5C_8855
chr2	131094725	-	C	179	43	0.24	0	0	T	136	M	0.184	0.308	0	0	0	157.824	cccaggtggcCcgggttcgac	m5C_8869
chr2	131094726	-	C	172	76	0.442	0	0	T	96	M	0.37	0.517	0	0	0	561.97	acccaggtggCccgggttcga	m5C_8877
chr2	131094733	-	C	130	31	0.238	0	0	T	99	M	0.173	0.319	0	0	0	107.497	ggttttcaccCaggtggcccg	m5C_8862
chr2	131094734	-	C	127	43	0.339	0	0	T	84	M	0.262	0.425	0	2.2E-16	0	225.403	tggttttcacCcaggtggccc	m5C_8879
chr2	131094735	-	C	126	37	0.294	0	0	T	89	M	0.221	0.378	0	0	0	163.673	ctggttttcaCccaggtggcc	m5C_8858
chr2	131094737	-	C	123	51	0.415	0	0	T	72	M	0.331	0.503	0	3.3E-15	0	338.072	tcctggttttCacccaggtgg	m5C_8882
chr2	131094745	-	C	133	43	0.323	0	0	T	90	M	0.25	0.407	0	0	0	214.778	gttaggattcCtggttttcac	m5C_8870
chr2	131094746	-	C	133	37	0.278	0	0	T	96	M	0.209	0.36	0	0	0	154.722	ggttaggattCctggttttca	m5C_8876
chr2	131094757	-	C	124	26	0.21	0	0	T	98	M	0.147	0.29	0	0	0	76.5949	tatggtctagCggttaggatt	m5C_8884
chr2	131094771	-	C	82	19	0.232	0	0	T	63	M	0.154	0.334	0	8.4E-14	0	58.3883	ttcagcagttCccatatggtc	m5C_8860
chr2	133010726	-	C	148	35	0.236	0	0	T	113	M	0.175	0.311	0	0	0	122.666	ctgagcgtcaCttgccaatca	m5C_12249
chr2	133010728	-	C	494	107	0.217	0	0	T	387	M	0.183	0.255	0	0	0	390.625	gtctgagcgtCacttgccaat	m5C_12526
chr2	133010736	-	C	650	201	0.309	0	0	T	449	M	0.275	0.346	0	0	0	1105.13	ctatgcctgtCtgagcgtcac	m5C_12208
chr2	133010738	+	C	42	15	0.357	0	0	T	27	M	0.23	0.508	0	2.8E-07	0	68.967	gacgctcagaCaggcatagcc	m5C_12138
chr2	133010740	-	C	652	152	0.233	0	0	T	500	M	0.202	0.267	0	0	0	614.973	agggctatgcCtgtctgagcg	m5C_12291
chr2	133010751	-	C	336	96	0.286	0	0	T	240	M	0.24	0.336	0	0	0	460.886	gggttcctccCagggctatgc	m5C_12463
chr2	133010752	-	C	326	78	0.239	0	0	T	248	M	0.196	0.288	0	0	0	306.007	cgggttcctcCcagggctatg	m5C_12197
chr2	133010756	-	C	271	60	0.221	0	0	T	211	M	0.176	0.275	0	0	0	211.267	ggcccgggttCctcccagggc	m5C_12315
chr2	133010762	-	C	179	42	0.235	0	0	T	137	M	0.179	0.302	0	0	0	149.969	acttgcggccCgggttcctcc	m5C_12497
chr2	133010845	-	C	67	17	0.254	0	0	T	50	M	0.165	0.369	0	1.8E-10	0	56.0585	gctcctgtgtCaatgaacaac	m5C_12388
chr2	133010851	-	C	120	6	0.6	110	0.917	G	110	M	0.313	0.832	1.2E-12	0.00146	1.4E-12	22.3851	cactcagctcCtgtgtcaatg	m5C_12354
chr2	133010852	-	C	254	56	0.22	0	0	T	198	M	0.174	0.275	0	0	0	194.727	tcactcagctCctgtgtcaat	m5C_12412
chr2	133010854	-	C	368	115	0.312	0	0	T	253	M	0.267	0.362	0	0	0	614.754	gatcactcagCtcctgtgtca	m5C_12185
chr2	133010857	-	C	773	217	0.281	0	0	T	556	M	0.25	0.313	0	0	0	1085.83	gtggatcactCagctcctgtg	m5C_12518
chr2	133010859	-	C	1119	370	0.331	0	0	T	749	M	0.304	0.359	0	0	0	2247.44	tggtggatcaCtcagctcctg	m5C_12166

chr2	133011956	-	C	108	62	0.574	0	0	C	62	M	0.48	0.663	0	3.2E-11	0	595.014	aaagtcgtaaCaaagtttcgg	m5C_12180
chr2	133011961	-	C	132	85	0.644	0	0	C	85	M	0.559	0.72	0	2E-13	0	950.712	aagtaaaagtCgtaacaaagt	m5C_12451
chr2	133011982	-	C	37	26	0.703	0	0	C	26	M	0.542	0.825	0	0.00013	0	281.926	tcgaacttgaCtatctagagg	m5C_12485
chr2	133011987	-	C	32	20	0.625	0	0	C	20	M	0.453	0.771	0	4.3E-05	0	181.016	gacagtcgaaCttgactatct	m5C_12345
chr2	133012243	-	C	91	72	0.791	0	0	C	72	M	0.697	0.862	0	3E-09	0	1003.34	gctacactgaCtggctcagcg	m5C_12448
chr2	133012256	-	C	107	84	0.785	0	0	C	84	M	0.698	0.852	0	9.5E-10	0	1172.73	gggctgcacgCgcgctacact	m5C_12488
chr2	133012263	-	C	87	61	0.701	0	0	C	61	M	0.598	0.787	0	1.1E-08	0	729.713	atgtccagggCtgcacgcgcg	m5C_12406
chr2	133012268	-	C	48	22	0.458	0	0	T	26	M	0.326	0.597	0	2.3E-06	0	143.33	cttaaatgtcCagggctgcac	m5C_12332
chr2	133012269	-	C	45	25	0.556	0	0	C	25	M	0.412	0.691	0	4.5E-06	0	205.879	ccttaaatgtCcagggctgca	m5C_12398
chr2	133012278	-	C	35	20	0.571	0	0	C	20	M	0.409	0.72	0	4.3E-05	0	163.429	ctgtgatgccCttaaatgtcc	m5C_12225
chr2	133012279	-	C	37	21	0.568	0	0	C	21	M	0.409	0.713	0	5.2E-05	0	171.838	tctgtgatgcCcttaaatgtc	m5C_12475
chr2	133012280	-	C	34	19	0.559	0	0	C	19	M	0.395	0.711	0	3.4E-05	0	149.924	gtctgtgatgCccttaaatgt	m5C_12477
chr2	133012288	-	C	41	21	0.512	0	0	C	21	M	0.365	0.657	0	1.8E-06	0	153.234	aataacaggtCtgtgatgccc	m5C_12234
chr2	133012293	-	C	52	34	0.654	0	0	C	34	M	0.518	0.768	0	1.1E-06	0	352.262	tgagcaataaCaggtctgtga	m5C_12401
chr2	133012299	-	C	69	38	0.551	0	0	C	38	M	0.434	0.662	0	9.6E-08	0	329.677	tgagattgagCaataacaggt	m5C_12535
chr2	133012310	-	C	137	55	0.401	0	0	T	82	M	0.323	0.485	0	2.2E-16	0	355.46	gttcagccacCtgagattgag	m5C_12289
chr2	133012311	-	C	130	43	0.331	0	0	T	87	M	0.256	0.415	0	0	0	219.961	cgttcagccaCctgagattga	m5C_12532
chr2	133012313	-	C	136	30	0.221	0	0	T	106	M	0.159	0.297	0	0	0	95.4672	ggcgttcagcCacctgagatt	m5C_12461
chr2	133012314	-	C	137	31	0.226	0	0	T	106	M	0.164	0.303	0	0	0	101.827	tggcgttcagCcacctgagat	m5C_12428
chr2	133012317	-	C	142	31	0.218	0	0	T	111	M	0.158	0.293	0	0	0	98.1305	aagtggcgttCagccacctga	m5C_12250
chr2	133012321	-	C	142	29	0.204	0	0	T	113	M	0.146	0.278	0	0	0	84.753	ggacaagtggCgttcagccac	m5C_12413
chr2	133012328	-	C	95	56	0.596	1	0.011	C	56	M	0.495	0.689	0	2.3E-10	0	554.032	cttagagggaCaagtggcgtt	m5C_12405
chr2	133012338	-	C	40	9	0.225	0	0	T	31	M	0.123	0.375	6.4E-12	1.2E-08	7.6E-12	12.4041	ccccccaacttCttagagggac	m5C_12329
chr2	133012341	-	C	33	18	0.545	0	0	C	18	M	0.38	0.702	0	2.7E-05	0	136.748	catcccccaaCttcttagagg	m5C_12512
chr2	133012344	-	C	33	8	0.242	0	0	T	25	M	0.128	0.41	3.1E-11	6.8E-07	3.6E-11	10.7866	cggcatccccCaacttcttag	m5C_12521
chr2	133012345	-	C	33	16	0.485	0	0	T	17	M	0.325	0.648	0	1.7E-05	0	104.012	tcggcatcccCcaacttctta	m5C_12187
chr2	133012346	-	C	31	23	0.742	0	0	C	23	M	0.568	0.863	0	7.6E-05	0	261.066	gtcggcatccCccaacttctt	m5C_12296
chr2	133012348	-	C	32	7	0.219	0	0	T	25	M	0.11	0.388	8.5E-10	3.4E-07	9.5E-10	6.99971	cggtcggcatCccccaacttc	m5C_12322
chr2	133012351	-	C	35	15	0.429	0	0	T	20	M	0.28	0.591	0	1.2E-05	0	83.953	gagcggtcggCatcccccaac	m5C_12377
chr2	133012354	-	C	33	15	0.455	0	0	T	18	M	0.298	0.62	0	1.2E-05	0	89.5281	ctggagcggtCggcatccccc	m5C_12178
chr2	133012468	-	C	184	145	0.788	0	0	C	145	M	0.723	0.841	0	8.9E-16	0	2097.89	atagctctttCtcgattccac	m5C_12394
chr2	133012472	-	C	162	119	0.735	0	0	C	119	M	0.662	0.797	0	6.2E-15	0	1574.87	attgatagctCtttctcgatt	m5C_12227
chr2	133012474	-	C	161	120	0.745	0	0	C	120	M	0.673	0.806	0	7.1E-15	0	1614.89	agattgatagCtctttctcga	m5C_12277
chr2	133012485	-	C	98	55	0.561	0	0	C	55	M	0.463	0.655	0	1.9E-10	0	508.759	acaggattgaCagattgatag	m5C_12528
chr2	133012494	-	C	51	14	0.275	0	0	T	37	M	0.171	0.409	0	2.8E-09	0	47.9195	tggacacggaCaggattgaca	m5C_12242
chr2	133012498	-	C	52	12	0.231	0	0	T	40	M	0.137	0.361	4.4E-15	8.8E-10	5.7E-15	23.6371	agcctggacaCggacaggatt	m5C_12271
chr2	133012500	-	C	50	10	0.2	0	0	T	40	M	0.112	0.33	2.6E-12	2E-10	3.1E-12	13.024	ccagcctggaCacggacagga	m5C_12483
chr2	133012543	-	C	65	14	0.215	0	0	T	51	M	0.133	0.33	1.1E-16	3.2E-11	1.5E-16	29.6776	gtggagcctgCagcttaattt	m5C_12198
chr2	133012546	-	C	129	44	0.341	0	0	T	85	M	0.265	0.426	0	4.4E-16	0	233.14	ggagtggagcCtgcagcttaa	m5C_12449
chr2	133012547	-	C	152	63	0.414	0	0	T	89	M	0.339	0.494	0	0	0	427.402	aggagtggagCctgcagctta	m5C_12352
chr2	133012558	-	C	393	97	0.247	0	0	T	296	M	0.207	0.292	0	0	0	401.149	agggcaccacCaggagtggag	m5C_12169
chr2	133012559	-	C	389	88	0.226	0	0	T	301	M	0.187	0.27	0	0	0	329.891	aagggcaccaCcaggagtgga	m5C_12376
chr2	133012561	-	C	399	81	0.204	1	0.003	T	317	M	0.167	0.246	0	0	0	270.353	agaagggcacCaccaggagtg	m5C_12331
chr2	133012564	-	C	395	98	0.248	0	0	T	297	M	0.208	0.293	0	0	0	407.816	gacagaagggCaccaccagga	m5C_12168
chr2	133012572	-	C	316	124	0.392	0	0	T	192	M	0.34	0.447	0	0	0	843.617	aaggaattgaCagaagggcac	m5C_12537
chr2	133012586	-	C	503	109	0.217	0	0	T	394	M	0.183	0.255	0	0	0	398.753	aaagctgaaaCttaaaggaat	m5C_12471
chr2	133012592	-	C	465	180	0.387	0	0	T	285	M	0.344	0.432	0	0	0	1238.12	ggttgcaaagCtgaaacttaa	m5C_12283
chr2	133012615	-	C	70	23	0.329	0	0	T	47	M	0.23	0.445	0	4.3E-11	0	105.794	tttttgggttCcgaggggagt	m5C_12190
chr2	133012644	-	C	44	11	0.25	0	0	T	33	M	0.146	0.394	1.6E-14	4.4E-08	2E-14	22.132	gggcagcttcCaagaaaccag	m5C_12268
chr2	133012645	-	C	41	20	0.488	0	0	T	21	M	0.343	0.635	0	1.4E-06	0	137.017	tgggcagcttCcaagaaacca	m5C_12324
chr2	133012651	-	C	53	18	0.34	0	0	T	35	M	0.227	0.474	0	1.7E-08	0	81.6727	acccgctgggCagcttccaag	m5C_12402
chr2	133012656	-	C	99	36	0.364	0	0	T	63	M	0.276	0.462	0	2E-12	0	198.445	ccatgacccgCtgggcagctt	m5C_12491
chr2	133012659	-	C	113	42	0.372	0	0	T	71	M	0.288	0.464	0	8.4E-15	0	242.065	ttcccatgacCcgctgggcag	m5C_12311
chr2	133012666	-	C	156	44	0.282	0	0	T	112	M	0.217	0.357	0	0	0	191.251	ggtgttattcCcatgacccgc	m5C_12385
chr2	133012667	-	C	146	42	0.288	0	0	T	104	M	0.22	0.366	0	0	0	185.16	cggtgttattCccatgacccg	m5C_12505
chr2	133012677	-	C	230	52	0.227	1	0.004	T	177	M	0.178	0.286	0	0	0	184.68	ggcgatgtggCggtgttattc	m5C_12524
chr2	133012685	-	C	131	36	0.275	0	0	T	95	M	0.206	0.357	0	0	0	148.033	tgccaactggCgatgtggcgg	m5C_12292
chr2	133012704	-	C	50	19	0.38	0	0	T	31	M	0.259	0.518	0	2.4E-08	0	98.2779	taattccgacCataaacgatg	m5C_12304
chr2	133012705	-	C	51	22	0.431	0	0	T	29	M	0.305	0.567	0	6.5E-08	0	134.205	gtaattccgaCcataaacgat	m5C_12161
chr2	133012708	-	C	53	23	0.434	0	0	T	30	M	0.31	0.567	0	8.7E-08	0	142.371	gttgtaattcCgaccataaac	m5C_12245
chr2	133012709	-	C	68	24	0.353	0	0	T	44	M	0.25	0.472	0	3E-09	0	120.009	cgttgtaattCcgaccataaa	m5C_12276
chr2	133012719	-	C	420	122	0.29	0	0	T	298	M	0.249	0.336	0	0	0	607.835	gatcagatacCgttgtaattc	m5C_12221
chr2	133012720	-	C	434	144	0.332	0	0	T	290	M	0.289	0.377	0	0	0	832.733	cgatcagataCcgttgtaatt	m5C_12282
chr2	133012726	-	C	581	167	0.287	0	0	T	414	M	0.252	0.326	0	0	0	842.101	cgaagacgatCagataccgtt	m5C_12335
chr2	133012730	-	C	790	298	0.379	3	0.004	T	489	M	0.345	0.413	0	0	0	2058.77	ggttcgaagaCgatcagatac	m5C_12393
chr2	133012736	-	C	888	228	0.257	2	0.002	T	658	M	0.23	0.287	0	0	0	1047.16	gtcggaggttCgaagacgatc	m5C_12263
chr2	133012751	-	C	389	167	0.429	0	0	T	222	M	0.381	0.479	0	0	0	1272.69	taatcaagaaCgaaagtcgga	m5C_12349
chr2	133012757	-	C	153	92	0.601	0	0	C	92	M	0.522	0.675	0	1.6E-15	0	960.768	tttcattaatCaagaacgaaa	m5C_12357
chr2	133012764	-	C	45	34	0.756	0	0	C	34	M	0.613	0.858	0	2.2E-05	0	417.019	agaatgttttCattaatcaag	m5C_12211
chr2	133012804	-	C	106	41	0.387	0	0	T	65	M	0.3	0.482	0	2.4E-13	0	245.662	cttggactggCagtttagacc	m5C_12370
chr2	133012808	-	C	134	37	0.276	0	0	T	97	M	0.207	0.357	0	0	0	153.522	aattcttggaCtggcagttta	m5C_12182
chr2	133012814	-	C	148	34	0.23	0	0	T	114	M	0.169	0.304	0	0	0	115.13	aggtgaaattCttggactggc	m5C_12473
chr2	133012830	-	C	42	21	0.5	0	0	CT	21	M	0.355	0.645	0	1.8E-06	0	149.208	catattgcacCgctagaggtg	m5C_12177
chr2	133012831	-	C	39	19	0.487	0	0	T	20	M	0.339	0.638	0	3.4E-05	0	128.687	tcatattgcaCcgctagaggt	m5C_12273
chr2	133012840	-	C	49	12	0.245	0	0	T	37	M	0.146	0.381	1.9E-15	7.5E-08	2.4E-15	25.8006	cgggggcattCatattgcacc	m5C_12286
chr2	133012844	-	C	82	44	0.537	0	0	C	44	M	0.429	0.64	0	5.2E-10	0	377.912	cggccgggggCattcatattg	m5C_12297
chr2	133012850	-	C	361	78	0.216	0	0	T	283	M	0.177	0.261	0	0	0	275.681	gagggacggcCgggggcattc	m5C_12519
chr2	133012851	-	C	429	102	0.238	0	0	T	327	M	0.2	0.28	0	0	0	407.829	agagggacggCcgggggcatt	m5C_12514
chr2	133012854	-	C	675	343	0.508	0	0	C	343	M	0.47	0.546	0	0	0	3227.59	ttaagagggaCggccgggggc	m5C_12275
chr2	133012870	-	C	1261	379	0.301	0	0	T	882	M	0.276	0.326	0	0	0	2091.21	ggaactgaggCcatgattaag	m5C_12431
chr2	133012876	-	C	1157	294	0.254	0	0	T	863	M	0.23	0.28	0	0	0	1351.58	gttttcggaaCtgaggccatg	m5C_12270
chr2	133012881	-	C	860	295	0.343	1	0.001	T	564	M	0.312	0.376	0	0	0	1843.32	tgttggttttCggaactgagg	m5C_12403
chr2	133012897	-	C	103	37	0.359	0	0	T	66	M	0.273	0.455	0	6.7E-14	0	202.145	gaccacagttCtattttgttg	m5C_12493
chr2	133012938	-	C	141	64	0.454	0	0	T	77	M	0.374	0.536	0	1.1E-16	0	478.761	cccaagccgcCtggatactgc	m5C_12366
chr2	133012942	-	C	158	46	0.291	0	0	T	112	M	0.226	0.366	0	0	0	207.856	caggcccaagCcgcctggata	m5C_12204
chr2	133012946	-	C	285	92	0.323	0	0	T	193	M	0.271	0.379	0	0	0	498.993	aaagcaggccCaagccgcctg	m5C_12301
chr2	133012947	-	C	305	96	0.315	0	0	T	209	M	0.265	0.369	0	0	0	509.205	caaagcaggcCcaagccgcct	m5C_12334
chr2	133012948	-	C	300	70	0.233	0	0	T	230	M	0.189	0.284	0	0	0	264.638	tcaaagcaggCccaagccgcc	m5C_12438
chr2	133012998	-	C	60	33	0.55	0	0	C	33	M	0.425	0.669	0	8.8E-07	0	280.445	agctgagtgtCctgtggcccg	m5C_12330
chr2	133013065	-	C	56	37	0.661	0	0	C	37	M	0.53	0.771	0	1.8E-06	0	392.194	agcgggtgggCtagcgagcca	m5C_12248
chr2	133013073	-	C	148	73	0.493	0	0	T	75	M	0.414	0.573	0	8.9E-16	0	604.281	atcttgggagCgggtgggcta	m5C_12420
chr2	133013081	-	C	208	101	0.486	0	0	T	107	M	0.419	0.553	0	0	0	845.439	gtagttgaatCttgggagcgg	m5C_12267
chr2	133013092	-	C	271	150	0.554	0	0	C	150	M	0.494	0.612	0	0	0	1481.94	ttaaaaagctCgtagttgaat	m5C_12408
chr2	133013094	-	C	271	140	0.517	0	0	C	140	M	0.457	0.575	0	0	0	1280.42	agttaaaaagCtcgtagttga	m5C_12387
chr2	133013105	-	C	222	113	0.509	0	0	C	113	M	0.444	0.574	0	0	0	1002.66	aaagttgttgCagttaaaaag	m5C_12235
chr2	133013123	-	C	338	142	0.434	11	0.033	T	185	M	0.382	0.488	0	0	0	1083.74	gctccaatagCgtatattaaa	m5C_12381
chr2	133013129	-	C	491	187	0.382	1	0.002	T	303	M	0.34	0.425	0	0	0	1270.47	attccagctcCaatagcgtat	m5C_12247
chr2	133013130	-	C	504	265	0.526	0	0	C	265	M	0.482	0.569	0	0	0	2555.49	aattccagctCcaatagcgta	m5C_12469
chr2	133013132	-	C	594	267	0.449	0	0	T	327	M	0.41	0.49	0	0	0	2189.09	gtaattccagCtccaatagcg	m5C_12365
chr2	133013135	-	C	687	341	0.496	0	0	T	346	M	0.459	0.534	0	0	0	3131.04	gtggtaattcCagctccaata	m5C_12264
chr2	133013136	-	C	750	373	0.497	0	0	T	377	M	0.462	0.533	0	0	0	3443.94	cgtggtaattCcagctccaat	m5C_12246

chr2	133013146	-	C	1254	556	0.443	0	0	T	698	M	0.416	0.471	0	0	0	4627.02	tgccagcagcCgtggtaattc	m5C_12450
chr2	133013147	-	C	1282	515	0.403	4	0.003	T	763	M	0.376	0.43	0	0	0	3877.03	gtgccagcagCcgtggtaatt	m5C_12176
chr2	133013150	-	C	1288	436	0.339	0	0	T	852	M	0.313	0.365	0	0	0	2730.93	ctggtgccagCagccgtggta	m5C_12455
chr2	133013153	-	C	1314	452	0.344	0	0	T	862	M	0.319	0.37	0	0	0	2881.87	agtctggtgcCagcagccgtg	m5C_12347
chr2	133013154	-	C	1277	502	0.393	0	0	T	775	M	0.367	0.42	0	0	0	3681.44	aagtctggtgCcagcagccgt	m5C_12422
chr2	133013160	-	C	1100	459	0.417	0	0	T	641	M	0.388	0.447	0	0	0	3566.15	gagggcaagtCtggtgccagc	m5C_12262
chr2	133013165	-	C	826	239	0.29	1	0.001	T	586	M	0.26	0.322	0	0	0	1241.72	aattggagggCaagtctggtg	m5C_12236
chr2	133013176	-	C	288	166	0.578	1	0.003	C	166	M	0.521	0.634	0	0	0	1728.39	taatgaggatCaattggaggg	m5C_12419
chr2	133013190	-	C	95	54	0.568	0	0	C	54	M	0.468	0.663	0	1.6E-10	0	505.527	cactttaaatCttttaatgag	m5C_12326
chr2	133013231	-	C	49	23	0.469	0	0	T	26	M	0.337	0.606	0	2.9E-06	0	155.035	caatacaggaCtctttctagg	m5C_12336
chr2	133013241	-	C	60	13	0.217	0	0	T	47	M	0.131	0.336	1.1E-15	1.6E-11	1.4E-15	25.5122	cgaaaaataaCaatacaggac	m5C_12205
chr2	133013251	-	C	75	46	0.613	0	0	C	46	M	0.5	0.715	0	1.9E-08	0	460.159	gaggtagtgaCgaaaaataac	m5C_12508
chr2	133013296	-	C	95	27	0.284	0	0	T	68	M	0.203	0.382	0	7.2E-14	0	109.773	tcaaggaaggCagcagacact	m5C_12417
chr2	133013305	-	C	95	30	0.316	0	0	T	65	M	0.231	0.415	0	2.5E-13	0	138.619	ctaccacattCaaggaaggca	m5C_12342
chr2	133013311	-	C	77	17	0.221	0	0	T	60	M	0.143	0.325	0	2.2E-12	0	48.5054	aaatggctacCacattcaagg	m5C_12434
chr2	133013312	-	C	83	22	0.265	0	0	T	61	M	0.182	0.369	0	4.4E-13	0	80.0989	gaaatggctaCcacattcaag	m5C_12426
chr2	133013315	-	C	65	21	0.323	0	0	T	44	M	0.222	0.444	0	1E-09	0	93.2557	tgagaaatggCtaccacattc	m5C_12520
chr2	133013344	-	C	34	8	0.235	0	0	T	26	M	0.124	0.4	4.2E-11	6.8E-07	4.8E-11	10.3259	aatcagggttCaattctggag	m5C_12299
chr2	133013359	-	C	57	13	0.228	0	0	T	44	M	0.138	0.352	4.4E-16	1.6E-09	5.8E-16	27.6258	ccacgggtgaCagggaatcag	m5C_12529
chr2	133013366	-	C	153	56	0.366	0	0	T	97	M	0.294	0.445	0	0	0	329.093	atggtgaccaCgggtgacagg	m5C_12318
chr2	133013382	-	C	94	40	0.426	0	0	T	54	M	0.33	0.526	0	6.4E-12	0	264.354	agtcgctgtgCctatcatggt	m5C_12192
chr2	133013411	-	C	32	15	0.469	0	0	T	17	M	0.309	0.636	0	1.2E-05	0	92.6074	gaatgtctgcCctatcaactt	m5C_12339
chr2	133013412	-	C	33	10	0.303	0	0	T	23	M	0.174	0.473	1.6E-14	2E-06	2E-14	23.982	cgaatgtctgCcctatcaact	m5C_12319
chr2	133013415	-	C	41	13	0.317	0	0	T	28	M	0.196	0.47	0	1.2E-07	0	50.8675	attcgaatgtCtgccctatca	m5C_12184
chr2	133013422	-	C	42	15	0.366	1	0.024	T	26	M	0.236	0.519	0	2.8E-07	0	70.7689	atgacccattCgaatgtctgc	m5C_12220
chr2	133013448	+	C	61	16	0.262	0	0	T	45	M	0.168	0.384	0	1.1E-10	0	53.8837	ccacggggggCgtgcgattgg	m5C_12140
chr2	133013460	+	C	35	7	0.2	0	0	T	28	M	0.1	0.359	1.9E-09	3.4E-07	2.1E-09	6.13884	tgcgattggcCcgaggttatc	m5C_12048
chr2	133013461	+	C	35	10	0.286	0	0	T	25	M	0.163	0.451	3.4E-14	2E-06	4.2E-14	21.9991	gcgattggccCgaggttatcc	m5C_12128
chr2	133013644	-	C	35	10	0.286	0	0	T	25	M	0.163	0.451	3.4E-14	2E-06	4.2E-14	21.9991	ttcttttggtCgctggctcct	m5C_12510
chr2	133013652	-	C	48	22	0.458	0	0	T	26	M	0.326	0.597	0	2.3E-06	0	143.33	agttatggttCttttggtcgc	m5C_12285
chr2	133013663	-	C	34	15	0.441	0	0	T	19	M	0.289	0.605	0	1.2E-05	0	86.6497	ctcattaaatCagttatggtt	m5C_12429
chr2	133013762	+	C	46	35	0.761	0	0	C	35	M	0.621	0.861	0	2.5E-05	0	434.452	ataggatcaaCcaggtaggta	m5C_12086
chr2	133013763	+	C	45	34	0.756	0	0	C	34	M	0.613	0.858	0	2.2E-05	0	417.019	taggatcaacCaggtaggtag	m5C_11932
chr2	133015196	-	C	37	10	0.27	0	0	T	27	M	0.154	0.43	6.7E-14	2E-06	8.3E-14	20.2815	tttggggtacCggatcccccg	m5C_12313
chr2	133015197	-	C	37	21	0.568	0	0	C	21	M	0.409	0.713	0	5.2E-05	0	171.838	ctttggggtaCcggatccccc	m5C_12237
chr2	133015220	-	C	130	41	0.315	0	0	T	89	M	0.242	0.4	0	0	0	198.261	ctctagcgatCtgagaggtgt	m5C_12171
chr2	133038365	-	C	318	77	0.245	4	0.013	T	237	M	0.201	0.296	0	0	0	309.393	aatgggttcgCcccgagagag	m5C_12172
chr2	133038367	-	C	316	91	0.288	0	0	T	225	M	0.241	0.34	0	0	0	438.31	ggaatgggttCgccccgagag	m5C_12244
chr2	133038379	-	C	313	64	0.204	0	0	T	249	M	0.164	0.253	0	0	0	209.282	gcagggcgccCtggaatgggt	m5C_12272
chr2	133038447	-	C	62	15	0.242	0	0	T	47	M	0.152	0.362	0	6E-11	0	45.7373	ggtaatgtgaCtgaccctgga	m5C_12243
chr2	133038458	-	C	73	24	0.329	0	0	T	49	M	0.232	0.443	0	6.4E-11	0	111.333	gcgtccagtgCggtaatgtga	m5C_12202
chr2	133038463	-	C	57	23	0.404	0	0	T	34	M	0.286	0.533	0	8.7E-08	0	131.64	atgaggcgtcCagtgcggtaa	m5C_12522
chr2	133038503	-	C	44	35	0.795	0	0	C	35	M	0.655	0.888	0	2.5E-05	0	458.494	ggttcagatcCccgaatccag	m5C_12407
chr2	133038531	-	C	33	23	0.697	0	0	C	23	M	0.527	0.826	0	7.6E-05	0	242.243	ttgccctcagCtgattggaaag	m5C_12427
chr2	133038914	-	C	33	26	0.788	0	0	C	26	M	0.622	0.893	0	0.00013	0	323.69	gggcccatacCcggccgttga	m5C_12207
chr2	133038989	-	C	359	219	0.61	0	0	C	219	M	0.559	0.659	0	0	0	2446.94	ttggaatccgCtaaggagtgt	m5C_12266
chr2	133038991	-	C	467	278	0.595	0	0	C	278	M	0.55	0.639	0	0	0	3058.94	agttggaatcCgctaaggagt	m5C_12240
chr2	133038992	-	C	523	379	0.725	0	0	C	379	M	0.685	0.761	0	0	0	5191.16	aagttggaatCcgctaaggag	m5C_12452
chr2	133039007	-	C	561	420	0.749	0	0	C	420	M	0.711	0.783	0	0	0	5973.73	ggatggtggcCatggaagttg	m5C_12500
chr2	133039008	-	C	563	376	0.668	0	0	C	376	M	0.628	0.706	0	0	0	4721.98	aggatggtggCcatggaagtt	m5C_12392
chr2	133039019	-	C	367	259	0.708	1	0.003	C	259	M	0.659	0.752	0	0	0	3414.07	atatagacagCaggatggtgg	m5C_12490
chr2	133039022	-	C	299	234	0.783	0	0	C	234	M	0.732	0.826	0	0	0	3427.77	ttgatatagaCagcaggatgg	m5C_12252
chr2	135894202	+	C	41	20	0.488	0	0	T	21	M	0.343	0.635	0	1.4E-06	0	137.017	tccaactgcaCttatgtatgt	m5C_8982
chr2	136618958	+	C	42	12	0.286	0	0	T	30	M	0.172	0.436	2.2E-16	7.5E-08	2.9E-16	32.2467	atacacatgcCtgtctgggtg	m5C_9181
chr2	136618962	+	C	45	11	0.244	0	0	T	34	M	0.142	0.387	2.1E-14	4.4E-08	2.7E-14	21.4112	acatgcctgtCtgggtgctgc	m5C_9192
chr2	136691503	-	C	68	21	0.309	0	0	T	47	M	0.212	0.426	0	1E-09	0	88.9234	tgatgctgttCggcctgaggc	m5C_9203
chr2	136691508	-	C	51	14	0.275	0	0	T	37	M	0.171	0.409	0	2.8E-09	0	47.9195	ctggatgatgCtgttcggcct	m5C_9217
chr2	136757496	+	C	32	16	0.5	0	0	CT	16	M	0.336	0.664	0	1.7E-05	0	107.618	tcaggatgaaCgtctgttggt	m5C_9190
chr2	145187560	-	C	34	11	0.324	0	0	T	23	M	0.191	0.492	4.4E-16	3.1E-06	5.8E-16	32.3088	gacacaggttCtgaaacagat	m5C_8961
chr2	145187566	-	C	36	10	0.278	0	0	T	26	M	0.158	0.44	4.8E-14	2E-06	5.9E-14	21.1115	gtagtggacaCaggttctgaa	m5C_8962
chr2	145187568	-	C	36	12	0.333	0	0	T	24	M	0.202	0.497	0	4.6E-06	0	48.5146	atgtagtggaCacaggttctg	m5C_8966
chr2	149583257	-	C	63	22	0.349	0	0	T	41	M	0.243	0.472	0	1.5E-09	0	107.034	aatggtgcaaCttcctcctct	m5C_9158
chr2	149583272	-	C	249	51	0.205	0	0	T	198	M	0.159	0.259	0	0	0	162.544	tgcagtacctCcaggaatggt	m5C_9170
chr2	149583274	-	C	241	54	0.224	0	0	T	187	M	0.176	0.281	0	0	0	190.066	attgcagtacCtccaggaatg	m5C_9167
chr2	149583289	-	C	59	20	0.339	0	0	T	39	M	0.231	0.466	0	3.4E-08	0	92.5499	atcgagtaacCtggtattgca	m5C_9147
chr2	149639322	+	C	40	31	0.775	0	0	C	31	M	0.625	0.877	0	1.4E-05	0	387.479	ggtgaactcaCtggaatgggg	m5C_9137
chr2	149639397	-	C	107	24	0.224	0	0	T	83	M	0.156	0.312	0	2.2E-16	0	74.6949	cacatcacccCataaacaaat	m5C_9171
chr2	149639398	-	C	109	45	0.413	0	0	T	64	M	0.325	0.507	0	7.4E-13	0	292.428	tcacatcaccCcataaacaaa	m5C_9163
chr2	149639405	-	C	107	30	0.28	0	0	T	77	M	0.204	0.372	0	4.7E-15	0	122.41	gacgggctcaCatcaccccat	m5C_9160
chr2	149639407	-	C	111	39	0.351	0	0	T	72	M	0.269	0.444	0	3.1E-15	0	209.718	tagacgggctCacatcacccc	m5C_9169
chr2	149639409	-	C	103	81	0.786	0	0	C	81	M	0.698	0.855	0	6.4E-10	0	1130.3	tttagacgggCtcacatcacc	m5C_9165
chr2	157257293	+	C	115	31	0.27	0	0	T	84	M	0.197	0.357	0	1.1E-16	0	122.07	ggatgtagctCagtggtagag	m5C_9432
chr2	157257306	+	C	88	20	0.227	0	0	T	68	M	0.152	0.325	0	1.5E-13	0	60.8865	tggtagagcgCgcgcttcgca	m5C_9426
chr2	157257308	+	C	77	18	0.234	0	0	T	59	M	0.153	0.34	0	4E-12	0	55.1862	gtagagcgcgCgcttcgcatg	m5C_9431
chr2	157257310	+	C	67	20	0.299	0	0	T	47	M	0.202	0.417	0	6.9E-10	0	80.9254	agagcgcgcgCttcgcatgtg	m5C_9434
chr2	157257669	-	C	103	24	0.233	0	0	T	79	M	0.162	0.323	0	2.2E-16	0	77.7	gttcgattccCggccaatgca	m5C_9444
chr2	157257683	-	C	88	23	0.261	0	0	T	65	M	0.181	0.362	0	7.2E-13	0	83.2283	cgcgggaggcCcgggttcgat	m5C_9443
chr2	157257704	-	C	126	36	0.286	0	0	T	90	M	0.214	0.37	0	0	0	154.145	tggtagaattCtcgcctgcca	m5C_9446
chr2	157257717	-	C	128	46	0.359	0	0	T	82	M	0.281	0.445	0	6.7E-16	0	258.949	attggtggttCagtggtagaa	m5C_9447
chr2	157257728	-	C	88	31	0.352	0	0	T	57	M	0.261	0.456	0	1.7E-11	0	161.548	ttgctataagCattggtggtt	m5C_9445
chr2	162226651	+	C	45	12	0.267	0	0	T	33	M	0.16	0.41	5.6E-16	7.5E-08	7.3E-16	29.2263	tccagtgttcCaagctaaaat	m5C_9452
chr2	162226678	+	C	75	17	0.227	0	0	T	58	M	0.147	0.333	0	2.2E-12	0	49.8482	tatgttgaagCagacaggaag	m5C_9454
chr2	168931670	-	C	35	25	0.714	0	0	C	25	M	0.549	0.837	0	0.00011	0	274.724	ggagtgacgaCgagatggatg	m5C_9354
chr2	169088578	-	C	35	11	0.314	0	0	T	24	M	0.186	0.48	6.7E-16	3.1E-06	8.7E-16	30.9686	tgtgtggtctCtggaggtcct	m5C_9351
chr2	169088591	-	C	36	20	0.556	0	0	C	20	M	0.396	0.705	0	4.3E-05	0	158.323	ttaccaggggCactgtgtggt	m5C_9343
chr2	169088597	-	C	32	8	0.25	0	0	T	24	M	0.133	0.421	2.3E-11	6.8E-07	2.6E-11	11.2828	tggatcttacCaggggcactg	m5C_9359
chr2	169088602	-	C	34	14	0.412	0	0	T	20	M	0.264	0.578	0	9.2E-06	0	73.8241	aactgtggatCttaccagggg	m5C_9357
chr2	170493615	+	C	31	24	0.774	0	0	C	24	M	0.602	0.886	0	9.1E-05	0	288.899	ccaggaagatCaagaagtaaa	m5C_9497
chr2	175104975	-	C	35	12	0.343	0	0	T	23	M	0.208	0.508	0	4.6E-06	0	49.9956	ggaggggtgaaCtggtccaggt	m5C_9951
chr2	175670529	+	C	101	54	0.535	0	0	C	54	M	0.438	0.629	0	6.4E-12	0	472.927	aggggcagctCagtggcagag	m5C_9933
chr2	177015060	+	C	63	15	0.238	0	0	T	48	M	0.15	0.356	0	6E-11	0	44.981	ctatatatacCctgtagaacc	m5C_9595
chr2	177015069	+	C	133	39	0.293	0	0	T	94	M	0.223	0.376	0	0	0	173.582	ccctgtagaaCcgaatttgtg	m5C_9597
chr2	177015070	+	C	129	31	0.24	0	0	T	98	M	0.175	0.321	0	0	0	108.359	cctgtagaacCgaatttgtgt	m5C_9598
chr2	177065910	-	C	56	18	0.321	0	0	T	38	M	0.214	0.452	0	1.7E-08	0	77.043	gtgacagtcaCggaggataag	m5C_9611
chr2	180728997	+	C	73	19	0.26	0	0	T	54	M	0.173	0.371	0	6.9E-12	0	65.8998	gggtatagctCagtggggagc	m5C_9614
chr2	180729007	+	C	68	34	0.5	0	0	CT	34	M	0.384	0.616	0	4.2E-08	0	261.377	cagtggggagCatttgactgc	m5C_9615
chr2	183179142	+	C	56	14	0.25	0	0	T	42	M	0.155	0.377	0	2.8E-09	0	43.4474	accaggttgtCtaaggagggg	m5C_9570
chr2	183179157	+	C	80	22	0.278	1	0.013	T	57	M	0.192	0.386	0	2.8E-11	0	84.3394	gaggggtgaaCtggaccaggt	m5C_9558
chr2	188361650	-	C	49	10	0.204	0	0	T	39	M	0.115	0.336	2.1E-12	2.4E-08	2.4E-12	13.4191	aaatcagaatCgatttgaaag	m5C_9658
chr2	197053968	-	C	44	24	0.545	0	0	C	24	M	0.401	0.683	0	3.6E-06	0	192.316	ggaagcactgCtggttcagtg	m5C_9815
chr2	198256921	-	C	64	14	0.219	0	0	T	50	M	0.135	0.334	1.1E-16	3.2E-11	1.5E-16	30.1591	tagaactggtCatagaggaag	m5C_9760

chr2	198256926	-	C	84	35	0.417	0	0	T	49	M	0.317	0.523	0	5.6E-11	0	222.003	cagtgtagaaCtggtcataga	m5C_9759
chr2	198273300	-	C	31	11	0.355	0	0	T	20	M	0.211	0.531	1.1E-16	3.1E-06	1.5E-16	37.0595	taaagatactCctgggcatgg	m5C_9775
chr2	201237905	+	C	65	29	0.446	0	0	T	36	M	0.332	0.567	0	1.3E-08	0	192.382	ctacgttggtCtgatggtagt	m5C_9850
chr2	201237928	+	C	41	9	0.22	0	0	T	32	M	0.12	0.367	8.4E-12	1.2E-08	9.9E-12	11.9621	gttaccagaaCttattaacat	m5C_9825
chr2	201726508	-	C	49	20	0.408	0	0	T	29	M	0.282	0.548	0	1.4E-06	0	112.86	ggagcagcagCagtcataaaa	m5C_9864
chr2	203141187	+	C	97	45	0.464	0	0	T	52	M	0.368	0.563	0	2.3E-11	0	331.152	cttcttggaaCtgaatctaag	m5C_10097
chr2	203141226	+	C	60	17	0.283	0	0	T	43	M	0.185	0.408	0	1.8E-10	0	62.9227	gtcactaccaCtgagacaacg	m5C_10075
chr2	203142864	+	C	84	20	0.238	0	0	T	64	M	0.16	0.339	0	1.5E-13	0	63.8983	cttattggaaCtgaatttaag	m5C_10087
chr2	203142879	+	C	53	23	0.434	0	0	T	30	M	0.31	0.567	0	8.7E-08	0	142.371	tttaagtgatCtgactcattc	m5C_10103
chr2	203142883	+	C	58	24	0.414	0	0	T	34	M	0.296	0.542	0	1.2E-07	0	142.214	agtgatctgaCtcattcgtca	m5C_10068
chr2	203142892	+	C	58	24	0.414	0	0	T	34	M	0.296	0.542	0	1.2E-07	0	142.214	actcattcgtCactaccactg	m5C_10081
chr2	203142897	+	C	57	21	0.368	0	0	T	36	M	0.255	0.498	0	4.8E-08	0	107.198	ttcgtcactaCcactgagaca	m5C_10095
chr2	203142900	+	C	55	14	0.255	0	0	T	41	M	0.158	0.383	0	2.8E-09	0	44.2735	gtcactaccaCtgagacaaca	m5C_10078
chr2	203157791	+	C	39	11	0.282	0	0	T	28	M	0.165	0.438	3.1E-15	3.1E-06	4E-15	26.4001	atgatgatttCtatttgtttg	m5C_10088
chr2	203157802	+	C	41	13	0.317	0	0	T	28	M	0.196	0.47	0	1.2E-07	0	50.8675	tatttgtttgCctgatttcct	m5C_10071
chr2	203157811	+	C	37	16	0.432	0	0	T	21	M	0.287	0.591	0	1.7E-05	0	91.7488	gcctgatttcCttttggataa	m5C_10089
chr2	204637874	-	C	40	12	0.3	0	0	T	28	M	0.181	0.454	1.1E-16	7.5E-08	1.5E-16	34.6049	tgcttagtagCtgtggaggaa	m5C_9902
chr2	206232921	+	C	55	11	0.2	0	0	T	44	M	0.116	0.324	2.9E-13	4.4E-10	3.6E-13	15.9231	agtggtggatCtttaagaaaa	m5C_11353
chr2	206991521	-	C	33	10	0.303	0	0	T	23	M	0.174	0.473	1.6E-14	2E-06	2E-14	23.982	aagtaaggaaCagattggaag	m5C_11360
chr2	207026635	+	C	370	82	0.222	0	0	T	288	M	0.182	0.267	0	0	0	298.943	aaatcaccatCtttcggctga	m5C_11338
chr2	207026669	+	C	400	92	0.23	0	0	T	308	M	0.191	0.274	0	0	0	352.255	ttgcttttttCtgattaagcc	m5C_11351
chr2	207026968	+	C	203	54	0.266	0	0	T	149	M	0.21	0.331	0	0	0	226.779	agctactggtCtgcagctgtt	m5C_11347
chr2	207026974	+	C	204	52	0.255	0	0	T	152	M	0.2	0.319	0	0	0	208.01	tggtctgcagCtgttcttatg	m5C_11335
chr2	207026979	+	C	204	63	0.309	0	0	T	141	M	0.249	0.375	0	0	0	314.302	tgcagctgttCttatggtagc	m5C_11337
chr2	207026998	+	C	108	23	0.213	0	0	T	85	M	0.146	0.299	0	1.1E-16	0	67.3019	gcagttgtggCattcctctgt	m5C_11346
chr2	207027003	+	C	90	23	0.256	0	0	T	67	M	0.177	0.354	0	1E-14	0	81.3033	tgtggcattcCtctgtgggaa	m5C_11345
chr2	207027034	+	C	53	20	0.377	0	0	T	33	M	0.259	0.512	0	3.4E-08	0	103.744	aacacaaacaCctctttctta	m5C_11336
chr2	207027035	+	C	56	25	0.446	0	0	T	31	M	0.324	0.576	0	1.5E-07	0	161.933	acacaaacacCtctttcttag	m5C_11355
chr2	207027041	+	C	48	22	0.458	0	0	T	26	M	0.326	0.597	0	2.3E-06	0	143.33	acacctctttCttagcaaaac	m5C_11344
chr2	207027079	+	C	40	9	0.225	0	0	T	31	M	0.123	0.375	6.4E-12	1.2E-08	7.6E-12	12.4041	gtgtgacagaCacaagatgta	m5C_11354
chr2	211452832	+	C	33	9	0.273	0	0	T	24	M	0.151	0.442	7.5E-13	1.2E-06	9E-13	16.4418	tagctgtagaCtgtgggatta	m5C_10224
chr2	211452847	+	C	32	12	0.375	0	0	T	20	M	0.229	0.547	0	4.6E-06	0	55.0408	ggattaaaaaCaatgtaatcc	m5C_10233
chr2	211454901	+	C	38	12	0.316	0	0	T	26	M	0.191	0.475	0	4.6E-06	0	45.8026	ggattttgatCgcgggaggac	m5C_10183
chr2	211465305	+	C	67	36	0.537	0	0	C	36	M	0.419	0.651	0	6.5E-08	0	301.797	gagaggtgtgCtcaaggaata	m5C_10196
chr2	211465307	+	C	61	15	0.246	0	0	T	46	M	0.155	0.367	0	6E-11	0	46.5196	gaggtgtgctCaaggaatatg	m5C_10219
chr2	211518752	+	C	31	14	0.452	0	0	T	17	M	0.292	0.622	0	9.2E-06	0	81.6522	gtgacaggagCacccagtggt	m5C_10201
chr2	211518764	+	C	101	33	0.327	0	0	T	68	M	0.243	0.423	0	1.6E-14	0	160.431	cccagtggtgCtgacaaaatt	m5C_10178
chr2	211518768	+	C	118	30	0.254	0	0	T	88	M	0.184	0.34	0	0	0	110.569	gtggtgctgaCaaaatttgtt	m5C_10238
chr2	211518787	+	C	333	86	0.258	0	0	T	247	M	0.214	0.308	0	0	0	368.412	ttgaaggggcCcgagaagtag	m5C_10195
chr2	211518788	+	C	342	75	0.219	0	0	T	267	M	0.179	0.266	0	0	0	268.044	tgaaggggccCgagaagtaga	m5C_10254
chr2	211518805	+	C	488	136	0.279	1	0.002	T	351	M	0.241	0.321	0	0	0	656.23	tagaaatggaCgctgttggca	m5C_10252
chr2	211518807	+	C	484	126	0.261	1	0.002	T	357	M	0.224	0.302	0	0	0	563.736	gaaatggacgCtgttggcaaa	m5C_10241
chr2	215178504	-	C	33	21	0.636	0	0	C	21	M	0.466	0.778	0	5.2E-05	0	195.787	ggtcattatcCtgaatgtggt	m5C_10021
chr2	215711782	+	C	41	19	0.463	0	0	T	22	M	0.321	0.613	0	1E-06	0	121.813	gtatgggttgCtctgagggac	m5C_10009
chr2	215711784	+	C	43	13	0.302	0	0	T	30	M	0.186	0.451	0	1.2E-07	0	48.3648	atgggttgctCtgagggacct	m5C_10004
chr2	217364687	+	C	34	15	0.441	0	0	T	19	M	0.289	0.605	0	1.2E-05	0	86.6497	gatgaagagaCgagctgtggg	m5C_10155
chr2	217364691	+	C	32	8	0.25	0	0	T	24	M	0.133	0.421	2.3E-11	6.8E-07	2.6E-11	11.2828	aagagacgagCtgtggggatc	m5C_10132
chr2	217366104	+	C	43	15	0.349	0	0	T	28	M	0.224	0.498	0	2.8E-07	0	67.2552	catcagaagaCtgaaggagtt	m5C_10150
chr2	217366122	+	C	45	13	0.289	0	0	T	32	M	0.177	0.434	0	1.2E-07	0	46.0978	gttgaaagacCagtagacgct	m5C_10151
chr2	219130371	-	C	33	23	0.697	0	0	C	23	M	0.527	0.826	0	7.6E-05	0	242.243	actgtggcctCccagcccagc	m5C_10437
chr2	219130374	-	C	32	22	0.688	0	0	C	22	M	0.514	0.82	0	6.3E-05	0	226.305	gagactgtggCctcccagccc	m5C_10453
chr2	219130380	-	C	33	12	0.364	0	0	T	21	M	0.222	0.534	0	4.6E-06	0	53.2487	agacctgagaCtgtggcctcc	m5C_10444
chr2	219340093	+	C	135	35	0.259	0	0	T	100	M	0.193	0.339	0	0	0	134.905	gtaatcccagCggtggatcac	m5C_10432
chr2	219340103	+	C	114	31	0.272	0	0	T	83	M	0.199	0.36	0	1.1E-16	0	123.184	cggtggatcaCctgaggtcag	m5C_10431
chr2	226452098	+	C	35	8	0.229	0	0	T	27	M	0.121	0.39	5.6E-11	6.8E-07	6.4E-11	9.89691	gaagatgaagCagcaaaggaa	m5C_10058
chr2	228625163	-	C	75	44	0.587	0	0	C	44	M	0.474	0.691	0	1.3E-08	0	416.824	acgaggtggcCgagtggttaa	m5C_10411
chr2	228625164	-	C	74	32	0.432	0	0	T	42	M	0.326	0.546	0	8.5E-10	0	208.422	gacgaggtggCcgagtggtta	m5C_10408
chr2	230045489	-	C	294	191	0.65	0	0	C	191	M	0.594	0.702	0	0	0	2267.22	cggagatgggCgtagtggagt	m5C_10576
chr2	230045499	-	C	361	239	0.662	0	0	C	239	M	0.612	0.709	0	0	0	2924.29	tccggagtggCggagatgggc	m5C_10591
chr2	230045507	-	C	280	163	0.582	0	0	C	163	M	0.524	0.638	0	0	0	1707.07	tccccgaatcCggagtggcgg	m5C_10570
chr2	230045508	-	C	247	122	0.494	0	0	C	125	M	0.432	0.556	0	0	0	1054.44	atccccgaatCcggagtggcg	m5C_10583
chr2	230045513	-	C	129	27	0.209	0	0	T	102	M	0.148	0.287	0	0	0	79.9307	ttcagatcccCgaatccggag	m5C_10600
chr2	230045514	-	C	109	36	0.33	0	0	T	73	M	0.249	0.423	0	4.8E-14	0	179.34	gttcagatccCcgaatccgga	m5C_10573
chr2	230045515	-	C	102	35	0.343	0	0	T	67	M	0.258	0.439	0	3.4E-14	0	180.743	ggttcagatcCccgaatccgg	m5C_10605
chr2	230045516	-	C	84	19	0.226	0	0	T	65	M	0.15	0.326	0	8.4E-14	0	56.9464	gggttcagatCcccgaatccg	m5C_10588
chr2	230045521	-	C	35	21	0.6	0	0	C	21	M	0.436	0.744	0	5.2E-05	0	183.004	gagtcgggttCagatccccga	m5C_10606
chr2	230045537	+	C	45	35	0.778	0	0	C	35	M	0.637	0.875	0	2.5E-05	0	446.113	gactccctttCgatccgctga	m5C_10533
chr2	230045569	-	C	35	27	0.771	0	0	C	27	M	0.61	0.879	0	0.00015	0	329.305	tccgaagggaCaggcaatggc	m5C_10568
chr2	230045577	-	C	73	58	0.795	0	0	C	58	M	0.688	0.871	0	1.2E-07	0	798.383	agcgccgttcCgaagggacag	m5C_10563
chr2	230045585	-	C	86	65	0.756	0	0	C	65	M	0.655	0.834	0	1.9E-08	0	851.98	gatgggcgagCgccgttccga	m5C_10580
chr2	230045672	-	C	39	19	0.487	0	0	T	20	M	0.339	0.638	0	3.4E-05	0	128.687	agagaggggcCcgtgccttgg	m5C_10569
chr2	230045684	-	C	50	10	0.2	0	0	T	40	M	0.112	0.33	2.6E-12	2E-10	3.1E-12	13.024	gggttcgcccCgagagagggg	m5C_10593
chr2	230045685	-	C	52	18	0.346	0	0	T	34	M	0.232	0.482	0	1.7E-08	0	83.343	tgggttcgccCcgagagaggg	m5C_10566
chr2	230045689	-	C	51	31	0.608	0	0	C	31	M	0.471	0.73	0	5.9E-07	0	291.926	ggaatgggttCgccccgagag	m5C_10596
chr2	231183792	+	C	152	39	0.257	0	0	T	113	M	0.194	0.331	0	0	0	151.122	acactgggtgCtgcaggtcaa	m5C_10487
chr2	231736682	-	C	58	40	0.69	0	0	C	40	M	0.562	0.794	0	2.9E-06	0	449.574	tcaggacaggCcctatctctc	m5C_10565
chr2	231736691	-	C	69	30	0.435	0	0	T	39	M	0.324	0.552	0	1.7E-08	0	194.596	ctaagtgactCaggacaggcc	m5C_10571
chr2	232320151	-	C	33	17	0.515	0	0	C	17	M	0.352	0.675	0	2.1E-05	0	119.742	tagaggaggcCgaggaggatt	m5C_14990
chr2	232320152	-	C	33	10	0.303	0	0	T	23	M	0.174	0.473	1.6E-14	2E-06	2E-14	23.982	gtagaggaggCcgaggaggat	m5C_14984
chr2	232320317	-	C	31	17	0.548	0	0	C	17	M	0.378	0.708	0	2.1E-05	0	128.424	ttgtagacttCaacagtgagg	m5C_15021
chr2	232320528	-	C	32	11	0.344	0	0	T	21	M	0.204	0.517	2.2E-16	3.1E-06	2.9E-16	35.1442	gtatatttagCtgtattgtga	m5C_14977
chr2	232321158	-	C	166	38	0.229	0	0	T	128	M	0.172	0.299	0	0	0	130.389	aagttctgatCcagctgtgtc	m5C_14980
chr2	232321163	-	C	220	78	0.355	0	0	T	142	M	0.294	0.42	0	0	0	459.144	tagtcaagttCtgatccagct	m5C_15035
chr2	232321169	-	C	254	70	0.276	0	0	T	184	M	0.224	0.334	0	0	0	314.01	tctagatagtCaagttctgat	m5C_15022
chr2	232321180	-	C	254	72	0.285	1	0.004	T	181	M	0.233	0.343	0	0	0	334.843	aagaaaattcCtctagatagt	m5C_15020
chr2	232321181	-	C	253	70	0.278	1	0.004	T	182	M	0.226	0.336	0	0	0	316.579	caagaaaattCctctagatag	m5C_14981
chr2	232321191	-	C	264	69	0.261	0	0	T	195	M	0.212	0.318	0	0	0	292.635	gatgaaatctCaagaaaattc	m5C_15001
chr2	232321214	-	C	402	94	0.234	1	0.002	T	307	M	0.196	0.278	0	0	0	367.71	gactgattacCtgagaaataa	m5C_14983
chr2	232321222	-	C	391	102	0.261	0	0	T	289	M	0.22	0.307	0	0	0	448.435	gatatgatgaCtgattacctg	m5C_14995
chr2	232325090	-	C	1249	325	0.26	0	0	T	924	M	0.237	0.285	0	0	0	1538.14	ttgtagtgtgCtgatgtgctg	m5C_15004
chr2	232325111	-	C	1310	304	0.232	0	0	T	1006	M	0.21	0.256	0	0	0	1276.82	atactgaaacCtgatgttaca	m5C_15027
chr2	232325441	-	C	190	44	0.232	0	0	T	146	M	0.177	0.297	0	0	0	156.002	acgacgacgaCgaagatgatg	m5C_15015
chr2	232325447	-	C	138	34	0.246	0	0	T	104	M	0.182	0.324	0	0	0	123.76	aggatgacgaCgacgacgaag	m5C_15008
chr2	232325459	-	C	81	33	0.407	0	0	T	48	M	0.307	0.516	0	3.1E-11	0	202.594	atgatgaggaCgaggatgacg	m5C_14989
chr2	232325493	-	C	58	31	0.534	0	0	C	31	M	0.408	0.657	0	5.9E-07	0	252.96	aagaacgtggCtgaggatgaa	m5C_14988
chr2	232326378	-	C	31	11	0.355	0	0	T	20	M	0.211	0.531	1.1E-16	3.1E-06	1.5E-16	37.0595	atgacgaggaCgaggatgagg	m5C_14987
chr2	232326384	-	C	35	10	0.286	0	0	T	25	M	0.163	0.451	3.4E-14	2E-06	4.2E-14	21.9991	aggaggatgaCgaggacgagg	m5C_15005
chr2	232326408	-	C	61	33	0.541	0	0	C	33	M	0.417	0.66	0	3.4E-08	0	275.377	aggatgatgaCagtgaggagg	m5C_15025
chr2	232327984	-	C	37	9	0.243	0	0	T	28	M	0.134	0.401	2.7E-12	1.2E-06	3.2E-12	13.907	gctcctcctcCaaaggaggta	m5C_15006
chr2	232327987	-	C	33	10	0.303	0	0	T	23	M	0.174	0.473	1.6E-14	2E-06	2E-14	23.982	atggctcctcCtccaaaggag	m5C_15002
chr2	232573440	+	C	35	7	0.2	0	0	T	28	M	0.1	0.359	1.9E-09	3.4E-07	2.1E-09	6.13884	ccgtagacacCagctccgaaa	m5C_14916

chr2	232573454	+	C	134	46	0.343	0	0	T	88	M	0.268	0.427	0	0	0	246.805	tccgaaatcaCcaccaaggtg	m5C_14939
chr2	232573455	+	C	136	37	0.272	0	0	T	99	M	0.204	0.352	0	0	0	151.179	ccgaaatcacCaccaaggtga	m5C_14932
chr2	232573457	+	C	150	37	0.247	0	0	T	113	M	0.185	0.321	0	0	0	136.59	gaaatcaccaCcaaggtgagg	m5C_14950
chr2	232573458	+	C	152	43	0.283	0	0	T	109	M	0.217	0.359	0	0	0	186.902	aaatcaccacCaaggtgaggc	m5C_14952
chr2	232576060	+	C	186	43	0.234	2	0.011	T	141	M	0.178	0.3	0	0	0	153.406	ttttgaaggaCttaaaggaga	m5C_14936
chr2	232576092	+	C	347	103	0.297	0	0	T	244	M	0.251	0.347	0	0	0	517.465	gtggaagaggCagaaaatgga	m5C_14923
chr2	232576110	+	C	34	15	0.441	0	0	T	19	M	0.289	0.605	0	1.2E-05	0	86.6497	ggaagagacgCccctgctaac	m5C_14918
chr2	232576618	+	C	79	41	0.519	0	0	C	41	M	0.411	0.626	0	7.1E-09	0	336.617	aaatggggagCaggaggctga	m5C_14972
chr2	232576625	+	C	101	44	0.436	0	0	T	57	M	0.343	0.533	0	5.7E-13	0	301.891	gagcaggaggCtgacaatgag	m5C_14914
chr2	232576629	+	C	123	29	0.238	1	0.008	T	93	M	0.171	0.321	0	0	0	99.1265	aggaggctgaCaatgaggtag	m5C_14959
chr2	232576641	+	C	250	91	0.364	0	0	T	159	M	0.307	0.425	0	0	0	558.434	atgaggtagaCgaagaagagg	m5C_14974
chr2	233681615	+	C	47	25	0.532	0	0	C	25	M	0.392	0.667	0	4.5E-06	0	196.161	gaaatgagggCaaaacgggaa	m5C_14965
chr2	233681620	+	C	31	10	0.323	0	0	T	21	M	0.186	0.499	7.1E-15	2E-06	9E-15	26.2725	gagggcaaaaCgggaagagga	m5C_14930
chr2	233684666	+	C	34	27	0.794	0	0	C	27	M	0.632	0.897	0	0.00015	0	341.287	ggaagaaaggCggaagcagga	m5C_14940
chr2	233684672	+	C	35	8	0.229	0	0	T	27	M	0.121	0.39	5.6E-11	6.8E-07	6.4E-11	9.89691	aaggcggaagCaggaagaatt	m5C_14917
chr2	233697652	+	C	31	8	0.258	0	0	T	23	M	0.137	0.432	1.7E-11	6.8E-07	1.9E-11	11.8182	gaagaggaggCagccagactc	m5C_14942
chr2	233697662	+	C	42	11	0.262	0	0	T	31	M	0.153	0.411	8.4E-15	4.4E-08	1.1E-14	23.69	cagccagactCcggcatgagg	m5C_14921
chr2	233697663	+	C	44	10	0.227	0	0	T	34	M	0.128	0.37	5.7E-13	2.4E-08	6.8E-13	15.7274	agccagactcCggcatgagga	m5C_14938
chr2	234184421	+	C	98	24	0.245	0	0	T	74	M	0.17	0.339	0	1.7E-14	0	81.8153	tgtcacggtcCctttgttgcc	m5C_10704
chr2	234184422	+	C	97	30	0.309	0	0	T	67	M	0.226	0.407	0	2.5E-13	0	135.624	gtcacggtccCtttgttgccc	m5C_10694
chr2	234184437	+	C	31	8	0.258	0	0	T	23	M	0.137	0.432	1.7E-11	6.8E-07	1.9E-11	11.8182	ttgccctcaaCtcccagcagc	m5C_10711
chr2	234184533	+	C	35	22	0.629	0	0	C	22	M	0.463	0.768	0	6.3E-05	0	203.881	gtgtgtgtttCtgagagtgat	m5C_10717
chr2	234184627	+	C	77	27	0.351	0	0	T	50	M	0.253	0.462	0	1.9E-10	0	136.882	ggtgcatgatCtcaagttttc	m5C_10691
chr2	234184629	+	C	81	20	0.247	0	0	T	61	M	0.166	0.351	0	1.5E-13	0	66.3608	tgcatgatctCaagttttcaa	m5C_10734
chr2	234184648	+	C	53	17	0.321	0	0	T	36	M	0.211	0.455	0	1.1E-08	0	71.713	aatctgagacCtcctaaggag	m5C_10692
chr2	234197346	+	C	78	22	0.282	0	0	T	56	M	0.194	0.39	0	2.8E-11	0	85.4709	ggtaaaaggtCtgagtcctca	m5C_10716
chr2	234197353	+	C	44	23	0.523	0	0	C	23	M	0.379	0.662	0	2.9E-06	0	174.502	ggtctgagtcCtcagatcccc	m5C_10725
chr2	234197552	+	C	65	17	0.262	0	0	T	48	M	0.17	0.38	0	1.8E-10	0	57.8614	gtgctggtatCtgtggctatg	m5C_10714
chr2	234197568	+	C	63	20	0.317	0	0	T	43	M	0.216	0.44	0	6.9E-10	0	86.3461	ctatgatctgCcttgttcaag	m5C_10723
chr2	234197569	+	C	66	22	0.333	0	0	T	44	M	0.232	0.453	0	1.5E-09	0	101.888	tatgatctgcCttgttcaagc	m5C_10706
chr2	234197586	+	C	53	12	0.226	0	0	T	41	M	0.135	0.355	5.8E-15	8.8E-10	7.3E-15	22.9888	aagctgagacCtctgattgaa	m5C_10713
chr2	234288594	-	C	40	26	0.65	0	0	C	26	M	0.495	0.779	0	5.5E-06	0	257.429	taccgctggcCtgaaggcagt	m5C_10738
chr2	234417218	+	C	61	28	0.459	0	0	T	33	M	0.34	0.583	0	9.8E-09	0	190.471	tctatgttgtCtgagtgttgc	m5C_10709
chr2	234417228	+	C	44	14	0.318	0	0	T	30	M	0.2	0.466	0	1.9E-07	0	55.9976	ctgagtgttgCtcttttttgg	m5C_10724
chr2	237061809	-	C	204	65	0.319	0	0	T	139	M	0.259	0.385	0	0	0	336.108	atggggtggtCtgagtgcagt	m5C_10356
chr2	238503230	+	C	62	25	0.403	0	0	T	37	M	0.29	0.527	0	4.1E-09	0	145.123	aggatgtggcCtgagtgcccg	m5C_10618
chr2	238503237	+	C	36	9	0.25	0	0	T	27	M	0.138	0.411	2E-12	1.2E-06	2.4E-12	14.4765	ggcctgagtgCccgcccaggg	m5C_10612
chr2	238671633	+	C	36	10	0.278	0	0	T	26	M	0.158	0.44	4.8E-14	2E-06	5.9E-14	21.1115	gtgggagaggCagcagtgact	m5C_10615
chr2	238671642	+	C	31	10	0.323	0	0	T	21	M	0.186	0.499	7.1E-15	2E-06	9E-15	26.2725	gcagcagtgaCtcaggttgaa	m5C_10619
chr2	238671644	+	C	33	9	0.273	0	0	T	24	M	0.151	0.442	7.5E-13	1.2E-06	9E-13	16.4418	agcagtgactCaggttgaaga	m5C_10616
chr2	240315168	-	C	74	34	0.459	0	0	T	40	M	0.351	0.572	0	1.4E-09	0	238.498	cagtgatgagCcgggccaact	m5C_11014
chr2	240315179	-	C	231	55	0.238	0	0	T	176	M	0.188	0.297	0	0	0	206.51	agtgcgtgagCcagtgatgag	m5C_11013
chr2	242179157	-	C	39	9	0.231	0	0	T	30	M	0.126	0.383	4.9E-12	1.2E-06	5.7E-12	12.8739	ccaggtcttgCgggagattgc	m5C_10915
chr2	242202179	-	C	32	18	0.562	0	0	C	18	M	0.393	0.718	0	2.7E-05	0	141.571	gtcaggaaagCtggatgctgt	m5C_10912
chr20	397573	+	C	86	31	0.36	0	0	T	55	M	0.267	0.466	0	1.7E-11	0	165.52	gttgatactgCagtgagccaa	m5C_62850
chr20	397591	+	C	89	62	0.697	0	0	C	62	M	0.595	0.782	0	1.3E-08	0	737.337	caaggtagcaCtactgcactc	m5C_62852
chr20	397594	+	C	66	13	0.2	1	0.015	T	52	M	0.121	0.313	3.8E-15	1.6E-11	4.8E-15	22.646	ggtagcactaCtgcactccag	m5C_62851
chr20	397611	+	C	44	12	0.273	0	0	T	32	M	0.163	0.418	4.4E-16	7.5E-08	5.8E-16	30.1147	ccagcctgggCcacagaaaga	m5C_62859
chr20	397626	+	C	42	13	0.31	0	0	T	29	M	0.191	0.46	0	1.2E-07	0	49.5844	gaaagagagcCtgtgtcttaa	m5C_62853
chr20	397632	+	C	42	12	0.286	0	0	T	30	M	0.172	0.436	2.2E-16	7.5E-08	2.9E-16	32.2467	gagcctgtgtCttaaaattaa	m5C_62840
chr20	1542931	-	C	55	16	0.291	0	0	T	39	M	0.188	0.421	0	7.3E-09	0	60.0673	gatcacttggCttgtgtgctg	m5C_62879
chr20	2443618	-	C	233	61	0.262	0	0	T	172	M	0.21	0.322	0	0	0	255.644	agcaggattaCtctgagatcc	m5C_66030
chr20	2443657	-	C	298	98	0.329	0	0	T	200	M	0.278	0.384	0	0	0	544.865	ataaccttgaCtgaagctgat	m5C_66032
chr20	2443662	-	C	290	62	0.214	0	0	T	228	M	0.171	0.265	0	0	0	211.43	gagatataacCttgactgaag	m5C_66046
chr20	2446453	-	C	32	9	0.281	0	0	T	23	M	0.156	0.454	5.3E-13	1.2E-06	6.4E-13	17.1983	caaagaactcCaaacaagcag	m5C_66065
chr20	2633437	+	C	32	8	0.25	0	0	T	24	M	0.133	0.421	2.3E-11	6.8E-07	2.6E-11	11.2828	ggaacgggttCcggcagacgc	m5C_65962
chr20	2633438	+	C	32	8	0.25	0	0	T	24	M	0.133	0.421	2.3E-11	6.8E-07	2.6E-11	11.2828	gaacgggttcCggcagacgct	m5C_65978
chr20	2634884	+	C	825	169	0.205	0	0	T	656	M	0.179	0.234	0	0	0	604.026	cgaatcaaatCtgtcaatccc	m5C_66007
chr20	2634892	+	C	825	212	0.257	0	0	T	613	M	0.228	0.288	0	0	0	968.104	atctgtcaatCccctgagtgc	m5C_66009
chr20	2634893	+	C	824	172	0.209	0	0	T	652	M	0.182	0.238	0	0	0	627.358	tctgtcaatcCcctgagtgca	m5C_65990
chr20	2634895	+	C	791	205	0.259	1	0.001	T	585	M	0.23	0.291	0	0	0	943.579	tgtcaatcccCtgagtgcaat	m5C_65993
chr20	2634915	+	C	683	175	0.256	0	0	T	508	M	0.225	0.29	0	0	0	787.183	tcactgatgtCtccatgtctc	m5C_65986
chr20	2635181	+	C	40	9	0.225	0	0	T	31	M	0.123	0.375	6.4E-12	1.2E-08	7.6E-12	12.4041	agttagggtaCaactgccaga	m5C_65999
chr20	2635184	+	C	46	10	0.217	0	0	T	36	M	0.123	0.356	9.7E-13	2.4E-08	1.2E-12	14.7309	tagggtacaaCtgccagactg	m5C_66011
chr20	2635188	+	C	45	15	0.333	0	0	T	30	M	0.214	0.479	0	2.8E-07	0	64.0761	gtacaactgcCagactggagg	m5C_66004
chr20	2635719	+	C	68	17	0.25	0	0	T	51	M	0.162	0.364	0	1.8E-10	0	55.1986	tgtaggcctcCtggtgcttac	m5C_65964
chr20	2635725	+	C	71	25	0.352	0	0	T	46	M	0.251	0.468	0	9.3E-11	0	125.613	cctcctggtgCttaccacagg	m5C_65969
chr20	2635736	+	C	73	27	0.429	10	0.137	T	36	M	0.314	0.551	0	7.5E-09	0	169.542	ttaccacaggCtgtgttctta	m5C_66012
chr20	2635747	+	C	81	26	0.321	0	0	T	55	M	0.229	0.429	0	2.7E-12	0	119.299	tgtgttcttaCactgactgta	m5C_65998
chr20	2635753	+	C	90	22	0.244	0	0	T	68	M	0.167	0.342	0	5.8E-15	0	73.6237	cttacactgaCtgtatagaaa	m5C_66019
chr20	2635780	+	C	92	37	0.402	0	0	T	55	M	0.308	0.504	0	2.7E-12	0	227.808	gtagagtaaaCctaccccata	m5C_66008
chr20	2635786	+	C	64	34	0.531	0	0	C	34	M	0.411	0.648	0	4.2E-08	0	279.289	taaacctaccCcatatacacc	m5C_65966
chr20	2635796	+	C	50	26	0.52	0	0	C	26	M	0.385	0.652	0	5.5E-06	0	200.26	ccatatacacCtcagctcagg	m5C_66022
chr20	2635814	+	C	175	37	0.214	2	0.011	T	136	M	0.159	0.281	0	0	0	117.908	aggccctgtgCctggtctgta	m5C_66021
chr20	2635815	+	C	187	58	0.31	0	0	T	129	M	0.248	0.38	0	0	0	287.962	ggccctgtgcCtggtctgtat	m5C_65985
chr20	2635820	+	C	218	108	0.495	0	0	T	110	M	0.43	0.561	0	0	0	928.148	tgtgcctggtCtgtattgtga	m5C_65973
chr20	2636748	+	C	52	11	0.212	0	0	T	41	M	0.122	0.34	1.4E-13	4.4E-10	1.7E-13	17.3019	tgggggatcaCggtgatggct	m5C_65987
chr20	2636757	+	C	119	34	0.286	0	0	T	85	M	0.212	0.373	0	4.4E-16	0	144.328	acggtgatggCtgaccagggc	m5C_66002
chr20	2636767	+	C	128	27	0.211	0	0	T	101	M	0.149	0.29	0	0	0	80.5745	ctgaccagggCtccctgacct	m5C_65961
chr20	2636769	+	C	124	30	0.242	0	0	T	94	M	0.175	0.324	0	0	0	105.029	gaccagggctCcctgacctat	m5C_65958
chr20	2636771	+	C	125	37	0.296	0	0	T	88	M	0.223	0.381	0	0	0	165.038	ccagggctccCtgacctatac	m5C_65982
chr20	2636775	+	C	132	46	0.348	0	0	T	86	M	0.273	0.433	0	0	0	250.723	ggctccctgaCctatacaggc	m5C_65975
chr20	2636785	+	C	99	23	0.232	0	0	T	76	M	0.16	0.325	0	1E-14	0	73.6414	cctatacaggCctctgctatg	m5C_65971
chr20	2636786	+	C	99	29	0.296	1	0.01	T	69	M	0.215	0.393	0	1.7E-13	0	124.486	ctatacaggcCtctgctatgg	m5C_65994
chr20	2636791	+	C	98	33	0.337	0	0	T	65	M	0.251	0.435	0	7.5E-13	0	165.594	caggcctctgCtatgggggtg	m5C_65981
chr20	2636806	+	C	115	33	0.287	0	0	T	82	M	0.212	0.375	0	2.2E-16	0	140.068	ggggtgatggCcagtcctggt	m5C_65989
chr20	2636811	+	C	98	34	0.347	0	0	T	64	M	0.26	0.445	0	1.1E-12	0	176.857	gatggccagtCctggtgtctg	m5C_66015
chr20	2636812	+	C	99	22	0.222	0	0	T	77	M	0.152	0.314	0	5.8E-15	0	66.6952	atggccagtcCtggtgtctga	m5C_66001
chr20	2637613	+	C	2888	705	0.245	5	0.002	T	2178	M	0.229	0.261	0	0	0	3231.64	tgagcctgacCttgtagaatg	m5C_65972
chr20	2637636	+	C	2499	523	0.209	1	0	T	1975	M	0.194	0.226	0	0	0	2027.82	ggcaaaaaaaCtgatttaatg	m5C_65968
chr20	3064532	+	C	33	9	0.273	0	0	T	24	M	0.151	0.442	7.5E-13	1.2E-06	9E-13	16.4418	ttctctggcgCaatggatagc	m5C_66005
chr20	5326782	-	C	195	43	0.221	0	0	T	152	M	0.168	0.284	0	0	0	144.509	gatcacctggCtggtgtgtcg	m5C_62989
chr20	5326789	-	C	204	58	0.284	0	0	T	146	M	0.227	0.35	0	0	0	263.139	agtggtggatCacctggctgg	m5C_62990
chr20	5870785	+	C	36	24	0.667	0	0	C	24	M	0.503	0.798	0	9.1E-05	0	241.602	ggtgctgatcCtgtgatttcc	m5C_62970
chr20	5870796	+	C	43	17	0.395	0	0	T	26	M	0.264	0.544	0	5.6E-07	0	89.6414	tgtgatttccCcaaatgtggg	m5C_62950
chr20	5870797	+	C	46	17	0.37	0	0	T	29	M	0.245	0.514	0	5.6E-07	0	83.3801	gtgatttcccCaaatgtggga	m5C_62960
chr20	6195497	+	C	37	8	0.216	0	0	T	29	M	0.114	0.372	9.7E-11	6.8E-07	1.1E-10	9.12273	atggtgctgtCaaggctatgg	m5C_62929
chr20	7963007	-	C	38	14	0.378	1	0.026	T	23	M	0.241	0.539	0	9.2E-06	0	67.3786	gaagtagagcCtgaggaggct	m5C_62935
chr20	7963008	-	C	36	14	0.389	0	0	T	22	M	0.248	0.551	0	9.2E-06	0	69.397	ggaagtagagCctgaggaggc	m5C_62934
chr20	7963023	-	C	38	27	0.711	0	0	C	27	M	0.552	0.83	0	0.00015	0	298.31	cggtgtgaccCgggaggaagt	m5C_62941
chr20	7963024	-	C	37	11	0.297	0	0	T	26	M	0.175	0.458	1.4E-15	3.1E-06	1.9E-15	28.5508	acggtgtgacCcgggaggaag	m5C_62940

chr20	7963135	-	C	54	16	0.296	0	0	T	38	M	0.191	0.428	0	7.3E-09	0	61.2393	aagatcttggCgatgaggatg	m5C_62943
chr20	9984926	-	C	40	19	0.475	0	0	T	21	M	0.329	0.625	0	1E-06	0	125.154	tagtactctgCattgtgaaac	m5C_63111
chr20	9984929	-	C	60	28	0.467	0	0	T	32	M	0.346	0.591	0	3.1E-07	0	193.915	ggttagtactCtgcattgtga	m5C_63121
chr20	9984931	-	C	72	27	0.375	0	0	T	45	M	0.272	0.49	0	1.9E-10	0	146.983	gtggttagtaCtctgcattgt	m5C_63120
chr20	9984947	-	C	101	54	0.535	0	0	C	54	M	0.438	0.629	0	6.4E-12	0	472.927	cagctgtgatCgtatagtggt	m5C_63113
chr20	9984954	-	C	45	12	0.267	0	0	T	33	M	0.16	0.41	5.6E-16	7.5E-08	7.3E-16	29.2263	cttccttcagCtgtgatcgta	m5C_63110
chr20	11874835	-	C	57	15	0.263	0	0	T	42	M	0.166	0.39	0	4.6E-09	0	49.9369	gtcgaccctgCtggccttcct	m5C_63445
chr20	11874839	-	C	58	35	0.603	0	0	C	35	M	0.475	0.719	0	1.3E-06	0	332.451	tttggtcgacCctgctggcct	m5C_63447
chr20	13408757	-	C	50	29	0.58	0	0	C	29	M	0.442	0.706	0	3.9E-07	0	256.553	ttaagcaattCaggataggag	m5C_63074
chr20	16651310	-	C	258	63	0.244	0	0	T	195	M	0.196	0.3	0	0	0	246.668	tctccactccCactgcttcac	m5C_64647
chr20	16651317	-	C	251	66	0.263	0	0	T	185	M	0.212	0.321	0	0	0	280.305	tgttccttctCcactcccact	m5C_64610
chr20	16651322	-	C	255	55	0.216	0	0	T	200	M	0.17	0.27	0	0	0	186.584	ttctttgttcCttctccactc	m5C_64668
chr20	16651358	-	C	263	70	0.273	7	0.027	T	186	M	0.222	0.331	0	0	0	311.483	gtgtttacaaCtaattgatca	m5C_64664
chr20	16651361	-	C	264	57	0.216	0	0	T	207	M	0.171	0.269	0	0	0	194.415	gtggtgtttaCaactaattga	m5C_64652
chr20	17640938	-	C	62	15	0.242	0	0	T	47	M	0.152	0.362	0	6E-11	0	45.7373	aaaggaaagaCcaagaaaaag	m5C_64611
chr20	17943353	-	C	46	19	0.413	0	0	T	27	M	0.283	0.557	0	1E-06	0	107.496	aatctgatcaCttgctgcagc	m5C_64643
chr20	17943355	-	C	353	115	0.326	0	0	T	238	M	0.279	0.376	0	0	0	641.679	aaaatctgatCacttgctgca	m5C_64638
chr20	17943360	-	C	579	160	0.277	1	0.002	T	418	M	0.242	0.315	0	0	0	774.094	tggtgaaaatCtgatcacttg	m5C_64670
chr20	17943382	-	C	404	95	0.235	0	0	T	309	M	0.196	0.279	0	0	0	373.176	agacccatctCacaaagatga	m5C_64661
chr20	17943388	-	C	318	85	0.267	0	0	T	233	M	0.222	0.319	0	0	0	376.794	cctctgagacCcatctcacaa	m5C_64625
chr20	17943398	-	C	270	94	0.348	0	0	T	176	M	0.294	0.407	0	0	0	552.371	gggacggggcCctctgagacc	m5C_64660
chr20	17943404	-	C	272	91	0.337	2	0.007	T	179	M	0.283	0.395	0	0	0	515.586	tcagcagggaCggggccctct	m5C_64650
chr20	17943433	-	C	120	47	0.392	0	0	T	73	M	0.309	0.481	0	1E-15	0	290.451	tggcaggctaCcggtaagaat	m5C_64645
chr20	17943436	-	C	110	32	0.291	0	0	T	78	M	0.214	0.382	0	2.2E-16	0	137.11	tgttggcaggCtaccggtaag	m5C_64641
chr20	17943440	-	C	92	22	0.239	0	0	T	70	M	0.164	0.336	0	5.8E-15	0	71.9621	tgtgtgttggCaggctaccgg	m5C_64659
chr20	17943502	-	C	32	15	0.469	0	0	T	17	M	0.309	0.636	0	1.2E-05	0	92.6074	gaacttgaggCtatgtagcct	m5C_64613
chr20	17943516	-	C	53	24	0.453	0	0	T	29	M	0.327	0.585	0	1.2E-07	0	156.755	agttcctgttCtaggaacttg	m5C_64667
chr20	17943522	-	C	70	18	0.257	0	0	T	52	M	0.169	0.37	0	4E-12	0	60.9449	agcctcagttCctgttctagg	m5C_64623
chr20	17943538	-	C	165	33	0.2	0	0	T	132	M	0.146	0.268	0	0	0	96.4293	cactgaccttCttccaagcct	m5C_64618
chr20	17943542	-	C	221	61	0.276	0	0	T	160	M	0.221	0.338	0	0	0	269.971	gctgcactgaCcttcttccaa	m5C_64619
chr20	17943546	-	C	267	74	0.277	0	0	T	193	M	0.227	0.334	0	0	0	335.833	gtgcgctgcaCtgaccttctt	m5C_64646
chr20	17943551	-	C	306	72	0.235	0	0	T	234	M	0.191	0.286	0	0	0	275.371	gctgagtgcgCtgcactgacc	m5C_64635
chr20	17943562	-	C	334	123	0.368	0	0	T	211	M	0.318	0.421	0	0	0	783.033	tttatccattCgctgagtgcg	m5C_64651
chr20	17943567	-	C	346	70	0.202	0	0	T	276	M	0.163	0.248	0	0	0	228.701	ttcagtttatCcattcgctga	m5C_64665
chr20	18246389	-	C	34	9	0.265	0	0	T	25	M	0.146	0.431	1.1E-12	1.2E-06	1.3E-12	15.7394	ggagcttgctCcatccactcc	m5C_63419
chr20	18246391	-	C	71	25	0.352	0	0	T	46	M	0.251	0.468	0	9.3E-11	0	125.613	taggagcttgCtccatccact	m5C_63420
chr20	18246395	-	C	141	34	0.241	0	0	T	107	M	0.178	0.318	0	0	0	121.038	ggaataggagCttgctccatc	m5C_63411
chr20	18246414	-	C	82	26	0.317	0	0	T	56	M	0.226	0.424	0	2.7E-12	0	117.769	attttttgagCagggagatgg	m5C_63418
chr20	22758609	-	C	75	20	0.267	0	0	T	55	M	0.18	0.376	0	1.1E-11	0	71.9047	ctgtaccaccCtgaatgctcc	m5C_64203
chr20	22758614	-	C	67	22	0.328	0	0	T	45	M	0.228	0.447	0	1.5E-09	0	100.281	tatggctgtaCcaccctgaat	m5C_64201
chr20	22758619	-	C	72	19	0.264	0	0	T	53	M	0.176	0.376	0	6.9E-12	0	66.8558	tggcctatggCtgtaccaccc	m5C_64182
chr20	23286065	+	C	53	12	0.226	0	0	T	41	M	0.135	0.355	5.8E-15	8.8E-10	7.3E-15	22.9888	cctctgggaaCggtgcacaca	m5C_64170
chr20	23286072	+	C	52	28	0.538	0	0	C	28	M	0.405	0.667	0	3.1E-07	0	226.819	gaacggtgcaCacagcactgt	m5C_64156
chr20	23345941	+	C	40	8	0.2	0	0	T	32	M	0.105	0.348	2.1E-10	5.5E-09	2.3E-10	8.13503	aggaggaggaCgaagaagggg	m5C_64155
chr20	23436442	+	C	35	18	0.514	0	0	C	18	M	0.356	0.67	0	2.7E-05	0	128.047	cttggttgatCtcgtcagtgg	m5C_64149
chr20	24229713	+	C	38	9	0.237	0	0	T	29	M	0.13	0.392	3.7E-12	1.2E-06	4.3E-12	13.3739	ctgcccagccCagcaggaaca	m5C_64027
chr20	24565092	-	C	32	8	0.25	0	0	T	24	M	0.133	0.421	2.3E-11	6.8E-07	2.6E-11	11.2828	cagaactggcCcccgttgccac	m5C_64050
chr20	24565097	-	C	31	14	0.452	0	0	T	17	M	0.292	0.622	0	9.2E-06	0	81.6522	acctgcagaaCtggccccgtt	m5C_64030
chr20	26188853	-	C	33	8	0.242	0	0	T	25	M	0.128	0.41	3.1E-11	6.8E-07	3.6E-11	10.7866	ggtgggatccCgcggccgtgt	m5C_63730
chr20	26188855	-	C	32	16	0.5	0	0	CT	16	M	0.336	0.664	0	1.7E-05	0	107.618	gcggtgggatCccgcggccgt	m5C_63712
chr20	26188870	-	C	37	17	0.459	0	0	T	20	M	0.31	0.616	0	2.1E-05	0	105.53	gccctcgtgtCtgtggcggtg	m5C_63753
chr20	26188875	-	C	34	13	0.382	0	0	T	21	M	0.239	0.55	0	6.7E-06	0	62.1399	ggaccgccctCgtgtctgtgg	m5C_63714
chr20	26189012	-	C	36	13	0.361	0	0	T	23	M	0.225	0.524	0	6.7E-06	0	58.436	gaatcacgagCgatgtggccc	m5C_63744
chr20	26189016	-	C	54	18	0.333	0	0	T	36	M	0.222	0.466	0	1.7E-08	0	80.0685	acgagaatcaCgagcgatgtg	m5C_63723
chr20	26189025	-	C	98	45	0.459	0	0	T	53	M	0.364	0.558	0	2.3E-11	0	327.533	ggaggatggaCgagaatcacg	m5C_63763
chr20	26189036	-	C	68	33	0.485	0	0	T	35	M	0.371	0.602	0	3.4E-08	0	244.534	gggaccgggtCggaggatgga	m5C_63715
chr20	26189041	-	C	52	23	0.442	0	0	T	29	M	0.316	0.577	0	8.7E-08	0	145.336	ggagcgggacCgggtcggagg	m5C_63709
chr20	26189042	-	C	51	11	0.216	0	0	T	40	M	0.125	0.346	1.1E-13	4.4E-10	1.4E-13	17.8061	cggagcgggaCcgggtcggag	m5C_63710
chr20	26189047	-	C	73	23	0.315	0	0	T	50	M	0.22	0.429	0	4.3E-11	0	101.216	tctcccggagCgggaccgggt	m5C_63722
chr20	26189053	-	C	81	40	0.494	0	0	T	41	M	0.388	0.6	0	2.1E-10	0	310.175	cgccggtctcCcggagcggga	m5C_63737
chr20	26189054	-	C	82	26	0.317	0	0	T	56	M	0.226	0.424	0	2.7E-12	0	117.769	gcgccggtctCccggagcggg	m5C_63713
chr20	26189060	-	C	94	25	0.266	0	0	T	69	M	0.187	0.363	0	2.9E-14	0	93.5574	gggggcgcgcCggtctcccgg	m5C_63717
chr20	26189065	-	C	77	35	0.467	2	0.026	T	40	M	0.358	0.578	0	1.9E-09	0	250.709	acggtgggggCgcgccggtct	m5C_63747
chr20	26189322	-	C	46	21	0.457	0	0	T	25	M	0.322	0.598	0	1.8E-06	0	135.049	cgcggctcgtCgcctactgtg	m5C_63738
chr20	26189325	-	C	50	17	0.34	0	0	T	33	M	0.224	0.478	0	5.6E-07	0	76.285	gagcgcggctCgtcgcctact	m5C_63716
chr20	26189327	-	C	65	34	0.523	0	0	C	34	M	0.404	0.64	0	4.2E-08	0	274.58	ccgagcgcggCtcgtcgccta	m5C_63741
chr20	26189336	-	C	64	39	0.609	0	0	C	39	M	0.487	0.719	0	1.2E-07	0	379.795	cggtggtggcCgagcgcggct	m5C_63745
chr20	26189346	-	C	57	19	0.333	0	0	T	38	M	0.225	0.463	0	2.4E-08	0	85.4758	ccggccaccgCggtggtggcc	m5C_63757
chr20	26189349	-	C	51	20	0.392	0	0	T	31	M	0.27	0.529	0	3.4E-08	0	108.109	ccgccggccaCcgcggtggtg	m5C_63718
chr20	26189351	-	C	47	10	0.213	0	0	T	37	M	0.12	0.349	1.3E-12	2.4E-08	1.5E-12	14.271	ttccgccggcCaccgcggtgg	m5C_63725
chr20	26189355	-	C	31	11	0.355	0	0	T	20	M	0.211	0.531	1.1E-16	3.1E-06	1.5E-16	37.0595	agggttccgcCggccaccgcg	m5C_63751
chr20	26189803	-	C	41	24	0.585	0	0	C	24	M	0.434	0.722	0	3.6E-06	0	208.161	tccgaccggtCgtgtgtgggt	m5C_63762
chr20	26189989	-	C	103	50	0.485	0	0	T	53	M	0.391	0.581	0	2.6E-12	0	391.189	gggttcgaggCggttgagtga	m5C_63743
chr20	26189994	-	C	113	37	0.327	0	0	T	76	M	0.248	0.418	0	1.4E-15	0	183.392	gtgtgggggttCgaggcggttg	m5C_63721
chr20	26190006	-	C	144	80	0.556	0	0	C	80	M	0.474	0.634	0	3.6E-15	0	758.399	gggagctcgtCggtgtggggt	m5C_63750
chr20	26190009	-	C	154	61	0.396	0	0	T	93	M	0.322	0.475	0	0	0	393.193	tgagggagctCgtcggtgtgg	m5C_63764
chr20	26190011	-	C	143	59	0.413	0	0	T	84	M	0.335	0.495	0	0	0	395.553	cctgagggagCtcgtcggtgt	m5C_63734
chr20	26190020	-	C	110	32	0.291	0	0	T	78	M	0.214	0.382	0	2.2E-16	0	137.11	cgcgtgggtcCtgagggagct	m5C_63755
chr20	26190021	-	C	104	43	0.413	0	0	T	61	M	0.324	0.51	0	4.3E-13	0	278.251	gcgcgtgggtCctgagggagc	m5C_63727
chr20	26190028	-	C	73	21	0.288	0	0	T	52	M	0.197	0.4	0	1.8E-11	0	82.5367	ggtgtccgcgCgtgggtcctg	m5C_63728
chr20	26190032	-	C	45	10	0.222	0	0	T	35	M	0.125	0.363	7.4E-13	2.4E-08	8.9E-13	15.2157	cctcggtgtcCgcgcgtgggt	m5C_63719
chr20	26190033	-	C	34	9	0.265	0	0	T	25	M	0.146	0.431	1.1E-12	1.2E-06	1.3E-12	15.7394	gcctcggtgtCcgcgcgtggg	m5C_63720
chr20	26190456	-	C	116	46	0.397	0	0	T	70	M	0.312	0.488	0	2.8E-14	0	287.244	gaggggcggaCattcgtggcg	m5C_63729
chr20	26190460	-	C	204	56	0.275	0	0	T	148	M	0.218	0.339	0	0	0	244.007	gggcgaggggCggacattcgt	m5C_63761
chr20	26190467	-	C	314	136	0.433	0	0	T	178	M	0.379	0.488	0	0	0	1032.1	ccgcgacgggCgaggggcgga	m5C_63735
chr20	26190471	-	C	316	92	0.291	0	0	T	224	M	0.244	0.343	0	0	0	448.587	cgagccgcgaCgggcgagggg	m5C_63759
chr20	30386271	+	C	32	10	0.312	0	0	T	22	M	0.18	0.486	1.1E-14	2E-06	1.3E-14	25.0836	aggtagaagcCcagaaagccc	m5C_64512
chr20	30386297	+	C	45	20	0.444	0	0	T	25	M	0.309	0.588	0	1.4E-06	0	123.755	ttggaggaggCcagactacag	m5C_64502
chr20	30386298	+	C	39	16	0.41	0	0	T	23	M	0.271	0.566	0	1.7E-05	0	86.6531	tggaggaggcCagactacagg	m5C_64521
chr20	30516948	+	C	36	16	0.444	0	0	T	20	M	0.295	0.604	0	1.7E-05	0	94.5313	atcctccccacCttgatcttcc	m5C_64471
chr20	31032431	-	C	36	12	0.333	0	0	T	24	M	0.202	0.497	0	4.6E-06	0	48.5146	agtggtggatCatcaggtaga	m5C_64534
chr20	31825602	-	C	34	17	0.5	0	0	CT	17	M	0.341	0.659	0	2.1E-05	0	115.835	agggtctggtCcaggggcacg	m5C_64547
chr20	32664995	+	C	33	12	0.364	0	0	T	21	M	0.222	0.534	0	4.6E-06	0	53.2487	gggggaagcaCggacccgaga	m5C_64094
chr20	32664999	+	C	35	14	0.4	0	0	T	21	M	0.256	0.564	0	9.2E-06	0	71.5414	gaagcacggaCccgagacgac	m5C_64055
chr20	32665000	+	C	32	12	0.375	0	0	T	20	M	0.229	0.547	0	4.6E-06	0	55.0408	aagcacggacCcgagacgacg	m5C_64068
chr20	32665006	+	C	33	23	0.697	0	0	C	23	M	0.527	0.826	0	7.6E-05	0	242.243	ggacccgagaCgacggcgatg	m5C_64091
chr20	32665025	+	C	61	40	0.656	0	0	C	40	M	0.53	0.763	0	1.4E-07	0	424.396	tgaggaagggCtcctgacaca	m5C_64061
chr20	32665032	+	C	59	19	0.322	0	0	T	40	M	0.217	0.449	0	2.4E-08	0	82.4122	gggctcctgaCacacagcgag	m5C_64086
chr20	32665036	+	C	54	22	0.407	0	0	T	32	M	0.287	0.54	0	6.5E-08	0	126.183	tcctgacacaCagcgaggaag	m5C_64057
chr20	34076144	-	C	41	10	0.244	0	0	T	31	M	0.138	0.393	2.4E-13	2.4E-08	2.9E-13	17.4472	gggtatagctCagtggcagag	m5C_63967
chr20	34076146	-	C	40	10	0.25	0	0	T	30	M	0.142	0.402	1.8E-13	2.4E-08	2.2E-13	18.0914	gcgggtatagCtcagtggcag	m5C_63966

chr20	34312998	-	C	68	24	0.353	0	0	T	44	M	0.25	0.472	0	3E-09	0	120.009	aagaattcgaCcaagggattt	m5C_63971
chr20	34313009	-	C	63	21	0.333	0	0	T	42	M	0.229	0.456	0	1E-09	0	96.3877	cagctggcggCaagaattcga	m5C_63986
chr20	34313012	-	C	62	14	0.226	0	0	T	48	M	0.14	0.344	0	3.2E-11	0	39.0742	atgcagctggCggcaagaatt	m5C_63993
chr20	34319979	-	C	52	20	0.385	0	0	T	32	M	0.265	0.52	0	3.4E-08	0	105.881	gaagtagagaCagagaaagga	m5C_63969
chr20	34319990	-	C	74	19	0.257	0	0	T	55	M	0.171	0.367	0	6.9E-12	0	64.9708	ggaacggaagCgaagtagaga	m5C_64007
chr20	34319996	-	C	66	23	0.348	0	0	T	43	M	0.245	0.469	0	2.1E-09	0	112.588	aagtaaggaaCggaagcgaag	m5C_63995
chr20	34441259	-	C	32	8	0.25	0	0	T	24	M	0.133	0.421	2.3E-11	6.8E-07	2.6E-11	11.2828	tgagttatctCaattgatggt	m5C_63994
chr20	34441279	-	C	31	11	0.355	0	0	T	20	M	0.211	0.531	1.1E-16	3.1E-06	1.5E-16	37.0595	tcaggctggtCtgaaagtagt	m5C_64004
chr20	35500277	-	C	43	10	0.233	0	0	T	33	M	0.132	0.377	4.3E-13	2.4E-08	5.2E-13	16.2683	tcaaacatggCtgttgcaatg	m5C_63992
chr20	37053867	-	C	48	18	0.375	0	0	T	30	M	0.252	0.516	0	7.7E-07	0	90.7743	ctatcccttcCaaactctgga	m5C_67931
chr20	37053871	-	C	47	10	0.213	0	0	T	37	M	0.12	0.349	1.3E-12	2.4E-08	1.5E-12	14.271	actcctatccCttccaaactc	m5C_67962
chr20	37053872	-	C	45	12	0.267	0	0	T	33	M	0.16	0.41	5.6E-16	7.5E-08	7.3E-16	29.2263	cactcctatcCcttccaaact	m5C_67908
chr20	37053883	-	C	86	33	0.384	0	0	T	53	M	0.288	0.489	0	3.1E-11	0	190.088	tttgaagggtCcactcctatc	m5C_67913
chr20	37053898	-	C	93	19	0.204	0	0	T	74	M	0.135	0.297	0	8.9E-16	0	51.2526	aagcgcttccCcgtgtttgaa	m5C_67911
chr20	37053905	-	C	130	36	0.277	0	0	T	94	M	0.207	0.359	0	0	0	149.216	tcaatgaaagCgcttccccgt	m5C_67951
chr20	37053914	-	C	199	42	0.211	0	0	T	157	M	0.16	0.273	0	0	0	134.488	ggagaggaatCaatgaaagcg	m5C_67961
chr20	37053943	-	C	668	149	0.223	1	0.001	T	518	M	0.193	0.257	0	0	0	576.371	tgcctttgccCtggtcattga	m5C_67921
chr20	37053954	-	C	671	137	0.205	4	0.006	T	530	M	0.176	0.238	0	0	0	483.517	tgatcgtgggCtgcctttgcc	m5C_67968
chr20	37055957	-	C	129	31	0.24	0	0	T	98	M	0.175	0.321	0	0	0	108.359	ctgaagctttCacacaactcc	m5C_67929
chr20	37055967	-	C	142	31	0.22	1	0.007	T	110	M	0.159	0.295	0	0	0	98.8482	catcccgaaccCtgaagctttc	m5C_67938
chr20	37055969	-	C	137	32	0.235	1	0.007	T	104	M	0.172	0.313	0	0	0	110.006	cccatccgaaCcctgaagctt	m5C_67937
chr20	37055974	-	C	118	32	0.271	0	0	T	86	M	0.199	0.358	0	2.2E-16	0	127.444	cctatcccatCcgaaccctga	m5C_67909
chr20	37055977	-	C	134	35	0.261	0	0	T	99	M	0.194	0.341	0	0	0	135.949	caccctatccCatccgaaccc	m5C_67912
chr20	37055983	-	C	135	30	0.222	0	0	T	105	M	0.16	0.299	0	0	0	96.1969	agggtccaccCtatcccatcc	m5C_67958
chr20	37055987	-	C	225	58	0.258	0	0	T	167	M	0.205	0.319	0	0	0	237.829	ttggagggtcCaccctatccc	m5C_67959
chr20	37056002	-	C	264	55	0.208	0	0	T	209	M	0.164	0.261	0	0	0	180.07	aggttgtcccCgtgtttggag	m5C_67960
chr20	37056005	-	C	282	101	0.359	1	0.004	T	180	M	0.306	0.417	0	0	0	617.251	aagaggttgtCcccgtgtttg	m5C_67916
chr20	37056019	-	C	320	72	0.225	0	0	T	248	M	0.183	0.274	0	0	0	263.036	gagaggaacgCtgaaagaggt	m5C_67900
chr20	37056051	-	C	571	137	0.24	0	0	T	434	M	0.207	0.277	0	0	0	566.398	gctgcctgtgCctaggtcatt	m5C_67974
chr20	37056056	-	C	562	160	0.285	0	0	T	402	M	0.249	0.323	0	0	0	796.632	cataggctgcCtgtgcctagg	m5C_67975
chr20	37058382	-	C	51	12	0.235	0	0	T	39	M	0.14	0.368	3.3E-15	8.8E-10	4.3E-15	24.3305	gggagaggaaCtgccgagagc	m5C_67954
chr20	37058411	-	C	162	48	0.304	4	0.025	T	110	M	0.237	0.379	0	0	0	227.949	ctgcccgtgcCctggtcattg	m5C_67924
chr20	37058417	-	C	159	61	0.384	0	0	T	98	M	0.312	0.461	0	0	0	380.217	catgagctgcCcgtgccctgg	m5C_67905
chr20	37058421	-	C	167	32	0.212	16	0.096	T	119	M	0.154	0.284	0	0	0	98.7534	tgatcatgagCtgcccgtgcc	m5C_67897
chr20	37060009	-	C	106	25	0.236	0	0	T	81	M	0.165	0.325	0	4.4E-16	0	82.5797	ctgcttgtgtCttggttattg	m5C_67941
chr20	37060019	-	C	171	82	0.482	1	0.006	T	88	M	0.408	0.557	0	0	0	669.876	tgattacaggCtgcttgtgtc	m5C_67891
chr20	37062511	-	C	38	14	0.368	0	0	T	24	M	0.234	0.527	0	9.2E-06	0	65.4752	ggagctttctCacacctcctt	m5C_67901
chr20	37062523	-	C	54	14	0.259	0	0	T	40	M	0.161	0.389	0	2.8E-09	0	45.1318	ctttcaaactCtggagctttc	m5C_67950
chr20	37062529	-	C	61	15	0.246	0	0	T	46	M	0.155	0.367	0	6E-11	0	46.5196	ctgtcccttttCaaactctgga	m5C_67903
chr20	37062533	-	C	59	22	0.373	0	0	T	37	M	0.261	0.5	0	6.5E-08	0	114.769	gctcctgtccCtttcaaactc	m5C_67949
chr20	37062539	-	C	59	25	0.424	0	0	T	34	M	0.306	0.551	0	1.5E-07	0	153.063	gggtccgctcCtgtccctttc	m5C_67943
chr20	37062540	-	C	59	13	0.22	0	0	T	46	M	0.134	0.341	8.9E-16	1.6E-09	1.2E-15	26.1299	agggtccgctCctgtcccttt	m5C_67895
chr20	37062545	-	C	93	29	0.312	0	0	T	64	M	0.227	0.412	0	1.7E-13	0	131.502	tttggagggtCcgctcctgtc	m5C_67942
chr20	37062559	-	C	103	24	0.233	0	0	T	79	M	0.162	0.323	0	2.2E-16	0	77.7	agtgcttcccCgtgtttggag	m5C_67899
chr20	37062574	-	C	163	38	0.233	0	0	T	125	M	0.175	0.304	0	0	0	132.863	aaagaaatcgCggaaagtgct	m5C_67966
chr20	37062605	-	C	1021	248	0.243	0	0	T	773	M	0.218	0.27	0	0	0	1079.25	tgcctgtgccCtggtcattga	m5C_67945
chr20	37062607	-	C	1006	205	0.204	0	0	T	801	M	0.18	0.23	0	0	0	738.141	gctgcctgtgCcctggtcatt	m5C_67892
chr20	37062616	-	C	1023	208	0.203	0	0	T	815	M	0.18	0.229	0	0	0	747.932	tgatcgtgggCtgcctgtgcc	m5C_67964
chr20	37076729	+	C	56	31	0.554	0	0	C	31	M	0.424	0.676	0	5.9E-07	0	262.961	ttttctgcgcCtgcattcgta	m5C_67865
chr20	37076752	+	C	109	27	0.248	0	0	T	82	M	0.176	0.336	0	1.1E-15	0	95.1394	tgatcacgggCtgcccgtgtc	m5C_67864
chr20	37076755	+	C	107	30	0.28	0	0	T	77	M	0.204	0.372	0	4.7E-15	0	122.41	tcacgggctgCccgtgtcctg	m5C_67830
chr20	37076756	+	C	110	23	0.209	0	0	T	87	M	0.144	0.294	0	0	0	66.0388	cacgggctgcCcgtgtcctgg	m5C_67884
chr20	37076757	+	C	102	33	0.324	0	0	T	69	M	0.241	0.419	0	1.6E-14	0	158.781	acgggctgccCgtgtcctggt	m5C_67837
chr20	37076762	+	C	118	26	0.22	0	0	T	92	M	0.155	0.303	0	0	0	80.6176	ctgcccgtgtCctggtcattg	m5C_67883
chr20	37076763	+	C	110	27	0.245	0	0	T	83	M	0.175	0.334	0	0	0	94.2425	tgcccgtgtcCtggtcattgg	m5C_67863
chr20	37076768	+	C	99	30	0.303	0	0	T	69	M	0.221	0.4	0	2.5E-13	0	132.757	gtgtcctggtCattggtagtg	m5C_67876
chr20	37076783	+	C	50	18	0.36	0	0	T	32	M	0.241	0.499	0	1.7E-08	0	86.8988	gtagtgcaggCagaggaaatg	m5C_67834
chr20	37076806	+	C	33	14	0.424	0	0	T	19	M	0.272	0.592	0	9.2E-06	0	76.259	ggaaaggttgCtgtgtttgga	m5C_67827
chr20	37078018	+	C	89	32	0.36	0	0	T	57	M	0.268	0.463	0	2.3E-11	0	171.249	tatacacctgCattcaaaaat	m5C_67833
chr20	37078038	+	C	92	29	0.315	0	0	T	63	M	0.229	0.416	0	1.7E-13	0	133.002	tgatcacgggCtgcctgtgct	m5C_67858
chr20	37078042	+	C	82	30	0.366	0	0	T	52	M	0.27	0.474	0	1.2E-11	0	161.871	cacgggctgcCtgtgctctgg	m5C_67843
chr20	37078047	+	C	87	31	0.356	0	0	T	56	M	0.264	0.461	0	1.7E-11	0	163.51	gctgcctgtgCtctggtcatc	m5C_67823
chr20	37078049	+	C	90	20	0.222	0	0	T	70	M	0.149	0.318	0	1.7E-15	0	59.4849	tgcctgtgctCtggtcatcaa	m5C_67838
chr20	37078054	+	C	88	19	0.216	0	0	T	69	M	0.143	0.313	0	8.4E-14	0	54.2667	gtgctctggtCatcaataacg	m5C_67882
chr20	37078057	+	C	88	32	0.364	0	0	T	56	M	0.271	0.468	0	2.3E-11	0	173.307	ctctggtcatCaataacgcag	m5C_67832
chr20	39482849	+	C	38	9	0.237	0	0	T	29	M	0.13	0.392	3.7E-12	1.2E-06	4.3E-12	13.3739	caggtgtgttCagggcggtat	m5C_64429
chr20	39482862	+	C	38	12	0.316	0	0	T	26	M	0.191	0.475	0	4.6E-06	0	45.8026	ggcggtatggCtgtaggcttc	m5C_64445
chr20	39482869	+	C	36	16	0.444	0	0	T	20	M	0.295	0.604	0	1.7E-05	0	94.5313	tggctgtaggCttctcataca	m5C_64452
chr20	39652884	+	C	121	49	0.405	0	0	T	72	M	0.322	0.494	0	1.9E-15	0	315.289	tgtgcagtatCtgttcttacc	m5C_64428
chr20	39652889	+	C	110	49	0.445	0	0	T	61	M	0.356	0.539	0	6.6E-14	0	348.856	agtatctgttCttaccagttt	m5C_64444
chr20	39652894	+	C	102	29	0.284	0	0	T	73	M	0.206	0.378	0	2.9E-15	0	119.392	ctgttcttacCagtttaatat	m5C_64430
chr20	39652905	+	C	67	44	0.657	0	0	C	44	M	0.537	0.759	0	2.8E-07	0	472.845	agtttaatatCtgatacatcc	m5C_64450
chr20	39652972	+	C	60	12	0.2	0	0	T	48	M	0.118	0.318	3.3E-14	7.6E-12	4.2E-14	19.13	ggaataggagCttgctctgtc	m5C_64427
chr20	39652976	+	C	48	10	0.208	0	0	T	38	M	0.117	0.343	1.6E-12	2.4E-08	1.9E-12	13.8343	taggagcttgCtctgtccact	m5C_64441
chr20	39652978	+	C	50	12	0.24	0	0	T	38	M	0.143	0.374	2.4E-15	8.8E-10	3.1E-15	25.0697	ggagcttgctCtgtccactct	m5C_64443
chr20	39652982	+	C	46	16	0.348	0	0	T	30	M	0.227	0.492	0	4E-07	0	72.5786	cttgctctgtCcactctacac	m5C_64425
chr20	39652983	+	C	40	10	0.25	0	0	T	30	M	0.142	0.402	1.8E-13	2.4E-08	2.2E-13	18.0914	ttgctctgtcCactctacaca	m5C_64426
chr20	39652985	+	C	32	15	0.469	0	0	T	17	M	0.309	0.636	0	1.2E-05	0	92.6074	gctctgtccaCtctacacatt	m5C_64442
chr20	39652999	+	C	38	10	0.263	0	0	T	28	M	0.15	0.42	9.4E-14	2E-06	1.2E-13	19.5037	acacattggcCtggattgcag	m5C_64433
chr20	39653013	+	C	565	121	0.214	0	0	T	444	M	0.182	0.25	0	0	0	441.218	attgcagtacCtccaggaatg	m5C_64449
chr20	39653029	+	C	496	116	0.234	0	0	T	380	M	0.199	0.273	0	0	0	461.102	gaatggtgcaCcctctctacc	m5C_64439
chr20	39653031	+	C	97	31	0.32	0	0	T	66	M	0.235	0.418	0	3.7E-13	0	145.815	atggtgcaccCtctctaccac	m5C_64447
chr20	43129141	-	C	62	15	0.242	0	0	T	47	M	0.152	0.362	0	6E-11	0	45.7373	ggctgggggCtgcaggaggc	m5C_64329
chr20	43129149	-	C	71	28	0.394	0	0	T	43	M	0.289	0.511	0	2.6E-10	0	161.802	ttggccttggCtggggggctg	m5C_64313
chr20	43129154	-	C	72	28	0.389	0	0	T	44	M	0.285	0.504	0	2.6E-10	0	159.407	atttcttggcCttggctgggg	m5C_64326
chr20	43514434	+	C	31	15	0.484	0	0	T	16	M	0.32	0.652	0	1.2E-05	0	95.91	gcaggcaccgCtgccgccgcc	m5C_64258
chr20	43514441	+	C	36	10	0.278	0	0	T	26	M	0.158	0.44	4.8E-14	2E-06	5.9E-14	21.1115	ccgctgccgcCgcctgagtag	m5C_64290
chr20	43514443	+	C	36	11	0.306	0	0	T	25	M	0.18	0.469	1E-15	3.1E-06	1.3E-15	29.7077	gctgccgccgCctgagtagtg	m5C_64281
chr20	43514444	+	C	33	11	0.333	0	0	T	22	M	0.198	0.504	2.2E-16	3.1E-06	2.9E-16	34.0074	ctgccgccgcCtgagtagtgg	m5C_64272
chr20	43530409	+	C	117	25	0.214	0	0	T	92	M	0.149	0.296	0	0	0	74.5642	gaagaagcagCagatgggcaa	m5C_64252
chr20	43632699	+	C	99	29	0.293	0	0	T	70	M	0.212	0.389	0	1.7E-13	0	123.172	gttggttagtCcgagtgcagt	m5C_64288
chr20	43632736	+	C	32	12	0.375	0	0	T	20	M	0.229	0.547	0	4.6E-06	0	55.0408	tgatcacaacCagttttttct	m5C_64297
chr20	43951005	+	C	40	11	0.275	0	0	T	29	M	0.161	0.428	4.3E-15	3.1E-06	5.5E-15	25.4502	tgcctggcttCtgtaatgtag	m5C_64263
chr20	43951023	+	C	57	20	0.351	0	0	T	37	M	0.24	0.481	0	3.4E-08	0	96.001	tagtggttatCacattcgcct	m5C_64285
chr20	47895494	+	C	469	110	0.235	0	0	T	359	M	0.198	0.275	0	0	0	436.581	taaatgatgaCttcacttttt	m5C_64698
chr20	47895506	+	C	471	155	0.33	1	0.002	T	315	M	0.289	0.374	0	0	0	895.321	tcactttttttCcccatcagat	m5C_64715
chr20	47895508	+	C	471	103	0.219	0	0	T	368	M	0.184	0.258	0	0	0	378.444	acttttttccCcatcagatcg	m5C_64739
chr20	47895509	+	C	452	110	0.243	0	0	T	342	M	0.206	0.285	0	0	0	453.361	cttttttcccCatcagatcga	m5C_64727
chr20	47895517	+	C	452	182	0.403	0	0	T	270	M	0.358	0.449	0	0	0	1304.74	cccatcagatCgacaatgctg	m5C_64703
chr20	47895529	+	C	457	115	0.253	2	0.004	T	340	M	0.215	0.295	0	0	0	494.496	acaatgctgaCgtcttatatt	m5C_64677
chr20	47895532	+	C	440	100	0.227	0	0	T	340	M	0.191	0.269	0	0	0	381.148	atgctgacgtCttatatttg	m5C_64712

chr20	47895553	+	C	387	241	0.623	0	0	C	241	M	0.573	0.67	0	0	0	2764.09	ccagttagttCtgatacatcg	m5C_64721
chr20	47896873	+	C	254	99	0.391	1	0.004	T	154	M	0.333	0.453	0	0	0	659.777	tatatgatgaCttagcttttt	m5C_64689
chr20	47896878	+	C	256	72	0.281	0	0	T	184	M	0.23	0.339	0	0	0	330.797	gatgacttagCtttttcccc	m5C_64679
chr20	47896885	+	C	256	111	0.434	0	0	T	145	M	0.374	0.495	0	0	0	830.969	tagctttttCcccgacagat	m5C_64688
chr20	47896886	+	C	251	90	0.359	0	0	T	161	M	0.302	0.42	0	0	0	543.199	agcttttttCcccgacagatc	m5C_64736
chr20	47896887	+	C	250	110	0.442	1	0.004	T	139	M	0.381	0.504	0	0	0	839.154	gctttttccCcgacagatcg	m5C_64691
chr20	47896888	+	C	250	71	0.284	0	0	T	179	M	0.232	0.343	0	0	0	329.012	ctttttcccCgacagatcga	m5C_64694
chr20	47896891	+	C	245	84	0.343	0	0	T	161	M	0.286	0.404	0	0	0	480.911	ttttccccgaCagatcgacta	m5C_64687
chr20	47896896	+	C	228	92	0.404	0	0	T	136	M	0.342	0.468	0	0	0	629.161	cccgacagatCgactatgttg	m5C_64708
chr20	47896899	+	C	189	56	0.298	1	0.005	T	132	M	0.237	0.367	0	0	0	265.527	gacagatcgaCtatgttgatc	m5C_64723
chr20	47896909	+	C	189	48	0.254	0	0	T	141	M	0.197	0.321	0	0	0	189.349	ctatgttgatCtaacttttct	m5C_64705
chr20	47896913	+	C	188	56	0.301	2	0.011	T	130	M	0.24	0.37	0	0	0	268.488	gttgatctaaCttttctaagc	m5C_64684
chr20	47896918	+	C	183	47	0.257	0	0	T	136	M	0.199	0.325	0	0	0	187.047	tctaacttttCtaagccagtt	m5C_64713
chr20	47896923	+	C	180	66	0.367	0	0	T	114	M	0.3	0.439	0	0	0	395.652	cttttctaagCcagtttctgt	m5C_64728
chr20	47896924	+	C	184	54	0.293	0	0	T	130	M	0.232	0.363	0	0	0	251.041	ttttctaagcCagtttctgtc	m5C_64734
chr20	47896930	+	C	180	78	0.438	2	0.011	T	100	M	0.367	0.512	0	0	0	573.111	aagccagtttCtgtctgatat	m5C_64730
chr20	47896934	+	C	169	57	0.337	0	0	T	112	M	0.27	0.411	0	0	0	308.162	cagtttctgtCtgatatgcca	m5C_64678
chr20	47897249	+	C	209	51	0.244	0	0	T	158	M	0.191	0.307	0	0	0	194.567	cagcttctttCcccatcagat	m5C_64710
chr20	47897250	+	C	208	43	0.207	0	0	T	165	M	0.157	0.267	0	0	0	135.241	agcttctttcCccatcagatc	m5C_64717
chr20	47897252	+	C	208	47	0.226	0	0	T	161	M	0.174	0.287	0	0	0	163.93	cttctttcccCatcagatcga	m5C_64700
chr20	47897264	+	C	184	57	0.31	0	0	T	127	M	0.247	0.38	0	0	0	282.071	tcagatcgacCctgttgatct	m5C_64704
chr20	47897265	+	C	188	60	0.319	0	0	T	128	M	0.257	0.389	0	0	0	308.048	cagatcgaccCtgttgatctc	m5C_64718
chr20	47897273	+	C	196	51	0.26	0	0	T	145	M	0.204	0.326	0	0	0	207.884	ccctgttgatCtctacactat	m5C_64719
chr20	47897297	+	C	179	61	0.341	0	0	T	118	M	0.275	0.413	0	0	0	335.919	ccagttttgtCtgatgcattg	m5C_64722
chr20	55063820	+	C	36	10	0.278	0	0	T	26	M	0.158	0.44	4.8E-14	2E-06	5.9E-14	21.1115	tacccatcaaCagactgaggg	m5C_64770
chr20	55088437	+	C	47	10	0.213	0	0	T	37	M	0.12	0.349	1.3E-12	2.4E-08	1.5E-12	14.271	tgtggacgtgCtgaagacaag	m5C_64760
chr20	55317542	+	C	31	22	0.71	0	0	C	22	M	0.534	0.839	0	6.3E-05	0	234.992	agagcttgatCctggttttga	m5C_64775
chr20	55496568	-	C	39	11	0.282	0	0	T	28	M	0.165	0.438	3.1E-15	3.1E-06	4E-15	26.4001	ccaggcttgcCtgagtgctct	m5C_64786
chr20	57329296	+	C	37	11	0.297	0	0	T	26	M	0.175	0.458	1.4E-15	3.1E-06	1.9E-15	28.5508	agaaagctggCcacagtgatc	m5C_64818
chr20	57329307	+	C	31	24	0.774	0	0	C	24	M	0.602	0.886	0	9.1E-05	0	288.899	cacagtgatcCgagagacagc	m5C_64830
chr20	57570779	-	C	35	10	0.286	0	0	T	25	M	0.163	0.451	3.4E-14	2E-06	4.2E-14	21.9991	tgaccagcacCtataaggatg	m5C_64839
chr20	57570791	-	C	43	9	0.209	0	0	T	34	M	0.114	0.352	1.4E-11	1.2E-08	1.6E-11	11.1522	ggctgaggatCgtgaccagca	m5C_64842
chr20	57570799	-	C	36	11	0.306	0	0	T	25	M	0.18	0.469	1E-15	3.1E-06	1.3E-15	29.7077	gagaggctggCtgaggatcgt	m5C_64853
chr20	57603834	-	C	40	15	0.375	0	0	T	25	M	0.242	0.53	0	2.8E-07	0	72.6683	catgttatggCagattgaaaa	m5C_64845
chr20	57603844	-	C	40	18	0.45	0	0	T	22	M	0.307	0.602	0	2.7E-05	0	110.538	tgtgtgggcaCatgttatggc	m5C_64850
chr20	57603846	-	C	41	11	0.268	0	0	T	30	M	0.157	0.419	6E-15	4.4E-08	7.6E-15	24.5536	gatgtgtgggCacatgttatg	m5C_64838
chr20	60963534	+	C	73	18	0.247	0	0	T	55	M	0.162	0.356	0	4E-12	0	58.3356	tggagagaatCacagatgtgg	m5C_65477
chr20	60963536	+	C	71	30	0.423	0	0	T	41	M	0.315	0.538	0	4.8E-10	0	188.717	gagagaatcaCagatgtggaa	m5C_65478
chr20	61448315	+	C	33	13	0.394	0	0	T	20	M	0.247	0.563	0	6.7E-06	0	64.1755	ggggcttgtgCaggcgggggc	m5C_65481
chr20	61448738	-	C	37	24	0.649	0	0	C	24	M	0.488	0.782	0	9.1E-05	0	234.042	ctccctccggCggcgggaaca	m5C_65513
chr20	62540331	+	C	60	19	0.317	0	0	T	41	M	0.213	0.442	0	4.5E-10	0	80.9617	aggggagataCcatgatcgcg	m5C_64862
chr20	62540338	+	C	77	24	0.312	0	0	T	53	M	0.219	0.422	0	6.4E-11	0	105.25	ataccatgatCgcgaaggtgg	m5C_64858
chr20	62540340	+	C	74	18	0.243	0	0	T	56	M	0.16	0.352	0	4E-12	0	57.5149	accatgatcgCgaaggtggtt	m5C_64870
chr20	62648134	+	C	32	15	0.469	0	0	T	17	M	0.309	0.636	0	1.2E-05	0	92.6074	gtcatgcgtgCcgtgattggg	m5C_64864
chr21	9825638	+	C	33	14	0.424	0	0	T	19	M	0.272	0.592	0	9.2E-06	0	76.259	ggtggccccgCctggaccga	m5C_67277
chr21	9825639	+	C	31	17	0.548	0	0	C	17	M	0.378	0.708	0	2.1E-05	0	128.424	gtggccccgcCtggaccgaa	m5C_67176
chr21	9825645	+	C	48	16	0.333	0	0	T	32	M	0.217	0.475	0	4E-07	0	69.3651	ccgcctgggaCcgaacccggc	m5C_67117
chr21	9825652	+	C	51	14	0.275	0	0	T	37	M	0.171	0.409	0	2.8E-09	0	47.9195	ggaccgaaccCggcaccgcct	m5C_67056
chr21	9825658	+	C	40	11	0.275	0	0	T	29	M	0.161	0.428	4.3E-15	3.1E-06	5.5E-15	25.4502	aacccggcacCgcctcgtggg	m5C_67023
chr21	9825660	+	C	41	15	0.366	0	0	T	26	M	0.236	0.519	0	2.8E-07	0	70.7689	cccggcaccgCctcgtggggc	m5C_67103
chr21	9825661	+	C	38	9	0.237	0	0	T	29	M	0.13	0.392	3.7E-12	1.2E-06	4.3E-12	13.3739	ccggcaccgcCtcgtggggcg	m5C_67244
chr21	9825663	+	C	32	9	0.281	0	0	T	23	M	0.156	0.454	5.3E-13	1.2E-06	6.4E-13	17.1983	ggcaccgcctCgtggggcgcc	m5C_67069
chr21	9825720	+	C	43	23	0.535	0	0	C	23	M	0.389	0.675	0	2.9E-06	0	179.011	ccggcgcgcgCcttggggacc	m5C_67151
chr21	9825721	+	C	46	10	0.217	0	0	T	36	M	0.123	0.356	9.7E-13	2.4E-08	1.2E-12	14.7309	cggcgcgcgcCttggggaccg	m5C_67127
chr21	9825741	+	C	33	15	0.469	1	0.03	T	17	M	0.309	0.636	0	1.2E-05	0	92.6074	gggttggtggCgcccccgcgtg	m5C_67120
chr21	9825743	+	C	34	21	0.618	0	0	C	21	M	0.45	0.761	0	5.2E-05	0	189.171	gttggtggcgCccccgcgtggg	m5C_67116
chr21	9825768	+	C	34	12	0.353	0	0	T	22	M	0.215	0.521	0	4.6E-06	0	51.5705	ggtgggcttcCcggagggttc	m5C_67196
chr21	9825769	+	C	33	7	0.212	0	0	T	26	M	0.107	0.378	1.1E-09	3.4E-07	1.2E-09	6.69165	gtgggcttccCggagggttcc	m5C_67018
chr21	9825796	+	C	57	14	0.246	0	0	T	43	M	0.152	0.371	0	2.8E-09	0	42.6516	cggcctgcggCgcgtgcgggg	m5C_67021
chr21	9825798	+	C	77	20	0.26	0	0	T	57	M	0.175	0.367	0	1.1E-11	0	69.9563	gcctgcggcgCgtgcggggga	m5C_67228
chr21	9825802	+	C	83	25	0.301	0	0	T	58	M	0.213	0.407	0	1.8E-12	0	106.553	gcggcgcgtgCgggggaggag	m5C_67166
chr21	9825814	+	C	72	28	0.389	0	0	T	44	M	0.285	0.504	0	2.6E-10	0	159.407	ggggaggagaCggttccgggg	m5C_67275
chr21	9825819	+	C	59	27	0.458	0	0	T	32	M	0.337	0.583	0	2.5E-07	0	181.986	ggagacggttCcgggggaccg	m5C_66984
chr21	9825820	+	C	63	19	0.302	0	0	T	44	M	0.202	0.424	0	4.5E-10	0	76.9026	gagacggttcCgggggaccgg	m5C_67215
chr21	9825827	+	C	49	15	0.306	0	0	T	34	M	0.195	0.445	0	2.8E-07	0	58.546	ttccgggggaCcggccgcgac	m5C_66985
chr21	9825831	+	C	37	20	0.541	0	0	C	20	M	0.384	0.69	0	4.3E-05	0	153.535	gggggaccggCcgcgactgcg	m5C_67005
chr21	9825832	+	C	36	12	0.333	0	0	T	24	M	0.202	0.497	0	4.6E-06	0	48.5146	ggggaccggcCgcgactgcgg	m5C_67246
chr21	9826150	+	C	37	17	0.459	0	0	T	20	M	0.31	0.616	0	2.1E-05	0	105.53	ccgtcccgggCgtcggcgtcg	m5C_67163
chr21	9826159	+	C	33	8	0.242	0	0	T	25	M	0.128	0.41	3.1E-11	6.8E-07	3.6E-11	10.7866	gcgtcggcgtCggggagagcc	m5C_67113
chr21	9826181	+	C	34	11	0.324	0	0	T	23	M	0.191	0.492	4.4E-16	3.1E-06	5.8E-16	32.3088	gtcctccccgCgtggcgtcgc	m5C_67201
chr21	9826189	+	C	73	27	0.37	0	0	T	46	M	0.268	0.485	0	1.9E-10	0	144.844	cgcgtggcgtCgcccccgttcg	m5C_67278
chr21	9826192	+	C	70	18	0.257	0	0	T	52	M	0.169	0.37	0	4E-12	0	60.9449	gtggcgtcgcCcccgttcggcg	m5C_67016
chr21	9826193	+	C	74	18	0.243	0	0	T	56	M	0.16	0.352	0	4E-12	0	57.5149	tggcgtcgccCcgttcggcgc	m5C_67224
chr21	9826207	+	C	85	28	0.329	0	0	T	57	M	0.239	0.435	0	5.8E-12	0	133.715	tcggcgcgcgCgtgcgcccga	m5C_67194
chr21	9826213	+	C	84	20	0.238	0	0	T	64	M	0.16	0.339	0	1.5E-13	0	63.8983	cgcgcgtgcgCccgagcgcgg	m5C_66964
chr21	9826214	+	C	80	18	0.225	0	0	T	62	M	0.147	0.328	0	4.5E-14	0	53.0391	gcgcgtgcgcCcgagcgcggc	m5C_66968
chr21	9826226	+	C	71	19	0.268	0	0	T	52	M	0.179	0.381	0	6.9E-12	0	67.8401	gagcgcggccCggtggtccct	m5C_67218
chr21	9826235	+	C	58	24	0.414	0	0	T	34	M	0.296	0.542	0	1.2E-07	0	142.214	ccggtggtccCtcccggacag	m5C_67102
chr21	9826239	+	C	63	16	0.254	0	0	T	47	M	0.163	0.373	0	1.1E-10	0	52.0969	tggtccctccCggacaggcgt	m5C_67156
chr21	9826247	+	C	76	19	0.25	0	0	T	57	M	0.166	0.358	0	6.9E-12	0	63.1894	cccggacaggCgttcgtgcga	m5C_67220
chr21	9826251	+	C	102	29	0.284	0	0	T	73	M	0.206	0.378	0	2.9E-15	0	119.392	gacaggcgttCgtgcgacgtg	m5C_67146
chr21	9826265	+	C	130	29	0.223	0	0	T	101	M	0.16	0.302	0	0	0	92.8281	cgacgtgtggCgtgggtcgac	m5C_66986
chr21	9826272	+	C	98	45	0.459	0	0	T	53	M	0.364	0.558	0	2.3E-11	0	327.533	tggcgtgggtCgacctccgcc	m5C_67089
chr21	9826275	+	C	74	24	0.324	0	0	T	50	M	0.229	0.437	0	6.4E-11	0	109.747	cgtgggtcgaCctccgccttg	m5C_67274
chr21	9826276	+	C	71	30	0.423	0	0	T	41	M	0.315	0.538	0	4.8E-10	0	188.717	gtgggtcgacCtccgccttgc	m5C_67256
chr21	9826278	+	C	58	13	0.224	0	0	T	45	M	0.136	0.347	6.7E-16	1.6E-09	8.7E-16	26.8192	gggtcgacctCcgccttgccg	m5C_67078
chr21	9826414	+	C	33	15	0.455	0	0	T	18	M	0.298	0.62	0	1.2E-05	0	89.5281	ccttggccgtCgtgtggcgtg	m5C_66983
chr21	9826421	+	C	40	18	0.45	0	0	T	22	M	0.307	0.602	0	2.7E-05	0	110.538	cgtcgtgtggCgtgtgccacc	m5C_67159
chr21	9826428	+	C	36	13	0.361	0	0	T	23	M	0.225	0.524	0	6.7E-06	0	58.436	tggcgtgtgcCacccctgcgc	m5C_67165
chr21	9826431	+	C	40	14	0.35	0	0	T	26	M	0.221	0.505	0	1.9E-07	0	61.9761	cgtgtgccacCcctgcgcccg	m5C_67227
chr21	9826436	+	C	34	10	0.294	0	0	T	24	M	0.168	0.462	2.3E-14	2E-06	2.9E-14	22.9564	gccacccctgCgcccgcgccc	m5C_66961
chr21	9826439	+	C	35	15	0.429	0	0	T	20	M	0.28	0.591	0	1.2E-05	0	83.953	acccctgcgcCcgcgcccgcc	m5C_66959
chr21	9826445	+	C	41	13	0.317	0	0	T	28	M	0.196	0.47	0	1.2E-07	0	50.8675	gcgcccgcgcCcgccggcggg	m5C_67179
chr21	9826452	+	C	54	30	0.556	0	0	C	30	M	0.424	0.68	0	4.8E-07	0	254.252	cgcccgccggCggggctcgga	m5C_67006
chr21	9826457	+	C	55	33	0.6	0	0	C	33	M	0.468	0.719	0	8.8E-07	0	308.963	gccggcggggCtcggagccgg	m5C_66999
chr21	9826459	+	C	43	29	0.674	0	0	C	29	M	0.525	0.795	0	9.7E-06	0	304.592	cggcggggctCggagccgggc	m5C_66966
chr21	9826465	+	C	37	21	0.568	0	0	C	21	M	0.409	0.713	0	5.2E-05	0	171.838	ggctcggagcCgggcttcggc	m5C_66991
chr21	9826512	+	C	42	12	0.286	0	0	T	30	M	0.172	0.436	2.2E-16	7.5E-08	2.9E-16	32.2467	ggtgcgcgggCgctgcggccg	m5C_67045
chr21	9826514	+	C	47	15	0.319	0	0	T	32	M	0.204	0.462	0	2.8E-07	0	61.1856	tgcgcgggcgCtgcggccgca	m5C_67035
chr21	9826517	+	C	49	15	0.306	0	0	T	34	M	0.195	0.445	0	2.8E-07	0	58.546	gcgggcgctgCggccgcacgg	m5C_67155
chr21	9826521	+	C	49	13	0.265	0	0	T	36	M	0.162	0.403	0	1.2E-07	0	42.1489	gcgctgcggcCgcacggcgcg	m5C_66979

chr21	9826525	+	C	40	20	0.5	0	0	CT	20	M	0.352	0.648	0	1.4E-06	0	140.797	tgcggccgcaCggcgcgactg	m5C_67142
chr21	9826528	+	C	38	10	0.263	0	0	T	28	M	0.15	0.42	9.4E-14	2E-06	1.2E-13	19.5037	ggccgcacggCgcgactgtcc	m5C_67020
chr21	9826530	+	C	38	10	0.263	0	0	T	28	M	0.15	0.42	9.4E-14	2E-06	1.2E-13	19.5037	ccgcacggcgCgactgtcccc	m5C_67251
chr21	9826637	+	C	31	9	0.29	0	0	T	22	M	0.161	0.466	3.7E-13	1.2E-06	4.4E-13	18.0154	ccgccgcccgCgggcgccggc	m5C_66953
chr21	9826643	+	C	37	11	0.297	0	0	T	26	M	0.175	0.458	1.4E-15	3.1E-06	1.9E-15	28.5508	cccgcgggcgCcggccgcgcg	m5C_67178
chr21	9826647	+	C	41	19	0.463	0	0	T	22	M	0.321	0.613	0	1E-06	0	121.813	cgggcgccggCcgcgcgcgcg	m5C_67147
chr21	9826650	+	C	41	9	0.22	0	0	T	32	M	0.12	0.367	8.4E-12	1.2E-08	9.9E-12	11.9621	gcgccggccgCgcgcgcgcgc	m5C_67068
chr21	9826658	+	C	37	10	0.27	0	0	T	27	M	0.154	0.43	6.7E-14	2E-06	8.3E-14	20.2815	cgcgcgcgcgCgcgcgtggcc	m5C_67170
chr21	9826726	+	C	55	30	0.545	0	0	C	30	M	0.415	0.67	0	4.8E-07	0	249.135	cgcgggcgggCgcgacgaaga	m5C_66997
chr21	9826742	+	C	56	38	0.679	0	0	C	38	M	0.548	0.786	0	2.1E-06	0	416.65	gaagaagcgtCgcgggtctgt	m5C_67173
chr21	9826755	+	C	54	21	0.389	0	0	T	33	M	0.27	0.522	0	4.8E-08	0	113.575	gggtctgtggCgcggggcccc	m5C_67048
chr21	9826939	+	C	49	29	0.592	0	0	C	29	M	0.452	0.718	0	9.7E-06	0	262.433	ggcgccgggcCcgtcctcgcg	m5C_67010
chr21	9826940	+	C	48	16	0.333	0	0	T	32	M	0.217	0.475	0	4E-07	0	69.3651	gcgccgggccCgtcctcgcga	m5C_66974
chr21	9826944	+	C	52	13	0.25	0	0	T	39	M	0.152	0.382	1.1E-16	1.6E-09	1.5E-16	31.5947	cgggcccgtcCtcgcgaggcc	m5C_66969
chr21	9826946	+	C	49	17	0.347	0	0	T	32	M	0.229	0.487	0	5.6E-07	0	77.9423	ggcccgtcctCgcgaggcccc	m5C_67186
chr21	9826954	+	C	49	14	0.286	0	0	T	35	M	0.178	0.424	0	1.9E-07	0	49.9784	ctcgcgaggcCccccggccgg	m5C_66956
chr21	9826958	+	C	61	16	0.262	0	0	T	45	M	0.168	0.384	0	1.1E-10	0	53.8837	cgaggcccccCggccggccgt	m5C_67063
chr21	9826962	+	C	63	20	0.317	0	0	T	43	M	0.216	0.44	0	6.9E-10	0	86.3461	gccccccggcCggccgtccgg	m5C_67183
chr21	9826976	+	C	69	25	0.362	0	0	T	44	M	0.259	0.48	0	4.1E-09	0	129.478	cgtccggccgCgtcggggcct	m5C_67100
chr21	9826979	+	C	63	23	0.365	0	0	T	40	M	0.257	0.489	0	2.1E-09	0	118.29	ccggccgcgtCggggcctcgc	m5C_67143
chr21	9826984	+	C	61	19	0.311	0	0	T	42	M	0.209	0.436	0	4.5E-10	0	79.5616	cgcgtcggggCctcgccgcgc	m5C_67280
chr21	9826985	+	C	62	17	0.274	0	0	T	45	M	0.179	0.396	0	1.8E-10	0	60.7951	gcgtcggggcCtcgccgcgct	m5C_67109
chr21	9826987	+	C	61	15	0.246	0	0	T	46	M	0.155	0.367	0	6E-11	0	46.5196	gtcggggcctCgccgcgctct	m5C_67073
chr21	9827004	+	C	50	12	0.24	0	0	T	38	M	0.143	0.374	2.4E-15	8.8E-10	3.1E-15	25.0697	ctctacctacCtacctggttg	m5C_67114
chr21	9827017	+	C	1412	287	0.203	0	0	T	1125	M	0.183	0.225	0	0	0	1050.91	cctggttgatCctgccagtag	m5C_67273
chr21	9827018	+	C	1401	369	0.263	0	0	T	1032	M	0.241	0.287	0	0	0	1778.49	ctggttgatcCtgccagtagc	m5C_67264
chr21	9827039	+	C	1522	315	0.207	1	0.001	T	1206	M	0.187	0.228	0	0	0	1181.16	atatgcttgtCtcaaagatta	m5C_67167
chr21	9827052	+	C	1409	284	0.202	2	0.001	T	1123	M	0.182	0.224	0	0	0	1032.05	aaagattaagCcatgcatgtc	m5C_66960
chr21	9827057	+	C	1475	295	0.2	0	0	T	1180	M	0.18	0.221	0	0	0	1064.23	ttaagccatgCatgtctaagt	m5C_66972
chr21	9827062	+	C	1685	375	0.223	0	0	T	1310	M	0.203	0.243	0	0	0	1525.01	ccatgcatgtCtaagtacgca	m5C_67169
chr21	9827076	+	C	2954	682	0.232	8	0.003	T	2264	M	0.217	0.247	0	0	0	2954.76	gtacgcacggCcggtacagtg	m5C_67205
chr21	9827077	+	C	3101	892	0.288	1	0	T	2208	M	0.272	0.304	0	0	0	4853.83	tacgcacggcCggtacagtga	m5C_67040
chr21	9827090	+	C	3482	758	0.218	2	0.001	T	2722	M	0.204	0.232	0	0	0	3098.97	tacagtgaaaCtgcgaatggc	m5C_67096
chr21	9827100	+	C	2062	551	0.267	1	0	T	1510	M	0.249	0.287	0	0	0	2740.5	ctgcgaatggCtcattaaatc	m5C_67237
chr21	9827102	+	C	1762	440	0.25	0	0	T	1322	M	0.23	0.27	0	0	0	2024.57	gcgaatggctCattaaatcag	m5C_66981
chr21	9827110	+	C	1231	247	0.201	0	0	T	984	M	0.179	0.224	0	0	0	885.367	ctcattaaatCagttatggtt	m5C_67027
chr21	9827121	+	C	635	211	0.332	0	0	T	424	M	0.297	0.37	0	0	0	1252.29	agttatggttCctttggtcgc	m5C_67219
chr21	9827122	+	C	641	235	0.367	0	0	T	406	M	0.33	0.405	0	0	0	1551.97	gttatggttcCtttggtcgct	m5C_66975
chr21	9827129	+	C	845	327	0.387	1	0.001	T	517	M	0.355	0.421	0	0	0	2322.71	ttcctttggtCgctcgctcct	m5C_67271
chr21	9827131	+	C	838	310	0.37	0	0	T	528	M	0.338	0.403	0	0	0	2095	cctttggtcgCtcgctcctct	m5C_67019
chr21	9827133	+	C	869	344	0.397	3	0.003	T	522	M	0.365	0.43	0	0	0	2512.31	tttggtcgctCgctcctctcc	m5C_67099
chr21	9827135	+	C	870	386	0.444	1	0.001	T	483	M	0.411	0.477	0	0	0	3176.55	tggtcgctcgCtcctctccta	m5C_67255
chr21	9827137	+	C	928	347	0.374	1	0.001	T	580	M	0.344	0.406	0	0	0	2385.63	gtcgctcgctCctctcctact	m5C_67132
chr21	9827138	+	C	978	412	0.421	0	0	T	566	M	0.391	0.452	0	0	0	3219.28	tcgctcgctcCtctcctactt	m5C_67135
chr21	9827140	+	C	1287	432	0.336	0	0	T	855	M	0.31	0.362	0	0	0	2681.75	gctcgctcctCtcctacttgg	m5C_67081
chr21	9827142	+	C	1518	422	0.278	0	0	T	1096	M	0.256	0.301	0	0	0	2160.99	tcgctcctctCctacttggat	m5C_66963
chr21	9827143	+	C	1769	530	0.3	0	0	T	1239	M	0.279	0.321	0	0	0	2954.33	cgctcctctcCtacttggata	m5C_67137
chr21	9827146	+	C	2418	673	0.279	2	0.001	T	1743	M	0.261	0.297	0	0	0	3513.68	tcctctcctaCttggataact	m5C_67030
chr21	9827155	+	C	3607	1091	0.303	1	0	T	2515	M	0.288	0.318	0	0	0	6279.24	acttggataaCtgtggtaatt	m5C_67189
chr21	9827166	+	C	3775	1233	0.327	1	0	T	2541	M	0.312	0.342	0	0	0	7692.15	tgtggtaattCtagagctaat	m5C_67024
chr21	9827172	+	C	3753	1378	0.367	0	0	T	2375	M	0.352	0.383	0	0	0	9698.2	aattctagagCtaatacatgc	m5C_66970
chr21	9827178	+	C	3798	1090	0.288	12	0.003	T	2696	M	0.274	0.303	0	0	0	5966.67	agagctaataCatgccgacgg	m5C_67248
chr21	9827182	+	C	4044	924	0.229	3	0.001	T	3117	M	0.216	0.242	0	0	0	3991.1	ctaatacatgCcgacgggcgc	m5C_67182
chr21	9827183	+	C	4066	1204	0.296	3	0.001	T	2859	M	0.282	0.311	0	0	0	6802.34	taatacatgcCgacgggcgct	m5C_67270
chr21	9827186	+	C	4374	1871	0.429	10	0.002	T	2493	M	0.414	0.443	0	0	0	15496.4	tacatgccgaCgggcgctgac	m5C_66976
chr21	9827190	+	C	4505	1146	0.255	3	0.001	T	3356	M	0.242	0.267	0	0	0	5547.6	tgccgacgggCgctgaccccc	m5C_67015
chr21	9827192	+	C	4749	1445	0.304	2	0	T	3302	M	0.291	0.318	0	0	0	8423.63	ccgacgggcgCtgaccccctt	m5C_66977
chr21	9827196	+	C	4813	1048	0.218	1	0	T	3764	M	0.206	0.23	0	0	0	4325.19	cgggcgctgaCccccttcgcg	m5C_67164
chr21	9827197	+	C	4980	1429	0.287	1	0	T	3550	M	0.275	0.3	0	0	0	7848.3	gggcgctgacCccccttcgcgg	m5C_67130
chr21	9827198	+	C	5082	1073	0.211	3	0.001	T	4006	M	0.2	0.223	0	0	0	4297.49	ggcgctgaccCccttcgcggg	m5C_67070
chr21	9827199	+	C	5242	1132	0.216	0	0	T	4110	M	0.205	0.227	0	0	0	4641.63	gcgctgacccCcttcgcgggg	m5C_67157
chr21	9827203	+	C	5941	1344	0.226	3	0.001	T	4594	M	0.216	0.237	0	0	0	5802.7	tgacccccttCgcgggggga	m5C_67253
chr21	9827205	+	C	6502	1580	0.249	147	0.023	T	4775	M	0.238	0.259	0	0	0	7525.55	acccccttcgCggggggggatg	m5C_67029
chr21	9827237	+	C	3004	626	0.208	1	0	T	2377	M	0.194	0.223	0	0	0	2432.72	cagatcaaaaCcaacccggtc	m5C_66965
chr21	9827241	+	C	2361	493	0.209	0	0	T	1868	M	0.193	0.226	0	0	0	1901.93	tcaaaaccaaCccggtcagcc	m5C_67238
chr21	9827242	+	C	2241	493	0.22	3	0.001	T	1745	M	0.204	0.238	0	0	0	2007.52	caaaaccaacCcggtcagccc	m5C_67002
chr21	9827243	+	C	2036	477	0.234	1	0	T	1558	M	0.217	0.253	0	0	0	2065.44	aaaaccaaccCggtcagcccc	m5C_67013
chr21	9827250	+	C	1372	327	0.239	3	0.002	T	1042	M	0.217	0.262	0	0	0	1419.34	acccggtcagCccctctccgg	m5C_66971
chr21	9827253	+	C	1200	255	0.213	1	0.001	T	944	M	0.19	0.237	0	0	0	971.302	cggtcagcccCtctccggccc	m5C_67085
chr21	9827257	+	C	1036	279	0.269	0	0	T	757	M	0.243	0.297	0	0	0	1356.95	cagcccctctCcggccccggc	m5C_67038
chr21	9827258	+	C	1023	230	0.225	0	0	T	793	M	0.2	0.251	0	0	0	921.394	agcccctctcCggccccggcc	m5C_67247
chr21	9827260	-	C	71	25	0.352	0	0	T	46	M	0.251	0.468	0	9.3E-11	0	125.613	ccggccggggCcggagagggg	m5C_67404
chr21	9827261	+	C	1146	248	0.217	1	0.001	T	897	M	0.194	0.241	0	0	0	960.765	ccctctccggCcccggccggg	m5C_67245
chr21	9827262	+	C	1209	323	0.267	0	0	T	886	M	0.243	0.293	0	0	0	1569.7	cctctccggcCccggccgggg	m5C_67126
chr21	9827263	+	C	1252	305	0.244	0	0	T	947	M	0.221	0.268	0	0	0	1345.9	ctctccggccCcggccggggg	m5C_67107
chr21	9827264	+	C	1272	410	0.322	0	0	T	862	M	0.297	0.349	0	0	0	2437.13	tctccggcccCggccgggggg	m5C_67007
chr21	9827265	-	C	145	78	0.538	0	0	C	78	M	0.457	0.617	0	2.4E-15	0	712.686	gccccccggCcggggccggag	m5C_67374
chr21	9827266	-	C	154	64	0.416	0	0	T	90	M	0.341	0.495	0	0	0	436.131	cgcccccggCcggggccgga	m5C_67345
chr21	9827267	+	C	1567	389	0.248	0	0	T	1178	M	0.227	0.27	0	0	0	1769.86	ccggccccggCcgggggggcgg	m5C_67198
chr21	9827268	+	C	1657	611	0.369	0	0	T	1046	M	0.346	0.392	0	0	0	4226.12	cggccccggcCgggggggcgg	m5C_67241
chr21	9827269	-	C	173	67	0.387	0	0	T	106	M	0.318	0.462	0	0	0	425.976	gcccgcccccCggccggggcc	m5C_67366
chr21	9827270	-	C	177	107	0.605	0	0	C	107	M	0.531	0.674	0	0	0	1136.34	cgcccgccccCcggccggggc	m5C_67332
chr21	9827271	-	C	174	94	0.54	0	0	C	94	M	0.466	0.613	0	0	0	876.281	gcgcccgcccCccggccgggg	m5C_67442
chr21	9827272	-	C	172	76	0.442	0	0	T	96	M	0.37	0.517	0	0	0	561.97	ggcgcccgccCcccggccggg	m5C_67303
chr21	9827273	-	C	187	66	0.353	0	0	T	121	M	0.288	0.424	0	0	0	380.206	cggcgcccgcCccccggccgg	m5C_67338
chr21	9827274	-	C	181	68	0.376	0	0	T	113	M	0.308	0.448	0	0	0	419.435	ccggcgcccgCcccccggccg	m5C_67434
chr21	9827275	+	C	2306	835	0.364	13	0.006	T	1458	M	0.345	0.384	0	0	0	5756.46	ggccggggggCgggcgccggc	m5C_67234
chr21	9827276	-	C	178	81	0.455	0	0	T	97	M	0.384	0.528	0	0	0	621.464	cgccggcgccCgcccccggc	m5C_67281
chr21	9827277	-	C	169	82	0.485	0	0	T	87	M	0.411	0.56	0	0	0	674.082	ccgccggcgcCcgcccccgg	m5C_67415
chr21	9827278	-	C	179	98	0.547	0	0	C	98	M	0.474	0.619	0	0	0	929.695	gccgccggcgCccgccccccg	m5C_67352
chr21	9827279	+	C	2796	925	0.332	12	0.004	T	1859	M	0.315	0.35	0	0	0	5827.51	ggggggcgggCgccggcggct	m5C_67232
chr21	9827280	-	C	172	67	0.39	0	0	T	105	M	0.32	0.464	0	0	0	428.573	aagccgccggCgcccgccccc	m5C_67342
chr21	9827281	+	C	3105	959	0.309	1	0	T	2145	M	0.293	0.325	0	0	0	5618.69	ggggcgggcgCcggcggcttt	m5C_66958
chr21	9827282	+	C	3190	963	0.302	1	0	T	2226	M	0.286	0.318	0	0	0	5513.88	gggcgggcgcCggcggctttg	m5C_67105
chr21	9827283	-	C	172	87	0.506	0	0	C	87	M	0.432	0.58	0	0	0	751.312	ccaaagccgcCggcgcccgcc	m5C_67417
chr21	9827284	-	C	167	77	0.461	0	0	T	90	M	0.387	0.537	0	0	0	596.285	accaaagccgCcggcgcccgc	m5C_67355
chr21	9827285	+	C	3534	918	0.26	4	0.001	T	2612	M	0.246	0.275	0	0	0	4513.84	cgggcgccggCggctttggtg	m5C_67202
chr21	9827286	-	C	157	72	0.459	0	0	T	85	M	0.383	0.537	0	0	0	550.906	tcaccaaagcCgccggcgccc	m5C_67379
chr21	9827287	-	C	146	77	0.527	0	0	C	77	M	0.447	0.607	0	2E-15	0	688	gtcaccaaagCcgccggcgcc	m5C_67341
chr21	9827288	+	C	3777	1599	0.423	1	0	T	2177	M	0.408	0.439	0	0	0	13041.1	gcgccggcggCtttggtgact	m5C_66988
chr21	9827292	-	C	104	45	0.433	0	0	T	59	M	0.342	0.529	0	7.4E-13	0	307.394	ctagagtcacCaaagccgccg	m5C_67412

chr21	9827293	-	C	95	55	0.579	0	0	C	55	M	0.478	0.673	0	1.9E-10	0	526.344	tctagagtcaCcaaagccgcc	m5C_67336
chr21	9827295	-	C	97	50	0.515	0	0	C	50	M	0.417	0.612	0	7.1E-11	0	417.33	tatctagagtCaccaaagccg	m5C_67330
chr21	9827297	+	C	4610	1306	0.283	1	0	T	3303	M	0.271	0.297	0	0	0	7066.33	gctttggtgaCtctagataac	m5C_67036
chr21	9827299	+	C	4529	1073	0.237	0	0	T	3456	M	0.225	0.25	0	0	0	4823.36	tttggtgactCtagataacct	m5C_67101
chr21	9827302	-	C	41	20	0.488	0	0	T	21	M	0.343	0.635	0	1.4E-06	0	137.017	ccgaggttatCtagagtcacc	m5C_67389
chr21	9827307	+	C	4105	880	0.214	2	0	T	3223	M	0.202	0.227	0	0	0	3558.5	ctctagataaCctcgggctga	m5C_67212
chr21	9827308	+	C	4135	1050	0.254	2	0	T	3083	M	0.241	0.268	0	0	0	5061.28	tctagataacCtcgggctgat	m5C_66954
chr21	9827310	+	C	4048	1015	0.251	0	0	T	3033	M	0.238	0.264	0	0	0	4823.87	tagataacctCgggctgatcg	m5C_67122
chr21	9827314	+	C	3948	1165	0.295	3	0.001	T	2780	M	0.281	0.31	0	0	0	6553.82	taacctcgggCtgatcgcacg	m5C_66982
chr21	9827316	-	C	138	85	0.616	0	0	C	85	M	0.533	0.693	0	2E-13	0	905.586	ggcgtgcgatCagcccgaggt	m5C_67324
chr21	9827319	+	C	3878	905	0.234	3	0.001	T	2970	M	0.22	0.247	0	0	0	3990.96	tcgggctgatCgcacgccccc	m5C_67231
chr21	9827320	-	C	190	98	0.516	0	0	C	98	M	0.445	0.586	0	0	0	872.44	ggggggcgtgCgatcagcccg	m5C_67381
chr21	9827323	+	C	3729	844	0.226	0	0	T	2885	M	0.213	0.24	0	0	0	3598.62	gctgatcgcaCgccccccgtg	m5C_67052
chr21	9827324	-	C	246	133	0.541	0	0	C	133	M	0.478	0.602	0	0	0	1272.08	ccacggggggCgtgcgatcag	m5C_67361
chr21	9827326	+	C	3554	753	0.212	0	0	T	2801	M	0.199	0.226	0	0	0	2993.23	gatcgcacgcCccccgtggcg	m5C_66967
chr21	9827327	+	C	3572	931	0.261	2	0.001	T	2639	M	0.247	0.275	0	0	0	4592.51	atcgcacgccCcccgtggcgg	m5C_67067
chr21	9827328	+	C	3518	722	0.205	3	0.001	T	2793	M	0.192	0.219	0	0	0	2777.89	tcgcacgcccCccgtggcggc	m5C_67204
chr21	9827329	+	C	3525	781	0.222	9	0.003	T	2735	M	0.209	0.236	0	0	0	3259.81	cgcacgccccCcgtggcggcg	m5C_66995
chr21	9827330	+	C	3677	921	0.251	1	0	T	2755	M	0.237	0.265	0	0	0	4361.88	gcacgcccccCgtggcggcga	m5C_67249
chr21	9827331	-	C	344	222	0.645	0	0	C	222	M	0.593	0.694	0	0	0	2634.88	gtcgccgccaCggggggcgtg	m5C_67294
chr21	9827333	-	C	353	236	0.669	0	0	C	236	M	0.618	0.716	0	0	0	2916.32	tcgtcgccgcCacggggggcg	m5C_67305
chr21	9827334	-	C	360	243	0.677	1	0.003	C	243	M	0.627	0.723	0	0	0	3046.49	gtcgtcgccgCcacgggggc	m5C_67386
chr21	9827335	+	C	4110	965	0.235	9	0.002	T	3136	M	0.223	0.249	0	0	0	4295.74	ccccccgtggCggcgacgacc	m5C_67128
chr21	9827336	-	C	363	239	0.658	0	0	C	239	M	0.608	0.705	0	0	0	2907.12	gggtcgtcgcCgccacggggg	m5C_67354
chr21	9827337	-	C	364	230	0.632	0	0	C	230	M	0.581	0.68	0	0	0	2673.45	tgggtcgtcgCcgccacgggg	m5C_67375
chr21	9827338	+	C	4117	823	0.201	20	0.005	T	3274	M	0.189	0.213	0	0	0	3109.18	cccgtggcggCgacgacccat	m5C_67272
chr21	9827339	-	C	361	258	0.715	0	0	C	258	M	0.666	0.759	0	0	0	3436.71	aatgggtcgtCgccgccacgg	m5C_67364
chr21	9827342	-	C	361	252	0.698	0	0	C	252	M	0.649	0.743	0	0	0	3270.05	tcgaatgggtCgtcgccgcca	m5C_67318
chr21	9827345	+	C	3804	929	0.244	3	0.001	T	2872	M	0.231	0.258	0	0	0	4292.17	cggcgacgacCcattcgaacg	m5C_67043
chr21	9827354	+	C	2825	586	0.208	2	0.001	T	2237	M	0.193	0.223	0	0	0	2262.21	cccattcgaaCgtctgcccta	m5C_67074
chr21	9827357	+	C	2263	498	0.22	0	0	T	1765	M	0.203	0.238	0	0	0	2026.61	attcgaacgtCtgccctatca	m5C_67190
chr21	9827362	+	C	1564	342	0.219	0	0	T	1222	M	0.199	0.24	0	0	0	1360.39	aacgtctgccCtatcaacttt	m5C_67243
chr21	9827366	+	C	1275	287	0.225	0	0	T	988	M	0.203	0.249	0	0	0	1165.33	tctgccctatCaactttcgat	m5C_67148
chr21	9827374	-	C	144	108	0.75	0	0	C	108	M	0.673	0.814	0	3.7E-13	0	1454.55	cgactaccatCgaaagttgat	m5C_67371
chr21	9827381	-	C	132	94	0.712	0	0	C	94	M	0.63	0.782	0	8.2E-13	0	1183.92	ggcacggcgaCtaccatcgaa	m5C_67299
chr21	9827385	+	C	568	125	0.22	0	0	T	443	M	0.188	0.256	0	0	0	469.855	atggtagtcgCcgtgcctacc	m5C_67034
chr21	9827390	+	C	628	167	0.266	0	0	T	461	M	0.233	0.302	0	0	0	777.774	agtcgccgtgCctaccatggt	m5C_67138
chr21	9827394	+	C	754	166	0.22	0	0	T	588	M	0.192	0.251	0	0	0	637.581	gccgtgcctaCcatggtgacc	m5C_67131
chr21	9827395	+	C	799	170	0.213	0	0	T	629	M	0.186	0.242	0	0	0	631.709	ccgtgcctacCatggtgacca	m5C_67177
chr21	9827404	+	C	1444	430	0.299	7	0.005	T	1007	M	0.276	0.323	0	0	0	2374.62	ccatggtgacCacgggtgacg	m5C_67188
chr21	9827406	+	C	1655	500	0.303	7	0.004	T	1148	M	0.282	0.326	0	0	0	2816.8	atggtgaccaCgggtgacggg	m5C_67158
chr21	9827413	+	C	2621	1035	0.399	26	0.01	T	1560	M	0.38	0.418	0	0	0	7869.45	ccacgggtgaCggggaatcag	m5C_67139
chr21	9827428	+	C	3867	1077	0.279	2	0.001	T	2788	M	0.265	0.293	0	0	0	5702.61	aatcagggttCgattccggag	m5C_67080
chr21	9827433	+	C	3441	907	0.264	0	0	T	2534	M	0.249	0.279	0	0	0	4519.3	gggttcgattCcggagaggga	m5C_67260
chr21	9827434	+	C	3407	939	0.276	1	0	T	2467	M	0.261	0.291	0	0	0	4900.48	ggttcgattcCggagagggag	m5C_67000
chr21	9827445	+	C	2886	1009	0.35	2	0.001	T	1875	M	0.333	0.367	0	0	0	6713.18	ggagagggagCctgagaaacg	m5C_67141
chr21	9827446	+	C	2733	924	0.338	0	0	T	1809	M	0.321	0.356	0	0	0	5924.55	gagagggagcCtgagaaacgg	m5C_67028
chr21	9827454	+	C	2053	631	0.307	0	0	T	1422	M	0.288	0.328	0	0	0	3631.67	gcctgagaaaCggctaccaca	m5C_67265
chr21	9827457	+	C	1764	468	0.266	2	0.001	T	1294	M	0.246	0.287	0	0	0	2297.98	tgagaaacggCtaccacatcc	m5C_67213
chr21	9827460	+	C	1497	364	0.243	2	0.001	T	1131	M	0.222	0.266	0	0	0	1619.06	gaaacggctaCcacatccaag	m5C_67229
chr21	9827461	+	C	1471	489	0.332	0	0	T	982	M	0.309	0.357	0	0	0	3020.23	aaacggctacCacatccaagg	m5C_67208
chr21	9827463	+	C	1356	388	0.286	0	0	T	968	M	0.263	0.311	0	0	0	2038.63	acggctaccaCatccaaggaa	m5C_66973
chr21	9827466	+	C	1344	508	0.378	0	0	T	836	M	0.352	0.404	0	0	0	3580.74	gctaccacatCcaaggaaggc	m5C_66978
chr21	9827467	+	C	1402	436	0.311	0	0	T	966	M	0.287	0.336	0	0	0	2505.24	ctaccacatcCaaggaaggca	m5C_67077
chr21	9827476	+	C	2143	486	0.227	0	0	T	1657	M	0.21	0.245	0	0	0	2036.86	ccaaggaaggCagcaggcgcg	m5C_67050
chr21	9827479	+	C	2240	456	0.204	6	0.003	T	1778	M	0.188	0.221	0	0	0	1713.82	aggaaggcagCaggcgcgcaa	m5C_67037
chr21	9827483	+	C	2242	1115	0.497	0	0	T	1127	M	0.477	0.518	0	0	0	10629.3	aggcagcaggCgcgcaaatta	m5C_66992
chr21	9827485	+	C	2133	431	0.202	2	0.001	T	1700	M	0.186	0.22	0	0	0	1601.08	gcagcaggcgCgcaaattacc	m5C_67150
chr21	9827487	+	C	1998	536	0.268	0	0	T	1462	M	0.249	0.288	0	0	0	2672.48	agcaggcgcgCaaattaccca	m5C_67057
chr21	9827494	+	C	1170	330	0.282	0	0	T	840	M	0.257	0.309	0	0	0	1696.28	gcgcaaattaCccactcccga	m5C_67279
chr21	9827495	+	C	1135	311	0.274	0	0	T	824	M	0.249	0.301	0	0	0	1547.88	cgcaaattacCcactcccgac	m5C_67033
chr21	9827496	+	C	968	366	0.378	0	0	T	602	M	0.348	0.409	0	0	0	2548.02	gcaaattaccCactcccgacc	m5C_67161
chr21	9827498	+	C	942	194	0.208	11	0.012	T	737	M	0.184	0.236	0	0	0	712.031	aaattacccaCtcccgacccg	m5C_67082
chr21	9827500	+	C	961	245	0.255	1	0.001	T	715	M	0.229	0.284	0	0	0	1120.35	attacccactCccgacccggg	m5C_67206
chr21	9827501	+	C	1036	238	0.23	1	0.001	T	797	M	0.205	0.257	0	0	0	977.426	ttacccactcCcgacccgggg	m5C_67207
chr21	9827502	+	C	1084	303	0.28	0	0	T	781	M	0.254	0.307	0	0	0	1536.93	tacccactccCgacccgggga	m5C_67154
chr21	9827506	+	C	1244	406	0.33	12	0.01	T	826	M	0.304	0.356	0	0	0	2467.37	cactcccgacCcggggaggta	m5C_67086
chr21	9827507	+	C	1279	319	0.249	0	0	T	960	M	0.226	0.274	0	0	0	1444.91	actcccgaccCggggaggtag	m5C_67263
chr21	9827521	+	C	1131	570	0.504	1	0.001	C	570	M	0.475	0.534	0	0	0	5418.5	gaggtagtgaCgaaaaataac	m5C_67129
chr21	9827531	+	C	661	336	0.508	0	0	C	336	M	0.47	0.546	0	0	0	3160.22	cgaaaaataaCaatacaggac	m5C_67064
chr21	9827536	+	C	508	199	0.393	1	0.002	T	308	M	0.351	0.436	0	0	0	1396.82	aataacaataCaggactcttt	m5C_67216
chr21	9827541	+	C	451	159	0.353	0	0	T	292	M	0.31	0.398	0	0	0	985.387	caatacaggaCtctttcgagg	m5C_67046
chr21	9827543	+	C	421	158	0.375	0	0	T	263	M	0.33	0.422	0	0	0	1043.96	atacaggactCtttcgaggcc	m5C_67259
chr21	9827547	+	C	498	129	0.26	1	0.002	T	368	M	0.223	0.3	0	0	0	575.244	aggactctttCgaggccctgt	m5C_67059
chr21	9827552	+	C	643	259	0.403	0	0	T	384	M	0.366	0.441	0	0	0	1893.68	tctttcgaggCcctgtaattg	m5C_67153
chr21	9827553	+	C	663	210	0.317	0	0	T	453	M	0.282	0.353	0	0	0	1186.39	ctttcgaggcCctgtaattgg	m5C_67185
chr21	9827554	+	C	686	222	0.324	0	0	T	464	M	0.29	0.36	0	0	0	1286.14	tttcgaggccCtgtaattgga	m5C_67197
chr21	9827572	+	C	324	96	0.296	0	0	T	228	M	0.249	0.348	0	0	0	478.458	ggaatgagtcCactttaaatc	m5C_67230
chr21	9827574	+	C	278	135	0.486	0	0	T	143	M	0.427	0.544	0	0	0	1154.13	aatgagtccaCtttaaatcta	m5C_67055
chr21	17729698	-	C	56	29	0.518	0	0	C	29	M	0.39	0.643	0	3.9E-07	0	226.259	gtggggattaCctttacatga	m5C_65029
chr21	17729714	-	C	60	26	0.433	0	0	T	34	M	0.316	0.559	0	1.9E-07	0	164.175	acttacacgaCtcttagtggg	m5C_65026
chr21	17911432	+	C	43	9	0.209	0	0	T	34	M	0.114	0.352	1.4E-11	1.2E-08	1.6E-11	11.1522	acccgtagatCcgatcttgtg	m5C_65011
chr21	17911433	+	C	43	11	0.256	0	0	T	32	M	0.149	0.402	1.2E-14	4.4E-08	1.5E-14	22.8887	cccgtagatcCgatcttgtgg	m5C_65018
chr21	17911437	+	C	39	22	0.564	0	0	C	22	M	0.41	0.707	0	6.3E-05	0	180.292	tagatccgatCttgtggtgaa	m5C_65017
chr21	18827105	-	C	56	14	0.25	0	0	T	42	M	0.155	0.377	0	2.8E-09	0	43.4474	gcccatgcagCacgaaaatgt	m5C_64958
chr21	18827113	-	C	354	91	0.257	0	0	T	263	M	0.214	0.305	0	0	0	390.054	gattcccggcCcatgcagcac	m5C_64963
chr21	18827124	-	C	356	106	0.298	0	0	T	250	M	0.253	0.347	0	0	0	535.545	ggcccgggttCgattcccggc	m5C_64954
chr21	18827130	-	C	394	84	0.213	0	0	T	310	M	0.176	0.256	0	0	0	295.052	gcgggaggccCgggttcgatt	m5C_64956
chr21	18827131	-	C	398	89	0.224	0	0	T	309	M	0.185	0.267	0	0	0	330.074	cgcgggaggcCcgggttcgat	m5C_64964
chr21	18827132	-	C	393	94	0.239	0	0	T	299	M	0.2	0.284	0	0	0	375.367	acgcgggaggCccgggttcga	m5C_64969
chr21	18827150	-	C	701	182	0.26	0	0	T	519	M	0.229	0.293	0	0	0	831.904	gtagaattctCgcctgccacg	m5C_64955
chr21	18827152	-	C	745	175	0.236	2	0.003	T	568	M	0.206	0.267	0	0	0	722.5	tggtagaattCtcgcctgcca	m5C_64961
chr21	18827165	-	C	941	228	0.242	0	0	T	713	M	0.216	0.271	0	0	0	984.969	atgggtggttCagtggtagaa	m5C_64957
chr21	18827176	-	C	434	90	0.207	0	0	T	344	M	0.172	0.248	0	0	0	309.381	tctatcggtgCatgggtggtt	m5C_64959
chr21	20717609	+	C	39	13	0.333	0	0	T	26	M	0.206	0.49	0	6.7E-06	0	53.6452	tagcgggggaCtgcattaata	m5C_64887
chr21	21957198	-	C	35	20	0.571	0	0	C	20	M	0.409	0.72	0	4.3E-05	0	163.429	aaagcgtcgaCagcgtagaag	m5C_64915
chr21	24654192	-	C	37	20	0.541	0	0	C	20	M	0.384	0.69	0	4.3E-05	0	153.535	agtgtagtatCtgttcttatc	m5C_64981
chr21	24654204	-	C	37	10	0.27	0	0	T	27	M	0.154	0.43	6.7E-14	2E-06	8.3E-14	20.2815	tggctgagatCaagtgtagta	m5C_64980
chr21	25816300	-	C	35	23	0.657	0	0	C	23	M	0.492	0.792	0	7.6E-05	0	226.098	ggtgtgggaaCttgttcttgt	m5C_64984
chr21	26197468	-	C	50	23	0.46	0	0	T	27	M	0.33	0.596	0	2.9E-06	0	151.659	gggactgagaCatggaaattg	m5C_65195
chr21	26197474	-	C	57	33	0.579	0	0	C	33	M	0.45	0.698	0	8.8E-07	0	296.867	gacaccgggaCtgagacatgg	m5C_65224

chr21	27354660	-	C	36	10	0.278	0	0	T	26	M	0.158	0.44	4.8E-14	2E-06	5.9E-14	21.1115	agagaatgtcCcaggtaagtc	m5C_65181
chr21	27394280	-	C	324	83	0.256	0	0	T	241	M	0.212	0.306	0	0	0	351.407	atgacgaggaCgatgaggatg	m5C_65196
chr21	27394286	-	C	283	107	0.378	0	0	T	176	M	0.324	0.436	0	0	0	692.469	ccgatgatgaCgaggacgatg	m5C_65223
chr21	27394295	-	C	186	67	0.36	0	0	T	119	M	0.295	0.431	0	0	0	394.891	aagaagaagcCgatgatgacg	m5C_65203
chr21	27394296	-	C	178	43	0.242	0	0	T	135	M	0.185	0.309	0	0	0	158.738	gaagaagaagCcgatgatgac	m5C_65186
chr21	27394317	-	C	114	26	0.228	0	0	T	88	M	0.161	0.313	0	0	0	83.5431	gaagaagtggCtgaggtggaa	m5C_65191
chr21	27394335	-	C	72	24	0.333	0	0	T	48	M	0.235	0.448	0	6.4E-11	0	112.966	gtagaagtagCagaggaggaa	m5C_65185
chr21	30365348	+	C	65	21	0.323	0	0	T	44	M	0.222	0.444	0	1E-09	0	93.2557	ccacgcctgtCtgagtgatgt	m5C_65083
chr21	30415801	+	C	62	28	0.452	0	0	T	34	M	0.334	0.575	0	9.8E-09	0	187.149	ggatgaagatCaagatagtga	m5C_65089
chr21	33040810	+	C	75	26	0.347	0	0	T	49	M	0.249	0.459	0	1.3E-10	0	129.387	atgacttgggCaaaggtggaa	m5C_65054
chr21	33040833	+	C	76	19	0.25	0	0	T	57	M	0.166	0.358	0	6.9E-12	0	63.1894	gaagaaagtaCaaagacagga	m5C_65049
chr21	33749502	-	C	43	11	0.256	0	0	T	32	M	0.149	0.402	1.2E-14	4.4E-08	1.5E-14	22.8887	aagtgggttgCacaatagata	m5C_65080
chr21	33749564	-	C	35	14	0.4	0	0	T	21	M	0.256	0.564	0	9.2E-06	0	71.5414	ccagagcaaaCtgtagccctg	m5C_65073
chr21	33749568	-	C	38	29	0.763	0	0	C	29	M	0.608	0.87	0	0.00019	0	352.599	catgccagagCaaactgtagc	m5C_65081
chr21	33749578	-	C	40	9	0.231	1	0.025	T	30	M	0.126	0.383	4.9E-12	1.2E-06	5.7E-12	12.8739	catagtacccCatgccagagc	m5C_65070
chr21	33749598	-	C	82	21	0.256	0	0	T	61	M	0.174	0.36	0	2.6E-13	0	73.0945	gcccgtccccCtggacctctc	m5C_65079
chr21	33749601	-	C	88	29	0.33	0	0	T	59	M	0.24	0.433	0	8.4E-12	0	139.361	tgggcccgtcCccctggacct	m5C_65074
chr21	33749602	-	C	94	35	0.372	0	0	T	59	M	0.281	0.473	0	1.5E-12	0	197.001	atgggcccgtCccccctggacc	m5C_65078
chr21	34351140	+	C	82	35	0.427	0	0	T	47	M	0.325	0.535	0	5.6E-11	0	227.792	gtacaataggCcacagggcgg	m5C_66073
chr21	36113129	+	C	34	8	0.235	0	0	T	26	M	0.124	0.4	4.2E-11	6.8E-07	4.8E-11	10.3259	aggtcagcagCatacgcctgc	m5C_65230
chr21	38588075	+	C	45	12	0.267	0	0	T	33	M	0.16	0.41	5.6E-16	7.5E-08	7.3E-16	29.2263	accaggttgcCtaaggaggtg	m5C_65108
chr21	38588090	+	C	53	12	0.226	0	0	T	41	M	0.135	0.355	5.8E-15	8.8E-10	7.3E-15	22.9888	gaggtgtgaaCcggcccagat	m5C_65119
chr21	38588091	+	C	53	20	0.377	0	0	T	33	M	0.259	0.512	0	3.4E-08	0	103.744	aggtgtgaacCggcccagatc	m5C_65127
chr21	38588096	+	C	51	16	0.314	0	0	T	35	M	0.203	0.45	0	7.3E-09	0	65.048	tgaaccggccCagatcagaaa	m5C_65115
chr21	40505777	-	C	33	13	0.394	0	0	T	20	M	0.247	0.563	0	6.7E-06	0	64.1755	aggcctgcagCctaggtggca	m5C_65682
chr21	40646349	-	C	43	12	0.279	0	0	T	31	M	0.167	0.427	2.2E-16	7.5E-08	2.9E-16	31.457	gagatttgagCagttagaatg	m5C_65685
chr21	40646369	-	C	39	22	0.564	0	0	C	22	M	0.41	0.707	0	6.3E-05	0	180.292	gatggaacagCacggatttgg	m5C_65683
chr21	40646372	-	C	43	12	0.279	0	0	T	31	M	0.167	0.427	2.2E-16	7.5E-08	2.9E-16	31.457	agagatggaaCagcacggatt	m5C_65680
chr21	40717085	-	C	53	12	0.226	0	0	T	41	M	0.135	0.355	5.8E-15	8.8E-10	7.3E-15	22.9888	aacggggaaaCgaagactgag	m5C_65693
chr21	40717093	-	C	68	16	0.235	0	0	T	52	M	0.15	0.349	0	1.1E-10	0	48.11	ctgcggaaaaCggggaaacga	m5C_65676
chr21	40717100	-	C	59	15	0.254	0	0	T	44	M	0.161	0.378	0	4.6E-09	0	48.1675	gacttacctgCggaaaacggg	m5C_65672
chr21	40717103	-	C	48	18	0.375	0	0	T	30	M	0.252	0.516	0	7.7E-07	0	90.7743	gaagacttacCtgcggaaaac	m5C_65677
chr21	40717104	-	C	50	16	0.32	0	0	T	34	M	0.208	0.458	0	7.3E-09	0	66.4257	agaagacttaCctgcggaaaa	m5C_65688
chr21	43795839	-	C	31	12	0.387	0	0	T	19	M	0.237	0.562	0	4.6E-06	0	56.9589	ggactggatcCacgagcagat	m5C_65269
chr21	43795840	-	C	31	9	0.29	0	0	T	22	M	0.161	0.466	3.7E-13	1.2E-06	4.4E-13	18.0154	tggactggatCcacgagcaga	m5C_65270
chr21	43795846	-	C	31	12	0.387	0	0	T	19	M	0.237	0.562	0	4.6E-06	0	56.9589	ccttcctggaCtggatccacg	m5C_65255
chr21	44273826	-	C	39	9	0.231	0	0	T	30	M	0.126	0.383	4.9E-12	1.2E-06	5.7E-12	12.8739	tggacgcccgCagacagcagt	m5C_65337
chr21	45451446	+	C	32	10	0.312	0	0	T	22	M	0.18	0.486	1.1E-14	2E-06	1.3E-14	25.0836	tgtgcctttcCtcccaggagt	m5C_65302
chr21	45744024	+	C	51	14	0.275	0	0	T	37	M	0.171	0.409	0	2.8E-09	0	47.9195	gtgatgagctCagatggggag	m5C_65320
chr21	45744036	+	C	44	27	0.614	0	0	C	27	M	0.466	0.743	0	6.7E-06	0	251.766	gatggggagaCtgaggcggga	m5C_65315
chr21	45878989	+	C	81	33	0.407	0	0	T	48	M	0.307	0.516	0	3.1E-11	0	202.594	gccacgctgcCtgagtgtcgc	m5C_65288
chr21	45878999	+	C	73	20	0.274	0	0	T	53	M	0.185	0.386	0	1.1E-11	0	73.9652	ctgagtgtcgCcatgctcgga	m5C_65311
chr22	18274171	+	C	31	21	0.677	0	0	C	21	M	0.501	0.814	0	5.2E-05	0	210.591	caagatgctgCcggaacttcc	m5C_65696
chr22	18291852	-	C	38	8	0.211	0	0	T	30	M	0.111	0.363	1.3E-10	6.8E-07	1.4E-10	8.77246	caggtggtctCcacgccacac	m5C_65748
chr22	18291854	-	C	38	12	0.316	0	0	T	26	M	0.191	0.475	0	4.6E-06	0	45.8026	tgcaggtggtCtccacgccac	m5C_65738
chr22	18291862	-	C	42	20	0.476	0	0	T	22	M	0.334	0.623	0	1.4E-06	0	133.437	ggttctggtgCaggtggtctc	m5C_65770
chr22	18291878	-	C	38	21	0.553	0	0	C	21	M	0.397	0.699	0	5.2E-05	0	166.765	tctacacaagCtaggtggttc	m5C_65765
chr22	18291884	-	C	39	10	0.256	0	0	T	29	M	0.146	0.411	1.3E-13	2E-06	1.6E-13	18.7753	ctggcgtctaCacaagctagg	m5C_65761
chr22	18291887	-	C	41	10	0.244	0	0	T	31	M	0.138	0.393	2.4E-13	2.4E-08	2.9E-13	17.4472	agcctggcgtCtacacaagct	m5C_65746
chr22	18291894	-	C	36	12	0.333	0	0	T	24	M	0.202	0.497	0	4.6E-06	0	48.5146	ggcccggagcCtggcgtctac	m5C_65762
chr22	18291901	-	C	39	8	0.205	0	0	T	31	M	0.108	0.355	1.6E-10	6.8E-07	1.8E-10	8.44384	ctagcaaggcCcggagcctgg	m5C_65745
chr22	18291907	-	C	37	8	0.216	0	0	T	29	M	0.114	0.372	9.7E-11	6.8E-07	1.1E-10	9.12273	tgctaactagCaaggcccgga	m5C_65737
chr22	18291911	-	C	38	8	0.211	0	0	T	30	M	0.111	0.363	1.3E-10	6.8E-07	1.4E-10	8.77246	ttgatgctaaCtagcaaggcc	m5C_65772
chr22	19032771	+	C	44	14	0.318	0	0	T	30	M	0.2	0.466	0	1.9E-07	0	55.9976	aaagattaggCtggtctgaag	m5C_65694
chr22	19032776	+	C	131	35	0.267	0	0	T	96	M	0.199	0.349	0	0	0	139.18	ttaggctggtCtgaaggtagt	m5C_65714
chr22	19032796	+	C	116	26	0.224	0	0	T	90	M	0.158	0.308	0	0	0	82.0542	tgagtcatctCgattgattgt	m5C_65699
chr22	19934459	-	C	40	8	0.2	0	0	T	32	M	0.105	0.348	2.1E-10	5.5E-09	2.3E-10	8.13503	gggactctgtCtcaaaaaaaa	m5C_65751
chr22	19934463	-	C	37	10	0.27	0	0	T	27	M	0.154	0.43	6.7E-14	2E-06	8.3E-14	20.2815	cagtgggactCtgtctcaaaa	m5C_65750
chr22	19934475	-	C	36	12	0.333	0	0	T	24	M	0.202	0.497	0	4.6E-06	0	48.5146	gcctgagcaaCacagtgggac	m5C_65771
chr22	19934487	-	C	33	8	0.242	0	0	T	25	M	0.128	0.41	3.1E-11	6.8E-07	3.6E-11	10.7866	cactgcactcCagcctgagca	m5C_65722
chr22	20113930	+	C	204	43	0.211	0	0	T	161	M	0.16	0.272	0	0	0	137.963	tctttgcagaCtcactctgca	m5C_65603
chr22	20113936	+	C	204	58	0.284	0	0	T	146	M	0.227	0.35	0	0	0	263.139	cagactcactCtgcatctgac	m5C_65590
chr22	20113946	+	C	217	63	0.29	0	0	T	154	M	0.234	0.354	0	0	0	294.83	ctgcatctgaCtatacttcca	m5C_65623
chr22	20113951	+	C	190	70	0.368	0	0	T	120	M	0.303	0.439	0	0	0	424.299	tctgactataCttccaggcgg	m5C_65611
chr22	20113955	+	C	156	39	0.25	0	0	T	117	M	0.189	0.323	0	0	0	147.116	actatacttcCaggcgggtgc	m5C_65618
chr22	20113959	+	C	139	36	0.259	0	0	T	103	M	0.193	0.338	0	0	0	139.205	tacttccaggCgggtgctttt	m5C_65612
chr22	20113965	+	C	120	30	0.25	0	0	T	90	M	0.181	0.334	0	0	0	108.658	caggcgggtgCttttctgtc	m5C_65606
chr22	20113975	+	C	92	27	0.293	0	0	T	65	M	0.21	0.393	0	7.2E-14	0	113.517	ctttttctgtCtgccagataa	m5C_65600
chr22	20113979	+	C	92	24	0.261	0	0	T	68	M	0.182	0.359	0	1.7E-14	0	87.3701	ttctgtctgcCagataaacat	m5C_65627
chr22	20113992	+	C	69	21	0.304	0	0	T	48	M	0.208	0.421	0	1E-09	0	87.5678	ataaacattcCagggtgctgt	m5C_65609
chr22	20114005	+	C	75	24	0.329	2	0.027	T	49	M	0.232	0.443	0	6.4E-11	0	111.333	ggtgctgtggCcgcctcacgt	m5C_65608
chr22	20114018	+	C	97	26	0.268	0	0	T	71	M	0.19	0.364	0	4.6E-14	0	98.7866	cctcacgtatCcagagtgatg	m5C_65599
chr22	20114032	+	C	102	21	0.206	0	0	T	81	M	0.139	0.294	0	0	0	58.2909	agtgatgcagCtccctgggga	m5C_65620
chr22	20114034	+	C	100	20	0.2	0	0	T	80	M	0.133	0.289	0	0	0	53.3464	tgatgcagctCcctggggaca	m5C_65586
chr22	20114035	+	C	97	32	0.33	0	0	T	65	M	0.244	0.428	0	5.3E-13	0	156.403	gatgcagctcCctggggacac	m5C_65624
chr22	20114036	+	C	103	37	0.359	0	0	T	66	M	0.273	0.455	0	6.7E-14	0	202.145	atgcagctccCtggggacaca	m5C_65589
chr22	20114043	+	C	94	23	0.245	0	0	T	71	M	0.169	0.34	0	1E-14	0	77.7094	tccctggggaCacaggtgctt	m5C_65626
chr22	20228628	+	C	53	17	0.321	0	0	T	36	M	0.211	0.455	0	1.1E-08	0	71.713	gggggctgccCgggcagcctg	m5C_65592
chr22	20228632	+	C	90	26	0.289	0	0	T	64	M	0.205	0.39	0	4.6E-14	0	106.818	gctgcccgggCagcctgattg	m5C_65615
chr22	21804686	-	C	72	24	0.333	0	0	T	48	M	0.235	0.448	0	6.4E-11	0	112.966	gtgggcggccCggtgcccccg	m5C_65651
chr22	21804687	-	C	117	69	0.59	0	0	C	69	M	0.499	0.675	0	5.2E-12	0	688.817	ggtgggcggcCcggtgccccc	m5C_65643
chr22	21804691	-	C	178	74	0.416	0	0	T	104	M	0.346	0.489	0	0	0	511.864	agtgggtgggCggcccggtgc	m5C_65631
chr22	22718118	+	C	46	14	0.304	0	0	T	32	M	0.191	0.448	0	1.9E-07	0	53.4231	gtgctgtaggCttgtccttga	m5C_65548
chr22	24349663	+	C	74	36	0.486	0	0	T	38	M	0.376	0.598	0	2.4E-09	0	270.804	gctaagtaagCtattgggccc	m5C_66517
chr22	24349671	+	C	71	23	0.324	0	0	T	48	M	0.227	0.439	0	4.3E-11	0	104.222	agctattgggCccataccctg	m5C_66515
chr22	24349672	+	C	69	19	0.275	0	0	T	50	M	0.184	0.39	0	4.5E-10	0	69.8987	gctattgggcCcatacccctga	m5C_66500
chr22	24349673	+	C	66	41	0.621	0	0	C	41	M	0.501	0.729	0	1.7E-07	0	410.478	ctattgggccCataccctgaa	m5C_66503
chr22	24349679	+	C	47	28	0.596	0	0	C	28	M	0.453	0.724	0	8.1E-06	0	253.914	ggcccataccCtgaaaatgtt	m5C_66523
chr22	25587651	+	C	32	15	0.469	0	0	T	17	M	0.309	0.636	0	1.2E-05	0	92.6074	tggtgcttgaCtgagtgctgc	m5C_66532
chr22	26854297	-	C	35	25	0.714	0	0	C	25	M	0.549	0.837	0	0.00011	0	274.724	agttgtctgaCctgcgttcag	m5C_66142
chr22	26854317	-	C	34	27	0.794	0	0	C	27	M	0.632	0.897	0	0.00015	0	341.287	gatgacacggCtgtgcacaga	m5C_66138
chr22	26854320	-	C	34	7	0.206	0	0	T	27	M	0.103	0.368	1.4E-09	3.4E-07	1.6E-09	6.4054	catgatgacaCggctgtgcac	m5C_66136
chr22	26854322	-	C	38	14	0.368	0	0	T	24	M	0.234	0.527	0	9.2E-06	0	65.4752	atcatgatgaCacggctgtgc	m5C_66135
chr22	27296723	+	C	39	12	0.308	0	0	T	27	M	0.186	0.464	0	4.6E-06	0	44.5578	cttgccaaagCttgtagattt	m5C_66123
chr22	28452262	-	C	37	15	0.405	0	0	T	22	M	0.263	0.565	0	1.2E-05	0	79.0389	gagggggtgaaCtgacccaggt	m5C_65829
chr22	29664292	+	C	64	22	0.344	0	0	T	42	M	0.239	0.466	0	1.5E-09	0	105.261	gagagggagaCggacggttgag	m5C_65805
chr22	29664307	+	C	59	29	0.492	0	0	T	30	M	0.368	0.616	0	3.9E-07	0	213.693	gttgagagaaCgaggaggaag	m5C_65794
chr22	29695816	+	C	56	16	0.286	0	0	T	40	M	0.184	0.415	0	7.3E-09	0	58.9396	aagaggaggaCgtggaggacc	m5C_65784
chr22	29729170	-	C	36	28	0.778	0	0	C	28	M	0.619	0.883	0	0.00017	0	346.724	aagaagttgaCtgaaggggct	m5C_65855
chr22	29729182	-	C	31	11	0.355	0	0	T	20	M	0.211	0.531	1.1E-16	3.1E-06	1.5E-16	37.0595	aactgagcctCtaagaagttg	m5C_65856
chr22	29729184	-	C	33	11	0.333	0	0	T	22	M	0.198	0.504	2.2E-16	3.1E-06	2.9E-16	34.0074	caaactgagcCtctaagaagt	m5C_65842

chr22	29729203	-	C	33	20	0.606	0	0	C	20	M	0.437	0.753	0	4.3E-05	0	174.733	tgagcacgctCatgatgagca	m5C_65827
chr22	29729214	-	C	32	8	0.25	0	0	T	24	M	0.133	0.421	2.3E-11	6.8E-07	2.6E-11	11.2828	attcttcttcCtgagcacgct	m5C_65828
chr22	29729215	-	C	33	7	0.212	0	0	T	26	M	0.107	0.378	1.1E-09	3.4E-07	1.2E-09	6.69165	gattcttcttCctgagcacgc	m5C_65854
chr22	29729218	-	C	34	7	0.206	0	0	T	27	M	0.103	0.368	1.4E-09	3.4E-07	1.6E-09	6.4054	gatgattcttCttcctgagca	m5C_65819
chr22	29729221	-	C	33	21	0.636	0	0	C	21	M	0.466	0.778	0	5.2E-05	0	195.787	cctgatgattCttcttcctga	m5C_65846
chr22	30975188	-	C	92	40	0.435	0	0	T	52	M	0.338	0.537	0	6.4E-12	0	270.491	gatgcagaggCtggttcagaa	m5C_65887
chr22	32435688	+	C	102	31	0.304	0	0	T	71	M	0.223	0.399	0	7.1E-15	0	138.336	gtatctccatCaagctgcagg	m5C_67585
chr22	32435692	+	C	127	52	0.409	0	0	T	75	M	0.328	0.496	0	4.4E-15	0	340.92	ctccatcaagCtgcaggagga	m5C_67577
chr22	36204757	+	C	39	11	0.282	0	0	T	28	M	0.165	0.438	3.1E-15	3.1E-06	4E-15	26.4001	atgagctgggCgtggggtgtg	m5C_66396
chr22	36204780	+	C	37	8	0.216	0	0	T	29	M	0.114	0.372	9.7E-11	6.8E-07	1.1E-10	9.12273	cttgtagtccCagctagatgg	m5C_66399
chr22	36236451	-	C	70	19	0.271	0	0	T	51	M	0.181	0.385	0	6.9E-12	0	68.854	ggagagactgCcagagaggaa	m5C_66494
chr22	36236454	-	C	79	28	0.354	0	0	T	51	M	0.258	0.464	0	2.6E-10	0	144.453	gggggagagaCtgccagagag	m5C_66480
chr22	36236479	-	C	35	12	0.343	0	0	T	23	M	0.208	0.508	0	4.6E-06	0	49.9956	tgggagggagCtggagaaaga	m5C_66460
chr22	36681270	-	C	40	15	0.375	0	0	T	25	M	0.242	0.53	0	2.8E-07	0	72.6683	taaggtcaagCtgcaggagat	m5C_66427
chr22	36681274	-	C	38	8	0.211	0	0	T	30	M	0.111	0.363	1.3E-10	6.8E-07	1.4E-10	8.77246	agcttaaggtCaagctgcagg	m5C_66439
chr22	36691094	-	C	68	27	0.397	0	0	T	41	M	0.289	0.516	0	7.5E-09	0	156.214	ggtgaacatcCtgaagaagac	m5C_66481
chr22	36691115	-	C	43	24	0.558	0	0	C	24	M	0.411	0.696	0	3.6E-06	0	197.315	caggtcaaaaCgtgagcagga	m5C_66452
chr22	36691120	-	C	35	27	0.771	0	0	C	27	M	0.61	0.879	0	0.00015	0	329.305	ctgtccaggtCaaaacgtgag	m5C_66472
chr22	37903808	-	C	62	24	0.387	0	0	T	38	M	0.276	0.512	0	3E-09	0	132.405	gtacgaggccCtgggccagtt	m5C_66451
chr22	37903809	-	C	70	23	0.329	0	0	T	47	M	0.23	0.445	0	4.3E-11	0	105.794	tgtacgaggcCctgggccagt	m5C_66433
chr22	37903810	-	C	69	20	0.29	0	0	T	49	M	0.196	0.406	0	6.9E-10	0	78.4637	gtgtacgaggCcctgggccag	m5C_66459
chr22	37903821	-	C	32	11	0.344	0	0	T	21	M	0.204	0.517	2.2E-16	3.1E-06	2.9E-16	35.1442	gcccagcctgCgtgtacgagg	m5C_66415
chr22	38891869	-	C	49	14	0.286	0	0	T	35	M	0.178	0.424	0	1.9E-07	0	49.9784	ggcaaatgttCtagattgaag	m5C_67497
chr22	38891889	-	C	50	14	0.28	0	0	T	36	M	0.175	0.417	0	1.9E-07	0	48.9272	agcaggtggcCgatgactatg	m5C_67508
chr22	38891890	-	C	49	11	0.224	0	0	T	38	M	0.13	0.359	6.6E-14	4.4E-08	8.1E-14	18.8888	cagcaggtggCcgatgactat	m5C_67526
chr22	38891897	-	C	33	13	0.394	0	0	T	20	M	0.247	0.563	0	6.7E-06	0	64.1755	gcaagtacagCaggtggccga	m5C_67503
chr22	39709822	-	C	40	8	0.2	0	0	T	32	M	0.105	0.348	2.1E-10	5.5E-09	2.3E-10	8.13503	gagaacagccCtgtggtcagg	m5C_67540
chr22	39709841	-	C	2158	496	0.23	0	0	T	1662	M	0.213	0.248	0	0	0	2108.8	tccttgttgcCttccttctga	m5C_67551
chr22	39709873	-	C	2726	587	0.216	3	0.001	T	2136	M	0.201	0.231	0	0	0	2354.24	ggcacagcgcCcgagacagac	m5C_67531
chr22	39711217	-	C	32	9	0.281	0	0	T	23	M	0.156	0.454	5.3E-13	1.2E-06	6.4E-13	17.1983	tgagaacagcCttgtggtcag	m5C_67559
chr22	39711235	-	C	1581	354	0.224	0	0	T	1227	M	0.204	0.245	0	0	0	1444.63	tctctactgcCttccttctga	m5C_67495
chr22	39711242	-	C	1772	368	0.208	0	0	T	1404	M	0.189	0.227	0	0	0	1394.2	gaaccattctCtactgccttc	m5C_67527
chr22	39715076	-	C	1506	309	0.206	3	0.002	T	1194	M	0.186	0.227	0	0	0	1148.99	aaactgtgtgCtgattgtcac	m5C_67535
chr22	39715097	-	C	1476	312	0.213	9	0.006	T	1155	M	0.193	0.234	0	0	0	1201.22	aacttattgaCgggcggacag	m5C_67547
chr22	39918095	+	C	125	30	0.24	0	0	T	95	M	0.174	0.322	0	0	0	104.16	tagtttagagCtgggcagtga	m5C_67493
chr22	39918100	+	C	151	49	0.325	0	0	T	102	M	0.255	0.403	0	0	0	249.884	tagagctgggCagtgaagtgg	m5C_67489
chr22	39918117	+	C	190	58	0.305	0	0	T	132	M	0.244	0.374	0	0	0	283.245	gtggatatcaCtgaaggagat	m5C_67482
chr22	39918619	+	C	37	19	0.514	0	0	C	19	M	0.359	0.666	0	3.4E-05	0	136.4	tgaggatagtCaggagcgtca	m5C_67469
chr22	39918625	+	C	34	16	0.471	0	0	T	18	M	0.315	0.633	0	1.7E-05	0	100.644	tagtcaggagCgtcaatgtgc	m5C_67449
chr22	39918628	+	C	31	15	0.484	0	0	T	16	M	0.32	0.652	0	1.2E-05	0	95.91	tcaggagcgtCaatgtgcttg	m5C_67467
chr22	40762482	+	C	36	11	0.306	0	0	T	25	M	0.18	0.469	1E-15	3.1E-06	1.3E-15	29.7077	cctgttaaaaCcatatgaaag	m5C_66569
chr22	41226861	-	C	45	10	0.222	0	0	T	35	M	0.125	0.363	7.4E-13	2.4E-08	8.9E-13	15.2157	tcgagaagagCatgagagagc	m5C_66595
chr22	41226870	-	C	43	10	0.233	0	0	T	33	M	0.132	0.377	4.3E-13	2.4E-08	5.2E-13	16.2683	taagaaggctCgagaagagca	m5C_66578
chr22	41226872	-	C	42	11	0.262	0	0	T	31	M	0.153	0.411	8.4E-15	4.4E-08	1.1E-14	23.69	gttaagaaggCtcgagaagag	m5C_66576
chr22	41226885	-	C	48	23	0.479	0	0	T	25	M	0.345	0.617	0	2.9E-06	0	158.567	aagaatagaaCgagttaagaa	m5C_66582
chr22	41226901	-	C	33	9	0.273	0	0	T	24	M	0.151	0.442	7.5E-13	1.2E-06	9E-13	16.4418	agcgagagatCaaagaaagaa	m5C_66575
chr22	41461620	-	C	133	45	0.338	0	0	T	88	M	0.263	0.422	0	0	0	237.13	gttgtcagagCttactaacat	m5C_66589
chr22	41461642	-	C	329	91	0.277	0	0	T	238	M	0.231	0.327	0	0	0	420.509	ttggctggtcCgatggtagtg	m5C_66579
chr22	41461643	-	C	320	100	0.312	0	0	T	220	M	0.264	0.365	0	0	0	528.383	cttggctggtCcgatggtagt	m5C_66604
chr22	41461648	-	C	158	33	0.209	0	0	T	125	M	0.153	0.279	0	0	0	100.824	ggcttcttggCtggtccgatg	m5C_66580
chr22	41650445	-	C	97	22	0.229	1	0.01	T	74	M	0.156	0.323	0	5.8E-15	0	68.8547	gaagaggaagCagaagaagag	m5C_66592
chr22	41650486	-	C	58	27	0.466	0	0	T	31	M	0.343	0.592	0	2.5E-07	0	185.392	atgacgaggaCgaggaggagg	m5C_66590
chr22	41650492	-	C	35	17	0.486	0	0	T	18	M	0.33	0.644	0	2.1E-05	0	112.18	gcagtgatgaCgaggacgagg	m5C_66585
chr22	42961101	-	C	36	12	0.333	0	0	T	24	M	0.202	0.497	0	4.6E-06	0	48.5146	aacaagctctCaaggtccatt	m5C_66808
chr22	42961109	-	C	81	46	0.568	0	0	C	46	M	0.459	0.67	0	7.9E-10	0	422.623	aacctccaaaCaagctctcaa	m5C_66805
chr22	42961113	-	C	80	20	0.25	0	0	T	60	M	0.168	0.355	0	1.5E-13	0	67.2244	ctagaacctcCaaacaagctc	m5C_66839
chr22	42961114	-	C	78	17	0.218	0	0	T	61	M	0.141	0.322	0	2.2E-12	0	47.8608	gctagaacctCcaaacaagct	m5C_66793
chr22	42961116	-	C	78	21	0.269	0	0	T	57	M	0.183	0.377	0	1.8E-11	0	77.009	ctgctagaacCtccaaacaag	m5C_66838
chr22	42961117	-	C	81	23	0.284	0	0	T	58	M	0.197	0.39	0	7.2E-13	0	90.7515	cctgctagaaCctccaaacaa	m5C_66843
chr22	42961128	-	C	80	16	0.2	0	0	T	64	M	0.127	0.3	0	1.1E-14	0	40.6488	agctgcgctcCcctgctagaa	m5C_66815
chr22	42961133	-	C	79	18	0.228	0	0	T	61	M	0.149	0.332	0	4E-12	0	53.736	cgagtagctgCgctcccctgc	m5C_66803
chr22	43011260	+	C	45	12	0.267	0	0	T	33	M	0.16	0.41	5.6E-16	7.5E-08	7.3E-16	29.2263	atgccttaaaCttatgagtaa	m5C_66760
chr22	43011296	+	C	121	34	0.281	0	0	T	87	M	0.209	0.367	0	0	0	141.845	ggggtgacgcCcgaatcctca	m5C_66769
chr22	43011303	+	C	80	26	0.325	0	0	T	54	M	0.232	0.434	0	2.7E-12	0	120.869	cgcccgaatcCtcactgctaa	m5C_66751
chr22	43011305	+	C	64	31	0.484	0	0	T	33	M	0.366	0.604	0	2.1E-08	0	227.127	cccgaatcctCactgctaatg	m5C_66785
chr22	43011310	+	C	76	16	0.211	0	0	T	60	M	0.134	0.315	0	1.2E-12	0	42.8641	atcctcactgCtaatgtgaga	m5C_66768
chr22	43011344	+	C	371	105	0.283	0	0	T	266	M	0.24	0.331	0	0	0	503.129	gggtaaaggtCgccctcaagg	m5C_66774
chr22	43011350	+	C	430	89	0.207	0	0	T	341	M	0.171	0.248	0	0	0	305.02	aggtcgccctCaaggtgaccc	m5C_66773
chr22	43011359	+	C	682	154	0.226	0	0	T	528	M	0.196	0.259	0	0	0	603.718	tcaaggtgacCcgcctacttt	m5C_66783
chr22	43011363	+	C	778	163	0.21	0	0	T	615	M	0.182	0.24	0	0	0	594.548	ggtgacccgcCtactttgcgg	m5C_66779
chr22	43011379	+	C	1367	363	0.266	2	0.001	T	1002	M	0.243	0.29	0	0	0	1765.45	tgcgggatgcCtgggagttgc	m5C_66748
chr22	43011393	+	C	1280	395	0.309	1	0.001	T	884	M	0.284	0.335	0	0	0	2244.53	gagttgcgatCtgcccgacct	m5C_66752
chr22	43011396	+	C	1165	320	0.275	0	0	T	845	M	0.25	0.301	0	0	0	1598.84	ttgcgatctgCccgaccttat	m5C_66749
chr22	43011397	+	C	1051	312	0.297	0	0	T	739	M	0.27	0.325	0	0	0	1684.92	tgcgatctgcCcgaccttatt	m5C_66754
chr22	43011401	+	C	111	30	0.27	0	0	T	81	M	0.196	0.36	0	0	0	117.821	atctgcccgaCcttattcacg	m5C_66780
chr22	44305522	+	C	48	11	0.229	0	0	T	37	M	0.133	0.365	5E-14	4.4E-08	6.2E-14	19.4712	gctggtgcttCcaaggggccc	m5C_66660
chr22	44546548	+	C	98	22	0.224	0	0	T	76	M	0.153	0.317	0	5.8E-15	0	67.3998	ctcggatgatCctcagtggtc	m5C_66663
chr22	44546549	+	C	100	36	0.36	0	0	T	64	M	0.273	0.458	0	4.8E-14	0	196.352	tcggatgatcCtcagtggtct	m5C_66648
chr22	44546551	+	C	104	22	0.212	0	0	T	82	M	0.144	0.3	0	0	0	63.3825	ggatgatcctCagtggtctgg	m5C_66657
chr22	44546558	+	C	95	20	0.211	0	0	T	75	M	0.141	0.303	0	1.7E-15	0	56.2483	cctcagtggtCtggggtgcag	m5C_66654
chr22	44546570	+	C	76	29	0.382	0	0	T	47	M	0.281	0.494	0	3.6E-10	0	162.73	ggggtgcaggCttcaaacctg	m5C_66655
chr22	44546573	+	C	63	21	0.333	0	0	T	42	M	0.229	0.456	0	1E-09	0	96.3877	gtgcaggcttCaaacctgtag	m5C_66647
chr22	44546578	+	C	47	21	0.447	0	0	T	26	M	0.314	0.588	0	1.8E-06	0	131.925	ggcttcaaacCtgtagctgtc	m5C_66646
chr22	44546595	+	C	32	18	0.562	0	0	C	18	M	0.393	0.718	0	2.7E-05	0	141.571	tgtctagtgaCagagtggttc	m5C_66659
chr22	46263602	+	C	39	14	0.359	0	0	T	25	M	0.227	0.516	0	9.2E-06	0	63.6773	catccgcggcCccgggccctt	m5C_66862
chr22	46263603	+	C	37	19	0.514	0	0	C	19	M	0.359	0.666	0	3.4E-05	0	136.4	atccgcggccCcgggcccttt	m5C_66886
chr22	46263604	+	C	40	8	0.2	0	0	T	32	M	0.105	0.348	2.1E-10	5.5E-09	2.3E-10	8.13503	tccgcggcccCgggcccttttc	m5C_66891
chr22	46508659	+	C	45	13	0.289	0	0	T	32	M	0.177	0.434	0	1.2E-07	0	46.0978	tagtttggggCtctgccctgc	m5C_66910
chr22	46508665	+	C	39	9	0.231	0	0	T	30	M	0.126	0.383	4.9E-12	1.2E-06	5.7E-12	12.8739	ggggctctgcCctgctatggg	m5C_66908
chr22	46508666	+	C	37	15	0.405	0	0	T	22	M	0.263	0.565	0	1.2E-05	0	79.0389	gggctctgccCtgctatggga	m5C_66900
chr22	46508669	+	C	41	10	0.244	0	0	T	31	M	0.138	0.393	2.4E-13	2.4E-08	2.9E-13	17.4472	ctctgccctgCtatgggataa	m5C_66909
chr22	46509610	+	C	88	19	0.216	0	0	T	69	M	0.143	0.313	0	8.4E-14	0	54.2667	agtgatgttgCccctcggaag	m5C_66861
chr22	46509611	+	C	83	18	0.217	0	0	T	65	M	0.142	0.317	0	4.5E-14	0	51.0532	gtgatgttgcCcctcggaaga	m5C_66897
chr22	46509612	+	C	80	20	0.25	0	0	T	60	M	0.168	0.355	0	1.5E-13	0	67.2244	tgatgttgccCctcggaagat	m5C_66919
chr22	46509615	+	C	75	23	0.307	0	0	T	52	M	0.214	0.418	0	4.3E-11	0	98.3789	tgttgcccctCggaagataac	m5C_66866
chr22	51108270	+	C	148	41	0.277	0	0	T	107	M	0.211	0.354	0	0	0	173.234	gctaattgatCacaaccagcc	m5C_66689
chr22	51108276	+	C	66	16	0.242	0	0	T	50	M	0.155	0.358	0	1.1E-10	0	49.629	tgatcacaacCagccagttac	m5C_66713
chr3	869140	+	C	61	15	0.246	0	0	T	46	M	0.155	0.367	0	6E-11	0	46.5196	gctggctttgCcagtagctga	m5C_10638
chr3	869147	+	C	54	12	0.222	0	0	T	42	M	0.132	0.349	7.5E-15	8.8E-10	9.6E-15	22.3614	ttgccagtagCtgactgtaga	m5C_10642
chr3	3477579	+	C	33	9	0.273	0	0	T	24	M	0.151	0.442	7.5E-13	1.2E-06	9E-13	16.4418	gagagtgacgCtcagatggat	m5C_10819
chr3	4712454	+	C	38	9	0.237	0	0	T	29	M	0.13	0.392	3.7E-12	1.2E-06	4.3E-12	13.3739	gtctcttccaCtggagagaat	m5C_10668

chr3	4856840	+	C	49	11	0.224	0	0	T	38	M	0.13	0.359	6.6E-14	4.4E-08	8.1E-14	18.8888	gacacttttgCtgacctgagg	m5C_10679
chr3	4856860	+	C	75	20	0.267	0	0	T	55	M	0.18	0.376	0	1.1E-11	0	71.9047	gagtgagaagCagaagaagga	m5C_10664
chr3	7596254	-	C	58	27	0.466	0	0	T	31	M	0.343	0.592	0	2.5E-07	0	185.392	tgatatttagCggtggattac	m5C_10768
chr3	10342944	-	C	49	10	0.204	0	0	T	39	M	0.115	0.336	2.1E-12	2.4E-08	2.4E-12	13.4191	cgagcagtgaCaagacaggtg	m5C_10793
chr3	10342950	-	C	48	12	0.25	0	0	T	36	M	0.149	0.388	1.3E-15	7.5E-08	1.7E-15	26.6338	gcagaacgagCagtgacaaga	m5C_10786
chr3	10342963	-	C	52	12	0.231	0	0	T	40	M	0.137	0.361	4.4E-15	8.8E-10	5.7E-15	23.6371	tgacagagggCcagcagaacg	m5C_10802
chr3	12552595	-	C	34	17	0.5	0	0	CT	17	M	0.341	0.659	0	2.1E-05	0	115.835	atgctgtaggCttaaaacaag	m5C_12950
chr3	12552602	-	C	46	19	0.413	0	0	T	27	M	0.283	0.557	0	1E-06	0	107.496	atactggatgCtgtaggctta	m5C_12959
chr3	12552609	-	C	57	15	0.263	0	0	T	42	M	0.166	0.39	0	4.6E-09	0	49.9369	cctgggaataCtggatgctgt	m5C_12965
chr3	12552618	-	C	47	10	0.213	0	0	T	37	M	0.12	0.349	1.3E-12	2.4E-08	1.5E-12	14.271	tggagactgcCtgggaatact	m5C_12946
chr3	12552619	-	C	52	16	0.308	0	0	T	36	M	0.199	0.443	0	7.3E-09	0	63.7266	gtggagactgCctgggaatac	m5C_12969
chr3	12552622	-	C	33	8	0.242	0	0	T	25	M	0.128	0.41	3.1E-11	6.8E-07	3.6E-11	10.7866	ggagtggagaCtgcctgggaa	m5C_12935
chr3	12625313	-	C	40	10	0.25	0	0	T	30	M	0.142	0.402	1.8E-13	2.4E-08	2.2E-13	18.0914	ccctatggggCggggccgcag	m5C_12951
chr3	12881818	-	C	89	18	0.202	0	0	T	71	M	0.132	0.297	0	4.5E-14	0	47.4972	agacactctgCgacagtgttc	m5C_12962
chr3	12881846	-	C	104	21	0.202	0	0	T	83	M	0.136	0.289	0	0	0	57.1344	ccagcttgctCcgtagctggc	m5C_12953
chr3	12881848	-	C	99	20	0.202	0	0	T	79	M	0.135	0.292	0	1.7E-15	0	53.9025	tcccagcttgCtccgtagctg	m5C_12961
chr3	12881857	-	C	146	39	0.267	0	0	T	107	M	0.202	0.344	0	0	0	157.558	ccattccattCccagcttgct	m5C_12941
chr3	12881861	-	C	174	42	0.241	0	0	T	132	M	0.184	0.31	0	0	0	154.413	gcacccattcCattcccagct	m5C_12928
chr3	12881868	-	C	221	48	0.217	0	0	T	173	M	0.168	0.276	0	0	0	161.196	ggcagaggcaCccattccatt	m5C_12948
chr3	12881880	-	C	275	79	0.287	0	0	T	196	M	0.237	0.343	0	0	0	374.478	gaaagcaagcCtggcagaggc	m5C_12931
chr3	12881912	-	C	1153	267	0.232	0	0	T	886	M	0.208	0.257	0	0	0	1111.45	gcctagtattCtgccagcttc	m5C_12957
chr3	12881920	-	C	1275	268	0.211	2	0.002	T	1005	M	0.189	0.234	0	0	0	1013.14	tggagagtgcCtagtattctg	m5C_12934
chr3	12881921	-	C	1292	259	0.2	0	0	T	1033	M	0.18	0.223	0	0	0	929.998	ctggagagtgCctagtattct	m5C_12964
chr3	12881936	-	C	1361	346	0.254	1	0.001	T	1014	M	0.232	0.278	0	0	0	1605.29	ctcctgggatCgcatctggag	m5C_12954
chr3	13708676	+	C	35	22	0.629	0	0	C	22	M	0.463	0.768	0	6.3E-05	0	203.881	gaacatgcccCtgatcaccct	m5C_12921
chr3	15549511	+	C	66	26	0.394	0	0	T	40	M	0.285	0.515	0	5.6E-09	0	148.215	attggtggttCagtggtagaa	m5C_11061
chr3	15549528	+	C	31	7	0.226	0	0	T	24	M	0.114	0.398	6.4E-10	3.4E-07	7.2E-10	7.33205	agaattctcgCctcccacatt	m5C_11063
chr3	15780090	+	C	52	14	0.269	0	0	T	38	M	0.168	0.403	0	2.8E-09	0	46.9527	atcgcttgagCccaggagttc	m5C_11089
chr3	15780091	+	C	51	23	0.451	0	0	T	28	M	0.323	0.586	0	8.7E-08	0	148.43	tcgcttgagcCcaggagttct	m5C_11078
chr3	15780100	+	C	51	15	0.294	0	0	T	36	M	0.187	0.43	0	4.6E-09	0	56.1257	cccaggagttCtgggctgtag	m5C_11095
chr3	15780105	+	C	50	15	0.3	0	0	T	35	M	0.191	0.438	0	4.6E-09	0	57.3102	gagttctgggCtgtagtgcgc	m5C_11072
chr3	15780113	+	C	46	11	0.239	0	0	T	35	M	0.139	0.379	2.8E-14	4.4E-08	3.5E-14	20.7303	ggctgtagtgCgctatgccga	m5C_11090
chr3	15780120	+	C	35	15	0.429	0	0	T	20	M	0.28	0.591	0	1.2E-05	0	83.953	gtgcgctatgCcgatcgggtg	m5C_11056
chr3	20234319	-	C	55	14	0.255	0	0	T	41	M	0.158	0.383	0	2.8E-09	0	44.2735	atattagtgtCactaaggttg	m5C_11330
chr3	20234359	-	C	105	26	0.248	0	0	T	79	M	0.175	0.338	0	6.7E-16	0	90.9733	ttaggctggtCtgatggtagt	m5C_11333
chr3	20234364	-	C	48	10	0.208	0	0	T	38	M	0.117	0.343	1.6E-12	2.4E-08	1.9E-12	13.8343	tatgtttaggCtggtctgatg	m5C_11322
chr3	21591514	-	C	34	11	0.324	0	0	T	23	M	0.191	0.492	4.4E-16	3.1E-06	5.8E-16	32.3088	ggttaggattCagctctctca	m5C_11332
chr3	21591529	-	C	65	20	0.312	1	0.015	T	44	M	0.212	0.434	0	6.9E-10	0	84.9236	ccctcgtggtCtagtggttag	m5C_11329
chr3	22423842	+	C	67	16	0.239	0	0	T	51	M	0.153	0.353	0	1.1E-10	0	48.8576	gttgtcaaggCtgaaaaaagc	m5C_11031
chr3	23315329	+	C	42	10	0.238	0	0	T	32	M	0.135	0.385	3.2E-13	2.4E-08	3.9E-13	16.8407	gtgtttacaaCcaactgatca	m5C_11032
chr3	23315338	+	C	45	9	0.2	0	0	T	36	M	0.109	0.338	2.3E-11	1.2E-08	2.7E-11	10.4289	accaactgatCacagccagtt	m5C_11037
chr3	23315365	+	C	47	11	0.234	0	0	T	36	M	0.136	0.372	3.8E-14	4.4E-08	4.7E-14	20.0856	ttctttgttcCttttctactc	m5C_11029
chr3	23315376	+	C	44	9	0.205	0	0	T	35	M	0.112	0.345	1.8E-11	1.2E-08	2.1E-11	10.7806	ttttctactcCcactgcttca	m5C_11033
chr3	23315392	+	C	44	15	0.341	0	0	T	29	M	0.219	0.489	0	2.8E-07	0	65.6269	cttcacttgaCtagccatata	m5C_11042
chr3	23934710	-	C	31	10	0.323	0	0	T	21	M	0.186	0.499	7.1E-15	2E-06	9E-15	26.2725	gtgactgttaCagatcggaaa	m5C_11045
chr3	23934729	-	C	36	16	0.444	0	0	T	20	M	0.295	0.604	0	1.7E-05	0	94.5313	gaaagtaagaCtgtgggaggt	m5C_11049
chr3	24664471	+	C	71	39	0.549	0	0	C	39	M	0.434	0.66	0	4.7E-09	0	338.527	tctcttgtttCctggtcaacg	m5C_11189
chr3	24664472	+	C	70	35	0.5	0	0	CT	35	M	0.386	0.614	0	1.9E-09	0	270.169	ctcttgtttcCtggtcaacgt	m5C_11195
chr3	24664477	+	C	69	27	0.391	0	0	T	42	M	0.285	0.509	0	7.5E-09	0	153.798	gtttcctggtCaacgtactaa	m5C_11186
chr3	24664480	+	C	66	14	0.212	0	0	T	52	M	0.131	0.325	2.2E-16	3.2E-11	2.9E-16	28.6601	tcctggtcaaCgtactaaagc	m5C_11193
chr3	24664484	+	C	57	29	0.509	0	0	C	29	M	0.383	0.634	0	3.9E-07	0	221.906	ggtcaacgtaCtaaagctata	m5C_11192
chr3	27526649	-	C	61	15	0.246	0	0	T	46	M	0.155	0.367	0	6E-11	0	46.5196	cccagctactCaggaggctga	m5C_11409
chr3	27526654	-	C	84	20	0.238	0	0	T	64	M	0.16	0.339	0	1.5E-13	0	63.8983	gtagtcccagCtactcaggag	m5C_11418
chr3	27526658	-	C	110	47	0.427	0	0	T	63	M	0.339	0.521	0	3.8E-14	0	318.492	gtctgtagtcCcagctactca	m5C_11411
chr3	27526659	-	C	109	31	0.284	0	0	T	78	M	0.208	0.375	0	7.1E-15	0	129.073	cgtctgtagtCccagctactc	m5C_11407
chr3	27526666	-	C	117	25	0.214	0	0	T	92	M	0.149	0.296	0	0	0	74.5642	tggcgcgcgtCtgtagtccca	m5C_11415
chr3	27526671	-	C	114	29	0.254	0	0	T	85	M	0.183	0.341	0	0	0	106.344	cgtggtggcgCgcgtctgtag	m5C_11410
chr3	27526673	-	C	115	41	0.357	0	0	T	74	M	0.275	0.447	0	6E-15	0	225.434	ggcgtggtggCgcgcgtctgt	m5C_11408
chr3	27526685	-	C	115	30	0.261	0	0	T	85	M	0.189	0.348	0	0	0	113.564	aaaaagtttgcCgggcgtggtg	m5C_11413
chr3	30646375	+	C	50	11	0.22	0	0	T	39	M	0.128	0.352	8.5E-14	4.4E-10	1.1E-13	18.3342	ggcttgaagaCacgttcaagg	m5C_11236
chr3	33144658	+	C	62	14	0.226	0	0	T	48	M	0.14	0.344	0	3.2E-11	0	39.0742	aacaggggacCaccaggttgc	m5C_11434
chr3	33144661	+	C	103	28	0.272	0	0	T	75	M	0.195	0.365	0	1.8E-15	0	109.36	aggggaccacCaggttgccta	m5C_11443
chr3	33144688	+	C	245	52	0.212	0	0	T	193	M	0.166	0.268	0	0	0	172.316	ggtgaattggCcaaggttgga	m5C_11449
chr3	33144689	+	C	287	82	0.286	0	0	T	205	M	0.237	0.341	0	0	0	387.939	gtgaattggcCaaggttggaa	m5C_11435
chr3	33185672	+	C	53	13	0.25	1	0.019	T	39	M	0.152	0.382	1.1E-16	1.6E-09	1.5E-16	31.5947	ataggatttgCtgagagtttg	m5C_11445
chr3	35225121	-	C	45	11	0.244	0	0	T	34	M	0.142	0.387	2.1E-14	4.4E-08	2.7E-14	21.4112	ggtcaggaacCtgtgtgcatg	m5C_11285
chr3	35225122	-	C	55	19	0.345	0	0	T	36	M	0.234	0.477	0	2.4E-08	0	88.7776	aggtcaggaaCctgtgtgcat	m5C_11290
chr3	35225128	-	C	57	24	0.421	0	0	T	33	M	0.302	0.55	0	1.2E-07	0	144.9	agtaaaaggtCaggaacctgt	m5C_11281
chr3	36966194	-	C	46	14	0.304	0	0	T	32	M	0.191	0.448	0	1.9E-07	0	53.4231	gaggagtgtcCttagggcaac	m5C_11659
chr3	38278383	+	C	41	10	0.244	0	0	T	31	M	0.138	0.393	2.4E-13	2.4E-08	2.9E-13	17.4472	aggatggaggCtgggagtgga	m5C_12994
chr3	38691349	-	C	31	21	0.677	0	0	C	21	M	0.501	0.814	0	5.2E-05	0	210.591	tgtgtccccсCtccgagcggg	m5C_13043
chr3	38691350	-	C	34	13	0.382	0	0	T	21	M	0.239	0.55	0	6.7E-06	0	62.1399	gtgtgtccccCctccgagcgg	m5C_13031
chr3	38691351	-	C	35	27	0.771	0	0	C	27	M	0.61	0.879	0	0.00015	0	329.305	ggtgtgtcccCcctccgagcg	m5C_13040
chr3	38691353	-	C	33	14	0.424	0	0	T	19	M	0.272	0.592	0	9.2E-06	0	76.259	ctggtgtgtcCcccctccgag	m5C_13054
chr3	38691354	-	C	33	14	0.424	0	0	T	19	M	0.272	0.592	0	9.2E-06	0	76.259	gctggtgtgtCccccctccga	m5C_13036
chr3	38691363	-	C	35	16	0.457	0	0	T	19	M	0.305	0.618	0	1.7E-05	0	97.4905	ccggcgggcgCtggtgtgtcc	m5C_13022
chr3	39449899	+	C	211	46	0.218	0	0	T	165	M	0.168	0.278	0	0	0	154.201	tattaaagctCagggtggagg	m5C_12987
chr3	39449910	+	C	204	48	0.235	0	0	T	156	M	0.182	0.298	0	0	0	175.018	agggtggaggCcagtcttggc	m5C_12990
chr3	39449911	+	C	195	59	0.303	0	0	T	136	M	0.242	0.37	0	0	0	286.049	gggtggaggcCagtcttggct	m5C_12997
chr3	39449920	+	C	208	58	0.279	0	0	T	150	M	0.222	0.343	0	0	0	257.91	ccagtcttggCtcatgaactt	m5C_13017
chr3	39449922	+	C	199	75	0.377	0	0	T	124	M	0.312	0.446	0	0	0	468.727	agtcttggctCatgaacttct	m5C_13003
chr3	39449928	+	C	198	44	0.222	0	0	T	154	M	0.17	0.285	0	0	0	149.522	ggctcatgaaCttctgagtgt	m5C_13016
chr3	39449931	+	C	156	40	0.256	0	0	T	116	M	0.194	0.33	0	0	0	155.455	tcatgaacttCtgagtgtcgg	m5C_12993
chr3	39449949	+	C	75	21	0.28	0	0	T	54	M	0.191	0.39	0	1.8E-11	0	80.2327	cggaagtgtgCtatatcaatg	m5C_13009
chr3	39449955	+	C	62	16	0.258	0	0	T	46	M	0.166	0.379	0	1.1E-10	0	52.9752	tgtgctatatCaatggcagga	m5C_12996
chr3	39449961	+	C	54	14	0.259	0	0	T	40	M	0.161	0.389	0	2.8E-09	0	45.1318	atatcaatggCaggattttcg	m5C_13015
chr3	39452562	+	C	893	211	0.237	1	0.001	T	681	M	0.21	0.266	0	0	0	885.463	attgttagagCttggagttga	m5C_13005
chr3	39452575	+	C	768	212	0.276	0	0	T	556	M	0.246	0.309	0	0	0	1041.34	ggagttgaggCtactgactgg	m5C_13002
chr3	39452586	+	C	543	128	0.236	0	0	T	415	M	0.202	0.273	0	0	0	517.017	tactgactggCcgatgaactc	m5C_13014
chr3	39452594	+	C	416	86	0.207	0	0	T	330	M	0.171	0.248	0	0	0	293.405	ggccgatgaaCtcgcaagtgt	m5C_12995
chr3	39452615	+	C	267	58	0.217	0	0	T	209	M	0.172	0.271	0	0	0	199.48	aggtagtgtgCtacatgaggg	m5C_13007
chr3	39452641	+	C	168	44	0.263	1	0.006	T	123	M	0.203	0.335	0	0	0	178.218	ttttcgctaaCaccacaaggg	m5C_13004
chr3	39452644	+	C	157	36	0.229	0	0	T	121	M	0.17	0.301	0	0	0	122.743	tcgctaacacCacaagggtct	m5C_13012
chr3	39452653	+	C	119	27	0.229	1	0.008	T	91	M	0.162	0.312	0	0	0	87.6343	ccacaagggtCtctggcccaa	m5C_13001
chr3	39452660	+	C	148	44	0.297	0	0	T	104	M	0.23	0.375	0	0	0	201.999	ggtctctggcCcaatgagtgg	m5C_13018
chr3	39452686	+	C	281	62	0.221	0	0	T	219	M	0.176	0.273	0	0	0	218.368	gatagtaattCttgctacaag	m5C_13010
chr3	39452693	+	C	262	55	0.21	0	0	T	207	M	0.165	0.263	0	0	0	181.478	attcttgctaCaagtataaca	m5C_13006
chr3	40250625	+	C	34	12	0.353	0	0	T	22	M	0.215	0.521	0	4.6E-06	0	51.5705	agtttggcgtCaatatggtga	m5C_11532
chr3	40250637	+	C	63	35	0.556	0	0	C	35	M	0.433	0.672	0	5.3E-08	0	303.236	atatggtgacCtcccgggagc	m5C_11515
chr3	40250639	+	C	66	25	0.379	0	0	T	41	M	0.271	0.499	0	4.1E-09	0	135.746	atggtgacctCccgggagcgg	m5C_11540
chr3	40250640	+	C	61	29	0.475	0	0	T	32	M	0.355	0.598	0	1.3E-08	0	206.076	tggtgacctcCcgggagcggg	m5C_11528

chr3	40935800	+	C	34	14	0.412	0	0	T	20	M	0.264	0.578	0	9.2E-06	0	73.8241	ttatgtttgcCtgagcaacag	m5C_11530
chr3	45049010	+	C	49	18	0.367	0	0	T	31	M	0.247	0.507	0	7.7E-07	0	88.7939	ttgtgacgtgCatgaggaaag	m5C_11779
chr3	45730484	-	C	304	63	0.207	0	0	T	241	M	0.165	0.256	0	0	0	208.485	ctcgcgatgtCtgttttgcca	m5C_11810
chr3	45730532	-	C	170	41	0.241	0	0	T	129	M	0.183	0.311	0	0	0	150.097	acgcgtctgaCtacggatcag	m5C_11804
chr3	45730541	-	C	158	33	0.209	0	0	T	125	M	0.153	0.279	0	0	0	100.824	caatggataaCgcgtctgact	m5C_11806
chr3	45730551	-	C	204	43	0.211	0	0	T	161	M	0.16	0.272	0	0	0	137.963	gccagtggcgCaatggataac	m5C_11800
chr3	45784270	+	C	38	12	0.316	0	0	T	26	M	0.191	0.475	0	4.6E-06	0	45.8026	ttatgctgatCggatgtccgc	m5C_11784
chr3	47703142	-	C	32	13	0.406	0	0	T	19	M	0.255	0.577	0	6.7E-06	0	66.3505	aaatgtagaaCcatagaattt	m5C_11880
chr3	48129014	-	C	34	9	0.265	0	0	T	25	M	0.146	0.431	1.1E-12	1.2E-06	1.3E-12	15.7394	agggtgccgtCagcttggctg	m5C_12777
chr3	48136332	-	C	32	9	0.281	0	0	T	23	M	0.156	0.454	5.3E-13	1.2E-06	6.4E-13	17.1983	tgtgaatagcCactgcactcc	m5C_12728
chr3	48136343	-	C	41	10	0.244	0	0	T	31	M	0.138	0.393	2.4E-13	2.4E-08	2.9E-13	17.4472	ggggtcgcacCtgtgaatagc	m5C_12730
chr3	48136346	-	C	48	11	0.229	0	0	T	37	M	0.133	0.365	5E-14	4.4E-08	6.2E-14	19.4712	agtggggtcgCacctgtgaat	m5C_12704
chr3	48136371	-	C	38	14	0.368	0	0	T	24	M	0.234	0.527	0	9.2E-06	0	65.4752	ggtcaaaactCccgtgctgat	m5C_12694
chr3	48330084	+	C	40	18	0.462	1	0.025	T	21	M	0.316	0.614	0	2.7E-05	0	113.64	tgaggctggtCtgaaggtagt	m5C_12666
chr3	48642186	-	C	52	36	0.692	0	0	C	36	M	0.557	0.801	0	1.5E-06	0	401.257	tggcagtgccCtggagaagga	m5C_12679
chr3	48642197	-	C	40	8	0.2	0	0	T	32	M	0.105	0.348	2.1E-10	5.5E-09	2.3E-10	8.13503	aagaatcggcCtggcagtgcc	m5C_12757
chr3	48642198	-	C	40	8	0.2	0	0	T	32	M	0.105	0.348	2.1E-10	5.5E-09	2.3E-10	8.13503	aaagaatcggCctggcagtgc	m5C_12707
chr3	48642201	-	C	35	8	0.229	0	0	T	27	M	0.121	0.39	5.6E-11	6.8E-07	6.4E-11	9.89691	aacaaagaatCggcctggcag	m5C_12773
chr3	48643122	-	C	55	24	0.436	0	0	T	31	M	0.314	0.567	0	1.2E-07	0	150.592	aaaaattagcCtgattatggg	m5C_12725
chr3	48643135	-	C	101	21	0.208	0	0	T	80	M	0.14	0.297	0	0	0	58.8869	ggatgaaggtCagaaaaatta	m5C_12724
chr3	49057638	-	C	49	20	0.408	0	0	T	29	M	0.282	0.548	0	1.4E-06	0	112.86	acgatcactcCcgttgagtgg	m5C_12726
chr3	49057639	-	C	54	15	0.278	0	0	T	39	M	0.176	0.409	0	4.6E-09	0	52.85	cacgatcactCccgttgagtg	m5C_12732
chr3	49057641	-	C	49	30	0.612	0	0	C	30	M	0.472	0.736	0	1.2E-05	0	283.474	gacacgatcaCtcccgttgag	m5C_12692
chr3	49057643	-	C	56	17	0.304	0	0	T	39	M	0.199	0.433	0	1.1E-08	0	67.6606	atgacacgatCactcccgttg	m5C_12748
chr3	49057647	-	C	54	12	0.222	0	0	T	42	M	0.132	0.349	7.5E-15	8.8E-10	9.6E-15	22.3614	tggaatgacaCgatcactccc	m5C_12750
chr3	49058107	-	C	46	24	0.522	0	0	C	24	M	0.381	0.659	0	3.6E-06	0	183.058	ccaaaagcagCtgttgtctcc	m5C_12706
chr3	49058110	-	C	49	19	0.388	0	0	T	30	M	0.264	0.528	0	1E-06	0	100.429	atcccaaaagCagctgttgtc	m5C_12746
chr3	49058116	-	C	54	28	0.519	0	0	C	28	M	0.389	0.646	0	3.1E-07	0	217.568	aacggaatccCaaaagcagct	m5C_12788
chr3	49058117	-	C	55	18	0.327	0	0	T	37	M	0.218	0.459	0	1.7E-08	0	78.5264	caacggaatcCcaaaagcagc	m5C_12717
chr3	49058118	-	C	53	33	0.623	0	0	C	33	M	0.488	0.741	0	8.8E-07	0	322.124	gcaacggaatCccaaaagcag	m5C_12786
chr3	49135867	-	C	42	10	0.238	0	0	T	32	M	0.135	0.385	3.2E-13	2.4E-08	3.9E-13	16.8407	tgcagacgggCagatgctgga	m5C_12761
chr3	49135871	-	C	50	32	0.64	0	0	C	32	M	0.501	0.759	0	7.2E-07	0	320.901	gacctgcagaCgggcagatgc	m5C_12781
chr3	49135889	-	C	63	13	0.206	0	0	T	50	M	0.125	0.322	2.4E-15	1.6E-11	3.1E-15	23.6977	tgtagagagtCtggaggtgac	m5C_12764
chr3	50152909	+	C	137	63	0.46	0	0	T	74	M	0.379	0.543	0	2.4E-15	0	477.075	tgaggaggagCtggtggagag	m5C_11685
chr3	50153384	+	C	31	10	0.323	0	0	T	21	M	0.186	0.499	7.1E-15	2E-06	9E-15	26.2725	cgagcaggagCtggaagcctt	m5C_11735
chr3	50153391	+	C	38	10	0.263	0	0	T	28	M	0.15	0.42	9.4E-14	2E-06	1.2E-13	19.5037	gagctggaagCcttggagcta	m5C_11686
chr3	50562006	+	C	47	15	0.319	0	0	T	32	M	0.204	0.462	0	2.8E-07	0	61.1856	taaggccatgCcaccctgaac	m5C_11736
chr3	50562009	+	C	46	16	0.348	0	0	T	30	M	0.227	0.492	0	4E-07	0	72.5786	ggccatgccaCcctgaacgtg	m5C_11695
chr3	50562011	+	C	48	24	0.5	0	0	CT	24	M	0.364	0.636	0	3.6E-06	0	174.668	ccatgccaccCtgaacgtgcc	m5C_11677
chr3	50562020	+	C	45	9	0.2	0	0	T	36	M	0.109	0.338	2.3E-11	1.2E-08	2.7E-11	10.4289	cctgaacgtgCctgatctcgt	m5C_11729
chr3	50562026	+	C	46	25	0.543	0	0	C	25	M	0.402	0.678	0	4.5E-06	0	200.9	cgtgcctgatCtcgtctgaga	m5C_11731
chr3	50795962	+	C	31	8	0.258	0	0	T	23	M	0.137	0.432	1.7E-11	6.8E-07	1.9E-11	11.8182	ccaccctgaaCgcgccgatct	m5C_11741
chr3	51426364	+	C	35	26	0.743	0	0	C	26	M	0.579	0.858	0	0.00013	0	301.238	gcacagtggaCctgaagaagc	m5C_11734
chr3	51426377	+	C	36	20	0.556	0	0	C	20	M	0.396	0.705	0	4.3E-05	0	158.323	gaagaagctcCgagttaaaga	m5C_11698
chr3	51426401	+	C	31	14	0.452	0	0	T	17	M	0.292	0.622	0	9.2E-06	0	81.6522	gaagaagattCtggatgactg	m5C_11696
chr3	51728482	-	C	53	24	0.453	0	0	T	29	M	0.327	0.585	0	1.2E-07	0	156.755	gtgctgtaggCttttggccta	m5C_11766
chr3	51728489	-	C	70	26	0.371	0	0	T	44	M	0.268	0.489	0	5.6E-09	0	139.207	ataccgggtgCtgtaggcttt	m5C_11760
chr3	51728495	-	C	77	20	0.26	0	0	T	57	M	0.175	0.367	0	1.1E-11	0	69.9563	cctggaatacCgggtgctgta	m5C_11764
chr3	51728496	-	C	72	24	0.333	0	0	T	48	M	0.235	0.448	0	6.4E-11	0	112.966	gcctggaataCcgggtgctgt	m5C_11762
chr3	51728504	-	C	63	29	0.46	0	0	T	34	M	0.343	0.582	0	1.3E-08	0	198.99	gggagactgcCtggaataccg	m5C_11753
chr3	51728508	-	C	47	13	0.277	0	0	T	34	M	0.169	0.418	0	1.2E-07	0	44.0347	ggatgggagaCtgcctggaat	m5C_11759
chr3	52302356	-	C	127	93	0.732	0	0	C	93	M	0.649	0.802	0	1.2E-11	0	1207.68	gtagtttgtaCagtttgaggg	m5C_16502
chr3	52521090	+	C	33	9	0.273	0	0	T	24	M	0.151	0.442	7.5E-13	1.2E-06	9E-13	16.4418	atgtgggaggCttgggagacc	m5C_16420
chr3	52722929	+	C	152	38	0.25	0	0	T	114	M	0.188	0.324	0	0	0	142.803	ggtcaacattCttcataccaa	m5C_16447
chr3	52722948	+	C	162	46	0.284	0	0	T	116	M	0.22	0.358	0	0	0	202.533	aaagtgaagaCatgagatcca	m5C_16456
chr3	52722960	+	C	139	47	0.338	0	0	T	92	M	0.265	0.42	0	0	0	248.893	tgagatccaaCtctgagctca	m5C_16458
chr3	52722962	+	C	143	70	0.49	0	0	T	73	M	0.409	0.571	0	4.4E-16	0	572.501	agatccaactCtgagctcacc	m5C_16455
chr3	52722967	+	C	92	21	0.228	0	0	T	71	M	0.154	0.324	0	3.1E-15	0	64.8567	caactctgagCtcaccctgtt	m5C_16441
chr3	52724785	+	C	647	152	0.241	17	0.026	T	478	M	0.209	0.276	0	0	0	636.856	gatgagtgtaCaaaatcttga	m5C_16442
chr3	52724822	+	C	572	142	0.248	0	0	T	430	M	0.215	0.285	0	0	0	609.483	attacaagatCcaactctgat	m5C_16416
chr3	52725408	+	C	384	165	0.44	9	0.023	T	210	M	0.391	0.491	0	0	0	1289.04	aatgaagaaaCtaaaattggt	m5C_16430
chr3	52725419	+	C	390	89	0.228	0	0	T	301	M	0.189	0.272	0	0	0	336.995	taaaattggtCttagtattga	m5C_16439
chr3	52725451	+	C	358	160	0.447	0	0	T	198	M	0.396	0.499	0	0	0	1268.03	tgagatccaaCtctgatcttg	m5C_16432
chr3	52725453	+	C	370	191	0.516	0	0	C	191	M	0.465	0.567	0	0	0	1777.79	agatccaactCtgatcttgcc	m5C_16426
chr3	52725458	+	C	275	60	0.218	0	0	T	215	M	0.173	0.271	0	0	0	208.118	caactctgatCttgccctaaa	m5C_16427
chr3	52726781	+	C	1485	411	0.277	1	0.001	T	1073	M	0.255	0.3	0	0	0	2094.33	taaactggatCtgactgactg	m5C_16449
chr3	52726789	+	C	1517	540	0.356	1	0.001	T	976	M	0.332	0.381	0	0	0	3590.84	atctgactgaCtgtgctgagt	m5C_16428
chr3	52726794	+	C	1528	505	0.33	0	0	T	1023	M	0.307	0.354	0	0	0	3104.36	actgactgtgCtgagtctgtt	m5C_16429
chr3	52726813	+	C	1165	280	0.24	0	0	T	885	M	0.217	0.266	0	0	0	1213.44	ttcaatccaaCcctgagcttc	m5C_16418
chr3	52726820	+	C	628	186	0.296	0	0	T	442	M	0.262	0.333	0	0	0	973.878	caaccctgagCttcatgttct	m5C_16421
chr3	58095769	+	C	39	28	0.718	0	0	C	28	M	0.562	0.835	0	0.00017	0	314.857	gtgtgatgccCgggtgttaag	m5C_12794
chr3	59246316	+	C	44	30	0.682	0	0	C	30	M	0.534	0.8	0	1.2E-05	0	320.657	cttggtgactCagtggcagaa	m5C_12800
chr3	65330388	+	C	32	7	0.219	0	0	T	25	M	0.11	0.388	8.5E-10	3.4E-07	9.5E-10	6.99971	cactttgtagCttagcaggca	m5C_12567
chr3	68708231	-	C	78	17	0.218	0	0	T	61	M	0.141	0.322	0	2.2E-12	0	47.8608	cgacaggtggCacggagaatt	m5C_12827
chr3	69077098	-	C	32	12	0.375	0	0	T	20	M	0.229	0.547	0	4.6E-06	0	55.0408	gaaagttgaaCaagaaaggaa	m5C_12818
chr3	69153662	+	C	36	22	0.611	0	0	C	22	M	0.449	0.752	0	6.3E-05	0	197.402	caagaacaaaCtggagaataa	m5C_12814
chr3	73160226	+	C	282	81	0.287	0	0	T	201	M	0.238	0.343	0	0	0	384.849	tatccgaggaCaatatattaa	m5C_13127
chr3	73160270	+	C	152	49	0.322	0	0	T	103	M	0.253	0.4	0	0	0	248.17	ggaataggagCttgctctctc	m5C_13130
chr3	73160274	+	C	68	40	0.588	0	0	C	40	M	0.47	0.697	0	1.4E-07	0	375.69	taggagcttgCtctctccact	m5C_13134
chr3	73160276	+	C	53	17	0.321	0	0	T	36	M	0.211	0.455	0	1.1E-08	0	71.713	ggagcttgctCtctccactcc	m5C_13136
chr3	75732210	-	C	72	19	0.264	0	0	T	53	M	0.176	0.376	0	6.9E-12	0	66.8558	gaaattcagtCtctgaacaga	m5C_14056
chr3	99328405	-	C	51	35	0.686	0	0	C	35	M	0.55	0.797	0	1.3E-06	0	384.809	ccctggtggcCtagtggctaa	m5C_13126
chr3	100100507	-	C	41	10	0.244	0	0	T	31	M	0.138	0.393	2.4E-13	2.4E-08	2.9E-13	17.4472	ggagagaaatCtgatgaagat	m5C_13251
chr3	100504568	-	C	36	10	0.278	0	0	T	26	M	0.158	0.44	4.8E-14	2E-06	5.9E-14	21.1115	gtggttaaggCaatagactag	m5C_13240
chr3	100504581	-	C	37	20	0.541	0	0	C	20	M	0.384	0.69	0	4.3E-05	0	153.535	tagttgtggcCgagtggttaa	m5C_13233
chr3	102878890	+	C	64	17	0.266	0	0	T	47	M	0.173	0.385	0	1.8E-10	0	58.8072	gtgtgctgacCcctgtgattt	m5C_13159
chr3	102878902	+	C	60	19	0.317	0	0	T	41	M	0.213	0.442	0	4.5E-10	0	80.9617	ctgtgatttcCcaaatgtgg	m5C_13152
chr3	102878903	+	C	63	18	0.286	0	0	T	45	M	0.189	0.407	0	2.9E-10	0	68.051	tgtgatttccCaaatgtggg	m5C_13157
chr3	107743290	+	C	38	19	0.5	0	0	CT	19	M	0.348	0.652	0	3.4E-05	0	132.429	gaagatgtagCcttcctttga	m5C_13287
chr3	108107939	+	C	55	24	0.436	0	0	T	31	M	0.314	0.567	0	1.2E-07	0	150.592	caaaacgtgaCctcagaccag	m5C_13758
chr3	108107940	+	C	56	19	0.339	0	0	T	37	M	0.229	0.47	0	2.4E-08	0	87.0952	aaaacgtgacCtcagaccaga	m5C_13753
chr3	108107942	+	C	57	21	0.368	0	0	T	36	M	0.255	0.498	0	4.8E-08	0	107.198	aacgtgacctCagaccagacg	m5C_13750
chr3	108107947	+	C	55	16	0.291	0	0	T	39	M	0.188	0.421	0	7.3E-09	0	60.0673	gacctcagacCagacgctgcc	m5C_13748
chr3	108107951	+	C	46	15	0.326	0	0	T	31	M	0.209	0.47	0	2.8E-07	0	62.5973	tcagaccagaCgctgccacca	m5C_13754
chr3	108107953	+	C	31	7	0.226	0	0	T	24	M	0.114	0.398	6.4E-10	3.4E-07	7.2E-10	7.33205	agaccagacgCtgccaccaag	m5C_13743
chr3	116301506	-	C	175	60	0.343	0	0	T	115	M	0.277	0.416	0	0	0	331.9	gaggggtgagCtggcccaggt	m5C_13312
chr3	116301529	-	C	36	12	0.333	0	0	T	24	M	0.202	0.497	0	4.6E-06	0	48.5146	agggaaccacCaggttgccca	m5C_13318
chr3	119248302	+	C	84	55	0.655	0	0	C	55	M	0.548	0.748	0	4.2E-09	0	603.17	aactcacctgCcaaatcgact	m5C_13482
chr3	119248303	+	C	40	22	0.55	0	0	C	22	M	0.398	0.693	0	6.3E-05	0	175.247	actcacctgcCaaatcgacta	m5C_13459
chr3	120118782	-	C	38	13	0.342	0	0	T	25	M	0.212	0.501	0	6.7E-06	0	55.1518	ggggcccagaCccagacagag	m5C_13428
chr3	120118788	-	C	43	18	0.419	0	0	T	25	M	0.284	0.567	0	7.7E-07	0	102.184	cagaagggggCccagacccag	m5C_13414

chr3	121151185	-	C	34	18	0.529	0	0	C	18	M	0.367	0.685	0	2.7E-05	0	132.251	aaaataggcgCcagctgggga	m5C_13379
chr3	121372891	-	C	100	23	0.23	0	0	T	77	M	0.158	0.322	0	1.1E-16	0	72.8785	gggattgtgtCtgtgagtagc	m5C_13439
chr3	121372908	-	C	39	25	0.641	0	0	C	25	M	0.484	0.773	0	0.00011	0	242.089	cagtgctgatCagtagtggga	m5C_13381
chr3	121412732	-	C	33	8	0.242	0	0	T	25	M	0.128	0.41	3.1E-11	6.8E-07	3.6E-11	10.7866	atagatgaagCtaagaaatgg	m5C_13420
chr3	121413611	-	C	34	18	0.529	0	0	C	18	M	0.367	0.685	0	2.7E-05	0	132.251	aaggagacagCagaagaagag	m5C_13406
chr3	121413614	-	C	34	10	0.294	0	0	T	24	M	0.168	0.462	2.3E-14	2E-06	2.9E-14	22.9564	ctaaaggagaCagcagaagaa	m5C_13441
chr3	121435747	-	C	45	12	0.267	0	0	T	33	M	0.16	0.41	5.6E-16	7.5E-08	7.3E-16	29.2263	agcagaagcaCaaagcagaaa	m5C_13392
chr3	121435763	-	C	36	11	0.306	0	0	T	25	M	0.18	0.469	1E-15	3.1E-06	1.3E-15	29.7077	cggtatagtgCtttggagcag	m5C_13391
chr3	122215313	-	C	54	19	0.352	0	0	T	35	M	0.238	0.485	0	2.4E-08	0	90.5268	agaaggactgCagttacgaaa	m5C_13534
chr3	123210867	-	C	67	15	0.224	0	0	T	52	M	0.141	0.337	0	6E-11	0	42.1908	ggtttagatgCtggagagttg	m5C_13542
chr3	124456651	+	C	35	23	0.657	0	0	C	23	M	0.492	0.792	0	7.6E-05	0	226.098	gctggagattCtcgagcagca	m5C_13548
chr3	124602911	-	C	58	31	0.534	0	0	C	31	M	0.408	0.657	0	5.9E-07	0	252.96	ccatgcctgcCtgggtgttcc	m5C_13565
chr3	124602915	-	C	62	20	0.323	0	0	T	42	M	0.22	0.446	0	6.9E-10	0	87.8173	agggccatgcCtgcctgggtg	m5C_13571
chr3	127125760	-	C	40	18	0.45	0	0	T	22	M	0.307	0.602	0	2.7E-05	0	110.538	agggatgggaCgtggtgtagt	m5C_13663
chr3	127305084	+	C	31	9	0.29	0	0	T	22	M	0.161	0.466	3.7E-13	1.2E-06	4.4E-13	18.0154	tcctgcatgaCttctggtggc	m5C_13615
chr3	127707115	+	C	50	28	0.56	0	0	C	28	M	0.423	0.688	0	8.1E-06	0	236.912	ggtggagcagCctggcagtcc	m5C_13594
chr3	127793921	-	C	54	20	0.37	0	0	T	34	M	0.254	0.504	0	3.4E-08	0	101.693	gaatagtgcaCcccctttggg	m5C_13654
chr3	127793955	-	C	45	10	0.222	0	0	T	35	M	0.125	0.363	7.4E-13	2.4E-08	8.9E-13	15.2157	tccatgcatcCacctggtatt	m5C_13644
chr3	127793973	-	C	39	12	0.308	0	0	T	27	M	0.186	0.464	0	4.6E-06	0	44.5578	ggaccttgctCtttccactcc	m5C_13656
chr3	127793975	-	C	54	34	0.63	0	0	C	34	M	0.496	0.746	0	1.1E-06	0	337.465	taggaccttgCtctttccact	m5C_13653
chr3	127793979	-	C	70	14	0.2	0	0	T	56	M	0.123	0.308	4.4E-16	2.8E-13	5.8E-16	26.447	ggaataggacCttgctcttc	m5C_13651
chr3	127793980	-	C	77	19	0.594	45	0.584	G	41	M	0.423	0.745	0	3.4E-05	0	160.587	tggaataggaCcttgctcttt	m5C_13658
chr3	127793998	-	C	775	180	0.233	1	0.001	T	594	M	0.204	0.264	0	0	0	734.977	atttttggagCagggagatgg	m5C_13643
chr3	127794023	-	C	51	17	0.333	0	0	T	34	M	0.22	0.47	0	1.1E-08	0	74.6972	tatctgaggaCagtatgttaa	m5C_13639
chr3	128369406	-	C	46	16	0.348	0	0	T	30	M	0.227	0.492	0	4E-07	0	72.5786	gctcgaggccCggctggcgca	m5C_14346
chr3	128852994	-	C	59	12	0.203	0	0	T	47	M	0.12	0.323	2.6E-14	8.8E-10	3.3E-14	19.6113	gagagaggctCggctggcaag	m5C_14305
chr3	128852996	-	C	57	21	0.368	0	0	T	36	M	0.255	0.498	0	4.8E-08	0	107.198	gagagagaggCtcggctggca	m5C_14321
chr3	128890069	-	C	39	11	0.282	0	0	T	28	M	0.165	0.438	3.1E-15	3.1E-06	4E-15	26.4001	tgcaaggagcCcaagagagag	m5C_14322
chr3	128890077	-	C	39	16	0.41	0	0	T	23	M	0.271	0.566	0	1.7E-05	0	86.6531	ccaaggactgCaaggagccca	m5C_14311
chr3	128890080	-	C	35	12	0.343	0	0	T	23	M	0.208	0.508	0	4.6E-06	0	49.9956	ttgccaaggaCtgcaaggagc	m5C_14300
chr3	128976440	+	C	31	8	0.258	0	0	T	23	M	0.137	0.432	1.7E-11	6.8E-07	1.9E-11	11.8182	atccgggtggCcagcaagcag	m5C_14263
chr3	128976444	+	C	32	7	0.219	0	0	T	25	M	0.11	0.388	8.5E-10	3.4E-07	9.5E-10	6.99971	gggtggccagCaagcagctgg	m5C_14257
chr3	128993745	+	C	35	10	0.286	0	0	T	25	M	0.163	0.451	3.4E-14	2E-06	4.2E-14	21.9991	aacttcgaagCagcctgggat	m5C_14284
chr3	128993749	+	C	60	19	0.317	0	0	T	41	M	0.213	0.442	0	4.5E-10	0	80.9617	tcgaagcagcCtgggatgagg	m5C_14287
chr3	129116097	-	C	32	10	0.312	0	0	T	22	M	0.18	0.486	1.1E-14	2E-06	1.3E-14	25.0836	attccattccCagcttgctcc	m5C_14323
chr3	129116099	-	C	31	9	0.29	0	0	T	22	M	0.161	0.466	3.7E-13	1.2E-06	4.4E-13	18.0154	ccattccattCccagcttgct	m5C_14298
chr3	129116103	-	C	37	8	0.216	0	0	T	29	M	0.114	0.372	9.7E-11	6.8E-07	1.1E-10	9.12273	gcacccattcCattcccagct	m5C_14310
chr3	129116104	-	C	35	10	0.286	0	0	T	25	M	0.163	0.451	3.4E-14	2E-06	4.2E-14	21.9991	ggcacccattCcattcccagc	m5C_14349
chr3	129116110	-	C	50	10	0.2	0	0	T	40	M	0.112	0.33	2.6E-12	2E-10	3.1E-12	13.024	ggcagaggcaCccattccatt	m5C_14342
chr3	129116112	-	C	51	13	0.255	0	0	T	38	M	0.155	0.389	1.1E-16	1.6E-09	1.5E-16	32.2431	ctggcagaggCacccattcca	m5C_14338
chr3	129116118	-	C	60	13	0.217	0	0	T	47	M	0.131	0.336	1.1E-15	1.6E-11	1.4E-15	25.5122	gcaagcctggCagaggcaccc	m5C_14308
chr3	129116144	-	C	269	62	0.231	1	0.004	T	206	M	0.185	0.285	0	0	0	229.235	ctgccagcttCggaaagggag	m5C_14297
chr3	129116154	-	C	364	98	0.269	0	0	T	266	M	0.226	0.317	0	0	0	443.445	gcctagtattCtgccagcttc	m5C_14340
chr3	129116173	-	C	411	86	0.209	0	0	T	325	M	0.173	0.251	0	0	0	297.047	gggatcgcatCtggagactgc	m5C_14309
chr3	131198017	-	C	32	9	0.281	0	0	T	23	M	0.156	0.454	5.3E-13	1.2E-06	6.4E-13	17.1983	acagtgtatgCaagaataatt	m5C_13736
chr3	131198035	-	C	39	12	0.308	0	0	T	27	M	0.186	0.464	0	4.6E-06	0	44.5578	catgtccaggCaggggggaca	m5C_13738
chr3	131198039	-	C	54	11	0.204	0	0	T	43	M	0.118	0.329	2.3E-13	4.4E-10	2.8E-13	16.3622	tgctcatgtcCaggcaggggg	m5C_13734
chr3	131198040	-	C	53	27	0.509	0	0	C	27	M	0.379	0.639	0	2.5E-07	0	204.57	gtgctcatgtCcaggcagggg	m5C_13739
chr3	131198052	-	C	45	20	0.444	0	0	T	25	M	0.309	0.588	0	1.4E-06	0	123.755	ccttgcctgaCtgtgctcatg	m5C_13737
chr3	131198061	-	C	53	11	0.208	0	0	T	42	M	0.12	0.335	1.8E-13	4.4E-10	2.2E-13	16.8213	actcgtagacCttgcctgact	m5C_13735
chr3	131947985	-	C	53	23	0.434	0	0	T	30	M	0.31	0.567	0	8.7E-08	0	142.371	gagcatttgaCtgcagatcaa	m5C_13742
chr3	131948003	-	C	114	26	0.228	0	0	T	88	M	0.161	0.313	0	0	0	83.5431	gggtgtagctCagtggtagag	m5C_13741
chr3	132038636	-	C	31	15	0.484	0	0	T	16	M	0.32	0.652	0	1.2E-05	0	95.91	gggttatataCacacagcagg	m5C_14627
chr3	132289133	+	C	32	10	0.312	0	0	T	22	M	0.18	0.486	1.1E-14	2E-06	1.3E-14	25.0836	tgagttggtcCaagtgttgtg	m5C_14620
chr3	133204741	-	C	57	12	0.211	0	0	T	45	M	0.125	0.333	1.6E-14	8.8E-10	2E-14	20.643	cttgtctggtCttggaagcta	m5C_14631
chr3	133572417	+	C	39	29	0.744	0	0	C	29	M	0.589	0.854	0	0.00019	0	341.725	atgcagctggCagagtgcccc	m5C_14618
chr3	134653800	+	C	47	14	0.298	0	0	T	33	M	0.187	0.44	0	1.9E-07	0	52.223	ttggcccagaCaggtgtagtt	m5C_13696
chr3	134653811	+	C	40	20	0.5	0	0	CT	20	M	0.352	0.648	0	1.4E-06	0	140.797	aggtgtagttCcatgtctcta	m5C_13692
chr3	139303130	-	C	64	15	0.234	0	0	T	49	M	0.147	0.351	0	6E-11	0	44.2493	atgaccatccCcgatagagga	m5C_13820
chr3	139303131	-	C	68	18	0.265	0	0	T	50	M	0.174	0.38	0	2.9E-10	0	62.8186	gatgaccatcCccgatagagg	m5C_13821
chr3	139303135	-	C	114	46	0.404	0	0	T	68	M	0.318	0.495	0	2.8E-14	0	292.577	agaggatgacCatccccgata	m5C_13811
chr3	139303152	-	C	132	33	0.25	0	0	T	99	M	0.184	0.33	0	0	0	121.38	gctgcgtgctCggctgaagag	m5C_13822
chr3	142743623	+	C	31	9	0.29	0	0	T	22	M	0.161	0.466	3.7E-13	1.2E-06	4.4E-13	18.0154	agcgggggacCaggaaaacat	m5C_13828
chr3	142772620	+	C	32	13	0.406	0	0	T	19	M	0.255	0.577	0	6.7E-06	0	66.3505	tgagtgtactCcgacaaggaa	m5C_13833
chr3	142772624	+	C	31	22	0.71	0	0	C	22	M	0.534	0.839	0	6.3E-05	0	234.992	tgtactccgaCaaggaaggaa	m5C_13834
chr3	144268174	+	C	41	23	0.561	0	0	C	23	M	0.41	0.701	0	2.9E-06	0	188.785	tgctgtcaggCatggtatcag	m5C_13853
chr3	148221290	-	C	33	8	0.242	0	0	T	25	M	0.128	0.41	3.1E-11	6.8E-07	3.6E-11	10.7866	gctgagtggtCtaagattctg	m5C_13942
chr3	148221299	-	C	37	14	0.378	0	0	T	23	M	0.241	0.539	0	9.2E-06	0	67.3786	ggtagcatggCtgagtggtct	m5C_13925
chr3	149086944	-	C	126	46	0.365	0	0	T	80	M	0.286	0.452	0	6.7E-16	0	263.268	gatgactgggCaagaagaaag	m5C_13922
chr3	149086955	-	C	111	34	0.306	0	0	T	77	M	0.228	0.397	0	4.4E-16	0	155.196	agaaaggctaCgatgactggg	m5C_13928
chr3	149086980	-	C	33	10	0.303	0	0	T	23	M	0.174	0.473	1.6E-14	2E-06	2E-14	23.982	tactagggatCatgtgtttaa	m5C_13919
chr3	149212583	+	C	37	22	0.595	0	0	C	22	M	0.435	0.737	0	6.3E-05	0	191.337	gagaaggaggCtgctaagaag	m5C_13905
chr3	150905894	+	C	49	14	0.286	0	0	T	35	M	0.178	0.424	0	1.9E-07	0	49.9784	ctgtctacggCcataccaccc	m5C_14019
chr3	150905903	+	C	61	14	0.233	1	0.016	T	46	M	0.144	0.354	0	3.2E-11	0	40.4304	gccataccacCctgaacgcgc	m5C_14011
chr3	150905909	+	C	69	16	0.232	0	0	T	53	M	0.148	0.344	0	1.1E-10	0	47.3849	ccaccctgaaCgcgcccgatc	m5C_14007
chr3	150905911	+	C	78	20	0.256	0	0	T	58	M	0.173	0.363	0	1.1E-11	0	69.0212	accctgaacgCgcccgatctc	m5C_14016
chr3	150905913	+	C	80	24	0.3	0	0	T	56	M	0.211	0.408	0	1.1E-12	0	101.109	cctgaacgcgCccgatctcgt	m5C_14010
chr3	150905914	+	C	76	22	0.293	1	0.013	T	53	M	0.202	0.404	0	2.8E-11	0	89.056	ctgaacgcgcCcgatctcgtc	m5C_14014
chr3	150905915	+	C	76	28	0.368	0	0	T	48	M	0.269	0.481	0	2.6E-10	0	150.501	tgaacgcgccCgatctcgtct	m5C_14005
chr3	150905919	+	C	86	24	0.279	0	0	T	62	M	0.195	0.382	0	1.1E-12	0	93.737	cgcgcccgatCtcgtctgatc	m5C_14001
chr3	150905921	+	C	91	20	0.22	0	0	T	71	M	0.147	0.315	0	1.7E-15	0	58.8081	cgcccgatctCgtctgatctc	m5C_14002
chr3	150905924	+	C	88	32	0.364	0	0	T	56	M	0.271	0.468	0	2.3E-11	0	173.307	ccgatctcgtCtgatctcgga	m5C_14008
chr3	150905929	+	C	92	20	0.217	0	0	T	72	M	0.145	0.312	0	1.7E-15	0	58.1465	ctcgtctgatCtcggaagcta	m5C_14020
chr3	150905931	+	C	86	18	0.209	0	0	T	68	M	0.137	0.307	0	4.5E-14	0	49.211	cgtctgatctCggaagctaag	m5C_14018
chr3	150905942	+	C	96	24	0.25	0	0	T	72	M	0.174	0.345	0	1.7E-14	0	83.5865	ggaagctaagCagggtcgggc	m5C_14025
chr3	150905948	+	C	109	44	0.404	0	0	T	65	M	0.316	0.498	0	5.7E-13	0	278.399	taagcagggtCgggcctggtt	m5C_14000
chr3	150905953	+	C	120	33	0.275	0	0	T	87	M	0.203	0.361	0	2.2E-16	0	133.998	agggtcgggcCtggttagtac	m5C_13998
chr3	150905963	+	C	108	45	0.421	1	0.009	T	62	M	0.331	0.515	0	7.4E-13	0	298.234	ctggttagtaCttggatggag	m5C_14009
chr3	152917640	-	C	35	18	0.514	0	0	C	18	M	0.356	0.67	0	2.7E-05	0	128.047	tggtgacctcCtgggagggga	m5C_13901
chr3	152917643	-	C	38	10	0.263	0	0	T	28	M	0.15	0.42	9.4E-14	2E-06	1.2E-13	19.5037	atatggtgacCtcctgggagg	m5C_13895
chr3	152917644	-	C	37	9	0.243	0	0	T	28	M	0.134	0.401	2.7E-12	1.2E-06	3.2E-12	13.907	aatatggtgaCctcctgggag	m5C_13899
chr3	155378883	+	C	35	17	0.486	0	0	T	18	M	0.33	0.644	0	2.1E-05	0	112.18	atgataccttCcctggtggtc	m5C_14170
chr3	155378884	+	C	40	15	0.375	0	0	T	25	M	0.242	0.53	0	2.8E-07	0	72.6683	tgataccttcCctggtggtct	m5C_14176
chr3	155378885	+	C	50	23	0.46	0	0	T	27	M	0.33	0.596	0	2.9E-06	0	151.659	gataccttccCtggtggtcta	m5C_14174
chr3	155378893	+	C	177	76	0.429	0	0	T	101	M	0.359	0.503	0	0	0	545.253	ccctggtggtCtagtggttag	m5C_14182
chr3	155378908	+	C	182	72	0.398	1	0.005	T	109	M	0.329	0.471	0	0	0	474.225	ggttaggattCggcactctca	m5C_14173
chr3	155378911	+	C	167	77	0.461	0	0	T	90	M	0.387	0.537	0	0	0	596.285	taggattcggCactctcatat	m5C_14179
chr3	155378913	+	C	74	51	0.689	0	0	C	51	M	0.577	0.783	0	4.3E-08	0	588.144	ggattcggcaCtctcatatac	m5C_14183
chr3	157129620	-	C	58	29	0.5	0	0	CT	29	M	0.375	0.625	0	3.9E-07	0	217.721	ttgtatgactCtcagtgggta	m5C_14079
chr3	160122548	+	C	40	10	0.25	0	0	T	30	M	0.142	0.402	1.8E-13	2.4E-08	2.2E-13	18.0914	actctagcagCacgtaaatat	m5C_14482
chr3	160122550	+	C	38	10	0.263	0	0	T	28	M	0.15	0.42	9.4E-14	2E-06	1.2E-13	19.5037	tctagcagcaCgtaaatattg	m5C_14474

chr3	160122562	+	C	34	12	0.353	0	0	T	22	M	0.215	0.521	0	4.6E-06	0	51.5705	taaatattggCgtagtgaaat	m5C_14486
chr3	160137300	+	C	41	19	0.463	0	0	T	22	M	0.321	0.613	0	1E-06	0	121.813	gaaaaaaaatCtggcaggatt	m5C_14470
chr3	160137304	+	C	44	20	0.455	0	0	T	24	M	0.317	0.599	0	1.4E-06	0	126.82	aaaaatctggCaggattccag	m5C_14460
chr3	160137311	+	C	57	16	0.281	0	0	T	41	M	0.181	0.408	0	7.3E-09	0	57.8536	tggcaggattCcaggaatata	m5C_14467
chr3	160232979	-	C	272	59	0.217	0	0	T	213	M	0.172	0.27	0	0	0	203.027	tgttgtatgaCtggaaatttt	m5C_14491
chr3	160283823	+	C	39	24	0.615	0	0	C	24	M	0.459	0.751	0	9.1E-05	0	220.314	gtctcccggtCggacgctcgc	m5C_14465
chr3	160283833	+	C	34	10	0.294	0	0	T	24	M	0.168	0.462	2.3E-14	2E-06	2.9E-14	22.9564	cggacgctcgCagctgcgttc	m5C_14473
chr3	168269672	+	C	59	20	0.339	0	0	T	39	M	0.231	0.466	0	3.4E-08	0	92.5499	tgaaatcaagCgtgggtgaga	m5C_14425
chr3	168269684	+	C	49	30	0.612	0	0	C	30	M	0.472	0.736	0	1.2E-05	0	283.474	tgggtgagacCtggtgcagaa	m5C_14394
chr3	168269690	+	C	48	22	0.458	0	0	T	26	M	0.326	0.597	0	2.3E-06	0	143.33	agacctggtgCagaacgggaa	m5C_14378
chr3	169482398	-	C	125	26	0.208	0	0	T	99	M	0.146	0.287	0	0	0	75.9632	ctcacacatgCagttcgcttt	m5C_14432
chr3	169482404	-	C	185	53	0.286	0	0	T	132	M	0.226	0.355	0	0	0	239.771	actcggctcaCacatgcagtt	m5C_14440
chr3	169482413	-	C	197	43	0.219	1	0.005	T	153	M	0.167	0.282	0	0	0	143.751	gcacccaggaCtcggctcaca	m5C_14453
chr3	169482434	-	C	141	33	0.237	2	0.014	T	106	M	0.174	0.315	0	0	0	115.062	attccctgagCtgtgggacgt	m5C_14455
chr3	169482439	-	C	109	26	0.239	0	0	T	83	M	0.168	0.327	0	6.7E-16	0	87.5137	gcgcgattccCtgagctgtgg	m5C_14435
chr3	169482440	-	C	112	31	0.277	0	0	T	81	M	0.202	0.366	0	1.1E-16	0	125.473	ggcgcgattcCctgagctgtg	m5C_14456
chr3	169482441	-	C	106	22	0.208	0	0	T	84	M	0.141	0.294	0	0	0	62.148	cggcgcgattCcctgagctgt	m5C_14454
chr3	169482453	-	C	47	16	0.34	0	0	T	31	M	0.222	0.483	0	4E-07	0	70.9352	agtccccgcgCgcggcgcgat	m5C_14436
chr3	169482457	-	C	35	17	0.486	0	0	T	18	M	0.33	0.644	0	2.1E-05	0	112.18	agcgagtcccCgcgcgcggcg	m5C_14447
chr3	169482459	-	C	32	8	0.25	0	0	T	24	M	0.133	0.421	2.3E-11	6.8E-07	2.6E-11	11.2828	ggagcgagtcCccgcgcgcgg	m5C_14442
chr3	169490023	+	C	132	42	0.318	0	0	T	90	M	0.245	0.402	0	0	0	205.656	gtgggggtttcCgtagtgtagt	m5C_14411
chr3	169710768	+	C	50	13	0.26	0	0	T	37	M	0.159	0.396	0	1.6E-09	0	41.2656	ggaactggaaCagcaaacaga	m5C_14401
chr3	169710771	+	C	48	16	0.333	0	0	T	32	M	0.217	0.475	0	4E-07	0	69.3651	actggaacagCaaacagatgg	m5C_14403
chr3	172347107	-	C	43	17	0.395	0	0	T	26	M	0.264	0.544	0	5.6E-07	0	89.6414	aggacatggtCtgagtctcgc	m5C_14713
chr3	172347113	-	C	50	12	0.24	0	0	T	38	M	0.143	0.374	2.4E-15	8.8E-10	3.1E-15	25.0697	agggagaggaCatggtctgag	m5C_14711
chr3	181540668	-	C	154	58	0.377	0	0	T	96	M	0.304	0.455	0	0	0	352.611	ataccgggtgCtgtagggttg	m5C_14548
chr3	181540674	-	C	145	37	0.255	0	0	T	108	M	0.191	0.332	0	0	0	141.464	ctgggaatacCgggtgctgta	m5C_14566
chr3	181540675	-	C	154	44	0.286	0	0	T	110	M	0.22	0.362	0	0	0	193.829	cctgggaataCcgggtgctgt	m5C_14550
chr3	181540684	-	C	85	27	0.318	0	0	T	58	M	0.228	0.423	0	4E-12	0	123.336	gggagaccgcCtgggaatacc	m5C_14543
chr3	181540687	-	C	68	24	0.353	0	0	T	44	M	0.25	0.472	0	3E-09	0	120.009	catgggagacCgcctgggaat	m5C_14544
chr3	181540688	-	C	60	24	0.4	0	0	T	36	M	0.286	0.526	0	1.2E-07	0	137.133	gcatgggagaCcgcctgggaa	m5C_14545
chr3	183169671	+	C	42	19	0.452	0	0	T	23	M	0.312	0.601	0	1E-06	0	118.648	tacaatatagCtgtgaagtgc	m5C_14782
chr3	183171611	+	C	179	41	0.229	0	0	T	138	M	0.174	0.296	0	0	0	142.324	gaggcaggatCtagttacatt	m5C_14826
chr3	183171626	+	C	149	42	0.282	0	0	T	107	M	0.216	0.359	0	0	0	181.291	tacattgtagCtgtgaagtgc	m5C_14817
chr3	183171636	+	C	62	42	0.677	0	0	C	42	M	0.554	0.78	0	2E-07	0	465.09	ctgtgaagtgCtgcattgtct	m5C_14783
chr3	183171639	+	C	48	13	0.271	0	0	T	35	M	0.166	0.41	0	1.2E-07	0	43.0711	tgaagtgctgCattgtctttg	m5C_14827
chr3	183354244	+	C	40	9	0.225	0	0	T	31	M	0.123	0.375	6.4E-12	1.2E-08	7.6E-12	12.4041	ccgctgcggcCgtccccgggg	m5C_14824
chr3	183354247	+	C	39	20	0.513	0	0	C	20	M	0.362	0.661	0	4.3E-05	0	144.796	ctgcggccgtCcccggggagc	m5C_14798
chr3	183804640	-	C	45	10	0.222	0	0	T	35	M	0.125	0.363	7.4E-13	2.4E-08	8.9E-13	15.2157	tagtcagttaCagacggaatt	m5C_14837
chr3	183804652	-	C	73	15	0.205	0	0	T	58	M	0.129	0.312	0	6E-13	0	38.6006	gttgattgttCatagtcagtt	m5C_14840
chr3	184039793	+	C	141	48	0.34	0	0	T	93	M	0.267	0.422	0	0	0	256.676	gcaggagaagCtgagagtgag	m5C_14863
chr3	184042681	+	C	61	22	0.361	0	0	T	39	M	0.252	0.486	0	1.5E-09	0	110.765	ggaacgaggaCgcctgaagga	m5C_14873
chr3	184043496	+	C	505	129	0.255	0	0	T	376	M	0.219	0.295	0	0	0	565.932	cctctgatgaCttcctgttag	m5C_14867
chr3	184043509	+	C	525	118	0.227	5	0.01	T	402	M	0.193	0.265	0	0	0	455.484	cctgttagtgCcacgtgtctg	m5C_14879
chr3	184043512	+	C	516	165	0.32	0	0	T	351	M	0.281	0.361	0	0	0	927.249	gttagtgccaCgtgtctgggc	m5C_14880
chr3	184043522	+	C	541	155	0.287	0	0	T	386	M	0.25	0.326	0	0	0	775.053	cgtgtctgggCcactgagaca	m5C_14860
chr3	184043523	+	C	549	113	0.206	0	0	T	436	M	0.174	0.242	0	0	0	393.485	gtgtctgggcCactgagacac	m5C_14861
chr3	184043525	+	C	557	129	0.232	0	0	T	428	M	0.198	0.268	0	0	0	512.063	gtctgggccaCtgagacacca	m5C_14882
chr3	184043544	+	C	615	166	0.27	1	0.002	T	448	M	0.237	0.307	0	0	0	785.96	catgatggaaCtgaggatctg	m5C_14883
chr3	184043552	+	C	530	193	0.364	0	0	T	337	M	0.324	0.406	0	0	0	1251.79	aactgaggatCtgaggaaggg	m5C_14877
chr3	184078242	-	C	31	8	0.258	0	0	T	23	M	0.137	0.432	1.7E-11	6.8E-07	1.9E-11	11.8182	gaggggccaaCccttgggatg	m5C_14901
chr3	184366126	-	C	114	31	0.272	0	0	T	83	M	0.199	0.36	0	1.1E-16	0	123.184	acctgtcatgCgggagacggg	m5C_14909
chr3	185141149	-	C	49	14	0.286	0	0	T	35	M	0.178	0.424	0	1.9E-07	0	49.9784	gccagtgactCagcagttact	m5C_14900
chr3	185141151	-	C	48	20	0.417	0	0	T	28	M	0.288	0.557	0	1.4E-06	0	115.398	tagccagtgaCtcagcagtta	m5C_14890
chr3	186302249	+	C	40	11	0.275	0	0	T	29	M	0.161	0.428	4.3E-15	3.1E-06	5.5E-15	25.4502	gatcaccaggCcaggagcgaa	m5C_16359
chr3	186302256	+	C	57	28	0.491	0	0	T	29	M	0.366	0.617	0	3.1E-07	0	205.05	aggccaggagCgaagctatgg	m5C_16396
chr3	186502599	+	C	1247	268	0.215	0	0	T	979	M	0.193	0.239	0	0	0	1034.54	gaaatgatggCaatcatcttt	m5C_16351
chr3	186502603	+	C	1286	283	0.222	10	0.008	T	993	M	0.2	0.245	0	0	0	1131.13	tgatggcaatCatctttcggg	m5C_16326
chr3	186502610	+	C	1299	286	0.221	2	0.002	T	1011	M	0.199	0.244	0	0	0	1137.07	aatcatctttCgggactgacc	m5C_16371
chr3	186502615	+	C	1364	497	0.365	1	0.001	T	866	M	0.339	0.391	0	0	0	3374.6	tctttcgggaCtgacctgaaa	m5C_16362
chr3	186502620	+	C	1356	396	0.292	0	0	T	960	M	0.268	0.317	0	0	0	2126.11	cgggactgacCtgaaatgaag	m5C_16398
chr3	186502644	+	C	909	266	0.293	0	0	T	643	M	0.264	0.323	0	0	0	1404.34	atactcattgCtgatcacttg	m5C_16323
chr3	186504121	+	C	143	47	0.329	0	0	T	96	M	0.257	0.409	0	0	0	241.622	aatgcaggtaCtgttacaata	m5C_16310
chr3	186504156	+	C	119	53	0.445	0	0	T	66	M	0.359	0.535	0	1.8E-13	0	380.729	tgttgtcgttCcccctgctta	m5C_16346
chr3	186504158	+	C	121	25	0.207	0	0	T	96	M	0.144	0.287	0	0	0	72.0228	ttgtcgttccCcctgcttaaa	m5C_16325
chr3	186504159	+	C	121	25	0.207	0	0	T	96	M	0.144	0.287	0	0	0	72.0228	tgtcgttcccCctgcttaaag	m5C_16393
chr3	186504172	+	C	110	29	0.264	0	0	T	81	M	0.19	0.353	0	0	0	110.364	gcttaaagcaCttgatgcata	m5C_16388
chr3	186504184	+	C	110	35	0.321	1	0.009	T	74	M	0.241	0.414	0	3.4E-14	0	168.58	tgatgcataaCtctgtctacc	m5C_16309
chr3	186504190	+	C	117	33	0.282	0	0	T	84	M	0.208	0.37	0	2.2E-16	0	137.575	ataactctgtCtaccttcatt	m5C_16381
chr3	186504193	+	C	125	29	0.232	0	0	T	96	M	0.167	0.313	0	0	0	96.6667	actctgtctaCcttcattccg	m5C_16337
chr3	186504194	+	C	127	31	0.244	0	0	T	96	M	0.178	0.326	0	0	0	110.126	ctctgtctacCttcattccgt	m5C_16368
chr3	186504197	+	C	131	63	0.481	0	0	T	68	M	0.397	0.566	0	2.4E-15	0	500.377	tgtctaccttCattccgtagt	m5C_16397
chr3	186504201	+	C	131	50	0.382	0	0	T	81	M	0.303	0.467	0	0	0	302.984	taccttcattCcgtagtaaga	m5C_16390
chr3	186504218	+	C	81	19	0.241	2	0.025	T	60	M	0.16	0.345	0	6.9E-12	0	60.6938	aagacagagaCgcttggcttc	m5C_16311
chr3	186504228	+	C	71	31	0.437	0	0	T	40	M	0.327	0.552	0	6.4E-10	0	203.025	cgcttggcttCagacattttc	m5C_16314
chr3	186504465	+	C	54	17	0.315	0	0	T	37	M	0.207	0.447	0	1.1E-08	0	70.309	cattattttaCcttcttgtat	m5C_16399
chr3	186504466	+	C	64	13	0.203	0	0	T	51	M	0.123	0.317	3E-15	1.6E-11	3.9E-15	23.1722	attattttacCttcttgtata	m5C_16320
chr3	186504469	+	C	71	17	0.239	0	0	T	54	M	0.155	0.35	0	2.2E-12	0	52.7708	attttaccttCttgtataagc	m5C_16389
chr3	186504481	+	C	157	56	0.357	0	0	T	101	M	0.286	0.434	0	0	0	320.321	tgtataagcaCtgtgctaaaa	m5C_16392
chr3	186504486	+	C	159	45	0.283	0	0	T	114	M	0.219	0.358	0	0	0	196.884	aagcactgtgCtaaaattgca	m5C_16327
chr3	186504499	+	C	130	36	0.277	0	0	T	94	M	0.207	0.359	0	0	0	149.216	aaattgcagaCactaggacca	m5C_16365
chr3	186504507	+	C	74	26	0.351	0	0	T	48	M	0.252	0.465	0	1.3E-10	0	131.238	gacactaggaCcatgtcttgg	m5C_16322
chr3	186504581	+	C	53	12	0.226	0	0	T	41	M	0.135	0.355	5.8E-15	8.8E-10	7.3E-15	22.9888	attgtaaagaCtggggtggac	m5C_16375
chr3	186504594	+	C	73	20	0.274	0	0	T	53	M	0.185	0.386	0	1.1E-11	0	73.9652	gggtggacctCtttcttaatg	m5C_16328
chr3	186504613	+	C	58	16	0.276	0	0	T	42	M	0.178	0.402	0	7.3E-09	0	56.8071	tgtccaatgtCctttgtctta	m5C_16332
chr3	186504640	+	C	63	33	0.524	0	0	C	33	M	0.403	0.642	0	3.4E-08	0	265.783	ggtgcaatatCtcattagacc	m5C_16354
chr3	186505099	+	C	899	184	0.205	1	0.001	T	714	M	0.18	0.233	0	0	0	661.603	aagcaggattCagactacaat	m5C_16334
chr3	186505114	+	C	770	205	0.266	0	0	T	565	M	0.236	0.299	0	0	0	968.547	tacaatatagCtgctaagtgc	m5C_16340
chr3	186505124	+	C	636	155	0.244	2	0.003	T	479	M	0.213	0.279	0	0	0	659.151	ctgctaagtgCtgtgttgtcg	m5C_16316
chr3	186505138	+	C	319	64	0.201	0	0	T	255	M	0.16	0.248	0	0	0	205.26	gttgtcgttcCcccttgcttaa	m5C_16350
chr3	186505187	+	C	164	33	0.201	0	0	T	131	M	0.147	0.269	0	0	0	97.0335	ctgctattctCctgtagcagc	m5C_16348
chr3	186505188	+	C	165	33	0.2	0	0	T	132	M	0.146	0.268	0	0	0	96.4293	tgctattctcCtgtagcagcc	m5C_16352
chr3	186505197	+	C	99	27	0.273	0	0	T	72	M	0.195	0.368	0	7.2E-14	0	105.15	cctgtagcagCcagggacgct	m5C_16335
chr3	186505212	+	C	100	41	0.41	0	0	T	59	M	0.319	0.508	0	2.4E-13	0	261.311	gacgcttggtCtcatacatgt	m5C_16347
chr3	186505218	+	C	83	29	0.349	0	0	T	54	M	0.256	0.457	0	8.4E-12	0	148.225	tggtctcataCatgttgatta	m5C_16387
chr3	186505458	+	C	93	33	0.355	0	0	T	60	M	0.265	0.456	0	7.5E-13	0	174.984	ccaacttgggCtatttggaag	m5C_16391
chr3	186505478	+	C	34	11	0.324	0	0	T	23	M	0.191	0.492	4.4E-16	3.1E-06	5.8E-16	32.3088	gagtaaaagaCcacactccac	m5C_16315
chr3	186505479	+	C	34	26	0.765	0	0	C	26	M	0.6	0.876	0	0.00013	0	312.018	agtaaaagacCacactccaca	m5C_16394
chr3	192691879	-	C	46	14	0.304	0	0	T	32	M	0.191	0.448	0	1.9E-07	0	53.4231	tattggagggCaagtctggtg	m5C_14730
chr3	193458365	-	C	78	26	0.333	0	0	T	52	M	0.239	0.444	0	1.3E-10	0	124.137	gagggtgaaCtggctgaagt	m5C_14738
chr3	194422834	-	C	39	9	0.231	0	0	T	30	M	0.126	0.383	4.9E-12	1.2E-06	5.7E-12	12.8739	cctgtagcccCagctacttgg	m5C_15359

chr3	194422836	-	C	44	9	0.205	0	0	T	35	M	0.112	0.345	1.8E-11	1.2E-08	2.1E-11	10.7806	tgcctgtagcCccagctactt	m5C_15345
chr3	194422843	-	C	48	20	0.417	0	0	T	28	M	0.288	0.557	0	1.4E-06	0	115.398	tggtgcgtgcCtgtagcccca	m5C_15348
chr3	194422844	-	C	49	10	0.204	0	0	T	39	M	0.115	0.336	2.1E-12	2.4E-08	2.4E-12	13.4191	gtggtgcgtgCctgtagcccc	m5C_15375
chr3	194422848	-	C	47	15	0.319	0	0	T	32	M	0.204	0.462	0	2.8E-07	0	61.1856	ggtggtggtgCgtgcctgtag	m5C_15372
chr3	195800915	-	C	50	13	0.26	0	0	T	37	M	0.159	0.396	0	1.6E-09	0	41.2656	accgagtctcCagtgagggag	m5C_15368
chr3	195800916	-	C	63	23	0.365	0	0	T	40	M	0.257	0.489	0	2.1E-09	0	118.29	aaccgagtctCcagtgaggga	m5C_15358
chr3	195800924	-	C	70	14	0.2	0	0	T	56	M	0.123	0.308	4.4E-16	2.8E-13	5.8E-16	26.447	ctggcaggaaCcgagtctcca	m5C_15360
chr3	195800934	-	C	70	43	0.614	0	0	C	43	M	0.497	0.72	0	2.4E-07	0	427.553	gtgtgagagaCtggcaggaac	m5C_15365
chr3	195903769	-	C	37	21	0.568	0	0	C	21	M	0.409	0.713	0	5.2E-05	0	171.838	ccggaggattCaacctggtgg	m5C_15339
chr3	196214350	-	C	32	7	0.219	0	0	T	25	M	0.11	0.388	8.5E-10	3.4E-07	9.5E-10	6.99971	ggaaaaaagaCaggcagaaaa	m5C_15063
chr4	1976378	+	C	71	24	0.338	0	0	T	47	M	0.239	0.454	0	6.4E-11	0	114.647	cactaaggctCggtcctctcc	m5C_15122
chr4	1976383	+	C	74	21	0.284	0	0	T	53	M	0.194	0.395	0	1.8E-11	0	81.3683	aggctcggtcCtctccacgtg	m5C_15093
chr4	1976385	+	C	74	17	0.23	0	0	T	57	M	0.149	0.337	0	2.2E-12	0	50.548	gctcggtcctCtccacgtggt	m5C_15121
chr4	1976388	+	C	68	26	0.382	0	0	T	42	M	0.276	0.501	0	5.6E-09	0	143.568	cggtcctctcCacgtggtcct	m5C_15091
chr4	1976397	+	C	77	24	0.312	0	0	T	53	M	0.219	0.422	0	6.4E-11	0	105.25	ccacgtggtcCtgacctgtcc	m5C_15085
chr4	1976416	+	C	57	14	0.246	0	0	T	43	M	0.152	0.371	0	2.8E-09	0	42.6516	cctctgtgagCaagagaaaca	m5C_15103
chr4	1976445	+	C	37	11	0.297	0	0	T	26	M	0.175	0.458	1.4E-15	3.1E-06	1.9E-15	28.5508	ttgggggtgtCctgtctcagt	m5C_15117
chr4	1976452	+	C	37	8	0.216	0	0	T	29	M	0.114	0.372	9.7E-11	6.8E-07	1.1E-10	9.12273	tgtcctgtctCagtggacaca	m5C_15114
chr4	3319243	-	C	76	17	0.224	0	0	T	59	M	0.145	0.329	0	2.2E-12	0	49.1676	ggggccgtgtCtgctcgagct	m5C_15312
chr4	3319249	-	C	73	17	0.233	0	0	T	56	M	0.151	0.342	0	2.2E-12	0	51.2678	gcctccggggCcgtgtctgct	m5C_15316
chr4	3319254	-	C	32	9	0.281	0	0	T	23	M	0.156	0.454	5.3E-13	1.2E-06	6.4E-13	17.1983	gcgctgcctcCggggccgtgt	m5C_15297
chr4	3526677	-	C	116	43	0.371	0	0	T	73	M	0.288	0.461	0	1.2E-14	0	247.904	acggcttggaCgaagatgggg	m5C_15294
chr4	3526686	-	C	128	26	0.203	0	0	T	102	M	0.143	0.281	0	0	0	74.1294	taaagcttgaCggcttggacg	m5C_15324
chr4	3526691	-	C	89	31	0.348	0	0	T	58	M	0.257	0.452	0	1.7E-11	0	159.633	gaaactaaagCttgacggctt	m5C_15327
chr4	8394826	+	C	46	17	0.37	0	0	T	29	M	0.245	0.514	0	5.6E-07	0	83.3801	tgggagaccgCctgggaatag	m5C_15447
chr4	12975456	-	C	87	28	0.322	0	0	T	59	M	0.233	0.426	0	5.8E-12	0	130.483	ccatgcctgcCtgagtgctgg	m5C_16009
chr4	12975460	-	C	85	23	0.271	0	0	T	62	M	0.188	0.373	0	7.2E-13	0	86.2936	ggatccatgcCtgcctgagtg	m5C_16012
chr4	13338843	+	C	59	25	0.424	0	0	T	34	M	0.306	0.551	0	1.5E-07	0	153.063	cgaagaaaagCccaagatcga	m5C_16000
chr4	13578543	-	C	34	15	0.441	0	0	T	19	M	0.289	0.605	0	1.2E-05	0	86.6497	gagcaggagtCtgatgaggaa	m5C_16039
chr4	20678301	+	C	39	27	0.692	0	0	C	27	M	0.536	0.814	0	0.00015	0	289.324	agtgggggaaCaaaaaaaaaa	m5C_15419
chr4	24578222	-	C	73	20	0.29	4	0.055	T	49	M	0.196	0.406	0	6.9E-10	0	78.4637	agatagagagCgagagaggga	m5C_15587
chr4	24578249	-	C	48	18	0.375	0	0	T	30	M	0.252	0.516	0	7.7E-07	0	90.7743	agatcgagacCgagaacgtga	m5C_15598
chr4	24578250	-	C	48	11	0.229	0	0	T	37	M	0.133	0.365	5E-14	4.4E-08	6.2E-14	19.4712	aagatcgagaCcgagaacgtg	m5C_15590
chr4	24578255	-	C	32	18	0.562	0	0	C	18	M	0.393	0.718	0	2.7E-05	0	141.571	gtctaaagatCgagaccgaga	m5C_15589
chr4	27310050	-	C	41	12	0.293	0	0	T	29	M	0.176	0.445	1.1E-16	7.5E-08	1.5E-16	33.7134	acaggaaggcCtgagtgtctg	m5C_22925
chr4	27371402	-	C	48	13	0.271	0	0	T	35	M	0.166	0.41	0	1.2E-07	0	43.0711	ggggtcatgcCtgcactatgg	m5C_22928
chr4	37956474	+	C	64	22	0.344	0	0	T	42	M	0.239	0.466	0	1.5E-09	0	105.261	tatcatatgaCtcttagcagt	m5C_15538
chr4	39697103	-	C	51	11	0.216	0	0	T	40	M	0.125	0.346	1.1E-13	4.4E-10	1.4E-13	17.8061	cgctgtagtcCcaactacttg	m5C_15698
chr4	39697111	-	C	58	13	0.224	0	0	T	45	M	0.136	0.347	6.7E-16	1.6E-09	8.7E-16	26.8192	gtggcgtgcgCtgtagtccca	m5C_15704
chr4	39697117	-	C	61	32	0.525	0	0	C	32	M	0.402	0.645	0	2.7E-08	0	257.004	ggtgtggtggCgtgcgctgta	m5C_15703
chr4	41652415	+	C	39	10	0.256	0	0	T	29	M	0.146	0.411	1.3E-13	2E-06	1.6E-13	18.7753	tataggtagaCgggaaagtca	m5C_15656
chr4	41682072	+	C	66	20	0.303	0	0	T	46	M	0.206	0.422	0	6.9E-10	0	82.2155	tgggaaaaggCccaaaaggag	m5C_15655
chr4	41682073	+	C	68	16	0.235	0	0	T	52	M	0.15	0.349	0	1.1E-10	0	48.11	gggaaaaggcCcaaaaggagg	m5C_15638
chr4	41682074	+	C	63	17	0.279	2	0.032	T	44	M	0.182	0.402	0	1.8E-10	0	61.8406	ggaaaaggccCaaaaggaggt	m5C_15661
chr4	41682098	+	C	34	19	0.559	0	0	C	19	M	0.395	0.711	0	3.4E-05	0	149.924	agaggaagaaCgcagatacta	m5C_15632
chr4	41682100	+	C	39	10	0.256	0	0	T	29	M	0.146	0.411	1.3E-13	2E-06	1.6E-13	18.7753	aggaagaacgCagatactatg	m5C_15646
chr4	41684374	+	C	46	15	0.326	0	0	T	31	M	0.209	0.47	0	2.8E-07	0	62.5973	gggaaactgtCaagatgaaaa	m5C_15649
chr4	53578789	+	C	45	9	0.2	0	0	T	36	M	0.109	0.338	2.3E-11	1.2E-08	2.7E-11	10.4289	cgcccggtgtCcccacgagct	m5C_15777
chr4	53578794	+	C	99	42	0.424	0	0	T	57	M	0.332	0.523	0	1.1E-11	0	278.468	ggtgtccccaCgagctttgcc	m5C_15791
chr4	53578803	+	C	112	30	0.268	0	0	T	82	M	0.195	0.357	0	0	0	116.727	acgagctttgCccgcctcgag	m5C_15780
chr4	53578807	+	C	117	30	0.256	0	0	T	87	M	0.186	0.342	0	0	0	111.549	gctttgcccgCctcgagctcc	m5C_15794
chr4	53578810	+	C	117	37	0.316	0	0	T	80	M	0.239	0.405	0	1.4E-15	0	176.834	ttgcccgcctCgagctcctct	m5C_15786
chr4	53578816	+	C	106	48	0.453	0	0	T	58	M	0.361	0.548	0	1.6E-12	0	346.921	gcctcgagctCctctgctccc	m5C_15768
chr4	53578824	+	C	98	26	0.265	0	0	T	72	M	0.188	0.36	0	4.6E-14	0	97.7371	ctcctctgctCccggcggcca	m5C_15799
chr4	53578826	+	C	84	20	0.238	0	0	T	64	M	0.16	0.339	0	1.5E-13	0	63.8983	cctctgctccCggcggccaga	m5C_15776
chr4	53578838	+	C	77	31	0.403	0	0	T	46	M	0.3	0.514	0	6.4E-10	0	186.135	gcggccagaaCggaggggtag	m5C_15771
chr4	53578851	+	C	58	28	0.483	0	0	T	30	M	0.359	0.608	0	3.1E-07	0	201.197	aggggtagagCagccctcggc	m5C_15787
chr4	53578854	+	C	50	12	0.24	0	0	T	38	M	0.143	0.374	2.4E-15	8.8E-10	3.1E-15	25.0697	ggtagagcagCcctcggcggc	m5C_15802
chr4	53578856	+	C	43	10	0.233	0	0	T	33	M	0.132	0.377	4.3E-13	2.4E-08	5.2E-13	16.2683	tagagcagccCtcggcggccc	m5C_15792
chr4	53578858	+	C	45	24	0.533	0	0	C	24	M	0.391	0.671	0	3.6E-06	0	187.571	gagcagccctCggcggcccgg	m5C_15801
chr4	53578885	+	C	66	22	0.333	0	0	T	44	M	0.232	0.453	0	1.5E-09	0	101.888	ggcggcggtgCccgtcccggg	m5C_15795
chr4	53578891	+	C	117	29	0.248	0	0	T	88	M	0.178	0.333	0	0	0	103.517	ggtgcccgtcCcggggctgcg	m5C_15775
chr4	53578892	+	C	119	46	0.393	2	0.017	T	71	M	0.309	0.484	0	2.8E-14	0	284.65	gtgcccgtccCggggctgcgc	m5C_15774
chr4	53578897	+	C	151	38	0.252	0	0	T	113	M	0.189	0.326	0	0	0	143.781	cgtcccggggCtgcgcgaggc	m5C_15800
chr4	53578913	+	C	94	20	0.213	0	0	T	74	M	0.142	0.306	0	1.7E-15	0	56.8671	gaggcacaggCgccgcgccca	m5C_15767
chr4	53579413	+	C	42	11	0.262	0	0	T	31	M	0.153	0.411	8.4E-15	4.4E-08	1.1E-14	23.69	agcaccctttCctgtgccctt	m5C_15773
chr4	53579421	+	C	40	11	0.275	0	0	T	29	M	0.161	0.428	4.3E-15	3.1E-06	5.5E-15	25.4502	ttcctgtgccCttttaaggtt	m5C_15778
chr4	53579435	+	C	38	9	0.237	0	0	T	29	M	0.13	0.392	3.7E-12	1.2E-06	4.3E-12	13.3739	taaggttgacCcagtgcttta	m5C_15779
chr4	53579441	+	C	34	7	0.206	0	0	T	27	M	0.103	0.368	1.4E-09	3.4E-07	1.6E-09	6.4054	tgacccagtgCtttaagaggc	m5C_15769
chr4	53579485	+	C	36	18	0.5	0	0	CT	18	M	0.345	0.655	0	2.7E-05	0	124.107	ctccataaaaCccagagaaga	m5C_15789
chr4	53579506	+	C	87	21	0.241	0	0	T	66	M	0.164	0.341	0	2.6E-13	0	68.729	gactggaaagCtcctctttgg	m5C_15770
chr4	53579508	+	C	95	21	0.221	0	0	T	74	M	0.149	0.314	0	3.1E-15	0	62.7364	ctggaaagctCctctttggat	m5C_15784
chr4	53579509	+	C	100	30	0.3	0	0	T	70	M	0.219	0.396	0	4.7E-15	0	131.369	tggaaagctcCtctttggatc	m5C_15781
chr4	53579511	+	C	114	23	0.202	0	0	T	91	M	0.138	0.285	0	0	0	63.6501	gaaagctcctCtttggatcct	m5C_15796
chr4	53579531	+	C	151	39	0.258	0	0	T	112	M	0.195	0.333	0	0	0	152.158	tgtctggagtCacaactgaac	m5C_15793
chr4	53579536	+	C	146	61	0.418	0	0	T	85	M	0.341	0.499	0	0	0	415.919	ggagtcacaaCtgaaccagaa	m5C_15798
chr4	57976247	-	C	70	15	0.214	0	0	T	55	M	0.134	0.324	0	6E-13	0	40.3158	cgtgaagagcCgcaagaggcg	m5C_15990
chr4	57976248	-	C	71	16	0.225	0	0	T	55	M	0.144	0.335	0	1.2E-12	0	45.9986	gcgtgaagagCcgcaagaggc	m5C_15988
chr4	57976270	-	C	46	14	0.304	0	0	T	32	M	0.191	0.448	0	1.9E-07	0	53.4231	tactgcgcgcCgggcatggag	m5C_15977
chr4	57976271	-	C	47	20	0.426	0	0	T	27	M	0.295	0.567	0	1.4E-06	0	118.054	gtactgcgcgCcgggcatgga	m5C_15981
chr4	71388489	-	C	31	7	0.226	0	0	T	24	M	0.114	0.398	6.4E-10	3.4E-07	7.2E-10	7.33205	gagggagtccCaaagcaggtt	m5C_15907
chr4	71514735	-	C	38	18	0.474	0	0	T	20	M	0.325	0.627	0	2.7E-05	0	116.924	gtcccagctaCttgggaggct	m5C_15899
chr4	71514742	-	C	83	33	0.398	0	0	T	50	M	0.299	0.505	0	3.1E-11	0	197.398	gcgtgtagtcCcagctacttg	m5C_15913
chr4	71514743	-	C	78	19	0.244	0	0	T	59	M	0.162	0.349	0	6.9E-12	0	61.5034	tgcgtgtagtCccagctactt	m5C_15920
chr4	71514751	-	C	89	24	0.27	0	0	T	65	M	0.188	0.37	0	1.1E-12	0	90.4412	tgtggtggtgCgtgtagtccc	m5C_15905
chr4	71514766	-	C	39	14	0.359	0	0	T	25	M	0.227	0.516	0	9.2E-06	0	63.6773	caaaaattagCtgggtgtggt	m5C_15911
chr4	71554845	+	C	33	8	0.242	0	0	T	25	M	0.128	0.41	3.1E-11	6.8E-07	3.6E-11	10.7866	ccggcagagtCaacaggaggc	m5C_15878
chr4	71554848	+	C	47	29	0.617	0	0	C	29	M	0.474	0.742	0	9.7E-06	0	275.073	gcagagtcaaCaggaggcaga	m5C_15874
chr4	71554855	+	C	53	12	0.226	0	0	T	41	M	0.135	0.355	5.8E-15	8.8E-10	7.3E-15	22.9888	caacaggaggCagaggaggag	m5C_15866
chr4	76587390	-	C	44	20	0.455	0	0	T	24	M	0.317	0.599	0	1.4E-06	0	126.82	tcaggttagtCtgatggtagt	m5C_16291
chr4	76654445	-	C	124	63	0.508	0	0	C	63	M	0.421	0.594	0	6.6E-14	0	530.66	ccctggtgggtCtagtggttag	m5C_16267
chr4	76654453	-	C	47	10	0.213	0	0	T	37	M	0.12	0.349	1.3E-12	2.4E-08	1.5E-12	14.271	catgaagtccCtggtggtcta	m5C_16274
chr4	76654455	-	C	31	10	0.323	0	0	T	21	M	0.186	0.499	7.1E-15	2E-06	9E-15	26.2725	agcatgaagtCcctggtggtc	m5C_16295
chr4	81409368	-	C	51	20	0.392	0	0	T	31	M	0.27	0.529	0	3.4E-08	0	108.109	cagtggggggCtgtagtgttt	m5C_15996
chr4	83348406	-	C	32	21	0.656	0	0	C	21	M	0.483	0.796	0	5.2E-05	0	202.905	gcaacagcaaCaacaaaaagg	m5C_16119
chr4	83348415	-	C	39	9	0.231	0	0	T	30	M	0.126	0.383	4.9E-12	1.2E-06	5.7E-12	12.8739	atataggcagCaacagcaaca	m5C_16137
chr4	83817043	-	C	74	18	0.243	0	0	T	56	M	0.16	0.352	0	4E-12	0	57.5149	ctgttcacatCatgatttgtt	m5C_16127
chr4	83817053	-	C	73	17	0.233	0	0	T	56	M	0.151	0.342	0	2.2E-12	0	51.2678	ttttccactgCtgttcacatc	m5C_16120
chr4	83819330	-	C	49	14	0.286	0	0	T	35	M	0.178	0.424	0	1.9E-07	0	49.9784	cataatgaacCgttgtggatta	m5C_16138
chr4	83819347	-	C	47	22	0.468	0	0	T	25	M	0.333	0.608	0	2.3E-06	0	146.658	cttttcactgCtgttcacata	m5C_16118
chr4	83819357	-	C	50	17	0.34	0	0	T	33	M	0.224	0.478	0	5.6E-07	0	76.285	gtgattaaacCttttcactgc	m5C_16129

chr4	86021941	+	C	73	20	0.274	0	0	T	53	M	0.185	0.386	0	1.1E-11	0	73.9652	ccagacacatCcaaatgaggt	m5C_16742
chr4	86021956	+	C	220	46	0.211	2	0.009	T	172	M	0.162	0.27	0	0	0	149.12	tgaggtgctgCatgtggcagt	m5C_16757
chr4	86021967	+	C	219	52	0.237	0	0	T	167	M	0.186	0.298	0	0	0	193.353	atgtggcagtCtgcctttta	m5C_16752
chr4	86021971	+	C	206	54	0.262	0	0	T	152	M	0.207	0.326	0	0	0	223.371	ggcagtctgcCtttttaaaaa	m5C_16744
chr4	88813737	+	C	61	23	0.377	0	0	T	38	M	0.266	0.503	0	2.1E-09	0	122.426	aagaaaagccCaagatcaaag	m5C_16176
chr4	90626424	+	C	34	10	0.294	0	0	T	24	M	0.168	0.462	2.3E-14	2E-06	2.9E-14	22.9564	gtggactattCtgtctcttgg	m5C_16160
chr4	94076210	+	C	118	44	0.373	0	0	T	74	M	0.291	0.463	0	1.6E-14	0	256.019	ccttggtggtCtagtggttag	m5C_16790
chr4	94076225	+	C	121	39	0.322	0	0	T	82	M	0.246	0.41	0	0	0	191.58	ggttaggattCggctctctca	m5C_16786
chr4	94076228	+	C	119	60	0.513	2	0.017	C	60	M	0.423	0.602	0	9E-13	0	507.95	taggattcggCtctctcaaat	m5C_16798
chr4	94076230	+	C	48	21	0.438	0	0	T	27	M	0.307	0.577	0	1.8E-06	0	128.944	ggattcggctCtctcaaataa	m5C_16775
chr4	100929952	+	C	75	23	0.307	0	0	T	52	M	0.214	0.418	0	4.3E-11	0	98.3789	agtctgtggcCcagtggttgg	m5C_16553
chr4	106405953	+	C	41	10	0.244	0	0	T	31	M	0.138	0.393	2.4E-13	2.4E-08	2.9E-13	17.4472	gtggcgtcgaCaaaagaacca	m5C_16806
chr4	106405961	+	C	40	16	0.4	0	0	T	24	M	0.263	0.554	0	1.7E-05	0	84.314	gacaaaagaaCcatcgaaaaa	m5C_16809
chr4	106405985	+	C	39	29	0.744	0	0	C	29	M	0.589	0.854	0	0.00019	0	341.725	gagaaggaggCtgctgagatg	m5C_16808
chr4	106891353	+	C	60	25	0.417	0	0	T	35	M	0.301	0.543	0	1.5E-07	0	150.32	tgtgttggaaCgggatttaca	m5C_16802
chr4	106891364	+	C	46	11	0.239	0	0	T	35	M	0.139	0.379	2.8E-14	4.4E-08	3.5E-14	20.7303	gggatttacaCacactggagg	m5C_16805
chr4	107924469	-	C	53	39	0.736	0	0	C	39	M	0.604	0.836	0	2.5E-06	0	471.269	tcctgatgtgCagatcggtca	m5C_16831
chr4	107924478	-	C	181	90	0.497	0	0	T	91	M	0.425	0.569	0	0	0	765.385	ttgtagtggtCctgatgtgca	m5C_16834
chr4	108134293	-	C	47	24	0.511	0	0	C	24	M	0.372	0.647	0	3.6E-06	0	178.763	gatgaaagggCcctgtgataa	m5C_16663
chr4	112905827	+	C	166	55	0.331	0	0	T	111	M	0.264	0.406	0	0	0	290.673	acatgcttgtCtgagtgttgt	m5C_16846
chr4	114341633	+	C	60	12	0.2	0	0	T	48	M	0.118	0.318	3.3E-14	7.6E-12	4.2E-14	19.13	tttgtttgtgCtttcccctgg	m5C_16931
chr4	114341637	+	C	42	15	0.357	0	0	T	27	M	0.23	0.508	0	2.8E-07	0	68.967	tttgtgctttCccctggaaaa	m5C_16933
chr4	119200355	+	C	680	136	0.2	0	0	T	544	M	0.172	0.232	0	0	0	466.908	ctccatgtatCtttgggacct	m5C_17779
chr4	119200371	+	C	707	154	0.218	0	0	T	553	M	0.189	0.25	0	0	0	582.01	gacctgtcagCcgtggcagtc	m5C_17790
chr4	119200372	+	C	713	160	0.224	0	0	T	553	M	0.195	0.256	0	0	0	624.975	acctgtcagcCgtggcagtct	m5C_17788
chr4	119200404	+	C	129	31	0.242	1	0.008	T	97	M	0.176	0.323	0	0	0	109.236	catggaagagCatatccttgt	m5C_17782
chr4	119200409	+	C	110	32	0.291	0	0	T	78	M	0.214	0.382	0	2.2E-16	0	137.11	aagagcatatCcttgtttatt	m5C_17771
chr4	119200410	+	C	96	24	0.253	1	0.01	T	71	M	0.176	0.348	0	1.7E-14	0	84.5012	agagcatatcCttgtttattg	m5C_17786
chr4	119200431	+	C	75	32	0.438	2	0.027	T	41	M	0.33	0.552	0	8.5E-10	0	211.489	gcaaagctgtCaccatttaat	m5C_17774
chr4	119200434	+	C	76	24	0.316	0	0	T	52	M	0.222	0.427	0	6.4E-11	0	106.707	aagctgtcacCatttaattgg	m5C_17772
chr4	122605923	-	C	78	36	0.462	0	0	T	42	M	0.355	0.571	0	2.4E-09	0	255.832	attgcaggcaCagatgaggag	m5C_17161
chr4	122607447	-	C	87	27	0.31	0	0	T	60	M	0.223	0.414	0	4E-12	0	120.361	ctatgaaaggCttgggtaaat	m5C_17169
chr4	122607467	-	C	52	18	0.346	0	0	T	34	M	0.232	0.482	0	1.7E-08	0	83.343	tgcagaaactCttcggaaggc	m5C_17164
chr4	125354320	+	C	52	13	0.25	0	0	T	39	M	0.152	0.382	1.1E-16	1.6E-09	1.5E-16	31.5947	agtggtggacCacttgactct	m5C_17482
chr4	129003722	-	C	47	15	0.319	0	0	T	32	M	0.204	0.462	0	2.8E-07	0	61.1856	atccttggagCaggggagggg	m5C_17002
chr4	129191383	-	C	50	12	0.24	0	0	T	38	M	0.143	0.374	2.4E-15	8.8E-10	3.1E-15	25.0697	atggatgtgtCtgaatgtgtg	m5C_17001
chr4	136917086	-	C	46	16	0.348	0	0	T	30	M	0.227	0.492	0	4E-07	0	72.5786	gcgctttcccCtgagaaaaaa	m5C_16943
chr4	136917104	-	C	61	20	0.328	0	0	T	41	M	0.223	0.453	0	6.9E-10	0	89.3398	tagtgggggaCtgcattcgcg	m5C_16945
chr4	141789379	-	C	42	15	0.357	0	0	T	27	M	0.23	0.508	0	2.8E-07	0	68.967	tagtaggaccCaagggaaagg	m5C_17102
chr4	141789381	-	C	45	9	0.205	1	0.022	T	35	M	0.112	0.345	1.8E-11	1.2E-08	2.1E-11	10.7806	gatagtaggaCccaagggaaa	m5C_17084
chr4	151773581	-	C	33	12	0.364	0	0	T	21	M	0.222	0.534	0	4.6E-06	0	53.2487	tcagagatgcCagaattcttg	m5C_17395
chr4	152025000	+	C	557	218	0.391	0	0	T	339	M	0.352	0.433	0	0	0	1533.53	aaaaaagtttCggtcccagat	m5C_17895
chr4	152025004	+	C	554	168	0.303	0	0	T	386	M	0.266	0.343	0	0	0	895.221	aagtttcggtCccagatgatg	m5C_17879
chr4	152025017	+	C	545	189	0.347	0	0	T	356	M	0.308	0.388	0	0	0	1164.35	agatgatggcCagtgataaca	m5C_17901
chr4	152025029	+	C	454	99	0.218	0	0	T	355	M	0.183	0.258	0	0	0	361.407	gtgataacaaCatttttctga	m5C_17899
chr4	152025036	+	C	363	126	0.348	1	0.003	T	236	M	0.301	0.399	0	0	0	758.071	caacatttttCtgatgttccc	m5C_17887
chr4	154565581	-	C	46	10	0.217	0	0	T	36	M	0.123	0.356	9.7E-13	2.4E-08	1.2E-12	14.7309	gctgagggggCccaccaaagg	m5C_17458
chr4	155711617	+	C	95	22	0.232	0	0	T	73	M	0.158	0.326	0	5.8E-15	0	69.6061	tgcagtacctCcaggtatggt	m5C_17435
chr4	159866843	-	C	37	8	0.216	0	0	T	29	M	0.114	0.372	9.7E-11	6.8E-07	1.1E-10	9.12273	gtcattatgtCtgtggctggt	m5C_17302
chr4	160862010	+	C	43	16	0.372	0	0	T	27	M	0.244	0.521	0	4E-07	0	78.0036	agtggtggatCaatacataaa	m5C_17326
chr4	163648218	-	C	46	17	0.37	0	0	T	29	M	0.245	0.514	0	5.6E-07	0	83.3801	gtcccagttaCttgggaggct	m5C_17383
chr4	163648224	-	C	51	27	0.529	0	0	C	27	M	0.395	0.659	0	2.5E-07	0	213.425	cctgtagtccCagttacttgg	m5C_17384
chr4	163648225	-	C	50	16	0.32	0	0	T	34	M	0.208	0.458	0	7.3E-09	0	66.4257	gcctgtagtcCcagttacttg	m5C_17380
chr4	163648226	-	C	50	18	0.36	0	0	T	32	M	0.241	0.499	0	1.7E-08	0	86.8988	tgcctgtagtCccagttactt	m5C_17363
chr4	163648233	-	C	47	17	0.362	0	0	T	30	M	0.24	0.505	0	5.6E-07	0	81.4845	gtggggtgcCtgtagtccca	m5C_17379
chr4	163648247	-	C	42	20	0.476	0	0	T	22	M	0.334	0.623	0	1.4E-06	0	133.437	aaaagctgggCgtggtgggtg	m5C_17365
chr4	165044035	-	C	34	25	0.735	0	0	C	25	M	0.569	0.854	0	0.00011	0	284.413	tgatagtgggCatccttgtcg	m5C_17644
chr4	166256889	+	C	34	16	0.471	0	0	T	18	M	0.315	0.633	0	1.7E-05	0	100.644	aaagaataagCtgtagtctag	m5C_17530
chr4	166256910	+	C	78	35	0.449	0	0	T	43	M	0.343	0.559	0	1.9E-09	0	240.339	ttggtcagagCtagttgttta	m5C_17528
chr4	167839569	-	C	47	15	0.319	0	0	T	32	M	0.204	0.462	0	2.8E-07	0	61.1856	cagggctgggCctggttagta	m5C_17541
chr4	170788454	-	C	31	12	0.387	0	0	T	19	M	0.237	0.562	0	4.6E-06	0	56.9589	gatgaggcggCtgcgcggcca	m5C_17598
chr4	174253301	-	C	242	61	0.252	0	0	T	181	M	0.202	0.31	0	0	0	245.861	aagaacgaacCagaagatgag	m5C_17868
chr4	174253319	-	C	79	17	0.215	0	0	T	62	M	0.139	0.318	0	2.2E-12	0	47.2331	ccaacaggctCaaagaagaag	m5C_17867
chr4	174253325	-	C	52	16	0.308	0	0	T	36	M	0.199	0.443	0	7.3E-09	0	63.7266	ggcaggccaaCaggctcaaag	m5C_17871
chr4	174253329	-	C	31	11	0.355	0	0	T	20	M	0.211	0.531	1.1E-16	3.1E-06	1.5E-16	37.0595	ccctggcaggCcaacaggctc	m5C_17865
chr4	178273502	+	C	32	15	0.469	0	0	T	17	M	0.309	0.636	0	1.2E-05	0	92.6074	agaggcggagCccatggaaaa	m5C_17610
chr4	186049905	+	C	36	12	0.333	0	0	T	24	M	0.202	0.497	0	4.6E-06	0	48.5146	tggtgggggaCtgcgcctgcg	m5C_17683
chr4	186049908	+	C	34	18	0.529	0	0	C	18	M	0.367	0.685	0	2.7E-05	0	132.251	tgggggactgCgcctgcgagg	m5C_17686
chr4	186049914	+	C	33	8	0.242	0	0	T	25	M	0.128	0.41	3.1E-11	6.8E-07	3.6E-11	10.7866	actgcgcctgCgaggccttct	m5C_17678
chr4	186337233	-	C	114	58	0.509	0	0	C	58	M	0.418	0.599	0	5.8E-13	0	485.138	actggctggtCtgagggtagt	m5C_17694
chr5	144305	-	C	51	14	0.275	0	0	T	37	M	0.171	0.409	0	2.8E-09	0	47.9195	ttcagctggtCtgaaggtagt	m5C_19779
chr5	301708	+	C	38	10	0.263	0	0	T	28	M	0.15	0.42	9.4E-14	2E-06	1.2E-13	19.5037	tcgagtgctgCtgtgtgtgta	m5C_19760
chr5	891787	-	C	35	8	0.229	0	0	T	27	M	0.121	0.39	5.6E-11	6.8E-07	6.4E-11	9.89691	gaaggagaagCatctggacga	m5C_19798
chr5	3188109	+	C	31	8	0.258	0	0	T	23	M	0.137	0.432	1.7E-11	6.8E-07	1.9E-11	11.8182	ccgcgtctgtCtgggcgtcgc	m5C_17818
chr5	3188114	+	C	31	9	0.29	0	0	T	22	M	0.161	0.466	3.7E-13	1.2E-06	4.4E-13	18.0154	tctgtctgggCgtcgcaggag	m5C_17811
chr5	6600137	-	C	35	8	0.229	0	0	T	27	M	0.121	0.39	5.6E-11	6.8E-07	6.4E-11	9.89691	gactgaggaCagagagcagg	m5C_18069
chr5	6600145	-	C	36	15	0.417	0	0	T	21	M	0.271	0.578	0	1.2E-05	0	81.421	aatgacgtgaCtgagggacag	m5C_18077
chr5	6600233	-	C	54	18	0.333	0	0	T	36	M	0.222	0.466	0	1.7E-08	0	80.0685	gatgatggggCtggaggtatt	m5C_18068
chr5	6633025	-	C	83	19	0.229	0	0	T	64	M	0.152	0.33	0	8.4E-14	0	57.6583	gaggatggcgCcgagggtggt	m5C_18066
chr5	6633037	-	C	43	10	0.233	0	0	T	33	M	0.132	0.377	4.3E-13	2.4E-08	5.2E-13	16.2683	ccggaggacgCggaggatggc	m5C_18073
chr5	6943746	+	C	31	8	0.258	0	0	T	23	M	0.137	0.432	1.7E-11	6.8E-07	1.9E-11	11.8182	ggcggtaggtCcctggcgata	m5C_18049
chr5	10254825	+	C	34	13	0.382	0	0	T	21	M	0.239	0.55	0	6.7E-06	0	62.1399	ggagatgtgaCtgtaactaat	m5C_22334
chr5	10254909	+	C	33	9	0.273	0	0	T	24	M	0.151	0.442	7.5E-13	1.2E-06	9E-13	16.4418	ctgtccaagtCtcaggatgat	m5C_22378
chr5	10256094	+	C	33	9	0.273	0	0	T	24	M	0.151	0.442	7.5E-13	1.2E-06	9E-13	16.4418	ttagaagaagCggagcaattg	m5C_22381
chr5	10256099	+	C	33	24	0.727	0	0	C	24	M	0.558	0.849	0	9.1E-05	0	267.754	agaagcggagCaattgctaga	m5C_22370
chr5	11082845	+	C	62	23	0.371	0	0	T	39	M	0.262	0.495	0	2.1E-09	0	120.322	aggccatgttCcgcagcgcag	m5C_22359
chr5	11082846	+	C	68	16	0.235	0	0	T	52	M	0.15	0.349	0	1.1E-10	0	48.11	ggccatgttcCgcagcgcagt	m5C_22349
chr5	11082863	+	C	105	31	0.295	0	0	T	74	M	0.216	0.388	0	7.1E-15	0	134.208	cagtggccacCgcgcacacca	m5C_22343
chr5	14359530	+	C	35	18	0.514	0	0	C	18	M	0.356	0.67	0	2.7E-05	0	128.047	tgagacggtgCtgcagcagct	m5C_18263
chr5	14359539	+	C	63	14	0.222	0	0	T	49	M	0.137	0.339	0	3.2E-11	0	38.4297	gctgcagcagCtggacgaggc	m5C_18258
chr5	14359544	+	C	65	23	0.354	0	0	T	42	M	0.249	0.475	0	2.1E-09	0	114.426	agcagctggaCgaggcgcagt	m5C_18275
chr5	14359549	+	C	64	19	0.297	0	0	T	45	M	0.199	0.418	0	4.5E-10	0	75.639	ctggacgaggCgcagtcgcag	m5C_18276
chr5	14359551	+	C	62	18	0.29	0	0	T	44	M	0.192	0.413	0	2.9E-10	0	69.2043	ggacgaggcgCagtcgcagat	m5C_18253
chr5	14359555	+	C	49	29	0.592	0	0	C	29	M	0.452	0.718	0	9.7E-06	0	262.433	gaggcgcagtCgcagatggag	m5C_18279
chr5	14464116	+	C	47	15	0.319	0	0	T	32	M	0.204	0.462	0	2.8E-07	0	61.1856	tggctgtgagCtgcaggactg	m5C_18256
chr5	14464124	+	C	47	17	0.362	0	0	T	30	M	0.24	0.505	0	5.6E-07	0	81.4845	agctgcaggaCtgagtcacagg	m5C_18272
chr5	14464131	+	C	49	18	0.367	0	0	T	31	M	0.247	0.507	0	7.7E-07	0	88.7939	ggactgagcaCaggttggagt	m5C_18268
chr5	14464143	+	C	33	16	0.485	0	0	T	17	M	0.325	0.648	0	1.7E-05	0	104.012	ggttggagtcCtggagttttg	m5C_18261
chr5	16463773	-	C	114	44	0.386	0	0	T	70	M	0.302	0.478	0	1.6E-14	0	265.501	gagcaggatgCagaggaggaa	m5C_18156
chr5	16463793	-	C	80	18	0.225	0	0	T	62	M	0.147	0.328	0	4.5E-14	0	53.0391	aagcaatggaCgatgtggtgg	m5C_18171
chr5	16463800	-	C	41	9	0.243	4	0.098	T	28	M	0.134	0.401	2.7E-12	1.2E-06	3.2E-12	13.907	gatactgaagCaatggacgat	m5C_18158

chr5	16465163	-	C	33	14	0.424	0	0	T	19	M	0.272	0.592	0	9.2E-06	0	76.259	aggaagaggaCctggatggag	m5C_18176
chr5	16465195	-	C	87	20	0.23	0	0	T	67	M	0.154	0.329	0	1.5E-13	0	61.6125	ggcaaagcagCaggaggagga	m5C_18163
chr5	16465198	-	C	81	51	0.63	0	0	C	51	M	0.521	0.727	0	2.1E-09	0	531.252	gttggcaaagCagcaggagga	m5C_18174
chr5	16623172	-	C	87	23	0.264	0	0	T	64	M	0.183	0.366	0	7.2E-13	0	84.2255	cgattgttcgCagtcagttac	m5C_18173
chr5	16623174	-	C	101	30	0.297	0	0	T	71	M	0.217	0.392	0	4.7E-15	0	130.009	accgattgttCgcagtcagtt	m5C_18166
chr5	16623206	-	C	136	48	0.353	0	0	T	88	M	0.278	0.436	0	2.1E-09	0	266.565	cctggctggtCtgaaggtagt	m5C_18165
chr5	17275370	+	C	35	11	0.314	0	0	T	24	M	0.186	0.48	6.7E-16	3.1E-06	8.7E-16	30.9686	acaatgtgaaCgacgagaaag	m5C_18152
chr5	17275373	+	C	39	15	0.385	0	0	T	24	M	0.249	0.541	0	1.2E-05	0	74.6735	atgtgaacgaCgagaaagcca	m5C_18125
chr5	17275394	+	C	86	26	0.302	0	0	T	60	M	0.215	0.406	0	2.7E-12	0	112.026	aggagaaagaCaagaaggccg	m5C_18106
chr5	17275402	+	C	78	24	0.308	0	0	T	54	M	0.216	0.417	0	6.4E-11	0	103.833	gacaagaaggCcgagggcgcg	m5C_18120
chr5	17275403	+	C	83	18	0.217	0	0	T	65	M	0.142	0.317	0	4.5E-14	0	51.0532	acaagaaggcCgagggcgcgg	m5C_18103
chr5	17275409	+	C	71	16	0.225	0	0	T	55	M	0.144	0.335	0	1.2E-12	0	45.9986	aggccgagggCgcggcgacgg	m5C_18092
chr5	17275411	+	C	83	26	0.313	0	0	T	57	M	0.224	0.419	0	2.7E-12	0	116.279	gccgagggcgCggcgacggaa	m5C_18140
chr5	17275414	+	C	43	17	0.395	0	0	T	26	M	0.264	0.544	0	5.6E-07	0	89.6414	gagggcgcggCgacggaagag	m5C_18112
chr5	17275538	+	C	38	9	0.237	0	0	T	29	M	0.13	0.392	3.7E-12	1.2E-06	4.3E-12	13.3739	agaaggagggCgagaaggacg	m5C_18115
chr5	17275547	+	C	41	31	0.756	0	0	C	31	M	0.607	0.862	0	1.4E-05	0	376.069	gcgagaaggaCgcggcggctg	m5C_18131
chr5	17275552	+	C	36	20	0.571	1	0.028	C	20	M	0.409	0.72	0	4.3E-05	0	163.429	aaggacgcggCggctgccaag	m5C_18142
chr5	17275555	+	C	41	20	0.488	0	0	T	21	M	0.343	0.635	0	1.4E-06	0	137.017	gacgcggcggCtgccaaggag	m5C_18091
chr5	17275559	+	C	61	23	0.377	0	0	T	38	M	0.266	0.503	0	2.1E-09	0	122.426	cggcggctgcCaaggaggagg	m5C_18138
chr5	17275570	+	C	65	13	0.2	0	0	T	52	M	0.121	0.313	3.8E-15	1.6E-11	4.8E-15	22.646	aaggaggaggCcccgaaggcg	m5C_18102
chr5	17275572	+	C	70	16	0.229	0	0	T	54	M	0.146	0.34	0	1.2E-12	0	46.6814	ggaggaggccCcgaaggcgga	m5C_18108
chr5	17275573	+	C	70	23	0.329	0	0	T	47	M	0.23	0.445	0	4.3E-11	0	105.794	gaggaggcccCgaaggcggag	m5C_18113
chr5	17275584	+	C	68	18	0.265	0	0	T	50	M	0.174	0.38	0	2.9E-10	0	62.8186	gaaggcggagCccgagaagac	m5C_18124
chr5	17275585	+	C	69	31	0.449	0	0	T	38	M	0.338	0.566	0	2.1E-08	0	209.366	aaggcggagcCcgagaagacg	m5C_18129
chr5	17275586	+	C	68	14	0.206	0	0	T	54	M	0.127	0.316	2.2E-16	3.2E-11	2.9E-16	27.787	aggcggagccCgagaagacgg	m5C_18134
chr5	17275733	+	C	34	13	0.382	0	0	T	21	M	0.239	0.55	0	6.7E-06	0	62.1399	cggcccctgcCgccggggagg	m5C_18096
chr5	17275736	+	C	42	11	0.262	0	0	T	31	M	0.153	0.411	8.4E-15	4.4E-08	1.1E-14	23.69	cccctgccgcCggggaggagc	m5C_18109
chr5	17275746	+	C	40	20	0.5	0	0	CT	20	M	0.352	0.648	0	1.4E-06	0	140.797	cggggaggagCccagcaagga	m5C_18119
chr5	17275747	+	C	42	17	0.405	0	0	T	25	M	0.27	0.555	0	5.6E-07	0	91.945	ggggaggagcCcagcaaggag	m5C_18135
chr5	17275748	+	C	48	14	0.292	0	0	T	34	M	0.182	0.432	0	1.9E-07	0	51.076	gggaggagccCagcaaggagg	m5C_18133
chr5	17275751	+	C	55	13	0.236	0	0	T	42	M	0.144	0.363	2.2E-16	1.6E-09	2.9E-16	29.2353	aggagcccagCaaggaggaag	m5C_18110
chr5	17275767	+	C	33	11	0.333	0	0	T	22	M	0.198	0.504	2.2E-16	3.1E-06	2.9E-16	34.0074	ggaaggggaaCccaaaaagac	m5C_18128
chr5	17275768	+	C	35	9	0.257	0	0	T	26	M	0.142	0.421	1.5E-12	1.2E-06	1.7E-12	15.0859	gaaggggaacCcaaaaagact	m5C_18148
chr5	25189531	-	C	40	12	0.3	0	0	T	28	M	0.181	0.454	1.1E-16	7.5E-08	1.5E-16	34.6049	ttgatgtcttCtgggttgatg	m5C_18035
chr5	25189534	-	C	39	15	0.385	0	0	T	24	M	0.249	0.541	0	1.2E-05	0	74.6735	aatttgatgtCttctgggttg	m5C_18037
chr5	25190102	-	C	52	13	0.25	0	0	T	39	M	0.152	0.382	1.1E-16	1.6E-09	1.5E-16	31.5947	agctggaagaCctgagaagga	m5C_18036
chr5	25190117	-	C	62	14	0.226	0	0	T	48	M	0.14	0.344	0	3.2E-11	0	39.0742	ggttagatgaCatggagctgg	m5C_18042
chr5	27478218	+	C	34	23	0.676	0	0	C	23	M	0.508	0.809	0	7.6E-05	0	233.876	ttgtcagaaaCcatgactaga	m5C_18184
chr5	27478219	+	C	35	7	0.2	0	0	T	28	M	0.1	0.359	1.9E-09	3.4E-07	2.1E-09	6.13884	tgtcagaaacCatgactagaa	m5C_18188
chr5	32174363	-	C	43	9	0.209	0	0	T	34	M	0.114	0.352	1.4E-11	1.2E-08	1.6E-11	11.1522	ggactgcggcCacaggcagga	m5C_18636
chr5	32174364	-	C	49	24	0.49	0	0	T	25	M	0.356	0.625	0	3.6E-06	0	170.759	gggactgcggCcacaggcagg	m5C_18642
chr5	32174376	-	C	52	20	0.385	0	0	T	32	M	0.265	0.52	0	3.4E-08	0	105.881	cggggtgtttCggggactgcg	m5C_18652
chr5	32395284	-	C	144	39	0.271	0	0	T	105	M	0.205	0.349	0	0	0	159.827	agaagaggagCgttggagaat	m5C_18658
chr5	32395299	-	C	95	32	0.337	0	0	T	63	M	0.25	0.437	0	5.3E-13	0	159.866	ctggcgaagaCgagaagaaga	m5C_18653
chr5	32395305	-	C	143	42	0.302	4	0.028	T	97	M	0.232	0.383	0	0	0	194.868	ggagtactggCgaagacgaga	m5C_18651
chr5	32395309	-	C	156	65	0.417	0	0	T	91	M	0.342	0.495	0	0	0	444.877	aggaggagtaCtggcgaagac	m5C_18654
chr5	33445451	+	C	36	8	0.222	0	0	T	28	M	0.117	0.381	7.4E-11	6.8E-07	8.4E-11	9.49676	tgaagagaagCaaaaggaagg	m5C_18585
chr5	33445477	+	C	95	50	0.526	0	0	C	50	M	0.427	0.624	0	7.1E-11	0	426.852	aaaagaagaaCaaagaaggat	m5C_18591
chr5	33445488	+	C	97	22	0.227	0	0	T	75	M	0.155	0.32	0	5.8E-15	0	68.1195	aaagaaggatCtggagatgga	m5C_18590
chr5	34802401	+	C	31	16	0.516	0	0	C	16	M	0.348	0.68	0	1.7E-05	0	111.488	gccataccacCctgaacacgc	m5C_18468
chr5	34824178	+	C	32	10	0.312	0	0	T	22	M	0.18	0.486	1.1E-14	2E-06	1.3E-14	25.0836	cggactgcagCaaaagagatg	m5C_18464
chr5	34929946	+	C	46	15	0.326	0	0	T	31	M	0.209	0.47	0	2.8E-07	0	62.5973	ctatgaggcgCtgggggtgcg	m5C_18460
chr5	34929955	+	C	62	13	0.21	0	0	T	49	M	0.127	0.326	1.8E-15	1.6E-11	2.3E-15	24.3221	gctgggggtgCggcgcgacgc	m5C_18457
chr5	34929960	+	C	59	34	0.576	0	0	C	34	M	0.449	0.694	0	1.1E-06	0	305.553	gggtgcggcgCgacgccagcg	m5C_18451
chr5	34929965	+	C	53	18	0.34	0	0	T	35	M	0.227	0.474	0	1.7E-08	0	81.6727	cggcgcgacgCcagcgaggag	m5C_18458
chr5	34929966	+	C	51	11	0.216	0	0	T	40	M	0.125	0.346	1.1E-13	4.4E-10	1.4E-13	17.8061	ggcgcgacgcCagcgaggagg	m5C_18471
chr5	34936290	+	C	34	11	0.333	1	0.029	T	22	M	0.198	0.504	2.2E-16	3.1E-06	2.9E-16	34.0074	aaatgattgcCaaggaagaac	m5C_18445
chr5	34936307	+	C	39	20	0.513	0	0	C	20	M	0.362	0.661	0	4.3E-05	0	144.796	gaactagaatCtgtgttagag	m5C_18463
chr5	36604703	+	C	35	18	0.514	0	0	C	18	M	0.356	0.67	0	2.7E-05	0	128.047	tggtgaattaCttcccttctc	m5C_18348
chr5	37206375	-	C	36	16	0.444	0	0	T	20	M	0.295	0.604	0	1.7E-05	0	94.5313	ggcatataaaCttggagactg	m5C_18377
chr5	37206383	-	C	38	13	0.342	0	0	T	25	M	0.212	0.501	0	6.7E-06	0	55.1518	gtgtggttggCatataaactt	m5C_18369
chr5	37396638	+	C	78	21	0.269	0	0	T	57	M	0.183	0.377	0	1.8E-11	0	77.009	gaagaagaagCagaggaagaa	m5C_18334
chr5	40832776	-	C	139	35	0.252	0	0	T	104	M	0.187	0.33	0	0	0	130.886	ttgatttcagCatttaactga	m5C_18718
chr5	40832806	-	C	171	36	0.212	1	0.006	T	134	M	0.157	0.279	0	0	0	113.088	aaataaatgtCtgaacatatg	m5C_18688
chr5	40834325	-	C	32	7	0.219	0	0	T	25	M	0.11	0.388	8.5E-10	3.4E-07	9.5E-10	6.99971	accaccggaaCtggtcgaatg	m5C_18707
chr5	40835299	-	C	37	10	0.27	0	0	T	27	M	0.154	0.43	6.7E-14	2E-06	8.3E-14	20.2815	tggtctcggcCgcagaagcga	m5C_18710
chr5	40835300	-	C	37	13	0.351	0	0	T	24	M	0.218	0.512	0	6.7E-06	0	56.7461	ctggtctcggCcgcagaagcg	m5C_18698
chr5	40835303	-	C	36	9	0.25	0	0	T	27	M	0.138	0.411	2E-12	1.2E-06	2.4E-12	14.4765	tttctggtctCggccgcagaa	m5C_18690
chr5	40835305	-	C	35	8	0.229	0	0	T	27	M	0.121	0.39	5.6E-11	6.8E-07	6.4E-11	9.89691	tctttctggtCtcggccgcag	m5C_18694
chr5	44827468	+	C	42	16	0.381	0	0	T	26	M	0.25	0.532	0	4E-07	0	79.9985	ggaagtgtgaCtaagctcgaa	m5C_18665
chr5	44827475	+	C	34	16	0.471	0	0	T	18	M	0.315	0.633	0	1.7E-05	0	100.644	tgactaagctCgaagaaagga	m5C_18672
chr5	52158138	+	C	55	19	0.345	0	0	T	36	M	0.234	0.477	0	2.4E-08	0	88.7776	tcagaatggtCgagcggtcta	m5C_18814
chr5	52158146	+	C	52	11	0.212	0	0	T	41	M	0.122	0.34	1.4E-13	4.4E-10	1.7E-13	17.3019	gtcgagcggtCtaaggcgctg	m5C_18810
chr5	52183676	+	C	36	17	0.472	0	0	T	19	M	0.32	0.63	0	2.1E-05	0	108.752	gcccgggggtgCccgaagagga	m5C_18790
chr5	52183678	+	C	36	10	0.294	2	0.056	T	24	M	0.168	0.462	2.3E-14	2E-06	2.9E-14	22.9564	ccgggggtgccCgaagaggagt	m5C_18801
chr5	52183698	+	C	38	10	0.263	0	0	T	28	M	0.15	0.42	9.4E-14	2E-06	1.2E-13	19.5037	ttaaaaaagtCatggttattg	m5C_18796
chr5	55073313	+	C	153	40	0.261	0	0	T	113	M	0.198	0.336	0	0	0	158.61	ggccataccaCcctgaacgtg	m5C_18982
chr5	55073315	+	C	155	53	0.342	0	0	T	102	M	0.272	0.42	0	0	0	288.203	ccataccaccCtgaacgtgtc	m5C_18993
chr5	55073330	+	C	99	24	0.242	0	0	T	75	M	0.169	0.335	0	1.7E-14	0	80.9576	cgtgtccgatCtcgtctgatc	m5C_18994
chr5	55073332	+	C	81	22	0.272	0	0	T	59	M	0.187	0.377	0	4.4E-13	0	82.1642	tgtccgatctCgtctgatctt	m5C_19007
chr5	55073335	+	C	56	28	0.5	0	0	CT	28	M	0.373	0.627	0	3.1E-07	0	209.057	ccgatctcgtCtgatcttgga	m5C_18986
chr5	55073340	+	C	46	13	0.283	0	0	T	33	M	0.173	0.425	0	1.2E-07	0	45.0425	ctcgtctgatCttggaagcta	m5C_18980
chr5	55073348	+	C	38	12	0.316	0	0	T	26	M	0.191	0.475	0	4.6E-06	0	45.8026	atcttggaagCtaagcagggt	m5C_19001
chr5	55204542	+	C	55	19	0.345	0	0	T	36	M	0.234	0.477	0	2.4E-08	0	88.7776	accatcattcCcaggaatggc	m5C_18971
chr5	55204556	+	C	63	36	0.571	0	0	C	36	M	0.449	0.686	0	6.5E-08	0	323.002	gaatggcgtgCctggcttaaa	m5C_18974
chr5	55204557	+	C	56	18	0.321	0	0	T	38	M	0.214	0.452	0	1.7E-08	0	77.043	aatggcgtgcCtggcttaaag	m5C_18984
chr5	55468992	+	C	60	17	0.283	0	0	T	43	M	0.185	0.408	0	1.8E-10	0	62.9227	tcgaggaccaCctggtctgtg	m5C_18995
chr5	55470810	-	C	39	10	0.256	0	0	T	29	M	0.146	0.411	1.3E-13	2E-06	1.6E-13	18.7753	gttatcagaaCttattcacat	m5C_19013
chr5	55470833	-	C	62	18	0.29	0	0	T	44	M	0.192	0.413	0	2.9E-10	0	69.2043	ccaggctggtCtgatggtaga	m5C_19012
chr5	62059744	+	C	33	15	0.455	0	0	T	18	M	0.298	0.62	0	1.2E-05	0	89.5281	acactgggtgCtgtaggacca	m5C_18767
chr5	63975645	+	C	34	20	0.588	0	0	C	20	M	0.422	0.736	0	4.3E-05	0	168.885	gtggacagtcCagctggtaga	m5C_18766
chr5	71146743	+	C	64	17	0.266	0	0	T	47	M	0.173	0.385	0	1.8E-10	0	58.8072	gaaatttcttCcagtgcggta	m5C_19532
chr5	71146744	+	C	111	27	0.243	0	0	T	84	M	0.173	0.331	0	0	0	93.3623	aaatttcttcCagtgcggtaa	m5C_19566
chr5	71146749	+	C	220	54	0.247	1	0.005	T	165	M	0.194	0.308	0	0	0	209.719	tcttccagtgCggtaacgtga	m5C_19495
chr5	71146755	+	C	373	133	0.357	0	0	T	240	M	0.31	0.406	0	0	0	823.661	agtgcggtaaCgtgaccgatc	m5C_19549
chr5	71146760	+	C	424	110	0.259	0	0	T	314	M	0.22	0.303	0	0	0	484.007	ggtaacgtgaCcgatcccgga	m5C_19536
chr5	71146761	+	C	408	143	0.351	1	0.002	T	264	M	0.307	0.399	0	0	0	876.754	gtaacgtgacCgatcccggag	m5C_19538
chr5	71146762	-	C	56	41	0.732	0	0	C	41	M	0.604	0.83	0	3.3E-06	0	495.323	tctccgggatCggtcacgtta	m5C_19621
chr5	71146765	+	C	451	146	0.324	0	0	T	305	M	0.282	0.368	0	0	0	823.987	cgtgaccgatCccggagaagc	m5C_19497
chr5	71146766	+	C	470	178	0.379	0	0	T	292	M	0.336	0.423	0	0	0	1196.23	gtgaccgatcCcggagaagcc	m5C_19523
chr5	71146767	+	C	467	140	0.3	0	0	T	327	M	0.26	0.343	0	0	0	728.007	tgaccgatccCggagaagccg	m5C_19502

chr5	71146775	+	C	659	252	0.382	0	0	T	407	M	0.346	0.42	0	0	0	1744.22	cccggagaagCcggcgggagc	m5C_19520
chr5	71146776	+	C	672	223	0.332	0	0	T	449	M	0.297	0.368	0	0	0	1325.9	ccggagaagcCggcgggagcc	m5C_19550
chr5	71146779	+	C	684	260	0.38	0	0	T	424	M	0.345	0.417	0	0	0	1791.42	gagaagccggCgggagccccg	m5C_19537
chr5	71146785	+	C	577	223	0.386	0	0	T	354	M	0.348	0.427	0	0	0	1550.41	ccggcgggagCcccggggaga	m5C_19530
chr5	71146786	+	C	555	247	0.445	0	0	T	308	M	0.404	0.487	0	0	0	1996.83	cggcgggagcCccggggagag	m5C_19556
chr5	71146787	+	C	544	165	0.304	1	0.002	T	378	M	0.267	0.344	0	0	0	880.016	ggcgggagccCcggggagagt	m5C_19539
chr5	71146788	+	C	534	192	0.36	0	0	T	342	M	0.32	0.401	0	0	0	1228.75	gcgggagcccCggggagagtt	m5C_19509
chr5	71146801	+	C	88	66	0.75	0	0	C	66	M	0.65	0.829	0	2.2E-08	0	858.485	ggagagttctCttttctttgt	m5C_19543
chr5	71146806	+	C	85	64	0.753	0	0	C	64	M	0.652	0.832	0	1.7E-08	0	834.115	gttctcttttCtttgtgaagg	m5C_19554
chr5	71146820	-	C	36	27	0.75	0	0	C	27	M	0.589	0.862	0	0.00015	0	318.218	ccagggcgccCtgcccttcac	m5C_19578
chr5	71146821	-	C	44	28	0.636	0	0	C	28	M	0.489	0.762	0	8.1E-06	0	273.65	tccagggcgcCctgcccttca	m5C_19620
chr5	71146822	-	C	47	37	0.787	0	0	C	37	M	0.651	0.88	0	3.3E-05	0	481.708	ttccagggcgCcctgcccttc	m5C_19610
chr5	71146823	+	C	99	36	0.364	0	0	T	63	M	0.276	0.462	0	2E-12	0	198.445	aagggcagggCgccctggaat	m5C_19547
chr5	71146825	+	C	128	51	0.405	2	0.016	T	75	M	0.323	0.492	0	3.3E-15	0	329.565	gggcagggcgCcctggaatgg	m5C_19511
chr5	71146826	+	C	160	50	0.314	1	0.006	T	109	M	0.247	0.39	0	0	0	247.394	ggcagggcgcCctggaatggg	m5C_19515
chr5	71146827	+	C	214	67	0.313	0	0	T	147	M	0.255	0.378	0	0	0	341.309	gcagggcgccCtggaatgggt	m5C_19505
chr5	71146829	-	C	40	18	0.45	0	0	T	22	M	0.307	0.602	0	2.7E-05	0	110.538	gaacccattcCagggcgccct	m5C_19618
chr5	71146830	-	C	41	31	0.756	0	0	C	31	M	0.607	0.862	0	1.4E-05	0	376.069	cgaacccattCcagggcgccc	m5C_19583
chr5	71146834	-	C	38	28	0.737	0	0	C	28	M	0.58	0.85	0	0.00017	0	324.749	ggggcgaaccCattccagggc	m5C_19571
chr5	71146835	-	C	41	19	0.463	0	0	T	22	M	0.321	0.613	0	1E-06	0	121.813	cggggcgaacCcattccaggg	m5C_19585
chr5	71146839	+	C	228	71	0.311	0	0	T	157	M	0.255	0.374	0	0	0	361.871	ggaatgggttCgccccgagag	m5C_19560
chr5	71146840	-	C	39	17	0.436	0	0	T	22	M	0.293	0.59	0	2.1E-05	0	99.6354	tctctcggggCgaacccattc	m5C_19589
chr5	71146841	+	C	241	100	0.415	0	0	T	141	M	0.355	0.478	0	0	0	709.082	aatgggttcgCcccgagagag	m5C_19518
chr5	71146842	+	C	229	73	0.319	0	0	T	156	M	0.262	0.382	0	0	0	382.28	atgggttcgcCccgagagagg	m5C_19541
chr5	71146843	+	C	224	52	0.232	0	0	T	172	M	0.182	0.292	0	0	0	188.917	tgggttcgccCcgagagaggg	m5C_19507
chr5	71146844	+	C	220	58	0.266	2	0.009	T	160	M	0.212	0.328	0	0	0	245.705	gggttcgcccCgagagagggg	m5C_19542
chr5	71146845	-	C	40	19	0.475	0	0	T	21	M	0.329	0.625	0	1E-06	0	125.154	gcccctctctCggggcgaacc	m5C_19575
chr5	71146847	-	C	47	36	0.766	0	0	C	36	M	0.628	0.864	0	2.9E-05	0	451.983	gggcccctctCtcggggcgaa	m5C_19644
chr5	71146849	-	C	62	41	0.661	0	0	C	41	M	0.537	0.767	0	1.7E-07	0	440.483	acgggcccctCtctcggggcg	m5C_19595
chr5	71146851	-	C	66	43	0.652	0	0	C	43	M	0.531	0.755	0	2.4E-07	0	456.763	gcacgggcccCtctctcgggg	m5C_19586
chr5	71146852	-	C	64	44	0.688	0	0	C	44	M	0.566	0.788	0	2.8E-07	0	498.145	ggcacgggccCctctctcggg	m5C_19606
chr5	71146853	-	C	68	45	0.662	0	0	C	45	M	0.543	0.763	0	3.3E-07	0	489.033	aggcacgggcCcctctctcgg	m5C_19649
chr5	71146854	-	C	70	46	0.657	0	0	C	46	M	0.54	0.758	0	1.9E-08	0	497.162	aaggcacgggCccctctctcg	m5C_19597
chr5	71146855	+	C	77	58	0.753	0	0	C	58	M	0.646	0.836	0	1.2E-07	0	749.899	gagagaggggCccgtgccttg	m5C_19527
chr5	71146856	+	C	85	57	0.679	1	0.012	C	57	M	0.573	0.769	0	5.8E-09	0	652.973	agagaggggcCcgtgccttgg	m5C_19521
chr5	71146857	+	C	94	56	0.596	0	0	C	56	M	0.495	0.689	0	2.3E-10	0	554.032	gagaggggccCgtgccttgga	m5C_19555
chr5	71146858	-	C	82	59	0.72	0	0	C	59	M	0.614	0.805	0	8E-09	0	724.694	ttccaaggcaCgggcccctct	m5C_19628
chr5	71146860	-	C	81	59	0.728	0	0	C	59	M	0.623	0.813	0	8E-09	0	734.956	ctttccaaggCacgggcccct	m5C_19601
chr5	71146888	+	C	133	104	0.782	0	0	C	104	M	0.704	0.844	0	3.4E-12	0	1465.16	gttctggcggCatccggtgag	m5C_19551
chr5	71146891	+	C	126	97	0.77	0	0	C	97	M	0.689	0.835	0	2.1E-11	0	1336.69	ctggcggcatCcggtgagctc	m5C_19557
chr5	71146905	+	C	90	55	0.611	0	0	C	55	M	0.508	0.705	0	4.2E-09	0	558.605	tgagctctcgCtggcccttga	m5C_19499
chr5	71146909	+	C	70	50	0.714	0	0	C	50	M	0.599	0.807	0	3.7E-08	0	599.496	ctctcgctggCccttgaaaat	m5C_19503
chr5	71146911	+	C	54	34	0.63	0	0	C	34	M	0.496	0.746	0	1.1E-06	0	337.465	ctcgctggccCttgaaaatcc	m5C_19548
chr5	71158139	+	C	32	15	0.469	0	0	T	17	M	0.309	0.636	0	1.2E-05	0	92.6074	gattatctggCatcacccctg	m5C_19519
chr5	72379014	-	C	195	58	0.297	0	0	T	137	M	0.238	0.365	0	0	0	275.72	aacggtgcacCcatggccagg	m5C_19898
chr5	72379015	-	C	217	87	0.401	0	0	T	130	M	0.338	0.467	0	0	0	588.093	gaacggtgcaCccatggccag	m5C_19903
chr5	72379022	-	C	364	81	0.223	0	0	T	283	M	0.183	0.268	0	0	0	296.168	cttccaggaaCggtgcaccca	m5C_19906
chr5	74063440	-	C	38	9	0.237	0	0	T	29	M	0.13	0.392	3.7E-12	1.2E-06	4.3E-12	13.3739	agcggtggatCaaaaagaaaa	m5C_18961
chr5	74063448	-	C	43	18	0.419	0	0	T	25	M	0.284	0.567	0	7.7E-07	0	102.184	ggactcttagCggtggatcaa	m5C_18969
chr5	76376267	-	C	35	19	0.543	0	0	C	19	M	0.382	0.695	0	3.4E-05	0	145.12	ctgtgctggcCgacatttgtg	m5C_19166
chr5	76376268	-	C	37	10	0.27	0	0	T	27	M	0.154	0.43	6.7E-14	2E-06	8.3E-14	20.2815	gctgtgctggCcgacatttgt	m5C_19171
chr5	76376277	-	C	40	11	0.275	0	0	T	29	M	0.161	0.428	4.3E-15	3.1E-06	5.5E-15	25.4502	aggggcagcgCtgtgctggcc	m5C_19192
chr5	76376336	-	C	44	20	0.455	0	0	T	24	M	0.317	0.599	0	1.4E-06	0	126.82	ttaagacctcCaaaatcgaag	m5C_19180
chr5	76376337	-	C	44	9	0.205	0	0	T	35	M	0.112	0.345	1.8E-11	1.2E-08	2.1E-11	10.7806	gttaagacctCcaaaatcgaa	m5C_19194
chr5	76376339	-	C	54	13	0.241	0	0	T	41	M	0.146	0.369	2.2E-16	1.6E-09	2.9E-16	29.8003	cggttaagacCtccaaaatcg	m5C_19187
chr5	76376340	-	C	54	31	0.574	0	0	C	31	M	0.442	0.697	0	5.9E-07	0	273.807	ccggttaagaCctccaaaatc	m5C_19169
chr5	76376356	-	C	153	35	0.229	0	0	T	118	M	0.169	0.301	0	0	0	118.531	cccgtgctgcCttccaccggt	m5C_19191
chr5	76376357	-	C	165	50	0.303	0	0	T	115	M	0.238	0.377	0	0	0	238.046	ccccgtgctgCcttccaccgg	m5C_19190
chr5	76376360	-	C	178	51	0.287	0	0	T	127	M	0.225	0.357	0	0	0	229.659	ttgccccgtgCtgccttccac	m5C_19177
chr5	76376364	-	C	182	75	0.412	0	0	T	107	M	0.343	0.485	0	0	0	514.671	agttttgcccCgtgctgcctt	m5C_19174
chr5	76376375	-	C	278	94	0.339	1	0.004	T	183	M	0.286	0.397	0	0	0	537.918	gaaggtgaggCagttttgccc	m5C_19182
chr5	76376389	-	C	313	64	0.204	0	0	T	249	M	0.164	0.253	0	0	0	209.282	atgtggaggaCtgagaaggtg	m5C_19186
chr5	78466076	-	C	65	14	0.215	0	0	T	51	M	0.133	0.33	1.1E-16	3.2E-11	1.5E-16	29.6776	actcccactgCttcacttgac	m5C_19366
chr5	78466082	-	C	63	19	0.302	0	0	T	44	M	0.202	0.424	0	4.5E-10	0	76.9026	ttctccactcCcactgcttca	m5C_19372
chr5	78466083	-	C	64	14	0.219	0	0	T	50	M	0.135	0.334	1.1E-16	3.2E-11	1.5E-16	30.1591	cttctccactCccactgcttc	m5C_19350
chr5	78466085	-	C	65	14	0.215	0	0	T	51	M	0.133	0.33	1.1E-16	3.2E-11	1.5E-16	29.6776	tccttctccaCtcccactgct	m5C_19371
chr5	78466093	-	C	69	26	0.377	0	0	T	43	M	0.272	0.495	0	5.6E-09	0	141.353	ttcttttgttcCttctccactc	m5C_19370
chr5	78466094	-	C	67	19	0.284	0	0	T	48	M	0.19	0.401	0	4.5E-10	0	72.0865	tttcttttgttCcttctccact	m5C_19373
chr5	78466101	-	C	67	17	0.254	0	0	T	50	M	0.165	0.369	0	1.8E-10	0	56.0585	ttacagatttCtttgttcctt	m5C_19347
chr5	78466118	-	C	72	15	0.208	0	0	T	57	M	0.131	0.316	0	6E-13	0	39.1558	taattgatcaCaaccagttac	m5C_19353
chr5	78516884	+	C	56	13	0.232	0	0	T	43	M	0.141	0.358	4.4E-16	1.6E-09	5.8E-16	28.1397	tgggaaggaaCtacaagacag	m5C_19334
chr5	78516887	+	C	53	18	0.34	0	0	T	35	M	0.227	0.474	0	1.7E-08	0	81.6727	gaaggaactaCaagacagttg	m5C_19326
chr5	78516892	+	C	52	14	0.269	0	0	T	38	M	0.168	0.403	0	2.8E-09	0	46.9527	aactacaagaCagttgctaaa	m5C_19327
chr5	78610216	+	C	62	16	0.258	0	0	T	46	M	0.166	0.379	0	1.1E-10	0	52.9752	gaagagaaaaCtgaagaggtg	m5C_19336
chr5	79205155	-	C	140	28	0.2	0	0	T	112	M	0.142	0.274	0	0	0	79.6061	gcttacatgaCtcttagtggt	m5C_19374
chr5	79298925	+	C	59	26	0.441	0	0	T	33	M	0.322	0.567	0	1.9E-07	0	167.18	tggttaactcCtgggatggag	m5C_19331
chr5	79948139	+	C	33	14	0.424	0	0	T	19	M	0.272	0.592	0	9.2E-06	0	76.259	aatttgaaatCtggttaggct	m5C_19313
chr5	81573624	-	C	37	8	0.216	0	0	T	29	M	0.114	0.372	9.7E-11	6.8E-07	1.1E-10	9.12273	tagtcaccgaCgagaccagaa	m5C_19041
chr5	81573628	-	C	42	15	0.357	0	0	T	27	M	0.23	0.508	0	2.8E-07	0	68.967	tccgtagtcaCcgacgagacc	m5C_19031
chr5	81573636	-	C	41	11	0.268	0	0	T	30	M	0.157	0.419	6E-15	4.4E-08	7.6E-15	24.5536	taggaagctcCgtagtcaccg	m5C_19056
chr5	81573637	-	C	40	13	0.325	0	0	T	27	M	0.201	0.48	0	1.2E-07	0	52.2192	ctaggaagctCcgtagtcacc	m5C_19040
chr5	81573639	-	C	41	14	0.341	0	0	T	27	M	0.216	0.495	0	1.9E-07	0	60.3641	tgctaggaagCtccgtagtca	m5C_19046
chr5	82360128	-	C	99	24	0.242	0	0	T	75	M	0.169	0.335	0	1.7E-14	0	80.9576	gcttctcattCcttgggagca	m5C_19394
chr5	82360132	-	C	101	30	0.297	0	0	T	71	M	0.217	0.392	0	4.7E-15	0	130.009	gtctgcttctCattccttggg	m5C_19402
chr5	82360134	-	C	104	36	0.346	0	0	T	68	M	0.262	0.442	0	4.8E-14	0	188.406	atgtctgcttCtcattccttg	m5C_19392
chr5	82360137	-	C	104	26	0.255	2	0.019	T	76	M	0.18	0.347	0	6.7E-16	0	93.7536	gaaatgtctgCttctcattcc	m5C_19406
chr5	84311147	-	C	73	15	0.205	0	0	T	58	M	0.129	0.312	0	6E-13	0	38.6006	gtgggatgtgCttgtgaatag	m5C_19134
chr5	85916513	+	C	47	13	0.277	0	0	T	34	M	0.169	0.418	0	1.2E-07	0	44.0347	gaggtgcagcCtctggaagtg	m5C_19122
chr5	85916515	+	C	53	12	0.226	0	0	T	41	M	0.135	0.355	5.8E-15	8.8E-10	7.3E-15	22.9888	ggtgcagcctCtggaagtgga	m5C_19108
chr5	85916527	+	C	57	24	0.421	0	0	T	33	M	0.302	0.55	0	1.2E-07	0	144.9	ggaagtggatCaaactagaac	m5C_19107
chr5	85916531	+	C	51	16	0.314	0	0	T	35	M	0.203	0.45	0	7.3E-09	0	65.048	gtggatcaaaCtagaactcat	m5C_19120
chr5	85916539	+	C	39	16	0.41	0	0	T	23	M	0.271	0.566	0	1.7E-05	0	86.6531	aactagaactCatatgccata	m5C_19117
chr5	90770142	-	C	129	33	0.256	0	0	T	96	M	0.188	0.337	0	0	0	124.306	gactatttggCctgtgtgttc	m5C_19227
chr5	92956462	-	C	44	11	0.25	0	0	T	33	M	0.146	0.394	1.6E-14	4.4E-08	2E-14	22.132	gggctgagcgCtgccagtcag	m5C_20119
chr5	92956464	-	C	43	13	0.302	0	0	T	30	M	0.186	0.451	0	1.2E-07	0	48.3648	gcgggctgagCgctgccagtc	m5C_20128
chr5	93018930	-	C	61	37	0.607	0	0	C	37	M	0.481	0.719	0	7.9E-08	0	356.066	aagatgatgaCgatgatgatg	m5C_20125
chr5	97065640	+	C	35	15	0.429	0	0	T	20	M	0.28	0.591	0	1.2E-05	0	83.953	tagaggggttCtgtagtgtag	m5C_19970
chr5	97065658	+	C	61	25	0.41	0	0	T	36	M	0.295	0.535	0	4.1E-09	0	147.676	tagcagtcatCacactcatct	m5C_19955
chr5	97065660	+	C	60	14	0.233	0	0	T	46	M	0.144	0.354	0	3.2E-11	0	40.4304	gcagtcatcaCactcatctca	m5C_19957
chr5	99384599	-	C	41	25	0.61	0	0	C	25	M	0.457	0.743	0	4.5E-06	0	228.633	ataagaactgCtaactcatac	m5C_19426
chr5	99384602	-	C	41	24	0.585	0	0	C	24	M	0.434	0.722	0	3.6E-06	0	208.161	ctcataagaaCtgctaactca	m5C_19447

chr5	99384610	-	C	42	23	0.548	0	0	C	23	M	0.399	0.688	0	2.9E-06	0	183.765	cgagaaagctCataagaactg	m5C_19437
chr5	99384612	-	C	39	24	0.615	0	0	C	24	M	0.459	0.751	0	9.1E-05	0	220.314	accgagaaagCtcataagaac	m5C_19444
chr5	99384645	-	C	40	11	0.275	0	0	T	29	M	0.161	0.428	4.3E-15	3.1E-06	5.5E-15	25.4502	aatctgataaCagaggctcac	m5C_19431
chr5	99384652	-	C	46	17	0.37	0	0	T	29	M	0.245	0.514	0	5.6E-07	0	83.3801	gattgtgaatCtgataacaga	m5C_19435
chr5	111497203	+	C	86	28	0.326	0	0	T	58	M	0.236	0.43	0	5.8E-12	0	132.079	tgcccattcaCtttggaaact	m5C_19834
chr5	111497228	+	C	95	21	0.221	0	0	T	74	M	0.149	0.314	0	3.1E-15	0	62.7364	aatgtggtgtCaaaaaaggcg	m5C_19836
chr5	111497237	+	C	78	17	0.218	0	0	T	61	M	0.141	0.322	0	2.2E-12	0	47.8608	tcaaaaaaggCgtaaattaaa	m5C_19846
chr5	111497248	+	C	80	16	0.2	0	0	T	64	M	0.127	0.3	0	1.1E-14	0	40.6488	gtaaattaaaCgctttgcagc	m5C_19852
chr5	111497250	+	C	91	42	0.462	0	0	T	49	M	0.363	0.563	0	1.1E-11	0	304.712	aaattaaacgCtttgcagcct	m5C_19840
chr5	111497264	+	C	109	25	0.229	0	0	T	84	M	0.16	0.317	0	4.4E-16	0	80.2272	gcagccttttCctgcccttaa	m5C_19848
chr5	111497268	+	C	121	37	0.306	0	0	T	84	M	0.231	0.393	0	0	0	170.732	ccttttcctgCccttaaattt	m5C_19855
chr5	111497269	+	C	115	30	0.261	0	0	T	85	M	0.189	0.348	0	0	0	113.564	cttttcctgcCcttaaatttg	m5C_19861
chr5	111497284	+	C	159	32	0.201	0	0	T	127	M	0.146	0.27	0	0	0	93.6458	aatttgatacCtttggtgtag	m5C_19858
chr5	111497298	+	C	254	152	0.598	0	0	C	152	M	0.537	0.657	0	0	0	1632.8	ggtgtaggagCtgcataagta	m5C_19837
chr5	111497310	+	C	229	65	0.284	0	0	T	164	M	0.229	0.345	0	0	0	298.204	gcataagtaaCagttgctgct	m5C_19832
chr5	114937987	+	C	38	17	0.447	0	0	T	21	M	0.301	0.603	0	2.1E-05	0	102.497	cccggacgcgCgtgagtcggg	m5C_20332
chr5	114937994	+	C	59	27	0.458	0	0	T	32	M	0.337	0.583	0	2.5E-07	0	181.986	gcgcgtgagtCgggggcccgc	m5C_20337
chr5	114938000	+	C	55	18	0.327	0	0	T	37	M	0.218	0.459	0	1.7E-08	0	78.5264	gagtcgggggCccgcagaggc	m5C_20335
chr5	114938002	+	C	45	10	0.222	0	0	T	35	M	0.125	0.363	7.4E-13	2.4E-08	8.9E-13	15.2157	gtcgggggccCgcagaggcct	m5C_20336
chr5	115110020	-	C	141	35	0.248	0	0	T	106	M	0.184	0.326	0	0	0	128.965	aatggtgcatCccctttgggg	m5C_20345
chr5	115110035	-	C	197	44	0.223	0	0	T	153	M	0.171	0.286	0	0	0	150.302	tacagtacctCcaggaatggt	m5C_20344
chr5	115110052	-	C	103	23	0.223	0	0	T	80	M	0.154	0.313	0	1.1E-16	0	70.682	acccattgacCtggtattaca	m5C_20353
chr5	122990705	+	C	34	25	0.735	0	0	C	25	M	0.569	0.854	0	0.00011	0	284.413	gggaagagccCagcattgaat	m5C_20181
chr5	122990781	-	C	41	26	0.634	0	0	C	26	M	0.481	0.764	0	5.5E-06	0	250.226	ttgggcccccCatgagcatca	m5C_20225
chr5	122990782	-	C	38	25	0.658	0	0	C	25	M	0.499	0.788	0	0.00011	0	249.459	cttgggccccCcatgagcatc	m5C_20213
chr5	122990784	-	C	39	28	0.718	0	0	C	28	M	0.562	0.835	0	0.00017	0	314.857	gacttgggccCcccatgagca	m5C_20235
chr5	122990785	-	C	42	32	0.762	0	0	C	32	M	0.615	0.865	0	1.6E-05	0	393.422	ggacttgggcCccccatgagc	m5C_20224
chr5	122990786	-	C	45	29	0.644	0	0	C	29	M	0.498	0.768	0	9.7E-06	0	289.046	aggacttgggCccccatgag	m5C_20221
chr5	122990787	+	C	205	67	0.327	0	0	T	138	M	0.266	0.394	0	0	0	356.867	tcatggggggCccaagtcctt	m5C_20205
chr5	122990788	+	C	208	51	0.245	0	0	T	157	M	0.192	0.308	0	0	0	195.531	catggggggcCcaagtccttc	m5C_20187
chr5	122990789	+	C	219	66	0.301	0	0	T	153	M	0.244	0.365	0	0	0	322.675	atgggggccCaagtccttct	m5C_20178
chr5	122990794	+	C	240	124	0.517	0	0	C	124	M	0.454	0.579	0	0	0	1125.13	gggcccaagtCcttctgattg	m5C_20169
chr5	122990795	+	C	236	86	0.364	0	0	T	150	M	0.306	0.428	0	0	0	525.686	ggcccaagtcCttctgattga	m5C_20184
chr5	122990798	+	C	278	147	0.529	0	0	C	147	M	0.47	0.587	0	0	0	1382.11	ccaagtccttCtgattgaggc	m5C_20156
chr5	122990808	+	C	341	120	0.352	0	0	T	221	M	0.303	0.404	0	0	0	727.496	ctgattgaggCccagctcatg	m5C_20202
chr5	122990809	+	C	342	138	0.404	0	0	T	204	M	0.353	0.456	0	0	0	973.902	tgattgaggcCcagctcatgg	m5C_20191
chr5	122990810	+	C	320	109	0.341	0	0	T	211	M	0.291	0.394	0	0	0	634.083	gattgaggccCagctcatgga	m5C_20166
chr5	122990813	+	C	280	103	0.368	0	0	T	177	M	0.314	0.426	0	0	0	645.845	tgaggcccagCtcatggacgg	m5C_20173
chr5	122990815	+	C	263	94	0.357	0	0	T	169	M	0.302	0.417	0	0	0	567.626	aggcccagctCatggacggtg	m5C_20168
chr5	122990821	+	C	321	156	0.486	0	0	T	165	M	0.432	0.54	0	0	0	1347.2	agctcatggaCggtgtgaggc	m5C_20180
chr5	122990831	+	C	312	119	0.381	0	0	T	193	M	0.329	0.436	0	0	0	783.647	cggtgtgaggCtggtagtggt	m5C_20186
chr5	122990842	+	C	128	34	0.266	0	0	T	94	M	0.197	0.348	0	0	0	133.793	tggtagtggtCcccagtgtgc	m5C_20195
chr5	122990843	+	C	113	37	0.327	0	0	T	76	M	0.248	0.418	0	1.4E-15	0	183.392	ggtagtggtcCccagtgtgcc	m5C_20185
chr5	122990844	+	C	94	29	0.309	0	0	T	65	M	0.224	0.408	0	1.7E-13	0	130.036	gtagtggtccCcagtgtgcca	m5C_20194
chr5	126848462	-	C	49	10	0.204	0	0	T	39	M	0.115	0.336	2.1E-12	2.4E-08	2.4E-12	13.4191	aaatgccctgCagaagatatg	m5C_20010
chr5	126848465	-	C	52	16	0.308	0	0	T	36	M	0.199	0.443	0	7.3E-09	0	63.7266	ggtaaatgccCtgcagaagat	m5C_20021
chr5	126848467	-	C	52	21	0.404	0	0	T	31	M	0.282	0.539	0	4.8E-08	0	118.27	ttggtaaatgCcctgcagaag	m5C_20008
chr5	126889582	+	C	40	10	0.25	0	0	T	30	M	0.142	0.402	1.8E-13	2.4E-08	2.2E-13	18.0914	tggtaagcctCctgctcggaa	m5C_20000
chr5	126889583	+	C	47	20	0.426	0	0	T	27	M	0.295	0.567	0	1.4E-06	0	118.054	ggtaagcctcCtgctcggaac	m5C_20002
chr5	126889594	+	C	73	16	0.219	0	0	T	57	M	0.14	0.327	0	1.2E-12	0	44.6912	tgctcggaacCgtgtgagtgg	m5C_20004
chr5	126889626	+	C	90	20	0.222	0	0	T	70	M	0.149	0.318	0	1.7E-15	0	59.4849	gagagatggtCagatggaaga	m5C_19993
chr5	130495216	-	C	42	10	0.238	0	0	T	32	M	0.135	0.385	3.2E-13	2.4E-08	3.9E-13	16.8407	aatgaaggttCagatggtgga	m5C_20100
chr5	130500902	-	C	33	11	0.333	0	0	T	22	M	0.198	0.504	2.2E-16	3.1E-06	2.9E-16	34.0074	ggagagaggcCgagatggcag	m5C_20112
chr5	131516484	+	C	47	16	0.34	0	0	T	31	M	0.222	0.483	0	4E-07	0	70.9352	gggtatagctCagtggtagag	m5C_20089
chr5	131516495	+	C	41	18	0.439	0	0	T	23	M	0.299	0.59	0	7.7E-07	0	107.604	agtggtagagCatttgactgc	m5C_20094
chr5	132203216	+	C	84	25	0.298	0	0	T	59	M	0.21	0.402	0	1.8E-12	0	105.223	acatgggggaCtgaagagttc	m5C_20415
chr5	132328816	-	C	32	10	0.312	0	0	T	22	M	0.18	0.486	1.1E-14	2E-06	1.3E-14	25.0836	ttctccactcCcattgtttca	m5C_20458
chr5	132328821	-	C	31	7	0.226	0	0	T	24	M	0.114	0.398	6.4E-10	3.4E-07	7.2E-10	7.33205	gttccttctcCactcccattg	m5C_20466
chr5	132328835	-	C	31	7	0.226	0	0	T	24	M	0.114	0.398	6.4E-10	3.4E-07	7.2E-10	7.33205	ttacagatttCtttgttcctt	m5C_20429
chr5	132328854	-	C	31	10	0.323	0	0	T	21	M	0.186	0.499	7.1E-15	2E-06	9E-15	26.2725	actaattgatCacaaccagtt	m5C_20486
chr5	132427032	+	C	39	15	0.385	0	0	T	24	M	0.249	0.541	0	1.2E-05	0	74.6735	aatgaggagcCaatggaaaca	m5C_20417
chr5	132427046	+	C	45	16	0.356	0	0	T	29	M	0.232	0.502	0	4E-07	0	74.3005	ggaaacagatCagaatgcaaa	m5C_20411
chr5	132427053	+	C	36	27	0.75	0	0	C	27	M	0.589	0.862	0	0.00015	0	318.218	gatcagaatgCaaaggaggaa	m5C_20402
chr5	133561743	-	C	41	13	0.317	0	0	T	28	M	0.196	0.47	0	1.2E-07	0	50.8675	agctctggagCctcagcgagc	m5C_20421
chr5	133561749	-	C	45	10	0.222	0	0	T	35	M	0.125	0.363	7.4E-13	2.4E-08	8.9E-13	15.2157	gagccgagctCtggagcctca	m5C_20481
chr5	133561751	-	C	46	11	0.239	0	0	T	35	M	0.139	0.379	2.8E-14	4.4E-08	3.5E-14	20.7303	gagagccgagCtctggagcct	m5C_20431
chr5	134259800	+	C	113	34	0.301	0	0	T	79	M	0.224	0.391	0	4.4E-16	0	152.327	tggttgtagcCcgtgcaagaa	m5C_20728
chr5	134259801	+	C	119	25	0.21	0	0	T	94	M	0.147	0.292	0	0	0	73.2714	ggttgtagccCgtgcaagaat	m5C_20725
chr5	134262280	-	C	70	35	0.5	0	0	CT	35	M	0.386	0.614	0	1.9E-09	0	270.169	ccttatttacCgagaaagcgc	m5C_20739
chr5	134262281	-	C	90	40	0.444	0	0	T	50	M	0.346	0.547	0	6.4E-12	0	276.924	cccttatttaCcgagaaagcg	m5C_20743
chr5	134262289	-	C	101	42	0.416	0	0	T	59	M	0.325	0.513	0	3.2E-13	0	272.605	ctcacgacccCttatttaccg	m5C_20736
chr5	134262290	-	C	108	52	0.481	0	0	T	56	M	0.39	0.575	0	4.1E-12	0	405.093	gctcacgaccCcttatttacc	m5C_20742
chr5	134262291	-	C	105	42	0.4	0	0	T	63	M	0.311	0.496	0	3.2E-13	0	261.597	ggctcacgacCccttatttac	m5C_20748
chr5	134262292	-	C	106	35	0.33	0	0	T	71	M	0.248	0.424	0	3.4E-14	0	173.585	aggctcacgaCcccttattta	m5C_20752
chr5	134262295	-	C	100	46	0.46	0	0	T	54	M	0.366	0.557	0	2.9E-11	0	336.358	cagaggctcaCgaccccttat	m5C_20775
chr5	134262299	-	C	107	78	0.729	0	0	C	78	M	0.638	0.804	0	4.2E-10	0	995.134	acaacagaggCtcacgacccc	m5C_20765
chr5	134262305	-	C	77	39	0.506	0	0	C	39	M	0.397	0.615	0	4.7E-09	0	309.815	aatctgacaaCagaggctcac	m5C_20753
chr5	134262308	-	C	68	46	0.676	0	0	C	46	M	0.558	0.776	0	3.8E-07	0	513.767	gtgaatctgaCaacagaggct	m5C_20762
chr5	134262312	-	C	50	26	0.52	0	0	C	26	M	0.385	0.652	0	5.5E-06	0	200.26	gattgtgaatCtgacaacaga	m5C_20756
chr5	134705810	-	C	31	12	0.387	0	0	T	19	M	0.237	0.562	0	4.6E-06	0	56.9589	agctggctggCaatgcagcga	m5C_20773
chr5	135416190	-	C	458	96	0.21	0	0	T	362	M	0.175	0.249	0	0	0	335.658	cttactgaccCttttatgcaa	m5C_20738
chr5	135416200	-	C	579	161	0.278	0	0	T	418	M	0.243	0.316	0	0	0	782.859	cccgcgggtgCttactgaccc	m5C_20770
chr5	135416209	-	C	574	150	0.261	0	0	T	424	M	0.227	0.299	0	0	0	681.156	ggttcgagacCcgcgggtgct	m5C_20754
chr5	135416215	-	C	571	125	0.22	2	0.004	T	444	M	0.188	0.256	0	0	0	469.014	tgctggggttCgagacccgcg	m5C_20735
chr5	135416223	-	C	510	114	0.224	0	0	T	396	M	0.19	0.262	0	0	0	432.094	catctctgtgCtggggttcga	m5C_20755
chr5	135416238	-	C	401	85	0.212	0	0	T	316	M	0.175	0.255	0	0	0	297.155	gactttctatCtgtccatctc	m5C_20772
chr5	135416250	-	C	401	104	0.259	0	0	T	297	M	0.219	0.304	0	0	0	455.271	ctcctcatgcCggactttcta	m5C_20767
chr5	135416260	-	C	403	100	0.249	1	0.002	T	302	M	0.209	0.293	0	0	0	418.017	aagcggttacCtcctcatgcc	m5C_20757
chr5	135416282	-	C	330	78	0.236	0	0	T	252	M	0.194	0.285	0	0	0	302.207	ctacccgggtCggagttagct	m5C_20749
chr5	135416287	-	C	78	16	0.205	0	0	T	62	M	0.13	0.308	0	1.2E-12	0	41.727	cactcctaccCgggtcggagt	m5C_20750
chr5	135416288	-	C	33	14	0.424	0	0	T	19	M	0.272	0.592	0	9.2E-06	0	76.259	acactcctacCcgggtcggag	m5C_20751
chr5	136983270	-	C	32	16	0.5	0	0	CT	16	M	0.336	0.664	0	1.7E-05	0	107.618	gagggaccgaCtggctgggcc	m5C_20296
chr5	136983274	-	C	45	17	0.378	0	0	T	28	M	0.251	0.524	0	5.6E-07	0	85.3668	gcccgagggaCcgactggctg	m5C_20311
chr5	136983281	-	C	43	9	0.209	0	0	T	34	M	0.114	0.352	1.4E-11	1.2E-08	1.6E-11	11.1522	tgccctggccCgagggaccga	m5C_20277
chr5	136983282	-	C	46	26	0.565	0	0	C	26	M	0.422	0.698	0	5.5E-06	0	219.685	ctgccctggcCcgagggaccg	m5C_20273
chr5	136983283	-	C	43	27	0.628	0	0	C	27	M	0.479	0.756	0	6.7E-06	0	258.442	cctgccctggCccgagggacc	m5C_20320
chr5	137893113	-	C	33	7	0.212	0	0	T	26	M	0.107	0.378	1.1E-09	3.4E-07	1.2E-09	6.69165	gagaaatatgCtgaagaagac	m5C_20319
chr5	137894662	-	C	63	34	0.54	0	0	C	34	M	0.418	0.657	0	4.2E-08	0	284.165	tgtgactgagCatgatacatc	m5C_20302
chr5	137894667	-	C	69	23	0.333	0	0	T	46	M	0.234	0.451	0	2.1E-09	0	107.414	ctttctgtgaCtgagcatgat	m5C_20321
chr5	137894677	-	C	73	21	0.288	0	0	T	52	M	0.197	0.4	0	1.8E-11	0	82.5367	tatgtggtgaCtttctgtgac	m5C_20307
chr5	137894698	-	C	87	18	0.207	0	0	T	69	M	0.135	0.304	0	4.5E-14	0	48.6261	acacatcactCtgaagaaaag	m5C_20290

chr5	137894724	-	C	46	17	0.37	0	0	T	29	M	0.245	0.514	0	5.6E-07	0	83.3801	atatttgtgcCgtgatgtatt	m5C_20287
chr5	137896735	-	C	561	132	0.235	0	0	T	429	M	0.202	0.272	0	0	0	533.454	aatgactgagCacaaacctgt	m5C_20303
chr5	137896740	-	C	743	169	0.227	0	0	T	574	M	0.199	0.259	0	0	0	671.813	aaactaatgaCtgagcacaaa	m5C_20269
chr5	137896758	-	C	819	344	0.42	0	0	T	475	M	0.387	0.454	0	0	0	2660.3	tctgaaagaaCgtgtggaaaa	m5C_20286
chr5	137896767	-	C	715	170	0.238	0	0	T	545	M	0.208	0.27	0	0	0	707.237	acacatcattCtgaaagaacg	m5C_20276
chr5	138611888	+	C	407	123	0.302	0	0	T	284	M	0.26	0.349	0	0	0	638.657	aggcagctgcCtaggttgtct	m5C_21399
chr5	138611918	+	C	246	61	0.249	1	0.004	T	184	M	0.199	0.307	0	0	0	242.764	gcaagtgttaCactgctggga	m5C_21400
chr5	138611920	+	C	229	53	0.231	0	0	T	176	M	0.182	0.29	0	0	0	192.409	aagtgttacaCtgctgggaga	m5C_21430
chr5	138611935	+	C	206	49	0.238	0	0	T	157	M	0.185	0.3	0	0	0	181.157	gggagaacagCagccaatagc	m5C_21446
chr5	138611938	+	C	192	41	0.215	1	0.005	T	150	M	0.162	0.278	0	0	0	133.134	agaacagcagCcaatagctgg	m5C_21395
chr5	138611939	+	C	197	64	0.325	0	0	T	133	M	0.263	0.393	0	0	0	337.108	gaacagcagcCaatagctggt	m5C_21387
chr5	138611945	+	C	168	44	0.262	0	0	T	124	M	0.201	0.333	0	0	0	177.121	cagccaatagCtggttggcat	m5C_21405
chr5	138611989	+	C	110	26	0.236	0	0	T	84	M	0.167	0.324	0	0	0	86.6896	cttgtgttgaCtaccccagga	m5C_21428
chr5	138612002	+	C	119	28	0.235	0	0	T	91	M	0.168	0.319	0	0	0	94.1382	ccccaggatgCcagcatagtt	m5C_21417
chr5	138614505	+	C	1017	253	0.249	2	0.002	T	762	M	0.224	0.277	0	0	0	1131.55	tcatgtggtgCctgtgatggt	m5C_21422
chr5	138614522	+	C	490	111	0.227	0	0	T	379	M	0.192	0.266	0	0	0	425.525	tggtgttacaCtgttggaaga	m5C_21451
chr5	138614538	+	C	218	48	0.22	0	0	T	170	M	0.17	0.28	0	0	0	163.474	gaagagcaaaCactgtcttta	m5C_21424
chr5	138614560	+	C	67	28	0.418	0	0	T	39	M	0.307	0.537	0	9.8E-09	0	172.156	tgaggtttggCtccaagcact	m5C_21444
chr5	138614562	+	C	65	22	0.338	0	0	T	43	M	0.235	0.46	0	1.5E-09	0	103.547	aggtttggctCcaagcactgt	m5C_21411
chr5	138614567	+	C	68	25	0.368	0	0	T	43	M	0.263	0.486	0	4.1E-09	0	131.501	tggctccaagCactgttttgg	m5C_21396
chr5	138614569	+	C	89	24	0.27	0	0	T	65	M	0.188	0.37	0	1.1E-12	0	90.4412	gctccaagcaCtgttttggtg	m5C_21409
chr5	138614586	+	C	62	16	0.258	0	0	T	46	M	0.166	0.379	0	1.1E-10	0	52.9752	ggtgttgtagCtgagtacctt	m5C_21440
chr5	138614593	+	C	70	16	0.229	0	0	T	54	M	0.146	0.34	0	1.2E-12	0	46.6814	tagctgagtaCctttgggcag	m5C_21392
chr5	138614613	+	C	150	30	0.2	0	0	T	120	M	0.144	0.271	0	0	0	86.304	gtgttttgcaCctctgagagt	m5C_21441
chr5	138614614	+	C	163	42	0.258	0	0	T	121	M	0.197	0.33	0	0	0	165.186	tgttttgcacCtctgagagtg	m5C_21394
chr5	138614616	+	C	194	51	0.263	0	0	T	143	M	0.206	0.329	0	0	0	210.097	ttttgcacctCtgagagtgga	m5C_21416
chr5	138614631	+	C	420	100	0.238	0	0	T	320	M	0.2	0.281	0	0	0	399.701	agtggaatgaCtcctgtggag	m5C_21449
chr5	138614634	+	C	467	112	0.24	0	0	T	355	M	0.203	0.281	0	0	0	455.449	ggaatgactcCtgtggagttg	m5C_21432
chr5	138658591	+	C	52	12	0.231	0	0	T	40	M	0.137	0.361	4.4E-15	8.8E-10	5.7E-15	23.6371	gaaaaaggaaCcatcagataa	m5C_21437
chr5	138658592	+	C	62	13	0.21	0	0	T	49	M	0.127	0.326	1.8E-15	1.6E-11	2.3E-15	24.3221	aaaaaggaacCatcagataaa	m5C_21412
chr5	138658604	+	C	75	28	0.373	0	0	T	47	M	0.273	0.486	0	2.6E-10	0	152.633	tcagataaagCtgtgaaaaaa	m5C_21408
chr5	139063111	-	C	48	29	0.604	0	0	C	29	M	0.463	0.73	0	9.7E-06	0	268.599	gggtgcagctCagctggcctg	m5C_21484
chr5	139063113	-	C	53	17	0.321	0	0	T	36	M	0.211	0.455	0	1.1E-08	0	71.713	gtgggtgcagCtcagctggcc	m5C_21462
chr5	139140570	+	C	39	20	0.513	0	0	C	20	M	0.362	0.661	0	4.3E-05	0	144.796	ccctggtggtCtagtggttag	m5C_21434
chr5	139283551	+	C	43	18	0.419	0	0	T	25	M	0.284	0.567	0	7.7E-07	0	102.184	gtctgatcagCcgccgccttt	m5C_21435
chr5	140038651	+	C	45	20	0.444	0	0	T	25	M	0.309	0.588	0	1.4E-06	0	123.755	agagcgagagCgagaacgaga	m5C_20671
chr5	140038657	+	C	60	22	0.367	0	0	T	38	M	0.256	0.493	0	1.5E-09	0	112.731	agagcgagaaCgagatcggga	m5C_20623
chr5	140038663	+	C	50	10	0.2	0	0	T	40	M	0.112	0.33	2.6E-12	2E-10	3.1E-12	13.024	agaacgagatCgggaacgaga	m5C_20684
chr5	140038669	+	C	61	19	0.311	0	0	T	42	M	0.209	0.436	0	4.5E-10	0	79.5616	agatcgggaaCgagagcgaga	m5C_20637
chr5	140038681	+	C	84	22	0.268	2	0.024	T	60	M	0.184	0.373	0	4.4E-13	0	81.1184	agagcgagagCgggaccgaga	m5C_20667
chr5	140038686	+	C	78	37	0.474	0	0	T	41	M	0.367	0.584	0	3E-09	0	271.854	gagagcgggaCcgagagagag	m5C_20677
chr5	140084084	+	C	35	10	0.286	0	0	T	25	M	0.163	0.451	3.4E-14	2E-06	4.2E-14	21.9991	gttttgaggtCaacaagaaga	m5C_20628
chr5	140084087	+	C	34	11	0.324	0	0	T	23	M	0.191	0.492	4.4E-16	3.1E-06	5.8E-16	32.3088	ttgaggtcaaCaagaagaaga	m5C_20675
chr5	140090867	+	C	176	40	0.227	0	0	T	136	M	0.172	0.295	0	0	0	137.259	tccgggctggCtttagctcag	m5C_20661
chr5	140090878	+	C	176	49	0.278	0	0	T	127	M	0.217	0.349	0	0	0	213.114	tttagctcagCggttacttcg	m5C_20680
chr5	140090884	+	C	174	52	0.299	0	0	T	122	M	0.236	0.371	0	0	0	245.209	tcagcggttaCttcgacagtt	m5C_20673
chr5	140090887	+	C	169	39	0.232	1	0.006	T	129	M	0.175	0.302	0	0	0	136.283	gcggttacttCgacagttctt	m5C_20670
chr5	140090895	+	C	151	38	0.252	0	0	T	113	M	0.189	0.326	0	0	0	143.781	ttcgacagttCtttaattgaa	m5C_20632
chr5	140090907	+	C	125	26	0.208	0	0	T	99	M	0.146	0.287	0	0	0	75.9632	ttaattgaaaCaagcaacctg	m5C_20660
chr5	140090911	+	C	144	53	0.368	0	0	T	91	M	0.294	0.449	0	0	0	311.287	ttgaaacaagCaacctgtctg	m5C_20651
chr5	140090934	+	C	191	51	0.268	1	0.005	T	139	M	0.21	0.336	0	0	0	214.667	ttgttcgagaCccgcgggcgc	m5C_20642
chr5	140090938	+	C	176	36	0.206	1	0.006	T	139	M	0.152	0.272	0	0	0	109.768	tcgagacccgCgggcgctctc	m5C_20666
chr5	140090942	+	C	174	43	0.247	0	0	T	131	M	0.189	0.316	0	0	0	162.504	gacccgcgggCgctctccagt	m5C_20655
chr5	140090944	+	C	158	42	0.266	0	0	T	116	M	0.203	0.34	0	0	0	170.598	cccgcgggcgCtctccagtcc	m5C_20688
chr5	140090946	+	C	167	69	0.413	0	0	T	98	M	0.341	0.489	0	0	0	470.942	cgcgggcgctCtccagtcctt	m5C_20676
chr5	140090954	+	C	163	53	0.325	0	0	T	110	M	0.258	0.4	0	0	0	273.464	ctctccagtcCttttacctct	m5C_20662
chr5	140098523	+	C	644	136	0.211	0	0	T	508	M	0.181	0.244	0	0	0	493.453	ctggctttagCtcagcggtta	m5C_20647
chr5	140098552	+	C	654	131	0.2	0	0	T	523	M	0.171	0.233	0	0	0	449.124	acattgtaacCacctctctgg	m5C_20649
chr5	140098555	+	C	659	151	0.229	0	0	T	508	M	0.199	0.263	0	0	0	599.987	ttgtaaccacCtctctgggtg	m5C_20689
chr5	140098575	+	C	841	227	0.27	0	0	T	614	M	0.241	0.301	0	0	0	1094.19	ggttcgagacCcgcgggtgct	m5C_20626
chr5	140098584	+	C	780	245	0.315	3	0.004	T	532	M	0.284	0.349	0	0	0	1389.74	cccgcgggtgCtttccagctc	m5C_20631
chr5	140098592	+	C	582	149	0.257	3	0.005	T	430	M	0.223	0.294	0	0	0	665.767	tgctttccagCtcttttactg	m5C_20674
chr5	140098594	+	C	557	186	0.334	0	0	T	371	M	0.296	0.374	0	0	0	1101.2	ctttccagctCttttactgct	m5C_20630
chr5	140105750	+	C	127	28	0.22	0	0	T	99	M	0.157	0.3	0	0	0	88.016	tctgggctggCtttagctcag	m5C_20682
chr5	140105758	+	C	126	43	0.341	0	0	T	83	M	0.264	0.428	0	2.2E-16	0	227.277	ggctttagctCagcggttact	m5C_20664
chr5	140105772	+	C	134	31	0.231	0	0	T	103	M	0.168	0.31	0	0	0	104.182	ggttacttcgCgtgtcatcaa	m5C_20652
chr5	140105788	+	C	129	27	0.209	0	0	T	102	M	0.148	0.287	0	0	0	79.9307	atcaaaccacCtctctgggtt	m5C_20619
chr5	140105792	+	C	126	40	0.317	0	0	T	86	M	0.243	0.403	0	1.1E-16	0	194.089	aaccacctctCtgggttgttc	m5C_20616
chr5	140105808	+	C	120	43	0.358	0	0	T	77	M	0.278	0.447	0	2.2E-16	0	239.217	tgttcgagacCcgcgggcgct	m5C_20629
chr5	140105817	+	C	102	23	0.228	1	0.01	T	78	M	0.157	0.319	0	1.1E-16	0	72.1313	cccgcgggcgCtctccagccc	m5C_20620
chr5	140105821	+	C	103	29	0.282	0	0	T	74	M	0.204	0.375	0	2.9E-15	0	118.183	cgggcgctctCcagccctctt	m5C_20639
chr5	140105822	+	C	90	23	0.256	0	0	T	67	M	0.177	0.354	0	1E-14	0	81.3033	gggcgctctcCagccctctta	m5C_20663
chr5	140105825	+	C	81	40	0.494	0	0	T	41	M	0.388	0.6	0	2.1E-10	0	310.175	cgctctccagCcctcttactg	m5C_20643
chr5	140105827	+	C	77	19	0.247	0	0	T	58	M	0.164	0.354	0	6.9E-12	0	62.335	ctctccagccCtcttactgct	m5C_20634
chr5	140105829	+	C	70	14	0.2	0	0	T	56	M	0.123	0.308	4.4E-16	2.8E-13	5.8E-16	26.447	ctccagccctCttactgctga	m5C_20635
chr5	141248101	+	C	60	12	0.2	0	0	T	48	M	0.118	0.318	3.3E-14	7.6E-12	4.2E-14	19.13	agtggtgggtCaccctgacag	m5C_20672
chr5	141248103	+	C	52	18	0.346	0	0	T	34	M	0.232	0.482	0	1.7E-08	0	83.343	tggtgggtcaCcctgacagtt	m5C_20636
chr5	141774000	-	C	138	47	0.341	0	0	T	91	M	0.267	0.423	0	0	0	250.78	gggttcgattCcctgaggagg	m5C_20700
chr5	141774005	-	C	141	41	0.291	0	0	T	100	M	0.222	0.37	0	0	0	182.174	gactggggttCgattccctga	m5C_20691
chr5	141774013	-	C	138	55	0.399	0	0	T	83	M	0.321	0.482	0	2.2E-16	0	352.745	acgcggagaCtggggttcga	m5C_20717
chr5	141774029	-	C	82	25	0.305	0	0	T	57	M	0.216	0.411	0	1.8E-12	0	107.916	agtatccccgCctgtcacgcg	m5C_20709
chr5	141774033	-	C	66	15	0.227	0	0	T	51	M	0.143	0.342	0	6E-11	0	42.8553	ggtgagtatcCccgcctgtca	m5C_20697
chr5	141774034	-	C	64	26	0.406	0	0	T	38	M	0.295	0.529	0	5.6E-09	0	153.175	tggtgagtatCcccgcctgtc	m5C_20704
chr5	147661605	+	C	37	9	0.243	0	0	T	28	M	0.134	0.401	2.7E-12	1.2E-06	3.2E-12	13.907	gcagaagtgaCaggaagaaat	m5C_20365
chr5	149212518	-	C	34	20	0.588	0	0	C	20	M	0.422	0.736	0	4.3E-05	0	168.885	agtaggtgtgCatgtagcgta	m5C_21142
chr5	149477975	+	C	87	25	0.287	0	0	T	62	M	0.203	0.39	0	1.8E-12	0	101.428	aataggctgtCtgagtgcagt	m5C_21131
chr5	149477994	+	C	87	23	0.264	0	0	T	64	M	0.183	0.366	0	7.2E-13	0	84.2255	gtggtgtttaCaacaaattga	m5C_21127
chr5	149477997	+	C	78	26	0.333	0	0	T	52	M	0.239	0.444	0	1.3E-10	0	124.137	gtgtttacaaCaaattgatca	m5C_21110
chr5	149478006	+	C	31	8	0.258	0	0	T	23	M	0.137	0.432	1.7E-11	6.8E-07	1.9E-11	11.8182	acaaattgatCacaaccagtt	m5C_21109
chr5	149758914	+	C	53	17	0.321	0	0	T	36	M	0.211	0.455	0	1.1E-08	0	71.713	cagaggaggaCtcagggagca	m5C_21097
chr5	149758916	+	C	51	11	0.216	0	0	T	40	M	0.125	0.346	1.1E-13	4.4E-10	1.4E-13	17.8061	gaggaggactCagggagcagt	m5C_21118
chr5	149787635	-	C	72	26	0.361	0	0	T	46	M	0.26	0.476	0	1.3E-10	0	135.104	ctggttgatcCtgccagcaga	m5C_21135
chr5	149787636	-	C	67	25	0.373	0	0	T	42	M	0.267	0.493	0	4.1E-09	0	133.59	cctggttgatCctgccagcag	m5C_21184
chr5	149823866	-	C	32	11	0.344	0	0	T	21	M	0.204	0.517	2.2E-16	3.1E-06	2.9E-16	35.1442	ggggggtcgcCgtggtcgccg	m5C_21134
chr5	149823869	-	C	36	11	0.306	0	0	T	25	M	0.18	0.469	1E-15	3.1E-06	1.3E-15	29.7077	gaaggggggtCgccgtggtcg	m5C_21140
chr5	149825182	-	C	44	16	0.364	0	0	T	28	M	0.238	0.511	0	4E-07	0	76.1067	gtatgaagatCgggcggattg	m5C_21173
chr5	149826481	-	C	43	10	0.233	0	0	T	33	M	0.132	0.377	4.3E-13	2.4E-08	5.2E-13	16.2683	taaaggcagaCcgagatgaat	m5C_21155
chr5	149826485	-	C	125	32	0.256	0	0	T	93	M	0.188	0.339	0	0	0	120.042	aaggtaaaggCagaccgagat	m5C_21192
chr5	149826506	-	C	138	43	0.314	1	0.007	T	94	M	0.242	0.396	0	0	0	208.236	tgccgtgtgaCtggtgggatg	m5C_21186
chr5	149826513	-	C	85	38	0.447	0	0	T	47	M	0.346	0.553	0	1.3E-10	0	262.91	aaccatctgcCgtgtgactgg	m5C_21187
chr5	149826520	-	C	50	22	0.44	0	0	T	28	M	0.312	0.577	0	6.5E-08	0	137.113	ccagggaaacCatctgccgtg	m5C_21145

chr5	149826521	-	C	52	22	0.423	0	0	T	30	M	0.299	0.558	0	6.5E-08	0	131.419	cccagggaaaCcatctgccgt	m5C_21157
chr5	149827287	-	C	71	21	0.296	0	0	T	50	M	0.202	0.41	0	1.8E-11	0	84.9775	aatggcacctCgaaaggggaa	m5C_21161
chr5	149827292	-	C	72	18	0.25	0	0	T	54	M	0.164	0.361	0	4E-12	0	59.1801	tcagaaatggCacctcgaaag	m5C_21147
chr5	149829275	-	C	58	18	0.31	0	0	T	40	M	0.206	0.438	0	1.7E-08	0	74.239	agtctggagaCgacgtgcagg	m5C_21151
chr5	149829282	-	C	45	16	0.356	0	0	T	29	M	0.232	0.502	0	4E-07	0	74.3005	ggtgtggagtCtggagacgac	m5C_21168
chr5	149874015	-	C	44	33	0.75	0	0	C	33	M	0.606	0.854	0	1.9E-05	0	399.69	actcaggaggCtgaagtggga	m5C_21150
chr5	149874756	-	C	118	37	0.314	0	0	T	81	M	0.237	0.402	0	1.4E-15	0	175.268	cccggctactCgggaggctga	m5C_21149
chr5	149874758	-	C	120	29	0.242	0	0	T	91	M	0.174	0.326	0	0	0	100.837	gtcccggctaCtcgggaggct	m5C_21191
chr5	149874765	-	C	126	27	0.214	0	0	T	99	M	0.152	0.294	0	0	0	81.8938	gcctgtagtcCcggctactcg	m5C_21189
chr5	149874793	-	C	58	14	0.241	0	0	T	44	M	0.15	0.365	0	2.8E-09	0	41.8845	caaaaattagCtgggcgtggt	m5C_21158
chr5	150519225	-	C	31	9	0.29	0	0	T	22	M	0.161	0.466	3.7E-13	1.2E-06	4.4E-13	18.0154	ggatcaggggCcttctgagca	m5C_22070
chr5	153135020	+	C	89	21	0.236	0	0	T	68	M	0.16	0.334	0	2.6E-13	0	67.1258	cacaggagttCtgggctgtag	m5C_20608
chr5	153135025	+	C	100	22	0.22	0	0	T	78	M	0.15	0.311	0	0	0	66.0052	gagttctgggCtgtagtgtgt	m5C_20607
chr5	156814276	-	C	59	19	0.322	0	0	T	40	M	0.217	0.449	0	2.4E-08	0	82.4122	tcctggtggcCtagtggttag	m5C_20566
chr5	157403856	-	C	247	68	0.275	0	0	T	179	M	0.223	0.334	0	0	0	303.771	atttttggagCagggagatag	m5C_20563
chr5	158478403	-	C	78	25	0.321	0	0	T	53	M	0.227	0.43	0	9.3E-11	0	113.74	ggaatgcggcCcgaggcgggg	m5C_20900
chr5	159435459	+	C	275	59	0.215	0	0	T	216	M	0.17	0.267	0	0	0	200.759	tgaggttggtCtgagtgcagt	m5C_20864
chr5	159435523	+	C	67	22	0.328	0	0	T	45	M	0.228	0.447	0	1.5E-09	0	100.281	gtttcttctcCactcccactg	m5C_20868
chr5	159435525	+	C	63	13	0.206	0	0	T	50	M	0.125	0.322	2.4E-15	1.6E-11	3.1E-15	23.6977	ttcttctccaCtcccactgtt	m5C_20863
chr5	159435528	+	C	54	11	0.204	0	0	T	43	M	0.118	0.329	2.3E-13	4.4E-10	2.8E-13	16.3622	ttctccactcCcactgtttga	m5C_20866
chr5	159437849	+	C	41	19	0.463	0	0	T	22	M	0.321	0.613	0	1E-06	0	121.813	aaaaacatgtCggatgaagag	m5C_20884
chr5	159437863	+	C	42	10	0.238	0	0	T	32	M	0.135	0.385	3.2E-13	2.4E-08	3.9E-13	16.8407	tgaagagaaaCaggtaagtat	m5C_20857
chr5	166802240	-	C	34	18	0.529	0	0	C	18	M	0.367	0.685	0	2.7E-05	0	132.251	gtggggagggCtcacagatgc	m5C_20827
chr5	167987919	-	C	60	14	0.233	0	0	T	46	M	0.144	0.354	0	3.2E-11	0	40.4304	cagcattgtaCagggctatga	m5C_20823
chr5	168193668	+	C	74	26	0.351	0	0	T	48	M	0.252	0.465	0	1.3E-10	0	131.238	tggaagggcaCcattatggac	m5C_20785
chr5	172116526	-	C	33	8	0.242	0	0	T	25	M	0.128	0.41	3.1E-11	6.8E-07	3.6E-11	10.7866	cacacgtgaaCatctctgatt	m5C_21072
chr5	172197126	-	C	66	34	0.515	0	0	C	34	M	0.397	0.632	0	4.2E-08	0	270.031	gaagtaagaaCtggcaaaggc	m5C_21050
chr5	172197150	-	C	41	11	0.268	0	0	T	30	M	0.157	0.419	6E-15	4.4E-08	7.6E-15	24.5536	agtaggtgtcCctgccacagg	m5C_21075
chr5	172198182	-	C	32	17	0.531	0	0	C	17	M	0.364	0.691	0	2.1E-05	0	123.927	gacatttgggCtgtgtgtgcg	m5C_21076
chr5	172447743	+	C	203	58	0.286	0	0	T	145	M	0.228	0.351	0	0	0	264.48	cagcagtagtCagccatccgg	m5C_21012
chr5	172447761	+	C	176	46	0.261	0	0	T	130	M	0.202	0.331	0	0	0	185.879	cggacagaacCgttcctgtga	m5C_21040
chr5	172447894	+	C	73	22	0.301	0	0	T	51	M	0.208	0.414	0	2.8E-11	0	91.6188	gctaagcattCactttggctg	m5C_20999
chr5	172447896	+	C	76	21	0.276	0	0	T	55	M	0.188	0.386	0	1.8E-11	0	79.1284	taagcattcaCtttggctgct	m5C_21027
chr5	172447917	+	C	213	63	0.296	0	0	T	150	M	0.239	0.36	0	0	0	300.559	tagttctagtCtgggagcaga	m5C_21016
chr5	172461752	+	C	37	16	0.432	0	0	T	21	M	0.287	0.591	0	1.7E-05	0	91.7488	gcaagacaaaCaagactccag	m5C_21035
chr5	172461759	+	C	89	28	0.315	0	0	T	61	M	0.228	0.417	0	5.8E-12	0	127.405	aaacaagactCcagtggggtg	m5C_20984
chr5	172461772	+	C	193	62	0.321	0	0	T	131	M	0.259	0.39	0	0	0	321.663	gtggggtggtCagtaggagag	m5C_21034
chr5	172461789	+	C	190	39	0.205	0	0	T	151	M	0.154	0.268	0	0	0	120.078	agagcacgttCagagggaaga	m5C_21013
chr5	173043881	-	C	48	28	0.583	0	0	C	28	M	0.443	0.712	0	8.1E-06	0	247.974	gaaggcctggCggggaaggcc	m5C_21062
chr5	173043885	-	C	50	15	0.3	0	0	T	35	M	0.191	0.438	0	4.6E-09	0	57.3102	cggggaaggcCtggcggggaa	m5C_21070
chr5	173315446	+	C	40	17	0.425	0	0	T	23	M	0.285	0.578	0	5.6E-07	0	96.9312	gcagcggcgaCagcagaggag	m5C_21017
chr5	173315449	+	C	53	12	0.226	0	0	T	41	M	0.135	0.355	5.8E-15	8.8E-10	7.3E-15	22.9888	gcggcgacagCagaggaggaa	m5C_21014
chr5	174541801	-	C	57	18	0.316	0	0	T	39	M	0.21	0.445	0	1.7E-08	0	75.6149	caattattccCcatgaccgag	m5C_20962
chr5	174541802	-	C	75	26	0.347	0	0	T	49	M	0.249	0.459	0	1.3E-10	0	129.387	gcaattattcCccatgaccga	m5C_20956
chr5	174541803	-	C	93	44	0.473	0	0	T	49	M	0.375	0.574	0	1.8E-11	0	329.769	tgcaattattCcccatgaccg	m5C_20960
chr5	174541811	-	C	121	45	0.372	0	0	T	76	M	0.291	0.461	0	5.6E-16	0	261.872	ttggggattgCaattattccc	m5C_20966
chr5	174541832	-	C	67	19	0.284	0	0	T	48	M	0.19	0.401	0	4.5E-10	0	72.0865	gaaccctattCgtgatgggga	m5C_20973
chr5	174542098	-	C	71	54	0.761	0	0	C	54	M	0.65	0.845	0	6.9E-08	0	701.552	ggtggtggtgCatggccattc	m5C_20943
chr5	174542113	-	C	102	40	0.392	0	0	T	62	M	0.303	0.489	0	1.8E-13	0	242.379	tttctcgattCtgtgggtggt	m5C_20959
chr5	174542118	-	C	87	32	0.368	0	0	T	55	M	0.274	0.473	0	2.3E-11	0	175.415	agctctttctCgattctgtgg	m5C_20951
chr5	174542124	-	C	49	29	0.592	0	0	C	29	M	0.452	0.718	0	9.7E-06	0	262.433	attgacagctCtttctcgatt	m5C_20941
chr5	174542126	-	C	41	26	0.634	0	0	C	26	M	0.481	0.764	0	5.5E-06	0	250.226	agattgacagCtctttctcga	m5C_20949
chr5	176130367	+	C	65	27	0.415	0	0	T	38	M	0.304	0.537	0	7.5E-09	0	163.944	tgattcctagCggtggactcg	m5C_21264
chr5	177019580	+	C	37	12	0.324	0	0	T	25	M	0.196	0.485	0	4.6E-06	0	47.1194	ctcaaggaaaCtaccggacgc	m5C_21280
chr5	177019583	+	C	42	9	0.214	0	0	T	33	M	0.117	0.359	1.1E-11	1.2E-08	1.3E-11	11.5454	aaggaaactaCcggacgcagc	m5C_21307
chr5	177019584	+	C	42	20	0.476	0	0	T	22	M	0.334	0.623	0	1.4E-06	0	133.437	aggaaactacCggacgcagct	m5C_21283
chr5	177019588	+	C	44	10	0.227	0	0	T	34	M	0.128	0.37	5.7E-13	2.4E-08	6.8E-13	15.7274	aactaccggaCgcagctgtat	m5C_21299
chr5	177019601	+	C	45	15	0.333	0	0	T	30	M	0.214	0.479	0	2.8E-07	0	64.0761	agctgtatgaCaagcagcggg	m5C_21255
chr5	177019605	+	C	31	9	0.29	0	0	T	22	M	0.161	0.466	3.7E-13	1.2E-06	4.4E-13	18.0154	gtatgacaagCagcgggagga	m5C_21257
chr5	177021236	+	C	33	9	0.273	0	0	T	24	M	0.151	0.442	7.5E-13	1.2E-06	9E-13	16.4418	acgagtgcgaCagctggtgga	m5C_21291
chr5	177021248	+	C	40	19	0.475	0	0	T	21	M	0.329	0.625	0	1E-06	0	125.154	gctggtggaaCaagtggagca	m5C_21281
chr5	177021257	+	C	33	10	0.303	0	0	T	23	M	0.174	0.473	1.6E-14	2E-06	2E-14	23.982	acaagtggagCagatccagaa	m5C_21290
chr5	177770496	-	C	52	30	0.577	0	0	C	30	M	0.442	0.701	0	4.8E-07	0	265.165	ttctaaaaagCctgtagctgg	m5C_21342
chr5	178696025	-	C	60	18	0.3	0	0	T	42	M	0.199	0.425	0	2.9E-10	0	71.6328	ccgagtaatcCaggacaggct	m5C_21975
chr5	178696026	-	C	59	15	0.254	0	0	T	44	M	0.161	0.378	0	4.6E-09	0	48.1675	cccgagtaatCcaggacaggc	m5C_21979
chr5	178772190	+	C	42	20	0.476	0	0	T	22	M	0.334	0.623	0	1.4E-06	0	133.437	gccgcacctaCctggggggtc	m5C_21877
chr5	178772191	+	C	41	16	0.39	0	0	T	25	M	0.257	0.543	0	4E-07	0	82.099	ccgcacctacCtggggggtcg	m5C_21938
chr5	178772200	+	C	74	30	0.405	0	0	T	44	M	0.301	0.519	0	4.8E-10	0	180.542	cctggggggtCggcggcggcg	m5C_21911
chr5	178772203	+	C	73	22	0.301	0	0	T	51	M	0.208	0.414	0	2.8E-11	0	91.6188	ggggggtcggCggcggcggcg	m5C_21840
chr5	178772206	+	C	68	36	0.529	0	0	C	36	M	0.412	0.643	0	6.5E-08	0	296.933	gggtcggcggCggcggcgagc	m5C_21924
chr5	179045007	-	C	310	97	0.313	0	0	T	213	M	0.264	0.367	0	0	0	511.855	ggattttgggtCagatagattt	m5C_21964
chr5	179045051	-	C	51	11	0.216	0	0	T	40	M	0.125	0.346	1.1E-13	4.4E-10	1.4E-13	17.8061	gctatggaggCtatgatgatt	m5C_21954
chr5	179132785	+	C	32	7	0.219	0	0	T	25	M	0.11	0.388	8.5E-10	3.4E-07	9.5E-10	6.99971	tgaggatgacCttgacgatgt	m5C_21853
chr5	179143204	+	C	34	10	0.294	0	0	T	24	M	0.168	0.462	2.3E-14	2E-06	2.9E-14	22.9564	aaatccttcaCgtgaaattga	m5C_21867
chr5	179143234	+	C	37	16	0.432	0	0	T	21	M	0.287	0.591	0	1.7E-05	0	91.7488	agaccggaagCccgaggattg	m5C_21907
chr5	179143235	+	C	43	10	0.233	0	0	T	33	M	0.132	0.377	4.3E-13	2.4E-08	5.2E-13	16.2683	gaccggaagcCcgaggattgg	m5C_21928
chr5	179146739	+	C	50	10	0.2	0	0	T	40	M	0.112	0.33	2.6E-12	2E-10	3.1E-12	13.024	agatgatgagCctgagtacgt	m5C_21884
chr5	179146740	+	C	42	11	0.262	0	0	T	31	M	0.153	0.411	8.4E-15	4.4E-08	1.1E-14	23.69	gatgatgagcCtgagtacgta	m5C_21866
chr5	179146751	+	C	48	15	0.312	0	0	T	33	M	0.199	0.453	0	2.8E-07	0	59.8365	tgagtacgtaCctgatccaga	m5C_21872
chr5	179146752	+	C	55	23	0.418	0	0	T	32	M	0.297	0.55	0	8.7E-08	0	136.793	gagtacgtacCtgatccagac	m5C_21856
chr5	179146762	+	C	113	27	0.239	0	0	T	86	M	0.17	0.325	0	0	0	91.6506	ctgatccagaCgcagagaaac	m5C_21914
chr5	179146772	+	C	229	50	0.218	0	0	T	179	M	0.17	0.276	0	0	0	169.72	cgcagagaaaCctgaggattg	m5C_21886
chr5	179149990	+	C	40	9	0.225	0	0	T	31	M	0.123	0.375	6.4E-12	1.2E-08	7.6E-12	12.4041	atggatggggCctgaagaaag	m5C_21885
chr5	179149991	+	C	37	16	0.444	1	0.027	T	20	M	0.295	0.604	0	1.7E-05	0	94.5313	tggatggggcCtgaagaaagc	m5C_21843
chr5	179150001	+	C	39	13	0.333	0	0	T	26	M	0.206	0.49	0	6.7E-06	0	53.6452	ctgaagaaagCtgctgatggg	m5C_21942
chr5	179150004	+	C	42	16	0.381	0	0	T	26	M	0.25	0.532	0	4E-07	0	79.9985	aagaaagctgCtgatggggct	m5C_21837
chr5	179150662	+	C	130	32	0.246	0	0	T	98	M	0.18	0.327	0	0	0	115.262	gtttttcagcCaggcgttgtg	m5C_21861
chr5	179150666	+	C	168	68	0.405	0	0	T	100	M	0.333	0.48	0	0	0	453.519	ttcagccaggCgttgtggggc	m5C_21943
chr5	179150676	+	C	218	56	0.257	0	0	T	162	M	0.203	0.319	0	0	0	227.855	cgttgtggggCagatgatcga	m5C_21842
chr5	179150684	+	C	204	42	0.206	0	0	T	162	M	0.156	0.267	0	0	0	131.106	ggcagatgatCgaggcagctg	m5C_21841
chr5	179151689	+	C	190	43	0.226	0	0	T	147	M	0.173	0.291	0	0	0	148.421	tataagaaaaCtgatgcacct	m5C_21897
chr5	179151703	+	C	473	114	0.241	0	0	T	359	M	0.205	0.282	0	0	0	466.615	tgcacctcaaCcggatgtgaa	m5C_21925
chr5	179151704	+	C	514	175	0.34	0	0	T	339	M	0.301	0.382	0	0	0	1052.87	gcacctcaacCggatgtgaag	m5C_21865
chr5	179151747	+	C	385	162	0.421	0	0	T	223	M	0.372	0.471	0	0	0	1206.85	aggaaaaggaCaagggagatg	m5C_21937
chr5	179151781	+	C	90	40	0.444	0	0	T	50	M	0.346	0.547	0	6.4E-12	0	276.924	agaagagaaaCttggtaagaa	m5C_21904
chr5	179153689	+	C	99	21	0.212	0	0	T	78	M	0.143	0.303	0	3.1E-15	0	60.1164	agaagagaaaCagaaaagtga	m5C_21869
chr5	179153702	+	C	157	47	0.299	0	0	T	110	M	0.233	0.375	0	0	0	219.222	aaaagtgatgCtgaagaagat	m5C_21940
chr5	179153720	+	C	95	25	0.263	0	0	T	70	M	0.185	0.36	0	2.9E-14	0	92.532	gatggtggcaCtgtcagtcaa	m5C_21935
chr5	179153724	+	C	95	30	0.316	0	0	T	65	M	0.231	0.415	0	2.5E-13	0	138.619	gtggcactgtCagtcaagagg	m5C_21844
chr5	179260186	+	C	41	12	0.293	0	0	T	29	M	0.176	0.445	1.1E-16	7.5E-08	1.5E-16	33.7134	ttgagggcgcCacgcagtctc	m5C_21847
chr5	179260194	+	C	46	18	0.391	0	0	T	28	M	0.264	0.535	0	7.7E-07	0	95.0149	gccacgcagtCtctggcggag	m5C_21887

chr5	179260200	+	C	44	19	0.432	0	0	T	25	M	0.297	0.578	0	1E-06	0	112.794	cagtctctggCggagcagatg	m5C_21858
chr5	180524075	+	C	87	20	0.233	1	0.011	T	66	M	0.156	0.332	0	1.5E-13	0	62.356	agccggtttcCgtagtgtagt	m5C_24291
chr5	180524092	+	C	98	28	0.286	0	0	T	70	M	0.206	0.382	0	1.1E-13	0	115.184	tagtggttatCacgttcgcct	m5C_24284
chr5	180524100	+	C	40	8	0.2	0	0	T	32	M	0.105	0.348	2.1E-10	5.5E-09	2.3E-10	8.13503	atcacgttcgCctcacacgcg	m5C_24298
chr5	180600654	+	C	41	11	0.268	0	0	T	30	M	0.157	0.419	6E-15	4.4E-08	7.6E-15	24.5536	atgcaggtttCcgtagtgtag	m5C_24296
chr5	180600672	+	C	56	20	0.357	0	0	T	36	M	0.245	0.488	0	3.4E-08	0	97.8256	tagtggttatCacgttcgcct	m5C_24282
chr5	180600674	+	C	42	15	0.357	0	0	T	27	M	0.23	0.508	0	2.8E-07	0	68.967	gtggttatcaCgttcgcctca	m5C_24311
chr5	180600678	+	C	39	11	0.282	0	0	T	28	M	0.165	0.438	3.1E-15	3.1E-06	4E-15	26.4001	ttatcacgttCgcctcacacg	m5C_24262
chr5	180600680	+	C	36	9	0.25	0	0	T	27	M	0.138	0.411	2E-12	1.2E-06	2.4E-12	14.4765	atcacgttcgCctcacacgcg	m5C_24264
chr5	180600681	+	C	35	8	0.229	0	0	T	27	M	0.121	0.39	5.6E-11	6.8E-07	6.4E-11	9.89691	tcacgttcgcCtcacacgcga	m5C_24270
chr5	180600697	+	C	46	16	0.348	0	0	T	30	M	0.227	0.492	0	4E-07	0	72.5786	cgcgaaaggtCcccggttcga	m5C_24292
chr5	180600700	+	C	48	14	0.292	0	0	T	34	M	0.182	0.432	0	1.9E-07	0	51.076	gaaaggtcccCggttcgaaac	m5C_24265
chr5	180600721	+	C	46	10	0.217	0	0	T	36	M	0.123	0.356	9.7E-13	2.4E-08	1.2E-12	14.7309	cgggcggaaaCacctccttct	m5C_24276
chr5	180600723	+	C	42	20	0.476	0	0	T	22	M	0.334	0.623	0	1.4E-06	0	133.437	ggcggaaacaCctccttctgc	m5C_24273
chr5	180600724	+	C	50	26	0.52	0	0	C	26	M	0.385	0.652	0	5.5E-06	0	200.26	gcggaaacacCtccttctgct	m5C_24258
chr5	180614706	+	C	46	12	0.261	0	0	T	34	M	0.156	0.403	6.7E-16	7.5E-08	8.7E-16	28.4116	aaagaggtagCgtggccgagc	m5C_24279
chr5	180614712	+	C	51	22	0.431	0	0	T	29	M	0.305	0.567	0	6.5E-08	0	134.205	gtagcgtggcCgagcggtcta	m5C_24278
chr5	180614720	+	C	54	14	0.259	0	0	T	40	M	0.161	0.389	0	2.8E-09	0	45.1318	gccgagcggtCtaaggcgctg	m5C_24295
chr5	180614748	+	C	31	13	0.419	0	0	T	18	M	0.264	0.592	0	6.7E-06	0	68.6798	gctccagtctCttcgggggcg	m5C_24260
chr5	180614757	+	C	46	21	0.467	1	0.022	T	24	M	0.329	0.609	0	1.8E-06	0	138.327	tcttcgggggCgtgggttcga	m5C_24267
chr5	180614770	+	C	50	11	0.22	0	0	T	39	M	0.128	0.352	8.5E-14	4.4E-10	1.1E-13	18.3342	gggttcgaatCccaccgctgc	m5C_24307
chr5	180614781	+	C	52	11	0.212	0	0	T	41	M	0.122	0.34	1.4E-13	4.4E-10	1.7E-13	17.3019	ccaccgctgcCagtttgtggt	m5C_24283
chr5	180615423	-	C	45	11	0.244	0	0	T	34	M	0.142	0.387	2.1E-14	4.4E-08	2.7E-14	21.4112	cgaaaccgggCggaaacatgc	m5C_24392
chr5	180615457	-	C	43	13	0.302	0	0	T	30	M	0.186	0.451	0	1.2E-07	0	48.3648	tcacgttcgcCtaacacgcga	m5C_24351
chr5	180615458	-	C	42	9	0.214	0	0	T	33	M	0.117	0.359	1.1E-11	1.2E-08	1.3E-11	11.5454	atcacgttcgCctaacacgcg	m5C_24379
chr5	180615469	-	C	118	43	0.364	0	0	T	75	M	0.283	0.454	0	1.2E-14	0	243.483	gtgtagtggtCatcacgttcg	m5C_24336
chr5	180615860	-	C	52	23	0.442	0	0	T	29	M	0.316	0.577	0	8.7E-08	0	145.336	aaatcccggaCgagcccactt	m5C_24334
chr5	180615871	-	C	50	33	0.66	0	0	C	33	M	0.522	0.776	0	8.8E-07	0	344.214	gtcccgggttCaaatcccgga	m5C_24390
chr5	180615896	-	C	47	12	0.255	0	0	T	35	M	0.153	0.395	1E-15	7.5E-08	1.3E-15	27.4561	atgattctcgCtttgggtgcg	m5C_24356
chr5	180615898	-	C	56	17	0.304	0	0	T	39	M	0.199	0.433	0	1.1E-08	0	67.6606	gtatgattctCgctttgggtg	m5C_24324
chr5	180615900	-	C	52	15	0.288	0	0	T	37	M	0.183	0.423	0	4.6E-09	0	54.9894	gggtatgattCtcgctttggg	m5C_24347
chr5	180615914	-	C	56	13	0.232	0	0	T	43	M	0.141	0.358	4.4E-16	1.6E-09	5.8E-16	28.1397	gctcgttggtCtaggggtatg	m5C_24322
chr5	180615921	-	C	50	10	0.2	0	0	T	40	M	0.112	0.33	2.6E-12	2E-10	3.1E-12	13.024	acagccggctCgttggtctag	m5C_24352
chr5	180618607	-	C	36	24	0.667	0	0	C	24	M	0.503	0.798	0	9.1E-05	0	241.602	cctgggaggcCagagcctggc	m5C_24388
chr5	180618608	-	C	35	12	0.343	0	0	T	23	M	0.208	0.508	0	4.6E-06	0	49.9956	tcctgggaggCcagagcctgg	m5C_24325
chr5	180618734	-	C	32	18	0.562	0	0	C	18	M	0.393	0.718	0	2.7E-05	0	141.571	ggggttagagCactggtcttg	m5C_24353
chr5	180618746	-	C	32	10	0.312	0	0	T	22	M	0.18	0.486	1.1E-14	2E-06	1.3E-14	25.0836	ctccatagctCaggggttaga	m5C_24316
chr5	180618748	-	C	31	18	0.581	0	0	C	18	M	0.408	0.736	0	2.7E-05	0	146.757	ggctccatagCtcaggggtta	m5C_24345
chr5	180618754	-	C	31	8	0.258	0	0	T	23	M	0.137	0.432	1.7E-11	6.8E-07	1.9E-11	11.8182	acagccggctCcatagctcag	m5C_24339
chr5	180633880	+	C	115	34	0.296	0	0	T	81	M	0.22	0.385	0	4.4E-16	0	149.564	ggatgtagctCagtggtagag	m5C_24257
chr5	180633897	+	C	108	43	0.402	1	0.009	T	64	M	0.314	0.497	0	4.3E-13	0	269.983	agagcgcatgCtttgcatgta	m5C_24299
chr5	180633902	+	C	63	20	0.317	0	0	T	43	M	0.216	0.44	0	6.9E-10	0	86.3461	gcatgctttgCatgtatgagg	m5C_24312
chr5	180633913	+	C	48	20	0.417	0	0	T	28	M	0.288	0.557	0	1.4E-06	0	115.398	atgtatgaggCcccgggttcg	m5C_24286
chr5	180633914	+	C	50	23	0.46	0	0	T	27	M	0.33	0.596	0	2.9E-06	0	151.659	tgtatgaggcCccgggttcga	m5C_24308
chr5	180633915	+	C	49	12	0.245	0	0	T	37	M	0.146	0.381	1.9E-15	7.5E-08	2.4E-15	25.8006	gtatgaggccCcgggttcgat	m5C_24271
chr5	180633916	+	C	48	20	0.417	0	0	T	28	M	0.288	0.557	0	1.4E-06	0	115.398	tatgaggcccCgggttcgatc	m5C_24293
chr5	180633922	+	C	54	16	0.296	0	0	T	38	M	0.191	0.428	0	7.3E-09	0	61.2393	gccccgggttCgatccccggc	m5C_24277
chr5	180633926	+	C	55	16	0.291	0	0	T	39	M	0.188	0.421	0	7.3E-09	0	60.0673	cgggttcgatCcccggcatct	m5C_24300
chr5	180634756	+	C	96	26	0.271	0	0	T	70	M	0.192	0.367	0	4.6E-14	0	99.859	tcatcgggcgCccggctagct	m5C_24269
chr5	180634767	+	C	148	35	0.236	0	0	T	113	M	0.175	0.311	0	0	0	122.666	ccggctagctCagtcggtaga	m5C_24285
chr5	180634786	+	C	45	26	0.578	0	0	C	26	M	0.433	0.71	0	5.5E-06	0	225.163	gagcatgagaCtcttaatctc	m5C_24268
chr5	180634788	+	C	44	13	0.295	0	0	T	31	M	0.182	0.442	0	1.2E-07	0	47.2039	gcatgagactCttaatctcag	m5C_24272
chr5	180634826	+	C	32	10	0.312	0	0	T	22	M	0.18	0.486	1.1E-14	2E-06	1.3E-14	25.0836	ccacgttgggCggcaactggt	m5C_24281
chr5	180634829	+	C	36	16	0.444	0	0	T	20	M	0.295	0.604	0	1.7E-05	0	94.5313	cgttgggcggCaactggtcgt	m5C_24275
chr5	180634832	+	C	35	8	0.229	0	0	T	27	M	0.121	0.39	5.6E-11	6.8E-07	6.4E-11	9.89691	tgggcggcaaCtggtcgtttt	m5C_24301
chr5	180634837	+	C	32	16	0.5	0	0	CT	16	M	0.336	0.664	0	1.7E-05	0	107.618	ggcaactggtCgttttagtcc	m5C_24306
chr5	180645277	-	C	35	10	0.286	0	0	T	25	M	0.163	0.451	3.4E-14	2E-06	4.2E-14	21.9991	cgaaaccgggCggaaacaaga	m5C_24340
chr5	180645337	-	C	38	15	0.395	0	0	T	23	M	0.256	0.553	0	1.2E-05	0	76.7936	catctgtttcCgtagtgtagt	m5C_24350
chr5	180645338	-	C	40	16	0.4	0	0	T	24	M	0.263	0.554	0	1.7E-05	0	84.314	gcatctgtttCcgtagtgtag	m5C_24318
chr5	180648987	-	C	49	13	0.265	0	0	T	36	M	0.162	0.403	0	1.2E-07	0	42.1489	tcgagccccaCgttgggcgta	m5C_24333
chr5	180648996	-	C	55	28	0.509	0	0	C	28	M	0.381	0.636	0	3.1E-07	0	213.226	gtcgtggggttCgagccccacg	m5C_24363
chr5	180649012	-	C	79	24	0.304	0	0	T	55	M	0.213	0.412	0	6.4E-11	0	102.453	gactcttaatCtcagggtcgt	m5C_24327
chr5	180649020	-	C	82	31	0.378	0	0	T	51	M	0.281	0.486	0	1.7E-11	0	174.085	gagcatgagaCtcttaatctc	m5C_24344
chr5	180649035	-	C	96	24	0.25	0	0	T	72	M	0.174	0.345	0	1.7E-14	0	83.5865	ctagctcagtCggtagagcat	m5C_24341
chr5	180649048	-	C	59	14	0.237	0	0	T	45	M	0.147	0.36	0	2.8E-09	0	41.1446	catggccgccCggctagctca	m5C_24342
chr5	180649050	-	C	57	13	0.228	0	0	T	44	M	0.138	0.352	4.4E-16	1.6E-09	5.8E-16	27.6258	gacatggccgCccggctagct	m5C_24315
chr5	180663992	-	C	38	8	0.211	0	0	T	30	M	0.111	0.363	1.3E-10	6.8E-07	1.4E-10	8.77246	ggcaggtgacCattggcacac	m5C_24376
chr5	180663993	-	C	39	18	0.462	0	0	T	21	M	0.316	0.614	0	2.7E-05	0	113.64	tggcaggtgaCcattggcaca	m5C_24373
chr5	180668824	-	C	534	128	0.24	0	0	T	406	M	0.205	0.278	0	0	0	525.908	tcctgcaattCtgaagggatt	m5C_24395
chr5	180668832	-	C	608	177	0.291	0	0	T	431	M	0.256	0.328	0	0	0	907.69	aggacatgtcCtgcaattctg	m5C_24335
chr5	180668833	-	C	601	205	0.342	1	0.002	T	395	M	0.305	0.381	0	0	0	1249.81	gaggacatgtCctgcaattct	m5C_24357
chr5	180668855	-	C	630	126	0.2	0	0	T	504	M	0.171	0.233	0	0	0	429.974	cagccaatccCcaagtgggag	m5C_24326
chr5	180670311	-	C	293	72	0.246	0	0	T	221	M	0.2	0.298	0	0	0	287.91	ttctgagctgCcgcccggccc	m5C_24389
chr5	180670325	-	C	1401	418	0.304	27	0.019	T	956	M	0.28	0.329	0	0	0	2344.71	aatccagaggCtgtttctgag	m5C_24386
chr5	180670332	-	C	1559	388	0.25	4	0.003	T	1167	M	0.229	0.272	0	0	0	1774.28	gtgctgaaatCcagaggctgt	m5C_24378
chr5	180670339	-	C	1548	329	0.213	3	0.002	T	1216	M	0.193	0.234	0	0	0	1271.62	agtgtcggtgCtgaaatccag	m5C_24382
chr5	180670344	-	C	1499	369	0.246	1	0.001	T	1129	M	0.225	0.269	0	0	0	1661.8	atctgagtgtCggtgctgaaa	m5C_24314
chr5	180670352	-	C	1467	297	0.203	1	0.001	T	1169	M	0.183	0.224	0	0	0	1085.87	aacatgccatCtgagtgtcgg	m5C_24384
chr5	180670365	-	C	1468	341	0.232	1	0.001	T	1126	M	0.212	0.255	0	0	0	1442.76	cggtgatgacCccaacatgcc	m5C_24375
chr5	180670885	-	C	45	23	0.511	0	0	C	23	M	0.37	0.65	0	2.9E-06	0	170.219	tgcaaggcggCggcaggagag	m5C_24323
chr5	180670888	-	C	36	23	0.639	0	0	C	23	M	0.476	0.775	0	7.6E-05	0	218.844	cactgcaaggCggcggcagga	m5C_24320
chr6	2948888	-	C	46	11	0.239	0	0	T	35	M	0.139	0.379	2.8E-14	4.4E-08	3.5E-14	20.7303	atggacgaggCtggacatgat	m5C_21527
chr6	3148980	-	C	47	30	0.638	0	0	C	30	M	0.495	0.76	0	1.2E-05	0	297.214	ttgataagcaCtggctaattc	m5C_21508
chr6	4428210	+	C	663	149	0.225	0	0	T	514	M	0.195	0.258	0	0	0	579.91	tacggccataCcaccctgaac	m5C_23540
chr6	4428222	+	C	1332	282	0.212	0	0	T	1050	M	0.191	0.234	0	0	0	1075.08	accctgaacgCccccgatctc	m5C_23526
chr6	4428223	+	C	1357	17	0.266	1293	0.953	G	1293	M	0.173	0.385	0	1.8E-10	0	58.8072	ccctgaacgcCcccgatctcg	m5C_23547
chr6	4428224	+	C	1444	398	0.28	23	0.016	T	1023	M	0.257	0.304	0	0	0	2048.53	cctgaacgccCccgatctcgt	m5C_23533
chr6	4428225	+	C	1472	394	0.27	15	0.01	T	1063	M	0.248	0.294	0	0	0	1956.11	ctgaacgcccCcgatctcgtc	m5C_23548
chr6	4428226	+	C	1504	384	0.256	2	0.001	T	1118	M	0.234	0.278	0	0	0	1798.96	tgaacgccccCgatctcgtct	m5C_23521
chr6	4428230	+	C	1643	436	0.265	0	0	T	1207	M	0.245	0.287	0	0	0	2132.77	cgcccccgatCtcgtctgatc	m5C_23542
chr6	4428232	+	C	1714	443	0.259	2	0.001	T	1269	M	0.239	0.28	0	0	0	2113.75	cccccgatctCgtctgatctc	m5C_23546
chr6	4428235	+	C	1817	519	0.286	1	0.001	T	1297	M	0.265	0.307	0	0	0	2755.71	ccgatctcgtCtgatctcgga	m5C_23531
chr6	4428240	+	C	2014	603	0.3	2	0.001	T	1409	M	0.28	0.32	0	0	0	3377.77	ctcgtctgatCtcggaagcta	m5C_23541
chr6	4428242	+	C	2139	574	0.269	3	0.001	T	1562	M	0.25	0.288	0	0	0	2874.07	cgtctgatctCggaagctaag	m5C_23528
chr6	4428248	+	C	2571	911	0.355	5	0.002	T	1655	M	0.337	0.374	0	0	0	6135.43	atctcggaagCtaagcagggt	m5C_23537
chr6	4428253	+	C	2956	937	0.319	15	0.005	T	2004	M	0.302	0.336	0	0	0	5659.58	ggaagctaagCagggtcgggc	m5C_23520
chr6	4428259	+	C	3485	1161	0.341	80	0.023	T	2244	M	0.325	0.357	0	0	0	7551.93	taagcagggtCgggcctggtt	m5C_23534
chr6	4428263	+	C	3833	1310	0.342	5	0.001	T	2518	M	0.327	0.357	0	0	0	8576.57	cagggtcgggCctggttagta	m5C_23527
chr6	4428264	+	C	3944	1359	0.345	3	0.001	T	2582	M	0.33	0.36	0	0	0	8973.58	agggtcgggcCtggttagtac	m5C_23518
chr6	4428274	+	C	5014	1467	0.293	3	0.001	T	3544	M	0.28	0.306	0	0	0	8224.58	ctggttagtaCttggatggga	m5C_23514
chr6	4428287	+	C	5676	2078	0.366	1	0	T	3597	M	0.354	0.379	0	0	0	14700.9	ggatgggagaCcgcctgggaa	m5C_23529

chr6	4428288	+	C	5665	1984	0.351	5	0.001	T	3676	M	0.338	0.363	0	0	0	13420	gatgggagacCgcctgggaat	m5C_23530
chr6	4428290	+	C	5730	1762	0.308	1	0	T	3967	M	0.296	0.32	0	0	0	10421.9	tgggagaccgCctgggaatac	m5C_23535
chr6	4428291	+	C	5721	1927	0.337	4	0.001	T	3790	M	0.325	0.349	0	0	0	12522.6	gggagaccgcCtgggaatacc	m5C_23538
chr6	4428300	+	C	4981	1478	0.297	5	0.001	T	3498	M	0.284	0.31	0	0	0	8409.52	cctgggaataCcgggtgctgt	m5C_23543
chr6	4428301	+	C	4917	1720	0.35	5	0.001	T	3192	M	0.337	0.364	0	0	0	11590.9	ctgggaatacCgggtgctgta	m5C_23544
chr6	4428307	+	C	4430	1698	0.384	4	0.001	T	2728	M	0.369	0.398	0	0	0	12545.6	ataccgggtgCtgtaggctta	m5C_23549
chr6	4428314	+	C	3022	1475	0.488	0	0	T	1547	M	0.47	0.506	0	0	0	13873.6	gtgctgtaggCttactttgtg	m5C_23524
chr6	7581122	+	C	49	20	0.408	0	0	T	29	M	0.282	0.548	0	1.4E-06	0	112.86	gaaagagctgCagctgcagaa	m5C_21587
chr6	7581134	+	C	50	10	0.2	0	0	T	40	M	0.112	0.33	2.6E-12	2E-10	3.1E-12	13.024	gctgcagaagCagaaggtgga	m5C_21570
chr6	7581203	+	C	36	24	0.667	0	0	C	24	M	0.503	0.798	0	9.1E-05	0	241.602	gaggaagaagCtggaggaaga	m5C_21592
chr6	7583125	+	C	33	20	0.606	0	0	C	20	M	0.437	0.753	0	4.3E-05	0	174.733	aaggaggaggCtattaggaag	m5C_21594
chr6	7610857	+	C	40	10	0.25	0	0	T	30	M	0.142	0.402	1.8E-13	2.4E-08	2.2E-13	18.0914	aagaaagataCaagtgaaaat	m5C_21576
chr6	8086720	-	C	40	12	0.3	0	0	T	28	M	0.181	0.454	1.1E-16	7.5E-08	1.5E-16	34.6049	gggctgctatCtgcctcataa	m5C_21552
chr6	8086724	-	C	40	8	0.2	0	0	T	32	M	0.105	0.348	2.1E-10	5.5E-09	2.3E-10	8.13503	tttggggctgCtatctgcctc	m5C_21554
chr6	8086727	-	C	39	10	0.256	0	0	T	29	M	0.146	0.411	1.3E-13	2E-06	1.6E-13	18.7753	tggttttgggGctgctatctgc	m5C_21553
chr6	8086745	-	C	91	33	0.371	2	0.022	T	56	M	0.278	0.475	0	3.1E-11	0	183.305	caggccccaaCctatccatgg	m5C_21558
chr6	8086760	-	C	116	34	0.293	0	0	T	82	M	0.218	0.382	0	4.4E-16	0	148.219	taattgaggtCtgcgcaggcc	m5C_21561
chr6	10222301	+	C	63	30	0.476	0	0	T	33	M	0.358	0.597	0	1.7E-08	0	214.692	gttggcattcCctttgacaag	m5C_21749
chr6	10222308	+	C	44	16	0.364	0	0	T	28	M	0.238	0.511	0	4E-07	0	76.1067	ttccctttgaCaaggatggaa	m5C_21743
chr6	10250974	-	C	34	13	0.382	0	0	T	21	M	0.239	0.55	0	6.7E-06	0	62.1399	gctgtgtgtaCatgcacacgc	m5C_21763
chr6	10250983	-	C	38	14	0.368	0	0	T	24	M	0.234	0.527	0	9.2E-06	0	65.4752	atttacttggCtgtgtgtaca	m5C_21764
chr6	13214340	+	C	54	15	0.278	0	0	T	39	M	0.176	0.409	0	4.6E-09	0	52.85	tttcccagggCgaggcttatc	m5C_21780
chr6	13214345	+	C	81	25	0.309	0	0	T	56	M	0.219	0.416	0	1.8E-12	0	109.315	cagggcgaggCttatccatcg	m5C_21803
chr6	13214350	+	C	91	27	0.297	0	0	T	64	M	0.213	0.397	0	7.2E-14	0	114.822	cgaggcttatCcatcgcactc	m5C_21788
chr6	13214358	+	C	93	26	0.28	0	0	T	67	M	0.199	0.378	0	4.6E-14	0	103.221	atccatcgcaCtccggatatg	m5C_21785
chr6	13214360	+	C	91	28	0.308	0	0	T	63	M	0.222	0.409	0	1.1E-13	0	124.469	ccatcgcactCcggatatgct	m5C_21806
chr6	13214440	+	C	43	10	0.233	0	0	T	33	M	0.132	0.377	4.3E-13	2.4E-08	5.2E-13	16.2683	actgtgcttgCgctttcccct	m5C_21796
chr6	13214446	+	C	42	19	0.452	0	0	T	23	M	0.312	0.601	0	1E-06	0	118.648	cttgcgctttCccctgaaaaa	m5C_21778
chr6	13214447	+	C	42	13	0.31	0	0	T	29	M	0.191	0.46	0	1.2E-07	0	49.5844	ttgcgctttcCcctgaaaaaa	m5C_21779
chr6	13214448	+	C	43	16	0.372	0	0	T	27	M	0.244	0.521	0	4E-07	0	78.0036	tgcgctttccCctgaaaaaaa	m5C_21792
chr6	13615746	+	C	52	21	0.457	6	0.115	T	25	M	0.322	0.598	0	1.8E-06	0	135.049	tggaggaagaCgaggaagggg	m5C_21802
chr6	13615758	+	C	78	33	0.434	2	0.026	T	43	M	0.329	0.546	0	1.1E-09	0	216.881	aggaaggggaCgatgagtttg	m5C_21794
chr6	15680423	-	C	69	20	0.29	0	0	T	49	M	0.196	0.406	0	6.9E-10	0	78.4637	gcctgtgttgCtagaggtgaa	m5C_21650
chr6	17722018	-	C	53	12	0.226	0	0	T	41	M	0.135	0.355	5.8E-15	8.8E-10	7.3E-15	22.9888	gtgctgtagaCttaaaaaaaa	m5C_21670
chr6	17722025	-	C	104	49	0.471	0	0	T	55	M	0.378	0.566	0	2.1E-12	0	370.404	ataccgggtgCtgtagactta	m5C_21687
chr6	17722031	-	C	102	26	0.255	0	0	T	76	M	0.18	0.347	0	6.7E-16	0	93.7536	ctgggaatacCgggtgctgta	m5C_21688
chr6	17731114	-	C	62	15	0.242	0	0	T	47	M	0.152	0.362	0	6E-11	0	45.7373	agtgtagtatCtgttctgatc	m5C_21684
chr6	17731133	-	C	71	21	0.296	0	0	T	50	M	0.202	0.41	0	1.8E-11	0	84.9775	ggcctttggCtaggatcaag	m5C_21686
chr6	17731140	-	C	33	20	0.606	0	0	C	20	M	0.437	0.753	0	4.3E-05	0	174.733	acttcttggcCttttggctag	m5C_21677
chr6	18249927	-	C	82	22	0.268	0	0	T	60	M	0.184	0.373	0	4.4E-13	0	81.1184	aaaagaaaaaCaaggaagagt	m5C_21717
chr6	18264099	-	C	82	39	0.557	12	0.146	C	39	M	0.441	0.668	0	1.2E-07	0	343.85	acgaggacgaCgaggaggagg	m5C_21723
chr6	18264102	-	C	76	42	0.553	0	0	C	42	M	0.441	0.659	0	8.7E-09	0	370.443	aggacgaggaCgacgaggagg	m5C_21725
chr6	18264108	-	C	88	19	0.216	0	0	T	69	M	0.143	0.313	0	8.4E-14	0	54.2667	aggaagaggaCgaggacgacg	m5C_21721
chr6	18264123	-	C	72	42	0.583	0	0	C	42	M	0.468	0.69	0	8.7E-09	0	393.183	gagaggagagCgaggaggaag	m5C_21729
chr6	18264137	-	C	48	15	0.312	0	0	T	33	M	0.199	0.453	0	2.8E-07	0	59.8365	aatgcccggtCccagagagga	m5C_21727
chr6	18264142	-	C	47	14	0.298	0	0	T	33	M	0.187	0.44	0	1.9E-07	0	52.223	cccgaaatgcCcggtcccaga	m5C_21718
chr6	18836426	+	C	40	9	0.225	0	0	T	31	M	0.123	0.375	6.4E-12	1.2E-08	7.6E-12	12.4041	atggttagcaCtctggactct	m5C_21714
chr6	18836428	+	C	42	9	0.214	0	0	T	33	M	0.117	0.359	1.1E-11	1.2E-08	1.3E-11	11.5454	ggttagcactCtggactctga	m5C_21708
chr6	18836445	+	C	38	8	0.211	0	0	T	30	M	0.111	0.363	1.3E-10	6.8E-07	1.4E-10	8.77246	ctgaatccagCgatccgagtt	m5C_21709
chr6	18836449	+	C	36	14	0.389	0	0	T	22	M	0.248	0.551	0	9.2E-06	0	69.397	atccagcgatCcgagttcaaa	m5C_21704
chr6	18836450	+	C	39	13	0.333	0	0	T	26	M	0.206	0.49	0	6.7E-06	0	53.6452	tccagcgatcCgagttcaaat	m5C_21713
chr6	18836461	+	C	35	10	0.286	0	0	T	25	M	0.163	0.451	3.4E-14	2E-06	4.2E-14	21.9991	gagttcaaatCtcggtggaac	m5C_21711
chr6	18836463	+	C	38	13	0.342	0	0	T	25	M	0.212	0.501	0	6.7E-06	0	55.1518	gttcaaatctCggtggaacct	m5C_21699
chr6	22349248	+	C	62	16	0.258	0	0	T	46	M	0.166	0.379	0	1.1E-10	0	52.9752	tagcctccagCggtggatcat	m5C_22007
chr6	22349256	+	C	56	13	0.232	0	0	T	43	M	0.141	0.358	4.4E-16	1.6E-09	5.8E-16	28.1397	agcggtggatCattaggataa	m5C_21997
chr6	25287735	-	C	41	12	0.293	0	0	T	29	M	0.176	0.445	1.1E-16	7.5E-08	1.5E-16	33.7134	ggggtgtttaCaactaattga	m5C_22614
chr6	25287754	-	C	48	13	0.271	0	0	T	35	M	0.166	0.41	0	1.2E-07	0	43.0711	catggctggtCtgagtgtagg	m5C_22618
chr6	26056157	-	C	32	15	0.469	0	0	T	17	M	0.309	0.636	0	1.2E-05	0	92.6074	gccactgtaaCcaagaaagtg	m5C_54559
chr6	26056176	-	C	52	11	0.212	0	0	T	41	M	0.122	0.34	1.4E-13	4.4E-10	1.7E-13	17.3019	agcgaagaagCcggccgcggc	m5C_54666
chr6	26056194	-	C	79	24	0.304	0	0	T	55	M	0.213	0.412	0	6.4E-11	0	102.453	taagaaaacaCgcgaagaagc	m5C_54328
chr6	26056207	-	C	82	31	0.378	0	0	T	51	M	0.281	0.486	0	1.7E-11	0	174.085	cgaagaagagCgctaagaaaa	m5C_54314
chr6	26056228	-	C	57	16	0.281	0	0	T	41	M	0.181	0.408	0	7.3E-09	0	57.8536	aggcggctggCggcgcaactc	m5C_54416
chr6	26056232	-	C	61	23	0.377	0	0	T	38	M	0.266	0.503	0	2.1E-09	0	122.426	aagaaggcggCtggcggcgca	m5C_54594
chr6	26056244	-	C	46	16	0.348	0	0	T	30	M	0.227	0.492	0	4E-07	0	72.5786	gccaagaagcCcaagaaggcg	m5C_54444
chr6	26056256	-	C	45	22	0.489	0	0	T	23	M	0.35	0.63	0	2.3E-06	0	153.81	ccagttggggCagccaagaag	m5C_54576
chr6	26124073	-	C	147	42	0.286	0	0	T	105	M	0.219	0.363	0	0	0	183.852	aggcagtgacCaaagcgcaga	m5C_54285
chr6	26124080	-	C	116	40	0.345	0	0	T	76	M	0.265	0.435	0	4.3E-15	0	211.645	tccaagaaggCagtgaccaaa	m5C_54454
chr6	26124089	-	C	49	18	0.367	0	0	T	31	M	0.247	0.507	0	7.7E-07	0	88.7939	aagaagggctCcaagaaggca	m5C_54577
chr6	26156990	+	C	39	17	0.436	0	0	T	22	M	0.293	0.59	0	2.1E-05	0	99.6354	aaaaggcaggCgcggccaagg	m5C_54046
chr6	26156992	+	C	36	9	0.25	0	0	T	27	M	0.138	0.411	2E-12	1.2E-06	2.4E-12	14.4765	aaggcaggcgCggccaaggcc	m5C_53880
chr6	26157010	+	C	44	10	0.227	0	0	T	34	M	0.128	0.37	5.7E-13	2.4E-08	6.8E-13	15.7274	gccaagaagcCagcaggagcg	m5C_53792
chr6	26157019	+	C	69	15	0.217	0	0	T	54	M	0.136	0.328	0	6E-11	0	40.9219	ccagcaggagCggcgaagaag	m5C_53817
chr6	26157031	+	C	43	31	0.721	0	0	C	31	M	0.573	0.833	0	1.4E-05	0	355.314	gcgaagaagcCcaagaaggcg	m5C_53816
chr6	26158475	+	C	132	34	0.258	0	0	T	98	M	0.191	0.338	0	0	0	129.59	agaagaaggaCgggaagaagc	m5C_53787
chr6	26158485	+	C	127	44	0.346	0	0	T	83	M	0.269	0.433	0	4.4E-16	0	236.989	cgggaagaagCgcaagcgcag	m5C_54117
chr6	26158493	+	C	107	31	0.29	0	0	T	76	M	0.212	0.382	0	7.1E-15	0	131.59	agcgcaagcgCagccgcaagg	m5C_53922
chr6	26199846	+	C	40	14	0.35	0	0	T	26	M	0.221	0.505	0	1.9E-07	0	61.9761	aggcggtgacCaaggcgcaga	m5C_53901
chr6	26199851	+	C	48	12	0.25	0	0	T	36	M	0.149	0.388	1.3E-15	7.5E-08	1.7E-15	26.6338	gtgaccaaggCgcagaagaag	m5C_53963
chr6	26205143	+	C	54	18	0.333	0	0	T	36	M	0.222	0.466	0	1.7E-08	0	80.0685	ggtctacgcgCtgaagagaca	m5C_54165
chr6	26250371	-	C	47	10	0.213	0	0	T	37	M	0.12	0.349	1.3E-12	2.4E-08	1.5E-12	14.271	ttcagagccaCcaacaatttc	m5C_54385
chr6	26250374	-	C	47	17	0.362	0	0	T	30	M	0.24	0.505	0	5.6E-07	0	81.4845	cttttcagagCcaccaacaat	m5C_54517
chr6	26250402	-	C	73	18	0.247	0	0	T	55	M	0.162	0.356	0	4E-12	0	58.3356	gggtgtgcgcCaacttaaacc	m5C_54260
chr6	26250405	-	C	84	23	0.274	0	0	T	61	M	0.19	0.377	0	7.2E-13	0	87.3664	tgagggtgtgCgccaacttaa	m5C_54321
chr6	26250427	-	C	40	10	0.25	0	0	T	30	M	0.142	0.402	1.8E-13	2.4E-08	2.2E-13	18.0914	ggagagagggCataagttgta	m5C_54193
chr6	26250729	-	C	31	20	0.645	0	0	C	20	M	0.469	0.789	0	4.3E-05	0	187.791	ccaccggtggCgtgaagaagc	m5C_54398
chr6	26251966	+	C	32	10	0.312	0	0	T	22	M	0.18	0.486	1.1E-14	2E-06	1.3E-14	25.0836	tggcaagaagCgtaaacgcag	m5C_54066
chr6	26271149	-	C	31	12	0.387	0	0	T	19	M	0.237	0.562	0	4.6E-06	0	56.9589	ttaagagccgCctactttcac	m5C_54225
chr6	26271151	-	C	31	9	0.29	0	0	T	22	M	0.161	0.466	3.7E-13	1.2E-06	4.4E-13	18.0154	ttttaagagcCgcctactttc	m5C_54244
chr6	26271162	-	C	32	10	0.312	0	0	T	22	M	0.18	0.486	1.1E-14	2E-06	1.3E-14	25.0836	taaaaaggctCttttaagagc	m5C_54610
chr6	26271178	-	C	33	13	0.394	0	0	T	20	M	0.247	0.563	0	6.7E-06	0	64.1755	gtaattctatCcaacttaaaa	m5C_54496
chr6	26271182	-	C	32	14	0.438	0	0	T	18	M	0.282	0.607	0	9.2E-06	0	78.8622	ggcagtaattCtatccaactt	m5C_54605
chr6	26271194	-	C	42	12	0.286	0	0	T	30	M	0.172	0.436	2.2E-16	7.5E-08	2.9E-16	32.2467	tagaggtttcCgggcagtaat	m5C_54650
chr6	26271195	-	C	45	25	0.556	0	0	C	25	M	0.412	0.691	0	4.5E-06	0	205.879	gtagaggtttCcgggcagtaa	m5C_54651
chr6	26286748	+	C	69	14	0.203	0	0	T	55	M	0.125	0.312	3.3E-16	3.2E-11	4.4E-16	27.0622	gtgtgcctggCagaacagcag	m5C_53917
chr6	26286769	+	C	345	85	0.246	0	0	T	260	M	0.204	0.294	0	0	0	346.57	agtggcgcagCggaagcgtgc	m5C_53971
chr6	26286784	+	C	336	85	0.253	0	0	T	251	M	0.209	0.302	0	0	0	356.09	gcgtgctgggCccataaccca	m5C_53832
chr6	26286786	+	C	309	98	0.317	0	0	T	211	M	0.268	0.371	0	0	0	524.848	gtgctgggccCataacccaga	m5C_53868
chr6	26286791	+	C	285	62	0.218	0	0	T	223	M	0.174	0.269	0	0	0	215.229	gggcccataaCccagaggtcg	m5C_54042
chr6	26286792	+	C	294	72	0.245	0	0	T	222	M	0.199	0.297	0	0	0	286.905	ggcccataacCcagaggtcga	m5C_53856
chr6	26286817	+	C	117	52	0.444	0	0	T	65	M	0.358	0.535	0	1.4E-13	0	371.9	tcgaaaccatCctctgctagg	m5C_54137
chr6	26286818	+	C	102	28	0.275	0	0	T	74	M	0.197	0.368	0	1.8E-15	0	110.477	cgaaaccatcCtctgctaggt	m5C_54108

chr6	26286820	+	C	104	21	0.202	0	0	T	83	M	0.136	0.289	0	0	0	57.1344	aaaccatcctCtgctaggtcc	m5C_53972
chr6	26286830	+	C	90	20	0.222	0	0	T	70	M	0.149	0.318	0	1.7E-15	0	59.4849	ctgctaggtcCttttttttc	m5C_53959
chr6	26313345	-	C	52	20	0.385	0	0	T	32	M	0.265	0.52	0	3.4E-08	0	105.881	gctatatggcCgcatatattt	m5C_54192
chr6	26313359	-	C	74	20	0.27	0	0	T	54	M	0.182	0.381	0	1.1E-11	0	72.9204	cgaaaccatcCtctgctatat	m5C_54371
chr6	26313369	-	C	86	26	0.302	0	0	T	60	M	0.215	0.406	0	2.7E-12	0	112.026	gtcgatggatCgaaaccatcc	m5C_54226
chr6	26313386	-	C	113	27	0.239	0	0	T	86	M	0.17	0.325	0	0	0	91.6506	gggcccataaCccagaggtcg	m5C_54268
chr6	26313393	-	C	119	24	0.202	0	0	T	95	M	0.139	0.283	0	0	0	66.9316	gcgtgctgggCccataaccca	m5C_54539
chr6	26313398	-	C	116	44	0.379	0	0	T	72	M	0.296	0.47	0	1.6E-14	0	260.673	cggaagcgtgCtgggcccata	m5C_54233
chr6	26319363	-	C	34	9	0.265	0	0	T	25	M	0.146	0.431	1.1E-12	1.2E-06	1.3E-12	15.7394	gactccagatCagaaggttgc	m5C_54626
chr6	26319375	-	C	36	8	0.222	0	0	T	28	M	0.117	0.381	7.4E-11	6.8E-07	8.4E-11	9.49676	ggtagcgcgtCtgactccaga	m5C_54198
chr6	26319396	-	C	42	10	0.238	0	0	T	32	M	0.135	0.385	3.2E-13	2.4E-08	3.9E-13	16.8407	tccgtgacctCgtggcgcaac	m5C_54526
chr6	26323137	+	C	34	10	0.294	0	0	T	24	M	0.168	0.462	2.3E-14	2E-06	2.9E-14	22.9564	actgactagtCtggtgttatt	m5C_53837
chr6	26327822	+	C	95	33	0.347	0	0	T	62	M	0.259	0.447	0	7.5E-13	0	171.102	tatctgtagtCgtggccgagt	m5C_53900
chr6	26327827	+	C	106	31	0.292	0	0	T	75	M	0.214	0.385	0	7.1E-15	0	132.886	gtagtcgtggCcgagtggtta	m5C_53788
chr6	26327828	+	C	106	29	0.274	0	0	T	77	M	0.198	0.365	0	2.9E-15	0	114.7	tagtcgtggcCgagtggttaa	m5C_53877
chr6	26327868	+	C	72	24	0.333	0	0	T	48	M	0.235	0.448	0	6.4E-11	0	112.966	attggggtctCcccgcgcagg	m5C_54093
chr6	26327869	+	C	71	15	0.211	0	0	T	56	M	0.132	0.32	0	6E-13	0	39.7273	ttggggtctcCccgcgcaggt	m5C_53855
chr6	26327870	+	C	62	29	0.468	0	0	T	33	M	0.349	0.59	0	1.3E-08	0	202.47	tggggtctccCcgcgcaggtt	m5C_54069
chr6	26327871	+	C	64	27	0.422	0	0	T	37	M	0.309	0.544	0	7.5E-09	0	166.696	ggggtctcccCgcgcaggttc	m5C_54057
chr6	26327873	+	C	69	19	0.275	0	0	T	50	M	0.184	0.39	0	4.5E-10	0	69.8987	ggtctccccgCgcaggttcga	m5C_53848
chr6	26327886	+	C	51	15	0.3	1	0.02	T	35	M	0.191	0.438	0	4.6E-09	0	57.3102	aggttcgaatCctgccgacta	m5C_53846
chr6	26327890	+	C	49	11	0.224	0	0	T	38	M	0.13	0.359	6.6E-14	4.4E-08	8.1E-14	18.8888	tcgaatcctgCcgactacggc	m5C_53882
chr6	26327891	+	C	49	10	0.204	0	0	T	39	M	0.115	0.336	2.1E-12	2.4E-08	2.4E-12	13.4191	cgaatcctgcCgactacggca	m5C_53829
chr6	26327897	+	C	48	10	0.208	0	0	T	38	M	0.117	0.343	1.6E-12	2.4E-08	1.9E-12	13.8343	ctgccgactaCggcagtgggt	m5C_53863
chr6	26327900	+	C	45	13	0.289	0	0	T	32	M	0.177	0.434	0	1.2E-07	0	46.0978	ccgactacggCagtgggtttt	m5C_53948
chr6	26330523	-	C	94	21	0.223	0	0	T	73	M	0.151	0.318	0	3.1E-15	0	63.4276	tgctagggagCgttttgtgga	m5C_54352
chr6	26330541	-	C	53	21	0.396	0	0	T	32	M	0.276	0.531	0	4.8E-08	0	115.875	tggatcgaaaCcatcctctgc	m5C_54434
chr6	26330562	-	C	67	26	0.388	0	0	T	41	M	0.28	0.508	0	5.6E-09	0	145.854	ggcccataacCcagaggtcga	m5C_54307
chr6	26330569	-	C	67	24	0.358	0	0	T	43	M	0.254	0.478	0	3E-09	0	121.91	cgtgctgggcCcataacccag	m5C_54353
chr6	26330570	-	C	68	33	0.485	0	0	T	35	M	0.371	0.602	0	3.4E-08	0	244.534	gcgtgctgggCccataaccca	m5C_54624
chr6	26330575	-	C	68	28	0.412	0	0	T	40	M	0.303	0.53	0	9.8E-09	0	169.444	cggaagcgtgCtgggcccata	m5C_54376
chr6	26330590	-	C	72	35	0.486	0	0	T	37	M	0.374	0.599	0	1.9E-09	0	262.027	agcagagtggCgcagcggaag	m5C_54324
chr6	26331682	-	C	32	15	0.469	0	0	T	17	M	0.309	0.636	0	1.2E-05	0	92.6074	gttcaaatcaCgtcggggtca	m5C_54310
chr6	26331695	-	C	39	10	0.256	0	0	T	29	M	0.146	0.411	1.3E-13	2E-06	1.6E-13	18.7753	cagaaggttgCgtgttcaaat	m5C_54599
chr6	26331711	-	C	58	15	0.259	0	0	T	43	M	0.163	0.384	0	4.6E-09	0	49.0362	gcgtctgactCcagatcagaa	m5C_54638
chr6	26331731	-	C	75	17	0.227	0	0	T	58	M	0.147	0.333	0	2.2E-12	0	49.8482	cctcgtggcgCaacggtagcg	m5C_54635
chr6	26331733	-	C	52	16	0.308	0	0	T	36	M	0.199	0.443	0	7.3E-09	0	63.7266	gacctcgtggCgcaacggtag	m5C_54488
chr6	26331740	-	C	46	13	0.283	0	0	T	33	M	0.173	0.425	0	1.2E-07	0	45.0425	tcccggagacCtcgtggcgca	m5C_54545
chr6	26521447	+	C	249	61	0.245	0	0	T	188	M	0.196	0.302	0	0	0	238.755	tcaggatggcCgagcggtcta	m5C_53994
chr6	26521455	+	C	210	63	0.301	1	0.005	T	146	M	0.243	0.367	0	0	0	306.515	gccgagcggtCtaaggcgctg	m5C_54147
chr6	26521461	+	C	169	38	0.248	16	0.095	T	115	M	0.187	0.322	0	0	0	141.838	cggtctaaggCgctgcgttca	m5C_53771
chr6	26521484	+	C	106	23	0.217	0	0	T	83	M	0.149	0.305	0	1.1E-16	0	68.6143	tcgcagtctcCctggaggcg	m5C_54064
chr6	26521485	+	C	112	23	0.205	0	0	T	89	M	0.141	0.289	0	0	0	64.8224	cgcagtctccCctggaggcgt	m5C_53961
chr6	26521501	+	C	154	37	0.24	0	0	T	117	M	0.18	0.314	0	0	0	132.926	ggcgtgggttCgaatcccact	m5C_53869
chr6	26521507	+	C	148	44	0.297	0	0	T	104	M	0.23	0.375	0	0	0	201.999	ggttcgaatcCcactcctgac	m5C_54112
chr6	26521513	+	C	152	36	0.237	0	0	T	116	M	0.176	0.31	0	0	0	126.911	aatcccactcCtgacagttcg	m5C_53845
chr6	26533146	-	C	52	11	0.212	0	0	T	41	M	0.122	0.34	1.4E-13	4.4E-10	1.7E-13	17.3019	ccagcggggcCtgttgtagtt	m5C_54345
chr6	26533152	-	C	53	20	0.377	0	0	T	33	M	0.259	0.512	0	3.4E-08	0	103.744	cgaatcccagCggggcctgtt	m5C_54664
chr6	26533155	-	C	52	18	0.346	0	0	T	34	M	0.232	0.482	0	1.7E-08	0	83.343	gttcgaatccCagcggggcct	m5C_54367
chr6	26533156	-	C	52	22	0.423	0	0	T	30	M	0.299	0.558	0	6.5E-08	0	131.419	ggttcgaatcCcagcggggcc	m5C_54274
chr6	26533214	-	C	44	14	0.318	0	0	T	30	M	0.2	0.466	0	1.9E-07	0	55.9976	catggtggctCcgtggcttag	m5C_54656
chr6	26533216	-	C	44	9	0.205	0	0	T	35	M	0.112	0.345	1.8E-11	1.2E-08	2.1E-11	10.7806	ggcatggtggCtccgtggctt	m5C_54414
chr6	26537753	+	C	100	32	0.32	0	0	T	68	M	0.237	0.417	0	1.1E-14	0	151.482	gataacgcgtCtgactacgga	m5C_53911
chr6	26537765	+	C	79	25	0.316	0	0	T	54	M	0.224	0.425	0	9.3E-11	0	112.226	gactacggatCagaagattcc	m5C_53984
chr6	26537774	+	C	85	23	0.271	0	0	T	62	M	0.188	0.373	0	7.2E-13	0	86.2936	tcagaagattCcaggttcgac	m5C_54101
chr6	26537781	+	C	65	19	0.292	0	0	T	46	M	0.196	0.412	0	4.5E-10	0	74.4164	attccaggttCgactcctggc	m5C_54085
chr6	26537787	+	C	64	23	0.359	0	0	T	41	M	0.253	0.482	0	2.1E-09	0	116.326	ggttcgactcCtggctggctc	m5C_53841
chr6	26537791	+	C	74	31	0.419	0	0	T	43	M	0.313	0.533	0	6.4E-10	0	194.21	cgactcctggCtggctcggtg	m5C_54073
chr6	26537795	+	C	81	36	0.444	0	0	T	45	M	0.341	0.553	0	7.4E-11	0	245.66	tcctggctggCtcggtgtaag	m5C_54097
chr6	26537812	+	C	75	33	0.44	0	0	T	42	M	0.333	0.553	0	1.1E-09	0	219.986	taagcagggtCgttttacaac	m5C_54098
chr6	26538286	+	C	171	56	0.327	0	0	T	115	M	0.262	0.401	0	0	0	293.002	tagtggggttttCcgtagtgtag	m5C_54103
chr6	26538287	+	C	174	39	0.224	0	0	T	135	M	0.169	0.292	0	0	0	131.444	agtgggtttcCgtagtgtagt	m5C_54084
chr6	26538304	+	C	364	100	0.275	1	0.003	T	263	M	0.232	0.324	0	0	0	464.11	tagtggttatCacgttcgcct	m5C_54000
chr6	26538310	+	C	355	119	0.335	0	0	T	236	M	0.288	0.386	0	0	0	685.678	ttatcacgttCgcctcacacg	m5C_53898
chr6	26538312	+	C	346	72	0.208	0	0	T	274	M	0.169	0.254	0	0	0	242.839	atcacgttcgCctcacacgcg	m5C_54161
chr6	26538313	+	C	342	85	0.249	0	0	T	257	M	0.206	0.297	0	0	0	349.686	tcacgttcgcCtcacacgcga	m5C_53944
chr6	26538315	+	C	344	99	0.288	0	0	T	245	M	0.242	0.338	0	0	0	480.147	acgttcgcctCacacgcgaaa	m5C_54179
chr6	26538317	+	C	326	66	0.202	0	0	T	260	M	0.162	0.249	0	0	0	214.389	gttcgcctcaCacgcgaaagg	m5C_53809
chr6	26538330	+	C	165	54	0.327	0	0	T	111	M	0.26	0.402	0	0	0	281.142	gcgaaaggtcCccggttcgaa	m5C_54017
chr6	26538331	+	C	161	38	0.236	0	0	T	123	M	0.177	0.307	0	0	0	134.566	cgaaaggtccCcggttcgaaa	m5C_53834
chr6	26538332	+	C	152	37	0.243	0	0	T	115	M	0.182	0.317	0	0	0	134.733	gaaaggtcccCggttcgaaac	m5C_54025
chr6	26553754	+	C	97	23	0.237	0	0	T	74	M	0.164	0.331	0	1E-14	0	75.2163	agtggtagagCgcatgcttcg	m5C_54155
chr6	26553760	+	C	75	28	0.373	0	0	T	47	M	0.273	0.486	0	2.6E-10	0	152.633	agagcgcatgCttcgcatgta	m5C_54048
chr6	26553777	+	C	31	7	0.226	0	0	T	24	M	0.114	0.398	6.4E-10	3.4E-07	7.2E-10	7.33205	tgtatgaggtCccgggttcga	m5C_53824
chr6	26553801	+	C	32	12	0.375	0	0	T	20	M	0.229	0.547	0	4.6E-06	0	55.0408	ccggcatctcCaagttgtttt	m5C_53967
chr6	26554360	+	C	84	18	0.214	0	0	T	66	M	0.14	0.313	0	4.5E-14	0	50.424	ggccggttagCtcagttggtt	m5C_54148
chr6	26554406	+	C	43	27	0.628	0	0	C	27	M	0.479	0.756	0	6.7E-06	0	258.442	gtcgcgggttCgatccccgta	m5C_53851
chr6	26554413	+	C	54	19	0.352	0	0	T	35	M	0.238	0.485	0	2.4E-08	0	90.5268	gttcgatcccCgtacgggcca	m5C_54087
chr6	26554417	+	C	58	14	0.241	0	0	T	44	M	0.15	0.365	0	2.8E-09	0	41.8845	gatccccgtaCgggccaactt	m5C_53858
chr6	26554421	+	C	57	15	0.263	0	0	T	42	M	0.166	0.39	0	4.6E-09	0	49.9369	cccgtacgggCcaactttgtt	m5C_54105
chr6	26554425	+	C	54	16	0.296	0	0	T	38	M	0.191	0.428	0	7.3E-09	0	61.2393	tacgggccaaCtttgttttct	m5C_53885
chr6	26556775	+	C	180	38	0.211	0	0	T	142	M	0.158	0.276	0	0	0	119.964	ttcgggtgagCccggctagct	m5C_54107
chr6	26556777	+	C	181	45	0.249	0	0	T	136	M	0.191	0.316	0	0	0	172.184	cgggtgagccCggctagctca	m5C_53826
chr6	26556780	+	C	218	48	0.22	0	0	T	170	M	0.17	0.28	0	0	0	163.474	gtgagcccggCtagctcagtc	m5C_53977
chr6	26556805	+	C	223	51	0.231	2	0.009	T	170	M	0.18	0.291	0	0	0	183.707	gagcatgagaCtcttaatctc	m5C_53815
chr6	26556829	+	C	214	54	0.252	0	0	T	160	M	0.199	0.315	0	0	0	214.767	gtcgtgggttCgagccccacg	m5C_53789
chr6	26556834	+	C	209	55	0.266	2	0.01	T	152	M	0.21	0.33	0	0	0	231.214	gggttcgagcCcccacgttggg	m5C_53879
chr6	26556847	+	C	177	45	0.256	1	0.006	T	131	M	0.197	0.325	0	0	0	177.236	acgttgggcgCtctggttttc	m5C_54007
chr6	26556862	+	C	32	21	0.656	0	0	C	21	M	0.483	0.796	0	5.2E-05	0	202.905	gttttcaatcCtaaaatgttt	m5C_54008
chr6	26569086	+	C	73	20	0.274	0	0	T	53	M	0.185	0.386	0	1.1E-11	0	73.9652	tcgtgtgaatCcttcgatagc	m5C_53993
chr6	26569090	+	C	70	32	0.457	0	0	T	38	M	0.346	0.573	0	8.5E-10	0	221.264	gtgaatccttCgatagctcag	m5C_54049
chr6	26569098	+	C	71	22	0.31	0	0	T	49	M	0.214	0.425	0	2.8E-11	0	94.3341	ttcgatagctCagttggtaga	m5C_54146
chr6	26569110	+	C	57	15	0.263	0	0	T	42	M	0.166	0.39	0	4.6E-09	0	49.9369	gttggtagagCggaggactgt	m5C_54122
chr6	26569117	+	C	54	18	0.333	0	0	T	36	M	0.222	0.466	0	1.7E-08	0	80.0685	gagcggaggaCtgtagttggc	m5C_53781
chr6	26569127	+	C	58	20	0.345	0	0	T	38	M	0.236	0.473	0	3.4E-08	0	94.2437	ctgtagttggCtgtgtcctta	m5C_54095
chr6	26569151	+	C	41	12	0.293	0	0	T	29	M	0.176	0.445	1.1E-16	7.5E-08	1.5E-16	33.7134	atccttaggtCgctggttcga	m5C_53912
chr6	26569153	+	C	43	20	0.465	0	0	T	23	M	0.325	0.611	0	1.4E-06	0	130.043	ccttaggtcgCtggttcgaat	m5C_54111
chr6	26569159	+	C	41	12	0.293	0	0	T	29	M	0.176	0.445	1.1E-16	7.5E-08	1.5E-16	33.7134	gtcgctggttCgaatccggct	m5C_53814
chr6	26569165	+	C	52	17	0.327	0	0	T	35	M	0.215	0.462	0	1.1E-08	0	73.1745	ggttcgaatcCggctcgaagg	m5C_54047
chr6	26577332	+	C	42	9	0.214	0	0	T	33	M	0.117	0.359	1.1E-11	1.2E-08	1.3E-11	11.5454	cgttccgtgtCcttcgatagc	m5C_53946
chr6	26577342	+	C	84	19	0.226	0	0	T	65	M	0.15	0.326	0	8.4E-14	0	56.9464	ccttcgatagCtcagttggta	m5C_54152

chr6	26577344	+	C	83	35	0.422	0	0	T	48	M	0.321	0.529	0	5.6E-11	0	224.86	ttcgatagctCagttggtaga	m5C_53909
chr6	26577370	+	C	66	26	0.394	0	0	T	40	M	0.285	0.515	0	5.6E-09	0	148.215	ggactgtaggCtcattaagca	m5C_53979
chr6	26577397	+	C	37	10	0.27	0	0	T	27	M	0.154	0.43	6.7E-14	2E-06	8.3E-14	20.2815	ccttaggtcgCtggttcgaat	m5C_53797
chr6	26577408	+	C	43	20	0.465	0	0	T	23	M	0.325	0.611	0	1.4E-06	0	130.043	tggttcgaatCcggctcggag	m5C_53874
chr6	26577409	+	C	43	10	0.233	0	0	T	33	M	0.132	0.377	4.3E-13	2.4E-08	5.2E-13	16.2683	ggttcgaatcCggctcggagg	m5C_53831
chr6	26577421	+	C	48	9	0.205	4	0.083	T	35	M	0.112	0.345	1.8E-11	1.2E-08	2.1E-11	10.7806	gctcggaggaCggtagttttg	m5C_53893
chr6	26595117	+	C	34	9	0.265	0	0	T	25	M	0.146	0.431	1.1E-12	1.2E-06	1.3E-12	15.7394	gatagctcagCtggtagagcg	m5C_54180
chr6	26988128	+	C	57	18	0.316	0	0	T	39	M	0.21	0.445	0	1.7E-08	0	75.6149	actgggtgctCcagtggcgca	m5C_54183
chr6	26988141	+	C	76	54	0.711	0	0	C	54	M	0.6	0.8	0	6.9E-08	0	648.435	gtggcgcaatCggttagcgcg	m5C_54150
chr6	26988148	+	C	74	24	0.324	0	0	T	50	M	0.229	0.437	0	6.4E-11	0	109.747	aatcggttagCgcgcggtact	m5C_53825
chr6	26988157	+	C	73	20	0.274	0	0	T	53	M	0.185	0.386	0	1.1E-11	0	73.9652	gcgcgcggtaCttatatggca	m5C_54099
chr6	26988187	+	C	167	34	0.204	0	0	T	133	M	0.15	0.271	0	0	0	101.666	cgagtgatgcCgaggttgtga	m5C_53990
chr6	26988201	+	C	214	59	0.276	0	0	T	155	M	0.22	0.339	0	0	0	259.815	gttgtgagttCgagcctcacc	m5C_53867
chr6	26988211	+	C	225	64	0.284	0	0	T	161	M	0.229	0.347	0	0	0	293.757	cgagcctcacCtggagcattg	m5C_54045
chr6	27059532	+	C	35	16	0.457	0	0	T	19	M	0.305	0.618	0	1.7E-05	0	97.4905	gctcgttggtCtaggggtatg	m5C_53878
chr6	27065095	+	C	54	25	0.521	6	0.111	C	25	M	0.383	0.655	0	4.5E-06	0	191.645	gacgaggtggCcgagtggtta	m5C_53827
chr6	27100116	-	C	31	12	0.387	0	0	T	19	M	0.237	0.562	0	4.6E-06	0	56.9589	tcaaccctaaCggctctttta	m5C_54479
chr6	27100122	-	C	32	9	0.281	0	0	T	23	M	0.156	0.454	5.3E-13	1.2E-06	6.4E-13	17.1983	aaactctcaaCcctaacggct	m5C_54229
chr6	27100452	-	C	49	16	0.327	0	0	T	33	M	0.212	0.466	0	4E-07	0	67.8634	agaagaaagaCggcaagaagc	m5C_54500
chr6	27100477	-	C	77	42	0.545	0	0	C	42	M	0.435	0.652	0	8.7E-09	0	365.172	tccaagaaggCggtgactaag	m5C_54406
chr6	27100485	-	C	48	26	0.542	0	0	C	26	M	0.403	0.674	0	5.5E-06	0	209.512	aaaagggctcCaagaaggcgg	m5C_54668
chr6	27107274	+	C	34	14	0.412	0	0	T	20	M	0.264	0.578	0	9.2E-06	0	73.8241	gaaggtgttcCtggagaacgt	m5C_53986
chr6	27115321	+	C	52	20	0.385	0	0	T	32	M	0.265	0.52	0	3.4E-08	0	105.881	aactggaaaaCaaaggctctt	m5C_54149
chr6	27115327	+	C	54	11	0.204	0	0	T	43	M	0.118	0.329	2.3E-13	4.4E-10	2.8E-13	16.3622	aaaacaaaggCtcttttcaga	m5C_54164
chr6	27115329	+	C	53	12	0.226	0	0	T	41	M	0.135	0.355	5.8E-15	8.8E-10	7.3E-15	22.9888	aacaaaggctCttttcagagc	m5C_54053
chr6	27115334	+	C	53	12	0.226	0	0	T	41	M	0.135	0.355	5.8E-15	8.8E-10	7.3E-15	22.9888	aggctcttttCagagccattc	m5C_53873
chr6	27115340	+	C	52	23	0.442	0	0	T	29	M	0.316	0.577	0	8.7E-08	0	145.336	ttttcagagcCattctacact	m5C_54184
chr6	27144993	-	C	139	33	0.237	0	0	T	106	M	0.174	0.315	0	0	0	115.062	gtacgggccaCttccgtgggt	m5C_54504
chr6	27144996	-	C	157	61	0.389	0	0	T	96	M	0.316	0.467	0	0	0	385.303	cccgtacgggCcacttccgtg	m5C_54665
chr6	27145000	-	C	151	41	0.272	0	0	T	110	M	0.207	0.347	0	0	0	169.666	gatccccgtaCgggccacttc	m5C_54546
chr6	27145004	-	C	150	31	0.207	0	0	T	119	M	0.15	0.278	0	0	0	92.7446	gttcgatcccCgtacgggcca	m5C_54397
chr6	27145007	-	C	151	34	0.225	0	0	T	117	M	0.166	0.298	0	0	0	112.772	cgggttcgatCcccgtacggg	m5C_54405
chr6	27145011	-	C	150	51	0.34	0	0	T	99	M	0.269	0.419	0	0	0	274.413	gtcgcgggttCgatccccgta	m5C_54191
chr6	27145028	-	C	79	18	0.228	0	0	T	61	M	0.149	0.332	0	4E-12	0	53.736	gtgctaataaCgccaaggtcg	m5C_54658
chr6	27145035	-	C	66	14	0.212	0	0	T	52	M	0.131	0.325	2.2E-16	3.2E-11	2.9E-16	28.6601	gagcgtggtgCtaataacgcc	m5C_54289
chr6	27145042	-	C	71	15	0.214	1	0.014	T	55	M	0.134	0.324	0	6E-13	0	40.3158	ttggttagagCgtggtgctaa	m5C_54606
chr6	27145055	-	C	70	17	0.243	0	0	T	53	M	0.158	0.355	0	2.2E-12	0	53.5559	ccggttagctCagttggttag	m5C_54210
chr6	27145064	-	C	62	19	0.306	0	0	T	43	M	0.206	0.43	0	4.5E-10	0	78.2094	ccaaggaggcCggttagctca	m5C_54361
chr6	27177638	+	C	33	8	0.242	0	0	T	25	M	0.128	0.41	3.1E-11	6.8E-07	3.6E-11	10.7866	gctgtgatggCcgagtggtta	m5C_53779
chr6	27181618	-	C	133	40	0.301	0	0	T	93	M	0.229	0.383	0	0	0	183.445	ggctcggaggCttattttctt	m5C_54277
chr6	27181626	-	C	141	30	0.213	0	0	T	111	M	0.153	0.287	0	0	0	91.979	tcctggctggCtcggaggctt	m5C_54276
chr6	27181630	-	C	144	39	0.271	0	0	T	105	M	0.205	0.349	0	0	0	159.827	cgactcctggCtggctcggag	m5C_54318
chr6	27181634	-	C	134	29	0.216	0	0	T	105	M	0.155	0.294	0	0	0	89.9704	ggttcgactcCtggctggctc	m5C_54241
chr6	27181647	-	C	146	33	0.226	0	0	T	113	M	0.166	0.3	0	0	0	109.371	tcagaagattCtaggttcgac	m5C_54227
chr6	27181685	-	C	190	52	0.274	0	0	T	138	M	0.215	0.341	0	0	0	223.854	gggccagtggCgcaatggata	m5C_54215
chr6	27181691	-	C	190	39	0.205	0	0	T	151	M	0.154	0.268	0	0	0	120.078	aacgacgggcCagtggcgcaa	m5C_54313
chr6	27182976	+	C	198	59	0.298	0	0	T	139	M	0.239	0.365	0	0	0	281.559	atggataacgCgtctgactac	m5C_54035
chr6	27183000	+	C	199	55	0.276	0	0	T	144	M	0.219	0.342	0	0	0	240.82	tcagaagattCtaggttcgac	m5C_54113
chr6	27183007	+	C	209	64	0.306	0	0	T	145	M	0.248	0.372	0	0	0	317.05	attctaggttCgactcctggc	m5C_54118
chr6	27183010	+	C	218	75	0.344	0	0	T	143	M	0.284	0.409	0	0	0	426.248	ctaggttcgaCtcctggctgg	m5C_54139
chr6	27183017	+	C	235	47	0.2	0	0	T	188	M	0.154	0.256	0	0	0	144.635	cgactcctggCtggctcgggt	m5C_53794
chr6	27183023	+	C	228	95	0.417	0	0	T	133	M	0.355	0.482	0	0	0	673.684	ctggctggctCgggtgttaat	m5C_53939
chr6	27183034	+	C	201	83	0.413	0	0	T	118	M	0.347	0.482	0	0	0	576.222	gggtgttaatCttggcttttt	m5C_54123
chr6	27198338	-	C	58	21	0.362	0	0	T	37	M	0.251	0.491	0	4.8E-08	0	105.23	gccccactccCggtaagcgtt	m5C_54646
chr6	27198340	-	C	59	22	0.373	0	0	T	37	M	0.261	0.5	0	6.5E-08	0	114.769	gagccccactCccggtaagcg	m5C_54356
chr6	27198342	-	C	53	11	0.208	0	0	T	42	M	0.12	0.335	1.8E-13	4.4E-10	2.2E-13	16.8213	tcgagccccaCtcccggtaag	m5C_54634
chr6	27198346	-	C	62	19	0.306	0	0	T	43	M	0.206	0.43	0	4.5E-10	0	78.2094	gggttcgagcCccactcccgg	m5C_54581
chr6	27198347	-	C	53	20	0.377	0	0	T	33	M	0.259	0.512	0	3.4E-08	0	103.744	tgggttcgagCcccactcccg	m5C_54550
chr6	27198351	-	C	51	14	0.275	0	0	T	37	M	0.171	0.409	0	2.8E-09	0	47.9195	cgcgtgggttCgagccccact	m5C_54422
chr6	27198359	-	C	59	16	0.271	0	0	T	43	M	0.174	0.396	0	7.3E-09	0	55.7978	caggtgtccgCgtgggttcga	m5C_54442
chr6	27198361	-	C	67	18	0.269	0	0	T	49	M	0.177	0.385	0	2.9E-10	0	63.7994	gacaggtgtcCgcgtgggttc	m5C_54598
chr6	27198377	-	C	39	8	0.205	0	0	T	31	M	0.108	0.355	1.6E-10	6.8E-07	1.8E-10	8.44384	gacttaagatCcaatggacag	m5C_54555
chr6	27198385	-	C	55	13	0.236	0	0	T	42	M	0.144	0.363	2.2E-16	1.6E-09	2.9E-16	29.2353	aggcgttggaCttaagatcca	m5C_54450
chr6	27198392	-	C	71	20	0.282	0	0	T	51	M	0.19	0.395	0	1.1E-11	0	76.1478	gtggttaaggCgttggactta	m5C_54642
chr6	27248099	-	C	41	9	0.22	0	0	T	32	M	0.12	0.367	8.4E-12	1.2E-08	9.9E-12	11.9621	tagtggttatCacgttcgcct	m5C_54418
chr6	27248117	-	C	35	12	0.343	0	0	T	23	M	0.208	0.508	0	4.6E-06	0	49.9956	gtcagggcttCtgtagtgtag	m5C_54280
chr6	27258410	+	C	104	27	0.26	0	0	T	77	M	0.185	0.351	0	1.1E-15	0	99.8939	cacaggttttcCgtggtgtagt	m5C_54039
chr6	27258427	+	C	126	26	0.206	0	0	T	100	M	0.145	0.285	0	0	0	75.3419	tagtggttatCacattcgcct	m5C_53899
chr6	27261700	+	C	691	168	0.243	0	0	T	523	M	0.213	0.276	0	0	0	714.397	ttagagcgtgCtgctactaat	m5C_53935
chr6	27261719	+	C	714	146	0.205	1	0.001	T	567	M	0.177	0.236	0	0	0	516.161	atgccagggtCgaggtttcga	m5C_53966
chr6	27261731	+	C	687	165	0.24	0	0	T	522	M	0.21	0.274	0	0	0	692.112	aggtttcgatCcccgtacggg	m5C_53982
chr6	27261743	+	C	657	152	0.231	0	0	T	505	M	0.201	0.265	0	0	0	610.208	ccgtacgggcCtttggctttt	m5C_53850
chr6	27261749	+	C	476	154	0.324	0	0	T	322	M	0.283	0.367	0	0	0	871.823	gggcctttggCtttttccccc	m5C_54181
chr6	27263236	+	C	40	18	0.45	0	0	T	22	M	0.307	0.602	0	2.7E-05	0	110.538	atggttagcaCtctggactct	m5C_53938
chr6	27263245	+	C	49	17	0.347	0	0	T	32	M	0.229	0.487	0	5.6E-07	0	77.9423	actctggactCtgaatccggt	m5C_54141
chr6	27263251	+	C	49	10	0.204	0	0	T	39	M	0.115	0.336	2.1E-12	2.4E-08	2.4E-12	13.4191	gactctgaatCcggtaatccg	m5C_54106
chr6	27263252	+	C	50	22	0.44	0	0	T	28	M	0.312	0.577	0	6.5E-08	0	137.113	actctgaatcCggtaatccga	m5C_53847
chr6	27263259	+	C	41	22	0.537	0	0	C	22	M	0.387	0.679	0	2.3E-06	0	170.483	atccggtaatCcgagttcaaa	m5C_53915
chr6	27263260	+	C	40	10	0.25	0	0	T	30	M	0.142	0.402	1.8E-13	2.4E-08	2.2E-13	18.0914	tccggtaatcCgagttcaaat	m5C_54186
chr6	27263273	+	C	38	10	0.263	0	0	T	28	M	0.15	0.42	9.4E-14	2E-06	1.2E-13	19.5037	gttcaaatctCggtggaacct	m5C_53947
chr6	27263282	+	C	38	9	0.237	0	0	T	29	M	0.13	0.392	3.7E-12	1.2E-06	4.3E-12	13.3739	tcggtggaacCttggatttag	m5C_54140
chr6	27265785	+	C	170	62	0.365	0	0	T	108	M	0.296	0.439	0	0	0	367.137	gacgaggtggCcgagtggtta	m5C_53844
chr6	27265786	+	C	166	45	0.271	0	0	T	121	M	0.209	0.343	0	0	0	188.287	acgaggtggcCgagtggttaa	m5C_54096
chr6	27265809	+	C	60	12	0.2	0	0	T	48	M	0.118	0.318	3.3E-14	7.6E-12	4.2E-14	19.13	cgatggactgCtaatccattg	m5C_54028
chr6	27265814	+	C	48	20	0.417	0	0	T	28	M	0.288	0.557	0	1.4E-06	0	115.398	gactgctaatCcattgtgctc	m5C_54171
chr6	27265815	+	C	40	14	0.35	0	0	T	26	M	0.221	0.505	0	1.9E-07	0	61.9761	actgctaatcCattgtgctct	m5C_53782
chr6	27271593	-	C	44	12	0.273	0	0	T	32	M	0.163	0.418	4.4E-16	7.5E-08	5.8E-16	30.1147	acccagaggtCaatggatcga	m5C_54572
chr6	27271600	-	C	45	22	0.489	0	0	T	23	M	0.35	0.63	0	2.3E-06	0	153.81	gcccgtaaccCagaggtcaat	m5C_54670
chr6	27271602	-	C	60	36	0.6	0	0	C	36	M	0.474	0.714	0	6.5E-08	0	341.034	gggcccgtaaCccagaggtca	m5C_54432
chr6	27271607	-	C	66	17	0.258	0	0	T	49	M	0.167	0.374	0	1.8E-10	0	56.9456	gtgctgggccCgtaacccaga	m5C_54357
chr6	27271608	-	C	76	17	0.224	0	0	T	59	M	0.145	0.329	0	2.2E-12	0	49.1676	cgtgctgggcCcgtaacccag	m5C_54509
chr6	27271609	-	C	88	41	0.466	0	0	T	47	M	0.365	0.569	0	2.6E-10	0	299.548	gcgtgctgggCccgtaaccca	m5C_54560
chr6	27271614	-	C	133	36	0.271	0	0	T	97	M	0.202	0.352	0	0	0	145.722	cggaagcgtgCtgggcccgta	m5C_54417
chr6	27271618	-	C	130	37	0.285	0	0	T	93	M	0.214	0.367	0	0	0	158.435	gcagcggaagCgtgctgggcc	m5C_54520
chr6	27271627	-	C	133	53	0.398	0	0	T	80	M	0.319	0.483	0	2.2E-16	0	338.421	cagagtggtgCagcggaagcg	m5C_54409
chr6	27300776	-	C	34	9	0.265	0	0	T	25	M	0.146	0.431	1.1E-12	1.2E-06	1.3E-12	15.7394	tggatcgaaaCcatcctctgc	m5C_54655
chr6	27300797	-	C	47	12	0.255	0	0	T	35	M	0.153	0.395	1E-15	7.5E-08	1.3E-15	27.4561	ggcccataacCcagaggtcga	m5C_54623
chr6	27300798	-	C	45	10	0.222	0	0	T	35	M	0.125	0.363	7.4E-13	2.4E-08	8.9E-13	15.2157	gggcccataaCccagaggtcg	m5C_54306
chr6	27300803	-	C	58	19	0.328	0	0	T	39	M	0.221	0.456	0	2.4E-08	0	83.9159	gtgctgggccCataacccaga	m5C_54383
chr6	27300804	-	C	57	30	0.526	0	0	C	30	M	0.399	0.65	0	4.8E-07	0	239.507	cgtgctgggcCcataacccag	m5C_54585
chr6	27300823	-	C	63	18	0.286	0	0	T	45	M	0.189	0.407	0	2.9E-10	0	68.051	cagagtggcgCagcggaagcg	m5C_54212

chr6	27300825	-	C	60	17	0.283	0	0	T	43	M	0.185	0.408	0	1.8E-10	0	62.9227	agcagagtggCgcagcggaag	m5C_54516
chr6	27302810	-	C	98	24	0.245	0	0	T	74	M	0.17	0.339	0	1.7E-14	0	81.8153	gagcatcagaCttttaatctg	m5C_54484
chr6	27302817	-	C	188	46	0.245	0	0	T	142	M	0.189	0.311	0	0	0	173.645	gtcggtagagCatcagacttt	m5C_54640
chr6	27302825	-	C	205	70	0.341	0	0	T	135	M	0.28	0.409	0	0	0	391.999	gtagctcagtCggtagagcat	m5C_54447
chr6	27302829	-	C	194	53	0.273	0	0	T	141	M	0.215	0.34	0	0	0	228.272	ctgggtagctCagtcggtaga	m5C_54201
chr6	27302840	-	C	121	25	0.207	0	0	T	96	M	0.144	0.287	0	0	0	72.0228	ccctaaggagCctgggtagct	m5C_54363
chr6	27446640	+	C	37	10	0.27	0	0	T	27	M	0.154	0.43	6.7E-14	2E-06	8.3E-14	20.2815	ccattggggtCtccccgcgca	m5C_53936
chr6	27446642	+	C	35	17	0.486	0	0	T	18	M	0.33	0.644	0	2.1E-05	0	112.18	attggggtctCcccgcgcagg	m5C_53860
chr6	27446647	+	C	41	27	0.659	0	0	C	27	M	0.505	0.784	0	6.7E-06	0	272.968	ggtctccccgCgcaggttcga	m5C_53957
chr6	27446665	+	C	33	15	0.455	0	0	T	18	M	0.298	0.62	0	1.2E-05	0	89.5281	cgaatcctgcCgactacggga	m5C_54023
chr6	27446668	+	C	31	10	0.323	0	0	T	21	M	0.186	0.499	7.1E-15	2E-06	9E-15	26.2725	atcctgccgaCtacgggatgt	m5C_53836
chr6	27447480	+	C	827	169	0.204	0	0	T	658	M	0.178	0.233	0	0	0	602.543	gtgagtatccCcgcctgtcac	m5C_53910
chr6	27447483	+	C	925	192	0.208	2	0.002	T	731	M	0.183	0.235	0	0	0	702.982	agtatccccgCctgtcacgcg	m5C_53897
chr6	27447499	+	C	1210	351	0.29	0	0	T	859	M	0.265	0.316	0	0	0	1861.77	acgcgggagaCcggggttcga	m5C_53889
chr6	27447512	+	C	1027	249	0.243	1	0.001	T	777	M	0.217	0.27	0	0	0	1082.89	gggttcgattCcccgacgggg	m5C_53991
chr6	27447513	+	C	1030	211	0.205	0	0	T	819	M	0.181	0.231	0	0	0	765.189	ggttcgattcCccgacgggga	m5C_53818
chr6	27463598	+	C	58	34	0.586	0	0	C	34	M	0.458	0.704	0	1.1E-06	0	311.428	cagctgtagtCgtggccgagt	m5C_54006
chr6	27463603	+	C	60	13	0.217	0	0	T	47	M	0.131	0.336	1.1E-15	1.6E-11	1.4E-15	25.5122	gtagtcgtggCcgagtggtta	m5C_54050
chr6	27463604	+	C	60	21	0.35	0	0	T	39	M	0.242	0.476	0	1E-09	0	101.504	tagtcgtggcCgagtggttaa	m5C_53930
chr6	27471550	+	C	504	105	0.208	0	0	T	399	M	0.175	0.246	0	0	0	367.813	gtgagtatccCcgcctgtcac	m5C_54124
chr6	27471569	+	C	746	179	0.24	0	0	T	567	M	0.211	0.272	0	0	0	754.245	acgcgggagaCcggggttcga	m5C_53810
chr6	27471582	+	C	648	157	0.242	0	0	T	491	M	0.211	0.277	0	0	0	662.147	gggttcgattCcccgacgggg	m5C_53777
chr6	27471584	+	C	646	138	0.214	0	0	T	508	M	0.184	0.247	0	0	0	507.168	gttcgattccCcgacggggag	m5C_54041
chr6	27473677	-	C	62	32	0.516	0	0	C	32	M	0.394	0.636	0	2.7E-08	0	252.462	tagtcgtggcCgagtggttaa	m5C_54262
chr6	27487341	+	C	44	12	0.273	0	0	T	32	M	0.163	0.418	4.4E-16	7.5E-08	5.8E-16	30.1147	actctggactCtgaatccagc	m5C_54032
chr6	27487348	+	C	42	11	0.262	0	0	T	31	M	0.153	0.411	8.4E-15	4.4E-08	1.1E-14	23.69	actctgaatcCagcgatccga	m5C_54052
chr6	27487351	+	C	44	30	0.682	0	0	C	30	M	0.534	0.8	0	1.2E-05	0	320.657	ctgaatccagCgatccgagtt	m5C_54061
chr6	27515532	-	C	204	49	0.24	0	0	T	155	M	0.187	0.303	0	0	0	182.986	tcggtggaacCtgctcgaaga	m5C_54591
chr6	27515543	-	C	254	68	0.268	0	0	T	186	M	0.217	0.325	0	0	0	295.149	gagttcaagtCtcggtggaac	m5C_54592
chr6	27515559	-	C	221	52	0.235	0	0	T	169	M	0.184	0.295	0	0	0	191.554	ctgaatccagCgatccgagtt	m5C_54286
chr6	27515562	-	C	199	56	0.281	0	0	T	143	M	0.224	0.348	0	0	0	250.346	actctgaatcCagcgatccga	m5C_54236
chr6	27515576	-	C	191	41	0.215	0	0	T	150	M	0.162	0.278	0	0	0	133.134	ggttagcactCtggactctga	m5C_54588
chr6	27521233	-	C	33	10	0.303	0	0	T	23	M	0.174	0.473	1.6E-14	2E-06	2E-14	23.982	actagaaaccCattggggtct	m5C_54607
chr6	27521262	-	C	55	15	0.273	0	0	T	40	M	0.173	0.402	0	4.6E-09	0	51.8418	tagtcgtggcCgagtggttaa	m5C_54283
chr6	27521268	-	C	58	10	0.208	10	0.172	T	38	M	0.117	0.343	1.6E-12	2.4E-08	1.9E-12	13.8343	tgtctgtagtCgtggccgagt	m5C_54441
chr6	27551223	-	C	148	65	0.439	0	0	T	83	M	0.362	0.52	0	1.1E-16	0	470.305	gaagcagttgCttttgattac	m5C_54618
chr6	27551246	-	C	473	134	0.283	0	0	T	339	M	0.245	0.326	0	0	0	655.425	gttcgattccCcgacggggag	m5C_54389
chr6	27551261	-	C	530	179	0.338	1	0.002	T	350	M	0.299	0.38	0	0	0	1071.66	acgcgggagaCcggggttcga	m5C_54204
chr6	27551270	-	C	634	351	0.554	0	0	C	351	M	0.515	0.592	0	0	0	3613.36	ccgtctgtcaCgcgggagacc	m5C_54551
chr6	27551303	-	C	381	110	0.289	0	0	T	271	M	0.245	0.336	0	0	0	540.095	aagtcttcctCgttagtatag	m5C_54354
chr6	27559590	-	C	54	15	0.278	0	0	T	39	M	0.176	0.409	0	4.6E-09	0	52.85	ttcaggcgaaCttcagaagtt	m5C_54485
chr6	27559603	-	C	54	29	0.537	0	0	C	29	M	0.406	0.663	0	3.9E-07	0	235.51	gttcaagtccCtgttcaggcg	m5C_54308
chr6	27559618	-	C	36	18	0.5	0	0	CT	18	M	0.345	0.655	0	2.7E-05	0	124.107	atctgaggttCcagggttcaa	m5C_54644
chr6	27559634	-	C	71	19	0.268	0	0	T	52	M	0.179	0.381	0	6.9E-12	0	67.8401	gagcatcagaCttttaatctg	m5C_54217
chr6	27559641	-	C	104	25	0.24	0	0	T	79	M	0.168	0.331	0	4.4E-16	0	84.2263	gtcggtagagCatcagacttt	m5C_54507
chr6	27559649	-	C	99	35	0.354	0	0	T	64	M	0.266	0.452	0	1.5E-12	0	186.513	atagctcagtCggtagagcat	m5C_54521
chr6	27559663	-	C	110	23	0.209	0	0	T	87	M	0.144	0.294	0	0	0	66.0388	tgtagagagcCtggatagctc	m5C_54424
chr6	27560607	-	C	37	11	0.297	0	0	T	26	M	0.175	0.458	1.4E-15	3.1E-06	1.9E-15	28.5508	cgaaaccatcCtctgctaggt	m5C_54562
chr6	27560617	-	C	89	37	0.416	0	0	T	52	M	0.319	0.52	0	9.7E-11	0	235.989	gtcgatggatCgaaaccatcc	m5C_54355
chr6	27560625	-	C	92	20	0.22	1	0.011	T	71	M	0.147	0.315	0	1.7E-15	0	58.8081	acccagaggtCgatggatcga	m5C_54372
chr6	27560632	-	C	112	48	0.429	0	0	T	64	M	0.341	0.521	0	5E-14	0	327.158	gcccataaccCagaggtcgat	m5C_54478
chr6	27560633	-	C	117	47	0.402	0	0	T	70	M	0.317	0.492	0	3.8E-14	0	298.326	ggcccataacCcagaggtcga	m5C_54476
chr6	27560650	-	C	122	39	0.322	1	0.008	T	82	M	0.246	0.41	0	0	0	191.58	gcagcggaagCgtgctgggcc	m5C_54461
chr6	27560659	-	C	131	35	0.267	0	0	T	96	M	0.199	0.349	0	0	0	139.18	cagagtggcgCagcggaagcg	m5C_54505
chr6	27560672	-	C	132	67	0.508	0	0	C	67	M	0.423	0.591	0	6E-15	0	567.205	ctgaaggggaCagcagagtgg	m5C_54659
chr6	27570426	-	C	31	12	0.387	0	0	T	19	M	0.237	0.562	0	4.6E-06	0	56.9589	tctaaggcgcCagactcaagt	m5C_54466
chr6	27570435	-	C	35	12	0.343	0	0	T	23	M	0.208	0.508	0	4.6E-06	0	49.9956	gccgagtggtCtaaggcgcca	m5C_54426
chr6	27570443	-	C	57	14	0.246	0	0	T	43	M	0.152	0.371	0	2.8E-09	0	42.6516	tcaggatggcCgagtggtcta	m5C_54293
chr6	27570444	-	C	54	17	0.315	0	0	T	37	M	0.207	0.447	0	1.1E-08	0	70.309	gtcaggatggCcgagtggtct	m5C_54380
chr6	27570452	-	C	55	21	0.382	0	0	T	34	M	0.265	0.514	0	4.8E-08	0	111.366	cagtgcgtgtCaggatggccg	m5C_54388
chr6	27573468	-	C	36	10	0.278	0	0	T	26	M	0.158	0.44	4.8E-14	2E-06	5.9E-14	21.1115	ttcctgtgttCgggtcttctg	m5C_54617
chr6	27573479	-	C	37	8	0.216	0	0	T	29	M	0.114	0.372	9.7E-11	6.8E-07	1.1E-10	9.12273	aagcttactgCttcctgtgtt	m5C_54261
chr6	27573482	-	C	44	16	0.364	0	0	T	28	M	0.238	0.511	0	4E-07	0	76.1067	ctcaagcttaCtgcttcctgt	m5C_54240
chr6	27573490	-	C	63	13	0.206	0	0	T	50	M	0.125	0.322	2.4E-15	1.6E-11	3.1E-15	23.6977	gcgccagactCaagcttactg	m5C_54458
chr6	27573505	-	C	70	16	0.229	0	0	T	54	M	0.146	0.34	0	1.2E-12	0	46.6814	gccgagtggtCtaaggcgcca	m5C_54325
chr6	27573513	-	C	74	36	0.486	0	0	T	38	M	0.376	0.598	0	2.4E-09	0	270.804	tcaggatggcCgagtggtcta	m5C_54472
chr6	27618696	-	C	51	22	0.431	0	0	T	29	M	0.305	0.567	0	6.5E-08	0	134.205	agtggttaccCttcttacgtc	m5C_54648
chr6	27618697	-	C	50	20	0.4	0	0	T	30	M	0.276	0.538	0	1.4E-06	0	110.433	aagtggttacCcttcttacgt	m5C_54427
chr6	27618718	-	C	36	15	0.417	0	0	T	21	M	0.271	0.578	0	1.2E-05	0	81.421	ggatcaaaacCaggcggaaac	m5C_54199
chr6	27618732	-	C	60	27	0.45	0	0	T	33	M	0.331	0.575	0	2.5E-07	0	178.705	cgcgaaaggtCcctggatcaa	m5C_54627
chr6	27618740	-	C	60	14	0.233	0	0	T	46	M	0.144	0.354	0	3.2E-11	0	40.4304	gcctaacacgCgaaaggtccc	m5C_54614
chr6	27618748	-	C	121	30	0.248	0	0	T	91	M	0.18	0.332	0	0	0	107.728	tcacgttcgcCtaacacgcga	m5C_54486
chr6	27618775	-	C	325	95	0.292	0	0	T	230	M	0.246	0.344	0	0	0	466.478	agggttgtttCcgtagtgtag	m5C_54208
chr6	27618786	-	C	63	15	0.238	0	0	T	48	M	0.15	0.356	0	6E-11	0	44.981	ctcccaccagCagggttgttt	m5C_54494
chr6	27636364	+	C	61	21	0.344	0	0	T	40	M	0.237	0.47	0	1E-09	0	99.7388	attcgtgtggCcggttagctc	m5C_53923
chr6	27636372	+	C	60	12	0.2	0	0	T	48	M	0.118	0.318	3.3E-14	7.6E-12	4.2E-14	19.13	ggccggttagCtcagtcggct	m5C_54013
chr6	27636378	+	C	59	22	0.373	0	0	T	37	M	0.261	0.5	0	6.5E-08	0	114.769	ttagctcagtCggctagagcg	m5C_53955
chr6	27636387	+	C	49	26	0.531	0	0	C	26	M	0.394	0.663	0	5.5E-06	0	204.779	tcggctagagCgtggtgctaa	m5C_54062
chr6	27636422	+	C	37	8	0.216	0	0	T	29	M	0.114	0.372	9.7E-11	6.8E-07	1.1E-10	9.12273	cgggttcgatCcccgtacggg	m5C_54170
chr6	27636434	+	C	37	13	0.351	0	0	T	24	M	0.218	0.512	0	6.7E-06	0	56.7461	ccgtacgggcCactggatgcc	m5C_54077
chr6	27636455	+	C	118	29	0.246	0	0	T	89	M	0.177	0.331	0	0	0	102.608	atagtggggcCcttcacttac	m5C_54182
chr6	27636465	+	C	104	26	0.252	1	0.01	T	77	M	0.178	0.344	0	6.7E-16	0	92.8081	ccttcacttaCctgaagggag	m5C_54162
chr6	27636466	+	C	106	38	0.358	0	0	T	68	M	0.274	0.453	0	9.4E-14	0	207.951	cttcacttacCtgaagggagg	m5C_53871
chr6	27636477	+	C	86	19	0.221	0	0	T	67	M	0.146	0.319	0	8.4E-14	0	55.5742	tgaagggaggCctttttaattt	m5C_53796
chr6	27636478	+	C	81	17	0.21	0	0	T	64	M	0.135	0.311	0	2.3E-14	0	46.026	gaagggaggcCttttaattta	m5C_53795
chr6	27638343	-	C	38	28	0.737	0	0	C	28	M	0.58	0.85	0	0.00017	0	324.749	ggctggctcgCttgtgctctc	m5C_54207
chr6	27638345	-	C	46	28	0.609	0	0	C	28	M	0.465	0.736	0	8.1E-06	0	260.157	ctggctggctCgcttgtgctc	m5C_54425
chr6	27638347	-	C	45	26	0.578	0	0	C	26	M	0.433	0.71	0	5.5E-06	0	225.163	tcctggctggCtcgcttgtgc	m5C_54394
chr6	27638351	-	C	47	28	0.596	0	0	C	28	M	0.453	0.724	0	8.1E-06	0	253.914	cgactcctggCtggctcgctt	m5C_54258
chr6	27638355	-	C	44	27	0.614	0	0	C	27	M	0.466	0.743	0	6.7E-06	0	251.766	ggttcgactcCtggctggctc	m5C_54209
chr6	27638356	-	C	46	28	0.609	0	0	C	28	M	0.465	0.736	0	8.1E-06	0	260.157	aggttcgactCctggctggct	m5C_54358
chr6	27638361	-	C	44	26	0.591	0	0	C	26	M	0.444	0.723	0	5.5E-06	0	230.929	attctaggttCgactcctggc	m5C_54470
chr6	27640299	-	C	167	46	0.277	1	0.006	T	120	M	0.215	0.35	0	0	0	197.477	ctgtgatggcCgagtggttaa	m5C_54563
chr6	27648411	+	C	98	25	0.255	0	0	T	73	M	0.179	0.35	0	2.9E-14	0	89.5867	acgaggtttcCgtagtgtagt	m5C_53875
chr6	27648428	+	C	135	53	0.393	0	0	T	82	M	0.314	0.477	0	2.2E-16	0	333.139	tagtggttgtCacgtttgtct	m5C_53859
chr6	27655970	+	C	129	41	0.323	2	0.016	T	86	M	0.248	0.408	0	1.1E-16	0	203.156	gtcatccggcCggttagctca	m5C_54172
chr6	27655979	+	C	130	72	0.554	0	0	C	72	M	0.468	0.637	0	4.1E-13	0	674.021	ccggttagctCagttggttag	m5C_53887
chr6	27655992	+	C	141	54	0.383	0	0	T	87	M	0.307	0.465	0	0	0	331.402	ttggttagagCgtggtgctaa	m5C_54089
chr6	27655999	+	C	130	41	0.315	0	0	T	89	M	0.242	0.4	0	0	0	198.261	gagcgtggtgCtaataacgcc	m5C_53813
chr6	27656009	+	C	115	25	0.217	0	0	T	90	M	0.152	0.301	0	0	0	75.9035	ctaataacgcCaaggtcgcgg	m5C_53804
chr6	27656017	+	C	118	54	0.458	0	0	T	64	M	0.37	0.547	0	2.3E-13	0	400.129	gccaaggtcgCgggttcgatc	m5C_54080

chr6	27656028	+	C	73	16	0.219	0	0	T	57	M	0.14	0.327	0	1.2E-12	0	44.6912	gggttcgatcCccgtactggc	m5C_53926
chr6	27656038	+	C	68	20	0.294	0	0	T	48	M	0.199	0.411	0	6.9E-10	0	79.6755	cccgtactggCcaagtattct	m5C_53916
chr6	27656047	+	C	57	22	0.386	0	0	T	35	M	0.271	0.516	0	6.5E-08	0	119.075	gccaagtattCtctgtggctt	m5C_54094
chr6	27656049	+	C	55	27	0.491	0	0	T	28	M	0.364	0.619	0	2.5E-07	0	196.433	caagtattctCtgtggctttt	m5C_53890
chr6	27721186	-	C	63	22	0.349	0	0	T	41	M	0.243	0.472	0	1.5E-09	0	107.034	cgaaaccgggCggaaacaaag	m5C_54378
chr6	27745702	+	C	149	43	0.289	0	0	T	106	M	0.222	0.366	0	0	0	190.811	ggcccataacCcagaggtcga	m5C_54163
chr6	27745728	+	C	49	10	0.204	0	0	T	39	M	0.115	0.336	2.1E-12	2.4E-08	2.4E-12	13.4191	ctaaaccatcCtctgctaaag	m5C_54130
chr6	27745746	+	C	44	13	0.302	1	0.023	T	30	M	0.186	0.451	0	1.2E-07	0	48.3648	aagaaggggtgCttttttttt	m5C_53996
chr6	27759204	-	C	32	10	0.312	0	0	T	22	M	0.18	0.486	1.1E-14	2E-06	1.3E-14	25.0836	aagaacttggCcccatggtgt	m5C_54296
chr6	27763638	-	C	65	37	0.569	0	0	C	37	M	0.448	0.682	0	7.9E-08	0	331.762	gtgggaccttCttggctcttt	m5C_54333
chr6	27763641	-	C	65	15	0.231	0	0	T	50	M	0.145	0.346	0	6E-11	0	43.5411	tcggtgggacCttcttggctc	m5C_54341
chr6	27763642	-	C	66	20	0.303	0	0	T	46	M	0.206	0.422	0	6.9E-10	0	82.2155	ctcggtgggaCcttcttggct	m5C_54515
chr6	27763650	-	C	60	20	0.333	0	0	T	40	M	0.227	0.459	0	6.9E-10	0	90.9164	gttcaaatctCggtgggacct	m5C_54464
chr6	27763652	-	C	60	16	0.267	0	0	T	44	M	0.171	0.39	0	1.1E-10	0	54.824	gagttcaaatCtcggtgggac	m5C_54221
chr6	27763657	-	C	61	18	0.295	0	0	T	43	M	0.196	0.419	0	2.9E-10	0	70.3975	gatccgagttCaaatctcggt	m5C_54604
chr6	27763672	-	C	50	14	0.28	0	0	T	36	M	0.175	0.417	0	1.9E-07	0	48.9272	gactttgaatCcagcgatccg	m5C_54224
chr6	27776385	+	C	40	17	0.425	0	0	T	23	M	0.285	0.578	0	5.6E-07	0	96.9312	aagtagaagcCtggattagtt	m5C_53902
chr6	27776404	+	C	45	18	0.4	0	0	T	27	M	0.27	0.545	0	7.7E-07	0	97.2889	tttgcagcaaCtcaatcccaa	m5C_53988
chr6	27778265	+	C	31	20	0.645	0	0	C	20	M	0.469	0.789	0	4.3E-05	0	187.791	gggcttgagtCtcaaggactc	m5C_54038
chr6	27805719	-	C	31	20	0.645	0	0	C	20	M	0.469	0.789	0	4.3E-05	0	187.791	agtagcggggCtggagcagtt	m5C_54495
chr6	27806856	+	C	35	10	0.286	0	0	T	25	M	0.163	0.451	3.4E-14	2E-06	4.2E-14	21.9991	cagtctcggcCcacaccccaa	m5C_53919
chr6	27806862	+	C	34	12	0.353	0	0	T	22	M	0.215	0.521	0	4.6E-06	0	51.5705	cggcccacacCccaaaggctc	m5C_53925
chr6	27834618	-	C	41	17	0.415	0	0	T	24	M	0.278	0.566	0	5.6E-07	0	94.3715	aggaagctggCgtgtgaaaac	m5C_54419
chr6	27834856	-	C	39	8	0.205	0	0	T	31	M	0.108	0.355	1.6E-10	6.8E-07	1.8E-10	8.44384	gcgaaaaaggCagtgaagaag	m5C_54421
chr6	27834871	-	C	48	16	0.333	0	0	T	32	M	0.217	0.475	0	4E-07	0	69.3651	aagaaggctgCaggggcgaaa	m5C_54657
chr6	27834874	-	C	53	22	0.415	0	0	T	31	M	0.293	0.549	0	6.5E-08	0	128.747	gccaagaaggCtgcaggggcg	m5C_54340
chr6	27860531	-	C	32	14	0.438	0	0	T	18	M	0.282	0.607	0	9.2E-06	0	78.8622	caagtaagggCtgaactttaa	m5C_54238
chr6	27870273	-	C	40	15	0.375	0	0	T	25	M	0.242	0.53	0	2.8E-07	0	72.6683	ccatcctctgCtatttttttt	m5C_54602
chr6	27870276	-	C	40	8	0.2	0	0	T	32	M	0.105	0.348	2.1E-10	5.5E-09	2.3E-10	8.13503	aaaccatcctCtgctattttt	m5C_54436
chr6	27870278	-	C	49	18	0.367	0	0	T	31	M	0.247	0.507	0	7.7E-07	0	88.7939	cgaaaccatcCtctgctattt	m5C_54628
chr6	27870279	-	C	53	19	0.358	0	0	T	34	M	0.243	0.493	0	2.4E-08	0	92.3467	tcgaaaccatCctctgctatt	m5C_54323
chr6	27870282	-	C	73	20	0.274	0	0	T	53	M	0.185	0.386	0	1.1E-11	0	73.9652	ggatcgaaacCatcctctgct	m5C_54654
chr6	27870283	-	C	80	26	0.325	0	0	T	54	M	0.232	0.434	0	2.7E-12	0	120.869	tggatcgaaaCcatcctctgc	m5C_54232
chr6	27870288	-	C	82	24	0.293	0	0	T	58	M	0.205	0.399	0	1.1E-12	0	98.5258	gtcgatggatCgaaaccatcc	m5C_54259
chr6	27870304	-	C	65	18	0.277	0	0	T	47	M	0.183	0.396	0	2.9E-10	0	65.8564	ggcccataacCcagaggtcga	m5C_54294
chr6	27870312	-	C	60	17	0.283	0	0	T	43	M	0.185	0.408	0	1.8E-10	0	62.9227	gcgtgctgggCccataaccca	m5C_54231
chr6	27870317	-	C	74	23	0.311	0	0	T	51	M	0.217	0.423	0	4.3E-11	0	99.7772	cggaagcgtgCtgggcccata	m5C_54337
chr6	27870321	-	C	75	15	0.2	0	0	T	60	M	0.125	0.304	0	6E-13	0	37.5361	gcagcggaagCgtgctgggcc	m5C_54399
chr6	27870678	-	C	159	56	0.352	0	0	T	103	M	0.282	0.429	0	0	0	316.109	gcaacgatgaCgtttttattct	m5C_54567
chr6	27870698	-	C	203	50	0.246	0	0	T	153	M	0.192	0.31	0	0	0	192.11	gggttcgattCccggccaatg	m5C_54590
chr6	27870709	-	C	424	103	0.249	11	0.026	T	310	M	0.21	0.293	0	0	0	432.815	gcgggaggccCgggttcgatt	m5C_54483
chr6	27870710	-	C	417	109	0.268	11	0.026	T	297	M	0.228	0.314	0	0	0	496.348	cgcgggaggcCcgggttcgat	m5C_54632
chr6	27870711	-	C	419	110	0.272	14	0.033	T	295	M	0.231	0.317	0	0	0	507.281	acgcgggaggCccgggttcga	m5C_54218
chr6	27870720	-	C	431	313	0.726	0	0	C	313	M	0.682	0.766	0	0	0	4270.95	tcgcctgccaCgcgggaggcc	m5C_54279
chr6	27870727	-	C	454	93	0.205	0	0	T	361	M	0.17	0.244	0	0	0	316.703	agaattctcgCctgccacgcg	m5C_54269
chr6	27870729	-	C	450	99	0.22	0	0	T	351	M	0.184	0.261	0	0	0	364.685	gtagaattctCgcctgccacg	m5C_54248
chr6	27870731	-	C	453	115	0.254	0	0	T	338	M	0.216	0.296	0	0	0	496.729	tggtagaattCtcgcctgcca	m5C_54391
chr6	27870744	-	C	492	113	0.23	0	0	T	379	M	0.195	0.269	0	0	0	439.991	attggtggttCagtggtagaa	m5C_54206
chr6	27870762	-	C	37	21	0.568	0	0	C	21	M	0.409	0.713	0	5.2E-05	0	171.838	ttcttttgtaCgcgaggcatt	m5C_54566
chr6	28115587	+	C	41	19	0.463	0	0	T	22	M	0.321	0.613	0	1E-06	0	121.813	atgaggggcaCaggtaggata	m5C_27094
chr6	28180825	+	C	316	105	0.333	1	0.003	T	210	M	0.284	0.387	0	0	0	595.472	gacgaggtggCcgagtggtta	m5C_27044
chr6	28180869	+	C	102	27	0.265	0	0	T	75	M	0.189	0.358	0	1.1E-15	0	101.932	gtgctctgcaCacgtgggttc	m5C_27066
chr6	28180871	+	C	99	32	0.323	0	0	T	67	M	0.239	0.42	0	5.3E-13	0	153.087	gctctgcacaCgtgggttcga	m5C_26979
chr6	28180885	+	C	99	20	0.202	0	0	T	79	M	0.135	0.292	0	1.7E-15	0	53.9025	ggttcgaatcCcatcctcgtc	m5C_27100
chr6	28180892	+	C	134	35	0.261	0	0	T	99	M	0.194	0.341	0	0	0	135.949	atcccatcctCgtcggctgca	m5C_27082
chr6	28180898	+	C	163	45	0.276	0	0	T	118	M	0.213	0.349	0	0	0	191.878	tcctcgtcggCtgcatagcaa	m5C_27135
chr6	28180906	+	C	165	44	0.267	0	0	T	121	M	0.205	0.339	0	0	0	180.453	ggctgcatagCaagccttttg	m5C_26975
chr6	28180911	+	C	155	54	0.348	0	0	T	101	M	0.278	0.426	0	0	0	300.096	catagcaagcCttttgttcca	m5C_27124
chr6	28446456	-	C	31	15	0.484	0	0	T	16	M	0.32	0.652	0	1.2E-05	0	95.91	tggtctaagaCgctggattaa	m5C_27302
chr6	28446471	-	C	51	19	0.373	0	0	T	32	M	0.253	0.51	0	2.4E-08	0	96.2173	ggtagcgtggCcgagtggtct	m5C_27234
chr6	28510908	-	C	78	20	0.256	0	0	T	58	M	0.173	0.363	0	1.1E-11	0	69.0212	attgaggggtCgaatcccttc	m5C_27330
chr6	28510936	-	C	161	43	0.267	0	0	T	118	M	0.205	0.34	0	0	0	176.084	gataaggcgtCtgacttcgga	m5C_27369
chr6	28510958	-	C	67	23	0.343	0	0	T	44	M	0.241	0.463	0	2.1E-09	0	110.809	catgggaccaCgtggcctaat	m5C_27159
chr6	28510961	-	C	49	21	0.429	0	0	T	28	M	0.3	0.567	0	1.8E-06	0	126.097	agccatgggaCcacgtggcct	m5C_27227
chr6	28557182	+	C	133	34	0.258	1	0.008	T	98	M	0.191	0.338	0	0	0	129.59	ggttagcactCtggactttga	m5C_27001
chr6	28557187	+	C	138	34	0.246	0	0	T	104	M	0.182	0.324	0	0	0	123.76	gcactctggaCtttgaatcca	m5C_27062
chr6	28557203	+	C	89	20	0.225	0	0	T	69	M	0.15	0.322	0	1.5E-13	0	60.1775	atccagcaatCcgagttcgaa	m5C_27125
chr6	28557210	+	C	58	13	0.224	0	0	T	45	M	0.136	0.347	6.7E-16	1.6E-09	8.7E-16	26.8192	aatccgagttCgaatctcggt	m5C_27105
chr6	28557217	+	C	31	15	0.484	0	0	T	16	M	0.32	0.652	0	1.2E-05	0	95.91	gttcgaatctCggtgggacct	m5C_27127
chr6	28557225	+	C	32	13	0.406	0	0	T	19	M	0.255	0.577	0	6.7E-06	0	66.3505	ctcggtgggaCctttcaaagg	m5C_27004
chr6	28565113	-	C	55	39	0.709	0	0	C	39	M	0.579	0.812	0	2.5E-06	0	451.3	ctcgtcgtttCtggaggtttt	m5C_27314
chr6	28565121	-	C	150	75	0.5	0	0	CT	75	M	0.421	0.579	0	0	0	631.483	atcccatcctCgtcgtttctg	m5C_27352
chr6	28565123	-	C	165	43	0.261	0	0	T	122	M	0.2	0.332	0	0	0	171.67	gaatcccatcCtcgtcgtttc	m5C_27303
chr6	28565124	-	C	159	38	0.239	0	0	T	121	M	0.179	0.311	0	0	0	136.313	cgaatcccatCctcgtcgttt	m5C_27141
chr6	28565127	-	C	164	37	0.226	0	0	T	127	M	0.168	0.295	0	0	0	124.574	gttcgaatccCatcctcgtcg	m5C_27275
chr6	28565129	-	C	166	51	0.307	0	0	T	115	M	0.242	0.381	0	0	0	246.91	gggttcgaatCccatcctcgt	m5C_27324
chr6	28565134	-	C	166	48	0.291	1	0.006	T	117	M	0.227	0.364	0	0	0	217.913	cgcgtgggttCgaatcccatc	m5C_27197
chr6	28565144	-	C	161	36	0.224	0	0	T	125	M	0.166	0.294	0	0	0	119.601	gtgctctgcaCgcgtgggttc	m5C_27336
chr6	28565151	-	C	125	25	0.2	0	0	T	100	M	0.139	0.279	0	0	0	69.6492	atccattgtgCtctgcacgcg	m5C_27286
chr6	28565158	-	C	129	60	0.465	0	0	T	69	M	0.381	0.551	0	3.3E-14	0	457.559	actgctaatcCattgtgctct	m5C_27283
chr6	28565188	-	C	274	55	0.201	0	0	T	219	M	0.158	0.252	0	0	0	173.346	gacgaggtggCcgagtggtta	m5C_27146
chr6	28565196	-	C	216	65	0.301	0	0	T	151	M	0.244	0.365	0	0	0	316.776	tagcatgtgaCgaggtggccg	m5C_27315
chr6	28574943	+	C	76	40	0.526	0	0	C	40	M	0.416	0.635	0	5.8E-09	0	332.415	gggggtgtagCtcagtggtag	m5C_26952
chr6	28574956	+	C	74	16	0.216	0	0	T	58	M	0.138	0.323	0	1.2E-12	0	44.0651	agtggtagagCgcgtgcttag	m5C_27104
chr6	28574973	+	C	80	33	0.412	0	0	T	47	M	0.311	0.522	0	3.1E-11	0	205.298	ttagcatgtaCgaggtcccgg	m5C_27108
chr6	28574979	+	C	114	27	0.237	0	0	T	87	M	0.168	0.323	0	0	0	90.8181	tgtacgaggtCccgggttcaa	m5C_27053
chr6	28574980	+	C	128	41	0.32	0	0	T	87	M	0.246	0.405	0	1.1E-16	0	201.498	gtacgaggtcCcgggttcaat	m5C_26960
chr6	28574981	+	C	141	42	0.298	0	0	T	99	M	0.229	0.378	0	0	0	191.992	tacgaggtccCgggttcaatc	m5C_27052
chr6	28574987	+	C	146	40	0.274	0	0	T	106	M	0.208	0.351	0	0	0	166.497	gtcccgggttCaatccccggc	m5C_26971
chr6	28574991	+	C	131	43	0.328	0	0	T	88	M	0.254	0.413	0	0	0	218.206	cgggttcaatCcccggcacct	m5C_27014
chr6	28574992	+	C	129	46	0.357	0	0	T	83	M	0.279	0.442	0	6.7E-16	0	256.842	gggttcaatcCccggcacctc	m5C_27077
chr6	28574994	+	C	129	26	0.202	0	0	T	103	M	0.141	0.279	0	0	0	73.5376	gttcaatcccCggcacctcca	m5C_26990
chr6	28574997	+	C	138	44	0.319	0	0	T	94	M	0.247	0.401	0	0	0	217.268	caatccccggCacctccacca	m5C_27038
chr6	28575000	+	C	131	33	0.252	0	0	T	98	M	0.185	0.333	0	0	0	122.34	tccccggcacCtccaccagtt	m5C_27119
chr6	28575002	+	C	134	29	0.216	0	0	T	105	M	0.155	0.294	0	0	0	89.9704	cccggcacctCcaccagtttt	m5C_27132
chr6	28575003	+	C	120	33	0.275	0	0	T	87	M	0.203	0.361	0	2.2E-16	0	133.998	ccggcacctcCaccagttttg	m5C_26951
chr6	28575005	+	C	118	35	0.297	0	0	T	83	M	0.222	0.384	0	6.7E-16	0	155.165	ggcacctccaCcagttttggg	m5C_27000
chr6	28575006	+	C	120	28	0.233	0	0	T	92	M	0.167	0.317	0	0	0	93.3266	gcacctccacCagttttggga	m5C_27072
chr6	28611270	+	C	37	10	0.27	0	0	T	27	M	0.154	0.43	6.7E-14	2E-06	8.3E-14	20.2815	tatgaggtccCgggttcgatc	m5C_27020
chr6	28611280	+	C	37	19	0.514	0	0	C	19	M	0.359	0.666	0	3.4E-05	0	136.4	cgggttcgatCcccggcatct	m5C_27091
chr6	28611281	+	C	36	8	0.222	0	0	T	28	M	0.117	0.381	7.4E-11	6.8E-07	8.4E-11	9.49676	gggttcgatcCccggcatctc	m5C_27113

chr6	28611289	+	C	36	12	0.333	0	0	T	24	M	0.202	0.497	0	4.6E-06	0	48.5146	tccccggcatCtccaataggt	m5C_27099
chr6	28611291	+	C	37	11	0.297	0	0	T	26	M	0.175	0.458	1.4E-15	3.1E-06	1.9E-15	28.5508	cccggcatctCcaataggtat	m5C_27137
chr6	28615986	-	C	58	12	0.207	0	0	T	46	M	0.123	0.328	2.1E-14	8.8E-10	2.6E-14	20.1159	cccagcggggCcttagggtgt	m5C_27308
chr6	28615994	-	C	53	20	0.377	0	0	T	33	M	0.259	0.512	0	3.4E-08	0	103.744	gttcgaatccCagcggggcct	m5C_27168
chr6	28615996	-	C	50	16	0.32	0	0	T	34	M	0.208	0.458	0	7.3E-09	0	66.4257	gggttcgaatCccagcggggc	m5C_27381
chr6	28616001	-	C	40	16	0.4	0	0	T	24	M	0.263	0.554	0	1.7E-05	0	84.314	atcctgggttCgaatcccagc	m5C_27356
chr6	28616008	-	C	41	11	0.268	0	0	T	30	M	0.157	0.419	6E-15	4.4E-08	7.6E-15	24.5536	acaggagatcCtgggttcgaa	m5C_27322
chr6	28616009	-	C	44	12	0.273	0	0	T	32	M	0.163	0.418	4.4E-16	7.5E-08	5.8E-16	30.1147	aacaggagatCctgggttcga	m5C_27353
chr6	28616017	-	C	45	13	0.289	0	0	T	32	M	0.177	0.434	0	1.2E-07	0	46.0978	gtctcgtaaaCaggagatcct	m5C_27173
chr6	28616023	-	C	38	17	0.447	0	0	T	21	M	0.301	0.603	0	2.1E-05	0	102.497	gcgcctgtctCgtaaacagga	m5C_27217
chr6	28626021	-	C	266	97	0.365	0	0	T	169	M	0.309	0.424	0	0	0	599.702	cgatccccagCatctccagtg	m5C_27321
chr6	28626025	-	C	262	55	0.21	0	0	T	207	M	0.165	0.263	0	0	0	181.478	ggttcgatccCcagcatctcc	m5C_27290
chr6	28626027	-	C	265	65	0.245	0	0	T	200	M	0.197	0.3	0	0	0	256.571	cgggttcgatCcccagcatct	m5C_27231
chr6	28626056	-	C	209	64	0.306	0	0	T	145	M	0.248	0.372	0	0	0	317.05	agagcgcatgCttagcatgca	m5C_27176
chr6	28626062	-	C	292	60	0.214	11	0.038	T	221	M	0.17	0.265	0	0	0	203.568	agtggtagagCgcatgcttag	m5C_27368
chr6	28626073	-	C	412	107	0.26	0	0	T	305	M	0.22	0.304	0	0	0	470.214	ggatgtagctCagtggtagag	m5C_27210
chr6	28626075	-	C	412	101	0.245	0	0	T	311	M	0.206	0.289	0	0	0	416.295	ggggatgtagCtcagtggtag	m5C_27337
chr6	28641603	-	C	33	11	0.333	0	0	T	22	M	0.198	0.504	2.2E-16	3.1E-06	2.9E-16	34.0074	aaggcgatcaCgtagattttg	m5C_27325
chr6	28641650	-	C	35	11	0.314	0	0	T	24	M	0.186	0.48	6.7E-16	3.1E-06	8.7E-16	30.9686	gcatgcttcgCatgtatgagg	m5C_27233
chr6	28641652	-	C	33	17	0.515	0	0	C	17	M	0.352	0.675	0	2.1E-05	0	119.742	gcgcatgcttCgcatgtatga	m5C_27183
chr6	28641659	-	C	36	10	0.278	0	0	T	26	M	0.158	0.44	4.8E-14	2E-06	5.9E-14	21.1115	tggtagagcgCatgcttcgca	m5C_27386
chr6	28641672	-	C	88	36	0.409	0	0	T	52	M	0.312	0.514	0	7.4E-11	0	224.831	ggatgtagctCagtggtagag	m5C_27245
chr6	28641674	-	C	88	34	0.386	0	0	T	54	M	0.291	0.491	0	4.2E-11	0	198.165	ggggatgtagCtcagtggtag	m5C_27239
chr6	28663710	-	C	49	24	0.49	0	0	T	25	M	0.356	0.625	0	3.6E-06	0	170.759	ctggtacctcCatttttcctt	m5C_27174
chr6	28663726	-	C	73	35	0.479	0	0	T	38	M	0.369	0.592	0	1.9E-09	0	258.141	gtccctggttCaatccctggt	m5C_27156
chr6	28663734	-	C	77	23	0.299	0	0	T	54	M	0.208	0.408	0	4.3E-11	0	95.6973	tgtacgaggtCcctggttcaa	m5C_27186
chr6	28663746	-	C	76	24	0.316	0	0	T	52	M	0.222	0.427	0	6.4E-11	0	106.707	gcatgcttcgCatgtacgagg	m5C_27343
chr6	28663748	-	C	65	28	0.431	0	0	T	37	M	0.318	0.552	0	9.8E-09	0	177.852	gcgcatgcttCgcatgtacga	m5C_27225
chr6	28663751	-	C	66	14	0.212	0	0	T	52	M	0.131	0.325	2.2E-16	3.2E-11	2.9E-16	28.6601	agagcgcatgCttcgcatgta	m5C_27139
chr6	28663757	-	C	64	29	0.453	0	0	T	35	M	0.337	0.574	0	1.3E-08	0	195.63	agtggtagagCgcatgcttcg	m5C_27185
chr6	28697115	+	C	114	25	0.219	0	0	T	89	M	0.153	0.304	0	0	0	76.5914	agtggtagagCgcgtgcttcg	m5C_26982
chr6	28697121	+	C	91	25	0.275	0	0	T	66	M	0.194	0.374	0	2.9E-14	0	96.7753	agagcgcgtgCttcgcatgta	m5C_26991
chr6	28697124	+	C	93	19	0.204	0	0	T	74	M	0.135	0.297	0	8.9E-16	0	51.2526	gcgcgtgcttCgcatgtacga	m5C_26957
chr6	28697138	+	C	38	19	0.5	0	0	CT	19	M	0.348	0.652	0	3.4E-05	0	132.429	tgtacgaggcCcccgggttcga	m5C_26984
chr6	28697139	+	C	33	7	0.212	0	0	T	26	M	0.107	0.378	1.1E-09	3.4E-07	1.2E-09	6.69165	gtacgaggccCcgggttcgac	m5C_26959
chr6	28703218	-	C	104	21	0.202	0	0	T	83	M	0.136	0.289	0	0	0	57.1344	gggttcgatcCccagcacttc	m5C_27357
chr6	28703219	-	C	107	28	0.262	0	0	T	79	M	0.188	0.352	0	1.8E-15	0	105.11	tgggttcgatCcccagcactt	m5C_27390
chr6	28703230	-	C	75	28	0.373	0	0	T	47	M	0.273	0.486	0	2.6E-10	0	152.633	tcatgaggctCtgggttcgat	m5C_27232
chr6	28703243	-	C	33	12	0.364	0	0	T	21	M	0.222	0.534	0	4.6E-06	0	53.2487	gtatgcttaaCattcatgagg	m5C_27138
chr6	28703248	-	C	50	11	0.22	0	0	T	39	M	0.128	0.352	8.5E-14	4.4E-10	1.1E-13	18.3342	agagcgtatgCttaacattca	m5C_27179
chr6	28703254	-	C	86	25	0.291	0	0	T	61	M	0.205	0.394	0	1.8E-12	0	102.662	agtggtagagCgtatgcttaa	m5C_27378
chr6	28703265	-	C	130	31	0.238	0	0	T	99	M	0.173	0.319	0	0	0	107.497	gggtgtagctCagtggtagag	m5C_27222
chr6	28710730	-	C	33	18	0.545	0	0	C	18	M	0.38	0.702	0	2.7E-05	0	136.748	ccttcgtggtCgtgttcttac	m5C_27198
chr6	28710736	-	C	37	18	0.486	0	0	T	19	M	0.334	0.641	0	2.7E-05	0	120.406	cgagtcccttCgtggtcgtgt	m5C_27187
chr6	28710740	-	C	34	10	0.294	0	0	T	24	M	0.168	0.462	2.3E-14	2E-06	2.9E-14	22.9564	ggttcgagtcCcttcgtggtc	m5C_27382
chr6	28710746	-	C	38	8	0.211	0	0	T	30	M	0.111	0.363	1.3E-10	6.8E-07	1.4E-10	8.77246	attgagggttCgagtcccttc	m5C_27282
chr6	28715516	+	C	39	22	0.564	0	0	C	22	M	0.41	0.707	0	6.3E-05	0	180.292	ttgcgtctacCcagggcctgg	m5C_27060
chr6	28715548	+	C	188	40	0.213	0	0	T	148	M	0.16	0.277	0	0	0	128.255	ggtagaacatCagacttttaa	m5C_27068
chr6	28715552	+	C	130	50	0.385	0	0	T	80	M	0.305	0.47	0	2.4E-15	0	305.438	gaacatcagaCttttaatctg	m5C_27010
chr6	28715560	+	C	46	19	0.413	0	0	T	27	M	0.283	0.557	0	1E-06	0	107.496	gacttttaatCtgacggtgca	m5C_27081
chr6	28715564	+	C	68	19	0.279	0	0	T	49	M	0.187	0.396	0	4.5E-10	0	70.9757	tttaatctgaCggtgcagggt	m5C_27059
chr6	28715581	+	C	121	39	0.322	0	0	T	82	M	0.246	0.41	0	0	0	191.58	gggttcaagtCcctgttcagg	m5C_26945
chr6	28715582	+	C	116	36	0.31	0	0	T	80	M	0.233	0.399	0	1E-15	0	168.027	ggttcaagtcCctgttcaggc	m5C_26970
chr6	28715583	+	C	120	38	0.317	0	0	T	82	M	0.24	0.404	0	0	0	182.573	gttcaagtccCtgttcaggcg	m5C_27033
chr6	28715588	+	C	119	32	0.269	0	0	T	87	M	0.197	0.355	0	2.2E-16	0	126.331	agtccctgttCaggcgaaata	m5C_27065
chr6	28726143	-	C	259	88	0.34	0	0	T	171	M	0.285	0.399	0	0	0	501.253	cccggcacctCcaaatggtgg	m5C_27203
chr6	28726158	-	C	257	54	0.21	0	0	T	203	M	0.165	0.264	0	0	0	177.943	gccccgggttCgatccccggc	m5C_27145
chr6	28726165	-	C	280	69	0.246	0	0	T	211	M	0.2	0.3	0	0	0	275.462	gtgtgaggccCcgggttcgat	m5C_27366
chr6	28726166	-	C	284	71	0.25	0	0	T	213	M	0.203	0.303	0	0	0	288.546	tgtgtgaggcCccgggttcga	m5C_27262
chr6	28726167	-	C	286	68	0.238	0	0	T	218	M	0.192	0.29	0	0	0	261.261	atgtgtgaggCcccgggttcg	m5C_27361
chr6	28726183	-	C	290	74	0.255	0	0	T	216	M	0.208	0.308	0	0	0	308.466	agagcacatgCtttgcatgtg	m5C_27342
chr6	28757564	-	C	125	39	0.312	0	0	T	86	M	0.237	0.398	0	0	0	185.178	gtcccgggttCgatccccggc	m5C_27339
chr6	28757571	-	C	145	56	0.386	0	0	T	89	M	0.311	0.467	0	0	0	348.174	gtatgaggtcCcgggttcgat	m5C_27169
chr6	28757595	-	C	141	29	0.206	0	0	T	112	M	0.147	0.28	0	0	0	85.3718	agtggtagagCgcatgctttg	m5C_27218
chr6	28763685	-	C	31	9	0.29	0	0	T	22	M	0.161	0.466	3.7E-13	1.2E-06	4.4E-13	18.0154	tattccccaaCtttaaatttt	m5C_27305
chr6	28763691	-	C	32	8	0.25	0	0	T	24	M	0.133	0.421	2.3E-11	6.8E-07	2.6E-11	11.2828	ccatgatattCcccaacttta	m5C_27350
chr6	28763701	-	C	45	14	0.311	0	0	T	31	M	0.195	0.457	0	1.9E-07	0	54.6799	aagaagcggtCcatgatattc	m5C_27296
chr6	28763705	-	C	44	17	0.386	0	0	T	27	M	0.257	0.534	0	5.6E-07	0	87.4514	cagtaagaagCggtccatgat	m5C_27298
chr6	28763718	-	C	48	11	0.229	0	0	T	37	M	0.133	0.365	5E-14	4.4E-08	6.2E-14	19.4712	ttccccgggaCaccagtaaga	m5C_27346
chr6	28763723	-	C	43	12	0.279	0	0	T	31	M	0.167	0.427	2.2E-16	7.5E-08	2.9E-16	31.457	ttttcttcccCgggacaccag	m5C_27384
chr6	28763724	-	C	36	12	0.333	0	0	T	24	M	0.202	0.497	0	4.6E-06	0	48.5146	gttttcttccCcgggacacca	m5C_27277
chr6	28763726	-	C	40	12	0.3	0	0	T	28	M	0.181	0.454	1.1E-16	7.5E-08	1.5E-16	34.6049	gtgttttcttCcccgggacac	m5C_27340
chr6	28763729	-	C	37	19	0.514	0	0	C	19	M	0.359	0.666	0	3.4E-05	0	136.4	gccgtgttttCttccccggga	m5C_27263
chr6	28763742	-	C	49	16	0.327	0	0	T	33	M	0.212	0.466	0	4E-07	0	67.8634	ccagtacctcCaggccgtgtt	m5C_27230
chr6	28763751	-	C	53	15	0.288	1	0.019	T	37	M	0.183	0.423	0	4.6E-09	0	54.9894	gttcgatcccCagtacctcca	m5C_27323
chr6	28763783	-	C	97	49	0.505	0	0	C	49	M	0.407	0.603	0	5.8E-11	0	399.226	agagcgcgtgCttagcatgca	m5C_27241
chr6	28763800	-	C	242	56	0.231	0	0	T	186	M	0.183	0.288	0	0	0	204.643	gggtatagctCagtggtagag	m5C_27332
chr6	28770525	-	C	65	22	0.338	0	0	T	43	M	0.235	0.46	0	1.5E-09	0	103.547	tgcctcctcaCatttttctat	m5C_27372
chr6	28770529	-	C	71	24	0.338	0	0	T	47	M	0.239	0.454	0	6.4E-11	0	114.647	cctctgcctcCtcacattttt	m5C_27280
chr6	28770530	-	C	72	21	0.292	0	0	T	51	M	0.199	0.405	0	1.8E-11	0	83.7392	acctctgcctCctcacatttt	m5C_27348
chr6	28770532	-	C	70	32	0.457	0	0	T	38	M	0.346	0.573	0	8.5E-10	0	221.264	agacctctgcCtcctcacatt	m5C_27196
chr6	28770533	-	C	76	16	0.211	0	0	T	60	M	0.134	0.315	0	1.2E-12	0	42.8641	cagacctctgCctcctcacat	m5C_27383
chr6	28770538	-	C	71	55	0.775	0	0	C	55	M	0.665	0.856	0	8E-08	0	731.331	agcgacagacCtctgcctcct	m5C_27307
chr6	28770539	-	C	73	17	0.233	0	0	T	56	M	0.151	0.342	0	2.2E-12	0	51.2678	aagcgacagaCctctgcctcc	m5C_27212
chr6	28770561	-	C	80	20	0.25	0	0	T	60	M	0.168	0.355	0	1.5E-13	0	67.2244	tggtttccctCtggcagttct	m5C_27226
chr6	28770584	-	C	56	14	0.25	0	0	T	42	M	0.155	0.377	0	2.8E-09	0	43.4474	cgatccccgaCacctccaagt	m5C_27155
chr6	28770587	-	C	61	22	0.361	0	0	T	39	M	0.252	0.486	0	1.5E-09	0	110.765	gttcgatcccCgacacctcca	m5C_27242
chr6	28770594	-	C	45	12	0.267	0	0	T	33	M	0.16	0.41	5.6E-16	7.5E-08	7.3E-16	29.2263	ggcctcggttCgatccccgac	m5C_27373
chr6	28770601	-	C	45	26	0.578	0	0	C	26	M	0.433	0.71	0	5.5E-06	0	225.163	tgtatgaggcCtcggttcgat	m5C_27278
chr6	28770618	-	C	188	45	0.239	0	0	T	143	M	0.184	0.305	0	0	0	165.577	agagcgcatgCtttgcatgta	m5C_27285
chr6	28770624	-	C	246	71	0.289	0	0	T	175	M	0.236	0.348	0	0	0	334.534	agtggtagagCgcatgctttg	m5C_27171
chr6	28775614	-	C	39	28	0.718	0	0	C	28	M	0.562	0.835	0	0.00017	0	314.857	tcccgggtttCggcagttgca	m5C_27333
chr6	28775621	-	C	33	22	0.667	0	0	C	22	M	0.496	0.802	0	6.3E-05	0	218.273	ggttcaatccCgggtttcggc	m5C_27213
chr6	28775622	-	C	33	17	0.515	0	0	C	17	M	0.352	0.675	0	2.1E-05	0	119.742	tggttcaatcCcgggtttcgg	m5C_27194
chr6	28775633	-	C	53	14	0.264	0	0	T	39	M	0.164	0.396	0	2.8E-09	0	46.0242	ctaaaggtccCtggttcaatc	m5C_27392
chr6	28775634	-	C	57	29	0.509	0	0	C	29	M	0.383	0.634	0	3.9E-07	0	221.906	tctaaaggtcCctggttcaat	m5C_27389
chr6	28775635	-	C	54	11	0.204	0	0	T	43	M	0.118	0.329	2.3E-13	4.4E-10	2.8E-13	16.3622	atctaaaggtCcctggttcaa	m5C_27258
chr6	28775643	-	C	53	12	0.226	0	0	T	41	M	0.135	0.355	5.8E-15	8.8E-10	7.3E-15	22.9888	gactgaagatCtaaaggtccc	m5C_27152
chr6	28775651	-	C	75	16	0.213	0	0	T	59	M	0.136	0.319	0	1.2E-12	0	43.4563	gagcgttagaCtgaagatcta	m5C_27228
chr6	28775680	-	C	132	29	0.22	0	0	T	103	M	0.158	0.298	0	0	0	91.3768	tcacggtggcCgagatagctc	m5C_27349
chr6	28775681	-	C	116	32	0.276	0	0	T	84	M	0.203	0.363	0	2.2E-16	0	129.73	atcacggtggCcgagatagct	m5C_27216

chr6	28779859	-	C	52	19	0.365	0	0	T	33	M	0.248	0.501	0	2.4E-08	0	94.242	gttcaatcccCaatacctcca	m5C_27236
chr6	28779861	-	C	52	18	0.346	0	0	T	34	M	0.232	0.482	0	1.7E-08	0	83.343	gggttcaatcCccaatacctc	m5C_27326
chr6	28779866	-	C	55	11	0.2	0	0	T	44	M	0.116	0.324	2.9E-13	4.4E-10	3.6E-13	15.9231	gtcctgggttCaatccccaat	m5C_27253
chr6	28779873	-	C	55	11	0.2	0	0	T	44	M	0.116	0.324	2.9E-13	4.4E-10	3.6E-13	15.9231	gcacgaggtcCtgggttcaat	m5C_27297
chr6	28779880	-	C	58	13	0.224	0	0	T	45	M	0.136	0.347	6.7E-16	1.6E-09	8.7E-16	26.8192	ttagcatgcaCgaggtcctgg	m5C_27341
chr6	28779882	-	C	55	12	0.218	0	0	T	43	M	0.129	0.344	9.8E-15	8.8E-10	1.2E-14	21.7648	gcttagcatgCacgaggtcct	m5C_27192
chr6	28779908	-	C	77	22	0.286	0	0	T	55	M	0.197	0.395	0	2.8E-11	0	86.6333	gggtatagctCagcggtagag	m5C_27248
chr6	28785019	-	C	215	62	0.288	0	0	T	153	M	0.232	0.352	0	0	0	287.621	cgatccccgaCacctccaagc	m5C_27288
chr6	28785025	-	C	217	79	0.364	0	0	T	138	M	0.303	0.43	0	0	0	478.605	cgggttcgatCcccgacacct	m5C_27371
chr6	28785038	-	C	234	64	0.274	0	0	T	170	M	0.22	0.334	0	0	0	282.103	atgtatgaggCctcgggttcg	m5C_27189
chr6	28785054	-	C	126	36	0.286	0	0	T	90	M	0.214	0.37	0	0	0	154.145	agagcgcatgCtttgcatgta	m5C_27328
chr6	28795249	-	C	59	13	0.22	0	0	T	46	M	0.134	0.341	8.9E-16	1.6E-09	1.2E-15	26.1299	gggtatagctCagtggtagag	m5C_27170
chr6	28806222	-	C	41	17	0.415	0	0	T	24	M	0.278	0.566	0	5.6E-07	0	94.3715	ccggcacctcCattcctttg	m5C_27157
chr6	28806226	-	C	41	10	0.244	0	0	T	31	M	0.138	0.393	2.4E-13	2.4E-08	2.9E-13	17.4472	atccccggcaCctccattcct	m5C_27252
chr6	28806228	-	C	42	14	0.333	0	0	T	28	M	0.21	0.484	0	1.9E-07	0	58.8344	caatccccggCacctccattc	m5C_27388
chr6	28806233	-	C	42	14	0.333	0	0	T	28	M	0.21	0.484	0	1.9E-07	0	58.8344	gggttcaatcCccggcacctc	m5C_27148
chr6	28806246	-	C	46	11	0.239	0	0	T	35	M	0.139	0.379	2.8E-14	4.4E-08	3.5E-14	20.7303	tgcacgaggcCccgggttcaa	m5C_27367
chr6	28806247	-	C	45	13	0.289	0	0	T	32	M	0.177	0.434	0	1.2E-07	0	46.0978	atgcacgaggCcccgggttca	m5C_27259
chr6	28806254	-	C	36	9	0.25	0	0	T	27	M	0.138	0.411	2E-12	1.2E-06	2.4E-12	14.4765	gcttagcatgCacgaggcccc	m5C_27220
chr6	28806280	-	C	77	22	0.286	0	0	T	55	M	0.197	0.395	0	2.8E-11	0	86.6333	gggtgtagctCagtggtagag	m5C_27256
chr6	28831457	-	C	39	9	0.231	0	0	T	30	M	0.126	0.383	4.9E-12	1.2E-06	5.7E-12	12.8739	cctccagcttCatttttctcc	m5C_27387
chr6	28831460	-	C	40	11	0.275	0	0	T	29	M	0.161	0.428	4.3E-15	3.1E-06	5.5E-15	25.4502	gcacctccagCttcatttttc	m5C_27200
chr6	28831463	-	C	44	20	0.455	0	0	T	24	M	0.317	0.599	0	1.4E-06	0	126.82	ccggcacctcCagcttcattt	m5C_27376
chr6	28831473	-	C	54	20	0.37	0	0	T	34	M	0.254	0.504	0	3.4E-08	0	101.693	ggttcaatccCcggcacctcc	m5C_27205
chr6	28831485	-	C	119	33	0.277	0	0	T	86	M	0.205	0.364	0	2.2E-16	0	135.17	cacgaggcccCgggttcaatc	m5C_27295
chr6	28831486	-	C	114	23	0.202	0	0	T	91	M	0.138	0.285	0	0	0	63.6501	gcacgaggccCcgggttcaat	m5C_27265
chr6	28831487	-	C	113	25	0.221	0	0	T	88	M	0.155	0.306	0	0	0	77.2919	tgcacgaggcCccgggttcaa	m5C_27319
chr6	28831488	-	C	120	35	0.292	0	0	T	85	M	0.218	0.378	0	0	0	152.47	atgcacgaggCcccgggttca	m5C_27320
chr6	28831493	-	C	163	33	0.202	0	0	T	130	M	0.148	0.271	0	0	0	97.6453	ttagcatgcaCgaggccccgg	m5C_27312
chr6	28831521	-	C	429	103	0.24	0	0	T	326	M	0.202	0.283	0	0	0	416.313	gggtgtagctCagtggtagag	m5C_27235
chr6	28831523	-	C	415	111	0.267	0	0	T	304	M	0.227	0.312	0	0	0	504.289	gggggtgtagCtcagtggtag	m5C_27391
chr6	28849189	+	C	44	11	0.324	10	0.227	T	23	M	0.191	0.492	4.4E-16	3.1E-06	5.8E-16	32.3088	atggataaggCgctgattcc	m5C_27040
chr6	28849192	+	C	36	19	0.528	0	0	C	19	M	0.37	0.68	0	3.4E-05	0	140.622	gataaggcgtCgattccgga	m5C_27027
chr6	28849198	+	C	37	18	0.486	0	0	T	19	M	0.334	0.641	0	2.7E-05	0	120.406	gcgtctgattCcggatcagaa	m5C_27024
chr6	28849220	+	C	35	10	0.286	0	0	T	25	M	0.163	0.451	3.4E-14	2E-06	4.2E-14	21.9991	attgaggggttCgagtcccttc	m5C_27133
chr6	28849226	+	C	39	20	0.513	0	0	C	20	M	0.362	0.661	0	4.3E-05	0	144.796	ggttcgagtcCcttcgtggtc	m5C_26986
chr6	28849227	+	C	41	19	0.463	0	0	T	22	M	0.321	0.613	0	1E-06	0	121.813	gttcgagtccCttcgtggtcg	m5C_26963
chr6	28849230	+	C	37	19	0.514	0	0	C	19	M	0.359	0.666	0	3.4E-05	0	136.4	cgagtcccttCgtggtcgtcg	m5C_26962
chr6	28849236	+	C	38	12	0.316	0	0	T	26	M	0.191	0.475	0	4.6E-06	0	45.8026	ccttcgtggtCgtcgttttgc	m5C_27012
chr6	28849239	+	C	40	11	0.275	0	0	T	29	M	0.161	0.428	4.3E-15	3.1E-06	5.5E-15	25.4502	tcgtggtcgtCgttttgcgtt	m5C_26968
chr6	28864011	-	C	335	90	0.269	0	0	T	245	M	0.224	0.319	0	0	0	403.217	ggttcgaatcCcacttctgac	m5C_27163
chr6	28864034	-	C	347	82	0.236	0	0	T	265	M	0.195	0.284	0	0	0	319.229	ttctggtctcCaatggaggcg	m5C_27316
chr6	28864035	-	C	322	67	0.208	0	0	T	255	M	0.167	0.256	0	0	0	224.19	attctggtctCcaatggaggc	m5C_27165
chr6	28864055	-	C	224	55	0.246	0	0	T	169	M	0.194	0.306	0	0	0	213.151	aagcttcctcCgcggtgggga	m5C_27334
chr6	28864058	-	C	239	62	0.259	0	0	T	177	M	0.208	0.318	0	0	0	257.87	gctaagcttcCtccgcggtgg	m5C_27215
chr6	28864059	-	C	253	74	0.292	0	0	T	179	M	0.24	0.351	0	0	0	355.012	agctaagcttCctccgcggtg	m5C_27254
chr6	28864071	-	C	441	111	0.252	0	0	T	330	M	0.213	0.294	0	0	0	473.876	gcgccagactCaagctaagct	m5C_27201
chr6	28864073	-	C	480	146	0.304	0	0	T	334	M	0.265	0.347	0	0	0	772.92	aggcgccagaCtcaagctaag	m5C_27318
chr6	28864080	-	C	586	133	0.229	4	0.007	T	449	M	0.196	0.264	0	0	0	522.038	tggtctaaggCgccagactca	m5C_27175
chr6	28864086	-	C	663	133	0.201	0	0	T	530	M	0.172	0.233	0	0	0	457.213	gccgagtggtCtaaggcgcca	m5C_27274
chr6	28864094	-	C	680	190	0.279	0	0	T	490	M	0.247	0.314	0	0	0	938.588	tcaggatggcCgagtggtcta	m5C_27363
chr6	28908840	+	C	385	77	0.201	1	0.003	T	307	M	0.164	0.243	0	0	0	251.834	gtcaggatggCcgagtggtct	m5C_27021
chr6	28908841	+	C	389	105	0.27	0	0	T	284	M	0.228	0.316	0	0	0	479.255	tcaggatggcCgagtggtcta	m5C_27031
chr6	28908855	+	C	358	78	0.223	9	0.025	T	271	M	0.183	0.27	0	0	0	285.377	tggtctaaggCgccagactca	m5C_27028
chr6	28908857	+	C	363	73	0.202	1	0.003	T	289	M	0.164	0.246	0	0	0	238.79	gtctaaggcgCcagactcaag	m5C_26987
chr6	28908858	+	C	359	72	0.201	0	0	T	287	M	0.162	0.245	0	0	0	233.862	tctaaggcgcCagactcaagc	m5C_27129
chr6	28908868	+	C	267	61	0.231	3	0.011	T	203	M	0.184	0.286	0	0	0	224.834	cagactcaagCttggcttcct	m5C_26974
chr6	28908877	+	C	238	56	0.235	0	0	T	182	M	0.186	0.293	0	0	0	208.177	gcttggcttcCtcgtgttgag	m5C_27061
chr6	28908899	+	C	349	78	0.223	0	0	T	271	M	0.183	0.27	0	0	0	285.377	attctggtctCcaatggaggc	m5C_27048
chr6	28908900	+	C	357	73	0.204	0	0	T	284	M	0.166	0.249	0	0	0	242.205	ttctggtctcCaatggaggcg	m5C_27115
chr6	28908917	+	C	326	66	0.202	0	0	T	260	M	0.162	0.249	0	0	0	214.389	ggcgtgggttCgaatcccact	m5C_27123
chr6	28908929	+	C	292	70	0.24	0	0	T	222	M	0.194	0.292	0	0	0	272.076	aatcccacttCtgacacaact	m5C_27037
chr6	28908938	+	C	256	84	0.328	0	0	T	172	M	0.274	0.388	0	0	0	459.512	tctgacacaaCtatcttattc	m5C_27080
chr6	28909379	-	C	46	18	0.391	0	0	T	28	M	0.264	0.535	0	7.7E-07	0	95.0149	tcggtggaacCtttcatttct	m5C_27284
chr6	28909380	-	C	51	20	0.392	0	0	T	31	M	0.27	0.529	0	3.4E-08	0	108.109	ctcggtggaaCctttcatttc	m5C_27161
chr6	28909388	-	C	55	23	0.418	0	0	T	32	M	0.297	0.55	0	8.7E-08	0	136.793	gttcaaatctCggtggaacct	m5C_27289
chr6	28909390	-	C	59	24	0.407	0	0	T	35	M	0.291	0.534	0	1.2E-07	0	139.626	gagttcaaatCtcggtggaac	m5C_27299
chr6	28909395	-	C	52	27	0.519	0	0	C	27	M	0.387	0.649	0	2.5E-07	0	208.901	gatccgagttCaaatctcggt	m5C_27181
chr6	28909401	-	C	53	22	0.415	0	0	T	31	M	0.293	0.549	0	6.5E-08	0	128.747	tccagcgatcCgagttcaaat	m5C_27209
chr6	28909402	-	C	52	15	0.288	0	0	T	37	M	0.183	0.423	0	4.6E-09	0	54.9894	atccagcgatCcgagttcaaa	m5C_27164
chr6	28909406	-	C	49	20	0.408	0	0	T	29	M	0.282	0.548	0	1.4E-06	0	112.86	ctgaatccagCgatccgagtt	m5C_27224
chr6	28909409	-	C	54	17	0.315	0	0	T	37	M	0.207	0.447	0	1.1E-08	0	70.309	actctgaatcCagcgatccga	m5C_27365
chr6	28909410	-	C	53	17	0.321	0	0	T	36	M	0.211	0.455	0	1.1E-08	0	71.713	gactctgaatCcagcgatccg	m5C_27206
chr6	28909416	-	C	54	17	0.315	0	0	T	37	M	0.207	0.447	0	1.1E-08	0	70.309	actctggactCtgaatccagc	m5C_27338
chr6	28909418	-	C	53	21	0.396	0	0	T	32	M	0.276	0.531	0	4.8E-08	0	115.875	gcactctggaCtctgaatcca	m5C_27273
chr6	28911404	-	C	91	19	0.209	0	0	T	72	M	0.138	0.303	0	8.9E-16	0	52.4171	aatcccaccgCtgccagtgtg	m5C_27374
chr6	28911406	-	C	82	30	0.366	0	0	T	52	M	0.27	0.474	0	1.2E-11	0	161.871	cgaatcccacCgctgccagtg	m5C_27267
chr6	28911407	-	C	90	37	0.411	0	0	T	53	M	0.315	0.514	0	2.7E-12	0	233.197	tcgaatcccaCcgctgccagt	m5C_27214
chr6	28911409	-	C	94	38	0.404	0	0	T	56	M	0.311	0.505	0	3.7E-12	0	236.132	gttcgaatccCaccgctgcca	m5C_27143
chr6	28911411	-	C	89	30	0.337	0	0	T	59	M	0.247	0.44	0	1.2E-11	0	148.456	gggttcgaatCccaccgctgc	m5C_27167
chr6	28911416	-	C	152	32	0.211	0	0	T	120	M	0.153	0.282	0	0	0	98.0846	ggcgtgggttCgaatcccacc	m5C_27393
chr6	28911424	-	C	259	83	0.32	0	0	T	176	M	0.267	0.38	0	0	0	442.573	tcttcgggggCgtgggttcga	m5C_27345
chr6	28911442	-	C	32	21	0.656	0	0	C	21	M	0.483	0.796	0	5.2E-05	0	202.905	tggattaaggCtccagtctct	m5C_27264
chr6	28912357	+	C	84	22	0.262	0	0	T	62	M	0.18	0.365	0	4.4E-13	0	79.1047	cagttgcctcCttagcgcagt	m5C_27017
chr6	28912374	+	C	83	17	0.205	0	0	T	66	M	0.132	0.304	0	2.3E-14	0	44.8792	cagtaggcagCgcgtcagtct	m5C_27032
chr6	28912376	+	C	81	27	0.333	0	0	T	54	M	0.24	0.441	0	4E-12	0	129.755	gtaggcagcgCgtcagtctca	m5C_27097
chr6	28912391	+	C	58	16	0.276	0	0	T	42	M	0.178	0.402	0	7.3E-09	0	56.8071	gtctcataatCtgaaggtcct	m5C_26973
chr6	28912411	+	C	42	16	0.381	0	0	T	26	M	0.25	0.532	0	4E-07	0	79.9985	tgagttcgaaCctcagagggg	m5C_27050
chr6	28912428	+	C	102	51	0.5	0	0	CT	51	M	0.405	0.595	0	3.3E-12	0	412.838	gggggcaaggCgctgttttg	m5C_27120
chr6	28912431	+	C	105	48	0.457	0	0	T	57	M	0.365	0.552	0	1.6E-12	0	350.452	ggcaaggcgtCtgttttgcca	m5C_27083
chr6	28918807	+	C	193	44	0.228	0	0	T	149	M	0.174	0.292	0	0	0	153.507	ttgcagggagCccggatagct	m5C_27075
chr6	28918808	+	C	198	59	0.319	13	0.066	T	126	M	0.256	0.389	0	0	0	302.112	tgcagggagcCcggatagctc	m5C_27109
chr6	28918830	+	C	176	38	0.216	0	0	T	138	M	0.162	0.282	0	0	0	122.768	gtcggtagagCatcagacttt	m5C_27008
chr6	28918837	+	C	140	30	0.214	0	0	T	110	M	0.154	0.289	0	0	0	92.6561	gagcatcagaCttttaatctg	m5C_27002
chr6	28918877	+	C	34	11	0.324	0	0	T	23	M	0.191	0.492	4.4E-16	3.1E-06	5.8E-16	32.3088	cctgttcgggCgggagtggtg	m5C_27067
chr6	28918889	+	C	81	45	0.556	0	0	C	45	M	0.447	0.659	0	6.4E-10	0	402.546	ggagtggtggCttttagtacc	m5C_27074
chr6	28921036	-	C	156	54	0.346	0	0	T	102	M	0.276	0.424	0	0	0	298.085	gggcagctgcCattttgtccc	m5C_27309
chr6	28921059	-	C	124	27	0.218	0	0	T	97	M	0.154	0.298	0	0	0	83.2571	gtcctgagttCgaacctcaga	m5C_27394
chr6	28921067	-	C	119	45	0.378	0	0	T	74	M	0.296	0.468	0	2.1E-14	0	266.51	atctgaaggtCctgagttcga	m5C_27294
chr6	28921075	-	C	103	39	0.379	0	0	T	64	M	0.291	0.475	0	1.3E-13	0	226.922	gtctcataatCtgaaggtcct	m5C_27255
chr6	28921083	-	C	112	26	0.232	0	0	T	86	M	0.164	0.318	0	0	0	85.0872	gcgcgtcagtCtcataatctg	m5C_27190

chr6	28921104	-	C	111	27	0.243	0	0	T	84	M	0.173	0.331	0	0	0	93.3623	gcctccttagCgcagtaggca	m5C_27291
chr6	28921112	-	C	112	26	0.232	0	0	T	86	M	0.164	0.318	0	0	0	85.0872	agttgtctgcCtccttagcgc	m5C_27351
chr6	28921113	-	C	109	25	0.229	0	0	T	84	M	0.16	0.317	0	4.4E-16	0	80.2272	tagttgtctgCctccttagcg	m5C_27301
chr6	28921116	-	C	59	16	0.271	0	0	T	43	M	0.174	0.396	0	7.3E-09	0	55.7978	cactagttgtCtgcctcctta	m5C_27379
chr6	28949438	-	C	35	22	0.629	0	0	C	22	M	0.463	0.768	0	6.3E-05	0	203.881	tgagagcgctCggttttttgt	m5C_27154
chr6	28949440	-	C	32	10	0.312	0	0	T	22	M	0.18	0.486	1.1E-14	2E-06	1.3E-14	25.0836	catgagagcgCtcggtttttt	m5C_27237
chr6	28949442	-	C	32	21	0.656	0	0	C	21	M	0.483	0.796	0	5.2E-05	0	202.905	ggcatgagagCgctcggtttt	m5C_27287
chr6	28949450	-	C	55	14	0.255	0	0	T	41	M	0.158	0.383	0	2.8E-09	0	44.2735	cgggtttcggCatgagagcgc	m5C_27211
chr6	28949453	-	C	48	13	0.271	0	0	T	35	M	0.166	0.41	0	1.2E-07	0	43.0711	tcccgggtttCggcatgagag	m5C_27276
chr6	28949460	-	C	42	15	0.357	0	0	T	27	M	0.23	0.508	0	2.8E-07	0	68.967	ggttcgatccCgggtttcggc	m5C_27223
chr6	28949461	-	C	43	13	0.302	0	0	T	30	M	0.186	0.451	0	1.2E-07	0	48.3648	tggttcgatcCcgggtttcgg	m5C_27260
chr6	28949462	-	C	46	32	0.696	0	0	C	32	M	0.552	0.809	0	1.6E-05	0	353.242	ctggttcgatCccgggtttcg	m5C_27270
chr6	28949466	-	C	35	14	0.4	0	0	T	21	M	0.256	0.564	0	9.2E-06	0	71.5414	gtccctggttCgatcccgggt	m5C_27335
chr6	28949472	-	C	32	12	0.375	0	0	T	20	M	0.229	0.547	0	4.6E-06	0	55.0408	ctaaaggtccCtggttcgatc	m5C_27199
chr6	28949473	-	C	31	11	0.355	0	0	T	20	M	0.211	0.531	1.1E-16	3.1E-06	1.5E-16	37.0595	tctaaaggtcCctggttcgat	m5C_27151
chr6	28949509	-	C	65	13	0.2	0	0	T	52	M	0.121	0.313	3.8E-15	1.6E-11	4.8E-15	22.646	cgaaatagctCagttgggaga	m5C_27243
chr6	28949511	-	C	64	14	0.219	0	0	T	50	M	0.135	0.334	1.1E-16	3.2E-11	1.5E-16	30.1591	gccgaaatagCtcagttggga	m5C_27293
chr6	28949519	-	C	71	28	0.394	0	0	T	43	M	0.289	0.511	0	2.6E-10	0	161.802	tgggcggagcCgaaatagctc	m5C_27193
chr6	28949520	-	C	56	12	0.214	0	0	T	44	M	0.127	0.338	1.3E-14	8.8E-10	1.6E-14	21.1897	gtgggcggagCcgaaatagct	m5C_27300
chr6	28949987	+	C	1308	290	0.222	0	0	T	1018	M	0.2	0.245	0	0	0	1160.19	ccctggtggtCtagtggttag	m5C_27006
chr6	28950007	+	C	1437	340	0.237	4	0.003	T	1093	M	0.216	0.26	0	0	0	1468.52	ggattcggcgCtctcaccgcc	m5C_27111
chr6	28950009	+	C	1445	311	0.215	1	0.001	T	1133	M	0.195	0.237	0	0	0	1212.53	attcggcgctCtcaccgccgc	m5C_26964
chr6	28950022	+	C	1527	333	0.218	0	0	T	1194	M	0.198	0.239	0	0	0	1319.24	accgccgcggCccgggttcga	m5C_27130
chr6	28950023	+	C	1550	328	0.212	0	0	T	1222	M	0.192	0.233	0	0	0	1259.55	ccgccgcggcCcgggttcgat	m5C_27005
chr6	28950024	+	C	1567	362	0.231	2	0.001	T	1203	M	0.211	0.253	0	0	0	1528.3	cgccgcggccCgggttcgatt	m5C_27047
chr6	28950036	+	C	1560	445	0.285	0	0	T	1115	M	0.263	0.308	0	0	0	2344.24	ggttcgattcCcggtcaggga	m5C_26989
chr6	28950037	+	C	1549	377	0.245	11	0.007	T	1161	M	0.224	0.267	0	0	0	1691.05	gttcgattccCggtcagggaa	m5C_27013
chr6	28990183	+	C	52	12	0.231	0	0	T	40	M	0.137	0.361	4.4E-15	8.8E-10	5.7E-15	23.6371	ggacagatctCttgccaggga	m5C_26948
chr6	28990196	+	C	49	17	0.347	0	0	T	32	M	0.229	0.487	0	5.6E-07	0	77.9423	gccagggaatCcaagatggtg	m5C_27134
chr6	29817934	+	C	351	77	0.219	0	0	T	274	M	0.179	0.266	0	0	0	276.04	gatgtggtgaCgtcgcgttct	m5C_27102
chr6	29817943	+	C	304	111	0.365	0	0	T	193	M	0.313	0.421	0	0	0	694.868	acgtcgcgttCtcccgggcgg	m5C_27126
chr6	29817947	+	C	233	48	0.206	0	0	T	185	M	0.159	0.263	0	0	0	152.687	cgcgttctccCgggcggggcc	m5C_27063
chr6	29817951	+	C	103	28	0.272	0	0	T	75	M	0.195	0.365	0	1.8E-15	0	109.36	ttctcccgggCggggccggag	m5C_27055
chr6	29912332	+	C	44	12	0.273	0	0	T	32	M	0.163	0.418	4.4E-16	7.5E-08	5.8E-16	30.1147	gcctggttctCcttggagctg	m5C_26983
chr6	29912340	+	C	68	32	0.471	0	0	T	36	M	0.357	0.588	0	2.7E-08	0	228.307	ctccttggagCtgtgatcact	m5C_26966
chr6	29912347	+	C	135	49	0.363	0	0	T	86	M	0.287	0.447	0	0	0	280.946	gagctgtgatCactggagctg	m5C_27034
chr6	29912362	+	C	214	67	0.313	0	0	T	147	M	0.255	0.378	0	0	0	341.309	gagctgtggtCgctgccgtga	m5C_27085
chr6	29912364	+	C	206	48	0.233	0	0	T	158	M	0.18	0.295	0	0	0	173.27	gctgtggtcgCtgccgtgatg	m5C_27039
chr6	29912367	+	C	205	74	0.361	0	0	T	131	M	0.298	0.429	0	0	0	441.548	gtggtcgctgCcgtgatgtgg	m5C_27029
chr6	29912389	+	C	112	25	0.223	0	0	T	87	M	0.156	0.309	0	0	0	78.0053	ggaggaagagCtcaggtggag	m5C_26961
chr6	30550282	+	C	54	23	0.426	0	0	T	31	M	0.303	0.558	0	8.7E-08	0	139.525	gctgaaaaaaCaggtaagacc	m5C_26238
chr6	30552124	+	C	118	34	0.288	0	0	T	84	M	0.214	0.376	0	4.4E-16	0	145.602	ggagatggcgCaggggacacg	m5C_26194
chr6	30552131	+	C	71	24	0.338	0	0	T	47	M	0.239	0.454	0	6.4E-11	0	114.647	gcgcaggggaCacgggcaaag	m5C_26207
chr6	30692115	+	C	50	14	0.28	0	0	T	36	M	0.175	0.417	0	1.9E-07	0	48.9272	tcagcagtacCaggatgccac	m5C_26213
chr6	30692123	+	C	92	36	0.391	0	0	T	56	M	0.298	0.493	0	2E-12	0	214.455	accaggatgcCaccgcagaag	m5C_26222
chr6	30692126	+	C	106	51	0.481	0	0	T	55	M	0.388	0.575	0	3.3E-12	0	396.117	aggatgccacCgcagaagagg	m5C_26199
chr6	30717491	-	C	109	50	0.459	0	0	T	59	M	0.368	0.552	0	2.6E-12	0	368.17	cctgattcagCggcggatcac	m5C_26363
chr6	30955068	+	C	34	11	0.324	0	0	T	23	M	0.191	0.492	4.4E-16	3.1E-06	5.8E-16	32.3088	ccagtgggacCagcacagcca	m5C_26218
chr6	31172131	-	C	48	18	0.375	0	0	T	30	M	0.252	0.516	0	7.7E-07	0	90.7743	aggtgcagtcCtgggaggaac	m5C_26308
chr6	31172132	-	C	49	10	0.204	0	0	T	39	M	0.115	0.336	2.1E-12	2.4E-08	2.4E-12	13.4191	caggtgcagtCctgggaggaa	m5C_26314
chr6	31238883	-	C	38	9	0.237	0	0	T	29	M	0.13	0.392	3.7E-12	1.2E-06	4.3E-12	13.3739	ccgcagatacCtggagaacgg	m5C_26409
chr6	31238892	-	C	42	9	0.214	0	0	T	33	M	0.117	0.359	1.1E-11	1.2E-08	1.3E-11	11.5454	ggagtggctcCgcagatacct	m5C_26358
chr6	31322910	-	C	94	22	0.234	0	0	T	72	M	0.16	0.329	0	5.8E-15	0	70.3741	gtggtcgctgCtgtgatgtgt	m5C_26428
chr6	31322913	-	C	81	34	0.42	0	0	T	47	M	0.318	0.528	0	4.2E-11	0	216.449	gctgtggtcgCtgctgtgatg	m5C_26435
chr6	31324566	-	C	43	9	0.209	0	0	T	34	M	0.114	0.352	1.4E-11	1.2E-08	1.6E-11	11.1522	caggaggggcCggagtattgg	m5C_26334
chr6	31324567	-	C	44	22	0.5	0	0	CT	22	M	0.358	0.642	0	2.3E-06	0	157.659	gcaggaggggCcggagtattg	m5C_26380
chr6	31324576	-	C	37	16	0.432	0	0	T	21	M	0.287	0.591	0	1.7E-05	0	91.7488	gtggatagagCaggaggggcc	m5C_26371
chr6	31324588	-	C	40	19	0.475	0	0	T	21	M	0.329	0.625	0	1E-06	0	125.154	gccgcgggcgCcgtggataga	m5C_26384
chr6	31324590	-	C	36	16	0.444	0	0	T	20	M	0.295	0.604	0	1.7E-05	0	94.5313	gagccgcgggCgccgtggata	m5C_26447
chr6	31324596	-	C	32	12	0.375	0	0	T	20	M	0.229	0.547	0	4.6E-06	0	55.0408	agagaggagcCgcgggcgccg	m5C_26366
chr6	31504152	-	C	168	80	0.476	0	0	T	88	M	0.402	0.551	0	0	0	643.275	tgactgatggCtctgggttcc	m5C_26374
chr6	31504159	-	C	277	86	0.31	0	0	T	191	M	0.259	0.367	0	0	0	445.283	tcacccatgaCtgatggctct	m5C_26345
chr6	31504164	-	C	288	74	0.257	0	0	T	214	M	0.21	0.31	0	0	0	310.667	atctatcaccCatgactgatg	m5C_26301
chr6	31504168	-	C	295	59	0.2	0	0	T	236	M	0.158	0.249	0	0	0	186.842	tcttatctatCacccatgact	m5C_26448
chr6	31504172	-	C	285	96	0.337	0	0	T	189	M	0.284	0.394	0	0	0	546.168	cgactcttatCtatcacccat	m5C_26332
chr6	31504182	-	C	266	147	0.553	0	0	C	147	M	0.493	0.611	0	0	0	1448.1	agtgcaaggaCgactcttatc	m5C_26422
chr6	31504188	-	C	266	60	0.226	0	0	T	206	M	0.179	0.279	0	0	0	215.339	cacctcagtgCaaggacgact	m5C_26331
chr6	31504198	-	C	258	52	0.202	0	0	T	206	M	0.157	0.255	0	0	0	163.427	gaaacaggagCacctcagtgc	m5C_26430
chr6	31504204	-	C	267	62	0.232	0	0	T	205	M	0.186	0.286	0	0	0	230.116	ttatttgaaaCaggagcacct	m5C_26338
chr6	31508886	-	C	361	88	0.244	0	0	T	273	M	0.202	0.291	0	0	0	356.093	tcacccttgaCtgatggctgc	m5C_26398
chr6	31508899	-	C	389	137	0.352	0	0	T	252	M	0.306	0.401	0	0	0	839.465	tgaccctcatCtatcaccctt	m5C_26400
chr6	31508906	-	C	414	107	0.258	0	0	T	307	M	0.219	0.303	0	0	0	467.888	gcagtgatgaCcctcatctat	m5C_26329
chr6	31508922	-	C	368	92	0.25	0	0	T	276	M	0.208	0.297	0	0	0	383.63	aaggtgagcgCtttgcgcagt	m5C_26385
chr6	31508924	-	C	365	91	0.249	0	0	T	274	M	0.208	0.296	0	0	0	378.01	gaaaggtgagCgctttgcgca	m5C_26425
chr6	31508935	-	C	329	87	0.264	0	0	T	242	M	0.22	0.315	0	0	0	382.273	gttttcttttCgaaaggtgag	m5C_26437
chr6	31590870	+	C	651	141	0.217	1	0.002	T	509	M	0.187	0.25	0	0	0	527.201	cctacaaaggCgtgtctgtgg	m5C_26241
chr6	31590875	+	C	653	211	0.323	0	0	T	442	M	0.288	0.36	0	0	0	1216.96	aaaggcgtgtCtgtggttccc	m5C_26261
chr6	31590883	+	C	634	152	0.24	1	0.002	T	481	M	0.208	0.275	0	0	0	633.781	gtctgtggttCcctgtctttg	m5C_26230
chr6	31590884	+	C	607	123	0.203	0	0	T	484	M	0.173	0.236	0	0	0	424.532	tctgtggttcCctgtctttgg	m5C_26262
chr6	31590885	+	C	614	167	0.272	0	0	T	447	M	0.238	0.309	0	0	0	795.881	ctgtggttccCtgtctttgga	m5C_26282
chr6	31590889	+	C	602	152	0.252	0	0	T	450	M	0.219	0.289	0	0	0	667.07	ggttccctgtCtttggacacg	m5C_26203
chr6	31590952	+	C	70	17	0.243	0	0	T	53	M	0.158	0.355	0	2.2E-12	0	53.5559	ggccagcttgCttcttgcagg	m5C_26172
chr6	31590963	+	C	37	10	0.27	0	0	T	27	M	0.154	0.43	6.7E-14	2E-06	8.3E-14	20.2815	ttcttgcaggCtcatgatcaa	m5C_26245
chr6	31590965	+	C	32	9	0.281	0	0	T	23	M	0.156	0.454	5.3E-13	1.2E-06	6.4E-13	17.1983	cttgcaggctCatgatcaacc	m5C_26271
chr6	31590975	+	C	36	10	0.278	0	0	T	26	M	0.158	0.44	4.8E-14	2E-06	5.9E-14	21.1115	catgatcaacCaatctcacat	m5C_26209
chr6	31694924	-	C	44	9	0.205	0	0	T	35	M	0.112	0.345	1.8E-11	1.2E-08	2.1E-11	10.7806	gctgaataggCagtagttggg	m5C_26390
chr6	31694933	-	C	47	29	0.617	0	0	C	29	M	0.474	0.742	0	9.7E-06	0	275.073	atagagaatgCtgaataggca	m5C_26294
chr6	31746653	-	C	34	7	0.206	0	0	T	27	M	0.103	0.368	1.4E-09	3.4E-07	1.6E-09	6.4054	gtgttgacaaCtaattgatcg	m5C_26313
chr6	31746674	-	C	57	16	0.281	0	0	T	41	M	0.181	0.408	0	7.3E-09	0	57.8536	tgggctggtcCgagtgcagtg	m5C_26352
chr6	31746675	-	C	53	24	0.453	0	0	T	29	M	0.327	0.585	0	1.2E-07	0	156.755	ctgggctggtCcgagtgcagt	m5C_26438
chr6	31803052	+	C	533	110	0.206	0	0	T	423	M	0.174	0.243	0	0	0	383.197	tgatgatgacCccaggtaact	m5C_26174
chr6	31803074	+	C	530	138	0.26	0	0	T	392	M	0.225	0.299	0	0	0	620.544	ttgagtgtgtCgctgatgcca	m5C_26220
chr6	31804873	+	C	1220	253	0.208	3	0.002	T	964	M	0.186	0.232	0	0	0	941.29	atttcacagaCtagagtctcc	m5C_26221
chr6	31804880	+	C	1183	256	0.217	1	0.001	T	926	M	0.194	0.241	0	0	0	993.471	agactagagtCtccgatgctg	m5C_26186
chr6	31804882	+	C	1174	344	0.293	0	0	T	830	M	0.268	0.32	0	0	0	1841.7	actagagtctCcgatgctggt	m5C_26175
chr6	31804901	+	C	1078	224	0.208	2	0.002	T	852	M	0.185	0.233	0	0	0	828.702	gtcatgatgtCaaaactaagt	m5C_26265
chr6	31804906	+	C	1050	243	0.231	0	0	T	807	M	0.207	0.258	0	0	0	1005.66	gatgtcaaaaCtaagttctga	m5C_26191
chr6	31805141	+	C	39	17	0.447	1	0.026	T	21	M	0.301	0.603	0	2.1E-05	0	102.497	catgcttagaCagtgattcct	m5C_26254
chr6	31805149	+	C	50	20	0.4	0	0	T	30	M	0.276	0.538	0	1.4E-06	0	110.433	gacagtgattCctgcaacttg	m5C_26257
chr6	31805150	+	C	47	17	0.362	0	0	T	30	M	0.24	0.505	0	5.6E-07	0	81.4845	acagtgattcCtgcaacttga	m5C_26249
chr6	31805161	+	C	58	19	0.328	0	0	T	39	M	0.221	0.456	0	2.4E-08	0	83.9159	tgcaacttgaCcttcaggctg	m5C_26244

chr6	31805162	+	C	52	19	0.365	0	0	T	33	M	0.248	0.501	0	2.4E-08	0	94.242	gcaacttgacCttcaggctgg	m5C_26181
chr6	31805169	+	C	31	11	0.355	0	0	T	20	M	0.211	0.531	1.1E-16	3.1E-06	1.5E-16	37.0595	gaccttcaggCtgggagaggt	m5C_26211
chr6	31805259	+	C	137	30	0.219	0	0	T	107	M	0.158	0.295	0	0	0	94.7485	taatgatgatCtggttggaca	m5C_26178
chr6	31805275	+	C	134	27	0.201	0	0	T	107	M	0.142	0.277	0	0	0	76.8604	ggacaagagtCtctgagcttt	m5C_26285
chr6	31805289	+	C	131	44	0.336	0	0	T	87	M	0.261	0.42	0	0	0	229.414	gagcttttctCtgaggatctt	m5C_26225
chr6	31805297	+	C	116	34	0.293	0	0	T	82	M	0.218	0.382	0	4.4E-16	0	148.219	ctctgaggatCtttgaaccca	m5C_26180
chr6	31805306	+	C	101	26	0.257	0	0	T	75	M	0.182	0.35	0	6.7E-16	0	94.7186	tctttgaaccCacctgatcca	m5C_26255
chr6	31922398	-	C	36	10	0.278	0	0	T	26	M	0.158	0.44	4.8E-14	2E-06	5.9E-14	21.1115	ggatcgagatCgagaccggga	m5C_26311
chr6	31922428	-	C	31	18	0.581	0	0	C	18	M	0.408	0.736	0	2.7E-05	0	146.757	agacagagagCgggacaggga	m5C_26408
chr6	31922441	-	C	32	17	0.531	0	0	C	17	M	0.364	0.691	0	2.1E-05	0	123.927	gggatcgagaCagagacagag	m5C_26339
chr6	31922446	-	C	33	19	0.576	0	0	C	19	M	0.408	0.728	0	3.4E-05	0	155.067	agaccgggatCgagacagaga	m5C_26347
chr6	31922476	-	C	53	22	0.415	0	0	T	31	M	0.293	0.549	0	6.5E-08	0	128.747	ggacagagacCgagatcggga	m5C_26396
chr6	31922477	-	C	48	14	0.292	0	0	T	34	M	0.182	0.432	0	1.9E-07	0	51.076	gggacagagaCcgagatcggg	m5C_26344
chr6	31922483	-	C	40	17	0.425	0	0	T	23	M	0.285	0.578	0	5.6E-07	0	96.9312	ggaaccgggaCagagaccgag	m5C_26357
chr6	31922488	-	C	47	10	0.213	0	0	T	37	M	0.12	0.349	1.3E-12	2.4E-08	1.5E-12	14.271	tgagaggaacCgggacagaga	m5C_26349
chr6	31922489	-	C	45	17	0.378	0	0	T	28	M	0.251	0.524	0	5.6E-07	0	85.3668	atgagaggaaCcgggacagag	m5C_26419
chr6	31922632	-	C	39	29	0.744	0	0	C	29	M	0.589	0.854	0	0.00019	0	341.725	agaactaggaCcagatggaga	m5C_26323
chr6	31922638	-	C	35	19	0.543	0	0	C	19	M	0.382	0.695	0	3.4E-05	0	145.12	acttcgagaaCtaggaccaga	m5C_26423
chr6	31922644	-	C	37	17	0.459	0	0	T	20	M	0.31	0.616	0	2.1E-05	0	105.53	tgatcgacttCgagaactagg	m5C_26328
chr6	31924709	-	C	35	8	0.229	0	0	T	27	M	0.121	0.39	5.6E-11	6.8E-07	6.4E-11	9.89691	ctgtgagtgaCaggggaaatg	m5C_26405
chr6	32148244	+	C	40	21	0.525	0	0	C	21	M	0.375	0.671	0	5.2E-05	0	157.488	ccaggaaggcCtggggaggag	m5C_22142
chr6	32148269	+	C	33	12	0.364	0	0	T	21	M	0.222	0.534	0	4.6E-06	0	53.2487	gaagtctgtgCatagatggga	m5C_22122
chr6	33171842	+	C	42	17	0.405	0	0	T	25	M	0.27	0.555	0	5.6E-07	0	91.945	ctaaatggggCcatgaagaag	m5C_22117
chr6	33171843	+	C	41	13	0.317	0	0	T	28	M	0.196	0.47	0	1.2E-07	0	50.8675	taaatggggcCatgaagaagg	m5C_22145
chr6	33243748	+	C	37	13	0.351	0	0	T	24	M	0.218	0.512	0	6.7E-06	0	56.7461	ccaggtggaaCgtgtgatcac	m5C_22136
chr6	33243756	+	C	33	16	0.485	0	0	T	17	M	0.325	0.648	0	1.7E-05	0	104.012	aacgtgtgatCaccattatgc	m5C_22133
chr6	33243758	+	C	37	11	0.297	0	0	T	26	M	0.175	0.458	1.4E-15	3.1E-06	1.9E-15	28.5508	cgtgtgatcaCcattatgcag	m5C_22108
chr6	33243759	+	C	35	18	0.514	0	0	C	18	M	0.356	0.67	0	2.7E-05	0	128.047	gtgtgatcacCattatgcaga	m5C_22113
chr6	34231421	-	C	33	7	0.212	0	0	T	26	M	0.107	0.378	1.1E-09	3.4E-07	1.2E-09	6.69165	gagatcttcgCgggaagaagg	m5C_22244
chr6	34231434	-	C	34	8	0.25	2	0.059	T	24	M	0.133	0.421	2.3E-11	6.8E-07	2.6E-11	11.2828	aagatcaaggCtcgagatctt	m5C_22252
chr6	35920666	+	C	36	23	0.639	0	0	C	23	M	0.476	0.775	0	7.6E-05	0	218.844	tggtgtgcgcCtgtagtccca	m5C_22224
chr6	35920673	+	C	34	10	0.294	0	0	T	24	M	0.168	0.462	2.3E-14	2E-06	2.9E-14	22.9564	cgcctgtagtCccagctactc	m5C_22219
chr6	35920674	+	C	33	9	0.273	0	0	T	24	M	0.151	0.442	7.5E-13	1.2E-06	9E-13	16.4418	gcctgtagtcCcagctactca	m5C_22222
chr6	35920675	+	C	36	9	0.25	0	0	T	27	M	0.138	0.411	2E-12	1.2E-06	2.4E-12	14.4765	cctgtagtccCagctactcag	m5C_22212
chr6	36564715	+	C	39	10	0.256	0	0	T	29	M	0.146	0.411	1.3E-13	2E-06	1.6E-13	18.7753	ccccgagatgCagctgatgca	m5C_22294
chr6	36564729	+	C	64	17	0.266	0	0	T	47	M	0.173	0.385	0	1.8E-10	0	58.8072	tgatgcagtcCgagagctaga	m5C_22296
chr6	36564735	+	C	60	27	0.45	0	0	T	33	M	0.331	0.575	0	2.5E-07	0	178.705	agtccgagagCtagatggaag	m5C_22284
chr6	37880354	+	C	44	22	0.5	0	0	CT	22	M	0.358	0.642	0	2.3E-06	0	157.659	tgtagttggtCcaagtgttgt	m5C_22268
chr6	39926563	-	C	31	8	0.258	0	0	T	23	M	0.137	0.432	1.7E-11	6.8E-07	1.9E-11	11.8182	gatgttgaagCgaggacgtgg	m5C_22426
chr6	41986159	-	C	31	12	0.387	0	0	T	19	M	0.237	0.562	0	4.6E-06	0	56.9589	attcgtgaagCgttccgtatt	m5C_22462
chr6	41986185	-	C	32	13	0.406	0	0	T	19	M	0.255	0.577	0	6.7E-06	0	66.3505	ggcccctgcgCagggatgaca	m5C_22452
chr6	42074288	+	C	60	14	0.233	0	0	T	46	M	0.144	0.354	0	3.2E-11	0	40.4304	gtgctgaagaCgctgtgttct	m5C_22742
chr6	42074297	+	C	65	38	0.585	0	0	C	38	M	0.463	0.696	0	9.6E-08	0	352.175	acgctgtgttCtctgggctgg	m5C_22705
chr6	42529285	-	C	116	33	0.284	0	0	T	83	M	0.21	0.372	0	2.2E-16	0	138.811	tagtggtgagCatagttacct	m5C_22758
chr6	42981006	-	C	49	13	0.265	0	0	T	36	M	0.162	0.403	0	1.2E-07	0	42.1489	gggattggagCagtgaggagc	m5C_22802
chr6	43701643	-	C	41	14	0.341	0	0	T	27	M	0.216	0.495	0	1.9E-07	0	60.3641	ggaagcttgtCtaatctttcc	m5C_22800
chr6	43720662	+	C	42	27	0.643	0	0	C	27	M	0.492	0.77	0	6.7E-06	0	265.497	gggagaccacCttggaatact	m5C_22723
chr6	43720671	+	C	65	16	0.246	0	0	T	49	M	0.158	0.363	0	1.1E-10	0	50.4251	ccttggaataCtgggtgctgt	m5C_22719
chr6	43720678	+	C	64	28	0.438	0	0	T	36	M	0.323	0.559	0	9.8E-09	0	180.845	atactgggtgCtgtaggcttt	m5C_22707
chr6	43720685	+	C	50	16	0.32	0	0	T	34	M	0.208	0.458	0	7.3E-09	0	66.4257	gtgctgtaggCttttggcctt	m5C_22706
chr6	43738717	+	C	35	11	0.314	0	0	T	24	M	0.186	0.48	6.7E-16	3.1E-06	8.7E-16	30.9686	ggagccgcagCcggaggaggg	m5C_22747
chr6	43738718	+	C	55	30	0.545	0	0	C	30	M	0.415	0.67	0	4.8E-07	0	249.135	gagccgcagcCggaggagggg	m5C_22718
chr6	44218068	+	C	671	164	0.247	8	0.012	T	499	M	0.216	0.282	0	0	0	708.592	gatgatgaggCagaggaagag	m5C_22547
chr6	44219797	+	C	38	18	0.474	0	0	T	20	M	0.325	0.627	0	2.7E-05	0	116.924	aacggggcttCgaggtggtat	m5C_22523
chr6	44219814	+	C	32	9	0.281	0	0	T	23	M	0.156	0.454	5.3E-13	1.2E-06	6.4E-13	17.1983	gtatatatgaCcgagcccatt	m5C_22529
chr6	44219821	+	C	45	20	0.444	0	0	T	25	M	0.309	0.588	0	1.4E-06	0	123.755	tgaccgagccCattgacgagt	m5C_22521
chr6	44219848	+	C	43	13	0.302	0	0	T	30	M	0.186	0.451	0	1.2E-07	0	48.3648	tgcagcagctCaaggaatttg	m5C_22516
chr6	44219870	+	C	107	26	0.243	0	0	T	81	M	0.172	0.332	0	6.7E-16	0	89.2099	tgggaagagcCtggtctcagt	m5C_22552
chr6	44219875	+	C	132	34	0.258	0	0	T	98	M	0.191	0.338	0	0	0	129.59	agagcctggtCtcagttacca	m5C_22539
chr6	44219877	+	C	150	40	0.267	0	0	T	110	M	0.202	0.343	0	0	0	161.896	agcctggtctCagttaccaag	m5C_22525
chr6	44219883	+	C	224	52	0.232	0	0	T	172	M	0.182	0.292	0	0	0	188.917	gtctcagttaCcaaggaggt	m5C_22553
chr6	44219884	+	C	254	70	0.276	0	0	T	184	M	0.224	0.334	0	0	0	314.01	tctcagttacCaaggagggtc	m5C_22524
chr6	44219894	+	C	496	126	0.254	0	0	T	370	M	0.218	0.294	0	0	0	548.636	caaggagggtCtggagctgcc	m5C_22533
chr6	44219900	+	C	591	155	0.263	1	0.002	T	435	M	0.229	0.3	0	0	0	709.328	gggtctggagCtgcctgagga	m5C_22538
chr6	44219904	+	C	699	165	0.236	0	0	T	534	M	0.206	0.269	0	0	0	680.017	ctggagctgcCtgaggatgag	m5C_22520
chr6	44221250	+	C	33	8	0.242	0	0	T	25	M	0.128	0.41	3.1E-11	6.8E-07	3.6E-11	10.7866	gaagtggcagCagaggaaccc	m5C_22526
chr6	44221265	+	C	33	9	0.273	0	0	T	24	M	0.151	0.442	7.5E-13	1.2E-06	9E-13	16.4418	gaacccaatgCtgcagttcct	m5C_22528
chr6	52129198	-	C	87	31	0.356	0	0	T	56	M	0.264	0.461	0	1.7E-11	0	163.51	tgaaagctgcCatggggtgag	m5C_22892
chr6	52132676	-	C	469	97	0.207	0	0	T	372	M	0.173	0.246	0	0	0	334.882	ggaccaggagCagaagaggaa	m5C_22893
chr6	52132683	-	C	493	109	0.222	1	0.002	T	383	M	0.187	0.26	0	0	0	407.845	gccaagaggaCcaggagcaga	m5C_22900
chr6	52132691	-	C	439	122	0.278	0	0	T	317	M	0.238	0.322	0	0	0	580.875	ggagaaaagcCaagaggacca	m5C_22887
chr6	52132714	-	C	285	79	0.285	8	0.028	T	198	M	0.235	0.341	0	0	0	371.692	gaatcagagaCagaagatgaa	m5C_22898
chr6	52132736	-	C	155	51	0.329	0	0	T	104	M	0.26	0.406	0	0	0	265.179	acgtaagaagCgaagtgagga	m5C_22895
chr6	52833838	+	C	36	12	0.333	0	0	T	24	M	0.202	0.497	0	4.6E-06	0	48.5146	aaaaaaaaagCccgcagctgg	m5C_22867
chr6	52833840	+	C	37	14	0.378	0	0	T	23	M	0.241	0.539	0	9.2E-06	0	67.3786	aaaaaaagccCgcagctgggc	m5C_22839
chr6	52860641	+	C	43	12	0.279	0	0	T	31	M	0.167	0.427	2.2E-16	7.5E-08	2.9E-16	31.457	gctagaacctCcaaacaagct	m5C_22846
chr6	52860642	+	C	40	14	0.35	0	0	T	26	M	0.221	0.505	0	1.9E-07	0	61.9761	ctagaacctcCaaacaagctc	m5C_22864
chr6	52860646	+	C	48	19	0.396	0	0	T	29	M	0.27	0.537	0	1E-06	0	102.678	aacctccaaaCaagctctcaa	m5C_22836
chr6	52860650	+	C	108	34	0.315	0	0	T	74	M	0.235	0.407	0	2.3E-14	0	159.708	tccaaacaagCtctcaaggtc	m5C_22833
chr6	52860652	+	C	120	37	0.308	0	0	T	83	M	0.233	0.396	0	0	0	172.217	caaacaagctCtcaaggtcca	m5C_22872
chr6	52860654	+	C	134	43	0.321	0	0	T	91	M	0.248	0.404	0	0	0	213.104	aacaagctctCaaggtccatt	m5C_22840
chr6	52860660	+	C	220	74	0.336	0	0	T	146	M	0.277	0.401	0	0	0	410.276	ctctcaaggtCcatttgtagg	m5C_22871
chr6	52860661	+	C	248	78	0.316	1	0.004	T	169	M	0.261	0.376	0	0	0	407.187	tctcaaggtcCatttgtagga	m5C_22859
chr6	52860675	+	C	525	136	0.259	0	0	T	389	M	0.223	0.298	0	0	0	607.693	tgtaggagaaCgtagggtagt	m5C_22862
chr6	52860686	+	C	652	212	0.325	0	0	T	440	M	0.29	0.362	0	0	0	1230.92	gtagggtagtCaagcttccaa	m5C_22855
chr6	52860690	+	C	737	176	0.24	3	0.004	T	558	M	0.21	0.272	0	0	0	740.256	ggtagtcaagCttccaagact	m5C_22844
chr6	52860693	+	C	725	228	0.314	0	0	T	497	M	0.282	0.349	0	0	0	1284.72	agtcaagcttCcaagactcca	m5C_22866
chr6	52860694	+	C	737	211	0.287	1	0.001	T	525	M	0.255	0.32	0	0	0	1076.89	gtcaagcttcCaagactccag	m5C_22843
chr6	52860699	+	C	773	211	0.273	1	0.001	T	561	M	0.243	0.306	0	0	0	1025.71	gcttccaagaCtccagacaca	m5C_22875
chr6	52860701	+	C	746	279	0.376	3	0.004	T	464	M	0.341	0.411	0	0	0	1905.06	ttccaagactCcagacacatc	m5C_22856
chr6	52860702	+	C	753	218	0.29	0	0	T	535	M	0.258	0.323	0	0	0	1125.96	tccaagactcCagacacatcc	m5C_22868
chr6	52860706	+	C	861	229	0.269	10	0.012	T	622	M	0.24	0.3	0	0	0	1100.96	agactccagaCacatccaaat	m5C_22876
chr6	52860708	+	C	899	188	0.209	1	0.001	T	710	M	0.184	0.237	0	0	0	691.877	actccagacaCatccaaatga	m5C_22853
chr6	52860711	+	C	998	266	0.267	0	0	T	732	M	0.24	0.295	0	0	0	1276.98	ccagacacatCcaaatgaggc	m5C_22873
chr6	52860712	+	C	1068	225	0.211	0	0	T	843	M	0.187	0.236	0	0	0	842.742	cagacacatcCaaatgaggcg	m5C_22850
chr6	52860721	+	C	1524	384	0.252	1	0.001	T	1139	M	0.231	0.275	0	0	0	1773.83	ccaaatgaggCgctgcatgtg	m5C_22870
chr6	52860726	+	C	1623	387	0.238	0	0	T	1236	M	0.218	0.26	0	0	0	1690.01	tgaggcgctgCatgtggcagt	m5C_22845
chr6	52860737	+	C	1608	516	0.321	1	0.001	T	1091	M	0.299	0.344	0	0	0	3082.76	atgtggcagtCtgcctttctt	m5C_22851
chr6	52860741	+	C	1478	426	0.288	0	0	T	1052	M	0.266	0.312	0	0	0	2263.83	ggcagtctgcCtttcttttga	m5C_22842
chr6	52860745	+	C	1343	410	0.305	0	0	T	933	M	0.281	0.33	0	0	0	2306.17	gtctgcctttCttttgaccca	m5C_22849
chr6	56336875	-	C	32	16	0.5	0	0	CT	16	M	0.336	0.664	0	1.7E-05	0	107.618	tcaatgatgcCttggacagac	m5C_22666

chr6	56969703	+	C	54	12	0.222	0	0	T	42	M	0.132	0.349	7.5E-15	8.8E-10	9.6E-15	22.3614	aaggatttgaCcgtttaatag	m5C_22648
chr6	56969723	+	C	85	31	0.365	0	0	T	54	M	0.27	0.471	0	1.7E-11	0	167.581	gggatggggaCagaatgcaga	m5C_22647
chr6	56969730	+	C	84	35	0.417	0	0	T	49	M	0.317	0.523	0	5.6E-11	0	222.003	ggacagaatgCagatcaggaa	m5C_22650
chr6	64423340	+	C	49	16	0.327	0	0	T	33	M	0.212	0.466	0	4E-07	0	67.8634	gagacagaagCcaagacaagg	m5C_22807
chr6	64423341	+	C	51	14	0.275	0	0	T	37	M	0.171	0.409	0	2.8E-09	0	47.9195	agacagaagcCaagacaagga	m5C_22809
chr6	64423352	+	C	81	19	0.235	0	0	T	62	M	0.156	0.338	0	8.4E-14	0	59.1371	aagacaaggaCagagacagaa	m5C_22805
chr6	64423367	+	C	75	24	0.32	0	0	T	51	M	0.225	0.432	0	6.4E-11	0	108.206	acagaaaaagCagggaggaag	m5C_22803
chr6	69375061	-	C	39	8	0.205	0	0	T	31	M	0.108	0.355	1.6E-10	6.8E-07	1.8E-10	8.44384	caccgagaacCtacaaaatac	m5C_22697
chr6	69375062	-	C	52	21	0.404	0	0	T	31	M	0.282	0.539	0	4.8E-08	0	118.27	gcaccgagaaCctacaaaata	m5C_22699
chr6	69375080	-	C	59	18	0.305	0	0	T	41	M	0.203	0.431	0	1.7E-08	0	72.9125	tttctctgaaCgtgtagagca	m5C_22700
chr6	70183134	-	C	57	23	0.404	0	0	T	34	M	0.286	0.533	0	8.7E-08	0	131.64	ccggagaagcCagctggagcc	m5C_23189
chr6	70183135	-	C	54	26	0.481	0	0	T	28	M	0.354	0.611	0	1.9E-07	0	184.048	cccggagaagCcagctggagc	m5C_23179
chr6	70183143	-	C	61	31	0.508	0	0	C	31	M	0.386	0.629	0	2.1E-08	0	239.335	tgactgatccCggagaagcca	m5C_23176
chr6	70183144	-	C	60	37	0.617	0	0	C	37	M	0.49	0.729	0	7.9E-08	0	362.729	gtgactgatcCcggagaagcc	m5C_23185
chr6	70183145	-	C	61	15	0.246	0	0	T	46	M	0.155	0.367	0	6E-11	0	46.5196	cgtgactgatCccggagaagc	m5C_23191
chr6	70183150	-	C	58	17	0.293	0	0	T	41	M	0.192	0.42	0	1.1E-08	0	65.2053	ggtaacgtgaCtgatcccgga	m5C_23174
chr6	70183155	-	C	53	19	0.358	0	0	T	34	M	0.243	0.493	0	2.4E-08	0	92.3467	agtgtggtaaCgtgactgatc	m5C_23175
chr6	72086668	-	C	41	19	0.463	0	0	T	22	M	0.321	0.613	0	1E-06	0	121.813	gctgtttactCtttctgcctt	m5C_22973
chr6	72086677	-	C	60	17	0.283	0	0	T	43	M	0.185	0.408	0	1.8E-10	0	62.9227	tgggagaaggCtgtttactct	m5C_22971
chr6	72086688	-	C	43	18	0.419	0	0	T	25	M	0.284	0.567	0	7.7E-07	0	102.184	agtaagaaagCtgggagaagg	m5C_22974
chr6	72113257	-	C	35	7	0.2	0	0	T	28	M	0.1	0.359	1.9E-09	3.4E-07	2.1E-09	6.13884	atgtttgcagCtgcctactgc	m5C_22969
chr6	72113260	-	C	41	9	0.22	0	0	T	32	M	0.12	0.367	8.4E-12	1.2E-08	9.9E-12	11.9621	cggatgtttgCagctgcctac	m5C_22987
chr6	72113270	-	C	53	23	0.434	0	0	T	30	M	0.31	0.567	0	8.7E-08	0	142.371	ggctttcagtCggatgtttgc	m5C_22982
chr6	72113278	-	C	34	8	0.235	0	0	T	26	M	0.124	0.4	4.2E-11	6.8E-07	4.8E-11	10.3259	cacagatgggCtttcagtcgg	m5C_22975
chr6	72126249	-	C	31	10	0.323	0	0	T	21	M	0.186	0.499	7.1E-15	2E-06	9E-15	26.2725	tggagagttgCtgtagaagaa	m5C_22985
chr6	73549645	+	C	83	66	0.795	0	0	C	66	M	0.696	0.868	0	2.2E-08	0	919.041	atgggtggttCggtggtagaa	m5C_22958
chr6	75851866	-	C	35	13	0.371	0	0	T	22	M	0.232	0.537	0	6.7E-06	0	60.2306	tagaggtggaCagatcagaga	m5C_23041
chr6	75852974	-	C	37	16	0.432	0	0	T	21	M	0.287	0.591	0	1.7E-05	0	91.7488	aagaggatgtCaaagaggtag	m5C_23040
chr6	75950972	-	C	33	11	0.333	0	0	T	22	M	0.198	0.504	2.2E-16	3.1E-06	2.9E-16	34.0074	ggctcttcgtCagattgggca	m5C_23038
chr6	75953435	-	C	32	20	0.625	0	0	C	20	M	0.453	0.771	0	4.3E-05	0	181.016	gcggaatctgCtggtgaggag	m5C_23054
chr6	75963007	-	C	31	9	0.29	0	0	T	22	M	0.161	0.466	3.7E-13	1.2E-06	4.4E-13	18.0154	gtggggtacaCaggaaaaacc	m5C_23036
chr6	76183456	-	C	140	30	0.214	0	0	T	110	M	0.154	0.289	0	0	0	92.6561	gtgctttcccCttaaaaaaag	m5C_23149
chr6	76183459	-	C	166	37	0.223	0	0	T	129	M	0.166	0.292	0	0	0	123.028	ttcgtgctttCcccttaaaaa	m5C_23143
chr6	76183463	-	C	173	40	0.231	0	0	T	133	M	0.175	0.299	0	0	0	139.711	tgcgttcgtgCtttccccctta	m5C_23145
chr6	76183467	-	C	181	42	0.235	2	0.011	T	137	M	0.179	0.302	0	0	0	149.969	ggtctgcgttCgtgctttccc	m5C_23153
chr6	76183474	-	C	188	58	0.309	0	0	T	130	M	0.247	0.378	0	0	0	286.372	tagtgggggtCtgcgttcgtg	m5C_23152
chr6	76183496	-	C	40	9	0.225	0	0	T	31	M	0.123	0.375	6.4E-12	1.2E-08	7.6E-12	12.4041	aactgagttgCataatttgtg	m5C_23146
chr6	76183517	-	C	188	47	0.25	0	0	T	141	M	0.194	0.316	0	0	0	181.915	gtggtttcccCaaatgtggga	m5C_23133
chr6	76183519	-	C	189	41	0.217	0	0	T	148	M	0.164	0.281	0	0	0	134.583	ctgtggtttcCcccaaatgtgg	m5C_23132
chr6	76183547	-	C	91	34	0.374	0	0	T	57	M	0.281	0.476	0	1.1E-12	0	191.249	atccattgcaCtctggatgtg	m5C_23139
chr6	76183554	-	C	34	12	0.353	0	0	T	22	M	0.215	0.521	0	4.6E-06	0	51.5705	gaggctcatcCattgcactct	m5C_23154
chr6	79912052	-	C	56	13	0.232	0	0	T	43	M	0.141	0.358	4.4E-16	1.6E-09	5.8E-16	28.1397	ggaggaaaagCaggaagctgg	m5C_23081
chr6	82188158	-	C	42	15	0.357	0	0	T	27	M	0.23	0.508	0	2.8E-07	0	68.967	tctgagtcgtCtggacatttc	m5C_23309
chr6	82188161	-	C	41	25	0.61	0	0	C	25	M	0.457	0.743	0	4.5E-06	0	228.633	aggtctgagtCgtctggacat	m5C_23308
chr6	86387008	-	C	76	22	0.289	0	0	T	54	M	0.2	0.4	0	2.8E-11	0	87.8279	ctgagatggaCcgttataagg	m5C_24556
chr6	86387018	-	C	1865	422	0.226	0	0	T	1443	M	0.208	0.246	0	0	0	1754.31	gatctggcttCtgagatggac	m5C_24543
chr6	86387021	-	C	2084	482	0.232	4	0.002	T	1598	M	0.214	0.25	0	0	0	2063.94	acggatctggCttctgagatg	m5C_24553
chr6	86387025	-	C	2138	442	0.207	2	0.001	T	1694	M	0.19	0.225	0	0	0	1682.1	ttttacggatCtggcttctga	m5C_24550
chr6	86387053	-	C	2063	498	0.241	0	0	T	1565	M	0.223	0.26	0	0	0	2225.28	acctgaacttCtgttgaaaaa	m5C_24515
chr6	86387061	-	C	2083	434	0.208	0	0	T	1649	M	0.191	0.226	0	0	0	1661.84	tatcccgaacCtgaacttctg	m5C_24557
chr6	86387062	-	C	2067	568	0.275	4	0.002	T	1495	M	0.256	0.295	0	0	0	2913.65	ttatcccgaaCctgaacttct	m5C_24514
chr6	86387073	-	C	2004	482	0.241	0	0	T	1522	M	0.222	0.26	0	0	0	2143.11	ctgtgatgatCttatcccgaa	m5C_24542
chr6	86387313	-	C	670	140	0.209	0	0	T	530	M	0.18	0.241	0	0	0	503.642	gattcggcttCtgagattaag	m5C_24516
chr6	86387316	-	C	762	179	0.235	0	0	T	583	M	0.206	0.266	0	0	0	738.134	acggattcggCttctgagatt	m5C_24523
chr6	86387325	-	C	776	177	0.228	0	0	T	599	M	0.2	0.259	0	0	0	707.828	aaaataagtaCggattcggct	m5C_24547
chr6	86387342	-	C	734	154	0.21	0	0	T	580	M	0.182	0.241	0	0	0	560.256	agctgataacCtgaagaaaaa	m5C_24528
chr6	86387350	-	C	708	142	0.201	0	0	T	566	M	0.173	0.232	0	0	0	490.526	tatcccgaagCtgataacctg	m5C_24538
chr6	86387357	-	C	673	156	0.232	2	0.003	T	515	M	0.202	0.266	0	0	0	630.565	tgaaacctatCccgaagctga	m5C_24555
chr6	88555552	+	C	100	29	0.29	0	0	T	71	M	0.21	0.385	0	2.9E-15	0	121.885	gaggggtgaaCcggcccgggt	m5C_23371
chr6	88555553	+	C	101	45	0.446	0	0	T	56	M	0.352	0.543	0	7.4E-13	0	317.14	aggggtgaacCggcccgggtt	m5C_23367
chr6	88555557	+	C	104	33	0.317	0	0	T	71	M	0.236	0.412	0	1.6E-14	0	155.582	gtgaaccggcCcgggttggaa	m5C_23372
chr6	88555569	+	C	72	18	0.254	1	0.014	T	53	M	0.167	0.366	0	4E-12	0	60.0495	gggttggaaaCggagcaggtc	m5C_23369
chr6	88555574	+	C	43	13	0.302	0	0	T	30	M	0.186	0.451	0	1.2E-07	0	48.3648	ggaaacggagCaggtcaaaac	m5C_23363
chr6	89773278	-	C	46	22	0.478	0	0	T	24	M	0.341	0.619	0	2.3E-06	0	150.148	taggagcatgCtccatccact	m5C_23391
chr6	89773282	-	C	445	161	0.362	0	0	T	284	M	0.319	0.407	0	0	0	1025.6	ggaataggagCatgctccatc	m5C_23382
chr6	89773301	-	C	1898	402	0.212	0	0	T	1496	M	0.194	0.231	0	0	0	1559.85	atttttggagCagggagatgg	m5C_23380
chr6	89773326	-	C	965	215	0.226	12	0.012	T	738	M	0.2	0.253	0	0	0	860.85	tgtctgaggaCaatatattaa	m5C_23381
chr6	89773333	-	C	402	86	0.214	0	0	T	316	M	0.177	0.257	0	0	0	303.836	cgtcctttgtCtgaggacaat	m5C_23377
chr6	89773343	-	C	143	31	0.217	0	0	T	112	M	0.157	0.291	0	0	0	97.4233	atatctgataCgtcctttgtc	m5C_23374
chr6	89773349	-	C	89	24	0.27	0	0	T	65	M	0.188	0.37	0	1.1E-12	0	90.4412	agtttaatatCtgatacgtcc	m5C_23389
chr6	97362789	-	C	92	32	0.348	0	0	T	60	M	0.258	0.449	0	5.3E-13	0	165.361	gggttctgggCtgtagtgtgc	m5C_23245
chr6	100060611	+	C	85	29	0.341	0	0	T	56	M	0.249	0.447	0	8.4E-12	0	144.546	gccagcctgcCtgagtgctgc	m5C_23474
chr6	105586839	+	C	32	16	0.5	0	0	CT	16	M	0.336	0.664	0	1.7E-05	0	107.618	ggtttgaatcCcccctctcct	m5C_23343
chr6	106868588	+	C	45	9	0.2	0	0	T	36	M	0.109	0.338	2.3E-11	1.2E-08	2.7E-11	10.4289	tctggctggtCtgagtgcagt	m5C_23483
chr6	106868607	+	C	43	19	0.442	0	0	T	24	M	0.304	0.589	0	1E-06	0	115.646	gtggtgtttaCaactaattga	m5C_23489
chr6	106902760	+	C	230	66	0.287	0	0	T	164	M	0.232	0.349	0	0	0	306.733	ttacagatttCtttgttcctt	m5C_23490
chr6	106902767	+	C	187	50	0.269	1	0.005	T	136	M	0.21	0.337	0	0	0	210.255	tttctttgttCcttctccact	m5C_23497
chr6	106902768	+	C	180	43	0.239	0	0	T	137	M	0.182	0.306	0	0	0	156.92	ttctttgttcCttctccactc	m5C_23506
chr6	106902773	+	C	165	47	0.285	0	0	T	118	M	0.221	0.358	0	0	0	208.198	tgttccttctCcactcccact	m5C_23505
chr6	106902800	+	C	66	29	0.439	0	0	T	37	M	0.326	0.559	0	1.3E-08	0	189.242	cttgactagcCttaagaaaaa	m5C_23491
chr6	107280236	-	C	31	10	0.323	0	0	T	21	M	0.186	0.499	7.1E-15	2E-06	9E-15	26.2725	gtagtgtgtgCagtgtctcag	m5C_23509
chr6	108306951	-	C	47	11	0.234	0	0	T	36	M	0.136	0.372	3.8E-14	4.4E-08	4.7E-14	20.0856	tcactctcccCaggcgctgtc	m5C_23731
chr6	108306952	-	C	54	13	0.241	0	0	T	41	M	0.146	0.369	2.2E-16	1.6E-09	2.9E-16	29.8003	gtcactctccCcaggcgctgt	m5C_23728
chr6	108306954	-	C	70	15	0.214	0	0	T	55	M	0.134	0.324	0	6E-13	0	40.3158	aggtcactctCcccaggcgct	m5C_23713
chr6	108306956	-	C	69	23	0.333	0	0	T	46	M	0.234	0.451	0	2.1E-09	0	107.414	ggaggtcactCtccccaggcg	m5C_23746
chr6	108306958	-	C	79	38	0.481	0	0	T	41	M	0.374	0.589	0	3.7E-09	0	284.465	tgggaggtcaCtctccccagg	m5C_23706
chr6	108306960	-	C	115	28	0.243	0	0	T	87	M	0.174	0.329	0	0	0	97.531	cctgggaggtCactctcccca	m5C_23712
chr6	108306969	-	C	178	80	0.449	0	0	T	98	M	0.378	0.523	0	0	0	605.115	tgtggatatcCtgggaggtca	m5C_23708
chr6	109626704	+	C	69	21	0.304	0	0	T	48	M	0.208	0.421	0	1E-09	0	87.5678	tcaagctggtCtgagtgcagt	m5C_23689
chr6	109626726	+	C	56	12	0.214	0	0	T	44	M	0.127	0.338	1.3E-14	8.8E-10	1.6E-14	21.1897	gtgtttacaaCtaattgatca	m5C_23696
chr6	109626735	+	C	36	15	0.417	0	0	T	21	M	0.271	0.578	0	1.2E-05	0	81.421	actaattgatCacaaccagtt	m5C_23705
chr6	109626737	+	C	31	12	0.387	0	0	T	19	M	0.237	0.562	0	4.6E-06	0	56.9589	taattgatcaCaaccagttac	m5C_23693
chr6	109626740	+	C	31	7	0.226	0	0	T	24	M	0.114	0.398	6.4E-10	3.4E-07	7.2E-10	7.33205	ttgatcacaaCcagttacaga	m5C_23678
chr6	110074413	-	C	31	7	0.226	0	0	T	24	M	0.114	0.398	6.4E-10	3.4E-07	7.2E-10	7.33205	cgtctgatctCggaagctaag	m5C_23442
chr6	110074415	-	C	32	11	0.344	0	0	T	21	M	0.204	0.517	2.2E-16	3.1E-06	2.9E-16	35.1442	ctcgtctgatCtcggaagcta	m5C_23429
chr6	110074425	-	C	35	10	0.286	0	0	T	25	M	0.163	0.451	3.4E-14	2E-06	4.2E-14	21.9991	cacgcccgatCtcgtctgatc	m5C_23441
chr6	110074429	-	C	36	8	0.222	0	0	T	28	M	0.117	0.381	7.4E-11	6.8E-07	8.4E-11	9.49676	tgaacacgccCgatctcgtct	m5C_23437
chr6	110074431	-	C	37	12	0.324	0	0	T	25	M	0.196	0.485	0	4.6E-06	0	47.1194	cctgaacacgCccgatctcgt	m5C_23458
chr6	110074435	-	C	33	14	0.424	0	0	T	19	M	0.272	0.592	0	9.2E-06	0	76.259	ccaccctgaaCacgcccgatc	m5C_23435
chr6	110074440	-	C	33	10	0.303	0	0	T	23	M	0.174	0.473	1.6E-14	2E-06	2E-14	23.982	ccataccaccCtgaacacgcc	m5C_23439
chr6	110074444	-	C	35	12	0.343	0	0	T	23	M	0.208	0.508	0	4.6E-06	0	49.9956	acggccatacCaccctgaaca	m5C_23454

chr6	110074445	-	C	37	14	0.378	0	0	T	23	M	0.241	0.539	0	9.2E-06	0	67.3786	tacggccataCcaccctgaac	m5C_23434
chr6	110074449	-	C	37	9	0.243	0	0	T	28	M	0.134	0.401	2.7E-12	1.2E-06	3.2E-12	13.907	cgtctacggcCataccaccct	m5C_23460
chr6	110074450	-	C	35	13	0.371	0	0	T	22	M	0.232	0.537	0	6.7E-06	0	60.2306	tcgtctacggCcataccaccc	m5C_23452
chr6	111280000	+	C	32	9	0.281	0	0	T	23	M	0.156	0.454	5.3E-13	1.2E-06	6.4E-13	17.1983	cgagcggagtCcagaggacgg	m5C_23426
chr6	111280001	+	C	33	12	0.364	0	0	T	21	M	0.222	0.534	0	4.6E-06	0	53.2487	gagcggagtcCagaggacgga	m5C_23423
chr6	111804357	-	C	59	12	0.203	0	0	T	47	M	0.12	0.323	2.6E-14	8.8E-10	3.3E-14	19.6113	tgattcccggCggtggcggca	m5C_23430
chr6	111918099	+	C	37	13	0.351	0	0	T	24	M	0.218	0.512	0	6.7E-06	0	56.7461	cacgcctggcCtgggggcatg	m5C_23425
chr6	116600380	-	C	87	40	0.46	0	0	T	47	M	0.359	0.564	0	2.1E-10	0	287.175	atagaagtggCggaggaggat	m5C_23617
chr6	119505644	+	C	62	14	0.226	0	0	T	48	M	0.14	0.344	0	3.2E-11	0	39.0742	tggagtaaggCtgtctgcttg	m5C_23994
chr6	121984765	+	C	47	12	0.255	0	0	T	35	M	0.153	0.395	1E-15	7.5E-08	1.3E-15	27.4561	gaggctgcagCctaggtggca	m5C_23754
chr6	123066572	+	C	170	36	0.212	0	0	T	134	M	0.157	0.279	0	0	0	113.088	agcaggttgcCtaaggagggg	m5C_23671
chr6	123066587	+	C	278	80	0.288	0	0	T	198	M	0.238	0.344	0	0	0	380.365	gaggggtggaCcggcccaggt	m5C_23667
chr6	123066591	+	C	226	51	0.226	0	0	T	175	M	0.176	0.284	0	0	0	179.532	ggtggaccggCccaggtcgga	m5C_23659
chr6	123066593	+	C	233	68	0.292	0	0	T	165	M	0.237	0.353	0	0	0	322.626	tggaccggccCaggtcggaaa	m5C_23668
chr6	125584072	+	C	39	10	0.256	0	0	T	29	M	0.146	0.411	1.3E-13	2E-06	1.6E-13	18.7753	tggcaggaggCtcccggcgga	m5C_23779
chr6	125584075	+	C	37	10	0.27	0	0	T	27	M	0.154	0.43	6.7E-14	2E-06	8.3E-14	20.2815	caggaggctcCcggcggacca	m5C_23775
chr6	131191075	-	C	38	17	0.447	0	0	T	21	M	0.301	0.603	0	2.1E-05	0	102.497	gcagcagcagCagtgagagtg	m5C_23826
chr6	131191078	-	C	36	17	0.472	0	0	T	19	M	0.32	0.63	0	2.1E-05	0	108.752	gcagcagcagCagcagtgaga	m5C_23827
chr6	131191084	-	C	32	8	0.25	0	0	T	24	M	0.133	0.421	2.3E-11	6.8E-07	2.6E-11	11.2828	ctgagagcagCagcagcagca	m5C_23835
chr6	131276401	-	C	39	11	0.282	0	0	T	28	M	0.165	0.438	3.1E-15	3.1E-06	4E-15	26.4001	cacaggaagaCaaattagaag	m5C_23833
chr6	131276409	-	C	37	28	0.757	0	0	C	28	M	0.599	0.866	0	0.00017	0	335.341	aaaagaaacaCaggaagacaa	m5C_23829
chr6	131276411	-	C	40	13	0.325	0	0	T	27	M	0.201	0.48	0	1.2E-07	0	52.2192	gtaaaagaaaCacaggaagac	m5C_23821
chr6	133136483	+	C	807	175	0.217	0	0	T	632	M	0.19	0.247	0	0	0	664.289	taccccttcaCtccttttatg	m5C_24985
chr6	133137962	+	C	452	144	0.319	0	0	T	308	M	0.277	0.363	0	0	0	798.661	tgacaactggCtccctctact	m5C_24944
chr6	133137964	+	C	461	130	0.282	0	0	T	331	M	0.243	0.325	0	0	0	631.414	acaactggctCcctctactga	m5C_24980
chr6	133137971	+	C	503	125	0.249	0	0	T	378	M	0.213	0.288	0	0	0	531.86	gctccctctaCtgaactgcca	m5C_24982
chr6	133137976	+	C	507	131	0.258	0	0	T	376	M	0.222	0.298	0	0	0	582.152	ctctactgaaCtgccatgagg	m5C_24951
chr6	133137979	+	C	493	105	0.213	0	0	T	388	M	0.179	0.251	0	0	0	376.177	tactgaactgCcatgaggaaa	m5C_24950
chr6	133137990	+	C	520	151	0.29	0	0	T	369	M	0.253	0.331	0	0	0	764.114	catgaggaaaCtgccatgtca	m5C_24960
chr6	133138001	+	C	465	166	0.358	1	0.002	T	298	M	0.315	0.402	0	0	0	1047.38	tgccatgtcaCccttctgatt	m5C_24948
chr6	133138006	+	C	447	178	0.399	1	0.002	T	268	M	0.355	0.445	0	0	0	1262.74	tgtcacccttCtgattacagc	m5C_24945
chr6	133138365	+	C	212	43	0.203	0	0	T	169	M	0.154	0.262	0	0	0	132.624	tacaagccagCcaatgaatct	m5C_24963
chr6	133138377	+	C	225	60	0.267	0	0	T	165	M	0.213	0.328	0	0	0	255.785	aatgaatctgCttacctgatt	m5C_24976
chr6	133138401	+	C	189	39	0.206	0	0	T	150	M	0.155	0.27	0	0	0	120.73	tttgtgcagaCatactttaaa	m5C_24939
chr6	133138405	+	C	143	34	0.238	0	0	T	109	M	0.175	0.314	0	0	0	119.289	tgcagacataCtttaaaaact	m5C_24953
chr6	133138414	+	C	114	27	0.237	0	0	T	87	M	0.168	0.323	0	0	0	90.8181	actttaaaaaCtggcaatagt	m5C_24946
chr6	133138430	+	C	97	21	0.216	0	0	T	76	M	0.146	0.308	0	3.1E-15	0	61.3984	atagtaaagcCatgttacgag	m5C_24978
chr6	133138450	+	C	94	22	0.234	0	0	T	72	M	0.16	0.329	0	5.8E-15	0	70.3741	gccttaaggaCattgaagtcg	m5C_24973
chr6	133138468	+	C	140	39	0.279	0	0	T	101	M	0.211	0.358	0	0	0	164.568	tcgttaaggtCcctgagaatg	m5C_24962
chr6	133138486	+	C	109	25	0.231	1	0.009	T	83	M	0.162	0.319	0	4.4E-16	0	80.9963	atggctataaCaaatcttagt	m5C_24958
chr6	134578240	+	C	97	22	0.227	0	0	T	75	M	0.155	0.32	0	5.8E-15	0	68.1195	ctggctggtaCttggatggga	m5C_23892
chr6	134578253	+	C	178	64	0.36	0	0	T	114	M	0.293	0.432	0	0	0	374.664	ggatgggagaCtgtttgggaa	m5C_23886
chr6	134578267	+	C	214	70	0.327	0	0	T	144	M	0.268	0.393	0	0	0	374.885	ttgggaatacCaggtgctgta	m5C_23882
chr6	134578273	+	C	202	58	0.287	0	0	T	144	M	0.229	0.353	0	0	0	265.834	ataccaggtgCtgtaggcttc	m5C_23883
chr6	134578280	+	C	174	72	0.414	0	0	T	102	M	0.343	0.488	0	0	0	494.273	gtgctgtaggCttcgctgggc	m5C_23884
chr6	140222447	+	C	33	22	0.667	0	0	C	22	M	0.496	0.802	0	6.3E-05	0	218.273	tttcacttggCtttttacaat	m5C_23938
chr6	144537694	+	C	202	45	0.223	0	0	T	157	M	0.171	0.285	0	0	0	153.779	accaggatggCcgagtggtta	m5C_24187
chr6	144537695	+	C	200	43	0.215	0	0	T	157	M	0.164	0.277	0	0	0	140.797	ccaggatggcCgagtggttaa	m5C_24191
chr6	144537708	+	C	89	42	0.472	0	0	T	47	M	0.372	0.575	0	3.3E-10	0	312.076	gtggttaaggCgttggactta	m5C_24190
chr6	144537715	+	C	49	19	0.388	0	0	T	30	M	0.264	0.528	0	1E-06	0	100.429	aggcgttggaCttaagatcca	m5C_24171
chr6	145503883	+	C	55	21	0.382	0	0	T	34	M	0.265	0.514	0	4.8E-08	0	111.366	atggttagcaCtctgggcttt	m5C_24173
chr6	145503885	+	C	56	13	0.232	0	0	T	43	M	0.141	0.358	4.4E-16	1.6E-09	5.8E-16	28.1397	ggttagcactCtgggctttga	m5C_24177
chr6	145503890	+	C	55	21	0.382	0	0	T	34	M	0.265	0.514	0	4.8E-08	0	111.366	gcactctgggCtttgaatcca	m5C_24189
chr6	145784449	-	C	67	34	0.507	0	0	C	34	M	0.391	0.623	0	4.2E-08	0	265.632	tgttctgaccCctgtgacttc	m5C_24210
chr6	145784451	-	C	81	19	0.235	0	0	T	62	M	0.156	0.338	0	8.4E-14	0	59.1371	gatgttctgaCccctgtgact	m5C_24195
chr6	145784455	-	C	80	25	0.312	0	0	T	55	M	0.222	0.421	0	1.8E-12	0	110.751	tctggatgttCtgacccctgt	m5C_24207
chr6	145784464	-	C	61	17	0.279	0	0	T	44	M	0.182	0.402	0	1.8E-10	0	61.8406	ccattgcactCtggatgttct	m5C_24193
chr6	145784466	-	C	47	23	0.489	0	0	T	24	M	0.353	0.628	0	2.9E-06	0	162.266	atccattgcaCtctggatgtt	m5C_24192
chr6	148939869	+	C	60	33	0.55	0	0	C	33	M	0.425	0.669	0	8.8E-07	0	280.445	tggtggaccaCaggactctga	m5C_24063
chr6	150639786	+	C	67	19	0.284	0	0	T	48	M	0.19	0.401	0	4.5E-10	0	72.0865	gctctgtggcCtagtggcagg	m5C_24446
chr6	151426777	-	C	48	25	0.521	0	0	C	25	M	0.383	0.655	0	4.5E-06	0	191.645	ccctggaggtCtagtggctag	m5C_24479
chr6	151620060	-	C	49	14	0.286	0	0	T	35	M	0.178	0.424	0	1.9E-07	0	49.9784	taggctggtcCgatggtagtg	m5C_24475
chr6	151620061	-	C	47	11	0.234	0	0	T	36	M	0.136	0.372	3.8E-14	4.4E-08	4.7E-14	20.0856	ttaggctggtCcgatggtagt	m5C_24486
chr6	159191860	-	C	133	49	0.368	0	0	T	84	M	0.291	0.453	0	0	0	285.384	agatgatgcgCgagaaggagg	m5C_24797
chr6	159191862	-	C	122	32	0.262	0	0	T	90	M	0.192	0.347	0	0	0	123.106	gcagatgatgCgcgagaagga	m5C_24800
chr6	159191890	-	C	114	32	0.281	0	0	T	82	M	0.206	0.369	0	2.2E-16	0	132.1	ggagagaaacCgtggagagag	m5C_24787
chr6	160210504	-	C	32	14	0.438	0	0	T	18	M	0.282	0.607	0	9.2E-06	0	78.8622	tcgctgccgtCaagatggagg	m5C_24436
chr6	160210507	-	C	32	10	0.312	0	0	T	22	M	0.18	0.486	1.1E-14	2E-06	1.3E-14	25.0836	gtatcgctgcCgtcaagatgg	m5C_24441
chr6	160210523	-	C	35	9	0.257	0	0	T	26	M	0.142	0.421	1.5E-12	1.2E-06	1.7E-12	15.0859	aagcagaattCgacgtgtatc	m5C_24443
chr6	163210074	-	C	61	26	0.426	0	0	T	35	M	0.31	0.551	0	5.6E-09	0	161.278	actccctggtCtagtggttag	m5C_24662
chr6	163210081	-	C	32	10	0.312	0	0	T	22	M	0.18	0.486	1.1E-14	2E-06	1.3E-14	25.0836	agcatgcactCcctggtctag	m5C_24644
chr6	163337965	+	C	32	7	0.219	0	0	T	25	M	0.11	0.388	8.5E-10	3.4E-07	9.5E-10	6.99971	gccaaacttaCggtttctagt	m5C_24632
chr6	166478908	+	C	35	17	0.486	0	0	T	18	M	0.33	0.644	0	2.1E-05	0	112.18	ctcacagtttCcatgcagacc	m5C_24560
chr6	166478917	+	C	37	28	0.757	0	0	C	28	M	0.599	0.866	0	0.00017	0	335.341	tccatgcagaCcccctgaaga	m5C_24572
chr6	166478919	+	C	35	19	0.543	0	0	C	19	M	0.382	0.695	0	3.4E-05	0	145.12	catgcagaccCcctgaagagg	m5C_24563
chr6	166478920	+	C	35	17	0.486	0	0	T	18	M	0.33	0.644	0	2.1E-05	0	112.18	atgcagacccCctgaagaggg	m5C_24565
chr6	166557905	+	C	62	24	0.387	0	0	T	38	M	0.276	0.512	0	3E-09	0	132.405	ggattatttgCtccttctgag	m5C_24564
chr7	606819	-	C	63	16	0.254	0	0	T	47	M	0.163	0.373	0	1.1E-10	0	52.0969	gggtacagttCagctggcatt	m5C_25798
chr7	611304	-	C	113	31	0.274	0	0	T	82	M	0.201	0.363	0	1.1E-16	0	124.318	gcagctggacCccgggagccg	m5C_25793
chr7	1062607	-	C	55	14	0.255	0	0	T	41	M	0.158	0.383	0	2.8E-09	0	44.2735	cctgtgagcgCctcgacgaca	m5C_25799
chr7	1314568	+	C	31	23	0.742	0	0	C	23	M	0.568	0.863	0	7.6E-05	0	261.066	ctgtccggggCtgcatgtgtg	m5C_25758
chr7	1314571	+	C	31	23	0.742	0	0	C	23	M	0.568	0.863	0	7.6E-05	0	261.066	tccggggctgCatgtgtgtgt	m5C_25764
chr7	1756286	+	C	55	37	0.673	0	0	C	37	M	0.541	0.782	0	1.8E-06	0	400.362	acccctggggCtgcatgcgtg	m5C_25728
chr7	1756289	+	C	54	37	0.685	0	0	C	37	M	0.553	0.793	0	1.8E-06	0	408.897	cctggggctgCatgcgtgtca	m5C_25749
chr7	1756293	+	C	49	33	0.673	0	0	C	33	M	0.534	0.788	0	1.9E-05	0	352.301	gggctgcatgCgtgtcatagc	m5C_25722
chr7	1988327	+	C	34	13	0.382	0	0	T	21	M	0.239	0.55	0	6.7E-06	0	62.1399	aaagggcgggCggcctgccgt	m5C_25731
chr7	1988331	+	C	32	12	0.375	0	0	T	20	M	0.229	0.547	0	4.6E-06	0	55.0408	ggcgggcggcCtgccgtggcc	m5C_25740
chr7	1988335	+	C	32	7	0.219	0	0	T	25	M	0.11	0.388	8.5E-10	3.4E-07	9.5E-10	6.99971	ggcggcctgcCgtggccaggg	m5C_25753
chr7	2102749	+	C	40	20	0.5	0	0	CT	20	M	0.352	0.648	0	1.4E-06	0	140.797	gtttcttacaCtgggactgag	m5C_24831
chr7	2102761	+	C	45	27	0.6	0	0	C	27	M	0.455	0.73	0	6.7E-06	0	245.439	gggactgagaCacgggaactt	m5C_24838
chr7	2102763	+	C	47	26	0.553	0	0	C	26	M	0.412	0.686	0	5.5E-06	0	214.475	gactgagacaCgggaacttgc	m5C_24837
chr7	2464323	+	C	57	32	0.561	0	0	C	32	M	0.433	0.682	0	7.2E-07	0	276.978	actgtgctgtCcgagtgttgt	m5C_24851
chr7	4167837	+	C	41	22	0.537	0	0	C	22	M	0.387	0.679	0	2.3E-06	0	170.483	ccacgcttgtCtcagtgctgc	m5C_25006
chr7	4678841	-	C	111	68	0.613	0	0	C	68	M	0.52	0.698	0	4.3E-12	0	706.747	ggaactagctCaaatggtaga	m5C_25048
chr7	4678843	-	C	115	54	0.47	0	0	T	61	M	0.381	0.56	0	2.3E-13	0	411.279	ggggaactagCtcaaatggta	m5C_25026
chr7	5568041	-	C	35	8	0.229	0	0	T	27	M	0.121	0.39	5.6E-11	6.8E-07	6.4E-11	9.89691	ggacttcgagCaagagatggc	m5C_25049
chr7	5568045	-	C	34	12	0.353	0	0	T	22	M	0.215	0.521	0	4.6E-06	0	51.5705	ccctggacttCgagcaagaga	m5C_25023
chr7	5568048	-	C	33	12	0.364	0	0	T	21	M	0.222	0.534	0	4.6E-06	0	53.2487	tcgccctggaCttcgagcaag	m5C_25047
chr7	6056527	+	C	165	39	0.236	0	0	T	126	M	0.178	0.307	0	0	0	138.838	tggattatgaCaggcccatcc	m5C_25095
chr7	6056531	+	C	132	29	0.22	0	0	T	103	M	0.158	0.298	0	0	0	91.3768	ttatgacaggCccatcccct	m5C_25105
chr7	6056540	+	C	97	29	0.299	0	0	T	68	M	0.217	0.396	0	1.7E-13	0	125.828	gcccatccccCtggacctctc	m5C_25102

chr7	6056548	+	C	54	19	0.352	0	0	T	35	M	0.238	0.485	0	2.4E-08	0	90.5268	ccctggacctCtcatagtgcc	m5C_25106
chr7	6056557	+	C	37	20	0.541	0	0	C	20	M	0.384	0.69	0	4.3E-05	0	153.535	tctcatagtgCcccatgccag	m5C_25100
chr7	6056559	+	C	37	13	0.351	0	0	T	24	M	0.218	0.512	0	6.7E-06	0	56.7461	tcatagtgccCcatgccagag	m5C_25080
chr7	6056565	+	C	37	8	0.216	0	0	T	29	M	0.114	0.372	9.7E-11	6.8E-07	1.1E-10	9.12273	tgccccatgcCagagcaaact	m5C_25084
chr7	6056570	+	C	38	10	0.263	0	0	T	28	M	0.15	0.42	9.4E-14	2E-06	1.2E-13	19.5037	catgccagagCaaactgtggc	m5C_25103
chr7	6068565	-	C	37	14	0.378	0	0	T	23	M	0.241	0.539	0	9.2E-06	0	67.3786	cagactggacCaacagaaacg	m5C_25120
chr7	6068575	-	C	40	17	0.425	0	0	T	23	M	0.285	0.578	0	5.6E-07	0	96.9312	cagaagaacaCagactggacc	m5C_25134
chr7	6068577	-	C	41	27	0.659	0	0	C	27	M	0.505	0.784	0	6.7E-06	0	272.968	tacagaagaaCacagactgga	m5C_25115
chr7	6252363	-	C	33	11	0.333	0	0	T	22	M	0.198	0.504	2.2E-16	3.1E-06	2.9E-16	34.0074	aaggctggtcCgaaggtagtg	m5C_25121
chr7	6252364	-	C	32	10	0.312	0	0	T	22	M	0.18	0.486	1.1E-14	2E-06	1.3E-14	25.0836	gaaggctggtCcgaaggtagt	m5C_25113
chr7	6523644	-	C	37	13	0.351	0	0	T	24	M	0.218	0.512	0	6.7E-06	0	56.7461	acctggcggcCatcgtcatcc	m5C_25131
chr7	6523657	-	C	34	17	0.5	0	0	CT	17	M	0.341	0.659	0	2.1E-05	0	115.835	ggggacctgtCccacctggcg	m5C_25142
chr7	6523669	-	C	32	18	0.562	0	0	C	18	M	0.393	0.718	0	2.7E-05	0	141.571	ttccggctgaCtggggacctg	m5C_25136
chr7	16640047	-	C	32	14	0.438	0	0	T	18	M	0.282	0.607	0	9.2E-06	0	78.8622	aacaaagggaCaagcagaaca	m5C_24901
chr7	21421027	+	C	68	18	0.265	0	0	T	50	M	0.174	0.38	0	2.9E-10	0	62.8186	ggtggctttcCcagggtgaga	m5C_25194
chr7	21421028	+	C	72	23	0.319	0	0	T	49	M	0.223	0.434	0	4.3E-11	0	102.697	gtggctttccCagggtgagat	m5C_25191
chr7	21421099	+	C	46	19	0.413	0	0	T	27	M	0.283	0.557	0	1E-06	0	107.496	ggaaacttggCtgcataattt	m5C_25207
chr7	21948088	-	C	44	18	0.409	0	0	T	26	M	0.277	0.556	0	7.7E-07	0	99.6755	ggcaaagcatCtttggtgagc	m5C_25212
chr7	22852730	-	C	59	21	0.356	0	0	T	38	M	0.246	0.483	0	4.8E-08	0	103.333	aagatgtgtgCtctggctcgg	m5C_25251
chr7	22896268	+	C	154	33	0.214	0	0	T	121	M	0.157	0.286	0	0	0	103.52	attttatgtgCttctgctgtg	m5C_25217
chr7	22896271	+	C	171	62	0.363	0	0	T	109	M	0.294	0.437	0	0	0	364.893	ttatgtgcttCtgctgtgatg	m5C_25227
chr7	23531433	-	C	32	10	0.312	0	0	T	22	M	0.18	0.486	1.1E-14	2E-06	1.3E-14	25.0836	tccactcccaCtgcttcactt	m5C_25242
chr7	23531436	-	C	33	7	0.212	0	0	T	26	M	0.107	0.378	1.1E-09	3.4E-07	1.2E-09	6.69165	ttctccactcCcactgcttca	m5C_25246
chr7	23531455	-	C	35	9	0.257	0	0	T	26	M	0.142	0.421	1.5E-12	1.2E-06	1.7E-12	15.0859	ttatagatttCttcgttcctt	m5C_25236
chr7	23531468	-	C	34	11	0.324	0	0	T	23	M	0.191	0.492	4.4E-16	3.1E-06	5.8E-16	32.3088	tgatcacaacCagttatagat	m5C_25240
chr7	23531505	-	C	31	11	0.355	0	0	T	20	M	0.211	0.531	1.1E-16	3.1E-06	1.5E-16	37.0595	aagggccggtCtgagtgcagt	m5C_25253
chr7	25989547	-	C	61	19	0.311	0	0	T	42	M	0.209	0.436	0	4.5E-10	0	79.5616	cactacagaaCtttgtctcta	m5C_25157
chr7	25989552	-	C	60	22	0.367	0	0	T	38	M	0.256	0.493	0	1.5E-09	0	112.731	cagtgcactaCagaactttgt	m5C_25161
chr7	25989555	-	C	61	19	0.311	0	0	T	42	M	0.209	0.436	0	4.5E-10	0	79.5616	agtcagtgcaCtacagaactt	m5C_25151
chr7	25989557	-	C	62	20	0.323	0	0	T	42	M	0.22	0.446	0	6.9E-10	0	87.8173	gaagtcagtgCactacagaac	m5C_25166
chr7	26231830	-	C	31	10	0.323	0	0	T	21	M	0.186	0.499	7.1E-15	2E-06	9E-15	26.2725	tggcagaagaCaccagagcag	m5C_25389
chr7	26233292	-	C	58	19	0.328	0	0	T	39	M	0.221	0.456	0	2.4E-08	0	83.9159	gaggtagcccCggttatggag	m5C_25380
chr7	26233293	-	C	62	27	0.435	0	0	T	35	M	0.319	0.559	0	7.5E-09	0	172.489	ggaggtagccCcggttatgga	m5C_25381
chr7	26233295	-	C	60	16	0.267	0	0	T	44	M	0.171	0.39	0	1.1E-10	0	54.824	ttggaggtagCcccggttatg	m5C_25374
chr7	26236201	-	C	53	27	0.509	0	0	C	27	M	0.379	0.639	0	2.5E-07	0	204.57	gcaggaagttCagagttctag	m5C_25394
chr7	26237053	-	C	32	19	0.594	0	0	C	19	M	0.423	0.745	0	3.4E-05	0	160.587	agcaaaagatCaagaggattt	m5C_25392
chr7	26237061	-	C	45	19	0.422	0	0	T	26	M	0.29	0.567	0	1E-06	0	110.08	atcctgcaagCaaaagatcaa	m5C_25386
chr7	26237065	-	C	41	24	0.585	0	0	C	24	M	0.434	0.722	0	3.6E-06	0	208.161	agggatcctgCaagcaaaaga	m5C_25382
chr7	26237068	-	C	41	20	0.488	0	0	T	21	M	0.343	0.635	0	1.4E-06	0	137.017	atgagggatcCtgcaagcaaa	m5C_25385
chr7	26246006	+	C	31	8	0.258	0	0	T	23	M	0.137	0.432	1.7E-11	6.8E-07	1.9E-11	11.8182	gggaaaaaaaCagaatggaaa	m5C_25366
chr7	27209157	-	C	69	45	0.652	0	0	C	45	M	0.534	0.754	0	3.3E-07	0	481.029	aggtagtttcCtgttgttggg	m5C_25373
chr7	29297329	-	C	31	19	0.613	0	0	C	19	M	0.438	0.763	0	3.4E-05	0	166.53	caggatatcgCcaggccgagt	m5C_25334
chr7	29720419	-	C	37	8	0.216	0	0	T	29	M	0.114	0.372	9.7E-11	6.8E-07	1.1E-10	9.12273	ggtgcagtgcCtgagggagta	m5C_25320
chr7	29962148	-	C	43	15	0.349	0	0	T	28	M	0.224	0.498	0	2.8E-07	0	67.2552	gtaaatacagCagctagtggg	m5C_25336
chr7	29962151	-	C	41	10	0.244	0	0	T	31	M	0.138	0.393	2.4E-13	2.4E-08	2.9E-13	17.4472	tatgtaaataCagcagctagt	m5C_25342
chr7	29962169	-	C	43	19	0.442	0	0	T	24	M	0.304	0.589	0	1E-06	0	115.646	agatgatgaaCagaagtgtat	m5C_25321
chr7	33028231	+	C	39	12	0.308	0	0	T	27	M	0.186	0.464	0	4.6E-06	0	44.5578	gattgtggtcCcgcctcacct	m5C_25421
chr7	33028232	+	C	41	15	0.366	0	0	T	26	M	0.236	0.519	0	2.8E-07	0	70.7689	attgtggtccCgcctcacctg	m5C_25422
chr7	33028234	+	C	41	13	0.317	0	0	T	28	M	0.196	0.47	0	1.2E-07	0	50.8675	tgtggtcccgCctcacctggg	m5C_25426
chr7	33028235	+	C	43	27	0.628	0	0	C	27	M	0.479	0.756	0	6.7E-06	0	258.442	gtggtcccgcCtcacctgggg	m5C_25420
chr7	33028237	+	C	41	22	0.537	0	0	C	22	M	0.387	0.679	0	2.3E-06	0	170.483	ggtcccgcctCacctggggta	m5C_25410
chr7	33028240	+	C	38	13	0.342	0	0	T	25	M	0.212	0.501	0	6.7E-06	0	55.1518	cccgcctcacCtggggtatgg	m5C_25418
chr7	33217361	+	C	55	12	0.218	0	0	T	43	M	0.129	0.344	9.8E-15	8.8E-10	1.2E-14	21.7648	agtctatggcCataccaccct	m5C_25419
chr7	33217365	+	C	55	15	0.273	0	0	T	40	M	0.173	0.402	0	4.6E-09	0	51.8418	tatggccataCcaccctgaat	m5C_25409
chr7	33217370	+	C	56	15	0.268	0	0	T	41	M	0.17	0.396	0	4.6E-09	0	50.8715	ccataccaccCtgaatgtgcc	m5C_25427
chr7	36438903	+	C	69	33	0.478	0	0	T	36	M	0.365	0.594	0	3.4E-08	0	240.692	gaggagagggCtgaactcaag	m5C_25616
chr7	36438908	+	C	57	28	0.491	0	0	T	29	M	0.366	0.617	0	3.1E-07	0	205.05	gagggctgaaCtcaagattgg	m5C_25647
chr7	36458994	+	C	53	19	0.358	0	0	T	34	M	0.243	0.493	0	2.4E-08	0	92.3467	caagcattagCagaaagcagc	m5C_25618
chr7	42977009	+	C	60	12	0.2	0	0	T	48	M	0.118	0.318	3.3E-14	7.6E-12	4.2E-14	19.13	tgcaaggagaCtgcagaaatc	m5C_25548
chr7	42977012	+	C	60	33	0.55	0	0	C	33	M	0.425	0.669	0	8.8E-07	0	280.445	aaggagactgCagaaatcaga	m5C_25558
chr7	42977019	+	C	54	12	0.222	0	0	T	42	M	0.132	0.349	7.5E-15	8.8E-10	9.6E-15	22.3614	ctgcagaaatCagacgacaga	m5C_25566
chr7	42977023	+	C	48	10	0.208	0	0	T	38	M	0.117	0.343	1.6E-12	2.4E-08	1.9E-12	13.8343	agaaatcagaCgacagatagg	m5C_25554
chr7	43917654	-	C	58	15	0.259	0	0	T	43	M	0.163	0.384	0	4.6E-09	0	49.0362	tgaggagtgtCagaaagcgaa	m5C_25591
chr7	43918631	-	C	40	11	0.275	0	0	T	29	M	0.161	0.428	4.3E-15	3.1E-06	5.5E-15	25.4502	gacgccaggcCtgtggagaag	m5C_25582
chr7	44471236	+	C	41	23	0.561	0	0	C	23	M	0.41	0.701	0	2.9E-06	0	188.785	ttgagtgctgCcactagaaac	m5C_28068
chr7	44524826	-	C	78	39	0.5	0	0	CT	39	M	0.392	0.608	0	4.7E-09	0	305.504	aaggcaggaaCttgaagagaa	m5C_28158
chr7	44620706	-	C	43	9	0.209	0	0	T	34	M	0.114	0.352	1.4E-11	1.2E-08	1.6E-11	11.1522	tcagaaggagCaggattacca	m5C_28140
chr7	44620715	-	C	44	23	0.523	0	0	C	23	M	0.379	0.662	0	2.9E-06	0	174.502	ggaacagattCagaaggagca	m5C_28092
chr7	45024981	-	C	293	88	0.3	0	0	T	205	M	0.251	0.355	0	0	0	441.254	aaatttcagaCaatgacttcc	m5C_28111
chr7	45024985	-	C	312	66	0.212	0	0	T	246	M	0.17	0.26	0	0	0	224.228	atagaaatttCagacaatgac	m5C_28159
chr7	45025007	-	C	262	52	0.202	5	0.019	T	205	M	0.158	0.256	0	0	0	164.078	ctgctccatcCagagcaaggt	m5C_28178
chr7	45025034	-	C	411	85	0.208	2	0.005	T	324	M	0.171	0.25	0	0	0	291.224	agttttgaaaCggtttgaggc	m5C_28120
chr7	45025084	-	C	1244	360	0.29	1	0.001	T	883	M	0.265	0.315	0	0	0	1908.61	gtgcttgggtCtgcggtgacc	m5C_28186
chr7	45144010	-	C	36	16	0.444	0	0	T	20	M	0.295	0.604	0	1.7E-05	0	94.5313	cacagaaacaCtgtgactcat	m5C_28173
chr7	45144020	-	C	47	12	0.255	0	0	T	35	M	0.153	0.395	1E-15	7.5E-08	1.3E-15	27.4561	agaaggctgcCacagaaacac	m5C_28093
chr7	45144024	-	C	59	19	0.322	0	0	T	40	M	0.217	0.449	0	2.4E-08	0	82.4122	gcccagaaggCtgccacagaa	m5C_28096
chr7	45144033	-	C	58	19	0.328	0	0	T	39	M	0.221	0.456	0	2.4E-08	0	83.9159	taggcactggCccagaaggct	m5C_28114
chr7	45144039	-	C	107	41	0.387	1	0.009	T	65	M	0.3	0.482	0	2.4E-13	0	245.662	gcatggtaggCactggcccag	m5C_28105
chr7	45144053	-	C	160	34	0.212	0	0	T	126	M	0.156	0.282	0	0	0	106.243	agtacctgtcCtatgcatggt	m5C_28132
chr7	45144054	-	C	175	61	0.349	0	0	T	114	M	0.282	0.422	0	0	0	343.929	cagtacctgtCctatgcatgg	m5C_28088
chr7	45144058	-	C	203	70	0.345	0	0	T	133	M	0.283	0.413	0	0	0	396.017	aattcagtacCtgtcctatgc	m5C_28150
chr7	45144070	-	C	212	50	0.236	0	0	T	162	M	0.184	0.297	0	0	0	183.719	gcagccgtgtCaaattcagta	m5C_28103
chr7	45144075	-	C	210	49	0.233	0	0	T	161	M	0.181	0.295	0	0	0	177.608	tgcctgcagcCgtgtcaaatt	m5C_28152
chr7	45144076	-	C	199	45	0.226	0	0	T	154	M	0.174	0.289	0	0	0	156.163	atgcctgcagCcgtgtcaaat	m5C_28171
chr7	45144117	-	C	56	17	0.304	0	0	T	39	M	0.199	0.433	0	1.1E-08	0	67.6606	ttggacagaaCggtggagaga	m5C_28187
chr7	45144122	-	C	60	13	0.217	0	0	T	47	M	0.131	0.336	1.1E-15	1.6E-11	1.4E-15	25.5122	gccctttggaCagaacggtgg	m5C_28139
chr7	45144129	-	C	49	14	0.286	0	0	T	35	M	0.178	0.424	0	1.9E-07	0	49.9784	atggtgagccCtttggacaga	m5C_28110
chr7	45144130	-	C	47	36	0.766	0	0	C	36	M	0.628	0.864	0	2.9E-05	0	451.983	tatggtgagcCctttggacag	m5C_28101
chr7	45144131	-	C	51	12	0.235	0	0	T	39	M	0.14	0.368	3.3E-15	8.8E-10	4.3E-15	24.3305	ctatggtgagCcctttggaca	m5C_28134
chr7	45144509	-	C	465	93	0.2	0	0	T	372	M	0.166	0.239	0	0	0	309.07	tggctgtggaCagtgctccag	m5C_28107
chr7	45144532	-	C	313	86	0.275	0	0	T	227	M	0.228	0.327	0	0	0	392.611	accctgggacCtggggataag	m5C_28149
chr7	45144533	-	C	292	73	0.25	0	0	T	219	M	0.204	0.303	0	0	0	297.543	taccctgggaCctggggataa	m5C_28130
chr7	45144544	-	C	164	41	0.25	0	0	T	123	M	0.19	0.321	0	0	0	155.769	cctgggtcacCtaccctggga	m5C_28148
chr7	45144554	-	C	87	21	0.241	0	0	T	66	M	0.164	0.341	0	2.6E-13	0	68.729	gctcatgggcCctgggtcacc	m5C_28190
chr7	45144561	-	C	70	17	0.243	0	0	T	53	M	0.158	0.355	0	2.2E-12	0	53.5559	ctccacagctCatgggccctg	m5C_28183
chr7	45144568	-	C	75	21	0.28	0	0	T	54	M	0.191	0.39	0	1.8E-11	0	80.2327	cagcaagctcCacagctcatg	m5C_28138
chr7	45144569	-	C	74	21	0.284	0	0	T	53	M	0.194	0.395	0	1.8E-11	0	81.3683	acagcaagctCcacagctcat	m5C_28129
chr7	45144571	-	C	76	19	0.25	0	0	T	57	M	0.166	0.358	0	6.9E-12	0	63.1894	ccacagcaagCtccacagctc	m5C_28106
chr7	45144578	-	C	97	22	0.227	0	0	T	75	M	0.155	0.32	0	5.8E-15	0	68.1195	atggctgccaCagcaagctcc	m5C_28168
chr7	45144584	-	C	138	37	0.268	0	0	T	101	M	0.201	0.348	0	0	0	148.906	gcccagatggCtgccacagca	m5C_28116
chr7	45144591	-	C	166	36	0.217	0	0	T	130	M	0.161	0.286	0	0	0	115.893	ggggctggccCagatggctgc	m5C_28124

chr7	45144592	-	C	165	59	0.358	0	0	T	106	M	0.288	0.433	0	0	0	340.369	gggggctggcCcagatggctg	m5C_28170
chr7	45144593	-	C	169	53	0.314	0	0	T	116	M	0.248	0.387	0	0	0	263.367	cgggggctggCccagatggct	m5C_28189
chr7	45144617	-	C	150	47	0.313	0	0	T	103	M	0.245	0.391	0	0	0	229.875	attcagtgccCgtttctgtca	m5C_28131
chr7	45144618	-	C	167	40	0.24	0	0	T	127	M	0.181	0.31	0	0	0	144.889	aattcagtgcCcgtttctgtc	m5C_28157
chr7	45144619	-	C	158	35	0.222	0	0	T	123	M	0.164	0.292	0	0	0	114.666	aaattcagtgCccgtttctgt	m5C_28174
chr7	45144642	-	C	32	8	0.25	0	0	T	24	M	0.133	0.421	2.3E-11	6.8E-07	2.6E-11	11.2828	ggctgtagccCtgcagtcaag	m5C_28192
chr7	45308259	-	C	46	18	0.391	0	0	T	28	M	0.264	0.535	0	7.7E-07	0	95.0149	tgatgctgtgCcttttttttcg	m5C_28175
chr7	45493494	-	C	39	13	0.333	0	0	T	26	M	0.206	0.49	0	6.7E-06	0	53.6452	agaatgtggtCtgagtccagg	m5C_28108
chr7	45952686	-	C	92	42	0.457	0	0	T	50	M	0.359	0.558	0	1.1E-11	0	301.161	gtaggagggaCagagagacag	m5C_28118
chr7	45952702	-	C	67	17	0.254	0	0	T	50	M	0.165	0.369	0	1.8E-10	0	56.0585	gctgcggcccCgtggggtagg	m5C_28135
chr7	45952705	-	C	59	14	0.237	0	0	T	45	M	0.147	0.36	0	2.8E-09	0	41.1446	ttgctgcggcCccgtggggt	m5C_28094
chr7	51267008	-	C	71	27	0.38	0	0	T	44	M	0.276	0.497	0	1.9E-10	0	149.186	cctagctcacCagactgatgt	m5C_26094
chr7	51267013	-	C	32	9	0.281	0	0	T	23	M	0.156	0.454	5.3E-13	1.2E-06	6.4E-13	17.1983	ctactcctagCtcaccagact	m5C_26100
chr7	53843905	+	C	43	18	0.419	0	0	T	25	M	0.284	0.567	0	7.7E-07	0	102.184	cttcctctatCccaggacaat	m5C_25597
chr7	53843912	+	C	149	41	0.275	0	0	T	108	M	0.21	0.352	0	0	0	172.028	tatcccaggaCaatatattaa	m5C_25593
chr7	53843937	+	C	460	131	0.285	0	0	T	329	M	0.245	0.328	0	0	0	643.09	atttttggagCagggagatgg	m5C_25595
chr7	55223571	+	C	43	25	0.581	0	0	C	25	M	0.433	0.716	0	4.5E-06	0	216.642	gcctgtggggCcgacagctat	m5C_25679
chr7	56128169	+	C	190	48	0.253	0	0	T	142	M	0.196	0.319	0	0	0	188.32	aaaggcatggCcgaatactgt	m5C_25921
chr7	56128170	+	C	197	121	0.614	0	0	C	121	M	0.545	0.679	0	0	0	1318.1	aaggcatggcCgaatactgtg	m5C_25907
chr7	56128200	+	C	86	22	0.256	0	0	T	64	M	0.175	0.357	0	4.4E-13	0	77.189	gtagtttacaCagccagacac	m5C_25927
chr7	56128208	+	C	64	18	0.281	0	0	T	46	M	0.186	0.401	0	2.9E-10	0	66.9356	cacagccagaCaccatgcaaa	m5C_25924
chr7	56128221	+	C	33	7	0.212	0	0	T	26	M	0.107	0.378	1.1E-09	3.4E-07	1.2E-09	6.69165	catgcaaaagCagtcttccct	m5C_25906
chr7	56128225	+	C	34	10	0.294	0	0	T	24	M	0.168	0.462	2.3E-14	2E-06	2.9E-14	22.9564	caaaagcagtCttccctttag	m5C_25899
chr7	56128228	+	C	33	9	0.273	0	0	T	24	M	0.151	0.442	7.5E-13	1.2E-06	9E-13	16.4418	aagcagtcttCcctttagaat	m5C_25914
chr7	57075605	+	C	49	12	0.245	0	0	T	37	M	0.146	0.381	1.9E-15	7.5E-08	2.4E-15	25.8006	ccgtgttcctCtctgccggcc	m5C_25910
chr7	64791646	+	C	110	36	0.327	0	0	T	74	M	0.247	0.419	0	1E-15	0	177.631	aggatttagaCtatgatatag	m5C_25879
chr7	64791657	+	C	63	24	0.381	0	0	T	39	M	0.271	0.504	0	3E-09	0	130.163	tatgatatagCtgttaagtgc	m5C_25874
chr7	68527705	+	C	32	13	0.406	0	0	T	19	M	0.255	0.577	0	6.7E-06	0	66.3505	gtgggaggccCccagcgcccc	m5C_26118
chr7	72892164	-	C	43	18	0.419	0	0	T	25	M	0.284	0.567	0	7.7E-07	0	102.184	tgaagaacaaCggaaagaata	m5C_26054
chr7	73089099	+	C	55	17	0.309	0	0	T	38	M	0.203	0.44	0	1.1E-08	0	68.9592	gggagtgagaCccctctgtgt	m5C_26045
chr7	73508610	-	C	36	10	0.278	0	0	T	26	M	0.158	0.44	4.8E-14	2E-06	5.9E-14	21.1115	gcggtgatgaCtcacccgagg	m5C_26067
chr7	75010922	+	C	42	15	0.357	0	0	T	27	M	0.23	0.508	0	2.8E-07	0	68.967	tggagctggcCcgagtgcagt	m5C_26460
chr7	75573142	+	C	31	13	0.419	0	0	T	18	M	0.264	0.592	0	6.7E-06	0	68.6798	gtaaatgcttCcaagatatga	m5C_26452
chr7	75573143	+	C	33	7	0.212	0	0	T	26	M	0.107	0.378	1.1E-09	3.4E-07	1.2E-09	6.69165	taaatgcttcCaagatatgag	m5C_26451
chr7	75573160	+	C	40	9	0.225	0	0	T	31	M	0.123	0.375	6.4E-12	1.2E-08	7.6E-12	12.4041	tgagcgaatgCtatagaaatt	m5C_26453
chr7	75573181	+	C	86	35	0.407	0	0	T	51	M	0.309	0.513	0	5.6E-11	0	216.503	gcaggaaagtCcaaagggctg	m5C_26450
chr7	75573189	+	C	108	33	0.306	0	0	T	75	M	0.227	0.398	0	1.6E-14	0	149.557	gtccaaagggCtgcgcgtctc	m5C_26458
chr7	75573200	+	C	122	44	0.361	0	0	T	78	M	0.281	0.449	0	4.4E-16	0	247.194	tgcgcgtctcCtgtggctcag	m5C_26456
chr7	75573219	+	C	102	22	0.216	0	0	T	80	M	0.147	0.305	0	0	0	64.6672	agtcttatttCatacctgcaa	m5C_26457
chr7	80963010	+	C	31	14	0.452	0	0	T	17	M	0.292	0.622	0	9.2E-06	0	81.6522	tgcggtaattCcagctccaat	m5C_26796
chr7	80963011	+	C	36	12	0.333	0	0	T	24	M	0.202	0.497	0	4.6E-06	0	48.5146	gcggtaattcCagctccaata	m5C_26800
chr7	80963014	+	C	36	14	0.389	0	0	T	22	M	0.248	0.551	0	9.2E-06	0	69.397	gtaattccagCtccaatagtg	m5C_26806
chr7	80963016	+	C	54	14	0.259	0	0	T	40	M	0.161	0.389	0	2.8E-09	0	45.1318	aattccagctCcaatagtgta	m5C_26830
chr7	80963017	+	C	65	19	0.292	0	0	T	46	M	0.196	0.412	0	4.5E-10	0	74.4164	attccagctcCaatagtgtat	m5C_26793
chr7	80963038	+	C	540	112	0.207	0	0	T	428	M	0.175	0.244	0	0	0	392.75	attaaagttgCtgcagttaaa	m5C_26809
chr7	80963041	+	C	643	201	0.313	0	0	T	442	M	0.278	0.349	0	0	0	1117.44	aaagttgctgCagttaaaaag	m5C_26808
chr7	80963052	+	C	999	313	0.314	1	0.001	T	685	M	0.286	0.343	0	0	0	1787.87	agttaaaaagCccgtagttgg	m5C_26799
chr7	80963054	+	C	1059	288	0.272	0	0	T	771	M	0.246	0.3	0	0	0	1417.04	ttaaaaagccCgtagttggat	m5C_26814
chr7	80963065	+	C	1073	438	0.409	1	0.001	T	634	M	0.38	0.438	0	0	0	3324.7	gtagttggatCtcgggagcag	m5C_26828
chr7	80963073	+	C	562	128	0.228	0	0	T	434	M	0.195	0.264	0	0	0	499.203	atctcgggagCaggcgtgagg	m5C_26818
chr7	80963077	+	C	39	16	0.41	0	0	T	23	M	0.271	0.566	0	1.7E-05	0	86.6531	cgggagcaggCgtgaggtccg	m5C_26820
chr7	80963096	+	C	271	74	0.273	0	0	T	197	M	0.223	0.329	0	0	0	330.729	cgctgcgaggCgagccaccgc	m5C_26815
chr7	80963100	+	C	260	98	0.377	0	0	T	162	M	0.32	0.437	0	0	0	627.614	gcgaggcgagCcaccgcccgt	m5C_26790
chr7	80963103	+	C	213	82	0.385	0	0	T	131	M	0.322	0.452	0	0	0	528.434	aggcgagccaCcgcccgtccc	m5C_26829
chr7	80963104	+	C	211	44	0.209	0	0	T	167	M	0.159	0.268	0	0	0	140.068	ggcgagccacCgcccgtcccc	m5C_26807
chr7	80963107	+	C	159	56	0.352	0	0	T	103	M	0.282	0.429	0	0	0	316.109	gagccaccgcCcgtccccacc	m5C_26794
chr7	80963111	+	C	71	15	0.211	0	0	T	56	M	0.132	0.32	0	6E-13	0	39.7273	caccgcccgtCcccacccgtt	m5C_26823
chr7	80963129	-	C	50	11	0.22	0	0	T	39	M	0.128	0.352	8.5E-14	4.4E-10	1.1E-13	18.3342	gagggggcgcCgagagttaac	m5C_26856
chr7	80963130	-	C	54	29	0.537	0	0	C	29	M	0.406	0.663	0	3.9E-07	0	235.51	cgagggggcgCcgagagttaa	m5C_26848
chr7	80963132	-	C	58	21	0.362	0	0	T	37	M	0.251	0.491	0	4.8E-08	0	105.23	atcgaggggCgccgagagtt	m5C_26844
chr7	80963140	-	C	50	14	0.28	0	0	T	36	M	0.175	0.417	0	1.9E-07	0	48.9272	gctagagcatCgagggggcgc	m5C_26846
chr7	81936348	+	C	55	27	0.491	0	0	T	28	M	0.364	0.619	0	2.5E-07	0	196.433	agcctgtggcCtagtggctag	m5C_26831
chr7	88898041	+	C	37	10	0.27	0	0	T	27	M	0.154	0.43	6.7E-14	2E-06	8.3E-14	20.2815	gtgtacccatCagatctgggg	m5C_26571
chr7	89234925	-	C	37	24	0.649	0	0	C	24	M	0.488	0.782	0	9.1E-05	0	234.042	gggttgcaggCagctgtactg	m5C_26577
chr7	97114005	+	C	46	15	0.326	0	0	T	31	M	0.209	0.47	0	2.8E-07	0	62.5973	gtagtagtatCatgcaagatc	m5C_26725
chr7	97249098	+	C	53	11	0.208	0	0	T	42	M	0.12	0.335	1.8E-13	4.4E-10	2.2E-13	16.8213	tcaggctgtgCaaaaaaaaaa	m5C_26730
chr7	97481517	-	C	60	22	0.367	0	0	T	38	M	0.256	0.493	0	1.5E-09	0	112.731	tggatggggaCtgtgggtaga	m5C_26754
chr7	98479340	+	C	361	90	0.249	0	0	T	271	M	0.207	0.296	0	0	0	373.459	atgagtaataCtggctggagc	m5C_28414
chr7	98479344	+	C	348	85	0.244	0	0	T	263	M	0.202	0.292	0	0	0	343.509	gtaatactggCtggagcccca	m5C_28400
chr7	98479350	+	C	329	122	0.371	0	0	T	207	M	0.32	0.424	0	0	0	781.77	ctggctggagCcccaaagagg	m5C_28412
chr7	98479363	+	C	252	89	0.356	2	0.008	T	161	M	0.299	0.417	0	0	0	532.639	caaagaggcaCgtgtgtgtgt	m5C_28428
chr7	98479398	+	C	50	14	0.28	0	0	T	36	M	0.175	0.417	0	1.9E-07	0	48.9272	atatgcttgtCagtgcatgca	m5C_28431
chr7	98479478	+	C	35	12	0.343	0	0	T	23	M	0.208	0.508	0	4.6E-06	0	49.9956	tctattaggcCatgacagtca	m5C_28438
chr7	98479483	+	C	39	21	0.538	0	0	C	21	M	0.386	0.684	0	5.2E-05	0	161.991	taggccatgaCagtcaaactg	m5C_28445
chr7	98479487	+	C	48	30	0.625	0	0	C	30	M	0.484	0.748	0	1.2E-05	0	290.175	ccatgacagtCaaactgataa	m5C_28417
chr7	98479491	+	C	51	25	0.49	0	0	T	26	M	0.359	0.623	0	1.5E-07	0	179.286	gacagtcaaaCtgataagatc	m5C_28441
chr7	98479501	+	C	47	15	0.319	0	0	T	32	M	0.204	0.462	0	2.8E-07	0	61.1856	ctgataagatCtgattgcttc	m5C_28402
chr7	99056237	-	C	37	13	0.351	0	0	T	24	M	0.218	0.512	0	6.7E-06	0	56.7461	gacgggttctCtggggctgtt	m5C_28482
chr7	99691197	-	C	48	38	0.792	0	0	C	38	M	0.657	0.883	0	3.8E-05	0	499.63	ttgtctcggtCtgacagtgcc	m5C_28486
chr7	99691201	-	C	54	16	0.296	0	0	T	38	M	0.191	0.428	0	7.3E-09	0	61.2393	gcacttgtctCggtctgacag	m5C_28452
chr7	99691203	-	C	54	38	0.704	0	0	C	38	M	0.572	0.809	0	2.1E-06	0	434.509	ttgcacttgtCtcggtctgac	m5C_28474
chr7	99691208	-	C	54	12	0.222	0	0	T	42	M	0.132	0.349	7.5E-15	8.8E-10	9.6E-15	22.3614	gggcattgcaCttgtctcggt	m5C_28493
chr7	99691448	-	C	209	100	0.478	0	0	T	109	M	0.412	0.546	0	0	0	823.49	aagtgctgttCgtgcaggtag	m5C_28456
chr7	99691625	-	C	39	12	0.308	0	0	T	27	M	0.186	0.464	0	4.6E-06	0	44.5578	gtacttgctgCtccagcaggg	m5C_28478
chr7	99691628	-	C	74	30	0.405	0	0	T	44	M	0.301	0.519	0	4.8E-10	0	180.542	tgggtacttgCtgctccagca	m5C_28471
chr7	99691632	-	C	81	29	0.358	0	0	T	52	M	0.262	0.467	0	8.4E-12	0	152.096	actgtgggtaCttgctgctcc	m5C_28475
chr7	99691641	-	C	82	19	0.232	0	0	T	63	M	0.154	0.334	0	8.4E-14	0	58.3883	tgctaccgcaCtgtgggtact	m5C_28496
chr7	99691645	-	C	48	12	0.25	0	0	T	36	M	0.149	0.388	1.3E-15	7.5E-08	1.7E-15	26.6338	tccgtgctacCgcactgtggg	m5C_28480
chr7	99691675	-	C	189	77	0.407	0	0	T	112	M	0.34	0.479	0	0	0	523.411	aaagtgctgaCagtgcagata	m5C_28484
chr7	99691679	-	C	193	43	0.223	0	0	T	150	M	0.17	0.287	0	0	0	146.049	ggctaaagtgCtgacagtgca	m5C_28491
chr7	99719953	+	C	31	12	0.387	0	0	T	19	M	0.237	0.562	0	4.6E-06	0	56.9589	ggtgctggagCtggggcaggt	m5C_28425
chr7	99719959	+	C	33	21	0.636	0	0	C	21	M	0.466	0.778	0	5.2E-05	0	195.787	ggagctggggCaggtgctgga	m5C_28408
chr7	99719965	+	C	33	19	0.576	0	0	C	19	M	0.408	0.728	0	3.4E-05	0	155.067	ggggcaggtgCtggatacagg	m5C_28406
chr7	99751561	+	C	32	11	0.344	0	0	T	21	M	0.204	0.517	2.2E-16	3.1E-06	2.9E-16	35.1442	gaggcagaacCgaggtcggga	m5C_28415
chr7	100867242	+	C	43	24	0.558	0	0	C	24	M	0.411	0.696	0	3.6E-06	0	197.315	ctgtgtgagcCtgggcattcc	m5C_26650
chr7	100867247	+	C	41	10	0.244	0	0	T	31	M	0.138	0.393	2.4E-13	2.4E-08	2.9E-13	17.4472	tgagcctgggCattcccagag	m5C_26642
chr7	100867252	+	C	40	9	0.225	0	0	T	31	M	0.123	0.375	6.4E-12	1.2E-08	7.6E-12	12.4041	ctgggcattcCcagaggaggaa	m5C_26658
chr7	102782156	-	C	60	29	0.483	0	0	T	31	M	0.362	0.607	0	1.3E-08	0	209.814	ggcatatgggCataggtaagc	m5C_26639
chr7	102782164	-	C	80	18	0.231	2	0.025	T	60	M	0.151	0.336	0	4E-12	0	54.4514	gaagggcaggCatatgggcat	m5C_26635
chr7	102782176	-	C	69	16	0.232	0	0	T	53	M	0.148	0.344	0	1.1E-10	0	47.3849	cttggcctgcCggaagggcag	m5C_26640
chr7	104309751	+	C	102	52	0.51	0	0	C	52	M	0.414	0.605	0	4.1E-12	0	430.777	tgaggaaggaCcaggaaggaa	m5C_26901

chr7	104309834	+	C	33	12	0.364	0	0	T	21	M	0.222	0.534	0	4.6E-06	0	53.2487	gtccaggagaCtgagggaaca	m5C_26900
chr7	104309843	+	C	34	8	0.235	0	0	T	26	M	0.124	0.4	4.2E-11	6.8E-07	4.8E-11	10.3259	actgagggaaCagagaccact	m5C_26896
chr7	104309849	+	C	34	7	0.206	0	0	T	27	M	0.103	0.368	1.4E-09	3.4E-07	1.6E-09	6.4054	ggaacagagaCcactggaagg	m5C_26907
chr7	104309850	+	C	33	8	0.242	0	0	T	25	M	0.128	0.41	3.1E-11	6.8E-07	3.6E-11	10.7866	gaacagagacCactggaagga	m5C_26908
chr7	104309852	+	C	33	25	0.758	0	0	C	25	M	0.59	0.872	0	0.00011	0	294.875	acagagaccaCtggaaggagt	m5C_26910
chr7	111928970	+	C	88	30	0.341	0	0	T	58	M	0.25	0.445	0	1.2E-11	0	150.234	ccagacacatCcaaatgaagt	m5C_26865
chr7	111928985	+	C	244	52	0.213	0	0	T	192	M	0.166	0.269	0	0	0	173.04	tgaagtgctgCatgtggcagt	m5C_26863
chr7	111928996	+	C	266	68	0.256	0	0	T	198	M	0.207	0.311	0	0	0	281.457	atgtggcagtCtgcctttctt	m5C_26869
chr7	111929000	+	C	258	71	0.275	0	0	T	187	M	0.224	0.333	0	0	0	318.499	ggcagtctgcCtttcttaaaa	m5C_26878
chr7	111929004	+	C	242	116	0.479	0	0	T	126	M	0.417	0.542	0	0	0	967.931	gtctgcctttCttaaaaaaaa	m5C_26864
chr7	115890251	+	C	34	9	0.265	0	0	T	25	M	0.146	0.431	1.1E-12	1.2E-06	1.3E-12	15.7394	caaggaaaagCagccagtagc	m5C_27412
chr7	115890254	+	C	34	9	0.265	0	0	T	25	M	0.146	0.431	1.1E-12	1.2E-06	1.3E-12	15.7394	ggaaaagcagCcagtagcagg	m5C_27408
chr7	115890255	+	C	32	8	0.25	0	0	T	24	M	0.133	0.421	2.3E-11	6.8E-07	2.6E-11	11.2828	gaaaagcagcCagtagcaggc	m5C_27415
chr7	119646110	-	C	48	26	0.542	0	0	C	26	M	0.403	0.674	0	5.5E-06	0	209.512	aggtggttttCccagggtgaa	m5C_27526
chr7	126149007	+	C	108	30	0.278	0	0	T	78	M	0.202	0.369	0	4.7E-15	0	121.229	tggagggcatCaccaggagtg	m5C_27680
chr7	126149009	+	C	107	22	0.206	0	0	T	85	M	0.14	0.292	0	0	0	61.5486	gagggcatcaCcaggagtggt	m5C_27684
chr7	128005745	+	C	542	124	0.229	0	0	T	418	M	0.195	0.266	0	0	0	484.593	tgatcattgaCacttcgaatg	m5C_28329
chr7	128005747	+	C	693	182	0.263	1	0.001	T	510	M	0.232	0.297	0	0	0	842.934	atcattgacaCttcgaatgca	m5C_28298
chr7	128005750	+	C	787	197	0.25	0	0	T	590	M	0.221	0.282	0	0	0	871.977	attgacacttCgaatgcattt	m5C_28290
chr7	128005765	+	C	891	271	0.304	0	0	T	620	M	0.275	0.335	0	0	0	1489.63	gcatttgtggCtctgggttcc	m5C_28282
chr7	128005767	+	C	890	259	0.291	1	0.001	T	630	M	0.262	0.322	0	0	0	1359.33	atttgtggctCtgggttcctc	m5C_28327
chr7	128005775	+	C	232	50	0.216	0	0	T	182	M	0.167	0.273	0	0	0	167.469	ctctgggttcCtcccaggct	m5C_28295
chr7	128005778	+	C	150	49	0.327	0	0	T	101	M	0.257	0.405	0	0	0	251.622	tgggttcctcCcagggctatt	m5C_28310
chr7	128005784	+	C	32	20	0.625	0	0	C	20	M	0.453	0.771	0	4.3E-05	0	181.016	cctcccagggCtattaaaaaa	m5C_28320
chr7	128033373	-	C	68	26	0.382	0	0	T	42	M	0.276	0.501	0	5.6E-09	0	143.568	ccatgcctgcCtgagctgtcc	m5C_28338
chr7	128033377	-	C	68	21	0.309	0	0	T	47	M	0.212	0.426	0	1E-09	0	88.9234	tgggccatgcCtgcctgagct	m5C_28347
chr7	128337569	+	C	41	11	0.268	0	0	T	30	M	0.157	0.419	6E-15	4.4E-08	7.6E-15	24.5536	ctggttagtaCttggatggga	m5C_28323
chr7	128337583	+	C	119	30	0.252	0	0	T	89	M	0.183	0.337	0	0	0	109.605	gatgggagatCgcctggaaat	m5C_28280
chr7	128337585	+	C	130	43	0.331	0	0	T	87	M	0.256	0.415	0	0	0	219.961	tgggagatcgCctggaaatac	m5C_28318
chr7	128337586	+	C	129	37	0.287	0	0	T	92	M	0.216	0.37	0	0	0	159.713	gggagatcgcCtggaaatacc	m5C_28325
chr7	128337595	+	C	142	43	0.303	0	0	T	99	M	0.233	0.383	0	0	0	200.602	cctggaaataCcgggtgctgt	m5C_28330
chr7	128337596	+	C	138	48	0.348	0	0	T	90	M	0.273	0.43	0	0	0	262.519	ctggaaatacCgggtgctgta	m5C_28292
chr7	128337602	+	C	139	40	0.288	0	0	T	99	M	0.219	0.368	0	0	0	175.212	ataccgggtgCtgtaggtttt	m5C_28314
chr7	128337759	+	C	41	14	0.341	0	0	T	27	M	0.216	0.495	0	1.9E-07	0	60.3641	tgccataccaCcctgaacgcg	m5C_28317
chr7	128337760	+	C	39	8	0.205	0	0	T	31	M	0.108	0.355	1.6E-10	6.8E-07	1.8E-10	8.44384	gccataccacCctgaacgcgc	m5C_28322
chr7	128337761	+	C	41	9	0.22	0	0	T	32	M	0.12	0.367	8.4E-12	1.2E-08	9.9E-12	11.9621	ccataccaccCtgaacgcgcc	m5C_28293
chr7	128337771	+	C	41	20	0.488	0	0	T	21	M	0.343	0.635	0	1.4E-06	0	137.017	ctgaacgcgcCgatctcgtc	m5C_28326
chr7	128337778	+	C	42	13	0.31	0	0	T	29	M	0.191	0.46	0	1.2E-07	0	49.5844	cgcccgatctCgtctgatctc	m5C_28309
chr7	128407545	+	C	31	14	0.452	0	0	T	17	M	0.292	0.622	0	9.2E-06	0	81.6522	tactgatgagCcagaatgggt	m5C_28321
chr7	128407587	+	C	126	31	0.246	0	0	T	95	M	0.179	0.328	0	0	0	111.031	tgttgagtttCgggataagaa	m5C_28319
chr7	129410288	-	C	112	67	0.598	0	0	C	67	M	0.506	0.684	0	3.6E-12	0	677.529	agaactcacaCtggtgaggta	m5C_28367
chr7	129410290	-	C	151	60	0.397	0	0	T	91	M	0.323	0.477	0	0	0	387.333	gtagaactcaCactggtgagg	m5C_28358
chr7	129410292	-	C	150	60	0.4	0	0	T	90	M	0.325	0.48	0	0	0	390.05	tggtagaactCacactggtga	m5C_28350
chr7	129410294	-	C	155	50	0.323	0	0	T	105	M	0.254	0.4	0	0	0	254.047	aatggtagaaCtcacactggt	m5C_28360
chr7	129410305	-	C	142	51	0.359	0	0	T	91	M	0.285	0.441	0	0	0	290.612	ccgtttttggCaatggtagaa	m5C_28357
chr7	129414810	-	C	35	7	0.2	0	0	T	28	M	0.1	0.359	1.9E-09	3.4E-07	2.1E-09	6.13884	tggtagaattCactgtgaaca	m5C_28356
chr7	129474869	-	C	33	10	0.303	0	0	T	23	M	0.174	0.473	1.6E-14	2E-06	2E-14	23.982	ggaggaggcgCtgaaagaaca	m5C_28362
chr7	130824925	+	C	67	28	0.418	0	0	T	39	M	0.307	0.537	0	9.8E-09	0	172.156	tggtactcacCtgtgggtgct	m5C_27618
chr7	130824934	+	C	120	66	0.55	0	0	C	66	M	0.461	0.636	0	3E-12	0	608.274	cctgtgggtgCttcttagtct	m5C_27611
chr7	132570452	-	C	31	12	0.387	0	0	T	19	M	0.237	0.562	0	4.6E-06	0	56.9589	atgcattctaCaaagaacagc	m5C_27757
chr7	134552521	+	C	46	25	0.543	0	0	C	25	M	0.402	0.678	0	4.5E-06	0	200.9	acttagaaggCaaaagaggga	m5C_27999
chr7	134617957	+	C	31	14	0.452	0	0	T	17	M	0.292	0.622	0	9.2E-06	0	81.6522	gaaaatgaaaCtaccgagaag	m5C_27949
chr7	134618069	+	C	134	44	0.328	0	0	T	90	M	0.255	0.412	0	0	0	224.046	ctgaagaaaaCaagaaagaag	m5C_27968
chr7	134618081	+	C	128	47	0.367	0	0	T	81	M	0.289	0.453	0	1E-15	0	271.362	agaaagaagaCaaggaaaaagg	m5C_27990
chr7	134618112	+	C	63	14	0.222	0	0	T	49	M	0.137	0.339	0	3.2E-11	0	38.4297	agaggagaagCcaaagcgagg	m5C_27978
chr7	134632419	+	C	62	26	0.419	0	0	T	36	M	0.305	0.543	0	5.6E-09	0	158.482	ggctttggagCtggaggaact	m5C_27967
chr7	134632430	+	C	106	37	0.349	0	0	T	69	M	0.265	0.444	0	6.7E-14	0	196.126	tggaggaactCaagaaaaaga	m5C_28002
chr7	134632460	+	C	205	76	0.371	0	0	T	129	M	0.308	0.439	0	0	0	467.489	gaaggaaggtCctggaggagg	m5C_27956
chr7	134632461	+	C	196	82	0.418	0	0	T	114	M	0.352	0.488	0	0	0	576.501	aaggaaggtcCtggaggagga	m5C_27947
chr7	134632476	+	C	90	20	0.222	0	0	T	70	M	0.149	0.318	0	1.7E-15	0	59.4849	ggaggaagagCagaggaggaa	m5C_27980
chr7	134632488	+	C	140	32	0.229	0	0	T	108	M	0.167	0.305	0	0	0	106.762	gaggaggaagCaggaggaagc	m5C_27950
chr7	134632499	+	C	152	44	0.289	0	0	T	108	M	0.223	0.366	0	0	0	196.477	aggaggaagcCgatcgaaaac	m5C_27965
chr7	134632509	+	C	142	32	0.225	0	0	T	110	M	0.164	0.301	0	0	0	105.21	cgatcgaaaaCtcagagagga	m5C_27963
chr7	134635140	+	C	67	30	0.448	0	0	T	37	M	0.335	0.566	0	1.7E-08	0	200.857	gaagaggaggCtaaaggaaga	m5C_27988
chr7	138100232	+	C	45	12	0.267	0	0	T	33	M	0.16	0.41	5.6E-16	7.5E-08	7.3E-16	29.2263	catggctggtCcaatgatagt	m5C_27590
chr7	138100255	+	C	39	14	0.359	0	0	T	25	M	0.227	0.516	0	9.2E-06	0	63.6773	gttatcagaaCttattaacat	m5C_27573
chr7	139025456	+	C	69	14	0.203	0	0	T	55	M	0.125	0.312	3.3E-16	3.2E-11	4.4E-16	27.0622	gccccagtggCctaatggata	m5C_27591
chr7	139025457	+	C	71	20	0.282	0	0	T	51	M	0.19	0.395	0	1.1E-11	0	76.1478	ccccagtggcCtaatggataa	m5C_27564
chr7	139025477	+	C	88	23	0.261	0	0	T	65	M	0.181	0.362	0	7.2E-13	0	83.2283	aggcattggcCtcctaagcca	m5C_27583
chr7	139025480	+	C	92	26	0.283	0	0	T	66	M	0.201	0.382	0	4.6E-14	0	104.393	cattggcctcCtaagccaggg	m5C_27581
chr7	139025507	+	C	124	44	0.355	0	0	T	80	M	0.276	0.442	0	4.4E-16	0	243.008	ggttcgagtcCcatctggggt	m5C_27576
chr7	139025508	+	C	138	29	0.21	0	0	T	109	M	0.15	0.286	0	0	0	87.2836	gttcgagtccCatctggggtg	m5C_27570
chr7	139025527	+	C	138	64	0.464	0	0	T	74	M	0.383	0.547	0	3E-15	0	489.823	tggcctgtgaCttttgtcctt	m5C_27584
chr7	139470115	-	C	33	9	0.273	0	0	T	24	M	0.151	0.442	7.5E-13	1.2E-06	9E-13	16.4418	gttataatgcCgaggttgtga	m5C_27598
chr7	140086589	-	C	35	8	0.235	1	0.029	T	26	M	0.124	0.4	4.2E-11	6.8E-07	4.8E-11	10.3259	ataccgggtgCtgtaggcttt	m5C_27927
chr7	140086595	-	C	35	11	0.314	0	0	T	24	M	0.186	0.48	6.7E-16	3.1E-06	8.7E-16	30.9686	ctgggaatacCgggtgctgta	m5C_27930
chr7	140086596	-	C	36	12	0.333	0	0	T	24	M	0.202	0.497	0	4.6E-06	0	48.5146	cctgggaataCcgggtgctgt	m5C_27925
chr7	140086605	-	C	33	7	0.212	0	0	T	26	M	0.107	0.378	1.1E-09	3.4E-07	1.2E-09	6.69165	gggacactgcCtgggaatacc	m5C_27924
chr7	140486765	+	C	36	14	0.389	0	0	T	22	M	0.248	0.551	0	9.2E-06	0	69.397	ccgatcttgtCtgatcctgga	m5C_27901
chr7	141304708	-	C	31	9	0.29	0	0	T	22	M	0.161	0.466	3.7E-13	1.2E-06	4.4E-13	18.0154	aatatgtgatCccagattgga	m5C_27943
chr7	141427866	+	C	38	8	0.211	0	0	T	30	M	0.111	0.363	1.3E-10	6.8E-07	1.4E-10	8.77246	tccggatgtgCtgacccctgt	m5C_27876
chr7	141427871	+	C	38	8	0.211	0	0	T	30	M	0.111	0.363	1.3E-10	6.8E-07	1.4E-10	8.77246	atgtgctgacCcctgtgatat	m5C_27914
chr7	141692326	-	C	33	21	0.636	0	0	C	21	M	0.466	0.778	0	5.2E-05	0	195.787	cccaaggcagCaaatgtctaa	m5C_27929
chr7	141692335	-	C	33	26	0.788	0	0	C	26	M	0.622	0.893	0	0.00013	0	323.69	cacaatggccCcaaggcagca	m5C_27942
chr7	146008819	+	C	39	14	0.359	0	0	T	25	M	0.227	0.516	0	9.2E-06	0	63.6773	attggctgatCtgagtgcagt	m5C_27720
chr7	148135536	+	C	58	13	0.224	0	0	T	45	M	0.136	0.347	6.7E-16	1.6E-09	8.7E-16	26.8192	agagtggggaCtgccaggttg	m5C_33300
chr7	148135540	+	C	59	28	0.475	0	0	T	31	M	0.353	0.6	0	3.1E-07	0	197.488	tggggactgcCaggttgccta	m5C_33290
chr7	148135547	+	C	59	12	0.203	0	0	T	47	M	0.12	0.323	2.6E-14	8.8E-10	3.3E-14	19.6113	tgccaggttgCctaaggaggg	m5C_33287
chr7	148638653	+	C	1220	245	0.201	0	0	T	975	M	0.179	0.224	0	0	0	878.542	gcttgactagCttgctgtttt	m5C_33298
chr7	148638657	+	C	1054	282	0.268	0	0	T	772	M	0.242	0.295	0	0	0	1363.22	gactagcttgCtgttttgcac	m5C_33281
chr7	148660414	+	C	408	147	0.36	0	0	T	261	M	0.315	0.408	0	0	0	926.723	tctggctggtCcgatggtagt	m5C_33305
chr7	148660415	+	C	413	130	0.315	0	0	T	283	M	0.272	0.361	0	0	0	706.835	ctggctggtcCgatggtagtg	m5C_33297
chr7	148660470	+	C	447	112	0.251	1	0.002	T	334	M	0.213	0.293	0	0	0	477.378	agttggtataCaaccccccac	m5C_33310
chr7	148660483	+	C	440	116	0.264	1	0.002	T	323	M	0.225	0.307	0	0	0	522.38	ccccccactgCtaaatttgac	m5C_33307
chr7	148660497	+	C	264	71	0.269	0	0	T	193	M	0.219	0.325	0	0	0	311.047	atttgactggCtttttaccat	m5C_33303
chr7	148663329	-	C	36	8	0.222	0	0	T	28	M	0.117	0.381	7.4E-11	6.8E-07	8.4E-11	9.49676	gcctgggcaaCatagtgagac	m5C_33373
chr7	148663341	-	C	37	14	0.378	0	0	T	23	M	0.241	0.539	0	9.2E-06	0	67.3786	agttcgagacCagcctgggca	m5C_33374
chr7	148680890	+	C	286	64	0.225	1	0.003	T	221	M	0.18	0.276	0	0	0	230.35	ttgatcacaaCcagttacaga	m5C_33289
chr7	148680911	+	C	286	63	0.22	0	0	T	223	M	0.176	0.272	0	0	0	221.925	tttctttgttCcttctccact	m5C_33285
chr7	148680912	+	C	285	80	0.281	0	0	T	205	M	0.232	0.336	0	0	0	370.745	ttctttgttcCttctccactc	m5C_33293
chr7	148680918	+	C	292	66	0.226	0	0	T	226	M	0.182	0.277	0	0	0	239.964	gttccttctcCactcccactg	m5C_33295

chr7	148680944	+	C	152	38	0.25	0	0	T	114	M	0.188	0.324	0	0	0	142.803	cttgactagcCttttgctgtg	m5C_33311
chr7	148684237	-	C	145	29	0.2	0	0	T	116	M	0.143	0.272	0	0	0	82.9494	ctgcacttgaCtagtctttta	m5C_33379
chr7	148684242	-	C	300	63	0.21	0	0	T	237	M	0.168	0.26	0	0	0	211.329	cactactgcaCttgactagtc	m5C_33334
chr7	148684257	-	C	360	78	0.217	0	0	T	282	M	0.177	0.262	0	0	0	276.464	tcttcccccCttctcactac	m5C_33365
chr7	148684261	-	C	363	86	0.237	0	0	T	277	M	0.196	0.283	0	0	0	337.243	ctactctttcCccccttctca	m5C_33347
chr7	148684271	-	C	385	80	0.209	2	0.005	T	303	M	0.171	0.252	0	0	0	273.849	actccttgttCtactctttcc	m5C_33332
chr7	148684277	-	C	450	94	0.209	0	0	T	356	M	0.174	0.249	0	0	0	326.878	gatcgaactcCttgttctact	m5C_33377
chr7	148684278	-	C	445	94	0.211	0	0	T	351	M	0.176	0.252	0	0	0	330.624	agatcgaactCcttgttctac	m5C_33337
chr7	148684280	-	C	483	115	0.239	1	0.002	T	367	M	0.203	0.279	0	0	0	466.209	acagatcgaaCtccttgttct	m5C_33345
chr7	148684289	-	C	647	132	0.204	1	0.002	T	514	M	0.175	0.237	0	0	0	462.082	cagtcagttaCagatcgaact	m5C_33321
chr7	148684295	-	C	715	150	0.21	0	0	T	565	M	0.182	0.241	0	0	0	544.608	tgttcacagtCagttacagat	m5C_33336
chr7	148684299	-	C	700	170	0.244	2	0.003	T	528	M	0.213	0.277	0	0	0	724.778	tgattgttcaCagtcagttac	m5C_33333
chr7	148684315	-	C	754	161	0.214	2	0.003	T	591	M	0.186	0.245	0	0	0	599.787	agtgagttatCtcaattgatt	m5C_33346
chr7	148684332	-	C	718	234	0.326	0	0	T	484	M	0.293	0.361	0	0	0	1369.49	ttggctggtcCgaaggtagtg	m5C_33357
chr7	148684333	-	C	711	216	0.304	1	0.001	T	494	M	0.272	0.339	0	0	0	1172.93	attggctggtCcgaaggtagt	m5C_33320
chr7	148700871	-	C	35	8	0.229	0	0	T	27	M	0.121	0.39	5.6E-11	6.8E-07	6.4E-11	9.89691	ctgaggtctgCggaaggtggg	m5C_33378
chr7	148700874	-	C	37	17	0.459	0	0	T	20	M	0.31	0.616	0	2.1E-05	0	105.53	ggcctgaggtCtgcggaaggt	m5C_33313
chr7	148700881	-	C	40	30	0.75	0	0	C	30	M	0.598	0.858	0	1.2E-05	0	358.834	ctttgaaggcCtgaggtctgc	m5C_33325
chr7	148700882	-	C	38	8	0.211	0	0	T	30	M	0.111	0.363	1.3E-10	6.8E-07	1.4E-10	8.77246	gctttgaaggCctgaggtctg	m5C_33352
chr7	148702324	-	C	40	14	0.35	0	0	T	26	M	0.221	0.505	0	1.9E-07	0	61.9761	tcaatgccgcCatcctggacg	m5C_33324
chr7	148702327	-	C	36	12	0.333	0	0	T	24	M	0.202	0.497	0	4.6E-06	0	48.5146	atgtcaatgcCgccatcctgg	m5C_33341
chr7	148702328	-	C	32	11	0.344	0	0	T	21	M	0.204	0.517	2.2E-16	3.1E-06	2.9E-16	35.1442	gatgtcaatgCcgccatcctg	m5C_33318
chr7	148702333	-	C	32	11	0.344	0	0	T	21	M	0.204	0.517	2.2E-16	3.1E-06	2.9E-16	35.1442	gggaggatgtCaatgccgcca	m5C_33344
chr7	149388313	-	C	57	13	0.228	0	0	T	44	M	0.138	0.352	4.4E-16	1.6E-09	5.8E-16	27.6258	gagcatttgaCtgcagatcaa	m5C_33314
chr7	149388331	-	C	92	25	0.272	0	0	T	67	M	0.191	0.37	0	2.9E-14	0	95.6782	ggatatagctCaggggtagag	m5C_33366
chr7	149388346	-	C	37	13	0.351	0	0	T	24	M	0.218	0.512	0	6.7E-06	0	56.7461	ctttgtggccCatggggatat	m5C_33364
chr7	149388347	-	C	37	14	0.378	0	0	T	23	M	0.241	0.539	0	9.2E-06	0	67.3786	actttgtggcCcatggggata	m5C_33359
chr7	149388348	-	C	38	14	0.368	0	0	T	24	M	0.234	0.527	0	9.2E-06	0	65.4752	aactttgtggCccatggggat	m5C_33327
chr7	150763862	+	C	36	10	0.278	0	0	T	26	M	0.158	0.44	4.8E-14	2E-06	5.9E-14	21.1115	agtgctgggcCttggccaagc	m5C_27775
chr7	150763867	+	C	37	12	0.324	0	0	T	25	M	0.196	0.485	0	4.6E-06	0	47.1194	tgggccttggCcaagcccggc	m5C_27782
chr7	150763872	+	C	33	7	0.212	0	0	T	26	M	0.107	0.378	1.1E-09	3.4E-07	1.2E-09	6.69165	cttggccaagCccggcactgg	m5C_27771
chr7	150763874	+	C	36	12	0.333	0	0	T	24	M	0.202	0.497	0	4.6E-06	0	48.5146	tggccaagccCggcactggtg	m5C_27766
chr7	150763879	+	C	37	10	0.27	0	0	T	27	M	0.154	0.43	6.7E-14	2E-06	8.3E-14	20.2815	aagcccggcaCtggtgggcaa	m5C_27779
chr7	150763887	+	C	38	10	0.263	0	0	T	28	M	0.15	0.42	9.4E-14	2E-06	1.2E-13	19.5037	cactggtgggCaaaggagtga	m5C_27770
chr7	150935541	+	C	105	30	0.286	0	0	T	75	M	0.208	0.378	0	4.7E-15	0	124.842	gaggaggaagCcctggagggg	m5C_27781
chr7	150935543	+	C	106	32	0.302	0	0	T	74	M	0.223	0.395	0	1.1E-14	0	142.517	ggaggaagccCtggaggggct	m5C_27769
chr7	150935552	+	C	98	34	0.347	0	0	T	64	M	0.26	0.445	0	1.1E-12	0	176.857	cctggaggggCtggaggtgat	m5C_27763
chr7	151722307	-	C	32	10	0.312	0	0	T	22	M	0.18	0.486	1.1E-14	2E-06	1.3E-14	25.0836	ccactgccctCcagcctgggc	m5C_27787
chr7	151722309	-	C	31	8	0.258	0	0	T	23	M	0.137	0.432	1.7E-11	6.8E-07	1.9E-11	11.8182	agccactgccCtccagcctgg	m5C_27800
chr7	151722311	-	C	46	13	0.283	0	0	T	33	M	0.173	0.425	0	1.2E-07	0	45.0425	atagccactgCcctccagcct	m5C_27805
chr7	151722317	-	C	68	19	0.279	0	0	T	49	M	0.187	0.396	0	4.5E-10	0	70.9757	ctgtgaatagCcactgccctc	m5C_27789
chr7	151722344	-	C	93	19	0.204	0	0	T	74	M	0.135	0.297	0	8.9E-16	0	51.2526	ctgtgctgatCggtagcggga	m5C_27799
chr7	152290066	-	C	355	124	0.349	0	0	T	231	M	0.302	0.4	0	0	0	747.856	atactgggtgCtgtaggtgtg	m5C_27849
chr7	152290073	-	C	344	91	0.265	0	0	T	253	M	0.221	0.314	0	0	0	401.689	cctgggaataCtgggtgctgt	m5C_27839
chr7	152290082	-	C	202	59	0.292	0	0	T	143	M	0.234	0.358	0	0	0	275.788	gggagactgcCtgggaatact	m5C_27853
chr7	152290083	-	C	187	60	0.321	0	0	T	127	M	0.258	0.391	0	0	0	309.76	tgggagactgCctgggaatac	m5C_27838
chr7	152290086	-	C	158	59	0.376	1	0.006	T	98	M	0.304	0.454	0	0	0	358.547	ggatgggagaCtgcctgggaa	m5C_27851
chr7	155122445	-	C	36	8	0.222	0	0	T	28	M	0.117	0.381	7.4E-11	6.8E-07	8.4E-11	9.49676	ccttcccctggCtgtgttgga	m5C_28251
chr7	155978903	-	C	40	8	0.2	0	0	T	32	M	0.105	0.348	2.1E-10	5.5E-09	2.3E-10	8.13503	ggtctgagtgCagtggtgttt	m5C_28257
chr7	155978910	-	C	39	28	0.718	0	0	C	28	M	0.562	0.835	0	0.00017	0	314.857	ctctgctggtCtgagtgcagt	m5C_28242
chr7	157314002	-	C	47	12	0.255	0	0	T	35	M	0.153	0.395	1E-15	7.5E-08	1.3E-15	27.4561	ccatgtctgcCcgagcgtctc	m5C_29511
chr7	158526934	-	C	31	11	0.355	0	0	T	20	M	0.211	0.531	1.1E-16	3.1E-06	1.5E-16	37.0595	ggtatgacctCacggaagatg	m5C_28608
chr8	1998415	-	C	31	7	0.226	0	0	T	24	M	0.114	0.398	6.4E-10	3.4E-07	7.2E-10	7.33205	ccaggacggcCgagtggccca	m5C_28528
chr8	6926163	-	C	169	72	0.426	0	0	T	97	M	0.354	0.501	0	0	0	509.672	ggattcggtgCttttaccgct	m5C_28277
chr8	6926168	-	C	215	46	0.214	0	0	T	169	M	0.164	0.274	0	0	0	151.256	ggttgggattCggtgctttta	m5C_28270
chr8	6926183	-	C	242	84	0.347	0	0	T	158	M	0.29	0.409	0	0	0	487.099	ccctggtggtCtagtggttgg	m5C_28279
chr8	6926193	-	C	96	22	0.229	0	0	T	74	M	0.156	0.323	0	5.8E-15	0	68.8547	ttcaaagttcCcctggtggtc	m5C_28271
chr8	8090678	-	C	52	11	0.212	0	0	T	41	M	0.122	0.34	1.4E-13	4.4E-10	1.7E-13	17.3019	tgggctggtcCagcctggtag	m5C_28552
chr8	11357052	+	C	37	17	0.459	0	0	T	20	M	0.31	0.616	0	2.1E-05	0	105.53	gtggcttgtgCtgaaggctgc	m5C_28671
chr8	11357059	+	C	33	8	0.242	0	0	T	25	M	0.128	0.41	3.1E-11	6.8E-07	3.6E-11	10.7866	gtgctgaaggCtgcataagaa	m5C_28668
chr8	11770847	+	C	34	12	0.353	0	0	T	22	M	0.215	0.521	0	4.6E-06	0	51.5705	ctgccactacCcgggtttgat	m5C_28655
chr8	11770861	+	C	82	17	0.207	0	0	T	65	M	0.134	0.307	0	2.3E-14	0	45.4454	gtttgatttcCggtcagggaa	m5C_28665
chr8	12570076	+	C	49	13	0.265	0	0	T	36	M	0.162	0.403	0	1.2E-07	0	42.1489	tctaagttccCctggtggtct	m5C_28850
chr8	12570085	+	C	202	61	0.303	1	0.005	T	140	M	0.244	0.37	0	0	0	297.8	ccctggtggtCtagtggttgg	m5C_28849
chr8	12570103	+	C	197	57	0.289	0	0	T	140	M	0.231	0.356	0	0	0	262.8	tgggatttggCgctctcactg	m5C_28854
chr8	12570105	+	C	168	35	0.208	0	0	T	133	M	0.154	0.276	0	0	0	107.648	ggatttggcgCtctcactgct	m5C_28853
chr8	12570109	+	C	99	38	0.384	0	0	T	61	M	0.294	0.482	0	3.7E-12	0	223.505	ttggcgctctCactgctgctg	m5C_28862
chr8	12570111	+	C	79	17	0.215	0	0	T	62	M	0.139	0.318	0	2.2E-12	0	47.2331	ggcgctctcaCtgctgctgcc	m5C_28861
chr8	12570114	+	C	39	11	0.282	0	0	T	28	M	0.165	0.438	3.1E-15	3.1E-06	4E-15	26.4001	gctctcactgCtgctgcccgc	m5C_28858
chr8	13110617	+	C	68	20	0.294	0	0	T	48	M	0.199	0.411	0	6.9E-10	0	79.6755	tgggttaactCctggactggt	m5C_28856
chr8	13110623	+	C	82	37	0.451	0	0	T	45	M	0.348	0.559	0	9.7E-11	0	257.602	aactcctggaCtggtgtaaga	m5C_28859
chr8	14190071	+	C	44	17	0.386	0	0	T	27	M	0.257	0.534	0	5.6E-07	0	87.4514	tgaggctggtCtgaaggtagt	m5C_28627
chr8	14190089	+	C	51	12	0.235	0	0	T	39	M	0.14	0.368	3.3E-15	8.8E-10	4.3E-15	24.3305	agtgagttatCtcaattgatt	m5C_28628
chr8	16786915	-	C	44	11	0.25	0	0	T	33	M	0.146	0.394	1.6E-14	4.4E-08	2E-14	22.132	ttctgagggaCagagagtgag	m5C_28578
chr8	17352732	-	C	31	10	0.323	0	0	T	21	M	0.186	0.499	7.1E-15	2E-06	9E-15	26.2725	cgcggtggctCagggcggttg	m5C_28576
chr8	17352734	-	C	31	10	0.323	0	0	T	21	M	0.186	0.499	7.1E-15	2E-06	9E-15	26.2725	gtcgcggtggCtcagggcggt	m5C_28573
chr8	17352742	-	C	32	8	0.25	0	0	T	24	M	0.133	0.421	2.3E-11	6.8E-07	2.6E-11	11.2828	tcctgggagtCgcggtggctc	m5C_28577
chr8	20066990	+	C	63	17	0.27	0	0	T	46	M	0.176	0.39	0	1.8E-10	0	59.7846	ccggatggcaCaaagagaagt	m5C_28711
chr8	21456079	-	C	32	13	0.406	0	0	T	19	M	0.255	0.577	0	6.7E-06	0	66.3505	gtccctggagCgggttgtgcc	m5C_28738
chr8	22102490	-	C	185	67	0.362	0	0	T	118	M	0.296	0.434	0	0	0	397.12	gttgagagggCgaaaaaggat	m5C_29435
chr8	22576063	+	C	144	54	0.375	0	0	T	90	M	0.3	0.456	0	0	0	324.153	gcctgggtgcCtgtctgagtg	m5C_29394
chr8	22576067	+	C	175	70	0.4	0	0	T	105	M	0.33	0.474	0	0	0	462.442	gggtgcctgtCtgagtgttgc	m5C_29401
chr8	22576077	+	C	152	31	0.204	0	0	T	121	M	0.148	0.275	0	0	0	91.4894	ctgagtgttgCccgggatggc	m5C_29384
chr8	23147875	+	C	57	17	0.298	0	0	T	40	M	0.195	0.427	0	1.1E-08	0	66.4101	gaaggagagtCtgttggagaa	m5C_29408
chr8	23147890	+	C	75	26	0.347	0	0	T	49	M	0.249	0.459	0	1.3E-10	0	129.387	ggagaagaggCtggtggcaga	m5C_29414
chr8	23147897	+	C	81	28	0.346	0	0	T	53	M	0.251	0.454	0	5.8E-12	0	140.686	aggctggtggCagaggaggaa	m5C_29388
chr8	23147913	+	C	63	35	0.556	0	0	C	35	M	0.433	0.672	0	5.3E-08	0	303.236	aggaagaggaCgaagaggagg	m5C_29412
chr8	25165165	+	C	78	22	0.282	0	0	T	56	M	0.194	0.39	0	2.8E-11	0	85.4709	tggtgatgtgCctcctgctta	m5C_28796
chr8	25165169	+	C	81	21	0.259	0	0	T	60	M	0.176	0.364	0	2.6E-13	0	74.0352	gatgtgcctcCtgcttaattt	m5C_28792
chr8	25165185	+	C	75	16	0.213	0	0	T	59	M	0.136	0.319	0	1.2E-12	0	43.4563	aatttctgaaCcagctttcct	m5C_28803
chr8	25994486	+	C	51	11	0.216	0	0	T	40	M	0.125	0.346	1.1E-13	4.4E-10	1.4E-13	17.8061	agatggaagaCtgcctgggaa	m5C_28812
chr8	25994489	+	C	63	21	0.333	0	0	T	42	M	0.229	0.456	0	1E-09	0	96.3877	tggaagactgCctgggaatac	m5C_28808
chr8	25994490	+	C	64	15	0.234	0	0	T	49	M	0.147	0.351	0	6E-11	0	44.2493	ggaagactgcCtgggaatact	m5C_28811
chr8	25994506	+	C	59	25	0.424	0	0	T	34	M	0.306	0.551	0	1.5E-07	0	153.063	atactgggtgCtgtaggctta	m5C_28789
chr8	25994513	+	C	56	21	0.375	0	0	T	35	M	0.26	0.506	0	4.8E-08	0	109.242	gtgctgtaggCttaaaaagaa	m5C_28809
chr8	32872739	+	C	37	8	0.216	0	0	T	29	M	0.114	0.372	9.7E-11	6.8E-07	1.1E-10	9.12273	gctaaataagCtgttgggccc	m5C_30325
chr8	32872747	+	C	38	25	0.658	0	0	C	25	M	0.499	0.788	0	0.00011	0	249.459	agctgttgggCccataccca	m5C_30335
chr8	32872748	+	C	39	19	0.487	0	0	T	20	M	0.339	0.638	0	3.4E-05	0	128.687	gctgttgggcCcataccccaa	m5C_30326
chr8	33371091	+	C	1086	223	0.206	2	0.002	T	861	M	0.183	0.231	0	0	0	814.909	cattacccgtCtgacctgaac	m5C_30319
chr8	33371096	+	C	818	198	0.242	0	0	T	620	M	0.214	0.273	0	0	0	847.243	cccgtctgacCtgaacttcaa	m5C_30336
chr8	34386131	-	C	31	15	0.484	0	0	T	16	M	0.32	0.652	0	1.2E-05	0	95.91	ggggtcagccCacccggccag	m5C_28848

chr8	41660874	+	C	43	11	0.256	0	0	T	32	M	0.149	0.402	1.2E-14	4.4E-08	1.5E-14	22.8887	cctggctggtCtgagtgcagt	m5C_29245
chr8	41660893	+	C	32	8	0.25	0	0	T	24	M	0.133	0.421	2.3E-11	6.8E-07	2.6E-11	11.2828	gtgatgtttaCaactaattgg	m5C_29246
chr8	41660896	+	C	32	7	0.219	0	0	T	25	M	0.11	0.388	8.5E-10	3.4E-07	9.5E-10	6.99971	atgtttacaaCtaattggtca	m5C_29249
chr8	41697605	-	C	35	12	0.343	0	0	T	23	M	0.208	0.508	0	4.6E-06	0	49.9956	gcgctccagcCtgggcaatgt	m5C_29281
chr8	41697614	-	C	42	13	0.31	0	0	T	29	M	0.191	0.46	0	1.2E-07	0	49.5844	atagccactgCgctccagcct	m5C_29279
chr8	41697617	-	C	44	14	0.318	0	0	T	30	M	0.2	0.466	0	1.9E-07	0	55.9976	tgaatagccaCtgcgctccag	m5C_29272
chr8	41697619	-	C	43	19	0.442	0	0	T	24	M	0.304	0.589	0	1E-06	0	115.646	tgtgaatagcCactgcgctcc	m5C_29261
chr8	41697652	-	C	53	20	0.377	0	0	T	33	M	0.259	0.512	0	3.4E-08	0	103.744	aactcctgtgCtgatcggtag	m5C_29276
chr8	41697658	-	C	52	20	0.385	0	0	T	32	M	0.265	0.52	0	3.4E-08	0	105.881	ggtcaaaactCctgtgctgat	m5C_29274
chr8	41697665	-	C	42	11	0.262	0	0	T	31	M	0.153	0.411	8.4E-15	4.4E-08	1.1E-14	23.69	tggagcaggtCaaaactcctg	m5C_29267
chr8	41982260	-	C	156	38	0.244	0	0	T	118	M	0.183	0.317	0	0	0	139.021	tttttggcggCgggaggggcg	m5C_29284
chr8	41982263	-	C	168	70	0.417	0	0	T	98	M	0.345	0.492	0	0	0	482.71	tttttttggCggcgggaggg	m5C_29263
chr8	51982880	+	C	38	8	0.211	0	0	T	30	M	0.111	0.363	1.3E-10	6.8E-07	1.4E-10	8.77246	gtgtgaattgCaggatgtatt	m5C_29043
chr8	52630753	-	C	49	17	0.347	0	0	T	32	M	0.229	0.487	0	5.6E-07	0	77.9423	ttggggtctgCttgggacaat	m5C_29198
chr8	52630771	-	C	88	18	0.205	0	0	T	70	M	0.133	0.3	0	4.5E-14	0	48.055	ctgcctgtggCtgtggtgttg	m5C_29183
chr8	53554965	-	C	31	13	0.419	0	0	T	18	M	0.264	0.592	0	6.7E-06	0	68.6798	gctgtggaaaCagcagatgaa	m5C_29165
chr8	53554977	-	C	33	8	0.242	0	0	T	25	M	0.128	0.41	3.1E-11	6.8E-07	3.6E-11	10.7866	tcagatagatCcgctgtggaa	m5C_29182
chr8	55311404	-	C	45	18	0.4	0	0	T	27	M	0.27	0.545	0	7.7E-07	0	97.2889	tttttttggCggcgggggag	m5C_29309
chr8	56986427	-	C	367	119	0.326	2	0.005	T	246	M	0.28	0.376	0	0	0	666.319	tgttgagtaaCggtgataatt	m5C_29696
chr8	56986443	-	C	477	132	0.277	0	0	T	345	M	0.238	0.319	0	0	0	629.601	gaggaggtacCtattgtgttg	m5C_29669
chr8	56987049	-	C	33	13	0.394	0	0	T	20	M	0.247	0.563	0	6.7E-06	0	64.1755	gaggaagacgCggtcgtaagg	m5C_29684
chr8	59337326	-	C	34	8	0.235	0	0	T	26	M	0.124	0.4	4.2E-11	6.8E-07	4.8E-11	10.3259	agcagaaaagCaagaggaaag	m5C_29330
chr8	59337334	-	C	34	9	0.265	0	0	T	25	M	0.146	0.431	1.1E-12	1.2E-06	1.3E-12	15.7394	tgaagaagagCagaaaagcaa	m5C_29332
chr8	59504808	+	C	70	17	0.243	0	0	T	53	M	0.158	0.355	0	2.2E-12	0	53.5559	tccttgatgtCtagtggttag	m5C_29316
chr8	59504830	+	C	102	32	0.314	0	0	T	70	M	0.232	0.409	0	1.1E-14	0	148.37	atttggtgctCtcactgcagc	m5C_29317
chr8	59504837	+	C	71	26	0.366	0	0	T	45	M	0.264	0.482	0	1.3E-10	0	137.124	gctctcactgCagcagcctgg	m5C_29324
chr8	60368452	+	C	39	11	0.282	0	0	T	28	M	0.165	0.438	3.1E-15	3.1E-06	4E-15	26.4001	tagtctatggCcataacaccc	m5C_29654
chr8	60368453	+	C	38	11	0.289	0	0	T	27	M	0.17	0.448	2.1E-15	3.1E-06	2.7E-15	27.4488	agtctatggcCataacaccct	m5C_29646
chr8	60368462	+	C	36	14	0.389	0	0	T	22	M	0.248	0.551	0	9.2E-06	0	69.397	ccataacaccCtgaacgcgcc	m5C_29650
chr8	60368471	+	C	38	15	0.395	0	0	T	23	M	0.256	0.553	0	1.2E-05	0	76.7936	cctgaacgcgCccgatctcgt	m5C_29643
chr8	60368479	+	C	33	11	0.333	0	0	T	22	M	0.198	0.504	2.2E-16	3.1E-06	2.9E-16	34.0074	cgcccgatctCgtctgatctc	m5C_29655
chr8	60368482	+	C	34	10	0.294	0	0	T	24	M	0.168	0.462	2.3E-14	2E-06	2.9E-14	22.9564	ccgatctcgtCtgatctcaga	m5C_29652
chr8	67025603	+	C	57	15	0.263	0	0	T	42	M	0.166	0.39	0	4.6E-09	0	49.9369	gtacacgtccCttcgatagct	m5C_29584
chr8	67025612	+	C	86	23	0.267	0	0	T	63	M	0.185	0.369	0	7.2E-13	0	85.247	ccttcgatagCtcagctggta	m5C_29593
chr8	67025614	+	C	80	16	0.2	0	0	T	64	M	0.127	0.3	0	1.1E-14	0	40.6488	ttcgatagctCagctggtaga	m5C_29582
chr8	67025626	+	C	89	18	0.202	0	0	T	71	M	0.132	0.297	0	4.5E-14	0	47.4972	gctggtagagCggaggactgt	m5C_29603
chr8	67025639	+	C	49	16	0.327	0	0	T	33	M	0.212	0.466	0	4E-07	0	67.8634	aggactgtagCtacttcctca	m5C_29583
chr8	67025642	+	C	45	25	0.556	0	0	C	25	M	0.412	0.691	0	4.5E-06	0	205.879	actgtagctaCttcctcagca	m5C_29598
chr8	67025661	+	C	66	19	0.288	0	0	T	47	M	0.193	0.406	0	4.5E-10	0	73.2329	caggagacatCcttaggtcgc	m5C_29595
chr8	67025669	+	C	91	20	0.22	0	0	T	71	M	0.147	0.315	0	1.7E-15	0	58.8081	atccttaggtCgctggttcga	m5C_29587
chr8	67834714	-	C	217	110	0.507	0	0	C	110	M	0.441	0.573	0	0	0	969.876	aattttaagaCtgagatgcca	m5C_29626
chr8	67834757	-	C	289	71	0.246	0	0	T	218	M	0.2	0.298	0	0	0	283.421	tactttttgcCgtttacccag	m5C_29619
chr8	67834758	-	C	282	60	0.214	1	0.004	T	221	M	0.17	0.265	0	0	0	203.568	ttactttttgCcgtttaccca	m5C_29614
chr8	67834765	-	C	288	78	0.271	0	0	T	210	M	0.223	0.325	0	0	0	347.529	acttaaattaCtttttgccgt	m5C_29617
chr8	68858368	-	C	33	14	0.424	0	0	T	19	M	0.272	0.592	0	9.2E-06	0	76.259	gtagtcccagCtactcggagg	m5C_29558
chr8	68858372	-	C	49	20	0.408	0	0	T	29	M	0.282	0.548	0	1.4E-06	0	112.86	gcctgtagtcCcagctactcg	m5C_29573
chr8	68858373	-	C	46	10	0.217	0	0	T	36	M	0.123	0.356	9.7E-13	2.4E-08	1.2E-12	14.7309	tgcctgtagtCccagctactc	m5C_29560
chr8	68858380	-	C	59	14	0.237	0	0	T	45	M	0.147	0.36	0	2.8E-09	0	41.1446	tggtgtgtgcCtgtagtccca	m5C_29580
chr8	68858381	-	C	58	17	0.293	0	0	T	41	M	0.192	0.42	0	1.1E-08	0	65.2053	gtggtgtgtgCctgtagtccc	m5C_29565
chr8	68858394	-	C	57	12	0.211	0	0	T	45	M	0.125	0.333	1.6E-14	8.8E-10	2E-14	20.643	aatagccgggCgggtggtgtg	m5C_29561
chr8	70602288	-	C	87	58	0.667	0	0	C	58	M	0.562	0.757	0	6.8E-09	0	652.409	ggcgccccccCgtacccgcaa	m5C_30559
chr8	70602289	-	C	94	65	0.691	0	0	C	65	M	0.592	0.776	0	1.1E-09	0	769.784	tggcgcccccCcgtacccgca	m5C_30504
chr8	70602290	-	C	106	59	0.557	0	0	C	59	M	0.462	0.648	0	1.8E-11	0	544.803	ctggcgccccCccgtacccgc	m5C_30544
chr8	70602291	-	C	116	92	0.793	0	0	C	92	M	0.711	0.857	0	1.7E-10	0	1307.46	cctggcgcccCcccgtacccg	m5C_30542
chr8	70602423	-	C	233	76	0.329	2	0.009	T	155	M	0.272	0.392	0	0	0	412.897	acctactatcCagcgaaacca	m5C_30515
chr8	70602432	-	C	305	106	0.348	0	0	T	199	M	0.296	0.403	0	0	0	628.145	actgtccctaCctactatcca	m5C_30510
chr8	70602593	-	C	119	95	0.798	0	0	C	95	M	0.717	0.861	0	2.5E-10	0	1363.1	ggtgttgacgCgatgtgattt	m5C_30536
chr8	70602595	-	C	123	95	0.772	0	0	C	95	M	0.691	0.838	0	1.6E-11	0	1312.28	cgggtgttgaCgcgatgtgat	m5C_30589
chr8	70602608	-	C	94	75	0.798	0	0	C	75	M	0.706	0.867	0	4.6E-09	0	1058.62	caaaggcccgCggcgggtgtt	m5C_30540
chr8	80976767	-	C	33	10	0.303	0	0	T	23	M	0.174	0.473	1.6E-14	2E-06	2E-14	23.982	cgatcagtgcCacagagaccc	m5C_29727
chr8	81177906	-	C	86	43	0.5	0	0	CT	43	M	0.397	0.603	0	4.1E-10	0	341.083	gacagctaccCtggagaagag	m5C_29745
chr8	81177908	-	C	92	21	0.228	0	0	T	71	M	0.154	0.324	0	3.1E-15	0	64.8567	gtgacagctaCcctggagaag	m5C_29749
chr8	81177914	-	C	93	27	0.29	0	0	T	66	M	0.208	0.389	0	7.2E-14	0	112.24	caggcagtgaCagctaccctg	m5C_29721
chr8	81177920	-	C	77	19	0.268	6	0.078	T	52	M	0.179	0.381	0	6.9E-12	0	67.8401	aagtaccaggCagtgacagct	m5C_29722
chr8	81177934	-	C	31	9	0.29	0	0	T	22	M	0.161	0.466	3.7E-13	1.2E-06	4.4E-13	18.0154	acgaggttggCtggaagtacc	m5C_29720
chr8	89045625	-	C	36	9	0.25	0	0	T	27	M	0.138	0.411	2E-12	1.2E-06	2.4E-12	14.4765	ttgattccagCggtggatcag	m5C_29846
chr8	91523440	+	C	32	12	0.375	0	0	T	20	M	0.229	0.547	0	4.6E-06	0	55.0408	tgatgggagaCcacctgggaa	m5C_29864
chr8	91523441	+	C	38	16	0.421	0	0	T	22	M	0.279	0.578	0	1.7E-05	0	89.1273	gatgggagacCacctgggaat	m5C_29861
chr8	91523444	+	C	43	13	0.302	0	0	T	30	M	0.186	0.451	0	1.2E-07	0	48.3648	gggagaccacCtgggaatacc	m5C_29866
chr8	91523453	+	C	47	12	0.255	0	0	T	35	M	0.153	0.395	1E-15	7.5E-08	1.3E-15	27.4561	cctgggaataCcgggtgctat	m5C_29867
chr8	91523454	+	C	48	16	0.333	0	0	T	32	M	0.217	0.475	0	4E-07	0	69.3651	ctgggaatacCgggtgctata	m5C_29863
chr8	91523460	+	C	44	22	0.5	0	0	CT	22	M	0.358	0.642	0	2.3E-06	0	157.659	ataccgggtgCtataggcttg	m5C_29860
chr8	91523467	+	C	34	14	0.412	0	0	T	20	M	0.264	0.578	0	9.2E-06	0	73.8241	gtgctataggCttggccaggc	m5C_29865
chr8	92607342	+	C	67	26	0.388	0	0	T	41	M	0.28	0.508	0	5.6E-09	0	145.854	tcgaaggaggaCgaccatcctg	m5C_29877
chr8	92607350	+	C	66	48	0.727	0	0	C	48	M	0.61	0.82	0	5.1E-07	0	585.192	gacgaccatcCtgatagagga	m5C_29888
chr8	94738567	+	C	39	18	0.462	0	0	T	21	M	0.316	0.614	0	2.7E-05	0	113.64	cttaatttgaCtgtcttgatt	m5C_30039
chr8	96281942	-	C	57	14	0.246	0	0	T	43	M	0.152	0.371	0	2.8E-09	0	42.6516	gtggttaaggCgatggactag	m5C_29951
chr8	96281955	-	C	66	25	0.379	0	0	T	41	M	0.271	0.499	0	4.1E-09	0	135.746	tagtcgtggcCgagtggttaa	m5C_29946
chr8	96281961	-	C	68	19	0.279	0	0	T	49	M	0.187	0.396	0	4.5E-10	0	70.9757	agccagtagtCgtggccgagt	m5C_29939
chr8	96281968	-	C	39	11	0.282	0	0	T	28	M	0.165	0.438	3.1E-15	3.1E-06	4E-15	26.4001	ttcacacagcCagtagtcgtg	m5C_29945
chr8	97506163	+	C	32	17	0.531	0	0	C	17	M	0.364	0.691	0	2.1E-05	0	123.927	tgaacctcggCacgggaaagg	m5C_29919
chr8	97506165	+	C	34	7	0.206	0	0	T	27	M	0.103	0.368	1.4E-09	3.4E-07	1.6E-09	6.4054	aacctcggcaCgggaaaggag	m5C_29910
chr8	98784548	+	C	76	20	0.263	0	0	T	56	M	0.177	0.372	0	1.1E-11	0	70.9171	ggctgattgtCtgaaggtagt	m5C_30110
chr8	98784580	+	C	33	8	0.242	0	0	T	25	M	0.128	0.41	3.1E-11	6.8E-07	3.6E-11	10.7866	attgattgttCatggtcagtt	m5C_30089
chr8	99054400	-	C	44	9	0.205	0	0	T	35	M	0.112	0.345	1.8E-11	1.2E-08	2.1E-11	10.7806	tcaggacaggCtaaacattcg	m5C_30144
chr8	99054425	-	C	57	14	0.246	0	0	T	43	M	0.152	0.371	0	2.8E-09	0	42.6516	tctcgctgttCtgattttgta	m5C_30134
chr8	99054432	-	C	57	24	0.421	0	0	T	33	M	0.302	0.55	0	1.2E-07	0	144.9	cgaatattctCgctgttctga	m5C_30151
chr8	99054434	-	C	60	14	0.233	0	0	T	46	M	0.144	0.354	0	3.2E-11	0	40.4304	tgcgaatattCtcgctgttct	m5C_30146
chr8	99057237	-	C	40	10	0.25	0	0	T	30	M	0.142	0.402	1.8E-13	2.4E-08	2.2E-13	18.0914	tacaagcagaCtctgaagatg	m5C_30140
chr8	99057253	-	C	38	8	0.211	0	0	T	30	M	0.111	0.363	1.3E-10	6.8E-07	1.4E-10	8.77246	gaagtacgtcCtggggtacaa	m5C_30152
chr8	99057254	-	C	40	8	0.2	0	0	T	32	M	0.105	0.348	2.1E-10	5.5E-09	2.3E-10	8.13503	ggaagtacgtCctggggtaca	m5C_30148
chr8	99057587	-	C	33	16	0.485	0	0	T	17	M	0.325	0.648	0	1.7E-05	0	104.012	agatggtggcCgcaaagaaga	m5C_30133
chr8	101197373	+	C	87	35	0.407	1	0.011	T	51	M	0.309	0.513	0	5.6E-11	0	216.503	catggctggtCtgagtacagt	m5C_30263
chr8	101197395	+	C	87	18	0.207	0	0	T	69	M	0.135	0.304	0	4.5E-14	0	48.6261	gtgtttacaaCtaattgatca	m5C_30273
chr8	104438326	+	C	82	30	0.366	0	0	T	52	M	0.27	0.474	0	1.2E-11	0	161.871	aatggatgggCcaggctatgg	m5C_30216
chr8	110594869	-	C	161	48	0.298	0	0	T	113	M	0.233	0.373	0	0	0	223.536	gatgatattaCctgaagtcca	m5C_30189
chr8	110594886	-	C	163	74	0.454	0	0	T	89	M	0.379	0.531	0	0	0	561.647	atgatgatgaCgatgatgatg	m5C_30190
chr8	111946931	-	C	39	16	0.41	0	0	T	23	M	0.271	0.566	0	1.7E-05	0	86.6531	ggaaaaaaggCgggagaagcc	m5C_30188
chr8	115870037	-	C	39	11	0.282	0	0	T	28	M	0.165	0.438	3.1E-15	3.1E-06	4E-15	26.4001	tagtggtgagCatggctgcct	m5C_30160
chr8	117861255	-	C	51	13	0.255	0	0	T	38	M	0.155	0.389	1.1E-16	1.6E-09	1.5E-16	32.2431	gaagatgcatCagggggcgat	m5C_30424
chr8	117862913	-	C	51	12	0.235	0	0	T	39	M	0.14	0.368	3.3E-15	8.8E-10	4.3E-15	24.3305	gttagaacttCtgccagaaaa	m5C_30425

chr8	117864909	-	C	35	7	0.2	0	0	T	28	M	0.1	0.359	1.9E-09	3.4E-07	2.1E-09	6.13884	taccagaagaCcttagaaaaa	m5C_30412
chr8	117864916	-	C	36	21	0.583	0	0	C	21	M	0.422	0.729	0	5.2E-05	0	177.24	ccgcttgtacCagaagacctt	m5C_30408
chr8	117870670	-	C	53	11	0.208	0	0	T	42	M	0.12	0.335	1.8E-13	4.4E-10	2.2E-13	16.8213	agcagttcagCttgaatcaga	m5C_30411
chr8	117870678	-	C	51	14	0.275	0	0	T	37	M	0.171	0.409	0	2.8E-09	0	47.9195	tgtggcccagCagttcagctt	m5C_30405
chr8	117870682	-	C	50	13	0.26	0	0	T	37	M	0.159	0.396	0	1.6E-09	0	41.2656	tcgatgtggcCcagcagttca	m5C_30422
chr8	120874962	+	C	62	17	0.274	0	0	T	45	M	0.179	0.396	0	1.8E-10	0	60.7951	gctggaggatCgcttgagtcc	m5C_30679
chr8	120874964	+	C	68	45	0.662	0	0	C	45	M	0.543	0.763	0	3.3E-07	0	489.033	tggaggatcgCttgagtccag	m5C_30673
chr8	120874971	+	C	63	32	0.508	0	0	C	32	M	0.388	0.627	0	2.7E-08	0	248.081	tcgcttgagtCcaggagttct	m5C_30668
chr8	120874993	+	C	37	15	0.405	0	0	T	22	M	0.263	0.565	0	1.2E-05	0	79.0389	ggctgtagtgCgctatagcga	m5C_30661
chr8	124169469	-	C	62	20	0.323	0	0	T	42	M	0.22	0.446	0	6.9E-10	0	87.8173	acacggggcaCaataattttg	m5C_30811
chr8	124169471	-	C	74	18	0.243	0	0	T	56	M	0.16	0.352	0	4E-12	0	57.5149	tcacacggggCacaataattt	m5C_30814
chr8	124169476	-	C	86	35	0.407	0	0	T	51	M	0.309	0.513	0	5.6E-11	0	216.503	gatcctcacaCggggcacaat	m5C_30800
chr8	124169478	-	C	84	25	0.298	0	0	T	59	M	0.21	0.402	0	1.8E-12	0	105.223	tcgatcctcaCacggggcaca	m5C_30802
chr8	124169480	-	C	90	48	0.533	0	0	C	48	M	0.431	0.633	0	4.6E-11	0	413.779	gttcgatcctCacacggggca	m5C_30799
chr8	124169482	-	C	89	25	0.281	0	0	T	64	M	0.198	0.382	0	1.8E-12	0	99.0468	gagttcgatcCtcacacgggg	m5C_30807
chr8	124169483	-	C	88	32	0.364	0	0	T	56	M	0.271	0.468	0	2.3E-11	0	173.307	tgagttcgatCctcacacggg	m5C_30793
chr8	124169487	-	C	85	41	0.482	0	0	T	44	M	0.379	0.587	0	2.6E-10	0	310.949	gtcgtgagttCgatcctcaca	m5C_30784
chr8	124169495	-	C	86	25	0.291	0	0	T	61	M	0.205	0.394	0	1.8E-12	0	102.662	atctgaaggtCgtgagttcga	m5C_30809
chr8	124169503	-	C	74	38	0.514	0	0	C	38	M	0.402	0.624	0	3.7E-09	0	305.375	gtctcataatCtgaaggtcgt	m5C_30790
chr8	124169509	-	C	56	42	0.75	0	0	C	42	M	0.623	0.845	0	3.9E-06	0	523.38	gcgtcagtctCataatctgaa	m5C_30796
chr8	124169511	-	C	56	25	0.446	0	0	T	31	M	0.324	0.576	0	1.5E-07	0	161.933	gcgcgtcagtCtcataatctg	m5C_30783
chr8	124169532	-	C	101	21	0.208	0	0	T	80	M	0.14	0.297	0	0	0	58.8869	gcctcgttagCgcagtaggta	m5C_30804
chr8	124169540	-	C	83	29	0.349	0	0	T	54	M	0.256	0.457	0	8.4E-12	0	148.225	tactaggtgcCtcgttagcgc	m5C_30817
chr8	125785386	-	C	40	20	0.5	0	0	CT	20	M	0.352	0.648	0	1.4E-06	0	140.797	ttggtgctctCaaagtacaag	m5C_30797
chr8	125785388	-	C	72	40	0.556	0	0	C	40	M	0.441	0.665	0	5.8E-09	0	352.697	atttggtgctCtcaaagtaca	m5C_30813
chr8	125785390	-	C	115	55	0.478	0	0	T	60	M	0.389	0.569	0	2.9E-13	0	428.065	ggatttggtgCtctcaaagta	m5C_30801
chr8	125785410	-	C	224	92	0.411	0	0	T	132	M	0.348	0.476	0	0	0	640.91	ccctagtggtCtagtggtcag	m5C_30787
chr8	125785418	-	C	103	37	0.359	0	0	T	66	M	0.273	0.455	0	6.7E-14	0	202.145	aggagcatccCtagtggtcta	m5C_30795
chr8	125785419	-	C	99	42	0.424	0	0	T	57	M	0.332	0.523	0	1.1E-11	0	278.468	aaggagcatcCctagtggtct	m5C_30781
chr8	125785420	-	C	83	33	0.398	0	0	T	50	M	0.299	0.505	0	3.1E-11	0	197.398	caaggagcatCcctagtggtc	m5C_30805
chr8	126283015	+	C	39	10	0.256	0	0	T	29	M	0.146	0.411	1.3E-13	2E-06	1.6E-13	18.7753	accaggttgcCtaaggagggg	m5C_30596
chr8	126283030	+	C	40	12	0.3	0	0	T	28	M	0.181	0.454	1.1E-16	7.5E-08	1.5E-16	34.6049	gaggggtgaaCgggcccaggt	m5C_30606
chr8	126283035	+	C	32	10	0.312	0	0	T	22	M	0.18	0.486	1.1E-14	2E-06	1.3E-14	25.0836	gtgaacgggcCcaggttgaaa	m5C_30593
chr8	126289487	-	C	37	18	0.486	0	0	T	19	M	0.334	0.641	0	2.7E-05	0	120.406	gggagggaagCctaggtgagg	m5C_30618
chr8	128136440	+	C	35	16	0.457	0	0	T	19	M	0.305	0.618	0	1.7E-05	0	97.4905	ggtggtggatCccaggtagtc	m5C_30959
chr8	128241285	-	C	42	23	0.548	0	0	C	23	M	0.399	0.688	0	2.9E-06	0	183.765	tgactgctgaCtcctaattca	m5C_30989
chr8	128241310	-	C	38	20	0.526	0	0	C	20	M	0.373	0.675	0	4.3E-05	0	149.035	agaagattagCaggtagtaga	m5C_31028
chr8	128241439	-	C	34	17	0.5	0	0	CT	17	M	0.341	0.659	0	2.1E-05	0	115.835	taagaagctgCacataagatg	m5C_30996
chr8	128241442	-	C	45	10	0.222	0	0	T	35	M	0.125	0.363	7.4E-13	2.4E-08	8.9E-13	15.2157	ctttaagaagCtgcacataag	m5C_31018
chr8	128241469	-	C	123	31	0.252	0	0	T	92	M	0.184	0.335	0	0	0	113.838	agggagttgaCagaggtgtga	m5C_31025
chr8	129011490	+	C	256	67	0.262	0	0	T	189	M	0.212	0.319	0	0	0	283.648	atgtgggagaCttgactgcat	m5C_30967
chr8	131090561	-	C	44	10	0.227	0	0	T	34	M	0.128	0.37	5.7E-13	2.4E-08	6.8E-13	15.7274	gaccctgtctCaaaaaaaaag	m5C_30659
chr8	131090568	-	C	48	11	0.229	0	0	T	37	M	0.133	0.365	5E-14	4.4E-08	6.2E-14	19.4712	acagcaagacCctgtctcaaa	m5C_30650
chr8	131090569	-	C	46	24	0.522	0	0	C	24	M	0.381	0.659	0	3.6E-06	0	183.058	gacagcaagaCcctgtctcaa	m5C_30654
chr8	131090577	-	C	42	10	0.238	0	0	T	32	M	0.135	0.385	3.2E-13	2.4E-08	3.9E-13	16.8407	tccggggcgaCagcaagaccc	m5C_30657
chr8	131090592	-	C	40	11	0.275	0	0	T	29	M	0.161	0.428	4.3E-15	3.1E-06	5.5E-15	25.4502	cacctctgcaCtccatccggg	m5C_30655
chr8	131090599	-	C	41	10	0.244	0	0	T	31	M	0.138	0.393	2.4E-13	2.4E-08	2.9E-13	17.4472	atgatcgcacCtctgcactcc	m5C_30651
chr8	131090600	-	C	43	14	0.326	0	0	T	29	M	0.205	0.475	0	1.9E-07	0	57.3807	catgatcgcaCctctgcactc	m5C_30649
chr8	131090602	-	C	41	14	0.341	0	0	T	27	M	0.216	0.495	0	1.9E-07	0	60.3641	gtcatgatcgCacctctgcac	m5C_30660
chr8	131090604	-	C	41	16	0.39	0	0	T	25	M	0.257	0.543	0	4E-07	0	82.099	gagtcatgatCgcacctctgc	m5C_30652
chr8	131090621	-	C	43	14	0.326	0	0	T	29	M	0.205	0.475	0	1.9E-07	0	57.3807	gaaattgaggCtgcagtgagt	m5C_30647
chr8	133497751	-	C	67	21	0.313	0	0	T	46	M	0.215	0.432	0	1E-09	0	90.3218	aacgtggatcCtgggaggaat	m5C_30832
chr8	133497752	-	C	63	15	0.238	0	0	T	48	M	0.15	0.356	0	6E-11	0	44.981	taacgtggatCctgggaggaa	m5C_30836
chr8	133497759	-	C	54	11	0.204	0	0	T	43	M	0.118	0.329	2.3E-13	4.4E-10	2.8E-13	16.3622	cctaccttaaCgtggatcctg	m5C_30835
chr8	136786016	+	C	133	31	0.233	0	0	T	102	M	0.169	0.312	0	0	0	104.992	acatttcccaCctcgatcaga	m5C_30701
chr8	136786017	+	C	163	33	0.202	0	0	T	130	M	0.148	0.271	0	0	0	97.6453	catttcccacCtcgatcagaa	m5C_30712
chr8	136786019	+	C	185	68	0.368	0	0	T	117	M	0.301	0.439	0	0	0	409.96	tttcccacctCgatcagaagg	m5C_30703
chr8	136786023	+	C	242	90	0.372	0	0	T	152	M	0.313	0.434	0	0	0	564.215	ccacctcgatCagaaggactt	m5C_30705
chr8	136786031	+	C	472	261	0.553	0	0	C	261	M	0.508	0.597	0	0	0	2651.05	atcagaaggaCttgggccccc	m5C_30715
chr8	136786037	+	C	610	344	0.565	1	0.002	C	344	M	0.525	0.604	0	0	0	3613.37	aggacttgggCccccccatgag	m5C_30723
chr8	136786038	+	C	637	380	0.597	0	0	C	380	M	0.558	0.634	0	0	0	4240.64	ggacttgggcCccccatgagt	m5C_30726
chr8	136786039	+	C	650	376	0.578	0	0	C	376	M	0.54	0.616	0	0	0	4061.9	gacttgggccCcccatgagtg	m5C_30729
chr8	136786040	+	C	665	417	0.627	0	0	C	417	M	0.59	0.663	0	0	0	4917.94	acttgggcccCccatgagtgg	m5C_30730
chr8	136786041	+	C	695	400	0.576	0	0	C	400	M	0.538	0.612	0	0	0	4307.81	cttgggccccCcatgagtggc	m5C_30725
chr8	136786042	+	C	689	366	0.531	0	0	C	366	M	0.494	0.568	0	0	0	3615.15	ttgggcccccCatgagtggcg	m5C_30709
chr8	136786051	+	C	660	350	0.534	5	0.008	C	350	M	0.496	0.572	0	0	0	3472.42	ccatgagtggCgctggggagt	m5C_30721
chr8	136786053	+	C	647	326	0.504	0	0	C	326	M	0.465	0.542	0	0	0	3034.59	atgagtggcgCtggggagtgg	m5C_30710
chr8	136786066	+	C	526	326	0.62	0	0	C	326	M	0.578	0.66	0	0	0	3765.68	gggagtgggtCttccgtacgc	m5C_30722
chr8	136786069	+	C	528	316	0.6	1	0.002	C	316	M	0.557	0.641	0	0	0	3521.58	agtgggtcttCcgtacgccac	m5C_30717
chr8	136786070	+	C	501	284	0.568	1	0.002	C	284	M	0.524	0.611	0	0	0	2977.6	gtgggtcttcCgtacgccaca	m5C_30719
chr8	136786074	+	C	486	259	0.535	2	0.004	C	259	M	0.491	0.579	0	0	0	2541.24	gtcttccgtaCgccacatttc	m5C_30713
chr8	136786076	+	C	488	257	0.527	0	0	C	257	M	0.482	0.571	0	0	0	2479.05	cttccgtacgCcacatttcga	m5C_30700
chr8	136786077	+	C	437	221	0.506	0	0	C	221	M	0.459	0.552	0	0	0	2028.78	ttccgtacgcCacatttcgaa	m5C_30699
chr8	136786079	+	C	364	188	0.516	0	0	C	188	M	0.465	0.567	0	0	0	1749.31	ccgtacgccaCatttcgaata	m5C_30708
chr8	141950754	+	C	45	28	0.622	0	0	C	28	M	0.476	0.749	0	8.1E-06	0	266.726	atactgggtgCtgtaggctta	m5C_31224
chr8	142233886	-	C	38	10	0.263	0	0	T	28	M	0.15	0.42	9.4E-14	2E-06	1.2E-13	19.5037	ggcacggtctCtcgtgagtgc	m5C_31085
chr8	142233888	-	C	41	20	0.488	0	0	T	21	M	0.343	0.635	0	1.4E-06	0	137.017	ggggcacggtCtctcgtgagt	m5C_31091
chr8	142233892	-	C	42	21	0.5	0	0	CT	21	M	0.355	0.645	0	1.8E-06	0	149.208	tctgggggcaCggtctctcgt	m5C_31093
chr8	143784551	+	C	31	7	0.226	0	0	T	24	M	0.114	0.398	6.4E-10	3.4E-07	7.2E-10	7.33205	cagtgctccgCtggttgtgca	m5C_31063
chr8	143784563	+	C	35	14	0.4	0	0	T	21	M	0.256	0.564	0	9.2E-06	0	71.5414	ggttgtgcagCgatggagaga	m5C_31062
chr8	144073051	-	C	34	11	0.324	0	0	T	23	M	0.191	0.492	4.4E-16	3.1E-06	5.8E-16	32.3088	ctttggggacCgggtgttgca	m5C_31372
chr8	146015178	-	C	50	27	0.54	0	0	C	27	M	0.404	0.67	0	6.7E-06	0	218.153	tgctgagggcCtcaataaagt	m5C_30863
chr8	146015179	-	C	49	12	0.245	0	0	T	37	M	0.146	0.381	1.9E-15	7.5E-08	2.4E-15	25.8006	gtgctgagggCctcaataaag	m5C_30864
chr8	146015186	-	C	74	37	0.5	0	0	CT	37	M	0.389	0.611	0	3E-09	0	287.804	agaactagtgCtgagggcctc	m5C_30886
chr8	146015192	-	C	65	23	0.354	0	0	T	42	M	0.249	0.475	0	2.1E-09	0	114.426	agaaagagaaCtagtgctgag	m5C_30847
chr8	146015216	-	C	33	8	0.242	0	0	T	25	M	0.128	0.41	3.1E-11	6.8E-07	3.6E-11	10.7866	tccggggaacCaagactgtgc	m5C_30858
chr8	146015227	-	C	53	28	0.528	0	0	C	28	M	0.397	0.656	0	3.1E-07	0	222.095	gactggacgtCtccggggaac	m5C_30871
chr8	146015242	-	C	45	27	0.6	0	0	C	27	M	0.455	0.73	0	6.7E-06	0	245.439	cattgctgccCgccggactgg	m5C_30860
chr8	146015243	-	C	44	22	0.5	0	0	CT	22	M	0.358	0.642	0	2.3E-06	0	157.659	tcattgctgcCcgccggactg	m5C_30874
chr8	146015244	-	C	38	17	0.447	0	0	T	21	M	0.301	0.603	0	2.1E-05	0	102.497	ctcattgctgCccgccggact	m5C_30883
chr8	146016815	-	C	31	10	0.323	0	0	T	21	M	0.186	0.499	7.1E-15	2E-06	9E-15	26.2725	cgtgtgctgcCtggaggagaa	m5C_30884
chr8	146016816	-	C	32	10	0.312	0	0	T	22	M	0.18	0.486	1.1E-14	2E-06	1.3E-14	25.0836	tcgtgtgctgCctggaggaga	m5C_30877
chr8	146017493	-	C	37	16	0.432	0	0	T	21	M	0.287	0.591	0	1.7E-05	0	91.7488	gatccgtggaCagaggaaggg	m5C_30854
chr8	146017499	-	C	107	47	0.439	0	0	T	60	M	0.349	0.534	0	1.3E-12	0	328.003	ccgtgtgatcCgtggacagag	m5C_30873
chr8	146017509	-	C	109	30	0.275	0	0	T	79	M	0.2	0.366	0	4.7E-15	0	120.071	tcgccatgggCcgtgtgatcc	m5C_30878
chr8	146017515	-	C	89	19	0.213	0	0	T	70	M	0.141	0.31	0	8.4E-14	0	53.6358	gacccgtcgcCatgggccgtg	m5C_30857
chr8	146017522	-	C	73	22	0.301	0	0	T	51	M	0.208	0.414	0	2.8E-11	0	91.6188	cccccaggacCcgtcgccatg	m5C_30894
chr8	146017753	-	C	64	18	0.281	0	0	T	46	M	0.186	0.401	0	2.9E-10	0	66.9356	cgctggtgaaCaggtaggtca	m5C_30880
chr8	146017761	-	C	61	16	0.262	0	0	T	45	M	0.168	0.384	0	1.1E-10	0	53.8837	ttcggcgcgCtggtgaacag	m5C_30869
chr8	146017766	-	C	59	12	0.203	0	0	T	47	M	0.12	0.323	2.6E-14	8.8E-10	3.3E-14	19.6113	cctctttcggCcgcgctggtg	m5C_30868
chr8	146017769	-	C	50	18	0.36	0	0	T	32	M	0.241	0.499	0	1.7E-08	0	86.8988	tttcctctttCggccgcgctg	m5C_30862

chr8	146017773	-	C	36	10	0.278	0	0	T	26	M	0.158	0.44	4.8E-14	2E-06	5.9E-14	21.1115	cgtgtttcctCtttcggccgc	m5C_30870
chr9	2162768	-	C	190	50	0.263	0	0	T	140	M	0.206	0.33	0	0	0	205.683	gaatgatgcaCccccttaaga	m5C_31547
chr9	2162785	-	C	187	51	0.273	0	0	T	136	M	0.214	0.341	0	0	0	218.228	tactgcagtaCctccaggaat	m5C_31549
chr9	2162799	-	C	107	27	0.252	0	0	T	80	M	0.18	0.342	0	1.1E-15	0	96.9857	acacatcgacCtggtactgca	m5C_31545
chr9	9442074	+	C	50	11	0.22	0	0	T	39	M	0.128	0.352	8.5E-14	4.4E-10	1.1E-13	18.3342	ggcgcggtggCgcgtgcctgt	m5C_31207
chr9	9442080	+	C	52	14	0.269	0	0	T	38	M	0.168	0.403	0	2.8E-09	0	46.9527	gtggcgcgtgCctgtggtccc	m5C_31206
chr9	9442088	+	C	51	11	0.216	0	0	T	40	M	0.125	0.346	1.1E-13	4.4E-10	1.4E-13	17.8061	tgcctgtggtCccagctactc	m5C_31201
chr9	9442089	+	C	53	16	0.302	0	0	T	37	M	0.195	0.435	0	7.3E-09	0	62.4581	gcctgtggtcCcagctactcg	m5C_31212
chr9	9442096	+	C	50	15	0.3	0	0	T	35	M	0.191	0.438	0	4.6E-09	0	57.3102	gtcccagctaCtcgggaggct	m5C_31197
chr9	9442098	+	C	51	16	0.314	0	0	T	35	M	0.203	0.45	0	7.3E-09	0	65.048	cccagctactCgggaggctga	m5C_31211
chr9	17054061	+	C	39	13	0.333	0	0	T	26	M	0.206	0.49	0	6.7E-06	0	53.6452	gggagtgggaCcactgggttg	m5C_31271
chr9	17054064	+	C	41	12	0.293	0	0	T	29	M	0.176	0.445	1.1E-16	7.5E-08	1.5E-16	33.7134	agtgggaccaCtgggttgcct	m5C_31281
chr9	17054073	+	C	54	14	0.259	0	0	T	40	M	0.161	0.389	0	2.8E-09	0	45.1318	actgggttgcCtaaggagggg	m5C_31280
chr9	17054088	+	C	49	18	0.367	0	0	T	31	M	0.247	0.507	0	7.7E-07	0	88.7939	gaggggtgaaCtggcccaggc	m5C_31275
chr9	17054092	+	C	41	11	0.268	0	0	T	30	M	0.157	0.419	6E-15	4.4E-08	7.6E-15	24.5536	ggtgaactggCccaggctgga	m5C_31273
chr9	17054094	+	C	46	13	0.283	0	0	T	33	M	0.173	0.425	0	1.2E-07	0	45.0425	tgaactggccCaggctggaaa	m5C_31270
chr9	17578937	-	C	41	9	0.22	0	0	T	32	M	0.12	0.367	8.4E-12	1.2E-08	9.9E-12	11.9621	ggatgtggctCagcggcaccc	m5C_31290
chr9	17578939	-	C	44	16	0.364	0	0	T	28	M	0.238	0.511	0	4E-07	0	76.1067	ggggatgtggCtcagcggcac	m5C_31283
chr9	19030081	+	C	45	21	0.467	0	0	T	24	M	0.329	0.609	0	1.8E-06	0	138.327	tgatggggagCcacagggctg	m5C_31679
chr9	19063673	-	C	328	81	0.247	0	0	T	247	M	0.203	0.296	0	0	0	329.491	tcgtaacggaCagatacgggg	m5C_31730
chr9	19063697	-	C	255	54	0.213	2	0.008	T	199	M	0.167	0.268	0	0	0	180.826	atgaccgggaCgaattgagtg	m5C_31717
chr9	19063736	-	C	145	32	0.221	0	0	T	113	M	0.161	0.295	0	0	0	102.966	tacttgtcacCatgcctccct	m5C_31727
chr9	19063752	-	C	210	45	0.214	0	0	T	165	M	0.164	0.275	0	0	0	147.764	ggctgaaataCtgctctactt	m5C_31721
chr9	19063760	-	C	237	73	0.309	1	0.004	T	163	M	0.254	0.371	0	0	0	370.547	acaaattgggCtgaaatactg	m5C_31713
chr9	19063769	-	C	279	61	0.219	0	0	T	218	M	0.174	0.271	0	0	0	212.449	ggctgatacaCaaattgggct	m5C_31708
chr9	19063771	-	C	285	87	0.305	0	0	T	198	M	0.255	0.361	0	0	0	443.144	gaggctgataCacaaattggg	m5C_31728
chr9	19063777	-	C	279	80	0.287	0	0	T	199	M	0.237	0.342	0	0	0	378.961	aattgggaggCtgatacacaa	m5C_31731
chr9	19200406	-	C	43	18	0.419	0	0	T	25	M	0.284	0.567	0	7.7E-07	0	102.184	aggaaaaagcgCcaggaacaaa	m5C_31736
chr9	19200408	-	C	44	9	0.205	0	0	T	35	M	0.112	0.345	1.8E-11	1.2E-08	2.1E-11	10.7806	taaggaaaagCgccaggaaca	m5C_31707
chr9	19200419	-	C	45	9	0.2	0	0	T	36	M	0.109	0.338	2.3E-11	1.2E-08	2.7E-11	10.4289	atgaaggaggCtaaggaaaag	m5C_31703
chr9	19376347	-	C	31	11	0.355	0	0	T	20	M	0.211	0.531	1.1E-16	3.1E-06	1.5E-16	37.0595	tgcgaagagaCgcagactttc	m5C_31710
chr9	19376362	-	C	114	30	0.263	0	0	T	84	M	0.191	0.351	0	0	0	114.599	gcgccaggaaCaaattgcgaa	m5C_31725
chr9	19376368	-	C	122	27	0.221	0	0	T	95	M	0.157	0.303	0	0	0	84.6667	ggagaagcgcCaggaacaaat	m5C_31705
chr9	19376382	-	C	105	35	0.337	1	0.01	T	69	M	0.253	0.432	0	3.4E-14	0	177.091	gtttaggaggCtaaggagaag	m5C_31729
chr9	19376504	-	C	78	19	0.244	0	0	T	59	M	0.162	0.349	0	6.9E-12	0	61.5034	aacttttggcCaagagaatga	m5C_31732
chr9	19376529	-	C	49	23	0.469	0	0	T	26	M	0.337	0.606	0	2.9E-06	0	155.035	aaagaagaggCtgcagaatat	m5C_31733
chr9	19376549	-	C	32	18	0.562	0	0	C	18	M	0.393	0.718	0	2.7E-05	0	141.571	agcagcgtacCaagaaaaata	m5C_31724
chr9	21160250	-	C	31	9	0.29	0	0	T	22	M	0.161	0.466	3.7E-13	1.2E-06	4.4E-13	18.0154	cccactgcttCacttgactag	m5C_32819
chr9	21160256	-	C	32	11	0.344	0	0	T	21	M	0.204	0.517	2.2E-16	3.1E-06	2.9E-16	35.1442	tccactcccaCtgcttcactt	m5C_32831
chr9	21160285	-	C	31	13	0.419	0	0	T	18	M	0.264	0.592	0	6.7E-06	0	68.6798	caaccagttaCagattccttt	m5C_32842
chr9	21160292	-	C	33	14	0.424	0	0	T	19	M	0.272	0.592	0	9.2E-06	0	76.259	tccatcacaaCcagttacaga	m5C_32841
chr9	21160297	-	C	54	13	0.241	0	0	T	41	M	0.146	0.369	2.2E-16	1.6E-09	2.9E-16	29.8003	actaatccatCacaaccagtt	m5C_32823
chr9	21160306	-	C	70	14	0.2	0	0	T	56	M	0.123	0.308	4.4E-16	2.8E-13	5.8E-16	26.447	gtgtttacaaCtaatccatca	m5C_32836
chr9	21160328	-	C	72	20	0.278	0	0	T	52	M	0.188	0.39	0	1.1E-11	0	75.0406	ctgggctggtCtgagtgcagt	m5C_32835
chr9	21512133	-	C	34	7	0.206	0	0	T	27	M	0.103	0.368	1.4E-09	3.4E-07	1.6E-09	6.4054	cctgctatgcCaacatattgc	m5C_32847
chr9	21512150	-	C	33	9	0.273	0	0	T	24	M	0.151	0.442	7.5E-13	1.2E-06	9E-13	16.4418	agctgttgaaCtgggaacctg	m5C_32837
chr9	21512158	-	C	158	50	0.316	0	0	T	108	M	0.249	0.393	0	0	0	249.024	gctggcatagCtgttgaactg	m5C_32821
chr9	21512163	-	C	187	57	0.305	0	0	T	130	M	0.243	0.374	0	0	0	277.377	aagatgctggCatagctgttg	m5C_32822
chr9	21699345	+	C	84	19	0.226	0	0	T	65	M	0.15	0.326	0	8.4E-14	0	56.9464	gaggctgaggCtggaggatcg	m5C_32790
chr9	21699354	+	C	109	24	0.22	0	0	T	85	M	0.153	0.307	0	2.2E-16	0	73.2781	gctggaggatCgcttgagtcc	m5C_32797
chr9	21699356	+	C	110	59	0.536	0	0	C	59	M	0.444	0.627	0	1.8E-11	0	523.356	tggaggatcgCttgagtccag	m5C_32791
chr9	21699372	+	C	113	37	0.327	0	0	T	76	M	0.248	0.418	0	1.4E-15	0	183.392	tccaggagttCtgggctgtag	m5C_32809
chr9	21699387	+	C	85	55	0.647	0	0	C	55	M	0.541	0.74	0	4.2E-09	0	595.235	ctgtagtgtgCtatgcgtatc	m5C_32814
chr9	21699477	+	C	35	13	0.371	0	0	T	22	M	0.232	0.537	0	6.7E-06	0	60.2306	gaggggtgaaCtggcccaggt	m5C_32802
chr9	21699482	+	C	37	8	0.216	0	0	T	29	M	0.114	0.372	9.7E-11	6.8E-07	1.1E-10	9.12273	gtgaactggcCcaggtcggaa	m5C_32786
chr9	21699483	+	C	35	12	0.343	0	0	T	23	M	0.208	0.508	0	4.6E-06	0	49.9956	tgaactggccCaggtcggaaa	m5C_32792
chr9	22397541	+	C	31	7	0.226	0	0	T	24	M	0.114	0.398	6.4E-10	3.4E-07	7.2E-10	7.33205	aattgcaggaCatgtcttcat	m5C_31521
chr9	24905733	+	C	53	24	0.453	0	0	T	29	M	0.327	0.585	0	1.2E-07	0	156.755	ctcagtgtggCtgcttaaaat	m5C_31450
chr9	32811225	+	C	42	11	0.262	0	0	T	31	M	0.153	0.411	8.4E-15	4.4E-08	1.1E-14	23.69	tggttgatcaCtatccccttc	m5C_32013
chr9	33026973	+	C	83	43	0.518	0	0	C	43	M	0.412	0.622	0	4.1E-10	0	354.471	aggaaggatgCagagagaaag	m5C_31993
chr9	33264406	-	C	33	10	0.303	0	0	T	23	M	0.174	0.473	1.6E-14	2E-06	2E-14	23.982	tgacccggagCgaggagttga	m5C_32062
chr9	33934301	-	C	176	43	0.244	0	0	T	133	M	0.187	0.313	0	0	0	160.599	gagactgagtCtttccacagc	m5C_32077
chr9	33934313	-	C	258	61	0.236	0	0	T	197	M	0.189	0.292	0	0	0	230.203	gtggacagcaCtgagactgag	m5C_32034
chr9	33934364	-	C	71	16	0.225	0	0	T	55	M	0.144	0.335	0	1.2E-12	0	45.9986	ctggaaaagaCaatgatgttt	m5C_32016
chr9	33952781	-	C	223	45	0.202	0	0	T	178	M	0.154	0.259	0	0	0	138.935	atttaactacCtgagtttcct	m5C_32028
chr9	33952809	-	C	239	64	0.268	0	0	T	175	M	0.216	0.327	0	0	0	276.021	aagcacatatCtgatgattcc	m5C_32060
chr9	33952820	-	C	252	60	0.238	0	0	T	192	M	0.19	0.294	0	0	0	227.627	tggtaatttcCaagcacatat	m5C_32072
chr9	33952821	-	C	240	48	0.2	0	0	T	192	M	0.154	0.255	0	0	0	148.126	gtggtaatttCcaagcacata	m5C_32017
chr9	34282554	-	C	42	9	0.214	0	0	T	33	M	0.117	0.359	1.1E-11	1.2E-08	1.3E-11	11.5454	catgcattgaCttggtagtgc	m5C_34852
chr9	34282565	-	C	49	24	0.49	0	0	T	25	M	0.356	0.625	0	3.6E-06	0	170.759	tctgtccgctCcatgcattga	m5C_34790
chr9	34282567	-	C	46	11	0.239	0	0	T	35	M	0.139	0.379	2.8E-14	4.4E-08	3.5E-14	20.7303	gctctgtccgCtccatgcatt	m5C_34780
chr9	34282569	-	C	55	20	0.364	0	0	T	35	M	0.249	0.496	0	3.4E-08	0	99.7214	ttgctctgtcCgctccatgca	m5C_34807
chr9	34282570	-	C	55	39	0.709	0	0	C	39	M	0.579	0.812	0	2.5E-06	0	451.3	cttgctctgtCcgctccatgc	m5C_34811
chr9	34282574	-	C	66	15	0.227	0	0	T	51	M	0.143	0.342	0	6E-11	0	42.8553	ggagcttgctCtgtccgctcc	m5C_34859
chr9	34282576	-	C	67	36	0.537	0	0	C	36	M	0.419	0.651	0	6.5E-08	0	301.797	taggagcttgCtctgtccgct	m5C_34865
chr9	35622087	-	C	35	9	0.257	0	0	T	26	M	0.142	0.421	1.5E-12	1.2E-06	1.7E-12	15.0859	aagtcacataCtgtggcaagg	m5C_34837
chr9	35657751	-	C	1164	425	0.365	0	0	T	739	M	0.338	0.393	0	0	0	2872.55	ctcagcgcggCtgttttttgt	m5C_34796
chr9	35657761	-	C	1379	279	0.202	0	0	T	1100	M	0.182	0.224	0	0	0	1015.32	ggggctcattCtcagcgcggc	m5C_34827
chr9	35657772	-	C	1222	253	0.207	0	0	T	969	M	0.185	0.231	0	0	0	937.389	accaaccacaCggggctcatt	m5C_34798
chr9	35657788	-	C	964	254	0.263	0	0	T	710	M	0.237	0.292	0	0	0	1202.21	cagtgcgtgtCcgcgcaccaa	m5C_34818
chr9	35657813	-	C	327	106	0.324	0	0	T	221	M	0.276	0.377	0	0	0	584.532	cgtcagctccCtctagttacg	m5C_34815
chr9	35657823	-	C	286	84	0.294	0	0	T	202	M	0.244	0.349	0	0	0	409.809	gggcgtcatcCgtcagctccc	m5C_34800
chr9	35657830	-	C	419	115	0.274	0	0	T	304	M	0.234	0.319	0	0	0	538.035	ggcgcgggggCgtcatccgtc	m5C_34816
chr9	35657838	-	C	507	232	0.458	0	0	T	275	M	0.415	0.501	0	0	0	1924.25	agcggcgtggCgcgggggcgt	m5C_34830
chr9	35657843	-	C	493	189	0.383	0	0	T	304	M	0.342	0.427	0	0	0	1290.89	cgctgagcggCgtggcgcggg	m5C_34810
chr9	35657853	-	C	297	100	0.337	0	0	T	197	M	0.285	0.392	0	0	0	570.684	aagcgtatccCgctgagcggc	m5C_34843
chr9	35657868	-	C	65	20	0.308	0	0	T	45	M	0.209	0.428	0	6.9E-10	0	83.5475	ccaaagtccgCcaagaagcgt	m5C_34765
chr9	35657891	-	C	40	9	0.225	0	0	T	31	M	0.123	0.375	6.4E-12	1.2E-08	7.6E-12	12.4041	gtagacattcCccgcttccca	m5C_34772
chr9	35657892	-	C	41	15	0.366	0	0	T	26	M	0.236	0.519	0	2.8E-07	0	70.7689	cgtagacattCcccgcttccc	m5C_34860
chr9	35657914	-	C	338	82	0.243	0	0	T	256	M	0.2	0.291	0	0	0	327.939	gagagtgccaCgtgcatacgc	m5C_34808
chr9	35657933	-	C	694	160	0.231	0	0	T	534	M	0.201	0.263	0	0	0	642.387	agtccccggaCctcgggcaga	m5C_34779
chr9	35657939	-	C	867	185	0.214	4	0.005	T	678	M	0.188	0.243	0	0	0	696.656	ggggaaagtcCccggacctcg	m5C_34797
chr9	35657956	-	C	1330	271	0.204	3	0.002	T	1056	M	0.183	0.227	0	0	0	994.017	cctcccctttCcgcctagggg	m5C_34845
chr9	35657961	-	C	1720	470	0.273	0	0	T	1250	M	0.253	0.295	0	0	0	2375.55	ctgttcctccCcttttccgcct	m5C_34820
chr9	35657962	-	C	1806	392	0.217	0	0	T	1414	M	0.199	0.237	0	0	0	1557.44	tctgttcctcCcctttccgcc	m5C_34829
chr9	35657963	-	C	1900	478	0.252	0	0	T	1422	M	0.233	0.272	0	0	0	2223.48	ctctgttcctCccctttccgc	m5C_34802
chr9	35657965	-	C	2003	442	0.221	3	0.001	T	1558	M	0.203	0.24	0	0	0	1797.7	gactctgttcCtcccctttcc	m5C_34789
chr9	35657966	-	C	2038	491	0.241	1	0	T	1546	M	0.223	0.26	0	0	0	2189.52	ggactctgttCctcccctttc	m5C_34821
chr9	35657971	-	C	2443	502	0.205	0	0	T	1941	M	0.19	0.222	0	0	0	1906.9	actggaggactCtgttcctccc	m5C_34849
chr9	35657992	-	C	3654	797	0.218	4	0.001	T	2853	M	0.205	0.232	0	0	0	3271.74	ggcctgtatcCtaggctacac	m5C_34767
chr9	35657999	-	C	3681	1027	0.279	1	0	T	2653	M	0.265	0.294	0	0	0	5439.4	tgctgaaggcCtgtatcctag	m5C_34806

chr9	35685281	-	C	37	10	0.27	0	0	T	27	M	0.154	0.43	6.7E-14	2E-06	8.3E-14	20.2815	gaggagagggCtgaggtggcc	m5C_34864
chr9	35685522	-	C	39	18	0.462	0	0	T	21	M	0.316	0.614	0	2.7E-05	0	113.64	gaaaaccgggCcatgaaggat	m5C_34792
chr9	35685526	-	C	41	14	0.341	0	0	T	27	M	0.216	0.495	0	1.9E-07	0	60.3641	catcgaaaacCgggccatgaa	m5C_34841
chr9	35685527	-	C	36	14	0.389	0	0	T	22	M	0.248	0.551	0	9.2E-06	0	69.397	tcatcgaaaaCcgggccatga	m5C_34828
chr9	35685533	-	C	31	9	0.29	0	0	T	22	M	0.161	0.466	3.7E-13	1.2E-06	4.4E-13	18.0154	tgaaggtcatCgaaaaccggg	m5C_34775
chr9	35685536	-	C	32	12	0.375	0	0	T	20	M	0.229	0.547	0	4.6E-06	0	55.0408	gaatgaaggtCatcgaaaacc	m5C_34854
chr9	35685671	-	C	50	10	0.2	0	0	T	40	M	0.112	0.33	2.6E-12	2E-10	3.1E-12	13.024	ctggaggaggCcgagaaggcg	m5C_34850
chr9	35689172	-	C	37	10	0.27	0	0	T	27	M	0.154	0.43	6.7E-14	2E-06	8.3E-14	20.2815	ccaggagaaaCtggagcaggc	m5C_34799
chr9	35689181	-	C	40	19	0.475	0	0	T	21	M	0.329	0.625	0	1E-06	0	125.154	gaaggaggccCaggagaaact	m5C_34847
chr9	35689182	-	C	39	10	0.256	0	0	T	29	M	0.146	0.411	1.3E-13	2E-06	1.6E-13	18.7753	tgaaggaggcCcaggagaaac	m5C_34824
chr9	35689194	-	C	31	24	0.774	0	0	C	24	M	0.602	0.886	0	9.1E-05	0	288.899	attctgaatcCgtgaaggagg	m5C_34794
chr9	35689195	-	C	33	13	0.394	0	0	T	20	M	0.247	0.563	0	6.7E-06	0	64.1755	tattctgaatCcgtgaaggag	m5C_34766
chr9	36204107	+	C	80	26	0.325	0	0	T	54	M	0.232	0.434	0	2.7E-12	0	120.869	aaagaaaaggCaataaaggag	m5C_31597
chr9	36204134	+	C	103	22	0.214	0	0	T	81	M	0.145	0.302	0	0	0	64.0184	gaatggtatgCaagacaggac	m5C_31593
chr9	72803488	-	C	315	75	0.238	0	0	T	240	M	0.194	0.288	0	0	0	291.59	ggtgcaccctCtttggggaat	m5C_32102
chr9	72803492	-	C	349	76	0.218	0	0	T	273	M	0.178	0.264	0	0	0	270.048	gaatggtgcaCcctctttggg	m5C_32100
chr9	72803508	-	C	160	39	0.244	0	0	T	121	M	0.184	0.316	0	0	0	143.318	attgcagtacCtccaggaatg	m5C_32109
chr9	72803539	-	C	109	47	0.431	0	0	T	62	M	0.342	0.525	0	1.3E-12	0	321.6	gggataggagCttactctgtc	m5C_32099
chr9	75778427	+	C	38	11	0.289	0	0	T	27	M	0.17	0.448	2.1E-15	3.1E-06	2.7E-15	27.4488	tgaatgaagaCttggctgatt	m5C_31657
chr9	75778432	+	C	45	14	0.311	0	0	T	31	M	0.195	0.457	0	1.9E-07	0	54.6799	gaagacttggCtgattcagat	m5C_31658
chr9	75778444	+	C	38	13	0.342	0	0	T	25	M	0.212	0.501	0	6.7E-06	0	55.1518	gattcagatgCcagggtaagg	m5C_31650
chr9	75780033	+	C	47	12	0.255	0	0	T	35	M	0.153	0.395	1E-15	7.5E-08	1.3E-15	27.4561	tcttgacaggCcttgtatgaa	m5C_31655
chr9	75780034	+	C	57	22	0.386	0	0	T	35	M	0.271	0.516	0	6.5E-08	0	119.075	cttgacaggcCttgtatgaag	m5C_31651
chr9	77518001	-	C	89	25	0.281	0	0	T	64	M	0.198	0.382	0	1.8E-12	0	99.0468	ggttcgattcCccaacgggga	m5C_31762
chr9	77518007	-	C	90	20	0.222	0	0	T	70	M	0.149	0.318	0	1.7E-15	0	59.4849	gaccggggttCgattccccaa	m5C_31770
chr9	77518014	-	C	140	30	0.214	0	0	T	110	M	0.154	0.289	0	0	0	92.6561	cgcgggagacCggggttcgat	m5C_31768
chr9	77518015	-	C	145	40	0.276	0	0	T	105	M	0.21	0.354	0	0	0	167.688	acgcgggagaCcggggttcga	m5C_31764
chr9	77518024	-	C	154	119	0.773	0	0	C	119	M	0.7	0.832	0	1E-13	0	1666.89	ctgcctgtcaCgcgggagacc	m5C_31766
chr9	77518030	-	C	147	39	0.265	0	0	T	108	M	0.201	0.342	0	0	0	156.447	gtatccctgcCtgtcacgcgg	m5C_31761
chr9	77518036	-	C	142	32	0.225	0	0	T	110	M	0.164	0.301	0	0	0	105.21	tggtgagtatCcctgcctgtc	m5C_31767
chr9	77518057	-	C	33	8	0.242	0	0	T	25	M	0.128	0.41	3.1E-11	6.8E-07	3.6E-11	10.7866	aggcggtcctCgttagtatgg	m5C_31763
chr9	78150950	+	C	40	28	0.7	0	0	C	28	M	0.546	0.819	0	8.1E-06	0	305.59	tgtgctggagCatgtggccta	m5C_31849
chr9	79186673	-	C	34	10	0.294	0	0	T	24	M	0.168	0.462	2.3E-14	2E-06	2.9E-14	22.9564	cgaggccaacCgaggctccgc	m5C_31933
chr9	79186674	-	C	36	8	0.222	0	0	T	28	M	0.117	0.381	7.4E-11	6.8E-07	8.4E-11	9.49676	ccgaggccaaCcgaggctccg	m5C_31920
chr9	79186677	-	C	34	8	0.235	0	0	T	26	M	0.124	0.4	4.2E-11	6.8E-07	4.8E-11	10.3259	tatccgaggcCaaccgaggct	m5C_31885
chr9	79186678	-	C	33	9	0.273	0	0	T	24	M	0.151	0.442	7.5E-13	1.2E-06	9E-13	16.4418	ctatccgaggCcaaccgaggc	m5C_31893
chr9	79186679	+	C	89	29	0.326	0	0	T	60	M	0.237	0.429	0	8.4E-12	0	137.714	cctcggttggCctcggatagc	m5C_31850
chr9	79186684	-	C	40	20	0.5	0	0	CT	20	M	0.352	0.648	0	1.4E-06	0	140.797	gaccggctatCcgaggccaac	m5C_31876
chr9	79186688	-	C	58	18	0.31	0	0	T	40	M	0.206	0.438	0	1.7E-08	0	74.239	gggggaccggCtatccgaggc	m5C_31887
chr9	79186689	+	C	97	21	0.216	0	0	T	76	M	0.146	0.308	0	3.1E-15	0	61.3984	cctcggatagCcggtccccccg	m5C_31831
chr9	79186690	+	C	95	40	0.421	0	0	T	55	M	0.327	0.522	0	6.4E-12	0	261.389	ctcggatagcCggtcccccgc	m5C_31796
chr9	79186691	-	C	87	20	0.23	0	0	T	67	M	0.154	0.329	0	1.5E-13	0	61.6125	ggcgggggacCggctatccga	m5C_31932
chr9	79186692	-	C	91	35	0.385	0	0	T	56	M	0.291	0.487	0	1.5E-12	0	203.885	aggcgggggaCcggctatccg	m5C_31912
chr9	79186694	+	C	87	23	0.264	0	0	T	64	M	0.183	0.366	0	7.2E-13	0	84.2255	gatagccggtCccccgcctgt	m5C_31840
chr9	79186695	+	C	77	28	0.364	0	0	T	49	M	0.265	0.475	0	2.6E-10	0	148.43	atagccggtcCcccgcctgtc	m5C_31814
chr9	79186696	+	C	77	20	0.26	0	0	T	57	M	0.175	0.367	0	1.1E-11	0	69.9563	tagccggtccCccgcctgtcc	m5C_31817
chr9	79186697	+	C	77	42	0.545	0	0	C	42	M	0.435	0.652	0	8.7E-09	0	365.172	agccggtcccCcgcctgtccc	m5C_31832
chr9	79186698	+	C	67	32	0.478	0	0	T	35	M	0.363	0.595	0	2.7E-08	0	232.001	gccggtccccCgcctgtcccc	m5C_31803
chr9	79186699	-	C	129	54	0.419	0	0	T	75	M	0.337	0.505	0	7.5E-15	0	363.995	cggggacaggCgggggaccgg	m5C_31875
chr9	79186700	+	C	61	18	0.295	0	0	T	43	M	0.196	0.419	0	2.9E-10	0	70.3975	cggtcccccgCctgtccccgc	m5C_31852
chr9	79186701	+	C	64	33	0.516	0	0	C	33	M	0.396	0.634	0	3.4E-08	0	261.237	ggtcccccgcCtgtccccgcc	m5C_31847
chr9	79186703	-	C	130	52	0.4	0	0	T	78	M	0.32	0.486	0	4.4E-15	0	332.616	ccggcggggaCaggcgggggga	m5C_31940
chr9	79186705	+	C	51	33	0.647	0	0	C	33	M	0.51	0.764	0	8.8E-07	0	336.506	ccccgcctgtCcccgccggcg	m5C_31795
chr9	79186706	+	C	57	28	0.491	0	0	T	29	M	0.366	0.617	0	3.1E-07	0	205.05	cccgcctgtcCccgccggcgg	m5C_31842
chr9	79186707	+	C	55	36	0.655	0	0	C	36	M	0.523	0.766	0	1.5E-06	0	376.226	ccgcctgtccCcgccggcggg	m5C_31835
chr9	79186708	+	C	45	26	0.578	0	0	C	26	M	0.433	0.71	0	5.5E-06	0	225.163	cgcctgtcccCgccggcgggc	m5C_31836
chr9	79186709	-	C	102	34	0.333	0	0	T	68	M	0.249	0.429	0	2.3E-14	0	169.571	ggcccgccggCggggacaggc	m5C_31870
chr9	79186710	+	C	32	24	0.75	0	0	C	24	M	0.579	0.867	0	9.1E-05	0	277.887	cctgtccccgCcggcgggccg	m5C_31794
chr9	79186712	-	C	85	49	0.576	0	0	C	49	M	0.47	0.676	0	1.4E-09	0	460.954	ggcggcccgcCggcggggaca	m5C_31871
chr9	79186713	-	C	82	27	0.329	0	0	T	55	M	0.237	0.437	0	4E-12	0	128.088	gggcggcccgCcggcggggac	m5C_31881
chr9	79186715	-	C	69	19	0.275	0	0	T	50	M	0.184	0.39	0	4.5E-10	0	69.8987	cggggcggccCgccggcgggg	m5C_31869
chr9	79186783	+	C	242	49	0.203	1	0.004	T	192	M	0.157	0.259	0	0	0	154.2	aaacggggtgCggccggaaag	m5C_31821
chr9	79186786	+	C	230	65	0.283	0	0	T	165	M	0.228	0.344	0	0	0	296.865	cggggtgcggCcggaaaggcg	m5C_31790
chr9	79186795	+	C	202	64	0.32	2	0.01	T	136	M	0.259	0.388	0	0	0	331.858	gccggaaaggCggccgccccc	m5C_31856
chr9	79186798	+	C	194	40	0.206	0	0	T	154	M	0.155	0.269	0	0	0	124.182	ggaaaggcggCcgccccctcg	m5C_31843
chr9	79186799	+	C	193	39	0.202	0	0	T	154	M	0.151	0.264	0	0	0	118.162	gaaaggcggcCgccccctcgc	m5C_31811
chr9	79186801	+	C	144	41	0.285	0	0	T	103	M	0.217	0.363	0	0	0	178.232	aaggcggccgCccccctcgccc	m5C_31801
chr9	79186802	+	C	76	25	0.329	0	0	T	51	M	0.234	0.441	0	9.3E-11	0	116.896	aggcggccgcCccctcgcccg	m5C_31792
chr9	79186873	-	C	32	20	0.625	0	0	C	20	M	0.453	0.771	0	4.3E-05	0	181.016	aaccccgaccCagaagcaggt	m5C_31896
chr9	79186913	+	C	77	49	0.636	0	0	C	49	M	0.525	0.735	0	3.1E-08	0	514.324	tcgttcgatgCgatctattga	m5C_31830
chr9	79186928	+	C	121	89	0.736	0	0	C	89	M	0.651	0.806	0	6.9E-12	0	1158.04	tattgaaagtCagccctcgac	m5C_31855
chr9	79186931	+	C	116	76	0.655	0	0	C	76	M	0.565	0.735	0	1.7E-11	0	858.728	tgaaagtcagCcctcgacaca	m5C_31793
chr9	79186932	+	C	116	92	0.793	0	0	C	92	M	0.711	0.857	0	1.7E-10	0	1307.46	gaaagtcagcCctcgacacaa	m5C_31829
chr9	79186933	+	C	115	76	0.661	0	0	C	76	M	0.57	0.741	0	1.7E-11	0	866.999	aaagtcagccCtcgacacaag	m5C_31798
chr9	79186935	+	C	119	88	0.739	0	0	C	88	M	0.654	0.81	0	1E-10	0	1151.09	agtcagccctCgacacaaggg	m5C_31800
chr9	79186938	+	C	112	73	0.652	0	0	C	73	M	0.56	0.734	0	1E-11	0	817.381	cagccctcgaCacaagggttt	m5C_31823
chr9	79186940	+	C	112	84	0.75	0	0	C	84	M	0.662	0.821	0	5.8E-11	0	1112.87	gccctcgacaCaagggtttgt	m5C_31858
chr9	89037857	-	C	870	253	0.291	0	0	T	617	M	0.262	0.322	0	0	0	1323.69	aatggtgcacCaaaaaaaaaa	m5C_32405
chr9	89037858	-	C	821	237	0.289	0	0	T	584	M	0.259	0.321	0	0	0	1226.31	gaatggtgcaCcaaaaaaaaa	m5C_32415
chr9	89037889	-	C	861	206	0.24	1	0.001	T	654	M	0.212	0.269	0	0	0	874.296	atgcattgacCtggtattgca	m5C_32409
chr9	89037916	-	C	32	10	0.312	0	0	T	22	M	0.18	0.486	1.1E-14	2E-06	1.3E-14	25.0836	ggaataggagCttaccccatc	m5C_32407
chr9	95054795	-	C	68	22	0.324	0	0	T	46	M	0.224	0.442	0	1.5E-09	0	98.7248	gtgatttatgCatggaggctg	m5C_32685
chr9	95054807	-	C	123	31	0.252	0	0	T	92	M	0.184	0.335	0	0	0	113.838	catagcaatcCtgtgatttat	m5C_32712
chr9	95054808	-	C	140	30	0.214	0	0	T	110	M	0.154	0.289	0	0	0	92.6561	ccatagcaatCctgtgattta	m5C_32704
chr9	95054812	-	C	151	31	0.205	0	0	T	120	M	0.149	0.277	0	0	0	92.1127	tgggccatagCaatcctgtga	m5C_32693
chr9	95054817	-	C	175	70	0.402	1	0.006	T	104	M	0.332	0.477	0	0	0	465.231	ttcgatgggcCatagcaatcc	m5C_32732
chr9	95054818	-	C	182	39	0.214	0	0	T	143	M	0.161	0.279	0	0	0	125.504	attcgatgggCcatagcaatc	m5C_32715
chr9	95054825	-	C	209	57	0.273	0	0	T	152	M	0.217	0.337	0	0	0	247.216	ccagtggattCgatgggccat	m5C_32721
chr9	95054834	-	C	353	103	0.292	0	0	T	250	M	0.247	0.341	0	0	0	508.422	gacagctctcCagtggattcg	m5C_32689
chr9	95054837	-	C	360	80	0.222	0	0	T	280	M	0.182	0.268	0	0	0	291.737	atggacagctCtccagtggat	m5C_32695
chr9	95054839	-	C	401	82	0.206	2	0.005	T	317	M	0.169	0.248	0	0	0	276.771	gcatggacagCtctccagtgg	m5C_32725
chr9	95054848	-	C	585	121	0.207	1	0.002	T	463	M	0.176	0.242	0	0	0	426.611	tgctgttgtgCatggacagct	m5C_32708
chr9	95054872	-	C	461	116	0.252	0	0	T	345	M	0.214	0.293	0	0	0	496.89	tgtccctgccCtgtggttgct	m5C_32726
chr9	95054874	-	C	270	58	0.215	0	0	T	212	M	0.17	0.268	0	0	0	197.209	tctgtccctgCcctgtggttg	m5C_32687
chr9	95703490	+	C	44	21	0.477	0	0	T	23	M	0.338	0.621	0	1.8E-06	0	141.77	tgaaagctggCcacagcgacc	m5C_32671
chr9	96938675	+	C	157	35	0.223	0	0	T	122	M	0.165	0.294	0	0	0	115.419	gtgattttacCctgttcagga	m5C_32345
chr9	96938676	+	C	153	33	0.216	0	0	T	120	M	0.158	0.287	0	0	0	104.217	tgattttaccCtgttcaggag	m5C_32340
chr9	96938681	+	C	146	33	0.226	0	0	T	113	M	0.166	0.3	0	0	0	109.371	ttaccctgttCaggagataac	m5C_32325
chr9	96938691	+	C	129	40	0.312	1	0.008	T	88	M	0.239	0.397	0	1.1E-16	0	190.923	caggagataaCtatacaatct	m5C_32317
chr9	96941138	+	C	59	15	0.254	0	0	T	44	M	0.161	0.378	0	4.6E-09	0	48.1675	tagtaggttgCatagttttag	m5C_32334
chr9	97109699	-	C	68	24	0.353	0	0	T	44	M	0.25	0.472	0	3E-09	0	120.009	aggaggctgaCaatgaggtag	m5C_32373

chr9	97109703	-	C	68	27	0.397	0	0	T	41	M	0.289	0.516	0	7.5E-09	0	156.214	gagcaggaggCtgacaatgag	m5C_32364
chr9	97847549	+	C	34	7	0.206	0	0	T	27	M	0.103	0.368	1.4E-09	3.4E-07	1.6E-09	6.4054	agattaaaatCacattgccag	m5C_32356
chr9	97847551	+	C	31	11	0.355	0	0	T	20	M	0.211	0.531	1.1E-16	3.1E-06	1.5E-16	37.0595	attaaaatcaCattgccaggg	m5C_32348
chr9	97847556	+	C	65	32	0.492	0	0	T	33	M	0.375	0.611	0	2.7E-08	0	239.767	aatcacattgCcagggattac	m5C_32310
chr9	97847557	+	C	68	43	0.632	0	0	C	43	M	0.514	0.737	0	2.4E-07	0	441.657	atcacattgcCagggattacc	m5C_32316
chr9	97847566	+	C	65	25	0.385	0	0	T	40	M	0.276	0.506	0	4.1E-09	0	137.974	ccagggattaCcacgcaacca	m5C_32358
chr9	97847567	+	C	54	17	0.315	0	0	T	37	M	0.207	0.447	0	1.1E-08	0	70.309	cagggattacCacgcaaccac	m5C_32343
chr9	97847804	+	C	47	13	0.277	0	0	T	34	M	0.169	0.418	0	1.2E-07	0	44.0347	tggctaagttCtgcacctgaa	m5C_32360
chr9	98865449	-	C	34	11	0.324	0	0	T	23	M	0.191	0.492	4.4E-16	3.1E-06	5.8E-16	32.3088	agaggctggtCcgatggtagt	m5C_32301
chr9	100619995	+	C	48	22	0.458	0	0	T	26	M	0.326	0.597	0	2.3E-06	0	143.33	cggagacgcgCgagggttggg	m5C_32620
chr9	100767431	+	C	47	19	0.404	0	0	T	28	M	0.276	0.547	0	1E-06	0	105.031	aggaggaggaCgaaggtgagt	m5C_32565
chr9	100773572	+	C	50	19	0.38	0	0	T	31	M	0.259	0.518	0	2.4E-08	0	98.2779	atgaggaagaCgaggacgatg	m5C_32624
chr9	100773578	+	C	46	13	0.283	0	0	T	33	M	0.173	0.425	0	1.2E-07	0	45.0425	aagacgaggaCgatgaggatg	m5C_32585
chr9	100773650	+	C	119	41	0.345	0	0	T	78	M	0.265	0.434	0	6E-15	0	217.481	gggatgaggaCgacgatgaag	m5C_32578
chr9	100773662	+	C	97	22	0.227	0	0	T	75	M	0.155	0.32	0	5.8E-15	0	68.1195	acgatgaagtCagtgaggagg	m5C_32575
chr9	100774718	+	C	42	29	0.69	0	0	C	29	M	0.54	0.809	0	9.7E-06	0	313.048	agaatttggaCttgatgaaga	m5C_32610
chr9	100843182	+	C	35	11	0.314	0	0	T	24	M	0.186	0.48	6.7E-16	3.1E-06	8.7E-16	30.9686	cgcagtggctCtgggggccaa	m5C_32622
chr9	100843189	+	C	51	33	0.647	0	0	C	33	M	0.51	0.764	0	8.8E-07	0	336.506	gctctgggggCcaaggtgttg	m5C_32580
chr9	100843190	+	C	49	21	0.429	0	0	T	28	M	0.3	0.567	0	1.8E-06	0	126.097	ctctgggggcCaaggtgttgg	m5C_32607
chr9	100843203	+	C	83	25	0.301	0	0	T	58	M	0.213	0.407	0	1.8E-12	0	106.553	ggtgttggaaCgtcacataac	m5C_32623
chr9	100843208	+	C	86	19	0.221	0	0	T	67	M	0.146	0.319	0	8.4E-14	0	55.5742	tggaacgtcaCataactttgg	m5C_32627
chr9	101018581	-	C	47	14	0.298	0	0	T	33	M	0.187	0.44	0	1.9E-07	0	52.223	catgggtttcCgcagcgcagc	m5C_32644
chr9	104314845	+	C	31	15	0.484	0	0	T	16	M	0.32	0.652	0	1.2E-05	0	95.91	agaacgagaaCgagaaaggag	m5C_32466
chr9	104314872	+	C	60	24	0.4	0	0	T	36	M	0.286	0.526	0	1.2E-07	0	137.133	ggagagggaaCgagaaagaga	m5C_32465
chr9	111754688	-	C	673	212	0.315	0	0	T	461	M	0.281	0.351	0	0	0	1191.63	ctgagcgttgCttaaaaaaaa	m5C_40057
chr9	111754698	-	C	843	250	0.297	0	0	T	593	M	0.267	0.328	0	0	0	1333.53	ctatgcatgtCtgagcgttgc	m5C_40081
chr9	111754717	-	C	882	207	0.235	0	0	T	675	M	0.208	0.264	0	0	0	860.754	ccctgggttcCtcccggggct	m5C_40082
chr9	111754718	-	C	903	195	0.216	0	0	T	708	M	0.19	0.244	0	0	0	742.332	gccctgggttCctcccggggc	m5C_40065
chr9	111754768	-	C	92	33	0.359	0	0	T	59	M	0.268	0.461	0	7.5E-13	0	176.992	atgcaggataCattgacacac	m5C_40071
chr9	111754775	-	C	157	37	0.236	0	0	T	120	M	0.176	0.308	0	0	0	130.305	atgtgcaatgCaggatacatt	m5C_40049
chr9	112296098	-	C	32	9	0.281	0	0	T	23	M	0.156	0.454	5.3E-13	1.2E-06	6.4E-13	17.1983	ggggaaagaaCgagggatggg	m5C_32535
chr9	113018720	-	C	31	9	0.29	0	0	T	22	M	0.161	0.466	3.7E-13	1.2E-06	4.4E-13	18.0154	actccagcagCcaagatggtg	m5C_32540
chr9	114133923	-	C	35	12	0.343	0	0	T	23	M	0.208	0.508	0	4.6E-06	0	49.9956	caaggcctggCtggaagaacg	m5C_32552
chr9	116140465	-	C	34	17	0.5	0	0	CT	17	M	0.341	0.659	0	2.1E-05	0	115.835	ggttaggattCcaggctctta	m5C_32758
chr9	116140480	-	C	39	21	0.538	0	0	C	21	M	0.386	0.684	0	5.2E-05	0	161.991	ccctggtggtCtagtggttag	m5C_32780
chr9	116171145	-	C	89	68	0.764	0	0	C	68	M	0.666	0.84	0	2.9E-08	0	905.844	atgatgaagaCgaagaaaggc	m5C_32782
chr9	116171157	-	C	82	30	0.366	0	0	T	52	M	0.27	0.474	0	1.2E-11	0	161.871	gggatgaggaCaatgatgaag	m5C_32774
chr9	116171169	-	C	57	12	0.211	0	0	T	45	M	0.125	0.333	1.6E-14	8.8E-10	2E-14	20.643	aagacaagagCagggatgagg	m5C_32760
chr9	116171194	-	C	54	12	0.222	0	0	T	42	M	0.132	0.349	7.5E-15	8.8E-10	9.6E-15	22.3614	gacaaaaaaaCagactcggaa	m5C_32775
chr9	116171202	-	C	79	35	0.443	0	0	T	44	M	0.339	0.553	0	1.9E-09	0	237.073	aggacaaagaCaaaaaaacag	m5C_32776
chr9	116171208	-	C	75	13	0.2	10	0.133	T	52	M	0.121	0.313	3.8E-15	1.6E-11	4.8E-15	22.646	agaagaaggaCaaagacaaaa	m5C_32763
chr9	116171227	-	C	61	29	0.475	0	0	T	32	M	0.355	0.598	0	1.3E-08	0	206.076	aaggaggcctCagagcaaaag	m5C_32784
chr9	116171229	-	C	60	12	0.2	0	0	T	48	M	0.118	0.318	3.3E-14	7.6E-12	4.2E-14	19.13	agaaggaggcCtcagagcaaa	m5C_32754
chr9	123171473	-	C	31	18	0.581	0	0	C	18	M	0.408	0.736	0	2.7E-05	0	146.757	agaaagaaggCagcgagaagg	m5C_33027
chr9	123171487	-	C	36	11	0.306	0	0	T	25	M	0.18	0.469	1E-15	3.1E-06	1.3E-15	29.7077	aaatgaaaggCtgcagaaaga	m5C_33025
chr9	123851340	+	C	39	10	0.256	0	0	T	29	M	0.146	0.411	1.3E-13	2E-06	1.6E-13	18.7753	gggggtatagCttagtggtag	m5C_33013
chr9	123965604	+	C	77	25	0.325	0	0	T	52	M	0.231	0.435	0	9.3E-11	0	115.296	atgaaccggcCcaggtcggag	m5C_33005
chr9	123965610	+	C	81	26	0.321	0	0	T	55	M	0.229	0.429	0	2.7E-12	0	119.299	cggcccaggtCggagatggag	m5C_33012
chr9	123965631	+	C	37	22	0.595	0	0	C	22	M	0.435	0.737	0	6.3E-05	0	191.337	caggtcaaaaCtcccgtgctg	m5C_33017
chr9	125642511	-	C	43	16	0.372	0	0	T	27	M	0.244	0.521	0	4E-07	0	78.0036	actgtggaatCtgaagacact	m5C_32984
chr9	125642520	-	C	46	10	0.217	0	0	T	36	M	0.123	0.356	9.7E-13	2.4E-08	1.2E-12	14.7309	gatactcctaCtgtggaatct	m5C_32985
chr9	125642526	-	C	48	13	0.271	0	0	T	35	M	0.166	0.41	0	1.2E-07	0	43.0711	atcactgataCtcctactgtg	m5C_32987
chr9	125642537	-	C	49	14	0.286	0	0	T	35	M	0.178	0.424	0	1.9E-07	0	49.9784	aaatttaattCatcactgata	m5C_33000
chr9	125642556	-	C	55	15	0.273	0	0	T	40	M	0.173	0.402	0	4.6E-09	0	51.8418	agggcagattCtgaggtgaaa	m5C_32992
chr9	127454770	+	C	33	8	0.242	0	0	T	25	M	0.128	0.41	3.1E-11	6.8E-07	3.6E-11	10.7866	acattcaacgCtgtcggtgag	m5C_33157
chr9	127454774	+	C	33	16	0.485	0	0	T	17	M	0.325	0.648	0	1.7E-05	0	104.012	tcaacgctgtCggtgagtttg	m5C_33161
chr9	127620200	-	C	35	24	0.686	0	0	C	24	M	0.52	0.814	0	9.1E-05	0	249.696	cggtcaaggcCtgaggggcgc	m5C_33195
chr9	127620210	-	C	73	23	0.315	0	0	T	50	M	0.22	0.429	0	4.3E-11	0	101.216	cggaagtacgCggtcaaggcc	m5C_33182
chr9	127620212	-	C	69	26	0.377	0	0	T	43	M	0.272	0.495	0	5.6E-09	0	141.353	tgcggaagtaCgcggtcaagg	m5C_33223
chr9	127620223	-	C	73	20	0.274	0	0	T	53	M	0.185	0.386	0	1.1E-11	0	73.9652	gctgtacccgCtgcggaagta	m5C_33226
chr9	127620227	-	C	71	20	0.282	0	0	T	51	M	0.19	0.395	0	1.1E-11	0	76.1478	agcggctgtaCccgctgcgga	m5C_33229
chr9	127620265	-	C	114	37	0.325	0	0	T	77	M	0.246	0.415	0	1.4E-15	0	181.707	cgaggagaacCtgaagaccaa	m5C_33211
chr9	127620266	-	C	117	50	0.427	0	0	T	67	M	0.341	0.518	0	8.5E-14	0	341.427	acgaggagaaCctgaagacca	m5C_33169
chr9	127620275	-	C	125	32	0.256	0	0	T	93	M	0.188	0.339	0	0	0	120.042	tcaacaagcaCgaggagaacc	m5C_33178
chr9	127620284	-	C	95	42	0.442	0	0	T	53	M	0.346	0.542	0	1.1E-11	0	290.993	gccgccggctCaacaagcacg	m5C_33227
chr9	127620289	-	C	78	30	0.385	0	0	T	48	M	0.284	0.496	0	4.8E-10	0	170.693	catgcgccgcCggctcaacaa	m5C_33190
chr9	127620293	-	C	59	24	0.407	0	0	T	35	M	0.291	0.534	0	1.2E-07	0	139.626	gtgccatgcgCcgccggctca	m5C_33173
chr9	127623826	-	C	65	15	0.231	0	0	T	50	M	0.145	0.346	0	6E-11	0	43.5411	aggccaagatCaaggctcgag	m5C_33215
chr9	127623833	-	C	52	13	0.25	0	0	T	39	M	0.152	0.382	1.1E-16	1.6E-09	1.5E-16	31.5947	tgcgcgcaggCcaagatcaag	m5C_33213
chr9	127624202	-	C	51	29	0.569	0	0	C	29	M	0.433	0.695	0	3.9E-07	0	250.937	gcgttggcggCttgtgcagca	m5C_33177
chr9	127624205	-	C	62	21	0.339	0	0	T	41	M	0.233	0.463	0	1E-09	0	98.0345	gcggcgttggCggcttgtgca	m5C_33210
chr9	127624214	-	C	42	19	0.452	0	0	T	23	M	0.312	0.601	0	1E-06	0	118.648	gagcgggcggCggcgttggcg	m5C_33172
chr9	127624217	-	C	40	16	0.4	0	0	T	24	M	0.263	0.554	0	1.7E-05	0	84.314	tcggagcgggCggcggcgttg	m5C_33192
chr9	127624221	-	C	38	9	0.237	0	0	T	29	M	0.13	0.392	3.7E-12	1.2E-06	4.3E-12	13.3739	tcccctcggagCgggcggcggc	m5C_33193
chr9	128001348	-	C	50	31	0.62	0	0	C	31	M	0.482	0.741	0	5.9E-07	0	298.531	gaaactccggCgcgaggtaga	m5C_32862
chr9	128001352	-	C	38	12	0.316	0	0	T	26	M	0.191	0.475	0	4.6E-06	0	45.8026	tgcagaaactCcggcgcgagg	m5C_32905
chr9	128001360	-	C	33	10	0.303	0	0	T	23	M	0.174	0.473	1.6E-14	2E-06	2E-14	23.982	tagagctgtgCagaaactccg	m5C_32882
chr9	128001382	-	C	32	9	0.281	0	0	T	23	M	0.156	0.454	5.3E-13	1.2E-06	6.4E-13	17.1983	gcaaagatgtCaggaaagaca	m5C_32912
chr9	128001391	-	C	37	18	0.486	0	0	T	19	M	0.334	0.641	0	2.7E-05	0	120.406	agaagacgggCaaagatgtca	m5C_32883
chr9	128001395	-	C	34	26	0.765	0	0	C	26	M	0.6	0.876	0	0.00013	0	312.018	aaaaagaagaCgggcaaagat	m5C_32902
chr9	128003320	-	C	33	11	0.333	0	0	T	22	M	0.198	0.504	2.2E-16	3.1E-06	2.9E-16	34.0074	gacgtgggcaCggtggtcggc	m5C_32872
chr9	128003322	-	C	51	29	0.569	0	0	C	29	M	0.433	0.695	0	3.9E-07	0	250.937	aggacgtgggCacggtggtcg	m5C_32910
chr9	128003328	-	C	46	23	0.5	0	0	CT	23	M	0.361	0.639	0	2.9E-06	0	166.146	agaaggaggaCgtgggcacgg	m5C_32886
chr9	128003357	-	C	48	31	0.646	0	0	C	31	M	0.504	0.766	0	1.4E-05	0	312.721	cagcgcggcgCgggccgagga	m5C_32884
chr9	128003364	-	C	40	17	0.425	0	0	T	23	M	0.285	0.578	0	5.6E-07	0	96.9312	tgctgctcagCgcggcgcggg	m5C_32892
chr9	128003574	-	C	72	16	0.222	0	0	T	56	M	0.142	0.331	0	1.2E-12	0	45.3354	gggtgtttcgCgagtgtgaga	m5C_32869
chr9	128003576	-	C	71	33	0.465	0	0	T	38	M	0.354	0.58	0	1.1E-09	0	233.365	tggggtgtttCgcgagtgtga	m5C_32904
chr9	128003590	-	C	50	11	0.22	0	0	T	39	M	0.128	0.352	8.5E-14	4.4E-10	1.1E-13	18.3342	acagattgacCtattggggtg	m5C_32909
chr9	128003603	-	C	44	10	0.227	0	0	T	34	M	0.128	0.37	5.7E-13	2.4E-08	6.8E-13	15.7274	caagacagcaCagacagattg	m5C_32907
chr9	128003608	-	C	33	10	0.303	0	0	T	23	M	0.174	0.473	1.6E-14	2E-06	2E-14	23.982	ccggccaagaCagcacagaca	m5C_32865
chr9	130210780	-	C	404	115	0.285	0	0	T	289	M	0.243	0.331	0	0	0	558.531	tgggaacagcCtagaaatgaa	m5C_33679
chr9	130210781	-	C	531	136	0.256	0	0	T	395	M	0.221	0.295	0	0	0	600.679	atgggaacagCctagaaatga	m5C_33724
chr9	130210784	-	C	652	182	0.279	0	0	T	470	M	0.246	0.315	0	0	0	895.727	agcatgggaaCagcctagaaa	m5C_33742
chr9	130210799	-	C	727	230	0.316	0	0	T	497	M	0.284	0.351	0	0	0	1304.57	tgactctgtgCcgaaagcatg	m5C_33660
chr9	130210843	-	C	356	83	0.233	0	0	T	273	M	0.192	0.28	0	0	0	319.074	atagtaaaccCcagcttagga	m5C_33706
chr9	130210844	-	C	364	114	0.313	0	0	T	250	M	0.268	0.363	0	0	0	610.359	gatagtaaacCccagcttagg	m5C_33691
chr9	130210856	-	C	477	186	0.39	0	0	T	291	M	0.347	0.434	0	0	0	1291.63	cagtggtgagCtgatagtaaa	m5C_33713
chr9	130210886	-	C	511	134	0.262	0	0	T	377	M	0.226	0.302	0	0	0	605.597	ctgaccaggtCtctgttggct	m5C_33671
chr9	130210891	-	C	555	126	0.227	0	0	T	429	M	0.194	0.264	0	0	0	489.184	tgcacctgacCaggtctctgt	m5C_33714
chr9	130210896	-	C	574	121	0.211	1	0.002	T	452	M	0.18	0.246	0	0	0	434.947	gccactgcacCtgaccaggtc	m5C_33695
chr9	130210907	-	C	547	120	0.22	1	0.002	T	426	M	0.187	0.256	0	0	0	448.967	ctcagccaccCgccactgcac	m5C_33744

chr9	130438090	+	C	31	18	0.581	0	0	C	18	M	0.408	0.736	0	2.7E-05	0	146.757	taggacctggCcatgggcaca	m5C_33608
chr9	130883501	-	C	37	11	0.297	0	0	T	26	M	0.175	0.458	1.4E-15	3.1E-06	1.9E-15	28.5508	gttcgatgacCtgatgcagca	m5C_33688
chr9	130883512	-	C	45	10	0.222	0	0	T	35	M	0.125	0.363	7.4E-13	2.4E-08	8.9E-13	15.2157	gggctggatgCgttcgatgac	m5C_33667
chr9	130883519	-	C	45	13	0.289	0	0	T	32	M	0.177	0.434	0	1.2E-07	0	46.0978	gatggaggggCtggatgcgtt	m5C_33662
chr9	131102362	-	C	36	10	0.278	0	0	T	26	M	0.158	0.44	4.8E-14	2E-06	5.9E-14	21.1115	cgagtcccacCagagtcgaat	m5C_33712
chr9	131102363	-	C	33	19	0.576	0	0	C	19	M	0.408	0.728	0	3.4E-05	0	155.067	tcgagtcccaCcagagtcgaa	m5C_33681
chr9	131102365	-	C	38	10	0.263	0	0	T	28	M	0.15	0.42	9.4E-14	2E-06	1.2E-13	19.5037	gttcgagtccCaccagagtcg	m5C_33731
chr9	131102366	-	C	36	9	0.25	0	0	T	27	M	0.138	0.411	2E-12	1.2E-06	2.4E-12	14.4765	ggttcgagtcCcaccagagtc	m5C_33687
chr9	131102367	-	C	38	11	0.289	0	0	T	27	M	0.17	0.448	2.1E-15	3.1E-06	2.7E-15	27.4488	gggttcgagtCccaccagagt	m5C_33737
chr9	131102372	-	C	37	12	0.324	0	0	T	25	M	0.196	0.485	0	4.6E-06	0	47.1194	gttgtgggttCgagtcccacc	m5C_33684
chr9	131102401	-	C	31	9	0.29	0	0	T	22	M	0.161	0.466	3.7E-13	1.2E-06	4.4E-13	18.0154	ctagctgagcCtagtgtggtc	m5C_33722
chr9	131102411	-	C	42	20	0.476	0	0	T	22	M	0.334	0.623	0	1.4E-06	0	133.437	cattggacttCtagctgagcc	m5C_33696
chr9	131102441	-	C	52	12	0.231	0	0	T	40	M	0.137	0.361	4.4E-15	8.8E-10	5.7E-15	23.6371	gcagccggctCtgtggcgcaa	m5C_33728
chr9	131331668	+	C	43	9	0.209	0	0	T	34	M	0.114	0.352	1.4E-11	1.2E-08	1.6E-11	11.1522	acctattggtCaatgaccatg	m5C_33643
chr9	131331674	+	C	47	16	0.34	0	0	T	31	M	0.222	0.483	0	4E-07	0	70.9352	tggtcaatgaCcatggtgatg	m5C_33652
chr9	131331675	+	C	45	9	0.2	0	0	T	36	M	0.109	0.338	2.3E-11	1.2E-08	2.7E-11	10.4289	ggtcaatgacCatggtgatgg	m5C_33631
chr9	131331688	+	C	43	13	0.302	0	0	T	30	M	0.186	0.451	0	1.2E-07	0	48.3648	ggtgatgggaCtgactttgag	m5C_33576
chr9	131331692	+	C	44	15	0.341	0	0	T	29	M	0.219	0.489	0	2.8E-07	0	65.6269	atgggactgaCtttgaggcag	m5C_33599
chr9	131392277	+	C	36	16	0.444	0	0	T	20	M	0.295	0.604	0	1.7E-05	0	94.5313	agaggatgctCagacaggcct	m5C_33657
chr9	131392281	+	C	34	10	0.294	0	0	T	24	M	0.168	0.462	2.3E-14	2E-06	2.9E-14	22.9564	gatgctcagaCaggcctgcag	m5C_33612
chr9	131451532	+	C	33	9	0.273	0	0	T	24	M	0.151	0.442	7.5E-13	1.2E-06	9E-13	16.4418	gaggaggaggCggctcgggag	m5C_33629
chr9	131451566	+	C	46	10	0.217	0	0	T	36	M	0.123	0.356	9.7E-13	2.4E-08	1.2E-12	14.7309	ctggctggatCgccgagcgcg	m5C_33586
chr9	131451568	+	C	44	11	0.25	0	0	T	33	M	0.146	0.394	1.6E-14	4.4E-08	2E-14	22.132	ggctggatcgCcgagcgcgag	m5C_33621
chr9	131455933	+	C	88	19	0.216	0	0	T	69	M	0.143	0.313	0	8.4E-14	0	54.2667	acgaaacgttCgagtcaaacg	m5C_33595
chr9	131455938	+	C	84	19	0.226	0	0	T	65	M	0.15	0.326	0	8.4E-14	0	56.9464	acgttcgagtCaaacgcagaa	m5C_33620
chr9	131455954	+	C	67	20	0.299	0	0	T	47	M	0.202	0.417	0	6.9E-10	0	80.9254	cagaataaagCcagcaggaag	m5C_33575
chr9	131455955	+	C	74	27	0.37	1	0.014	T	46	M	0.268	0.485	0	1.9E-10	0	144.844	agaataaagcCagcaggaaga	m5C_33580
chr9	131456180	+	C	38	13	0.342	0	0	T	25	M	0.212	0.501	0	6.7E-06	0	55.1518	ttaaggttccCgatatggatg	m5C_33579
chr9	131965197	-	C	54	26	0.481	0	0	T	28	M	0.354	0.611	0	1.9E-07	0	184.048	gcgctggtggCtccgcccctc	m5C_33702
chr9	131965204	-	C	54	29	0.537	0	0	C	29	M	0.406	0.663	0	3.9E-07	0	235.51	ggggcgggcgCtggtggctcc	m5C_33719
chr9	131965206	-	C	52	11	0.212	0	0	T	41	M	0.122	0.34	1.4E-13	4.4E-10	1.7E-13	17.3019	gcggggcgggCgCtggtggct	m5C_33739
chr9	133333808	+	C	34	22	0.647	0	0	C	22	M	0.479	0.785	0	6.3E-05	0	210.82	aggatgtcagCagggagtttg	m5C_34329
chr9	133348124	-	C	41	10	0.244	0	0	T	31	M	0.138	0.393	2.4E-13	2.4E-08	2.9E-13	17.4472	ggtggctggcCtgagtgttgt	m5C_34353
chr9	133377344	+	C	36	17	0.472	0	0	T	19	M	0.32	0.63	0	2.1E-05	0	108.752	ggacctggggCacagagcagg	m5C_34336
chr9	133981324	-	C	77	16	0.208	0	0	T	61	M	0.132	0.311	0	1.2E-12	0	42.2879	acttgggaggCtgaggctgga	m5C_34354
chr9	133981339	-	C	79	31	0.392	0	0	T	48	M	0.292	0.503	0	6.4E-10	0	181.119	cctgtagtccCagctacttgg	m5C_34376
chr9	133981341	-	C	77	31	0.403	0	0	T	46	M	0.3	0.514	0	6.4E-10	0	186.135	tgcctgtagtCccagctactt	m5C_34371
chr9	133981348	-	C	74	21	0.284	0	0	T	53	M	0.194	0.395	0	1.8E-11	0	81.3683	tggtgcgtgcCtgtagtccca	m5C_34352
chr9	133981368	-	C	31	14	0.452	0	0	T	17	M	0.292	0.622	0	9.2E-06	0	81.6522	aaaaaattagCtgggtgtggt	m5C_34373
chr9	134183195	+	C	37	15	0.405	0	0	T	22	M	0.263	0.565	0	1.2E-05	0	79.0389	gtcagtgtggCtgagtggcct	m5C_33099
chr9	134183204	+	C	34	11	0.324	0	0	T	23	M	0.191	0.492	4.4E-16	3.1E-06	5.8E-16	32.3088	gctgagtggcCtgagtgagga	m5C_33060
chr9	134365945	+	C	33	19	0.576	0	0	C	19	M	0.408	0.728	0	3.4E-05	0	155.067	atgaagaggaCagtgactgag	m5C_33064
chr9	134365951	+	C	32	14	0.438	0	0	T	18	M	0.282	0.607	0	9.2E-06	0	78.8622	aggacagtgaCtgagagacca	m5C_33079
chr9	135125718	+	C	34	10	0.294	0	0	T	24	M	0.168	0.462	2.3E-14	2E-06	2.9E-14	22.9564	tggcttggccCagcaggaaca	m5C_33082
chr9	135894974	+	C	37	9	0.243	0	0	T	28	M	0.134	0.401	2.7E-12	1.2E-06	3.2E-12	13.907	gcggtggcatCgacaaaagaa	m5C_33089
chr9	135894977	+	C	50	17	0.34	0	0	T	33	M	0.224	0.478	0	5.6E-07	0	76.285	gtggcatcgaCaaaagaacca	m5C_33049
chr9	135895009	+	C	223	88	0.395	0	0	T	135	M	0.333	0.46	0	0	0	585.68	gagaaggaggCtgctgagatg	m5C_33055
chr9	135895012	+	C	191	46	0.241	0	0	T	145	M	0.186	0.306	0	0	0	170.835	aaggaggctgCtgagatggga	m5C_33075
chr9	135895211	+	C	50	13	0.26	0	0	T	37	M	0.159	0.396	0	1.6E-09	0	41.2656	actgtgctgtCctgattgttg	m5C_33043
chr9	135895246	+	C	188	41	0.218	0	0	T	147	M	0.165	0.282	0	0	0	135.319	gaatttgaagCtggtatctcc	m5C_33067
chr9	135895253	+	C	214	66	0.308	0	0	T	148	M	0.25	0.373	0	0	0	330.484	aagctggtatCtccaagaatg	m5C_33045
chr9	135895256	+	C	211	63	0.299	0	0	T	148	M	0.241	0.363	0	0	0	303.508	ctggtatctcCaagaatgggc	m5C_33083
chr9	135895266	+	C	205	56	0.273	0	0	T	149	M	0.217	0.338	0	0	0	242.777	caagaatgggCagacccgaga	m5C_33113
chr9	136216316	+	C	148	40	0.27	0	0	T	108	M	0.205	0.347	0	0	0	164.164	ccaccaagatCgctgatgcac	m5C_33488
chr9	136216318	+	C	141	33	0.234	0	0	T	108	M	0.172	0.31	0	0	0	113.377	accaagatcgCtgatgcacca	m5C_33499
chr9	136216324	+	C	82	21	0.256	0	0	T	61	M	0.174	0.36	0	2.6E-13	0	73.0945	atcgctgatgCaccaacaccc	m5C_33500
chr9	136216974	+	C	266	75	0.282	0	0	T	191	M	0.231	0.339	0	0	0	346.924	aatttcttggCctgaaattac	m5C_33482
chr9	136216975	+	C	257	113	0.44	0	0	T	144	M	0.38	0.501	0	0	0	859.556	atttcttggcCtgaaattact	m5C_33493
chr9	136216999	+	C	191	44	0.23	0	0	T	147	M	0.176	0.295	0	0	0	155.161	aagagtaaaaCcgagcttttt	m5C_33472
chr9	136217358	+	C	239	59	0.247	0	0	T	180	M	0.196	0.305	0	0	0	231.854	aagtgcgaatCcatgagcttt	m5C_33484
chr9	136217365	+	C	235	66	0.281	0	0	T	169	M	0.227	0.341	0	0	0	299.997	aatccatgagCtttttaaccc	m5C_33514
chr9	136217705	+	C	70	14	0.2	0	0	T	56	M	0.123	0.308	4.4E-16	2.8E-13	5.8E-16	26.447	gaacttttgcCaatgatggtt	m5C_33469
chr9	136217724	+	C	150	62	0.413	0	0	T	88	M	0.338	0.493	0	0	0	418.689	ttaagaatttCttcacctgaa	m5C_33479
chr9	136217730	+	C	153	49	0.32	0	0	T	104	M	0.252	0.398	0	0	0	246.48	atttcttcacCtgaataaacc	m5C_33486
chr9	136217756	+	C	162	35	0.216	0	0	T	127	M	0.16	0.286	0	0	0	111.752	gtcagcattgCatctgaggca	m5C_33480
chr9	136217759	+	C	161	46	0.286	0	0	T	115	M	0.222	0.36	0	0	0	203.838	agcattgcatCtgaggcaaaa	m5C_33489
chr9	136882061	+	C	39	27	0.692	0	0	C	27	M	0.536	0.814	0	0.00015	0	289.324	ggtcttgaagCtgaggggggc	m5C_33466
chr9	137029571	-	C	37	16	0.432	0	0	T	21	M	0.287	0.591	0	1.7E-05	0	91.7488	ggcatctaacCatcgttttttt	m5C_33535
chr9	137029576	-	C	37	18	0.486	0	0	T	19	M	0.334	0.641	0	2.7E-05	0	120.406	ttcgtggcatCtaaccatcgt	m5C_33520
chr9	137029579	-	C	36	15	0.417	0	0	T	21	M	0.271	0.578	0	1.2E-05	0	81.421	tacttcgtggCatctaaccat	m5C_33570
chr9	137029587	-	C	36	24	0.667	0	0	C	24	M	0.503	0.798	0	9.1E-05	0	241.602	ttgccacctaCttcgtggcat	m5C_33530
chr9	137029591	-	C	36	8	0.222	0	0	T	28	M	0.117	0.381	7.4E-11	6.8E-07	8.4E-11	9.49676	ggcattgccaCctacttcgtg	m5C_33518
chr9	137029594	-	C	31	10	0.323	0	0	T	21	M	0.186	0.499	7.1E-15	2E-06	9E-15	26.2725	taaggcattgCcacctacttc	m5C_33539
chr9	137029608	-	C	31	18	0.581	0	0	C	18	M	0.408	0.736	0	2.7E-05	0	146.757	aacacgcataCggttaaggca	m5C_33531
chr9	137029657	-	C	141	29	0.206	0	0	T	112	M	0.147	0.28	0	0	0	85.3718	aaggttagcaCtccccttgac	m5C_33542
chr9	137314987	+	C	37	10	0.27	0	0	T	27	M	0.154	0.43	6.7E-14	2E-06	8.3E-14	20.2815	gaggggcgtgCtgactggggc	m5C_33501
chr9	137314991	+	C	36	13	0.361	0	0	T	23	M	0.225	0.524	0	6.7E-06	0	58.436	ggcgtgctgaCtggggctggt	m5C_33462
chr9	137623458	-	C	34	16	0.471	0	0	T	18	M	0.315	0.633	0	1.7E-05	0	100.644	cgatctccgaCggggagctgg	m5C_33516
chr9	138489375	+	C	44	9	0.205	0	0	T	35	M	0.112	0.345	1.8E-11	1.2E-08	2.1E-11	10.7806	tcagggaattCttttggtttg	m5C_33950
chr9	139565125	+	C	51	34	0.667	0	0	C	34	M	0.53	0.78	0	1.1E-06	0	360.183	agtaaataatgCgccgtccacg	m5C_33930
chr9	139620564	-	C	178	63	0.354	0	0	T	115	M	0.287	0.427	0	0	0	362.182	ggtttatggcCtgacattact	m5C_33987
chr9	139620582	-	C	170	53	0.312	0	0	T	117	M	0.247	0.385	0	0	0	261.756	ccagtgggcaCtgagaatggt	m5C_33963
chr9	139620591	-	C	177	42	0.237	0	0	T	135	M	0.181	0.305	0	0	0	151.715	gctcgggaacCagtgggcact	m5C_33979
chr9	139620605	-	C	149	36	0.242	0	0	T	113	M	0.18	0.316	0	0	0	129.55	tttctacactCagtgctcggg	m5C_33984
chr9	139620609	-	C	160	38	0.237	0	0	T	122	M	0.178	0.309	0	0	0	135.434	gcgatttctaCactcagtgct	m5C_33967
chr9	139620618	-	C	198	59	0.298	0	0	T	139	M	0.239	0.365	0	0	0	281.559	gagaaagtggCgatttctaca	m5C_34013
chr9	139620639	-	C	302	83	0.275	0	0	T	219	M	0.228	0.328	0	0	0	377.732	tgcctgtggcCtcggggcaaa	m5C_34004
chr9	139620640	-	C	311	117	0.376	0	0	T	194	M	0.324	0.431	0	0	0	758.59	gtgcctgtggCctcggggcaa	m5C_34011
chr9	139620647	-	C	421	104	0.247	0	0	T	317	M	0.208	0.29	0	0	0	433.145	ctctgctgtgCctgtggcctc	m5C_33955
chr9	139620657	-	C	384	83	0.216	0	0	T	301	M	0.178	0.26	0	0	0	295.306	cagacagttgCtctgctgtgc	m5C_33971
chr9	139620670	-	C	698	162	0.233	2	0.003	T	534	M	0.203	0.266	0	0	0	657.337	cctgttggcaCcagacagtt	m5C_33980
chr9	139620679	-	C	738	211	0.286	0	0	T	527	M	0.254	0.32	0	0	0	1073.91	tgtcctggacCtgttggcacc	m5C_33954
chr9	139620686	-	C	567	138	0.243	0	0	T	429	M	0.21	0.28	0	0	0	579.234	cggatgctgtCctggacctgt	m5C_33985
chr9	139620700	-	C	162	50	0.309	0	0	T	112	M	0.243	0.384	0	0	0	242.63	gagcggctgcCctgcggatgc	m5C_33969
chr9	139621199	-	C	62	19	0.306	0	0	T	43	M	0.206	0.43	0	4.5E-10	0	78.2094	gtctcacactCctgttttgtg	m5C_33996
chr9	139621205	-	C	289	66	0.228	0	0	T	223	M	0.184	0.28	0	0	0	242.517	ttgatagtctCacactcctgt	m5C_33994
chr9	139621233	-	C	455	103	0.226	0	0	T	352	M	0.19	0.267	0	0	0	392.027	gcgttctgccCcgtgaacgtt	m5C_33959
chr9	139621252	-	C	417	87	0.209	0	0	T	330	M	0.172	0.25	0	0	0	299.941	gaggctatgtCtacgctcagc	m5C_34018
chr9	139621303	-	C	1340	301	0.226	9	0.007	T	1030	M	0.204	0.249	0	0	0	1230.96	gcagctgtggCtgccgtgtca	m5C_33953
chr9	139908208	-	C	32	7	0.219	0	0	T	25	M	0.11	0.388	8.5E-10	3.4E-07	9.5E-10	6.99971	gatgcccactCctgagcttcc	m5C_33968
chr9	139908210	-	C	34	18	0.529	0	0	C	18	M	0.367	0.685	0	2.7E-05	0	132.251	atgatgcccaCtcctgagctt	m5C_33992

chr9	139908213	-	C	37	19	0.514	0	0	C	19	M	0.359	0.666	0	3.4E-05	0	136.4	ctgatgatgcCcactcctgag	m5C_33960
chrM	8	-	C	34	8	0.235	0	0	T	26	M	0.124	0.4	4.2E-11	6.8E-07	4.8E-11	10.3259	GGTGATAgacctgtgatC	m5C_38977
chrM	35	-	C	31	8	0.258	0	0	T	23	M	0.137	0.432	1.7E-11	6.8E-07	1.9E-11	11.8182	tggagagctcCcgtgagtggt	m5C_38493
chrM	38	-	C	32	9	0.281	0	0	T	23	M	0.156	0.454	5.3E-13	1.2E-06	6.4E-13	17.1983	gcatggagagCtcccgtgagt	m5C_39091
chrM	94	-	C	74	16	0.216	0	0	T	58	M	0.138	0.323	0	1.2E-12	0	44.0651	gctccagcgtCtcgcaatgct	m5C_38503
chrM	97	-	C	81	27	0.333	0	0	T	54	M	0.24	0.441	0	4E-12	0	129.755	ccggctccagCgtctcgcaat	m5C_38717
chrM	103	-	C	82	24	0.293	0	0	T	58	M	0.205	0.399	0	1.1E-12	0	98.5258	ggtgctccggCtccagcgtct	m5C_38905
chrM	106	-	C	78	26	0.333	0	0	T	52	M	0.239	0.444	0	1.3E-10	0	124.137	tagggtgctcCggctccagcg	m5C_38479
chrM	109	-	C	86	26	0.302	0	0	T	60	M	0.215	0.406	0	2.7E-12	0	112.026	acatagggtgCtccggctcca	m5C_39028
chrM	124	-	C	66	28	0.424	0	0	T	38	M	0.312	0.544	0	9.8E-09	0	174.957	aagacagataCtgcgacatag	m5C_39095
chrM	130	-	C	55	17	0.309	0	0	T	38	M	0.203	0.44	0	1.1E-08	0	68.9592	gaatcaaagaCagatactgcg	m5C_38645
chrM	143	-	C	62	25	0.403	0	0	T	37	M	0.29	0.527	0	4.1E-09	0	145.123	tagaatgaggCaggaatcaaa	m5C_39083
chrM	228	-	C	136	40	0.294	0	0	T	96	M	0.224	0.376	0	0	0	179.233	ttattatgtcCtacaagcatt	m5C_38506
chrM	229	-	C	156	37	0.237	0	0	T	119	M	0.177	0.31	0	0	0	131.167	attattatgtCctacaagcat	m5C_38764
chrM	260	-	C	3074	884	0.288	3	0.001	T	2187	M	0.272	0.304	0	0	0	4810.97	tggaaagcggCtgtgcagaca	m5C_39076
chrM	263	-	C	3101	649	0.209	1	0	T	2451	M	0.195	0.224	0	0	0	2536.25	gtgtggaaagCggctgtgcag	m5C_38834
chrM	324	-	C	225	62	0.277	1	0.004	T	162	M	0.222	0.339	0	0	0	275.691	gtgctgtggcCagaagcgggg	m5C_38548
chrM	325	-	C	290	130	0.448	0	0	T	160	M	0.392	0.506	0	0	0	1019.42	agtgctgtggCcagaagcggg	m5C_38945
chrM	331	-	C	379	152	0.401	0	0	T	227	M	0.353	0.451	0	0	0	1072.95	tgtttaagtgCtgtggccaga	m5C_38746
chrM	368	-	C	1394	502	0.36	0	0	T	892	M	0.335	0.386	0	0	0	3366.73	tgttagggttCtttgttttttg	m5C_38890
chrM	382	-	C	1743	736	0.423	2	0.001	T	1005	M	0.4	0.446	0	0	0	5884.11	tctggttaggCtggtgttagg	m5C_38480
chrM	391	-	C	1751	533	0.304	0	0	T	1218	M	0.283	0.326	0	0	0	3019.89	aatttgaaatCtggttaggct	m5C_38832
chrM	594	+	C	86	33	0.384	0	0	T	53	M	0.288	0.489	0	3.1E-11	0	190.088	tgtagcttacCtcctcaaagc	m5C_38123
chrM	604	+	C	84	17	0.202	0	0	T	67	M	0.13	0.3	0	2.3E-14	0	44.327	ctcctcaaagCaatacactga	m5C_38417
chrM	611	+	C	91	23	0.253	0	0	T	68	M	0.175	0.351	0	1E-14	0	80.3739	aagcaatacaCtgaaaatgtt	m5C_38204
chrM	626	+	C	74	41	0.562	1	0.014	C	41	M	0.448	0.67	0	7.1E-09	0	367.012	aatgtttagaCgggctcacat	m5C_38277
chrM	630	+	C	75	54	0.72	0	0	C	54	M	0.61	0.809	0	6.9E-08	0	658.358	tttagacgggCtcacatcacc	m5C_38396
chrM	637	+	C	75	15	0.2	0	0	T	60	M	0.125	0.304	0	6E-13	0	37.5361	gggctcacatCaccccataaa	m5C_38197
chrM	641	+	C	69	16	0.232	0	0	T	53	M	0.148	0.344	0	1.1E-10	0	47.3849	tcacatcaccCcataaacaaa	m5C_38349
chrM	642	+	C	64	14	0.219	0	0	T	50	M	0.135	0.334	1.1E-16	3.2E-11	1.5E-16	30.1591	cacatcacccCataaacaaat	m5C_38126
chrM	1614	+	C	124	39	0.315	0	0	T	85	M	0.239	0.401	0	0	0	186.738	cagagtgtagCttaacacaaa	m5C_38296
chrM	1621	+	C	119	26	0.234	8	0.067	T	85	M	0.165	0.321	0	0	0	85.8809	tagcttaacaCaaagcaccca	m5C_38385
chrM	1626	+	C	118	26	0.22	0	0	T	92	M	0.155	0.303	0	0	0	80.6176	taacacaaagCacccaactta	m5C_38318
chrM	1654	+	C	80	19	0.237	0	0	T	61	M	0.158	0.341	0	8.4E-14	0	59.9053	gagatttcaaCttaacttgac	m5C_38263
chrM	1665	+	C	81	33	0.407	0	0	T	48	M	0.307	0.516	0	3.1E-11	0	202.594	ttaacttgacCgctctgagct	m5C_38340
chrM	1751	+	C	38	29	0.763	0	0	C	29	M	0.608	0.87	0	0.00019	0	352.599	aaagtataggCgatagaaatt	m5C_38271
chrM	2476	+	C	71	49	0.69	0	0	C	49	M	0.575	0.786	0	3.1E-08	0	563.655	gtaaaaggaaCtcggcaaacc	m5C_38356
chrM	2478	+	C	60	42	0.7	0	0	C	42	M	0.575	0.801	0	2E-07	0	482.925	aaaaggaactCggcaaacctt	m5C_38305
chrM	2481	+	C	50	16	0.32	0	0	T	34	M	0.208	0.458	0	7.3E-09	0	66.4257	aggaactcggCaaaccttacc	m5C_38173
chrM	2685	+	C	103	40	0.388	0	0	T	63	M	0.3	0.485	0	1.8E-13	0	239.888	gtgaaattgaCctgcccgtga	m5C_38156
chrM	2686	+	C	104	23	0.221	0	0	T	81	M	0.152	0.31	0	1.1E-16	0	69.979	tgaaattgacCtgcccgtgaa	m5C_38216
chrM	2689	+	C	118	26	0.222	1	0.008	T	91	M	0.156	0.306	0	0	0	81.3295	aattgacctgCccgtgaagag	m5C_38273
chrM	2690	+	C	121	53	0.438	0	0	T	68	M	0.353	0.527	0	5.8E-15	0	374.042	attgacctgcCcgtgaagagg	m5C_38408
chrM	2691	+	C	135	29	0.215	0	0	T	106	M	0.154	0.291	0	0	0	89.2833	ttgacctgccCgtgaagaggc	m5C_38288
chrM	2701	+	C	157	66	0.42	0	0	T	91	M	0.346	0.499	0	0	0	456.685	cgtgaagaggCgggcatgaca	m5C_38369
chrM	2705	+	C	148	47	0.318	0	0	T	101	M	0.248	0.396	0	0	0	233.113	aagaggcgggCatgacacagc	m5C_38161
chrM	2708	-	C	291	164	0.567	2	0.007	C	164	M	0.51	0.623	0	0	0	1672.27	cttgctgtgtCatgcccgcct	m5C_38887
chrM	2710	+	C	135	41	0.304	0	0	T	94	M	0.232	0.386	0	0	0	190.609	gcgggcatgaCacagcaagac	m5C_38237
chrM	2712	+	C	97	49	0.505	0	0	C	49	M	0.407	0.603	0	5.8E-11	0	399.226	gggcatgacaCagcaagacga	m5C_38346
chrM	2715	+	C	83	51	0.614	0	0	C	51	M	0.507	0.712	0	2.1E-09	0	517.037	catgacacagCaagacgagaa	m5C_38247
chrM	2720	+	C	42	31	0.738	0	0	C	31	M	0.589	0.847	0	1.4E-05	0	365.372	cacagcaagaCgagaagaccc	m5C_38133
chrM	2763	+	C	35	27	0.771	0	0	C	27	M	0.61	0.879	0	0.00015	0	329.305	gcaaacagtaCctaacaaacc	m5C_38397
chrM	2764	+	C	34	24	0.706	0	0	C	24	M	0.538	0.832	0	9.1E-05	0	258.388	caaacagtacCtaacaaaccc	m5C_38107
chrM	2823	+	C	39	21	0.538	0	0	C	21	M	0.386	0.684	0	5.2E-05	0	161.991	gttggggcgaCctcggagcag	m5C_38383
chrM	2824	+	C	41	24	0.585	0	0	C	24	M	0.434	0.722	0	3.6E-06	0	208.161	ttggggcgacCtcggagcaga	m5C_38283
chrM	2826	+	C	43	20	0.465	0	0	T	23	M	0.325	0.611	0	1.4E-06	0	130.043	ggggcgacctCggagcagaac	m5C_38330
chrM	2831	+	C	45	16	0.356	0	0	T	29	M	0.232	0.502	0	4E-07	0	74.3005	gacctcggagCagaacccaac	m5C_38141
chrM	2836	+	C	47	30	0.638	0	0	C	30	M	0.495	0.76	0	1.2E-05	0	297.214	cggagcagaaCccaacctccg	m5C_38315
chrM	2837	+	C	51	21	0.412	0	0	T	30	M	0.288	0.548	0	4.8E-08	0	120.768	ggagcagaacCcaacctccga	m5C_38322
chrM	2838	+	C	51	15	0.294	0	0	T	36	M	0.187	0.43	0	4.6E-09	0	56.1257	gagcagaaccCaacctccgag	m5C_38108
chrM	2841	+	C	51	16	0.314	0	0	T	35	M	0.203	0.45	0	7.3E-09	0	65.048	cagaacccaaCctccgagcag	m5C_38105
chrM	2842	+	C	47	13	0.277	0	0	T	34	M	0.169	0.418	0	1.2E-07	0	44.0347	agaacccaacCtccgagcagt	m5C_38102
chrM	3012	-	C	343	259	0.755	0	0	C	259	M	0.707	0.798	0	0	0	3661.9	gctgcaccatCgggatgtcct	m5C_38575
chrM	3135	+	C	56	35	0.625	0	0	C	35	M	0.494	0.74	0	1.3E-06	0	345.836	tacgaaaggaCaagagaaata	m5C_38390
chrM	3150	+	C	64	20	0.312	0	0	T	44	M	0.212	0.434	0	6.9E-10	0	84.9236	gaaataaggcCtacttcacaa	m5C_38145
chrM	3246	+	C	117	28	0.239	0	0	T	89	M	0.171	0.324	0	0	0	95.8045	gatggcagagCccggtaatcg	m5C_38169
chrM	3247	+	C	116	49	0.426	1	0.009	T	66	M	0.34	0.517	0	6.6E-14	0	332.745	atggcagagcCcggtaatcgc	m5C_38365
chrM	3248	+	C	120	30	0.25	0	0	T	90	M	0.181	0.334	0	0	0	108.658	tggcagagccCggtaatcgca	m5C_38229
chrM	3255	+	C	123	34	0.276	0	0	T	89	M	0.205	0.361	0	0	0	139.447	gcccggtaatCgcataaaact	m5C_38138
chrM	3257	+	C	120	38	0.317	0	0	T	82	M	0.24	0.404	0	0	0	182.573	ccggtaatcgCataaaactta	m5C_38294
chrM	3271	+	C	120	28	0.233	0	0	T	92	M	0.167	0.317	0	0	0	93.3266	aaacttaaaaCtttacagtca	m5C_38185
chrM	3276	+	C	92	20	0.217	0	0	T	72	M	0.145	0.312	0	1.7E-15	0	58.1465	taaaactttaCagtcagaggt	m5C_38158
chrM	3455	-	C	37	12	0.324	0	0	T	25	M	0.196	0.485	0	4.6E-06	0	47.1194	atggcgtcagCgaagggttgt	m5C_39073
chrM	3694	-	C	151	34	0.225	0	0	T	117	M	0.166	0.298	0	0	0	112.772	gtgcgccgatCagggcgtagt	m5C_38453
chrM	3699	-	C	151	40	0.265	0	0	T	111	M	0.201	0.34	0	0	0	160.786	tcgcagtgcgCcgatcagggc	m5C_38768
chrM	3706	-	C	133	29	0.218	0	0	T	104	M	0.156	0.296	0	0	0	90.6681	ctactgctcgCagtgcgccga	m5C_38542
chrM	4333	-	C	34	7	0.206	0	0	T	27	M	0.103	0.368	1.4E-09	3.4E-07	1.6E-09	6.4054	tctcatagtcCtagaaataag	m5C_39046
chrM	4340	-	C	35	11	0.314	0	0	T	24	M	0.186	0.48	6.7E-16	3.1E-06	8.7E-16	30.9686	gttcgattctCatagtcctag	m5C_38523
chrM	4342	-	C	37	10	0.27	0	0	T	27	M	0.154	0.43	6.7E-14	2E-06	8.3E-14	20.2815	gggttcgattCtcatagtcct	m5C_38692
chrM	4359	-	C	48	11	0.229	0	0	T	37	M	0.133	0.365	5E-14	4.4E-08	6.2E-14	19.4712	tttggattctCagggatgggt	m5C_38979
chrM	4414	+	C	48	12	0.25	0	0	T	36	M	0.149	0.388	1.3E-15	7.5E-08	1.7E-15	26.6338	gtaaggtcagCtaaataagct	m5C_38274
chrM	4427	+	C	46	13	0.283	0	0	T	33	M	0.173	0.425	0	1.2E-07	0	45.0425	aataagctatCgggcccatac	m5C_38182
chrM	4431	+	C	52	23	0.442	0	0	T	29	M	0.316	0.577	0	8.7E-08	0	145.336	agctatcgggCccatacccg	m5C_38332
chrM	4432	+	C	51	11	0.216	0	0	T	40	M	0.125	0.346	1.1E-13	4.4E-10	1.4E-13	17.8061	gctatcgggcCcatacccga	m5C_38306
chrM	4437	+	C	53	14	0.264	0	0	T	39	M	0.164	0.396	0	2.8E-09	0	46.0242	cgggcccataCcccgaaaatg	m5C_38336
chrM	4439	+	C	54	14	0.259	0	0	T	40	M	0.161	0.389	0	2.8E-09	0	45.1318	ggcccataccCcgaaaatgtt	m5C_38379
chrM	4468	+	C	35	12	0.343	0	0	T	23	M	0.208	0.508	0	4.6E-06	0	49.9956	ccttcccgtaCtaattaatcc	m5C_38150
chrM	4854	-	C	31	9	0.29	0	0	T	22	M	0.161	0.466	3.7E-13	1.2E-06	4.4E-13	18.0154	tgagaagaagCaggccggatg	m5C_38498
chrM	4921	-	C	147	51	0.347	0	0	T	96	M	0.275	0.427	0	0	0	280.27	agaaggcttaCgtttagtgag	m5C_38714
chrM	4925	-	C	147	73	0.497	0	0	T	74	M	0.417	0.576	0	8.9E-16	0	608.665	gaggagaaggCttacgtttag	m5C_38775
chrM	4960	-	C	42	23	0.548	0	0	C	23	M	0.399	0.688	0	2.9E-06	0	183.765	caactgcctgCtatgatggat	m5C_38923
chrM	4963	-	C	34	15	0.441	0	0	T	19	M	0.289	0.605	0	1.2E-05	0	86.6497	cctcaactgcCtgctatgatg	m5C_38638
chrM	4964	-	C	31	15	0.484	0	0	T	16	M	0.32	0.652	0	1.2E-05	0	95.91	acctcaactgCctgctatgat	m5C_38995
chrM	5826	+	C	33	11	0.333	0	0	T	22	M	0.198	0.504	2.2E-16	3.1E-06	2.9E-16	34.0074	cacctcggagCtggtaaaaag	m5C_38342
chrM	5838	-	C	126	33	0.262	0	0	T	93	M	0.193	0.345	0	0	0	127.377	ggggttaggcCtctttttacc	m5C_38611
chrM	5840	+	C	34	9	0.265	0	0	T	25	M	0.146	0.431	1.1E-12	1.2E-06	1.3E-12	15.7394	taaaaagaggCctaacccctg	m5C_38297
chrM	5841	+	C	32	17	0.531	0	0	C	17	M	0.364	0.691	0	2.1E-05	0	123.927	aaaaagaggcCtaacccctgt	m5C_38155
chrM	6461	-	C	66	25	0.379	0	0	T	41	M	0.271	0.499	0	4.1E-09	0	135.746	taggacggatCagacgaagag	m5C_38747
chrM	6466	-	C	86	54	0.628	0	0	C	54	M	0.522	0.723	0	3.5E-09	0	564.123	gtgattaggaCggatcagacg	m5C_38554
chrM	6478	-	C	98	35	0.357	0	0	T	63	M	0.269	0.456	0	1.5E-12	0	188.52	agtaggactgCtgtgattagg	m5C_38557
chrM	6481	-	C	95	61	0.642	0	0	C	61	M	0.542	0.731	0	5.7E-10	0	661.134	agaagtaggaCtgctgtgatt	m5C_38426

chrM	6505	-	C	61	21	0.35	1	0.016	T	39	M	0.242	0.476	0	1E-09	0	101.504	gcagctaggaCtgggagagat	m5C_38678
chrM	6511	-	C	97	54	0.557	0	0	C	54	M	0.458	0.652	0	1.6E-10	0	494.161	atgccagcagCtaggactggg	m5C_38576
chrM	6514	-	C	94	28	0.298	0	0	T	66	M	0.215	0.397	0	1.1E-13	0	120.312	gtgatgccagCagctaggact	m5C_38820
chrM	6517	-	C	102	39	0.382	0	0	T	63	M	0.294	0.479	0	1.3E-13	0	229.275	atagtgatgcCagcagctagg	m5C_38892
chrM	6518	-	C	96	38	0.396	0	0	T	58	M	0.304	0.496	0	3.7E-12	0	230.912	tatagtgatgCcagcagctag	m5C_38708
chrM	6538	-	C	71	35	0.493	0	0	T	36	M	0.38	0.607	0	1.9E-09	0	266.035	aggttgcggtCtgttagtagt	m5C_38689
chrM	6542	-	C	62	15	0.242	0	0	T	47	M	0.152	0.362	0	6E-11	0	45.7373	gttgaggttgCggtctgttag	m5C_38522
chrM	7228	-	C	35	10	0.286	0	0	T	25	M	0.163	0.451	3.4E-14	2E-06	4.2E-14	21.9991	tcggggtagtCcgagtaacgt	m5C_38675
chrM	7549	-	C	34	12	0.353	0	0	T	22	M	0.215	0.521	0	4.6E-06	0	51.5705	ttaactttgaCaaagttatga	m5C_38492
chrM	7555	-	C	31	10	0.323	0	0	T	21	M	0.186	0.499	7.1E-15	2E-06	9E-15	26.2725	tataatttaaCtttgacaaag	m5C_38994
chrM	7567	-	C	32	11	0.344	0	0	T	21	M	0.204	0.517	2.2E-16	3.1E-06	2.9E-16	35.1442	taggatttagCctataattta	m5C_38852
chrM	7703	-	C	35	8	0.229	0	0	T	27	M	0.121	0.39	5.6E-11	6.8E-07	6.4E-11	9.89691	aaagggcataCaggactagga	m5C_39064
chrM	7707	-	C	45	9	0.2	0	0	T	36	M	0.109	0.338	2.3E-11	1.2E-08	2.7E-11	10.4289	aggaaaagggCatacaggact	m5C_38461
chrM	7831	-	C	78	28	0.359	0	0	T	50	M	0.261	0.47	0	2.6E-10	0	146.414	gtaaaggatgCgtagggatgg	m5C_38620
chrM	7848	-	C	86	32	0.372	0	0	T	54	M	0.277	0.478	0	2.3E-11	0	177.575	ttgacctcgtCtgttatgtaa	m5C_39074
chrM	7851	-	C	88	36	0.409	0	0	T	52	M	0.312	0.514	0	7.4E-11	0	224.831	tcgttgacctCgtctgttatg	m5C_38623
chrM	7853	-	C	88	35	0.398	0	0	T	53	M	0.302	0.502	0	5.6E-11	0	211.271	gatcgttgacCtcgtctgtta	m5C_38942
chrM	7860	-	C	90	26	0.289	0	0	T	64	M	0.205	0.39	0	4.6E-14	0	106.818	agggagggatCgttgacctcg	m5C_38786
chrM	9330	-	C	54	14	0.259	0	0	T	40	M	0.161	0.389	0	2.8E-09	0	45.1318	gtatgaggagCgttatggagt	m5C_38738
chrM	9343	-	C	38	23	0.605	0	0	C	23	M	0.447	0.744	0	7.6E-05	0	205.701	gttagtaggcCtagtatgagg	m5C_38735
chrM	9860	-	C	115	57	0.496	0	0	T	58	M	0.406	0.586	0	4.7E-13	0	462.726	gcggatgaagCagatagtgag	m5C_38514
chrM	9869	-	C	123	59	0.48	0	0	T	64	M	0.393	0.567	0	2.6E-14	0	464.147	tattagtggCggatgaagca	m5C_38584
chrM	11100	-	C	387	175	0.452	0	0	T	212	M	0.403	0.502	0	0	0	1411.62	atgattagttCtgtggctgtg	m5C_38499
chrM	11127	-	C	43	15	0.349	0	0	T	28	M	0.224	0.498	0	2.8E-07	0	67.2552	agtgtggtttCgaagaagata	m5C_38946
chrM	11247	-	C	57	20	0.351	0	0	T	37	M	0.24	0.481	0	3.4E-08	0	96.001	taaattagtgCgatgagtagg	m5C_38772
chrM	11519	-	C	33	12	0.364	0	0	T	21	M	0.222	0.534	0	4.6E-06	0	53.2487	tgtgttttgtCaggggggttga	m5C_38613
chrM	11532	-	C	45	16	0.356	0	0	T	29	M	0.232	0.502	0	4E-07	0	74.3005	aaggggtaggCtatgtgtttt	m5C_38782
chrM	11763	-	C	36	8	0.222	0	0	T	28	M	0.117	0.381	7.4E-11	6.8E-07	8.4E-11	9.49676	tgagtgcgttCgtagtttgag	m5C_38504
chrM	11799	-	C	78	26	0.333	0	0	T	52	M	0.239	0.444	0	1.3E-10	0	124.137	gtttgaagtcCttgagagagg	m5C_38511
chrM	11903	-	C	40	13	0.325	0	0	T	27	M	0.201	0.48	0	1.2E-07	0	52.2192	tggttactagCacagagagtt	m5C_38815
chrM	11915	-	C	52	25	0.481	0	0	T	27	M	0.351	0.613	0	1.5E-07	0	175.519	atcaggagaaCgtggttacta	m5C_38840
chrM	11950	-	C	64	17	0.266	0	0	T	47	M	0.173	0.385	0	1.8E-10	0	58.8072	tatgttgagtCctgtaagtag	m5C_38656
chrM	12010	-	C	44	9	0.205	0	0	T	35	M	0.112	0.345	1.8E-11	1.2E-08	2.1E-11	10.7806	ggtgagtgagCcccattgtgt	m5C_38578
chrM	12182	+	C	78	22	0.282	0	0	T	56	M	0.194	0.39	0	2.8E-11	0	85.4709	aatctgacaaCagaggcttac	m5C_38344
chrM	12188	+	C	66	42	0.636	0	0	C	42	M	0.516	0.742	0	2E-07	0	433.267	acaacagaggCttacgacccc	m5C_38170
chrM	12215	+	C	273	90	0.33	0	0	T	183	M	0.277	0.387	0	0	0	497.893	accgagaaagCtcacaagaac	m5C_38215
chrM	12217	+	C	281	60	0.214	0	0	T	221	M	0.17	0.265	0	0	0	203.568	cgagaaagctCacaagaactg	m5C_38413
chrM	12219	+	C	284	123	0.433	0	0	T	161	M	0.377	0.491	0	0	0	926.783	agaaagctcaCaagaactgct	m5C_38209
chrM	12225	+	C	290	127	0.438	0	0	T	163	M	0.382	0.495	0	0	0	970.302	ctcacaagaaCtgctaactca	m5C_38350
chrM	12228	+	C	290	143	0.493	0	0	T	147	M	0.436	0.55	0	0	0	1247.04	acaagaactgCtaactcatgc	m5C_38230
chrM	12234	+	C	264	60	0.227	0	0	T	204	M	0.181	0.282	0	0	0	217.012	actgctaactCatgcccccat	m5C_38353
chrM	12238	+	C	253	74	0.292	0	0	T	179	M	0.24	0.351	0	0	0	355.012	ctaactcatgCccccatgtct	m5C_38142
chrM	12239	+	C	251	134	0.534	0	0	C	134	M	0.472	0.595	0	0	0	1265.24	taactcatgcCcccatgtcta	m5C_38256
chrM	12240	+	C	244	109	0.447	0	0	T	135	M	0.386	0.509	0	0	0	840.709	aactcatgccCccatgtctaa	m5C_38181
chrM	12241	+	C	237	135	0.57	0	0	C	135	M	0.506	0.631	0	0	0	1366.11	actcatgcccCcatgtctaac	m5C_38360
chrM	12242	+	C	233	92	0.395	0	0	T	141	M	0.334	0.459	0	0	0	615.07	ctcatgccccCatgtctaaca	m5C_38098
chrM	12247	+	C	215	67	0.312	0	0	T	148	M	0.253	0.376	0	0	0	339.664	gcccccatgtCtaacaacatg	m5C_38291
chrM	12254	+	C	179	45	0.251	0	0	T	134	M	0.194	0.32	0	0	0	174.17	tgtctaacaaCatggctttct	m5C_38132
chrM	12259	+	C	149	31	0.208	0	0	T	118	M	0.151	0.28	0	0	0	93.3852	aacaacatggCtttctcaact	m5C_38211
chrM	12265	+	C	90	38	0.422	0	0	T	52	M	0.325	0.525	0	3.7E-12	0	247.321	atggctttctCaacttttaaa	m5C_38113
chrM	12731	-	C	31	11	0.355	0	0	T	20	M	0.211	0.531	1.1E-16	3.1E-06	1.5E-16	37.0595	ttagcggtaaCtaagattagt	m5C_38441
chrM	12819	-	C	52	12	0.231	0	0	T	40	M	0.137	0.361	4.4E-15	8.8E-10	5.7E-15	23.6371	ggcatctgctCgggcgtatca	m5C_38508
chrM	12836	-	C	59	17	0.288	0	0	T	42	M	0.188	0.414	0	1.1E-08	0	64.0436	tgaatggctgCtgtgttggca	m5C_38633
chrM	12941	-	C	42	13	0.31	0	0	T	29	M	0.191	0.46	0	1.2E-07	0	49.5844	tttagaagggCtatttgttgt	m5C_39012
chrM	12963	-	C	32	18	0.562	0	0	C	18	M	0.393	0.718	0	2.7E-05	0	141.571	tggggtgaggCttggattagc	m5C_38680
chrM	13596	-	C	53	13	0.245	0	0	T	40	M	0.149	0.376	2.2E-16	1.6E-09	2.9E-16	30.3876	gctataggcgCttgtcaggga	m5C_38646
chrM	13598	-	C	42	19	0.452	0	0	T	23	M	0.312	0.601	0	1E-06	0	118.648	gtgctataggCgcttgtcagg	m5C_38452
chrM	13605	-	C	33	8	0.242	0	0	T	25	M	0.128	0.41	3.1E-11	6.8E-07	3.6E-11	10.7866	tattcgagtgCtataggcgct	m5C_38484
chrM	14161	-	C	62	27	0.435	0	0	T	35	M	0.319	0.559	0	7.5E-09	0	172.489	tgagattgctCgggggaatag	m5C_38814
chrM	14163	-	C	49	16	0.327	0	0	T	33	M	0.212	0.466	0	4E-07	0	67.8634	attgagattgCtcgggggaat	m5C_38629
chrM	14686	-	C	1272	268	0.211	0	0	T	1004	M	0.189	0.234	0	0	0	1013.94	ggttgtagtcCgtgcgagaat	m5C_38844
chrM	14699	-	C	1130	284	0.251	0	0	T	846	M	0.227	0.277	0	0	0	1288.83	tatcattggtCgtggttgtag	m5C_38920
chrM	15429	-	C	31	10	0.323	0	0	T	21	M	0.186	0.499	7.1E-15	2E-06	9E-15	26.2725	ccgagggcgtCtttgattgtg	m5C_38527
chrM	15467	-	C	1653	791	0.479	0	0	T	862	M	0.455	0.503	0	0	0	7190.5	ggttaatgtCattaaggaga	m5C_38793
chrM	15489	-	C	1456	1117	0.768	1	0.001	C	1117	M	0.745	0.789	0	0	0	16650.2	cctaggaggtCtggtgagaat	m5C_38699
chrM	15498	-	C	1090	524	0.481	0	0	T	566	M	0.451	0.51	0	0	0	4728.5	tctgggtcgcCtaggaggtct	m5C_38895
chrM	15499	-	C	1017	260	0.256	0	0	T	757	M	0.23	0.283	0	0	0	1194.95	gtctgggtcgCctaggaggtc	m5C_38989
chrM	15501	-	C	951	530	0.557	0	0	C	530	M	0.526	0.589	0	0	0	5571.05	ttgtctgggtCgcctaggagg	m5C_38816
chrM	15507	-	C	749	301	0.402	0	0	T	448	M	0.367	0.437	0	0	0	2211.41	gtataattgtCtgggtcgcct	m5C_38424
chrM	15522	-	C	161	60	0.373	0	0	T	101	M	0.302	0.45	0	0	0	362.119	aagggggttggCtagggtataa	m5C_38861
chrM	15968	-	C	573	123	0.215	1	0.002	T	449	M	0.183	0.251	0	0	0	450.98	gagttaaagaCtttttctctg	m5C_38661
chrM	15976	+	C	40	22	0.55	0	0	C	22	M	0.398	0.693	0	6.3E-05	0	175.247	aagtctttaaCtccaccatta	m5C_38339
chrM	15979	+	C	44	21	0.477	0	0	T	23	M	0.338	0.621	0	1.8E-06	0	141.77	tctttaactcCaccattagca	m5C_38378
chrM	15981	+	C	40	12	0.3	0	0	T	28	M	0.181	0.454	1.1E-16	7.5E-08	1.5E-16	34.6049	tttaactccaCcattagcacc	m5C_38154
chrM	15982	+	C	42	28	0.667	0	0	C	28	M	0.516	0.79	0	8.1E-06	0	288.692	ttaactccacCattagcaccc	m5C_38392
chrM	15991	+	C	31	12	0.387	0	0	T	19	M	0.237	0.562	0	4.6E-06	0	56.9589	ccattagcacCcaaagctaag	m5C_38178
chrM	15992	+	C	31	12	0.387	0	0	T	19	M	0.237	0.562	0	4.6E-06	0	56.9589	cattagcaccCaaagctaaga	m5C_38317
chrM	16068	+	C	32	19	0.594	0	0	C	19	M	0.423	0.745	0	3.4E-05	0	160.587	caagtattgaCtcacccatca	m5C_38363
chrM	16073	+	C	32	10	0.312	0	0	T	22	M	0.18	0.486	1.1E-14	2E-06	1.3E-14	25.0836	attgactcacCcatcaacaac	m5C_38327
chrM	16391	-	C	121	25	0.207	0	0	T	96	M	0.144	0.287	0	0	0	72.0228	tcaagggaccCctatctgagg	m5C_38848
chrM	16392	-	C	129	26	0.202	0	0	T	103	M	0.141	0.279	0	0	0	73.5376	gtcaagggacCcctatctgag	m5C_38636
chrM	16400	-	C	78	45	0.577	0	0	C	45	M	0.466	0.68	0	1.6E-08	0	419.592	ggatggtggtCaagggacccc	m5C_38702
chrM	16414	-	C	34	10	0.294	0	0	T	24	M	0.168	0.462	2.3E-14	2E-06	2.9E-14	22.9564	attgatttcaCggaggatggt	m5C_38884
chrM	16457	-	C	49	18	0.367	0	0	T	31	M	0.247	0.507	0	7.7E-07	0	88.7939	gttatgggccCggagcgagga	m5C_38751
chrM	16458	-	C	55	23	0.418	0	0	T	32	M	0.297	0.55	0	8.7E-08	0	136.793	tgttatgggcCcggagcgagg	m5C_38854
chrM	16459	-	C	58	13	0.224	0	0	T	45	M	0.136	0.347	6.7E-16	1.6E-09	8.7E-16	26.8192	gtgttatgggCccggagcgag	m5C_38984
chrM	16472	-	C	75	38	0.507	0	0	C	38	M	0.396	0.617	0	3.7E-09	0	300.946	tagctaccccCaagtgttatg	m5C_38954
chrM	16474	-	C	84	22	0.262	0	0	T	62	M	0.18	0.365	0	4.4E-13	0	79.1047	tttagctaccCccaagtgtta	m5C_38948
chrM	16475	-	C	85	45	0.529	0	0	C	45	M	0.424	0.632	0	6.4E-10	0	381.906	ctttagctacCccaagtgtt	m5C_39087
chrM	16479	-	C	100	58	0.58	0	0	C	58	M	0.482	0.672	0	1.5E-11	0	559.193	ttcactttagCtacccccaag	m5C_39086
chrM	16485	-	C	120	37	0.308	0	0	T	83	M	0.233	0.396	0	0	0	172.217	atacagttcaCtttagctacc	m5C_38902
chrM	16487	-	C	111	34	0.306	0	0	T	77	M	0.228	0.397	0	4.4E-16	0	155.196	ggatacagttCactttagcta	m5C_38664
chrM	16518	-	C	180	43	0.239	0	0	T	137	M	0.182	0.306	0	0	0	156.92	ctttatggccCtgaagtagga	m5C_38833
chrM	16528	-	C	174	50	0.287	0	0	T	124	M	0.225	0.359	0	0	0	225.281	gctatttaggCtttatggccc	m5C_38988
chrX	2641365	+	C	87	35	0.402	0	0	T	52	M	0.306	0.507	0	5.6E-11	0	213.855	gaaaaggaggCagtgatggtg	m5C_33420
chrX	5301635	+	C	93	23	0.247	0	0	T	70	M	0.171	0.344	0	1E-14	0	78.5777	ggtgaggggtCgggatcctgt	m5C_33400
chrX	5301641	+	C	89	32	0.36	0	0	T	57	M	0.268	0.463	0	2.3E-11	0	171.249	gggtcgggatCctgtgattgc	m5C_33396
chrX	5301642	+	C	88	21	0.239	0	0	T	67	M	0.162	0.337	0	2.6E-13	0	67.9179	ggtcgggatcCtgtgattgca	m5C_33403
chrX	9360809	-	C	44	27	0.614	0	0	C	27	M	0.466	0.743	0	6.7E-06	0	251.766	caccctggctCttatttgctg	m5C_33859
chrX	9414518	+	C	31	7	0.226	0	0	T	24	M	0.114	0.398	6.4E-10	3.4E-07	7.2E-10	7.33205	agagaagtttCtctggatgtg	m5C_33831
chrX	9842715	-	C	72	25	0.347	0	0	T	47	M	0.248	0.462	0	9.3E-11	0	123.766	gcggtgtttaCaaataattag	m5C_33842

chrX	10253796	-	C	41	26	0.634	0	0	C	26	M	0.481	0.764	0	5.5E-06	0	250.226	gtggttatcaCgttcacctga	m5C_33786
chrX	10253798	-	C	57	34	0.596	0	0	C	34	M	0.467	0.714	0	1.1E-06	0	317.54	tagtggttatCacgttcacct	m5C_33781
chrX	10253815	-	C	61	33	0.541	0	0	C	33	M	0.417	0.66	0	3.4E-08	0	275.377	gcatggtttcCgtagtgtagt	m5C_33788
chrX	10253816	-	C	40	12	0.3	0	0	T	28	M	0.181	0.454	1.1E-16	7.5E-08	1.5E-16	34.6049	tgcatggtttCcgtagtgtag	m5C_33787
chrX	11027316	+	C	76	19	0.25	0	0	T	57	M	0.166	0.358	0	6.9E-12	0	63.1894	agactcaagtCtgcttgagtg	m5C_33770
chrX	11027330	+	C	77	18	0.234	0	0	T	59	M	0.153	0.34	0	4E-12	0	55.1862	ttgagtgtcgCttctagtttt	m5C_33763
chrX	13243707	+	C	31	13	0.419	0	0	T	18	M	0.264	0.592	0	6.7E-06	0	68.6798	gcttgggttgCaggttgactt	m5C_34057
chrX	13243715	+	C	33	22	0.667	0	0	C	22	M	0.496	0.802	0	6.3E-05	0	218.273	tgcaggttgaCttctctctct	m5C_34037
chrX	16773164	+	C	36	10	0.278	0	0	T	26	M	0.158	0.44	4.8E-14	2E-06	5.9E-14	21.1115	aacagcaggcCgcagggaagg	m5C_33883
chrX	16773166	+	C	34	8	0.235	0	0	T	26	M	0.124	0.4	4.2E-11	6.8E-07	4.8E-11	10.3259	cagcaggccgCagggaaggag	m5C_33879
chrX	16875804	-	C	34	18	0.529	0	0	C	18	M	0.367	0.685	0	2.7E-05	0	132.251	atcctgatctCagattaagag	m5C_33906
chrX	16875806	-	C	35	7	0.2	0	0	T	28	M	0.1	0.359	1.9E-09	3.4E-07	2.1E-09	6.13884	taatcctgatCtcagattaag	m5C_33900
chrX	16875811	-	C	35	9	0.257	0	0	T	26	M	0.142	0.421	1.5E-12	1.2E-06	1.7E-12	15.0859	gaatgtaatcCtgatctcaga	m5C_33893
chrX	16888180	-	C	34	17	0.5	0	0	CT	17	M	0.341	0.659	0	2.1E-05	0	115.835	cgacccgacgCaagatggcga	m5C_33894
chrX	16888411	-	C	32	11	0.344	0	0	T	21	M	0.204	0.517	2.2E-16	3.1E-06	2.9E-16	35.1442	aagtctcgttCcaagtttgga	m5C_33903
chrX	16888415	-	C	33	12	0.364	0	0	T	21	M	0.222	0.534	0	4.6E-06	0	53.2487	tgaaaagtctCgttccaagtt	m5C_33907
chrX	17201191	+	C	33	8	0.242	0	0	T	25	M	0.128	0.41	3.1E-11	6.8E-07	3.6E-11	10.7866	atgtggatatCttgggagact	m5C_33889
chrX	17356711	-	C	31	17	0.548	0	0	C	17	M	0.378	0.708	0	2.1E-05	0	128.424	ggtgttggtgCggagagggtc	m5C_33917
chrX	18414172	-	C	38	13	0.342	0	0	T	25	M	0.212	0.501	0	6.7E-06	0	55.1518	ttgatcacaaCcagctgtaga	m5C_34314
chrX	18693030	-	C	224	46	0.205	0	0	T	178	M	0.158	0.263	0	0	0	145.025	ccagtggaacCatagccgtaa	m5C_34297
chrX	18693041	-	C	340	78	0.229	0	0	T	262	M	0.188	0.277	0	0	0	293.108	gggttcgagcCccagtggaac	m5C_34303
chrX	18693053	-	C	500	104	0.208	0	0	T	396	M	0.175	0.246	0	0	0	363.409	gcagaaggtcCtgggttcgag	m5C_34296
chrX	18693054	-	C	501	132	0.263	0	0	T	369	M	0.227	0.304	0	0	0	598.761	cgcagaaggtCctgggttcga	m5C_34301
chrX	18693074	-	C	469	113	0.241	1	0.002	T	355	M	0.205	0.282	0	0	0	463.04	gttatcacgtCtgctttacac	m5C_34323
chrX	19377112	+	C	46	12	0.261	0	0	T	34	M	0.156	0.403	6.7E-16	7.5E-08	8.7E-16	28.4116	ggatggtgaaCagcaatcttg	m5C_34294
chrX	19377119	+	C	49	15	0.306	0	0	T	34	M	0.195	0.445	0	2.8E-07	0	58.546	gaacagcaatCttgccagtgt	m5C_34287
chrX	19377123	+	C	45	11	0.244	0	0	T	34	M	0.142	0.387	2.1E-14	4.4E-08	2.7E-14	21.4112	agcaatcttgCcagtgtggaa	m5C_34277
chrX	19377137	+	C	48	16	0.333	0	0	T	32	M	0.217	0.475	0	4E-07	0	69.3651	tgtggaagaaCtaaaggtaca	m5C_34286
chrX	22002444	+	C	31	15	0.484	0	0	T	16	M	0.32	0.652	0	1.2E-05	0	95.91	gactgtatccCggtactgaag	m5C_34177
chrX	22002449	+	C	34	13	0.382	0	0	T	21	M	0.239	0.55	0	6.7E-06	0	62.1399	tatcccggtaCtgaagaggta	m5C_34206
chrX	22002462	+	C	32	19	0.594	0	0	C	19	M	0.423	0.745	0	3.4E-05	0	160.587	aagaggtacgCcaaagaaggg	m5C_34186
chrX	23685779	+	C	48	11	0.229	0	0	T	37	M	0.133	0.365	5E-14	4.4E-08	6.2E-14	19.4712	ttcctgctgcCggctggagct	m5C_34211
chrX	23685798	+	C	83	34	0.41	0	0	T	49	M	0.31	0.517	0	4.2E-11	0	210.887	ctgtgcagggCtgggagacag	m5C_34210
chrX	23802249	+	C	35	10	0.286	0	0	T	25	M	0.163	0.451	3.4E-14	2E-06	4.2E-14	21.9991	agtgtttaagCtgcttaagta	m5C_34195
chrX	24091256	+	C	73	27	0.37	0	0	T	46	M	0.268	0.485	0	1.9E-10	0	144.844	cataggatccCtgtcaacagg	m5C_34420
chrX	24762587	+	C	35	9	0.257	0	0	T	26	M	0.142	0.421	1.5E-12	1.2E-06	1.7E-12	15.0859	agtttcttctCaaggtgaaga	m5C_34423
chrX	24762616	+	C	42	14	0.333	0	0	T	28	M	0.21	0.484	0	1.9E-07	0	58.8344	tgtaaatgtcCtacagaaata	m5C_34412
chrX	24762637	+	C	49	18	0.367	0	0	T	31	M	0.247	0.507	0	7.7E-07	0	88.7939	tagtagctttCtgttcttcct	m5C_34424
chrX	24762642	+	C	47	17	0.362	0	0	T	30	M	0.24	0.505	0	5.6E-07	0	81.4845	gctttctgttCttcctttgca	m5C_34433
chrX	24762646	+	C	48	16	0.333	0	0	T	32	M	0.217	0.475	0	4E-07	0	69.3651	tctgttcttcCtttgcaagta	m5C_34438
chrX	24762651	+	C	53	17	0.321	0	0	T	36	M	0.211	0.455	0	1.1E-08	0	71.713	tcttcctttgCaagtaaaaag	m5C_34414
chrX	24762683	+	C	51	15	0.294	0	0	T	36	M	0.187	0.43	0	4.6E-09	0	56.1257	ccactactgaCatttatatat	m5C_34431
chrX	29885575	-	C	73	32	0.438	0	0	T	41	M	0.33	0.552	0	8.5E-10	0	211.489	ggtggaatggCaaaaaagggc	m5C_34470
chrX	30156371	+	C	53	28	0.528	0	0	C	28	M	0.397	0.656	0	3.1E-07	0	222.095	tgaaaagaatCtagcatttat	m5C_34474
chrX	34234109	+	C	64	35	0.547	0	0	C	35	M	0.426	0.663	0	5.3E-08	0	298.014	tgagcagtaaCaggtctgtga	m5C_34234
chrX	34234114	+	C	76	51	0.671	0	0	C	51	M	0.559	0.766	0	4.3E-08	0	570.625	agtaacaggtCtgtgatgctc	m5C_34231
chrX	34234122	+	C	91	44	0.484	0	0	T	47	M	0.384	0.585	0	1.8E-11	0	337.574	gtctgtgatgCtcttagatgt	m5C_34230
chrX	34234124	+	C	89	46	0.517	0	0	C	46	M	0.415	0.618	0	7.9E-10	0	381.344	ctgtgatgctCttagatgtct	m5C_34237
chrX	34234133	+	C	88	45	0.511	0	0	C	45	M	0.409	0.613	0	6.4E-10	0	367.788	tcttagatgtCtggggctgca	m5C_34232
chrX	34234139	+	C	80	49	0.613	0	0	C	49	M	0.503	0.712	0	3.1E-08	0	492.876	atgtctggggCtgcatgcatg	m5C_34235
chrX	34234142	+	C	78	29	0.372	0	0	T	49	M	0.273	0.483	0	3.6E-10	0	158.302	tctggggctgCatgcatgcca	m5C_34238
chrX	34234146	+	C	42	10	0.238	0	0	T	32	M	0.135	0.385	3.2E-13	2.4E-08	3.9E-13	16.8407	gggctgcatgCatgccacagt	m5C_34233
chrX	38864990	+	C	47	32	0.681	0	0	C	32	M	0.538	0.796	0	1.6E-05	0	344.527	agtggtggaaCattactggat	m5C_34559
chrX	41782197	-	C	44	11	0.25	0	0	T	33	M	0.146	0.394	1.6E-14	4.4E-08	2E-14	22.132	acgagctgtgCgaggtgatcg	m5C_34633
chrX	44260875	+	C	43	9	0.209	0	0	T	34	M	0.114	0.352	1.4E-11	1.2E-08	1.6E-11	11.1522	gcggtgtttaCaactaattga	m5C_34496
chrX	45606439	-	C	42	25	0.595	0	0	C	25	M	0.445	0.73	0	4.5E-06	0	222.47	tactgggtctCtgatggcatc	m5C_34510
chrX	45606447	-	C	72	55	0.764	0	0	C	55	M	0.654	0.847	0	8E-08	0	719.411	catctggctaCtgggtctctg	m5C_34511
chrX	45606454	-	C	79	61	0.772	0	0	C	61	M	0.668	0.851	0	1.8E-07	0	815.362	gcagctacatCtggctactgg	m5C_34515
chrX	45606457	-	C	80	47	0.588	0	0	C	47	M	0.478	0.689	0	2.2E-08	0	449.356	tcagcagctaCatctggctac	m5C_34527
chrX	45606460	-	C	40	21	0.525	0	0	C	21	M	0.375	0.671	0	5.2E-05	0	157.488	taatcagcagCtacatctggc	m5C_34506
chrX	45606481	-	C	38	19	0.5	0	0	CT	19	M	0.348	0.652	0	3.4E-05	0	132.429	cagtgtagatCctgtctttcg	m5C_34520
chrX	45606491	-	C	42	21	0.5	0	0	CT	21	M	0.355	0.645	0	1.8E-06	0	149.208	gctcagtagcCagtgtagatc	m5C_34528
chrX	45606492	-	C	37	10	0.27	0	0	T	27	M	0.154	0.43	6.7E-14	2E-06	8.3E-14	20.2815	ggctcagtagCcagtgtagat	m5C_34525
chrX	48386857	+	C	36	18	0.5	0	0	CT	18	M	0.345	0.655	0	2.7E-05	0	124.107	gagtggtggaCcaggctcgaa	m5C_35706
chrX	48386858	+	C	38	10	0.263	0	0	T	28	M	0.15	0.42	9.4E-14	2E-06	1.2E-13	19.5037	agtggtggacCaggctcgaac	m5C_35702
chrX	48386862	+	C	35	9	0.257	0	0	T	26	M	0.142	0.421	1.5E-12	1.2E-06	1.7E-12	15.0859	gtggaccaggCtcgaacactg	m5C_35705
chrX	48386864	+	C	34	17	0.5	0	0	CT	17	M	0.341	0.659	0	2.1E-05	0	115.835	ggaccaggctCgaacactggc	m5C_35698
chrX	48759667	+	C	41	19	0.463	0	0	T	22	M	0.321	0.613	0	1E-06	0	121.813	acaaggtagaCagagagagag	m5C_35723
chrX	48759698	+	C	56	13	0.232	0	0	T	43	M	0.141	0.358	4.4E-16	1.6E-09	5.8E-16	28.1397	ggaacgggatCgggaccgcgg	m5C_35715
chrX	48759703	+	C	56	35	0.625	0	0	C	35	M	0.494	0.74	0	1.3E-06	0	345.836	gggatcgggaCcgcgggtatg	m5C_35720
chrX	48759715	+	C	59	46	0.78	0	0	C	46	M	0.659	0.866	0	6.6E-06	0	605.977	gcgggtatgaCaaggcagacc	m5C_35679
chrX	48976134	+	C	54	18	0.333	0	0	T	36	M	0.222	0.466	0	1.7E-08	0	80.0685	taggcctccaCggggaccgcc	m5C_35699
chrX	48976140	+	C	49	36	0.735	0	0	C	36	M	0.597	0.838	0	2.9E-05	0	430.11	tccacggggaCcgcctcataa	m5C_35672
chrX	49009077	+	C	128	58	0.453	0	0	T	70	M	0.37	0.539	0	2.1E-14	0	428.628	ctgtagtgtgCtatgctgatt	m5C_35712
chrX	49454029	-	C	46	18	0.391	0	0	T	28	M	0.264	0.535	0	7.7E-07	0	95.0149	gtgaaaacacCtgaggaaggt	m5C_35773
chrX	49454032	-	C	53	21	0.396	0	0	T	32	M	0.276	0.531	0	4.8E-08	0	115.875	gaggtgaaaaCacctgaggaa	m5C_35745
chrX	49767791	+	C	39	20	0.513	0	0	C	20	M	0.362	0.661	0	4.3E-05	0	144.796	gagtgtaggaCcgttggcatc	m5C_35729
chrX	49767792	+	C	38	15	0.395	0	0	T	23	M	0.256	0.553	0	1.2E-05	0	76.7936	agtgtaggacCgttggcatct	m5C_35677
chrX	49773587	+	C	116	30	0.259	0	0	T	86	M	0.188	0.345	0	0	0	112.548	atccttggaaCctaggtgtga	m5C_35695
chrX	49773601	+	C	44	18	0.409	0	0	T	26	M	0.277	0.556	0	7.7E-07	0	99.6755	ggtgtgagtgCtatttcagtg	m5C_35725
chrX	49774355	+	C	38	11	0.289	0	0	T	27	M	0.17	0.448	2.1E-15	3.1E-06	2.7E-15	27.4488	tcctttgtccCtgggtgagag	m5C_35678
chrX	50374827	+	C	86	20	0.233	0	0	T	66	M	0.156	0.332	0	1.5E-13	0	62.356	ggctaggattCggcgctttca	m5C_34537
chrX	50374832	+	C	76	23	0.303	0	0	T	53	M	0.211	0.413	0	4.3E-11	0	97.0195	ggattcggcgCtttcataaaa	m5C_34534
chrX	50374836	+	C	51	26	0.51	0	0	C	26	M	0.377	0.641	0	1.9E-07	0	195.94	tcggcgctttCataaaatctc	m5C_34542
chrX	53353774	+	C	46	24	0.522	0	0	C	24	M	0.381	0.659	0	3.6E-06	0	183.058	tgaggctggtCcaaaggcagt	m5C_35845
chrX	53353794	+	C	47	15	0.319	0	0	T	32	M	0.204	0.462	0	2.8E-07	0	61.1856	tgagttatctCaattggttgt	m5C_35849
chrX	53423455	-	C	47	17	0.362	0	0	T	30	M	0.24	0.505	0	5.6E-07	0	81.4845	gccaagaagtCggaagtgaat	m5C_35896
chrX	53439019	-	C	60	21	0.35	0	0	T	39	M	0.242	0.476	0	1E-09	0	101.504	ggagaaggctCggcaggagtt	m5C_35933
chrX	53439021	-	C	58	26	0.448	0	0	T	32	M	0.327	0.575	0	1.9E-07	0	170.298	gtggagaaggCtcggcaggag	m5C_35893
chrX	53440301	-	C	38	11	0.289	0	0	T	27	M	0.17	0.448	2.1E-15	3.1E-06	2.7E-15	27.4488	ggagctggcgCaggagtatga	m5C_35947
chrX	53440303	-	C	37	22	0.595	0	0	C	22	M	0.435	0.737	0	6.3E-05	0	191.337	ggggagctggCgcaggagtat	m5C_35878
chrX	53440307	-	C	37	20	0.541	0	0	C	20	M	0.384	0.69	0	4.3E-05	0	153.535	ttctggggagCtggcgcagga	m5C_35876
chrX	53459001	-	C	58	24	0.414	0	0	T	34	M	0.296	0.542	0	1.2E-07	0	142.214	ccagaatgaaCcagaccaggg	m5C_35952
chrX	53459010	-	C	53	21	0.396	0	0	T	32	M	0.276	0.531	0	4.8E-08	0	115.875	tgagatgggcCagaatgaacc	m5C_35910
chrX	53459011	-	C	56	13	0.232	0	0	T	43	M	0.141	0.358	4.4E-16	1.6E-09	5.8E-16	28.1397	gtgagatgggCcagaatgaac	m5C_35926
chrX	53583238	-	C	43	9	0.209	0	0	T	34	M	0.114	0.352	1.4E-11	1.2E-08	1.6E-11	11.1522	atattaggccCcaattagaag	m5C_35891
chrX	53583239	-	C	42	12	0.286	0	0	T	30	M	0.172	0.436	2.2E-16	7.5E-08	2.9E-16	32.2467	gatattaggcCccaattagaa	m5C_35940
chrX	53583240	-	C	43	11	0.256	0	0	T	32	M	0.149	0.402	1.2E-14	4.4E-08	1.5E-14	22.8887	ggatattaggCcccaattaga	m5C_35948
chrX	53589116	-	C	72	32	0.444	0	0	T	40	M	0.335	0.559	0	8.5E-10	0	214.649	ggatgatagtCaggatgaaga	m5C_35923
chrX	53600857	-	C	61	16	0.262	0	0	T	45	M	0.168	0.384	0	1.1E-10	0	53.8837	ctgaggaaggCaaacagaaag	m5C_35909
chrX	53600867	-	C	59	19	0.322	0	0	T	40	M	0.217	0.449	0	2.4E-08	0	82.4122	gaccgggcctCtgaggaaggc	m5C_35912

chrX	53600874	-	C	45	17	0.378	0	0	T	28	M	0.251	0.524	0	5.6E-07	0	85.3668	agaaggggacCgggcctctga	m5C_35864
chrX	53611209	-	C	31	7	0.226	0	0	T	24	M	0.114	0.398	6.4E-10	3.4E-07	7.2E-10	7.33205	tggacaggaaCtagagaagac	m5C_35924
chrX	53616728	-	C	70	35	0.5	0	0	CT	35	M	0.386	0.614	0	1.9E-09	0	270.169	tcgggaaaagCaggaggagga	m5C_35936
chrX	53616737	-	C	84	23	0.274	0	0	T	61	M	0.19	0.377	0	7.2E-13	0	87.3664	acggaaagctCgggaaaagca	m5C_35873
chrX	53616739	-	C	82	35	0.427	0	0	T	47	M	0.325	0.535	0	5.6E-11	0	227.792	gaacggaaagCtcgggaaaag	m5C_35888
chrX	54840811	+	C	85	32	0.376	0	0	T	53	M	0.281	0.483	0	2.3E-11	0	179.79	gtggggtgtgCtcagagcagg	m5C_35043
chrX	54840813	+	C	84	34	0.405	0	0	T	50	M	0.306	0.512	0	4.2E-11	0	208.212	ggggtgtgctCagagcagggg	m5C_35021
chrX	54840818	+	C	81	20	0.247	0	0	T	61	M	0.166	0.351	0	1.5E-13	0	66.3608	gtgctcagagCagggggccca	m5C_35024
chrX	54840825	+	C	80	28	0.354	1	0.013	T	51	M	0.258	0.464	0	2.6E-10	0	144.453	gagcagggggCccaaagaatg	m5C_35014
chrX	54840837	+	C	56	15	0.268	0	0	T	41	M	0.17	0.396	0	4.6E-09	0	50.8715	caaagaatggCtcctctgttt	m5C_35028
chrX	54840839	+	C	46	10	0.217	0	0	T	36	M	0.123	0.356	9.7E-13	2.4E-08	1.2E-12	14.7309	aagaatggctCctctgtttac	m5C_35027
chrX	54840842	+	C	47	18	0.383	0	0	T	29	M	0.258	0.526	0	7.7E-07	0	92.8457	aatggctcctCtgtttacaac	m5C_35035
chrX	54840852	+	C	31	10	0.323	0	0	T	21	M	0.186	0.499	7.1E-15	2E-06	9E-15	26.2725	ctgtttacaaCacacccaaca	m5C_35022
chrX	54840868	+	C	31	10	0.323	0	0	T	21	M	0.186	0.499	7.1E-15	2E-06	9E-15	26.2725	caacaggaatCtggggtcatt	m5C_35032
chrX	54840892	+	C	39	8	0.205	0	0	T	31	M	0.108	0.355	1.6E-10	6.8E-07	1.8E-10	8.44384	atgagggcgtCaaacttgtgg	m5C_35034
chrX	55206608	-	C	33	9	0.273	0	0	T	24	M	0.151	0.442	7.5E-13	1.2E-06	9E-13	16.4418	tggttacaccCttcccatact	m5C_35059
chrX	55206610	-	C	34	11	0.333	1	0.029	T	22	M	0.198	0.504	2.2E-16	3.1E-06	2.9E-16	34.0074	gttggttacaCccttcccata	m5C_35058
chrX	55206628	-	C	324	86	0.265	0	0	T	238	M	0.22	0.316	0	0	0	378.921	ggcccatactCcgaaaatgtt	m5C_35047
chrX	55206630	-	C	355	92	0.259	0	0	T	263	M	0.216	0.307	0	0	0	398.035	cgggcccataCtccgaaaatg	m5C_35057
chrX	55206634	-	C	381	295	0.774	0	0	C	295	M	0.73	0.813	0	0	0	4305.12	ctatcgggccCatactccgaa	m5C_35056
chrX	55206635	-	C	387	131	0.339	1	0.003	T	255	M	0.294	0.388	0	0	0	770.099	gctatcgggcCcatactccga	m5C_35049
chrX	55206636	-	C	390	200	0.515	2	0.005	C	200	M	0.466	0.565	0	0	0	1863.31	agctatcgggCccatactccg	m5C_35048
chrX	55206640	-	C	387	129	0.333	0	0	T	258	M	0.288	0.382	0	0	0	743.574	aataagctatCgggcccatac	m5C_35052
chrX	55206656	-	C	354	78	0.22	0	0	T	276	M	0.18	0.266	0	0	0	281.255	agagtaaggtCagctaaataa	m5C_35053
chrX	57001147	-	C	90	20	0.222	0	0	T	70	M	0.149	0.318	0	1.7E-15	0	59.4849	gtgggggtgtgCtgattggtcg	m5C_34672
chrX	57147688	-	C	56	20	0.357	0	0	T	36	M	0.245	0.488	0	3.4E-08	0	97.8256	cgatccccggCggtggacgcc	m5C_34686
chrX	64738033	-	C	38	16	0.432	1	0.026	T	21	M	0.287	0.591	0	1.7E-05	0	91.7488	aaaatgatgaCcaagaggagg	m5C_34933
chrX	64749685	-	C	58	13	0.224	0	0	T	45	M	0.136	0.347	6.7E-16	1.6E-09	8.7E-16	26.8192	tgagagagacCtgggagttgg	m5C_34932
chrX	64956685	+	C	142	29	0.204	0	0	T	113	M	0.146	0.278	0	0	0	84.753	gaagaagaagCgtgaaatggc	m5C_34919
chrX	64956695	+	C	207	50	0.242	0	0	T	157	M	0.188	0.304	0	0	0	188.288	cgtgaaatggCagagaaggag	m5C_34920
chrX	64956721	+	C	221	75	0.339	0	0	T	146	M	0.28	0.404	0	0	0	420.238	gaagattgaaCgggagaagga	m5C_34922
chrX	64956736	+	C	38	11	0.289	0	0	T	27	M	0.17	0.448	2.1E-15	3.1E-06	2.7E-15	27.4488	gaaggaggagCtgatggagag	m5C_34893
chrX	64956748	+	C	58	30	0.517	0	0	C	30	M	0.392	0.641	0	4.8E-07	0	234.972	gatggagaggCtgaagcagat	m5C_34929
chrX	64956759	+	C	36	15	0.417	0	0	T	21	M	0.271	0.578	0	1.2E-05	0	81.421	tgaagcagatCgaggaacaga	m5C_34914
chrX	67580924	+	C	178	64	0.36	0	0	T	114	M	0.293	0.432	0	0	0	374.664	aagggctggtCtgagggtagt	m5C_34946
chrX	67580962	+	C	84	19	0.226	0	0	T	65	M	0.15	0.326	0	8.4E-14	0	56.9464	tgttcacagtCagttacaaat	m5C_34950
chrX	67988145	-	C	40	15	0.375	0	0	T	25	M	0.242	0.53	0	2.8E-07	0	72.6683	tgtctgaggcCtagaattcag	m5C_34954
chrX	67988152	-	C	135	42	0.311	0	0	T	93	M	0.239	0.394	0	0	0	200.889	ccacgtctgtCtgaggcctag	m5C_34953
chrX	67988156	-	C	142	29	0.204	0	0	T	113	M	0.146	0.278	0	0	0	84.753	ggggccacgtCtgtctgaggc	m5C_34952
chrX	67988159	-	C	136	41	0.301	0	0	T	95	M	0.231	0.383	0	0	0	189.149	gctggggccaCgtctgtctga	m5C_34955
chrX	67988162	-	C	126	46	0.365	0	0	T	80	M	0.286	0.452	0	6.7E-16	0	263.268	ggagctggggCcacgtctgtc	m5C_34956
chrX	68892332	+	C	73	26	0.356	0	0	T	47	M	0.256	0.471	0	1.3E-10	0	133.143	agtctacggcCataccaccct	m5C_35267
chrX	68892336	+	C	75	15	0.2	0	0	T	60	M	0.125	0.304	0	6E-13	0	37.5361	tacggccataCcaccctgaat	m5C_35250
chrX	68892337	+	C	75	16	0.213	0	0	T	59	M	0.136	0.319	0	1.2E-12	0	43.4563	acggccatacCaccctgaatg	m5C_35275
chrX	68892340	+	C	74	21	0.284	0	0	T	53	M	0.194	0.395	0	1.8E-11	0	81.3683	gccataccacCctgaatgagc	m5C_35261
chrX	68892341	+	C	78	20	0.256	0	0	T	58	M	0.173	0.363	0	1.1E-11	0	69.0212	ccataccaccCtgaatgagcc	m5C_35272
chrX	68892361	+	C	101	34	0.337	0	0	T	67	M	0.252	0.433	0	2.3E-14	0	171.336	ctgatctcgtCtgatcttgga	m5C_35260
chrX	68892366	+	C	114	49	0.43	0	0	T	65	M	0.343	0.522	0	6.6E-14	0	335.846	ctcgtctgatCttggaagcta	m5C_35274
chrX	68892374	+	C	136	49	0.36	0	0	T	87	M	0.284	0.444	0	0	0	278.779	atcttggaagCtaagcagggt	m5C_35276
chrX	68892379	+	C	165	75	0.455	0	0	T	90	M	0.38	0.531	0	0	0	570.697	ggaagctaagCagggtcgggc	m5C_35273
chrX	68892385	+	C	196	71	0.362	0	0	T	125	M	0.298	0.432	0	0	0	423.443	taagcagggtCgggcctggtt	m5C_35248
chrX	68892389	+	C	199	71	0.357	0	0	T	128	M	0.294	0.425	0	0	0	416.79	cagggtcgggCctggttagta	m5C_35245
chrX	68892390	+	C	202	93	0.46	0	0	T	109	M	0.393	0.529	0	0	0	731.054	agggtcgggcCtggttagtac	m5C_35264
chrX	68892400	+	C	295	94	0.319	0	0	T	201	M	0.268	0.374	0	0	0	504.018	ctggttagtaCttggatggga	m5C_35281
chrX	68892413	+	C	382	102	0.268	2	0.005	T	278	M	0.226	0.315	0	0	0	461.746	ggatgggagaCcacctgggaa	m5C_35278
chrX	68892414	+	C	405	116	0.286	0	0	T	289	M	0.245	0.332	0	0	0	567.374	gatgggagacCacctgggaat	m5C_35279
chrX	68892416	+	C	405	147	0.363	0	0	T	258	M	0.318	0.411	0	0	0	933.807	tgggagaccaCctgggaatac	m5C_35270
chrX	68892417	+	C	402	165	0.41	0	0	T	237	M	0.363	0.459	0	0	0	1199.31	gggagaccacCtgggaatacc	m5C_35257
chrX	68892426	+	C	383	154	0.402	0	0	T	229	M	0.354	0.452	0	0	0	1090.9	cctgggaataCcgggtgctgt	m5C_35255
chrX	68892427	+	C	385	136	0.353	0	0	T	249	M	0.307	0.402	0	0	0	835.49	ctgggaatacCgggtgctgta	m5C_35258
chrX	68892433	+	C	385	146	0.379	0	0	T	239	M	0.332	0.429	0	0	0	969.945	ataccgggtgCtgtaggcttt	m5C_35253
chrX	68892440	+	C	324	145	0.448	0	0	T	179	M	0.394	0.502	0	0	0	1143.52	gtgctgtaggCtttggccggg	m5C_35259
chrX	70516440	+	C	37	13	0.351	0	0	T	24	M	0.218	0.512	0	6.7E-06	0	56.7461	gactgtggagCccatggacca	m5C_35090
chrX	70516804	+	C	33	10	0.303	0	0	T	23	M	0.174	0.473	1.6E-14	2E-06	2E-14	23.982	gcagcaggacCaagtggaccg	m5C_35089
chrX	70516813	+	C	70	18	0.257	0	0	T	52	M	0.169	0.37	0	4E-12	0	60.9449	ccaagtggacCgcaacatcaa	m5C_35079
chrX	70516818	+	C	89	20	0.225	0	0	T	69	M	0.15	0.322	0	1.5E-13	0	60.1775	tggaccgcaaCatcaaggagg	m5C_35076
chrX	70516829	+	C	170	44	0.259	0	0	T	126	M	0.199	0.329	0	0	0	174.967	atcaaggaggCtcgtgagaag	m5C_35081
chrX	70516840	+	C	214	89	0.416	0	0	T	125	M	0.352	0.483	0	0	0	626.386	tcgtgagaagCtggagatgga	m5C_35082
chrX	70516894	+	C	33	12	0.364	0	0	T	21	M	0.222	0.534	0	4.6E-06	0	53.2487	gctaatgagaCagggtgagtc	m5C_35071
chrX	70517240	+	C	49	19	0.388	0	0	T	30	M	0.264	0.528	0	1E-06	0	100.429	tgatgaggcgCcaagaagaac	m5C_35092
chrX	70517253	+	C	46	32	0.696	0	0	C	32	M	0.552	0.809	0	1.6E-05	0	353.242	agaagaacttCggaggatgga	m5C_35075
chrX	71596851	-	C	64	16	0.25	0	0	T	48	M	0.16	0.368	0	1.1E-10	0	51.2473	tattgcagtaCctccagaaat	m5C_35115
chrX	71596865	-	C	64	17	0.266	0	0	T	47	M	0.173	0.385	0	1.8E-10	0	58.8072	atgcatcaacCtggtattgca	m5C_35102
chrX	71596869	-	C	44	16	0.364	0	0	T	28	M	0.238	0.511	0	4E-07	0	76.1067	ctccatgcatCaacctggtat	m5C_35112
chrX	73438432	-	C	31	12	0.387	0	0	T	19	M	0.237	0.562	0	4.6E-06	0	56.9589	tataatacaaCctgctaagtg	m5C_34980
chrX	76287233	+	C	52	14	0.269	0	0	T	38	M	0.168	0.403	0	2.8E-09	0	46.9527	cctgggaataCtgggtgttgt	m5C_35209
chrX	76287247	+	C	50	27	0.54	0	0	C	27	M	0.404	0.67	0	6.7E-06	0	218.153	gtgttgtaggCtttttaaata	m5C_35216
chrX	76954090	-	C	33	24	0.727	0	0	C	24	M	0.558	0.849	0	9.1E-05	0	267.754	ggaagtaactCtgatatgatg	m5C_35239
chrX	77380449	+	C	32	11	0.344	0	0	T	21	M	0.204	0.517	2.2E-16	3.1E-06	2.9E-16	35.1442	gtggaatggtCctgtgggggt	m5C_35202
chrX	77381871	+	C	39	14	0.359	0	0	T	25	M	0.227	0.516	0	9.2E-06	0	63.6773	agtggggcagCagcagtggag	m5C_35228
chrX	90633635	-	C	52	20	0.385	0	0	T	32	M	0.265	0.52	0	3.4E-08	0	105.881	gccgttcggaCtggtgtaaga	m5C_35348
chrX	91714970	+	C	38	12	0.316	0	0	T	26	M	0.191	0.475	0	4.6E-06	0	45.8026	agcctggagaCtgagaaccgt	m5C_35344
chrX	100667738	+	C	44	29	0.659	0	0	C	29	M	0.511	0.781	0	9.7E-06	0	296.605	atggatatggCtttgggtctg	m5C_35314
chrX	102508655	-	C	32	12	0.375	0	0	T	20	M	0.229	0.547	0	4.6E-06	0	55.0408	gcttgaaaggCttagggaaga	m5C_35398
chrX	102612570	+	C	48	25	0.521	0	0	C	25	M	0.383	0.655	0	4.5E-06	0	191.645	gaagaataagCaagaaagaaa	m5C_35374
chrX	102612644	+	C	49	10	0.204	0	0	T	39	M	0.115	0.336	2.1E-12	2.4E-08	2.4E-12	13.4191	gaaggaaaacCagaaaatgag	m5C_35381
chrX	102841796	+	C	124	50	0.403	0	0	T	74	M	0.321	0.491	0	2.4E-15	0	321.049	taaaggaaagCagagagtga	m5C_35368
chrX	102841797	+	C	127	66	0.52	0	0	C	66	M	0.433	0.605	0	1.2E-13	0	572.216	aaaggaaagcCagagagtgag	m5C_35371
chrX	102841830	+	C	42	15	0.357	0	0	T	27	M	0.23	0.508	0	2.8E-07	0	68.967	gaaggaaagtCagagagggag	m5C_35356
chrX	102841883	+	C	53	16	0.302	0	0	T	37	M	0.195	0.435	0	7.3E-09	0	62.4581	agagggaaaaCcagagataga	m5C_35370
chrX	102841884	+	C	51	22	0.431	0	0	T	29	M	0.305	0.567	0	6.5E-08	0	134.205	gagggaaaacCagagatagag	m5C_35382
chrX	102841901	+	C	71	21	0.296	0	0	T	50	M	0.202	0.41	0	1.8E-11	0	84.9775	agagggaaagCcagagagtga	m5C_35363
chrX	102841902	+	C	65	22	0.338	0	0	T	43	M	0.235	0.46	0	1.5E-09	0	103.547	gagggaaagcCagagagtgaa	m5C_35383
chrX	102931722	-	C	33	11	0.333	0	0	T	22	M	0.198	0.504	2.2E-16	3.1E-06	2.9E-16	34.0074	ccagaaagaaCaagcagaaga	m5C_35388
chrX	103551230	-	C	46	10	0.217	0	0	T	36	M	0.123	0.356	9.7E-13	2.4E-08	1.2E-12	14.7309	cacagtgatcCtcagtggtgt	m5C_35403
chrX	106957267	-	C	51	13	0.255	0	0	T	38	M	0.155	0.389	1.1E-16	1.6E-09	1.5E-16	32.2431	gcaggggacaCagatgggaga	m5C_36028
chrX	107920280	-	C	31	10	0.323	0	0	T	21	M	0.186	0.499	7.1E-15	2E-06	9E-15	26.2725	ggagactggtCtgaaggtagt	m5C_36021
chrX	108297350	+	C	60	41	0.683	0	0	C	41	M	0.558	0.787	0	1.7E-07	0	457.281	aagaatttttCgcgaaggccc	m5C_35517
chrX	108297352	+	C	67	32	0.478	0	0	T	35	M	0.363	0.595	0	2.7E-08	0	232.001	gaatttttcgCgaaggcccgc	m5C_35543
chrX	108297358	+	C	116	53	0.457	0	0	T	63	M	0.369	0.547	0	1.8E-13	0	391.225	ttcgcgaaggCccgcggcggg	m5C_35527
chrX	108297359	+	C	113	56	0.496	0	0	T	57	M	0.405	0.586	0	3.7E-13	0	453.669	tcgcgaaggcCcgcggcgggt	m5C_35549

chrX	108297360	+	C	115	63	0.548	0	0	C	63	M	0.457	0.636	0	1.7E-12	0	575.546	cgcgaaggccCgcggcgggtg	m5C_35563
chrX	108297362	+	C	121	61	0.504	0	0	C	61	M	0.416	0.592	0	4.2E-14	0	507.884	cgaaggcccgCggcgggtgtt	m5C_35551
chrX	108297365	+	C	123	66	0.537	0	0	C	66	M	0.449	0.622	0	1.2E-13	0	592.269	aggcccgcggCgggtgttgat	m5C_35577
chrX	108297377	+	C	139	72	0.518	0	0	C	72	M	0.436	0.599	0	1.7E-14	0	627.201	ggtgttgatgCgatgtgattt	m5C_35556
chrX	108297714	-	C	37	21	0.568	0	0	C	21	M	0.409	0.713	0	5.2E-05	0	171.838	gcagggccggCagaccccgcc	m5C_35643
chrX	108297717	-	C	51	27	0.529	0	0	C	27	M	0.395	0.659	0	2.5E-07	0	213.425	cccgcagggcCggcagacccc	m5C_35661
chrX	108297718	-	C	50	33	0.66	0	0	C	33	M	0.522	0.776	0	8.8E-07	0	344.214	gcccgcagggCcggcagaccc	m5C_35651
chrX	108297723	-	C	63	26	0.413	0	0	T	37	M	0.3	0.536	0	5.6E-09	0	155.783	cggcggcccgCagggccggca	m5C_35601
chrX	108297725	-	C	86	42	0.488	0	0	T	44	M	0.386	0.592	0	3.3E-10	0	323.823	accggcggccCgcagggccgg	m5C_35587
chrX	108297726	-	C	86	62	0.721	0	0	C	62	M	0.618	0.805	0	1.3E-08	0	766.633	caccggcggcCcgcagggccg	m5C_35602
chrX	108297727	-	C	86	63	0.733	0	0	C	63	M	0.631	0.815	0	1.5E-08	0	794.49	tcaccggcggCccgcagggcc	m5C_35652
chrX	108297730	-	C	101	74	0.74	1	0.01	C	74	M	0.646	0.816	0	2.3E-10	0	956.507	atttcaccggCggcccgcagg	m5C_35640
chrX	108297733	-	C	104	60	0.577	0	0	C	60	M	0.481	0.667	0	2.2E-11	0	577.076	ggtatttcacCggcggcccgc	m5C_35666
chrX	108297734	-	C	105	64	0.61	0	0	C	64	M	0.514	0.697	0	4.6E-11	0	657.821	tggtatttcaCcggcggcccg	m5C_35631
chrX	108297736	-	C	121	91	0.752	0	0	C	91	M	0.668	0.82	0	9.3E-12	0	1216.06	agtggtatttCaccggcggcc	m5C_35608
chrX	108297790	-	C	31	11	0.355	0	0	T	20	M	0.211	0.531	1.1E-16	3.1E-06	1.5E-16	37.0595	cccctcagggCtcgccccccc	m5C_35646
chrX	108297797	-	C	70	20	0.286	0	0	T	50	M	0.193	0.401	0	1.1E-11	0	77.2883	gcgagagcccCtcagggctcg	m5C_35616
chrX	108297798	-	C	71	37	0.521	0	0	C	37	M	0.407	0.633	0	3E-09	0	301.075	agcgagagccCctcagggctc	m5C_35645
chrX	108297800	-	C	99	27	0.273	0	0	T	72	M	0.195	0.368	0	7.2E-14	0	105.15	gaagcgagagCccctcagggc	m5C_35592
chrX	108297801	+	C	42	33	0.786	0	0	C	33	M	0.641	0.883	0	1.9E-05	0	422.795	ccctgaggggCtctcgcttct	m5C_35541
chrX	108297803	+	C	41	27	0.659	0	0	C	27	M	0.505	0.784	0	6.7E-06	0	272.968	ctgaggggctCtcgcttctgg	m5C_35559
chrX	108297805	+	C	43	28	0.651	0	0	C	28	M	0.502	0.776	0	8.1E-06	0	280.96	gaggggctctCgcttctggcg	m5C_35531
chrX	108297807	+	C	46	27	0.587	0	0	C	27	M	0.443	0.717	0	6.7E-06	0	239.432	ggggctctcgCttctggcgcc	m5C_35554
chrX	108297810	+	C	46	28	0.609	0	0	C	28	M	0.465	0.736	0	8.1E-06	0	260.157	gctctcgcttCtggcgccaag	m5C_35530
chrX	108297814	+	C	39	25	0.641	0	0	C	25	M	0.484	0.773	0	0.00011	0	242.089	tcgcttctggCgccaagcgcc	m5C_35537
chrX	108297816	+	C	41	22	0.537	0	0	C	22	M	0.387	0.679	0	2.3E-06	0	170.483	gcttctggcgCcaagcgcccg	m5C_35542
chrX	108297817	+	C	36	21	0.583	0	0	C	21	M	0.422	0.729	0	5.2E-05	0	177.24	cttctggcgcCaagcgcccgg	m5C_35565
chrX	108297821	+	C	38	13	0.342	0	0	T	25	M	0.212	0.501	0	6.7E-06	0	55.1518	tggcgccaagCgcccggccgc	m5C_35569
chrX	108297826	-	C	46	11	0.239	0	0	T	35	M	0.139	0.379	2.8E-14	4.4E-08	3.5E-14	20.7303	tgcgtgcggcCgggcgcttgg	m5C_35615
chrX	111904892	+	C	207	97	0.469	0	0	T	110	M	0.402	0.537	0	0	0	779.513	gatgactggtCtgaaggtagt	m5C_35456
chrX	111904910	+	C	205	42	0.205	0	0	T	163	M	0.155	0.265	0	0	0	130.45	agtgagttgtCtcaattgatt	m5C_35447
chrX	116256125	-	C	32	12	0.375	0	0	T	20	M	0.229	0.547	0	4.6E-06	0	55.0408	ttcatgggtgCtggtctttcc	m5C_35494
chrX	118557790	+	C	32	10	0.312	0	0	T	22	M	0.18	0.486	1.1E-14	2E-06	1.3E-14	25.0836	gatgtgctgaCccctgcgatt	m5C_36053
chrX	118557791	+	C	34	8	0.235	0	0	T	26	M	0.124	0.4	4.2E-11	6.8E-07	4.8E-11	10.3259	atgtgctgacCcctgcgattt	m5C_36038
chrX	118557796	+	C	33	8	0.242	0	0	T	25	M	0.128	0.41	3.1E-11	6.8E-07	3.6E-11	10.7866	ctgacccctgCgatttcccca	m5C_36055
chrX	118557803	+	C	36	16	0.444	0	0	T	20	M	0.295	0.604	0	1.7E-05	0	94.5313	ctgcgatttcCccaaatgtgg	m5C_36043
chrX	118557805	+	C	33	8	0.242	0	0	T	25	M	0.128	0.41	3.1E-11	6.8E-07	3.6E-11	10.7866	gcgatttcccCaaatgtggga	m5C_36042
chrX	118557848	+	C	41	11	0.268	0	0	T	30	M	0.157	0.419	6E-15	4.4E-08	7.6E-15	24.5536	tagtgggggaCtgtgttcgcg	m5C_36047
chrX	118557855	+	C	43	11	0.256	0	0	T	32	M	0.149	0.402	1.2E-14	4.4E-08	1.5E-14	22.8887	ggactgtgttCgcgctttccc	m5C_36033
chrX	118557859	+	C	40	11	0.275	0	0	T	29	M	0.161	0.428	4.3E-15	3.1E-06	5.5E-15	25.4502	tgtgttcgcgCtttcccttga	m5C_36058
chrX	118557864	+	C	41	22	0.537	0	0	C	22	M	0.387	0.679	0	2.3E-06	0	170.483	tcgcgctttcCcttgagaaaa	m5C_36035
chrX	118723395	-	C	34	19	0.559	0	0	C	19	M	0.395	0.711	0	3.4E-05	0	149.924	tagaaaaagaCggaaggaaga	m5C_36078
chrX	118921320	-	C	53	24	0.453	0	0	T	29	M	0.327	0.585	0	1.2E-07	0	156.755	ttgacatggaCaattctggga	m5C_36082
chrX	118921326	-	C	60	23	0.383	0	0	T	37	M	0.271	0.51	0	8.7E-08	0	124.605	gacagattgaCatggacaatt	m5C_36079
chrX	118921334	-	C	67	26	0.388	0	0	T	41	M	0.28	0.508	0	5.6E-09	0	145.854	aaaatcatgaCagattgacat	m5C_36070
chrX	118921371	-	C	52	15	0.288	0	0	T	37	M	0.183	0.423	0	4.6E-09	0	54.9894	ccgtgttaatCatttttgatt	m5C_36084
chrX	118921380	-	C	66	18	0.273	0	0	T	48	M	0.18	0.39	0	2.9E-10	0	64.8115	aaagaaaagcCgtgttaatca	m5C_36080
chrX	118921396	-	C	69	18	0.261	0	0	T	51	M	0.172	0.375	0	2.9E-10	0	61.8675	tgccattatcCtgctgaaaga	m5C_36075
chrX	118921403	-	C	75	31	0.413	0	0	T	44	M	0.309	0.526	0	6.4E-10	0	191.441	gaagtactgcCattatcctgc	m5C_36071
chrX	118921407	-	C	73	24	0.329	0	0	T	49	M	0.232	0.443	0	6.4E-11	0	111.333	tgtggaagtaCtgccattatc	m5C_36081
chrX	122990987	-	C	32	8	0.25	0	0	T	24	M	0.133	0.421	2.3E-11	6.8E-07	2.6E-11	11.2828	gtggtgtttaCaaccaattga	m5C_35831
chrX	123191735	+	C	55	15	0.273	0	0	T	40	M	0.173	0.402	0	4.6E-09	0	51.8418	tctgagagatCcagaggagga	m5C_35819
chrX	123191736	+	C	58	18	0.31	0	0	T	40	M	0.206	0.438	0	1.7E-08	0	74.239	ctgagagatcCagaggaggat	m5C_35827
chrX	125715081	+	C	368	80	0.217	0	0	T	288	M	0.178	0.262	0	0	0	285.256	aggaaggatgCgcgagggccg	m5C_36109
chrX	125715083	+	C	374	99	0.265	0	0	T	275	M	0.223	0.312	0	0	0	440.647	gaaggatgcgCgagggccgga	m5C_36105
chrX	125715089	+	C	217	70	0.323	0	0	T	147	M	0.264	0.387	0	0	0	369.51	tgcgcgagggCcggaacgccg	m5C_36100
chrX	125715090	+	C	172	54	0.314	0	0	T	118	M	0.249	0.387	0	0	0	269.245	gcgcgagggcCggaacgccgg	m5C_36095
chrX	125715237	+	C	57	13	0.228	0	0	T	44	M	0.138	0.352	4.4E-16	1.6E-09	5.8E-16	27.6258	cgagtcccccCcccggcggtg	m5C_36102
chrX	125715238	+	C	95	27	0.284	0	0	T	68	M	0.203	0.382	0	7.2E-14	0	109.773	gagtccccccCccggcggtga	m5C_36093
chrX	125715240	+	C	140	40	0.286	0	0	T	100	M	0.217	0.365	0	0	0	173.911	gtcccccccCggcggtgaac	m5C_36111
chrX	125715243	+	C	241	91	0.378	0	0	T	150	M	0.319	0.44	0	0	0	580.142	ccccccccggCggtgaacggg	m5C_36107
chrX	125715250	+	C	379	144	0.38	0	0	T	235	M	0.333	0.43	0	0	0	957.646	cggcggtgaaCggggaggagg	m5C_36106
chrX	125715261	+	C	285	94	0.33	0	0	T	191	M	0.278	0.386	0	0	0	522.302	ggggaggaggCgggaacccaa	m5C_36110
chrX	125715267	+	C	110	58	0.527	0	0	C	58	M	0.435	0.618	0	5.8E-13	0	504.177	gaggcgggaaCccaagaagcg	m5C_36103
chrX	125715268	+	C	110	51	0.464	0	0	T	59	M	0.373	0.556	0	1.1E-13	0	380.714	aggcgggaacCcaagaagcgg	m5C_36094
chrX	125715276	+	C	101	31	0.307	0	0	T	70	M	0.225	0.403	0	7.1E-15	0	139.77	acccaagaagCggggcgcgcc	m5C_36101
chrX	125715281	+	C	83	33	0.398	0	0	T	50	M	0.299	0.505	0	3.1E-11	0	197.398	agaagcggggCgcgccgagca	m5C_36108
chrX	125715286	+	C	40	18	0.45	0	0	T	22	M	0.307	0.602	0	2.7E-05	0	110.538	cggggcgcgcCgagcagggat	m5C_36099
chrX	128570515	-	C	54	33	0.611	0	0	C	33	M	0.478	0.73	0	8.8E-07	0	315.401	aatacgggtgCtgtaggcatt	m5C_35964
chrX	128986077	+	C	41	21	0.512	0	0	C	21	M	0.365	0.657	0	1.8E-06	0	153.234	agaagaaggaCaaagacaaaa	m5C_35956
chrX	128986083	+	C	38	9	0.237	0	0	T	29	M	0.13	0.392	3.7E-12	1.2E-06	4.3E-12	13.3739	aggacaaagaCaaaaaaacag	m5C_35958
chrX	128986091	+	C	36	11	0.306	0	0	T	25	M	0.18	0.469	1E-15	3.1E-06	1.3E-15	29.7077	gacaaaaaaaCagactcggaa	m5C_35959
chrX	133303577	-	C	57	24	0.421	0	0	T	33	M	0.302	0.55	0	1.2E-07	0	144.9	ttgtcccggcCtgtggaagaa	m5C_36469
chrX	133303578	-	C	52	11	0.212	0	0	T	41	M	0.122	0.34	1.4E-13	4.4E-10	1.7E-13	17.3019	cttgtcccggCctgtggaaga	m5C_36463
chrX	133303581	-	C	56	38	0.679	0	0	C	38	M	0.548	0.786	0	2.1E-06	0	416.65	gcacttgtccCggcctgtgga	m5C_36481
chrX	133303582	-	C	56	28	0.5	0	0	CT	28	M	0.373	0.627	0	3.1E-07	0	209.057	tgcacttgtcCcggcctgtgg	m5C_36475
chrX	133303583	-	C	53	16	0.302	0	0	T	37	M	0.195	0.435	0	7.3E-09	0	62.4581	ttgcacttgtCccggcctgtg	m5C_36479
chrX	133303588	-	C	54	11	0.204	0	0	T	43	M	0.118	0.329	2.3E-13	4.4E-10	2.8E-13	16.3622	aagtattgcaCttgtcccggc	m5C_36461
chrX	133680364	-	C	41	10	0.244	0	0	T	31	M	0.138	0.393	2.4E-13	2.4E-08	2.9E-13	17.4472	tttccgctgcCagggtaagtc	m5C_36477
chrX	133680365	-	C	40	11	0.275	0	0	T	29	M	0.161	0.428	4.3E-15	3.1E-06	5.5E-15	25.4502	gtttccgctgCcagggtaagt	m5C_36473
chrX	133680370	-	C	42	16	0.381	0	0	T	26	M	0.25	0.532	0	4E-07	0	79.9985	gtattgtttcCgctgccaggg	m5C_36471
chrX	133680385	-	C	32	20	0.625	0	0	C	20	M	0.453	0.771	0	4.3E-05	0	181.016	gatcggtgctCtggggtattg	m5C_36466
chrX	133680396	-	C	43	10	0.233	0	0	T	33	M	0.132	0.377	4.3E-13	2.4E-08	5.2E-13	16.2683	ctgcagtgagCgatcggtgct	m5C_36460
chrX	133680406	-	C	76	37	0.487	0	0	T	39	M	0.378	0.597	0	3E-09	0	279.598	gggaacagttCtgcagtgagc	m5C_36474
chrX	133680411	-	C	82	20	0.244	0	0	T	62	M	0.164	0.347	0	1.5E-13	0	65.5191	gcagcgggaaCagttctgcag	m5C_36480
chrX	133680417	-	C	76	29	0.382	0	0	T	47	M	0.281	0.494	0	3.6E-10	0	162.73	gccctagcagCgggaacagtt	m5C_36464
chrX	135593205	+	C	36	12	0.333	0	0	T	24	M	0.202	0.497	0	4.6E-06	0	48.5146	tttgaaaagaCagaagatggg	m5C_36289
chrX	135961360	-	C	214	70	0.327	0	0	T	144	M	0.268	0.393	0	0	0	374.885	aagttctgagCtttctgttta	m5C_36295
chrX	135961375	-	C	312	108	0.346	0	0	T	204	M	0.296	0.401	0	0	0	638.331	tttttgtcctCtaagaagttc	m5C_36309
chrX	135961377	-	C	324	77	0.238	0	0	T	247	M	0.195	0.287	0	0	0	299.607	tatttttgtcCtctaagaagt	m5C_36302
chrX	135961378	-	C	316	84	0.266	0	0	T	232	M	0.22	0.317	0	0	0	369.832	gtatttttgtCctctaagaag	m5C_36321
chrX	135961397	-	C	415	119	0.287	0	0	T	296	M	0.245	0.332	0	0	0	583.927	gatcgtctgaCatgataatgt	m5C_36303
chrX	135961401	-	C	415	104	0.251	0	0	T	311	M	0.211	0.294	0	0	0	439.554	cattgatcgtCtgacatgata	m5C_36320
chrX	135961404	-	C	410	98	0.239	0	0	T	312	M	0.2	0.283	0	0	0	392.558	ttgcattgatCgtctgacatg	m5C_36300
chrX	135961429	-	C	153	40	0.261	0	0	T	113	M	0.198	0.336	0	0	0	158.61	aaaaatataagCtatgatgaat	m5C_36310
chrX	136677555	+	C	31	12	0.387	0	0	T	19	M	0.237	0.562	0	4.6E-06	0	56.9589	gtatggtggcCtcttgggagc	m5C_36207
chrX	139006371	-	C	57	26	0.456	0	0	T	31	M	0.334	0.584	0	1.9E-07	0	173.536	agtgggggagCcaggaagtat	m5C_36264
chrX	139615214	+	C	45	17	0.378	0	0	T	28	M	0.251	0.524	0	5.6E-07	0	85.3668	aggtggtatgCttaatacatg	m5C_36248
chrX	139615225	+	C	31	13	0.419	0	0	T	18	M	0.264	0.592	0	6.7E-06	0	68.6798	ttaatacatgCaccttttcat	m5C_36255
chrX	142654191	-	C	32	10	0.312	0	0	T	22	M	0.18	0.486	1.1E-14	2E-06	1.3E-14	25.0836	ttgtatgtttCctgtggcctc	m5C_36349
chrX	146958991	+	C	42	30	0.714	0	0	C	30	M	0.564	0.828	0	1.2E-05	0	338.595	atcctatggtCtagtggctag	m5C_36217

chrX	149887118	+	C	50	10	0.2	0	0	T	40	M	0.112	0.33	2.6E-12	2E-10	3.1E-12	13.024	tggaaataagCtggcacagat	m5C_36381
chrX	151128153	-	C	60	20	0.333	0	0	T	40	M	0.227	0.459	0	6.9E-10	0	90.9164	gcagaggaaaCtgagactttg	m5C_36278
chrX	153063824	+	C	45	20	0.444	0	0	T	25	M	0.309	0.588	0	1.4E-06	0	123.755	gaggtgctggCtgcggcgatc	m5C_36801
chrX	153063830	+	C	41	9	0.22	0	0	T	32	M	0.12	0.367	8.4E-12	1.2E-08	9.9E-12	11.9621	ctggctgcggCgatcggcctt	m5C_36802
chrX	153628649	+	C	506	112	0.221	0	0	T	394	M	0.187	0.26	0	0	0	419.654	gactgagttcCtttcctcatg	m5C_36785
chrX	153628654	+	C	540	114	0.212	2	0.004	T	424	M	0.179	0.248	0	0	0	409.189	agttcctttcCtcatggggac	m5C_36798
chrX	153628695	+	C	671	152	0.227	0	0	T	519	M	0.196	0.26	0	0	0	597.251	acagcagcttCcttggtagtg	m5C_36846
chrX	153628714	+	C	633	132	0.209	1	0.002	T	500	M	0.179	0.242	0	0	0	472.493	tgtacgcagcCtgttggttgt	m5C_36831
chrX	153628733	+	C	391	102	0.261	0	0	T	289	M	0.22	0.307	0	0	0	448.435	gtatgggttgCtctaagggac	m5C_36828
chrX	153628735	+	C	398	84	0.211	0	0	T	314	M	0.174	0.254	0	0	0	292.025	atgggttgctCtaagggacct	m5C_36810
chrX	153628743	+	C	387	95	0.245	0	0	T	292	M	0.205	0.291	0	0	0	389.954	ctctaagggaCcttggagaca	m5C_36837
chrX	153628752	+	C	339	76	0.224	0	0	T	263	M	0.183	0.272	0	0	0	278.198	accttggagaCaggcctttca	m5C_36823
chrX	153628756	+	C	236	70	0.297	0	0	T	166	M	0.242	0.358	0	0	0	338.756	tggagacaggCctttcaggtg	m5C_36796
chrX	153674876	+	C	32	19	0.594	0	0	C	19	M	0.423	0.745	0	3.4E-05	0	160.587	gaagaggaggCggccatgtat	m5C_36779
chrX	153712628	-	C	34	9	0.265	0	0	T	25	M	0.146	0.431	1.1E-12	1.2E-06	1.3E-12	15.7394	gacagaggccCaatgggtgaa	m5C_36909
chrX	153712629	-	C	35	14	0.4	0	0	T	21	M	0.256	0.564	0	9.2E-06	0	71.5414	ggacagaggcCcaatgggtga	m5C_36887
chrX	153712630	-	C	35	16	0.457	0	0	T	19	M	0.305	0.618	0	1.7E-05	0	97.4905	tggacagaggCccaatgggtg	m5C_36890
chrX	154003280	+	C	56	15	0.268	0	0	T	41	M	0.17	0.396	0	4.6E-09	0	50.8715	gcacggcagaCagttatccct	m5C_36352
chrX	154003288	+	C	60	12	0.2	0	0	T	48	M	0.118	0.318	3.3E-14	7.6E-12	4.2E-14	19.13	gacagttatcCctttctagtc	m5C_36373
chrX	154003289	+	C	60	18	0.3	0	0	T	42	M	0.199	0.425	0	2.9E-10	0	71.6328	acagttatccCtttctagtct	m5C_36363
chrX	154003293	+	C	56	17	0.304	0	0	T	39	M	0.199	0.433	0	1.1E-08	0	67.6606	ttatccctttCtagtctggct	m5C_36359
chrX	154003298	+	C	62	13	0.21	0	0	T	49	M	0.127	0.326	1.8E-15	1.6E-11	2.3E-15	24.3221	cctttctagtCtggctcgtgg	m5C_36365
chrX	154003311	+	C	71	22	0.31	0	0	T	49	M	0.214	0.425	0	2.8E-11	0	94.3341	gctcgtgggaCtctagaggga	m5C_36350
chrX	154003328	+	C	41	10	0.244	0	0	T	31	M	0.138	0.393	2.4E-13	2.4E-08	2.9E-13	17.4472	gggagtcagtCtgcaacagta	m5C_36364
chrX	154003350	+	C	47	21	0.447	0	0	T	26	M	0.314	0.588	0	1.8E-06	0	131.925	gtggtgagttCttctgtccag	m5C_36357
chrX	154003357	+	C	44	15	0.341	0	0	T	29	M	0.219	0.489	0	2.8E-07	0	65.6269	gttcttctgtCcagcgtcagt	m5C_36370
chrX	154003358	+	C	45	24	0.533	0	0	C	24	M	0.391	0.671	0	3.6E-06	0	187.571	ttcttctgtcCagcgtcagta	m5C_36369
chrX	154003361	+	C	51	12	0.235	0	0	T	39	M	0.14	0.368	3.3E-15	8.8E-10	4.3E-15	24.3305	ttctgtccagCgtcagtattt	m5C_36366
chrX	154003364	+	C	51	12	0.235	0	0	T	39	M	0.14	0.368	3.3E-15	8.8E-10	4.3E-15	24.3305	tgtccagcgtCagtattttga	m5C_36358
chrX	154003381	+	C	53	13	0.245	0	0	T	40	M	0.149	0.376	2.2E-16	1.6E-09	2.9E-16	30.3876	ttgatggtggCtttagacttg	m5C_36360
chrY	8862341	+	C	33	10	0.303	0	0	T	23	M	0.174	0.473	1.6E-14	2E-06	2E-14	23.982	tgggggtgcgCtggtggactc	m5C_36404
chrY	10035052	+	C	38	16	0.421	0	0	T	22	M	0.279	0.578	0	1.7E-05	0	89.1273	ccaaagggcgCtgaccccctt	m5C_42627
chrY	10035057	+	C	43	19	0.442	0	0	T	24	M	0.304	0.589	0	1E-06	0	115.646	gggcgctgacCcccttcgcgg	m5C_42599
chrY	10035059	+	C	52	11	0.212	0	0	T	41	M	0.122	0.34	1.4E-13	4.4E-10	1.7E-13	17.3019	gcgctgacccCcttcgcgggg	m5C_42579
chrY	10035060	+	C	51	16	0.314	0	0	T	35	M	0.203	0.45	0	7.3E-09	0	65.048	cgctgaccccCttcgcgggga	m5C_42584
chrY	10035065	+	C	52	16	0.308	0	0	T	36	M	0.199	0.443	0	7.3E-09	0	63.7266	accccccttcgCggggaagatg	m5C_42553
chrY	10035080	+	C	39	11	0.282	0	0	T	28	M	0.165	0.438	3.1E-15	3.1E-06	4E-15	26.4001	aagatgcgtgCatttatcaga	m5C_42646
chrY	10035265	+	C	127	30	0.236	0	0	T	97	M	0.171	0.317	0	0	0	102.463	ccacggtgacCatgggtgatg	m5C_42571
chrY	10035289	+	C	155	64	0.413	0	0	T	91	M	0.338	0.492	0	0	0	433.167	aatcagggttCcactctgaag	m5C_42529
chrY	10035290	+	C	134	35	0.261	0	0	T	99	M	0.194	0.341	0	0	0	135.949	atcagggttcCactctgaaga	m5C_42519
chrY	10035315	+	C	32	11	0.344	0	0	T	21	M	0.204	0.517	2.2E-16	3.1E-06	2.9E-16	35.1442	gcctgagaaaCagctaccaca	m5C_42512
chrY	10035322	+	C	35	8	0.229	0	0	T	27	M	0.121	0.39	5.6E-11	6.8E-07	6.4E-11	9.89691	aaacagctacCacattcaagg	m5C_42632
chrY	10035328	+	C	70	19	0.271	0	0	T	51	M	0.181	0.385	0	6.9E-12	0	68.854	ctaccacattCaaggaaggca	m5C_42637
chrY	10035340	+	C	122	25	0.205	0	0	T	97	M	0.143	0.285	0	0	0	71.4143	aggaaggcagCaggtgcaaaa	m5C_42643
chrY	10035456	+	C	520	264	0.51	2	0.004	C	264	M	0.467	0.552	0	0	0	2464.12	ttaggaggatCcattggaggg	m5C_42516
chrY	10035457	+	C	574	117	0.204	0	0	T	457	M	0.173	0.239	0	0	0	404.577	taggaggatcCattggagggc	m5C_42589
chrY	10035467	+	C	683	215	0.315	0	0	T	468	M	0.281	0.351	0	0	0	1208.62	cattggagggCaagtctggtg	m5C_42576
chrY	10035472	+	C	568	177	0.312	0	0	T	391	M	0.275	0.351	0	0	0	973.155	gagggcaagtCtggtgccagc	m5C_42618
chrY	10035478	+	C	290	76	0.262	0	0	T	214	M	0.215	0.316	0	0	0	326.498	aagtctggtgCcagcagccac	m5C_42526
chrY	10035479	+	C	253	110	0.435	0	0	T	143	M	0.375	0.496	0	0	0	825.271	agtctggtgcCagcagccaca	m5C_42547
chrY	10035482	+	C	192	75	0.391	0	0	T	117	M	0.324	0.461	0	0	0	486.607	ctggtgccagCagccacagta	m5C_42523
chrY	10035485	+	C	86	22	0.256	0	0	T	64	M	0.175	0.357	0	4.4E-13	0	77.189	gtgccagcagCcacagtaatt	m5C_42601
chrY	10035486	+	C	36	10	0.278	0	0	T	26	M	0.158	0.44	4.8E-14	2E-06	5.9E-14	21.1115	tgccagcagcCacagtaattc	m5C_42611
chrY	10035584	-	C	47	25	0.532	0	0	C	25	M	0.392	0.667	0	4.5E-06	0	196.161	aggcaaggggCggggacggtc	m5C_42707
chrY	10035599	-	C	92	27	0.293	0	0	T	65	M	0.21	0.393	0	7.2E-14	0	113.517	cgaggggggtgCtgagaggcaa	m5C_42682
chrY	10035669	+	C	126	28	0.222	0	0	T	98	M	0.158	0.302	0	0	0	88.7373	ttagactgttCaaagcaggcc	m5C_42639
chrY	10035679	+	C	151	68	0.45	0	0	T	83	M	0.373	0.53	0	0	0	507.535	caaagcaggcCtgagccacct	m5C_42511
chrY	10035684	+	C	132	27	0.205	0	0	T	105	M	0.145	0.281	0	0	0	78.0597	caggcctgagCcacctagata	m5C_42551
chrY	10035685	+	C	100	28	0.28	0	0	T	72	M	0.201	0.375	0	1.1E-13	0	112.782	aggcctgagcCacctagatat	m5C_42545
chrY	10035775	+	C	1053	294	0.28	2	0.002	T	757	M	0.253	0.308	0	0	0	1490.2	agagggatggCtgggggcatt	m5C_42515
chrY	10035782	+	C	1075	265	0.247	0	0	T	810	M	0.222	0.273	0	0	0	1174.91	tggctgggggCattcgtattg	m5C_42510
chrY	10035786	+	C	1077	319	0.296	0	0	T	758	M	0.27	0.324	0	0	0	1720.61	tgggggcattCgtattgtgcc	m5C_42563
chrY	10035795	+	C	924	186	0.202	3	0.003	T	735	M	0.177	0.229	0	0	0	659.515	tcgtattgtgCcactagaggt	m5C_42598
chrY	10035796	+	C	920	237	0.258	2	0.002	T	681	M	0.231	0.287	0	0	0	1094.51	cgtattgtgcCactagaggtg	m5C_42543
chrY	10035812	+	C	992	231	0.233	0	0	T	761	M	0.208	0.26	0	0	0	959.214	aggtgaaattCttggactggt	m5C_42621
chrY	10035818	+	C	797	200	0.251	0	0	T	597	M	0.222	0.282	0	0	0	888.336	aattcttggaCtggtgcagtt	m5C_42647
chrY	10036071	+	C	190	40	0.211	0	0	T	150	M	0.159	0.274	0	0	0	126.868	aagggcaccaCcaggagtgga	m5C_42604
chrY	10036083	+	C	100	27	0.27	0	0	T	73	M	0.193	0.364	0	1.1E-15	0	104.055	aggagtggagCctgcagctta	m5C_42532
chrY	10036084	+	C	75	17	0.227	0	0	T	58	M	0.147	0.333	0	2.2E-12	0	49.8482	ggagtggagcCtgcagcttaa	m5C_42596
chrY	10036100	+	C	44	19	0.432	0	0	T	25	M	0.297	0.578	0	1E-06	0	112.794	cttaatttgaCtcaatatggg	m5C_42552
chrY	10036102	+	C	48	27	0.562	0	0	C	27	M	0.423	0.693	0	6.7E-06	0	228.284	taatttgactCaatatggaa	m5C_42592
chrY	10036114	+	C	261	66	0.253	0	0	T	195	M	0.204	0.309	0	0	0	269.262	atatgggaaaCctcacccggc	m5C_42531
chrY	10036115	+	C	297	93	0.313	0	0	T	204	M	0.263	0.368	0	0	0	489.285	tatgggaaacCtcacccggcc	m5C_42624
chrY	10036117	+	C	324	85	0.262	0	0	T	239	M	0.217	0.313	0	0	0	369.63	tgggaaacctCacccggcccg	m5C_42641
chrY	10036119	+	C	335	94	0.281	0	0	T	241	M	0.235	0.331	0	0	0	442.139	ggaaacctcaCccggcccgga	m5C_42514
chrY	10036120	+	C	344	87	0.254	2	0.006	T	255	M	0.211	0.303	0	0	0	367.37	gaaacctcacCcggcccggac	m5C_42524
chrY	10036121	+	C	346	130	0.376	0	0	T	216	M	0.326	0.428	0	0	0	848.427	aaacctcaccCggcccggaca	m5C_42633
chrY	10036124	+	C	353	91	0.258	0	0	T	262	M	0.215	0.306	0	0	0	391.187	cctcacccggCccggacatgg	m5C_42588
chrY	10036125	+	C	360	130	0.361	0	0	T	230	M	0.313	0.412	0	0	0	814.321	ctcacccggcCcggacatgga	m5C_42634
chrY	10036126	+	C	376	118	0.314	0	0	T	258	M	0.269	0.362	0	0	0	634.856	tcacccggccCggacatggac	m5C_42635
chrY	10036130	+	C	351	130	0.37	0	0	T	221	M	0.322	0.422	0	0	0	835.922	ccggcccggaCatggacagga	m5C_42521
chrY	10036136	+	C	239	91	0.381	0	0	T	148	M	0.322	0.444	0	0	0	585.198	cggacatggaCaggattgaca	m5C_42629
chrY	10036145	+	C	86	36	0.419	0	0	T	50	M	0.32	0.524	0	7.4E-11	0	230.409	acaggattgaCagattgatag	m5C_42557
chrY	10036260	-	C	106	77	0.726	0	0	C	77	M	0.635	0.802	0	3.6E-10	0	977.517	ttgggggtcgCgtaactagtt	m5C_42724
chrY	10036274	-	C	135	93	0.689	0	0	C	93	M	0.606	0.761	0	7.1E-13	0	1128.05	gggatgctgaCcgcttggggg	m5C_42727
chrY	10036582	+	C	305	148	0.485	0	0	T	157	M	0.43	0.541	0	0	0	1271.88	tttagtgaggCccttggatcg	m5C_42566
chrY	10036583	+	C	300	222	0.74	0	0	C	222	M	0.688	0.786	0	0	0	3052.73	ttagtgaggcCcttggatcgg	m5C_42636
chrY	10036584	+	C	293	187	0.638	0	0	C	187	M	0.582	0.691	0	0	0	2175.72	tagtgaggccCttggatcggc	m5C_42550
chrY	10036591	+	C	287	191	0.666	0	0	C	191	M	0.609	0.718	0	0	0	2326.57	gcccttggatCggccccgccg	m5C_42517
chrY	10036595	+	C	242	159	0.657	0	0	C	159	M	0.595	0.714	0	0	0	1892.67	ttggatcggcCccgccgggtc	m5C_42607
chrY	10037788	+	C	2871	609	0.212	0	0	T	2262	M	0.198	0.227	0	0	0	2406.24	gatcactcggCtcctgcgttg	m5C_42590
chrY	10037843	+	C	7244	1634	0.226	15	0.002	T	5595	M	0.217	0.236	0	0	0	7076.5	aattgcaggaCacattgatca	m5C_42578
chrY	10037849	-	C	37	20	0.541	0	0	C	20	M	0.384	0.69	0	4.3E-05	0	153.535	tgtcgatgatCaatgtgtcct	m5C_42702
chrY	10037852	+	C	5483	1600	0.292	2	0	T	3881	M	0.28	0.304	0	0	0	8960.97	acacattgatCatcgacactt	m5C_42600
chrY	10037855	+	C	4574	996	0.218	0	0	T	3578	M	0.206	0.23	0	0	0	4104.14	cattgatcatCgacacttcga	m5C_42541
chrY	10037856	-	C	65	29	0.446	0	0	T	36	M	0.332	0.567	0	1.3E-08	0	192.382	ttcgaagtgtCgatgatcaat	m5C_42697
chrY	10037858	+	C	3596	832	0.231	0	0	T	2764	M	0.218	0.245	0	0	0	3625.45	tgatcatcgaCacttcgaacg	m5C_42577
chrY	10037860	+	C	2883	843	0.293	3	0.001	T	2037	M	0.276	0.31	0	0	0	4659.69	atcatcgacaCttcgaacgca	m5C_42631
chrY	10037863	+	C	2320	465	0.201	2	0.001	T	1853	M	0.185	0.217	0	0	0	1718.67	atcgacacttCgaacgcactt	m5C_42608
chrY	10037867	+	C	1999	480	0.24	0	0	T	1519	M	0.222	0.259	0	0	0	2130.28	acacttcgaaCgcacttgcgg	m5C_42568
chrY	10037869	+	C	1715	344	0.201	0	0	T	1371	M	0.182	0.22	0	0	0	1254.29	acttcgaacgCacttgcggcc	m5C_42562

chrY	10037871	+	C	1507	372	0.247	1	0.001	T	1134	M	0.226	0.269	0	0	0	1680.64	ttcgaacgcaCttgcggcccg	m5C_42612
chrY	10037878	+	C	741	230	0.31	0	0	T	511	M	0.278	0.345	0	0	0	1279.39	gcacttgcggCccgggttcct	m5C_42575
chrY	10037879	+	C	685	153	0.223	0	0	T	532	M	0.194	0.256	0	0	0	592.902	cacttgcggcCcgggttcctc	m5C_42513
chrY	10037880	+	C	626	147	0.235	1	0.002	T	478	M	0.204	0.27	0	0	0	598.668	acttgcggccCgggttcctcc	m5C_42537
chrY	10037896	+	C	456	92	0.203	3	0.007	T	361	M	0.169	0.243	0	0	0	310.245	cctcccagggCtatgcctgtc	m5C_42645
chrY	10037902	+	C	431	122	0.283	0	0	T	309	M	0.243	0.327	0	0	0	591.929	agggctatgcCtgtctgagct	m5C_42565
chrY	10037906	+	C	433	99	0.229	0	0	T	334	M	0.192	0.27	0	0	0	379.308	ctatgcctgtCtgagctttgc	m5C_42535
chrY	10037911	+	C	403	84	0.208	0	0	T	319	M	0.172	0.251	0	0	0	288.327	cctgtctgagCtttgcttgcc	m5C_42560
chrY	10037916	+	C	294	67	0.228	0	0	T	227	M	0.184	0.279	0	0	0	246.067	ctgagctttgCttgccaatca	m5C_42615
chrY	13208643	-	C	45	9	0.2	0	0	T	36	M	0.109	0.338	2.3E-11	1.2E-08	2.7E-11	10.4289	gtcagctggcCtgagtgctgt	m5C_36422
chrY	13954420	+	C	35	10	0.286	0	0	T	25	M	0.163	0.451	3.4E-14	2E-06	4.2E-14	21.9991	atcttgtggtCcagctgttag	m5C_36420
chrY	16637806	+	C	43	10	0.233	0	0	T	33	M	0.132	0.377	4.3E-13	2.4E-08	5.2E-13	16.2683	ccccacgaagCcggaattgcc	m5C_36493
chrY	16637807	+	C	44	22	0.5	0	0	CT	22	M	0.358	0.642	0	2.3E-06	0	157.659	cccacgaagcCggaattgcca	m5C_36496
chrY	16637815	+	C	39	31	0.795	0	0	C	31	M	0.645	0.892	0	0.00025	0	399.688	gccggaattgCcagtttgtgt	m5C_36499
chrY	20358976	+	C	82	26	0.317	0	0	T	56	M	0.226	0.424	0	2.7E-12	0	117.769	ttatgtttgtCtgtgcgctgc	m5C_36508
chrY	20358986	+	C	75	27	0.36	0	0	T	48	M	0.261	0.473	0	1.9E-10	0	140.75	ctgtgcgctgCccccagaggt	m5C_36516
chrY	28420721	-	C	72	17	0.236	0	0	T	55	M	0.153	0.346	0	2.2E-12	0	52.0084	atttggtgctCtcgctctctg	m5C_36644
chrY	28420723	-	C	70	17	0.243	0	0	T	53	M	0.158	0.355	0	2.2E-12	0	53.5559	ggatttggtgCtctcgctctc	m5C_36640
chrY	28420743	-	C	73	27	0.37	0	0	T	46	M	0.268	0.485	0	1.9E-10	0	144.844	ctctggtggtCtagtggttag	m5C_36641
