# Supplementary material for: Aberrant NSUN2-mediated m5C modification of H19 lncRNA is associated with poor differentiation of hepatocellular carcinoma
Source: Oncogene. 2020 Sep 25;39(45):6906–19. doi: 10.1038/s41388-020-01475-w (PMC7644462; doi:10.1038/s41388-020-01475-w)
Supplement: Supplementary file 5 — Additional file 3B [file 41388_2020_1475_MOESM5_ESM.pdf]

SeqID	refPos	refStran	refBase	cov	C_count	methRa	mut_cou	mutRat	CalledB	CB_cou	state	c95_CI_	c95_CI_	p_value_	p_value_	p_value_	score	seqContext	candidateName
chr1	892380	-	C	36	10	0.278	0	0	T	26	M	0.158	0.44	5E-14	2E-06	5E-14	21.112	gaggatggagCggaggaagga	m5C_65
chr1	2835877	-	C	56	21	0.375	0	0	T	35	M	0.26	0.506	0	5E-08	0	109.24	ggagacggtgCagggaaacag	m5C_666
chr1	3409584	-	C	33	11	0.333	0	0	T	22	M	0.198	0.504	2E-16	3E-06	2E-16	34.007	ggtggtgactCtggacgtgtg	m5C_671
chr1	3409586	-	C	35	21	0.6	0	0	C	21	M	0.436	0.744	0	5E-05	0	183	tcggtggtgaCtctggacgtg	m5C_659
chr1	6673481	-	C	31	12	0.387	0	0	T	19	M	0.237	0.562	0	5E-06	0	56.959	tcggcgggcgCgggggcggga	m5C_519
chr1	8925499	-	C	31	20	0.645	0	0	C	20	M	0.469	0.789	0	4E-05	0	187.79	gctggctacaCtgataaggtg	m5C_1303
chr1	11968263	+	C	49	18	0.367	0	0	T	31	M	0.247	0.507	0	8E-07	0	88.794	ctaaagatttCcgtggagaga	m5C_108
chr1	11968264	+	C	55	16	0.291	0	0	T	39	M	0.188	0.421	0	7E-09	0	60.067	taaagatttcCgtggagagaa	m5C_112
chr1	11968276	+	C	130	60	0.462	0	0	T	70	M	0.378	0.547	0	3E-14	0	453.81	tggagagaaaCgagtgtgagt	m5C_100
chr1	11968287	+	C	82	44	0.537	0	0	C	44	M	0.429	0.64	0	5E-10	0	377.91	gagtgtgagtCtgaaaccaat	m5C_124
chr1	11968293	+	C	65	42	0.646	0	0	C	42	M	0.525	0.751	0	2E-07	0	440.79	gagtctgaaaCcaattttttg	m5C_115
chr1	11968307	+	C	61	36	0.59	0	0	C	36	M	0.465	0.705	0	6E-08	0	334.8	ttttttgaggCcttgcgtttc	m5C_105
chr1	11968312	+	C	68	37	0.544	0	0	C	37	M	0.427	0.657	0	8E-08	0	315.66	tgaggccttgCgtttcttagc	m5C_109
chr1	11968317	+	C	67	37	0.552	0	0	C	37	M	0.434	0.665	0	8E-08	0	320.84	ccttgcgtttCttagcagggc	m5C_113
chr1	11968322	+	C	55	32	0.582	0	0	C	32	M	0.45	0.703	0	7E-07	0	288.21	cgtttcttagCagggcttatt	m5C_107
chr1	11968327	+	C	61	35	0.574	0	0	C	35	M	0.449	0.69	0	5E-08	0	314.27	cttagcagggCttattttaag	m5C_106
chr1	12306131	+	C	38	10	0.263	0	0	T	28	M	0.15	0.42	9E-14	2E-06	1E-13	19.504	tccaggctgaCttgtgtggag	m5C_331
chr1	15755130	+	C	32	11	0.344	0	0	T	21	M	0.204	0.517	2E-16	3E-06	2E-16	35.144	aggaggagatCaaggcagagc	m5C_93
chr1	15755135	+	C	49	12	0.245	0	0	T	37	M	0.146	0.381	2E-15	7E-08	2E-15	25.801	gagatcaaggCagagcaggag	m5C_92
chr1	15755140	+	C	55	11	0.2	0	0	T	44	M	0.116	0.324	3E-13	4E-10	3E-13	15.923	caaggcagagCaggaggaaag	m5C_95
chr1	16134098	-	C	35	16	0.457	0	0	T	19	M	0.305	0.618	0	2E-05	0	97.49	tccggagatcCtgaggaggag	m5C_784
chr1	16134107	-	C	31	17	0.548	0	0	C	17	M	0.378	0.708	0	2E-05	0	128.42	cttaccgaatCcggagatcct	m5C_782
chr1	16861780	-	C	338	82	0.243	0	0	T	256	M	0.2	0.291	0	0	0	327.94	gattcccggtCagggaattgt	m5C_827
chr1	16861784	-	C	337	114	0.338	0	0	T	223	M	0.29	0.39	0	0	0	660.83	gttcgattccCggtcagggaa	m5C_817
chr1	16861785	-	C	326	115	0.353	0	0	T	211	M	0.303	0.406	0	0	0	696.62	ggttcgattcCcggtcaggga	m5C_799
chr1	16861786	-	C	335	119	0.355	0	0	T	216	M	0.306	0.408	0	0	0	728	gggttcgattCccggtcaggg	m5C_800
chr1	16861799	-	C	338	138	0.408	0	0	T	200	M	0.357	0.461	0	0	0	985.87	accgccgcggCccgggttcga	m5C_785
chr1	16861802	-	C	308	97	0.316	1	0.003	T	210	M	0.267	0.37	0	0	0	517.02	ttcaccgccgCggcccgggtt	m5C_816
chr1	16861804	-	C	303	121	0.399	0	0	T	182	M	0.346	0.455	0	0	0	836.8	ctttcaccgcCgcggcccggg	m5C_807
chr1	16861805	-	C	286	92	0.322	0	0	T	194	M	0.27	0.378	0	0	0	497.19	gctttcaccgCcgcggcccgg	m5C_805
chr1	16861807	-	C	258	86	0.333	0	0	T	172	M	0.279	0.393	0	0	0	479.24	gcgctttcacCgccgcggccc	m5C_812
chr1	16861808	-	C	251	72	0.287	0	0	T	179	M	0.234	0.346	0	0	0	337.6	ggcgctttcaCcgccgcggcc	m5C_810
chr1	16861810	-	C	235	68	0.289	0	0	T	167	M	0.235	0.35	0	0	0	319.79	tcggcgctttCaccgccgcgg	m5C_789
chr1	16861814	-	C	199	65	0.327	0	0	T	134	M	0.265	0.395	0	0	0	344.88	ggattcggcgCtttcaccgcc	m5C_811
chr1	16861816	-	C	186	73	0.392	0	0	T	113	M	0.325	0.464	0	0	0	474.72	taggattcggCgctttcaccg	m5C_795
chr1	16861819	-	C	173	56	0.324	0	0	T	117	M	0.258	0.397	0	0	0	289.48	ggctaggattCggcgctttca	m5C_802
chr1	16861834	-	C	104	24	0.231	0	0	T	80	M	0.16	0.32	0	2E-16	0	76.926	ccctggtggtCtagtggctag	m5C_806
chr1	16861842	-	C	32	7	0.219	0	0	T	25	M	0.11	0.388	8E-10	3E-07	9E-10	6.9997	ctgtgtgtccCtggtggtcta	m5C_796
chr1	17004798	-	C	39	8	0.205	0	0	T	31	M	0.108	0.355	2E-10	7E-07	2E-10	8.4438	gcctcccatgCgggagacccg	m5C_820
chr1	17004802	-	C	40	22	0.55	0	0	C	22	M	0.398	0.693	0	6E-05	0	175.25	tctcgcctccCatgcgggaga	m5C_783
chr1	17004806	-	C	81	30	0.37	0	0	T	51	M	0.273	0.479	0	1E-11	0	163.99	gaattctcgcCtcccatgcgg	m5C_804
chr1	17004807	-	C	95	43	0.453	0	0	T	52	M	0.356	0.553	0	1E-11	0	306.44	agaattctcgCctcccatgcg	m5C_825
chr1	17004809	-	C	103	60	0.583	0	0	C	60	M	0.486	0.673	0	2E-11	0	583.2	gtagaattctCgcctcccatg	m5C_818
chr1	17004811	-	C	124	78	0.629	0	0	C	78	M	0.541	0.709	0	1E-12	0	844.47	tggtagaattCtcgcctccca	m5C_792
chr1	17004835	-	C	77	36	0.468	0	0	T	41	M	0.36	0.578	0	2E-09	0	259.42	tccagagaggCgttggtggtt	m5C_788
chr1	17052083	+	C	31	19	0.613	0	0	C	19	M	0.438	0.763	0	3E-05	0	166.53	tagtggttatCatgtttgcct	m5C_709
chr1	17199089	+	C	156	37	0.237	0	0	T	119	M	0.177	0.31	0	0	0	131.17	ccctggtggtCtagtggctag	m5C_729
chr1	17199104	+	C	214	79	0.373	2	0.009	T	133	M	0.31	0.439	0	0	0	490.37	ggctaggattCggcgctttca	m5C_778
chr1	17199107	+	C	229	86	0.376	0	0	T	143	M	0.315	0.44	0	0	0	542.42	taggattcggCgctttcaccg	m5C_680
chr1	17199109	+	C	240	81	0.34	2	0.008	T	157	M	0.283	0.403	0	0	0	458.62	ggattcggcgCtttcaccgcc	m5C_724
chr1	17199113	+	C	265	101	0.381	0	0	T	164	M	0.325	0.441	0	0	0	656	tcggcgctttCaccgccgcgg	m5C_757
chr1	17199115	+	C	277	95	0.343	0	0	T	182	M	0.29	0.401	0	0	0	550.14	ggcgctttcaCcgccgcggcc	m5C_698
chr1	17199116	+	C	284	132	0.465	0	0	T	152	M	0.408	0.523	0	0	0	1076.2	gcgctttcacCgccgcggccc	m5C_712
chr1	17199118	+	C	284	74	0.261	0	0	T	210	M	0.213	0.315	0	0	0	315.16	gctttcaccgCcgcggcccgg	m5C_705
chr1	17199119	+	C	292	107	0.369	2	0.007	T	183	M	0.315	0.426	0	0	0	675.13	ctttcaccgcCgcggcccggg	m5C_727
chr1	17199121	+	C	293	119	0.409	2	0.007	T	172	M	0.354	0.466	0	0	0	842.5	ttcaccgccgCggcccgggtt	m5C_764
chr1	17199124	+	C	295	136	0.472	7	0.024	T	152	M	0.415	0.53	0	0	0	1129.6	accgccgcggCccgggttcga	m5C_696
chr1	17199132	+	C	290	82	0.283	0	0	T	208	M	0.234	0.337	0	0	0	383.81	ggcccgggttCgattcccggc	m5C_776
chr1	17199137	+	C	302	87	0.29	2	0.007	T	213	M	0.242	0.344	0	0	0	420.32	gggttcgattCccggccaggg	m5C_762
chr1	17199138	+	C	299	121	0.407	2	0.007	T	176	M	0.353	0.464	0	0	0	854.39	ggttcgattcCcggccaggga	m5C_694
chr1	17199139	+	C	287	102	0.358	2	0.007	T	183	M	0.304	0.415	0	0	0	621.11	gttcgattccCggccagggaa	m5C_758
chr1	17199143	+	C	288	101	0.353	2	0.007	T	185	M	0.3	0.41	0	0	0	606.07	gattcccggcCagggaattgt	m5C_689
chr1	17439797	-	C	55	17	0.309	0	0	T	38	M	0.203	0.44	0	1E-08	0	68.959	ccctggtggtCtagtggttag	m5C_798
chr1	21012796	+	C	47	17	0.362	0	0	T	30	M	0.24	0.505	0	6E-07	0	81.484	agggagagaaCagggaagaga	m5C_74
chr1	21103142	-	C	42	16	0.381	0	0	T	26	M	0.25	0.532	0	4E-07	0	79.998	aaagggggaaCctgagaatga	m5C_86
chr1	25554662	-	C	33	15	0.455	0	0	T	18	M	0.298	0.62	0	1E-05	0	89.528	gcagaagatgCagaaagatgg	m5C_175
chr1	28358600	-	C	47	30	0.638	0	0	C	30	M	0.495	0.76	0	1E-05	0	297.21	ggctgtagtgCgctatgctga	m5C_2374
chr1	28358608	-	C	42	29	0.69	0	0	C	29	M	0.54	0.809	0	1E-05	0	313.05	gagtcctgggCtgtagtgcgc	m5C_2375
chr1	28358613	-	C	38	29	0.763	0	0	C	29	M	0.608	0.87	0	0.0002	0	352.6	cccaggagtcCtgggctgtag	m5C_2343
chr1	28833879	+	C	34	12	0.353	0	0	T	22	M	0.215	0.521	0	5E-06	0	51.571	actgattgtcCaacgtggata	m5C_2288
chr1	28833882	+	C	47	33	0.702	0	0	C	33	M	0.56	0.813	0	2E-05	0	369.75	gattgtccaaCgtggatacac	m5C_2292
chr1	28833892	+	C	77	36	0.468	0	0	T	41	M	0.36	0.578	0	2E-09	0	259.42	cgtggatacaCccgggaggtc	m5C_2306
chr1	28833893	+	C	73	40	0.548	0	0	C	40	M	0.434	0.657	0	6E-09	0	347.39	gtggatacacCcgggaggtca	m5C_2291
chr1	28833902	+	C	45	14	0.311	0	0	T	31	M	0.195	0.457	0	2E-07	0	54.68	cccgggaggtCactctccccg	m5C_2293
chr1	28834048	+	C	45	10	0.222	0	0	T	35	M	0.125	0.363	7E-13	2E-08	8E-13	15.216	gtcctgtcgaCaattactggg	m5C_2266
chr1	28834054	+	C	81	21	0.259	0	0	T	60	M	0.176	0.364	0	3E-13	0	74.035	tcgacaattaCtggggagaca	m5C_2297
chr1	28834063	+	C	131	29	0.221	0	0	T	102	M	0.159	0.3	0	0	0	92.097	actggggagaCaaaccatgca	m5C_2283
chr1	28834068	+	C	145	39	0.269	0	0	T	106	M	0.203	0.346	0	0	0	158.68	ggagacaaacCatgcaggaaa	m5C_2274
chr1	28834082	+	C	102	42	0.412	0	0	T	60	M	0.321	0.509	0	3E-13	0	269.77	caggaaacagCcttctagagc	m5C_2304
chr1	28834083	+	C	93	25	0.269	0	0	T	68	M	0.189	0.367	0	3E-14	0	94.606	aggaaacagcCttctagagca	m5C_2287
chr1	28835075	+	C	42	18	0.429	0	0	T	24	M	0.291	0.578	0	8E-07	0	104.82	agcattccaaCgtggataccc	m5C_2271
chr1	28835083	+	C	79	26	0.329	0	0	T	53	M	0.236	0.439	0	1E-10	0	122.48	aacgtggataCcctgggaggt	m5C_2278
chr1	28835245	+	C	54	19	0.352	0	0	T	35	M	0.238	0.485	0	2E-08	0	90.527	tggacaatgaCtggggagaca	m5C_2299
chr1	28835258	+	C	77	46	0.597	0	0	C	46	M	0.486	0.7	0	2E-08	0	446.91	gggagacaaaCcatgcaggaa	m5C_2270
chr1	28905265	-	C	38	9	0.237	0	0	T	29	M	0.13	0.392	4E-12	1E-06	4E-12	13.374	ggactgagacCagctcctagg	m5C_2340
chr1	28905266	-	C	42	31	0.738	0	0	C	31	M	0.589	0.847	0	1E-05	0	365.37	gggactgagaCcagctcctag	m5C_2333
chr1	28905272	-	C	77	23	0.299	0	0	T	54	M	0.208	0.408	0	4E-11	0	95.697	ggatatgggaCtgagaccagc	m5C_2321
chr1	28905283	-	C	97	27	0.278	0	0	T	70	M	0.199	0.375	0	7E-14	0	107.41	atgagaaatgCggatatggga	m5C_2338
chr1	28905298	-	C	81	17	0.21	0	0	T	64	M	0.135	0.311	0	2E-14	0	46.026	agtggacatcCatggatgaga	m5C_2350
chr1	28905302	-	C	85	17	0.2	0	0	T	68	M	0.129	0.297	0	2E-14	0	43.788	tttgagtggaCatccatggat	m5C_2379
chr1	28905316	-	C	89	58	0.652	0	0	C	58	M	0.548	0.743	0	7E-09	0	636.02	aggatgaaacCtaatttgagt	m5C_2356

chr1	28906280	-	C	87	18	0.207	0	0	T	69	M	0.135	0.304	0	4E-14	0	48.626	tagtttcagaCaggtttcaga	m5C_2369
chr1	28906284	-	C	100	40	0.4	0	0	T	60	M	0.309	0.498	0	6E-12	0	247.52	gggctagtttCagacaggttt	m5C_2317
chr1	28906291	-	C	104	30	0.288	0	0	T	74	M	0.21	0.382	0	5E-15	0	126.09	ggcatgtgggCtagtttcaga	m5C_2325
chr1	28906299	-	C	76	23	0.303	0	0	T	53	M	0.211	0.413	0	4E-11	0	97.019	tggtatatggCatgtgggcta	m5C_2355
chr1	28906996	-	C	43	14	0.326	0	0	T	29	M	0.205	0.475	0	2E-07	0	57.381	tgtggctggtCatagccatgg	m5C_2372
chr1	28907007	-	C	69	27	0.391	0	0	T	42	M	0.285	0.509	0	7E-09	0	153.8	tttccaagggCtgtggctggt	m5C_2342
chr1	28907014	-	C	72	28	0.389	0	0	T	44	M	0.285	0.504	0	3E-10	0	159.41	cagcatgtttCcaagggctgt	m5C_2328
chr1	28975167	+	C	41	9	0.22	0	0	T	32	M	0.12	0.367	8E-12	1E-08	9E-12	11.962	gctctgcgtgCggaatcgaca	m5C_2289
chr1	28975173	+	C	41	11	0.268	0	0	T	30	M	0.157	0.419	6E-15	4E-08	7E-15	24.554	cgtgcggaatCgacatcaaga	m5C_2302
chr1	28975234	+	C	38	18	0.474	0	0	T	20	M	0.325	0.627	0	3E-05	0	116.92	gatcgttggtCccggcgccct	m5C_2273
chr1	28975235	+	C	34	16	0.471	0	0	T	18	M	0.315	0.633	0	2E-05	0	100.64	atcgttggtcCcggcgccctt	m5C_2282
chr1	28975236	+	C	35	8	0.229	0	0	T	27	M	0.121	0.39	6E-11	7E-07	6E-11	9.8969	tcgttggtccCggcgcccttt	m5C_2303
chr1	28975239	+	C	37	15	0.405	0	0	T	22	M	0.263	0.565	0	1E-05	0	79.039	ttggtcccggCgccctttctt	m5C_2290
chr1	29793170	+	C	61	25	0.41	0	0	T	36	M	0.295	0.535	0	4E-09	0	147.68	gttcaggacaCtgttgacaac	m5C_2311
chr1	35650115	-	C	32	12	0.375	0	0	T	20	M	0.229	0.547	0	5E-06	0	55.041	ggaactccagCaggatatggt	m5C_552
chr1	35653642	-	C	49	19	0.388	0	0	T	30	M	0.264	0.528	0	1E-06	0	100.43	gattcgtcaaCgtgagatgga	m5C_570
chr1	35653675	-	C	114	29	0.254	0	0	T	85	M	0.183	0.341	0	0	0	106.34	ggaggaacgaCgtagaagaga	m5C_569
chr1	35653690	-	C	75	23	0.307	0	0	T	52	M	0.214	0.418	0	4E-11	0	98.379	tatttctaggCaagaggagga	m5C_576
chr1	38435347	-	C	95	19	0.2	0	0	T	76	M	0.132	0.291	0	9E-16	0	50.139	aggagaagagCgagaagaaga	m5C_629
chr1	38435364	-	C	80	20	0.25	0	0	T	60	M	0.168	0.355	0	2E-13	0	67.224	cgcaagcaagCcaggacagga	m5C_616
chr1	38435368	-	C	78	20	0.256	0	0	T	58	M	0.173	0.363	0	1E-11	0	69.021	acagcgcaagCaagccaggac	m5C_628
chr1	38435372	-	C	63	16	0.254	0	0	T	47	M	0.163	0.373	0	1E-10	0	52.097	atgtacagcgCaagcaagcca	m5C_614
chr1	38435374	-	C	56	19	0.339	0	0	T	37	M	0.229	0.47	0	2E-08	0	87.095	aaatgtacagCgcaagcaagc	m5C_619
chr1	40538448	-	C	33	10	0.303	0	0	T	23	M	0.174	0.473	2E-14	2E-06	2E-14	23.982	ggattaagagCaagaagttgg	m5C_435
chr1	41270699	+	C	74	26	0.351	0	0	T	48	M	0.252	0.465	0	1E-10	0	131.24	gaggggtgaaCtggcccaggt	m5C_411
chr1	41270705	+	C	71	23	0.324	0	0	T	48	M	0.227	0.439	0	4E-11	0	104.22	tgaactggccCaggtcagaaa	m5C_423
chr1	43162445	+	C	34	10	0.294	0	0	T	24	M	0.168	0.462	2E-14	2E-06	3E-14	22.956	gcaaaattacCagaatagtga	m5C_444
chr1	43162471	+	C	101	27	0.267	0	0	T	74	M	0.191	0.361	0	1E-15	0	102.98	gggaaaagaaCgagggatcgg	m5C_443
chr1	45241548	+	C	289	65	0.225	0	0	T	224	M	0.181	0.276	0	0	0	234.73	tgtatgatgaCaactcggtaa	m5C_1445
chr1	45241551	+	C	295	76	0.259	1	0.003	T	218	M	0.212	0.311	0	0	0	321.93	atgatgacaaCtcggtaatgc	m5C_1449
chr1	45241553	+	C	304	81	0.266	0	0	T	223	M	0.22	0.319	0	0	0	356.22	gatgacaactCggtaatgctg	m5C_1456
chr1	45241561	+	C	288	67	0.234	2	0.007	T	219	M	0.189	0.287	0	0	0	253.13	ctcggtaatgCtgcatactcc	m5C_1447
chr1	45241564	+	C	292	81	0.277	0	0	T	211	M	0.229	0.331	0	0	0	371.28	ggtaatgctgCatactcccga	m5C_1442
chr1	45241570	+	C	300	81	0.27	0	0	T	219	M	0.223	0.323	0	0	0	361.1	gctgcatactCccgagtgcgc	m5C_1429
chr1	45241578	+	C	296	78	0.265	2	0.007	T	216	M	0.218	0.319	0	0	0	340.24	ctcccgagtgCgcggtgggga	m5C_1468
chr1	45241592	+	C	328	86	0.262	0	0	T	242	M	0.218	0.312	0	0	0	374.18	gtggggaagcCaaccttggag	m5C_1446
chr1	45241595	+	C	339	74	0.218	0	0	T	265	M	0.178	0.265	0	0	0	262.86	gggaagccaaCcttggagagc	m5C_1427
chr1	45241596	+	C	346	123	0.355	0	0	T	223	M	0.307	0.407	0	0	0	754.96	ggaagccaacCttggagagct	m5C_1423
chr1	45241605	+	C	276	63	0.228	0	0	T	213	M	0.183	0.281	0	0	0	230.17	ccttggagagCtgagcgtgcg	m5C_1452
chr1	45241610	+	C	169	55	0.327	1	0.006	T	113	M	0.261	0.402	0	0	0	287.07	gagagctgagCgtgcgaccgg	m5C_1463
chr1	45242184	+	C	91	44	0.484	0	0	T	47	M	0.384	0.585	0	2E-11	0	337.57	gaaaaagaatCcttaggcgtg	m5C_1424
chr1	45242185	+	C	81	39	0.481	0	0	T	42	M	0.376	0.589	0	2E-10	0	293.28	aaaaagaatcCttaggcgtgg	m5C_1425
chr1	45242191	+	C	54	43	0.796	0	0	C	43	M	0.671	0.882	0	4E-06	0	577.04	aatccttaggCgtggttgtgg	m5C_1464
chr1	45242202	+	C	58	42	0.724	0	0	C	42	M	0.598	0.822	0	4E-06	0	502.28	gtggttgtggCcgtcttggtc	m5C_1469
chr1	45242203	+	C	55	36	0.655	0	0	C	36	M	0.523	0.766	0	2E-06	0	376.23	tggttgtggcCgtcttggtca	m5C_1436
chr1	45242206	+	C	55	38	0.691	0	0	C	38	M	0.56	0.797	0	2E-06	0	425.38	ttgtggccgtCttggtcacct	m5C_1444
chr1	45242212	+	C	52	39	0.75	0	0	C	39	M	0.618	0.848	0	2E-06	0	481.99	ccgtcttggtCacctgtgtgc	m5C_1434
chr1	45242214	+	C	54	35	0.648	0	0	C	35	M	0.515	0.762	0	1E-06	0	360.39	gtcttggtcaCctgtgtgcca	m5C_1430
chr1	45242215	+	C	51	33	0.647	0	0	C	33	M	0.51	0.764	0	9E-07	0	336.51	tcttggtcacCtgtgtgccac	m5C_1461
chr1	45242222	+	C	53	37	0.698	0	0	C	37	M	0.565	0.805	0	2E-06	0	417.83	cacctgtgtgCcacttgccaa	m5C_1462
chr1	45242223	+	C	56	35	0.625	0	0	C	35	M	0.494	0.74	0	1E-06	0	345.84	acctgtgtgcCacttgccaat	m5C_1476
chr1	45242225	+	C	53	31	0.585	0	0	C	31	M	0.451	0.707	0	6E-07	0	279.58	ctgtgtgccaCttgccaatgc	m5C_1450
chr1	45242229	+	C	55	32	0.582	0	0	C	32	M	0.45	0.703	0	7E-07	0	288.21	gtgccacttgCcaatgcaagg	m5C_1470
chr1	45242230	+	C	53	32	0.604	0	0	C	32	M	0.469	0.724	0	7E-07	0	300.42	tgccacttgcCaatgcaagga	m5C_1451
chr1	45242235	+	C	54	33	0.611	0	0	C	33	M	0.478	0.73	0	9E-07	0	315.4	cttgccaatgCaaggacttgt	m5C_1433
chr1	45242241	+	C	52	35	0.673	0	0	C	35	M	0.538	0.785	0	1E-06	0	376.29	aatgcaaggaCttgtcatagt	m5C_1474
chr1	45242246	+	C	50	34	0.68	0	0	C	34	M	0.542	0.792	0	2E-05	0	368.49	aaggacttgtCatagttacac	m5C_1477
chr1	45242254	+	C	48	27	0.562	0	0	C	27	M	0.423	0.693	0	7E-06	0	228.28	gtcatagttaCactgactgtt	m5C_1480
chr1	45242256	+	C	48	29	0.604	0	0	C	29	M	0.463	0.73	0	1E-05	0	268.6	catagttacaCtgactgttgc	m5C_1458
chr1	45242260	+	C	52	35	0.673	0	0	C	35	M	0.538	0.785	0	1E-06	0	376.29	gttacactgaCtgttgcctcc	m5C_1432
chr1	45243516	+	C	52	17	0.327	0	0	T	35	M	0.215	0.462	0	1E-08	0	73.174	ctccagccttCtcgtgatgaa	m5C_1438
chr1	45243518	+	C	52	21	0.404	0	0	T	31	M	0.282	0.539	0	5E-08	0	118.27	ccagccttctCgtgatgaaaa	m5C_1440
chr1	45243529	+	C	103	43	0.417	0	0	T	60	M	0.327	0.514	0	4E-13	0	281.12	gtgatgaaaaCtctgtccagt	m5C_1453
chr1	45243531	+	C	106	33	0.311	0	0	T	73	M	0.231	0.405	0	2E-14	0	152.51	gatgaaaactCtgtccagttc	m5C_1439
chr1	45243535	+	C	108	34	0.315	0	0	T	74	M	0.235	0.407	0	2E-14	0	159.71	aaaactctgtCcagttctgct	m5C_1479
chr1	45243536	+	C	114	59	0.518	0	0	C	59	M	0.427	0.607	0	7E-13	0	503.56	aaactctgtcCagttctgcta	m5C_1428
chr1	45243541	+	C	105	32	0.305	0	0	T	73	M	0.225	0.398	0	1E-14	0	143.94	ctgtccagttCtgctactgaa	m5C_1467
chr1	45243544	+	C	107	80	0.755	1	0.009	C	80	M	0.665	0.827	0	6E-10	0	1063.8	tccagttctgCtactgaaggg	m5C_1441
chr1	45243547	+	C	107	33	0.308	0	0	T	74	M	0.229	0.401	0	2E-14	0	151.02	agttctgctaCtgaagggaga	m5C_1426
chr1	45243568	+	C	71	21	0.296	0	0	T	50	M	0.202	0.41	0	2E-11	0	84.978	gagatgagagCcttttaggct	m5C_1471
chr1	45243569	+	C	70	25	0.357	0	0	T	45	M	0.255	0.474	0	4E-09	0	127.52	agatgagagcCttttaggctg	m5C_1460
chr1	45243577	+	C	57	41	0.719	0	0	C	41	M	0.592	0.819	0	3E-06	0	485.19	gccttttaggCtgaggaaggc	m5C_1459
chr1	45244077	+	C	52	15	0.288	0	0	T	37	M	0.183	0.423	0	5E-09	0	54.989	gtgatgaaaaCtttgtccagt	m5C_1472
chr1	45244083	+	C	49	13	0.265	0	0	T	36	M	0.162	0.403	0	1E-07	0	42.149	aaaactttgtCcagttctgct	m5C_1455
chr1	45244084	+	C	51	13	0.255	0	0	T	38	M	0.155	0.389	1E-16	2E-09	1E-16	32.243	aaactttgtcCagttctgcta	m5C_1473
chr1	45244089	+	C	49	14	0.286	0	0	T	35	M	0.178	0.424	0	2E-07	0	49.978	ttgtccagttCtgctactgac	m5C_1435
chr1	45244122	+	C	70	40	0.571	0	0	C	40	M	0.455	0.681	0	6E-09	0	363.82	taaagtgtgtCtgaggagaca	m5C_1437
chr1	52306043	-	C	34	12	0.353	0	0	T	22	M	0.215	0.521	0	5E-06	0	51.571	gattctggagCtgaaatagaa	m5C_1048
chr1	65330598	-	C	39	14	0.359	0	0	T	25	M	0.227	0.516	0	9E-06	0	63.677	ggaaaataaaCacaagaagga	m5C_899
chr1	76253577	+	C	45	15	0.333	0	0	T	30	M	0.214	0.479	0	3E-07	0	64.076	ctttcaaggtCaatgatgtgt	m5C_1091
chr1	76253591	+	C	117	30	0.256	0	0	T	87	M	0.186	0.342	0	0	0	111.55	gatgtgttggCatgtattatc	m5C_1094
chr1	76253601	+	C	116	52	0.448	0	0	T	64	M	0.361	0.539	0	1E-13	0	375.31	catgtattatCtgaatctatt	m5C_1084
chr1	76253613	+	C	79	24	0.304	0	0	T	55	M	0.213	0.412	0	6E-11	0	102.45	gaatctattgCtgatgtgtaa	m5C_1083
chr1	76253646	+	C	55	16	0.291	0	0	T	39	M	0.188	0.421	0	7E-09	0	60.067	tctagaattaCtctgagacct	m5C_1079
chr1	91852821	-	C	565	222	0.393	0	0	T	343	M	0.354	0.434	0	0	0	1569.5	attcaccaagCgttggattgt	m5C_4083
chr1	91852984	-	C	59	43	0.729	0	0	C	43	M	0.604	0.826	0	4E-06	0	519.46	accgtgaaagCggggcctcac	m5C_4045
chr1	91852993	-	C	48	28	0.583	0	0	C	28	M	0.443	0.712	0	8E-06	0	247.97	cgaatacagaCcgtgaaagcg	m5C_4043
chr1	91852997	-	C	66	42	0.636	0	0	C	42	M	0.516	0.742	0	2E-07	0	433.27	agtacgaataCagaccgtgaa	m5C_4051
chr1	91853017	-	C	220	78	0.355	0	0	T	142	M	0.294	0.42	0	0	0	459.14	ctcgcttgatCttgattttca	m5C_4027
chr1	91853023	-	C	246	105	0.427	0	0	T	141	M	0.367	0.489	0	0	0	769.88	caaaagctcgCttgatcttga	m5C_4022
chr1	91853033	-	C	144	90	0.625	0	0	C	90	M	0.544	0.7	0	2E-14	0	978.56	agcagaagggCaaaagctcgc	m5C_4046
chr1	91853041	-	C	80	62	0.775	0	0	C	62	M	0.672	0.853	0	1E-08	0	833.44	tcccgtggagCagaagggcaa	m5C_4037

chr1	91853088	-	C	111	77	0.694	0	0	C	77	M	0.603	0.772	0	2E-11	0	928.09	aacggtaacgCaggtgtccta	m5C_4018
chr1	91853090	-	C	92	72	0.783	0	0	C	72	M	0.688	0.855	0	3E-09	0	990.62	caaacggtaaCgcaggtgtcc	m5C_4032
chr1	93302861	+	C	55	16	0.291	0	0	T	39	M	0.188	0.421	0	7E-09	0	60.067	atgatgatatCccactaactg	m5C_1364
chr1	93302863	+	C	58	12	0.207	0	0	T	46	M	0.123	0.328	2E-14	9E-10	2E-14	20.116	gatgatatccCactaactgag	m5C_1368
chr1	93302865	+	C	56	14	0.25	0	0	T	42	M	0.155	0.377	0	3E-09	0	43.447	tgatatcccaCtaactgagca	m5C_1369
chr1	93302869	+	C	57	15	0.263	0	0	T	42	M	0.166	0.39	0	5E-09	0	49.937	atcccactaaCtgagcagtca	m5C_1371
chr1	93302874	+	C	58	13	0.224	0	0	T	45	M	0.136	0.347	7E-16	2E-09	7E-16	26.819	actaactgagCagtcagtagt	m5C_1352
chr1	93302878	+	C	58	12	0.207	0	0	T	46	M	0.123	0.328	2E-14	9E-10	2E-14	20.116	actgagcagtCagtagttggt	m5C_1359
chr1	93302899	+	C	58	25	0.431	0	0	T	33	M	0.312	0.559	0	2E-07	0	155.91	cctttggttgCatatgatgcg	m5C_1355
chr1	93302908	+	C	64	49	0.766	0	0	C	49	M	0.649	0.853	0	6E-07	0	635.69	gcatatgatgCgataattgtt	m5C_1367
chr1	93302920	+	C	76	16	0.211	0	0	T	60	M	0.134	0.315	0	1E-12	0	42.864	ataattgtttCaagacgggac	m5C_1360
chr1	93302925	+	C	80	23	0.287	0	0	T	57	M	0.2	0.395	0	7E-13	0	91.939	tgtttcaagaCgggactgatg	m5C_1361
chr1	93302930	+	C	75	53	0.707	0	0	C	53	M	0.596	0.798	0	6E-08	0	631.33	caagacgggaCtgatggcagc	m5C_1353
chr1	93306289	+	C	31	11	0.355	0	0	T	20	M	0.211	0.531	1E-16	3E-06	1E-16	37.059	caaactcgatCactagctctg	m5C_1363
chr1	93306310	+	C	32	12	0.375	0	0	T	20	M	0.229	0.547	0	5E-06	0	55.041	cgtgatgtggCagaagcgaag	m5C_1372
chr1	93981875	-	C	38	17	0.447	0	0	T	21	M	0.301	0.603	0	2E-05	0	102.5	gagcatttgaCtgcagatcaa	m5C_1373
chr1	93981894	-	C	51	37	0.725	0	0	C	37	M	0.591	0.829	0	2E-06	0	436.99	gggtatagctCaggtggtaga	m5C_1382
chr1	93981896	-	C	54	23	0.426	0	0	T	31	M	0.303	0.558	0	9E-08	0	139.53	gggggtatagCtcaggtggta	m5C_1383
chr1	94058374	-	C	46	15	0.326	0	0	T	31	M	0.209	0.47	0	3E-07	0	62.597	ctttgggcctCactgcccaga	m5C_1205
chr1	108113330	-	C	34	17	0.5	0	0	CT	17	M	0.341	0.659	0	2E-05	0	115.83	tccggagtggCggagatggac	m5C_1745
chr1	108113484	-	C	31	20	0.645	0	0	C	20	M	0.469	0.789	0	4E-05	0	187.79	cttcaaaggcCgaagtggaga	m5C_1715
chr1	108113565	-	C	136	34	0.25	0	0	T	102	M	0.185	0.329	0	0	0	125.64	gtcttgaagcCcagggctggg	m5C_1743
chr1	108113566	-	C	151	84	0.556	0	0	C	84	M	0.477	0.633	0	3E-16	0	800.73	agtcttgaagCccagggctgg	m5C_1733
chr1	108113573	-	C	163	76	0.466	0	0	T	87	M	0.391	0.543	0	0	0	594.83	tgcggtgagtCttgaagccca	m5C_1711
chr1	108113581	-	C	172	62	0.36	0	0	T	110	M	0.292	0.435	0	0	0	362.68	agggccgctgCggtgagtctt	m5C_1748
chr1	108113584	-	C	159	68	0.428	0	0	T	91	M	0.353	0.505	0	0	0	480.58	aggagggccgCtgcggtgagt	m5C_1718
chr1	108113586	-	C	144	43	0.299	0	0	T	101	M	0.23	0.378	0	0	0	197.7	gtaggagggcCgctgcggtga	m5C_1737
chr1	108113587	-	C	122	56	0.459	0	0	T	66	M	0.373	0.547	0	1E-14	0	417.98	agtaggagggCcgctgcggtg	m5C_1742
chr1	109642907	+	C	48	21	0.438	0	0	T	27	M	0.307	0.577	0	2E-06	0	128.94	tgtgagtggaCgcgtgagtgt	m5C_1695
chr1	111991798	-	C	31	14	0.452	0	0	T	17	M	0.292	0.622	0	9E-06	0	81.652	gagttggaacCgtggagatgc	m5C_1286
chr1	120543995	-	C	126	93	0.738	0	0	C	93	M	0.655	0.807	0	1E-11	0	1218.5	cgtagcagtcCtgatgtgcaa	m5C_1585
chr1	120543996	-	C	124	61	0.492	0	0	T	63	M	0.406	0.579	0	4E-14	0	494.73	ccgtagcagtCctgatgtgca	m5C_1601
chr1	120544000	-	C	225	168	0.747	0	0	C	168	M	0.686	0.799	0	0	0	2305	aggtccgtagCagtcctgatg	m5C_1592
chr1	120544006	-	C	235	91	0.387	0	0	T	144	M	0.327	0.451	0	0	0	595.58	tggtggaggtCcgtagcagtc	m5C_1588
chr1	120544019	-	C	181	138	0.762	0	0	C	138	M	0.695	0.819	0	3E-16	0	1919.2	ccagaggaaaCtctggtggag	m5C_1580
chr1	145277346	+	C	59	47	0.797	0	0	C	47	M	0.677	0.88	0	8E-06	0	636.68	cagggcaaagCcagaggaaac	m5C_1846
chr1	145277347	+	C	72	54	0.75	0	0	C	54	M	0.639	0.836	0	7E-08	0	690.19	agggcaaagcCagaggaaact	m5C_1854
chr1	145277356	+	C	98	63	0.643	0	0	C	63	M	0.544	0.731	0	8E-10	0	685.76	ccagaggaaaCtctggtggag	m5C_1850
chr1	145277358	+	C	101	66	0.653	0	0	C	66	M	0.557	0.739	0	6E-11	0	734.68	agaggaaactCtggtggaggt	m5C_1840
chr1	145277370	+	C	96	62	0.646	0	0	C	62	M	0.546	0.734	0	7E-10	0	677.34	ggtggaggtcCgtagcagtcc	m5C_1856
chr1	145277375	+	C	99	63	0.636	0	0	C	63	M	0.538	0.724	0	8E-10	0	678.08	aggtccgtagCagtcctgatg	m5C_1849
chr1	145277379	+	C	94	25	0.266	0	0	T	69	M	0.187	0.363	0	3E-14	0	93.557	ccgtagcagtCctgatgtgca	m5C_1838
chr1	145277418	+	C	109	87	0.798	0	0	C	87	M	0.713	0.863	0	1E-09	0	1241.1	ggtgtaggggCgaaagactaa	m5C_1852
chr1	145277434	+	C	108	84	0.778	0	0	C	84	M	0.691	0.846	0	9E-10	0	1160.2	actaattgaaCcatctagtag	m5C_1842
chr1	145277435	+	C	51	40	0.784	0	0	C	40	M	0.654	0.875	0	3E-06	0	522.99	ctaattgaacCatctagtagc	m5C_1843
chr1	145395523	-	C	51	15	0.294	0	0	T	36	M	0.187	0.43	0	5E-09	0	56.126	ccacgttgggCgctgtgtttt	m5C_1919
chr1	145395530	-	C	60	18	0.3	0	0	T	42	M	0.199	0.425	0	3E-10	0	71.633	tcgagccccaCgttgggcgct	m5C_1901
chr1	145395533	-	C	62	17	0.274	0	0	T	45	M	0.179	0.396	0	2E-10	0	60.795	ggttcgagccCcacgttgggc	m5C_1860
chr1	145395535	-	C	61	18	0.295	0	0	T	43	M	0.196	0.419	0	3E-10	0	70.398	tgggttcgagCcccacgttgg	m5C_1869
chr1	145395539	-	C	63	29	0.46	0	0	T	34	M	0.343	0.582	0	1E-08	0	198.99	gtcgtgggttCgagccccacg	m5C_1912
chr1	145395547	-	C	58	35	0.603	0	0	C	35	M	0.475	0.719	0	1E-06	0	332.45	atctcagggtCgtgggttcga	m5C_1878
chr1	145395555	-	C	36	28	0.778	0	0	C	28	M	0.619	0.883	0	0.0002	0	346.72	gactcttaatCtcagggtcgt	m5C_1900
chr1	145457073	-	C	31	13	0.419	0	0	T	18	M	0.264	0.592	0	7E-06	0	68.68	gaagtaacttCagaggaggat	m5C_1875
chr1	146556212	-	C	74	47	0.635	0	0	C	47	M	0.521	0.736	0	2E-08	0	490.04	gtgggaagctCgactgcataa	m5C_1545
chr1	149194111	-	C	39	13	0.333	0	0	T	26	M	0.206	0.49	0	7E-06	0	53.645	ttcgcgctttCccctgaattt	m5C_2058
chr1	149194115	-	C	47	18	0.4	2	0.043	T	27	M	0.27	0.545	0	8E-07	0	97.289	tgcgttcgcgCtttcccctga	m5C_2049
chr1	149194117	-	C	44	9	0.205	0	0	T	35	M	0.112	0.345	2E-11	1E-08	2E-11	10.781	actgcgttcgCgctttcccct	m5C_2041
chr1	149194126	-	C	51	21	0.412	0	0	T	30	M	0.288	0.548	0	5E-08	0	120.77	tagtggggaCtgcgttcgcg	m5C_2042
chr1	150193024	-	C	42	11	0.262	0	0	T	31	M	0.153	0.411	8E-15	4E-08	9E-15	23.69	aaacgagatgCtgaagacgat	m5C_1963
chr1	150193031	-	C	46	12	0.261	0	0	T	34	M	0.156	0.403	7E-16	7E-08	7E-16	28.412	gaagaggaaaCgagatgctga	m5C_1962
chr1	150199001	-	C	37	18	0.486	0	0	T	19	M	0.334	0.641	0	3E-05	0	120.41	gaagcaggttCagagttggga	m5C_1951
chr1	150199007	-	C	37	20	0.541	0	0	C	20	M	0.384	0.69	0	4E-05	0	153.54	gaagatgaagCaggttcagag	m5C_1972
chr1	150199079	-	C	89	49	0.551	0	0	C	49	M	0.447	0.65	0	1E-09	0	438.32	gctggtccacCggaaggatat	m5C_1958
chr1	150199082	-	C	94	38	0.404	0	0	T	56	M	0.311	0.505	0	4E-12	0	236.13	gaagctggtcCaccggaagga	m5C_1954
chr1	150199083	-	C	89	18	0.202	0	0	T	71	M	0.132	0.297	0	4E-14	0	47.497	tgaagctggtCcaccggaagg	m5C_1961
chr1	150199123	-	C	74	34	0.63	20	0.27	C	34	M	0.496	0.746	0	1E-06	0	337.47	ttaaagatggCgatgaagatg	m5C_1950
chr1	153591391	-	C	54	11	0.204	0	0	T	43	M	0.118	0.329	2E-13	4E-10	3E-13	16.362	gaagaaagacCtgaagatcag	m5C_1769
chr1	153591392	-	C	57	36	0.632	0	0	C	36	M	0.502	0.745	0	2E-06	0	361.28	agaagaaagaCctgaagatca	m5C_1768
chr1	153591417	-	C	37	19	0.514	0	0	C	19	M	0.359	0.666	0	3E-05	0	136.4	ggggagctggCcaaggaaatc	m5C_1764
chr1	153591421	-	C	33	23	0.697	0	0	C	23	M	0.527	0.826	0	8E-05	0	242.24	gattggggagCtggccaagga	m5C_1771
chr1	153634963	-	C	40	22	0.55	0	0	C	22	M	0.398	0.693	0	6E-05	0	175.25	tatgagaagcCaccagagaag	m5C_1763
chr1	153634984	-	C	41	18	0.439	0	0	T	23	M	0.299	0.59	0	8E-07	0	107.6	gtaacaccttCagaaaaggct	m5C_1766
chr1	155889719	-	C	89	39	0.438	0	0	T	50	M	0.34	0.542	0	2E-10	0	265.04	gctgtggacaCtgaaggggt	m5C_2134
chr1	155895839	-	C	42	19	0.452	0	0	T	23	M	0.312	0.601	0	1E-06	0	118.65	tgaagtaagaCtttgctgata	m5C_2124
chr1	155895853	-	C	82	34	0.415	0	0	T	48	M	0.314	0.523	0	4E-11	0	213.63	aaggctgagtCtgatgaagta	m5C_2149
chr1	155895859	-	C	84	20	0.241	1	0.012	T	63	M	0.162	0.343	0	2E-13	0	64.698	actaagaaggCtgagtctgat	m5C_2135
chr1	155895868	-	C	83	28	0.337	0	0	T	55	M	0.245	0.444	0	6E-12	0	137.11	aactggaggaCtaagaaggct	m5C_2159
chr1	155895876	-	C	34	11	0.324	0	0	T	23	M	0.191	0.492	4E-16	3E-06	5E-16	32.309	ttgatgacaaCtggaggacta	m5C_2126
chr1	156713636	-	C	51	21	0.412	0	0	T	30	M	0.288	0.548	0	5E-08	0	120.77	gcagaaaaccCtgaaggagag	m5C_1825
chr1	156713637	-	C	50	28	0.571	1	0.02	C	28	M	0.433	0.7	0	8E-06	0	242.31	ggcagaaaacCctgaaggaga	m5C_1827
chr1	156713638	-	C	53	21	0.396	0	0	T	32	M	0.276	0.531	0	5E-08	0	115.87	aggcagaaaaCcctgaaggag	m5C_1829
chr1	160874836	+	C	35	19	0.543	0	0	C	19	M	0.382	0.695	0	3E-05	0	145.12	agaatgtggtCtgagtgattt	m5C_2436
chr1	161413106	+	C	34	9	0.265	0	0	T	25	M	0.146	0.431	1E-12	1E-06	1E-12	15.739	atgggtggttCagtggtagaa	m5C_2487
chr1	161420468	+	C	40	14	0.35	0	0	T	26	M	0.221	0.505	0	2E-07	0	61.976	gccatcggtgCatgggtggtt	m5C_2464
chr1	161420479	+	C	86	41	0.477	0	0	T	45	M	0.374	0.581	0	3E-10	0	307.05	atgggtggttCagtggtagaa	m5C_2439
chr1	161420492	+	C	65	34	0.523	0	0	C	34	M	0.404	0.64	0	4E-08	0	274.58	tggtagaattCtcgcctgcca	m5C_2448
chr1	161420494	+	C	55	24	0.436	0	0	T	31	M	0.314	0.567	0	1E-07	0	150.59	gtagaattctCgcctgccacg	m5C_2473
chr1	161420496	+	C	61	34	0.557	0	0	C	34	M	0.433	0.675	0	4E-08	0	294.46	agaattctcgCctgccacgcg	m5C_2462
chr1	161420497	+	C	56	25	0.446	0	0	T	31	M	0.324	0.576	0	2E-07	0	161.93	gaattctcgcCtgccacgcgg	m5C_2435
chr1	161450368	+	C	106	70	0.66	0	0	C	70	M	0.566	0.744	0	1E-10	0	792.43	ataggtggttCagtggtagaa	m5C_2486
chr1	161450381	+	C	91	64	0.703	0	0	C	64	M	0.603	0.787	0	9E-10	0	771.53	tggtagaattCttgcctgcca	m5C_2455
chr1	161450385	+	C	90	65	0.722	0	0	C	65	M	0.622	0.804	0	1E-09	0	808.66	agaattcttgCctgccacgca	m5C_2441

chr1	161450386	+	C	83	50	0.602	0	0	C	50	M	0.495	0.701	0	2E-09	0	494.85	gaattcttgcCtgccacgcag	m5C_2444
chr1	161450389	+	C	56	27	0.482	0	0	T	29	M	0.357	0.61	0	2E-07	0	192.61	ttcttgcctgCcacgcaggag	m5C_2475
chr1	161450390	+	C	52	25	0.481	0	0	T	27	M	0.351	0.613	0	2E-07	0	175.52	tcttgcctgcCacgcaggagg	m5C_2479
chr1	161450394	+	C	47	25	0.532	0	0	C	25	M	0.392	0.667	0	4E-06	0	196.16	gcctgccacgCaggaggccca	m5C_2456
chr1	161493636	-	C	94	66	0.702	0	0	C	66	M	0.603	0.785	0	1E-09	0	796.26	ggccaatgcaCtttcattttt	m5C_2560
chr1	161493638	-	C	109	32	0.294	0	0	T	77	M	0.216	0.385	0	1E-14	0	138.42	ccggccaatgCactttcattt	m5C_2520
chr1	161493643	-	C	117	29	0.248	0	0	T	88	M	0.178	0.333	0	0	0	103.52	gattcccggcCaatgcacttt	m5C_2543
chr1	161493644	-	C	120	41	0.342	0	0	T	79	M	0.263	0.43	0	1E-16	0	215.58	cgattcccggCcaatgcactt	m5C_2545
chr1	161493647	-	C	117	49	0.419	0	0	T	68	M	0.333	0.509	0	7E-14	0	326.71	gttcgattccCggccaatgca	m5C_2542
chr1	161493648	-	C	124	58	0.468	0	0	T	66	M	0.382	0.555	0	2E-14	0	443.37	ggttcgattcCcggccaatgc	m5C_2552
chr1	161493649	-	C	120	27	0.225	0	0	T	93	M	0.159	0.308	0	0	0	86.125	gggttcgattCccggccaatg	m5C_2491
chr1	161493654	-	C	118	38	0.322	0	0	T	80	M	0.244	0.411	0	2E-15	0	185.81	ggcccgggttCgattcccggc	m5C_2510
chr1	161493669	-	C	105	44	0.419	0	0	T	61	M	0.329	0.515	0	6E-13	0	289.67	gcctgccacgCgggaggcccg	m5C_2503
chr1	161493673	-	C	102	27	0.265	0	0	T	75	M	0.189	0.358	0	1E-15	0	101.93	tctcgcctgcCacgcgggagg	m5C_2549
chr1	161493674	-	C	106	28	0.264	0	0	T	78	M	0.19	0.355	0	2E-15	0	106.14	ttctcgcctgCcacgcgggag	m5C_2556
chr1	161493677	-	C	106	35	0.33	0	0	T	71	M	0.248	0.424	0	3E-14	0	173.58	gaattctcgcCtgccacgcgg	m5C_2511
chr1	161493678	-	C	108	29	0.269	0	0	T	79	M	0.194	0.359	0	3E-15	0	112.49	agaattctcgCctgccacgcg	m5C_2494
chr1	161493680	-	C	104	30	0.288	0	0	T	74	M	0.21	0.382	0	5E-15	0	126.09	gtagaattctCgcctgccacg	m5C_2537
chr1	161493682	-	C	101	42	0.416	0	0	T	59	M	0.325	0.513	0	3E-13	0	272.61	tggtagaattCtcgcctgcca	m5C_2555
chr1	161493695	-	C	83	21	0.253	0	0	T	62	M	0.172	0.356	0	3E-13	0	72.177	attggtggttCagtggtagaa	m5C_2535
chr1	161493706	-	C	38	9	0.237	0	0	T	29	M	0.13	0.392	4E-12	1E-06	4E-12	13.374	ctgacagtagCattggtggtt	m5C_2551
chr1	161500904	+	C	39	19	0.487	0	0	T	20	M	0.339	0.638	0	3E-05	0	128.69	accgggtacgCgttggtggta	m5C_2488
chr1	161500925	+	C	89	39	0.443	1	0.011	T	49	M	0.344	0.547	0	2E-10	0	268.27	tagtggtgagCatagctgcct	m5C_2447
chr1	161500930	+	C	98	60	0.612	0	0	C	60	M	0.513	0.703	0	5E-10	0	615.94	gtgagcatagCtgccttccaa	m5C_2468
chr1	161500933	+	C	96	42	0.438	0	0	T	54	M	0.343	0.537	0	1E-11	0	287.76	agcatagctgCcttccaagca	m5C_2445
chr1	161500934	+	C	94	42	0.447	0	0	T	52	M	0.35	0.547	0	1E-11	0	294.3	gcatagctgcCttccaagcag	m5C_2449
chr1	161500937	+	C	94	45	0.479	0	0	T	49	M	0.381	0.579	0	2E-11	0	342.51	tagctgccttCcaagcagttg	m5C_2461
chr1	161500938	+	C	94	52	0.553	0	0	C	52	M	0.453	0.65	0	1E-10	0	470.67	agctgccttcCaagcagttga	m5C_2472
chr1	161500942	+	C	93	43	0.462	0	0	T	50	M	0.365	0.563	0	1E-11	0	313.51	gccttccaagCagttgacccg	m5C_2474
chr1	161582507	+	C	54	30	0.556	0	0	C	30	M	0.424	0.68	0	5E-07	0	254.25	aaaccggggaCgcgttggtgg	m5C_2434
chr1	161582530	+	C	80	17	0.212	0	0	T	63	M	0.137	0.314	0	2E-14	0	46.622	tagtggtgagCacagctgcct	m5C_2454
chr1	161582539	+	C	43	23	0.535	0	0	C	23	M	0.389	0.675	0	3E-06	0	179.01	gcacagctgcCtttcaagcag	m5C_2466
chr1	161582554	+	C	43	12	0.279	0	0	T	31	M	0.167	0.427	2E-16	7E-08	2E-16	31.457	aagcagttaaCgcgggttcga	m5C_2471
chr1	161582568	+	C	43	11	0.256	0	0	T	32	M	0.149	0.402	1E-14	4E-08	1E-14	22.889	ggttcgattcCcgggtaacga	m5C_2483
chr1	166975289	+	C	382	294	0.77	0	0	C	294	M	0.725	0.809	0	0	0	4262.1	tgagagactgCctgggaatac	m5C_2710
chr1	166975299	+	C	481	365	0.759	0	0	C	365	M	0.719	0.795	0	0	0	5246.1	cctgggaataCtgggtgctgt	m5C_2718
chr1	166975306	+	C	473	375	0.793	0	0	C	375	M	0.754	0.827	0	0	0	5655	atactgggtgCtgtaggcttt	m5C_2720
chr1	171461724	+	C	41	9	0.22	0	0	T	32	M	0.12	0.367	8E-12	1E-08	9E-12	11.962	taacaggttgCctgaggaggg	m5C_1991
chr1	171461725	+	C	43	16	0.372	0	0	T	27	M	0.244	0.521	0	4E-07	0	78.004	aacaggttgcCtgaggagggg	m5C_1992
chr1	171501770	+	C	31	21	0.677	0	0	C	21	M	0.501	0.814	0	5E-05	0	210.59	agaaaaagaaCgggagcgtga	m5C_2011
chr1	171501776	+	C	46	22	0.478	0	0	T	24	M	0.341	0.619	0	2E-06	0	150.15	agaacgggagCgtgagaaaga	m5C_1993
chr1	171501800	+	C	48	38	0.792	0	0	C	38	M	0.657	0.883	0	4E-05	0	499.63	tgaaaaagaaCaagaacagga	m5C_1994
chr1	171501806	+	C	60	12	0.2	0	0	T	48	M	0.118	0.318	3E-14	8E-12	4E-14	19.13	agaacaagaaCaggagcgaga	m5C_2004
chr1	173833976	-	C	43	17	0.395	0	0	T	26	M	0.264	0.544	0	6E-07	0	89.641	tcgctcctatCtgatgtatct	m5C_3662
chr1	173833980	-	C	39	16	0.41	0	0	T	23	M	0.271	0.566	0	2E-05	0	86.653	tctttcgctcCtatctgatgt	m5C_3612
chr1	173833983	-	C	42	17	0.405	0	0	T	25	M	0.27	0.555	0	6E-07	0	91.945	aagtctttcgCtcctatctga	m5C_3631
chr1	173833985	-	C	42	18	0.429	0	0	T	24	M	0.291	0.578	0	8E-07	0	104.82	ttaagtctttCgctcctatct	m5C_3633
chr1	173833989	-	C	41	17	0.415	0	0	T	24	M	0.278	0.566	0	6E-07	0	94.372	aatattaagtCtttcgctcct	m5C_3630
chr1	173834000	-	C	42	14	0.333	0	0	T	28	M	0.21	0.484	0	2E-07	0	58.834	cgctgatgagCaatattaagt	m5C_3600
chr1	173834008	-	C	40	17	0.425	0	0	T	23	M	0.285	0.578	0	6E-07	0	96.931	cagactaacgCtgatgagcaa	m5C_3581
chr1	173834010	-	C	44	18	0.409	0	0	T	26	M	0.277	0.556	0	8E-07	0	99.676	agcagactaaCgctgatgagc	m5C_3669
chr1	173834014	-	C	52	17	0.327	0	0	T	35	M	0.215	0.462	0	1E-08	0	73.174	gttcagcagaCtaacgctgat	m5C_3599
chr1	173834018	-	C	53	16	0.302	0	0	T	37	M	0.195	0.435	0	7E-09	0	62.458	catagttcagCagactaacgc	m5C_3651
chr1	173834021	-	C	55	16	0.291	0	0	T	39	M	0.188	0.421	0	7E-09	0	60.067	taacatagttCagcagactaa	m5C_3613
chr1	173834028	-	C	54	14	0.259	1	0	T	40	M	0.161	0.389	0	3E-09	0	45.132	atgatgataaCatagttcagc	m5C_3611
chr1	173834496	-	C	67	25	0.373	0	0	T	42	M	0.267	0.493	0	4E-09	0	133.59	taatcttaaaCtgaaacagta	m5C_3570
chr1	173834502	-	C	80	49	0.613	0	0	C	49	M	0.503	0.712	0	3E-08	0	492.88	ggagattaatCttaaactgaa	m5C_3593
chr1	173834526	-	C	138	47	0.341	0	0	T	91	M	0.267	0.423	0	0	0	250.78	gggaatctctCtgagaaagaa	m5C_3666
chr1	173834528	-	C	140	47	0.336	0	0	T	93	M	0.263	0.417	0	0	0	247.03	atgggaatctCtctgagaaag	m5C_3579
chr1	173834769	-	C	101	21	0.208	0	0	T	80	M	0.14	0.297	0	0	0	58.887	acaaaggtaaCactgaagaac	m5C_3585
chr1	173834786	-	C	109	32	0.294	0	0	T	77	M	0.216	0.385	0	1E-14	0	138.42	ctgaaatgagCatgtagacaa	m5C_3668
chr1	173834796	-	C	114	24	0.211	0	0	T	90	M	0.146	0.294	0	0	0	69.961	aatgtctgacCtgaaatgagc	m5C_3620
chr1	173834797	-	C	112	37	0.33	0	0	T	75	M	0.25	0.422	0	1E-15	0	185.11	aaatgtctgaCctgaaatgag	m5C_3617
chr1	173834808	-	C	109	25	0.229	0	0	T	84	M	0.16	0.317	0	4E-16	0	80.227	tgatgttgatCaaatgtctga	m5C_3626
chr1	173835107	-	C	144	65	0.451	0	0	T	79	M	0.372	0.533	0	1E-16	0	484.15	ctctaactgaCtaaaggcatt	m5C_3618
chr1	173835111	-	C	196	72	0.367	0	0	T	124	M	0.303	0.437	0	0	0	436.33	ttagctctaaCtgactaaagg	m5C_3615
chr1	173835115	-	C	219	77	0.352	0	0	T	142	M	0.291	0.417	0	0	0	448.78	ttaattagctCtaactgacta	m5C_3595
chr1	173835117	-	C	239	99	0.414	0	0	T	140	M	0.354	0.478	0	0	0	700.15	tcttaattagCtctaactgac	m5C_3635
chr1	173835126	-	C	255	86	0.337	0	0	T	169	M	0.282	0.397	0	0	0	485.08	acatgaaggtCttaattagct	m5C_3604
chr1	173835135	-	C	288	115	0.399	0	0	T	173	M	0.344	0.457	0	0	0	792.18	gctgactgaaCatgaaggtct	m5C_3589
chr1	173835140	-	C	324	121	0.373	0	0	T	203	M	0.323	0.427	0	0	0	780.59	caaatgctgaCtgaacatgaa	m5C_3652
chr1	173835144	-	C	351	72	0.205	0	0	T	279	M	0.166	0.25	0	0	0	239.31	taagcaaatgCtgactgaaca	m5C_3567
chr1	173835150	-	C	354	83	0.236	2	0.006	T	269	M	0.194	0.283	0	0	0	322.79	tgatgataagCaaatgctgac	m5C_3572
chr1	173835166	-	C	95	37	0.389	0	0	T	58	M	0.298	0.49	0	3E-12	0	220.18	gtaaatgcagCctggatgatg	m5C_3590
chr1	173835449	-	C	83	21	0.253	0	0	T	62	M	0.172	0.356	0	3E-13	0	72.177	tactatctgtCtgatgtatct	m5C_3642
chr1	173835784	-	C	115	41	0.357	0	0	T	74	M	0.275	0.447	0	6E-15	0	225.43	ccaccattatCttaactgagg	m5C_3628
chr1	173835790	-	C	121	38	0.314	0	0	T	83	M	0.238	0.401	0	0	0	181	tcttaaccacCattatcttaa	m5C_3619
chr1	173835793	-	C	122	54	0.443	0	0	T	68	M	0.358	0.531	0	8E-15	0	386.18	ggatcttaacCaccattatct	m5C_3656
chr1	173835799	-	C	128	62	0.484	0	0	T	66	M	0.4	0.57	0	5E-14	0	495.41	tgatgaggatCttaaccacca	m5C_3637
chr1	173835815	-	C	136	36	0.265	0	0	T	100	M	0.198	0.345	0	0	0	142.39	ctcttgagtgCtagaatgatg	m5C_3622
chr1	173835839	-	C	125	50	0.4	0	0	T	75	M	0.318	0.488	0	2E-15	0	318.34	acaatgatgaCagtttatttg	m5C_3659
chr1	173835848	-	C	47	17	0.362	0	0	T	30	M	0.24	0.505	0	6E-07	0	81.484	ggatgtgccaCaatgatgaca	m5C_3609
chr1	173835850	-	C	46	12	0.261	0	0	T	34	M	0.156	0.403	7E-16	7E-08	7E-16	28.412	ttggatgtgcCacaatgatga	m5C_3573
chr1	173836024	-	C	142	37	0.261	0	0	T	105	M	0.195	0.338	0	0	0	144.56	aaattctattCtgaggcttta	m5C_3629
chr1	173836037	-	C	168	39	0.232	0	0	T	129	M	0.175	0.302	0	0	0	136.28	gaagggatttCtgaaattcta	m5C_3592
chr1	173836065	-	C	194	52	0.268	0	0	T	142	M	0.211	0.334	0	0	0	219.09	gcctgtgatgCtttaagagta	m5C_3605
chr1	173836073	-	C	89	29	0.326	0	0	T	60	M	0.237	0.429	0	8E-12	0	137.71	tggtctcagcCtgtgatgctt	m5C_3665
chr1	173836821	-	C	155	59	0.381	0	0	T	96	M	0.308	0.459	0	0	0	363.4	gccaaccgctCtgatggtggc	m5C_3574
chr1	173836823	-	C	172	44	0.256	0	0	T	128	M	0.196	0.326	0	0	0	172.87	atgccaaccgCtctgatggtg	m5C_3580
chr1	173836825	-	C	185	67	0.362	0	0	T	118	M	0.296	0.434	0	0	0	397.12	gaatgccaacCgctctgatgg	m5C_3623
chr1	173836826	-	C	187	47	0.251	0	0	T	140	M	0.195	0.318	0	0	0	182.92	tgaatgccaaCcgctctgatg	m5C_3632
chr1	173836829	-	C	190	84	0.442	0	0	T	106	M	0.373	0.513	0	0	0	627.19	tgatgaatgcCaaccgctctg	m5C_3587

chr1	173836830	-	C	189	77	0.407	0	0	T	112	M	0.34	0.479	0	0	0	523.41	atgatgaatgCcaaccgctct	m5C_3607
chr1	173836852	-	C	168	41	0.244	0	0	T	127	M	0.185	0.314	0	0	0	151.94	ttggtagggaCatctgagagt	m5C_3638
chr1	173836868	-	C	151	35	0.232	0	0	T	116	M	0.172	0.305	0	0	0	120.15	tctgatgaagCctgtgttggt	m5C_3649
chr1	173836879	-	C	70	16	0.229	0	0	T	54	M	0.146	0.34	0	1E-12	0	46.681	cgtgccctgcCtctgatgaag	m5C_3627
chr1	173836880	-	C	67	19	0.284	0	0	T	48	M	0.19	0.401	0	5E-10	0	72.087	tcgtgccctgCctctgatgaa	m5C_3598
chr1	185276170	-	C	64	30	0.469	0	0	T	34	M	0.352	0.589	0	2E-08	0	211.05	agaatggagaCagtctggaag	m5C_2699
chr1	185276185	-	C	68	34	0.5	0	0	CT	34	M	0.384	0.616	0	4E-08	0	261.38	agcgtagcatCtgggagaatg	m5C_2688
chr1	185276193	-	C	56	18	0.321	0	0	T	38	M	0.214	0.452	0	2E-08	0	77.043	ctgggtgcagCgtagcatctg	m5C_2687
chr1	185276196	-	C	57	18	0.316	0	0	T	39	M	0.21	0.445	0	2E-08	0	75.615	caactgggtgCagcgtagcat	m5C_2700
chr1	202129921	-	C	65	26	0.4	0	0	T	39	M	0.29	0.521	0	6E-09	0	150.65	tcgggaaggcCtggcccctca	m5C_2609
chr1	202129922	-	C	65	23	0.354	0	0	T	42	M	0.249	0.475	0	2E-09	0	114.43	gtcgggaaggCctggcccctc	m5C_2615
chr1	204475656	+	C	77	23	0.299	0	0	T	54	M	0.208	0.408	0	4E-11	0	95.697	ttcgggagcgCccggatagct	m5C_3023
chr1	204475658	+	C	74	42	0.568	0	0	C	42	M	0.454	0.674	0	9E-09	0	381.47	cgggagcgccCggatagctca	m5C_3022
chr1	205687439	-	C	37	20	0.541	0	0	C	20	M	0.384	0.69	0	4E-05	0	153.54	gatgaagggtCtgaagatgaa	m5C_3035
chr1	205689662	-	C	192	102	0.531	0	0	C	102	M	0.461	0.601	0	0	0	939.92	tgaggaagaaCaagaagagga	m5C_3040
chr1	205689675	-	C	216	67	0.31	0	0	T	149	M	0.252	0.375	0	0	0	338.03	aagatgtgggCagtgaggaag	m5C_3037
chr1	205689690	-	C	95	31	0.326	0	0	T	64	M	0.24	0.426	0	4E-13	0	149.04	gagagatgctCatggaagatg	m5C_3041
chr1	205689692	-	C	82	21	0.256	0	0	T	61	M	0.174	0.36	0	3E-13	0	73.094	gagagagatgCtcatggaaga	m5C_3038
chr1	205698702	-	C	90	20	0.222	0	0	T	70	M	0.149	0.318	0	2E-15	0	59.485	tctgatgatgCaggtaagttt	m5C_3070
chr1	205698724	-	C	44	16	0.364	0	0	T	28	M	0.238	0.511	0	4E-07	0	76.107	tgattactcaCagtttcagga	m5C_3030
chr1	212454147	+	C	50	32	0.64	0	0	C	32	M	0.501	0.759	0	7E-07	0	320.9	ggatttggtgCtctctcctgc	m5C_4522
chr1	212454149	+	C	33	22	0.667	0	0	C	22	M	0.496	0.802	0	6E-05	0	218.27	atttggtgctCtctcctgcag	m5C_4521
chr1	223943237	+	C	36	19	0.528	0	0	C	19	M	0.37	0.68	0	3E-05	0	140.62	aggatgaggaCgaggaggatg	m5C_2778
chr1	225706984	-	C	31	15	0.484	0	0	T	16	M	0.32	0.652	0	1E-05	0	95.91	gagactggaaCgggagaggca	m5C_2761
chr1	225706999	-	C	48	32	0.667	0	0	C	32	M	0.525	0.783	0	2E-05	0	336.26	acgagagaggCtggagagact	m5C_2771
chr1	225707230	-	C	36	22	0.611	0	0	C	22	M	0.449	0.752	0	6E-05	0	197.4	ggagctggagCgggaaaggct	m5C_2765
chr1	225707236	-	C	32	11	0.344	0	0	T	21	M	0.204	0.517	2E-16	3E-06	2E-16	35.144	gcaaaaggagCtggagcggga	m5C_2757
chr1	226349320	-	C	80	32	0.4	0	0	T	48	M	0.3	0.51	0	8E-10	0	191.75	ggaagagaaaCgtaggagaga	m5C_2812
chr1	226349338	-	C	84	62	0.738	0	0	C	62	M	0.635	0.82	0	1E-08	0	787.58	agaacgtctgCaaaaggagga	m5C_2818
chr1	226349341	-	C	76	56	0.737	0	0	C	56	M	0.628	0.823	0	9E-08	0	703.57	aagagaacgtCtgcaaaagga	m5C_2817
chr1	226349344	-	C	55	43	0.782	0	0	C	43	M	0.656	0.871	0	4E-06	0	564.42	agaaagagaaCgtctgcaaaa	m5C_2825
chr1	226349365	-	C	33	11	0.333	0	0	T	22	M	0.198	0.504	2E-16	3E-06	2E-16	34.007	gcgaaggcggCgtgaagagga	m5C_2828
chr1	229673634	+	C	42	32	0.762	0	0	C	32	M	0.615	0.865	0	2E-05	0	393.42	gaattctcacCtgctaattca	m5C_2864
chr1	236431200	+	C	35	17	0.486	0	0	T	18	M	0.33	0.644	0	2E-05	0	112.18	taggagcttgCtccatccact	m5C_3155
chr1	236431223	+	C	94	20	0.213	0	0	T	74	M	0.142	0.306	0	2E-15	0	56.867	tcgcatcgacCtggtattgca	m5C_3150
chr1	236431237	+	C	184	56	0.304	0	0	T	128	M	0.242	0.374	0	0	0	271.52	tattgcagtaCttcaggaacg	m5C_3158
chr1	236431254	+	C	130	31	0.238	0	0	T	99	M	0.173	0.319	0	0	0	107.5	aacggtgcacCcctccggggg	m5C_3146
chr1	239847440	+	C	51	32	0.627	0	0	C	32	M	0.49	0.747	0	7E-07	0	313.76	atgggtggttCagtggtagaa	m5C_2931
chr1	240964260	+	C	32	10	0.312	0	0	T	22	M	0.18	0.486	1E-14	2E-06	1E-14	25.084	tagtgggtctCtggtggggca	m5C_2967
chr1	241793554	-	C	42	21	0.5	0	0	CT	21	M	0.355	0.645	0	2E-06	0	149.21	aaaagtagtaCatggggagat	m5C_2982
chr1	242247840	-	C	39	10	0.256	0	0	T	29	M	0.146	0.411	1E-13	2E-06	1E-13	18.775	tcaggacggcCgagtggtctg	m5C_2964
chr1	242247841	-	C	35	15	0.429	0	0	T	20	M	0.28	0.591	0	1E-05	0	83.953	gtcaggacggCcgagtggtct	m5C_2962
chr1	245021336	-	C	71	40	0.563	0	0	C	40	M	0.448	0.673	0	6E-09	0	358.17	accaaagggg Cctgaagagaa	m5C_3006
chr1	245021345	-	C	46	20	0.435	0	0	T	26	M	0.302	0.578	0	1E-06	0	120.84	agttagaggaCcaaaggggcc	m5C_3001
chr1	245027364	-	C	149	32	0.215	0	0	T	117	M	0.156	0.287	0	0	0	100.12	cggccggcggCgatgaagagg	m5C_3005
chr1	245027367	-	C	151	70	0.464	0	0	T	81	M	0.386	0.543	0	0	0	540.3	ccgcggccggCggcgatgaag	m5C_3002
chr1	245027370	-	C	148	86	0.581	0	0	C	86	M	0.501	0.658	0	1E-14	0	860.9	aggccgcggcCggcggcgatg	m5C_2997
chr1	245027374	-	C	110	37	0.336	0	0	T	73	M	0.255	0.429	0	1E-15	0	188.64	caggaggccgCggccggcggc	m5C_3008
chr1	245027376	-	C	105	75	0.714	0	0	C	75	M	0.622	0.792	0	3E-10	0	932.27	agcaggaggcCgcggccggcg	m5C_3009
chr1	245027377	-	C	96	26	0.271	0	0	T	70	M	0.192	0.367	0	5E-14	0	99.859	gagcaggaggCcgcggccggc	m5C_3000
chr1	249168121	+	C	54	20	0.37	0	0	T	34	M	0.254	0.504	0	3E-08	0	101.69	gctttctggtCtccggatgga	m5C_3483
chr1	249168123	+	C	62	24	0.387	0	0	T	38	M	0.276	0.512	0	3E-09	0	132.41	tttctggtctCcggatggagg	m5C_3491
chr1	249168448	+	C	68	18	0.265	0	0	T	50	M	0.174	0.38	0	3E-10	0	62.819	atcatgtcttCcctggtggtc	m5C_3462
chr1	249168449	+	C	70	31	0.443	0	0	T	39	M	0.332	0.559	0	6E-10	0	206.15	tcatgtcttcCctggtggtct	m5C_3485
chr1	249168450	+	C	68	34	0.5	0	0	CT	34	M	0.384	0.616	0	4E-08	0	261.38	catgtcttccCtggtggtcta	m5C_3493
chr1	249168458	+	C	167	108	0.647	0	0	C	108	M	0.572	0.715	0	1E-15	0	1234.8	ccctggtggtCtagtggttag	m5C_3481
chr1	249168473	+	C	178	97	0.545	0	0	C	97	M	0.472	0.616	0	0	0	914.92	ggttaggattCggcgctctca	m5C_3464
chr1	249168476	+	C	178	93	0.522	0	0	C	93	M	0.449	0.595	0	0	0	835.87	taggattcggCgctctcaccg	m5C_3466
chr1	249168478	+	C	177	87	0.492	0	0	T	90	M	0.419	0.565	0	0	0	728.78	ggattcggcgCtctcaccgcc	m5C_3489
chr1	249168480	+	C	174	88	0.506	0	0	C	88	M	0.432	0.579	0	0	0	760.57	attcggcgctCtcaccgccgc	m5C_3470
chr1	249168482	+	C	176	104	0.591	0	0	C	104	M	0.517	0.661	0	0	0	1075.5	tcggcgctctCaccgccgcgg	m5C_3488
chr1	249168484	+	C	168	88	0.527	1	0.006	C	88	M	0.451	0.601	0	0	0	794.59	ggcgctctcaCcgccgcggcc	m5C_3465
chr1	249168485	+	C	163	76	0.466	0	0	T	87	M	0.391	0.543	0	0	0	594.83	gcgctctcacCgccgcggccc	m5C_3492
chr1	249168487	+	C	137	44	0.321	0	0	T	93	M	0.249	0.403	0	0	0	218.92	gctctcaccgCcgcggcccgg	m5C_3468
chr1	249168488	+	C	125	52	0.416	0	0	T	73	M	0.333	0.504	0	4E-15	0	346.69	ctctcaccgcCgcggcccggg	m5C_3474
chr1	249168490	+	C	107	54	0.505	0	0	C	54	M	0.411	0.598	0	6E-12	0	444.35	ctcaccgccgCggcccgggtt	m5C_3476
chr1	249168493	+	C	100	44	0.44	0	0	T	56	M	0.347	0.538	0	6E-13	0	305.11	accgccgcggCccgggttcga	m5C_3473
chr1	249168506	+	C	52	12	0.231	0	0	T	40	M	0.137	0.361	4E-15	9E-10	5E-15	23.637	gggttcgattCccggtcagga	m5C_3460
chr1	249168507	+	C	51	19	0.373	0	0	T	32	M	0.253	0.51	0	2E-08	0	96.217	ggttcgattcCcggtcaggaa	m5C_3486
chr1	249168508	+	C	50	11	0.22	0	0	T	39	M	0.128	0.352	9E-14	4E-10	9E-14	18.334	gttcgattccCggtcaggaaa	m5C_3463
chr10	1759668	-	C	43	11	0.256	0	0	T	32	M	0.149	0.402	1E-14	4E-08	1E-14	22.889	aagaaaagaaCagaaaagaaa	m5C_20544
chr10	5794938	-	C	61	34	0.557	0	0	C	34	M	0.433	0.675	0	4E-08	0	294.46	ctctggcggtCtagtggttag	m5C_20674
chr10	17272665	+	C	38	8	0.211	0	0	T	30	M	0.111	0.363	1E-10	7E-07	1E-10	8.7725	ggaggagatgCttcagagaga	m5C_20634
chr10	19887384	+	C	37	26	0.703	0	0	C	26	M	0.542	0.825	0	0.0001	0	281.93	agaatgcagtCtgagtggttt	m5C_20701
chr10	32699342	-	C	63	27	0.429	0	0	T	36	M	0.314	0.551	0	7E-09	0	169.54	cttgggaatgCagtttacagt	m5C_20937
chr10	32699352	-	C	93	59	0.634	0	0	C	59	M	0.533	0.725	0	4E-10	0	628.95	agttgggttgCttgggaatgc	m5C_20938
chr10	37555528	-	C	51	30	0.588	0	0	C	30	M	0.452	0.712	0	5E-07	0	270.99	agttgtgcagCggaagcgtgc	m5C_21529
chr10	37555531	-	C	53	16	0.302	0	0	T	37	M	0.195	0.435	0	7E-09	0	62.458	cagagttgtgCagcggaagcg	m5C_21530
chr10	66499062	+	C	52	33	0.635	0	0	C	33	M	0.499	0.752	0	9E-07	0	329.15	tcctggtggtCtcgtggttag	m5C_21015
chr10	69767605	-	C	33	18	0.545	0	0	C	18	M	0.38	0.702	0	3E-05	0	136.75	accaggttgcCtaaggagggg	m5C_21056
chr10	69767613	-	C	35	7	0.2	0	0	T	28	M	0.1	0.359	2E-09	3E-07	2E-09	6.1388	ggggggccacCaggttgccta	m5C_21062
chr10	69767614	-	C	33	10	0.303	0	0	T	23	M	0.174	0.473	2E-14	2E-06	2E-14	23.982	tggggggccaCcaggttgcct	m5C_21057
chr10	70098955	+	C	31	19	0.613	0	0	C	19	M	0.438	0.763	0	3E-05	0	166.53	ggaatgatggCtttgatgaca	m5C_21117
chr10	70514949	+	C	50	14	0.28	0	0	T	36	M	0.175	0.417	0	2E-07	0	48.927	gtgtgtaaatCctattccatt	m5C_21132
chr10	70514950	+	C	47	16	0.34	0	0	T	31	M	0.222	0.483	0	4E-07	0	70.935	tgtgtaaatcCtattccattg	m5C_21122
chr10	70514955	+	C	51	17	0.333	0	0	T	34	M	0.22	0.47	0	1E-08	0	74.697	aaatcctattCcattgctgaa	m5C_21138
chr10	70514956	+	C	50	13	0.26	0	0	T	37	M	0.159	0.396	0	2E-09	0	41.266	aatcctattcCattgctgaaa	m5C_21140
chr10	70514961	+	C	49	13	0.265	0	0	T	36	M	0.162	0.403	0	1E-07	0	42.149	tattccattgCtgaaatgcag	m5C_21131
chr10	70514969	+	C	50	17	0.34	0	0	T	33	M	0.224	0.478	0	6E-07	0	76.285	tgctgaaatgCagtgtggaac	m5C_21123
chr10	70514979	+	C	51	13	0.255	0	0	T	38	M	0.155	0.389	1E-16	2E-09	1E-16	32.243	cagtgtggaaCacaatgaact	m5C_21143
chr10	70514988	+	C	43	25	0.581	0	0	C	25	M	0.433	0.716	0	4E-06	0	216.64	acacaatgaaCtgaactcttc	m5C_21139
chr10	70514993	+	C	44	23	0.523	0	0	C	23	M	0.379	0.662	0	3E-06	0	174.5	atgaactgaaCtcttccttga	m5C_21142

chr10	101590102	+	C	36	26	0.722	0	0	C	26	M	0.56	0.842	0	0.0001	0	291.24	tgactatgggCtgatatccag	m5C_21534
chr10	101590109	+	C	35	11	0.314	0	0	T	24	M	0.186	0.48	7E-16	3E-06	7E-16	30.969	gggctgatatCcagtgtggaa	m5C_21535
chr10	103124607	-	C	76	25	0.329	0	0	T	51	M	0.234	0.441	0	9E-11	0	116.9	gaacggtgcaCtcgctccggg	m5C_21616
chr10	103124620	-	C	113	26	0.23	0	0	T	87	M	0.162	0.316	0	0	0	84.308	gcagtacctcCaggaacggtg	m5C_21639
chr10	103124624	-	C	115	40	0.348	0	0	T	75	M	0.267	0.439	0	4E-15	0	213.58	tattgcagtaCctccaggaac	m5C_21628
chr10	103124655	-	C	67	28	0.418	0	0	T	39	M	0.307	0.537	0	1E-08	0	172.16	cttgctccgtCcactccatgc	m5C_21643
chr10	103124659	-	C	76	21	0.276	0	0	T	55	M	0.188	0.386	0	2E-11	0	79.128	ggagcttgctCcgtccactcc	m5C_21631
chr10	103124684	-	C	131	65	0.496	0	0	T	66	M	0.412	0.581	0	4E-15	0	535.47	atttttggagCagggagatgg	m5C_21634
chr10	103577786	-	C	41	13	0.317	0	0	T	28	M	0.196	0.47	0	1E-07	0	50.868	cggccggggggCaggaggatgg	m5C_21647
chr10	103577792	-	C	43	17	0.395	0	0	T	26	M	0.264	0.544	0	6E-07	0	89.641	tggcagcggcCgggggcagga	m5C_21630
chr10	103577796	-	C	45	13	0.289	0	0	T	32	M	0.177	0.434	0	1E-07	0	46.098	gaggtggcagCggccgggggc	m5C_21617
chr10	103923338	+	C	31	10	0.323	0	0	T	21	M	0.186	0.499	7E-15	2E-06	8E-15	26.273	agtagcggtaCtcagccagac	m5C_21608
chr10	105154061	-	C	97	58	0.598	0	0	C	58	M	0.498	0.69	0	3E-10	0	578.18	tggcgtcggtCgtggtagata	m5C_21499
chr10	105154065	-	C	117	47	0.402	0	0	T	70	M	0.317	0.492	0	4E-14	0	298.33	ggcgtggcgtCggtcgtggta	m5C_21502
chr10	105154068	-	C	117	43	0.368	0	0	T	74	M	0.286	0.458	0	1E-14	0	245.67	ctcggcgtggCgtcggtcgtg	m5C_21487
chr10	105154073	-	C	111	33	0.297	0	0	T	78	M	0.22	0.388	0	2E-16	0	145.34	atcgactcggCgtggcgtcgg	m5C_21483
chr10	105154078	-	C	44	11	0.25	0	0	T	33	M	0.146	0.394	2E-14	4E-08	2E-14	22.132	tgccaatcgaCtcggcgtggc	m5C_21484
chr10	120797810	-	C	77	39	0.506	0	0	C	39	M	0.397	0.615	0	5E-09	0	309.81	gacaaggaccCtgagagagaa	m5C_21872
chr10	120797811	-	C	73	20	0.274	0	0	T	53	M	0.185	0.386	0	1E-11	0	73.965	tgacaaggacCctgagagaga	m5C_21890
chr10	120797832	-	C	60	20	0.333	0	0	T	40	M	0.227	0.459	0	7E-10	0	90.916	taatcaagatCgggaggagaa	m5C_21891
chr10	120797838	-	C	99	26	0.263	0	0	T	73	M	0.186	0.357	0	5E-14	0	96.71	cagagataatCaagatcggga	m5C_21909
chr10	120797848	-	C	82	46	0.561	0	0	C	46	M	0.453	0.663	0	8E-10	0	416.97	aagaaagggaCagagataatc	m5C_21878
chr10	120797866	-	C	68	40	0.588	0	0	C	40	M	0.47	0.697	0	1E-07	0	375.69	gtgaatgggaCagagaaaaag	m5C_21911
chr10	120797926	-	C	45	26	0.578	0	0	C	26	M	0.433	0.71	0	6E-06	0	225.16	aagaaaaagcCagagaggaga	m5C_21905
chr10	120797927	-	C	52	25	0.481	0	0	T	27	M	0.351	0.613	0	2E-07	0	175.52	aaagaaaaagCcagagaggag	m5C_21887
chr10	120801508	-	C	36	21	0.583	0	0	C	21	M	0.422	0.729	0	5E-05	0	177.24	ttagtcaagcCaggtaaaatt	m5C_21881
chr10	120802244	-	C	35	14	0.4	0	0	T	21	M	0.256	0.564	0	9E-06	0	71.541	ggacaggtctCatagaagaga	m5C_21903
chr10	120809363	-	C	219	78	0.356	0	0	T	141	M	0.296	0.422	0	0	0	461.33	agaacgagaaCggcgtagaga	m5C_21902
chr10	120809369	-	C	239	83	0.347	0	0	T	156	M	0.29	0.41	0	0	0	481.01	aattgaagaaCgagaacggcg	m5C_21908
chr10	120809393	-	C	116	50	0.431	0	0	T	66	M	0.345	0.522	0	9E-14	0	344.55	gaaaaaacgcCaaagggagtt	m5C_21877
chr10	120809394	-	C	103	33	0.32	0	0	T	70	M	0.238	0.416	0	2E-14	0	157.17	ggaaaaaacgCcaaagggagt	m5C_21889
chr10	120809396	-	C	90	24	0.267	0	0	T	66	M	0.186	0.366	0	2E-14	0	89.394	aaggaaaaaaCgccaaaggga	m5C_21882
chr10	120817632	-	C	31	7	0.226	0	0	T	24	M	0.114	0.398	6E-10	3E-07	7E-10	7.3321	ccagaaagtgCggaaggctga	m5C_21894
chr10	120817659	-	C	37	16	0.432	0	0	T	21	M	0.287	0.591	0	2E-05	0	91.749	agaattggaaCagagggaagc	m5C_21874
chr10	131239934	+	C	53	27	0.509	0	0	C	27	M	0.379	0.639	0	2E-07	0	204.57	aacaggtgtgCggtggggaga	m5C_21715
chr10	135062936	-	C	44	17	0.386	0	0	T	27	M	0.257	0.534	0	6E-07	0	87.451	tgcagagtggCctggcaggtg	m5C_22444
chr11	532453	-	C	31	19	0.613	0	0	C	19	M	0.438	0.763	0	3E-05	0	166.53	gaaggaaggaCggaagcaagg	m5C_21841
chr11	568115	-	C	41	13	0.317	0	0	T	28	M	0.196	0.47	0	1E-07	0	50.868	gtgacagcggCtgatctgtgc	m5C_21830
chr11	568121	-	C	44	17	0.386	0	0	T	27	M	0.257	0.534	0	6E-07	0	87.451	gtgcgtgtgaCagcggctgat	m5C_21829
chr11	812631	+	C	58	12	0.207	0	0	T	46	M	0.123	0.328	2E-14	9E-10	2E-14	20.116	gcccctgctgCaggtaaagtgg	m5C_21794
chr11	812793	+	C	73	38	0.521	0	0	C	38	M	0.408	0.631	0	4E-09	0	309.94	aaggaggagtCtgaagagtca	m5C_21790
chr11	833461	+	C	34	12	0.353	0	0	T	22	M	0.215	0.521	0	5E-06	0	51.571	gcggggtcggCggcggcggtg	m5C_21811
chr11	833464	+	C	35	17	0.486	0	0	T	18	M	0.33	0.644	0	2E-05	0	112.18	gggtcggcggCggcggtgacc	m5C_21786
chr11	833467	+	C	33	21	0.636	0	0	C	21	M	0.466	0.778	0	5E-05	0	195.79	tcggcggcggCggtgacctca	m5C_21816
chr11	833473	+	C	33	19	0.576	0	0	C	19	M	0.408	0.728	0	3E-05	0	155.07	gcggcggtgaCctcattgcag	m5C_21806
chr11	2016650	-	C	42	15	0.357	0	0	T	27	M	0.23	0.508	0	3E-07	0	68.967	tacgagtgtgCgtgagtgtga	m5C_22286
chr11	2017542	-	C	42	17	0.405	0	0	T	25	M	0.27	0.555	0	6E-07	0	91.945	acggcgaggaCagaggaggcg	m5C_22371
chr11	2017551	-	C	117	29	0.248	0	0	T	88	M	0.178	0.333	0	0	0	103.52	tccggtgtgaCggcgaggaca	m5C_22379
chr11	2017894	-	C	40	14	0.35	0	0	T	26	M	0.221	0.505	0	2E-07	0	61.976	aatgagctctCaggagggagg	m5C_22285
chr11	2017896	-	C	43	28	0.651	0	0	C	28	M	0.502	0.776	0	8E-06	0	280.96	tgaatgagctCtcaggaggga	m5C_22370
chr11	2017925	-	C	31	16	0.516	0	0	C	16	M	0.348	0.68	0	2E-05	0	111.49	ccagactaggCgaggcgggcg	m5C_22327
chr11	2017930	-	C	45	33	0.733	0	0	C	33	M	0.59	0.84	0	2E-05	0	389.14	cgagaccagaCtaggcgaggc	m5C_22337
chr11	2017934	-	C	41	17	0.415	0	0	T	24	M	0.278	0.566	0	6E-07	0	94.372	ggggcgagacCagactaggcg	m5C_22284
chr11	2017940	-	C	37	11	0.297	0	0	T	26	M	0.175	0.458	1E-15	3E-06	2E-15	28.551	gggcatggggCgagaccagac	m5C_22366
chr11	2018037	-	C	80	23	0.287	0	0	T	57	M	0.2	0.395	0	7E-13	0	91.939	cggagagggcCcacagtggac	m5C_22376
chr11	2018038	-	C	86	19	0.221	0	0	T	67	M	0.146	0.319	0	8E-14	0	55.574	gcggagagggCccacagtgga	m5C_22383
chr11	2018187	-	C	43	18	0.419	0	0	T	25	M	0.284	0.567	0	8E-07	0	102.18	aggcagtgctCggggagttgc	m5C_22358
chr11	2018194	-	C	52	17	0.327	0	0	T	35	M	0.215	0.462	0	1E-08	0	73.174	ggaagacaggCagtgctcggg	m5C_22320
chr11	2018206	-	C	57	29	0.509	0	0	C	29	M	0.383	0.634	0	4E-07	0	221.91	ggctggagagCgggaagacag	m5C_22356
chr11	2018222	-	C	55	11	0.2	0	0	T	44	M	0.116	0.324	3E-13	4E-10	3E-13	15.923	atgccggagcCtcgagggctg	m5C_22319
chr11	2018223	-	C	43	17	0.395	0	0	T	26	M	0.264	0.544	0	6E-07	0	89.641	tatgccggagCctcgagggct	m5C_22294
chr11	2018229	-	C	36	20	0.556	0	0	C	20	M	0.396	0.705	0	4E-05	0	158.32	caggcctatgCcggagcctcg	m5C_22288
chr11	2018324	-	C	33	20	0.606	0	0	C	20	M	0.437	0.753	0	4E-05	0	174.73	gcaggggacaCaggacagagg	m5C_22316
chr11	2018333	-	C	69	16	0.232	0	0	T	53	M	0.148	0.344	0	1E-10	0	47.385	agggacatggCaggggacaca	m5C_22334
chr11	2018338	-	C	80	46	0.575	0	0	C	46	M	0.466	0.677	0	8E-10	0	428.44	cagacagggaCatggcagggg	m5C_22295
chr11	2018344	-	C	70	43	0.614	0	0	C	43	M	0.497	0.72	0	2E-07	0	427.55	ctgggtcagaCagggacatgg	m5C_22269
chr11	2018348	-	C	60	15	0.25	0	0	T	45	M	0.158	0.372	0	6E-11	0	47.329	aggcctgggtCagacagggac	m5C_22308
chr11	2018354	-	C	49	17	0.347	0	0	T	32	M	0.229	0.487	0	6E-07	0	77.942	taccccaggcCtgggtcagac	m5C_22268
chr11	2018355	-	C	37	29	0.784	0	0	C	29	M	0.628	0.886	0	0.0002	0	364.27	gtaccccaggCctgggtcaga	m5C_22378
chr11	2018464	-	C	62	14	0.23	1	0.016	T	47	M	0.142	0.349	0	3E-11	0	39.741	gccgagactgCgcaaggctgg	m5C_22373
chr11	2018467	-	C	67	15	0.224	0	0	T	52	M	0.141	0.337	0	6E-11	0	42.191	ggagccgagaCtgcgcaaggc	m5C_22342
chr11	2018472	-	C	61	35	0.574	0	0	C	35	M	0.449	0.69	0	5E-08	0	314.27	cggggggagcCgagactgcgc	m5C_22368
chr11	2018473	-	C	49	35	0.714	0	0	C	35	M	0.576	0.822	0	3E-05	0	403.14	tcgggggagCcgagactgcg	m5C_22298
chr11	2018482	-	C	35	19	0.543	0	0	C	19	M	0.382	0.695	0	3E-05	0	145.12	gacatcttctCgggggggagcc	m5C_22270
chr11	2018484	-	C	39	18	0.462	0	0	T	21	M	0.316	0.614	0	3E-05	0	113.64	gtgacatcttCtcggggggag	m5C_22296
chr11	2018487	-	C	37	18	0.486	0	0	T	19	M	0.334	0.641	0	3E-05	0	120.41	aaggtgacatCttctcggggg	m5C_22324
chr11	2018663	-	C	37	9	0.243	0	0	T	28	M	0.134	0.401	3E-12	1E-06	3E-12	13.907	tagctggcagCagcgggcagg	m5C_22291
chr11	2018666	-	C	33	15	0.455	0	0	T	18	M	0.298	0.62	0	1E-05	0	89.528	gtgtagctggCagcagcgggc	m5C_22297
chr11	2018716	-	C	41	23	0.561	0	0	C	23	M	0.41	0.701	0	3E-06	0	188.79	acgaggggagCtggtgggcag	m5C_22341
chr11	2018725	-	C	69	41	0.594	0	0	C	41	M	0.476	0.702	0	2E-07	0	390.61	gctggttggaCgaggggagct	m5C_22375
chr11	2018734	-	C	65	21	0.323	0	0	T	44	M	0.222	0.444	0	1E-09	0	93.256	gacgtggcagCtggttggacg	m5C_22326
chr11	2018742	-	C	74	56	0.757	0	0	C	56	M	0.648	0.84	0	9E-08	0	725.69	ggttacaggaCgtggcagctg	m5C_22304
chr11	2018794	-	C	308	88	0.286	0	0	T	220	M	0.238	0.339	0	0	0	419.13	tgagctagggCtggaaagaag	m5C_22351
chr11	2018800	-	C	299	117	0.391	0	0	T	182	M	0.338	0.448	0	0	0	790.21	tgggcctgagCtagggctgga	m5C_22314
chr11	2018805	-	C	268	59	0.22	0	0	T	209	M	0.175	0.274	0	0	0	206.13	gacgatgggcCtgagctaggg	m5C_22293
chr11	2018806	-	C	267	150	0.562	0	0	C	150	M	0.502	0.62	0	0	0	1505.5	ggacgatgggCctgagctagg	m5C_22365
chr11	2018813	-	C	212	79	0.373	0	0	T	133	M	0.31	0.439	0	0	0	490.37	cagctggggaCgatgggcctg	m5C_22323
chr11	2018820	-	C	151	35	0.232	0	0	T	116	M	0.172	0.305	0	0	0	120.15	gcgacatcagCtggggacgat	m5C_22283
chr11	2018823	-	C	126	45	0.357	0	0	T	81	M	0.279	0.444	0	6E-16	0	250.96	agggcgacatCagctggggac	m5C_22309
chr11	2018826	-	C	80	25	0.312	0	0	T	55	M	0.222	0.421	0	2E-12	0	110.75	gacagggcgaCatcagctggg	m5C_22282
chr11	2018829	-	C	79	40	0.506	0	0	C	40	M	0.398	0.614	0	6E-09	0	318.7	gcagacagggCgacatcagct	m5C_22362
chr11	2018834	-	C	42	18	0.429	0	0	T	24	M	0.291	0.578	0	8E-07	0	104.82	atcgtgcagaCagggcgacat	m5C_22321

chr11	2018845	-	C	49	20	0.408	0	0	T	29	M	0.282	0.548	0	1E-06	0	112.86	ggcgcccaggCatcgtgcaga	m5C_22279
chr11	2018850	-	C	61	21	0.344	0	0	T	40	M	0.237	0.47	0	1E-09	0	99.739	gagtaggcgcCcaggcatcgt	m5C_22346
chr11	2018851	-	C	58	19	0.328	0	0	T	39	M	0.221	0.456	0	2E-08	0	83.916	ggagtaggcgCccaggcatcg	m5C_22380
chr11	2018853	-	C	68	17	0.25	0	0	T	51	M	0.162	0.364	0	2E-10	0	55.199	gtggagtaggCgcccaggcat	m5C_22350
chr11	2018875	-	C	93	54	0.581	0	0	C	54	M	0.479	0.676	0	2E-10	0	517.44	gggcctgaggCcagtgaggag	m5C_22305
chr11	2018881	-	C	91	54	0.593	0	0	C	54	M	0.491	0.689	0	2E-10	0	529.95	ctggtggggcCtgaggccagt	m5C_22387
chr11	2018882	-	C	91	35	0.385	0	0	T	56	M	0.291	0.487	0	1E-12	0	203.88	gctggtggggCctgaggccag	m5C_22377
chr11	2018891	-	C	62	45	0.726	0	0	C	45	M	0.604	0.821	0	3E-07	0	543.67	cgaggcagggCtggtggggcc	m5C_22347
chr11	2018919	-	C	94	69	0.734	0	0	C	69	M	0.637	0.813	0	2E-09	0	878.81	ggagtgatgaCgggtggaggg	m5C_22382
chr11	2018931	-	C	65	15	0.231	0	0	T	50	M	0.145	0.346	0	6E-11	0	43.541	tggagtctggCaggagtgatg	m5C_22367
chr11	2019000	-	C	47	20	0.426	0	0	T	27	M	0.295	0.567	0	1E-06	0	118.05	gaggggcaacCaggggaagat	m5C_22310
chr11	2019004	-	C	41	24	0.585	0	0	C	24	M	0.434	0.722	0	4E-06	0	208.16	aaccgaggggCaaccaggga	m5C_22275
chr11	3178797	+	C	37	28	0.757	0	0	C	28	M	0.599	0.866	0	0.0002	0	335.34	ggggtggctcCacaggccagc	m5C_22261
chr11	3810678	-	C	117	36	0.308	0	0	T	81	M	0.231	0.396	0	1E-15	0	166.53	ggaataaagaCagagatgtga	m5C_22384
chr11	4142910	+	C	36	8	0.222	0	0	T	28	M	0.117	0.381	7E-11	7E-07	8E-11	9.4968	aagaagaacaCaggaaaggaa	m5C_21759
chr11	4159503	+	C	69	21	0.304	0	0	T	48	M	0.208	0.421	0	1E-09	0	87.568	taaggagaagCtaaaagataa	m5C_21761
chr11	4159525	+	C	173	37	0.214	0	0	T	136	M	0.159	0.281	0	0	0	117.91	gaaaaggtatCaaaagaggaa	m5C_21771
chr11	4159596	+	C	329	87	0.264	0	0	T	242	M	0.22	0.315	0	0	0	382.27	agatgaatgtCtgatgtgtgg	m5C_21763
chr11	4159609	+	C	357	91	0.255	0	0	T	266	M	0.212	0.303	0	0	0	386.69	atgtgtggatCctgaggaaag	m5C_21764
chr11	4159610	+	C	336	93	0.277	0	0	T	243	M	0.232	0.327	0	0	0	430.91	tgtgtggatcCtgaggaaaga	m5C_21770
chr11	7256443	+	C	57	31	0.544	0	0	C	31	M	0.416	0.666	0	6E-07	0	257.86	tggggggcagCttattcggcc	m5C_22017
chr11	7256452	+	C	56	24	0.429	0	0	T	32	M	0.308	0.559	0	1E-07	0	147.69	gcttattcggCcatcaatttt	m5C_22015
chr11	8705781	+	C	69	18	0.261	0	0	T	51	M	0.172	0.375	0	3E-10	0	61.868	gtcatcgaggCtagagtcacg	m5C_22114
chr11	8705788	+	C	62	13	0.21	0	0	T	49	M	0.127	0.326	2E-15	2E-11	2E-15	24.322	aggctagagtCacgcttgggt	m5C_22102
chr11	8705790	+	C	65	18	0.277	0	0	T	47	M	0.183	0.396	0	3E-10	0	65.856	gctagagtcaCgcttgggtat	m5C_22108
chr11	8705792	+	C	61	18	0.295	0	0	T	43	M	0.196	0.419	0	3E-10	0	70.398	tagagtcacgCttgggtatcg	m5C_22111
chr11	8705804	+	C	52	18	0.346	0	0	T	34	M	0.232	0.482	0	2E-08	0	83.343	tgggtatcggCtattgcctga	m5C_22110
chr11	8705811	+	C	42	13	0.31	0	0	T	29	M	0.191	0.46	0	1E-07	0	49.584	cggctattgcCtgagtgtgct	m5C_22112
chr11	10530739	-	C	72	49	0.681	0	0	C	49	M	0.566	0.777	0	3E-08	0	554.75	cttgaccgctCtgagccaaac	m5C_21998
chr11	10530743	-	C	76	57	0.75	0	0	C	57	M	0.642	0.834	0	1E-07	0	732.14	tcaacttgacCgctctgagcc	m5C_22004
chr11	10530744	-	C	72	49	0.681	0	0	C	49	M	0.566	0.777	0	3E-08	0	554.75	ctcaacttgaCcgctctgagc	m5C_21996
chr11	10530749	-	C	77	54	0.701	0	0	C	54	M	0.592	0.792	0	7E-08	0	638.82	ttcaactcaaCttgaccgctc	m5C_21989
chr11	10530754	-	C	73	42	0.575	0	0	C	42	M	0.461	0.682	0	9E-09	0	387.23	gagatttcaaCtcaacttgac	m5C_21992
chr11	10530757	-	C	73	41	0.562	0	0	C	41	M	0.448	0.67	0	7E-09	0	367.01	taggagatttCaactcaactt	m5C_22003
chr11	18425324	+	C	37	18	0.486	0	0	T	19	M	0.334	0.641	0	3E-05	0	120.41	agataaggaaCagtggaaaga	m5C_22510
chr11	21022435	-	C	66	51	0.773	0	0	C	51	M	0.658	0.857	0	8E-07	0	671.47	ataccgggtgCtgtaggctgt	m5C_22039
chr11	35684961	+	C	45	14	0.311	0	0	T	31	M	0.195	0.457	0	2E-07	0	54.68	gagagtgagtCggaggaagag	m5C_22500
chr11	35684997	+	C	43	22	0.733	13	0.302	C	22	M	0.556	0.858	0	6E-05	0	244.43	gagagcgagaCagaggaagag	m5C_22491
chr11	35685022	+	C	96	52	0.542	0	0	C	52	M	0.442	0.638	0	1E-10	0	460.01	aggatgagagCgatgaggaga	m5C_22492
chr11	35828474	+	C	48	19	0.396	0	0	T	29	M	0.27	0.537	0	1E-06	0	102.68	agatggatgaCtggaaaaagg	m5C_22502
chr11	45848213	-	C	31	22	0.71	0	0	C	22	M	0.534	0.839	0	6E-05	0	234.99	ggtggtggtgCatggccattc	m5C_22673
chr11	45848228	-	C	51	11	0.216	0	0	T	40	M	0.125	0.346	1E-13	4E-10	1E-13	17.806	tttctcaattCtgtgggtggt	m5C_22684
chr11	45848235	-	C	73	40	0.548	0	0	C	40	M	0.434	0.657	0	6E-09	0	347.39	atagctctttCtcaattctgt	m5C_22682
chr11	45848239	-	C	123	68	0.553	0	0	C	68	M	0.465	0.638	0	2E-13	0	632	attgatagctCtttctcaatt	m5C_22688
chr11	45848241	-	C	115	63	0.548	0	0	C	63	M	0.457	0.636	0	2E-12	0	575.55	agattgatagCtctttctcaa	m5C_22668
chr11	45848252	-	C	140	84	0.6	0	0	C	84	M	0.517	0.677	0	8E-15	0	868.94	acaggattgaCagattgatag	m5C_22669
chr11	45848261	-	C	131	75	0.573	0	0	C	75	M	0.487	0.654	0	3E-14	0	730.39	tggacacggaCaggattgaca	m5C_22695
chr11	45848265	-	C	110	55	0.5	0	0	CT	55	M	0.408	0.592	0	3E-13	0	448.97	agcctggacaCggacaggatt	m5C_22686
chr11	45848267	-	C	106	52	0.491	0	0	T	54	M	0.397	0.584	0	4E-12	0	413.3	ccagcctggaCacggacagga	m5C_22674
chr11	45848272	-	C	85	38	0.447	0	0	T	47	M	0.346	0.553	0	1E-10	0	262.91	ctcacccagcCtggacacgga	m5C_22689
chr11	45848273	-	C	82	36	0.439	0	0	T	46	M	0.337	0.547	0	7E-11	0	242.45	cctcacccagCctggacacgg	m5C_22692
chr11	45848276	-	C	56	25	0.446	0	0	T	31	M	0.324	0.576	0	2E-07	0	161.93	aaacctcaccCagcctggaca	m5C_22687
chr11	45848277	-	C	48	24	0.5	0	0	CT	24	M	0.364	0.636	0	4E-06	0	174.67	gaaacctcacCcagcctggac	m5C_22675
chr11	45848278	-	C	50	24	0.48	0	0	T	26	M	0.348	0.615	0	1E-07	0	167.03	ggaaacctcaCccagcctgga	m5C_22671
chr11	45848280	-	C	48	21	0.438	0	0	T	27	M	0.307	0.577	0	2E-06	0	128.94	cgggaaacctCacccagcctg	m5C_22691
chr11	45848282	-	C	41	20	0.488	0	0	T	21	M	0.343	0.635	0	1E-06	0	137.02	cacgggaaacCtcacccagcc	m5C_22696
chr11	45848283	-	C	42	21	0.5	0	0	CT	21	M	0.355	0.645	0	2E-06	0	149.21	acacgggaaaCctcacccagc	m5C_22690
chr11	46265717	+	C	33	23	0.697	0	0	C	23	M	0.527	0.826	0	8E-05	0	242.24	atgggctggtCcgagtgcagt	m5C_22539
chr11	46265718	+	C	33	16	0.485	0	0	T	17	M	0.325	0.648	0	2E-05	0	104.01	tgggctggtcCgagtgcagtg	m5C_22535
chr11	46265739	+	C	32	12	0.375	0	0	T	20	M	0.229	0.547	0	5E-06	0	55.041	gtgtttgcaaCtaactgatca	m5C_22550
chr11	46784009	-	C	51	26	0.51	0	0	C	26	M	0.377	0.641	0	2E-07	0	195.94	gccatggtatCaggtgataca	m5C_22582
chr11	46784031	-	C	63	38	0.603	0	0	C	38	M	0.48	0.715	0	1E-07	0	364.65	tgatgagttgCacactggtgg	m5C_22555
chr11	57094201	-	C	34	13	0.382	0	0	T	21	M	0.239	0.55	0	7E-06	0	62.14	agaacaagagCaaaaagaaga	m5C_22785
chr11	57094207	-	C	39	16	0.41	0	0	T	23	M	0.271	0.566	0	2E-05	0	86.653	cgggagagaaCaagagcaaaa	m5C_22790
chr11	57095222	-	C	32	12	0.375	0	0	T	20	M	0.229	0.547	0	5E-06	0	55.041	agaaggcaggCgagatctgga	m5C_22784
chr11	57097820	-	C	77	44	0.571	0	0	C	44	M	0.46	0.676	0	1E-08	0	404.89	aggaggaaggCaagatccggg	m5C_22788
chr11	58346746	+	C	31	22	0.71	0	0	C	22	M	0.534	0.839	0	6E-05	0	234.99	gggggacggaCaagccccgat	m5C_22598
chr11	58346751	+	C	32	19	0.594	0	0	C	19	M	0.423	0.745	0	3E-05	0	160.59	acggacaagcCccgatgccgg	m5C_22600
chr11	58346753	+	C	31	9	0.29	0	0	T	22	M	0.161	0.466	4E-13	1E-06	4E-13	18.015	ggacaagcccCgatgccgggg	m5C_22599
chr11	58379201	+	C	47	21	0.447	0	0	T	26	M	0.314	0.588	0	2E-06	0	131.93	ggatgaggaaCctccaaggaa	m5C_22619
chr11	58379204	+	C	43	12	0.279	0	0	T	31	M	0.167	0.427	2E-16	7E-08	2E-16	31.457	tgaggaacctCcaaggaagtg	m5C_22613
chr11	58379205	+	C	36	8	0.222	0	0	T	28	M	0.117	0.381	7E-11	7E-07	8E-11	9.4968	gaggaacctcCaaggaagtga	m5C_22607
chr11	61732280	-	C	34	17	0.5	0	0	CT	17	M	0.341	0.659	0	2E-05	0	115.83	ccaacttgcgCaagatgggag	m5C_23321
chr11	61732298	-	C	36	16	0.444	0	0	T	20	M	0.295	0.604	0	2E-05	0	94.531	aattgggtgaCcacgtgacca	m5C_23311
chr11	61732554	-	C	53	19	0.358	0	0	T	34	M	0.243	0.493	0	2E-08	0	92.347	ggagagcgggCtgaatgcaat	m5C_23319
chr11	61732558	-	C	54	19	0.352	0	0	T	35	M	0.238	0.485	0	2E-08	0	90.527	actgggagagCgggctgaatg	m5C_23317
chr11	61915997	+	C	54	30	0.556	0	0	C	30	M	0.424	0.68	0	5E-07	0	254.25	ggagcagctgCagagggaact	m5C_23297
chr11	62400707	-	C	33	16	0.485	0	0	T	17	M	0.325	0.648	0	2E-05	0	104.01	gaaagatgagCcaggagcctg	m5C_26337
chr11	62400721	-	C	31	19	0.613	0	0	C	19	M	0.438	0.763	0	3E-05	0	166.53	caggggaaggCagagaaagat	m5C_26322
chr11	62609092	-	C	35	23	0.657	0	0	C	23	M	0.492	0.792	0	8E-05	0	226.1	ggtgcactctCccttcggggа	m5C_26381
chr11	62609096	-	C	299	155	0.518	0	0	C	155	M	0.462	0.574	0	0	0	1431.8	gaacggtgcaCtctcccttcg	m5C_26321
chr11	62609098	-	C	325	120	0.369	0	0	T	205	M	0.319	0.423	0	0	0	764.58	aggaacggtgCactctccctt	m5C_26257
chr11	62609103	-	C	379	194	0.512	0	0	C	194	M	0.462	0.562	0	0	0	1791.3	cttccaggaaCggtgcactct	m5C_26256
chr11	62609109	-	C	405	141	0.348	0	0	T	264	M	0.303	0.396	0	0	0	855.52	gcagtacttcCaggaacggtg	m5C_26285
chr11	62609110	-	C	407	151	0.371	0	0	T	256	M	0.325	0.419	0	0	0	982.97	tgcagtacttCcaggaacggt	m5C_26308
chr11	62609113	-	C	417	154	0.369	0	0	T	263	M	0.324	0.417	0	0	0	999.07	tattgcagtaCttccaggaac	m5C_26345
chr11	62609118	-	C	418	149	0.357	1	0.002	T	268	M	0.313	0.404	0	0	0	932.18	cctggtattgCagtacttcca	m5C_26354
chr11	62609127	-	C	412	215	0.522	0	0	C	215	M	0.474	0.57	0	0	0	2036.6	acgcatcgacCtggtattgca	m5C_26258
chr11	62609128	-	C	416	199	0.481	2	0.005	T	215	M	0.433	0.529	0	0	0	1723.1	cacgcatcgaCctggtattgc	m5C_26378
chr11	62609131	-	C	360	191	0.531	0	0	C	191	M	0.479	0.582	0	0	0	1829.6	ctccacgcatCgacctggtat	m5C_26294
chr11	62609134	-	C	343	149	0.434	0	0	T	194	M	0.383	0.487	0	0	0	1141.2	ccactccacgCatcgacctgg	m5C_26351
chr11	62609136	-	C	313	152	0.486	0	0	T	161	M	0.431	0.541	0	0	0	1309.5	gtccactccaCgcatcgacct	m5C_26352

chr11	62609138	-	C	312	160	0.513	0	0	C	160	M	0.458	0.568	0	0	0	1464.1	ccgtccactcCacgcatcgac	m5C_26353
chr11	62609139	-	C	314	177	0.564	0	0	C	177	M	0.508	0.617	0	0	0	1799.7	tccgtccactCcacgcatcga	m5C_26252
chr11	62609141	-	C	305	203	0.666	0	0	C	203	M	0.611	0.716	0	0	0	2480.1	gctccgtccaCtccacgcatc	m5C_26283
chr11	62609143	-	C	283	168	0.594	0	0	C	168	M	0.536	0.649	0	0	0	1799.4	ttgctccgtcCactccacgca	m5C_26249
chr11	62609144	-	C	280	174	0.621	0	0	C	174	M	0.563	0.676	0	0	0	1960.4	cttgctccgtCcactccacgc	m5C_26243
chr11	62609147	-	C	251	188	0.749	0	0	C	188	M	0.692	0.799	0	0	0	2601.5	gagcttgctcCgtccactcca	m5C_26218
chr11	62609148	-	C	237	148	0.624	0	0	C	148	M	0.561	0.684	0	0	0	1661.4	ggagcttgctCcgtccactcc	m5C_26360
chr11	62609150	-	C	232	158	0.681	0	0	C	158	M	0.619	0.738	0	0	0	1954.5	taggagcttgCtccgtccact	m5C_26226
chr11	62609154	-	C	199	128	0.643	0	0	C	128	M	0.575	0.706	0	0	0	1470.8	ggaataggagCttgctccgtc	m5C_26241
chr11	62609198	-	C	221	162	0.733	0	0	C	162	M	0.671	0.787	0	0	0	2174.3	tatccgaggaCaatatattaa	m5C_26335
chr11	62609204	-	C	203	114	0.562	0	0	C	114	M	0.493	0.628	0	0	0	1123.6	gtcctctatcCgaggacaata	m5C_26233
chr11	62609205	-	C	203	116	0.571	0	0	C	116	M	0.503	0.638	0	0	0	1166.1	cgtcctctatCcgaggacaat	m5C_26348
chr11	62609209	-	C	206	153	0.743	0	0	C	153	M	0.679	0.798	0	0	0	2077.6	gatacgtcctCtatccgagga	m5C_26228
chr11	62609211	-	C	189	141	0.746	0	0	C	141	M	0.679	0.803	0	4E-16	0	1916.2	ctgatacgtcCtctatccgag	m5C_26347
chr11	62609212	-	C	188	121	0.644	0	0	C	121	M	0.573	0.709	0	0	0	1386.4	tctgatacgtCctctatccga	m5C_26230
chr11	62609215	-	C	172	119	0.692	0	0	C	119	M	0.619	0.756	0	3E-16	0	1473.9	atatctgataCgtcctctatc	m5C_26229
chr11	62620390	-	C	99	23	0.232	0	0	T	76	M	0.16	0.325	0	1E-14	0	73.641	aaggaactgtCtgagggatga	m5C_26300
chr11	62620394	-	C	100	33	0.333	1	0.01	T	66	M	0.248	0.431	0	8E-13	0	163.84	ccagaaggaaCtgtctgaggg	m5C_26380
chr11	62620403	-	C	93	43	0.462	0	0	T	50	M	0.365	0.563	0	1E-11	0	313.51	agatttgttcCagaaggaact	m5C_26242
chr11	62620404	-	C	87	23	0.264	0	0	T	64	M	0.183	0.366	0	7E-13	0	84.226	aagatttgttCcagaaggaac	m5C_26368
chr11	62620429	-	C	137	62	0.453	0	0	T	75	M	0.372	0.536	0	2E-15	0	460.83	ctgggactggCtgggggagaa	m5C_26377
chr11	62620433	-	C	138	83	0.601	0	0	C	83	M	0.518	0.679	0	1E-13	0	860.01	cccactgggaCtggctggggg	m5C_26245
chr11	62620439	-	C	137	51	0.372	0	0	T	86	M	0.296	0.456	0	1E-16	0	301.75	ggtgaacccaCtgggactggc	m5C_26291
chr11	62620442	-	C	119	33	0.277	0	0	T	86	M	0.205	0.364	0	2E-16	0	135.17	aaaggtgaacCcactgggact	m5C_26269
chr11	62620443	-	C	113	45	0.398	0	0	T	68	M	0.313	0.49	0	2E-14	0	281.47	taaaggtgaaCccactgggac	m5C_26305
chr11	62620801	-	C	89	27	0.303	0	0	T	62	M	0.218	0.405	0	4E-12	0	117.53	attttctgagCtgtgagcctg	m5C_26332
chr11	62620806	-	C	184	56	0.304	0	0	T	128	M	0.242	0.374	0	0	0	271.52	aaggtattttCtgagctgtga	m5C_26248
chr11	62620820	-	C	238	65	0.273	0	0	T	173	M	0.22	0.333	0	0	0	286.57	caatgtgtgaCtgaaaggtat	m5C_26270
chr11	62620830	-	C	224	85	0.379	0	0	T	139	M	0.318	0.445	0	0	0	541.37	ccagtctgatCaatgtgtgac	m5C_26232
chr11	62620835	-	C	228	74	0.325	0	0	T	154	M	0.267	0.388	0	0	0	395.35	ccgccccagtCtgatcaatgt	m5C_26340
chr11	62620839	-	C	231	74	0.32	0	0	T	157	M	0.264	0.383	0	0	0	390.03	aataccgcccCagtctgatca	m5C_26341
chr11	62620840	-	C	231	63	0.273	0	0	T	168	M	0.219	0.334	0	0	0	276.4	gaataccgccCcagtctgatc	m5C_26343
chr11	62620841	-	C	228	71	0.311	0	0	T	157	M	0.255	0.374	0	0	0	361.87	tgaataccgcCccagtctgat	m5C_26237
chr11	62620842	-	C	224	63	0.281	0	0	T	161	M	0.226	0.343	0	0	0	285.32	ttgaataccgCcccagtctga	m5C_26317
chr11	62620844	-	C	233	74	0.318	0	0	T	159	M	0.261	0.38	0	0	0	386.56	agttgaatacCgccccagtct	m5C_26214
chr11	62620845	-	C	239	71	0.298	1	0.004	T	167	M	0.244	0.359	0	0	0	346.15	gagttgaataCcgccccagtc	m5C_26312
chr11	62620862	-	C	100	26	0.26	0	0	T	74	M	0.184	0.354	0	7E-16	0	95.704	tgctgctcacCagtgatgagt	m5C_26314
chr11	62620863	-	C	94	26	0.277	0	0	T	68	M	0.196	0.374	0	5E-14	0	102.08	ttgctgctcaCcagtgatgag	m5C_26220
chr11	62621143	-	C	292	114	0.39	0	0	T	178	M	0.336	0.447	0	0	0	766.59	actctgaaatCtgattttctg	m5C_26365
chr11	62621150	-	C	345	120	0.348	0	0	T	225	M	0.299	0.4	0	0	0	718.78	tgttgagactCtgaaatctga	m5C_26293
chr11	62621152	-	C	357	130	0.364	0	0	T	227	M	0.316	0.415	0	0	0	821.4	gctgttgagaCtctgaaatct	m5C_26325
chr11	62621161	-	C	377	128	0.34	0	0	T	249	M	0.294	0.389	0	0	0	751.5	tgatgacttgCtgttgagact	m5C_26282
chr11	62621165	-	C	368	145	0.394	0	0	T	223	M	0.345	0.445	0	0	0	1001.8	cggctgatgaCttgctgttga	m5C_26304
chr11	62621172	-	C	361	152	0.421	0	0	T	209	M	0.371	0.473	0	0	0	1128.5	tctcgttcggCtgatgacttg	m5C_26219
chr11	62621175	-	C	354	187	0.528	0	0	C	187	M	0.476	0.58	0	0	0	1781.1	gaatctcgttCggctgatgac	m5C_26262
chr11	62621179	-	C	347	142	0.409	0	0	T	205	M	0.359	0.462	0	0	0	1018.9	catggaatctCgttcggctga	m5C_26344
chr11	62621181	-	C	353	165	0.467	0	0	T	188	M	0.416	0.52	0	0	0	1372.8	tacatggaatCtcgttcggct	m5C_26234
chr11	62621189	-	C	358	157	0.439	0	0	T	201	M	0.388	0.49	0	0	0	1218.5	tgatgacttaCatggaatctc	m5C_26209
chr11	62621193	-	C	355	133	0.375	0	0	T	222	M	0.326	0.426	0	0	0	866.87	tttgtgatgaCttacatggaa	m5C_26208
chr11	62621387	-	C	47	19	0.404	0	0	T	28	M	0.276	0.547	0	1E-06	0	105.03	aatacatgaaCacctgagaaa	m5C_26299
chr11	62621393	-	C	52	22	0.423	0	0	T	30	M	0.299	0.558	0	7E-08	0	131.42	agtgaaaataCatgaacacct	m5C_26336
chr11	62621404	-	C	51	19	0.373	0	0	T	32	M	0.253	0.51	0	2E-08	0	96.217	ctatgaccgaCagtgaaaata	m5C_26213
chr11	62621407	-	C	50	28	0.56	0	0	C	28	M	0.423	0.688	0	8E-06	0	236.91	tcactatgacCgacagtgaaa	m5C_26329
chr11	62621414	-	C	52	12	0.231	0	0	T	40	M	0.137	0.361	4E-15	9E-10	5E-15	23.637	aactagctcaCtatgaccgac	m5C_26216
chr11	62621416	-	C	54	23	0.426	0	0	T	31	M	0.303	0.558	0	9E-08	0	139.53	caaactagctCactatgaccg	m5C_26255
chr11	62621418	-	C	56	32	0.571	0	0	C	32	M	0.441	0.692	0	7E-07	0	282.48	atcaaactagCtcactatgac	m5C_26363
chr11	62621422	-	C	54	11	0.204	0	0	T	43	M	0.118	0.329	2E-13	4E-10	3E-13	16.362	atgaatcaaaCtagctcacta	m5C_26238
chr11	62621426	-	C	55	15	0.273	0	0	T	40	M	0.173	0.402	0	5E-09	0	51.842	tatgatgaatCaaactagctc	m5C_26374
chr11	62622495	-	C	55	42	0.764	0	0	C	42	M	0.637	0.856	0	4E-06	0	534.67	tgtcatcttaCtactgagaag	m5C_26278
chr11	62622499	-	C	55	41	0.745	0	0	C	41	M	0.617	0.842	0	3E-06	0	505.92	gtgatgtcatCttactactga	m5C_26273
chr11	62622512	-	C	57	41	0.732	1	0.018	C	41	M	0.604	0.83	0	3E-06	0	495.32	gctgaactttCaagtgatgtc	m5C_26265
chr11	62622516	-	C	52	36	0.692	0	0	C	36	M	0.557	0.801	0	2E-06	0	401.26	tatggctgaaCtttcaagtga	m5C_26376
chr11	62622532	-	C	41	26	0.634	0	0	C	26	M	0.481	0.764	0	6E-06	0	250.23	cacaaaatgaCaagcatatgg	m5C_26330
chr11	62622540	-	C	35	19	0.543	0	0	C	19	M	0.382	0.695	0	3E-05	0	145.12	atgatgaacaCaaaatgacaa	m5C_26254
chr11	62622542	-	C	33	18	0.545	0	0	C	18	M	0.38	0.702	0	3E-05	0	136.75	ccatgatgaaCacaaaatgac	m5C_26281
chr11	62622772	-	C	231	119	0.515	0	0	C	119	M	0.451	0.579	0	0	0	1073.3	acagaaaattCtgagtagcac	m5C_26231
chr11	62622781	-	C	264	100	0.379	0	0	T	164	M	0.322	0.439	0	0	0	644.81	gtggagaaaaCagaaaattct	m5C_26277
chr11	62622802	-	C	230	108	0.47	0	0	T	122	M	0.406	0.534	0	0	0	877.17	ctctctctttCtgatggatta	m5C_26211
chr11	62622806	-	C	228	99	0.434	0	0	T	129	M	0.371	0.499	0	0	0	735.55	tgaactctctCtttctgatgg	m5C_26306
chr11	62622808	-	C	226	99	0.438	0	0	T	127	M	0.375	0.503	0	0	0	742.37	actgaactctCtctttctgat	m5C_26224
chr11	62622810	-	C	230	102	0.443	0	0	T	128	M	0.381	0.508	0	0	0	776.68	gaactgaactCtctctttctg	m5C_26327
chr11	62622812	-	C	231	137	0.593	0	0	C	137	M	0.529	0.654	0	0	0	1448.6	acgaactgaaCtctctctttc	m5C_26302
chr11	62622817	-	C	246	98	0.398	0	0	T	148	M	0.339	0.461	0	0	0	664.85	attttacgaaCtgaactctct	m5C_26210
chr11	62622821	-	C	251	106	0.422	0	0	T	145	M	0.363	0.484	0	0	0	769.19	gatgattttaCgaactgaact	m5C_26206
chr11	62622835	-	C	109	57	0.523	0	0	C	57	M	0.43	0.614	0	1E-11	0	490.19	gaagttgctaCggggatgatt	m5C_26276
chr11	62622838	-	C	55	17	0.309	0	0	T	38	M	0.203	0.44	0	1E-08	0	68.959	gtggaagttgCtacggggatg	m5C_26289
chr11	62623047	-	C	68	21	0.309	0	0	T	47	M	0.212	0.426	0	1E-09	0	88.923	ggataaataaCtctgaggaga	m5C_26357
chr11	62623062	-	C	56	20	0.357	0	0	T	36	M	0.245	0.488	0	3E-08	0	97.826	tactgagctcCgtgaggataa	m5C_26328
chr11	62623065	-	C	59	23	0.397	1	0.017	T	35	M	0.281	0.525	0	9E-08	0	129.21	ctgtactgagCtccgtgagga	m5C_26313
chr11	62623070	-	C	59	17	0.288	0	0	T	42	M	0.188	0.414	0	1E-08	0	64.044	cagacctgtaCtgagctccgt	m5C_26367
chr11	62623075	-	C	62	18	0.29	0	0	T	44	M	0.192	0.413	0	3E-10	0	69.204	tttcacagacCtgtactgagc	m5C_26264
chr11	62623076	-	C	64	19	0.297	0	0	T	45	M	0.199	0.418	0	5E-10	0	75.639	ttttcacagaCctgtactgag	m5C_26296
chr11	62623080	-	C	66	21	0.318	0	0	T	45	M	0.218	0.438	0	1E-09	0	91.765	gaccttttcaCagacctgtac	m5C_26303
chr11	62623082	-	C	63	17	0.27	0	0	T	46	M	0.176	0.39	0	2E-10	0	59.785	aggaccttttCacagacctgt	m5C_26215
chr11	62623087	-	C	63	36	0.571	0	0	C	36	M	0.449	0.686	0	6E-08	0	323	tgatgaggacCttttcacaga	m5C_26331
chr11	62623088	-	C	67	16	0.242	1	0.015	T	50	M	0.155	0.358	0	1E-10	0	49.629	atgatgaggaCcttttcacag	m5C_26372
chr11	62648535	+	C	38	20	0.526	0	0	C	20	M	0.373	0.675	0	4E-05	0	149.03	ggaggtggagCtgaatgagtt	m5C_26152
chr11	62659095	-	C	58	29	0.5	0	0	CT	29	M	0.375	0.625	0	4E-07	0	217.72	ccctggtggtCtagtggttag	m5C_26225
chr11	64027637	+	C	31	10	0.323	0	0	T	21	M	0.186	0.499	7E-15	2E-06	8E-15	26.273	gacctgctgaCcgtgaggatg	m5C_25928
chr11	64027638	+	C	34	9	0.265	0	0	T	25	M	0.146	0.431	1E-12	1E-06	1E-12	15.739	acctgctgacCgtgaggatga	m5C_25974
chr11	65190685	+	C	31	23	0.742	0	0	C	23	M	0.568	0.863	0	8E-05	0	261.07	atcaggaaggCaggcagtggg	m5C_25945
chr11	65266721	+	C	36	11	0.306	0	0	T	25	M	0.18	0.469	1E-15	3E-06	1E-15	29.708	aatatgaagaCttagaagagt	m5C_25953

chr11	65266734	+	C	33	14	0.424	0	0	T	19	M	0.272	0.592	0	9E-06	0	76.259	agaagagtagCatgaggaagg	m5C_26014
chr11	65267103	+	C	609	157	0.261	7	0.011	T	445	M	0.227	0.297	0	0	0	713.78	taggatgaaaCaatttggaga	m5C_26030
chr11	65267137	+	C	875	394	0.45	0	0	T	481	M	0.418	0.483	0	0	0	3290.8	aagtggaaaaCtggaagacag	m5C_25987
chr11	65267145	+	C	849	232	0.274	1	0.001	T	616	M	0.245	0.305	0	0	0	1135.2	aactggaagaCagaagtacgg	m5C_26038
chr11	65267153	+	C	589	214	0.364	1	0.002	T	374	M	0.326	0.404	0	0	0	1395.5	gacagaagtaCgggaaggcga	m5C_25949
chr11	65267161	+	C	659	157	0.245	19	0.029	T	483	M	0.214	0.28	0	0	0	670.58	tacgggaaggCgaagaaaaga	m5C_25977
chr11	65267301	+	C	51	17	0.333	0	0	T	34	M	0.22	0.47	0	1E-08	0	74.697	gcaaaatgtaCaaacttagaa	m5C_25933
chr11	65267667	+	C	58	17	0.293	0	0	T	41	M	0.192	0.42	0	1E-08	0	65.205	ttaaaaaaaaCtaaggcagaa	m5C_26048
chr11	65267961	+	C	250	69	0.276	0	0	T	181	M	0.224	0.334	0	0	0	309.53	gactgaggagCaagcgagcaa	m5C_26008
chr11	65267965	+	C	280	68	0.243	0	0	T	212	M	0.196	0.296	0	0	0	267.01	gaggagcaagCgagcaagcag	m5C_26039
chr11	65267976	+	C	319	92	0.288	0	0	T	227	M	0.241	0.34	0	0	0	444.25	gagcaagcagCagttcgtggt	m5C_25935
chr11	65267981	+	C	348	85	0.244	0	0	T	263	M	0.202	0.292	0	0	0	343.51	agcagcagttCgtggtgaaga	m5C_25940
chr11	65268012	+	C	293	62	0.212	0	0	T	231	M	0.169	0.262	0	0	0	209.21	gtccaggagcCagtgcgattt	m5C_26009
chr11	65268017	+	C	338	89	0.263	0	0	T	249	M	0.219	0.313	0	0	0	390.19	ggagccagtgCgatttggtga	m5C_26043
chr11	65268034	+	C	288	63	0.219	0	0	T	225	M	0.175	0.27	0	0	0	220.35	gtgaaggaagCtaggaagaag	m5C_26005
chr11	65268054	+	C	46	17	0.37	0	0	T	29	M	0.245	0.514	0	6E-07	0	83.38	ggaaggagcgCtaacgatttg	m5C_26004
chr11	65268058	+	C	49	32	0.653	0	0	C	32	M	0.513	0.771	0	2E-05	0	328.39	ggagcgctaaCgatttggtgg	m5C_25999
chr11	65268074	+	C	66	22	0.333	0	0	T	44	M	0.232	0.453	0	1E-09	0	101.89	ggtggtgaagCtaggaaaaag	m5C_25914
chr11	65268090	+	C	56	15	0.268	0	0	T	41	M	0.17	0.396	0	5E-09	0	50.871	aaaaggattcCaggaaggagc	m5C_26012
chr11	65268439	+	C	41	21	0.512	0	0	C	21	M	0.365	0.657	0	2E-06	0	153.23	tgagggcagaCtgccaagtcc	m5C_25929
chr11	65268449	+	C	39	27	0.692	0	0	C	27	M	0.536	0.814	0	0.0001	0	289.32	ctgccaagtcCtggagaaata	m5C_26035
chr11	65268671	+	C	49	18	0.367	0	0	T	31	M	0.247	0.507	0	8E-07	0	88.794	ggatgtgtaaCtgaggcgggg	m5C_25944
chr11	65270538	+	C	49	30	0.612	0	0	C	30	M	0.472	0.736	0	1E-05	0	283.47	gagttgggatCaagtggattg	m5C_26036
chr11	65270560	+	C	53	42	0.792	0	0	C	42	M	0.665	0.88	0	4E-06	0	558.95	ggaggctgtgCtgtgtgccaa	m5C_25938
chr11	65270568	+	C	33	26	0.788	0	0	C	26	M	0.622	0.893	0	0.0001	0	323.69	tgctgtgtgcCaatgtttcgt	m5C_26050
chr11	65270996	+	C	32	11	0.344	0	0	T	21	M	0.204	0.517	2E-16	3E-06	2E-16	35.144	gcatgatgtgCtgttagaatc	m5C_26006
chr11	65271247	+	C	93	25	0.269	0	0	T	68	M	0.189	0.367	0	3E-14	0	94.606	aggatttgagCggaagaacga	m5C_25962
chr11	65271255	+	C	106	30	0.283	0	0	T	76	M	0.206	0.375	0	5E-15	0	123.61	agcggaagaaCgaatgtaact	m5C_26001
chr11	65273299	+	C	74	24	0.324	0	0	T	50	M	0.229	0.437	0	6E-11	0	109.75	ggagaaataaCatgttcaaga	m5C_26040
chr11	65273330	+	C	240	58	0.242	0	0	T	182	M	0.192	0.3	0	0	0	222.55	tgggtgggaaCatgtaacttg	m5C_25931
chr11	65273337	+	C	243	114	0.469	0	0	T	129	M	0.407	0.532	0	0	0	928.77	gaacatgtaaCttgtagactg	m5C_25994
chr11	65273345	+	C	51	31	0.608	0	0	C	31	M	0.471	0.73	0	6E-07	0	291.93	aacttgtagaCtggagaagat	m5C_26049
chr11	65661664	+	C	51	26	0.51	0	0	C	26	M	0.377	0.641	0	2E-07	0	195.94	catggtggccCagtggtagga	m5C_26010
chr11	66234545	+	C	34	10	0.294	0	0	T	24	M	0.168	0.462	2E-14	2E-06	3E-14	22.956	caggcgggccCggcgggcgc	m5C_22747
chr11	66234546	+	C	36	12	0.333	0	0	T	24	M	0.202	0.497	0	5E-06	0	48.515	aggcgggcccCggcgggcgcg	m5C_22748
chr11	66234549	+	C	33	16	0.485	0	0	T	17	M	0.325	0.648	0	2E-05	0	104.01	cgggccccggCgggcgcgggg	m5C_22758
chr11	70918775	-	C	35	18	0.514	0	0	C	18	M	0.356	0.67	0	3E-05	0	128.05	ctgagagagaCggggtcacac	m5C_22811
chr11	75380203	+	C	43	33	0.767	0	0	C	33	M	0.623	0.868	0	2E-05	0	410.88	cacctgctgtCgtcagcttgc	m5C_23400
chr11	75380206	+	C	39	28	0.718	0	0	C	28	M	0.562	0.835	0	0.0002	0	314.86	ctgctgtcgtCagcttgcgct	m5C_23406
chr11	85195023	+	C	31	20	0.645	0	0	C	20	M	0.469	0.789	0	4E-05	0	187.79	cccgggtcttCcaggagtcgg	m5C_24945
chr11	85195024	+	C	33	21	0.636	0	0	C	21	M	0.466	0.778	0	5E-05	0	195.79	ccgggtcttcCaggagtcggg	m5C_24960
chr11	85195038	+	C	124	86	0.694	0	0	C	86	M	0.608	0.768	0	4E-12	0	1045.1	agtcgggttgCttgggaatgc	m5C_24923
chr11	85195048	+	C	180	93	0.517	0	0	C	93	M	0.444	0.589	0	0	0	825.99	cttgggaatgCagcccaaagc	m5C_24929
chr11	85195051	+	C	182	71	0.392	1	0.005	T	110	M	0.324	0.465	0	0	0	460.19	gggaatgcagCccaaagcggg	m5C_24928
chr11	85195052	+	C	177	84	0.475	0	0	T	93	M	0.402	0.548	0	0	0	675.92	ggaatgcagcCcaaagcgggt	m5C_24973
chr11	85195053	+	C	189	89	0.471	0	0	T	100	M	0.401	0.542	0	0	0	713.83	gaatgcagccCaaagcgggtg	m5C_24969
chr11	85195058	+	C	181	104	0.605	9	0.05	C	104	M	0.53	0.675	0	0	0	1102.5	cagcccaaagCgggtggtaaa	m5C_24936
chr11	85195069	+	C	106	59	0.557	0	0	C	59	M	0.462	0.648	0	2E-11	0	544.8	gggtggtaaaCtccatttaag	m5C_24975
chr11	85195072	+	C	71	31	0.437	0	0	T	40	M	0.327	0.552	0	6E-10	0	203.02	tggtaaactcCatttaaggct	m5C_24927
chr11	85195081	+	C	109	36	0.33	0	0	T	73	M	0.249	0.423	0	5E-14	0	179.34	ccatttaaggCtaaataccgg	m5C_24925
chr11	85195088	+	C	138	51	0.37	0	0	T	87	M	0.294	0.453	0	1E-16	0	299.45	aggctaaataCcggcacgaga	m5C_24966
chr11	85195089	+	C	152	42	0.276	0	0	T	110	M	0.211	0.352	0	0	0	177.58	ggctaaatacCggcacgagac	m5C_24954
chr11	85195092	+	C	160	53	0.333	1	0.006	T	106	M	0.265	0.41	0	0	0	280.64	taaataccggCacgagaccga	m5C_24958
chr11	85195094	+	C	180	76	0.422	0	0	T	104	M	0.352	0.495	0	0	0	535.7	aataccggcaCgagaccgata	m5C_24967
chr11	85195099	+	C	249	124	0.498	0	0	T	125	M	0.436	0.56	0	0	0	1082.3	cggcacgagaCcgatagtcaa	m5C_24934
chr11	85195100	+	C	252	123	0.488	0	0	T	129	M	0.427	0.55	0	0	0	1050.5	ggcacgagacCgatagtcaac	m5C_24956
chr11	85195107	+	C	399	155	0.391	3	0.008	T	241	M	0.345	0.44	0	0	0	1068.3	gaccgatagtCaacaagtacc	m5C_24971
chr11	85195110	+	C	480	188	0.392	0	0	T	292	M	0.349	0.436	0	0	0	1312.3	cgatagtcaaCaagtaccgta	m5C_24942
chr11	85195116	+	C	629	276	0.439	0	0	T	353	M	0.4	0.478	0	0	0	2210.7	tcaacaagtaCcgtaagggaa	m5C_24983
chr11	85195117	+	C	705	314	0.446	1	0.001	T	390	M	0.41	0.483	0	0	0	2572.9	caacaagtacCgtaagggaaa	m5C_24955
chr11	85195139	+	C	1611	763	0.474	0	0	T	848	M	0.449	0.498	0	0	0	6856.8	ttgaaaaagaaCtttgaagaga	m5C_24941
chr11	85195155	+	C	537	241	0.449	0	0	T	296	M	0.407	0.491	0	0	0	1962.9	agagagagtaCaagagggcgt	m5C_24968
chr11	85195163	+	C	641	386	0.602	0	0	C	386	M	0.564	0.639	0	0	0	4352.5	tacaagagggCgtgaaaccgt	m5C_24980
chr11	85195170	+	C	479	159	0.332	0	0	T	320	M	0.291	0.375	0	0	0	926.19	gggcgtgaaaCcgttaagagg	m5C_24952
chr11	85195171	+	C	459	126	0.275	0	0	T	333	M	0.236	0.317	0	0	0	593.92	ggcgtgaaacCgttaagaggt	m5C_24961
chr11	85195195	+	C	57	29	0.509	0	0	C	29	M	0.383	0.634	0	4E-07	0	221.91	tgggtcaggtCcgcgcagtcc	m5C_24939
chr11	85195196	+	C	73	23	0.315	0	0	T	50	M	0.22	0.429	0	4E-11	0	101.22	gggtcaggtcCgcgcagtccg	m5C_24962
chr11	85195198	+	C	102	41	0.41	2	0.02	T	59	M	0.319	0.508	0	2E-13	0	261.31	gtcaggtccgCgcagtccgcc	m5C_24951
chr11	85195200	+	C	109	32	0.294	0	0	T	77	M	0.216	0.385	0	1E-14	0	138.42	caggtccgcgCagtccgcccg	m5C_24949
chr11	85195204	+	C	143	44	0.308	0	0	T	99	M	0.238	0.388	0	0	0	209.35	tccgcgcagtCcgcccggagg	m5C_24976
chr11	85195207	+	C	153	32	0.209	0	0	T	121	M	0.152	0.28	0	0	0	97.425	gcgcagtccgCccggaggatt	m5C_24926
chr11	85195209	+	C	165	48	0.291	0	0	T	117	M	0.227	0.364	0	0	0	217.91	gcagtccgccCggaggattca	m5C_24959
chr11	85195221	+	C	96	27	0.281	0	0	T	69	M	0.201	0.378	0	7E-14	0	108.58	gaggattcaaCccggtggcgg	m5C_24950
chr11	85195222	+	C	95	42	0.442	0	0	T	53	M	0.346	0.542	0	1E-11	0	290.99	aggattcaacCcggtggcgga	m5C_24953
chr11	85195229	+	C	40	18	0.45	0	0	T	22	M	0.307	0.602	0	3E-05	0	110.54	aacccggtggCggatctttcc	m5C_24957
chr11	85195241	-	C	37	12	0.324	0	0	T	25	M	0.196	0.485	0	5E-06	0	47.119	gaacggggggCgggaaagatc	m5C_24989
chr11	85195248	-	C	40	20	0.5	0	0	CT	20	M	0.352	0.648	0	1E-06	0	140.8	tcgggaggaaCgggggcggg	m5C_25021
chr11	86632101	-	C	41	22	0.537	0	0	C	22	M	0.387	0.679	0	2E-06	0	170.48	gtaggaggagCtgaacaggga	m5C_24550
chr11	86632115	-	C	43	20	0.465	0	0	T	23	M	0.325	0.611	0	1E-06	0	130.04	taatgttgaaCggagtaggag	m5C_24548
chr11	91818577	-	C	60	36	0.6	0	0	C	36	M	0.474	0.714	0	6E-08	0	341.03	ccctggtggtCtagtagttag	m5C_23288
chr11	93463794	-	C	44	11	0.25	0	0	T	33	M	0.146	0.394	2E-14	4E-08	2E-14	22.132	tcaaagagggCttatgaggct	m5C_24447
chr11	93464672	-	C	42	26	0.619	0	0	C	26	M	0.468	0.75	0	6E-06	0	243.43	cctttctgaaCatccatttac	m5C_24430
chr11	93464677	-	C	89	49	0.551	0	0	C	49	M	0.447	0.65	0	1E-09	0	438.32	aatggcctttCtgaacatcca	m5C_24421
chr11	93464681	-	C	94	25	0.266	0	0	T	69	M	0.187	0.363	0	3E-14	0	93.557	tgaaaatggcCtttctgaaca	m5C_24386
chr11	93464682	-	C	97	27	0.278	0	0	T	70	M	0.199	0.375	0	7E-14	0	107.41	atgaaaatggCctttctgaac	m5C_24404
chr11	93464696	-	C	118	43	0.368	1	0.008	T	74	M	0.286	0.458	0	1E-14	0	245.67	tctgaaggggCtgaatgaaaa	m5C_24406
chr11	93464705	-	C	121	60	0.496	0	0	T	61	M	0.408	0.584	0	3E-14	0	489.95	ttcaactgctCtgaaggggct	m5C_24445
chr11	93464707	-	C	118	24	0.203	0	0	T	94	M	0.141	0.285	0	0	0	67.516	tgttcaactgCtctgaagggg	m5C_24444
chr11	93464710	-	C	117	26	0.222	0	0	T	91	M	0.156	0.306	0	0	0	81.33	aaatgttcaaCtgctctgaag	m5C_24391
chr11	93464713	-	C	118	70	0.593	0	0	C	70	M	0.503	0.678	0	6E-12	0	704.21	gcgaaatgttCaactgctctg	m5C_24448
chr11	93464722	-	C	118	71	0.602	0	0	C	71	M	0.512	0.685	0	7E-12	0	726.33	tgatgatgggCgaaatgttca	m5C_24395
chr11	93466402	-	C	35	26	0.743	0	0	C	26	M	0.579	0.858	0	0.0001	0	301.24	aaacttaattCtgacagagct	m5C_24382

chr11	93466415	-	C	37	26	0.703	0	0	C	26	M	0.542	0.825	0	0.0001	0	281.93	cgggcatgaaCtaaaacttaa	m5C_24436
chr11	93466421	-	C	39	28	0.718	0	0	C	28	M	0.562	0.835	0	0.0002	0	314.86	tgataacgggCatgaactaaa	m5C_24415
chr11	93466436	-	C	46	35	0.761	0	0	C	35	M	0.621	0.861	0	3E-05	0	434.45	tgttcaactgCtgaatgataa	m5C_24400
chr11	93466439	-	C	46	35	0.761	0	0	C	35	M	0.621	0.861	0	3E-05	0	434.45	aactgttcaaCtgctgaatga	m5C_24420
chr11	93466447	-	C	47	36	0.766	0	0	C	36	M	0.628	0.864	0	3E-05	0	451.98	atgaatttaaCtgttcaactg	m5C_24409
chr11	93466720	-	C	62	43	0.694	0	0	C	43	M	0.57	0.794	0	2E-07	0	490.48	gcctgcacatCgttggaaacg	m5C_24413
chr11	93466723	-	C	63	44	0.698	0	0	C	44	M	0.576	0.798	0	3E-07	0	507.23	gtagcctgcaCatcgttggaa	m5C_24422
chr11	93466729	-	C	59	40	0.678	0	0	C	40	M	0.551	0.783	0	3E-06	0	440.84	tttcctgtagCctgcacatcg	m5C_24419
chr11	93466735	-	C	37	19	0.514	0	0	C	19	M	0.359	0.666	0	3E-05	0	136.4	ggggcttttcCtgtagcctgc	m5C_24402
chr11	93466736	-	C	37	20	0.541	0	0	C	20	M	0.384	0.69	0	4E-05	0	153.54	tggggcttttCctgtagcctg	m5C_24433
chr11	93466741	-	C	45	20	0.444	0	0	T	25	M	0.309	0.588	0	1E-06	0	123.75	cccgatggggCttttcctgta	m5C_24385
chr11	93466749	-	C	38	18	0.474	0	0	T	20	M	0.325	0.627	0	3E-05	0	116.92	aggtctatccCgatggggctt	m5C_24428
chr11	93466751	-	C	37	18	0.486	0	0	T	19	M	0.334	0.641	0	3E-05	0	120.41	tgaggtctatCccgatggggc	m5C_24429
chr11	102100518	+	C	58	39	0.672	0	0	C	39	M	0.544	0.779	0	2E-06	0	424.51	caaatgtggaCcttggaacac	m5C_24618
chr11	102100526	+	C	82	29	0.354	0	0	T	53	M	0.259	0.462	0	8E-12	0	150.14	gaccttggaaCactggaagga	m5C_24623
chr11	102100548	+	C	86	26	0.302	0	0	T	60	M	0.215	0.406	0	3E-12	0	112.03	atggaatgaaCatagaaggag	m5C_24619
chr11	108593985	+	C	40	19	0.475	0	0	T	21	M	0.329	0.625	0	1E-06	0	125.15	aagaagaagaCgatgaagaag	m5C_24569
chr11	118622539	-	C	75	27	0.36	0	0	T	48	M	0.261	0.473	0	2E-10	0	140.75	ggaaagggaCaaggaaggaa	m5C_24717
chr11	119181097	-	C	44	20	0.455	0	0	T	24	M	0.317	0.599	0	1E-06	0	126.82	gggcctcctgCagggcagcag	m5C_24707
chr11	119181101	-	C	43	19	0.442	0	0	T	24	M	0.304	0.589	0	1E-06	0	115.65	agatgggcctCctgcagggca	m5C_24702
chr11	120356317	+	C	56	26	0.464	0	0	T	30	M	0.34	0.593	0	2E-07	0	176.9	ggaagtgagaCggggaaggga	m5C_24587
chr11	122022988	-	C	33	18	0.545	0	0	C	18	M	0.38	0.702	0	3E-05	0	136.75	tagatccgaaCttgtggtatt	m5C_24801
chr11	122930045	-	C	34	17	0.5	0	0	CT	17	M	0.341	0.659	0	2E-05	0	115.83	gatgtctgagCgagtgatagt	m5C_24792
chr11	122930050	-	C	38	18	0.474	0	0	T	20	M	0.325	0.627	0	3E-05	0	116.92	ccttggatgtCtgagcgagtg	m5C_24813
chr11	122930059	-	C	41	20	0.488	0	0	T	21	M	0.343	0.635	0	1E-06	0	137.02	caacaccttcCttggatgtct	m5C_24790
chr11	122930063	-	C	42	20	0.476	0	0	T	22	M	0.334	0.623	0	1E-06	0	133.44	atggcaacacCttccttggat	m5C_24807
chr11	122930066	-	C	39	20	0.513	0	0	C	20	M	0.362	0.661	0	4E-05	0	144.8	atgatggcaaCaccttccttg	m5C_24818
chr11	122930069	-	C	39	18	0.474	1	0.026	T	20	M	0.325	0.627	0	3E-05	0	116.92	ctaatgatggCaacaccttcc	m5C_24802
chr11	122930106	-	C	48	34	0.708	0	0	C	34	M	0.568	0.818	0	2E-05	0	386.38	gattgttaaaCattcgtagtt	m5C_24808
chr11	122930127	-	C	33	9	0.273	0	0	T	24	M	0.151	0.442	8E-13	1E-06	8E-13	16.442	aggtgactcgCtgtgatgagt	m5C_24798
chr12	970269	+	C	43	11	0.256	0	0	T	32	M	0.149	0.402	1E-14	4E-08	1E-14	22.889	gaaacgagagCagcggcagtt	m5C_25139
chr12	970284	+	C	78	43	0.551	0	0	C	43	M	0.441	0.657	0	1E-08	0	379.34	gcagttggtaCgggaggagca	m5C_25130
chr12	970293	+	C	84	35	0.417	0	0	T	49	M	0.317	0.523	0	6E-11	0	222	acgggaggagCaagaaaaaaa	m5C_25122
chr12	3563416	+	C	40	11	0.275	0	0	T	29	M	0.161	0.428	4E-15	3E-06	5E-15	25.45	cttctgactcCctgacgggga	m5C_24871
chr12	3563421	+	C	39	16	0.41	0	0	T	23	M	0.271	0.566	0	2E-05	0	86.653	gactccctgaCggggagcctg	m5C_24877
chr12	3563428	+	C	32	20	0.625	0	0	C	20	M	0.453	0.771	0	4E-05	0	181.02	tgacggggagCctgcggatgg	m5C_24866
chr12	6878771	+	C	41	10	0.244	0	0	T	31	M	0.138	0.393	2E-13	2E-08	3E-13	17.447	ctttgcaggaCctgaaggaga	m5C_25211
chr12	6879098	+	C	39	14	0.359	0	0	T	25	M	0.227	0.516	0	9E-06	0	63.677	aggaggagaaCggggctgagg	m5C_25207
chr12	6879124	+	C	106	29	0.274	0	0	T	77	M	0.198	0.365	0	3E-15	0	114.7	gaagaagaaaCtgccgaggat	m5C_25210
chr12	6879127	+	C	114	53	0.465	0	0	T	61	M	0.376	0.556	0	2E-13	0	398.55	gaagaaactgCcgaggatgga	m5C_25191
chr12	6879128	+	C	129	35	0.271	0	0	T	94	M	0.202	0.354	0	0	0	141.42	aagaaactgcCgaggatggag	m5C_25200
chr12	6976778	+	C	47	30	0.638	0	0	C	30	M	0.495	0.76	0	1E-05	0	297.21	ggaagatgaaCgggcggaagc	m5C_25199
chr12	6976782	+	C	48	31	0.646	0	0	C	31	M	0.504	0.766	0	1E-05	0	312.72	gatgaacgggCggaagcagag	m5C_25193
chr12	6976788	+	C	35	26	0.743	0	0	C	26	M	0.579	0.858	0	0.0001	0	301.24	cgggcggaagCagagtctggg	m5C_25190
chr12	17143740	+	C	37	16	0.432	0	0	T	21	M	0.287	0.591	0	2E-05	0	91.749	gagaaggaggCtgcttagatg	m5C_25069
chr12	17143743	+	C	35	17	0.486	0	0	T	18	M	0.33	0.644	0	2E-05	0	112.18	aaggaggctgCttagatggga	m5C_25067
chr12	19877513	+	C	79	27	0.342	0	0	T	52	M	0.247	0.452	0	2E-10	0	133.22	agtggggggaCtgtgtttgcg	m5C_25149
chr12	19877524	+	C	82	22	0.268	0	0	T	60	M	0.184	0.373	0	4E-13	0	81.118	tgtgtttgcgCtttccctgtg	m5C_25148
chr12	20704372	+	C	57	37	0.649	0	0	C	37	M	0.519	0.76	0	2E-06	0	384.37	acaggattgaCagattgatag	m5C_26967
chr12	20704391	+	C	278	97	0.349	0	0	T	181	M	0.295	0.407	0	0	0	572.87	agctctttctCgattccgtgg	m5C_26953
chr12	20704396	+	C	442	280	0.633	0	0	C	280	M	0.588	0.677	0	0	0	3290.5	tttctcgattCcgtgggtggt	m5C_26963
chr12	20704397	+	C	468	162	0.367	27	0.058	T	279	M	0.324	0.413	0	0	0	1048.7	ttctcgattcCgtgggtggtg	m5C_26945
chr12	20704435	+	C	1043	520	0.499	0	0	T	523	M	0.468	0.529	0	0	0	4870.1	gttggtggagCgatttgtctg	m5C_26966
chr12	20704499	+	C	155	78	0.503	0	0	C	78	M	0.425	0.581	0	1E-16	0	663.61	acgcgaccccCgagcgggtct	m5C_26950
chr12	21791334	-	C	48	19	0.396	0	0	T	29	M	0.27	0.537	0	1E-06	0	102.68	tgggaactgaCaatgatagtg	m5C_27016
chr12	21791338	-	C	49	28	0.571	0	0	C	28	M	0.433	0.7	0	8E-06	0	242.31	gaaatgggaaCtgacaatgat	m5C_27023
chr12	21796993	-	C	59	15	0.254	0	0	T	44	M	0.161	0.378	0	5E-09	0	48.168	ctgcaggagtCcgtcagcaag	m5C_26987
chr12	21797000	-	C	45	24	0.533	0	0	C	24	M	0.391	0.671	0	4E-06	0	187.57	gtggtaactgCaggagtccgt	m5C_27007
chr12	21797003	-	C	40	14	0.35	0	0	T	26	M	0.221	0.505	0	2E-07	0	61.976	gtagtggtaaCtgcaggagtc	m5C_27010
chr12	22079792	+	C	42	17	0.405	0	0	T	25	M	0.27	0.555	0	6E-07	0	91.945	ccagtgattcCagaccagatg	m5C_25403
chr12	22079797	+	C	41	16	0.39	0	0	T	25	M	0.257	0.543	0	4E-07	0	82.099	gattccagacCagatgtgcat	m5C_25401
chr12	27908142	+	C	74	29	0.392	0	0	T	45	M	0.289	0.506	0	4E-10	0	167.42	aaaagtaagaCtgaagcagac	m5C_25533
chr12	27908148	+	C	66	14	0.212	0	0	T	52	M	0.131	0.325	2E-16	3E-11	2E-16	28.66	aagactgaagCagacatggaa	m5C_25521
chr12	31479284	+	C	33	20	0.606	0	0	C	20	M	0.437	0.753	0	4E-05	0	174.73	agctccgcggCgcgggaaacg	m5C_25270
chr12	34356839	+	C	32	22	0.688	0	0	C	22	M	0.514	0.82	0	6E-05	0	226.31	tctgggaatgCcgggtgctgt	m5C_25325
chr12	34356840	+	C	31	23	0.742	0	0	C	23	M	0.568	0.863	0	8E-05	0	261.07	ctgggaatgcCgggtgctgta	m5C_25324
chr12	34356846	+	C	32	19	0.594	0	0	C	19	M	0.423	0.745	0	3E-05	0	160.59	atgccgggtgCtgtaggcttt	m5C_25315
chr12	42707688	-	C	50	39	0.78	0	0	C	39	M	0.648	0.872	0	2E-06	0	505.11	gctcctgaacCagaagaagaa	m5C_25364
chr12	42707689	-	C	52	21	0.404	0	0	T	31	M	0.282	0.539	0	5E-08	0	118.27	agctcctgaaCcagaagaaga	m5C_25365
chr12	42707694	-	C	45	11	0.244	0	0	T	34	M	0.142	0.387	2E-14	4E-08	2E-14	21.411	aagaaagctcCtgaaccagaa	m5C_25355
chr12	42707697	-	C	39	18	0.462	0	0	T	21	M	0.316	0.614	0	3E-05	0	113.64	aaaaagaaagCtcctgaacca	m5C_25363
chr12	45581244	-	C	69	18	0.261	0	0	T	51	M	0.172	0.375	0	3E-10	0	61.868	caagttggtcCgagtgttgtg	m5C_25343
chr12	45581245	-	C	63	28	0.444	0	0	T	35	M	0.328	0.567	0	1E-08	0	183.94	ccaagttggtCcgagtgttgt	m5C_25342
chr12	46320751	-	C	40	9	0.225	0	0	T	31	M	0.123	0.375	6E-12	1E-08	7E-12	12.404	ggtcccagagCagagaacgag	m5C_25388
chr12	49048295	-	C	31	12	0.387	0	0	T	19	M	0.237	0.562	0	5E-06	0	56.959	tccagtggccCtgactgaaga	m5C_25874
chr12	49231357	-	C	42	23	0.548	0	0	C	23	M	0.399	0.688	0	3E-06	0	183.76	ggaagggcggCagaagatccg	m5C_25877
chr12	49231385	-	C	42	12	0.286	0	0	T	30	M	0.172	0.436	2E-16	7E-08	2E-16	32.247	aacgggagacCaatggaaatg	m5C_25870
chr12	49333527	-	C	32	14	0.438	0	0	T	18	M	0.282	0.607	0	9E-06	0	78.862	tgatcgggagCgagtaaatga	m5C_25894
chr12	49333533	-	C	34	8	0.235	0	0	T	26	M	0.124	0.4	4E-11	7E-07	4E-11	10.326	cagcaatgatCgggagcgagt	m5C_25885
chr12	49666979	+	C	56	13	0.232	0	0	T	43	M	0.141	0.358	4E-16	2E-09	5E-16	28.14	gcagatagtgCtgacggagag	m5C_25864
chr12	50571162	-	C	36	22	0.611	0	0	C	22	M	0.449	0.752	0	6E-05	0	197.4	cctggcaaaaCaaagaatcta	m5C_25444
chr12	53433101	+	C	47	35	0.745	0	0	C	35	M	0.605	0.847	0	3E-05	0	423.44	acggagggaaCagagaccact	m5C_25835
chr12	53433107	+	C	56	15	0.268	0	0	T	41	M	0.17	0.396	0	5E-09	0	50.871	ggaacagagaCcactggaagg	m5C_25845
chr12	53433110	+	C	57	37	0.649	0	0	C	37	M	0.519	0.76	0	2E-06	0	384.37	acagagaccaCtggaaggagt	m5C_25830
chr12	53433121	+	C	57	27	0.474	0	0	T	30	M	0.35	0.601	0	2E-07	0	188.93	tggaaggagtCagataggtat	m5C_25836
chr12	56320745	+	C	38	29	0.763	0	0	C	29	M	0.608	0.87	0	0.0002	0	352.6	gaaggcggtcCggggggcggg	m5C_25646
chr12	56320752	+	C	33	21	0.636	0	0	C	21	M	0.466	0.778	0	5E-05	0	195.79	gtccggggggCggggcaaagg	m5C_25636
chr12	56572601	-	C	37	20	0.541	0	0	C	20	M	0.384	0.69	0	4E-05	0	153.54	atggagacgaCgggcaaggtg	m5C_25658
chr12	57037500	-	C	45	31	0.689	0	0	C	31	M	0.543	0.805	0	1E-05	0	336.88	tttcgttcttCtgagtttgct	m5C_25672
chr12	57037503	-	C	43	30	0.698	0	0	C	30	M	0.549	0.814	0	1E-05	0	329.37	gactttcgttCttctgagttt	m5C_25661
chr12	57037511	-	C	41	29	0.707	0	0	C	29	M	0.555	0.824	0	1E-05	0	322.02	tacgttctgaCtttcgttctt	m5C_25730
chr12	57037515	-	C	49	36	0.735	0	0	C	36	M	0.597	0.838	0	3E-05	0	430.11	tgagtacgttCtgactttcgt	m5C_25712

chr12	57037519	-	C	48	32	0.667	0	0	C	32	M	0.525	0.783	0	2E-05	0	336.26	ctgatgagtaCgttctgactt	m5C_25682
chr12	57108175	-	C	37	8	0.216	0	0	T	29	M	0.114	0.372	1E-10	7E-07	1E-10	9.1227	taaagcaaaaCagagtcggag	m5C_25687
chr12	58112019	+	C	79	52	0.658	0	0	C	52	M	0.548	0.753	0	5E-08	0	570.43	tgatccagaaCggcagagaga	m5C_25489
chr12	58112022	+	C	75	44	0.587	0	0	C	44	M	0.474	0.691	0	1E-08	0	416.82	tccagaacggCagagagagat	m5C_25490
chr12	58190322	+	C	32	18	0.6	2	0.062	C	18	M	0.423	0.754	0	3E-05	0	152.35	agactttgtgCggtttgaatg	m5C_25491
chr12	58388801	-	C	32	20	0.625	0	0	C	20	M	0.453	0.771	0	4E-05	0	181.02	gccatctgctCatccccattg	m5C_25496
chr12	58388806	-	C	32	18	0.562	0	0	C	18	M	0.393	0.718	0	3E-05	0	141.57	gagttgccatCtgctcatccc	m5C_25503
chr12	58388810	-	C	34	9	0.265	0	0	T	25	M	0.146	0.431	1E-12	1E-06	1E-12	15.739	ctgggagttgCcatctgctca	m5C_25499
chr12	58388820	-	C	37	10	0.27	0	0	T	27	M	0.154	0.43	7E-14	2E-06	7E-14	20.282	tgcgggatgcCtgggagttgc	m5C_25507
chr12	58388821	-	C	37	15	0.405	0	0	T	22	M	0.263	0.565	0	1E-05	0	79.039	ttgcgggatgCctgggagttg	m5C_25506
chr12	62997488	+	C	59	30	0.508	0	0	C	30	M	0.384	0.632	0	5E-07	0	230.61	gtagtttgtgCtgttggtcgg	m5C_26090
chr12	76443543	-	C	59	17	0.288	0	0	T	42	M	0.188	0.414	0	1E-08	0	64.044	ggtgaagaagCggatgaggta	m5C_25779
chr12	90024332	-	C	31	20	0.69	2	0.065	C	20	M	0.508	0.827	0	0.001	0	203.08	ttacttggagCtggaggtgaa	m5C_25786
chr12	93750936	-	C	43	32	0.744	0	0	C	32	M	0.598	0.851	0	2E-05	0	382.47	ccctgatggtCtagtggctag	m5C_26122
chr12	96429859	+	C	163	40	0.245	0	0	T	123	M	0.186	0.317	0	0	0	148.56	ggttcgattcCccgacgggga	m5C_26474
chr12	98850879	+	C	32	19	0.594	0	0	C	19	M	0.423	0.745	0	3E-05	0	160.59	aggggagcgcCgcgtcaccag	m5C_26421
chr12	98897306	+	C	57	22	0.386	0	0	T	35	M	0.271	0.516	0	7E-08	0	119.08	tggttagtatCcccgcctgtc	m5C_26420
chr12	98897307	+	C	54	19	0.352	0	0	T	35	M	0.238	0.485	0	2E-08	0	90.527	ggttagtatcCccgcctgtca	m5C_26424
chr12	98897308	+	C	57	19	0.333	0	0	T	38	M	0.225	0.463	0	2E-08	0	85.476	gttagtatccCcgcctgtcac	m5C_26410
chr12	98897309	+	C	58	24	0.414	0	0	T	34	M	0.296	0.542	0	1E-07	0	142.21	ttagtatcccCgcctgtcacg	m5C_26427
chr12	98897311	+	C	59	25	0.424	0	0	T	34	M	0.306	0.551	0	2E-07	0	153.06	agtatccccgCctgtcacgcg	m5C_26425
chr12	98897312	+	C	51	12	0.235	0	0	T	39	M	0.14	0.368	3E-15	9E-10	4E-15	24.331	gtatccccgcCtgtcacgcgg	m5C_26429
chr12	98897327	+	C	86	25	0.291	0	0	T	61	M	0.205	0.394	0	2E-12	0	102.66	acgcgggagaCcggggttcaa	m5C_26422
chr12	98897328	+	C	83	64	0.771	0	0	C	64	M	0.67	0.848	0	2E-08	0	857.49	cgcgggagacCggggttcaat	m5C_26426
chr12	98897335	+	C	85	17	0.2	0	0	T	68	M	0.129	0.297	0	2E-14	0	43.788	gaccggggttCaattccccga	m5C_26414
chr12	98897340	+	C	84	32	0.381	0	0	T	52	M	0.284	0.488	0	2E-11	0	182.06	gggttcaattCcccgacgggg	m5C_26417
chr12	98897341	+	C	84	20	0.238	0	0	T	64	M	0.16	0.339	0	2E-13	0	63.898	ggttcaattcCccgacgggga	m5C_26416
chr12	104325338	+	C	31	22	0.71	0	0	C	22	M	0.534	0.839	0	6E-05	0	234.99	gtggatggtaCagtagaagag	m5C_26813
chr12	104325352	+	C	70	23	0.329	0	0	T	47	M	0.23	0.445	0	4E-11	0	105.79	agaagaggatCtgggtaaaag	m5C_26803
chr12	104325374	+	C	191	77	0.403	0	0	T	114	M	0.336	0.474	0	0	0	517.67	agagaaggatCaaggacggat	m5C_26801
chr12	104341102	+	C	34	14	0.412	0	0	T	20	M	0.264	0.578	0	9E-06	0	73.824	gaagaagagcCcgaagaagaa	m5C_26814
chr12	104341114	+	C	45	21	0.467	0	0	T	24	M	0.329	0.609	0	2E-06	0	138.33	gaagaagaacCtgaagagaca	m5C_26805
chr12	104341123	+	C	45	22	0.489	0	0	T	23	M	0.35	0.63	0	2E-06	0	153.81	cctgaagagaCagcagaagac	m5C_26812
chr12	104720177	+	C	97	33	0.34	0	0	T	64	M	0.254	0.439	0	8E-13	0	167.39	ggcttagaaaCcgtaggggtg	m5C_26806
chr12	106573499	-	C	32	24	0.75	0	0	C	24	M	0.579	0.867	0	9E-05	0	277.89	gactccagatCaggcgcaatg	m5C_26449
chr12	106573504	-	C	34	27	0.794	0	0	C	27	M	0.632	0.897	0	0.0001	0	341.29	tgtgtgactcCagatcaggcg	m5C_26447
chr12	110867827	-	C	31	23	0.742	0	0	C	23	M	0.568	0.863	0	8E-05	0	261.07	ggggcagaccCaaaaaactgg	m5C_26648
chr12	112460375	+	C	47	33	0.702	0	0	C	33	M	0.56	0.813	0	2E-05	0	369.75	agatgagtgaCgggaagaagg	m5C_26569
chr12	117908958	+	C	33	8	0.242	0	0	T	25	M	0.128	0.41	3E-11	7E-07	3E-11	10.787	tctgtgtctcCcctcatcccc	m5C_26519
chr12	120729574	-	C	73	17	0.233	0	0	T	56	M	0.151	0.342	0	2E-12	0	51.268	ttgacagtctCtacggagact	m5C_26728
chr12	120729576	-	C	71	17	0.239	0	0	T	54	M	0.155	0.35	0	2E-12	0	52.771	ttttgacagtCtctacggaga	m5C_26716
chr12	120729590	-	C	72	21	0.292	0	0	T	51	M	0.199	0.405	0	2E-11	0	83.739	tcggcattggCaatttttgac	m5C_26726
chr12	120729615	-	C	72	19	0.264	0	0	T	53	M	0.176	0.376	0	7E-12	0	66.856	cccgccatgaCgacttgaaat	m5C_26699
chr12	120729621	-	C	42	21	0.5	0	0	CT	21	M	0.355	0.645	0	2E-06	0	149.21	caataccccgCcatgacgact	m5C_26705
chr12	123466284	+	C	122	66	0.541	0	0	C	66	M	0.453	0.627	0	1E-13	0	597.51	agtacaaggaCaagaggagga	m5C_26920
chr12	123466318	+	C	74	25	0.338	0	0	T	49	M	0.24	0.451	0	9E-11	0	120.23	gaggaagaagCtgaagaagaa	m5C_26922
chr12	125406302	-	C	38	16	0.421	0	0	T	22	M	0.279	0.578	0	2E-05	0	89.127	ccggcatctcCattgtagttt	m5C_27155
chr12	125406303	-	C	40	29	0.725	0	0	C	29	M	0.572	0.839	0	1E-05	0	331.56	cccggcatctCcattgtagtt	m5C_27152
chr12	125406305	-	C	39	24	0.615	0	0	C	24	M	0.459	0.751	0	9E-05	0	220.31	tccccggcatCtccattgtag	m5C_27156
chr12	125406308	-	C	37	24	0.649	0	0	C	24	M	0.488	0.782	0	9E-05	0	234.04	cgatccccggCatctccattg	m5C_27148
chr12	125406311	-	C	41	19	0.463	0	0	T	22	M	0.321	0.613	0	1E-06	0	121.81	gttcgatcccCggcatctcca	m5C_27171
chr12	125406312	-	C	41	18	0.439	0	0	T	23	M	0.299	0.59	0	8E-07	0	107.6	ggttcgatccCcggcatctcc	m5C_27166
chr12	125406313	-	C	39	19	0.487	0	0	T	20	M	0.339	0.638	0	3E-05	0	128.69	gggttcgatcCccggcatctc	m5C_27149
chr12	125406318	-	C	41	26	0.634	0	0	C	26	M	0.481	0.764	0	6E-06	0	250.23	gccccgggttCgatccccggc	m5C_27164
chr12	125406324	-	C	36	23	0.639	0	0	C	23	M	0.476	0.775	0	8E-05	0	218.84	tatgaggcccCgggttcgatc	m5C_27129
chr12	125406326	-	C	38	18	0.474	0	0	T	20	M	0.325	0.627	0	3E-05	0	116.92	tgtatgaggcCccgggttcga	m5C_27123
chr12	127650600	-	C	31	19	0.613	0	0	C	19	M	0.438	0.763	0	3E-05	0	166.53	ggcggccgccCtctcgcccgt	m5C_28021
chr12	127650601	-	C	32	20	0.625	0	0	C	20	M	0.453	0.771	0	4E-05	0	181.02	aggcggccgcCctctcgcccg	m5C_28056
chr12	127650604	-	C	47	26	0.553	0	0	C	26	M	0.412	0.686	0	6E-06	0	214.48	gaaaggcggcCgccctctcgc	m5C_28059
chr12	127650605	-	C	50	31	0.62	0	0	C	31	M	0.482	0.741	0	6E-07	0	298.53	ggaaaggcggCcgccctctcg	m5C_28062
chr12	127650608	-	C	50	7	0.259	23	0.46	G	23	M	0.132	0.447	2E-10	3E-05	2E-10	8.9677	gccggaaaggCggccgccctc	m5C_28028
chr12	127650616	-	C	113	46	0.407	0	0	T	67	M	0.321	0.499	0	3E-14	0	295.32	ggggcgcggcCggaaaggcgg	m5C_28011
chr12	127650617	-	C	109	40	0.367	0	0	T	69	M	0.282	0.461	0	2E-13	0	225.96	cggggcgcggCcggaaaggcg	m5C_28040
chr12	127650620	-	C	120	43	0.358	0	0	T	77	M	0.278	0.447	0	2E-16	0	239.22	aaacggggcgCggccggaaag	m5C_28073
chr12	127650627	-	C	89	42	0.472	0	0	T	47	M	0.372	0.575	0	3E-10	0	312.08	tcctgggaaaCggggcgcggc	m5C_28006
chr13	42030115	-	C	60	18	0.383	13	0.217	T	29	M	0.258	0.526	0	8E-07	0	92.846	tggtctagtgCttaggattcg	m5C_26912
chr13	42030121	-	C	58	38	0.655	0	0	C	38	M	0.527	0.764	0	2E-06	0	400.27	ccctggtggtCtagtgcttag	m5C_26916
chr13	45912861	-	C	35	19	0.543	0	0	C	19	M	0.382	0.695	0	3E-05	0	145.12	ctctattggaCtaccgtgagg	m5C_27799
chr13	46549602	-	C	55	27	0.491	0	0	T	28	M	0.364	0.619	0	2E-07	0	196.43	ggagagagagCgagaacggga	m5C_27479
chr13	46948618	-	C	695	269	0.388	1	0.001	T	425	M	0.352	0.424	0	0	0	1894.2	atttttggagCagggacatgg	m5C_27477
chr13	46948643	-	C	712	205	0.288	0	0	T	507	M	0.256	0.322	0	0	0	1049.1	tatccgaggaCaatatattaa	m5C_27476
chr13	46948657	-	C	176	61	0.347	0	0	T	115	M	0.28	0.419	0	0	0	341.89	tctgatatgtCctctatccga	m5C_27480
chr13	58458610	-	C	44	22	0.5	0	0	CT	22	M	0.358	0.642	0	2E-06	0	157.66	ccctggtggtCtaatggttag	m5C_27092
chr13	60413570	-	C	44	17	0.386	0	0	T	27	M	0.257	0.534	0	6E-07	0	87.451	tctgtagaaaCgctggaaaag	m5C_27745
chr13	60761957	-	C	34	21	0.618	0	0	C	21	M	0.45	0.761	0	5E-05	0	189.17	caaggctggtCtgatggtagt	m5C_27733
chr13	64346805	-	C	83	44	0.53	0	0	C	44	M	0.424	0.634	0	5E-10	0	372.95	tataaactctCaataatggac	m5C_27191
chr13	73765780	+	C	32	10	0.312	0	0	T	22	M	0.18	0.486	1E-14	2E-06	1E-14	25.084	ttggcattggCaatttttgac	m5C_27292
chr13	73765794	+	C	33	21	0.636	0	0	C	21	M	0.466	0.778	0	5E-05	0	195.79	ttttgacagtCtctatggaga	m5C_27295
chr13	89494706	+	C	57	41	0.719	0	0	C	41	M	0.592	0.819	0	3E-06	0	485.19	attggtggttCagtagtagaa	m5C_27376
chr13	89494721	+	C	51	39	0.765	0	0	C	39	M	0.632	0.86	0	2E-06	0	493.25	gtagaattctCgcctgccaac	m5C_27380
chr13	89494724	+	C	42	26	0.619	0	0	C	26	M	0.468	0.75	0	6E-06	0	243.43	gaattctcgcCtgccaactat	m5C_27375
chr13	97537407	-	C	38	12	0.316	0	0	T	26	M	0.191	0.475	0	5E-06	0	45.803	gatgatgaggCagaggaagag	m5C_28252
chr13	110567332	-	C	31	14	0.452	0	0	T	17	M	0.292	0.622	0	9E-06	0	81.652	ctttcccttcCccagcacact	m5C_27836
chr13	110567333	-	C	31	14	0.452	0	0	T	17	M	0.292	0.622	0	9E-06	0	81.652	tctttcccttCcccagcacac	m5C_27828
chr13	110567336	-	C	33	14	0.424	0	0	T	19	M	0.272	0.592	0	9E-06	0	76.259	ttctctttccCttccccagca	m5C_27833
chr13	110567337	-	C	34	14	0.412	0	0	T	20	M	0.264	0.578	0	9E-06	0	73.824	attctctttcCcttccccagc	m5C_27824
chr13	110567338	-	C	34	14	0.412	0	0	T	20	M	0.264	0.578	0	9E-06	0	73.824	cattctctttCccttccccag	m5C_27830
chr13	110567348	-	C	34	13	0.382	0	0	T	21	M	0.239	0.55	0	7E-06	0	62.14	gtgttgccgtCattctctttc	m5C_27829
chr13	110567351	-	C	33	14	0.424	0	0	T	19	M	0.272	0.592	0	9E-06	0	76.259	actgtgttgcCgtcattctct	m5C_27843
chr13	110997031	-	C	38	19	0.5	0	0	CT	19	M	0.348	0.652	0	3E-05	0	132.43	agtatgtggcCtgagcgaggc	m5C_27838
chr13	114239019	-	C	31	17	0.548	0	0	C	17	M	0.378	0.708	0	2E-05	0	128.42	gccggcgtgaCgtgcgcgcgg	m5C_28627
chr14	20811276	-	C	41	19	0.463	0	0	T	22	M	0.321	0.613	0	1E-06	0	121.81	tgaggtacccCgcaggggacc	m5C_28302

chr14	20811292	-	C	71	24	0.338	0	0	T	47	M	0.239	0.454	0	6E-11	0	114.65	agttcaatggCtgaggtgagg	m5C_28319
chr14	20811313	-	C	53	14	0.264	0	0	T	39	M	0.164	0.396	0	3E-09	0	46.024	agactcacggCcagcgaagtg	m5C_28312
chr14	20811324	-	C	61	17	0.279	0	0	T	44	M	0.182	0.402	0	2E-10	0	61.841	gagcttggaaCagactcacgg	m5C_28276
chr14	20811331	-	C	41	19	0.463	0	0	T	22	M	0.321	0.613	0	1E-06	0	121.81	tttgccggagCttggaacaga	m5C_28321
chr14	20811337	-	C	38	10	0.263	0	0	T	28	M	0.15	0.42	9E-14	2E-06	1E-13	19.504	gcctcctttgCcggagcttgg	m5C_28318
chr14	20811352	-	C	56	13	0.232	0	0	T	43	M	0.141	0.358	4E-16	2E-09	5E-16	28.14	aggggcccggCggatgcctcc	m5C_28271
chr14	20811356	-	C	57	17	0.298	0	0	T	40	M	0.195	0.427	0	1E-08	0	66.41	ggggaggggcCcggcggatgc	m5C_28293
chr14	20811377	-	C	32	23	0.719	0	0	C	23	M	0.546	0.844	0	8E-05	0	251.28	atgccgtggaCcccgcccttc	m5C_28289
chr14	20811393	-	C	222	170	0.766	0	0	C	170	M	0.706	0.817	0	0	0	2399.8	tcagactgggCaggagatgcc	m5C_28258
chr14	20811398	-	C	208	66	0.317	0	0	T	142	M	0.258	0.383	0	0	0	340.37	cgaggtcagaCtgggcaggag	m5C_28270
chr14	20811408	-	C	116	37	0.319	0	0	T	79	M	0.241	0.408	0	1E-15	0	178.43	gggctccgcgCgaggtcagac	m5C_28279
chr14	20811410	-	C	80	33	0.412	0	0	T	47	M	0.311	0.522	0	3E-11	0	205.3	cggggctccgCgcgaggtcag	m5C_28299
chr14	20811413	-	C	76	37	0.487	0	0	T	39	M	0.378	0.597	0	3E-09	0	279.6	gaacggggctCcgcgcgaggt	m5C_28291
chr14	20811415	-	C	57	41	0.719	0	0	C	41	M	0.592	0.819	0	3E-06	0	485.19	gagaacggggCtccgcgcgag	m5C_28303
chr14	20811428	-	C	31	21	0.677	0	0	C	21	M	0.501	0.814	0	5E-05	0	210.59	aggtgagttcCcagagaacgg	m5C_28268
chr14	20811429	-	C	32	10	0.312	0	0	T	22	M	0.18	0.486	1E-14	2E-06	1E-14	25.084	gaggtgagttCccagagaacg	m5C_28311
chr14	20811471	-	C	43	9	0.209	0	0	T	34	M	0.114	0.352	1E-11	1E-08	2E-11	11.152	gggccagaggCggccctaaca	m5C_28278
chr14	20811477	-	C	48	18	0.375	0	0	T	30	M	0.252	0.516	0	8E-07	0	90.774	agactagggcCagaggcggcc	m5C_28287
chr14	20811484	-	C	57	27	0.474	0	0	T	30	M	0.35	0.601	0	2E-07	0	188.93	aggtctgagaCtagggccaga	m5C_28313
chr14	20811527	-	C	66	16	0.242	0	0	T	50	M	0.155	0.358	0	1E-10	0	49.629	gagctgagtgCgtcctgtcac	m5C_28310
chr14	20811534	-	C	170	94	0.553	0	0	C	94	M	0.478	0.626	0	1E-16	0	898.36	gggccacgagCtgagtgcgtc	m5C_28262
chr14	20811538	-	C	208	60	0.288	0	0	T	148	M	0.231	0.353	0	0	0	277.4	agtggggccaCgagctgagtg	m5C_28322
chr14	20811540	-	C	217	75	0.346	0	0	T	142	M	0.286	0.411	0	0	0	428.29	tcagtggggcCacgagctgag	m5C_28317
chr14	20811541	-	C	238	126	0.529	0	0	C	126	M	0.466	0.592	0	0	0	1174.4	atcagtggggCcacgagctga	m5C_28267
chr14	20811549	-	C	363	190	0.523	0	0	C	190	M	0.472	0.574	0	0	0	1793.8	ggaagctcatCagtggggcca	m5C_28256
chr14	20811552	-	C	377	95	0.252	0	0	T	282	M	0.211	0.298	0	0	0	400.55	gagggaagctCatcagtgggg	m5C_28301
chr14	20811554	-	C	408	214	0.525	0	0	C	214	M	0.476	0.573	0	0	0	2037.5	cggagggaagCtcatcagtgg	m5C_28273
chr14	20811564	-	C	386	147	0.381	0	0	T	239	M	0.334	0.43	0	0	0	981.33	tcccatagggCggagggaagc	m5C_28255
chr14	21679971	-	C	31	16	0.516	0	0	C	16	M	0.348	0.68	0	2E-05	0	111.49	agcaaacaagCaggtaaatgg	m5C_28297
chr14	24606632	+	C	38	12	0.316	0	0	T	26	M	0.191	0.475	0	5E-06	0	45.803	gcggaagaaaCagcaggaggc	m5C_27666
chr14	31602811	-	C	45	11	0.244	0	0	T	34	M	0.142	0.387	2E-14	4E-08	2E-14	21.411	gagaattaaaCagatggggaa	m5C_27860
chr14	31602826	-	C	48	19	0.396	0	0	T	29	M	0.27	0.537	0	1E-06	0	102.68	agggtggagaCatgtgagaat	m5C_27858
chr14	39649914	+	C	39	12	0.308	0	0	T	27	M	0.186	0.464	0	5E-06	0	44.558	atagaggaagCaggagaggaa	m5C_28091
chr14	39649991	+	C	40	11	0.275	0	0	T	29	M	0.161	0.428	4E-15	3E-06	5E-15	25.45	agaacaaaaaCaggaaatgga	m5C_28100
chr14	50320335	-	C	55	40	0.727	0	0	C	40	M	0.598	0.827	0	3E-06	0	478.14	gaccctgcctCttttgagttc	m5C_28453
chr14	50320337	-	C	54	38	0.704	0	0	C	38	M	0.572	0.809	0	2E-06	0	434.51	gagaccctgcCtcttttgagt	m5C_28529
chr14	50320342	-	C	62	47	0.77	1	0.016	C	47	M	0.651	0.858	0	4E-07	0	611.81	atagtgagacCctgcctcttt	m5C_28477
chr14	50320343	-	C	60	43	0.717	0	0	C	43	M	0.592	0.815	0	2E-07	0	509.4	catagtgagaCcctgcctctt	m5C_28554
chr14	50320353	-	C	65	47	0.723	0	0	C	47	M	0.604	0.817	0	4E-07	0	567.94	gcctgggcaaCatagtgagac	m5C_28538
chr14	50320356	-	C	65	47	0.723	0	0	C	47	M	0.604	0.817	0	4E-07	0	567.94	ccagcctgggCaacatagtga	m5C_28486
chr14	50320361	-	C	67	51	0.761	0	0	C	51	M	0.647	0.847	0	8E-07	0	659.67	gcactccagcCtgggcaacat	m5C_28488
chr14	50320362	-	C	69	51	0.739	0	0	C	51	M	0.625	0.828	0	8E-07	0	637.39	tgcactccagCctgggcaaca	m5C_28437
chr14	50320365	-	C	67	49	0.731	0	0	C	49	M	0.615	0.823	0	6E-07	0	602.52	cactgcactcCagcctgggca	m5C_28535
chr14	50320366	-	C	66	48	0.75	2	0.03	C	48	M	0.632	0.84	0	5E-07	0	606.56	ccactgcactCcagcctgggc	m5C_28540
chr14	50320368	-	C	62	44	0.71	0	0	C	44	M	0.587	0.808	0	3E-07	0	516.67	agccactgcaCtccagcctgg	m5C_28551
chr14	50320370	-	C	64	45	0.703	0	0	C	45	M	0.582	0.801	0	3E-07	0	524.07	atagccactgCactccagcct	m5C_28548
chr14	50320373	-	C	73	54	0.74	0	0	C	54	M	0.629	0.827	0	7E-08	0	679.22	tgaatagccaCtgcactccag	m5C_28568
chr14	50320375	-	C	70	52	0.743	0	0	C	52	M	0.63	0.831	0	5E-08	0	654.93	tgtgaatagcCactgcactcc	m5C_28470
chr14	50320376	-	C	71	52	0.732	0	0	C	52	M	0.619	0.821	0	5E-08	0	644.24	ctgtgaatagCcactgcactc	m5C_28559
chr14	50320389	-	C	72	54	0.75	0	0	C	54	M	0.639	0.836	0	7E-08	0	690.19	agtgggattgCgcctgtgaat	m5C_28474
chr14	50329296	-	C	36	16	0.457	1	0.028	T	19	M	0.305	0.618	0	2E-05	0	97.49	gcactccagcCtgagcaacat	m5C_28484
chr14	50329297	-	C	38	20	0.526	0	0	C	20	M	0.373	0.675	0	4E-05	0	149.03	tgcactccagCctgagcaaca	m5C_28520
chr14	50329300	-	C	38	18	0.474	0	0	T	20	M	0.325	0.627	0	3E-05	0	116.92	cactgcactcCagcctgagca	m5C_28514
chr14	50329301	-	C	43	21	0.488	0	0	T	22	M	0.346	0.632	0	2E-06	0	145.39	ccactgcactCcagcctgagc	m5C_28509
chr14	50329305	-	C	37	19	0.514	0	0	C	19	M	0.359	0.666	0	3E-05	0	136.4	atagccactgCactccagcct	m5C_28490
chr14	50329308	-	C	38	17	0.447	0	0	T	21	M	0.301	0.603	0	2E-05	0	102.5	tgaatagccaCtgcactccag	m5C_28476
chr14	50329310	-	C	42	25	0.595	0	0	C	25	M	0.445	0.73	0	4E-06	0	222.47	tgtgaatagcCactgcactcc	m5C_28531
chr14	50329311	-	C	42	20	0.476	0	0	T	22	M	0.334	0.623	0	1E-06	0	133.44	ctgtgaatagCcactgcactc	m5C_28550
chr14	50329321	-	C	42	25	0.595	0	0	C	25	M	0.445	0.73	0	4E-06	0	222.47	gggatcgcgcCtgtgaatagc	m5C_28481
chr14	50329322	-	C	45	28	0.622	0	0	C	28	M	0.476	0.749	0	8E-06	0	266.73	tgggatcgcgCctgtgaatag	m5C_28442
chr14	50329324	-	C	34	26	0.765	0	0	C	26	M	0.6	0.876	0	0.0001	0	312.02	agtgggatcgCgcctgtgaat	m5C_28541
chr14	50329326	-	C	32	22	0.688	0	0	C	22	M	0.514	0.82	0	6E-05	0	226.31	gtagtgggatCgcgcctgtga	m5C_28503
chr14	50329407	-	C	34	14	0.412	0	0	T	20	M	0.264	0.578	0	9E-06	0	73.824	cgggggaccaCcaggttgcct	m5C_28508
chr14	50329410	-	C	32	13	0.419	1	0.031	T	18	M	0.264	0.592	0	7E-06	0	68.68	gagcgggggaCcaccaggttg	m5C_28468
chr14	50329423	-	C	31	21	0.677	0	0	C	21	M	0.501	0.814	0	5E-05	0	210.59	ggtgacctccCgggagcgggg	m5C_28560
chr14	50329427	-	C	34	13	0.382	0	0	T	21	M	0.239	0.55	0	7E-06	0	62.14	atatggtgacCtcccgggagc	m5C_28466
chr14	50329428	-	C	33	12	0.364	0	0	T	21	M	0.222	0.534	0	5E-06	0	53.249	aatatggtgaCctcccgggag	m5C_28565
chr14	50329439	-	C	51	18	0.353	0	0	T	33	M	0.236	0.49	0	2E-08	0	85.084	agttcggcatCaatatggtga	m5C_28566
chr14	50329442	-	C	51	18	0.353	0	0	T	33	M	0.236	0.49	0	2E-08	0	85.084	ctaagttcggCatcaatatgg	m5C_28534
chr14	50329445	-	C	65	30	0.462	0	0	T	35	M	0.346	0.581	0	2E-08	0	207.54	gcactaagttCggcatcaata	m5C_28447
chr14	50329452	-	C	62	28	0.452	0	0	T	34	M	0.334	0.575	0	1E-08	0	187.15	ggtgtccgcaCtaagttcggc	m5C_28530
chr14	50329454	-	C	63	34	0.54	0	0	C	34	M	0.418	0.657	0	4E-08	0	284.17	cgggtgtccgCactaagttcg	m5C_28479
chr14	50329456	-	C	54	27	0.5	0	0	CT	27	M	0.371	0.629	0	2E-07	0	200.42	atcgggtgtcCgcactaagtt	m5C_28518
chr14	50329457	-	C	56	38	0.679	0	0	C	38	M	0.548	0.786	0	2E-06	0	416.65	gatcgggtgtCcgcactaagt	m5C_28491
chr14	50329464	-	C	59	42	0.712	0	0	C	42	M	0.586	0.812	0	4E-06	0	492.4	ctatgccgatCgggtgtccgc	m5C_28502
chr14	50329468	-	C	66	37	0.561	0	0	C	37	M	0.441	0.674	0	8E-08	0	326.21	tgcgctatgcCgatcgggtgt	m5C_28460
chr14	50329469	-	C	73	40	0.548	0	0	C	40	M	0.434	0.657	0	6E-09	0	347.39	gtgcgctatgCcgatcgggtg	m5C_28467
chr14	50329474	-	C	70	37	0.529	0	0	C	37	M	0.413	0.641	0	8E-08	0	305.78	ctgtagtgcgCtatgccgatc	m5C_28545
chr14	50329476	-	C	67	47	0.701	0	0	C	47	M	0.583	0.798	0	4E-07	0	548.44	ggctgtagtgCgctatgccga	m5C_28510
chr14	50329484	-	C	65	37	0.569	0	0	C	37	M	0.448	0.682	0	8E-08	0	331.76	gagttctgggCtgtagtgcgc	m5C_28544
chr14	50329489	-	C	67	36	0.537	0	0	C	36	M	0.419	0.651	0	6E-08	0	301.8	cccaggagttCtgggctgtag	m5C_28501
chr14	50329497	-	C	53	26	0.491	0	0	T	27	M	0.361	0.621	0	2E-07	0	187.84	cgcttgagccCaggagttctg	m5C_28519
chr14	50329498	-	C	51	23	0.451	0	0	T	28	M	0.323	0.586	0	9E-08	0	148.43	tcgcttgagcCcaggagttct	m5C_28473
chr14	50329505	-	C	33	22	0.667	0	0	C	22	M	0.496	0.802	0	6E-05	0	218.27	gggaggatcgCttgagcccag	m5C_28528
chr14	50329507	-	C	36	21	0.583	0	0	C	21	M	0.422	0.729	0	5E-05	0	177.24	gtgggaggatCgcttgagccc	m5C_28526
chr14	58706669	-	C	127	99	0.78	0	0	C	99	M	0.7	0.843	0	3E-11	0	1385.6	ctagctcagtCggtagagcat	m5C_28231
chr14	58706675	-	C	126	100	0.794	0	0	C	100	M	0.715	0.855	0	3E-11	0	1429.6	gcccggctagCtcagtcggta	m5C_28225
chr14	58706682	-	C	64	45	0.703	0	0	C	45	M	0.582	0.801	0	3E-07	0	524.07	aacaggcgccCggctagctca	m5C_28234
chr14	58706684	-	C	67	46	0.687	0	0	C	46	M	0.568	0.785	0	4E-07	0	522.52	gtaacaggcgCccggctagct	m5C_28229
chr14	66480007	-	C	147	55	0.374	0	0	T	92	M	0.3	0.455	0	0	0	330.07	gggaaaagaaCgagggatcgg	m5C_28362
chr14	66480033	-	C	48	16	0.333	0	0	T	32	M	0.217	0.475	0	4E-07	0	69.365	gcaaaattacCagaatagtga	m5C_28363
chr14	73569957	+	C	49	19	0.388	0	0	T	30	M	0.264	0.528	0	1E-06	0	100.43	tgagaaagaaCggagagaaag	m5C_28886

chr14	73576100	+	C	107	28	0.262	0	0	T	79	M	0.188	0.352	0	2E-15	0	105.11	gaaatggaagCagatgaacga	m5C_28891
chr14	73576108	+	C	106	57	0.538	0	0	C	57	M	0.443	0.63	0	1E-11	0	505.21	agcagatgaaCgagataggaa	m5C_28876
chr14	96851069	+	C	326	153	0.469	0	0	T	173	M	0.416	0.524	0	0	0	1272.4	atttttggagCagggagatag	m5C_28955
chr14	102549471	-	C	61	36	0.59	0	0	C	36	M	0.465	0.705	0	6E-08	0	334.8	ctggaacttcCagaggatgaa	m5C_29625
chr14	102549472	-	C	54	16	0.296	0	0	T	38	M	0.191	0.428	0	7E-09	0	61.239	cctggaacttCcagaggatga	m5C_29636
chr14	102549475	-	C	49	21	0.429	0	0	T	28	M	0.3	0.567	0	2E-06	0	126.1	aggcctggaaCttccagagga	m5C_29631
chr14	102549482	-	C	35	7	0.2	0	0	T	28	M	0.1	0.359	2E-09	3E-07	2E-09	6.1388	ccaaagaaggCctggaacttc	m5C_29629
chr14	102551211	-	C	36	11	0.306	0	0	T	25	M	0.18	0.469	1E-15	3E-06	1E-15	29.708	gatgttggttCtgatgaggaa	m5C_29634
chr14	102551279	-	C	223	53	0.238	0	0	T	170	M	0.187	0.298	0	0	0	197.73	aaaaggaagaCaaagaagaag	m5C_29619
chr14	102551306	-	C	140	32	0.229	0	0	T	108	M	0.167	0.305	0	0	0	106.76	aagaagtaagCgatgatgagg	m5C_29622
chr14	102551323	-	C	34	18	0.529	0	0	C	18	M	0.367	0.685	0	3E-05	0	132.25	ggagaaggaaCgtgataaaga	m5C_29632
chr14	102551697	-	C	241	65	0.27	0	0	T	176	M	0.218	0.329	0	0	0	282.9	cttggaggaaCgaagaataaa	m5C_29613
chr14	102551714	-	C	85	24	0.282	0	0	T	61	M	0.198	0.386	0	1E-12	0	94.89	gaagaccaaaCtgagtacttg	m5C_29614
chr14	102551718	-	C	49	18	0.367	0	0	T	31	M	0.247	0.507	0	8E-07	0	88.794	gaaagaagacCaaactgagta	m5C_29615
chr14	102551719	-	C	50	14	0.28	0	0	T	36	M	0.175	0.417	0	2E-07	0	48.927	tgaaagaagaCcaaactgagt	m5C_29627
chr14	105179578	+	C	31	10	0.323	0	0	T	21	M	0.186	0.499	7E-15	2E-06	8E-15	26.273	gcggcgaaggCagagaggagg	m5C_29083
chr15	41988398	+	C	31	12	0.387	0	0	T	19	M	0.237	0.562	0	5E-06	0	56.959	ggtgagtgccCagaaggggtc	m5C_29254
chr15	45010017	+	C	36	10	0.278	0	0	T	26	M	0.158	0.44	5E-14	2E-06	5E-14	21.112	tctaggagggCtggcaactta	m5C_29328
chr15	45010021	+	C	33	19	0.576	0	0	C	19	M	0.408	0.728	0	3E-05	0	155.07	ggagggctggCaacttagagg	m5C_29315
chr15	45010024	+	C	31	17	0.548	0	0	C	17	M	0.378	0.708	0	2E-05	0	128.42	gggctggcaaCttagaggtgg	m5C_29310
chr15	53140881	-	C	52	13	0.25	0	0	T	39	M	0.152	0.382	1E-16	2E-09	1E-16	31.595	gtggggggtcCggatatctgc	m5C_29647
chr15	55652580	-	C	38	9	0.237	0	0	T	29	M	0.13	0.392	4E-12	1E-06	4E-12	13.374	aaacaaggaaCagatggaaaa	m5C_29407
chr15	64008585	-	C	48	31	0.646	0	0	C	31	M	0.504	0.766	0	1E-05	0	312.72	aatggaagaaCaggctgagag	m5C_29782
chr15	65588459	+	C	186	110	0.591	0	0	C	110	M	0.52	0.66	0	0	0	1143.1	agaggaacaaCtctgagtctt	m5C_29761
chr15	65588487	+	C	173	103	0.595	0	0	C	103	M	0.521	0.666	0	0	0	1073.1	tttttgaggCcttgctttgg	m5C_29776
chr15	65588492	+	C	165	70	0.424	0	0	T	95	M	0.351	0.501	0	0	0	491.96	tgaggccttgCtttggcaagg	m5C_29751
chr15	66794400	-	C	36	15	0.417	0	0	T	21	M	0.271	0.578	0	1E-05	0	81.421	cttaattggtCcgtgtttctg	m5C_29573
chr15	66794410	-	C	33	13	0.394	0	0	T	20	M	0.247	0.563	0	7E-06	0	64.175	tgagattccaCttaattggtc	m5C_29575
chr15	66795588	-	C	117	91	0.778	0	0	C	91	M	0.694	0.844	0	2E-10	0	1263.6	ctcggatgttCtgatgctgtt	m5C_29563
chr15	66795598	-	C	118	86	0.729	0	0	C	86	M	0.642	0.801	0	8E-11	0	1104.8	acatgattttCtcggatgttc	m5C_29576
chr15	69072774	-	C	212	55	0.261	1	0.005	T	156	M	0.206	0.324	0	0	0	226.69	aggtagatgaCgaggaagatg	m5C_29711
chr15	69072792	-	C	196	74	0.378	0	0	T	122	M	0.313	0.447	0	0	0	462.72	aaggttataaCgatggagagg	m5C_29701
chr15	69076763	-	C	33	9	0.273	0	0	T	24	M	0.151	0.442	8E-13	1E-06	8E-13	16.442	cgtggagggcCtggatgatga	m5C_29712
chr15	70961196	-	C	37	18	0.486	0	0	T	19	M	0.334	0.641	0	3E-05	0	120.41	gaagaaaggtCacagagatgg	m5C_30387
chr15	70961219	-	C	44	19	0.432	0	0	T	25	M	0.297	0.578	0	1E-06	0	112.79	tgaaatggagCgagagaagaa	m5C_30388
chr15	72491725	-	C	87	24	0.276	0	0	T	63	M	0.193	0.378	0	1E-12	0	92.612	cccctggagcCagatggcaag	m5C_30754
chr15	72491726	-	C	165	90	0.545	0	0	C	90	M	0.469	0.62	0	0	0	844.75	gcccctggagCcagatggcaa	m5C_30774
chr15	72491732	-	C	169	44	0.26	0	0	T	125	M	0.2	0.331	0	0	0	176.04	ctggaggcccCtggagccaga	m5C_30789
chr15	72491733	-	C	167	37	0.222	0	0	T	130	M	0.165	0.29	0	0	0	122.27	actggaggccCctggagccag	m5C_30769
chr15	72491747	-	C	166	35	0.211	0	0	T	131	M	0.156	0.279	0	0	0	108.98	aggaggaatgCtggactggag	m5C_30776
chr15	73212641	-	C	45	28	0.622	0	0	C	28	M	0.476	0.749	0	8E-06	0	266.73	tcagtgctctCaaatgttaat	m5C_30771
chr15	73212665	-	C	236	176	0.746	0	0	C	176	M	0.687	0.797	0	0	0	2416.8	ccctcgtggtCtagtggttag	m5C_30786
chr15	73212673	-	C	102	70	0.686	0	0	C	70	M	0.591	0.768	0	1E-10	0	827.2	cctcgtctccCtcgtggtcta	m5C_30794
chr15	73212674	-	C	82	54	0.659	0	0	C	54	M	0.551	0.752	0	4E-09	0	594.95	ccctcgtctcCctcgtggtct	m5C_30783
chr15	73212675	-	C	74	26	0.351	0	0	T	48	M	0.252	0.465	0	1E-10	0	131.24	tccctcgtctCcctcgtggtc	m5C_30762
chr15	74682179	-	C	75	36	0.48	0	0	T	39	M	0.371	0.591	0	2E-09	0	266.9	gccctggtgaCgggggggccc	m5C_29675
chr15	74682186	-	C	33	8	0.242	0	0	T	25	M	0.128	0.41	3E-11	7E-07	3E-11	10.787	ccatggtgccCtggtgacggg	m5C_29680
chr15	83424779	+	C	51	32	0.627	0	0	C	32	M	0.49	0.747	0	7E-07	0	313.76	tagaacaggtCttgtttgcaa	m5C_31768
chr15	90226451	-	C	35	21	0.6	0	0	C	21	M	0.436	0.744	0	5E-05	0	183	aggccaagaaCggagaaggga	m5C_30122
chr15	90226458	-	C	35	21	0.6	0	0	C	21	M	0.436	0.744	0	5E-05	0	183	agagatgaggCcaagaacgga	m5C_30113
chr15	91187006	+	C	74	30	0.405	0	0	T	44	M	0.301	0.519	0	5E-10	0	180.54	aggaggagaaCcctgaaggag	m5C_30099
chr15	91187008	+	C	70	41	0.586	0	0	C	41	M	0.469	0.694	0	2E-07	0	384.42	gaggagaaccCtgaaggagag	m5C_30094
chr15	96289141	+	C	43	18	0.419	0	0	T	25	M	0.284	0.567	0	8E-07	0	102.18	atttttggagCagagagatgg	m5C_29859
chr15	99610024	+	C	34	7	0.206	0	0	T	27	M	0.103	0.368	1E-09	3E-07	2E-09	6.4054	ttgagagaagCggcctgagtg	m5C_29985
chr16	686735	-	C	35	24	0.686	0	0	C	24	M	0.52	0.814	0	9E-05	0	249.7	gggcggcgcaCttagtttttt	m5C_30211
chr16	686737	-	C	37	15	0.405	0	0	T	22	M	0.263	0.565	0	1E-05	0	79.039	ccgggcggcgCacttagtttt	m5C_30205
chr16	686739	-	C	44	17	0.386	0	0	T	27	M	0.257	0.534	0	6E-07	0	87.451	tcccgggcggCgcacttagtt	m5C_30210
chr16	686742	-	C	40	15	0.375	0	0	T	25	M	0.242	0.53	0	3E-07	0	72.668	gattcccgggCggcgcactta	m5C_30176
chr16	686746	-	C	45	16	0.356	0	0	T	29	M	0.232	0.502	0	4E-07	0	74.3	gttcgattccCgggcggcgca	m5C_30206
chr16	686747	-	C	43	32	0.744	0	0	C	32	M	0.598	0.851	0	2E-05	0	382.47	ggttcgattcCcgggcggcgc	m5C_30201
chr16	686748	-	C	42	21	0.5	0	0	CT	21	M	0.355	0.645	0	2E-06	0	149.21	gggttcgattCccgggcggcg	m5C_30188
chr16	686753	-	C	44	25	0.568	0	0	C	25	M	0.422	0.703	0	4E-06	0	211.12	gacccgggttCgattcccggg	m5C_30204
chr16	686761	-	C	47	25	0.532	0	0	C	25	M	0.392	0.667	0	4E-06	0	196.16	attcttgcgaCccgggttcga	m5C_30199
chr16	686764	-	C	45	26	0.578	0	0	C	26	M	0.433	0.71	0	6E-06	0	225.16	cccattcttgCgacccgggtt	m5C_30184
chr16	686768	-	C	42	19	0.452	0	0	T	23	M	0.312	0.601	0	1E-06	0	118.65	gattcccattCttgcgacccg	m5C_30187
chr16	686772	-	C	38	17	0.447	0	0	T	21	M	0.301	0.603	0	2E-05	0	102.5	gcaagattccCattcttgcga	m5C_30195
chr16	686773	-	C	34	7	0.206	0	0	T	27	M	0.103	0.368	1E-09	3E-07	2E-09	6.4054	tgcaagattcCcattcttgcg	m5C_30182
chr16	686774	-	C	34	13	0.382	0	0	T	21	M	0.239	0.55	0	7E-06	0	62.14	atgcaagattCccattcttgc	m5C_30186
chr16	686781	-	C	33	7	0.212	0	0	T	26	M	0.107	0.378	1E-09	3E-07	1E-09	6.6917	tggtatcatgCaagattccca	m5C_30197
chr16	686785	-	C	38	9	0.237	0	0	T	29	M	0.13	0.392	4E-12	1E-06	4E-12	13.374	gtagtggtatCatgcaagatt	m5C_30191
chr16	686800	-	C	33	7	0.212	0	0	T	26	M	0.107	0.378	1E-09	3E-07	1E-09	6.6917	gacagcgccgCtggtgtagtg	m5C_30173
chr16	11990523	-	C	39	14	0.359	0	0	T	25	M	0.227	0.516	0	9E-06	0	63.677	ggaaagtgccCatgaaatgat	m5C_30230
chr16	11990524	-	C	41	17	0.415	0	0	T	24	M	0.278	0.566	0	6E-07	0	94.372	aggaaagtgcCcatgaaatga	m5C_30231
chr16	11990525	-	C	37	12	0.324	0	0	T	25	M	0.196	0.485	0	5E-06	0	47.119	gaggaaagtgCccatgaaatg	m5C_30233
chr16	22207102	-	C	72	38	0.528	0	0	C	38	M	0.414	0.639	0	4E-09	0	314.64	gtagcgtggcCgagtggtcta	m5C_30340
chr16	22207103	-	C	72	34	0.472	0	0	T	38	M	0.361	0.586	0	1E-09	0	245.66	ggtagcgtggCcgagtggtct	m5C_30336
chr16	22207108	-	C	69	31	0.449	0	0	T	38	M	0.338	0.566	0	2E-08	0	209.37	cgggaggtagCgtggccgagt	m5C_30331
chr16	28856114	-	C	66	29	0.439	0	0	T	37	M	0.326	0.559	0	1E-08	0	189.24	gatggtggaaCtggtggaact	m5C_30615
chr16	33963193	+	C	47	36	0.766	0	0	C	36	M	0.628	0.864	0	3E-05	0	451.98	atcttgggagCgggcgggcag	m5C_31319
chr16	33963197	+	C	44	35	0.795	0	0	C	35	M	0.655	0.888	0	3E-05	0	458.49	tgggagcgggCgggcagtccg	m5C_31423
chr16	33963251	+	C	101	79	0.782	0	0	C	79	M	0.692	0.852	0	5E-10	0	1093.6	tgcctcttggCgccccctcga	m5C_31362
chr16	33963253	+	C	103	75	0.728	0	0	C	75	M	0.635	0.805	0	3E-10	0	952.78	cctcttggcgCccccctcgatg	m5C_31240
chr16	33963254	+	C	101	72	0.713	0	0	C	72	M	0.618	0.792	0	2E-10	0	890.09	ctcttggcgcCccctcgatgc	m5C_31248
chr16	33963256	+	C	104	75	0.721	0	0	C	75	M	0.628	0.798	0	3E-10	0	942.41	cttggcgcccCctcgatgctc	m5C_31308
chr16	33963259	+	C	61	29	0.475	0	0	T	32	M	0.355	0.598	0	1E-08	0	206.08	ggcgccccctCgatgctctta	m5C_31400
chr16	33963264	+	C	48	20	0.417	0	0	T	28	M	0.288	0.557	0	1E-06	0	115.4	cccctcgatgCtcttagctga	m5C_31410
chr16	33963325	+	C	33	15	0.455	0	0	T	18	M	0.298	0.62	0	1E-05	0	89.528	gtgttcaaagCaggtccaagc	m5C_31315
chr16	33963331	+	C	34	15	0.441	0	0	T	19	M	0.289	0.605	0	1E-05	0	86.65	aaagcaggtcCaagccgcctg	m5C_31445
chr16	33963335	+	C	38	18	0.474	0	0	T	20	M	0.325	0.627	0	3E-05	0	116.92	caggtccaagCcgcctggata	m5C_31305
chr16	33963336	+	C	41	20	0.488	0	0	T	21	M	0.343	0.635	0	1E-06	0	137.02	aggtccaagcCgcctggatac	m5C_31416
chr16	33963338	+	C	44	24	0.545	0	0	C	24	M	0.401	0.683	0	4E-06	0	192.32	gtccaagccgCctggataccg	m5C_31226
chr16	33963339	+	C	44	27	0.614	0	0	C	27	M	0.466	0.743	0	7E-06	0	251.77	tccaagccgcCtggataccgc	m5C_31230

chr16	33963347	+	C	58	35	0.603	0	0	C	35	M	0.475	0.719	0	1E-06	0	332.45	gcctggatacCgcagctagga	m5C_31353
chr16	33963349	+	C	64	40	0.625	0	0	C	40	M	0.503	0.733	0	1E-07	0	402	ctggataccgCagctaggaat	m5C_31313
chr16	33963352	+	C	77	53	0.688	0	0	C	53	M	0.578	0.781	0	6E-08	0	612.68	gataccgcagCtaggaataat	m5C_31314
chr16	33963373	+	C	113	88	0.779	0	0	C	88	M	0.694	0.845	0	1E-10	0	1221	ggaataggacCgcggttctat	m5C_31300
chr16	33963423	+	C	48	38	0.792	0	0	C	38	M	0.657	0.883	0	4E-05	0	499.63	ttaagagggaCagctgggggc	m5C_31325
chr16	33963426	+	C	46	13	0.283	0	0	T	33	M	0.173	0.425	0	1E-07	0	45.043	agagggacagCtgggggcatt	m5C_31238
chr16	33963535	+	C	187	65	0.348	0	0	T	122	M	0.283	0.418	0	0	0	367.94	gaatgaaagtCggaggttcga	m5C_31273
chr16	33963543	+	C	178	135	0.758	0	0	C	135	M	0.691	0.815	0	4E-15	0	1864.4	gtcggaggttCgaagacgatc	m5C_31333
chr16	33963549	+	C	167	130	0.783	1	0.006	C	130	M	0.714	0.839	0	3E-14	0	1857.5	ggttcgaagaCgatcagatac	m5C_31387
chr16	33963666	+	C	67	45	0.672	0	0	C	45	M	0.553	0.772	0	3E-07	0	497.32	tttttaggttCcggggggagt	m5C_31311
chr16	33963667	+	C	76	50	0.658	0	0	C	50	M	0.546	0.755	0	4E-08	0	545.96	ttttaggttcCggggggagta	m5C_31229
chr16	33963684	+	C	77	58	0.753	0	0	C	58	M	0.646	0.836	0	1E-07	0	749.9	agtacggttgCaaagctgaaa	m5C_31259
chr16	33963689	+	C	53	34	0.642	0	0	C	34	M	0.507	0.757	0	1E-06	0	344.7	ggttgcaaagCtgaaacttaa	m5C_31430
chr16	33963734	+	C	62	43	0.694	0	0	C	43	M	0.57	0.794	0	2E-07	0	490.48	aggagtggagCctgcggctta	m5C_31446
chr16	33963735	+	C	59	41	0.695	0	0	C	41	M	0.569	0.797	0	3E-06	0	466.2	ggagtggagcCtgcggcttaa	m5C_31377
chr16	33963738	+	C	58	40	0.69	0	0	C	40	M	0.562	0.794	0	3E-06	0	449.57	gtggagcctgCggcttaattt	m5C_31356
chr16	33963741	+	C	56	43	0.768	0	0	C	43	M	0.642	0.859	0	4E-06	0	552.39	gagcctgcggCttaatttgac	m5C_31293
chr16	33964508	+	C	97	26	0.268	0	0	T	71	M	0.19	0.364	0	5E-14	0	98.787	gccctgagagCcggagaactc	m5C_31337
chr16	33964509	+	C	108	44	0.407	0	0	T	64	M	0.319	0.502	0	6E-13	0	281.13	ccctgagagcCggagaactcg	m5C_31324
chr16	33964516	+	C	137	32	0.234	0	0	T	105	M	0.171	0.311	0	0	0	109.18	agccggagaaCtcgggaggga	m5C_31253
chr16	33964518	+	C	137	78	0.569	0	0	C	78	M	0.486	0.649	0	6E-14	0	757.61	ccggagaactCgggagggaga	m5C_31343
chr16	33964962	+	C	35	27	0.771	0	0	C	27	M	0.61	0.879	0	0.0001	0	329.3	cgcgttctggCgcggaggttt	m5C_31431
chr16	33965201	+	C	40	23	0.575	0	0	C	23	M	0.422	0.715	0	3E-06	0	194.1	gtgcaccgtgCtgtgggggcg	m5C_31455
chr16	33965210	+	C	119	27	0.227	0	0	T	92	M	0.161	0.31	0	0	0	86.873	gctgtgggggCgggaatcccc	m5C_31264
chr16	33965217	+	C	157	61	0.389	0	0	T	96	M	0.316	0.467	0	0	0	385.3	gggcgggaatCcccgggcgcc	m5C_31367
chr16	33965219	+	C	153	51	0.333	0	0	T	102	M	0.264	0.411	0	0	0	268.8	gcgggaatccCcgggcgcccg	m5C_31280
chr16	33965220	+	C	149	42	0.282	0	0	T	107	M	0.216	0.359	0	0	0	181.29	cgggaatcccCgggcgcccgt	m5C_31369
chr16	33965224	+	C	141	76	0.539	0	0	C	76	M	0.457	0.619	0	2E-15	0	694.31	aatccccgggCgcccgtgggg	m5C_31278
chr16	33965226	+	C	140	35	0.25	0	0	T	105	M	0.186	0.328	0	0	0	129.92	tccccgggcgCccgtggggtg	m5C_31432
chr16	33965227	+	C	131	58	0.443	0	0	T	73	M	0.361	0.528	0	7E-16	0	418.21	ccccgggcgcCcgtggggtgc	m5C_31331
chr16	47539114	+	C	34	15	0.441	0	0	T	19	M	0.289	0.605	0	1E-05	0	86.65	gggtaagaagCccggcttgct	m5C_30556
chr16	47539115	+	C	34	16	0.471	0	0	T	18	M	0.315	0.633	0	2E-05	0	100.64	ggtaagaagcCcggcttgctg	m5C_30546
chr16	47539116	+	C	36	26	0.722	0	0	C	26	M	0.56	0.842	0	0.0001	0	291.24	gtaagaagccCggcttgctgg	m5C_30555
chr16	47539119	+	C	34	15	0.441	0	0	T	19	M	0.289	0.605	0	1E-05	0	86.65	agaagcccggCttgctggcgt	m5C_30561
chr16	47539123	+	C	32	15	0.469	0	0	T	17	M	0.309	0.636	0	1E-05	0	92.607	gcccggcttgCtggcgtgtgg	m5C_30554
chr16	47539156	+	C	37	18	0.486	0	0	T	19	M	0.334	0.641	0	3E-05	0	120.41	aatgtgagtgCctagtgggcc	m5C_30557
chr16	47539157	+	C	36	19	0.528	0	0	C	19	M	0.37	0.68	0	3E-05	0	140.62	atgtgagtgcCtagtgggccg	m5C_30544
chr16	49854070	+	C	39	23	0.59	0	0	C	23	M	0.434	0.729	0	8E-05	0	199.72	gaaggatggaCagtgagctga	m5C_30890
chr16	50132200	+	C	35	11	0.314	0	0	T	24	M	0.186	0.48	7E-16	3E-06	7E-16	30.969	agtgggttatCaggacttatt	m5C_30637
chr16	50132205	+	C	35	22	0.629	0	0	C	22	M	0.463	0.768	0	6E-05	0	203.88	gttatcaggaCttattaacat	m5C_30638
chr16	57697472	-	C	33	9	0.273	0	0	T	24	M	0.151	0.442	8E-13	1E-06	8E-13	16.442	ggacgaggtgCtgcccgagct	m5C_30718
chr16	57697479	-	C	37	19	0.514	0	0	C	19	M	0.359	0.666	0	3E-05	0	136.4	tgcggctggaCgaggtgctgc	m5C_30713
chr16	58633481	+	C	32	20	0.625	0	0	C	20	M	0.453	0.771	0	4E-05	0	181.02	gaggctggtcCaggcgcagtg	m5C_31075
chr16	58633502	+	C	31	10	0.323	0	0	T	21	M	0.186	0.499	7E-15	2E-06	8E-15	26.273	gtgtttacaaCtaattgatca	m5C_31066
chr16	58804587	+	C	34	7	0.206	0	0	T	27	M	0.103	0.368	1E-09	3E-07	2E-09	6.4054	tggagactgcCaggctgccta	m5C_31072
chr16	58804595	+	C	46	16	0.348	0	0	T	30	M	0.227	0.492	0	4E-07	0	72.579	gccaggctgcCtaaggagggg	m5C_31069
chr16	58804610	+	C	41	26	0.634	0	0	C	26	M	0.481	0.764	0	6E-06	0	250.23	gaggggtgaaCtggcccaggt	m5C_31065
chr16	58804614	+	C	34	8	0.235	0	0	T	26	M	0.124	0.4	4E-11	7E-07	4E-11	10.326	ggtgaactggCccaggtcagt	m5C_31078
chr16	67663332	+	C	31	19	0.613	0	0	C	19	M	0.438	0.763	0	3E-05	0	166.53	gataattgtgCtggcccagat	m5C_30851
chr16	67663337	+	C	34	11	0.324	0	0	T	23	M	0.191	0.492	4E-16	3E-06	5E-16	32.309	ttgtgctggcCcagatggcgt	m5C_30848
chr16	67663338	+	C	34	19	0.559	0	0	C	19	M	0.395	0.711	0	3E-05	0	149.92	tgtgctggccCagatggcgta	m5C_30846
chr16	67663345	+	C	37	12	0.333	1	0.027	T	24	M	0.202	0.497	0	5E-06	0	48.515	gcccagatggCgtagaggggg	m5C_30857
chr16	69814947	+	C	37	21	0.568	0	0	C	21	M	0.409	0.713	0	5E-05	0	171.84	acaggttggtCctgtggtagt	m5C_31167
chr16	69814948	+	C	36	16	0.444	0	0	T	20	M	0.295	0.604	0	2E-05	0	94.531	caggttggtcCtgtggtagtg	m5C_31159
chr16	69814965	+	C	34	17	0.5	0	0	CT	17	M	0.341	0.659	0	2E-05	0	115.83	agtgggttatCagaacttatt	m5C_31172
chr16	69814970	+	C	31	17	0.548	0	0	C	17	M	0.378	0.708	0	2E-05	0	128.42	gttatcagaaCttattaacat	m5C_31169
chr16	70296395	-	C	41	23	0.561	0	0	C	23	M	0.41	0.701	0	3E-06	0	188.79	ggtgatggctCtgcgcaggga	m5C_31011
chr16	70812154	-	C	32	18	0.562	0	0	C	18	M	0.393	0.718	0	3E-05	0	141.57	gaattctcgcCtgccacgcgg	m5C_31027
chr16	70812157	-	C	31	11	0.367	1	0.032	T	19	M	0.219	0.545	0	3E-06	0	48.122	gtagaattctCgcctgccacg	m5C_31012
chr16	70822609	+	C	73	57	0.781	0	0	C	57	M	0.673	0.86	0	1E-07	0	767.48	attggtggttCagtggtagaa	m5C_30990
chr16	70822626	+	C	72	50	0.694	0	0	C	50	M	0.58	0.789	0	4E-08	0	580.46	agaattctcgCctgccatgcg	m5C_30996
chr16	70822627	+	C	66	43	0.652	0	0	C	43	M	0.531	0.755	0	2E-07	0	456.76	gaattctcgcCtgccatgcgg	m5C_30983
chr16	70822630	+	C	37	19	0.514	0	0	C	19	M	0.359	0.666	0	3E-05	0	136.4	ttctcgcctgCcatgcgggcg	m5C_30976
chr16	70822631	+	C	34	18	0.529	0	0	C	18	M	0.367	0.685	0	3E-05	0	132.25	tctcgcctgcCatgcgggcgg	m5C_30993
chr16	70823422	+	C	97	67	0.691	0	0	C	67	M	0.593	0.774	0	1E-09	0	794.56	attggtggttCagtggtagaa	m5C_30967
chr16	70823435	+	C	88	59	0.67	0	0	C	59	M	0.567	0.76	0	8E-09	0	668.97	tggtagaattCtcgcctgcca	m5C_30992
chr16	70823437	+	C	84	56	0.667	0	0	C	56	M	0.561	0.758	0	5E-09	0	627.79	gtagaattctCgcctgccacg	m5C_30985
chr16	70823439	+	C	59	28	0.475	0	0	T	31	M	0.353	0.6	0	3E-07	0	197.49	agaattctcgCctgccacgcg	m5C_30988
chr16	70823440	+	C	48	23	0.479	0	0	T	25	M	0.345	0.617	0	3E-06	0	158.57	gaattctcgcCtgccacgcgg	m5C_30975
chr16	70823443	+	C	32	10	0.312	0	0	T	22	M	0.18	0.486	1E-14	2E-06	1E-14	25.084	ttctcgcctgCcacgcgggag	m5C_30980
chr16	74339527	+	C	55	23	0.418	0	0	T	32	M	0.297	0.55	0	9E-08	0	136.79	gaaagaagggCaggagaaaga	m5C_30955
chr16	74339544	+	C	73	34	0.466	0	0	T	39	M	0.356	0.579	0	1E-09	0	242.02	aagaagagagCaaaaaggata	m5C_30958
chr16	74339565	+	C	99	46	0.465	0	0	T	53	M	0.37	0.562	0	3E-11	0	340	ggaaagaggaCaaggagaaag	m5C_30950
chr16	85391728	-	C	42	12	0.286	0	0	T	30	M	0.172	0.436	2E-16	7E-08	2E-16	32.247	ctgtggctggCgggagtggga	m5C_31059
chr16	87902981	-	C	75	31	0.413	0	0	T	44	M	0.309	0.526	0	6E-10	0	191.44	cgccggcggcCgaggagaagg	m5C_31120
chr16	87902982	-	C	79	43	0.544	0	0	C	43	M	0.435	0.65	0	1E-08	0	374.08	gcgccggcggCcgaggagaag	m5C_31117
chr16	87902985	-	C	85	27	0.318	0	0	T	58	M	0.228	0.423	0	4E-12	0	123.34	gcggcgccggCggccgaggag	m5C_31132
chr16	87902988	-	C	85	20	0.235	0	0	T	65	M	0.158	0.336	0	2E-13	0	63.118	ctagcggcgcCggcggccgag	m5C_31128
chr16	87902991	-	C	88	25	0.284	0	0	T	63	M	0.2	0.386	0	2E-12	0	100.22	gcgctagcggCgccggcggcc	m5C_31115
chr16	87903000	-	C	66	24	0.364	0	0	T	42	M	0.258	0.484	0	3E-09	0	123.87	aagcggcgcgCgctagcggcg	m5C_31119
chr16	89351179	-	C	33	22	0.667	0	0	C	22	M	0.496	0.802	0	6E-05	0	218.27	atcgctgaagCcagtgaggaa	m5C_31682
chr16	89351187	-	C	35	22	0.629	0	0	C	22	M	0.463	0.768	0	6E-05	0	203.88	agtgtggaatCgctgaagcca	m5C_31681
chr16	89351199	-	C	34	13	0.382	0	0	T	21	M	0.239	0.55	0	7E-06	0	62.14	tctgagggctCcagtgtggaa	m5C_31687
chr16	89627838	+	C	51	26	0.51	0	0	C	26	M	0.377	0.641	0	2E-07	0	195.94	tggcctcagtCgcgtgatgac	m5C_31670
chr16	89627840	+	C	55	26	0.473	0	0	T	29	M	0.347	0.602	0	2E-07	0	180.4	gcctcagtcgCgtgatgacat	m5C_31647
chr16	89627848	+	C	149	87	0.584	0	0	C	87	M	0.504	0.66	0	1E-14	0	876.28	cgcgtgatgaCattctccgga	m5C_31660
chr16	89627852	+	C	152	81	0.533	0	0	C	81	M	0.454	0.61	0	2E-16	0	735.07	tgatgacattCtccggaatcg	m5C_31674
chr16	89627854	+	C	153	105	0.686	0	0	C	105	M	0.609	0.754	0	1E-14	0	1278.8	atgacattctCcggaatcgct	m5C_31648
chr16	89627855	+	C	153	89	0.582	0	0	C	89	M	0.502	0.657	0	9E-16	0	894.4	tgacattctcCggaatcgctg	m5C_31640
chr16	89627861	+	C	150	91	0.607	0	0	C	91	M	0.527	0.681	0	1E-15	0	958.71	tctccggaatCgctgtacggc	m5C_31653
chr16	89627863	+	C	146	89	0.61	0	0	C	89	M	0.529	0.685	0	2E-14	0	940.94	tccggaatcgCtgtacggcct	m5C_31656
chr16	89627868	+	C	135	77	0.57	0	0	C	77	M	0.486	0.651	0	5E-14	0	748.53	aatcgctgtaCggccttgatg	m5C_31659

chr16	89627871	+	C	147	106	0.721	0	0	C	106	M	0.644	0.787	0	3E-13	0	1364.6	cgctgtacggCcttgatgaaa	m5C_31644
chr16	89627872	+	C	147	81	0.551	0	0	C	81	M	0.47	0.629	0	4E-15	0	761.94	gctgtacggcCttgatgaaag	m5C_31666
chr16	89627883	+	C	143	74	0.517	0	0	C	74	M	0.436	0.598	0	1E-15	0	645.58	ttgatgaaagCacatttgaac	m5C_31655
chr16	89627885	+	C	141	80	0.567	0	0	C	80	M	0.485	0.646	0	4E-15	0	775.81	gatgaaagcaCatttgaaccc	m5C_31651
chr16	89627893	+	C	143	87	0.608	0	0	C	87	M	0.527	0.685	0	1E-14	0	916.21	cacatttgaaCccttttccat	m5C_31671
chr16	89627894	+	C	133	92	0.692	0	0	C	92	M	0.609	0.764	0	6E-13	0	1120.2	acatttgaacCcttttccatc	m5C_31652
chr16	89627895	+	C	139	87	0.626	0	0	C	87	M	0.543	0.702	0	3E-13	0	944.97	catttgaaccCttttccatct	m5C_31657
chr16	89627900	+	C	139	96	0.691	0	0	C	96	M	0.61	0.761	0	1E-12	0	1170.3	gaacccttttCcatctgattg	m5C_31658
chr16	89627901	+	C	133	80	0.602	0	0	C	80	M	0.517	0.681	0	8E-14	0	826.52	aacccttttcCatctgattgc	m5C_31646
chr16	89627904	+	C	114	75	0.658	0	0	C	75	M	0.567	0.739	0	1E-11	0	850.4	ccttttccatCtgattgctga	m5C_31663
chr17	1368040	-	C	38	15	0.395	0	0	T	23	M	0.256	0.553	0	1E-05	0	76.794	cgtcttagacCatagaggttg	m5C_31999
chr17	1368041	-	C	40	17	0.425	0	0	T	23	M	0.285	0.578	0	6E-07	0	96.931	ccgtcttagaCcatagaggtt	m5C_31993
chr17	1368047	-	C	46	20	0.435	0	0	T	26	M	0.302	0.578	0	1E-06	0	120.84	ggaaggccgtCttagaccata	m5C_31991
chr17	1437431	-	C	31	14	0.452	0	0	T	17	M	0.292	0.622	0	9E-06	0	81.652	ggacgacattCgaaggatgga	m5C_31987
chr17	1437435	-	C	33	14	0.424	0	0	T	19	M	0.272	0.592	0	9E-06	0	76.259	ccatggacgaCattcgaagga	m5C_32005
chr17	2236259	-	C	65	30	0.462	0	0	T	35	M	0.346	0.581	0	2E-08	0	207.54	gaggaggaatCtcaggtaagg	m5C_31839
chr17	2319545	-	C	41	21	0.512	0	0	C	21	M	0.365	0.657	0	2E-06	0	153.23	ctgctgaagcCgcggcatttc	m5C_31838
chr17	2593832	-	C	32	12	0.375	0	0	T	20	M	0.229	0.547	0	5E-06	0	55.041	agcccgactgCgggagaaggg	m5C_31862
chr17	2593835	-	C	31	10	0.323	0	0	T	21	M	0.186	0.499	7E-15	2E-06	8E-15	26.273	gggagcccgaCtgcgggagaa	m5C_31846
chr17	4448417	-	C	49	29	0.592	0	0	C	29	M	0.452	0.718	0	1E-05	0	262.43	aagaggagagCgaggggggagg	m5C_31962
chr17	4448447	-	C	52	21	0.404	0	0	T	31	M	0.282	0.539	0	5E-08	0	118.27	aaggtgaggaCaacagaagct	m5C_31957
chr17	6356314	-	C	40	10	0.25	0	0	T	30	M	0.142	0.402	2E-13	2E-08	2E-13	18.091	gaaaggaagaCagagagagag	m5C_32174
chr17	7218281	-	C	41	22	0.537	0	0	C	22	M	0.387	0.679	0	2E-06	0	170.48	gcgcaagcggCagggtgagcc	m5C_32159
chr17	7478041	+	C	43	14	0.326	0	0	T	29	M	0.205	0.475	0	2E-07	0	57.381	tgtccctgacCtgggtagagt	m5C_32145
chr17	7480130	+	C	34	8	0.235	0	0	T	26	M	0.124	0.4	4E-11	7E-07	4E-11	10.326	ggaagagctgCtctgtgatgg	m5C_32140
chr17	7480145	+	C	86	28	0.326	0	0	T	58	M	0.236	0.43	0	6E-12	0	132.08	tgatggagccCatgcgtgtca	m5C_32094
chr17	7480149	+	C	86	20	0.233	0	0	T	66	M	0.156	0.332	0	2E-13	0	62.356	ggagcccatgCgtgtcatctg	m5C_32133
chr17	7480157	+	C	90	46	0.511	0	0	C	46	M	0.41	0.612	0	3E-11	0	376.75	tgcgtgtcatCtgagcctctg	m5C_32082
chr17	7480162	+	C	68	43	0.632	0	0	C	43	M	0.514	0.737	0	2E-07	0	441.66	gtcatctgagCctctggcttc	m5C_32085
chr17	7480174	+	C	37	14	0.378	0	0	T	23	M	0.241	0.539	0	9E-06	0	67.379	tctggcttccCtgccagtgca	m5C_32074
chr17	7480177	+	C	42	17	0.405	0	0	T	25	M	0.27	0.555	0	6E-07	0	91.945	ggcttccctgCcagtgcagcc	m5C_32090
chr17	7480254	+	C	50	15	0.3	0	0	T	35	M	0.191	0.438	0	5E-09	0	57.31	aaggatcagtCtttgtactct	m5C_32080
chr17	7480261	+	C	53	21	0.396	0	0	T	32	M	0.276	0.531	0	5E-08	0	115.87	agtctttgtaCtctgagagca	m5C_32108
chr17	7809273	+	C	36	18	0.5	0	0	CT	18	M	0.345	0.655	0	3E-05	0	124.11	gtggctggtgCgggacctgag	m5C_32097
chr17	7809279	+	C	39	21	0.538	0	0	C	21	M	0.386	0.684	0	5E-05	0	161.99	ggtgcgggacCtgaggggcaa	m5C_32113
chr17	7809287	+	C	37	11	0.297	0	0	T	26	M	0.175	0.458	1E-15	3E-06	2E-15	28.551	acctgaggggCaagactgaga	m5C_32105
chr17	8029076	+	C	35	13	0.371	0	0	T	22	M	0.232	0.537	0	7E-06	0	60.231	attggtggttCagtggtagaa	m5C_31712
chr17	8029089	+	C	34	18	0.529	0	0	C	18	M	0.367	0.685	0	3E-05	0	132.25	tggtagaattCtcgcctgcca	m5C_31705
chr17	8029091	+	C	33	11	0.333	0	0	T	22	M	0.198	0.504	2E-16	3E-06	2E-16	34.007	gtagaattctCgcctgccacg	m5C_31700
chr17	8029093	+	C	33	12	0.364	0	0	T	21	M	0.222	0.534	0	5E-06	0	53.249	agaattctcgCctgccacgcg	m5C_31718
chr17	8090194	+	C	109	50	0.459	0	0	T	59	M	0.368	0.552	0	3E-12	0	368.17	gacgaggtggCcgagtggtta	m5C_31702
chr17	8090195	+	C	113	36	0.319	0	0	T	77	M	0.24	0.409	0	1E-15	0	172.7	acgaggtggcCgagtggttaa	m5C_31698
chr17	8090208	+	C	49	14	0.286	0	0	T	35	M	0.178	0.424	0	2E-07	0	49.978	gtggttaaggCgatggactgc	m5C_31706
chr17	16343352	+	C	69	42	0.609	0	0	C	42	M	0.491	0.715	0	2E-07	0	412.22	tgcttgactgCtctgatgaaa	m5C_33009
chr17	16343354	+	C	78	49	0.628	0	0	C	49	M	0.517	0.727	0	3E-08	0	506.96	cttgactgctCtgatgaaatc	m5C_33044
chr17	16343364	+	C	215	159	0.74	0	0	C	159	M	0.677	0.794	0	0	0	2153	ctgatgaaatCactaatagga	m5C_33020
chr17	16343366	+	C	222	127	0.572	0	0	C	127	M	0.506	0.635	0	0	0	1286	gatgaaatcaCtaataggaag	m5C_33050
chr17	16343379	+	C	235	139	0.591	0	0	C	139	M	0.528	0.652	0	0	0	1466.9	ataggaagtgCcgtcagaagc	m5C_33047
chr17	16343380	+	C	231	122	0.528	0	0	C	122	M	0.464	0.592	0	0	0	1131.7	taggaagtgcCgtcagaagcg	m5C_33058
chr17	16343383	+	C	237	116	0.489	0	0	T	121	M	0.426	0.553	0	0	0	989.45	gaagtgccgtCagaagcgata	m5C_33025
chr17	16343389	+	C	238	123	0.517	0	0	C	123	M	0.454	0.58	0	0	0	1115.8	ccgtcagaagCgataactgac	m5C_33027
chr17	16343395	+	C	235	132	0.562	0	0	C	132	M	0.498	0.624	0	0	0	1314.1	gaagcgataaCtgacgaagac	m5C_33036
chr17	16343399	+	C	237	148	0.627	1	0.004	C	148	M	0.564	0.686	0	0	0	1669	cgataactgaCgaagactact	m5C_33038
chr17	16343405	+	C	235	163	0.694	0	0	C	163	M	0.632	0.749	0	0	0	2060.2	ctgacgaagaCtactcctgtc	m5C_33048
chr17	16343408	+	C	227	138	0.613	2	0.009	C	138	M	0.548	0.675	0	0	0	1513.3	acgaagactaCtcctgtctga	m5C_33018
chr17	16343410	+	C	229	132	0.576	0	0	C	132	M	0.512	0.639	0	0	0	1350.8	gaagactactCctgtctgatt	m5C_33063
chr17	16343411	+	C	215	153	0.712	0	0	C	153	M	0.648	0.768	0	0	0	1982.2	aagactactcCtgtctgattg	m5C_33013
chr17	16343415	+	C	182	129	0.709	0	0	C	129	M	0.639	0.77	0	1E-16	0	1648.7	ctactcctgtCtgattgcagt	m5C_33064
chr17	16344539	+	C	47	16	0.34	0	0	T	31	M	0.222	0.483	0	4E-07	0	70.935	aaaaagatatCaaatgatgaa	m5C_33023
chr17	16344552	+	C	172	40	0.233	0	0	T	132	M	0.176	0.301	0	0	0	140.55	atgatgaaatCacccaaaata	m5C_33046
chr17	16344554	+	C	177	72	0.409	1	0.006	T	104	M	0.339	0.483	0	0	0	488.37	gatgaaatcaCccaaaatagc	m5C_33022
chr17	16344555	+	C	173	63	0.364	0	0	T	110	M	0.296	0.438	0	0	0	373.12	atgaaatcacCcaaaatagct	m5C_33061
chr17	16344556	+	C	168	46	0.274	0	0	T	122	M	0.212	0.346	0	0	0	195.04	tgaaatcaccCaaaatagctg	m5C_33017
chr17	16344564	+	C	169	73	0.432	0	0	T	96	M	0.36	0.507	0	0	0	525.02	cccaaaatagCtggaattacc	m5C_33035
chr17	16344573	+	C	171	72	0.421	0	0	T	99	M	0.35	0.496	0	0	0	503.4	gctggaattaCcggcagattg	m5C_33033
chr17	16344574	+	C	170	62	0.365	0	0	T	108	M	0.296	0.439	0	0	0	367.14	ctggaattacCggcagattgt	m5C_33043
chr17	16344577	+	C	179	55	0.307	0	0	T	124	M	0.244	0.378	0	0	0	268.75	gaattaccggCagattgtgta	m5C_33055
chr17	16344596	+	C	226	70	0.31	0	0	T	156	M	0.253	0.373	0	0	0	354.27	tagtggtgaaCctatggtttt	m5C_33057
chr17	16344597	+	C	220	61	0.277	0	0	T	159	M	0.222	0.34	0	0	0	271.24	agtggtgaacCtatggttttc	m5C_33052
chr17	16344607	+	C	163	72	0.442	0	0	T	91	M	0.368	0.518	0	0	0	529.48	ctatggttttCtgaagatatc	m5C_33054
chr17	18150121	-	C	42	23	0.548	0	0	C	23	M	0.399	0.688	0	3E-06	0	183.76	ctgtggagtaCgaggaggagg	m5C_34167
chr17	18150131	-	C	40	27	0.675	0	0	C	27	M	0.52	0.799	0	0.0001	0	280.89	tactgggtgcCtgtggagtac	m5C_34170
chr17	18965433	+	C	58	21	0.362	0	0	T	37	M	0.251	0.491	0	5E-08	0	105.23	agaacgcggtCtgagtggttt	m5C_34085
chr17	18967241	-	C	50	23	0.46	0	0	T	27	M	0.33	0.596	0	3E-06	0	151.66	agaacgcggtCtgagtggttt	m5C_34148
chr17	19015741	-	C	128	47	0.367	0	0	T	81	M	0.289	0.453	0	1E-15	0	271.36	agaacgcggtCtgagtggttt	m5C_34154
chr17	19015747	-	C	131	39	0.298	0	0	T	92	M	0.226	0.381	0	0	0	176.34	agggagagaaCgcggtctgag	m5C_34130
chr17	19015841	-	C	32	10	0.312	0	0	T	22	M	0.18	0.486	1E-14	2E-06	1E-14	25.084	ttctcctgagCgtgaagccgg	m5C_34140
chr17	19015846	-	C	37	9	0.243	0	0	T	28	M	0.134	0.401	3E-12	1E-06	3E-12	13.907	gcgttttctcCtgagcgtgaa	m5C_34164
chr17	19015849	-	C	37	25	0.676	0	0	C	25	M	0.515	0.804	0	0.0001	0	257.32	gtagcgttttCtcctgagcgt	m5C_34156
chr17	19091397	+	C	32	19	0.594	0	0	C	19	M	0.423	0.745	0	3E-05	0	160.59	gtgtagagcaCcgaaaaccac	m5C_34107
chr17	19091407	+	C	41	10	0.244	0	0	T	31	M	0.138	0.393	2E-13	2E-08	3E-13	17.447	ccgaaaaccaCgaggaagaga	m5C_34113
chr17	19091423	+	C	52	28	0.538	0	0	C	28	M	0.405	0.667	0	3E-07	0	226.82	agagaggtagCgttttctcct	m5C_34098
chr17	19091429	+	C	53	16	0.302	0	0	T	37	M	0.195	0.435	0	7E-09	0	62.458	gtagcgttttCtcctgagcgt	m5C_34084
chr17	19091437	+	C	52	12	0.231	0	0	T	40	M	0.137	0.361	4E-15	9E-10	5E-15	23.637	ttctcctgagCgtgaagccgg	m5C_34104
chr17	19091537	+	C	531	190	0.358	0	0	T	341	M	0.318	0.399	0	0	0	1209.2	agaacgcggtCtgagtggttt	m5C_34105
chr17	19093480	-	C	95	23	0.242	0	0	T	72	M	0.167	0.337	0	1E-14	0	76.86	ccgaaaaccaCgaggaagaga	m5C_34134
chr17	19093482	-	C	95	36	0.379	0	0	T	59	M	0.288	0.479	0	2E-12	0	207.29	caccgaaaacCacgaggaaga	m5C_34160
chr17	19093483	-	C	95	31	0.326	0	0	T	64	M	0.24	0.426	0	4E-13	0	149.04	gcaccgaaaaCcacgaggaag	m5C_34169
chr17	19093490	-	C	98	21	0.214	0	0	T	77	M	0.145	0.305	0	3E-15	0	60.751	gtgtagagcaCcgaaaaccac	m5C_34137
chr17	19093492	-	C	98	23	0.235	0	0	T	75	M	0.162	0.328	0	1E-14	0	74.42	acgtgtagagCaccgaaaacc	m5C_34162
chr17	19093501	-	C	83	20	0.241	0	0	T	63	M	0.162	0.343	0	2E-13	0	64.698	tttctctgaaCgtgtagagca	m5C_34172
chr17	19093506	-	C	60	12	0.2	0	0	T	48	M	0.118	0.318	3E-14	8E-12	4E-14	19.13	agaagtttctCtgaacgtgta	m5C_34181

chr17	19093508	-	C	41	12	0.293	0	0	T	29	M	0.176	0.445	1E-16	7E-08	1E-16	33.713	agagaagtttCtctgaacgtg	m5C_34165
chr17	19764205	+	C	38	30	0.789	0	0	C	30	M	0.637	0.889	0	0.0002	0	381.92	gaattctcgcCtcccacgcag	m5C_34123
chr17	20135081	+	C	35	9	0.257	0	0	T	26	M	0.142	0.421	1E-12	1E-06	2E-12	15.086	gtagtgtgatCaagctggagg	m5C_32242
chr17	20135085	+	C	34	17	0.5	0	0	CT	17	M	0.341	0.659	0	2E-05	0	115.83	tgtgatcaagCtggaggaaca	m5C_32247
chr17	20135101	+	C	36	13	0.361	0	0	T	23	M	0.225	0.524	0	7E-06	0	58.436	gaacagaagtCagacctggag	m5C_32251
chr17	20135118	+	C	31	10	0.323	0	0	T	21	M	0.186	0.499	7E-15	2E-06	8E-15	26.273	ggagaggcagCtgaagactct	m5C_32249
chr17	20135125	+	C	31	21	0.677	0	0	C	21	M	0.501	0.814	0	5E-05	0	210.59	cagctgaagaCtctgaccaag	m5C_32253
chr17	27047600	+	C	69	25	0.362	0	0	T	44	M	0.259	0.48	0	4E-09	0	129.48	aatctcctgaCacttgtgatg	m5C_32632
chr17	27047602	+	C	70	21	0.3	0	0	T	49	M	0.205	0.415	0	2E-11	0	86.253	tctcctgacaCttgtgatgtc	m5C_32613
chr17	27047623	+	C	68	24	0.353	0	0	T	44	M	0.25	0.472	0	3E-09	0	120.01	ttcaaaggaaCcactgatgca	m5C_32622
chr17	27047624	+	C	67	17	0.254	0	0	T	50	M	0.165	0.369	0	2E-10	0	56.058	tcaaaggaacCactgatgcac	m5C_32614
chr17	27050450	+	C	152	42	0.276	0	0	T	110	M	0.211	0.352	0	0	0	177.58	gtcctctgggCtaatgatgga	m5C_32599
chr17	27050466	+	C	376	111	0.295	0	0	T	265	M	0.251	0.343	0	0	0	558.03	atggaaaaatCattattggaa	m5C_32601
chr17	27050502	+	C	373	126	0.338	0	0	T	247	M	0.292	0.387	0	0	0	735.02	aaaggaaccaCtgaagtgccg	m5C_32615
chr17	28444120	+	C	140	28	0.2	0	0	T	112	M	0.142	0.274	0	0	0	79.606	ggctgaggggCagagagcgag	m5C_32406
chr17	31149420	-	C	131	93	0.71	0	0	C	93	M	0.627	0.781	0	7E-13	0	1166.4	gcgctggacgCgttatccctc	m5C_32469
chr17	31149422	-	C	124	68	0.548	0	0	C	68	M	0.461	0.633	0	2E-13	0	626.49	cggcgctggaCgcgttatccc	m5C_32501
chr17	31149427	-	C	143	72	0.503	0	0	C	72	M	0.423	0.584	0	7E-16	0	608.45	acggacggcgCtggacgcgtt	m5C_32493
chr17	31149429	-	C	152	84	0.553	0	0	C	84	M	0.473	0.629	0	3E-16	0	795.07	ggacggacggCgctggacgcg	m5C_32461
chr17	31149432	-	C	194	113	0.582	0	0	C	113	M	0.512	0.65	0	0	0	1157.4	aacggacggaCggcgctggac	m5C_32481
chr17	31149436	-	C	147	38	0.437	60	0.408	A	60	M	0.337	0.541	0	1E-10	0	256.44	gacgaacggaCggacggcgct	m5C_32482
chr17	31149440	-	C	672	324	0.482	0	0	T	348	M	0.445	0.52	0	0	0	2880.8	ggaagacgaaCggacggacgg	m5C_32503
chr17	31149444	-	C	843	308	0.365	0	0	T	535	M	0.334	0.398	0	0	0	2054.6	gggaggaagaCgaacggacgg	m5C_32451
chr17	31149459	-	C	969	569	0.587	0	0	C	569	M	0.556	0.618	0	0	0	6326.4	cgggagaggcCgggagggagg	m5C_32452
chr17	31149460	-	C	940	491	0.523	1	0.001	C	491	M	0.491	0.555	0	0	0	4820.8	gcgggagaggCcgggagggag	m5C_32443
chr17	31149469	-	C	581	283	0.487	0	0	T	298	M	0.447	0.528	0	0	0	2528.1	ccgtggtcggCgggagaggcc	m5C_32457
chr17	31149472	-	C	470	348	0.74	0	0	C	348	M	0.699	0.778	0	0	0	4864.7	tgcccgtggtCggcgggagag	m5C_32487
chr17	31149478	-	C	154	52	0.338	0	0	T	102	M	0.268	0.415	0	0	0	278.44	caccactgccCgtggtcggcg	m5C_32490
chr17	31149479	-	C	133	47	0.353	0	0	T	86	M	0.277	0.438	0	0	0	260.66	ccaccactgcCcgtggtcggc	m5C_32491
chr17	31149480	-	C	110	36	0.327	0	0	T	74	M	0.247	0.419	0	1E-15	0	177.63	cccaccactgCccgtggtcgg	m5C_32464
chr17	31149524	-	C	372	82	0.22	0	0	T	290	M	0.181	0.265	0	0	0	297.3	accgaagaagCggcgcgccga	m5C_32480
chr17	31149532	-	C	728	371	0.51	0	0	C	371	M	0.473	0.546	0	0	0	3512.2	aggcgggaacCgaagaagcgg	m5C_32468
chr17	31149533	-	C	740	374	0.505	0	0	C	374	M	0.469	0.541	0	0	0	3511.5	gaggcgggaaCcgaagaagcg	m5C_32453
chr17	31149539	-	C	694	391	0.563	0	0	C	391	M	0.526	0.6	0	0	0	4115.3	ggggaggaggCgggaaccgaa	m5C_32492
chr17	31149605	-	C	36	18	0.5	0	0	CT	18	M	0.345	0.655	0	3E-05	0	124.11	gcggccgcctCcgcggttccc	m5C_32483
chr17	31149607	-	C	54	18	0.333	0	0	T	36	M	0.222	0.466	0	2E-08	0	80.068	gcgcggccgcCtccgcggttc	m5C_32436
chr17	31149608	-	C	54	36	0.667	0	0	C	36	M	0.534	0.778	0	2E-06	0	384.2	ggcgcggccgCctccgcggtt	m5C_32460
chr17	31149610	-	C	52	17	0.327	0	0	T	35	M	0.215	0.462	0	1E-08	0	73.174	tcggcgcggcCgcctccgcgg	m5C_32497
chr17	31149611	-	C	57	18	0.316	0	0	T	39	M	0.21	0.445	0	2E-08	0	75.615	ctcggcgcggCcgcctccgcg	m5C_32448
chr17	31149614	-	C	81	43	0.531	0	0	C	43	M	0.423	0.636	0	4E-10	0	364.01	cggctcggcgCggccgcctcc	m5C_32456
chr17	31149616	-	C	93	61	0.656	0	0	C	61	M	0.555	0.745	0	6E-10	0	676.99	cccggctcggCgcggccgcct	m5C_32435
chr17	31149619	-	C	95	43	0.453	0	0	T	52	M	0.356	0.553	0	1E-11	0	306.44	gggcccggctCggcgcggccg	m5C_32442
chr17	31149621	-	C	80	18	0.225	0	0	T	62	M	0.147	0.328	0	4E-14	0	53.039	acgggcccggCtcggcgcggc	m5C_32472
chr17	31149624	-	C	76	50	0.658	0	0	C	50	M	0.546	0.755	0	4E-08	0	545.96	gccacgggccCggctcggcgc	m5C_32455
chr17	31149625	-	C	72	35	0.486	0	0	T	37	M	0.374	0.599	0	2E-09	0	262.03	ggccacgggcCcggctcggcg	m5C_32474
chr17	31149626	-	C	69	28	0.406	0	0	T	41	M	0.298	0.524	0	1E-08	0	166.82	gggccacgggCccggctcggc	m5C_32473
chr17	31149630	-	C	55	11	0.2	0	0	T	44	M	0.116	0.324	3E-13	4E-10	3E-13	15.923	cggcgggccaCgggcccggct	m5C_32476
chr17	31149632	-	C	41	16	0.39	0	0	T	25	M	0.257	0.543	0	4E-07	0	82.099	gccggcgggcCacgggcccgg	m5C_32462
chr17	31149633	-	C	43	27	0.628	0	0	C	27	M	0.479	0.756	0	7E-06	0	258.44	ggccggcgggCcacgggcccg	m5C_32458
chr17	31149663	-	C	44	24	0.545	0	0	C	24	M	0.401	0.683	0	4E-06	0	192.32	ggggcccgcgCggccaccccc	m5C_32498
chr17	31149665	-	C	49	16	0.327	0	0	T	33	M	0.212	0.466	0	4E-07	0	67.863	ccggggcccgCgcggccaccc	m5C_32445
chr17	31149667	-	C	48	27	0.562	0	0	C	27	M	0.423	0.693	0	7E-06	0	228.28	caccggggccCgcgcggccac	m5C_32502
chr17	31149674	-	C	45	16	0.356	0	0	T	29	M	0.232	0.502	0	4E-07	0	74.3	gccgccccacCggggcccgcg	m5C_32465
chr17	31149677	-	C	41	32	0.78	0	0	C	32	M	0.633	0.88	0	2E-05	0	405.09	gtggccgcccCaccggggccc	m5C_32441
chr17	31149679	-	C	36	14	0.389	0	0	T	22	M	0.248	0.551	0	9E-06	0	69.397	gggtggccgcCccaccggggc	m5C_32499
chr17	33478193	-	C	659	462	0.701	0	0	C	462	M	0.665	0.735	0	0	0	6144.8	aggattccctCagtaatggcg	m5C_35677
chr17	33478195	-	C	676	484	0.716	0	0	C	484	M	0.681	0.749	0	0	0	6590.5	ccaggattccCtcagtaatgg	m5C_35657
chr17	33478196	-	C	657	459	0.699	0	0	C	459	M	0.662	0.732	0	0	0	6081.5	accaggattcCctcagtaatg	m5C_35634
chr17	33478197	-	C	657	464	0.706	0	0	C	464	M	0.67	0.74	0	0	0	6220.3	aaccaggattCcctcagtaat	m5C_35690
chr17	33478205	-	C	636	501	0.788	0	0	C	501	M	0.754	0.818	0	0	0	7557.9	aagaaactaaCcaggattccc	m5C_35679
chr17	33478223	-	C	679	459	0.676	0	0	C	459	M	0.64	0.71	0	0	0	5874.2	tatttgtcagCggaggaaaag	m5C_35648
chr17	33478226	-	C	674	416	0.617	0	0	C	416	M	0.58	0.653	0	0	0	4825.2	gcatatttgtCagcggaggaa	m5C_35678
chr17	33478235	-	C	929	463	0.499	1	0.001	T	465	M	0.467	0.531	0	0	0	4322.8	tgaatttaagCatatttgtca	m5C_35682
chr17	33478246	-	C	936	384	0.41	0	0	T	552	M	0.379	0.442	0	0	0	2912.1	tggcgacccgCtgaatttaag	m5C_35662
chr17	33478248	-	C	944	395	0.418	0	0	T	549	M	0.387	0.45	0	0	0	3060.1	cgtggcgaccCgctgaattta	m5C_35665
chr17	33478249	-	C	940	418	0.445	0	0	T	522	M	0.413	0.477	0	0	0	3454.4	acgtggcgacCcgctgaattt	m5C_35681
chr17	33478250	-	C	937	439	0.469	1	0.001	T	497	M	0.437	0.501	0	0	0	3839	gacgtggcgaCccgctgaatt	m5C_35649
chr17	33478253	-	C	934	439	0.473	5	0.005	T	490	M	0.441	0.505	0	0	0	3868.7	tcagacgtggCgacccgctga	m5C_35661
chr17	33478258	-	C	938	450	0.483	6	0.006	T	482	M	0.451	0.515	0	0	0	4058	tcagatcagaCgtggcgaccc	m5C_35700
chr17	33478262	-	C	863	386	0.447	0	0	T	477	M	0.414	0.481	0	0	0	3199.2	gacctcagatCagacgtggcg	m5C_35698
chr17	33478267	-	C	834	366	0.439	0	0	T	468	M	0.406	0.473	0	0	0	2968.4	cccgcgacctCagatcagacg	m5C_35645
chr17	33478269	-	C	804	321	0.4	1	0.001	T	482	M	0.366	0.434	0	0	0	2352.4	ggcccgcgacCtcagatcaga	m5C_35680
chr17	33478270	-	C	764	348	0.455	0	0	T	416	M	0.42	0.491	0	0	0	2926.6	aggcccgcgaCctcagatcag	m5C_35639
chr17	33478273	-	C	349	114	0.327	0	0	T	235	M	0.28	0.378	0	0	0	637.4	tcgaggcccgCgacctcagat	m5C_35650
chr17	33478275	-	C	304	79	0.26	0	0	T	225	M	0.214	0.312	0	0	0	337.78	gatcgaggccCgcgacctcag	m5C_35703
chr17	33478296	-	C	40	11	0.275	0	0	T	29	M	0.161	0.428	4E-15	3E-06	5E-15	25.45	gtgtggggccCaagtccttct	m5C_35676
chr17	33478297	-	C	61	13	0.213	0	0	T	48	M	0.129	0.331	1E-15	2E-11	2E-15	24.887	cgtgtggggcCcaagtccttc	m5C_35686
chr17	33478307	-	C	144	30	0.208	0	0	T	114	M	0.15	0.282	0	0	0	90.006	cggcgccgctCgtgtggggcc	m5C_35699
chr17	33478314	-	C	146	34	0.233	0	0	T	112	M	0.172	0.308	0	0	0	116.76	cactccccggCgccgctcgtg	m5C_35667
chr17	33478315	+	C	37	27	0.73	0	0	C	27	M	0.57	0.846	0	0.0001	0	307.92	acgagcggcgCcggggagtgg	m5C_35601
chr17	33478316	+	C	39	31	0.795	0	0	C	31	M	0.645	0.892	0	0.0003	0	399.69	cgagcggcgcCggggagtggg	m5C_35629
chr17	33478320	-	C	160	36	0.225	0	0	T	124	M	0.167	0.296	0	0	0	120.37	aagacccactCcccggcgccg	m5C_35663
chr17	33478322	-	C	145	43	0.297	0	0	T	102	M	0.228	0.375	0	0	0	196.28	ggaagacccaCtccccggcgc	m5C_35695
chr17	33478324	-	C	141	59	0.418	0	0	T	82	M	0.34	0.501	0	0	0	401.48	acggaagaccCactccccggc	m5C_35696
chr17	33478325	-	C	160	71	0.444	0	0	T	89	M	0.369	0.521	0	0	0	523.95	tacggaagacCcactccccgg	m5C_35651
chr17	33478326	-	C	157	75	0.478	0	0	T	82	M	0.401	0.555	0	0	0	601.56	gtacggaagaCccactccccg	m5C_35664
chr17	33478328	+	C	52	39	0.75	0	0	C	39	M	0.618	0.848	0	2E-06	0	481.99	gggagtgggtCttccgtacgc	m5C_35615
chr17	33478331	+	C	50	29	0.58	0	0	C	29	M	0.442	0.706	0	4E-07	0	256.55	agtgggtcttCcgtacgccac	m5C_35604
chr17	33478332	+	C	47	24	0.511	0	0	C	24	M	0.372	0.647	0	4E-06	0	178.76	gtgggtcttcCgtacgccaca	m5C_35612
chr17	33478336	+	C	47	28	0.596	0	0	C	28	M	0.453	0.724	0	8E-06	0	253.91	gtcttccgtaCgccacatttc	m5C_35619
chr17	33478338	+	C	47	24	0.511	0	0	C	24	M	0.372	0.647	0	4E-06	0	178.76	cttccgtacgCcacatttccc	m5C_35603
chr17	33478339	+	C	42	20	0.476	0	0	T	22	M	0.334	0.623	0	1E-06	0	133.44	ttccgtacgcCacatttcccg	m5C_35623

chr17	33478341	+	C	51	25	0.49	0	0	T	26	M	0.359	0.623	0	2E-07	0	179.29	ccgtacgccaCatttcccgcg	m5C_35593
chr17	34151156	+	C	34	16	0.485	1	0.029	T	17	M	0.325	0.648	0	2E-05	0	104.01	aggtagagggCgtgggggata	m5C_32217
chr17	34151170	+	C	37	26	0.703	0	0	C	26	M	0.542	0.825	0	0.0001	0	281.93	ggggatatgaCaaggatggaa	m5C_32216
chr17	37009131	-	C	39	18	0.462	0	0	T	21	M	0.316	0.614	0	3E-05	0	113.64	ttgcttcaatCggtggtgtga	m5C_32304
chr17	37009135	-	C	37	11	0.297	0	0	T	26	M	0.175	0.458	1E-15	3E-06	2E-15	28.551	ttgtttgcttCaatcggtggt	m5C_32334
chr17	37009196	-	C	37	9	0.243	0	0	T	28	M	0.134	0.401	3E-12	1E-06	3E-12	13.907	attgagccgtCaagaaagggg	m5C_32323
chr17	37009200	-	C	39	13	0.333	0	0	T	26	M	0.206	0.49	0	7E-06	0	53.645	acctattgagCcgtcaagaaa	m5C_32329
chr17	37009208	-	C	43	14	0.326	0	0	T	29	M	0.205	0.475	0	2E-07	0	57.381	ggctaatgacCtattgagccg	m5C_32307
chr17	37017996	-	C	35	13	0.371	0	0	T	22	M	0.232	0.537	0	7E-06	0	60.231	gggtatagctCaggggtagag	m5C_32324
chr17	37017998	-	C	35	12	0.343	0	0	T	23	M	0.208	0.508	0	5E-06	0	49.996	gggggtatagCtcaggggtag	m5C_32333
chr17	37023908	+	C	33	8	0.242	0	0	T	25	M	0.128	0.41	3E-11	7E-07	3E-11	10.787	gggggtatagCtcagtggtag	m5C_32285
chr17	37023910	+	C	36	15	0.417	0	0	T	21	M	0.271	0.578	0	1E-05	0	81.421	gggtatagctCagtggtagag	m5C_32275
chr17	37023921	+	C	35	24	0.686	0	0	C	24	M	0.52	0.814	0	9E-05	0	249.7	agtggtagagCatttgactgc	m5C_32290
chr17	37360816	+	C	57	18	0.316	0	0	T	39	M	0.21	0.445	0	2E-08	0	75.615	accaaggaagCacgcaagcgc	m5C_32295
chr17	37360836	+	C	44	13	0.295	0	0	T	31	M	0.182	0.442	0	1E-07	0	47.204	ccgtgaagagCgcctccaggc	m5C_32270
chr17	37360842	+	C	39	12	0.308	0	0	T	27	M	0.186	0.464	0	5E-06	0	44.558	agagcgcctcCaggccaagaa	m5C_32266
chr17	38282517	+	C	31	10	0.323	0	0	T	21	M	0.186	0.499	7E-15	2E-06	8E-15	26.273	caagctgtttCagggctatga	m5C_32347
chr17	38399482	+	C	42	19	0.452	0	0	T	23	M	0.312	0.601	0	1E-06	0	118.65	tggggctggtCtgatggtagt	m5C_32359
chr17	38399500	+	C	38	13	0.342	0	0	T	25	M	0.212	0.501	0	7E-06	0	55.152	agtgggttatCagagcttatt	m5C_32366
chr17	38399505	+	C	35	17	0.486	0	0	T	18	M	0.33	0.644	0	2E-05	0	112.18	gttatcagagCttattaacat	m5C_32343
chr17	39624284	+	C	34	11	0.324	0	0	T	23	M	0.191	0.492	4E-16	3E-06	5E-16	32.309	cgtcctccatCtgaggacaac	m5C_32365
chr17	39624291	+	C	40	11	0.275	0	0	T	29	M	0.161	0.428	4E-15	3E-06	5E-15	25.45	catctgaggaCaacatattaa	m5C_32360
chr17	40489528	-	C	31	8	0.258	0	0	T	23	M	0.137	0.432	2E-11	7E-07	2E-11	11.818	gaggagctggCtgactggaag	m5C_32590
chr17	40489532	-	C	41	11	0.268	0	0	T	30	M	0.157	0.419	6E-15	4E-08	7E-15	24.554	ggacgaggagCtggctgactg	m5C_32576
chr17	40489539	-	C	53	42	0.792	0	0	C	42	M	0.665	0.88	0	4E-06	0	558.95	ctctcacggaCgaggagctgg	m5C_32565
chr17	40489545	-	C	41	24	0.585	0	0	C	24	M	0.434	0.722	0	4E-06	0	208.16	agaaaactctCacggacgagg	m5C_32568
chr17	40489547	-	C	40	24	0.6	0	0	C	24	M	0.446	0.737	0	4E-06	0	214.06	gcagaaaactCtcacggacga	m5C_32556
chr17	40574893	-	C	37	22	0.595	0	0	C	22	M	0.435	0.737	0	6E-05	0	191.34	tcaagcacagCtggaggagcg	m5C_32577
chr17	41400535	+	C	57	39	0.684	0	0	C	39	M	0.555	0.79	0	2E-06	0	433.06	cgcgtgtgtgCggagacgaga	m5C_32525
chr17	41400541	+	C	79	36	0.48	4	0.051	T	39	M	0.371	0.591	0	2E-09	0	266.9	tgtgcggagaCgagagatcga	m5C_32510
chr17	41400563	+	C	116	24	0.207	0	0	T	92	M	0.143	0.289	0	0	0	68.717	gagataaagaCagaagacaaa	m5C_32539
chr17	41400570	+	C	98	29	0.296	0	0	T	69	M	0.215	0.393	0	2E-13	0	124.49	agacagaagaCaaagagatag	m5C_32546
chr17	41464613	-	C	128	44	0.344	0	0	T	84	M	0.267	0.43	0	4E-16	0	235.05	tgcagtacctCcaggaatggg	m5C_32571
chr17	41464616	-	C	138	48	0.348	0	0	T	90	M	0.273	0.43	0	0	0	262.52	tattgcagtaCctccaggaat	m5C_32583
chr17	41464630	-	C	89	25	0.281	0	0	T	64	M	0.198	0.382	0	2E-12	0	99.047	acgcgtcgacCtggtattgca	m5C_32559
chr17	41464634	-	C	68	19	0.279	0	0	T	49	M	0.187	0.396	0	5E-10	0	70.976	ctccacgcgtCgacctggtat	m5C_32558
chr17	42088964	-	C	71	29	0.408	0	0	T	42	M	0.302	0.525	0	4E-10	0	174.98	gagtgggagaCagaggaagga	m5C_33000
chr17	46133870	+	C	33	15	0.455	0	0	T	18	M	0.298	0.62	0	1E-05	0	89.528	gagatggaggCgagcaggaca	m5C_32693
chr17	46133874	+	C	33	14	0.424	0	0	T	19	M	0.272	0.592	0	9E-06	0	76.259	tggaggcgagCaggacacctg	m5C_32697
chr17	46133879	+	C	34	19	0.559	0	0	C	19	M	0.395	0.711	0	3E-05	0	149.92	gcgagcaggaCacctgggcag	m5C_32698
chr17	46133891	+	C	32	13	0.406	0	0	T	19	M	0.255	0.577	0	7E-06	0	66.35	cctgggcaggCgagggcgcgg	m5C_32691
chr17	46657275	-	C	74	55	0.743	0	0	C	55	M	0.633	0.829	0	8E-08	0	696.8	cctgtagatcCgaatttgtgt	m5C_32719
chr17	46657285	-	C	36	22	0.611	0	0	C	22	M	0.449	0.752	0	6E-05	0	197.4	ctgtatatacCctgtagatcc	m5C_32717
chr17	46657286	-	C	36	23	0.639	0	0	C	23	M	0.476	0.775	0	8E-05	0	218.84	tctgtatataCcctgtagatc	m5C_32703
chr17	46685176	-	C	59	17	0.288	0	0	T	42	M	0.188	0.414	0	1E-08	0	64.044	gaggaagagaCatgagaaagg	m5C_32709
chr17	46685188	-	C	83	22	0.265	0	0	T	61	M	0.182	0.369	0	4E-13	0	80.099	ggagaaagggCagaggaagag	m5C_32714
chr17	48462587	-	C	59	28	0.475	0	0	T	31	M	0.353	0.6	0	3E-07	0	197.49	actgcggaagCgggagaaggc	m5C_32926
chr17	48462596	-	C	73	35	0.479	0	0	T	38	M	0.369	0.592	0	2E-09	0	258.14	ggagcgggaaCtgcggaagcg	m5C_32946
chr17	48462602	-	C	63	20	0.317	0	0	T	43	M	0.216	0.44	0	7E-10	0	86.346	agctcaggagCgggaactgcg	m5C_32941
chr17	48462608	-	C	42	25	0.595	0	0	C	25	M	0.445	0.73	0	4E-06	0	222.47	taaggaagctCaggagcggga	m5C_32950
chr17	48823123	+	C	31	20	0.645	0	0	C	20	M	0.469	0.789	0	4E-05	0	187.79	tcgtgatgagCgtctaaaaaa	m5C_32903
chr17	48823126	+	C	31	11	0.355	0	0	T	20	M	0.211	0.531	1E-16	3E-06	1E-16	37.059	tgatgagcgtCtaaaaaagga	m5C_32912
chr17	48823168	+	C	54	27	0.5	0	0	CT	27	M	0.371	0.629	0	2E-07	0	200.42	agaaaaagaaCgggagagaga	m5C_32892
chr17	55868719	-	C	118	91	0.771	0	0	C	91	M	0.688	0.838	0	2E-10	0	1251.4	tcagttggtcCtgagagatgg	m5C_32754
chr17	55868720	-	C	120	95	0.792	0	0	C	95	M	0.711	0.855	0	2E-11	0	1350	gtcagttggtCctgagagatg	m5C_32761
chr17	55868746	-	C	97	75	0.773	0	0	C	75	M	0.68	0.845	0	5E-09	0	1020.6	ttattgtgaaCagcagttgaa	m5C_32755
chr17	57918642	+	C	119	94	0.79	0	0	C	94	M	0.708	0.853	0	2E-10	0	1331.5	ggtagcttatCagactgatgt	m5C_33323
chr17	57918656	+	C	63	49	0.778	0	0	C	49	M	0.661	0.863	0	6E-07	0	647.66	ctgatgttgaCtgttgaatct	m5C_33324
chr17	58160993	-	C	34	15	0.441	0	0	T	19	M	0.289	0.605	0	1E-05	0	86.65	tggatggagaCtacaggggat	m5C_32781
chr17	62223457	+	C	309	95	0.308	1	0.003	T	213	M	0.259	0.362	0	0	0	493.02	tgatgacattCcaattaaagc	m5C_33164
chr17	62223458	+	C	299	65	0.218	1	0.003	T	233	M	0.175	0.268	0	0	0	227.48	gatgacattcCaattaaagca	m5C_33150
chr17	62223481	+	C	334	72	0.216	1	0.003	T	261	M	0.175	0.264	0	0	0	252.53	tgttagactgCtgacgcgggt	m5C_33171
chr17	62223485	+	C	379	102	0.271	2	0.005	T	275	M	0.228	0.318	0	0	0	465.51	agactgctgaCgcgggtgatg	m5C_33170
chr17	62223500	+	C	393	100	0.254	0	0	T	293	M	0.214	0.3	0	0	0	427.82	ggtgatgcgaaCtggagtctga	m5C_33161
chr17	62223512	+	C	206	46	0.223	0	0	T	160	M	0.172	0.285	0	0	0	158.05	ggagtctgagCctgcccgagc	m5C_33158
chr17	62223513	+	C	174	55	0.316	0	0	T	119	M	0.252	0.389	0	0	0	276.78	gagtctgagcCtgcccgagcg	m5C_33148
chr17	62223817	+	C	45	12	0.267	0	0	T	33	M	0.16	0.41	6E-16	7E-08	6E-16	29.226	gcctttttaaCcgcgagcgac	m5C_33162
chr17	62223818	+	C	45	12	0.267	0	0	T	33	M	0.16	0.41	6E-16	7E-08	6E-16	29.226	cctttttaacCgcgagcgaca	m5C_33154
chr17	73030524	-	C	34	25	0.735	0	0	C	25	M	0.569	0.854	0	0.0001	0	284.41	cctggggtgtCgagaggggct	m5C_33280
chr17	73030558	-	C	46	34	0.739	0	0	C	34	M	0.597	0.844	0	2E-05	0	406.24	cctcctaagcCagggattgtg	m5C_33274
chr17	73030559	-	C	34	17	0.5	0	0	CT	17	M	0.341	0.659	0	2E-05	0	115.83	gcctcctaagCcagggattgt	m5C_33278
chr17	73035328	-	C	56	20	0.357	0	0	T	36	M	0.245	0.488	0	3E-08	0	97.826	tctcaaaggcCaggattgtag	m5C_33294
chr17	73035329	-	C	51	11	0.216	0	0	T	40	M	0.125	0.346	1E-13	4E-10	1E-13	17.806	ctctcaaaggCcaggattgta	m5C_33270
chr17	74222322	-	C	45	16	0.356	0	0	T	29	M	0.232	0.502	0	4E-07	0	74.3	gtggatatcaCtgaagaagat	m5C_33468
chr17	74554900	+	C	72	19	0.264	0	0	T	53	M	0.176	0.376	0	7E-12	0	66.856	taaagttatcCctgtctgaaa	m5C_33415
chr17	74554917	+	C	75	27	0.36	0	0	T	48	M	0.261	0.473	0	2E-10	0	140.75	gaaatggtatCttttgtgagg	m5C_33423
chr17	74554932	+	C	99	30	0.303	0	0	T	69	M	0.221	0.4	0	3E-13	0	132.76	gtgaggaggtCtgacttgctg	m5C_33411
chr17	74557241	+	C	39	22	0.564	0	0	C	22	M	0.41	0.707	0	6E-05	0	180.29	aaagaaaggcCtttctgtgtg	m5C_33431
chr17	74557245	+	C	54	21	0.389	0	0	T	33	M	0.27	0.522	0	5E-08	0	113.58	aaaggcctttCtgtgtggaat	m5C_33416
chr17	74557264	+	C	65	30	0.462	0	0	T	35	M	0.346	0.581	0	2E-08	0	207.54	atttgaatatCtgaaactcag	m5C_33439
chr17	76220332	+	C	41	23	0.561	0	0	C	23	M	0.41	0.701	0	3E-06	0	188.79	acggggtgaaCttcaggtgga	m5C_33666
chr17	76220351	+	C	40	14	0.35	0	0	T	26	M	0.221	0.505	0	2E-07	0	61.976	gatgaggagaCagaatagagt	m5C_33683
chr17	76794999	-	C	31	9	0.29	0	0	T	22	M	0.161	0.466	4E-13	1E-06	4E-13	18.015	aagagtttgaCcgagggaagg	m5C_33720
chr17	77809498	-	C	49	28	0.571	0	0	C	28	M	0.433	0.7	0	8E-06	0	242.31	acggcaggagCagctgatggg	m5C_33714
chr17	77809507	-	C	53	33	0.623	0	0	C	33	M	0.488	0.741	0	9E-07	0	322.12	acccagggaaCggcaggagca	m5C_33694
chr17	79803787	-	C	37	20	0.541	0	0	C	20	M	0.384	0.69	0	4E-05	0	153.54	actttgaagaCgtggcttttg	m5C_33121
chr18	252571	-	C	65	22	0.338	0	0	T	43	M	0.235	0.46	0	1E-09	0	103.55	tagaagaaggCgaaatgggag	m5C_33227
chr18	7301141	-	C	35	20	0.571	0	0	C	20	M	0.409	0.72	0	4E-05	0	163.43	tgcctctgacCtgggtagagt	m5C_33312
chr18	7301142	-	C	33	14	0.424	0	0	T	19	M	0.272	0.592	0	9E-06	0	76.259	atgcctctgaCctgggtagag	m5C_33304
chr18	7301146	-	C	35	8	0.229	0	0	T	27	M	0.121	0.39	6E-11	7E-07	6E-11	9.8969	gtcaatgcctCtgacctgggt	m5C_33313
chr18	8476926	-	C	99	54	0.545	0	0	C	54	M	0.448	0.64	0	2E-10	0	483.31	caggagggtgCgtgggctggg	m5C_33388
chr18	19203898	+	C	34	9	0.265	0	0	T	25	M	0.146	0.431	1E-12	1E-06	1E-12	15.739	aaaagggaagCtggtaagttt	m5C_33732

chr18	19209026	+	C	68	15	0.221	0	0	T	53	M	0.138	0.333	0	6E-11	0	41.547	ccttcagttgCaggaagaggc	m5C_33737
chr18	44790111	+	C	32	10	0.312	0	0	T	22	M	0.18	0.486	1E-14	2E-06	1E-14	25.084	atcgcatagcCcagccggatc	m5C_33815
chr18	47017661	-	C	32	20	0.625	0	0	C	20	M	0.453	0.771	0	4E-05	0	181.02	ttaataaattCtgagcagcca	m5C_34022
chr18	47017679	-	C	39	27	0.692	0	0	C	27	M	0.536	0.814	0	0.0001	0	289.32	ttggatttacCgtgaaaatta	m5C_34016
chr18	47017691	-	C	38	27	0.711	0	0	C	27	M	0.552	0.83	0	0.0001	0	298.31	cttaggacacCtttggattta	m5C_34010
chr18	47017692	-	C	38	28	0.737	0	0	C	28	M	0.58	0.85	0	0.0002	0	324.75	tcttaggacaCctttggattt	m5C_33989
chr18	47017701	-	C	39	27	0.692	0	0	C	27	M	0.536	0.814	0	0.0001	0	289.32	tgatgactttCttaggacacc	m5C_34006
chr18	47811112	-	C	59	33	0.559	0	0	C	33	M	0.433	0.678	0	9E-07	0	285.71	gagaagaagtCtgagaagaag	m5C_33994
chr18	47811128	-	C	31	12	0.387	0	0	T	19	M	0.237	0.562	0	5E-06	0	56.959	gcatgtgaagCgtcgggagaa	m5C_33978
chr18	48810123	-	C	41	19	0.463	0	0	T	22	M	0.321	0.613	0	1E-06	0	121.81	tagtgggggaCtgtgtgtgtg	m5C_34439
chr18	57022805	-	C	32	9	0.281	0	0	T	23	M	0.156	0.454	5E-13	1E-06	6E-13	17.198	caaaagcggcCtttgagaact	m5C_34469
chr18	58072215	+	C	74	39	0.527	0	0	C	39	M	0.415	0.637	0	5E-09	0	323.52	gagtgatgttCagacaggtga	m5C_33899
chr18	58072219	+	C	67	34	0.507	0	0	C	34	M	0.391	0.623	0	4E-08	0	265.63	gatgttcagaCaggtgaaaag	m5C_33909
chr18	70999774	+	C	34	26	0.765	0	0	C	26	M	0.6	0.876	0	0.0001	0	312.02	ccagcagagcCagatctgagc	m5C_34462
chr18	70999779	+	C	32	15	0.469	0	0	T	17	M	0.309	0.636	0	1E-05	0	92.607	agagccagatCtgagctggga	m5C_34457
chr19	926319	+	C	67	31	0.463	0	0	T	36	M	0.349	0.581	0	2E-08	0	216.12	ggtgggggtcCcggactggga	m5C_34366
chr19	3539410	-	C	32	21	0.656	0	0	C	21	M	0.483	0.796	0	5E-05	0	202.91	cggaggggaaCaggaagagct	m5C_34280
chr19	3539425	-	C	42	20	0.476	0	0	T	22	M	0.334	0.623	0	1E-06	0	133.44	tgagcggccaCtggccggagg	m5C_34261
chr19	3539428	-	C	36	13	0.361	0	0	T	23	M	0.225	0.524	0	7E-06	0	58.436	cagtgagcggCcactggccgg	m5C_34286
chr19	3539660	-	C	32	17	0.531	0	0	C	17	M	0.364	0.691	0	2E-05	0	123.93	aagacagaagCagagaccggg	m5C_34292
chr19	3976158	-	C	58	24	0.414	0	0	T	34	M	0.296	0.542	0	1E-07	0	142.21	gaaaggaacaCgcgggtagat	m5C_34283
chr19	3976160	-	C	50	16	0.32	0	0	T	34	M	0.208	0.458	0	7E-09	0	66.426	gggaaaggaaCacgcgggtag	m5C_34281
chr19	3977953	-	C	31	13	0.419	0	0	T	18	M	0.264	0.592	0	7E-06	0	68.68	tgggacgtggCtgaggcccgc	m5C_34276
chr19	3977958	-	C	45	17	0.378	0	0	T	28	M	0.251	0.524	0	6E-07	0	85.367	acgagtgggaCgtggctgagg	m5C_34298
chr19	4423372	+	C	48	13	0.271	0	0	T	35	M	0.166	0.41	0	1E-07	0	43.071	agaagagaaaCggttaagaga	m5C_34608
chr19	4446369	-	C	43	22	0.512	0	0	C	22	M	0.368	0.654	0	2E-06	0	161.71	cggaccaaggCcatgcgggag	m5C_34663
chr19	4510607	-	C	301	147	0.488	0	0	T	154	M	0.432	0.545	0	0	0	1271.3	gacgtggcgaCgttcacccaa	m5C_34664
chr19	4724112	+	C	103	55	0.534	0	0	C	55	M	0.438	0.627	0	8E-12	0	481.98	agcatagctgCcttccaagca	m5C_34633
chr19	4724113	+	C	101	36	0.356	0	0	T	65	M	0.27	0.454	0	5E-14	0	194.3	gcatagctgcCttccaagcag	m5C_34621
chr19	4724117	+	C	99	36	0.364	0	0	T	63	M	0.276	0.462	0	2E-12	0	198.44	agctgccttcCaagcagttga	m5C_34620
chr19	5600214	-	C	33	7	0.212	0	0	T	26	M	0.107	0.378	1E-09	3E-07	1E-09	6.6917	agcctgaagaCattaagaagg	m5C_34643
chr19	5600221	-	C	36	19	0.528	0	0	C	19	M	0.37	0.68	0	3E-05	0	140.62	gaaaaaaagcCtgaagacatt	m5C_34670
chr19	6589226	-	C	39	11	0.282	0	0	T	28	M	0.165	0.438	3E-15	3E-06	3E-15	26.4	aggagaataaCagagaggaag	m5C_34348
chr19	6589254	-	C	34	10	0.294	0	0	T	24	M	0.168	0.462	2E-14	2E-06	3E-14	22.956	gaaagaaagcCagagagagga	m5C_34342
chr19	6589255	-	C	33	21	0.636	0	0	C	21	M	0.466	0.778	0	5E-05	0	195.79	agaaagaaagCcagagagagg	m5C_34347
chr19	8793673	-	C	38	29	0.763	0	0	C	29	M	0.608	0.87	0	0.0002	0	352.6	gaattctcgcCtgccacaatg	m5C_34317
chr19	8793691	-	C	110	87	0.791	0	0	C	87	M	0.706	0.856	0	9E-11	0	1228	atcagtggttCagtggtagaa	m5C_34312
chr19	10670331	-	C	34	10	0.294	0	0	T	24	M	0.168	0.462	2E-14	2E-06	3E-14	22.956	aggatgagcgCagaaaggaga	m5C_34758
chr19	11170485	+	C	40	20	0.5	0	0	CT	20	M	0.352	0.648	0	1E-06	0	140.8	ggcagaaaatCgagaaggagg	m5C_34750
chr19	11170500	+	C	39	19	0.487	0	0	T	20	M	0.339	0.638	0	3E-05	0	128.69	aggaggatgaCagtgaaggcg	m5C_34729
chr19	11306387	-	C	36	19	0.528	0	0	C	19	M	0.37	0.68	0	3E-05	0	140.62	gagacagagaCgtggagagac	m5C_34794
chr19	11306393	-	C	50	13	0.26	0	0	T	37	M	0.159	0.396	0	2E-09	0	41.266	cgcagagagaCagagacgtgg	m5C_34784
chr19	11306415	-	C	34	7	0.206	0	0	T	27	M	0.103	0.368	1E-09	3E-07	2E-09	6.4054	aggacagagaCgtggagagag	m5C_34779
chr19	11553280	+	C	58	17	0.293	0	0	T	41	M	0.192	0.42	0	1E-08	0	65.205	aaggaggaagCtgagaagcca	m5C_34741
chr19	11553289	+	C	57	36	0.632	0	0	C	36	M	0.502	0.745	0	2E-06	0	361.28	gctgagaagcCagagagagag	m5C_34751
chr19	12814428	-	C	130	73	0.562	0	0	C	73	M	0.476	0.644	0	2E-14	0	694.51	gggatgtattCgtactgtctg	m5C_34569
chr19	12814442	-	C	127	28	0.22	0	0	T	99	M	0.157	0.3	0	0	0	88.016	actgatgaccaCattgggatgt	m5C_34548
chr19	12814444	-	C	129	52	0.403	0	0	T	77	M	0.322	0.489	0	4E-15	0	335.34	tgactgtgacCacattgggat	m5C_34547
chr19	12814445	-	C	128	44	0.344	0	0	T	84	M	0.267	0.43	0	4E-16	0	235.05	atgactgtgaCcacattggga	m5C_34550
chr19	12814460	-	C	124	25	0.202	0	0	T	99	M	0.14	0.281	0	0	0	70.228	gagcaataccCggggatgact	m5C_34544
chr19	12817265	-	C	68	52	0.765	0	0	C	52	M	0.651	0.85	0	9E-07	0	677.5	ctggctgatcCtgagttgccc	m5C_34541
chr19	12817266	-	C	69	24	0.348	0	0	T	45	M	0.246	0.466	0	3E-09	0	118.17	actggctgatCctgagttgcc	m5C_34602
chr19	12817271	-	C	84	33	0.393	0	0	T	51	M	0.295	0.5	0	3E-11	0	194.9	ttcgtactggCtgatcctgag	m5C_34585
chr19	12817275	-	C	88	34	0.386	0	0	T	54	M	0.291	0.491	0	4E-11	0	198.17	cgtattcgtaCtggctgatcc	m5C_34545
chr19	12817279	-	C	82	29	0.354	0	0	T	53	M	0.259	0.462	0	8E-12	0	150.14	atcgcgtattCgtactggctg	m5C_34530
chr19	12817285	-	C	87	45	0.517	0	0	C	45	M	0.414	0.619	0	6E-10	0	372.37	tgatttatcgCgtattcgtac	m5C_34599
chr19	12817287	-	C	83	31	0.373	0	0	T	52	M	0.277	0.481	0	2E-11	0	171.86	actgatttatCgcgtattcgt	m5C_34537
chr19	12817296	-	C	88	33	0.375	0	0	T	55	M	0.281	0.479	0	3E-11	0	185.51	tgatgtggaaCtgatttatcg	m5C_34584
chr19	12817310	-	C	92	33	0.359	0	0	T	59	M	0.268	0.461	0	8E-13	0	176.99	acacctgtgaCtgttgatgtg	m5C_34561
chr19	12817316	-	C	81	36	0.444	0	0	T	45	M	0.341	0.553	0	7E-11	0	245.66	gtgatgacacCtgtgactgtt	m5C_34577
chr19	12817317	-	C	84	58	0.69	0	0	C	58	M	0.585	0.779	0	7E-09	0	678.73	agtgatgacaCctgtgactgt	m5C_34598
chr19	12817319	-	C	72	29	0.403	0	0	T	43	M	0.297	0.518	0	4E-10	0	172.38	gaagtgatgaCacctgtgact	m5C_34564
chr19	13041089	-	C	112	58	0.518	0	0	C	58	M	0.426	0.608	0	6E-13	0	494.47	ggctgagaagCtgggggagaa	m5C_34600
chr19	13054554	+	C	35	19	0.543	0	0	C	19	M	0.382	0.695	0	3E-05	0	145.12	gaaggacaaaCaggacgagga	m5C_34516
chr19	13054559	+	C	47	19	0.404	0	0	T	28	M	0.276	0.547	0	1E-06	0	105.03	acaaacaggaCgaggacgaga	m5C_34526
chr19	13054572	+	C	40	23	0.575	0	0	C	23	M	0.422	0.715	0	3E-06	0	194.1	ggagcagaggCttaaggagga	m5C_34518
chr19	13054643	+	C	78	32	0.41	0	0	T	46	M	0.308	0.521	0	8E-10	0	197	atgatgaggaCaaagatgagg	m5C_34523
chr19	13054673	+	C	171	86	0.503	0	0	C	86	M	0.429	0.577	0	0	0	737.44	atgaggaggaCaaggaggaag	m5C_34529
chr19	13065108	-	C	44	13	0.295	0	0	T	31	M	0.182	0.442	0	1E-07	0	47.204	ggaggaaaaaCagaaacggaa	m5C_34571
chr19	13065123	-	C	47	11	0.234	0	0	T	36	M	0.136	0.372	4E-14	4E-08	4E-14	20.086	gcgcaagcgcCtcaaggagga	m5C_34582
chr19	13065124	-	C	41	12	0.293	0	0	T	29	M	0.176	0.445	1E-16	7E-08	1E-16	33.713	agcgcaagcgCctcaaggagg	m5C_34572
chr19	13947419	-	C	35	17	0.486	0	0	T	18	M	0.33	0.644	0	2E-05	0	112.18	atcacattgcCagggatttcc	m5C_34559
chr19	14524009	-	C	33	16	0.485	0	0	T	17	M	0.325	0.648	0	2E-05	0	104.01	atgtggaaaaCgatcttttgg	m5C_34698
chr19	16197255	+	C	34	12	0.353	0	0	T	22	M	0.215	0.521	0	5E-06	0	51.571	gatagaaaacCgggccatgaa	m5C_35554
chr19	17515204	-	C	47	20	0.426	0	0	T	27	M	0.295	0.567	0	1E-06	0	118.05	ggcccaaggaCaaaagaaagt	m5C_35573
chr19	17515210	-	C	37	10	0.27	0	0	T	27	M	0.154	0.43	7E-14	2E-06	7E-14	20.282	agagaaggccCaaggacaaaa	m5C_35579
chr19	22877645	-	C	52	36	0.692	0	0	C	36	M	0.557	0.801	0	2E-06	0	401.26	gttggtggagCgatttgtctg	m5C_34501
chr19	24184682	-	C	35	24	0.686	0	0	C	24	M	0.52	0.814	0	9E-05	0	249.7	ctgaagttttCctaaggataa	m5C_35110
chr19	24184692	-	C	51	39	0.765	0	0	C	39	M	0.632	0.86	0	2E-06	0	493.25	ctggttccctCtgaagttttc	m5C_35120
chr19	24184694	-	C	52	40	0.769	0	0	C	40	M	0.639	0.863	0	3E-06	0	510.93	agctggttccCtctgaagttt	m5C_35241
chr19	24184696	-	C	48	22	0.458	0	0	T	26	M	0.326	0.597	0	2E-06	0	143.33	gtagctggttCcctctgaagt	m5C_35217
chr19	24184702	-	C	52	40	0.769	0	0	C	40	M	0.639	0.863	0	3E-06	0	510.93	catctagtagCtggttccctc	m5C_35239
chr19	24184750	-	C	56	32	0.571	0	0	C	32	M	0.441	0.692	0	7E-07	0	282.48	gcaaatcggtCatctgatctg	m5C_35207
chr19	24184754	-	C	87	48	0.552	0	0	C	48	M	0.447	0.652	0	1E-09	0	429.35	acgtgcaaatCggtcatctga	m5C_35226
chr19	24184759	-	C	98	41	0.418	0	0	T	57	M	0.326	0.517	0	8E-12	0	266.98	tcctgacgtgCaaatcggtca	m5C_35233
chr19	24184763	-	C	112	59	0.527	0	0	C	59	M	0.435	0.617	0	7E-13	0	513.27	gtggtcctgaCgtgcaaatcg	m5C_35201
chr19	24184767	-	C	101	49	0.485	0	0	T	52	M	0.39	0.581	0	2E-12	0	382.22	tgtagtggtcCtgacgtgcaa	m5C_35195
chr19	24184768	-	C	99	57	0.576	0	0	C	57	M	0.477	0.668	0	3E-10	0	544.2	ctgtagtggtCctgacgtgca	m5C_35204
chr19	24184778	-	C	70	16	0.229	0	0	T	54	M	0.146	0.34	0	1E-12	0	46.681	tggtgggggtCtgtagtggtc	m5C_35135
chr19	24184789	-	C	40	29	0.725	0	0	C	29	M	0.572	0.839	0	1E-05	0	331.56	agaggaaactCtggtgggggt	m5C_35125
chr19	24184791	-	C	34	24	0.706	0	0	C	24	M	0.538	0.832	0	9E-05	0	258.39	cgagaggaaaCtctggtgggg	m5C_35155
chr19	24184806	-	C	36	26	0.722	0	0	C	26	M	0.56	0.842	0	0.0001	0	291.24	ctgggcagggCgaagcgagag	m5C_35242

chr19	24184811	-	C	32	14	0.438	0	0	T	18	M	0.282	0.607	0	9E-06	0	78.862	tatgcctgggCagggcgaagc	m5C_35206
chr19	24184918	-	C	46	26	0.565	0	0	C	26	M	0.422	0.698	0	6E-06	0	219.69	cctgaggcctCtccagtttgc	m5C_35179
chr19	24184920	-	C	44	11	0.25	0	0	T	33	M	0.146	0.394	2E-14	4E-08	2E-14	22.132	atcctgaggcCtctccagttt	m5C_35146
chr19	24184921	-	C	47	17	0.362	0	0	T	30	M	0.24	0.505	0	6E-07	0	81.484	gatcctgaggCctctccagtt	m5C_35121
chr19	24184956	-	C	123	46	0.374	0	0	T	77	M	0.294	0.462	0	7E-16	0	270.03	aaggtgaaggCtgatgcgctg	m5C_35111
chr19	24184971	-	C	203	68	0.335	0	0	T	135	M	0.274	0.402	0	0	0	372.16	cactgtggcgCaatgaaggtg	m5C_35209
chr19	24184973	-	C	165	54	0.327	0	0	T	111	M	0.26	0.402	0	0	0	281.14	gccactgtggCgcaatgaagg	m5C_35215
chr19	24184981	-	C	55	37	0.673	0	0	C	37	M	0.541	0.782	0	2E-06	0	400.36	gcacgaaagcCactgtggcgc	m5C_35108
chr19	24184988	-	C	67	20	0.299	0	0	T	47	M	0.202	0.417	0	7E-10	0	80.925	ggggctcgcaCgaaagccact	m5C_35182
chr19	24184990	-	C	64	17	0.266	0	0	T	47	M	0.173	0.385	0	2E-10	0	58.807	caggggctcgCacgaaagcca	m5C_35229
chr19	24184994	-	C	47	13	0.277	0	0	T	34	M	0.169	0.418	0	1E-07	0	44.035	gagtcaggggCtcgcacgaaa	m5C_35186
chr19	24185132	-	C	63	37	0.587	0	0	C	37	M	0.464	0.7	0	8E-08	0	343.47	gctgtcgggtCtggggggact	m5C_35227
chr19	24185141	-	C	48	26	0.542	0	0	C	26	M	0.403	0.674	0	6E-06	0	209.51	gctggttgtgCtgtcgggtct	m5C_35200
chr19	24185476	-	C	48	11	0.229	0	0	T	37	M	0.133	0.365	5E-14	4E-08	5E-14	19.471	aaaggcccggCagggaaggtg	m5C_35143
chr19	24185479	-	C	51	22	0.431	0	0	T	29	M	0.305	0.567	0	7E-08	0	134.2	tggaaaggccCggcagggaag	m5C_35123
chr19	24185480	-	C	57	22	0.386	0	0	T	35	M	0.271	0.516	0	7E-08	0	119.08	ctggaaaggcCcggcagggaa	m5C_35145
chr19	24185481	-	C	57	18	0.316	0	0	T	39	M	0.21	0.445	0	2E-08	0	75.615	gctggaaaggCccggcaggga	m5C_35196
chr19	24185490	-	C	216	71	0.329	0	0	T	145	M	0.27	0.394	0	0	0	382.73	tctgggacggCtggaaaggcc	m5C_35136
chr19	24185493	-	C	259	91	0.351	0	0	T	168	M	0.296	0.411	0	0	0	538.3	ggctctgggaCggctggaaag	m5C_35119
chr19	24185499	-	C	301	136	0.452	0	0	T	165	M	0.397	0.508	0	0	0	1078.7	gtgactggctCtgggacggct	m5C_35132
chr19	24185501	-	C	292	95	0.325	0	0	T	197	M	0.274	0.381	0	0	0	520.93	ccgtgactggCtctgggacgg	m5C_35222
chr19	24185505	-	C	249	57	0.229	0	0	T	192	M	0.181	0.285	0	0	0	206.44	tgcgccgtgaCtggctctggg	m5C_35171
chr19	24185510	-	C	150	41	0.273	0	0	T	109	M	0.208	0.35	0	0	0	170.84	ggtgctgcgcCgtgactggct	m5C_35192
chr19	24185511	-	C	130	31	0.238	0	0	T	99	M	0.173	0.319	0	0	0	107.5	tggtgctgcgCcgtgactggc	m5C_35224
chr19	24185513	-	C	97	34	0.351	0	0	T	63	M	0.263	0.45	0	1E-12	0	178.78	tgtggtgctgCgccgtgactg	m5C_35212
chr19	24185524	-	C	51	12	0.235	0	0	T	39	M	0.14	0.368	3E-15	9E-10	4E-15	24.331	tgcatttccaCtgtggtgctg	m5C_35115
chr19	24185526	-	C	38	11	0.289	0	0	T	27	M	0.17	0.448	2E-15	3E-06	2E-15	27.449	gatgcatttcCactgtggtgc	m5C_35240
chr19	24185527	-	C	40	17	0.425	0	0	T	23	M	0.285	0.578	0	6E-07	0	96.931	agatgcatttCcactgtggtg	m5C_35245
chr19	24185790	-	C	163	47	0.288	0	0	T	116	M	0.224	0.362	0	0	0	210.85	gggcgtgaaaCcgttaagact	m5C_35174
chr19	24185797	-	C	173	71	0.41	0	0	T	102	M	0.34	0.485	0	0	0	482.55	ttcaagagggCgtgaaaccgt	m5C_35141
chr19	24185812	-	C	37	12	0.444	10	0.27	T	15	M	0.276	0.627	0	0.0002	0	66.205	actttgaagaCgcagttcaag	m5C_35202
chr19	24185821	-	C	530	228	0.43	0	0	T	302	M	0.389	0.473	0	0	0	1772.4	ttgaaaagaaCtttgaagacg	m5C_35221
chr19	24185844	-	C	131	29	0.221	0	0	T	102	M	0.159	0.3	0	0	0	92.097	tcaacaagtaCtgtaagggaa	m5C_35237
chr19	24185973	-	C	62	25	0.403	0	0	T	37	M	0.29	0.527	0	4E-09	0	145.12	cggtgtgaggCcattagcagc	m5C_35199
chr19	24185990	-	C	82	41	0.5	0	0	CT	41	M	0.394	0.606	0	3E-10	0	323.27	cccagccagcCtgtggacggt	m5C_35139
chr19	24185991	-	C	54	19	0.352	0	0	T	35	M	0.238	0.485	0	2E-08	0	90.527	gcccagccagCctgtggacgg	m5C_35189
chr19	24187261	-	C	46	17	0.37	0	0	T	29	M	0.245	0.514	0	6E-07	0	83.38	aagaatgcagCcagctgtgag	m5C_35168
chr19	24187264	-	C	38	12	0.316	0	0	T	26	M	0.191	0.475	0	5E-06	0	45.803	atgaagaatgCagccagctgt	m5C_35103
chr19	36066545	+	C	92	52	0.565	0	0	C	52	M	0.463	0.662	0	1E-10	0	481.85	ttgatatagaCagcaggacgg	m5C_35330
chr19	36066548	+	C	106	61	0.575	0	0	C	61	M	0.48	0.665	0	3E-11	0	586.04	atatagacagCaggacggtgg	m5C_35319
chr19	36066553	+	C	95	72	0.758	0	0	C	72	M	0.663	0.833	0	3E-09	0	954.48	gacagcaggaCggtggccatg	m5C_35311
chr19	36540055	+	C	267	80	0.3	0	0	T	187	M	0.248	0.357	0	0	0	396.54	tggagctggtCcgagtgttgt	m5C_35327
chr19	36540056	+	C	266	57	0.215	1	0.004	T	208	M	0.17	0.268	0	0	0	193.66	ggagctggtcCgagtgttgtg	m5C_35316
chr19	38795565	+	C	37	28	0.757	0	0	C	28	M	0.599	0.866	0	0.0002	0	335.34	aggagaagggCaagaaggaga	m5C_35411
chr19	39340555	-	C	36	12	0.333	0	0	T	24	M	0.202	0.497	0	5E-06	0	48.515	gcggctggagCagaggcagca	m5C_35421
chr19	39340561	-	C	34	14	0.412	0	0	T	20	M	0.264	0.578	0	9E-06	0	73.824	gcgtcggcggCtggagcagag	m5C_35425
chr19	39340564	-	C	37	10	0.27	0	0	T	27	M	0.154	0.43	7E-14	2E-06	7E-14	20.282	gcggcgtcggCggctggagca	m5C_35438
chr19	39340570	-	C	38	23	0.605	0	0	C	23	M	0.447	0.744	0	8E-05	0	205.7	ggagaagcggCgtcggcggct	m5C_35440
chr19	39340573	-	C	31	12	0.387	0	0	T	19	M	0.237	0.562	0	5E-06	0	56.959	ggcggagaagCggcgtcggcg	m5C_35439
chr19	39877126	-	C	37	15	0.429	2	0.054	T	20	M	0.28	0.591	0	1E-05	0	83.953	aagctgggggCtcaggtaaat	m5C_35434
chr19	43910975	-	C	58	46	0.793	0	0	C	46	M	0.672	0.877	0	7E-06	0	618.52	gtttaaaactCaaaaggtcag	m5C_34912
chr19	45381734	+	C	45	10	0.222	0	0	T	35	M	0.125	0.363	7E-13	2E-08	8E-13	15.216	tcccatggaaCcagatggcaa	m5C_34933
chr19	45381735	+	C	47	14	0.298	0	0	T	33	M	0.187	0.44	0	2E-07	0	52.223	cccatggaacCagatggcaag	m5C_34934
chr19	45381742	+	C	53	20	0.377	0	0	T	33	M	0.259	0.512	0	3E-08	0	103.74	aaccagatggCaaggatgagg	m5C_34928
chr19	45981916	-	C	33	15	0.455	0	0	T	18	M	0.298	0.62	0	1E-05	0	89.528	gctggggtgCaggcttcaaa	m5C_34957
chr19	45981924	-	C	37	16	0.432	0	0	T	21	M	0.287	0.591	0	2E-05	0	91.749	cctcagtggtCtggggtgcag	m5C_34963
chr19	45981933	-	C	39	19	0.487	0	0	T	20	M	0.339	0.638	0	3E-05	0	128.69	ccggatgatcCtcagtggtct	m5C_34938
chr19	45981934	-	C	38	8	0.211	0	0	T	30	M	0.111	0.363	1E-10	7E-07	1E-10	8.7725	cccggatgatCctcagtggtc	m5C_34962
chr19	46142272	-	C	51	12	0.235	0	0	T	39	M	0.14	0.368	3E-15	9E-10	4E-15	24.331	acacggcctgCagagaggcag	m5C_35502
chr19	46142275	-	C	54	33	0.611	0	0	C	33	M	0.478	0.73	0	9E-07	0	315.4	agcacacggcCtgcagagagg	m5C_35499
chr19	46142279	-	C	52	27	0.519	0	0	C	27	M	0.387	0.649	0	2E-07	0	208.9	gcaaagcacaCggcctgcaga	m5C_35480
chr19	46142281	-	C	51	22	0.431	0	0	T	29	M	0.305	0.567	0	7E-08	0	134.2	gagcaaagcaCacggcctgca	m5C_35472
chr19	47858242	-	C	37	19	0.514	0	0	C	19	M	0.359	0.666	0	3E-05	0	136.4	gggtaccagcCcctggccctg	m5C_35497
chr19	48427137	+	C	59	12	0.203	0	0	T	47	M	0.12	0.323	3E-14	9E-10	3E-14	19.611	ccacatgggtCggaaaaaagg	m5C_35931
chr19	48448273	+	C	41	11	0.275	1	0.024	T	29	M	0.161	0.428	4E-15	3E-06	5E-15	25.45	gcctggtccaCatgggtcgga	m5C_35899
chr19	49120041	-	C	37	12	0.324	0	0	T	25	M	0.196	0.485	0	5E-06	0	47.119	aacaagacggCcgtggttgtg	m5C_35964
chr19	49120044	-	C	37	25	0.676	0	0	C	25	M	0.515	0.804	0	0.0001	0	257.32	gaaaacaagaCggccgtggtt	m5C_35968
chr19	49120049	-	C	35	13	0.371	0	0	T	22	M	0.232	0.537	0	7E-06	0	60.231	gccgggaaaaCaagacggccg	m5C_35963
chr19	49120057	-	C	39	27	0.692	0	0	C	27	M	0.536	0.814	0	0.0001	0	289.32	gcttcctggcCgggaaaacaa	m5C_35971
chr19	49120058	-	C	38	24	0.649	1	0.026	C	24	M	0.488	0.782	0	9E-05	0	234.04	agcttcctggCcgggaaaaca	m5C_35973
chr19	49993889	+	C	32	22	0.688	0	0	C	22	M	0.514	0.82	0	6E-05	0	226.31	gtgatgagaaCttctcccact	m5C_35918
chr19	49993892	+	C	31	24	0.774	0	0	C	24	M	0.602	0.886	0	9E-05	0	288.9	atgagaacttCtcccactcac	m5C_35881
chr19	49993894	+	C	32	22	0.688	0	0	C	22	M	0.514	0.82	0	6E-05	0	226.31	gagaacttctCccactcacat	m5C_35888
chr19	49993896	+	C	34	25	0.735	0	0	C	25	M	0.569	0.854	0	0.0001	0	284.41	gaacttctccCactcacattc	m5C_35894
chr19	49993898	+	C	31	20	0.645	0	0	C	20	M	0.469	0.789	0	4E-05	0	187.79	acttctcccaCtcacattcga	m5C_35945
chr19	49994177	+	C	77	18	0.234	0	0	T	59	M	0.153	0.34	0	4E-12	0	55.186	ccatgatgttCcgcaactacc	m5C_35887
chr19	49994221	+	C	91	49	0.538	0	0	C	49	M	0.437	0.637	0	6E-11	0	427.83	gcagcactggCtgagacgcca	m5C_35932
chr19	49994227	+	C	78	18	0.231	0	0	T	60	M	0.151	0.336	0	4E-12	0	54.451	ctggctgagaCgccagtccag	m5C_35948
chr19	49994444	+	C	90	29	0.322	0	0	T	61	M	0.235	0.424	0	8E-12	0	136.11	cagatgatgtCcttatctcac	m5C_35896
chr19	49994452	+	C	94	35	0.372	0	0	T	59	M	0.281	0.473	0	1E-12	0	197	gtccttatctCacgatggtct	m5C_35917
chr19	49994464	+	C	115	30	0.261	0	0	T	85	M	0.189	0.348	0	0	0	113.56	cgatggtctgCggatgtccct	m5C_35929
chr19	49994471	+	C	104	22	0.212	0	0	T	82	M	0.144	0.3	0	0	0	63.383	ctgcggatgtCcctgtgggaa	m5C_35939
chr19	49994472	+	C	101	23	0.228	0	0	T	78	M	0.157	0.319	0	1E-16	0	72.131	tgcggatgtcCctgtgggaat	m5C_35920
chr19	49994494	+	C	96	20	0.208	0	0	T	76	M	0.139	0.3	0	2E-15	0	55.643	gcgacaatgcCaatggcttag	m5C_35921
chr19	49994511	+	C	50	11	0.22	0	0	T	39	M	0.128	0.352	9E-14	4E-10	9E-14	18.334	ttagctgatgCcaggaggctt	m5C_35942
chr19	51302709	-	C	47	22	0.468	0	0	T	25	M	0.333	0.608	0	2E-06	0	146.66	acccgggggtCtgaggggccc	m5C_35257
chr19	51302716	-	C	47	20	0.426	0	0	T	27	M	0.295	0.567	0	1E-06	0	118.05	ctttgacaccCgggggtctga	m5C_35283
chr19	51302717	-	C	49	24	0.49	0	0	T	25	M	0.356	0.625	0	4E-06	0	170.76	cctttgacacCcgggggtctg	m5C_35289
chr19	51302718	-	C	46	20	0.435	0	0	T	26	M	0.302	0.578	0	1E-06	0	120.84	acctttgacaCccgggggtct	m5C_35251
chr19	51302732	-	C	47	26	0.553	0	0	C	26	M	0.412	0.686	0	6E-06	0	214.48	cggtgcccccCgggacctttg	m5C_35266
chr19	51302734	-	C	46	18	0.391	0	0	T	28	M	0.264	0.535	0	8E-07	0	95.015	cacggtgcccCccgggacctt	m5C_35298
chr19	51302735	-	C	48	20	0.417	0	0	T	28	M	0.288	0.557	0	1E-06	0	115.4	acacggtgccCcccgggacct	m5C_35262

chr19	51302737	-	C	46	24	0.545	2	0.043	C	24	M	0.401	0.683	0	4E-06	0	192.32	ggacacggtgCcccccgggac	m5C_35261
chr19	51302757	-	C	41	23	0.561	0	0	C	23	M	0.41	0.701	0	3E-06	0	188.79	tgggctccgaCtgccactgag	m5C_35280
chr19	51302760	-	C	39	19	0.487	0	0	T	20	M	0.339	0.638	0	3E-05	0	128.69	cactgggctcCgactgccact	m5C_35268
chr19	51302761	-	C	41	18	0.439	0	0	T	23	M	0.299	0.59	0	8E-07	0	107.6	gcactgggctCcgactgccac	m5C_35253
chr19	51302763	-	C	42	20	0.476	0	0	T	22	M	0.334	0.623	0	1E-06	0	133.44	cagcactgggCtccgactgcc	m5C_35290
chr19	51302770	-	C	40	16	0.4	0	0	T	24	M	0.263	0.554	0	2E-05	0	84.314	tgatgtccagCactgggctcc	m5C_35294
chr19	51305603	-	C	93	31	0.333	0	0	T	62	M	0.246	0.434	0	4E-13	0	152.41	cctttgacacCtgggggtctg	m5C_35277
chr19	51305604	-	C	96	51	0.531	0	0	C	51	M	0.432	0.628	0	9E-11	0	440.8	acctttgacaCctgggggtct	m5C_35291
chr19	51305606	-	C	93	23	0.247	0	0	T	70	M	0.171	0.344	0	1E-14	0	78.578	ggacctttgaCacctgggggt	m5C_35270
chr19	51305612	-	C	103	52	0.505	0	0	C	52	M	0.41	0.599	0	4E-12	0	426.27	accccaggacCtttgacacct	m5C_35271
chr19	51305613	-	C	103	31	0.301	0	0	T	72	M	0.221	0.395	0	7E-15	0	136.93	caccccaggaCctttgacacc	m5C_35295
chr19	51305619	-	C	84	25	0.298	0	0	T	59	M	0.21	0.402	0	2E-12	0	105.22	acagtgcaccCcaggaccttt	m5C_35252
chr19	51305623	-	C	68	20	0.294	0	0	T	48	M	0.199	0.411	0	7E-10	0	79.675	ggacacagtgCaccccaggac	m5C_35288
chr19	51305639	-	C	48	22	0.458	0	0	T	26	M	0.326	0.597	0	2E-06	0	143.33	ctctgatcacCcctgaggaca	m5C_35279
chr19	53284658	+	C	46	25	0.543	0	0	C	25	M	0.402	0.678	0	4E-06	0	200.9	ggaggaaggaCgtgtggctga	m5C_35014
chr19	55390060	-	C	292	199	0.682	0	0	C	199	M	0.626	0.732	0	0	0	2491.5	tcggaatctgCtaaggagtgt	m5C_35864
chr19	55390063	-	C	290	191	0.659	0	0	C	191	M	0.602	0.711	0	0	0	2300.7	aagtcggaatCtgctaaggag	m5C_35875
chr2	1607499	-	C	54	23	0.426	0	0	T	31	M	0.303	0.558	0	9E-08	0	139.53	tggggatcggCgggggatcc	m5C_3079
chr2	26357845	+	C	36	9	0.25	0	0	T	27	M	0.138	0.411	2E-12	1E-06	2E-12	14.476	atgtagatatCagcagtggag	m5C_3786
chr2	26357848	+	C	47	20	0.426	0	0	T	27	M	0.295	0.567	0	1E-06	0	118.05	tagatatcagCagtggaggag	m5C_3767
chr2	26357865	+	C	41	18	0.439	0	0	T	23	M	0.299	0.59	0	8E-07	0	107.6	ggaggcgtgaCaggctggaag	m5C_3781
chr2	27274092	+	C	66	44	0.667	0	0	C	44	M	0.547	0.768	0	3E-07	0	480.97	gggggattagCtcaaatggta	m5C_3782
chr2	27274094	+	C	66	43	0.652	0	0	C	43	M	0.531	0.755	0	2E-07	0	456.76	gggattagctCaaatggtaga	m5C_3787
chr2	27603889	-	C	33	23	0.697	0	0	C	23	M	0.527	0.826	0	8E-05	0	242.24	gcggctgcccCtcgccccctc	m5C_3806
chr2	27603891	-	C	36	9	0.25	0	0	T	27	M	0.138	0.411	2E-12	1E-06	2E-12	14.476	tcgcggctgcCcctcgccccc	m5C_3798
chr2	27603895	-	C	36	26	0.722	0	0	C	26	M	0.56	0.842	0	0.0001	0	291.24	ggcgtcgcggCtgcccctcgc	m5C_3795
chr2	27603898	-	C	36	9	0.25	0	0	T	27	M	0.138	0.411	2E-12	1E-06	2E-12	14.476	gcgggcgtcgCggctgcccct	m5C_3807
chr2	27606873	-	C	92	28	0.304	0	0	T	64	M	0.22	0.405	0	1E-13	0	123.05	ctgaagaggaCgatgaagaag	m5C_3810
chr2	27606892	-	C	59	36	0.61	0	0	C	36	M	0.483	0.724	0	2E-06	0	347.52	gaggatgacaCcgaggaggct	m5C_3800
chr2	27606894	-	C	66	40	0.606	0	0	C	40	M	0.485	0.715	0	1E-07	0	388.39	atgaggatgaCaccgaggagg	m5C_3812
chr2	27606954	-	C	46	21	0.457	0	0	T	25	M	0.322	0.598	0	2E-06	0	135.05	ggcaggaatgCagcgaggaag	m5C_3802
chr2	27607546	-	C	107	26	0.243	0	0	T	81	M	0.172	0.332	0	7E-16	0	89.21	aagaggaagaCagtgaggtaa	m5C_3813
chr2	29136544	+	C	53	29	0.547	0	0	C	29	M	0.415	0.673	0	4E-07	0	240.43	tgtgatgatgCcttaatattg	m5C_4123
chr2	29136561	+	C	49	27	0.551	0	0	C	27	M	0.413	0.681	0	7E-06	0	223.1	attgtggtttCgactcactga	m5C_4122
chr2	29136566	+	C	49	27	0.551	0	0	C	27	M	0.413	0.681	0	7E-06	0	223.1	ggtttcgactCactgagagta	m5C_4130
chr2	29136568	+	C	49	27	0.551	0	0	C	27	M	0.413	0.681	0	7E-06	0	223.1	tttcgactcaCtgagagtaaa	m5C_4128
chr2	36647854	+	C	37	25	0.676	0	0	C	25	M	0.515	0.804	0	0.0001	0	257.32	gcagagaaggCatagggacat	m5C_4167
chr2	36647862	+	C	34	23	0.676	0	0	C	23	M	0.508	0.809	0	8E-05	0	233.88	ggcatagggaCatggggtaag	m5C_4170
chr2	45508265	+	C	39	30	0.769	0	0	C	30	M	0.617	0.874	0	0.0002	0	369.98	gacgcggcggCtgatggtgag	m5C_3525
chr2	47507895	-	C	52	41	0.788	0	0	C	41	M	0.66	0.878	0	3E-06	0	540.93	tcgcctgacaCaattacaacg	m5C_3845
chr2	54871721	+	C	67	22	0.328	0	0	T	45	M	0.228	0.447	0	1E-09	0	100.28	gaaaaagcaaCaggcaagtgg	m5C_3891
chr2	54886309	+	C	45	20	0.444	0	0	T	25	M	0.309	0.588	0	1E-06	0	123.75	actggaagtgCgcagacagca	m5C_3899
chr2	54886315	+	C	48	18	0.375	0	0	T	30	M	0.252	0.516	0	8E-07	0	90.774	agtgcgcagaCagcaagagga	m5C_3870
chr2	54886318	+	C	47	17	0.362	0	0	T	30	M	0.24	0.505	0	6E-07	0	81.484	gcgcagacagCaagaggaaga	m5C_3875
chr2	55277338	-	C	71	33	0.465	0	0	T	38	M	0.354	0.58	0	1E-09	0	233.36	agcccgaggaCgaggaggaag	m5C_3908
chr2	55277345	-	C	39	14	0.359	0	0	T	25	M	0.227	0.516	0	9E-06	0	63.677	gtgagggagcCcgaggacgag	m5C_3910
chr2	71654044	+	C	32	8	0.25	0	0	T	24	M	0.133	0.421	2E-11	7E-07	2E-11	11.283	cgcttggtaaCtgtggatgaa	m5C_4438
chr2	72949096	+	C	31	17	0.548	0	0	C	17	M	0.378	0.708	0	2E-05	0	128.42	atgcatcaacCtggcattgca	m5C_4553
chr2	72949105	+	C	52	12	0.231	0	0	T	40	M	0.137	0.361	4E-15	9E-10	5E-15	23.637	cctggcattgCagtacctcca	m5C_4550
chr2	72949110	+	C	56	13	0.232	0	0	T	43	M	0.141	0.358	4E-16	2E-09	5E-16	28.14	cattgcagtaCctccaggaat	m5C_4548
chr2	72949113	+	C	57	25	0.439	0	0	T	32	M	0.318	0.567	0	2E-07	0	158.86	tgcagtacctCcaggaatggt	m5C_4557
chr2	72949114	+	C	59	12	0.203	0	0	T	47	M	0.12	0.323	3E-14	9E-10	3E-14	19.611	gcagtacctcCaggaatggtg	m5C_4547
chr2	86259406	-	C	45	11	0.244	0	0	T	34	M	0.142	0.387	2E-14	4E-08	2E-14	21.411	caaggagaagCaggaggagga	m5C_4494
chr2	86363024	+	C	49	29	0.592	0	0	C	29	M	0.452	0.718	0	1E-05	0	262.43	ggggtacggaCctcagctgag	m5C_4476
chr2	115695115	-	C	56	29	0.518	0	0	C	29	M	0.39	0.643	0	4E-07	0	226.26	ggcgtgaaacCttaagggtaa	m5C_5659
chr2	115695116	-	C	81	37	0.457	0	0	T	44	M	0.353	0.565	0	1E-10	0	261.02	gggcgtgaaaCcttaagggta	m5C_5656
chr2	115695123	-	C	406	322	0.793	0	0	C	322	M	0.751	0.83	0	0	0	4836.7	ttcaagagggCgtgaaacctt	m5C_5658
chr2	115695131	-	C	440	158	0.359	0	0	T	282	M	0.316	0.405	0	0	0	997.49	agagagagttCaagagggcgt	m5C_5660
chr2	115695147	-	C	113	42	0.372	0	0	T	71	M	0.288	0.464	0	8E-15	0	242.07	ttggaaagaaCtttgaagaga	m5C_5657
chr2	131058804	+	C	43	29	0.674	0	0	C	29	M	0.525	0.795	0	1E-05	0	304.59	ttggtggtgaCgtcggacact	m5C_5199
chr2	133010728	-	C	36	9	0.25	0	0	T	27	M	0.138	0.411	2E-12	1E-06	2E-12	14.476	gtctgagcgtCacttgccaat	m5C_6788
chr2	133010731	-	C	46	10	0.217	0	0	T	36	M	0.123	0.356	1E-12	2E-08	1E-12	14.731	cctgtctgagCgtcacttgcc	m5C_6833
chr2	133010736	-	C	50	24	0.48	0	0	T	26	M	0.348	0.615	0	1E-07	0	167.03	ctatgcctgtCtgagcgtcac	m5C_6592
chr2	133010740	-	C	48	25	0.521	0	0	C	25	M	0.383	0.655	0	4E-06	0	191.64	agggctatgcCtgtctgagcg	m5C_6868
chr2	133010857	-	C	135	84	0.622	0	0	C	84	M	0.538	0.7	0	2E-13	0	904.05	gtggatcactCagctcctgtg	m5C_6839
chr2	133010859	-	C	172	125	0.727	0	0	C	125	M	0.656	0.788	0	9E-16	0	1639.3	tggtggatcaCtcagctcctg	m5C_6656
chr2	133010861	-	C	184	119	0.647	0	0	C	119	M	0.575	0.712	0	0	0	1369.3	agtggtggatCactcagctcc	m5C_6671
chr2	133010876	-	C	230	102	0.443	0	0	T	128	M	0.381	0.508	0	0	0	776.68	ccttgtatgaCttttagtggt	m5C_6805
chr2	133012252	-	C	78	59	0.756	0	0	C	59	M	0.651	0.838	0	1E-07	0	767.71	tgcacgcgcgCtacactgact	m5C_6678
chr2	133012254	-	C	76	55	0.724	0	0	C	55	M	0.614	0.812	0	8E-08	0	675.67	gctgcacgcgCgctacactga	m5C_6664
chr2	133012256	-	C	84	60	0.714	0	0	C	60	M	0.61	0.8	0	9E-09	0	731.97	gggctgcacgCgcgctacact	m5C_6590
chr2	133012258	-	C	83	60	0.723	0	0	C	60	M	0.618	0.808	0	9E-09	0	742.06	cagggctgcaCgcgcgctaca	m5C_6731
chr2	133012260	-	C	77	51	0.662	0	0	C	51	M	0.551	0.758	0	4E-08	0	562.27	tccagggctgCacgcgcgcta	m5C_6706
chr2	133012268	-	C	66	39	0.591	0	0	C	39	M	0.47	0.701	0	1E-07	0	366.98	cttaaatgtcCagggctgcac	m5C_6587
chr2	133012269	-	C	59	32	0.542	0	0	C	32	M	0.417	0.663	0	7E-07	0	266.61	ccttaaatgtCcagggctgca	m5C_6628
chr2	133012278	-	C	71	38	0.535	0	0	C	38	M	0.42	0.646	0	4E-09	0	319.5	ctgtgatgccCttaaatgtcc	m5C_6828
chr2	133012279	-	C	84	59	0.702	0	0	C	59	M	0.598	0.79	0	8E-09	0	705.06	tctgtgatgcCcttaaatgtc	m5C_6710
chr2	133012280	-	C	82	59	0.72	0	0	C	59	M	0.614	0.805	0	8E-09	0	724.69	gtctgtgatgCccttaaatgt	m5C_6791
chr2	133012288	-	C	92	62	0.674	0	0	C	62	M	0.573	0.761	0	7E-10	0	710.31	aataacaggtCtgtgatgccc	m5C_6804
chr2	133012293	-	C	105	82	0.781	0	0	C	82	M	0.693	0.849	0	7E-10	0	1136	tgagcaataaCaggtctgtga	m5C_6604
chr2	133012299	-	C	115	90	0.783	0	0	C	90	M	0.699	0.848	0	1E-10	0	1257.8	tgagattgagCaataacaggt	m5C_6829
chr2	133012485	-	C	149	113	0.758	0	0	C	113	M	0.684	0.82	0	7E-13	0	1545.2	acaggattgaCagattgatag	m5C_6641
chr2	133012494	-	C	70	33	0.471	0	0	T	37	M	0.359	0.587	0	1E-09	0	236.97	tggacacggaCaggattgaca	m5C_6545
chr2	133012498	-	C	59	25	0.424	0	0	T	34	M	0.306	0.551	0	2E-07	0	153.06	agcctggacaCggacaggatt	m5C_6794
chr2	133012500	-	C	55	20	0.364	0	0	T	35	M	0.249	0.496	0	3E-08	0	99.721	ccagcctggaCacggacagga	m5C_6855
chr2	133012505	-	C	47	19	0.404	0	0	T	28	M	0.276	0.547	0	1E-06	0	105.03	ctcacccagcCtggacacgga	m5C_6826
chr2	133012506	-	C	51	20	0.392	0	0	T	31	M	0.27	0.529	0	3E-08	0	108.11	actcacccagCctggacacgg	m5C_6620
chr2	133012558	-	C	33	7	0.212	0	0	T	26	M	0.107	0.378	1E-09	3E-07	1E-09	6.6917	agggcaccacCaggagtggag	m5C_6556
chr2	133012559	-	C	37	23	0.622	0	0	C	23	M	0.461	0.759	0	8E-05	0	212.06	aagggcaccaCcaggagtgga	m5C_6774
chr2	133012561	-	C	45	20	0.444	0	0	T	25	M	0.309	0.588	0	1E-06	0	123.75	agaagggcacCaccaggagtg	m5C_6553
chr2	133012562	-	C	46	20	0.435	0	0	T	26	M	0.302	0.578	0	1E-06	0	120.84	cagaagggcaCcaccaggagt	m5C_6573
chr2	133012564	-	C	44	23	0.523	0	0	C	23	M	0.379	0.662	0	3E-06	0	174.5	gacagaagggCaccaccagga	m5C_6607

chr2	133012744	-	C	155	57	0.368	0	0	T	98	M	0.296	0.446	0	0	0	337.31	gaacgaaagtCggaggttcga	m5C_6817
chr2	133012851	-	C	51	39	0.765	0	0	C	39	M	0.632	0.86	0	2E-06	0	493.25	agaggacggCcgggggcatt	m5C_6842
chr2	133013105	-	C	99	65	0.657	0	0	C	65	M	0.559	0.743	0	1E-09	0	726.38	aaagttgttgCagttaaaaag	m5C_6596
chr2	133013422	-	C	31	10	0.323	0	0	T	21	M	0.186	0.499	7E-15	2E-06	8E-15	26.273	atgacccattCgaatgtctgc	m5C_6538
chr2	133014841	-	C	50	10	0.2	0	0	T	40	M	0.112	0.33	3E-12	2E-10	3E-12	13.024	accgtgtttgCgtggcacggg	m5C_6540
chr2	133014856	-	C	44	14	0.318	0	0	T	30	M	0.2	0.466	0	2E-07	0	55.998	cgaccggtgcCgcgaccgtg	m5C_6795
chr2	133014857	-	C	39	24	0.615	0	0	C	24	M	0.459	0.751	0	9E-05	0	220.31	gcgaccggtgCcgacgaccgt	m5C_6780
chr2	133015207	-	C	38	12	0.316	0	0	T	26	M	0.191	0.475	0	5E-06	0	45.803	agaggtgtgcCtttggggtac	m5C_6782
chr2	133015208	-	C	38	27	0.711	0	0	C	27	M	0.552	0.83	0	0.0001	0	298.31	gagaggtgtgCctttggggta	m5C_6694
chr2	133015220	-	C	74	34	0.459	0	0	T	40	M	0.351	0.572	0	1E-09	0	238.5	ctctagcgatCtgagaggtgt	m5C_6572
chr2	133032466	-	C	31	9	0.29	0	0	T	22	M	0.161	0.466	4E-13	1E-06	4E-13	18.015	attagccaggCctggtggcgt	m5C_6663
chr2	133037880	-	C	56	40	0.714	0	0	C	40	M	0.585	0.816	0	3E-06	0	468.2	tgactctggaCacaagctggg	m5C_6589
chr2	133037885	-	C	71	55	0.775	0	0	C	55	M	0.665	0.856	0	8E-08	0	731.33	ggtggtgactCtggacacaag	m5C_6864
chr2	133037887	-	C	78	38	0.487	0	0	T	40	M	0.379	0.596	0	4E-09	0	288.41	gtggtggtgaCtctggacaca	m5C_6703
chr2	133038365	-	C	47	10	0.213	0	0	T	37	M	0.12	0.349	1E-12	2E-08	1E-12	14.271	aatgggttcgCcccgagagag	m5C_6566
chr2	133038367	-	C	50	17	0.34	0	0	T	33	M	0.224	0.478	0	6E-07	0	76.285	ggaatgggttCgccccgagag	m5C_6773
chr2	133038441	-	C	42	14	0.333	0	0	T	28	M	0.21	0.484	0	2E-07	0	58.834	gtgactgaccCtggagaagca	m5C_6536
chr2	133038442	-	C	40	14	0.35	0	0	T	26	M	0.221	0.505	0	2E-07	0	61.976	tgtgactgacCctggagaagc	m5C_6594
chr2	133038447	-	C	48	12	0.25	0	0	T	36	M	0.149	0.388	1E-15	7E-08	1E-15	26.634	ggtaatgtgaCtgaccctgga	m5C_6680
chr2	133038458	-	C	39	9	0.231	0	0	T	30	M	0.126	0.383	5E-12	1E-06	5E-12	12.874	gcgtccagtgCggtaatgtga	m5C_6652
chr2	133038509	-	C	44	35	0.795	0	0	C	35	M	0.655	0.888	0	3E-05	0	458.49	gagttgggttCagatccccga	m5C_6815
chr2	133038877	-	C	37	15	0.405	0	0	T	22	M	0.263	0.565	0	1E-05	0	79.039	atgggagcggCaggggcagta	m5C_6617
chr2	133038880	-	C	69	26	0.377	0	0	T	43	M	0.272	0.495	0	6E-09	0	141.35	tggatgggagCggcaggggca	m5C_6810
chr2	133038989	-	C	609	420	0.69	0	0	C	420	M	0.652	0.725	0	0	0	5475.3	ttggaatccgCtaaggagtgt	m5C_6801
chr2	133038991	-	C	676	452	0.669	0	0	C	452	M	0.632	0.703	0	0	0	5715.9	agttggaatcCgctaaggagt	m5C_6534
chr2	133038992	-	C	735	524	0.713	0	0	C	524	M	0.679	0.744	0	0	0	7117.8	aagttggaatCcgctaaggag	m5C_6542
chr2	133039007	-	C	828	554	0.669	0	0	C	554	M	0.636	0.7	0	0	0	7050.4	ggatggtggcCatggaagttg	m5C_6646
chr2	133039008	-	C	837	512	0.613	2	0.002	C	512	M	0.58	0.646	0	0	0	5936.1	aggatggtggCcatggaagtt	m5C_6768
chr2	133039019	-	C	597	334	0.559	0	0	C	334	M	0.519	0.599	0	0	0	3469.5	atatagacagCaggatggtgg	m5C_6559
chr2	133039022	-	C	472	253	0.536	0	0	C	253	M	0.491	0.581	0	0	0	2484	ttgatatagaCagcaggatgg	m5C_6618
chr2	133039177	-	C	31	19	0.613	0	0	C	19	M	0.438	0.763	0	3E-05	0	166.53	ggtaagaagcCtggctggctg	m5C_6699
chr2	133039178	-	C	32	9	0.281	0	0	T	23	M	0.156	0.454	5E-13	1E-06	6E-13	17.198	gggtaagaagCctggctggct	m5C_6519
chr2	136691493	-	C	51	32	0.627	0	0	C	32	M	0.49	0.747	0	7E-07	0	313.76	cggcctgaggCagaaggagaa	m5C_4817
chr2	136691499	-	C	51	18	0.353	0	0	T	33	M	0.236	0.49	0	2E-08	0	85.084	gctgttcggcCtgaggcagaa	m5C_4816
chr2	136691500	-	C	49	19	0.388	0	0	T	30	M	0.264	0.528	0	1E-06	0	100.43	tgctgttcggCctgaggcaga	m5C_4814
chr2	136691503	-	C	52	20	0.385	0	0	T	32	M	0.265	0.52	0	3E-08	0	105.88	tgatgctgttCggcctgaggc	m5C_4819
chr2	136691508	-	C	49	17	0.347	0	0	T	32	M	0.229	0.487	0	6E-07	0	77.942	ctggatgatgCtgttcggcct	m5C_4818
chr2	149583272	-	C	52	14	0.269	0	0	T	38	M	0.168	0.403	0	3E-09	0	46.953	tgcagtacctCcaggaatggt	m5C_4836
chr2	149583275	-	C	53	13	0.245	0	0	T	40	M	0.149	0.376	2E-16	2E-09	2E-16	30.388	tattgcagtaCctccaggaat	m5C_4834
chr2	169020359	-	C	34	20	0.588	0	0	C	20	M	0.422	0.736	0	4E-05	0	168.89	aatacattgtCaaccgaggag	m5C_4980
chr2	211518768	+	C	40	15	0.375	0	0	T	25	M	0.242	0.53	0	3E-07	0	72.668	gtggtgctgaCaaaatttgtt	m5C_5514
chr2	211518786	+	C	144	35	0.243	0	0	T	109	M	0.18	0.319	0	0	0	126.19	gttgaaggggCccgagaagta	m5C_5523
chr2	211518788	+	C	160	34	0.212	0	0	T	126	M	0.156	0.282	0	0	0	106.24	tgaaggggccCgagaagtaga	m5C_5521
chr2	211518805	+	C	239	101	0.423	0	0	T	138	M	0.362	0.486	0	0	0	730.59	tagaaatggaCgctgttggca	m5C_5511
chr2	211518807	+	C	232	83	0.358	0	0	T	149	M	0.299	0.421	0	0	0	496.1	gaaatggacgCtgttggcaaa	m5C_5520
chr2	211518814	+	C	167	48	0.287	0	0	T	119	M	0.224	0.36	0	0	0	215.21	acgctgttggCaaagatggaa	m5C_5512
chr2	213403502	+	C	31	12	0.387	0	0	T	19	M	0.237	0.562	0	5E-06	0	56.959	tcggtgtgtgCgcttggcgct	m5C_5377
chr2	230045489	-	C	194	135	0.696	0	0	C	135	M	0.628	0.756	0	0	0	1695.2	cggagatgggCgtagtggagt	m5C_6023
chr2	230045499	-	C	314	213	0.678	0	0	C	213	M	0.625	0.728	0	0	0	2661.6	tccggagtggCggagatgggc	m5C_6021
chr2	230045507	-	C	300	200	0.667	0	0	C	200	M	0.612	0.718	0	0	0	2446	tccccgaatcCggagtggcgg	m5C_6016
chr2	230045508	-	C	276	180	0.652	0	0	C	180	M	0.594	0.706	0	0	0	2139.3	atccccgaatCcggagtggcg	m5C_6029
chr2	230045513	-	C	179	109	0.609	0	0	C	109	M	0.536	0.677	0	0	0	1168.2	ttcagatcccCgaatccggag	m5C_6049
chr2	230045514	-	C	166	113	0.681	0	0	C	113	M	0.606	0.747	0	3E-15	0	1370.5	gttcagatccCcgaatccgga	m5C_6017
chr2	230045515	-	C	155	95	0.613	0	0	C	95	M	0.534	0.686	0	3E-15	0	1015.3	ggttcagatcCccgaatccgg	m5C_6014
chr2	230045516	-	C	152	113	0.743	0	0	C	113	M	0.669	0.806	0	4E-14	0	1511	gggttcagatCcccgaatccg	m5C_6012
chr2	230045521	-	C	133	91	0.684	0	0	C	91	M	0.601	0.757	0	5E-13	0	1093.8	gagtcgggttCagatccccga	m5C_6051
chr2	230045527	-	C	120	85	0.708	0	0	C	85	M	0.622	0.782	0	7E-11	0	1056.6	gaaaggagtCgggttcagat	m5C_6047
chr2	230045577	-	C	87	68	0.782	0	0	C	68	M	0.684	0.855	0	3E-08	0	930.11	agcgccgttcCgaagggacag	m5C_6045
chr2	230045578	-	C	91	57	0.626	0	0	C	57	M	0.524	0.719	0	3E-10	0	597.08	gagcgccgttCcgaagggaca	m5C_6036
chr2	230045582	-	C	105	73	0.695	0	0	C	73	M	0.602	0.775	0	2E-10	0	878.33	gggcgagcgcCgttccgaagg	m5C_6050
chr2	230045583	-	C	97	62	0.639	0	0	C	62	M	0.54	0.728	0	7E-10	0	669.59	tgggcgagcgCcgttccgaag	m5C_6028
chr2	230045585	-	C	103	58	0.563	0	0	C	58	M	0.467	0.655	0	1E-11	0	541.44	gatgggcgagCgccgttccga	m5C_6035
chr2	230045589	-	C	109	68	0.624	0	0	C	68	M	0.53	0.709	0	9E-11	0	721.01	gagagatgggCgagcgccgtt	m5C_6027
chr2	230045601	-	C	90	64	0.711	0	0	C	64	M	0.61	0.795	0	9E-10	0	781.26	tcagtcggtcCtgagagatgg	m5C_6015
chr2	230045602	-	C	83	46	0.554	0	0	C	46	M	0.447	0.656	0	8E-10	0	411.47	gtcagtcggtCctgagagatg	m5C_6041
chr2	230045606	-	C	67	43	0.642	0	0	C	43	M	0.522	0.746	0	2E-07	0	449.08	acgggtcagtCggtcctgaga	m5C_6031
chr2	230045673	-	C	46	21	0.457	0	0	T	25	M	0.322	0.598	0	2E-06	0	135.05	gagagaggggCccgtgccttg	m5C_6033
chr2	231183792	+	C	41	23	0.561	0	0	C	23	M	0.41	0.701	0	3E-06	0	188.79	acactgggtgCtgcaggtcaa	m5C_5941
chr2	231736691	-	C	34	16	0.471	0	0	T	18	M	0.315	0.633	0	2E-05	0	100.64	ctaagtgactCaggacaggcc	m5C_6026
chr2	232322446	-	C	65	46	0.708	0	0	C	46	M	0.588	0.804	0	4E-07	0	540.99	aagcagggaaCagagatcgat	m5C_7360
chr2	232322467	-	C	66	36	0.545	0	0	C	36	M	0.426	0.66	0	6E-08	0	306.83	gcagagaaaaCctttgaagaa	m5C_7368
chr2	232322488	-	C	61	15	0.246	0	0	T	46	M	0.155	0.367	0	6E-11	0	46.52	gaatttaagaCagaagctgat	m5C_7387
chr2	232325082	-	C	61	15	0.246	0	0	T	46	M	0.155	0.367	0	6E-11	0	46.52	tgctgatgtgCtgtgtataga	m5C_7371
chr2	232325090	-	C	306	90	0.294	0	0	T	216	M	0.246	0.347	0	0	0	442.57	ttgtagtgtgCtgatgtgctg	m5C_7365
chr2	232325102	-	C	316	93	0.294	0	0	T	223	M	0.247	0.347	0	0	0	458.99	cctgatgttaCattgtagtgt	m5C_7391
chr2	232325111	-	C	308	99	0.321	0	0	T	209	M	0.272	0.376	0	0	0	538.06	atactgaaacCtgatgttaca	m5C_7376
chr2	232325125	-	C	301	110	0.365	0	0	T	191	M	0.313	0.421	0	0	0	688.73	aacaaagggaCttaatactga	m5C_7389
chr2	232325447	-	C	244	61	0.253	3	0.012	T	180	M	0.202	0.312	0	0	0	246.91	aggatgacgaCgacgacgaag	m5C_7390
chr2	232325450	-	C	194	63	0.326	1	0.005	T	130	M	0.264	0.395	0	0	0	332.92	acgaggatgaCgacgacgacg	m5C_7377
chr2	232325459	-	C	148	38	0.259	1	0.007	T	109	M	0.195	0.335	0	0	0	147.83	atgatgaggaCgaggatgacg	m5C_7380
chr2	232325493	-	C	57	39	0.684	0	0	C	39	M	0.555	0.79	0	2E-06	0	433.06	aagaacgtggCtgaggatgaa	m5C_7375
chr2	232326408	-	C	84	17	0.202	0	0	T	67	M	0.13	0.3	0	2E-14	0	44.327	aggatgatgaCagtgaggagg	m5C_7393
chr2	232326444	-	C	44	15	0.341	0	0	T	29	M	0.219	0.489	0	3E-07	0	65.627	gcaagaatgcCaagaaggaag	m5C_7392
chr2	232326445	-	C	43	10	0.233	0	0	T	33	M	0.132	0.377	4E-13	2E-08	5E-13	16.268	ggcaagaatgCcaagaaggaa	m5C_7364
chr2	232576092	+	C	74	31	0.419	0	0	T	43	M	0.313	0.533	0	6E-10	0	194.21	gtggaagaggCagaaaatgga	m5C_7312
chr2	232576618	+	C	31	20	0.645	0	0	C	20	M	0.469	0.789	0	4E-05	0	187.79	aaatggggagCaggaggctga	m5C_7335
chr2	232576629	+	C	42	21	0.5	0	0	CT	21	M	0.355	0.645	0	2E-06	0	149.21	aggaggctgaCaatgaggtag	m5C_7313
chr2	233390151	+	C	49	21	0.429	0	0	T	28	M	0.3	0.567	0	2E-06	0	126.1	agggcaggggCccgggctgga	m5C_7330
chr2	233390153	+	C	49	10	0.204	0	0	T	39	M	0.115	0.336	2E-12	2E-08	2E-12	13.419	ggcaggggccCgggctggaga	m5C_7334
chr2	233681615	+	C	65	37	0.569	0	0	C	37	M	0.448	0.682	0	8E-08	0	331.76	gaaatgagggCaaaacggaaa	m5C_7348
chr20	2443651	-	C	40	15	0.375	0	0	T	25	M	0.242	0.53	0	3E-07	0	72.668	ttgactgaagCtgatgatgag	m5C_36028
chr20	2443657	-	C	43	16	0.372	0	0	T	27	M	0.244	0.521	0	4E-07	0	78.004	ataaccttgaCtgaagctgat	m5C_36024

chr20	2634884	+	C	41	9	0.22	0	0	T	32	M	0.12	0.367	8E-12	1E-08	9E-12	11.962	cgaatcaaatCtgtcaatccc	m5C_35995
chr20	2634902	+	C	32	13	0.406	0	0	T	19	M	0.255	0.577	0	7E-06	0	66.35	cccctgagtgCaatcactgat	m5C_35991
chr20	2634906	+	C	33	13	0.394	0	0	T	20	M	0.247	0.563	0	7E-06	0	64.175	tgagtgcaatCactgatgtct	m5C_35998
chr20	2634908	+	C	32	12	0.375	0	0	T	20	M	0.229	0.547	0	5E-06	0	55.041	agtgcaatcaCtgatgtctcc	m5C_36009
chr20	2634915	+	C	34	12	0.353	0	0	T	22	M	0.215	0.521	0	5E-06	0	51.571	tcactgatgtCtccatgtctc	m5C_35992
chr20	2634917	+	C	32	14	0.438	0	0	T	18	M	0.282	0.607	0	9E-06	0	78.862	actgatgtctCcatgtctctg	m5C_35987
chr20	2634918	+	C	32	13	0.406	0	0	T	19	M	0.255	0.577	0	7E-06	0	66.35	ctgatgtctcCatgtctctga	m5C_36001
chr20	2634923	+	C	35	16	0.457	0	0	T	19	M	0.305	0.618	0	2E-05	0	97.49	gtctccatgtCtctgagcaat	m5C_35984
chr20	2634925	+	C	31	11	0.355	0	0	T	20	M	0.211	0.531	1E-16	3E-06	1E-16	37.059	ctccatgtctCtgagcaatgc	m5C_35996
chr20	2637602	+	C	205	57	0.278	0	0	T	148	M	0.221	0.343	0	0	0	252.2	gatgaactgtCtgagcctgac	m5C_36015
chr20	2637607	+	C	196	72	0.367	0	0	T	124	M	0.303	0.437	0	0	0	436.33	actgtctgagCctgaccttgt	m5C_35988
chr20	2637608	+	C	193	47	0.245	1	0.005	T	145	M	0.189	0.31	0	0	0	178.01	ctgtctgagcCtgaccttgta	m5C_36002
chr20	2637613	+	C	196	47	0.24	0	0	T	149	M	0.185	0.304	0	0	0	174.27	tgagcctgacCttgtagaatg	m5C_35997
chr20	2637628	+	C	150	39	0.283	12	0.08	T	99	M	0.214	0.363	0	0	0	167.05	agaatggaggCaaaaaaactg	m5C_36011
chr20	2637636	+	C	158	57	0.361	0	0	T	101	M	0.29	0.438	0	0	0	330.61	ggcaaaaaaaCtgatttaatg	m5C_35993
chr20	2637649	+	C	107	24	0.224	0	0	T	83	M	0.156	0.312	0	2E-16	0	74.695	atttaatgagCctgatccaat	m5C_35990
chr20	2637650	+	C	107	32	0.299	0	0	T	75	M	0.221	0.392	0	1E-14	0	141.13	tttaatgagcCtgatccaata	m5C_36003
chr20	2637656	+	C	52	21	0.404	0	0	T	31	M	0.282	0.539	0	5E-08	0	118.27	gagcctgatcCaataaagcca	m5C_36005
chr20	3765604	-	C	43	25	0.581	0	0	C	25	M	0.433	0.716	0	4E-06	0	216.64	gtggggaggaCtctgattcag	m5C_36022
chr20	9089476	+	C	37	21	0.568	0	0	C	21	M	0.409	0.713	0	5E-05	0	171.84	ggggaaaagcCtagtgagaaa	m5C_35765
chr20	9089493	+	C	36	16	0.444	0	0	T	20	M	0.295	0.604	0	2E-05	0	94.531	gaaagcaaagCcaattgcagg	m5C_35768
chr20	17469759	+	C	59	23	0.39	0	0	T	36	M	0.276	0.517	0	9E-08	0	126.86	agcacaagtgCacgtgttagg	m5C_36445
chr20	17469761	+	C	78	43	0.551	0	0	C	43	M	0.441	0.657	0	1E-08	0	379.34	cacaagtgcaCgtgttaggac	m5C_36448
chr20	17469771	+	C	100	62	0.62	0	0	C	62	M	0.522	0.709	0	3E-11	0	647.4	cgtgttaggaCctgaaagatg	m5C_36449
chr20	17469772	+	C	101	78	0.772	0	0	C	78	M	0.681	0.843	0	4E-10	0	1063	gtgttaggacCtgaaagatgg	m5C_36443
chr20	17601423	-	C	46	30	0.652	0	0	C	30	M	0.508	0.773	0	1E-05	0	304.62	tgcagaagagCgtggaggagg	m5C_36475
chr20	17601434	-	C	49	14	0.286	0	0	T	35	M	0.178	0.424	0	2E-07	0	49.978	gctcagagacCtgcagaagag	m5C_36462
chr20	17601435	-	C	50	18	0.36	0	0	T	32	M	0.241	0.499	0	2E-08	0	86.899	tgctcagagaCctgcagaaga	m5C_36455
chr20	17640938	-	C	45	17	0.378	0	0	T	28	M	0.251	0.524	0	6E-07	0	85.367	aaaggaaagaCcaagaaaaag	m5C_36470
chr20	17943355	-	C	66	17	0.258	0	0	T	49	M	0.167	0.374	0	2E-10	0	56.946	aaaatctgatCacttgctgca	m5C_36460
chr20	18246414	-	C	33	11	0.333	0	0	T	22	M	0.198	0.504	2E-16	3E-06	2E-16	34.007	attttttgagCagggagatgg	m5C_35813
chr20	20536858	-	C	32	14	0.438	0	0	T	18	M	0.282	0.607	0	9E-06	0	78.862	ggacttggggCtgagggagcg	m5C_36131
chr20	23345941	+	C	56	20	0.357	0	0	T	36	M	0.245	0.488	0	3E-08	0	97.826	aggaggaggaCgaagaagggg	m5C_36158
chr20	26188847	-	C	55	14	0.255	0	0	T	41	M	0.158	0.383	0	3E-09	0	44.274	atcccgcggcCgtgttttcct	m5C_36328
chr20	26188848	-	C	53	12	0.226	0	0	T	41	M	0.135	0.355	6E-15	9E-10	6E-15	22.989	gatcccgcggCcgtgttttcc	m5C_36339
chr20	26188851	-	C	58	30	0.517	0	0	C	30	M	0.392	0.641	0	5E-07	0	234.97	tgggatcccgCggccgtgttt	m5C_36334
chr20	26188864	-	C	40	19	0.475	0	0	T	21	M	0.329	0.625	0	1E-06	0	125.15	gtgtctgtggCggtgggatcc	m5C_36350
chr20	26188870	-	C	31	22	0.71	0	0	C	22	M	0.534	0.839	0	6E-05	0	234.99	gccctcgtgtCtgtggcggtg	m5C_36317
chr20	26188882	-	C	38	19	0.5	0	0	CT	19	M	0.348	0.652	0	3E-05	0	132.43	cgccgcgggaCcgccctcgtg	m5C_36364
chr20	26188887	-	C	40	17	0.425	0	0	T	23	M	0.285	0.578	0	6E-07	0	96.931	cggggcgccgCgggaccgccc	m5C_36325
chr20	26188892	-	C	40	24	0.6	0	0	C	24	M	0.446	0.737	0	4E-06	0	214.06	ccaggcggggCgccgcgggac	m5C_36358
chr20	26188897	-	C	38	16	0.421	0	0	T	22	M	0.279	0.578	0	2E-05	0	89.127	gcgtcccaggCggggcgccgc	m5C_36304
chr20	26189012	-	C	184	59	0.321	0	0	T	125	M	0.257	0.391	0	0	0	303.82	gaatcacgagCgatgtggccc	m5C_36321
chr20	26189018	-	C	228	58	0.254	0	0	T	170	M	0.202	0.315	0	0	0	234.61	ggacgagaatCacgagcgatg	m5C_36319
chr20	26189025	-	C	278	77	0.277	0	0	T	201	M	0.228	0.332	0	0	0	350.63	ggaggatggaCgagaatcacg	m5C_36301
chr20	26189036	-	C	201	70	0.348	0	0	T	131	M	0.286	0.416	0	0	0	400.12	gggaccgggtCggaggatgga	m5C_36312
chr20	26189041	-	C	187	42	0.225	0	0	T	145	M	0.171	0.29	0	0	0	143.37	ggagcgggacCgggtcggagg	m5C_36330
chr20	26189042	-	C	184	55	0.299	0	0	T	129	M	0.237	0.369	0	0	0	261.17	cggagcgggaCcgggtcggag	m5C_36320
chr20	26189047	-	C	235	66	0.281	0	0	T	169	M	0.227	0.341	0	0	0	300	tctcccggagCgggaccgggt	m5C_36363
chr20	26189052	-	C	170	35	0.206	0	0	T	135	M	0.152	0.273	0	0	0	106.35	gccggtctccCggagcgggac	m5C_36353
chr20	26189053	-	C	149	40	0.268	0	0	T	109	M	0.204	0.345	0	0	0	163.02	cgccggtctcCcggagcggga	m5C_36356
chr20	26189056	-	C	117	51	0.436	0	0	T	66	M	0.349	0.526	0	1E-13	0	356.49	gcgcgccggtCtcccggagcg	m5C_36335
chr20	26189061	-	C	116	39	0.336	0	0	T	77	M	0.257	0.426	0	3E-15	0	200.24	tgggggcgcgCcggtctcccg	m5C_36302
chr20	26189063	-	C	115	31	0.27	0	0	T	84	M	0.197	0.357	0	1E-16	0	122.07	ggtgggggcgCgccggtctcc	m5C_36342
chr20	26189065	-	C	105	39	0.371	0	0	T	66	M	0.285	0.467	0	1E-13	0	222.36	acggtgggggCgcgccggtct	m5C_36352
chr20	26189074	-	C	51	32	0.627	0	0	C	32	M	0.49	0.747	0	7E-07	0	313.76	aagcgtcccaCggtgggggcg	m5C_36343
chr20	26189327	-	C	82	27	0.329	0	0	T	55	M	0.237	0.437	0	4E-12	0	128.09	ccgagcgcggCtcgtcgccta	m5C_36349
chr20	26189330	-	C	90	19	0.211	0	0	T	71	M	0.14	0.306	0	9E-16	0	53.019	tggccgagcgCggctcgtcgc	m5C_36354
chr20	26189337	-	C	121	52	0.43	0	0	T	69	M	0.345	0.519	0	4E-15	0	358.85	gcggtggtggCcgagcgcggc	m5C_36345
chr20	26189351	-	C	58	24	0.414	0	0	T	34	M	0.296	0.542	0	1E-07	0	142.21	ttccgccggcCaccgcggtgg	m5C_36361
chr20	26189961	-	C	55	19	0.345	0	0	T	36	M	0.234	0.477	0	2E-08	0	88.778	cgcgcccctcCcacgcgggga	m5C_36331
chr20	26189964	-	C	53	17	0.321	0	0	T	36	M	0.211	0.455	0	1E-08	0	71.713	agacgcgcccCtcccacgcgg	m5C_36322
chr20	26189965	-	C	54	19	0.352	0	0	T	35	M	0.238	0.485	0	2E-08	0	90.527	gagacgcgccCctcccacgcg	m5C_36351
chr20	26189966	-	C	53	13	0.25	1	0.019	T	39	M	0.152	0.382	1E-16	2E-09	1E-16	31.595	cgagacgcgcCcctcccacgc	m5C_36329
chr20	26189967	-	C	51	11	0.216	0	0	T	40	M	0.125	0.346	1E-13	4E-10	1E-13	17.806	acgagacgcgCccctcccacg	m5C_36324
chr20	26189969	-	C	55	13	0.236	0	0	T	42	M	0.144	0.363	2E-16	2E-09	2E-16	29.235	agacgagacgCgcccctccca	m5C_36347
chr20	26189971	-	C	80	34	0.425	0	0	T	46	M	0.323	0.534	0	4E-11	0	219.34	tgagacgagaCgcgcccctcc	m5C_36340
chr20	26189976	-	C	78	45	0.577	0	0	C	45	M	0.466	0.68	0	2E-08	0	419.59	ttgagtgagaCgagacgcgcc	m5C_36336
chr20	26189989	-	C	249	119	0.478	0	0	T	130	M	0.417	0.54	0	0	0	991.69	gggttcgaggCggttgagtga	m5C_36348
chr20	26189994	-	C	236	83	0.352	0	0	T	153	M	0.294	0.415	0	0	0	487.36	gtgtggggttCgaggcggttg	m5C_36305
chr20	26190009	-	C	286	66	0.232	1	0.003	T	219	M	0.186	0.284	0	0	0	246.01	tgagggagctCgtcggtgtgg	m5C_36323
chr20	26190020	-	C	168	92	0.548	0	0	C	92	M	0.472	0.621	0	0	0	868.72	cgcgtgggtcCtgagggagct	m5C_36303
chr20	26190021	-	C	167	70	0.419	0	0	T	97	M	0.347	0.495	0	0	0	485.75	gcgcgtgggtCctgagggagc	m5C_36355
chr20	26190033	-	C	40	9	0.225	0	0	T	31	M	0.123	0.375	6E-12	1E-08	7E-12	12.404	gcctcggtgtCcgcgcgtggg	m5C_36344
chr20	26190120	-	C	34	11	0.324	0	0	T	23	M	0.191	0.492	4E-16	3E-06	5E-16	32.309	ctggagcgctCcgggttgtcc	m5C_36359
chr20	26190131	-	C	36	20	0.556	0	0	C	20	M	0.396	0.705	0	4E-05	0	158.32	ttgcggtgctCctggagcgct	m5C_36346
chr20	26190456	-	C	83	31	0.373	0	0	T	52	M	0.277	0.481	0	2E-11	0	171.86	gaggggcggaCattcgtggcg	m5C_36333
chr20	26190460	-	C	193	77	0.399	0	0	T	116	M	0.332	0.469	0	0	0	512.05	gggcgaggggCggacattcgt	m5C_36366
chr20	26190467	-	C	232	62	0.267	0	0	T	170	M	0.214	0.328	0	0	0	265.89	ccgcgacgggCgaggggcgga	m5C_36307
chr20	26190471	-	C	222	55	0.248	0	0	T	167	M	0.196	0.309	0	0	0	215.13	cgagccgcgaCgggcgagggg	m5C_36362
chr20	26190481	-	C	65	19	0.292	0	0	T	46	M	0.196	0.412	0	5E-10	0	74.416	tggccgtgtcCgagccgcgac	m5C_36306
chr20	26190482	-	C	39	10	0.256	0	0	T	29	M	0.146	0.411	1E-13	2E-06	1E-13	18.775	ctggccgtgtCcgagccgcga	m5C_36318
chr20	32665025	+	C	42	9	0.214	0	0	T	33	M	0.117	0.359	1E-11	1E-08	1E-11	11.545	tgaggaagggCtcctgacaca	m5C_36207
chr20	32665027	+	C	42	13	0.31	0	0	T	29	M	0.191	0.46	0	1E-07	0	49.584	aggaagggctCctgacacaca	m5C_36223
chr20	32665032	+	C	39	10	0.256	0	0	T	29	M	0.146	0.411	1E-13	2E-06	1E-13	18.775	gggctcctgaCacacagcgag	m5C_36219
chr20	32665034	+	C	35	12	0.343	0	0	T	23	M	0.208	0.508	0	5E-06	0	49.996	gctcctgacaCacagcgagga	m5C_36224
chr20	32665036	+	C	41	10	0.244	0	0	T	31	M	0.138	0.393	2E-13	2E-08	3E-13	17.447	tcctgacacaCagcgaggaag	m5C_36221
chr20	34319990	-	C	80	22	0.275	0	0	T	58	M	0.189	0.381	0	3E-11	0	83.238	ggaacggaagCgaagtagaga	m5C_36269
chr20	34319996	-	C	69	17	0.246	0	0	T	52	M	0.16	0.36	0	2E-10	0	54.365	aagtaaggaaCggaagcgaag	m5C_36268
chr20	37053938	-	C	125	25	0.2	0	0	T	100	M	0.139	0.279	0	0	0	69.649	ttgccctggtCattgatagtg	m5C_36853
chr20	37053943	-	C	130	37	0.285	0	0	T	93	M	0.214	0.367	0	0	0	158.43	tgcctttgccCtggtcattga	m5C_36843
chr20	37053951	-	C	135	35	0.259	0	0	T	100	M	0.193	0.339	0	0	0	134.91	tcgtgggctgCctttgccctg	m5C_36879

chr20	37053954	-	C	137	60	0.438	0	0	T	77	M	0.358	0.522	0	1E-15	0	429.23	tgatcgtgggCtgcctttgcc	m5C_36898
chr20	37053960	-	C	137	34	0.248	0	0	T	103	M	0.183	0.327	0	0	0	124.69	cgaaagtgatCgtgggctgcc	m5C_36865
chr20	37053977	-	C	65	18	0.277	0	0	T	47	M	0.183	0.396	0	3E-10	0	65.856	tattttgtccCtgtattcgaa	m5C_36916
chr20	37053978	-	C	60	14	0.233	0	0	T	46	M	0.144	0.354	0	3E-11	0	40.43	ctattttgtcCctgtattcga	m5C_36873
chr20	37053979	-	C	61	26	0.426	0	0	T	35	M	0.31	0.551	0	6E-09	0	161.28	cctattttgtCcctgtattcg	m5C_36887
chr20	37056044	-	C	33	11	0.333	0	0	T	22	M	0.198	0.504	2E-16	3E-06	2E-16	34.007	gtgcctaggtCattgatagtg	m5C_36868
chr20	37056056	-	C	33	9	0.273	0	0	T	24	M	0.151	0.442	8E-13	1E-06	8E-13	16.442	cataggctgcCtgtgcctagg	m5C_36891
chr20	37056057	-	C	32	10	0.312	0	0	T	22	M	0.18	0.486	1E-14	2E-06	1E-14	25.084	tcataggctgCctgtgcctag	m5C_36905
chr20	37056060	-	C	32	7	0.219	0	0	T	25	M	0.11	0.388	8E-10	3E-07	9E-10	6.9997	tgatcataggCtgcctgtgcc	m5C_36846
chr20	37056066	-	C	32	11	0.344	0	0	T	21	M	0.204	0.517	2E-16	3E-06	2E-16	35.144	cgaaagtgatCataggctgcc	m5C_36906
chr20	37056077	-	C	32	23	0.719	0	0	C	23	M	0.546	0.844	0	8E-05	0	251.28	gctcctgcatCcgaaagtgat	m5C_36863
chr20	37056080	-	C	31	7	0.226	0	0	T	24	M	0.114	0.398	6E-10	3E-07	7E-10	7.3321	tctgctcctgCatccgaaagt	m5C_36866
chr20	37062600	-	C	157	48	0.306	0	0	T	109	M	0.239	0.382	0	0	0	229.46	gtgccctggtCattgatagtg	m5C_36899
chr20	37062605	-	C	167	98	0.587	0	0	C	98	M	0.511	0.659	0	2E-16	0	1001.6	tgcctgtgccCtggtcattga	m5C_36875
chr20	37062607	-	C	154	34	0.221	0	0	T	120	M	0.163	0.293	0	0	0	110.51	gctgcctgtgCcctggtcatt	m5C_36882
chr20	37062612	-	C	154	48	0.312	0	0	T	106	M	0.244	0.389	0	0	0	234.11	cgtgggctgcCtgtgccctgg	m5C_36913
chr20	37062613	-	C	155	38	0.245	0	0	T	117	M	0.184	0.319	0	0	0	139.95	tcgtgggctgCctgtgccctg	m5C_36860
chr20	37062616	-	C	154	45	0.292	0	0	T	109	M	0.226	0.368	0	0	0	203.52	tgatcgtgggCtgcctgtgcc	m5C_36911
chr20	37062622	-	C	151	50	0.331	0	0	T	101	M	0.261	0.41	0	0	0	261.07	cgaaagtgatCgtgggctgcc	m5C_36869
chr20	37062632	-	C	142	45	0.317	0	0	T	97	M	0.246	0.397	0	0	0	221.45	cacctgtattCgaaagtgatc	m5C_36892
chr20	37062640	-	C	48	10	0.208	0	0	T	38	M	0.117	0.343	2E-12	2E-08	2E-12	13.834	cttttttgcaCctgtattcga	m5C_36902
chr20	39653012	+	C	124	30	0.242	0	0	T	94	M	0.175	0.324	0	0	0	105.03	gattgcagtaCctccaggaat	m5C_36278
chr20	39653016	+	C	152	32	0.212	1	0.007	T	119	M	0.154	0.284	0	0	0	98.753	gcagtacctcCaggaatggtg	m5C_36271
chr20	39653029	+	C	105	34	0.324	0	0	T	71	M	0.242	0.418	0	2E-14	0	164.49	gaatggtgcaCcctctctacc	m5C_36272
chr20	39653030	+	C	96	36	0.375	0	0	T	60	M	0.285	0.475	0	2E-12	0	205	aatggtgcacCctctctacca	m5C_36274
chr20	43530409	+	C	58	21	0.362	0	0	T	37	M	0.251	0.491	0	5E-08	0	105.23	gaagaagcagCagatgggcaa	m5C_36583
chr20	55063820	+	C	51	30	0.588	0	0	C	30	M	0.452	0.712	0	5E-07	0	270.99	tacccatcaaCagactgaggg	m5C_36384
chr20	55088444	+	C	31	9	0.29	0	0	T	22	M	0.161	0.466	4E-13	1E-06	4E-13	18.015	gtgctgaagaCaaggatggag	m5C_36376
chr20	57570385	-	C	34	17	0.5	0	0	CT	17	M	0.341	0.659	0	2E-05	0	115.83	caactgtgggCaataaatatgg	m5C_36618
chr20	57570395	-	C	31	15	0.484	0	0	T	16	M	0.32	0.652	0	1E-05	0	95.91	ttgtatttggCaactgtgggc	m5C_36616
chr20	60963536	+	C	34	13	0.382	0	0	T	21	M	0.239	0.55	0	7E-06	0	62.14	gagagaatcaCagatgtggaa	m5C_37060
chr21	9825711	+	C	32	13	0.406	0	0	T	19	M	0.255	0.577	0	7E-06	0	66.35	ccgcgtccccCggcgcgcgcc	m5C_38149
chr21	9825714	+	C	38	10	0.263	0	0	T	28	M	0.15	0.42	9E-14	2E-06	1E-13	19.504	cgtcccccggCgcgcgccttg	m5C_38080
chr21	9825718	+	C	43	27	0.628	0	0	C	27	M	0.479	0.756	0	7E-06	0	258.44	ccccggcgcgCgccttgggga	m5C_38150
chr21	9825720	+	C	44	15	0.341	0	0	T	29	M	0.219	0.489	0	3E-07	0	65.627	ccggcgcgcgCcttggggacc	m5C_38244
chr21	9825721	+	C	46	29	0.63	0	0	C	29	M	0.486	0.755	0	1E-05	0	281.88	cggcgcgcgcCttggggaccg	m5C_38012
chr21	9825729	+	C	45	13	0.289	0	0	T	32	M	0.177	0.434	0	1E-07	0	46.098	gccttggggaCcgggttggtg	m5C_38091
chr21	9825730	+	C	36	10	0.278	0	0	T	26	M	0.158	0.44	5E-14	2E-06	5E-14	21.112	ccttggggacCgggttggtgg	m5C_37928
chr21	9825778	+	C	42	22	0.524	0	0	C	22	M	0.377	0.666	0	2E-06	0	165.98	ccggaggggttCcgggggtcgg	m5C_37933
chr21	9825779	+	C	44	10	0.233	1	0.023	T	33	M	0.132	0.377	4E-13	2E-08	5E-13	16.268	cggaggggttcCgggggtcggc	m5C_38025
chr21	9825786	+	C	36	22	0.611	0	0	C	22	M	0.449	0.752	0	6E-05	0	197.4	ttccgggggtCggcctgcggc	m5C_38070
chr21	9825789	+	C	33	9	0.273	0	0	T	24	M	0.151	0.442	8E-13	1E-06	8E-13	16.442	cgggggtcggCctgcggcgcg	m5C_38132
chr21	9825790	+	C	37	9	0.243	0	0	T	28	M	0.134	0.401	3E-12	1E-06	3E-12	13.907	gggggtcggcCtgcggcgcgt	m5C_37987
chr21	9825793	+	C	50	16	0.32	0	0	T	34	M	0.208	0.458	0	7E-09	0	66.426	ggtcggcctgCggcgcgtgcg	m5C_38046
chr21	9825796	+	C	61	23	0.377	0	0	T	38	M	0.266	0.503	0	2E-09	0	122.43	cggcctgcggCgcgtgcgggg	m5C_37907
chr21	9825798	+	C	79	23	0.291	0	0	T	56	M	0.203	0.399	0	4E-11	0	93.159	gcctgcggcgCgtgcgggga	m5C_37970
chr21	9825802	+	C	90	44	0.489	0	0	T	46	M	0.388	0.59	0	2E-11	0	341.62	gcggcgcgtgCgggggaggag	m5C_38193
chr21	9825814	+	C	109	30	0.275	0	0	T	79	M	0.2	0.366	0	5E-15	0	120.07	ggggaggagaCggttccgggg	m5C_38210
chr21	9825819	+	C	70	24	0.343	0	0	T	46	M	0.242	0.46	0	6E-11	0	116.38	ggagacggttCcgggggaccg	m5C_38121
chr21	9825820	+	C	63	13	0.206	0	0	T	50	M	0.125	0.322	2E-15	2E-11	3E-15	23.698	gagacggttcCgggggaccgg	m5C_38045
chr21	9825827	+	C	45	20	0.444	0	0	T	25	M	0.309	0.588	0	1E-06	0	123.75	ttccgggggaCcggccgcgac	m5C_38014
chr21	9825828	+	C	55	16	0.291	0	0	T	39	M	0.188	0.421	0	7E-09	0	60.067	tccgggggacCggccgcgact	m5C_38037
chr21	9825831	+	C	52	30	0.577	0	0	C	30	M	0.442	0.701	0	5E-07	0	265.16	gggggaccggCcgcgactgcg	m5C_37997
chr21	9825832	+	C	44	13	0.295	0	0	T	31	M	0.182	0.442	0	1E-07	0	47.204	ggggaccggcCgcgactgcgg	m5C_38172
chr21	9825840	+	C	34	9	0.265	0	0	T	25	M	0.146	0.431	1E-12	1E-06	1E-12	15.739	gccgcgactgCggcggcggtg	m5C_38158
chr21	9825861	+	C	40	10	0.25	0	0	T	30	M	0.142	0.402	2E-13	2E-08	2E-13	18.091	gtgggggggagCcgcggggatc	m5C_38219
chr21	9825862	+	C	53	34	0.642	0	0	C	34	M	0.507	0.757	0	1E-06	0	344.7	tgggggggagCgcggggatcg	m5C_38235
chr21	9825864	+	C	66	15	0.227	0	0	T	51	M	0.143	0.342	0	6E-11	0	42.855	gggggagccgCggggatcgcc	m5C_38155
chr21	9825871	+	C	82	26	0.317	0	0	T	56	M	0.226	0.424	0	3E-12	0	117.77	ccgcggggatCgccgaggggcc	m5C_38180
chr21	9825874	+	C	93	40	0.43	0	0	T	53	M	0.334	0.532	0	6E-12	0	267.39	cggggatcgcCgaggggccggt	m5C_37991
chr21	9825880	+	C	66	14	0.212	0	0	T	52	M	0.131	0.325	2E-16	3E-11	2E-16	28.66	tcgccgagggCcggtcggccg	m5C_38242
chr21	9825881	+	C	58	30	0.517	0	0	C	30	M	0.392	0.641	0	5E-07	0	234.97	cgccgagggcCggtcggccgc	m5C_38187
chr21	9825900	+	C	68	18	0.265	0	0	T	50	M	0.174	0.38	0	3E-10	0	62.819	gccccgggtgCcgcgcggtgc	m5C_37996
chr21	9825901	+	C	73	30	0.411	0	0	T	43	M	0.305	0.526	0	5E-10	0	183.19	ccccgggtgcCgcgcggtgcc	m5C_38015
chr21	9825903	+	C	104	33	0.317	0	0	T	71	M	0.236	0.412	0	2E-14	0	155.58	ccgggtgccgCgcggtgccgc	m5C_38125
chr21	9825910	+	C	130	44	0.338	0	0	T	86	M	0.263	0.423	0	0	0	231.26	ccgcgcggtgCcgccggcggc	m5C_38151
chr21	9825911	+	C	129	37	0.287	0	0	T	92	M	0.216	0.37	0	0	0	159.71	cgcgcggtgcCgccggcggcg	m5C_38251
chr21	9825914	+	C	140	40	0.286	0	0	T	100	M	0.217	0.365	0	0	0	173.91	gcggtgccgcCggcggcggtg	m5C_38237
chr21	9825917	+	C	152	33	0.217	0	0	T	119	M	0.159	0.289	0	0	0	104.92	gtgccgccggCggcggtgagg	m5C_38166
chr21	9825929	+	C	150	51	0.34	0	0	T	99	M	0.269	0.419	0	0	0	274.41	gcggtgaggcCccgcgcgtgt	m5C_38021
chr21	9825930	+	C	159	85	0.538	1	0.006	C	85	M	0.46	0.614	0	4E-16	0	782.44	cggtgaggccCcgcgcgtgtg	m5C_38040
chr21	9825935	+	C	147	47	0.32	0	0	T	100	M	0.25	0.399	0	0	0	234.77	aggccccgcgCgtgtgtcccg	m5C_37930
chr21	9825943	+	C	67	20	0.299	0	0	T	47	M	0.202	0.417	0	7E-10	0	80.925	cgcgtgtgtcCcggctgcggt	m5C_37957
chr21	9825944	+	C	68	19	0.279	0	0	T	49	M	0.187	0.396	0	5E-10	0	70.976	gcgtgtgtccCggctgcggtc	m5C_38094
chr21	9825947	+	C	80	28	0.35	0	0	T	52	M	0.255	0.459	0	6E-12	0	142.54	tgtgtcccggCtgcggtcggc	m5C_38071
chr21	9825958	+	C	55	23	0.418	0	0	T	32	M	0.297	0.55	0	9E-08	0	136.79	tgcggtcggcCgcgctcgagg	m5C_38228
chr21	9825962	+	C	37	11	0.297	0	0	T	26	M	0.175	0.458	1E-15	3E-06	2E-15	28.551	gtcggccgcgCtcgaggggtc	m5C_38075
chr21	9825964	+	C	36	8	0.222	0	0	T	28	M	0.117	0.381	7E-11	7E-07	8E-11	9.4968	cggccgcgctCgaggggtccc	m5C_37990
chr21	9825972	+	C	40	19	0.475	0	0	T	21	M	0.329	0.625	0	1E-06	0	125.15	ctcgaggggtCcccgtggcgt	m5C_38069
chr21	9826083	+	C	31	9	0.29	0	0	T	22	M	0.161	0.466	4E-13	1E-06	4E-13	18.015	accgggtcggCgcgtcccccg	m5C_37905
chr21	9826088	+	C	38	9	0.237	0	0	T	29	M	0.13	0.392	4E-12	1E-06	4E-12	13.374	gtcggcgcgtCccccgggtgc	m5C_38005
chr21	9826089	+	C	38	10	0.263	0	0	T	28	M	0.15	0.42	9E-14	2E-06	1E-13	19.504	tcggcgcgtcCcccgggtgcg	m5C_38195
chr21	9826092	+	C	39	17	0.436	0	0	T	22	M	0.293	0.59	0	2E-05	0	99.635	gcgcgtccccCgggtgcgcct	m5C_37998
chr21	9826193	+	C	33	12	0.364	0	0	T	21	M	0.222	0.534	0	5E-06	0	53.249	tggcgtcgccCcgttcggcgc	m5C_37959
chr21	9826198	+	C	33	18	0.545	0	0	C	18	M	0.38	0.702	0	3E-05	0	136.75	tcgccccgttCggcgcgcgcg	m5C_38123
chr21	9826201	+	C	40	18	0.45	0	0	T	22	M	0.307	0.602	0	3E-05	0	110.54	ccccgttcggCgcgcgcgtgc	m5C_38254
chr21	9826205	+	C	46	15	0.326	0	0	T	31	M	0.209	0.47	0	3E-07	0	62.597	gttcggcgcgCgcgtgcgccc	m5C_38188
chr21	9826211	+	C	52	13	0.25	0	0	T	39	M	0.152	0.382	1E-16	2E-09	1E-16	31.595	cgcgcgcgtgCgcccgagcgc	m5C_38098
chr21	9826214	+	C	40	8	0.2	0	0	T	32	M	0.105	0.348	2E-10	5E-09	2E-10	8.135	gcgcgtgcgcCcgagcgcggc	m5C_37951
chr21	9826225	+	C	57	31	0.544	0	0	C	31	M	0.416	0.666	0	6E-07	0	257.86	cgagcgcggcCcggtggtccc	m5C_38222
chr21	9826226	+	C	50	10	0.2	0	0	T	40	M	0.112	0.33	3E-12	2E-10	3E-12	13.024	gagcgcggccCggtggtccct	m5C_38011
chr21	9826238	+	C	95	25	0.263	0	0	T	70	M	0.185	0.36	0	3E-14	0	92.532	gtggtccctcCcggacaggcg	m5C_37931

chr21	9826239	+	C	106	27	0.255	0	0	T	79	M	0.181	0.345	0	1E-15	0	97.936	tggtccctccCggacaggcgt	m5C_38055
chr21	9826247	+	C	122	32	0.262	0	0	T	90	M	0.192	0.347	0	0	0	123.11	cccggacaggCgttcgtgcga	m5C_38117
chr21	9826255	+	C	206	48	0.233	0	0	T	158	M	0.18	0.295	0	0	0	173.27	ggcgttcgtgCgacgtgtggc	m5C_38247
chr21	9826258	+	C	258	97	0.376	0	0	T	161	M	0.319	0.436	0	0	0	619.04	gttcgtgcgaCgtgtggcgtg	m5C_38078
chr21	9826265	+	C	234	97	0.415	0	0	T	137	M	0.353	0.479	0	0	0	685.37	cgacgtgtggCgtgggtcgac	m5C_37974
chr21	9826272	+	C	134	28	0.209	0	0	T	106	M	0.149	0.285	0	0	0	83.278	tggcgtgggtCgacctccgcc	m5C_38152
chr21	9826276	+	C	65	29	0.446	0	0	T	36	M	0.332	0.567	0	1E-08	0	192.38	gtgggtcgacCtccgccttgc	m5C_37962
chr21	9826414	+	C	55	19	0.345	0	0	T	36	M	0.234	0.477	0	2E-08	0	88.778	ccttggccgtCgtgtggcgtg	m5C_38111
chr21	9826421	+	C	57	18	0.316	0	0	T	39	M	0.21	0.445	0	2E-08	0	75.615	cgtcgtgtggCgtgtgccacc	m5C_38029
chr21	9826427	+	C	49	18	0.367	0	0	T	31	M	0.247	0.507	0	8E-07	0	88.794	gtggcgtgtgCcacccctgcg	m5C_38230
chr21	9826428	+	C	51	12	0.235	0	0	T	39	M	0.14	0.368	3E-15	9E-10	4E-15	24.331	tggcgtgtgcCaccccctgcgc	m5C_38022
chr21	9826449	+	C	36	12	0.333	0	0	T	24	M	0.202	0.497	0	5E-06	0	48.515	ccgcgcccgcCggcggggctc	m5C_38089
chr21	9826452	+	C	40	14	0.35	0	0	T	26	M	0.221	0.505	0	2E-07	0	61.976	cgcccgccggCggggctcgga	m5C_38213
chr21	9826459	+	C	38	21	0.553	0	0	C	21	M	0.397	0.699	0	5E-05	0	166.77	cggcggggctCggagccgggc	m5C_38056
chr21	9826508	+	C	62	20	0.323	0	0	T	42	M	0.22	0.446	0	7E-10	0	87.817	gaccggtgcgCgggcgctgcg	m5C_37981
chr21	9826514	+	C	76	27	0.355	0	0	T	49	M	0.257	0.467	0	2E-10	0	138.79	tgcgcgggcgCtgcggccgca	m5C_38058
chr21	9826520	+	C	76	20	0.263	0	0	T	56	M	0.177	0.372	0	1E-11	0	70.917	ggcgctgcggCcgcacggcgc	m5C_38190
chr21	9826521	+	C	55	18	0.327	0	0	T	37	M	0.218	0.459	0	2E-08	0	78.526	gcgctgcggcCgcacggcgcg	m5C_38248
chr21	9826523	+	C	56	20	0.357	0	0	T	36	M	0.245	0.488	0	3E-08	0	97.826	gctgcggccgCacggcgcgac	m5C_38205
chr21	9826572	+	C	35	18	0.514	0	0	C	18	M	0.356	0.67	0	3E-05	0	128.05	ctctcgctcgCcgcccggacg	m5C_38186
chr21	9826577	+	C	44	22	0.5	0	0	CT	22	M	0.358	0.642	0	2E-06	0	157.66	gctcgccgccCggacgtcggg	m5C_38168
chr21	9826581	+	C	52	13	0.25	0	0	T	39	M	0.152	0.382	1E-16	2E-09	1E-16	31.595	gccgcccggaCgtcggggccg	m5C_37992
chr21	9826584	+	C	57	30	0.526	0	0	C	30	M	0.399	0.65	0	5E-07	0	239.51	gcccggacgtCggggccgccc	m5C_38170
chr21	9826589	+	C	60	32	0.533	0	0	C	32	M	0.409	0.654	0	3E-08	0	261.72	gacgtcggggCcgccccgcgg	m5C_38109
chr21	9826593	+	C	69	28	0.406	0	0	T	41	M	0.298	0.524	0	1E-08	0	166.82	tcggggccgcCccgcggggcg	m5C_38241
chr21	9826594	+	C	62	32	0.516	0	0	C	32	M	0.394	0.636	0	3E-08	0	252.46	cggggccgccCcgcggggcgg	m5C_38197
chr21	9826595	+	C	63	39	0.619	0	0	C	39	M	0.496	0.729	0	1E-07	0	386.56	ggggccgcccCgcggggcggg	m5C_38209
chr21	9826597	+	C	62	24	0.387	0	0	T	38	M	0.276	0.512	0	3E-09	0	132.41	ggccgccccgCggggcgggcg	m5C_37912
chr21	9826643	+	C	45	12	0.267	0	0	T	33	M	0.16	0.41	6E-16	7E-08	6E-16	29.226	cccgcgggcgCcggccgcgcg	m5C_37935
chr21	9826644	+	C	48	23	0.479	0	0	T	25	M	0.345	0.617	0	3E-06	0	158.57	ccgcgggcgcCggccgcgcgc	m5C_38156
chr21	9826647	+	C	43	13	0.302	0	0	T	30	M	0.186	0.451	0	1E-07	0	48.365	cgggcgccggCcgcgcgcgcg	m5C_38189
chr21	9826648	+	C	44	10	0.227	0	0	T	34	M	0.128	0.37	6E-13	2E-08	6E-13	15.727	gggcgccggcCgcgcgcgcgc	m5C_38127
chr21	9826650	+	C	55	18	0.327	0	0	T	37	M	0.218	0.459	0	2E-08	0	78.526	gcgccggccgCgcgcgcgcgc	m5C_38020
chr21	9826654	+	C	66	20	0.303	0	0	T	46	M	0.206	0.422	0	7E-10	0	82.215	cggccgcgcgCgcgcgcgcgt	m5C_38065
chr21	9826658	+	C	61	22	0.361	0	0	T	39	M	0.252	0.486	0	1E-09	0	110.77	cgcgcgcgcgCgcgcgtggcc	m5C_38079
chr21	9826671	+	C	40	8	0.2	0	0	T	32	M	0.105	0.348	2E-10	5E-09	2E-10	8.135	gcgtggccgcCggtccctccc	m5C_37995
chr21	9826675	+	C	37	8	0.216	0	0	T	29	M	0.114	0.372	1E-10	7E-07	1E-10	9.1227	ggccgccggtCcctcccggcc	m5C_38110
chr21	9826722	+	C	79	50	0.633	0	0	C	50	M	0.523	0.731	0	4E-08	0	522.77	tcctcgcgggCgggcgcgacg	m5C_38103
chr21	9826726	+	C	120	36	0.3	0	0	T	84	M	0.225	0.387	0	0	0	162.18	cgcgggcgggCgcgacgaaga	m5C_38006
chr21	9826728	+	C	126	33	0.262	0	0	T	93	M	0.193	0.345	0	0	0	127.38	cgggcgggcgCgacgaagaag	m5C_38211
chr21	9826731	+	C	129	37	0.287	0	0	T	92	M	0.216	0.37	0	0	0	159.71	gcgggcgcgaCgaagaagcgt	m5C_38131
chr21	9826744	+	C	65	19	0.292	0	0	T	46	M	0.196	0.412	0	5E-10	0	74.416	agaagcgtcgCgggtctgtgg	m5C_38105
chr21	9826749	+	C	62	32	0.516	0	0	C	32	M	0.394	0.636	0	3E-08	0	252.46	cgtcgcgggtCtgtggcgcgg	m5C_37975
chr21	9826757	+	C	40	23	0.575	0	0	C	23	M	0.422	0.715	0	3E-06	0	194.1	gtctgtggcgCggggccccgg	m5C_38137
chr21	9826931	+	C	31	9	0.29	0	0	T	22	M	0.161	0.466	4E-13	1E-06	4E-13	18.015	gcttgcggggCgccgggcccg	m5C_37911
chr21	9826933	+	C	32	21	0.656	0	0	C	21	M	0.483	0.796	0	5E-05	0	202.91	ttgcggggcgCcgggcccgtc	m5C_37965
chr21	9826934	+	C	32	9	0.281	0	0	T	23	M	0.156	0.454	5E-13	1E-06	6E-13	17.198	tgcggggcgcCgggcccgtcc	m5C_37955
chr21	9827007	+	C	204	48	0.235	0	0	T	156	M	0.182	0.298	0	0	0	175.02	tacctacctaCctggttgatc	m5C_37927
chr21	9827008	+	C	213	64	0.3	0	0	T	149	M	0.243	0.365	0	0	0	310.89	acctacctacCtggttgatcc	m5C_38179
chr21	9827017	+	C	510	201	0.394	0	0	T	309	M	0.353	0.437	0	0	0	1417.7	cctggttgatCctgccagtag	m5C_38206
chr21	9827018	+	C	502	191	0.38	0	0	T	311	M	0.339	0.424	0	0	0	1295.2	ctggttgatcCtgccagtagc	m5C_38042
chr21	9827021	+	C	523	178	0.34	0	0	T	345	M	0.301	0.382	0	0	0	1071.7	gttgatcctgCcagtagcata	m5C_38113
chr21	9827022	+	C	518	185	0.357	0	0	T	333	M	0.317	0.399	0	0	0	1173.2	ttgatcctgcCagtagcatat	m5C_37963
chr21	9827028	+	C	508	235	0.463	0	0	T	273	M	0.42	0.506	0	0	0	1972.5	ctgccagtagCatatgcttgt	m5C_38092
chr21	9827034	+	C	419	153	0.366	1	0.002	T	265	M	0.321	0.413	0	0	0	983.06	gtagcatatgCttgtctcaaa	m5C_37932
chr21	9827039	+	C	381	197	0.517	0	0	C	197	M	0.467	0.567	0	0	0	1839.8	atatgcttgtCtcaaagatta	m5C_37917
chr21	9827041	+	C	339	136	0.401	0	0	T	203	M	0.35	0.454	0	0	0	953.07	atgcttgtctCaaagattaag	m5C_38229
chr21	9827052	+	C	172	109	0.634	0	0	C	109	M	0.56	0.702	0	0	0	1219.8	aaagattaagCcatgcatgtc	m5C_37982
chr21	9827053	+	C	166	108	0.655	1	0.006	C	108	M	0.579	0.723	0	1E-15	0	1251.1	aagattaagcCatgcatgtct	m5C_38081
chr21	9827057	+	C	164	117	0.713	0	0	C	117	M	0.64	0.777	0	5E-15	0	1497.5	ttaagccatgCatgtctaagt	m5C_38200
chr21	9827062	+	C	183	138	0.754	0	0	C	138	M	0.687	0.811	0	3E-16	0	1895.9	ccatgcatgtCtaagtacgca	m5C_37937
chr21	9827069	+	C	196	145	0.74	0	0	C	145	M	0.674	0.796	0	0	0	1955.1	tgtctaagtaCgcacggccgg	m5C_37952
chr21	9827073	+	C	211	151	0.716	0	0	C	151	M	0.651	0.772	0	0	0	1967	taagtacgcaCggccggtaca	m5C_37920
chr21	9827076	+	C	199	129	0.648	0	0	C	129	M	0.58	0.711	0	0	0	1495.5	gtacgcacggCcggtacagtg	m5C_38142
chr21	9827077	+	C	186	140	0.753	0	0	C	140	M	0.686	0.809	0	4E-16	0	1920.8	tacgcacggcCggtacagtga	m5C_38253
chr21	9827082	+	C	198	119	0.601	0	0	C	119	M	0.532	0.667	0	0	0	1265	acggccggtaCagtgaaactg	m5C_38083
chr21	9827090	+	C	261	198	0.759	0	0	C	198	M	0.703	0.807	0	0	0	2784.7	tacagtgaaaCtgcgaatggc	m5C_38162
chr21	9827093	+	C	250	164	0.656	0	0	C	164	M	0.595	0.712	0	0	0	1952.1	agtgaaactgCgaatggctca	m5C_37948
chr21	9827102	+	C	202	137	0.678	0	0	C	137	M	0.611	0.739	0	0	0	1674.1	gcgaatggctCattaaatcag	m5C_37976
chr21	9827199	+	C	325	242	0.747	1	0.003	C	242	M	0.697	0.791	0	0	0	3372.8	gcgctgacccCcttcgcgggg	m5C_37925
chr21	9827200	+	C	314	228	0.726	0	0	C	228	M	0.674	0.772	0	0	0	3074.7	cgctgaccccCttcgcggggg	m5C_37979
chr21	9827203	+	C	311	155	0.498	0	0	T	156	M	0.443	0.554	0	0	0	1373.9	tgaccccccttCgcgggggga	m5C_37929
chr21	9827205	+	C	318	140	0.47	20	0.063	T	158	M	0.414	0.526	0	0	0	1158.9	acccccttcgCgggggggatg	m5C_37958
chr21	9827216	+	C	603	161	0.267	1	0.002	T	441	M	0.234	0.304	0	0	0	752.32	gggggggatgCgtgcatttat	m5C_37989
chr21	9827220	+	C	559	203	0.363	0	0	T	356	M	0.324	0.404	0	0	0	1316.8	gggatgcgtgCatttatcaga	m5C_37924
chr21	9827227	+	C	432	117	0.271	0	0	T	315	M	0.231	0.315	0	0	0	540.73	gtgcatttatCagatcaaaac	m5C_38086
chr21	9827232	+	C	271	122	0.45	0	0	T	149	M	0.392	0.51	0	0	0	956.62	tttatcagatCaaaaccaacc	m5C_37994
chr21	9827237	+	C	210	122	0.581	0	0	C	122	M	0.513	0.646	0	0	0	1252.6	cagatcaaaaCcaacccggtc	m5C_37914
chr21	9827238	+	C	199	117	0.588	0	0	C	117	M	0.519	0.654	0	0	0	1213.3	agatcaaaacCaacccggtca	m5C_38178
chr21	9827241	+	C	191	121	0.634	0	0	C	121	M	0.563	0.699	0	0	0	1362.9	tcaaaaccaaCccggtcagcc	m5C_37916
chr21	9827242	+	C	174	121	0.695	0	0	C	121	M	0.623	0.759	0	4E-16	0	1508.6	caaaaccaacCcggtcagccc	m5C_38177
chr21	9827243	+	C	175	99	0.566	0	0	C	99	M	0.492	0.637	0	0	0	973.45	aaaaccaaccCggtcagcccc	m5C_37926
chr21	9827247	+	C	161	36	0.409	73	0.453	G	73	M	0.312	0.514	0	7E-11	0	224.83	ccaacccggtCagcccctctc	m5C_37973
chr21	9827250	+	C	133	102	0.767	0	0	C	102	M	0.688	0.831	0	3E-12	0	1403.9	acccggtcagCccctctccgg	m5C_37910
chr21	9827255	+	C	135	33	0.244	0	0	T	102	M	0.18	0.323	0	0	0	118.59	gtcagcccctCtccggccccg	m5C_38024
chr21	9827261	+	C	146	97	0.664	0	0	C	97	M	0.584	0.736	0	7E-14	0	1133.8	ccctctccggCcccggccggg	m5C_38030
chr21	9827267	+	C	168	94	0.56	0	0	C	94	M	0.484	0.632	0	1E-16	0	909.83	ccggccccggCcggggggcgg	m5C_37972
chr21	9827275	+	C	218	141	0.647	0	0	C	141	M	0.581	0.707	0	0	0	1639.3	ggccgggggggCgggcgccggc	m5C_37967
chr21	9827279	+	C	273	149	0.55	2	0.007	C	149	M	0.49	0.608	0	0	0	1461.1	ggggggcgggCgccggcggct	m5C_38196
chr21	9827281	+	C	315	173	0.549	0	0	C	173	M	0.494	0.603	0	0	0	1709.2	ggggcgggcgCcggcggcttt	m5C_38074
chr21	9827282	+	C	302	162	0.536	0	0	C	162	M	0.48	0.592	0	0	0	1555.4	gggcgggcgcCggcggctttg	m5C_38231
chr21	9827285	+	C	326	191	0.586	0	0	C	191	M	0.532	0.638	0	0	0	2031.2	cgggcgccggCggctttggtg	m5C_37964

chr21	9827288	+	C	339	211	0.622	0	0	C	211	M	0.57	0.672	0	0	0	2404.2	gcgccggcggCtttggtgact	m5C_38066
chr21	9827297	+	C	460	315	0.685	0	0	C	315	M	0.641	0.726	0	0	0	4037.9	gctttggtgaCtctagataac	m5C_37977
chr21	9827299	+	C	474	326	0.688	0	0	C	326	M	0.645	0.728	0	0	0	4203.3	tttggtgactCtagataacct	m5C_37983
chr21	9827307	+	C	483	322	0.667	0	0	C	322	M	0.623	0.707	0	0	0	4015.1	ctctagataaCctcgggctga	m5C_38087
chr21	9827308	+	C	498	347	0.697	0	0	C	347	M	0.655	0.736	0	0	0	4546	tctagataacCtcgggctgat	m5C_38099
chr21	9827310	+	C	529	352	0.665	0	0	C	352	M	0.624	0.704	0	0	0	4393.9	tagataacctCgggctgatcg	m5C_38224
chr21	9827314	+	C	523	351	0.671	0	0	C	351	M	0.63	0.71	0	0	0	4420.8	taacctcgggCtgatcgcacg	m5C_38031
chr21	9827319	+	C	563	339	0.602	0	0	C	339	M	0.561	0.642	0	0	0	3804.5	tcgggctgatCgcacgccccc	m5C_37968
chr21	9827321	+	C	602	324	0.538	0	0	C	324	M	0.498	0.578	0	0	0	3228.8	gggctgatcgCacgccccccg	m5C_38250
chr21	9827323	+	C	612	395	0.645	0	0	C	395	M	0.607	0.682	0	0	0	4793.1	gctgatcgcaCgccccccgtg	m5C_38153
chr21	9827325	+	C	552	248	0.451	2	0.004	T	302	M	0.41	0.493	0	0	0	2032.6	tgatcgcacgCcccccgtggc	m5C_38082
chr21	9827326	+	C	570	266	0.467	0	0	T	304	M	0.426	0.508	0	0	0	2266.7	gatcgcacgcCccccgtggcg	m5C_38238
chr21	9827327	+	C	640	228	0.357	2	0.003	T	410	M	0.321	0.395	0	0	0	1464.4	atcgcacgccCcccgtggcgg	m5C_38232
chr21	9827328	+	C	637	185	0.29	0	0	T	452	M	0.257	0.327	0	0	0	949.09	tcgcacgcccCccgtggcggc	m5C_38167
chr21	9827329	+	C	640	201	0.314	0	0	T	439	M	0.279	0.351	0	0	0	1122.8	cgcacgccccCcgtggcggcg	m5C_37960
chr21	9827330	+	C	644	177	0.276	2	0.003	T	465	M	0.243	0.312	0	0	0	858.61	gcacgcccccCgtggcggcga	m5C_37923
chr21	9827335	+	C	651	187	0.288	2	0.003	T	462	M	0.255	0.324	0	0	0	952.27	ccccccgtggCggcgacgacc	m5C_38008
chr21	9827338	+	C	660	205	0.312	2	0.003	T	453	M	0.277	0.348	0	0	0	1137.1	cccgtggcggCgacgacccat	m5C_37918
chr21	9827341	+	C	645	167	0.259	0	0	T	478	M	0.227	0.294	0	0	0	756.87	gtggcggcgaCgacccattcg	m5C_38023
chr21	9827344	+	C	609	152	0.25	0	0	T	457	M	0.217	0.285	0	0	0	659.25	gcggcgacgaCccattcgaac	m5C_38207
chr21	9827345	+	C	598	190	0.318	1	0.002	T	407	M	0.282	0.357	0	0	0	1072.2	cggcgacgacCcattcgaacg	m5C_38140
chr21	9827350	+	C	543	122	0.225	0	0	T	421	M	0.192	0.262	0	0	0	467.45	acgacccattCgaacgtctgc	m5C_38048
chr21	9827357	+	C	380	94	0.247	0	0	T	286	M	0.207	0.293	0	0	0	388.51	attcgaacgtCtgccctatca	m5C_38147
chr21	9827360	+	C	236	48	0.203	0	0	T	188	M	0.157	0.259	0	0	0	150.7	cgaacgtctgCcctatcaact	m5C_38128
chr21	9827361	+	C	184	43	0.234	0	0	T	141	M	0.178	0.3	0	0	0	153.41	gaacgtctgcCctatcaactt	m5C_38183
chr21	9827362	+	C	141	40	0.284	0	0	T	101	M	0.216	0.363	0	0	0	172.63	aacgtctgccCtatcaacttt	m5C_38161
chr21	9827366	+	C	119	78	0.655	0	0	C	78	M	0.566	0.735	0	2E-11	0	883.62	tctgccctatCaactttcgat	m5C_38243
chr21	9827373	+	C	135	42	0.311	0	0	T	93	M	0.239	0.394	0	0	0	200.89	tatcaactttCgatggtagtc	m5C_38234
chr21	9827383	+	C	61	30	0.492	0	0	T	31	M	0.371	0.614	0	2E-08	0	222.36	cgatggtagtCgccgtgccta	m5C_38122
chr21	9827385	+	C	71	41	0.577	0	0	C	41	M	0.462	0.685	0	7E-09	0	378.43	atggtagtcgCcgtgcctacc	m5C_38192
chr21	9827386	+	C	71	30	0.423	0	0	T	41	M	0.315	0.538	0	5E-10	0	188.72	tggtagtcgcCgtgcctacca	m5C_38226
chr21	9827390	+	C	72	39	0.542	0	0	C	39	M	0.427	0.652	0	5E-09	0	333.37	agtcgccgtgCctaccatggt	m5C_38090
chr21	9827391	+	C	72	38	0.528	0	0	C	38	M	0.414	0.639	0	4E-09	0	314.64	gtcgccgtgcCtaccatggtg	m5C_38017
chr21	9827394	+	C	86	38	0.442	0	0	T	48	M	0.342	0.547	0	1E-10	0	259.64	gccgtgcctaCcatggtgacc	m5C_38175
chr21	9827395	+	C	100	47	0.47	0	0	T	53	M	0.375	0.567	0	1E-12	0	352.6	ccgtgcctacCatggtgacca	m5C_38114
chr21	9827403	+	C	149	63	0.423	0	0	T	86	M	0.346	0.503	0	0	0	436.48	accatggtgaCcacgggtgac	m5C_38249
chr21	9827404	+	C	157	63	0.406	2	0.013	T	92	M	0.332	0.485	0	0	0	418.69	ccatggtgacCacgggtgacg	m5C_38104
chr21	9827406	+	C	188	91	0.484	0	0	T	97	M	0.414	0.555	0	0	0	752.83	atggtgaccaCgggtgacggg	m5C_38223
chr21	9827413	+	C	294	168	0.571	0	0	C	168	M	0.514	0.627	0	0	0	1728	ccacgggtgaCggggaatcag	m5C_37984
chr21	9827421	+	C	346	91	0.263	0	0	T	255	M	0.219	0.312	0	0	0	399.31	gacggggaatCagggttcgat	m5C_38160
chr21	9827428	+	C	423	220	0.52	0	0	C	220	M	0.473	0.567	0	0	0	2079.1	aatcagggttCgattccggag	m5C_37908
chr21	9827433	+	C	357	223	0.625	0	0	C	223	M	0.573	0.673	0	0	0	2557.1	gggttcgattCcggagaggga	m5C_37946
chr21	9827434	+	C	374	266	0.711	0	0	C	266	M	0.663	0.755	0	0	0	3528.9	ggttcgattcCggagagggag	m5C_38003
chr21	9827476	+	C	152	121	0.796	0	0	C	121	M	0.725	0.852	0	1E-13	0	1754.7	ccaaggaaggCagcaggcgcg	m5C_38041
chr21	18827143	-	C	48	14	0.292	0	0	T	34	M	0.182	0.432	0	2E-07	0	51.076	tctcgcctgcCacgcgggagg	m5C_36709
chr21	18827147	-	C	100	37	0.37	0	0	T	63	M	0.282	0.468	0	7E-14	0	208.55	gaattctcgcCtgccacgcgg	m5C_36715
chr21	18827148	-	C	126	61	0.484	0	0	T	65	M	0.399	0.571	0	4E-14	0	486.33	agaattctcgCctgccacgcg	m5C_36714
chr21	18827150	-	C	139	68	0.489	0	0	T	71	M	0.408	0.571	0	7E-15	0	554.23	gtagaattctCgcctgccacg	m5C_36720
chr21	18827152	-	C	166	101	0.608	0	0	C	101	M	0.533	0.679	0	3E-16	0	1075.7	tggtagaattCtcgcctgcca	m5C_36704
chr21	18827165	-	C	259	128	0.494	0	0	T	131	M	0.434	0.555	0	0	0	1110.7	atgggtggttCagtggtagaa	m5C_36707
chr21	18827176	-	C	108	43	0.398	0	0	T	65	M	0.311	0.492	0	4E-13	0	267.34	tctatcggtgCatgggtggtt	m5C_36719
chr21	26197468	-	C	34	19	0.559	0	0	C	19	M	0.395	0.711	0	3E-05	0	149.92	gggactgagaCatggaaattg	m5C_37284
chr21	26197474	-	C	38	22	0.579	0	0	C	22	M	0.422	0.721	0	6E-05	0	185.65	gacaccgggaCtgagacatgg	m5C_37298
chr21	26946306	+	C	37	24	0.649	0	0	C	24	M	0.488	0.782	0	9E-05	0	234.04	taatgctaatCgtgatagggg	m5C_37261
chr21	27354674	-	C	31	16	0.516	0	0	C	16	M	0.348	0.68	0	2E-05	0	111.49	ggccaagcacCgagagagaat	m5C_37281
chr21	27354681	-	C	31	13	0.419	0	0	T	18	M	0.264	0.592	0	7E-06	0	68.68	ggcttgaggcCaagcaccgag	m5C_37276
chr21	27354682	-	C	32	18	0.562	0	0	C	18	M	0.393	0.718	0	3E-05	0	141.57	aggcttgaggCcaagcaccga	m5C_37287
chr21	27394280	-	C	283	77	0.272	0	0	T	206	M	0.224	0.327	0	0	0	344.26	atgacgaggaCgatgaggatg	m5C_37294
chr21	27394286	-	C	242	79	0.326	0	0	T	163	M	0.27	0.388	0	0	0	427.36	ccgatgatgaCgaggacgatg	m5C_37306
chr21	27394295	-	C	149	51	0.342	0	0	T	98	M	0.271	0.422	0	0	0	276.34	aagaagaagcCgatgatgacg	m5C_37271
chr21	27394296	-	C	117	33	0.282	0	0	T	84	M	0.208	0.37	0	2E-16	0	137.58	gaagaagaagCcgatgatgac	m5C_37283
chr21	40717093	-	C	86	37	0.43	0	0	T	49	M	0.331	0.536	0	1E-10	0	244.79	ctgcggaaaaCggggaaacga	m5C_37173
chr21	40717100	-	C	74	39	0.527	0	0	C	39	M	0.415	0.637	0	5E-09	0	323.52	gacttacctgCggaaaacggg	m5C_37178
chr21	40717108	-	C	36	22	0.611	0	0	C	22	M	0.449	0.752	0	6E-05	0	197.4	ctaaagaagaCttacctgcgg	m5C_37170
chr22	20228628	+	C	45	9	0.2	0	0	T	36	M	0.109	0.338	2E-11	1E-08	2E-11	10.429	gggggctgccCgggcagcctg	m5C_37209
chr22	24001056	-	C	50	32	0.64	0	0	C	32	M	0.501	0.759	0	7E-07	0	320.9	aatgaatgaaCaagatgaggt	m5C_37339
chr22	29664292	+	C	52	27	0.519	0	0	C	27	M	0.387	0.649	0	2E-07	0	208.9	gagagggagaCggacgttgag	m5C_37589
chr22	32795677	-	C	35	15	0.429	0	0	T	20	M	0.28	0.591	0	1E-05	0	83.953	gggctgaagaCaaggagcact	m5C_37419
chr22	32795690	-	C	38	20	0.526	0	0	C	20	M	0.373	0.675	0	4E-05	0	149.03	gaagggtatgCctgggctgaa	m5C_37421
chr22	36236450	-	C	45	30	0.667	0	0	C	30	M	0.521	0.786	0	1E-05	0	312.42	gagagactgcCagagaggaag	m5C_37639
chr22	36236451	-	C	56	20	0.357	0	0	T	36	M	0.245	0.488	0	3E-08	0	97.826	ggagagactgCcagagaggaa	m5C_37648
chr22	36236454	-	C	56	32	0.571	0	0	C	32	M	0.441	0.692	0	7E-07	0	282.48	gggggagagaCtgccagagag	m5C_37671
chr22	36681186	-	C	31	12	0.387	0	0	T	19	M	0.237	0.562	0	5E-06	0	56.959	gctggaggagCagctggacaa	m5C_37650
chr22	36681270	-	C	37	19	0.514	0	0	C	19	M	0.359	0.666	0	3E-05	0	136.4	taaggtcaagCtgcaggagat	m5C_37636
chr22	36681282	-	C	39	19	0.487	0	0	T	20	M	0.339	0.638	0	3E-05	0	128.69	gaacaaggagCttaaggtcaa	m5C_37664
chr22	36745110	-	C	43	34	0.791	0	0	C	34	M	0.648	0.886	0	2E-05	0	440.6	catcgtggagCtggtggagaa	m5C_37658
chr22	36745121	-	C	46	26	0.565	0	0	C	26	M	0.422	0.698	0	6E-06	0	219.69	ggcgaagaggCcatcgtggag	m5C_37632
chr22	39708983	-	C	55	37	0.673	0	0	C	37	M	0.541	0.782	0	2E-06	0	400.36	acagggaccaCtgaagaaaga	m5C_37816
chr22	39708985	-	C	59	36	0.61	0	0	C	36	M	0.483	0.724	0	2E-06	0	347.52	ccacagggacCactgaagaaa	m5C_37818
chr22	39709834	-	C	51	12	0.235	0	0	T	39	M	0.14	0.368	3E-15	9E-10	4E-15	24.331	tgccttccttCtgagaacagc	m5C_37832
chr22	39709837	-	C	54	14	0.259	0	0	T	40	M	0.161	0.389	0	3E-09	0	45.132	tgttgccttcCttctgagaac	m5C_37806
chr22	39709838	-	C	52	18	0.346	0	0	T	34	M	0.232	0.482	0	2E-08	0	83.343	ttgttgccttCcttctgagaa	m5C_37815
chr22	39709841	-	C	55	24	0.436	0	0	T	31	M	0.314	0.567	0	1E-07	0	150.59	tccttgttgcCttccttctga	m5C_37826
chr22	39709850	-	C	54	19	0.352	0	0	T	35	M	0.238	0.485	0	2E-08	0	90.527	cggaaccgttCcttgttgcct	m5C_37834
chr22	39709854	-	C	58	12	0.207	0	0	T	46	M	0.123	0.328	2E-14	9E-10	2E-14	20.116	actgcggaacCgttccttgtt	m5C_37830
chr22	39709860	-	C	61	17	0.283	1	0.016	T	43	M	0.185	0.408	0	2E-10	0	62.923	agacagactgCggaaccgttc	m5C_37844
chr22	39709863	-	C	64	13	0.203	0	0	T	51	M	0.123	0.317	3E-15	2E-11	3E-15	23.172	ccgagacagaCtgcggaaccg	m5C_37804
chr22	39709867	-	C	87	26	0.302	1	0.011	T	60	M	0.215	0.406	0	3E-12	0	112.03	gcgcccgagaCagactgcgga	m5C_37813
chr22	39709873	-	C	126	33	0.262	0	0	T	93	M	0.193	0.345	0	0	0	127.38	ggcacagcgcCcgagacagac	m5C_37835
chr22	39709881	-	C	150	30	0.2	0	0	T	120	M	0.144	0.271	0	0	0	86.304	gtgcgctgggCacagcgcccg	m5C_37808
chr22	39709886	-	C	167	56	0.335	0	0	T	111	M	0.268	0.41	0	0	0	300.32	ggaatgtgcgCtgggcacagc	m5C_37824
chr22	39709888	-	C	166	36	0.217	0	0	T	130	M	0.161	0.286	0	0	0	115.89	ctggaatgtgCgctgggcaca	m5C_37845

chr22	39709899	-	C	178	37	0.209	1	0.006	T	140	M	0.156	0.275	0	0	0	115.17	agtgatgaggCctggaatgtg	m5C_37833
chr22	39709910	-	C	78	22	0.282	0	0	T	56	M	0.194	0.39	0	3E-11	0	85.471	ctcagctgttCagtgatgagg	m5C_37842
chr22	39711269	-	C	72	32	0.444	0	0	T	40	M	0.335	0.559	0	8E-10	0	214.65	ggtacagcgcCcgaatcggac	m5C_37819
chr22	39711275	-	C	71	21	0.296	0	0	T	50	M	0.202	0.41	0	2E-11	0	84.978	gcgctgggtaCagcgcccgaa	m5C_37809
chr22	39711284	-	C	107	28	0.262	0	0	T	79	M	0.188	0.352	0	2E-15	0	105.11	tcagagtgagCgctgggtaca	m5C_37825
chr22	39711293	-	C	153	43	0.281	0	0	T	110	M	0.216	0.357	0	0	0	185.63	tgatgaggctCagagtgagcg	m5C_37807
chr22	39711295	-	C	158	48	0.304	0	0	T	110	M	0.237	0.379	0	0	0	227.95	gttgatgaggCtcagagtgag	m5C_37801
chr22	39711306	-	C	67	31	0.463	0	0	T	36	M	0.349	0.581	0	2E-08	0	216.12	cccagctgttCgttgatgagg	m5C_37802
chr22	39715062	-	C	173	77	0.445	0	0	T	96	M	0.373	0.52	0	0	0	574.45	ttgtcacgttCtgatttggct	m5C_37810
chr22	39715066	-	C	203	69	0.34	0	0	T	134	M	0.278	0.407	0	0	0	383.99	ctgattgtcaCgttctgattt	m5C_37822
chr22	39715068	-	C	230	65	0.283	0	0	T	165	M	0.228	0.344	0	0	0	296.86	tgctgattgtCacgttctgat	m5C_37836
chr22	39715076	-	C	253	59	0.233	0	0	T	194	M	0.185	0.289	0	0	0	218.68	aaactgtgtgCtgattgtcac	m5C_37837
chr22	39715083	-	C	244	58	0.238	0	0	T	186	M	0.189	0.295	0	0	0	218.8	cggacagaaaCtgtgtgctga	m5C_37817
chr22	39715097	-	C	233	133	0.571	0	0	C	133	M	0.507	0.633	0	0	0	1347.6	aacttattgaCgggcggacag	m5C_37814
chr22	39715105	-	C	235	47	0.2	0	0	T	188	M	0.154	0.256	0	0	0	144.63	agatgatgaaCttattgacgg	m5C_37811
chr22	39715116	-	C	93	39	0.419	0	0	T	54	M	0.324	0.521	0	5E-12	0	252.89	gcagaaaccaCagatgatgaa	m5C_37805
chr22	39918100	+	C	46	14	0.304	0	0	T	32	M	0.191	0.448	0	2E-07	0	53.423	tagagctgggCagtgaagtgg	m5C_37799
chr22	39918117	+	C	76	20	0.263	0	0	T	56	M	0.177	0.372	0	1E-11	0	70.917	gtggatatcaCtgaaggagat	m5C_37788
chr22	41226885	-	C	39	11	0.282	0	0	T	28	M	0.165	0.438	3E-15	3E-06	3E-15	26.4	aagaatagaaCgagttaagaa	m5C_37751
chr22	41461643	-	C	56	31	0.554	0	0	C	31	M	0.424	0.676	0	6E-07	0	262.96	cttggctggtCcgatggtagt	m5C_37759
chr22	43011333	+	C	49	19	0.388	0	0	T	30	M	0.264	0.528	0	1E-06	0	100.43	aatttttgagCgggtaaaggt	m5C_37694
chr22	43011348	+	C	33	13	0.394	0	0	T	20	M	0.247	0.563	0	7E-06	0	64.175	aaaggtcgccCtcaaggtgac	m5C_37683
chr22	43011363	+	C	44	27	0.614	0	0	C	27	M	0.466	0.743	0	7E-06	0	251.77	ggtgacccgcCtactttgcgg	m5C_37688
chr22	43011379	+	C	127	41	0.325	1	0.008	T	85	M	0.25	0.411	0	1E-16	0	204.84	tgcgggatgcCtgggagttgc	m5C_37701
chr22	43011393	+	C	138	40	0.29	0	0	T	98	M	0.221	0.37	0	0	0	176.53	gagttgcgatCtgcccgacct	m5C_37702
chr22	43011396	+	C	131	57	0.435	0	0	T	74	M	0.353	0.521	0	4E-16	0	402.72	ttgcgatctgCccgaccttat	m5C_37689
chr22	43011397	+	C	116	91	0.784	0	0	C	91	M	0.701	0.85	0	2E-10	0	1276.2	tgcgatctgcCcgaccttatt	m5C_37684
chr22	43579085	-	C	33	19	0.576	0	0	C	19	M	0.408	0.728	0	3E-05	0	155.07	gacgaggcagCccgggaggtg	m5C_37727
chr22	43579088	-	C	35	22	0.629	0	0	C	22	M	0.463	0.768	0	6E-05	0	203.88	gaggacgaggCagcccgggag	m5C_37733
chr22	43579093	-	C	52	39	0.75	0	0	C	39	M	0.618	0.848	0	2E-06	0	481.99	aggaggaggaCgaggcagccc	m5C_37714
chr3	12881931	-	C	42	27	0.643	0	0	C	27	M	0.492	0.77	0	7E-06	0	265.5	gggatcgcatCtggagagtgc	m5C_5809
chr3	12881936	-	C	36	14	0.389	0	0	T	22	M	0.248	0.551	0	9E-06	0	69.397	ctcctgggatCgcatctggag	m5C_5810
chr3	12881943	-	C	37	11	0.297	0	0	T	26	M	0.175	0.458	1E-15	3E-06	2E-15	28.551	ccttgacctcCtgggatcgca	m5C_5808
chr3	15094934	-	C	31	12	0.387	0	0	T	19	M	0.237	0.562	0	5E-06	0	56.959	agaccaagagCaataaggaga	m5C_5927
chr3	18543289	+	C	37	12	0.324	0	0	T	25	M	0.196	0.485	0	5E-06	0	47.119	gttttggttgCtgctgtttgt	m5C_5829
chr3	21591529	-	C	48	37	0.787	1	0.021	C	37	M	0.651	0.88	0	3E-05	0	481.71	ccctcgtggtCtagtggttag	m5C_6122
chr3	22423842	+	C	41	20	0.488	0	0	T	21	M	0.343	0.635	0	1E-06	0	137.02	gttgtcaaggCtgaaaaaagc	m5C_6132
chr3	33144658	+	C	32	9	0.281	0	0	T	23	M	0.156	0.454	5E-13	1E-06	6E-13	17.198	aacaggggacCaccaggttgc	m5C_8318
chr3	33144660	+	C	49	11	0.224	0	0	T	38	M	0.13	0.359	7E-14	4E-08	7E-14	18.889	caggggaccaCcaggttgcct	m5C_8319
chr3	33144669	+	C	144	44	0.306	0	0	T	100	M	0.236	0.385	0	0	0	207.84	accaggttgcCtaaggagggg	m5C_8315
chr3	33144688	+	C	168	45	0.268	0	0	T	123	M	0.207	0.339	0	0	0	185.97	ggtgaattggCcaaggttgga	m5C_8314
chr3	33144689	+	C	192	40	0.208	0	0	T	152	M	0.157	0.271	0	0	0	125.51	gtgaattggcCaaggttggaa	m5C_8337
chr3	33185660	+	C	38	20	0.526	0	0	C	20	M	0.373	0.675	0	4E-05	0	149.03	tgaaaagagcCaataggattt	m5C_8336
chr3	33185672	+	C	68	45	0.662	0	0	C	45	M	0.543	0.763	0	3E-07	0	489.03	ataggatttgCtgagagtttg	m5C_8302
chr3	39452562	+	C	46	14	0.304	0	0	T	32	M	0.191	0.448	0	2E-07	0	53.423	attgttagagCttggagttga	m5C_6197
chr3	39452575	+	C	34	10	0.294	0	0	T	24	M	0.168	0.462	2E-14	2E-06	3E-14	22.956	ggagttgaggCtactgactgg	m5C_6201
chr3	48614446	-	C	31	10	0.323	0	0	T	21	M	0.186	0.499	7E-15	2E-06	8E-15	26.273	aacctgggaCcctggagaag	m5C_7965
chr3	48642186	-	C	39	19	0.487	0	0	T	20	M	0.339	0.638	0	3E-05	0	128.69	tggcagtgccCtggagaagga	m5C_7958
chr3	48642187	-	C	42	24	0.571	0	0	C	24	M	0.422	0.709	0	4E-06	0	202.59	ctggcagtgcCctggagaagg	m5C_7959
chr3	48642197	-	C	33	23	0.697	0	0	C	23	M	0.527	0.826	0	8E-05	0	242.24	aagaatcggcCtggcagtgcc	m5C_7950
chr3	48642201	-	C	33	8	0.242	0	0	T	25	M	0.128	0.41	3E-11	7E-07	3E-11	10.787	aacaaagaatCggcctggcag	m5C_7978
chr3	49135878	-	C	38	16	0.421	0	0	T	22	M	0.279	0.578	0	2E-05	0	89.127	tggaggtgacCtgcagacggg	m5C_7977
chr3	50152909	+	C	58	39	0.672	0	0	C	39	M	0.544	0.779	0	2E-06	0	424.51	tgaggaggagCtggtggagag	m5C_6932
chr3	50152921	+	C	41	13	0.317	0	0	T	28	M	0.196	0.47	0	1E-07	0	50.868	ggtggagagaCttgagagtga	m5C_6929
chr3	50314507	+	C	34	24	0.706	0	0	C	24	M	0.538	0.832	0	9E-05	0	258.39	acaggctgggCtgctggaggt	m5C_6933
chr3	52724816	+	C	38	26	0.684	0	0	C	26	M	0.525	0.809	0	0.0001	0	273.23	tgaaaaattaCaagatccaac	m5C_7436
chr3	52724828	+	C	32	25	0.781	0	0	C	25	M	0.612	0.89	0	0.0001	0	306.22	agatccaactCtgatttcagc	m5C_7411
chr3	52726761	+	C	46	12	0.261	0	0	T	34	M	0.156	0.403	7E-16	7E-08	7E-16	28.412	aaatgtgaagCaaatgatgat	m5C_7415
chr3	52726775	+	C	120	54	0.45	0	0	T	66	M	0.364	0.539	0	2E-13	0	393.03	tgatgataaaCtggatctgac	m5C_7435
chr3	52726781	+	C	123	42	0.341	0	0	T	81	M	0.264	0.429	0	2E-16	0	221.42	taaactggatCtgactgactg	m5C_7433
chr3	52726785	+	C	123	42	0.341	0	0	T	81	M	0.264	0.429	0	2E-16	0	221.42	ctggatctgaCtgactgtgct	m5C_7425
chr3	52726789	+	C	133	93	0.699	0	0	C	93	M	0.617	0.771	0	7E-13	0	1146.9	atctgactgaCtgtgctgagt	m5C_7442
chr3	52726794	+	C	143	105	0.734	0	0	C	105	M	0.656	0.8	0	2E-13	0	1378.5	actgactgtgCtgagtctgtt	m5C_7410
chr3	52726800	+	C	140	48	0.343	0	0	T	92	M	0.269	0.425	0	0	0	258.59	tgtgctgagtCtgttcaatcc	m5C_7439
chr3	52726805	+	C	141	58	0.411	0	0	T	83	M	0.334	0.494	0	0	0	386.89	tgagtctgttCaatccaaccc	m5C_7444
chr3	52726809	+	C	137	55	0.401	0	0	T	82	M	0.323	0.485	0	2E-16	0	355.46	tctgttcaatCcaaccctgag	m5C_7417
chr3	52726810	+	C	136	53	0.39	0	0	T	83	M	0.312	0.474	0	2E-16	0	330.56	ctgttcaatcCaaccctgagc	m5C_7419
chr3	52726813	+	C	127	76	0.598	0	0	C	76	M	0.511	0.68	0	8E-13	0	777.46	ttcaatccaaCcctgagcttc	m5C_7430
chr3	52726814	+	C	127	79	0.622	0	0	C	79	M	0.535	0.702	0	1E-12	0	845.78	tcaatccaacCctgagcttca	m5C_7424
chr3	52726815	+	C	114	57	0.5	0	0	CT	57	M	0.41	0.59	0	5E-13	0	467.08	caatccaaccCtgagcttcat	m5C_7426
chr3	52726820	+	C	71	35	0.493	0	0	T	36	M	0.38	0.607	0	2E-09	0	266.03	caaccctgagCttcatgttct	m5C_7412
chr3	73160251	+	C	168	66	0.393	0	0	T	102	M	0.322	0.468	0	0	0	425.29	gttttttggagCagggagatgg	m5C_7216
chr3	99328405	-	C	49	27	0.551	0	0	C	27	M	0.413	0.681	0	7E-06	0	223.1	ccctggtggcCtagtggctaa	m5C_7185
chr3	101931074	+	C	46	11	0.239	0	0	T	35	M	0.139	0.379	3E-14	4E-08	3E-14	20.73	tgtgtgggggCttcaacccca	m5C_7240
chr3	108107939	+	C	67	32	0.478	0	0	T	35	M	0.363	0.595	0	3E-08	0	232	caaaacgtgaCctcagaccag	m5C_7267
chr3	108107940	+	C	70	32	0.457	0	0	T	38	M	0.346	0.573	0	8E-10	0	221.26	aaaacgtgacCtcagaccaga	m5C_7261
chr3	108107942	+	C	71	37	0.521	0	0	C	37	M	0.407	0.633	0	3E-09	0	301.07	aacgtgacctCagaccagacg	m5C_7263
chr3	108107947	+	C	61	32	0.525	0	0	C	32	M	0.402	0.645	0	3E-08	0	257	gacctcagacCagacgctgcc	m5C_7262
chr3	108107951	+	C	62	38	0.613	0	0	C	38	M	0.488	0.724	0	1E-07	0	371.24	tcagaccagaCgctgccacca	m5C_7264
chr3	116301500	-	C	83	22	0.265	0	0	T	61	M	0.182	0.369	0	4E-13	0	80.099	tgagctggccCaggttagaaa	m5C_7285
chr3	116301502	-	C	92	22	0.239	0	0	T	70	M	0.164	0.336	0	6E-15	0	71.962	ggtgagctggCccaggttaga	m5C_7305
chr3	116301506	-	C	107	41	0.383	0	0	T	66	M	0.297	0.478	0	2E-13	0	243.24	gaggggtgagCtggcccaggt	m5C_7304
chr3	116301521	-	C	64	13	0.203	0	0	T	51	M	0.123	0.317	3E-15	2E-11	3E-15	23.172	accaggttgcCcaaggagggg	m5C_7286
chr3	120115630	-	C	40	31	0.775	0	0	C	31	M	0.625	0.877	0	1E-05	0	387.48	aggtggctggCtaggaaggga	m5C_7541
chr3	120115634	-	C	45	27	0.6	0	0	C	27	M	0.455	0.73	0	7E-06	0	245.44	atgaaggtggCtggctaggaa	m5C_7529
chr3	121412732	-	C	41	24	0.585	0	0	C	24	M	0.434	0.722	0	4E-06	0	208.16	atagatgaagCtaagaaatgg	m5C_7534
chr3	127379412	+	C	50	11	0.22	0	0	T	39	M	0.128	0.352	9E-14	4E-10	9E-14	18.334	ggtagagaaaCaagaggagga	m5C_7588
chr3	127707115	+	C	47	31	0.66	0	0	C	31	M	0.517	0.778	0	1E-05	0	320.36	ggtggagcagCctggcagtcc	m5C_7604
chr3	127793998	-	C	221	51	0.231	0	0	T	170	M	0.18	0.291	0	0	0	183.71	atttttggagCagggagatgg	m5C_7626
chr3	128890069	-	C	40	9	0.225	0	0	T	31	M	0.123	0.375	6E-12	1E-08	7E-12	12.404	tgcaaggagcCcaagagagag	m5C_7683
chr3	128890070	-	C	39	23	0.59	0	0	C	23	M	0.434	0.729	0	8E-05	0	199.72	ctgcaaggagCccaagagaga	m5C_7700
chr3	128890086	-	C	32	10	0.312	0	0	T	22	M	0.18	0.486	1E-14	2E-06	1E-14	25.084	gccacattgcCaaggactgca	m5C_7691

chr3	131948003	-	C	37	20	0.541	0	0	C	20	M	0.384	0.69	0	4E-05	0	153.54	gggtgtagctCagtggtagag	m5C_7719
chr3	134076387	-	C	31	19	0.613	0	0	C	19	M	0.438	0.763	0	3E-05	0	166.53	aggaggaggaCcccaggggag	m5C_7779
chr3	140216289	+	C	37	18	0.486	0	0	T	19	M	0.334	0.641	0	3E-05	0	120.41	tgcttcttgaCcgaaccacct	m5C_7807
chr3	140216290	+	C	36	12	0.333	0	0	T	24	M	0.202	0.497	0	5E-06	0	48.515	gcttcttgacCgaaccacctg	m5C_7809
chr3	144268166	+	C	43	11	0.256	0	0	T	32	M	0.149	0.402	1E-14	4E-08	1E-14	22.889	gagcaggatgCtgtcaggcat	m5C_7730
chr3	144268174	+	C	39	27	0.692	0	0	C	27	M	0.536	0.814	0	0.0001	0	289.32	tgctgtcaggCatggtatcag	m5C_7732
chr3	149086944	-	C	43	19	0.442	0	0	T	24	M	0.304	0.589	0	1E-06	0	115.65	gatgactgggCaagaagaaag	m5C_7892
chr3	149086949	-	C	43	14	0.326	0	0	T	29	M	0.205	0.475	0	2E-07	0	57.381	gctacgatgaCtgggcaagaa	m5C_7897
chr3	149095313	-	C	33	18	0.545	0	0	C	18	M	0.38	0.702	0	3E-05	0	136.75	gaagtgtgcaCgatgcatcgg	m5C_7908
chr3	149212583	+	C	41	16	0.39	0	0	T	25	M	0.257	0.543	0	4E-07	0	82.099	gagaaggaggCtgctaagaag	m5C_7874
chr3	149212586	+	C	33	13	0.394	0	0	T	20	M	0.247	0.563	0	7E-06	0	64.175	aaggaggctgCtaagaaggga	m5C_7879
chr3	155378883	+	C	32	21	0.656	0	0	C	21	M	0.483	0.796	0	5E-05	0	202.91	atgataccttCcctggtggtc	m5C_8678
chr3	155378908	+	C	94	74	0.787	0	0	C	74	M	0.694	0.858	0	4E-09	0	1027.2	ggttaggattCggcactctca	m5C_8682
chr3	169384360	-	C	42	12	0.286	0	0	T	30	M	0.172	0.436	2E-16	7E-08	2E-16	32.247	ccggttgctgCtgggtgtttg	m5C_8743
chr3	169384363	-	C	41	16	0.39	0	0	T	25	M	0.257	0.543	0	4E-07	0	82.099	cgaccggttgCtgctgggtgt	m5C_8742
chr3	169384369	-	C	41	11	0.268	0	0	T	30	M	0.157	0.419	6E-15	4E-08	7E-15	24.554	cgagcgcgacCggttgctgct	m5C_8746
chr3	169710762	+	C	37	8	0.222	1	0.027	T	28	M	0.117	0.381	7E-11	7E-07	8E-11	9.4968	aaaagaggaaCtggaacagca	m5C_8728
chr3	169710768	+	C	42	9	0.214	0	0	T	33	M	0.117	0.359	1E-11	1E-08	1E-11	11.545	ggaactggaaCagcaaacaga	m5C_8734
chr3	169710771	+	C	40	10	0.303	7	0.175	T	23	M	0.174	0.473	2E-14	2E-06	2E-14	23.982	actggaacagCaaacagatgg	m5C_8727
chr3	169710775	+	C	39	15	0.385	0	0	T	24	M	0.249	0.541	0	1E-05	0	74.673	gaacagcaaaCagatggggat	m5C_8733
chr3	184039729	+	C	38	10	0.263	0	0	T	28	M	0.15	0.42	9E-14	2E-06	1E-13	19.504	ttccccagctCaggaggagga	m5C_8406
chr3	184039793	+	C	257	96	0.374	0	0	T	161	M	0.317	0.434	0	0	0	608	gcaggagaagCtgagagtgag	m5C_8407
chr3	184042109	+	C	51	18	0.353	0	0	T	33	M	0.236	0.49	0	2E-08	0	85.084	tgagaagaagCaaaaagagat	m5C_8408
chr3	184042675	+	C	41	26	0.634	0	0	C	26	M	0.481	0.764	0	6E-06	0	250.23	ggcagaggaaCgaggacgcct	m5C_8405
chr3	184042684	+	C	45	11	0.244	0	0	T	34	M	0.142	0.387	2E-14	4E-08	2E-14	21.411	acgaggacgcCtgaaggaaga	m5C_8420
chr3	186502599	+	C	142	98	0.69	0	0	C	98	M	0.61	0.76	0	8E-14	0	1195.4	gaaatgatggCaatcatcttt	m5C_8505
chr3	186502603	+	C	165	111	0.673	0	0	C	111	M	0.598	0.74	0	2E-15	0	1327.4	tgatggcaatCatctttcggg	m5C_8513
chr3	186502610	+	C	167	122	0.731	0	0	C	122	M	0.659	0.792	0	1E-14	0	1607	aatcatctttCgggactgacc	m5C_8528
chr3	186502615	+	C	163	124	0.761	0	0	C	124	M	0.69	0.82	0	1E-14	0	1710.5	tctttcgggaCtgacctgaaa	m5C_8497
chr3	186502619	+	C	164	121	0.738	0	0	C	121	M	0.666	0.799	0	8E-15	0	1610.7	tcgggactgaCctgaaatgaa	m5C_8512
chr3	186502620	+	C	161	118	0.733	0	0	C	118	M	0.66	0.795	0	5E-15	0	1557	cgggactgacCtgaaatgaag	m5C_8507
chr3	186502649	+	C	61	25	0.41	0	0	T	36	M	0.295	0.535	0	4E-09	0	147.68	cattgctgatCacttgattat	m5C_8493
chr3	186505099	+	C	77	49	0.636	0	0	C	49	M	0.525	0.735	0	3E-08	0	514.32	aagcaggattCagactacaat	m5C_8520
chr3	186505106	+	C	75	22	0.293	0	0	T	53	M	0.202	0.404	0	3E-11	0	89.056	attcagactaCaatatagctg	m5C_8485
chr3	186505114	+	C	70	30	0.429	0	0	T	40	M	0.319	0.545	0	5E-10	0	191.61	tacaatatagCtgctaagtgc	m5C_8482
chr3	186505124	+	C	68	24	0.353	0	0	T	44	M	0.25	0.472	0	3E-09	0	120.01	ctgctaagtgCtgtgttgtcg	m5C_8481
chr3	186505133	+	C	43	25	0.581	0	0	C	25	M	0.433	0.716	0	4E-06	0	216.64	gctgtgttgtCgttccccctg	m5C_8488
chr3	193458365	-	C	43	19	0.442	0	0	T	24	M	0.304	0.589	0	1E-06	0	115.65	gaggggtgaaCtggctgaagt	m5C_8231
chr3	195903765	-	C	87	41	0.471	0	0	T	46	M	0.37	0.575	0	3E-10	0	303.25	aggattcaacCtggtggtggg	m5C_8657
chr3	195903766	-	C	86	19	0.221	0	0	T	67	M	0.146	0.319	0	8E-14	0	55.574	gaggattcaaCctggtggtgg	m5C_8665
chr3	195903769	-	C	79	23	0.291	0	0	T	56	M	0.203	0.399	0	4E-11	0	93.159	ccggaggattCaacctggtgg	m5C_8656
chr4	2397458	-	C	31	22	0.71	0	0	C	22	M	0.534	0.839	0	6E-05	0	234.99	gtgttgggtcCggccacctct	m5C_8457
chr4	12975460	-	C	44	12	0.273	0	0	T	32	M	0.163	0.418	4E-16	7E-08	5E-16	30.115	ggatccatgcCtgcctgagtg	m5C_8549
chr4	13578543	-	C	41	13	0.317	0	0	T	28	M	0.196	0.47	0	1E-07	0	50.868	gagcaggagtCtgatgaggaa	m5C_8551
chr4	24578222	-	C	34	18	0.529	0	0	C	18	M	0.367	0.685	0	3E-05	0	132.25	agatagagagCgagagaggga	m5C_8647
chr4	69202874	-	C	45	15	0.333	0	0	T	30	M	0.214	0.479	0	3E-07	0	64.076	agaatatgaaCaggatgagag	m5C_8998
chr4	70296621	-	C	52	27	0.519	0	0	C	27	M	0.387	0.649	0	2E-07	0	208.9	cgagagtggaCggcagcagcc	m5C_9079
chr4	70296635	-	C	53	14	0.264	0	0	T	39	M	0.164	0.396	0	3E-09	0	46.024	ctgttgccggCagtcgagagt	m5C_9096
chr4	70296639	-	C	48	19	0.396	0	0	T	29	M	0.27	0.537	0	1E-06	0	102.68	caggctgttgCcggcagtcga	m5C_9099
chr4	70296669	-	C	146	109	0.747	0	0	C	109	M	0.67	0.81	0	4E-13	0	1461.3	atggatggcgCtggagcgttg	m5C_9093
chr4	70296671	-	C	136	57	0.419	0	0	T	79	M	0.34	0.503	0	4E-16	0	387.06	aaatggatggCgctggagcgt	m5C_9080
chr4	70296685	-	C	116	85	0.733	0	0	C	85	M	0.646	0.805	0	7E-11	0	1097.7	tcaactagccCtgaaaatgga	m5C_9094
chr4	70296686	-	C	110	87	0.791	0	0	C	87	M	0.706	0.856	0	9E-11	0	1228	atcaactagcCctgaaaatgg	m5C_9097
chr4	70296687	-	C	106	84	0.792	0	0	C	84	M	0.706	0.859	0	9E-10	0	1185.6	aatcaactagCcctgaaaatg	m5C_9082
chr4	70296735	-	C	38	25	0.658	0	0	C	25	M	0.499	0.788	0	0.0001	0	249.46	ccatggaagtCagaatccgct	m5C_9105
chr4	76654445	-	C	58	22	0.379	0	0	T	36	M	0.266	0.508	0	7E-08	0	116.88	ccctggtggtCtagtggttag	m5C_10144
chr4	88813736	+	C	46	13	0.283	0	0	T	33	M	0.173	0.425	0	1E-07	0	45.043	gaagaaaagcCcaagatcaaa	m5C_9120
chr4	107249377	+	C	51	21	0.412	0	0	T	30	M	0.288	0.548	0	5E-08	0	120.77	gaaaagaaagCgaaagagaaa	m5C_9475
chr4	107924469	-	C	76	55	0.724	0	0	C	55	M	0.614	0.812	0	8E-08	0	675.67	tcctgatgtgCagatcggtca	m5C_9483
chr4	107924477	-	C	238	155	0.651	0	0	C	155	M	0.589	0.709	0	0	0	1825.1	tgtagtggtcCtgatgtgcag	m5C_9486
chr4	110476768	+	C	31	19	0.613	0	0	C	19	M	0.438	0.763	0	3E-05	0	166.53	tgaaggctgtCaagtatatgg	m5C_9384
chr4	122605923	-	C	37	15	0.405	0	0	T	22	M	0.263	0.565	0	1E-05	0	79.039	attgcaggcaCagatgaggag	m5C_9590
chr4	122605925	-	C	33	9	0.273	0	0	T	24	M	0.151	0.442	8E-13	1E-06	8E-13	16.442	aaattgcaggCacagatgagg	m5C_9591
chr4	122607457	-	C	50	14	0.28	0	0	T	36	M	0.175	0.417	0	2E-07	0	48.927	cttcggaaggCtatgaaaggc	m5C_9582
chr4	127569076	+	C	41	20	0.488	0	0	T	21	M	0.343	0.635	0	1E-06	0	137.02	ccttagctgtCtgtgaggtct	m5C_9465
chr4	129191383	-	C	31	16	0.516	0	0	C	16	M	0.348	0.68	0	2E-05	0	111.49	atggatgtgtCtgaatgtgtg	m5C_9308
chr4	163917817	-	C	64	16	0.25	0	0	T	48	M	0.16	0.368	0	1E-10	0	51.247	ttgttggggggCggggtggtag	m5C_9616
chr4	166256910	+	C	50	37	0.74	0	0	C	37	M	0.604	0.841	0	2E-06	0	447.3	ttggtcagagCtagttgttta	m5C_9796
chr4	174253301	-	C	59	33	0.559	0	0	C	33	M	0.433	0.678	0	9E-07	0	285.71	aagaacgaacCagaagatgag	m5C_9786
chr4	174253302	-	C	31	12	0.387	0	0	T	19	M	0.237	0.562	0	5E-06	0	56.959	gaagaacgaaCcagaagatga	m5C_9785
chr5	6600233	-	C	101	62	0.614	0	0	C	62	M	0.516	0.703	0	3E-11	0	640.34	gatgatggggCtggaggtatt	m5C_10321
chr5	6600248	-	C	38	13	0.342	0	0	T	25	M	0.212	0.501	0	7E-06	0	55.152	gcttcattatCtcaggatgat	m5C_10302
chr5	11082863	+	C	31	13	0.419	0	0	T	18	M	0.264	0.592	0	7E-06	0	68.68	cagtggccacCgcgcacacca	m5C_10558
chr5	14758653	-	C	55	16	0.291	0	0	T	39	M	0.188	0.421	0	7E-09	0	60.067	gtggacgagtCggtggggagc	m5C_10178
chr5	14758658	-	C	55	14	0.255	0	0	T	41	M	0.158	0.383	0	3E-09	0	44.274	accatgtggaCgagtcggtgg	m5C_10201
chr5	14758666	-	C	42	22	0.524	0	0	C	22	M	0.377	0.666	0	2E-06	0	165.98	taaactgcacCatgtggacga	m5C_10174
chr5	14758667	-	C	31	11	0.355	0	0	T	20	M	0.211	0.531	1E-16	3E-06	1E-16	37.059	ataaactgcaCcatgtggacg	m5C_10192
chr5	16463773	-	C	40	20	0.5	0	0	CT	20	M	0.352	0.648	0	1E-06	0	140.8	gagcaggatgCagaggaggaa	m5C_10831
chr5	16463793	-	C	60	25	0.417	0	0	T	35	M	0.301	0.543	0	2E-07	0	150.32	aagcaatggaCgatgtggtgg	m5C_10821
chr5	16465195	-	C	40	18	0.45	0	0	T	22	M	0.307	0.602	0	3E-05	0	110.54	ggcaaagcagCaggaggagga	m5C_10825
chr5	16465203	-	C	37	8	0.216	0	0	T	29	M	0.114	0.372	1E-10	7E-07	1E-10	9.1227	aagaagttggCaaagcagcag	m5C_10812
chr5	16703074	-	C	74	24	0.324	0	0	T	50	M	0.229	0.437	0	6E-11	0	109.75	aaagaagaaaCaggaagagga	m5C_10806
chr5	17275736	+	C	51	25	0.49	0	0	T	26	M	0.359	0.623	0	2E-07	0	179.29	cccctgccgcCggggaggagc	m5C_10793
chr5	17275746	+	C	54	36	0.667	0	0	C	36	M	0.534	0.778	0	2E-06	0	384.2	cggggaggagCccagcaagga	m5C_10797
chr5	25190102	-	C	32	20	0.625	0	0	C	20	M	0.453	0.771	0	4E-05	0	181.02	agctggaagaCctgagaagga	m5C_10014
chr5	25190110	-	C	34	22	0.647	0	0	C	22	M	0.479	0.785	0	6E-05	0	210.82	tgacatggagCtggaagacct	m5C_10015
chr5	31401583	-	C	31	12	0.387	0	0	T	19	M	0.237	0.562	0	5E-06	0	56.959	ggaaagaggagCatcaagagag	m5C_10155
chr5	32395284	-	C	44	13	0.295	0	0	T	31	M	0.182	0.442	0	1E-07	0	47.204	agaagaggagCgttggagaat	m5C_10467
chr5	32395305	-	C	40	11	0.275	0	0	T	29	M	0.161	0.428	4E-15	3E-06	5E-15	25.45	ggagtactggCgaagacgaga	m5C_10471
chr5	32395309	-	C	42	12	0.286	0	0	T	30	M	0.172	0.436	2E-16	7E-08	2E-16	32.247	aggaggagtaCtggcgaagac	m5C_10473
chr5	32395329	-	C	43	13	0.302	0	0	T	30	M	0.186	0.451	0	1E-07	0	48.365	aatgaggaagCaaatgcagaa	m5C_10476
chr5	33445451	+	C	32	9	0.281	0	0	T	23	M	0.156	0.454	5E-13	1E-06	6E-13	17.198	tgaagagaagCaaaaggaagg	m5C_10459

chr5	33445465	+	C	85	37	0.435	0	0	T	48	M	0.335	0.541	0	1E-10	0	247.87	aggaaggaggCaaaaagaaga	m5C_10439
chr5	33445477	+	C	113	62	0.549	0	0	C	62	M	0.457	0.637	0	1E-12	0	566.46	aaaagaagaaCaaagaaggat	m5C_10458
chr5	34937728	+	C	32	21	0.656	0	0	C	21	M	0.483	0.796	0	5E-05	0	202.91	gaagctaaagCaggccaagtg	m5C_10238
chr5	68562552	+	C	38	16	0.421	0	0	T	22	M	0.279	0.578	0	2E-05	0	89.127	aaaaaaggaaCcgatggatat	m5C_10482
chr5	71146744	+	C	36	18	0.5	0	0	CT	18	M	0.345	0.655	0	3E-05	0	124.11	aaatttcttcCagtgcggtaa	m5C_10959
chr5	71146749	+	C	146	66	0.452	0	0	T	80	M	0.374	0.533	0	2E-16	0	493.13	tcttccagtgCggtaacgtga	m5C_10953
chr5	71146755	+	C	243	89	0.369	2	0.008	T	152	M	0.311	0.432	0	0	0	553.33	agtgcggtaaCgtgaccgatc	m5C_10932
chr5	71146760	+	C	309	109	0.353	0	0	T	200	M	0.302	0.408	0	0	0	657.44	ggtaacgtgaCcgatcccgga	m5C_10936
chr5	71146761	+	C	327	140	0.428	0	0	T	187	M	0.376	0.482	0	0	0	1051.8	gtaacgtgacCgatcccggag	m5C_10987
chr5	71146765	+	C	355	206	0.582	1	0.003	C	206	M	0.53	0.632	0	0	0	2183.3	cgtgaccgatCccggagaagc	m5C_11000
chr5	71146766	+	C	358	174	0.486	0	0	T	184	M	0.435	0.538	0	0	0	1512.7	gtgaccgatcCcggagaagcc	m5C_10981
chr5	71146767	+	C	370	175	0.473	0	0	T	195	M	0.423	0.524	0	0	0	1479.2	tgaccgatccCggagaagccg	m5C_10964
chr5	71146775	+	C	394	216	0.548	0	0	C	216	M	0.499	0.597	0	0	0	2155	cccggagaagCcggcgggagc	m5C_10931
chr5	71146776	+	C	405	186	0.459	0	0	T	219	M	0.411	0.508	0	0	0	1530.2	ccggagaagcCggcgggagcc	m5C_10933
chr5	71146779	+	C	385	189	0.491	0	0	T	196	M	0.441	0.541	0	0	0	1668.1	gagaagccggCgggagccccg	m5C_10939
chr5	71146785	+	C	212	129	0.608	0	0	C	129	M	0.541	0.672	0	0	0	1396.9	ccggcgggagCcccggggaga	m5C_10937
chr5	71146786	+	C	214	152	0.71	0	0	C	152	M	0.646	0.767	0	0	0	1964.5	cggcgggagcCccggggagag	m5C_10985
chr5	71146787	+	C	208	118	0.567	0	0	C	118	M	0.499	0.633	0	0	0	1178.5	ggcgggagccCcggggagagt	m5C_10940
chr5	71146788	+	C	213	133	0.627	1	0.005	C	133	M	0.561	0.69	0	0	0	1491.1	gcgggagcccCggggagagtt	m5C_10960
chr5	71146799	+	C	133	103	0.774	0	0	C	103	M	0.696	0.837	0	3E-12	0	1434.3	ggggagagttCtcttttcttt	m5C_10980
chr5	71146801	+	C	133	98	0.737	0	0	C	98	M	0.656	0.804	0	1E-12	0	1286	ggagagttctCttttctttgt	m5C_10996
chr5	71146806	+	C	54	25	0.463	0	0	T	29	M	0.337	0.594	0	2E-07	0	168.45	gttctcttttCtttgtgaagg	m5C_10963
chr5	71146855	+	C	60	17	0.283	0	0	T	43	M	0.185	0.408	0	2E-10	0	62.923	gagagaggggCccgtgccttg	m5C_10989
chr5	71146856	+	C	57	40	0.702	0	0	C	40	M	0.573	0.805	0	3E-06	0	458.68	agagaggggcCcgtgccttgg	m5C_10951
chr5	71146857	+	C	57	15	0.263	0	0	T	42	M	0.166	0.39	0	5E-09	0	49.937	gagaggggccCgtgccttgga	m5C_10982
chr5	71146861	+	C	81	50	0.617	0	0	C	50	M	0.508	0.716	0	2E-09	0	508.41	ggggcccgtgCcttggaaagc	m5C_10968
chr5	71146862	+	C	84	26	0.31	0	0	T	58	M	0.221	0.415	0	3E-12	0	114.83	gggcccgtgcCttggaaagcg	m5C_10969
chr5	71146871	+	C	112	49	0.438	0	0	T	63	M	0.349	0.53	0	7E-14	0	342.23	ccttggaaagCgtcgcggttc	m5C_10975
chr5	71146874	+	C	118	40	0.339	0	0	T	78	M	0.26	0.428	0	4E-15	0	207.88	tggaaagcgtCgcggttctgg	m5C_10966
chr5	71146876	+	C	122	52	0.426	0	0	T	70	M	0.342	0.515	0	4E-15	0	355.73	gaaagcgtcgCggttctggcg	m5C_10958
chr5	71146881	+	C	108	40	0.37	0	0	T	68	M	0.285	0.464	0	2E-13	0	228.17	cgtcgcggttCtggcggcatc	m5C_11002
chr5	71146885	+	C	113	61	0.54	0	0	C	61	M	0.448	0.629	0	1E-12	0	546.72	gcggttctggCggcatccggt	m5C_10934
chr5	71146888	+	C	93	54	0.581	0	0	C	54	M	0.479	0.676	0	2E-10	0	517.44	gttctggcggCatccggtgag	m5C_10992
chr5	71146891	+	C	94	52	0.553	0	0	C	52	M	0.453	0.65	0	1E-10	0	470.67	ctggcggcatCcggtgagctc	m5C_10986
chr5	71146892	+	C	88	54	0.614	0	0	C	54	M	0.509	0.709	0	4E-09	0	549.92	tggcggcatcCggtgagctct	m5C_10984
chr5	71146901	+	C	97	57	0.588	0	0	C	57	M	0.488	0.68	0	3E-10	0	556.49	ccggtgagctCtcgctggccc	m5C_10943
chr5	71146903	+	C	88	44	0.5	0	0	CT	44	M	0.398	0.602	0	5E-10	0	350.01	ggtgagctctCgctggccctt	m5C_10990
chr5	71146905	+	C	64	47	0.734	0	0	C	47	M	0.615	0.827	0	4E-07	0	578.26	tgagctctcgCtggcccttga	m5C_10961
chr5	71146909	+	C	47	29	0.617	0	0	C	29	M	0.474	0.742	0	1E-05	0	275.07	ctctcgctggCccttgaaaat	m5C_10949
chr5	71146910	+	C	52	27	0.519	0	0	C	27	M	0.387	0.649	0	2E-07	0	208.9	tctcgctggcCcttgaaaatc	m5C_10973
chr5	71146911	+	C	48	15	0.312	0	0	T	33	M	0.199	0.453	0	3E-07	0	59.837	ctcgctggccCttgaaaatcc	m5C_10942
chr5	71146920	+	C	33	10	0.303	0	0	T	23	M	0.174	0.473	2E-14	2E-06	2E-14	23.982	ccttgaaaatCcgggggagag	m5C_10978
chr5	71146921	+	C	33	22	0.667	0	0	C	22	M	0.496	0.802	0	6E-05	0	218.27	cttgaaaatcCgggggagagg	m5C_10945
chr5	90606809	-	C	35	10	0.286	0	0	T	25	M	0.163	0.451	3E-14	2E-06	4E-14	21.999	gaaaggagggCggaaggaaag	m5C_10880
chr5	114937994	+	C	31	8	0.258	0	0	T	23	M	0.137	0.432	2E-11	7E-07	2E-11	11.818	gcgcgtgagtCgggggcccgc	m5C_11122
chr5	115110094	-	C	85	21	0.247	0	0	T	64	M	0.168	0.348	0	3E-13	0	70.411	atttttggagCtagtagatgg	m5C_11134
chr5	122518448	+	C	49	36	0.735	0	0	C	36	M	0.597	0.838	0	3E-05	0	430.11	ggcttaaattCtgtgtatatg	m5C_11273
chr5	122990634	+	C	33	18	0.545	0	0	C	18	M	0.38	0.702	0	3E-05	0	136.75	tgaatttaagCatattagtca	m5C_11245
chr5	122990643	+	C	47	31	0.66	0	0	C	31	M	0.517	0.778	0	1E-05	0	320.36	gcatattagtCagtggaggaa	m5C_11266
chr5	122990787	+	C	63	42	0.667	0	0	C	42	M	0.544	0.771	0	2E-07	0	456.68	tcatggggggCccaagtcctt	m5C_11261
chr5	122990789	+	C	69	48	0.696	0	0	C	48	M	0.579	0.792	0	5E-07	0	556	atggggggccCaagtccttct	m5C_11259
chr5	122990831	+	C	167	91	0.545	0	0	C	91	M	0.469	0.619	0	0	0	853.98	cggtgtgaggCtggtagtggt	m5C_11263
chr5	132427041	+	C	36	11	0.306	0	0	T	25	M	0.18	0.469	1E-15	3E-06	1E-15	29.708	ccaatggaaaCagatcagaat	m5C_11322
chr5	134099691	+	C	31	9	0.29	0	0	T	22	M	0.161	0.466	4E-13	1E-06	4E-13	18.015	agatgacagaCggtctagaag	m5C_11553
chr5	137896733	-	C	53	35	0.66	0	0	C	35	M	0.526	0.773	0	1E-06	0	368.16	tgactgagcaCaaacctgttg	m5C_11482
chr5	137896740	-	C	110	84	0.764	0	0	C	84	M	0.676	0.833	0	6E-11	0	1136	aaactaatgaCtgagcacaaa	m5C_11481
chr5	137896747	-	C	115	85	0.739	0	0	C	85	M	0.652	0.811	0	7E-11	0	1108.5	gtgtggaaaaCtaatgactga	m5C_11475
chr5	138378337	-	C	42	17	0.405	0	0	T	25	M	0.27	0.555	0	6E-07	0	91.945	aaggaggggggCagagatggag	m5C_11503
chr5	138378355	-	C	34	14	0.412	0	0	T	20	M	0.264	0.578	0	9E-06	0	73.824	agtgcactggCaaaattcaag	m5C_11500
chr5	138614648	+	C	34	9	0.265	0	0	T	25	M	0.146	0.431	1E-12	1E-06	1E-12	15.739	ggagttgatcCtagtctgggt	m5C_11494
chr5	138658591	+	C	58	19	0.328	0	0	T	39	M	0.221	0.456	0	2E-08	0	83.916	gaaaaaggaaCcatcagataa	m5C_11486
chr5	138658604	+	C	56	35	0.625	0	0	C	35	M	0.494	0.74	0	1E-06	0	345.84	tcagataaagCtgtgaaaaaa	m5C_11489
chr5	140038686	+	C	36	18	0.5	0	0	CT	18	M	0.345	0.655	0	3E-05	0	124.11	gagagcgggaCcgagagagag	m5C_11377
chr5	140038687	+	C	38	11	0.289	0	0	T	27	M	0.17	0.448	2E-15	3E-06	2E-15	27.449	agagcgggacCgagagagaga	m5C_11336
chr5	148808561	+	C	53	41	0.774	0	0	C	41	M	0.645	0.865	0	3E-06	0	528.68	cactgtagctCaggaagagag	m5C_11816
chr5	150005000	+	C	38	9	0.237	0	0	T	29	M	0.13	0.392	4E-12	1E-06	4E-12	13.374	tggggtctggCtgtgccggtg	m5C_11663
chr5	153135020	+	C	43	30	0.698	0	0	C	30	M	0.549	0.814	0	1E-05	0	329.37	cacaggagttCtgggctgtag	m5C_11431
chr5	153135025	+	C	44	35	0.795	0	0	C	35	M	0.655	0.888	0	3E-05	0	458.49	gagttctgggCtgtagtgtgt	m5C_11429
chr5	156814276	-	C	40	18	0.45	0	0	T	22	M	0.307	0.602	0	3E-05	0	110.54	tcctggtggcCtagtggttag	m5C_11677
chr5	157403856	-	C	72	39	0.542	0	0	C	39	M	0.427	0.652	0	5E-09	0	333.37	atttttggagCagggagatag	m5C_11673
chr5	161501959	+	C	36	9	0.25	0	0	T	27	M	0.138	0.411	2E-12	1E-06	2E-12	14.476	tacctgatttCcgtaggcaga	m5C_11590
chr5	167987919	-	C	32	13	0.406	0	0	T	19	M	0.255	0.577	0	7E-06	0	66.35	cagcattgtaCagggctatga	m5C_11722
chr5	167987926	-	C	32	13	0.406	0	0	T	19	M	0.255	0.577	0	7E-06	0	66.35	gatcaagcagCattgtacagg	m5C_11718
chr5	172197122	-	C	73	33	0.452	0	0	T	40	M	0.343	0.566	0	1E-09	0	226.48	taagaactggCaaaggcatgg	m5C_11777
chr5	172197126	-	C	82	32	0.39	0	0	T	50	M	0.292	0.498	0	2E-11	0	186.78	gaagtaagaaCtggcaaaggc	m5C_11775
chr5	172197143	-	C	52	11	0.212	0	0	T	41	M	0.122	0.34	1E-13	4E-10	2E-13	17.302	gtccctgccaCagggaagaag	m5C_11774
chr5	172197145	-	C	43	9	0.209	0	0	T	34	M	0.114	0.352	1E-11	1E-08	2E-11	11.152	ggtgtccctgcCacagggaaga	m5C_11770
chr5	172197146	-	C	34	10	0.294	0	0	T	24	M	0.168	0.462	2E-14	2E-06	3E-14	22.956	ggtgtccctgCcacagggaag	m5C_11763
chr5	172461772	+	C	41	12	0.293	0	0	T	29	M	0.176	0.445	1E-16	7E-08	1E-16	33.713	gtggggtggtCagtaggagag	m5C_11725
chr5	172461783	+	C	51	12	0.235	0	0	T	39	M	0.14	0.368	3E-15	9E-10	4E-15	24.331	agtaggagagCacgttcagag	m5C_11731
chr5	172461785	+	C	47	10	0.213	0	0	T	37	M	0.12	0.349	1E-12	2E-08	1E-12	14.271	taggagagcaCgttcagaggg	m5C_11734
chr5	172461789	+	C	50	30	0.6	0	0	C	30	M	0.462	0.724	0	5E-07	0	277.09	agagcacgttCagagggaaga	m5C_11745
chr5	173043881	-	C	87	62	0.713	0	0	C	62	M	0.61	0.797	0	1E-08	0	756.6	gaaggcctggCggggaaggcc	m5C_11754
chr5	173043885	-	C	89	25	0.281	0	0	T	64	M	0.198	0.382	0	2E-12	0	99.047	cggggaaggcCtggcggggaa	m5C_11772
chr5	174542113	-	C	68	23	0.338	0	0	T	45	M	0.237	0.457	0	2E-09	0	109.08	tttctcgattCtgtgggtggt	m5C_11950
chr5	174542118	-	C	40	8	0.2	0	0	T	32	M	0.105	0.348	2E-10	5E-09	2E-10	8.135	agctctttctCgattctgtgg	m5C_11949
chr5	174542120	-	C	37	17	0.459	0	0	T	20	M	0.31	0.616	0	2E-05	0	105.53	acagctctttCtcgattctgt	m5C_11939
chr5	179045033	-	C	40	8	0.2	0	0	T	32	M	0.105	0.348	2E-10	5E-09	2E-10	8.135	attacaatggCtataatgatg	m5C_12280
chr5	179146762	+	C	89	38	0.427	0	0	T	51	M	0.329	0.531	0	1E-10	0	250.29	ctgatccagaCgcagagaaac	m5C_12258
chr5	179146764	+	C	112	38	0.339	0	0	T	74	M	0.258	0.431	0	2E-15	0	196.25	gatccagacgCagagaaacct	m5C_12247
chr5	179146773	+	C	157	49	0.312	0	0	T	108	M	0.245	0.388	0	0	0	239.94	gcagagaaacCtgaggattgg	m5C_12268
chr5	179147414	+	C	212	105	0.495	0	0	T	107	M	0.429	0.562	0	0	0	900.2	gggatgaagaCatggatggag	m5C_12222

chr5	179150661	+	C	34	10	0.294	0	0	T	24	M	0.168	0.462	2E-14	2E-06	3E-14	22.956	tgtttttcagCcaggcgttgt	m5C_12263
chr5	179150662	+	C	31	22	0.71	0	0	C	22	M	0.534	0.839	0	6E-05	0	234.99	gtttttcagcCaggcgttgtg	m5C_12245
chr5	179150666	+	C	50	15	0.3	0	0	T	35	M	0.191	0.438	0	5E-09	0	57.31	ttcagccaggCgttgtggggc	m5C_12235
chr5	179150676	+	C	60	25	0.417	0	0	T	35	M	0.301	0.543	0	2E-07	0	150.32	cgttgtggggCagatgatcga	m5C_12246
chr5	179151695	+	C	96	24	0.25	0	0	T	72	M	0.174	0.345	0	2E-14	0	83.586	aaaactgatgCacctcaaccg	m5C_12226
chr5	179151704	+	C	243	72	0.296	0	0	T	171	M	0.242	0.357	0	0	0	349.08	gcacctcaacCggatgtgaag	m5C_12266
chr5	179151747	+	C	224	90	0.402	0	0	T	134	M	0.34	0.467	0	0	0	611.57	aggaaaaggaCaagggagatg	m5C_12242
chr5	179151781	+	C	38	29	0.763	0	0	C	29	M	0.608	0.87	0	0.0002	0	352.6	agaagagaaaCttggtaagaa	m5C_12230
chr5	179153689	+	C	45	9	0.2	0	0	T	36	M	0.109	0.338	2E-11	1E-08	2E-11	10.429	agaagagaaaCagaaaagtga	m5C_12240
chr5	179252180	+	C	34	18	0.529	0	0	C	18	M	0.367	0.685	0	3E-05	0	132.25	gtgtgaatttCctgaagaacg	m5C_12248
chr5	179252181	+	C	39	29	0.744	0	0	C	29	M	0.589	0.854	0	0.0002	0	341.72	tgtgaatttcCtgaagaacgt	m5C_12260
chr5	180668824	-	C	42	33	0.786	0	0	C	33	M	0.641	0.883	0	2E-05	0	422.8	tcctgcaattCtgaagggatt	m5C_12180
chr5	180668829	-	C	43	29	0.674	0	0	C	29	M	0.525	0.795	0	1E-05	0	304.59	acatgtcctgCaattctgaag	m5C_12170
chr5	180668833	-	C	44	30	0.682	0	0	C	30	M	0.534	0.8	0	1E-05	0	320.66	gaggacatgtCctgcaattct	m5C_12157
chr5	180668838	-	C	40	21	0.525	0	0	C	21	M	0.375	0.671	0	5E-05	0	157.49	ggagtgaggaCatgtcctgca	m5C_12179
chr5	180670314	-	C	154	54	0.351	0	0	T	100	M	0.28	0.429	0	0	0	302.13	tgtttctgagCtgccgcccgg	m5C_12185
chr5	180670325	-	C	287	106	0.369	0	0	T	181	M	0.316	0.427	0	0	0	669.01	aatccagaggCtgtttctgag	m5C_12172
chr5	180670331	-	C	306	73	0.239	0	0	T	233	M	0.194	0.289	0	0	0	283.58	tgctgaaatcCagaggctgtt	m5C_12155
chr5	180670339	-	C	311	106	0.341	0	0	T	205	M	0.29	0.395	0	0	0	615.62	agtgtcggtgCtgaaatccag	m5C_12173
chr5	180670344	-	C	324	69	0.213	0	0	T	255	M	0.172	0.261	0	0	0	237.2	atctgagtgtCggtgctgaaa	m5C_12156
chr5	180670352	-	C	345	139	0.403	0	0	T	206	M	0.352	0.455	0	0	0	979.91	aacatgccatCtgagtgtcgg	m5C_12183
chr5	180670355	-	C	339	96	0.283	0	0	T	243	M	0.238	0.333	0	0	0	456.69	cccaacatgcCatctgagtgt	m5C_12175
chr5	180670365	-	C	358	140	0.391	0	0	T	218	M	0.342	0.443	0	0	0	957.39	cggtgatgacCccaacatgcc	m5C_12176
chr5	180670366	-	C	364	82	0.225	0	0	T	282	M	0.185	0.271	0	0	0	303.98	gcggtgatgaCcccaacatgc	m5C_12181
chr5	180670375	-	C	154	34	0.221	0	0	T	120	M	0.163	0.293	0	0	0	110.51	ccgggcggggCggtgatgacc	m5C_12177
chr6	13615848	+	C	36	28	0.778	0	0	C	28	M	0.619	0.883	0	0.0002	0	346.72	tgcgggagacCgtgcgcaggt	m5C_12186
chr6	18249916	-	C	44	11	0.25	0	0	T	33	M	0.146	0.394	2E-14	4E-08	2E-14	22.132	aaggaagagtCttcagatgat	m5C_12105
chr6	18264099	-	C	100	51	0.51	0	0	C	51	M	0.413	0.606	0	9E-11	0	421.75	acgaggacgaCgaggaggagg	m5C_12100
chr6	18264102	-	C	90	49	0.544	0	0	C	49	M	0.442	0.643	0	6E-11	0	433.01	aggacgaggaCgacgaggagg	m5C_12103
chr6	18264108	-	C	89	29	0.326	0	0	T	60	M	0.237	0.429	0	8E-12	0	137.71	aggaagaggaCgaggacgacg	m5C_12114
chr6	18264123	-	C	42	10	0.238	0	0	T	32	M	0.135	0.385	3E-13	2E-08	3E-13	16.841	gagaggagagCgaggaggaag	m5C_12099
chr6	26124066	-	C	68	20	0.294	0	0	T	48	M	0.199	0.411	0	7E-10	0	79.675	gaccaaagcgCagaagaaaga	m5C_12997
chr6	26124068	-	C	74	29	0.392	0	0	T	45	M	0.289	0.506	0	4E-10	0	167.42	gtgaccaaagCgcagaagaaa	m5C_12960
chr6	26124073	-	C	68	15	0.221	0	0	T	53	M	0.138	0.333	0	6E-11	0	41.547	aggcagtgacCaaagcgcaga	m5C_12958
chr6	26158475	+	C	35	17	0.486	0	0	T	18	M	0.33	0.644	0	2E-05	0	112.18	agaagaaggaCgggaagaagc	m5C_12950
chr6	26158485	+	C	34	18	0.529	0	0	C	18	M	0.367	0.685	0	3E-05	0	132.25	cgggaagaagCgcaagcgcag	m5C_12918
chr6	26250416	-	C	35	15	0.429	0	0	T	20	M	0.28	0.591	0	1E-05	0	83.953	ataagttgtaCtgagggtgtg	m5C_12982
chr6	26538319	+	C	52	39	0.75	0	0	C	39	M	0.618	0.848	0	2E-06	0	481.99	tcgcctcacaCgcgaaaggtc	m5C_12908
chr6	27447478	+	C	72	27	0.375	0	0	T	45	M	0.272	0.49	0	2E-10	0	146.98	tggtgagtatCcccgcctgtc	m5C_12932
chr6	27447479	+	C	76	45	0.592	0	0	C	45	M	0.48	0.696	0	2E-08	0	431.81	ggtgagtatcCccgcctgtca	m5C_12890
chr6	27447480	+	C	75	26	0.347	0	0	T	49	M	0.249	0.459	0	1E-10	0	129.39	gtgagtatccCcgcctgtcac	m5C_12898
chr6	27447481	+	C	72	15	0.208	0	0	T	57	M	0.131	0.316	0	6E-13	0	39.156	tgagtatcccCgcctgtcacg	m5C_12914
chr6	27447484	+	C	95	24	0.253	0	0	T	71	M	0.176	0.348	0	2E-14	0	84.501	gtatccccgcCtgtcacgcgg	m5C_12953
chr6	27447488	+	C	116	47	0.405	0	0	T	69	M	0.32	0.496	0	4E-14	0	301.05	ccccgcctgtCacgcgggaga	m5C_12853
chr6	27447507	+	C	135	29	0.215	0	0	T	106	M	0.154	0.291	0	0	0	89.283	gaccggggttCgattccccga	m5C_12917
chr6	27447513	+	C	136	31	0.228	0	0	T	105	M	0.165	0.305	0	0	0	102.6	ggttcgattcCcgacgggga	m5C_12940
chr6	27447514	+	C	137	33	0.241	0	0	T	104	M	0.177	0.319	0	0	0	116.8	gttcgattccCcgacggggag	m5C_12952
chr6	27471549	+	C	31	17	0.548	0	0	C	17	M	0.378	0.708	0	2E-05	0	128.42	ggtgagtatcCccgcctgtca	m5C_12887
chr6	27471550	+	C	33	14	0.424	0	0	T	19	M	0.272	0.592	0	9E-06	0	76.259	gtgagtatccCcgcctgtcac	m5C_12913
chr6	27471551	+	C	32	9	0.281	0	0	T	23	M	0.156	0.454	5E-13	1E-06	6E-13	17.198	tgagtatcccCgcctgtcacg	m5C_12854
chr6	27471553	+	C	46	11	0.239	0	0	T	35	M	0.139	0.379	3E-14	4E-08	3E-14	20.73	agtatccccgCctgtcacgcg	m5C_12934
chr6	27471554	+	C	54	12	0.222	0	0	T	42	M	0.132	0.349	8E-15	9E-10	8E-15	22.361	gtatccccgcCtgtcacgcgg	m5C_12942
chr6	27471558	+	C	72	26	0.361	0	0	T	46	M	0.26	0.476	0	1E-10	0	135.1	ccccgcctgtCacgcgggaga	m5C_12945
chr6	27471583	+	C	80	17	0.212	0	0	T	63	M	0.137	0.314	0	2E-14	0	46.622	ggttcgattcCcgacgggga	m5C_12920
chr6	27471584	+	C	72	17	0.236	0	0	T	55	M	0.153	0.346	0	2E-12	0	52.008	gttcgattccCcgacggggag	m5C_12871
chr6	27551272	-	C	33	8	0.242	0	0	T	25	M	0.128	0.41	3E-11	7E-07	3E-11	10.787	ccccgtctgtCacgcgggaga	m5C_12981
chr6	27834874	-	C	35	12	0.343	0	0	T	23	M	0.208	0.508	0	5E-06	0	49.996	gccaagaaggCtgcaggggcg	m5C_12984
chr6	28180817	+	C	32	22	0.688	0	0	C	22	M	0.514	0.82	0	6E-05	0	226.31	ctttgtgtgaCgaggtggccg	m5C_13607
chr6	28574987	+	C	34	25	0.735	0	0	C	25	M	0.569	0.854	0	0.0001	0	284.41	gtcccgggttCaatccccggc	m5C_13623
chr6	28574992	+	C	32	23	0.719	0	0	C	23	M	0.546	0.844	0	8E-05	0	251.28	gggttcaatcCccggcacctc	m5C_13601
chr6	28574993	+	C	34	26	0.765	0	0	C	26	M	0.6	0.876	0	0.0001	0	312.02	ggttcaatccCcggcacctcc	m5C_13650
chr6	28574994	+	C	34	25	0.735	0	0	C	25	M	0.569	0.854	0	0.0001	0	284.41	gttcaatcccCggcacctcca	m5C_13608
chr6	28574997	+	C	35	27	0.771	0	0	C	27	M	0.61	0.879	0	0.0001	0	329.3	caatccccggCacctccacca	m5C_13665
chr6	28574999	+	C	35	22	0.629	0	0	C	22	M	0.463	0.768	0	6E-05	0	203.88	atccccggcaCctccaccagt	m5C_13641
chr6	28575000	+	C	31	23	0.742	0	0	C	23	M	0.568	0.863	0	8E-05	0	261.07	tccccggcacCtccaccagtt	m5C_13636
chr6	28575003	+	C	34	24	0.706	0	0	C	24	M	0.538	0.832	0	9E-05	0	258.39	ccggcacctcCaccagttttg	m5C_13619
chr6	28575006	+	C	33	24	0.727	0	0	C	24	M	0.558	0.849	0	9E-05	0	267.75	gcacctccacCagttttggga	m5C_13629
chr6	28726189	-	C	39	29	0.744	0	0	C	29	M	0.589	0.854	0	0.0002	0	341.72	atggtagagCacatgctttg	m5C_13675
chr6	28726200	-	C	40	13	0.325	0	0	T	27	M	0.201	0.48	0	1E-07	0	52.219	gggtgtagctCagtggtagag	m5C_13679
chr6	28831510	-	C	54	38	0.704	0	0	C	38	M	0.572	0.809	0	2E-06	0	434.51	agtggtagagCgcgtgcttag	m5C_13685
chr6	28831521	-	C	59	37	0.627	0	0	C	37	M	0.5	0.739	0	2E-06	0	369.65	gggtgtagctCagtggtagag	m5C_13723
chr6	28831523	-	C	57	34	0.596	0	0	C	34	M	0.467	0.714	0	1E-06	0	317.54	gggggtgtagCtcagtggtag	m5C_13695
chr6	28918816	+	C	31	8	0.258	0	0	T	23	M	0.137	0.432	2E-11	7E-07	2E-11	11.818	gcccggatagCtcagtcggta	m5C_13613
chr6	28918818	+	C	31	10	0.323	0	0	T	21	M	0.186	0.499	7E-15	2E-06	8E-15	26.273	ccggatagctCagtcggtaga	m5C_13657
chr6	28950007	+	C	95	22	0.232	0	0	T	73	M	0.158	0.326	0	6E-15	0	69.606	ggattcggcgCtctcaccgcc	m5C_13614
chr6	28950013	+	C	97	24	0.247	0	0	T	73	M	0.172	0.342	0	2E-14	0	82.691	ggcgctctcaCcgccgcggcc	m5C_13639
chr6	28950014	+	C	101	23	0.228	0	0	T	78	M	0.157	0.319	0	1E-16	0	72.131	gcgctctcacCgccgcggccc	m5C_13660
chr6	28950017	+	C	103	41	0.398	0	0	T	62	M	0.309	0.495	0	2E-13	0	253.24	ctctcaccgcCgcggcccggg	m5C_13611
chr6	28950022	+	C	107	51	0.477	0	0	T	56	M	0.384	0.57	0	3E-12	0	392.15	accgccgcggCccgggttcga	m5C_13634
chr6	28950036	+	C	93	22	0.237	0	0	T	71	M	0.162	0.332	0	6E-15	0	71.159	ggttcgattcCcggtcaggga	m5C_13666
chr6	29817934	+	C	179	94	0.525	0	0	C	94	M	0.452	0.597	0	0	0	850.19	gatgtggtgaCgtcgcgttct	m5C_13630
chr6	29817937	+	C	181	39	0.215	0	0	T	142	M	0.162	0.281	0	0	0	126.22	gtggtgacgtCgcgttctccc	m5C_13615
chr6	29817943	+	C	140	72	0.514	0	0	C	72	M	0.432	0.596	0	7E-16	0	622.4	acgtcgcgttCtcccgggcgg	m5C_13659
chr6	29817945	+	C	111	26	0.234	0	0	T	85	M	0.165	0.321	0	0	0	85.881	gtcgcgttctCccgggcgggg	m5C_13599
chr6	29817946	+	C	84	19	0.226	0	0	T	65	M	0.15	0.326	0	8E-14	0	56.946	tcgcgttctcCcgggcggggc	m5C_13600
chr6	29817947	+	C	78	25	0.321	0	0	T	53	M	0.227	0.43	0	9E-11	0	113.74	cgcgttctccCgggcggggcc	m5C_13651
chr6	29912364	+	C	36	9	0.25	0	0	T	27	M	0.138	0.411	2E-12	1E-06	2E-12	14.476	gctgtggtcgCtgccgtgatg	m5C_13663
chr6	29912389	+	C	35	26	0.743	0	0	C	26	M	0.579	0.858	0	0.0001	0	301.24	ggaggaagagCtcaggtggag	m5C_13656
chr6	30522941	-	C	38	10	0.263	0	0	T	28	M	0.15	0.42	9E-14	2E-06	1E-13	19.504	agagagacagCaggaggagg	m5C_12607
chr6	30545873	+	C	38	29	0.763	0	0	C	29	M	0.608	0.87	0	0.0002	0	352.6	agcgagatacCcgaaaggca	m5C_12578
chr6	30545886	+	C	55	20	0.364	0	0	T	35	M	0.249	0.496	0	3E-08	0	99.721	aaaaggcaggCggaagaagga	m5C_12579
chr6	30552122	+	C	72	16	0.222	0	0	T	56	M	0.142	0.331	0	1E-12	0	45.335	gaggagatggCgcaggggaca	m5C_12558

chr6	30552124	+	C	68	22	0.324	0	0	T	46	M	0.224	0.442	0	1E-09	0	98.725	ggagatggcgCaggggacacg	m5C_12596
chr6	30552131	+	C	32	12	0.375	0	0	T	20	M	0.229	0.547	0	5E-06	0	55.041	gcgcaggggaCacgggcaaag	m5C_12577
chr6	30671412	-	C	41	10	0.244	0	0	T	31	M	0.138	0.393	2E-13	2E-08	3E-13	17.447	tgcagagaagCcagggaagga	m5C_12636
chr6	30671420	-	C	46	21	0.457	0	0	T	25	M	0.322	0.598	0	2E-06	0	135.05	gaagatactgCagagaagcca	m5C_12618
chr6	30671423	-	C	46	22	0.478	0	0	T	24	M	0.341	0.619	0	2E-06	0	150.15	gaagaagataCtgcagagaag	m5C_12634
chr6	31803052	+	C	123	46	0.374	0	0	T	77	M	0.294	0.462	0	7E-16	0	270.03	tgatgatgacCccaggtaact	m5C_12557
chr6	31803061	+	C	117	27	0.231	0	0	T	90	M	0.164	0.315	0	0	0	88.409	ccccaggtaaCtcttgagtgt	m5C_12588
chr6	31803076	+	C	117	32	0.274	0	0	T	85	M	0.201	0.361	0	2E-16	0	128.58	gagtgtgtcgCtgatgccatc	m5C_12585
chr6	31803082	+	C	120	29	0.242	0	0	T	91	M	0.174	0.326	0	0	0	100.84	gtcgctgatgCcatcaccgca	m5C_12561
chr6	31803086	+	C	117	31	0.265	0	0	T	86	M	0.193	0.351	0	1E-16	0	119.9	ctgatgccatCaccgcagcgc	m5C_12559
chr6	31803088	+	C	116	26	0.224	0	0	T	90	M	0.158	0.308	0	0	0	82.054	gatgccatcaCcgcagcgctc	m5C_12553
chr6	31803096	+	C	114	30	0.265	1	0.009	T	83	M	0.193	0.354	0	0	0	115.65	caccgcagcgCtctgaccgcc	m5C_12580
chr6	31803103	+	C	84	37	0.44	0	0	T	47	M	0.339	0.547	0	1E-10	0	251.03	gcgctctgacCgccccctcgg	m5C_12586
chr6	31804867	+	C	114	67	0.588	0	0	C	67	M	0.496	0.674	0	4E-12	0	664.56	atgatgatttCacagactaga	m5C_12582
chr6	31804869	+	C	117	61	0.521	0	0	C	61	M	0.432	0.61	0	1E-12	0	526.57	gatgatttcaCagactagagt	m5C_12595
chr6	31804873	+	C	106	80	0.755	0	0	C	80	M	0.665	0.827	0	6E-10	0	1063.8	atttcacagaCtagagtctcc	m5C_12563
chr6	31804880	+	C	84	59	0.702	0	0	C	59	M	0.598	0.79	0	8E-09	0	705.06	agactagagtCtccgatgctg	m5C_12575
chr6	31804882	+	C	83	55	0.663	0	0	C	55	M	0.556	0.755	0	4E-09	0	611.33	actagagtctCcgatgctggt	m5C_12602
chr6	31804883	+	C	84	32	0.381	0	0	T	52	M	0.284	0.488	0	2E-11	0	182.06	ctagagtctcCgatgctggtc	m5C_12572
chr6	31804888	+	C	62	40	0.656	1	0.016	C	40	M	0.53	0.763	0	1E-07	0	424.4	gtctccgatgCtggtcatgat	m5C_12570
chr6	31804893	+	C	53	34	0.642	0	0	C	34	M	0.507	0.757	0	1E-06	0	344.7	cgatgctggtCatgatgtcaa	m5C_12560
chr6	31804901	+	C	50	32	0.64	0	0	C	32	M	0.501	0.759	0	7E-07	0	320.9	gtcatgatgtCaaaactaagt	m5C_12593
chr6	31804906	+	C	54	32	0.604	1	0.019	C	32	M	0.469	0.724	0	7E-07	0	300.42	gatgtcaaaaCtaagttctga	m5C_12556
chr6	31804913	+	C	50	29	0.58	0	0	C	29	M	0.442	0.706	0	4E-07	0	256.55	aaactaagttCtgactcattt	m5C_12597
chr6	31804917	+	C	47	28	0.596	0	0	C	28	M	0.453	0.724	0	8E-06	0	253.91	taagttctgaCtcatttaggg	m5C_12565
chr6	31922470	-	C	37	18	0.486	0	0	T	19	M	0.334	0.641	0	3E-05	0	120.41	agaccgagatCgggagcggga	m5C_12632
chr6	33287796	-	C	33	12	0.364	0	0	T	21	M	0.222	0.534	0	5E-06	0	53.249	gaggaagaagCagcagcaggt	m5C_12324
chr6	39463178	-	C	38	28	0.737	0	0	C	28	M	0.58	0.85	0	0.0002	0	324.75	caggatgctgCtgaggaagtt	m5C_12351
chr6	41902812	-	C	34	19	0.559	0	0	C	19	M	0.395	0.711	0	3E-05	0	149.92	ctggaggtggCtgagcccctg	m5C_12450
chr6	43738275	+	C	33	12	0.364	0	0	T	21	M	0.222	0.534	0	5E-06	0	53.249	aaagaggtagCaagagctcca	m5C_12393
chr6	43738281	+	C	31	12	0.387	0	0	T	19	M	0.237	0.562	0	5E-06	0	56.959	gtagcaagagCtccagagaga	m5C_12390
chr6	43738718	+	C	58	26	0.448	0	0	T	32	M	0.327	0.575	0	2E-07	0	170.3	gagccgcagcCggaggagggg	m5C_12394
chr6	44218040	+	C	213	55	0.27	9	0.042	T	149	M	0.213	0.334	0	0	0	234.72	ggagaaggaaCgagagaagga	m5C_13037
chr6	44218068	+	C	505	201	0.406	10	0.02	T	294	M	0.364	0.45	0	0	0	1462	gatgatgaggCagaggaagag	m5C_13045
chr6	44219903	+	C	349	75	0.215	0	0	T	274	M	0.175	0.261	0	0	0	262.55	tctggagctgCctgaggatga	m5C_13042
chr6	44219904	+	C	373	93	0.249	0	0	T	280	M	0.208	0.296	0	0	0	387.12	ctggagctgcCtgaggatgag	m5C_13036
chr6	52132683	-	C	188	60	0.319	0	0	T	128	M	0.257	0.389	0	0	0	308.05	gccaagaggaCcaggagcaga	m5C_12726
chr6	52132692	-	C	121	54	0.446	0	0	T	67	M	0.361	0.535	0	8E-15	0	389.57	aggagaaaagCcaagaggacc	m5C_12724
chr6	52132720	-	C	68	15	0.221	0	0	T	53	M	0.138	0.333	0	6E-11	0	41.547	gaggatgaatCagagacagaa	m5C_12722
chr6	56557786	-	C	35	26	0.743	0	0	C	26	M	0.579	0.858	0	0.0001	0	301.24	ttggggcaaaCaatgaaaatg	m5C_12682
chr6	64423352	+	C	32	9	0.281	0	0	T	23	M	0.156	0.454	5E-13	1E-06	6E-13	17.198	aagacaaggaCagagacagaa	m5C_12755
chr6	64423358	+	C	34	14	0.412	0	0	T	20	M	0.264	0.578	0	9E-06	0	73.824	aggacagagaCagaaaaagca	m5C_12753
chr6	64423367	+	C	38	16	0.421	0	0	T	22	M	0.279	0.578	0	2E-05	0	89.127	acagaaaaagCagggaggaag	m5C_12748
chr6	70183134	-	C	56	20	0.357	0	0	T	36	M	0.245	0.488	0	3E-08	0	97.826	ccggagaagcCagctggagcc	m5C_12796
chr6	70183135	-	C	54	32	0.593	0	0	C	32	M	0.46	0.713	0	7E-07	0	294.19	cccggagaagCcagctggagc	m5C_12800
chr6	70183143	-	C	64	27	0.422	0	0	T	37	M	0.309	0.544	0	7E-09	0	166.7	tgactgatccCggagaagcca	m5C_12786
chr6	70183144	-	C	61	17	0.279	0	0	T	44	M	0.182	0.402	0	2E-10	0	61.841	gtgactgatcCcggagaagcc	m5C_12793
chr6	70183145	-	C	57	34	0.596	0	0	C	34	M	0.467	0.714	0	1E-06	0	317.54	cgtgactgatCccggagaagc	m5C_12801
chr6	70183150	-	C	64	24	0.375	0	0	T	40	M	0.267	0.497	0	3E-09	0	128	ggtaacgtgaCtgatcccgga	m5C_12803
chr6	73549645	+	C	49	31	0.633	0	0	C	31	M	0.493	0.753	0	1E-05	0	305.45	atgggtggttCggtggtagaa	m5C_12810
chr6	76599944	+	C	43	12	0.279	0	0	T	31	M	0.167	0.427	2E-16	7E-08	2E-16	31.457	gtgaagaagaCgaaaaacgtc	m5C_13118
chr6	76599954	+	C	39	13	0.333	0	0	T	26	M	0.206	0.49	0	7E-06	0	53.645	cgaaaaacgtCgaagaaagga	m5C_13115
chr6	86387061	-	C	263	209	0.795	0	0	C	209	M	0.742	0.839	0	0	0	3100.7	tatcccgaacCtgaacttctg	m5C_13282
chr6	86387062	-	C	259	207	0.799	0	0	C	207	M	0.746	0.843	0	0	0	3089.4	ttatcccgaaCctgaacttct	m5C_13276
chr6	86387068	-	C	274	214	0.781	0	0	C	214	M	0.728	0.826	0	0	0	3117.4	atgatcttatCccgaacctga	m5C_13280
chr6	86387073	-	C	268	209	0.78	0	0	C	209	M	0.726	0.825	0	0	0	3036.7	ctgtgatgatCttatcccgaa	m5C_13289
chr6	86387313	-	C	42	13	0.31	0	0	T	29	M	0.191	0.46	0	1E-07	0	49.584	gattcggcttCtgagattaag	m5C_13286
chr6	86387316	-	C	55	38	0.691	0	0	C	38	M	0.56	0.797	0	2E-06	0	425.38	acggattcggCttctgagatt	m5C_13294
chr6	86387319	-	C	53	30	0.566	0	0	C	30	M	0.433	0.69	0	5E-07	0	259.59	agtacggattCggcttctgag	m5C_13291
chr6	86387325	-	C	52	15	0.288	0	0	T	37	M	0.183	0.423	0	5E-09	0	54.989	aaaataagtaCggattcggct	m5C_13292
chr6	86387343	-	C	48	21	0.438	0	0	T	27	M	0.307	0.577	0	2E-06	0	128.94	aagctgataaCctgaagaaaa	m5C_13281
chr6	86387350	-	C	41	20	0.488	0	0	T	21	M	0.343	0.635	0	1E-06	0	137.02	tatcccgaagCtgataacctg	m5C_13283
chr6	86387361	-	C	41	11	0.268	0	0	T	30	M	0.157	0.419	6E-15	4E-08	7E-15	24.554	atgatgaaacCtatcccgaag	m5C_13272
chr6	86387362	-	C	37	9	0.243	0	0	T	28	M	0.134	0.401	3E-12	1E-06	3E-12	13.907	aatgatgaaaCctatcccgaa	m5C_13287
chr6	109103258	+	C	31	9	0.29	0	0	T	22	M	0.161	0.466	4E-13	1E-06	4E-13	18.015	ggtgtgttggCaaggaggga	m5C_13394
chr6	111280000	+	C	43	11	0.256	0	0	T	32	M	0.149	0.402	1E-14	4E-08	1E-14	22.889	cgagcggagtCcagaggacgg	m5C_13251
chr6	111280008	+	C	36	11	0.306	0	0	T	25	M	0.18	0.469	1E-15	3E-06	1E-15	29.708	gtccagaggaCggagaagacg	m5C_13244
chr6	123066572	+	C	33	8	0.242	0	0	T	25	M	0.128	0.41	3E-11	7E-07	3E-11	10.787	agcaggttgcCtaaggaggg	m5C_13386
chr6	123066587	+	C	85	43	0.506	0	0	C	43	M	0.402	0.61	0	4E-10	0	345.43	gaggggtggaCcggcccaggt	m5C_13384
chr6	123066588	+	C	84	27	0.321	0	0	T	57	M	0.231	0.427	0	4E-12	0	124.88	aggggtggacCggcccaggtc	m5C_13383
chr6	123066592	+	C	81	19	0.235	0	0	T	62	M	0.156	0.338	0	8E-14	0	59.137	gtggaccggcCcaggtcggaa	m5C_13382
chr6	123066593	+	C	79	20	0.253	0	0	T	59	M	0.17	0.359	0	1E-11	0	68.111	tggaccggccCaggtcggaaa	m5C_13385
chr6	133137955	+	C	74	55	0.743	0	0	C	55	M	0.633	0.829	0	8E-08	0	696.8	tacatgatgaCaactggctcc	m5C_14526
chr6	133137958	+	C	71	55	0.775	0	0	C	55	M	0.665	0.856	0	8E-08	0	731.33	atgatgacaaCtggctccctc	m5C_14518
chr6	133137962	+	C	69	51	0.739	0	0	C	51	M	0.625	0.828	0	8E-07	0	637.39	tgacaactggCtccctctact	m5C_14545
chr6	133137964	+	C	74	55	0.743	0	0	C	55	M	0.633	0.829	0	8E-08	0	696.8	acaactggctCcctctactga	m5C_14547
chr6	133137966	+	C	75	56	0.747	0	0	C	56	M	0.638	0.831	0	9E-08	0	714.44	aactggctccCtctactgaac	m5C_14523
chr6	133137968	+	C	72	56	0.778	0	0	C	56	M	0.669	0.858	0	9E-08	0	749.38	ctggctccctCtactgaactg	m5C_14500
chr6	133137971	+	C	75	55	0.733	0	0	C	55	M	0.624	0.82	0	8E-08	0	686.06	gctccctctaCtgaactgcca	m5C_14505
chr6	133137979	+	C	76	57	0.75	0	0	C	57	M	0.642	0.834	0	1E-07	0	732.14	tactgaactgCcatgaggaaa	m5C_14551
chr6	133137980	+	C	79	57	0.722	0	0	C	57	M	0.614	0.808	0	1E-07	0	700.16	actgaactgcCatgaggaaac	m5C_14535
chr6	133137990	+	C	81	56	0.691	0	0	C	56	M	0.584	0.781	0	5E-09	0	654.1	catgaggaaaCtgccatgtca	m5C_14510
chr6	133137993	+	C	70	53	0.757	0	0	C	53	M	0.645	0.842	0	6E-08	0	683.75	gaggaaactgCcatgtcaccc	m5C_14549
chr6	133137994	+	C	71	53	0.746	0	0	C	53	M	0.634	0.833	0	6E-08	0	672.53	aggaaactgcCatgtcaccct	m5C_14521
chr6	133137999	+	C	70	52	0.743	0	0	C	52	M	0.63	0.831	0	5E-08	0	654.93	actgccatgtCacccttctga	m5C_14504
chr6	133138001	+	C	74	58	0.784	0	0	C	58	M	0.677	0.862	0	1E-07	0	785.62	tgccatgtcaCccttctgatt	m5C_14528
chr6	133138003	+	C	73	56	0.767	0	0	C	56	M	0.658	0.849	0	9E-08	0	737.32	ccatgtcaccCttctgattac	m5C_14541
chr6	133594084	+	C	37	29	0.784	0	0	C	29	M	0.628	0.886	0	0.0002	0	364.27	ccctagtgggCcacttttggt	m5C_14537
chr6	133594098	+	C	50	39	0.78	0	0	C	39	M	0.648	0.872	0	2E-06	0	505.11	ttttggtaagCagaactggca	m5C_14498
chr6	134578240	+	C	33	26	0.788	0	0	C	26	M	0.622	0.893	0	0.0001	0	323.69	ctggctggtaCttggatggga	m5C_13505
chr6	136597551	-	C	67	29	0.433	0	0	T	38	M	0.321	0.552	0	1E-08	0	186.2	aaagggagggCagagggagaa	m5C_14210
chr6	136597566	-	C	31	18	0.581	0	0	C	18	M	0.408	0.736	0	3E-05	0	146.76	gagaaaggatCagagaaaggg	m5C_14202

chr6	139308568	-	C	35	14	0.4	0	0	T	21	M	0.256	0.564	0	9E-06	0	71.541	cggcgggggCtggttcctcg	m5C_13552
chr6	139308579	-	C	34	11	0.324	0	0	T	23	M	0.191	0.492	4E-16	3E-06	5E-16	32.309	tccttggggcCcggcgggggg	m5C_13549
chr6	139308580	-	C	34	13	0.382	0	0	T	21	M	0.239	0.55	0	7E-06	0	62.14	ttccttggggCccggcgggg	m5C_13561
chr6	144537695	+	C	47	25	0.532	0	0	C	25	M	0.392	0.667	0	4E-06	0	196.16	ccaggatggcCgagtggttaa	m5C_13753
chr6	150639786	+	C	39	8	0.205	0	0	T	31	M	0.108	0.355	2E-10	7E-07	2E-10	8.4438	gctctgtggcCtagtggcagg	m5C_13780
chr6	151426777	-	C	36	13	0.361	0	0	T	23	M	0.225	0.524	0	7E-06	0	58.436	ccctggaggtCtagtggctag	m5C_13787
chr6	159191860	-	C	97	32	0.33	0	0	T	65	M	0.244	0.428	0	5E-13	0	156.4	agatgatgcgCgagaaggagg	m5C_14092
chr6	159191890	-	C	105	33	0.314	0	0	T	72	M	0.233	0.408	0	2E-14	0	154.03	ggagagaaacCgtggagagag	m5C_14108
chr6	159191891	-	C	100	33	0.333	1	0.01	T	66	M	0.248	0.431	0	8E-13	0	163.84	aggagagaaaCcgtggagaga	m5C_14094
chr6	159191912	-	C	62	22	0.355	0	0	T	40	M	0.247	0.479	0	1E-09	0	108.87	cagctggaaaCagagaagaaa	m5C_14104
chr6	160525725	+	C	35	20	0.571	0	0	C	20	M	0.409	0.72	0	4E-05	0	163.43	gaagaagagaCagatgagaat	m5C_13902
chr6	160525740	+	C	32	18	0.562	0	0	C	18	M	0.393	0.718	0	3E-05	0	141.57	gagaatgaaaCagagtggctg	m5C_13900
chr6	166365102	-	C	48	36	0.75	0	0	C	36	M	0.612	0.851	0	3E-05	0	440.75	aaggctggagCcagatggaag	m5C_13982
chr6	166365108	-	C	46	16	0.348	0	0	T	30	M	0.227	0.492	0	4E-07	0	72.579	gaaaggaaggCtggagccaga	m5C_13972
chr6	166400055	-	C	41	18	0.439	0	0	T	23	M	0.299	0.59	0	8E-07	0	107.6	gagagagagaCtgagagaggg	m5C_13975
chr7	1314568	+	C	35	18	0.514	0	0	C	18	M	0.356	0.67	0	3E-05	0	128.05	ctgtccggggCtgcatgtgtg	m5C_14880
chr7	1314571	+	C	38	20	0.526	0	0	C	20	M	0.373	0.675	0	4E-05	0	149.03	tccggggctgCatgtgtgtgt	m5C_14890
chr7	26233292	-	C	31	16	0.516	0	0	C	16	M	0.348	0.68	0	2E-05	0	111.49	gaggtagcccCggttatggag	m5C_14333
chr7	26233293	-	C	31	14	0.452	0	0	T	17	M	0.292	0.622	0	9E-06	0	81.652	ggaggtagccCcggttatgga	m5C_14326
chr7	26233295	-	C	34	15	0.441	0	0	T	19	M	0.289	0.605	0	1E-05	0	86.65	ttggaggtagCcccggttatg	m5C_14338
chr7	26236054	-	C	34	17	0.5	0	0	CT	17	M	0.341	0.659	0	2E-05	0	115.83	aaatttcggaCcaggaccagg	m5C_14331
chr7	26236058	-	C	36	18	0.5	0	0	CT	18	M	0.345	0.655	0	3E-05	0	124.11	gtggaaatttCggaccaggac	m5C_14329
chr7	44608551	-	C	31	10	0.323	0	0	T	21	M	0.186	0.499	7E-15	2E-06	8E-15	26.273	attcggaggCaagattgaag	m5C_15993
chr7	45024985	-	C	35	18	0.514	0	0	C	18	M	0.356	0.67	0	3E-05	0	128.05	atagaaatttCagacaatgac	m5C_15989
chr7	45025074	-	C	34	14	0.412	0	0	T	20	M	0.264	0.578	0	9E-06	0	73.824	ctgcggtgacCctatgcattc	m5C_15970
chr7	45025084	-	C	34	18	0.529	0	0	C	18	M	0.367	0.685	0	3E-05	0	132.25	gtgcttgggtCtgcggtgacc	m5C_15973
chr7	45025095	-	C	36	13	0.361	0	0	T	23	M	0.225	0.524	0	7E-06	0	58.436	aagcctccagCgtgcttgggt	m5C_15990
chr7	45025101	-	C	36	19	0.528	0	0	C	19	M	0.37	0.68	0	3E-05	0	140.62	ggtagcaagcCtccagcgtgc	m5C_15977
chr7	45952686	-	C	56	26	0.464	0	0	T	30	M	0.34	0.593	0	2E-07	0	176.9	gtaggagggaCagagagacag	m5C_15991
chr7	53843937	+	C	248	76	0.306	0	0	T	172	M	0.252	0.366	0	0	0	383.63	atttttggagCagggagatgg	m5C_14425
chr7	55275228	+	C	33	10	0.303	0	0	T	23	M	0.174	0.473	2E-14	2E-06	2E-14	23.982	gagcacagagCagggaggttg	m5C_14609
chr7	55275242	+	C	37	10	0.27	0	0	T	27	M	0.154	0.43	7E-14	2E-06	7E-14	20.282	gaggttgggtCctgcctgagg	m5C_14617
chr7	55275243	+	C	36	17	0.472	0	0	T	19	M	0.32	0.63	0	2E-05	0	108.75	aggttgggtcCtgcctgagga	m5C_14619
chr7	55275247	+	C	33	16	0.485	0	0	T	17	M	0.325	0.648	0	2E-05	0	104.01	tgggtcctgcCtgaggagacc	m5C_14618
chr7	75959222	-	C	36	16	0.444	0	0	T	20	M	0.295	0.604	0	2E-05	0	94.531	gaagtggccaCcggagagaaa	m5C_14720
chr7	80963038	+	C	53	37	0.698	0	0	C	37	M	0.565	0.805	0	2E-06	0	417.83	attaaagttgCtgcagttaaa	m5C_15058
chr7	80963067	+	C	66	13	0.203	2	0.03	T	51	M	0.123	0.317	3E-15	2E-11	3E-15	23.172	agttggatctCgggagcaggc	m5C_15064
chr7	80963073	+	C	42	18	0.429	0	0	T	24	M	0.291	0.578	0	8E-07	0	104.82	atctcgggagCaggcgtgagg	m5C_15067
chr7	80963129	-	C	40	30	0.75	0	0	C	30	M	0.598	0.858	0	1E-05	0	358.83	gagggggcgcCgagagttaac	m5C_15107
chr7	80963130	-	C	38	23	0.605	0	0	C	23	M	0.447	0.744	0	8E-05	0	205.7	cgagggggcgCcgagagttaa	m5C_15110
chr7	80963132	-	C	37	22	0.595	0	0	C	22	M	0.435	0.737	0	6E-05	0	191.34	atcgaggggCgccgagagtt	m5C_15108
chr7	80963140	-	C	41	16	0.39	0	0	T	25	M	0.257	0.543	0	4E-07	0	82.099	gctagagcatCgagggggcgc	m5C_15100
chr7	97481517	-	C	32	15	0.469	0	0	T	17	M	0.309	0.636	0	1E-05	0	92.607	tggatggggaCtgtgggtaga	m5C_15368
chr7	99691444	-	C	67	19	0.284	0	0	T	48	M	0.19	0.401	0	5E-10	0	72.087	gctgttcgtgCaggtagtgtg	m5C_15304
chr7	99691448	-	C	70	45	0.643	0	0	C	45	M	0.526	0.745	0	3E-07	0	473.29	aagtgctgttCgtgcaggtag	m5C_15306
chr7	99691453	-	C	72	15	0.208	0	0	T	57	M	0.131	0.316	0	6E-13	0	39.156	ctccaaagtgCtgttcgtgca	m5C_15323
chr7	99691460	-	C	33	8	0.242	0	0	T	25	M	0.128	0.41	3E-11	7E-07	3E-11	10.787	cctgggggctcCaaagtgctgt	m5C_15322
chr7	99691675	-	C	56	40	0.714	0	0	C	40	M	0.585	0.816	0	3E-06	0	468.2	aaagtgctgaCagtgcagata	m5C_15319
chr7	99691679	-	C	53	17	0.321	0	0	T	36	M	0.211	0.455	0	1E-08	0	71.713	ggctaaagtgCtgacagtgca	m5C_15318
chr7	100482634	+	C	32	14	0.438	0	0	T	18	M	0.282	0.607	0	9E-06	0	78.862	agagagcggcCaggctgagga	m5C_15194
chr7	100482638	+	C	31	19	0.613	0	0	C	19	M	0.438	0.763	0	3E-05	0	166.53	agcggccaggCtgaggaggag	m5C_15201
chr7	102782156	-	C	48	28	0.583	0	0	C	28	M	0.443	0.712	0	8E-06	0	247.97	ggcatatgggCataggtaagc	m5C_15147
chr7	102782176	-	C	50	21	0.42	0	0	T	29	M	0.294	0.558	0	5E-08	0	123.37	cttggcctgcCggaagggcag	m5C_15148
chr7	102782177	-	C	48	22	0.458	0	0	T	26	M	0.326	0.597	0	2E-06	0	143.33	ccttggcctgCcggaagggca	m5C_15146
chr7	102782181	-	C	38	14	0.368	0	0	T	24	M	0.234	0.527	0	9E-06	0	65.475	aacaccttggCctgccggaag	m5C_15144
chr7	129410288	-	C	40	30	0.75	0	0	C	30	M	0.598	0.858	0	1E-05	0	358.83	agaactcacaCtggtgaggta	m5C_15594
chr7	129410290	-	C	58	42	0.724	0	0	C	42	M	0.598	0.822	0	4E-06	0	502.28	gtagaactcaCactggtgagg	m5C_15577
chr7	129410292	-	C	60	45	0.75	0	0	C	45	M	0.628	0.842	0	3E-07	0	564.91	tggtagaactCacactggtga	m5C_15582
chr7	134851421	-	C	37	11	0.297	0	0	T	26	M	0.175	0.458	1E-15	3E-06	2E-15	28.551	agcaggagccCtgaggaggag	m5C_15838
chr7	134851423	-	C	39	18	0.462	0	0	T	21	M	0.316	0.614	0	3E-05	0	113.64	gtagcaggagCcctgaggagg	m5C_15839
chr7	139094341	+	C	41	19	0.463	0	0	T	22	M	0.321	0.613	0	1E-06	0	121.81	agaaaagaaaCcaggaacggc	m5C_15726
chr7	139094348	+	C	71	23	0.324	0	0	T	48	M	0.227	0.439	0	4E-11	0	104.22	aaaccaggaaCggctgaaacg	m5C_15729
chr7	139094351	+	C	69	28	0.406	0	0	T	41	M	0.298	0.524	0	1E-08	0	166.82	ccaggaacggCtgaaacgaag	m5C_15732
chr7	139094357	+	C	74	26	0.351	0	0	T	48	M	0.252	0.465	0	1E-10	0	131.24	acggctgaaaCgaagagaaga	m5C_15720
chr7	141304706	-	C	36	8	0.222	0	0	T	28	M	0.117	0.381	7E-11	7E-07	8E-11	9.4968	tatgtgatccCagattggatg	m5C_15484
chr7	141304708	-	C	37	10	0.27	0	0	T	27	M	0.154	0.43	7E-14	2E-06	7E-14	20.282	aatatgtgatCccagattgga	m5C_15476
chr7	148638588	+	C	104	32	0.308	0	0	T	72	M	0.227	0.402	0	1E-14	0	145.38	acagttggtcCgagtgttgtg	m5C_16248
chr7	148638627	+	C	109	32	0.294	0	0	T	77	M	0.216	0.385	0	1E-14	0	138.42	ttaacattgtCtccccccaca	m5C_16252
chr7	148638630	+	C	111	44	0.396	0	0	T	67	M	0.31	0.489	0	2E-14	0	273.09	acattgtctcCccccacaacc	m5C_16249
chr7	148638649	+	C	189	59	0.312	0	0	T	130	M	0.25	0.381	0	0	0	295.47	ccgcgcttgaCtagcttgctg	m5C_16247
chr7	148638657	+	C	162	55	0.34	0	0	T	107	M	0.271	0.415	0	0	0	298.16	gactagcttgCtgttttgcac	m5C_16251
chr7	148684289	-	C	55	38	0.691	0	0	C	38	M	0.56	0.797	0	2E-06	0	425.38	cagtcagttaCagatcgaact	m5C_16269
chr7	148684295	-	C	69	43	0.623	0	0	C	43	M	0.505	0.728	0	2E-07	0	434.49	tgttcacagtCagttacagat	m5C_16260
chr7	148684299	-	C	76	47	0.618	0	0	C	47	M	0.506	0.719	0	2E-08	0	475.65	tgattgttcaCagtcagttac	m5C_16253
chr7	148684301	-	C	76	48	0.632	0	0	C	48	M	0.519	0.731	0	3E-08	0	498.48	attgattgttCacagtcagtt	m5C_16263
chr7	148684313	-	C	64	32	0.5	0	0	CT	32	M	0.381	0.619	0	3E-08	0	243.85	tgagttatctCaattgattgt	m5C_16266
chr7	148684315	-	C	64	28	0.438	0	0	T	36	M	0.323	0.559	0	1E-08	0	180.85	atgagttatCtcaattgatt	m5C_16257
chr7	148684332	-	C	64	30	0.469	0	0	T	34	M	0.352	0.589	0	2E-08	0	211.05	ttggctggtcCgaaggtagtg	m5C_16256
chr7	148684333	-	C	68	51	0.75	0	0	C	51	M	0.636	0.838	0	8E-07	0	648.32	attggctggtCcgaaggtagt	m5C_16276
chr7	158456915	-	C	70	46	0.657	0	0	C	46	M	0.54	0.758	0	2E-08	0	497.16	ctccagaggaCgaggaggaag	m5C_15713
chr7	158456926	-	C	34	19	0.559	0	0	C	19	M	0.395	0.711	0	3E-05	0	149.92	agtgagagagCctccagagga	m5C_15676
chr8	1847065	-	C	34	22	0.647	0	0	C	22	M	0.479	0.785	0	6E-05	0	210.82	gatcctagacCagacacagac	m5C_17134
chr8	1847072	-	C	34	19	0.559	0	0	C	19	M	0.395	0.711	0	3E-05	0	149.92	ttcatgtgatCctagaccaga	m5C_17127
chr8	6926168	-	C	73	44	0.638	4	0.055	C	44	M	0.52	0.741	0	3E-07	0	457.42	ggttgggattCggtgcttta	m5C_15945
chr8	6926183	-	C	118	54	0.458	0	0	T	64	M	0.37	0.547	0	2E-13	0	400.13	ccctggtgggtCtagtggttgg	m5C_15937
chr8	6926191	-	C	57	23	0.404	0	0	T	34	M	0.286	0.533	0	9E-08	0	131.64	caaagttcccCtggtggtcta	m5C_15944
chr8	6926192	-	C	56	22	0.393	0	0	T	34	M	0.276	0.524	0	7E-08	0	121.35	tcaaagttccCctggtggtct	m5C_15938
chr8	6926193	-	C	53	23	0.434	0	0	T	30	M	0.31	0.567	0	9E-08	0	142.37	ttcaaagttcCcctggtggtc	m5C_15942
chr8	12570085	+	C	39	29	0.744	0	0	C	29	M	0.589	0.854	0	0.0002	0	341.72	ccctggtggtCtagtggttgg	m5C_15778
chr8	12570103	+	C	38	29	0.763	0	0	C	29	M	0.608	0.87	0	0.0002	0	352.6	tgggatttggCgctctcactg	m5C_15780
chr8	12570105	+	C	36	26	0.722	0	0	C	26	M	0.56	0.842	0	0.0001	0	291.24	ggatttggcgCtctcactgct	m5C_15781
chr8	22102490	-	C	94	37	0.394	0	0	T	57	M	0.301	0.495	0	3E-12	0	222.66	gttgagagggCgaaaaaggat	m5C_16138

chr8	22576077	+	C	33	11	0.333	0	0	T	22	M	0.198	0.504	2E-16	3E-06	2E-16	34.007	ctgagtgttgCccgggatggc	m5C_16114
chr8	23147913	+	C	44	34	0.773	0	0	C	34	M	0.63	0.872	0	2E-05	0	428.45	aggaagaggaCgaagaggagg	m5C_16111
chr8	30432335	-	C	32	19	0.594	0	0	C	19	M	0.423	0.745	0	3E-05	0	160.59	tgtatgtgaaCagtggtggag	m5C_16301
chr8	33371030	+	C	51	15	0.294	0	0	T	36	M	0.187	0.43	0	5E-09	0	56.126	attgggtgttCatacgcttgt	m5C_16191
chr8	33371034	+	C	53	19	0.358	0	0	T	34	M	0.243	0.493	0	2E-08	0	92.347	ggtgttcataCgcttgtgtga	m5C_16185
chr8	33371036	+	C	50	13	0.26	0	0	T	37	M	0.159	0.396	0	2E-09	0	41.266	tgttcatacgCttgtgtgaga	m5C_16195
chr8	33371051	+	C	39	13	0.333	0	0	T	26	M	0.206	0.49	0	7E-06	0	53.645	gtgagatgtgCcacccttgaa	m5C_16192
chr8	33371052	+	C	40	16	0.4	0	0	T	24	M	0.263	0.554	0	2E-05	0	84.314	tgagatgtgcCacccttgaac	m5C_16201
chr8	33371054	+	C	37	15	0.405	0	0	T	22	M	0.263	0.565	0	1E-05	0	79.039	agatgtgccaCccttgaacct	m5C_16187
chr8	33371055	+	C	40	15	0.375	0	0	T	25	M	0.242	0.53	0	3E-07	0	72.668	gatgtgccacCcttgaacctt	m5C_16197
chr8	33371056	+	C	37	19	0.514	0	0	C	19	M	0.359	0.666	0	3E-05	0	136.4	atgtgccaccCttgaaccttg	m5C_16198
chr8	33371062	+	C	39	16	0.41	0	0	T	23	M	0.271	0.566	0	2E-05	0	86.653	cacccttgaaCcttgttacga	m5C_16200
chr8	33371063	+	C	42	15	0.357	0	0	T	27	M	0.23	0.508	0	3E-07	0	68.967	acccttgaacCttgttacgac	m5C_16189
chr8	33371070	+	C	60	20	0.339	1	0.017	T	39	M	0.231	0.466	0	3E-08	0	92.55	aaccttgttaCgacgtgggca	m5C_16178
chr8	33371073	+	C	68	23	0.338	0	0	T	45	M	0.237	0.457	0	2E-09	0	109.08	cttgttacgaCgtgggcacat	m5C_16193
chr8	33371079	+	C	73	31	0.425	0	0	T	42	M	0.318	0.539	0	6E-10	0	197.06	acgacgtgggCacattacccg	m5C_16184
chr8	33371081	+	C	78	26	0.333	0	0	T	52	M	0.239	0.444	0	1E-10	0	124.14	gacgtgggcaCattacccgtc	m5C_16186
chr8	33371086	+	C	81	29	0.358	0	0	T	52	M	0.262	0.467	0	8E-12	0	152.1	gggcacattaCccgtctgacc	m5C_16203
chr8	33371087	+	C	81	40	0.494	0	0	T	41	M	0.388	0.6	0	2E-10	0	310.18	ggcacattacCcgtctgacct	m5C_16179
chr8	33371088	+	C	83	22	0.265	0	0	T	61	M	0.182	0.369	0	4E-13	0	80.099	gcacattaccCgtctgacctg	m5C_16188
chr8	33371091	+	C	75	38	0.507	0	0	C	38	M	0.396	0.617	0	4E-09	0	300.95	cattacccgtCtgacctgaac	m5C_16181
chr8	33371095	+	C	75	34	0.453	0	0	T	41	M	0.346	0.566	0	1E-09	0	235.07	acccgtctgaCctgaacttca	m5C_16194
chr8	33371096	+	C	59	23	0.39	0	0	T	36	M	0.276	0.517	0	9E-08	0	126.86	cccgtctgacCtgaacttcaa	m5C_16182
chr8	40449638	+	C	99	33	0.333	0	0	T	66	M	0.248	0.431	0	8E-13	0	163.84	gggtgtggaaCatcagttagt	m5C_16335
chr8	41917311	+	C	40	17	0.425	0	0	T	23	M	0.285	0.578	0	6E-07	0	96.931	gatggagtgaCagtattacta	m5C_16326
chr8	46951851	-	C	39	19	0.487	0	0	T	20	M	0.339	0.638	0	3E-05	0	128.69	ctcggatagcCggtcctctgc	m5C_16423
chr8	46951852	-	C	43	22	0.512	0	0	C	22	M	0.368	0.654	0	2E-06	0	161.71	cctcggatagCcggtcctctg	m5C_16432
chr8	46951859	-	C	44	23	0.523	0	0	C	23	M	0.379	0.662	0	3E-06	0	174.5	tggttggcctCggatagccgg	m5C_16428
chr8	46951861	-	C	44	22	0.5	0	0	CT	22	M	0.358	0.642	0	2E-06	0	157.66	cttggttggcCtcggatagcc	m5C_16433
chr8	46951862	-	C	44	23	0.523	0	0	C	23	M	0.379	0.662	0	3E-06	0	174.5	ccttggttggCctcggatagc	m5C_16422
chr8	56755213	-	C	69	20	0.29	0	0	T	49	M	0.196	0.406	0	7E-10	0	78.464	atcgtccgacCtgggtatagg	m5C_16505
chr8	56755214	-	C	62	40	0.645	0	0	C	40	M	0.521	0.753	0	1E-07	0	416.65	catcgtccgaCctgggtatag	m5C_16510
chr8	56986427	-	C	38	29	0.763	0	0	C	29	M	0.608	0.87	0	0.0002	0	352.6	tgttgagtaaCggtgataatt	m5C_16502
chr8	67834714	-	C	40	21	0.525	0	0	C	21	M	0.375	0.671	0	5E-05	0	157.49	aattttaagaCtgagatgcca	m5C_16742
chr8	67834736	-	C	58	41	0.707	0	0	C	41	M	0.58	0.808	0	3E-06	0	475.49	ctgaggttgtCtttgaagaaa	m5C_16746
chr8	67834746	-	C	54	23	0.426	0	0	T	31	M	0.303	0.558	0	9E-08	0	139.53	gtttacccagCtgaggttgtc	m5C_16747
chr8	67834749	-	C	54	32	0.593	0	0	C	32	M	0.46	0.713	0	7E-07	0	294.19	gccgtttaccCagctgaggtt	m5C_16744
chr8	67834750	-	C	53	26	0.491	0	0	T	27	M	0.361	0.621	0	2E-07	0	187.84	tgccgtttacCcagctgaggt	m5C_16734
chr8	67834751	-	C	57	24	0.421	0	0	T	33	M	0.302	0.55	0	1E-07	0	144.9	ttgccgtttaCccagctgagg	m5C_16743
chr8	67834757	-	C	57	36	0.632	0	0	C	36	M	0.502	0.745	0	2E-06	0	361.28	tactttttgcCgtttacccag	m5C_16737
chr8	67834758	-	C	55	24	0.436	0	0	T	31	M	0.314	0.567	0	1E-07	0	150.59	ttactttttgCcgtttaccca	m5C_16735
chr8	67834765	-	C	51	32	0.627	0	0	C	32	M	0.49	0.747	0	7E-07	0	313.76	acttaaattaCtttttgccgt	m5C_16733
chr8	67834774	-	C	55	25	0.455	0	0	T	30	M	0.33	0.585	0	2E-07	0	165.13	acaatgatgaCttaaattact	m5C_16745
chr8	68070802	+	C	50	29	0.58	0	0	C	29	M	0.442	0.706	0	4E-07	0	256.55	tgaagaggaaCaggaaaagaa	m5C_16528
chr8	69218664	-	C	43	25	0.581	0	0	C	25	M	0.433	0.716	0	4E-06	0	216.64	ggaggtggagCatgagcacag	m5C_16530
chr8	70602423	-	C	318	183	0.575	0	0	C	183	M	0.521	0.629	0	0	0	1905.3	acctactatcCagcgaaacca	m5C_17994
chr8	70602432	-	C	444	189	0.426	0	0	T	255	M	0.381	0.472	0	0	0	1438.3	actgtccctaCctactatcca	m5C_17949
chr8	98725892	+	C	33	10	0.303	0	0	T	23	M	0.174	0.473	2E-14	2E-06	2E-14	23.982	aacctaaggtCtcagatgatg	m5C_16926
chr8	100218905	-	C	45	29	0.644	0	0	C	29	M	0.498	0.768	0	1E-05	0	289.05	ggttttagacCagatgtttaa	m5C_16860
chr8	110594886	-	C	31	12	0.387	0	0	T	19	M	0.237	0.562	0	5E-06	0	56.959	atgatgatgaCgatgatgatg	m5C_17575
chr8	117862909	-	C	46	10	0.217	0	0	T	36	M	0.123	0.356	1E-12	2E-08	1E-12	14.731	gaacttctgcCagaaaaagag	m5C_16989
chr8	125785419	-	C	76	56	0.737	0	0	C	56	M	0.628	0.823	0	9E-08	0	703.57	aaggagcatcCctagtggtct	m5C_17222
chr8	125785420	-	C	68	46	0.676	0	0	C	46	M	0.558	0.776	0	4E-07	0	513.77	caaggagcatCcctagtggtc	m5C_17206
chr8	142233886	-	C	41	21	0.512	0	0	C	21	M	0.365	0.657	0	2E-06	0	153.23	ggcacggtctCtcgtgagtgc	m5C_17264
chr8	142233892	-	C	40	30	0.75	0	0	C	30	M	0.598	0.858	0	1E-05	0	358.83	tctgggggcaCggtctctcgt	m5C_17258
chr8	142233894	-	C	41	10	0.244	0	0	T	31	M	0.138	0.393	2E-13	2E-08	3E-13	17.447	cctctgggggCacggtctctc	m5C_17250
chr8	144997043	-	C	49	18	0.367	0	0	T	31	M	0.247	0.507	0	8E-07	0	88.794	ggacaaggagCagatggcgca	m5C_17426
chr8	144997050	-	C	57	29	0.509	0	0	C	29	M	0.383	0.634	0	4E-07	0	221.91	tgcaggaggaCaaggagcaga	m5C_17421
chr8	144997058	-	C	50	16	0.32	0	0	T	34	M	0.208	0.458	0	7E-09	0	66.426	gcggcggctgCaggaggacaa	m5C_17419
chr9	4793080	-	C	71	38	0.535	0	0	C	38	M	0.42	0.646	0	4E-09	0	319.5	tgcgcgctctCcgaagagacg	m5C_17305
chr9	17578939	-	C	47	20	0.426	0	0	T	27	M	0.295	0.567	0	1E-06	0	118.05	ggggatgtggCtcagcggcac	m5C_18508
chr9	19063673	-	C	31	18	0.581	0	0	C	18	M	0.408	0.736	0	3E-05	0	146.76	tcgtaacggaCagatacgggg	m5C_17487
chr9	21512158	-	C	82	28	0.341	0	0	T	54	M	0.248	0.449	0	6E-12	0	138.88	gctggcatagCtgttgaactg	m5C_17855
chr9	21512163	-	C	95	51	0.537	0	0	C	51	M	0.437	0.634	0	9E-11	0	445.84	aagatgctggCatagctgttg	m5C_17848
chr9	35657751	-	C	93	44	0.473	0	0	T	49	M	0.375	0.574	0	2E-11	0	329.77	ctcagcgcggCtgttttttgt	m5C_18100
chr9	35657754	-	C	103	49	0.476	0	0	T	54	M	0.382	0.571	0	2E-12	0	374.26	attctcagcgCggctgttttt	m5C_18137
chr9	35657761	-	C	110	24	0.218	0	0	T	86	M	0.151	0.304	0	0	0	72.59	ggggctcattCtcagcgcggc	m5C_18101
chr9	35657765	-	C	108	51	0.472	0	0	T	57	M	0.381	0.566	0	3E-12	0	388.26	acacggggctCattctcagcg	m5C_18106
chr9	35657767	-	C	99	31	0.313	0	0	T	68	M	0.23	0.41	0	4E-13	0	142.73	ccacacggggCtcattctcag	m5C_18135
chr9	35657774	-	C	71	25	0.352	0	0	T	46	M	0.251	0.468	0	9E-11	0	125.61	gcaccaaccaCacggggctca	m5C_18121
chr9	35657780	-	C	64	28	0.438	0	0	T	36	M	0.323	0.559	0	1E-08	0	180.85	gtccgcgcacCaaccacacgg	m5C_18128
chr9	35657785	-	C	64	28	0.438	0	0	T	36	M	0.323	0.559	0	1E-08	0	180.85	tgcgtgtccgCgcaccaacca	m5C_18125
chr9	35657787	-	C	62	32	0.516	0	0	C	32	M	0.394	0.636	0	3E-08	0	252.46	agtgcgtgtcCgcgcaccaac	m5C_18142
chr9	35657838	-	C	47	24	0.511	0	0	C	24	M	0.372	0.647	0	4E-06	0	178.76	agcggcgtggCgcgggggcgt	m5C_18113
chr9	35657843	-	C	58	33	0.569	0	0	C	33	M	0.441	0.688	0	9E-07	0	291.18	cgctgagcggCgtggcgcggg	m5C_18109
chr9	35657851	-	C	42	11	0.262	0	0	T	31	M	0.153	0.411	8E-15	4E-08	9E-15	23.69	gcgtatcccgCtgagcggcgt	m5C_18102
chr9	35657914	-	C	41	11	0.268	0	0	T	30	M	0.157	0.419	6E-15	4E-08	7E-15	24.554	gagagtgccaCgtgcatacgc	m5C_18126
chr9	35657930	-	C	77	19	0.247	0	0	T	58	M	0.164	0.354	0	7E-12	0	62.335	ccccggacctCgggcagagag	m5C_18129
chr9	35657937	-	C	52	19	0.365	0	0	T	33	M	0.248	0.501	0	2E-08	0	94.242	ggaaagtcccCggacctcggg	m5C_18104
chr9	35657938	-	C	51	21	0.412	0	0	T	30	M	0.288	0.548	0	5E-08	0	120.77	gggaaagtccCcggacctcgg	m5C_18112
chr9	35657973	-	C	92	26	0.286	1	0.011	T	65	M	0.203	0.386	0	5E-14	0	105.59	acactgaggaCtctgttcctc	m5C_18119
chr9	35657980	-	C	241	86	0.357	0	0	T	155	M	0.299	0.419	0	0	0	514.35	aggctacacaCtgaggactct	m5C_18122
chr9	35657987	-	C	468	131	0.28	0	0	T	337	M	0.241	0.322	0	0	0	631.83	gtatcctaggCtacacactga	m5C_18115
chr9	35657993	-	C	715	169	0.236	0	0	T	546	M	0.207	0.269	0	0	0	698.59	aggcctgtatCctaggctaca	m5C_18110
chr9	35657999	-	C	831	305	0.367	1	0.001	T	525	M	0.335	0.401	0	0	0	2045.6	tgctgaaggcCtgtatcctag	m5C_18132
chr9	35658000	-	C	831	179	0.215	0	0	T	652	M	0.189	0.245	0	0	0	675.89	gtgctgaaggCctgtatccta	m5C_18140
chr9	35658007	-	C	821	216	0.263	0	0	T	605	M	0.234	0.294	0	0	0	1011.4	gtggttcgtgCtgaaggcctg	m5C_18136
chr9	79186661	+	C	35	13	0.371	0	0	T	22	M	0.232	0.537	0	7E-06	0	60.231	tggggcatcgCcgcggagcct	m5C_18242
chr9	79186662	+	C	43	21	0.488	0	0	T	22	M	0.346	0.632	0	2E-06	0	145.39	ggggcatcgcCgcggagcctc	m5C_18238
chr9	79186669	+	C	59	38	0.644	0	0	C	38	M	0.517	0.754	0	2E-06	0	392.58	cgccgcggagCctcggttggc	m5C_18241
chr9	79186670	+	C	60	31	0.517	0	0	C	31	M	0.393	0.638	0	6E-07	0	243.71	gccgcggagcCtcggttggcc	m5C_18236
chr9	79186672	+	C	62	40	0.645	0	0	C	40	M	0.521	0.753	0	1E-07	0	416.65	cgcggagcctCggttggcctc	m5C_18247

chr9	79186679	+	C	54	20	0.37	0	0	T	34	M	0.254	0.504	0	3E-08	0	101.69	cctcggttggCctcggatagc	m5C_18231
chr9	79186680	+	C	52	36	0.692	0	0	C	36	M	0.557	0.801	0	2E-06	0	401.26	ctcggttggcCtcggatagcc	m5C_18249
chr9	79186682	+	C	59	27	0.458	0	0	T	32	M	0.337	0.583	0	2E-07	0	181.99	cggttggcctCggatagccgg	m5C_18244
chr9	79186683	-	C	31	14	0.452	0	0	T	17	M	0.292	0.622	0	9E-06	0	81.652	accggctatcCgaggccaacc	m5C_18262
chr9	79186688	-	C	48	11	0.229	0	0	T	37	M	0.133	0.365	5E-14	4E-08	5E-14	19.471	gggggaccggCtatccgaggc	m5C_18265
chr9	79186691	-	C	64	31	0.484	0	0	T	33	M	0.366	0.604	0	2E-08	0	227.13	ggcgggggacCggctatccga	m5C_18305
chr9	79186692	-	C	68	22	0.324	0	0	T	46	M	0.224	0.442	0	1E-09	0	98.725	aggcgggggaCcggctatccg	m5C_18280
chr9	79186699	-	C	84	52	0.619	0	0	C	52	M	0.512	0.716	0	2E-09	0	532.64	cggggacaggCgggggaccgg	m5C_18324
chr9	79186703	-	C	81	33	0.407	0	0	T	48	M	0.307	0.516	0	3E-11	0	202.59	ccggcggggaCaggcgggggga	m5C_18294
chr9	79186709	-	C	56	22	0.393	0	0	T	34	M	0.276	0.524	0	7E-08	0	121.35	ggcccgccggCggggacaggc	m5C_18298
chr9	79186712	-	C	44	18	0.409	0	0	T	26	M	0.277	0.556	0	8E-07	0	99.676	ggcggcccgcCggcggggaca	m5C_18278
chr9	89037857	-	C	190	56	0.295	0	0	T	134	M	0.234	0.363	0	0	0	262.63	aatggtgcacCaaaaaaaaaa	m5C_17826
chr9	89037858	-	C	156	80	0.513	0	0	C	80	M	0.435	0.59	0	2E-16	0	696.04	gaatggtgcaCcaaaaaaaaa	m5C_17825
chr9	89037860	-	C	182	40	0.22	0	0	T	142	M	0.166	0.285	0	0	0	132.6	aggaatggtgCaccaaaaaaa	m5C_17824
chr9	89037890	-	C	111	29	0.261	0	0	T	82	M	0.189	0.35	0	0	0	109.33	catgcattgaCctggtattgc	m5C_17827
chr9	97109699	-	C	55	20	0.364	0	0	T	35	M	0.249	0.496	0	3E-08	0	99.721	aggaggctgaCaatgaggtag	m5C_18381
chr9	97109703	-	C	59	29	0.492	0	0	T	30	M	0.368	0.616	0	4E-07	0	213.69	gagcaggaggCtgacaatgag	m5C_18375
chr9	100773572	+	C	100	33	0.33	0	0	T	67	M	0.246	0.427	0	2E-14	0	162.12	atgaggaagaCgaggacgatg	m5C_19793
chr9	100773578	+	C	82	30	0.366	0	0	T	52	M	0.27	0.474	0	1E-11	0	161.87	aagacgaggaCgatgaggatg	m5C_19791
chr9	100773650	+	C	111	26	0.234	0	0	T	85	M	0.165	0.321	0	0	0	85.881	gggatgaggaCgacgatgaag	m5C_19789
chr9	100773653	+	C	107	35	0.327	0	0	T	72	M	0.246	0.421	0	3E-14	0	171.88	atgaggacgaCgatgaagtca	m5C_19788
chr9	100774718	+	C	56	35	0.625	0	0	C	35	M	0.494	0.74	0	1E-06	0	345.84	agaatttggaCttgatgaaga	m5C_19792
chr9	104314872	+	C	37	17	0.459	0	0	T	20	M	0.31	0.616	0	2E-05	0	105.53	ggagagggaaCgagaaagaga	m5C_18178
chr9	104328738	-	C	33	12	0.364	0	0	T	21	M	0.222	0.534	0	5E-06	0	53.249	tgcaggtttcCgtagcgtagt	m5C_18188
chr9	111754688	-	C	276	103	0.373	0	0	T	173	M	0.318	0.432	0	0	0	655.56	ctgagcgttgCttaaaaaaaa	m5C_19384
chr9	111754693	-	C	318	70	0.22	0	0	T	248	M	0.178	0.269	0	0	0	249.31	catgtctgagCgttgcttaaa	m5C_19377
chr9	111754698	-	C	330	108	0.327	0	0	T	222	M	0.279	0.38	0	0	0	602.4	ctatgcatgtCtgagcgttgc	m5C_19378
chr9	111754703	-	C	347	95	0.274	0	0	T	252	M	0.23	0.323	0	0	0	436.1	cggggctatgCatgtctgagc	m5C_19344
chr9	111754708	-	C	374	91	0.243	0	0	T	283	M	0.203	0.289	0	0	0	368.7	cctcccggggCtatgcatgtc	m5C_19357
chr9	111754713	-	C	387	83	0.214	0	0	T	304	M	0.176	0.258	0	0	0	292.97	gggttcctccCggggctatgc	m5C_19356
chr9	111754714	-	C	391	102	0.261	0	0	T	289	M	0.22	0.307	0	0	0	448.43	tgggttcctcCcggggctatg	m5C_19360
chr9	111754715	-	C	385	131	0.34	0	0	T	254	M	0.295	0.389	0	0	0	772.16	ctgggttcctCccggggctat	m5C_19346
chr9	111754717	-	C	375	146	0.389	0	0	T	229	M	0.341	0.44	0	0	0	996.72	ccctgggttcCtcccggggct	m5C_19381
chr9	111754718	-	C	383	101	0.264	0	0	T	282	M	0.222	0.31	0	0	0	448.6	gccctgggttCctcccggggc	m5C_19375
chr9	111754725	-	C	335	112	0.334	0	0	T	223	M	0.286	0.386	0	0	0	640.51	acttgtggccCtgggttcctc	m5C_19341
chr9	111754726	-	C	325	92	0.283	0	0	T	233	M	0.237	0.334	0	0	0	435.81	cacttgtggcCctgggttcct	m5C_19359
chr9	111754727	-	C	316	74	0.234	0	0	T	242	M	0.191	0.284	0	0	0	282.46	gcacttgtggCcctgggttcc	m5C_19347
chr9	111754734	-	C	173	45	0.26	0	0	T	128	M	0.2	0.33	0	0	0	180.41	gttgaatgcaCttgtggccct	m5C_19342
chr9	111754736	-	C	159	33	0.208	0	0	T	126	M	0.152	0.277	0	0	0	100.17	acgttgaatgCacttgtggcc	m5C_19383
chr9	111754801	-	C	945	272	0.288	0	0	T	673	M	0.26	0.318	0	0	0	1413.7	aagaatgcagCtagctgtggg	m5C_19365
chr9	111754804	-	C	1126	301	0.267	0	0	T	825	M	0.242	0.294	0	0	0	1458.6	atgaagaatgCagctagctgt	m5C_19352
chr9	111754816	-	C	1552	479	0.309	0	0	T	1073	M	0.286	0.332	0	0	0	2741.3	gcttgtgtgtCgatgaagaat	m5C_19355
chr9	111754825	-	C	1414	365	0.258	0	0	T	1049	M	0.236	0.282	0	0	0	1722.8	gattgcttggCttgtgtgtcg	m5C_19343
chr9	111754830	-	C	1305	531	0.407	0	0	T	774	M	0.381	0.434	0	0	0	4041.5	tggtggattgCttggcttgtg	m5C_19358
chr9	111754845	-	C	992	262	0.264	0	0	T	730	M	0.238	0.292	0	0	0	1245.2	ttttatgactCttagtggtgg	m5C_19348
chr9	111754847	-	C	646	200	0.31	0	0	T	446	M	0.275	0.346	0	0	0	1100.6	ctttttatgaCtcttagtggt	m5C_19382
chr9	116171145	-	C	31	18	0.581	0	0	C	18	M	0.408	0.736	0	3E-05	0	146.76	atgatgaagaCgaagaaaggc	m5C_18445
chr9	116171157	-	C	34	12	0.353	0	0	T	22	M	0.215	0.521	0	5E-06	0	51.571	gggatgaggaCaatgatgaag	m5C_18436
chr9	127620265	-	C	41	21	0.512	0	0	C	21	M	0.365	0.657	0	2E-06	0	153.23	cgaggagaacCtgaagaccaa	m5C_18658
chr9	127620266	-	C	40	11	0.275	0	0	T	29	M	0.161	0.428	4E-15	3E-06	5E-15	25.45	acgaggagaaCctgaagacca	m5C_18649
chr9	127620275	-	C	39	18	0.462	0	0	T	21	M	0.316	0.614	0	3E-05	0	113.64	tcaacaagcaCgaggagaacc	m5C_18647
chr9	127623819	-	C	68	29	0.426	0	0	T	39	M	0.316	0.545	0	1E-08	0	183.26	gatcaaggctCgagatcttcg	m5C_18670
chr9	127623821	-	C	59	17	0.288	0	0	T	42	M	0.188	0.414	0	1E-08	0	64.044	aagatcaaggCtcgagatctt	m5C_18665
chr9	131456179	+	C	38	13	0.342	0	0	T	25	M	0.212	0.501	0	7E-06	0	55.152	tttaaggttcCcgatatggat	m5C_18561
chr9	135894974	+	C	41	24	0.585	0	0	C	24	M	0.434	0.722	0	4E-06	0	208.16	gcggtggcatCgacaaaagaa	m5C_19009
chr9	135894985	+	C	90	20	0.222	0	0	T	70	M	0.149	0.318	0	2E-15	0	59.485	gacaaaagaaCcattgaaaaa	m5C_19015
chr9	135894986	+	C	90	28	0.311	0	0	T	62	M	0.225	0.413	0	1E-13	0	125.92	acaaaagaacCattgaaaaat	m5C_19007
chr9	135895009	+	C	173	93	0.538	0	0	C	93	M	0.463	0.61	0	0	0	861.67	gagaaggaggCtgctgagatg	m5C_19038
chr9	135895012	+	C	153	56	0.366	0	0	T	97	M	0.294	0.445	0	0	0	329.09	aaggaggctgCtgagatggga	m5C_19024
chr9	135895253	+	C	31	9	0.29	0	0	T	22	M	0.161	0.466	4E-13	1E-06	4E-13	18.015	aagctggtatCtccaagaatg	m5C_19030
chr9	135895255	+	C	32	10	0.312	0	0	T	22	M	0.18	0.486	1E-14	2E-06	1E-14	25.084	gctggtatctCcaagaatggg	m5C_19021
chr9	136905268	-	C	32	23	0.719	0	0	C	23	M	0.546	0.844	0	8E-05	0	251.28	gaaggagaagCacaaagtgaa	m5C_18913
chr9	139565125	+	C	60	46	0.767	0	0	C	46	M	0.646	0.856	0	7E-06	0	593.98	agtaataatgCgccgtccacg	m5C_18794
chr9	139620679	-	C	33	10	0.303	0	0	T	23	M	0.174	0.473	2E-14	2E-06	2E-14	23.982	tgtcctggacCtgttggcacc	m5C_18828
chr9	139621283	-	C	39	27	0.692	0	0	C	27	M	0.536	0.814	0	0.0001	0	289.32	acatctgtgtCattaggtggc	m5C_18830
chr9	139621300	-	C	59	28	0.475	0	0	T	31	M	0.353	0.6	0	3E-07	0	197.49	gctgtggctgCcgtgtcacat	m5C_18825
chr9	139621317	-	C	69	16	0.232	0	0	T	53	M	0.148	0.344	0	1E-10	0	47.385	cccctagaggCgttgcagctg	m5C_18833
chr9	139621325	-	C	67	20	0.299	0	0	T	47	M	0.202	0.417	0	7E-10	0	80.925	ggacactgccCctagaggcgt	m5C_18834
chrM	247	-	C	215	62	0.288	0	0	T	153	M	0.232	0.352	0	0	0	287.62	tgcagacattCaattgttatt	m5C_23822
chrM	251	-	C	311	68	0.219	0	0	T	243	M	0.176	0.268	0	0	0	239.76	gctgtgcagaCattcaattgt	m5C_24009
chrM	260	-	C	498	183	0.367	0	0	T	315	M	0.326	0.411	0	0	0	1194.2	tggaaagcggCtgtgcagaca	m5C_24070
chrM	263	-	C	601	170	0.283	0	0	T	431	M	0.248	0.32	0	0	0	844.29	gtgtggaaagCggctgtgcag	m5C_24134
chrM	275	-	C	556	124	0.223	0	0	T	432	M	0.19	0.259	0	0	0	472.16	gttatgatgtCtgtgtggaaa	m5C_24137
chrM	324	-	C	96	41	0.427	0	0	T	55	M	0.333	0.527	0	8E-12	0	272.91	gtgctgtggcCagaagcgggg	m5C_24039
chrM	325	-	C	95	31	0.326	0	0	T	64	M	0.24	0.426	0	4E-13	0	149.04	agtgctgtggCcagaagcggg	m5C_24246
chrM	331	-	C	97	35	0.361	0	0	T	62	M	0.272	0.46	0	1E-12	0	190.57	tgtttaagtgCtgtggccaga	m5C_24124
chrM	368	-	C	173	85	0.491	0	0	T	88	M	0.418	0.565	0	0	0	710.32	tgttagggttCtttgtttttg	m5C_24036
chrM	382	-	C	265	157	0.595	1	0.004	C	157	M	0.535	0.652	0	0	0	1678.4	tctggttaggCtggtgttagg	m5C_24064
chrM	2521	-	C	81	63	0.778	0	0	C	63	M	0.676	0.855	0	1E-08	0	851.56	actggtgatgCtagaggtgat	m5C_23789
chrM	2530	-	C	85	65	0.765	0	0	C	65	M	0.664	0.842	0	2E-08	0	863.61	gcctctaataCtggtgatgct	m5C_23907
chrM	2536	-	C	71	55	0.775	0	0	C	55	M	0.665	0.856	0	8E-08	0	731.33	ggcggtgcctCtaatactggt	m5C_24031
chrM	2538	-	C	77	56	0.727	0	0	C	56	M	0.619	0.814	0	9E-08	0	693.04	caggcggtgcCtctaatactg	m5C_23928
chrM	2539	-	C	80	60	0.75	0	0	C	60	M	0.645	0.832	0	9E-09	0	774.18	gcaggcggtgCctctaatact	m5C_23944
chrM	2708	-	C	384	276	0.719	0	0	C	276	M	0.672	0.761	0	0	0	3708.2	cttgctgtgtCatgcccgcct	m5C_24128
chrM	3012	-	C	296	230	0.777	0	0	C	230	M	0.726	0.821	0	0	0	3340.7	gctgcaccatCgggatgtcct	m5C_23959
chrM	3241	+	C	50	34	0.68	0	0	C	34	M	0.542	0.792	0	2E-05	0	368.49	gttaagatggCagagcccggt	m5C_23724
chrM	3246	+	C	45	29	0.644	0	0	C	29	M	0.498	0.768	0	1E-05	0	289.05	gatggcagagCccggtaatcg	m5C_23543
chrM	3248	+	C	49	33	0.673	0	0	C	33	M	0.534	0.788	0	2E-05	0	352.3	tggcagagccCggtaatcgca	m5C_23490
chrM	3255	+	C	51	34	0.667	0	0	C	34	M	0.53	0.78	0	1E-06	0	360.18	gcccggtaatCgcataaaact	m5C_23592
chrM	3257	+	C	47	31	0.66	0	0	C	31	M	0.517	0.778	0	1E-05	0	320.36	ccggtaatcgCataaaactta	m5C_23440
chrM	3264	+	C	50	34	0.68	0	0	C	34	M	0.542	0.792	0	2E-05	0	368.49	tcgcataaaaCttaaaacttt	m5C_23705
chrM	3271	+	C	49	34	0.694	0	0	C	34	M	0.555	0.805	0	2E-05	0	377.21	aaacttaaaaCtttacagtca	m5C_23542

chrM	3276	+	C	49	33	0.673	0	0	C	33	M	0.534	0.788	0	2E-05	0	352.3	taaaactttaCagtcagaggt	m5C_23643
chrM	3288	+	C	47	36	0.766	0	0	C	36	M	0.628	0.864	0	3E-05	0	451.98	gtcagaggttCaattcctctt	m5C_23541
chrM	6264	+	C	40	29	0.725	0	0	C	29	M	0.572	0.839	0	1E-05	0	331.56	tagtggaggcCggagcaggaa	m5C_23645
chrM	6461	-	C	35	22	0.629	0	0	C	22	M	0.463	0.768	0	6E-05	0	203.88	taggacggatCagacgaagag	m5C_23804
chrM	6466	-	C	92	44	0.478	0	0	T	48	M	0.379	0.579	0	2E-11	0	333.63	gtgattaggaCggatcagacg	m5C_24087
chrM	6481	-	C	97	60	0.619	0	0	C	60	M	0.519	0.709	0	5E-10	0	622.95	agaagtaggaCtgctgtgatt	m5C_23799
chrM	6505	-	C	79	33	0.418	0	0	T	46	M	0.315	0.528	0	1E-09	0	208.07	gcagctaggaCtgggagagat	m5C_24027
chrM	6511	-	C	66	38	0.576	0	0	C	38	M	0.456	0.688	0	1E-07	0	346.26	atgccagcagCtaggactggg	m5C_23770
chrM	6514	-	C	47	13	0.277	0	0	T	34	M	0.169	0.418	0	1E-07	0	44.035	gtgatgccagCagctaggact	m5C_24030
chrM	6517	-	C	63	36	0.571	0	0	C	36	M	0.449	0.686	0	6E-08	0	323	atagtgatgcCagcagctagg	m5C_24126
chrM	6518	-	C	71	35	0.493	0	0	T	36	M	0.38	0.607	0	2E-09	0	266.03	tatagtgatgCcagcagctag	m5C_23878
chrM	6542	-	C	64	43	0.672	0	0	C	43	M	0.55	0.774	0	2E-07	0	472.99	gttgaggttgCggtctgttag	m5C_23833
chrM	7638	-	C	113	24	0.212	0	0	T	89	M	0.147	0.297	0	0	0	70.6	ataagctcttCtatgataggg	m5C_24103
chrM	7641	-	C	134	28	0.209	0	0	T	106	M	0.149	0.285	0	0	0	83.278	gtgataagctCttctatgata	m5C_24303
chrM	9134	-	C	35	20	0.571	0	0	C	20	M	0.409	0.72	0	4E-05	0	163.43	acagcgatttCtaggatagtc	m5C_23940
chrM	9143	-	C	36	21	0.583	0	0	C	21	M	0.422	0.729	0	5E-05	0	177.24	attaaggcgaCagcgatttct	m5C_23754
chrM	9146	-	C	38	23	0.605	0	0	C	23	M	0.447	0.744	0	8E-05	0	205.7	tggattaaggCgacagcgatt	m5C_23932
chrM	9158	-	C	34	20	0.588	0	0	C	20	M	0.422	0.736	0	4E-05	0	168.89	aaaacgtaggCttggattaag	m5C_24214
chrM	9308	-	C	41	27	0.659	0	0	C	27	M	0.505	0.784	0	7E-06	0	272.97	gaagtgaaatCacatggctag	m5C_24125
chrM	9330	-	C	47	32	0.681	0	0	C	32	M	0.538	0.796	0	2E-05	0	344.53	gtatgaggagCgttatggagt	m5C_23965
chrM	9343	-	C	47	15	0.319	0	0	T	32	M	0.204	0.462	0	3E-07	0	61.186	gttagtaggcCtagtatgagg	m5C_24185
chrM	9923	+	C	31	10	0.323	0	0	T	21	M	0.186	0.499	7E-15	2E-06	8E-15	26.273	gaagccgccgCctgatactgg	m5C_23685
chrM	11100	-	C	506	284	0.561	0	0	C	284	M	0.518	0.604	0	0	0	2940.7	atgattagttCtgtggctgtg	m5C_23797
chrM	11247	-	C	128	48	0.375	0	0	T	80	M	0.296	0.461	0	1E-15	0	284.09	taaattagtgCgatgagtagg	m5C_24010
chrM	11274	-	C	87	38	0.437	0	0	T	49	M	0.337	0.541	0	1E-10	0	256.44	tttagtgagcCtagggtgttg	m5C_23829
chrM	11275	-	C	75	23	0.307	0	0	T	52	M	0.214	0.418	0	4E-11	0	98.379	gtttagtgagCctagggtgtt	m5C_24083
chrM	12584	-	C	65	26	0.4	0	0	T	39	M	0.29	0.521	0	6E-09	0	150.65	gagaagtagtCtagtttgaag	m5C_23746
chrM	13580	-	C	65	46	0.708	0	0	C	46	M	0.588	0.804	0	4E-07	0	540.99	agggaggtagCgatgagagta	m5C_23765
chrM	13591	-	C	57	33	0.579	0	0	C	33	M	0.45	0.698	0	9E-07	0	296.87	aggcgcttgtCagggaggtag	m5C_23825
chrM	13596	-	C	51	20	0.392	0	0	T	31	M	0.27	0.529	0	3E-08	0	108.11	gctataggcgCttgtcaggga	m5C_24111
chrM	13598	-	C	46	23	0.5	0	0	CT	23	M	0.361	0.639	0	3E-06	0	166.15	gtgctataggCgcttgtcagg	m5C_23919
chrM	14161	-	C	76	33	0.434	0	0	T	43	M	0.329	0.546	0	1E-09	0	216.88	tgagattgctCgggggaatag	m5C_23830
chrM	14163	-	C	69	33	0.478	0	0	T	36	M	0.365	0.594	0	3E-08	0	240.69	attgagattgCtcgggggaat	m5C_23834
chrM	14682	-	C	124	31	0.25	0	0	T	93	M	0.182	0.333	0	0	0	112.89	gtagtccgtgCgagaataatg	m5C_23910
chrM	14686	-	C	125	57	0.456	0	0	T	68	M	0.371	0.543	0	2E-14	0	423.28	ggttgtagtcCgtgcgagaat	m5C_23836
chrM	14687	-	C	120	46	0.383	0	0	T	74	M	0.301	0.473	0	3E-14	0	277.14	tggttgtagtCcgtgcgagaa	m5C_23982
chrM	14946	-	C	35	25	0.714	0	0	C	25	M	0.549	0.837	0	0.0001	0	274.72	gtgatgtgggCgattgatgaa	m5C_23820
chrM	15315	-	C	36	20	0.556	0	0	C	20	M	0.396	0.705	0	4E-05	0	158.32	gctagggctgCaataatgaag	m5C_24189
chrM	15324	-	C	34	12	0.353	0	0	T	22	M	0.215	0.521	0	5E-06	0	51.571	tggagtgctgCtagggctgca	m5C_23990
chrM	15467	-	C	1181	524	0.444	0	0	T	657	M	0.416	0.472	0	0	0	4355.3	gtgttaatgtCattaaggaga	m5C_23826
chrM	15498	-	C	811	423	0.522	0	0	C	423	M	0.487	0.556	0	0	0	4121.5	tctgggtcgcCtaggaggtct	m5C_23767
chrM	15501	-	C	658	426	0.647	0	0	C	426	M	0.61	0.683	0	0	0	5198.5	ttgtctgggtCgcctaggagg	m5C_24230
chrM	15507	-	C	503	215	0.43	3	0.006	T	285	M	0.387	0.474	0	0	0	1665.4	gtataattgtCtgggtcgcct	m5C_23983
chrM	15522	-	C	80	51	0.637	0	0	C	51	M	0.528	0.734	0	2E-09	0	538.67	aaggggttggCtagggtataa	m5C_24247
chrM	16440	-	C	36	18	0.5	0	0	CT	18	M	0.345	0.655	0	3E-05	0	124.11	aggagagtagCactcttgtgc	m5C_24296
chrX	1554623	-	C	35	15	0.429	0	0	T	20	M	0.28	0.591	0	1E-05	0	83.953	ctggagaagcCggtggacaag	m5C_18676
chrX	1554624	-	C	39	18	0.462	0	0	T	21	M	0.316	0.614	0	3E-05	0	113.64	tctggagaagCcggtggacaa	m5C_18674
chrX	2641383	+	C	53	21	0.396	0	0	T	32	M	0.276	0.531	0	5E-08	0	115.87	gtggaggcagCcacaggaaag	m5C_18681
chrX	2641384	+	C	49	16	0.327	0	0	T	33	M	0.212	0.466	0	4E-07	0	67.863	tggaggcagcCacaggaaaga	m5C_18686
chrX	5301641	+	C	38	20	0.526	0	0	C	20	M	0.373	0.675	0	4E-05	0	149.03	gggtcgggatCctgtgattgc	m5C_18701
chrX	5301642	+	C	41	13	0.317	0	0	T	28	M	0.196	0.47	0	1E-07	0	50.868	ggtcgggatcCtgtgattgca	m5C_18708
chrX	10096686	+	C	42	27	0.643	0	0	C	27	M	0.492	0.77	0	7E-06	0	265.5	agctggagtaCacggaggagg	m5C_18762
chrX	10096688	+	C	37	8	0.216	0	0	T	29	M	0.114	0.372	1E-10	7E-07	1E-10	9.1227	ctggagtacaCggaggaggag	m5C_18763
chrX	10096701	+	C	40	23	0.575	0	0	C	23	M	0.422	0.715	0	3E-06	0	194.1	aggaggaggtCctggagatgg	m5C_18758
chrX	10096702	+	C	39	22	0.564	0	0	C	22	M	0.41	0.707	0	6E-05	0	180.29	ggaggaggtcCtggagatgga	m5C_18765
chrX	17168035	-	C	65	20	0.308	0	0	T	45	M	0.209	0.428	0	7E-10	0	83.548	atttgattgtCcaatgcaagt	m5C_19068
chrX	18693030	-	C	91	59	0.648	0	0	C	59	M	0.546	0.739	0	4E-10	0	644.35	ccagtggaacCatagccgtaa	m5C_19307
chrX	18693031	-	C	93	67	0.72	0	0	C	67	M	0.622	0.801	0	1E-09	0	833.31	cccagtggaaCcatagccgta	m5C_19308
chrX	18693039	-	C	103	77	0.748	0	0	C	77	M	0.656	0.822	0	4E-10	0	1010	gttcgagcccCagtggaacca	m5C_19311
chrX	18693040	-	C	103	77	0.748	0	0	C	77	M	0.656	0.822	0	4E-10	0	1010	ggttcgagccCcagtggaacc	m5C_19312
chrX	18693041	-	C	107	70	0.654	0	0	C	70	M	0.56	0.738	0	1E-10	0	784.21	gggttcgagcCccagtggaac	m5C_19314
chrX	18693042	-	C	103	69	0.67	0	0	C	69	M	0.574	0.753	0	1E-10	0	792.69	tgggttcgagCcccagtggaa	m5C_19313
chrX	18693046	-	C	98	64	0.653	0	0	C	64	M	0.555	0.74	0	9E-10	0	709.96	gtcctgggttCgagccccagt	m5C_19304
chrX	18693062	-	C	135	43	0.319	0	0	T	92	M	0.246	0.401	0	0	0	211.46	gctttacacgCagaaggtcct	m5C_19294
chrX	18693064	-	C	113	49	0.434	0	0	T	64	M	0.346	0.526	0	7E-14	0	339.01	ctgctttacaCgcagaaggtc	m5C_19291
chrX	18693066	-	C	111	32	0.288	0	0	T	79	M	0.212	0.379	0	2E-16	0	135.82	gtctgctttaCacgcagaagg	m5C_19298
chrX	18693071	-	C	95	51	0.537	0	0	C	51	M	0.437	0.634	0	9E-11	0	445.84	atcacgctgCtttacacgca	m5C_19299
chrX	18693074	-	C	99	55	0.556	0	0	C	55	M	0.457	0.65	0	2E-10	0	503.16	gttatcacgtCtgctttacac	m5C_19309
chrX	18693077	-	C	104	28	0.269	0	0	T	76	M	0.193	0.362	0	2E-15	0	108.27	gtggttatcaCgtctgcttta	m5C_19305
chrX	18693079	-	C	104	29	0.279	0	0	T	75	M	0.202	0.372	0	3E-15	0	117	tagtggttatCacgtctgctt	m5C_19310
chrX	18693096	-	C	112	28	0.25	0	0	T	84	M	0.179	0.338	0	0	0	100.24	gtagaggttcCatagtgtagt	m5C_19300
chrX	18693097	-	C	61	24	0.393	0	0	T	37	M	0.281	0.519	0	3E-09	0	134.73	cgtagaggttCcatagtgtag	m5C_19306
chrX	30156371	+	C	61	29	0.475	0	0	T	32	M	0.355	0.598	0	1E-08	0	206.08	tgaaaagaatCtagcatttat	m5C_19117
chrX	34234109	+	C	46	18	0.391	0	0	T	28	M	0.264	0.535	0	8E-07	0	95.015	tgagcagtaaCaggtctgtga	m5C_19141
chrX	34234114	+	C	78	25	0.321	0	0	T	53	M	0.227	0.43	0	9E-11	0	113.74	agtaacaggtCtgtgatgctc	m5C_19132
chrX	34234139	+	C	84	21	0.25	0	0	T	63	M	0.17	0.352	0	3E-13	0	71.283	atgtctggggCtgcatgcatg	m5C_19140
chrX	34234146	+	C	69	23	0.333	0	0	T	46	M	0.234	0.451	0	2E-09	0	107.41	gggctgcatgCatgccacagt	m5C_19133
chrX	50374827	+	C	38	28	0.737	0	0	C	28	M	0.58	0.85	0	0.0002	0	324.75	ggctaggattCggcgctttca	m5C_19328
chrX	50374832	+	C	32	22	0.688	0	0	C	22	M	0.514	0.82	0	6E-05	0	226.31	ggattcggcgCtttcataaaa	m5C_19331
chrX	53423442	-	C	53	33	0.623	0	0	C	33	M	0.488	0.741	0	9E-07	0	322.12	aagtgaatgaCaagaatcatg	m5C_19830
chrX	53423455	-	C	37	20	0.571	2	0.054	C	20	M	0.409	0.72	0	4E-05	0	163.43	gccaagaagtCggaagtgaat	m5C_19867
chrX	53430711	-	C	37	12	0.324	0	0	T	25	M	0.196	0.485	0	5E-06	0	47.119	gaagatgaacCaggttggtag	m5C_19869
chrX	53438731	-	C	32	10	0.312	0	0	T	22	M	0.18	0.486	1E-14	2E-06	1E-14	25.084	tctggaagaaCggaagaaagt	m5C_19845
chrX	53589116	-	C	112	39	0.348	0	0	T	73	M	0.266	0.44	0	3E-15	0	207.75	ggatgatagtCaggatgaaga	m5C_19842
chrX	53589141	-	C	38	11	0.289	0	0	T	27	M	0.17	0.448	2E-15	3E-06	2E-15	27.449	acagcagtggCagtaacgagg	m5C_19852
chrX	53590720	-	C	46	17	0.37	0	0	T	29	M	0.245	0.514	0	6E-07	0	83.38	gatctgggaaCagtacaatta	m5C_19834
chrX	53616737	-	C	90	19	0.211	0	0	T	71	M	0.14	0.306	0	9E-16	0	53.019	acggaaagctCgggaaaagca	m5C_19854
chrX	53616739	-	C	87	42	0.483	0	0	T	45	M	0.381	0.586	0	3E-10	0	319.81	gaacggaaagCtcgggaaaag	m5C_19844
chrX	57001147	-	C	57	23	0.404	0	0	T	34	M	0.286	0.533	0	9E-08	0	131.64	gtggggtgtgCtgattggtcg	m5C_19386
chrX	63041801	+	C	35	18	0.514	0	0	C	18	M	0.356	0.67	0	3E-05	0	128.05	tttgggatggCtgggaaggct	m5C_19571
chrX	64956685	+	C	145	51	0.352	0	0	T	94	M	0.279	0.432	0	0	0	284.32	gaagaagaagCgtgaaatggc	m5C_19418
chrX	64956721	+	C	172	39	0.227	0	0	T	133	M	0.171	0.295	0	0	0	133.02	gaagattgaaCgggagaagga	m5C_19401

chrX	64956736	+	C	47	15	0.319	0	0	T	32	M	0.204	0.462	0	3E-07	0	61.186	gaaggaggagCtgatggagag	m5C_19398
chrX	67580924	+	C	38	23	0.605	0	0	C	23	M	0.447	0.744	0	8E-05	0	205.7	aagggctggtCtgagggtagt	m5C_19470
chrX	67580942	+	C	37	11	0.297	0	0	T	26	M	0.175	0.458	1E-15	3E-06	2E-15	28.551	agtgagttatCtcaactgatt	m5C_19471
chrX	67580944	+	C	31	14	0.452	0	0	T	17	M	0.292	0.622	0	9E-06	0	81.652	tgagttatctCaactgattgt	m5C_19476
chrX	70516821	+	C	39	10	0.256	0	0	T	29	M	0.146	0.411	1E-13	2E-06	1E-13	18.775	accgcaacatCaaggaggctc	m5C_19611
chrX	70516840	+	C	75	48	0.64	0	0	C	48	M	0.527	0.739	0	3E-08	0	505.92	tcgtgagaagCtggagatgga	m5C_19622
chrX	80370383	-	C	32	23	0.719	0	0	C	23	M	0.546	0.844	0	8E-05	0	251.28	agaaaagaaaCaggagatgga	m5C_19722
chrX	102841797	+	C	102	42	0.412	0	0	T	60	M	0.321	0.509	0	3E-13	0	269.77	aaaggaaagcCagagagtgag	m5C_19750
chrX	102841901	+	C	44	15	0.341	0	0	T	29	M	0.219	0.489	0	3E-07	0	65.627	agagggaaagCcagagagtga	m5C_19747
chrX	108297350	+	C	57	30	0.526	0	0	C	30	M	0.399	0.65	0	5E-07	0	239.51	aagaatttttCgcgaaggccc	m5C_20350
chrX	108297352	+	C	76	42	0.553	0	0	C	42	M	0.441	0.659	0	9E-09	0	370.44	gaatttttcgCgaaggcccgc	m5C_20311
chrX	108297358	+	C	117	62	0.53	0	0	C	62	M	0.44	0.618	0	1E-12	0	545.56	ttcgcgaaggCccgcggcggg	m5C_20325
chrX	108297359	+	C	130	91	0.7	0	0	C	91	M	0.616	0.772	0	5E-13	0	1121.9	tcgcgaaggcCcgcggcgggt	m5C_20321
chrX	108297360	+	C	131	70	0.534	0	0	C	70	M	0.449	0.618	0	1E-14	0	628.84	cgcgaaggccCgcggcgggtg	m5C_20300
chrX	108297362	+	C	132	86	0.652	0	0	C	86	M	0.567	0.727	0	2E-13	0	975.22	cgaaggcccgCggcgggtgtt	m5C_20343
chrX	108297365	+	C	154	116	0.753	0	0	C	116	M	0.68	0.815	0	7E-14	0	1576.5	aggcccgcggCgggtgttgat	m5C_20290
chrX	111904912	+	C	41	11	0.268	0	0	T	30	M	0.157	0.419	6E-15	4E-08	7E-15	24.554	tgagttgtctCaattgattgt	m5C_19992
chrX	118975164	-	C	36	26	0.722	0	0	C	26	M	0.56	0.842	0	0.0001	0	291.24	aagaaaaagaCaaagagaaga	m5C_19945
chrX	125715081	+	C	206	58	0.282	0	0	T	148	M	0.225	0.347	0	0	0	260.5	aggaaggatgCgcgagggccg	m5C_19961
chrX	125715083	+	C	188	92	0.489	0	0	T	96	M	0.419	0.56	0	0	0	770.66	gaaggatgcgCgagggccgga	m5C_19963
chrX	125715089	+	C	107	56	0.523	0	0	C	56	M	0.43	0.616	0	1E-11	0	481.12	tgcgcgagggCcggaacgccg	m5C_19969
chrX	125715090	+	C	90	53	0.589	0	0	C	53	M	0.486	0.685	0	3E-09	0	514.77	gcgcgagggcCggaacgccgg	m5C_19964
chrX	125715238	+	C	42	19	0.452	0	0	T	23	M	0.312	0.601	0	1E-06	0	118.65	gagtccccccCccggcggtga	m5C_19968
chrX	125715239	+	C	48	20	0.417	0	0	T	28	M	0.288	0.557	0	1E-06	0	115.4	agtcccccccCcggcggtgaa	m5C_19957
chrX	125715240	+	C	58	22	0.379	0	0	T	36	M	0.266	0.508	0	7E-08	0	116.88	gtccccccccCggcggtgaac	m5C_19959
chrX	125715243	+	C	87	45	0.517	0	0	C	45	M	0.414	0.619	0	6E-10	0	372.37	ccccccccggCggtgaacggg	m5C_19960
chrX	125715250	+	C	150	86	0.573	0	0	C	86	M	0.493	0.65	0	1E-14	0	848.52	cggcggtgaaCggggaggagg	m5C_19962
chrX	125715261	+	C	143	32	0.224	0	0	T	111	M	0.163	0.299	0	0	0	104.45	ggggaggaggCgggaacccaa	m5C_19970
chrX	125715268	+	C	56	18	0.321	0	0	T	38	M	0.214	0.452	0	2E-08	0	77.043	aggcgggaacCcaagaagcgg	m5C_19958
chrX	128788242	+	C	66	26	0.394	0	0	T	40	M	0.285	0.515	0	6E-09	0	148.21	gggcgtgggaCacgcggcgca	m5C_20112
chrX	128788249	+	C	65	28	0.431	0	0	T	37	M	0.318	0.552	0	1E-08	0	177.85	ggacacgcggCgcacacctgt	m5C_20108
chrY	10036071	+	C	217	57	0.263	0	0	T	160	M	0.209	0.325	0	0	0	237.82	aagggcaccaCcaggagtgga	m5C_23038
chrY	10036083	+	C	68	37	0.544	0	0	C	37	M	0.427	0.657	0	8E-08	0	315.66	aggagtggagCctgcagctta	m5C_23142
chrY	10036084	+	C	51	14	0.275	0	0	T	37	M	0.171	0.409	0	3E-09	0	47.92	ggagtggagcCtgcagcttaa	m5C_23043
chrY	10036260	-	C	89	69	0.775	0	0	C	69	M	0.678	0.85	0	3E-08	0	935.95	ttgggggtcgCgtaactagtt	m5C_23178
chrY	10036584	+	C	82	48	0.585	0	0	C	48	M	0.477	0.686	0	1E-09	0	458.16	tagtgaggccCttggatcggc	m5C_23116
chrY	10036591	+	C	67	39	0.582	0	0	C	39	M	0.463	0.693	0	1E-07	0	360.9	gcccttggatCggccccgccg	m5C_23099
chrY	10036595	+	C	50	35	0.7	0	0	C	35	M	0.562	0.809	0	1E-06	0	393.75	ttggatcggcCccgccgggtc	m5C_23032
chrY	10037757	+	C	105	31	0.295	0	0	T	74	M	0.216	0.388	0	7E-15	0	134.21	ccaaattgacCttgtatgact	m5C_23140
chrY	10037766	+	C	2263	826	0.365	1	0	T	1436	M	0.346	0.385	0	0	0	5708.7	ccttgtatgaCttttagcggt	m5C_23108
chrY	10037773	+	C	3520	1402	0.399	3	0.001	T	2115	M	0.383	0.415	0	0	0	10727	tgacttttagCggtggatcac	m5C_23105
chrY	10037781	+	C	3653	1409	0.386	0	0	T	2244	M	0.37	0.402	0	0	0	10428	agcggtggatCactcggctcc	m5C_23069
chrY	10037783	+	C	3703	1476	0.399	1	0	T	2226	M	0.383	0.415	0	0	0	11307	cggtggatcaCtcggctcctg	m5C_23137
chrY	10037785	+	C	3687	1459	0.396	0	0	T	2228	M	0.38	0.412	0	0	0	11090	gtggatcactCggctcctgcg	m5C_23055
chrY	10037788	+	C	3674	1498	0.408	1	0	T	2175	M	0.392	0.424	0	0	0	11746	gatcactcggCtcctgcgttg	m5C_23087
chrY	10037790	+	C	3449	1432	0.415	2	0.001	T	2015	M	0.399	0.432	0	0	0	11430	tcactcggctCctgcgttgat	m5C_23083
chrY	10037794	+	C	3419	1313	0.384	3	0.001	T	2103	M	0.368	0.401	0	0	0	9668.8	tcggctcctgCgttgatgaag	m5C_23056
chrY	10037809	+	C	2917	1161	0.398	0	0	T	1756	M	0.38	0.416	0	0	0	8832.7	atgaagaatgCagctagctgt	m5C_23155
chrY	10037812	+	C	2814	1094	0.389	0	0	T	1720	M	0.371	0.407	0	0	0	8115.8	aagaatgcagCtagctgtgag	m5C_23074
chrY	10037816	+	C	2599	884	0.34	1	0	T	1714	M	0.322	0.359	0	0	0	5698.1	atgcagctagCtgtgagaatt	m5C_23076
chrY	10037838	+	C	1285	685	0.533	0	0	C	685	M	0.506	0.56	0	0	0	6928.6	atgtgaattgCaggacacatt	m5C_23054
chrY	10037843	+	C	1090	664	0.609	0	0	C	664	M	0.58	0.638	0	0	0	7700.7	aattgcaggaCacattgatca	m5C_23163
chrY	10037845	+	C	859	539	0.627	0	0	C	539	M	0.595	0.659	0	0	0	6410.2	ttgcaggacaCattgatcatc	m5C_23094
chrY	10037852	+	C	687	495	0.721	0	0	C	495	M	0.686	0.753	0	0	0	6789.5	acacattgatCatcgacactt	m5C_23107
chrY	10037916	+	C	34	27	0.794	0	0	C	27	M	0.632	0.897	0	0.0001	0	341.29	ctgagctttgCttgccaatca	m5C_23037
